# Exploiting Gene Families for Phylogenomic Analysis of Myzostomid Transcriptome Data

**DOI:** 10.1371/journal.pone.0029843

**Published:** 2012-01-20

**Authors:** Stefanie Hartmann, Conrad Helm, Birgit Nickel, Matthias Meyer, Torsten H. Struck, Ralph Tiedemann, Joachim Selbig, Christoph Bleidorn

**Affiliations:** 1 Department of Bioinformatics, Institute of Biochemistry and Biology, University of Potsdam, Potsdam, Germany; 2 University of Leipzig, Institute for Biology II, Molecular Evolution and Systematics of Animals, Leipzig, Germany; 3 Max Planck Institute for Evolutionary Anthropology, Department of Evolutionary Genetics, Leipzig, Germany; 4 Zoological Research Museum Alexander Koenig, Bonn, Germany; 5 Department of Evolutionary Biology, Institute of Biochemistry and Biology, University of Potsdam, Potsdam, Germany; Biodiversity Insitute of Ontario - University of Guelph, Canada

## Abstract

**Background:**

In trying to understand the evolutionary relationships of organisms, the current flood of sequence data offers great opportunities, but also reveals new challenges with regard to data quality, the selection of data for subsequent analysis, and the automation of steps that were once done manually for single-gene analyses. Even though genome or transcriptome data is available for representatives of most bilaterian phyla, some enigmatic taxa still have an uncertain position in the animal tree of life. This is especially true for myzostomids, a group of symbiotic (or parasitic) protostomes that are either placed with annelids or flatworms.

**Methodology:**

Based on similarity criteria, Illumina-based transcriptome sequences of one myzostomid were compared to protein sequences of one additional myzostomid and 29 reference metazoa and clustered into gene families. These families were then used to investigate the phylogenetic position of Myzostomida using different approaches: Alignments of 989 sequence families were concatenated, and the resulting superalignment was analyzed under a Maximum Likelihood criterion. We also used all 1,878 gene trees with at least one myzostomid sequence for a supertree approach: the individual gene trees were computed and then reconciled into a species tree using gene tree parsimony.

**Conclusions:**

Superalignments require strictly orthologous genes, and both the gene selection and the widely varying amount of data available for different taxa in our dataset may cause anomalous placements and low bootstrap support. In contrast, gene tree parsimony is designed to accommodate multilocus gene families and therefore allows a much more comprehensive data set to be analyzed. Results of this supertree approach showed a well-resolved phylogeny, in which myzostomids were part of the annelid radiation, and major bilaterian taxa were found to be monophyletic.

## Introduction

The need for resolving the evolutionary relationships of all life on Earth has never been more important than in today's times of high-throughput data. Knowing how organisms are related to each other allows to put biological phenomena into evolutionary perspective, and it is a crucial prerequisite for comparative analyses that aim to analyze and integrate the wealth of currently available data. The current flood of sequence data from model and non-model organisms is a great starting point for resolving the tree of life. However, these data also present us with new challenges with regard to data quality, the selection of data for subsequent analysis, and the automation of steps that were once done carefully for single-gene analyses.

In the current study, we use next generation sequence data to analyze the controversial phylogenetic position of Myzostomida, a group of enigmatic marine invertebrate organisms. Myzostomids are either ectocommensals or endosymbionts of echinoderms [1]. The myzostomid bodyplan is highly adapted to its parasitic lifestyle and co-evolution with its hosts has been demonstrated [2]. These taxa are non-model systems for which only limited sequence data were available so far. Furthermore, the Myzostomida are an example of non-model taxa whose phylogenetic position is contentious [3], [4], and placing Myzostomida within the tree of life has been difficult since their first description in 1827. Initially classified as trematode flatworms, they have been allied with crustaceans, pentastomids, acanthocephalans, and annelids [3]. Problems of placing this taxon are credited to their aberrant morphology, but even molecular systematic studies led to highly incongruent results. Several studies that used single or only a few selected genes have supported different hypotheses. Whereas mitochondrial genes, myosin II, and hox genes suggest that Myzostomida are relatives of Annelida [3], [5], the phylogenetic analysis of ribosomal protein genes, ribosomal RNA genes, and Elongation-factor 1 supports Myzostomida as relatives of Platyzoa (Platyhelminthes/flatworms and Syndermata) [3], [6]. Large-scale studies that included a broad taxon sampling have not been able to resolve this question unambiguously, either. A phylogenetic analysis of up to 150 genes from a set of 71 Metazoa, mainly derived from EST-libraries, group Myzostomida within a flatworm/Syndermata clade, but the authors regard the position of this taxon as highly unstable [7] and exclude it from final analyses. Hejnol [8] recovered myzostomids as part of Annelida, using on the same EST-data as Dunn et al. [7]. In another recent phylogenomic study, Myzostomida were also first considered but then excluded because of insufficient data [9]. Finally, in a phylogenomic analysis addressing annelid relationships [10], the question of whether myzostomids are an annelid ingroup was dependent on model-choice.

Selecting genes for subsequent analysis is a critical but difficult step in phylogenomics. Generally, genes are selected using predefined sets that are assumed to be orthologs [11] or using clustering approaches [7]. Selected genes are aligned, concatenated into a superalignment (also called supermatrix), and then used for phylogenetic analysis. Both of these strategies exclude a substantial amount of available information from the analyses, and they also may be biased towards selecting highly conserved genes. It was shown, for example, that the highly conserved ribosomal proteins frequently used for phylogenomic analyses can contain a phylogenetic signal that is incongruent with other marker genes [4], [10]. In contrast to superalignment analyses, supertree approaches that are based on reconciliation of gene family trees make use of much more of the available data because they allow to include paralogs. Gene duplications and losses can be inferred by comparison (reconciliation) of a gene tree with a known species tree [12]–[14]. During this process, the reconciliation cost is computed as the total number of duplications and gene losses that are required to reconcile a gene tree with its species tree. Tree reconciliation can also be applied to infer a species tree from a set of gene trees: gene tree parsimony (GTP) [15], [16] allows to reconcile multiple gene trees by proposing a species tree that would require the minimal number of duplications and/or losses. Gene tree parsimony is therefore an approach suited for the analysis of species trees, given a set of multigene families; recently it was successfully applied to deep level phylogenomic studies of plants [17] and animals [18].

In this study, we specifically target the phylogenetic position of Myzostomida as a taxonomic group that has been considered problematic in previous studies. We will use this particular phylogenetic real-world problem to compare the applicability and usefulness of both superalignment and supertree approaches. To this end, we sequenced the transcriptome of *M. cirriferum* using Illumina technology. This data was combined with a small set of EST contigs derived from Sanger sequencing. We used the combined data in an analysis pipeline that was designed to maximize the amount of information available for analysis from two myzostomids. The pipeline we developed is adapted to the practical problems of large-scale approaches that rely on ESTs and thus incomplete data. We analyzed a superalignment that consisted of 989 concatenated gene alignments and also performed a Gene Tree Parsimony analysis of 1,878 individual gene trees. In contrast to the superalignment approach, GTP allowed us to analyze a much more comprehensive data set. The results show that multi-locus nuclear gene families contain a strong phylogenetic signal, and that GTP is well suited to exploit this information. Using GTP, myzostomids are shown to be related to annelids.

## Methods

### Obtaining Myzostoma sequences

Individuals of *Myzostoma cirriferum* were collected from its host *Antedon bifida* (Echinodermata, Crinoidea) sampled in Morgat (France). Around one hundred male and female stages of the protandric hermaphroditic *Myzostoma* species were pooled in RNAlater. RNA was extracted using TRIZOL (Sigma, USA) and subsequently purified using the RNeasy MinElute Cleanup Kit (Qiagen, Germany). mRNA was converted into double stranded cDNA following Illuminas mRNA-Seq sample preparation guide (Part #1004898). Briefly, mRNA was isolated from total RNA using Dynal oligo(dT) beads (Invitrogen, Germany) and fragmented (ca. 200 bp) using divalent cationic ions. First strand synthesis was performed with random hexamer primers, using Superscript II polymerase (Invitrogen, Germany). Subsequent second strand synthesis was carried out with DNA Pol I polymerase (Invitrogen, Germany). Double stranded cDNA was blunt end repaired and converted into multiplex sequencing libraries following a previously published protocol [19]. Libraries were pooled and sequenced according to the manufacturer's instructions for single read multiplex experiments with 76 cycles paired-end on the Genome Analyzer IIx platform (v4 sequencing chemistry and v4 cluster generation kit). Raw sequences were analyzed with IBIS 1.1.2 [20]. Paired-end reads from a single cluster were merged if at least 11 bp were overlapping [21]. From this data, reads with more than 5 bases below a quality score of 15 and reads with low complexity were removed.

### Sequence assembly

A total of 16,327,304 reads were obtained as described, and CLC Genomics Workbench 4.0 was used to assemble these into 53,097 contigs with a mean length of 394 nucleotides (Table S1). Of these, we selected contigs that were at least 400 nucleotides in length for further analysis. We combined this data set with 2,900 *Myzostoma cirriferum* EST-contigs that were derived from Sanger sequencing [4], resulting in a final data set of 18,477 sequences that were at least 400 nucleotides in length.

**Figure 1:**
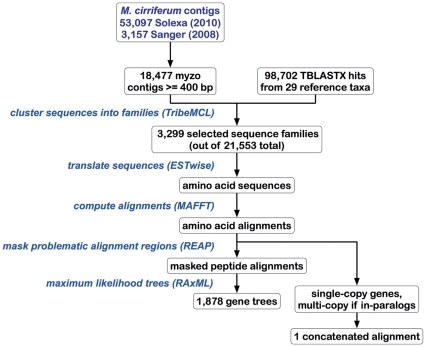
Outline of the analysis pipeline used in this study. We used existing sequence analysis software and customized perl scripts for this study.

A diagram outlining the pipeline analyzing these data is shown in Figure 1 and is described in more detail below. Freely available software as well as custom Perl scripts that made use of BioPerl [22] were used for its implementation.

### Annotating myzostomid sequences with Gene Ontology terms

Manually curated protein sequences in the UniProt-SwissProt data base were used to annotate myzostomid sequences with Gene Ontology IDs. Each myzostomid contig was compared against the UniProt sequences using BLAST [23]. Gene Ontology GO IDs of hits with E-values of 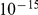 were then transferred to the corresponding myzostomid sequence.

### Obtaining sequences from other taxa

For the remaining taxa used in this study, we retrieved sequence data from the Joint Genome Institute and the National Center for Biotechnology Information. Fully sequenced genomes were available for *Daphnia pulex*, *Drosophila melanogaster*, *Caenorhabditis elegans*, *Capitella teleta*, *Helobdella robusta*, and *Lottia gigantea*. Other taxa were chosen if at least 1,500 EST contigs and/or predicted gene sequences were available. As the only exception we also included *Myzostoma seymourcollegiorum*, for which 571 EST contigs are publicly available. Full taxonomic names, number of sequences available for each taxon, and data sources are given in Table 1.

**Table 1:** Taxa used in this study.

Taxon	Phylum	Group	Sequences	Source
*Alvinella pompejana*	Annelida	Lophotrochozoa	25723	NCBI
*Capitella teleta*	Annelida	Lophotrochozoa	32415	JGI
*Helobdella robusta*	Annelida	Lophotrochozoa	23432	JGI
*Hirudo medicinalis*	Annelida	Lophotrochozoa	10582	NCBI
*Lumbricus rubellus*	Annelida	Lophotrochozoa	9085	NCBI
*Pomatoceros lamarckii*	Annelida	Lophotrochozoa	2702	NCBI
*Tubifex tubifex*	Annelida	Lophotrochozoa	8008	NCBI
*Terebratalia transversa*	Brachiopoda	Lophotrochozoa	1902	NCBI
*Gnathostumula peregrina*	Gnathostumulidae	Lophotrochozoa	2108	NCBI
*Pedicellina cernua*	Kamptozoa	Lophotrochozoa	2348	NCBI
*Aplysia califonica*	Mollusca	Lophotrochozoa	59801	NCBI
*Crassostrea virginica*	Mollusca	Lophotrochozoa	4556	NCBI
*Euprymna scolopes*	Mollusca	Lophotrochozoa	27036	NCBI
*Lottia gigantea*	Mollusca	Lophotrochozoa	23851	JGI
*Myzostoma cirriferum*	Myzostomida	Lophotrochozoa	35132	(this study)
*Myzostoma seymourcollegiorum*	Myzostomida	Lophotrochozoa	571	NCBI
*Cerebratulus lacteus*	Nermertea	Lophotrochozoa	1676	NCBI
*Dugesia japonica*	Platyhelminthes	Lophotrochozoa	3912	NCBI
*Macrostomum lignano*	Platyhelminthes	Lophotrochozoa	5534	NCBI
*Paraplanocera sp.*	Platyhelminthes	Lophotrochozoa	1485	NCBI
*Schistosoma mansoni*	Platyhelminthes	Lophotrochozoa	33704	NCBI
*Schmidtea mediterranea*	Platyhelminthes	Lophotrochozoa	15530	NCBI
*Taenia solium*	Platyhelminthes	Lophotrochozoa	6542	NCBI
*Brachionus plicatilis*	Rotifera	Lophotrochozoa	18828	NCBI
*Boophilus microplus*	Arthropoda	Ecdysozoa	14586	NCBI
*Daphnia pulex*	Arthropoda	Ecdysozoa	30907	JGI
*Drosophila melanogaster*	Arthropoda	Ecdysozoa	19841	HGSC
*Tribolium castaneum*	Arthropoda	Ecdysozoa	28381	NCBI
*Caenorhabditis elegans*	Nematoda	Ecdysozoa	28336	SI
*Trichinella spiralis*	Nematoda	Ecdysozoa	8843	NCBI
*Xiphinema index*	Nematoda	Ecdysozoa	4824	NCBI

Full list of taxa used in this study. Number of sequences and data sources are also given for each taxon. Abbreviation for data sources are as follows: NCBI: National Center for Biotechnology Information; JGI: Joint Genome Institute; HGSC: Human Genome Sequencing Center;SUGD: Sea Urchin Genome Database; SI: Sanger Institute.

### Assigning gene families

The 18,477 myzostomid EST contigs were compared against all available sequences from 29 non-Myzostomid reference genomes using TBLASTX [23]. The resulting 98,702 hits with an E-value of at least 1e-10 were then, together with the sequences of the two myzostomids, clustered into sequence (gene) families using TribeMCL [24] with an inflation value of 5.

### Translation of DNA sequences and computing sequence alignments

Because most sequences in our dataset are EST contigs, they were translated into amino acid sequences using ESTwise [25], which is specifically designed to address common problems of EST sequences, such as sequencing errors and indels. We generated for this step one set of reference amino acid sequences for each sequence family. Reference sequences for translation were identified using BLASTX searches of family members against a sequence database that contained protein sequences of reference taxa for which a full genome sequence was available as well as protein sequences of the fungal, invertebrate, and vertebrate divisions of UniProt [26], [27]. The best hits, if they had an E-value of less than 1e-40, were used as a database for ESTwise-aided translations of the family members into amino acid sequences. The amino acid sequences were retrieved from the ESTwise result, and sequences for which no ESTwise result was available were discarded from the sequence family. For each sequence family, we computed a multiple sequence alignment with the software MAFFT [28]. Alignments have been deposited at http://datadryad.org/.

### Concatenating alignments

We identified all families in which each taxon was represented by exactly one sequence. We also identified all families in which all sequences for a given taxon represented the full clade, in which case the sequence with the shortest branch length was chosen as the representative of the taxon. We included this latter set of trees because in-paralogs [29] do not affect the species tree. A total of 989 gene families fulfilled these criteria. All sequences from a given taxon from the 989 sequence families were concatenated, resulting in a superalignment of 306,257 alignment columns. We then removed all columns with 70% gaps or more, leaving 90,630 columns. RAxML v7.2.3 (PROTCATWAG) was used to infer a phylogeny from the masked alignment and to compute 100 bootstrap replicates [30]. Leaf stability of the taxa was assessed using the software Phyutility [31].

### Gene tree parsimony analysis

The software REAP [32] was used to remove alignment columns with more than 70% gaps from the individual sequence alignments. Masked alignments were then used as input for inference of rooted Maximum Likelihood trees and bootstrap replicates using RAxML v7.2.3 [30]. If present in the alignment, an ecdysozoan sequence was used for outgroup-rooting, otherwise a platyzoan sequence was used. Trees were outgroup-rooted, in order of preference, with a nematode or arthropod sequence. If ecdysozoa were not represented in the gene family, the tree was left unrooted. The 1,878 gene trees that contained myzostomid EST-contigs were reconciled into a species tree using Gene tree parsimony (GTP) [33], [34], as implemented in the software DupTree [35]. We used the ‘-r opt’ option that examines alternate gene tree rootings when optimizing reconciliation costs for the species tree. To evaluate support, we combined all bootstrap trees that were computed for the ML gene trees; this data set was then used to compute a species tree using DupTree.

## Results and Discussion

### Myzostomid data set

The 16,327,304 *M. cirriferum* sequence reads were clustered into 53,097 contigs with a mean length of 394 nt. Calculating the N50 weighted median statistic shows that 50% of the entire assembly comprised 14,265 contigs of at least 734 nt. Only contigs that were at least 400 nt in length were used for further analysis; these were combined with 2,900 *M. cirriferum* EST-contigs that were derived from Sanger sequencing [4]. The final data set used in this study consisted of 18,477 sequences that were at least 400 nucleotides in length. Their median length was 603 nt, suggesting that in most cases they represent only partial gene sequences.

**Figure 2:**
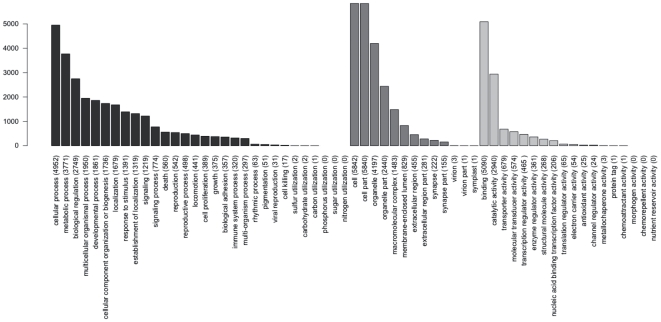
GO annotation of myzostomid sequences used in this study. The number of genes that are annotated with terms of the three GO hierarchies ‘molecular function’ (dark gray), ‘cellular component’ (gray), and ‘biological process’ (light gray) are listed.

We were able to annotate 6,574 of the myzostomid contigs with Gene Ontology-IDs. Within each of the three top-level GO-branches ‘molecular function’, ‘biological process’, and ‘cellular component’, we collapsed all annotations at different levels of specificity for a given myzostomid sequence. The distribution of GO-IDs is shown in Figure 2 and illustrates that in all three main branches of the GO hierarchy, most functional gene categories are represented in the myzostomid data set.

Neither juveniles nor trochophora stages were included in the library, and a surprising result was therefore the high number of developmental genes found in adult-based mRNA-seq. A total of 1,802 myzostomid contigs were annotated with GO-terms in the branch “developmental process”, which includes the terms “anatomical structure development”, “embryo development”, “multicellular organismal development”, “anatomical structure morphogenesis”, “regulation of developmental process”, “developmental growth”, and “anatomical structure formation involved in morphogenesis”. This included several members of the *hox*-, *wnt*-, and *fox*-gene families, which are usually only rarely encountered in adult-stage transcriptome libraries. One might speculate that expression of these genes play a role in the transition from male to female stages in this protandric hermaphrodite. Another hypothesis might be that these genes are already expressed in the fertilized eggs, even though cleavage starts after egg laying [36]. *In situ* hybridization expression studies of developmental genes in adult stages will help to clarify this question in the future.

### Assigning and processing of gene families

As described above, the 18,477 myzostomid contigs were clustered into sequence families with 98,702 BLAST hits from 29 taxa and 571 *M. seymourcollegiorum* contigs using the software TribeMCL. Clustering resulted in a total of 21,553 sequence families, a third of which contained three or more sequences. A large number of myzostomid sequences in our dataset therefore are singletons or in families of size two. Comparisons with the UniRef90 database did not suggest contamination with taxa outside of those included in our analyses. Many of these sequences may therefore represent myzostomid-specific genes or non-coding genes, including long ncRNAs. Alternatively, singletons and very small families may also be the result of using a relatively high inflation parameter of 5 for the clustering step.

For further analysis we selected sequence families that contained at least one myzostomid sequence and had at least three members. In addition, selected families were required to have also at least one annelid and platyhelminth sequence, or one trochozoan and one platyzoan sequence. A total of 3,299 families fulfilled these criteria. Sequences were translated, and a multiple sequence alignment was computed for each family. It was shown that EST-based data are challenging for phylogenetic analysis, but that alignment masking improves the phylogenetic accuracy of these data [32]. Because a large proportion of the sequence data used in our study is based on EST data, we consider alignment masking to be an important prerequisite for computing phylogenies.

### Concatenated alignments

**Figure 3:**
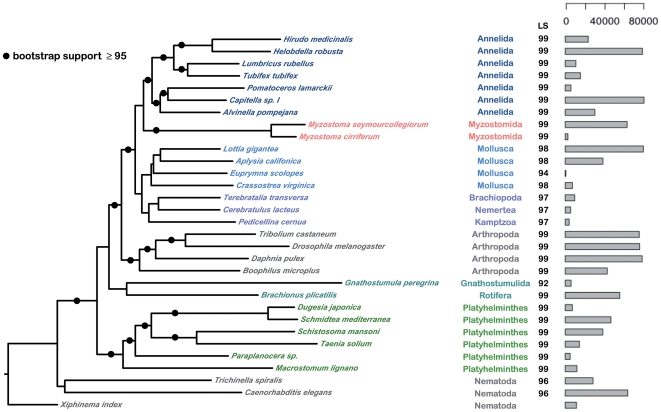
Maximum likelihood tree computed from a superalignment of 989 concatenated single-gene alignments. Names of taxa and tree branches are colored according to their taxonomic groups. The results of a leaf stability test (LS) is shown next to the taxon and lineage names. Also shown is a barplot indicating the number of columns of the superalignment (after alignment masking) with data for each of the taxa.

To potentially increase the phylogenetic signal and to overcome possible stochastic errors of single-gene analyses, we combined several of the individual alignments into a single superalignment as described above. The best ML tree computed from the reduced superalignment of 31 taxa (Figure 3) placed the two myzostomids as sister taxon of annelids.

Surprisingly, lophotrochozoans are not monophyletic in our analyses, and arthropods appear closer related to the included trochozoans (Annelida, Mollusca, Brachiopoda, Nemertea, Kamptozoa) with high bootstrap support. However, this is most likely an effect of taxon sampling, which was optimized to investigate the position of myzostomids, and of attraction of long branching flatwormns to the root of the tree consisting of long branched nematodes. Although the myzostomids are also long-branched, they are not attracted by the root and instead group with annelids.

Motivated by the suggestion that genome-scale data sets may be able to “resolve incongruence in molecular phylogenies” [37], anywhere from a handful [38] to more than hundred [7], [39] individual gene families have been combined into a single superalignment for the purpose of placing problematic taxa onto the tree of life. In many cases this strategy proved to be successful, but these phylogenomic approaches are also known to introduce bias that potentially cause systematic errors, e.g. from non-phylogenetic signals such as compositional bias [40]. In addition, superalignments often contain large amounts of missing data due to incomplete taxon sampling, and even after alignment masking, 15 of the 31 taxa in our data set were represented in less than 20% of the columns in the superalignment. Finally, the approach of concatenating multiple genes into a single alignment requires strict orthologs and thus severely limits the amount of data that is available for analysis. In our study, only about half of the gene families with a myzostomid representative (989 of 1,878) were used in the superalignment, leaving any phylogenetic signal present in the other half of the data set unexploited. In order to harness the information present in all gene families, we next used an approach that does not rely on the selection of orthologs and instead also makes use of multi-locus gene families.

### Gene tree parsimony

Many nuclear genes exist as multigene families that arose by duplication. Gene duplications and subsequent differential loss or divergence of duplicated genes in different lineages have led to complex patterns of orthology and paralogy in these gene families. In addition, incomplete sampling can present serious challenges for assigning orthology. For this reason, relatively few studies have used multigene families for resolving systematic relationships. In order to utilize the wealth of sequence data currently available for phylogenetic analyses, including the many multigene families, methods are needed that allow to deal with, or even directly address, the problems that paralogous sequences pose.

Gene Tree Parsimony (GTP) is a supertree approach that is specifically designed to address the complications presented by multi-locus data [41], [42]. This approach identifies evolutionary events that lead to incongruence between gene trees and species trees. Using a number of gene trees as input, GTP reconciles these by proposing a species tree that would require the minimal number of gene duplications and/or losses [42]–[45]. Thus, it aims to find the species tree that best represents the evolution of the source trees (gene trees) in a biologically meaningful way [46].

Gene tree parsimony was previously applied to a phylogenomic data set from seven plant taxa [47]. In this study, exhaustive search methods were used to infer a species tree under the GTP criterion, and the results demonstrated the utility of this approach for resolving organismal relationships using nuclear, EST-based sequence data. Fast heuristics for GTP have since been developed [48] and implemented in the software DupTree [35], now making this approach promising for data sets with large numbers of taxa. Recently, GTP was applied to almost 19,000 nuclear multigene families in order to resolve relationships of 136 plant taxa [17]. This study showed that EST-based multigene families contain a strong phylogenetic signal, but that large amounts of data may be required to obtain a resolved species tree.

**Figure 4:**
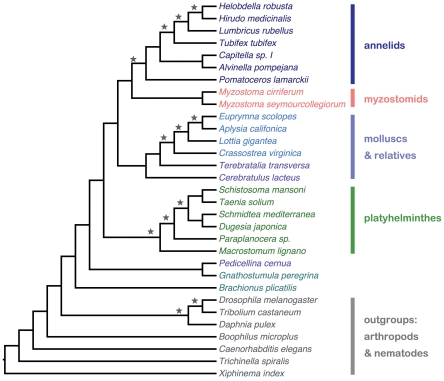
A reconciled tree inferred using gene tree parsimony. 1,878 individual gene trees were used to compute a species tree. Clades indicated with stars are also found in the bootstrap GTP approach (see text for details). Names of taxa and tree branches are colored according to their taxonomic groups.

We used the rooted likelihood trees for 1,878 individual sequence families to compute a species tree using GTP (Figure 4). The individual gene trees ranged in size from 4 to 338 sequences, with a mean (median) of 23 (14) sequences. Depending of the taxon representation for each gene family, arthropod or nematode sequences were used to outgroup-root the individual gene trees, 134 gene trees had neither arthropod nor nematode sequences and were left unrooted. Consequently, outgroups are not resolved in the GTP tree and Ecdysozoa appear to be paraphyletic. To assess confidence in the GTP species tree clades, we used all bootstrap data sets for all gene trees to compute a “bootstrap GTP species tree” using the DupTree software. Clades from the GTP species tree that were also found in the bootstrap GTP species tree are indicated with a star in Figure 4. There is currently, however, no established method for assessing confidence of GTP trees [17], [18].

Using GTP, we recovered monophyletic Lophotrochozoa, and major groups of this taxon (Annelida, Mollusca, Platyhelminthes) were also found to be monophyletic. As with the result of the superalignment approach, the GTP tree places the myzostomids as sister group of the annelids. This result is in congruence with a recent phylogenomic analysis of annelid relationships, in which all annelids included in the present study were recovered as part of the monopyletic Sedentaria [10]. The phylogenetic signal present in the entire data set, not only in gene families that can be included in a superalignment approach, therefore seem to be required to compute a resolved phylogeny of the 31 taxa used in this study.

Nuclear genes are often multi-locus in nature and have a history of duplications and losses. In the context of organismal evolution, these data have therefore presented problems for traditional phylogenetic analyses that rely on strictly orthologous loci. Supertree methods that can exploit gene families of orthologs and paralogs are not yet widely used for phylogenetic analysis, but GTP results in the current and other studies clearly show that multi-locus gene families are phylogenetically informative [17], [18]. These results are encouraging on two levels. First, applied to data from low-cost and high-throughput sequencing technologies, GTP has the potential to efficiently place non-model organisms on the tree of life. Second, the initial success of Gene Tree Parsimony will provide the impetus for further development and refinement of this approach [49].

### Conclusions

High-throughput sequencing technologies have radically changed the way data is analyzed, especially for non-model organisms. Instead of using experimentally targeted markers for phylogenetic analysis, the availability of EST-data provided us with a somewhat random access to gene sequences, as exemplified here for the invertebrates *Myzostoma cirriferum* and *M. seymourcollegiorum*. Gene phylogenies, superalignments, and gene tree reconciliation are not new methods, but they are currently being applied to increasingly large data sets that often focus on non-model organisms. It is known that combining systematically biased data into superalignments can amplify the non-phylogenetic signal [40]. In contrast, the signal is decomposed in supertree analyses, where separate gene trees are computed before integrating their information into a species tree. In our study, especially gene tree parsimony turned out to be a fast and powerful method, and it allowed to include much of the available transcriptome data. Whether GTP is as powerful in other cases of hard-to-place and long-branched taxa remains to be investigated.

Our phylogenomic analyses presented here are in line with previous results from analyzing mitochondrial gene order [3] and support an annelid origin of myzostomids. These results are also in congruence with the morphology of these enigmatic protostomes, including the presence of a chaetae bearing trochophore-like larvae, chaetae, and the rope-ladder like organized nervous system [1]. However, to clarify the exact phylogenetic position of myzostomids a much broader taxon sampling and deeper EST-sequencing of the hyperdiverse Annelida is necessary.

## Supporting Information

Table S1 **De novo assembly of the Illumina reads.** Assembly computed with the CLC Genomic Workbench 4.0 using default parameters.
(PDF)
