# Supplementary material for: Exploiting Gene Families for Phylogenomic Analysis of Myzostomid Transcriptome Data
Source: PLoS One. 2012 Jan 20;7(1):e29843. doi: 10.1371/journal.pone.0029843 (PMC3262807; doi:10.1371/journal.pone.0029843)
Supplement: Table S1 — De novo assembly of the Illumina reads. Assembly computed with the CLC Genomic Workbench 4.0 using default parameters. (PDF) [file pone.0029843.s001.pdf]

>ConsensusfromContig1 Average coverage: 45.53
CCGCTATTCTGAGTATGGCGCTGCAGTCCTGCTGCTTCTCATGGC
AACCTTTGCCCTGAT
TGCTCATTGGCTGGCCTGCATCTGGTATGCCATTGGCAATGTTGA
GCGTCCCACGCTTGA
ACCACCCAAAATTGGCTGGTTAGATCAGCTGGCTCGGACGACCA
ATCAACATTACATAAA
CGATACATCCGGAGGACCTTCCATCAGGTCCAAATACATCACCG
CCCGAGATCGGAAGAG
CGTCGTGTAGGGAAAGA
>ConsensusfromContig2 Average coverage: 38.24
TTTTTATCGCGTGTTTTCTGCTTATTTTAGAGCTTTTTACGAGTTT
ATAACGCCACTCAT
TATTGGTCGAATATGTGTATAGTATGTATTCTCATAGCATGCTTTA
TTTGATTCTATTTT
ATATCAGAGGTATTCAAACATTTTGATGGAGATTTAAATTCTTGT
ATAATGTCAATTTGA
ATTAAAATGCATATAATTTATGAATGTTGAGCTCCAATTTATCTT
TGCCAGGTGTTTA
>ConsensusfromContig3 Average coverage: 4.93
GGGAAATTTCATCCAATTAATTGGATTTTATATTTCAGACCTTTTT
GATACCGATATTTT
TCAGTTTATCCTGAATTCTAGTGACCAGAACATGGGTGAAAAAA
TGAAATTAATCAGAGG
CCTAGTGACCTAATTAATTACCAGCAGAGCAAATTAAAACGTGGG
AACTACTCGGATTCA
AAACAAGCACATTCTCAGCTTTCGAATGATGTATAAA
>ConsensusfromContig4 Average coverage: 9.94
GCTTATTCAGCGGAAAAATATCGTACGTTTTTGTTTACGTTTTAA
TCGAATGTTTGTGGA
AATTTTTTCAGTTATCTTTAAACTTCATGACATCGAACGATTCACC
ATTTCATACATTAA
ATTTCTTGATGCAATTGTGAGCAAAAGGAACCGAGATAAATACAG
ATTGTTACCGTGGTT
TATACCGAACTGAGAAGTAACTCTAGATTAGCC
>ConsensusfromContig5 Average coverage: 12.61
CCTACACGACGCTCTTCCGATCTCCAGCATGGAGAAGAATGAGAA
ATAGCAGGTATTTAT
TGCAGTAGAATGCCACCTCTAACGGCGTATACAGCTTTTCCGTAG
GTTTGTATTTTATCT
TCCCATTATTATTAATTGATTCGAATAATTTGTCTAGGTTTGTTTT
TCTATTGATGTTAC

AATCAGCGCGAATGAGCATTTTGAAGATAGCGAGTGAACATGCA
TCTATACAATTCGATA
GCATTGCGTATATAAGAGCAG
>ConsensusfromContig6 Average coverage: 6.12
GAATGAAGATTGCTTGCATTAGTATGCAAATTTAATTAGAATATG
ACCAGAATTGGTGAC
CTCTGGTGGTAGATAATAACAATCGATATATAAAACTTTACTCGGT
GTCTATTTTTTCTT
ATTTATCGTCATCGTCGTCGTTGTGATTGTTATCGCTCTCGTCGTTG
TTCTCTTTTTTGT
AGTGGAAATAGACAAAAATGTTTCGATCAACACCTGATTTGGATT
TTAATACGAATTATC
ATGTGTTAACAACTAAACCCGAATTAAAACGTGTCAAT
>ConsensusfromContig7 Average coverage: 7.64
ACAGGATACTTGATATCGCCTACCGCATAGAACTGTTTTTTGGCC
ACATGTATTGTGAGG
TAGTCGTGAATAGTCGACGAGAAGCTACTTTTACCGCCAAACGGT
GCCGGGGCTGTTGCG
TTGAAGTACCGACCGTCTCGATTGTCCAACCGCAGCTCGGCTGAA
CTTCCCTGACGCTCA
ATCTTCACGGTGTGCCATGCTCCATCAGAAACGTTGACCCAAGAG
AGAATAATATCCGAC
ACCCCAGATCG
>ConsensusfromContig8 Average coverage: 7.43
CATGACGCTCTTCCGAAATTGCGAAACGAACTGATAACACAGTTG
GTTATAAAACGATGT
ATGTCGACGAAAAGAAAGAACGTAGAACAAACAATGGCAAAAAC
AATATTGAAAATAACA
CACAAAGTACTGAGATTAAAGAACTGACAACAGCGCAACCGTTG
AAAATTATCTCAAATT
CAACACCTCCATTAACAAAGATCCAGATCGGAAGAGCACACGT
>ConsensusfromContig9 Average coverage: 44.23
CATAGGAAATGTTGAAGCGAATTCCATCACTCGGCCTTTCAGCTC
TTAAATATCCATTCC
AGGCTTGAGCTTTTACATTTTTTACCTGATTCTAATTCATGTTTTG
AGAAATCTCACTCA
TTTTATCTTCTACAAATAGTCTAGTGAATTTTTAAAAGTGTATGC
GGTAGCCAGTAGATG
ATATGAAGTTGTTATAACTATTATATCAGCCAAGGTGTTTTCTTTA
G
>ConsensusfromContig10 Average coverage: 12.39
GTCCAAACATACTTGACTTTCTGATCCCTCTTCATCTGGCGCAGG

GTATACATCAGAGAA
TTTATTTTACGGCATAGATGGTCATTGATATATATATTTGTCCTC
GGGAAAGTTCTATGA
ATGTCTTGAGCATAGATTCTTTTTCCGTCTTTGAATGCTTCCCTTT
TCGCGTTCATCAGT
GCACTCTTGTCCGATTTGTG
>ConsensusfromContig11 Average coverage: 5.63
TGCTCTTCCGATCTCTGGAATATTCTGAATCATTCTGCGGATGCCT
CAATTGTCTGAGAT
GTTCCAAAGCGCTATTATTGGTGTTCAAACAGTCAAAGTCACCCG
TTAGAGTACACTGAA
TAATGTGAAGACTATCAATGGCCTTCCCCTCGTTTGGAATTTCATT
CTTGTATGTTGAAT
TATTACTGAAATTCAAATCTTCACTATTCATTTCATGAGGTGTTCT
TTGATCGAAATTGT
CA
>ConsensusfromContig12 Average coverage: 5.65
AAACCAAGTTGCTGCTTTACTTTCTGAAAAACCAGTTTATTCGCT
ATTCTGAAAGAGAGT
GGAGGACTGCCATGCTGAGGCTGAATAACACTCCATTCGTAACCG
ACTCCCATCGCCCAA
TCCAGCAGCCATCTTCTTGGGAGAGAGGCGCTATCGAATGCTGAC
TTCAATTTCTCGTGA
ATTTTCTCCCATATCCTGGGCACACTCCCGAAGTTTGTAGGCTTCA
CTTCCAGTAGTGTT
TTTAACAATGAACCTGTGAAGAAATATTGCGCAGTTCGTGATTTA
AACAACTTTTAG
>ConsensusfromContig13 Average coverage: 5.77
ATTTTATATACATACATGATTTATAATTTCATACATACAGTCATA
ATATAAACATGAGAA
AAGGTGAAATGAATTTAAAGCGCACAGCGCCCATGTATTTATTAA
CAAATGGAGATCTAT
TCTAATTTCATATTCAGCTCGATTTGAATAGATTGAATGATACGA
AGACCTAAATAGGAA
TGAAAAGTTGAAAAGCGTGATTATCTAAAACGA
>ConsensusfromContig14 Average coverage: 8.30
TTATTCTATGATATANCAAGAATAGAGCATCACTAAATAGAATAA
TAATACGGTATATGA
ATCATTAGGATTCAGAGGTCATCCTGGTTTATTCAAACAGATTAA
AGCAATTCAAAGCGA
TAAATTTCGGATCGTCGTCATAACAATTATCCGGGTTTATATCCG
ATCCGAGATGATTCA

AATACCACGCCGACACCAAGCGGTCACATGAGATGTTGAAAATCC
ATAATATTTTATTGA
AACAAGCTCTTGAT
>ConsensusfromContig15 Average coverage: 14.30
TGGATGTTTTCGGTGATGGTGAAACTAGTGAATTCTTATCGAGAG
ACTGGCAAAGAATCG
AAAATACCTTCAACAAAATTGGCTATAGAGAAGGTTTACAGTCCA
ATCAAGATAGCTCTC
GGCAAATGGCATTTGATACAGGATTTTCCACAGCAGTTCATACTC
TGCGAAAAGTATCTT
ACATACAAGGACACGTTAGGGCACTTTCTCATCTTAAAGGTGTGT
CAAATACTAAGCAGG
CTTCGATCATCAAATTTTGTGAGCTGGTCATTAATTTTGTGAAACA
TTCGTTTTTCTGGT
CCAAGTGTGCT
>ConsensusfromContig16 Average coverage: 9.75
CTTTTTGAAAATTCTCAATATTTATGATGTTGGGTATAAAGATAC
ATTAAAATAATCGTT
ATATTCACAAATATATGCGTACACACACGTTTTGCTTCAATCTTT
CACAAGATGAAAGTG
CGAATTCTTATTCATGTTTTCGGACTAGATACTAAATACTCAAAT
TTGGATATAATGCTC
ACTTCGTTTAAAATATTATATATTTGATACAATAACAACAACAAT
CAAACGAACATGAAC
AAT
>ConsensusfromContig17 Average coverage: 9.26
GATCTCCCCCATTTCGATACCTTGAGCTATTATCACTGTATTGCG
GACAGGAAGCATGTA
TTTCTCTTGATTCGACATGACTCCTCCCCGTCACATCCGGTTGCTG
AGATGCCGGCTCTT
GGATGCGGCAACCGTTACTGCCGTTGGCCTGGTAGCAATCATAG
AGAGGAGTCATGGATA
TGTTTGCATCCTGATCCCAATCTTGGCCACAATGAACGCTCCCGC
GTGAAGGCGGAATGG
CAGTCCTGTTCTGCTCCAGCGAAGGCCGCCTTGAGCTCTGTGTTG
AATTCCCCAACTGCG
AATAGCAGCAAGGCTGAGTCCCGGGCCCGC
>ConsensusfromContig18 Average coverage: 5.50
CATCACACCCAAATCTCTATCATCATCCATTCTGGCGTTGAGCGA
TAAATCGGATACGGA
TACGTTGGTCGGTGTGTCATCGAGTAAGGACATGATGTTTTGTGC
CGATGGGAAATCTAT

CAAGTGCATCAGTACCCCATCGCTGGCTCACAAAAGAACATTAT
CCGAGGGAGAATCACT
GAATAACAATAAAATACGGACGTCATCGGCCGCCAACAGTAACG
CCCACTTGGG
>ConsensusfromContig19 Average coverage: 6.21
CATCCATCTGGCTTCTCGCCTTCTGCATAGCCGGGCCGCTCTTCGT
CGTCTCAATGTACG
ACCAGCAGTCTTCAATAACAAACACCAGCCCACCACAGTCAGAA
GGAGTGTATAAAGGCT
GCGGACCCGAAACGACTCTTTTCGTTTTGACTGCGACGATCCTGA
CCTTCTTTTTGCCGC
TCCTCATAATGACTCTTATGTATGTGTTGACGGTCA
>ConsensusfromContig20 Average coverage: 6.42
GTGAAAACGAAGGCTCAGCAATATCCATAAACTCCACTCAATCAA
ATCCGTTGATATCCT
CAACTGCTGCACGAGATTCGAGAAGAAATTCAGGTTCACGACAG
GTCAACGGAAACAAAA
CAACGCTCGTCAGTATTGATAGGTGAAGGTCATTCGAACACAAAA
TTATCCCATTTCAAA
CATCCTAAACGAAAGTATTACACATGATTTCGTATCATCCTTACA
AATCACTCTGCCCCA
AATCCAAATCTCC
>ConsensusfromContig21 Average coverage: 20.46
AATTCAAATGCACTTGACTCGAATTGGGACCAGAAGTTGGAGCA
AGCGAGATTAGAAACG
ACCGACAGTCAAAGTGATCGGAAAAACAAGGATGCTCATTTATC
GAATCTCAACGAAGAC
ATTATGCTGTCAGGTAAAGTTCAGTATTACATGGAATCTGGAAC
AACAACAATAGGGAGA
AAATCAGATGAAACCCCTCCTTCGATTGAACTTTCTGGATTAAGC
ATTTCAAAGCGCCAC
GCTGTGATCACAAAAGAGAAAAACGACTTCACAATCAAACCCGA
TGAAAAAGACGCCAAG
AT
>ConsensusfromContig22 Average coverage: 6.99
GTGGTTTTAAATGTATTAGTAATAACTGAATAACGTATGAATAAT
GTTTGAGTGAATCGA
TATCAACAAATGTTCAACTCTTGTGCGATGCAAAATTCATCAATT
TCGCCTTCGAAACTC
CCGGCCTTCCACGCACCGATAAACGTATTTGGTATTTTTGATTGG
AAATATCCACAACCA
CCATCAGACGCCACCGCATTAGTTTGTTTCTCTCCATTTACTGCAA

GTTCTATCTTCTTT
TCTGCTCTGTGAAACGTGGCCACGATCATACTCCATGTATTTCTTG
GTGCTTCAATTCCG
ATATCGAGGTAGTGAATACAACCTCCAATCTCATAAAAAAATTCG
AACCAGAATTTCCCG
GACACGTAATACAAACAAGTGAAAGGATATCCGCTCCCGATGAT
GTCACAGGTTCTTCGA
GATCGGAAGAGCGT
>ConsensusfromContig23 Average coverage: 6.53
TCCGATATCAAGGAGTTCGCAGATCCTGCATCACATGAAACTGG
TCCAGAACCAGCACCA
GGGCAATAGTAACCGGGAAGACATTGGACGCAAGACGACGTGTC
TAGGTCAAAAAAGCTA
CCGGCTGAACAGTTGTTTGAACAGGAGACGGAGCAGTTGACAGG
ACATTCCGTTTCACAG
ACACATGCGCCATTTGAATATTGGAGAAATGACGTAAAA
>ConsensusfromContig24 Average coverage: 26.32
TCCATATTCATTTTGAATTTAGTATCAAAATATTTCTATGCAAGAT
TTTATCTTCGAATA
TTTTTTCAATGCATCGTTTTTCATAATTTCACGTTATATTTTCATT
GGTTGCCTCGTTAA
ATTTGTTCGCCTTATCCATTGCTTTCTATTGGGCAAAAAGATGTA
TTTTAGTAGTGAAGA
TGTATCTAGTCTATTATTCTTTGTATGTCTCAATTTGTATATGAAC
CATACCATTCCCAA
ATCTTAATTCACGAGTGCCTTATATTCGATGTTATAATCTTTCATG
TTCAGAATACATCA
ATATTCAATTACTGAGAATTGTTCCAGTGATATTTTAGTTGTTTT
>ConsensusfromContig25 Average coverage: 9.13
GTGCTCTTCCGATCGTTGCAATAACAATTTGTATATGATATTAGTA
GTTTGAGCTAATTT
TCTTGTGAGAAATCTCTCAAAGTGGCGAAGCTGTGCGAAATTTGA
TTTGATACATAAAAC
AAATTGTTGCTTAGATTTCATTGAATTAATAACAGCTAATGAATA
ACAATTTGAGCAAAA
CTATTTTTTCTGAGGTATTTGAGTCAATACTAAACC
>ConsensusfromContig26 Average coverage: 8.11
TGGAGTTTGCTCCGGCTATCCAGCTGAGAGCGAAATCGTTGGCGA
AATTGTTGACAGGTG
TCGCCACACTTCCAGCAGAGTCGATGAGGTCGTTGGATGAGTCGT
CGTCGTAAAATCCAC
AAAGACCTTCTGTCGAATTGAAGTCTTCGGCTGAGGGAACAATC

CAGACGTTGAGAAAGT
CAACGTAAGACTTTTTGACGATGACTTTTGTGGAGCTTGGCAGGA
AA
>ConsensusfromContig27 Average coverage: 16.57
GGGGCAATGGCATGCCGAGCGGCTGGAGGTGGACTAGTTTGGGG
GATACCCTCCTAAACT
GGGCTAGCCTCCAGGTTATAGCTGAATATGTGGGCACAATATAC
CAAGAACCGGTGATAA
TGAAAGAGTTGTGTGTACAAGGAGATGATATTCTCTTCAAAACTA
GTCACCCTAGAATGA
TTTCTTACTTGGTAAGAGGTTATTCTGCTTTCGGTTATGAGGTCCA
TCCTCATAAAATGT
TTATTTCGAGGGAAACAGGGGAATTCTTACGTAGGATGATCACTC
CAATGGGGGCTTACG
GGAACATATCGCGCACATTTATAGGGATTTCTTACCGTAATCCAA
TCCAAGAAGATGGGT
TACATGTTTTGTTGCGTTTATATTCAAAACTTAGTCAGTATCATTT
GCTTTTCTTAAGGG
GATGTGGTAGCCGTTTGGGAGATATTAAAGGTTTGGTTGCGACTT
TGAGATTAGATGTTG
AGCAGAGTGGCGTTGAGTGGAGGAAGGCAGTGAGGTTCTTCTTAA
CACCTAGTTCTTTAC
ACGGGGGTGGGGTTTACAGGGGTAGTAGATTTGGGAAAGAGCTT
AGGAAACA
>ConsensusfromContig28 Average coverage: 6.96
CGACGCTCTTCCGATCTTTTCAATGGTACTTGAGGAAATTTAGAG
ATAATAAATGTACGA
CGAAGGTAGATACTGATAGTCCAAAATATTTATTTAAAATCCCAT
GTATGGGTGATGTTC
CCGTTAAATTTTCACCAATTATAATAAATCATCATTTCGCCTTTT
AAGATCGATCCAGCG
TTTTATTGAAATCGTCATGTATTTATTTATTCACATGTTTGGGAGA
CCAAGATAATTCCT
ATATCGGAG
>ConsensusfromContig29 Average coverage: 9.01
AAAGAATTTTACATTTTAAACTCTATACAAATTATACAAAATTGT
GTCTTTCAAATATTT
TTATCACCATCCAGGATGATATGTGAATTGCTGTGGGTTTGAAGG
AGGGTACCCAGGCTG
CTGCCTGATTGGAGGCCCTTTGGTTTGCTGTCCACCCCTGCCCCGC
CCAGCTTTTCCATT
GTGGGTGGGTGGTGGGGGTTGAGGAGGTGCTTGTGTAACAGCTGT

CATCTCGTACTGAAT
TTGCTCAATTTTGGAACTGAGGGAATCTATTCTCATATCTTTCAAC
TTTAGAAGATGTTC
AAGTCGCTGTATTTTTGCTTGCATGATCTCCACAGTGTGGCGTTTA
TCTTCAAGTTCCCT
ATTTTTTTCGTTGAGGATCGACTTTGGCACGAGGTCAGAAGGACG
CGATAATCCTGGCAA
GACAGGTGGCCTGGGGCCAAACCTTTGTTGTGGAGGCGTTACTTG
AGGCAAATATTCTGG
GTTAGCTATGAACATATCTTGTGAAGGCGCACCTAGAGTATAACC
GGCGTATGCCTGGTC
GGCCTCCGGACGGTCACTCCCCATA
>ConsensusfromContig30 Average coverage: 6.28
ATCGACGTCGATCGAAACCCCGAAGGCAGTAATAGGGTCAGGGT
CACTTTCATCAATCAA
AACTATGTTGGGCTGGATTTAGAACAAACATACGATCCTAAATTC
GAAGGAGTCATGAAT
ACGCCTGAAAATACAAAACCAATGACAAGTCAACAGAATTCGGG
TGCTCCTAACAACATA
TCGAATCCCTCTTGTTGTCAT
>ConsensusfromContig31 Average coverage: 13.08
CGCATGGCATTCCGCAATTTATGATTCAATAATCTACTATATCGAT
ATACTTTTTCATTT
GTTGGTTGTTACTTTCGTTCTATAATTCACATACCCACTTTATTTC
GAGTCGGCCTTACG
CAGCCTTACCAGCGTTTACGGGATAATCTTATGTTCACAAATATG
AAATTGTTTTGATGA
TGAAATATCGCGACCAGCTGGTTTCGAACCAGTGCTTGCTCGTGT
TGTC
>ConsensusfromContig32 Average coverage: 5.11
CTGGAAGTATCTATATAATAATATTAACATTCGACATCTTCAATG
TTATGATTGCTAGAT
TTTCGGTTGTGTGTCGTGTATATGGTCGCCTATCACCAGCTGTAG
GCAAAGAGATTGAGC
TTCAATGCAAGCAAGAATGTTTGTTAACATGAGCTGTCAAAAAAT
ATGATTTCGCACAGG
AACAAATGATTTTTTAATGTATTGCAAG
>ConsensusfromContig33 Average coverage: 6.27
CGCTCTTCCGATCTTCCAATTTAGCTTAGGAATTAATGGACTTGG
ATGAAGTGGAGCAAA
TAGAGCTCGAATTACCTGCAACAGTGCTCGCGAACAATACAACGT
TCAAAAATATTTTAT

TTCAGCATGTGGTGGAGATATTTTTAAAAAGGAATGCAAACTTCA
TTCATATCAGAAATC
TTCTTAGATACGGAACAATTGGTCATCTTCGCATGATGAACCGTG
AATCAATACAAATGA
TTGCTTTTAGGATATGTG
>ConsensusfromContig34 Average coverage: 9.83
TTCGATAATTCCATCTTCAGAATGGGTTAAAAATTTATTTCTTAT
ACATGATGGGATATT
GTTAATTACCTTAGGGATGTTATACCTAACGCATTTTTGTGTAGA
AACTAGTCTAGTACG
GATTATGGAAATTGAAGTGCAATTTCGTGTTTGATGGTCATGTAT
ATTGGACTGAAGTTG
AAAAGGCATATTGATGAAATATTCAGGAAGCTTAT
>ConsensusfromContig35 Average coverage: 9.02
CCGATCTCATGCATGCAATGATGTTCCAGTCTGGAAAGTAGTCGT
TACAGACACAATAAA
TGAAGGCAGTCACGGCCCTGGTCTGAAGGAAGAAACCTCACATAA
AGTTGTCTTTTCCAT
CGACGACAATGACTATGACTACGCACAATGGGCTTCCTGGTCGCC
GTGCAGCGTCACTTG
TGGAACGGGTCACCAGACAAGAGAACCGCTCTGCCGAACATCTG
AGAGGCGCAACAGCAA
GAAATGTCGCTTTCAGTCTCTGGATGCCCGCCCTTGTCAAATGCC
CATATGCTCTGATGC
GCTCCCGGACCAGACTGGTGTGGATTGTGGAGATCCGGGCGACG
TGGAGCATGCCAAGCG
GACCGGTGGAGGTACTGCACCC
>ConsensusfromContig36 Average coverage: 9.77
CTACTCTCTCGTTTCCCAGGTTTTTGTGTTTTATTTGTTCAAGTAT
CGAAAACACTTTCC
ATGCGGTTTTCGATATGTTAGAATGATAAAACTTGGTTTCTATGA
ATTCTTATGAATGCT
CATATTAAGATTTATTTCCGTATAAATAGACATGTCTACCAATGA
GCTTCGTTGAAATCA
CTAACCTGTTTTCAAATAGTGTCTTTTCACACAAACTATCAATTTA
AATAT
>ConsensusfromContig37 Average coverage: 9.05
ATATTCACTGCGTTTGAGAGGTCTCAAAGACTCGTTAGAGTATGA
TATCCAATGGAATAG
AGTTCGTGTAACTTCGCACACTGAACGGTTTGACCTGGAGGAAG
ACACAAACCCCAAGAT
CTGTCTCTTCGAAAGTGTTTATAGAAATAATTCAGATACAGAACA

AGAGTATTCCTTCAC
AGGTTACCGACAAACACAGACTCATGTGGAAACATCTGTAC
>ConsensusfromContig38 Average coverage: 4.62
TAACAGCCAGTCTTACAAAGGGTTATCATGATGAAGCTGATCTG
AAGGAAACAAGGCAAA
GATCGAAAAGCGAATCACGGCTGCATGGATCAGAAAAAAGAAAG
CGATCCACCGTTCCAT
CCTCTTCAACGTATTACTCTGAAGTATCCACGAGTGCCGACTCAT
TATCTGAAGCATCTG
ACAGCATTGAGTATTCACGTCACGAATCTCTGA
>ConsensusfromContig39 Average coverage: 6.33
TAGGTCACAAAAATCATAGAAATATGCGTTTAAAACTGATTTAAA
AGTATTCGATTTTTC
CGAAATATTCTATTTTCCCACGGAATTACCCGCCTATCTGCTATCT
CCATTCACTCTCGC
TTCGCATTTTTGGGGTAGAGTAACCGTTAAATTCTTTCAATGCAA
ATAAATGAATATTTG
ATTATCAGTGCCTTAACGATAATGTATATTCGCCAGCATTCAGCAA
CTTATTTGCTTTTA
AAATCGAATCTCAAAACA
>ConsensusfromContig40 Average coverage: 9.65
ACTTCCCTCTAGATCACAAATGGCTTGCAAAGCCCTATCATGGCC
TTTTATCGCTAAATA
GTGTGTCGGTGTACGTTTGAAATTATTGGTTATGTTAGGGTTCGC
GCCGTGGTCCAAGAG
ACTGATAACTGCTCCATCAAATCCTTCGAATGATGCAAGATGCAT
AGCTGTATCTCCCTG
GTCATCTTGTATATCTGTCCTCGTCTCTTTCCTACGTGAAATCTCA
CTTCCGATTCTGGA
CTGTAGTAGATTATTAAATCCACAATTTTTATGGCAGCAATAATG
CAATGCGGTATGTCC
AGATAAATCTGTGTAATTAATGTCCGCGTTAGCGTTTAGCAAAGT
ATCGATTATGAGATT
AGGACACTCGCTGTATTTAGCAGCTTTGAGAAGTGGAGTTTCCAT
ATTGGAGCCAATAAA
ATTAGTATTCGCA
>ConsensusfromContig41 Average coverage: 10.73
CCGATCTCCAGTGTTCAATAATTTGGAGAGTTGCCGAATCTGTAC
TCATAGTAGTTATTA
TCGTGGACAGTTGAAAATTAGCATCGTTGCCGTGCTGTAATAATT
TCAATTGTGATCCTT
CATAAAGACCAAGTCAGTGCCATCATCGGCATCAGTGAATTGAC

AGTTGAATGCATAATG
TACCTACATTGAAATGTTATTATGCCAATTA
>ConsensusfromContig42 Average coverage: 7.68
CGTGGTTGTCCTTTGGCACAAACTGCTGTGGTCTGTCACTCCATT
TCCCCTTGAAAGAAT
GCTGAGCAAGAGTCTCGTTTGAGCAGACGGCCATTCCTGGGTAG
ATGATCGAGCCGGCGA
ATGAGGGGCATTCCTTGAAAGGAATAGCTCGCTCCCTGACGTCG
ATTGGAATGAAGGTGC
TCTTGTTTGTTGTTGTGCTTTCCATCTTGCCTGGATCCAGAATCTG
TTTTTGAACCCGCT
CGTTTTCCTCCTTCAATCTTTTCGCTCTCATCAAATCTTCGATATT
ATGACTTGAGTAAT
CTGATCTATACATTGACTTTTCTGCCATTTTTCCGGATGGCAACCC
AAGGTTGTGTTCCA
GTTTCCTCTGTTCCGCCTTGCTTATTTCCTTCAGATC
>ConsensusfromContig43 Average coverage: 12.62
CAAATAGATTCGATCAATCATTGTAACAAGATGATATCAAAATAG
ATCCTAATTAATAGT
TGAACGCCCATATGGCTTATATGAGGAATCGGAGCATTGTAAAAA
AATCGTGAATGCTTG
AATATTATTCAGTGTGAATCTATGAATTCACCATATATTCGATGC
CATATACCTAGAATG
ATATGAAATGTCTGAATGTGGTCAGTCAGACCTGAATCATATTCT
AAACGATGAGTGA
>ConsensusfromContig44 Average coverage: 6.32
CTTCCATCATCAAAATATATCTGGCATCGGAATGCATTGATTTGT
TGAGACAAAATAAAT
AATCGCTTTTTTCTTTTTCGAATATTGAGCCCATGTTGAAATGTCG
TTTTTCGAATCTTG
TCCATATTTCCACCACTGAAGACAAGTTCTTCACTTCAACAAAAT
TCAACGGGTCCTCCT
CGACGTTGCAGACGACAATCTTTGTTGTGAGCTCCTTTTCATTTC
GTTGAATATCCAAGA
ATTTTGAAACGACTTGCGATACGTAATATGGATTTATAGATGTTTT
TTCCTTTTTCAAAC
GATTCACAGATATAATGGGTACTATTATATCTATCTTTTTCGAATT
GATTTTTTCCATTT
GTTGCGTTTTAGAATTGTG
>ConsensusfromContig45 Average coverage: 8.19
TTTCGAAATATTTCACGTAGAATAATTAAATTCTGAAAACCGTTC
ATTTTATATAATATT

TTAAATACTAGTGGTGCGACACATTGAGGGTTTTGTGGTCGATTT
TGTTGAACCAATTTC
CAAACAACGCACAATTTCATGCACACAACGGTCACTTGTTCTATA
ATAGATTGGATAAAA
TAAATCCAAATAACCAAACTTAACATCCCTATAATTAATGTCAAG
TATTCCTTTTTAATA
GGAGATCGT
>ConsensusfromContig46 Average coverage: 11.39
TCTTATTACATATCACGCATTCAAATCGGATATAATAACCTTTCA
GTCGAACATCAGAAT
CTTCTAATTTCCGACTCAAACACTTATTGCAACGCCAACAGGTCG
TGACTGACGTATCTG
TTTCACTGAAACCATACTCGTAATAGCTGTATTCACAGTATTCGC
AACAAAATGAGACCT
TGTAAATCCGGTGAGCTGTGCCGGGTATGA
>ConsensusfromContig47 Average coverage: 7.05
CCGGTGTATGGTGTATATTTTTCTTTATACGAATTCATTCCCCAAT
ATCTGAAACTGTAT
TTGGGCATTGAACGAAATGTTCTTGTAGATGCGACCGTTGGTGGT
TTTGCCGGGTCCTTT
ACGTGGTTCATGGTGTATCCAATCGATGTCATCAAATCTAAGTAC
CAAGCTGACCTGAAG
GGTTCTTATAAGAGTGCCACGGACTGTTTCTTGAAAACCGGAAG
AGGAGCCGACTGGAAG
ATATTCTATCGCGGACTCTCCGTGTGTCTGATTAGGGGACTACCG
AACAACGCTGCGATA
TTTGGTGTCTACGGTTTGATAATGCGACATGTTCAAGCTCATGTT
GTCATTGAGA
>ConsensusfromContig48 Average coverage: 5.39
TGATTGGATCGTATTATCATTATGTTTGTGGTGGAGATATTTTTA
ATATTATGAAGCAAT
AGTTACTATTAATATGGCTGTAAAACGTTATACTTTGATGAAAGT
ATTGATAGAAGACAT
GCGGTTTTAACACATGATCCCGAGATTGGATGCTTCTACGTCAAA
GACCTTGCCTCAGTT
CACGGTACATTCGTCAATGAAACACGAGCTCCGGAACAGGAATA
TTATTTGATCAAAGAG
GAAGATATAATTCGCTTCGGTTCTGATACCGCACCCCACTTCCGA
GTGCACCACGGCCGA
CTCAAGGACATCGAATTGAAAATGGCTTCTGGACTAATCCTGCCC
ACGGATTTACCCAAC
TGGGCCACTCG

>ConsensusfromContig49 Average coverage: 28.94
ACCAAGCTTCTCGTGATTGATCAAAAGTTATTTCCGTATATTAAC
TTATATCAGTCTTAA
AATTTGGGGGTCTCACAATACAGGACACTCAAGCGCTGATAATTA
TTTTCCGGTAATATA
AATTGAATGGCGTGTCTAGATTATTGTGTATCTTATTTTCGAACA
GATAACCACTTAAAG
ATGCAATTCTCGATAAAAATGAGTTTATTATTCTGGCATTTCTCTG
AACAACT
>ConsensusfromContig50 Average coverage: 4.01
TCGCCTATTATTATGATTTTTCTAAACTGTTTGGTTTATCATATTA
CATTATTTTATAGA
TTATGTTCAGGCTCTGAAAAAAAGAAGAATTGTTCGTTATATCAC
GCATGCGCCAATGAA
TATTACTTATACACATGGATTCACGAAATAGGAGCATGTGTTTAT
ATTAAAATATAAGCA
GTAACAGAGATATGCGTAGGGTGTAGA
>ConsensusfromContig51 Average coverage: 6.92
GAGATCATACAATCTTGCTCGCAATCTTCAATATTCCAAGATCCA
GTTGAACCGCATGGA
GTGATTTTGAATATCTGTTACAATCATAATGTGTCGAAAAAAATC
CTGGCTCAGCACAAG
GACCTTTCAACTCATGATGAACAAACTTTCACTTCAGTTGAATCG
TTAGCAGACAGTATT
TGTTCTCTCGAAGAATTGTCA
>ConsensusfromContig52 Average coverage: 10.66
CAGATCATAATTACAAAGTTTATTGCAAACACTGTCGAAAACAAA
TTTGAATTAAATAGC
GAATTGAAACCCGATTGTTCACATTATGAACGCGTTGATATGGTG
TTAGTATTTACAACA
CAAAAATACGAAGGCAAGCAAGGTATAACAAGGAATTCACATTC
TGAGTAACATTCAGAA
TTCCGATCATATAAATTACAAAAATATATTATGTTAAAAATCACG
AATGAAAAATTAC
>ConsensusfromContig53 Average coverage: 9.27
GTCCTCTTATACCCAATACTTATCGTCATGTAAAACGGGATTTGT
TTTCCGGCAGTGTTG
CAAAAGCGCAGGATCCGCCTCTTGCCAGTTGACCCCCACATGACC
TCTTTGTAGGGTGTT
TGCGGAAGATTGCGAAACTCCATTGGCTTGAGGATTTATAGGAAC
GCCCACACTCGTGTT
TGGCAGTACAGGATATGAGCCACTCACAGGCAGTGTTGAGCTAG

AATGAACATGACGGTG
ATTCGAAAATCCTATTGG
>ConsensusfromContig54 Average coverage: 11.56
AGGAGCTTTGTGTTTTTGCCAGCGTGGTACTGGATGAATACACAA
GGTCGGAGTGGTCGC
TCAGGATAATAATATCTGATTCCTTCTTGTATGTCTTGGGCCCACT
TTGTTGAAATGTTC
TCACTTGGGTTACCAAACATTATTTCCTTCTCTCTATAACAACCGT
TATGCTTTCTCACT
AAAACATGACAACGGAATCCTTCACACACTCCTTCTCCAGGTCCT
TGTTTGTTGTTTCGA
AAGCGTTTGACCTGTACACCAGATCGGAAGAGCGTCGTGTAGG
>ConsensusfromContig55 Average coverage: 2.88
TGGCGATCTTCCATTCTATTCCCTTTTTAGATATCTCCATATCTGC
CAAATCAAAGTACT
TTCATTCCTCTCAAAAGTGAAGTGTGAATATATACCACCGCGCAT
GGCTGATTATGAATA
CATCCGAAAGAAAAGAATTCCATTTCGTTTTTAAAGTCGGGATTG
AAAAGAATACATGTG
GCGGCTTTGTGATTAATTCGAGGTGACAAAT
>ConsensusfromContig56 Average coverage: 8.47
TCACAAAAATCTTATAACATCAACGCCTCCTGACCGTTTGATCGT
TGAATCAAAACTATG
GCGTATATTGTTCGCATGTTTATTATATAATATTTATTGATGTTGA
TCGAATAAGCGTCA
GTAAATTCTGACGCTTTTTTAAAAATTCAATTATCTTGTGTGGTCA
TTGTTGACAGTGGT
GACATTGACGTGGGAATCCATTGAGATTGGTTGATTAAACGTATA
ATAGAGATATTGAAA
TGTTAATCCCGAATATAAACGCGACAGTACAGTTATTTGAAAAAA
TTTATCAATATTAAC
AAGGTTAGTGTGAACTATGTTCTGTTGCCATCTTCTCTTCTGCTCG
AAACTAAAATGTTC
CCAAATTATCATTCGTACAAATTTATCATAACATTCAGCATCA
>ConsensusfromContig57 Average coverage: 5.42
TCTTAATTCAAAATGAAATGCATATTTCGTACAAAATTGTCAAAC
AGCATTCTCAGTCGT
ATGAGGACTTACTACATAAATACTAGATCTGAATAAAAAACACAA
GTTTCTATCCCATTA
AAATTTGATTAAATTATTATTCAAATAAAGAATACAAAATGAAT
AAGGGATGGCCAAGTA
CAAATTGCGTGCAACAATCTGACTGTACTCAT

>ConsensusfromContig58 Average coverage: 6.56
ATCCGTCAATTACACCAAAAACATGGAGTTTCCCGCGGTGACAAT
CTGCAACGACAACCT
TTTCCGAATCACGGCGGCTGCAGAGGGGGCTGGCTGGACAGCG
TTGATAGGCTCATAAA
TCCTTCGACGAATCTAACGACACCCACTATTTGTGATAGCGACGA
GTACACTTGTGGCAC
AGGGGAATGCATATTTTCGGACCGCCTCTGTGACACATTTCCTGA
TTGCGCAGACTCGAG
CGATGAGACTTCATGTAGTTTTTGTACAGCCTTCGTAATCTCCAGC
TCAACTGGTCTGTT
CCAACAACAACATCAACAAGTGGATCTTTC
>ConsensusfromContig59 Average coverage: 8.18
TGTCTGATAGCTCTTCAGTGTCATTGCTCCCCCTTATCGCTGCTTC
CCAACCGACTTCTA
CAGCAACGTCGGCCGCTAATCGTCCATCCACTCTGTCTCGTCCGT
TCGATAATACTCATG
TCGGATCGAAGCGTTCGTTAGATCACTCCCCTCATGGGCTGTTGG
ATCGCAATATCTTCG
AACTTGTGGGTGGACCTGATGACCGTCCCCGAAGTATTTCGCCAA
AGGCTTGTCCTGAAT
TGTCATCTCTTAAGCTGCCACGGAAAATCAGATGTAAGGGGCGGC
CGAAGGGCTCTGGTT
TGACAGCAATCGGCTTGGCTCGAAAGAAGCACAAGCTCCATCCAG
GTCCGGTGCAGTTTC
TGGAGAAACCTCTCCAGGATAGGCAGCGAATTATTCTGCGATGG
CTGGCCGATGAAGGTG
CTGCTGACCGAGCACTAGG
>ConsensusfromContig60 Average coverage: 5.00
GCGGAATCCGGTATCGAGTACGCATTCGTCGAGTATGACATATAT
TACCGCGAATGTGGG
GGTGAGTGTTGCGTTCACGAGTTTCCTCCGGTGAGCACAGGGAAT
GATGGGCTTGGATTT
GATTGGATGGAAAAACCGGAAAGGATAGAGTTCAAAGCTTGCAT
GATCAACGGGACCTTC
ACCTCAGCCTGTGACTCAACCAGGACCATTGGCCTCCAGTTTCTC
TACAGAGATTCA
>ConsensusfromContig61 Average coverage: 5.82
GGTAGGAACTGGAGAATTCATTCTCACCACTGAGCAGGAAATGTT
CGTCATAAATGGTCA
AAAGACTAAATATTGCATGGGAAATGTTGGTGATTGTCGCGAATG
CAAGGATACTCTCAC

GTCATTCGAGTGTACAATCGTGCCGTGTCTGGATTCATTCGATAT
TCAGAGCCATAAGCA
TGTGAGAGGAGGAGTTTTGATCGCTGGAACATTCACCAA
>ConsensusfromContig62 Average coverage: 9.53
GCAGAACAAATTTGAAAGTTGATCGTTTGTAACAAGTTGGATTTA
AATATATAATTCGCT
GAGCTATTTCATTAATGCCACTTATGATACGATGTTTGATTTAATA
ATGTCCATATATGT
AAATCCACATCATAAAACTTCGTTTCATTCAATTCGTAACATGAC
TAAAAATAGGATGAG
TGTTTTATTCTGATTCTTTACTATTTATTCTATCAGACAAAATGCT
GACAAACACCAGCT
AAAAATAGTTATATACGTCAATGCTGTTATCATTATTCAGGATTC
ATAAGACTGAGATGG
AGAAATCATTTCTAATACAACAAACTATATAGCAAT
>ConsensusfromContig63 Average coverage: 10.04
TTTACGTTTGCACTGTTCTGTACGAAACTGGCTGTATGAGAAACA
TCATTTTCCGCTGAT
ATAACCAGGCTCTTTTCTTCTAATGTATGTCTTGAAAACGAAGCA
GAAATCTCTGCAGTC
GTTGCAAAATTTTGTGCTACATTTGTCTCGTGGTCTTTTTGCGAAT
CAGAAACATTCAGT
TCGTTTTCTTGTTTTTCGAGATCGGAA
>ConsensusfromContig64 Average coverage: 18.08
AATTTTCTCATTTGATGTAACAAAGAAGACGTGTGCGAATAACAT
CAATACACATGTTGG
TTCATGTAGTTTTGTAATATTTTTGAAAGGACGGATAGGGGAAAA
ATGATTTTCCACATC
TTGAAGCGCATTGAAAATTTCAAGTAATTTCTCAGATTCCTCAAA
ACCTGTAACAATTGT
ATTGAATATATCAGGTTCTATATATTTGAAATTATTTAGTACAATA
GTTAATTCTGTTTT
AGTATTACTAATTAAATTACTCGAGATTGGATGAAATAGAA
>ConsensusfromContig65 Average coverage: 4.85
ATAGGTCCATCAGTTTCAAAGAACTTGTTGTCTGCGTACGATCAT
CCTCAAGTTGTACAC
TCTTATATCGCAAAAGAGCTGCAATTAGGTCGCATCTCCGGCCCA
TTCAATTCAATACCT
TCATCCAACCTGGTAGTAAGCCCCATAGGCGTTGTGCCTAAGAA
AACGGAGGGCAAATTC
AGAGTCATTCAGAATCTTAGCGCACCTAGGGGCTCCTCTGTCAAC
GATACCATTCCATTA

AATAGCAGACAGGTACAGTATTCATCTATCGGCGATGCACCGGTC
CTTGTTGCCCCAATG
GCCGTTTCACCTTTTTTTGCC
>ConsensusfromContig66 Average coverage: 10.88
TGTATTGAACAGTAAAGATATCAATTGTGATATTGAAAACTCAAC
GTAATATTTCTTTCT
TATAAATATCCAAAGGAGCGTTCGAAACCGAACAAGCCAGCATAA
TTTCACCCAGTCTTG
AAACGTGAGCGAGGTTGGCTGAATAACGCCGTTTTTGTTTGATCT
GAGATGCTATTCAAT
TTTTAAATAGATTTTATATAGGATTGCGAGAT
>ConsensusfromContig67 Average coverage: 5.35
AGTGCATGACTGCGAAAACTCGAACTTTAATAGATTTGAAGGAAC
GTTTTCCACGCTGTT
TTGGTCTATTTTCGGCATGGGGAATCGCGAAGCTCCGAACATTGA
CCGGGAAATGATATA
CGTCGGGGACAAAACTCAGCAAGGAATTATCTTCAGCAGCAAAT
ATGCTGAGTTGTTCGG
CTACCTGCTATTCTGGGCGTTTCTGGCCTGCACTGTGATTGTCATG
TTGAACATGCTCAT
TGC
>ConsensusfromContig68 Average coverage: 7.15
AAAATATTAGACGAATATAGATCTTTCTTCATGGATCTTCACCCC
TTCATGAAATTGGTC
GTAGTTGATGTACATCGACGCTTGATCAGGGCCATTGACGATTGG
ATGAACAGTTTCAGA
CTCCCTTTCTGGGTTGTCTACTTTGCCTGGGTCTACCTGACCCTCT
CTGCAATCATGGCG
ACCTGCCTCATGTTCATGAAGGGGCTTCGATTCGGGTTCACAGAC
CGGCAGAGGACACGT
CTGAAATACC
>ConsensusfromContig69 Average coverage: 4.94
CCGATAGATGACTCCATCCCTGATCGAGATTCGTTATCCAAGTCA
TCTTCGCTGATCGGA
CAGAATCTTGTTAAGCGCTTCTCTAGATCGTTGCACTTGCGCAAA
AGAGATTGTATTTCG
CTCTCCTTTGTCTCACGAAAAATCTTGAACTGCTTTTGGATCTTGT
CCCGGGCTTTCTCG
ATTGTGTCAGCTCGGACTAGCGCTTGGCGACCCTGATCTAGGGCC
GAATCCCTTTCGCGA
TTGAGACGCTTACACATCTTTTGTAGCGCCGTCAATTCCGATCGA
AGGCGCTTTGTCTCC

AGTTCATACTTTGAC
>ConsensusfromContig70 Average coverage: 5.97
GGTTCCATTGCCATCCCAACGAACTCATCAGGATTGCTGATGATG
AACGAGTCTTGATCA
TACAAGGGGCTGTACGGGTCGATTGTGTTCGCTATAGGCCGGTAC
CACAATCTCAAGTTT
AGCATTTGCCTATCATGACATTGCTCAGTGAAAAAGTCATTCATG
ATCTCCAATTCTGTG
ATGAGCTCTTTGTATATGCGAAAA
>ConsensusfromContig71 Average coverage: 11.25
TTTTTATATTCATGGGATATAATTGTTCATTAGAGTCCAAACGCT
TAGCAACCAATCCGG
TATACTTTGAACGATCACACGTTTCTGAGATGTTCTGCTGTGTATT
TCTGAGCTGTGAGT
AACCATGAATCGGTTCGCTGTGATTCCCATACACAGATTTTTGTA
TCCGCCAATCATTCC
CCAAATGGGATTTCCCATTAAAAGGGTTATACAACCCGTCGCGT
TGGAAAAATGGTGAAC
CCAAGCTTTGTCCGCAATAACCTCTTCTGCTTTGACCCATTCCACT
CAGCATAGATTTTC
CGGTCCGACAATATCTACAGCCAGATCGATGTGCTTCCGCCGGGC
AATTGAAAATCTCAT
TTCGAGAGGGAAACATGCATCCCACACCGGGTTTAGTTGGAATTT
CTTGTGAGTCCGAAA
GATATGTATTCATCCCATCATTTGCATATTGAGGAGAAAACATGC
ATCCGAAACCGGGTT
TAACTTTTGAATTAAGACGATTAATATTCATACTGTTACTA
>ConsensusfromContig72 Average coverage: 11.08
CGATCTCTAAAACCCAATCATGAATCCAGCAATTATTGTTACTTC
TTCGGAACCAAGTAT
CAGAGCTGGAAAGCCAGTCAGACTTATTGCGCCCAGCTGCACCA
AGGAAGCAGCCTGGTT
TGGATCCACGATCAAGTGGAGAATGCGTATATTCAGAAGAACAT
GATTCGATCGGAATAT
TGGACTGGGCTCTATTCATCTTCAAAGACAGACGAGTGGATATG
GTTAAACGGGGCCGAA
GTATCGTACATCAACGTCGAGAAAGATCTTGTCCCCAAAGACTCT
TGTGCTGTTTTGACT
GGAGAGACACGCTGGCGAACTTCTGTCTGTATTGCTGGAAGGAGT
TATGCGTTGTGCAAG
ATGAACAAACTACCTGCATTTGATATCCCCAACATCACAAATGTG
GAATGTCCTGATTCT

TCTTGGGTGAAACGAGGCCAAAGCTGCTATCTATTCCAGCCAGAT
GAGTTGGTCAATTGG
GAAGAAGCTCAGAGTATTTGCTTGACCAGAGGTGGTTCAAAAAG
CCAGCTGGTGAATAT
>ConsensusfromContig73 Average coverage: 8.60
ACCATCATCATAACTTATTGAGCTCTTGTTCTCAAACTGTGCAAT
CACTTTATCAGCCAC
CGTATAATCGATAGAGGTAAATGAAAAATGACAAATATTGGTTAA
TATCTATTTCACAAC
CACTACTTGAAGAAGTGTGGTAAAATGTGTTAAAAAAAGCTAGC
GAAACAACAGAATCAT
CACTTGTCAAACAACCATCAATACAAAAATTTTCAGAATTGTGCT
CATAGGTTGAAACCT
TTATCGAATCCCAAGATCGG
>ConsensusfromContig74 Average coverage: 31.39
CATACCTGCTGCTGACAAACAATCTTCTGTTTTGATAAAGCCTGT
TCTGGAGTCCCCACA
ATCTGGATTCAAGCAAGCTTTCGTAAGAAAATCAACAATACCTGC
TCCTTACAAACAATC
TTCTGTTGCGCAAACACCTAATCTGGAGTCCTCGCATGCCAAATC
TGAGCAACCATTTGT
TACAACATTTCCCGCTCCTGACAACCAATCTTCTGCTTTTCCATC
ACCTCAGCCAAAATC
CAAATATTGCCAATATAAGGATCAACATCTAAAAAAACTCGAGC
CTGACGTGAATGTGAG
ATCGGTAATAGAAGCAGGGACTGAATCGAATGTAAATGGGATTC
GTAAATTCTCTTCGAA
TTCATCCGAAATCTACGACAATAACTTCGCGAATATTAACCTATA
GGAA
>ConsensusfromContig75 Average coverage: 11.04
GTTACTTTTTCGTCTTCTGCAACCTAGAAACAAATTACTGCTCCA
AATACGAGCCCTGCG
AAAATGGGGGCGAGTGCATAAACGGCGAACCGGGCAATTATCAG
TGCGAGTGCCCCCCTG
AGTTCAACGGGACCAACTGTGAAATGGCCGTCCTTGATTGTCGCC
ATCGCCCCTGCCAAA
ATCAGGGGGTCTGCAATAGCGACGGATCTTGTGGCTGCCCACCAG
GCTTCTCTGGGCTCC
ATTGCGAGAGTAACGCCACCTCGTGCGAAGAAAGTCCGTGTCGC
AATGACGGACTCTGCA
AAGTAGATGGAAATTCCTACGTCTGTGAATGTCTTCAGGGGTTCT
CTGGAATAAACTGCG

AACTCAATGATGACGATTGTTCTTCCATGCCTTGT
>ConsensusfromContig76 Average coverage: 9.00
CTCGAACCTGTCACTCAGCCATTCTAGAAGGTTCTAATTGCGTTC
ATAACGCCTTCCCGA
CCCTAGTGGCGAACCTCGGGTCACTCAAGTGGGGGCTCAAGGACT
TAAGCTTTTTAAGAC
CCTCTTCTTTGTCTATTTGTTGTGTTAGGCATGCTGTATATTATAG
TGTGAATTGGAGAA
AATCGTTAACAATTAGACGTTAATTTTTACATGCCATTCAGAAAT
GTATCGAAG
>ConsensusfromContig77 Average coverage: 6.85
TGATTATAAGGTCTAGAGCTCCTCCTTTAATGAGCCTCGTTGAAA
TAATTATAAAGATAT
GAAATTTTGAAAAATATTTTAATGGGGATTTAAGGGGGGAGGCT
AACCCCCCGAGAGCAA
CAAACGGTGAAATTACCGGGCTTGTCGCCTCATCATCATATGTCT
CGCCGAATTGTCTGA
GGGTTAGTGCGGTAATATTTTAGTACACGGGTCGCGAGC
>ConsensusfromContig78 Average coverage: 10.59
CTCATAGTCATTTGGTTATGAACAAATTTGAAAATATAATTTGCT
TCAAACATTGTGATG
TGAATAAAGCTATATGTGTTATTGATATTCCCGAATGCTGCCCAC
TATGTGGCGAAAACA
TGTTCGAATGGGGAAGAAGAATTTACCCTTATATCATTAATAACC
CCTTAAAAAGATTCA
AGGAAAATCCGATTTCAATAC
>ConsensusfromContig79 Average coverage: 8.95
GGAAGTGACGCTGCATATCAATTATCTCCTCGACGTCATATCCGG
TGTAGTTGACCAATC
CGGAGAAGACACCAGGAACGAGAAGCTTGTTGAGGTCTGGGCTA
GATTTTGGGACGCACT
GGTCCTCGCCAGGGGTGGGCTGGAAGGCTGGGGCATCGTTGAGA
TTCAGGATCTCAAACG
TGAAATTGTAGACTCTATCCTGCACCAGGCATCCATCAGCCTCCA
GACTATCTCTCAAAT
CCGCCCTCTGCGATGTCGTGAAGGTGGGCCTCCATTTCAGAATGA
GATCTATGCAAGGCC
TCGTAGCTTTGATGCTGTACAGATTGACGTTGCATGCAGAGTTCA
GTCCTGTCGTTGACT
CCATTTGAAACGCTCCATCATCAGTCATTATTCCTATGTCTGGAGT
TATATATTTCTTTA
CTCGTCCGAAATTCC

>ConsensusfromContig80 Average coverage: 29.67
ATATCAGTGGAGAAGGACTACTCTTATAGGAATACAAAGAGCGC
TTAGCCTTTTGAAGAG
GGCTTCTTGTAGGAGTGCGTTTCAATCTTTTATTCGATATTAAAA
ACGATGTAGATCCCA
ATCGCTTCATTCTGCTCTTTCGCTTATCGCTTTGAGATTCATATGT
TTTGAAAGAGCTGT
TGAGAGCAACATCTGAACTAGTATTACAAGAATCAGTTGGAGTCG
CAGGAATATATCCTT
CATCAAT
>ConsensusfromContig81 Average coverage: 7.39
TGAAACATTCTTATATCATGAAATAGATGCGGGTACCTCAACACA
AAAAAGGTCTACGAT
CTAAAAGACAGGCAAACTGCCTAACCAATATATCACGCAAGAATT
TTCAAGTGACATTCC
TCAACTTTAATGTGTGATGACCCGTCTAGTAAGAGTGATCCTAAA
CATTATGATGTAATC
AAATAAATATAAAAATTTCA
>ConsensusfromContig82 Average coverage: 6.74
GCTCTTCCGATCTGTGCAAACAAAAAATAAATCATAAAATATTTA
TGAAACTCAAATTTT
TGACATTTAATTCGTTGATGAAAAACAATCCTTTCAAACGAAACA
CCATTTCATCATATC
TCGAAAATGGATTGCAAATTCAGGAATATAAGCCCAAAATTATTT
CTTTATGCAACTTTA
TTTTTTCTAGTACTTTTAAGTGCATTTCATCCTTCAATTAGGGTAC
ACGTGCTGTCAATT
TCTGAAACTCCAAAAAAG
>ConsensusfromContig83 Average coverage: 10.67
TGGTAATAACTTATCATTTTTTTCTGTCTTTCTATATTAAAGTTCT
CTTCAGATGGAATC
CTACTTTCAATAGGTAAGTGGAATGTGTTGGCTACCCAGCAGATT
GTATTTGCGTATTCT
CGATGGCTTATTGTGAATTGGGCTGGACACCAGCATGCAATTGGG
CTTCCAACAAACTGC
TTCGTGCTGACAATGAAGGA
>ConsensusfromContig84 Average coverage: 19.55
TTTTGGTCACGCCCAATTCCACGAATATCTTTCCTAGGCGAAATA
TCTCATCATTCGTGA
GCAGCTGGGGGCGTGGCGTTAGATCGACACCCTCATCAGGCATG
CAATACATCGCAAATT
GCATTTCTCAGTCAGAGAAATTCTGAGATAAGAATGTTTTCGTTT

AAACGTATCTGTTAA
CCAATCAGTTTCTTGTGGAAGTG
>ConsensusfromContig85 Average coverage: 8.87
CACTGTTATTTTTTTGTTCATGTCCAAAACAATATTTAGTCGTTAT
ATATTTTCTTGGTT
TATCCGGAAGTTTTCTGTACAATGGTGGTCTCGGTTTGAAGTATA
TAGCGTTGGGTGACG
TGCTTGTTTTTATGACCTTTGGTCCTGTTACAGTTCTATTCGCCTA
TCTCACACAATGTG
GCAAACTTTCGATTATTCCCGTGCTCTATGGAATACCACTCGCTT
TACACATCACACTGA
T
>ConsensusfromContig86 Average coverage: 12.26
CGCGCCAGGCTTTCACCAATAGGGAGCCGTATATGAGCGTGAAA
GAGAAAGAAAGAATCC
ATGTGACAGTCCGGCAGAACAGAGTGTCCAGTAACTGAGGGTCT
TTTGTAACAGCTGGAA
GCACACTCAATGGCAGAGACATAAAAGACATTGCCGACCCAAGC
ACGACGGCTAAGTTGA
TTGAGGGACTGGACATTTTCACAAGTCTGTTGTGACGCCAATTCA
CATTGAAGATGGCGA
ACACAATACAAGCGACAACCCCGAATCCAGACAACACAAGGGCA
GCGATGAAAACTG
>ConsensusfromContig87 Average coverage: 18.54
CCGAAATCATATCAGATACGGAGGAGAAATCTGATTGTGTATTGA
TGCTCGCAGCTAGGT
GGAAATGAAGAGGAGTTTTGGCAGTGACATCAGAGTTTGCATTA
TTAACACTCGGAATGA
TGGAGGAAGCGGCTCTGAAGGCCAGCCCAGTTTTATCTGTTGGGG
GATTGCAGTTCATCT
TGTGAGATGCATGGACTCCTCGAGAGCTTATCAGTTGC
>ConsensusfromContig88 Average coverage: 6.70
GAGAAGCGATACGATACCCGTATAGCCTCTGATTGCGTTTGATAT
GTCTCGGAACAAAAA
ATTCAGAAGCTTTCACAATAGGATCCAAAATATTCAATCTGCACG
ACATTATATAAAATG
AAATAAATTGTCACCACCACCATCTTGTGAAATAGTGGATTGTAA
AATTAATATCAGAAC
CGCTTTTCTTCGATGAAAACGTAAAATGATTAAAA
>ConsensusfromContig89 Average coverage: 21.58
CTTCCGATCTCGACGATGGTGCGAATGGCCGCGTCACCTACGCTC
TGATCTCAGCAGCAG

TAGAGTCCCGGGGGTTTTTCAAGATCGATTCAAATACCGGGGCTT
TATCGGTTGTCAAGA
AGCTAGACCGCGAATCCTCGCCTTCTCACCGAATCACCGTCCGCG
CTTCTGACCACGGCC
AGCCGCATTCTATGTGGTCGGACATTTTTGTCAACATTCGCGTGG
GAGACGTCAATGACA
ACGCCCCTACCTTTGTTTTTCAACGGACTTCGGACGCTCTGCTCTC
TGCTTCAACGGGTG
CTATTTCAGGCGCTCAGTACGCTTCCGTGCCGGAGAATTCACCTG
TCGATTCTCTTGTAC
TCAAAATGAATGCTATCGATCCTGATATGGGCAAAAACGGCGAGT
T
>ConsensusfromContig90 Average coverage: 4.36
CGAGTAACTTATTTTTGAAAAACTATTTCCACAAAATGAATAGTG
GATGGTTATTCAATC
AAGGTCATATTGTGTGAAACACATTTGTTCGGAATCATTTTGTGA
TTATTATAATGATAA
CAAGGGGATAACAATTCGTAAATACATATTTCATATATGTTGAGC
GAGAATTGCACACAT
TAAAACAAATAGAAATACCAAGTAGAATAACATACGCATACGCC
ATGCCGTTGAAAACAA
AAATAAG
>ConsensusfromContig91 Average coverage: 16.61
CCTATTGGGCAGAGGACAGGCAGTTAGCAGCAAGGACTTCTCTA
TGGGGAGATATGCAAG
AGATAGGGTCAGAAAGCGAGCCTGGGGGCGAACGCGATAAAGAA
CATTCCTGTGCTCCAC
CAAACTTTCAGAGTGTTGCTGTTATGCATGATGACTCAATGGAGT
CCTGCCCCTTCGGAA
GTGGACCTTACAGAGAACTCACATTATCAAAGTCTAGACCATCTC
A
>ConsensusfromContig92 Average coverage: 12.86
CGGCCGAGAGGGCTGCTCGGATGTGATATGACACTTCATAGTCC
ATGTGGGCCCAAACGA
GGATGCATAGAAGAGCCATGCCGGAGACCCATAATACGAAGTAA
AGTATGAACGAGGCAC
ATTTGAAACACACTGAGCAATTAACCATTCTTCTTCTCTTATACT
CAATATATGTAGCAG
AATAATTAGTGAATATTTCGTATTCTATTTTTCATCATTATAATGA
TAGCGCTTTGGTAG
TTTG
>ConsensusfromContig93 Average coverage: 9.30

ATTCTTCTTAGTTATTTATAAAATTTATTCTTGATTAAAATAGAAT
GAATAAATTGGTAT
TGGACCTTGAATAACTGGTGTTTTTCGTCAAATTAATATGGTAGA
ATAGTTTTAGTTATA
ACTGTTTGGCCTGAAGAAGGTTATCTTAGATGTACTCCCATTTGG
AATAATTCTCTACTA
AATCTGAAGTTATGTACTTAATAATTGGAACCTA
>ConsensusfromContig94 Average coverage: 6.51
CTCCAATACGCGTACATCCATCAGCCATATTGAACGCCTTGTGTG
AGCAGACACCTTGAC
CTGTAGCGATATATATTAATAACCATTGTGATAATTTTTCAATGC
CATGAAACATATCTT
CCAGATTCCATTCTATATTTTCTGCAATCAATATTGTATTTCGTTC
CATTCCAATGGCCA
GTTTCAGAATACAGTCAGACACCGTTTCGATTCCCTCATCTGCAT
TGTCACAGTACGATT
TCAATTTTGCCAGTCCATCAACACACAAATCT
>ConsensusfromContig95 Average coverage: 11.54
CCCTGGCAAGGGACTTGAAGGGCAGTTGTGTCAACAACAGCTGAC
TTGTATCCCATCGCG
GAGAAGCAGATCAGAGGTGCGCTACAGCTCTCACGCTTGAAATGA
GTCACGCTTTCTTCA
TCCTTGAGGTAAAGGTCATCGCGAACCTTGTGCAGGAAGCAGCGC
TTGGTGTTGAAGTTG
AAGTCGACGGCTTTGCAGAAGCCGATGGTCATGCAATGACTCTTG
CAGTCCTCTACGTCA
TCGATGGTCGGAAT
>ConsensusfromContig96 Average coverage: 4.36
TTCGCAGAATCTTTCTTATCCATCATTGACTTCGAAGCCAGGTAT
CGATCCTTGACCGAA
TTCCATTTTCTCATCATCTTGTGTTTTTCAACCATTGGCTGAACTT
CCTGAATCTTGGTC
ACATCAGAGGAATCCGAGTTGATTATCTTCTCAAACTCTTTAGAA
TCTGATTTGGTACCG
CTAACATGAAGCTCGCTATCGAGCCCTC
>ConsensusfromContig97 Average coverage: 9.29
GGTAATGAATTCACGGCTGGAGATGAATTTGACATTTTGGAATTT
GAAGAATCTGTAACA
AAGCATGAATCTAAAGAGTTATCAAGATTGTGGTATGATTGGTCT
TCGTATTGGTATTCG
GGATGGATTGTTAATACAACTGATAAATGGGTGTGTCACCGACGT
TATCCAACTTACGAG

AATATGTATATATCTGAGTCTGATAATAGATTGTTAACAACCCAC
ACTGGATATACATTG
AAAATTTGTGAAGAGAATTGTTATATCGTGAATGGAGTATCTTAT
TATATCTTAGAGATC
GGAAGAG
>ConsensusfromContig98 Average coverage: 14.02
CAAAAAAAAACTTTTATTATTGACTAGCTCGTTATGGTTCATTTA
TCTCATCAAATTAAC
TTTAATTAAAATCATACATGCTAGTTGGAACAATTTTCAACTATT
TGATACCCCACAGTT
GCTTCAATTAACGCGATACAGTGGTACATTGTTGGTAGAGAAGAT
GTTCTATAAAAATCT
GTAATTTATTAAAATGTAAACATGGGAATAATAGACATTAACAG
AGCATGCAAATGAAAT
TAACACAATTTTCGACATACAAATGAATATGAACACAAACACACT
CAATTGTGGTAATTT
CATTAACA
>ConsensusfromContig99 Average coverage: 9.47
GACGTGTGCTCTTCCGATCTTCCGGATGTCTCGGCGAAACGATTT
TATGGCTTCGACTCG
AATGACGTACTTCTCCAATGCTACAGCCTTGATTCTTGAGGCCGT
CAAGAATTTTTCTGA
TGCTTTGTTGTAATTCTCCATGGCAACCCTAACTCCATCCTCATC
AGCAGTTGCAGAGAA
TCTGAGGAGTGCGTTGGATGCAGTATTCTCGTTCTCTC
>ConsensusfromContig100 Average coverage: 7.75
TCACTTTCGCCTACTCCAAGGAGCTCACTTCACGCCACTGGCCCT
GCGAAGCAATGATAA
AACAAAGCAAATATTCAAGATACAGTTCATGATGGGGGAGAATG
CAATAATCATAGATAC
GTTCGATTTCACGGGAAATATGGTACAGAACAAGAAAGAGACTG
AAGTCAGAATGGGCAC
CTGGATAGATCGGAAGAGCGTCGTGTAG
>ConsensusfromContig101 Average coverage: 7.07
TCCGATCTCAAACTTGGCTTTGGGTAAACCAGCATTCACTACAAA
TGGGGATACGGATGG
ACCTTATACGGCGAATAGCAGTGTAGACGGGCTGAATAATCTTTA
TTTCGGACGCACAAG
CGGCCATGGGTCGATTCTGCCATGGTTTTTCGTAGATCTGGGAGA
GAGCTATGCGGTTCA
TGCAGTTGTAGTCTACAATATACACCATTGTTGTCAAGAACGTTG
GCATGACGTCAATAT

ATCTCTGACTGCAAGCGACCCTCGAGGTTCCGTCACTCGCACCGA
TGTCGAATTGGACTA
TTCCTCTGCGGTGACCTGCGGCCATCATGCGGGAGCCATGAGCGG
ACCTGCTGCTACGAG
CTC
>ConsensusfromContig102 Average coverage: 7.10
CTCGCCCAGATGGTCTGTAGGGGCGTGAAGGATGTAAGGGCGCT
CGGGGTGCATGACTAC
GTTGAGTTGATATCTTTCTGGTCGGCCATCTGGCGGATGATGCTG
GTCGCCGGGCCAGAC
CAACTCTTCCATCTCGATTCCCCTGCTGTAGCTCCAGGCACCTTT
CGTGAGCCATCGTGA
TTTCCTATTTTTACTCTCCTTTTCAGGAACTTTATTGACAATTTCG
AAGTTGACATGTTT
TGGCTTTCCTTC
>ConsensusfromContig103 Average coverage: 9.45
ATCTGGCTTGGGCACGATCTTTTGTCAGGCATCGTACTACACAAT
AGATGGGCGGCAGGT
TCTATTTGTGTAGCACAATTGCTGACTCCGTCTATTCATGGAGTG
AACGCAAGAGATTTT
GAAGTTGATTGTCTTGACACATAAAATTTTTAACCGTCATGACTA
ATTCTTTGTCTAAAA
TTTCTTGGTTTATATGGTCCTCCACTTGCGTTCGTTCTTTG
>ConsensusfromContig104 Average coverage: 14.80
GAGGTGTATGTTCAAAAAGCTCTTACAAATTCTAGGAAAATGGAT
CAAATGGGGATTTTT
AAATATAATAGTATTGCCGACACTGTTGTAATTTACAGTGGAGTT
GATTTTGTTACGAAT
ATGAGTGAAGAAGATGTATCTTATTCTGAAAAAGGAAATTGTATG
GACGGTGATTTACTT
GAGAGATTAGTTAACTCTGCGATGGAAAATGAGCAACATGTGCAA
ATGTTTTTAAACTTC
AAAAGAAAACGAAAGTTTTGTTTAGGGAAAAGTCTGTGTCATAT
TGCCACAAAAACAAAA
GTGAGTTGCAGAGGTGTACATGGAACCCTTGTGGAAATATTATTT
GACACAATATTCACT
TCATTCCGCAGTTCAATTGTTTTTAAAATTCTTATATATGTTGGAA
TAATACATGAATAT
ATAGACTATTTGAGAGGAAAGGTTTTTCTTGACACGGAAGATAAA
ATGACTATTCATGAC
TTAAAAA
>ConsensusfromContig105 Average coverage: 8.23

GTTATAATGCAGATTCAGAACTCGGTATATTTCTGCATCAGATGT
GACTATAAGGTCTTC
AGGATTCTGAATCCCTTCTGAAAGAGCCTGCCGCGTATTTTTC
CATTTGAATGTAGAA
GAATTCTCTGAAATTGCTGAACCATTTAACGAGCTGTGCTGTATT
GTTTTTGTTGAAAAG
CACATCCGGGAAGTACATCTTCAAGATGGCGGACGATGGATATC
TCACGAAGAAGAACAT
GAGCTTGGCTTTGCGCAGGTGCATCGGAGTCAACGTGCTGGT
>ConsensusfromContig106 Average coverage: 5.62
CAATGTTCTTGATGTTATCTGAAAGCAGCTCATGCGTCAACTCGA
GGTCTCTCTCGCTTG
ATATAATCAATGTGCCTTTTTGAATGCCATATTTTTGGTAGAAAT
ATGCATCAGAAGAAT
CAAACACAAGATGAGTGATCAATTGCCTTTTAAGTTCTGTTTCGT
ATTGACAGGGTCTTG
GCTGAGATTCAAACGGTGTAATGTCTTCGTCCATTGCACATATCTT
CGTATATTGTTTCT
CATTTTTG
>ConsensusfromContig107 Average coverage: 12.33
TTAGAACTAGTTCTGTTGAAAACTGTATGAACAATTGTCATCATT
TCAGCTCTCGAGAAA
ATACAAATATTATTTCTATGAAGTGATCCAAATTATTGCAAAATT
AATATGTTTATTGCG
AAAATAATGAATATAATTAAAATCAATACTGTCGAAAAATATTG
AAATATTCAGAACATA
GCAAAAGTCGCGAGAATTGATGAA
>ConsensusfromContig108 Average coverage: 8.89
CTGAGATTTGTTCGGTTCGTAATTTTGTATTTGTTTCTAAGATATA
CGAAAGTCGTCTCA
ACTATTTTACAATCGAATTGTGTCGATTGACCTATGGCAAGCTGT
CTGGTTGAAGGGCCA
AGTAGAACAGGCGTTGGGGTACATGGTTTGGACGGAATTCTTTGC
TGGTTGTGTACACAT
TTGGTAGAGTCAGGACTCCGAAGAATTATTTCTGAGTTCGCTGTT
CTGTACATCTCTGCG
ATCCACAGAGCATTGCAGTCGCAAATAATTGGGTTGTGCGAAAGA
TCTATTTCCAATACA
CTTCTCTGATGTTGCTTACATATGCTGCTGAGAAGGTCAAAAGAT
ATGGACTGTATTGAA
TTGTTTCGGAGTGATATATTTTGCAAGCTAGGCAAATCACGGAAA
GCTGTGGAA

>ConsensusfromContig109 Average coverage: 4.59
TTGGAATCGCTCTGTGGGAAGAGAACTGCCGACCAGAAATACTC
GAGATGCAGTTTAGCA
GACTCTGGCAATTGGGAAGGAGGTTGAATGGAGGTGGCAGAACG
TCTGCTTCAAGGTAAT
CCAACATTCGACAAGTTCTGTTGTACAAAAATGATACTTCGTCTT
CGAAGTTATAACACT
GGCTATAAGTCTCATGGAAAACGCCAGATCGGA
>ConsensusfromContig110 Average coverage: 15.38
AAGTTAGGGAAGGTAACTGCATATAAGTATCTAGGTGTGTGGGT
TAATGATAAGGGTAAC
ATGGAGAGGCAGATTGAGGAAGTGGGTAAGAAGGTAAATGGGG
CAGTGGTGGGACTGAAG
AAAATGATTGGAAGTGAATATGGTATGGCGGTGATGGAACTGAG
AATTTTATTATATAGA
ACAACAGTTATTCCATTAATGACATATATGATGGAGGGATGGAAA
A
>ConsensusfromContig111 Average coverage: 7.46
ACACGACGCTCTTCCGATCTGCCGGTTTTATCCCGCAGCTGTTTCT
CTTCTGCGATTGCG
ATCGCATAAGTTCCTGTGCTTTTTATTGCCAGAGCTGCCGAAAAT
ACAATGTAGCTGAAG
GACACCAAACGGCAAATTGTTCTTGAACATAAATTCTTCTTGTCA
CTCTCAGCATTCTAT
AAAAGATGATGAAAACATATCGCG
>ConsensusfromContig112 Average coverage: 8.10
CAGACTGTTTGAGCAGAGGAGACGATAAGAACAGAATTGTTTTAT
TCCGACTGTTAACAC
TATACTGACTCATCGGATTCCATGATACGACAAATCGTATGATGT
GCTGGACGATAATGT
ATACGAAAATCTACCCCGAACATCTCGTCAAATGCGCAATCATAG
CTCACCACTTTTTGG
GTGCCACTTCAACCCTGACCAGATATTGTTATCAAGTGAAT
>ConsensusfromContig113 Average coverage: 8.18
CTAGCCTCCAGCTACATCACCCATCTCCACAATATGCTGGTCGTT
GGGTCTGAAGCAAAC
GAGCTGACTTGTACTTCCAGAGTTTCCGCTCTGAATTCTACTGAG
CACAGAAATTTGATG
TCAGAATCACCCATGATGGTTGACTCAAAGCCGCAAATCCTCGTC
AAAAGACCTGTGTGT
ACGTTTTGCATGAGTGCCATGAAGTATAAACATCATGTTCGCCGA
AATATGGTGATGAAC

ACGTACCCACCAAGATGAGTATTTTATTTCTATTATTATATTATTT
AGAAATT
>ConsensusfromContig114 Average coverage: 5.29
TCTCACAGATGATTTGATGTGGCTGGAGCCTCCAACGTCAGACGA
ATTTGCAGATGTGAT
CTATAATTTGACAACACTCGGTTTGTCTGACCAAAGCGATGATAA
ATATCGGAAGCGGAT
CAAACATTGGTTCACCGCTTTCGAATCAGATATGACCAAATCCTG
GAATAGTGCCAAACA
ATTCTATATCAATCCAGATAGTTTGGATTGCATGTTACTTCATGGA
TCTTTGAATATGTA
TGGATATATATTACGACCTGATTTTGTTCGTGACAATATTGAAGG
GATGCGAGATAGATG
CGCATTTTTGCAAGAAACAGTTTCGATGGAATTGTGTGTTGATCT
ACTGGTCAAACTGAA
ATCGTACTGTGACGCCTGGAATGATGATATAGAAACAGTATTTGA
CTGTTTG
>ConsensusfromContig115 Average coverage: 10.95
GGTGCTCTTGCCGCAAATATCAAACTGGCGGGTATCTCAATTAAT
CCCAAGAATATTACA
TGCACCACTGATAATAGTGCGTTTCGTGTGGTACCCATATTTGAG
TACGAGTTTTCATTG
GTGACTGCATCTAAATTGGATCGAGAAGAGCAGGACTCTCATGAT
ATCACCATCGTTTGT
TTTCACATTTCCCAAATCATAATTGAATTGTCAGAGCGAAAGATT
ACTGTAGAAGTCATA
GATATCAATGACAATAGTCCTTTATTCCAATACAAAATTTACTAC
GCTGAATTAGA
>ConsensusfromContig116 Average coverage: 5.88
CTCTTCCGATCTCTTGGCATTTGTTTATATTCTGATTATCTATCAAT
TTCTTTTTCTCCA
TTTTTTTCGGATTTTTGTTTGTATCAGGCATGAAATAAAGCGCATT
CATTTCTTTGTGGC
TTATATCATCACTTGCGGCGTTCACAATGTCTTGTTGTTCAACCAA
GTCGTACTTCAATT
GTGTTGAATCTGGGAGGCCAGAGAGAAG
>ConsensusfromContig117 Average coverage: 4.77
CTCCGGTGAAGTGGACTGTGCAGAAGCACAAAAACTCATAACCA
TGACCAGTGCTTGTCC
ATGTACATGTTCCGCCATTCAAGCAAGGATCCGGGATACAAGGGT
TTTTGGTTTTTGTCG
ATTGTTCAGGACTGGTTGCCTGGGTTTCGGTGGGTTGTTCAGGAT

TGGTCACAAATTTTG
GGATGAAAACACCTCCGGCTTGAATTGAGTGGAAGCAATTGATCT
TGTTTTCTGTGAAGA
TTGTAGTTCTTGCCATCATGCA
>ConsensusfromContig118 Average coverage: 9.67
GCGCTCCACAGCGATGATCTGGATGCTTTCGAATCTGAGTTAGCG
CTGATGGAGCGCTGG
TGTGAAGATGAGGATACGGAGATCCCGCCATTTCCTGAGAATGT
GACAGACGATCTGGAT
ATGCTCGATTTGGTGGAATGGCGCTCCAAATATCAGCACAAGAA
TCACGTCGCGCACAGC
ATGGTGGAACGACGGAAGCTGGTCTTTGAGATGATGAACACGCT
GCTACCCAGGCTATGC
GTCGAGGATGCTAAGGCGCTTGATGTCGCTCTATCAACGGTTCTC
AGCGGTGTTGTCAAT
CGAGCCGTTAAGATTCAAGGAAGATTGCGAGACCTGGAATATCA
AATCTCGCGCAGATGC
CGGATGGAACGAGAGATGGTCGAGAGTTTGTC
>ConsensusfromContig119 Average coverage: 9.94
GTAACATCTAATAAAAAGAAAATTTATTGATTTATTCTTCGAAAG
TGAATCAAATAAATC
TCAATTAATCATCAACAGAAATCTCAGCATCTTCGGTGATCTTAT
GGAGTGACGCAAGAC
TCTGTTCTGACTTCACGAATGCTTGGTTTTGGAATTCTTCTTCGAA
TTTGAACTCTTCTT
CGACCACTTCGTCTACTTTGCTTCTGGCTGCCATTCGAAGCTCAAT
GAATTTTTC
>ConsensusfromContig120 Average coverage: 7.05
CGGTGACGTAGGTGGTGGCGTCACTGTCAAGGACGGCGGATCAG
TTACCATGGGAACTAT
CGTTGTCGTGGTAACCGTTGGTGGCACGGTAGCAACCGGAGGTTT
AGCCTTGGGTCTGCT
GTCGTAGTTGTATAGACAGCCAAGAGGATGATAACTCTGGATTG
GTCCAGTCATGAAAGA
GTTGAACCCGATCTGGGAGAGGTACCTTCGAACCCTTGACTCATG
CCGCATCCGGAAGTG
CTGGATGTTGCGTGCGATGACGAAGAGTTCATTCCCGAGGAGGC
TGGAGACGATGGCGTA
GTCGTACTCTGCGGTCCCACTGCGGGGCATGACGTCCAGCACCCA
ATAACTGTTTCCGAA
CGGGTTTCCGCTATCCTTCAGCGTAAACTTCC
>ConsensusfromContig121 Average coverage: 7.42

ATGTTAACTTATTTTTATATATAAACGAAAATATGTTAAAAAGTC
AATCTGAGAGGCAGT
GTGATACAGAGATGTATTATTTTACTTGATGCTTTTTTGAAAAAC
TATACTGCATGATTA
ATTCAAAACAAGATATTCGAAAATATTACGTGAAACCGTTTTGAG
GAATGAGATTTATAT
TTAATATCTCAATATAATTTTTT
>ConsensusfromContig122 Average coverage: 12.09
CAGGATAGTAAATAGTACGAGCAGCCGGGTTGTTTCCCATCCTAA
ATATGAGTGCTGATA
GTCGGTATAACATTATTTTTATCGTCATTTCCATAATCGAAAGCAC
TGTTTGCGTTCATT
CCAGATTCATGTGGATTTTGATCTTTTTTATATTTGAGGTTTTCCA
TCACAGTTGTTCCA
GCTTTGGTCACCAAGAGAAAGAATACAAACCCGAATAAGATAAC
ACAGACCGAAATTATG
ATCAATTCTTTCGAAAGAGTGACAAGAATGAAGGCAGTGATCAA
GGCAACCGTGTCAATG
ATGTAAACAAGTGA
>ConsensusfromContig123 Average coverage: 19.53
TCTTTCCCTACACGACGCTCTTCCGATCTTCGCGGCCATACTCTAA
TTCATTGACTGGTG
ACAATATCACGAAATTTACGAGGTTGTTGATGCATGGTATTATCA
ATATGGTCCTCGTGC
TATTGAATGATTCCAAATAGATTTATTTCTGCTATAAACGTGGCCT
ATCTTTCAGATATT
CGCCAAATTCAAAGGGTTTGTCATATGAATATATTCG
>ConsensusfromContig124 Average coverage: 9.81
TCAGACCCTGTGCGTTTTTCTGGAGAGCCGAGAGGTCGAGATATC
GCGAGTTCCAATCCT
GCGCGAGCTGTTCTTCCTCTTGCTCGTTGAGCCAAACCAACTCTG
TGACGCATGACTGAA
GAACGTCTATCAAGCTGAGCAGACATTTCTCCTTTCGCGAAGAAA
CCTCCTTAAGATTTG
AGTATGAAGCTTCAAGTTTGGCCAAATAGTTGTTGAAAACTTTAA
TTTCGCCAGCTTCAA
GTTGAGTCCTTCCTTTCTTGCAATCTTCAATAGATCTTTTCATGTG
GAGAATCTCAGCAT
GGACACTTCTGTGTCGTTGAAGTTCGGTTTGGGCCTCTTCTAGATC
CGATCCGTATTTCG
AATTCTCCAAAAGCGCCTCTTGACTCTTAATCCACTCGAGGCACT
TTTGAACCAATTGGA

AGGCCTCGTGGTTTTCAACCAATCGCGTTTCAGAAACAACTTCTG
TCTCTCTCGAGACGT
TGACAACTCTCTCTGTTCCTCTCTCGACGCGATCCATGGACGAGA
AGCTGGGATTGAGCG
AGCCGAACCCGAGAGAACCCGCTCCCATGTCCGGGGAATCCGCCG
TCAAGTCTCGCTCTA
CCGATGCGTAGAGGTCCGCCCAACGCCGTTTCAGGCTGTTGACTT
GTCTGGTAAGATGTT
CGGCATTGAGAAATCTTCCCTCTCTCAGAGATCGACAATCTTGCT
CTAGTTGTGTTATCG
AGTCTTGAACAGAGCGAAGTGCCCTTCTCAACTGATCCCCCACGA
TGGGTCCGTCTCTGC
TGGAACCAGACCGAAGAACCTCCTGCATAGCCTCATTCTTCCTTC
TTTGCACATCTTCAA
TGTGGTGATTGCAGTTTTGACATTGCCTTTCAATTCCTTCTGCCAA
GCGACTCATTCGAT
CAACTCTCGATATCTCGGACTGAATGGCCATGTCTTTTTCTTGGA
CGGCAGAAGTGAA
>ConsensusfromContig125 Average coverage: 4.41
GAACAATTGGATGACAGAAATAACAAAAACACTGAAATTCAGTT
CGGGACCACGTGAGAC
TCTGAAAGCCAGATGGAGTGATTTAATTGAACTATACGAACACG
AGAAAAATAGTTTAAT
AACTCTGTCAGACCTCACAAAATCTGCTGTCATGCCATCCAATAT
TCAGAAACAAAGCGT
TCCCCTCGCGTTGAAAGTTTTCAGCGAGAAGACCTCGTCAGTTCT

>ConsensusfromContig126 Average coverage: 5.48
ACTAAGAACATGACTTGTCACAAAGATCATAGTTTGCATGTTCGA
CCCCAAAGTTGGAAT
CCAATAATTGACGAAGAATATTATTATCGACGGAAGAACAGCAG
TTATTAGGTAGATAAT
TGGCACAGCTACCAAGTATGATGTTTTGGATGAAAAAAATGAAG
AGAGGAAATATGCGAG
CAAAAGATTCAGAGGAATTGCATGGGAGAGAAGAATTACGAGAT
GCCAGGCACCGGACAA
GGTTCTGAAACTCTCGCTCCCAATTCCGAATTCGAAGAAGACGAT

>ConsensusfromContig127 Average coverage: 9.76
CGCCGCTTCTCACATGAGTAAGTCTGTTCCACCGCTTATGAACGT
AAAGCCATTTCAGTA
CACTAATATTCCAATACTACCGGGTTTGTTTGCAAAGAAACCAGA

TCCTATTCAAACTTT
TTCTCCATGAACACATCCAGCCCACCCTTAGGTCTCGTGAATATC
AAGCCATACCCAGCT
GCATAAGTCCTGCAAGCCGTGCTGATTTCTCTGTACTTGAAAGCC
AGACAGCTCCTGTCT
CGGGTGCAGATGAGGGCGCATTGGCTGAAACTCTTTCCCGTCACC
CACAAGGTATTTTCG
ATCTCAGAACTGAAACCTGGTGTAGCATGAAATAGATCGGAAGA
GCACACGTCTG
>ConsensusfromContig128 Average coverage: 4.67
TGTCACTTGTCACTTCTCGATTGCACATTTGATGATTTGTGTTCAC
GGGGTTTGAGTCCC
GTTTGCTGATCATAAGTTTCAAGTAATGAACTCTTGATGATTGTCT
GTTCGGAAATTTTC
TACTGAATGATTGCAGTATGTTGAGAATAATGGTTTTGTCGAAAT
TATATTTCAAAATAC
CCTCCATTATTGTTATGGTTAACG
>ConsensusfromContig129 Average coverage: 6.47
CACGGCTACCTATGTGTAAAACAATCTCAAAATATGCGTAGTGAA
TGCTTTCAAAATATA
TTTTATACATTTCACATTAGATGCCCAATTCCAACCTAATGTTAA
CGTAAACTAGTGTTA
TGCAAGTGATCTCTGTGATAAATAATGCAATTTTATATGTTGTGT
TCAAACGTTTAGAAT
GTAATGTGTTTAGTATGTTCATCGTATGTCATCACAGATGTAG
>ConsensusfromContig130 Average coverage: 8.08
TCTCCAATTGAGTAGATATCGCATTCGGTCCTGTCTTCGTCCGAA
TTGTCACCACAATCG
CTCTCGAGATCACACTGCCAAACCTGTGGGATGCACTTCCCTTCA
GCACATGTAAACTCG
TTTGAGTAACATCCGTCCTCTTTTGTGGAGATTTCATGCGGTATC
CAATACACGTAATAT
TCGTGTAAGGGTGAGTTACTGATGAAATAGTCACAATAATCATGA
AATACGTGTAGCATT
TCACAGTACGGATCAGCCTTGCAATCTTCGAAGCATACAATTTTC
GGAATATGATCTGTA
TATTTGCTGCCATACTGATTCGCCATCGCAGTGGGTGATTGACCG
AACATAAAAACATCA
ACGGGCGGCTTTTCCAATTGGGTGGTTTTCG
>ConsensusfromContig131 Average coverage: 5.76
ACAAGTGATTTGGATTATTGATTTTTGTTTGCCTTGCAGTGCACA
TATAACCGGGTGTAA

ATGTATTTGGCTTTTCTCTGCTCAATTTATCTGCGATTTTCTATAG
TATTCGCATTTGTA
GATTTCTTTGATTTCTTGGTGTCTGTGGTCTCTGTTCATTTCATGA
AAATGTTCTACTGT
CTCCGCTTCATTGGTGCATAAGGAACATTCTGCTTTCACCTCCATA
TTGAATTGATGCTT
TATACTATGTATTTTGAAGAAACCAGCCCTGGGCAAA
>ConsensusfromContig132 Average coverage: 7.76
GTGCTCAAATATGAATTCGAAAGGCTCATAGAAGACTTTTGAAAA
TTTCATCTTGTTTTT
TGTTGCTGTTACAAATAATACACTATAATTTCCCAAAACGTATTC
AGCCACAATCATTAC
AAGCCAGTTTTGCTGTTCAAATTTGTTCTCGGTAAAATAATTCAT
AATTTCACCTTACAA
AATCCAAAATTCACACATCGAATTTTATTGTCGATCATAAATACA
AAAT
>ConsensusfromContig133 Average coverage: 11.85
GTGAAACATAAAAAGAATCATTTTATCATCATCACATGAATGCCG
ATAATAAAACTAATC
CAGTAAAAGACATTTTTTAGCGAGTGGAAAGGCCTTTCTATGCTG
TTCCACTACTTGCAG
AATGAACTGCTTCTGCTCTTCGTTGTAGCTTAGGGCAGAATTATA
CGACTCCATCAATGC
CACTCCTCGTTCTGCCAGATCATTAGTGACATTAAG
>ConsensusfromContig134 Average coverage: 7.10
GGATGTGGACGGAAGAAGTGTGATGCACACAGCCGCAGAACTCG
GATCAGTGGCTTCAGG
AACCCTTATACTTGAAACTGCTGGAAAGCGATGTCTGGAATTAAG
AGATTCAAGCAATCG
AACCCCTCTGCTAATAGCAACGATAGGTGGACATGGCGAAATGGT
CAACTTTCTCCTTTC
AAAAGGGGCGGACGTGGCGAGTGTTGATGAGAACGGTGCGACGG
CGTGGGACTACGCATG
CTCGAG
>ConsensusfromContig135 Average coverage: 7.91
CACGACGCTCTTCCGATCTTGACCGTTCTCCATGAGCTGGGCGAC
GAATGTGGAGGAGAG
ATGGTTCACAGATCCCCCGCATTCTGATATTATGGAAGTTTCTAG
AAGCTCTATGATTCG
TTCGCCAGCCTCGATCCATCCCACTCGGAGGGCGGGGCTTAAAAT
TTTCGACATAGTGCA
GTTGGAAATAACGTGATTTTCACTGCCGGGAACCCGATCAGTGTA

TCTGAAGAG
>ConsensusfromContig136 Average coverage: 20.46
GACACAAGACTCGATGCCAGGTGCTCTACAGTCCTAAATCAATG
GACGGGATGGGAATGG
CAGATGGTGAGGGAATTGAGCGCCTTTGGGCGATTATCGGGCGT
CTATCGAAAATCACGA
AGGAGATGTCTCCGGAGAATCGGATCGATTTTCTCGCCGATGGAC
TTCTCTATCGTGAGA
AACAGATTCGCGACAAATTGGGT
>ConsensusfromContig137 Average coverage: 3.93
TCACAGTAAGAATCAAAATATCTCAATATATGAGATTTTGGAAC
AATCGTGGCAATAAGG
ACTTGTGAAGACTCTACGCATGTGTTTCATAATAGAAATTTTCGT
TCAGTATCTGAGCGC
GGCTGACGGTGTAGCGTGATGCTTTATCCGCCATTCATGATGTAC
GATTTTTGAATAATC
ACAATCAGTTTACAATCAATTAGAGTATGT
>ConsensusfromContig138 Average coverage: 5.32
CCGATATCATGGCGGATTGTTCGTTTTTTCAAAGCAGTAATATTTT
TATTAATTTGAGAC
TACAAAGCTATTATATCTCTCATTAATATGATTTGTTATGTTTTTA
TTGTGAACTAACTC
TTCACTCGGCAGCTAATGGTCGTATAGGAATCCGGGAAAAGCCT
CAAGTCCCTCAGAACA
ATCACGGAAGCTAAAAATCTCCTTGTCGCCACAATGATTC
>ConsensusfromContig139 Average coverage: 6.41
CGCTATCTACACCGGTGGGCGAGTCGGTATAGCAAAAGCCACAAT
CCCATTGGCTGATGC
AGGCATCGCATGACCCGAGGTTGTCGCATTTAGAAATGGCAGGA
CTTTGAGAGATGACTC
TTGGCGAATTGGCAGACGAGAGGTGAAAGCTTGTGGCTATCCATG
TCAGGCTCACAATTG
TTCCAATCAGACTAGCGAAGACGAAAGTACGTCTGCTGAAACGT
TCAACAATGTAGAATC
C
>ConsensusfromContig140 Average coverage: 6.92
TATTATCCGATAGAACCTTCCCGCGAGTTTGCTCACATGATAAAG
TATACAAAAGATTAT
ACGAATCATTTTCGGCACTTCTGTAATCACCCAATCCTGAAGTTT
CATGAATATTCGAAG
ATGAAGCATTGTCAACATTCGACTTGGAAGATCCGTTGTTCGAGT
GCGCTATACCAGCAC

CCGTAGAGCTATTGATGGTAAAATTCTGCTTAGAATCAGTGGAAG
TTTT
>ConsensusfromContig141 Average coverage: 7.08
TTATTGAGATGAAGAAAAAGACGTTGTGGCATCTCTCAGTCTAT
GACAACGAACACAAGA
TGCGGCAACTCTATGTCAAGTTTTGTGAGAAATTACCAGCCTACA
ACTGCAAATTATATT
CACTTCGCTTCTCGAGTGCCATATCCAAAAAACGAACGAATTGTC
TCCTGGGTGTTTCCA
GCAACAGAGTGGTTCTGCTGCATCAGAA
>ConsensusfromContig142 Average coverage: 10.70
TGAATTTCGAGCAGTTCTTTTTCAATATTGTTGTAGCTGTCTCTTA
TATCTTTTGTTTTA
TAAATTTGAATGAAGGGCACACGAGGTTGCGATATTTTGTATTTT
ATTCGATTGTTTACT
TAGAAAATGTTGCTATGGTTTCTGTTTGGTATTGGCGAACTCTAT
CATCAAATATTTGGT
ATAACGAATTGGTTGTCATTCATGTTTGTGGATGGTTTTTCATTTG
TGTATTTTTACAAA
TATTGTATTTTATTGTTTTTATTCCCA
>ConsensusfromContig143 Average coverage: 21.42
TGAATATGAAACGTAAAGCACACAATGATAGGAATTAATAAGAT
ACATGTGTTCATGGAT
ATTCTGGGATATTGAAATAAAAAAAGAGCAATCGAAGTAACATCT
AGTGTTTCATTTATA
TTTATCACACTATAATAAATATGATTTGTAGATAATGGGATATCA
GTGGTTCCTCATGAT
AGGAAATATTAGGAGGAATGAATTCCTGATATGAGTCATATTTAT
CAATGATCATTCATA
TGATCTATGACAATCGAGCGAACATGCGATGAAACATACATGAT
AAGACCCAGATATATA
ATATATTGTGTATGATATCATTGTATTCTAATAAATAACCATGAC
AATG
>ConsensusfromContig144 Average coverage: 10.03
CGCAAGTCGCGCCCTTACTCCAACAGAACGACGGTATTCGCAAA
TAGAAAGAGAAGCCCT
AGCAATAGTATTCGGGGCCGAAAAATTTCGAACATATGTGCTTGG
TCATCATTTTTATAT
CATATCGGATCACAAGCCTCTAATTCCATTATTCAACAAAGTTGG
ATCAAACCCTCCACC
AAGAATTGAGCGACTTCTTATGCGATTACAAATTTATCAATACAC
GATAATGTACAGACC

AGGAGCAGAAAATCCAGCAGATTATACTAGCAGACATCCAATCC
CGTCAACACAAGATAA
TCATGAAGAAGAACTGGAAGAGCACATTGCATTCATATTCAAGA
ACACTACTCCCAAAAC
TATGACTTCTCAAGAGATCAAGGATGAGTCCCTAAAAGACCATG
AAATTGTAGAAGCGAT
AAAATCTCTG
>ConsensusfromContig145 Average coverage: 11.88
CATCAATCGTGTTGCCATTGGAAACAAAAATAAACCGCTACAAAG
TCAAATCGCCCCTTG
ACGACTATTCAAATCCGGGCTACGTCAGAGATTCGGGCGATATA
TCAGGGAATAACCGCC
AACAACAGTACGTCGTCAAAACAAACGAAAGAGAGCTGTGCTCT
GCGAATATGTTCATTT
TAATCGTCCCCAAGGCAACATCACATTCTCCGCCAGGGGCGTTAG
AATCCGGCAGATCGG
AAGAGCCC
>ConsensusfromContig146 Average coverage: 10.21
GCCGTATCTCCCAATGAAAACACGCAGCGGTCCATTCGAATGTTT
ACTGTTGTCAGACCG
TATGCAGACGATGAAAAAGGCAGAGAAAGTTTGTTGGTGTCGTTG
ACTGATTCTAACGCC
CCTGGAAGAGAATACCAGGTTGCCTTGGGAACGATTAAAATGAA
CATATTCGCAGAGCAC
AGCTCTCTTTCGTTTGTTTTGACGACGTACTGTTGTTGGCGGTTTTT
TTC
>ConsensusfromContig147 Average coverage: 7.68
AGAGGAAGATACTGACTGGAGCATTCTGCAGAAAGTAAATAACC
TCATGTGCCAGATAAC
CAATAGGATGCCCACGGCGGAACACATAGATCATCGCGAGGGAT
ATATCCACCACACGAT
AGCGTCGGATCATTTGATCCCGGCCATGGAAGTCCTGGACGAAT
TCAAGGCTGAAGATAA
GATTCATTCTTATAGCGTCATGCCCGTATCTGTCGAACATACGTA
CCGCATAAATGGCCG
CCGCACTTTTGTTTTCAGGGTTCTCATTAAGTAAATTTGTTTAGGT
TATAAATCACAATA
CGGG
>ConsensusfromContig148 Average coverage: 10.95
TAGTGATATGTTTTGATAACAAATTATTACGTGAATAAAAATACA
AATGGGCATAAAAGC
TATATCCCGGACATGAGGTATTCCAAAATGTACCATTTTTAGAAT

TTGTTGAAACGAGAT
CTTCCAGACAAGAAGTATACCAAAATATACCTTTTTTAGAATAAT
TGCTGAAAATGTCTT
CAACAATCTTTAAAACAACATAAGAAAAC
>ConsensusfromContig149 Average coverage: 4.11
GTGTGCTCTTCCGATCTCTGATATTGATCACAATTTGATGCTTTTG
TTCCCTCATGTGCT
TTTTCGGTAATCTCAGGATGAGATGTACCTACCAATGTTTTCTGA
CATAGTTTTTGGCCA
TTGTTCATGTTGTCAACTACATTCCCACCAATGGTTCGATATGATA
TTCCTGCTTTTGTC
AATACATAAAAATTACTTTCACTGGCGATTATCATGGAAACTGAA
GGCTGACCATCTATA
TCATCGATATACTCAGGTTGTGCGGATTCATAAGATCG
>ConsensusfromContig150 Average coverage: 5.03
CCGGTCGTCTGGTTCTTCCTCACCATCCTTTTTGACATCGTCAGGC
TCATCTCCATCATC
TTCTTTTACAACGTCAGCCATTTCACCGTCATCATCCTTCTCATCT
TCTGATTCTCCAGC
ATTTCATTCTCCGCCACCTCTGACCAACATCTTCTCTGATGGGCA
GAGATTGAATGATGC
GTACATCAACAACAAACATAATATTCCAGCTATCAATATTTACAG
TGCTTTCTTGCGAGA
GTATTATTCAAGAATGTCACATCTCTACAAACGACAACAAGATT
ATGAGATCGGAAGAGC
GT
>ConsensusfromContig151 Average coverage: 26.33
ATAAATTAAAAGAACCAATATAGGCAAACACTTTCAAATCAATAT
TTTAATATTTTTATG
ACACATAGCAGAATAAAGCAAATATAATGATGAAATAACAAAGG
AATGATACAGGGAAAC
ATCTTACTAATTCGTTTCTTCATTGAAAGGTATGTCATGTCCAAA
AACAAATCTGCTAAC
TATGGCAGATTGTACGATATGAAATAGATTCGAGCAGT
>ConsensusfromContig152 Average coverage: 8.37
CTCCATTTGTTATCATGCATAACCCTAATATGCATTTTATTGCGC
ATGCTCCAAAGACAA
ACAGCTTCTTCCTGTGCACGCCAGCGGATAGAAATGGGAATAAG
TCGGAAACCGAAGTGA
TGAATCCTTCCACGTCGACGAACTGACTATCAAGTCCAAGCAGAA
TAATCATTATGAAGA
AGAAGACAGACCACACAGGGGCGGCAGGCAGTGTTGAAAGAGCC

TTTGGATATACAACAA
ACGCAAGACCTGGGCCCCCAAC
>ConsensusfromContig153 Average coverage: 9.98
GTGATATTTGGAAATAACTTTTCAACTGGCTCCCAGATTGGTAAC
ATATCTTCCGAACTA
AAATTTGGTGTTTCGGTTGGTAGTGAAAATTTTGTATTTTTCACAA
ACTTTGGGTATAAT
TCGAAGAATGCTGACGACAATGTACAAAACTCGGATATATTAACT
TTTTGTAACGGTGGT
TTAACGTTTATAATCGCCATTTGCGTATCATTACAAACCTGCTTTA
AGCGTATTTCTTGC
TTTGTCGAAATCAT
>ConsensusfromContig154 Average coverage: 48.60
CACGCTGACAATCAGCTGCCGTTCGATTTATCACTGCTTGCTCAA
CAATTGAGGCCATGC
TGACATGATTCTTGTCGGCCTTATCAGTTAACAATGCCTGCTGCTC
TTCTCTCTTCTGCT
TTAGCATATTGCGCAGTTTGCGAGATCTCTCCAGCAGATTTTCAT
AATCATCAGGAACCG
GATTACTCCTTCTTGGAGACACTTCTCTTGCAGA
>ConsensusfromContig155 Average coverage: 6.50
ATCAACACTGATCGTGTCCATGTCTCCAATGGATGTTACAGACTC
TGGTGGGTTCGAATG
GATATCCAGAATTTTCCGATTGGAGCTCAGCCAAGAACCAAGATT
TTCTAATTCTTCCAT
GAAATATTTCGTTCGCTCCAGCTTACACCTCAATTCGCTCTCGTGC
CTTTTAAAATCCGT
CAAACCCTCATTCAACTGTTTCTCCGAGATCGG
>ConsensusfromContig156 Average coverage: 14.91
GTGAAACACAGAAAACCTTCATTATTAGAATTAATTTTTTATTGG
AAAAATAAATATTAT
GGATGCAATTTTCGAATACGTATACTATATCACATTGCTATATCG
CCATTTGGAAATCAT
CATTCATTATTTTGAGAGTATAATGTTGATTATCATTATGGATAA
TATTGACGAGTGCTA
TTTTGCTGTACAATGTAGTATTAGGATAATTTTGTGTCAAGATGAT
CT
>ConsensusfromContig157 Average coverage: 18.50
CATTACCACAAAATCAAATGCTAAATCAAATAAAAAACATTTGCA
ATCATCATGTATCAC
TCAAAGTTGCGAATGTAAGATTGGTAATATAACTAATATAGAAAA
AGATGCCTGGCATTT

AATAAAAGGTAAAGCCTCTGTATATTCTATACGAGGTTTAAGAC
CATACATGGAAGATAG
ATTTAATATAATAGATAAACTGGCAAATACAAATATATCATT
>ConsensusfromContig158 Average coverage: 11.27
TCCGATCTGAAAAATTGTGCGAGTGATATTTCAATACATAACAAA
ATATGGAGCAGTACA
CAATATTTGACTGTCTATGCTCATAATGTTAAAGGATTGGATAAC
CTCAGATATTTTGAA
TTGTCTGATTATTTGACCTCTTATGCAGGTGTAACATTAAAATAT
GTTGGTAATTACAAA
CATGAGATTATTAAAAAAATAATTGAAAAGAAAGAGAATTTTTA
TCGCACAAAACAAAGA
AATCAATCATTCTATTCATCTGAAATACTAAAGCAGTCTATGAGA
TATCAGAAACTATTC
GAATCCATTTGTGAATGGAGTTTTCAGCAGTGGCAGCCCAAAACT
CTATACAAGTCTTCA
ATAGTAATGAAATTAGGTTCAGTTTTGCTCGTGAACACTGTACCA
TCTTTATCGCAACAT
CCTTTGACGTGGATGGATATGTTCAAAAGTGACAGTAAAAAGA
>ConsensusfromContig159 Average coverage: 8.18
TGCTCTTCCGATCTTCAATTCAGTAGAGAAGTAGATTCTGATGCT
AGATGTGTTCATTTG
GTTGTGCCTTCAAGAGGTGGGTACGGGTTATGGACTACCGAAGG
ATGTCGAAAACTTGAC
CAGTCATTATCTGAAGTTCAATGCTCATGTGCGCACGTCGATGGA
ATATTTGCCGTACGC
TCATCTCCTAAGCCTGGAAGTCGATTCAATTCATTGCTT
>ConsensusfromContig160 Average coverage: 9.50
AAATTGTACACAAACCTCATGCATGTTTTTTACTTGGAATAACGA
GACAACTCAATGCTT
GGTTTGCAGAAAGGGACGCTACAGGAGAACAATCGGTCATAGAA
ATGAAGAGTTATACAT
CCCATATGATTCCAAGAGGAACCGTACAACTGGTGTGGGATGCA
AAACCATCGTCAACCT
CGATTCATATGATTGCAAAGATAAAGATATCCGTATCAAAATGA
ACAGCATCACTGGGGG
CAACACCACTACTGATGACGGGGATATTACACGGGTCGGGTCCGG
TGTTAGTGTTATCAA
AGACGGAGAAAAAGGAAAAGTTTTTCACTTCAG
>ConsensusfromContig161 Average coverage: 13.10
AGTTCTTCTTCGAGAACTTTCCGAGTATATCTCATCGAATCCATAT
CACTATTGGATTTG

GAGACAATTCTTTGGTAATAATTCTCAATCGTTACGCATTGATCC
TGAATGTGATTCTTA
CAGAACTCCTGGTAGATTTTTTCTTCTTGCCACTGATACATCCAGA
AATGTATCGCTCTT
GAGCATATTTCAATCACAGTTTCAGGTCTCAATCCAGCCAAGATC
ATGGATTTGTATTTC
TCCGACGGCTGGAGGTGAGATTTCACAATTTCGAAATCTTCTATA
AGTTTTTGATCGCAG
GCTGGACATTCTTTCTTCATATGTAGCTTGAAAATTCTATCCCCA
CAACCTTCGCAAAAT
AAATGGCAGCATGAAGTGGCCCATGCATAATTGGCGATCGGCCT
CCGGCAATCTCGGAGG
TTGCAGTATATGGTTGAATCGAGTACCATAACGAAAGCCCAAGC
AGACGTAAAAT
>ConsensusfromContig162 Average coverage: 25.39
ATTACTTTCTTACCCATCACATTAATCGCTGTATTTCTTATTTGAA
AATCATGACGCTTC
TACTCAATACAACTGTTCAAGCCTCATGCAGTGCTCTAGGATACT
TGGATGGTGACAGTT
ACCAGAAAGAGCCTGATTGTTTACAAACCATTCGAGAGTTGATTC
GGTTCCTCAAGAGAG
ATGATGAGAGTTGTTCGATAAGGCGACAACTAGGTCAGAGTGAG
GTCGTCCGAAATGACC
TACTTCCTATCATCAAGTACCA
>ConsensusfromContig163 Average coverage: 4.06
TATATCAAAATGATGGATTGTTGAGTAGATTCAACAAAACTAAAT
ATGGTAACACAATGC
CTTCTTGTCTGTCAGCAATTAAAATTTAAGATTTGTACAATTAGA
ATAGCTCGATGCAAA
GATGGTAAACATACGATCCCAATAATGTGCAAATATTTGAATTCA
ACATCTTCATTTTTG
GATGATTTTTTCAAGAGATCGAGTTTTTCTGACATTTTGATTGGCT
TCG
>ConsensusfromContig164 Average coverage: 6.97
TTCCGATCTGCCATTTCTTCTTTATTATCAGCCAATAGAATCTCTA
TTGTTTTTCTATAA
GATGTGATGAGAACATGTATCTGTGATTGCGGAAGTGGGTCATGA
AGAACTGCGGACTTT
TGACCACCGAATCCTGCTTCAAGGTCTCCAGGAGTCGGCTCGTAA
ATGGTCTCAAATGCC
CCAGTTTCACTTCTTGGATATATCTCGCGTTTTTCAGATGTTGCC
AAAGAGGCAAGGTGA

AGAACGTGAAGTCTTCTAGAGTGTCTGAGCGCACGACTG
>ConsensusfromContig165 Average coverage: 7.82
TTTCAGGCGTGTGCTCTTCCGATCTGGACAACCGACTGGATTGGA
TCTGTCGAGACTGGA
GATATTCCGATATGGATAGAATATTGGCTCCTCCTCATATGTGGA
GGAATTCCATGGCAG
GTTTACTTTCAAAGGGTATTGAGCTCCAAATCTGCGAAGAATGCA
CAAATTCTGTCGTTT
GTAGCGGGTGCAGGATGCATTTTCATGGCAGGAGCAGCCGGTAA
TGACGTGGGCGGTGG
>ConsensusfromContig166 Average coverage: 8.90
ACTGTAACCTTTATCGGATCATAAACCAGACTGATTTAAAATATA
ATTACTTCTCATATA
AACAGCAAACTGTATGCATTTTCAGATATGAACTTTCACGATGTA
CCTTATCTATAAGGA
GGGGTAATGAAATGATATTAATTAATGTTTGTATATGTTCACTTA
TATGTTACTATGGGT
ACTTTTAAATTGTGTCAGAGTCTGTCAAGGATTCACCAAGCCCGC
CAAGATCTGTTCCCT
CTGAAATCTGTGTTCGTATTTGAAATGGTGCGTGACAATCTGTCA
AAGATTACAAACCAT
GTTCATTCCATTTGATGTATATCTTATTTTATGGATTTAGAGGCCG
AAATTTGTTTGTAT
TATGGACTTAACTCGTATGGGCATGCGCATGTACAGCGTTCACTC
ACGAATATTGATGAA
TTTCAT
>ConsensusfromContig167 Average coverage: 3.28
TTGAGAATTATCATCCATCGTGCCATCTATTTCATTTAATGTTTTT
ATCTCAGCTCTTCC
ACCGGGCCCACCTTCAAAATCTTCTTCATTTGAATCTGAAGTATT
CACCATTCTCAAAAC
ATCATTATTCTGTTTACTATTATTCATCGAATCGATTGAACTATGA
TTGTTGAAAGTGCG
TGGGAATCGCTCAGCTGCTGTTCC
>ConsensusfromContig168 Average coverage: 10.99
ACCTTGGCCAATACGATGTCTGGACTTATTCATAGTATAATCACA
AAAAGCAAGAAACCT
ACAGTGATTAACGCACTAGGGACTTTCATGTGCGTTTCTGGTGCA
ATTTTGTCGAATATT
TCTGGAGTCCAATCAGCAACTAGTCCTTCGAACGGGATGAATATA
TCAGCTTTTGTCGGA
TATAGTCTGATGGCTGTAACAGCCGGGCTTTTCTTTACGTTTAGAT

GCGTCTTTACCCCC
CCGTACTTGG
>ConsensusfromContig169 Average coverage: 7.28
GGAACGAGTGGCCGTCACTGAGTCACTCTATGTACCACAATGTAC
AAAATGCTATAAATT
TGGGCATGCAAAAAATTCATGCAAATCACCAACTTTCACATGTAA
ACACTGTCATGAAAA
TCATGAATCAAAATCATGTACCAAAAAAGAACAACAAATTTGTT
CAAACTGTGTCGGAAA
CAACTTAGAAATCACAAATCCACACGATGCTAGTGATAGGGCATG
TCCATGCT
>ConsensusfromContig170 Average coverage: 12.15
ACGCAGCAAACTTTGTGGAAGTGATCCATACCGACTCTGACACA
CTTGGGACTGATGAAA
GACTTGGCCATGTTGATTTTTATGTGGAAGGTGGCGCGTCTCAGC
CTGGTTGCGAACATT
TGTCTGGTTGCGATCATCTAAGAGCCTTTCAACTGTTTTTCTGGTC
TTTCGAAAAGACTT
GCCTAGTAGGTAGTGGCAAATGCCAAGGAAAATCTTGGAAAAAG
AGAAAGGGGTGTCATA
CGCCAAGGTTCGTGAACTGGAAACAATATCTCGGTTTGGGGGCA
TGTGAGGACGATTATG
ACCCGGGGATGCACATCGTAGTGACTCTCATGAAAGCACCGTTTT
GTGTGTGGCCAGAGA
AAATCAAGATAGTTGATATTTAAAGATCGGAAGAG
>ConsensusfromContig171 Average coverage: 7.48
TCGTCGGAATCGGAGACCATCTCCACTACATTGACTTCAGACGGT
ATATGCCAAACGAGT
ACCGTGTCAAAATGGCAGCGATGCGGTTGAATGACCCCGTCAATG
AGATGCCTATTCCAT
ATGACCTTGACTTACTCGAGGCCATGACCAGAGATGATGTCAAG
CGACTCAAAGGACCTA
GATCATCATACAAATTCACAGA
>ConsensusfromContig172 Average coverage: 6.68
AAATAATTCACAGAGTGAATAATTTGCATAATACACTTCCATATT
TTTAAAACATCAACT
CGGTGTGATGTTAGTCACCTGTCATTCTTAAGTAAAGTGTCGAAG
ATCCGAATCCATTAT
ATGGTCCCAATCAGAAGCGTAGCCAGACAAATTTCCGGAGGGGG
ACAATTTTTTAAAGGG
GGTCAAAATCATCAAAATCATTGATCACATAATCATAGCACATA
ATCAAAACCAGCAGGT

TGAAATCTATGATACAGCTGATTATAAAT
>ConsensusfromContig173 Average coverage: 11.19
CTTACTATTTGTGATATCAGAGCTATTATACATTGATTTTTCGAA
GTAAAATGCATTTT
TAACAATTTCGGGATACTTTTTGTTTAAAAATGTTTATCACACTCA
TCGGCTCGCATCTA
GAACATTTTCTGCAATGTTATTCACAATATGGAATAGATATACAC
ATGAGACAAGTTGCT
ATTCTTGATAAAACTCTTGATTTGTTGAACCAATCATTGTAATAAC
CAGTCTTGACTTAT
TTATAATCAGCTGT
>ConsensusfromContig174 Average coverage: 8.06
CAGAGTACAACGAGCGGAAAAGAGGTTGGAAGCAATGTGCAAC
AAGATACAGGAAAAACA
AAAAAATCGTCAGTTAAATTGACTTTATCACCGCCTACACTACGG
AAAAATACACAGCGC
CCTCTACTCAGGAATCCTTCTGGCCGAGAAGAGTTTTTCAAAAAT
TCACAGGATGAGCAC
CTGGTAACGTCTGATTCGAAACAAGAGTCAACGTCAAGTAAATT
ATCACAAAAA
>ConsensusfromContig175 Average coverage: 6.38
CCAACTAATGATTCTTTTTTCAAGAAGGCCCTGTCTTTATCCGGT
AGATTATTTAATAAA
TCCCAAACGGGATTTTCTGAGAAAGAAAATAATAACAGTGATGA
TGATAAAATTTCCAAC
GACAGTTCATCTGAATCAAGTGAGGTATCTCTGGAGACTGGTGCT
TTTTCAGCGTTAGAA
ACATACGAAAGACTCTATGATGCAAAA
>ConsensusfromContig176 Average coverage: 12.20
ACAAAATGGCCTTCTCGGGGGCAGGTCTCTCTTTGGAAACACACC
TGGTATGGGCTCAAA
TGACGCCGGTCAAATCGGAACCATGAGTCCCGAAATGAATCGAC
CCCAAAACGGCGACTT
CGGATACACCCAGGACGAGAGAGGGTCCAACGACGCAACGGGTG
GCAGGGTAATGGCTGG
GGATGCCACGACGCCCAACATGCCCAAACAGAACAAGTTGCCCC
AGGGACCAAGCCGAAT
CAAGACAACCATTCCTGATAGCGGTTGTCCATACGGTCTCAAGAA
GTACCCCGGAAAGTC
CCACTACGGCGGTGACCTAATTCCAACCATCGATGACGTGGAG
>ConsensusfromContig177 Average coverage: 15.03
ACCCTCTTTCCCAACACGACGCTCTTCCGATCTCCTGTTGTTGCCT

CCTTGGTTTCCATT
CTTAACTGACGATAACCTGTTCGACACCCTATAACACGACATTTTC
TACCCATTTTTTCA
GTGAGATTCACTTATTCTCAATATATTTCCTGTTTGTTTTCATCGCT
TTTTTTATCTGAT
CTTTTATACAAAAAAAGGTCTGTG
>ConsensusfromContig178 Average coverage: 6.60
AAGATAGGCGTATGATGGACACAAACCGTGGCTATCAACAATAT
CAAGTCCAAAGAAATG
GACCACAAAGAGTTAGACCTCTACTAGACACACCAGATAGACAA
CCAAGAAACGTAAAAG
GGACAGATCCCAGATCCAGATTTCCTAGTAGGAATCCCGCGCCT
AGACGCGATCCGAGAA
GTCAGAATGACATACCACCACATTTATTAGATAATCCCCAATACG
ATAAACAGGGGCGTT
TGGGAAACTAGAGGGTCGACGTTATTATAATGAGGCTGAATACGT

>ConsensusfromContig179 Average coverage: 9.03
TTGATATAGAGGATATCCCAGTTTTCCCGAAGAAACAGTTTTCAA
TGTGTCTTCCAGAGG
TGTTTGATTACATTAGGACGAACTTTCCAGAAAGAAACTCTGTGA
TTTTATGTGGAATCG
AGGCTCATGTATGCATCCAAAACACATGCCTTGACCTTCTCGAAT
CTGGCCTTGACGTCC
ACCTCCCTGTCGATGCCGTTTCGTCGAGGAGTCAAGTGGACAGAA
TGTTCGCTCTTCAAC
GCATGAAACAAGCTGGCGCGTTTCTGTCAACTTCAGAAAGCATAA
TCTTATCTCTATGCA
AAGGGTCAGACCACCCTCAATTCAAGGATGTTCAAAAGGTCATT
TGGGACACAGCCCCTG
ACTCTGGGCTGTTGGGTATG
>ConsensusfromContig180 Average coverage: 10.10
ATAAAAGAAGATGTAAGGCAGAAGCACATATTTGATCAGGCCGG
GATGGTCATTCGTGGT
CTGGAGGCGAGTGACGCTGGGAGGTACGAGTGCAGGGTTTCACT
TGGTGCACAACTAGAA
ACAAGGCTCATCACTCTCAATGTCGAATATGCTCCCAAAATCACC
AAGCCTCCTCAAGAC
GCTGCTCTCCTCATGGGTGCAACAGCAGAATTTGAGTGTCAAGC
CGTGGGAGATCGGAAG
AGCACACGT
>ConsensusfromContig181 Average coverage: 4.75

CAGCACTTGATTTGGTAAAACAAAGCAAAACATGTCGAACTTAT
CCGGAGCAACTTCACA
TAAAATGACTTTGAAGAAATGCGTGGGTACTGCCACCTGGTTGAT
GCCGATAACTTCATA
CTTCACGAAAAATTTCCCATTTTCTTCCATTGGTAGGAAAAGTGG
TCCCGTGCAGACATA
TACATTTGGAAAAAATTTGACCAAGTGTCTCGTAAATTTTTCCAA
AGTGTTCCAGGCATC
GCGATTAAAGCCTTTCCCCACCTGGGGCGCAACATTCGACAGTCG

>ConsensusfromContig182 Average coverage: 3.14
CTGATATTGCTTTTAATCAACGGCATATGAACATGTTATACGGCA
TGCTCAAATTACATT
TTTGAAATATGATCATATCAACAAGGTTTGAAATCATGATTGAAT
GAGTTTTAAAACATG
CTTGATAAAATACAAATATGCAAAAAAGAACATCGAAATGAACA
ATTAAAATCATGTTGG
AAATTCAAACTAGCACATGGAAAATTT
>ConsensusfromContig183 Average coverage: 8.74
GCAATTTTAGGGATAGGTACAAACAACATCTTATGATTACTATCA
ACGTAGATCCGCTCT
ATCATTTCCGGGACCTTTACTGTTCTGGAAAGATCGCAGCTAATC
GAATTCCCATCAAAT
TCGACATTAGAATGTTCCGTGAGATGCAAGGTGTGAGAACGGAG
ATAATTCAAAGTTCTA
TTAGAAGATTCATCGAGCACTTTCTCATCGCGAACATTGAAAGCC
GGCTCCGAATCCGG
>ConsensusfromContig184 Average coverage: 6.45
CTTGGTTCTGGACTTCATTATTTTTGCCGGGTCACCGACATTCGCC
GAGTTCCGTTTAAA
TGGGAAAGCGGTTGGTCAGGGAGTTGGAGCCGTTCTGCACCTGA
CAAGGTCTCTGAAGGA
GGGAACCCGTCTATATTGTGATAGATACTTCACAACATTGGCCCT
GGCAAGTCATCTGCT
CGAAAAAAAAATCTATCTAACCGGAACAATGAAGAA
>ConsensusfromContig185 Average coverage: 3.87
CGAGCCCACATAGTCCTCTGTCGTCGATGTAAATTGGGGACGGCT
TCTTCTACTTCTTAA
TTAACGATATTATAGGTGTCATCACCAATTTCCACTTACGTTTGCT
TCTCTTTCTTGAAA
CATGGGACGAACTCACATAGTCCTCTGTCGTCGATGTAAATTGGG
GACGGC

>ConsensusfromContig186 Average coverage: 5.17
TATATACCATCATCAAACCGGCAGTACTAGAAACCACTTTGGCTG
CACAATTGTATCGAA
TTGAATCTGAATCATGGGCTGTCCAATTTTGGTTTAATTATGCTG
TACACACAGTAACAC
TGACATATAATGGAACTGAATATTTGAACGAGATAAACCAAGAT
CTGAAGAGGCCAAGTT
GGTTTCAAATTGCAGTTGTCACACAAAACAGAATGGTGGT
>ConsensusfromContig187 Average coverage: 6.77
ATTTAATTCCCAAGCAAATTATTTTAAATGTGTTTCTTACTATTG
AAAAAGCTATATGTG
CAGGTTTAAAAAATTATTGTCTCACCAAATCACGTAATAATAGAT
AAGTCCATCACCACG
ATAACGTGTACGGTTCAAGTCGATTGCCGAACCCTCGATGACGTC
ATGATTGAATCACCT
ACAGGTCGGAGGTGAGCCTCATCAGCCCTCTGAGGTCGATTCCTG
TTGCCTATTTTATGC
CGTT
>ConsensusfromContig188 Average coverage: 6.64
CTCGTCTGTGCCCTCTGCATTAAATTGTCCATCTGGAATCTCGGA
CTCCGCACCAACGAA
CAAATTCGGGTTAAAGCCAGCTCCGCGTTTCCAGATCCTGCAAAC
ATTCCCACCAAAGAG
TTCCCACATCAAGCAGCCTTTGCAATAACCAGCACATCCATCGAT
ACCGTTTGTTGAGAT
TTTGTATTTAATATATATATAATATTCTCCGCCTTGAACTCCAACA
TATCCGTTCGCC
>ConsensusfromContig189 Average coverage: 29.51
TGGCTGCTTCGTGGGCGGCTGTGACGTTGGTGCGAACCGCCGTCT
TGTTAGGGTCAGAGC
CAGTTCTCAGCAATGCTGCGACGACGACAGATGAGCCAGCAGCT
GCCGCTAGCATCAATG
GTGTTTTTTTGCGGATAGGCATAAAGCAGTCCGGGTTCGCTGCAT
ACATCGTCAGCAGAA
GACAAAACGCTGGGTTATTTTTCGC
>ConsensusfromContig190 Average coverage: 12.20
CCGATCTCTTCCGACTAATTTGATAAAATCACAAACTGAAAATGA
GCTATTGGTAGATAA
ATTAAGTATGTCAGATAATTATTCTTGGCAGTGTTTTTGGCAGCT
GAGCGCCTACATATT
TGTTTGTGTCTATTGCTCTTCTTTAGCATTAACCTCAAATTTGACTG
TCCTTATTTTCTT

GTAGGCTTTATTACCGAAAAAACAGATACATGCTAGCACTACAA
TACAACTTAGAACCAA
AATAGTGCAAGTTGGTATTAAGCTAGCGATTTCAGATTCTGGACT
TGGCA
>ConsensusfromContig191 Average coverage: 1,478.60
GCAATGTGACTGGAGTTCAGACGTGTGCTCTTCCGATCTCAAAA
AACAAAGCAGACGCCC
GGAAGGAAAAGCCGCGCAAGTGCAGAGTATTGGGCAACAGCAGG
GATCTTATGGTGGCGG
AATGAGCCAGTACGATAGCGGTAATGCGTATGGCGGATATGGAA
CCAGCAACAATAACAA
CTACAACAACAATAATAACTACAATAATGGCAATATGAATGGAA
ATATGAATAACAACAT
GAATAGTAAT
>ConsensusfromContig192 Average coverage: 8.21
GGGGAGATAACATTCGACAGTTCATTATGTTCAAGATCAAATGT
AACACAGCGGAAGTCA
GATGTTGACATAAATAAACGCTCTATTGTTGAATATTCAAATCAC
AATGAATCGAATGCA
ACAGGGGTCAAATCAGATGATGACTCGAATGTACGCCCAATAGTT
GAATATTCATTGGAT
AGTGACCCGCATGTGACGCAAGTGAAGTCCCATATTGAAACGAA
TGAGCAA
>ConsensusfromContig193 Average coverage: 6.50
ATTACAACCTCTCAAAAACACCAGAATTCACAATCAATAGCAGA
AAAATGTAAAACCCAT
GAGGTAAAACAAAGAGAGAAAAAAAAGTAAATATATAATCTTTA
GTGGAATGAAATTAAT
AACCAAAAACATAAACTGGCAATGAATGAATTATAAATAAAACA
CTGAAATACCATGTTA
ATATTCCTGAACTAAATTCTGGAGACTTTGATGACAGAAAA
>ConsensusfromContig194 Average coverage: 5.80
AGCAATTCTCTGAAGTACGTTATTCTTCCGTAGGCGACGCTGTAA
AGCTAATAACATCAT
TCGGGTCGGTGGTTTATATGGCTAAAGTCGATATAAAAAATGCTT
TTCGTCTAATCCCAG
TCAGTCCCTCATTCCGGCAATTTCTTGGTATGTCGTGGCGAAATCA
CTTGTATTTCGATA
AATGTCTGCCGATGGGTCTTTCAGAAAGCTGTAGACTATTTGAAA
AATTTGCAAGCGCTG
TTGAATGGATAGCAAGGCACCATTTCGATGTGCCAAATATTCTAC
ATTACCCTGATGATT

TTATACCGGCGAAAA
>ConsensusfromContig195 Average coverage: 5.31
CTTCATAGATTACTAATCATTTATTCAATCAAATTGCCCTCTACC
AATAAATTAATTGCA
ATCATTTCACACACTTATTCTTTATCAGCTCCTGGTGACCAATAA
TGATACGAGAAACGA
AAATCCTAATACTATTAGACAAGGCCATCATACATATGAGTTTGG
TTGCTATGCATTTGT
TCACTGTCGGTTTGGTGCTTTTTACCGATCGGCCAACTAATCAATG
ATTGTGTATCATTT
CATATTATCTTATATATGAAAATCAATTATATTACAAGCGGAATT
ATATTATATCGAAA
>ConsensusfromContig196 Average coverage: 17.28
TCCCTACACGACGCTCTTCCGATCTCGATCCCAATATTCCAAAGT
GCCATTGTTTTCGCA
GGAAATATCGAATCACACGATGGAGGCGCATTATTTGTAGACACC
GGAGATATATCGTTC
CACACGAGTTTCATCACACCTGTTTTTCGATTCTAGCTGATTCA
TACAGAAAGCATTTC
TCTCCTCGAAACAACGCTACGGG
>ConsensusfromContig197 Average coverage: 4.88
TGTAAGATGACTGCGCACAGCCTGCTCACCGTGTGCTTCTCGGAC
AATTTGTAATTTCGT
CATATCGCCATTGAAAAAGTTTCCCAAACGAACAGAATGGTGAG
TGAAGAATGCATCCTG
AGTATAATTCTTGGAAAATGTTTGAGAGACCCCATTGATGAATAC
TTTCAGCATTCTGTT
TTGTGTGACAACTGCAATGTGAAACCAACTTGGCCTTTTCAGATCT
TGGTTTATCTCGTT
CAAATATTCAGTTCCATTATATGTCAGTGTTAC
>ConsensusfromContig198 Average coverage: 26.28
AATGAATCCTGATTCCAAAGAATGAATTATCCTTTTAGCATCCTT
GATTTATCCCGAAGC
GAATATCACAAACTATCGAAACTTATTTTTTATACACGGAAAATTT
TGTCTCTCTCTTTT
GACTAAGTTCAAGCTCCCGATAGAAATAAAAAGAACCAGGTGCA
TGAAGTCCTACCAAGA
TCCCGTTGTGTAAAAATTCCGAGTCATATGCAAGGGTGTCCTTAG
ATCCTGAGATCCAGT
GGACC
>ConsensusfromContig199 Average coverage: 7.79
AAATGGGAAGCGCCCTTCACTTCGATGGATCGACAAGTTCTTACA

TTGATCTTGGATCAC
ACGAGGATAGCATTTTGGGGAATGAGTTGATAACGGATAGAGATC
TGGTCTATGACCTCT
GGTACTACCACGATGCATCCAGGCAATACGGATACCTTCTCGGTA
GTGGAGACTACTCTC
GTCGGCTGGGTGGGGGGTTCCCTACCCCACTCCGGCTCCTTGTT
>ConsensusfromContig200 Average coverage: 5.37
ACTGCCGAAGAAATTGAAGCAAGAAAAAAACTCAAGAAAGAAAT
GAAAAAGTTTTCTGCA
GAGAAAACGAGCGAGGAGAAATCAGCTGAATTCAGAGCCTTTGA
AGAAATGCAATCTAGA
ATTCGAAATGCAGTCGTCAGCACAAAAGAAAAGAATGCAGAGCT
GGAAACAAAAATTGAC
GAAACAATACAAACAGTGGCTTCATCGGATTCACCCTGGACTGT
TTGGGGCGATACAAAA
GACTTTGAAACACAA
>ConsensusfromContig201 Average coverage: 10.99
TACATAATCTAATTGTATCTATTGAATGATGAGATCTTGGAGAAA
TAAGATGAAAGCTTT
CGAAATGTGATTTTACTCTCACATAGATATTTGTACACCGAAAAT
CTTGTTTACACGGAT
TTTAAAACAAGAAATTTTTGTCAAGCCTTTAGACACAAAAGTTAA
ACATATTCCCAACTC
CATTTATTTGCCATGGAATATACAATGTTTTTCACATCGCAATAAC
TCTGCCATGTTTTC
AAATGAACATATCAGATAAAATCGCGTGAAGGAACAAAATTTAA
ACAGAGGTTTTGTTTA
TTAACTCAATGTTTTCTTAAATTCAATATGTTAATTTTTTTCATGC
GTTCAACTTTGGAC
TCAAGTTATTTAAAAAATGCCGGCTTGTTACTGA
>ConsensusfromContig202 Average coverage: 89.03
TATACTGCGGTATATAAATGCTTTCATCAGCGTCTTCTATGAATC
GTATGGGCTGTTACT
GATGTATAGTGTGATTGACGCATTAACCCAAGGTGGCGTTGTAGT
GAATCTTTATCTTTT
CGTTTGGGATGTATTCGGTGGCAAACCCGAGATGGCAAGAAGAAT
CGCTGCATTTCAAGT
GTCCAGATCGGAAGAGCACACGTCTGAACTCCA
>ConsensusfromContig203 Average coverage: 17.58
GGCTGGTCGGGCCTTTCAGCCATTCCTTGCTCGGCCTTGTAGAAT
GTGTTCAAATAATGA
TTCGTGGGCTGATCTTTGAATCCAAGCATCCTAAGAGTCCATATG

CCCATATGAGGGCCG
TCTTCAGCATACTGTGTCAAATAATCATGCTTTTTCAGTTCCTTCC
AGATCCACGGATGT
CCGTCAACAGGGCGCGCTCCAGGCTTGCCTCTCCGTGCTTCAGGA
AGATTCCACTCCATT
TTCCCAGTCAACATTGGTAGCAATGCCTGAGGTGTCCCATCGCCG
GCTATGTTGTATCCC
TCCAAGACTGTCCCGCCCATCACCCGAGTGAAGTATTCATGTGTC
T
>ConsensusfromContig204 Average coverage: 6.64
CTCAATCACAGTGTGGTTTGATATAACCATTATTTATTGCATCCC
TGCCATCTGAAAACA
GTGACCTCGACTGTTCGGCCCCATTTACCGCTCGTGTTCAGAATAT
TTTTCTGTAATTCA
TAGCGAATCTTAATTTTCTCAGTTATTTCATTCATTTTGCTGTTGT
GTCAAAAACAGAAA
ATTTTAATGATTATTTATATAATAAAATAAGTGATGGAAAAGTG
AATCCTAAGCAACGA
CGGG
>ConsensusfromContig205 Average coverage: 18.66
GTGATCGCAAATGGACTGAGGAAGACTAATGATTGTGTCATTCAT
TGGATGTTACATTAA
TAATTTATCTTCAATCAAAAGATCTTGATGATTGGTGTCGTTTTTT
AATATAGACGTTCG
AACTGTGTTTTTAAAATGAGATTGTTTTTACGAGATGGAAAATAT
TCAGAATATTGTTAT
TGTTGTTATCCGTAGTTCCCGTGATTGAAGTTAGCATGCATGAGT
CATTAAGTCGGGATA
AGTCTTGTTACTTCCGAATGATCGACATAAATTTTGTTCCGAGAA
CAACTAAAACTGGGA
GATCGGAA
>ConsensusfromContig206 Average coverage: 13.96
CAGCGTTTCATTTTATCATTTTTTATATTTGCGTTACGAAACATTA
TTTTAAAGTTTTCA
ATTGCTTTCCCGTATAATACATCGTTCTCTTCAGTCAATTTGCTAA
TTTCATTCCATTGT
TGATTGGTCCACATTGTACTAGAAAAAATTGTTTTCCACAACATT
TGATGGTTATTCATT
CTTGTTTCATTTCCTTCAAGAAGCGCTGCGACACCACCGCTGGCTG
ATCCTATCCATTCT
AATTGAGCATTAAATATTTTCGAGTGTAAAGATAATAGACTATTC
GCTAACCATTCAACC

ATGGTTTCA
>ConsensusfromContig207 Average coverage: 4.09
CGCAACTTCCCCAACATTGCTCTTGCATCGTCTTGGTATGATGTTC
AAGTATCCTATTTC
CTGAGTCCTATCGATGAATTGAAGATTTGAAATGCTGCCTGCACG
CTCTTTGAGATACCA
TAACCTGAGACCTTTGATGTAAAAGGCTGTTTTCCCTGTTGAGGA
AGTCATAGGAGAATC
TGACTGAACATCTTCCAAGCCTGATATTCGTTTTTCC
>ConsensusfromContig208 Average coverage: 8.99
GGCGATTCTCTCACGAACATTCATGAACAAGAAAAAGTATGTTG
GGAATGACAAGATGCT
CTTCTCTGACGAAATGTTCTCCATTGACCCCTTGGAGGGAAGTGT
GTGGCCCAACTCGAA
GATGGAAATCTCTGTGGTCTTCAAACCAAGTGAAGCCAAGGAAT
ATAACAGCACAGCTTT
CTGTGAGATAACTGGAAGAGAATCTCGCTTGCCTCTGAGGATGA
CAGGACAAGGGAAGGG
ACCGAAGATAAGATTTTCTCTGGACGAACTAGAAATGGGGAAAAT
TTTCGTCAAT
>ConsensusfromContig209 Average coverage: 9.31
CTCTTCCGATCTAGAATTTTCAATATCATCATTGCGAGCCCTTAAC
TCATCTTTGTTATA
ACTTTTCGCATCGTATATCGGAATTTCTTCCGAATTCAATTCATCA
GTGCTGGACTCATT
CCCACTTGATGCTAATTTGAATTTACTATCAAAAGACACTTCAGG
CTTTTTCTCGTACAA
AACGTCAATTGAGCTCTTTGTGGACCATGGACTTTTCTTTAAACT
TTCTGAGAGAAAATC
TATATTCCCCTTTGGAATAGGGGTCAGTCTTTTTAATTCTTCGACA
GATATGACATTAAT
TCGTCGGTTGATGTTCTCCCGTTC
>ConsensusfromContig210 Average coverage: 14.67
GTGACATCTTTTTCGAAATGACCAGTTACATTTATATCACCCCATC
TATCGGATAATTTT
TCACCCAATAGATGGCACTCTTCATTGCAGATATCCGTATTTCTGT
TTTCGACGCATTCT
ATTGTCGATGCAGAACTATAACTTCCAGATCGTTTCCTAGAAATG
TCTCTAAAAGAGTGA
ATAGAATCTTGACTGTTAAGTGAG
>ConsensusfromContig211 Average coverage: 22.99
ATTTTTTATTGATCAGGTATTCGAAATAAATAGAATCGCAATGCG

AATCGAATAAAATGG
CAAATATATTCAATAGTCTGATTTGAGTTAATTGAAGCTAGTGCA
CGCATACAGATATAT
AATTGGTATCGTATATTATATAGTCGCATGAAAATCAAGTTAAAA
CTCAGATCAAGACTG
CCTGCATCATAAAAACAAAGGCAAAGAGATTAGTTATATCTCAT
AGACTAATAGATTCAA
ACAAAAGAAACAATACTTTGCGTTACAAAACTAATGTGCAACAA
TAACGAAATATTCAAA
GGATACTATTAACCAATAATGAGTCATCTGAATCCGAATACACGA
TTTGTAAGTATTTC
>ConsensusfromContig212 Average coverage: 26.92
ATTTTGTATTGGCAGATCAACTGATTCCATGCATCGTATTTCGTA
AAATTGAAAGATATG
AAATCATAATCTCGAATGTGAATGCCTTTCATATGTTGGAAAATA
CCATGTAGTCTTCCA
ATCTTAATGCCTGAAAAAATATCATGGAATATATAGATTCGAGTT
ACGTTTCAAAAAATA
TTTCAAACAGGACGAGATGATTCATGGTAGTTCATTGGAATTTTT
GACTTCTCACTTAAA
ATTGTTATGAGAATTAATAACAAAAAACACTGGCCAGATGTAGGG
GTTCTTAGACTTGAT
TGGAATTTTTCTAAACACAAATGACACTGAACAATTTGAAAAAAG
TCAAGTTGTGTGAGA
TCTTGTCGGACCAAAATCCTTATCTGATATGCATGGATAGACAAC
ATATTCTATCAATTT
TCCA
>ConsensusfromContig213 Average coverage: 4.72
GTCAAAATAAAAAATGTTTGCTTGACAACGTGACCCTACGTCGGC
CATCGCAGGTGTGGT
TGATATAAACATAAGCATTCAATTCAGTTCTTGGATATTCTGAAA
AGCTATAACACTCAA
TAAGATTGGATCTGATTGACGCCTTTTGGCAGCCATTTAACGCGA
TAACCGAGCTATAAA
AGGCACTCAAATTCATTTCGG
>ConsensusfromContig214 Average coverage: 14.65
TATCCACGACTCCTCATCTTCTGCAACAGTTGCGCCACCTCTTGG
GGGGAGAGGGCGTGG
CAATTCGCCATCGGAGTTTATCTCGTTAATTTCGGATCTGGAAAT
CTGACCCTAACCGCC
ATCTACGGATTTCTGAGTTGTGGCTCCCTCTTTCTTTTTGGGGGTA
ATCTGGGCAGCTGG

GTGGATCGAAACCCCAGGCTCTACTGTGTG
>ConsensusfromContig215 Average coverage: 10.66
CTCTTCCGATCTGAAATTCTCTCACATTTTCTTTTTGATAGCACCA
AGATGTTTCACACT
TGCAATCTGTGCTGTGTTTAGGATAAATTTTCAAAGAGTTCAATT
AAGTTTTCCCAACAA
TACCGTGAAGGATCCAATACGATCAAACAAACGAATTCGATTATC
TGGTCTCATACATGT
CGCGTATCCAGAAATATTCTTCGGACTGCCTTCAGCCATCTTTCTT
CTCAACTATAACAT
ACCCTCATATCATTTCGGCTCGGATTCTGACCTCTATGATCAAGA
TCCTGGATGGAGGGA
ATACTTCTCAGCTGCTTCGATCTTCATGACTCTGAATACACTTTAT
GTGATATCTTCGGC
TCTTCGTGCTGTGGCGTACTGCTCTTGTGGCGACATTTTCAAAAC
TGCAGATCGGAA
>ConsensusfromContig216 Average coverage: 64.19
GCGGTTTCCGAAGCAGAGGGAGACATCAGTTCGTATTCCTGCGTC
ACCGTACCATCAGAA
AGAATTTCCAGCGAGAGACGGTTGTCATCGAGAGAAACTGAAAC
TCTTTCAGTGTCGAGA
AACCAAAAATCGTCTTCGGATGACTCGACAACCATAGTCTCTTTA
CTCTGAACACTCAAC
ACTTCGCTGCAAGTTTCATCCCCAGAGTCGGTTG
>ConsensusfromContig217 Average coverage: 7.11
CGACAATTTTTCAATGGATTAATGAATAAAGATGCGATCTACGCA
AATACAATGCAGATA
GAAAACAAGGTGTGAGCCCCAAAAAAGATCGAGTTATCAATCCA
CTCCGCTTTGTGGAAG
ACGGCCAATCAGAATCTGCGGATAACGACGTTTTCACAGAGAAA
ATGAATGAGTTTGATG
AAAAAGGGTGGGCCCATATACACCAGATCGGAA
>ConsensusfromContig218 Average coverage: 11.17
TCCCTCTCCCCTCTGAATGACGTTCGTTTTTCGGCCTACCGAACG
GCGCTGAAATTGCGG
GCGCTTCAGAGAAAACTTTCCATGGACTTAGTCAATTTGCGGCTG
TTGGCGGAGGTGTTC
GAAGGGCAGGGGCTGGTATCGGGGCCGGAGGATGCGTGGATAGA
CGCGGCGGATATGGCA
TCATGCACCGTCGCCATCTACGAGACTGTCGCTCTCCGCTACGCA
GCCGAA
>ConsensusfromContig219 Average coverage: 7.77

AAGCCATCTTCGCGGCCTTTCTCCGAGTATACAACTTCTGTCGAG
CACAGAATATCGTAC
AAGTGTGGCTTTTCCGCATTCAATTGCGCCGCCACACTAGCGCCA
TCTACAAAGCAATTT
TCTCCGCCAGCGGATTTATCGACTTGTTTCAGACAGTGTAGAAAT
TGGACCCCAGGTGGG
GATGTGTAGTAAACTACATCCGTATGTAG
>ConsensusfromContig220 Average coverage: 9.96
TGCAAAGCCTTATGATAATTGATATGAGTTTTGATTAAAATGTGA
TCTTTCTAAAAAGAG
AAATGATTGGTATTTAGAAAAAAATATCGACCTAATTTAATTATT
CATTAACTTAGGCCT
TCTTACCTACAGACACCTAAATTATATTCATTTTTGAGGTAACGA
TATGGGTATGTCCGG
TTATATTAGAGGTTGTCCTT
>ConsensusfromContig221 Average coverage: 8.14
CTAATATTAAATACTATGACGATAATTATGAATGTAATAATGGC
AAAAAATAGGATTAGA
ATCGTCGTTATATGTTATAACATGTAATATCACATGCGAATTATC
TTGTGTAATATAAGC
AAAAACCGAACTCTCCCCAAATATTATCACTATCTTCTACATTTT
AAACCTTCCATGAAA
CAATAATTTTAAATAATAAATCAGATTAAG
>ConsensusfromContig222 Average coverage: 14.17
TTCGACTGTGGCCACTTTAGAATTTGTGAGAGGAATTTTTGTGGC
TATATTTGCAATTAT
CGGTGGAATAGTGGCGGATTACTCATGTTCAAGAGTATTCGTGAT
GCTAGTAGCCTTTCT
CGGAGTAGTATCTCTCTTCTGTTGTGCCTTTGTCACTTCATATATC
AGTTTACTGATTTT
TTATGGAGCAATCTTCGGAATGTGCATAGGAATGCTCGAAAGCCC
ATTCATGTCAATCAT
GGGTAAATGGTTCACTGTATACCGAGACTT
>ConsensusfromContig223 Average coverage: 5.51
TTTAGCTCTTCGTAGTATTCTTCCCATATTTATTTGATATTTTCTGA
ATGTGTTGTGTTT
GTGCCATTCAGATTTTGTCGAAGTTTAATCATCCGATAATTCACG
AATAAGTTTATTGAC
ATTATGAGCATTGCGTAAATGTTTTCTGGGCCTGTATGATTTCTCC
TATATATTGATTTC
CGTCTTTCTGCGATGCTTAGGGCGACCCAAGATCGGAAG
>ConsensusfromContig224 Average coverage: 11.44

TCACGAATAAAAAATCTATTGAAAAAGTGTTCGAAGTAATACAA
TAGAAAAGTTGAATTA
ACAGCAACCATTAACATTGTTTATGTGATCACCATGGTTCGTCCT
ATCGCTACTTGAAAT
CTCTTGGATATTGGTGCAAATTATACTTGAAAATATAGTCAGATG
AGAAGAATTTCTATA
TGAATTTTATTTATCACGAATAGCAGATTGAAACAATACAAA
>ConsensusfromContig225 Average coverage: 16.37
CCTTTTTGGGACCACAGCGGAGGGTCGAGACTCCCATAAGGCAG
AAGACCATTCTCCAAA
CACGCCAAAAATGAGATGAGGTGACCTCCAGCTGGGAAGTTCAA
GGGCGGGTATTGGACG
CCATCTTGTCCCACCATGGCTATTGTTCCGCCCCGATCACTTTGT
TTGTGGCAATGAAGA
TAAACGATTTCGTCAAGACAGACGCAAAGAGCCAGGTTCCAGTAG
AAACTGTCTTCTTGA
GAGACAGCTTTGGGTTCCTCGCCCCCCTTCATCAAGAGGTTTGGG
GTCCACTTGAGAACA
AGGGCGCAGTCAGGTGTCTGGTGTAGGGAGA
>ConsensusfromContig226 Average coverage: 7.14
GGAAAATTTTCTATCAAAAAATGAGATTTAATTTGAATTATTCGT
CAATATTGTAATTGA
TGTAGAAATAGTAAATTTTTTAGAAATGTTGAGGTCAATTTTTTTC
ATTGTATTATTTTT
GTTGTATAGCCTACTCTATTGTTGTGTGAATTAAATTTATTCTCAT
GGGTTGACCGTTCA
TAAATATATAAGTATGACTAGTT
>ConsensusfromContig227 Average coverage: 7.36
ATGGGGAGGCAACTTAATAGAACGGTTCCTGCAGTACTGTAGCA
AAGTAATATTCCAGGT
CTTATCTCATATTCACAAATTCATTAAATGACATTTTTAAAGGTCT
GGTCATGTGTTTAT
GTTACATAATTTGATTAATAAGTGTTGGTTAGAGTTAGAAAACCA
CGCTTTCGTTGCGAG
ACAGTGCATAATTCTAAACTGGTGAAATTTCACAACTCACATC
>ConsensusfromContig228 Average coverage: 9.44
CTCTTCCGATCTTGTGGCATCTAGTAGTTCCAAAATAAACTATGT
TGTTATATGGATGAA
ATTTAGATGAATTATCATGAATCATACATTGTGTATTTAAATCAC
ATAAAACCAGCACCA
ACTTCTCAGTGCTCATGGTGATTTAATGAGTTCTAAAACTTTATGT
CATTGATTTGGTTT

CATCTGATACAGACTCTACTCATCACATGAATATTTTTTGTCAGT
AAATGAATCTGTCCC
AAAAC
>ConsensusfromContig229 Average coverage: 11.17
CTGTTGCTTGCTTTTCAAAATTTCGTCACTGACTGGATTTCCGTCT
GTCTCACTCAATTG
TTCAATAATTAATTCCAATGAAGGCTTTTGAAAAATTTTTGGAGG
CATCTGACAGAAAGG
TGTTGATGAGCAAATTTTATTAATCATCACACTGATTTCTCTGTT
CGCTGGAAATCCGAT
TGACCTTTTTTATGAGTCATTACAAGACCAAAAATTAGCACTAGA
ATCATATAGAATATA
GTCGTTGTTCAGTCTACATTCTTTTGTAATATAATTTTT
>ConsensusfromContig230 Average coverage: 7.80
ATCTCTACAAGGGATTCCCCTCAATATCCTCCCGAACCTGCAAAA
TTTGAGCAACCCTAA
CATGAACCTTAATCAAAAACCTGTTGATAATAATCTGAACACGAT
CCAACCAATGGTTAT
TCAGAACCTTCAAAATACTGGGGGATCTGTTTTTGATGCGGATGG
TTCAAATCTTAAAAC
CGCTTCACGACAAATAGCTCCGAACTCAAGCGGAGGAATACAAC
AACAACTCATTCTGAA
CTCAATGAATCCTTCCGCGTCTGGC
>ConsensusfromContig231 Average coverage: 12.82
TTCCGATATCTACAAACGGGGATCTTCTCCCAAGGTTCGGTTTGA
CGATACTGTGAGCGA
TATCGAAACAGCTTCTGTGATTTCGCGAACACTCTCGGCACAAGA
CATTGCGAGAGCCTC
TTCAGCCCTTTCCCAGAAAGAAAGAACTCCCAAATTATCAAAGTT
ATCAAATCCGAAGAG
GATGGATAGAAACTTTTTTCTAAAGCAACGCTCGTCAAGTTTAAC
AAATGTTGCATCATT
GAAATCTCCAGCAATGCAAAGGCCTACGAGAAAAACAACACGTA
AGGACATATTTCATCC
ACATTTACAAGATAGGGATTCAAATAATATAGAGGCTGCTGCGAA
GAATCCTGGTTCTGG
TATCATAACAAACATGGAATCTGGGCATACGCGTCG
>ConsensusfromContig232 Average coverage: 9.42
TCGATAGGTGGCCACGCAGTGTCAGAGACAACTTTAGTCCACCAG
ATTCGCTCCAATTCC
AAACAAAGACAACTGGTTGATCTTTACATATCTCAAGGAATACGA
ATTCACCGTCTTTGG

ACAACATAGTTGCAGTGTTACTCTGCGAAAGAACTGGCACAAGAT
TTTTATAGATATTTG
TTTTATGAGAAAATGCCTTTGATGGCGTAAAGAAAG
>ConsensusfromContig233 Average coverage: 7.17
TAACAACTACTTTTCAAAGATGTATTTGACTGTGATAACTCAATC
AGCTCAGCATTATTT
ATCACAGTGGAATTAGTTGTGATCTTTATTGTTGTTGTTTTCGCTT
CGGTTACTGAGAAT
GCACAACCCATTTGGAAATCATGAAATTATTATTATAATGATATC
TGAAAACATTGATAG
AATTGATAAGTCAGATTAGTTGTGATCTGTTT
>ConsensusfromContig234 Average coverage: 4.49
CTTTTATGAATTAATTGAGTGTTAGTGTCTTATCGGCTAATATTGA
ATGTATATTGTGCC
AACATATTTCTTATAATGAAACTTGCACTTACGCGTAAGTTCTCA
GAGCTTTCATTAGGC
TCCCACTTGCGCCTAAAATATAGGTTTCGTTCCATCCCAATAATA
CATATGAAAAATACA
CTGAAAGCAGACCATGCTTCTGAAGAAAGGAGTTGAATGATTCTC
TATCGGAATGCTTCA
ATGTTCCTAGTGACGAGTAAATTTTGGGGTG
>ConsensusfromContig235 Average coverage: 7.60
GAAAAGTCATGTATTGTTGGGAGAATGGGTACTTGACTATCCGTA
AATGATGTTGATACA
TTTGTCGCGTCATTTTGTTGAGAATTACTTATACTCGAAAAGTGTT
GTATTGTTGTGAAA
GTTGGTTCTTGACTTCCAGTGAAGTGTGTTGATAAATTTTCTGCGT
CTTTTTGATTACCT
GTACTTAAAAAGTCCTGTTGTGTTGTAGAAGTAAGTTCTTGACTC
ACCATTTGTGCTGGT
TGGCACGTATGACCAGGAACGTGTCCTCGATATTTATATTCTGG
>ConsensusfromContig236 Average coverage: 10.35
TGGTGAAGCTCGCAATGACCACGCCCACAAAGAGAGTTAAGCCA
ATCATACAGCCGATGA
ACACAAAAACGTGGATGAAGATGGCGTGGCTGATTCCAACTCGT
TGTACGATGACGTCAC
GAACTTCCAACCACCCTTCAAGAGAAAGGACCTCAAAAAGAGCC
AGCATTCCGTTGCCGA
TGTTGTCAAAGTTGAAGTTTCGGGG
>ConsensusfromContig237 Average coverage: 5.45
CGGAATTAATAATTAATAGTGTACTTCAATCATATATGAATGAAT
GAAAAAAATGCTTAT

GATGTACTGATACGGAACAAATGTAATTTGGGATACTCCTTATGA
GAGATTCATTATGAA
ATTGTCATCAAATCTCGGATGAATACTGTGTCAATTTTTTCAAATC
AATCATTTATATCT
AAAATTTTAGGATCCTAACTATATTCATG
>ConsensusfromContig238 Average coverage: 15.38
GATGCAGTAACCGTATCATTCTCGTAAGTAATTTTCGTTGAATTTG
CTTTGGTTTGTGAA
ACAGTTGTATAAATTTCATTGGTTATATTATAACCAGTTATGTTTA
CTGCTGGTGACGTT
GATGTTGAATAATATACAGGGGATGTCAGAGAACTGTATAACGT
TGTGAAGTTCAGAGTA
GAATTTAAACTGACTTCAGTGTCGTAATCTGAGCTTGTGTTTTGT
GCAACAGTTG
>ConsensusfromContig239 Average coverage: 7.85
TTTTTTAATTATCATATATATGAGGACATAGCAAATACGATTCAT
CCCAGAATAATTTTC
ATATCAGCACTTTCGGTTCTATATGAGGACATCGAAAATATAATA
AGTCCCACAAATGAA
GTGATATTAATATAAGTTGGTAAATATATCATCAAATGACAACTG
TCAACACTTTCGATT
GTATATTATGACAGGTGAGGACATATCGTTTATCCCACAAATAAA
ATAGTATATTCATAT
TAGTTAGTAAATAAATCAT
>ConsensusfromContig240 Average coverage: 16.05
CCGAGACAAAAACATTTATGGTCGAAGTTGCAGTAGGCGCAGCTC
TTCTGCTCATCTTCA
TTCTCGTCATCATCATCTGCATCTGTTGTCATCATCATGGAGATA
GTAATGAAGCCAAGA
AAGATTCAGACCAGACGGACAAAGACGTGTCTAGTATCGTTTCA
GATACGTGGGTGGAAA
CAGATCAAGAAGCGGCGGATAAAACAGATGCGGAAGATGAGAAT
GAGTCGGTGTTCGAGG
GAAACAAGATTGAAATCAGAATAGAAGATGAAGACGGATCGGTT
TTCGAAAGCAAGGAAA
CAGAGCCTAAATCGATAGAAGATAAATCTATATTGTGTTCAATGG
AGGCCAACTTGCCCG
GTGAAGAGTATATCGACTACCCATAACATTTGTGCTGATGTTTCG
TATATTTATTGCATA
CCTGAGATCGG
>ConsensusfromContig241 Average coverage: 10.96
CTGCGACAGAGGCGCTGTGAGACCCTCTCTCAACAAATCAGTATG

TTTTGGTGACGAGTA
CACAGGGCTCGATGAGTATTCAAGCTTGATAGAATGGTTTTGAGG
GTTCAGAGTGCTACC
CATTGTGAGACCATCGTGTTTGTGTCCGCCTTTTTGAGGAGAAGG
GCTTCCATTGACGCT
GAAGCCACTGTCAGATGATGAGGAGAAGCTGTAAGGAGTCGACA
GAGGATATTGCGCGGA
TTTTGGTGTAGCGTAGTTGGATGAGAGATCAGTGCTTGGCCAGTG
AACGCCTGGAGGGCT
TATGCCCAGATTTCCCTCCTTCATTTCCGCCTCTGGTTTACTGCTT
TCGCTCGTGGAGCT
CAATTTATCGGAGTCCATATCTGCCATGTGCTTTGGAGAGATATCG
TCTTGTTTTGCATC
TCCTTCGGTCTTTGTGGGGGTTTTGATCTTGCAGCTTC
>ConsensusfromContig242 Average coverage: 5.95
TGTAAATGATACTCATAATCGTAGTATAACTGCATTTGGTTATCA
TATTGCTAGACGAGA
AATATTTATCGGATACGAAGATGGAAAAATACGAGTACTTGACG
GTGAATCGGGCGGATG
GTTGGCTACACACACTGACCATAATGGCTGGCTGAACTGCTTTCA
TTATTGGGACGAACC
CAAACTATTGATGTCATCTGCAAATGACGAAACAATTCGACTCT
GGGGGGCGGGTGGTGG
AGTCT
>ConsensusfromContig243 Average coverage: 13.17
AAATGTTAACATTAAATTTGTTTGCATTTTTATTTATACTTCTAGA
TTTTTGTCAAAATC
ATAGCGTACAAGTTGACATACAACGGTGTCTTGCACATGACACCC
TGATCAGCTTCGACA
AAGACAGAAAAGCTTCTGAAAAATATGTCAGAAAATATTATAAA
GGTGACACGAGTTTCT
CAGACAACTCGTACTCAGGCGATGGTGGTCTCATTGATGAGGATT
TGCGCCTGTATCACT
ATACAAATGTTGAATTAAATGTGACAACCCCCTTCGATTGTTCGC
TCAAAGCATGGAGCC
TTGGGATCTGGATTCGATTTACCAAACCTATGCAATACACAGCTG
TGTTTCGTTCATCCA
CATTCAGTATGGGATATAGAAAAGATCAAGACGGTTGGTACCTAG
ATGTTCGACATTATG
GCACCAGAAAATGCCCTGCAAAAGCTAGAATATCTGTACATTTT
GAGGCCAACAATGAAT
GGAGACGAGATCGGAAGAGCAC

>ConsensusfromContig244 Average coverage: 15.66
TTTTTTTTTTTTTTTTTTTTTTTTTTTTTTTCAATGTTCATTATTCAT
TGAAGAAAATTT
ACAAAACTTACGAGATAACCAAACTAGATCAACTGGTATGGTCT
ATGACAAACATCTCGA
TATTAAATGAGACGGTTCATTAATTTATCCGAAAGTTCTTGATTC
ACATAAAATATATAA
AATTTTGACATATTACTTGAAAC
>ConsensusfromContig245 Average coverage: 10.59
AGAGCAACACCAAAAAGAGCCAGCAAAAGTACTCCATTCCCAGC
AAACGTTGCAGAGATC
AATGCAGAGATCATTGTTAGACATCCCAGAGAGACCACGTTTCTG
TACCCAAGACAATCA
GCCAATGGGACTGCAAGGAATCCAAATATTGCAGGAAATGATAC
TTGAAAACTCAGTATA
AAAGAGAGGTAGGTGGAATTTTCTCCAAAAGTACCCAAAAGATC
GCCAT
>ConsensusfromContig246 Average coverage: 7.01
AACAATACTAGCGTTCACAGAGGCATATTCTCTCATTTTAAAAAA
AATTTAGTCACCTTG
TTTGAGTGAATCATTTTATCTCGAAATTTAATTTTCTTAAATGATA
CCAGCTGTTTAGTA
TGGAAATCATTTGTTACACATTCAATAATATTATTATCGATCACT
AGAATGAGTGGACTG
ATCAATTCATAATTTATTGA
>ConsensusfromContig247 Average coverage: 8.92
TTTCAGATTCAAAATAGTTACGTCTCTTTTGAAGTCTGCCCACAAT
CGGCATTTTTGGAT
GTGATGAACCGAAATCATCGGCACTTGTGTTCCGCGAGACCGGG
AAGCGAACGACATCGG
AACCACGAGATGCCGTCCACTTTCGCGTCGACACGCCCTCGTCGT
TCAATTCAACGGGTG
CGACCGGGTTCAATTTCAAG
>ConsensusfromContig248 Average coverage: 8.27
ATAATTTAATTTATGACATGCAATAATCGCATTTGTTTTTGCTCGT
AAATCACTCTGGTA
ATCGCATATTATACTTGAATATATTTCGGTTAATACAATCAAGAG
TGGAAGATGACATTT
CGATCGTGATTTCTGATAAACATCTGTTTCAACACTTGATACACG
CGGATTTGTTTGACA
CCATATAGTAGAATATCTAGGAGAGGAGTGATGATAAAACTGATT
AACACATTCTTTAAT

TTTAGGGATAATTCTACCACAATTGATTGACATATCTGCGATAACT
TGTTCAATTTGTT
>ConsensusfromContig249 Average coverage: 4.10
TTTTCATAATTTTTATACTGGTTCGTATATCCTAATTATTTTATTT
ATGATAACAAACAG
TAAAAAAGTAGTGCTGTTTTTGACATTATCTCAGGTGTATTTATG
ACCCTGATCATGTTG
ATAAAATCTTGGGTGCTATTTTCCACTCGGGCATACATCTGAAAT
TTTAGCTTCAAATTC
AATTTGGATGTTTTGCAATATTT
>ConsensusfromContig250 Average coverage: 6.30
CATGATTTAATAAATTTTTCTTCGTAGTCATAAAATGATATTTCGA
ATGATTATATTATA
TTGGATAGAGTCAGTGATGAAAAACAATGAATCAAATGATGGTA
AGCTGTGGCATCGAAT
AGAAAATAATTTAAATAATTTCACATTTCCAAAATATATGATATT
TACAGACTTACAAAA
TTTTGTTTGAATACAATGGAGCTCTCCC
>ConsensusfromContig251 Average coverage: 8.40
CAGTTTTGTTCGAGCGAATAAGGTCATAAAACCAAATACAGATGA
AAGTTTACTTTGGTG
GTGTGAAATTGTCACAATTATATTTATAAAACATGGATCATGAGG
GATGCTAAACAATCT
ACTTTCATATTCTGGTCACTTATTCAATTTTTTATGGCAGTGTGCA
ATTGATAAATCAGT
AGTTTATACAAACACAGTTGGTTATATACATATAATTGAGCAATT
TATGGTACTGATTAA
TTGCAAATATATTATATTGTTAAAT
>ConsensusfromContig252 Average coverage: 12.44
GAATTCCAGAACATTGCAAAAGAGGCTGGAATATATATTTACAAA
ATTCTGTATATAATC
ATGAAGAAAATAAACATAGGACGATTTTACAGAGAAAATAAGTC
AAAATGCTCACAAGAA
AACAAGTTTGGTGAAATAGTAATAAAATCAAATGAAACATGCAA
ATGAAAAGTAGCAATA
AAGAAACGTGCAATCCCTGAAAATAATGAGATTTGATGATTGATA
TCCAAAGCTGATTAT
TCCGAGAAAAAAAAGTATAAATTTGAAGTTAGGCAGACTCTGAG
AGTATTAAGAGAAATT
TTAAAACAAACACCCCGGGAAGACATATACTAGTGAACATTCAC
GAAAAGCCATAGAATT
TAGAAATAACATTAAAGCTGGAAAACACGATTACGAATAAATAA

AAATTGAAATCTAGAG
TGATAAAATACCCCAATGAAATTATGAAAAACTTGAAATACATG
AGAATACTGAGAATGA
ACTGGTATACGATCTTGCGAAATTCCACCAAGAACATAAAAATCA
TTTTAGTCTCATGAG
>ConsensusfromContig253 Average coverage: 5.16
ATAAACATGTTCCCTGTGCTCTGAAATTTGAATCGAGAAATAATT
CATGGAATAAAATAC
TAAGAAGAAAACTCGCGAATAATAACGTTGTTTCAAGGTTGATGG
ATCTCGGCCCAGTGT
TTTTCAGAAATATGAACATATTCTTCGTGTCCTGCAATAAAATTG
AAAATGTAAACTTCT
CATGAAACACAATTCTTTGAAGAAAACATTAATGTTCTTACGAGC
TGGATGAATGTT
>ConsensusfromContig254 Average coverage: 5.31
TAGAAAATGGCTCAAGAAAGGAAAGTGTTGATATGTATCCTTCG
GGTCTTTCAAGAACAA
CATGTTGATTAAGGCCACATTGAGTTTCGGACAAGACATCAACTT
TTTTGCACTCAAATT
GTGCATCCAAAAAGCTAGTGGCTCGACCACATGGTTTAACTTTGA
AAACAAGTTTAACCA
TGGGGAAGTATTCATTCTTTTCGT
>ConsensusfromContig255 Average coverage: 6.64
TAATTTCGTCTTATATACGGAGCATCGCCAAAATTAAAGATGCCA
TACATTGAATCCCGA
TAAAAAAATTCTGAATTTCTAAACTGAGATAGAGAAATTATTTTC
AGAGCACCATAACAA
TATCCACTAACCTGACCTCAACAAAGCACATACCTTTTATACCAG
ATGTCTTTCAATGTA
CTGAAAGCATTATCACACATATGACCTTAATACATGATTGAC
>ConsensusfromContig256 Average coverage: 14.10
CCGCCGATTCAGGATTGCGTTCCTGGCAGCTGTGTAGGTCGGAGT
TTTTCTCATTTGACC
CCAAGTGACCTGGCGGAAACAGACGTTGATGGAACAATTCAGCT
CACTTGCAATGAAAGA
TATGTATTTGAAAAGGGTACCAAAAATAAAATAAGTACTCACACT
TGTGATACTAATGGG
CAATGGAATCCGCCGATTCAGGATTGCGTTC
>ConsensusfromContig257 Average coverage: 10.45
AAATAGTCAAAATCACAAGTATGAAATGCCATTCATGAAAAATTT
GCTGATTATCAATGA
AGAATGTCTATATTTGGACTCTCGACTCTAGTGAGACCGCTATGT

TTATGATGAAAATAT
GCACTTGTCGATAGCAAAAATCATGAAGAATCAATAAAAGACAA
GTGATCAACAATAAAA
TACAATATAATCAGCAAACCACCATGATTCAATCACAAATTTTTA
GTGAAACCAATTTTT
TTCATATATCGGAACTTTGTTTCGCGTTTTACTCAGTTTGCACTTT
TTGGTCAATTTCAT
GACACTTAAAAATCTTCCACAATTTCTGTCATATGTACAACAATG

>ConsensusfromContig258 Average coverage: 7.75
ATCAAAACTTGGCAGAAGGCACTTCCTGAATGATTAACTGGAAAA
CCACACCTTTTTCGG
TGTTACATCAATTAAATTCCAAAACATCAAGGACGATTAATTCAG
CTTACGGAAATGGAT
TATGATGTGCCCAAAGCCAGCACAAACCGTTCAATAGTTTTAAAA
TTATAGAGGAGCTAT
GGACAGCCAATGGCATAAGCCAGCTTGTTATGTAGACCTGCCCAC
ATACTAGATATGCTC
AACCAGGGCTTTCATTCATCAATTTGAGTCACTTTATTTATTATCT
GGTCCATCTGTCTC
ACGACTGTGTCCCCGATGTATTTCATGATCTAAAATCAAAACGAA
TTTCTCAATTCAAGA
ACGTTTTTCCTCACATAAATGAGACTCACAAATTAATCAAAACAG
TTTGATCAAG
>ConsensusfromContig259 Average coverage: 5.52
TCGATTTTTTTTCCACAGCACAAAATCATTTTTTACGAGAGGCTG
TAATGTTTTGCACG
TATTTGATAGAGATAATAAATCTCTGGTATTGCAAAATGAAAAA
ATAAGATGTAGGGCAG
TGCTAGGTAGGCTTTCAAAACATATCGCACTTCTTGACATTCGAA
TAAAGAAGAATGAGA
GCTCCCTGTCCAGATGTAAAAATATTTCTGAGCGGTAACTAAACG
A
>ConsensusfromContig260 Average coverage: 10.42
GAAGTTCTCTGCGGAAGACTGATGCTGACAAATTTAATAACGAGA
ACTCATTTTGTTTAG
ATCGAAATGAGTATAGAAGTTCTTTGCGGTACCGAACATCTAGCC
CTATGAACTCAATGC
GGTATAGATCCCCAAGTCCACTCGACCTCTCACTCCGATATAGGT
CACCGAGCCCTATTC
GAAGTTTTTTCAGGGAATCGAAAACTGAACAGTCCAGACCGACA
TGCACGCCATTTCAAA

GCCGTGTAAGAAACCTTTCTAATATTGGTGCTAAAAATGACAGAG
AAAATTCGATATGTG
ATAGAAAATCGTTTAATACGCA
>ConsensusfromContig261 Average coverage: 10.14
CCCCGACGCTCTTCCGATCTCTCGCAGCGCCGTCAATCGGTTGCG
CCTCTGGAACATAAT
CTGTTGCCATTTCGACGCATTCATCCGTATCCTCTTTCCCCTCAGC
TGAGAATTCTTCGT
CATAATTAATCTCAGCGCGCAGCCCCGTATCACTGCGCCGACTCT
GCCTCGACGACGACC
GAAGCTGCTGCGTACCGCACCGCTCTTCGTCGAGGTCGTATGATA
GCTTGGAGTCGCTGC
TGCACAGCCGTTTCGATGAGCGACTATAAAA
>ConsensusfromContig262 Average coverage: 8.49
GAATTTTTTCACGAGTTTGAGTCATTTTTGAACGAAATCAAACCT
AATAAAAACTGTTTA
TTTTGTGGAGATTTTAATATAGATATGTCAACTGACACGAAAAAT
GCTAGTGATCTCATT
CAAAACTTTCTTTCTTATTCATTTTATCCAGCCATCTTAGGACCAA
CACGGGTCACATTA
TCTTCATCAACATTAATCGACAACATATTTCTTTCGGACCAAACG
GCATGCCAATCAGCA
TGCATAACATCAAACATGTCTGATCATTATATATGTTTTATATCT
ATCGCATCTCAAGTA
TCAAATAGTGTCAATGAATCAAAGCTCC
>ConsensusfromContig263 Average coverage: 14.64
GTAAAGTAGAAAACAATACAATAGTCAAGAGTAAAGTAGAATGA
GCAAAGATGATTATTA
TGTCATTATGATAAAACCACTATGAGAAAAGATCGTCAAATATT
ATGGAAGACAGATATA
GAATATTAGTCTTAACATTAGTCACAAATATGATTTACTCATAGA
TTCGATAAGCAGAAC
CGAAAATAGAAATCAACTATAAATACAGTGCTGTCATCTCCATTT
TATAGCC
>ConsensusfromContig264 Average coverage: 11.76
GTTTGCATTCACATTCGGAACTTTGATGGGTTCATGGAGTACAAA
GAGGTATGGAAGGAA
GGGCAGCATCCTCAGAATCGCAATTTGCAACGTTCTGGTCTGTGT
GATGATGTCGTTGTC
AAAAATGTTCACCTCATACGAGCTGTTGATCGCTGGACGATTCCT
GAATGGACTTCTCAT
CGGAAACTATTTCGTGTCAAGCGGAAT

>ConsensusfromContig265 Average coverage: 4.97
TCAGAAAACCAAGTCGGCTGGCGACGCTCGGGCTCAGATATCAA
GTACTTCAGGAATAAT
TACAAACCGAATTATGGGCGGGGCGAACGATATAGTCTTACGTG
GACATGTCAATTCAAG
TACACAAGGGATACCTGCTATTTCGCCCATTGCTACCCATATACA
TACACCGACTTACAG
GAATACCTGAACAGTATTTTG
>ConsensusfromContig266 Average coverage: 6.46
CTATATCGGCCGAGTGAAAGACGACCAATTCGTGACTTATATTTT
CGACTTCAATTTCAA
CAAAGGAAAAATAAAATCAGTCGAACTTTCCAACATCGGAAACGC
GACTATTCCATCTCT
GACTAAACTACGCAAATATGTGCTTGCAACCGTAATCGATGTCA
ACAACGAAGTGAAACG
GCTGCAAGGATGTTCTCGTC
>ConsensusfromContig267 Average coverage: 4.98
TGCACACCAAAAATATGGTCAAAAACATAACTACTTTCCAGTCTG
GCATTGGCATTATTA
TTATGATTTTTATTGATCATTTGAAAGTGGTTAAAGTGTGCATAA
AATACTCATTAGGGT
TTTGAATCACATCGGTTATGTCGAGTGAACAAATTGGATTGTGCT
AAAATTTGAGCGATT
AATAAAGTGAAACTTAACAAAGGACAGGTGTAAATAAAATTATTA
TGTTTTAT
>ConsensusfromContig268 Average coverage: 4.37
ATTGTCTCTGACTATGAATATTCTCCGTTGGAACTCGAAAGCTGC
ACTGCCCCCTGCCGG
TACAGAGAGCGCTCATCTGGTCGTGTACGTCTGCTGCGCACGTTG
AAGAGTGGTATTTGT
GCTTGATGCAACGGCTGGATTGTCGCCCGTATTTGGCTTCTGCTA
TGAAAATTGCGAAGC
CAGACGGACAGCTGATGTTGAGATTCTGGTAGTTGCAGAAGGTTT
CAGGTCCTTTAATAA
TCGTATCGTCTC
>ConsensusfromContig269 Average coverage: 5.85
ATCAACTATCGTCTCAAAATGGAAATCCTTGATGAATCATATTCA
AAACATCCATGTTCA
TAATGACCCTGGTTTCCCCGAATGTTTGCACGGAGAAATGTCTCC
AGAATCGTATGACTG
GTTGGAAACAGGAGGAGTCGCTGCAGCAAAATTAGAGAATTTGG
TGGCACGGAAAACGTT

CATAAAAGATGTATTCAAGCTATCCTCTAGCAGTCAGACTTCAAA
TTTGGAATCTTTCCA
TAACGTGATGATCCGGTACGCCCCGAAAAATACGAGTTTCTCGTT
CATGGGCATGGAATG
CCGGTTAGCCTTATCTCAAAATTTAGCATAAAAAATGAGCATTAG
GAT
>ConsensusfromContig270 Average coverage: 8.20
TCGAAGATTTCTTTGACACGAGCCATAAAAGAATCAAGAACTCCT
CTATGCTCTGACGTC
ATATCTCCAGGGGTGATTCGCTTTAGGACCTGGGTGATTTCAGAG
AGGATTGGTTCCACT
CTAGTTTCGTGCATTCCTTGCATGAAACCCAACAGAATTTCGACA
AAGACACTGCTCGAC
TGCAGAGAACCATCGCCGATCTCATGCTTTCTGTTAT
>ConsensusfromContig271 Average coverage: 5.32
TAAAAACAGAAAATATCACATAATTTCAAGAAAGAATATGAATC
ATACAAAATTTCATGG
TAAACCTACATGTTTGTATACAGGGTACACACGAACGGATATGA
TACAAAAAGAGATTCA
AAAACATACCTGCAACTCAACATTACGAAATCAGATGAACAAATC
AATTTGTTTTCAATG
TAATATCAGTCATGAAAAAAGTCAAGTAAATATACATCACACATT
CTTGTATAGCA
>ConsensusfromContig272 Average coverage: 8.81
GGCTTCACAGGAGAGATCTGCCAATTGGAAGCTGAAATCCATGA
ACCATGCAATGGGCTC
GGGGAGCTTGTTTGTGAATGTGCCGAAGGCCATTTTGGACCTCAA
TGTGTGAACAGTTGC
TGTTCAAATGAACATGGGAAATGCCGTCGACATGGAGAGAATTG
GGTATGCTGGTGCCCT
TCACCCCAGGGTGTCGAGCAAATAACAGGACTCGCATGTACAGC
CCCCTAATCTGGATAA
TCGGTTAAAGTCTAGCAGGATGTCTTCATCACGCGTTGGATCTGG
T
>ConsensusfromContig273 Average coverage: 13.89
GTTTCAGAACGAGTACGGAGGGTTATGGTCACCTGACCTTCTTTC
TGGCTCAAGGACCTC
CTAGTAATCTTGTTGAAATACAGGAGCCCGTAAGATCCATTGAG
GCGAAGGGGCATATCC
ATGGAAACAGCGCCGTGGCCTTCTTCGCAAGTTCTCCCATCAAAT
CCGGGCTGACATGCG
CACAAGAAGTTATCCAGCTGCGAATCACAACGACCGCCATTTTTA

CAGGGGTTCAAGGCC
GCCAGAGGGCAGGGGTCACCGTCGTATGGGGTCACACTTTGAGTT
GATA
>ConsensusfromContig274 Average coverage: 12.66
ACAAAGTCCCTTTACATGAATGTTATATGAGATATATATTGAAAT
TTTAAACCATAGTAG
ACTTCGAATATACCAAAATTGGAAATTCATTGATGATATGATGAT
TAAAAAAAATATTGA
AGTAGTAAAATCATAATAATTACTAACCTACTGGAGGTCCATTAG
TTGTTGATCAAACAT
GCAAATTTTATTATACATCAATCAAAAAAGA
>ConsensusfromContig275 Average coverage: 5.50
ATTTTATCGACGTTCATAAAGATGTCAATAAATTATCAGGACAAC
TTGAACACACTCCCA
ACTGTTTCGAGTCGACCAAAACAGCAATGGAAGTTCCAATCGTGC
GTGATTTTGTGAGAA
AAACTTCCACCAACGACAAAATTGAAACAGTCAAGCGTCTGATA
GAAGAAATGAAAGATG
AAGTCATTGATAATCTCAAAGAGACACAGTGGTTGGACGAGAAC
CG
>ConsensusfromContig276 Average coverage: 7.91
TTCGTTTTTGATTCTAGTAATATTTTTTTCTCTCTTCTTGGTTTCTG
AAGTATTATTTAC
TGTTGATTTTTTAACATTCCGCAGGTCTCTCAACTTAGGTCTCAAT
TTTTTCACAATTGC
TGTTCGTGCAACTAAACCAAAAATCAAGCTTGGCAGGTATGTTGC
TGCATGCATTGCGAA
GAAGGATACACCAAATTTCAGAATCACTTGTGCTGGCACACCAA
ACTCCAAAATATGAAT
GATCATTGTGACTAGAATCGCAACGAACAAAGCCGATAATTTTTT
GCAGCGTAACAT
>ConsensusfromContig277 Average coverage: 9.86
CAATCCACTGACTACTCGTCCAAAAATATTCGAGACACCGAAAA
ATGTGATGAGACAAGC
ACTGGCGGAGAGTGAGTACCCAAGGCTGGTACTGAAGTCAATCA
TATAGAAAATAGGCAC
GTAATACCCCATGCTTGCAAGAAACGAGCTGAGACATACCAATAC
AAAAGCAATATTTTT
GAATAATGTGTAATCGAATATTTTTTTCAGAGCATCAATTGTCGAG
TCTTTACT
>ConsensusfromContig278 Average coverage: 7.71
TCCAACTTCAACCAGATTGTTCTCTCCAGAATTGCGCAGGACAGA

GCTTTTCTATGCCCC
ATCATAGAAATGACGAATGCCTTCAGAGAACATCATCACAGTGTA
TATCTATATCTGAAT
GGTTATCGAGCTTCTATTGAACAAACAGCAGATTGGATTGGGGTC
ATCCATCATTCTGAT
ATACAGTGGCTTTTCGGTCACCCTCTGAATCCCGGATCGAATTAT
ACGGATTCTGAAGTA
GAAGCGAGCATACAAATAATGAAATTCTTAACGAATTTTGCACG
ATCAGGAGTTGCACAG
TTCGGAATCACCGACACGTGGCCAAAAGCAGACGTTCAACTGTTG
CGTTATATGGAGTGG
GCGGAGACTGGAGAATTCGCGGTGGGGCGTGGCTTCTATCGAGAC
GATTATTGTAGCTTC
TGGCAGTCTTTCATGGATGACCTCGAAAGTGTTGACCCCAGGATT
GATTCGGGAGATCGG
>ConsensusfromContig279 Average coverage: 12.43
CTACACGACGCTCTTCCGATCTCAAAGTTCCACGCTCTCATGCAG
TTGCAAAACCAGTTA
CACATAACAACAGCGGCACATTCATCTCACTCAGCCGCGAAAACC
CTTCGTATGAGATTC
ACTACAGAAGCAGCAAAAATGATTCTTTGATCATGTGGGTGATCC
GATCTTCCAGCTGGA
ATCTCCTGCCGCAGATAAAGTTCACTTCTCTTGGTTCTCTGAAAT
CTGACAAATGC
>ConsensusfromContig280 Average coverage: 11.26
AGGCTCCGACCACTCGCTAGTTTTAGCTATTCTTGATATAGCGAT
ACCAAACAGATCTAG
ATATGCTCCGAGAAAAAACGAAAAACGCTATGATTTAAGAAAAC
TAAAGATACCTGCCGT
GGCGAGACGATACAAAGTGGAAATAGGGGGAGATTCTCGGCACT
ACAAGAAGAGACTGAG
CCTCTTGAATTGTATGAAAAATTTAAGAACATA
>ConsensusfromContig281 Average coverage: 4.65
AACAACCTCATGTTCGGGTAAAGACTTGTGAAAGCATCGAGGTT
AAAGGTCGATACGATG
GCATTGTCGACACAAATAAAAGCTCTTACTTTGAGGCTGACAGC
AGCAAACGAAGAATGC
GGAATATGTACATCCGAGCATTGAGATTATGGGTGGTTCCGAAG
CGAGCTGAGGAGTCCA
GCGTCCCGCAACAAATTGGATACGTC
>ConsensusfromContig282 Average coverage: 10.31
ATCTTGACAGACATCGACCTCATCCAAGAGGCTGTCAAAGACAC

CAAACAAGTCAAATGT
CTACAGCAAAAAATGCACCAAGCTCTGATCGAGTACTATAAATAC
CGTGGCCTGTACAAT
GAAGCCGAAATGCAGAAACTAAGCGAAGAAACTTGTAAGAACGC
AAATAAAGTGGAAATT
CTTCGGGAAAAGATGGACAAATCTCTGAATAAACTCACACAATTT
CTCGACACTAAAGTT
GGCCATGAGCCCGTGAAGGATCT
>ConsensusfromContig283 Average coverage: 5.72
GCGGGGAACAACCGTGAATCCTTCGCAGTGTCGCGTTACTTTCGG
GTCTTCGACAGAAGA
CAATGCAATTTTGCCATTATTCATTTTCACCTGAAAATCGCCTTT
CAGAATCCACGGTAG
ACTTTCCTCTGGAGCAATGAGATCTTTTATTTTTAGACCTTCTGA
GTCAATCGCACAAAT
TTTCTTAATCGTCGTCGCGTCTTTTTCTTCAGGGAATTCTCTCTGA
TGTCCTTCGTAGAT
TTTTGTGCAAACACCTTTAAATTCTTGATTTCGA
>ConsensusfromContig284 Average coverage: 6.51
AGCATTTATAAGCCAAGTTCACCATAACTAGGATGACGCAGATTG
GGATGATTCCATAGA
AATAAAGCTTAATATTGAAAGCCTTTTTTAGCAACACAATAAATA
AAACAATCCCGACGG
TGAATATGATAAGAAAGATGCTGCTGAGACCCGCCCCACCCCGC
GCCGCTGCTATAAGGA
CTAGGAGAAAGATGAAAGGGGTCCATATCACCCTCATCAAGTCCG
AGCGAATTGTTCGTT
TGGATTTTTCCTCGAATTTTGTCGAAGCCCAGCGCACGGAGATCC
AAAATGCCCATAAAT
ACACCATCTTGAGCAAGTATTGATAGCCATTTCCACCAAAATCTC
TACCTATCAAAACTG
TGGTTATTACCCCAAATCGAGAAAAGATATAATGATAAAACAGTT
TG
>ConsensusfromContig285 Average coverage: 9.14
ACGACGCTCTTCCGATCTAAAAGCCTTACATCTGCAATATTTGCC
AAAAATCCTTCGCGG
GAACAGACTATCTGCGCACACATATCAGGAGCGAACATGATAAA
ATTAAAAAGCCGGCAA
CATGCGAAGTATGCGATAAGACTTTTCTAGACAAACGTCGTCTAA
AGCGTCATGTGGTCA
TCCACAGTGAGCCCAAGTTCGAATGCAAGGAATGCAAAAAAATG
TTCCTATCTCCGGGAG

ATCTCAGCAGACACCGTTTTTTGCATACAGGAGAAAGACCATACT
CCTGTGTTCTGTGCA
ATAAGAAGTTCTCACGAAATTATAATCTCAGGGCTCACCAACGTG
TACATGGCGACATGA
CTGTGGCTGTGAGAGCATATTACAAGTATACGAA
>ConsensusfromContig286 Average coverage: 6.55
CCGATGTGGCTTTGATAAATGGACCAAGAGCATCAAAACATGTTT
TCCGAGGTGCTTCAT
TGGTGAACGGTAGAAATTTCCCTATAACATTCAGGATTTCTTCTG
GAGCAATGATTGAAA
AATAATACTTGCCACCATATTGCCAAAGATAATCCAATTCATCGT
CCCGAGGAAGATCAT
CCGCTTCACCTGCATGGCTATTGCAATACGCATGTGTGTTGATCA
AGAATTCCGAAGTCA
GAACAG
>ConsensusfromContig287 Average coverage: 222.21
ACTGGAGTTCAGACGTGTGCTCTTCCGATCTGCCATTTCTATTTC
GGTACCGCAACAAAA
ACGTGTCGTATCAAGCAAGTATCATCGACCGTTTTGGGTGAATGA
TTCTCTACCCGGATG
CTCGACATTAATAGCACAGAATTTAGATGGCTTCAAAAAGATTGA
TCTATGATGTTTTAA
ACATTAATACGACTCGTTGCCTAGCATTTATAGTCCATTTTGTGTT
TATC
>ConsensusfromContig288 Average coverage: 13.02
CTTGTGCTCTTCCGATCTCTGTTGCAAACGGATCGAATTTCAGAA
ACCAGCAGATCGAAA
TTGTGATCAATGTTCCTGGAGCTAATTACAGACAAACATTTCCAA
AGAATATAGCTGAAC
CTATTCTGTCTGTCAACCATAGTTTTAATACAACACTTGGCGGCA
ATACTTCATGTTCGG
CCATAAATTCAATAGGTCAAAAAACCGTTTTCGAAAATTTTTCTGT
TTTT
>ConsensusfromContig289 Average coverage: 11.09
CCCACAATAAGTTTTTATATCTTCTCGAAATTTCTGCATGATTCG
TTCAGTGAAGAAGAT
AGAACACAAATGAACTGATAGACAAAAAAACAAACTGGGTCAAT
ATATTTAGATTTAGAC
ATCGCAACGTATAACTTTAGCATTAAAACATGACCTCATATTAAT
ATTTTACATGACCAG
GTGAATTTGTTATTGAATGTTATGAAACATGAACTTTTCCCGAAT
AATAATTCCAATGCA

ACACAGAAATATTTTTGGTAAACATAAAACGTGCATTTAAAAGGC
A
>ConsensusfromContig290 Average coverage: 4.23
TCGAAAACGGGCACTNCAGCTCAACATTTTGTCCAGAAAAAAATA
TTTCGCCAGCCTCTC
GACAAATTGAATGGACTTTGTGATTGGTCCTCTCGGTTCGCCATG
AGTCCTGCTCCATAA
CCAGCGATGTTTCCGATAACCCAAGAGTGGCAACATGTTTCATA
AGAGAAATAATAAAGA
AACGTTTCTTGGATGACATTTTTGTACACAGGGCGTCCAGCTATTG
TTTCAGCCTCTCGT
GTATATTCGCCTTTAAC
>ConsensusfromContig291 Average coverage: 11.49
GCCAAGAGCGATTGTCTTCCTTGGCTCTGATGACTATTCATTATG
ATCTGGAAATTGATT
TCGAGGAAGTTGTTGAACTATTCTGCCAACGAAATCCAAGAAAAC
TGCAGATGGATACTT
TATTGAGATGATTTCATTTTCAAAATTATTATTTTACTTATGTGGC
GATTTTATTATGTA
AATAAATTGTTGAATATTATTGGTTCAGATTTTTAAAAATGCGTA
GTA
>ConsensusfromContig292 Average coverage: 7.14
AAACACAGAAGGTATGTTCCGAGAAGACACTTTTCCGATAACTC
TGATGAAGAACGTGAT
TCTTCACTCGTGGATGAGATTATAACGAAGAGATTGGGAAGACT
CTACGAACTACGAAGA
GGATTGGGCTTTGAGGAGCCCAGAGGTCACGGGCCCCAAATGAT
CGAGCGAGAAGGTCAA
GGAGATAAAGATCAACAGAATGATCCAAAGCTTGATCGAAAGAG
ACTGGAAAGCAAACCG
AAAATAAGCAAGAGGCCACCTAGAACTAAAGCTGACAAGCCTTA
CATTCCCTACAGGAAT
GATCTGCCGGAAACTGAGCTCCC
>ConsensusfromContig293 Average coverage: 3.64
AAAGAGGAAACCAATGGAGATTGGATCTTTGTCTACCAAACGAA
TGTGCTAATCAAAACC
CCCCACACAAATGTATTACAACGCAAGAAATGAAAACTGAATGT
GAAAGAGATAACAATG
GTAGAGTTAGTCGGCCAACTGGATAACTAAGTGACCAAAAGCTG
AATGTGTTGACGAACT
AAAGAATTTCTTTGGCTTGGGAATAAAC
>ConsensusfromContig294 Average coverage: 8.00

GTTTTACTGCATTCCTCACATTTGGAGATTTCACCTGAGGCGCTA
CACTCACAACACATT
TTTGAATCAATTTCGCCGGTCGCATCATTTCTGGGACAACTTGAA
ACATTACCATGGCGC
GGTATATTAACTTGGAGACTTTCATTTGGCTTAAGTTGAAGAAAT
CCAACAAGTGTTATG
TTGGTAGGGAAGCTGTCCACGCCGACTGACCGAACTCTGTGAAG
AACGCGGCACTGAGGG
CATTTTATTGTTGAGGTTGATCTGTCGATGAGCTGTATCAAGCAT
GGCGTTTTGCAGAAA
GTGTGTTGGCAGGGCAGAATTTTTGGCTCTTTGAATATTTTCATTT
TCATATGCG
>ConsensusfromContig295 Average coverage: 6.87
ACGCTCTTCCGATCTCGTTTCCATTTGACGATAATTTATGTTTTAA
TTTTGATAAGCAGA
CAACCCTACAACGAGCTGCCTCGATTTCGCGTTCGGTCAGGGCAC
TTCGGTCACGGTTGG
TCGCCATATTGGAAGAACAGCAAAGACGTGTTATGATGAACCAAA
TGACTGCTTGTACAT
ACATTATCGAACTTGAAATGAGT
>ConsensusfromContig296 Average coverage: 7.32
GGTTTTTAAATTGATTTTGATCCTAAATATAATCTCAGTCTTCCGT
TTTTATATATTGGT
TTTTAACTTAATTTGGAGATTTTCATTATTTCATCAATTGCATCTT
TGATTCAAATTCTA
GGTTAAGCCGTTTAATGAATGAGAAATAAAATTCACACCAATGAT
ACAATTTTGTGTGTA
ATTTGTTTAAATTTTCTTCAAAGCTACATCTAAATTCGTTCTGCTT
TGGAGAAAAAT
>ConsensusfromContig297 Average coverage: 10.92
CGGAAACAGAATTTGAACTCTGAAAAAATAGGTCAAAAACCTCTT
CATCCTTTAATTTAT
GATAACATGCGTGATAATGAGTGATCTTTTCATGAAATTTTTCTTC
CTTAATATCTTCGT
CAGTCTTGAAAGCTATATCCACTGTAGCAAACAAGTTACCTTTAA
AATCAAATATTTTTT
CAAAATTTTTAGAAAACGGAATAAGTTGTATTGTTGATTTTGTCTC
ATCTCTTGTTTTTA
TATTTTCG
>ConsensusfromContig298 Average coverage: 27.43
GCACTCTTCACGCAGGCCCTTCTATGGACACGAAGTATTTTGCTG
TCAAAACAGTGAAAG

AATGCGTAGCTCAAGACTACGAACCGATCATTGGAAATAATGGT
GGAAACGAAGTCACTA
CTAAGAGTCATAACTTCGTTCTACCAGAACCCGGATTGAAAGAAG
ATACTCATGAAATCG
GCAGATCGGAAGAGCGTCGTGTAGGG
>ConsensusfromContig299 Average coverage: 17.06
AACATGCGGTGGCACTTGCATCTTCACATTTGGAGCCAGACTTCA
GCATTTTGCACTCGC
ACACATACTTCGCGTCACCGCCTGCGAGACTGTCGTAGCACACTC
CATTCGCAGCCGAAC
ACTCTGTTGCAGCAGTTCTAGTAGCGCATGACCCGTCGTTAACTG
TCAAAGCTTGAATTG
AAGAGACAAATGCAGCGAAAACCACAATTTCATTAAGTGGCTTCA
TTGTTGGGGCAGTTT
TCGTTAATCTTTTCCAGCATTGTCTTTCAG
>ConsensusfromContig300 Average coverage: 11.35
ATGGCGTTTTAATGGATTCAATGGAGTATTTTAAACGTTTACTG
GCTTCTTCTAAAGGC
ATTTCTTCTGAGTCGTCGCTATATTCTCTGTTTGTTCGTGTTTCCTT
AATGTTACGTTTC
AGGGCTCTTTTATGTACTTCTCTCATTTCGACGTCGTTATGGATGT
TTGAATCTGGAGTT
GGTGTCGCATTGCTATTTAGAGGAGGAATTGGATTTGGT
>ConsensusfromContig301 Average coverage: 16.70
TCAATTCCGATGCGATTCATGAATTTTTTCGTAATTCTTTTCCGGT
CTCGAAGAATATCA
TTTGTTGAAACTTATGATTGAACAATCACAGATGAATTTTATTTCA
TGAGCATTTCTATT
GTATAGTTGATCACTAGTTGATGGACATGAACGAGTCAGATATGT
GACACATATGATTAT
ATAATAATTATAATAAATACTAGCATTTTTA
>ConsensusfromContig302 Average coverage: 7.08
ACTGGCAGAGATTTTTTGATACTCTGTTCTGAATCAGAAATTTCC
GAATAACTTTCAAGA
ATATTGCATAAAGTTTGATCACATATCATTTTGTAGCCAATAATT
TGGTTCTCTCGATGA
TGCGCATAACTCTTAATCTACAATTACAACATGATACTGATTCCCC
ATGCTGATTTTCTT
TAATGCGTTTTCAGTAAAGTTATT
>ConsensusfromContig303 Average coverage: 13.46
CGAGATGCCGCTGAGGATGAAGTTGAAGAGTATTGGTTTGTATCC
AATCGCTGGTTCGCG

AAGGGAGAAGATGATGGAAAGATCGTGAGAGAACTGCTGCCGA
CCGATGAGAGTGGAAAA
CCTCTCGACACTGGACTGAAAGAGATAAATTACATCGTGAAGGTT
TTCACCGGAGATATT
TTCGGAGCTGGTACTGACGCCAATGTCTTCGTGAACATGACAGGA
GAATTCGGAGACACT
GGAGAGCGCCAGCTCAAAAACTCAGACCACGTCAATAAGTTCGA
ACGTAATCAGACTGAT
GTGTTCGAACTGAATGCCGTAGAACTGGGTCTCATCAGAAAGGT
CAAGATACGTCACGAC
AACAAAGGGGGCGGGGCCGCTTGGTTTCTGGGTCACGTGGAGGT
TGAAAATCCAAGCACC
AAGAAAATATTCTTCTTTCCATGCCAACGTTGGCTGGCGACCAA
TGAGGACGACGGACAA
ATCGCGCGCGAGCTTGTTCCAGTTGACCCTGCTTTGAAGAAAAAA
CTTTCCCGCCAAAAT
TCAAAAGCGATTCGCGATGAA
>ConsensusfromContig304 Average coverage: 12.34
CATCTTCGCCAATCAATGTAGTTACATTGAGATCATGATGCCTTG
TGTGTGATTATCTGT
GATTTCATCTGCGGAGGATGGGCGATAGATTTTCTGAAATTCGAG
ATCATCTTTCAGATT
GACCGATGATTATCGAGTTGATCCAATCAATGAAGCTGGTCACTC
GCGTATAAATCCCGT
ACTTGCCCCGCTCCCCACACCCCTCGCCGAAGCTCGTTACTCC
>ConsensusfromContig305 Average coverage: 7.09
TCTACAATCCACTGAGTGACGATGTTTCTAATGGAATGATGAAAG
AAAACTCGCTACTGA
GTGATAGAACTTCGGCCACCCCAAGAAGTAATCCCACAGCTAGGT
CGAATGCAGTTTCCA
GATCTGAGTCTTTCTACAAGAGAGCTGGAATTCTCTCGAGGAAGC
CTGCTGAGAGAACAT
TGACTTATGAGTTGAGAGAAGCCCATAGAGCAAAGTACGTCCGA
TCGAACGCCCCGCGCT
GGTTTGAACGAGAAGCTCGAATTGAAGATTTATTTGCTGGATCAT
AAGATTCTCATGGCA
ATCAGTGGGATATAATAGGTTCATCATTATTTTGTTATATAAAGT
CGTATACCAAGACAC
TTACATGCG
>ConsensusfromContig306 Average coverage: 7.97
CTTTAGCAAGGCAGGAGCACGAAAGTCTGGTTATGGCGACTGTAG
ACGTGTAGTTTATTG

GAGGTGTAATCGGAGTGGAAAGTATAAGAGTAAGCGTCGTGCCA
TATATGGACCTCATAA
ACGGACACTCAAGATGGATGGAGTGTGTTGTGCTTATATAAGAC
AAGTGATTAACCCCGA
CCAAAATTTTATAGAAGTAGAGAGATCGGAAGAGC
>ConsensusfromContig307 Average coverage: 25.73
GATCTTGGCAGACGGTTCAATGAGCAAAGATGATCCAGATATTC
CAGATACTGGAGCAGT
TTTCACTTTTGGCAGAAGTAAATTTGCAGATAATGTTGCAAACAA
ATTCTGGATTAGAAA
TGATAGAGTCGCTGATGTTTCTTGCGGCGATGGTCATACAGCCTT
GGTTGCAGAGAGCGG
TCGTGTGTTTACTTTCGGCACCAACGAATGGGGTCAGCTAGGACT
TGGCCACACAAGAGC
ATTGAGATCGGAAGAGCACACGTCTGAACT
>ConsensusfromContig308 Average coverage: 9.25
CTCATATAGGATTAGTATGGAACCGATCTATCTTTCTACCTCCCT
GGAAACCTTCCAAGA
CTATTTAAAAACTTTCCAATATTCTTGGTTTTCCTTCATGTAGAAA
TCATATTGTAAATC
CGCTGACACAAGATGATAAGTTGATTGGGTCTCGTCCACATTCAT
ATTCTATAAATGACA
TTAACATTCTTTTTGCAAATAGCTCCG
>ConsensusfromContig309 Average coverage: 6.86
TGTTGATGCAAAACATCGAGTACTTGCCGAATGCTCTACAAGGCA
GCACATCTACGATGT
ACGCGATCATGAATGAAAGGAGTCTTGAGACATACCGGTCGGTC
TTCTTCGACGAGAATG
CATTCAACGGTACTTTGGCGAGCAAGCATTCAATATGGCTCCAA
CACACAAAAATGAATA
ATACCAATCTGCAGGCATTC
>ConsensusfromContig310 Average coverage: 14.36
TTTGCATTTGCTTTTCAAATTTTTCAAATTTAACGCTCAAATTTCG
ATCTACGTTACGGT
TATCTTTATTTTTATTAGTAGTTGTACGATTTTCTTGATGTTTTGCA
ACCAAATTTTTTC
TGAACCCTTCAAAACCAATGCATTTTTCACTTTGCTCTTCATCGGT
TTCAGATACATAAT
TCTGCTCTTCGTGAACATTCTGTGGATGCTC
>ConsensusfromContig311 Average coverage: 14.34
AGTAGATTTCATTACGTAACAAATGTTATATTTTAAAAAGATATC
TTTTTCAAAATGTAG

ATTTCCTACGTAAAAAATTCTGCTTTTGGGGAGTGCCAACATCAA
CCCCATCCCAGTGCG
CCTCAGCAGACTTTCTGTCAGGCACCTCTTGCTCTAGAGCTATAA
TTTCCAAACTGATAT
AATAAAATAATCTAGAGTGTTAGGTGATTTCAAAATAAACGTACA
CATGTGGAATCTTTG
ATGAATATTTCAAATAATTTGAAGTCTTCGAATGATTGGCCGGAT
A
>ConsensusfromContig312 Average coverage: 11.61
TACGGTCAGAATTAAGTGAGCCAAATTCAGTAAAGACAGAAGGT
TAACATATACTAACTA
TTAACATTATAGGATGGAATGCTTCACGAGGAGATTGTGAGTTTT
CGAATCGTTAGAAAA
ATGAGTTTTGTATGGTAAAAGGAAATTGTGGCTTATATAATAAA
ACGAGTGATAAGGTGA
ACCTCGAAAGTTCTATGAGATTGTTTGCGTCAAAATGTATCATAA
GGAGATGAAATAAAT
GAAAATTCGAAATTATATGAATGAAAATTAAGGGAGCGCCC
>ConsensusfromContig313 Average coverage: 7.53
AGATCACTTTCATGAATCACGAACTCTCTGCCTTTCGATAAAGGC
GAGGCTTCCCTCTTA
TGAGCCGACGGTTGGCTTGGTTTTGAACTGGAATGAATGGGCTTA
GGCTTTGAGGCAAAG
CTTGGGGATCTATTCGTGCCGGCAGTTGAGGGTCTGAGATTATCA
GTGGTTTCAATCGGG
ATTGTGTCACGCCGGATAAATGGGTGGTGAGGAGTTGATTTTGAG
ACGTTGGAATACAGT
CCAGGAGATTCG
>ConsensusfromContig314 Average coverage: 6.07
GAAAACTTGAACAAAATATTGGAAACAGGCAGATTTTCAATATT
TGATATGGAAATAAAA
AACTCGAATGTTGAATATAGGATGAAGAAAGCGATGCAAAAATGC
TTTGTGTTCTTAATT
TTTGATTTTCATGCATATTATTCAAATGATCGTATAAAACAGGGT
TTCGTATATTTGAAA
TCAAAATCACGAAAGCCGAAACCG
>ConsensusfromContig315 Average coverage: 6.37
TGTATCAGAAAAGTGCATCTAGTATCATTTTGTATAATTCTGAGA
AGTACTGCTGATATC
ATCAGTCTGATTACGACTACCGAGTTGGAGATCATGATCTACATC
GTTGGATCAGCGTTG
ATGGATGGAATATACTTCCCAAACATGTTCTGTGTGATGCGGGAA

TTCACGTACGATGAG
CCAGCGCAAATGAAAAACGCTATAACGCTTCATCTGACATGCCA
GGCTATGGGTTGGATG
TTTGGATGCGGCTTCACAGGCCAGCTTCTCCGCATCACAGGTGGT

>ConsensusfromContig316 Average coverage: 7.35
CAGGCGGCAATCATAAAAACAGAATCGCAGATCCAAAATACAGC
CTCCGATCCCGATGCA
GAACATGACCCTGAGATGTCCTTCAGGCAATCGTCAATGGGTGAA
GGTCAACTTGAATCA
ACACTAGAGCCGTCCTTGACCGAATATGAGACTCTTCTTAGAGAC
ACGCTCAAATCAGTC
TTAACAAAGCACAGAGGGAGCGAGCAGACAGTCCCAGAAGACGC

>ConsensusfromContig317 Average coverage: 9.44
ATGTAACACTTGCGCATGAGGGGTCGTGTGAGGCTGTTAATATTG
ATGATGAATGTGACC
TCTCAACTTGTCGCTTTGGTGGACGATGTGGTGATAGAAATGGCA
TATCCAAATGTTTGT
GTGAATTCTCTTGCTCTTCGAAACATAGGTCTATGGTTTGTGGGA
CAAATGGTGTCACTT
ATGAAAATGAATGCAGTCTTCGAGCAGCTGCTTGTAGTCATCAAA
GTGACATTGCGATTC
AGAGCAAAGGACCCTGTGACTTACTTCTACGTACGTTCTGCGACG
GAGACCCGGCCGTAA
CAAATCCCACCACCGGCCGAACAGAGCTGTGTATCGGCTCGGGAA
ACTCCTGTCCATCGT
CCTCGCATTGCCATCGGGCGCTCGGCATGGCCGCTTGCTGTCCTG
ATGTCACCGAAGCCA
CGCGCTGCATGGAAACAAAGTTCGGATGCTGTTTGGATGGATTCA
CTGCTGCGCTTGGGC
CTGATA
>ConsensusfromContig318 Average coverage: 9.03
TCTTCCGATCTCTCTGCCATCGCCGCCAGCTTGGTCTGTACCATAG
CCTGCTTCCCCTCA
ACACTCTCTGGTTCTTCTGCATTTGACCCTCCAGCTTTTCGATATT
TCTTGTTGAGCTTG
TACCGGTTTTGAATGGACATCTCGAATTGGACCGCATGGGGGTTG
ACAATGTTGGCAGGT
TTTTCTCGATCCTGCCATTGGTATATGAACAAGCCATTGTGTGTC
GATGTTGACTGCCAG
CTGTAGATGTAACCTTCAGTCTCCTCACAAAAGAGACTTATGATA

TCTCCCACGCACAGA
AAGTCACTCATGGTGCCGGCCTACGCGGAACCACCTCCTGCTTCC
TCACGGCTTCATCAA
TTATTGTATTCTATGTCGATTCTATACTTTAATGATTTATGAAAAC

>ConsensusfromContig319 Average coverage: 9.17
ATGAAACTTCATTCGGTCCTGTCTGTATTGTATAAAATGAAGAAA
GTAGTTATTTTGTAA
AATGCAAACATGAGTTATACAGATAATTCATCATAGCATGCCAGA
TGTTGTCACAAATTG
AAACTATGAACAAATTGCCATTTTAATTCGCATTGAATTTGAAAG
AAAACGGTTCGATAA
TAATTTATTTGATTTGTGGCTTCCATCATAATCAATCAATCTGACG
AATAAATGTTTTTG
ATTGATACGATTTTTGAAAAAAG
>ConsensusfromContig320 Average coverage: 4.28
CTTCCGATCTGCAACCTTTGCTCCCAATCTGATTAGCCTGTCTTGA
TACTTTCTTTTTTA
TTGTCTTGGAGCGTTTGCTTTTCCATATCATCATTACCCAGGATGG
AATTGATATTTCTG
AAGTCATGGCGTTTTTTGGTATTGTTTTAAATATGAAAATTTATG
AGAATATTATGTTCA
AAATTATTCCGGATACAGTACATGCCATAATTGATTTGTATGATG
AGCCATCA
>ConsensusfromContig321 Average coverage: 7.08
TACACGACGCTCTTCCGATCTCAGCGTCCCGTAAAAGGCTGGGCC
GAATCGTTTGAATAA
AATGATCTAATCTCGCTTCTCTTTTCCTGCTGAAAGCTAGCTCCTG
GTTCCACAATTCCC
ATATTGCCAGGCCCAAACTGTACATGTCTGAAGATTTCTTGTAAA
TCTCTCCTCTAAGAA
CCTCTGCGGGAAGGTATACATGGGAGTTGGCGGGAATGTCTGCAG
CTGTTGGAATTTCTG
CCACGCGGGGTTCACCAAGGTTTACTAGTTTCACCACTTCTCTTG
AAGTTGTAATCATTA
TCGTATCGAGTGATAGCTCCAGAGACA
>ConsensusfromContig322 Average coverage: 23.81
TTTTTTTTTGTGTATGATAATAATACTTGGAAAAGTAAAACGTTT
ATATCAATAACTAGA
AATTCACGACTAGAATAATTTCGAGTCTACATTTTCTATTTACAA
AATTAAAATCAAATC
TCACCCAGATTCGCAATAAAAAACAATTGAGTTTAATACAGGCAT

ACTTTGTAAGATTAT
CAGATTATAAAACTCCGATTCATTCATTGATCAAGATCTTGGTGT
A
>ConsensusfromContig323 Average coverage: 10.86
ACAACTTATCTGAAACTTACGCTTACCTAGACGATATTACTATTT
GTGGTAAAACCCAGG
AGGAGCATGATCATAATCTAGCGAAGTTCAGAAAGGTAGTAGAG
AAATATAACATCCAAC
TCAATCCTGCCAAATGCTCTTTTTCTACTTCTGTTGTAAAAATTCT
GGGATATGAGATTT
CTGGGGGGGAGCTACGCCCGGATCCTGAGAGACTGCGGCCCCTCC
TTGAATACCCACTTC
CTTCCAACAAGAAGGAGTTACAACGCCTGGTCGGCATGTTTTCGT
ACCACAGCAAGTGGA
TTCCAAACTTCTCACAAAAACTCAGACCCTTAATAACCGAGGAAT
TCCCGCTCCAAAATG
ATGCCTGTGTAGCGATTAAAAGCTTAACTGATAGTATCACTAAGG
CTACACGATTTTCCA
TTGACGAGAACGCGCCTTTCACTGTAGAGACAGACGCAAGTGAT
CATGCGATAGCG
>ConsensusfromContig324 Average coverage: 10.22
ATGAGCTCAAGTTTTTTCACCCAAAAACCACCCCCAATGTCGAGG
TGTTGAGAACTTTGA
TGCTCTCTGCTCGCATCCCCATGTTCCTACAGCCTGGAAAGCTAA
AGACTGGTAAATCAA
CACATTACTACATTGATGGTGGTGTACTTTGCAACTTCCCGATTTT
CGCATTCGATGGCT
GGAAGCTTTCAATGAAGCCGAATGACTCGTTCTTTCTAAAAATAA
ATTCTGGGGAGAATC
CATTCAGAGGCTACAACAGCAAAACCCTTGGCTTTATGACTTTC
AGTGCAGGGGAGAACG
AAATCTATATGGCCAACTTTCTCGCCAGGTACGACTGCATGGGTA
TTCAACGTCCGGACA
CAAAATACAGCAAGGAGATCGGAAGAGCG
>ConsensusfromContig325 Average coverage: 9.99
TGTTTATATTATATTCAGAGCAATCCGATATTTTATGTCAACTGAT
TACCATTTGATTAT
CGAATAAAACTGAAAATGGCTGACGTCCGAGTTTCCAGAGCGCCT
AGTTCCATTGGAGGT
AAACTGACCCTGCCCGCAATCGAACATCCAATGTCAAGGATGTCT
AAACCCGAATATCTC
GACGTCCGCGGCTCCAGATCTGGATCTGTCATGGAGATTGGGGTC

ACCCC
>ConsensusfromContig326 Average coverage: 7.39
AAACTATTTTACGAGGCCCCATTGGTCACAGATGGTCACGTGACT
GGATGGCGCACGTGG
TGGATGCCCAATAGGAAGTTCCCTGTCGATATGGCTGGATTTGCT
ATTGGCTTAGATCTT
ATTCGTGATAACAGCGAGATAGTCTTCCAGAATGACGCATGCGT
GGGATGTGTGGAGACT
GCGTTTATCGAACAATTCGTGTCTTCCCTAAAAGAATTGGAGCCT
CTCGCTGATGATTGC
TCGAAAATT
>ConsensusfromContig327 Average coverage: 12.73
TTTATTCTTAATATTTGATAACATAGCCTGTTTGTTTGTAATTTCT
GCTGTTTGACAAAT
ATCAATGAACTGTTGCATTGCTATTGGAGTTTTCGATAGGTCGGTT
TGTGTTCTGATATT
ATCAACACCAATTACTGATGAACCTCTGCTGTTTTCGTCTGTGCA
TACACTGACAAAAGT
TTAAATCTTCGCTAAACTTACGCACGTCCGATAGAGATTCTCTGA
ATAGTTC
>ConsensusfromContig328 Average coverage: 5.45
ATCAGATCCGATTCTCGAGGAAATTTCACATCAGTTTCGTTCTTG
AGATGTAAACAATAA
TTTTCATAGCGTTGAAGTCGTGATTATTTATATACCTCGGATAAT
TAGATTGTCAAAAGT
AATATATTTTTGGGAAAAATTTATTTGCTTGCTATTCGACAGCTT
CGAAAATATCTGAAT
ATAGTGAAAGTTCAAGAAATCTCAAAATCAGACTTAAAAAAAT
>ConsensusfromContig329 Average coverage: 15.71
GGAAGTCTGCAACAAGAAGTTCTCCGTCATCTGATTGGTAGTCA
GCACGGAAGGACAGAG
TTTTATCTGATGCAAATAAGCCCGAGCCGACATAATTGTTGGCGG
GGTCATGCAAATTAC
TGCTGAGCATATGAACCCCAGTTCCAATAGCGAAACCTTGATGA
AAAGTAGTTGCTGAGA
GATGAGGGTCTTGGTTCATCCAGGCAGTC
>ConsensusfromContig330 Average coverage: 8.25
TATCGATTATGAATAATCATCTGTGAATATCTAGGAGGCAGTAAT
AATGGTGTTTTAGTA
CGCATTGTCATGTTTCATACCTGTCGTTCTTCTTTTTATAATCATG
ATGTTTGAAGATTT
TACTCAAAGACCGATTGATGTAGCTGTTGTTTGCTTATTTATTGA

TCTATCATGTGGGGC
GTATGTGGTTTATTTCAAATTTTTGTATTATAAATACCTATATATG
TGCCTGG
>ConsensusfromContig331 Average coverage: 15.17
GGCATTTTCTGTACAATGAACATTATGTAAAATAATACATTTGCA
TCAAAACATGAAATG
CATCTGTCGGTAAGTACTAGAGAACAAAAAAACAAAAAGGACAT
TACATACAGTTAAATA
TCATGTGACCATCATATGATTGTGAGAAACAAAGCTCAATGGAT
GAGAAATAAAGGACTA
CGATATATCATAGAGTGATATACACACAATGAGACACTTCG
>ConsensusfromContig332 Average coverage: 9.24
AGCCAACAGTTGGCCTCGATCCGTTATTGAGACGGAAGATATGG
GATCACTTGAAGGGTC
TCACTCTGAAGAATCCTGTCTCAGTGATCCTCATAACTCATTATG
CCAATGAAGCCGAGA
GAGCAGACCAGGTAGGAATAATGCATAATGGTCAAATGATAAAA
AAAGGGAAACCAGACA
TTTTAATGAATGAAGAAAATGTTGACACATTAGAAAAAGTCTTCA
TTCACACCGCTGGTA
GAAACAGGTCTTTATGCACTTCAACTGAAAATTCTTGTGCAATTG
ATCCCCCAAACGCTG
AAATAAATCT
>ConsensusfromContig333 Average coverage: 5.82
ATAATCATTTGAGTTCACACGGATTTCTGGAAGATGAGACATATT
CAAGAGTGGGCCTAT
TAGATGATACTCAGAGTGAAGTCGAAATCGGTTGTGATACTCAAT
GGTTGGAAATAGTTG
TGGAAAATAAGAAATATTGTACATATATTTATACTTTGTGTTGGA
ATAATCAAGGAACTC
CAGGAAAACCCGGCAATTGTACCTCGAAAGAACAGGTTTTGTTTA
TTTTCAAGAATATAA
AAAAGATATTTGTGGCTTTTCATAATCATTTCAATTTATCTGAGG
ATGATGGTGGTG
>ConsensusfromContig334 Average coverage: 5.56
CTTTTCTAAATTATTTTTCAGGACTATTCTTTTTCGAAATGCTGTT
GTTTCGGAGATATC
ATATGGTGAGCCATTTCTAGAATCATCATCTCTTTGATTTCCAGA
CATAGGTGATGGCCT
TGTTGCTTTGAAGTTCGCATCAGTATTCTGTCTTTGAGACGTTGT
TGGCAATCGAGGCAG
TGAATTCGTCCCCCGATTTTCTCTTAATCTTTCAATAGATAGGTTC

TGAATGTCTCCCAT
TTGTTCC
>ConsensusfromContig335 Average coverage: 7.35
CCGTGGTCGAGCGAGACGTTCATCACGATCTCTCTGATGGCCAAA
CGGGTATTCTTGCAC
TCTTTGAAGGTGTAATGTGTTCCAGAAGTTTCGGCAATCAAAGCA
TTGCACGATTCCTGT
TCGGGATGAAGTGTGCAAGTGCCGTTCGTGTAAAAGTCCACCGCC
TTGCACAATTGGCTG
ACTTCGCATGTTTGAAGACAGTCCTTTGCATTTGCAACAGGGATC
GGGCTGCCCCCTGTT
ATTGCGTAGCC
>ConsensusfromContig336 Average coverage: 14.26
TCAGACGTGTGCTCTTCCGATCTGGGTATTATAAACATGATGCAT
AATAAGAATGTATAA
CATGAGGGAAGCTTTGGCCATTCGTACGAAATGAGGGCGTGCCCT
CACTTTGTTTGTGAA
GACAGACAGGCAGGCTAGTGGATGCGAAACTTTTTATGAACGAG
ACGACATGAAAACATC
AAGATACCGCGTATCGTCAATCCAAAAGTTCAGTAGCAGCAACA
GAAATAGGAAAGTGGG
TCATCTTCAATTTACATCGTATGAACTGAATAGAATTGAGTGATT
TTTTCGTAATAGAAA
TCAAGAAACCGTTTG
>ConsensusfromContig337 Average coverage: 11.62
TTCATCTGTCAAATCACTTTTAAAGTCAAAACATGAATCTCCTCC
GATCTCATCACAGCT
AAGCAAAGTTTCGTTATCATCCCGCGAGAAGGAGGCTGATTCTGC
CGTATTCCATTCTAA
GATGAATTTGAGTAAACGCATCAAACGGGTCCAGTCATTTGGTTT
CCGTCGTAGGGAAAG
TAGAAAAACCCGAGCTAAGAAGGGTTTGAAGAGTTTGAATGAAG
ACCCGCGGCTGTCGAC
TCTATCCATCCCTGATGCCCTGTCGAGATCTGGGGACGACCGACC
AATTCCACCGCCAAG
GAAAAAGCGCGGGAAGCATGGAGGGATGGAGGAAAAGTCCGTCG
ATGAAGACTATGGCAT
GAATGTTTACTCATCC
>ConsensusfromContig338 Average coverage: 9.41
CAAGAGCCATGTCCTCGAGGCCAGTATCAATCCGCTCGTTTATCC
TCTTCTTGTGAACAA
TGCTTACAGGGTTTTTATTGCAATACTACTGGAACCATTGAAATT

GAGATATGTCCGAAA
GGACATTATTGCCCGATCGGAAGCGTCGTACCGATACCATGTCCT
ACTGGAACCTTTGTT
GACGAATTTGGGCGTTACGAAGAGTCTCATTGCACTATATGCACA
CCTGGTTATGCATGT
GATACCGTCGGACTAGCAGTACCCTTGACAATGTGCACTGCAGGA
TTCTACTGTCGTTCC
GGTGCCAATATATCCAATCCTATCAATGAAGAATTCGGTGACGTA
TGTCCCCCCGGCTAT
TTCTGCGAAGAAGGGACGTCAGATTATTTGACATCGCCCTGTGAG
AACGGAACATACTCA
AACTCGAGTGGTAATCAATCACCAAATGACTGTATCCCATGTGAT
GCTGGTTTGGTGTGC
TCCGGAACGGCTCTGCTGGAACCAAATGGGATTTGC
>ConsensusfromContig339 Average coverage: 10.23
GTTGGCAGATCCATCATTGTGACAGATCTATCAATAGAATCTGCC
TTTTTGAGATCCACC
GTTGATTCTATAATCGACATTTGATCCTGGGCCATTCTTTTTCTTC
TCTCTTCGATCTCT
GTTTTCTTTTTCCGATCCTCTATTCGTTGGACAACAACCGCTTGTA
GAATCTCGCTGAAG
TTAATTGGCCCATCGCTTTCATTCAATTTCCCTTTCGCCATTATGC
ATTCGAAAAGTTCT
TTCATCATTGCTGGTTTCCAAATTATTCCGAA
>ConsensusfromContig340 Average coverage: 4.65
GATTATTACCTGATCGGTGATATGTCATTTTATGAGGGGAATTTG
TTTGTTTGGGAATAT
CAGAAGGTCACCCCCTATGAGTCATCAACAATCCATGGAATAGA
GATCTTCATGAAGAAC
ATCACTGCAGTATACGACATCTCGCTGACAATTCCTAAGGAGCAT
TTTGCGACGGCTGTG
GCTTCACAGTCGAACTATGAATACGATCCCTGCTTGACTAAAC
>ConsensusfromContig341 Average coverage: 6.82
TTAAATTTCACATCCTCGTAGAGCAATGAAATGAACACATCATG
AACAACAAAAATGCAA
ATTGGTTGTTTCGAAATAACAACAATCACAACCAGAAATAAAAAA
AACTACCACTTTATG
ACCAGCAAAAATGTCTGTTTTGTGTTTTCAACAGTCCCCATCTAG
CACTGAAAATATAAA
CACAATGAACAATAGTATAATCAGCAGGGGGGATAAATATGCGG
GGATCCTCCATTCGAT
GTGT

>ConsensusfromContig342 Average coverage: 10.81
ATGTAGAATTGTGTATCAGCCATATAATGATCCTTCAAACGCTTGG
TTTACTCTTTATGT
ATACCAGATTAGGCGTATCTGGGGAAATAAATAATTCACCACCAG
TTATCACTCCCGACC
GACCTGTTCTTCTCAGTTACTACTCACTACTATACTATGATATAC
CAGAAGAAGATCGTA
TAGACATGTTGCCGGTTTTTGCGGATAGGGGCATATATTATTTGT
CCACTTATTCTGACA
GTTTCAAGATCGCTTGCAATA
>ConsensusfromContig343 Average coverage: 7.39
GTCAGTAACACTTCTTTCTGTTCCCAGTAAATATTCTGAGATTGG
GTTTCAATGGCCAGC
GTCATGACAGGTAAACTTTTTTTTGGAATAATCCAGTGCATCGCT
TGAACATAAATACCA
TTCCAGCTGCTCAACACACACTGGATCTTGATTGGTTCGCTATCT
TGGTCAACATCATTC
ATTGTGAAGTCTAATCGAATCCCATTGAACACACTGATCGGAAGA
G
>ConsensusfromContig344 Average coverage: 11.24
ATTTTTTCATGATATTATACATACTCATATTAGTATCGCATTTTGT
TCTGATAATTAATG
CAATTGACAGATCTTGGGACTGATAGGCATCAGTGATGAAATCGG
TTATATTTTATCTCG
TGTTATAGAGTTATACATTATATTCTAAATTTGCTAATCCATGTA
TAACCTCATCGATAA
TGTTGAAAGGATCTTCATATTCATGTGCAGTGTAT
>ConsensusfromContig345 Average coverage: 8.17
CGAGCGAATCACAATGCGGTCGCATAATCTGCCTGGCCGGTCGTA
GAATCTCGCGATTAT
CGTGTAGTTTTTGATCGAAATAATTGTGCGACCGGCTAAAAAGTT
ATATATTTTATTTGC
GATAGCAATCAAATGAAGTTTTGCATACATTATGTGAAATGACT
CTGGAAGCCAAAAATA
ATGTTTTTTTTCTCAATTTCGAAATATCACAAGAT
>ConsensusfromContig346 Average coverage: 9.20
CTTTCCCTACACGACGCTCTTCCGATCTCTTAGAGATCTAGCCTCT
CCACTGGGTTTCGT
ATACGTGTAGAGCGTCTTAAACCAGGCTCCGCAGGATCAAGAGA
GGGTCCAGGACTGTTG
ACAGGCACAGGGGTCGAAGGGGCAGGACTATCCTCAACTTGATTG
TGTTCCTCTGGGTTA

CTAGAAAGATGCGGAGGAGGAGAATGCTCTTCAGGATCAGGAGG
CGTAGTGCGAGGAAGT
GG
>ConsensusfromContig347 Average coverage: 6.88
ATGTATACCCAAAGCTCAAGCAGTTTTGCAGAGACACCTATGGCC
TAGAATTCCAGCTGA
TGGATCTTCGATGGGGAATAAGGGAAGAAGCACACGACGACCAG
ACGATCATCGACTTCT
GCCTCCAAGAAATTGACAACTGCAGGGCGACATCTATCGGGCCTA
ACTTTGTCGCCTTCG
TGGGTCAGAAGTATGGCTTCCGCCCCTTCCCTCCTGCTATTGCCG
CCAGCGAATTCGAGG
CTC
>ConsensusfromContig348 Average coverage: 7.22
GGACCGGACATAAAATGGAATCATAGAATAATACAATTTCATAA
TTGGACCGGAATAAGG
AATGAATACTTTAGATCGGATCGAAATTAAGAGCGACATTTTTGG
TCCATGCCTAGCACT
GGTGCGAATATTTCCGCTGTATTTCGATATTCCCGTTTCGTCTAAT
CCTCGCATCGTCTT
ATTCCGGTTGAAGCTTTTTGAGCATCGCATCAACATGAATCTGGC

>ConsensusfromContig349 Average coverage: 10.38
GCAGGGTTTAATATTTTATTTAAATCACTCATTAATAGCTTCCTA
TCTACCGTGTCAAAA
GCTTTGCTCATATCGAGCATTATTATATTTGTGCTCTCGTGGTCTG
TTGTTAAGGCAGTT
TCAATAAGTGTTTTTATGGCGAATATTTGCTCTGTAGAGCTCCTG
CCCTGTAGGTAGGCT
GCCTGATCGATGGATATTTTTTTGGATAGTTTTTCTGCTGTTCGAT
TTATCATAATAATG
GCTAATATTTTCCTTAGG
>ConsensusfromContig350 Average coverage: 8.21
CGTGACTCCGATTCGAGTAGGGCGACTAGAAGCCACGCCCCCAG
AGCGGGGTCTCGGAGA
CGGTCCTGCCGCCGACCCGAGCGGATTCGAAGAAAAGTTGTAGC
AAGCCGATAATGATGA
TGTAGATGATGAAAGCATGCGGTAATTGGATGCGACGCTAGTATT
TATCGGCGCTACACT
ACCGACGACACTACCCCTAGTGTGGGCACCACCGCCCCTTCCCTC
GGCCGGCG
>ConsensusfromContig351 Average coverage: 5.05

GCATAGAAGACGACCAAGAGGATGAAAAAGACGATAGCGATTCT
TTCCCCATTTTGAAGC
AGAAACTGAATGAAACAACTGGACGAGATCAGACTATCTCAAGA
GATGGTATCAAAAACG
AAAGAGACGTACTGAAATCTGATGACGCTGTTGAGAAAACGCAA
CATGAGAGTTCAGTTG
AGTCTAATAGACAGAACACAGCTCAAACTGTAACA
>ConsensusfromContig352 Average coverage: 8.27
GCTCTTCCGATCTGCCAACTTCTTATGATCATACTCGAATCTGAA
ATGATACCACTGGTT
TGGATATGCTCTGGTTTGAACATGCAAACTTTTTATACGATCAGG
CCTTTGGATCTTGAT
ATTAAATGTGATAGCAGATTCGAAACTCGAATATCGGGACCGTTC
TGAAGTTCCAAAAAT
GAATCCTCCGTGTTTATCAGCATCACGTTTGCACCATCCGTCGAT
CACAAGATCTCTGTT
CGTCACAGCTGGGTCTGC
>ConsensusfromContig353 Average coverage: 11.93
TCATCCTCCTCATCAATATCTGTTTGAGTCAGTTTCTGATGCGCAG
AAATCTTCACTTCA
TTTATCCTTCGACCGCAAGCTGGAGCATTTCTACTGTCGCCAAGA
GGGGAAATTTGTGTA
TTTGATAATGAAGCAGATTCTGAAGATTCATCAGCATAATAAGT
AGATACAACAGCCGCA
CGCTGCGTCTCCATGCTGTTGTCAGCAA
>ConsensusfromContig354 Average coverage: 8.23
GTCTCGTCACGGAGTGCAAAAAAGAGAATGGTGTATATAAATGG
TCGAAGCCCGATGCCG
CTTGCATCGACAGCACCAAAAGCAATGCCAGCAGCAACAACAGC
GACAGCACCAACCACC
CCTGCATGAACTCTGCGAAGAAAGAGATACTTTTCTTTGACATGG
GCTTCAACTGCATTT
GCACCAAGAAATGGTCTGGGCCCCGCTGCGACAAACCTAACA
>ConsensusfromContig355 Average coverage: 6.67
GGCACTGGGTGGAAGGGACTGGCCACCAGCCACCATTCCGACAT
TCTGGTAGCAGCTGTC
GTATGTTCCCACGTCGATGGGGAAAGGTTTTACTTTGATGTATCC
GCTGCAGTGGATTAC
CTTGTACCCACCGGTTGTGAGACCAGCATTCCGTTTGGCCAGTAC
ACATTTCATTCGGAT
GAAGAACGAACGCTCCACTTCATATTCTCTGATGACATGTGTTGG
GAATGGTGGCTTTAC

TGTGAGAATCGCAGCCATCTCGTCATGATCAGCAGGATGCACGTA
TTCATAGATGCTGTT
TCC
>ConsensusfromContig356 Average coverage: 4.87
TATTTGTGTGATAATATGTGATATTATTTATGTATTTGATAGTGTA
TCACTGAGTTTATG
AGTCGATTGTTCAAATACTATGGATTAGAAAATATACGGTGATAC
AAATTGGATAAGTCA
TTTGTTTTTATATCCAGAAAAAATACAATTTAATGTATATGCAAT
TGAAAAATAATGTTT
CACCATTTTTTAACGTTTTTTAAATAGTCTACTTTATTCTACC
>ConsensusfromContig357 Average coverage: 5.74
ACGTGTGCTCTTCCGATCTCTCACTTGTCTCCGCGTTCAAGGTCAA
TTTGAAGGCGTGTT
CGGATCCGGATCGTTCCACTCTTTCGGATCTCGCTCGGATTTCTG
CGGCGTTGAATGAAT
AGCAGTTCCCGTAATCGTAGGAAAATGTATGTCGGAAATCCTTGT
AGGAGCATAATTCCC
CATTAAAAGAACATTCGAGAAGCATGTCATGTATTTGATGTCCATT
CGCTTTTCTTGTTT
CTAAGGAATGATCATTCGCAAATGTGTCTTTAACTTGATTCCGCA
AAACAACATAGTCGT
CTCTTTCCGATTCTCCGGGTCGCGCATGCGTCAGCCAACTTTCGA
ATTCGTCAGAAGGT
>ConsensusfromContig358 Average coverage: 3.76
ACCAATTCGGGATCTACATTCGAGATCACAGTTGATACGAACAGA
TGCATTGGGGACATT
TATTATTGCTTTTCGGACATTTCTTAATGACTTTTGGACATTTCTC
TCTGCTTTTCGGAC
ATGTCTTTGATTCGGGGACATTTCTCATTGCTTCTCGGACATTTCT
TAATGACTTTTGGA
CATTTCTCATTGCTTCTCGG
>ConsensusfromContig359 Average coverage: 5.58
CAACGATGATCCTGAAAAAAGCAGATGAAGAAGTAGACAAAGTC
AGACTTCCTCATGGTG
AGATGCTTGACATGATCGATGACTATGAGACAAGACATCCGAAC
ATGGGAAATACTCTGA
AGACGGTGTTTGAGAACGTTCGACGTCCCCATAACATATGGCCT
GGTGATGCTGAAGGGG
TCCATGAAAAAATGAATCTTCAATGAAAGATTTTGTGCTACAAAA
AATAGTTTGCTGAAA
TGAATTTTGGTTCGCTATTTCCAATAGAAGAAAACGTGTTGGTTTT

TTTCTCATTTTCAG
TTCGCACGAATAATTTC
>ConsensusfromContig360 Average coverage: 14.25
TTCGTAAACAAGTTTTTCCTTTGTATCCTCTTCGGCATATGCATTC
GAACCAGTCCACCC
CTCTCTTTATGTCTTCTTTTGTTCTTTGGTTATTTCGAAGGCAGTG
GCCGTTATCACAAT
TACTTCGGTCACATTTTGGTCTTGGATCTGGATCTTCATTGTATCT
AGCGAAGATATTTG
GGGATTTGAGTACGATATAGAAAATTATATGGCACAAACAATTCA
TTGGTTTATACCACC
TGCTGTGATACAGCAGAAAAATTGAT
>ConsensusfromContig361 Average coverage: 9.25
GCTGCCACCACAAGTGTATTAATACGCCGGGCAGCTACCGGTGC
GAGTGCGCGTATGGAT
ACAAACTGGATCACAACCAAAAATCCTGCCGCGACTTCAACGAG
TGCCTGGAGAAGACAC
ACCAGTGCGAACACGCCTGCTTCAACACGAACGGATCTTACGTG
TGCGCCTGCCGGGAGG
GGTTCTCCATCAACTCGGATGGGTACCACTGCGAGATCGGAAGAG
C
>ConsensusfromContig362 Average coverage: 7.19
GATCTTGGGCAGTTGCGGATTGTAACTGGTCCAGTGTATACAGAC
AACAATGATCTAAAT
CACATGTCGGATTACAACTAGTCGCATAACTTCACTAATCTTGTT
ACCGGGTGATCAATC
ATTTATCCCTGAGTCAATGTTAACATGAACATATCGAGGCATGTT
TATGGTGTGGGCTGA
CAAAGGTTTTCGTTTTATGTTGCAGGTTGGCAGAAAGCGAAGTGT
TGTTGTCGAGGCAGC
CTCTGGAGAAACTCTGTCCGCCGAGATGCTTGAGCAGATCAAAGT
AGCGGTTGCAACTGC
GGTGGCTGCTGCTGTTCCACCCCAACCTGACGCCCCCTCCGATGC
GGTTTCCG
>ConsensusfromContig363 Average coverage: 7.55
TAGAATTTACCATAAACTATAGTAGAAAGAATCGAGTTGTCAAAG
GGAAGTTTCGTTTGA
AAGAAAACAGAATGATTATGAGACATAAGACCGGCAATTCACGC
TACTGCTACTATTATA
AGTTTATTATAATCAAGGTGTCACATAGACTTCACATAATAAACT
TAACACCTCGATTGC
TCGATGTATTTTAGGAAACTAGGTTATTTAAAGAAATATAACT

>ConsensusfromContig364 Average coverage: 16.66
TCCCGAGGCGACTGAGACCATAGAACCTGAAATCGGTCCAGCAT
CCGGCTCCGGGGATTC
AACTTGGATTTCTGATCCAATTGGCGATCCTCTCACCAATCTGAC
ATCATCGGATTCCTC
AACTACAGTTCCTTGATCTACACAACCTGATGTCTTCAATCGCTC
GAATATATAACGCGG
ATTTTATTCAAATTTTTCATTCATCTCACTCTATTCTATTTTTCCGA
CCACCGCATTATT
GATCTTCC
>ConsensusfromContig365 Average coverage: 8.89
CTGACTCGACTGCCGAATTTTCCCAGAGAGATTTTAATCCTATAG
AATCAATTCCTAATA
ATAATGTGCAAGAAGTCTTGAGCATGGAAACTGCAGATAATGAA
TTGGTTGAGAATCAAA
ATGTGAACGATCAAACAGAGAACTTTGACTCGGATACTGTTTCAA
CGGAAAAAATTGTTC
ACCCGAATATGACTGCTATGAAAACCGAAT
>ConsensusfromContig366 Average coverage: 47.58
GAAAGCTTCGTTTCATCGCCCAGTTTTTGTTACAAATTTAAAATTC
AACTATATTCCAAT
TATCGTAATTTCTGTGAGAAATATTTTGAAATTACATATTTGCATC
TCCGGACTCTGTCA
TAGTGTGTGAGGTTTACGGTCTCGCATCTTCAACTAAAGCATATA
ATAAGCAGATCGGAA
AGCACACGTCTGAACTCCAGTCACATT
>ConsensusfromContig367 Average coverage: 5.90
GTATACCTTATCCTCCCGTGGAAACCTATGCTATGTCTACAGCCG
TAATCCCGAACCACC
TCAGTTTTTCAATTATGAAAATGTCTTCTATGAAAATTCACCAGT
GAACATGACAGCACG
AGAAAACAGAGGGAAAACTTCAAATATCGCTGAATGCCGAGACT
TGTGTGAATCTGACGA
GAGCTGTGTTAGAGTTTCGTTCCATCTT
>ConsensusfromContig368 Average coverage: 8.37
ACAAAACATGCGTACTGGCAGTGAAAACTCAACTCTCAATTGAT
GGAGCCTGTAAATAAA
CTTTAAAAGCGTTAGAGCGCCATCGCCCCATAAGCCGGATCTGGG
CGTCAGACTTCCCCT
CTAACATTGCTAAGGACGCACCGCCGATTCGAAAACTATGCGGCT
TGTAATTAGCCGGGT
CCAGCCCTGCCGCTCGAAGACACGCCTGAAAAACCTCGCAAAAG

CGGGCGCTATGAATCG
GACGGTGTCGTTCGTCCCGG
>ConsensusfromContig369 Average coverage: 10.40
CTTTCATATTTACGTTTTTACATGATTGAGAGATGAAGCAATATT
GACAAATGATTTGCC
AAACTATTGATGAGCTTATATTCCCACCATTCCAAATTATGTATA
AAAAGTATTTATTTT
GCTCAAATTTGTTGGGATTTGTGATATTGGGAAAATATTGTCTCT
GTTGTTGATAAACCT
GAATCCATTCTACGGTGCAAAATTCGAGCGTCTTGTTCAACATAG
ATTAATTCTATTTTG
TTAGAAGTGCTGTTTGTAGTTTTTAGTTTATGCTAGTTTTTGTCAT
TTTATGATATTACT
GCAAACCCAG
>ConsensusfromContig370 Average coverage: 4.93
GTGCATTTTATTTTTTCACGTAAAAATTGAACTATTTTCACAGAT
TATCACTCAAACCGA
TCCATCACAAAATCAAGCATTATTCTTCTGATCTTAGCATCAAATG
TCAGTGTTTTGTTT
TATTCGTTATTGTGAAAACTCACGTCATTCGAAACTGTATTATTAT
GTTTGGTACTGTAG
AAATCTGAGATTATTTCCACATACTTACAGAATCA
>ConsensusfromContig371 Average coverage: 7.87
ATATTTAATAATGCAAGTCTAGAATGGTGCAGGGCTATTTTGTGC
AGAATAGATTTATGA
AATGTGTTACATGAAGTGTGGTATCAAAAGTATTCAGAAGAAAA
ACAAGCGGGAGAGCGT
ATATAACAGCAAAAATATAACGCCACAGATTTCACCGACATTTTT
GAGATTTCTGGCAGC
TTTTCCATGTATTCGTGCCGCAGCAATATCCCCCCGTTGGTTGGCA
TTGAGGGTTTCTAC
GGACTTCCATATTCCGAAAATCCCAATTGGACAACAGCAGAACAA
CGTCACTAGGATATT
CCAAATAAGATTATCTTCCTGAATATGGCCCTCTAC
>ConsensusfromContig372 Average coverage: 13.18
ATAATGGTTGCATTTCGAATGATGATTTTTCATTAAATTAGACGT
AAATGTTTGATTCAT
CAAGATGTAATTTATGCTTATATATGAGAAGTAATAAAGTACTCA
ATATCGTTTAAAAAC
GGTTCATTGAAACCATGAAATCAGTTTGAAATTTATCATATCATG
ACCCCTTTATAAGGG
TTGTATCGGCTTGACTGAAAAGCAATAATTAATATCAGTTCATTTT

ACTCTCATCTATGT
TTAATCGCAAGTATAGTTTTAATTGAAGTGAAATCGAATAGATAT
GAAATAAATCTCGAA
ACTACTT
>ConsensusfromContig373 Average coverage: 10.54
CGTGCCTGGATTTTTTTGTACAAGAATATATGTTGGAAGTATGGA
GAAGTATATTATTAT
TACTATCAAAGACAATCAGCATTGATAATTTTCTCAGATCCATCA
TACTTATAAGCAAAC
TGGTAGTAATATATAAAATCACATTGTTACAATTTCTCGACACAA
TATTATAATGAAACA
ATTAAATAAAAACGTTCAAAAACAAACAATATCAAACAATTACG
AAATACGATTAAATCA
ACTTGTAAAAAAC
>ConsensusfromContig374 Average coverage: 11.30
CTTAAGTGTACACGCAAAAACGGTGAATGCTCGAAAACTTAAGA
TAAGTTTGTAATAAAC
ACTTCACACATGCGTGACAAATGATGATTAAAACAGTAGTAAAA
CAAGGATACAAAATTT
TATGTGTTGGTAATTCAGTTACATGGAAAACATGCAGTGTATTAC
AAATAGTGTTATGAG
CGGGGAGATATTTGTTTTTAACGTATTCA
>ConsensusfromContig375 Average coverage: 5.24
CCAGAAAGTACAGCAGAACTCTTTGTTGACATCGCCACAGCAGT
AGCCGAGGAGCTCAGA
AGTGCCATTGTGGGGGCAGATAAACCACCTGTAGAACTGCGGCTC
TTTTATACGTGGAAA
TGTATCAATCTTGCGTTTGAACTTGCGATCATTTTCACAGATAGA
GGGCTCCCTGGCGGT
ATTTTGTCGAAAAATAGACGACTGGATATAGTATTCATACCCGGT
GGAAAG
>ConsensusfromContig376 Average coverage: 8.92
GAAAGAGAGATTGAACGGTCCCACATATTTTCCCACATTCTTCA
GGAACGGCAGAGTAGA
GCAGATATGCAGCAGGGATGCGATGATAAGCAATTCGTCTGTTG
CCACTCAGGAGCCAAA
TGAACACATCTGGAAGACTGTGCTGTGGGTCTTCCACGAGAGAA
CGAAGTCTGGCCTGAA
GAGATACCATGGATCGGAATTTGTCTTTTACATTATCCGTGTTCA
GTGCAGCTTTCAAGG
ATCGAGCCATCATTCCTATTTGCTCGATTTCGCGATGGCATAGCC
TCATCCGTTCCCGGT

CAAGTCTGG
>ConsensusfromContig377 Average coverage: 9.25
GTGCGTAAACAGAGTTGTCCTCCAATCAATGACATGTATTCTACT
GATGGCAAATAGTTT
ATTATTTGGTTTCGTCCGGTCGTATCCGGTTCTCATTCTATGCGGC
GCTGCTGAAGGACT
TCTAATAAATGCGATGATAACCAACATTCCCCCGGCTTTTGGTGA
CATCGTGGGAGACGT
CGGGTTCCCCTTTATGGCTGGGTTGGCTGACTTGTTCATTGGATCC
TTTTGCATCAGTGC
TGTACCAACAGCAAATTTCTTATACAATATCACCGGGGTTTACGA
TGGGATATTCTACGC
GCAAGCCATACTTTTTCCAATCGGAGCAATTGCGGCT
>ConsensusfromContig378 Average coverage: 13.52
AGAGTCATTTGTTAGAATCGTCCTTGAATCCTGTGACCCTGTGAC
GTCGGATAAGGTCAG
AATTTCGTCATCGTCGGTGTATCCGTCCATCGTCATCTCAAAATC
CGCGTAGGGGAGCAT
GACCTCGCCACTGTACTTTCTCAGCGAGGGATCCGTGGAGCCAAT
CTCCCCATCGACTCC
ATCTTGAGAGGAACCTGACCTTCCAGTCACCCAGCGACGCAACGC
CAGCTCCGTTGTTTC
TGACTTG
>ConsensusfromContig379 Average coverage: 11.40
TACTTTATGAATTCATTATAAAGTATAATCACCACGAGAACCCCG
TAGTTTTCCTCATTT
GAATCGCAAATTATCCAGTATTTAACTCGTTTTGAATCATTATAA
CTAGAACTCACATTT
TTATCATGAGAAGCAACATAAAACAAAAATGTTCGTTATGTAAAT
CATTCTCATGGTTAC
CAATCGAGTTAAAATCGAAATTAGTTGATAAACAGAAACATGAC
GATACACAGCATTTTC
TT
>ConsensusfromContig380 Average coverage: 4.76
GTCGCTTCATCCTAGGAGTGTTTAAACTTAGACTCAGTTCAGCCA
AAAAGCTTTCAGACT
GACACCGCGCTTTAGTTTAGTGTCGTTCAGTGCATTCACACGTGG
TCGGCAGAAGAACGG
TTTCAAAGCGAGGGGTCAGCATCATTCCTGGGCCGAGGTCTCGAC
TGCATTTATCGCTGA
CGAGAACTTTCTCCGTTGAATTGACGACCGCGCAAGATCGGAAG
>ConsensusfromContig381 Average coverage: 13.95

GAAATATGAACCAGACTTATGACTCATTCACTTAGAACAAACCAC
TAGCTGTCTTGATAT
AAATTCTGATATTCGGACGGCTTTGAGTGAGATCGGATTCTTCGT
GCTCATATAAATATT
TCACTCAATGCATATGACATAAGCAATTATTTTTCGTGAACCACA
GTCTTTATACAGAAA
TAACAATCACATGTTGAATATAATCAATCTTCATTTTTATTGAAT
ATATCTGAATGGA
>ConsensusfromContig382 Average coverage: 9.20
ATCATTGTTCATATTTTTTGTAGCGAGCAATGATATTTCTCTTTTA
CCAAAAATAATCTC
AAAAGTTAGCCGTGGTATAATTTTTAGACAAGGTCCCCATATTTT
GAATGGTGTCACTAA
ATTTAAACACACATTCACAGTGAAAGTACCAGTTATTAAGTTTGC
TACATTACCTACATT
ACCATGTGACACAGTAGCAATGAAAGCACTTGGATGTGAAGCCA
ATAATAATCTCACA
>ConsensusfromContig383 Average coverage: 5.21
CCAGTGATAGAAAAGGCTGTTGAGACGAAGCGCTGGAAGAGAG
CAGTAAAAAACAGCGGA
GAGTCCAGATATCTTCGCGAGCAGTTGGCTTACAGCACAAGAAA
CTAGATACAAGCCAGG
CAATGTTGTGATCATGGGGTCCCACTTCTCGTAACTCCCATTGCA
GTATGAGACAGCTTG
AGGAGCATGGAAAATTTCATCCATGTATGCGG
>ConsensusfromContig384 Average coverage: 5.59
CGGATATCAGATTCTTTTATTTTTGAGCATTTTAGGTGATTACTGT
CATACTAGTGAATG
TCAAACAGATCGAAGAGAGTATTTCAAAATACAATCACGCTGTCA
ATGAGTTTATATGAA
ACTTATGGTTTACAATGGAGGTATCGCAAAATCCCAGGAAAACA
CCGAATAAGGTGCGGC
ACGCTTTCAGATGCTACATGTGAACAAAGATTTATAAAGTATT
>ConsensusfromContig385 Average coverage: 12.67
CTTGTAACAATCATCCCAAAACGCTGAGGCCGAAAACTTCTCTCG
TAGAACAACCGTGCC
CCCAACAGAAAATGTCGCGAAAGTCGACATGACAAGACCCATGA
TATGGTAGAGAGGCAG
TGTGACATACATGACATCTGTGCTATTCAGACGGAGACTTACCTT
GAATACCGTCAAGGT
CAAGGCTATCTTCTTATGTTGTATCACTACAGGTTTTGAAAGACC
CGTCGTTCCAGATGT

ATATATGAATAGGCAAGTGTTTTC
>ConsensusfromContig386 Average coverage: 6.04
AAAAATAACAATAATAACCGTTAAAAAGTGAGAAAAGAGAGAAT
ACATATTATTACAGAG
TGTCATCTTCTACTGTTTCCTCTCTCTTAGGGCTTTGATTGTCTTG
GATTCGGATATCGC
TTTCGAAAATGTGTTGAATAATGATTAATAAGTGTATATAAGTTAT
AATATTTATTTTTC
TGTAATGTTTAATTGGGAAA
>ConsensusfromContig387 Average coverage: 9.61
ACGCTATCATTTCGTTCTGCTGTTCCGTGTTGAGATGGACTTCAGC
GCAATGTTTCGTCT
TATTCACTTGCTTTCCTGTCTTGTTGACCTGAGATGCTAAGACATT
GACATCAATTGGAT
TCTCCTCTGAAGAATTTTTGATAGCATCTTCTATGCCTTTCTTTTG
CTCAAGGCTGAAAT
AAGATGCGACCTGCGACAGAT
>ConsensusfromContig388 Average coverage: 11.14
CAGCAGAAAGAAAAGCCTTTCAAGATGATTCCTCGGGATATCGAA
CATCTTCTTAAGGCC
CGTGACATGGAATCAGATGCCTGATACGGGCTCTTAAATTATTCG
CCATTATTCTTCAGT
CATTAGCCACTCCTCTTCAGAAACGTGTCATCCCCGTGAAGCCATT
TGTCATTCTTTTTC
GAATATTTGTCACTCTTTCTCAGATAGATAGATGTTATCGAAGAA
TAAAAAAAATTCTTA
CGGATTATATGCTCATAATTGAGATATTTTTCATTTTTTTGAAAAG
TTGC
>ConsensusfromContig389 Average coverage: 7.81
CTCCGACACAAAACGAGATTCGAACATTAACTGCGGGAATACTT
TGGCGAGGTTGAGGTC
TCCAAAGACGGCCAATACAAATCAACGTGACAGTCCATTGAACC
AGCCGACGAGCTACCC
CACAAATGCAGAGGAGCTGGACCAGCAAAGAGTTTCCCTCATAGA
CTTGTTCTCTCTCGA
AGGCCCTAAAGCGATTTATTCTGATGCCGTAGATCGGAAGAGCGT
CG
>ConsensusfromContig390 Average coverage: 10.18
AAACACTTTCCATAAAATCATAAATTCAGCGCGTATTATCAGAAT
TCATCATTGTCCTTT
TTCAGAAAAAATATTCTCACAGATGAGCAGAAAGAAGTTCAAATC
CATCAAGAGTGTTTT

TTTCTGTGTGTCTTCCATCTTGAGAACTAGCAGTTTACCGCGCTTC
GCCAGGGTTCAACT
ATGTGTGGATTCGGTTCATG
>ConsensusfromContig391 Average coverage: 4.21
CGGGAATATATTTTTATTCTCAATGTGATTTTATGCGGAGCGAAT
AATCAACTATATACA
AGAATTTGTTGATGCAAAATAATGGATTAGTTCATTTTTGGGATA
TTTTGATAAAGTAAC
CGGATTAAAACCCAGAGTGAAACAAATTCTTTCCTCAGATATATT
CAAATTTTGATTACA
AGTAGAACTGCCTATTTCGAGGTGTTTGGAGAAG
>ConsensusfromContig392 Average coverage: 6.78
TCAATATTTACCTGAGGATATAATATATACACTAATCTTGCCCCC
AGCGTTTGGTAATTT
AGTTCATTCTACGGGAGTACCTAGGAATTTGACTTACTCATCGAA
TTTTACACAGGCTGA
TATAGACGCAAATTCAATTGTGTACACATTAACGGCGAACAACTT
GGATGGCGTTCACGA
AGATAATTTTGAATTCAACGTTTCAATTCCCGGAAAAGTTTTGGG
CGGTCGAGAGTTTCT
GATAACATATCTTCCATATCAAGAGAAAATGCCGGCGGTTTTGA
ATAAAGGTCTCAAAAA
TGTTATTGAAGGGGGTGAAAAGCTTATCACGACAGGTGTATTGTA
CGTGGGCG
>ConsensusfromContig393 Average coverage: 9.58
ACCGCGTATGCGAGAGATGCGGGGTTTATGACGCTGAGAGCGGC
GTGTGTAGAAGGTGTG
CTGTACTCTTCCGAGAAGTGACTAGAAGGCCTGATCACAGGAGG
AGATTCTGGGCGTGGC
CTATGGAGAATATCTTTGAGGGAATTTATGATGTAGAGCCCGAGT
GCTACAGGTATGACG
TAACGCCTTGCTACGACCATGTCGAATGCCAAGGCAA
>ConsensusfromContig394 Average coverage: 8.92
GTTTTAATAGAGTAGATGAATCACAATGAAATAATTATATGATAC
GAAGCGCTGCTTCTT
GACGAGCATGTCTGCTTGAGTTATATACAATTTCAATAACCTTCT
GCACTGCATTTTCCA
TAATTAGCATATTTTGATTGGATTATACAAATAAGGGTCACAGTT
TTTCGAGTTAAGACT
CATGATCTGCCGTTGTAACAGTTTCGACAGCCATG
>ConsensusfromContig395 Average coverage: 6.46
ATGAACTTGCCCCTCAAACGGAACCAGGGTTACGTCATGAAGTAC

TTCCTCGAGTCGAGA
TCAGAGGTGGCGAGTGTTTTGGATCAGCCGCAAGAAAAGTGGAT
GGCGCTTTTGAAGGAA
TCGAACAGTGAACCTCTCATTCATCTCATGAGTCTGGTGGATTTG
CTTGCTACGTGCGCA
GAGGTTGGACGTACCTTAGGATCCTACGTTCTTTGCCGACACCGA

>ConsensusfromContig396 Average coverage: 6.79
GCCGACTCTCGAACTGGCGGTCTCATCGAAAGAAATCATTCTTGG
TCAAACATTCTCATT
AGAATGTGTTGTGAAGGACGCATCTCCATGGCCGAATTTGGACGA
GATTATGTGGTACAA
ACAGGAGCTATCCAACAACGTGCCTGATGAAAACGCTGTCGACG
ATATTTACCAACAAGA
AATGATCGGACCCATATACACGTTCGATGAACTCAAGCGTGAAG
ACGTAGACAATGGACT
CAAGTGTCGAGCTGGTGATACATACAGTTCCTGGTACATGCTCAT
CATAAAACGACCGTT
TTTATTCACGACGATCGGCGGCGAATCACCAGTTACTGTTCCCAA
AGGTGATAAATACAA
CTTGAAAGGCCCTTTTACAGATGG
>ConsensusfromContig397 Average coverage: 13.12
TTATCTTCTAGATTCATTTTTATATCATTATCTAATGCCCAAACAA
AATTCTCAATGATC
ATCAACTCTTTAAGTGATTCAAAATCATTAATGTCGCATGAACTC
AACCATTTATTGAAT
GCTATTCTCATTCGGTCTGATAATTCTACATAAGTTTCCCCTTGCC
TTTGCCTACAGGTT
CTAAATCGTTTTTTATATTCACATGGTACTAAATCGTACGCTGTGA
GTATCTCCCTTTTC
AAAATATCGTAATTTGCACTACTTGCATGATTCAGTGAAATGTAT
GTTTGCCTAGCCTTA
CCTGTGAATATTGACTGTAATAATACTGGCCAAATGTTTTTAGAC
CATTCCATATTTATG
GCAACCTTCTCAAAATGGATAAAAAAGATATCTACATTATTCTCA
TCAAATTTCGGTAGC
AACCGCAATTTGTTACTCAGATTATTCATTTCGTTATTCTGACCCC
CATTATAAACGTTA
CCA
>ConsensusfromContig398 Average coverage: 6.71
TGTTATGAGAAATTCACAGGAGAACTGTTCAGGATCCAATAAATG
ACTTGTTTCTTCTGT

ATGATGTTTTGGTTTTATCGGATGATCTAGGTCTATTTTTTCAGGA
TTGCATTTAATTAA
ATAAGTACCATGTCCCCAAATAACGACCGATGAGCCCAATATTG
ACATTGAAGATAAACT
GTGCTTGGTATAGGTTGTATGCTTATACCATTTAAAATCATTTTC
GCCACCTAATAAAGT
GTGCAAGTGAAATAAATTCCACCGCTTTATACATT
>ConsensusfromContig399 Average coverage: 7.45
ATCTTGTCCGCACTATCTACCGTCATATTATGCTTCGGCACAAAA
GGCTGTGCAAAATCC
ACATGCTCGGGCTTCTTGAAGTCAGCTCCGGTCATCGAATCCGCA
GCGACAACGCCGCTT
GGTACGAGGAACCCGCCTTCGGGGCCTCGGTCACCGCAGCGCGTT
CGTGGCTTGACGGTG
TGGTGTTTAAAATGCTGCTTGCTCGTCGTGTCGC
>ConsensusfromContig400 Average coverage: 3.94
AATATGTAATTGAATATCTACCTGCCTCCACGACCCATTAGATGG
TGTACATTCCTCTTC
TCTCCCATCAGCTTCCTGAGCTTCTCGAGCAGAGCCATCTTTGATT
TGTCTTTGGCTTTG
GCCAAACAGTCTGCTGCCCCTTCCTCCGCAAGACTCTGCAAACTC
TGTCCAGACTTTCTT
TGCAACCGCTTGACCGCTGCCTCGTCTTTTCTCTCCATGGCCAAG
ACGAGAT
>ConsensusfromContig401 Average coverage: 8.13
CGATCACCAAACCCAATGCCATCCCACAAATACCCGTTAGAAAA
CAGACCAGGAACGCGC
CCATAAATTGGGACCCATCAGTAGCCCCTAAAGCATAGAGAGTT
GATACGCCGAACACAC
TCTGTATCAGTATAAGAATGCTGGTGAATAAAACTTGACTGAGAA
ATATTTCGTTGAAGT
TCACACCATATGTCTTCAAGCGAAATAGCATCCCAGATGATTTTTC
TTTTATTATCATCA
TCGATGTTTGAC
>ConsensusfromContig402 Average coverage: 13.35
GTGGATCTTTCGGTCGGTACGGCTCCCCATGGGCCGGTAGAAAAT
CGCTGACTGCACTTT
CTGATGTAGCGACTCCAACAAAAACAAATGAAACCAAAACTGCG
TCTGATGAGCAGGTGA
CACCAAAAGCGGTTGAAATCACGACAACACAGGTGGAAGACAAC
GCACAAAATGAAGATG
TAAAAGCTGTTAAAATTTCAAAAATTAAAGCTGATGATGAATTCA

TGATAGTGGACCCGA
AAGATGAAGCGCAATATGAATATGTTTACGAAGACGAAGATGAA
GCGTCAATAAAAGAAA
AACAAACAAAGGATACAAAAATAAAAACCTCTTCTGTTGAGATC
GGAAGA
>ConsensusfromContig403 Average coverage: 8.64
GTAGCATGTCTCTATACGTACGTTCGCATGAATTCAATTGAAGGC
ATTTGCTCGACCCAC
CCTTGCAAGAATCGCGGGAATGCCACGATAATCAGAATGGGACC
TATTGTTGCTCTTGC
ATCAACAATTTTTCTGGGAAAACTGCGAAATTTGCGACTGTATA
AAGGGTCAATTGTGT
GTGCTAAACGAGACAGTGGGATGTGAATGTCCCGACGGCA
>ConsensusfromContig404 Average coverage: 46.41
AAAATGGGTTTCAAAAATATGAAAGTCTCTTGTCCAGGGGGTGGA
CGCATTGACCATTCT
CCTGCAAATAAGACCATCAAAATATATGGTTACTCAATGGGGTTT
GGCCGGGCTGATCAT
CAACTTACCGAAGATATATTACTGAAAAAATATCCTGACTATAAA
ATATCATGGGAAAAT
GATGGTTACTGATGGATGATGATTGATAGATTATTCGAA
>ConsensusfromContig405 Average coverage: 5.52
ATTTGATTAATAACATAACAAAAGTTATTAAACTATGAACTATTC
ATCAGATTCATTTCC
ACTCAAGTCGCATTCCTCGCCGTTCATGCAGTTAACACATTGGCA
CAAATCTGTGCATTG
AAGTCCTTCTTTTTGACAAGAGCATCTAGCAGATTGACAACCTGTT
TTGCATTTGCATTT
AACAGAATTGAGAAGGCTGCATGGAGCAATTGTCCCTG
>ConsensusfromContig406 Average coverage: 6.91
CCGAGGTTTGTGGAAGCGACGGGAATACGTATATGAATGAATGC
GAGTTGAAGGTATCAT
CTTGTAGGAAGAAGAAATTGATAAAAGTCGAATCATCCGGTGCA
TGTGATGACTGTGAAT
CTGTCCGATGCCGACACGGAGCCGTTTGCATCAAAGGTCGATGCG
AATGTATTACCAGCT
GTCCGATAGATGACAGTCCTGTATGTGGATCTGATGATGTAACAT
ATGCAAATGAATGCG
AACTTTATCGAGAGGCATGTCAAAGAGAGCATCATGTAACACTT
GCGCATGAGAGA
>ConsensusfromContig407 Average coverage: 6.09
GAGGAGTAGTCGGCTGGTAGAATTGGTTATTCCATATACAAGAAC

CATTGAAGATATTGC
TGACATCGGAATGCCTAGTTTTGTTGCTCGATGCGGAGTGAAAGT
TATTGTGGTGAAATC
TGCAAGTGAGATGAGAAAAGACCCGATGCAGGCAATGATGATAG
AAGGCTGCCTTTTTAT
TTCTGTATCCGCATTCATTCCGCAGCTAAATCTGTTTTTGAA
>ConsensusfromContig408 Average coverage: 6.96
ATCTCACGAGTAGACTTATGCATACACCTTGTACGAAGAGCAAG
AAGCGATATACCAGAA
TATAAATCAGTCGTCCTTTAGCTTAAAATATTAGTAGAGACGCAA
CATCAAGATACGAGC
CTTAAGAAATATGTAGAGGACAAAAAGTTCATGAGGTTTAATCTG
TGTCAAGAAACATTC
TATGCTATAATAATTACTTTGAATGCAACACTGATTTTATTCATA
TATCACAGATGAAGA
CACGATACAGATCTTAAACGAATAACTTCGAAACGCAATGCAGG
AATACTATTTAGATAA
AGTACTAGATAATGA
>ConsensusfromContig409 Average coverage: 8.12
TCTTTTTCACGATCATGAAACATTGACAATCTTTTCCCTTTATATA
CTAAATAATTTGAG
CTCAAAATAAAAACACCTTTACGAATAGGCGTAATTGAGGTTTGA
TCACTGATTCGTTTA
TAGAATAATTTGCGAGTAGGTGTGTTTAAGGACTTCACATTGATT
CGTTCACGAAAAAA
TCTTCACTTCCAACAATTAATATTTTTTCGTTTTGATCACTAACTTT
TTGTGTTACTTTT
GATTCGCTAAGTAATTGAAAGGCTTCCTCATCGGTTCCTTTCATG
GGTCGCTTCGAAGAA
TATATCGCATCAAATAACAATTTGGATGAATTTTGTTTTGTAATTT
TTCGTAACTGATTA
ACTATAATACTAA
>ConsensusfromContig410 Average coverage: 6.16
TTCATATTTTTGTCTAACGAATTGACATGGTTTATATAGTTCAGGA
GATCCTTTTCAGTA
TGTGTCCAAATCATAAATATATCATCAAGGTACCTGTAATAAACT
AGTGGCTGTTTTTCA
AACTTTACAATACTTTTCTCAAGTTCCGCCAAATATTCATCTGCC
AGAATTGGTGAAAAA
CTCTTACCCATACTTATGCCGTGAACTTGTTTATAATAGTTTTCAT
CAAATCTGAATGAA
TTGTTGTACAATTGTATCTCTAATAGTTTGATAATATAATCAGAA

>ConsensusfromContig411 Average coverage: 8.16
CGCCTGCCAGAACAACGCTATCATCACTGTTGGCAACAAGGGCT
TTTGTAAAAAAGACGA
ACTGTGCGAGAACCATAAATGCAAGCACTACGGGATGTGTCGTG
TTCAGAAGGGAGACCC
TGTCTGTGTGTGCCCCGCTTGTGGCTCCGCTTTTGACCCAGTCTGT
GGCTCCGACCTCAA
ACTTTATTCCAGTGAATGCGATTTGAAAGCAGCATCATGCGAAGA
GCAAGTTCTGATCAC
CATCATCTCCAGGGATAAATGCCGAGATGCTTGCGATACGGTGAC
ATGTGATTTCTACGC
ATTTTGTGAGCCTGACGAGTTTCTTGAACCAACTTGCCAATGTCC
AACATCATGTATCTC
GGTTCATGCCGAGGTTTGTGGAAGCGACGAGATCGGA
>ConsensusfromContig412 Average coverage: 7.26
ATTAAATCTGTTTCGATGCTATTTCTCGCTGTTAAAATGTTATTTA
TGTGAGGATTATTT
TTAGTGATCATGAGAATGAAGTACATTAAAATAAACGTGCTCGAT
GCCATTATTTTTGTC
AATTCGTTGTCATCAATGAACCGCACTTCCTCGGATGAACCGTAC
GGGATAACTTTAATG
ATAATTGAACCTGTTTAATTGAATATCTTGAATATA
>ConsensusfromContig413 Average coverage: 5.65
AACAGCGCTCCGGATTCTGGAGAACATCGAAGTTCTCAAAATCC
CGAACCTCGGAATCGA
GGAGAGATACCAAAATCTACTGGAAGCATTCGAAGCAGATTTGG
AGAGAGTTGGGAATAT
GTACACCAAGAATAACAACGATCCTCCGATCATGAGGAATATGC
CTCCGATGGCAGGTCG
CATTGAATGGGCCCGCCAACTATCGAATGGACTTGAAGTGAAAA
TGCAGGCCCTAGCCAA
ACATGACAATATTATAAAGACGGAAAAGGGGAAGAAAGTCGTTA
AATTCTAAG
>ConsensusfromContig414 Average coverage: 42.65
GTAAAACTGTAACAGAGTGTCCACATGTGAATTTACTGACAGATG
CTGAATTTAATTCTG
CAAAAGAGTTTAATTTATTTGACCATTTCTGCACAATGAATTCTG
AAATAATAGCGCTCT
ATGCAATTTCGTGTATAATAATTACATTTATTGTTTGGTTCTTCGT
ATTTAGAAAATCAC
ATAAACGTGACTCTGCTCTCTTTGTTG
>ConsensusfromContig415 Average coverage: 6.76

GCGGGCTGCGATGCCAAATAAACATTGATTTAACCCGCCCAAAAT
TTATTCACGCAGTTC
GATTTGCTATTTGCTGAACACTAATGAAGCATGTAAAATATCGAT
TGAGCCAGGCAGGAC
GAAAAAAACACCTATTTTGATCGATCTACATCGTTGAATAAAAGG
CAGATTTTAATTCGA
TGAACTAAATTTTCACATACTTCGG
>ConsensusfromContig416 Average coverage: 4.38
TGGGTCATCACATGCTTCCCTCCCACCACCCCCTCCTGCCGACTC
TCATGGGCAATGGAC
AGAGCATGCAAAACTCTTCTCAAAACCCTATGGTAAGCAGTCATC
TTTATCGAATGGATA
ACTCAAGCATGTACGCGGGGGGAATGAGTGGACACAACGGAATT
CAGAACGGGGTGAACA
GTTCCTCTGGGCAACTCTGTGCCCCCCAAAACGGGGGTCCGAACA
ACGACCCCTCCACGT
GCGGAAATATGAGCTCAACAGATCGGAAGAGCGT
>ConsensusfromContig417 Average coverage: 12.48
GGACCTCAGATGAGTCGGAGCGGGGAGATGCTGCAATACATCGA
AAGCCTCGGGAAATAC
AAGATGACTTTTATCGAGTATTCCGACCTCATCTCTGACCCAATC
AGCGCTACTGAAGCG
GCGCTTAGTGAGATCGGAATTCCGAGAAATGCTTCCAGGCTACAA
TTGTATGACATGTAT
TACAAGCAGAACCCTCAGCACAAGAA
>ConsensusfromContig418 Average coverage: 4.80
TGTCTCTCATGGGAACAATTCGATAACTCACCACAGCCATCAGCA
AGCGTTTCCGATTTG
GTATTCGGGTCGTTGAATTTTGAGTACTCGTATACATTATTGACA
ATGGCATTGTGTTCC
TTCTGGCATTCCCAAGGATTCTCACGAGCAGCCGAGCCATTATTC
TTTAAAATAAGATGA
TCAGCATCACGTCCAAAGCG
>ConsensusfromContig419 Average coverage: 17.65
GTTCAGACGTGTGCTCTTCCGATCTCCGCCTACCAAGAGAAAAAA
AGTGTCGAACGTGAA
AAATACTGACATAAAAGCACAACGTGAGAAGGAGAGAAATGAG
AAGAAACGAAACAAAGA
GCGAGAAGCTGCACTGAAGAGATGCCAACGAGATGTGCTCAAGA
TGGCAAGGCCTGGAGA
CTGCATCAAGGCGCTGACAGTGATCATGGATGTTGAGGTCG
>ConsensusfromContig420 Average coverage: 6.28

TTCCGATCTATCCGCTCATGGTGTTAATAAATTAGTGATATTGCT
ATGTCTGTTATAAAG
ACATGTGAGATGGAAAAAATACCCGTTCACGAAATAAATATGGA
TATAAAATGAATGGGC
GATATTAACAAGTCGAGCAAATTCGATAAAATTCGTAATATTGCT
CATCGACTTACATAG
GGTACTCGAACAAACCAAATATGAAAA
>ConsensusfromContig421 Average coverage: 10.71
CCGAATTTGAAGTACGTGAATTCGCCGTTTCCTTCATACGGAGTT
TTGTGCTGTGCCTCG
AAGTAGTTTTGAGTCTCGATAACGCCATTGATCATCAACCATGCG
AATGTCCCATTGAAG
ATGGCCGTGACATCATATGTGTGTCCTGCTTCGAGAATTCCATTCC
CAGTGTCTTCTATT
CGTGCATAGTATTCCTTGGTGTTTGTCTTAACCATGAAACGGAGT
CTTGGGCTGGTGCGG
AAGTATAGCAGTGTTATTCCCCACGTATCATATCGG
>ConsensusfromContig422 Average coverage: 7.31
CGCGCATCTTTGAAATCACCATCCCGCTTATGAATTCTGTCTCTCT
CATGGGAAATTTGT
GTATCTACATTTAAATATTTATTCAAATGGCCGATAAGCCCGATG
TGGATACGACGGCGG
TAGACCTTGAACTTGAACGCGTCCTTGACACCGAACGTGGTAATG
GATCTCAGAGATACG
AAATCATTCACGTGGAAGAAAAACATGGTTTCTTCCAGGCTGCCA
TTCCAAAGGTGCCAC
TCCCCCTGGCCCTGCTGCTTCTGTTCTTCAATTGCTTTCTGCCTGG
CATCGGGACAATGC
TGTC
>ConsensusfromContig423 Average coverage: 8.88
TTTCTTCTTCGGCGTGGACATCACCAATGACATCGGCTCGGTTTG
TTCGGGTTCGGATGG
CGAACTGAAGATATTTTTGAAATAGGATCCCTGAAGATGTGAAG
GTATCTGTGACGAAGA
GAAATACGGCGGAGGAGGAAAGATAGATTGGTGGGATTGCTGGT
CAACAGAATTTCTGAA
CAAGCCATCACCATTTTGACTGTCTGGTTTTTCAGGCATGTTTAT
AGACGATCGCCC
>ConsensusfromContig424 Average coverage: 7.29
CTTCCTACTTCGCTGCCAATTGTTCGATTTTCTCTCATTGTAAAGA
AATATTTTGATTGG
TTGAAAACTGGTGGCGAATCATTTGTATCAATTAGAGTGATGATA

ACTTCAGCTTCGGAA
GAAGATGGCGGCGAGCCCCGATCTGATACTGCGACCTTGAACTC
GAGGCTGGTGTGCATC
TCGTAATCGAAGGACTTCATAGCTTTGATGATTCCATTGTCTTTGT
TTATCTCTATGAAA
TCTGAATAATC
>ConsensusfromContig425 Average coverage: 10.82
CCGCAACGGCCTTGAGAATTCTGCTCCCGGCCTTGAGGGCAAATG
GTCCTCTGTATCTGA
AGGTCGTCTCTCTACCTGCGAATTTGCGTTGAAAGGGGTTGGGTT
TGGCCCCATCCGTGG
AAAAATACACCTTCGCGCCATCCGTTTCTGAATTGATTGATAAGT
AGGTAGAAGTATTGA
GATAGGTGTGTGGAAGCCCAATACTGGAATGTCTCAATGG
>ConsensusfromContig426 Average coverage: 9.97
CTCCCCGAAATGCATTTAGGTGCAGCGTTGAGCGAAAGTTTTATA
GAGGTAGAGCTACTG
ATTGGATGCGGGGGCTTCACCGCCTACCAATTCCTGACAAACTCC
GAATGCTATAAAATG
TTACTCAGCAGTGAGGGCATGGGTGCTAAGGTCCATGTCCGAGA
GGGAAAGAACCCAGAC
CATCAGCTAAGGTCCCCAAATATATGTTAAGTTGAACTAACGAGG
TGAAACTGCTTAGAC
AGCTAGGATGTTGGCTTGGAAGCAGCCATTCATTTAAAGAGTGCG
TAACAGCTCACTAGT
CGAGCGGTTTTGCATGGATAATAATCGGGCATAAACATATTACCG
AAGCTATGGATTTAT
ACTAAGTATAAGTGGTAGGGGAGCATTCTATTTGCGCCGAAG
>ConsensusfromContig427 Average coverage: 10.49
TGTTATTAAAATGTTCCTCATCTACAAGGACAATGTTATGAGATCA
TTTACTTCTGATTT
TGTAGAAAAATGTTTGTAGAATCAATATCACCAGATTAGATTATA
AAATTCGAACGAATC
GTCGTTAAACATATCCTTTGTAGCCATTTATAATTTGCAAATGTA
AATTCTCAAAAAATT
GCATTTCAGAAGGTTGAAGCCAGAAATT
>ConsensusfromContig428 Average coverage: 7.93
CTGTCCCAACAGATAATGTTGCGATTTGTACACCGGCCTGTTTCA
AGAGCACCGCTTCAG
GAACCACACGATCCGCCATTATGTTGGTTCCACCGTCGGAAATTA
GGAGCATTTTGTTCT
CGTTTCCCGTTCTTTCTCCACCACTTCGCACGAAGAGCTGCTCTC

GGGCCATGCGAAGTC
CGTCTGAGGTGTTGGTTCGGTCATCCAAGAATGGGGCTTGACCAA
TAGCAAGCATGGTCT
CGTAAGCGCTTGTGTAAGCATCAAAGCCAAATCTGAGACTGGCAT
CATAGCTGTACGTTG
CCATGGCAATTCTGAAAGA
>ConsensusfromContig429 Average coverage: 6.68
TGAATATTTATGAATAAGGATTTTCTAAATTCTAAGAAATTTTAA
AATGATATCCGAGGA
GACCCTCAATGATATTCCTGAGTTAAACGAGCCCCCTTCGTACTC
CATGTCAATCAACGC
CATCAACAGTCAGATGATCGCAAACAACTGCAAGCCATGGGAGA
GTTTTGAGGCAGACGA
TATGTATGACTTTTACGAAAACCGCAAAGAATCAAGTGTCACAGA
TCGTCATGTT
>ConsensusfromContig430 Average coverage: 7.69
GTGAGGGCGGAATTCATCAGCGAGCCCAGAATGATTCGCCTCAC
AGTCAATGAAAACACG
GAAGAGAAACGAGACGCCAGAAAATTCGGAGGGAAATGCGACAG
TGACCACTTTGTGGGA
TTCGGAGAGCCCCAAAACATCCTCATTGGAGTTGGTTTCTACGGT
CACATGGACGAGCTA
TGCATGTCTGAGAATCTGAACCTTTGCTAGACTATTCATACTTGT
TAATATTGCACCAAG
CTAAATATTAATATTCAGGGTGATTTGCTGATAAAGCGTTGAATA
C
>ConsensusfromContig431 Average coverage: 12.26
TTGTAAAGGTGCTTAAATTAAACAGCTTACATAGCATTGAAATGA
TAATTCATGCTTAAT
TTGTATTTTCGAATGATGTGCGTAATTATCTAAATTCATAATATTC
CGCATGTTTTTTTA
TCCATATAATATATTCACAAACAAATTCATTGTTTACGATGCAAG
TTCACATATTTTGAT
AAAAGACTCCAATTCATCATATTGTAAATATTTCGAATGTTCATG
ATCTAAACCATAATC
GAAATTATTTTGTACTAAACTCTTGCGAGTTTTC
>ConsensusfromContig432 Average coverage: 4.49
GGTTGACGACACAGTTCTTATGACGAGTGGTGCTCAATATTATGC
ATTCCGCAAATCTAC
AATGACCCTGGTGAAGACAGGATCATTCGATACAATAAGTCGCGC
CACTGCGTCTTTTAA
TCAAAATGGAGAAATCTGGATAGCAGACGATACAAACATGTATA

TCTACGATGCTTATGC
TCAGCAGATAAAAAATATGAGACTGGACTCGTTCTTTGGTTGCCC
AGGCAGAATTATTAC
TTTGAATATTTCTTGCCCACTTTGTTTTTGATATTTTT
>ConsensusfromContig433 Average coverage: 13.22
GCGATGTTTTTGAAGAAGAATTCCACAGCTTCTTCGGCAGCCGAT
ATTGACCCTTCTCGA
TTGTACCATTCTTCCCAAATAGTATGGTCGGCCACAAAATCCATC
TCCACTTCATAATCT
GGGTTGGAGCTTTGTTTTCCAACATTTCTCAGTGCCAACAACGGT
TTCGAGTTATTGAAA
CCAAGTGCAATGTCATTCATTGGGCTGAAATCCTGTTTCATTTTT
CGGATCACATGGCCC
TTTGCTGTG
>ConsensusfromContig434 Average coverage: 9.60
CGATCTCGCCTTCTGAGTGACTGATTGTTGTACCTTTCTGAGTTTT
TTGCTGTTTCTCTT
TGTTGGCATATTTCCATGTTTTCGGAAAATTAATGTGATCAATTTG
CCTATTTGACATCT
GATTGGGAGCATCTTTCAAGCCAGATTGCCTTTTGGTCGTCTGCT
GCTTGGAAGACAAAA
TGATGTCAGAATCTAATTGCTTCAGGGATGCCATTCGTTGTTTCA
TCATGGTCCCGTGAT
TCAATTGTC
>ConsensusfromContig435 Average coverage: 17.79
TTCAGACGTGTGCTCTTCCGATCTCGAATCCTGATCGATAGCTGA
GGACTCATATCCGAG
ACTATCCTCACGTTGCCTGGTCTTGCTCTCCTCTTCTAGTGTCATG
ACGGAATATTGTAT
ATGCTGTATATAAGCTTTCATCTGCTTATCGCCATTTTCTGTTTGT
ATCCACACCTGACA
GACTGAACCACATTGCAGCATTTCTCGCCTTTCTGGAACAATTTCG
TCCATCCATTCCTC
TTTTACTTGTTCACTAACGACATCTGCCGCCACATTCCGGTACGT
GGTTGGAATGATTGA
TTTTGCAATCCG
>ConsensusfromContig436 Average coverage: 8.43
CGCGAGTCAGATGTGGTCAAGCGACCAGGACACAGACAAGAGTTA
TTCTGTCCTCTCTGA
TATGACTTCATCGAGATTCAAAGCCAAGTTTCAGTCAATTAACGA
AGCATCAGAAGACCA
CGGGGATGGTGCTCTCTCCAAATGCACCAACTGTGGGGCCTGCTC

CGAAGGCACAACTCC
GTTCAAAGCAAGAAATACAGTAGATGGAGAAAAGATGGCGGCTG
AATCCAAAATGAATTC
ACCACCAAGAGTATTGAATGATTCTTTGAAGAAGGCTTTGCAAA
GAGAGAAACATTTGAT
TCAGAGTTTCGAGTCACAAGCTTCATTCGTAGAAGTTGGGGGAA
AGATGATTAAGATAGC
TGCACCTCGCTATGTATCAGACATTGTTGGCAAAAAAAAACCACC
AAAGTGTCATCACAC
GGTGGATTGGCTAAAA
>ConsensusfromContig437 Average coverage: 3.30
GGCATTTCTGTTTTTGTAGAACGCGGGGTTGTCAACTCGAGATAC
ATGGGCGTAGCCAGT
TGGGGATTAAATCCATACAGATGCTGTCTATTAACACTTCGTGTT
TAGAATAACATGTTT
CAAAACTCAATAAAAATTGCATTGATACGGATGAGAGATTTTGTA
GAGATTTTTCGATCG
CGTGATATAGTAACACAGCATTGTGAT
>ConsensusfromContig438 Average coverage: 14.33
GCGGGAATAGTAAAAAGATTGGCACTGATACTAAGCGAAGAGTA
AATATGAGCCGTCGTA
AACCTAAAGGTCCGACGATGTTTGAGATCGTCAAAGAAGGAAAG
GTGGCAATTGTTACTG
TTGTAGACTCGTGGATCACTCAGTACAAAGAAGCACATGATGTTG
GCATCAGAAACCTGC
TCCAGTTCTTTTTTCATAGCACTGGCTGCCGTGGCTTCATCGCGCC
TCATATGTATTCCA
TGGATCACACAGATATCATCAAGCTGATGACTGAGAACT
>ConsensusfromContig439 Average coverage: 8.62
AAGTTTGTAAGAGTAAAATCTCATTCCGAGGTATTCAGTATTAAT
TTTGAAAACATTACG
GCATATCCAATAGCAGATAATAATATACTTGACCACACTAATCAA
AATTAGATTACTCAT
TTATGAGCGTGTCTTTTGCATGCAACACGAGTCACGGCCTTGTTT
TTATCACTGAATTGA
TTCAAGTTTGAAATGTCTAAAAAATAAT
>ConsensusfromContig440 Average coverage: 15.61
ACCTAAACTTATTTCTGACACTTCCCTTCCCCTGCATGATATTTCT
CATTTTTGGTTTTG
CCAAATTCTCTCTCAACCTTCTATACCCTTTTTTTGTCATCACTTT
TCTTTTACGAACTA
TCCTGATTTCATCCCATTCCATTTTCATCTCCATCTCTATCACATT

ATGGTCAGAATATA
TCTCCTTATCTTTTGTCAATCTGTATGGTGC
>ConsensusfromContig441 Average coverage: 4.21
GTGAACCATGGTGAAAACGTGTACTTTATATTCTCCAAAATATAC
AGATTTATGGAGGCT
CTCATCCAGGAAAGATTTTAGAGGAACGTTAAACACAGTTTCGAC
TTCATCATCGTTATA
AATTGGTCTAAATTTTGCAGAAACTAAACAAACCACAGAATAAA
CATTGTACCCAGTGAG
TAGCAAAAGAGGCGGAAATTCAAAAATGACCTTGACATCCGATT
GTGGTAATCCAAT
>ConsensusfromContig442 Average coverage: 12.94
CAAATATTCTCCTGGTGGGTGGTTCTACAAATATCACATGACACT
TCAAAAGTCTTTTGG
TTCAATTTGCATCGGCAATTCTCATCGGACACGAGATTTTTGGTT
CTGGTTTCACCACCA
CAACAAAGATGGCGGCACCTATTTTCGCTTCTTTGTATTATCGTT
CCGTTTCGCCAGGAA
ATCTTTTGACACACCCGACCAGCCGTTCCGAAGGTTTTGTATTTG
CTGTTACGCCGGCAG
AAGTTAGGTGAAGGATTCAAAAAGACAAGATCGTGTTGGGTGGGT
GTTGACCTCATTTCT
CTCGCTGGCTTTAATCCGCGAAGATCATTGGTTGATGTAACGAGG
AAGGCGGAGTCAAAT
TTGGCTAGAAGGCTGTGACTGGTCGAGCGAAGAGATGGGAGGGA
GTTGTAGCAAGACTTG
TAAGTGCATGACCCCGATGGACCGTGGCATTGACACCGCTTTGAC
CTT
>ConsensusfromContig443 Average coverage: 41.30
GAGTTCAGACGTGTGCTCTTCCGATCTGTTGTGTCTGATTTCGCGC
CCTTTTCCTCAGCC
ACTGCCTCATTGGCCGCTGGTGTGGTTTCAGATGCCGCCGGTTCT
GACGCCGCCGGTTCG
GGTTTGGGACTCGCTGGTTCCGGTTCAGTCTGTTCGGTGACTCCG
TTGTGGGGAGCATCG
TTCTGCTCGGTGGTGGTGGGAGTGGGCTCCTCAATTAGTGCCCCC
TTTGAA
>ConsensusfromContig444 Average coverage: 6.09
TCCATGCCACACATGTCACCTGACCTTGATGACCTTCAAGGCGGG
CTACGAGATCCATTT
TTGATCCCTGAAGGCTGAATCGTCTTATGTAGACGAAAGTATCAC
TCCCTCCATATGCTA

TCTGTTTTTTTCTGCTGGAGAATGCCATGTCGAGTATGGCCCCAT
CTGCAGCCAATTCCA
CATGCTCAAACCGCGTAGAGGAATCACGGAATACGTCTCGTAATA
ATCCGTTCTCAACAT
CCCAAA
>ConsensusfromContig445 Average coverage: 4.22
ATCCCATTATCCCCCTGAATCTACGCGTCTATTAAAACAATGTAC
TGAGCAAACCCCTCT
TTATCTTCGTCCTAAATAGACCATCAATTGATTTTGGGGTCGGCA
TTCTCTGGAACAACT
CTATTCAGATGACTCGGACATTTCTAAAGCTAAGATGCGGCGCTC
CTCGTCAATCCCAAA
AACAGACTCCCGACGAAATCGTTAACAAATTGATACACATAATTC
TTTAATGAAGATGAC
TCATATGGTTGAAAAAT
>ConsensusfromContig446 Average coverage: 4.87
ATCAGGCCTACTCTGTGTCGATTTGATAATGCAACCAATGATTGT
GAACACGAAGAAAAC
TGCAAGAACGCACACTGTTATTGAAATCCCTGCTTCCCAATTTGC
AGATTCTCCTGTGAG
CAGAATGAGTTGACAAAGATCTCCTGCTCGAGTGGAGGGACAGT
CACAGATAGTTGTATT
TTCATCAGAAGAGCAAACGCCC
>ConsensusfromContig447 Average coverage: 6.68
TCTTCCGATCTCTTCTAGAGGCGCTCCAGAGCCCCCAAATTCTAA
GAGAATCCCTTCGTT
ATTAGGCTCAAAACAGATGTCTTCACCAATCAGCTCAATAAAGA
CGATGGAATCAAAACA
AACAAAAAACATTGATTCGCCCCCGAATATTGTTTCATCTGCTCC
TCCAAAACCCATGGC
GAGTTTATCTGTTCCTTTGATTAACTTGGAGACAGTCAG
>ConsensusfromContig448 Average coverage: 6.26
GAATACAAAAGGATTTCACTTATCAGCACATAGTCAGGTACCATC
ATCGCCACGGTTCGG
AACAGCACCTTTAGATTATCCGGAAGCTCAGCTCGACCCGCATAT
CCTGGATTCATCGTG
ATGAACAGATTACAGCTCGGGTCGAGGGTTATTGTGACACCCTCG
AATTCGAACTTGGAT
TTCGATTCGACCAAGGCTCGC
>ConsensusfromContig449 Average coverage: 6.37
TTGTTACACAACTGGTAGAAGAAGGAAAAAATCATCGGAAAAAA
AAACAAAACATCGTGA

AAATTTCCGATTTTTAAAGTCCCGATTTTTCAGTCCAGCACCTAAT
AAATTATTGTGATT
TGTTATATTCAAGCAGTTTAGGATGTGCTGAGTAATAATAAATTG
ATCTCAACTTTGCAT
TCAACCTTTTTCTTTGTTCATTTTGTGAAATT
>ConsensusfromContig450 Average coverage: 10.10
CCAGCTTTAGTTAATGTTCAAGATACCATAAGAAATAGATCTAGA
ACTTCATCGAAAGAA
AGAATTCGCCGGACTGAAAAATCGACGGAGAGAAGTCGGTCTCG
ACCGCCCTCTATAATC
GTCAATACCGACGAACAAAATCAGAAAAGTAGGTCACGAACTTT
ATCACATGAGAGAAGA
AGAAATGCTGCAGAGCGTTCAG
>ConsensusfromContig451 Average coverage: 8.75
CGCTTTTGAAGAGCCTGACATCACATTGACAAGAATTCGCCTTCC
ATTCTGCCAGTATTT
CAGAAATCGCATCAGAACGTTCTTCTGTCTTTGAAGAGAATTGAT
GATGAGTTGCCAAAG
CGCCCCAAGCGACCAATGATTCTTTTGAAATCATTTTAAATAAGA
GAAAGGGAATTCCAT
CCGGCAAAGAACTGCTCCTCAACGAAAA
>ConsensusfromContig452 Average coverage: 4.94
ATCCCGCGATACTTCGTGATGGTAATCCGAAAGAAAGCGGAATC
AAGCACGGGAGCAACC
GGAAAGTACCTTCTCGCATGCGTCGAATGTTGCCTCAAATGTGTC
GAGCTCCTCCTGAAG
TTCATCAGCAACAATGCCTACGTGGTTGTGGCAGTTGAGGGACAG
AATTTCTTCGGAGCA
GCCAAGCGCGCCGTGTTTCTT
>ConsensusfromContig453 Average coverage: 6.52
GTGCATCCGTTGTACAGTTTCTGTTGGATGAGGGCGGGGCATATG
CGCTCGAACTCTGAG
TACGATAAGCCATCAGTGGAGATGTTCAAGGGGAAAGCCTCTGCC
ATCTTGGCCGCTGTA
TAACACTGGGTGATCGTGGCATCCCTCAGAGTGCTGTGTTGATGT
GCATCAGGATTACCA
TCAGAGAAGACTGTGATCTCGATAGGTTTGGCGCCATCTTCTGAC
GGATATCCGATCTCC
AGTGCTGCCATCATTGTTGCCATATCACCAGCAGTGAGTATGTTA
GCCTCGGCGCCTACG
CGGCTGTAGACGTCTTTGATGAAGTGTCTTTCAAGTGGAAGAACT
CCCGG

>ConsensusfromContig454 Average coverage: 20.94
GCAACCAATAAATTTGTTACAAAACAGTGCACGTATACCACGTTT
AAATCAGGATCATCA
TACTTTGATAAGGAATAGAAGATGGAAACACACCCTGAACAATG
TGCAAGTATTGTGACA
GAGAGAATACAGCAAATAATGTCTTCGGAATATCAAAGATCCTC
GAATCGTCACGAAAAT
GTTCCTATTGTTGAGCTTCAGTCGCTGACTCTACGACCATATCTGG
A
>ConsensusfromContig455 Average coverage: 5.99
GGAAGAGAGAGACAACAACAGGGATGATGCCTTGGCCAAGAAAA
AGGAGATTGCACTTCA
AAGAAGAAAATTCGACCGTCAAATGCGGGACGGGCGCGGAAGA
GGGAACATGCGATGTT
GAAGAGCATTCATGGAAGAGAGACGGCCCTGACCCCTGATGAGG
TAGAGGAGAGCGAATG
TCCATCTGTGGACCCGGCCAGAGAGGAATATGAAGTACTCGCACA
ACT
>ConsensusfromContig456 Average coverage: 7.16
ACGGTATGTTTTCATTGAATTGTATTTGTTGGATTGAGTTTTATTT
CATCATTTAACTGA
TAATTTTGATACTTGTTTTGCAGTTAGATTTAATGGTACTACTTA
AAAAATTATACTGGA
AAATACAGAATGCTCTTCGTTCTTTGATCGAAGGATAAATAGGCA
ATCAAACTCTTATTA
GAATTTCGTGATAAGACCAATATCAATGAATGATAAAAACGTCA
TCTTAAATGAGTGGCG
TTCCTCTGCTGTTCTTGAAAGGGTTA
>ConsensusfromContig457 Average coverage: 15.30
AGAATGGCTCAGAATACAACAAATGTATCCCAGTCGAGGGAGTT
GATGTTAAAGTCATAT
CACAAAAAAGCCCATGAAGATATAAAAAGTATGGCTGAAAATTA
CCAAACGATAATTAAA
CTATCCAAAGTTGCTACACCTTCTGCAAATGACAAGGCTTTGAG
CAAGGCTGAGGAAGAA
AATGCTCTTAGTTGGCAAACCAGAGCTGAGCAGGACATGTATGA
GATGCAAGTCAGAGCT
TCAAATATAGTCCGAGCTGGTAGCTCTCTGCTAAGT
>ConsensusfromContig458 Average coverage: 9.64
GTTTTATTCAATACTTCAACTAATTTAACTCGATAGTTATTTGAG
AAAAAGTGATTGGTT
CACCATTCAATTACATAAGGCCGACTCAAATTGCACATTCATTCC

TCTTCACATATACAT
ATTTAATGACTAAAAATCCAATATCATGTTTACAACTTTAATATA
TTCAACAGCAAAAGC
TAGACAAAACGGGTGTATTTTTCGCAAGGATTTATATGATTATA
TTTCACGA
>ConsensusfromContig459 Average coverage: 9.42
TCGAAGTGTGTTAGCACAATGATAATGTATTTTGTTTCTTAATGA
AAGCTCACATAATGA
ATTTTGACAGCGGTCATATTTATATCTGGTCGCAGTCAGGACAAA
TTGGTGGAGATTCCA
ACAATCTATTTTCTGCATACCGCGCGAGGGGAGTACCAAGAGTG
AATACGGTTGAGAGCT
ATATATACTTCACATGACTTGATTATGACA
>ConsensusfromContig460 Average coverage: 10.66
TCCTCGACTTCCTCAGTAGCCGGGAGCTCTTCTGCCTGAGAATAT
TCGTAGTTCATCATG
GCGAAAAGAAGCATTTGCTGGGATTTCACGACAGCACCAACGGC
CTCTGCCTCAAATCCA
TTATCAGGAAGTTCATCCATATCTTTCATTGCAGATATTACTTTTT
TCTTCAGCTCTATA
GCAGCATCGGAAGGTTTCGGTTCATCCATTTTTTGCTTCCAGATCG
G
>ConsensusfromContig461 Average coverage: 6.82
TCCAGGAAAGAAAGATTACGTCAGTGTTGTTGGGGATAATGGCA
GAAAAAGAATTTTAAA
AAGGCTCATACTTTGTAATCTCCAAGACCTATATGGGAAATGGCG
ATCAGATCACGAGCA
GGCCACTACAAAAATAGGATTGACATCTTTCATATCATTGAGACC
GAAGCATTGCTTTTT
CGCAGGGGCGTCGGGTTCACATACCGTTTGTGTATGCATCCACCA
TCAAAACCCGAAATT
GAAATTAATGTCCCTGGATCGAAAATTGAAAACGACAAAATTTAT
GGACTGCATTTTGTA
TGAGAAAATTTTTTATCCAAATTCATTT
>ConsensusfromContig462 Average coverage: 6.53
GATGAATTCAAACTTCAGCATGATAAATAATGAAAAAATCTCCA
ACACATGACACCGAGA
GCAGCGAGCAGTGGTAGACGACCAGGTGGTAGGAGGGAGGGCG
AAGAAGAGAAGTCAGTA
TCCTGCATAGCAGTCTGGCATTTCTTCCCCATGAACCCAGCATCG
CAAAGGCAGACGGCA
GTCCTCCCATGGTTCTGACATTGGCCACGCCCACTGCAGCGCTCT

CCATCACATACGTTG
CCAACCACTGCTAGAATTGATAAGACTCATAAGGTGCTATTTCA
AAAACATAGAAACAAT
>ConsensusfromContig463 Average coverage: 5.28
GTTGCGAGATCATGGGCCTCTCTCAATGCCAAATTATTCATTTTG
CGGATAACTGAGCCT
TTGAAAATTCTTTTTTTCCTCTCTTCAGTTCTTCTTAGTGGTTCATC
CTCTGGCGATCTC
GCGATGATGCAATCTTTCGAGCAAACTTTTGTTGTGCCAAGATAT
GAATTTTTTATGCGC
ATCGGGACATTTGCACGATTTTTGATGCAAGACTTCTCTTTTTTGT
AGCTGGCATTTGAC
GAGAAATTTAAGAC
>ConsensusfromContig464 Average coverage: 7.73
ACCGTTCCTTCGTAGTTTGAAACGCAGATCAGACTTGTCCTCTGA
CTGAAAACCTCTGTG
CTGTTTTTGACATATACATGACAGCATAATATTTTTCTTACAAGG
TAATGTGTGTCCAGG
TGCATGGCCATTTCCAGAGAATTCATAAACTGATCATTTTCTCCG
AAGACCAGGCTCTTC
ATAATTGATTTTTCAACAGATCGGAAGACA
>ConsensusfromContig465 Average coverage: 11.73
ATCTCTTCACCAAGGCCGCAAATACTTCTGGCTTTTTTCGTATGGA
CTTCAATTTCACGG
TTCTTGGTTCAGTCGTAGTCGGTTCATCTTCAGGGTTTTCCATCTT
ATAAGAGATCATTC
CACAATCGCTCACTGCTTTTTTGATCGTCTTGAGGCAAAAATAAC
TGATTTGCTTCACAC
GAGCCAAAACTATTCCAGACTTGTTATGTATAGGCTCCAATTCCT
TGAG
>ConsensusfromContig466 Average coverage: 10.62
CCTTGCGAGTTACCCCGGCTCTGGGAATACCTGGTTTCGACATCT
CTTACAGGAAAGCTC
TGGTATCTTGACTGGGAGCGTTTTCAAGACGAAGTTTCTGCACAA
CTATTTCCCCGGCGA
ACATATCAGCAATGAAAGTGTTCTCGTAGTAAAGACTCACACCCC
GACATCATCTCAGAA
ACCCACAAAACCCTACACAAAGACAGTCCTTCTCATCC
>ConsensusfromContig467 Average coverage: 14.80
AGACGTGTGCTCTTCCGATCTCAGAAAGCGAACAGGAAATGAAC
AGTAAAAATATTAATC
AGGAAATCAAATCGAGTTCAAGTTATAGCAACAATAATAACTAC

AACCAATTCATGAAGA
GTCACATGACTCAGGAGATGAGCCGAGAGGTTAGCAATCAAGGC
CAAACCATTTCGGTGG
ATATGAGAAAGTCTCATTCAGCCAAAAACATTCCATCCGTACCA
GCGTCAGCGAACA
>ConsensusfromContig468 Average coverage: 9.31
TCAAATATCTGAACTTTTTTCAATGCAACAATAGTCTCATCAGCTT
TGCACTTAGCCTTG
TAGACTTCTGAGAATTGGCCCTTGCCAATTTTTTTCTCGACATCA
AAACAATCGCAATTA
CTGTACTTTGGATCATTGTCAGAAATAATTGAAGCCACTTCTGCA
TCCATAGTCGTGTAT
CAAAAAGTAAATCAGAACTAGTTACGAGGAATTTTCTAG
>ConsensusfromContig469 Average coverage: 11.48
CAAGTCTTTCATACTCGGCTGGACGAACAACATCAGAAGAAGCG
ACAAACGATTCTCGAC
AATCAATTCGCGCCACTGGTGTTGATTCCTGTTGTCATTCTAATTG
CTTTTCTCAACGGC
TCCATCTACTGGCTCTGCTGCGTCAAACGTCGCATCTTGAACGTC
TGCTGTCCGTACGCC
TGTCCTTGCTGCGCATGCTTTAACCCCGATGAACGATCTCGAAAC
ATCATTAACCGATAC
GCGCCGGGAATTTACATCGACAGTCGAGGAAA
>ConsensusfromContig470 Average coverage: 6.43
CTCAGTCATATCGGCCCAGTTAAAGATTTATGTACTGGCACCCAT
TTGAGCCCGGACGTA
TTAGATGCAATCAACAAGCGAATTGCTGATAAGGAGGGAGACAT
CACCGCGCAAGAGCTA
ATGCAAGACTTCCATATTGGCAGAAATACTCTTCAAACTGTGATT
GAATGTTTGAAATAT
ATCACCACGGGAGATATGCC
>ConsensusfromContig471 Average coverage: 10.83
GCGGAAAACCCCAGCAATTACGTCCTTGTCGTGGAGAACATCACG
TCTGCCTGCGCCTTT
ATTCCCTCGGAAGAGCCGACGACGTCCACAGCAGATGGCAGCAC
CTGGTCTCAGCTGATC
GAGAGAAACGTGCTTGCCGACCACGAGATAGTCTTCGAGGCAGC
TGGTCTCAGCAATGGT
GGAAAACTTCTTTTGACGCACAAAAGCAAGATTGCTGAAGAACG
AAAAATGAAGAAAAAT
AGCAGAAAATCAACTATAAACCACAGAGAGGAATCGATAACGAC
CTTAACAGCATGAACG

AAATTGTTTTATCTTGTGATAGCAGATCGGAAG
>ConsensusfromContig472 Average coverage: 8.17
CCCAATTCCAAGTAAATTTTTCGATTTCACCATCTGATTTCTTTTT
CGGTTTCCGCATCT
TTTGGGAGCGATGACTCGGACATATTCGGGCCGTCGCTGTTCTTC
ACTATCGCTGCTCTG
TGACTCGGAATAACATCGAAAAGTCCGCCGCAGCGTATCTCTAGC
CTCTTCAGAATCCGC
ACTGAATGTACTCTCTTCACATCTCCTTTCTTTGTTTCTATACAACT
TTG
>ConsensusfromContig473 Average coverage: 305.79
TGACTGGAGTTCAGACGTGTGCTCTTCCGATCTGTCCCATTGTCCG
ACATCTTCTCCACC
AAGCTGACCACTGTACTTGAATCGATTTTGAGATTTTCGCTCACA
TCTGCACTCTGCACA
AAATCTTCTGTTTGTAAATTTTCAACGCACTTGAGTTGTTTATTAG
CCAGCTCGATTATC
GAACCCCTTGAAAAATGAGGAGGGTATATAAGATGCGGCGTCTG
ATGGTCATGTG
>ConsensusfromContig474 Average coverage: 9.01
ATTCGTTCGAGGATGCGAGAGATATGCTCGAAATATATGAGAAC
TTGATAACAGACAACA
AGTTATTCGAGACGGCAGAACATCATCAACACAAAGCAAAGAAA
CGTCTCGCGAGCGCAC
GCAAAGATGGACCTGCATCTTCGTGCACCAACAGCGAGCTTCTCC
TCAACTCGGTGGACA
GCCGCTGGTCGCAGATGTCTTCCAGAATCCTCCTGCACCAAAAAC
AGCTCCAGAACGTCG
TCGCTCATTGGTCTCAGCTCGATTCTTCGGCGATGCGTCTTGAGC
AATGGCTGGCTGCAG
CACAGACCTCTGTGGCAGATGGAAGTACAAAT
>ConsensusfromContig475 Average coverage: 13.53
AGACGTGTGCTCTTCCGATTGAAAACATAAATAGAAAGGCAGAG
CGTGTTGAGCTCATTA
GATAATTCATTTGGCGATTCCTCACGAATGTTCGGAAACGTTTCG
TTGTTAACATTTTCA
AAGTGATTTCGATTTTAAAACGATGCAGCTATAAAATGATCACTA
TTCTAGGAAGGAAAA
GAAGAAGCATCACTGAATTGGTTGTTTGTTTATTTTGATTTTGCA
GCTTCAACTCGAGCT
GTCAATCCGAATTTCTTGGATTTAGATC
>ConsensusfromContig476 Average coverage: 6.47

CTTCACCAGATACCCATCCCCCGCCAGTAATGTGGTGATCAAATA
TCCGCCCTCGTTTTC
CGGAACTTCAACGTTGTACGTCCTCTGAGTGAAGATGGGAAACAC
GTCCTGGGCGTCCCT
GATATTGACCGTGACCTGGACGAAGCTGGTCCGACTGCTGCTGCC
CCCATCCCGAGCTCC
GACGATCAGGGCGTGGCTAGTTTCGTCCTCGTAGTCCAATCCGAC
CGATGTCCGAAGCTG
ACCACTCGTTTCGTCGATAACGA
>ConsensusfromContig477 Average coverage: 11.70
CCTCCTTATATCCTATCGTGAAACCACGCCCACATTTACACATAG
CACCTTAATGTAATT
TTTTCGCTTTTAATCCAATTCCTCGATATCATAATTGTCAATTTCA
ACAAGTTTATCATA
ATCATCATCATCGTTTACAACGTTTGCGATAAGATCGTCAATTTC
AACTAATACACCATT
GTTATAATCATCGTTATCATTTACAAGATCGTCAACTATATCTTCC
ATTTTCTGTTTAAA
ACTAGCCTCTAATTCATCAGAATCATCAAGATCGTAATCGTAATCG
TAGTTTTCTTCTTC
TTCAATTTCATTAGAATTGAATTCTAACGAATTTACATATCCACC
AATTGTTATGAGATC
AGACCATGGATTACTAAGAATCGATTTAATAGTATTATGATTTGA
TACATTCTCATTGCT
TTTTCCAAT
>ConsensusfromContig478 Average coverage: 5.03
CATCAACAAAGTTTATTCAGTCAAAATTGGCATGCCTCTACTGTG
GATTATTGTGCAAAA
TTTTATTCAGATAAGAATTTGTATGCCTTTAGTGCTGATTATCAT
CAGCAAGACTAATCC
AATCAAAGTCGTTTTGCCCTAGTGGTGTATTATCAATGAAACAAT
CAGAATGTTACGTTA
CCCTCTAAGTATTGTTGTGAATACTGGCTATGAGTATCATCAAAA
AAATTC
>ConsensusfromContig479 Average coverage: 13.49
TTGGATGGCCGACAGAGATGCATACGATGAGTTGTCAGGTTTTAA
CATCGTCAGAGTTTC
GACAACACTTCAAGAAACTGTGGTCAAATCTCTTCCGATTTCGGA
GAATGAAGCAATATG
GACTTATTCAATGGCATGGCTGGGAACTGGGATTTACATGGTATC
CATCCAGGCTGAGGC
GACTGAGACTAACAGAATAAGCATCGATAAACTGACCGTTGACTA

TTGTCATTTATTCGC
TGAACAAGAGTGCAAACGCACCAAGACAGGCGTGGATTACATCG
GCTACCGAAATACTTC
TTCATCAGGAACAGCTTGCATTCCATGGAGCGAAGCCGCTTCGTT
TCTGAACTCTCAAGT
TTCCATGCAATGGGAGTTCCCTGATCCTAGCATTGCTGAAGCATT
GAATTATTGCAGAAA
TCCAGTTTCTAAAGAAGGAGGGCCGTGGTGTTTTGTTGCCGACCC
AAGGATGACCAATGA
CTCCACGGAATATTGCTCATACG
>ConsensusfromContig480 Average coverage: 7.08
CCGCAGAACATACCCCCTCAGATACTACAGACTCGGGGACAGAC
AGGAGGAGAAACTCTC
TACCGCCCAAACGTGTTTCCCAGGACACTCAGCTCCCCACCTGCG
TATCAAGGACCTTTC
AGTTACCAAGATCAGTATCTTGCCTCTGAAATACCGCAGACTCAG
GGATACCCAGGCGCA
GGGACACCTGTATATGGAAGGC
>ConsensusfromContig481 Average coverage: 3.54
GGAAAAGATCAAAATATGATATCAGATGTGTTTTATACTAAGATA
TATTTAAAATCCCCC
TGTTTCGTAGTATGGTTTCCATCGTGTGACGTGTTTAGCTGGTTTT
ATACTCTAATCAGA
TCTCTTTTGGGTTAATTTTACGATAAAGTCTTTGTTGAGAATCGAG
AGCCTTGTTTCGAT
ATTTCAATTCGTATGGATATTTATCATTAGA
>ConsensusfromContig482 Average coverage: 7.32
AAGCTGTCCAAATGAACTGGCACAGGTAGATCATCTGATTGCGCC
ATTGGTGATACTTGA
TATTCGGGCTTTTTTGCATTGAAGCCAATCAGAGTCGTCAAACAT
AAATAACACTCAGTG
ACGTGATCTCTCGGCTAACGCCAGATCATAGGTATGGAAAATGGC
GTTTTATTCCTCTTT
CCTTTGGTCCTTAGCTGCAAAGTTTCCAAACAATATTTACATGCT
ATATGTGGAGCCCAT
GATTTGTCCAGATG
>ConsensusfromContig483 Average coverage: 17.53
GATACTCTGGAATGCTATCACAGTCATTGCATCACAGATTGTTCG
ACAAATTTCGAGAGA
TTTAGCAAATAATCTCAAATACTTATTACCGCCTTTGGGTATATT
TCACGGATCGAAGGA
TATGTACGAAAGTTGGGAAACAAGAAAACCTTATGGAGATGAAG

GCTGTAGCAGCGTGAA
TTCAAGGAAAAATTGCTATGA
>ConsensusfromContig484 Average coverage: 9.69
TGTTCACATCGTGGGCGTGGTATCAAATTGTCTGAGTAATTTTGC
GTAGTTCAGAATGCT
TCTATATAAAAAGACGAAGTTTGCAAATAAGTGCAGTGAGAAGA
GAGATACGCTTAATAA
GTTATTAACTCTACAATTATGTGGAAAAGTGAGCAAGACTGTCG
CAGGACAATCCCGACG
TATTTCTATAGATTTATGAATTCCACAGATCGGAAGAG
>ConsensusfromContig485 Average coverage: 10.18
ACTATATCATATGTTCCGATAAGGCTAAATTGATGGAACATAACA
TCTGATCAGTATCAC
CATTAAGAGAGTCAATAAGTTTTGGAGTTATAACAGATTTAGACA
TTTCCACAGAGTTGG
GAATTTGTTTTCTGATTGGTTTGTTCACTACATTACTTTTATGTTT
TATGGAGTAACAAT
AATTTAAAGATAATAAACTGTGGTCACTGATATTTTCCGGTACTG
GTATAATATCCAGAC
CAGAAATATTTGATGAAAATTCAGTGGGTGTAATAATTAAGTCTA
GG
>ConsensusfromContig486 Average coverage: 11.67
TTTCATCAAACCGTATAATCTATTATGAAAATCCCGAAGCCTCTT
GTATATCAATTTATC
TGTATTCATTGGTGATGATTTAATCATCGATATCTTCCTTCCCTGT
CACGAAATACGTGA
GCACCTTCATCCCAGTCTTCAATTCCGTCATTCCTCTTTCGACTGT
CACAAATCCCGAAC
TTTCTAGATGCTTTTTCGTCAC
>ConsensusfromContig487 Average coverage: 7.00
TTCGACCTTGGAAGGTGGCTTCCCGTGTTTGGTGAAGGTGACCAA
AGCGTAAGGTAGGAT
ATGACGTGGCCGCAATGCCGTATTCCCATTGGTTGCGTTGGTCTC
AAATATATAAACCTG
CGTCCTATCAAATTTCGTCTGCAGAGAATCACCAATAGAGGGCTG
AAGGGCGCTCACGTG
ACAGTCGTAGCCGGGGGTAGGTCCGATGAACTCTGATGAAGTTG
AAGGCCGAGGGATGGC
CGATGTTTTACGCCCCAACATATATTTGCATAGGAGTAAACGGA
GGACAGTGGTAGCAGG
ATACCTGATTAGCATGTGTTGGATGCTGACATCAGGTCGTGTTCC
GATGTAGACACCCAA

GTGGCTGTACCCAAGAC
>ConsensusfromContig488 Average coverage: 8.80
TGCTTTGTGATGGAAAGAGTATTCTTTGGCAAAATGAAGCGATTC
GCATGAGACTCAATT
ACAGAAAAAATGACAAAAATTTTGATGTCAAACAATTTCTATTCA
ATCACTTTAGGAAAA
AGAAACAGGAGCAAATGCGACGTCTCATGGCACCCATTCTACCC
TACACGAATCTACCGA
GACATACTGAGAAAATCCTAAACGATTCAAATGTGGTTTGGAAGA
TTTCCTTACAGGACA
GC
>ConsensusfromContig489 Average coverage: 5.84
AAGGCCTGATGGGGGAAGACGGCGGAGGGACTAAGTACGGATCG
CTCCCCTACGTCGGAA
ATCATGTTGAGTCGACATTTTGCTACATCAAGCGTCATTTGAGGA
TCGTTTACTACTGGT
TGGGTTTTCCTGGGGAAGAGTCAAGGCCAAACTCAAAAACGGCC
AAGAGTGCCATCTTGT
TTGTGTGTGGAATGCTGTTATCTGAAATCGCCCTCTGTATGGTGAT
TGTCTA
>ConsensusfromContig490 Average coverage: 5.18
TGAATAACTTATCGTTTTTCTCCAATATGCAAATTAACCGTTTTTG
TTTTGTTTGTATTC
TAGTTGTTTGTTATTCGTGTGAAAAAATACATGATATTTTATCATT
TCAATAAAATCCAT
TGATAGCTCACATATTATTTATGTTTCTCTGATACGTAACTGCCAT
ATATATCACGTTTT
GCTTCACCCAAGTCATTATTAC
>ConsensusfromContig491 Average coverage: 6.92
GGAATATTAGGAACATCAAGTGCTTCGTATGCTTAAGTGAAAATT
ATCACTATTACATAG
TTTAATAAACGAAGTGTATGCAGTTACATCGATGTAACTTAATTC
CGAGCAATACATGGT
TACATCGATGGAACTGAAGTCCGAAAGATACAGGGTTACATCAA
TGCATCTCGACATGAA
GATTCACACAAATAATTCATCTACAAATCCATAATCC
>ConsensusfromContig492 Average coverage: 6.75
ATGGAGAATTGTTCTTCTCTATTCTTTCTTATCATAATCATTTTAA
TGGAATCAACAACG
GGGAGGTGGATTTACAAGAGCCACCCAGATGAAGCTGCTATCGA
ATGTGAGTGTCATGGA
GAGCATTGTGAACCATTTGGAATAAGCTGTCCATCAGGAAAGTG

TTTGAGGGTGCGAGGC
ACTTGTGGTGGAGTCGAAGAATATCAGGTAGGATGCGCTGATAA
AAATGACC
>ConsensusfromContig493 Average coverage: 6.86
TAAATATACACGAAACATACAATACGCAATTCGCAAACGAAGAA
TCATACACTAATATGG
TGATTGGAACTCACAGCGATGGTGCAGGCGCTAAGTTCATCGGTA
CTTTGTATGAGATGA
AGCTTTATGACGAAACGGTTTTGACTAATTGGGACGTCGATAGAC
TGTATAAGAACACGC
CTAGTAGCTGTCAGGACAAACCAGATCTGGAGCCTGTCTTCACA
GAGACTGGAGCGAAGT
TGACTGCCAATTGCG
>ConsensusfromContig494 Average coverage: 9.35
TTCCGATCTGTTTTAACTGAAGATTTTGATCCTGATCAAATCGAA
ACAACTGATGTTGAT
TTCAACAAAGTTGCAACAGGAACATGTGCTCATAATAACCTGCTG
ATGGAGAATTTTAAC
ACCGTTAGAGCAGGAGCCGAATTGCTCCTGGAGTCCACTGAAGA
AGGTTCTGCGAAAGAT
TTTGATTCTGATCAAATTGAAACAAAAGATGTTGATCCGATGAAT
GTCGCCACATACTCA
AATGTTCTTGATAACCAGCCATCAGAAAACTCACACATCGTTGTT
GCAGAAACTAGATTG
GCTCAGTACTCAACCGAGATCGGAAGAGCACAC
>ConsensusfromContig495 Average coverage: 345.58
AATCAATAAAACCGGAAGCACTTGAATCAAAGGTCATGCACATTT
TACGAACGTCGTTCA
TGGATTTTGACATCGCATTTCTGAATTTCGCCTCTACTTGATTCAG
GCTGAGATGGTTCA
AGCTTTTTCTCTCTTCATGGATGACATTCATTTGAGGAACGTTTC
GGTTAACGATCACCG
GCATTACATTTCCTTGGCCCTGGCGTGAGATCGGAAGAGCGTCGT
GTAGGGAAAGAGTGT
AGATCTCGGTG
>ConsensusfromContig496 Average coverage: 7.58
GTTGCGTCGCCAGTAAGAGTGTATTCCACATCGCTGTAGGGCGCC
TGGATATCTGGATCG
ATGGCGATGACCCGACTGAATGAAGAGGAGAGCGGCTCGTTGTC
TTGGATGGTGCTCGCG
TATTCGGCGGGGTCAAAAGAAGGGGCATTCAGATTCCGATTGAC
GGTCAGGGAAACGATG

GACTCTGATATCTTCCCTCCGCCGTCCGTCGCTTGCACTCGAATC
AGATAGGACAAATCG
GTGTCTTCACTGAGACTGCGTTTGATTGATATTTCACCCGTATTT
CGATCGATCCTGAAG
AACCGTGAGGCCGTATTATCGCCCACGATTTCATACGTCACGACA
TTGAATGGTGCCGTT
GTATCTTTG
>ConsensusfromContig497 Average coverage: 15.45
CCGATCTAAGATACGTCAGAGGCGGTACTTGCGATATGCATGGA
AATTCACTGAAACAAC
AAGAATGTAAAGGATCTTCTTTCTTTGTTGGACAGTCGGTAATTT
GAAAATTTGTTTGTG
GATCATCAGACATGTAGAAACCATGTCTTCTAAGAATGTCCATAA
CATTTTCAAGATATT
CGGTCCGACGGAATGCCTTATAAATATCATCTAGGATCATTTTAT
AGGATGCCATATCGC
>ConsensusfromContig498 Average coverage: 6.57
GAACTTTTGGCCCAAAATTTAGATGGGTTTAGTGCCTATCATGTT
CGGTAAATCAGTATT
TCTTCTGTTATTTTTTATCGTGTCATCGGGAGGAGAGACCTGTAT
ACAGAAGATGGACGC
GGTTATGAGTGACCCAACACTGATTAAAATTGCATCTCATAATTA
CGAAACGGCCGCGGT
GTGTCTCGACTACAAATCTGGGATTTTAGATCGGAAGA
>ConsensusfromContig499 Average coverage: 6.49
CAAACAGCTAGAAAAATGTTGTGAGCTAAGATCTTACTCACCATA
ACTATGGAAATAGTC
TTTTTCTGAAATAATACGATAATTGTTTTCGGTTTCAAGACTAAT
AAGTTAGTAACTGAC
ATTTTGGATCGTTCAAACACAACAAATGACGTTTTCAAAGAACAT
AATTTAGGAACTGTT
AAATATTCATAAACATCGAATACAAGTAC
>ConsensusfromContig500 Average coverage: 6.26
AAGTTGCCTCAAAAACCCTGTTGAAAGATCACTTCAAATTCACCT
TCGTGAGGCACCCTC
TTGATAGAGTGCTGTCAGCCTACCGTGACAAATTCCTGACTTTTA
ATAAGAGAACGTGTT
TTTTCCAAACTGTTTATGGAAAGTACATTGTGATGAACTATCGCA
AACCGAAGCCTCATG
AATCTAAGTCGAATGGCAGAGATGTAACATTCCCAGAG
>ConsensusfromContig501 Average coverage: 7.55
GGATATATATCCAATCGGATTATCGGACTGGTATGCTCACGATGG

TTTGAAAATTGGAAA
TCCAGGTGGATGGTCGGCCAAACCTGATACTCGTTGTGAAATCGA
GCGTTCGCCCTATTT
TATTCGTTGCAGTTTTCATCTATCTCCCAGAGATGAGCGTTGACT
GGCAGTTTTTGACGC
CAACAAGTGTGGTGCGTGTGCGTCTCAATTATCG
>ConsensusfromContig502 Average coverage: 12.05
CATAACAAGAACTTTACTCGACGCAGATATCAATTTCATTCCATA
ACATTCCGGGACGCT
GAAAACCCTTCGCAAATCTTGCAAGAAAGTCGACGTCGGTGAAG
ATAGAGAGACCTGGAC
GGCAAATTTAGGCTCTCGCTTTCTGGGAGAACTCGTTGGCGGTTT
GTTGATATCAGAACA
AATGACTGAACGAATGCAATCATTTTCATCAAGTTTCTGTGGCTT
AGATTTATTCTGGGA
ATGCGAGCA
>ConsensusfromContig503 Average coverage: 7.44
TCCACATTAATATTTCGACAAACTTTATTTTCAGAATAGAGCTACC
GTTTTTTTTATTTG
TGATTTTTAATCGATATAATAGGAGCTATATTATCGATAAATATC
AGTTACCGGACACAA
TTCAAATATGGAGGAGTTGGTGATTTTTAATAATGTTATAGTGGC
TATATTTGACACGAA
GGGTAGGTAATTTTGAATTTTCGTCAAGTTACGACTTTGCAGAAA
CAATGTCCAGATTAA
GATAAAGTCTGAAAGTGACGATTACCCCATGTTTATG
>ConsensusfromContig504 Average coverage: 6.87
AATTCATCTGATAGCAATTTAATCTTCGGTTGTAATGATTTGCCTT
TCATAGAATTTTTA
ACAGAAAAAATATATAATGGGAATGTAAATACGGATCTTAGAAT
GAATGTTGACAAGATA
ATGGAACTCCACCATTCCAGTCCTGATAAATCATGAATAAAATCT
ATATATGATTTTGCA
AATGAAATTGGCATAAAATCTGTAGTTAAATAACTTTCAGCAAAT
GTTC
>ConsensusfromContig505 Average coverage: 5.62
TACAGTCAGTGCATAATGGCTTGTCCATGCATGATTCTTTCTTTTG
ACCTTTCCTGATGA
ACAATTCTTCCAGAGGGTCCAGGCTGACCTCTCCATCTCCCGATT
GGCAGATTCTGCAAG
TGTCCAGAAAGGCGTTCCAGAGAAAGGCAGGCATTCCAGCCTTC
ACTGCAAATGCCGCAC

ATTCTTCCTGAGAGGTCTTCT
>ConsensusfromContig506 Average coverage: 6.07
TGGGCATGGATATTATAAGCCTTATCATATATATTGGTTTGGGTAT
TTTTCAATTCCACT
CTTGTTAATCTTAGCAAATACATATACCCGGATGCTGAACTATTG
CATAATCATTATACT
GTACTTTCCATAGACACCAATGTTATTTGCATGTAGAGAGTCATT
TGCGTCGGCAAATCG
TGTATTCCCAAATCATTCCATCAATCAGCAGAAACTCTCTATAAT
GACAG
>ConsensusfromContig507 Average coverage: 7.46
CTCTTCCGATCACCCCAATATAAATAAGGAATGAGAAGCCTGAC
AAGCCCAAAAGACCCA
GCACTTGGAACTGTCGCATTATCTGTTATGCTGGAGTATTGATCT
CACTCATCTCAAGAT
ATGAACCATGGGATTGAGGAATCTGGGTGATGGACGACAGGTTT
TCATACCCATCAGCAG
TTTCATACCCATCCGCTGGTATAACGTTCATTGTGCTGGAGTCGTG
TTTTCCACGGATTT
TGCTTGGATTT
>ConsensusfromContig508 Average coverage: 4.92
CCCAAACTTCCAGAATGTACGAAATGCGTAAGGTTTCAACTCTTA
TGTTTTATACATGTA
TATAGAAGTGACCGTCAATGTTTTCCCATGTATTTTGTTCACTGTT
ATTTGATATAAAAG
GAATATCACATGATACTACTCGAGATCAGTTTTATTATGAGGCAC
AGATCTCATATGGTG
TGACAAGCATTCCAGCTCGTTTGATTGTTCATTTCCATTTATTGGA
TAACATTTACATAT
CTCATAAAGGTTTCAAAATTGTCGTCAGTTTTTTTGGGAAAATAA
GTACAAAACATTTTT
ATTATCTTATATTACGATATGAAACATATGTGATGCATAATAAGA

>ConsensusfromContig509 Average coverage: 14.59
ATTTTTAAAAAGGTCACTCCCATCTGAATCATCTAACAGATTGGA
GAGACACGGGATGTT
GCTTGCCTTTTTTGCGCGCCCCCCACTGCCAATGTAACACGAAGT
GTCACGAGGGGCGCT
TTGGCGTCTGCTTAGGAGTATATTATTTTGATCATGATTATTAGTA
GATTTTAAATTGTA
AGAGGTTGAGTTAACCCCTGGTTTGATTTTATTATTCCCATCAGC
TGATTCAATGA

>ConsensusfromContig510 Average coverage: 21.98
AAGATATGAACGGCAACAACCACGCACATAATACCGAGGAAAC
AAGCAAAATAGTGAGC
TGGAATGAGTTGCCTGTTGGTTCAATGATCACAAACAATATTAAG
CGTACGTAAAACGAA
AATATATAGGCAGGTTATTACAGCCGCACAGTAACTTAATTATAC
TGTCTAATCTTCGAT
AATCTTCCACGAATTTCCAG
>ConsensusfromContig511 Average coverage: 5.99
ACGCTCTTCCGATCTTTTGATGTTCCAAATTGAGCCTTTTTCATAA
GCATTTCTCGTAGC
TTCTGTTTTGTGACAGATGTGGTTTCACTCCCTGGTTTCTTCACTT
GCATCTCGCTTGCG
ATCTTGCTCGGAGTCATGGCTGGTTGCTCCATGACTGCACCAGTT
GTGACTTCATCAATT
GGCCGATTTGTTGAGGTCCCACTTCGAGCCTTGCCCAGAGTCTCGT
CTGGTTTCTTCTTG
GTTGTGTCAGTTGTGGCGATATTTTTTGACCGCTTTGCCGGTGCCC
CACTTG
>ConsensusfromContig512 Average coverage: 9.00
TCCATGAGTTTTTAAATCGCATATAAATAAAACTTTTTTAATAGG
ATCCCACTTCATCAT
GTCAATAATGCCACAATATACTATTCCATCGAAACTCCAATTAAA
TCGAAATTCACTCAC
TCGATGATGGTCTATCAAATGTTTCGTATATTCGATGAGATTCAT
CAATTTACATTCGTA
AGGAGTGTTATTGTTTGTTTTTGTC
>ConsensusfromContig513 Average coverage: 8.25
TCGCAATAAAGACCCATGCAACGCGCACCACTCCGGCACATCCGG
TCCACGGGTCCAATT
CGCGCCGTCGGTGATTGAACTATCTTCACCAGGATGTCCGAAAGA
TCCCCAAGAATCTGC
ACGCTGCTCCTTGACGCACTACGACTCACAGCCCCTGTGCCGCTT
ACCAGAAGAAGGGTG
TACGTCGTCATTTGGCAATCCGGACACTCTCATACCAGAAACTGG
AACAGAATCTCCTCA
GAGAAACCCAGATTGCCCGGAGTGTTCATTGTCAACTTTTTC
>ConsensusfromContig514 Average coverage: 8.50
GGAATATACACATATCGATTTAGATGATATTTCGAAAAGTATCAC
GGATATAAGATGAAC
ATAAAAAACAAATTATCACAAACGAACTCATAAGCACGTATGTA
TTTGAAGATACCACAA

ATCTTATTTCAACCATAAACTAGCATTGATAGATCGATGAAAATA
GCTTATACTGGGAAG
ACCCAAATGAACTAATAATACACATGATTGAAATGCGAAATAAT
AG
>ConsensusfromContig515 Average coverage: 7.82
CTTTTAAAGATTTATGTTTGCAGTTACGTTGAAGGTGCCGTGTATC
ACCTTCCATTTGAG
GAATATGTGAATAGTACAGTACTCGGGGATATAAATTTAATAGC
AAAACGATTTGAAAAT
AATAATCATGGCGAATTGCGAAAAAATGATGCACTCGAAATAGTT
GATGGTTACTGCATT
AATTCTGTAAAAATCAGAGATCCCACTTCGCACTATCTTGCTGTG
GATAAAACACATGAC
AGCTGTCTTGGCACGATATCAAAATGTCAAGTGGGACACATACGA
TTTTTTATCAGGTCT
GATAGTATCACAGACAAATGCGGCCTAGCTTTGGCTACAGGAAT
GCGTGACCCAGACCCC
AAACGGCAACTGAAAGTTTATGTTTGTAATGAAGGCATTATTGTC
ATTC
>ConsensusfromContig516 Average coverage: 8.50
ATGATATTTAAATGATGATTGAATATTTTTTCTGATACACAGGGT
GGGAAACCTATATGC
GAAATGAATATTTTTGCTAATTTAGGGACCGCTACTTCAAATTAG
TCTCTCATTTCGACA
TAATTTTCTAATAAGTTTCTTCACAAAGTATCAGTCATTTTGAAAC
CAAACGACTTTGTT
ATACAGAGAAGAGTCGAATTAAGGATTATTCTAT
>ConsensusfromContig517 Average coverage: 435.01
CCGAGTTGGGTATCTATCCGGCCGTAGATCCTCTTGATTCCTCGT
CAAGAATTCTCGATC
CCAATGTTGTTGGAACGGAGCATTATGAATGTGCCCGTGCAGTAC
AGAAGGTTTTGCAGA
ACTTTCGATCTTTGCAAGATATCATTGCTATCCTTGGTATGGATG
AACTCTCGGAAGAAG
ACAAATTGACTGTGGCCAGAGCACGAAAGGCGCAGAAGTTTATG
TCCCAGCCATTTCAAG
TGGCCGAGGTGTTCACTGGTTCC
>ConsensusfromContig518 Average coverage: 6.12
GTTATTCATTATCGGTTTCAAACAAAACTTTTATTGATATAAAGG
TGTGCATGCTGAAAT
TCATCACATCCAGCCATAGAAATATACACTCATTTGCGTGTTCTG
TATTTTAGAGGTCCT

CGATGTTCATTAACATAACAACAAGATATTAATTCACGAAACTAG
AAAACATTTATTTGA
TACTACAAATATGAGAATGATAG
>ConsensusfromContig519 Average coverage: 7.81
AATAATAACATGGTATAAAAGCACGAATTCCAATATATTGAATGT
TTTCTCTTCTCGCAC
ATATTATAAATCTGAGATTGTGTTTCATGCAAAATAGTGAACAAG
AACAATAAACGCACT
GAAAGAATTTTAGAAAACTAGCAATAATTTTCAGCCGATTTTTAG
AACAATTGCAGGTAA
TGTTATAATCACCCTAATACACTGTTTTGA
>ConsensusfromContig520 Average coverage: 7.15
AAAATATGATACTCAAGGAAAGACATCAATCGTATCTATTGAAA
ACGTAAAAAGTACATA
AGGATACTGGAGATAAATCAACTGTTTCGGCTAAACATTTTCATA
AATTCGGGCTGGAAT
ATACTGACACACATTACTGCAAAGAAGGACAGAAAAAGGAGTCC
CAGAAGAATTTTCATG
GTGGCATCGATGATGGTCTTCCTCGATTCAGCATTGTCATAAAAC
GACGGCTGTGTTATA
CTCACGCGCAGCCAATTCAGAGTGATGGCTTTAGCATTTATATTT
ACATTAGCCTGACAT
TCCCAGATTCCTGCATCGGACAAGCTCATCTTATGTATGGTGAAC
GAATCCGCAATCTCC
AATATTGAGTCGGTTATCTCTATGCGTATAGTACCATTGTGTAGC
CATCTTGTAGGAAAT
GTAGAGAGATGATCAGGTATATGAATAAGATTGCAGACAAATTCA
AATTCTCTGTTAACG
TGTGCTTCAATTGTCGGCTCATTGGATTTGACAATCAAAGCATAG
GAGGCAACGACTCTT
GTGAAATCGGCGGTGTGTTCGAGCACACAGGAGTATACTGCTGT
GTCGACGGCACGTAAA
TTATTTATGTAAAGATTACTTGAAATGAATTGC
>ConsensusfromContig521 Average coverage: 12.80
AAATGAAAATGCAAATCACGATACAATCCTTCAAAATTCAGTCAG
TGTCATCCACAACAT
TGAAAATTATAGTGAAAATAGAAACAGTGATTTTCATGACCAGA
ATCAAGACAAAGATAA
TGCTATTGAAATTGAAAATCCAGGTGATAAGTATATAATTTCTCG
AAATTCAGTTAATGT
CATACATAATATCGACAACAGCATACCTGTAGAGATTGATAATC
GTGATCAAAATAAAGA

TA
>ConsensusfromContig522 Average coverage: 11.71
CAATAATCGAAGGACGCTCAGCCACCATTTTGGCATCTCTCACAG
TTTTCCCAGCCGGGA
CGAATGAAGTGAGAACCAGTAGGCATGACGGTCTGGGCGTAGAA
GGTTTTCGACTCCAAA
GTCCTCTGGTTCTTCACCTTAATTGAGTCCATAATCCAAGCATCCT
CAGTGCCTTGTTTG
ATCACGAGGATGAGATTCAAAGTTCCGACATCTTTATCTTCAAAA
ACGAAGTCATCCGTG
TGTCCTGCTTCCATATCATTATGGCCTGGATTGTTAAGATGATGAT
CAGCTGTGTTTGTA
ACGGATCCAAGGAGACGGATGAGAACTCTTTCATCGGTCCCAGCT
CCCAAATTACTGCTT
GTCGTAACTGTTATTCTGTACGTCGCATTTCCACGATAAAGACATT
CTCCGTCCTCTACT
CGACATGGC
>ConsensusfromContig523 Average coverage: 7.22
GAGAAATGTTGATAAAAAATCAATTATCCCTTCATTTTAAATCCA
GAATTTCTTTTATAC
TGTATTCATACAAGTTTGGGTGGTATGATGAATCATAATTATAAA
TAGCAATTGCTCATG
TGAAATTCCAAAAGCTTATAATATATCATACTCTTTCTTTGATCG
TAAATTTAAAATAAA
AGGCATACATTTGATCGCTT
>ConsensusfromContig524 Average coverage: 20.04
CGGATAGCATAAACAATGTTGAAAATTTCAACGCAAATATAAACA
ATGCTCATGAATTAT
ATGAGGCACGGACGGTATTGACCTAATTTAAGCCCACACTATGAA
ATGTTTTAATAGAAT
TAAAGCACGCAAGGGCGAAAAGTCACCGAATAACATTTTGTCGA
TGACACAATATTGCAT
TGAAATCATTTAACGTATTTCACATAAAATTTACTAAAATGAAA
>ConsensusfromContig525 Average coverage: 6.69
CCTTCATCGACCAGCCATCGCAGAATAACTCAGATAACTCGTGGT
TATGTGAGTCAGAAA
GATGCTTTATCTCAAGACTCTGTCCATCAGCTGAAGCCCTGACTT
TGATGACAGCAGAAC
AGCCTTTCTGAAATGTTTTCGATTGACGCACTCCCTCACCGCGTGC
CTTGAAATTCCTCC
CGCCATTTATGCAGTTGTAATTTATCTCATAATACTTAAGTGATT
CTTTCAAATCACGGG

AAACTATTTTTCTTGCATACGCTATTGTTTTTGAATTTGGC
>ConsensusfromContig526 Average coverage: 14.08
AGATGAAAACAAATTGTAACCATTATACATCATAGATTTGTTTTT
GTCAACATCTTGAAG
TATAAATGAATATCCACATTGTTCAATTGATACTATCTCACTTGAT
AACTTTTTATGACC
GAGTGAATGAATTTCTTTATTCCCAGCTGTGTTCTTTACACAAAT
GTGAATGGTATGCCA
TTCAATAGCCATTTGGACTAAATGCTCATCACATGATGATACAGA
TACATATTCACGAGC
TAGCATTGATTCTGTAA
>ConsensusfromContig527 Average coverage: 4.79
AATCAATGCATTATAAGGTTGACCATCAGAGGCCCAAAACACAA
AGGATCGAATTTGAAT
TTAAAGTTTATATGCAGAGGGGTTTATATTATGAGGACGCAATGT
GAGTTTGAAATTGTT
CGCATTCTGGTGAAAATCTGCACTCATACTCAAATTTATCGGAAT
GTTAACCATATTCAG
TGGCTCAGCCCGCTCGAAATCATTTGAAATTTGGTGCTTAGAAGC
ACGTGCGTAGCGGAG
TATAATAAGTTAAAATAATACTTCGTGCATGATCACTCTGACATTT
AGGGTTTTTTTTAC
A
>ConsensusfromContig528 Average coverage: 10.75
GTTGTGCAGTTGACACCTCTATCATGATATCCTGCTATTTGCGTC
ACAGGAACATCCCCT
CTCATCAAATAAATCTCATCTATCATATAAGAGATGCTCGAATCA
TTATTTCGTGTTACA
TCTCCGAAATCAATCGATGTTATCTCAGTCTGGAACAGCAGATTT
TCCGTTGGGATGTTA
CCAAGAAGTTCTCCGTCCTGGTAAACTGCTGCTCCTCTCTTGGCC
GGTTTGAATTTCAGA
GCAATATGCGCCCAAACCCCT
>ConsensusfromContig529 Average coverage: 6.10
CTCTTCCGATCCCTCCATTCTTTGGCTGTCGAATTTTGGCGGTAAT
TGCATCTTCGGTAT
GCAATATGTACAGCCGATATATACAGATGTGCAAACTTATGTTCA
TTGCCAACCTTCGCT
TCCTCCATTCTAGCTACGACTCTGACCATACCCTCGAACTTTGTTA
TCTGGTGATCTCAA
CATTACCTAAAATGTTTATTAAAACCGGCTGGCCCA
>ConsensusfromContig530 Average coverage: 4.56

GTTTTACCTTACCCGTTTGCCTCCATTTTCCAGGCCAATAGGGGT
GCTACTATTATGGGA
CACGATGACGTCTCCGATACCATGGTCTTGGGGGTCGCCCAAGGG
TCTACCATCATTATA
GGGTCCCAGACTATGCCGAGGCGAATTATATTTGTCACCTCTGAG
ACTACTGCCTTTGAA
AACACGGAATTCTTTGCCGATAACAATACCGAAATACGGCCTGA
AATGCCCCG
>ConsensusfromContig531 Average coverage: 10.88
CTCCAGTCTTTGTAGAAAATTGGAACCATACGAATGAAAGACGG
TGACAACAGCATGCCA
AATACAAACAACGCAAAGAATGACCAGAGACAAAAGAACGTATT
TATCCATATATGTGAG
GTATGATATCTTGGGCAGACTCTGATTGATGACGAATTTGAAGG
CGACGGAAGTGAGAAG
CAAGGTGAAGGACAATTGAAGCCGGTTTTGAGGCAGTTCCGGCG
AGACGGCAAATGTTGC
GAATGATAAGGCGCTGATGAAAAACATGACGAGAAAAACGTTCC
AATAGAAGTAGCCGGG
CCTCCGCGCCGCTCGACACGTGACCGAG
>ConsensusfromContig532 Average coverage: 9.82
CACAAATGATAATTTATATGTTCATCATATTTCTATATCATCTTGT
TTTTCGAGTTCGTT
GATTTTCTTCACCAATTTGTTAGCGATTTTCCAAATATTATTTTTGC
TTTCCCACCTTTT
CATCAATTTCTTGATATCTTCATCCTCCTTTTTTCCATATTCTTCA
ATCACGTGATCCAA
TTCAGAGTCCATTTTCTCCACTATATCTTCCAGCTGTGTTT
>ConsensusfromContig533 Average coverage: 10.40
ACAATGCTTAATTACACAGAATTCAACCTACGAAATGCGACTTTC
ATTTTCGGCCACGAT
GAAGACAGAGTTGTGTTGCCTCTTCTGCTTTCATTCGTCGGAATT
TGTGCCCTTGCAGCA
AATATTCTAATAATTATGTCAATCATAGGCCACCACAGGATGAGG
ACAGGTCCCAACATG
ATGCTCCTCAATATGGCTTCTTCAGACACTCTCCTAACGCTTTTTG
TAATCCCAATGGCC
ATTCTCAATCATGCAATGCC
>ConsensusfromContig534 Average coverage: 5.64
AACGACAATAACATATCCTTATTTACGATGAAAACCAAAGTTATG
ACTCACCATATAGAA
TTATGCAATGGCACAAAGGAAAGAAATTTCACTTGGTTTCAAGTC

TACGGAGGTCTAGGA
CCGGGTAAGAAAATACAAGTTATTAGCTCTGATCCTTCCCCCAAG
GTGTTCAAATGCGAA
CGTTTGGATTTTTTCATTCATGATAATACCATCAAAGTTAAAGCC
TCGAACCCAAATGAT
GACATTGCCAATTTTCCGAAAAAGAAAATCAAAAATGCGAATTA
GTTTGCAGGATGATA
>ConsensusfromContig535 Average coverage: 4.74
TGTTCATATAATAGGAGCTTCAGGACTTTCTTTTATATATTTCCTG
GTTTCTATTCGAAA
ATTCCTCATGTAAAACGCTAGTTGTCGCTTTAATGTCGAATATCA
TATATGATAGTTTGA
GGGTTTCGATAAATCCATATAATACAATATTTTAATAGAGGCCAT
ATAATTTGAAATCTA
ATCATTTCACGACACATTTAAAGCAATTAAAATTATATACTGC
>ConsensusfromContig536 Average coverage: 14.73
TCAACATCAATCGATTATCAACCTTTTGTTATATTTGCTAAATGAT
TATCTATGTATTCA
ATCTATCACGATGTCTCGTTTTTCATAAATTTCAGAGTGAGAAAG
TCAGATAGCCTTAAT
ATATAATCATAAATCAATATATGGATATTTTTGGACATTCCAAAA
TACGTGTGTGACATT
CCACTGGACCGAAAAATGAACGAAAATTGAACGGTTTCATTTATT
TTTCT
>ConsensusfromContig537 Average coverage: 7.63
TTACATGGATGTTGATTACGATTTGACATACCGTTGTATGATGGA
TTTTATTGGCGATTG
ATTGGTCAGAAAGATCTTCGCTTTTGAATCTTTCTTTTCTGATTGA
GAGTTCTGATCAGT
ATTTTTTATTGGTATGGTCGATCTCCGGTCGATAAACTGCCAAAA
AATCTGGTTGTTGAG
CATCTCTCAAATTTAATATTTTA
>ConsensusfromContig538 Average coverage: 10.27
GTAGATTCTCATGAGATTGAATGAGTATCAAAATCACATCCTGTT
CTCGCTATATGATTG
CATGGGAGATATTTATTTGAAAGTGGGTGTTCTTGGGTCTATTTA
CGATATTTACGATAG
TATCGAAATTTTGTTTGTTGATATATGATTTTCACAAGATACTTTT
TTGAGTAGAGTAGA
TGATGCTTGGGAGTTTTTTCACTATGTATTTATTTGCG
>ConsensusfromContig539 Average coverage: 7.09
CCGATCTCGAGTTTAGATTACTGAAAACTGTCCTGATAGATTTTTC

AAGGCTTCTTCGTT
CAGGATCAAAGTCAAGGATGAGTGGCAAAATCAATTTATTGTTCA
CATCTTGGGATTCAA
GCAATGTTTCTCGCTTCATTTTTGATACGGTTTCCACACATTTACG
AATCATACACATTG
GATACCCGCATGACAAAAACTCATACACGAGTTGACGGAGTCCG
ACGTGGAACAACTCAT
CGGATGAG
>ConsensusfromContig540 Average coverage: 6.77
CCGAAACGTGTGATTTTATGGGCCGCCCCTCGAACAAGGTCGTCC
GCGTTTTACAGGGCT
ATGTCGAATAAATCAAGGACAGAGGTTCACTTTGAGCCGTATGGT
GAAGCCTATTACCGC
GGACCAGGCAAAACATCAGACAGATCTGCGCCGGAGATTTTGTT
TGATGAAAAGTATTCC
ACCGTCAAACAAAATCTTGAGAATAATCCGCCAGATGTGGACCTA
GTATTTGTCAAAGAC
ATGGCTTATTTAGATCGGAAGAGCGT
>ConsensusfromContig541 Average coverage: 6.26
ATTTGAAAGGACGGTTTTTAATAAACATTCGGGATATTACAATTT
TTTGTGGAAATAGTG
ACAGATGACAGATTGAATTATTATCGCCCATACTAACAACATTTT
TCATATATTTTTTGC
TTACTTATAATACTTTACTTGTGTATACATATATACCTCTAATATC
ATATATACATCTAA
ACATATATCTCTATTATAGAAACGATAACTAGGTAAG
>ConsensusfromContig542 Average coverage: 5.20
CAGCTTACATTTGTAACGATTCCCCATTCATTATAACTCCAAAAC
GAAACAATGGGACAT
TAACAGATGATGAAATAGCAAATAACACTAGAATTTGCAGAGGA
CGAGTGATAATTGAAA
ACACATTCGGCAGACTCAAATGCCGTTTCAGAAGGATGCAGGAC
TTGCAGAATGTTGGGC
TTCTCATGAAAGTGCGGCTTATAATATCAGCATGTACTTTGCACA
ATATGTGAGATCGGA
AGAGCGTCGT
>ConsensusfromContig543 Average coverage: 8.20
TTCAGACTCAAATCACATATGGTTCAGCACAAGCACAATAAGATG
TACAAATGTGTCGTG
GCCAGCTGTGGAGATCTTTTCCCAGACCTAGAAGCCTTCCTCGAG
CACACCAAGCGACAT
GACGGACACATGACCTATCGTTGCCATACGTGTGACACGGAATG

CGCCGATCTGAATGAG
CTGAACGTCCATCAATTCACGCACACGATCTGCCCCTACAGAAGA
CCCAATATCCAAAAC
AAGCGCTACAAATGTCCGAAATGC
>ConsensusfromContig544 Average coverage: 7.89
CGAGTGTCGAAGGCTACCGTCTCTCCTGGTTCAAAGTACAACCGG
CGGAAACTTCACAGA
ACGACACTTCAGTACACTACGAATTTTCAGAAAGAGAGTCACCCC
TCATAAAAGTCGTCA
ACAAGCGACCCACATGGTTTGCATTGGATAGTTTAGACGCGGCAT
CTTCTTATATGGTCA
GAATCGCCGCCTACTCATCTGCAGGCGCAGGGCCCAAAGCCAGTC
ACATCTTTAGCACTC
CAACAATACGAGCGCGC
>ConsensusfromContig545 Average coverage: 11.02
CTCTACTTCTATGCCTCTAATTGATAAAGATTGCTACTGTTTAATA
TATGCACGGTTCTC
GAAATTTGCGCGGACTGTTCCATCTCAGAGGTTCATAAATTAATA
TATGGCATTGGATAT
CATGTGCTCATATTTTATAAACAAAAGAGAATTTTTAGGATTTCA
TCTTGAAATGTATTA
TAACAGATTTACGTCACAATAGCAGAAAAATCC
>ConsensusfromContig546 Average coverage: 3.82
CAATCATTGAAGGAGATAAAAAGTCCAGTAACATACATTGAATA
AGATAAAAAGACAAGG
AAAAGAAACACGAGAATATTTGTCGATGATATTAACATCCCTGAT
TCTTGGAATTCTACG
CTTCCAGTCAGCTGCTCTCCGTTTGATATTGACAAGAGTGGAAGG
GCGATGGTTTCTGAC
TGTGGCAGGCTTTCGCCCCCCACACCG
>ConsensusfromContig547 Average coverage: 6.65
AAAGTTTATATTATATAGCAGAACATAATCAAAAAGCCTGAATAT
GTCAACTGGGAGTCT
CAGTCGACCGGCTTCAAGGACTGTAGCAATGGATGATGCCCCAA
AGACAGCGTCCAACGC
AGATTCATCTAGAAAGAGATCTGTAGCAGATACAGACCCTCATTC
CGCAGCTACATCAGG
TATGCTGGAATCGAGGACCGGAGCAGAACTGACAAATGTTCTGA
C
>ConsensusfromContig548 Average coverage: 4.91
AGACTTTTTTGGCCCTTGCCTTTTCCTTATTTTGAGGGCAGGGTCA
AGTTCTATTGTGTT

AAGCAATGTCTGTTTAGCATTCTCAATCGTTTTTTCATCATAAAA
ATCCAAACATAGTTT
GGTGATCATAAGGGTGGGCATAATTTTCATTTTATTTTTTGCAAA
GCACAGTAGTTCATT
AATAACTATTATATTGGACGAAGCGGATTTGTCCACCATGATTGA
TTACC
>ConsensusfromContig549 Average coverage: 16.05
TTCAGACGTGTGCTCTTCCGATCTGTAACGATGGGTACAAGTCTT
CATCTATAGAAATGA
CGGGCATGGAACCTGCCATTCATGATTCCCAAAAAGGACTATTTG
ATCGCCGGCCGAACA
TGCAAGCAACTGCCCAGACGCTGGCGAACACTGGATCAGTCTGA
TCATAACTGCAACATA
CGTGGAACTCGGTTTAAACATCTGGGAAGGACACATACAAGATT
AGGC
>ConsensusfromContig550 Average coverage: 5.84
TGTCAACACATCATGGCGGATTCCCCACTCATAACTCTAGTTCAA
GAAAATGCTGCCGCG
ATCAGTTGTCCCATTTACGGGAAATTCAAATTCAAGTACAACAAC
AATCGTGCAGGATTT
TGCGGGAACCCGATATCTCATGCATATTCATGTGCTGGTAGTTCT
GTGTTCAGGCTCTCA
TTCAAGAAATGCAGCCACATTTTACCCTTCCAGTACTCAGAGTTG
GATGTCAAATGTA
>ConsensusfromContig551 Average coverage: 6.80
AAAAAACTCAATGTATATCTAGAATGTTTTATTTTATCATAGACA
CTGTCAAAAGTTTCA
ATGAGAATGGGTCTTCAGCGGAGGAGTCTGACAATTCTTCCGATT
CGGACTATGTCCCTG
ATTCAACAGAAAGCGAGGACGAATATGTCCCATCTATCACAGTCT
ACCACAAACAGCTCT
TGTTGGATCAATCATCTCAGCAAATGGAAAATGAC
>ConsensusfromContig552 Average coverage: 8.46
ACTCCGAGCAGAATGAAATTTTAAAGTTAAATAATTTGGTTCATG
ATCACAATAAATAGG
AAGAATACTGTTCATGTATTGCATAAATAATGATGCGTTTCTGTA
TACTCTACGTTACAT
TCGAACAAACGCACAACTCTCCAGTGAACCTGTTTCTATACTCAA
AGCAGATTAACATTT
AAATATGAACTGATTTGATACACAAGCAAGTTTTTTAT
>ConsensusfromContig553 Average coverage: 16.97
CCTCTATGAACGACTGGTGTGAAGAACAAGCCATCTTCATCCTGC

TTGCATTTGTGTTTG
ACACTTGAGACGGTATTGATGCAAAGCTGAACGAGGCATTGAAC
GGCTTCTGATGCCAGC
GGGGAGCTGTGGTCGTACTTATAAATGATGACAATGAGTGAAGT
GAGCCAGGCATATCTG
AAATGACTATCCAGAAATGCCAGAGTGTAGTTTGGCGGTTGAGAG
TCGCTCGC
>ConsensusfromContig554 Average coverage: 9.44
CTTGCAATATTCATTTCAATATGATAACGACTTTATGACAAATAG
ACACTAATGGCACAT
TTAATTTCTGTTACTGTCTCAGCGTATTTCTGTCGTGAGCTCTTCT
GGATATTCTTCTGT
TTCAATTTCATGCTGTTCAATCGGTAATTCCACATAAAGTTCATG
GGATGGAACTGCGGG
GTTCTTTCGAGATCTTATTTCTGAGCCGGATGAATGTGCCACATTG
GGCTCTCTTTCATG
TTCGACTGAATTCATATTAACATCCTCTGTTTCATTCAC
>ConsensusfromContig555 Average coverage: 8.61
ATCAAGGGCCGGCAGCCCCCCTCCCAGAAGAGCACCCCCTGGCG
GCGGTCTGCAGATTGG
ATTTCGACTGCTGGCATCGTGTGTCGATATAGGTGGCGACCAAGG
TTTGAACGTGAGATG
GCGGCACTGGTCTGCTCCGTGACTTGAATTATAGACCAAAGATGG
CACTGCTGAGTTGTC
CAAATCATTTTTCTTTGAATTCGGAGATCTTAGTGAGCCCGGC
>ConsensusfromContig556 Average coverage: 8.00
TCTGGTTCTTTATAATGTGGTTTACGGCAGCAGTTCAGCTGCTTTG
GTCGCAATGATTTC
GATTGTTCCGTATGAAGTTATTGGAGCGGAACGAATAACTGGGTC
ATTCGGAATTATGTA
CTTCTTTGCTGGGATTTCAACATTCTGCGGACCGCCGCTGACCGG
TCATATAAAGGAAGT
TTCCAGCTCTTTCGTTTCCAGTTTTGT
>ConsensusfromContig557 Average coverage: 15.28
AGATTGAGTGACAAAATCAATAGAAGTTGAAGAAAGAGCAGGGG
CAAGGAAAATTAACTA
GGAAAAGTGAAAAGTGGCTGAAGAAAAGGAAGAATTGATATAA
TAAAGAAAATCATGACA
AATTTTCCAACCTATCCCGGAACATTCTAAATATATTAATGTCCG
GTTTCTGTAATGACC
ATTGCTCGATTAAATCTGCCCTGCTGTCTCTCAGTAATGCTGACG
CGAACTGGTAAAATT

AATATA
>ConsensusfromContig558 Average coverage: 7.44
ATGTTAACTTACCTGTCATACAACAACAATTAATCACGATTTTTA
TAAGGGGATTAGGAT
CAAGGGGGCTTCAAGAAAAAATATTTAGGTCAAATAGCGAAACC
TTGGGGAGGGCCGCAG
CACTTGCGAAACACGAAGAAAATAGTCACACAAGATTTAATACAC
AGTTTTCGTTAACTG
AACCAATGGACGTCGATCACACGAACAATAGCAAACCAAGGTGC
AATCATTGTAAAAGAG
GACACCTCACTAGGGATTGTGTTATAGATACATTATCACCGAACG
AAGACGAACATGTTA
ATGTTATGTATAGACTAGACCGTAGACCCGATATCATCTGTTATG
GATGTGGATTACCTG
GTCACATCAAACGTTATTGTAGAAATTCACAATCATGGGACATCA
ATTACCATAAGGGAT
TCAATTACAATTTTGCGCCTAGAATGAGGCAAGATAGGCGTATGA
TGGACAC
>ConsensusfromContig559 Average coverage: 7.83
AAGAAGCCCTCGAAACGCTTCAGGACCACAATGACGTCATTTGT
GGGATGGGCGATCAGT
TATTGGAAGAGGCTCAGCGTCTTCTTGACCTTCTATCGCTTCCGG
TCAAGAACTCGTTTG
GCAAGGACGTTTCTCCGGATAACCAAGGTCACGTGCAGCATGTC
AAAGAGCTGCGGGACA
GACTGATCGGGCGCCGGTCCAACGTTGAGAGCCTCTCCGATCTCA
GGAGGATTAATCTAC
AGCAGATT
>ConsensusfromContig560 Average coverage: 25.46
AAACAGGAGCAAAAGACGCCATAGGAGCAGAAGAGCAGAGTGAC
GGGAATCATTCCATCT
GCGAGAAAATCCAAAATGGCCGGAATCCCGCCCAGGGCCGATGA
CACGCCCAGAATAAAG
AGGCGGTTCGCTCTCGGCCAATCAGCGATCTTGCTCACGATCAGA
CGAACAATGATACCG
GAAATTGCATCAATGGAGGGAAGCCAGAG
>ConsensusfromContig561 Average coverage: 4.37
CCCACCCCCCCCGTAGCCACAGCAGCTAAGCCCTTCACTGATCTG
GGTGTCTCTGTTGAA
ACCACGGATAATCCAGACTATGATCCGCTCGTGACGCTTGTAACC
AAATTGTCGACGATT
GAGCCTGCGGTGGCATGTTACGTCGATACATCAAAGATGGCGCTC

ACTCTTTACGTTCCG
GGAAATCTCATAAAGGTTGAATTTGAAAATTGCGTTCGGTTCGGA
ATTGGTATGATGATT
GGACTGCCC
>ConsensusfromContig562 Average coverage: 8.08
GCCACTCTGCAATCTTTCCAGTTGCATCCGAGAACATGGTCCATG
TCTGCGAGCGGTGAC
TTTGCGTGGCGCAAACGACGGCGTTCAGGAAGGAAGGGGTTCGT
GGAAGACTCAGTTTAT
CAATGAGACACAAGTCGATCGGCTTGTGCAAAAAGGTGAAGAGG
CTCATGTCTCGGAACA
GAACACGTCTTCCCTCTTTCACCCCGTGACCCTTGCAGATTGACA
TAATGTCACCGACTA
TTTTTGTCCTCAGATCACGATTGAAAGGCGAACAATA
>ConsensusfromContig563 Average coverage: 6.41
GATCTCGGATCCCAGCGGAGGGATTCATACGCGTTTTTTATCAGA
AATGCATATATATCC
AGGTATTAAAATTTTCTGTTTTGACAAGGCTTACTTTTGACGCAT
AATAAAAACATGAAA
CCGATCCGGTTCCCGATGAAACTCATAATTAGTTAAATAAACGA
GTGTGAATGAGTGGGA
AATATAGTGCCATTGACCCAGTTTCGTGCGGCG
>ConsensusfromContig564 Average coverage: 19.16
GCAATGCAGCTATTAACAATATACGTGTGCCGGTAGGCTAGTCTT
GACACGCTTTTCTCC
GGTGGCTCCAAATCAGGCCTTCTCAATGCAATGACGTAGAAACCC
GTCATAACGATTGTG
CTCGTCGTTCCGATGCTGAACAACATCCAATTGAAACAGAGAAT
CAGAGGAGGCCAGAAC
TCTCTTTCCTCATCAAACAGGAGCGCATCTGTACTCAGAGACTGG
GCACTTTTGGTCCTG
ACCTGGAGTCGACCTTTGTCTTCACTT
>ConsensusfromContig565 Average coverage: 7.78
GCACGGTAATAAGATGCCCTCAAAACGAGTTTCTTTCTAACGTTT
CTAACAAAGCAAGTT
TTGTGAAATTATTGGCAGGACATATTTCTTCATATGACATCCATG
TGGTAGAGTGTCGCG
GAGACGCAGATTTCGAGATCGTAAAAGCTGCACTGAGTGAGTCC
AAGTCCGGAAAAACAA
CAGCTATTGTGGGTAGTGATACCGATCTCTTGGTGATGCTTTTGC
ATCATAGAGATGTTG
ATGACAAACTATTCATGCTGCAATCCGGTGTCACAATAAATATTG

AACAAATACAGGTCG
GATTGGGTCCGCTCAAGAGTGTCATTCTGTTTATCCACGCAATTG
CAGGCTGTGATACAA
C
>ConsensusfromContig566 Average coverage: 38.91
TGTCTAACAGGTTTTTCAGAGAAGAACTATCGTCTTTATCTTGTT
AATGAATGGCACAAA
GAGCATTATTGCGATATCATTGGTGAGAAGACTCTGTTCGTGTCC
AACGGTGGTGAGTGT
TATCAGTTCAATCGGTCTACTGATGGATCAATGAATGTCCAAACT
CCGTCGTTTCTACAA
GCTGACCACGAGGAAGCAAATACTCTGATAACATTTCAGATCGG
AAGAGCACACGTCTGA
ACTCC
>ConsensusfromContig567 Average coverage: 9.25
TTGTCATAGGAACACGGAAAATATATATACAACTGACTGAAATCA
ATCTAGAATTATTCT
CATAATTTGGTGGATATTAAACGCACAATTCTTATTTATAAAGAA
TTTACAAAGAGGTTC
CCATGAGGAATCTCTGATATAGTTTTGATATATTTAGCATTATCA
TTTTATGGATACCAA
TCATATTTTCAAATTGATAAAAACAGTTTAATCACCAAGT
>ConsensusfromContig568 Average coverage: 7.22
TTCCGATCTTTTTAATGAATATCGATATAATAGCTGTCGGTAGAA
TTAGGCAGAATCCGA
TCCCTATAACTATTGCAGCTATTACACCAGGATCGGTCGAATCACT
TGTGTCCTTTTTTT
GAGCAATCGTACTAAATACTACATTCGATGCGTTCGTTTTAATTC
TGACTGAAGAGTAAC
TCATCTTAACATAATCTTGAAGAGACCGCGAAACGATAAAGT
>ConsensusfromContig569 Average coverage: 8.66
TGAAAAAGTATGCGATTGGACCCGACAGCGTATAGCCAACTGCA
AACACACAACCCATAG
CAGACAAGGCATGTGGACGTGTTTTGTTGGTTGTTATCTCAGAAA
CTACTACAAACGCAC
TCATGTACATAACTGTACCAAGACACATGTGGAGTACCCTAAAT
ATTCCGAAGAAAAGCG
GACTCGTAGAGAAGATGCTTACGAGCAAAGAGAATATCCATAGA
ACGCCACATATCCGAA
GAGATTTC
>ConsensusfromContig570 Average coverage: 9.49
CTTTCTCCTTCAAGTATTGGGTCAATTCATCTTCGTTCGAGTCTGA

GATGAGACCGTTTA
TGAAGTAATGTCGTCTAGGGGGAAGTTTTGGTATTGTACGCAAAC
GTGTATTATCATGTC
CCACAATTCGAGTCCCCTTAACGCTAGGTATAGGGGTACGTTTCG
GTTGTTCCTTCGAGA
TAACATTGAGCACAGGCTTATTGACCGCTAGCGCATTGGTCATCA
ATTTTATGTCTCCGA
CTAGGTCAGCCTTGGTATTCATTTGTTTATTCTCGCTATGACCTGT
GACCTCATTGGATT
CAAATTTTTCCAAAATCAGACTTTTTA
>ConsensusfromContig571 Average coverage: 4.94
CACGGCATTTAGTTAAGCACTCCTTGTACAGCCCGCTTCTGCAAG
CACCCGTGATTGACA
ATCTTGATCCAGGGATGTCACTTGTACATATTTTTCAAGCTGACA
AAATAGAATACATTG
ATCGTGGCACAGATCATCATAATGCACGGGTGGTACAACACAATC
TCTCTCCAAAATATG
TCTCCATGAGTGTGCGAATTTTCA
>ConsensusfromContig572 Average coverage: 4.04
TTCATCAGCAATCATTGATAATATTTGCCTTAAGCGGATTATAGC
CTGATATAAAGGAGC
CTGATTTCGATTCTGTAACAGGCTTCGTACGTCTATTTGCGAAAT
TTTAATGGTACAAGC
TAAGGACGTCTAAAGTTCCAGAGTCACAAACTGAGATCATTCCCA
GATCCAAGTCGATCC
TACCTCAAACTTGTTTGATATATGAACAAG
>ConsensusfromContig573 Average coverage: 15.93
CACATGTGAGTATTGCCGCGTGGACTAATCACGAAAAAGTCATTC
TAATGTAACTCTATC
GGTAACATCATGTGACCCCAAATTACAGAGGTCGCTCCCTATTGG
ATAATCCATCAACAT
GATTGACGCCTCTCCTGAGATTATCTACCTCTCCAGAAGGTGGCA
CCACACGTCAAGTGT
CTGGGGGGCGGGGAGATCGGAAGAGCGTCGTGTAGGGAA
>ConsensusfromContig574 Average coverage: 13.47
TCGACGCTGAAATCAATCGATACTTCGAGCTCCTGGCAATTGGAG
TTTTACTTGGTACAC
CTCTGATTATTGCAATCAAGTCTTTTTTAAAATTCCGAACACCATT
CCGCCGTGAATTGT
TATTGTGGTTCGCTGTTGTGACATATGTTATCCTCCTGGCAATGA
GCTACGGCAGAGCAG
ACTTGCTGGAGCTTCGGAGGTCCTTCACGCCATTTCTCTACAGCA

AAGTTCCCGCCCCA
>ConsensusfromContig575 Average coverage: 8.51
AATGTCTCAAGTTTGTGCGAAATCAAGAAGTTCCCGCCATTGATT
GCAAAATTGGTCCGC
GAAACACGTTGAATCAAGTGACGCCTTGGATAGATGGCTCTATG
ATATACGGAAGCACAA
TTGACGAGATGAGGCCGATTCGAGACTTCACAGATCCAGGACGCG
GCCGCATGGCTGTTT
CGCCTCACCCGAAAGGAGCCGATTACCAACCAATGCTTCCCACA
GATGAAGTCGAGACTT
GTACTGGGAC
>ConsensusfromContig576 Average coverage: 6.88
CGATCTTGTATTTGTACACTGTTCGATCTCAATGACTCGATAAAT
GTCGACGGATTATCG
TCAACGTAAGCTTGTAGGGCGTCTCTGCAGCTTGTAAGAGCTCCG
ATTTTTGCATAAGCT
GCTTGTACATCAGATGATGGTATGCCACCAGATTCAAAATCGGTG
CAAAGCGCAGCTTTA
TCAGAGCCATGACCAACGACTTTGATCCAGGTAGCATCATTTACA
ATCCCATCAAACTGT
TCTGAACATGTCTGTGCCTCGCTTGTTGCCAAAAGCACTGCTGTT
ATAACAAGCTTGTTT
ATGATTTGTAACGACATGGTGTTC
>ConsensusfromContig577 Average coverage: 6.85
TGCTCTTCCGATCTAAAATTGACAAAATGGACATTATATCTGAGG
AGAAGTAATTGAAAT
CTATTAATAGCAGCATATTATTGTGTGACCTTGATAATCTGCTTG
GAGCTGGACCAGAAT
ACGGATTAGAAATTCAAAATATTTAGTATTATAAAATGCCTGATA
ATTTTTTAAACACAT
ACCACTTCCTTCTCGTACATTCATCAATCATGAATGCATTGTAAAT
TATCTATAATGTAT
TTGATCGATATTTTTCTACAATTATTTCTCGCCAATTTCGCAGGTG
AGGCCAGT
>ConsensusfromContig578 Average coverage: 10.58
CACCACAATACCAGATGCAGAACCATCAAATATAGATTTTCGAAT
GTTTTTAACAAATTC
GGTATTGAATACTTCTTTTGGTTTCACTTGTGTGAACGACGCTGA
GGTTATGAAGATCAT
ATCTGAATTACCAAACAAAAAAATCGAAGGGGTTTGATAACATTCA
CATTACTCACCTGAA
AAATATCAAAACTATCATTTCCCCATATATCTCCGATCTTTTCAA

TAGATGTATAAGTAC
CGGAACATTCCCAGATATGCTTAAAGTTGGCAAA
>ConsensusfromContig579 Average coverage: 10.67
GAGAAATCAGCGTAAACATACTTTCTTTCCAAATTAATTTATTTA
GTGCATGTGTACCAA
GAGATTCTCGGGTACACATCTTACCTACTTCCCAAGAAACAGCAG
ATCCATCATTAGAAA
AAAATATTGATCATTATCGTATCACTCACCCTCACTTCCCAAATA
AGCTGAACGTCTCTA
GGCTTTCCTTCATTTAAAATCGCGAGTTTT
>ConsensusfromContig580 Average coverage: 13.15
TTTTTGGGACATATATACTATTTTTACACAATTATTTACACGTTTT
GTTGATGATTTTTC
ATTAAAGGTGGCGCCAGTGACGCTTTCGCATTCAATGTATTTATTC
AATGCTTATTTGTT
TGTTGTTCGATTTCATTGTTTTGTTCTGTTATGTTTCCGAAATATC
ATCTACTATCATGC
TATTCGCAATATATGAATTTGTTAAAATCTGTTTAATGAGAGTTA
ATATGTTTAAAAAAA
A
>ConsensusfromContig581 Average coverage: 9.39
TCGACACGTGTTTCGAAGAGTCCCCCTTTAATATTGATTGGATAA
CTTTATTCAGCTTAG
CATCTTTCTGTTGTTCCTTTGGCATGTCGAGCTTAAATTCATCTAG
TTTCAAGTCCGTCT
CTGGCTCGGACATCTCTTCCATTTTGTTGAAGTCTTGAATTTGATC
GCGATTAGTGTTAA
TCATATCAACATACATTGGT
>ConsensusfromContig582 Average coverage: 7.00
GATAAACTCAGGCCGCCTTACGTAGGAAGCGTTTCAGCTTCACCG
AGTCTGAATAAGTTC
ACGAGGCAATCTCAGACCAAGGATAACTGCAGAGTGTCGAGATCA
GGCAGCTTTTCTGTT
TCGCCTAGCTGCAAAATGTCATCAGGAGCTTATCCCAGCGAAACT
CCGAAGATGGGCTCG
GCGTGGGATCTGGGCTCAAACGAGTGGAGTTTGCGGAGACTCACT
CGGTCCTCTAGCGTA
ATCCAACTCAGAAAGATGAGTGATGGCCGGAAGACGCAAATCGT
TCTTCTAGACGATCG
>ConsensusfromContig583 Average coverage: 4.91
GTTAGCGTCTGGATGATTCTGGCCTTTGATGCCGCCGACTTTGATA
CTTCCTCTTTTAGC

TTCTCTGTTTCTTTGAGACGAGATTGGAGGGAAGCACACTGGTCG
TCTAGATTCTTCATC
TTTGCTGTCACTTCTTTATGCCATTCTCTTTGCTTGGTCTCGTGGTT
TTTATTGACCTCC
TTCAATGCTGTCACCTCTTTATGCCATTCTCTTTGCTTGGTCT
>ConsensusfromContig584 Average coverage: 11.34
TGACAAAATACTTGCAATAACCATTATTTTCACTTTCTAGATACAT
TTTTTGTTGGGCAA
CAGATTTTGAAGCGCCGAAAATTGATCTCTGATAATTGATCGCTC
ACCGCTATTGAACAA
AATACTCTCTGCGGATATTCTACTCGTAATAATATCGATTGTTCA
TCAGGATGTGAGCAC
GTTGAGAATCAAATCATCAAACGTTGAATCAACAGTTCAAAACAT
GCTTAGTGATATGTA
TATTGATGGTGTTCATTATGTTAAATGAGAAGGTTTACACCACTT
ACTTATGCAAAAACC
TGAATATCACATTCTCATCCTCCGA
>ConsensusfromContig585 Average coverage: 11.58
CCATACAAGACAAAATGTGATACCAAAAATGATCAAAGGCCGGG
TAGTGTTGATACCGTT
CCAAATAGAGGATCCAAACGGCCGAAGAGGCGTAATAGAATTCA
TAGCGCAATTGAAGCA
ACCATTGTACCAGCATCTGACTCATTCATGTCAAAAACAAGTGAG
AGTGATGTCAGTACA
AATGAGGAGATTCCAGTTTTCAAGTTTGAAGAAAATTCCGATGGC
CTCAATCGTAATACA
AATACCGACCCAACCATGCATGACAGCTCACAGGATCTAGATTAT
GATGAACTCCTTGAA
AAAGCTGATAGAGATTTTGC
>ConsensusfromContig586 Average coverage: 8.52
CTCTTCCGATCTCCCTCTGATAAGAAAGAAGCAATTGCCAGGAAA
ATTGACGGTAGGACT
GTAGATCAATCGAAATCATTTGTTAGCGTATATAGAGGACCGTAC
GTACAAGTCGCATCT
CATTTGATTACGGCGATCGACACCCTTTTACTTTCGCGGCTGAGT
ATGAGACATTTAAAT
GAGACTCAGCAGAAAATTATAGAACGTCATGAAAGAGAGAG
>ConsensusfromContig587 Average coverage: 14.67
TCTTTCTTTTCAATCTTTCAATAATATATTCGCTAGTTTGTGATCA
AAATTTTTTATGTT
TGAGATTCTGCAATTCCATTTCGAATTCCTGTATTTTTTGTCGTCT
TTTGGCGCGATAAT

TGCGCTTTTGCAGTCTCCAATAGAGTCGTTTCCTCTCCATAAGCG
CCGAGGAATCGAAAG
AAGCCAAGGACTCATTAAGATACTCATCATTAGCGCCATGCAGAT
AAGCATTGCTCTGGC
TCATATATTGTTGCGTGGGA
>ConsensusfromContig588 Average coverage: 5.49
ATTGATCCAAGTCTTTTGAGAGGTCCGGTTTCGAATGTTTAGGCT
TCGAATCGTATAATC
CCATACCCTTTTTGAAGTTGCTCGAAACACAATCACAAAATTTCA
GAGATCGCCCAACAC
GCTGAACGGACTTGGATGTCATGTTGCCACGGAGACCACGTAGAG
CATCTTTGGCGATCA
TATTCATAAACTCTAGCGCCAGATCACCGGGAACATTACATCCC
>ConsensusfromContig589 Average coverage: 11.14
CGAAATAATGTTCAAATAAGCGTTTTATTAACCATTTACTAAGAT
TATTCAACCTGCAGT
GTGTTCTGCATGGTGATTAAATTTTCTGAAGAGTGTTATTCTACA
CACTTATCTAATGCG
GCCTGATATGATTTTTCATCAACATATGCGGTTTGTAATCACTTA
TATGAAGCCAATATA
GCGCCTTAACTGTCTGAGTTCCTATATAATTATACCTGACAATTTT
TTTAGTAATCTGCT
TTATTAACAGATATATATTTTTATATAGA
>ConsensusfromContig590 Average coverage: 18.08
CCAGAACTCCCTCGAGCGCCACTGCGACAAACCAAGCTCAGCTG
CCATCTGGCGGGCGAC
GTACTGAATCTTTGTTGAGCCACTTCTCCCCACCCTCCCTTTATCA
TCGAAGGGTTTTAC
GACACTGAAGCAATGTTGTTCAAGCTGCTGATTATAAGTTAGCTTC
TTCGTCTTCTTCTT
TTGAAGAAGTGGAGTCTTTGGCAAAAGAGTGTACAGCTTCCGATG
ATATTCTTGAAGTGC
TGTCGCGGCATATTTCTTGTAGAATAATCCAGGGGTATTTTTGAC
GGGAAAGTAATCATC
>ConsensusfromContig591 Average coverage: 10.35
TCCGAGCATTTCATCCAAAGACAAGACATCTTCACCAGTGTCCGT
CGTTTGTTCCTTCAA
AATTTCACCCGTGCACCTGAGCCAGTCTTCTATAACCATAAGATT
TGCTTCCCGATCAAC
TCTCACTCTCTTGGAATTCTCGAGAACGCGTTCCAAAACTTCGAT
TTTGGACATAACAGA
TTCTTGAGCTGTTTTCGCAGATCGGA

>ConsensusfromContig592 Average coverage: 6.53
GGGCAGAGGCTTCTTGTTAAAATCAGCTCGAGTTCTGCTGACGTG
TTCAAATTTTTCGGT
GGGTGGGCGGTACTTGGCCTCTCTGGTCAGAGTAGCAGGGGGAG
GATGTTTCATGGGGTC
AACGCCCATGTACTGTGCCCTCTGCTCCGTAACAAAGTCAATCCT
GGCTCCCCGCCGACC
GATGCCGGAAAATGCTTCTCGATTCGGTATGATAT
>ConsensusfromContig593 Average coverage: 10.91
CTCGTTTTTATCTACATTCAGAGCCAGTGATGTCTTACGCCCATAT
GTTAGCTCCTTGGC
GCTATAAGGCCGCGGCTTGACTCCACTGGGCGGGCGCAAATGACT
TCGTACTTTGGCGCT
CTCTATACACCGCCGTGGCTGGCGTCTTAGAGCAGTCTCCTGGAC
TTTGTTGAATGCATC
TGGTTTATTGGTGTGCCATGCATGATTCCGTACTTTTGAAATGAT
GAGTGGGGGTTCGAC
>ConsensusfromContig594 Average coverage: 9.33
CATTATAGTAGATTGTAATTTTCATTTCTTCAATAATTTATCCATTA
TTTCTTATTTATT
TCTCTGGAGAAGGGAGCTAACGGTCCAGCAACACTATAAGAGTCC
CTATTTGATAATATC
CAAAATGGTAAAAATATGATACACACTTCAATACTAGACACTTCT
TCTTGAGAAGTAATT
CGAAATTATTTTTCGGAACATAAT
>ConsensusfromContig595 Average coverage: 14.78
GATTTTCTACGTTATATGAAGTATATACGAAATTCAAAATGAGAA
ATGTGTTTCTGCAAA
CGGTCTGAGCTCTGCTTATTTTTCTTCATTGGATTCGTATTGATTA
CCAGCTATGTTTAC
ACCATCCTACTCTTCTTTCTCACATATACTCATGCCCTTATGGACG
AAGACCTTGATTTT
AGATTGAAAAATAATATAAG
>ConsensusfromContig596 Average coverage: 7.22
TCCGGACAGTAGTGACCCTCTGGACAAGGTTTATGGAAATACCCC
GATTGACCAGGACTG
TTACAGTAGTATCCTGCGGCACAATCAATGCAGTCCGAAGGTCTG
CTTGAGCCATTCAAG
GGGTTATACGTTCCGGGCAAGCAGGGGAAGAACTGGAGTCCATC
GCAATAGCCCCCCGTC
TCACACACGCCATCTGCTGGCCGGGGGGTCACTGACCCACTGCT
ACAGGTGAACCCGGGG

TCACAGGGGCGGCAGAAGGCCGAAGCGTCTACGGAATACGTCCC
TGGGAGACATTCCGTT
TTCTTTGAGGCTTGAGGTGCAGCACAGATCGGAAGA
>ConsensusfromContig597 Average coverage: 23.98
AGATGTATTCTTCTATAAATCAAAATTTCCACCATACATAGCTTGA
TATATATTTCTGAT
ATTCCGAGGTTTCAAAACGAACTAATCAATCAGAATAATGCTTAT
GCTTACAGATCGTAT
GAAAATTCCATTAAAAGTCCTCATCACTATTATTTTCTAATTCTCC
GATTTTCCCAAAAA
CCTCCATTTTAATAAGAATTAATTCATTATTTGATGGTTTTAACTT
TTGA
>ConsensusfromContig598 Average coverage: 9.04
GAAAGATTTCTTGATAAGATAGAATTCGTTGAAAGCAACAGAACT
GATTCATTTTACAAC
AGAATGCATCATGTGCAATGAGAGGAATCGGTACCACCTTGGCTC
ATATTAACATGTGAT
GTATATAGTTTCAGAAAATAATGAAAATCCCATCCGTAACCGAAC
CTAATTGGACAGCAT
TTGAACTTTTATCGTCTGTTTTAAACATGCAGTTACAGGGGGGAT
GAATGCGCATCGGTG
TCATCTTCTGCTTTGAACAAAATCAACATATTCAATTTCCAATCAT
TACCAAACCCT
>ConsensusfromContig599 Average coverage: 12.63
AATACGGATGAGAAAATCAATTTATATAGATCAAATAAAATCA
TAAATATTCGATTGCG
TTTGAAAGAATACATCATCGCAAAATCTGAAAAACAGATGCAATT
GTTTATGAAAGTTGT
TCACCTCAAAGAAAAAAAAATAACTTATCTGAACCAAGGTCCGA
CTGAACTGATATCTCA
TTTGGTAGATCATCTTCTTGGATTAGATG
>ConsensusfromContig600 Average coverage: 4.94
CAAAGGTTCAATTCAGGCTGTTGAATGAGAAACTATCAGTGCAT
GGGATGGAAGAACCTC
CACGAGAAATCGGCGGCAGACAGAGAGTTCCGATGGAACACAAG
ACCCTTATGTTCCTTT
GGTACATGGCGAACAGGAATTCTTTTCGTGAGATAAGTGATAAAT
TCAACGTATCACAAA
GTACTGCAGATCGGAAGAGCGTC
>ConsensusfromContig601 Average coverage: 6.61
TGTGGTTCGAACGGGCGCTGGGTACCGCCCATCACCCGCTGCATC
CGAAAACAATGCGAT

GAATTGAAAATTGCCAATTCGAGCCTCAAAGATGGTGTACAGGG
GGTGACAAAAGATGCT
CACATTGTTACATGTCAGTATGGATATGTGATTCGATCCGGTGTA
CATCTCATACAACAA
GACAATTTTTATATCGTGTGCGAAGACACAGGAGAATGGTCTAGA
GTATACTCATGTGTT
TTGC
>ConsensusfromContig602 Average coverage: 6.44
GGTTCTTTCTCCTCTTTTTCGCAAGGGGTCTTGCGCCTCTAGGTCC
CTTCGTAGTCATGA
TATACAAGATGTGTGTCACTGACATCTTCAGATTTGTGATCATTTT
CGGTATTTTCCTGC
TCCAGTTTTCACAGGGGTTTTACTTTTTATTTCGCCACATATCGGA
TAGTAATATATCTA
CATTCAGAACGCCCTTCGGAACTGTCATGGCACTTTTCAAAATGA
CCCTCGGAGACTTCG
CGTATGATGAATTCGACCAAACAAGAGAGCCAGCCCTGGCCAAA
TTGATGTTCGTAA
>ConsensusfromContig603 Average coverage: 18.93
CGTCAACAACATTCTTTATCTTTTGATGAATTGCATAGTTCTGTC
ATAAGTCAAAAAACA
TATGAAAAACTCCTACCGCAATATAAATCACACTCCCTTAACTCT
GCTTATTATAATGCA
AAAAATTTACTAGTGTTCAATACAAGTTTACTCGACATTGCCATG
TATGGTCGTAGTCTC
AATATCTTTGATACATACACACCATTATTTGTTCTTGCGGCGAGA
CATGTATTTGATAAA
CCTCGAATTTTGATTGAAACACCTGATCCAAGAAATATTGATCTA
GTGAAAGTTGGTTCC
AGACCTTTGTATCAAATCACAGAGGAGCGTGAAATATGGGACAA
AACTGCAAAATGGTTA
TACGCATTACCCTCTTCTAGAATCTTAAGAGATTATCACAATCGT
TTTGCTGATGCGATT
AAACGATATAAAGTAGTGAATGTCTTACATCTACAACAAAGCATA
TATCATGCTAACACC
ACAGTCCAACTCGGAAACGATTTTACAGAAAAATGCTTTAGCGA
TGAAAAATATGAATAT
CAAACCGCCAGATCGGAAGAGCACACGTCTG
>ConsensusfromContig604 Average coverage: 5.46
TCTTCCGATCTCCGGATGTTCCTTCAACTGACCGTTCAGTTGACCA
TCGCAAATACTTGT
TTGATCAGTCCCCGCAGTATCCCTGAAAAGCTCATGGCCGATGAA

GCATCGTGGAACATT
AGCTGGGTGATCACAAACCCCATTGACTTCGTTAAAGAAAAGGC
CCGCAGTGCATGGCAC
CGTCCTTTCCAGTCTGTTTTGACAAAT
>ConsensusfromContig605 Average coverage: 16.20
ATAACGGTTTGTATATGAACATATCATACCGTACATCGATGTAAA
CTTTTCTATACAATG
TAAATTTTGTAGTTTTATATTTCAGCTGAATGTGCAATTAAACTGC
CTTATCATTTGTAT
CATAAAGTGTATGATAGTAGTGTTCCAACATGTGCCTGATCACGA
ATGATATGATCTTCA
TGCAATATTCTGTGTTCATTTTAAAATGTGAATATTCCTTTCTATT
TTGTATACAAAAAT
CTGTGATGA
>ConsensusfromContig606 Average coverage: 6.15
GGCAGATCAAATTTGTGATAGTTCGAACGCAACTCGAATCAGTTT
TATCCCCCAATATTT
GAGGGAATACGATGCAATAAAAGGTGTCGAGACTGTGAAAGAAA
TTGTATCTCGCTTTAT
CACGGATTACGATACAACAAGCCTTCTGAGACCTGTTGCAGATGT
TGTAATTGATTTCTG
GACAGAACAGATTGAGCTTCAATTGAGAATTCTGG
>ConsensusfromContig607 Average coverage: 39.28
CAATTATGAGTTGTTCAAAATTGATCTTGATACATTCCTTGATATT
TCACAGTATTCAGT
CGTGACTAATAATATTACTCATCTGACAACTCAATAAGATTTTGT
GATCACTTCATGCAT
AACGTATGTTACAATATAAAACAACATTAATAAAACCTTGATGTC
GTGTATAATATAACT
CCTAAATGCGCTGGGCAGATCGGAAGAGCACACGTCTGAACTCC
AGT
>ConsensusfromContig608 Average coverage: 8.32
TTTGCAATCGAAGGTTTTAATACATCTATTGAGACATCATGTCTT
GCCTTGAAGAGGCTC
AAGAAACCTGTCCGACTTCTGAACAGCAAACAATGATGCTACAG
ATGACTGGAACTGCTC
TACAGGCCATACTGAATCGACACAGATTGATACTTCTCGCACACG
AGTCCATTGTTCTGT
CGGAGATTTATAAAAATCAATGTAAACAATATGGATTCGAGACGT
CTCATCTTTATCTCC
G
>ConsensusfromContig609 Average coverage: 18.48

GATCTCCCACAACCGTTCGAGATTTCTGCTTCAGTTTTCTGACGT
TTGGTTGAAAGAGCT
GGACTTTCAAGTAGACGTATATAGGTGTCACAATTTTTCCGTGGT
TGATCTTCAGACGTG
TGCTCAGGCGCGCTTCTACTTTGCTGCAGGACGGCATGAAATTGA
GACAAACATTCAATC
CGCAGATCGGAAGAGCACACGTCTG
>ConsensusfromContig610 Average coverage: 6.05
TACTGCTTTCAAATCGGCCAACGGGGATTTGACTGCGAGAAAAG
CTGCGTAAATAGAACC
AGCACTGACGCCAGCAAACCTTTTTATTCGCTCAAGAATTCCAAC
TGACTCTAGAATCTG
AAGTGCGCCGATATATGCCATTCCCTTCGTGCCTCCGCCTTCGAA
TACCAGATTTTCAAA
CGGGGGGAGGTCCGACCCAAATCGTGCCTTTAAGCTCAAAGCTCT
C
>ConsensusfromContig611 Average coverage: 4.80
AGGGGTGCCAGGAGAGACAGCATGAGACTGGCGGCGCTGTAGCT
AGACCGGAGGAGACTG
TAGTGGAACCAGACGGATCGGAGGAGACTGCGATGTGCCGGAGG
AGATCGGAGCGAAGCG
AAGTTGATGAACAAGAGTGAAGTGAATCGAAAGACGTGATATTC
CATGGTCCAACGACCA
GTATATGTGTGTGGTAGGATCGCT
>ConsensusfromContig612 Average coverage: 11.28
GTTGAAACATTTTCTAACCATTGCAGAAATGGCTGGTCTCCATAA
AACAATCACAGTAGT
GCGTAATCAATGCATGACTTAACTTTTCGTAGTTATGGTTCAACG
AAAGGCTCTCTGACT
ATCAGATATATCGAGATATTCTAAAATTATGGTCTCAAATTGGAA
AATTATTTATAATGC
TCACAGAATTACACAGGGAATAATTTGTCAGACAA
>ConsensusfromContig613 Average coverage: 8.77
CGGAATCATTGGGTTTAAAGGGTCCGCAGGCGGTCAATTAAGTC
AGAGGTGAAATCCCAT
CGCTCAACGATGGAACTGCCTTTGATACTGGTTGACTTGAGTTAT
ACGGAAGTAGATAGA
ATAAGTAGTGTAGCGGTGAAATGCATAGATATTACTTAGAATAC
CGATTGCGAAGGCAGT
CTACTACGTATATACTGACGCTCATGGACGAAAGCGTGGGGAGC
GAACAGGATTAGATAC
CCTGGTA

>ConsensusfromContig614 Average coverage: 16.64
GCTACCGAGGCTGTTCCGTCACTATTGGATGAGCCCTTCATCATC
TGCGACTTCGGATGT
GCCGACGCCGGCCCGAGTCTTCAACTCATCTCTGATGTCATTGAT
TGCGTCCGCGAGAAG
AATGGAGGGGTCTACCGATCTGGGTTTTCCACGAGGACCAACCA
ACCAATGATTTCAAT
GCCGTTGTTAATAGGGTTCATGG
>ConsensusfromContig615 Average coverage: 11.18
TGGCTTATTTTAAAGGATTTTTCGACACCTGTTCTGTACTGGTTTC
ACTATTAATATGAA
ATTTTACGGCTGATTCATATGACCATAAAAAAGCCTAGTTGAAAA
TGTCTGCCCTCTATC
GTTAAATCCCTGGCGACGGCCATACACCGATATTGTATGTTACAA
TATAAAGCAGTTTGA
AACAAATGTACGCAGACTTATTAATTAGTCTA
>ConsensusfromContig616 Average coverage: 7.28
GCACAACCTGTGGTCATGGCCAACAAACAAGGGAAAGAGTGTGT
GACAACCCGGTCCCCA
AAAACGGTGGAAACTCATGCAGTGGGCCGAGCGTTGCTAAGCGG
ATATGCGACAACGCTG
GTGTTCCTTGTCAACAGCCTAAAAATTCTTCGACGACCCCTTCGT
CAACCACAACAACCC
AAAAAGCATTGAAATCCAATAATGCCACAAAGTCTCCTGCATTCA
C
>ConsensusfromContig617 Average coverage: 6.57
CTTTTCCCGCCTTTGCCCCAAAAGCCAAATAAACATCTTCGAAAT
AAAAAAACGGCACAC
TTTCCGAGGTCTCAACAAGGATCGGTATAAATTTTGACGGAACGG
TATATGAAGGTCCGA
AGCATAAACGTGGATACTTAGAAGGAGTGTAAGAATAATGAGGA
GCTCTATATTCCTCGT
TTTTATCACGATGCGGCTCCTCAGAATCAGAACATC
>ConsensusfromContig618 Average coverage: 5.58
CAGATGTAAATGATGAGATACCAAGACTTTCGTGTCGAGCCATA
AAGAGTACACCCACTA
ATAAACTTTCAACAGCCGAAATGACTAATTCTACAGACTGCAAAG
GGTTTCCGAATTCTT
CCAAAACGACTGCTCGAGAAGGAGAAAAAAATCCAGTGAAACAA
AACCATTCCTACAACA
GGCACGGTGCTATGATTCCCAGAGAAAGAAACATGCTGAATGAT
GTTATGAAGCGAAAC

>ConsensusfromContig619 Average coverage: 11.46
CTCCGAAACAGAAAAACCGGCAAAGAGAAAAGTAGCCAAAAATA
AAACGCCCGAAATTCC
TGCGCAGTGCGACATCTGTGAGCGAGTCTTGGTTAGCGAGAAGG
CGTTGAGACAACACCA
CAAGAGGTTCCATGAGGCTGTGAAGAGGTTTTTCTGTGATGTGTG
TGGAAAGGGGTTCAA
AGGAAAGTACTCTCTGGTGAAACATACTCATGTGCACACAGGCG
AGAAACCTTATGAATG
CACGCTCTGTGATGCGGCTTTCAGTCGCGCATTCAACCGCAGAAA
ACACATGCTAACACA
TACTGG
>ConsensusfromContig620 Average coverage: 6.77
CTTTTATGGTGTGATTCTTGTTGGCCGAAGGGTTTTTATTTGAAAT
AATTTTAATATATT
ACTCAATTAATCACTCCATCAGAGCTATTTCAAACTTGGCAGCAT
TTCACCTCGATATAT
ATCAATAATTGTTTTATTATTTCATTCTCGCTTGTTTCGGAGCGGA
ATAAATCTTTCTTA
CATCTTCGTTCCCTCCTTCACTTTGTTTATTGAAGTTTTCACAAGA
TTCGTAGAAATTTA
TTTCACTCAACTCGAAAAATACTTTTTGTTATGCAGC
>ConsensusfromContig621 Average coverage: 3.88
GGAATACAGGAAAAAATGAAAGAGCAAAACCAAAAAGAGTATAC
GGGTGAATGCATGTTG
AAAAATGAAACATATCTAACGTTTTGCTTGAATTAGTTCTTAATT
TAAGTATCAGGTTAA
ATGGGGACGAAACAGTGCAATGAATGGTCCGTATTTTGGTTGTTT
TTGCGATCGAACTCC
GCGTTGTGTTATATCAGGATACA
>ConsensusfromContig622 Average coverage: 7.06
ATAAAACACATTTTGACATAATGATGGAAACGAATGGCACGGCT
AAATACCGGTACGAGG
ACGAAGGGGATGATAGTTCACTCCGAAGACCTGCCTTTAGCTTG
GAGACCGTCAGAAAGT
CTCGCAAGCTGATCGTTTTTATTGTTTTTGTTGCGCTCTTCTTGGA
TAACATGCTTCTCA
CCACTGTTGTGCCAATCATACCCAATTTTTTATTCGCACGCGATTC
CTCGAAATTTGTTA
AAAAGTACTCCCATTCAAATAAAACTTCTGGCATATCCAACATGA
CGAATTCGGAGATAC
TGAAAATAATATGCCCCTCAATCTATGACGAAAAACACGGAGAA

GATTCGAGCAGTGATT
CTCAAGG
>ConsensusfromContig623 Average coverage: 6.43
AGAAATAGGAAATAATAGAGTTTTTTACTATCATGATCATTGCC
ACCATCTGTGAGTAA
CTTAAAAAAATCTAAAGACATGATGGTATTGTTTGTGAGAGAATG
TATCTCATTATGAGA
ATTATTGTTGATTTTAACAATCGGATATACACCGGTATTATGAACA
TAAATTTTAATTTT
CGAAAAATTGTGTTTCCCTCTTTTCGTTTCTTGTGTAATTTTTTTGT
GAGTTTA
>ConsensusfromContig624 Average coverage: 10.30
AAGAAATATAATTTCGATTATTCTAATAATGTTCTTGTTAAATTA
CCGATGGCAGTAGTG
TTTGATCATAAAAATTGTAGGCATAGTCATTGATTGCGATCTTTGA
ATTCCTTGTTCTTT
CATAAATAATTGGCTGGCCCAATGGCGATCAATTTCGTCTCATTT
TATGAGTGATTTGTG
TTCTGCATACTAAACGTTTTATACCATTGTTTTCATCAAGTGCCG
>ConsensusfromContig625 Average coverage: 5.67
GACTCTGATAAATCTTTTGAAACCCCAACAAACCGACACGGTTC
AATCAACACAGGGAAC
TCAAACTGACTGTTCTGAACTTCTCGGTTTCGATATGAAAGCTAT
ATCAAAGCCGAAACC
CACCACCAATGTTGTTATGAATGCACCAAGTAACACTGCGTCCAC
TGAACTGACACATGT
CAAAACAGCCCCAAAGAAAGAAAGCCCAAAAATAAACTGTGTTG
TTATGAATGCACCTAG
TAACACTGCGTCCACTGAACTGACACATGTCA
>ConsensusfromContig626 Average coverage: 3.85
CTTCATATAGGGTCTATGATGTAGGTCTATCCATTAGAGTTTCTC
CCACATCCGATAGTA
CATGCCAAGCAACAGCGTTTGATGCGAGGATAATAGTGGAATGG
GATGAAAACAACAGAC
GGTGCAAACATTATGGAGCTGTTGATAAAAATCCTGGTAACCAAT
TCATTCTCCCTGGTA
CTGCAGCCAGGGATCATACAGAATTCCGTATATACAACTGTGAAT
ACTCC
>ConsensusfromContig627 Average coverage: 5.89
AGTGTTCATTGTCAACTTTTTCTCCGACAAATCAAGCTAATAGTT
ATTCAAGTTATACCC
CGAATTTTGAAAATAGTCTTCCTCCTTACCCCGGTTTGTCCACAT

CAATGACTCCATCAC
TTCCATTAAGACAGCCCTTATCAGTCAATTTTCATACACATAGTT
CAACAACTCCCCGCT
GTAGAGTTACCCCTTTAGGTCAG
>ConsensusfromContig628 Average coverage: 12.04
TATGTATAGTCAAAACTTCAAAAACATTAATGACCATATAAAAAT
TGAGATAGATGAATT
TATGAGTAAAGATATAGAAAATAAGATGATTCTTGGTGATTTTAA
TGGCCATATTCAGGG
CATTGGCAAACATCCTACAAATTCAAATGGAAAAATTGCTCTTG
AAATAGCAGAAAACCA
CAAAATGACTATACTTAACCTAGAGGAAAATGTGAT
>ConsensusfromContig629 Average coverage: 4.05
ATGCATAGTGTGACAATGTCTAGTAATATAAATTCGAGAGTATCG
ATACAATGCGAATCA
TTGAGGCCATTTAGTGCCGAATAACTTCAAAAACAAGTGACATA
CATGGAATGTGAGAAA
GATTTATAATCTCGCTCAACTCATTAATGATAATTAATGATATTC
TGATCACATACTACT
TAGCATTATGTTCAATAACCACCAAAACAATCTCTCATTTGAGAA
TGTGGAACTGTATAG
GTGAATGATGGTCAATTTTGCCTCGGAATGATTCAAACCA
>ConsensusfromContig630 Average coverage: 7.25
CGCGAACATTAAGAACCTCAATCTGTGGTGAGATCAACGATTAA
TAAAGCGGATTAACGA
TTAATCAAATCAGCGAAAAGCCGGTTTGCTTGTATTTGAACTATT
CAATTTCATGATCAC
AATAAAGGGGTTGCACAAACAATTCAATGAATAGAAAAAAGGTG
ATAGCATGGTTACATG
CCGATCAGCTACCGGCTGGTCAACCATGGAGGTAAAGCCTCGAAA
TTGACTAATGTTTCA
AATACATTCAGCAGTATTCTTAGTTTCTTAAAAAATGTCGAAAAT
ATGAGATATTCAACA
TATAATTCTAAAATACCATGAATTCACACGACATAACCGGTTTAC
TTAC
>ConsensusfromContig631 Average coverage: 6.77
CTCTATATCGGCCCTTGAGGAGGCTGACTCCATGGATTGCTGCAG
TATCCCCAACCAGGA
GCGCCACGATCCCGATCCTCACTCGATCCGCCCTGATCCCATGAA
TTGCCGCAGAACATT
CCCGAGCCAGAAGCGACACGATCCTGATCTTCACCCCATCCGGCC
TGTTGCTGTTGCCAG

CGGTGAGGGCCCAATCCTTCCAGATGCCGACTCTCTTTCATATACC
TCCCTCTCTCCTTC
TGTGAATTTCTGTAGAATACCAACAATGAATATGTATCTTCAATT
TTGTGCTGTGTATAT
GTGCACACTTG
>ConsensusfromContig632 Average coverage: 8.22
TGCGCATATGGAGAGATGTTAATGGGGATTAGGAGATATCACAG
GTATAATTGCGACTCG
TATCGGAGCCATAGAATGGTCACAGCACCTTGAATAAAAAGTGTT
GTTTTTGTACCCTGC
AAAGTTTTGCTCTTTACCACTCCGTTTATCCGACTTTTACGGTCAA
ATATACAAACCGCT
GAAACTTTACGAGATCAGTG
>ConsensusfromContig633 Average coverage: 7.13
GACAGATATACGCCAGCACCGGGACCTCATCAGCGTGTTCGCCG
AGTTCGAACTGCGATG
TCTCGATAGCGACGTAAGCGAGTGGGATGGGACCCTCCCCCAAGC
TGCTCTCACCATTCT
TCATCAGCACGCTATTCAAGGAGACATCACTCAATTCCAGCAAGC
GATATGTTGGTGGGT
AGCCCTCAGTCGGAAGCAAC
>ConsensusfromContig634 Average coverage: 41.46
CTTTCCCTACACGACGCTCTTCCGATCTCTCGGACACAATGGAAG
AAACGCGACTTCGAA
TCCCGGTTGCTTTGTGGTCTCATGAGTATAGGATTAGAGAATGAA
GGATATCCTGAACGT
AGCAATCTTTCTTGTTAATTTTGATCAAAATTGTTGGACTGAAGC
CAAGTTTAAATATGT
GTGCGATGAAAATCACTTATGAAATCTATATTTTTTCATTTGTGTG
ACGATCCTTA
>ConsensusfromContig635 Average coverage: 5.37
GTTTTCAATGACAAGTCATTGAACAACCTTGTGATATTTCGTATC
TTGGTAGAAGCCCTC
GAAAAGGTACGAATTCATATCAACACTGTCCATCGATGTCTGATG
CAATGCGCTATGGAT
GAGGACTGCATGCTGGCTAGAACTACATACTTCACGGATAGCAAA
ATCAATTGTTACCAC
GTCATTCGTCGAGAAAACGCATTCATCCAGAATATCGGCTGGGG
GAGGAT
>ConsensusfromContig636 Average coverage: 5.65
GGTGCTCTTCCGATCAGCTTGCTTCTCGGAGCCGCGTTTTTGTCAT
GTATTCTCATCATT

AGAAGGAACCGCGCCGATGCCCGGAAAACAATGTCACACATTCG
TGAAATCGAGGCCAAA
ACGGGTGACGGAAAATGGGAGGATGAAGCCCCCAGCACTTTCCC
CCACCCACACTTTCAG
ATGAGGGAGAAGCTTATGTCGGAAACACTTTACGACTATGACGC
CCCCTCCCAAAACCCA
CACGACAAATTCATCACTCAAATACTCATTCAGGAAAACCATCAT
CCGGATTGTATTTAC
CAAAGCACCGCCCAGGTTAAGAACAACATGTCACCATTCAACAA
CGTGTGATACCCTGCC
GAATCTAGCCGAGATCG
>ConsensusfromContig637 Average coverage: 5.04
CTTCCGATCTCGCAATTATATGATATGTCCATAACACCTGATCTT
ATACAATCGAGTAAT
ATTGGTAGGTCTTTGCTCGTTCTTGCGGCGCTAGTTCTTTGTGGAG
TATTATTTTTTCGT
AAAGACGCAGTTGTTTGACCACCACAATTATATGAAGAAGGTGAA
GAATAATAATTGTTT
TTTAACGATACACTTCTTACTCTGTGAG
>ConsensusfromContig638 Average coverage: 9.54
GCGGAATATAATATCGTGCCTGTTGTATAGAGTGGCGCCAGATAT
ACAGAACTTGTGCCG
AAGAAATCATACATAGATGTCTCCGCATTTAGATCAATAAAACAT
TCAGCTAGGTATTTG
AAATATCTGGTAAATCATCCTATGGAGTATGCATCCTACTTTGAA
TGGAGAACTGACTGG
AAACTTCTGCCACAAGGTCAAGTCATCCAGAGAGAAATCGAAAA
GATTTGTGATGATGTT
AACAATGATAATTTGAAGCGTGAGACGATTAACGATTTCAGTAA
AATATGGAACAGACAG
CTGGCGTGTCCAAGAAAACAAA
>ConsensusfromContig639 Average coverage: 8.41
GCTGAACATATCCATACAAATAGCCATCTGATATGAGGACAAATT
GAACTTGACAACGTT
GCTTGAAATATAACCTGGAGGAAAAGAAGTTATGTTGGGCGCTTG
ATCACGGTAATTCTT
CCGCCCAGTAGTTGAATCTAAATTTCGTTCCATCATGAACGTATT
TTCCATAAATGATTG
ATATTTGATCTCCGTCTGAATAAATCACTTCGTCTTCACAACATGA
TTTCATTCCAGCGT
AACCATCAGTTCCGATTTCAATCCAATAGTCCGAACACTCCGAAT
CGCAGCTCAAATCGA

ATCCCTCAAGAAATTTCACACTAATCCTGGGATTTTAGATCGGA
AGAGCACACGTCTGA
>ConsensusfromContig640 Average coverage: 9.89
CTTTGCCCTCGTTCAGGGTGGTCACCGCCTGTTCTTCGGCCTTGAC
ACACTTTATGAACT
TGGCAGCCTCCATGTTGATTGATTCTATTTCCGGTTGCATGGATTT
CAGCTCATTGAATA
TCGTCGCGCATCGTTTGGCGTTGAAATCCGTCTGTTCATGCGATA
GCGATCCAACCTGGG
ATTCGATTTGCTTCAATTTCTTCGAAGCTATTTTTGATTCGGATT
GGAAAGACTTAAGAC
TTTTGCTGGCAGAGGACAAAACCCCGGACCTTAAATCTGTGTGAT

>ConsensusfromContig641 Average coverage: 5.86
ATTCGAACCTAATTGATCAATCTAGCATTGTTCTATCTCATTCCCG
ATACAAATTTCGTC
CAGAATTCCTTTAAAGGGATAACCAGTATGACCACTCCATGTGCC
TAAGTAAACCTTCCG
GGCTACATTTTGTTTAGTACAGCCTTCTGCAGAAGTATCGGTATC
AACGAGATTCCCATC
AATGAACATTTTCAGCTCGAAACGCTTATGGCTGTATTCCACCCT
AACATGGTACCATTT
GTTCCCTACTGTTCCTGATTTACGAACATCAAATTGTCTGATGCA
ATTGGGAGTTCCTGT
GTAGAACTCTGCTCGAATATAATAACCGAACCACAAGA
>ConsensusfromContig642 Average coverage: 6.63
CAAAATGTTTCCTGAGGCCAGATGTCTAATTTGACAATTCATGAT
ATGTGCATTGAATCT
CACGTTTTGAACGAAGGTTCTACATTACTACAGTTTGTTTTTAAC
GAAACTTGACATGGA
AACTATTATAGATCATTCCTAGATACATACATTTTGATCGTATGA
AAACCACGGACTATG
ACAAGTATTGAGCCCATAAGTGTTTGAATTTCCAGATCGGA
>ConsensusfromContig643 Average coverage: 17.59
AATGACCGCGATCGCACCTTCCTGAAGCAAGAGGCTCTCCAGGC
CAAATTGAGATCTGGA
ATCCAGCTATGGTTGGAAGATTTCGCTGATGTAGATTCTGAGCAA
AGCAGCAATGTCAGA
TACGATCAATCAGAAAGTCTATTGGCTCAACCAATATCAGCATG
CCCGGAAATAAAACGC
ATACCTGAGAACGAATGCTATTCCATGTCCAACTCATGTGAAAA
TGACGAAGAATGTGAG

ATCGGAAGAGCAC
>ConsensusfromContig644 Average coverage: 5.92
CTCTTCCGATCTGTGGATTTGTGCAGATGGCATTTGAATTCTTTGT
CGGTGCTATCGAAT
ATCGGGTGGAGATCGTACATGAAGACATCACCTTCGGCACAAGC
AACGGGGACGTCCTGA
CATTTTCTCGAGAATGCAAGTTTGCAATGATTCAAGGCGTAACAG
TTCGATCCGCAACAT
TGGTCTCCACTACTTTTTCCATCTTTGCAAGCAGTATGGTTCAGAC

>ConsensusfromContig645 Average coverage: 4.51
TTTAGAAATGTATGGATCGATCAAAAGGAATGGTTCGGAACGTCA
TGAATTTTTGGGAGA
CAACAATACAAAAACATTCAATAAGGAGCCGAGTGGTCAATCGA
TGAAGCAGTCTTCGTC
AGTGAGTATCGCCCTGCGGAGAAACTCATGGGTCATGGCACCGAT
GACTTGCATCTCAAC
TGATGAGGAGTCGGATGGTGAAAAC
>ConsensusfromContig646 Average coverage: 6.60
TCGTTCTCATATTTGATATTTTTAGCCCATTTTGCACAGGTTTTGT
TATCTCGGAAAATT
GTTTTTTGAAATTATTGCTGTTATCTTTATCTGGCTTGGTATGTAC
ACCCTTTTTTTGAG
CAAAAGAGTGTTCATATTCAATTTAGGTTTCACGAGGCTGCTCGA
GGAGAGAAAAGTGCT
GGATGTTGAGACTGGAATATCCGGTACGGAGGCCAGAGTGTTGG
GCAAGGAGACCGGAAT
ATTAGGCAGTGATGCATGGAAGAGAAGCACGTGTTCCTGTTTTAC
TAGACGGTGCCTTCA
CGATTTCTGA
>ConsensusfromContig647 Average coverage: 6.92
ATTTGATCTGTTAACAGAGACATTGGTCTGCCATCTATCGGATTT
TTGCCTCAAATTCGC
AAACAATATTTGAGTGCTTTTCAAATAGAGAACTGATTCATCGGA
TCAAATTTCCCCCAA
AATTTCAAAAACGTACGATAAATATGCTTTCAGCTTCAGAATTGT
CAGCCTTTTTACTGG
GATATTACCCACTATAACTCTGTATGTTTGCCAATTGATGTTATC
GTGGAGCATGAGACT
GCTCAATGATTTCATGGAGTCTCCCTGTCGAAGAGCAATATTCCA
CGCGTTTCACACAAT
TGGTTTCATCTCACATCAAGCATACGACTTGCTCTCGTTTGATCT

ATGTGAACGTGAAAC
ATTGCGAAAATTTCCTAGAGAATTATTTCCCAAACTACATTGTCA
ATAAAAT
>ConsensusfromContig648 Average coverage: 7.96
AATTTTACAAAATTATAAATTATGTAAAAATCACGAATATACTAA
TACTTATTTTGAATT
TCAACTTCTTTTTGTCTTAGGATAGGTCATTTAAATCACTGAAGTT
ATATTTGTATGACA
ACTCTAATGTTGTTTGTTAGATTAAAACACAGAAGCGCATCGAAC
GAACATACCATTCCA
TATACCACCACCACCATCAGTTTTTTT
>ConsensusfromContig649 Average coverage: 5.08
AACAGTGAAGAGCGAAAAATAGGGTGAAAGATGGAAAGAGGGG
TGTAGTGGAGTAGAATA
GAATAAGAAGAATGAGGGGACATGTGATGTTCATAAAGTGAGTT
TGTGGGAAAGGTATGG
ATTGGTGGTGAAAAAAACAGTGAAGAGCGAAAAATAGGGTGATT
GATGGAAAAAGGATGT
AGTGGAGTGGAATAGAATAAGAAGAATGAG
>ConsensusfromContig650 Average coverage: 8.51
CGCAAACACTCTCATGTTTTTTGCCCTTGATCATATTTATCCAGAT
TGGCAAGCTTTCGA
CCTGCAATTGGTTTTCAATATTTATCCGGTGATGACAAAAGACTC
GCTGCTTACTTCGCA
TGCCATTTCCACGAAGGTCACTGATCCAGATGACATCTCTCAATA
CTTCGACAGTATTTC
CTACGATAAAGGCATGGCGATACTACGACTGCTTCGAGGATTCCT
CGGATGGCACGAGTT
TCAGTTAGGATTACAGA
>ConsensusfromContig651 Average coverage: 4.41
CCATTCTCCATTCATATATAGGTTGGAAAACCGTTCGATAAAGCC
AGCAGTATTTAGTTG
ATTCGAAAAATTAGTTCAATGATTGGAGAGCGCGTTTTCAATACT
GGATCAGTCTAAAAT
GTAGAAAGGGATATTCTCATTGCTAACTCAAATTAAACTTTGCTT
CAAACTCTCTACTTC
AATTGCCCACAATGTGAACACATA
>ConsensusfromContig652 Average coverage: 6.65
TTTTAAATCACAGTTTAGATACCCTTCCTTTTCATTACCCCAATA
GCGGAACACATGGAA
GATAGATGAATCCTGGTTGAACTGTCACACGTGGTCTTTTTTATA
CAATAAGAAAGCCGA

CAGCAAAGGGACATGCAATTTAAGACATAAACTATCACTACATC
GACCCCACTGGCAACA
TCCACCCCTGCCTCTCACAAAGGATAATTATTCGCATCCAATCAA
TCGGTCATAAATGTC
TCATGTGATCTCTTCC
>ConsensusfromContig653 Average coverage: 8.62
CTATCAGAAGCCTTAAAGGCCCAGAGGAATCAAATAAAGGAAGA
AACCACACAGGAAATA
GCAAACGCTCGCCATGAAATATATAAACAATTCCAAAACGAATT
TCAATCACGAACAAAT
TCAGCAATTGAACAAGCTCTGAGTAAACGAAATGACGAAAACAA
AAAAGACTGCCAGAAA
TTGGAGAAGAATATTCGAGAAGAGTGTTCCAATGAG
>ConsensusfromContig654 Average coverage: 9.24
GCGATTCAATATCATCATCATGATTTGGCCATGTCATCGCAATAT
CAACCAGATTCATAT
ATGAGAACTATGACTTCGTCTATATAAACGGTTATATATCTGAGA
AATCAGGTCACGATC
ATCGAAAATTTGACGACCACTCAATAAGGAAATATTATCGGTAGG
AAGATTCTTGGTGTA
TGGGGTTAGAAGCTCGCATCCGATTCCATGCATCATGAATTTTCT
ATGGAGACCTAAAAG
TGAGATCGCGA
>ConsensusfromContig655 Average coverage: 6.60
TATGTGATTATTTGCTGAAAACCGATAAAATAAAACGAATGATA
GACTAACTGCGATAGA
TGAAGCAATCTATCTCGATCACTAGATTGTTTAAGCTTATCATTA
TTGGAATAACCATTG
TATGAAATTCTAACGATTGTAACACGCATAACCGATGCAAATAA
AAAAATAGAATGAATT
AAACAGTAGTAACAGAGAGCAAAGGGTTATGCGGCTTTTGCAAT
G
>ConsensusfromContig656 Average coverage: 6.76
CGGCCAGCTCCCTCCGATCAAACCTTTTGATCTTCGACAAGGAGC
TACAAGACCGATCTT
GCCTTTCTCTCCTGTTGGAATTATAAATCCTGCTTCTCTATCAGC
CACCAGCAGCCAGAA
TATGTTCAGTCTCCCAAAACCCACGATTCAACCAACATCTTTAGC
CTACTCGCGCGAGTC
TCTGCCAAATCCGATAAACTTAGCCGTCTCTTCAACGAGTCCTCT
CACAAATACTCCTCT
CAATCGACTTCAGACCATGCAGCCTCATGATTTTCGTCGGGAAAA

AATCAGCCCCAATCC
AATGCTGACTCCAACTAAGTCAAAGCCTCA
>ConsensusfromContig657 Average coverage: 7.94
CATTGGACCACATATCTCCACAAGCCGTCCCCCAGACATCCTCAT
AGAAAATTTCAACCA
ATCCGTCATGATACACGCCCTCTTTGGAGGTGGAGTCGCTCGAGA
AACGAACTTGTATTT
CACACGAAACTGAGAGGATCAATTTAAATATTGAAATTAATGTTG
TCCTGGACAATATCA
TTTTTGAGGAAATGGGTTAGAATTCAAATGTATAAATGTTTGGAA
TGACCGTTTTTCTTG
GAAGCAGT
>ConsensusfromContig658 Average coverage: 9.34
TACGCTCTCAATTGCAGGAATCAGAAATGCTTTTTGCAGGCGGAA
ATATTGGCTCAGTGA
GAAGTTTGCCATGTCCATGTGTCTGAACATCACAATAACCTTAGC
TGGGGAGGTTCCGCT
GAACAGCGTCGCGGCGGCAAGAATGAGATTTCCAGCTGGCGACG
GCCCAATCATGTCCTG
CGACTGCCAAGAAAAGTCATGACCAGCCGCACAAAGGCAATCGC
AGAATACCAT
>ConsensusfromContig659 Average coverage: 6.42
TGATACTGGACGTTGTCTCGGTTAGCCTTCATCCAAAACCTCAAT
GTGAATCCATTTGTA
CAACTCTCACCCCTCAACCAGCAGCCGTGCTGTCCCATGTCGATT
CTCAGACGTCTTGCA
GGTGTCCCATCGAAGAAAATTCCATCTACAGTCGATAAAGTTGGG
CCCGTCCACGATGGG
AGAAGGCTGGCGCTGCCGCTGAATTCAAGAAGATTTTCTCTTAAG
CCAAAATAGAATCTG
AGCCAATCGTTATACAGATCGGAAGAGC
>ConsensusfromContig660 Average coverage: 7.38
GTTGGCTTTTGAATCAATGATGACATCATGGATCGTATGCTCCCC
GGGTGAGCTGTAATT
CAATACTGGATATCCAGATCGGATGCCAATTGACAGCATCTGCAA
AGCGAAATCTGTTCT
GTCAGATATACTGATTATGTCGAGATCCGATGATTTAGTTTTAAA
AACGATACTCAGTAT
GACAGACAGAGACGACATTCTTAAATCCTGA
>ConsensusfromContig661 Average coverage: 10.13
CGCCAGTTGGGATGAGAGGATTGGCACATCCCATCACATTATAT
TTGAAAAGAAATAGAG

GCAATTTGCTAGATTGGACAATCGGGGCGACTTGCATGACAAAA
GCGTTATCTCGCGAAG
TGTAAATGTTGCGTTGTCGAATGTCCCTCTTATCTGTGCATATTGA
TTGTCGAAACGTTT
CCCCGAGGTCTTTGATGTGAATTTCGACAAAACAATTCTCGCTCC
ACTGAGAATTGTATG
AAGATGAGGAGGAGCTCATCTGAGACAGCGACAAAAACTCGACGT
TTATTATTTGTCCTT
TTTCGGCCTGAATACGCCACGTTATTGTTTTGCTT
>ConsensusfromContig662 Average coverage: 6.63
CCATGATTGCCAGCTTCCGTGATAAAAGAATGATATGGATCCTAT
TGTTCTACATATGAA
ACCTACTACATATAAAATCCCTAACTTGGTTCACAAGCCATGAGG
CTGTACCCATGATTG
CCAGCCTCCGTGATAAAAGAGTGATATGGATCCTTTTGTTCTACA
TATGAAACATACTAC
ATATAAAATCCCTAGCTTGGTTCA
>ConsensusfromContig663 Average coverage: 5.56
AAATATCATAAATAGAAATGCGATGCATATCAAACAGGTATTTT
CAGGAGAAACGTCCGA
TTTGCGAGGCCTCAACTCCTGCTTCATCTAGATTGTTTTGGTAAG
GTTATAAGAATGGAA
TCGTTGCAATTTGTGCCAGAATAAAGTGTGAGAATATGGTAATGT
GTTGTTATTTGAAAA
TCGGGTTTTGGTATGAAGAATATCCAATATTTTCTTTGTATTTCAT
TGATGTGAAA
>ConsensusfromContig664 Average coverage: 11.88
TCATACTTCGAAAGTAGAATTCATTTGGATAAAGTCAATGTACCA
ACCACAATTTCTGAA
AAGAGTGTGGGAGAGTCTGAATTCAGCAGAGAATCTAATTCCGAT
AATATCTTTAATTCC
GAATCCGTGGCTCCACCCTCTGAAGTTCATCTGGAAGAGTCTGTT
TTCATCAGAGAATAC
AATTCTTTACCTGTCGTTCATCAACAACCTTCAGATTCTGCAGCCA
ATCTTGAAGATCTT
TGGGGAGTTGAAGAAAACGATGATGATGAATCTATTTTCGACGGA
CTTGGTGATATGTAG
CCCTGTCTCATCTGACTCATTTTCTTCTCTTAGCTTTCATGAAAAC
GCGAGATCGGA
>ConsensusfromContig665 Average coverage: 5.90
GATTTTTTCATTGAAAATGGTGAATGTCTCCACCTTCAGAACACC
GGTAGATGCCACAAA

GTCTCCTATTGGAGGTACTTTTAATAGAAATGGCTCAGCTTTTGT
AATTGTCCCGTCAAT
GTTATTTGTTATTGTCTTATTTCCATCCATCTTCGGGGGCTGTCTT
TCTAGGTCATCCTT
AGCTCTTCGGAGCTCTTGTATCACAACATCCATTGATT
>ConsensusfromContig666 Average coverage: 8.50
GCAATATTGAACCTGAAACTTTGGCGACAAATGTGTCCTCCTGGT
TACTTGAGGTGAGTT
TCTCATACCAGAAAATGACAATAAGCACAGTTACCAAGAGTAAT
ACCAAAAACGCAGAGA
TCCGAAATATAACTATAGGTTTGATTTCCATGTCTTCTTTCACAA
GAATACAGTGTCGTT
TGTAATTTTATTCATTACATATTTT
>ConsensusfromContig667 Average coverage: 6.45
ATTTGCATAAAAAGTATATCATTTTAAATCCCTTAATTTAGTACG
TGCGGTTATACTCGA
AATTCGATTTTTCGACATTGGCTTGATACTCTTAGATCGCATCTCA
TGTAAATTTCTCAT
TATAAAAGTTATCACGTGAAATACAGCACTTAAAATGAACTTAAT
AATTTTTTAAGAATG
AGGGTCTTGTACTTATTTTCACCCCAAACAATCATTTTGCATCGA
ATTGGACCCTTGAAC
TTTGATGTAATTTCTTGCTAGCCAACATACCATACTCATGGCATCT
TCATTACGAATTTC
GAACTTGTTTCAATTCTTATTCCGGAATTGCCCCTCATCACAAACG

>ConsensusfromContig668 Average coverage: 5.50
CTGAAATTCGCCGTTATAAAGTTTTGCAAATCAAATGAACCGTGA
AATCGATAATTGTTC
ATCAAACTTGGCGCCAAATAATTCATTGAAACTTTTTCCAGCTGC
AGCAACAAGAGAAAC
GGCGACAGATGGCCGAATCAATAAAGGATGGAGACAACGAATAT
TGAGAGGCATGACACA
GGCCATGTGATTTCGGATGTAAACAAAACAGATGCGGAAATAAA
GTGAATCTCAACAAGG
AATCAGAACAGCATAATTATAT
>ConsensusfromContig669 Average coverage: 6.22
ATCATGATCTAGATCTTTGAATTGTATGATTGATGTTTTGGCAAT
TTATTAAAATGAAGA
GATCCTCAAGATTTTACACAGTATGCAAAAACTAGTGGGGTACCC
AGTTCCGCATGTTCA
GCCACGGCTTATTTTGGTATATGTTGATATTCATACAATACTCAT

ACAATAATTTACACA
ATAGCGACTATTGGGGTAACCAGCTCCGCAC
>ConsensusfromContig670 Average coverage: 12.87
AACTCTCTGAGTTCTATTGTCCTATGTGTTGTGTCGAACTATTAT
AAATCAAATGGTACA
TATTTTCACATGTCCCAGCACTAACTCTCTGAGTTCTATTGTCCTA
TGTGTTGTGTCGAA
CTATTATAAATCGAATGATGGAGTTATGTGAAATTCTATATTATG
GTTGAGAGAGAAAAG
TTTTGAGGTCAAACGATTCGGACCTGTCGATAGAGTGAATCTTAT
TGTCTTAAATTC
>ConsensusfromContig671 Average coverage: 4.12
ATCTCTCTACAATCTTCAACACTATGTGTGACTCATGAAGTGAAC
AATCTGGGTTTCTTG
TTCGATGATTGTTTATCACTAACGTCTCAAATTAATGCAACCGTA
AAATCATGTAATCTT
CAAATTCGTAATTTATATCTCATTAAAAAATATCTGAATCGCAAT
AACTTATTATCATTA
ATACACAGTCTTGTGTTCTCTA
>ConsensusfromContig672 Average coverage: 339.61
TTTTTTTTTTTTTTTTTTTTTTTTTTTTTTTTTTTTTTTTTGATAT
TTATTTTTTAT
AAATCACGCAGCCCAATTGCAATATCAAACAAAGCTCGATCTCAT
GCAATAATATAGCAT
AATAGGAATGCATGAAAAATGAGAGAATTTTGAAACAAGAAATC
AAGTTAATTATGCCAG
TTAAATTTGACCATAACAATTGCCTATTATCA
>ConsensusfromContig673 Average coverage: 5.45
CTTTGAGCTCATCTTTATCTGTCCCCCGTGTTCTTTTTTCCACTCC
TCGAAATTCTAGAC
CTGTCCTAATCCCTCCATCACGTCATTCCCGCCCTCCACCTTCTAT
CATTTCATCTTCGT
CTTCCCGCCTTCTTGTTTCTGTTCTTCGGAATTCTGGTCCGGCTTC
TATTCCAACGATAC
GCTCATCCCGCCCTTCTTCTATCATTTCATTTTCTTCTGTCACAAG
CCCTCCTGTTTCCA
CTCTCAGAAGTAACTTACCTTCTACTCCCATGATACGTCATTCTCG
CCCTCACACTCCTT
TCGGCCCCT
>ConsensusfromContig674 Average coverage: 11.66
TGGTTTGTTTGCATTCATTCAACGAACTTGATTTGTTGGAATGAA
TGATGATGATGAGCA

GAAAATTAGATGAACACCGACCATACTACGAGTCTGTCTGCTAAA
ATTTGCAAACAGTCT
AAATAAAATATACGAAACTATTGAATACACATATCTCTATTCTTC
GCATGGGTTAATTTT
CTCTAATTACTTCGACACTCGAGATGTGTTGCTTTCATTCTTACA
AAGAAGATCGGAAGA
GCACACGTCTGAA
>ConsensusfromContig675 Average coverage: 16.23
AAAAAAATTATGTTCATAACTATAACTCTTATAAATCTCATATGA
GTGCTAAGTGAAATG
TGAATTAACAAAAATGAGTGCTTCATATTCCGCGATTTTATAGAA
AACTTTCGAAGGCTC
AAAATTTGATTATATGTAATTGAAGATTTGTGACAAAATGTGAGC
AAATTCTTGTCGAGG
TGAATGCAGTAAAAAGTCGGCATTTTCATTCTGAGTAAAATGAGT
TCAAAGTA
>ConsensusfromContig676 Average coverage: 7.57
TGAATTTCTATTAAAACTTCTCTTGAATGAGTCTTAAGCCGTGTTA
TTATCATGTATCCT
TCTGATTAAAATTTTGTTTGATATGTTCATTCTTAAGCTCTGATCT
TGATGAACAATAAA
TACCTAATAATATACCTAGTCATGAAAGACTGTATAGAATGAAG
GAATAGATCGACAATT
CGATGATAGACCAATGTTTCCTGACATCATTCGAATATTCTTATAT
TAAATATTTGTTAT
TGATTTTATGAATGTTACTTAAGCA
>ConsensusfromContig677 Average coverage: 8.36
CTCCGCCCCCGGACATCCAGCCCAACACTCGAGCGCCTACCACAA
GACACTCTACGGACC
TTTTGGTGGGGCCCCCTCACAAGACCTCTTCTCAAATTATCATCA
ACAGTTTCCACAAGC
TTTCAAGCATCACACACACCATCCTCCACCTTCTCATCATCACCA
GGCTAGTCCTCTTCA
ATCTTCATCAATCGGCGCTTCAGCGACCTCGGCCAGAAACGGCGT
CGGCGC
>ConsensusfromContig678 Average coverage: 293.82
TCTACACTCTTTCCCTACACGACGCTCTTCCGATCTGGCACAATC
AACACAGAACGACTT
CACGAGCAATGCGGGGATTCTTTCATATTCTCCGATGCTTTCCTA
ATCCAAAAATCCTCA
TGAAAAGCCGTGGTTCTCTTCTTTGTTCGCATCCGTTCTTTTTTTA
CAATCTCAAATATA

TATGTGATATCCTCAATTTGTATTCATGTGTTCACCG
>ConsensusfromContig679 Average coverage: 1,968.66
TTTGTTTACATCCAGTAACACAATCAAAGCAGGAGTTAGGCTGCT
GCATGGACAATATTT
GATGACACCTGCGCTTGCCATCTCAGCTTTTGATGAGTGGAATCA
CTTTGCTCTGACCTA
CTTACCCCAAGACCTCAAACTTTATGTCAACGGTCGCTATGGCAG
CGGTGAATCTGTAGA
AACAAGAGTTGAAGCTAGATCGGAAGAGCACACGTCTGAACTCC
AGTCACATTGCTAATC
TC
>ConsensusfromContig680 Average coverage: 8.52
ATAAAGTCTCTGAAACAATTCAGGAGGAATCAGCAAATGATTCT
GGATTCGTGAGAGCAG
ACTCGCCGACTTCCTTCTGTGGTTCATCGTGCGCTGAGCGATTCT
CAAAGAAAAACCATG
GATACTACATCGCCCTCTACAACTTCGATGCAAGGGAAGAAAAT
GATGCGAGTATAACAC
GTGGCGAATTTGTACAGGTCCTCAACATGGACGACAACGACTGG
ACCTGGGTCAGCAAAT
CCAACGGAGTCGAAGGGTTTGTTCCTCGCAGCTTTCTCTGTCCAG
CGGAAGCACAAAGTG
AACGTTGTGGTCTTGGCTCTAC
>ConsensusfromContig681 Average coverage: 6.87
GTGAATTCGGCCAAACTTTAATCGTCATTCAACTAAATCGTCCAT
TACATCAATCGTCCA
TGATCGTGTCAACATTCTCCGATCTCGTCAGCATTTCGAGAATGA
GCGGCGAAAGAGAAG
ATTTCAAGTCTGTGCGCAAATTGAAAATTCTTTCAGGGCATTGCA
CGGATATTGAACGCA
AATCTGTCATCCGAGCCAAGATGGCCGTCAAGGCATTCTGCTTCC
CGG
>ConsensusfromContig682 Average coverage: 6.04
AGGAAGAGTTGACCTGAACTGCCCAGATTCATCAGATTTGGCAAG
TCTACAATGCTCCTT
CTCATCATGATACATTCTGTAGGAATCTGGACTCGAATACTTCAG
AGAAAATCCAGGAAA
ACTTTCGATAAAACTCCCAATTTTTGATCTAGCTGGAGACATGAT
TGATGGCTCTGGATG
TCTTATAGTGGCTTCTTCAAAAAATTGACTGCGCCGCCTGTTCAC
CGATTTCTCAAAAAC
AGGCAAATCGGAATGAATATTCGATTCTGTTGGTTCTCTT

>ConsensusfromContig683 Average coverage: 6.73
GGAAACAGACAAGGCATGCGTTTAGAGTCTTCAGTTCCGTATACT
CGAATTTGATGTTCA
CGTGGGCAGAGGATTTGATATGGAAAGGTGTGAAAATGCCTCTC
ACGACCGAAGATCATT
TGAACATCAAGATAATGAGCGAGCACACGAAAGGCTGCAACTTT
ATTGAAACGTGGGATG
AATCGTCGAACAGGATTAATAAAGAGAAATCAAA
>ConsensusfromContig684 Average coverage: 8.17
CAGTAAATGTAAATTTATTGGCAAGATGTTTGCTTGGTTTGTACT
GTTATGTTCAATGCA
ATCATTTCAGGTTTCACCGAGGGTTTCAGGATCCCTTCTACACTTT
CAGAATTTTCTAAT
CAATCATCAAATTTTTTACAGCGAATCAGAACCCTGATTTAGTC
ATGTCAATAATTAAG
GATGAGATTTGTGCAAAAAGAGTGGCTGGCCCCTTTGACACCCCG
CCATACTCAATTTAT
>ConsensusfromContig685 Average coverage: 4.85
GTTGCTGTCTGAAAAACGGAACTGTGTTCTGGGCGAGGGGAGTT
GAGGTTGACCCAGAAA
CAAGAGAAATTGTCAGCAACTGCGGGGGGAGGTCAGAAACTGCG
CAGCACTACATCGAGT
ACATCAGACCCTGGATTGTTATGATTCTCGTAAACGCCATTCCAT
TGTGCGCTATTGTCC
TGTTTAATTCATTCATTGTTATGATTCTCGTAAACGCCAT
>ConsensusfromContig686 Average coverage: 13.93
AAAAAGTGGCTGTACAAAAGCGGACAAAATTCGAAATTAAATTT
GAGCTCGCCAAACAAA
CAATATTTTGCAGAGTATGGACAAGATGCGATTGTTGATAAGCA
TAAAAACCGCAAAACA
AATGGGTTTTACATCGAATGTGGTGCAGCAGATGGTGAATTTATT
TCAAACACACTGTTT
CTGGAAATAGAGCGCAATTGGACAGGCCTACTGTTGGAGCCCGA
T
>ConsensusfromContig687 Average coverage: 5.43
GCGGTTAGTGTGACATGTTCTTTGTGAGATCTCTGCCAAAATGGCT
CCTATTCTACTGTT
TTCAATTTGCCCTCCTGCATTCGAGAATATGGCCAATTGATCTTC
AGAACCCAGATCCCT
ATTTTCTACCAGGCTTTCATCATGCTCAGATGGGGTTCGACTGATA
GTTTTGCATTTTTT
TGCATTCTCTCTTAGTGATCTTGTCGTGTCTTTTGAGCTGATGTTT

TTCGTCTCTGAAAT
AGTATCAAGTTTTGATAAAGATAGTACACTCATTTTCCCAATATT
TTTACAAACTTCAGA
AATATTTTTTGCATTGAATTCATCACCG
>ConsensusfromContig688 Average coverage: 9.98
CTATAAAAAAAATCATGTGAAATGCAAGATAGAAATGTCAATAT
AAATTTGACCTGTGAG
TCATTCAAAGGCACTGAAGAAGATGAATCAAAGCAGCTGTCTGA
CGATGATTCAATTGTA
AAGCAAGTTTGTATAGACAATGGTGTCAATTTCAATCGCATTCAT
CCGGATGGTCTCTGC
ACACCACGGCTGGAAATGTTTTTCGGGGATTATCCTAAAATTGTT
GGCATCAACTACTTC
CCCAATCTGCGAGA
>ConsensusfromContig689 Average coverage: 8.68
TTCGATTTGTTTACTTTCTTTATTGTTTTTAAAGTGAGACAATAAT
TCACATGTGTTTGG
TACATCCATGGGATAATCAAAAATGAATAAATACGGTGATATCAC
TCCTAATCGAGTTTC
AATACAGTTGTACATTATCTTGCTTCATTAATAACATTCGAGGTC
ACTCCCTAGTTACCA
ATATCCAGGTCAATCATCCAAAACTATGTCAACTAAAGTCAAGGC
AACTTGTGGTTCGTG
CGGGTGATGAAATTACAAGTTTAATATAT
>ConsensusfromContig690 Average coverage: 11.61
GTTTCACGTTAATAATATTTTTCAAAAGTTTATTGGCAATTTTTTG
ATGATGGATGATAA
TCATATCTTAGATGCAGTTAATTATAACTCTGTTAAGTATGTGAT
ATTCAAGACTTACTC
TGTTGTTGATCTGGTTAACTAAATATTGAGTATATACGCTATAGA
ATGTGACAACGATTC
AAAATATTAAAGTATTTAACTTGGTGATGCTAAATATATCAAAAC
TATATCAGAGATTCC
T
>ConsensusfromContig691 Average coverage: 7.51
CTTCCGATCTGCAGAATGCTGGTATTTTTTGGATGGAAACGGGAT
CAAAATAGATATAAA
TTTGCCAGATTCGTGCGCCAGCAATCCCTGCACAAACCCACATGA
GGCAATTTGCACCAA
AACTAATCGATTTGGCCACACATGCAGCCCGGATCTGAATGATCC
TTGCGGTTCCAGACC
TTGCGATGGCAAGCCAAAACGCGATTGCAAGCCTAATAGCTCTGG

CGGTCACGAATGTTT
TGCTGGCCGGAAAACCGAAGCCGC
>ConsensusfromContig692 Average coverage: 10.87
GATCTTTCAGAACCAGCACTTTTCTGTTTCAGATTTTCCGAAGCA
AAGTTTTTGATTGGA
ATGCGGTTGATCTTGCTGTGGAATTTTCCCGGATCTTCATCCCACT
GATGCTGGAATTCT
TTGAAGCATTTTCCCCCTCTATTCAATGTGTTAAGAAGGTACTTTC
TTGTTTCATCTTTC
GCCACCTGCCCCGTTGCAATGTTGACAAGTTGTAGAGGAGAATTG
TCGGAAAATGGATTA
CAGAACTCGTCAATTCTGCTGCTCAGGGCCTTTTTGTGGTTATTGT
CCTTTTTCACTCTT
GAGGGACGACATTGACTGGTTGTCA
>ConsensusfromContig693 Average coverage: 6.13
GGATGGATCATTTTTGAGAAAGGAAAAGGCGCAATCGAGAATTG
TTGTACTGAGAAATCA
TTGAACGAAAATACATTTGCCTACGTCAAAGAAGAATTGACTGC
AAATAGGAAGAGGCTT
GGAGATGTGCAAGGAAATATCGGAAAAGTGGGAAATAAGAAAGC
ATGCTTCGATTTATGC
AGATCTAATGATGATTGTGTATTCGCTAGCTTTGAGAATCAGACA
GATGAAATATGTACT
TGGTATCAAACGCAGGAGCCGAGTCCGAAACAGATTGCT
>ConsensusfromContig694 Average coverage: 17.49
GCTGCGTCATCCAGGAAGGTCTTTCGTTCTATGACATCATTTCCG
AAGAACTTATCTTGA
ATTATTTGTTGTCGAGTGCCGAGACATATGCACGAAAAAATAAGC
AATCTTAAAATCATC
TCCATTTATTGATTCCTTGTATAACGTATCATCGAAATACTGATTT
GATAAATAATTTCT
ATAATAATTTATGAAAAAAAAACACTTTTTAAAATGGCTCAATT
GAAAGACAAAA
>ConsensusfromContig695 Average coverage: 5.52
GAAACAATGAAATATATACGTTCAAAGCGTTACAGATATGCCAG
TGTGCAAGAATTATGG
AACTTGTACAGGAATGTGCAATCTTTTTGCCACAATCATTGATGG
AGAGCCCAATTTCAC
ATGTTCCTGCCCACTCGGTGTTTCTGGTGTTCGATGCGAAGAATC
GAGTCCGTATGACAA
TCACAATATGGATCCACTCGGAACACAACCCGGTGGAAAATG
>ConsensusfromContig696 Average coverage: 10.11

TGTTGGTTGAAGGCAGTGGCGGTTCTTCTAGGCTGGAGAGAAGT
AGGCAGAAGATCGTTG
AAATCAGATGAATGCAGTAAGTTAGATGTGGATGAAACATCTTCT
ATCCTAGAAGATGTC
ATCAGAGATTGTTGTATGGGATTTTGAATGATTCCGCTGTGATCC
AAAGATGATGTGAGG
TTTGCGAAAGATTTGTAGAACGAGGACGACTCCCCGCTTCTCTGC
CCATCGG
>ConsensusfromContig697 Average coverage: 6.71
GTTTTTTCCCAGGTACGTAGATCTTGAATTTTCTGCAATACAGCTT
TGGCACACCTTTCT
TTCTCATTTCGTCTGTTATATTATCAGGCTCCAACACAATTAACTT
ATGCTCCTCATTGT
ATCTCAAATAAATTCCGCCGTCCGGGAAAGAAAGAATGTCTCCCT
GTACAACATCCACTT
CTAAAGTCTGCAGTCCTTCTTTATTTGCCTTGCACAGCTTTTGAAT
GTTCTGCAATTCAT
AGACTTTGGAAGGTTTTGCATCAGATTTCTTAAGTTCACTCTCTTC
TGCG
>ConsensusfromContig698 Average coverage: 7.58
CTTTGGCCCATGACTGATCAGAAGCGAGTGCTGGGTAGTGGCGAG
AGCGATTTTAAGGTT
CTTGACGCTGATGCTACCGGTAATTCTGGTATGGTCCTCCTCGTG
AGGGTCCATGTGTTG
TGGCAATATACCAATGCATCATTTTAATTTAACATCATTTACTCCA
TTAATTCTAATTCG
ACCAATTTGTATGACAAAGTTAGGGAGGCCGGGTATTTTCTGAAA
AATCTCATTAGCGTA
GCGTTGGATGACCTTGAATAGAAACGGTACTGGGGTCAATCGAG
CGCTACGTGTGAGTAA
TCAGCGGAACCTAATCGCTCTAAAACACTGAGAGCCGGTGGTGC
CCGATAGCGGCTTGGT
ATTTGCCCTCCTTGGCAAAT
>ConsensusfromContig699 Average coverage: 22.81
ATAGTGTGAGAATTCTTTTAAGTACAGATTCCAAGTGCGAAGTTT
GAAGAAACTCGGAAA
ATCACCAATGTGCTGAAAAAAACGAGCCAATCCCATGATTTGGAG
GGTGAACACTTTTTT
CATGTCTTCACTGGCCTGATTTGGATGAAAATAAACCACAGGATC
TGTGAATTCAGAATC
GTCTTTTTCCATGACATTTTTGTCATAAATAAATAAAATATCACTG
TCTAGATTCA

>ConsensusfromContig700 Average coverage: 7.56
GACTCGGTCTCGAGAAAACCACCTCTTGTCAGAACTCGCAATTTG
GATGTTGCCTTCAAC
ATTTTATTTTTGGTTTCAGATTTCTCATTTCCTTTGCTTCCCAACG
TTCCTTCCAAAGAC
TTTTTAAGAATATCACAGGCTTTTTCTACAAGAGCTTCTTTGGCA
CATCGTTCGAATATA
TAGGTAGCAGGCATGATTATGAGAAGTGCAATTATGGATGATAG
GAAAAAGGACGTAACT
TCGAAAAGGTCGAGTGTATAAATGTCACTGAAAGTCGGTGGTTTT
AAAACTCCACATTCA
AGATCGGAAG
>ConsensusfromContig701 Average coverage: 29.62
CAGAGATTTCTAGAGCTCATCCAAGCTCATGTGTGATATCGATGA
ATTTTGAAAAGAATT
TGCCGCTTCCCTTAAAGATATTTGGCAAGAATTTCACAAGAGGCA
GCTGTGGGTCCACAA
TTAATGTATTCACGAGAGTGTAACTGAAGATCCCACAATGTATCT
TTACGTCGAACACTC
TGCAGATCGGAAGAGCACACGTCTGAAC
>ConsensusfromContig702 Average coverage: 725.69
TGTGACTGGAGTTCAGACGTGTGCTCTTCCGATCTGACGAAACCG
AGGTGCGATAAAACG
AAAATGTTCATAAGAACAATAAGAACCATGAAACACATGATTATT
CATTTGAATCAATTA
CAAAAAGTCAGACCTGCTGACGTGCCGAAGCATAAAATAATATT
CTAAATAATAAAAAAC
AAAAATATATAAAACGAATAAATTCACAATAGAATCGAAGTCAC
GAGACGTGAGTGTCGA
A
>ConsensusfromContig703 Average coverage: 8.15
TTATTTCATTTTTTATTGTCCCCAATCTTTGCATTTGAAACGCACG
AAATGTGTCTAAGA
GACTCATGGAAGATGGAATGTGAATTTCAACATGTCCTTGACATT
GAATCGGCTCTATTC
GGAAGGATGAAGGTCGGGAAATGCATAGGTCAAGGTTCCTCAGA
TATTGGATGTGCTCTA
GATGTGACAGATAGGGTGAAGGTCGGAAAGTGTATAGGCC
>ConsensusfromContig704 Average coverage: 6.93
GTTTGAAATGTTCTGTTATTCGAGGAACTTGTCAGATGTCACGAG
ATGAGTTGTTCTTGA
ATATCTGTGCCTTTGTGTCATAGCTTCATACGATGGCAATCAAATC

GGTGTTGTCTCATT
GAAGTGGTACACAATCTATACGCCGTCGGAAAATGGGATGAGATT
CCATTAATCAATTCC
TAAGCAACATTGTTTCTTGATCTGATATTTTTTCAATTTCTACAG
AAGACAAGAAAGCGT
TTTTTCAGGATCCAACCTCAGGATTGTACAAAAACCATGATAATG
TAAATATGTTATAAG
AATTATTCGAATGATACACTTACAAGTTTTTATCCCAT
>ConsensusfromContig705 Average coverage: 13.55
CGGGTTTCAGTTTATATCAAAACAACAACGAAAATCGTGGGTGCT
TCGGTTTACATGAAT
ATCAGTTATATATATTTTTTAGATCATCGAAAATAGATTTACTGA
AACAGAGCATGGTTA
TAAAAGAGAAATTTATAAGCATTATTCAAATATATGAAAACTAG
GGTGAACAATGTCATA
CACCAACTTCTTTGAATTGCTGCGAAGCTGGTTTATTTACTTCTTT
CAGGAACTTCCGCA
GACGACCTGAAGGGCCAAGGCCATACACGAGAGGCATTGATTGG
CCGCTTTTTCGCGCGA
AGATCGAATG
>ConsensusfromContig706 Average coverage: 5.02
GTTTATTTCTGGGGATGTTTATCACAGGATGAAGCAATTTATCTG
ACCTACTTCATGGGT
ATCACAACACTTTCTGTGGCATTGTCAGTAACGGTCATCAAGGTC
CACTACAAAGCTCCC
GAAGCACACCCTTCTAGATTCACAAGAATTCTCGTCTTCCATTAT
ATAGCCAGAGTCGTC
TTCTGCGACACGACTGGAAAGTATACAGAGATTCTGAG
>ConsensusfromContig707 Average coverage: 6.86
AGACTATATATGGTGAAAAGATTGGGGTGCTCCTTAGCTCATCTC
GCCAGTCTTTGAATG
TCTTCAAAGGGTAAATTGGACATGTGGGATTTTCTCTTCTTTTTG
ACCTTCCTTCTCCT
AACTTGAGCCTCGCCTCACTTCATATATCATCAAGAATATGGATA
GAGACACAATATACC
ACTCTTTCAATCTTGCCTCACTTCATCCCATATGCTCTTCGAATG
AAGCACGGCACTGCA
TTCCCCCCATTCTTCAATCTTGTCTCACTCTATATTTCCCCTTCAA
CGTAGATAGAACCC
ATTAGTAGACC
>ConsensusfromContig708 Average coverage: 5.56
GTTTCTAAACCGGATCGTGATTTCATCTATAAGCATGTCGGGCAT

TCAAAGGAGATCAAT
CAGCATGTATAATAGGCGCCATTGGGGTCGGGCGAAATAACTTT
TGTCGGAACGAGACTC
CCGATTATAGATATGGGTGAGTCTTAGATGATGTTTGCTTATCAC
GGATCGATAAAATCT
CTTATAACTCATCAACCTCTAAACCACTAAATGCATCCGAGGGGC
GCATAAA
>ConsensusfromContig709 Average coverage: 6.12
GGATTCCGCTGGAGCCGATCCCGATAGAATTTAATCCCGCTCGAT
TTATTCCTGCCGGCC
AAAGCCGTGCTGCTAATCCGCCTCTCGATGAATTCCAAGCTAGTT
CGCTGGCAAGAATGG
CACGTTGCGTCGCAAGATCTATCGGTGACAAGTCTTGGTGAATTT
TTGAAAACCTATTTT
CGGCACATTGATAGATCGGAAGAG
>ConsensusfromContig710 Average coverage: 3.60
TGGCGGAATAATCTCTGCAAGTAGGAGAATGAACACAGTGAGTG
AAAGCAAAATTGAAAT
ACAAAGAGCAATTTTCTCCCCACTGTCAGAGGGAAGGTAAAAAA
CGAGAACAGTCAAAAA
CGAGATCGCAACACAAGGAATGATGAGGTTGACTGTATGGAAGAG
AGTTTTTCTCCTCAT
GGTAACGTTGAATGTGATGTCTGTGAATGGCTCATC
>ConsensusfromContig711 Average coverage: 7.88
ATATCTGTCAACATAATGCCAACCAACTGAATTCTGCTGCAAATC
TGCCGGAAGACGAAA
AAGAGGAGATACTTGCTTAATCTGCACGACATTTGGGGCATGTG
AAGATCGAAAGAGAAT
TCTATAGAGATGTTTGCCGAGTTTCAAAAGATATACTGGCAAATG
CCTGGCTCGGCAGAC
ACACACCATGTTCACTTGATGCCACAGTCCACTACAGCTTTG
>ConsensusfromContig712 Average coverage: 114.27
TCAACGATTCGTCCTGCGGCCACCGTGACTGAGCCACCAGAACA
GGGAATTGTGCCAGGA
CCGCAAAGTTCTTCGCACGGGCAAGGGAAGGTGCAGTCAGCCAC
CACAGCGACGGAATTC
AAGACAATGCCGAAAAGCTCTTCACATGCGAAAGAAGGTGTGAA
GGCCACAACAATGACT
GCAGCGAAGAGATCGGAAGAGCACACGTCTGAACTCCA
>ConsensusfromContig713 Average coverage: 5.35
CGCATTTTTATTTCTTCCTTCCTAATCGATCGAATAATATTCTGCC
AGAATTTGTTTGCA

AAACCATTAAACCTATATTTGTTGACCTATCTGATCCAGTTCTCTT
GTGAAAATGTCTCC
ATGACTCAAAATGGCAATGAGTCCCTCCACAATCTCATTTGAGAG
CGGTGCCCATAGACA
AATTTTGCCCGTAAGACGAGGCTCGAAATTGCAGGGGAGGCTTCT

>ConsensusfromContig714 Average coverage: 7.62
TCTTCCGATCTCTCAATTTCTGGTTCGTATGACCTCCATATACGTC
CGTCGTCAATCCCA
ACGTCAAAGTATTGATTAATCGGAAAAATATTTGTTCTGCTATTC
TCACTCTCAGTTTCA
TTAAGTGCTGTCACGATTATTTTTTCAACACACCAAATTGCTCCTT
CTTCTTCGAAATCA
TGTCCAAACTGTATATAAAGAAGTTTTTTGAGATCAGTACACCTG
ACAGTGAACTCGTCG
ATACTACCGTCCTTGAA
>ConsensusfromContig715 Average coverage: 12.33
TTTTTTTTTTTTTTTTTTTAATATGACGAGTATTATCAGAAATGAA
AGTCACACACAATT
GGTGGCTAAATACAGTTTGTTATTCAAGCCGGAATACGAAATAAA
TATTAAGCTTTAAAT
CTGAACACATAAAACAAGATCAATGATTGAGATTACGGGTGAAG
GTAAAAAATTAAATGA
GAATTTCACACACTACAAAATCTAAAGTCTCAAAAATCCAGCTTA
AGGCATGACTAGGTA
AATTACAAACTATGTACACATGATAAATGAAAATGTCATGAAGA
GAATAATGAACCAAAT
GGATGGTATATAA
>ConsensusfromContig716 Average coverage: 4.64
AAAGCATTGGATGGTGTTTATCTTGTCAAACTCTGGGTTCACGAA
GTCATCAATTCTTTC
ACAAATACTCCTGGTGTTCTTGAAGATAAAACATGGCTGAATGAA
ACACTGAAAGATATT
GTGGTAAATAGGTTTTGTTCTCGACAATCTTTTGATGAATCGAAA
ACAACTGACCATCAA
GAGAATAATTTTATAAATCGTTTACCGAATCAGATCACCACTCCT
GCTAAAACAAATAAT
AT
>ConsensusfromContig717 Average coverage: 7.20
CGCATTAGTTCGAGTTTTGTTATTATAATCATTCTACCTTTACGAA
TTTGTTTGTTCTGG
TTTTAATCCTCCATTATATGCCACATATATGCATAAGCAATGGCTT

GGTTCTCTTGTCTC
ATCCTCATCATCTCATGGCATTCATCATACAGCTCAGACGTCTAT
GATTCAGCAGCCGTG
TCCGATTTCAGAGACAACTCCATAAACAATGGTGCCAAACGTCTT
TATAACGACAGATCG
GAAGAG
>ConsensusfromContig718 Average coverage: 10.09
CATGTGCTTTTGCGGACCACGCTTTATTTTATTCACCATGTTTCAT
GTATTCAATGTGTC
CAATGTGAGACTTTTTTGTTTCTGTTGTCGTCATTCAATGTTATTC
TGAGAACGTTATTG
AGGCACGAGCTTATTTTAAGTCATTTAATTCAATTCAATTATGCC
CGATATATTCTACAT
AAGTTGTTATGATTCTATATATTT
>ConsensusfromContig719 Average coverage: 7.69
GATCTATCTGATCCATCTTTTGATATCATAAATAGATTTTATGGT
CGAGAAACCTGTCAA
ATACATTATATATCACGGGCGATTCAGAGAATTTGAGACGACAA
GGCATAAAATATGTAA
TATTGAATAAAATAGAATTGACCGGGTAATCAAATCAACACTCGT
TAAAATCTTTTGAGA
TCTGCTAGTACGATAGTGATTACATATATTGCATTCAATCGTGGT
CAGATCTTCAATCGA
TATTGTTCAGTAAATTAGAAAAATAATAAAAGAGCGAATGAACA
TGCGAATAAGGTTAAT
C
>ConsensusfromContig720 Average coverage: 4.53
AGGCAAACAGTGTGTACTTCCATAAGAAAAGATTGTATTGCCTG
TCGGAAGAGGAAAAAC
TTTACTGCTTGATCGAAACAAAATGGGAGCTAAGCGAGCTCAAGG
GTTTTTACCACAAGA
CAGGCTAGATGAATGATAGATGATAGATATGGCGTCTATTCGTAT
GTAGATAATATATTG
GAATGTATTTGTTTTTGTGATTCG
>ConsensusfromContig721 Average coverage: 6.75
GTATCTTTCGCTCTGATTATGACGGTGAAAATCGGATGCAGACTG
AAGCATCTCTCGAAT
ATCCTACGATGGCTCTCAGTGAAATCGTGAACATTTTCTACGTAC
AGCATGTCACCACCG
GAGATTCATACCTCGAATTGTTCAGACCGGCTACAAATACTGTAC
ACATCACAGATGGAA
GAGCACACGTCTGAACTCCA

>ConsensusfromContig722 Average coverage: 7.03
GTGTATAAATAGGGATGATTATTCGATTCTAGTCATATTATCTCT
CTTCAACAATGGATT
TCAAATGTACCTTTTGTAAAAGAAATGTTTCGGGAAGACAGCAGG
CTTTACAGTGCGACA
AATGTGAAAAGTGGCAACATAGAAAATGTGCAACCGGAATAACT
ATTCAACAATATTGGG
AGATGGCCAGAACGGAAACGAATTGGAAATGCTGCAAATGCATG
GAGC
>ConsensusfromContig723 Average coverage: 12.30
TCCGATCTATCGAGCATACTTGTCGTGCTCATATTTCTCCTAGTA
GGAGGGATAATAATC
TACATATACTCATGCGACTCACAAGCTGGAGGAGATGCTTTGGA
AAGGACCCCAGAAAAC
TTTGTATCATGGACAAACTCTTCTTCAACCGCATCATGCTCAGAG
AGCGTATGAAATAGC
GTGATAATATCCATGGTTCCTTACTTCATTCCTACAGCGCCACCTA
TCACCGC
>ConsensusfromContig724 Average coverage: 12.09
TGAAGTATTCATCGATTGGCGACGCAATTGAGTTTATAAAATCCA
CTCAATGCTCATCAT
ACTTAGGCAAGATGGACATCAAGTCCGCCTTCCGGATCGTACCGA
TACATCCTGATGACC
GCCATCTCTTGGGTTTTTTAATAAATGGCATCTATTATTTTGATAC
ATGTTTACCACAAG
GTTTGGCCATGAGCTGTAAAATTTTTCAAGATCTTAGTAATGCGT
TGACATGGATTGCCA
CTCATAAATTTAAATGTTGT
>ConsensusfromContig725 Average coverage: 11.46
TTTGTCGCTACATTCAAATGAATCTTCCCTCTTTTTAGCTGACTGA
TATTTTATATAATG
TGAAATGTTATGAAACTGTTAAGCTACCGACTCGATCAGAGAATA
TACGGCTTCATCTTA
TTTCATTATTAAACATGTAATGTGAACCACAGTGAAAAAATAACT
TCATAATTATTTCAA
AATCTACAGCATCACGAACCCAAGCAATTATTTCGAAGGTACTCC
AACCTTCCAATTCAA
AGTCGCGTTATTGTGTTTGTATAAGATAACAAACATTGGTGTATT
TCAAAAATATCCTTC
ACAAAATTTACACAGCGTTTTACAACGTCCGTCATGTGGACCTGT
AGTTCAATCCTTGAC
AGGGGGCTGTTCATACCAAAGAATTGGA

>ConsensusfromContig726 Average coverage: 7.82
TGTATGTGGGTTTTTCTGCCCTCTTACATCTTTAAATAGAACTTTG
CATGGTGATTAAAT
GAGATGAAGGTCCCTGTAATCTCAATTACTGATATAAATATTCTG
AATTTTCTGGAGAGC
CACCTATAGCAAAATAGAATGGAACATAGCTGGCGCTGCTCGATA
TTTAATGTTCCCGAT
ATGGAGTATGCGGAAGAAAAGGGAGAGTAGAGGCGGGTGACAA
GATCACAGAAGCGAGCT
CAGAAGCCGATAGTTATAAAATAAGCCTCAATAAGAAGTATCAA
TAA
>ConsensusfromContig727 Average coverage: 5.72
CCATAAATGAATATATAAATTCATGCACCATGTACATAGATTTTC
ATCCGTCATTTGACA
TAAACATGTCACTTCCTATTGCCTCGAAAGCCTCATTTTGGTAGA
ATACACGAAAGAACT
TTTAAGCACAATTGAACCACGAAAATAAATTTGTAGGTAATTTAA
TATAACATCAAATTA
ACTTCAATGAAACTGCTTTTACTAAAACAAAC
>ConsensusfromContig728 Average coverage: 3.80
GGAAAATAGAATAATTAGGTTACAACTTTGCACGCATACAGAAA
TTTCAAAGATGCATGC
ATATCAATAATGACATGTCCGGTAATAAACACTCGGAAGGTGGA
AGACAGATTATTCAAA
ACCCCAGCATGGTTAACCCCTAATCACCCATGAAAGCATATTCGA
ACATTTTGATTTTAC
AGACTCGCATTCATCATCACTGCTGTAATCAC
>ConsensusfromContig729 Average coverage: 10.37
GCGTTGTGTATGGAGGTTGCATAGCTGCATTCATATCAATGACGT
CAATAGTATTGTTCA
AAGTCATCGGAGCGGAACGGTTGACTGGATCCTTCGGGATTGTAC
TCTTCTTCCAGGGCA
TGGCTGCGTTTTGTGGCCCTCCTCTTTCGAGTTATATCAAAACGAT
GTCGACTTCTTTTT
CTGTTTGCTTCATATTTTCTGGCGGTTTCATGTTCGTTGCTGGTTT
GGTTTGCGTTCCGA
TAAAACTTATCCAACAATGGGAGAAGAAAAGAGAGAGATCGGAA

>ConsensusfromContig730 Average coverage: 5.10
TTCCGCCCCTTCTGTGGCCACGCCTTCGGGGGGATATGACGTCAT
ACATCGTTCAACGGG
AAGCCAATGGGATCATCGTTCAGTATTGGTATCATCGACAGTTCA

TCGCCGCTGCACAGC
GAAGATACTTATCAGACAAAGCGGAAACCAGATCTATCCATTCTT
TGCTGGCGGACTACT
TCTTGGGGACATGGGGAGGGGGCAGGCCCAATGCTT
>ConsensusfromContig731 Average coverage: 5.49
AAAATATATTTGGTAAAAAAATAGTTAGTAACAAACAGTAAGTA
ATCAATGTCTTGAGAT
TACAACATGAAAGACAATTTGGATTTTCTCAATCCAAACTCATCA
GAACCGCGTGTGCAT
TCTCCTGTTGACCGGCCTCCGATTTGTGATCGCTGTGATGATCTAG
GATCTGTCTTCTTA
AATATCAATTGCCATACATGCATTGAAAAAATCACAGAATTTGAT
ATTCCACAACTGTTT
GCAATCATACGACAATGGACGATACATACTCAACAGTTCATGCG
GAAAT
>ConsensusfromContig732 Average coverage: 6.00
TTCGATCATTCCTTTCAACGAATGAGATGGTCCAGCGTTTTGTACA
GTTGTTTTTTTTTA
AACCGGCCAGATCATATGTATACGGTTTGAATGGGAGTTTTCCAA
AAGTACCAAGTTCCT
CATTTGAAGTGGATAAAATGCGATTCAGAATCCGTGTTTTTTTTAA
TTTTTCCCCCGGTG
TGCGGCACCTGATTCCACCCAAACTATGAAAGGTATTATGGCCGG
CCAAGAGTCTGATGT
TGAAATCTGCATTATCA
>ConsensusfromContig733 Average coverage: 6.36
AGAGATCTAGATCATAAAATCCTTGTGATTCCAAATATGGCATTT
AAAATATATGAATAT
CTGAAATTAAATCACCCTCATTAGCATATTCACAATTTTCAAAAA
AATCTCAGTACGCTT
GAAAATCAATGGATCAACATGTAATTTATGGTGTAACATCCATCA
ATATGGGGGGTGGGT
TTCCTGAACATCAGTAGTACAGTACAGTGTAAAAAAATTATCTCA
TGTTGAGTATATAAA
AATTATTTTAAAAAACTTTAATGATTTCCAATGAACACAAATAAG
ACACATAACCTAAAT
TATATTTATTTTTTTTTATTT
>ConsensusfromContig734 Average coverage: 8.14
GCACGACCTGCTAGTCACAGGGTAGGCAATTTTCAGCGGTAGAG
AAGAATCTGAATTAGC
TGAGAGCCTATTTTTTCTGAAAAAATCATCGTAGTGGTACCCAAGT
CTTCTGTTATTTTC

CATAACCTCGTATCTTTTTTTCCAGAACTCCATCGTCGCTGCATCT
TTCTTCATCAAAGA
TTTCATTTCAGCATTTTTTTTCTCGAGATCTTCGAGTTTATCGTTAT
TTGTTTTCGTTTG
TGCTTCCCAAAATATGATTTGCTTTTGTTTTTCTGCTAGTTCTTTA
TTGAAATCCGTTCT
CAGCGTCTCCAATCGCTGGTCAGAGGAAATTGCCATAGTGTCACT
GAGATTTGCAGATCG
GAAGGCACACGTCTGAA
>ConsensusfromContig735 Average coverage: 7.88
GAAATGGATGCAGCTCGCTCTCGTGTTGTGCATCAAAAAACGTAG
TTCATCCAGAAATTT
CACAAGTGACTAAGCCGAACACGTCAACTCTAGAAACAGACAAC
AATATATTGGCCAATG
GAACCGCACCAAAATCAATTTTGTTAAAACGACAACAGAAACAA
AAGGTTTATTCGAATG
ACACACGTCGAAGAAATTCAATCACCAAGAATATAACACATACC
GCG
>ConsensusfromContig736 Average coverage: 6.25
GTAAAATCTGATCAAATATAAAAACATAAAGAGAGATGACATGT
AAACATATTACGAAAT
TTCGACAAATTTTGGCATAATAACAAGTATGTGAAAGAAAACAC
CCGGCCAAATAATACT
TTTAGGCCTCGATTCGAGTAAGTAACTTTCTTTTTTAAATAATTGA
GATTCGATTCCATT
ATTTATCATATTGGAAAGCATAATTATTATAGCAATTTCGTGGCA
CTGAG
>ConsensusfromContig737 Average coverage: 11.86
GTTTTTGTTTTGTTCGCAACTTTTTTCAACTTTTGACAAAAGTTTT
GTCAATAAGTAAAA
TGATAATTGGTTTCACTGCGTGAATTAGATCAGACTGGCTTATTTA
TCCTTTTTGATGGA
TCCCAAATTATCGAATTGAGTTGTTCGACTAACTTATTGTCGTTC
CACCGAAGAACGGTC
ATAAATCTTTTATAAAAAAATATTGAGTTTTTTTTCAGTTGGTTTT
CTTCTCATAAATCT
CCGA
>ConsensusfromContig738 Average coverage: 15.23
GATCGGCTGTAACCCTTGGCAACGTGGACGACATGATCAACAGA
GAGCCCGACAACGATC
ATCAGATTTCCACTTTCCAGCATACCAAGTTTCCATCCGGCGAGA
GGGATGGTTGCTGCA

ACCATGAGGACATTGACGCAGATGCAGAATGTGGCGATTGCCCC
GACGGAGAGGCTGCCT
GTGGCGAAGATGAGGATGGG
>ConsensusfromContig739 Average coverage: 8.77
AGATTCTGATCTTCACCTGATCCGATTGTTTACCATTTATTTGTAT
ACCTATATGTGATT
TATACAGGTTTGTTTCGCTTTTATTTTCGGATCGTCTTTGATATTT
TTACGTCAAAATTC
AGCAAATTGTGATCCTCCGCTTTCTGCCAGTTTAAATCATTCGTGT
GTTCATATTATTGG
AGCAATTTAATGTGAGCGGGAATTTACCACACGTTTCTATATTTC
ACAATGAGTATATCA
AATTTCAATGATCAAGT
>ConsensusfromContig740 Average coverage: 6.14
GGTTGATATGCCTAATCTTTAATTTTTTAATATTCCATATTGACTT
GAAATCAATCATCG
GCGAATACAGACCCTACAACGCGGCGGAGATCCAAACAACGTCA
TGTGAGACTCCAACAA
TTGTGGCTTCAGTGTTTCACTGTGGTCTGATTGCTTCTGCAGCGA
AGAGGCACACCTTCA
GTTACAACACTAATGAGAGA
>ConsensusfromContig741 Average coverage: 7.93
CTGGTTTCATTTTTCACACAATACTTTGTACATCTTCGCCAGTCA
AGAGGCTTCACCAGA
TGAAGGCATTTCCGATCGTCTGCTTTTTCTGCTTCCTCGGAGGATT
CGCACCAAACCTCT
CTTGTTTGCTCGCCTGGCGATCCGCAATCCTCACTGTTGCAAAGA
CTCCAATGGTCTGCT
TCCCAAGAATAGGATGCCGTTGCCAAGTCGAGTCGAAGCAAGAT
CATCCATGACACGAAA
AAACTTGATATGTT
>ConsensusfromContig742 Average coverage: 7.70
TGTTATGATGAATTTGGTGTTATCTCAAACCATATTATTTTAATTA
GACATTGGTGCATG
TCGCACTATATTTTGCTTTCAGGATTTTTGAGAGCTGACGGTTCTA
GATTTTTTTAACGA
CATCCAATATCACATTCTTCACTCTTTTCTTGGTTTTAGAATCGTC
GGATATTGAACTTC
TATGTTTTCGTTTTGAATGACGTTTCAATTCATAACGAAAACAAA
AACCTTTGTCACAAA
GTTTGCAAATATGTGCTTTTTTCTTCGAATGCCGAATTGTGTGTCT
TTTCAAATAGTCTC

TTGATCGAAAAGTCTTATCACAAAATTCACAACTATATAGATCAT
AAAAACACCTTCCAG
GTGTTAGCGAATCATCTAACATAACGATATTATTGGTAACC
>ConsensusfromContig743 Average coverage: 9.08
CGTACCTAGGATATGCCAGTGGATACATAGCTGAAAAACTCGAA
CCAGAGTGTGAATTCT
CATTTCGAGTAAAATTCAGCAAAGAAGGCGAGGAAGGGATCTGG
AGTGAGATTAGAACCG
TTTCCACATCCAAGGAGCCGATCAATGGAGAGCTTATCCACAGG
AGTGTCCTGAGAGGTG
ATGAAGTGGGGCTCATGGCTATCGTAGACAGATTACATCACCAA
GGGACCAG
>ConsensusfromContig744 Average coverage: 4.65
TTTGCATTGGAATAGTGACAATACTCTTCGTATTCATTTTGATAG
CTGCTATAGTCATAG
TCAGGCGGCGTCAGAACAAATCGGACCGCAACAGTTCAACATGT
CAATGGTTTCGGTCAA
GTCCAAGATCGTCGCCCTCTTCTGTCGCCGTTAAACCGAATAATT
ACATGGACAGACAAA
GCAAGTTTCGCAATCAAAATATGATCCAAGGCGACCAAATGAAT
GAAGACAAGTATATGC
AACGACAGACGCTATCTGGAAACATGGCGTTTCCCGCCAATGGCT
CT
>ConsensusfromContig745 Average coverage: 5.53
CCGTTGCATTGCGTCCAAACTCTAATCTGAAAAAATCAGGGATAA
TTGCTCGTATATCTG
TGGATAATCCGCTGGACGTAAGATGAATATCTGGATGAGGAGTG
TGGCGCATGTGGCGCT
AGGCCTTCAGAGACACATTAGCCATGGGTGGGAGGAAGCAGGGA
TCGAGGAGGAGCAAGT
GGCGGCCTCCAGAGAGTATAATCTAATCGCCAGAATAAAAGATC
AATTTGGAAGCGAGAT
CAAAAGATTTTGAGCTTGTTGGATGGAATCCAATAAAATCCTACC
GCAGATTCCAG
>ConsensusfromContig746 Average coverage: 7.81
ATGAGTTTGACTTTGAACTCATTTTACGCAAAATGAAAAAGCCGA
CTTGTTACTGATTTT
CCCATGGCGCCTCTCATATATAGAAATTATAGGCACATAAACATC
CAAAAAAGATTTAAA
ATTTGAGACCAGCGCTTTCTTCATTGAATATTTTGTCGCATTCATG
TGTTTTATTTTGAA
CTAGTTTCACCTTAATGAATATTCCGACTTTGTACTGAATTTCTTG

TGTCGTCTCTCATA
TGCTGTCG
>ConsensusfromContig747 Average coverage: 10.17
GGGAAACATGGCGATGAATGCGGAGAAGCCATCGACCACGCAGA
CGATTGCACTGCTTTA
CAAAGTACATTCATTTTGAACATGTGCAAATGCAATCGTGGATTC
TTTGGTTCGACCTGC
TCGAGAGCTCTCAGGTGTGCTGACCCCCCTTGTAAGAATGAAGGT
GAATGTGTACCTCAT
TCCAGTCTCGGCGATACCTGCACGTGC
>ConsensusfromContig748 Average coverage: 6.13
GTAAGAGTCAAAAGACACGCAAGCAGCAGGACTTAGTCTGTTCG
AAGATCCACTGAGGTT
GAGGTCGGGATGTAAAGGCTCTCCTTTCACTGTGCTGGTGAGGTT
CTGAATTCTTGTGTC
GAGAGTATTGATGCTGTCGACGAGGTATCTCTGGTTCTGCTTGAG
TGAATTGATCATGTC
GAATCTGAAGCTAGAGAGATCTTGCTTCAGCTCTTCGATCTCACC
CTCCACGACGGACAT
CTCCT
>ConsensusfromContig749 Average coverage: 6.46
ATGACCAACACTTGAAATTGTTCGCTTCACGTTATGAAAAAGATA
AATATATTGGGATGT
CTGAACAAATCACGATATCTGCAAACAGAAGACTTGCTTTGCTTC
ACATAACAGTATTCA
TTTGCCAAGGTGATACGGAGCTGGATGAACGGACATGGTCGCAG
TTAAAGAAGAGAAAAG
AGACGGTCAAGCCTACAAAAGTCCCTGATAAGCCAGTTGTTTCC
GATATAACAGCTAAAG
AGGGCGTTACACTCATAAAACACGAA
>ConsensusfromContig750 Average coverage: 7.43
GAAAATTTGTTCAATATGGGCATGGTGAAACAATTGATTGTAACG
ATATTTGGCACAAAT
ATCAATAAGAAAGTAATGGGGCTCATGTATATGGAATAATATGG
TACATACCATTGGAAA
GTCGGAGTGAGGCTAATGTACAATATATTTAGGAATATGAAATAT
GATAGGTTCTCAACA
AGTTGCCAATAGGTTTATAAGGACGTGAATCGTTG
>ConsensusfromContig751 Average coverage: 4.65
GGTAGATTGATCTTCAGAGCAACTTCCTCACGCATGAAGATGTCT
GGGTAACGCGTCTTC
TGAAAGAGGGACTCCAAGATGTCCAGCTGTGCTCGGGTGAAGGTC

GTTCGCTCTCGCCTC
TGCTTTCTCTGCATATTTTGATAGTTCATGGCAGATTGAATTTCC
ACGCCAGTTGAAGAT
AATCCACCCATACCATAAGCCGTGAGTCCGCCTTTAGGTTGTCCA
CCCCCCGGTACACCA
GAT
>ConsensusfromContig752 Average coverage: 4.60
AAAGAATTGGTAATATAGCAAGGTATTTTGTTACTGTCGAACCAA
AACTTGTTTTTTGAA
TCACATAATATGAACCAATATCTCATCCTCGTATCTTTCGGTTCA
AATCCGCAACTCTAG
TTGATCCTATGTACGGAGTCAACTTTGGTGTGTATACATAACGGA
GACCTCTGAGAGTGG
GCCTATAAGTTATGTAATCATCACTTCTATATGTC
>ConsensusfromContig753 Average coverage: 7.52
AACGGAAAAAAAATTCATCGTTTGCGAGAGCAAGCTTATGGTAC
TATTTCAAAGGTGCCG
AAGATGTGGCAGCGATCAACCTAAAAATTACCGCAAATCAATCGG
TACCGCCATTATCAT
TCATCAAGAATGCAAAGACTGTGGTTCAGTAACGAGATGGCAAA
ATCAAGATTTTGAAGG
AAAGATGCCCGTCCTGAATATTATTATGGCTGCCGCGATATTATT
CACAGGCAGCATAGC
TGCACAGGCCCTACGAATTTTCGAATTCATGAACATTGGAAAAAT
AGGGAAGACTTCATT
CTTCGAAATACAGGCGAAATTCCTATATCCAACAATCGCCATGA
AATTCGAGGAACAAAC
AAGTTGTTTAATGAAGAGGATTCACAGGCAGCATAGCTGC
>ConsensusfromContig754 Average coverage: 4.77
GAGTTCAGCCTTGTGCTCTTCCGATCTTGTCAATCGAGAGGGTGT
CTGCCGATCCAAGGT
GCCTCTGTTGTATCCGTGCGACTCTCAAGATATCTGCGAAGATGC
GAATTCGGAGTGCAT
TGGTGGGAAATGCACTCCCACAGCCATATATTTTATCAGTGAAAA
TAGCTGCAAGCCTCG
AGGGTATTTAGAAACACCGTGTATTGAATCTGACAGTAGTTGTTA
CGATATCAA
>ConsensusfromContig755 Average coverage: 5.93
TGAATATAGAAATAAAATAGGGAAACAAGTTATATACGATGGGA
ATATGTCATGAGTGAG
TATACTGATTTTGAGCAAATCTGAGATGTTTAGTGGCGTTTCAAA
AGCGAATCGATAGTC

TACTTGATATAAGTATGGGTCATCAGTATAAAACATAAAGAATTC
GGAACTACAATCCGT
CAGTGAATTCGTTATTTCCGTAACCTCCCTGTATACAAACTAATA
GGTCTTTGCAGCAAA
ATGGATAAAGGAGAAACTGCATAACTAGTCGCAACTAGTATGCA
TTAACACACCGATTCA
ACATAACGTTACGAAAATCATTTCAACGAGATAAACACACTAACC
CGACTTCACTTTAAC
>ConsensusfromContig756 Average coverage: 21.87
TCAGACGTGTGCTCTTCCGATCTTCTGAGTACACTTTCCATTACTT
ACTGGTTTCTGTAG
AGCGGCATTATTAACCCTTTTCATAACAACATCATTATTGACATGT
TCTTTAGAGCTTTC
GAATATTTGTGAGGTATTGTCTTCATGATTAGTATATATATCTCGT
TCTTTATTTGCTGA
ATTTTCCAGTGATATATCTCCAAACCTTTTACATATCGGTTTGTTA
TTTCCATGTTTTCT
TGATATAACATCGGCTGCCTCACCTTTTTT
>ConsensusfromContig757 Average coverage: 13.58
GGGATTTGGATTATTCGAATAGGTTACACAGGGTCAAGGGAGAAT
CGTTGTGTTGATGCA
TCTGTTGATAGACATCAGTCGATTGCAAACCACACAAATAACACA
TGTTGGGAAAATCAT
TAAATGCAGAACAGGAACATTAGCGGCCCAATTCCTTAGGGCAGT
GTATTTTGGTTCGGG
GCCCGTTCTATGAGATCGGAAGAGCGTCGTGTAGGGAAAGA
>ConsensusfromContig758 Average coverage: 6.54
GCGAAACTCAATTTTTTCATATAGTTATCATCGACAATGCCATCG
AATATCCGTTCAACG
ACCTCTTCACTCATAGTATCATCAACTGGCATCAGACCCAAACTA
GCTACAGGTTGAGTT
TCTTGAGTAAAGCTGTTGTTTTTGTCAGATTGCAGAGATATTTCA
GAAGTCTCAATTTCA
TTTTCATCCTCCTCCTGGCTCTCGACAACGTGAACATTCTCTTCTT
CACAAATTTTTGGA
TCGACGATGAAAGAGTCATTTTCTAATATTGATCCTTGGATAGAT
GTACCATCAGATTGA
TCGACTGATAAAGTGTCTCCAGAACCACCAGGA
>ConsensusfromContig759 Average coverage: 17.07
TTCAGGTATGTACTCACTCCCCAATTTTCGGTGAAAATTACATCA
GCAATATACCCCCAA
AGACCGTCTACAGTACAGCTCCTGTACAAACGCGTATATCATGAA

AAGAAAATACATTAT
CGCCATCAGAAGTCCGGCGATTTTCTTAAGACGGAATTTGAGGG
AGCCAAAAATCACGAG
GGTTATGACGAGAATGATGAGAAGGATGAATCCAAACTTTGCAT
GTGGAACTATACCCAT
CACTCCTGTGCCAAGGTCGTTCCATTCATCATTGTC
>ConsensusfromContig760 Average coverage: 9.33
GGAGAAGTGTTACTGTCCCGGTCAAACTCATGGGACTCTATGCGG
TGATACTGCGTTGCC
GAGTCCATGCACTTCTGACACGTGCAGAAATGGTGGTACATGTAC
TGTACCGAGCTCGGC
GGCAACATGTAGCTGTACCGATGGATGGACTGGAACTACCTGTC
AAGAAGCGGTTTGCCC
GACCAGCGATTGTGGGACAACTGGCGTCTGTGCAATTCTGAACAA
CAGTCCGATATGCTA
TTGCCCCGCAGGATTCACTGGAACTTCCTGTACAACAGCTGTCGA
TGAATGCAACCCGAA
TCCATGTCTCAACGGCGGTACGTGTGCATCAGGACCAACCTGTAC
ATATTTATCTTGAT
>ConsensusfromContig761 Average coverage: 4.44
GAAGATAGTTAGAAAACCCTTTTACAATATCATTGATATATTTTTT
CATTTCCATGAATT
CAACGTTAGACGCATGGTTGGTTTTTATCATGTATAAGGATAGTA
CTGATACGGGAGTTG
GGTGAATAAGTCGTGTTATTTTATCAATAGTCGCATTATGATAAT
CACTAAGGATTGATT
TTAAAACGCTTACTCCTGGA
>ConsensusfromContig762 Average coverage: 18.13
ATCTATGACGTGCTAGGCGTGAAATAATAGAAGCCATATTTTGTA
TTAGATTTTTATAGA
TCATTATTTCTTATATAAACCTGCTATTTGTAAGATTCATTTATAT
GTAGTTCTAGAAAA
ATAAGAAATGACTATATTACCGAAGAAAAAACAGCAGAGGGACA
AAGGAGAGAATGATCC
AAACGAACATTCCTCCATATCGAGAATTCATCCATC
>ConsensusfromContig763 Average coverage: 6.11
TCTGCTTGTTATTGGTTTTCTGGAGATTCGCCACCCTACCACAGA
AAAACTGGAAGAATT
TCCCAATGACGCTGCGAGCTCTCGACACTCCATGATCAAAGATGC
GCTCCTTGTTTGTAC
GCCACGTGCCCGGAAACGCCAGGAAGTTGGTTGTTGTTGTTGTGG
GAAACCTCATGCATA

ATGGAGAGAAGAAATCCCAAATCGCTGGCAGAGTCTGACGGATT
ATTCTGTGGAGCATTT
TCAGACTCTGGATTGAAGTCCATGACGCTTGAGTCT
>ConsensusfromContig764 Average coverage: 6.95
GGCGGAGGGTGGATTACAACCCTGTGTATTACTAGCACCCCGCCA
GCTGATCAATTGTGT
TTACATAAAGTTTCAATATTGTTGTTTTCGAAATGGTCACTCTGA
TAAAGAAAAAATCTT
CAAATAAAAGAAGGTTTACCGGTTCCTGCTGCGTGGCATATGGAT
GCAATAAATGGTCTA
GTCAATCACAACATATGTATATATTTCCCACAAATGAAGCTAGGC
GTTCAAGATGGATCC
AAGCACTGAGAGGAGAGAACTGGACCCCTCGTTACGAGAGACTG
TGCAATAATCATTTCA
TATCAGGTGAAATGTTGAGATTTTTCACTCAAAA
>ConsensusfromContig765 Average coverage: 10.84
CTCCGCTACCATCGGTGAAAGAAGCTTGCAGATGAGATCCACTGA
GCGAGTAACTGCATT
CAAACGTGCAAGTCTTTTCGGGTCTCCCTTTGATATGACCACGAT
CCAATCTCGTGTGAA
TGCTATTTTGTAGGTTGTGCTTGCAATATTTGCGATGACTGCTGT
TGTGATGCAAAGTAG
ATCGGAAGAGCACACGTCTGAACTC
>ConsensusfromContig766 Average coverage: 5.46
TCCAATGTTCGGGCTGCCCGCAACTACATTGACATGAACGGAGC
TGAAGTGGACGAGGAG
GCGCAGGAGCTGCTCTCCAATCTGCGTGACGTTCTGGTTCCGGCC
AAGATGGACCCCGAT
AACGTCACGAGATTTGACGTCGAATGGAGAGGAAAAGATGGGAT
GGAAGTAGAGTTTCTT
GGTGACCATCTTCACAATTTCGG
>ConsensusfromContig767 Average coverage: 4.73
ATCGATATATAAAGTGTTGTTCGAGACACTATTGAATGCATCTAT
TGCTCAGGTAGATAA
CACATTATTTGATTACTTATAGTATTGCCAACTACGGAAGCAAAC
AAAACTCTCTGGCCG
CTTCCTCTATCTATCGATATATAAAGTGTTGTTCGAGACACTATTG
AATGCATCTATTGC
TCAGGTAGAGAACACATTGCTCGATTAC
>ConsensusfromContig768 Average coverage: 5.63
CGAGGCACACTCATTTTTCTGATATGTCGAAAGAGTGAAACACAT
TGTCTAGAATTGACA

TCTCGTGTAAAATTAGTTTAATGATACGAAAAACCAAATCACGTT
AATAATATCAGCATG
AGAGCGATTTGAAGCGTACATGAGTGGAAAAAGTGAAGCACGTA
CAACAATCCATAAGAA
TTCGAAATTCATGCTGTAAATATCAGATTATTTATTTCAAAATAT
GTGTCAAGAAATAAC
ATGGTAAAAAATTGTATCAAAGGGTACAAATACTGCTCTTTAATG
TAGGGAATACATTA
>ConsensusfromContig769 Average coverage: 9.89
ACAATCTATGGTTAAAAATGCGTATGTTTGTATTTTATTTTCCTTG
TTTTAAGTTGACCT
ACTTATGAAAAGGGCGTGGCCTATATATTCTTCGACTTTTTTCATC
ATGCTCCTCGTCTA
AATTAGCCTTTATTGGTTTATAAATATGAAAATCGGATGAATGAG
ACTTGTTAAATACGA
TTTTATCATGTATTATTGGGTTTTCAGGCCGCATTGATGTGATATT
AATAGTTTTATTCA
TGGTGTTGAGCCTTGACATTTGCCAA
>ConsensusfromContig770 Average coverage: 7.09
CTCGGGACCAGAAAGAATGTGAAGAGAGGCTGGAGTTGATAGAG
ATAATGGCCACGCTCA
AGAGCGAGCTACTCCAGTTGAAAATGCCTCCTGGCGGCTACCGAT
CAAGCAGTATCTCCA
GCTCATCGTCTGCTCCTGCTATGATTAGAGATTACAACGGTCAAA
ACTCAGGTCCTGAGT
CTCTCTCAGCCAGAAAAAACACAACTTTATCTGCTCCTGACTATC
CAAATCCTCTACCTT
CTTTACCCATGTCGTCTGCTTTATCAGATTCTGCATCAGGTATAAA
GATCG
>ConsensusfromContig771 Average coverage: 4.17
CCTCCCCCCCACCACCCGCCACTATCAGCTGGACGGTAACTTCCA
GCTGGTACGCAAGAA
ATCGGCTGGGAAAATGCGACGAGATCCGCTCATCCCTGGAATGTT
CCTGGATCAATTTTC
AGTCGACTCTTTCGTTGAAGGCTATAACGATAGACCATCTAAATC
ATCATGCAGCAACTT
CCAGGCCCTTCGGAAGCTTCG
>ConsensusfromContig772 Average coverage: 5.12
AAAAACTATCCAGAATAAGATTCAAAACTGAGCAGTCTCGTAAGT
GATTTTTGGTGGCTT
GAGAGACTGTGAGCCAGATGCAGTCTGAAAGCCTTTTCTTGTGCA
GGTGGTGTGTGGATT

AATTGCAATGTGTTTCTTGCGTGTGAGAGCCAGATTCTGTCTTGC
CTTGAATTATGAATT
TAGCGGAAGATTAAAAGCAGC
>ConsensusfromContig773 Average coverage: 14.42
GTCGGTATCATAAGACAGGTTATCTTTGGAACAACTACCTCGATG
CGAAATATCTACATG
AGTATTATTTCTCACATCAGAATCTTTATCAAATTTGGTAAATTTC
TGTGGAGTATTTTC
ATTTGAAATATTCAATTGTTCATCGTATTCTTCTGGATCCATAATT
TTATAAATCCTTAT
GCCTTTCGAAAGTAGTTAAAAACAGGCAACCTTGTTATTAATTTT
TATCACTCGC
>ConsensusfromContig774 Average coverage: 11.22
GACGTGTGCTCTTCCGATCTGTAAATCTACGCCCTCGTCCAATTC
GAGCGAGCAATACTT
ATTGATATACTTTCTGATATTCTTTTCCGAACTGCCATTTGGTTCA
TTCAATTCTTTAAT
TGCTTTGATTATCAATTTTCTCAGGTCTGTTCTTCTCGACACAGCA
ATTATTCGAGATTT
GAAATTAAAAAACTGTTTGGGATCAGTGTAGGAGGATACACCATT

>ConsensusfromContig775 Average coverage: 5.45
CCAGTGGTGACGCCGATGCGCTTCTTGCTCCCCACCGAACGAAGA
CCGTGTCCAAAACGG
GGGCGGGGACGAAATTCGATAGGGATTTGCTTGGTGATGCCATCT
ATATCACCCCCCCTA
TCCCGCCCACTTCGGAGTAAAGAGCTCGTCGTACCCATCGCTCAC
AAATCCTTGCCTCCC
CTAGAATGGTATTTTTGTTTG
>ConsensusfromContig776 Average coverage: 6.05
CTACAGAAGACCAGAAGGAAAATCTTGGACGGAGTCGAAAGCAC
CAGTGAACACCAGACG
TTATGAGAAGCCATATGTACCCCCACGTGTTGAGAGAACAGTGA
TCGAACCAATAAAAGG
AGTCTCTTCAACCAACTATTACGGTGGAGCTCGCAAGAGGACCAG
CAGCATTAGCTTTTT
CGATACCATGCTACCAACTGCCAACACTGGAGATCGGAAGA
>ConsensusfromContig777 Average coverage: 9.29
GGCAGTTATCGTGGAATGGAAAACTCACGAGTTTACTTTCGCGAC
GTTCAAGAACCGTGG
AGAGCTTCTGCTCAGAGGGGACGCCACAAGCGAAGTTGTTTCACT
GATGGAGGATTCTTT

GGTTGCCCTTCACTCGCTGCTTAGCAACCGGTACAACGCCCCATT
CAAGAAGTCCATCCA
GCTCTGGGTGCAAAACCTCTCAAACTGCGTCGAAATCATTGAAAA
CTGGATGACGGTTCA
AAATCTCTGGGTATATCTGGAAGCTGTTTTTGTTGGTGGGGATAT
CGCCAAGCAAATGCC
CAAGGAAGCGAAACGGTTCTCCAACATTGACAA
>ConsensusfromContig778 Average coverage: 4.86
CGAACGTACTCGAAATAAGCATAGCTCAATCGAATTCAAGCAATT
TGGAAATGCTGTGCC
ATGAGAATATGAGAAAGCGCCAATCCTGGCCATGGATCTCAACA
GAAAACATTTCACCAA
TGGATTGTGGACTTCAAGATAGTTACAGTTTCTCGACACATCTCA
ATGGAACGCGGAAGC
TGTGCCAGCGGGAATGGGTCCTATCCAAGTTGGATATAGAGTGC
ATCCGAGGAGAGGGGC
TCATCTTCACATTCCCGCAGCAGAACTGCATTCCATTCAGAAACC
AGTACACAGTTTACA
GTACCCGATGCTGGGGATCTTGGGCTGTAG
>ConsensusfromContig779 Average coverage: 7.17
CCGGAATCCATCAACCTATTGCCGAACATATGTCAAAGTTCTGGA
AAAAGATACTTCAAG
AAATCTACAGGCATGTGGGGGCAGGGTACACCTGCCGCTACATC
ACGTTACGGACTCTAA
TTCTATCAGAATATATGCTATAAAAGGTGCTATCGATCAACACTT
TTTAGTCAAGTTCAA
AGTAATGGGATGCCGTCAACCGAAAATACCAGAGAACAGCTACC
TCATTTTGGAGAGGGA
CAAAGCCTTCATCAGATG
>ConsensusfromContig780 Average coverage: 5.42
TTTGAATTGGTAGATGATCATACCACGAGCGGTACTCTGTATCGC
ATCCTTCTTGGTGAG
ACGGAACTGAATCTTGATCCAGTTGTTGTTGCCGGTGGTGTTGTT
GTTGACGGTTACTTC
TCCAAGTTCAAAACCTTCAACATTCTTCAGAACATTCTCGTAATT
GTACGAGTCATCAGC
AAACATGACGATTATGGTACACTTCTGTTCCCAGATCGCATGCCA

>ConsensusfromContig781 Average coverage: 10.16
GCCAGATTTGTGGGGGCACTGATCCTTGTTGTGAGTGAAATCATG
GCAGTGTTGACACTG
ATTGACAAAGAATCGGTCAAAAATTTTTATTGAAGAAAGGCCAAC

AAAAATCTTATCATC
ACTTGATTTGATGAATGACCTCACACGAGGAGACACTTGGAACAC
CGCTGTTTTCCCATT
TCTGGCAGATTTTATAAAAAGCAGTTTGAAATCATCTGA
>ConsensusfromContig782 Average coverage: 6.18
TCTGTCGATGCGTCAAGATATCTTCTAGATTCAAGATCTGAGTAA
TGGCGTCGCATTGAG
TCTCTACTGATTTGTGATGATATTTCGGTGGAATTGCAGCGTTTTA
CTGAAGAGTGTTTT
CGTAGCGTCGAGTGTATTTTTCGCCGACGAATTGAATCACTCGAG
CTGGATTGTGGTAGG
CAAGAAGTCGAACTGTTTTTAGA
>ConsensusfromContig783 Average coverage: 7.46
TACAGACCGTGGAATAGCTCATCATTTGTTATTTTAATTAAGTCA
TTCCTGAAAGAAATT
CCTGGATGTTATTTTGTCGGTGATATCAAATTTGATTCAAAAGGA
AACATCAAAGGTACA
CGAATTCATGAAGAAATATCTTTACTTGACAGCACAGAAAAGATC
GATGATTTTCTAACT
TCGATAAGGCAATCGATTGCGGAGATCG
>ConsensusfromContig784 Average coverage: 5.22
CTATTCCATTGATTGCCAAATATCTTTTATTCACATTCATCATGAA
CATTGTAACAATTC
TTATAACTGTGATCATTATAAACTGGAACTTCAGAACGCCCCGA
ACACACAGAATGGGGA
AATTAGCCCGTATGATTTTCCTGAATTATTTGCCTCGAATTTTAT
TGATGAAACGGCCAA
ATCACAATCAGAGATGGTCGAAGAAGTATTACACTCGACAAAAC
AGTCTTCTGGGTGAAA
TGAACTCATCACTTAAAACAGAACGAGGGGCACCGCCCCCTCATA
ACTCTATGCCATCAT
ATTCCTCTGC
>ConsensusfromContig785 Average coverage: 11.90
ATTTCCTATACATATTTCAAAAAAACATAGAGGGTATATTTTGAA
TGAAAATTATGGGTT
GAAAACCATTGGAAATTATAATGTAACTATTCGAAACCCTCCAAG
CGTTTTTGTTTGTTC
TGAACAATATTTTCTACAGGGAGCACAAAATTGAATAACATACTA
ATAAGGCATAAATTT
AAGCTTATGTGAAGACGCCCCCGTAGGTACAGCGATGCC
>ConsensusfromContig786 Average coverage: 4.62
ATCGCGTCTCCACATTTTTTGGATGTGTGCCCGAGGTTGATTGTA

GAATTTCAACCTAGC
GGAAAATGTACGAATCAGTGTTATGATCATTCTGTTTAGAGGTAA
TAACTTTTATTTATA
TTGAAGAAACATCCTAATGATGCGTCCCATGTCTTCACCTATTTTC
TATCTCAAGTCTTT
ATTCATTTTTCCATATTGTCTTTT
>ConsensusfromContig787 Average coverage: 20.06
ATTAAGTCTCTTTTCGAATTATTGGCCCTGAAAAGGGCCGTTAAT
ACAAAGATCAAGATT
CTTTACTTGGTCTTCTGGCTTTTCTTGGGCAAAAGCACTGCCTGG
ATGTTAGGCAACACA
CCACCCTGAGCAATGGTCACACCCGACAAGAGTCTGTTCAACTCT
TCGTCGTTACGGATG
GCCAATTGAAGATGACGAGGAATGATTCGAGTCTTCTTGTTGTCT
CGTGCAGCATTTCCT
GCCAATTCAAGCACCTCAGCGGCCAGGTACTCCATAACCGCAGC
AAGGTACACTGGGGCA
CCAGCTCCGATTCTCTCAGCATAGTTACCCTTGCGGAGATGTCTG
TGAATACGACCGACT
GGGAATTGAAGTCC
>ConsensusfromContig788 Average coverage: 8.25
GCGACGCTTTCGACTCGCCATGTCGTACCTTCCTTCATGCGAATT
TCCGAGTAAGAATCG
CTCATCATGGCAATGAGTAAGTTTAATAGAATGACAACAGTCAG
TAAGACGTAGACTACA
TATACGGCTTTCACATATAGAGACCTTTGCCCGGCTGCGGAAAAC
TTCTCATCGACATTA
TCCGCAAGTATGTCGCCAGATCCGATCATTGTGCCGAAGGTGTG
GTAGAAGATGGAACGA
GTGTTTTCAAAGTCCTGTGAAGTGAGTGGGGCAATTTTATAAAGA
GCCGAAAATCCAAAT
CCAAATCCAAGTAGAATAAAACTGTATAAAATGAAGAACCGCAC
AATGTCTCTGAGATCG
GAAGAG
>ConsensusfromContig789 Average coverage: 15.40
GTTGTTTTTGTGGAATGTTCGTTTGTTGCGTATTCGAAACGGAAG
TAGTTTGAATGGCCG
AGGCACAACAAATTACCGTTTTCTAGCACCAGGGGGCGCCCGAC
GAAGCGACTATTGATG
TGGGAGAGAAGGGCGATTGGATAGAATACGGCATCCCCATTGGA
CGTAACTTCGATTCGA
CAATGGCGGCCTTCGATTCCCAGACCTTGAACTCTGACCTCGGTA

TACTGTCCTTGACCT
TGATCGTCACTTTTCAAAGAGCCGATAAGTGTATATCCTGCCTGA
ATAGGCTAAAATATT
TTGCACAAACAAGATCGGAAG
>ConsensusfromContig790 Average coverage: 5.44
CAGACATCGAACTAAGTTCGAATGATGATTCGAAAGAGAAAGTA
GCTGGCCCTTTCTCAG
ATTATGAGAAATTGAAAAAAGACGAGCAGTCAAATCACAAAAAT
GACATTAAACCCTTTG
ATGCTCAAGTTGAATCGAACTTATCATTTGACATTACCTTACCTG
AAATTTCATCAACTA
ATTGTGATATAACGGGAATCGAAGAGACACGATTTGATATTACTG
ACGATTTA
>ConsensusfromContig791 Average coverage: 5.86
TAGCATCTTTCACACAATTTCCTATTTTCATTCTGCCATATTTGGC
CCATTGAAGAAATA
TAACACTGCCGTGACGACATTCTGGCGTAAAACTTTCCGTGTCAC
AGACCTCGTTTGATA
CATTTTCTTTCAACTTCACAAGTTTGACGGAATATAAACATGAGC
AAAGTACAATAACTT
TTATGTGAAAATTCACCAGAACTTCCATTATCTGAATGAATAGTT
TATCAAATATGAATT
G
>ConsensusfromContig792 Average coverage: 8.41
CAGGCATTGAACATGGTCCTCGGATAGCTCTTGACTATATATGGG
GTCAGCATCTGGTTT
TGCCTTGCATATCAACGGCCAAACCTCACACATATAATTGGAAAT
CCCGCAGCATTGTGA
GGCAAACTTCAAATGAACTTTTTGTCCTGGAGAATGGAGACTTGG
TATTACGAAATTGGT
ATGCACTACCTGATGGTTTGGCGGTCACAGTTCAAG
>ConsensusfromContig793 Average coverage: 5.99
TATAAATGAAATTTTGCATACAATTATTCTCTCAAGTAATACATG
TTTTGATTTTAAAAA
TATTTGTCGTTATGAAAATGTGCTTCTTCATATTACGCTTGCCAAT
GTATAATATTCAGG
CCTAATTCATAATATTTCACCGTTTATGACCTGCTTGCGATGTGA
AGATATAGTTTCAGT
GAAAATATGAGACTTAATGAG
>ConsensusfromContig794 Average coverage: 13.33
CGAGATCCAAACAATGGCTGCCGATGGCCCCAAAGCACGGCCTT
GCCTTGTGGGGCGTGC

TTGAGAAGCAGCTTTCTATGGAAGTTGATCACAGTTCATTCAATC
GACAAGAATTGCCAG
ATATAACTCATAAGGACTGACGATACTATGATGCTCTTAGTTTGG
TTGTATGGTCTTGTT
GCGTCCATATCTGCCTTCGATGAAATCCC
>ConsensusfromContig795 Average coverage: 6.53
AAAGACTGAAAAACACTTTTTTGAGTAATTTGTAAAAAAAAAATT
TTCCATGTTTTTTTT
TTCAATGAAAAAACTTTTCAGAGTTAGGAGCTATTACAAAAAGTT
TTGTATTGTCTTTGC
AACCATAAGATTCTCCATAGTTCATAATGTAGATTTAGATAGCTTC
TATTTTCATATTCG
ACGTCGCAATGTCTCGCTGAGACGGTTGAAACTTTTTTGGCTCAG
AGCTCTGCTCACTAA
ATAATAGTGATGTAAACAAAGGGTAGTTTTTTATTTTAGTTTTGG
CTATAGAATCATTTA
AATACCTTAAGAGTCAAAATAAAT
>ConsensusfromContig796 Average coverage: 8.55
AAGTGTCTCAGGTGATTTCACTGTTTTGACGGCTGTTGATTCGAT
TGGTATTACCGAGCT
TTCAACAGATTCCTCTAACTGTGGTATACCTATTTCTAGAACACGT
TCGGTTTTTGAAGT
AGTTGAAATTTCCTTTGAAAGATATTGCTCAACCCGTTTCTGAGCC
GTCGTCTGTATTGT
AGAAGTCAAACTTGATTCAGCAACAGTACTTGGTGCCTCTTCAGT
TAAGAGTTTCAGTTC
CGGAACTTCAACATCTGTGACTTGAGGATCTACATTTGTTGGAAA
CGGTTTTGTCAATTG
AGATGAAACGACATCCACA
>ConsensusfromContig797 Average coverage: 4.78
CTCAAATATTCAAATGAGTCATATGATTGATTGTAATGCCGTCTC
AAACGGTATAATCGG
TATATAAAAAGGCAGCTCCCATTAACAACTAGGTGTTACATCAAT
GCCATGTTCTTGTAT
CCACTGGTATCCATGAACAAGCTCAATGTGAACCCACTGCCTTGC
TCAAGGTGCACACGA
ATGGGACATAAAAAATGATGTTCTTATATGCAGAATATTAAAGCC
TTCAAGGTTTCAATT
GGCCAAAGCAATTAGATTCATGTCAATATTTTGTATTTTCCATAAA
GTTGTTC
>ConsensusfromContig798 Average coverage: 4.27
CTCAACAATTTATAAAATTGAATTCAAAATTCATGATGAAGAAGG

ACCCTCGAGATTGTA
CATATTTGGAACCGACACTGTCATTGTATCGCAAACACGAATCCC
GATTGAGATCACCGA
AATTGGGGTTCAACCGCTCTTGGAATCAAACGATGTGGAGTCCT
CAGAAAATGTCAAGGA
TTTCATGAAAACAATGAATGGTATCATGTA
>ConsensusfromContig799 Average coverage: 14.68
GGAATTTCTCTTTATTCCCCCCGCATCAAAAACGACACAAAAACG
ACACACAATAATGCG
AGGAGAAACATAATTCATATGAACATGTAATGGTGATAATAATA
ACTCCAATCCCACGCC
CCGTACCGGGGATAAATACGACATAAAGAATAAAGGCATATACG
CCATAAGTGTTAATTA
GTGATAGCTGTGGAAGTGAG
>ConsensusfromContig800 Average coverage: 6.11
GGGCAACCGATCAAGGGGGAGTGTGAAAAACTGCTGATAGAAAT
TATCATACAGTGAAAA
TTCTGAGAGTGCAACAATATTTAGTTTCTTAACTAGATATAAATT
CGCGATATCACGTTA
AAACGTGCGAGGCAGAGGAACGGGCTTGCAATATATAGTTTTAA
AATGAATTTTTATGGA
ACATGCATCCTGTCATTCATACATAATATTCAACAAAATTTACAC
AAAAATTGTGTAGAA
AAATACATGGTACTTG
>ConsensusfromContig801 Average coverage: 12.58
TTTCCCTACACGACGCTCTTCCGATCTGCCCAATACATCCAGAGT
GAAAATCTTGGCCAC
AATTTCGGAAAATTCGGAATTGAATTCAGTTTTTGAGTTGTTGTA
TAATATAGTGAATCG
TATCAACGCGCCAAACTGTATGAAGTCTATGATAATTCTTAGAGA
AACATCGCGAGTGTT
CTTTTCGCTTTCACCTCGTCTTTTTGAATGATGAATTCTGGTAGTT
GACCACATCTTTAC
TTTTGTGAAACAATAGTTTATGGACGAGCTAATTTCCCATTCGAA
AAGAAAAATCAAGAC
ATATATCAAATCATATAATGAGATAACAAGATAATGCGTAGCATT
ATGAGAGCATTTCTT
ATTCGAAACACAAGTGGATCCAAAAAGTGTTAGGCTGTG
>ConsensusfromContig802 Average coverage: 10.04
GTGATAAGAAGGTGTCATGAAATTGTCTTCAGATGACATGTAATG
TCTATCCCATCTTGC
TGGATACTCGCTTTCTGGACCGTCCTGGTAGAAAAAATGAGGTCT

GGAATATTCCTCCTC
ATATCGCCTCTGTGGTTCTCTTTCCTTATGGAAATTCCTTCTTTTGT
GATTATGCTTTCT
TGGTTTGTATCCTGATTCGGCTTGGCTGAACTGATTGAAATCTTTA
TG
>ConsensusfromContig803 Average coverage: 14.54
ACAAATATCAGACGCATTCAAAATATATAACCATTATAGAGTCC
TCAAAAAACAACTTGG
TATCTTTGAATATCCATAATTTTATGTCCATGAGTCATGCATATGC
TCTCCGAATTCGCA
AACTCAGCATTTTCATCCTCTTTTTTTCTAATCCCATTTTCCCCATC
CTTATCCACATTC
TCTTGCAATTATGATATCGATAGATTCAAGAACCCGCATCCCCAG
TCTGATTTTCATTCA
TCAGAGATACTTCGGCAGATCGGAAGAGCGTCGTGTAGG
>ConsensusfromContig804 Average coverage: 8.02
GTTTGTCAATTGTTTTTGGGACGAGCCTACGAAATATTTCTGAAA
TGAACATTCTGGTAT
ATTTGCTCAAAACACTGTTCTGTATCTCATACGTTCAATGCATAA
GTTCGAGTTTGAAAA
AGCCATCCACATGTCGCCGAATTAGTTATTATACACGGGAATCTA
AACAGGAATGCGATG
TCGAAGGCTTGGTCGGAGTGGCAGGGCCTCCAGAACAGCCTGAG
AACATCACTCTACTCA
T
>ConsensusfromContig805 Average coverage: 4.71
TACACGACGCTCTTCNCATCTCGGACGTGGAATTCCCCTTTGAGG
AATACCGGTAACATG
CTTTTTCCGATCCACATGATGTGATGTCTATTGGTGGCAGTTTGG
CTGATATTGGCCTGG
AGTCTATGTATCTGGGACTTGCCTGGTTGTTGTGAAGCATAGTCA
CCCTAAACTTAATGC
CCCCTTTAAAGATCGGAAGAGCACACG
>ConsensusfromContig806 Average coverage: 3.05
AATAGGGCAGATGATAGTTACATGGATTCGAATGAATGTTTAAAT
GTCGTCAGATTTAAT
GGCACGCAACTCGAATTTATTTCATGGATATTCTCCTCATATTTT
AAAAAAATTTGAGAT
TGACAATGAAATGGTGATGAGAAAAGCGTACTATGAATTGGAAT
AATGGAAAAGCTTTCG
TGGATTTTTTCACTGGAGATCAACAATACTCTGACAGATGATCGA
ACATCAAAAATATAA

TTATTTAAA
>ConsensusfromContig807 Average coverage: 5.00
TTTCAGTTCATATATTACCTGGCATTTTATATGAATAATGAAATA
ACATAAGCAAGATAG
TATGTATTCATTCAATTGTGAGTAGTCAACAAATAATCGGCGAGG
AAACGCAGTTTGCGA
TATTGCCATTGTTTGGCAATAAGTCTCTTTTGGTCATCAGTAAAT
GATTTGAAGAACAAA
AAGCCACAACAAAATAGTTCGATATGTGACCACATGGACACAAG

>ConsensusfromContig808 Average coverage: 7.90
AGGACGACCCAGGAGAATGGCGACCTTCAACTCTTCATTCAAGA
CTTGGAGAACAAACTT
CGAGATGCTCTGACGAGACGCGATGTGGCAGAGCACGGCCGTGG
AGCGGAGAGTGAATCG
CGCGAGTTGACTGAACAAAGAAATGCAAAATTGATCAAAGAAAT
GGCGCTCTTGCATGAG
AAAGTGAACGTCATGAATTTGAATTCAAGAACAGCAAAG
>ConsensusfromContig809 Average coverage: 6.75
ATCACACTGGCGTATTAATATAAACAAACAAACGAAGATAAATC
AGCCATAATCATAAAT
TCTGAGAGAGGGATTATGGTTTCTTGTCTTCCAGACCAAAACAA
TGAGCCAGAAACAGAC
CCCGCTCGCGATGGCATTGAGAGCGCATTGGAGGATGAGGTAGTC
CTTGATGGTGGTTGA
GACAACACTATTGCAGCGACTGAGGTTTTCATAGACGTAA
>ConsensusfromContig810 Average coverage: 12.97
AGTGCGAGAGAAGAGAATGATGGAATAACATTTGGAAAAAATCT
GGATGGCTTCGTGGAT
GATTTCTCCTTTGTTCCTGTGGCGATCTCTGTGTTCGAAATGGAG
AAAATGGCATTCTGA
GATTGTATCGGTTACACTTTCTGTTAATATGGATTCATTATCATAC
TCCTTTGCTGAAAA
CTATTTCTATCACGTGGAATCAGTAACATTTATATATTATGATTGT
TGTATCTTTTAACT
GCGC
>ConsensusfromContig811 Average coverage: 7.91
GTTTCAAACCCAACATCCAATTCTTCCTTAAAAACGTTGTACGAG
TTAGTTGATATAATT
ATTTGCTGTTATTACATAATTCGTTAGATGCCATAAAGTACGGTC
CATGCGTAAAATTTT
TGTTCATTCAGTAGCACACTACTCATCCCCATGATACACTCGGTA

TCTGCTAAAGTTAAT
TAAATCTATCTTAATATAATTTATCAAATCCTG
>ConsensusfromContig812 Average coverage: 4.58
AGAAAGAAAAGCCAATGTAAGAAGTTAGTAATATGCGCTTTATAA
ATTTGACACCCTCAT
CACACATATCGTTTTCAATAAATTACAATATCAAGAAAATGATCA
AATCATGAATATGAG
AGAAACGCGAATCTGTCATTAAACGCTCACTATTTATTATAAATA
ACAGCCTTCTTCAAA
TCGAGTGAATACTTACATTTCTATTGAATACTCAATCTTTTTAATA
TAAGACTCTTAGGC
AAACTCCGTAGTGAAATTTCATATGTTATTGCACTAGCTTTTAAT
TTTTGGACTCTAAAA
GTTGGGTTTTCAA
>ConsensusfromContig813 Average coverage: 17.30
TAAATCCCGTTTCATTTTTCGGATCACATGGCCCTTTCCTGTGTTG
TCCAATTCCACATT
GATGCCTCTCAGTTCGAATGCACCGTGAAAAGTTCTCTTTCCATTT
TCGTGTGTTCTGAC
ACCAATCGCAGCGTTACTCCTTTTGTCCGTGTAGTAAGCATGCCTG
TCTTTGTTGTTGTT
GCGTTTCTCGACTTCTCTATCATCGCCAACGTAGAAAACTTCAAT
GCCGCGGACGTTTAA
ATTTCTGCGCAGATTCAGCCTCTTGATGAGGGCGCCACTTTCAGC
ACCTCTGACTTCCAT
GGTCATGAGGCCAGGATGTTCCCGCTTGGAGTTGGAGGAGA
>ConsensusfromContig814 Average coverage: 4.33
ATTTTAACACCTCCAGCCTTTAATTGATCAAAAAAATCACATTTTG
ATTTGCTTAATATC
AGTTAATTTAATCCCGTGCGATATTTTCTTTATCGAAATTCAAATC
TTGGAATGTTCTTA
ATCACCGGGCGTTAACACAAATAAAAACACTTGGGAAAAATCGT
ACATCACACTATGAAA
GGGGAATTTTAAGAGTGGGTGGCGCACAATCTAGGGTATGGATT
CGAATATTCCGGCAAA
CTTGTGTAATAATAAAGGAGACACACGAATAAAAATAAA
>ConsensusfromContig815 Average coverage: 7.89
CCACATTTCAACATATAACACATAATCTAAGGTTATTTTAGATCA
CGGAGCTGATTACAG
AATTATCTTGAATTTAAAGTTAAACCATAATGTTACATTGACCCA
TGTCTTGTGACAATA
ACGTTCGCAATATGACGAACAAATGAGATTCTTGCATTGAGCTGC

TTTCAATTATAAATA
TGATAACACGCCCACATAAATAGATG
>ConsensusfromContig816 Average coverage: 5.06
TCTTGACAACGGTTTCCACCGGCACCGCTGCGACAGTTGCAGCTT
GTGCTCCGTGCTGTT
GCGACACATGTCCCACCGTTCTGGCATGTTATTGCGGAGCAGGCA
TGACTTGCAGATTCG
TCCCCGAAGTAACACGTGTGACCACCTGTTCCAACTGCATTGTTC
CTGCAGTCTCGATTC
TTCGGTTCGTTATCGCAAGGATCTCTGG
>ConsensusfromContig817 Average coverage: 5.89
CCCAAATTAGAATTATCGAGCCCATAAACAACAAAAACAAGTTAA
GCATCTTGGCATTTT
CATTCGTATAGAACTGATTGATTGTGTAGAATATATCAAAAACAT
ATAAAATCATTGCGA
GATATTGCACTAGTATTATTGTATACTGTTGGCTAGAAGCCTGTA
AATAATCTCTATGAG
AAAACAATTTTCTCGCAAATTTGGAAATATTTTCATGTGTCAAAT
AAATGAATATACACG
CACCACGGTGATAGAAT
>ConsensusfromContig818 Average coverage: 10.96
TACGTGTCATGTATAAACCCGACTTTAATGAGATTACTTGATATA
TTGAGAAAATATAAT
CCGATATGAGATATTTATGAAGAATACTACTCAAGACACAATGA
ACGAGCAATGAGAGTT
ACACATTTCCGTATGAAATAAAATTCAATGAGATTATTTAAGACA
TAAAACCCGATCTTA
TATAACGAGATGTTTGTAACGGGTATTAATCCGAAAATATCATCC
CACAACATTTCTAGT
CTTTCTCATTATGTTTAAAATAATCAAATGGTATAATTCATACAT
CCGAAGTATGAGTA
>ConsensusfromContig819 Average coverage: 6.82
GCTCTTCATAGTTATGGGTAGGTGTATGGAAAAATCGCTATTGTT
ATTAAACGTCAGTTC
AAACTTCAATTCAAATTTTGCGAACAGGCCATTTCTCAACAGTTG
CTGGAGGTTCAATCT
TGTCCAGCACATAATATGTTGTCTGATAAATATACCAGTGAAATC
AACAATCGCTCAACA
TCTTAAAAAAAGTAGTGTTCTAGAAATTAACATGCTTATTGCCAC
TCATTCTTCGAGTTT
TATTGAGGATTGAAAGGTCGA
>ConsensusfromContig820 Average coverage: 16.36

TGGGAATTCCAGTGGAAGGGGTCTGCTCATCGCCTGACGTTGCAT
CGTTAATCATGTTAG
AATGGTGGCCAGGTGATGCTTCAACTCTATCGTGCGGGTCAGTG
CAAACAACAAACGAAG
TGTTATCTACATTCCCATCGTGAGTTTCACCACTTTTGTTATGATC
TACATTTGAAGAAT
GAGAAGGAAGAGAAATTTGGTTAGGTAAAGCATTAGAAGAATCA
CCATTATCCCTCTCAC
GATCCTT
>ConsensusfromContig821 Average coverage: 12.34
AGTTCAGACGTGTGCTCTTCCGATCTGCATCCATAGCGAAAGCTG
GAGCCCATTTGGGAC
ACGCAACATATCCTGCGTTCATTCATCCGTCTTGTAGAATTGGTCG
GTTTTGCATTTAGC
TCATTTTCTGTTGCCAGATTATATTTGAAAACTTTCAACCACTCA
TAGGTACCACTAGAG
AGTTCGCCGCCCTGTAGAACTTCATCAATAACATCCACAAAGTAG
CTTTTTTGGCCAATG
AGAGAAGCGAGAAGCATTACTAATCTTTGGCGAGTCAAACTATTC
ATTTTCACAGCATGA
GAATACCACATGATTTTACTTAGCACCTTTAGGTCTTTCAAACAC
GTCCTCCTCAGACTT
TTAATTTTATTGCGATCATTCGTATCCAATGAATGGATCATGCATT
GTGTCCATGTGACT
GCGTCTGCGATGGATATGCAGCGGTTTGGATACTCAAAAAGCTG
ACAGCCTTCAGCGGCA
TTAAAAGATATTGCAGAAGTAA
>ConsensusfromContig822 Average coverage: 5.74
GCCACACTGATTCGAAACAGATCGGAAAATTAGTAAACAAGATTT
TCTGATTTAGTATGT
GTAAATCAGTACTCTTTTCTTCTATCGAAACTCCACAGAATGCAT
AATAACGCTGCCGAG
ATTTAGAGCCAGCACACAGTTATTCAACATTGATCAATCGAAGGT
CAATTATCAGGGGAC
CGAAATAAGGGTGGCATAAAATGGTTATACTCACAAAAATGTTG
CTTATGAACGAGCTAT
TTTGAATGCGCATAGATAACACTAAGTGCCAAAG
>ConsensusfromContig823 Average coverage: 9.86
GCCATTGGTGACGCGCCAGTATGGGTGTAGTCGCCACTCTACCGT
GAGAGACAAGTCTGT
ACGTTATACGTAGTTTGAAGAATTCTGACAAAAATATGTCAAAGC
CAGTATGTCCAGGTG

CTCTGTTATTGATGACATTCAGTTCTCCATAGTTCACATACATCAT
GAAACTTATTGAGT
GATAGTTACATTGCTTCACGTTCC
>ConsensusfromContig824 Average coverage: 6.04
ACAACATTGACACATCTGCTAGTGATAAACCCGAATATAGATTTC
TGTCATTGCCGATTG
AGATGCACTGGTTTTCCATTGGTCTTCATTCCGGAATTATAACGA
CAACAAAACGAATTG
ATCGGGATTCAATGTTTGATCCAGATGAAAGGTCCATAATCAAAA
TTTCTGTTATCATGA
CTACACATAAGGCAAATGTGATAAAAATAGAAATAGAAATAAAT
GATATTGATGACAACA
GACCAAAGTTTCCAC
>ConsensusfromContig825 Average coverage: 8.99
CCGCCGCAAAATTTCAATCAAGCCCGGCTGCTCGCAACGGATGAA
TTTTAACATCTCGTT
CGAATGACATTTAATGTTCGTTAACGTGAAAAACTGTCTCGCTTG
TGATTGAGACCATCA
AAGATCAAACCCGTGAGCGTTGGATCGAAATAATTAACGACTCT
AAACCAAAGTGCAAAC
AGTTTATCAATCAATTCGATGGCGACTATATCATATATAACAACAT
TTGTTTGTTTGTTC
TGTTCTATATCCCATGGATTAAATAATGAGCCCGGCGCAGTACA
GAAGGAAAAGCTGC
>ConsensusfromContig826 Average coverage: 11.99
AGGTAGGGGTCATTGAGGATCGACGTAACAATATGCAATTTTTCC
GAAAGTTGATTTTCA
CCCTTTGGAATTTTCTCCCTCTGCTGGATCGACATTTTCTTCGTCA
GACCATTTTCGACA
CTCCAATCGGCAACGAGTTGTATCTTTTGGATTTTTGTGACTTCG
AAGAAATTGAGTTCG
TAATTCTTCCGAAAACCATTTTCATCAAATTGCACTGTTCCTGTTC
TCCCATTAGCCAAA
TACACCTTCTTCATGTTTGCGAGCAGTTCATCCCCATAGCGGGAA
G
>ConsensusfromContig827 Average coverage: 6.19
CAATGAATGGAGCGGTTTTTTGATCAATATTTTTTGGCATTTTGGA
GAATTATTTTACTT
CGACTGATCATTCTTGGCCGTAAGTTATGTATTCTACAAGATTCA
AAACTAAAAAAAAGT
TTAAATATCGCCTTCTGGGCCGCGATGGAAGGTCTAGAATTGTGT
CTACACAATTCGCAC

GAACAGCAACAAGGACAGAGGAA
>ConsensusfromContig828 Average coverage: 8.29
ATACTCCTCTGTTTGCTTTCCATAAAACGCCAGTTTCATTATAAAT
ATTGGCTCTTTCGT
ATTTTCTGCTGTTTTCTCGGGTGTCTGTTCAGGTGTTGGTGTTTCC
AGGATTTTGCTTAG
TGCTCTATTAATGAGTGGTTCATAAAACAATGGAGGGTATTGGTT
TTTCTCCAGCATTTG
TTTGCCTATTTGAAGGCTTTCATGAAATAATTTCCACGATGAACA
CGCTCTGAATATTCG
ATGAACCATTCCTGACACTACCGATTTTTTGTATTGTTTTGGCGC
TGGTGCGTGAAAATT
CATG
>ConsensusfromContig829 Average coverage: 6.68
CAATAACCACATTACCTTGAACTCTATAACATTGTTTCAACATCG
AAATGCAAGCCACAT
ACTCTTTCATTGGCTGCTGGATATAAGTCTTCATTATTTTGACCCG
TTCTTCTAGAAACG
GTAAAAACACGTCCATTTCGTTCTTGAGTTTGTACTCTATGAGTGC
TGTGTCGTATGTCT
TAGCTGATTGCTGAAGAATTTCAAAGTATTTTGCGGGATCGTGAG
ATGTGCTACAATTCT
CACACTTCACTCTGTCTTGTTTCTTCTTTACTTGAACTGACAAAAC
GCTTTCGCAGACAT
AGCATTTTAGAATCGGGATACCGGAAGGCAGAGCTTTCAGGACAG
GCCAATTTTCATCAC
ACGCGTGACAGCAGCATTCGAAAAAGTACTGATCTCTAAGGGCTA
TTATTCTCTGACTTT
TGGATGTTAGATAGTATATGAAGCCAT
>ConsensusfromContig830 Average coverage: 12.55
GAAGCGGAACCCGTGTGCAACCGAAAATGTGCGGATAATGCGAC
GATGAACCACGATAAC
TGTGTATGTACCTGTGTTGATGGTTCTACTGGGGCCAATTGTGAG
GTGCAAGTGATGTGT
GACAAGCCCTGTCAGCATGGAACCCACGACCCTGGATGCACCAAA
TGTATTTGCGACACT
GGCTTCAGCGGGCATACTTGTGCAAGTCGACGTGGTTTTATTACC
ATGCAGTTCAGAGAT
ATCACGCTTGAAAACG
>ConsensusfromContig831 Average coverage: 5.15
CAGATGGATGTCGTTATTGTGAATATGATCTCGCTTTATTCCGATT
TTCATTGATTAAAA

TCAATGCGCAATATAAAATAGCAGTGACTTTACTATCGATGAGGA
TTCGCTCAATAATAG
TCCTTTTGGGAGCCTTTGAGCAAAGCATCTATATCTTACAAGTTT
AAAAATTATTTGTGA
ATATGAAACGACACGGCCACTTTAAAATTCAG
>ConsensusfromContig832 Average coverage: 11.26
GTGGCTTCTTACCACTGCACCTGCAACACAGCTGGATATGTGCTC
GGCTCTGACGGCAAG
ACTTGTAGAGATGTGAATGAATGCGACGATAACAACGGAGGCTG
TAACGAGATTTGCATG
AACACGTTGGGTTCGTACGAGTGCCTTTGTTCTCCTGGGTACACA
CTCGATGTTGACGGC
AGGCATTGCTTAGATTTCAATGAATGCAAATATCTGGAAGGCAT
CAAATGCGAACATAAC
TGCACGAACGTGGCGGGGTCATACAAATGCCTCTGTCGACCCGGA
TTCGTGCTCAACATT
GACGCCAAATCATGCGACGATGAAAATGAGTGCAAAAGTGGGCA
TCATGCGTGCAGTCAG
ATCTGCGTGAATAACTATGGCTCTTACTCATGCGCTTGCCATCAT
GGCTACTTCCTCGAC
AACAACGGAAA
>ConsensusfromContig833 Average coverage: 5.66
GTTGACCGACTGCGGATTTGCGAATTTCGATGGTGGGAACGACAT
GTGCGACCTGCTGCT
GGTCAACAGCGTGGCCGACTCCACTGCCGCGGCGTCAGAGTCAAG
TCTGGTGTCTGCGCT
GTTCCCTATCAGCGATTGGCTGCTCGTATTCAGAGTTTCGGCTGG
CAGTGGGATAAATGT
CAAGGACACTTGGCTCAGCAGTGCTGGTATCAACGAAGGAAATG
AAGCATTCCGCACCAC
AGACTCTGAGATCGGAAG
>ConsensusfromContig834 Average coverage: 6.96
GGTTTTTATTCTTGCCGAATGATATTAGAGAATAAAAATACTAAAT
TCTTCTAAATTTTT
GTCTTTCTGTTGATCAGTATTTCACGTTGATTGTTTTATGAATCTG
ACGCTTGCATCATA
TGACACGGCTATGTGCATCAGTTTTGTGTTCCATTTGTTTACCTGT
TATTGATGCTTACA
CAGATGTAATCATTCAATTTGTTGATAATATTGACTTTTTTTGTAG
ATATTTATATTATT
TGTCATCGCCTAAATCTGTTTCAAA
>ConsensusfromContig835 Average coverage: 13.33

TGGAGGTTCACTGAGCGAATAATACTTGAGAAAATCATACAGCAT
GTTATCTGCAATCAT
ATCGTCTTCCAAAACAAGAATACGTTTATTTTTGAATGCCTGCAA
CGACTCATTGAGACA
ATATACGTAATCTGTTCTCTGCTTTTCGAAAATTCCCATAACGGA
AAAATGGGAATCGGT
TGCATTGAATCTTCGCCGAACTTTGAAATACTTTGATAAGAGCTG

>ConsensusfromContig836 Average coverage: 5.80
GCGAAACGATGTATCCAAGAAAAAGGCTCATTATATAATATGCTA
ATACAATTCTACCAC
ACTCGCGACTAGTGGGCTGGAATTGACATTTATAGACTTTACGT
GAAAACATGAGAAAAA
TTCTGATTTATACGCATATACACATATGCCTGTAAATAATGTGGTC
AAATCTATTTTTTT
GCTGTGATAACGACAAGTTTGAGATATTTTGTATTTTTAGACATT
TCAAACGAAG
>ConsensusfromContig837 Average coverage: 2.91
CAAGGATGAGCGTCTCTCTCAGTGAGTAAAATACTAATCAAATCG
TATTTATGATAAAAT
ATATAAATGAATATCATTTGAGAACTGCTCTAATTGTGATAACTG
TATCACAGAGCTGGG
ATCTAACCGTTGGCTTCGATCTGTATTGTTGCCGTGAGTATTTGTT
AAATTTATATTAGG
ATCAGTTTCGATTGAATGATGAGATGAAT
>ConsensusfromContig838 Average coverage: 7.43
GGTATTCTTCGAAGTGTAAGAATTCATAAGTGACGAGGCATTAGA
AGTCACGTGAGATCT
CGCCTGAACCATTTGCTTCTCCTGAAACTTGTCTCTCCATCTCGC
GTAAATAATGAAAAG
AACGATAACAATAACAGTTCCGATGACAATAGTTAGACAAACCA
CGACGACAGAAACAAG
AATTAGGTCATGGAGTCGCTCCTCATCTACGGATGACCCACTTTCT
GTTGTTTTTAAATT
TTTTGA
>ConsensusfromContig839 Average coverage: 10.04
TAGTACATCATCATATGAACAACATCGCAATATATTGTATAAGAC
ATATCATAAAAGTTC
TGAACAGCAACATATAACTGCAAGGAAGAAAGTAGCTGCAAGCC
ATGTGCTGACTGATAA
ACCTGGGAATAAAACTATCAAAAAGAGCGATAGCATTCATTGGG
TGTAAAATACAACAGA

AAACCTTATGCAATAGAAATAGTGATAAGCGATATGTATACTTCA
TAACTTATTCTAAAA
AATATTAAAATAATCTGCGTATTAATACTGGAATCACCGGAAATC
AATAGTGC
>ConsensusfromContig840 Average coverage: 8.53
TCGGTGCTAATTCTATTTATATTATTGGTAGGATATAACCATGGT
TCTGAAAAAATTATA
TCGCAATACTCAGTTTTGAACTCTGTATTTAGTGTTTTCAAGTCC
TCAAAGTAGTATACA
TTATTCTGTAATGTTTTTTGAGTTTATAGCCTTGTGATGATCTGCG
CTCGGATTTTTTTT
GATCAATGTTGAATCATGTGATCAGATGCAG
>ConsensusfromContig841 Average coverage: 3.95
ACCTTGGTGAGCCGGTTGTAATAGTCCTTGAACCTCGCTTCTTGG
GCGATTATTCTATTG
GCTGATTCAGTTCCTCGAATATTCATGGCCAACAAGCATCGCGTT
TCGCGCATCAATTCA
AGAACGAGAGGATCAGCATTAACGACGATGAAGGAGTGGACGTG
TCTGGCGAGGAGGGGC
GCCTTGCAGCTCTCTCTCACCATCTCCAGTCTTTGTCTCCATTGCT
CAAGCCAA
>ConsensusfromContig842 Average coverage: 7.68
ATAATTTCAAATTTTATATTAACTATTTTTTTTAATATCGTCATCG
AATACATATTTATA
TTTTGCAGAGGCACATCGGCCTCTGCCTCCCCCTCAAATTTTAAT
GAGGCGCCGATGTCT
CCAGCGGAGATCTTTTTAAAGGGGACTTTAGGGTCCCCTTTGAGC
TCATACCATAATTTT
CGAATTTTTTCTCGGGCCCTCATTCTGAGGACCCGCCTCGACCCT
CCTTGGCTCAG
>ConsensusfromContig843 Average coverage: 5.68
CGAGCGTGTGCGACCAACTTATACACACGAAGCGCTCGCTCTTCT
GTTGGAGAAAGGCTT
CATTCATCATATCATTTCCCAAAACACAGACGGGCTGCACAAACT
TTCGGGGATTCCTCG
GGAAAAAATTTCAGAGCTACACGGAAACAGTTTTGTAGAAAAAT
GCGAGAAGTGTGGAGC
GCGATACGAGCGAACCTTCGCCTGCAGGCCAACAGCAGACGTCA
ACGCTAAGATATGTCA
ACATTGCCACGTGACTCATCGAACAGGGAGGGTGTGCGAAAGAA
AGGGTTGCGACGGCCT
TCTTACAAACACTC

>ConsensusfromContig844 Average coverage: 9.21
ATGATACTCTCTTGAATTTCTCCATTTTCGATAATTCCGAGATGC
AAAAAGTAAGCTTCA
ACTACGAGCTGCTGAACGAAGAATGGTCTCACGTCGTGGTGGCA
GTGAATGCCTTTGAAA
GAACTCTCAGTCTACGCCTCAATGGAGTAGCAATGCCTCAGAGAT
TCTTGAGTTTCCAGC
CACACTTCGAGCAAATGGAGAAGGTTCATG
>ConsensusfromContig845 Average coverage: 5.50
GTTTTGATAAGCTATCATTATAAAATTGAAATTTATAATGTTTGG
ATCACTCGCAAAACT
GAAACAAAGCAATGCTCTCGAATATGAACTTATTCCTGGATCTGA
TAATCAACAATTTGA
AAATTATACGAACAGCTCAATATTTGCGAAAAGAAAGATTATTG
GTGGACAACAAGAAGT
ATTCACGACATCGGAAGCAATTGATGCAATGGGTTTCGGAAAATT
CCAAATAATGCTCAC
ACTCATAATAGGATTGGCCTGGTTGGCTGATGCGATGGAGATTAT
GGTTTTGAGCATTCT
>ConsensusfromContig846 Average coverage: 6.18
GGGGGAGTCCTGCAATAGAGATGAAGTAACAAGTGCTATATCTG
GCGAGGGTGGAGTCGA
TGAACTTAGTGATACTCCCTGCGGAAAACAAACTGTTGATACAG
ATAAAACTACGGATGC
AATAAGTGCTGATAATGAAGTCGATGATGTTCTTGATATAACCAG
CAAAACACAGCCTTT
GGACAGATCGGAAGAGCGTCGT
>ConsensusfromContig847 Average coverage: 9.80
ATGAATCCTCATACCTCAACAAGAAATAAACATGACTAGTGTATG
GCAATTTGTTTTCGA
TATTCTATAGAGTTAGACGGCACTCCCTACTACAGATAAAATTTG
AATAAAACAATCTAA
ATAATAAATAATTAAGTCAAAATCTCTGAATTGAAAGTCATGATT
GATCAAAAATCATAA
ATTGTGAACGAATTCTACGATTTCAAGAAAATAAATTAAAC
>ConsensusfromContig848 Average coverage: 6.64
CAATAGTGTCATCGAGGCTGTTATCCGAAAGCTGATTATCTGCCA
AAGTATTGCTGCATC
TTTCGCAAACCCGTGGAGATCTGCCGGGTCCGCGGGATAGGCTC
TCTTTGTGAATTCAT
CATGATTAAGAATGCGCTCGATGGCACCACCATGGTACTCTGGCC
TTTCGCGTTGCCATG

CCCAATGATTCGGAGATCGGAAGAGCGTCGTGTA
>ConsensusfromContig849 Average coverage: 6.30
TGCGAGATCGAAAGCAAAACTTTCTATTCATAAAAATCAGAATA
CAACATACATATTGAA
GCGATCTTCAAAGCAGCTCATGCCTTCAATAATTGAACGAAACCA
AGATCTTCGAACAGT
TATTTCTGCTATGTCACTGGATGTGGGAAATTTGAATAAAATTTC
TGAAGAAATCCAAAG
GAATGCATCAGACACCATTGAGAAATTGAAAAGAGTAATCAGTA
CT
>ConsensusfromContig850 Average coverage: 17.34
TTCTGCGCAGTGAGCTCCGCAGTGAAGGCACGCGAGAAATACA
TGAAGTATACCTTTC
TTTCAGCGCACAAGTGTCTCAAGGCTGCCGGTCAGCGCTTAGGTG
CATCAGCGCTAATCT
GTCATATTCAGCGCGTATCCGGTGAAGTTACGCCCGTGACATCGA
ACGAACTTCCTTCGC
GCTCCGGTCGAAAATTTCCTCAAACGCCGACCGTCAAACCCAGTC
AGTACGTTCTCACCC
TGGGGAACGTCGGCGATATCGAAGCGGTCGTGTGTCGACGCGGCG
GGGCCCTTTCCCCCC
CCCCCAAAATTCACGCCGGAATGCCC
>ConsensusfromContig851 Average coverage: 5.76
GATATGAATCGACGAAAATTTAAAGATACGATGCGATAGTCCAA
TGCGTGGATTTGATGG
GAGCAGCGAAATTGGGAAGAACCGAACGTAGATATCTCCAAACT
AACCGGCGGGTTAATA
TTGTTAACCTAATTTATGAGCAGCACATGCCTTGCCGGGCATTGT
GGCAGACTGGCACAG
GAGCGTGAGATAAAGACTTTTTTTAACGAAACGCTCATATTAATG
CAACCTCA
>ConsensusfromContig852 Average coverage: 6.34
AAGCTTCTACTCCATGTTTCCGTGACGACAAAGATGTTGAAACCT
GTTACTGCGATAAGG
GATTCTTTGGAAACGACTGTTCACAAGCTATCACACAAGCTAGTG
AATGTGGTGATAATG
GTGTACTTAGAGGAAGTGAATGCGTTTGCAAAAATGGTTTTGTCG
GCTCGGTCTGTAATA
AAGCCATACAATGTCTGCCTATCTCGCAAAGTGATTTTCTCGTAA
AAT
>ConsensusfromContig853 Average coverage: 683.68
TAAAACCCACAAACTAAGCTCATACGACGTTGAATAAAAGATAT

ACCAAAAAGTGAAGAT
GAAAATTGTATGTCAACACTTGTGAATATAGCACAAAAACACAA
AATCAATAACATGAAA
AGCAATGATTTGATAACAACAATTGCCAGACATCGTGATGAAGA
AAGGAATAAAATTGAA
AGTTCAGAAGATATATCAAACAT
>ConsensusfromContig854 Average coverage: 12.01
ATCTGGGGGGAGATTTTACTGATTTCGTTTTGATGTTTTTTGGGA
AGAGGCTGCTTTTAA
GATGTGTCGACGGAAAATGCTTTCTCTGAACTCTCATCCAATCCTG
CTCTTTTTTACGCT
CAACTGAGCACCACGTTTCTCGATAGTTGCCAAAGCTTTCAGCAT
CAAAAGACACACTTT
TGTTATCAGACATTATTTATTATTAAATAAAT
>ConsensusfromContig855 Average coverage: 17.12
TCGCATCAAATTCGGTTGATCTCTGGATTTTGACACCATCTGTGA
ATCCTCTCAAGGCGA
ACGCGAGGGTGGCTCCACACTTGGGCGAGGCATGTCTGTCCCCA
AACACGACGAGGACAG
CTTCGGCCGTTTTCCCGTTGCTGGGACGAACAAGAAAACGACTCT
TGTAGGCCACATCCA
TTTCGAGGGCGGACCGAGCTGGAACAACAACTGAGGCATTTCCTT

>ConsensusfromContig856 Average coverage: 7.17
GGAAGATATTCAAAAGTTGATGAGAGAAACTGAGCTTAAAATTG
AAAAAGATCTCGAATC
GACTTTATCAGCGGAAATAGATTTACGAGAGATGGTTGCACCGT
CGAGTGAACAAAGAAA
ATCTAATTTCAGACACAAGCGCCAAAACTCAGATCCTGGACTTGC
CCAGTTCTCACCCGT
TGACGAGCCCGACTCAACCTTTGATTACGATATCGATAAACGCCT
TGGAATTGATATTGC
TAATGCTGCTTCTGAAATATACCGGGATATGGCATACCGATCTGA
CCGTATATATCACTC
GAG
>ConsensusfromContig857 Average coverage: 6.05
CAGCACTGCTTTTTGTTCAAGATCGCTCACAGATCCTGTTTGCAC
CTCTGCTCGAACCGA
CGCTCTCGTTCTGTGCCCGGGGTCGAGGATCAAGTTCACTCCTAA
CAACGGCATAACGGA
ATGGAATACGGGAGATGCATTGGCCAATATGTTCGCTTACACGGC
TGTAGAATGCTCTGC

CCAACCAAATAACACGGCCTCATTTGAAACTGTTGCCCCTATTTG
CTGTGATGATGAATG
TTTCGGGCGCGGTGGTTCC
>ConsensusfromContig858 Average coverage: 9.40
CGAAGATTTCAGAATAATAGCATACATCCAAGACCAGGCGATTA
AACTTTCACAGCTCGA
TACTCAGAAAATCGTGCACTTCTTTTCCTGGTTCATGTTCGATCC
GGGTTTACAATTGAA
GTTCTCAGCGAAATATTTGATATTAGCCGCAAAACCATTGACCCT
TATTTGAGGTATACT
GTGAGGCTCCCCCCATGCATTTTTCTCCTCATCTGTATGAACTGAA
CAGTGTTT
>ConsensusfromContig859 Average coverage: 7.63
CTATCATGAGTAAATCAGCGGTTAAAGATCCTCCAGTTCGATCTG
CTACGGACTTTTTCA
GCTTGACAGCCCCCCAGATTGAAGAACGTACCCCTCTCCCGCTAT
CAGCTCCGAGTTCTC
CAAAATTGAACGACAGACTCTCACCGAAAGTAGAAGAACATGAC
TCATGCTATAGTGGCT
CCCCCTCGGAATCACCCGAGCCTCCTGAGTCGCGAGATCGGAAGA
GC
>ConsensusfromContig860 Average coverage: 5.99
TTCCGATACATAATTGGTCAATGCATATCTCTCTCCTGACTATGC
GAATTAAATATGAAT
GTAGAGCCTGAATGTTAACTGTTTTTTGATAATCAGCCTGATTCT
CAGCTTCAATTCCGA
TAAAGCTTCATCCATACGCCCATTTTTAATCAATTTTATGAGCCTT
CGTTTATTTGTACA
TCAATGATGAGAATCTGCTGAATGATTCAATTTGAAATGAAAATG
AAAAATGTGCACTCG
>ConsensusfromContig861 Average coverage: 6.18
CGCCGCCACGACCGCCGCCGCGTATCCCACACCTTTGAAAAGCGG
GCATATTTTCCAAAC
AGCGATTCCGCCACTGCTGAAATATTGACCTAGGGCAAGTTCGA
GGAAGAACATGGGAAT
GCCGGCGAAGAAGAGGGTCAGCAGGTAAGGAATGAGAAAGGCTC
CTCCACCATTCTTGTA
CGCCAGGTAAGGAAATCGCCAGACATTACCCAGGCCAATGGAAT

>ConsensusfromContig862 Average coverage: 9.45
GAAAACTGCAGGCATTTAATTCTTGTAGTCAGTTTTCTTCAATTCA
ATAGAAAGTTTTTT

TGTATGATTCTTCTAGATATAAAAATATTTAAGAAATTAAATTCC
TGGAATACGAAACCT
TTTAGTCCGAATATCGATTCAGCTTCATGTAGCATATTATTTCCA
AAGTTGAAATGTTAT
AATAAAATGAACTCGGTGCATGATCGGCATATTGCATTTGATGAT
ATTGGTGGTTTTTGA
ATATGTATTCGACGAGCATTTCTTGTTTAATTTTTAACTTCAATAA
T
>ConsensusfromContig863 Average coverage: 10.54
CTGACATCTATTGACAATGACTCACAATTTCGATTTTTTGAAATT
TCGCCAAGAAGACTT
TGCCCTCTCGTCTTCCCGATTGGCTCTCTATTTTCATTGTAGTTTA
TGATATCCAATAAT
GTTGCGTATACATCAGAACTGGTTGTAATTCGTTTAGCGTTCTCCC
GCAAATTTTTCTCG
AATGTTGGGTATTTTTCAAATA
>ConsensusfromContig864 Average coverage: 6.04
CGTGTGCTCTTCCGATCTCAGCCTCAGATATATAATCTGTGCGAG
AAGAGAAAACATTCA
ATATATTGGAACTCGTGCTTTTATACCATGTTATTATTTTTTCGAT
ATTTTCCGGATAAC
TTATGGTCTATTCGGATTGATTGTTTGAAATATTTGTGCCATTTCT
TTGAATGTAAAAAC
CGAATTCTTATACATTTCTGTTTATCTATATAGCCTGAGGCT
>ConsensusfromContig865 Average coverage: 5.14
CTTGGTTGTGCTTCCGAGTTGCCATTTGATAAGAAAGAGGTTTCT
AACGAATGCAGAAAC
TTTCAATAAAGAAAGTGTGTCACACCAAGCGTATGGGCCTGCTCA
TAGTTGTACAATGAC
TCCAAAAGATCTCCCGGAATTCACCGAAACGGCCGTGAGACTTAC
AAACCCTGGTGGTAT
GTTAGTCCAGAGTGATTGAGGCAAGACGTTACTTC
>ConsensusfromContig866 Average coverage: 5.35
GGCGATTTTTGATCAATACTTTGATCCTCGTATAAATGGGAGTTT
CTTTAGTCGATATAA
TTTTATATTTTGTGTTTGAGAGTCCATTTTTCCTGTAAGAATCTGG
TGAGTATATAACCG
ATTTGAACCTCTTAGGATTTATAGGATTGCTTTTATCTCGAACGTG
TTTCAGCATTCTAA
ATTTGGCAACTGCACTTGGTGGACGGAAGTACGGGATCCCAG
>ConsensusfromContig867 Average coverage: 4.22
GAATCCAATCCCCAACCACATCCTCAACCATCTCTATGAGCGAG

ATGAGAGAGTAAAATC
ATCTCAACTACCTCAGTGGACTAATATCAAACTAGTACAAAGTCC
CCACAAAGACATAAT
CCAAAGAATTGTGTATCTGAAGACAGGAAACCGTTACGTTATTG
TAAGCAAAGAAGGATG
TGTCTCTCTGTATGACAGTGACATCAATCTGCAACGAACCATGA
AAGCGACGACGGAGAG
TTGCCACGCGCG
>ConsensusfromContig868 Average coverage: 7.06
ACGACGCTCTTCCGATCTGTGGTGGTGGTGGTGGCGGACCC
GTGTCAGGCTCCCGC
GAACACGTTGGCCGCCGTTGATATGTAACGCGCTGTTTATAAGCA
TAAAATTCAAACTCT
TTGGTACGAATTGGATAAACTCCACTCTGAAAAATTGAACTTTGG
ATCTTCCGTTCTTAC
GGCAGGGCATCAGATCATGTGCTCTATGGGGGTGGTAAAAAAAA

>ConsensusfromContig869 Average coverage: 4.59
CTGTTGAAAAGTTTTATGAAGATGTATCATTCTCCTCTGATTTCG
CACTTATGAACATCA
TAAGCCTGAAAAAGTTCCTACAAACTCTGAATCTTAGAAGAATCC
GCAATCCAGCGGTAT
TCAGAACATGGCCCTCCTATCCGGATCGTGGTGCATCCTATGACT
CTCTACGTAACGCCT
TCTACACAGTTGTAGATGCGCTGGTCGGATTCAATGCAAATTTAC
CATATTTAGCGTATG
GAAATATTGGATTCGTAGTTGGTCACGAGATTGGACATGGTTTGG
ATACAGCGG
>ConsensusfromContig870 Average coverage: 9.06
ATATTCAGAGGGTCATACATGACATCACTTTGTATTTTCACATCTG
TTTACAATATTGCC
TCAAAATACAATACTTCAACATATGAATTATATCCTTTTTGCAGA
CAATTCTATATCATG
TATTAGATTCATAACATTTACATGAGATTTGAACACTTGTTGACT
GCTTACAATAATAAT
AAAACTTACTCCCATATGAATATGATACTTCATGCATGTTTTTGA
TAAGCTG
>ConsensusfromContig871 Average coverage: 5.30
CGTCCGATAGATGGTACTTCTCGCTCTGAAAGAAGTCGTAGAGAA
GCTCTCGATTCTTCT
TGAGGTTGTTTTTACCGCCAGCGTGCGGAAAATTGAAGATGATAA
AATCGAAAGTTTGCG

AAACGGAAGTTTGGTGAATTTTCGTTGCATCCACCTCGAATAAGA
CCTTGCCAGTTATCT
GGGATCACTCATGTTCATGTTCCGACTCCAATATCAACC
>ConsensusfromContig872 Average coverage: 16.79
TTCCCTACACGACGCTCTTCCGATCTAATCACCAATGTCCGCTCC
GATTGATTCTGGAGG
TTCGTCCGTGGTCGATGAAGCCAGCGACGATAGAGAACCAGCTGT
TGAACCATCTCCTTC
GTATGAGTAGAGGCGGAGAGAGTCGAAGGGGAGAAGCGAAGGGT
CACGATCCGCTTCCTC
TAAACGCCCCTCCACGAAGGAGGTCAGATCTTTCCCTGAGCTCA
>ConsensusfromContig873 Average coverage: 4.95
GCTCTTCCGATCTCTGCATGTCACCCCTCTCTCTCCTGTAGCCCA
GGTCGCACGAGTAAG
TGATTACACTGCTGAAGAATGTCGAGCTTCCGCTGCGAGTCGCGT
AGTCAACGTGCGGCG
GGTTCAAGCAATCAATAGCGGTGCATTTGGGTTCGATACCGCTCC
AGGCGCCTGAGGCCT
CGCAGGTCCGCACAAGGTCGCCCGAGGCCAATTCGTAGCCTTCGT
CGCAC
>ConsensusfromContig874 Average coverage: 9.47
GGCAATACATTTCTTAACATAATGACTGCATATCACATACAAATT
GATTGTTAGATTATT
ATTACTAAGACCATTGACCGATCAATAACGCTATGAATCCATACT
TCAATAACCTGTAAC
ACCAGACGAAGATGAATTTAAAAATTCGGGGATTCAATATTATTT
CTCAGCATAAATGCT
AATTCATAACGAATGAGCATAATTGTATTGAATATACAATAATA
>ConsensusfromContig875 Average coverage: 4.05
CACAACACTAACTAAACAATCACCTCTTGGGCCTGATCACATTGT
GGTGACTCCTCGTTA
TAGATCTTCAGCGGAAAGCTTCTAGGTCGGTGTCCCTCTTCTATT
TAAGTACCAATTCTC
CTTTCACATATATTCATTAACCTTAACTAACACAACACTAACTAA
AGCATCACCTCTTGG
CCCTGATCACATTGTGGTGACTGCTC
>ConsensusfromContig876 Average coverage: 5.71
GCAGGCAGCCTCTCCCTTCTTTTTTAGTACAGCATTCAAGGAGCT
GCAGCATAATGCTTC
GACACCTTTCGATTCCTTGTCACATGAAGAGCATGTGCACCAGTT
AAATAATTGAGGTGT
CATTTCATCATCACTTTCACTATCACTGGTGCTTGAATACACCATT

TCTGCATGATTAAA
ATTAGCAAGAGGCTCAAATTGGTAAGATTGAACGTCCATTTTGCG
AAATCATAACATTGA
TTGACCACGTGACAAAATTTTAGAACAGATCGGA
>ConsensusfromContig877 Average coverage: 6.51
TTCGCCTCAAGAAGCTTCTGAAAACGGCTCTCCATTAACAGTAGC
CGACACCTGTAGAAA
TCGAGAGGACCTGCCTTCCAGCTCCACAGGATCCGAGTCAAGACC
TTTTGCTAAGTCGAG
GCGGGATTATGAGGTTCATCAGTTTTTGCGGTTGATGAAAGAGC
GCGATGGAGAGAGTAC
ATCCTCGGGGGAATTTTCCAATGCTGACAGTGGCAGAGGGGCAA
GTGAAGAAGGTACAGA
ACACCACGCTGGCACCAGCATTCACAACAAATCTGCCAATGGGT
GCAGCATAGGTGGTCG
AGGGGGTCACCGTAGAATCCGCGGCAATCGCAATAAAGACCCAT
GCAACGCGCACCACTC
CGGTACATCCGGTCCACGGGTCCAATTCGC
>ConsensusfromContig878 Average coverage: 8.67
TTTTAGTATTCAAGTTTTATATTATCAATAACAAAGTGAGTGCAT
AATCGGCGCAACTAT
GCAATTCGGATTTTAAATGTTTTAGGCATTTATGGACTTTATAC
ATTGGAATATATTTA
CATACGTAAGCGAAAAATAACTCACATTGAATTTGCATAAGCAAT
CTGATCAATTTGGGT
CTTGTACAGATATAATTTTAAAAGCTCATTGTATCACGAATAAAA
A
>ConsensusfromContig879 Average coverage: 4.70
GGCCAACGCAATCAAATCGGGATCTGCCAAGGCGTTGGTCAGTC
TGCTGGAGCAATCATC
AGTAGAATCTTCGTCAACGCCATATAAGAGACAATAAGCAGTTG
GATTGCTGAGGAAAGA
TTGTATTTGTACAATATTTGATTTCAATGATTCAATAAATGTCGA
CGGATTATCGTCAAC
GTAAGCTTGT
>ConsensusfromContig880 Average coverage: 6.68
CTCTTCCGATCTGACGCCACGTCCAGTCTTTCAGCGGACTTCGAC
CGAGTACAATCTCGT
CAACCCAAAAGGCAGCGTCGTAGGACCCGTGATCAATCCGGTCT
TTGGGAAACCTCCTGA
GAACACGAATAGTCAAAAGATAATCAAAGACATTCCCCTATACTC
TTACAATCACCCGTT

CATGACCACGTACGGGAGATACTTCAACAGATTTGCGAGA
>ConsensusfromContig881 Average coverage: 23.68
TTTTTTTTTTTTTTTTTTTTTTTTAATTGTGTAATATCTCATCAGAA
ACTCTGGATATGA
TTTGATTGCGTATTCAAAATGAACCCTCTTTCTCCAGTCGAAGTA
GAAATATTGAATACA
AAAAAGATATAATAATTATAATTAACCATGATCGGACACTTTATA
CATCCTTATGTATAT
AAATGCAAATTTAATTTATGAA
>ConsensusfromContig882 Average coverage: 9.81
AAAGATTTGTAATTCTTGTTCATGCCTCTTTTGTCGTACTAAAAA
AATAGTGCGTATATA
TCTCGTATTGCTGTTTTGATTTCTGCGTTGAATCTTGAAGCGTTCG
TGCATGGTGATGAC
TTTTTGCAAGCGACGTGCAAACCAATGCAATTATTACCAAAAAAC
TGATAACAGACTCCA
TAGCCATTTGGTGCACACATACTGAACCCA
>ConsensusfromContig883 Average coverage: 9.19
CCTGTTGAGCCATGTATAGCTTTTGAATCTTCGGGAACATAAAAT
CAGAATTGAATTTCC
CATTGTCGACGATGTTCACGAATCGCATGTAGTCATCCTGTTGAG
CCATGTATAGCTTTT
GTGCATATTTTCTTTGACGCGTTTCTTTTTCCAATTTTTGACTCAC
ATCATCAAGTTTCT
GTTTTAGTCTAGCATATGTTTTATCACTGT
>ConsensusfromContig884 Average coverage: 4.37
ATTCATGTTGTTCAGTTTGTGTGTATTTTATTTGACTGAAAGTTTA
ATTTTCGCTTCAAA
ACAATACATTCTTATCTTATATATTCAAGATTTATTCGGATTTTC
ATCGAATAGGAGGCA
ATATGCTATATCTCGAAAATAAGTCAGTAAACATGTTGACATTTT
TAAATTCAGGTATTT
TTAATTCACCAGGATTTCCTTATGATT
>ConsensusfromContig885 Average coverage: 1,379.97
TTGGAAGCTCGAACGGCACAAGATCGCCATCTGTACGCGACTTC
ATCGCCAAATGCGAGA
AGCTGAATGACGTATCTATAAAAGGGCGCGGCCACAACAGCGAT
CTGTATGACACTTGTA
ACTCTTCGTTGCTGTCGTTCGGATTGGACAATACAGAGATGGACC
GAGATGCGAGTACCT
TCAAAACAGAGATCGGAAGAGCACACGTCTGAACTCCAGTCACA
TTGCT

>ConsensusfromContig886 Average coverage: 5.86
AGTTAAGGAACTTGTTCACAATCGGATGTATTCGGTCAACCGTGA
GGACAGGATTTCAGC
CGCTCGACAGCTAGGACATTTGAAATGCGGAGATCCGCTTGTTTT
GGGGGCTTTGGAGGA
ACGACTGAAATGTGATGAAGACAAAAGGGTCAAATATGAAGCGA
CTAAAAGCCTCATGAT
GCTAGGTCTATTCACCGACA
>ConsensusfromContig887 Average coverage: 6.73
AGTTCGAATGAGTATGACGCGGATGCACCAAAGAAATTTTCTGAT
AAATCATCAAATAAT
ATTAATGGATATTCACCCGACGCATCTTTGAAGGCCTCTTACAAA
ACATCAGCGAATATT
TATACTATCAATGGGCCCATGAGAGTTTCAAAGACGATGATAGAT
GTATATGACTCTCCA
TATAAGCATG
>ConsensusfromContig888 Average coverage: 8.51
GCAAAATATATGGCAACTGCTTCGCGGTAATTGTAGTCGATTATT
GGAAATGTGACATGA
GCTCGAAAAGAATCATATGCGAGATAGAAGTTTGGAAACCTGCA
GGACCACTAGTCAGAT
TTCATTATTATTTGCGATATAATGATAAGTGGAGAAAGAATAAAG
AAGTTGGGACCTTAT
GGGATTGCGAAGGGGGAAAATATAAGGTCGTGATAAGGATGGAG
CTCCCTAAAGGAACGC
AGAAAGTGGAACTCAATGTCAAACTACAGGATGTGGC
>ConsensusfromContig889 Average coverage: 24.90
AATTTTAACAAAATGTTATAAGGGATATGTGCATGAATGGATACT
TATAGAAATAATTAT
ATAGCTGTTCAAACTAGGAGCATTTAATTTTGAGTATTTTCGTCGA
ATCCATTTTCTCTT
CTCTGACGCAGTAAGAAAATAATCCAAAGCCATGGCAAGCAGCC
CCGTATGATCCATTGG
AATTGACAGCGACAACCGCGCCACTGAAAGAAGGAACTTTAGCC
GTAATTCTCG
>ConsensusfromContig890 Average coverage: 9.08
GCTCTTCCGATCTGGGAAGTGTCACGCAGTTTTGGAGGAGATAGA
CGTAATCTTTTTTCC
ACTTGTCCAACTCTTGAGTTAAATATTCATGCCTCCATAATGACC
TTGTCCATAAAAGAC
GCTGCGTGTGCCTTTCTTGGGCCATTCTTCGAATTTCGTCATTGAT
CAGCCTATTGTGAT

GCTTGAGATTCGATATTTCCTGTTTTGATGTCTTTGAAGTATCTGT
GAATAGTTTCCGAA
TATTGG
>ConsensusfromContig891 Average coverage: 7.49
GTTTGCAGATTATAGTTACAAGTATTTTCGCTGAATCACATGATG
TAATAATGAGAGTTG
TATTGTTGTTGTTATTTGTGCTGACATACTTTGAAGAAGCTTTAGC
ATCTGTGTGCTATA
AAGGAGTTCGTATAAAAGCAGATGACCATGATCTCTTCTGCAATA
AATCGACGACGTCTG
ATAGGGACGCTTGCATTAAATTAGCAGACGACAAGCAAGCAAAA
CAGGTCTACTTC
>ConsensusfromContig892 Average coverage: 6.27
GTCGTAGTATCGTACGACCTATCCGTAATATAAATATAGCCTTTG
ATTTGAATCTCATTT
CAGGTGAAAATTCCAATGAATCGCCACAATCACTTATTCGTGATC
CGCCTTTCCCGGACA
AATTGTGGCTATCAAAATAATCTCTCAACAAAAGTGAATAACGAG
CATGGCTTTCAAATT
TCAGAAATATAATGTTCATAATCTTGTTAC
>ConsensusfromContig893 Average coverage: 4.84
CTTATATAATACGACTATGTTATTTGCAATTTTTGAGAACCTTGA
AAACCGAGTAAATTC
ATAAGGCATGAAGCACAAAAGTGGGGGCCCATATACGCCCTACCC
TTCTTACGCTCCATA
GATACTCTATCTCAGCATACCTTGTATATATTGACTCGAATAAGG
AATAATATCCTTCAA
TAAGAAATTCAACCATAAAATAATAA
>ConsensusfromContig894 Average coverage: 6.99
ACCCGAACAAGAAACCAGATATTTTCCCCATAATGCTCTTGTCAT
CTTCTCCTGTTACAT
CTCGTTTGTTTTCAGCAGTATTTCCATGGTCGGCACTTCCTTTGTG
AAGAAATCCTTTTG
TTGAACTGCTATCTTTTTTTGCAGTTTTCATCAGATTTGTTTTAGA
TCCCTCTACTGCAG
GAACCTCTTCATAATCCGAAATTTTGAATTTTCTCA
>ConsensusfromContig895 Average coverage: 7.90
ACGCTCTTCCGATCTCGCCATCGCTGCTGTTGAATTTTTTCCTATA
AATATAATAAGAAT
TTTGTATCTGCGGTATAATGATTCTTCAAGCATTCGGTTTATTTTT
TATGTGTATCAGAG
CATGTATATCTGAAGAAATAAATAAATCACCACCTCTCATCACGC

CTGAACGACCAGCTC
TTGTTAATTACATGCAGAATTATAAAGCAACTGA
>ConsensusfromContig896 Average coverage: 5.39
ATGTATTTATTGATGCATATATTATATACAGTTGTTATGCCGGTGC
TTGATTGTTTTTGA
TTTGCCAATCTCTTATCATCAATACACATGACGTCATGTGTCCTAT
GTTATTCATATTGA
ATCATCGTTTCACTCGATCTCAAAGATATATTATTGTTAAGTTTTG
CTATTTATGTTCCT
TTATGCTTAAATTATTATCATGAAGATGGGAG
>ConsensusfromContig897 Average coverage: 4.43
TCCGATCTTGGGAGAAGACTATAGGAGATATAATGAGCGTATGG
ATGAGAAAATGCGGAG
TGGGGGTAAAAGTTTTCTGATTCAGCAAGCATAATATATGAATC
TGACGAAAGGCATGAA
CCGGCTGAAAAAATTAAGTCGTTATGGAATGCAGTTCCAGCTTGA
GAGCTGGATAATACT
GCTGCTATCTCATCAAAATGTGTTATCAACCTAGCATTTAG
>ConsensusfromContig898 Average coverage: 10.96
CATTCGTGAAAATTCTCGAATTACTTATAAAATAATTGGCGATTC
CGTTACGATTCTAGA
AGTGGATGAAAGCGGTGATATATATGCTAGAGTTTCGTTTGATTT
TGAGCAGGTTCACTT
ATTTCAGTGTACATTGATAGCTACAGACAACGGTTCGCCTGCTCA
TAATGACACAGCCAC
ATTAACCATCCGTATACTCAGATCGGAAGAGCACACGTCTGAA
>ConsensusfromContig899 Average coverage: 11.23
GAAGAACAATTCGAACGGATTTTCCGATATCGCCGCTTTGCGTTC
GATTATTCGCATCAA
ACTGAGATGAAACTTGAGCGCATTTCCGAAAATTCGCAGTGATTT
CGTCTGAAATTTCGT
CAATTCTCGAGTCTACACTAGTCGTTCCAGAACTGTACGAACCAC
CGGGAAGAAAATTGA
AAGCGGTCGATGGGAAAGAATAATCGCTTGAAGAGGTCCTGTTT
CTCAGGTCTGAAGAAC
T
>ConsensusfromContig900 Average coverage: 8.98
GCGGCATACTCGATGGAAATATTTTTCACGTCGTTGGTGGTACGG
CTCGGGCTTCTGAAA
CTGTTGCTATAAATTCTGAATTATCTTCTCTCAAACTGCCTGAAC
GAATTCGATGTAAAG
GTCGACCGAAGGGCACGGGGATGACAGTGATAGGCTTGGCACGA

AAAAAAAGGAAACTTG
CTCCTGGTCCCGTTCAGTTTCTGGAGCAACCACTTCAAAATAGGC
AAAAAGTCATGCTAC
GATGGCTAGTC
>ConsensusfromContig901 Average coverage: 2,078.93
CGTCGCTAAATCCATTAATGTTCGGTCGGGAAAGCGAGCACATCC
GGAAGTATAGCCGTC
CGGAGTCAGTCCCCGGTAGAGATAAGCATCCGATGGAATTCCCA
CCGAGAATATTATCCA
AAAGGTTTGATGAATCCAAAGCACCGTTCGGATCCGCCAGATCG
GAAGAGCACACGTCTG
AACTCCAGTCACATTGCTAA
>ConsensusfromContig902 Average coverage: 10.76
CTGAAATGTCCTCTCCAGGGCGCAGCCTGGGCAATTTATATGGAG
ACTCTGGGAAGCCCA
GACGACAGAGTCCCCCTCTCGCCCTTACTGATGTATTCCGAAGGA
AAGCAGAGCTATACG
TTCGGCATCAGCTTGGGTGCAGGAGCTGCCAGAAAGAAGTACGTG
TTGACATCTGACTGC
CTTAGGGGCTCCACTCCGGATTTGGATGAGGTTTACTCCCTAGCC
TTCTCTTCTCCCGAA
AATACCCACAAGGCAGTGGGGTAGTGCCGGGTCCTATGCACCCTA
TGGT
>ConsensusfromContig903 Average coverage: 6.82
GACATTGTCTTATCAGATCGAATATAATATTCATACGTATCAATTT
TTGATACGACTTTC
GCCGATGAAATTTCTGCGTCAGACTTTGGAGATGAATTGCCTCCG
AAATCTTTCTCGATC
TTCAAATCCCTGGTATGCACTCGTATAAATTTTTTTTCCGACTTTT
CTTTTGGCTCCATA
TTTGACATATTCGTAAATTCCTGGTTATTGCGGTCTTTTCAACAGT
AACTTGTGAATTAT
AA
>ConsensusfromContig904 Average coverage: 5.24
CATGACCTTGGTTCTCATGACCTAGATGCGAATCGACCTTCCGAC
CTTCCTTGTCTTCAA
ATTGAAGTGATTTCGGTATGTTCTTCCGACTGAACACCTTGGTAT
CTTCCAAAAGCATCG
CTCTACTTCGAATGCTCACTTTACCATCGCTCTCATTTTCCGAAAT
CTTTTTGCAAAAAT
CCTCTCCATTATCCGCAACAGTTTCTGTCGGTGGACAAGTCCTCAT
ACCC

>ConsensusfromContig905 Average coverage: 7.77
ATCTCAAAACTATTGTTTGGGAGCGTTCTGTGCATTGTATCTATA
TCACCGGCTCCATCA
TTGAAATCCATATTCGCAGTGTTTTTCATAATTTCTTTTGCTTCTT
CTGAGGTTGGTATA
TCTTGTTGCCGATTTAAATGCTTTCTCCTTGGAGCAGGAGACACA
TGACTGTCCAAAGAC
TTTTCTGGCGCAACGGGAGCCTGAGATCG
>ConsensusfromContig906 Average coverage: 5.34
GAAGTCTGCATGCAATTGTTGTTGAACCCATCAAGCAAAATTCCA
TTTCCGACCAACGCG
CCATCATTAACAATTTGATTTTTGTTCTGTTTTTTATACGATGATT
TCAAATGTCCGTGA
ACGATCAAAACTTTATCTTTCGCTCGATCGCACCATTTTCTAATTT
TTGTATAAACATTA
AGCGGGATGGAGTATTTCGAGTTTTGGTAAGCAATCCTACACCAC
TGTTTTGTGAGGAGA
AATGATGCACATCCTTT
>ConsensusfromContig907 Average coverage: 9.01
GCTCCAATGACATTTAACTGTTGACATTTTCGCCTGAACACTCCAG
CATTTGTAGTTTTT
TCTTGCTCAGTTGGCCAGCAAATATATTCAGCTGCAAATTGACAT
AGCGATTACGCAGCA
GGATTTATAACCGTAAAAGCCGTACTTTGTGAAACGCTTAATTTA
TCGCCTATTCCACGA
AAAGAATTCCTGTGCACCAAGTACCAAAGATACATGAGTGT
>ConsensusfromContig908 Average coverage: 6.68
CTGCTATGAAGACACTAAAAAAAGATGCAAATAAAAAGGATTCA
CTGCCGCAAGATGGCA
GCACTGCGATTGAAAAAACTTCGAAAGCTTCAACAGTAACATAC
AAGAAAAGGAGAAGAC
CTCTAATTCGTACATATTTGAAGGAATTATTCCGACCCCATAGCC
AGGAAGAAAAAAATA
CGTCATTAAGGAGTTTGCAGAACTCAATCATCACACAAAAAACA
GATGCAAATCTAGAGG
ATTTGCCGCTAGATCGTACCACCGCGGAGGCAAGAGCTTTAAAAG

>ConsensusfromContig909 Average coverage: 5.69
CTTCCGATCTTTGGAATCGTATGCACTACTGCGATATAAGTGCCG
ATGGTCAAGAGGCTC
ATCACAAGTAATTTTGTTGCCCATGCCGCGATTATCCTAGTCCTAG
GCTTTGCCTTCTTT

TTGTGCTCTTGCCATAGCTCTGTCAGTGTAGGTTTCAAGGGCTCT
ATAAGGATCTCAATG
TTTCCACTTGACTGACGCATTCGTAAGTCAACATCTTGCATTTTCT
CATGCAGTTTTGCT
CGAAAGGCAGATTATTCAGTTCGTGCG
>ConsensusfromContig910 Average coverage: 7.14
AAAAATCCAAAAAAGATGACAAGACAGCGTGTATCAGCTGAACT
CGATTGGAAATTCACG
AATGTCTATAAAATTCTTCATAAACAATAATCGATGACTTCATAA
TTCCTAATGTTCGTA
GAATCGTGATCGTATATTAGACTCTGCTTGTCGCATCTTTAGTCG
ATCTGTATCAAGACA
GTGTCTGGAGAGGATGTTATAAATGGCACAACCCCGATGGAA
>ConsensusfromContig911 Average coverage: 10.69
TCTCGATCAGAGAAATACGTACACAATTTTGTTATTGATGTCGAA
CTCGATAAAAGGTAT
AAACATGCTGCTTCTAACATTTTAAAAGCTGGATGGTTTGTTTAT
AAGAACAATAAAAGT
GGACGGATGCGCATTGTGAGGCGGTATCAACGAAAATTGTTGAA
AGCCATACATACGATG
AGGGAAATAAAGCAAGAGCAGCGAAAACTGGCAGATAGCTCCGT
TTCCCTGCTGGAGACA
TATGCTCTGCAAACAACCTCTCAAGACTACATCGAGCGAATCGA
CACAGCACAAACTGCG
ATGGAAGAGCGTGTGGCTTCCATGGAACGATCTCTAAATGCCATA
AATTCAAAACTTG
>ConsensusfromContig912 Average coverage: 15.61
GAAAAATACTAATCTGAAACCTTTCGCGCCACTGAAGAATTGGAA
CGACACCATCTATCT
TGAATTGAATAACGCCCACATGGCTGTTCTGGATCCCACTGAATT
CAGAGATTACCATAT
GGAGGTACATGTGGTAAATGGTGAAAAAAGCCCATTCTCTTGCGA
TTGCTGCAACTTGGA
GCTATTCAAAACCTATACACAAAACCAAGAAACAGATCGGAAGA
GCGTCGTGTAGGG
>ConsensusfromContig913 Average coverage: 5.09
TTGCATTCATTGTGTGTCAATTGATAACTGCAGCTCAAACAGATA
TCTACGAAGTATGGA
TTCGAACATCCAGATGGGAAAATGACGGGACAGATGATCATGAT
CATGGAGAAATTCGTC
TGTTCGGCGAAGATATACAGCGAACACAAAAACATTATCTCAGT
GATGATTTTGAAAGGA

ATGCGATAGACTCCTTCAAGTT
>ConsensusfromContig914 Average coverage: 3.02
CAAATATCAGTGTTCCAAAAATGGAATCTCGTACTTTTTGTGAAA
TTCAAATTTTCTGTA
AATGAATGTTTTTCAAATGTTGACAATGGCTCTACGTCCTGATTCA
GGTTTTTGGTATTC
GGGCACTACGTGTTGTGTGCACGACTCGAGAATGTCACATCTTCA
TACAAAAGTGAATTA
GCCATGACCGTCACGATCTTGATTGCC
>ConsensusfromContig915 Average coverage: 5.16
GGCAGAAGGAAGTAAGCGAAGTGATTTTTGTCTAGCGGAGAGCA
ACGGTATTATGAGTTT
AAAACCATCACACCCATACTACTTCCAAGTACAGGCCCAAATGGA
AATTCTTGATTTCGA
AAAATGCGATTTTGTTGTGTGGTCACCATCTTCAATCCACATTGA
GACTATCGACAGGGA
CAGTGAGTTTTGGAAAGACTGCCTGGAAAAATGCTCAAAATTCTA

>ConsensusfromContig916 Average coverage: 9.30
CTTCGAAAGCCCCTCTTCGTACCCAAGTCTGGCTCAAACGATCAC
GTCTTACAGTCCCCC
TCCGTCTGATTGCAGTTCTGATGGAGCCATTGAGGAGCTCATAAA
TGCAGCACAATTCGC
TGAAGACCAACCCCGCGGAAATCTCACCGACTGTGCTTTTGACCT
CAAGAATGGTGACGC
CCTTGACCTGACAGTTCAAGGTCTTCAGCTCCGAGATCGGA
>ConsensusfromContig917 Average coverage: 8.06
ACACGACGCTCTTCCGATCTGCAGATATGAAACAACAATTGACTG
ATGGTGCGTTTTATT
TCAATAGTGATGATACTAATTATCCTTTATCCCTATTCAAACTCA
ATACGAATACCAGAT
CAATTGATCATGAAATCCGTTTGTACAGTGGAGACTTATTCATTA
TACGTGCTGTTTTCA
GTTCCTAAATTATCGTATGAAAAATATTTAACAAGAAAATCAGTG
AGGCTTGGAAAAACT
AGGCGAGATAAACTGGGTATTAGAACACATCGTTGAAATTAAATT
AATAATTGTTATTAA
GTGGGGGTGAGTCATCACACAATTCAC
>ConsensusfromContig918 Average coverage: 5.83
ATTTTTTCGACATGATTTGAGGATCTCATTACGACTTAAGCTCAA
AACTGAATATATCTA
TTTTTAGACTGTTGTTTCATCAGCATCTGAGTGTGTGAATAATTTA

TTATAATTGCAATT
CCCATAGAATGTCATGATAGAGGCATTTCTTTTAATAGTCTTTTTA
CGTTTCAAGGTTCC
AATATCTTTACGGGTGCCTGCTCCTTAAATCTCAAACTATGTCTC
AAACTTAATATATGA
ATAAACTCCCTATTCGAGTTGTTAAATGTTGCA
>ConsensusfromContig919 Average coverage: 15.25
CCGATTGTTGCAGCATCTGAGTTAGTGAGTTTCGATGGCTTCTTA
TTACGAATAATTTGT
CTGATTTTATTCTTTAGATCAATAAATGAATTTTTCTTCTCAATAT
GAGGATTGGGGGTT
TCTGCTGAACCTGAAGACGTTGATGCAGATGTGCCCGGAAGGCCA
GGCCAAGTATCTTCA
ACATAATCACATGCAATGGAATCTTCCAGATCGGAAGAGCACAC
GTCTGA
>ConsensusfromContig920 Average coverage: 4.15
TTCGATTCTTTAGTTTCAGGTTGGATAATGGGATTTGATAAAATA
AAGTCGATGGATAAA
AAACACATAAAATGATCCGATAATCCTTCTTCGATTACTCCAGAG
ATTACACTAAGAGTC
GAGGTATAAAATATATTGTCTATGAGAGACGAATATCTGTTTGAT
ACATGAGTTGGTAAA
GTTATGGAAGGAATGAGTGAAAA
>ConsensusfromContig921 Average coverage: 25.58
CTTTCCCTACACGACGCTCTTCCGATCTCGAAAAGCCTCTTTTTAT
CATCATGGATTCAC
TTGACTTGCTCTCCACAACAGATAACGCCCATAGCCTCTCGTGGC
TGCCTTTCGAACTTC
CACCATATGTCAAGATGATTGTTTCCACCGTCACAGAGCGTGAAG
TCATCGTTAAAAGCC
TTAGGCGATTTATGCCGTCCAACGACCAATATATAATTGTCGACT
GTCTAGGGGAGGAGT
TGGCAATGAGCGCACTTGAG
>ConsensusfromContig922 Average coverage: 14.48
TTGGAAGTTTTGCCGTCTCTGTGCTTCACGTGATTCTGCATTGAA
GCGACGTGGGAGTGA
GCGATCGGGGGGAGCTCGGTGAATCTTGGGGTCATGACCCGGAG
CACCGATTCCAGTCAT
CCAAGCCATCCTGCCACTGAAGAGTCGACCGGAATATCCGCTGGA
GACTGTGCCCCCGTG
GAAGGCAGAAGAGTACCTGTTGGAAGTGTCGTATTCAGAGACAT
CGAAAGAGTCCTCATC

GGTCGTGCTTGCAATCCTCTTTTTGCATCTGAACCTTGAACTCCGC
AA
>ConsensusfromContig923 Average coverage: 7.62
CTCGGAGCGAACCATGATGAACCTCCGATTATCAGCTCTCTCATA
TCAACCTTTGGATAT
CGTAACGCTCAAATGGGTGGACATGCCGGCTGTGCGCTCTTCTTAT
TTTTATCTTCGTTT
TCAACCCAAGTCTGGCACCTTTACGCAACTCTTGGACTTGGCATC
GGTTTATTCATTTGC
TGCCAATATTACGTCGGTTACGTGTTCACCGCCGAACATTTCAAG
T
>ConsensusfromContig924 Average coverage: 4.16
TTATTTTTTGTGATCCGCATGAGCCGGTGTTAGAAATTTTTTTTAT
TTGACCAAGATTTT
CATTCAGGTAAATTTTGATCGTTTCGCTTTTATGGTCGAATATTGC
TCTAACATGATGCC
ATTGGCCGATTGATGCTGGTGTTCTGACACTTATTCGTCTGGTGC
AACCTGGTGTGCCCG
AGATCGGAAGAGCGTCGTGT
>ConsensusfromContig925 Average coverage: 735.54
ATGTGACTGGAGTTCAGACGTGTGCTCTTCCGATCTCACGATGCC
AACCAGCCCTTGAAG
TGGCGCTCCATAGCGGCACGCTTCGATGCGGATCCCATATTGACA
GTCGGCAGAATCTTG
GCGGGGCGGATGTAACGGACGAAGTCTCTCAATTCGGTAAAGCTG
CTGTGTTCGCTGTAC
GGGATAGATCGGAAGAGCGTC
>ConsensusfromContig926 Average coverage: 5.26
ATCTGGAACAGTCGTCGCCTATGAGAGGCCAGCCTCTTTTCGAGA
CATCTTGAGTGTCGT
CTGACTGATATTCGAGAGGGTAGGATGACGCGATGTGAGTGATA
GTAGCTATCACTAATG
ATAGGATGAATAGGATCTGGTATAATCTGAACATTGCTGATTTCA
TTGTCTTCTGGTATG
GAAAAACCTTCTAGTTGATAAATTAAAGTTTTT
>ConsensusfromContig927 Average coverage: 7.53
GTGGAAAAATTAACAGATTCATTCGTTAAAAAGTTCATCAATTAT
TTATTAAACTGTTGG
AATTTTTTCAGATGGCCTCTATTGCAGGGAATCCAGGCATGATTT
TAAAAACTCACGACT
TTATTGACGTACTATTAGTGAGGATTCTAAGGTTGGGGATGATA
AAGAATGGGGATCAGA

TGACCAAAACATCCCAGCAAGCCGTATTCTAATACGAG
>ConsensusfromContig928 Average coverage: 5.31
CCTAATAATCGCAATGACAACAACACGTCGTTGAATGAATCTAGA
ATATTCGAAGATCGC
CGTAAAGTTATTGTTGAAACTACTCCTCGTAATGAGAGGCTTTCA
TTAAAAGAACGTCTT
CGGCAAAATAGATCAAATATGCAAATGATGTTGAATTCTTCAGCT
TCGGTTATGGGATCA
AGGACAAATTTGAATTCTTCTTTTTCGTCTTGTGTTTCGTTGAACG
TTCCTCGGATCAGT
GTTTCAGAGCACAGAAGAGGGAAGACCGAATCTAAGCAAGTTAA
TAACGACACCTCTTTT
TGTTTTGGGAGGAGTGAGTATAGA
>ConsensusfromContig929 Average coverage: 12.82
CCAGTACCTGTTGGGCTTTCTAGAACTGCGTTCTGTCTAGATTTC
AAGGCTTCAATCACT
TTCTTCATATAAGTTTCTTGGCATGAATATGGAGTAAATGGAAAA
TTGACCTGTACGCCA
TTAACACTCAAAAAAGGCATTTCACAAGAATGTTGTATTCGAAAT
CATATCTCAGAAAAA
TATAATCTTGAATTTTTTTCTGAGCGCGCCATAAA
>ConsensusfromContig930 Average coverage: 10.47
GTTGTTTATGAGGAATATTAATCTTACTATCCTATTATTTACCAAA
AATTTCTCTTCACT
GGTATACAGAATCCTATTCAAAGCTTATTCTAGAATGCCGGTCAT
TCTAAGTGGTTATTT
CTCAGGAATGCTTACAAGTTTGTGCTTCGAGATTTGTTATAAAAA
CTGGTAGAAATTATT
TTCCAGATGGATTTATGTTTTATTATTGTTATTATGAATGTAGC
>ConsensusfromContig931 Average coverage: 2,016.12
TTCGTGCTCATCGACGCATGCGAGATTATGAACAACTTAAGGGTC
TTCGCATTCACACGT
TATATCTCGATACGACATATTGCGATTCACAGTATGCTTTTCCTC
TGCAAGACGAGGCAA
TATCAGCAACACTGAAGATTGTCAAAAACTCTGATCCTAACACTG
CTTTCGTAGTTGGAA
CTTACACCATTGGAAAAGAGAGGATTTTCAGATCGGAAGAGCAC
ACGTCTGAACTCCAGT
CACATTGCT
>ConsensusfromContig932 Average coverage: 7.64
CTCATCGTTCTTCACTATTTCCATCATATCACTATGGCAACGATTA
CTTGTTGCTTGTCA

ATATTTGAGTTATCAGCACTGTACAGATGGTACGCCACTATCAAT
CTCTTCATTCATTCC
TTCATGTACCCATACTACACTGCCACGGCCGCCAAGATATACCCA
CCAAGATGGATCAGT
AAGGCAATCACGACAGTACAGATTGTGCAGTTCGTTTTGGGGGTT
CCCATGGTCTATTTG
G
>ConsensusfromContig933 Average coverage: 5.16
ATTCAGAAAATCTCTGATTCCAGTACCGAAAATTAAATACACATC
ACCATCGAAATCCGT
CTCGTGATATGACCACACAAGTTGTTTGATAAATATATCAACAGT
TTATATTTATTTATG
TTAACCGGACAACCAAATTAACAAATACGTGTTGATATACTCTCA
TCGGAAATCACTTTT
CTCACTCCAACAGACCAAATATCGAC
>ConsensusfromContig934 Average coverage: 7.39
ATTTATACTCTAAGACGCCTTGAGCTGATTGGAAACCCAGTTCAT
CAAGAGGACAACTAC
CGCCTCATCATCGCCTACTATCTGCCGTGCGTCACTCACCTGGAC
GATCAAGAGATTACG
AATGATGAAAGAAAGCTTGCTATGAAACTCTTCGATAGCACTGGT
GTTCAACTGAATCGC
GATATTGCTTTTGGGAGAAGACAAATTCACAAAAGTCCACAACG
GCTGAGACACAATCTC
AAACTAGCACCAGCTCCCGACTTTATTGGAAACAGCCTTTGGCGA
AACAACCCACCTTTG
CCTGACGAAAATGAAGCAGTATCAAGGCGTGGCTTACGACGTTC
>ConsensusfromContig935 Average coverage: 11.37
CTTTCACATCCAAATTGCGTTTCAGCGTGTATCTTGTAAAGATGA
TAATTAGAATTTTGT
TGAAAGATATGAATTGTTGATAATGATAATGCATTCTTTCCAGAC
AGTTTTCTATATTTC
CTTTGCAGTTTCATCCATAGTGACTCATTATTCAGAATGAAAGATT
TCATTATTGAATTC
ACATTTATATAGAAATCAAATCCTATTTTTCACATTTATATTGTG
TTCATCATCAGGGGA
ATGAAATCAGTAATATTATATTTT
>ConsensusfromContig936 Average coverage: 10.28
AGGCTGTCAAAAACAGCCTTTGTGTGGTGCCACTCGCCTCTAGAG
ACCGTTGTTATGTAG
CCCATGTCTTTTAGGCAACCCTTGCTCATGGTAAAGAATTCAAAT
AACAATAAACCATGA

CCAACATACAATTGTCTCGAGTTTCGCCCACCCCCTATGATAAAA
GCATAGCCGGCGTGA
TAGAGATCGGTCTTAAACCACGCACTAATCTC
>ConsensusfromContig937 Average coverage: 7.26
ATTGTTTTTCCTATCACTCAAATTGGAATAAAGTACCCTTGTGCA
TTATTCAAACAGCT
GATCATTTTGGTTAGAATCTCGAATTTTTCTAATAATTAAACCTT
TATCACAAAATGATA
ATCATGATTTGGAACATATCTGCTATTTTAAAATTCATACACCGA
GTCAATTATATCACT
CTGTATAATTTTTTTCTCAATCATTTTGTTCTCTTATGTAGCTGAT
TTATGTGCACTG
>ConsensusfromContig938 Average coverage: 13.00
TGAAAATACATTGTTTTAGAAAACCAAGTGGCAAGGCTGAATGC
AGTATTGATCGATTA
CCAAAAAAATGACATCGGCAACCACTCCAGGAATGATGAGGCAG
ATAGGAATATTATTGA
TTTACCATGGCTTGTGGATGAAGGTTTAATGACACCGTTATTATG
GGAATATGATAAAGC
TATAGAGGAAATCACGATAAATTTAGAGTCTACGCAGAAAGAAC
GTGATAACTTGCAATC
CGGTTTGAAAGATGTCATTCACGAGAATAAAAAGCTTACTGAAG
AGATGAAAGAACTCGT
TGGGACGAGTTTGAAGGAGAGCAGTGGCGATCCATTCAGTGATA
ATGACGTCATTAACAA
TCTCCAGCGACAGATTGAATTGGCGA
>ConsensusfromContig939 Average coverage: 4.11
CACGACGCTCTTCCGATCTCACAGTTGTAGAGGGAAGCCAGGACG
ATAGTCATCTCGGAT
CTTCCGGGGCTCCTGCCGACCTTGCCCTGGATGGGTGGACGCATC
AGGGCGTTTCCACGC
TCGCCGGTGTCAACGTGAACAAGTACGTCAAGATGGGCCCTCAA
GGGGTTGACGCTGCAA
CTGGAGCCAATTACACCGCCCTCTACCAGACTGGCATGTACCCAG
ATCAG
>ConsensusfromContig940 Average coverage: 3.50
AAAGAAGTTGGTTTAGGAATAACAAACAATAAACAATCGAATAC
TGGAAGACCAGAATTG
GATGGAAATGTCAAAAAAATGATTCTAGAGTTCTACCTAAAAAT
GATATAACATGACAGG
CTCCAGGACAAGATATATGCTGATGTCACTGGCAGAGGCGCATCA
AGTTTTTGTTGAAGA

GAATGACGAAATTTTTTGGTGCCTCCAGACATATCCTTCTTTGAT
TGTCGTCAGAGTCAA
CCGTCGATCCCTTCATATTTCAA
>ConsensusfromContig941 Average coverage: 9.69
CGGGAGTCTTTTATCGAAAGAAAATAGATATAAAAGTCGACGGTA
CTGTTTGTCAGTAGC
AAATAGTAGCTGAAAAATACTGCTAGCTTATGGAGTTCACCTAA
ATCCAGAAAAGTTGAC
GTTAGGTACCTCAAATATTGTGGCGTTGTCAGTACTAAGTAGGTG
ATCGTAACAATGATC
AGCATGACTGTTATTCCTTTGCTCTCTGACTTAGTATTTGAATGCC

>ConsensusfromContig942 Average coverage: 5.52
GCGACTGCTGCTTCACATATTTCGCGAGTATGTCTGGAGTCAAAC
TCTGTGTTTACATTC
TTTTTGGATTATTCATCTCAAATTTTGCAAAAGAAGAAGAATATA
CAAAAGAATTGTGCT
TGAATGAGAAATGGCAGCTTGAATGCGATTCGAATGAAATCCTA
AAAATAAACTCAGCGA
GACTTGGGAGGTTCAAGGTGGGAAGGTGCAACAAAAATCACG
>ConsensusfromContig943 Average coverage: 7.48
CTGTAAAACTACAACAACGCGTTTGGTTTTGAGCCGCGATAATGC
AAGACTTACTTTCTA
TTTTTATGCAGACGCAGGCTGCGGCTACCGTTCATATTTGATAGT
ATTTATATCGGACAT
AATTTCTGATGAATGGTTCCATGTCAGAGCAACATACTCTCATGT
TTCAGATAAACTCGA
GATTTTTAAAAATGGAAATTTGGAACAATCCGCAGGAATATACAA
CATAGCGTGGTTTGG
GCAACTTGACCCATTTGAATTAT
>ConsensusfromContig944 Average coverage: 7.11
CTACGCCCTCTCAAGGTCGATGACTGTATCAATGCGAGGTTCTCT
TGATGTCCAGTCACA
ACAAGTCGGAGCGTCGTCATCTTCTTCATTTAGCGCCGACGCAAG
AGGACCCCTAGCTCG
TTGCGCATCTCCGCTTGCGCTTGTTGCCCGCACACTCAAATATCGC
CCATTCCGTTTGGC
ATTGCTGTAGCGCGCACAAAAACGGCAACAACAGAAGAAACATG
AGAGGAAGCCTTCGCA
AGGTCCTTTCCGCTTCCGTGCTTG
>ConsensusfromContig945 Average coverage: 6.56
GCTTTGGCAGATAGAAGAATATGGGCGATTTCTGGAAGTTTTTT

TGAACGGCATAATGG
AGGGGCGTGGTCCTTGTTTCATTGGCTATATCGACATTTGCCCCT
TTCTCAACAAGCATC
CGAACTGCTGTCACATGACCATGTGCACAGCAGTAATGCAGGGGC
GTATTCCGTTTGTGA
TCGATTGTATCCAATTTAAC
>ConsensusfromContig946 Average coverage: 4.39
GAATTTTTGGTTTTAAAAGCAGTTCATGATAATTACCTTTTATAAT
AGTATTCTAAATGT
AATACGCTTTGACCCACATCGTTGTCTGTTTGTCCGCATCTGTTTT
TCACATCTGTTTCT
CTGTGTGATTCCTCTCCTCGTCAAGGCTGTTTGTTTATAGTGCATC
GATTATTGTGACGA
TCAAGTTCATTTATGACTAGTATT
>ConsensusfromContig947 Average coverage: 11.05
CTAGTATATTTATTGATAACTCCAAATGTTATAGAAGAAGAAGAG
ATGAAGCGTTTTGCT
TATGATTGTCTATTTTATACTCTCAGAGGAAATGACAACGAGGAT
AAACAAAGTTATCTT
CAAGGAAAGGATGTTCACGATAGAATTCTAAACACAGTAAATCT
GTGGGATTCCTACACG
TCATTAACTGCAGATGAAAGTTTGGAAAATCTCGTACGTAAAAA
AGAGAATGTCGACGCG
ATGAGAATAAAACAAACTTGTATAAAAATAAGCGAAAAAATTTC
TGAGGGGAAATTATAT
AAACCTCTAAGAAATATAGAAGAAGAAGAAGATCAAGCACAAA
CACAAGAAGAGGAAGAA
GAAGAA
>ConsensusfromContig948 Average coverage: 5.66
TGGCTTTTGACATGAATTTTTATTGATAGCTTTGACATAATGGAA
AAACGACCTGAGGCA
ATACGATGTACGTTGCAAGGATGCCGGTGTGATAGCTACACCCCC
AATAAAGTCGTTCTA
CGGTCATGTGACTCCTGCAAACATGGCTGGGTTCATCATGCTCTG
GAAAAGCTTGGATAC
CGTTCCCCTTTCCAAACCGG
>ConsensusfromContig949 Average coverage: 14.85
ACAACATAAATAAATACTAAACAGGTTAAAATTTCAGGTAAAGG
TATCATAATATAAATC
AAACACTTTCTATAAATGAATCAAACACAGCTAAGGTGATTCATG
CCCATAAATCATGCA
GTTATAGAATGCAGAAAGATGTACGGGACCAAGTTGAATAATTAT

TCAATGTGTATCAAT
CTACATCTACTATCTGAAAAAACATCCCCCCATTAAAATTTTGAT
ACAGTTATGATGCCT
TCTCAAATTATAATTTCAAAAAATACAATGCAGCAATATTTTTTT
AAAACTATAAAATGC
TGTCTGGAAATAGAAAA
>ConsensusfromContig950 Average coverage: 7.43
CGTTGTCCAACTTGCAGTTTGCGCAGCTTTTCTTCGAGGGGTCTT
GTAGGTTTGAGCAGT
CACGCTTCGAATGGTTTCCGGCACAGAATGAACATCTTTCGGAAT
GTTTGCATTCAGAGG
CGATATGATTGAATGCTGAACATTTGAAACACTGCTTGATGTGGT
AATGCGCATACACAA
AACACGCACTACCGAAACCAATGAAAAGTCGAGCTTTTTCCATAA
CCAATCTATAAATTT
TTGTCGAACACTTAAAGATCCAGTCATTCAACCCAGATCTGTTTTT
GTATTTCTTAATCA
CCTCAAGATTTTCATTTTCCAAAGA
>ConsensusfromContig951 Average coverage: 7.54
TCTGGCGGCACCAGCGTGGAGACAGGCTGCGATGTTTTCAAGAT
GGTCGAGAAGAAGATC
GCCGGTGACACTTTCCATGTTGAATCGAGATGTTTCAATGTTTGC
CCAATTCCTCATGCC
AGCTTCACAAGTACATTCGATGATGGATGAGAGAGTGTCCGTAC
AAGACGAGCAATAGAA
TGCTGTCGAGCATTGCTTTTGTTTCACACCCTTCTCGAGGAACAAC
T
>ConsensusfromContig952 Average coverage: 62.23
TTTAAAAATCATGATATTTGTTTTTCGAGAATCAGATATGATTTTT
TTTATCAGAATTTT
TCACCCACAGCCGCCCTTTCCAAGCCATTGGTTACTTTTTTAGAC
GGAATCACGATGGGA
GGAGCGATTTTATTCTTTGGACATGCTGGATACAAGATTTCAACA
CAGAAAACCTTGTTT
GCCATCCCAGAAACTGCAATTGGATACTTTCCTGATTGCGGCAG
ATCGGAAGAGCACACG
TCTGAACTCCAG
>ConsensusfromContig953 Average coverage: 4.95
CGACCCTTTACGTGTACGGATGTCATCAACAATCGATTAGTTTTG
TCCACGTCGAAGAAT
AACTCGCACTCAAAGTCTCTATACGCAGATGATAGGCTAGGACAG
CCTTGACTCATGAGA

ACCATGAAATTCTTCGAACGACACTCAAATGTGCTCCAATCAGAG
AGCGTTGCTCTGCAC
CTCCCGCCATCAGACGCCACAAGAGCTTCGACTAGAAGCGACCC
CTGAATGCCGCAAGTG
TTGAGAGATGCGGTATTGGTTGGGAAAAGTAGCTTGTAGGCTTT
>ConsensusfromContig954 Average coverage: 8.42
GATCTAAGATTCAATAGCAGTATCGAATCTACACAGTTGTGATGG
ATCATTTTAAATAAG
GTTTCATCAACAAGACTTATTATTGAGAAGAACAATATGGTTCCT
ATGTACAAGTTTCAA
GATTTACCCTATCGAATGAACATGACAGTTTTAGGAGACGACTA
GATGAAGACAGCTAAA
TGAAACACGACGGATTTGTTGAAACACCGAAAATTGTCCCGTGAA
CATTGAGCAAATTTC
GGATTATTTTTATATATATTCATGTTAAAAAATTCATGAACGATTA
TTTATTCG
>ConsensusfromContig955 Average coverage: 8.32
GATCTGTGTATTCAGAAGTATTAAAGCAATTTTATCCCAACTGA
AGGCTAAGAGAGAGAA
AAACTCAATACATCGCATTCCGGTGTGTGACAAAAACTTACATA
TATCATGGAAAGGCAA
ATTTCCTCTGAATGACATTAAGCAAATAAAAAATTACTACAGATG
CTCGGTGAACGATGT
AATGACAGCAGCCCTCAGCGGTGCTTTGAGAAATTACTTCTTGAA
GTATTTTGAAAACGC
AGTAAATATAACAGCTGGAATTCCTGTCAGTCTTCGAACACCGG
>ConsensusfromContig956 Average coverage: 4.48
GACAACCCAATAGAGGAGAGTGTGCATGTACTATCCATCGAGCG
GGCTGGAGGAAATTTA
GGAAATCAAAAGCTCTTCTGGCAAATATACGGGCCAAATGACCG
AAACTGTACCTTACTG
TCTACACTCGACATTTATCATTTGGATGCGAATGGCGATCGTGTT
ACTGGAGGGAACCTT
GTTTGGCTTGATGGTGAAAATGGACTCAAATTCATTGAGAT
>ConsensusfromContig957 Average coverage: 7.03
CAATTTCCATCAGAATCAATGATGAGATTTGTATTGTTTTTTCGT
CGTAAAAATATAGAT
GGATGCTGGACATACTTGGAAATGAAATATAGTCTAGTATTTTCA
TATCGGGAAGGATAT
TCCTATCGCTAATCAATCTGATTGAATTATATGACGCCGTAATCA
AAATGTAACCTCATT
TTGTTTCATGAGTTATTTCACATTGGTTCTATTTAGCGATGAAATT

TTGTGTTCTAGT
>ConsensusfromContig958 Average coverage: 4.12
TTTTTTTCAAGAAAACTGCCAACATGTTTTTTTCTTTTTCGGGGAT
AACTCTTAATGTGG
CATTCCTGTGTCCAACAATATGAGGACCGAAAGTCATGAGCTGTG
CTCCTATGTAGCTGT
TGTAAGCAGGCATCTCGGGCTCATTCCAGTAGAACATTTCTGGGT
CAGCTTTGAATAATT
TTCCTAGCAAAGTTGAGCCAGATCTCAAATACGTATACAAGATGA
TAACTTTGGGATAAG
AAACAGCTTTTTCTGTTCCACTCAATTTCTT
>ConsensusfromContig959 Average coverage: 8.14
TCCGACAGCTCAATGTATTCATTATATTATTGGAGAGTGATGTGT
GACGTGGAGATCATA
GCAATAGAGTTTCGGCCGCATGCCTTCACGATGAGGAGTTAAAG
AGTCAGCCAAAGCACT
GTTCCAATTCTTTGCGATCTCCGTATGCCCTAATTCTGATCATCCC
CGAAAAAATTTGTG
ATAGAAACTCATTTTTGTGGATATCACTCAAATAGAATGTGCAAA
ACTGTATAAACTTTA
TTAATTACTACAATATATTCCTAATATGAATACGTGGATTATTTTA
CATTCTTTCCTGTT
TCTATTTTTGCTATACTATACTGGAATAATGCGATCGGTAGCAGA
GGCCTCGC
>ConsensusfromContig960 Average coverage: 4.59
CTCAGAACCTCATTGGAAACAGGAGCAAATGGTGGCCTAAAACT
CACAATCAACATTGAG
GAAGCACAATATATGATGGGGCCTCAACTCGACACAGGTGTTAAG
GTTGTCCTTCATGAT
CAATCTGACGTGCCCTTTGTGCGTGACCTGGGATTCACAGTGAGT
CCTGGAGTTCACGCC
CTCGTTGTAGCGAAACCGAGTGAGGTACGCAATTTA
>ConsensusfromContig961 Average coverage: 8.67
ATAATGCAAACGGAGTAAACATGCGAAAAAAACTGTTGCATATT
AAAGCTTGGCACGACT
TTTGCATTAATAAAAAAATTCACAAGTAACATATGAGTTAAATTAA
AGCGTACACATAACT
AATAGTAGTGCATGTGAAAGTATAGGCGAATCAATGCAAAAAAA
ACAGTAATTTACTAGA
ATATAAGCCTACCAGATGATGCTTATGGATATTCAG
>ConsensusfromContig962 Average coverage: 14.53
CGTCTTGATCGCTGTGATACGAATCACTCGCGCTGTACGTCCCATC

TTGTTTCTTCCTTC
TCCTGCGAACATGTCTTGTTCCTCCAGCGCTATGAACGCTTTTGTA
GCTAAAGTCACTTT
CACTATTACTATTCCTCCGACCGTCTGCAAAGGCTTTTTCTTCTC
TTTTATCGCAGCGA
CTTTTCGTTTCTGTCTTCTGTTTTTGCGTCTGGCTTTACCATCTTCA
TCTT
>ConsensusfromContig963 Average coverage: 5.21
CGGCGATATTCACAGGGCGGAAGCCTTGGACACTAAATTCACAT
AAAAAAAACAGAGAAA
AGTAATCAAGATGGTTACGACAGATCTTTGGTCCACGCTGTGCAT
ATAAAACAACCTGGA
GCGCACCAGACAGAACGAAATCTGCCAGTACATACGTGTGCGTG
CACCCAATGCTTTACA
CAATGGGGTACCTTATTTCGCATTGTTCGGAATCAGGCAGTCTAA
CCTGAGCATTCGACC
AAACCACGGCTACTCTCCCCACCACGCCGCGCGGTTTCATACGGG
GAAACGTTACCGTTT
AGATTAGACGAGTTCATAGATAAGTGACCGCCAATATTGCGCTAC
CAGCCGTATCATTAA
TGTACATGGTGGGTAAGCAACCTAACGTATCAAGTGTGGAAGTTC
T
>ConsensusfromContig964 Average coverage: 8.33
TTTTCATTCTTGTCCGAACGTCCGCGTTTGGGATTAACACCTATA
CTGCGTGCACCATGC
ATAAATCATGTATAGCAACCCATTAACCGACTGGTCGCTAAGAC
ACAGCAGGTAAATATA
TCAGGGTGTGATCCCCGGATCTGTGGTGTATAATTCTATTGAAAC
CATTTGGAAGGTGAC
GGTCTATTAAATCGATAATACAAAAAGAACTTACAAATTCTTCAA
CAACTCACAACTTTG
ACCGTTTTCTG
>ConsensusfromContig965 Average coverage: 4.16
GGCAAATGCATCGGGGTATCCCGCCTTCTTCTATGCAGACACCAC
CCCCTTGGCACGAGT
CAGCTTCGCATTCCAGAAAGGGGTCTGGTTTGGAACAGTTGCTAC
GTTTGTTATCCGGGA
GCTGACAGACGCATGTGAATTCTTTTGGGTTTTGGGGTTCGCACA
TGGCCATAGGGTGAC
AGGTATGCCCCTCGCATGCTCCGATTATATATCCAGGAATCTGCC
AGAGGTCGTAATAGA
TAAAATCGGGAGCCAGTACAAGGCCCGTCAC

>ConsensusfromContig966 Average coverage: 9.49
ATCTCCGTAAATTCGAAGTGTCGCTCCTCAACTCTAATACCATTT
TCGGAAGCTTTACTG
AAAAGATCTTTGATTCGCTGTTCGTCCTTTGAGAATGATGACTTG
TAAAAGGGTTTGTTT
TGATGCGATTCATCAACTCCAGCCAAAATAGTGGCATAAGAATGG
TCAATGTTATTGGCG
CGAAGAAGGTAGGATAAGTCCATTGTCCACGTGCTTGAACCGATT
CCAATGTCTTGCATT
TGTTTAGTCAAATTTCC
>ConsensusfromContig967 Average coverage: 8.39
GATGAATTATGTATTAAATTGAAGGTAATCATTCAATTTCGCGCC
TGATGAACCCATTAT
AGTTAGGAGTGGCTGTTCACAAGTGAATAAGTGAACTCTGCTCAG
CACACACCGAGTTGC
TCTGCGATGCAGAGTTCATCCATCAGTCCATGAAAATTCACAGTT
GTGCGGTCGTCATTC
GGCTCAGCACCGATCACCACTGGACGGTCGGGTTTCTTCTGCTCG
TAACACGTATTTTCA
TCATCAGAAAAGCTTTCAGAATCACCGATTGCCACCTCAGAGTCT
GTCCCGACGAGGGTT
CCATTGAGAAAGAGGTGGATCTCCTTTTCCATGCTGTCAAACTGA
GCCCTGACGTGATTC
CAAACTCCTTTCGCAACCGGTGTACGAATGAACAGATGGTTCAGA
CAGCCGTCTTGCACG
AGGTAGAATTTGAAATCCAGCTTCTCTGAAGACGCACTGTAGATC
AGGTTTCTTGTGTAG
AATCCAC
>ConsensusfromContig968 Average coverage: 7.45
CTCTTCCGATCTCGGAATCAAACAGAGTGGGAAAAGTTTACTGTT
GTACAACCATTTCTG
ATATATTCGAGTAGTCACATTTCTGAATATTATTTTTCCATTAATG
TTTTCAGCCTCCAG
CCGTGCCGGGCACCATGAGGCCAAATCAGGCGAAGAAGGCCTAA
GGCAGCCCAGAGGAAG
GGGCGCGATCCAAAGCTCAAGTGGAAGGCTTCTCTATGCGCA
>ConsensusfromContig969 Average coverage: 8.30
TATCCTATCACTTACCAATCTATTCAATCAAGATACAAGGCCCAA
GAAGAGAAGTTCAAT
AACTTCTACGTAGGGGTCAATCGTCGAACCCCAAACCAAGGCAAT
GCCTCCTGCATTTCA
GGGCTGTATGGCCTTCGAATCCATGACAGAGTATTGAACAAAAC

CGAGATACTGGATATA
CAAGCGAAGGAAAATGAAGTTTGCGTATCAGTTGAAGATTGTTT
GGCGATATTTGAAAGA
AATATTAGCGCTCATATAATGATTCATGGATCAGGTCTTTGTAAA
ATGTATACACCCATG
CATCCAGTGTTTGCTTACTATAAATTCTACTTCCAATCCAG
>ConsensusfromContig970 Average coverage: 7.60
GAAAGTCTAAAGATCAGCTCTGGCAGCTTTGATGGAAAGACCCT
GCCAAGCGAGACTGGA
ACCGTTACTGGAACTGCAACTCTTCCTAGATACCAGCCTACTTTT
CTGCACCAGCCAAGA
TCAGGTGAACAAGCGTTGAAGCCTCCGCCAAGCGTTCGACCGAA
GACTAATAAAGAGACT
GGAACCGTTACTGGAACTGCAACTC
>ConsensusfromContig971 Average coverage: 7.75
TGTATTCATAATGATGAAAATTATCAACCCATACGAAAATAAGAT
ATTTCAGTTAATAAA
AATAGTGATCTAGTCACTAGTAGAGGCTCCAGGACCCTATTGCAA
TTAATTGTGATAAAG
TAGTGAACATATAATTTGAGCTAAATATAACACAAGTTTGTACCT
CGGTTGTATCGTAGC
AGAAAAAAATGAAATAAAAATTCAGGGTGATGTGAAATAACTAA
TATTCA
>ConsensusfromContig972 Average coverage: 8.50
GCAGATTATTAGATGTTCTTCGATTTAAAATTAACTCATATTGAA
ATTATCTCCCAAACT
CAAACCTTGTATTTCAGGTTTCTACGCCTTTCCCCTGTCAGTTTTT
CAAAAGCATTACTG
TTAAGTTCAGCATCAATGCGTCCGAATTTGAAGTACGCGAATTCG
CCGTTTCCTTCATAC
GGAGGTTTGTGCTGTATCTCGAAGTAGTTTTGAGTCTCG
>ConsensusfromContig973 Average coverage: 5.75
TCTGCCTGTAGTTCCTCACCAACATTGGTCCATTTTGTTGCTTTCT
TGAAGGGCTCTCCT
TTACCGATTCCAGTCTTTCTTTCTCTTTGCATATTTCTTTCCAATC
GTATAAGAACCGTA
GAGTTAAACCAACGATAACAGCTGTTACGTACAGTACGATATTCC
ACATTTCAAAATTAA
TCCATGCGATTTCACTGCCAAGCAGCTTCTCCAAACTGTTTGTTTC
CATCAT
>ConsensusfromContig974 Average coverage: 5.82
CCTCGAAATAAAGTTTCATATCTTTTACATCGCATATTCGAACAG

GAGAGTGAATGCACA
CAACGTCAGCGTCAGCATGGAGAAACCCGGCATCACAGAGCTTC
CTGGCTGCAGCAAAGC
TGGTTTGCAACTCAAGGTCGGTTCTTCTCCAGCAAGAAATGCGAT
CGCCCCCGTCAGGGC
TTCGGGTGCCCCCTCAAGCACGACACTCGCAGTTGAGCATTGGGC
AAAGCTGTTCACACA
AGTCCTGTAGATAGCCTTGTCCCCCAGATCG
>ConsensusfromContig975 Average coverage: 5.57
TGACAATATCGATAATTTTATGCTTCAAAGCTCCGAAAATCATGG
AATTGATGAAAATTA
CCAAAAATATCACAAAATAAGCAGAGAGACAAATGAGCAAGCAA
ATTCGCAATTACAAAA
ACATGAATATGATTCAAAGAGCACAGAACTGTGTAAAATCAATAC
TTTTCGAGATCGTTG
TGAATTGAAACAAGAAAAGAATCATCAATCTGATGG
>ConsensusfromContig976 Average coverage: 4.82
ATCAAATGGTACGATAGGTTCAAATGCGTTCGATATGAATGAAA
ATGAGAGTATACACAA
ATAACAAGGCATCATGAAAACACGGTTCATATGTATCTAGATCTG
AATTGAATGACTGCT
CGTTGCCGACGTATTTTCAATAGTTTACCAAACAATATTCGTACA
TGTAATATTGCAATA
CCTTCAGACACAATATGACGGATTGTTATTATTATACTTTCAC
>ConsensusfromContig977 Average coverage: 10.86
CTCAGATAATGTTGTGGAATCTTTTGAAGTTTTCAAAGAAGAAAA
TATTTCTGAAGGCGA
GAGTGAATTGAAACATAACATTTTGCCAGAAATCGATTCACAAAA
GCAACTTGATTTATA
CTCGACAAAAGAAGAACACATCAAAGAAGAAGACCTCGAATTAG
ATACTAATTTTGATCA
CAGTACATTTCACCCGGAAAATTTTAAGGCTGACAAAATTGAGAA
AAATCGTTATAAATC
AACCGA
>ConsensusfromContig978 Average coverage: 6.21
GCTCTTCCGATCTGTTTGATACACTTTTTAAAGGCGTCAGAACTC
CAGCGCCCTTGCGCT
CTTATTTGGGCATCCGAAAACCTGCGACTTGCTGCCATTGTTGCA
CCGCCGATTCCGAAA
CTATGGCTCCTATAACGCAAGCTGTCCACGCCACATAGTTTGAAG
GTTTTCTGAAGAGCG
TCGTCAACCTGCTTCTTTTCAACATCC

>ConsensusfromContig979 Average coverage: 9.35
GACCGAATAAAACATTAGAAATAAAATTGACTTTAGAAACTATTA
TATGAAAACCATTTA
CACATTTAAGAATCAGTATCGCAATCATCCGTTCCCGCATCGTCG
AATTCTGAATTAGCC
CCAAAATCAGACACACAACGATCAGACGTGAAGACTGAACCAGA
AAACTCAGACATGTTT
CTTGTCGTAACTGTATAGGAACTTGGTTCGCC
>ConsensusfromContig980 Average coverage: 6.38
CCGGGATTTAGGAAACCGGGATAAATGAGCAGTTCTGTATTTCG
ACCTGGGGAAACACCT
TCCGCACAGCCTTCCATGTGGATTTCTAGGAATCGGGAACGACCC
TTCAGAAGATGGATG
CATCACCTCAATCTGAACTTTAGCATAGTCGTCTTTTTTCCGCCAA
ACATCTTAATTATT
ATCGAGGGGACTTGTTTCGTCAGATCGG
>ConsensusfromContig981 Average coverage: 9.03
ATATTTTGTTCAAAAAGAAGAAAAAATGTTGAAGGTGATGAGAA
CGCTACAACTGAAAAA
TCTGGAATCAAACGGATATTCAATGATGATTCCACAACAGAGCA
GCCAGTGAGAGGAGGC
AAGAGGAAAACAAGGAAAAATGTTGAACATGGACAGAAACGAA
ATAAAGGCGGTGGTGGT
GGTGGGGGGGGACACCCCCCCCCCCACCC
>ConsensusfromContig982 Average coverage: 5.62
CCGGCCCCGAAAGTAGTAGGTTGTTCAAAGACTGACCCTTGGAT
TCAGCGGCACAGTCGA
AAACTACTCTAATTTTACCAGGCTTGTTCGTATGTCTAACCCCGT
GGTGAGGCAAATACC
ATGTCCTTTTTCCTTGATGCTTGTGTTCCACCACCTCTGCATATCC
CTTTTCCACGTACT
CGTTCATCGCTGCAACATATTGATCTCTATACTCATAATCTCTTGC
AAATCGTTTACCCA
GAGATTCTAA
>ConsensusfromContig983 Average coverage: 6.17
AGAAATCATTCAGACCATTGATGACTACTGATGTCAAATACTCAA
ATCAATCAAATGATG
AATCTGTCTCAGAACTAAACTCAGCCAAACAGGAGAACGTTTCTT
CTTCTATGATAACAA
TCGATGTACAGAAAGTGGAGGATGGTACAATTCCAGAAAAACAC
GTTGCTCCACAAATGG
AAAGAGTGATTGATGAAGTGCCTAGATCGGAA

>ConsensusfromContig984 Average coverage: 7.52
TAAATCGACTGTCGCAACAGCAACAACCACTCTTGCACCTAGTAG
AAGTACTGATTCACC
ACAACAGGAAGAAAAAACAACCCTTGGAATACCCATAAAATTAT
TATGGATCCCATATTT
ACTGTTATTCATGTTCCTGATAGGAATTGTCGTTGTTTCATTCTAC
AAATATCATCAGCG
AAATGGTCACAGATATCATCAGAAAGCTGAGTCACCACTTACGC
TCAAGAACCGCTACGA
ACCTGGCGCAGCCACCACGCAGGTCGAACTCTGTGATATGATCTC
AGGAGTGCCTCCAGC
CAATCAT
>ConsensusfromContig985 Average coverage: 9.49
AAGTGAGTTCTATTCCCCGGCGTCGCCTCTTGCCTGGCTCGGGGA
TGCGGTCCAGCATCC
GAGCAGTGGTCGAATCATCGGCGGTTTCGAAAGGAGAAAGAAA
TTTAATGAATTTGACA
GGAAATAGTAGAGATATGGAAGCGTTCATAAAGTATTCCAACCA
GCTGAAAGTTGTTCAA
GTGTTATGACTTTTGTTCTAAGTAATAATAGTAGCAAACTAACGA
TAAGTTTTGTAAGCG
TAGTAGTGTTAGAATAATCAAAACAAAGTGCTTATTTT
>ConsensusfromContig986 Average coverage: 3.99
TTTTTGTATATGATAAATGATTTATTAATGTTTATGTTTTCTATTT
ACCATGCATCACAG
CCTAAAAGTATCAGAATGAAATGTGAAAATCAACTCACAACTTTT
GTGAATCTCATCTTG
GTGAACAGTAATGGTTTCAGAAGTTTTTGATTCGAATTATTATAT
TTATTACTTGTGGAG
ATATTTGATTTAAAGATTCATT
>ConsensusfromContig987 Average coverage: 10.55
AATAAGACAATCTCGCGCCCAGACACTCTTCTACCGAAAATGAA
AACCAAACTGTAGAAA
ACTTCACTGCTCAAGCTCCTGGAGAGTATCAACTTCAATTGGATT
GGGACCGCCCCCTGC
TGACCTACTTTAATCAAACTACATCCATAGATATTCTCAAATGGG
CTTATGAAATCGAGT
GCTTTCCTGCCGTTTCTAATGTGCCAACCTTGACCCGCACCATCA
AGATCG
>ConsensusfromContig988 Average coverage: 6.25
AATATTTGATAAAAATAAATACAGAATCGTGTTTCTTTCTGTCAA
TTATACATACAACAT

GTGTAAACATTTTTTTGTCAATCCATATTCATATGAAAATCTATTA
TTCACTCGTAATAC
AAGTAACACGAATGAATTCAGATCCTTCCATTTGCTGTTTTTCAT
GACTTTTTCAAAATG
TTATATCAAACAGCCGAATAGGTTGATATATTTAGATCGGAAGAG
CGTC
>ConsensusfromContig989 Average coverage: 122.00
GACTATTTCGCTGACTGGACTGGTTAGATTTTTGCTGAATGAGTTC
CACCCCTGGCTTTC
CTCCTCGATGGTCTGTTGTCGTAACAAATAAGTGAGTTGATTTTGT
TGATTTGTTTTGAA
GAACAGCTTGATTGTTAGGACTATTATTATATAGCGTTGATTGTTT
TTCATTTGTAGATT
GCTCTTCTTTCTCCTCCAAATCTTGTGGTTTTCGCTTACATCGCCT
ACAGCACATGCGAC
AAAGAAGGTATGTGCCTCTGTAGAGAAACCGGAAAGCCTTCGCCA
TGAACCTTCCCATGT
TGGATAGCGTTAAAAGAGTCAGAAGATCGGAAGAGCACACGTCT
GAACTCCAGTC
>ConsensusfromContig990 Average coverage: 4.29
CAAAACTGAAGGAAACTCCTCGTTCCATACACGCACTTAGCAAG
AGGGCCAGAACGAAGA
TGACCCAACAGCTGACCACGATGATGTAAACACTTGGGTTGGTG
TAGAAAGCACGCAGAG
CCTCTAGACCTGTCAGTAGATAAACGAAGTTGGGTAGATAACTCC
TTCTTCCAATAAGTA
ACAGTGTGTGTTTTTCGCAACTGCAAA
>ConsensusfromContig991 Average coverage: 8.65
AAAGAATCATGTACGGAAGCAGTTGATGCTGATGTGATTGAAGC
TCCAGGTGTGTCAGAT
GAAGAGCTGCATCTTGCTTTCTTCTGCATTTTTTTCCGCTGCCGTC
TAAGTCTCTTTTTA
CGTTCATATGTTTCGTCTTCTTCTGGGTCGTCAAGTTCTTCTGCGG
ATATTTCGCGACGT
TGCTCATCCCCTGATTCATCTTCGAATACGT
>ConsensusfromContig992 Average coverage: 6.96
ATCATGTCAATTATTTTGAAAAAAATGTTTACAATACGATAATTA
CGTTTGACATTGATG
CCGCATGGAATTTCACGGATACATGCACGATAAACGATTCGCACT
TGCATTATTTTAACA
GCGATTAGCAAATTAAATTTCAGTAGGGTATATCTAGCGTTTTTAT
CGCTGTTTTCATTT

GCGTTTTCAATTCTAGCTCCATCTTTTTC
>ConsensusfromContig993 Average coverage: 8.36
TTCGGTAGTTTTCGGTCATTGAAAATTATTTACACATCCAGAAAA
ATCGACTAAGGTTGA
AAATAACTGTCTATAAAACCAGAAGTTCTTTTCTTGGTTATACAA
TGAGACAATGAATAC
AATTCGTAATTTTATAATCGATAAGCACAACATTTATATCGGAAT
TCTAAAACGATATAC
ACGCAACATACAAAACAATCC
>ConsensusfromContig994 Average coverage: 32.26
TCTTTCCCTACACGACGCTCTTCCGATCTGGCCGATGTATATGAC
ATGTTCAAGATATAA
CAATGGCCAAGTTTGGTAGTGTGAAAAACTACTTTGAAGCTCTTT
CTCTAATTTCTTTAA
TTTCTTGACTTTCCCTTTTTATCATGGATAACACTTGTTCTTTGTA
CGTAGTGCATGATG
GTGGTGAATATGGATATTTGAATTTTGGTTTTATATCTTGAATTTT
TCTGGGTTTGATCG
GCGGTTTTCTCTTATGTTTCGGTGCTAACCATGTAACTGGTTGTAT
TTTTTCTTTATAGT
TTTGAAGATGCTTCGCCAATGTGTCTTTTGCGCGATCCAGTGTAG
GGTCCCAGTATGGCA
CCACGTCATTCCCAA
>ConsensusfromContig995 Average coverage: 6.82
CTTCAACTTCCGAGCAAAGTCCATGACCGCAACCGGATATTGGA
CAGGGATCACAATGCG
GCATGTGCCGATTTCCTTGAATGCATGCTATTTCTAAATCAGTAC
ATTGAATCGTAATAG
CAGCCTCACCACCTTCTGGAACCCACACGCGTCCGACTCCAGGCT
TGAAGTAAGTATTTC
CAGTGTACGGAATGCTGGGATAAAA
>ConsensusfromContig996 Average coverage: 5.32
CGTTGACTAGATAATCAATGTATTGAATAGAAATGTATAAGATT
CAATGAAGAATGTGAG
ACACGTTATATTATCAAGAGTTTTTCAATGTATCATTAGCAAAAC
TAAAATGAGAAACGA
AACACGTATTCTCAATTAGCTAATCTCATGTCAAGACGATACAGG
TTCACTTGAAAATGT
CAAGTTTACAGAGTAATTGT
>ConsensusfromContig997 Average coverage: 16.70
CACTAATACGCTGATAGAATTTGATTTGTTTGGTGACTGCTTCAA
ATCATCAATCCATTG

AAAAAAATTCGAATGAATTATTCTTGGATAACAGAATGCCATGAT
CACAAACATTACTTT
GTACCAATTTCTCACCATACGATACATGTGTAAGATCATTTAACA
CTTCTAACATCTTTC
TTTATCATCAACATAACTTCACTCTCTTTTATCATCCCTGTGTTTTT
CTTCATCTGTTTC
GCCTTCTCTAATTCCGGTGGCACCAGGTCTTTATCATCAACATAGC
TCCAATCTCTTTCA
TCATTCCTGTGTTTTTCTTTATTGTTTCGCCTTTCTTAATTCCGGT
GGCACCTGAGGTGG
GATAAGGCTCTTATGAATGTATGATACTAGATTTGATATTTTATTT
CAGTTTGGCATCTG
CATTATTTTACTTATACATTATCGTTTAAGTTGGAAATTTTGAGGT
AAGATTCGGCTTTA
TATCATGTATGATAAGGGATATGCAAGT
>ConsensusfromContig998 Average coverage: 6.64
AAGACGCATGGGCAAAGACCGGTGCGACACGGACCCGTCGCTAG
GCACGACACGGGATGA
TCCAATCTTGATCCGCTTGGCGACAATCAAAAATCTGCAAGCACA
ATGGTTCACGCTCAA
ACACAATGGAGATGTATTGATTGTAAAAGTATTGTTTATTCAGAA
TCTATTTTAAGATCT
AAGCAATTGACGCCTCTGAAATTTTTGTCAGTAA
>ConsensusfromContig999 Average coverage: 7.01
ACCGAATTTACGATTTGATTTTTTTGAAGTCCATGATGGCTCAAA
GTTATGAGTCGTCGC
CATTGTTTGAATCCAACGTCTTCTCCGGTTTATTCTGTAGTATGCC
ACGTTTTGAGTATG
CCAAGTTCCAAGTGTGAGCCAAAGTAGACGAGGTCCATGTTCGGT
ACTTTATGGCTCTAA
GCCAACGGTGATGTATCAACCCGGCGCACAGTTTAATGTCATGTT
CGGGACGTTGACGTT
TCAGATTACCGAAGTAATCATTGGATGTCATATCCAAGATAGTTT
CGTGTCCACCACGGG
AATATCCATACCCTACTTTGC
>ConsensusfromContig1000 Average coverage: 4.82
ATGTATTCTATTCGTAATAATGATCAGAATGAAATTCATTTCATGA
TAAATTATTTCATT
TGAATTTTCAGTTTCTACCTTGAGTGATCAAATATGTTGAATGATC
ACAAATTTCGTATT
GCCCATTTGAATAATCTGTTCACAAACATCATAATATTTCAGATT
TCTCATATATGAAAA

CTCAGTATATCAAATAATGTT
>ConsensusfromContig1001 Average coverage: 7.25
TCGCCCTATTTTGTTATTCCGCTATATAGTACATATTGCTATATGA
ATTTTGTGTATTTA
TTCTGTTATTTCGCTTTTCAATAATTATTGATAAATGAAGTTTCTG
TGAATTCACTGAAG
GTCAAATATAACGTTGGAATTGGAATAGAAATGTATTCAACATAA
TTCACAATCCCAATC
TTTGTCATCTGTAACATTGTTCAATAGTACGAATCGACCAGAAGG
CTTTGCGAGGATTGA
ACCGGACCTCCG
>ConsensusfromContig1002 Average coverage: 7.62
CGGTGGAAAAGTATATCCGGGAATTCCAGAATCTGATACCATTAT
CAAATTTTACCAGTA
GAAAAAGAGCCGATCGAATTCTCAGAAAGGCTTACTAATACGAA
TCTGCGAGGAAGTGCT
ATTCTCCGGGTGGGGCAGTGATTGGGTTGGTGAAAGACTCTCAGT
GTGGTAACCGAATCA
ATACTATAGTGAGATGACATTTGAAAAAAT
>ConsensusfromContig1003 Average coverage: 6.31
CCTTCATTTAGGTTTTATCTGAAAAAAAGAACCCTGATTTTGAGA
CATCTGGAATTCAAG
AAAAAAGAATTGAACGCATTGATAAGGCAATAACATCGGAAAAT
TCGATTTTTCATGATC
CCGAAATATGAAATTTTTCTTACATGACAAAAGAAGTATCAAATA
TTCATCAAAAAACCC
ACATCAAAAATATAATGGAATATTTT
>ConsensusfromContig1004 Average coverage: 7.81
GCTCTTCCGATCTGCTTCAAACTTCATTTCCTTCTTTATTTCGAAA
TCACTGATGATGGA
ATGAGAGTGCTCGTTGAATACATTCCTTACACCATCCTCCATTGA
GCAATCAGGGGGAGA
CGTCGCTTCAGATCTGAGGGGGTCTGTCCCCTCTTGTGCTTCTCCG
TCGATGTCTTTTTC
ACCACGTTTCAAAAATTTTGGATTCAGTTGCTTTCGTCTTGACAT
AATAGGCGAGAGATC
GG
>ConsensusfromContig1005 Average coverage: 6.57
TCCCTCGACGATGAAGTGAGTTTCAAACAGGTTGATGAGTTAGAA
CAACCCCTGTTCAGA
AGGACCTCACCTGTTGTTCAACCCAAGACATTTGAAGCTATCATG
TCCCTCCCCCGCATC

GAACCAATGAAATTTCACGGTGACCCTTTAAAGTTTCAACTTTTC
AAAGCGAATTTCAGA
GCCTTGGTAGATACAAAGGATTTATCGGACGGCCATAAATTAGA
TCGGAAGAGCGTCGTG
TAGGG
>ConsensusfromContig1006 Average coverage: 3.40
ATAGTTTCTGCTTGCTTATTCATAATCATATGCATATCACCCTAGA
TTGCTCATATTTCA
TAGTATTTACAGTACTTTCATCCATTGGGTATTTTACATGAAATAT
CGTCTTATTTGCGT
ACGAGCAAAGATTTCTGTTGATATCCTTTACTTATAATTTATTAT
ATGGTATTGATCGAA
TTAACAAAGACATCATTCGCT
>ConsensusfromContig1007 Average coverage: 10.58
CAATGGTTCAATTGGTTCCCGATACAAACTTGATCGATCATTCCT
TTGAAGTGATAATGG
CCGATCCTGATTGCGCCTATATATGTTTTGTGGGCCGATGGTTGTT
TTCTGCAGCCACCC
GGAGCGTTGTCGGTCTCAGCAAGTTTCCCATCAATGAAAATTTTC
AGCTCAATCCGCTTA
TGGTCAAATTCCACCCTGACGTGG
>ConsensusfromContig1008 Average coverage: 6.83
GTGATATCATTCCGATAGCTGGATTTGATACCAAAGATTTCCGAT
GTGAATCCAGCATCT
TGCACAAGTTCAGAATTCAGAGCAAAGTTGTAGTCAGTAGTGCTT
CGTTCGATCTTCAAT
TTACGGCAACTATTCCGATCTTTGGTAAACGTCACATCGAGATGA
TCCAAATCTACACAC
AATCCGTCCTGTGAGTCTTGCTCGATCTTATATGTGACATAGTCT
GAAGTTGCA
>ConsensusfromContig1009 Average coverage: 4.12
CCTTAGATCATTTTCTCTGCTAAAATACAAACGCTTAATCCTTCC
TCTCATAAAAGTGAC
ATCCGATTGATGAATTTTAAAGTCCGAAGTTATGGCATTTGAATC
GTTTTCCCATATGTA
CATCATTTCATTTATCTGACAAAAACTGTCAAACGACTCCAGGGC
ACCTGAAAGATTTGC
CTTATAATGATGCATCAGAGAAACAGTTTCATCCACGATATCTGT
TAGGACTT
>ConsensusfromContig1010 Average coverage: 6.25
ACGGCATATACATGTATTAGGAACTTCTACAAAACAGAATTTCAT
GCTGACTGCGACTGC

GACCTTTCCTGTATGAGCGAGCAATTTTTCTCAGAACTGTCTCAT
GCCAGACTAAGCGAG
GCGAATATTCCCAAGCAGAAAAAAATTCAAAAAATTCTCAATCA
TGCCATTGAAGTAAAA
CATCGTGTCGACCCTGAAATTGCCGAGAAAGATTCAAAAGTCCTG

>ConsensusfromContig1011 Average coverage: 5.86
TCGCATATGTATTTTTGGTATTGATGTGGGCCCTAGCATATTCTTT
GGTATAGTCACTAA
TAGCTTCCAGTATCTCAACTGTGAAAAAGAGTTTGAAGAACTTGA
GACCTGTTGTCATAT
GATTCACACTTTCACGGGTTATGAGTTCTTTGAATTGGCATCCTG
AAACTAGAGAATTTC
AGTGCTGCTGCATTTGGTGCACCATCATCGACATCATCATTAG
>ConsensusfromContig1012 Average coverage: 7.25
ACGTATCATCATTTTTTCCGTGATTAGATATAAAGTTTAAAATAT
TGTTACCATGATTAG
TGATGCTGTTATACGGTCGGCTTTTTATATGAACTTCCAAGCTATC
TGTGATGTTTGTGT
CTAGTCTATTTTTCTAATTGTTTTATTGCTATGTATTTTATATTGT
ATTTATATGCGGGA
CGGTCGATATGAGTAGGAAATCTCGCATTAAAATTCAGCA
>ConsensusfromContig1013 Average coverage: 8.02
TCAGAATTCAATTTTATTCAGATTTATTGAGAAGGCAAATGAGTA
GATTGGGTCAATCTA
TAATAAACAAATTCTAACCTGAAATTTATTGAATTTATTATATTGA
CCGTCCTTGGTCCT
TGTATACAATAGATAACAAAGAATAACATCTTGATAGAATGCAA
ACAAGCCTGTATTAGG
ACGTCAGAAAAGATGAGTAATGTTTATACGGACACAAGAAAAAC
CTATTACCATGGAAGC
TTGAGCCAGGAAAACATTCAATGTATT
>ConsensusfromContig1014 Average coverage: 11.18
ATTTGTACCAACACCATATTTGCGAAAACATTGTGAAATTTCAGA
AATTAACGGTAGTTT
TCCCTGAACTAGCCAAATTGAGTGAGTGTTTTTTCGAGTCTTCATT
TTTTTGGCGACGAC
AATGTCAGATGTCGAATGTCTTCGCCTCCTTTGCCGACGACAACT
CAACATCAACAAATC
TCTCCGGAAGCGCCAGATGGTGGAGTCGCCCAACACTACCAGGCT
CGCCAATTAATTTCG
CAATACTCTCCACTGGGTGGACATACAACCCCATTTCCTCCTCAT

CAAACGACGCTCCGG
CGGTCACCGATTCCACATCCCACCCAGCCTTGCACATC
>ConsensusfromContig1015 Average coverage: 9.17
AGACATTATTGATTCCAGAGTGCCTTGTGTATGTGAATTGAAAAA
TGATAATGAACATAA
TTTTCTGAAAAGTGAATATGAAGATACGCATATCACATCATTTCA
AAATGGCAAAGAGAC
CCACTCCATAGATAAAATGGCCTTTCCAACTAACTATTGTCCATC
TCCTACGGTATATCC
TAATCTGTTCACGACCAAAGACAAGAATCACATATGGGAGGATAC
CATATCTGCTTTCCA
TGACACTCGAAACATTTCATCCGATAACAATAATTCAAAATACTT
TTGTCGAAGCAGAGA
CAGTTTGTCTATGTTCGATTCGAAATATAACAAGATCAGGCCATC
TGACAACGACTATTT
GTTGTATGGCATTCGTAACGAACAGAATATTCCGAGCATATC
>ConsensusfromContig1016 Average coverage: 5.60
ATCGACAGACACAAAGGACGTGTAGAGCCAGAGGACTTTAGGGC
ATCATACATTGACATC
GCTGAATTCATAATCAAGAGTATCGCGCAGAGGAGTGTGAGGAA
TTGACTACGCCGCGAC
TTGCAGAATTCGCCCGAAGCAGAGCGAGTGTCCTCGGAACCGCC
ATCGCTGCTCCAAGCA
ACAAACGGACGAAGATATAAGCCAACAGATCGG
>ConsensusfromContig1017 Average coverage: 5.33
AACATGAAAATTAAAAGTAGAATAAAAGTCTCTCCGAATAACGTG
TTTCTTATTTCCACT
GAGAAAATAAGATTATTTTGTATGTTTTTGTCTTGAATTATATTTC
TTTCCTTGCCTACT
TTCCAAAAACAGAGAAAAATTACAAAAGAACAAAACTTCCTTTG
GAGACCAATGTCTCTC
AATAGAGATAAATTGGTCTTGTAGAAAAATCAATTTAATATAATA
TTCA
>ConsensusfromContig1018 Average coverage: 5.36
GAAAAAAACAGAACTTCCGTTGTCAGCAAATACTAGAAAAACTC
TAAATAAATTATTACA
AATTAATTTTTGTTTCCATATATTATTTCACACCAACAATATTTTT
CACGACTACAACAA
ATCAACATATATTTAATCTTTATGCCATAAAGTTTGATATATAAG
TATGCCAATAAGTGT
TGTACAGTACTAGCACATATG
>ConsensusfromContig1019 Average coverage: 5.22

CTCTGGATATTCAATAACTTTTGAAGACTTTGCTATTCTCCCAAA
AGCATCCAGAAGAAA
TGGCCACATGCTATTTTTGTCAAAGAATGGGGTCGTAACTTCCAA
GATAATAAAAACGTT
GAGATGGTAGTATCGCAGCTTGAAGAAGCATTTCAAAGTCTAAGC
TTAATATTCATTCAA
CACTGATGTTCACAAGTTTGCGTAAGTTATCACTCACTTTCAGAT
AAAAATTTATTTCTG
AGAATTTCATAAAGCCTACATCTGATCTATG
>ConsensusfromContig1020 Average coverage: 5.28
GAAGACTCGCTAGTGTGTAACATCATACCGAAATATTGGCCAATA
ATGAGATACGTCTTG
ATGAGCTCGACCTTTATACTCTTTGTTATTCCCATGGTGCTCATAA
CTGTCCTGTACGTC
CTTCTGGGAATCAAGCTCAAACACTCCATTCAAAGACAGGAGTC
CACAAGAGGCATGAAT
GCCACCAATGGGAACACCAGCTCC
>ConsensusfromContig1021 Average coverage: 11.15
TTTTGACACAATAGGTGGCTACCTGCATAGATTTTTTAGAATTCTC
CATTTTTGCCACCA
GATGTCGCTAGATGTATTGGAATTTTATAGATGGCGAAATTTGCT
GTTTTCTTGGAAATT
GTTTGAAGGGCGTTCGAAGGAGTTTTACGTATTTTTCGAAGGAGT
GCTTATTTTGTATTT
TAATGACATAGTTCGAGGTTTTA
>ConsensusfromContig1022 Average coverage: 4.24
TTTCGGTTCAACGACTTCGAATTGTTGAAGAAAAACAGACGATA
GAGCACGTTCACGAAG
ATATTTCTCAATGGATAGTCGGGCAGTGCCATGAGGAAATCACGC
AATGTCCAAGCTCCT
GAAAAGACTAGCGGACCAACCATCTCCTGAAGAAACGAAAAGAA
TTCAAGATTGAGAATG
AAGACATTAAAGATAGATGATGCTGATTGAGAACAAAATGCATA
AACAAATTCGATTTTT
TCATGCATTTTAAATTCATTTCTAAATATTTTGATATGAAATTCGT
ACACATATTTTGTT
GTAGTGCCATGATAAAAC
>ConsensusfromContig1023 Average coverage: 8.84
GCTGGGGGCTCCATTTCTCTCTCCCGCGACCTTGGTGTCTCAGCT
TCAATGCCAAGGGCG
AGGGGTGAGAGTTGGGAATCTGACGTCATCGATTCTTGGAGTGA
GGAAACCAGCTGTGCT

GCTTCCTCAAGCCGCCTAGGTTCTTGGTTGATGAAGTTTGGTTCCT
GCGATATTTTTCCA
GTGAAGAAGCCTCTTGTTCTTTCAGGACCAATCACAGGGGGCTGT
ACATGTGGCATTAAA
TCTTCTTTACCATCAGCCCAGTGATGATAAACATGAACATCCTTG
CTAGATCCTTCACCT
TTTGGAGGATAAGCCGAAGGCGCTTTCAGAACATTGTTAGCGTG
GGGCTGTCGAGGCCCG
CCCTTATTGAAGGCATAATTTGTTGTCTTCTTCACGTCTCCACCAA
TCAGATTCTCGTCC
TTCAAATCCTTTGTCCTGGTTTCCGATGCAGATTTTATAACA
>ConsensusfromContig1024 Average coverage: 11.02
TGTATTGAAAAATTTATTCAACAACTTCTGTTAAAATTTATAGCA
TTACTTCCACCACGA
TTTCAACAACCATTTGTTATAAATCTATTTCTTTGTTGTACCCCAT
GTCTCTGTGAGTAC
TTCCGCCCTTATTGGTGATAATCATGAAAATTCTGGTACTCCCCT
TCATGATAAATGCAA
TTTCTGAGATGTTTGAGAGGGCAAGTTACTACCTTATATCCGATA
ATATTCTGGAGATGC
TCAATG
>ConsensusfromContig1025 Average coverage: 7.11
AGAATTTGGATGTTGAAAAACCCTCTTCAGATCTTTATCCGGCAT
CAAGCAATGCTCCTG
ACTTGAATTCAGACTGTGGACTTTTCAGTTATCAGAAAAACAACC
TCTGTGCAAATGACA
ACTTTGATAGACTCAGCTCAAAATCCGATATTTCTCCTGCTGTTTC
GCTCATCTTGGACA
TGGATGAGTCCTCAGATACCCTCACTGGCTCCCCCTTCAGCACTG
AGGCGCATCCCAACT
GCCTCAATGCCATCAAACACAAGTCCTCAACAGCCGCAAAAATT
GCAGGATTAAGTAGCT
ACT
>ConsensusfromContig1026 Average coverage: 10.21
GAATTTGATGCTTCCAGCCTGTCTTTGTATTTGGATGGTAGGAAA
GAACCGAATATTAAG
TGGATTCGGCCTTTTGAAGTTCTTGGAGAAGGTCAGAAGCCTCGT
CTGCTCGTGGATGGC
CATTGTCGATTCGACATTGTGCAAGGAGAATTGTCAAATTGCTGG
TTTCTTTCTGCCTGT
GCTGCAGTCGCTCAGCAAAA
>ConsensusfromContig1027 Average coverage: 8.01

TTGGAATAGCTTGATATTTGATTTCAGTAGTGAATTACCATGCAA
TATGGCCAAAAATTT
CCCTGATGGTGAGAAAATTGCCTTGAAAGGTAAATTTTGCGACTC
GAATTTATGAGCAAA
TATTTCGCGGAGAGAATTCTTCCGAATGTCCCAGATCTTCAGTAC
GCCATCTTTAGATGC
AGAAGCGACGAGATTTTTCAT
>ConsensusfromContig1028 Average coverage: 4.88
TATACATGTTAACTATTTTTGTTTGAGATCCATCCGGGGATAAAA
TATCAATACATAAAT
ATTCCCAATATAATGATGAATTCATTGAGCATTTATTTTCAAAAAT
GATTGATTCTTTCA
CATAAATCACAAGACCTCCATGGGAGGAGCAATTAGAGATACTG
TTTAATAATGTATAAC
CAGGAATATCATATAAATTGATTGTAGTATCACTTAAAAACCATG
TCTCCTGGAGACAAA
C
>ConsensusfromContig1029 Average coverage: 5.67
CGGCCTTCTTTGCTCGTCTTTTTTAATTATATTCTGTTCATTTGTCT
GATTGTCAGAATT
ATTACTGGTAGTTTTTCTACTTTTACTAATTTTTGAATTCTTTTTAG
TACTATCATTTTC
GTCTAATGTGCTGGAAGATATGTGTTTATCGTTGATATTACTTTTA
TAGGAATCTGCATT
CGAATTCCCTGATAATGGTGGCAGTCTACGATTTTTTGAAGTCATT
TTTCCTTTATTATT
ATTTACATTCGATGTCAGGGAG
>ConsensusfromContig1030 Average coverage: 4.63
GGTTATTTGAGTTCTCCGCATAAGGATGTTCCTCATTTTGTGTTTC
GTATAAAGATATCG
ATAGATAATATATAAAGAGCATTGCAGAGAGGCAGTCTCCTTGGC
ATATGCCCATTTCTG
TTTTAAAATATTCACCCTTTTTGCCATCTATTTTTATTCTGATTTTA
GTGTTATTGATCA
AAATGTGAAATATGAAGTATTCTGCAGGGTTTAATATTTTATTTA
GATCACTCATTAATA
GCTTCCTATCTACCGTGTCGAAAGCTTTGCTCATATCG
>ConsensusfromContig1031 Average coverage: 6.72
ATTCCTGATATAATTTGTTCGTAAAAGTATCTGGCTAAAAGTCAA
CAAACCTCACGAGTG
TCTAAATCAAGTCCTATTATGTCAACACATCTGATAACCCTATTG
AGCCGATCAAATTAG

AAAATCGTATTTCACTCCTCTCGTATTGCCAGATGGTCTATTATT
AAAATGTCACAAACG
GAATGTTCACAATGAAATGCGTGACATGCAG
>ConsensusfromContig1032 Average coverage: 7.83
GATTTTGCGGGAGTCGATTCGTAAATACCTAAACCAGCCAACGTT
TTCCAACTGTAAATT
GTGTTCAGGAAGAGCTGCGTGTCCGAAGAGAAGTGTTTGGAAAA
TATGGAACGGCCCTTC
TTTTTCTAGCTGGCAGACGACTCGAGATTTCTCCAGCAGGAACTC
CTCCACACGACCACC
AGCAATTGAACCATTCTCATCAAAGGAAATTCGGAGGTACTTGGC
AAAACGACTTGAATT
CCCATTCATCACCGTCTTAGCATTCCCGAACTCTTGTAATATTAAG
TTAACCTTCTCAAT
ACGTGCTCCTAGCTCCATTTCTTCAGTTGGAGCTACTCTGAGCAG
A
>ConsensusfromContig1033 Average coverage: 8.17
GCGGCCGCTGACTAGTAAACAAAACCCAGGCACTTGCTTTGTAAA
ATTATTCGGGTTTAG
AGAATCACGTATCGCGATAATGATGTCAACCATTATCAACACAAC
TATTTCAAAACATTA
TAAAATATTCACAATGCTTTGTGAATGATCGTCATAATTTAATTTG
CATTAGTTTTGTAA
AACTCGATTAAAATTGAAAATCGTCTAAATTATAAATGTCAACAA
AG
>ConsensusfromContig1034 Average coverage: 7.27
CGAGCAACGGTGGTGTGAGCACGGCCAGCAGTCAGAATAAACGC
CGAGGATCGCTCGGCG
TTTCCCGCAAGCCTCGGCTACCTCGAGCGGTCTCGTCTTCATCGG
CAGACCGCAATTTTT
CCTCAATGGAAAAAGGACATTCGGCATATTCTAACATACTGATG
CGAGGGAATGAAGGAT
ATTATTTTCAAGACGAAAATCCGTATTCCAGTATTTCTCCGCCAG
ACAACAGCATGCATT
ACGTCAAGAAACCGAATGGATCGATCGCTTCGTCGAATTCGTCTC
TCAGTTCATTGAACC
GTGGACTTCTCGGTCGAGGTCTTTCGAGAATACGTTCCTTCTCAT
CTAGCGTCAGAAAAC
GGCAGCCATTCCTATCGGTCCGCAGCAACTCGACGGCGAGAGACT
TCCCTTCGACCGGCA
ACGGATACGTTCCACACTCGCTCTCGAGTTCGATGTTCTCGCGTT
ACGATAAACTAGAAA

CGCCATCCAGGCTGGCGGCGTCGACGAATTCCATGGGCGTCGC
>ConsensusfromContig1035 Average coverage: 10.78
TCCCCGTGGATACGCATAGATTGTTGTTGGCGCTGAAGGAATTTT
TGCAAGAACAGAATC
CGCTTTTGCATTCCGCGTTGTGGTCCAGACACTCGTCGTCTTCCA
TGCAGGATTCCGCCA
ACCTCCGCTTTTGCACGCAGACTCCAGCGCTATCGAAAGCATCCG
AATCGCAAATACAAA
TCCCTTTTCTGCATTCTGTGTTGGGGTCGGCGCAAACAGAGCCTG
G
>ConsensusfromContig1036 Average coverage: 6.64
CACGGGAATAAAACGCATGGGTAACATAATATCAAAAATCACAT
GCAATTGAAAAATTGA
TAGAAATAGAAGAAAGAGTGGTTCCGGGATTGCCTCATTGAATTA
TACCGAACATCTTAG
CCAAATGAAAAATGAGGTTTTGAGTCATTTCATTAAAAAAAATAG
CAATTCGTGTTGGTT
TTATGATCACAAATTCCAATAAAACATCGCAGTTTTGGTTTT
>ConsensusfromContig1037 Average coverage: 5.42
CTTCCGATCTGGCGGTTCCAGTAGAAAAGGCTGTACAGACAATAG
ATCGAGTGTATGCAT
CCTCAAGCTTATCAGCACCTTCGGTCGCCACAATGACATTAGCAA
TATAGAGAGAGCCAT
ACATATGAGTGGCATCTTCCTGACCAAACTTTATTTGTTTTATATG
ACTTTCTGTGAGAA
ATTCTGAATTAGTTTTTATTGA
>ConsensusfromContig1038 Average coverage: 5.06
CACAATTTCATCGAGCATTGCAACTGCGACATTCAGAGCTGGTGT
CTGCTTGAATCTCTG
CATGGTCCCAGTCATTGATGTTTCTTGACATCCGGCTACAACTAC
TTCGGCCATGTGAAG
TTGTTCGAGGTCCAATAATTTGTGGATGATGAGGAACCTTCCATG
TGAACCGGTTACACA
AGAGAGTGTAACAGCAGGTCCACTCAGATTTCCAGTGTGGTGTG
CGCATGTCAGGGCAGA
GGTGTA
>ConsensusfromContig1039 Average coverage: 6.74
CAATTTGAAGCCGACATACCGAAAGCTATCATCTCATGATTTTGC
GATTTCAGCTAAAGT
TTGAAAATATAAAAAACTTTAGAATTTCAAATTTTAATAAGTCAA
CCACGAAAATCGGGA
GTACACGATGACTGCATAGAACCCGTGCATAGCTGAGTGGTCTGC

CTGTACACATAATTC
AGGAACTTTCGTCTTTGTTTC
>ConsensusfromContig1040 Average coverage: 7.23
TGTTGCGACAGTCGATTTAACTAATGAATCATAACGGGGAGATTT
TCTGTGCGATTTTTT
ATAACGATCCGAGTGCTGATTGAGTCCGCCACTGCCTCTATTCGA
ATCAAGCAAAGATTC
GTTGACATGGGAATTTTTTGACAGTTGATCGACATCAGAATTCTG
ATATTGCCTAAGTTG
TAATGCCTTCCTGTGTGACTCATGTTCAGATCGTTTTATCCGCTGT
GTTCGTCTTCTAGG
CGGGTGATATTTGTATGTATCCTCTTTGATATAGCTCGGATTTGA
AACTACATCATTGAC
TATGCTATAGTAGTCATACGGAAGCCGTCTCGATTTAGA
>ConsensusfromContig1041 Average coverage: 17.96
GGGGCGAAAATTGTTCGCGCTATCAATGCATATGGTATTGAAATT
TTATGACATATTGGA
TACCTCTGTCCTTGGATGATAATTTCGCTATATTTTAAAGAATTTT
AAATTAAATTTCTA
GATCTCAGATAGGTAGGGTTAATGAATACTCCGGCCCTCTTCGGG
ATTATAACTGAACTA
TTTTCTCACAAAAAATATCTTATTGATATGTTCTAATCAGAGTAT
GTGCAAACGAAAATT
CAATCAAATTGTA
>ConsensusfromContig1042 Average coverage: 7.33
CTCATTCATTTCATTTTTTCATCAAACCATGATTTAAATTTCAAAA
TACTGGTGCTACAA
TGATAAAGGATTACGCTATTTCAAATTTGAAGCAATTTGTTTATAA
TTATGTTTGCCGTT
TTATTCGGGTTGAATTTATTCAATAATATATCAACATTTTGAATTT
GGTCATTTTAACCC
AGCCAATATCCGAAATGTGTATACTTATTCTTCTTATGTCCCA
>ConsensusfromContig1043 Average coverage: 7.00
GGCCTATCCTTGGTGTCAGCTTGACAATTGTGAAGAACGGAAAG
AAAAATAATTGGACTC
TGAACTCTGTTTGCGTGTATGACTTGATGGACAATATTCAATACA
ACTTTCATCACGGTG
CTAAAATAAAAATGCCTTCTGGACAAACATCAGTTTCTGTCGACA
TCACCGAGATGATGT
CAAAAAAGGATGAGTTCAAATCCATGATTGTGGAAAACTTGAGAA
CGCGCCATTTCTTCT
TCTCAACAATTGAAACCTTC

>ConsensusfromContig1044 Average coverage: 5.12
TTCATCTCATATACCCTATCTCTAGGGTTATAATCATCGGCCATG
TAGTGCCGGCTGATC
CTCGACTCCAGGTCCATGATTGTATCTTGGTATATTTTCATTGTTG
ATAGAGTTTTGTGA
ATGGCAGTGTAGGAGTCCGCACAATTGGCCTGATGAATATTGAAG
TATGTCAATGTTCCG
TTGAATTGTGATACGAAATCCGCGAGATCGGAAG
>ConsensusfromContig1045 Average coverage: 10.01
CCAAGTTGGAATCTTAACAAACCCAGCCATAAGTTTGGTCTTCCT
TAATTCCGCATTCCC
GTTCAGGGTCGCAAATAATGCTCATGCAAGGGTCTCTGTAGAACA
TGTGTCTCTGTTTCA
ATCCATCTGTATTTCCTGCATACAGCTCGCAGATGAAGTGTTCTT
GCTCATTATGGTTGA
TGGAGAAGTATTCAGCTGTGGTGCAGCCGGTCGTTTTTCTACACT
TTTTGAAGCATTGAT
CCTGACTGTTCACATTGGGAAATGAAGACATCGATTCTACGAAAG
TCTGATTCGGCGGAT
GTGATGTGTAAACAACACGTG
>ConsensusfromContig1046 Average coverage: 6.77
ACGATGTCGACGATTTCTAGAATCTTTGATGGTTTTGTTATCGTT
GGGCAGCGGTCTATA
CAATGCGCTCAAGGGAATACAGTTAAACACTATACCCGACAGTAT
CATGATAGTTCCTCG
CCACCCTATTGTCTCCAATAAAGGCTGTATCATAAGAGGCATAGC
GATCGTGCCGATTCC
AGTTCCAGAGAGACATATTCCTGTTGCCAATGCCCGCTTTTTGTT
GAAATACTGATGAAC
AACCATAATTGAGGGAAGATACATCAAGCCAAAGCTGAAACCAA
CACCAACACCCCATGT
CAATGTCAAGATGGGGATAGAATTGGAAAAACTGGCGAGAAGAA
ATGATAAACCGC
>ConsensusfromContig1047 Average coverage: 4.50
TCCATCCGCACAAGCCTCCGCACAGACTCTGCCCATAAAAAGGA
ATAAAACATCATCAAC
TGTGCGGAGCATTCAATTCCAAGATTCCGACACTGCTGAAAAAG
AGAAGGCAAAAGAGAT
AGTGGCGAGACGGAATGCTGCTGCTGAAACGTGGAAGCCTATAA
CACATATCGATAAATT
TCGCGAGAAGCGCAACAAAAAGAGAAAGGAAACGATTGGAAGCA
GCACGATTTTGA

>ConsensusfromContig1048 Average coverage: 4.66
GTCCATACATGATCAAAGATCATAATGGGGAGTGTCCAAACAAA
GAATGTGATAACCGTA
TGCTCATACCTACATCCGAAAATGTGATGGATGCTTTTTATTAAA
ATTTTATTTATTAA
AATTTTAAGCAACAACAAAGTTCTAGGTGCATAGACACCGGACA
CAATCCAAACACCGGT
GAGAATTCGCCACACTTAAAGATGCAAAAAACATCTCAATTCCTC
CGTATTCACAGAAAC
CATAAAATATATGTTTGGATTTTTTGCAAAC
>ConsensusfromContig1049 Average coverage: 3.65
CCTCTTCCGATATACATCATCCATTCTATCAATTTCACGGCTTGAT
TTGTCGAAAATAAC
AGCAATATGATGCCAGGTTTTAAAATCACAGGGAAATTTTATTCG
TTTCAATTCCTCAAA
TTCAGCAGATTTGAATTCCATTTGGCAGTTTGGATACATTTTTATT
TGATAAAACTTCGA
TCTATATATGAATGTAGGCT
>ConsensusfromContig1050 Average coverage: 4.49
TTTATCTTGATTTCAGAATGTGTGATCTAGCTGCACATCAAGTTCG
TTATTTCCTGTTAA
GCATTTAACATTTCACGACATATTCATTCATTCAATCTGCATTTGA
TATTGGTATGAATG
GAAACAATGTTTTAACAATTATAACTAGGAGTCTGCCTTCGCAGT
GAATCTACTTGCCTG
GTGGATCGAACTTCATCAAAAAAAATTCCAAAAAC
>ConsensusfromContig1051 Average coverage: 9.78
ATTGATTCGAAGTGCGTGCTTCACCAAACTTTCTGATCAAACTTTT
GTTGTTGTCAATTT
CATCTTGCATAGTTATTCCAGGTATCGTGATTTGAGAGTGACACTC
TTTTCGTTCATACA
TTGTGCTCCGTTTTTTCGGGCTTCTCTCTCGATTTGAATGTTCAGT
TTTCTGCATTCCAT
CTGAAGTTTTTGTATGAGATCTTTTCTTCTTAAGATTTCGTTTTTC
ATCTTCACCATGAT
GTTTCTCCTTTGTGTACATTCC
>ConsensusfromContig1052 Average coverage: 5.61
ATTCTGTAAGATCCTCTGGTGTTGTCCCGATGGCTGTAGAAGCCA
TAGAAGAAAACGGAA
TACTTAACATAGCCCCCGAAGTCGAAGAAAACCCCCAAATCCTTG
CCGTGTGGTTCGTCT
TCCAGGGGTATTTGCTTTGAACGATCTGACCCATAACTTCCACCA

ACAAGGATCTCTGGA
ACGATGACGAAGGAAATGATGAGAATTGAAGTGATGATATTAAT
CCAGAAGAGCCAACGA
AGAAAGATGAAGAAAGATGCAACAACTGACCCGAAATTCCCTTCA
ATGCGTTTTATTCGC
>ConsensusfromContig1053 Average coverage: 8.62
GTTTTGCTTGGTCATAAGCTTTGTCATCCGATAGCGTCTGTGTGCT
GCCATCTTTTTGGG
GCGATATTGAATTGCTGATTTTTATGACTTCGGTTTGCGTTGAATT
TTGTCGTAGTCGTT
TTGCGTCAATTTTCTGACTGATCAAATTACACTTTGGTTGAGATG
TATCCGCATCCGAGT
TTTCAATTTTTTCGGCCACATCTTTAGGTAGATCGTTGAGGTGAT
TTGGATCAGTTGAAA
ATGCGTTCGTCTGCTTTATAGTTATTTGAGTTGAATCACCAAAAA
AGCTCAGATTTCTAG
GAGTAAATTGAGAACTATGAATTTTTTGTTTCTC
>ConsensusfromContig1054 Average coverage: 6.87
TCCTATCATGATGTCGACCATCTGAAACTATAGATGCAGCGTACC
GTTTCAGCATAATTG
GCACGCTGTAGCTACGAAATTTACCAACATAAGCTTAATTGAAAG
ATAGATACAAGTCTG
TATAATGTGAAAGTTCTGATACTGAAACGCGGTCAATTCAATACA
ACTGACATTACAGGC
TTATTTTTGTGATGCATTTCCACCCGTATGTGCTCTTTTAAATCAA
ATCTTGAGCAGGTA
TATGGCTCATTCG
>ConsensusfromContig1055 Average coverage: 7.30
GGAAAAGGTCATGGTCAAGGGCAGTCTTCAGTCACCTTCAGCGT
AAAGGTCTCGAGAAGC
GATTTTTCATTCAAAAATACGTCACAAAACCAGACCGGAAGCAGT
TGGCGGCCATGTTGG
GCCTTTCGGATGCTCAGGTGAAAGTTTGGTTTCAAAACCGAAGAA
TGAAGTGGCGTCACC
ACGAACAAATAAAGATGCGGAGATCGGA
>ConsensusfromContig1056 Average coverage: 20.49
TCCCTACACGACGCTCTTCCGATCTGAGAGAACTCCGAGTGGCTT
ATTTACTTTGAATTT
AGTCACAAAACTTGCTATAACCAAAAACACCATGCTAACAAGCA
ATGCGACTGCGTAGGG
TGTGAAGTTCACAGTTAGGAGGACGTCCTGCATCGGACCCTTGGA
TTCAATAATACCAAG

GATAAACCATGGCAGTCCAGTGCAGATTAGAGTCTCAAATATCAC
AGATCCTATAGTGTT
TGACATGGCCATGTT
>ConsensusfromContig1057 Average coverage: 1,748.30
AGCAATGTGACTGGAGTTCAGACGTGTGCTCTTCCGATCTTCGGA
ACAAAGACTCGCAAA
GATCACATGCGAAGACGGCGTCAGCAGGAAAAATCCTTTTGTGT
TGAAGAATATGAAGAG
CCATGATGGACATAAAATAGCCGGAGACCTCTGTGATAAGAATA
CTTCCCTGAAAAAAAT
GAATCTGTTGGGATCATCAAGAAAGGTTTTCACAAGTGGTCGAA
GAAACTCAGATCGCT
>ConsensusfromContig1058 Average coverage: 6.58
TTTTTTCCCAATATTTTTATCAAAAATTCCACAAAGTGCAATAAT
TAACAACATAATAAT
TCGTTTACGCATTCTTCTCAAAAATTTTCTCCAATCGAATCCTAAT
TATAAGATCCTCAT
AAGTACTATCCCCCCAGAAATGGGTTGATGGACTGTCGACACTGC
GCTGTGCCATCTATG
TACATGCAAAAGAAGCGCTTCGCGCCATTACACCCCAGGTCACAA

>ConsensusfromContig1059 Average coverage: 7.61
GAAACACTACTTTCGGTGTTTCACCTCGGCCTCGCGAAGAGTCTT
TCGTCGAACCGCCCC
TGACACAGGAAGAAATCATGCAGAAGGCAGCAGAGGAATTGATT
TCACACTTTGAGCATC
TCAACCTTGAATCTATTCTGAAAGCGATCAAAATATCTCTTGAAG
CGCTCCGAAAGAATG
TAACAACGACGCATTCTCGTAAATATGGCTCTGAAACGGTAGCA
GAGACTGCGATCAACC
GTCCTCTTTTTGCCTGTGAGATGGCGCTCGAAATCCCGTCAATCA
GAATGAACCCTTCCT
TGGATGATGTCCAAAATACGGTCAACCAAGCCGCTCAGATCATT
TTGGGGGTCGCAAAAA
ACATTCGGAAGTGGATAAAGGAAATCCAGCCAGAAGAGACCGGC
GATGACCTCATCGACG
TTTCAGTCAGGTCGCAGAGTGCCC
>ConsensusfromContig1060 Average coverage: 6.16
GCGTTTATTTCATATCCGATTATATATTAATGAGTAGGCTTTCGAT
TTCGTTATAAATGG
AGTATTAATTTGTTTTTTGACTAGAATTGAATATTGAGTACTTAGA
GTTTGCTTTGTCTA

GCGTATCATTATTTTTCATTCGATTGTATTGTTTTCCGTTATGATTA
GGCTTTTGATTTT
GTTATAGATGGAGTATTGATTTGTTTTCTGACTAAAATTCAATAT
TGAGTACTCAGTGTT
TGCTTCATC
>ConsensusfromContig1061 Average coverage: 5.51
CGATCTCTTCGCTATTCCTTCGTGCCAAAAATAGCATTCTGTTTTG
CCCGTCGAGAGATT
GTATACTGTGAAGTTGTAGCAAGATAATCGCCGCTTGTAGAACAG
CTCGAAGTGTTGTGA
CTTTGGTAAAAACAGTACTTGCTGCAAATCAAAAGATGCTGCTAT
ATAGGTCTGATCTTC
TGGAGACTTTATTTTATCCGCCGATTTTATTTCGGGAACAAGATTT
TTCTCCAGAATATG
C
>ConsensusfromContig1062 Average coverage: 8.91
ACATCTCGGTTCGGATTCTCGGCACGGAAAGTGCACCAGGCTCCT
CTCATCATCGAAATC
GCCATCTGTCCAAGCATCCTTCAAATTCTTCACATCGCCATCTTT
TAAACAGATAGTGTT
CTCGCTAATAAACGACGGAATTGTTTCTTCGACCAAATCTGATAA
TATGTTATTATATTC
AGCTTTTTTCATGTCTACTTCAACCTCGAGCCAAATTTTTCTATTA
GAAATCGC
>ConsensusfromContig1063 Average coverage: 13.79
GATGTGATGGATAAAGAAATAACTGCATGCGCCGTGGCTGCTGGT
GAGCTTCCACTGATG
AGAGAAGCTATCGCGAAGGAGGTTCTGACCAAAGTTGTCTCGAC
GGAAGAGTTTCAGAAC
ATGCAGTTTTCACTGGAGAGTAGAGTTCTATCCGCTGGCGAATGG
ATGCTGGAAAATTTC
AACGAAACTTTCGTTTCGAATTTATTTCCGACGAGGGAAAGATC
GGAAGAGCA
>ConsensusfromContig1064 Average coverage: 9.96
CTTCCATCGAAATCGGCTTCGAGACTGTTCGTCGCCAATATCCTG
GAATTCTGGATAGTT
TCGACATAAGCTTCACGACACGTGATTCGAAGTGCAACATAGGTT
ATGGAATCAACCAAT
TATTCGATGTGTACAGCACAACGGGACTTAGTGTAGCATTTGGCC
CATTTTGCGATTATT
CTGCTGCACCTGTTGCGAGGCAAATGATGTATTATGATATTCCCA
TGGTAACCGCAGGAG

CTGTTGTTGATCATTTCGGAACGAATAAATTGATCGAGTATGGAA
CCCTTACGCGTGTTG
GCATGTACACACTCAACGAGTTGACGACATCAGTCATTCACATGT
TCCTCCAATTCGGAT
GGTCCAAAATGATTTT
>ConsensusfromContig1065 Average coverage: 3.20
CCGATCAGTAGAAACACGATGAGCAGGATTATTTCTTTGCCACT
CGAGAACAAGGTGTAA
GCCAACACCTGAAGTCCTTGATAGTGTCTTATCAGTCTGAAAACA
CGGAAAATCCTCACG
ATTCTGAAGGCAAGCAGGATCTGGAGCAGTGAGCCTGTCTCGGCG
TCTCTACCCACTGTT
TTTTCCACGCACACTTCTATGTAAAATGGAAAGA
>ConsensusfromContig1066 Average coverage: 5.30
TCCGATCTCAGAACTCCAAATTTCACTCTCGCGTTGGTAATCAAA
GACAACGGAGTCGAA
GAATCGTTATCCGTCATGCAATTATTATATATATTCACAGAGAAT
GTCGAAGAAGTGACT
CCAAACGAAAAAGTTCCTCGGCCAAACAAATACGACATTATGATC
ATAGTTCCCTTGTGT
GTAATTTCCTTGAGTATCTTATTGACATTAATTCCACTCATCTTCT
GCGCATTTCGTAGA
AAGCGGGAGCCTAAATATGTGACGCGTATTCATGAAGGTGTTCA
GCAAAACTATTACACA
GAC
>ConsensusfromContig1067 Average coverage: 6.28
ACTCGTACATAAGAAAACTCGTTCACTGGGCGAAAACCGTTGGGT
GAACTTTTAGAATTT
TAGACGTCTCGGAAATATTAATTTTCGCTCCCTTACATTCACACC
GTAGCACACAACCAC
TAGCTCTACGCGTGCTTAAAGAAATAACGGAAGGTGTCATCTTTT
CTCTGCGATGACTCA
TGATGGCCGTTGGACAATGGTAGTGCCTTGTTGATGTTTTAAATA
AGATAAGACCCAAAT
CTATCATGGC
>ConsensusfromContig1068 Average coverage: 4.04
ATAGGCGGTGTTAGTTTTAAGCTTCCCGTTAATGTACCCAATCGT
CTTTTCGATGAAGTA
TTATAAGAATTCAAACAGCGTTGGCGCTCAATGAGTTGGGTAGCA
TGAATAAGTTCAATG
TTGATTCAGCATTTGACGCCGTGAACAAGATCACCCCGATGATTT
GTCATCTGCTCGGCA

TGTTTTTCGCTGCGTATTCTTTCATCATTCTTCTACCAAACTGAC
>ConsensusfromContig1069 Average coverage: 10.61
AAGAAAAAATAGAAGCGAAATATCAGATGAAAATAGCCATGAAC
ATCGCTGCGTTCATGG
GACATCTCGACCTGGCTGTATGTCTTTTGAGGCAAGGAATAAGA
GCAGACGAACCCACAA
AAGAACATCCATGGAGGCAGTGGTGTTCAGAGACGAACACTCAT
ATCGACCGTCTTCGAA
CGCCATGCCATATTGCAGCAGAACGGGGTCACCTGGGGGTCCTTA
GAACCTTTATTAATC
ACAATCTGTGCTATGTTCTCGCGAGATCGGAAGAGCGTCGTGTAG
GGA
>ConsensusfromContig1070 Average coverage: 7.05
GAAGCCCGATTCATCGCCTATGAATAATGAAAATGACGATACATA
TACTTCAGACAATTG
TAAAAAACCATTAACACCGTCAAATCTTAATGTATGTAATTCCAT
CGTAGATAATTCATT
TAAAATTCGTGATACACAAAAGGATAATATAACTCGAAGTGTTC
ATAATCCCGTGCAAGA
AGTTGATTCACATGATGACGTAGTAGATGCTAGAGACTCTAGAGC
AGATATATCTGATCC
TTATATTATTCATGAAAATTCAAAACATAATTCACCAGACGACCT
ATAT
>ConsensusfromContig1071 Average coverage: 7.72
TTGGAAGACTGATTGATCTTTTGTTTTGGAATCTCCAAATCCAAA
AACGCTTAGAGAGTT
CGAATTCGGTGAAAGATACGGCGTTATAAATTTAGAAATTGAGGT
TAATACTTTTTGATA
GGGGTTAGGCTGTTTTGGACCTCGAATGCGGTGTAGGTTACTTCC
CTCAATAGTTTTTCT
TCCTTGCCATTCATTGCTGGCACTGTAATCAATCCCAATGACAAC
AGATAACTTTTCAAT
ACCGACTGACACAAAACCTTC
>ConsensusfromContig1072 Average coverage: 8.17
GTTGGGCCCAGTACGACGCAGGGGCAACGGGTCGCATCCATTAC
TCTGCTATGTACGAAA
TGCTCCGGAACATGGAACCTCCGGTTGGATTTGGGAAGAAGTGTC
CGTACAGACTTGCTT
ACAGGAAACTGATTCGGATGAACATGCCGGTGAGAAAGGACGAC
TTGACAGTCCACTTCA
AGACGACTCTTTTGGCTCTGATCCGCGAGTCCCTCAGCATCAAGA
TGACGACAGCGGATC

GGATGGACAAGGCGGATGACGCTCTCAGGGACACGGTCCGAAAG
CTCTGGCCCATCCAGG
CCAAGAAGATGCTCAATCTCCTCATCCCACCAAATAACGAGC
>ConsensusfromContig1073 Average coverage: 15.82
GTTTTGTTACAATATTTGTTCAACGCCAAGTAATGTTGCTTCGTAT
GTATAATCATGGTT
ATTATGAGATGATATAATTCATGTATAATAACCATGGTATAACTT
TCATATCAAGTTAAC
AATCCATTCCTTATGTTCCAAAACATGATGGTGCTGAGAAGTAAT
ACTAACTAAATAAAG
AAGAAAGAATTCGATAAGACATG
>ConsensusfromContig1074 Average coverage: 6.41
CAGGATGGAAAAAAGATAGACATTATTGCTTATTCATATTATTTG
ACGGTTTCCGATTCA
TATGGCCAGCAGACGACAAGTCACCACCACCGCCATCATCATTC
AAGTGCACTTGAAGAG
CTGTCAAGCAAAGATCGCATCAAAAGATTTGTGGACACATCAAA
AGGAGGACACATTTAT
TGCCTAAGGTTTGAACTATTCAAAGAAATACTTCCCACACGATCA
TTGGTTGATGCGTCG
TTTATCTATGGTTTATCTATCGTGCTTTATTG
>ConsensusfromContig1075 Average coverage: 8.71
AAATAAAGGTACCTCCCAAACTGCAATAATAAAAGTCAGTCCAT
CCATACGACTAGCAAT
CCTCAGTAATAATCAGAGAATTTATCTTGCAATGAATTCAATCAA
AGTTTTTGATCGCTT
CCACATTCATCAGTGCTATCACTGTCAGGGATTCAATCATTCTCA
TGATAAGTGTCCTTC
AAAAGATAGATCTCCAGTCTGTGCATTTTGCTCAGAAAAACACAA
AACTGAAAATTGCTG
TAAGAAAGAAGATTTAAATTGTATAAATTGCAGAATGCCAAATA
TGATGAGGTGAATCAT
GCCGCCTTCAGTTTCATTTGTCCAGA
>ConsensusfromContig1076 Average coverage: 3.92
CAACGAAGTCGTCAACCTCTTGCTGAGGGTGCAGGTAGAATGGC
GGAGTCGAATTCCGCA
AGCCCTCAACCTCTTTCCCAGCAGCAGTCTTGCGGACAAGCTGGA
AGTCGGCATCAAGGC
ATATCACTCGCTGGGGAGCCTCGTAACATGCTGGGCAGCTGGTG
GAATCGACGATTGGAG
CACGGATCCGCTTTGCCCGCCAGATCGGAAGGCAC
>ConsensusfromContig1077 Average coverage: 5.36

GTTCATCACATGATTGTGTTCTGAATTATTAAGTGCCCACGAATG
TGGTGAACCATGGAG
TTGAGTGGTTGTATCGGATTGATCGAGCTGATTTTTCATCTGATTC
TCGTTTCCATCATC
GCTGTAGTCAGCATCAATTTGCACTAATGTTGTTTTATTGTCATAA
TCATCGCTGTTGTA
TTCCTCGTCTGCTAGTTTTGACATTTTGATTTGAT
>ConsensusfromContig1078 Average coverage: 4.95
TTTAGATATAAGCTGTGGGATGGTTTAGTTCCATTACACTTTGA
AATATGTATATCATT
GGATGACTCGGATAGACTCGTGGAGCTAGAGAATAAAACGAAAG
CATTGAATGGATGCCC
GAGTGATGATGAAACTGTTAAATGGTACCCAGGATTGAGCGAAC
ACTCTCTCACAAAGCA
GCTTGATCAACAAGCGATCGTCGAGAAGTGGTTTTCCAAGCAAA
GAATCAATACTCAATA
TGAGGTTTTGTATGTTGCTGTTCGACTATGTTTCATATGTTCAGAA
CTGCATCCGGATAT
TATCAGAGGTATAACTGATGAAGATAACCCATTCAGTACGATGC
GAAACAATGGACTACA
TATAATGGAAGACTG
>ConsensusfromContig1079 Average coverage: 5.66
ATGAAAAGTGCGGAACTATCGTTATCGATAAGTACCCCGACCGTA
GAGTCGATTCTGCCG
TCTATTTCAGAGATATCGAAGATCCGGAAAAAGTTTCAACCGATT
ATCCGATTATGCGGT
CACAGGGGACATCTAGATTAGGCGGGAAGCGATCCAATAGCTGC
TCCGAATGCCTGCTGA
TGGGAGAGGGGTCATGCAAACTTCCTCCCG
>ConsensusfromContig1080 Average coverage: 7.40
CACGTTTTCCTATGATATTACCTCCAAATCACAGGTTTGTTGAAA
TGCTCATTCGTGACC
AGCATCGAAGCGCTTGCCACCTAGGGCTAGAAGCTGTTCTTTCGT
CCTTAAGGGAAAGAT
TTTGGATAATTAAGGGTCGAGTGGCCATAAAAAGAATCATAAGG
AGATGTGTGAGCTGTC
AACGGCATACAAAGCAATGCGAGACACAATTGATGGCTGATCTA
CCGGGATATCGTGTCG
TAGATGGCCTATCTCCATTTTCGAATTGCGGCGTAGACTTCTTCGG
TCCAATGTTTGTCA
AAAGAGGGAGGGGAACTGAAAAGCGATATGGTTGTCTTTTCACG
TGTATGGCATCGAGGG

CAATTCATGTTGAGATTACCA
>ConsensusfromContig1081 Average coverage: 5.77
TTGTACAGGGGTTATCTGGTAAACACCGACACTTTTGAGTCATCG
TTGATGAAGGTAGCC
TGCTCGATGGGTCCGGACTCCAACAGTTCTTCAAGGATATGCTTT
TTCGTTTTTGAGCCG
TTCTACTTTCTCATGAGGCCGCTGCATTTCTTTCTCCAGAGAGGT
CTGTCTGCGGAGCGA
TTGTCGTTGTACCCGTTGATTGCGGGATGCACG
>ConsensusfromContig1082 Average coverage: 5.24
CTGGCGTAGAGATCCTGACAGGCAGATCTGATTTTCTTCTGAGCA
AATGTCCAAACATTC
GGTGATAGAGAACGCGTAAAACGATTATGGGATCAATTATGAGC
GGCATGACTTTGAAGA
ACTTTTCTGCTAACGTTAATGAATTCATTGGGCCTGACGTTCCCA
CATTACTCTCCAAGG
ATTTTTTGCTATCATTAGACGGTGAAATCATATCATGACGGTGAT
TTTCAGAGAAGAACA
TATGAGCCACACCATCTCTTTCGCTTTTCAGTGGACTTGTAC
>ConsensusfromContig1083 Average coverage: 9.05
CAGAGATCGCAACTTGACTGGTATAGAGAGGGAATTGTGTGCAGC
GATCTCTTCGTTCCC
TAACCACTGCGCCAAGACAGCGATGCGATTCTTGGCATCTTCCCA
CTGCTCAAGTCTAGA
GCGAAGTCTGTCCTCCATCGTCTTAACCGACACCATCGCCCCTCG
AAGGGTGTCCTCGAC
AGGGAGCTTGGCGGGGCGGAAGTCTCCGGGTACTGGATTCGGGGG
TTCTTCCTGAAATTC
TTGACAA
>ConsensusfromContig1084 Average coverage: 4.47
GATCTGGAGCAGCAGCCCGAAATTTTTCGTCCATTTCCTCGGAAT
GATCAAATTGACATG
AAACGTGTACTCGTTGTCGAAACAAGAAAAGAAATATATGCAGT
GGTGCCAGATGAGCAG
AAATTGCACAAATGGGACAATGCAAATGAAGAGTGGGCGACATG
GAATGTTTTCCCCATT
ACCGATCAAACACGCGAATGGGCGGAGATCCGCG
>ConsensusfromContig1085 Average coverage: 5.88
TCTGACGAATGTGCTCGTGGCCATTCCTGTTCCACGTGCCCACAA
AGGGTAGATTTCAGA
GTTGATTGTCCAAGGCATTGCACTCATTCCAGGTGCAAAAAACAC
TATGTATGCGATGAG

CCCTGCACTGGTGACCCAGACATAGGGAGTAGGGCAGAAATCAT
ACGCCCACCATCTCCT
CTCCCCGCCTTCTTGATTTTGGCACCGCCCCTCTAAAGCAAGCCAT
GGATTCTTGTAATT
G
>ConsensusfromContig1086 Average coverage: 6.23
GGGCGATCTCTGTCTTGAAACCGCGGTATTGGTACCGTAGTTGCG
GGAAATAATGGTTGA
ACAGTTACTTTGGGGCGATGCCTGTCTTAGAAATGTGGTATTGGT
CCCGTAGTTGCGGGT
AATAATGGGTGTTTCCCCGGTTAGTCCATTCTTTTATCTCAGCTGT
TTATGTTGTGTGCA
AATTCAAAAATTGATTCTCATCTCAATATTATCG
>ConsensusfromContig1087 Average coverage: 6.60
TGTTTGACCAGCCCGACAACTCGTGATCGATTTGATTGTCTCACT
TCGTGTGTGGAAGAC
GGGGATTGTAAGCTGGCTACATTTTATGAATCTGATTACGATAGC
TCCGAAAACAAATGT
TACTGCACCATTTTTGAGTATGGATCCCAAAGTCAACTCAGCTAT
GCGATCAAATGGAAT
CCTTAATTTTGACTACATGGAT
>ConsensusfromContig1088 Average coverage: 5.91
AGCGGTTTCGGAGTAGTAGTTTAGTAGAAGTAGTATGAGCAGCC
GGATAAACAATCGATA
GTTTAGTGACAGCCTGGTTTAGCAGAAGATTCTTCGGCATTTTCT
GGTTCAACAGAAGAC
CCCTCGCCATTGTCTGGTTTAGCAGAAGATCCTTTGCCATTATCC
AGCTCAACAGGAGAC
CCATATTCGCAGACACCACCAGTGTATCCCTTTTCACAATCGCAT
TTAAATGTACCATGT
TGATTGACACATTTTCCATGATTCTTGCAAGG
>ConsensusfromContig1089 Average coverage: 7.99
GAAAAACCCTAATATATATCAACCATTATTATGGATTTTAAACAT
CATCTAGTCGCCACT
TGGTTTTGCTGGAGATTATTTATTCTCTTGAAATCTTCTTATTTTT
TTCAGAAAGTAGCT
CACTGGTTATGTCACTGTTATTAACCAAAATTCAAACTGAAAAAA
ATCAGTACATTGTAA
TCAATTTCACATAATATGATTCATAACTC
>ConsensusfromContig1090 Average coverage: 7.81
CTCAGTAAGCTCTCACATAAGTCTTGAGTTTCCATCATTACCCCAT
TTAGTTCTTCTGAA

TCCGAAACAGGTGAATCATAAAAAGTTATATTTACCTCTACAGAC
CTACCAAATGCACAA
CCTCTTGCTAGCTTTGGAACCAAATGATAGCATATTGGATTCAGG
GCACCGAGAATGGTT
ATCTTTTTCGGATTACTCATCGAACGAAATCTTTCTGTTTCTGCCT
CAAGATCTGCATTT
GAAGCTGAGTTTTCTTAGGC
>ConsensusfromContig1091 Average coverage: 6.69
AGTTTATCCACCTGTAGTTTGGGCAGAAATCAATCGCTTCCAACA
GCATATATGGGCACA
GGCAAAAGGGACAAAAGCAAATTCCGAGAGAAGAAATTGATATT
GCATTTTGATATTCGA
AATACGATCCTTGTAGCAGATTCGGTCACTAACATCAGTATTGAA
CAAGCCTTGAACAGC
TATTTGACAAGCGTTACATGGGGTCAGGAAGATCATCGTAAAGG
CTGGCAGTGGTGTGAC
AATTCTGAATTGCAGCTCCGACCTCCTTCATCAGGAATCATAAGT
TATTATAAGTACTTG
GAAACCAAA
>ConsensusfromContig1092 Average coverage: 3.22
CGTATTTTAAACAAATGTGATTTTTTAGAAAACAATTTGAGAAGT
GATTTAAATACACCC
CAAATACTCGCCGAAGAAAAAACGATCCAAATTAACAACAACGA
TATTAAAGACGTGGAT
ATTAACGATGTGGATGTTAACGAACATGATAATAATGGCAACGG
GGACGATGAAACTACC
ACTACCACAGATCGGAAGAGCGTCG
>ConsensusfromContig1093 Average coverage: 4.79
ATTATAGTTCGGGAAGCATATTGTGAATGGGAGTAAGATGATAA
CAGAAACTGGATAGTT
TCTTACTCAAAAACGAAAATTCTTTGAAACTGCTTTACACAACCA
AATTTTTCAACCAAC
GGTCTGCCCTTCTTTTTTCTCTCTGAATTCTTTGAACACTTTATTTC
TCACTTTCCAATC
AAAACGGCAGTCAGCCTGGAGATCGGA
>ConsensusfromContig1094 Average coverage: 10.67
CTTATGGGTGATGATTAGCAGAAACGCAGAGGTAACTTTTTGAT
AATGCGGTGAAAGAGA
AGGAATATTTTTAGTCAATTTAGGCCAATTTTCAGAAGTAACTCT
GATTTTGATCTTGAT
AATATGAAAATAGCAGAAGCAATTCACATAATTGATAGAAGTCCT
AATCTTAATAATTTT

TGAGTAATTTGTGACCTATTTTCATAGACAAAT
>ConsensusfromContig1095 Average coverage: 4.55
ACGCTCTTCCGATCTTGGCACTTAACCGAGGAGCCAGGTTATCGG
CTCAACCAGTCCAAA
GCATTCACCAAAAAAGTAGGCAATCATCTCCCCAAACTCTTGAAC
ATGGCTCTGTCGATG
GTCCAGTGCGTCAATGGCGTTCAAGAATGTCCTGAAAACGGCAGA
GTTCTCACACCAGTT
GTAGCAGACCTTCTGAAAATAGATCGGAAGA
>ConsensusfromContig1096 Average coverage: 5.64
CGCATAATAACGTGCAATATTGAACGATTATATAAGTTCGACAAT
ATGAACGTTGTCAAT
ATTTACATCGCTCGATGTTGCATAACCAACCGCTTTTATCCGCTAT
CAGATTTGTTATAC
ATTATTCAAAATTTTCTCCTGAAAAGCAGTTGAATCCATCACTTTA
TTTTGAATATATAT
CGAACTGCTGCAATCCAATGTACTGTACTCC
>ConsensusfromContig1097 Average coverage: 5.56
CTGTTCCGAGAACTCGTCCGAAAGATTCGATCACCATTCAATCTC
GGCCAAGATCAGGAA
CTCTTCTGCAAAGTTTCAAGAACCTCGTTCCTGGAACAAGACAA
GAACCACATCGAAGAA
GTGTCTCAACCTATTGTGGACAGAGCAAACAAAGAATCATCGTTT
ATTTGAAATGGGATG
AAAACCAATTTGCTACTATGATAAATGGATCAG
>ConsensusfromContig1098 Average coverage: 4.58
CGATCTCTTATCATATGGCTCAAATCATCAAGGCTTGACAGAAGG
CCCTGTTATGAAGAC
ATATTTGAAAATTCCGTTCAGGGATCATAGGACTAGTACTTGTGA
GCTGCCATAAGCTCG
TATCTGATAGTTTATCAGATGACAATAAGCTCAAATCATCGTCAT
CACGAATCTAAATAA
AAACAATTTTTCCACTGAAAATCTAGAT
>ConsensusfromContig1099 Average coverage: 5.70
TTTCCCTACACGACGCTCTTCGATCTTCGATGGGTCGATAGTTGAT
ATATTTATTCATAA
GGTCGGCCAATCCTTTTTCACGCTGAATATTCAAAAGCCTGAGAA
TAACGAGCAATCCAG
GCGCCAGAGGATCAACAAACTCTGCTCTGAGACAAGACTCCGCC
AGCTCCAAACCGGCGG
CGCAATTCAAAGTCTCAGAATAAATACAAACGAG
>ConsensusfromContig1100 Average coverage: 7.64

TCGAGCAGTGATTCTCAAGGAGTGAGAAGAGACCTGGTCGCTCA
GTACTACATTCCAGAA
CACCTGCAGCTTCTGCGGAGACATTGTGACCTAGAAAACACAAC
AGAAATAATCGCTAAA
ATTGGCAACTCAAAAGCCGACAAAGAAGCTCTCGTTCATAAATC
GTTGGCAGCAAAGGAA
AAGAAAGAGCTGGAAGAGGAAAGACACAAGACAATAATGGAAG
AGAATGTGCAAGTTGGA
CTAATGTTTGCATCAAAGTCAATTATGCAGCTCATCGCCAACCCC
TTCGTTGGACCCATC
ACCAATAAAATAGGGTACAGCATCCCCATGTTCACCGGTTTCGTT
ATCATGTTTGTCTCG
ACAATCATATTTGCGTTCGGGGAGACCTTCACCGTCCTTGTTGTT
GCAAGAACAGTCCAA
GGTCTGGGGT
>ConsensusfromContig1101 Average coverage: 4.47
GAGCCACAACAGAGCAGCAGGAGAGGACGAGGACGACGAAGATT
ATAGGGCTTGAGCTTG
ATATATTCAGGCACTGGGCATAAGCGAAAGATTCATCTCTAGAGT
TTTGGCAATGCGTGA
TTCCATCACAGGTCTGGTTTGTGTGAATGCAGATGCTGGGGGTAC
AGTAGAATGCGTTTG
ACCCCGTACATGATCCAACAAAGGTCATTGAGATCGGAAGAGCA
CACGTC
>ConsensusfromContig1102 Average coverage: 10.59
CCCATATCCAGCGGTATAATACGGAGCGAACGCATATCTTGTGTT
AAAATTTATAACTCT
GTCATGGATGATTATAACACATTTCAATGTCCCGAATCATGACAA
AATAAACACATGAAT
GAAATACGGTCACGTGTATATAACATTAGTTCGAAGATCTGTTAA
TCGGAGTGTTCACGC
TTATAAATAATAATGAGTCTTTCTCCCAGAATAAAAAAAAACAAT
AATACCGTAAAGTAT
CAAACACGGAAATTATGAAGGTGAATATTCTGAGTGAGGTAAAA
G
>ConsensusfromContig1103 Average coverage: 6.38
CTTTATTTTTAATTCATCCAATCGGAAAATATCAGCGAATAGATT
AATCTTCGAGGCAAG
TTGATTACTGATAAAATCATGGTTCGTAATATCGTCCAGGTATTG
AAGTTGAAGTAGTAC
TTTCATCGAGGAATAAGGAGAAAATATCATATTTTTATCATCGTG
TTTTTTAATCAGATG

TTGATACCATTCTATATTGACACGTTTTAATTCGGG
>ConsensusfromContig1104 Average coverage: 4.38
TCTCCTCAAATAACTGTCTTCTCTCCTTCTCCTCTATGCCGATAC
ACGGGTCAGAAGAAA
ATCGCCTTTCGCACTCTTCTCTAATTCGGTTCTGCTGTTCAGCTAT
TTGACGTTCATACG
CCGCTTGACGTTTTTTCGCGATTGCTATATTGTCGGCAATTTTTC
GTTGAATAGACAAAA
CCAGGTTCGCATGAGCAGCAGAATTCTTATTTTGCACACGGGAAA
TTCTGG
>ConsensusfromContig1105 Average coverage: 3.57
GTTTGCCATCTCCAACAACATTAAGAAAGCTTAGTGGAGTTGGAT
CTGTTAAAAGTGGGG
TGGCACCATCCAATTATTTGACTAAAAGGGCTGATTGTCTTTCTC
AGGTCGAAAAAAATG
TGGTCCTCATGATTGATGAAATATACTCTGCAAAACAAATTGAAT
TTTCTGGTTGCAAAC
TTATCGGTTTCACTTGAAAC
>ConsensusfromContig1106 Average coverage: 6.13
CATTTCTCAGTGCGCTCTACCGATCTAAGGAATTTAGTTAAAATT
GAAGGTATTGCATCC
ATAAAGTGCATGCTGCTAAGGCCGTATATTGATTGTTATTGCACA
AACACTGCCATATAC
ACTTTATCATCTCAACCAAAAGAGAGTTTTTCAGTTGTTTGGCAT
GACCAGAACCAGCAA
AACCATTTATCACGAAGAAAATTGAACCTCAGTTCTCTGGATTAA
ATATCAGAGGGG
>ConsensusfromContig1107 Average coverage: 8.56
TCAGACGTGTGCTCTTCCGATCTTAATTGGCGATTTATCTGCAAAT
GGTTCTTCTTCGAT
TGAATTATTTACATTTTCTCGTTTCTGTCTGGTGTTAATATTGATT
TGAGCCTTGTGGAA
GAAATCTGTTAATGAGCAGTTTGTGTCATCGTTGCGAGAATTGCT
TTCACCACAGAATTG
AGCGTTTTGTGAAAACAAGTCGGCCAAACCAAGAGGACTCAAAA
TCTCAGAAAAACTGTA
AGTTTGCCTTATTTTAAAAT
>ConsensusfromContig1108 Average coverage: 7.83
TTCTCCGAATCTTCAGAACCATTGTCGGGCTCCGGAGAACTAGGG
CTGCGATGTTCATTG
GACATATTGACCTCTGATGGATTTTCTTCCATGTGTGTCATGAATC
CCCATGTTTCATCC

CATTGTTTCAATCCCATTTTCTCTTTATGAACAGCCTCCTTCCATA
TACTATCTTGTGCA
ACTATGTTTGCTTTGTCAATATCGACTTGGGAAATATTTCGAACG
GGCTTGACTTGGGCA
GTGCTTACTTTCTCAGTGTGAACTGGAACGCATGACCGATGGAAC
TCACTTTTGTTATAT
CCTAGATGGGTACCAGAAGGCACACTTTGATCCATACCGGGTGAG
TTTTAAGATTTTCTT
CCTCTTCAACTCTTCAGCGGGAAAGATGCCGATCGAG
>ConsensusfromContig1109 Average coverage: 11.04
CGACCATTATTATATGTTTCATTTTTGATTGAAATTTCTTCACTCA
CTCAAAAAAATATG
AAATGCAAATTTTCTCAGTTATCATTGACCGCGTATTCAACTGGG
ATAACATACACTATT
AGAAAAACACAATAAAATTAACTTCCATCTCTGAAACCATATTAT
TCTGATTAACCATAT
ATTCCAAGTGAATAACAAAAACATCCATTCTTTTT
>ConsensusfromContig1110 Average coverage: 9.14
TTTGGCCAAAAATTCAACAAAATCGATCATGTCTACCTCAGTTTG
TATACTCCCATTAGT
ATTATTTTCAACAAAGTTGGGTTTTTCGCATCGTTTTTCTAATGTA
TTATTTGATTTTAT
CGCGTCATGACAGAAATCATTTATGAGCGTATATATTATTTTTAT
GATAGCCAAGCAAAG
ACCATTGTGATTATTT
>ConsensusfromContig1111 Average coverage: 23.44
CAAGATATTTGAAACAAGACTTTCAGACATCCTCATTTCCAAAAT
TTCATCATCTCAATA
AAACTCCAAAATGCAGATCAAGTAATATAAAACACCGAAAACTA
GACCAGAATATCAAAA
CGCTTCTAATGTTCCGTTATATAGCGACGAAAATGCCCATCGTCA
AGGCTTGAGTTCTGA
TATGAGCGACCGTGAAATGTTCAATATACCACATTCAAAATACC
AAGTGAAAGAAGGAA
>ConsensusfromContig1112 Average coverage: 7.52
TAGTCTTCTCAAAGCGATTTTCTCATTATCGCATCCGACTTCTCCA
ACATGATTTATCAA
GTCCCGCACATCTTCAGAGACACTGGTCATCATGTCCATCCCCGA
TACACAATCAGGCAA
GCACCTGGAGGCCGAGGTTGCTCCCCTCCGAAATAATTTTTTTCA
TCATAGTGGCCTTCA
AAGCCATCCTTCCGCTGCGGTTTCACTCGGTGAAATGGAAGGGTT

CACCGCTTGGGCCCG
CTCATCCCAGTCTCTTCTTTCTTCGAGAGGTAGAGCAGACCAGGC
CGACCCGACCCGACA
TCTTAAAAT
>ConsensusfromContig1113 Average coverage: 5.64
CCCTACACACCGCCCTTCCGATCTCCTCCATTGACTCCCTGGATC
ATCATGGACGGTTCT
GGTGAGGTAGAGTCGGCCCACTGCACGTGTATGGCAGGTCTCGG
GGAGGCATGTTCCCAC
ATTGGTGCGACACTCTTCTGGATAGATTGCATTGTTCGGATGGCG
AACGACGTTTCCTGT
ACATCTCAACAAAATCAATGGCTTCCACCAAGGCAAACTGACGAT
GGCAGTGCTGCTCTA
CCCCTCTACAAGATCAACTTCACGAATAC
>ConsensusfromContig1114 Average coverage: 3.88
TATATAGCTCATATAGAATTACGGGTTATGTTGATAATGTTGAGG
TACTTTTATTAGTGG
AAATTTGCCCTGAAGGTATATTTAAAAAAAACATATACAAAAAG
ATCTTCGCAGCATGGC
ACAAATTGATATTGTTTGCTGGAACTGTCGTGGCTTAAAATCGGC
ACGGAAATATAATGA
TGAACT
>ConsensusfromContig1115 Average coverage: 7.23
TGATGATTCTAACATCACTACTAACAATACTAACGACGATATTGA
AATGAAAAATACAAT
TAACAACAAATTATATGATCTTCTCCCTGAAAATTATTCGGATTTC
TCGGTATTTCCCAT
TTTATATTTAGTGTCAGTTGGCGCTGCTGGCACCGGAAAATCTGA
ATGTATGAAAACATT
ACAAACTAAAAATAGAGAGATTTTCACAACGAGTTCGACGAATA
TTGCATCGGAGAACTA
CACCAAAAGTCTTGAGGGTATGAGTCACCCACAGTCTCGAAATTT
TGAGAGTAATTGGAA
GACGTTAGTGAAAACTTTATCTATTAAATATCAACATAAAGAT
>ConsensusfromContig1116 Average coverage: 6.02
AAAACACACACTACATTCCAATACAGCATCGAATACCAACTCAG
GAGGATCTAAAAAATT
ATAAACCGAAAATAAGTTATTATCAATGGATACCATTAGTGTATA
TGTTTCAGGCCATGC
TGTGTTATATACCTGGTATTATGTGGCGTTTCCTCAATCAGCGTT
CTGGCATAGATGTCA
GCAGTCTCCTCGATGCATCAAGAGTGCTGCAAAGGACCACCCAC

GAGATCGGAA
>ConsensusfromContig1117 Average coverage: 5.73
TGACTGTACACGATTGACCTTGAACATCGATGATCGGTTGTCCTG
ATGTGGAATCATCGA
AGTGGTCTCCGGAAATCGTGATCTTGGTACCGCCCATTTCCGAAC
CGAAGGCCGGGCTAA
TGCCGGACACAACAGCATAAGATTGGAACATTGTGATTTTGTCCA
ATGCTGTCACCCTAT
TCACAAGAGATGTCGATCTTCCATAATCCAGA
>ConsensusfromContig1118 Average coverage: 9.85
TCTTCCGATCTGAACAATGTCATCTGCAGCGTCAAGAAAAGCAAT
TTTGCGCTGATATCA
AGAAAATGGCCCAAATGGGTCAGATGGATGCTGTGAGAGTTATG
GCAAAAGATATCGTGA
AAACGCGAAATCACTGTGTGAAGCTTGAAATGTTCAGAATGAGA
TTAGAAAACGATAGGA
TGAAAATTAGAAGATTAACCAAAATTGACGACATGGCTGAGG
>ConsensusfromContig1119 Average coverage: 16.58
CCGGAGTCTATGAACATTCGTGCCTACCACTTGTTATTACTTTAA
ATTAATTTATGATGG
ATGGAAAAGTAACTGCTATATTGGTTCTACTATTCTACTGATAAT
GATCTACTGTTCTAC
CGGTAATTATCTGCTATTTTCTGGTAATAATCTACTATTCTACTGG
TAATATTGAGTTGG
CTATGCTTCGACTACTTCGAGCTATA
>ConsensusfromContig1120 Average coverage: 12.97
GATGTCTTTCTGAAAAACATTTATGAATATTACTACAACTACAAT
TCAAGAATATTTTTT
GCATGTAAAGCAAAACCATTTCCATCAGCAATAATGAATCTTACT
TCATATTTGTATGAT
ATGACAAAACCTGTAACAATCTATCCCAGCAACGATTCGCTGGCA
TTGACATTCCTTGAC
ATTGAGATCATCAAAAAAACCACCACAACACCCAGTGTTGTGATT
CAAGTTTCTGAAATT
ACAAATCATTCACATGAAAAATCAAAAACAGCATCTCTGGTTTAT
ACTGATCAATCAAAT
AAAAATATTTCAAAATATTTTCTTACAACATTT
>ConsensusfromContig1121 Average coverage: 10.96
CCGACATTGATGCTGTATTCAAATATTCGATGAATAATCGATTTT
ACATAATCAAGGGAT
CCCAATATTGGCGAATCGCCCAGGGATTCGCAACGGTTGACCCTG
GCTACCCGAAATCGC

TGAGTCTGTGGAATCTGCCATTCGGAAGTGTTGACAGCGCTTTCC
TCGCCAGCAATGGAT
ACACCTACTTCTTCAGCGGGAATCAGTACACGTGTTACAGCAAAC
TCTTCGCAAGGAAAT
GCATCGCCGAGATCGGAAGAGCGTCGTGAAGG
>ConsensusfromContig1122 Average coverage: 5.81
ACTACATCGAAAAGGAAAATTTGTTTCTATGGTCAAATCAGCAGA
AAATTATGCTGGTCA
TTGATCGACTTGGTCTAAATTCGCTCGGTCATTTCGACCCAAAGA
GGAGAATTATAGAAT
ACATGATAGAAGATGAAGCAGCAAGCAAATTGGCTAACTTATCA
TTGAAAGAGTTCATAG
CCAGCGTCGGAGACCGTACTCCTTCGCCGGGGGGCGGTTCTGTG
GCGGCCGGAAGCAAGA
GTGGCGTGGTGGGGA
>ConsensusfromContig1123 Average coverage: 6.09
ATCGCCAAAGTTTGAATAGATTCTTTCAGAATTCTGACATCTTTTT
CACTGACTTTTTCT
CTATTACTTTTTAATCTATTCTATTACGTGTTTAATGTTTCCATTG
TGATTATCCTCATA
TCATAATATTTAGCAACGAAATCCTCATAATTTTTCATCAGCCAA
CTGAGCCGGCATTTG
TTGCATCAATGTTGTTGGCATCATCAACATATCATGAACATTTCA
GCACTATAATTGTTT
TTTATCGCAATATCAATGA
>ConsensusfromContig1124 Average coverage: 4.65
TGAGAAAGATATCTGTACGACCTGGGGGCAAAAAGAGTTTGATA
AAAGCAAACAAAATAT
TTATCTGAAAACCCCATATAGAGAAGAGCTTACTCCATACATCAT
TACCAGTATTCACGC
ATCCTTGATTTTGTAGTAAGATATATTTATTGAATGATCACCTAA
AGCTTGCGGTTTTGA
TCACAATTCATGAAAATGGTA
>ConsensusfromContig1125 Average coverage: 8.02
AAACTCGAAGATCATTTTCCGATGTGAGCACAGTTGTTAACTGCA
TCCATGATTCGTACA
ACCTCTTTGAAGTATTACCAGTCCATATCATTGCACCGTTTGTCTG
TTTATTCCAAGCAG
TAACTGTGAGATTATCATTACTAAGTTGCAGAGACGCACCCCAGG
CTCCATTTTTTGAAG
CTATCATATGAGATGGGATTTCGGAAAAC
>ConsensusfromContig1126 Average coverage: 3.92

CGGAGTCTTCAACCCCCATCCGCTGGACCAGCATCATGGAGGATT
ACCTCTCGGCCCGCT
TGATCCTAATCAACACAACAATCATCTTCATCATCCAGGGGGATC
CATACATCCTTCGAT
AATTGAATCATTAACCATGCATCATCACCGCCATCATAGTAATAT
TAATCATAGTAAATC
TCGGAATGCGACGATGATGGATTTATGCCGAGAT
>ConsensusfromContig1127 Average coverage: 4.74
TACTTTTGTGCAAAAAGACCGTCAAAAACATTCATGTTTTGTTTT
CCATACACTATATAA
TTGCACTACAGAAGCGTCTTTATCATTATGAATTATCTACTCGAC
GGCACAGAGTTGCGC
TTCAAAAGAAAGATTGAAAGATCTTTGAGACAATTGACTGGGACA
AGTTTCATCTCCGCC
AGTGTTCTACTCGATTCCAACGAGTTTA
>ConsensusfromContig1128 Average coverage: 7.59
TTAGCATTAATTTAGAAAGTATCAAGTTTAGATAGAAACGACTCA
TTATTGAATATTCAT
ACTGTGCTCTGATATGAGGCTATGGTGTGTTATAAAGGGACGGCC
AATAAAACTTTCCCA
GTCTTATCAATATAAGCTCGTTGTATCCTTCGTATCTATGAGAATC
ATAAAATTTATTAT
ATAACAAGTAGTGAACTCCTTTATGACATCCCATGCGATTTTTGC
A
>ConsensusfromContig1129 Average coverage: 4.05
ATATTTGGTGATATCATCGCAGTGGTGGCCGTGTGCTTGTAGTAC
AACTCGTTTCAGCTC
TTGAATCGAGGATTCAGATTTTATGATATAGGAGTTTATGGTTTG
TTCATCGCATAATGC
TGTTGAGTCCACTCTTACCGTCAACATAGATGGAGTTTTGTTGCC
AGTTCTCAAACGCCG
ACGAAAACTTCTGTACACGGAATGAACTGCGGCCGTGGCCATTGT
TGTCGATGCCTTTTT
T
>ConsensusfromContig1130 Average coverage: 4.14
AAATAAGTCCCTGCAATAGAAAAAATACGCCAAAATATAAACTT
CAAATCTTCAGCATTA
TATGCAAACAAGTTAATAAATCAACAATGAGATGAGAACAAAGA
ATACAAAGGAGAAAAA
ACCAACGACAAAAACACAGCAATTCATGATAAGACATTGTTCAA
CATCTATAATATGATA
TATTATCAAACATCAACACATTACCCTAAAGAATATTCCATATAT

A
>ConsensusfromContig1131 Average coverage: 11.28
CCACCGCCGTCGCGAAAACATTGATATTCAAATATAAAGGATCTA
ACTGCAAGTTGCGCG
AAAAACTTAACAAATCAGAATAGTATTCGATTCGCGGTATATATA
ACAATATAGTTGCGT
ATATAAGAATGGGAAAAAATATGGCGATACCAGCTAATCGAACC
AGACAGAACCACGTCT
TTCTGCAGCAAGAGTTTCTTCTGCAAGCCCA
>ConsensusfromContig1132 Average coverage: 78.18
TGGAGTTCAGACGTGTGCTCTTCCGATCTCACGGATTCTTCTGCA
ACTTACACGCTGATG
GGTCTCGCTATCTCTGATGGTCTCTACTGCCTCACCAGATTTCCTT
CATACTTTCTTCAC
GAGCAGCTTGCATATCGAGAGAGGAGCTTGGGGTACTTCTACACT
ATTTATCATGCTTAT
ATCAGTAATACTTTCGCGAGATCG
>ConsensusfromContig1133 Average coverage: 3.76
CGGAGATGTTGCCCTGAGAGCTTGTGGTTGAAGACGGACTTTTCG
GTTTGGATAGAGCTA
CCGGCTCTGGAGGTCTGGGTGATCTAGGGAGACCTGATAGAGAA
GATCTGTCTAAGACGA
ACAGCCGTTTGTACATTGGGAGAAATTCCCCGAAATCGATACTGT
TACTCATATCCACAT
CTGTGGATCGGAATTCTTCATTGACATATCGATCTATCATTTGCG
>ConsensusfromContig1134 Average coverage: 4,078.91
GATATTAGCAATGTGACTGGAGTTCAGACGTGTGCTCTTCCGATC
TGAGAGATTATCTTC
CGGCCGATTCTTTTCCGAAGGGTTTGGACGAAACGGTGGCCAAT
ATACGAGACAGTTGGG
AGTACTACTCTCTCAACATCCCAGCGAAAATCTTAGAGCTGAAGT
TTGGCGTGGCTGAGT
CTGAATTCCTTGAGATGTTTGCATCGGTCGAGAATCAGTTACAGA
TTGAAAGAGCGGAA
>ConsensusfromContig1135 Average coverage: 5.52
GTAACTGTCTCCCTGGCTACACCGGAGTCTTTTGTGCTATTGAGA
TCGCTGAGTGTGCAT
CAAACCCCTGTCAAAATGCTGGCACCTGTAATGAGATGGTCAAC
GGATACACGTGCAGCT
GCGTCACGAGCTGGATTGGAGAAAAATGCGAAACCAGGGAACCA
GCATGCAACTCCAGCC
CCTGTCAAAATGGCGGAACAGATCGGAAGAGCACAGG

>ConsensusfromContig1136 Average coverage: 7.47
CTATGTATACAAATAGGAGATAACGTACATATTGAGGAGTGGTG
AGAAAAATATAAGTCA
CGGTCACGATCAACAACATTCGACTAATCGTTGATCTGTCTTTCTT
GTGTTTATCCAGCT
TATTTTTATAATTTTTCCGCCAATTGAAAATCCCTAAAAAGCGCTC
TTTGTTTTCTGGAA
ATGGCATCACGAACATGTTGTGCAC
>ConsensusfromContig1137 Average coverage: 3.55
CCTCCGTTCCCTGCTGCAAATGCGTACCTGGAGTTGAATAGGGTG
TAGTTGAGGGCCCCT
CCAAGAACCGACATTATAACACCAGTAGGTACGACCCAAGAGAT
GTTACTGCTCATGGCT
TGACTGCTAAAAACCTGAGCGACTGCGTCGGATATGACAAACTG
TTCTGGCGACAGGACA
GTGAAATAAGCTGTGTTTATTGCAACAAAGAGAGATCGGAAG
>ConsensusfromContig1138 Average coverage: 12.53
ACTCTTTCCCTACTCGACGCTCTTCCGATCTCTTCAGTGAATAAGC
CGAATATTCTCATT
GTGAGCTATGACTCTGTTTCCAACCTAGCATGGCTGCGAAATCTA
CCAAAAACTCGGAAG
TATTTTGTCGAGAAATTAAACGGAATTGAACTTCAAAGGTACAA
CACAGCTGGGGATGGT
ACGCCAGCTGCCTTTCTCCCGATTTTAACAGGGAAAAAAGAAGAA
GATTTACCCAACTGC
TTGAGAGGGCGTGAAGCTGCAG
>ConsensusfromContig1139 Average coverage: 5.43
GTTCAAAACCAAAAACCAGATAACAACCAGCGAAACCTCTGTTA
TGATTACGGTTAAAAC
AATCTTGAGCTAATACGGATATTCTCGGAAAACACGCAGTTGTTA
TTGGCGCAATAGAAA
ATATGTGAGATGAGCCAAAGAGTTTCGCGAATTCAAGAAAATAC
ACGAAATAGTCGATCT
TTTTTTCAAAGAATCGGTTAACATAGAGTTGACACCCATG
>ConsensusfromContig1140 Average coverage: 5.23
AAACTACATATAGAAATATCAGCACAAAATGAAGTGATGTATCCT
ATTCAACCAATTTAT
CATCTGAAAAATAGATTCAGTTAAAAGTAGTACATGAACCCAAC
CCAGTACAAAATGTTC
AGGCAGAGAAATGTGAACGGGAAGAGCACTCTTGCGAGTGAGTC
GATTCGACTGACGCTG
ACAGGCCCTAGTTTGCCACATGATATCCGCTGC

>ConsensusfromContig1141 Average coverage: 6.74
TAGATCGCATGAGGGAAAATATTAAAAAGCGTTTGAATAAACAA
TATGTAAAAAATGATA
AATCGAAACAAGAAATATTACGATGTTTTGAGAAAGGTCAGAGA
GAATTCGATGCAATAA
TTCTTGATAAAAATAAATTATGCACAGCGATCAACGGCGTTTTTA
ATAAAATTAATAATA
TGACATATGACAATAATAATATTTTAATATTCTACGGAAGTACGG
AAGAAAACAGATTAT
ATCTCACTAATAAAATAACAG
>ConsensusfromContig1142 Average coverage: 12.70
TTACAAGTGATATTAAATTCCCCTGCATGGCTATTCTCTGCTGGAG
AAATCTTGACTTTT
TTCTTCTGAGTGTACTCAAGCTGGTACATATTGCCATCTGAACAT
ACGACATGGGTCTTA
TTATCGAACTCGAATACTCCGTTCTTTTTGATTTCTACATTCATCT
TTATAGTAGGACTG
CACCATTTGCCATGATAGGTTCCTTTTCCACTCTGTCTTTTAGAT
CGGAAGAGCACACGT
CTGAATTCCAGTCACATTGCTAAT
>ConsensusfromContig1143 Average coverage: 7.12
TTTTTTTCTGCGGGAAACAGTGGAGTTTTTTTGGGGCCCCACCGC
CACTGGCCTTGCTGT
GCACATGCGATCCATCACTCCAACCCTCAGTATCGCCGAATGTCC
CTACTGGGCAGGAAT
TCCGCTAACAGTGGCCGGTGTATCGGGAATATTTTACTGCATTCG
TACTCAGTCTGCATA
CTTTGGCAGCCTCACATCCCATGTTATGAAGTGC
>ConsensusfromContig1144 Average coverage: 5.76
AGATTATTTGAAACCACGGAGTTTTCCGAAAGAGAAGTGGTGGTT
TGAAATTCAATGGCT
CGACGATTCGATGTTTGACCTTTTCCTGCACCAAATCTATTGTAG
ATATACATGATCATG
ATCACTCCGATGAGACCAATCACCACCCTCCGTGAATTATATAAC
ATGGCAGCTCTCCAG
AATCTGACGTTGGCATTTTTTTCGAAAAACCCGATAAATTAGAAA
AATTTGAATAATTTT
CAACCATTG
>ConsensusfromContig1145 Average coverage: 14.22
GTGATATCACCTGTGCTATCGAGAGAGCATGGCTGCAATTCTCTC
GATATAGATTTTTCT
CTAGGTCTGTTGATGCCAAAAAGCTGAGAAATACGTTGATGTTAT

AAAGGGTTTTATGGG
AGCCGCTTGTGGAATAATAATCAATATATGGTTTGAATACATGAG
TGCAGTGCGTTAATT
TTCTCGTTTAAATTAGATAGACAATTACTAATTTCCTGAAGGCTT
CAAGAATGCAAATTT
TATAAATATCTTAGAACATTATCTTGTCTTTATAATAAGAAATAT
CATAAAATCAACAAA
CCTGCACTATCCCAAAAAC
>ConsensusfromContig1146 Average coverage: 5.95
CCATATTCATTTTATTCTCTTGCATTTGATAAATTCCCCCTATCAA
TAATTTAAGTTTTT
GATTATTGAACTTTATCCACATACGTTCCCCTAACTCATTTTCCTC
ATTGATTAACTTAA
CGCAATTTACTATACTATTATTAACCCCAATCATTATGCCTCCCCC
CTTTGTGTCCCTCT
CTCTTCTATATAACTTGTAACCCTCTACCCTGATATTATCTTGCTC
TCCTAGATGAGTTT
C
>ConsensusfromContig1147 Average coverage: 3.40
TCTTTGCCACTTGGATTATCCATTATGTCTACGATTTTCATCTTTA
TTCTCTACACCGAA
AGTCAACATATTCGACAATGCCAATACGAGTTTATCATAATTGTT
TCGTGGTTAATGATA
AACGTTTTTATTATATTAGAAATGCTCGTTTTTATCACTCCCAAAT
TAATGAGTAAAATT
AAACAACATGATTCTCTGATAAACGATT
>ConsensusfromContig1148 Average coverage: 6.23
GGACATTGCTTTAAAATACTGAGACAAGAAACATTCATAATGACA
CATTTCATATCAAGG
AATCATATTGGCGATTGTTCACAGACAGCAAGAAATACAATGATG
AATGTTCTAGTACAT
ATACCAAACTTTATGATGCTAGTAAATAATCATTGTGATGACAGG
TTTTCATTTTGAATG
ATAATTTTATTCACGATCAC
>ConsensusfromContig1149 Average coverage: 5.12
GTCAACATCATGAGTGATATTATATGAATTCAATTACAATCTCTA
CCACCAGCTCATAAT
ATAATATAAAATTTGTAGCTAATGCCAGTATGTACAAATTAAGAA
CTAATGACTAATGAG
TCGAACGAATACGTTTTCAGATGAAATTGCAAGATGGAACATGGT
TGAGATCTAATTGTA
CAAAATGCGAATTGATAAGCATGGAACC

>ConsensusfromContig1150 Average coverage: 4.35
GAAAAAAAAAAATGAAAATATTGTTTTGTCAAACTCTTTTATCAT
GCCACAAGTGAATCC
TAGATATAAACTTCAGTGGATTATTGATGAATAAAATCAGAAACA
ATTTTGCATAAAACC
TAGTGAGCCCTGAAAACACCGCATAAGGTGGAGCTAGCATTAAA
GACACCAAGAAACACA
TTCATGTGAAGCGAAAAAGATTCACT
>ConsensusfromContig1151 Average coverage: 8.87
GAGTATACAATTTCAGACCGATTCTTATTAATCTGATGAAACGAG
AGCTGGTCGGGAAAT
TCAGAGAGCAATCTGGCCTTCAATTTATAATTTGAATAAGACGAT
AAATCTAACTGATAA
TCTCTATCAGCTAATTCAGCAAACTCATTTCGCAAATCTGTCATC
CAGCATATTTGATTG
TCATTCAAAACTTTCTTCTTCATCACTGAATTGCAGAACTGAGTG
AAAG
>ConsensusfromContig1152 Average coverage: 12.71
CAGACGTGTGCTCTTCCGATCTCAAATATCATCTCTGTTTATTCCA
TGTGGGCCTCACCC
TCTGCGATGGTTGCCCAAGCGCTAGCATTGAGCCAACAAGGCAA
GCGACGTCCGAGAAGC
GAGAAGAAAAACATGCCAGACACGTTCAAGGATGACAAGTACTT
CGAACGGCGAAAGCGC
AACAACAACGCTGCTAAAAAGTCACGAGATGCCAGAAAGAACCG
AGAGGACGAAATTGCT
GTGCGGGCCAGCTTCCTT
>ConsensusfromContig1153 Average coverage: 6.24
GCTCTGTTGGCTGCACCCTGCATGAGCATGATCTCCTCTTTGAGT
GAATCAACCACGCGA
TATTCGTTCAGCACTTGCTCTGAAACCCTCTTCCAGATCCAGTTGA
CTTTGCAGCATTCA
TTCAAGGTCATTTCATCAAGATAGCCGAGGATTTTCTTCGATAAT
TGAACAGGAAGATCA
AGAACGAAATCCCGATGAATGCACATATGATCAAGGTTCATCTCA
G
>ConsensusfromContig1154 Average coverage: 7.39
CTGCTTCAAAACAGTTTTGAACATGTTTTTATATTTTATCTCTACA
CTCTATAAAGTAAA
TGCACAAATGCACACAATTTCATGGAATTGATTGTTCTTTCATGG
AATTGATTGTTCTAT
GTCTTGATCGCTATCGCAAGCGCTAGATTTTTGCAAGGAATTGTTT

CTTGATGTTTCTTG
GTAGCTAAAAAGTAGGGTCAATGCAATCACCAACCCCCTTGATAT
TGCGCTTTTGAAATC
TGGATGAAAAAAAAAATTTGGATGAAAACATACATCAAACGAAG
TGGGCTACAAACCATA
TGGGTTATCAACTTTACCCGCGA
>ConsensusfromContig1155 Average coverage: 10.57
AGATGAATGAGTTATGAATTTATGATCATCACATACTTTATTCTT
CAAAACAGTGAGAAT
GAATTGTGACTTTTCTTAGATTGCATCACGCTGTATCTATGATGAT
GATATTGCTCTTTC
TTCATGAGGTTGCAATCGAATGTGTCGTGATGTGTATTTTCATATT
GATTACCATACATT
GTGAAATGGTGATTTAGACAGTCGATTTTGGTATATTATATATTT
GTTATGATTGGTGTA
AAAATTGCAAGAGAAAATTTCTTTTGATATGTTTGTTCATTATGC
TAGAAGCGAATCATT
CATTCTTTTTGTTATATGTTTGAATGGCTATTCGTAAGCATCCCTC
GTAATTTCATTCAG
ATCATGTTCAAAACGGTCATTCAAAGACTGTGATTATATTTTTTTT
TAATTGGTTTTTGC
CCATG
>ConsensusfromContig1156 Average coverage: 6.07
AAATGGAGCCTCTGCAATGCTCATACCATTCTGTAACACCGATCT
TAATAGCATCCTTGA
TTTCTGTGCCCATGTCCTTATTGAAGGGACAACAACATCGATAGG
TCTGAGATTCGTAGA
ATGCTGATAGACATCTGAGCATTTGTTGGAGGTTCCATTTCGCCTG
TTTATTGTTGGTCG
GATAAAACTTTCTCAACTTCTGA
>ConsensusfromContig1157 Average coverage: 533.78
ATGTGACTGGAGTTCAGACGTGTGCTCTTCCGATCTAAATGTTTG
TGGTGTACCACAAGG
CAGTGTTTTAGCCCCAACCTTGTTCATAATATTTATTAATGATCTA
TTAACATCAATACA
AGATATGCAGGAGCGTAGCCCACGTAGTGTGGACATCTATCCTGC
TGCATATGCGGATGA
CACACTCATATCAGTGACAGCATCAAATCTTCCTTTTG
>ConsensusfromContig1158 Average coverage: 4.76
GGAAGAGAGTTTTTCTCCTCATGGTAACGTTGAATGTGATGTCTG
TGAATGGCTCATCGC
AGCACGTGTAGTACTTCTGTGTCTTCTTGGCGGCGACTCTGAGCA

AGTCCCACTCAACGC
TCCTGTAGAAGTCGGTGAGGTCGATTCCGTTGGTGATGACAGCGA
GGTGGTCGACTCCGC
GCGAACGGGAAAGGAAACGTCCGAAACCCCACCAAAACTGT
>ConsensusfromContig1159 Average coverage: 6.06
TGCAATTCAAGCGCTTCGGCGCTATGTGTTCAGATGATAATGAAC
GAGCCTCGAATTTGA
AAGAATTCTCGAGCTTGAGTGAGCCATGATGAAATGGACTTTTTC
GACTCTCCAAAGCGC
TACAAAGCGTAAAGGCAATAATGACCGAAATTCACTTAGAAGGA
AAGGGGCCCATCAGCG
CCAAAGTGTAGCGCTGAAGCTTCTAGGTCGTGATACAGAA
>ConsensusfromContig1160 Average coverage: 3.58
TACAGAATCAGCTGCACATTTTATTACCTCGCAACCCCCTTTGCT
ATCAGTCTCATAACC
AGTCTTACATGTACAAGAATAAGATCCAACAGTGTTCTCACAAGT
TGCAGTTAGTTTGTC
ACAATTCTCGTCAGGTTCAGCAGCACATTCATCTATGTCCTCGCA
ACCCCCATTGTCATC
AGTCTCATAACCAGTCTTACATGTA
>ConsensusfromContig1161 Average coverage: 10.39
TGTGTGTTTCATCAGTATTTTAGGCATATACGAACTCGGTATTTTT
TCACAAACACTGTC
GTCTTGTCCTTTCTCCTTTGGGGGCTCTCCAGAACAGCACAGTAT
TCTGATCAAACTGCT
TTGCGCATGTCGCATGACATGATTTGGTTGAAGAACTGCGCACGT
GACGCAATGCCGAGC
CGCAGCAACTCGTTTTGCGTGAAGAACCAGACAGCCCCCAAAAA
AGGCGAGTTGGTTGAT
GTAGCAGAAGAAGATGGCGACCCCTGTAAAAATGCAGAAGTTTC
TAACACTCAT
>ConsensusfromContig1162 Average coverage: 6.75
GTCACCACAAATTATGCACGACTATTATTTGTCAGTCTTATTCATT
CTTATATTTCCGAT
CACATCCGGTGTGTTCATTCATGTTCCCCACACCAGCAGCAGAGG
GGATCGGAGTGGCGT
TGATGGATATTCTTCAGAAGATGTTTGTTCCTCATTTAGTCGTCAA
TTAAAACTTTTTGA
TGAGACAAAAGGCCCTCTCGATGTGATGTTCACCAAACAAGAGTT
CACGATCATTTTTTT
ATTCAAAATCAATTC
>ConsensusfromContig1163 Average coverage: 42.45

GTTCAGACGTGTGCTCTTCCGATCTGCGTGTTTGAATTCTTGCAG
ATGTAGGGTAGGAGC
GTTATCACTAATCCCCATTATTTGCAGAAACTTTTGGAAGGTCGT
TTCCCCCAGAAACAA
ATTGAAGTCGACCAGACACTGCCTCAGATCCTTTGCTGTAATGAC
CCCTCTCTGCTTTCT
ATCAATTTTCTTGAAGAAATCGTCGATGGTTTCATACCGGCTCGT
GGCAATATCTTTGAA
GTT
>ConsensusfromContig1164 Average coverage: 4.25
CGAAGGAACGTGAAGCTGATCTCATCCACATCTTCACAAAAGGA
CTAAATAATGAAGCCG
TCAAACTAGCCGCTCTCAGATCAGGAAGAAAGAAATTTAGTGAA
GTGGTAGATATAGCCG
CGACCGAGGAGAAAATTCATAAACAGATCAAACGCTCCGATAAC
ACCAATGACCATTTTT
CATACGACCATAATCGTGATAAATACCGATGGAGACATGACGAT
GACAGGCATAAAAATG
GCAAATATTCTAG
>ConsensusfromContig1165 Average coverage: 6.95
TCGTTTCCAATTCCAGAGAGGCATGATGACATTGGTCCACCAAGA
ATCGGTCCTGGCTCC
TTAGCAGATGAAGTGTCTATCAATCAAGGCCCTTATATACCACAA
ACAGTCAACAATGGT
CTACACGTCGGAATTGTTGAGCCATGGCTGCCCACTCACATCGAG
AACACAATGCTTAAT
TACACAGAAT
>ConsensusfromContig1166 Average coverage: 17.46
TCGGCGTTCTTGGAAGCGGTTGGGTCAAAAGCTAAAATTGATTGG
CCCAGTTGAGGATCC
ACGAGGGGTGCACATTTAGCCCACGTTTCGAAATCTCCATTCAGA
GCTTTGTCAGGATAG
AACACAGCCAGAATTGCCTGAATGACGTTTATGATGACGCAGGG
AGGCCGGTCGTAACTT
CTGAGCTCCAAATAATACTTCTTGTTCATTCCAGCGACGCATTGG
CGTAACTCCATGAGC
ATAAGCCGGTCGAAGAGGACCTCGCTTCCTTCATCTTCCC
>ConsensusfromContig1167 Average coverage: 11.16
GAGGAACATCTGACTGGTTCGTGGTCACCACCAGCGCAGAAATGG
CCTTTGGGGCATTCA
TTGAGTACGGGGCTGGCCGTATCACTCGACGCGGGGCAATAGAA
ACCAGGATCGCATAAT

CCCGTCGGAGCCGTATTCCCGTACCCAGAGCAGTATGACCCCAA
GGAGCATGGGAGGCAG
TCCCCCACCGCTTGGGCCAGGGGGGC
>ConsensusfromContig1168 Average coverage: 4.57
TTTCTATCCAATTTTCAAGAATCTGAATGTACTTGATTTGACAGG
AAACCAACTTTTGTG
CAATTGTGAATTAATGTGGCTGAAAACGTTGATAATTCAATATTC
TCATGAATGGGATTT
TCAATGCACGTCTGCGTTTTATTATACGCCAGTTTTCGGTCACGG
AATCGGGGGACCATT
ACAATATGAGAAAATATCGAAGATCCCAGT
>ConsensusfromContig1169 Average coverage: 6.08
TCGAATACTAAATTGTTTAAATGCAAAAAAGCTTTGTGACACAAA
ACAGATAACTGACTT
CCACAAAAAGTTATGGACTGTGATCATTAATCAGATCATTGAAC
AGAGGTGCAGCTAACG
GAACAAGAATGTTTTGGACTCATGTGAGAAAATGGCCTTCATTGC
AAGTCATTTGGAATG
AATTTTTTCAGGGCACCAG
>ConsensusfromContig1170 Average coverage: 9.75
CGACAACAGTTCTGGCTTCTTGGTGGAGTAGCCCCACCAGCCGAT
AAAACTGTAGGCCGG
TGCCTACAGAAGTATTTAGCCGGTGGTGCCACCCCTCAACGTGAT
TATTCGTTCTGATGA
CCTCCCTATAGACATTCCATGAGGCGATCGGCCACACCGAAGAAT
TCATCCAGGTCCTCT
GGACATTCCGGAAAAAATCGAGAAATCTCAC
>ConsensusfromContig1171 Average coverage: 5.70
ATTCATTCATGCATTGTAAATAAGAGGGTCTATGGATAGAGCAGT
CACTTCCAATTAATC
TTTCATCACTTTCGTACTTTCCAGTCTGGGGATTTAAAAATCTTAG
TTATTATTGTGCAC
CATCCTTTTTTCCAAGCTGGCCGGTGTGTGGAAAGTAGGTTAGGT
TGGCTAGAACGGAAG
AGAACACCACTTGCACTCCC
>ConsensusfromContig1172 Average coverage: 4.18
ATAATTATTTCTTGGGAGCTTATTTTCTGAATTTCTTCCTCTAGCT
TGTGGTAAAATTCC
TCCGATTCATGTTCGTGAGCCAATGTGGGGGCATGAATCGAGTAT
ATGTGAGTTTTTATT
TCGGTATTGTGGAGTGTGGCTTTACACACATTTTTGAAGATATTT
TGAAAGGTAGGCCCT

AGTCTGTTATTTATAAGATCGGAAGAGCGTC
>ConsensusfromContig1173 Average coverage: 6.85
CACCACTGTCGATGATCTATCTGAAAAAGAGAACGAAACTTATG
GCGATCAGGAATTGGT
GGAGATTCCGAGGTGTATAGAGGATGAGCAGGTTTGTCTCCATCG
GCTCTGAGCATGTCG
ACGGGGGTTCGAATCTCTCGGTGATGGCCTTTCTTTTTCGAGCAT
CTCAATCATCCACGT
TTTTATGTTCTCTTCTACACAGA
>ConsensusfromContig1174 Average coverage: 4.37
GAATATTTGATGTCAGTGGACACTGGAGAGGTACTTGATATGGAG
GTTATGTCGAAATAT
TGCAATGTATGCCATGGGAAGCCTAACTCAGCGGTTTATTCATAC
GATATTAATTATTCG
GGTGCGAGCGGATCGATTGAACAAGAGGCAGCGAGAAGAATATA
TTGTCTATCAATCAAA
CTGAATAAGATAAGGTATATTGATTTCACCTATACAAGTCTTCTT
G
>ConsensusfromContig1175 Average coverage: 11.20
CCGCATTGAAGAGTTTTTGGGGCATATTTCGAAATCACTATCACT
ACTTGAACCGGGCTC
TTGCTGGATGAACTTGGGGCTTTTTATCGAAGACATTTTAATCGG
TCGATTTTTGGCGCC
AGAAAGCGGAGAAATGTTTTTGTTTTGTTTTTTTGCCATCGTCCC
AAGAGGCGAAACCTC
GACACGTCTTCTCTTCTTCGAGCATTTCGCTCCATCGACTGAAACT
GCA
>ConsensusfromContig1176 Average coverage: 8.21
AACTGCGAAGTACGTCACTTCGATAAGAGAATCCTCAAAAGTAC
TTAATGAAGACGAAAG
TGCTGGTATAACCATGAGAAACCAAACGAAAATACACTGGGCAG
TTGCGAATTTCACATT
GTAGTATTTGTAATATAATCTCGGATACGATAATGCAATGAAAAC
TTCAGCTATGATCAA
CGTCAGTCCGTTATTTGATGCTCCAATACTCACCATAAA
>ConsensusfromContig1177 Average coverage: 3.60
GTTCTGAAATACGTTTCTTTATGCTTACTTGTACTTATATACAGTA
ATGACTGCTATAGT
AATGTTCACAAACAATATCTGAAACTGAACAAAGCTTTATTCTC
AGAGGTTAAAGGACGG
ATGAAAAAAGACGTCGAAGAAGTAACAAGTCTTCCATGTTACCT
CCAAGAGAACAGAGGA

TTCAAAGCTGTTGAGAATAGCGATCAGGCT
>ConsensusfromContig1178 Average coverage: 9.57
CGAGTGAATACCATTGGGGGTAGAGCACTGGATGGGCTATGGGG
GCTTACCGCCTTACTG
ATCCTAACCAAACTCCGAATACCAATGAGTACTACTCGGCAGACA
CACTGTGGGTGCTAA
GGTCCATGGTGGAAAGGGAAACAGCCCTGACCGACAGCTAAGGT
CCCAAAATGATGGCTA
AGTGTGAAAGGATGTAGAAATCCCATGACAACCAGGAGGTTGGC
TTAGAAGCAGC
>ConsensusfromContig1179 Average coverage: 9.32
GGAGAAGTAGATCGGCCTTTAAACGGAATTGTAGATAAATTAGTT
GCTGAAGTTGTAGGT
GATATGGGCGAAGTTCTGGTTAGAGTAGTGGGCAAAGTTGCAGA
TCGAGTATTGGGAGAG
GTCGCGGGCAAAGTTCCGGATATAGTGGGTGTACTTTTTAGAACA
GTAGGCGAACTGTTA
AATGCAGTAGTTGGTGAGGTTCTTAATCGAGTTGTGATTTGCGTA
GATGTG
>ConsensusfromContig1180 Average coverage: 8.37
TGTCGACTTGAATTTGTTCTTTCTGGATTCTATCGTATTGCGCAGT
AGGTAAGTGTTATC
TGCAATACCATCGCCAGACCGAAATGCTTTTTGTCTCACGCTGATG
TTGTAGTGTTTTTC
GATGCGGGCGGCCAAGATCCTATGGAATAACCTACCAACAAGGG
GACCCATGGTGATTGG
TCGATACTTTCCTGGTTTGTCCGCATTTGCTGTTTTTGGAATTAAA
AC
>ConsensusfromContig1181 Average coverage: 3.70
CAATTCCAAAATATTTTGATTTTGTGATTCCTATTCCCAGCAAAC
CCTGAAAAAAATCAT
GAAAGTGTCAGTATGAAAACACCCGCAGAAAAGCGATGGCAGAC
AACCTCTGTAAATGCA
TTGAAAGCTAGAATGAATACGAATAATAATTCAATATGACATCA
AAACGTGAATCTCCCA
GTCGGTTATATATATGTATATTATTATACTTTTCAAGAA
>ConsensusfromContig1182 Average coverage: 6.34
TTTTTCAAATAGTCAGTAAGGCCCAATCAAATATTGTGGTACAAA
TAAAAATATTATCGC
AACATATAACCGGAATGAATATGCCACATATTTTCAGTATAGAAT
GATTGTTTGTGGATG
AATAATCCCATAAAAATCCCTTTAAACCTCTATACATAAATATGC

ATTTGAACATATGCA
CTTATTTTTCATTAGCAAGATCGGAAA
>ConsensusfromContig1183 Average coverage: 7.54
GGGGGCTAATCCGTGCGCTAAAAACGCCAAATATTTTGGTTTATT
AAAAAACCTTTTTAC
GTTAGCCACTTCGTCGCTAACCTGTTGTTCGCTAGCCCCCTCCCT
TGCCGTTTCAGCAC
AGCATCGCTGCTGAATGTGCACGGATTCGGTTTGATTGAATGGT
TCCCGTGTACGCTAT
CCCCCTCTCTTTCAGGTATTTGATGAGGGGGATCGATGTGAAAAA
TCTGTCCGCATACAC
TAGGGTCCCCCGAGCTAGTCCTGCTGTCAGTCTCATGACTG
>ConsensusfromContig1184 Average coverage: 5.19
CGACGGATATGGTCTGACCTTTCATATCTTATCACACATTTGGAG
CGAGCTGGGTTGATA
TTTTCTTATAACTGATTCGTCCTGAACTATGCCTTATGTCCTATA
ACATACTAGACGGGG
TTCCCACCATTCGCATGTCGTCGTATTTTATTTTATAACCCATATA
TACAAAATCCACAT
ATATTCTCCTTTATCCTATCACATGTTATATGCCA
>ConsensusfromContig1185 Average coverage: 10.07
AAGATAAAGAATGTTGTTGACGAGGCATCTAAATGTAATTGAAA
ACAGTCAGTGAACAAT
GAAGGATTTTTAAACATAGATTTCGGTGGTATGGTTTTGTCGAAT
GGCACTGTGATATTC
GCAACTTGAATTCCATATTTCAAAGTGTGAGTTTCAAATGTAGAA
GAACATTGCCAAGGT
TGGTCGTTTGAATAAACATCAGAGATATTTACGATAAAAGAACGA
TTTTGAATGTTGTTT
TCAATCACATTTTGACATTTGACTTTGAAACGAGAGTCGTTTTTTT
TCGGCAGTATAATC
ACACTATGTGTACAACCATTATCTTCTCTTATAATATCTGCTTGTA
ATTCTGTTTTGTTG
TTATAAATCTTGACAATATTTGGTGTTGTAGTTGCATTTCTAATCA
TATTTT
>ConsensusfromContig1186 Average coverage: 8.97
GACACATCATTTTTCACTGGGTCTGTTTTGATGATATCAGATTTGT
TTTGAATGATTTCT
GTACAACTACTCTGAGCTTCTTCATCGGAGGAAGATTTTTCGTCTT
CATCGCTTGTGTTG
AGCAAGGGACGTTCGGCCCCTCGTTTACCTCCAGTGGTTGTATTA
TTCATAAAAGAATTA

CATCACATGGTTTAACTTTCTTAGAATATTATATAATTGTGAATA
TATCTGATCAAAACA
TGTTGTGC
>ConsensusfromContig1187 Average coverage: 5.06
TCTATATCTAGAATCTATATTATTATATCTCTATCTATGCATCCAT
CGTACTTTCTATTC
TAACATTCAAACTGCTTTGTTGGTGGTGTTGCACATCCTAATATG
AATAATTTGATTGAG
AAATTCATCTTGTTACAAACTCAGGTGATATTGAGTTCATATCTGC
CGTTTTTTTGTAAT
CCATGAGAATTAATTTTAAACAACACATCGACTTTCAAATGGG
>ConsensusfromContig1188 Average coverage: 7.41
TACTAGGTAGATGAATCAGAATCAACATGGGGCTCGAAAAAACG
GCGATTCTTGCCATGC
CACTCGATATAACAACATCAGTAAACGCTCATTCATTTGTGGATC
TGCTTGAATTCAATC
ACGTTAGCTTCATTTTCACTGAAGTTAAGTCAAATTGCATCTTATT
ATATTTACGTTTTT
GTGAAGCCGTTCCAAATTTAGGGGGGG
>ConsensusfromContig1189 Average coverage: 5.35
GTGCTCTTCCGATCTATATGACACAAAATACCAAGGTTTGGACA
CACAAAGGGCAAATAC
CAACCCACTATAAGCGTGAATTCATAGAATGCATCGCATCCGATG
AAACCCATATCTTCT
CCGTCACTGAATGTCGGAACGTTGTGAAATACGACGTGGCTAAA
GGAGAATGGTCTCTGC
CAATGGGAACCCCTGTGATGCCTCTGAAGGAAGAACCCGAATGA
TTCTCGACGCAGGTGA
GC
>ConsensusfromContig1190 Average coverage: 8.40
TGTTGTCGCCTCCGTTTTACTGGTCTCCATAAAAGCTACTTATAC
GGAAAACGCTGATGC
TTGTCCAGCAACACCGACCACTGACCAGTGTGGTGCGCTAACGAA
TTTCTGCTATGATAG
AAAATTTCCAGTGGCGGCACAATACATATGCGAATGTCTTGGATC
CACTGGCGCAAAATG
TGCAACCCCCAAGACAGAGTGTACTGGGCC
>ConsensusfromContig1191 Average coverage: 4.06
TGGCAAGCGTCTGTATAATGAATCAGAACAGCTGTAAGAAGAAT
GAAGCACTTCATGTGG
ACTCAATCGGTTCATGGAATCTATTTTTCTGACTCGATGTGTCGG
AGGAAATTTCGTAGA

TCGATGTCGCATTTCTATTGAAAGTTCTTGAGTTGGATTGAAATG
GAATGTCATTAAAAA
TCATATCGAGATGGTGAGAAGCAAACAAAGAAACTATATGATGGT
GTTCATATATTTATG
GGTTTTG
>ConsensusfromContig1192 Average coverage: 19.67
CGCTCTCTGCATCATTTGTATCTTACTATTCGTCTTTAGCAGGAA
AATTAGCAAGAAAGT
TGGATTATCTAGAGGCCATGACTCTGAAAACGGTAGCGTTGGTGT
CGTGAACCCGATTTT
CGTGGCTGGACAAGAACTCAACTAAGGGGTACATGCAAGTCTGG
AGATATTCTCTGAGAA
TACAGATCGGAAGAGCACACGTCTGA
>ConsensusfromContig1193 Average coverage: 5.77
AGATAATGAAAAAGACTTGAACGAGCAGTGCCGTAAAGAAAAAA
GTCTTCGGATATTTTA
CAGTCAGCTTTGCAATTAGAATTGGATCTTTGTCTCTTTCTTCCTT
CTCTACAAGTTCAA
TGTCCGTATGGTCAGTACGCAGCTCTTCTATAACAATTTGTTCTTG
CATAGCTCCCTGTT
TTATATGATATGTATGAAGTGTCAATATATGAATCCGAA
>ConsensusfromContig1194 Average coverage: 3.54
CTCTTCCGATCTAGGTGATGTACAGCGATCTCCACAATGTGTCAT
CAGCAGATTCTCTAT
GACTGAGTGATAGAGCGCCCAATAGATTAACGTGTGATCCAACG
TTCAATCCACAAGTCG
GGAGATATTATCTGTGTAGATTCATCTATAGCCAGACAATATCTA
TACACTATATATCCT
TGTCTGGCACTTGTCAATAAAGGGTTGATATTTCAGACGCTAAAA
CAGCCCCA
>ConsensusfromContig1195 Average coverage: 5.47
GCAAATTTCTCCTACATCGCACGATTCATATTCGACACAGGTCGC
CAACCCGCAGAATGT
TCCCTCCCACCCATTTTCGCAATCAAGTTTATGAACTCTGTTGGCC
TCGCATGACTTGCA
ATGATTGGTCGTTTCCTCACAGGGGGCACATGTCGCTTTGTCGCA
GGTTGGGTAAATCCA
TCCGGTCTCGCAAATGCAGGTATCAC
>ConsensusfromContig1196 Average coverage: 5.19
CTAAAGGATCCTGTTTCAAAGAGAGGGGATGTTGTATGGTCAAAG
GCTTTGGTCCTTCCA
CGAGCTCAGTGCGGATTTTATGAGGACATTGACCCCTCAATATCA

AAACAAGCTGATTTC
ACTCTCAACATTCGAGAAAGTCTTCCCGTGAGATGCTTGGATCCA
GTCAAAAATTCGAAT
ATCAGCGGGGGCAAATCACCACAACATGATCCAGATTTTCTGATT
TATTTGCTTTGCTTT
CTGA
>ConsensusfromContig1197 Average coverage: 5.73
CAAGAGCATTTTCAACGGTATCCCATTATAGTCTATGCATTTTGA
CAGGTTTAATACCAA
TCCTATTAGAAATAAAGCGGAAAATATGCATGAAATCTATTATTC
AAAACAGATACCACC
ATTCTAGGGCAAGACGACATTTAGCACACTGGGAGTTTAATGCA
GATATCACAAAAGAAT
TTGCAAGATCGGAAGAGCGT
>ConsensusfromContig1198 Average coverage: 6.48
TCAATATCTAGGTGCCTTCTATTTTTCTTTCTATCCTTTTTCAATG
AAAATAAATGTTTA
TCAATAGCATGTTTATAAAATGAATGTTTGGCTCTTAACGCTGGT
AAATCTGGGATTTTA
AATTTTAAAAACTCTGGGCATATATCAAGTTCTACACAATGTTCA
AAGTACTTGATATCT
AAATCAAGTTTTAGATGCTTCTTTCTGAGTTTCTCAAGATTTCTAA
ATTGCTTCACAGCT
ATATGTCTTCTGTTTCCATATCGATGATAAATAGAT
>ConsensusfromContig1199 Average coverage: 14.49
ACTTGGATTCCACCAATGAAAATCAGCATGATCATTTAAACAAGG
TCTCGTTAATAATAA
ACATAATTTATTATAGTCATCTTTAGTTGGTGAAAGTCCCTCCAG
CACTGTTAAACACTC
AACAATTTCATCAACAGCAACCTCATGGTTTATATTATCTTCAGA
TTCAGATCGTAGACA
CAGAAGTTCAAAAAAATTTGTATTTTACAATAATAAACTTGAACAT
CTTCAAATTAAAATT
TGTC
>ConsensusfromContig1200 Average coverage: 10.39
ACACGACGCTCTTCCGATCTATCATTCTAGACATGAAAAAATGA
TAGAAGACAGGTAGAG
GAAACGTCCAGAGAAAAAAATAGCCCCTGAAGTCGAAGAATGTT
CAATAAAACAAAAAGT
TGAAACAAAATGCTTTATTGCGTGGAAACCTATTCCATTGATCAA
CGGATCCAATGCTTA
ATCTCTAGTAGGTGTGCTCACGGGGTCTGCGCTGGGGTTG

>ConsensusfromContig1201 Average coverage: 5.85
TCGCAATCACCATATCAAATGACCATGGTGACAGCACTCGTCAGT
CAGGAAAACTTGCTT
CATATATTTTAGGCTTGTCTCAAAAGCATGAGGCAGATGAGAGAG
TTCTGAAATATATGT
TGACAATAGCACATCATACAATACCATATTTGGAGCCGAACGAA
ACTGACATGTTCACAA
ACTCGGATATCGTACATGTTATGCAATTATTGCACTCATTTTCTC
CAGAGGAAGAT
>ConsensusfromContig1202 Average coverage: 8.27
TGAAATCATAATAATTGTGAAGCTTATGAATTCACTCATAGGGAC
TTATGATCACATCAC
ATGGTGTATTGAAAAACAACCTGAAATTGAGAAAATGTTTATTCA
TGATATTCACACATT
ATCTTTGGTAATATGATTGCACTTTATTTATCTCTTTTTGGCTCTC
ATTATTGGGTAATT
CATGAAAACCTCCTTTTATTTAGATGAAAATTAAAATGATTGCAT
ATAATCGAATATTCA
CAAGCCATTGTACGAA
>ConsensusfromContig1203 Average coverage: 5.42
GATGACCACAGAACTCTTAATATATCAAATAGAATCAATGATCGT
TGGATGGTCCAAAAC
TGAATAATTATGTTACAAAAACATTTTGTTGAGGAATAATATTTG
TTTAAATGTAAGTGA
AAATATTCGGGAGCAGGCGGGTAGATGAGTGACTGATTACTAAC
ATCAATAAATGCAAGC
GTCTGTTTGAATGAGTGCATATCAGATTTCAC
>ConsensusfromContig1204 Average coverage: 7.21
GCCTCAGTGGTTTTCAGTACTTCAGACACGGGGCGAACATGCCG
CAGTGGGGAATGGCGA
GAGTCTGACTTCAGTATGGTTCTTCTCTCACATTCTGCGCGAACT
GAGAATGTTTCTCAC
GTCTGCCAGTCTTCGCCTCTGGAACAAGGCGTCAACAGTTTGCTG
ATAAAAACATTCGAC
GATGGAATCATAGATGCGCCAGGCTCTCTTCAGAATGTAATTGTC
GAGATATTTGAAGAT
CACTACGTGGAAGGAGTACATGTGGGTAACTACAGAATCGCACG
TCAATGGCTGAAAATC
GAAGAGGCTCGAAGTCGGAGATCGGAAGAGCGGCGGG
>ConsensusfromContig1205 Average coverage: 7.62
GAAAATAATTAATTTAGTATTAAGGAGGCGCCTTCAGATCCTCCA
GAAAAGTCATTCCGG

ATCTAATAATGAATATCGGAATCTTAGATTGGATTCAAAATTTGA
TCTGAAAGTCAGATC
TATGGATCAGAACAGACAGGAGCATACTGAAAATCGAACATTTT
CAGCGCACTGACTGCG
CAGAGAATTTATTTATGGCATATGAA
>ConsensusfromContig1206 Average coverage: 7.58
CTGGAAAGACGGATGGCTGACAAATTTTGACCCTTTGCCATATAT
CATTTACTAAGTAGA
GAAATATTGCCTAATTGTTTCAGAATTTGGTTTGGATAGTTCGGA
CTAGTTATATCCTTG
GATTGTGTTCAGTTTTGGATTAGGTGGCGTATCCTTGAACCACGG
ATTGCTGACGAAACT
CATTGAGGCTCCGAGATCGGAAGAGCACACGTCT
>ConsensusfromContig1207 Average coverage: 5.70
CTTCACTTTACCCTTCTATGTCTCAATTATAGAAATCGGCTCTATT
GTATCATTCAACTT
GTTTGTTTGTTTCTTCTTGACTTTCCACCGCTGGATCAGAATCGG
GAAGCAAACAAAACC
CGCAGCGAGCATTAAAGATCCAGAGAGAAAGAATGACACTTCGT
ATGAATCTGTTCTCGT
TTTGATATAACCTGTTAATGCAGAACCGGAAAAATTAGCCACGCC
CTCGAAAA
>ConsensusfromContig1208 Average coverage: 7.21
TACTCGAAATCTTCGATGGCCCAATATTTGGTTTGAGAAATATTT
CACGGAGTAGGCGCG
AAAGGTTTCAAAAAATTTTAATACGTCTTCGGAAAGATAGTTGCG
AGAGATGTGATTATG
GCAAGAAGATAGCAGAAGAAGAAAACTCTGTCAAGCAAGACAGC
GACAATTCTCCATTCC
AGTTCTCTTCTGTGTTTCTCGTCTTTCATGTGAATTCTATGAATAA
GAG
>ConsensusfromContig1209 Average coverage: 4.51
GCCGCATACATACAATATTGATATCTAATATTCCATTCATTATTC
CCACATGGTCATGTC
ACTTTGTTAACCTACTAGTAATCACTAAGCCATTTTTCGATCGTTC
GCGTTCTGGCTTAT
TCAAATACATTTATTCTTTGATCGGTTTGATGGTTGACGAATTAA
AAATGTTCCATTACA
CCCTCACAACGTCTGTTATAAC
>ConsensusfromContig1210 Average coverage: 5.38
GATAAATTGAAGTTATGATATTCTATAATGAATAGGGTGGAGAT
TATTATAAAAAAAGAA

TTATTATGCTGGATATATTGATATTTCTTAATCGTTCAATCCATTG
AGTTTTACTCATTT
GGGGGAGTTGCTTCTTTTCTGTGAATATCAATAAGATTATATTTCT
GTTACTGTTTCAAT
CACGAATAATCCTTTTTTCAAATCCAGAAATTGGTTATCCTTATCC
AGCATTTTCCACTG
ATACTCACGACTAAATGAGAGTAGAAATGAAATGAGCAAAAATA
GTCTTCATCGGATAAT
AAGGTTATTTCGCAAAATTCGAGAATAT
>ConsensusfromContig1211 Average coverage: 12.00
ATTCTGAAATTTTATCAAACGCGTGATTTTGCTTTTTAAAAGAAA
TTTTATGACGACTAT
TGGAACTTTAATTTCATGATATATTTTCACATGGATGCATTGTTCT
ATCGCTTTTTTCAA
GGCCGTTTTTTGGTACTGTAAATACTCTGAAACGTGTGGAACATA
TTTTTATGAAACTTA
CAAAACTTTTATATTCAATATTTATACAG
>ConsensusfromContig1212 Average coverage: 9.45
CTTGTGGATTCTAAAAAGCTTTCAAGATGAGTTTGTTCATATTGA
TTGTAATTGAAACCT
TCGAGTGGCCTATCCTGCAGTAGTGTTATACTTTCGTTATTTGTG
ACATAGCGATATAAT
CATTTTTTTAAAAAGTTCATTTTTTATATATTAATTCTTCTGAAGG
CACAGTTATATTTT
AGTTTAGTTCAACTGTGAATATTGTTTTTGATATTTTTCAGATCGG
AAGAG
>ConsensusfromContig1213 Average coverage: 6.11
TGCTTCCGTCATGCGCTCGGCAACAAAATCGGATCCGGCCAATCC
ATCACAAAGCCTCGT
TCGCCGGATCTGTTACCCGAAGAGGTTCAAGCTAAATACCGACGC
TGTGAAATGGGGTTG
TCTGCATGAGAAGACTGCGAAAAGCGCGTACTTCAGACAAGCCA
GGGATGAACATAGCGA
CCTCGATATTCGTGACAGCGGCTTCTGCATATCCGAGATC
>ConsensusfromContig1214 Average coverage: 6.08
TTGACTCATTTCATCATTGTCTTTATTCAACAAAGCTGTCACAAAT
CACATTGATTATTT
CATATTTAACAAATTCGAACATGTTATCGAGCATAAAAGTAGATG
AAATCGATGGCTTTA
TTTTTTGAAATGTGTTATTTCTGTGCACAATACAGACTTCAACAT
AAATAAGAATTCTTC
ATTCAAATTGGGAAGGCCCAAT

>ConsensusfromContig1215 Average coverage: 7.04
AAAAAGTGCACGCTGTCTCCTAAACCACGGCCAATGAGCCTGTCG
GCTTTATCAGCCAGT
TCATCTTCCATTACAAGACTTCCAACTGGTGATATCGATCGGTCG
TTTTTCGACGCTGGT
GAAAAAAATACGCCACCTGTTTATTATACTCAATCAGGGATGAAA
CAATCAATTTGCTCG
AACTGTAAACAAAAATTTCTGAGAAATGATGTGCCTAAAGATAAT
C
>ConsensusfromContig1216 Average coverage: 4.25
CTGTTCATAATCTTTTTGTTGCAAGTCGTACAAGCTCAACTAAAA
GACACGTTGTATATT
CGTGTCCCAAAACAAGGAGGGATTGAAGAGAAACATTTCGAACCT
CTTAGTGTCGCCTTT
TTAGTATTTTTCGCCATGATCATTTTGATTCAGTTCATATCAATGC
TTTTCCATCGGTAT
GGTACCTTCTTGCATATCCTCGCATCGACGAGTTTGCGTTGCTGC
AATCAGG
>ConsensusfromContig1217 Average coverage: 5.94
CCTGAGAGAAACAAGAAAACTAACAAATCGAATATAAGAGCAGA
CTAGGAAGACTATATC
ATATCACGAAAGTTTTTGAAAAAAGCTTTCAGGTTCAGCGTGTTA
CACATCAATTAAACT
GAAAATTCAAGCGAGCATTAGGTAAAAAAAATCTAATATGAACA
GAAAATCGAATGTAAT
AATAGACTATAAAAGTTCATGGAAAATATCAAACATTTG
>ConsensusfromContig1218 Average coverage: 6.27
GTCTGGTGTTGCCAAGGGGTGTGCGGTCTTGGTTCATAAGGTCCA
TGAGCTCTTTGACCA
TTTGCTTGGATGCTATGATCATATTCTTTCGGATGATCTGTTCCGA
GGCATCTGCATGTT
GTCTGGCGATGTACTCAGCCGTCTGCCGAGATGGGAATTCTGTCT
CACAGAGGTAACCGA
AGTCTCGTGCTAGTCTAACGGCTTCTCCTTCGACGAGGGATGTGA
GAAGGGTGACGGTGG
CTGCCTTCCGC
>ConsensusfromContig1219 Average coverage: 5.25
CGGTTAGATAATGACTTGATTTATCAAATACAGGATAGTTATCAT
TATCGTCAATGATAT
TCACAAGTATATTGATAGTTTTGTAGTACTGCTGAGGCCTGATAG
CAACCGTCAATTTTT
CTTGGCAAACAGGATTTTGTTGACACATTGTCTCACGGTCAATCT

TCAGTCTAACACGGA
TTATTCCGGTATTTGGTTCTATGAGAAATGAACGATTCGAGGGAT
GAATATTATCGAAGA
AATGAAACTGCATTTCGTT
>ConsensusfromContig1220 Average coverage: 23.19
TTCCCTACACGACGCTCTTCCGATCTCGAACATTCAATCTACATGT
GTCGGATTTTGTTC
GATTACTCCACGTCAGCGAACTTTTGATACTATGAAATCTAGTTT
AAAACAGAAAATACA
GGAGAATAAAATTCAAAAAATTAAATTGAAGCAATTTAAAGATG
AAATCAGAAATGCAAA
ATCCAAAAAATTAACAAGATTGAAAATTCGCTTCGAC
>ConsensusfromContig1221 Average coverage: 4.01
ATCGCTTATCGCATTCTCAGATTTGAATAAACCTAAGCATAACCC
GCTCTGTGGTTTAGG
AGAGTATACAGGAGAAACACACATCACACACGAACTGAATTAGA
TATTTATATGATCAGC
ATGCTAAATTTCGAAAAATCCACCTATTTTTGAAGCACATGCTCA
GCCGCCCCTCATGTT
GTCCCTGACCATACTCTCAAGCATAGTATTGAGATTATTTCAATA
TAGTCAGTAACTTCT
CTTGAATTCT
>ConsensusfromContig1222 Average coverage: 5.98
GGCCTGATGAGCGGCTCTTGGCTTCGCAAGAAGAGAACTTGGTG
CATAAGGATCGTAAGA
CGTAAACGCAGAATGGATTGGATTGTATAACGTAGGTTGCGGGA
GGGAATTTGTTGGGAA
GTATCTGAAATCTTTGAGGGGATTCAGTTTATGAATATTGCTTTG
CTGTGATATGTTGAT
GATATTTTCTATGTAGGAAGGAAAAGGATTGAGATCACGAAAGC
CGCTGGATAGTGGAAT
GTGCGA
>ConsensusfromContig1223 Average coverage: 8.43
TAACTGCGTTATATAATATATTGGAACACAATAAATGACAGTGAA
ACCAAATATGGCTAC
GAGAACGGCCAATGTCTTTTTCTTGTTGCTCGTAGTGATATGATA
TTGAATCGGGTCGGA
TATGGCCTTATACCTGTCGTAGTTCATCAGCAGGATTAATATGGA
TGCAGATACGGTCAA
GACACTGAAAATATAACAATTGAGCTTGCATGTAAATGTGTCATA
CCCTCGGATATACAT
CGTTTCGAAAATTCCTGATGTTATCAAAATAAGCAAATCACAAAT

GGCCAGAGCTCTCAT
ATATTTCGAAAAATCTTTATTACATATCTTACTTTTGCTTTTGGTG
ATCACATATAGCGC
TAGAATATTACCGGGTATTCCAAAAATGAACAATAATATTGAATA
TTTATGTAAAAATGA
AATGAAATTTTC
>ConsensusfromContig1224 Average coverage: 7.07
GGTTAGTAAGAGTGCAACGCATGTAATTTCAAAGGGCTATGGTAT
TTGGCAGAAACTTTC
TATACTTAGTTGTAAAAACATCTGCCGGGACCTCCTAAGTATTTC
TTCCCGATTACCCAC
CTGAAATGATTGGATATGGCAAAATATTAATCCGATAAAATACA
AGAAAATAATATCCGA
AAACTTAGTCATATAATAATATCATCAATCAACTGTAGACCAGAA
ATGCTTACGCGGTGT
CTTGTGAGAGTGAGAGAGGGAATGAAACAAAAAAATCAGATTTT
GTATTTAATGCCGGAT
AAGGTTTGTATGCGAGGGAAAAAATAAA
>ConsensusfromContig1225 Average coverage: 6.30
TGCAAATGATCGATCTGGATGTTATGCTGTCTGGATGCCAGCAGA
GCTTTGGCAGCTCTA
CATGGATTTCCGTTGGTCCAGTGCTCTGCTAGCTGACCATTGACC
ACAAACTGAACATCA
TGAGCCAGATCTCTAGCATATTGAACACTGTTGCCAGCATTGTTT
CCACCAGCATTTCCT
GCTTTCTGTCTCATTGTTGTCCAGAGTTCCAGAAGAGACACAGCT
TCAACAGGCGTAATA
CCATTGAGGTACACAACTGCTGTGTCCAGCTGTCTCAGAATCTTA
AAGTCTTTTCTGAAC
TTAATGAAGTGGCTGTTTATGGCTCTAGCAGCATCTGCTTTTCCCT
CTCTGTTGACGTTG
TCATAAGTG
>ConsensusfromContig1226 Average coverage: 5.87
GCAGTATCATTATTATTCTATCATTATAAGTTATGACCAGTTCAG
TACAGCAGGTCGGAA
AAATTATAGAATTTTTCTGTCAAATTTTACCACGTTTTCTCTGCAT
CATTTTCTATGATT
AAAATCAAAACACTGAACACCTTTAAAAACTTTAAGGCGTTTGTT
TACGTTTTTTTCAGC
TGTTGCTAAATTGTCCTTTGCTTTTGCAAAATATTTTACTCAATTT
TCTTGCTGGATTTC
CAATTTTTTTTAATTTTCGTGTGAAATTCTCATGTCATATTCATAG

TGTGGGATTTTTCA
CAGATCGGAA
>ConsensusfromContig1227 Average coverage: 3.98
TATCATTTGTGAGAGGAGGAGCAACTGCGTAGAATTGATCGATTG
TAGTATCACCGATCA
TCATTTTTGTGTCCTGCGCCCTGGCAGTCGTTGTTGTTGAGGTCG
CCAGATTCCAAGCCA
TGGTCGGTAGCAGGATTCTTGAGAGTCGAGCAGGAAGCTTTGTC
ATGACAATATGGAAGG
TGAATTTGATGAAAGCTAACGCAGTTATGTTCTTAAAG
>ConsensusfromContig1228 Average coverage: 7.60
CCGATCTATAACATCTACAATATCTACAATTAAACCTTCTAGCTTC
ATCAGAATTATTTT
TTGCCTTCGATTCTTCAATCGGTTGATAATTCAAATGTTTTTCGTG
AAATTCAATATGAC
ATAATTTAGTAATGGTCATAAACTTAAACACAACATCTTCAATAT
CTGCCATACCTTCTA
GCTTCATCTGCCTCATTCCGTGCTTGCGCTTC
>ConsensusfromContig1229 Average coverage: 5.33
CAAGAATGCATCTTAAATATTGATATTGCTATCAGACCCAAGCGT
TATTTTCAAATAATA
AAAGTTCAAATAATTGTCAGAGATATAAATGACAACAGACCGCG
GTTTCCGGAAAGCAGC
TATCTACTTACAATTCCAGAAGATACAACAGTTGCCAGACAGTTC
ATCATCCCATCTGCT
GAGGATGCCGACAGTTCAGTATTTGGGATAAGTAAATATGTCCTC
ATGGATCCTTCGGAT
ACTTTCGTCTTAGCCGTCACCAACATCTCCAGTAATGATTTCGAA
TTGAAATTGTCGATA
TCAAAGCCGATTGACCGCGAAAAGACAGCAGAATATTTCCTCACA
TTGTTAGCGTCAGAT
AAAGGGAATCCTCCTCTATCTT
>ConsensusfromContig1230 Average coverage: 6.56
AGCAGATCCAATCCTATTGGAGACACTCGAATCTAGCCCATCCCA
TCGGTCAAGAGCTTC
CAGTGGGAAACGATGACCCCGCATCTAAGATATTACCCGCATTC
AGGAGCACGTGATAAG
AGCAGGATTATTGCATGTGATTGCCCGTATCACTCGTGAATGACC
TTGAATCCCTCGTGC
TCAGACCATTTTGTGGCCAATAGATCTCCAAAAATCTATTAGATC
GGA
>ConsensusfromContig1231 Average coverage: 91.64

CACTCTTTCCCTACACGACGCTCTTCCGATCTGGGGAACACAGGG
GCATGTGCTCCAGCC
CTTCATGAAACTCGAACTGTCTCGTTTTATATGCTCTGTACACATA
TTACACAATCGTCC
AGACGTACAGAAATCATCATTAACAGCTGAACTTTGAGATTATAT
CAAATTCAGGTGTAT
AACCACGGGGAAGGGGGTGATTAATTGCTTTTTTGTCTTTCATCG
GATGTC
>ConsensusfromContig1232 Average coverage: 5.86
CAAAAGCCGTTTTCCCGTGAATCGGTAACATTAAATCACAAACAA
TTGGCCGTCATCAGC
CCAATTTACTGAAAAGTAGCTGAAAACCAAATCTGTTGAATTAAG
TATCACCATCAGAAA
ACAAAACCCACCACGAAAGAATATTTATCACAAATACGAAAGCA
ATGTACAAGCTGTACA
AATGATACCACGCGATCAACAAAGCCCAATTCTCGCCAC
>ConsensusfromContig1233 Average coverage: 6.28
GCGGCCCTTTGAACTGATTCCCACATTGACATATTCCACTGTCGT
CGCAGTTATCCACAT
TTTTGCAAGAACGGGGATTGACGGCCGTGTCACAAACATTGGTTC
CATCGCATGTGTCGC
CACTCTTGCCCTGTTTGCATTCGCAGACATAATCGGAATTACTAG
TTGGAAGAGCGTATG
CGGTTCCCTCATTGTGACACTTAGCATTATTAGAT
>ConsensusfromContig1234 Average coverage: 4.36
CTATTTTGTGTCGCTTGGGAATATATTGGTCGCTGTGGGGGAGAA
AACGTTGTTGATTCA
AGTTCAAGTTTTGCTTTCGGTTGGGTGGAAAGTATGTCAATTTTCG
AGTTTTGGTTTTTG
ATGAGGGTAGCAATAAAGGTTTTAATCTCAGCTATATCATCCTTG
GATGTTAAAGGATTT
GAATTGATAGGAACAGAAAAAAGGTGG
>ConsensusfromContig1235 Average coverage: 7.31
GGCATCAAAACGTGTCCACGCTACAAGGCTTATAATTTAACAAAT
TTGAATCATGATCAA
GCGGAATTAGAAATGAAATTCGGTTTTATCGAGGTAAAAAGTAT
TGAAAAATCTGATGGT
GAAAGAGTTGAGCTGATCTGTAGTAATAAGGCAGCGAAGAACTA
CGCTGAGAATTTCCCG
TTTGCAAAAGTTGTATGGGTGAAGAATGGAGAGTCTCTACTGTTA
GATGTGAAGCGG
>ConsensusfromContig1236 Average coverage: 5.96

GTTTTCAAATATGATCTTATTTCAATGATTTTCACACATATCATTT
CTGGCCAAATGTTA
GTAGTGTATTTGTTCATAAATGTATAAAACATTATTGAACATCAA
ACATCATTTGTGATA
TCATGAAATTTATCCCAATCTGTACAATAAGATATCCACGGGTAT
AATAAGTGGTATCAT
ATATTTTTGTTTTGTCTGGT
>ConsensusfromContig1237 Average coverage: 8.70
GTATTCGTTGTTTCAAGGGGCGTTTTTGATTCAACCCTTTTCGTCT
ATCTACATATTTGA
ACAATAGAATTTCGTCCCGGGAATTCCGCGACACAGATCATTAAC
TTTGCGCGCAAAATT
CCGGAACTGTGCATCTGCGTTTTTCGAAAATTCAACAATCCTGTG
CCCCTATTCTAGCTG
CTAATCTATATTTGATTTCTTTCAGTCTAGTGTTTGGTCATT
>ConsensusfromContig1238 Average coverage: 2.96
ATTCGAAAGACTTATTTGATTCAAAAGTGTTCTCTGATTTCTAATA
TCTGTGTTCAATGT
TTTCGTCATCTCATTAAAACGGCTTTTCATCCCCAATATAAATATT
GTATTTTTGATGAT
CATATAGCACGCTGGTTGAAATTCTGATTAATGGCATTCGATCTT
TAAGGTGTGAGATGT
TGAAAATTATGCATCATCATTTCTT
>ConsensusfromContig1239 Average coverage: 3.22
CCCCAACAGAACAGGCGTGGAGGAACCTTATATATGTGGCAACC
ATTTCGATCGAAAGAC
GCGTATGAAAAACGGTGGATACAAACTACATAAAGATGCGAATC
CCCTGCCATTGACCCC
AGAGCAGTCGAAGAGAAGAAAGGAGCAACTCCAGAAAATAGATT
CAGGAGAGCTCCGTCC
AACACTGGGGTGGACCGATG
>ConsensusfromContig1240 Average coverage: 17.20
GAAACAGTGGCAGGCGAGGCGTATTGCAAGTCGGAAACCTCGAA
AAGCAGCGCGCAGTGT
TTTTGCAAACGTATTCTTTCTCCATTTGCGAGAGTCAAAAGCTTGT
TATCGTCCATGACT
GAATTCATGTTTTCGACCCACAGTGCATCCACATCACCATCGAAT
ACGATATATCTTCTT
TCATTTTTGTCCGTAGGTTTGTTTATTTCCCTGAAGATATTGGA
>ConsensusfromContig1241 Average coverage: 4.65
CCGGATTACACGAGCAATGATAAGATGTTAACGAGTCGTGACAC
TCAAGGCAGGAATTGT

CTTGGCAAGATTGAATTTTATGACCTTTTCTGACGTGCAGTTCAT
CATCAAACTCTACAG
CTTGTCCTCCACTCCAAGATTTGCATAGTTTGCAAGTTTTGAAAA
GATCGTTCCACAAGA
ATGCTGGCGTTCTGTACTTCAG
>ConsensusfromContig1242 Average coverage: 9.79
CCGATCTCTTCTTCCGATTTGAATCCGAGACCTAATTTTGGATGT
AAGAGACCTAATTTC
GAATTGTATCCGAAAGGTGAACAAAAAAAGTACATGGAAAGACC
GCAACCTTTGAATTGT
ACGTTGGGTGAGAAAAACTGGGTTAGGACAATTAATGGCACGTT
CATAATAGACGCAGAT
GCCATCTCAAAGTATGGCCCCATTACATGTGAGTATAAGCCGTTT
GTTCGTCAAAATGAT
GATTTCAAACCATCAATTCTTTTCCCTACTGCCTCTAT
>ConsensusfromContig1243 Average coverage: 12.44
GTAAACAAGATCCTAGAGAAGTCGATGATATTCCCGAAGACGATA
GGCCATTGTCTGCTC
GCTTGAAGCAGCATATGCAAAACGATTTGAGCTCACTTATTGCCG
ATGAAACATTATTTG
AAAAATATCTAGAAAATTTCTCACCGGATCAATTAAATAGTAAGT
ACGTCAAATACATAC
AAGAGTTGTTTTCAAAGCATAGATCGG
>ConsensusfromContig1244 Average coverage: 7.12
GCAACAGAACAAAATTTTCTACTGGGAATACCCGTAAAGTTTTTC
ATCTGCGGATGCATC
ATTTCCATATTTCCTTTTCTGGTCTACGCCCTGGTTTGTTGCTGTA
TACCAAAGAACAAT
AAAACAGACGGAAACGACAAACAAGAGAATGTAAACATAAATAT
TGACGTGGATCAGTGC
AGTGAGACTTTGGAATCAAGCTCTCAGAAATCAGAATCGGATTG
AGAAATTATACTCAAA
ATATA
>ConsensusfromContig1245 Average coverage: 5.81
CAAGTTTTGCGGAAAAATAACGATCTAGTAGCTCAACTCGAATCA
TCAAAATCTGATAAA
TTATTAATCAGTACCGAATTGAATGAGATGACAATCAGCTACCA
AAACTGCAGGGACGAA
TCATCTAAATTACAAGAAAAGGTTTTTCACCTCGAATCTGGCAAG
TCAAAACTGGAGATC
GGAAGGCGTCGTGTAGGGAAAGAGTGTAGATCTCGGT
>ConsensusfromContig1246 Average coverage: 6.53

CTCTTCCGATCTCTTCCAGAAGGGCGCCTGTGTCTCGATAGGAAG
TCCAACGGAATCCTT
GTCCCAGTTTCCCGACAGAATCAAGTTTGCGCAGGCGGATCTATT
TTTCTTAAAGAGGTT
TTGGATTTTTGCGTATTGCGCTCTTCTTTTGCGACGAGATGAAGC
TCGCGCAGAATCAGA
CGCATGTCGGGTGTTATTGTTGGGACCTCGATTACTA
>ConsensusfromContig1247 Average coverage: 12.10
ATTTTCCTTTATCGTGGTATGAAGTTCCAATAGTTTGTCGTAGTTT
TTAAAATGATCAAA
TTTTTGTATGTAAAAATCCATAAAATTTCCGTTTTGAGGAAATTGC
TTATCCTTGATTTT
GAAAAACTGTTCCGGATATGAATTCTTTGGATACAATAATTCCAG
ATCCGTATACATCTT
TATTTTTGAGATACCGAAGATTCCCAACCCAAGAATGACCAGGCT
CGTCAATAAGGCACA
CACCTTGAATGTTCTGGAGCGTAATAACGGTACATATTTCTTTCG
TATGAATGTATCAAA
TCGAGGTTCGGCCA
>ConsensusfromContig1248 Average coverage: 5.15
CAAATTAATGCAATATTATGTGATAGATATAATAAGTACAACTGT
AGGAAACTGTAATGA
CATAACTTTCACATTTCGTTAATTCTTTTTCGGTGCATGCCGCCCT
CTAAGATCTGTTCT
TTATCAACTATATAACTATGAGCAGCAACTGAACTAAACAGTAGC
GTTAAAATCCGTATA
ATTTCAAACCACGCATGCGAGTCTCACTCGCGACAGTCTTTGATC
ATTTTTTCTTAAGCG
>ConsensusfromContig1249 Average coverage: 5.42
CTCATCTATAATTTTTTCTTTCTGTTTTTTGTTAGTTTTAATTAACC
ATGACTAGGCATT
GAATTATAGGTTTTCGGAAAATTGAATATACTGTGATCTATTCCAA
TTCTATTTTGATTT
TTAGATTCATCTTTGTTCTCGGTACACACATATATTATAAATGGA
AGTGTTCAATGCGTT
GTACCAAAGTCTTGTTTCTTGTTATACTCAAATGTATTATACTGT
GTTTCCGGCACAGAA
TGAT
>ConsensusfromContig1250 Average coverage: 8.42
CAAAATTGTCCATTTTTTATATAGATAATTATATGTGAGTTCATG
TAAATAACAGAAAAT
GTTCTCCTTATTAACAATATTACACATCGTTGCAGCGATTGCGGT

GACAGTCTGCATTCA
TTTCACTTTAAACTCCAATATCACCATCACTGCATTTCCATGTGA
GAAAAATATAAATTG
TCATCGACAATACATTGGAGATGTTTCTATTAGAGATGGAAGTCA
TGTTATTTGCGCGGG
TGATGAGAAATGTTGTGAGAGATATAATTATCATAATTATATATC
AGAAATTTCAAATAC
CAGATCGGA
>ConsensusfromContig1251 Average coverage: 8.86
ATATTGGTGTCCCCCCTTTTTTTAATTTCCTTAAAAATAAAACAT
CCAAAACCCAGCCGA
TAATGTTCATTATTACAATTGCACGGATCAACTGTATATTAAGAG
CACTAAGGAATGGAC
TGCCTAGCACGCTGGACGGAGCAGTGTTCTGCAATGGAGAAGAA
ATTGGACCGATTACGA
ACAGAACCTCTCTCAACGAAGGAGAATAATATGGTGGCAACAAA
GCGCTTCATCCCATGG
CAGATCCGATAACGATGAACAATTCAAACAGGGAAGCCTTATGTC
ATATCTCACAGGATT
CGTATGAGATGAGGCGAGAACATTACGCCGATATAACCAGCAAT
ACAACTGACATCTGAT
GGTATGACAACCGACAACCAAAGCCATGACAACCAATTTTATACG
TACTTATA
>ConsensusfromContig1252 Average coverage: 4.50
CTTTGACAACTGCAAAATTGGTTAAAGGCTCTGACGCATGTGACA
CCACTAAATGTACAC
TAGCGGCAAACCGCAACTGCTTCGACATTCAAGATTCAGCCACCC
CCAAGCATGTATGCG
AGTGTAAGCTCAACAAAAACGGTGATTTGTGTGCGGCAGATCCGA
AAAATTCCTGTCCCG
ATGAAAATCCATGCATGAACGGGGGCACATGTGAAGACCATAGT
GGTT
>ConsensusfromContig1253 Average coverage: 6.11
AAATATTTATACTATTTCTATATAAATATGTTTCGAACATGAGGT
TAGGTTTATATAAGC
GTAATATTAACGTAACATTAATTTTCAATATGATAAATATGACAC
ATATTGTTGAACGAA
CACACGCAATGTGTCGGTGAAATCGCAGTGAGTATAACTAGCAA
AAGCACCAAACATAAA
AATTATTCAAGTAAGCAAGCCCGTGCAAGCAATCACACAACCCCG
TGAATTGTTACTA
>ConsensusfromContig1254 Average coverage: 13.52

GAAGACTGTTGTACGATGAGGTTGAAGCATCCGACAAAAGTTTG
TTTACAGTACAGACCA
ATCTGTCAAGTTTCCTCAGAGACATTCTGTATGAATTGCAGTGGC
CTTGAATCAGAACTT
GATAGCGCAGAGCATCCTTTGGATATTTGTTATGAAAGGATCACA
AGAAAATAATTTTTG
AAGAAAAACAAATTGTGATTTTTCGGATCTCACCCTTCC
>ConsensusfromContig1255 Average coverage: 4.05
TCTGGGATCAAGTTATTAGGGAATCCTCCAGAAGTGTTTATAAAC
AAATCTGGAAACGAG
TGAGCGAATCTGATACGGCGGGCTTATCGAGTTTAGAGGGATCCA
TGGCGAAACTTTTTG
AGCTTGTTTGCAATGAGGGTTATGCAACATTAACCGACCTCATCA
CATTTGCCACGTCAG
TTCATCCATGTGGGATGAGAAAAATG
>ConsensusfromContig1256 Average coverage: 725.70
ATGTGACTGGAGTTCAGACGTGTGCTCTTCCGATCTGAGAAAGG
CTGAAAAACAGAGTGA
TGAAACTGAAACTAATTATATCGACACAAAACGACCTCTTATTAA
TAAATACAGCTCAAA
TTCACAATTTGTATAACTCCTATTTTATATAAATCAGGCATGTTC
ATTTACAACAGCTGG
TGAGACGGCAAATATCAATTTATCATGAACTGCTAAACACCATGG
CTATACAGATAATAA
ATCAAGGTTACTGCCAAATGAAAAGAATAATATATGCC
>ConsensusfromContig1257 Average coverage: 7.98
TATCTTCCGTAACTATTAGTGATAACTGTACACCCTCCTCTTCCTC
TGCATTCTGATGAT
TTCCCGACAGAGTTGCAAATCTCCGATGCTTCATATGCTGTTAGA
TACCATGCCGAAACT
ACAGAAGATACCAAAACAATGGAAAACATGAGAATCAACGGCAT
GGTATTCATTTTTGAA
AGCGGTAGTGCCAGAAATTCAAACCTCC
>ConsensusfromContig1258 Average coverage: 4.64
AGAGTATTTCTTATCAAATCCGAAGAAGACACTACCTGGGGTGT
AGGAGTCGGAGGAAGA
ACGACAAGACGTCCAACGCTTAGACTCCATATACTTCTCGTTCAC
TTGCAGTCCGATTTT
TTTTTCTCCGCTGTAATACATCATCTTAAAATATACCCATTCCCCT
GTTAGTGCTGAGGT
CGAGGTCGTTATACTGATTGATTTTCTACAATCGCCAGTTTGATA
TGTGAATTGGAAAGA

>ConsensusfromContig1259 Average coverage: 6.09
CTTTCATCTACCTGTGAACAAAATTGTTATCGAGAAAAGTTATTC
ATACATTTTAGAATA
CATTTCGCATTTGTCGATTTACATGTGTAGCAATCATTAAAACTCG
ATATGTTCGGTTAT
CGTCATACACATTACTAATTCTTTCATTATCTACCTGCCCGATCA
CTGAATATGTATAAA
AGATGTCATTGTTGTCTGTTTTTTGTCATCTTGTAACGTTTATCGT
CTTGCCTCGC
>ConsensusfromContig1260 Average coverage: 3.22
GACACAATTAAATCTTGCTCCAGGTCATTGCATACATATGGCCAC
CTCAAAAGCTACAAT
ACTGACGGGCCATATAGCAAAATAACAGAGCGCGCAGATCACAA
TTTAGTGATGCTTGTG
AGAGAGTGCAAGAGTCTCAAAACACTTGTTATAAGAGAACGTGT
ATCTACATCAACATTA
TTAGTGATCATTGCAGAATCAAAGAGTTT
>ConsensusfromContig1261 Average coverage: 4.00
ATAACACCAAGGCAACAAAAGACCCGATATTTCGCAAGGTACTG
ATAATAACGCTGGTCT
TCAAAAATACACAGAAAATCACTTTCAAAGTCCATAATTTGTCTC
ATTCGGAGTTATATA
CTGAACACAAAACGACCTCGTAACAATAACACATATCCATCAGA
AGCGGAGCAGTATGGC
TTAGGCCGAATACAAAATCTAGAATCGTTGTCCGAGATC
>ConsensusfromContig1262 Average coverage: 5.75
TTTGGACGCAGTTGTCGGAAAGCTATTTCACTTTGAAGTGGACGC
AAGCACTGCTGGTTT
AGGGGAAGTCGAGGTAACTTTGCGATTGGGGAATACAAAAATGG
ATCACAAATTCACAGA
AAAGGCAACCGGAAAGTATCAAGTAAGCTTCATTCCTCGACAGCC
CGTTGTTCATCTGGC
CTACATCACCTTCGGAAAACAAACTGTG
>ConsensusfromContig1263 Average coverage: 8.86
GGTAATTGTTCCCTTCAAAAGATTCATCTAATTTATATTCGGACAT
TTGTTTCATAAAAT
CCCGATCAAAACTCCGGTCAAAACCCGGTCTCAATGATCCAACCT
CAAAGTTCCGAATTA
CATGTCGGGTTAAGATTTCAATCCTACTAAACTAATCTATCAGCT
CTCAGTAACTATCCA
ATATTTTGTAAATTAGTTTTTCCTTATAAATACAATAATTCATAAT
GATATGATTTTG

>ConsensusfromContig1264 Average coverage: 5.42
CGAGGGGAAAGTGGAGGGCCTTGCCTGCGCCTTTGCCGGTTCTTT
GGGTTCAGGTTCAGC
CACTACATCTTGCAGTGAAAGCATTGGAGTGATTGGTGTGACAA
AGGTTGGCAGTCGCAG
CGTCGCAGTCTATGGTGGCATCATCTTGATCACACTTGGCATCAT
AGAAAAGTTTGGGGC
GATTTTCGTCACTGTTCCAGACCCCGTTATTGGCGG
>ConsensusfromContig1265 Average coverage: 41.01
CAGGATGATATATTGCGCCTCATTCTACAAACCATGGAGAAAAA
GGGGGCGAGCCATACG
GTACTCGATCAACACGAGAATTCTCCGGCTCATTTAGCGGCCAAA
TACGGAAATATTACA
TGCTTGCAGATTCTAGTCGAGTTCGGCCAGGATGTGTGCAGCAC
AAATAGATCGGAAGAG
CGTCGTGTAGGGAAAGAGCGCAGACCCTCGTG
>ConsensusfromContig1266 Average coverage: 3.87
ATTAATACCATGACTGATCTTGGTGAATCCTGTATCATTTGTGGT
GATGATTTTGACATG
TCCAACCTGCTTCGTACAAAAAGACGTAAATTCACAGAAAAGGAA
TTTGAAATCTTGCTT
GGATCAAAATATTCTTCAACATATATTGCCGGTCTATTCATATGC
CATAGGTGTTATTCG
AATATAGATTCTTATCAGAC
>ConsensusfromContig1267 Average coverage: 5.36
AAAAAAGAAATTCAGATTCATTAGTTTTTTTAAATAAAAATGGC
AAATATTTGCGGATG
GTCCGTATTAATTTTATCGTTAGGAACCATTTCGGCTCTTCAAAA
ATACGAAAAATTCGA
AAAGCGCTCTGAGCCTATTGTGCCAAACTTTGATTCCCCGACGCC
GATCTACCGTAACTT
CACGACGATAAGATATGGAG
>ConsensusfromContig1268 Average coverage: 6.55
CACGACGCTCTTCCGATCTCAAATAGTGTTTATGAATATTTATCCC
TACTTTATCGATCC
GCTGAATCCACCTAAGCTCAGCCTCCTTACGTAATTTAGCTGATT
TGAAATCTGTTTGTA
AGATAACAAATCTCAAATCATCTAAACAATGATTATTTGATAAA
AAATGGCAAGATACTG
AAGAATTTTGTATATTTCTG
>ConsensusfromContig1269 Average coverage: 10.67
ACGCATTCCACTTTAATTGTCAGACCCTCTGCACAGCGGAAGAAG

ACTCTGCAACTGTTG
CCATAGACGCCGTCTTTTCGTTGACTACAGTCGAACACGTCGTTG
CCAAGGGAAACAGTT
TCGAATTTATTAGCTGGCATCAGTTTTGGAGTAGAATTCGACTTTG
TGACATTCTTAATT
TTCGCTTTTTTATTTGATTCAATTTTTGCTGATTTTGCGTCGTCTC
TCACAGAATGATAT
TTCCTCCGTTTCGAATGATTTCTCGATTCTTTCTTGGAGGGAACG
CCAATACCAAATTGC
TTCTCCAGTAATATATCAAGATCGTCTTTCACC
>ConsensusfromContig1270 Average coverage: 6.80
CGTTCAATAGATAAAGACATCATCACAGAAGAATATTTATTCCGC
ATTGACTGCTCCGTC
ATCAACGACTATCTTCTACCAAAACTCAGTTTAATGATCAAACAA
ATATCTTCTTTTGTC
ACAACAACTGCATGCGAATTGGCTTCTAACATCTGTACCGAGCTT
CAACACCTCATTATT
GACTTAAAGAACAAAAGCGATGAGGTTTCTAAATTCTCTGCATAT
GCCGACATTGTTAAA
CGGTGCGATGCAAATGTGTCAGAATTCGGCCAA
>ConsensusfromContig1271 Average coverage: 12.83
AAAATATGTCATTAATTAAAAAAATGTATTCAAAACACTTTCAGT
GAAATCCCGTTCAAA
GATTTGCCTTAATTCAAAGAGACATTATCGGTGAATAAATTGAAA
TTACTAATAGGTTGG
AGGTGAAAAAAAGAGAACTGATTCCAAATGTAGGCCATTCAAAC
CATTCAAAAACGTTTC
ACTCAAACCAAGAACTTCAAAGATACCTGAAAAATCGTAGAAAG
AAATACTACGCTAACA
AATCAA
>ConsensusfromContig1272 Average coverage: 7.92
CGTCAATAAAAACCAGTCGGAATACCAGAAATCGAAATGGATAA
TATGAAAATGTGAAAT
TATATCGAAAAAGAGTGTGAAAAGAGGTAGTAAAAACTATTAAA
CATGCATGTGTTTCTA
ATCATAGTGTAGCCAATCACATGGATACTCTTACATCCGTCAAAT
GGGACTTTCAAAAAA
TATAAAATGAGTATAAAATATATCTTCAATATTGAGAAGACAAG
TACTAAAAGCCATAAA
A
>ConsensusfromContig1273 Average coverage: 5.46
TCTGCAAAAAATGTCCAGTCTTATCAACTTCAAACTCCTTTGCAG

TAAATATTCCGCGCT
GTGTGTTTTCTCCAAGCAGAGATCTTGCCGAACATGCAGACTCAA
TCTTGACTCTATTGC
CAGGAGTTGAAGAATTCAAGGACCCTGATGGTATTTCATCCAATC
TCGATAGGCCAGATT
CGGGTTTCATCTGAGGCACAGTGATAGGGTCGACTTCTTGTTTGA
AATCTTCAGTAATTG
CCGCAGATCGGAAGAGCGT
>ConsensusfromContig1274 Average coverage: 5.94
GTTTTGGATGAGCCTGTTCGTATTTGTGACTGCAAATTGTATCAT
TACTTAGATAAGGCA
ATATGGACATTGAAAGATTACAAATATATAGCACAGTTTGATTCA
AGAGATTCAATCGAT
GGAAATGGGTGTTTAGATGAATCTGAACGAAAAGTTGAGACATG
CCAAGTCCCACCAAGT
GACAACAGCGCCCTCAAAAACAAGTTATCAAAGTACGAGGTTGTC
TCGTTCATTGGAGAT
TCTCATTCAAGAATGATATTTGTTAAAATTCTGCAGACCCAAGGA
TTGCCTACACCTCCT
TTTGATCCAGGCAAGAGAAACGCTTC
>ConsensusfromContig1275 Average coverage: 7.15
TTCTATAGATGAAGCNTTGTACAGATCGTTATGCTGCCCCCATCT
GGAGCTACTCGGCTC
GTCGTATCTCCCAGTCTTTTTGGCCTGGCTGATGAACTTGGTGTAT
CTTCGCATGAGTGT
TCGCACTTCCTTATCTGCTTCTATTTGCTGCTTTGTTCGAGGAATT
CGGAAGTCATACGC
GCAGCAGAAAACGACGACGCCAGAAGTGGCCAG
>ConsensusfromContig1276 Average coverage: 5.97
TCCGCGCATCCCCTGTATATCATCCCATTTGGATCCTGGCGTATG
AAGCATTTGGCCTGC
ACAATTCCTGTATAACATTGGCCGGCGTATCCTTGCCTTATGTGGT
CTCTGCAATTCTGT
CCCGCACAAGAATAGCATTGAAGAGAAGAACCCCCAGAAACCGG
TGTGGATTCTGCATAC
TTGACTAGGATTACAATCAGCACACATATATGAAACAATTTGAAA
TATAATCTAGCATTC
AACATCATGGAATGAAAC
>ConsensusfromContig1277 Average coverage: 5.17
TCCATGTCGGAATTAAATTTTCATATCATTTTCGTCTTATATACGG
AACATCGCCAATAT
TAAAGATTCCATACATTTAATCCTGATGAGATAAAATTGATGAAT

TTCTGAACTGAGATA
GAGAAATTAAATTCAGAGCACCACATCAATATCCACTAACCTGA
CCTCAACAAAGCACAT
ACCTTTTATACCAGATGTCCTTCAATGTAATGAA
>ConsensusfromContig1278 Average coverage: 7.24
CTCCACCCGCTCTCAGTAGAGAAGTTTTATCGGAGATTTTCGAGA
CTTTCTTCGGGTTCT
TTTTCTGCCCTCCTCCGATGTCCCCCAAAGTGGTGTTGCTCGTGAG
GCTGGTTCGACTTC
CGAGGTAGCTCTTGCTCCCGTATCGATTCTTGAGAGCTTGACCCTT
GCCCTTGGTTCTCG
ACGACCCCATCGATAGCCTTGACCTCTGAACC
>ConsensusfromContig1279 Average coverage: 4.71
GCTTGCATGGCCATGTCCGCAATAGCATACGAAGACGAGAAAGA
ACAGTGCGGGATTGTG
GATCAAGGGGGTATTGTGCCACTTCTTCGCCTTCTCAAAAGTTCT
AGGTCTAAACCGACG
GTTTTGGAGAAAGCGAATTCCCTCGTCTCTCATTTATGCATAGGG
GTAGCATTGACCGCC
AATAGACGTGTTCAAAAAGTTCTTGCGCACGAAGGGGCTGTGGA
>ConsensusfromContig1280 Average coverage: 94.50
TAGCAATTCATGTTTCAAAGTCAGTCAATAAATAGATCATGATAG
AATGCACATATTCTA
GCTATGTGGTGATGTAAGACCCATTATTATCATCTTAACCGATTA
TTATATATCGTTAAA
ATGTCATGGTTTATTGTAAATGTTATTGTTTATTTGGCACCTGCTC
ATATGAAGTTTTCC
GAGATCGGAAGAGCACACGTCTGAACTCCAG
>ConsensusfromContig1281 Average coverage: 4.48
GAAAATTTTCCCTTGAGAAAAGCCTTCAAAACCTTACAAGCCAC
AGACCGGGATATTGGG
TTAAACGGAGAAATATTATACGGTCTGAGCAGCTCAACGTTCAAT
GATTTCGGCCATATT
TTTCACATCAATAATCGATCAGGAAAAGTAGAGCTACGGCAAAA
TCTAGATTACGAGCAG
AAAACTTCGTATAGATTAGGTATCACTGCCTCAGATAGA
>ConsensusfromContig1282 Average coverage: 15.80
CTACACGACGCTCTTCCGATCTATTTCTTAAAATTTCAGCGGTAT
ACAAAACCTATCGGA
CAGCCAAATGATCGCGGGCCTATTTTCAATCGGTGAACTAAAGTT
TTGTAAAAATATGGG
CATTCAGGTAGATTGAGACCTCCAGGTTTCATAAATTTTTTCATC

ATATGAGGAATCTTA
GAAGGCAAAAGTCAGTACAATCATTGCCCAGCATCATCATTTATT
CGAA
>ConsensusfromContig1283 Average coverage: 8.82
ATTGATTCTGATGTTCGTCGTGGTGGCTAGATTACATGCTGTACG
CTATACAGTATTAAA
GGCCGACTCATTCCCGAAAATACTTTGTGAGCCTGTCGAAGGCCG
AGCGAGGACGCTGCT
AGAGTGTGTCTCGAAATCGTATGGATCCAGACCCGGGTTGATAG
GATACGAAGAAGGTGT
ATGTCACTTGTGCATTTTTGATCAATCGTCAACATTTGATGTG
>ConsensusfromContig1284 Average coverage: 8.86
GAACAAACTACGAGTATAAAGCGAACAAAGAGTGGATCGTCTTT
GGATTTCAATTCGAAC
AAAAAATCATCTCAAATACCTTCTCGAGTTCATGACATCACAATA
GATGAAGACGCTGAG
TACAACAATAGTCACTTGGTCCAAAACGCACAGCGACAAAATCG
AGAGAGATTCGATGAG
ATTGTAGTGGAGCATCAGGGAGA
>ConsensusfromContig1285 Average coverage: 9.12
AAACCAATTCGAATTTGCAGCGGCTCGTCTGAATAGTGGGCGATT
CTAAAATTTGTGACG
GCCTCACGGATAGCCAATGCCATTGAAGCAATTTGCGCCGCGTGC
TCGTGACCGTTTTTG
ATTGGTAGACCAGATACGACCATGTAGGCGTCACCAATTGTCTCC
ACCTTGTAAACATCG
AACATTTCCAGAATTTCGTCGAAAACACGATACAGATCGTTGAG
>ConsensusfromContig1286 Average coverage: 17.74
GATCGGATGTTCCTACGGTCAGGATCAGCTGAGGATTCACATTCG
AAACCCTGTACTGTA
TCCGTATGCCTTTGCTGAGGCTTGTCTGCATTGAGGATACCACAG
TATACATTTGGCTAC
TGTAATAAGCCCAGCTTGCAATTTCTACCTTGAATTCCAAATCGA
ATCCGCCGGGGCAAA
GACCGGGGTCTGCGATACATGGGTTTGGAGCTTCTTGAT
>ConsensusfromContig1287 Average coverage: 4.75
GTGAAGTATCATGTAAGTTACGAGAAAACGAGAGAAGGTTCAAA
CTCAACATGGAAACTG
CTAATGAATTCCAAATTGTAACAAATACGAGTTTCGACCGCGAGG
CACTCTATGAGGTTT
TCACATCCCTCGAATGCCGAGATGGTGCAGATCCACCGAGAATA
TCAACAAAAGAAATTG

TGGCAAAAATCATAGATGTGAATGATAATTATCCCGTATTCA
>ConsensusfromContig1288 Average coverage: 4.98
CCATGCTGGGCGTTACCTTCATTGCTGATCATCTACGACAGCAAG
ATGAGTTCAACAAGG
TGAAGCAAGATTGGCAGTTTGTAGCCATGGTTCTTGATCGCCTCT
TTCTATGGATTTTCG
CGGTGGCTTGCATCGTCGGCACCTTCGGGATCATCATTCAAGCAC
CGACTCTTTATGACA
CGCGCGTTCCACTCGGATAATCAAGATGGCGGAGAATAATTGAA
GAATAAAAAGACTGCA
CTAATATTACATTTAAA
>ConsensusfromContig1289 Average coverage: 17.88
TTGCCTACACGACGCTCTTCCGATCTCGAAGTTGTAGAAAAAGCA
GAAGATCTCGTGGAA
AAAGTTTGTGTTGGGCAGGTGACACTTACCATTGGATTGAACAAT
ATCAGTTGCAATGGC
ACACATTACTGCCGTTACATAATGATTGCTGGAGGACTGATGCCT
CTCTCGATCAACGAG
ATCGCTGTCGGAAGATACACATGTGCATATGCAGAAAGGAAATA
CGATCTATTGTCAGAT
GGGGGCAACGAAAATGTATAGAATTATAAGCTCGGGGATAAAAT
AAAT
>ConsensusfromContig1290 Average coverage: 6.02
AGTTCAGACGTGTGCTTTCCGATCTTCTGTATATCATGAGGATCC
GCATTGTGCGGAGAA
GCAGACGACATGTAAGTATTGTCGTCATCAGTATCTGCAAGGAGT
TTTCCGAGCGATGAT
ATGTAACCGTGAGAATTGGAGATTCGTTTTTTAAGACTAGAGAAT
CGGAAAATATGACGT
TTTCCTTTATTGAAATCATCTTCACGAGATCGG
>ConsensusfromContig1291 Average coverage: 5.79
ATGAAATGATAAAAATCATCCTATATCTGAACGCATGACGTCGTA
CTATCATTTCGAGAG
TGTATGTGAATAAATATCAGATGATAAAAACAAGAGAAAACAAC
CTTACAAACAACATTA
TTGTGAACAGAAGAAAAGATTAGAAAAATAGGATGAGGAATCGT
GCTCTCATCAATTCCA
TCTAATCGCCTAAACAATATCAAATCAT
>ConsensusfromContig1292 Average coverage: 8.31
TAGTAAGCGAATTTTGTGTTCGTAGGAAAACGTTTGAAGCATGTT
GTAGTATTAAACAGT
AGAATCTTGTTCGCTGCTGGGTCAGAAACAATATAATTATTCTTG

GAATCGATGCTGATT
CCAAACGGATATTCGAATTCTCCTTGACCAAGAGTTCGCAATTCC
TGCCCACTTGTGTTG
AAAACACGGATACAATTCACATCTGCAGAACAATCCGTGACAGC
GATGTCTAGGTTACGA
TCTAC
>ConsensusfromContig1293 Average coverage: 4.86
GTGTGCTCTTCCGATCTCTGCTCTCATCAGCTGTTCAGCTGCTTGA
ACGTGTCCGAACTC
GGCGGCCCGCATAACCGCTGACCTCCCTTGGAAGTCGACGACAT
CAGGATGGGCTCCATG
GGCCAACAGCACTTCGATGACCTCTGTATCATTTCTGGCTGCGGC
TAAACAGAGAGCGGT
AGTGCCCTCTTCAGGCTCATTGTAGTTGATCAGATGTGGGACACC
ACTGATGACAAGTTT
TTCCAGGGCCGGGAAATCTTTGTTTCTGGTTAGTTGGAGAACTCT
TGATACTTGTGAGAT
CTCTAGCCGTGTGCTAGCGACGGGCATTCTATGAGTTCAATACAG
AATAACGAATAATAG
CTGTTCCCATTTAAA
>ConsensusfromContig1294 Average coverage: 8.15
TTTTTCGTCGCTCTCTACTTCATTGTTTTGAAAAGTAATGCATCTT
TGGTTGATAGAGAT
TCATTGTTAAGCTGGGTGTACATAATTTTCGTGTATCTGTCTGTTG
TGTCAGTCTTACAC
ATGGAGACCCCAAAACTGCCATCAACATTTCTCAATATATTCCTA
ACAGTCCTACACAGT
GTTGTTGCGGGCAGTGGAGCGTATTTGAATGTGTTATCCCTAAAT
TATATCGAACCATGG
GAAATTATGATATGTCTTTCTTTGGAGCTGGTGTTTGG
>ConsensusfromContig1295 Average coverage: 7.04
ATGTGATTCATTTGAATAAAACAAACCGTGATATATCAGAACCA
CAATCAACTAAAATAA
ACAGTTCGATAAAAAGTAGAGATAACCTGCCCTTGTTTGCCACGA
ATTTCGAGCCCGGTA
AAAAACCTCACGGTAAAAATAGGTTGAATAATTATGAGAATAGC
TCTTATATCGAAGCGA
AGAAAACAAAATATAGTGAATTGATAAGACAATCTAACTATTAT
TCGAATAATG
>ConsensusfromContig1296 Average coverage: 6.84
CTTATGTATGCGGAAAATTTTTCATTTGGAAAATTGGTTCATTCTC
ATATATTTTCTTAT

ATTCTTTCATTGAAACCAACTTTTCATTATCAGACTGAAACATACT
GGTTTTTAGTAATG
ATCAGATACCCTCTTTTAGTATTATCTATTTTTTATAATATTATTTC
AGTGGATTTTTTT
AAACACATGTCGATCCAGCTCATA
>ConsensusfromContig1297 Average coverage: 12.73
TCAGACGTGTGCTCTTCCGATCTGTTTCAGTAAGAATTGGCGCTT
CATCCGACGTATCAT
GTACATGGGATTTCAGTTGAATGTCTATCTCGTCATCGCTATTTAC
TTCTGTTTTAATAT
CTGTTGGATTTAACTTCGAGTCGAAGAAATATTCAAACAGTGGTT
CATCGGGCGTTGCGT
TGACATCCGATTTTACATGGTTCATATCTGAGATATTC
>ConsensusfromContig1298 Average coverage: 5.02
CTAAATAACAATTTCATAGTCTCCATCAATGCAGAAAGTCATTTT
AGCAGAATTCAAAAA
CTAAACCTTCAAAATAATTCCATATTTGAAGTCAACGAAAATTTT
ACACGATCACTCCCA
GAGGATCTCAAGACACTCAATTTACAAGACAATAAAATAACAGA
TCTGGACGAAAACGCC
TTCTCAATGTTTACTTCTCTGGAGTCACTACTCATAAGCGGAAGA
>ConsensusfromContig1299 Average coverage: 4.77
GTGTGTGAATTATAGGAGTGATCTAATTTTAATATGGAAATGGAA
AAATTGCCATGAGAA
TATGTATAGTTACCATGTGATTGTTTGCATGACTGTCAATGAATT
ATTCTCTCAGCAATA
TCCGGATGCCCAACTCAATTCCTAATTAAACCGATTGTTTTGCTT
GTGAAACTCCGACGA
TCTAAAAAGCTTTCAGCTCCAAACAGAT
>ConsensusfromContig1300 Average coverage: 9.05
CCGATCCCATCACTAGAAACCGCAGAAATAGATCTATTATCAGG
AAAAGCCAGTAAACTC
TGAGGGCAAGTATTTCCATATCCATTACTTGACTGAAAATTGCCA
ATGCCATATCCATTG
AGACAGCTTTCGTATTGCAAATAAGGCAAGTGGGGAGGCTGAAT
CATCACATTCCCAGGA
CTGTAGAGGCTCATCAGAGAAGAATTCTGAGGCCGATAAATAGA
AGGTG
>ConsensusfromContig1301 Average coverage: 18.32
GGTTTCATTCCATCCTTGCGACTTGGGATGTCAAACGCGGTCCAT
TGTGATGCTGCATTC
CATTGAAAAAGACGGTACATCCCTGGATCAAACGAGAAAGCGAC

ATCTGGAGTTGAAGCC
GACAGCATCCATTCTGTATTGCAGCTTTCTCGTCTCATCAATTGAG
ATATGGTATTCTTG
AAGATCGCCATGCTTGAGAAGTGCTCCATGTTGTCACTGGTACCA
CTGAT
>ConsensusfromContig1302 Average coverage: 6.64
TCTGAATGAGTGGTTTGACTTCAATGTTTGATTAATACTAAAAAA
GAAAATTTGTTTAGT
ATAGGAGATGGATGAACAGGCCGGCTCCAAATTGACAGTATGGT
TATCGCTAGAGCACCT
AATATTTACGGAAATATTCATGGTCAAGGGAAAAACAATAATGT
CCATTTGTGAGCCAAA
TAGATTCGTGTACTAAACACATCACTTT
>ConsensusfromContig1303 Average coverage: 28.90
ATTCGTTGAGAATCTTCGACCTGGCAGTAATAACATTGAACTGCT
AAGCTGGCAACTTGT
TCTTTATGGCACAAAAGAATTAACGAACAAGAATTTTTTTTCAA
AACGAAAACGAAATAA
TTTATATGAGAGCAAAGTATCACATGAGTATGATGCTGTTTTGAA
ACGTTCATCATTGTC
GAGATCGGAAGAGCACACGTCTGAAAT
>ConsensusfromContig1304 Average coverage: 8.54
TCGGCATTGGAAATAACACCAGTGTTCCCATCCAGATATTTCAGT
GCTTGGATAGAAACG
TGTGAGCTCTGTTGTGGTGTTTCCTCTGAATGCTTGTCCGCCTTTT
ATATATCCATGATG
GTCGTCCGATGATATAGTGTAGCTAGCACATATGTTTCGTTCAAG
TTCATAAACCATAAC
GTTCAGGCTTCGGTCCGATTTGAACATTTTTTCACAATGCTCAGA
AGAGTCATCAAACTG
GCAACGATATCCGAATTCCTCCGTGATCACACATTTTGATGCTTC
TGGTTGAACGCATGG
ATTGCTACTACACAGGTCTAAAGTCACTGTTGCT
>ConsensusfromContig1305 Average coverage: 5.50
TAACAAAAACCCCAACAATCAAAGACACAACAACGACAGATACA
ATTATCGATACACACA
ACGCACACGCTTGGCGTTGAGATAGATAACAGCCCACTGGGCGC
TTGGGACCAGATTTCA
CTTTGACTTTAGGCTCAGATTTGAGTTGAGGTATTGTACTGATTG
GAATGTATTCCTCGC
AAAATGAAGATCCGTCTATAAATCGATGCTGATTCATTTTGTTGG
GTGACGACCGCACTC

AAGACACTGATAATGCAGTGAATACTCACAATCCAATGTTAATC
>ConsensusfromContig1306 Average coverage: 7.79
CCGGTCCCAAGGGAGATCCCGGATATCCCGGTAAACCCGGGTTC
CATGGTGAGCCCGGAA
TCCCTGGACCGGAGGGTCCCGGTGGCAAAAGGGGAGCTATGGGA
CCAGAGGGTCGGAGAG
GAGCCCGTGGGTCCAAGGGCGATAAAGGGGAGATAGGTCAAGAA
GGCCTTCCGGGACTTC
CAGGAGAAAACGGGTTTAAGGGGGAAGCAGGGATGCGTGGACT
GAAGGGG
>ConsensusfromContig1307 Average coverage: 5.85
TTCTCCCATTGTAGCTTTGAAGGATTTTTGCATCACTTCCAAAGG
GTTATTGGCCAGGTC
CTCATACTTCTGTGTATGAATTCTGTTCGGAAATGTTTCGGATAG
GCGACGTATTCCATG
CAGATCAGCAGTCATTTTTGCACAAAGAACTTTGGCTTCTTTTTC
AATATATTCAGAATG
ATTTTTTATCTGAAAAAGAATTTTCATAAATAAGCG
>ConsensusfromContig1308 Average coverage: 5.58
GATCTTTTTGAAGCATGATGATAATTCACTCAATATGTCTGGCGT
CCAAAATAACAAAAA
CAACGATAGAATGATGACGTCATCCAACGGGTTTAGGAATCCCAA
TTTTTCAAAACAAAC
CATCGCGCCACCTATAAGACACCAGGAATCGTGCATGGGATAGCA
TGCTTTTTTTGGGGG
GTTCTAGGCCGTGTGGGTAC
>ConsensusfromContig1309 Average coverage: 163.43
GTTTGTGTAGAGTGATTCTATGTCCAAAGAAACCAAAATACATTC
TGGGGATATATTCCG
ATGTGTCTTCAGTTTGGATAAAAAATCCCATGTGTCTTTAATATA
GGACTTGTGAGTAGA
ACAAATCGGTTTCAAGACATATGATAAGAAGCATCCCGTGAGGTA
TGATTCTGAATTGAC
ATCTGATACTATAGGTCTACCTGGTGGAATGTCTCAGATCGGAAG
AGCACACGTCTGAAC
TCCAGT
>ConsensusfromContig1310 Average coverage: 6.46
GTGTGCTCTTCCGATCTGGAAAAAGGAAAAAGATGTGGAAGGTG
GTATCCCGACCCAGAA
CAACTGATAAAACTATTGAAAATGTACGAAGATGTGGAGAAGGT
CGATAACACGAAAGAA
CAGACAGAAATTATGCAGAAAGTGTCGAAGGAGATCAATGTCGA

CATGAGAAGAATCAAG
TCATGGAGATACAGAGCAAGGGCAAAAAAGCAGCGTGATGAAG
AACAGAGAAAGATGAGC
CTCATCCTTTCAATCGACAAATCCAACCCAAGAAAACCAGCTTTG
AAGCTCCTTGGGATG
AGTCGGGATAAAGATCGTATTCTGAAATTACCGAGCCAACATCAG
AGTCTGA
>ConsensusfromContig1311 Average coverage: 5.28
CAATGTCATACGTTCTTCTGATGAAAGTCACTCCTGTCGTCTGCT
CCTTGAGAAGGTTCG
GCCTATCACTGAGCTTCTGCATTGTTTACGCGGCGCTGTTGACGA
AAACAAACAGAATCT
ACAGAATATTCGAGAACGCCAGTCGATCAAACAAGAAGCCATCT
TTGATCAGTCCGAAAT
CCCAGATAATGATAACATTGGGTC
>ConsensusfromContig1312 Average coverage: 65.51
CGTCGGTATGTTGACAAGATCCAACTCAGCTTTGAATTTCTGCCA
TTGTCGCTTCGCATC
CAAGAGATCGTACAGCATGACTGCCACGTCTTCAAATATTTCTGT
TCCCATCTTCAACTT
TTCCGCAGGGTCCTCATCGAGAGGGGGCGCACTCCTTGGCGGGG
AAAAGTACTCTAACAA
AGCGATATTCTGCAGATCGGAAGAGCACACGTCTGAACTCCA
>ConsensusfromContig1313 Average coverage: 7.28
CTTGCCAATCCTATCGAGAATCACTTGCCTTTTATTGAGGTTTTT
GAATATAATCCAAAA
CCTCAAAGTGAATCCGTTTTCACACGTATCCGGGTCCAGCCAGCA
TCCTTCTTGTGTTGT
GTCGATTGTGAAGTAATCCTGCTCCGAGGATCTGAAGCCTTTTGA
TCCA
>ConsensusfromContig1314 Average coverage: 7.25
GTGTCGGATATTCAGCCAGCACATGTCCGGGTATGCAAGTGAAAT
CAATACTTTGACCTG
GCTTTGCATCTTTAAATTGGTCTGAAGTGAAATTAGTTAATTGCT
GGCCTTGACAGACGT
GGGAAATGCATCCTGCATTTCTTACACAAACTGCCAGAGCGAGTA
TGTGCAGTGTCTCGG
ATAAAGACATGACCTCGATTTTCTAGAGTTTGTAAGTATCCCAGC
TCAACTTAGCCATCA
GAGGTTTCCCATATTTTCCCACTTCAATTGAAAGTTTCTCCAATAT
TCTGACAGTGTGTC
A

>ConsensusfromContig1315 Average coverage: 6.18
AATAATGACGAAACAAATGTCAGATTCCGAATCTGGACGCAATA
AATGCTAAATTTTAAA
ATTCAACGCGATGAATACCACATTTCGATTCTGGACGCAATGAAT
AGCACATTTCGAAAT
TAGACGCGAATAATGCCAAATCGAAAAACGTGATATAAAGAATG
CCAGATATCATGAACA
ATAAATTACAAGAGATGAACAAGAAAAAATAAG
>ConsensusfromContig1316 Average coverage: 5.34
GTGAAATGCTCTTTTTGTGCTGAAGCACATGATTCAAAAAATTGC
CAAAATAAAGGTTCA
TTAAAATGTTCTAACTGTGAGGTCACATCGGTTAATGATGGTAAC
CATACGTCTTTCTCA
TTAAATTGCCCTGAATTGATAAAACAGCGCAAGTTTCTATTGAAT
AGGACAAATTTTGAT
GTTGACCACGAAATAAATCTAACAGATCGGAAGAGCG
>ConsensusfromContig1317 Average coverage: 4.58
GCTCTTCCGATCTGCGATCTGTACGAGAACAAGCATTTTTGAAAG
AATTAGAAGAGTTGA
GGGCCGAGAATATATCCCTGAAGAACACTGTTGCCTCGCTTGAAT
CTGAGAACGCATCCT
TGGTACAGAACTGTGACTCACTTGCCAGGCAAATTGAGAGCGAG
GCCTATGATACAAATC
GAAGACTAGAAGAAATAGATTTCTGCCAGAGACTTAAGGAC
>ConsensusfromContig1318 Average coverage: 4.83
TGGCTAAAATTGACATTGAAAATGCTTTCCGTCTAATTCCGATTCA
TTCTTCTTTCTGGT
TTTTATTAGGGATCTCCTGGAGGGATAAGTTATATTTTGATAAAT
GCTTGCCAATGGGGC
TTTCAGAAAGCTGTAGTCTATTTGAGAAATTTGCATGTGCGCTCG
AATGGATAGCTAAGA
CACATTTTAATATCCCTCATATACTTCACTATCTTGATGAT
>ConsensusfromContig1319 Average coverage: 13.77
CTTCCATGCAATTGCTAAACAAATATGAACATAAAATCATTCGCG
AAAGCTCATATAACA
CGAAATTCCAAGACCGCAATTAATGAACACGATATCATAACCAA
ATACCACCAAAACATA
ACAAGATAATTTGAAATCCGCAAATAAATTTGAAGGATTTAATTC
CCCTTATAATCAAAC
GGAGTAATACATAAAATAATCCATATTTGGAATGAATCAGATAA
TCAGGAGCTCAATAAA
TACTAGAGAGCCAGTAACTA

>ConsensusfromContig1320 Average coverage: 6.52
CTTGGATACTGGCACACGGGTTTTTAATGGTGAAAACATCACAAC
TGATTCAATAGTTTT
CTTTGGTGATGAATACGAACGAATGTGCAGAAAATATCTCTACAG
AGCTTTCATTTTTGA
GTCAAGGTTTGTCAGAAATCCTGCCGTAATTTATGACAAACACA
GAGAAAAATATGATAG
AATGGTTGATGATTTGAATATAATGAAAGAAACACTTGTGAGCCA
AATCGATATAACCAT
CTCTGCGATTGATGACTTTATCAATACATCTTTGCATTTACTAAGA
CCATTTATCACCGA
ATTGAAAACATATATGATAA
>ConsensusfromContig1321 Average coverage: 11.68
ACATGCGGACATGAACGCAAGAAGCGCTTCTCAGCTACAGTCAT
GTCCACGTCAAATAGC
ATTCCTCTGAGATCCGCTGATTTGAAAGTTTTGTTCCATCCGAGT
GTGGAAGTTCGTGAT
AGAGTATTAGTGTCATTAAGAGAATCCAAGTACAACTGTCTGTCG
AGCCGACTCTCGATA
TCCATTGTATCCGACAGGGTGACTCC
>ConsensusfromContig1322 Average coverage: 5.58
GCTCTTCCGATCTGCACCTCAGAAGTCAAATGCATAGGGGAGCCT
CCCACACAAGCTATC
TAAATCCCAGGCTGCCCCCCATCATGGGATTTACTCCACTAAGAC
CGGTCTGGACTGGTA
TCCATGGAAATGCTCTCAACATCGAGAATGTGACCCCTGGTGACC
TCTCTCCTCAGGCTC
GCAGACATCGAGCAGCCCCTCAATACAATAACAGCATTGGTAGG
AGCAGCCCCTCAATAC
AATAGATCGGAAGAGCG
>ConsensusfromContig1323 Average coverage: 6.03
GCCTATTCCTGTCAATATATTCCAAAATATGAGGAAATTATATCC
CGTGGAAGATGAAGT
GAAAACGAGAGATATGATGTAAGTCAGAGGTATAATAGACAAGC
CATAGCATAGATACAA
AATTATGAGGCTGCCATCCGAATATGCTTCAACTTCGTATATCAT
GAACACGATGACGGC
AAAAAAACTGGAGACAACCAGCAGAGTATAGTCCCATACGAAAG
AAGAAATCCAGAAGAT
>ConsensusfromContig1324 Average coverage: 7.44
GTGATTCTCGACACGCGCTATCATTGGTATTATTCACGATATTGAT
TGCTATGTCATGTT

AGAACACGCTGTAATATATGTTAGCATTATTGGTTTCTTTCACTGC
TCGTACTTTTGATC
GTGATGCTCCTCACATCGCAATGCCAATATCACGATTAGAACAAT
CAACAGATAAATTAA
TGAAATTGATTTTTAATAAATTGTACGCAAAAATAATTATTGCTG
GCA
>ConsensusfromContig1325 Average coverage: 5.75
TCGATTTATGTCTCAGAATAATTATCAGAGCAGAGGTAGAATGAG
GCGGTCTTTGAGCAA
GCCCATGTGCTCCAGATGCCGTAACCACGAACATAAATCCATTCT
CAAGGGTCACAAGCA
CAAGTGCCCTTGGAAAACGTGCACTTGCAAGAAATGTGTGATAG
TGATCAATCGTCAGAA
AAACATGGCAGCCCAAGTGGCCTACAGACGAGGCCAAAAGACAT
CGTCAATTGTCTCCAG
TGCCGTTTCAGACCCAAAACCACTGGGCGCCCATC
>ConsensusfromContig1326 Average coverage: 8.46
TGTGACCATAACTGTAGCCTGACTACTGACAGCTTCATTTTCCGTA
AGTTTTTCTGCTCT
TTCATGATCGTTTTCTGTTTTATCTGTTTGAGGTTCTTGGCCTACC
GTGTCTGCAGTTGA
ATTCACTAAAGAGTGAGTTTCGTCATCAAAACTCTCCGTCACATG
TTTTGCCGATATCGA
ACTTCTCCTGCTTTCTTCGTTTATTCGGACCCGCTTTTCCTCTAAT
TTTGGCTTGTAGAT
A
>ConsensusfromContig1327 Average coverage: 9.55
TCCGATTCCATGAGGCCATCTCTGAACAAACCAAAGTCGAGTCAA
CGATTCTGCAAAACC
CCTTCAAATTATGAAGCGCCATTTCGTGTTCGACATCTGACATCG
CCGCGCAGCCATCTT
CGATAACCGTGACCCAGTAACCTAGATCTGCGGCATGACGTACA
CACGACTCGACGCACT
GATTCGTCAATACCCCAACAACGACCAATTGCTTTACTCCTAGAT
TCCTCAAAACAAAGA
GATCGGAAGA
>ConsensusfromContig1328 Average coverage: 6.78
CCCAACTCGCCCGCGACCTCCATGGCAATAAAGCACGGGCGGAT
AGACACGGTCGAGTGG
TTGATGCTCAATACAGAGCTTAAACACGAGTTATCAGCACGAACT
TCGTGTCGAACTCTC
TTGCATCATTCAGCAAAGTTTGGACAGGATGATATATTGCGCCTG

ATTCTACAAACCATG
GAGAAGAAGGGGGCAAGCCATACCGTTCT
>ConsensusfromContig1329 Average coverage: 5.27
AGAAAACGTCAGGAGCCGTAAAACAGCAATCAAACACCAGCAAG
TCAGTCTCTAAAACTG
TTCCACCACCCAACAAAAAGTTTCCGAACCACATGCAGGAGAAC
TAGTCGAAAAATCAAT
TCCAACAGGGAAACGTTGTTTCAAGTCGTCATTCCATCAAGAAGC
GTATTAGAATGAGAG
AAATTCTACTGGATGAGTTCTATCGAATTAGATTTGAGTATGTTA
GATTGGAGATCGGA
>ConsensusfromContig1330 Average coverage: 8.30
ATTGAATGTAGATATTCTACAATATTAAAAAAGAACGTAGACAGT
GTGTCAACGGTCTTT
ACTCGTGCCAAATATTCTTATGCCGTTTTATTTTTGTACGTGTGAA
ATTTATTGCTAAAT
GAGACACTCGCTTTTTTGGATGAATCGAAGAACGGAAACTTTTAT
TTATTTATAACTCCA
CTAATCGGATATATCGTTGTTGAAGAATTTGTTATTACTGATATG
TTCGCGAGT
>ConsensusfromContig1331 Average coverage: 12.22
GTTGATCTTTTATTGAAGTCACCACCGATCACAATGTTGGCAGAG
TTTAGTTGATCCAAA
AAGTCCACATCAATGAGCAGGTTTTGTGGTCTGTTGTAAAGTACA
GCGAATACTATCTTG
TCATTCTTCGTCTTCAGTTCAATAGCTATAGCTTCAGACTGGGTCT
GGAGAGTCGTTTCT
GAGTACACAAGACTTTTCCTTATCAGAATAGCGACTCCACCACCT
GTGGTTGGCCTGTCC
TTTCTCACGATATCGTACATTGGAAAACGTGGATCGCTGTGTACA
TTAAACCACGTCTCG
GTCAT
>ConsensusfromContig1332 Average coverage: 4.69
TCTTCCGATCTCAGCGATGGATAAAAGGTCATCTCACAAGAATCA
AATTTATGCTACACG
TGAAATTCAAACACAGCATGGTGAATTTGAATAACTCACATACA
AGGCGAGCGGATAGCA
GAGAGAGCATCTACATGAAACAAGGCTACATGGACAGGAATCCT
CAAATATCCACTGACC
GATGTGATCCACGAAAATTGAATTATGACACGTATCTCAGAAACC
GGAGACCTGGTTCAC
GAGCACTAACGCCAC

>ConsensusfromContig1333 Average coverage: 4.28
ACGTTCTTCGCAAACGACAAAAACTCCTTTCTGCATGATGTCACC
GAGAAAAACAAGATG
GTCAGATTGCATGGCGACGGTCATATATCCTATGGAATGAGGTTT
ACTACAACTTTGGCT
TGTATGATGGATTTGCACAATTATCCCTTGGATGAACAGAATTGT
ACGGTAGAAATTGAA
AGTTATGGCTAACCGACAAAAAATGTTGTTATGAAATGGAGAGG
GGAGAGTCTTATATC
>ConsensusfromContig1334 Average coverage: 4.54
AGCTGACAGAGGTGACAGTCCTGCTGCTGGTCGAGTTGATCCTTG
TGAGTTTGCCATGAT
AGGCACTGAAACCATTGAGCCGCGAATGGATCAGCATTGCGAAT
CATATGTCTGAAATGT
TAGCCAAGAGGATCTCGATCATAGGCACTAGAAAATCTGAACGAT
ACTTCACTCTCACCA
AGATCAAATTACTCCAAGATCGGAAGAGC
>ConsensusfromContig1335 Average coverage: 8.22
TCTTGCGTTCATGTCCGCATGTTGTTCTAATATCATCAAGAATGC
CCGCTGCAGAGATAC
TTGTTGACGCAGTGAACTTTGGAGTCATTCCGTTGTTTTACGAGT
ATGAAGGAACAACTC
TCGAATCGCTAATTTATCGGATTTCGGAGCTACTGGTAGGAAATC
AAGCCCAAAGTATTG
GGCTCATGTGCCAACATGGGTCTGGAGGAAGTCTTCACCTAGTTT
CAGGAGTCTCT
>ConsensusfromContig1336 Average coverage: 8.68
TCTTCCGATCTGGCCATTCTACATGCATCTCGAGAGTACGATTTA
TGTCATTAAGGATGA
ATTTTCTGTTGTCCCCAAACATCACAAGTGTGTAGTTTTTAAGCA
GTATGAATACATATA
TGTTTCACGAGATTATACGACAGTGAGAACGACTTTTCACATACA
TTGCACATGTATGGT
TTTTCACCGCTATGAATCAT
>ConsensusfromContig1337 Average coverage: 5.17
CTTCCGATCTGCGATAATGAGTACTGAACACGTGTCAAATAGGGG
TTTCACCAAATGAGT
TCAAACAACCGGTCGGGGTATCAATGCGCGTTATCGCATATCTCA
TCTACCGTCATGTAA
ACAAATTAGAAAATGACACCTTTCGGTCGCTGCTAATTCTATCAT
CCAGTTAATATATTT
GAAACAATATTCTAAAATCATAAC

>ConsensusfromContig1338 Average coverage: 5.90
ACAGAATGCAATTATTTTTAATCATAAAATAACCTGTGTGACCC
AATCCCTGTCAAGCT
TTAGACGGCCAGCCCGGGTTAATCATAAAATGACGTTTCTCGAGT
ATGCTGGTTGATCTT
GAGAAATCAAATTTTGAAAAAGTCGATCTCTATCTCTCCACTATT
TCGTGGGCCGTGGAA
CATTTGAGCTCCCGTATTATATTTTC
>ConsensusfromContig1339 Average coverage: 4.76
CAAAACAATAATAACATGTAGTCACGAGAATCACATTTAAATAG
GTTATTCAAATAAAAC
TGATAGCGTTCAATAACTAATTGCGTCTCCCCTGAAATCGATACC
AGGAGCTAGCTTTGG
ATTTATTTATTCATTAAAAGTCGCTACTGTGTCTGAAATGTGCGA
TATATTGAGTATCGC
TCCATGAGCTCGTAGCATCAGCGCTAGAATATTGCTGTA
>ConsensusfromContig1340 Average coverage: 5.47
AAATAAGTGGCATTAATTCGGCTCTGGCATTTGGGATGGCATCGA
TTGTTGTTTCTGTGA
CTGCTTCTGTGGGCTTTCGCAAAACACAGATGATAGGTAATGCTG
GTTGTGCCTTGTTTC
TGATGTTAGCTTCCTTTGCTTCTGACACCTGGCAATTGTATGCGA
CCCTTGGATTTGGAA
CAGGACTATTCTTGTGCGCTCAATT
>ConsensusfromContig1341 Average coverage: 9.07
AACGTCGATTTTTTGGCGAAGAATGATGTCAAGAATATCAGTACA
ACCATCTTCAGCAGC
ATAATGACAGGGTGTGTTGCCGAAAGTGTCTTTGGCGTCCGGATC
AACGTCTACAGATAG
GAGTTTGTTGAAGCACGCAACCTTGTGTCTTAAAACAGCAAAATG
AAGAGCTGTTCTTCG
GAGAGTTCCTGTAGTTTGATGAATGTCACATTTCATGTCACAAAG
GTATTGTAACATCCC
CGTGTATCCCTTATAGCTGACC
>ConsensusfromContig1342 Average coverage: 7.08
CGTTTATTGTGCTAACAGCGTTTGTATACACAAAGCTATACGGGA
GCATATTGGAGATAT
GTAGCTTCTGTATACGTTCAACCAAGCAGTTTCCATTTTCTTCAC
AAGAGTGTTCGCAAG
AGCAAAACAATTCTGTTTGTTTTTTGTCTTTAGCGGAATAAACTTC
TTCCATATCACGAT
TTGAATATCCAGTGGAAAAAACTTGTGATATAGTCTGCATCATTT

TTGAAATCAAGCCGG
AGTATTTTTCAGCATTCGGCACTTCTTTTACATCACCATGATCGTC
GATTTGAAGTTCGG
TGATGAACAATGCACAGCTTTTCACCGAGCCATCCCTCTCAGACT
CCGAGTGAAGACCGT
CAGGCAGGGAGGTCAGTTCCGCAGACAGGCGTTGCATGTTTTGTT
CGATCAGCAGAATCA
AGTGAGAGTTGAGCCAAAT
>ConsensusfromContig1343 Average coverage: 6.86
CTTGATTATGTAAAATCGATTATTCATAGAATATTTGAATACAGC
ATCGATGTCGGAAGG
AGCACCCATGATGATTTCGTTGATGAACCCGCTTCTTTTGAGAAC
AGAAGCGTCATCGAA
AATGTACAGCATTGCTCCAGCGAACACATAATGCTCTTGAATAGC
GTTGAGATATGTGAT
GTCATCGATTAGTGAAGCATCGACTCCGGGAAATATGGACGCCAT
TGCCCCTCCGGATAT
CGCCTCGGATGTGATGAAGTAGGCGAAGGAGCCCATGAATGCGA
GAGTTTGTCCTCTGTA
CATGGTCACAGCATCAAACTGCCCCACGCTGCAAGGATCTGGGG
CAGGAAGAGGGGCAGT
TCCCG
>ConsensusfromContig1344 Average coverage: 4.70
GTGAAATGTCAAACTTCAGCCCAAAAAGTTATAAATGAAGTCAAT
TACAAAACTGTCATT
TACGAAGAAGAAGAAAAAAATGATATATATGATGATGATGATGT
CACTTATGATCAGAAT
TCAATTGCCGATTTTGTCGTGAAGTTAAATGGACTCTGGACATAC
ACTCTGGGGGGACAG
TCAAAATTGGAAAAGAAATTAA
>ConsensusfromContig1345 Average coverage: 552.64
GAGATCTACACTCTTTCCCTACACGACGCTCTTCCGATCTCAAGG
ACCAAGAATGGCCCA
ACAAGGCCCCCACCGGTCAATGTATGGCAATAAACACTTTCTTA
AAGATCATGATGAAAA
TAAAGATGGCGTTGACGACCGCTACGATCTAAACAGGGACGGAA
AGTTGGATCATTCATC
AAATGTCGGCGTGCATCGGAACTAGTGCAGGAATGATATTTCTGT
TTTTTC
>ConsensusfromContig1346 Average coverage: 6.09
ATCTCGAGCTCACAAGATATAGACAGCGTGGACTGCTGGACTCG
GAAGAGGAGGCTGAGG

AGAAAGACTATTCCAAAGACTGGCACAGAGTGGCTGAAGTCGTTG
ACCGACTGTTCTTCT
GGACATTCCTTCTTGCTATCGTCGGCGTTTCCATTCTTCTCTTTCA
TCCTCTTGCCAAAA
CCAATAATTTAATTCCCTTCGATTAAAAC
>ConsensusfromContig1347 Average coverage: 4.86
GACAAAACAGAACAATTCGAAGTAAATAACATCACACATGAATG
GAAACAAAACCTAAAA
ATCCTTGGATCCTATATAGAAACTAAAAATGACATCTGTCACAG
AAAAAGAATGGCATGG
ATGACAATGCACAAACAGAAACACTTCTTCAAAAGCAATAGACT
GACAATCAATAGTAAA
CTCAAAATATACAGAACATACATTGCCAGCATTTTCCTTCACAAC
AGCGAAACCTGGACA
CTGAC
>ConsensusfromContig1348 Average coverage: 4.42
TGTGTCGTTGGGCTCTCTGGTATCTCTCGAAATAAGATGTGACGA
AGAGCTTGAACAAGA
TAATGCCTGGTTTCTGGACACGGTTTTGGTCGGCGAATTGCAATC
CACAAGACCTCGATC
ATTATTTGTATTCGACAATTGGTTCGACGGAACGGAATCTAACTA
TAGATTGAGTGTTGC
TACAGAAGAAAAGATCAAATCTCGAGAAGCAAAAATGTG
>ConsensusfromContig1349 Average coverage: 5.20
CCGATCTCGCCGGGACTTTTTGAAAACATGTATCAAACAAATGAA
TTGGTGAAAGTTCAA
CAAATATCAAACGTAATAAATAACAAAGAAAACCATGAAATTAG
GTACTGTAACAAATAT
GTCAGAATAAAATTGTAGAAGAACAGTGAATACGACGGGAGTAG
AGTAAAACATTGGCTA
TTCACAATAATATGAGAGTAAGTTATAGCTTAATACACATCCATA
ATTTAGATC
>ConsensusfromContig1350 Average coverage: 8.24
ACACCATTCTTCAATAGATTGAAGTAGTATTACAAGCTCGTAATA
TTTATAAACGCATGC
ACTTGATGAAACCATCTTCATGAGAAATCAATACCACTCATTCAG
CACATGAATTCGATT
AGAATTAAGCTCTCGATTAATTCATTATGAATATCATGATTTTTTC
AATTATTATCATTA
ATATTATGTTCAAAATATTG
>ConsensusfromContig1351 Average coverage: 5.04
CTTCCGATCTAAGATATAACGCTGGAGTCAGAATGTCAGAATATG

GGTTACCAAGACCCA
TTGTCGTGTCATCAATGTCGATGCCCGGAGGGTCTTGGGGGGAAA
TTCTGCACTGAGCCT
ATCACTCTCACTCACGGAGGACATCCGCTTTGTGGGGGTTTGGTC
ATCGCTAGTAACACA
ACACAGATGCTTACAAGTCCGGACTACGAGGAAGGGGCG
>ConsensusfromContig1352 Average coverage: 5.45
GCAAGAAAAGTATTTCAAGTCCAAACGCATCCAACAGAACTCTT
CCATGCCGAAATACGA
CAAGAAAAATTGTTGGGAAACTTTGAAAAATCGAGCATCGAATG
TGAAGGTCAAAGCGAC
GACGCGAGGCAGGACCAGCGTCTCATCCGTCCCTGAGAATGAGG
CGCCGGCGGTCGATAA
AATAAAAACCTCCCCCCCCTCTTCTGCTTAAA
>ConsensusfromContig1353 Average coverage: 8.92
CATAATTCAAACATATTCATATTGAGCCTCAGTGAGTTATCACAC
ACGTTTGAGATTTAG
AAAATTTGAATTAAAAAATGACAATTAGAATCACAGAGACAAAA
CAACATGACGAAAGCA
TACAACAAAGCAAGAGTATCGATGAAATTCGAGTTCGCTATATA
TTGAACGGAATGCATA
TGACGATATCGTGGAGTATAAATGTG
>ConsensusfromContig1354 Average coverage: 7.04
TGATCAAGTGAACCTATACAGTTTCATTTTTGTATTTCTACAGTGT
TCAACTGGCAGCTT
TTCATTTAAACTAGAGCGAGAAAAGGGAGCAGACGATTCGAACG
TCGGATGGGCGCCTTC
AATGTCTGCACTCTGTCCCAAAATCTCATGAAGCAGAGGAAGGTA
TAATCATGGCTGTCA
TGGTTCCACGAATACATGTTAAGTTGAAG
>ConsensusfromContig1355 Average coverage: 4.51
GTTTGTACAAAAATAGGAGCCGAGAACGCTTGAAATGGTACGATC
GGCGTCATCCGTCTT
CGTTCATATTTCGGTGCCCCCCCAAAAACAAGATTAGGGTTGTAC
ATAACATATTCGGGA
TACCGATACACGAGGGGGAACGTCGATATCATTGGGGCCCCTTT
GTAATCCAGAATGACA
AGTTTTGGAGAATATGATAAAAATATTGATC
>ConsensusfromContig1356 Average coverage: 5.87
CCGATCTGCGCAGGCAAGTTCTAAAAATTGCAAAAGTCTATGTCT
CAATAACATCGCTTG
CTTTCGAGTTCAAGTCATCCACAGATAATGGATCGCTTTTTCTTCT

TCATACGCGAACCT
CTGATTTCTTCTTGATTCGTGTGAGTGGGTTCAACGTATTATTGGA
ATTTGGCTTTGCAA
AAAAATTCACCAGGATCTTCTGCAAC
>ConsensusfromContig1357 Average coverage: 5.01
CACGATTGGCATTTGTTATAACTGTTATTTACAATTATTATTTAT
AAGAGCTTGAGCTA
ATAATGGTTTAATGTATTTATTCTATTATTTTTCAATGTGCCATTG
CTTCTTGTGCAATA
TATTTGTTCCTTTAATCGGTGAGGCGAAATTGTCGGTTTTATTGT
TACTGAAAATGAACG
TGAGAAGTCGTTTTTCATGATATTATTTCGATGTTTTGAGAACCA
AAGATC
>ConsensusfromContig1358 Average coverage: 6.67
ACTTCCATTTCCGATATCAACTTTTTGCCATTGGACTCTCGCCGG
ATGTTTAGAGGTGAC
AAAACACAAGAAAATGAGAACAACGACTCCTAGTGTGGAAATCC
ATCTTTGGACATCATC
TTTGACATCAAGGGCGAAAAAGAGGATGATAAGAATCACCACAA
TGGCCGATAAAGTGTA
TTTGAGAACATTTTTGACGATTTTATTATC
>ConsensusfromContig1359 Average coverage: 5.65
AGCCAACCTGACCGCGCAGATCTGCGCTTGTGGAACCAGATTCTC
CTGGCCGAAAGCTTG
GCGAGGAACACTTGTTGACAAACACAGCACTGGCGGAGTCGGTG
AGAAGACGAGCCTTGT
GCTGACACCTGCATTGGCCGCTTGTGGACTTAAGATCCCAATGGT
ATCCGGACGGGGGTT
GGGCCATACGGGTGGCACGATTGATAAACTCGAC
>ConsensusfromContig1360 Average coverage: 6.38
GAAAACTGAAAGCGCTGTTGGAATCAATTCTCATATATAACACAT
GCACATACAAGTCCG
GTATTGAACACCTCCAAACAAGTTTCATACACCGACGATATTTCA
TTACAATTTTGGATA
GCGAAATGATCTCGCATAGGAAAAGAGTCTTGGAAATATTTACG
ACGATCAAAGCAATGG
TAAATATTCATTCCATTTATTTTGGTGACTGGGTTTCGATCTGTTC
TCTGACACGGAAGA
>ConsensusfromContig1361 Average coverage: 5.45
AGAATATGCCTTTCATGGACTACAACTTCTTCTAATAGCAGGAAA
ACAATAAATAGCAAA
GCTTAGGCTACTATGTAATCGATAAAGACATTATTCCAGTTTCTA

TTCGCAACATACTAT
GGTTTTCTTTCACCTCATTTATGGCAATTTTCGACACACTCTGGTA
TCATCTCTATTCTA
GTCCGACCGTACTTAGATATTTG
>ConsensusfromContig1362 Average coverage: 35.52
TCAGCTAGAAAGAGATCTGTAGCAGATACAGACCCTCATTCCACA
GCTGCATCAGGTTTG
CTGGAATCGAGGACCGGAGCAGAACTGACAAATGTTCTGACCTC
TGATGAGGGTAATGCG
CCTGTTGAAGAAGGCGGAGACAATGAGTCCGAAACTGATGATGT
GCCCGAGGCGAAGGAT
CCGGCAGATCGGAAGAGCGTCGTGTAGGGAAAG
>ConsensusfromContig1363 Average coverage: 5.66
AGTGAAAAATGAGTATGATTTTTTTCCAATGTTTACACGACGTTA
GTCAAAATATTGATT
CATAACATTCTTTTCGCCCGTGATTGGTGATCGCCAGTCTATTCCT
GATCGAACTTAATT
ATTTACGGGTTTATATGTAAACTGCGTACTTTTTAACCAAGACCT
ATATTCGAAACATCC
ATGTAATAACGGATTCAGTCCTGCAGATCGGA
>ConsensusfromContig1364 Average coverage: 6.16
TTATTATCAGTATTACCGGTGGCTACATTACGTAATCTTACCTATA
TTAACACATTCTTT
GTTTACACAAGCAAACGTACATCACTGCTTGAGTCTTCGGTCTAA
TCCGACCCATTGCAC
TTCCTCGAGAGTACAATACCAGCAATATAAGAATGATGCATGGTA
CATGACACCATTCAT
AATTATCCAAATTTGCCACATGTGGCACTCTTCAAAATTCAATGT
TTCAATTAATAAACT
AATCATCA
>ConsensusfromContig1365 Average coverage: 5.67
ATGAAAAATAAACTACACCAATTGAATAAGCTATAAACTGATTCA
TCTTTCCTGTGGAGT
CGGTCTGTACTATGCCTGCAGAGGAGAACGGCAAAACACGGCAG
ACGAATTCATGTTCGG
AAATCGCAGAATGTCTGTCGTACCTGTCTCGATGTCTCTGGTTGC
CAGCTGTATGACTGC
TGTGACTTTACTGGGCAACCCCGCGGAAGTCTATGTCTATGGCTC
TGAGTAAATACTGAA
TGTTATGGCGTATATC
>ConsensusfromContig1366 Average coverage: 9.59
TGAAGCTTGCTGCGATATCTGGCCCGGCGTTATCGTAATGAAAAG

ATAATAACTCTCCAT
CGCATAATTCGTACCTTGCTGTTTCAGAAGCGATATGTTCAAAAC
CTTCCATATAGGGGG
CATTGTTATTTGTACGTCCAATCCCTCCCTGTCGAACGCAAACAA
GAGCGTAACGCTCCG
TGTGATGGTGATTAGTTGACGGCAAAGATGGGCGTGGTTTTCTGA
ATACATGAAATTCAC
ATGGAGCTGTTCGATTATTCATAATAGCAGTGGCGTGTGCTTTGA
AACCGATGTTATAAC
CAATAGCAAGCCCTGCCCCCAAACTCATGACCGGCACAAGAACT
TTGAG
>ConsensusfromContig1367 Average coverage: 11.81
AACGGACCAATTCTACCTCAACAGAATCTAATTTCTCCAGAAGCC
GGATTTCTGGATCGC
GATTGCAGAAGTCGAGAAGAGTGAAAGATAAACAAGAAATCCTT
TCCACTTCGGACTGCC
TAAATCCATTGAAATCTGGTCCCCGCTCCTCTATTGGTGATTTTA
AAGAGAAGTCGATCA
TATCAACAGCCCATACCCGCAGAGATTTAAGAAATC
>ConsensusfromContig1368 Average coverage: 5.98
CTGTCAGCCTATGTGGACTGTGTGAATGGGACATGGCAAGATGCT
CCCGCGATTTGCAAG
AAAACATGCAGTGAAGAACTCAAGAAAAGTTGGAGCACGAGTCA
TTACTCTCTCGTAGCC
ACCTTACCAGCTTTTGAGGCCAGAGTCTCGTGCACGAGATTTGGA
TATCGGTTTGAGGAT
GGGAGCGATGACGTCATCATAAATTGTGATGACAGTGGAGAGTG
GGCGGAGCTTCCTTCA
GAATGTGACTTTTCATGGTGGGCCATTCTGATGGTTGTTGGCATA
GTATGTGCGATCGCA
ATCATAATAATTGCCGTCATAGCG
>ConsensusfromContig1369 Average coverage: 13.92
TTTTTCGGCGGTCTGTGCGTTCTTTTGATCGGGGCGTGGTCGCGG
ATCGACCGGTGGAAA
TCCAGCTTCTGTCGGGTGCTGGCAGCGGACCCGGCGCTGTTCCTC
ATCATCGTCGGATGT
GTCATGGTTCTAGTCAGCTTCTTTGGGTGCTTTGGATCCATTCGAG
AAAATACTTGTCTT
CTCAGAACATTCTGCCTTGTTATATCCGT
>ConsensusfromContig1370 Average coverage: 8.47
ATCTCGTTTTCATAAGATACTTGAGTATATATGACGCCGGTTTCA
AAATGATACTCGGGC

ACGCCGAGAAAAGGTGGGATTTGAGTCTGGTAGCTTTCCCACTGA
CTTCTGCCTCACAAT
ACTTGCATGTGACTATTTTTCTTCCTTCTTCCGTGAAGCGCTTATC
AAAGGCCCCCCATA
TCGGGTCCCTGGGTCTACCACCAATATTCTTGCATGCATTCGGCC
CGGTGGATTCTGATG
TGCCTT
>ConsensusfromContig1371 Average coverage: 9.14
CTTAATCTATGATGATATATTTATTAGGATTCTCTTCATTATCACG
GATTCGTTTATATA
GAAACCTATCAACTCATTTTGATCACAATTTGAAACTTGCAAGAA
TAAATAAATGTGAAC
GACCACTTCATATCAAACCCGGATAATGTGTCGTAATAAAAAAA
TTGAGAAAAAACATAT
ATCAAAATTTGATCATACTATTTATATATTTTGAA
>ConsensusfromContig1372 Average coverage: 5.21
GGCGCCGGTTTCTGAGTGTCTTCATCGTACATTATGACGTCATAA
GGTCTTACTCCAACA
TCAAACTCCAGTTCGTGACGTTTAGTGACGCGGTTGAATACGATG
AGCTGATTGGCTCTC
TGGTCGGTCGCGAATATGTAGTTCTGATAAAGTGCGATTCCGAAG
AAATGAGCATTCTTT
TCTTCGAAGAGCGTGTTTTGGCGTCCGCCATCAAGATCGATCTCA
TTGA
>ConsensusfromContig1373 Average coverage: 7.28
TACTTGCTGCCGTGGTTATCAAATGTGAAATATCATACTGGCAAA
CGAAGTATCTCAATC
CCTGGCAGGGAACGTCTTTCCATACAGCTACACCAGTTGTAAAGT
CTAAGCTCTGAATGA
GACAGTCAGAGTAGGCGTGGTCTGATCTCGATTCCGACCAATAGG
TAGCGTCGAATGACT
CTTCAATAATGGGCAGACCATCGGTCGTCAGCCAATGT
>ConsensusfromContig1374 Average coverage: 10.97
CAATGTGACTGGAGTTCAGACGTGTGCTCTTCCGGTCATGCAAAT
TTTGTTTTCGAAGTT
CGTAGTTCACTTTGAGATTTTGTATGCCTTTCTCGTATTTCTTCTTG
AGTTCTGTTCGTT
TGTATGCGAATCCTAGCAGATTCGCGAATTGCTTTCTTGCAGCCC
ATTTATCGATTATGC
CTCCTGGACCATGAAGTTTTGCGAATTGAAGAATATATCCTTCCT
TTGCATCATCTTTGG
TAAAACCGGCATAAGAAAA

>ConsensusfromContig1375 Average coverage: 6.21
ATTTTTTTCTTTAATTTGTTTCGTAGAAATGGTGGTGCTTTACCTC
AATACGTCGTTCTG
ATACTATACACGAGCCAATTCGAAATTCAAAATTTATGAACGAA
AATAGCTAGAGCAATG
CGCGAATTGTCGAACTAGGGAGCTGTGAAAATGAATTAAGTGAA
GACGCCTCTAAATCAA
ATTAATCAAATGGGACGTAACTCTACGATTCCTAAAATCG
>ConsensusfromContig1376 Average coverage: 5.69
CGATTGGATTCATAATAATATTCGTATCACAATCGAATTGAAATT
TTGTAATGGTTTTCA
GTCACGCATAGGCGAAAGTTATCTCACGTGTCATATTAGAATCGT
CTGAGATGTCTTTTG
GATATAACAAAACCTCACGACAATGGACGGAAAAATCGTTATTC
TTCAGAGTTCCACCAA
TGGGACTAATGAGATTATCGATAAATCATTACCGAGTAGAAAATT
TCGAGTAAAAT
>ConsensusfromContig1377 Average coverage: 6.64
CAAGAATGACAACCCGGAAAATTCTGAGCCCGATGATGAGTATA
GATGTGGAGATTGCAA
CAGATTCTACACATCGAAAGAAGATTTCAGAAAACACAGAGTTA
GGCACTTGAGTCACAG
ACCTCATATTTGTGAATATTGCGGTAGAGGTTTTTGCTGGCCGTC
CGAATTGAAACGTCA
TAAACTGAAGCACGCTGGTCTGAAATCTTACAAATATGATTTCA
AGGGAGAGGAGCCATA
CATATGTATTCATTGCAGAGATACGTTCGCTTCCTCAGATGAGCT
AAAAGCGCATACACT
CACTCATGAGAGCGATAAACGTTTCAAATGCGAGGAATGTAACG
AAAAGTACTTTCTCGG
AAAGGATTTGAGGCTACATCAGAGAGTTCATTCGGGAGATCGGA
A
>ConsensusfromContig1378 Average coverage: 6.03
AACAAATTCTCATCCTCGGTATTAGGTTTTTTGTTTATATTGTCTG
AAAATTTTAAGAAA
TGTTTTCGAATCTCTTGATAGTATTCAAATGGCAGACATGGACCC
ATCGTTAGAGTCGTA
TTTGAAGAATTTGAATCGTCGACTAATTCAATTCTCGCATGTTTC
AGTGTTAAACCTTCA
TCAAAAGTTATCCCAATCAATCACACACTTGAAGATTCTCTCATG
CTATTGCA
>ConsensusfromContig1379 Average coverage: 4.39

GAAAAATTAGATTCCGTAGAAAAAAAACTGAAAAGAACTTATTA
GAAAAGATCAATATAT
CTCTAAAGGGTAAGACACATATCGCAAAGAAAGAAGAGAACATA
GCAAAATTGATAGAAA
AGAAAACAAAACTACGATCGGAAAAGATGAGATTGGAGAAAGA
AAATATATACAAGAGGC
AAACCAGTTTTATAAAACAAAAACTCATGGGTGATATGAAAAAA

>ConsensusfromContig1380 Average coverage: 4.25
CTTCCGATCTTTTTTTTTCCATCATTCACCCGAGCAATGATACAT
GAACGAGCCAGATT
TACATCAGTTGAGAATGTTCAGTAATAACGGCATCTATCGATAAT
CTTATTCATACACTA
GATGGCACTGCTGATTTGAACTATCCCGATAATATCAATTCAAAT
ATAATTGATTTCAAA
GCATGAAATTGGCTTCAAATGAATCGAATTAAAATTATT
>ConsensusfromContig1381 Average coverage: 696.99
CAATGTGACTGGAGTTCAGACGTGTGCTCTTCCGATCTGAAATTA
TTATTATTGGGGTGA
TAGATGGCCTCCCAAATAGCTGTCCCCCTTGAGCGTTATGAATTC
ACCGCCACGGGGTAT
TTGGGCGACAAACGGACACGATGCTTAAAAATCAGATACTCAT
AAGGGATGAGCTTGCA
AAGGCCAAGACTCGGGGATATACAAACTATCCAGGACGTAGC
>ConsensusfromContig1382 Average coverage: 5.19
CGACATCAAAGAGATTCATTGCACTGGGATCTTTCCCCATTAAAT
TATGATTGTCGTAAT
TGTAGATAGAAGTAGCATCCACGCTTCGTAATGTCGTATATGTTG
GGTGAGATGGAAGAA
GAGTTTGCAGCCGCGCAGGATCAGGTTCTGTCACCTGCATGGATG
CCAAATTTCTTTGGT
GAATGGCAGGAGAAAGAAAT
>ConsensusfromContig1383 Average coverage: 5.46
TTTGAATTTTAATCAGAGCAAATTCGTTCCTTCTTGCGTCCCCAAT
AATCAATTATGATA
TACCCTGCATAGTAGCCTGCTGGCATAGCACAGAACGTATTGTAT
GTTTGATGATAATGT
GATCAACGTTTTTCTGGGACCTTTGAAGTTTTGCGCAATCCTCTCA
TAATTGTTGGCACC
CCATAGCATCATACATGGTATCTATCGATTCTTATATATCCTGAA
GTGTGGTGCAAATTC
CGCTCACCTCCATGAATTTTCAATCCATTAATATCACGATTCAG

>ConsensusfromContig1384 Average coverage: 3.46
ACCGGTTTCATCGAATTTTTCGAGAATTATGGCTCCAATGAAATC
CTCATTATTCGTTAC
TTCATCAATCATTGTCGAAACTTTTTCAAATTCAACGAGTTTTAT
CATACTAAACTGGAG
ACTCTCGAGTTTCTCCTCCACATCAAATAATGATAAATTCTGATT
ACTTTGAACGATTGT
ATATCCCAGATCGGAAGAGC
>ConsensusfromContig1385 Average coverage: 191.69
TGACTGGAGTTCAGACGTGTGCTCTTCCGATCTGCATATGTCAAC
GATCGCGTAACCTAG
AATCGTTCGGTAGTTTATTAAATAAATATATTCATAAATATTTAT
TGGATACAATACCTG
ACCAGAGTAAAAGTCTTCGTTATATTGAGTAGATAGTTTATTGAA
TAAAGGATTTTGAAA
TCTTATTAACAGCAAATTCATGCTGATTGTATAC
>ConsensusfromContig1386 Average coverage: 4.32
TTTCGTTCATATCTCGATGGTTGGCATCGTCATCAAGCTTATGATG
ATGATTTTTATGTT
GTGGACGTCGTTCGAAGTAATGTTTTTTTCTACGGGAGTGTTTTG
TGGCCTGGAATTGTT
TGTGATTCATTGGTCCGGAATGTCTGAAGACACTCAAGTCCATGG
TAGGGATCTCGCTGC
AGTGGCGGCGTTCGTTGCTGTCTCCGTGGTAGATGCTGGACAGAA
GATACGCATCTGTGT
ATTTCTTTTCTCTGTTTTGATTAG
>ConsensusfromContig1387 Average coverage: 6.94
CCAGCTAGGCGATTTACCGTGTTAAGAAAACAAAATGTCGCTAAA
TTTAATTAATCCGTA
TGATCGTAGAAATTTCTGATGCACACCCTACTACTTTAAGTCTGG
TTACTATCATATCGA
AATATTAATATAGATTTACTCACTGGCGCCATATTCAAATCCACA
CATAACAGGAAGTTA
TCTCTGGAGATGGTGACTGCTGTATCCATAGACCTCC
>ConsensusfromContig1388 Average coverage: 5.68
CACTGCTTTCTGTGAAGATGCACTGGCACCGGTTCATCAACCAGC
AGTTGATGGTTCTGA
ACTTGCATCTTCTAACTTTATCCACGTGCTTCTGGGTGTCGTCTCC
TCAATTTTGTTCAT
CAGAAAGATGTGATTCTTGGCGCTTTTATAACGTTTTTTTTGAAGT
TATATTCTTGAGTG
CTATATAATAACAATCCTAG

>ConsensusfromContig1389 Average coverage: 6.22
GTTGACAATATGATGAAGCCCCACCCCCTGGAGATTTGAATGATG
ATGGTGGTGGGCGTG
GTGGATAGGTGGAGAACTGTCAGAGAAGCTGGAGTCTTTGTGCAG
GCTTTTGGCTCGATC
GTTCATCTTCTTTCGCTTCATCCGTCTGTTCTGGAACCAAACCTTC
ACCTGGCGTTCGGA
GAGGTTCAGCTTGCAGGAGATTTCCCAACGCTTCTGACGGGTGAT
GTAAGAGTTGCTCAG
AAACTCG
>ConsensusfromContig1390 Average coverage: 7.35
GTAACAACTTTTAATATTGCTCCAGATTTGACTGCATAATCCAGT
TGTTCTCTGACATCA
TGAGCTGTAAAATCTCCCGAACATTCGTTACGAGATTTCATATTC
AATGTACATAATATT
CTGTCGAAGCTTGGCCTCTGTTTTTGTGATTTGATCTTTTTGATGA
TATTTAAAAGCATT
GATATTGTATTATTATCAAACTTATCTGAATCACCGATTTTCC
>ConsensusfromContig1391 Average coverage: 8.76
GCTTATCGTATCGTGAAGATACAACAGAACCAGAATTTGTAGACA
TTTTTTGTTCATGTT
TCGTGGTGAGGTTCAATATCATTGGACTGAAAAACATGCATTCAT
CGTCTGGATGAGCAG
TAATAAAAAGAATCTTTCTATTTTTACAAAGCAATCTATTTCTGAA
TTTCCGTTCCTTAG
AATATATATGATAGGCAAAGTAGACAAAGGGCAAAATAAAGATT
ATATGCCACATGATTG
>ConsensusfromContig1392 Average coverage: 9.49
GTTGCGCCCTTCAACTTTTTTAATGCTGGAATCGATGGAAGCAGT
AAAGGTAGAATGTAA
TGCAGTTATTGATGAAAAACCGAAACTTTTATCTGGTGCCAATTC
TGTATTTTTGCAGCC
TGAAATAACTGACCAAGTAAAAACTGAGGATGATTCAGGAATAT
TTACGAAGATGGATGT
TTCCCAGCATACAATTATTCATGAAGTCCACAAAAACGGCAATA
AAAAAATAAGTGATAC
TGTG
>ConsensusfromContig1393 Average coverage: 4.57
CTTCATGCCAATATCACATTCTATTTTGGAAATGTCTTCAAGACC
GGATACATATATGTG
TAAGTAAAATGCTTCTTGTTATATCAAATCGCATAGCAATGTGAA
AGAAAGTGTTATATA

AGTGTTGTGAATACAATCATTGAATCGAGTTCACTAATTCACACT
TGACTAAATAAGAAG
ACATTTGCAAATGGCGAATAAAA
>ConsensusfromContig1394 Average coverage: 5.51
GTCATAGAAAGGGGGCTGCACACAAACAACTTGTAACGGATCAA
ATAGCTGCCATGAACA
ACATCGCAAATGTCCAACACCAGATGCCAGAGCAAGAAACTCCC
ATAGTGAACACCAGTT
TAAGTGAGGACAATAGCGATCCCAAATCATGGTTGCTCCACAACA
GCAATGTTGTGGACT
TCCATCATTCCATCATAGACAAA
>ConsensusfromContig1395 Average coverage: 7.27
TTAGACTTAATCAAAGTTATCATACATTTTATAAGTATAAGTCAT
AAAACTTCTTTCATA
TTCATGGACATTATTCAAGAAACAGAACCTGATGAAGGTTCGTTG
ATGGATTTTGATGTC
AGGACCCTGGCTTCAGCAACCGGATATGTTCTTGAATGTTTGAAC
ATCAAAGCTATGGTT
GACCTTGACATTTTACCTCGTCTAGTGTCATCTTCAAGGACGCAC
GCTGAGCAGTGGCGT
ATTCCATTCATTGACATCAATGGCGATCCATTTGCTTTACTCAAT
ATTATGAGGGCGTGG
TATGTTCAGTATAGACAGGGCAATTGTAATAAATGGTGCATATAT
ATGAATATCGATGGA
ACAGCCGTAAATGCTATCGCAAATCAGGTTGAAGTTTTATATAGT
AAATTTCAATTCAAG
GAGCCTCTGATATTCAATGAAGATACCGAAATGGTAAAAAGGGC
AGATCGGAAGAGCAC
>ConsensusfromContig1396 Average coverage: 5.82
ATCTCTTGTATTACTTTCCAGAAGAGAAACGCGAATCTAAAAGCT
TTGTCAACAAAGTTC
ACACACTCGAAGACGGCTCACAAATGCTGGAAATTCTAGTGAGA
GTCGGCCAGAAAATCC
GTCCATCTGACGTGAAAGTTCGAGTCTCTCTGAACAACGAGCTTC
TGATTTTGGCTCAGA
AAGACGCTCCTGTCAAAGGAGAGCCA
>ConsensusfromContig1397 Average coverage: 10.85
TCCAGGGCAACAATCATAAACAAAGAAAATCAAATATTGGATGG
CAGTGTTCTAAGAGTG
AAATGCGAGATGTCCATCGTATACGAGCATTCACGGGAAACTGT
TAAAACCGAGAGTGAT
CTGGTCTCCAGTTTGGGATGTCTGCTAGATTCTGGAGAGGACTCA

GATGTGACATTGATT
TCATCCGACGGCGAAAAATTCAAATGCCATAAATTCATGCTTTCT
ACCCGTTCTTCGGTA
TTCAATGCGATGTTCTCACACGATATGA
>ConsensusfromContig1398 Average coverage: 6.13
CCGATGTCTCAAGTATTTGTTGGCAAGAGGAGCTGATCCTAATGT
AGTGAATAACGACAG
TTTCACCCCTCTGCACCAAGCAGCCAGCGAGGGGTTAGAAGGCTG
TATTCGGGCTCTGAT
TGATTCAGGAGCAGAACTGTCCCCCGCAGATCACCGTGAGAACA
CTCCGCTAGAGCTGGC
CAAACTGTGGGGGCACAGAAACT
>ConsensusfromContig1399 Average coverage: 13.15
TGCACTTAAGACACTTTCAACCACATTAGGCCTCGAATTATAAGTT
ATTTTTATTTCTCT
CGAGAGTTATATTCTAAATATTGATTCATGTAATCACATATTATC
ATCCACTGTTAGATC
ATTATTATAACATTTCATCAAATGCAAAGGTGCTATTCTAATTGA
CTGCGGTGTCGTAGC
ATAACAATCTGGTTGACGATGTAAAGAGTTTCGGTACCAATTTCA
TCGAGACAGACTTTA
GTAGGGCATTCGAAAACATTACCTCGTTACATTGGTTGTCCAATC
AAGAATCC
>ConsensusfromContig1400 Average coverage: 6.53
GTCTTATCATTCAAGACTTCATCAGTGACTCACATAATAGAAAAG
ACCCAGCTTGCATAT
TCATGAGATGGATGGACCAATCAAATGTCCGGGGTCGCTCGAAAT
GCGTCAAAGTTTGGT
ACAAGGGTGCATTCAGCTCGAACTGTCTCATCGGACAAAATGGA
GAAACACTATATAACA
ATAGTTTTATGTGAATTTTACGATTCAAGCATCGAG
>ConsensusfromContig1401 Average coverage: 4.31
GTGCAAGCTAAATCCCCTCGCACTACTTCATTTCGCAACTGTTGG
TAGCGCTGTAGGATG
TCCTTCTCTTGATCGCAATCGAGAGATCTTTCGGGAGATGAAGTT
TTCTGCTCACTCAGA
TCCAGATCCTTCTTGATTTTATCCGAGATGAACTGAAAAATGCGT
AGTGCATCCATTTTG
TTGACAAAGTCTTTCAACTCTCGTCTCATTTTGGAAGTCGAACTG
AAATCAGCAGGACAG
AT
>ConsensusfromContig1402 Average coverage: 7.65

GATCTCAGAGCTGGTGTTGTTCCTGGTTTACAGATTTTGAAAACGT
CTCATTTCCGCTTA
GAATGACGCTGATTTGAGGATTTTCGGACCCTGTTCTAGAATTCA
TTTCTTTTCGAAGAC
TCATCAATTTTTGGACAAGTTGAGTAGAATCATATTCCTTAGATC
CTTCTCTGAAATCAC
ACAGCAAAGAATGGCACGGATAGCA
>ConsensusfromContig1403 Average coverage: 8.08
ACACGACGCTCTTCCGATCTGTTGGAGTGACTGCCATGCTCAAAT
ACATAAATCGCCGCC
CCCTACGGCGATTGTTACTCTGGCGCCAGTACGACCTACTTCTCT
GAATATTCGCGATCA
GCGTGTGAACTGGAATCTGATATTGATGACATTATCCGTTTTTGT
GGATGCAAGGCTCAT
TATATGCCGGATCAAAACAATACAGCCGCCGATGTTGTTCCTCGC
ATTCGCCCGCAACAA
TGTTATCA
>ConsensusfromContig1404 Average coverage: 6.54
CCGTTTACCAGAAGGAGAGAAGGTAACTCACTTCTGGATAGTAG
TTAAACTACCCGATGA
GCCAGCGATTTTCGCCTGGGAGAAAGAGGTGATCTCGAAGTCGA
CAGAGTCCGAAGCTGT
TTTTTCAAGAAGGCGGCAGAGAGTATCAGGAGACGAGACAGATC
GGGAGGAGACCAGAAA
CATCAGAGACCAGGAACGTCG
>ConsensusfromContig1405 Average coverage: 8.29
CTGAATCGGTGAAACTCTGACAGCATGCTCCTAAGGGCAGTGAG
TGAGACAGGAAACCGT
TTTTCCAAGATGGCCGACGTCTCGGACCTGAGCCAGGAACTGAG
GGAAAGAGCAAGGCTC
TCATATTCCTTGACCTTGCTGTTGCTCTCCAGGCTGCGATCTGAG
AATTTCCCAGCGGTG
AAATCGGGAGATCGGAAGAGCAC
>ConsensusfromContig1406 Average coverage: 5.90
CGACTCCCTGAACTTTGGTCTTCCCATCCGCCTGAACCCCGGGGG
AGTTGAGCAGAAGGC
GGACCAGACCTTCGTGTCCTGAATGGGCCGCCAGGTGGAGAGGA
GACAAACCATATTCAT
TGCCGAGTTCTCGAATGGCCCCGATGTTGCCCCCCTCCGGCTGCC
CGCTCACGATAACTG
CTGAAACAGAGCCGAGCATCTCC
>ConsensusfromContig1407 Average coverage: 8.56

GTACATGTTCACACAAATTATTTTCCTACATTAATGAATATGATG
AGACCCGTATACTGT
TCTTCGTATAGTCAGATTCATAGGGATCTTCATATATTGTATTTTT
ATTGATTGTATTTA
ATCGCAATGAAAGGCCCTATGCGGCTCATCGTAGAATTGTGTAGA
TAAATATTTGTATAT
AGACCATACTGAACTTATCC
>ConsensusfromContig1408 Average coverage: 5.67
GACAAGAAGTCATTCAACCTTCAGAAGTCGCCAGAAATTGCGTC
AAATAGGACATGGTCG
TTCATTTGGTCGTCGCCAAACCTCAGATTGCCCTGATTTGAAAAT
AGATCTGACATTTGT
CATCGAATCCTCGAGCGGGATGGGACAAGCCAACTGGAATAAGAT
CTTGACATTTTTGTC
GGAAACAGTTGAAAGATTCGAAATCGG
>ConsensusfromContig1409 Average coverage: 4.01
CACAGCACAAGTGTCAGGTTGAATGATCTCTTGAGGAGGCTCCTC
TTCTTCAGGTTCAAC
TACTATTTTTCTTTTTCTTTGCAATGAAGCAAACGACGTTCTCTG
CTGGGATGAGGACGT
CGAGGGATTGTTGGGAAGTGATGCCTTATTCGGTACGGCATCAGG
ATATATCTTCCATGT
GGCATTCTTGTACCCGTATTTCTGCATTGTTGGAGCA
>ConsensusfromContig1410 Average coverage: 151.71
GAAATCATTTATGATCCCCACGGGTTCTCGGTTCTCCACATCGCC
TGCATCCACGGTCGC
TTGGCTGCAATCCGATATTTGGTTGAAAAAAAATGTTTTGACGTT
AACATGAACAGTAAG
ACTGGATGGAGGCCGATACATCTGGTCATCTCTCGACAAAATGG
GGGCGAGATCGGAAGA
GCACACGTCTGAACTCCAGT
>ConsensusfromContig1411 Average coverage: 5.05
ATTTTTAAATCATTTGCATCAATTTCTGAAAAATCATTTTGTACA
AAATCATAGATGACG
TTCTCATTCATTGAACTAATACTTTTTGGGAAGTACATTGTGAAA
TTAAAAGGGAATCTG
CGTGTACCATTACCTGTTATTTCTGGATTATCCAAATTATCGAGA
AAATCATCATCAAAT
AAGTGAGAATAATCAAAAAGTATTCCATATGGCAGTAAA
>ConsensusfromContig1412 Average coverage: 5.36
GAAGAATATAAACCAAATGAGAGCAAAAAAGTCATTTCAAAGCA
AAGACATTCAATTGTC

ATTATCGATCCATGTAGCTGCAATACTCAGATTCTGCAAATAAAC
GAACGCAAACTGTTT
TCTGTTCACGAAAAACTGAACGCAGACGAAAAGAATTCAGCCGA
ATTAACATATTACACA
ATAACATCTTTCTCTACGACAGATAGCAAGAATAGGAATAAATAT
CTAAATACTTATTCT
>ConsensusfromContig1413 Average coverage: 6.22
GTATACAAATCTAGCTCCATCTTTCTCGAGGTTTTCTCCCGTCTCA
TGGGTCCAATTATC
GTGATTTGAGAAGAGATTGAAAATTAGAAAAAAATTTATTTAAG
ATTTCGAAGCCAGGAA
CATATCAAGATATGAAAAAAATTTGTGTACGAATGATGCATGAA
CGTAAACGTGCAATCC
GATTTATTCTATATACCATACCATTTTTTTGGAAAGTGAAC
>ConsensusfromContig1414 Average coverage: 5.25
ATATAATAGCACAATTACTTGAACCACGTTAACCACATAATTGCA
CTTATTCGAGTCTTC
GTTGAAAAGGAGTTGAGCATGATTATCTCTATGTGAAGGCATTCC
GAACATTCATTTCAT
CGAGATGTGACGTCACGAGTAGCAGAGGTGCCACACTGGTTGAG
ACAATCATGACGTATT
TATTATCATCGTAGCCTTCAAGATCAGCCAAG
>ConsensusfromContig1415 Average coverage: 5.99
GAATATTTGATTGTGGAATAACGTAAATTCCAACCGTAAGCAGA
AGTGAAATGATATAGA
GAATAAAACATCGCGAACCATATTTACTCGTGTGTTTCGGCTTAG
GCTTAAAATCTCTGT
TCGACCAGTGCTCTTTCCATTTATCCATAAGAGGCCTGCGTCTTC
GACAACAATTATCCA
TTCTAGAATGTGCAGATCGGAAGAGCGTCG
>ConsensusfromContig1416 Average coverage: 7.16
CTTTTGAGAGAGTTCGAAGCACGCTACTGTGTCACAGAATGCTTT
CTTACAGAGAGAGCC
ATAGTATCCGTTCTTGCATACGCAGAGATGTGGGGTCCCCTTGTC
GCAACTTCTCGTATT
TGATATGAGACATGGGTTTGAATCACAACTGATGTCTTCCATGCA
ATCTGTTCCGTGATA
TCCTGGCTCGCAATCAAGTACAGGTGCA
>ConsensusfromContig1417 Average coverage: 7.21
TTCCGATCTTGCACATCCTTGGAACGAGCTATCCACAAGAAATCT
CCCACCGGAAGTACT
CTGATGCAAACCTTGATCCTATTTCGCTCAAGCAGAGGAATTAGT

TTCGCTTTATCGGCG
CCAGTTCCATAATGCTCTCTGTTATCCACGCATAGAACAACATCG
AATGAGCGAGGGGGA
AAAGACGTCTGGTGGATGGGGGCAGAAACCTCTGAAGGCGAAGC
TGGTTT
>ConsensusfromContig1418 Average coverage: 5.61
TCCGATCTGCAGCCATCCTCATTGGAAAGAAAAAACAGTGGGAG
GCCCTACGTTCAGAGG
TGGCCCCTTATATTTTGCAGCGATTTGGGCGGTCAAACTCCTACT
GTGTGTGTGGCTTGG
AGGCAGCCTTTCAATGTCAAGACTGTTGCGTTGGGGAGTCGTTTT
GTGAAGGGTGTATAG
ACACCATACACTCCAGGCCACATCTCCATGTGATTGAAGAATGGA
TTAGGGACTACATTG
TGTGGAGAAAGGAGCGCTACA
>ConsensusfromContig1419 Average coverage: 5.17
CACAGCGACACATCCATGATGTTTCTCAATAGTAGCACGGCCTTC
GATACGAATGTGAAA
ATTTTCAGCAGGAAAAGTGTGGATTGCTCAGTTTTGAACAGAGAG
AAAATTCTTTCTGCA
AGTCCTATGTATACTTCTTCATCGCAAACCATATTTGAATTCGAA
TCTGAATATGAAGAA
AACGATGACTCATCCTTTGTCGTGAGGAAGTGCC
>ConsensusfromContig1420 Average coverage: 5.84
GTTTAAGTGTTCATTGTAAGCAATGATGGTTTTACACACGGACCT
TCGAATTCATTGGCT
AATTTCCCCGCGCCTGGACGAAGGGAGGGGGTTCTACGTGTCCAA
AATACCTGGTTGAAC
TACGCATAATTAATGACGCCAAGTGGACAAGGGAGGATCTGCATG
TTCAACATACCTGCT
TGACCTAGGCATACTTAATGACATAAACAGGGAC
>ConsensusfromContig1421 Average coverage: 6.88
CAGAACTGACAGCCAGGATAGAAGAGATCGCTCTGAGCAGAGTT
TCGTCAAGAGCTCCAA
GTCCAGAGCAGGAAGAAACAAATCTTATGCTGCAAGATGTCGAA
TTTGCGACTGCAGAAA
ATGGTGCGGAAGAACAAGAAGAAGATTTTGCCAACTTTGACCAT
GTAAGTATGGATGGCT
CTTTCAATGAACCAAATTCTGATGATGCCAATACGAAACCGATGC
GTG
>ConsensusfromContig1422 Average coverage: 6.86
GACGAAGAATCGTAGTCACCTGAATCTTCTTCATTTGGCGGCTTT

CCAATTGAATTCATA
TTCTCTATAAGAAAACACACTAATCGTTCGACTGTTATACATTCT
TTATCTGTGATGATG
ATCGGAAAGTATCTCCTTCCATTTTCAAAAATGAGCAAATTGCAA
ACAATATGCAGAGAC
TGGAGAAAGTGAATATACGAAATCTGTTCGACGGAG
>ConsensusfromContig1423 Average coverage: 5.54
CCAGGATTTCGACCAAGAAGAGCTGAAAACAGAACAAAGACTCA
TGAGCCTCGATATCAG
CGACTCTGGAATATCTGACCCCCAGAGCGATATCAGCGCTCGTAG
TGATGAAGAATTTTT
CACAGAAAATAATAAGACAAAGATGAGAAGACAATCAGACCATT
CCACAGTCTCCGGCAT
AAACTACAGAAGAAAAATGACCAACATCAACA
>ConsensusfromContig1424 Average coverage: 5.23
AGAATATCGCAGTTACTGCTACGAAATCGAATAATAAAGTCGTT
TCAAAACCAGCCATAA
GTACAAATATGAAAAGTAGGCCAAGCTTAGCATACCGTCACGATA
TACCACGCATTTTAA
AACCCATTCCTCATTCGAGCAATACATTGACAGTCGAAAGACGAA
GACAGCTTTTACAGC
AATTGAAATCCATGCATAAAAGCAATGAAGCATGGGCGAATAAA
CAAAAT
>ConsensusfromContig1425 Average coverage: 7.36
TCCGGCTGACAACATTTCATTGTAGTTGATGCTCGCATCTCTTTCA
TGAGTTGCATTTTT
ATGATTTTGAAGAAATATGTACCCATCTGTTCCGACGCTTTGTAC
ATGATCATTTTCAAA
AGTGTCATTTAATAAATTCGATCCGCTATACTTCTCCTTGTTCGAA
CCACGTAGTATACT
ATCCTTTTGTGTCACGTTTTCATCAAATTTTATGGAATTATTT
>ConsensusfromContig1426 Average coverage: 4.47
GCTCTGCTTCGTAAATCCACCGTAGTAGAGGAACGAATTGGGAGC
GTTTTCACCGAAGAA
GACTTTGTCATTCTTGGTAAGTTGGGTGTGGTTGCCCACGGTGGC
GAGTTAGTGCAGTGA
CTGATTCAGGGTCCAATAGTATCGAACGATAGGTGTAATAACTA
TCCGACACTAAAGGAC
CCTAAAATCAATCACTGCTATTTATATATACCTATTAATATGCGA
GTGAATGTATGTGTG
TATGCGTGTTATATATAATAACCCGCTTCCGC
>ConsensusfromContig1427 Average coverage: 6.80

TCTTCCGATCTCCAGGATACACAAACAAAAGATTAAAATCATCG
AACGTAATAGAACAAA
TAAAAATCAATTCTCAACCATAGTGTAAAATTCATTGCAAGAAGT
ATACCAAATATTCAA
GATCACATAGGAGAGATCTAAAAAAGGTAACGATAATTCAACGA
TCCCGTCTAAAAAATG
AAGGTTTTACCAGAAAATTTTCCACAAAATG
>ConsensusfromContig1428 Average coverage: 5.77
ATGACATTCATTCAGCTCGAATTCCACCCATTGAATAACATCTGC
TTGAGCTGCATCTGT
GAATTCTCCTGCCATTTCCTCAATCACGATAACATCGTTTTTATG
GTCGATGACGTCACA
TTCTAACTCCCTAATCTTTAACGTACATTCCTCAAAGCGGAATGCT
ATACTGACTCCTTT
TTCGGCATTACTCGAATCTGT
>ConsensusfromContig1429 Average coverage: 7.01
ATGATTCGATTCCTGCCTGAATAGAACTAGAATATGAAGTCATCT
TTTATCCAAGAAAAA
AATATTACGCCACAACCGCACCATACTCTTCCATCAATAAGGCAT
GATACCTTCATGAAT
ATCGACCCGCGCAATCTCAACGATAGCCAGAAATGACCAACATG
ATAATTAATCAGAGAT
CACCTCAAATGATAATCTCAAATAGTATTTAATTAACC
>ConsensusfromContig1430 Average coverage: 4.00
AGTACCTGTCACTATAATACCGGAGACTTGTGTTGGGGAATCCAG
CCGGACCTTCCACCA
ATTGTTAGAGGCGATCCCTGATTGGGCGCAAGAGAGCTCGTCGTC
ATCCATTGCCAGGCG
TCCGTAGCGACCATTCCCCACCGTGGATTGGAGGAAAGACACGC
AACAGATGGCTCCGGT
CATAGAAGCCATCAGGAGATATTTTAAAA
>ConsensusfromContig1431 Average coverage: 7.37
CGAAGAACCTTCAGTACAGAGAGGATAAACAAAACTGCTGAAGA
GCAAAACACTCAGCAC
GAAGAACACGGTGCTGGGCTCAAGAAAAGCTCGTCATTTATGTCT
CTTTCTCGGGGGAAG
AAGAAACGGTCCAAACAAAAACAAGAAGGCGAGTTGGGTGTGGA
ATCGCCGAACAACTTT
CGTCAAATCGGAAAGGTCAAGGAAGTTTGTCCCGACGGAAGTAG
GATGCTCGAGCTTAAC
AAG
>ConsensusfromContig1432 Average coverage: 5.44

GGGACATATTAATTACAATGTTGTAAATCACTAGGGAAATGTATA
CCATTCGATTCTATT
CAATAGAATATGAGGCTTCAGTAAACCCGTAATAAAATGTTTATA
AAATGATCATTATAT
ATCTAGTTATCACACATACTCGTGAATCGTTGTATTATAAAAAAG
TTTTCCACCCATAAC
ATAAAGTGTTGATCCTTTTCTGTTCGGATCGGGAGCCTGAATCGA
AATCCTAAATCCG
>ConsensusfromContig1433 Average coverage: 4.08
GTGTGATTGAACAAGTGATGGCAAGGTTTGAGGCTTTTTCAGGAT
TAACTGGATGTTTTT
ATATGTGCTTGACCTGTCGTGACCCAATTCTTTCGGCCTTCAACT
TCATGCAAGAGATGC
GGTCGTGCGCTTTGAAAGAACCAGAGATGAAGGAGCCTTATCTTG
CCTTAGCTGATCGTT
GCGAAATGATATCTTCTGGACTGATCGAATGCTGTTCGAATGATA
AGGAAATCGAAATCG
TGATGAATAGAGAATCAAAAT
>ConsensusfromContig1434 Average coverage: 8.47
CTTGAAAATAAAAACAGTTCAATCACGAATATAACTGCATACATA
TTGTTAAAAAACATA
ACAAAATGACCAGTGATCCTACTTACATGTCGGATAATAATGAC
GAAATAACAAATGATG
ATTCAAGAGAGACAATTCAGAAAGAACTCAGCGGCACAAAGCGC
CTTTCTCCCCCTGATG
GAGGCTGGGGATGGCTTATAGTATTCGCTTCCCTCATGGTTCAAC
TCGTTATCGATGGCA
TCACCACCTGCTTCGGCCTTTTTCTTGCCGATATCAAAA
>ConsensusfromContig1435 Average coverage: 6.32
TATAGCCAAATTGCAAGAAGTATTGCAGGCAATTTGTGCCCATAG
ACCTGATATCGGATA
TTGCCAAGGAATGAACTTCATAACTGCGGTTGCCCTTCTCTTCTT
GGACTCAGAGAAAGC
ATTCTGGTGTTTGGTCGCAGTTTCCGAAAAATACTTTTACCGGAA
CTATTTCGATGAGGT
TCTGACAGGAGCTCAAATCGATCAACAAGTCTTAACCACTCTGG
TGAAGGCAAAACTGCC
AAGGCTCTGGAGGCATCTCACAGCCATCGATGTTGACCTAGTTTC
ACTCACGCTCAACTG
GTTTCTGGCTCTCTTCTACGATACTGTACCGTTCGAGACCCTTCTC
CGCATTTGGGACTG
CTTTCTTCTGGAAGGAGACAAAGCTCTTTTCCGATTCACCATCGC

GCTTCTAAAGATCAA
CGAACAAGGACTTCTGGAGAGAAATGATACGATGGGGA
>ConsensusfromContig1436 Average coverage: 6.05
GTCTCTGTTTCGTACTGCTGAGCTCATGAGCACATAATTGCAGTC
AGCGGGATCATACAA
GTACCAGCTCGAGTCGTCCTGTATTGGATGGTCTGGCACGATGTC
ACGGTCAAAACGAAG
GACGTCTGTATGGTTGGTGTGACGAGGAACGTAAGATACAGGCGG
TCCTCCTCCTTCGAT
TCGCTCTTTTATTTTTTGCGTGCAG
>ConsensusfromContig1437 Average coverage: 6.19
GCTCCTTCGAGATCAAAACAATCGGGTATGATTTGATCAGGCAAC
TTATTTGGATCAATT
CTCAGACTTTTACACACTTCATGCTTGACAATGTCCGAATTTAAAA
ATTCCTTCGATTCT
ACACAACCCGAAGAGGATTTGAAAAAAAAATGATTGAAAAAAAA
AAATTTTGAAATGTTT
TTTATTTTTGTCATTACTTCCAAC
>ConsensusfromContig1438 Average coverage: 11.07
TTCTTGGCTGTGAATTCGTCAGCTGCGCATCGAACTCATGCGACA
TTGTGGAATGTGCAA
AAACACCGCCATCGCTGTTCCGGCCATAGTCACCTATGTCTACGA
ATTGATACTTCGCAT
CGCAAACAGCCATCAATACCACGCTGTTGAAGCCCTTGTAATTAT
AGAATTGCGATCCGG
CATTGTTGGGTAGTTGAATG
>ConsensusfromContig1439 Average coverage: 6.09
TGTCCCTCCTGTCCTGTCGAAATACAATTTTTGCCTGGAGTGTGTC
ATAATACAGTACTT
TTTCATGATAATTGGCTCAATATAGACATATTTCATGAATTTCTG
GCAGATCATCTCATC
GAGCAATTAATTTTTATTCTGAGTCTTGCCGTGAGATAATCAGAT
GAACAAAGGTAATCA
TATATGGACTTCAGGAGTTTTGTTATAAGCTCAGGTAGTCCATGT
CTATGTGTGAA
>ConsensusfromContig1440 Average coverage: 6.64
AGACGTGTGCTCTTCCGATCTTTTTATACGCTTCATTGGATACTT
TTGGATGCAGCAGC
TGAGTGTAAAGATCTGGAACATGAGAGGTCCATAATGAATGGCA
CACCTGAAGTCAATCG
TGCTTATATCCAATCTCTAGAAAGTATTCACCTGTTTGTATTCCTG
TTCGCGCCATTATT

TTCAAGTATTAAAGAAGAGAATTTGACAACTCTCAA
>ConsensusfromContig1441 Average coverage: 7.47
ATTTCTTTTTCGTTTTCTTCTTTGTTTTCTTGCCGTTTCTTGCTGCT
CGCATCTTTCTGG
CAATTACACATCTATGTATCGGTTTTTCATATGTTTCCATCGGCC
ATCCGACGTAATAGG
AGAAAACCCAAGACCTTGTCATTCCTTGTCCCAATTGAACAGCCT
GTAGATTTCTTCTCT
GCTCCATATTGCGTTGAGCAGCCCTCATTTTCTCAGTTTTCAGAT
>ConsensusfromContig1442 Average coverage: 7.39
TAAATTCTAAAACGTGTATTAATATGTAATATGGCTGGTACAAAC
AGTAAATTCCCACAT
TTGTTTTATCTGGGTGGTTTGATTTTATGCATTTTCGGATTCTCGC
TCATGTTTTGGTAT
GAGAATGGACGAATTACTGAAATTGAAAACCGACAAAAGATTAT
TCAAAATTTGATAACA
GAATTATTCATAAATGATGGAGG
>ConsensusfromContig1443 Average coverage: 5.14
GGGAATTCTCTGAGTACCACTATTTTGGCAACTCATACTCCAATA
TTTGGTAAACAACTG
AAAACTTCCCTTGACGAAAAATTTCTGGATGCTTCAATTTGATTT
TATACCGACAGCGAT
GACACAAACTGAATGTAACAGTGATGTTTTTTGGGATGAAATGTA
CGATATCAAATCAGC
TGGTGATTTTGGAGCACATGAGAAATATTCAAATGAGAAGA
>ConsensusfromContig1444 Average coverage: 3.21
AATTGTAAAGAGCCATTGGGCCAATTTGCGGAATGTGACTCCCGG
TGTGATGATGTACGC
GAGTTGCAAGTGTCAATTATGTCATGCCTCGAAGAAGGATCTCCT
TGTCTCCGACCTGGC
ATGACTGGTCCGGTCAGCCAGCTCTACAACATCAAAGACGTGCA
ATATATTATACGAGGA
TTGGGAGATACTGATGCTATGCG
>ConsensusfromContig1445 Average coverage: 9.19
GTTCGCTTGCAAATATTTTGGCAGCATATTTATAACAAGTTGTTTT
TTTCATATTTTATA
TTCGTTTTAAACCTATAATTTCAGGTAACACAAACGTGTTTTGCAT
ATCGTGCTTTGTTA
GTCGATACTTTGTTGTTTTCTTCGCATGAATTCGATCAACTGACAA
TCGTGTTTTTCTCT
GTATTCTTTAAATATTTGTAAAAAAATATATTCAATCGAAGTATA
ATGTGC

>ConsensusfromContig1446 Average coverage: 6.26
CGACGAAATAACCTGTGATTGGGCCGCTCAAAAGAATACCCATG
CCGATGGACAAATAGC
TCAACGAGAAGGCGTTGTTGATTTGTGCCTTGTCCAGAATGTCTA
TACTAGCAACAGCAA
TGTTGATATACGTAGAACCCCTTGCTATTCCATAAAGGACGGCAG
CGATAGACATTGAAG
TAAAGTCTGTACAAAAGAGATCGGAAGAGCAC
>ConsensusfromContig1447 Average coverage: 6.50
CAGACGACATCGAAGCAGACGATTCGGAACTGATCAGAGATGAC
GATGTACCGGAAAAAA
CTGAACTCTGCTGCAATAGCACTACTGAACCTCTGAAACCAGCAA
CTCGAGATAAGTTCT
ACCACTTCTTGCCGTTCGATGACCAAAAACCCATCCTCTACATCC
GAGGTGGTTTTGGGG
ATCATAGACGCCGCATGTACAACACCAACATCATCGAAAAAATT
GCAGATGCGTTGGATG
A
>ConsensusfromContig1448 Average coverage: 6.87
GCCCTCCTGCCCATCCTATACAAGATGACTCATTCTGGTACCTTA
ATTACTCAGATGAAG
AATACAAAGATATGAATCATTTGGTCAGATCAGCTGATATTGCAT
CGCTAAAGGCTGCAA
AGGAGAGTGGCATTAGTTTAGATTTGCCAGACAAGTTTTACAAGA
CGCCGCTTATGATAG
CATGTCTCCAAGCAGACGTCATTGTTGTGCAGTTTTTACTCAATA
ATGGTTGTGACGTTA
ACGCCGTGGATCAGTTTTACTGGACTCCGCTTCACTTCGCGGCAA
GTGCTGGAATTTTCG
ATGTTGTACGTCTGCTTCTCGAACATGAAGCAGATATTGAAGC
>ConsensusfromContig1449 Average coverage: 7.26
GCAGGAGATAACCATAACATTATCATATTCCACAATTCATTGGAA
GTTTTGAGGACAGAA
AAAAAGAATACTTACAGAATATGAGGGTAAAACAAGAGGAAAGG
GATGTCAGATAAAGTT
AAAACGTAACTTCACAACCCCCCAATAATGAATAAACAAAAATG
AATATTTACAAAAAGA
TTATATCACATAAAGCAATTCACTCGAATTTAAATAGTTGATAGT
CACAAACAAAAGTTT
TGTTAT
>ConsensusfromContig1450 Average coverage: 4.55
GCCACCCACTAGGAAGTGATTGCTTATTATGGACACTCGAATTAT

CTGCGATTGTCACAT
CATACTGAATGCATATTTAGAAAATTGTTGTTGAATTGAATAAGC
GACATTTTTGAGATG
AAATTTTATGTTTTTCTGCTCCTCGATTTCATATATTCGTCAAATG
TGGAGGCTGGGTGT
CGCGACAAGGGAGAGATCGGAGAGCGT
>ConsensusfromContig1451 Average coverage: 6.29
ATTGTCAGCTTTGTTGAGGCTCTGGATAAAATTTCAGTGATGTGT
TTTATGATGCATTGA
GCACAATATGTATTTTAGAGGATTCATATAATTTGAATCAATTTT
GTTTAAAAATGCTAA
TCCTATTCCTGGGCGTAGTACTTAACAGATCGATATTTTTTATAGT
TTTCATCAAACTCC
ACAGATCGATAGTTTTATGAAACATTACATGTACCTTCTCCAAAA

>ConsensusfromContig1452 Average coverage: 5.94
GCAGTTTTTAATTAAATAAAAGAATTGAACAGCAAAAATATATAA
GTTAATATCTTATTG
CAATACACATATAGAACTAGATTTAGCTGGAGGAGGTCGATCAAA
AAACTGTTGTCTTCT
TTTACAACCTTGTAACCAAATGCTTCGATCGAAATTTCCTCCACA
TAAAGGAATTCCATC
TTTACCCGGTGCGATATGGC
>ConsensusfromContig1453 Average coverage: 5.99
TTATATGGGTGAACATTGTGAAATGAATATAGATGATTGCTTTGC
TTCTTCATGTTTGAA
TGGTGCTACATGTGCGGATGGAATCAATGATTATACGTGCATTTG
TTTGCCTGGTTTTAT
CGGTGAAAACTGTGAAATGAATTATAACGATTGTGGATCACTGCC
ATGTTCAAATAATGG
AACTTGTCTGGATGGTATAAACAAATACACTTGTTCATGCAGCCC
TGGTTTCACTGGTGA
TAACTGTCAAACTGAATCATTGGATGTCGGGAGATCGGAAGAGC
G
>ConsensusfromContig1454 Average coverage: 2.63
GTAATGACCTTCGTTAGTTTCGCTAGAGTACACTGTGGAAAGTGT
CAAGTCCAGATCTGA
TCTATGAATATCTTAGTGATCAATAGCTCAGGATAAATATAAAGT
TCGTGCAAAGTACAG
AGAACAGACTTTTTGTATCACTCACTACGTTTCTGCAATACTGTCA
CACTTCCTGTTTGA
ATATTCAAAAACGTTAGTTCAC

>ConsensusfromContig1455 Average coverage: 5.99
TCGATCAATACAATAATATTTCTTACTTGTATGATGAAACAATTA
ATGCAATTGAAGCAT
CTGTACTCTCTGTTCCAGGGGACATGAATTCGATTTTATCAATGG
AATTATACGAAATCA
CAGAAAGCAATGTCTTCAAACAAATATTAAAAGCGACACTTGACG
ATGAATTCCTCATTG
ATGGACGATATTTCTTTTCCGATAACAACGCCAGAGGCTTGGGGT
TGTTTCTGGGATCGG
AA
>ConsensusfromContig1456 Average coverage: 3.64
TATGAGAATAAATCCTAAGAAGGAATGACTATTTATGCATGTCAT
CGCCTTTGTCTAGTT
TCATGTGTTTGAGATTTGGGTCGAGGAGGTTGGGGTTGAAGCCGG
ACCCTTGAGTCTGCG
GATAATTTTTCTCAACGAACTTGAGCATTTCTGTAAGATAGTTTT
GAATGGCTGTGAGAG
TGGCGACCATGGCGGGAGATCCGAA
>ConsensusfromContig1457 Average coverage: 7.86
ACACGACGCTCTTCCGATCTCGGCAGCATTGCGCCCAGCATTGAA
TGCAGTGCGTCCAAC
TCCCATTATCGTCAACATCGCCCTGGCACCACGCTCAATCGCCTC
GGCTGACAGCCTTGT
GGGACCACGATTGTTTCTGCTGATGGTAGGATTGGATGTTCTTCC
CAGGTCGACTGAGCG
ATTGGCCTTCTTCGGTGGATCGCGACGGCGCAGAAACGTCTCCCA
TTCCTCGCTCGGGGT
GGC
>ConsensusfromContig1458 Average coverage: 7.83
CTAACCCTTTTGATCCCCTTTGTTTAAAAACATGAAGGGGGGAAA
ACAACAAAAAAACCT
GCCACTTCGAGTCATGCATTTGGTCAAATCGATGGATTTGGTCGA
GTCGAGTCTACATCG
TCTCATGAAATTTCACCATTTATATTCATAAATTTTGCAAAAAGG
CAATTCCTCAGTAAA
TGAGATTATATCTTTCATCTCCCTAGTGTCTATATCATC
>ConsensusfromContig1459 Average coverage: 6.30
GGACCCCTACGATCACCCTCACATCGGAACACACATCGTCTACC
AGAAGAATGCCGATGG
CCAATCACGTCATACGGATTTCAACGCTTTCTTCATTAATCAGCA
ATACAAAAAGAAAAA
CAAGAAGAAACACCATAAAGTCTCAACACCTATCCCTATCCCAA

CGACGGGATTTGAACG
AACCAATGGCGTCCGACCTTTGGAAGTGCTCA
>ConsensusfromContig1460 Average coverage: 16.87
TTTTTTTGTTTCTCGAAATTTGAAATAAGCAAACCAATGTGATAT
ACTAATAAAAGTTGG
ATAATTGATCATATCTGTCGAAGTCCATTAAGAATTATTACCCCT
TTCAATTGATAACAT
AATGCGCAATACAATAATAGTGTTTTATGAGACATTTTCTTCACG
AGATATTTTCAATAC
ATTCCTACGCATACTGATTAATACTGGC
>ConsensusfromContig1461 Average coverage: 3.25
GATAACATCAATCATGATAACGGCAACAAGACCAGTGACCAAGA
GAGACATCCAAAAATT
TGTGAGAAGAAATATGGACACAAGACCAACGGCAGCAATGGCAC
TTCCCATCGCGATTAA
AAACTCTTTGAACATGATTGAATTTAATTCGTACACAAAGACCAT
ATTTGAATACATATA
AACCTGATCATCCGAAAATCCTACATCCGCA
>ConsensusfromContig1462 Average coverage: 11.95
TCGCCATCCCTTCAACATGACCAATCCCTTTCTCAACAAACACCT
CGTGATCCTCCCCAT
TCAGTCTCGTTCTCTCAATGCTGTGCTTCCCAGTCGACCAATACAC
TTTTCCTTGAATGG
GATCAACGGCGACTGCTAGAACCTCATCCTTTCCAGTTTCATGAG
CGAAGGGAATTTCTC
CAACCGGAGCGACGTCAAGGGAGA
>ConsensusfromContig1463 Average coverage: 12.31
CAGCATTGTTAATGAATACAAAAGCACATGCTTAATTTTTAAGTA
TAGTATTGATTTATT
CAGCATATGACGTTGTCACGTAAGCAAAAACGACATAATTCCAAC
TATATTAGCCAATAT
TGGAATATATGTTCACATTGCAATATTACAACCAGCACAACTATC
ACGAGAGACAACCAT
ACAAACAAATAAAAGCAGTTGACGTATGAAAAGAATATAGTAAT
AAAATTATAATGTTAA
GTTCTAACGTGC
>ConsensusfromContig1464 Average coverage: 12.92
TACACGACGCTCTTCCGATCTCGCAGACAAAGAGTTTTCCAGTCTT
TTTGTTTCTCCGTG
CATTGCCGCACCGCTTTTGAAATTCTCTCTACAGACATGGTGTGG
GGCCATGCCGTGAAA
TCATGGCATTTGCTAAGCCATGATTTGGGCACGATGGCCACCGAA

GTATCTTCATCAACG
AAGCTCACTCTCGCACACATTTTGTGTGTTAATAAAGTTG
>ConsensusfromContig1465 Average coverage: 5.48
TGATAATATATCATATAATCAGTCTGGACATACTTTGCCCCTAAT
CATGGTCACAGCATA
ACTGATGCTATCAATCGATTCAATTTTGTATCCGAAATCCGCAACT
TCTTCGACTTGTTT
TCGGTGTTCATCTAGCATATCTCCATCATCCAGAATCGCAACGAT
GAATATCTTTGTTCC
TGCTTGTTTTGCGTCTGTCACTATCGGAACCAC
>ConsensusfromContig1466 Average coverage: 5.24
GGAAAATAGCAAAGAGTGTAGATGACGACAACTGCGATAAGCAT
CTTGATAACTCCCTTC
CTCGCTTCAGCTGGATCATTTTTCCGAGATTGCCTGCGATTTGCT
TGGTCATAGGCAGCA
GAGTTTTGAAGATTTCTAGAGCTTGTATAGAGCTTCTTGCAGGTC
ACTCCATACAGTACG
ATTTGTATTGCGACAGGAAATGCGTACATCAAAATGAAATCCAC
ACCGTAAAC
>ConsensusfromContig1467 Average coverage: 5.62
CTTCCGATCTGCAGCATTAATAAATGATTCATCCATAACACAAAA
CTCTTCATAACAATT
AAAATCTGAATTCAGTTCAAGGTTTTCGAACGTAAGTGCATTGTA
TAAAAAGAAGTTTCC
ATACGATATGTGATGAAAAATTCATAGTAAATTCCGATAAGCCGA
TAAGTCATGGAGTAT
AAACACAAGACACTGTATATGGCGAACAAA
>ConsensusfromContig1468 Average coverage: 9.67
ATCTAGCAAAAGACAAATCAAAACATCTCATATCAGAGAGTCAA
TCAGAAATCAAAACTT
TATCAGCAAAATCCAATCCATCTTCCACACTTCCTTCTACACATA
TTTTCGATGGATCCA
CAACAGATTTCTCTACAACAGAAAACAACTTCTCGTATCACCATA
GTAACGACGTCAATT
ATTCTTATCACCATGGTAACGAGATGTCAACAAATGTATCAACAG
AAAGT
>ConsensusfromContig1469 Average coverage: 8.43
GGATAAATGAATAATAAATAGTTCGATATGAGAAAATGAGAAAT
TTCACGAATTATTTAG
GGTATTTTGTTCGAAATGGTGCTACTGAATTTCTCGATTTCTCCTG
CTTGTTTTTGTTGC
CATACTTCGATATTTTCCTGATACAGTTGAAAACAAACAGATCTA

TGATTTCAACAACTG
TCATCATGCTAGCCCCCAGCATCA
>ConsensusfromContig1470 Average coverage: 3.99
CGAAGAAACCATGATTATTGCGAAGAAGACAAGCAGTGTGTTGA
TTAGGTGCATGGCAGC
CGAATATATAAGATCCGATGATTAATATAAGTGCTGTTTTGATAT
AGGAATGTTTTCTCT
GATTATTATCGTGAACACAAGGTGCTGTTTATATGCATCGCACAC
CTTTTGCCATCAACC
ATGCCTCGTGACTCCGAAAATATCG
>ConsensusfromContig1471 Average coverage: 3.69
CGAGTACTGAGTCCCAATGTGGCCGGCCTCCGTCGCCTCCAGTCG
CGAATGATCTACGAC
TTTGGCAAAGGCGAAAATGATGTGAGTGCAGAGTCTGGGCTGGA
TGTCCTCTGGCTCGAA
CTTGCCCACGCCCTGTCTGTATTGCGAAGAGCTGCTGTAGTAGCA
AAAGATGACACGGCC
ACTTTTGTCATAGGCCTGCGCAGATCGGAAGAGCGCCG
>ConsensusfromContig1472 Average coverage: 4.27
GCACACTATAAAAAAATAATTTTCGACGCATTAATCGAGGGATTT
AAGCATACAGAATTG
GGTATTATCCGATCTCACCAGAGCTTTTGTTCCCATACCATAAAT
GACGGCATCCAATTC
AGGAATCTCAAACGATTCACACACCGTCTCAGTCAGGTCAGCGAT
ATGGAAGATATATTA
TAAAGCTGGAGGTTATCTCCTGCCAGTGCTCGCTGATTATAGTGA
AAGAACAAAGG
>ConsensusfromContig1473 Average coverage: 6.33
GCAGCATTGTTAAGACCACACACATTATGTTGATTTTTTATTGAC
TTTTTAGAACAAAAT
TGGAAAGTGGAACGTTCATAATAGGGTTCGCCAGTATTTGAGAA
TGGTGTATAAGTGGAA
TACGCCGCGGTATTTGAATTTTCATTTGAATATGAATGCATACTA
GAGGGCCAGTTGCAT
TCTTTTTTCTCTATTTTATGAATATTGTCATGACTGTTTCTATTCAT
TTCGTAATTATCT
CCAGTAATA
>ConsensusfromContig1474 Average coverage: 5.15
CGCAACTTTTCGTTTCTGAACTCCTCGTCATTGGCATTTCGAAGTA
GGGTACTGTTCATT
GAATGGCATGTTTGCCATAGATCATACCCTGGATTGGAGACCGCG
ATATTCGGAACAAGC

TCGTTTCGCGACTACAAGAATGTCGACCTGTCTGCCTTGTGGTCG
TCCGAACTTGAATCA
AAATAAATTTTCCAGATCGATGGTTTTGGATTTATCG
>ConsensusfromContig1475 Average coverage: 7.07
TTCATTTTCCTATTCTACCGATTACCTTCAGACTAATTAAACCTAC
AATATTCCGTGTTC
TAAATATTCATCGTGTTCAATCAATATCAAACCATAATTACCTTG
ATATTCATGAGAACT
GTCTTTATGTGATGCATTTTTTGCGTCTTATGGTTAATTTCGTGAA
AATTCATTTTCCTA
CCTCACTCATTGCCTCATTAACAATACTTACCATATTGTAGTTGAA
AAATT
>ConsensusfromContig1476 Average coverage: 11.77
GTATTGAAAACAATGATGATAAATCTTTAGACACAGAAGAAACA
AATAAAATTTCAATAA
CCCTCGCTATCGATGACCAACAATCATCACATACTGCTATAACCC
GTGAGTATATTGAAC
TTGATGTATCTGATTTTTTCCACCACTCATCTGAAAAAGCGAGAG
AATTAGAAGATGAAG
AAGCTGGAATGATTAGTCAAACGATAGAAAATAT
>ConsensusfromContig1477 Average coverage: 5.79
CTACAATGTGCCTTAATCTTAATTGTGTGTTTTCACCAGCAAGCA
ACACAATCTCATTTG
ACTAGTCATATTCAAATTTAATGAGTATGCCAATTGAATTCACCA
GCCAAATTTATTATT
GTGTCTTATTCTGATTCATCCCAATTATCATACAATGGTTTGAAT
CATTCCAAGGCAAAA
TTGACCATCATTCACCTATACAATTCCACA
>ConsensusfromContig1478 Average coverage: 4.03
CACGACGCTCTTCCGATCTTTCCTAAACATAAAACACAAAATAAG
CACCAAGCTTTCACA
TTGATCAAAATCTTCGATAAATAAGACAATGGACATAAACAATT
GACATACGCAGTTAAA
TGAGCAATCCTCATATTGATTCACAAAAAAAACATGAATTATAAA
CAGTTTTACGAAATA
AAAAAACCAAAGTGAATATTGG
>ConsensusfromContig1479 Average coverage: 6.54
ATAGCCGAATGTTTAAAACAGTCCATTAGATAGACAGTGATAGA
GATGACAAATAAATCC
AAGAAGGTATCTGTCTTTTTAAACACAGACCGAGAACCGGAACAA
CTTCTCAGTTTCAAG
TCCAGCAAAACGCTGTTGTATGAAAAAATTCAGGAAACAAGCGT

ACAGGTGACACAAAGC
TTCTATTCTATATCAAATTCGAAACTAACAGACATACTTCTAGAT
AATAAAATTCAACCT
TATCAGTATCAGCAGAAAATTTCTGTTTCACTGGAGCAAAAAGCG
CAACTATGACCGTTA
ATGTATCATAAAAATAGAGTCCAATAAGACCTTATTCGATGCAAT
TTGAGA
>ConsensusfromContig1480 Average coverage: 8.18
GCTCTTCCGATCTGACTGTCCCACTGGGTTCATCGGCGACACATG
CCAACATGAAGATCC
ATGCATGAGCAACCCATGTCCTCGAGGCGGACAGTGCTTTCGGAA
CTGGGATGGAACTTT
CCATTGCTATTGTGGACAGGCATATCAAGGAGTTACTTGCGACAA
GTGCGCCTGCAAGAA
CGGCGGGAGCTGCCAGGGTGTCTACAGGGAATGC
>ConsensusfromContig1481 Average coverage: 4.04
TGTGATTACTGATAAAAATAATAATTCGAACATTTCTAGTCAATC
ATTCAATAGTAAAAC
AGAAATAAAATTTCCACAATTTATAAAATATCTCACGCGCGAAA
TCTCGAGTGGCGCGAG
ACTGAATGAGCATTGGGAGAGAATAGTTTCCATGATTGATCCCTG
CTCGGTTCGCCTGGA
TTTTGTCGGAAAGTTCGAGAAGCTGAG
>ConsensusfromContig1482 Average coverage: 8.51
AGTTTGGACGTCATTTTTCTGGGTCGGCCGACAAGCGCCGAGAAG
CCCTGCCTCAGCAAA
TCCACCTACAGCGTCCGCGCGCTTTCCTACTGCGATTTGCACAAA
ATCCAGTTGCAAGAC
TTACAGGATGTTCTCGAGAAATATCCAGAGTTCGCGTCGCACTTT
TTACAGACTTTTCGG
GTCACTTTCAACCTCAAAAAGACAATCGTCAATTCGAACCATTTG
AATTCTTTGATACAA
CCGGGCGCCTCCATGAATAATATACCAGAAAT
>ConsensusfromContig1483 Average coverage: 10.33
GGAACTTGTGCGGCAATTGATTACAACGATAACGACCACAGCTG
TTGGTACCACACGGCG
GACACGGCATGTTCGGGTCTCAACTTTGGCCTTCGTATCACTCAC
GTTCGTTTCACGGAT
TGCAACAACCGCTGGAATCCTCCCTCCGCATCCGTTACGCTTCCT
AGTCGTACTTCAGCC
CGGCGTCATGCAAGAACTAACATAATTCCACCAACTTCTAATAGA
TCGAAAGAGCGTCGT

GT
>ConsensusfromContig1484 Average coverage: 6.29
AAGAACCATCTGGGCGTAGAAACGATAGTACAGGATGCTCAACTT
TTTTGTATACGGACC
TATAGATGGATTTCCATGGATCAGTGTTCGATTTCAAGCAATATT
CATGCCAACTGTTCA
GCTTAGATGCATTCATCTCGTCTATGTAAAGGTTACGCGCGCATC
TATAGATGTGAAGGT
CTTGCAAAGTGCGGGAATTTTGATAGTTTTTTC
>ConsensusfromContig1485 Average coverage: 6.39
CGAAATTATGGTCAAGTTAATTGGCGCAAATAGCCTCAAGGGAG
GTAAGGTCCTCCAGGA
GACGGACTTCGACAACCATGAGGCGTTGGACGGGGAGATGACCG
TGGACATGGCCACCAC
TGAGATGCACTCGACCTACCAGATCAGCGGAGGTCGAAGACTCG
TTTCTGAGAGTCAGGG
CGCCGCTCCTCCCGTGAACCCCGCCTACCTCACGGCGACGTTTGT
CGGAGAAGCAGAACG
TGTATTCT
>ConsensusfromContig1486 Average coverage: 6.60
ATGGAATCATTCATCTCATCATCAGTTTTGTCTGAAATTACGAATT
CAATTGTCTCCTGA
GTAACTACTTTTTCACTTCCTTCTTCCTTCAACTTCCGTGCGAGTT
CTTTTACCAACAGC
TTCTTCATTTCAGGACATCGCACAAACCGAGAAGTCAGAGGAAA
GCTGAGGGGAAATTCA
CTTGAATCTATTATCCAGTGTGTTTTATGATAGAGATTGGCTTTA
GCACGAATCAATTCT
TCTGCGCA
>ConsensusfromContig1487 Average coverage: 5.12
TCTCGAACATTTAGAAATTATCCGGTGAGTACTCCTATATCATTA
TCAGAATCTTTTGAA
AGAGCTTCGGTCCAAGCCAACACTCCACAAACTCATAATCAAGCA
TTCATAAAACATTCA
GTAAACAGCCCGACATCAATATCAGAAACAATTGAGAGACCTTT
GGCTTGGGGGAATAGT
CCAATGTCTGTATCAGAAACTTTTAAAAATCCTTCGTTTCAAGCA
ATTATTCCGCAAAC
>ConsensusfromContig1488 Average coverage: 6.96
TAAAACCACTATGAAAAATAGATATCTATTGCCATTGAAGCAGCA
TTTGCCACTTTTTAT
TTGTTTTTGTTTGTCTGTTGGGTGACCGTCGATACCAGAATGGTT

TATCGCCACAGAACT
CAGATCTGATCCAGTATCCAACAAATTCATTTCAAATGAAGTAG
GTGAAGAGCGAAGAGA
CTCGTTTTCACCAGCGGTGTGGACGAATACTTTTTCCAATGTATCA
ACATTTTCTCGTCT
CATTAGAGTTTCTGGCCTTCCCAATCTCAGCATTCGGCCATCATGC
ATTATTCCTACTTG
ATCTGCTCTCTCAGCTTCATTTGCATAATGAGTTATCAGGATCAC
GGAGACTGGATTTCT
GAAAACGAGACTTCTTAAATGATCCCATAATTTTCTCCTTAGGTA
TGGATCCATACCAAC
TGTTGGCTCGTCAAGCAGAATCAAAGATGGCTTGTGAAGCAGAG
AGATGGCGAACGACAC
GCGACGCTTCGCCC
>ConsensusfromContig1489 Average coverage: 7.55
CATAACAACCGTAAAGGCAGCATCAAAACTGATTCTGGAAAAC
CAAGTCCCGAGAAACT
GGCTCTGAGAAAAACATAGAAGAGGAGAAGTACTTTATACAGAG
GAATCCCCTCGGTTTG
GTTGGACTGCTCAACAAGATTTACGTGTTCGCCTTCACTTGGGGA
GTCGGGGGCAATCTG
AAGAGGTCTGATGATCTGGACGATGATTTGCGACCTGTCAAAGG
CGATAAAGACCTTGAC
CCCACATCGGTT
>ConsensusfromContig1490 Average coverage: 3.73
TCGGGAATGGAATCTAATTCCCATCGCATTATTATTTTCATTTTCT
CCATCCTCCTGCTC
TTCTGAATTTTTCTCTCCAATTCCTTTGTTACATAATCTCTTTTTTT
CGTTAGCTTCCAT
CGCATGTTCTCATCCCAATCATCATTTTCTTCATCTCCTGTTCTGC
ATATTCTCGTTCAA
CTTCTTTGTTTCATAATCTT
>ConsensusfromContig1491 Average coverage: 6.22
CCGATCTACCGAGGATTTGATTTTAACATGCTGATATGCAGTTGT
TTAGCCCATAAGACA
AAAACATTGGGATTAGGTCATATTCGCAGGTACACTTCATGTCCT
CTTGAATGGATTATC
TAATCATTGAATAATATCGTCATGAATTGAGATTCTAAGAACACT
TATTATCACATTAAA
ATTTGATGTAAATGGAAATACATTTTCTGATTGCCCCATATATAA
GTTATTCGTTTCGAA
CTGATAATTCATTCACCG

>ConsensusfromContig1492 Average coverage: 4.16
TTTAACCAGTGATGGGAAAAGGGAATCTTAAATTTGCATCCTTCA
ATACGCGGGGCCTGA
GGGGACAAAAATATGAACTCATTAAAAAAGATCTTTCAAATTACT
CCATAGATGTCCTGG
GTGTACAGGAGACTCATCTGGGAGATACTTTTGCCAATGACCTC
ACAATAGAAACTAAAG
AAAACCAAAAAATAACATATATCTTC
>ConsensusfromContig1493 Average coverage: 7.79
CTCTTCCGATCTGGAACATGAAACATGTTTTATCCATTCGCTAAGT
TTTTTGAATATGTT
TGTACATCAAATCAGGGAGCTTGAATCTACAGAGGTTGGACTTGA
ATTTTGTTTGCTTGT
CGATGCTTTTCTTTATTTTCTCGAAAAAACTTTCTATACAGAGCG
CCAATGTCGAAATGA
CAACCAAAAGACCGAGGATACCAAAGACTCCAAAAACGTCCTCA
A
>ConsensusfromContig1494 Average coverage: 7.55
TGATCAGCAAATGCAAGAGGCAATAAATGCTAAAAATCAAATAC
AACATTCAAGACAAAA
TCGTTCGAATCAATTGAGAGAAAATCACGTCGACCACAGAAACC
AAATGACGCCACCATT
CGCAATTTCCAGCCAATCCCAATGCAGGAGAATCCCACCCGAGCC
ACAATTGCGACCCCT
GGCCAACTATAATCCCAACTCTGTTTCCTCATCCATACCAAACAA
CTTCGGACACAATGT
AGCCAAAAAGCCGCA
>ConsensusfromContig1495 Average coverage: 5.02
TTTTTCTTGATTTGTTTATATTGATTGTTAATATAAATAACTGATA
TTTTAGGATCAGTA
TTGCATGCAAATACAGATAATGGATTGAAACGGTATGCCCAAAC
TACTAATACAGAACAT
TAACATAATTGTACTTGATCGTTCCGGCTTTCTTATTGAAGATATC
GTGCTTATGCAATG
TTCTGTATCCTGAATATGAAATGGAGAATTTTTGTTGCCTGCTTA
CCCTATTACACAGGT
ATAAATCAAGAAAAAGCACAAAAGAGTGATCTCGATGGAAG
>ConsensusfromContig1496 Average coverage: 5.40
TGATAACATCAGTAATTGATGAAATGATTAATTTTTTGAAAAATA
TCATGCTTCACATGA
AATCAAAATCTAAAACGATACCAGAGTTGCAAGAAGTAGTGAGT
AGTCATTCGCAGGTGA

CACATAAAGTGCATCAGATTGTAGAGCGCTATATACCTGATGCTA
TATCTAAAATATTAT
ATATGAAGAATCAAATACAAAATATGTACATCAACCTGATCGAAG
ACTTCTATGTTT
>ConsensusfromContig1497 Average coverage: 5.32
TTCTCAACAAGGGATGATCTGGAAAAGTCATCATGGAGAGTGCGG
CCCTTCCCTTATTGC
CGTGAATAGATGTCGTTAGATGTCTTCTTATCTCGTTCGAAAGAT
TATCATCAAAATCTG
GAATATTTCCTCTTCCAAACAAATCGTGTTTATCTATCACAAATA
TTCGTTCGCCGCTT
TCTTAATTTGACGATTGACCTAC
>ConsensusfromContig1498 Average coverage: 6.08
CTATTCTCTGCTCTGGATACTTCATGTGCCGTTTTTCAATTTGAAA
TGTGATGTACTTTT
TCAGCATTGGAATTTCTTTTCCTTTGGCATCGTTATACTTGAGAG
CCATATATAGAATAG
GGCGGCCCAGCTTGTCTTTGCCCTTGAATGATATCGGATTATTCTT
TAGTCTCTCGTTGT
AGTCAGCAAATGTCTCATCGGTTACGTCCC
>ConsensusfromContig1499 Average coverage: 5.10
AAATCTAATGAAAAAAGCAGACATTGGAAATTCTATCTAAGACA
CCTGCAATATAACTTG
GAGCATATAATTTATCTTAGAATTTCGAGGGGATTGACCCATACC
GAACCACAATATGAT
CAGACATGCAATAAGTTTTGTCATATACTAGCAGGTCACCCAGGT
ACACATCAACTTGAT
TGAACTATATCATCAGGTCGGC
>ConsensusfromContig1500 Average coverage: 11.37
TTTTATCACAGACAATTGAAAATATAATGTGTAAACAAGAAAAA
TCACGAGAAAACCAAA
TCTTATAAAAATTAAAATGAATTTCATCATCCCCTAGATACGTCC
TAGATCGATTAGCAT
TGTGTACTATATAATTAACTTAACGCTTTCAATTGTTCAGTAACG
CACATCAAAATTTGT
ATCATAATGAACTATTCTAAGTCATTTTCAATTATACTCTTGAACG
CAATTTTATGCTCA
GCTGAGCAAAGTTTGATTGTAAATGTGGC
>ConsensusfromContig1501 Average coverage: 4.33
GGATGCTGAAGCTTGGTTCGGGAGTTCGGAATTCTCCATCGAGAT
GATTGTGGACCACAG
CTCTGCAACATCTTTCTCATCAACTGATCCTCTTCTCTTCAAACCA

TCCTCCATCGGTTT
TCCAATTATAAAAGAGACTGCAATCCATCTGACTTATTTCGATAT
AAGCAACATGACTCT
TGAGAGCCCTGATATTTCCGGGGCCGAGGCACCCCTTAGTGG
>ConsensusfromContig1502 Average coverage: 3.68
TTGCAGAGACGAGTGCTGCCGTCAATTCAAGGCGGGGTATGGTAG
TTATTTTAATGGGCG
CTACCCTAGATTTCCCCATAACAAGTTCTACATGAATTTTTCCTT
TTGGGTCGACAAACC
TGACGTACGAACATTGTCCGTACCCCTCCGAACTTGCATCCGAAA
AATGATGTAGTTCCA
TTGCAGTTTGGTTCGAAAAAGGCGATGGCTTGTAGCAGCGACTGA
AAGTTATGTCTGCCG
AATGCTCCCTCTCCGTG
>ConsensusfromContig1503 Average coverage: 5.80
GGGACTCAGAAAACAACATACCAGAAAGAACTGCTCCTGAAAAC
TGGTCAATGGTACCAT
TTTCTTGCAAATTATCACTATGAAAAAGAGATTGAATTTTACGTT
GATGGCAGATTTATG
AAAACTAAATCAGTATCTACTAAAAGCGTCACCGAAACTACGATC
AACCAATATAACTTC
AAGTGGTGTAACAGCAATTGTTATGAGGAATATAGCT
>ConsensusfromContig1504 Average coverage: 4.40
CACGGATCTCATGTCCCATTCCAAAGAACTTCTCGGTCTTGTGAT
CCTGGGAATTGCGTG
TTCTCTTGCTCTATTCAGATGTCGGTGTGTTTTTGGCGATGATTTC
CGATCATATATTTC
AACTCTTGGTAACGTATATCTATTCGGATAGGGGTCAATTTGACG
ATGTTTTCTTGGTGT
CCAATAATTTTTCAAAATTCCTGGATTACTCGCTTGAATTTCGTTT
CTAGAATTTCGATA
GTTTTGATGCTCTCCTGGTCC
>ConsensusfromContig1505 Average coverage: 6.88
CCACATTGTTGCTTGCTATTATTCCTCACAAAATTATTTTATTCTG
CAGTTTGAAACGAA
CGCACTGAGACACTCGATCGCACAACTGAAGTAGAATTATGCAG
TCAAGCATAACGATCT
CGAGAAATTCGTGTGTTTATTTACATGAAAGGCGACAGTTAGCCA
CATGTTGATAATCTT
ACTTTCAAATCGATTCACTTAC
>ConsensusfromContig1506 Average coverage: 10.35
TTTTGAATGAATTTTATTCTTGAACAACAGCCAGCTTAGAGTTGA

ACATATTTGACTTTG
ATCCATTCTTTCTGCTCAATATTTTATTGGGCCATGTACCATAAA
ACTCATAAAATGCCA
GATATACTTTTAGTACCAATCGATAATTATACAGCTCGACCTGAT
TATAAAGAATATTTC
ACTCATGATGAACTCGCCATATTCTACGCG
>ConsensusfromContig1507 Average coverage: 3.21
TCTTCCGATCTAGCGCCTTCAGTTCTGGAGCCAGCCAGTACTGTCA
GCTCTTCTCCTACT
CGATAGGGGGGTCGATTAGTACCAATCAACAATCACAGGACGAA
CTGTATTTCGAGGAGA
CCGTGTTCCCGATTGTTGAAGACTATGAATTCGGGATCGACCCCT
CACTCATAGAAGAAC
AGACAACAGCAGGCTCTCTTGAACAAAAGCTACAGTCAACTGAT
TCAAACGAG
>ConsensusfromContig1508 Average coverage: 6.70
TGAGGAACAGCTTCGTTGGTGGTATGGTTTGGATGATCTTTTTCGT
ATTCTTCTTTCTCT
TCCAATGATTTTATATATCGACAGAGAAAGGCGTGCTGCATCTTA
CTGATTTTTGTCAAA
AATTGTCTTTTGAAATGCTCCCTCTGATTGGAGAACAGTTGAACA
GTATAAAATTTGTTT
TGAGCTTCGTCCTTGACCGGGGTCATCTGTATGATCCCGGGGACA
TGACATCCCAGGCCC
GTTTGCTGGTTCAC
>ConsensusfromContig1509 Average coverage: 5.81
CGAATTCCTCAAATAGTTCAAATTTATGGATGTGGAATCCTACTT
CAATTCGTCACGGAA
TCAATGTAATTATAACCCCGGAAGCGGTATGAAAAACGTCTTAA
ATTACATGGGAGGACT
AAATGGGGGTCAATACATCGATTTTCCCCATGGAACCAGCAGTTA
TGCTTTCGGCTCATC
TTTCGGGCAAAACAGTCACCGATACGGCTACAGCGGCGAGCCATA
TTCCAGTTCTTCAGC
TACAGAGCCAGCTGCATTTGGAGCCGGCTGTTTCGGTGGGGATGC
CCACGGTATGTCTGA
CGCCCATCATTCGCATACAGCTGCCGAGACTTTTCGTGGCGAGAC
CGCAACACAAAATGG
GTTCTACGCCGGCGG
>ConsensusfromContig1510 Average coverage: 4.14
GTTTGGGAGCACAAAATGGAGACCTGCATCGTACTGAAGCGTGCT
CTTCAGTCGATTTTT

TTGGCAGGTTGTTGCACCGTAGTGCTCTAGAGAGCTCTTCTTGTG
TTTGTAGCAAGGAGG
AAAGGGGCAACTGATTCGAAAGCAATCCACCAGTCATCTGCCTCT
CACCTGCTCTTTAAG
ATTTTTTGTCGCATAATCAATT
>ConsensusfromContig1511 Average coverage: 4.67
CACGGGCGCTTTCCACAGCCCCACGATAGCACTCGACGTGATCAG
TAACACACTGCTACG
ATATACAAGTAAGATACCTAACGCCAGAGTGAGGGCGGTGAATG
AGCCACTTCCCAAACA
AAACCACACTACGAATGGACAGGAAGCCAATAGAAAGATGCTGA
CCATCACGTCTTGTTT
ACTCATTTCGATGTTTACTCTTTCCTTGTGGTTTGCGTCTGTGAG
GCAAATGATAGAGCG
GCTGAGTAGATCGCGGCAGTTGAAGCTCATGATTGCTCACCCGTG
GACCTTTTGGAGTTC
TGTCTTGACAGCCAACTGCATCGAGAACCT
>ConsensusfromContig1512 Average coverage: 5.67
ATTTTGTATAAAATTAGATCAATTTTATTTGTATGCATTCGACGG
TTATACAATGACCCA
AAGCCGTGTATTTATTGTTACTACAATAGGAGCGTCTTTTTTCAC
TGTAACCAGGTAATA
ATATCAGACCAATGTCATCATCCTTGAACAAATACCAATTCGAGT
ATTCTAGCAAATAAT
CTCACTAGCAATTAGCAATCGACGTGAGATGATTGTTGAAAAATT
T
>ConsensusfromContig1513 Average coverage: 5.81
AAGCGAGGAGAAATGAAGAACAGAGCTCTGCTATCTAATATATTC
TAAATTCTGTATGTG
CCAGGTTTTGGACATGTGATATTCGAATATGGTTTTGTGTTTGAT
ATAGCATTAAATACG
TGCTGCTCAATATTTTGTGGATAAACACGTTGATCACTATTTCATG
ATTATACATTATTA
TTATTTTGTAATACTGATATCATCA
>ConsensusfromContig1514 Average coverage: 6.67
GTTTTCGTTGAATTGACCCGGCAATGTTTTGAGATGAGGCTTGTGG
TCTCGCTTCTTACT
GTGATAATTTGTCAAACGTTGAGTGCCAACTGAATGCCCCTTGTC
GAGAATGGGGAATGA
ATCTAGAAATAAAGATGCAAGAATGCGATATTGATACTCTAAAT
GGGACAACCAGCCCTC
CTGAGATGACGAAGAGATCGGAAGAG

>ConsensusfromContig1515 Average coverage: 5.88
CGCTCTTCCGATCTCTGGAGTATGCACTCCCGTTTAACATATGAA
TAACTGTGTTTGGAG
CATATATCGTCTTCCAAAGTTCTTCAATGCCACTGCCACTCATGA
TATAACCGATCGATC
CCATGAAAGACATTAACATGTGGAATCCTCCAAGTCTCACTACAA
CATTTTGTAGATCCT
CACAAGAAGCCACAATCGCC
>ConsensusfromContig1516 Average coverage: 5.67
AATTTTATCTTGACAGGTTTTGAATTCTTTGTCTATCTATAATTTT
TGTAATTCCATAAA
TTTCCATTTGTGATATATTTAATCGCTATAAATGAAATAATAGGG
GAATTTTGATTTCCG
GAACAAATAGGATTTCTGTATATTCGAATGAATGCTATTCTGAAT
ATATATTATTGAATC
ATGTATATTACCCTCTATAACACTTTCTTGTATAGCGCG
>ConsensusfromContig1517 Average coverage: 5.82
ATCATTGTTGTTACATGTTGAATACATATTTATAAAAAATAATAA
TGCCAAATTATTTTA
TTCACACTGAGAATGAAATGCAAAGAGATACTGAATTGAGATTT
CAATGAAATATGTGCC
TGAATTGGTTTTTTGTTTTTTAAATGTGATATTGAAAATGTGGGA
GTGAAGAAAATTCCA
TCCCACTATATTGTTAAAATTCAAGCAAAATGTTTGATATTTTTGC
TCTACTATTGTATT
GTATATGTTCG
>ConsensusfromContig1518 Average coverage: 4.28
CGAGCATGCATAGTGTATGTTCTTCATCGGCTGGGTTTTCGATAG
ATTCAAGTTTTGGCA
CAATAGATTCGAGAAAGTTGCGAGATGATTTATCAAAGGAAGGT
TCGATCACTCCAGTCG
GATCAGTTACAGAACAATCGAGGGATGACTGGGAAACAATAGCA
CCTCCGTTACCTGATA
ACATTCTTTCCGACCTCTCAAA
>ConsensusfromContig1519 Average coverage: 6.96
ACACGACGCTCTTCCGATCGTTTGACGATAATGAACGCTTCACGC
TTCGAGTCAACCTCC
CATTACTGGATTTCCCCCTCTGAATTATTTGTCTCTTCAGCGTGTT
TCTTGAGGGTATTA
TCTCCGCCACGTCGATATTCCCATGTTTCGAGCCGATTTCGATAC
AGGTTGAAATCAGGC
TCTTCAACCCTCCTCCTTCTATTGCTGAAATCAGCCTTAAATCTCT

TGTGACG
>ConsensusfromContig1520 Average coverage: 10.39
CTACACGACGCTCTTCCGCTCTTACGAATTCGAAAGTACTTTCTT
TATGATAAGTGCCGC
GAAATCTCAGATGAACGGAACGTAACAAATCAGATTGTCTCATTT
CTCGATTCTGAAAAC
CGTCCTTAAAAAGTCAGGATCACAACATGTTGTCTACTGGATTTC
TGAATCAGATCATAT
GTAACCAATGCTACCAATGGAATTCTTCCTTGTTCAACCGTCATC
AGAACGCGTACAGAT
CCCAGACAGCAAAAGTTTCAGTTTCTTGATGATGTTCCTCTCATC
TCATCAGGC
>ConsensusfromContig1521 Average coverage: 5.63
TTTAATTCTACACATCCATAACATCGTTTAAATGGCCTGTGAATTC
TTCAACTTCGTTCT
GTGGGGTGTGGGCGGAGCCATCATCTGCGTCGTCGGCATGATTGG
AAACTCCTTGTCCTT
CTCCGCATTTCAGCGGGACAGAAGAACGTCTGCTACAACCCTGCT
GCAATTTCTCGCAAC
CAGCGACTTCTTCTTGCTGCTCGCTGTCTTCGTTACAGACGCCTTA
CCCTACTCTTGAGA
TCGGAAGAGG
>ConsensusfromContig1522 Average coverage: 9.43
TATAAGGAGAATTCCGTCTCTACCCAGTTTTGAACGTATCCTGTC
ATCATCAGCGAACCG
AGGCAGCTGAACTTTTTGTGTGAACGTCCTCGTAGCTTCTCTACCA
ATTCCATCTTTATG
AGCTTGTACAATCACTTTATTTTCTAGTTTATCCACGACGACCGA
TACAGTATGTGCTGG
AAACTCACTAACGTCAACAATTGCTTTGAATATCGCTGACCCAGA
TGATGTATCAACAAA
ATTCGATCGCTTTTCGACATTCCTCCCTGTTGAATGATGAGTACTC
GCCTCAGACGTCTC
GCCTCCTATGCTGCCACTCATGAAAGAATCCGTCAAACGATCGAG
ATCCGATTTCCATTC
TGTTCGCTGCTCATCTAATTCTTTTTGAACTCGTGTCATAAACATA
TCATCTCTGGCATT
ATGTCCGCGAGTCTCGTATCCGTCATCGTATTGTCTTTGGTCAACA
TCATATTGATTATT
ATTCCTGTGGCTTTTATTAGATTTCCTGGTTGGTGTTG
>ConsensusfromContig1523 Average coverage: 4.06
GTCGAATGACTTATGAGGATGAACTGAACAATGTTCAGGACAAC

AGACAGGCTCAAAATA
GTACTTTCAACTGGAATTCAGAAAATAAATCGCGTCACATATTAC
CGCAGAGTAACAGTG
GTACATTATCAGAAAGCATCACAGGAAAAGATTTGAAAACAGAG
AGCCAAGAAGAGCCCC
ACAGTGAACAGCTCAACAAAGTGATTTGGTGTTGAATATGGCGTT
CGTATGTCTCATCAC
ATTGGCGGAAGTTGTTTAT
>ConsensusfromContig1524 Average coverage: 5.33
AAATAAACTTAAAACGCTGCATAATCCAATCCTATTTCTCAACAT
ACATACGAATCATTA
GTATCATCATCGACTACATGACACACATCACCATGAACAGAAATT
GAACATCACGTCACA
TCAAGCAATCTCACATTCTCACATATCCAAAACAAATAGCAGCA
GTGAGATCACACACGA
CATTCGAACAACACGGGGAGAAGGG
>ConsensusfromContig1525 Average coverage: 35.76
GAGTTCAGACGTGTGCTCTTCCGATCTGCGTGCGATGATGCCATT
GAGCTGTTTACAGAA
TAAACTTCACAAAAATAAACTTCACAAACATGTTTGATGATATCA
ATGAATGGTTTTGAG
CCCAGGAGAGGAAAAAATAGTAAAGACGATGGGGAAAAAAGA
GCTAAAGAAAAATCTTC
AAGATATAATGGAGTAAATC
>ConsensusfromContig1526 Average coverage: 5.62
ATGGAAGCCATGCTCATCTCTGGGTTTATCAAACTCATCAATTCT
AATTGCTCCCGTGAG
CTGTACAGTTCTGGATACGTTCCCGTCGATAATATTGCAGAGAGG
CGATTCGTGAACATA
TGATCGTTTTCACTGAGATTTTTATCAAGACCACAATCGGTTTGT
TCTTCAAAGCAGTT
ATCTTGTGAGAAGTCTTAACCGTATTTTGTAGTTCTAC
>ConsensusfromContig1527 Average coverage: 5.04
GATCTGGCGATCTCGTCATTGAACTGGATTGGTGTGGCGTTGTTC
CAAATCAGTTCTAGT
TCATCAGCCGTGTGACCATAACTCTCCAATCGCAGAGAACAAATT
TGACTGTCGAGAGGG
AATTTATCCAGATGCATGTGACACACCAGCGTTAATGTGTATCTC
GACCACACCTGAAAC
CCATGCTCGCCAATATTTTC
>ConsensusfromContig1528 Average coverage: 5.39
CTCGGATATAAAATTGATCAAAAAAAGAATGAGAGAAGAGCTTC

AAATAATAACAGCATC
TCGTCGAATGAGAATTGAAGAAACTTTGGCAATTGAAAATATGG
AGCGTGAGGTTGAAAC
GAGAAGAATGGAGAAGGAACGGTTGAAATCTGAGGCGATTGCA
AAAGAAAAGGAACGGCA
ACGTCTTGAGGAAGATATTCAAAAGTTGA
>ConsensusfromContig1529 Average coverage: 5.74
CACGACGCTCTTCCGATCTGCGAAGGAAAAACTAAACTTGGTCCG
TTATCGTTCAGATCT
CTCAAGCGAACAGTCACTGTACACGTGTCTGTCATACTGGGGTAG
TTGAGATTCGAAGCA
ATTATTTTCATAATGTGACGCTCTTCCCTCTCTCGATCTAGCACTG
CATTGCTGATTATA
GCTCCGTCATTGGACATGCGAAACGGATAGTAGTGTAATCCACTT
TGATCGATCGAGTAG
GTTATCA
>ConsensusfromContig1530 Average coverage: 3.97
AAAATATTCAAAAGCATGAAATACAATGAGTAGTTTCCCTTGTTT
TAGGATTCATTGTAG
CAAATATGGAACCCTTTTTAATAAGATATTGAACATGATTCTACC
AGCAAGGATAACCAA
CAAATTCTCGTGAAATAGTGAAAAATATTCGGGGGTACTGATAAA
CATGTGTAGGATCGT
ACAATTATCAAAATAAATGCTTTACAAATTGAGTATCAGATACG
CGAAAACGAACTCTAA
AATATATGGCGTGAGACTAATGAAGA
>ConsensusfromContig1531 Average coverage: 5.82
TAATGAAATAAAATGAATAATGTTTCTTTGTAAATATGCATTTAT
ACTCCATATGCAGAA
TATAGTATAATAAAATTGGTTTCATACGCAACACAGATTCAATTG
TTCAATTGAGAGGCC
ATGAATCAAACATTATACATGTCTCATTGCTTGTTATTGATTATG
AAATATGGTGCGAGT
GATATAAATAATATTATTCTCAATTAAAAGAACGAAATGGTTGTT
C
>ConsensusfromContig1532 Average coverage: 9.53
CGAAGAATCATTATTAAAACGTTCTTCGATATTTGGGGAATTCTC
TGAACTGATTTCGGA
AACAGGTTCTTGACCAATAATTTGACGAGGCATCCAGTCTTTGTC
ACTGACTTTCGGTAG
TGGGCCAACGGGTATTTTGCGCATATGAGACGACCCATTGAACAA
TATAGAGCCGCTTGA

TTGTGATTCCGTTAAGTTTTCTTCAG
>ConsensusfromContig1533 Average coverage: 6.71
CATAAATCTTCTTTGACTGAACCTGTGACCGCTGATGACATGGAA
AAAACCAAGTGGGAT
AGAATAACCATATTTCGCTTGATCTCCTTCAGTTATGTTGTCTAC
GCGACATCGCTTGGG
CTTAAGAGTATAGGGACTTATGCCATTGCAATTGCCGTGGAGAAA
GACTTGCCTGCAGAC
ACCGGTGCAAATGGTCTCCTGGCCGCTGGGCTAGCTGGGTTGCTC
ATCACTATGCCCGTT
GGAATGATTCTCAAATTAAAGAAATTGGCCAATTTCAGAATACAT
ATTTATTTATTCATT
CTCACTTGGC
>ConsensusfromContig1534 Average coverage: 5.62
AAATAGAATCACCGGAGGTAAACAACACAAATCGGATCAGGACA
AATGTGATATATCCAT
CCGACTCATGCTTTCCTTTCTGGCGTCACTGTTTCTCGCGGCCTTG
ACATTCACGTGGAT
GTTGCACGCCCTTGGATACACGCTCCTCACGACCCTCATATCTTCC
ATGTGTGTCTTCTT
CGTCGTTCTCGTCGCTCTCAGTGCCTCGC
>ConsensusfromContig1535 Average coverage: 5.96
CCACTCACACTCTGGAAATTGCAGCCATTGGTTATGATCGATTGA
AATGGAGCTTTGTAT
TGTTGAACACTCCGCATTGAGCAGACACTGCACCGTGTCAAATAC
TCCTACACGATCGGT
CGGGCTCTTCCATGATATTTTTTTGGACCTTACTTTTCAAGTCTAA
TTTTTTGGCAGACT
ATTTCGTGTGAAGATCGATAAGACATGTGCGAAAAACAATGACA
TATGTAGAAAAGATCT
CACAGGCACACACATCTCAAAATTCTAATCTCTAATTGTACGAAT
GGAATATTGTTGATT
TACTAGTCTCGCAATGGTCATCACCCTATTCATCCGAACATTGAC
ATGAGTATGCGTTTA
ACCCATCAATACATGTTGCACCGTTTTG
>ConsensusfromContig1536 Average coverage: 7.81
CAGACATTGGCCTGGATTCAGTGATAGGTTCGTCGCATTTAAATC
AGGCAGATGATGCCA
CATTTTTGACGTCAAAAGTGAGTCTCACAGTTCGCGAAAGGATTG
TTAATGGAAACATGG
TTGATTTGGCCATCCTGCTTCAAGGTGGAGAAGACGTGGAAGGC
GAGAAAAGGTCGGGCA

AGCTTTCCCAAGAACTTTCGATTGGGCAATGGACCACAGCCTTTC
TTATCTACATTGCCG
TCTACTG
>ConsensusfromContig1537 Average coverage: 4.14
TCGCGGTGTAAACAAATCCATCAGATGAGTCTGGCTTTTTCGGTG
AAAGAAATGGAACCA
TACGTACCCAAGTTACCAGGATTTGAGTGGCTTCGTCCCAGTATA
TCCACGAAAGACATT
GTATATATTGGCAACAGGGATCTGGATTTTTCTGAAAAATACGTT
TTGGACAAATTTAAT
ATCAAAAGTTTTCATGTGAGAGACAT
>ConsensusfromContig1538 Average coverage: 4.42
AGAATATCAATGAACAAAATCACCAGCAATACTATAACGGACCC
ATATAACAGGTCCCAT
ACTTCAAATTACTGGTCTTTGTCGTTGGATCACATTATACTGATG
GACCAGACGAATGAA
ACATTGAGCTGGATATCATGACATAAATGAAAAATTTGCAAGGA
GTATTTACAAAAAAAC
GAAATCAATTGGAAATATAATTTCT
>ConsensusfromContig1539 Average coverage: 4.16
CGACGTGTGCTCTTCCGATCTAAGCGATGTCGGGAGACTACAAGT
TAAATACATGAACAA
AGGCACTATGTAGTCATAATAACATTATAGAATAAAATTATCTCC
ACAACTGAGAATGGA
AGATTACAAGGTGCGATAATGAAATAATACTTTCTATAGTGAAAA
TAACATTATATAATA
AAGATATATTCATAAGAGATGTCG
>ConsensusfromContig1540 Average coverage: 6.71
ACGAGGTACAGTACTCACTCTTACTTTCAAAAACAACAAATCGGT
ATATGACCGTGGAGT
CGAGGCCGTTTTATATTCCATTGCTGAATTTGTTTTCAGAGAAATA
ATTTTTTTATCTTC
GGAATGTTCGGTACATGAATTCAAAGTCACAATATCACAACAAA
ATGAAACGAATAAGTT
ATCTATTGATACTTGCATTGAAACATCATTATCTGTTGAAAAAGA
TACGGG
>ConsensusfromContig1541 Average coverage: 5.07
CAAAACGTGAAAAATTGTACACTTTACTGTTTGGAATTGAACTGG
TTGGAACTTTGATGT
TATCACTAGTTGAAAAAGTCGATGAAAACAAACAAAAACAAATT
GAGCAAATTCGATTAA
TGAAAAGCATTGTGAAATCGCTATAAAACGTGAAAAACAAACAT

GAAAAAGTACAACATA
ATCTTCGGAAAATTTGAAAAACTGTAAACTAAAATATATC
>ConsensusfromContig1542 Average coverage: 4.77
GACATTACTGATATANAGGAAGGAGTTGAAGAGCCGGTTCATAAC
CATCTCCTACATCTA
AGTATGTATGACTTCTTATGGCATGATGATATGAAACAACAGCAT
CGAGAATTGTCGACG
AATGATCCGGATCTATATGCCACTGAGCGAGAAGTGAATCGTTT
GCTGGAGATGGGCAGG
AAGATCAATGCCATTCCCGAACAACTGGAGGTGGGCGCCGTTCGA
CTTCATACATC
>ConsensusfromContig1543 Average coverage: 6.74
CAAAAACTCAATATGCATTTCATTTAATATCTTATCTTCACTTTCG
TGATTCGAATTCTT
CAAACCCCGTGGTAGAAATAGGTATTCCAGAATTTTGCGTATATC
CAAGTTCAGAAACTC
ACACACGAAATTCATAAAAGTTAGGAGAAGGTAGCTCGACAATA
TAAGAATCGTTTGAAG
CATATTGGTGAATCAAATTTATTATTTGTCGAACATCAATGCTATT
AGATTTCAC
>ConsensusfromContig1544 Average coverage: 5.40
CGGAAATATAAAGAATCGAAAACGTATTACAACGAAGCCATCTT
CCGCCAGAATCTCTTG
AAGAATCCAAAGCAAAAGCTGCAGCTTTCAGAAAGTTTGGTTGG
AGAAGCAACGGTTCTG
TACCAAGAAGGCGACATGAAAGAAGCCAAGCGCTTTCTTCTACG
CGCATTGGAGTTAGGC
ACTGAAATTCTGGGTCATAACCATTA
>ConsensusfromContig1545 Average coverage: 6.17
ACAATTCCGCATTGTACATGCGCATTTGCTCCAGCCACGATGGTC
GAAAATCCATTCAAA
GATCCCGAGAAAGGCTTGTCCAACGGCCATGACACGAACCCAAT
GCAGAAGACCGGGCGG
GAGGAAAGTCGACAAAAGCAGAAGATTGAACAAAAACAGGAGCG
CTCTTTCGAAAGCCAG
CCTCACGAAAGCGTCATCCTTGCTCG
>ConsensusfromContig1546 Average coverage: 9.60
TCTCAAGCAATACCAGAGAAAGTTGCTCCGCCCAATTTATCATCG
AAAAGGAAGCACGTT
GAACTTTACCAGAAGCCAAAAGTAGAGAAAATCTCGCCGCAAAA
ACCCAAACCACCTATA
AAATATCCTAAGCCGGCCATACCGCCTCGCACGACATCTATAAAG

CCCGTTCAAAGCCCT
GTATACAAAAAACTGATGGACAAAATGGATCCTGTTGCCCTAAC
ACGGTCCATGGATGGA
CCATTATTCACACCGAGGAAACTTTATGCCGAGAAACCATCAAA
ACATCGAACTGTGGCA
CGGCCTTTATCTGACGAATATCGGCCTGACACGAAATTGCTGGCC
GCTCTCAAGATGCCG
GAAAGTGGGAGTGAATGAGAGACAAGCATTCGTTCATACAAACA
GGTTCTCAAAAAAAGA
ACTATTCTGGATTAAAT
>ConsensusfromContig1547 Average coverage: 4.50
GATAAAATGACTTTAAGGTGACATTTTTCTTAGATTGAACAATAG
GTAGACATGAGATAA
AATGACTTTAGGGAGACATATACCACAAAACATTGAAAAATAGG
TAGACAGTATGATAAA
ACAGTTTAAGGTGACATATCTCACTGAGCATTGACCAATAGGTAG
ACATATAATGACTTT
AAGGCGACATATCCCACAAGCAGTAATAATACAAGGTCATGTTTT
CAAACTTATATGACG
AATAATATGATTTTTGTGACCTCCACATCGC
>ConsensusfromContig1548 Average coverage: 4.64
TGCTCTTCCGATCTTTCACTTATTTGTTAAATATATTAAATCCTCT
TTTGAAAAATAATA
TGATAATAAACTTCTGTAAAGTCTACCGACCATCACAATTCATGT
TAACATGTGGATCAA
ATATCACAATAAAATAACAATGAGAGATACTCTCAAAAACATCAA
TATATGGATACATTT
CAGTGTATGAATACGTAATAAG
>ConsensusfromContig1549 Average coverage: 12.09
TATGCCGCTATCTCAGGAAGCCTGACTCTGTGCGACAAGCAAAGA
TTTCGTATATGCAGC
GGCAACAAGAGACGCAAACGGTTGCCAGCTCACGTTCTTTCTCCC
GTCTTCATAATCAGC
CATTCCTCGAATGATGGTGAAGTTGGTCATCTCACACTCTTGGAG
AGTCTCCAGAATAGA
TCGGAAGAGCACACGTCTGAA
>ConsensusfromContig1550 Average coverage: 5.45
ATAAACTAGAGCAATCACTTTATCCATTGAGTAATGCTGAGGTTT
GCTTCAGAGAAACCA
ACCATGATTCTCTCAGAACTTTATCAGACACCGCATCACAAGTGG
ACGAACACACAAATA
TCACAAACACTTTGAAAACAAACGCGGTAAACAATATCAGCAGA

CGGCCTAGTTTAAAAC
CCTCAGTCACTTCCTGTACGAAAATT
>ConsensusfromContig1551 Average coverage: 4.38
CTTAAAGTCCCGCATCTCGTTGGTTTAGAGTGTTGACTGTTTTTA
GAAGAGGAATAACTA
AAAAATTCTATCAAATACCACACCTCAATATAAGATGGCGTTTAC
GTCCGTGAGAGAGTT
TTCACAATTAGGTTTGACTTGTATCCTTTCAGAGCAGATGCTTAAA
CTTCAGATGTTTTT
AACGAAATGCGATTTTCAACGATTT
>ConsensusfromContig1552 Average coverage: 10.43
GGAGCAGTAATATTTGGTTGACTATAATCAGAAGTTTTATTAATG
AAGAAATGATAAGAT
TGCACAGGAGTCATCCCCAATCTCTCCCATCCACCATAACGAATG
CCAGCTTCTCGGAAT
ATTACAACAATATCTTCTGCCTTCACATTCAACAGCGGATCATAA
ACGACGATATATAAA
TTTTGAGCCATTATGGGATATTCCAAGTCATATGATATTATCTGC
TCGGATGATGTCAAA
ATCGGATTTGATGGAGATTC
>ConsensusfromContig1553 Average coverage: 4.98
GTCAGGTTTTATGTTTCTTTGAAATTATAAAAACGTTATAAGCGT
GTATCTTTCGCATGA
TTCGGAGTAATATTGGCTGACTATTCTCATGTTCTAACATATGCT
CTTGATGAATAACGG
TATAAAAATAAATCACACTTTAATAAATATTTGATAATGCACATA
GTGAAACTCAAAAAA
GGCTGCAAGAACAAATTAACCGAGGG
>ConsensusfromContig1554 Average coverage: 5.57
CAAATTTTAAATAAATTATTCTAATTATATAGTTCGGTGTATGCTT
CTCCACATCGGATT
CAAAGACTATGTTTTATATATTAATGGTACAGCTGCTCCAACTAT
AAATCAATAACGTGT
ACTCAGACACGAACTGGAATTTTTACGAAAACAGAATCCGGCGAT
ATTTTTGCAGTCTTG
TATCCTAATATTGTATAATGAATTGAG
>ConsensusfromContig1555 Average coverage: 8.03
CTTTTTTGAATATTAATCGGAATTAGGTTGAGCGAAGCATGGTAT
GATTTTTGAAGATTT
ATTTGAATGATTTTCAGATTATCTTTTCGAGTTGTAATGGGATTAC
TGCCCATAGTTAGT
TCTTGCTTTCAATTCTTCAATTTTTTGTACGTAGCAAGGGCATGTT

TTATCCGATGCTGA
ATGATTAGACGCCTCTGCGCGATAGACGTTGTCCAACTTGCAGTT
TGCGCAGCTTTTCTT
CGAGGGGTCTTGTAG
>ConsensusfromContig1556 Average coverage: 5.40
CAATTGAAATGAACCTCTTTAAGTTTTCTATATTCTTGTTTTTAAT
CGTTTATGAACATC
GGTCAAATTTCAGAACCACTTAGCGACATCGAAAAAGTGCGATGG
AAATTATCGATTACT
ACAGCGTGCTCGGCCTTCAAAGACCAGCAGACGAGGACAGCATC
AAAAGAGCTTATCATG
ACCTAAGCCGTAAATGGCAT
>ConsensusfromContig1557 Average coverage: 4.68
TCAAAATGAAGTTTATCAGCCAGACTTTCAAATTCTTGTTTGAAA
TTCACTACAACCACA
TTATCGATATAGTTGTCTTTGTTATGATCAAAATGAAGTTTATCA
GCCAGACTTTCAAAT
TGTTGTTCACTGCACGCAGGTTCTAATTTTATAAAACTGTTATTCG
ACATCTTTTGGAAA
TAACCTTATATGTATTTTTTTCCTTTGATTTCAAC
>ConsensusfromContig1558 Average coverage: 5.18
CAGCAACTGTGGGATTCCCGGCGTCAACACGAACCATATTCAGTA
TGGGATGCCGATTGG
ATCTTCTGGAGCCATGTTGAACGCTGGCCAAACACCGTCACATCT
ACCCCAATTCTACAA
CATGTACCAGAATATGCCATACCTGCTCAAAACACCCAGCCCCCC
ACAGAGCTCCAGATG
GAAGAGCTTCGTGTAGGGAAAG
>ConsensusfromContig1559 Average coverage: 10.63
CACAAAGCAAAAGTCGAATATTGTTTGTTGATGACCTTTGGATTG
GCAAATATTGCAGTT
TTCTCAAGTAGAAATCTTCATTCAATTTTCTTCATTCCATTGGTTA
TTGCAAAAAAATGT
CGGTCTCGAGAAAAACTGGAGAGGACACTGAAATAAACAAATCC
CATCAGAATGGCTGTA
ATCTCACTTATAGCATCGAAGATAGACCACCTCTCTACA
>ConsensusfromContig1560 Average coverage: 5.16
GGAGTCCTCGGCGTATTTGCTGTTCTATCGAGGGAGTGGAGGA
ATCCGAATGGAAAACA
GCCGAGCCATCTATACTTTATGCTTCTTGGATACGGAATGAGGCC
AAATGCTTTCCTTTT
GAAGACATAGCTGTATGCGATGTACTGGTCGCCTTGGAAGAAATA

CGTCCTGCCAAGTCT
CTCAAAGGCAGAATCAAAAGAATCAAACTCGAGTTTG
>ConsensusfromContig1561 Average coverage: 6.98
GGCTGTTGTAGACACAAGTTTTGTTCTGCTGAGCATAAATCACCT
GTCTGTTTTAATAAT
TGCAGTTCGGCAGTTGTTGGACTTTTGTTTCCTACAATGAACTCG
TCCATGGTGGCATGA
CTATCAGTTCCAATTACCCATGAGCTTTCCTTCGATATTGCCACA
AGAGGGAACTCATAT
TTTTTCCTCCCGTTTTGATACGATATAAACTTCGTCATTTGTGGAG
TGTATACAAACACA
TTATGTGACCATTCC
>ConsensusfromContig1562 Average coverage: 6.87
CGACGCTCTTCCGATCTGAGATAATGAATCTAGTAGATCTTGATG
GTAACCTTTATTGAC
ACAGTCTAAAATGAACCTGAAGATTGAAGTTCGCTCAGAGCAGG
TAGATGAAACTCTGCT
TCTTATCAGCCTGGAAATGTCCGGATTGAGCTTCCCTGTGAGAAA
TCTGTATTCCAGAAT
ATTGGACAATATTCTGATGTTTGGATGAGGAGAGTCCAGATTGTA
CCTTAAACAAAAGGA
GACTTACTGAATGAATCCTGTGAAATGACTTCTTTGAAACAGTAA
GCTTATTAAAATAGG
TCGGATATTAATTCGCAAATATGTCACAATTGAGAACCTTTGTTC
GAAAACGGGGATCT
>ConsensusfromContig1563 Average coverage: 6.17
TCTCTCTCCTCGGCCCTCCCTCCGTCTTTGACTCTCCCGACGCGT
CGATGGGGGCGGGGG
TCGCACGGCGTATTGCTGCTGACCCTCTTTGATACTTCGCCTTCG
GGGTTCAGCTTCCGG
TGTTTCGTTGGATCCATATTCTGCTCTTGGTCTTTCTCTTGTTTCA
CCCCGATGTGAAAC
TTCCGGAAGCTTCCTTCGACTCTCATCAATGTTATCTCCTCTGTAA
ACATCTTCTTCTCT
CTGACTTCCAATTTGCC
>ConsensusfromContig1564 Average coverage: 8.11
GTGAGCCTTTGCTACATTTCTTTTGGTTTGTCAAAAAGCGACCAG
AAGTTCTCGTTACAA
CTTATTACAACTACAACAGCAATGTGTTGATAGTATCAGTGATGG
CAGCAAGAAATCTCC
CTGCTGTTGACATAGGTGGCACAAGTGATCCATACTGCATGGTTA
CTCTTCTACCCTACA

AAAAAACAGCTCACTTCACCAAAATTCAGTTTTCAACTGTCAATC
CAGTTTGGAATTCGT
CGTTTTACTTTAAAAATATATCTGTTAATAATATCAGAAAGGCAG
TTCTTCTTATACGTA
TCATTGACTACGACGAAATGGCCAGGGCAGATCGGAA
>ConsensusfromContig1565 Average coverage: 5.91
CAACTATTTGTTTATTTTTCACAGAAACTCTACTTACACAAACAG
AAATTGTCTGCCTAT
CAGCGATATGTTTCGCAACAAAAAAAAGGATCTCGGACATATTCG
AAATTTTTTAAAGAC
TGTTCTCAGGCCTGGAAGGGTCTATGCAAGGAGGAGAGGGTGGA
ATTCGAGGGGGGGAT
GAGAGAAAAGAGAGGCCAAG
>ConsensusfromContig1566 Average coverage: 11.62
AATGACTCCAAAAGAGGCGCTACGTCAAGGATTTGTTCCGCCAA
AACCGAGGCGTCTGAT
TGGTTCACTGCTGCGAGATACTGCATCAGGAGTTTGGCGCTTTCT
GTCTTTCCAGCTCCA
CTTTCTCCGCTGATGACGATGACCTGGTTTTTGGATCCCTCCGCT
AGAACCTTCAGAGCT
GCGTTGCCAAGGGCAAACAAATGTGGTG
>ConsensusfromContig1567 Average coverage: 4.62
ATCTATTCAATCAATTAATTGGCTCAATTAATTGCAACTTATTCTT
ACCAAATTGTGTTT
TTGCAGCCCCAAGTCGTATAGAATACAATAATTCACCCCTTCATG
TAATTTTATTTCTGA
TATTCGGCTCCGATTCGAAAAATCATATGATGCATATATATGATG
AATACATATAGTTTC
ATAAATTAGCAGGCTATCAATCGT
>ConsensusfromContig1568 Average coverage: 6.08
GAAATAAATACTGTGTGCTGACTGAAAAACTATTACGAGATCATT
TTTCCGCGCTGATCT
TCTAGACTAGGCTAGATGACCTCCTATGGATGAGAGTGTGCCACA
TGACAACCTCATCTC
GTTCCTGTGCTATTGATTTCTCCCAGTGCTCCAACTGAGTGCCTT
TGGCGAACATGAACG
GACCAAGGCATACCCGGCCTAGATTTCTAGATCGA
>ConsensusfromContig1569 Average coverage: 6.45
TCTTCCGATCTGGCGGCTCGTGGAATGGCGTCATCTTCTCTTGAA
AATGCCATCCTGGCT
GACCCGGAGATCCGTGGAGACTGATAAATCATGGCTTTTTGGGTT
CCAGGGACGGTCGTC

GTGCGGATTCTATTATCGACAGTTGCGATTGCGATTATGGCGACC
CAGATGCCGTTGATC
ATGGCGACCTCATTATCGCGATGCGTGAACTTGTCCCAGAGGACT
TTATATTTCAGCTCA
TCGTTTTTAGTTTCGTTTTGATGCGTTTTCTGTTTTACTCA
>ConsensusfromContig1570 Average coverage: 4.84
GTAAAGTGGGATTTATGGAAATGTCATATCCATCATATGTATTTC
GATAACATTGGTTTT
TAGAGCCAACATGATGGGGTGAAAAATTTCTCTTGTAGTCTTCTT
GTTGCATTGTCATAA
GCGCAGAGCTTGCAATATTATTACTAGATCCAATATTATACATGT
AAGAGGATTGCGCAT
ATGAATGAATTGTATACGGATTTTTCTGGTATTACTCTC
>ConsensusfromContig1571 Average coverage: 4.56
CTGTTTAGAACGTTGACTTCAACGAAAAATGAGTTAACAAAACA
TAAATAAATCTAGAAC
TGAAACAAAATTGAATTCATGTCCAGCCATGAGCGTACCACTGAC
ATCTATTAGTAGTAC
TTTTCATTTAATAAAGGTGGCGGCACAATAAGCGATATTTGCATA
TGTAAACAAACAATT
CTCATTTCGAAACTGGAGATATTGAACACAGTATGGCG
>ConsensusfromContig1572 Average coverage: 6.97
CTGATTCGAAGGCGTCGATCGGCACGACTGTCAAATCGCTGTGA
CGGAAAGCAAGACCGA
TGACGTCATAAGAGACCATCGGGAATATCATATGCTACGCCGTA
TGGAATAAAGACACTG
AAGATATAATTATACAGAACATATGAAAAGTGTGACAAGTCAAT
CGCATCAAGACGACTG
ATTCGAAGGCGTCGATCGGCACGACTGTCAAATCGCTGTGACGG
AAACCAAGACCGATGA
CGTCATAAGAGACCATCGGGAATATCATATGCTACGC
>ConsensusfromContig1573 Average coverage: 10.43
TTTCAGGATTCATATCTATTCAACATGATTTTTCATTAACCACTGA
ACTTGAATATTTGG
GCTTCTGAAATTAGCGATCTTATTGTAATATTATCTTTTATGTAAA
GTTCGAGTTCCCAT
GTTTATTTAATTTCACAGGGGGACATCGGTAATCGCGACTCGGAA
GGCATTATTCCGAGA
ATAATTCATGACCTTTTCACTGATAATAACTCGCGAAAACACATC
GAGGTTAATATTAAA
CTCTGTCATTCAATAT
>ConsensusfromContig1574 Average coverage: 5.26

CGACAGAATAAGATTTTTATGTAACAACTATTTTGGTGGTGAATG
ATCACGGTGAGGCAG
TGCCTGCTGCCTTCAAATCACAAAGATTCACAAAATTTTTAATGT
TCATGGAGGCTCTGC
GTGATAGATGCGGCCCGATTCATAATTCAATTCTCATGAAACTCT
TATAATGCTTGGAAT
AAGACATTCCCTTAGCCTTGAACAAAATT
>ConsensusfromContig1575 Average coverage: 3.94
TGGCTTTACAGGATTGCACTGCAATGAAGTAGATGGATGCCATAA
TTCGCCATGCTCGAA
TGAAGGCGTCTGCTCGTCTTCTGGGGGTGTGTTCACATGTGACTG
TCGTGGGACTGGTTT
TGAGGGGCAGACGTGCACTGATGATGTGAATGAATGCGATAGCG
ATCCATGCCTCAACGG
AGGAAGTTGTGTCAACAAACAAAATTCGTATTCGTGTAATTGTAA
AAAG
>ConsensusfromContig1576 Average coverage: 6.63
ATGGGTTGAAATGCTTATGCGAAGTCTTAAAGATATAAAAGTAT
AGACAGGCGGACAAAC
GGAACAGCCAGAAAGCAGACACATAAGACTAAGATCTTCCTTATT
GATTTTCAAACAGTT
GGATATTAGTACCATTCTATATATCTCATCTGATGCAGGGGCTGC
CAACCATCATACCTG
TATCCTGCAAGTTACTGATGAGTCGTCGACGAACCCTTATCCCCG
TTGAGAAACCCGATG
TCACATCCCGA
>ConsensusfromContig1577 Average coverage: 4.48
CTGTAGAACCTCCGAAACTACTAAATAGCCGGAACATCACCAAA
TTGAAAGCTGACATCG
GAACAAATAGGCAAGAATATAACAACAAGCAGCGAAATTTGATG
CTGTCCCATTACTGCA
ACAGGCTGAACGATTTAATACATACCATCCGTAAATCCACTTTCA
CCGCAAAAATGACCA
CATTAGAACANAAAAACTTATGACCC
>ConsensusfromContig1578 Average coverage: 4.90
CGGGCGAATACCTCCCATCGTACAAAAGGGGTCGGACTCAGTTAG
TCACTGTCTTTCTTT
CACTCCGCTCTGGTCCATCAACTTCACTCCGGCACACCGCTGTCTC
CTTCGATCTTGTTT
GATTCAACCGCAGTCTCCTCCGATCCTTCTGGCTCCACTGCAGTCT
TCTCCGGTATATAT
CTTCGGCGCACCGCCAGATCGGAAG

>ConsensusfromContig1579 Average coverage: 6.02
CTTTTCTGACTAAATTATAAACTATATAGTAAATAATAGTCGGTC
AGAGGCATCTGGAGA
CATTTCAGCATCCACAAATTGCCATTTTGAATGTTTAGGTCGCGA
CCATTATTAGAGATG
AGCTACCCGAATCTGCAACGAATATCAAGATAGGCTCAACATTTC
CACACTTGTTGGCCT
ATTAAAAATGTAAAGGACAATTCCAAACTTATGG
>ConsensusfromContig1580 Average coverage: 7.36
TGCTCTTCCGATCTTGTATACAATAAAAAAACATTAAAAAATCA
GAAGGCACCACTGGAA
TAACTGTTTTGTCTGCAGTCACATATCTTTTTTTGGTTTTGGAAG
ATTTCAATGACTTT
CCCATTCTCTTTCTGTCTCTCAGCTGATTTTGGCTCTTTAGCGTAG
AGCCGGGCTCGAGT
CCCATTTTGGCCATCAGAACACTT
>ConsensusfromContig1581 Average coverage: 4.26
GGTGATTGAATTTGTTTGATCTTTTTTAATGATCAGTGAATTCTTT
GTCGCTTGGGAGTA
TGATGGTCGCTGTGGGGGAGAAAATGTTGATTCTCGCTCAAGTTG
TGCTATTGGTTGGTT
GCGAAGAAGGTCAATGTGTTTTGAGTTTTCACTCTGATTTTTAAT
GAGGGTAGCAATTGA
GGTTTTCATAATAGCTATCTCATTATTAGATGCTGACGGATTTGA
TTTGACAGAAAAAAG
>ConsensusfromContig1582 Average coverage: 5.22
TCTTCCGATCTGGAACATGCACACCACACTGCATTCACAACTAT
TGGGAGAGGGGGGGGC
ATAAAGGTAAAATAAATAAATCTAACATGTTTGAAAACAATCAAC
CTTCAGTTTTACATG
CGTTCTTACTTTTGCGCCAACTCGTGCATTTTATTCACTGAGAAAA
TTCGTTTGTTTCCT
CATTTTTCTGGCCATTATTA
>ConsensusfromContig1583 Average coverage: 6.53
GGGTTGATTGCTTTTTCGATCTTTGTGTCCTGCCGGATGCGAATT
CGGGGGGTTTCACCA
CCAGTTATTCTCATGAATATTCTCCCATTTCCATCTGCGTCCTCAA
TCGCCAAACTACAA
CCGAGGGTGTTACCGCAATCCTTGAATGTGGTATTGTCGATATTC
TCGATTCGGGCAGAG
TGCCAGACGTTGTCGTTTTCATCATTTTCTTCTCTTCCTGC
>ConsensusfromContig1584 Average coverage: 3.17

TTATACTTAGCTGTCAAAGAGGGACAATCTGATGTTGTGAAAATT
TTGTTGAAAAATAAT
TGTAATATCCGTGTCAATCCTAAAGACGATACGGAAATAAATATT
CTTCATGTTGCGGTA
TTATCAAGCAACCTAAACACTGTGTCGTCATTGGTAAATGCGGGT
TGTGGTGTGAATGAG
TTTACAATATCAGAGGAAAACGGTAAAATCAC
>ConsensusfromContig1585 Average coverage: 7.17
GCAACATGGACACAGCCGTGTTCCTCATTCGATATTACCCTGGTG
GTTTGTGGATCTCGA
AAGTGACGCAATCATATATTCGGTGACAATCTACAATCGGAATGA
TGATGGTGGCTGCTG
CCGATCAAGACTAAGAGACTTTTCCATATCTCTGACTGACACCGA
TCCGCGTGGTCCGGC
GACGGGTAGGAAAGCTGACCT
>ConsensusfromContig1586 Average coverage: 5.91
GCATTTGTTTTAAATGAGTTGTTGGTACATCTACGTTCTCGAAATA
CTATTGATATTGAT
CGGCACGTTTTCGTGAAATTCCGGTTTATGGCAATTTTGGGCATT
CAAGTAACTTTCAAG
CACTCTTTTGCGTGTATTATGTTTTAATTTCATATAAAATTTATA
CGGCGGTGGGGGGG
AGGAGGGTGGCCGCCTTATCATAATGA
>ConsensusfromContig1587 Average coverage: 5.86
ATTTGAAGCAATGAAAATTGGATATCAGTGTTTTATTGCAATTAT
TTCAGCAATATCCAG
TGCCAATGTTAATCTCGCTGAAGATTCATTTTTCGACGGCTCGTT
TACAGATCCTTCGAA
CGTTGCCGTCACATCAATACAGTTTGAACCTGAAATCCACGACCA
AAAAGAATGTTACAC
CAGATGTGTCCAGACTACTGATTGTACCTCTGCGCGTTTTGTGAG
GGCTGGCAGGACGCA
AGACGCACTCTGTTGGCTTCTAATACGTAGAGCGACCGAAGAAAA
CGCTTCCGGCGATTC
TACACCTG
>ConsensusfromContig1588 Average coverage: 5.76
AAAACAGCTATTACAAATATACAAACAACCAAATTTAAAAAAAT
TCTAGACGACAATTTT
TACCCTGGAATATTTGGAGAAATTGATTACATGTTTGGCATACAG
GCTGATTCTAACGAA
AAAACTCTTCCTGAATACAAGCAGATATTAAAAAAATTGAACGA
ACGTGAGGTTTATGAT

GTAACAACCCTCAAAAGATTGCAAGAAGCAGCACCAA
>ConsensusfromContig1589 Average coverage: 5.00
AAAATTCTTCAATATAAAATTTCGAACAATCTTATTTTCAGCCTC
ACATACATGGTTGCT
TGATTACTCCGATGGCGCAATCAACTTTATTGAAATCAGAGCCTG
ATGAGCCAGACACTC
CTTTCGAGTATCTATTCGAACAAAGCTCAAGAGACTTCGGAAAT
CAGACGATAAAACAAG
AATCATCAAAATTAGAACACATTTCAG
>ConsensusfromContig1590 Average coverage: 2.20
TCCGTGATACATAATTGGTGTCAACATTTTCAAGCTGAACAGGAG
TATAATTTCAAGCTG
GTCCTCATCGAGTCTCGACTGACCAACAATAAGCTCAAAGAGTTT
GACGTCATACTGAAC
ATTATCCCGACCGACAACTCGCCAAGGAGTGTTTATAATATCATC
GAGTTCACGAAGAAA
GATCAATTCGATATGAAAATCCTATCT
>ConsensusfromContig1591 Average coverage: 6.53
GCAAACCGATATTAAATGAGGAAATCTAAAATATGTTCAAAAATA
GACTTCATTGTAATC
AGACACTGGTTTATTCGTGTGGTTGTGATAATGAATGCATTCTTG
GTTAGGCATGTGGAA
CGTTGCTGAAATTGTGTTTAACTCTGACCCCAAAACTCTGTACTG
TTAACTGACCAACAT
AATGAAAAAAATAAAAACTGGTAAATTGAAACGGCTTCAAAATAG
CATATTTTTTTCTGT
GTTTATAATATATGTGTAATTCGA
>ConsensusfromContig1592 Average coverage: 16.82
GTTTTATTATCATTCTTTAGATCTGAACAATTTGTTTAATTTTTCT
AGATGAAAGTGTAC
TGTTTTGAATTTTACAACATGTTCTGTTGATCAAATAAATCAAAA
TAATTCCGCGATAGT
GAAGTTTTTATACAGTCGACAGAGGCTGCTTTTTTAGTACTTCTCA
ATCCCTTGTATCTG
TTGGAAGCACTGAATTTAATCATATTGTTTGAAAGAAGTACGCAT
TAAAG
>ConsensusfromContig1593 Average coverage: 4.53
CAAAAAAAAAACGAGTTGAACAAATGACTGCAACATGAAATATC
AAAGTCTCGAATTTAT
CTGTTAAATATATTGCTATTGATATAACTCATACTTTAGAAAATG
ATGCCCCCATGATAT
ATAAAACCCCGGTGGATATGATTAACAGAAAATTTCAGAAAAGT

ATAGAAGCTTCAATAT
GAAATTATTGACAAATATCTGGCATCCAATT
>ConsensusfromContig1594 Average coverage: 3.14
TTATAATGAACACATGTCTGAGATTAAATATCATATGGAAAAATC
ATCATACAATTCATA
TATTATGTCAATTTCCCCGATTTTTTATGGCGTTCCTTTCTCAGAC
GGCATGTGTGTCAT
CGACGTGGAGCTCGCTTGCTGTTCCGATAGCAGATCCGTCACCAC
CCCAGCTGTCGCATT
TTCTCCCAACATACGAGCAAATCTCG
>ConsensusfromContig1595 Average coverage: 8.44
CTGCTTTGTTTTTCTGAATGCAGTCAACTGCCGTTTTACTCTTCTT
AGCAAGCCTAGAGC
TGAATCCGAAGACTTTATTCGTGAAACCAGTACCCAGAATATAGT
CGTAGTTGACCCCTA
AATTGTATGTTTTCACGTTTTCGTCAGGAACTATCCGAAATGTGC
TGCAAGCATTAGACT
CTGGGTATATCAAAGATATGTCCAGATCCACCAGCGAGATCG
>ConsensusfromContig1596 Average coverage: 5.92
TGATGAAACGAAATTCCCAATTGATAGAGTGGAGTTGCGAGTTGA
CATTAGCATCCTGAG
TGTAACCATATGCGCGGATGAATGCATGAAAGATGTCGACTGCA
TGTCGGCAGAATATTA
TGTAGGACAATGCACACTGTATAGGACACCAGTGGACGGTGACA
CGGCTGGGGAATTTGT
TTTTTTGAATAAAAAAGCGTGGTG
>ConsensusfromContig1597 Average coverage: 20.02
TTCCCTACACGACGCTCTTCCGATCTGTGACGCCAAATACAAAAT
GGAGGTCATTTAATG
TTTTTTTTCGCTCAAGACGATTGTGAATACAAATAAAGCTATGCT
ATATTTATGAATCTA
ACATTTTTATTAATATATGTATGAAACGTTAGGTTTATAACTACAT
ACTTATTTCTTCAA
ATATAATAACAACCGAAGGA
>ConsensusfromContig1598 Average coverage: 6.52
ATCTGCGGCTTTATGAATGAGAGTACATCGTCATTATCACAATAA
CCAGTAGTTTTCACA
TATTTTTATCCTTAACTGCTTTATACTTGTCCACACGCGAAATTGA
CTCCCGTTAAGACA
TATAGAGAATGCGAGAAATAGGAAATTTGGGAATAAATCTCTAA
ATTCTAACGACAATGT
CGGAACGGTCCAGATACCTCATATTTGGGAAAAACAAGAAGGTA

CATGAAATTATGATAT
GATGAACATCCTAGAGATCGGAAGAG
>ConsensusfromContig1599 Average coverage: 4.14
CGACGAAGAGATCAATGAATATTCAAATAATATTGAGAATTGTGT
TCCATATTCTAGTTC
TGCATCTAAAATTCGATTCGTAGGCTCATGTAGAGATGGTAGTAT
TTGTGAAAGAAAATC
CATGCTAATCGATCGGTTATATCCCGATTTGAATATACCAGTTGT
TGACAAAGAAACAGA
CAATATTTACATAAATAAACAC
>ConsensusfromContig1600 Average coverage: 20.91
GTTCAGACGTGTGCTCTTCCGATCTCCAAACCTCATGTCCTATCA
AGTCAAAAGAATATC
GTTTATTTGCTTGAACTTGCTTTTAGATTTCATTGAAAGACTAGCA
TGTCCATCTTGCAA
TTCATGTGGAACCTTGAAGTCTACAGAATCTTTTTGTGGATTTGCT
TCCAATTTTAGTTC
TCAATTTGTGAAAATTTGGACCATATAGAGTCTCCAAACATCACA
AAAACTTGAAGATGC
TTCATTTGAAAGAT
>ConsensusfromContig1601 Average coverage: 6.20
TTTGTTTTTAAATTTCTTTGTCTATTCCGTTAAAAATATATGTAAA
ATAATATTTTCGGT
AAAGTCCTTGGCGTGAAACTTCATATTCTCATGAGATACCCTCCT
CACTCATAATTCAAT
TGTCCTCTATTTTTTTTTATTTACATATAACTGCAATATATTTGGG
TCATCTATGCAAGT
TGTATTCTGCAATTCCTTTGTTATTATTTATCCCG
>ConsensusfromContig1602 Average coverage: 5.73
GCGACTTCAAGAACTCTGGCATCGGCGAGGACGAGCCATACGAA
GACGCCTGGGAAGAGC
CGAAAAAGACGAGCATTGGGATGTGGGTGCCTGGTAAAGATCAC
TTCAAGGGCCAGATCG
AAAGGCTTTGCATTGCCGAGGACTTGGATCTATGCTAGATGATTA
TGATTTGTTGATATT
TCACTGTATGATTTCCATAAAAAAATATTTGCATACCAG
>ConsensusfromContig1603 Average coverage: 8.59
ATTTTGTATTATTTGTCAAAATTTCAAATTTCTTAGCTATGAATT
CACTGCGAAAATGAG
AAATCGCCAAACACATTTAATAATGGTATTACGATAGTTTATGTT
GTGAAGATGTATTCC
CAGGCTCTTGGCAATAATGGGTTTAATTTATTCAGACTTGCATTG

AATTTTTGTTGCCAA
ATGATTTATACATTATTTTTACGCAATTATACATGACATACATTTG
CTGTATATATGTGT
CAGAAC
>ConsensusfromContig1604 Average coverage: 3.71
TAACTTTATCAGAGTTTCACGAAATTTTTTTGAGGACAGTCATAC
CTTGATATAATTAGT
CCCAAAACCACACTAGCATATAAGTGAACATCCATCAACAGTGA
ATATGAAAAACATAAT
GTTAGATTTTCACCTCCTTCACCTGTATAGATTGTTATTAGTACAA
TTTCTTTTTGTTTG
GAATGAATATTTTTGTGCCGTTCATCGAAGCGATGGA
>ConsensusfromContig1605 Average coverage: 4.65
CACGCATTAAACGACTGTTATTATTCCACCTCATGGATGAATGAA
GGGTTTCTGATTGCG
TCATTATAGTACTCTGAGGAATTAGCACACAAATACTTTTATCAG
TCGTCCATGCCTACA
TCTACATTTTCAGATTGTTTCAGGATAGGTCTATCTTATCGCACCA
AGCTCAGTTTTTGT
CATTTGGGTGGGGATCTCGATACCGTCATGAG
>ConsensusfromContig1606 Average coverage: 3.94
AACAAGGTAACCAAGATAATATGACTCAATACAGTTTTTTTTCTC
ATGATTCTGCTTAGA
AAAAAAGCTGTTGTATGTATATGGCAAAATATTCTGGATATGATT
GACCTCAATTTCCAA
TGAAATGGAAGAATGTCTCCAGAAACATACCTGAAAGTCGCGAT
CAAAATTCAGGTAGAC
AATGAAATATTTGAAATTCGACGCCGATTAAA
>ConsensusfromContig1607 Average coverage: 8.78
AACCCTATACCGCTATGTGAAAAAAATACAATCGAATATCCAAC
CAAGATGTGGGTATAA
CCCCCACTCACGTGTATTCTCAGATGATCAGGAAAAAAGTCTGGC
AGATTACATAAAACG
AGCCTGCGATATATATTTCGGTCTTTCGCCAAGAGAAGTTAGGAA
ATTGGCATACACTTG
TGCAATAAAATATTCTCTACAATTTCCA
>ConsensusfromContig1608 Average coverage: 2.95
TGTTGGGCCGTTTCAAAGGCCGAGTTTCCTTTTCCAAATATGAGT
ACGACTTTCCCTTCG
TAGTCGGTTTGATTTATTGACATTTCATCGTAGTACGTGATGTGCT
CATTACCATCCATT
GCCAGCTTGTTTTGGGCAGCCAGGCCCGTCGCCAGAATCAGCGTT

CGAGCTCCATATTCA
TTGCCCCCTTGATCCGTCAAAATGAC
>ConsensusfromContig1609 Average coverage: 6.93
CGCGCTTCTGATTCGTAATTCGAATGCAATGTTGTTCGAGAACTA
AAATTGAATTCTGTT
TTAATAACCCGAATACTGCGAAATCCACCAAATGAATTCAATTTG
ACATTCATTCTTGTT
CCCTATTAAATGCAAATGAGGGCATTCATAGTGTCCAATTTTGTT
TATGTTGAAGATATA
ATTAACATTGAAGAAACCCGTCATGATCTTATCCCCCATGCAGTA
CAA
>ConsensusfromContig1610 Average coverage: 5.37
AAAATTGCTTTCATGGGTATCAAGAAAATTGAGCGCGTTCGCCAT
TACGTCCTCGTCGAA
TACACACATATGAAAAATACCAATTTAAATGTTTTCAAACCACTC
ATGGACAAAAATCTG
CACATAAGATTCAAAGAATGCAATATGACGGATATTGATCCCACG
CCTTTTGAGAATTAT
GAGGAGACCTGCGTGCTGCACTTCGAAGACAATCCTTGGTTTTGC
AGCTG
>ConsensusfromContig1611 Average coverage: 6.96
GAATAAACATGTATATATTCGAGATTCACTGACATTTGCTGCATT
ATTATCATCTTATAA
GATTTTATTCCATTATTTATATGATATGGAGAGATGCAAACTCAA
TTTGGGTAAATAGAA
TGTGCTTCAAAATTGTAATTACTTTTGTTTCCATGTTATATCATGT
GTGCTTACTATGAA
CAATGATTAGTATGAAAATTTCTTCTC
>ConsensusfromContig1612 Average coverage: 6.63
CTGGAAGTCTCTGCAATTGTCGTGCCACGTTCTCGACTCTTGTTC
GATGTGGTCGGTATA
GGGCATTCAAATACGACCTGTATTCCCGGAAAAACGAGAGCTTT
GGAAGATGATAAAACA
GATTAAGGGTCTGCGCAATGTAGCATCATAGACAACAAGTTCTT
ACAATGAATAATCGGG
CAGTAAATGTATACAGCTCGACTCTTCAGTTTCTGCAGACGAGGT
ACGACTTCATAGATC
GGAAGAGCACAC
>ConsensusfromContig1613 Average coverage: 10.81
TTTCCCTACACGACGCTCTTCCGATCTCACTACGGCAACACACGT
ATTATCAACACTGCA
AGTTTCGAGGCAGTTGTCCGGGCCTCTTATCGTAAGTTGTGCTAC

GTCCAGTGGGGTCTC
ATCGAAAACAATGAACCTGTGCGTGGGATTGCCATCCGTTAGATT
CAACAGCAAGCATTC
ATCTCCAATAAAAGTCAGTCTGAACCAAACTGCATCAAAGTCGTC
C
>ConsensusfromContig1614 Average coverage: 6.39
CGTATTCATATATCATTGAACTGGTTGATTGAAGAATATCAATTG
ACAGTCAGAAAAATA
TATCAAGAATCCATATGATATATTATTAATTATTCAGAACATATT
TAGCAATGCTGAGTA
TAGTTTATGAAAATTATTTGACCCCAAACCCACATAGAACTTAAT
ATATAATATACCTTG
AATAGTCATATTTCATAATTGTTACAAATGAATAATTC
>ConsensusfromContig1615 Average coverage: 7.17
CGGTCATCATGTTTTTGAGTTCGCTTTCTCGACGAAGATTTCCCTC
AATCTTTAAGAATT
TTTCTTTCAATTCAGACTCAACACCTTCTATATCTTTGAGCTGTTT
ATCCCAAAATTCGT
TTCTCGCACCATCACCGGTAACGGTTGACTCTTGCCTTTCTCCTGA
CTTCTTGTCCTTTC
CTCCCTTGCCACTGAGAAAATTCATGTTGAACTAGAAAGAAATA
TATGAGACAAATCGCG
ATGGGGACTAATACCAGGCCTTTCAAAAACCTAGTGAATAAAC
>ConsensusfromContig1616 Average coverage: 5.74
CAATGAGTTCACCTGTTCCTGCACACCTGGTCATACCGGCGGATT
ATGTCAAACAGATGT
CGATGATTGCGCGCCTGGGCTCTGTGAGAACGGCGGGATATGTAC
GGATGGTTTAGATAC
CTATATTTGTGTCTGTGCATCTGGTTTCACAGGACCACACTGCCG
GCTGAGTATTGATGA
TTGTGTGGATTCAATGTGTCTCAATGGCG
>ConsensusfromContig1617 Average coverage: 3.54
GGTTAATCCTGCCATGTTCAAGGTGGTGGTGGACTCGCCTCA
GGCATTCGAGAACTC
ATCCCAGGGAATCCAGAGATCTGGCTGACCCGTCCCAAAAACAG
TATTGTGCCACATCGA
GTACAGTTTGAAGATTCAGAACGACCAAGAAGTTATTTGGTGCCG
CCTATAGTGTCGAGT
GCTCTACTTCGAAGCCTGCTCAAAC
>ConsensusfromContig1618 Average coverage: 4.33
CGGTAGATTAATTATGATAAACAAAAAATATTCCAGTTTATTTAC
CATTTAATTCATTGT

AAGTTTTTCATTCGGGTTATTTATGAATTTTTCCCCATCCTTTATG
TTACTGTGTCACTA
CTTGTGATACCCTACACTTTATGGCGCATGCTCAATTGACCCATT
GTTTTATAACAATCG
ATTCTGATGTGGTAGGATGACACTATTAGGATCCACATGAAGGG
GACAAAAT
>ConsensusfromContig1619 Average coverage: 6.29
CTCAAAATATACGATTGTTCTGTTCAGATATATTTTATGCATTACA
AATTATATTTTCTA
TGTCTGACATTTTATTTCACAGATTTATCTTTTTCACGACGCCGTT
TCATAGTCGTTTTC
TTATATTTATTATGTTTGACATTGGTCTACTTATTATTCAACACAT
AGACCGCTTAATGA
GGGGAATAAATAAGATTCACTTCTATTTTCAGGCTTTTGTATCCG
>ConsensusfromContig1620 Average coverage: 3.80
ATAAAAAGTCATTACATGAATACATGTGAGATCTCAATTTAGCGA
GTTTGACAGAATCCT
TGATAGAATGTTTTTCTGCACGTTTCTGAGTTTTTTCATAATGCTC
TATCTGTTTTTCTG
CGATAATTATAGCTCTTTCGCAGTTCTTACGAGTGACGTTTTGTTT
GAGTAATTCCTCTT
CAGTCATCGCAAGATATAATAACAGTCCTG
>ConsensusfromContig1621 Average coverage: 6.14
CGATCTGGAACAGTGAATCTTTCTTGTCCAATTAGAGCTGAGTCC
TTCAATATGGTCTTC
ATGCACATGAGTTAAGAAGAATAATGTACAGTTTCTAATTTCATT
GGATCTCCATTTATC
AATAGCAATTGTCGTGTTTGGTATGATGAATCCAGGCATACTATA
TATAGGTTCAGCTAT
GTTTTTTGCAGTTTTCAATTTGAAAG
>ConsensusfromContig1622 Average coverage: 5.82
CTAAAATTTCAGTATGAATTAAAATATGTTTAAAACGGGATAGTT
TCAGAAACAAAAAAT
TTTAGCGAAAATTTGAAACTGGTACAGAAAAAAGAGTTGAAGAA
GAAAGTCGATTATTTG
GACTTTCACCCTCGGTCAAAACCAGAACAGCTAACCAAATCCTTG
CAATAAACAATGTCA
GTTTATAAGATTTACGCCTACATTCCCGTTTT
>ConsensusfromContig1623 Average coverage: 4.73
ACACGACGCTCTTCCGATCTCTGAGATTCTCTTGAATGGCGCTAT
ATTACCGGCATGTCC
ACATACATGCGCTCCGAGACTCACACCGGTACAGTACATGTCTGA

AGCGCTTTGACCCGT
GATATTTCTCATTTTTTCGATGAGGCCAGCAATCTCGGCGCCGAT
GGATCTGGTTGTGGC
AGCTGCAATCGGATATTTCTGATCTGCTCCAACTGACCAG
>ConsensusfromContig1624 Average coverage: 4.82
TCAAGACAGCACCTTGGGGGTGCCGGCAAGGAGGGAGAGGGGA
GAGGAGAGAGAGAGGAG
TTGACTCTGAAGGAGCAGGTGCGGTTAGACAGGAAGGAAGAGAT
CAGTCGGATAAAGGGG
ATTGGAAGAGGGGAGCGAAGAAGAGAGAAGCGGAGGCCATCGT
GCCAGACCGTATCGAAG
GCTTTAGTCAGGTCAAGGAAAACAGCAGTGGTGCTTAATCCTTGC
CTGACTTTCATTTGC
CT
>ConsensusfromContig1625 Average coverage: 4.34
ACAAAATCGAAAGCACGTTCAGGTTTAAATAACATGCAGAGAAT
CATATTTAGTGAATAT
ATAGCTTATTCAAAACAATCCCTCACTTGATACTAGAATGTGTTT
ATGAAGCACACGTGA
AAAATAACTCGTCCGATATATGAACAGATTAGTCATATTCATAAA
AATGAATATCAGCAG
AACTGGATACTTCATAAACATTATTTC
>ConsensusfromContig1626 Average coverage: 5.79
TTGGTGGAGATACAGAAAAGCATGAGCCTGGCTGAAGGCAACGA
ATCGACAGGAGTAGGG
GGCGAGGGGATAATGGGAAACTCAAGTTGTGGATTCAGGAACAT
CTTCGAGAAGGCTGAT
CAGAACCAGGATGACTACCTTTCCCTTGAGGAGCTTCTAGGGGTT
GTAGGAATTCCTGAT
GTTCATTTACATGAATCTGAAAAACATC
>ConsensusfromContig1627 Average coverage: 4.75
ATCTAAGGTGTTGAATTATTGCCCGAAAGATATGCGAGCAGATAT
ATGTGTGCACTTGAA
CAGAAAAGTTTTCAACGAGCATCCAGCTTTTCGATTGGCAAGTGA
TGGCTGTTTGAGGGC
ACTCGCAATGCATTTCACAACCACACACAGTGCTCCAGGTGATTT
GATATTTCACCAAGG
AGAAAGCCTCGATTCACTCTGCTTTATTGTGTCCGGGTCCTTAGA
AGTCATGCAAGATGA
CGAGGTCGTAGCG
>ConsensusfromContig1628 Average coverage: 4.53
ATTGTTTGCTATTTTCACACCGTCACTTACGTTATTCTATATCGAT

TGGCAAATATCTCC
GAAAAAAGCTTTTTCGATTTTGTTCTCAACTACCAGAAAGCCAAA
ATCATCATCGTACTG
ATATTTTTAGGTTCCTGTGCCGCATGTGCTACCATAGTACTTTTTG
AGAACTGTTCTATA
CACATATGTTCCCGGCAGCAGATCGGAAGAGC
>ConsensusfromContig1629 Average coverage: 4.34
AGAAAAATCAAGGAAATGCTCATCCCGTTAGAGCATAGTTCAAG
GTTACCAGCACATATA
CCTAGCAGATCGTCGCCGTAGTCTATATAGGATCCGTTCGTAAAT
TTAGCGACATTGTTC
ACACCGCACATATCCAACGTGCTTTCTAGAGTTCCTGTTACCACT
CCTGTATAATTCCCG
TCCACTAGATTTCCACTTTTGA
>ConsensusfromContig1630 Average coverage: 8.76
CAAAAAAATTGTAAACAAAGGGTTCATCTGTAATGGCGACGTCT
GAAAATTCGAAAGAGC
GTAAGCGAAAGTCTTATGGAGCCTGTTGTGTAGCTCCAGGTTGCA
GTAAACGGACATCTG
ATGTTAAGTATATGTATAGGTTTCCATCGGAAGCTAAAAATTCA
GAAAGACGAGCGAAAT
GGGTTGTTGCGATGAAGAGAGAAGGATGGC
>ConsensusfromContig1631 Average coverage: 6.73
GCTCGCAATATTCTCATTTTGTTCAATTATTCATGAATGCTGTAA
TGACGTGGCACTGGA
TTCAATCATTAACACACACAAACTTGGCATTACATTGATAAGCA
CAAGAAGGCCCAGACG
TATTTCTGAAGAGAATAGTATATGCGGAAATGGTTTACGATTCAA
GGGTGACTCTTGGTT
GAATTTCGCGAATGAAAATGAAATACATAATTCATTATTTGATGG
AGA
>ConsensusfromContig1632 Average coverage: 5.83
CACGACGCTCTTCCGATCTATAACATGTCCCGCCATTTTGATATTT
GCCTTACTGAGTCG
GTTTGTTTCAACAAATGTCAAAATGGCGGGACATGTCATCTGACA
GCAGACGGATTTCGA
TGCGAATGCCATGAATTTTTCATGGGCGACACATGCGAAGCACAA
AACTTCTGCCCAAGC
GGGATATGTAAGAACGGCGGGAGGTGAAG
>ConsensusfromContig1633 Average coverage: 7.31
AAATCCATGTGACATATAGCCCACTGATGGAATAGCACCAATTGT
ACGGTGGATATATTA

TTAAAATAGTCACAAGAGGTGTCACAATAAATGCAGAAACATGA
ATCAGATAAACACATG
TACAACTAAGAATGTTTCGATAGTCCACGTTTATCCCTTTCAGAA
ACGCCATAGATGAAT
TTTTCATGTGGAATTCCTCTATCTAGAGATCGGAAGAGCACACG
>ConsensusfromContig1634 Average coverage: 4.07
AAAGAGTAGAAATCCCTTCAAAACATTTCTTCAGATGTGGTTGGA
CTCGCAGAGGGTCTT
TGGTCTCGGAGAGTATTTCAAGAAGTTCTTCGTTCGACAAGAAAA
AAAATCTTGAAAAGT
ACAGCCTTTTCGTTTCCAGGTATTCATTAAGACCTTTTTGTATTTC
TTCCAGCATTGCCT
CTGATTCTTTCAACTGCTTTAATAAGCCATTTTTCTTCATCATATC
AAG
>ConsensusfromContig1635 Average coverage: 5.97
GTTCATTTAAACATTTGTAGTTTAAAAAAATATGAAAAATTCACA
GAACTTCAACTACTA
ATCAATGATCTGAATTACCCAGATGTGATTGCTATCACTGAAACC
TGGCTTAGTGATGTT
GAAATACCATTTGTTGAAATTGATGGGTATAACACAATCACCAA
AAATAGACAAACTAGA
GGAGGTGGGATACTGATATATCACAAATTGAAATATGGATGCAA
GAGA
>ConsensusfromContig1636 Average coverage: 6.13
TTTGAATAAGTATGGCACAATTCATCGTCGAACAATACTATAACA
AACATATTTTTCACC
TGAACATCAATAATTTTCATTCAGTTCTGAATCACAAATGATGGC
AATGGAATATAGTTA
AATAAAGTTTCACATTAAGGCACATAAAAGAATGGATATCGTGG
GGCAAAACTCTAAAAT
ACCAGATATAGCGAGTAAAATTTAGAATCATACATGTTGAATAT
ATCAAAAATGACTAAG
GCACACATAAAAAGTTAAAATTGTAAATCAGGATCTTTC
>ConsensusfromContig1637 Average coverage: 8.03
CGATTACCTGATTTGTGATGGAAATGACGACTGCGGAGATAGTA
GTGATGAGTCACCAGA
TCACACTTGTGAATGTACGGAAGACCAATTCCTCTGCTCGGACGG
TGAATGTATGAACAA
AGCGATTGTTTGCCATGGGATGGTACAATCATGTACGATCGATCC
TGCAAAATACAAGGA
ATGCGGAGAATGTTTGGAGAATCATTTCAAATGCAATAATGGAA
AATGTATTGGCGA

>ConsensusfromContig1638 Average coverage: 3.96
CCTTAGTGCAGTCCATGTGTGAACTCATGAACATTTTCTCTGTACC
ATGGCCCCCTTCCT
TCACGTTCCAGTCCCCCTTTGTTTTCCAGTGCTTCGTGGAACCGTC
TTCTCCTGATGCGT
TCTTCAACAGATTCTGAGACCAACCAACAATTTTGTACTCTTTCA
AAGCCAGTGTTCTTA
TGGGAGGCACAACATCGTCAGTAAGAATTTGTATCATCTGATTAT
CT
>ConsensusfromContig1639 Average coverage: 5.01
GGCGAGATGAAGCGTGATGCTACTAGTTTTCTGGATCCTATCATC
GCTGGATGCGACATT
GAATGAAGGGAGTTGAATACTTTTCTGCGATATGCTTTTGGCAGC
CATGGTCTTGGTATT
CCTGTTGAAGTATCGCATATTATATAAGAATTATCAGGTGAAAAA
TATGATCTAAGTATC
AGGTTTCATGTGGTTTAATCGAATTCCAATAACTTCCCATCTGCG
ATTTGAGCCTGTGC
>ConsensusfromContig1640 Average coverage: 4.82
CAGAATTATGGCTTTCGCATATCATTGCAAGTAGGAAAACTACGT
TCGTATTTGAGGAGC
AGAAGTCAAGGCAGGGATGTGGAGAGTTTAATCCCAGTTCAGAA
CGGTATATGGACAGAA
TATATCTACGTTTACCAGCAACCGTTCGCGGTGAAGATATATATC
GATAAAAAGTTGATT
GTTACGTCCGAGGGCAAATATAAAGGTGTGAATAGAGGATTGAG
TGATAAATTATCCATT
AGCTG
>ConsensusfromContig1641 Average coverage: 5.10
GACACATCTCTCCTCACTCCGCAATTGCCATGTCATACAGGTGAG
TTATTTGAGATCATT
CATATGTATTCAGAACACACTCCAACAGCCATATCTCATGACACA
TCTCTCCTCACTCCG
CAATTGCCATGTCATACAGGTGAGTTATTTGACATCATTCGTATG
TATTCAGAACACACT
CCTACAACCATATCTCATGACACA
>ConsensusfromContig1642 Average coverage: 5.40
ATATTCATAGTGTTTGTAATACCTTGTATTATATTGAATCAAGGTA
TTCTTCAGCCTATG
ATATTTTGTTCTATTATATAACAGCGATGCTGTCATAATTCATACA
TTATTTGAATATCT
TCTCATTTGTGCTGAGAGATGTGTATTTATTAGAAAGCGGAATTA

CAATTTGTGAAATTA
AGATATTCAAGTAATTACACTGCAAAATGTCTGATTAATATTATT
CAGATTACTTATACC
ATGGTTA
>ConsensusfromContig1643 Average coverage: 3.93
ATGACAATATTTTTCTTGGGACAGCCCAGAGTATCGCATGTCACT
GGGTATTGATTTTTC
TCGGTGTACATAGTCCATTATGTATTATGACAACCTGGGGTGGGA
TCTACCAACAAGTTT
GATACCGAAGCTTCAAAAATAACAGAAATTAAAGGTTGAACCGT
AACACGAAACGATATG
AAGATGGCTCATGAACCAACAAGATCTGGGCCCAATCAGTGTGG
AGATTCGA
>ConsensusfromContig1644 Average coverage: 12.09
CCCGTTCTTGCGAATTATACCAATATTTGCCCTGGTCCTCTATCA
TAGCACTATGAAAAT
CTGGAGAACACACTGAAGTAAGATTCTAAGCCCGAACATGTGCTT
CATATTCGCGTCGAT
GAGCCTGTCCTTTGACGAGGAATGACCCCTACAAATGTCAAGGT
CGAAAATTAAAAAATT
TACAACATATTGTGGCAAATATTAAATAATTTCTCGAAATAAATA
CGTCAAACGTCTAAA
AATTGAATGATCTTTTAAAATTT
>ConsensusfromContig1645 Average coverage: 5.92
TTTCTCTTATCTGGCTCCTTGAACCACATGTTCGCCACATATAAAT
CCTTCTCATCCTAG
ACCTCTAGCAGCCTCCGACCTTCTAAGTTCCTTTCTTCGACCCTAT
TTACACAATACTCC
CCATCAAATTCATCACTCCTCTCCCCGATTTTAAAATTCATCATT
CCCAAAATCGGAAGA
GAGTGGTGTAGGGAAAGAGG
>ConsensusfromContig1646 Average coverage: 5.48
CACGAGAACACAGTTGTTGTATGAAAAAGAAAAATACACGTGCA
GTTATATTGGGTATCA
TATTTCTCATTGGGATTAAAATTAGATTTATTAGTATCAGTAGAC
AAATCAGAGTCGATT
ATTGTTATTAGTCCATTTGCATCATTGATGTGATAAACCTCTAAC
ATTTTGCGAAAATAA
AAATAATACCTATTATAACTATACATTTT
>ConsensusfromContig1647 Average coverage: 6.26
GGCATTGTGGCACACCCGCATAACGAGAACGCGTTCATAAACTGC
TCTTCTGGCAGAGCA

TACGTTTTCAATTGCCCAGCAGGTCTTCAATTTGATGCTAAAATT
GGAGCTTGCAACTGG
CCCGTCTTAATTAACTAAGAAAAACTTGAGGAAAAACTGATGGA
ACTTTGGAATATGCGA
CCTGCAACAATGATATTAATAGATTGATA
>ConsensusfromContig1648 Average coverage: 14.90
AACTTGAGTGTGTTGCTCACCATAAGTTATTATTTTTCTGTGCTG
CCATGTCCGAGAGT
GGTGTGAACACTCTCAGTAGTCGTTTCACTACTCTTCGTTCCAAT
ATCAGTTAAGGTGGA
ATAGGAATTTGTGATGTGAGTGGAGGCTGAAACGAAGTTTGTCGT
TTGCTCATCTTGATT
TGATGTCGATATGCTTTCTTCAGTTTGACGATCGGAAGAGCACAC
GTCTGAACTCCAGT
>ConsensusfromContig1649 Average coverage: 4.55
ACGAAAGTTAACGCCCGGAATCAGATTAAGATTGTCTATTTTATT
AACAGAGTTACTAAT
CAAGACAGATACCGCGATTTTGGTATCTAAACATGTATGTTGC
TAAGTACCCTACCAC
GTGACATGAGTAGGATCCATTCAGCAGCTTTTCGGTAATTGTGAG
TTCGGTCACATCTTT
GGACTTCGCTGAGACCATTGCAGCCAAAGCAATCAGGCCA
>ConsensusfromContig1650 Average coverage: 2.98
TGCTCTTCCGATCTAAAAAGGTCTTCCGGGATTTCTGCTTGAGCC
CAGTCTAGTCCCAGC
AAGACCTCAGCTTTTCGGACTCAAATGCCTGCCGCGGATGAGCAA
CAGTCAGCACTTTTT
CTCCGAACGCTGCAGGCAGTTTTTAGCACAAGTTGGCATCGGCTT
GGAATATTTACATCG
CAAAGGCCTTGTGTCCTTGGAGGTATCACT
>ConsensusfromContig1651 Average coverage: 5.15
GGCATCCGCAGAAGGGCAAAGATTTTACATATTATAAGAGATCA
AAATGACTACCACAAA
CAGCATTATAAAGGAGACGAGAACATACGGCATCCGCAGAAGGG
CAAAGACAAACATAGG
GATAGGCACGGAGGTGGGGAAGGAGATTCTGTACAATCCGTTAG
GCAAGGTTCAGGTACG
TTCTAAAAGGTAAGGGTGCTGGATTTAT
>ConsensusfromContig1652 Average coverage: 3.92
CGAAATATAGTCGTATTTAGGGCGGGGCTGGAATCTCCAGTCAAA
GGGGTTTTCTGCAAA
AAAACTAGCAATTTCCCATATGACCCAATGATAGCTGTTATTATC

ACTGGGAATGATCAA
AGTGAAATTGAGGCTGAACTGGGCTGTGAAATCTTTGATCTGAAA
TATCTCTGATGGGGG
CCGATCCCTCATAATAATAGTTCATTGATTTCACCACTTGTATAT
TTTATAATGAAATAA
CTAGCTTGAGTCCCAG
>ConsensusfromContig1653 Average coverage: 4.87
TTTTAGTACTTCGAACTATGTCATATTTCTCAATCACTATAAACA
AATGTTAAAAAAACA
GTAGATTGCTACGGTAGGTGCGATTGATTCTGGTAATGTAGAAGG
GTAGCCATCTCTACC
GAACAAAGGCTCCTTCGTGATATTCAAGCCTATCCTACTACCTAT
TCGTTCTCAACCAGG
GAAATTTCAGTATGCGTTGAAAGTCTTATTCAAAT
>ConsensusfromContig1654 Average coverage: 6.79
TTTTCTCTCATAAAACCTCAACTTGTGAAGTTTCAAGTTATAATCG
TGTATAGCCTTTCG
TTGCTGCTTCGGTGATTTATTTGGCGAGAAGAATGGGATGTTTTGT
TTATTTCTTTTTCG
GACTTATTCAATTCGGATTGGGATTTGGATCAGAAACACATGAGC
TGTGCCTGATAGACC
ATTGGAGACTAGATTGCGAAG
>ConsensusfromContig1655 Average coverage: 5.29
ACTAATCATAATTCCAGCACCAATTGATAGGAAACTGAGTGAAAA
AGAGTTTTTCAGCTG
GGATTCTTTCATTATTTCTATACTTGCCAGAGCAAGTGCTGTCGAC
GTTGCACCTTTAGC
GAATCCATACAATGAACTGGCAATTGCGAGTGGCCAAAATTGTG
AAAATAATGCCAGCAG
CGTCGAAGCCGCAGCAAATGTAGCGAATGATAAGA
>ConsensusfromContig1656 Average coverage: 5.91
CCACAACGAAAACGTTCCATAAAAAACCGCCTGCAACTAGAGCA
TATCCGCGATGTAGAG
AAACCATTGCATCAGAGCGAATCTCTTCTGAAGGCTTGATGGCGC
TACAACTGAATGGCA
GGATGTCGTCGGTTATTCCATAGAAAGCATTTGAGAGACGATAAA
ATTCGCTGACCTCAC
TCATGTAATGGAATATTTGAATGAAATGAGAAC
>ConsensusfromContig1657 Average coverage: 6.67
AGATAATTTAATTGTGAGAAATGTTCTCTAAACTTCGAGTTAAAC
TAAGGCCTGTCCTGT
AGCTCCTCATCCAGTTCTTCTAAATTCTTGAGAAGAACCTCCCCT

ATGCTCACTATTGCA
AAAACAATCTACGTATTGCAAAGACCCTGAAAAACGCAAATGGC
AGGAGAAGCTTTGAAC
GCTAAATGTAGATATGAATATATCAAGTATCTCGATATTCAAATC
ATCTTGGAATTTAAT
AATATATTTGAATAAACGCCAATCCATTCGATTTCGCTTCTTTAAT
T
>ConsensusfromContig1658 Average coverage: 4.87
CAATGAAAAAGTCTAGAATTAAAACACCCATTTCAAACCAGTTG
TAACGATCAATTGCAG
TCTTGTTATTTTTCCGTGGCAATCTAAGCATTCATCCCATAACTGA
TTCGATCTGTCCGA
TGTACTTGTGAGAATCCTTCGGGTCAGATGATCAATGCATGGTTC
AAGTTATGCAATGAA
TGATTCTTCTTATATTCATTGCATGAATATTCGGATAACCAAATC
AACCAAAAATTTCTT
ACAACAGATCAGAATATTCGAAATCAAATCGTTTATTCACAAACT
TCACTTCTCGCCCAA
G
>ConsensusfromContig1659 Average coverage: 4.52
CAAAAAACAAGTTTACTCATAGTTTTCATCGTATTGTTATTGCCTC
ATTATAATTCAATA
TTTGGGTCATCATTTTGATTTCATAATTTTCGCGGCTGGACCCTAT
TTTTATACATTCCT
CTTCCCACCTCTTTACTCAGAGTCTCTCTCTCTCACCCTTATTTAT
AAATTTTGCCCCAA
TGTTGCTGTCGTATATCCTTTTACTTTATGAATGAATAGCAACATT
AACATTTGC
>ConsensusfromContig1660 Average coverage: 6.70
CCCGTTACTATGTCCCAAACCGAGGGGCCAGCTGTCTCTCTGATT
GGCCACGACGTCTAG
AGGGAATGGCATCCTCCAGGGGCACTTGCCCTCGTTCTGAAACAG
GGTCCGGACGTCATG
GGCATCGAAGATATGAACGACATCCAAAAGGTCGTGACTCTCTTT
GTATATTGGTCCGTA
CTTTTCGTAGTTCTCGAATCGATCAAGTTCCACG
>ConsensusfromContig1661 Average coverage: 5.77
ATTGAATGATAATTTACATTCCCAGTCAGCACAAATTGCATATAT
TGATTGTAAATTCCC
TACAACTTTATCTAAATTTCTATCACAAGCATGAAAAACAATATC
ATCAGCATATGCATG
TAAATTGAAAAACCTCTATCATGAGCCGTTTTCAAAAGACTATTA

GTGCTATTTCCTAAT
ACAATTTGTGGATATACATAATCACTTAAATACGA
>ConsensusfromContig1662 Average coverage: 7.18
GATCTGATTAATTTAGCTGAATCGAAAAAAGATACATTAGGAATT
CGGCAGCCATTCCTC
TCGCATGAAAAATTTGTATATCGGACCAAACGATATTTTCCCAAA
ATGCTTGATTTGTCT
ATTATAGATGGATTAAACAGTAATGAAATTTCTTCCAAACTAGAA
GATGCCCTCACCATA
TTAATAAGTAAAGGCGAGTGTAAAC
>ConsensusfromContig1663 Average coverage: 3.98
CGCTCTTTTGACCTTTATTTTTTAAACACACCCTCTAGAGATGAT
AGACAAGCGATAAAA
GGTTGTAAAAACAATGAATGGGAGTGGCTTAAACGAGTAGGAGA
AGTGTGATAAAAACTA
GAATAAATAATTTTTTACAACTATTTATATGTTTCTATGTGAATA
AAGCGATGTGTTAAA
GATTACAAGATGATTTTCGTTTTCATATTA
>ConsensusfromContig1664 Average coverage: 5.75
TACAGTGTCGCCCTCTAAGATATGATCAGGTGTCCTGCGACAGCC
AACGATGCACTCTAT
TTTTACCTCTTCGAACCGATAATGAATGTCGATGAAAAGATGTAA
GCTGGAGACAGAATT
TCCGAAATCATTAGAAACCAGACAAGAGTAATTTCCGGTTGAATT
CATTGACGATCGAAT
GCCATAACGAGATATGAGATCATCATCTGCCTGTCTTTCTTGAGT
AATAGTGTGTATACA
TAGC
>ConsensusfromContig1665 Average coverage: 4.24
CAAACTTGAATTTAATATGATAAAAAGCAATACAAAAAAAGTCA
TGTTATGGTCCGCACC
CCGTTCATGCTCATCGGCGTTTGCAAGAGCTATGTGTAATAAATC
AAACAGCAAAGTTTA
TTTCGAGCCGTATTTTGACGCATATTGGCGTGGTCCGGAAAGATT
CAATAATGCATATTC
ACCAGATGGTTTGAATGACGTCAAGAT
>ConsensusfromContig1666 Average coverage: 6.08
CATTCACACAATCATTTGAATCTACGAGACTTTCACTCTCTGATTT
AATTGCCGCATTCA
ATTTCAGATCACTAAGTAACGATCTTACTTTGAATGGTGGATCGA
ATTGGTTTGATAAGG
TGGGATTGGACAGCCCGAGATTGGACACGCCTACTTGATCCTCCC

GTAATTGTGAGAGAT
CTGGAGTTGATCGGTTTAACTTCGGATTAGGCAGCCGTGAGATCC
TCAGAAATGCTGGAT
GACGTAATTCCATTTATTCTAGGCAGTGAATAGATCGGAAGAGCA
CACGTCT
>ConsensusfromContig1667 Average coverage: 4.77
ATAGCCTCGTCAAGCTTGATCTGAATGCTAGAAATCACAAATCCA
CATGATGCTAGTGAT
AGGGCATGTCCATGCTATATCAAACAAATTGAAAGATTGAAAAAT
CGAATTACTTATTGT
TAATGATGGCTATATCCGACGATTATGTATTCGATCCAAACTGTC
TCTATTACACTGATG
ATGATTTTTGAGATCGGAAGAG
>ConsensusfromContig1668 Average coverage: 4.48
TTTACATGGTTGAAATATATGAACTCAACTGTTATTTGCTACATA
GCCTATATTTTCAAA
TTCCTCCAGATACTAAGACGTACATATTTTTATATGAAGTATTCC
GATTATCTGAAATGT
TGGAAGTTCTCTGCTAGTTGTTTGATTATATATTTACTCACAGAAT
ATAAGAGTTTTACT
AGATTAGAAATATCACGTATT
>ConsensusfromContig1669 Average coverage: 4.87
CTCAAATGATAATTCCATAAGATCGATAAACAGAGCTGTAAATG
ATAATGAAACTCAAAC
CATCGATGGAATAAATGCCACTGAAACAACCATCAAAGGTAACA
ATCATACATACGAGGA
TGGGAACAAAGAAATCATAGTCGATATTCATTGAAGGTACAAAAT
TTTGAAACCGTTGTT
CGATGTTTCATCACTGAGATCGGA
>ConsensusfromContig1670 Average coverage: 5.81
TCTCCATGCAATTCGCTTTCATATCATAACTTACATCTTGCTCAGA
CATATTTGTTACAA
AATCGACCCCACTGTAGATTATAACGGTATCGGCCACACTGTTAT
ACTCAAAATCCCCCC
ATTTGATCTAATTTCCTAGAATTTGTGAGAGATTTTATCACATATA
CTTCTCCGTCATCT
AAAAATTTTTCGATGGTCTTTGAATCTCTTCTTGCTCGAATATGAT
CTTCTCTT
>ConsensusfromContig1671 Average coverage: 6.84
TCTTTTCCGTATAGCTGTCAATCTTTGTGAATTATAAATTCTTTTG
AGGTATGTTGGTCC
TCCCGCTATCTCTTCGTGATATTCCTCTATTAATTGATCTTCCATT

CCATTTGTTGTCTG
TTCCTGTGAATCTTCCGAAAATTTCTTCCAGTTTTCAATTGCACTC
GAAATATTGTTCAT
GTACGCATCACAACTATTCATATCAATCACGAATGACACA
>ConsensusfromContig1672 Average coverage: 5.80
TTTCAGTATTTGTTTTAGAGAGCTAATTGATTGGTTGTCTCTAGTT
TTAAATTCCTTTCG
TACTAATAAAACTCTTTTTTTCGTAGATAGAAACTGACCTGACTT
GCGTCGGTCTGAACT
CAGATCGCGTAGGGCTTTAAAGGTCGAACAGACCTACTTTTAGAG
GTTTTTGCTCCTCTA
AGGTGCCCCGGTCCAACATCGAGATCGGAAGA
>ConsensusfromContig1673 Average coverage: 4.17
GCGTAATAAATTCAAGAGTAACAGTACATATTCGATAAAGAGTG
CCGTAACACCCCCAGA
GGTCACATTGTATATTGATCATATGAATTAGTAACACCCCAAGAA
GTCATGGTCCATATT
CAGCAAATAGTGGGGTAAAAACTTTTGAAGTCACAATATCGATTC
GTTAGACTTATTCCG
ATGATTGAAAACCAAGATTTTCCTGTTGGCTGGTTGGTTCC
>ConsensusfromContig1674 Average coverage: 3.63
AAAGTCGCGAATATATATATATATCTATCTATTAATAATATATTT
AGCCATACATGTATA
TAAATATATACAAATAATTATAATGCCATGATGCCGCCAGAGAAC
ACGAGCCTCGTGTAT
TATTTACATAATAATATAATATTTACAAAAACACGAAAAATTCGA
CAAAGACCGGTTTTT
ACTGAAAGAAAAAGGACCTTTCTCGAAGCGCG
>ConsensusfromContig1675 Average coverage: 3.92
TCTCAAGAACGCAACGCGACAAGAAGTATCCAAGGGAACCCGAA
ATGTTCATAGAAACTG
GAGACATGAAAATCCCAGTTCAAAGATTCAGGAGCCAACATAAA
GTATACAACGAGACAA
AACTGAGTCTTTATCGGTCGACTGGTACCAAAGATTTCGCAGTGC
AGCAGCTCAAAATGC
CTGGTTCTAGCGATGGAGGCTCACCAATACCGTAAGAAACATGA
>ConsensusfromContig1676 Average coverage: 5.74
GGAAACTACCATGTCTGAAAAATGCTGGTGGTGTTCAACCAGGAT
TTATTCGCTATTTAA
AAAATAATTCATTGTTAAAAAAAGTCTTACTTTCTAGTCCGGTTCA
CTAAATTTTTATCT
TTTTAAACATTTTTATTACATATATTATACTATACTTTGATCCGTC

TTCCGCGTGATCAC
GAATCACACAGGGATTTGTTTGAAAGAAAAAAGTTCTTGAAAAA
TATTGAACAAGGTAAT
>ConsensusfromContig1677 Average coverage: 4.16
CATCAATCGGAAATGCATTCCAGGAATCAAAAGAAAGTGTCCCAT
CACTTGTTCGAATTC
AGAGCTTTCCAAAGCATGTGAAACCTCACCAATCATGGCAATCCA
GTGGCCATATCGTAA
CGCTAGATGCGCATGGTGCGATGGCAGGTCAACAGAAAAGCAGG
GGTGTGAGAAAAATAA
ATTACCATTACCAATTTATATAGGAC
>ConsensusfromContig1678 Average coverage: 4.65
GATCTGGCACATTTACATCGGCTCAAAAGGCTCCTGAATCATGTT
TGACTCTGGATCCAC
GTAATTTGCATCGTGAAGAGTTTGATCCTAAGAAAAACAATTCTT
CGACAATAAACTGCT
CTCAGCCATCTAGTAAATATCAAGACGTTCGCCATACATCCGCTG
AATCGCTATCGTCAT
ATAGTAAAGACAGTTCATGTACGCTCGATTGTGATTCTGCAG
>ConsensusfromContig1679 Average coverage: 3.49
TGTTAAGATGCTTCCGGATACTGTAAAAAGATTCCATATTTTCGC
GGATAACTGCACTTC
CCAGAATAAAAATCGATATCTGGTGGCCTTTCTTTATTTCCTCGC
GCAAACAAGATTCGA
TTCGGTGAAAATTTCATACCCGATACCGGGGCACAGCAGGATGG
CATGCGACAGAGATTT
CGGGAGGATCGAAAAAAAAGAAGAAAAAGAATGATCAAATTCTGA
AGCCATCAGATTGGAC
GAAAATA
>ConsensusfromContig1680 Average coverage: 10.21
TCTTCAACAGTATTTATCCGACCCTGTTCTTCAGGCTGAGACTTCG
ACTCAATTGCTTCA
TGATTTTCAGATTTATTTTCTTTCAATTTAAATCGGTGAGTTTTTG
ATGGATCAAAACTT
GAAGCAAGCGGATTCCTATATTTTGATATTGAAGTGAATGATTTT
CCCAATACGATTAAC
TCATTATCATTGATTTTTCTCGTACCTCCCATTCTAAA
>ConsensusfromContig1681 Average coverage: 7.68
GCGTAATGTGAAACAGTTATAATATTTAAAATCAAAATGTTATGC
TTAGCAATCGCTTTA
TATCTTCCATACGAATTACTCGAAATCACACCCGTCTCAATAGAG
GCTGTATCCAGCCAT

ACTATTCGTTATATTTCGAATTCAGTCATTTGATTAAAATGGAATT
TTCTCATCATAATT
ATGGATCCGAAACTCTTATTCGTAGGCGATAAAATCATTCTTTCA
CTTTATTAAATAAGA
AC
>ConsensusfromContig1682 Average coverage: 3.42
ATGAGAGAGGCCCTTGGGGTCGATTTACGACGAATAGGAATGGTA
TGGCTACCTGCATTT
GTGCCTGGCTGTCTTGGTGTTTATTTGACAATAATTTTGACAGAG
AAATATTCAAAATGT
TACTGGTTACTACCCACAGTAGGATTGGCAATTGAAGGAATCAGC
TGCATTATCATTCAT
TTCTGCACCGCGTATTACCAAGTCATCGTATCGATTATGATG
>ConsensusfromContig1683 Average coverage: 4.27
GAAAAAACCATTCACATACTCATTCTCATCATTAGTGATATTCTC
AGTCATGTTTTATAA
GATAAAAAATTATTAAATTCATATAATATGGAGCAAAATGATGG
ATGATAAATAATGCAT
GGATATACGTAACTCCCCTTATGGCATATATAACTGAAACGTCAA
ATTGATGCAAACAAA
TATAGTGAAAAATTCATATTTCACTGCTGAATCAGGTT
>ConsensusfromContig1684 Average coverage: 4.57
TGGTCAAGACCTTCATCTTCAGTGCCAAGGGACGGGGCCACCAC
AGCCGAAAGCTAGATG
GATAAAAATTGTTGGCAACGATGAGATAATATTAAGTGACGACC
CTGATGAGAAAAACCT
GGTTATAAATGAGCAAGGCCTCACATTATTCGATGTTCAGAAAGA
TGATACAAGGGTATT
AACATGCATCATTGAGAACTCCTCTGGGTCTAAGCGGGCATCTGC
TGCTCTAATGGTCTT
TGATGAGACAAAAATCGCAATCGAACCCAGAAACAGATCGGAAG
AGCGTCGT
>ConsensusfromContig1685 Average coverage: 6.81
GGCGTGTGCTCTTCCGATCTTTTTGATGAGACATTGACAAGAGG
GGAGGTGAACGAGAAG
GACCTTGGGATATCTCCTCGCCTTATTTTTCTTGGTGACAATATG
TTGTCGGCCAAGAGG
TGGATGGTGTCTGTGGAGAACACGGTCATCATCACGGGGTCTGA
GGCGGTTGAGGGCATG
GCTTCTCTGTTCGCCACATTCTA
>ConsensusfromContig1686 Average coverage: 5.25
AACAGAGCTTGCATTGACAATAATCCCCATTAATGTGAAAGTGA

ACATCTCAATGACAAA
TCCAATACATTCTGTTGAGATTATCACAATTCCCATCATTGTATA
TATAGCGCAAGCAAC
TGTTATGATTCTCACACAAATTCTCAACGGATGTATATTTGTTGGA
TTTTTTGTAGACGT
GCCACATTCGTGTCGGCAGCTCTCTTTGTCATCATCACATATTAGA
CTTTCCAGTCCTAT
CTGATCATTGATAGAAGTCATTTTGCCTGCCATATAACTTCGACAT
TTT
>ConsensusfromContig1687 Average coverage: 5.05
TGTTCTATTCAAACCATCTGTTATGATGATGAATTCAAAAATGAT
ATTCGTTATCGACTT
TTTTTTAGATTAGTCTGTAATATATATATCCTATTCGTATCGAATC
ATACGCACCAATTA
TTGGTGATATGAGTCTGAACATCAATTTATAACTATCTCGTTTCAC
TTCATCTCATTTTT
TGTGTTGATCAATGTTATCACTTATTTTTTATACCATGATTTTG
>ConsensusfromContig1688 Average coverage: 7.37
CTCTTCCGATCTGTTAGAACTCCCGCGTCGCGTCATGCTAGAATG
GTTCTGCTTGGCGCC
GAATCCTGCGACTGTACTGTAGGTAGGAAATGTCCGGTGATTGGT
GCATTGATGATGATG
CTGAATGCATTTCCGAGGGGGCGGTAGTTGCATCTGTTGTGGTGA
TGGATGGTTTGGCGA
AAGTCCTTGACTCGCAAGACCCTGAC
>ConsensusfromContig1689 Average coverage: 24.40
AAAATTGCTATTTCATTTGTGTATACTTATTTCATCATCAGTTTCA
CTTGGGTTTTCAGA
TTTGAGTATTATTTATTTTTCTATTGTGGTTTTTGACTGTGATATG
TCATCTTATGCAGT
AGTTTATTTTCATTCGGTTATTAACATTACATATTCCCTAAAACA
TCATTGAAAAGCGCA
GATCGGAAGAGCACACGTCTGAA
>ConsensusfromContig1690 Average coverage: 5.81
TCAGATTGTAAAGGAATTGTGATCATTGTTCAAATATGATTTTTC
TCGTATATAAAAGAA
GCACCAGGATAGATTCATTCTCGATGGCCGTTTCTGAGAGATCAG
ATAGCGATGGAGTTG
GGAATCTCTGAAGGGAGCCACACCACGATGGGTTTTCGCTTGAGA
GCTGCATGCCGGTTT
CTGATCTGCTTCTCGATCTGCACCAGGTCCTCTTTGAATATCTTTT
GTGCTTCTTCAGCT

TGGGGTGTATAGAGGTATTTCACTTCGAA
>ConsensusfromContig1691 Average coverage: 4.56
CGAAACATGATTTTCCCAGTCAACACAGATATACTGAATGAACCA
AAGTGTATATTAGCT
CCATTGTAGAATTTTTTCAATATAGCAGACAGGATAATTGCTAGT
AATCTCGAGTTATAG
AATGTTAAAAAAATAATCCAACTAAATGCAGTGAAATTGGCAAC
AAGCAGCCACCAGAAA
TTAGATTCAGGTAATTCTTTTTCAATCCTTCGGATTATACTGTCAT
TACCT
>ConsensusfromContig1692 Average coverage: 4.80
TCAAGGAAGTAAACAAGGGAGAATACACAACAAAGATTGGTGAT
TACTCCGGGATAGTAA
CCGATCAGGATACAGATGTAATCAGAGATTCGGCGCTGATATAT
ATTGAAATGGACAGAG
ATTCTTCGACTCCACGTGTGAGATGTGGCTCAGGAGAGATAACTG
ATGTATATTGCGGTA
GAAAAGACAAAATAATAAGTTCAACAGAGAATAATGATGATTTG
AAACACGATATTCAGT
TAATG
>ConsensusfromContig1693 Average coverage: 4.68
GTTTTTTGTACCTAAGTTACGGCGAACTCATCAGGCCCAATTGAA
AGGTAGCATTTTACG
TACACATGCTGCGAATTCCTCTTCTCTTCTACGCTTCGAAAAACTG
TCATGATTTTTTAT
GTTTGTGAATATCTTTCGAAGCATCAATCTTTTGCACTCGCTCTA
GCATCACACACGCGT
ATAATACCTCAAAACGTAAATCATTTTTCGAAATT
>ConsensusfromContig1694 Average coverage: 3.65
TTCTCTTCCACTTCGCTGAATGTCAAAAAATCGCCCCAGAGGCCC
GGTGACATCACTTTT
CGAACGGTCAAATACGAGGTTTTGCTCATCATAGTTTTTAGGACT
GATAAAATTATCTCG
AATGGGGACATCCTGTTAGAAAAAGAACTATTGAACCAACCAAAA
TTAAATAGTTTCTTC
AAAGATTCAACCATGACTGATATGTGCC
>ConsensusfromContig1695 Average coverage: 5.62
TGTTACGTCTTGGAAGGAGATATTGTATTGGTGATGTTGAGGAGCT
TTCTTGATGTTTTT
AAACGCCAGATCATCAAACACGATTTCTCCGGTAGAAATAAATT
CGAACTCGTGCGCCGG
GATTCCTTCGAAAGCATTTCGCATGAATTCAATTTTGATTCTCAA

ATTATGAAAAAACAA
TTTTTTCAAATCGCGGAACCTGTTGAAAGAATGTGCAGAGACTGT
TCCGATGGAC
>ConsensusfromContig1696 Average coverage: 6.39
GAACTTTGAATGCTGGATGTGGACCATTGGCCTTGCTAAATGGCA
GAAGGTCGCTCAACG
GGATTGGACCAAGAGTGTTCGAGTGAACTCTGCGTTCATCAATTC
TGATGGCCTTCCCCA
ATGCCAGGATGAGAACGGGACTTTCTGGATTCTCAAAGACCCCG
AAGGAAATGATGAAAT
GATTTGGGAGGAGGCGGGAGCGACGACAGAATGGTTCCAGAGGT
CTCTGACCGTTGGACA
AG
>ConsensusfromContig1697 Average coverage: 4.95
CGCGATTTATAGTCAAGAGTTCTAGACCGTGGCAGATAGAAATTC
CTTGCGGCTATGCCT
CCTGAGGACAAAGTAGTCTCTGAGTCTTCTTTGAGAACTTCATGT
ATGTATCTAGCTCTA
GCCTCCGCGTACCAAGGATCCGTTATTTCTTCATCGTTGATTTCAG
GTTTCGTGCTTGTA
TTCCGAGCATTTATCAGATCCTTAAACCTTG
>ConsensusfromContig1698 Average coverage: 4.22
GCCTACTCAAGATTATTCTAGTCCACATGTTTGTCCACATCATAT
TCACACCAGTTACAG
GTGCTATGAAGATGGTGGGAGGAAATCTGAAAATTACTCAGATC
GTGTACATCAACAACT
GGTTGATGAGCGCCATGAATTTCTTCAACACAATTCTCTTGAACG
CAAATCTTGTAACGT
CCAAAAATGTTGCAAAAGTTCTACG
>ConsensusfromContig1699 Average coverage: 4.32
CGTACATTCATATTTTGATGTTGTTTTGTTGAGAACGAAAAACAA
AAGGAACGTCGGAAC
CAGTAGGTAAAATTGTTTGATGCTCACTTTCGATTTGTGTAGATTT
TGGGTGTGTGACTT
GTCAATGAAAAGACCTCGCAGTTTGAATGTTATAGCAGACGTACA
TTCATATTTTTGATG
TTGTTTTGTTGAGAGCGAAAAAC
>ConsensusfromContig1700 Average coverage: 3.02
TTTCAGAAACATTTTATAGCTGTCTAATGAAATGTGGATTCTAAA
TATATACAGCAGTTG
CATGTCGTATATAAAACTTCATTCAATTACGTGACATTTTCTGAG
TCTCAAAGATAAGAA

TCAATCCCATGTCAACGTAACTCTTCTGATGCCATAAAAAGGGTC
TAATTATTTTATACA
ATCCGAAATCTCAGTTATCC
>ConsensusfromContig1701 Average coverage: 2.85
GGGAGACAGGGGTCTTCTGGCCAGTATTCATTTTGCCACTTGTCT
GCCAAGCCGGCTTGA
AGAAGATGTTGTATGACTTCGTCGAATAGGGCCTTATAAGGAGTC
CCTTTCGGTAAGGCA
ATTCCATAACCAGATTGATACATGATTTCTTTCAAGAAGTGATAA
TTGCAAAAACCAAGA
GACCTCAGAACCACATCAATGACTTTGAGATCA
>ConsensusfromContig1702 Average coverage: 5.04
TTACATTCAATGGACCAATTTGGTTAATTTTGCTTTCACCACTACT
CTCGATATCAGTAC
ATGATTTCATTCTGCCACATGTCTTGGATTCACGCTTAGTGTCACT
ATCAATCAATACTT
CTTCTTTTGTGTCCTCCAAGTCCGAATGTTTCACGTCGATTACGTT
ATGACTAGTTTGAC
ATAAATTTGATATCTCCTCGAAATTTTTCTGGTCAGAACTATGTTT

>ConsensusfromContig1703 Average coverage: 5.59
CGGTTCCATTGAAAAACAAGGCTTTTCCGACCATACCATCTTCTA
CAGTAATGTCGCCAA
CAACAGTTCCAACAAACAATCCGTTGAGACTGGAAATATTCCCCG
AAGTAATGTTCGACA
TCGTTATCGTTGCACCATCTACACAATTATCTTCCTCGTTCCGACC
GTATCCCGCATCGC
ACTCTTGACAATTGAGTCCATCAAAAC
>ConsensusfromContig1704 Average coverage: 3.26
GCACAAGTTATGTTGTCGTCTGCTCTCTTACATGAATGCTCACAC
AGTGAGCCCTCTTGT
TTTTTAAGGTGTTCAACTTCAGTATTAGAGAGCGCTTTCTCGTGA
AATTTCAGGGAGTAC
ATAGCCGATACACAATTTGATAAAGCTAGGACGTTACTCGGTGC
GCCGAAGAAAAGTTCG
TGGAATGTATTTTGTGTATT
>ConsensusfromContig1705 Average coverage: 4.75
GAAAAAACAATCATAGAGTATCTTTCAATTATTCTCAAATATGTT
TGAATCACAAGACAT
TATTAAAAACCATCAATGTTTGCTCACTGAATGATACGAATGCGA
ATATGTGTTTGAGGC
CTTATACTGTGGCTTCATTTGAACCTCATCATATGTCAGTAACTC

GTAATAAATCTCATA
GTTATATACCTACATCAGAATGTGTGTTTTAGGGTCG
>ConsensusfromContig1706 Average coverage: 6.17
GATCTCTCGCTGATATTTTCATTCTTATCTTCGTCGAACAACGAAA
GCACTTGAGGTTTC
AATTCCCAATTTTCGCTGTTTATTGCGGCAGTGAATTCGTTCAAGT
TTATTTTCTCACTA
TCATCAGAGTCGAGCTCTTTGAACAATGCATCGGTTTTTCCATCA
GATTGATTCCCCCCT
AGTTTTCGGAGGGAAGCCTTCAGTTCATTCAAAGTGAGGAGATCG

>ConsensusfromContig1707 Average coverage: 6.52
CATATGGCATATATACATTTATCACAATAATCTTTTCCTTCTTTAG
TTTTATGGCATTAC
ACCAATCACAGTTCAATTTCAAAATTTCAACATCATGTGAAATGT
TTTTCTCCCAAAGTA
TTGCTACCCCTCCCCTCAGCCTACCTTTGTGTAGTTTCTCACCAT
CATCACAAGGGGAAG
AGGCAATTCCATCGAAGTTATTATTGATTGTTTTTATAATGTGTTC
CTGTTGTTTTGAAA
>ConsensusfromContig1708 Average coverage: 5.86
ATTGCTGCTTTCAAAAAATTCTCTATCGCTTCATATATAATTATTA
GTTGCAATAAATAT
TGTGTTCATTCCCTCAAAATACTTAAACGGTCTAGTGGGCATGCT
CTATCAGTCTAACAC
AAACATCCAAGATGAAGGCACTGTGAATTATTATTTTTAGATTTT
GAGGTATGAATAATG
GCTGAATAATATCAAGCATCAGGGGCGGCCGCACGAATTCATCAT
TTTGT
>ConsensusfromContig1709 Average coverage: 3.37
CAAATCGCTCGAGAGAAATGCGGCTTCAAGTTCATCTTTTGGAAT
CAAATTTCTCAATTC
TTTTAACCTCATTTTTGCGTTCATTTTCTCTGATTCGTATTGATGC
CCCAAGATTCTCTC
CTCGCTATCTGTATTGATGTCAACAGAGGAAATTGGTCTTAAAGA
CTGTCGACTGGTATG
ATGAACTATTGGGTCATTTATG
>ConsensusfromContig1710 Average coverage: 4.54
AGAAATTGCTCTAACCATTATGTTTTGACCATTGTTTCCGAAAGT
TCGTTCGAAAAGTTT
TTGAACTTGTATGAAAATTGTGGGGTTCGCATCGCATTTCACTGTT
TCTATCTCGATATT

GGTCGCCATGCCCACGATAAACACCAAAAAAATAAGTATTTTAG
GCACATATATGGACCA
CATGTTAAAATCAGACCGATTGGAATGATCGGATTTAT
>ConsensusfromContig1711 Average coverage: 3.42
GATAAAATCATCAAAATACAAAATGATACATTTCAAATATTCATT
CCAATGCGCAAAATT
TATCATATACAAAAGAGAACATCACAACTTGGTATCATCTATCAT
TGACTCTAAAGTGGT
GTCGAAAAAAATCAATTAAAATATACTCTATGTAAATGTGTTCAT
TCTATTCATAAAACT
ATTTTCCAAGATTCATTGTC
>ConsensusfromContig1712 Average coverage: 5.77
GCGAATATTCGAATTTCTAAGAATCAAAAGGATTAGTTATTCTCT
TTTTTTCGAGTATCA
GTCCAAGTATCTATTCCCGACAATACATTCAGCTTTCAAAAGTCA
AACAGCCAGTTTGAA
GTCTCGAATTCGTGGTGGGCCGGTGGTACTCTCGGGTGATTGCCG
AAATGACAGTCCTGG
CCATTGTGCGAAATACGGCACCG
>ConsensusfromContig1713 Average coverage: 7.12
ATGCCGAGGGATAGATCAGAAAGTATATATTCAAGAACTCTATC
GGTATGATGTGATAGC
GACTGTTGGTTCCCTGAAATTTATGTAGAAGGACTTGCATTGCTC
ACAATACTGTTTCAA
AACTGAATTGCCTGATATACAATTTTAAAATTACATGAAGACGAT
TATTAAAACAATGAA
CACAATACATTTTACACCCCGATGCTTCTCAGATAAAAGGTAACC
TTCATAGCAACATTT
CGTGTTATGCTTGAAACGTAATGTTCT
>ConsensusfromContig1714 Average coverage: 3.61
ATTTGATCAATATCAACATTCAACCCATTCTTCTTTGGATTCAGAT
GTAATAAACTTACT
GTGGCGAACATTTCATATTGACACGTAGTGAACTCCTATCAATGA
AACGCATGAAAATCA
ATCAGCAATGAGCCTCAAAATTTCAACCAACGAATGCATCTTAGC
TTGCGACGTAATATT
CGGATTTATGCATATCGGACAAAATTCAAAATAACAGGATTAAAA
C
>ConsensusfromContig1715 Average coverage: 4.40
CAGACATGGGCGACCCACCATTCAGCTTTGCATCCATATGCGAAA
GAACATTTAACAGAT
GTTTACCACAAGCTCCATTGGCTATATTCAACTATAACGCTACAA

TAGCTCGACACAAAA
TCATATTTTTCCCATTTGTGGATGGAATGTTCATAGGAAAATCTT
TTGGTGGCGTAAATG
TTTCAAATGGAATTCTCATATCGGCAAAAATGGTCCGGATGAGC
>ConsensusfromContig1716 Average coverage: 4.74
TTTCATCAATTCGTTTCGTTTCTTTCATGACCTAGTGACCTTATTA
GCATCCCCTATAGT
GTCTTATAACTTCGTCAACGACCTTGGATTAAAAGAGTCGATAAG
CGCATTAATTGCCAT
ACTGCACGTATTTAATAACGGCCGAGACATCAATGCTATTAATGA
AAAAGCCCATCGTAC
GGGTGCGACCCTGTATAAAACAGATCGGAAGAGCACAC
>ConsensusfromContig1717 Average coverage: 3.49
TTTATGAAACAAAACACCTTTTGATGAGCACATAGGGATCAGAAT
ATTTACAAAATACGA
AATAATACATGAATTGCACGTTACCGTTAGGGGAGCAGCTTCTAA
TATATTTTGTAATTG
AAAAGATTTTGTATTGTTAATAAAATGATGAATGAATTGCACGTT
ACCATGAGAAAATAA
GCTCTAGTATATTAAGTGAATAAAAA
>ConsensusfromContig1718 Average coverage: 6.74
CGACGCTCTTCCGATCTGCCGAACAGCTCGAAAAGTGCCATCAAC
GTACCACCGCTTTGA
TCTTGCCAACATTGATAGCTGTAGATCTGTGCTGAAAGCAACATG
GCGCTGATCCCCAGA
TTCAACATTAATCCACTTGAAGTTTACCGGAATATTTGCAGGGTC
GATGACGAAGTCAAG
TTCTGAGGGTTCTTCTGGAAGATGCTTTTGTCAGTGTCGATTTGC
CAGGCGTATGAGATG
GGAAGAGCACACGTCTGAACTCCAG
>ConsensusfromContig1719 Average coverage: 4.29
CCCATTCAACCTTGACATAACGAAATACACTTCCGACTCCGCCCA
CAGTGCCACGATGAG
GTCAAGGAGAATGGCGATGCGGTGTGTACGGATTTTCGCTCAAC
GTAGAACCTGATGAAC
AATGCTTGAGTAAGAGGCTTGAAGGAATGATTAGTTTCGAAATCA
CAAACACCCTCTTGT
AGAAGCCACTCCCCCGGAAGTACCATGCTTTTG
>ConsensusfromContig1720 Average coverage: 2.83
ACTCTAACACATTTCCACGAGACAGCCTTCAATGAATATGACTAA
TTATTTCTCGAATCA
ATTCTATGAATATGTGTTCTTTATCAGCATGTCATCCAGAAAAAG

AGACTCGAATGATTT
CATTTGTATTACCGACCGATAAGTTTGAGCTGAGCACAAGAAAAT
CGTGATTTACAACGC
TCAATGCTTTCGACATTGCTGAATGTTTCCGAAAA
>ConsensusfromContig1721 Average coverage: 3.85
TCATAATAAAGTTTGAATTTGTCCATGTTTTTGTCATCACCATGTG
TTCTGAATTTGATT
TTCACACCAGAATAAAAGTAGAGGGAATCCGGACACATTGTTTCA
TGGTTATATTCCCAA
TGCTTGACGGATGGTTGAACAGGAGAGTCGAGTCCCAGAGTATCA
TTCTCCTCCGATAGT
GTTGATCCAGACAGAACAAGTACGGAAGCTTTTAGG
>ConsensusfromContig1722 Average coverage: 4.79
CGGCATTGTTCGGGACTACCATGGTCCGTTCTGGCCTACTGATTA
CGGCCCTTTGCACCC
TGCACCATCTTTCATTCGCAACTACCAGTTTTCTGAAGAAAAGTT
GAGCATGCACGTTGA
AGAGTCTCGGAATACGGTGGAATCTCAATCAACGCCATATGTAG
GTCACTGGAGGGGCAT
TCATACTGTCTTTGATAACGAGAGGAGCCCGCGGTACACCATAAG
TCCT
>ConsensusfromContig1723 Average coverage: 6.07
TTTCTCTCTTATCCAATAATATCAAAGGCATGATGTCTGTTATTTC
AAACTCCCTCATGT
GGAATAACATGAATATGTTTGCAAATATTTGCTCTTGGAAATATA
AAGCACATGAATTAC
CGATTCAAAAACAAATGGAAATATTCACAGAGGTATACAAACCAA
TTTTACGCCGTCTAT
TTCTATCTCGTTTCAATTACGCACAAGCAAATATTTTTTCTCATTA
TAATTCCAAAAAAA
TACCTTTTTTCGCTCAACATAATTTCCGTTGCATAACGCCAAAAT
TTAAATACACAACAA
G
>ConsensusfromContig1724 Average coverage: 5.30
TTTTCAACTCACAAATATCTATGATTTAGGTCAAATAAAATCTCAG
TCGTTTATTCTTTT
ACATGATTAAAGACCTATTAGGGACATTGATAAAATGTGGTAGAT
AGTATTATCGCAATA
AACAATGTTCAAAATAAATCAAGTTATGTGCTATTGTTTTATACG
ACAATAACTCTCCCA
TTTAAATGAATCATCCAACTAACTTTTATTGGTCAATCTGGATCA
AGGGAATAATTTTGT

CCGA
>ConsensusfromContig1725 Average coverage: 3.70
GATTTTGTTCACAGTCATCATAGGGTTCATTCAAGCGCCTGCGGA
TTCTTGTGTTAAGTT
CAATAGAACTCATTTCATTCTGACTCCGACAGATTACGATTATAG
ATATGAAAATGCTGT
GCCAATTTATGATGACGGTCAGCAGAAAAAACTAGTACAAAGAT
CTGGAAGGGATACTAG
GAACAACAGAACTAGTGATTATACACACGAGACCGCAGATCGG
>ConsensusfromContig1726 Average coverage: 4.14
GATGAAATAATAATGAATCAATTGATCGCCTCCTATGATGACATA
ATCACCCAATTCAAT
CATGCAGAGGAAGTGCTGGAAAATTTGAAAAAAGATATAATAAT
ATCATATGAGCAATTA
TTCAAAAGAACTCAACTTAATCTGTCGACGTTGGACAAAATAAAC
TACGTTGTTGATCAT
TGTTTTATCAGAGGATTCGATGTACACAAAGAAAGGACCTTATTC
TGGCTACC
>ConsensusfromContig1727 Average coverage: 4.31
TACAAATTTATCATAACATTTAGCATCAAACCACTCAATGTATGT
GAAGGAATATTATTT
GAATATTATAGATTCAGCTATATTATCGGAATCATTGTTTTCGAG
AAAAAAAATCTCAAA
CATACGTTATCTACCTGACCAGGTCATACTGTTCTCATTTGCTTAT
TGTCGGAGTTTATG
AACGAAGTCGTTCCAGCTATATAGACTTCTGTAGATCGGAAGA
>ConsensusfromContig1728 Average coverage: 2.94
AAAACTTCAAGACTCATCCAGAAAAGTTTCACGTTCTGATAAATC
TATTGAGAAACTTAC
CACAATAAGTGATTCGCATATAACATACTTCGAAGAGAGTATAA
CAACGAAAGATACTCC
GCCTATTTCTAATGAAAATTCAATAACAGTTACTGACGTTAAAAC
CTCAAGTCATTCATC
TAATATCTCACCAAAGGAAGCGGAG
>ConsensusfromContig1729 Average coverage: 1,194.43
TAGCAATGTGACTGGAGTTCAGACGTGTGCTCTTCCGATCTGCCT
AATCGTCTTGTGAGG
CGTGTGAACGTCTGGCCACACTTGGTTTAATGATTATTTCACGAT
AATTTGCTCCGGGAA
TATCTTCAGGTAGAAATAGTCTTGCTCGTGCGCAAACATGTGTTA
CTCTAATTCAATACA
GAGTTTCCAGATGCTATATTCCTCA

>ConsensusfromContig1730 Average coverage: 149.07
ACTGGAGTTCAGACGTGTGCTCTTCCGATCTCAAAATCCGGCGAG
GAAGTAAACATTACT
TCAAAACATGGTTCTGGTGATATCCTCTGGGCTGAATGCAAAGAA
GGCTATAGTGGAATG
AATAATGCTGCTAAACTAACATGCATCGATAGAGGGGAATGGGTG
GGACCCGTTTTTCAT
ATATCTCCATGCGCAAAGATCATCTGCG
>ConsensusfromContig1731 Average coverage: 2.19
ACGAACTCGAAAATTTCAGAGAATAATATTATACATTAATTTACA
TGCGATAAAATAAAC
TTCAATCAATTCGCATGTCCATAATGTTCATTAACATTTTCATGTA
AATGAGTTACTCAC
ATTCATGCACACAGTAAAAAATAAGCTGCAGCAAGCAAGAGAGG
ATTTCACCATGATCAA
TTATAATTCAACGTATGCATGATCAAGG
>ConsensusfromContig1732 Average coverage: 3.65
CAAATGTTCGACATACGTTCGAAGTATACACCAGAGCAATACATT
CATTTTTGTCGCAAT
GTTGTATACAGCCCAGCAGACTATCGAATTTGTTTTTATCTCGGG
TCCTCATTGAAATTG
TATCTGTTCCATAAATCCTCGCAGTTGTCGTGTATGTTTCTCCTTT
GACAGTCTCATACA
GCTGGCATTTCCCATCCATGTAGAGCAC
>ConsensusfromContig1733 Average coverage: 3.23
TGTTTTCACATGGTGGCAGACTGAATTCATTGATGTTTTATGATT
TAATTGAATATTAGA
TCTTTCCAGAGCACCTCAAAATCTGGATTAGAACAAATCCAGTGT
TGATGTATCACTGGT
GTCTAACAAGTGATACTTTTTCACAGTGAGGGCCCCAGGGCGAA
TATGGCTAAAACAAAA
ATAGAGACGAAAACAAGTAATACACATCGAT
>ConsensusfromContig1734 Average coverage: 6.48
GTCTACAGAATCTTTTTGTGGATTTGCTTCAATAGAGTCTCCAAA
CATCACAAAAACTTG
AAAGATGCTTCATTTGAAATCAATCGTAGGTTCCCTTTGGCAATT
ATGAGTCTTGGCAAG
AATCACGTAGGAATGATTCATCACCGAGATAGAATTCACAAGGC
TACAATGGAAGTCACT
GAGGATTCAATGAGGTCTCTGCTGCTGCTTTGGAATCTAATGAAG
CCACAGTGTCTGTTG
ATGGAACATGGCAAAAGCGTGTATTTACCAGCCATAAT

>ConsensusfromContig1735 Average coverage: 3.80
CCCTCTCCAACTGAGCGTTCCTTGACTCGAGGTCGGCAAGCTTGC
CACGAAGTTCGGCAA
GTTGAGTACGGAGCCTCTTGGATTCCTCCTTTGTGTGTACTGTTTC
CATGTTCTGGCGGG
TAGCTCCTGTTCGGAATTCCTGGACCTTGAGGTTGTAGTATGTTTC
GAGCTCTCCCCTCA
AGGCGTCCATCTTCTCGTCG
>ConsensusfromContig1736 Average coverage: 5.16
CATGGCATCGCACCATCTCTTACGTTGGAGAAATTCTTTTGGGAA
ACTAAAAATGGAAGT
CTTTGGAATATTTAATTCTCCCTTTGTTTCATCGATTCATAACCAG
TTCGGCATCCTTTG
ACAGTGCATTTTCGACCCATTTTCATGTGAGACTCCTTTAATCATT
ACTACTAGCTCATT
TTCAGAGACATGAATATAAAAATAGAAAGTTTGAAGTAATAATG
AAC
>ConsensusfromContig1737 Average coverage: 6.64
CAAAGATGCCACTTGTCACAAAATTGTACATTATCGTGATAAATA
GCAGACATTCCGTTG
CCGTACTTGAATCGGCGCTATTTTTCAAAGGCATCAGTTGTCATG
AAAATGCACATTATG
GTAATTAAAAAACATCAATTGTCACGAAAATACAGACATTTGATC
ATCGTAGTTGAATAG
GCGGTATTTTTTAAAAACGTCA
>ConsensusfromContig1738 Average coverage: 3.47
CAGTTATTTATTTATTGGTTCGGCCTTTTTTAGTAAGTGGCCTTTTT
TATCTTTTATCTA
GAAAACTCGTCTACCAGTAGACGAGTTTTCCCGTAGATGAGTTTG
CTTTTTACCAAAATA
ATGGCTTGGGAGAAACTGTTTTTTATACCAGTATCTTATAATGAG
AATAGGCATGGTTAT
GGTTGCAGGTGAAGAGATGAATCAG
>ConsensusfromContig1739 Average coverage: 4.37
GGCCAATGCAGTGTGGTAGGACGTGGATTTGTCTGTTCGTGTTCT
TCGCTGTTTAGTGGG
ATTACATGTGAAGAAGCATCGTGCACTGACACATACTGTTCTAAT
TCTGGCATATGCTCA
AGCAGTGGGACGAGTTTAAGTTGCGCTTGTCCGTTCTGTTATATT
GGCGAAACCTGTTCG
ACATATCAGTTTACGGCGAAACATTTTCCATACGGGA
>ConsensusfromContig1740 Average coverage: 1,064.94

TTCCTGATTTGGATATAGACACATTTTGAAAGACCTGTTTGATTT
CCAAATCACCTGATC
TCATCTGAAACTCCACCTTGTGCGCATTGAAGACTCCTCATCATTC
TTCCACTGTCTCCA
ATAAAGATGGCGACGCTGGGCCATCACCCGGCGCCGCGTAGCAA
CCAGATCGGAAGAGCA
CACGTCTGAACTCCAGTCACATTGCT
>ConsensusfromContig1741 Average coverage: 3.15
GTCGTATCTAAATTGGGAATTTCCGTATCACAATATTCGTATGGG
AACACTGATAGGGTA
TATTTTTTTAGAGCGGTAATGGAGTCAGTGTGAGGACCATGAGTA
ACAAAATTATAGAAA
AAATAAGGGATGATCACCTTAGTTTTGTTATATTTCTCAATGACTG
TTCGCATGATCTTT
GTTCTTTCTAGCTTTGTTGAGAC
>ConsensusfromContig1742 Average coverage: 26.09
AAATGATAATATTATTTAAAAACTAGCATGTATAATTGATGATAC
CTCATTCTTATCAGA
ATCATGGTGTTTGAATCTATCATTGTCAGTCTCGTAAACAAATAT
CTAGGTGATTTCATA
GAAAATCTGAATACATCTCAACTGAATGTCGGACTTTGGGGAGGA
GATGTTCTTCTCGAG
GATTTGTATGTCAAGCAAAGCGCGCTGGATGAGCTCAATTTGCCG
GTTAAGGTGCTTCAA
GGAAAAATCGGGAAGCTCGTGCTAAAAATTCCATGGAAGAATAT
ATACACACATCCTGTA
ATTGCTTCGCTGGATGGGTTCTACGTATTGGCTGTTCCCAATGCC
GATATCAAATACGAC
GCGGAGCATGAGGCGAAGGCGGAGTGGGAAGCGAAAGAAGCAG
AACTGGTTCGTATCGAG
CAAGTCAAGAAAAAACTCAATGAACAAAAAACAAGTCTTGTCAC
AGAAACGAAAAACGAT
ACGTTTGCGGAGAAGTTGACTGCTCACATTATCAAGAATCTGGTC
GTACATGTCAATAAT
GTTCATATTCGCTTTGAAGACAAGTTTACGGACTGTCTTTCACCT
TTTAACGCGGGAATT
ATGCTTCGCCAACTGTCACTTGAGAGTACTGACAGTAGTTGGAAT
TCTGGCAAAACAGGC
GAAGGCACTGCAATTGTATATAAGCTATTGAAACTTGAAGGCCTG
TCTGTTTATTGGAAC
AGCGGAAGTGACCT
>ConsensusfromContig1743 Average coverage: 16.26

TTTATTTCATCGGCATACACCTCTCCGTATATCATGAAGTAAGGC
TTTAGAAAGACGTCT
CGGACGAGGGATATTCTGGGCGGTTCGTAGGGGTAAGTCAGGCT
CTGGCGGGCCACACCG
AACGAGGTAAGTACAACCAAGAGGATGAGGATGAAGTATACTGTG
TCTTTGATCATTTTG
CCAATCATCATGACGTAAGGCCCGAGATGCTTATTAACGGCGAA
GAGGTCCAGAATCCTG
AGATACCAATAGATGATGTTGAGGATGTAGAGGACACGCCCCTC
GGCCATCCTCTCGGGA
CCGGGCTGGAGTCGTAGGACCAATCCGATGATGAAGAGGAGGAT
CGCGAAGGAGTCTGCG
CAATTCCAGACCTCCGCGAAATGAACGAAGAGCTTCTGGCTGATT
TTCTTTGGTTCTGAG
ATGATTATTTCTCGGATCTTCTCAGATAGGAAGCTGAAGATGTAA
CAGATGATGAAGATT
TCGGGCCATGAGAGCCACCGCTCCATCTTCACAAGCACCACGTA
CGTGAAGAACACGATG
AAGACAACGTACGCCATCGTGTGGAGCCAGAATTTGGTGATGGG
GGCTGTGTAGAATTCG
TGGATCCTTTTTGAGAACCGCAACCGCCCTTCGTCCCTCACCGCA
TACCGTCGGCGGCCA
ATCA
>ConsensusfromContig1744 Average coverage: 24.65
TTACAAAGCTTTGTGGCCAGCCGGATTATTTGTATAAGAAATACG
ATCAAAACAGTTTTA
TCATAACGTTATAGATAACTTTAATGTTGAAATACAGTTATATGA
AACACAGGACAATAC
TATGGAACAGCCGAGAGCCCTGGAGATCGGGCCGAATATATATC
AAGACTGGATCGTTCC
CCGACGTGTGATGTGCGCCCTCTTTTGGCTAGGAATCCTTCTTTTG
TTCACTGGGATCAT
TCTCCAAGTGACGAGCAAACACAAGCAAATATCTCTGCAGTCGTG
GGGAATGGTTTTGCT
GGGTATTGGCTTCTGTTTACTTCTCATTTGCCTTCTCCTCTGCATG
CATGCTTATTGGGT
CATAACTGTTGCAGAGGAAGAACAGACGACCAAACGACGGGAAC
AGACTTATTCCCGAGG
AATGTCAAGCGAATCTGCAGCTGCTGAGGCAGAAGATGTGGCGA
ATCAGCGGTCTCATAG
TCTTGAGAGAACAACTTCCATTAGGCTGCCACCAAAACTGACTCC
ACATGACATAGAACC

TTACTCACTGACTCCCATTATATGTCGAGCTGCTCAGATGAGACG
AGAATTCAGTGAGCG
AGATAGCAGATATCTCGATAGTTGTGCCATCCCTGTTTCTGCCTT
TTACCAGCAAAGGAG
ATGTTCCGATGGGGAAGAAAGTGAAGTCTGGAAAACACAAATGC
CATTATTTCATCAAAA
CAGAAGCGACAGTGAGCACTTTTCAAGGTCAGTCAAAACAACTA
CAGCAGAGGTCACTCT
ATTTCCAGACGAGAAAAGTCCATATCTGCAGCGACACTTCAGTG
AAGATAGCAAAATGAA
TTATTCTACAAAAACAGACACGAAGTGGAGATCGAAAAACCACA
CAATGCCAAATACCCC
TCACACACAGCAAATACCAAATTCCAATGAGAAACGGGAGAAGG
CCTTGATGAGAAAGTT
TGCTAAAATTGAACTCGAAGCACAAAATGTTCTGCAACAAAGCT
GCGATGAGCTCAACAC
CCCTCCGCTAAACCTCGCTTCCATTCCACCCCCTCCCTCATATCAG
ATTGTCACTAGTAA
CCAAGCGCTTTTCCCGCCAACCATGAGTATTGCTAGTCCTGCTCA
TTCATGTGGTGTCTC
TATGACAACGTCAATAACAACACAAGATCAAACGAGGTCTCGTA
GTTCCTCGCAGAGAGC
GATGGATAGACGAGAGAGACAAAATATTGACCCAAATAAAATAG
CAATAGATTCACATCA
ATCACATCAGAATAGTCGGAAATTCAAGACGCAGAATTCGCAAA
ATTTCAATAAACAAAA
TAACTCAATTAACAATCAAGTCGGTTTCCGAAGAACACGGAATC
AAAAATGTATTGATAA
CTCGTATAGACATCAACATCCGACTGCATCAAATAATATCCGAT
ACAAATCAGAGGGAGA
GTTGATGGCAAATGATGACAACGACCTGACTTCCAATGTTAGTGT
ATACGATAATGTGAA
ATACGTGCATCCAGAACACACAGAAATCTCGACAAACGCAGTTGA
CAGATTCTCTCGATC
CAAGCAAGTTCAAAACCCCAGAGGAGACCAATCTCCAATCATTC
ATACCGAATTCAGATC
GGAAGAGCACACGTCTGAACTCCAGTC
>ConsensusfromContig1745 Average coverage: 36.19
AGAAATGAAAGATTTCGCACCCAACGACAATTTTGTATTCGAGCT
TTCGGAAATTGCGCT
TTATTTGATGGATTCAAGATCGAAACCTTGTGAGGCCGTGACTTC
AAATGCTGCTTCTGC

CGTATCGAGTGTTGTCTGTACAACGCCAAGGCCTGGAGAAGCTAC
AAAGCCATATCTTGT
TGAAAATGTCATCTCTAGGACGAGCCTTTCTTTCAGAGTCGAGCG
TAATTTTTTAAATAT
CGATAAACAATTGCCTCGAAGTCCTGCGGAGATATTCAAAATGG
AGATGAATGAAACTGA
GCAAATAAGAGATGAAACTCAATGTGAGACTCATATCGCGATCG
AAGGAACTGTGATCGA
TTCTTTCGAAGCCTTTGTCTCCAAACAGACTTATGAATATATGAT
GGAAGCAGTTGAGTT
GATGTCTGATATAAGCGAAAAGACAGATATGGAACGATATAATG
ACGAAATGAATTGTAC
GAGATTGCGTCATAACACTTCCTTCCCTATAATATCTGGGAATTC
ATCAATTCCATCTGG
TTCAGAAAATTCATCCTTCGTTTATGGTGAGTCTAAGAGCAGAGA
TCGAAAAGTATCGGA
GGATGGTGAAGACAATGAAAGCTTTGATGCTGAAGGAGGCTCTG
ACAAACATTCCTCGTC
CATGAGTGCTTCTGATCGGAAAACAGAACCTGAAAAAATCAAAG
AAGGAGGCAAACGGGA
GTTTTCAGATCAACCTGGTAATTCAGTTGCGCTATCGGTAGAGTTT
AACTTGCCTTTTAT
GTGCCTCCATATCACTAGAGACTTCGGTTCTGGTCAGCAGAGTTT
GGCCGACGTGGCTCT
TCGAGAGTTTTCCTTCGTTCTTCAAGACATACCGAAATCTCTTGT
TGCTGAAGAATTCAA
TGAGTCGGATTCTTTCAGTGAAGAAAATTACCTCAGCGGAAAAC
AGAACCAGAAAGCTGG
AAATTTCGATGATGGTGAAATTAACCCTCAAAATAGATTACGGGT
TCGAGTCGTGAGTGT
TTCCTTGCTGTCTCTTTCTGTCGAAGATCTGATGCAGCCAAAAGG
CTCAAAATACAGATA
TCTCGTTCTCTCTGACGCTCTGTGTGATTCTGATGTTAAACCAGA
GGATGTGGTATGTGC
CCTTCGCAAAACGGGTTCTTCAGAGCCAAGTGACAGAGCGGTAAC
AATCGAGCCCCTTTC
AACATCCCTGCCCCCCAACATGCTCGACCTCTCAGACTCCCCTGT
CTCTCGATCGCTCTC
GTTGCCAACTTTCTTCAGAAATACCGACGGCTTGTCTGATCGGTT
TCTGCCTACGAAAAA
ACGCGCTGATCGGCAGAGCATTAATCCTGGAACACCACCACCTTC
CGTCAATGAAATCAA

GAAATTGGTTCGGATCAGAATAATCGTTCCACTCAACGCGAAGGA
AGAAATTTCCTCTGA
TTTGGGGACATCTGTATCAGTGGCTCTAGAAAGCCTTCACCTTTG
CATCAACCTTGAAAC
ATGGGTGGTTCTCCTTGATCTTCTGGGACTGGGTTCAAAGATTCA
CGATTTGGATGAAGT
GGCTCATCGACAAGCCAGCCAGAAAGAAGGAAGTGAAGAAATTC
CGTCACCATCAGAGGC
GGCGATAGGTGGCATGTCTGTAAACGTCGAAATGAAATCTCTGAC
TGCTCTCCTCCATCA
ACACTATGACGGCGCCATCAATAGTCATAGCGCTGACGCTTGCGG
TGATTTCGACGCGGA
GGCGAGCGTCACAGGTCTGACTTTTAACATGCAAAGACAACCAT
TGCCCTCTGGCGGAGA
TATGCGCATCCGTGGACGGCTGAGTGGAATAAAGATTGAGGATG
TGTCAGCGGGGACTGG
AGGCATGTACCGCGAGAGACTGATGAATACGGGCTCGACTGCGCT
TTTCTTTGATGTGTA
CAAATACGGACTGCCCGATCCCCTCCTCGCCCGTGATAGCGACGC
TTCTCTCCACATCCG
GACGTCTTCGCTCTGCTACATTCACACGCAGCGTTTTGTTATTCAA
ATCATTGCCTTTTG
CAACCAGTTCCTTCAGATGCAGGATGTCATGGGACGCATGCGTGC
AGCTGCTGCCGGAAT
AAAGATATCGGAGTTTGCCACAACTGGAGCACGGGTGAAGCTGG
ATGTTGAGGCCGGTGC
ACCAATCATAGTTCTTCCGGTCAGCTCTTTTTCAAATCTCGCTCTC
GTCCTAGATTTGGG
TACACTACGAGTGACAAATAGATTTGATAGAGACGGAACAAAGG
GAACCATTTCTGGAAA
TGAGAAAACGAGAACCGGGAAATCAAAAACATGCCGATCATCAA
CAATGGATTCTTCCCA
AGGCGATGACTCGCACGTGTGCTTGCTGGATGTGATGCG
>ConsensusfromContig1746 Average coverage: 29.04
ATAGATATTCAAATAACCCTGAAACAAATGCTTAGGGATTTTATT
GGTATCAGGGTTAAT
CGTGCCAAGAAAAGCGGCATGGTCAATATAACCAGTAGTGTTAA
CAGTCGGGAGAGGAGT
GGCATTAACACCATCCTTCATGAACTTAATCCACTGTTCACCATA
AACGTGACGATGAGG
GACATAAAAAGTAAAAATGTCTACAGTAGAGTCAATAGCAAGGC
CACGACGCAATGGAGA

AAGACGGAGAGCGCCAACGGCGTCCATCTCGAAGGAGTCGCCAG
CGATAACCGGAGTAGT
TGAAATGGTAATAAGACGACCAATCTGACCAGCAAGGAAGCCAA
GATGGGAAAGGTCATG
CGGCATACGCTCGGCGCCAGTTTGAATATTAGACATAATTTATCC
TCAAGTAAGGGGCCG
AAGCCCCTGCAATTAAAATTGTTGACCACCTACATACCAAAGACG
AGCGCCTTTACGCTT
GCCTTTAGTACCTCGCAACGGCTGCGGACGACCAGGGCGAGCGCC
AGAACGTTTTTTACC
TTTAGACATTACATCACTCCTTCTGCACGTAATTTTTGACGCACGT
TTTCTTCTGCGTCA
GTAAGAACGTCAGTGTTTCCTGCGCGTACACGCAAGGTAAACGC
GAACAATTCAGCGGCT
TTAACCGGACGCTCGACGCCATTAATAATGTTTTCCGTAAATTCA
GCGCCTTCCATGATG
AGACAGGCCGTTTGAATGTTGACGGGATGAACATAATAAGCAAT
GACGGCAGCAATAAAC
TCAACAGGAGCAGGAAAGCGAGGGTATCCTACAAAGTCCAGCGT
ACCATAAACGCAAGCC
TCAACGCAGCGACGAGCACGAGAGCGGTCAGTAGCAATCCAAAC
TTTGTTACTCGTCAGA
AAATCGAAATCATCTTCGGTTAAATCCAAAACGGCAGAAGCCTG
AATGAGCTTAATAGAG
GCCAAAGCGGTCTGGAAACGTACGGATTGTTCAGTAACTTGACTC
ATGATTTCTTACCTA
TTAGTGGTTGAACAGCATCGGACTCAGATAGTAATCCACGCTCTT
TTAAAATGTCAACAA
GAGAATCTCTACCATGAACAAAATGTGACTCATATCTAAACCAGT
CCTTGACGAACGTGC
CAAGCATATTAAGCCACTTCTCCTCATCCAACGCGTCAGTTTTTG
ACAGAATCGTTAGTT
GATGGCGAAAGGTCGCAAAGTAAGAGCTTCTCGAGCTGCGCAAG
GATAGGTCGAATTTTC
TCATTTTCCGCCAGCAGTCCACTTCGATTTAATTCGTAAACAAGC
AGTAGTAATTCCTGC
TTTATCAAGATAATTTTTCGACTCATCAGAAATATCCGAAAGTGT
TAACTTCTGCGTCAT
GGAAGCGATAAAACTCTGCAGGTTGGATACGCCAATCATTTTTA
TCGAAGCGCGCATAAA
TTTGAGCAGATTTGTCGTCACAGGTTGCGCCGCCAAAACGTCGGC
TACAGTAACTTTTCC

CAGCCTCAATCTCATCTCTCTTTTTGCGTTCTGCTTCAATATCTGG
TTGAACGGCGTCGC
GTCGTAACCCAGCTTGGTAAGTTGGATTAAGCACTCCGTGGACAG
ATTTGTCATTGTGAG
CATTTTCATCCCGAAGTTGCGGCTCATTCTGATTCTGAACAGCTT
CTTGGGAAGTAGCGA
CAGCTTGGTTTTTAGTGAGTTGTTCCATTCTTTAGCTCCTAGACCT
TTAGCAGCAAGGTC
CATATCTGACTTTTTGTTAACGTATTTAGCCACATAGAAACCAAC
AGCCATATAACTGGT
AGCTTTAAGCGGCTCACCTTTAGCATCAACAGGCCACAACCAAC
CAGAACGTGAAAAAGC
GTCCTGCGTGTAGCGAACTGCGATGGGCATACTGTAACCATAAGG
CCACGTATTTTGCAA
GCTATTTAACTGGCGGCGATTGCGTACCCGACGACCAAAATTAGG
GTCAACGCTACCTGT
AGGAAGTGTCCGCATAAAGTGCACCGCATGGAAATGAAGACGGC
CATTAGCTGTACCATA
CTCAGGCACACAAAAATACTGATAGCAGTCGGCGTGTGAATCATT
AGCCTTGCGACCCTC
GGCAGCAAGAACCATACGACCAATATCACGAAAATAGTCACGCA
AAGCATTGGGATTATC
ATAAAACGCCTCTAATCGGTCGTCAGCCAACGTGAGAGTGTCAA
AAACGATAAACCAACC
ATCAGCATGAGCCTGTCGCATTGCATTCATCAAACGCTGAATAGT
AAAGCCTCTACGCGA
TTTCATAGTGGAGGCCTCCAGCAATCTTGAACACTCATCCTTAATA
CCTTTCTTTTTGGG
GTAATTATACTCATCGCGAATATCCTTAAGAGGGCGTTCAGCAGC
CAGCTTGCGGCAAAA
CTGCGTAACCGTCTTCTCGTTCTCTAAAAACCATTTTTCGTCCCCT
TCGGGGCGGTGGTC
TATAGTGTTATTAATATCAAGTTGGGGGAGCACATTGTAGCATTG
TGCCAATTCATCCAT
TAACTTCTCAGTAACAGATACAAACTCATCACGAACGTCAGAAGC
AGCCTTATGGCCGTC
AACATACATATCACCATTATCGAACTCAACGCCCTGCATACGAAA
AGACAGAATCTCTTC
CAAGAGCTTGATGCGGTTATCCATCTGCTTATGGAAGCCAAGCA
TTGGGGATTGAGAAAG
AGTAGAAATGCCACAAGCCTCAATAGCAGGTTTAAGAGCCTCGA
TACGCTCAAAGTCAAA

ATAATCAGCGTGACATTCAGAAGGGTAATAAGAACGAACCATAA
AAAAGCCTCCAAGATT
TGGAGGCATGAAAACATACAATTGGGAGGGTGTCAATCCTGACG
GTTATTTCCTAGACAA
ATTAGAGCCAATACCATCAGCTTTACCGTCTTTCCAGAAATTGTT
CCAAGTATCGGCAAC
AGCTTTATCAATACCATGAAAAATATCAACCACACCAGAAGCAG
CATCAGTGACGACATT
AGAAATATCCTTTGCAGTAGCGCCAATATGAGAAGAGCCATACCG
CTGATTCTGCGTTTG
CTGATGAACTAAGTCAACCTCAGCACTAACCTTGCGAGTCATTTC
TTTGATTTGGTCATT
GGTAAAATACTGACCAGCCGTTTGAGCTTGAGTAAGCATTTGGCG
CATAATCTCGGAAAC
CTGCTGTTGCTTGGAAAGATTGGTGTTTTCCATAATAGACGCAAC
GCGAGCAGTAGACTC
CTTCTGTTGATAAGCAAGCATCTCATTTTGTGCATATACCTGGTCT
TTCGTATTCTGGCG
TGAAGTCGCCGACTGAATGCCAGCAATCTCTTTTTGAGTCTCATT
TTGCATCTCGGCAAT
CTCTTTCTGATTGTCCAGTTGCATTTTAGTAAGCTCTTTTTGATTC
TCAAATCCGGCGTC
AACCATACCAGCAGAGGAAGCATCAGCACCAGCACGCTCCCAAG
CATTAAGCTCAGGAAA
TGCAGCAGCAAGATAATCACGAGTATCCTTTCCTTTATCAGCGGC
AGACTTGCCACCAAG
TCCAACCAAATCAAGCAACTTATCAGAAACGGCAGAAGTGCCAG
CCTGCAACGTACCTTC
AAGAAGTCCTTTACCAGCTTTAGCCATAGCACCAGAAACAAAAC
TAGGGGCGGCCTCATC
AGGGTTAGGAACATTAGAGCCTTGAATGGCAGATTTAATACCAGC
ATCACCCATGCCTAC
AGTATTGTTATCGGTAGCAAGCACATCACCTTGAATGCCACCGGA
GGCGGCTTTTTGACC
GCCTCCAAACAATTTAGACATGGCGCCACCAGCAAGAGCAGAAG
CAATACCGCCAGCAAT
AGCACCAAACATAAATCACCTCACTTAAGTGGCTGGAGACAAATA
ATCTCTTTAATAACC
TGATTCAGCGAAACCAATCCGCGGCATTTAGTAGCGGTAAAGTT
AGACCAAACCATGAAA
CCAACATAAACATTATTGCCCGGCGTACGGGGAAGGACGTCAAT
AGTCACACAGTCCTTG

ACGGTATAATAACCACCATCATGGCGACCATTCAAAGGATAAAC
ATCATAGGCAGTCGGG
AGGGTAGTCGGAACCGAAGAAGACTCAAAGCGAACCAAACAGGC
AAAAAATTTAGGGTCG
GCATCAAAAGCAATATCAGCACCAACAGAAACAACCTGATTAGC
GGCGTTGACAGATGTA
TCCATCTGAATGCAATGAAGAAAACCACCATTACCAGCATTAAC
CGTCAAACTATCAAAA
TATAACGTTGACGATGTAGCTTTAGGTGTCTGTAAAACAGGTGC
CGAAGAAGCTGGAGTA
ACAGAAGTGAGAACCAGCTTATCAGAAAAAAAGTTTGAATTATG
GCGAGAAATAAAAGTC
TGAAACATGATTAAACTCCTAAGCAGAAAACCTACCGCGCTTCGC
TTGGTCAACCCCTCA
GCGGCAAAAATTAAAATTTTTACCGCTTCGGCGTTATAACCTCAC
ACTCAATCTTTTATC
ACGAAGTCATGATTGAATCGCGAGTGGTCGGCAGATTGCGATAA
ACGGTCACATTAAATT
TAACCTGACTATTCCACTGCAACAACTGAACGGACTGGAAACACT
GGTCATAATCATGGT
GGCGAATAAGTACGCGTTCTTGCAAATCACCAGAAGGCGGTTCC
TGAATGAATGGGAAGC
CTTCAAGAAGGTGATAAGCAGGAGAAACATACGAAGGCGCATAA
CGATACCACTGACCCT
CAGCAATCTTAAACTTCTTAGACGAATCACCAGAACGGAAAACAT
CCTTCATAGAAATTT
CACGCGGCGGCAAGTTGCCATACAAAACAGGGTCGCCAGCAATA
TCGGTATAAGTCAAAG
CACCTTTAGCGTTAAGGTACTGAATCTCTTTAGTCGCAGTAGGCG
GAAAACGAACAAGCG
CAAGAGTAAACATAGTGCCATGCTCAGGAACAAAGAAACGCGGC
ACAGAATGTTTATAGG
TCTGTTGAACACGACCAGAAAACTGGCCTAACGACGTTTGGTCAG
TTCCATCAACATCAT
AGCCAGATGCCCAGAGATTAGAGCGCATGACAAGTAAAGGACGG
TTGTCAGCGTCATAAG
AGGTTTTACCTCCAAATGAAGAAATAACATCACGGTAACGCTGCA
TGAAGTAATCACGTT
CTTGGTCAGTATGCAAATTAGCATAAGCAGCTTGCAGACCCATAA
TGTCAATAGATGTGG
TAGAAGTCGTCATTTGGCGAGAAAGCTCAGTCTCAGGAGGAAGC
GGAGCAGTCCAAATGT

TTTTGAGATGGCAGCAACGGAAACCATAACGAGCATCATCTTGAT
TAAGCTCATTAGGGT
TAGCCTCGGTACGGTCAGGCATCCACGGCGCTTTAAAATAGCTGT
TATAGATATTCAAAT
AACCCTGAAACAAATGCTTAGGGATTTTATTGGTATCAGGGTTAA
TCGTGCCAAGAAAAG
CGGCATGGTCAATATAACCAGTAGTGTTAACAGTCGGGAGAGGA
GTGGCATTAACACCAT
CCTTCATGAACTTAATCCACTGTTCACCATAAACGTGACGATGAG
GGACATAAAAAGTAA
AAATGTCTACAGTAGAGTCAATAGCAAGGCCACGACGCAATGGA
GAAAGACGGAGAGCGC
CAACGGCGTCCATCTCGAAGGAGTCGCCAGCGATAACCGGAGTA
GTTGAAATGGTAATAA
GACGACCAATCTGACCAGCAAGGAAGCCAAGATGGGAAAGGTCA
TGCGGCATACGCTCGG
CGCCAGTTTGAATATTAGACATAATTTATCCTCAAGTAAGGGGCC
GAAGCCCCTGCAATT
AAAATTGTTGACCACCTACATACCAAAGACGAGCGCCTTTACGCT
TGCCTTTAGTACCTC
GCAACGGCTGCGGACGACCAGGGCGAGCGCCAGAACGTTTTTTAC
CTTTAGACATTACAT
CACTCCTTCTGCACGTAATTTTTGACGCACGTTTTCTTCTGCGTCA
GTAAGAACGTCAGT
GTTTCCTGCGCGTACACGCAAGGTAAACGCGAACAATTCAGCGGC
TTTAACCGGACGCTC
GACGCCATTAATAATGTTTTCCGTAAATTCAGCGCCTTCCATGAT
GAGACAGGCCGTTTG
AATGTTGACGGGATGAACATAATAAGCAATGACGGCAGCAATAA
ACTCAACAGGAGCAGG
AAAGCGAGGGTATCCTACAAAGTCCAGCGTACCATAAACGCAAG
CCTCAACGCAGCGACG
AGCACGAGAGCGGTCAGTAGCAATCCAAACTTTGTTACTCGTCA
GAAAATCGAAATCATC
TTCGGTTAAATCCAAAACGGCAGAAGCCTGAATGAGCTTAATAG
AGGCCAAAGCGGTCTG
GAAACGTACGGATTGTTCAGTAACTTGACTCATGATTTCTTACCT
ATTAGTGGTTGAACA
GCATCGGACTCAGATAGTAATCCACGCTCTTTTAAAATGTCAACA
AGAGAATCTCTACCA
TGAACAAAATGTGACTCATATCTAAACCAGTCCTTGACGAACGTG
CCAAGCATATTAAGC

CACTTCTCCTCATCCAACGCGTCAGTTTTTGACAGAATCGTTAGT
TGATGGCGAAAGGTC
GCAAAGTAAGAGCTTCTCGAGCTGCGCAAGGATAGGTCGAATTTT
CTCATTTTCCGCCAG
CAGTCCACTTCGATTTAATTCGTAAACAAGCAGTAGTAATTCCTG
CTTTATCAAGATAAT
TTTTCGACTCATCAGAAATATCCGAAAGTGTTAACTTCTGCGTCA
TGGAAGCGATAAAAC
TCTGCAGGTTGGATACGCCAATCATTTTTATCGAAGCGCGCATAA
ATTTGAGCAGATTTG
TCGTCACAGGTTGCGCCGCCAAAACGTCGGCTACAGTAACTTTTC
CCAGCCTCAATCTCA
TCTCTCTTTTTGCGTTCTGCTTCAATATCTGGTTGAACGGCGTCGC
GTCGTAACCCAGCT
TGGTAAGTTGGATTAAGCACTCCGTGGACAGATTTGTCATTGTGA
GCATTTTCATCCCGA
AGTTGCGGCTCATTCTGATTCTGAACAGCTTCTTGGGAAGTAGCG
ACAGCTTGGTTTTTA
GTGAGTTGTTCCATTCTTTAGCTCCTAGACCTTTAGCAGCAAGGTC
CATATCTGACTTTT
TGTTAACGTATTTAGCCACATAGAAACCAACAGCCATATAACTGG
TAGCTTTAAGCGGCT
CACCTTTAGCATCAACAGGCCACAACCAACCAGAACGTGAAAAA
GCGTCCTGCGTGTAGC
GAACTGCGATGGGCATACTGTAACCATAAGGCCACGTATTTTGCA
AGCTATTTAACTGGC
GGCGATTGCGTACCCGACGACCAAAATTAGGGTCAACGCTACCT
GTAGGAAGTGTCCGCA
TAAAGTGCACCGCATGGAAATGAAGACGGCCATTAGCTGTACCA
TACTCAGGCACACAAA
AATACTGATAGCAGTCGGCGTGTGAATCATTAGCCTTGCGACCC
TCGGCAGCAAGAACCA
TACGACCAATATCACGAAAATAGTCACGCAAAGCATTGGGATTA
TCATAAAACGCCTCTA
ATCGGTCGTCAGCCAACGTGAGAGTGTCAAAAACGATAAACCAA
CCATCAGCATGAGCCT
GTCGCATTGCATTCATCAAACGCTGAATAGTAAAGCCTCTACGCG
ATTTCATAGTGGAGG
CCTCCAGCAATCTTGAACACTCATCCTTAATACCTTTCTTTTTGGG
GTAATTATACTCAT
CGCGAATATCCTTAAGAGGGCGTTCAGCAGCCAGCTTGCGGCAAA
ACTGCGTAACCGTCT

TCTCGTTCTCTAAAAACCATTTTTCGTCCCCTTCGGGGCGGTGGTC
TATAGTGTTATTAA
TATCAAGTTGGGGGAGCACATTGTAGCATTGTGCCAATTCATCCA
TTAACTTCTCAGTAA
CAGATACAAACTCATCACGAACGTCAGAAGCAGCCTTATGGCCG
TCAACATACATATCAC
CATTATCGAACTCAACGCCCTGCATACGAAAAGACAGAATCTCTT
CCAAGAGCTTGATGC
GGTTATCCATCTGCTTATGGAAGCCAAGCATTGGGGATTGAGAA
AGAGTAGAAATGCCAC
AAGCCTCAATAGCAGGTTTAAGAGCCTCGATACGCTCAAAGTCA
AAATAATCAGCGTGAC
ATTCAGAAGGGTAATAAGAACGAACCATAAAAAAGCCTCCAAGA
TTTGGAGGCATGAAAA
CATACAATTGGGAGGGTGTCAATCCTGACGGTTATTTCCTAGACA
AATTAGAGCCAATAC
CATCAGCTTTACCGTCTTTCCAGAAATTGTTCCAAGTATCGGCAA
CAGCTTTATCAATAC
CATGAAAAATATCAACCACACCAGAAGCAGCATCAGTGACGACA
TTAGAAATATCCTTTG
CAGTAGCGCCAATATGAGAAGAGCCATACCGCTGATTCTGCGTTT
GCTGATGAACTAAGT
CAACCTCAGCACTAACCTTGCGAGTCATTTCTTTGATTTGGTCAT
TGGTAAAATACTGAC
CAGCCGTTTGAGCTTGAGTAAGCATTTGGCGCATAATCTCGGAAA
CCTGCTGTTGCTTGG
AAAGATTGGTGTTTTCCATAATAGACGCAACGCGAGCAGTAGACT
CCTTCTGTTGATAAG
CAAGCATCTCATTTTGTGCATATACCTGGTCTTTCGTATTCTGGC
GTGAAGTCGCCGACT
GAATGCCAGCAATCTCTTTTTGAGTCTCATTTTGCATCTCGGCAAT
CTCTTTCTGATTGT
CCAGTTGCATTTTAGTAAGCTCTTTTTGATTCTCAAATCCGGCGT
CAACCATACCAGCAG
AGGAAGCATCAGCACCAGCACGCTCCCAAGCATTAAGCTCAGGA
AATGCAGCAGCAAGAT
AATCACGAGTATCCTTTCCTTTATCAGCGGCAGACTTGCCACCAA
GTCCAACCAAATCAA
GCAACTTATCAGAAACGGCAGAAGTGCCAGCCTGCAACGTACCTT
CAAGAAGTCCTTTAC
CAGCTTTAGCCATAGCACCAGAAACAAAACTAGGGGCGGCCTCA
TCAGGGTTAGGAACAT

TAGAGCCTTGAATGGCAGATTTAATACCAGCATCACCCATGCCTA
CAGTATTGTTATCGG
TAGCAAGCACATCACCTTGAATGCCACCGGAGGCGGCTTTTTGAC
CGCCTCCAAACAATT
TAGACATGGCGCCACCAGCAAGAGCAGAAGCAATACCGCCAGCA
ATAGCACCAAACATAA
ATCACCTCACTTAAGTGGCTGGAGACAAATAATCTCTTTAATAAC
CTGATTCAGCGAAAC
CAATCCGCGGCATTTAGTAGCGGTAAAGTTAGACCAAACCATGA
AACCAACATAAACATT
ATTGCCCGGCGTACGGGGAAGGACGTCAATAGTCACACAGTCCT
TGACGGTATAATAACC
ACCATCATGGCGACCATTCAAAGGATAAACATCATAGGCAGTCG
GGAGGGTAGTCGGAAC
CGAAGAAGACTCAAAGCGAACCAAACAGGCAAAAAATTTAGGGT
CGGCATCAAAAGCAAT
ATCAGCACCAACAGAAACAACCTGATTAGCGGCGTTGACAGATG
TATCCATCTGAATGCA
ATGAAGAAAACCACCATTACCAGCATTAACCGTCAAACTATCAA
AATATAACGTTGACGA
TGTAGCTTTAGGTGTCTGTAAAACAGGTGCCGAAGAAGCTGGAG
TAACAGAAGTGAGAAC
CAGCTTATCAGAAAAAAAGTTTGAATTATGGCGAGAAATAAAAG
TCTGAAACATGATTAA
ACTCCTAAGCAGAAAACCTACCGCGCTTCGCTTGGTCAACCCCTC
AGCGGCAAAAATTAA
AATTTTTACCGCTTCGGCGTTATAACCTCACACTCAATCTTTTATC
ACGAAGTCATGATT
GAATCGCGAGTGGTCGGCAGATTGCGATAAACGGTCACATTAAAT
TTAACCTGACTATTC
CACTGCAACAACTGAACGGACTGGAAACACTGGTCATAATCATG
GTGGCGAATAAGTACG
CGTTCTTGCAAATCACCAGAAGGCGGTTCCTGAATGAATGGGAA
GCCTTCAAGAAGGTGA
TAAGCAGGAGAAACATACGAAGGCGCATAACGATACCACTGACC
CTCAGCAATCTTAAAC
TTCTTAGACGAATCACCAGAACGGAAAACATCCTTCATAGAAAT
TTCACGCGGCGGCAAG
TTGCCATACAAAACAGGGTCGCCAGCAATATCGGTATAAGTCAAA
GCACCTTTAGCGTTA
AGGTACTGAATCTCTTTAGTCGCAGTAGGCGGAAAACGAACAAG
CGCAAGAGTAAACATA

GTGCCATGCTCAGGAACAAAGAAACGCGGCACAGAATGTTTATA
GGTCTGTTGAACACGA
CCAGAAAACTGGCCTAACGACGTTTGGTCAGTTCCATCAACATCA
TAGCCAGATGCCCAG
AGATTAGAGCGCATGACAAGTAAAGGACGGTTGTCAGCGTCATA
AGAGGTTTTACCTCCA
AATGAAGAAATAACATCACGGTAACGCTGCATGAAGTAATCACGT
TCTTGGTCAGTATGC
AAATTAGCATAAGCAGCTTGCAGACCCATAATGTCAATAGATGTG
GTAGAAGTCGTCATT
TGGCGAGAAAGCTCAGTCTCAGGAGGAAGCGGAGCAGTCCAAAT
GTTTTTGAGATGGCAG
CAACGGAAACCATAACGAGCATCATCTTGATTAAGCTCATTAGGG
TTAGCCTCGGTACGG
TCAGGCATCCACGGCGCTTTAAAATA
>ConsensusfromContig1747 Average coverage: 9.90
CGCAACTGAAACGAATCCAGTCAATGGCCTGCGAGACAGATAGC
AGTGAAGTTGATGCAG
TTGGAATTCTCACCTCAGCTGACAGAACGACCTGGGCCAGAGAT
AGAGAAACTCTTATGG
GTATATGCGAAGGCAACAGAACTATTTTGAAGAAAATCGAGACGT
CGCTCTTCATTCTGT
GTTTGGACGATGAAGTGTCGCGAACAGCCAATCAGAATTCCAGC
AATCACCGAGACGACG
AATCTCTGGCTTGTCAGATGCTGCATGGTCAAGGCTCTCGCACTA
ATAGCTGCAACAGAT
GGTTCGACAAAACCATTCATTTCATCGTGGCCGAAGATGGAGCCT
ATGGGCTGCATTACG
AACATTCTCCTGCAGAGGGAGTTGCTGTTGTTCAACTCATTGAACA
TCTTCTCCGTTTTA
TGTCGGAATTTCCAAGTCGAAAATTTCAGCGTATGAATTCTATCG
GTGAAATCAAAAGTG
CAAGACTTCTGAATTTTGAAATAAATGATGAATTAAGAGGCAGA
ATTACAGAAGCAAAAG
TGAAACTTGATGAATCAATCGAGAAGATGGACATGTATGTTTTGA
GGTTCGAAAAGTTCG
GGAAAAACGCCGTAAAATCGAACAAGATGAGCCCTGATGCTTTC
ATACAGCTAGGCTTAC
AACTGACGTATTTCAGAATTTATGGAAAACTGGTTTCAACATACG
AAAGTGCTTCAATTC
GGCGGTATCGGTATGGACGAGTCGACAATATCCGGGCCTGTACCT
CACCGGCTCTGAAAT

GGGTCCAATCCATGGTCTCAAAAGAAACTTCGGATGAGGAGAAAC
TTCAACTTTTTGTGA
AAGCAATGAAGAAACAGACTGAAATTCTTGTTCAAAACATCCTG
GGAAAAGGATTCGATA
ACCATCTTTTGGGACTTCGGATGACTGCCGTGAATTCTGGATGGG
ATCTGCCGAGAATAT
TCTCAGATGACTCGTATCGAATCTCAAACCATTTCAAACTATCCA
CGAGTCAGGTTCAGA
CGTCGATGGATGCGTTCATGACATACGGTCCGGTCGTGGATGACG
GGTACGGCGTTTGTT
AAAATCCCCACGAAGATTGGATCCTTTT
>ConsensusfromContig1748 Average coverage: 17.19
TGTGAGACAACTGGACTTACGGCACCTACTGGTTCATGTCATGAA
GGCTACTACTGTCCT
GGTGGGCAGGGAGTTGGTTCACCTAATGGCCTTGAGTGTACTCCT
GGTCACTTTTGCCCA
TTGGGATCCAGTGCTGAGATTGCTTGTGATCCTGGCTTGTACCAG
GATCTGTTTGCTCAA
AGCATTTGTAAAGAGTGTCCTTCCGGCTTCTATTGCGATTCAACA
ATTCAAAACAATACT
CATTGCGCCCATGGTGTCCAAAACCCTACTCCATGTCCCGAGGGT
CATTATTGTCCCAAT
GGAACAAGATACGGAACTGAATTCCCGTGCGAAACGGAACATT
CAACAATCGAACTGGT
GTCATGAATGGCTCGGAATGCACTCCTTGTCTAGCCGGTTTTTAT
TGTGGATCTTTGGGC
CTCATCGACCCCACTGCAGAATGTTATGCGGGGTTCTATTGTATA
TCAGGAGCTTCTATC
CCGACTCCAGTTGACGGGGAAACGGAGATATTTGTCCGGCTGGC
TCTTTCTGCCCTGAA
GGCTCTGGTTTTCACGAATTATGTCCATCAAGTACATACAATCCA
ACATCAGGTATAACC
GCAGAGTCTGAGTGTACGGCATGTGAGCCTGGGCAGTTTTGCTCC
ACTCAAGGGTTGAGT
CAACCGACTGGTAATTGTACCGCAGGATATTTTTGCCCCGGCAAT
TCCTCAACACCCACT
GATTCAGATTGTACACAAGGTCATTATTGCCCCGAGGGATCAGCT
TTCCCTCTCCCATGT
CAAGAAGGCACGTACAGCAGTACGAGTCGAAACTCTGAATGCGA
AGTGTGTCCGATGAGT
TATTACTGTCTGTCGGAAACTGTCACTCCAACCATCTGCCCTGCTG
GTTATTTCTGCATC

GAAGGGACTGGAATGGACTGGAAACCATGTCCTCCAGGAACATT
TGGCGCTGCGACAGGA
CTTAGTGCATTGGACATGTGCTCCAATTGCACGGGTGGATTTTAC
TGCGGGGATTATAAC
GCCACGGGTGTAACAGGACCATGTTCGGCCGGTTATTACTGCAGA
TCGGGTTCTGATCAA
GAAATGCCTGATGGAAGCTCGGGAGATGCGGGTATCTGTCCCCCT
GGGTACTTCTGCCCA
TTCCAGACCTCGGACCCTATTCCTTGCCCGGCAGGGACTTTCAAC
AACGTCTCTGGATTG
GATGCCCAAGAAGACTGCCAATCGTGTCCCCCGGGTCTCTTCTGC
GAATATCCTGGCCTC
AGTCTACCCTCAGGCCTCTGTGATACGGGTTTCTATTGCTCCGGG
GGCTCAAATAGCTCG
AACCCAGAGTCGGGTACA
>ConsensusfromContig1749 Average coverage: 22.26
AACTGGAGAAACCAAAGTTGAAGAGAAAGAATTTTCTGATTCGG
TTCTTGACGAAATTGC
CCTCAATCTCCACCGAATAGACAAGGATGTTCTGCGCTGCGACCG
AAACTACGCGTTCTT
CACCTCTGAGAACCTCAACAAGCTACGCAACGTCATCGCCACATA
TGTGTGGAACAATTT
ATCTGTGGGATACGTACAAGGGATGTGTGATCTATGTGCCCCACT
TCTTGTGATCCTAGG
AGATGAATCGCTGACATTCGGATGTTTTCGGGAACTGATGAAACG
AATGTCGTCCAATTT
CCCACACGGAACGGCGATGGATCAGCACTTTGCCAACATGAGATC
TCTCATTCAGATTCT
CGACGGGGAGATGTTCGAACACATGGAGCAGAACGGAGACTTGAC
TCATTTCTATTTCTG
CTATCGCTGGTTTCTTCTTGACTTCAAAAGAGAACTTGTCTACGA
TGATATCTTCACGGT
GTGGGAGACGATCTGGGCGGCAGCTCGGGTCAGCTCGGGAAACTT
CATTCTGTTCGTGGC
TCTAGCACTCGTGGAGACTTATCGGGAGATTATCCTTGATAACAA
TATGGACTTCACGGA
TATCATCAAGTTCTTCAATGAAATGGCGGAGAGGCATGACGTGA
AACAGGTGATGCGAGT
CGCAAAAGATCTGGTCTTCAAACTCCAGAAGATGATTGACAATC
GCAATTAGGATGGCAG
CTATGACAACCAAAGAACAATCGCGGAACGGCCGTCCCTGTTGG
CATGACAACCAAACAT

TTACTTATTTTGTTATGGAAGTGTGCATCTACGAATTATTAGCGA
ATAGACGTACAAATT
CATTACATCACTGCGCAGAATATTGTATTTATGTTATATAGTGAA
GGTTTATTTTGAAGC
GTGTATCACTACAGAGGCAGATCAAAATTCGAAGTGTGTTGCAT
GAATGGAGAATATAAA
GTTGAATTGTATTCGTATGGTGGCTATTATTCTTTGTCGCAATAAA
ATGAACCTTTACTA
GAACTGTTGAAAATGGTGGAGC
>ConsensusfromContig1750 Average coverage: 11.69
TTCCGATCTCAGGAAGTAGCATCTAGTATAAAGAATCCCAATCCA
TGGGTAACCATCAAG
CCAAAGGTGGTTAAAAGGCAAGAACCCAAAATGGGACATGTTAC
CAATTGTGACCTGAAA
TCTGCTGGTGTGCGATTATTCAAATCTCAAGTGTTCGCAACACGA
TTTCACCCTAACACG
AAAGCCGAGGAGGTAACTACTTATTTAAAGAAAAAAATAAATAC
AGATGTTGTTGTTGAA
AAACTCACTACCAGGTTTCCTGAGCATTATTCATCGTTTAAAATA
ACAGCATATGTAAAA
AACCATGCCATATTGATGGATGAAACAATATGGCCAGAAGGAGTT
TTATTCAAGAGATTT
TTCAACAAAAGACCCACCAATGAAAATGGCAACATTTAACATCA
TAAGTTGGAACTGTCG
AGGACTAAAATTTCCTCTAAAATATAATTTCGAACTACTACAAAT
CATGAAATCATATAA
AATAATTTGTTTACAGGAAACCTGGTTACCTAAACAGGTAGACCT
AGACATAAAAAAGTT
CAATGGAGGATGTGCGGTGTCAAACTCACCAAATGATGACTCAC
AAGGCC
>ConsensusfromContig1751 Average coverage: 35.84
CCGGGCGAGAGGATGAAAACGATGGGTGAGAGTGGCGTACTTTG
CTCCCAAATGGCCTCG
AAACTTATCGTGGGGGGTGTTACGTACTTTTCGCCCATGACCTTG
GTGACGTATTCCGTC
ACGCCTCGGTAGATGCGATCGACGCGGAAGCAACGGAGAAGAGT
CAGTCTCTCGAAGTCG
TTCAACTTCTCCTCATATCCATCAGGAATGGTTGCAGACTCCGGA
GCATCCATATCAAAC
CACGCCTTCCACTGTCCTTTGTCCGCATCTAACTGGTCCAGCAGA
TTCCCCAGCGTCTCC
GGCATAGCCTGCGTCACCGTGACGCAATCCTCCCACGTTCCGTCC

GGAATCCACGCGTGC
TGTTTGGTGCGGGCGCTTTTCTCTAGGGCAACGTTCCCCTTGATG
AAGAAATCCAGTTGA
TTCTGCAGGACATTGCATCTGTCCATCTCCAGTTTGACTGTGATC
TGGAACGAGAACAGC
AACTTGTGTTTCTCGAAGATTCCCGTGCAGCCATAGTTGTAGACA
TTATGCGTGAGCGTA
TCCATGATATTTCTGAGCCTCTTCTGCAGATTTGTATCGGGCATGC
TTTTCCTCAACGAG
AAGATAAAGACATCCAAATAAGCAGCCAGGGAGTACTGGTACAT
AGAGTTGATGGTTGAC
ATCTCAGAGAGCACAAAGAACAGAATGGCGCCTCGTTTAGCCGC
CGGCCTGTAGCCGTCT
CTGAGCTTATCAATGTCTATAGC
>ConsensusfromContig1752 Average coverage: 12.53
CTCTTCCGATCTCTTTTCTTCGGACACATGAGTGGACCCCCGCTGT
CTCCTGCGCATGTA
TCTTTTCCACCTCTCTTTCTTCCCGCACACAATTGACTATCTGTAA
CTTCAAATTGGAAA
GCTCGATTACATCTCTTGTTTGAAATAATTGGAATTTGGACTTCT
TGTAAGCGTGTTGCG
CCATATATATGAGAATTTTTGGTTTTACCCCATCCGGTTACTGTAC
ATTCTCTTCTTTTG
AGGTAATCGCCACGTTTCTGAGGTAAACAGGCGTAAGCTAGCTGT
GACTCTACTGTTTTG
TTTATTTCAGAAATCTTTACAAGAGCTATGTCGTTTTCTATCGTGT
TTCTGTTGTAACCC
GGGTATATATAAGTATTTATAGGAACAACACGATGGCCCTGGTTC
TTCTCTTTGATGTTG
AATGCCAGGAGCCAGACGAAGAACTGCCGAGAAACATGTCTTCG
TTGAATGCAGTGCGCT
GCTGTTAAGACCCACATCGGACTCAGCAAAACGCCCCCGCAGAAG
TGCTCGCTTTGGTTG
ACAGTCGCCAGCGCCACCTGCCATGGCCAGCTTCCCCGCTGCGAC
CTCTGACCCCCTATG
ATGCGGGGCTGGGCAACATCTGACAGCTTGAAGCCACATGATGA
CACGTTGAGAATTGAC
GAGAGGGTGTCGAAGCGATCTCGGTGCTTTTTTGAACTTTTTGCA
TATTGTCGGAATGGT
TTACATCTGCTGCTACCTGGAATCGAACATCGCATCTCCTGGAACA
TTGTTTTGTTCTCG
CAGACCTGACTGAGAATGCAAACTCGCGAACGCTCTTTTTTGCAC

CTTTGCAAGCATGCT
GACCAGTTGGACCAATTGGAGTAGACGAGGTCAATCAGGATATCC
TCCAGTGTATATTTT
CCATTGAATTCAAGCTCTGTGGCGTTCCCATCTGCGATTCTGATA
TCTTTCTCAGGATAA
TCTGTGCATTTGCCTTTGTCGAAGGAGCAGTCTTTTGTTTCTAGAA
TAACTGATCCTTTG
CAGACAGCTGTGATGTAACAGCGTCTCCT
>ConsensusfromContig1753 Average coverage: 19.03
GATCCTGCTTCCTCCAAATACCGACACAAGACGACGGATAAAGA
CTACAAAACTCAAGAA
TCTTGTCCAGAAATGAGAGATACTGACGATAATAATGTTATCGTA
GAATCTTCAAAAAAT
GTCGACCGAGCGACCATGACAGAATTGTGTACAACGATGAAGAG
CCAAATTCTCTCAAGA
GCATTCTATGGATGGGTTTCGTACTGCCGTCAGTTGTCTTCGGTG
AGAACGCATCTCACA
GGGCTTGTAAAACCGATACTTGCGGAGGAAGCTCCGCATAATCCT
ACACCACTTGATCGC
CTCTCGTGGGAAAATTTATCTGAAAATAATGACGATCAATCAATA
AACCAAATATGTCGA
TGCGTCTACTACGGTGGATGTGATCACTCAATTCGTAAACAGGTC
TGGCCCTATCTGTTG
GGCCATTATATGTACGGGATGGACGCCTTACACCTGGAAGCTAA
ACAAAAGGAGACCAGA
GCTGAGTATGAAGCGACGGTTGAGCGCTGGAAGGCCGCTGAGGA
AATAATCAAAGAGAGA
GAAAAAGATACGCAATTGAAAAAGATCAAAGCCGACAAATTAAA
GCAGAGAAGCACACTA
GAGAGCGCCAAAGATGGTCGCGAGCATTCGAAAGCGACGGCACA
GGATTCGATTGAGGGG
AATCCTTGGCTCTCCGGCGGATCTTCTTTTCCGCCGCTCGCCTTGT
CGGCAGCTTCACTT
GCCGCGGAGGCAAAGTTGAAGGCAACTAAAGGATTAAAGGATTT
GTTTCCGTTCATGAGG
AATGACTCAGACCTTAGTAATGATGTCTTTTTGAGTCTCGATAAT
GAAG
>ConsensusfromContig1754 Average coverage: 34.25
TTGTATCATTATTTGTAGATTGAAAATTACATTCTGTATGTTTATT
ATTAACTAAACATC
TAGTACGAGAAATGCAGAGAAAAAAATTGAATAATGGTCTTTATT
TCAATAAATCTAATA

AACAACAGCTGATCTCATATATAAGTAGGAATGTGATTGGTCAAG
ATATGCATTTTATAG
GACCATTCGGCCCGAGAAAAATAATATTTTGCGATCACACCGCTT
CGGGCCATTCTCTGA
CCTTCATCGAGAATTATATAAAAAACTCAGTTCTGCCCAGCTACG
GGAACACGCACACGT
CGACCAGCTTCTCTTCCCTGCAAACTTCACTTTTTAGAGATGAAG
CAAGAAATATAATCA
GAAATGCATGTAACGCGAGCAGTTTCGATTCTGTCATTTTCGCCG
GGAGTGGAACCACTG
GTGCCATGCAAGTTTGTATCAACGCGTTGGGCTTTTCAGAGATGG
TTGTGTTCCTCTGTC
CATTCACACATCACTCTTCACTACTGCCATGGCGAGAACGGGATG
CAAAAATCGTACGGA
TTCCAGAATCACCAGTAGGAAAGATGGATCTGATTCAACTTGAG
AAGGAGCTGGATCAAT
GGCGTGAAACAGGGATCCCCATGGTCTGCTGCTTCTCTGCTGCTT
CCAACATCACTGGTA
TTCTGGCTGACGTCGATGTAACGGTCAGGCTGGTCCACAAATACG
GAGGAATCGTTATAT
GGGACTATGCTACGGCGGCTCCTCACGTGAATATAGACATGAATC
CTTCATCGGATTCCC
GTGGTGTAGTAGGGTCTTTCGCCAAGGACGCTGTTGTTTTCTCAC
CGCACAAGCTCATAG
GAGGAACACAAACACCAGGCGTTCTGATAATGAAGAAGAAACTT
TTTCGAAACTCTGTTC
CGAATGCACCAGGAGGAGGAACAGTGCTTTATGTCATGAAAGAC
GGACACAGGTATTTGG
ATAACCCCGAGGAGCGAGAGGAAGGAGGTACACCAGCGATCGTT
GAGTCCATAAGAGCAG
GGCTCGTCTTGCAGTTGCATAAGTCTGTGGGTCATAACAGCATCC
GTCGAATTGATGAGA
TGCGGGCGATAAAGACTATTATGGCATGGGATCGACTGCCTAATC
TAATAATGTTAGGGC
CGACATTGTTGGCAGAAAATGGGAAAACAAAATGTATGAAACTTC
CGATATTTTCATTTC
TGATCCGGCATGCAAAATCAGGACTCTATCTGCATTGCAATTTTG
TATGTGTTCTACTCA
ATGATCTGTTTGGCATTCAATCAAGAGGCGGATGTGCATGTGCTG
GTCCCTACGCACAGG
ACCTTCTTGGATTTGATGACGCCATAACTAAAGAATATGTGCAGA
TTATCGATAGGTATC

CTGCATACAAACCAGGTTTCAGTCGATTGAGTTTCAC
>ConsensusfromContig1755 Average coverage: 20.61
TTCGTTCTCAAAATTCCTGGCATTGGTTCCTATAGGAGCATCTTG
GACGCAATTTCGAAG
ATACTCAGCATCATCATTCGTGGTCATTCATTCCAAATCCAGCATT
TGTTCGATATTTGT
TCATTGGTGACACGTGGCTTCAATAAAGAGCGCGATCGGATGTA
TATGGCCAGAACCGTG
ACTTTCGAACTGACGAGTTGTCTGAAGCATCGTCAGCCCCTCCCG
GATAATAACTTACTC
AAGCTGATCCAGTGGGTTTTACTGGACGCGGGTGGAACGATTGCA
CCAGTGTCTTTTGTA
GAGGAGCTAGCTGACCCATTCTACAATGACAACAACGCTTGTCTT
GTCTCTGCGAATGCT
GCGGAGTGTATGCGGCAGCATCTTCAGGAGTGCATTGAGTTCATA
ACAGATCTCCACTCC
ATACCCAAGGCTAAGAACAATTCAAAAGCTCTCGTGGCGCCGAC
GGCATCGACGGGCTCA
GGGTCAGGTGTAGGAGTAGGAACAGGTGCCATATCGAACACATT
GGCATCCAGTGGGGTC
AGTTTGAATGAAGACACCCTTGGCGCCCACATGAAGGGCGGAAT
AGCACAATTTGTGGCG
CTTGAAA
>ConsensusfromContig1756 Average coverage: 15.74
TGACATCGCATAATGACCGCCACCATGGCTCTGGAAGACTTCAGA
TCAATAATTCTTCTC
CAACGAACGCTGATCAAGCCTCGCCAATATCGCTCGGTGCTAGA
AGCAACTGCAGCAGTG
AGCACAACGGAGCATCTCCATCAACCCCTAAGCCGAGAATTTGGT
CTCTTGCCGATGTCG
CTACTGGAAATCATTCGACAAACACAGATAATAACTCTTCAACTT
CACCAAATGCTTCAT
CTAACTTATCATCAACGCTACGCAGTCCTCTTCACTCCGAAACAT
CTCCAGCATCAAATT
CAATCCACATGCAGCATCAACATCACATGGAGAACTCTTCGCAAT
CCTCTTCAACTGCAC
CCTTCAGCCCGTCGGAGGCCGGAATTCACCACAGCGCATTCATCT
CTAGATCTTCTTCAT
TTCAATTCAGCGCCGCCGCTGCTGCAGCCGCTCTTCATCAGAGAT
CGATGGGGGTCATGA
CGCAAATTTCAACGCAGAGCAACCCGTTCTCTCCGTATGCGGCTG
CTGCTGCCAACCAAG

CTGCCATGCTCAAAAGCGTCGACTTTGGTCAACTGGCAAGCTACA
AACAATCTTCCGAAC
TAGGTCAACTTCATCACAACATTCAAGCTAATCTAGGTCAACTTT
CTGGCACTGCTCCTG
GCCTGAACGGATTCCGACCGTGGATCAATGGAGCCGGATACAGC
CCCAGCAGCTCCGCCA
TCCCCCAGTCTTCTGTTCAGTCGCAATCCCCGACAACTCCCCCAA
ATACAGCGTCTGGCA
TCTTAAGACCCTACCCAACACTCCCCACCGCCAACAGGACTGAGG
TTCATTGAAACTGTA
TCAAAAGTCATCCTCTTGTACCTTTTGTGAAATCTTATTATTCATC
ATAT
>ConsensusfromContig1757 Average coverage: 35.45
TTTTTTTTGTAGTTTCTAAATATTCAATCAGCTGTTTAAAATTTTT
GCGATCATAATGTA
TATATATATATATAATTAACACAGTCCTAAATTGCTCATGGCGAA
GTCGTAATCAATTGA
GATTACTCCATTATGTGTTTGGAATGCATACTCTGTACTTTTTGTG
AGAATTGATTTGTA
AAATTTTCGAGCGATTTTTCTCAATTTTGGTGACGTTGCACATAT
TGTAAATTTAGGCCA
AAGAACAGACCCATAGCTGTTTTTGAGACCCAAATAGCAGTGAAT
AACCCTTTTCAATAA
CTCGCTCATAATTGGAGCGGGTTCTGCAATGCTCTCATCTATCAA
GTGCAACTCTTCTGG
CCCGAATTTTGCCCGAACCTCTAAGAAACGTGGCAGGTCCAGAGT
CGTATCAAGGGTCCA
TGTACACTTTTCGCATTCTTCAATAGTCGTTAGCCGTGTTTTGATG
ATCTCCAGCTGATT
TCTCAAATCCTCATCTGATTTTGTGTGACTTGATCTGGTCTCCAGG
TTTTCACAAGCTAT
TGTTAAAAGGACTTTCAATTTATGCGGTGCCTGCTCTTTAAGAGCC
CTCCACATTTCTTC
GTCTCCAATAAAATCATTAATTGCTGCATAACTCACAAGATTTGA
TCTGGAATTAACGGG
AAAGTAAAGTAGACACCTGACGTAAAGCTCAAGAATTTTCAAAC
AATCTAGTTTGGATGA
GTATGACATATATTTTATATAATTTGAAATATTGTAATGGGAGTTT
TTTAATATCTGTTT
ATT
>ConsensusfromContig1758 Average coverage: 56.63
ATAAGCTGCAATAGCATGCCGAACTTCTTTGTATCTAATTATTTTC

ATGGTTATTAATTA
AAGAACATTCTTGTTGTTATCTATCCGATTCAATACAATAATGGGT
TTTATCTTCTCAGG
AAGTCAGTGTGATTTTTATTCAAGCAAAGTTATGTGCCAAACATA
ATTGTGTTACATTCT
CATCAGTGCGTTCTTTGTTTCTGTATTCATTCAAAATATTCCCAAT
CTTTAGACGAATCT
TCAATATGCATTTATTTGGGCTTAAGTGATTCCTTGTCAAGTGAT
TTGCGATAAAACATC
ATACTTTGTCATTTCAGGTATATTCTATCGATAGTTATGCAACTG
AGTCTCGAATCAGTC
TTCCTACGTTTCAAAACCAATGTTTGATTTAGTCTGATTTATAGAT
GTTATTTCGCCGTG
TCTCCCACATTGTTATATTGAAAATCCAACTCAGTTATATAATATT
TTATGTGATGTGTT
CTCAATATCTTTGAATAATAATAAGTCGCAATACCAGGAATCACC
CGGATAAACTAATCG
CCGGATTTTATATTTTATTATACAGGATGTTTTTTCCATTTTGACA
GATATAAATAATTC
TTCCGCAAAACCATCCGAAAAGCTTTGTAAGCTTATATTCAGCTC
GCAAAATTTGGTTCA
CTTTATTCATGTTGCATTTGTTTGAAAATATCTACTTAATTTTTGG
ATCTCTTTAGTGCC
AATATGAGTAGAGATCTGAAATCTCGTGCTCTCATTTGCAAAATA
CTTTAGGAAAAAAGA
TATTTTGTGTCATTAACTGAAAAGTGATCATAGAATACGCTTGGC
CAAAAACGATTCTGT
GATTTTTAAGATTTTGTTAAAGCTCAAGTAGATGCTCTTGGTTCTT
ATTTGGATCTCATT
GTTTATACCGCAATCCATGATTTTTTCGTTATCCTAGATGTTTTAT
TATAATCTTTCTTG
CCTTTGTTTTTCATATTTTAAGCTGGTGATCCTCTTTTGCCTCCAAA
TATTTATTTTGTT
TTCAATATAGACGGTTGCGACGTTCAATTTTTAAATTATATTGAT
ATCGTATCCATGATG
TACGAATTTCAATTATTATGTCATGAAGACCGGTTATGTGATTCG
AACAAATTTTCTTAA
TGATCAGTGATATATATTATTCTGTTGTCGGAATGTCCATGAAATT
TATTTATATACAAT
ATATCAATATATAAAAAGTTATTTAATTTGAAAATCATTACTATTT
TTCACTGTATTGTT
AGTTATAAATTTCTTCATAAATTTTTCTATTATTCCAGTTATAGGC

CGCAGAATTGTATA
GCTCACTTCAGTATTTATACAGTCTTGTATTCGTTCGTGTGCATTC
TGACAGTATTTTTT
GACATAAAAGTATCACTCGAATTTGCCGAAGGTCATTATTCTTAG
GACAATGCTATAACC
CAATTAGCATGGCGTTTTTCAACTAAGATACATACTTCTCGTTATT
CGTTATTTATTCTA
TGGCCCATAGGACATCTTCTGAGTTTGATATATTTATTCCGAATAT
TCTCATCTGTTTCA
GCATGCTAGTCCTTTTGGTTATTAACGTCCCATAACATACTATTGA
ATGTCATTTATATA
TCGTGATAAATTTCATATTCATTCCTGTGTTATCGGCCGATACGC
AGTAATATTCCTAAA
TTCGCCGATTATAATCTGTGGGATAGCATTTATGAGTGAATATAT
CAGAATATTTTATTC
CGTTCACATTTTGGAATTTTTTCACTTGCACATTTATAGTCATTCT
ACGCGTTCTGTATA
TAAGTGTGTTGTTATTCCAATATGTTATCTGTTGAGTACAATATAC
TATTTTATATTTGT
TCACCTTTGTCACGTAACA
>ConsensusfromContig1759 Average coverage: 16.22
ATCCAAGAAGTTCGTCTTCAAAAAGCAATGCAAGATCTTCAAAC
GGCTCAGAAGGCACTG
GATGAAAAGCAGAAGGAATTGGATATCGCCCAGGAAGAATACAA
CGTTGCTCTGAGAAAG
AAAGAGACTTTAGTCGCTGAGACCAATCGATGTCGGCGAAAGAT
GGAGGCAGCGAAATCT
CTGATCGACGGTTTGGCGGGAGAGAAGGAGAGATGGACACAGCA
AAGCAAACAATTCAGA
GAGCTCATAGGAAGATTGGCTGGCGATATTCTCCTGTGCACGGCC
TTCCTATCGTACGCT
GGTCCCTTCAATCAAGACTTTCGCCAGTCGCTCTTCAAAAGCTGG
TCGAGGGAATTGCGT
GGTCGAAAGGTTCCTTTCTCCGTCGAACTAGATGTCATCAGTATG
CTGACTGAACCCATT
ACTGTTGAGCAATGGAATCTAGAGGGGTTGCCCGTTGATGATCTT
TCTGTGCAAAACGGG
ATAATCGTTACTAAAGCCGCTCGCTATCCGCTCCTCATAGACCCC
CAGACTCAGGGCAAG
ACCTGGATCAGAACCAGGGAAACTCCGCATGGATTACAGGTGAC
GAATCTAAACCACAAA
TACTTTCGTCAACATTTGGAAGACGCTTTATCAATCGGGCGACCT

CTTTTGATAGAAGAT
GTGGTCGAGACGATAGACCCTATTTTGGACAACATCTTGGAGAAG
AATTTCATCAAAATT
GGCTCTACGCATAAGGTGAAAATTGGAGATCGTGAGGTGGATGT
GATGGATAATTTCCGC
CTGTATCTGAC
>ConsensusfromContig1760 Average coverage: 11.46
TTCTTCGTCTCTTTCGGAACTTTACTAGTATCTTTGAGTTCTTTTAG
TATTTTGGCTTGT
ATTTTAGCTTGGTTTTTTTGTTTATAGTATTTAACTCTTCTGATGA
TCCACTCGACGCCT
AGTCGAACTTCTTCTTGTATTAGGTTTATACACATCGTCACAAAA
GCGAGTCCGAAAAAT
AAATAGAATGTGGCGATTATAGATTTGAGTATCGAACTTTTGATA
GCATTTCCAGGAGTA
AAATCTCCAAAGCCTATAGTTGTAAGAGAGATGAAGCAAAAGTA
AAATCCTTCGAAAAGC
GTCCAACTCTTCTCGCCTTTTTCATCCCTCTCCAGGGTATGGAATA
TGGCGGCTCCCATA
GCGATGTAACCAAAGAAGAGAGTCAGTGTGACGGGCACTGGAAC
CCGCACTTCTTTCTGC
AGGTTTTTGATATCACTTTTGCGAATTGACATTTCCAAGGACGGT
CGGCGTTCATCCTGA
GCCATCATTTTCTTCCATCGCTTCCATTGACTTGTGACGAG
>ConsensusfromContig1761 Average coverage: 13.42
TAAGTCGGTGTCGTGACATGTCTTCTGGAAGCCGAGTTTCGATTT
CGTTTTCTAGAGCGA
TCTCTGGATATAACCGGTGATCTTTGTGATATACTCGATGCTGAT
ATTGTATTGAATCTG
CCACAAGACTCAACAGCTGAATTTGATCGGTTATCAGGCAGTGA
AGAGCTAGAATTGTCA
TTATTCGAGTCAGAACGCAAGATAACCCTATCTTCCTCGTCAGAG
TCTGTCTGTAAAGAA
TTCAGATACTGATGGAGCATATCCATATCGAGAGGGTCGTTCTCA
TTTGGAATGACATCT
TGAAACCTATTTTCAGGACCAGGAGATCGGCCATTTCTAATAGCA
TCTTGAAAATTTGAA
CTACTACACGAAATCTCGGACATAAATTGTTGCTCTGACGGTGCT
GGTTGATTATCACGT
TCATCGCTAGAATCCTTATAATGATTAGGATGATATCCATGGGAC
TCATTTATGTTTCTA
ACTCTGGCAACATCCTTACTTATTATCTGAATCTGATCGTTTGAG

ATAGCACCTGCAGAA
TTGAGAGAATTGTGGCTCGAGCGCGAGTTCTGTTTCCAGTTTGCC
GATGGAAGCTGTTGA
GCAGACATACACATCTCTTTTTGTCTATTAACGTTTGACTTATTTC
TTAGATTTCCTAGA
GACCTGCTCTTGATGAGATTTGCAAATTCTCGATTGATATGTGTTT
TTTTGTGAATCAAA
GCAGTACTTGACTGTGCCGAGGACTTACGTAGTGGTCTATTCTCG
GTCATTTCAGTGTAT
GCATAATCATCTGAAGAACCGAGAGAGCCCTGGTCTATCAATTT
ATCAGAAAAAGACCCC
GAGGCTTCCAGGTCACTTATATTCAACGATATATCCAACTCATCGT
ATCTCTCCTCTCCT
TCGTCAGATCCTTCGAAATCCCTGCTGGATATGTCGGTCATTTCC
TCTCTCAAGTTCAAA
TCTTGATAAGAAGGTTTTTTATCTACTGCTCCTAGACCAGAGTCC
GTTCGTAATGTCATA
TG
>ConsensusfromContig1762 Average coverage: 16.15
TCAGGTATATATATTTATCACTTCCCGTGCAATGAATTCCAAATCC
TATTATTTCTTCCT
CATGTGTAGAATATAAATCAGATTTTAGATACGTTGCAGTGTTGG
TGGTTAGGTGGCTAT
TAGTGAACTGAACATTGATTTGTATTTCTCTCACATAACCATCTA
TTGTAATAGGAATTA
TATAAAGTTTATCGTTTGTTGTAAAAAGATTATTCTCATTTGAAA
TCGTGTAACGACATC
CAACCAATGTATCGCTTTCGAAAGGTTGGATATAATTTAGTGAAC
GTGGAACACCCGTAT
TTGGAAATTTGGTATTATGCCATTGAAAATCTTTTTTGATCCATAA
ATTATTAATATCAT
CACCACATAAACACACCACATGGTAGATAAAATCGGGATTAACA
TCTTCTGAAAAATTAA
TTTCTGCTTTTACTGTATTTTCGGTCTTGATGATGTTTTTTATAAA
TTTAATTGGATAGT
TTTTATAAACAGCTCCATCAAAAAAACGTGATGATAATTGCACCA
TACCCGGATTACTCG
TTTCACATATATAATGTGTTCTATTTTTTTCATATAGATTACAATA
TCGATTTTGTTCTA
AAATGGAATAAAT
>ConsensusfromContig1763 Average coverage: 21.78
TGTTGTGCTGCTGTTTTTCTCCTCCGAATATCATCCAAAAAGCAC

ATGCATATGCGATGA
AGAACTGGCCGTAGAGAAGAGCGAACTTCATCACATCAGTCATCA
TGCCTCCAATGATTA
CAATAAACGGTCCAAGTGTAGAGAAAGCTCGCATATTTTTCATGA
TTCGTAGCCATAAGA
GAATTACTGTAATTGCGAGTATTCTGATATGCCAAAGTGCGAGAT
CGTTATCATGTTGAA
CAACGTCTATTATGTGGGTTATGAAGGACAGTAGAAGTAGTAGA
TACACCAACCAGTCAA
ACATATTCCACCAATCATTGAAATATCGGGGTTTGATATCCTCAA
GCTGAGATATTTCTT
GTCGTAAATATAGCTCTTCTTCCGGCCAGCGTGGATGCGCGTACG
CTATATCCTTCTCTA
TTTCTTCTTCTCTCCATGTCTCCCAATGATAATGAGCTTTTCTCGA
GCTTCTGAATTCTT
GAATTTCATCAAAAATTTGCCAGAATGTGAATAAAATAGCAATG
ATAAGAAGCAGAATAC
GGTACCAGTCTGAAGGCAATTCATAGGAATGCTTGAGATCATATT
GTTTTAATATACCTT
GAATACTCCAAATCACAGCGCCAAACACACTCAATAATAAATTG
AATATGGCTCTGGGCC
TGGCGAATTTGGACCACTTGGCAAGAATGAGGCGATTTATAGCA
GGGTGTGTAACGAGAT
CATATTGTCGATATCGTACTATCGCTTGAAGTGCGCTATGAGGGC
ATCCCGGCCGCTTAT
CATCTGCTTTTCTTGGCTCGAGCCAGTTCAGATAAAAATACTGTC
TGCGATTGGCACGGT
CGATCCGATGAAACTGTTCGAGACCAATTTTGGCAACGTTGGGCA
TTTTTGTGATCATCC
AAGCGATGGCTGATTTTCCGCTGTAATCATCCACCCCCGCAGGTG
CCGGGTCTTCTAACT
CTAATAATGTTTTCGCGGTTTCACTACGATCTAATTCTGCAGCAA
CATGAAGAGGAGTAC
GGCCCTTGAAGTCTGTGTTGGTTTTATATTCAGCTCCTGCTTTCAC
CAATTCTTTCAGTG
CTTCACTGGCATCGTTTTTAGCAGCAAAAAAATAAAGGAGAATGTC
GTTCACCCACTGTCA
ATGCTTCTAGATGAGCCCCATTTTCAATGAGAAAAGCGACCATAT
CTGCGTAATCAACGG
CCGCAGCAACATGTAGCGGCGTACGGCCGAAGTTGTCCGTCACGT
TGGCATTCCCGCCGT
TGTCAAT

>ConsensusfromContig1764 Average coverage: 12.88
AAAAGTTGGCGGCCGATCCGCCATGTGACTGAGTCACCCGCCAA
CACTTCATGCAAGGGG
TGATGGGCGTATGTAACGTTTGAATGCGTCGGAGTGCCATCTACC
AAGTACTCTAATTTG
GGAGTCCGACATACCTTGCTGGGCTGCGAACGTGGCACCACCTAT
TCGAAAACTGTGGCC
TTTTATGCTCATGCCCTGCAAATTTGAAAAAGCTAGTGCTTCGTG
CATAATCCCTTCTAC
ATATTTTTTGCTGCATGTGGTCAGGTCTGATTTGATGAAAAGAGG
TCCTGGCGCGCAACC
CCGAAAAGTGAGGTACGCGGACATGGCAGCAACCGGACACGCCG
GGCAACCTTCGCGCGA
TTTCAGGGGAACAACGTGACCGCGACCTCCTATGTTGTGCTTGAA
ATTGGTGAAAGTGAT
CTCGACTAGCTTTGGAGACCTGGAATCAGGGCACACTTTTATTGA
TGCTAATTGTATTAC
CGAAGAAGCGTCGATGGCATTCGCAGCGACAAGTTCTCCCAGTC
TCGCGAGGCCAAAAAA
AGCCGTGGAGAAAATAGCTCGAAGCAAGGCCTTTTCGAACAGATT
TTTTCCGACAAATCG
CAATGCATGGATCATCTTCAGCAGCAAAGGAAGTGTGACCGGGGA
TCTATTATCTGCA
>ConsensusfromContig1765 Average coverage: 13.11
CTCACCAGTTGTCCAACGATCAGGCCTTGAGCCAGAGCGCGGTCA
ATGGGCATGTCACAT
CCGTTTATGACGTCACGATATAGCCCTGCCTTCTGGTTTATGATG
CCGGCGAGTATAGCG
TCTTCGAGTGTCATCAATTCATCGGAGTTTGGGTATCGAACACCG
GTTATCTGGAATCGC
TTCTCTTCTTCTGTCACTTCTTCTATCGTTACATCCACAACGTTTTC
GTCTTCATCTTCG
ATGTGGATGCTGTCGAGTTTATCATCGCTTTGTTCATCCGGATGC
TGATCAAACTCCCCA
CTCACAATTGTCACTAGATGTTTATCAATTGCATCTGCTAAGGGA
ATGACGCTTCCATCC
GCGCAATGATATTCTCCTTTTATCTGGTCGAGGATTCCCTCTCGA
ATGGCCTCACTGACT
TTCATGGCTTGTCCAGAAGACGGATGCATTACAGCCTTCAGTCGA
TACGTCACGTTGTGC
GTTGAAGTTATCTTTTCATCTTCGATTTGAGAAACCGGTTTTGAAG
TCGATTCGATTTCG

GCCCTAATCAGACCTCCGTTGATGGCTTCGATCAAAGGAATTCTA
GAATTGGAGGAACTG
TCGATGTAGAAACCGTTTTGAACATCGAAAATCCCAGAGGA
>ConsensusfromContig1766 Average coverage: 13.44
TTAGCTTTCGCTTCCAAGCATACAGTATCGAAGCGATGCACAAAA
AATGCCCATGTGAGA
ATTGTTGTTCCTGGAAGGACAGTTTGTAATTTTTGCATGGCTAAC
AATATAGAGGCTCGA
TCGGCAGGAACAGTGTCGAATTGTTGGCAGATATATTTCATCATC
AACTGGAGTGACACT
TTGGGTAAAGATTCAAGTTCGGCAAGAGCCACCTGTACGACGTG
GGGGTGAATGTCATCC
ACACACCAGATCATGTTGACAAAAACGAAGGTGAGGGCTTTTTGA
AACTGATGATTGTTG
GAAAGAGTCGTTTCGTGATTCATCCCTCGAATCAAAATCCCGCAG
CGCTCAACTGTATTG
AATCTGATAGAATAGTCTGAGTCTCCAAAAGCCTTGACGATGATG
GTAAGAAATGCTTCG
GTGGCTGGCTGGAGAGAAATGACTGAGAGGAGAATAGGAACAG
AATCCGAAGACAGGGAA
CAATCCTCTGGCTTCAACGTGTCCCACATCTGAGGTATCAGCATT
TGTGTAGTAACGTGC
TGAAAAAACTCCATGTTATCTGTATAGGCAAAATCAAGTGCCCC
AGATTGAAGACAGAGC
ATAGATAGAGATTTCAAAGACAGCTGAGTCGCCTCGCTGTCGGCT
ACGTTCTCTAATTCT
TTCAGTAGAACATGAACAAGAAGCAAATATGGAGGAAGTGAAGT
CATTTCTGGCACAGTT
TTGTACAGATCTAGATCTATATTCCTGGGGTATGCGCTTTGATCTT
TACCTGTATGTCTA
CTTGTGTCATGCTTCTTCTCTGGTGCAATGAATGTTATCAGAGAG
ACAGCGCTCTGATAC
CAGCCAGTAAGACGATCGCATGCAGGGCAATAATCATCCTCATCC
TCTTTTTGCATATTC
AGATTTTCCTGGCCATCCGCTGGATCTATAACGAATTCTGGCTGGT
TTTTCTTACTAGAT
TCCTCTGAATTTTCGAATTTATCATTCATGAAATTCTCTTTGATGT
TGGACCCCACGTCA
CAGGAATGAATATCAGCTGTCCAATCAGCCTTCAGCATGGATGTT
AGCAAAAGCATTATG
TCTTGATTCGTTTCGTTTTTCAAGCCCATATTCGGAGCTATTTTTT
GCATATTTATCTGA

GCCTCTACAATATCCAGCATGATGATGAAGCATTCAAAGGTCGTA
GCGGCCCGCTGACCT
AGAATATCCGGTCCATCGAATACGAACATTGGACTGTCTCCTTCA
ATGGGTCCAGGGGCC
GGTGGAGGAGCTGGTAAGGGGCTTACATGAGGG
>ConsensusfromContig1767 Average coverage: 41.63
ATTTTTTGAGATGAAAACCTGCCAAAATGACCGACATCCGTTTTT
CAGTGGATGATGTCG
AAGGAAAATATTCCTGGCTCATGTGAAGAAAATCTCGAAGCTGA
ACTCATATCATTGGAA
GAAGCAGAAAATTTTGTCAATAACAAAACGCCAGAAAATAATCA
ACAGAGCGATAAAAGA
GTGACCAGTTCAAAGGAATCGATTATCCAAAGTGTGAGCGGAGAT
AATCTTTTGAAGTCA
ACCAATAGCGTACCTGGAACCCGAAAAGGCGCCAACAGTCCTTCG
CCATCATTATCTCGT
CCCAACAGTTCTGCCAGCACAAGACATTTGATCGAAACGGACTC
GACGAAAAGGACCAAC
GGAAAATCGGACCATTTTTTGACAGCGTCCGCAAGAAATTCCACC
ACGGAGGTTGTCGAC
ATAAGTAACTCCGTCATGGATGACGGGTTAAGGAACGACAACGG
TAAAAAGAGACACGAT
GGCTCGAAAGCAAATCTCCAGGATGATCACTACAATGCCCAAGA
TGACGCGCAGGAGAGC
CTTGGCGTATGTGGCTGGTTTCTGACCCTTCTTTCTTGGTTTGCTG
TTCTCTGTACAATC
CCTCTTTCCCTCTGCGTTTGCATGAAAGTCGTGCAAGAATACGAA
AGAGCTGTTATCTTC
AGACTTGGTCGACTCCTCTCAGGGGGTGCCAAGGGTCCTGGTATT
TTTTTTGTTCTGCCG
TGCATCGAAAGTTACACAAAAGTTGATCTAAGAACAGTCTCCTTC
GATATTCCACCACAA
GAGGTTTTGACGAAAGACAGCGTAACAGTATCCGTGGACGCGGTC
GTTTATTACAGAGTC
AACAACGCCACTGTTTCTGTGGCCAACGTTGAAAACGCCCACCAC
TCAACTCGACTCTTG
GCACAAACGACCCTTCGAAATGTTCTCGGCACAAAAATTCTCTCG
GATATTCTCAGCGAC
CGCGAGAGTATAAGCGCACTCATGCAGGAGTCACTGGACGACGC
AACAGCTTTATGGGGA
ATAAAAGTGGAAAGAGTTGAAATTAAAGACGTGCGGCTGCCGGT
TCAGCTGCAGCGGGCG

ATGGCAGCGGAGGCCGAAGCTGCCAGGGAGGCAAGGGCAAAGGC
CATAGCTGCTGACGGT
GAACACAAAGCGTCCTTCGCCTTGATGAAGGCTGCAGAGGTCAT
CCGAGACAACCCCATG
GCGCTGCAACTGCGCTATCTGCAGACGCTTCAGTCCATTTCTGCT
GAGAAGAACTCCACC
ATCTTGTTCCCCCTACCCATGGAGTTCATGAGGAGCCTCGTTCCC
TATGACGAGCAGAAG
AAAGATAAGTAGAGGGAGCT
>ConsensusfromContig1768 Average coverage: 8.52
CCTCTGTACATCCAGGATGATTTGAACTCAAATATAGCATCTCCT
GATATGGACTTGTCA
CTATGATGAATGTTCATTAATGTCTGGAGATAGACCATGACATTT
TGAAACTCAAGCTGT
AACTTTCTCACAGCACTTCCTGGCTCATTCTTTTTAGTGACGAAC
TCCACTATACAGCCA
AAATCGTCTGAAGTTCCCATTTCGTCTATTATGATTTGATCCAGCA
AAGTCTGATATTTT
CTTAGAATGAAAGTTTTTGTTATGTCCAGACAAAAGGAATTAGCT
GTGCTTCCATAAACA
TCACTAGTGCTATGGAGCATGCCTTGACCCATATCCCATGAAACT
TCAAAGTACGGGCGG
CCCTGCTTGAGATGCTCAAGAATCCATTTTTCTTCTTCAGTCAGA
GGAGTTGCTTTAAGT
TTCTGGCCACTGATGAGACCTGAGCCAGTTCTCTCCTTTTCCCTCT
CTTCATTTTCGATA
ATTATGACCTTTTTCATAACATGTTTGATGTAATCGTGATGGATCT
CAAATCTCTTGTAC
ATGTGTTTTAGCAGACCCAACTCAAGAACATCTTTATAGCCTCTG
ACTGGTAAACTTTTG
TACATTCCTCTAAATGCAGCTGCTGACATTGTGGGTCCCCATTTTG
ATCTGTCCCAACTC
ATGTACATCATATGATTTATTGTTTTGATCTTGTCATCACCCAGAC
TGATAGCATTCCTG
TCTTGAGATCTGCAAGCAATGTCCTTTATTCTGTGCTCGTTGGATT
TGTTGTTTAGACAT
GAATTTGATGACCCAGCCGATAATGCCTTAGACACACTTTCCACT
GACTTTATCTCCAAT
TTACTCTTCTGAGTTCCTATTGTCAGCTCTCTCTCACCTCCAACCT
GCTGCTTAGGCGCC
GTTCCCATCTCAATTGGTTCGGAGTTGTATTTGTATTTAGACTCTA
TAATGTCATATACT

CCTGCATCTGATGTGCTCAAACTAAGAAGCAGCATGTAGACTTGG
TCTGTTGTTATTTCT
CGTGTATTTTTGTACTTCATGAGATTTTTGCGATTACCATCTAGAA
AGCTCATTAGAGTT
TCTGCAGGCTTTGAACAAGTGAATACAGCATTGAGTATATCTACA
AACACACTGTTTCCT
TCTTCAATGATTTTCAAAATTGCATTTATCAGAGAGACTGAAGCTC
TCTCTCTTGCATTC
ATAGAATCACCTTCTTGAGTATGTTTCACTGCAAAACTGGAAAGA
ATGATTGAGTTGCAG
AATTTCGGGTGGCTGTCAAAGAAAGCTTTCATTTCACTAGTGGTC
TCATCTTTCAGATC
>ConsensusfromContig1769 Average coverage: 18.38
GTTTAATGTTAAATTTTATCATGACACTTTGTGATCATAAATCTTT
ATAGCACATAATAT
TTTCAATAGTAGCAAATTCATAAATCCGACAATATGAGCTCGGAA
ATTGAAGATCACATC
AGTAAACGTTATGATATTAAAAAAAGACTTGGGAAAGGTGCTTA
TGGCATCGTGTGGAAA
GCTATTGACAGGCGCTCTGGAGAAGTAGTAGCAGTCAAGAAAATA
TTTGACGCCTTTCGA
AATCAAACAGATGCACAGAGAACATTCAGAGAAATAATGTTTCT
GCAAGAGTTCGGTGAT
CATCCAAACATAATCAGGCTTCACAACGTCCTTCGAGCAGAGAA
CGACAAAGACATTTAT
CTAGTTTTCGAATTTATGGATACCGATCTGCACAATGTGATAAAG
CGCGGGAACATTTTG
AAAGATGTGCATAAAAGATTCATTATGTATCAGCTTCTCAAGGCT
ACCAAATATATTCAC
TCGGGTAACGTGATACATCGAGATCATAAGCCATCAAATGTTTTA
TTGGATGGCGAATGC
TTCTGCAAAGTATGCGATTTCGGGTTGGCGAGATCCCTAGCAAGC
ATTGAGCCTGGCGAT
CACACGGATCCGAACCTGACCGAATACGTGGCGACCCGGTGGTA
CCGAGCACCCGAGATA
TTGCTAGCATCACACAAATATACAAAGGGTGTTGATATGTGGAGT
TTGGGCTGTATCCTT
GGGGAAATGCTTCTAGGTAAACCACTTTTCCCGGGTTCCTCTACT
CTGAATCAGATTGAG
CGAATAATGTCAGCAATTCCAAAACCGACAAAGGAGGATATTGA
AAGTATGGATTCTGCT
TATGGAACTTCTGTCTTAGAAAAGGCTTGCAGAAAACCACATCGG

AGTCTAGATGATATG
CTCGTAGACTGTCCGGATGACGCTATAAGTCTTCTCAAGGCTTTA
GTTCAATTCAATCCG
CATAAGCGAATAACGGCTGATCAGGCGCTAAAGCATCCCTACGT
TAGAAGATTTCATAAC
CCACCGGAAGAAATCCGAATAGGTCGAGATATACTGCCCCCTTA
CAGCGACGATATTCAG
CTATCCGTTGAGGAATATAGAAGTAAATTGTACGAGATGATAAA
TGAAAAGAAGCTGGAG
CGACGAAGGCGTCGCAGAATAGAACGTGACGATCATGAAGGGGC
TGCTTTCCACGATAAG
CAAAAAACGCAGGAAACGTCAAAGTCGTGGGAATCGGAAGAGAG
CGTTGTGGTTGAGCAC
AACACGACCAACAAACAGATGCAACACAGCAACGAAATTCATCC
GATGACCTCAAAACAT
AATGCTGCACATATCGAAGACCCGTCCGTTGATGAATATTCAGAT
GACCACCCCAACTCA
ACACCAAGTGCCCCCGAATGCAGCCCAGTTGCGAAACCTGTGGT
AGCCAAGGGCGGGAGT
ATTCCAAATGGTCCAATGACCCGAAATGGGTGCGATGACAAGAC
TGCTACGAGGCAGAAA
TCTGGAAACATAGCATTTGGACGAGCAGCATACGCCAACTCAAA
CGTGGGGTCAAAAGTG
GGGTCGAGGTCGACCACACCTGTTCAGGATCGACCCAAATCACAG
TTTCTTGTTCGACAA
AAACAGGCAGATACAACCTATTCCCAGATACAGGCGAATAAAAA
TGCGAACGGCCGACAA
GCATCAGCCCCTTCTGTTTTGCGAAGCGGCATCGGGGGGCGAAC
>ConsensusfromContig1770 Average coverage: 12.89
GGGACTGTCCCCGCCACAAAAACGCATACCCAGAAAACGGATTC
AGCATGTTATGACTGC
GTTCACATCAAAGAGAAGCCAGTTCCTGCCACGTCTGTTGGAAT
AGAACGCAATGAATAC
GACGTTGCCACAACCGCCGACAAACTGTTAGCCAAAATTCGTGAT
GAAATGACGTTTCTC
AATAGCAACTGTGGAAGCGTCAAGAAGATGACAACCTTCAAATGC
CGATTCTGCCCATTC
GTGGCGTCAAATTTAAAGTATCTCTACAGCCACTACGAGTTTCAT
CAGCTTCTGCTGTCC
ACTCAAGCCCGCACAACACATCTTGAATGGATGGCTACATACCT
CCATGAACAGAGTCAA
AACTTGAATGGGTCTCTTTCTCTGCATTTAACATCATCTCCTCCGA

AAAAAGCTTCTTCG
CTCTGCACGAGCAGCAGTGGTGCTTTCAAACCTTACATTGCCAGC
CCCGGGAAACGCACC
AGCGTCTCTACAGATCCAAAACCAGCTATCTCTTTAACAGAAAAT
GTACATTCTCATTCT
GAATCCACAAAATCGAGCGAACCAAATCAGTTGGCTATTGATACA
TCCATTCCCATCAAT
AAATTGCCAAAAATATCGAACAAGTTACCAGAAAAAACTTCACA
GTCCGCATCTGGCTGT
CTCAAGTCTATAGTCAGAGCCCTCCTGATGGACTCTTCAAATCAG
AAGTCACAGTTAACA
GAGAAACATTCCAACTTCAAAATACACACTAATACGAATGAAAG
TACAACATTACCAGAG
AGCAATTTGATGGAAAGCAATAAAGAATTTGAATCGGAAAGCAC
ATATTCACATTCAAAT
AAACGAAAACGTGAGAATTCAGTAAGTGAGGTAAATAATAATTA
CAAGTCTGTTTT
>ConsensusfromContig1771 Average coverage: 24.17
GGTCAATGGCTGTACCTGCAGGCACTCAGTTTACCCATCAACAAG
TTCCAGTTCTTGCCG
TACACCGTCAACCTCAACTACCAAAACACGTTGCTAACGACCGC
AGAAGAAATGATGATT
GTTCTGATTGGACCATTTTTCGTCATCCTTGCTTTTGTGATCCTCG
TCATTCTCTCCTGG
GTCATTCAAAAACTCTTCAGCGCATTTCCTGATATTGGCTCGAAA
TTCGTGGTCGTGTTT
GGACTCCACACTCTTCTAGATCCGCTACTAATCCTCATTGTCGAT
GCTATTTTGATGAGA
TTCGAAGTTCGAGGTGATGATTCTCCAATCATGGATGCAGCAAAA
CTTTACTGCACTTCG
TCAAAACAGAAGGTTCTGGAACATCTGGCATTTTCATCACCGTTG
TTATCTACTTATTCA
CCATGTTTGGTGCCGGCACTGTTCTTTACATGTATTTTCTAAGAC
TTCACAACAACGGGA
GGATGCTCGATGTCTTTTGGCGTCTTCACGGTTCGGCAGATATTTT
TTTCATTCCGAATG
ACCTTGAAATCTCCAACATGGAATTGAGCTACATCGTACACAAG
TCTGAACAATGGCGAG
GAATCGAAGGGGAACGGAAAAAAACAGCTGTTTACGATTATATT
TGGCAGCAAGACACGT
TTCTGGAACGAATTTGGGATGACGACGAATGGGACGACTCGAAA
GAGGGGCGGGTCCAGG

AGGAAGTGACGACACATGTTTCAATTCATACAGTTCACTTGGATG
GACTTCGGGAGCTCT
ACAGGCATTTCCTGAGACTTCCAGACGGAGCAATTGTTGAGGTAT
TCGGTGATATAGCCG
CAAGTGGCCTTGGTCAAGAGCTCTCATCGGCTGTTTCTAAAGGAC
GCAGAGCATTCGAGA
TGAACTCTTCAGCATCTCTTCAGCAAGATGAGTGCCAGAACGCAG
GTCGTGAAACGGTCT
TCACAAACAGTGGCTTCGAACGAGAATTGACTCAAATAGACGCC
CAGTCCCGAACCTCCA
TGGCGGATCTTGATTCGGTATCAAAAAAAGATGTATAAAAATGGG
CTGTTCAACAGAATT
GAATGCAGACTGATCAAAATTAATCTACAAGGGATCCTCAAAATC
AATATTCAACATTCA
GAC
>ConsensusfromContig1772 Average coverage: 23.93
ATTTGAAAAAAAATGGCTTAAGACTCTTCATCGTAGAATTTAATA
AAACTATAACTGACA
GTGCAATCAAAATATAATTGAATTCTTGCAGTTTCCTAATTTACA
ACAATACATTTGATT
ATTGATCATACAAAATAACCAAAGATCGTTTGGTCTAATCATCAA
AATTGTTATGAGTCA
CTTCTGTACATCTTGTTTGTCCATTGTCCTTTCATAATACTCTTCT
GAGATATTTGTGGT
GTGATGTGTTCGTGGCGTCGCATTGCTCTATGTTTTCTTCTGAAAT
TCTCGATTTTGTAG
AGTATGGTGTAGACAATAATACCATATCCCGTTATCATGAAGAAT
ATTATGCCAATACCG
TAGACTATATTCAGTCGAGATAAATGTTTTGGAGTGTATTTCTCCT
GTTTTGTATCAATC
TGAAGTGAATAAATTTCCCCACGTATATCCTCGTCGTTGACGGCG
TACAATTCAATGATG
AGAATGGCATTTTTTGGCACATTTTTGACTACTCTACTACGTGAT
GAAGATGGCATGGTG
AAATTTATCCCGCCACCTCCTTTCGCACGAATGCCTGCTCTGTATC
TTAACAACCCTCTG
CAGTTTATTTTCAATGGAACGTTCCAGCTAAATTCTATGTCTAGTG
CATCGCCTCTTCGC
CTTTGCTCAGTAGAATTTATCAGTGGGGCCGTGGGTGCTGTACAT
TGAGTTGTCACGCCC
TCGAGCGTTTTCAATTCGAGTTTTTTTGGGCCCAGAATCCGGAAG
TCATAGGTAAGAGAT

TGGTTCAGGCCATTAACCAAAATCTTATACACATCTACTGAAGAA
CCACTGTCCCTGAAT
TTCACAATATCGCTCCACTCTGACCTTGTACGGTTGTCGAAATAC
TCCCTCGACTGAATC
TTGTATGTAATTTGTGAGGCCAAGGGGTCACCATCCTCAGGCAAC
AGTCTCTCCCATTGA
ATAACGACCGAGCTCAGGTTAACAGGGACTATCAATGTATTTTTC
AGGATAATTGAAGGA
CATTCTCCGGAAATGTCATCGCAAGATTTTTCTCCGGACAATCG
CATTCTGATTCA
>ConsensusfromContig1773 Average coverage: 15.77
TCTCTTCCGATCTGCGGTGGTACACCTCTACTGAATATACGAATG
AAAAGAGATCCTGGC
TCGAGTTTGGGATTCAGTATTCGTGGAGGGTCTGAGCATGGATTA
GGCACATTCGTCAGT
GAAGTGATAGAAGACTCGACCGCTGAAAAATGCGGATTGAGCTC
GGGAGATCAGATCTTG
GAGGTCAACGGCATATCCCTTGAAGCTGCGACCTCTTCAAATGCC
GTGAAAGTTCTCACT
TCATCTGCAACTCTTTCCATCACCCTAAGGCGCACCGGCAAGATC
CCTTCATTCACCTCT
GGTGAAGAAATGACATCATGGTTTGACTGCAACTCTGCTTGTATC
GTTTCCGGGGAGGTT
CGTTGTCATGGTGAGTGGCACGACGCCATGGATTCGCATAGGGG
CGAAATGGTCATTGAC
CGAAGAGTCCAACTGGCTACCGACACAGATTGCCGGCCCCTGGGT
TTCAACATCCGTGGG
GGAGCAGAATTCGGACTCGGTTTATTTGTCTCCAGAATTGACCCG
GTCGGGATGGCAAAG
GAGCGAGGCCTCAAAATGGGGGATCAAATCATTTCTGTCAACTCC
AATGATGTCGAATTC
GCTCGTCATGAAGATGTTGTAGATATCATTCGGAGTCAGAGCCCG
CTGGTTTTAGTTATT
CGAGATATTGGACGATATCCTGTCTACAAAGAAGTGTTTGCTGAA
TACACATGGGAGGAT
GTCTCACATAACACTGTTCCCGAGAGTGTTGTAACCAACGGGAGA
TTCACTCAAATTCCA
TTGGAAACAAGTGTTGAGTCAGACACAAAACCCTTTAGCGATGCA
GAAATTCCTTCCAGC
CCAGAAGCTTCCGGTGAGGCAAGACCAGAGAACCAGGCTTATATT
TTTAGCTCTTCCACA
CAATATTCCAGTCCAAAGTCTATCATCACTAAATCGAACAAGAAG

ATGATAAAGTCAGTC
TTGCCAGACAACGTGATTCACGAGAAAAAATCACCGATTAAACA
AATCAAGCCGTTCAAG
CGTGCGGGTAATTATAATCTTCTTACAAATAGCAGACGACACGAC
GATTCTGATAGTTGG
ATTGGTAAATTCGAATTTCTTGATGCATATAGTCTTGCTGGTTCT
GATTTCAATGGCGCA
GACGATAATCTCGATGAAGTTGAGAAATACACAAGTCTGGAGCG
TGATGACGCTCTCCGT
GACCCAGTCGAATCCAACACATTGAAAGCCAGGAAGAAAAAGAA
AAAAATCAAATCTCAA
GACAAGTCCAGACTGGGTGTTTCTGCACTGAAGAAAGAAATTCA
AAATGAAATGAAGGCA
ACGGATGAAGATGTGGAAGAGCTAAACTTCGTTCAGAAAGAGTG
GCTGATCCAACAGATC
GTATGCGATGGTCCGAGCAACGGCTTGACTGACAATGAAACCGA
TTCCTTGCGGAGAACT
CTTCACTCGTTTATGGCGGAAGATGATGACGTTGATAAATTATCA
AAAAAACTACTTGCT
CTTCTCTCTACTCCTCAGAAAATGGTCCTGTT
>ConsensusfromContig1774 Average coverage: 17.28
ATGGCACCATTTAATAGTGAGTTATACGACCCGTAGTAAACTAG
CAATGAAGATCGATAA
TGTGCTTGTGGGTACTGATACCAAACCCAGTAGAAACTGCGTTAC
TGCGCCAGATCCGCA
CAACGTGTACATAGGTGGAGACGATCGTCCGTTTCGAGGCACAAT
GGACGAATTTTGTAT
TTCCAATTCTTTGAATCAGTGTTAGGTTTTTCATGAGCGGATATT
AAGGGGGACCAGGAA
GGAATAGCTTAATAATAATATGGAATAAAAATGTTTTATAATTAG
GATCTTGTAACCGAA
AACTGTTTGTCAAAGTTCTAGTGTGCCATTTTTTAGTAGACATCA
CTTGCTGAGAGGATA
ATTTTTCAAATAATTCGTGAAATCCTAGATGTGGCACTGTTATCA
ATATTGATGATTATT
TTTGATGGAACATTTTTATGATTTGTATCATATATGAAGATTGAG
ACAGTATTCTTACAA
TCCAATGGCGAATTTACTATAATCATGAATAAACTTGTAATAATG
GATTCGGATTAAACT
GGAAGTGAAGTCTTATGGTAGTTTCTAATACTCGAGAATATTCAG
ATGCCG
>ConsensusfromContig1775 Average coverage: 15.89

ATCTATTAAAATCCTTAAAACGTATTGAAATGCCTTCACTGAAGA
CTTCAGCATCATTTT
CGAGACTACATGAAACAAGAAAAACTACATGCAGGTCAACAATA
AACAGCAAATACAGAC
AGATGATGTATGAGAACCTTATACTTCTTGGATTTGACAAGAATG
TCGGCAAGCACGAAC
GATTCAATGCTGAAATAGATATGTTTCAAGCGCATAACCGAAAA
GCATCTGAACACGTGA
CTCATTTTCTTTTCTCAAAACTCGATCCCGCTGGCTGTGCTGAGAA
GTTTAGAGACTGTT
GGCCTATATATGACAAGAAACTTGAAGTGGCATATCAGAATGCAT
GTCTGTCTTGGTGTG
ATGAACTCTACTCTAAGCATAGTGGGTCTGGTTTCCCCAAAGTGA
CGAAATATATTCTCA
TATCTCCAGACAGTCGAATGGAAAAATTCATATTTAATTTTTCCA
CCTTCGTAATACGGA
AAAAAATAGAATACGAGATCGGTTTATCAGACTTAAACGACAGTC
ACCCTCGCCCTGATC
TGAAATTACTAGCCTCTACAATCGGCAGCGTTCTACCCGCCTCAA
GATTGTTCGAATGCG
CTACAGAGCGCAACTGTAAACATTATCTTCGAACACTGGGGTGCT
CTCGTAAAGTTCAAT
TGGAATGGAGCACTTCTGCAAGTGAAGATGTATCCGGTTATCGGC
GTTTGCAAAGTCGAA
TAAAGGAGAAGAGGGAGGAGTTGTCTAAAAAGAAAGATCGAGCA
ATAAACGCAACTATTT
TCAGTAGCACCGCCTCACAATTTCGAAAACGTGATGAATCTGAT
GTCAAGGAAAAGCTAG
CAATAGTTCGAGACTTGTGGGGAGGACTGATATCTATCGCAAAAT
TAGTTGAAGATTCAT
TGCTTTTCTGTCATTCGTCATTCGATATTCCAAAGCTACTTCCTCT
ACAAGACGAGTCGA
ATGAACATTCGCCGAGTTTGAATTCTGAAGCGATTCTCGATGTGA
CCAAGTTGGATGTTA
GGGTCCCAGAT
>ConsensusfromContig1776 Average coverage: 17.40
GCATACCACCTGTAAATATCATAGTCTTCCCAGTCGCCTTGAAAA
CTTTCAACGTCAGCA
TCGTATCCACTGATTGAAGATTGTGCATCGTTCGTGGAGCTCGTC
AATGAACCGATGTTT
TCTTCAATTGTACCATTCGAGAACTCTTGCAGCAAATTTTTCACAT
CTGTGATAATTGAT

TTTGTCTCATTGACGGTCTGTTCTGGGATATCGTCGAATGTTGCTT
TACCCTGAATGACA
AGTGCAGTGAGATTCTGAGCGGTAACCTGGTCCATCAGACTCAGC
TCGGATGAGATGTCA
GGCAGCTGGTCTGGGTCGAAATCTAGAGAGAGGCTACTCGTGTC
GACCGAGGCACAGCAA
GATGCTGATATCTTGGCCGCATCTATATCATTCTTAAGAGATAGA
AGGTCATCAGCAAGC
TCCTGAGCAGCAACTTTTATTGATGCGACGCTGCTGTTGAGGTTG
TAGACGGCGGTAGAA
GAATCCTGAATCGTTTGGTCGAAGTCAAGAATTGTGTCAATCAAA
GTGTCGATGTTGAGG
TTAGAACTGGCTTTGTCTCGGAGTGGAATTCCCAAGGTCACTCCG
ATATTATCGAGGTCC
CTTGAGATGACTTCTGATGTATAATTGAAGGCATCACTAGCAACA
TAAGTTATTTCGTCT
CTTGTGTTGTTCAAAAAGACTTCTGTATTGGAAAGAGTCGTTGAT
GCAATTTCTGTCAAT
TTCTTAGCCGAGTTTGCCATCTGTTCGTTCGCCAGGTAAGCACAT
CCTGAACCAGCAAGG
AGAAAAACCGTGAAGACAAATAGGCAAACCCCCAAACAAGATTT
CCATTTCCCGTCCTCC
TTTTTAAACCGTGCGTTTCTACCCCCGCCACAATTGCCACAACAA
CGACAGGCGCAGAAG
CAAATACCAAATATGGGCATGATGATCACGTAGAGCAGGCCGAA
AACGATGAGGGTAACC
ATGCCTGCAGCGTACTTCATTATGTCAAACTTTTTCTCCAAAATA
TAGTCCATGTCGAAA
CCTTCATCCAGAATATGCTGTATGATCTCGTAAGGAGGGTCTCCG
GGAATTACTATATGG
ACAAAAGTTTTAGCGAGTGTGTACATGGGTTTCAGCCCCCCTGTT
TCGTAGGCCACTCCA
TCTTCATAGCTCTCTCCTGATGGGATGGAAGCCCATG
>ConsensusfromContig1777 Average coverage: 10.23
CAGAGGTCTGCTCTCCGTCGGCGGCGATGAGCTTAGCTTTTGCTT
GTCTCGTTGCCTCCG
CTTCTGCTGACATCGCCTTTTGTAAGTCATGTGGCAAACGAACGT
CTTTTAGCTCAACCC
TTTCGATGATGACCCCCCAGGGACTTGTAGCCTCATCGAGCATTC
CCTTCATCGTCTCTG
TTGTGGTTTCTCTGTCACTCAGAACCTCTGATAGGGTCTTAGTGC
CCAGAACGTTTCGAA

GAACAGTCTGAGCAATGAGTTGTGCCGATGCTTGATGATCTTCAA
CATTATTGACAGAAG
CCACAATATCGGATATATGATAGAACACGATCGCGTCTATGAGAG
CAGTGACTGAGTCTT
TAGTCAATATTTCTTGTGGAGGAATATCAAAAGAGACTGTCCTCA
TATCCACTTTAACAC
ATTCTTCCACACAAGGATAAACAAATATAACTCCTGGTCCTCTTG
GCTTCCTGATGAGTC
TCCCCAATCGAAACACAACCGCTTTTTCATATTCGTGCACCAAAT
GAAAACTGGTGAAGA
TGCAGATTGGAAAAAATACTAGAAAAATTATGAGCGAAAAAACA
TTTGCAATTATGATCT
CAGCAAGACCTTGTTCTTCA
>ConsensusfromContig1778 Average coverage: 15.54
GACGAAATGGCGATGTCTTCAAAGTCCAAAGTTCTTGTGGGCCGG
CCTTCCAGTATCAGG
GCAAAAGTTATCTCAACATTACGGCGAAAGCAGGTGTCAGCAAT
GGAATCTCCAACGAAA
CGTACGCGGACGATGGACTCACAATTTCTGTATTTGTGAATTATA
ATGGAGAGTGTGAAT
TCGTGCCGTACTGGATTTCATCAAAAAATGATGCATGGAACGCAA
TATTGGTAATCAATT
CACAATTTAGTGCTCTTAGAGTGGAGGCTAGGAACAAACGGTTTG
GTAGCGATTTTATGA
CGTGGAAAACATTAGAAAATAATCTCAGACAAATGCAATGGATG
AGCATCGTCGTTGTGC
TGACTTCGAAGAATGATCTTCAGGTTTATCTTGATGGAGAACCAC
ATTCAATGAGATTGA
CCTACAAAGTATCAGAAAAGAAACCAAGGACCGGATACATGGGA
GGAGTCATACAATTAG
GGAAACTAAATCAGTTCCACGAACCACTATACTGCACTGGGATG
ATCAGTGGTTTGAGAA
TCGTGCCGCATGTCGAAACTGAGAAAAGTATCAAAGATCACATA
AAAAAATGGAAAGCCG
ATAACTGCAATGGCGATTGATGGATGCGAGAAAATAAACGGCAG
AGA
>ConsensusfromContig1779 Average coverage: 13.62
TCGAGTTCCAATTAGGTCCAAGTTAAGTAAAGTTGGGTTTGATCA
GAATTGTGCGGATTG
TAGGCAGTACAAGGCCGACAATCCTTCCCCAAAACCTAAACCAG
AGGTTCCAACGAGTGT
GAAGAGCACCCAGACCATTTTTAGGATGGACTATACACCAATAC

AATGTGAAAGAGATTT
TTTCTGTAAAATGGAGCCAAACACATTTGATGAAGTTGCTGAAGA
GCCTTATATGACGAG
AGGGCAAGAAGATCAAAAGAAACTTCTCGGCATCAGGATTATTAC
ATGGTTCAATATTGT
ATGCTTACTTCCACTGTGCATAACTATGTTACAAGGGCCAGTGTT
TATGAGGGAAAATCT
CATGAAATGCTTTAAAAATTCAAATTTAGCTGGATGCGTTATTCC
AAAAATGAATTACAT
TCTTCCGAGTTTATATTTCCACCTGAAACCTCTGGCATGGTTACT
TACAGAGAAGTCCTT
ATACCTTGCATTTGTTATGACAATTTCCGACTTGGAATCTAAATTC
TGTCCTTCTAAAAA
ACATGATTCACCGAAACCAAAAAAAATCAGAAACAGTAGTTTTG
ATGTAGGTCCAAGTTC
AAAATTGAAAAAAAGACAAAAAGAAAGCAAGAAAAATAAGAAA
AGAAAAAACACTCGTTC
TAGTAAAAGAAACAAGACTTAACCTCAACCTCATGATTTTTAAT
ATCTGAGTATATTCG
AGATATTTCTTCAATTCAC
>ConsensusfromContig1780 Average coverage: 20.10
GATTTAGATTTGGAGACTCTTACAATCCCTTGTGATATCGCCGTG
TCGAAATCCACATTC
TGGCCGCTTTGCGTGTTGAGGAATTCTCCGCTGACGTCATTATAG
AGGCCCCGCTTGGTG
CCGTTAGAAAGACTGAACTCCTCGCCAGTTTGCAGATCAATGAC
AGCATCAATGGAGCGA
GAATCGAAGAGAGAATCCGTGAGAATCCGATATGTCTTTCGGACC
ATGGCGTACGATTCT
TGAGTGATGATTAATTTCTTGTTGATCGCTTCTTGGATCGAAAGA
ACATTCCCGCTGTCC
GCGTCAAAGAACGTCCCAGACTCTTGATTGACAGTTCCTCTTTCA
AAGGCTTCTTCCAGA
GATATCTTTTCTTTTGTTACAGGATCCATAACGCCCACAATCTCAG
CGCATTTCCTCTTC
TTGTCTTTGCTTATGATCTTTTCTTTGGAGATCACCTTATCAAGAA
TGGCGCCACTTATG
AAGCCAAGCTTATGAGCCTGATCAATGGGCATAGGCTCTCCCTGG
TCTTGCTTTATATAA
AGCCCATCTCTCGCGTTCACAATCCCCAAATCGACGGCTGAGCGA
AGAGATATTTTCGCA
TTTGACCTCGGATCGATGACGTAATCAACCTGGAATTTTATTTTTT

CTATCAGCGTCTCG
CGTTCAAGATGGCTCCGATTGCTTTGCCGATCTCTGCCAGTTGTTC
CCGTTATCTCACGC
GTGCGCACAAATTCAATAACATTACGATCCAACAACTCAGGGTC
GGTCATGGTGACGTAA
TGCGAAAGGCCGACGCCGTTTT
>ConsensusfromContig1781 Average coverage: 37.96
CTCTTCCGATCTTTTGGAAGAAGCTATTGTTACGGACAAGCACC
AAGAATTTATCATTA
TCATTTATCACATCATTTTTATAAAACTTATTGGTACTGAAATATT
TCAGGATTGTTATT
ACGGTATTCTTGGTCTCACTATTTTCATGTCAACTATTCCCATGGC
TTCTCTACTGCGTA
GTCTCAGGTCATTGCCTACCTATGCATGTGCCATTGAACAGAAAT
CTGCCGTTCTATGTT
CGAGATACGCAAGCTCCGAATCTGACACCAGGCATCAAATAGTA
GTCTCAAAGTCCACAG
ATATTTTTGAAAATCTTGCAATCGAAGACAAGATTTACAAATCCA
ACATCCTGCAAAATG
AGGCGGTGCTATTCTTATGGCGGAATGCACCCACGGTTGTGATTG
GTCGACATCAAAATC
CGTGGGTGGAGTGTAACATTCAATGGTGTCTCAGAAATGGGGTC
AAAATTGCAAGGAGAA
GCAGTGGAGGCGGCACCGTATATCATGATTTGGGAAATCTGTGTT
GCACATTTTTCACAT
CACGGGAGAGATATCTTCGAAAGAGGAACCTCCAGTTTATTGTG
GATTCTATCAAGAAGA
TGAGTTCGGTAGACTTGGAGGTGAACGAGCGAGATGATATCATG
ATGGATGGCAAACACA
AGATTTCGGGAACAGCTGCGAAATTGGATTCCAAAAGAGCTTACC
ATCACTTCACCCTTC
TTGTGGATACGAATACCACGGATCTGAAATCAGCACTGTCTTGTG
ATAATGAAGAAATAT
CGAGCAACGCCACGAAAAGCTTGCCTGCATCTGTCAACAACCTCA
ATCAAGTCGATTCTT
CGCTCAATACCGAAAAATTGTTGAAAGAATTCGTCTTGGAATACG
CTCGATCCAATAACA
TCGAGAATGTTCTCGAAGTGGATGGGTCGTACGGGTCAATTGAAG
ATTCCTGCAATCAAT
TTCGGTCCTGGGATTGGATCTATGGGAAAACACCATCTTTCACAA
GCTCCCAGTCTTTCA
ATACACAGGTATCAACTGATGATGATAGAAACGCGAGTGGAGAC

CGAATTTTTGTGAACT
TTAACCTCGATGTCAATGTCGTGAAGGGTCTTGTGACGTCCCTTT
CATGTACCCTCGGTG
GTTCCGAGCCAATGTCTCGATGGGAATTCAGAAACCTAGAGTGTC
AGATCCAAATGGCTC
TCAGTTTCGCGGCGGTCGGAAAACCTTTCTACTTCTCCTCCCCTG
AGCGGCCCCAAGGCT
ATCTGGTCGAGGGCCTGGACAACGTCAGCACGACAGAAGTATTC
CTCGGCACGGCGCAAC
CCTTGGCGGCGTCCGCGGTCACTTCCATTCTCTGTTGGCTCAAGC
AGTGCTTTTTGAAGT
CGTCGCGAGGTCTACCGGAATATCTCCGCTCTGTGAAACTTGATG
ACGTTAGAATCAAGG
GAAGGATTTAGGAATAAAAAATGTTCTGAATTTATCTTACATACC
GTAATTTGTTTTTAA
CTAGACCCTTGAGCTTTTGATACCTAGTAAGATGATCAAAAAACC
GGAATATCGTTTCTC
TAGAAAATTTAGTGATGTTCGAATCAGTCACAAATTTAGATTAGT
TAAATTTATTTATTC
TCAATTGCGCCCCTTAACTTGAATGTTGGAATAATTTGAAAATTAA
AGTGTTGTTTTTTT
TAATGAGGTTCTGATTTTTAATAAACCCTTCGAGTTGTGCATATA
CTAAGAAGAAGGATT
TGTGAAACATATTCCAGACATTCGACATTTACGGGCTCTTTAGGA
TGAAATAAGATACTC
ATGTATGTTCTGTGCCATAATATCAAAGTTGTAATTGTTTGTCGT
TGAACGTTCAAATAC
CATGTGGAGCGACGAATCAAGTGTATTTTTTTCATTTATTTCATT
ATGAATGTGATACAT
CCCTTCCTCTTTGGAGGTTTCGGAGGAAAGAATTCCATCAAATAA
CGGGTTAATTTTCCA
TTTATAAAAATCAGATGGAAGAAAATCGAACGGAATTATGTTACT
TTTCTCTATATTGCC
ATAAACAAAGCTTTTTTCAAGAAATTCTATAGCAACCTTGACAGG
AA
>ConsensusfromContig1782 Average coverage: 14.13
GCAAGCTAATTCAGAACAAAAGTACAGTGATTGAAGCGTTTGAA
ATTTCCACGCCAGGCC
CTCTTCAAATTTGAATTAATTTTTAAATTAGCTCTAAATTTGGAAA
TTTCTAATCGAATT
AACTTTTTTATGATACGGTATTGACTTTTCAAAATGCACTGAATG
ATCGTTCGGCATTAT

GTATGTCGGTCGTAATGTTAGAAAAAAACACAGTAATATGTTGA
AATTTGAAAAGAATGA
TCGTATATCTGTTGATATTCAGCCATTCAAACACCCTAGGTATGA
TAATTTTGAAATGTT
TTATCGTGATAGCTTATTTACGGACGCACATTTTCATACGAATTCT
CCCGAATTTTTTTC
TGATAGCAATCTATGTGTCGGGGAATAATATATTTTGTGACTTTG
ATAAAAGTCCGATAA
TATCACATGAGTGCATACACAGTATAAAACAAATCTACATCTCGA
TGCTTGCCAATGTTA
TT
>ConsensusfromContig1783 Average coverage: 8.06
CAAACACTCGGTTGTTAGAATCATCCTGACCCGAAAACCGAGGC
GAGAGTCTGATGAGTC
CTGAGGTGGAGCCGATATAAGACCAAATCGCTATCCTTTTGTGAT
AGGGAGATCTTCGAG
TCCAAAAGCCATCGATTGCTTCAGTTATTGTCATATCTTCCCAAAC
TTCTTTGCTGACAT
TTGAGGAGCGGCAGAAGAAAGAACTTGTGCGTGTCGTTGCAGAG
AAAAAGCTAGGAGTTT
GACAGAAGGCAAGAGTTGAGAGATACAGCGAAGAACAACTGGAA
TTCAACGAAGGCTTGT
TGGTATTGGTGAATTTTGAGTCGTATTTTCCTCCGTGTAAATCCG
AAAATAGTACATGAT
TGTATCGCTCATCAACATTTCTGATCCTCTTGGTGCAATTAGGATC
TGTGCTGTTGTTTT
TATTCTTGCTCAATGGCGCTCTTTTCGTAGCGAAGGGTGCTCGTG
GAAATTGATAATTCT
CATCAGATGCCATTGAAGCAAGTACAAGGACAAGAGAGGATTCAT
TTATTTTTGTGAAAT
GATAAGTAATAGAAGGCGCTGAGATCCACTTCAAGAAACCCTGGT
ATTGTATTGCGGCCC
TGGTCCTTCGGAAAACAATCGACCCTGAACTCCTGGAGGTCATGA
CACTCAAGGTCCTCG
AGAACTCGTTTGCGTCATCGTGGTGTGATTCAAGGTCACGCAGAT
GAAGAAACTTCCGTT
GACTTGTCCACTCTCTCACGTTCTCGAGAAAAGGGTGAACAAGAA
CGATGTCTGAACTTC
TTTCAAAGATAAAAGAGTAGGCATTTCCCC
>ConsensusfromContig1784 Average coverage: 15.53
TAAGGTTCTGTATGAATCGAAATGTACCAGGATCCTTCTTTGGTA
TCAAGCCTACAGGAC

TAATGTGGAACTTCTCGAAGGGCGGACTGTTGAAAGGGCCAGCT
ATACGACCCTCTGCAA
CTTCTGCGTCAACCATTGACTGCAGCAAATCAGGGTACTGTCCAG
CTGTCGAAAAGTTCC
TAGAGGATACAGAAAATTCAGTCATCGAGGAAGGGATTCTAAAT
CCACCTGTAAAGCCTG
AAATCAACTCATTCATCAATACAACATCATAACCTCTTAAATAAT
ATGATAGCCGATCAA
CATTGACAGGTGAATTGATAGTGGAAAGGCCACCAACTTTATGAA
AGCCCATGTTCTTTC
CAACTGGATTCGGTTTCGGAGCAGATGTTGATGGAGCACCAGGA
GGACGATAAATCTGCT
GCACTGTCTTTTGAGCGGGG
>ConsensusfromContig1785 Average coverage: 19.20
GCGGGAAGTCTTCGCCGCATGACTCTTTACAAAGCAGCTCTGCTT
CACCGGAATTATCTG
GCGAAATAACTCAACTGACGTACTCTCCGAAATCCATGCCTCTCC
AACGGGTGGATTCAT
CTCCGCAGCCGTACGCCTCAAAATCATCGCCACTGAGTGTAAATT
CAATGGTATCCTACG
AAAGACCACCATTGGTTACGGCCGGCTCCTGGCCTTCGCAATGGA
ACATGCCTTTTCGTG
GAATCCCCGACGGAATGCCACAGGACTATCCTTCGAACAGAATG
GTGGAGAGAAGTTCGG
GTGTCAAGTTAATAAACATTGAACCTAAAGAGGACGTTCTGTCTG
AAGTCGAGAACAATT
TTGAACCAAATTCTCCTAGAAAAGGTAATAGTGTAAAAACTATGT
TCAAAAAAAAGTTCC
AGCTTACTCGTTGGCGAAAGTACAGTAATACTATGAGTGAAAAAG
TGAAATCTGCATTAC
TGAATCGTTTTCCACCAAACTCAGATAAGGGTAAACAGAATACGC
GTTTCAGATGTTGTC
ATTGTGGGAAACGTTTCAAATATCGTGTTGATTGTAGAGATCACA
TACGCACAGAACACC
CTTCGATTACCCGACTTAACGTTGCTCTCAAGCGTTCAAATGCTA
ATCTCAACTCGATTT
CGAATCCTCTTGTCAACAAATGTATTGCGCTGAATGATAAAAAGG
ATTGTGGGGTCACGG
AACAGTTTGCGAAACTAGAAACAAAACTCGAAACCAGTGAGCTT
CTGAAGTCTGATGATA
AAATCTCAGAACAAGCAAACCGTGATGAAAATTGCCAAATGCAA
GCTTTCACCGTAGATC

ACTTCGACAGCATGTCGAAGACGTCTGGAAATCTACTGGCTAACT
CTATGGTCTATCTGC
CTGTCCTGAAGCATATTCAAGAACCCCTCACCCTCAAGCTCATGC
CTCTCCCATTGACAA
TGAACAAATGATGAACCAGTTGTGGATACGTCGTTACGTAGGT
GAACCCACGACAGTT
TCTCACGACCCCAAATGTGCCTCGATTTTCCACCCCAACGCAAAA
ACAAGCATTCTTGCG
GTTCGCTCACTATTCTTATCATGTTTTATGTCCAATAATCCTTTTC
GTTTTACTCTAAAA
CTCAGGTTAGCTCGGTGTTACCGCGAAGACTCTCATTCCCAAATT
TGTGATCCCATTTTG
ATATCAAAGCTTTCAAGAAAGTAGTATTAGATTATTGGAGCCTAT
AATCGTTCTTGGTGT
CATGGGGCTCTTCATTATCATCAATATTGTAAATATTATTCACATA
ACCTTTTTTTTATA
ACCGTCGAGATCGGAAGAGC
>ConsensusfromContig1786 Average coverage: 15.83
CAACTATCTATCTATATAATTCGAAAAAAAAGAATCCTACTATGG
TGTTATTGGCACTGA
ATGAAGATGATGCTAATGACTCCTTTGAAGAAGAAATTGACGAA
AATGTCACCACAACAG
AAACAGAGGACACAGATGTGCTCACTTCCTACAATGAGGCTTTG
CGGTTAGAAAACCTGG
GATTACTGAATGATGCCGAAGAGACATTCCGCTATATTTTGGAGA
CTGATGTCATTCAAG
ATGTTGAAGAAGAAGAAGAAAATCCCTCGCTCTCCAATCTTATCT
CCATTAAATACTCTT
GTTTCAAAAACCTTGGCGAGATTCATCAGAAATTGAACAATCTTG
ATGAAGCAATTGAGT
GGAATTTGAAGGCAGCTGATATGGGACGAGGAGACGCAACTTCC
TGGTTCAAAATCGGGA
TTTTGGCAGAAAAGGCGGATAACTTGAACCTGGCGAATGCTTCAT
ATCTCAGAGGATTGA
ATATCGACTCTAAACACTGGCCTTGCTTGAACGGAGCTGTGATCA
GCACCTTCTCTCTCG
ATCTCTTCGAAGATTGTGTAAAACTCTTGAAACTGGTTTTCGAAT
TGGATCCTGGTTATG
TCACTGGACTTGTTCTTCTCGATTCCATTCATGATCAGAATTTAA
TTAGCAAGCAATTCG
TTTCCAAGTATATTGACAGACAATTTGTCATGAATTCTGATTCCA
TTCAACTTGAAACGC

GGCAGAAGATTCTTCAAAAATGTTCAGATTTAAGACGCAATCATA
TTGAATCTGTGAAAC
CTCGAAAACTAGAAGTCCAACAACTGAATTTTGGTCATTCTGATT
TCACATGGGCGAACT
TAGGCTCCCTTATAACAGATGCCTTATCAATATTCTCTTCCAATAT
TTCTGAGGTTTCAA
ATGTCGTTGATATCTCTGAAATCTTCAAACAACCCATTTTTGAAT
CGAATTCTGAACGAA
ATATTATCCCAGAAGATACCATCAGACAACCCGCAATCAGTGGCG
ATACAACTTTTCCAT
CTAGTTATCTCGAAGGAAAATCAACAAATAATTTGTTTTCAAGTG
AGAAGCCATTTCAGA
GTCACGAGTCTATGGTAGTTGATACGGATGAATTTAAAGAAAAC
GAAGAAGAATTTTCAT
CCGAGACAGGGAATGTCGAATTACTTCTTAAATCATTGATCGACC
GTGTTTCTTTTGATG
ATTATTCTGAGATCGTAATTTCATCTAGAATTAATTTTGTTTCCG
ATGAGAGAGTCAGTT
CACAAAATTCTCCTATTACGGT
>ConsensusfromContig1787 Average coverage: 11.27
TGTGTCAAGAAGGTGAAGCGAGTTTTGTCTGCCAATGTCCCTCTG
CATGGAAAGGAAAAC
ACTGCCAGCTAGATGCAATTGATGACTGTGATGGAATCGATTGC
AAGAACAACGGCTCGT
GTATCGATATTGGTGCGGAGTTTGTATGTATTTGTGCGCCGGGCT
ATTCTGGAAATCTCT
GTGGAGCATACGTAGGTTCCAGTATGACCAACATCGTCGTTACG
AGTGATGACGAAGGAC
AAGATGATTTAAGATGCGGCAACACAATGCCTGGACCCAAGGGA
GGTCCAAATGCAGAAC
CTGAATATGAAATGCCATGCAAGAGTATAGAATATAGTTTAGCCA
AACTATCTGACGGTG
GAACCGTTATCATCAAAAGTAATCAAACCAAAACCTATGTCAAT
GAAACGATCACCAAAT
CTGTCACCATTCAGGGCGATCAGTTGGAAATGAAGACTACGGATG
TCAATCTTCGTATAA
TTGGTACTAAAGAAAAGAAAATCAAAATCACTCTCAAAAATGTC
AATGTGAACGGAAATA
TTTCGTTGTCTTATTCATCAAGTGATTTCATAAACGTTAATGT
>ConsensusfromContig1788 Average coverage: 5.47
TTTCGAATTGCTTTCTCGTTTCAAATTTCTATATTTTTCATTGTTGA
GTCTATCATAATT

TAAACTTCTTAGCGACAAACATCTTGTACTGAGCGTATTGTCAGT
AGGCGAGCAAAGATG
TGTTCCATAACTACTAACAGCGATACCGATAGTACTCTTTGTTCCA
CTTCGTTTCTTTAT
TATTTTATGCATTGATTTCCATATCAAAATGTTTATTACAAGCAG
ACTGATTGACGGAAA
CACTGAAATTAATAGTGAGTAAAAAATAGAAAAAAACACAACACC
TCTGCTGTCTTTATT
TCCGAACGCACATTCGCCGTCTATTCTCAGTTCGCTGTAGAATAT
GTGTGGAATGCAGAG
AACCAAGCAAATAAAAAACAAAACTGTGACTATTTTCCTCTTGTT
GGATTTAACATTCGT
TTGCGAGTTTGTTTTACAGTGCAGCACCGAATAGAGCCTACACAC
ATTGAGCATTATCAC
CAAACCAATACTAAAGGTCACGAAAACAGTAATCCCGTACGAAT
ATATTGAACATTCATG
TGGCGACATCTCATAACAAGTTGTGATAACTCGTATATAGAGGG
CGCCGATGAGAACGAT
AGTGTCAGAAACGCCCAGTAGCTTCATATAAAAGGCAAACGCGCT
GTCTTTGTGTTTTTC
AAGATTCATTATATGGATCGACATGAGATTACCTGGGATTCCGA
AGACAAGAAGCGGGAT
GATAAGCCATCTTATCCCCAGTACGTAGGTCGAGAATTCTGGCTC
ATTATGTGTTATGCT
TTTATTCATATTAAGTCGATGAATGAACTTTCAATGTATATCCAA
ACACTTCAGTTCGTA
GCTCT
>ConsensusfromContig1789 Average coverage: 9.98
CGATCTCGGTGTTTACAACTTCAGATTCGATTATCTGCCAATAGA
ATTGAATAATCATAT
AGATCATAAAATTGCGAATGCCTGGGATCTAAGGATGAAGCTGA
ATTCATTATATCCGA
TAAGAAAGAGGACGAAAGGACAAAACAAGTCGATCCACTATTTC
TGATATTTGAACTCCC
AGAAAACGAGAATATTCTAACAAAAAACCACCTTCGAAATATTC
AGATCATAGAAAACGA
ATTTTACAACGCCGCCGAGTTTCGAGAGAAATATTGTCATCTAGG
GAGTGACGGATGTAA
CCTACCCATTTCTATTTTAAGGTTTTTTGATGGGTCATTTGTTGAT
TTGGATTCTAGATT
ATACGACCCTTATTTCGAACGAATCCCAGATATAATGAGACTAGC
GCAATACGCGCTGGA

GATAAACGATTATCTACCATCACTTTTGAGCTTAGATTATTCAAT
AACAGCAAATGAAGT
TTACTCATCAACAACACGATCCATCATTTATTTTGGAGGTCCGCT
GGCAGGATTCAATAA
AACAACTGACAGAATGAGAGAACAAAATGAAATTTACTCATCAA
CAACACGATCCATCAT
TTAT
>ConsensusfromContig1790 Average coverage: 33.63
ATTCGAACGCCGAAGATATGCGCGCCATTGTCAAGTTTGATGTAA
TGAAATGCATCTATA
AATATCTCAAGACTTCATCATTTGATAGTTTCGACATCAAAGAGC
TTATAATGAACAACG
CCGACATACCATGTATTGTCGTTGATAGTGGATTCACTTTTGTAC
ACCCACGACGTGTGG
TTATCAACTTATTCGAAGAGAACCAAATCATTCCGTATCTCTATA
AATGTCCGACTGAGT
TAGGAGAATTTAAGGACCTTTTCATTTGCATGGGGTCAGTTCTTA
ATGCTGGTTTCGATC
AGTACGCCGCAGTGTTGAACGAAATTAGTTCGACAGTCGGCGTCA
ACAATAAGCTTCATC
CAAACGAGATGCGATCTGTGTTCAAAGCCATGCGAGGCCTCTTCG
ATTCACTTGACTGTC
TTTCCAATCGTTCTGAAATCTCTTCAGAAATTCTGCATCTTCCCA
GCAAATCCAGTACGC
TGGTTCCTTCGCATGAACTGGTCTTCAACGATGATCCCTTCTGCA
TGGATCGGATAAACT
CCGAAGAGTTCGATCATCCATTCATGATAAACCTCTCTGTGGACT
GCGGGATTTCGGCAT
CTCACTTGCTCCATCAAAATGAACTCATCGAGCTCATTCCTGCCC
ATATGAGACCGCTCA
AGCTTAGCTCTCTCGTTCACGAATGCCTCGAAGACTCCTGTAAAG
AAACGAGTATTTCTC
ACGGAATTGCCGAGAAATTGCGGCATCAGCTCCACTCGAGATCTT
TCGCAGCGGGAGTTC
ATCGCGTCGTCAACCATGAAAGAAATCGCGGTGGAGTTGCAAAC
GTCAGACGAACGCTAG
ATGTCGATGCTGTTCATGTGAAGATAAAGAACCTCAACGTCTATA
GCGTCAGCAAGGTCG
TGACCTATTTGACGTACAATGATTCCCGTATTGTAGGCAGTGAAA
TGGAATGTCAATGTT
TTGTTGAGAAACTTCAAAGGTCTGATGATAAATCTTCCGGGATT
GATGAAGAAAATGAAG

ATTCTGTCTCGTGGAATATTTATATTGAACAAACAGCGACTCTTA
ATGAAGAGTTATTGG
TGAGCGTCTCATTGGTTGTTGACAGAATTGTGGGCGGGCTTTTGA
GAAACTCCGTTCACT
ATCTGCAGCCAATCATCGGCTGTGCGCCTCACGCCATATCGAAAG
TTCTCGACAAACTCA
AAATCAGGCCTGATCATTCGTTCAATTTCAAAGAACCAACGCTCC
CGATTCCAGGAACTT
TCGTTCCCATTGAGGATCATCATCTCCTCAAGGAAGATTTTGGGA
AACATTTCGAGCAAG
GTGAATACGTCGGATTCGAAATTCAAGACGACGATACAGTGGCG
GGATTCACAGTCGTCT
ACGCTACTGTTATACGAAAGCTAGATCTTGATAAATGCGACCGG
GAGGGGAGTCCTAGC
>ConsensusfromContig1791 Average coverage: 65.67
CACTATATAACTGATGATATATTATGGAGCAGTTCAACAACAGT
AACAAAGAGAGAGCTG
TGCATTCATTTCCAAGCAAATTACATATAGATCAAGTATTTGACT
AAGTCAAATTAAAAA
TGATAACTCAACATCCCACGAGTAAAGATATCAATTATATAAATT
AAAATTCTTTGTTGA
AACAATAATAATTTGAATACAAAAGTAAAAACATGGTGAGTATCA
TATTCTTTTCTAAAC
CTCATATCTGTCCTATACTGATATTTAAAATTTATGGAATTTATTG
AGATTTTATCTTTT
TTCCCGAAGCGGTTGTGGTTGTCATCAACTTCTTCTTTCTTTGAGC
ATACATTTGCTGCA
TCAACATATACACCCCACATGGTATGACTATGAGATAAAATTGA
AGAAAATACGGGAAGT
AGAAAGCGAAATTGGCGCTGTTGGGTAGAGCAAGGCTGTAGCGG
CCAGATTTCTCGAAAC
CGGGAATGGAAGAAATGACGGAATAACCTTCACATATGCAGCCT
ATTGGGTATAGTGGAA
TCCATATGGAATAGCGAAGCCACGATATGATTCTCCATTCCTTCC
TGGTTATTTGCAGCA
TGTAGTACGGGTATCTTATCAGTTCCGTTGCACTCCAAGTCGCGA
ACAGCCACCAGACAA
CGTGTTTCTCCTGAACCGTGGGTTCTCCAAGAATTACGACGAATA
GAATGAAGTTTCGCC
CCGTAACCTGCATAAAAGCATTGAGGAACGATGTTTTCGTCCAC
CCAAGAAGGGCATGGA
ACATTTCCAGAACGGCGCAGGATTGGGTTATCATCATTGCGGGTC

CAGTGATTGTGAAGG
CTGAAGGCTTTGAGGCTTCGCCAAACTTGTAGTATCGAACACTG
AGGACAACGAACACGA
AAGTGTATCCCACCCACATGAACATGTTGTAGACGAAGAGATAGC
TTTCTTTCCATGATG
TGGTATTTTTGTTCATTCTCTTCTTGGCGGACATTTCATTCATCATT
TTATTCAGCTTAT
CCGGACCATAGTTTTGTGGCATGACATTATCGTCGCTCTCGACAT
CATCCTCATCGTCCA
TCCACAGATGGTCAAAGTCAATGAGGAGCCACGCCTCTTTTTTGG
CTCCGTATGTGAGGC
GTGGCCAACTCTCACCTTCACCTTTCTTCTTCAGTTTGATCTCAAC
ACAAGAGTCTTTGA
CCGAGAATACACTCTCATCAGCATCAACAGGATGATACAAATCA
ATACTGAATCCATAAG
TGGTCTCTCCTGTATGTCCTTTGCCAGAGCCTTCAAACTTGAGGTA
ATCTTCATTGATAT
CAATATTTGGGGTCTGACAATCTCTGAGTTCAACTTTCAAATAAA
TAAGTGATTTATCCT
GACCCCAATACACAAAAGGTTTACAAATCTGTGGTACAGCCATC
GATTAAAAATAAATAA
AAAAAACTGTGACTATTTGGATTTTGCACCCAGAGATTCCGCAGA
TCGGAAG
>ConsensusfromContig1792 Average coverage: 7.50
GGTGACTCTATCAGTTCATTTCTCACATTTGAGTAGGAAGTCTTGA
TGAATTTTTCTGGT
GTTTGCAACAAATCTGTCTGTATATTCTTTATTTTCAATGAACCAA
TTACCTTTTTATTC
ATGCATATGATGTGCATAATTATGTTATTTTTTACCCTTACGCCGC
CGTCCTTCTGGTCA
CGCCTCCAAATATAACCCCAAACATTGTAAGCAAGTGATTTTTGT
CGAAATCGGCTGCTA
ATTTTCAGGGTTATACAGGAGTTTTAGAGCGACTTTGCTGATCAA
TCAATTGATTTGCGA
ATTAATTATTCCGAAATGCTCTATGCCTTCTCCAAGATGCAATGA
TTGTGGGAGCTTTCA
GCATCAATCAACTCATTAATTTTCAAAAAGATGGCGGACAAACA
TAAATGACACACGAAC
AAACAAACAAACTGGATTCTAAATTCCATACAAAAATCACACCAG
GATCAGCTTTCATTG
AAATGTTTTTTTCATTGAATCATTGAAAAAAG
>ConsensusfromContig1793 Average coverage: 22.97

GGAGTTCCTTTCAGCGATTTGGCGATCAAAAACCAGGTCTTAGCG
GCCTGTTTATCATTG
TCTGGTCTTGATTCGAAGTCTTTTTTGAAGAATTCGATGGCTTCT
GAGAACATCTTCAAA
TCTCGGTAGGTTTCGCCGATGGAAAAGAATATTGGTCCGAGAGA
GGACGGCGGTTCGTCA
ACACTGAGGGATATCACTTTCTTGTAATAAATGATGGCTAATCTC
AGTAAACCGGATTTG
AACTTCGAGCAATGATCTCCAATCTGCTCAGCTTTCTTTCTCTTCG
CACTGTGCTCCGAT
AACATCTCAAACTCTTCGCAGAGCAAAGCGGCTCTGGCAATATGT
CTGTTGATGTGGCGT
TCATCAGAACTTGATCTGCAGAAGACAGATGCTTGCTTCAAGCAT
TTCAGCGAGTCTTTG
AATTCATTCACATGTAAGAGAACAATAGCCTTCAGCTGCAGCATC
TCCAATCTCAGAGCT
TTGGGAACTTGCTGGACCTCTTTGGAGATATTAGCTATGTGCTCCA
GTGCTTTCTTGTAT
TCCCCATGTTGGTGGTATGTGGCACATAGGTTGAACACACATCCT
ACCAGATCCTCATAC
AGGTAGTGGTTTTTTGAATTTTCCATTGAGAGTTTGTGGAATTTTT
CGCTCTCCATCATA
TCGTTGCGGAGCTCGTAGACAATGCCCAGATTTCTATGAATCCTC
GCTTTCATCTCGGGT
GCTCCATCGTTTCTCTGCAAAAAGTTGTCACACGTCTCCAAACTC
TCCCGAAGAGCACTT
TCTGAATCGCGTAGAGATTTTATAAACTTCGAGTAGCTTCCTCTG
ACCACTTCAGCCTGA
CATAGATGAGTTCTGCCCAGCGAAGCGAGTGCTCTCTGGACTTCA
ACAGGACTATTTAAC
TGTCTGGCTAAGTGAAGATGACTCTGTTGATGTGATATCGCCTCC
TGCAGATCCCCCCCT
TCAAATAACAAGTCTGCGATGGAGCCATGTGTTGATGCTACATCT
GAGATGTTTCCTCTT
TTGATATGGATAGCTAGTTCCTTTTGAAGCCTTTTCAGCTCCATAA
TTAATGTTTTTCCT
TCCTGCTCTGGATTGTTTTTAGGTTACGTCGTCAGGTACTTTCTTT
TATTTTAGAATCTA
AACGGGTGTTCATAAAGAAAAAAAATTATATATTGGTACGTGTCG
ACTAAAAATATATAT
ATTAACATCTTATTTCATTCAATTAATCAATTTCAG
>ConsensusfromContig1794 Average coverage: 37.51

TGTGGCTCAAAAAAGTATTAAATGGTGATTTGAAAAATTTGAATA
GTATTCAAAATATTA
GTAAAATAAGATTAGATGCATTACAATTAGCTATGATGAAAGAG
GAAAAAGAATATAATT
CAATATCCCAGGCCAAAGTGGAACTTGCACATTTGAATCAGTCTA
GTATGAATGGTATAG
TTGGGAAAATTTTGAATGAAGTTTATGAAGCTGCTAATGATTTAG
AAGATAAGTTGAATG
AACATATATTCGACAAAGCTGCTGATATTCCTGAACTGGCAGAA
GGCGGAAGTTTGGAGA
CTGTTATAAGGCTAGACACTTTGAATAAGAAACATGGTCCCCTT
GAAAAAGACAAGTCCG
ACAAAGGGTCGTCGGTAAATCTGGTTGATGTTCATGACAATCAGT
TTGTTTTATCCAAGA
GTCACGATTCAACAACGCCTTTCGATGATCAACGTTTGATATTCG
ACTTGGTCACCGTGA
TCTCCTACTGCTTTGTAGCGGGATTCCTATTCAGCTTTTTGGGCAT
TCCAGCAATGTTTG
GTCATATTGTGGTTGGAGTAATGACTGGGCCTTCTTGTTTTGGGGT
TTTAAAGTCTTTGG
TACAGCTGGAGACGCTCGGACAGTTCGGAGCCTTCTTTATATTAT
TCAGCGTTGGCCTTG
AACTATCTCCGAGCCGTATTCGAAAGGTTGCGCGCATATCGGTTG
CGGGCACGCCCTTAA
TGATGGCGGCGCTTGTACTTCCAGCTATTGTGATTGGCTCACTTG
TGGGCTCAGGGGCCA
ACCAATCGGCATTTATTGCATCTGCATTAGCTTTGTCAAGTACGC
CATTGGTTGTGAGGT
TTTTGAATGAAGCCAATGTGAAACAGCCAGGTGCATCTCCAAGGT
CCACAACTGCGTCCA
CTGAATTGCCTGGCTCTGCGAGCGCAACGGAATACAGCGACATGC
TCCTTGGAATTCTCG
TTATGCAAGATGTTCTCCTGGGCTTCATCATGGCTGCTCTGCCAA
ATCTCTCCTCAAGAG
ATTCGGTTGTTGGAACCAGCGTTGCTATGGAGTACTTCAAACTAT
GTCTCCACATCTTTG
GAAGTCTAGTTACTCTTCTCGGCGCCGCCTTCTTCATTTCGACATA
TTTTATTCCTGTCT
TATT
>ConsensusfromContig1795 Average coverage: 7.87
GCATTTCTTGAGCAGATGTATATTTTGGCGATCCCTGATTGGCTA
AAAGAGGCTGGTCGA

GAAGGTTGATTTTCTTCGGTGCTTGCTTTCGGCTCGTTCTGCCTTG
CTGGACATCTCTCA
CATAAGGTTTCTCGAATACGACTATAGGACGTTCCGACTCCGTAG
AATCGCCAAGATTTC
TCCGCTTAGATTTGGGCTTATCCTCGTCGACAATGACATACTGAG
ACATCGATCCCTCTG
GCGATCGAGCTGGTTTTTCATTTGACGTTTTTCTAATGTTTATATC
ATCAATCTCGTCAA
CTTTGTGCTCGATGATTGCGATAGGGTCTTTGTTGATTAAATTTT
CTATAGGTATCGAAA
CGTCCGTTTGCATATTGATTGGAATATTCTGTATCTTACTGGACTG
CGTGATTTTGTTTG
TATTAAAAGCTGGTATGGGATTATCCGCCAAGTCTATGTTACCTT
TATCACTCTCGTCAT
CGTGTATCAATTCCTCTACTGGTATTTTATACGACGACATCTGATG
CTCGTTGTGCTTTG
GAGGATCGATTATTTCTTTCTCTTGTGTGTCTAAAAATTCTTTAG
GATAATGTGAAACAT
TCACGATTGGAATTCGGTCGGGTAGCTTTGATGAAAGATTTGGCA
TTCCAGCTTTTTCAA
CCAGGTTGCTGCTGTGTTTCTCTGTCTTCAGTGAG
>ConsensusfromContig1796 Average coverage: 14.97
GTCCAGCTGATGCTCGAAAAAACGGATTTCTTCGGGTTCATAGGT
CATATTTCTATCTTT
ATCCACAAACTCGTGAAGGGGGCTCCAAGAAGAGCCGTTGTTGG
TTGAGATGTAGATGAT
GTTGGGTTGGCCGTTGACGTTTTGCCCCCTCTCCGTCAGGAACCTA
GCTCCGCTCTTATC
TCGCCGCAACCAATGCTCCGTTATGTAAGTTTGATTATCAGGAAT
TGCGACGCCATCACA
GACACAAAGAACGTTGAGGAGGTCAAGATATCGGTAGTTCTTGT
TGATGCACAACAGTCC
CACAAACATCTCGATCTGTTCCCTCGTTATTCGATCGACGATTTT
CCGGTTGTCACGAAT
CAATTCGACGATCATCTGAGCCACGTTCAATCCGATTTCGCCCTT
GACCGTAATTTGTGT
CTGGAAGAAGGCGATGTTTTTAGCGAAATAGAGGGCGTTCTTGCG
ACTATTTCCGATCAT
GTACGTATAGAGAACGTCGTAGGCGCTCTTGAAGACATGGGATA
GATGAACGTTGTCGAT
AGCGCCGTCTAGCGGGAATTGAAGGACCTTGACGAGAAGGTCAA
TTACCCGTAAATTCCT

CAATAATTTCTGTCTTTCTTTCGTAGCGATTCCTTTCAGAATCATG
AAGTTTTTCAGCTC
AGATAAACCTACAGTGATTTTATGGGCCTTTTTTGCATTTAGTATT
TTTCCCTCCTTTCT
ATCCAGAATAAGATTGAGCAAAAAAGGAACGCAGCCTCCGATAA
AGTTGAAGTTTCGCAT
AAGTGGCGCCTCCACGGATTGAATAGATCGGAAGAGCGTCGTGT
AGGGAAA
>ConsensusfromContig1797 Average coverage: 14.11
TCGGCAATGACCCAATTTCGAATGTTGACCCCAACTCGATTGACA
TGCGAACTGCAAGCC
CCTATCGAGTTCTTCCTCCTATTGGATCTTTTGATGAAGAGGAGG
AAGAAGAGGAGGAGG
AGTCAGCCGGTTATTCGAACAGCTACGCGGCGAATCACGAAAGA
TCAAAACGCAGGGCTG
AGTCTAGCTACCAGCCCTATACATTGAAAGACTACAAAAGATTA
CAACCTTCCACAAAAA
TGGGAGGTCTAGGGCCTGATATGAACGCTGCTAGAGGAAGGAAT
CAAAAACAGCAGAAGC
AGATAATTTACGCTCGTGAAATCGCCGAAAGGAACAAAATCAGG
CTTCAGGAAGCTTCAG
AAAAACAAAAGTCGAAACCAGCCGACGAAGACTCTGAGGAAAT
GAACACACCGAGCAAGA
GGCAGCTGGCGATTGAGTATTCGAGACGTGTTCCCAAACCGAAG
ATACCCCCAAAACCAC
AAAACCTATCCACCAATCGTACTAACCCCGATGAGAAATCCAACC
CTCATCACGCCCACA
ACAGTTCCCCTCTTAGAGAAGTACTTGGCTCCAGCCCATCGATGA
ACAGCGGCATACAGG
GAGACTTGACCTTCAGAATTGCTGAGTTGCCACTGGCGACTCCAG
AACCTGAGAGGCTGA
GAGAGCTGAAGGAACGGCACGAAGCAGATAAGTTGAGAGCTATG
GCGATTCGACAGAAAA
TGGGGGCTGTTATGTGAATAGGAGAAATTTGAAGTTTATAAATTA
TCAGTATTGTTACTA
TTGGCAATTAATC
>ConsensusfromContig1798 Average coverage: 14.18
CGGACCTCCAGCTTTATTGTGAAGTCGTTGCGAAGGTCACGGACG
AAATCGATTCTTGCA
GACAGCAGAAATCCTCATCAAATGAAGAGAGCCGCTGCCATTGC
CATGGAAACGTATTCG
CGGATTGAACGTGCGGCGCGAAGGGAAGGATGGAAGCGAGAG

ATTCACCAGTGATAAT
TTTCCCGCTTACGAGGGTAGCTTTCTGCCACCGGATCATTTCGCGA
CTCCTCCGGTCTCC
ATCCCGGTTCAAAACATCAATTTGGGGAATTCTAACAATGGAAAT
ACTGATCGTGGTTCT
ACGGCGTTTGCCCCACGCGGCCATCAAAGTTTCCTATCAGTACCG
CAGATAAAATTGACA
AGAAATGATACCGAAGCTTCTGTATCGCCATCTATTGATTTACAT
TCGACTTCTAGGGGG
GATAAAGGCTCTCCACATACGAGCATTCTTGAAAATGCGATGAA
CGGTTACCAGGTGGAA
AGAAAGTATCCTATTTCCTTGCCCATGTCTGTTGGAAATTATACG
CCGTCGTTATTCAAT
CATAATTTTGAGCCGATTAACCGCAATCTAACCGGTCTCGGATCT
GGGTCTCGGACGACA
ATAAGTGGCGTTCCACGACACCCATTCCGCGATACGAATTT
>ConsensusfromContig1799 Average coverage: 7.64
CTGCCACGAAACTCTGCCCACAGCCGGATGACATCGGAGTCCAC
GGAACGGAATCAGAAA
TTACAGCGGATATTTCCCTTCGCCATTGATGACTTTGATGCTCTA
TTCGGATGCTGGGAT
GCCAAAGAAGACTGGAACGATGGGGTCCTCACTTCTGCTTGGAG
GAAGGCAGCTAGGAAT
CAATGCACAACGTGGCTGTGTTTGGACGGACCTCTGAGCACTGTT
CTCAGTGAAAGTTTG
ATCAGCGCTTTAGATGATAAGAAACTCGTGCAACTGGCCAATGGT
GATTGCCTCCGAATA
ACTTCAAACATGAAACTAGTCTTTGAAACAGATTCTATCGAAAAC
AGCTCCCCTGCAATG
CTCGCCAAAACGGGCATGATCTATGTTGAAGAAGGCCTGATTGG
CTGGCGTCCAGTGGCG
AAAGCTTGGCTTAAAGGGAGGTCGCAGCAAGAAGTGAAGGTTCT
CCAGAAAAGTTTCGAT
AAAACAGTTGACCATGTCGCAAACTTTGTCGCAAACGAAGCAAA
GCCCGAACTGCGGCAG
AGTGGGGTGTCCATGTTCACGACTTGCATCGGGCTGCTATCGAGC
ATGCTCGCAGATAAC
GTGGAGATCGGAGGAGAGTTGCATATAGAGCGGATGTATCTCTTC
TGTCTCATGTGGTCT
TTTGGTTCTCTTCTCAATGAAACTGATCGCAGAGGTCTTCATCAAC
TACTCGCTTCTCTG
TCTACGGCATTTCCGGATGACGACCGAGATATTTGTTACTTCGAT

TATTACGTCGACGAA
TCCGGAGAATGGGACCCTTGGCATTCAAAAGTTCCCAACGCCTCT
TTCTCTAAGA
>ConsensusfromContig1800 Average coverage: 10.91
CTTCCCGGACTCAGCTAGAGACGGTCAATTAGATATACTCAAAGA
ATTCATTGGAAAAGC
TGATATTAACTCCAGGAATTTTGAAGGAGATACAGCATTGCATTT
GAGTACGCGACAGGG
TCATGAGCAATGTGTTATGTTTTTGCTGAAAAAGTGTTCTGCTGA
TAAACGAAATAACGA
TGGTCACACACTCCTGCACGTCGCAGCAAAATCTTTCAGCACACC
AGCGACGATAGTTGA
GTTAATTGTCCAGACTGTAGTCAGCGCCAACTCATGGAATCTCAT
CAATGCAACAGATGT
CAACAATAATACTGCTTTGCATCTCGCCGTGAAGAACGAGCGGG
CAGATATCATCACAAA
ATTGACCCCGATTTCGGTGAGGGCGAAGAACAAGGACGGAGACA
CAGCACTCCACATTGC
CGCAAGAATGAAGAATAATGAAATCATCAAAGGGATGCTGAAAT
GTAATGACGAAATCGA
TATAAATGAACCTAATAGCAATGGTAGTACCGTGTTACATCTTCT
TTGTGAATCAGGAGA
TGTTCACATCATCGATTTACTGCTTCAGAACGGAGCTGACCTTGC
CATTCAAAATAATGA
CGGAGATACCTGCTTGCATATCCTGACACATAAATCTGCCATATC
GAAAAGTGATGTTTA
TGTTGATGCATTTAAAACTATTGTTCAAAAACTGGCAAATTGGTG
GTGCACTAAACATGA
ACTGCACCTTCCGGATGGGGAATCGCACCTGTTCAAAACATTGCA
GAAAAAAGCATTCTT
TTATTTGCGAGATGAAATCATCAACAACGAAGGTTTGAATGTTTT
AGAATTGGCGACAGC
TCTAGGGTCTGTCAACATGATCAAAGCTATGATTCATGTACCTTT
TGTAACGATGGAGGT
GAGTAATGATGTATATCTATTTGACATCACAACCATCACACCACA
AACTTTAAAATCTCA
AGAAAACAAGTCATCAAGATCTTTCACTGGGATCAACCGAAAAG
TGAACGCAATGCCCAC
TGCAAATGATGTACTTCA
>ConsensusfromContig1801 Average coverage: 11.28
TCGGAATCGCCGACTGAGAGGAATTGTCCAATGTCGAAAATCCA
CAGCTTCATCTGAGAA

ATGATGCTGAAGATAAAGCGGATCAACCGTGAGAGCTCGAGTCAA
TTGTTTCTTGTCATC
AACATAAAACATTGAACAGTCGAAAGTGATTAACATCCACTTATT
CGGGTTGAAATTGAA
AGAATTTGCCAGCTCAATTCCTTTCATGCGATGCCTAAATTCAGG
GCAACAGAGAGCATT
ACCAGCATAGGCAGCGTCTACATGTAGATAAAGCGAGTACTCTTT
AGACACTTGACCTAG
TTCTTGGATATTGTCGAAGGAGCATACAGCCGTCGTGCCAAATG
TAGCACAGACGAAAAA
TGGTGTAAGTCCTTTAGCAAGATCCTCTTTGATTGCTCTACGAAG
GGTATCGCCCCTCAG
AGAATATTTCTCATCGACATCCAATTGCCGCACTTGGACCAAAGC
TATTATCCCTGCCTT
TTCCACGCATGAATGCGCTAATTTCGAGCAGTACGCGATGAGTTT
TTGCGGCTGAAAATT
GATACTCCGACGGTGCTCAAAATCGGTGATGTCATCCAAGAAAAT
ATTCGGGTTTTCATT
AAAATTTTCGGATAAAGATCTCATATCCACGCTATCAGTATGCCC
ATTAGTATTCTGCAG
CGTATCTTCAACTACTTGAGTTTTCGATGCCATCATTGCACGACT
GCGTGCCACAACGAC
CGCGAC
>ConsensusfromContig1802 Average coverage: 35.44
CCATCATCTCTGACATTGGGGGTACTAGAGACCTCTCAATGCGAG
GATCTAAGGCGGCCC
TTCGGATTATCCGGTCATCTATCAGGATTTTGTGTTTGCTGCGAG
GTTCGCGTGGGCCAT
CGACCGTGACCTTGATGGCCCTCTCGTAGATTGCAACTTGGATCG
GACTAGTGTAGATTG
TTATGGCGATGTTGAAACTTTTCCCTCTTCCACTCCTGCCAACAA
ATCGAAGATCATTGA
ATTTTGCGACTTGATTTTTCATATAAGCGGTCCCATTTCTAAGAT
CAGCACAGAAATTTT
CATCATTCCCAGCACTTAGCGTAACTCGTGTTCCATCTTTCACTT
CACCTAGTGCGACAA
CTTTAAAAGTAACTGGAAGTGTTTTATTTGATCTCCAATGTGATG
GCAAGACTGAGCAGA
CAAAATTGGGACAGCCAGTCCTCACAAGCTCACCAGGGTGTTCTT
TCAGTACAGCACTGA
GTGTTCTTTCTCCAGTCAAAATATCTGACATAATATTTGTTTTGGC
TTGCTTCAAAACAC

CCTTTGATTTGATAAAACCCGAGCTGTCTCCTCGGACATCTGTAG
ATATATGCATTAGAT
CATTAGATATGTCAAACGTGCGGTGTCTAAAGTGAGGATCACCAC
AATTTGAAATGTCCT
GTACAACCATCTGCGCTGAGTGTCAGTACGCAAATACCACACGT
ACCGCCGGCACGATGA
ATATATTCGAAAAACGAAGTAATTTATGACCACGCCTTTTCACGT
GCCCAAGGATGAGTC
ACTTGGAGAAACTGGAAGAGTAATGTGTAGATAATGAGTATTGTA
CGATCGTTATAATGC
TTTATGATCTCATTAACAATTGGATTATATTCTATTAATTGTTCAT
ATTCAGAA
>ConsensusfromContig1803 Average coverage: 27.94
CCCTTCACCTCCATCACGATCAACTCTATCTTATAGTTCAATATGA
CATCAATCTTGGAT
AAGTGAGGATCAGCTTCTTCTTTTGGCTCCGCCCTACTGGGCCCC
GGACCCATGTGGAAA
GGGCCATGATGAGCTCGATTGTATTTCCGTAATTTCTGAAGCGAA
TATTTGGAGTCTGTA
GAACCCTTAGCAACCGGTAAACTCTCCAAGTTGGCCATCAAGAG
ATTAATGGAAGATCGG
AGTTCTTCTAGGTACATGCTTTCCATTTCTTTCAAGACGAATTTTG
GGAATTTTCGCATC
TTATACATAGCCTCAGATGTTTGTAATCGTCTGTCAAGCTCTCTT
CTGACTTGGGAAGCC
TGTTCATCAGCATTATCCAACTGCATTGCATTATATAGAATTTGAT
GCTCATATTTCTTG
ATAGCTAGAATATTTTGAAACATGTCATATAACTGCTCTTTGCTC
AAAACTAATTCAGAT
GTTGATGTGGATATTCTCTGCGAAAATTTACGCGGATCTTCATCC
CCCCTATAGATTTGA
TCAAATTTAGTCATCCATGAAGCCAAAACAGTCTCTTTGCTCAAA
CCATTTATTTCTGGT
AAGTTTTTCATTCGTTTTTCAATATTATTTTTGAATACATCTCTAA
AGTCATTTGCAGAA
CATCCACCTCCCCTCACCATTCTTGCAA
>ConsensusfromContig1804 Average coverage: 21.75
AGAGCTCGAATTCCATATTTTTTCGAGAGCGAAATGACAAAAATG
TTCTTCAACTCTTTC
TCGCATGCTCTGTATTCCTTGTCGTTTCTGAATCTCACAAGATCT
GGACTTGGAGAGTGG
GGAAGATGAACCATGAAAAGATCGGGCAATACCAAAAATCGGAA

TCCCATGAGGTGCATC
TCCATAAGGTGCGAAACCTTATTCCAACCGAACCCTAGAAACCGC
TCGTCGTAATCAGGG
ACGGATCGCCGATGAGCCACGACGTACGGTTCAAAGTCGGCACT
CCACTCGACATAATAC
GGCTCTGAAGCTGTAATCCATTTTGAGTAATTGGTAGCGGAATGT
CCTTTGTCCCAAACG
TCAATTCTGAAGGGTTGTACGGCTTTTCCAGCTAACTGTTCCCTTA
GAGCCTCTTTGTTC
TCCGGCATTTCGAAGCGGTATAGTGTCGTCTCAAAGACAGGAATC
ACCCATAAGTCCTTG
AGTGAGGATGTCCCGCCTTCATTCTCAGCCAATCGAATCCTGATA
TCAGAATAAGATGTG
GGGCTAGGTACAAAATCGATATCTGAGAGGAACACGTGACTCGT
CCATGCGTATCTCAGC
GCGATATTTCGGAGGGCGTTAATTGGATAGAGCATGCCTTGTTTA
AAGACCACGTGATAT
CCAATATTCGTCCGAAACCGCAAAACATTCGAGTTTTCTACAAAT
GCGAGCAAATTTTCA
GCATCTGCCTCCGTCGTATAATAGGCGATGCTCATTGGTCCAAGC
CAAGATCGCGC
>ConsensusfromContig1805 Average coverage: 16.53
GTGGCAAGCTACTTTTATGATTGGCTGCTTAGCCGTTTCGTTCATT
ATTTTTTTGAAAAC
CTTATCAATAGAGTCGAACTTTTTGGCCTCTTCTGGAAGTTGGGA
CCTGATGTCTCCACC
TATGAATATACCCTCGAGATACATCCACTTTCGCTGCACAACCAT
CCAAACATCGAGGAC
TTCAGCGATGAGAGAGAGGCTTTTTTCCCAGGTCTGAACGGCGT
TGAAGAACGGACCGAC
GAATCGAGAGGCGGACATTCCCTGAAGCTGCATCGCATTGTCAT
CCAATGACTGAAGGAT
CTCTTCGCTTGTGGCCAGGATGTAACCCCGGTCAGAAGTCCCTTT
CATGTATTTGTTGAC
CGTGAACTTCATCGAGCCCCACGTCTCCTCAATCTCGCGAACACC
TTTTTCTATGCCCAA
CTCTTTGATTGCACTATTGACAATTTCCCCAATCGTGTCCGTGAA
GCGGTGCAGCTCCAT
CGCGAACACATTGGCCAGCGTAAAAGTATCCGGGTTCATCTCGAA
CTTCTGGCCCGTTTT
CGTCATCAAATCTTTCCAATGTCTGTCCCTAAGTGCCTCGTGCTT
GAGATCCACAAAGAG

AGGCAGGGCATCCCTGAAACCCTTCATTTTTTCTTCGAGCGTTTTT
CCGATCATCAAATT
CCGGACTTCTCGTGGAAGTTTTCTCAGCAGTTTGAGATATTGTTCG
ATTCCTTCTTGCAG
CATTTGAACATTCAGATTCGCCCAAAGCGTCTCCGCCCAACCCTC
GCGCGCTTTACTCTG
TTCCTCATAGAGATCGAAGAGGCTTTGCAGTCCTTTCATGATATT
TTGGACCTCCAAAAG
TTCCGGATACATTGTGATTGGAAGGTCGAATAGCTTCTCAGCATT
TGCCAACTCCAGTCT
CCTCAATTGCATATTTTCGATTTCTACTTTGTACTTCTTCATTAGC
ACCAAACCCCTATC
CAGATCAGGCCCCACACTCCCTGGTCCATTTTGCTGAAAGTCTTC
TAGGAACTTTCTCAT
ATCTTCTCCAAAATCACCAATTTGAACTTGGGTTATCTCCGTGAAT
TTTCTCTTGACGAC
AATGAGACTGGCATCGATGTTCTTCGACTTGAGAGTGAGTTCTTC
CCAGAGGCTGTTGAG
GCGCCTCGCTCTCTCCACCTCAGAAGGATCCGGGACTTTATTATA
GACATCAAGCGTTCT
ATATCTCTCCTGAATGTCTCTCATCCTATCTTCGACATCGAGAGAC
ATGTTTCGTATGTC
CGTTATCGTCGTCAAAACGAACTTCAGGTC
>ConsensusfromContig1806 Average coverage: 9.79
AAAGCGTTTGCTGTATTCGATAAATCAATCGCGGTGTTTGATGTG
AATACCTGCGAGCTG
CACAAAATTCGAAGAGATCTCCACGGAACTTTGATCACTGCTTGC
CTCTTTTTCAATCCT
CTCAAGTATCTGATCACTGCTGCAAAAGATGGATCAATAAAAGT
ATGGAGCGAGACTTGG
ATGTTACAGCTGGTGTTCGTCGGCCATTCTGGTTCGGTGCTAGCC
CTGGCCCTCTACCCC
CATGGCCCAACAATCCTCTCATCGGGGGAAGACCGAACTCTGAA
AGTCTGGAGTCTGGAA
ACTTGCGACGAAGTGGACTGTGTCAGGACGAAAGAGGTTGCAAG
AGACATTCATACTACG
ATGAGCAGAGATATTTTGTGTGTCATCAGCGAGACGGGTGCTGAT
TGGTGGATTGTCAGA
CATCTTCATACATTGCATACACACATTGGATATCGAATTCAGAAG
TTATTCACAACGGAT
CATCCGCTCATCTCACCGAGGTCCGTTCTCGCGTGTAGAGACTCC
AGCGTCAGAATTGTG

AGTCCGGCGACGGGGCACATCATAACAACCCACCTGGCGCCCCCA
CACCCCCTCATCGTC
GCCGCTGCGTACGCCATTGAAGAGGATCTGCTTTTCGCCGCA
>ConsensusfromContig1807 Average coverage: 14.49
CTCATTTTGCCAACATTGTGCGCAAAGGTCGTTTCATAACTTATG
GAACAATATTTTCAT
TCATTGGCTGCATATTGAGTGGAATAAGAGGCAGCACTTGGGTG
ATTGAGGAAGTCCAAA
TAAATGCTGAATATATGCATTCTTCCATGTGCTATGATCCGGCCA
ATGATGGTTGTTCTG
CTCTAAAGGAAATCTCCGCAGCACAGTCGAATTTGGTCCTTCATG
GATTCGGAATGTTTC
TGCAAGGAATCGGATCCGGGTTTTTCATGCCACTTGTGATCGGGT
ACATAATCTGCAATG
TACGATCAGACAAAATGGTACTGAGGGTTGGTATGGGTTTTGGA
GCAACATGGCTGGGAC
GAATAATTGGGACTTTACTAGCAGAGATTTTTGTTGCTATTTCAA
ATGGAACCAAAACCG
AAGCGACACTAACTGGGTTATTCTTCCTCTCAGTGTTTTATACTAT
CTCTGGATTTTTCA
CATTCTTCATACCTCAATGGATCCAAGTTCCGGAATGGGTTAAAG
ATAGGATCGCTTTCG
TAAAACAGAAAGAAGAAAAATGGGACCAAAAATTGAGAAGGAAG
ATTGAAAAGTTTAATC
CATATGATGGAAAAAAATTGGAAGTGAAAAAAATTC
>ConsensusfromContig1808 Average coverage: 12.14
AAACCATAGATGGAAATCCTGAACAACCTTAATAATGGATTGAAG
TATTTTTCTCACACG
AACGATGAAATATAGCAGAAAAAATTCACAAACAATCACAGTCC
GTCCACCCTTGCACAG
GAAATTAAACAATGCAATCTTCAGAGACTACTGTCTGTTAACACA
CTTTCTTCACTTAGG
AATATACCCTTCAACGCAGTCTCTCTCTGGCAGTGACAATCGCTA
ATAGATTTGACAATA
CCTATCAATTGAAAATGCGGGGATTGAAATTTTTCTTTCTAAAAT
AAACTGACAAAAATA
TTTTTAAAATTTACAACTGGAGTCAGTATCTATTTCATCACAATT
CATCTACAATAAACA
ATTAACTGAAATGATCTGGAGATAATTCACCGGGATAGATGTACA
ACCTCGATAATT
>ConsensusfromContig1809 Average coverage: 8.58
GTGCTCTTCCGATCTCTCGGCTCGAGTTTTTGCTGTACGTAGAATT

GGTCTGTGAACATC
CATCGGTAAGATTGTTTGAAATCGATTTTTGTGTTTGTTTTCCTCC
CTTATTGCAGTTGT
GCATTTATCGATAGGGATTTCAGGTATGAATTTTTCTAATATTTCG
AATTGCTCTCTGAA
GAACGATTTATCTGTAATTGGATTTGTTCTTGTGAGAAGGCGATA
AGTGATTTTCAAGTC
TTTATCAATGATGTTATAATTGGTTATAAGTGCCTCAAACAGAGT
GTTGTATATAAATAT
ATATTGTTTGAGAGTTCGTACCATAAGAATACGGCTCTTTCTGAG
TTTCTGAACGAATTT
GAACACATTCACTGCATTTTCTTCCTTGGCTTGCTCGAGCATAGA
GTCGACAGCGATAAA
AACTGCTGTTCTGCTGACACCCGTTCCGCAATGAACCAAGATCGG
ACTCTGAGAATCCTT
TTCGGCGTTTTTTATCTTCATCACGAAATGCAAAAAGGCAATCGA
ATCTTCTGGAACACC
GTGATCGGGCCATGCCGTGAAATGATATTGTAGAACTTCCTGTGT
TTTATTGCTCATGGA
ATTATAAACAAGGAAGGTACGAATACAAAATCCAGCATAATACC
GTGAGCTTCCTAACGT
CAAA
>ConsensusfromContig1810 Average coverage: 30.63
ATTTCTCCTCCGTATCTGATATGATTTCGGCTCCTAGGAACAATAA
AGATTCTGTATTTG
TTGTCAATTGGCTCAATAATAAGGAGTTTCAATTGACACACAAAA
TGGAACAAATTCTTG
CTGTTGCTAAAGAAAAGGAGGAGAAACGTGCTGCTATGGAAGAG
TATGATGACGGTGAGA
TGGATGATGATTGCGATGATGAGAGAAAGGATGTGACGTCCTCGG
CTGGTTCTCCTGAAT
CAGAAAAGTCTACCAATATGGCGGAAGGACGAAAGATCGCCCCC
AAATCTCTACCGAGAC
GTTTTTCAGCTGTCAATCAAGCGGCTCTCTCGAAGACTTCTACAAT
GTTTGTTCCTGCCA
ATGGAAGCATCAGCGCGACACAGTCTCTCTCATCCCTTGCATCTG
CTCTTCAATCTCCCA
CGCCTTCCGAAGCTGTCCAGTCGGCAGACAGAAAGGCTAATCCA
CACGCAGAAATGGACG
CTTGGCTCTCCCATCTCATTCGACAGTGGAACTCCAGTAGCGACCT
CTTGTATTGCTTTC
ATCCATCAGATGGCAGCCTCCTCATCTGGCTTGTCGATTGGCTAG

ATGAATTCCGCTGCG
GCATCTACAGACAGCCTCAGTTGAGTTTCTCATGTCGGATTCCCT
CCGCAATTCCGATAG
GTGACGCCACAACGATTTCTTCAGAAATAAAGCTTTTTTGTTGTC
CTTCGGAAATCCTCA
ATATCTTCAATCTGAACACGGCGGCCATGTCGGAATTGAGAAGG
GGCGTGGACGTTAACA
CAGAAGTCTGGCATCGACCAATGAACCGTGGCCTAGTAGACGCTG
ACACGCCTCTTTCTC
CCGCCCACATGATCACGAAACATTCAAACGGATCGCTCAACGCTT
GGAAAATGGCCTTCG
CCGAGGGGTCAAAGTTCAAGACTGTTCTCAATGTAACGTGGGTG
AGGCGAATAAGTGGGC
ATCGCTTCAGTACGAATAACGCCGTGTGTCACCCCGTCTTGCCCC
TCGTTCTCACCACGT
CACATCACAACGGATTGCCGCCCCCTTCAACGGAGTCAACGCCG
CCGCAGGTAGGCGATG
GAGGTTTATCGGAAAATACTCCAAAAAACGGGAAGCAGAAGAAT
GAACCCGATGAGACCC
AAACGGGTTTCTGCAGCGAACTAATCTTATGGCGCGTCGACCCCG
TCGGGCCTTTGGGCA
TCTCCGGAGGCATTGCGGAGTTGTCTCGGATCAACTCTCCATCTC
CTGCCTCCTTCTCAG
AGGTCGCTTGGTTTCCCACCCTTCTCCCGAGCTGGACCCTTGGCT
CTGTTAGTAAATCGC
CGAGCTCTCTCTTCGTTGCGTCCGACGGCTTCAGCTTATGTGTCT
ATCAGGCGGTTGTGG
ACGCCAGAACCATTCTCTCTTCGAAGTTCGAGAGGGAAGATGGC
GAATCCCAACATCCGA
ATATAGTCAGCATCCAATCCACGGCGCGACCTGGCTGCATAATCC
AAATGGATCATTTAA
AGGACGCAAAGCAGAATTGGTGTGGGATACAATTGATGCATGTTT
TCCAAGAGTCTTTGG
TGGTCGGACATTCAACCGATGATGAAGACTCGAGAGACATCAAC
CAGTCAGCCTCTGGCT
CCGACCCGGAGAAGACAGACTTCAGAGAGCGGTTCTATCTTGTTG
TCATGAACCGGTCTG
AAGATGAAGATGGCTCCACCGTCCACATGTGGAGAATCGATATTC
ACTTCGTTTCGTTCG
ATAATTTTGTCAATTTTGAATCGAATGGGAATTCCGAATGGGGAA
AAGATACTTTATCTG
GAGGACATTTTTTGCGCATCGACAGTGAAAAAGTGTGCGGCGAAC

GCCTTCCGGTTGACC
CCGGGCTCAAGGTCAACCATGTGTGTGTGGCAGCGGGTCATTTGA
GTTCAGCGGGAATCT
ATCCGGCATGCGTTGCTCCATATCTCTTCAGCACGGCTTGTTCGG
ACGGGAGAATCAGAT
TTTGGTGTTGCGAAGAAAATTCAAGCAGTTTTGTGCGTCGTCCAA
ATATTTCGTGGAAAG
AATGGCCAATGATTTTAAGTGATGAAGACAGTCATATAAAAATCT
CAGGTCATCCTGTGG
TGGTGAGTTGTGCTTACAGCGGCCGAGTTGCTATTGGTTATGTGT
CTGGAAGGTTAAGGT
CGAAGGTCAATGACCATAATCACCTCTACGTCAACTTTCATATCG
CCATCTATGAGTGCG
AATCTACAGGTGGATCTCAATGGATTCTCGAGGACATGATAGTGT
TGCGAGATTCGAGAA
TATCTTCCCAAAGCCTTCGAAGAGGAATTCACGAGCCGAGGACA
GATTCAACGCCAAAAC
CATCCAACGCCGAAAAGAGCGCCGCTCCTGTTATTTTGATGAGT
GACGAAAGAGGGCAGT
GCAGCG
>ConsensusfromContig1811 Average coverage: 15.91
TGTGGAGATATGTGGTCCAATGAGAATTGCATGGTCGTCTGCCGC
CAGCTGAAATTAGGT
GGCGGCAGAGTTATTGATGCAGAACAATACAAAGCCTCAAGAAT
TCTGCACCTGGCTATC
AACAACGTCACGTGTCTCGGATCTGAGCATTCATTGGATGCGTGT
GATTTCAGTCACTAT
GGTAACGGCCCCTGTACGAGATATGGCGTGGCGTGTAGCTGCGA
CATGAGACGTCTTGAA
GATGGTTTTCAGACAGTCTCGCATTGCGACAAAGCTGCGCACAGG
ATTCGTCCAGTCGAC
CCTAGCCAGCCTCATTATGGGCGTGTCGAAATTAAAGATCCCGCC
TCCAAAGAATGGTCG
ACTGTTTGTTCCACTGATTGGACAGAGAGCGAGGCGAAAGTATTT
TGTTCTTGCATGGGT
TACACGAGAAAACAGTTTTTCTCAAGTTACACCAGTTTTGCATTG
GGACACCTGAATTTG
ACGCTCCCCGTTGGTTATGACGCAATCACGTGCTCTGAAAGAGAC
TTTCTATTGGGTACC
AATATATGGGCTTGTCATCAGTCAACACCTCATGAAGCTGGGGG
ATGCGGCGGGAAAAGG
ACAGAACATATGAATGCAGCCGGCGTCATTTGCGACAGAGTCTTG

CCTTCTGA
>ConsensusfromContig1812 Average coverage: 12.82
AACAGATCTTGTTTCTCCACGAACTCGGAGCCCATTTTGTAGAGA
TGGAAGTACAGAATT
GTGGAAAATATGCAGGAGAGCGCAGCCAGTCCCCCTGCAACCAG
CATGGCCGTCCCTGGA
AATACGTTAACTGTAGAGGCGTAGAGAGGGTTCAGCGTGAGACC
TCCCACGGTGAGGGCC
AGTGTCTGCAAAAGGCTGACTCCGGCAAACAGAATACCCTGTCTT
TCTTGAACAACCATT
TTGGAGAGTAAGTAACGAGAAAGGGGCGCGACGACTCCCGTCAA
ACTGAGTGGGATGAGA
GCTAGAAACACAGCTTGACTAGAATGGCCAAGCCCAACTATGAC
ATAGAAAACAATAAGG
AAAAATCCGCCAATCACAATAGTCGCCGCGATGCTAAACCTCCA
TAGCAGGAGCGGCGCG
GCAAGGACGGCAGCGGAGGTTTTCATGAAGGTGGAGAGGCCGTT
GAAAATAGAAAATTGG
GTGGAAGACAGGTTGTATGGAGCGCCAAGGAGGACGTATTGCAT
TGTGTTGGATGCCCCG
ACCGATGGCAAAAAGTGTAGAAAGAGTGCTGTGAGACCGATTGA
TAAGGCAGTTCTATAC
ACACCAAAATCCCATTCAATGCTGCTCGATCCGTTAGAAGTATCA
GCATCAGCTATAAGT
TGCATTCTTGCCAATTTGTGAGTATAATATTTCCTATTACAACGA
ATCATGAATATAGAA
TAAAAAAAGGCCAAAGTAGATAGAATAATAGCAAATACGAAAGA
CCACACGTATCCGGCT
TTGTTTTTAATAATAAAAACAATGAGAACACTTGCGATAATAGAG
CCAGCTCCAATTGAA
ACATCCAACAAAACGAATTTCTGGATTCTTTTCGGTCCAGAAAAA
TTATCTGCAGCGAGA
GCGAATATTGAACCTATACATGTAGTAATGCCACCTGAGCAGTCA
TATAGAAAATTACAT
GCGAATATCCAATAAACATTGAGATTCAATGAAATGAGAAATAT
CCAGGCAAGATTCGAT
AGCGTCAGAGATACGAGCGACATGACCAGAACGTTTTTCCGGCCG
TGTTTATCCGAGTAA
AGACCGAGCAAAGGGGCAGAGATGGTCTGCACGAGACCAGAAGC
TGCTAAAAAATACATA
TTCAATATGGAAGACTTTCCACTGACTGAGTCATCTGATGAGATG
CTTTGAATGCCATGC

TCTGCCTCTTCCATTCGATGCTGGATGTA
>ConsensusfromContig1813 Average coverage: 11.98
CAAAAACAAAAACTGCCCCGATAATTGCTCGTGTTTCAGCACCCA
ATCTCCGCCTATGGA
AGTTTTGAATTGTTTTGGAAACCGTAGTAGCACACTACCCACTGG
AATTCCCGCAAATAC
CACCTTTGCTTCATTATCATTTTTTAAATTGCCATTTTTGAGTGG
AGAAGAGTTAAACGA
TTTCAAAAGTGTGACAACTTTAGAGTTTCTTGAAGTTGAGCTCAT
AATGTTGAATCAAAG
CACATTCGAAAAATTTGCTCAAATAAAGCAACTGATTTTTGAAGG
AGGTTCTATCGACAA
TATACAAGATGGAGCCTTCAACAATATGAAACTTGAACTATTAA
GAATCGAGAGAGTGAA
AGATTTGTATTTGAAGCCAAACACATTTTCAGGTTTGAGCGTGAA
AGAACTTTCTTTAAG
TGAAAATAATATGTATTATATCGACTATAATGTATTTGAACCACA
ACTTGTATGGAGTCT
TTCGAAATTGGATCTCTCTCGAAATCATCTTACGGAAATTGGTGA
AGAATTTTCGGTGCT
ATTTCCAGTTTTGGACTCCCTGGAATTGACTGGAAACGAATTGCT
ATGCAATTGCGACTT
GTTTTGGTTGAAGAAGATAATTGTTGCTTACAATAAGCAGTGGGA
CTTTAGCTGTAGTCT
CGTAGATGCAAATCTTGATGTGAAAAAGCTCATCTCTGATATTCC
ACAGAAAGATTTCGG
ATGCAACAAAAATGATGTGATGTCTAATCGAATATACAATGCATA
TGTCTGCCCTGAGAA
CTGCGGATGTGGGAGTGAGAGATTTGTTGATGTTGTTATTGAGGG
CGCTAACTGCTTCAG
TGAAAGTCTGAAAACCCTACCGAAAACATTCCCAAGAAGACTCG
AAAGTATTCACATTCA
AGCTGAGAATTTGGATAGTCCCATTTTGGAGAGGCGCAATATGAC
CGTTTTTTCACATTC
AACAATTTTTCGCCTAAATTTTTCTAATATTAAAAAAATTGCCAA
AAGTGCTTTCTCCGA
TTTTCGTAATATGCAATATGTCCGAATATGCGATCAACTACATGA
AATAGAGAGAAACGC
ATTTCAGTTTGCAAGACTCGACACTTTGGATCTTGGTTCGAACGA
AAATTTACTGCCGAC
TGATGATATGTTTGAAGGAATGGAATATCTGAAGAACCTCCGAAT
ACAGTCTTGTGGATG

GAAGTATATTCCTCGTCAAATGTTCAAACCAATCGAACGAACGCT
CAAAATTCTCGCTTT
GGGATACAACAAAATAA
>ConsensusfromContig1814 Average coverage: 14.62
AAAGCAGTCGGAGGTTTTTATCATCCATATTTCAAAACACATCAG
AATTGTGAACACAGA
AACGTATATGACATCCTCGATGAAAGTTTGACTATTTTTAACATG
AGAGATAGCAACAAA
ACCTATATACAGAATCGAAATTGAAACCATGCCCCAAATGAAAAC
ATGCCTGCTGTTCGA
ACGGCTAACATCTCGTTTCCAATTCACATACACTCTAACAGCTAT
GATGAACAAAACCAG
AAAAGAGATTATGAAGAGAATAACATGAAGTGAAGCTATGAGTA
ATATTGATGGAACAGC
GATGGAGATTGTGATAACTTGAGAGGAAACTTTCTGAGCGCCAC
CAGGCATTGGAAATCT
GATCCTGTCAGCAAAAACATTAAAGACATCACCGAGGAGTTTTGT
TGGGACATTTTGACA
TCGGAATAATCTATCACAGGCTGAATCGAGTTCTTCAC
>ConsensusfromContig1815 Average coverage: 8.88
ACTTTTTCATCCGAGATGAATCGGATGAACATGGAAGAAGCGCCC
TTTTCAGTGATGATC
GGCCCGGGGGTTATTCGTCCACAAATTCTCTCCATCAGAACATAA
TTTTGATTATCTGCA
TCTTCAAACGAGCGAGATGCGGTCTTTTCTGTTTCGAACACTGAA
TATATCTCCAGGTAA
TCGGTCTTACAATCCCCATTTCTGTCATCAATAATCATATCTTCG
AAGGTCAATCGAATG
CTCTCTCCAGCATTACCCAGCAGCTTGTAGAGACACGTCAGCTTC
ACTGGATATGTACTT
GGGTTCCTTGGCGAATTGAAGTACCCTGACTTCTGCACAGAGCTC
CTGTAGCGGAACTGA
CAGCCGTCTTCTGTCGCTCGTTCTCCAGCTATGGCGTAATCTGTG
CGAAATTGAAACTGG
GCAGAGAACCCTGTGCCGAGCTGGTCATCAGGAGAAGTATGCGT
ATCAAATTCAAGCAAA
AGTCTAGGGTTGGATGAAACGAGAGAAGCTGGAACTTTATTTCC
ACACATGACAGCATCG
GCATCTCCCCGCCCTGGTTTTTGACCTTTCAGATACACACGAATG
GATCCACTCGGGCAC
CCAGAATTGTATTTTGGAATGTCCATATGAGTGAATGTTAGTTCC
ACGTTTTCCAAATTC

ATTTGGTCTACGAGGCCATCCATGTAGTAGCGGCATGTGACGTTC
CTGGGATAGAGATTG
GGGAAGCCTGGGGATATGATGGTTCCATTGGATTCCCCATCACTC
ATAAATCTGATGTCA
CATTCGGTTCCGCCAATATGGTGTCCGTCAGGAAAATAGTCTAGT
TTGACGAATTTATCG
CTGATCTCGAAGTTGGCCATGAATCCGCGCATCATGATCTTCTTG
GCGTCGTTCAGCTGG
TCATAAGTGAGGTAGCTGGGCGTGGTTAGTCGGGGTCGACCCTCC
CCCGTTTCCAGTTCT
AAATAAAGGGAGTTGTAGGTGCTGAACATGACGACGTTATGCTG
TCGACCGCAGTACACT
CCGATTATCTCTGAATCCGAATCAGGTCCGTCATATACGCGTAC
>ConsensusfromContig1816 Average coverage: 13.70
AGCTGACGGCACCGGGCTTTCCGTGGTATTTAAATAGGGTTTAGT
CTAGTGGTTATGTGA
TTTTTAATGAAACCTTTGAAGACGTGTCGATATTTATTTTATCTG
AACCATCTATGCAAA
TCCAACAACTGCAATTTCAGTACAAAACTTACGAATAAATTGTTT
TCCAAAGTTTTTGTA
AATGCCTATGCCATAAACAATGATGTACATGAGCGAATATTTGGA
ATATTTGGTACTCTT
TAGGATATTGTTGTATTTTCTTCTCATCCCTACATATTCTGGGACT
CATTGAGCGATTGT
TCTGTTAAAAGTATTGTTTCGACCATTTCGAGTATTGTTCCATCA
CTAAGATTAGCAAAC
GATCGTCTAATAAGTTCTGTTCATTGCCTCATTATTATTCAATCCA
GTGTCTCTGTTCCA
TCTGATGAATATTCATTTATTTTAAGTGAAATCCAGATCATTGAC
ATGCTGATAAACAGT
GCTCGCAGGGGTTGTGTAGATGTTGCTATTCATTGACGGCGATTT
GTGACTTCAATAACG
TTTTCTGTTTATGATCGTAGTCCTCAAATGCTGAACGAGATTTTG
TCAATAGCTAAATTG
TCATGGTTGAACTTGAATGATATCTCATTTCTCTAATTAAGCAGCT
TATATAAAATCTCT
TGTATCACTCATTTGCCTTTCACGTCTCTTGTTTACATCCTCATCC
TGATTGATTAAGCT
TTTGGATATCATTGCACGTATCATTTCTCCATAACGTTGTGTCTGT
TGTAACCATCCTCT
GCTCAAATTCTTCATCATCTCATTCCGGTCCTAATTCGAATCTTAT
TCTCAACCCGATCC

ATTAGTAGCTTTCGTTCTCTATTCGTATTCTCCACATCATGCTCTTT
TCATCTCTTTTAG
CCCCAATTTGATTTGTGTTTTTGTCATCTCATTCACCAATCTTGCC
TCAAACTAAATCTA
CAGCCTTCATTGTTGGTAAGTGTTGCTTTTATGCGGAATACCTCCT
TTTGTTGTTAGGGC
TTCCGATCTAGATCGGGTAGGATGTCGTATTATGGTTGTGTCATT
TGTTTCAAAACTGCT
TGGTTGTTTTAAATGATGGAAGACGACTAGTAAAACGTGGTGCAC
GACCAATATGGACCT
AATATAGCAGCCAATATCATTTGCACACAAATTTATCGTTCTAGA
TTGAACTGTATATGA
CCAAGAAGATTAAACCGAGCGTATATCGATAGCAGTCAAGTTCCG
TTCCCCCATTACGAA
TCGCACCTCAGAAATATTAAAAAAACGGAATGTCGCCATATATAA
GCATGTGTATAGCTC
GAATAGGGTTAAAATGGATTGAACATGCCGTTTATAAGAATCAA
TAGAGATTATGAAGCA
GTATCAAATATTGGAATTCAAGGTTATTTTTAAAAGAATCTCAAT
GCAAC
>ConsensusfromContig1817 Average coverage: 10.59
GCAAGGTTTATATCTAACAGAACCACTTTTAAACTTAGTTCACAT
GTCTCAAAAGCCACA
CTTACGAGAGTAAATATAATAAATCATGGAGTGACCACGCTGAT
ACCAATCAACAATATA
TGGATAGATTAATAAAACACGAACAAAAAAGAATATAATCCAAA
TTCTCGCGTTGGGAAT
AATTCAGATGATATCAGGCATATTTCAATTCATGGGTGTCGAGAG
GCAAAGTGACATCAG
CACATGTGGATGTCAATACACTTGTGTCTTCCACTTCGCTGTCTTG
GTCAGCAGTCCATT
GAGGGTCCACCAGGTCACTCTTGTGTATCCCGATCTCCATTGCTT
CCACCATATTCATAA
CTCTCACATTTGGGTAGCGTTCTTTGAGGCCATCCTCCTGTTCTTG
TGTTATTTCTGGTA
CCTTTATGACATTCAGAGGACTGTTTTTCGGCGGATCTTGAACAA
TTGCGGGTTCTTCAA
TCTCCTCTTCCTCCTCGATGCTTTCATCGACAGATAAATACGTTCT
CTGAGTAACCTCAA
GGCCATCATTATTCTTCTCTGGTTCAATTGTTCTCGCATCGTTCTC
AATGTTATTCACTT
CTTCTGACTGTTCCTCACATATTATCAGCGTTTCACTTTTGTAATT

AGTATTCAAATTGT
GATTTCCCATTAGAGAGTTCGTTCTCCGGTGTGAAGGTTTTTT
>ConsensusfromContig1818 Average coverage: 11.78
ATGTTTTGGATTCATCATGAAACGCATTGCACACTGATTGTTCAT
GATTATCCGATGCCA
TTCCATTCTTGACACTATGTTCAATTAAATAATCGACACTTATGTT
TCCCGGGTCCGATG
TAGCATCGATATTGGATATCTCAACCGTTTTGATTTTGGTTGTTTT
GTGACCAAGTGCCG
CCTCTGAATCAATCAATTTATGTTTTTCTCGCACAGCATATCCGAC
ACCACCCATATGTT
TTAGTGCGTAACTGTTCTGCGTTTTTTTCTGTTCCTTGGTCGGCAT
GCTACGGTATGCAG
ATATTGGTCGTGAATGGGTACGAATATCCTTCACCAATTGACTAT
TTGGTGTCTTTGTTA
TTCCCGTGAATGGTCTTATTATTTTCGCGTTGGGCCATTGTATCTG
ATCATCCCAACTCA
ACGGCATGCGCTGATCTTCCTCAATAGCAGTGGATCCAGTGGCGT
TTGACCTCTGATCCT
TTTCAAATGCATTGCCGTTAACACCAATTATGACGTTGTGGTTTT
CAACTAGCAGTTGTG
CGACCTTCTCCCCAGTGGTGTTGTCAACAGTGAAATATTCCGGTT
CAAATTCTGGCCCAC
CGAATTCAGATTCCACTGGGCTTCGATCTTTCTCGCTGTGAGTAG
CTCTACTGTTGGATC
GAGTCAGAATAATGGCTGGCAAATCAGAGGTGCCATCTACGAAAC
TTTTATCCGGTCGGC
ATAAACTGTTCACAGATGGCGCCAAATCTCTACTTTCAACACGAG
AATATTCGCGAAGTT
TCTCCCGGATGATACTATAATCGTTTTTCAGCTTAGAAAGAGTTT
CGTGGAATTTCAACA
ATGAAAATCCTCTCCTACCGTCATAATTAGACAAATCTCTTTCGT
GAGAGGTCTTCGGCC
TCTTTGATTTTGGTCTTTGCTTGCTGCACTCCGAGTAGCCCTTCGC
GCTTTTCAGCCTCG
GAAATATTTCTATATTATTGCACTTATTCGAGCCGAATTC
>ConsensusfromContig1819 Average coverage: 23.33
TCTGTGCACCTCAGCATCCTTGAAGAGAGTTTGAAAGAGACTTTT
GTAAACAGGCTCATA
GGCGGCAGCAGATGTTTCGGATGACGTCACTATCTGCTGCAACCA
CTTCCGCCTCGTTTC
GCCGCGCACGCGTTCTGCGTAGGTTTTCTCGACGTTGCTAACGAG

AAAGTCAATGAAAGG
GTCGATGGTATTTTCGCCCGACATTTTATCAGCTGCGAAATGAAT
GAGTTCTCGAAATTC
CCGTCGTGTGACCTTCGGAATGTCCGTCGCGTCCTCCTCGATCTT
CCCGAGGTTCAAGCG
GGCGGAATCAATGGCCGTCTGCTCAGCACCGTCCTTAAACTTGTG
CATTATGATGAATAT
ATCCTCAACATTTAGCATTCCACTCCCATCATTATCCCACATGTC
GAAAAGATTATTAAG
CAGTGCTTTTCTCTTAGCCGCCTGTGCCGCTTGTGTGGCCTTGATT
AGTCTTTCCATCCG
CTCTTCTTCAGTTTCCTTATAGCCCTCCTGAACGAATTTCACCAG
TTGCCGGAAAACCAC
TGGAGATGGGTCCTCGCCAAGAAAAGACTCGACCAGATGCACGA
ATTGTGTCATGCGCAG
AGTATTCGCGTCAAAAGCAGACGCCTCCGAAAGGCTTCTTGATG
CTCGTGAAACAGAGGC
GTCTCCCTGGTCATCGTTTTCGGCATTGTCTTCTTTTGTGCCAACA
GCAAATGATACTCT
ACTTTGATGAGTGTTGGGAGCTGTGGGTGGGGCTTGTGATGCTGA
CCTATTTGCTTCATT
GGCAGAGGTTAGCCCCAAAAGATCCAAAAGTTCGGGGTCATTCAT
AAATTTATTCATGTG
ATTGTTTTCTTTGTCACCTTCTTGTGGTATCACTTCTCCTCTTTCGT
CCACAATTTGGTT
GTCATTGATGAACTGAGGCTCGCCATCTGCATCATCATCATTCAC
ATCTCCAAAAGATCG
GAAGAGCACACGTCTGAACTCC
>ConsensusfromContig1820 Average coverage: 13.39
CTGATATTGATAATTTGATAGTTAGTAAATTAAGTTCAATTAGAT
TTTCTCAAAAACCTT
CAAAGAATATTGTTGTGAGTGGTAAAAAACACAAAGGATCTTTT
TATGAAACAGATGATA
TTGCTGTGACTACAATAATGGCCACAAGATTATTTAACACATATG
TTTATATGTTGCAGA
ATCACAGTCTTGCAGAATTGAAAAACAACCAATGTCTCTTGTGGA
TTGAACATGACTTCC
AAAATATACAATATGAAGAAAGTAAAGGAGAGTGGAAAAAAGC
GAGAAATGGATCAAATT
AAACGAACCAATATACAAGAATTATATGTCTTTATACTGAAATTT
CGAAGAACGATCATA
TCAATATTTGGTGCCACACCTACGAACGAAAAGGAATTGAAAGAA

CATGTTGATTTTTTA
GTATGGAAATGGTGTGGAGATAAAAAATTAAATAAATTGACATGT
TATTTAATTGCAATG
ATACATAAAGAGGATGATATGGCAGATATAAACTTGAAGTGTTAT
CTTCTTCTTCAGAAC
CCTACCTATATATTCAACATGTCGTCACTGTCAGCTATATTCAAG
TTAGGAAGCGTTCCC
ATTCTTCCATTTCAACAGAATTCAACGGAATATTTATATAAAATT
ATCAGTACTATCAGA
GCTTGTTTATGGAATTTACATTCATCGAAAATAAAACCATTTTTC
TATTGGGTGAATAAC
AATTTGAAATGCAAATGTAATAGAACGTTTAATAGATTTATTTTT
GTTGGTAAATTGACG
AATGTGAAAAGAACATTGTCTACGGTTAAAAATAGCTTGATAAC
AGAGAGATATAAAAAT
GCTCAAAAAATCGATGATATTCTCAATTCAGAATCGTTATGCGTT
AACTACGTACGACGA
GTTGCTATTGCGGAGGATATAATAAATAACAAATTACCGTTTATG
ACAATGGATTCGATA
TGTCAAATAGCAGATATTTTGACGAAACATTTTGGTGATGTAACG
AAAGCACACGTGAGC
AACGACGTATTTAAAGATCCAGATTTGATATATATATTATCTCAA
GTAAAAACTGCATAT
GACGCAAGATATAAAACCAAATCAATTGAACTTCTTCCAGCTGAT
TGTTTATGTTATACA
AAATGTCGAGGAAGTATGACATATG
>ConsensusfromContig1821 Average coverage: 9.65
ATTTGGTCTCGACCTCCTCATGAGGCCTGATTCTGAGAAACCTTA
TCAGTCCCAAGCTGA
GAAATCTGTCAAATGGCGAGTATCGAAGGAAAAGGCCCAAAATA
CGCGATGGAGAGATTT
GATACCCGTCATTAAAGCAGAGATTTCATCAGGGCGTTTTCTATT
TGGTAACGACCGACT
TCCTCATTCGCTATCAATCAAATTTCACGATGCGAATGTGATATA
CACGACAAAACCGGC
ATCGACCCCATTTGACCTATTTATGCACCACGTGAGAAGTGACGT
GGAGAACTTGCAGGT
CATGGTCGTTCCCAGCCGAGGTTTCGTTGGAGAGTTGGATGAACC
CCCGCGGACAATGGG
AGCCGGTTTCAGCATTCTGAGAAGCAACAAAGTCAATATTTATTA
TTATATGGATGAACC
AGGTATGGTTCCTTGGGAGCCTGAGCGAGTTCTCTTGGCGGACG

GAGATGTACAAGCGAG
TCGTACTTTCCCTTGCTGGGGAACAGAGATCAAATGTGGGAGGGG
CACTTACCTCTGTTA
TGGACCCTGGGTGGACCGTCAAAAAGATCAGCTTTGGAACTTTTT
CTTCCCGCCGGATTT
TCAAAAGACATCCTCGCCGCCCGTTCCAGACCCAGGTCAAAAGCG
AGTTCATAACGAATT
CGAGTTCAAGTTAAGCGTTCAGCACGAAACTTCCTTCGACATCAT
CTTCACGCAGGCGGA
AGAGACCAAGACCTTCTACATCTCCACGCAAGCCGGCTCTTACCT
CGAATTTCGACTTCC
CTACTTGTGGGATGACACGAAGGGATACGGAACGAATATCAGAG
GCGCGTTCCTCGAAGC
GGAGGCTACCACTAGCCTCGCACTCAGACATCTAATAAAATGCG
AAACGCTAGAGTTTAG
CGTTAACGTCAAATATCTCAAAACCTGGAACGACCATCAGGATTG
GACTGTAAACTTGAC
GTTCAGCAAGTGCACCATCAATCTTCTTTACGC
>ConsensusfromContig1822 Average coverage: 15.48
AAATGTATTCTCCACTATCGGTCGGTAGAACATGGTCAATTTCAT
ATTTACGTTCAATAG
CGTTGGTTATAACTTGTCCATCCTTATACCAAATGTATGCCACATT
ATCATCTGGGTTCC
CTTTCGTAGATCCACATGTCAGAGTCAATTTATCATCTTCATCCAA
CTCATGTTCACCGT
CGACCGCAAGATCTTCATATGGATATTCAACTCTGATTACACGTT
CTTCTGTTACATTCG
CACCACTATGTAAATTCCATGCTGATACTTTCATACGTTTCCCATT
ATGACTCCTATCAA
ATCTGTAAGAGAGGCGGTCAGTTTCCGCCTCATCTAATAATTTTT
CCCCATCGAGTTCCC
AAGTCGATCCTCTTATTGTATTTGGATTACCACCCACCGGGATAA
TTTTCAACAAAACCA
TGGAATTTTCGATAAAATGGTTTCCTTTTATATCTTCGATTTCAAT
TTCTATACTGTCCA
ATTTCTCTATTTTCACCAATTCATATGTCTGAATGCATATTCCTTC
CCGAGATTCTATTG
TGCATGAATATTCACCAGCCCCTTGTGCTCTTTTCACATCAATTAT
ACGATCATTT
>ConsensusfromContig1823 Average coverage: 10.28
TCAAAAATAAAATTCATTCAAAAAATACTTTAAAATGACACTTTG
TTTCAGTAGAGTCGC

GATGGTTACTTATCACTTTCGCTCGTGATTCATTTATGAAAAACC
TGAATGAACTATAGA
AAGCAAACTTGAAGAAAGCAACAATAAGATTTACAAGCAGATCT
GAATTCATAAAGCTGA
CGAATTCGATGTCACAGTTGTAAACGAAATTCCACATTGGCTCAA
TAGTGAAGCTTTTCA
AATCATATAACGCATATTTTTCTTATATGTTGACCGCTATCGGAAC
TTCCCGAAATATTT
ATTGAAGTACCAAATAAGTATGGACTTAAGCTTTGAAATCGATA
AAGAATGGGAAAGTTT
TGTTTAATCAGTTTGATATTCAAAATTCACATATATAGCTGCGGC
AGGGTTCGCTCTAAA
TCAGACTTCCGGACTAGAATGAAAACAAAATAAATCTTAACATCA
CTCATTGATATATGA
TCATTTCGGGTCTAATAAAATCATTCTGATAAAAAAATGGAGAGT
GTTTTAATACTTC
>ConsensusfromContig1824 Average coverage: 8.52
GTATGAACGATGCTTTACAGAATCGCGAATATGATTGAATATTTC
CCAAAATTTTGAGTG
TTGATGTTAAAAGATTTGCAAAGAGTCGATGAATAAACACAAATT
CATCGTTTACTTCAA
CATCTTCAGACCGTCTTTTCTTGACGAGGATGGTATCATATCTCG
AAATTCATAGGGACA
ATGACCCGATCTGGAAATCCACATAAAACTTTGTGCTGATGAGAC
CCTAATTGGTATATT
ATCAACATCAATCATTCCGAACAGCAGCAGTGTTAATACTGATCC
CGTCATCCGCTATCA
TGTTGAGCCCATCTTCTTCAATTTCAAACCTTTGATATGTTTTTGT
GACCCTTTTTGAGT
CGTACACACAATTGTGCCGCGTTTCATCATGCAAATAGTGATCTT
TCCATCATCCTCATC
TTTAGTATGAACTCCCCCTATCATCTATGTCACTGTAAGAATCAT
AGTATCTCATCAAAA
GAATAAAGTGTTTTGACATTTAAATGCAATAATACAACAGATTTC
GA
>ConsensusfromContig1825 Average coverage: 16.36
CCTCAAGATGCCAGACGGTCAAGTGGTCGCCATCGAGACCGCCG
AAGAATTTCAAATCCT
GAACTCTCTCCTGAGTAAAGTATCTGCCTCTTCACTCATGCTCCA
GCCCGTCACGGAAGA
AGGAGCTGTGTCGTCTGCTCCGGTCATGTCTTCAACCACGGTCAC
GGCATCAAGCCAAGG

TCCAATTTCTACGACGTGGTCGTCAGGAGCTCGAAATAAACGGAT
GGCAGTTGTTACGGA
CTCAGAAACTCCCGCGAAATGCCTGAAGATCAGCGAGAATCAAC
GACTTCAAGAAACAGG
AGTTATGAATCAGGATTCTGAAGTCGACGCCAATAGAGCTGATA
AGCGGCCGTTAAGCGA
TGTTGAGGCTCATGAATCGTTCGGGATAAATTCCGGTGATAGTAC
CAGTATTGGCGACAT
GAAAGGAGACGGCTACAACTCATCTTTGTTACTGCAAGACTCCCG
AATGAATATTCCCTG
CGCCAAGGAATTTCCGCACAATTTCATGGCTACTTCAGAGG
>ConsensusfromContig1826 Average coverage: 14.72
CCTTGGAAATTGACTTTAGTGATTGTTTATGTATCAATTTTGGACT
TCTGTAAGATATTG
GTACATCTGATGCAACAAGCCGTGAGATTGGTTTTTTGCTTCTCTC
TATCTTATTCTCCT
TTTGTACACTCCGTTGATTGCATTTTATTAAATTAAAGATATTACT
GAGTTGTGATGATA
TTTCAGTTATGAAATTCTGAAAAAAATTCTGTTGCACTATACGCT
GTTCTACACTATCAT
CTTTTCTGGGAATTTCTGTTTCCATTGTGAATTTGCGACACGGTTT
AGGGGTCTCACGTT
GTATACTGAAATTCGTGCGTTTATTTTCATCTTTCTCAACGGCGTA
CACGCGTTTTGTAT
ATCTAATTGTTTCTGGGGAACAAATAATATTAGTTTTATCGATCT
GTGACGCTGCCGGTC
CAAATAGAACACTTCCAAATGCATCAGAACTTTTGGTACTCTTTTT
GTTTACAAACTTTT
GAGGCCTAATATGTTCTTCAGTTTTTTCCCAGCTTTCATCAAAAG
TATCTAATGAACCAT
TTCTAGTGTTTGGTTTATATTTTTTTATGGGTTCATATGTTTCATC
ATCAGAGAATTCAA
CCTGTTGATTTCCGAGAGGTTCTGGGAAAGATTTAGAATATGAAT
TTATTGACCTCACTG
TGTTTTGACGTTTATTCATGATGGGCTGAGCATCATTTTCGATAAC
AGATTCTGTATACT
TCGAAGCCTGTTCACTCTTCATAGGTTGACTAATGTCTTCGATTTG
CTCTCTGATAGGCA
TAGAATATGGACTAACCGATCGACTTGACCCATATCTGGTGTTAA
TTTCACTTCTTTTAG
GCTGATCTCTGTTAATGTATGGACCTGGGGGTTTCCAAGGTGGTC
TAACTGCTCTACTTG

ACCGATATCCAGTTTTCATCCAATCACTCATAGGTTGGGCTCTAT
TACCGATATTTATAC
CATCAGGTTTCGCATATGAGCCCCCAGATTTTCCCAACCGATTTT
CAGTGTCCATTGCGC
TGCTCTTAGACTGACTAATACTACCGATGTTCTGACCTACCGGTT
TGGAATGTAGACTAA
CTGATGTACTGGATCGATATTCAATGTTCTTTCCAGTGTTATCACT
TTCTCCCATATCCA
AACCTGTAGGCTTCGAATATGGAGCAGCTGTTTTACTTGACTCGG
GAATGCTCCTGTAGT
CTTTACACTGATTTTGTGTGAAATATTGTTGCTGATTATTAAACGG
TTTGTTTTCAGAAG
TTTTAAGAGGGAATTTGTCAACTCCATTCGCCTGACGAGAACCAG
TTATTGTTTCTGCCA
TTTTGTGTGTGTTGCTCTTCGTCGTTACTGCTTGCATATTTTTTCC
TGAAGAGATATCTT
CAGATAGGGGAAATGGTCTTCTAATAGTAGAAGCTATTATCTTAT
CCTTATTCGTGGACG
ATTTTTGCGTTGGTACAATAATTTTATACTCATGTTTTGCCTGGA
AAGACGGTAGATCCT
TGAGTTCAATCGTCTTAATCGAATCCTTTTGTTCAAAGTTACGCTT
GTGTTTTTCGTGGT
CATCTATATAGTCATGTTTTCGACTTAAATAGTGATCTATTTCACC
GTCCTCATTCAATT
CATTAATAATGGGAATTTTCTCGTCGTTTTTGAATTGATATATACG
TTTATATTTATCGG
TTTTACTGTTGTCCTGCTCATTTTTTTCTGTTTTTAATGACCCGAA
ATCCTTAAAAACAC
TATTTTTCCCATGCTTCTTCCAATCACCATCATCTAAATTCATGAA
CAAATTATCACTTT
TTTTGTTTTTGGGCTGATTTGGAGCAGGTCCCTTGTCCCCGTGTTT
CATTGAAGAGTTTG
CTGAGACTGCTCTTCTATATTTTTTGTCTCCATGATTCACGGGGAT
TCCGCGAATATTCG
GGTATTCCACATCACTACTGTCAACGTATAGTTCACGACGCTTTC
GGGATCTCCCCTCTC
TTTCAAATATATGGTGTTGGGTGTACAAAGGATATCTCTTCAGCT
CCATATCATTCATTC
GATAGCGGCCCAATGATGCTCGGATATTATTGAACGCAGATTTTG
TTAAACGTCTTCTCG
ATTCATACGACGGAAACGTCACCAGGTGTGGATTCTTCTTCAATA
TTGGAATCTGAGCTG

CTCTATGATACGACCGCTTTGACAGAAAATCATAGCCAGGA
>ConsensusfromContig1827 Average coverage: 10.07
ATTCGGTGAGGACATTATCAAGTCGATATTTCGTTCATTTTGCGA
GTCGAGCGATTCTTC
GGAGATGATATGTGACAGAACACCACAGAGTCTGTCCAAACCCTC
GCCGGTCGCTTCATT
GAGAAGGGTCGGAAAACACGCAAATCTAGCCAAGTCAATATTTTT
TCTATAGGCCAGTAT
CAGTTCAACAATAAATTGCTCTATTCGCGTTTGATTCAATCCGGA
GCATAGAAGCAAACT
GAAGGCGAAATTCAATATTCTGTTGTTTTCATCACTCAGCAGTTT
CGACATGTATGAAGT
TGGTAGAGAACAAACTAGTAGAGATAATTGGGAAAACAAATAAT
AATGCCAGTCGACGAA
AGTTTGGGGGAGCTCACTCGAATGCCTCGGAACTATTAGAGAGGC
CTCGTCCGCATATTT
CCACATCATGAGTTTTCGGAGCAATACCACACAGCCATTGTGTCG
CAGCATAGCATTTAA
ATCCAGAAGCCTCTTCATACTTTCACTATGTCTCTCCTCATCAGC
ATCCAGATACTCAAG
CACTTCGTACACCTTCTTCTCTTCATCATCCTCTTCATTCACCAAA
AGGCTTTCTATGCA
ATAAAAGTCAAACTGATTTGTTTGACTGATCTGACGTCGTGCAAT
AAAGCGCAAAATATT
CATTTGTTTTCTTTGCATTCTGAAATGTGAATAATTGAATTTTCCG
CGAAATTTAAATGC
CC
>ConsensusfromContig1828 Average coverage: 12.05
TGAAAATGTTATAATAATAATGATAACAATCATTTGTATTATGCC
CCTTCTTATAAGCGT
GTAACAAATTTCTGGGTGGAATTTTTGAGCACTCTCAGGCGAAAT
CAGGAGATTTTGGGG
CTTACAGCGCTCAGCAAAATGTCTGACTTCCAAGAGCGGCGCTAC
ATATTTGAATTCTGG
CTGACCGGTTCAGCCATCTTGATCGGCATGGTCGGCAATATCATC
AGTCTCTCTGTCCTG
AGGAGGATGGATCCAGCGTCTGTTGTGTTCTTCGCATTGATGATT
CTTGCCGTCTTCGAT
TCCTGCTTTCTTGCTAGTTGTGCTGCTGTTGTGGTTCCAGCTGGGT
TTTCAGTTTACCAC
AAACCAGAGGTGTTGAGGAAACGTGGTTTTGTGTATCTGCAGTAT
ATCGGCTGGCCTCTT

TACAACATTCTTGGACTAATTTCTATGTGGATAACTGGTTTTATTG
GGGTTCTTAGGTGC
GTTCAAATCTACTCACTTAGACGCAAGGCGTGGATAAACAAGAA
ATGGGTCATAAAGGTG
ATTTCTTTCATCACATTTTTGACGATATTAACTCAAATACCACTC
TATATCGAAAAAAGC
ATTGATCAGATGAAATATAACAAAGAGATTGCGTATGTTTTAAA
ACCAACAGAGCTCGCA
AAAACGTGGGCATACAAGTTCTACTACAAATTGATTTTCCAGTTC
TTGATGCGATGCATC
CCGATTTTAGCTACCGCAATATCTACGTTTTTCTTGTTAGGAAA
CTACTCCAGCATAAG
AGAAACCCAATAGGGTCAACCGCAAGAAGAGACAGCAATACAAA
TGCAGTTACATCTGTC
CTGATCATAATAGCCATTTTATTCACTCTTTGTCAAATCCCAATGA
TGTTCCGAAAACTT
TTTCACGAGATGGTTCTTCTACGAATAATCAGTTTGAATATAAAT
CCAGAGTTTGTTTAC
CTCATTTCAGATTGGCTTTGGGTTTTTACTCTTCAATCAATTTTCT
TATTTTTTATCCT
TTCATTCCAATGTTCAGAAGGCAGTTAGCTGATTTGTTCAGGATT
CAACGAAGAGGAAAT
TATAACCCGAATGATCAACTTGATAATTCTGTCAGAGATATTGAT
AGTAGTAAAATATTG
CTGTATTCACTGAAGACAGAAGCTGGCGATGAATAAATTTATATA
ATTTGATTATTTACT
ATCGCTT
>ConsensusfromContig1829 Average coverage: 18.16
AAAGAACACACCACCGCCGAAATCGCGTTCGATAAGCTCGTCAT
CGGTGATACCGGGAGC
TACGAATGTGGTGGTACAAATATAAACAATCTTCAAAAGAGTTC
TCCACTCGAAATAAAA
GTTCATAGTCAAGGAACTGTCGAGCTATCCATCAAGCCCAATCC
TTCGAGGACGACAAGA
ACGATGGGCACAACTGTCAACTGCGAATATAAGGGGTTCTTCGA
GTATGAAATAAAGCAA
TACGGTTACAGAAAAGATGAAGTTGTAATCGAGGACCAAATTCAA
TCTTCTATAAGCTTT
GCATCCGTCACTGCTGACGATGCCGGCACGTACCAATGCTTTGCG
ATAATGCAAAATGAT
GAGGAACTCAAGTCCAAAGATGTCGAACTCATTATAGAATACCCC
TATGAGTTGGAAGTT

GAAATAGATTGCAATAATAACTGTCCGATCAATCGGTCAGCTGTC
TTGACTTGTGAAATC
ACACGGCATGGACATCCTCGAGAGGCCAGCGTCCCATTCAGATG
GCAGAAAGAAGGAGTG
CCACAACCCTCACATAATGACAAGGAAAAGATCTTTTTGGGAGC
GCTATCAAAAAAGGAA
CATCATCTCAAGACTTATACGTGCTCCGTAGTAAATCGCGGGGGC
ATTGGAAGATCCCAG
GGATTCAAGATCACTGCGAACTACCCGGATCAAACTTGGCCACA
TATAAATAACGATGGC
TATAAGGCAGTTTACAACATTCTGCCGGGCGAGGAAATCACACTG
CAATGTCACATGTCC
GACGGTTATCCATCGAGTATCGGCGAAACTCCAGAATGGTTCAG
AGAGCCAACCACTGCT
GGTGCTATACAGGAAAATAAACTGGTAGAGGAAGACGAGCTCAA
AATAATAAATGATGCC
AATTCAGATATTGAAGGAAAGTACTATTGTAAGATGTCGCACGG
ATCTGACAAACACAAA
TTGGAAATACTGAAAATACAAAAAACGACAGTTGAATTTCAAAG
TACATCATATACAATA
ACTGAGTATGAAGACCTCCAAATCACACTTACTATAAAGAGAGA
TAATAGTTTTAAAACA
GTAGCAGACGTGAAGTTTCTTTGGAATAAGGAACGGATTCCGAAG
CGGCAATACTCTTTC
G
>ConsensusfromContig1830 Average coverage: 17.74
ACAAACATATGACTCAAAAACACTCACGAAGATTGAAAAAAAAT
TTATTATTAACAATAT
TTGTGAACACACTGTCCTGTTTACGAATGTTCATTCGATAAGAAA
CACTCTGGATGGAAA
AGGTGGTGTGTTGGGGAAAGAAGCGATCAAATATTTCAGCTCTTT
TGAATTAAAATTCAA
TGAAGATGAAAATACGAATGTGGCAAAAAAAAAAAATTTATATT
ATCTACTTACTGATAA
AAGACAATTAGATTTTATTCTATATTGGATTCACGCATCGTGTCA
TAGTGAACGTGAAGC
TTTCAGAATAAAACGCATGATAATGTGGGAAAAAGCCTTAAAGA
AAGCCGATAAACATCC
ACGTATAGATAGAGATAAACTATCTGTGAAACATTCGATAATTTT
AATCCATCTACATGA
GGAATTATATCGCCTTACGCGTGCCATAACAGATATTACAACTTA
CGGCAGTCTTCTAAA

TAATTTACTAACGAATAGCAATGCATTAACAGAAGGTATGAGTTC
AAATGGAGTGGCATT
CAAAAAACGATCATTTTTACGATTCACAGAAATATTTCTGATAA
AACCGAAAAACAAAAA
TAGCAATGAATTGCATATAAGTTGTGATTCTTTTGAATTTATATT
ACACGACGAACGAAA
ACGAAGACCATCGGATGCTCATATTAATGGTTTACTTCAAGCATT
TTGGGAAGTTACGAA
TGTTTGTTACATCGAATCTAATATTCATGCGTACTTTAATTTAAGC
AATACAATTCCATG
TACGATGCTTACTTTAAAAAATGAAAACAACAATATTGAAAATCT
GTTTATTAAACGACT
AGAATCATGGATCATAAGGCAAAAGAACAATAATATATTTTCTA
ACAATGATAATAAAAT
CAAAACAAAACTTCAAAAATTAGAAACTGTCGACGTTGTTGCGGA
TTACGAACATTCATG
TATAACAGAAATAAACGACAC
>ConsensusfromContig1831 Average coverage: 37.02
GTCAAAATTACAATGTCAGTTATATGGATGATATCAAATCATATG
ATAAGATAACCACAT
AGGAATGAACAAAAAATTAACCGACCAGGGCAGCTGTGAAACCT
AAACTAGTATCCATCC
AGTCTTGTCGCATGATTTCAGCAACTTCAGGAGTAGAATAGTCTT
CTTGAGGTTCTGAGT
CAAGCATTTGAGAAGAATGCATTTGAAATACTGGTGTAGGAGTTT
CCATAACCATATTTC
CCAGATCAGAATCAATGAGTTTAGTGAAGTCAAAATTAGCCAATT
CATAATGCCAATCTG
TCGGCAGTTGAATCAATTCACCCAAACTATCCAAATCAGCAAGTG
GATTAGATTGAATAA
CATCTGTAGTGGCAGTCTTGCAATCACCCGAGAGTGAATTTTGTA
TCACATATTTGGCAG
ATTTCATATCATTAAATTCAGGGAAAATAGAAACTGGAGTAACTG
GTGGTGATATAAGAT
GCGAGCAGGTATCACGTAACTGAGATATTGGTTGCTCAGTAGGA
ATTGATGAACATATGA
TGAGATGACTATGTTGATCGGGTTCAGTTTTGATCAATGTACTGT
CTGGCACATTGGAAC
AATTAGAAAATTTTTTACTGAAGATACCATCATCTGGATAAAGAC
TGCCATGCTCTGGGC
TAATAGGTGATATTGAACCAGAATTGGTTGGGACTTTAGCTGGAG
GTGTTAATTGACTGT

TTATTTGAGAAAAAGCCTGATAATTTGCTTTCATACTATCTTTAAA
TTTCTTGTCAATCG
TCAACTTCAGTTTCAATCGATTATTCGATAAGAGTGAGGTAGTTG
ATGAACCCACAAAGG
GTGCAGCCTCATATTCTGTTTTCAAATCAAAATTCAAGTTTGAGA
ACGATTTACAGTTGT
TGTTTTGCTCTGTATTTATCTGAAACGATGATGAAATTCTGTTTG
AAATCTGAGTAGAAC
TCAACTGTTCAATTGATTGTTTGGATGATCGATAACTTCGTGGCTT
TTCACTTTTCATGG
GAGTAGCCAAGCTTTTAGATCGTGATTTATTGCCTGAATCTTTAG
TTGACACAGATTTTA
ATTTTTTCTTGGGTCTATATTTGTAATCTGGGTATTCCTGAATATG
TAACAGTCTCAATC
TTTCTGCCTCCTGAACAAAAGGTTGCCTTTCCACATCACCCAACAT
TTTCCATCTTTTGC
CAAGTCTTTTGGATATTTCAGCATTGTGCATATCTGGCTGAACTTC
TGCGATTTTTCTTC
TCTCCATTTGGGACCATACCATAAAGGCATTCATGGGTCGCTTCA
CATGGGGTCCTCCAA
TTTTTTTACATCTGGTTGCATCAGTGTATGGAGTTGAAGATGTTG
GGTTAACCATCTGAG
AGCCAAATACACTATCTGCTGATGGTGATCCAAATTCCTCATCGC
TACTGTAATAACTTC
CACTACTGATGGAGGCAGTATATGCCGTTCGAGGTGAATGTTGCA
AATCTGTATCAAACA
TAATGTAGTTTAAGAGCGAAGCTCAACGAAAATAATAACTGTTTG
GAGACACATTTGTTT
TATAATTCATTTTACAACTTTAGCGGTATTTATT
>ConsensusfromContig1832 Average coverage: 10.94
GACAGACAACTTTCATTCAACTATTGATCATTGTATTTTGTTTTA
AAGTTATACCAGATG
AACTGTCCAGTGTGCTTCGAGGTATTCAAATATCCATTACTTCTTC
CTGGTTGTGGTCAC
ACATTATGCAAACATTGTGCGGAGAAGATTTATGAATCTACCGGT
AATGGATCGCTACAG
TGCCCTGAATGCAGAGTTGTATATATGTTGAATGTTGGTGGTGTC
AAAGCACTGCCCAAG
AATGTTGCCCTGCAACGGACAATTGATGAATTTACAGATAAATTC
CACAAGGAGTGCAGT
ACACATTCAGAGGAACTAACGAGCCTTTACTGCAAGACATGCGAG
GTTCATATCTGTCTT

AAGTGCTATTTTCCTTCCACTCAAGCTTCTACCAATGACCATATCG
CAACTGTTTCTCAG
ACGCATTCTGGCCATCTGGTTGAGACGACTCAAAATGCCTTTTCT
AATGAAAAAAATCAA
CTTCAATCTAAAATTGCACATTGCAAGACTCAAATATCGGAGGAG
AAAAAATATGTTGCC
GAGATGGAGGAAGCCAAATCCAAATTAGAGAAGATGTACGACCA
GAAAACGGGTGAAATC
GACAAATTCAAATCTTCTCTCATCGGGAACATCAAACGGGCTTCG
GGAAAACTCAAGTTC
ACTTTGGATGGAACGATGTGTCGGGATAAGGAGGCTTTAGAAAG
GACCCTCGAGGCCTCC
AAGGGCCGTATAGATCGATATGTAGCCATACAGAAGGAAATTGA
CAAATTTATGACACCC
GGAAAACTCGACCATGGGGATGTTAATACCCTGAAAGAGGCGAA
AAGATTCATGGAACGG
TTTGAAGAACACGTTGAACGTCCGTCGCCAGAAACATTTTTAAAG
ATTTCTGAATTCGAT
TGTGACCTGCAGAGATGTATTGATGATCCACACAAGGCCCTCTCA
TATCAAACAGAATCT
CCTACACCGCCACAAATCCTCAGATCGGAAGGCGTC
>ConsensusfromContig1833 Average coverage: 11.64
ATGACATCCAATGCGATTTTCGCAATATTTCTTTTTATTGTTGATG
ACCGCTGTATAATG
ACTGAAAAATTAATGGCAAAACGTGAATAATCGTATTATTACCTG
ATCAACTATGTAAAA
TTACACAGGTATGATTATTCTCGGATAATTTCCGTATTCTTTCAT
ATGTGAGGAGTAATA
TTTCGCGATCCATTAAAAACCGGTACTCATAACATCCAGCCATAA
AGAACCCAGTAGACA
AGATTGAAAATAAGGAAGGCAGCAGGAAAAAGGAATCTCGAAG
ACATATCAACGAAGACG
GCCTTGCTTGCGTACATCCTGGAGATGGCGTTGGTCGGGATAGGA
TGGTATGTTACCTTT
GTATGTTTTCCACTTTGATGAGAACTGCCTATGCCACCGCCTCCA
TTTCGATAGACTGCA
CAGCCCTGACACGATGGTGCGCCGAAATTGGATCCCTGCTGTGGC
ATTTCAAAACTGTTG
GGTTGGTTCCTCTCATGATGAGACGACGGTCCAGATTTGTTGGAT
GAAACGTCATCCTTT
TTTTGCTGTTTTTCTCTTCGCAAAAGAACATTGACAATGGAGTAC
TCAATAAGGCCTCCG

AAGACGAATAAGAGACATACGGACATCCACAGGTCGATGGCTTT
CACGTACGAAACTTTT
GGCATTCTGGCGCTGATTGCTGAGGCCTGGGTGGTCATGGTGAG
AACTGTGAGAAGACCA
AGACTGATTCTTGCCGGGATAGCATCGATGTCGATCCAAAATGA
AAGCCAGGAGAGAATC
ACGATAAGAAAACTCGGAATGTACGTTTGAAGCAGGTAGTATTCA
AATTTTCTATCGAAG
TGGAATTCAGCATAGAGACACGCGAATACTCCTGTCGTAAAACC
AGCAGTACAGTCACCA
ATTTCTTTATCCCTGATTTCGAATTCCGGCAATTCGAGCTTTTCAT
TAATTGACAACGTT
TCTTTATCACCTTTATAGACATGCCAAATGAACTTCAAATCATCC
GTTGTATAACCATAA
CTCCCAATGTTTATTCGGCATTTTTGGTCATCCAATGGATATTTTT
GTAACTGCATATAA
CAATCAAGACTGAGTGAAATTCTCTGACTGTACAGCAATTGACCA
TTAGGTTCCAGTCTC
ATGAGTCTGTTTGGTACTGTCATCTGATGAAGTTTGCCAGATTTTT
CGTTTCTAAAGAAT
AAGTCGGGCACCCAGAGCCTTTCCACGTCATTATATGTAAGAGCT
ATGGATTTATTGTGT
CTGGCGGCGTACTGGAGTCGCTCATCGCGCCACATTTGCCGTAAA
TACATAGTCACTGAG
AAATCCATATTGAAGTCATCAATGGCCCCTATGCTAAGAATATGA
ATACTCAAATTGCAA
ATCAGTGGTTGGCCTGACTCCACACCTGGCCGAATATTTTCGTTA
TAGTTCTCGAATAAT
TCTTCCATAATATCGGTGACACTGGTGTGTCCGGATGCGCTTTTCT
TTTTGTTTCCACGG
CCGGCATTGAGTCGATCCATTGTGCCAGATGAAAAAACGAAGAG
AAATAATAGAAGATAG
ATCGGAAAAGGGTGGG
>ConsensusfromContig1834 Average coverage: 9.20
CTTGCTTTGTCGAAATCATCCATGTGTTATCAATAATATGATGTG
CTATAGGTAATTTCG
CGCTAGTGTTTATTTTAACTCGGCAATATTTACTTATTTGATCCTT
CGTTTTGGTTAATA
GTTTTATTACACATTCTTCCGAAGTTTCCACTATCAAATGTGGTTT
GTTTAAAATACAGA
ATGTCGTTTGAGGCGCTGTGCATCTATTGAATTCGTCTATGTCCA
AAATGATGAAATGTG

TTTGTAGTGCATTCATTGCTACTGCGTGAATCCCTGTATCTATCTC
TGCTGTCAGTGAAA
CTGAGTCGTTAGTAGAATAAGGTACTGGTAGTGATTTAATCGAAT
AGACTACAAAGTTTT
CCCGCGCTCTAACTAGCGGTATCGTCATGAACATTAGTAATCCAT
TATTATGTGCAACGG
ATCCACATGATAAGTATCTAAAATATAACCAGATGTCCGTAATA
GGATCGAACGGAAGAT
CCAAATACGATGGTATTTGTTCTTTTAAATCGTGTAATATTCTTCT
TAATTCGTTAGGTT
GAATAATGTCGACCGACAGGTGTCCGTATGCCAGGGAATTAATCT
TCCGTTTAAGTGTTT
GTACGAATGATTTTGTTGTTCTAATGTCTTCTTTCGTTGCATCAAA
GAATGATTCGTATG
CTTGTGCTGCTAAGAGATATGTCTCTATCGTTTCAGTTTCTTTAAT
CATCGTCTCGGATA
TATTTTGATCGGCGTTCATATAGTCTATAAGATGATTAATCTTATT
TCGGTTTTTCTGCA
GTTCGTCGCGGGTAATATTTATTACAGATAATTCTTTCCCGACAAT
ATGTTGCATTACCT
CTTGATTCGACGCCATATCTTTTACCGCTCGCTTGATGACAGATA
TATCTGATTCGGATA
CGATCCCGAACAACCAACTTAATCCTTTCCCAATAATTGGTAATA
AACTCCTTTTTTGAC
GTGCGCGGATCGATATAATCTCTTTAAATGCATCTGAAATTTCAT
CATTGGTACTTCTAA
GATGTCGTATTTGTGATCTGACATTTTTTATTAGGGCATTGTTCGG
TTCAAATTCTTGTT
TAAG
>ConsensusfromContig1835 Average coverage: 9.41
GAGATCTGTTAGCTTTGGCCAGGGTTTGTGTTGCAGAGATTGTGG
CTCTGAGCAGACGAC
GCGTTTCTGGAAGCAATGGAGGATCTTTGTCGTCTACATCTAGGC
TTTCATCATCGGAGG
ATGAACTCGTAACATCAAAATCTGTGCCACTACATGCGAGGTCTG
TCAATTCATCGCCTT
CATCAAAATCATTGAAGGTGTCGTCGCTTGCCAGACCGGCCTCTT
CAAGCAAGTTTGATT
CAGATGCTCCGATCGAACTACATGAAGAAATCTTCAGAGCTGAT
GACCCAGATATGGCAG
AGACGGGCCTTGATATTTGACGGTCACTTTTCACCTGACCATTTTC
AGTGTTCTTGTGAT

AAAAAATCGTTTTAGAGGAAGAATGGGTTGAAAGCTTATCACTAT
CTGCTTTCATATTAT
TTGAATCAAATTTAGCATGGTTTGATTTTGCTGTGGATGTCGCACA
TTTCTCTTGTGGAT
GTATAGCATTACTGTGAGAAGAGTTATTGAAATTCTTGGAGCATT
GCAAATTTGGCATGT
CGTTACATACTTGCTTCGTAGCACGATTTTTAGCAGAAAGTACTC
GAGATATGGGACTCC
TTACTACATGATGTACAGCATCTATTTTTTTACGTTGAACGCTATT
CTGTATGGTAGACA
ATTCACATTTTTCATTAGATGAGTTATGTTTTAACATATTCTGAT
GATGTGAAGAAGAAT
CATTGTTT
>ConsensusfromContig1836 Average coverage: 10.83
TACTATCAGTGATTATTGCTCTCGGAGCAAATATATCAGCTTCGG
TAGCGAACGTTCTGG
CTGTGCTTGGAATCGGAGTTTTAATAACGATAATAGTGTCTGGTT
TCTTCTACGCCGACA
TCGACACAATTTTGAATGTTAGTAGAGGTGGAATATTTCCTTATG
GAGTTGGAGGAATGC
TGACAGGTGCATCTATAACAATATTCTGCTACACAGGATTTGAAT
ATGTCGCCGTTTCAG
CGGAAGAGACAATAAATCCAGCAAAAGCAATACCTGCCGCTCTG
TTAATCGCCTTACCAG
TGGTGACTGTGCTGTACATTCTTGCATCTGGTGTGCTGAGTGCTTT
GATGCCCTACTGGT
TGATTGATGTTGCAGCTCCATTCCCTGCAGCCTTTGCCTTCCATGG
TGTTCTCTGGGTGA
AGATTTTTTCGATTATTGGCTCTGGACTAAGCATGGTATCTGTTCT
GCTCGGCTGTGTTT
ATGGATTGCCGCGTTTGTTTTTCGCCATGGCAAGAGATGGTTTAC
TTCCGAGCATTTTTG
CAAGGGTCAATGGCACGACACAGACACCTCTTGCATCAACTTTAT
TTTGCATGGTGACAA
CGTGTATAATCGCGTCAATGTTCAAAATTGAAGCACTCGTTCAAA
CACAATCTCTTGGAT
TTATTTTAGCCCTGACATTACTGACAATCACGTTGCTGAGAATGA
GATACATGTCTGTTC
TCAACTATCCGTTTGCGCTGCATGAATTCGATATACCATCAGAGC
GACTTGCTCAGGCGG
AAGATATTACTCTGGGCTCTCCCGTGAAGACTCAATATGGGAATG
CTGGATATATCAAGC

ACATGTTTCAGAAATACAATTACATTTCCTCATTAAACTCCCAAA
ATATCATCAATATAA
CTGTAATTGGGATCGTCATATCTCTCTGCCTATGCGCACTCTGTTG
CCAGTATGCTTATT
TGGATTCGAGGATATACGTTTTATTGGGAATTTCAGTCCTCGTCCT
TCTGGTTTTCAATT
GCTTACTTTTGATGTTCGATAGCCTCAAAGATTTGCCGCATTTTAA
TATGCCGTTTGTTC
CTGTTTTGCCTGAAATCTGCATATTCCTGGATTTCGTCATGATCA
GCAGTCTACCAAGCA
ATACATTTCTCATCTTCGG
>ConsensusfromContig1837 Average coverage: 10.96
TGCGCATTATCATTTACATCCAGAACAAAAATATTAATTTGCATC
GTATTTGAGTAGGAG
ATTGCGCCATGATCACGAGCTCGCAAAGTTAGTACGTATGAAGCG
ATATTCTCTCTGTCC
AAAGTCTTGTTCAATTTGAGACTTATTTGAAATGAATTAACGTCG
ATATAAAATACATCT
TGAGATGGACTGGAAGTCAGGCTGTAAGATATTCTGGAGTTTTCA
CCAGCATCTAAATCT
ACAGCTGGCGGGATGGGAAATAGTTGATCTAGCTTTGTCGATTCG
AGAACACTTATGTCA
TAGAATTTTGTTGGAAATTCTGGTTTATTGTCGTTCTCGTCTATTA
TCTCGATTTTAACT
TTATATATTTCGAAGATTGTGCTGGTTTTTGCAATGATCGTTATTT
CAATTTCGCAAGTA
TCGGCGAAATTTGCATCGCATACACTTTCTCGATCAATTGACAAA
ACCGTTCTGAGAATT
CCAGATTGAGCTGAAATTTCGAAGTTGGTGTAAACGTGTTCTGCT
TGGTTGCTGCTGAAG
CTGAACTCGACATTTGCAAGCAGTGCAGAGGAATACCTCAGCCCT
ATTCCGACATCTGCA
CCGATATTTGCGATGAAGAGGTCGGGAGTATCGTTTTCTCTGATG
AAATAATTAAGTGGT
TGGATCTCCTGGAACAGCGCCTGATTTATAATAGAATATATTACT
ATATTGATGGATAAA
AGATTCGTCATCGCTTCGTGAATCCTGTCGTTTATGATAGTTGCTG
CATTAAACCGATTT
ATGGTTGCTCTGG
>ConsensusfromContig1838 Average coverage: 11.04
CTCGACAGCGTGGATGAAGAGCCAGACCCTCAGTCTCTGCACTAC
CTCTACAACTACGAA

AGGTCTCAGTTCGAGGGTGATATGTGCAAAAACCTTTACGACAAC
GTGTATAGCAATCCG
TACGGGTCAATCATTTGGACGTTGGTGAAACCATTGGTGAAAGGA
ACGCTTCTTTTCACA
CCCGACAACCCAACAACACGACATATCATAGACCAGGCGTTCTG
GCTGTATCGAGATTGG
ATAAATATTAGCACACTTTTGGGAAAATGGAGCGAGTCTATCAAT
AATATTGCATTCTTC
AATAAAACAATCGATTTCGACACCCTCAAGGCAACGTTAACACAA
CCCTTTGTCAACGAC
GCCATGCGGACATACGTGGGAATCTCAGGGAAAGTCATGATGGA
GAAATTGGAACAGATC
CTCAACCTCCCGGCTGGATCTGATGTGAATGCCTTGAACCTGGCC
TCCGAGTACCTATAC
AACGCCTCAATGTGCTTCGGGCTCCGGATTGAGGGTGTGAAGGA
TGAAAACGCTCTTGAA
GAGAGGGCAGAAGAATTACTCGAGACGAACAGTCTGCTAGCTGG
TGTTGTTTTCAACAAT
GTTCCTGACGGGCCAATTGAATCGGACCCGGAGACAGGGGAGAA
GATGCTGAACTTGCCC
ACGCATTTCAAGTACAAGATACGCATGGGCATCGAAAAT
>ConsensusfromContig1839 Average coverage: 15.35
AGCATCCCATTCAAGATTTCTCTTCGGATTACTTGATACAGATAA
AGGATTTTTCACAGG
ATAGATTAAAAAACATTGAAGATTTATTCGCAGATTGCGAAGAAC
AACTGCATCATAATC
CGTCAGAAACGTTGAAAGATTTTTCACACATCTATTTGACTACTG
ATTGTAAAAATGAAG
ATCACGCATTCGAAATACATTCAGATCTCCTCGCAAAAATAGAA
GCAGAAGACTTGAGAG
AAAGTGAGGAGTTTGCATCGGCCGAAGAGACGGATTTCGAGGTT
CAAACTCCAGATGGAA
AAAACACAAAGGCAGAAAAGAGCAGTTTTGTGAATGATGTGTAT
GCGAACAAAACAGAAT
CGATTACGAACAATCAGATTGAAACAGACTTAAAAGTCATTGATC
GCCGTCTGAAATCTC
AACCATCAAAGTTGATTGAGCAAGAAGACTCTGGCAACGAATCA
TCTGACACACCTGAGA
TAGCCTATGTGAACGGGGACAATATCGTTTCTGATTCTCGGGGTC
CTGTTATATCCGATA
TCAATGGCTCTGAGATAGTTTGTGATGCTTATGACGATATTACTA
TTGATGAAAGGCTGC

CTAAACCTATTATGATCGACTGGTCATTGCTGGACAATGAAGATG
CATCGGAAGAGCTCG
CCAAACAGCA
>ConsensusfromContig1840 Average coverage: 12.22
TTCAGAACTCGACGCAATATTTTTCCGGTTGCTGATTTAGGAACC
TCATCAATGAACAGA
ACTCCCCCGAACAATTGCTTATAGGACGCAAGTTTCTCCTTGATG
AAGTTTTCAATCTCA
TCTTCTGTAGCATTCTTCCCTTGTTGCAGAACAACAAATCCAGTCG
CTACTTCATCTCCG
GAAGATGTCGGAATGCCCACAACTGCCGCATCTTTAATGGCAGG
ATGAGTCATCAGAACA
TCCTCTAGTTCAGCCGGTGCGACTTGAAATCCTTTTTGTTTTATGA
GTTCTTTGACGCGG
TCAGTCACAAAAAATCTCCCAGATTCATCGTAAAAACCGACGTC
ACCCGTGAGCAGAAAT
CCATCAGATTGAATAGTTTTTGCTGTGGCTCCTTCGTTTTTCAAAT
ATCCTTTCATCACT
TGGGGTCCTCTGACACAAATCTCGCCTTCTTCATTCGGACCCAATG
CTTTTCTTGTTTGA
ACATCAACAACCTTTACTTCCGTGTTATGAATGATGAACCCGGCT
GCTTTAGAGATCGGA
A
>ConsensusfromContig1841 Average coverage: 10.69
GCCCGAAGTGTCGTCCGAAGGAAGCCCTTTGGATTCTCTAGAGAT
TTCATTATAAGCCAT
TTGGAGTAGTTTCAAAATTCTCAATGCAGCCCTATGCTCACTTTT
AGGCAATTGTTTGAG
ATTGCCAATGCTCATGTCAACAACCGCAACAGCCATTCCTTCGCT
GTCTCGCATCGGGAC
CGCCAGATGGCGCTCGCCGTAGGCATCTGCGGTAATGGACTCGGA
GGTTTCTACAGTCTT
GAAGAGATAATCTCTGAAAAGATTGTCCTTCCTCTCCAAGATTCC
CGGACTCTTTATTGA
GACCACGCCAGATGCTTCCTCAGAGCTGCCTTTCTCAAGCATTTT
CCTGAGGACGACGTC
TGGCTGTTTGTTTATGGTGTGGTTCTCTACAAGGTATACCGACAT
ATGTTTCATTGAAGG
CGACCGTCTGTGAATCCACGCCACTGCACTCTCTGTGATTCGTAGC
ATCTTTTGCCTCTC
GCTTACTTTGTGATAAGCGATGCTGAAAGCTTTAGCGATTCCCTG
GAAGAATTGTATTTC

ATGAGTGATGAATAAGGCACGGCTGTGAGGATCTCGAAGGGTGT
CGATAGAGAGGACGCC
AAAGACTCGTTTCTTTCTATCTTTCATTGGAACGACCACCAGAGA
ACCTTCGCGGCTATC
GGATGGTCTTTCGTCGTCCCAAAAGTGGACATTCCCGCGATCGG
AAAGGCGGCCGGGTGG
GGAAGGGG
>ConsensusfromContig1842 Average coverage: 10.76
CAAACTTTGGCCGACTTCTGCTTTCGAACAACGCCGGGTGTTCTT
TGGCCAGGTCACTCA
GCGCCTTGGCTATGACGTCATAGCCATCTTTGATCAAAAGCTCTC
TAAAATCAGAAACGG
GTTCATCTTCTTCGAAAGACCTTGTGGTCAACCCGAATCCGGAAA
TATTCATCCCTCCGT
GCTGGAATCTTGACATGTCTATTTCGTTTATGATGAAATCAGCGA
TGAGAAAATGGAAGG
TGCTTTGCACTTGAATAAGACCCAAGAACAATGTATCGATATCTG
CTTTCTTCAAATCCA
CCACGACTTTTCTAGATTCCGTGGAGTCCAACGCCATCTTAGATA
GTTTCGCCACGCCTT
GTTCAACTCCCTCCACAAACAAGACTGTAACCTTCATACTGATGT
CCTCGACCACAATCT
GTCGGTAGAGGGCGTCCAGTCGCAACAGACCCTCTCTAGAATCG
TAGAGGTAGTAAACGT
GGTTCCAGCCGTAATTGGCCATCGCATGGAGAAGGGGCTTGGCTG
AATCTGGTCTCATGA
AGATTTCAAATCCGGGGTCGC
>ConsensusfromContig1843 Average coverage: 12.49
CGCGTACGAATTTGTTTCTCCGCGCCGGCTACTTTGATTAATGAT
GCCGGTGTGATTGGC
CGTCGTAAATCGGCGTCGCCCGATCTTTCCCGGTCCATCGAGAGG
TCCTGAGGACGTGTG
GGAGCAGGGCGATTCAATCGGATGCGAACAGTACTTTCGCCAGAT
CCTTGGCTATTAGGA
TTTGATCTGATATGATCGCCAGTTGACTTTTCATTTATCGATGAA
TCAGTTGTGCTAGAA
ATAGATTTTGATGCAATTTGCCGTGTTGGCCTGCTATTTGCTCGAT
AACGAGTTTCGATT
GGTTGACTAGAATATAACAAACCTAATATATTTATATGTGAAAAA
TCTATAATATTGCTT
TTGGAATCAGTTTGGGTTATATCACTTAACAAATTTTGTAAAGAA
TGTCCATACTCTTCA

TTGCTCAACAGGACGATTTGATTTGAACTGTCGTTCGACCTGTTTG
TACTCTGTTGGTGT
ATGGAAGACATTAGGAGTGCGGGTAATAGTTTAATAAGTCGCTGG
TTATAATCACTTTCG
ATTTGAAGCCGTACAAGGTACCCAGTCATTTGAAAACACGTACC
GAATAAAATTCCGACG
ATAAACACAGACAACATAACCGAATAATGCTTTCGTTTCATTGTG
GTGAGGTTGAAGCAA
GTGGCAGACACCGCAGAACGTTCGATAATATTCAGAAAGTAGAT
GTATCCATTACACTAG
AGAGCGAATTCACACAAGTCAGCCGTGGCAAGATGGCGGCAGCT
CCTCAAGATTAATGAC
ATTCTCCGGAGCCGTTTACGTCTTCTCTTTCTTTGTACCGAGTGAA
ATTGCTTGTCGAGT
AATTAAATTTCCATTCCCTCTAATTATACTCAAATACAATGCTGA
GATCGGAAGAGC
>ConsensusfromContig1844 Average coverage: 24.27
AGCATTTAAAAATAAATTTTATTAAAATAAATTTCAACGTTTTCA
AAATAAATATTGAAA
ACGTCAATGTTGAAATCGATATTGAAATCGATAACACGATAAAAG
ATCTTGCAGCGAGTT
GTGATATTCCTGTAAATGTAAATACGAGTTATATATTAAGGATT
TAAAGGATTTCAAGG
GATGTTATACGTTCAGGTCATATCCCGATATTTGGAAGTTAAGTA
ATGTACTACAATATC
CTTCAATATCAGAAGCAGAATCTATGTTAGAAAATATCTCACAAC
TATCATTATCTTTTT
CTTCTTTGAATATATCTTTCGATGAATCTTCTCATCCTGTAACTAC
TTCAACTCCAATAA
TAAAACGAGTCCAAGCACTCTGTGGTAGTATTAGATTGAATAATC
TAGATATTTCGACGA
TAATAACAATGTCTACAACAACGGTAACAAAGGAAAAAAAAGTA
AAACCGGATGTTGATA
TTAAAGAATGTGACATTAAAGAATGTTCGTATAGGAATAATGTTG
TGTTTAATGTTGATG
ATATAT
>ConsensusfromContig1845 Average coverage: 23.19
CCTTGCGCGAGGGCATTTCTTGTAAGACCTTGCTTCACCTTTTCAT
CATGCGACATTCTA
TTATAATGATTCCCACCATTCAATACAATCCCTTTCAGTTTTCGTT
CATATTGGACACAA
CGATTCCGTATGCTATAAATCACAGTAGTCTCTTCTTTGAACAAA

CAGCTCCACTTCGTT
GATCTCGGGATGGAAATAGTGGTTCCGCTGATAACGGGTCGCACT
GGCAACGCCCTTGCT
TTTTCAGGAAACAGCTTCTCCCAGAGAAGAAAATTTGAGGGATTC
GTCGATTGAAAAAGT
GATGCGTACGTCTCCCATGTGTCTGCAGTGGGGAACACGCGGACC
CAGCCCCCTCGCCTC
ATCCCTTCCTCCCGGGCTAATCGCACCATTTTCATTTCGTCACTTG
ACCGAGAATTCGAT
CTCCGACTTGCAGCAGATAGGGGGCGCTCGTCATCGGCGCTCCAA
GATCGCATTCCGGAC
TTGAGTTGATGGGCGGACATAGGGCGTCGAGAAGCATTTGTTCGA
GCATTCTTGGAGCTG
TTTTGCTGGTTGTGTTTGGAACGTCGAACAATGGGATCTTGACAT
ATCAGACCAGTGAGG
CTAAACACATCTGAGATCATATTTGATTTGACTTTGAGATCCAGT
GGAGTATCACAGTTT
AACGATGGCGAGAGATTGACTTCAAGAACCCACGGCCTCAGGTC
TTCGTCAATGAAAATA
TCGAAGCCGTAAACCTCGTAGCAGTTGCCTCGATGCGGGACGAAC
ATTCTGCAGGCGGTT
GAAATTGTGAGTTCAACCGAGAGAAATGTTTTGATAATCACGTCC
TCAACCCGCATCATG
AGCGCTGCAGGGTCATGTCCTTGAGACCTCAGGTATCGCAGAAG
CGCCCCCAGAGACCAT
TTATTTCCGTAGTCCTCCACGTCTGGGTCGTCATTTGAAACATAAT
CACCGCTCTTCTTG
TTAACGCTGTAATTGGTGAGATGCATACAGTTGTTCTTCATACTCC
GTATGCTCTTCTCA
TACTTGACGGTTGCGAATCTCACGATTCCTTCCTCATATATGTAG
ATTATAAGTGGGTCA
TACGACGTCACTGATACATAAAGACGAACGTCGAATTTGAAACC
ATCAACAAGAAGAGGG
TTGCTCATATATCGAGATATGATAAGATTGTCGTCGAGTGAGACT
TGATCCGGGTGGTTT
ATCAGTGAAATTCCGCGACCTCTCGACAACGCTACGGGCTTAACT
ATCCAAATCCCTTTC
TCGCGCATGAAAGTCGTGCAAAAGTCTTGAAA
>ConsensusfromContig1846 Average coverage: 10.16
AAGCGGGTCCGATACCGTCAGTGTCTGCGAGAGGGGAAAGCAGT
GGATGAACGAATCTGC
GCCCCAGATATGTCCGAAGAAGACTTACGTCGATACATCATTGA

AGAACGAGAATGCCAG
AGTTCGTGTCCTGTGGATTGCATAGTCTCTTCCTGGACACAGTGG
TCAGCTTGCAACGCA
TCCTGTGGTGTTGGTTATCAGCATCGTGAGCGCTGGACAGTATTT
TCTCCTTCAAACGGT
GGGGCCGCGTGTCCCAAGCCTCTCTCCCAACGACGCCCCTGTGCC
TTTCGAGAGTGCTTT
GAGTGGCGCCCTGCCCCTTGGTCGGACTGCGATATCGAGGAAGGG
TTTTGTGGGTTCGGT
TCAATGACGCGTTTTCTTCGTTGCTATGACGACGAAGGAAGAATG
GTTGCTGATAGTTAC
TGCGTGGCGTCATGGAGGGCGGGAATTAAGGTCGTGAAATCCTGT
CATATTCCCTGCCCT
GGTGACTGTCACGTGACAGACTGGTCAGAATGGGGACCATGCCAT
CGACTTTGCTCCAAA
AATATGGAAGATTCAAATATTGGTATGCAATCCCGGAGTCGGTTC
ATCTATCTAGACCCC
CAACCGGAGGGCGAGCCGTGCCCCACTGATCTCATTCAGACCCG
GACCTGCAACGGGGG
CCTTGTGTCACATATGCGTGGGAGAATGATAGTACTGGTAGGAAA
ATCTGCGCCAATTCT
GAAGGCATTCAAGTGTTTGGAGCTTGCCCTGGCAGTGACGAAACA
GCCCCCCAACCATGT
CCAATCGTGTGCAGCATCCCTTTTTCCTCATGTTCACCGCAACCT
TTAAACACATATCGA
ACAAATCAAACATTTCCGAGGAGCAATGGCTTTCAATGCGTTTGC
AATGATGGGTTTTTC
CCTGTTTTCAAAAACGGTTCTCTGAAGAATTGCCTGATCGGAGGT
ACATCTTTACCTAGA
ACACATGCACCTCGTCAGCACTACTACGATTATAGAGTGACAGAT
AAATCTGACACCTAT
GTCCTTATCTATATCATCATTTGCGGGACGGGAATTTTCACTCTTA
TCACGCTATTCACA
ATTATATACTTATGGCGCCGAAGTATCACAGAGCGAATCCAACA
GCAACCTGGAGCGATA
TCGCGCTCGATCGGTACAGTTGACGACATCAAAGGAAAAGTCAC
TTGCAACGAAGTTCAA
ATCCTGATGCGAACTGGTGAGGTGACAGACCCTCACACCAAGCT
GATCATGAAAGCCAAC
GGCATGGAAAACGAAGAGACCATTGCCAGATACCACCACATCAA
CCTCCTGAACCAGCAG
AGGAATAGCAGCACTTGCCAAACCATCGATCTTC

>ConsensusfromContig1847 Average coverage: 14.27
TCAAGTGCTACGAATGTAGCAGCGCGAATCCCGGTGAGGAAGAT
TGCAGTGATGGCAATA
AAGTCAAAGACAGCCACCAAGAATGGCTGCAGGATTGCTCGGAA
CGATTCGCAGGTTACG
AGTATTGCCGGATACATATATTCAGTGTTCATGGGAACGAATATA
CCGCAAGAAGTTGTG
CTAGGAGAGTAGACGAAGAAAAACTGAACACCTGCCATACAATT
AAAGAAGGAGGCTTCA
GCCCTGCTTACGTCTGCGAGTGTGATCGCCCGAGGTGCAACAATG
CACACCGCACTGTTG
CTGTTGCACTCCCACATCTCATGTTCCTCGTTCTCTTCTTGTTTGT
ATGAGACTGATTAA
GTCAACACTTCACAATTGTTGTTGCACTCCCAACTCACGTTCCTTG
CTCTCTACTTGTTT
GTGTAAGATATCAGTCAACAATGAACACCACATAGTAGCTGTTGC
AATTTCTTGCACGTT
TGTGTGAGACTAATCATTTAACAACGCACACCACACCGTCGCCAT
GCACTCCCAATCCTG
TGTTTCGTTTCCTACTTGTTTATGTGAGACTCATCTGCCAACAATG
CGGACCACACTGTT
TCTGTTTCACCCCGACATCTCCTATTCTTCGCTCTGTACTTATTTT
TGTGATACTAGTCA
TTCAAGAATTAGACAGTACCAAAATTAAAAATGTGATTC
>ConsensusfromContig1848 Average coverage: 15.72
ACAGAATATAACATTTGTGATATAAAAAGTGAAAAACTAGCAAC
GGCACACAAGTAATAA
TAGTATTTAAAGGCACTTTTCAAGGCCGAAAATAATAAAAGTTAG
CGATATTATTATCAA
CTAATAATGAATTTCACACGCATGAGTTAACGCCAGCGTAAATTT
TTTTGTCATAACATA
TCAAAAGTCAATAAGGTTTTTATATACTAATTATTTGAGATTTTG
GGATGTTTCACGTCC
TTCGATAATGACATAAAACTCAAACAAATGAAATATAAATATAT
AAATTATAAATCAGCA
AAAATTGAGGGGCCAAGATTTGAGGGGGTTTGGCCCCCCTGATAT
CACAGATAAATTTCG
GGAACATTTCATAGAGCCTGATACTGAAATGTATTCTATTGATAA
ATGTCGGAAAACGAT
GATAATGAAAAAAATACTATTCCTGCCAGGACAACACATATGTAA
AATCATGAGAATATGA
GAAAAATTACTTATATCAATTCCTTCAAAAAAATGAAATCTATCAC

AGAAAACTAGTATAC
TTATTGCACACAAATTTAGTTTGTTAGAACTAGTTTATCATATCA
CAACTAATATTATAC
CCGCTGAACACTACTTAAAAGAAGACTATAATCTTTAACTCCACC
ATCTTGTCAGTCTGA
CCATCTAGGGTTTTCTATGTTATATATATATAATTTTTTTCAACAA
TAAACATAACAATA
TTTGTAAACTATGGTTGAAATCATA
>ConsensusfromContig1849 Average coverage: 11.23
TCTTTCGTGTAAATTTATTTTTTATATTAACATCAATTTCTTTTGTT
TCTTTTTGGCCAC
TGTTAACATATTTTGCTTCAGTTATAATTGTTCCGGTACGTGCACC
TGCTTGACATATAC
TCACTTCATTGATAATTTCTGCTTTATTTTTATCGTGTGTGAGAC
TGAGACCGGGGAAAT
TTGACAATAAGATATCTTTTACTGTTAATGGTTGTTTATCGTCGGT
TTTATCCGTTTCAT
AAAATAGATCATGGGAATAATAATTTTTAGATGAATATGGATGAT
CTGTAACGGTATCTC
GGAAAGCGCTTAAAAAATCTACATTATCGATAATTCCATCCATTT
CTAACATTTTAATGG
GCTTGCCTTCAAGATTTCGATTATTGGTTATGAAGAATTTCACGA
CATGTCCGATTGGTG
GTATAGTTTCGTTTTCTATCTTGTCAATGTGTTGCAATTTAACATC
ACAATTTGATATTT
TTGTTATTTCAAGCGCTATGTGATAAGGTAATTTTAAAAATTGTTT
ATTTTCATTAATTT
TTAATTCTGTACAGGACTCGTCGCTGTCGATCTCTGCTGTGATTC
CTGAAAAGACTATTT
TTCCACTCATTTTTAAAGAATAATGAATAAGGAAAGAGAAGAAG
TGGACGAAAAAGAAGA
ATGTGTATCACTATTGTTGGCAAATATGAAACAAGTATTAACTTC
TAAATTATGTAGTGA
ATTATTGAATGATGATCATAGCTTAATACGG
>ConsensusfromContig1850 Average coverage: 4.43
GTCATGATTAAATTGAAAACATCGATTGAATGAGGTCACTATCTC
ACTATCTAAGCCTGA
AAATTCAAAATTCGATGAATTTAGGTCGTAATCGAGAGAAAAAT
ATCGATGAAACTAGGT
CATAATAGAAATGGAAACATCGATTGAATTCGATTCGAACTGGA
TCAGAAATGAAATTAG
GTCATGATTAAATTGAAAACATCGATTGAATGAGGTCACTATCTC

ACTATCTAAGCCTGA
AAATTCAAAATTCGATGAATTTAGGTCGTAATCGAGAGAAAAAT
ATCGATGAAACTAGAA
CATAATAGAAATAGAAACATCGATTGAATACGATTCGAACTGAA
TCAG
>ConsensusfromContig1851 Average coverage: 13.51
GCTAGTTTCGGGGTTACATCAAGTTGATGTTGAAATTCAATATCG
AAATGTTGTGGCTTT
TATCCTTCAGTTTGGCAGATTGGATAAATGCGGTTTACATTGATC
TACCAATGAATAGCC
TCGACGGGAAACAGGTGCACGGGGAACTCGTTGGTACCATTTCAA
ATTACCCCTTCGGAG
TCATGAACGGGTATTGCCCATCTGACATCGCCATCCACCACCACG
ATTCCAATCTCGACC
TTGGCCACGCAGAAGACATGAGGGAAGAGTTTGATTTGACAGAA
GGCCAAGGCCAAGGAC
ACAGCGTATCGATGTGGTTGTTCCCTATGAGCATTTATAAAAAGC
AGGTATTGATTGGAA
ACGCAGGGAATTTTGGCACGACCGATGATACAAGAGGCAATGGT
TTCGAGCTCGGCATCC
AATATACATATGGAGATATCCGCGGTCATCGAATTTTCATCTCGT
ATTACCAGAATGGAA
AGAATATACGCACCATTGATCCAGCATCATACGTTCAGCTGACCC
TGGGAGCATGGAATC
ATATTTTAGTAACGTACAAAGTGATTGATACCTTCATCAGATGGA
AACTGTTTGTAGATG
GAACCTTGAGTTTCATCAGAAACGAGGATATAAGTGGCATAACAC
CGTCCTATTATAAGA
GAATAGAACCCCTCTCAATCGGCAAAATAGGTTATACAGCCTCTC
CAATAACTAGAGTTA
AAATATATGCATACGACGTATCGACCGACCATGACAGTATATAC
AATGCTGAGAAAACGT
GTCCAGTTGATTATGATTACGGTGATTATGATGCGAATGCCATAT
TGAATAAAGGATATC
TGGATTTGCCGATGACGCATTTACGTGGATCAGACATTTTGGGCG
ACATTGAAAGGAAAT
TCACAATTGTTCCACGTGGCACTCACAAGCACGATTCGCCTTCAG
TGATATATGCGTACG
GTGGATATTGCCCATCGGATTTGGCTTTCTATCAAGAGAAGGGTA
ATGTCAACTATGGAA
TGCTCGA
>ConsensusfromContig1852 Average coverage: 18.84

CAAATAAATAAAACTATAAGTATACTCTCGAATTGAATTTCTGCA
TTATATTAAAATTGT
AACAATGTCATCAATTAAAACTGTAGGTTTTGCTGGTTCCATGGT
TACGAAGTATGATGA
CTAAATCTGAATTGCATTCGTTCCAATAACATACATTACGCAATT
TAATATACACCACAT
AAAGAACTAAACAGGTGCATATAATCGACGAGCATCAAGAATTA
TTACATAAAAATCATG
ACATTTAAAAACAGGCATCTCATTCATACTCATTGGATAAATGCG
ATGTATACGATCAAA
GGCATGAAACAAAAGAATTGGAGTGGTTGATTTATTTGTTAAATT
AGTACAAACATTTAT
GAATATCTCATGGCCTTACTCAGCAAAAAATGGTTATTTGACAGG
CAATAAAAAAGTTTT
AAAATATCACAAACTTAAGGATTTAATTCCACTAATATGCAAACA
ATTTCTAATATTTAT
CATCTTAAAATCAACAAAAAATCAGCAAGAGTTGTACATCTGAG
CAATAACGGGATTATT
CACGTAATACTCTGGTTGAAGCCATTGGTATATATCTTTCGGGGA
TTATAAGTATATTAC
ATAGCCCGTTTGAAAACCCACAGTAAATGGTGTAAAATATAATAA
GACTTTTAATTCGCA
GTAATAATGTATATTCAATCATGACACGAAAATAAATGAAATTGT
ATGTACATAATGATG
CGAATATATCCGCTACGAAGCAGTCAGAAATAATTTTTCTGAGGT
TTAATAATGGTGAAG
ACCGTGAGCTGTCAATTTTGGTCATGGTAGCATAGTGTAATTGAT
CGCGAACAAAAGCAA
TCACCAGTATGGGCAGTATAGCACCAGTAGCCAAAATTCATCAAA
TATCCAATATCCACA
TGCGATAGACTTTATAATATAATCAATTATGAGGATGCTACGAGC
ATTTTATATCACATA
AAGTAATTTAGCATTGAATTACTAAGACATGATAAACATAACAGC
TTTGTTTTGCAATAT
CTAATTTT
>ConsensusfromContig1853 Average coverage: 7.76
ATTCCTTTCAAGATTTGTTTGATTCCATTAAAACACAAAACAATA
TTGACCATGAAAACA
TTGAATTCAAACTGATCAAAACATTCCGATCCGAGAGAGGGTCA
GACCACATAATCAGCT
GTGACCCATTTACCTTCGCGTGCTTGATAAAATCCCAGAAAGTAT
TCGTGGGATATCAAT

CTTATCCGTGTTATGAAAATCTACATATTCCGATCTGTGGAAAAT
GCTCCAAATTTCATT
TCACTCCATCATGTAAATCATCTACCAAATGTTGTGGCCAATGTT
CATCATTGGAACATA
TCAGTTCTGAATGTCCAAATGTCTCAAACCGTTTTACATGCCCTA
ATTGCTCTGGGCATC
ACAAGGCATCCGATAGGAACTGTGAATCTTTCAAGAAACAAATC
AAAATTATGAAAGATC
GTATCAATTATGGCTGAAATCTTT
>ConsensusfromContig1854 Average coverage: 12.81
CACGGTCCATATTATCTACCTGCTTCTCTGTTTGTATTCCTCCGAA
CCGTGCTTTTTGAC
GTATATCAATTCTTTCCTTGACTGTTTGACGATTTATCTTATCATC
AAATTGTTGTTTTT
CCAATTGTTTGATCCTATGTTGAATTTGTGTGCAAGACTCCTCAA
GTCTTTTGCGGAACA
TATCTGTATATTTTTCGTTCAACTCATTCAGTTTCGTGTCAGAATC
ATCAATTTGACTCA
AAGCAGCCCTGGTACCAGAATCGTTATCGTTATCAAGGGCGTGAA
GTAATGTATCCGTTT
GTCCGATAATATAATTGAGCTGTGCTTCAGCCATCTCTACCTGAA
ACTTAGACGGCATGA
CGTCCTTGGCCAACATCGCTAGGATTCTCTTCCGCTCCCTCCTTAA
TAGTTCCAGTTCAT
CAGAAAAAGCATCGTCTAAGTCCGTCTCTGTTGTGGAGTCCTTGG
TTTGAATCAAAGAGC
CCTGGGATGTCTGAGATGGCGAGAAAAGATACGGGAGACTGACA
GAATTCACAGATGTGT
TGACATCAATCGATATACAATTTACTGGGTCGGTCACACTGTTTTC
TAGCACTGTATTTT
GTATCTTCACATTAATTTCATGCAGAGTGTCATCCG
>ConsensusfromContig1855 Average coverage: 17.15
GATAGCCTTTTAGATCTGATTGGAGTTTCCTCAGTGTGTCTTTGT
ACAGAATCCCATGAC
CGACGGCCGTGATGAGAATGTTCATATGATAGAGTCCTCTCACCG
GGAGTAGCTCCATCT
GTTGATTCGGACGGAGATGGTGTTCTATTTGCTTCCTTTTTAATA
TAGGATATTCCAGAA
TCGCGGACAGTTGGAGAGCGGTTCCAGTCTCTCATCGGAAAAATT
GAAGACGCGCTTTTA
GATTCGGAGCTAATTTTGCCATATGCTGAGGAAGTCGGGGGCGA
GGAATGATATAGTTGC

AATGGTGATATTTTGGTTGGTGATGATGGACTCTCTTTATGATGA
GCCAGTACGGGTGAT
TCTCTCCGCTTTCGATGAGAATTCGGAGAGAGCCTTGGCGGATCA
AATCTCGTAGGAGTT
GACGGTATAGAAGACGATCCGCTGCCGTAATATTGTCTGTGAAAG
GCTTTCGTTTGATGA
ACATTTTGTGATAACAAATGAGGTAAATGACCAAGAGCGGTAGC
CGCACTCGTAGCGGTG
CTAAGAAAGTGCGGCTGAGGTAGATGGATTCTTGGTGTGGAAGG
ATGGCGATGCAAAGCA
GCCGTCGGTGATTCTGACTGATGATGATGTTGCTGTAGCTGGCTT
CCCATTCTCGCCAAA
ACAGCTGAGTATGAATGCTGCTCCGCCACTGCTGCTGCTGCTTCC
AAACTCAGCTGTGCG
CTTCCAGCATATGATCTACTACCTGGACTCACACCACGATGAGCT
GCGACCGATAGCTGA
TGATATAAAGCGGAGTACGGAGACAGAGCTGCGACGGCACCGAA
ATACGGCGAGCTGGGA
GAATATACAGACGAGACAGGCTGTCCAGATGCTGACGACAACTG
CGGATATAAGCTTGAT
CCAGACTCTAGCGCCACGGGTGTATAAAATCTTTGAAGGGATTC
TGTAAGCAGGAGCGGT
TGTCGAAAGTAGCCGGGCGAAATCGGAATCTGGCCGGTGTCGGG
TCGACCGGATGTGCCA
GATCGGAAGA
>ConsensusfromContig1856 Average coverage: 8.86
GATCTATTGCTCTTATTCTATTTGAGTCTAAAAGCAGACGTGTTA
TGCTGCTCGGAAGAC
CTTCTGGAATCTTCTGCAGAAGATTATTGTTAAGCCATAACTGAT
CTAGGTTTTTGGCAT
TGAGAAACGTCTGTGCAGAAATGTCCCGGATTGAGTTATTGTCAA
GGATTATGATGCGCA
GATTTCTCATGTTTGCTGCAAGATCGTTTTTTCGGATTTCAGAAAT
GTTGTTTCTCCTCA
GCTGAAGATATCCAATCGTCGGGTGGATATTTCGCGGAAGTTTG
TCAGTTTGCATTCGG
AGATATCGATGTAGTTGAAGTGACTGGACGAGAAAACGATATCA
TCAAAGTCTGTAATAG
AATTTCCTTTGAGGACTAGATTTGTCAGTGAAGTCAATTGCGAGA
AGGCAGCTGTAGAAA
TCTCTGATATTTGATTGTGGCTCAAATCCAGGATCTTGAGTGAAA
TAAGACCGCTGAAAG

TCGAGTTGGTGAGCTTCTTGATGAAGTTCCTCTGCAGGTTGAGTC
TTGTAAGTGCTGCTG
AGTTCTGAAAGAGGTTATCAGAGATTTCTCCCAGAATGTTGCCAG
CTAAGTTGAGCTCTG
AAAGCATTTCGCAGTTATGAAAAGTCTGTGCTGGTATTTCCGATA
TCCTGTTGTCGCTCA
GATCGAGGTTTTGCAGGCGGAAAAGTGGGTCAAAGGCTGTTGCTT
CAAAGGTGCTGATCT
GATTTCTGCTCAGATCAAGATCAGTTATGTATTTTGTTTTCGTGA
AAGTGTTCGAATCCA
GTCTCGTCAGTTTTGACCCTGAGAGGTCGAGTAAAGTCAC
>ConsensusfromContig1857 Average coverage: 5.58
CAATAATATTCAATATGAAGAAAGAATAATTTCGAAATGAGCGA
AGCTGTCAAAATGTCA
GCTTTCAGGCTGTTGACAGAATATAATATATTTCTTGGTCACGGT
TTAAAGTACATTGAG
AACTACTTGGATCCACCAAGTAGTGCAATAGATCTAGAAAATAGT
GACATTTCTAATAAT
GATCTTCAGCATTCTTTCCTTTCGTTTTCTTCATCAAGATCTTCTT
CCACTGGAGAAATT
GTTATGAACTCGGTTAATACAGATTCTCATGAATACAACACACGA
AAAAATTTACTATCA
AATTGCTCACATAATTCTTTGGCATTTTCTTGTTATCTGATTTATC
GTGCTGGTGCATAT
TTCAATTACTTGAAAGAGAGGGGTAATGTATATTCAGATGAAAT
GAAAAAAATCAATGAA
TTTGATTCAAGTTACTCTACTATTATCACTGAATTGACTGATTTC
AAAAAGTGTGGATAT
CTCATTAATGATAGACTAGAAGATATGTTTGTTGAAGAATCCTTT
ACATCGAAGATAAGT
TTATTAGTGTCTTTATCATTATTGAAATGCCAGTCTCTTGCTAATC
CACAGGCATGTCAA
TTTATTGCATTATCATTGAATAAGTGTTTGGAAGATTATCCAATA
TTTGGAATTGCATAT
GAACCAGAAGATTGTTTAAATGGCATTGAGGATTTGTTATGTGAT
TGGATTCTGAGTAAT
ACACTAACTAAGAATATAGCAGAAGATGTTATGTCTTCCCTAGTT
ACCTTTGTATGCATT
AGAGGTTCGCTGAGAAGTCTCGTTAGAGTAATAAACGTTTTGATC
ACATTA
>ConsensusfromContig1858 Average coverage: 7.86
TGGTGAGTGAATTATCTGTATCCCATGTCTCGATCTTGATTTTATA

TTCCCTAACTTCGA
AGTCGGGAATTTTGAATTTCGTGATTTGAAAATTTGATGAGACGC
ACTGCACTTCATCGT
TGATTATCTGGAATCCTTGTATGTCCGTGACATTTGCTGGGATGGT
TTTCTGTAGCCATT
TGAAAGTAACGATCAATTTCCTCGCATCCCTCATCCTCTCATAAA
TGATGAACGGTTTCT
TCTTTGTAACAGCGAAGAATCTTGTTTTGTAAGAAAGAGTCAGGC
AATCAGCTGGGAAAC
CTTCTTTGCCATCGATTTCAAAGGCTTTCACAGTATCACCGTCGG
CGGCGAAGCCGGATG
GCTTGGAGATCTCATGATAAGTCCAGCTCAGGTTCTTCGTGTCAA
AAAGAGAGTAACTCT
TCCCCGTGTCGTAGATCAGCGCAATAATTCCGTTCGTCAATGTGA
TGAGATCAGTTACGT
CCTTTGCAGAATTTGGAACCAAGGAAAATCTCTTCTTATTATCTTC
ATTCCGGATGTCTT
TGAACTCGAGAGATCGGAAG
>ConsensusfromContig1859 Average coverage: 13.73
TCACGAAATATCACATGATTAAGATGTTTTAGGATATGGTCGATG
GTGTGAGGGGACAGT
AGAAACAGACAAAAATGAATATAATTGTGCATTTATCATGAAAGT
TAACCGGCGGGTTTT
GAATTATTTTTTTAGGAGATCGACAATATTAAATATTGACGAATT
GTGAAAATTTGAACG
TCAATTTTGTCGAATTTTCAACATACCAAGATCCCAAACTGCTGT
CATAAAAGGCAGTCA
AGAGGTCCCAGATAACCTCATGATTAATTTTCTACCAGTTCAAAG
GATTGAATTCGTGTA
TATCGTGATATAATCTTTCACTTGATCCAGATGATCAGGTGAAAG
AAGAATTTTCAGTAA
ACCTTCGATGCTTGTGAAGATCTTCCCAGCGTCACCTTCCAACGG
GAGATGCGCATCAAC
TTTGGCCCCGATCAGCTTAGGGACAACCCAGTGACTTCGATCAAT
TCGATCCTTATATGG
ATATCGCTCTTGAAATACTACCGCGTCATTCCCTGATCTTTTAAT
CCAATCGGATGCCCA
CATAGAGACGATTTTCTGGTCGATGTACCAATTTTCCGATCCCTT
TGAAACGCTGTGTTG
TACAGATTCTTGTCCGAAGAGTCCGGAAATAAAT
>ConsensusfromContig1860 Average coverage: 16.61
CTTCATATTATCAATCATCAAGAGAGATTTCAGCTCCGTGAAAAC

TCGCACTCCGATATT
TCACCTCCCACGCATTCCCTTGCATTTCCTTGATGCGCCATTCGCT
CTCTGTTAGCCAGT
CTTTTATGGGATTGTAATTGGAATTATACCACTTCATGTTCGATC
TGATGAGAGCTAGGG
AGTTATGGGCTGCTTTGCTCGCGACGCCAGCTGGTGGGTTTTCTC
TATACAGCATTTCAA
GCTGCTCGTATTCGTATAAGTTATTTATAAGCGATGTTGTTGCGA
TGATGATGTCACGTA
AAAGAAAAGCATCAGTTCCAAATCTGCGGTTGATCTCTTTCCATT
TCGTAAACAAGAATT
TCAGTCCGACAGTCCTGCTGAGAGGAGTCTCTGCGAAACTCCTGA
AGACCGACCCCACGT
CCTGCATCCGGACCTTGGTGGAATCCAGGGACCACATTGCGTACC
GGTAAAGTAACCACT
GTTGCTGTGAGTAAGCCAGTGCTTGTCGCATTGTTGCAGCTTCGG
ATGCAACTTTTGTGG
TCATGGATTTGTTCCAGAGGAATTCCCAATCTTGTGCGGATCCCA
GTTTAATTCCAGTGG
AGAAAATGATGGAAGAAAAATCCGGTCGAAGTTGGTATCTTCTTT
CTTTCCAGTTCTTGA
ACTCGGTCTGTGCAGACGTGACGGCCTCTTCAACATTCATTGTCA
CCGCCATTTGGGCGA
TTAGTTGAACGAAATATCTTTTGAGTAGC
>ConsensusfromContig1861 Average coverage: 11.68
GAACGAAGTGAATAAGTTGGTATTGAACAAATCAAAGGAGTTGG
CTGGATCTACAAAAAG
TGTTACAATAGACAAAAATGTTGAATTGAATGAATATGACAACA
TCAAGAACAAAGGTGA
GGACATAGGTAATGTGAAGCACGATATGCTGGGTGTCCAGGACA
TTAGAAATATCAAAAA
TGTTTCGGATTTTTCGGAGGCTGGCGCAGAAGAGACGTTCAACAA
TATAGAAAAATTACA
AAAAGATTCGATGCAAGATCGAGTTTCATCGCCGAATGCTGAGAG
CAACTTGCCCAACTC
TGAGAAACACAAGTTGGTTCGTAGAAACACTACGTCTTTCACTGA
AAAATCGAAAAATTT
AGAAAATAAGGAAGATTCACAAGGAAAAACATCGACGCAAACC
GAAGCTGACACGAGAAA
TAGTAGAATATTTCGGAGGAAAAGTACGTCAGAACTGCTCACTA
GAAAAATCACATCATT
GGAAAATGATGGTGACGTGGATAATTTTGAAAAAAAAGATCTGA

TAAGAAGGATGACAGC
ATCATTCAAAAATGATGATTATGATGTCATAAACTCAGAGAATA
AGGGATTAACAAGTAA
GAAAACAACATTTTTTCAAAACGATGATGTCGAGAAACCAGAGA
ATGATGGATTGTCCGA
AAAGAAAACAGCGTCATTGAAAATTGATGACGACTTTGGAATTT
TGAAAAATGAGGAATT
TTTACGAAAACAAACAACAAAGTTTGAAAATACTGCTGTGGAGG
AGAATTTTGAGAATAA
AAAATTAACAAAGAAGAAAACAAAATTTTTGGAAAATACTG
>ConsensusfromContig1862 Average coverage: 14.89
CACAGATACTGGATGCCATCAATGTAGGGATTTCCATATACAAAT
CAATGATTGCCGATG
TTAATCTATAAGGAAAGTAATCTATGAGAAATCCGGTCATGAACA
TTGCCACAAGTGGCT
CTACCAACGTTTTTATTTTATCTATTTCGTGTTTAACTTTCCTCAC
AGAAACATCGGGTG
AATCTTCGGGAGAGCATGGCAGGCATTTTGTTATGAGCGCAACGT
GGTTGGACAGTTTGA
TTCGCTCAGTAGATTTTCTCATATTGAGTGGGAACATAAACGACA
CTCCATCGCAAGGTA
AATTCACCTTTGCACTCTCAACTGATATGGCTTTTGCGATAGCGG
TCAACATGATGTCGT
TTATTGTTGCGTTCATCATTTTTGATTTGAGTTTAACGTCTTTGAA
TAAAACCTTGTCTG
TAGCCACATGCAGTCTCTTCCCGTTGGGTTTTGGTCCATGAAATGC
ATT
>ConsensusfromContig1863 Average coverage: 11.28
TGTGCTCTTCCGATCTCTGATATATAACATCGGAGTGGGTTCAAT
TTGGATCAGGAATAT
ACTTTGCAACTTCTACAAGATGTCCACTGGCGAGGAAGAAAATTA
CCTGATGCAATTTCA
GAAACAATTGACGAACAGAAAAATCAAGCTTACGGCGACGCAAT
TCACTGATGTCTGGAA
CCATTACGACAAAGATGGAAACGGTTTTCTGGAAGGGACTGAGCT
CGATGGATTTCTACA
TCAATTTATTCAGACAAGTAGTGGCGGTGGACAGACAATCACGA
GCACGGAATACGCAGA
GATGAAAGTTATGTTTCTCGAGGCTTTCGACGCGAATGGAGACA
AGCGGATCGATATACG
AGAGATGGCGCAGGTTCTTCCTACGGACGAGAATTTTCTACTGCT
ATTTCGGAGAGAGAA

TCCGATAGAAAGTAGCGTTGAATTCATGCGGATATGGAGGAATTA
TGACTCTGACCATAG
CGGTTTCATCGAAGGTGATGAATTAAAGGCCTTTCTTCATGATCT
GCTAAGACTTGCCAA
CAGCACAATCCAGCTTTCAGAAGACACCTTGATCACTTACACAGA
TGCAATCTTGAGAAT
ATTCGATCGAAACAGCGATGGGAAGCTTCAATTGAGTGAAATGT
CAAAACTATTGCCGGC
CAAAGAAAATTTATTATGTCGGCCGATGTTTCAGGGATCACGGA
TAATTACAAGACGCGA
TGTCAATAGAGTTTTCGCTCTCTACGACAAAGATAAAAATGGGTT
TGTTGAAGATGAAGA
GCTGCTGGGTTTCATGAAAGACCTCATCGAACTTAATCAACTGGA
CTACGATACGGATGA
TCTGATCGCTTTCCGAAGGATCATTTTGGACCACTGCCACAACAA
AGACGGCAGGATCAG
CCGCTCGGAGCTCACCACAATCCTCATGACGCTGGCCTCACAGAG
TGGCAGCAGTTTATT
CGACTTCACTCAATTCTCCCCGAATGCGTTCAATTAAATTCACAT
TTGTTGAGCTTTGAA
AATGTCTTGTTATTATCTCGAATTGAAACATTTTTGTTGGTCCTTC
AAGTTTTTGTTTTA
GCGTCATGTGCGGCCTTTGTTTTTCATCTTGTGTTCTG
>ConsensusfromContig1864 Average coverage: 8.64
GCTCTTCCGATCTTTCTTGCCCACCCGGTCATCATTGCAACAACAC
AGCTGTTTCTCCAA
TGATTTGTCAGGCCGGCTTCTTTAGTATTCAAGGCTCCCATCAAT
GTTTCAATTGCCCAG
CAGGATATTACAGCTCCAATTCGGGGTCGTCGGAATGCACTCCAT
GTCAATCCGGGTACC
GCTGTACGGTTTCCATGCAAATCGAGTGCGGGATTGGTTACTATA
GTGATGCGGCTGCAG
CGTCCTGTACTCCTTGTCCGATGGGTCACAGCTGCCCTGGGACCA
CAGAGAGTTCCCCTA
CGCCTTGTGGACCAGGCGACTATCAGAATCTGATAGGTCAAGGGT
CCTGCTCGCAGTGTC
CGAAAGGTCACGCATGCCCCTTCAATGCGACCCAAAGTGGGATC
CAATGTTCGAATGGAA
CTTATCAATCATCGGTCGGACAGGAACAATGTGATATTTGCCCCG
CAGGTCACGCCTGTC
CGTCTGTTACCGCTGTTGCCGTGCAATGTAGTGCCGGAACTTTCA
GCGCGGATGGGCAAA

TGGTTTGCACGCCATGCGACCCCGGAACCTACACCAATGAGACAA
TGTCAACTTCATGCA
CCATCTGTCCTGCGGGAAGTGCATGTCCTGATTCTACGCAAGCAC
CAAATTTATGCAGCG
TAGGACAAACAAGTGGAGAAGGTCAGCAAGTTTGCGTTAATTGT
GAACCAGGTCAATACA
AAAACACAACTGGAGCTGGCTCTTGCCTATCATGCCCACTAGGCC
ATCAATGTCCAACGA
CAGACAATTCTGAGGTTTGCGGTGCTGGATATTACTCACTGGGCA
ATGTTGTCTCCTGCA
TTCCTTGTCCGGGTGGACATAGCTGCGTGAACCAGACAGCAGAAC
CCGTGGTCTGTGTTA
CAGGAACATTCAGCCCGAATGGGACCGCTGGATGCCTTGATTGTC
CCCCGGGTCATTATT
GCCCCTCCGATGTCCTCTCAGAAGCTATTCCTTGCGCTATAGGAT
CATTCCAGCCAAATG
CGTCGCAGGAAACTTGTGCAACTTGCC
>ConsensusfromContig1865 Average coverage: 7.68
GAAACAGAGATGTCAGTAAAAGCAATAAAGATAGATGAAGACAA
AGAGTTGGCACCGGTT
AGGTTAGAAAGAAGAGAAGGGAATATAAAAGAAATAATGTCAGA
CCATAATACGATCATG
ATAGAAATGGACTGGTCTTGGGATAGAGGAGTGAAGGAGTTAGA
AGAAAAAATCATGACA
AATGAGGCATGGAATCGATTTGAGAGAATGATGAAAGGTGAGAG
GTTCAGAATAGAGGAC
AGAGAAAGTATGGATATAAATGACAACCGAATGGGGGAACTAGA
TAGTTATTATGAAAAA
TGGAGCATGGATATTGACAAGATTAGAAAAAATTGTGAAAAAAA
GGTGAAAAGAAAACAT
AGAAGCAAGAAATTGAGAGAACTTTACAGAATAAAAAGAAAAA
TAAATAGGCAGATGAGA
GAGCAGAAGAAGAAAGGGTCAGACATATTATTTAATCAATTAAG
AGAGAGAAAAATAACA
ATGAATACACATATAATGAGAGAAG
>ConsensusfromContig1866 Average coverage: 9.38
TCTTCCGATCTCTGGACCCGATAAACCTGATGAGCAGCTCAATAT
CATATTTCAATTGAT
AAATAAAGGATAGACGTAAATGCTCAAGATAGAGATGGTAACA
CAGCTCTACATATCGC
TTGTGAGAACAATTGCGATCCGTCTGTGATTCGGCTGCTGGTTAA
GGTCGGATGTGATCC

TCTTCTGTTGAATAACAATGATCAAGACGCCCTTGACCTCTCTGA
AAACCAAACCACTCG
AGATATCATCGAACATTTCGATTTGGGGCTCTGGCGTGCTATAAG
CGAGAAAAACTCAGC
GAAAGTTAATTTGCTTTTGAAGTATTGGTGCAGAATTTCATATCG
GCGAGACGGCGTGAG
CCTAGTCAAACACATGAACGACGAGTATCCCAAAGGAATGCCTTT
CATTAGTAAATATAT
ATGGTTCAATAATGTGGCATACTCGGCAATGTCAGGGGACACTGA
AAAGCTCATCGAGTT
ACTGAAGAACGTTAACGATCATACGGATGAAATAAATGTGTGTGA
TCCTAAGTTTGTTTA
CATTGGCAAAGAAATTATTACCAATATGCCACTTCTGGGTGAGAC
ACTTTTATATAAACA
ATACAAAACTAGTAAATTAATTCTCA
>ConsensusfromContig1867 Average coverage: 17.06
CTCGAATGATGGCTGGACGACGGAATGTGGGGTGGATAGTTTGCT
TGGTTATGAAGACCC
ACCAGTGACTAACGAAAATCACACTCTTATAAGATTCACTGGTGA
TTATTTTGATGAGTA
CGAATATTATGAAAAATCCGAACAAGATATGGCTACTGTGATTAT
CCTCAGCCCACAACT
CTTCGAAAATTGCCCCACAAATCATCTGGATTTTCTAGAATTTAC
AAAACTATCAATCAG
GTATGTACAATCGAAAACTTTTGAAAGATTTCCTTGTTTGAAGAA
GCTAGGATTCCATAA
CCTGATGCTTGATATTGAGTTCCTTCCGCATGCTTTCAGTGGGTCT
TGTGCAGAATTAAT
TTCTTTTTTGAACTTCACGACTGACATTAGAGACCCATTAGTGTC
TTTTCACGAAAATGC
ATTTTCTGGTGTGATAAACGAACCAATACATCGGGTTTGGTTCGT
CAATGTTACTGTGAA
TCTGACAGACCTGAAGGTTTTCGAACCGCTAATGTCTCAAAAAGC
AAAACAGATTTGGTT
TGCTTTTGCTGGAACGAAATTGACGAAATTGGACCCATCCCCTCT
AGATGAGTGGGAATC
GAATCCTGAAACCTCTCATGAAATCTGTGACGTCAACTTGTGGTC
TGAAGATCCGGCTCC
GTGGTTTTGTGGCTGTGAGAATAAAAAATTGATCGAGTACATCA
AAATGATGCATGAACG
TTACCGCCTGAATAGAGCTGATTCTCGCGATTGGGTATGTAAAAG
TCCATCGGAGTTCGA

GGGTATCAAAT
>ConsensusfromContig1868 Average coverage: 28.47
CAGCCAATCATACGATGGTGCGGCAGTTATGAGTGGCCAAGTTA
ATGGCGTACGTACAAT
TGTACGCAGATCTTTTCCTCATGCTGCGTTTCTTCACTGCTTCGCC
CATAAATTGAATCT
TGTTTTGATGGATGGAAAGAATGTGGACGGATTAGATATTTTTTT
CTGTGCTGTTCGCAG
TTTTCATACCTTTTTTCACTCATCAACAAAGCGAACTTCGTTTCTA
CGTAGTTTTAACTG
CGGAAATGAAATGCAAGGAGGATCTGATACTAGGTGGCAGTATT
TGGAACGGGCAGTCAG
CTCTATAATGAATAATATGAATAATCTGTTTCAGTGTTTAAATAC
CATCGCAACGGAATG
GGTTCATGATGAAGCGAGCTTCAACGGGGCCAATGGACTGTTGAC
TCACTTGAAGAATTT
TCGTTTCATTTTTTTGCTTTGTGTATATTCAGATATTTTCCCTAAAA
CTGGTCTCCTCTT
TCAAAAATTGCAGAGCCCCGAAATAGATGCATTGACGGCGATAG
ATCGGAAGAGCACACG
TCTGAACTCCA
>ConsensusfromContig1869 Average coverage: 14.71
TTGAGTACATGTCATGAATGAGGTCAGAGGAGAAGAGAGACAGA
TGGAAGAAGCGGAATC
AAGTGACGAAACACTTGGGGAAGCCTTTTCTGGTCATTTGTTGCA
TCAAGCTGTAATTCA
CAATAACACAGAGTTGTTAACCTCACTTCTTGAGGGTGAGGAGAG
AGCTAATATCGACAT
ACCTGATCCCTTCGGTAGAACACCACTCTACAGAGCTGTAGACAG
TAATAGTGCTGCGTG
CTGTCAAATACTGCTAGATACTAAAGCTGATCCTAATATTAGTTG
TGGGCCATACTATGG
AGGAAATACTCCCTTGCACGCTGCGATCAAAGATGATCGGCCGGA
TATGATCCAGATGCT
CTTGGAGTATGGAGCTGATCTTTTCCTCAAAAACGTGGTCGGAGA
TACACCGCTAAAGAT
TGCGCGCTCCTCCAGGAATGTCATCAGCTCGGAATCTGTGGATCT
CATTCTGCTCGCAGA
AGAGCGATGTGGCCCGGAGAGACGCTTGCATTGCAAAAAATTTT
ATGAGTTGTGTGAGGC
TGGGGATGTGGATGAAGCGAAAGCAATCGCTGCCTACATTTCTC
GACGCAAGCTGAGAAT

GGTCGTCAATAATCCCAAGGCCCACCACGTGTCTCTCCTCCATTT
AGCATGTGAATTGGA
CAACGCCCCTCTTTTGGACTACGTCATATCTCTTTCTGCCACGAT
GCAAGTTCACCCGAT
GTCCGGTCACTCGCCGCTCCACGCTGCGTGTGATCGCGGCAGTCT
TGAATGCGTCGATCT
CCTTTTAAAGAATTTCCCGGAGCTCGTCCAATCACCGATGATCGA
TGGGACCACGCCTCT
TCACACGGCTGCAGGTTTGGGTCATGTTCAAATCGTCCGGAGAAT
TCTGAGCTGGCCTTA
TCGGCACTGCATGACTCAT
>ConsensusfromContig1870 Average coverage: 7.19
TGATTCTATGAACACCTTCAGATGTGCAAATTACAGTTTCGCCTC
GATATTCTGGGCATG
CTTTTGGAGTTTCAAGCTTATCGTCATCAATCATACACAATTTTTC
TGAACCAGCAGTTT
TATCAAGGTATTTATCAGCATAACAATTGATTGTTTTATTATCCC
TACATGAACAAGACA
TTGTATTTATATCTGAGCAAAAATCGTTGATTAGAAGAGTATCAT
TCAAACTCATTCTAC
AATTATTTAGAGATATCGTTGAATGGTTCGTCGCGTGTACATTCG
ATTGTTTGAATTCCC
TATCTCTCCAAATGTTACTCATGTCTGAGGCAAAAAGCATCGAAA
AATAGATGGCTCCAC
CGAAGAACATCTGTAGGGCGAACATGATTTTGTGGTTCTGAAAGT
AATCCGCCACCATCC
CAGATATGGGACGGATGACAAGTGTGACGAAGGTACTTGTTGCTG
CGATTTGAGATATTT
TCGATGTCGATAAACCTCTTTGTTTCAGAAATAATGCAACATATC
GCAGACCGGTACAAA
TTGCTCCGTACAAAAAAAAGTATGCGAACTTTGAGGGCAGCAACT
TTCTATTGATCTTCA
TCTTCAACAAATAGGATTTGTAGAACAAGTCAACAATTTAACTGA
ACTTCAGTTTTTAAT
ATAATGTTTTTGTTTATATTAGACACAAAAATTTGGAGCAATGTT
GTGGCAAAC
>ConsensusfromContig1871 Average coverage: 15.87
AAACATGTCCACACACGTACCACCATACAAACAAGGACTGGATTT
ACAATCATTGTCATC
CAGTATGTTGCTGTTGGCTCCACTGTTTTTGACACGACTCCAACG
AACGATATATCCAAT
TGTGTTATCTCCGCCTTTTAGAAAGTGAGAACATTGTCGTTGACA

TTTGGTCCACATGGC
AAAAATGCATTCTTTATTCATATTACATTCCTCACTGCAGTCTTTC
ATTATCTCAGCATT
TGACATTGTTATCGTGCCATGTACTGGTTTTGAGTGTGTATATTT
GAAAAAACCATGAAC
AGTACCATCAAACTTCACCAATTCGCACAAATTTTTCGCATATTC
ATACAGGATGACTGA
AAACTGGTTGAGAGAAAGAGCTATGCACGAGGAAATATCGGTCA
CTGTAACATACTTCCT
TGGAACACTCACGGTACAAGATTTTCCAGATTTCGGACAGCGGCA
AGTGTTGAACAAGTC
GAAATTTCGGCATTCGTCCGTCGTATCTATACATTCAGAGCCGAA
TTCTTCAGTGAGCAT
GCAATTTTCGTCATCGGTGCAGAATGAGCCCTCAGAGCATTTAGA
ATATGGGAATCTCAC
AGTCAATCGCGCACCGATTCCTTTGTTGTACATTGAACAGCCATA
TTGATCATAATTAAT
CATCCAGGTGG
>ConsensusfromContig1872 Average coverage: 14.69
TCTTCCGATCTGCGGGGTTGTCGCCGGTGTGACGTCAGCAGCCCC
CACAGCGTCTGGGAT
GCATCACCACCAAAGCATGAACAGCCTCAGCGGCGTTGCTAACA
GCGGAGAATGCTCCCA
AACAGCGTCTCTTCTCACCAGCAAACACACGATGAATAGAGGAG
GTCCTCTTCAACAGAA
CGACTCATCCCCAAACTCTTACCAGCGTCAGCAACGTCAGATGTT
CTATGACCAGCATCA
GCAACAACAGCATCTGTATCCACATCAGCAGTGGATGCTCGATA
CAGACCAGTTGAATGG
ACCGCTCTCTATGAATAACAACAGTCATCAGTATGTCAGCCAGC
AATCACGACAACGAGA
CGTTATCCCTATGAACGAAGGCATAGATGGGGAAAATGCTGCCC
ATCGTGGAGGAATAGG
AGGGAGTCTTTCGAGGTACTGGCGGGGCCTCAATCGATCGATGGC
CCCCCAGAATTCCGC
CGGAGGACAATCGAACATGAGCGGCGATGGAGCATCATCGTCAT
GCGCCTACCAACGACT
TGCCCAGACAGAAGGAGTCATACGGCGAGCACCTGAAAGCAGCG
GCGTATCATGCCACCC
TTCTATCACAGACTGGGCGGTCCCCATAAGTAGTCAGCCCTCAAA
TCCAGATGCTAGTTC
AACATCACTTTTAGCGGACTATAATCACCAAAGGCAAGCCGCGTC

ATATGTCACCGAAGC
AAATGGTAAAGGAAGCAATACGAGTACCTTCGATGATGACGGTCT
T
>ConsensusfromContig1873 Average coverage: 9.88
CCTATGCAGGTCGCCGAACGGTGAGCTAGTGCTGGATGAAATCG
TCATACAGCATGTAGG
AAAGATTTCTTTGAAGACAGAATGAATACATTTATTCTGTTTAGA
AGTTGTTGAAGTCAA
GACTACTGACTGTGCGGCTCTGAAGAATGCCATTTCATCCAAAGA
ATACGAAATACCCTT
CGTTTCACCATTGACTTGCTCTGGATCAAGATCGAATATATTCAG
CCGAGGATTCTCACG
ACACATTTCGCTCATTTTTTCTTGGCAAAATTCTTGAAAAAGAAT
GGCTGCGCGTTTGAC
GATATCGTTTATGACTTGTATTGAAAATACAAACAGACTCTTGTC
CTCCAGTGAATCGAT
GAGAGTCTGTAGCTTCTTGGCGAGTGTATTCCATCGCGGCATATT
GCATGCCAGGAGCAG
AGCTGGGATGATGGCCCCTCGGTCCGGTCTCGCAACCGTGATCCG
ACGCATTCCCACCTT
GAGGTTATCAGGCACTTCAGCCAAAGATTTCGGAAAGCGGGCAT
TGAAATTCATGAAGCA
GCCGTAATGTGAGTTGACGTCGACGAGGGTTCTATTGAAGAGGA
CAGCCCCAAGATTGAC
ACCGGTGAACAAGATTTCATTGAATGGTACATCATCATCTAATA
TAAAACGGGACAGAAT
ATCATCATTTTGATGCAACAGGCTCTGATTCGGATGGAGGCACTT
ATTGTGATCGTCACT
AGAATACGAAAGCACATTCTGATCTTCGAACGTCCAATGAGAAA
GTGTATGCAGCGTAGA
TGCGGAATTCCTACGACGTCTGCTTCTCCGCTCTGAAACCTCGCG
TGAGCCACTACAACT
GTGTTGGGAAAGTTCTTTTTGCCAATCCAGTATTGAGAGTGCCTT
GCTCAGTTTGTCAAA
ATGTTGACTCAAGATTGAGAGATGACCTGGCGAGAGTTGCTCTGT
GTGATTCATCTGAAA
CCAATAGCCTCCTTCGAGGGAACCGTGGAGTAACTGGATGATAG
AGCTCAAGGACACGCC
GGGTTCACAGGAAAGAGAAACGCAGTTCCGGCCCAAAGCCGAAG
CAAGAGTGGCCGTGAG
GGGATTGAGGCCGTCCGGAGTCACCAAGGCGACCGGCACATATG
ATCCGATGGCGGCGTT

TATGGCCAGGTGAACTCGGTCTGACTGAGCCCGAGGAATGAGGC
AACCCTCTTGCGGGCC
GTAATACTCGCATCCATAGCGAAAGCTGG
>ConsensusfromContig1874 Average coverage: 10.41
TCCACCTGGGATGCCGCAGAATGTGCCAATGAAAGGACCCACTC
TGGGAATGCCCATTGG
TGACCCTATGGGAGGAGCTAGACCTCCGACAATGGGTGGTCCAC
GGCTCATGGGGGGTCC
TAGACCAATGGGAGGGCCTGGAATGCTGCCCCCTCCCCGCCCCT
CGCCCCAATCAGGGGG
TGTGCTCGGGGGCTCATTTGGTTCATTTCTCTCGTCGATGAACAA
ACAAGTTTCAAGTGC
CTTAAACAATATCATGTCGAATCAGCAACAGTCTCAAATGCCACC
TCATGTACAGGACAA
CGAAGGCCTGGTGCGGTACACAAGTTCAACAGCGACGGGCAGTC
GGGGGCCCAGGGACTC
GACAGCGGTGCATTCCATTCACCCGGCGGCCGCAGAACGAGCTC
ACGCCGCGGCTGCTGT
TGCCCCAACACTTTCTGGCCCTCTATCCACATCAGGGGCGCCACC
CATCACCACCACTGC
CATCTTCACCAACACCTTTACTCAAAGCACAACTGCCAAACCTGT
CCAAAGCCAGGCGCC
CGTGACCAATAGCAATGGAACTTTCAAACAAACGCTCCAGGGAC
CTTCATTGCCGAAGAC
AACTGACCCATCTAAAAGAGAACCTGGTGCGAGAGGAGAGAACC
CTCAACGGACTGTACC
GCCTATCCCCCGCACCCAAAGACCTGATGGGTCCAACATGAGTA
ATAGAGACCCCCCTGA
AGGTCATCACCAGGGCAACGAATACTTGGACGGAGGTCCACCGC
AGC
>ConsensusfromContig1875 Average coverage: 7.39
CACAAAAATTGTGTGTACCCTCGAAAATGAAAACAAAAATTTACC
CATTGTTTAGAACAC
TGTAGGAATGACTGAAGGAAGAGGCAGAATGCTGTGGTTGGGCC
ATTTGTTCAGCGAATT
CAAGCGGAATGAAATCGTGGTGGTCATCCTCTCTCTCCTCGCTTC
AATAATCGGCAACGG
CTATATTCTGGCTTCGCCTATCCTCGTTCCATTGATTGCTCAAAA
CTTCAACAACACGAG
TCCAGCAACGTTAGGCTGGATAATGAGCATCGGGATCGGTAGTGG
GCACATCCTCTCGCC
GGCTCTTGGGGTAATCTACACCTTGTGTGGAATCCGCCTGATGTG

TGTCATGGCTGGATT
CATGGGTGCCATCGGGGCCCTCTTCAACAGCTTTTCGACGGATTC
TTTGCAACTCATCAT
CAGCTTCTCGTTTGTTCAAATTGTCGGCCTTCACACGTGCTCACAG
AACTTTTGCTTCCT
GTTCAGACATATGAATATATACAGAATGGTCACGATAAATCGGT
GGAGAACAACAGCAAA
CTTCCTCGGAATGCTGTTTTTCTCTCTTCTGATTCCGTTGGCAGTG
AAGTATATGGGCTG
GTCAGCAGGTATTCAGATCTCGTCATTGTGGTCGACTTGTGTGGC
TCTCTTCGCATTAAT
CCCTAACCTGGAAAGTTTCTCTAAGAGCACGCGAACTCTGCCGTT
GAGCAAGATATGGAG
GAGCTTCAAAAAAGACTCGCTGTCGACTCTCTAAGATCGGAAGA
G
>ConsensusfromContig1876 Average coverage: 11.02
GGATCATATAAACTGATATAATCATATGAAATACTGATAATGATT
ATTCTTCAAAGTAAT
TTCGACATTGCGTCGGCTAGCCGTACTTTTTCCCACAGTAAATGG
GTGTTCACAGTAATG
GCAATAAATTCCTTCATCGAGAATTTTCTTGATCAATTGCGAATA
TTCGATCCGAGCTAT
ATCGAAACTCTTCATCAAGCTTTCCTCATATTATGCCACCTCGTCA
TATATTTGTCTTTG
TTTGTCTGCATGTCGTCCCAATGCTCTCTCTCACTTGGTTTTGCCT
GATTCCCAATGATG
ACCTGGCCAATGGTCTCGTTGTGTGCCAACCTGTCTCGATGCATA
GCTGAAATGACGAAG
AAAATACTCCCTAATTGAGTGAAATCGACGGAGAATATAAAATA
TTCATTAAAAACTGGG
TTTGATTTGTTTTTCACAACTTTGGTCTTCTGTTTCGACTCCATGA
CTGCCTCATGACAT
TTCCATACTTTAACATACGAATGGATTTTCTTGACAATATTCGGA
GCAACAAAATTGGCA
CCTCTAATTATACCCACAACAATTTGATTTTTTTTGGATTCGTATA
AAAGTGATATTTCC
AATTGACCATTGTTTTTCACGGGTGGGATCGTGATTGGATACCAA
TTGGGGAAATCTCTA
TCAATATCGGCTCGTTTCAGTAGGATTCTGACAGTACCGATAACA
TCGTCCCTGGCCATT
TCATCGTAGTCAATAGATTGGAAGAGCACACGATGTAATTCCAA
>ConsensusfromContig1877 Average coverage: 8.42

TGTGAATATTTTAACGAGAGCAAAAAAAAGCGAAAAGATGAATG
TCACCGTCGTGCTGGC
ATAAAAAGAACTAGTATTCAGCGAAATAGCATTGAAATAAAAAC
ACTAAACATTAATACA
AGTGCTACTCAAAACCCCTGCCAATACATTTTCTGCAAAACCACA
AACGCGATATGGGTT
TTTTTGTGAAGCCAAGGCATCTCACGTGGCAATACGCAAGGCAGG
AATCACATCCGACTG
TTTCTCCATCAGTTAACTTTTGGAAGCACACACGACATCCGCAGA
AGTTAGATTGTTTCA
ATTGATCAACGAGGGTCGTGACGCACTTCCAAGCCGCAGCAGAA
AAGAAGGGTCTGATAA
TGCAGAGGTCGACTTGCGCCGAAATGATCACTGGAGAGATCATTG
ATGGTATTGCTTCGA
TGTCTTTGTCCTTTAGCAGAAAGTCATTTGATGATAGAGCGCGGT
CTGCAGCTCCTTCAC
AGACTAGCCATCGTAGCATGACTGAAACAAAATAATGATTACTT
ATCAAATATATAAAAC
AGCAAATAAGCTGATATTAAGACACGAGCTGACGCGAATGGGGT
GATGCTGGCCTGACAA
TCATGCTCAGGACTACCATCTACGAAGCGAGTGACATTGACTTTC
GTCGATACTTGTGCA
GCAATGCTGTCACGCTCCACGCGCTGCATTCTCAGAAACAAAGAA
AATCTCTCATGAACA
CCTCTAATGAACTGAATTTACTTATCACAGATTTCAATTTCTGGT
TCAGTGATTCCAGAC
GATTATTAGTCTCATTGGCATAATTACCCATCTGGGCCAACAACC
CACTGCTCAGATATC
CCGTGCCAGTTTTTATCAAAATACGACACTATTGCATGGCCACAA
ACCTGACGAAACTGA
ACGTAAATTTCATTATAACCATCGAGACAACGAGCATATGC
>ConsensusfromContig1878 Average coverage: 9.06
TCGGAACATACCTTCTGTGTTTTCTTCTTTCTACAACTGCACTTCT
TCTAGACCTATGTA
TTTTCATCTGGCAATCCAAGTTTTTTGTAATTTCTCTGACAGGACA
CTTATGATCATTAT
TATAAACTATGACTATAGGTCGGCGATTCATCTCATCCAGTTCAG
CTTGACGTCTTTCTC
TTATTCTTATCAATGCATCTCCGCTAGTTTGATTCTCAGCCTTTTC
CATGTAGAGCTGTC
GCTGTATATTTCTTTCTTTTTCATTCAACTGTACAGGAGGTATTCC
ATCAGTAGTTGTCT

GTGTGCTGGTGTGTCCAAGCGCGTGTATTGGTGTGTATTTATTCT
CCCCATACCTCGACG
GTAATCCAGCAGCATGTCTCAAGTGAGGAACGGTCGAGACACCT
GGACTGGGTATCCTAG
ATAAAGGACCGTATTGTGGCTGCTTGAGACAGAGAGAAGATTTG
TACACGAGATGTCTAT
CGTCTCCATATTGTCTTTTCCCCGAAGCTTTTCCTTGTTCTCCATC
GTAAGCACAACTTC
TATCGCGTGGATAATCTCCAGTGTGTGCATAATCGTACTCAGGAT
CGTTGTGGATATATC
CACGACATTTATAAGTACGATTGCATTCTCTTGTCGTATTGCAAAT

>ConsensusfromContig1879 Average coverage: 8.64
TGACAGGGTTTCGACTTTTGATATGTTCTCACTCAATAATACCGA
CGAGCAATGTCATGA
AAATATGAGGGACTCTAAATCTACATCAATAGCTGATGACTGTGC
TTTCAATACCTCATT
GTCAAACAGCACACTACGATGTGAATCATTCACAACTGAAACCTG
TCGATTAAATCTTAT
AGAAAATTCACCGACAGTCTATACAAATAGGAAGAGGATGATTA
GTGAGAATAGCGAAGA
AAGGAGTTCGATAATCAATGGGGCAATGATTCATGATGTTTTTAG
AATATCAAATATGGA
TGAAGTTGAACAAAATAAACCGGAGCTTGTTTTACTTGATTACAG
CAGAGGGAAATCTAT
GCCCTCCATTGATATCGACCCTTTGAATGATATTGCATTGCCATCT
ATTGTATGTTCAAA
ATCGCATCAACATATCATATTTAGGAGCTCAGATGATATAATTAA
ATCATGTAATACATC
TTTCAAAAAAGTGGAAAATGGTGAGAAAAGGAATAGTCAAG
>ConsensusfromContig1880 Average coverage: 7.53
CAGCCGCCGCTTGAAAACAGCCATGTGGGCCACAGGATGTGCGC
AAAAAAACGACATGAG
TGGCGCTAGGGGTTCTTTTGCGTGGGGGTACAGCCGCCAGATTTT
TATTATGTTATCCAG
TCCCGAGGAAATGAGTTGGTCCATTTTAGAATAGGCGACCATACT
CACAACACCCTTGAT
ACCATGTGCCTCAATGTCGAAAGTGATTTCTCCTGAATTCCAATC
ATAGACGCATATATG
GCCATTCCGACAGCCACCCAGTAATAATGTCTTATTGCTTTGAAT
CATGCTGCTATCTGC
ATTGCTCTCGACGGAGCCCGCTGTGGCTGATATGATACGAGACAT

CGCGGCCCATTTGTC
TTCGGTATCATCCATGGCTGGCACGTATTCGTATACCAACAGGCA
ATTGATGAAATATTC
CTCGTCCTTACAGCTCCAGGTAGAGAGAACCGAACATGGATTCGT
CGATGTTCGACATTT
GAGCAGACGACCATCTTCGTATGCGGCGAAAAGCAGATC
>ConsensusfromContig1881 Average coverage: 12.94
GAGACGTGCGTCCGACGCCAGGGTGCAAATGTCGACTTTGTTCTC
GTAGATCTTTTGAAG
TTGGCCGAAATTTCGTTCGTTGAGACGGTCGAGATCCCAGATTTT
GACGGCCGTGTGGAT
GTTGGATGCGGTGGCGACGAAATTAGGGGCGATAGTAGTTTGGG
GGTCAGAGCTATTGAT
TGAATGCTTCGGATTGAGAGAGGAGGTGATTGCCACAGGAGTGC
CGGAGGAGTTAATTTT
TGGATTGATGATTTCACATCTGTCTACACCAGAAATATTTGATTCA
TTATTTTTATCATT
TTGAATTTCCACCGATTCACAGATTTCGCTGCAGTTTCCACTAGA
TGATGATTTAGCGTT
ATTGATCGGGTTGCTAAGGACTGCAAATGGAAACTTATTGTTGCT
ATGGACCATAATGGC
GGCTAGGTGTGACCCTGACACTGCGTCCCAGACGTGGAGAAATTG
TCCATTTCCGGCAAC
GACAAATCTCGCATCGGACGTAAATTGGCAGCCAAACAGATAAGT
TTTTTCGTGGGCGTT
GGCTCGAATCACCATAGGCGATTGGTTGACCGTTGAAGAGCCATG
TCCTACGTTTGAACG
AATCGACGAATCAGAATTGGCGGTTAGAATAGAAGCAGACGGCG
GAGATGAGCTCAGTAA
TTGATTGGATCCAAATTTAGCTGACGATCTCCGCGATTGGTTGAG
CTGAAAAAAGGGGGT
GGAGTTCATCGACGGCGAATTATTGGATTCTTCAGAAGTATAGCA
ATACCTATTGGCTGC
CGCGATCTCTGAGGTGTTTTCGACCTTGGCCTTACTCCCTCGTTT
CCATGGAGACGATGA
AGGAACAGAAGACGCGATCGCTGGAGTAGCCTTGAACTTCTTTG
ATCTTTGAACAGAAGA
AGAAACAGAAGACGCCGACGAGAAGTGATCTGATTGGTTGCTTCT
GCCTTCGAGTGAATT
CGTGTTGACGAGAATCGAACTCATGATCTCCGTGTCGAGGATGA
TGAGATGACAGCGACG
GACGTTGAGCAAAACGACGAAGGGGTTAACGGGAGATATCGAAA

TCTCGGCTAATTCTCG
GCCGTAGTGAGGTCCTTCCGCGAACGAGACTTCCGGAAAATCTTT
CTCGTAATTGTACTC
AATAAGCATTTTCCCGAAAGACTGGC
>ConsensusfromContig1882 Average coverage: 7.89
TAGGAAGATCCTGCATCATCCACATTTAATATGCTATTAGAAATG
GCACTTACTTTAGAA
ATCCTTTCAACACTGTCACGATACATCGATTCGCAATCATGAACG
TCAAAGCGCACATCC
AGTTTATTCAATATTTCTTTGAAATGAGCCAGGCTTAGATTTCTAC
AATATCTCCAAATT
TGCGTCATATCCTGATCGTTATTTTCTAATTCAATGCAAATTCCGT
CAATTCTTTGTCTC
AAATAAGCATTATCGGGATTTTTCAATTGGTGATTAACATTTACA
TAAATATCACTCAAT
TCTCTGAGATTTAGTTGGTTATAATTTGAGGAAGAATGCTGTTCG
ATTCCTGAGCATAGT
ATTCCGAACTGTGTTCCCCAATCACCATAATAGTTCAATCGGGTC
ACATCATGTCCAAAT
GCTTCATTTATATTAGCGATGAAATTTCCAATGAGAGTAGACCTC
AGATGTCCATAATGA
AAACGTTTAGCTATATTGGGAGAACAATATTCGATTACTATCCTA
TTTTTAGGGCTGTTC
TCACATGATGTTAGATGTATAGGAACATCAAATTTTACCCTATTC
TCCCGAATATCCCGA
ATCACACAATCACAA
>ConsensusfromContig1883 Average coverage: 8.74
GCACTCCAAAGGCTCTTTTCGTCGTACGGACCAGTGGACTCTGGA
CCTCCCCGCGCTATC
CCTATCGTCCAATCCACGCTCACATTCCGAATCGCTTAGGCAATC
TGAGAGTCTTTTGTA
TTGCTCGATGCGATCGAACCGGCATGCCCACATGGGAGGAGTCAA
ACCTTCGTTATCACG
GATGGTTTTGTCAATTCCCTCATTTAGAAGAAGGTCGAGGCATTC
GGTCGTTGCAGTCGT
ACATGCAAAGTGAATCGGTGTCCGCCCGAGTTCATCGGAGCCCT
GCAGCGAACATCCGAA
ATCGCGCAAAAGTCTAAGCGCCTCACAATTGCCGCTATGAGCTGC
TACATGGGCTGCACT
CGAGCCATCAAATGCCGCATGGTTGGCATCTGCCCGAGTCTCTAA
CAGCGCCTGCGCAAT
GTCGAGGCGATTTGAGTTGATAGCATAAAAGAGGGCACTGCGCC

CGAGCGAGTCAATATC
ATTAACACAATTTGGATCGCTCTGTACAAGTGAGATTACGACTTG
GAAATTACCGGCCGC
TGTTTCTACGATGACCTTGGAAGACAAATCTCCACTACCAGCAGA
GGAACTGGCTCTCTT
CTGGAACAAGTTTGTCCATTTGAATCCGAAATGATCCGGGGTGTC
ATGAGATGGGGTCAT
GGGACTCAGACCACGCATGGACGCCGCAAGCATGTTATTGCTTCC
ACTTCTGGCAATGCA
ATGTCCACTGTCGGTCCTCTCAATGCCG
>ConsensusfromContig1884 Average coverage: 8.68
CAGACTTTTATGCAATGATCGGTTGAGACCGAATAGAGTCTAGAA
TCCTCATGAACAATA
TCCAAGTAGCAAATCGGTGCAGTGTGACCCCGCAGCAGTCCTGTC
GGTTTTGAGCAAACG
TATCTGTTCCACAAACGAATTATGCGATCCATCCCTCCAGTGACGA
TGATGTTTCTTTCT
CTGCAGAATGAGAAACATTTGACCCCCTTATGAACCCGGAACACA
TCTTGGTCCCCCTCC
AGACGGTGTTTGGCCACGGCCATCTGGAAGTACGACTTGGCTTTG
TTCCGCTCCAGGCCT
GGCTCTTTTGTCTCCCTCAGCTGCTGCTCGATCTGTACGCTACCCG
TCGTGTTTCCTATC
ACCATGGCTGAGTTTGGGTCATTCGAACAGGATATCACTTGGTTT
ATTTCTGGATAATAT
TTGATCTGTTGAACCCATTCTTCGTGAACTTTCCATCGGACCAAT
CGCAATGTACCATTC
TGCACGGCGACATCCACTGGCACACTCGCGATTCC
>ConsensusfromContig1885 Average coverage: 8.95
CTGCGTGTTGTGCTTGTATTTTTATTCTTCTGTTTTCAACACCAAA
CTCTGAACCACAAG
CCCTGTCTTTTTTTTCGAATGCCGCATGCTTTATCAACATATCTTG
GCGGAGTTGCTCAT
AAGTTGATGGCTGCATGTACGTCACACCATCCCGCATAACTTTAT
TGAAGTCGAATGAGT
GAGAGGCAAGAAAATCAATTGATGAAGCCTGGATATGGAAAACC
ACATTGTTCTTATGTT
GCATCCCGGGCGCCTTCGGAAACAGGTAGAAATTGTATGGAGAAA
CCTCATACTTATTTT
CTTTTTCGATGAACTTAAAGCAGCACAAGCCAAGTTGTACGAGG
GGAAAATTGATAACAT
CCTTGGCTGTATTCAGGAATCGCTCTCGTGCTGTATCGAACAGCA

TTGTTCTCGCATCAG
GGGACCTCAATCCAAAAAACTCTCCATCAAATGAGAGGAAAGTC
GCAGATTCAATATTGT
TCTGTATATTTGAAAATTCTTCAAGGAAATTGTGCTTTGTAATATT
CATCATCGATTAGT
TTGCAGTTTGTACGATTGTATTGTATTTGTTTCGATTTCGATTTCG
A
>ConsensusfromContig1886 Average coverage: 8.36
CTTCCGATCTCCCGTTGTGCGTGGCTGTTCCGTTTGACATGTCTTCT
TTTTCTTTTACTT
GGCGCCTTTGCAGAAAATGTGCTAAGACTGTTGCGATTATTATG
GAAGACAAAATGGCGA
TAGTGAGGACCAGTAAAGTGTAACTGACAGCCCCAACATGTATAT
CCTTGGATTCTGTTT
CTTTTTTGGGCAGCGCGGAATATGTGATGTTGAACCCAGCATCTG
ATGTGTCTTTGTTGG
TTCTGAATATGACAAGTAGGTAAGGGGTAGTGCTGATGATCTCAT
CTCCTATAGAGCCCG
GAAGACAAACCTTCTTGACCGTTCTTGACCACGAATTGGGACCGT
CCTTGATCTCCACAT
AGTCAAGGCCACATGTACCACTTGTTTGAAGTTGAAGGTCATCAA
CAATGATTCTGATGA
CCTCGTAGTATTTCTCCGTAGCGATCCTCCAGCTGC
>ConsensusfromContig1887 Average coverage: 6.22
TGACTATTTCGAGTTCACAGAGAGTGAGTGCGATGACTTGACAGA
TATGTATAGTTCACT
TATCAAAATAGTTGTGAGAGCAAATAGCTGCCCTGCATTTTGTG
AATTACAGAGGGGAGA
ATCCGATTTCATCGAGGATCAAATGAAGTTTACTTTTATGGAATA
CATCTCACCAGTGCA
TTTTGACACCGATGACATCATGCTTATCACAGAAGACTCTTTATG
GATACAATTCCCTTC
CGAAGACCAATTCGCGGGCGTTTTGTACAATTTCACCACTCTCGG
TCTCGCGGATCAGAG
CGACCATGTTTATCGGGCTCGTCTTGGGCACATGTACGAAGCATA
CGAATCAAGCCTCAG
AATAACTTGGGAAAGTGCGAAGACATTTTTCATCAAACAAGATGA
TTATCATCTCGATTG
TGGATTGACTCGTGAACTCATCAATTTGATTGGCTATTTGCTCAG
ACCAGATTTTCTGCA
AGTACACAGAATGATGAATGCGGAAAAAATCAAACAAAAATGTA
GATTTTTAACCGGCAC

AGGATTCATAGATTTGTGCATGAAAAGTTTGAAAAAATTGAAAG
ATGCTGCAGATGTTGA
TGATGATGTTGATGTCAAATC
>ConsensusfromContig1888 Average coverage: 4.81
TCGGAGACCATATTTTTCGTCTGTGCATGTCGGATCGGTAGATC
CTGCCTGTATCAACG
TTTCATAGCCATCTTTGCATACAGCAGTTAGTGTTGTTCCAAAATT
AAATTCACCGCTTC
CGACAACATTTTCATCGTTTTAAATTCTACACTGGTATCAATAT
TTTGAGAACTGATCG
TGCATTTCTTTATCGAACATGAAAAACTACTCAATATCGGAGACC
ATATTTTTCGTCTG
TGCATGTCGGATCGGTAGATCCTGCCTGTATCAACGTTTCATAGC
CATCTTTGCATACAG
CAGTTAGTGTTGTTCCAAAATTAAACTCACCGCTCACAACAACAT
TTCCATCGATTTTAA
ATTCTAC
>ConsensusfromContig1889 Average coverage: 24.29
AAAGAAAATACAAAGCATCATGGGGATCATTGCATATCCAGAAT
TTATAACGGATACCAC
TGCGATAGACATGCTTTATAGTCATGTATCAGCCAATCAGTCGTT
GCTTACTAACAATTT
GGAGCTGGAAAAATCGAAGAGTGCCTTGGAATTGGGCAGGATAA
AACATCCGGATTCTCG
AGTGCCTGAGTGGAAAGATTACCCCGGGAGGGGGTCGTGGTATA
GTCCAATATCGAATGC
TTACTTCTTGTCCATCGGTTTGCTGTTGACATCACGTCCGAATTTA
GCATATTTGGCATA
CGGTGGACTTGGACAAATGATCGGATATGAGATATCTAAAACTCT
TAACCTTGATAAAAT
AGGTTTGGATGTGAAGGAATATGACCAAAGGGCACAATGTTACA
TTGATTATTACAACAA
GTTTGAAGACCCTCAAAGAAATTCAAAGAAAAGTTCAAAGCGAT
GCGATCAGGGAAAATG
TCTGGACGTCGAAACTCGATACAATTATAGTAACTCGAACCGAGG
ATTGATGACACTCCA
ATCGAATATGGCCAGTAACGAAGGTGCAAGGGCAGCAATAAAAG
CATATAAAAAATTTGC
ATCAAAATCAAAATTAGGTGTGAAGATGTTGCCGGATTCAAAACA
TAGCCCCATGCAATT
GCTGTTTATTTCCATGGCACAGTCCTACTGTATTGAGCTGATGGA
AGGCGAGAGAAGAGA

CTGGATGAATAAACGGCCGATTTCAGTTCCGGAAAATGTTGTAAA
TGGACTCGCTTCCAA
CAATCCAGAATTTTCAAAAGCTTTCAAATGTAAAGCAGGATCTA
AGATGAGCCCCAAAAA
GAGATGCACCATGCTGTGAAAGTTCTATGAGAAGTTGAAAAATGG
TTTTGAATATTTATT
CGATGAACACATAGGATGTATATTATT
>ConsensusfromContig1890 Average coverage: 18.61
AAAGGTCACAGTAATCTTTTTTGCATACGAGATCATCCGAGACAT
GCTGCGGCTCCACTA
ACTGATGGCCTGGTTGGCATAAGGCCTGCATTTATTAATAAAATA
ATGTCGCGTATGAAT
GATAATGTTAAACAGAAGAGTTTGACAGATATGCTCAATTTTCTG
TGGGAATCTAATACG
AATAGCAGGATGTGTTTCGACAGTGTCTCTTGCCAAAAATGGCCT
GATTCCGCGCCATTC
CCTGTTCTCAGAGGCGGCAATGAATTCGTTGGGCAAACATACGA
TGAAAATGATGAAAGC
TGATCAAGATGATACGTCAAAGTGGTCAAATATGTACAACAATCC
CGATTGTGTTTCAAT
CGAGAATACAGACTTCTCAAGAGACGCTCTCCGTCGTGTTATCAT
CAAACGACCCCTCAT
TTGGTCTCAGGATTTTCACGTCACTCCAATCATGGATTTGAAAAG
CCTTCTTCGTCCAAT
CGGAGTGCGTTTTGTGGATAAAAGTCTTTCTTACTATTGTGGCCA
GACTGGCACTTGTGC
CAAAGGGCTCAAAATCATAACTCGAGAAAACGGGATGAGGCTCA
CTCCAGAAATCATAGC
TCAATTTTATAATTATTATAAAAACAGTGACGAGATGAAAGATGT
TGTGGCCTTTGTTTG
TACGTTACCCGTAGCCATGTGTGAGGCTTTTCTGCCATTTAATAA
AAGTATCATTGTCAT
AGCGAATACAAGATATGAACAAGGGCGGCCTGAGCCTGAAAAAT
GGCATTTATTGAACGA
AAATCTCAAGCGAATATCTGAATCGCCAAGATCTATTTTAGCCAC
GAACAATGTTTACGA
TAAAAGGTATATCTATTATTTCACTGGATTGAACGCCACTGTGATT
CCTCCTGCGTGTTT
ATATTTGAAAGATTCTTATAAACCGACGAAAACACAGTTTCTTGT
TCCTCCAATCCATAG
TACTGAACTTTCTGAAAAGTTTTTTCTTCAGTTCGACGAGTTGTC
GGTGCGATCTAGGTC

AAATATCCAAATCCAGTCGTTGAGACAAATGTACGCTCAGTACTT
GTTCAGTGATATCGC
ATCACATCCTGGAATCATATACATTCCATATCAGGTGTCGATGGT
GAGTCTGACAGAACA
CTACCGCATGAACATTCCCCTGTTCTTTCCCACGGTCGACCTTCT
CGCAGAGTGGCATGT
CCGCTATCAAGTCGTGCGTCAGCGAACGTGGTCTGGATACATGC
GAAAGCGTCTGAACGC
CTCAAACATTCCCGGACATTTCAAAACAATACCTGATCCAAATGA
TGATGTGGATTTCTC
AGCTGTTAGATATTGGCTCCAATATGCCGATTTTTATCAGTGGCC
GCATATAGTTTACTA
TAGTTCGGTGGAAGACTTACATGATAAACTTATCAAAGCCAATTT
GACAGAAATTAGTGA
AAATATGAAAGTGTACAATGATAAATCAGTGAAGCAATCTCGTTC
TATATGGTCCTATAT
TTTATCCAAAATTTTACCACCTTAATAATGATTACATTCCACATTG
C
>ConsensusfromContig1891 Average coverage: 14.01
GGTTTCATGTGGCTGATCGGTTTGCCCCAGCCCAGGTTGGCGACG
TGGTCGTGGTCGTAG
ACATAGCTGCGTATCTTGCTACGACAATACGCATCCCACTCATTC
CTGTCAAAAATAATT
CTGTTCAGCTTGTCATCTTTGGGCCACGTGTTGAGATGGTGGTCG
ATGATGTTGAGTTTG
GTGATCTGGTTGAGAAGATTCAGGAAATGGTCCCCGGGGATGTG
GATGACCCAGCAGCCC
CGACTGACCATTGCCACGTTCGTTGTGTTTTCAGGGTCGAGCATC
AATTCCCAGCCAAAA
ACATCATCTTCCACCAAACTGGCAACTTTGACATAAAGAAAGTTC
TCGTCGTCTTGACTT
TCATTTGCTATAGGCTTGTTGCAATGTTCGCATATATTTTTTTTCC
TCGGATACCCTTCG
TTCAAAAATTCATTTTCATTAGATTCAAGATTGAACTCTTCTTCTA
AATCAATCGTCTGC
TTTCCTGTCATCAAAGGCGGCAGTCTGAAGCTATCGCCATATGGT
TCAGTCTCTTCGGAA
GCGTCCGACTCGATTTCTGGGAAATTTGGAATCACGTCTGCATGA
CAATGGGTGTTATCG
TTAATATCGAGGCTTGCCACTGAAGACTTTCTTTGCAATAATTTT
TCACGTTCGAGACTC
GGAAAAGTTTCCTCTAGTGATGGTGTTTTCACCTGAGCTGTAGTTT

TGTTCGAATTATCC
AATTCACCATCAGTTTTCAATGATGTTGTTTTTTTCTGTAACAATT
TGTTTTTTTTCGAA
TTGCTTTTGTTTTTTGATGGAGTTGTTTGCTTTTTTAGCGATTCAT
TGCTGCCCGATTTG
TCGTCAACTTCAACATTTTCAAGCGATGTTGTTTTTATCTGTAACA
TTTTCTTATCACTA
AAATTTTCAGAATCGTCAATGGTTTTTAATGATGTTGTTGTCTTGT
TCTG
>ConsensusfromContig1892 Average coverage: 49.65
AGCAGTATTTTCAAACAACATTTTAACACTAAATCAACCAAAACA
TGGAATATTTTATAT
GTGTACTTATTTTGTATGAGAAGCCAGAATTTTCAAAGAGGAATT
CAGAGATCTTCCGGG
AAAATAACGGGGGAGCTCATCTGTATGTCTTCTTTAAGAGCCTCA
AGTTCACTCGAACCA
AGGGGCAGGCTCCACTCGTTCCCTAATGAAGCTGGGGGCAGATCC
ATCCCCGATTCCGCT
ATCGCAATTGCATCGAGATCGATTCCAACGCCATAACCTTCACCT
GTGCCAGGAGGAGGC
TGGTTCAAGGTCAGGTCATCCACGTGCGATTTTGCCATTTGTTTA
ACTAGAACGGTCAAG
GCCTCCGGATCGAAAGTGTTGTCGTATGTTGACATATGAGGTAGT
GGGACATGGTTTGTA
GTTTTAGGGAGCGATCTGTTATAATTGTTATATTTAGTATTGGAA
AAATTGCTTCGATCA
AATACGAAAGGGAGCTCGGAATTGTGGATAGATGAGGCAAATGA
GTTGCTTGATCCCTCC
ATGGAAATATGAGAAGGAACACCTCTGCTCGATACTTGGTTCCCA
TGGTGACCCGATTCA
CTAGTTATTGGGAAGCTTTGAAGATATGCATCCTTCAGGCAATCT
TTGACAACCAAGTGA
TTGGCATCATACGAAGGAGGGGTTGGCCGCATATTTTGCCCCATT
AAAGACGGCCGTGAC
CCCGCCATAACATTATTTTGTTTCTGTTGCGCGTATACAGCATAG
GCGTGAGTGGATGGT
GTCATGTCTTTCAAGAAATCCACAATTTTATTCATAGTCTGCGGAT
CTGGTTTCTTCCGG
ATTTTTCTCTGGGATTTCGGAGTGTTGCTGTTTGATCCGGTTTGG
GGAGAGATGGGTGCA
ATCTGAATCACATTTGACACTGCAGACGGTTCTGTTGCTGGTATGT
TTGTATTGTTTACA

AAAGAGGATGACGAAAAAGTGTGTTGGCGAAAAGCATCTAGTTG
AGTATTAGTAAGAGAA
GTATTCATCATGGTGCTATGACCCACACTCGCTGTATTATGTAGA
GGATGCTGTGCTCGA
TGAAAATAATTCCGATTTTCGGGTGTATTTGGCGCCGAGAAATAG
TCCAAATCAGTATGA
TTGGCCCCGACATAAGGTGATCTAACCCCCATATTATGAGAAGCA
AGAACAGCTTGGTAT
ACCGTGGGCGCCGGTCGCAATTGGGGATGGTGTCCAGAGAAATG
TACAGAGGCAGGATTC
GGAATTTGTGGTGAGATACTTACATTATGTGATCTATTACTTGCA
TCAGAGCTTCGATTT
ACTCCATTATTATCGTTCATCGAATTTTGATTATGAATTGCATTGT
TGAAATTATCATGG
TTGTTGTGTCTCATTCTTCTGTCCTGATACGTCATATTTTGGTGAT
GTAATGTATTTTCC
GTGTCTGCTACATCGTCATTCTGTTGAAAACGGCCTAAACTGTTG
TGTGTGGTGCTGGGG
TAACTATTTTGAGCCATGTGCTTATTCTGTAGCGAATGTTGTGGCA
TTTGATCTTTTGAA
TTTGTTCGATACGCAAAAGCGACTCCATCCATACCGGGTATGGAC
GCGCTCGACTGATTA
TCCTTCTCCAAATTCTGTTCATGTTGAGAGAACAGATGTCGTTTG
GACATGCTTTCGAGC
ATGGAGCTATTTCCACTGACGAAATCAATGGAATCTGTTGTCTTG
GAGTGGACAAAGTTA
CCATACGAATCATTGGAGGCGTACGCATGAGTATTATGAGAATA
TCCAGCGGAGATGGAA
TTCTCAATCCACTGGTTCGTTTTTGGATCGTAAGCATTAGAGGAG
ACATAGGAGCCAAAA
TGTGTATCATCATAAATCGGAGCGTCATGAAAAGACTCCGGAGA
CCAGTACGGAGACAGC
TGAGACCCGAACTTGCGATGATATCTCCCGTTATCCGAAGAAGTC
TTCCGTCGTAGTCGC
GTTTGTTTTTCTCGGGAATCAACGGAGATATGTTATTGGCATCC
GAAGAAATGTTGGAA
GAACTATTCGAAGAATTGATTGAACCCGAACGAGGAATAATCGG
CACAGAGTTGCTTCTG
TAATTTTCTGAAGATCTGAGAGGGTTCAGGGTTAGAACAGAAGC
TCCACCGGGATCAGAA
CCAGGAGACCAGTGACTATTATACGGATCTGAGAGGCAAGAAGA
CGCGGGACTTGTGACG

GAAGACGTTGTATCGCTCCCGGGAGATCTGGCGCTGTCGGACGAG
TTTTGATCTCGCATG
GCCGACCATCGATTGAGCATGCCTTTATCCTCATCCGTTATAAAC
TCTGAACCCGTCTGT
TTTTTCTGTTGAAGTTTCTTGAGTTTCTTGGCCTTTGCTTCTTCAAG
TCTCTGTTGCCTC
TTCTGTTGGCGCTCTTCTTTGCGAGATCGACACGTGGGAACCTGC
TTAGGGGTTTCCTTC
GTTCCATCCATTTCCCGTCGCTTTTGAGCATTTAAAATGGCCGCTT
TGATCAACGCTTTC
AGATTGACTGAATTTTCTTCAGAAGCAAC
>ConsensusfromContig1893 Average coverage: 23.85
GTAACAGGCGGAAACGCACTTGGTCAATCGCCGTTGATCTCTTA
CAACAACACCGCCGGA
TCCGAGCGCACATTTCCCGGAGATCGAAGTCTTTCTTTGGGTCAA
ATCCAGCCATATTCT
CCGGCCTCACAAAATCATTCTCCCGGTCATGAATATGATCTTGGT
TCACTTTCTGCATTG
CGGGGAAATACTAAAGGATTCTTTGCGAATCCATATCATTATAAT
GGAGTATTTTACAAC
AAGACGCAGAAATCTCATTCGATGGACTCAATGACATTCGCTCAG
AATAATGGATATTTC
TCAAACGAATTCGCAAACAATTACAGAAATATGCACGGATTTTCA
GCATTATACGATCAA
TCTGGCCATTCAAATATTCCAACTTCCAAATCTATAAATTCGAAC
CAGGACCAACGAAAT
AACCATTCAAATTTCATCTACCAGAATGGAACATCAACAAGCCC
TCAAAACGGAATCGAA
ACTCCATCAAAATTTTGCTTCCAAAAAAATGATAGCCACGAAGCA
ACTTCAGATGGTTCT
GAACTCGACTCTTGCCATTCTGGAAGCCCGATGAATGAAAGCAT
TAAAGAGGAAGACAAT
GATGACCCCCATTCTGGAAGCGACCACATTCCTCATGTCCTTGCC
CCTGGTTTCCACGGC
CCCTCAAGGAAGTGCCTTCTCTGGGCCTGCAAGGCTTGCAAACG
AAAAACAGTCACGATC
GACCGTCGCAAAGCCGCAACAATGCGGGAACGCCGTCGTCTGCG
GAAGGTGAATGAAGCC
TTTGAGGTCCTCAAGCAGAGGACATGCCAGAACCCAAGTCAGCG
ACTGCCCAAAGTCGAG
ATCCTCAGGAACGCCATTGATTACATCGAGAATCTGGAAGACAT
GCTCCATGGGAACAGA

CCCAATCTCCTGAAGAGACCTGGGGACTTTTCTTCTATTCGGTCA
GATCCTTCAGCAGGA
TTCATGAATCTTGTCTCCGGTCATTATTTCCATGAAAAAGAAACT
TCAACTCAAAACTTT
CCCGCGAATAACATGAAAAACGGTTACGACGTTCCAACGAGTAA
CCTCAACACCTGCACC
TCAACATCCGCCCCTCAGACCAACACGAGTAGCGTATCCAGTCTT
GACTGTCTCTCCCTC
ATTGTCGACAGCATAAAGGGGACAGCAGAAGGGGCAACCCCCGT
CCTCCTCTCCGATGAC
AATGACGTCATATGATCATGTTTCATTGCTTTGAAATTTATATTAT
ATTTTGTATTGTTA
AATGTGTGTTTTGTTCTGTCGTCAAGCATTGCTATGCCTTCTTGTC
AGTAGTAACTATAG
TTATAGTTTGTACTCGGTTTATGTATGAACAATTTTA
>ConsensusfromContig1894 Average coverage: 35.99
CAATCAACTGTTCACGATTGATGTTATTTGTGTTATTTTGCATAAA
AGAATTATTCTTGA
ATATTTTGTTATTGGCAGTGTTTTCATTTCTTTTGAACTTCATTAC
ACCTGCTTTGAAAC
TAGACTGATAATTAATGGGATTCGATCCCTCAATCTCCTGGCAAA
CCTTCATCAGATGAA
CTTTTTTGGGTGAAGAATTTATGCACTCTTTTTCAATAACCTCTTC
ACGTATCCGACTGT
TACTTGATTCCTCTAAAATATGCATCAGACGAACCCTTTTTGGTGA
ACATGTATTTGTTT
CGAAATCTTCTTCAATGCCTTTAGGAGGCGAAAAATTATCATCGT
CCACAGAAAAGCTGA
TTTTGCGCTTTTGTCCCCCATACCTTTGCATGAATGGAGATTTGA
GGATACCAACTGGCA
TATCAGATGAGCTTCTAGACATCATTCCATCAAGACTTTCGGACT
TATCGAATATCGATG
TAGTTTTTATTCGATTAGCAATGGATGGAGAGAAACAAGTGCTCA
CCCTCCTCGGGGAAA
ACATCCTCTCTGAAACATCTATTGAACGACAAGCTGATTGCGCGTT
TCTTGTATTTTTCG
ACGGCGTACTTCGTTTTAAAAAAGGAGCATTCTCTGTGAGAGTCT
CTTGTATGGCACCAT
GTTTTGAGAGCCGACATCTCACTCTTGAAGACGGCGATTGCAGAG
AGGTAAAACTTTTGG
ATGGAGTTACACATTTTGTAGGGGTATTTCTCCTACTTGGAGAAA
ATCCAAATTCTTGTT

TGCTATGATTATAAGATAATCTAGAACTATTTCTTGAAGGAGATT
GAAAACTGCTGCAAA
TTCTAGAAGGGGATTTTGTAGGAGTATTTTTATTCGGGGTTCTATT
ACTCTTTTTTGGCG
TTAATATCCGTGGATTGGTTACACCTGTTTCAAATTTAGTAGTGG
ATTGCGAAAATCCAC
CGAATCCTTTCACTGGTGAAAAGGAATCTATTTGAAATAGTTTTT
TTCTGGCAACCTGAG
GAGAAGTGCCTTTGAAGACTGTCTTCACGGCTTTTATCGGAGATG
GACTTTTGATGGCGT
CGAACAGACAAACGGGTTTGATCTTCCGACAGCTCGATCCGCTGC
TTTTGTGGGGAGATG
CCGTCTTTTGAGCGGTGACGTTCCTCAGCGAAAAGCAAACTTGTG
GACTCTCAATCTTTT
TCGAGCGCATTCTCTTTTCCAATTGGTCATATTTAGCCAGTTTCGC
CTCCCAGTTCTCGA
GTTTCTTGTCATCAGCAAAAGA
>ConsensusfromContig1895 Average coverage: 14.99
TTGCACCAACAGTGCTGGCGGTTACAGCTGTGAGTGTCTGGCTGG
CTTCTCCATCAATGT
CGACAAGAGAACCTGCAATGATATCAATGAATGTCAAAATGCGA
ACAAATGCTCGCAGAT
CTGTATCAACACCATCGGTTCATATACATGTGACTGCGAAGTGG
GATACAAGATAGACAG
CGATGGGCTCTCATGCATAGATAATAATGAGTGTTTGATTCAAAA
CGGTGGCTGTGAGCA
GTTCTGCAGCAATGAACTCGGCTCTTTCAAGTGTTCCTGCAGAGA
TGGCAACAAACTTGA
TATCGATATGAGATCTTGCGTCGGCCTAGATTTGTGCAGTGTCGA
TAATGGAGGTTGTGA
CCATACTTGCACGACAACGGCTACTGGTGCAACATGCAGTTGTTA
CAAGGGCTATCAACT
CGCTAACGATCTCCGAAAGTGCATAGACATAAACGAGTGTCAAA
ATGCCAACGGTGGATG
CAGCCAAGTTTGCACGAATAGCCTCGGCCTCTTTTCTTGCTCGTG
TAACTATGGCTATGA
ATTGAATGTCGACGGGAGAACGTGTGACGATATAAACGAATGTT
TGAGTGCGAATGGGGG
CTGTGTCCAGC
>ConsensusfromContig1896 Average coverage: 9.00
CGGGCAACAAAATGCCAGATGTCTATCCAGTCATCTCGAAAGAG
CAGATAGTGCCTAAAA

TTTTCTTTTTTCAGTTTTTCAAAGTTTGCATGTCCTTGATGTGGGT
TACCCTTGACAGAA
TTATACCTTCTGTTGTAGTCGGCAAATATCGAATCCAAAGGGTCT
CGCAATAATAGAACA
ACTTTTGTATAGTTTTTGGCTGCTGGCGTATTATCCACCGGCCGAT
GTGTTTTAACAACA
AGAGCGCGCGAATCTTTGCAAGTTTCGCCTGGGAAGTCTTTCCGG
AGTGAGCCATCGCCA
TAAACGCTTCCAGTCATGAGCCCTGTACTCTCTTGTAGAACATGA
CGCAACCATGTGTTT
CCAGCACCAGGAAAACTCGCCAGAGCATATACTTCAGTGGGAAC
GGGGTTCGAACGAAAA
ACCAGTTTTTCGTGGCATTTGGGCTTGTTGCTTATTATCTTTTGTGG
TTTCCTCACTTTT
GAATTG
>ConsensusfromContig1897 Average coverage: 12.76
CTCTAAATCTTGGGATGAGCGTTTTCCGCCATCAAAAAGCACGCG
CTCCAGAAGACCGAT
ACTGAAGGAACATAATTGGATCAAATTGTCACGTGAGGCCTTTG
AAATGGCCAATCGCAA
TAAAAGCAGACGCACAAGTCTCTCATGATTAGATCTCGTTTTTTT
GTCCGGAATTTCCGG
GTTTTTAGTCAACCAGGCGCTCAAAGTTTCTTGCTCGACATAAAA
TCGCTGCAAAATGTC
GGCTAAAAAGTTCTTCGAAATCTCCAATTTACATTTCGTGTCAGTT
TTCTCGGCTGTATA
ATCAGATATGACGTCACCCATCATGACCAAGAGGTTATTTATGTC
TTTCGGTAAATTTGT
AGACAATTCTGACATCGCATCCAAATTGCCATCCCGTCGCAATTT
AATTTCTTCGTTCAC
TTTTCGCACAAGCAATTTTTGTTGTTCCTCTACACCAAACCGTTGC
CACGCCTCCCCATG
ATCAGCAGCAGTGGCGTGGATGAGAGCCACTCTTCTCAACGCTTC
GTATTTCTCCTCCAG
CGACGTCATGAACACGTGATCGGAGGAGGATA
>ConsensusfromContig1898 Average coverage: 32.32
GAAATGGAAAATCAAAGCGGCATTGAAACAACTTTTGTGAAGCC
TGAAGCTGTGTATGTG
ATAAAAACAGTTATAAAAGATAAGGTGAAAAAGAGTAAGTGTTT
CATCAACATATGTAAA
TCTGACAAAGTTGAAAAGCCTTCAGGAAGGAAAGATTCTAATGA
GAATGGTAATGTTGGT

ATTATGTGGTCTTTACCACATTGTTTCTCTCCGGAACGGGAAGAA
AAAGATCAAGGAGGA
GCTCTATGTAAAACTTTTGATGTAGTGTTTCATAACGATACTTTG
CGAATGGCTGAAACT
AATTCACAATTTCGAAATTTGATTAACAGTACTGCAATAGAGGGT
ATTGAAAATAATTTT
GATCTCAAACTAGATAGAAAATTGAAATTTCCCAAAATGAAATGT
AAAGGTACTTTAGCT
TCAACTGTTATAAGAAAAAGAAAAACTGATGATAATAAATCAAC
TGTTGATCAAATCCCA
TCAATGGAATCTTTAGGTGACAAAATGCAGAATGATCTGGTGAA
CCTCAAAAAAGAAGCT
TGTCAAAATGGATCAGAGACAAATTGGATTGTGCCAACCTATAAA
CTCATTCATCAAACA
AATTCAAAAGTTTCTGATTATACTTATAGCAATGAGAGTCAAACA
TTAGCTCCAGAATAT
CTGATATACTCAATCAATCTACCAAATATCATGAATGTGAATCAG
CTGAATTTAGATATT
TTTGAATCCAGAATGAAGTTAGAAACTATACAAGAGGCTGCAACA
TGTTATCGTTTAATG
ATTGATTTTCCTTATAAGATTAATGAGGATGAAGGTTCTGCCAGA
TTTGATAAGAGTAAA
TCCCAACTGATAATTGAATTACCGGTTCTGAGAGATACAGCGAA
TGGAATCACACAAAGC
AAAATTGAACTATTGTCTGAAAGTTCTGAAAATCATTATGATGAG
ACTCATGTTGCTGAT
GAGAGCAATACCATTATAAATAATAATGGTGAAAATCTAGAATA
TAGTGAAATTTTGAAA
AATTATGATGTTGATGATGAGAGTAATACACTGAAAAATAAACAT
TGTCCCAATTTCTTA
ATAAAGCAATGTGAACTGGATATAACTGGAATTGTTGCCATTAAA
AATGTCGATATCAAA
TCTATAAAAATCTTCAAAAAAACCTCAGAAACAACAATATTTGAA
TGTCAATCTATTGGC
TCTGGTGGCTTTCCATTATCTCATCATTTGTACATTGAACATATTG
GCATTGACCTCATG
GATTGGAATTCAAATGATTTCATGGTTAATCTACGCTTCAGAATA
AATGAGAATATTCCA
TCTGACCA
>ConsensusfromContig1899 Average coverage: 14.33
CATCAATATTGTTTACATCAATTTGAAATGAATGATTTGTCGTTTT
ATATGTTTGAAATT

TGATGAAATCGCATATAAAATATCACAAACTTGAGAACCAGTCA
CCAACAGAGAATTCGC
TGGATTCCTCGACGATAATTAACCAATCAGAATTGACGCTAATTG
ACCAATCAAATAGCA
ATGCCACTCATCTGTCACCCCCACACCCGAATAGCATGTGCGAGT
CATCCAGAGTTTCAA
ATGATGGGACTGTAGAAGATAGTGAAGGCGCAGGACCCCTGACT
TGTCTAAGACTCTGCA
TAAACTCTTTATTTCTCAACTGCTCCAGAAAGCATTCTTATGACA
TATCACCAGATAATG
TTGTGAATATTACAAAAGATTCGTTCGAATATGATTATAACAATA
TAATGTACCGAAAGA
AGAAACTGTATGATGACGAACTAAATGGGAACCCGCCGTATGTG
ATTGATCAAGATTACG
ATGCAGAAAACGGAAACGTCGAAAATTGTCATAAAAAAGGAAAT
TTCGCACAGAACTCGA
AAATATTTAATCAGGATATGATTTCAGATTGTTCAGGGACTCCAG
TAGCTAATGAGGAGA
ACAACAATCGAGTAAATATTTGGAGAAAACGTTCCTGTGATGAT
AAAACTAGTGAATCCG
ATAGCATGTGTCAGAACGATTCAACTTCGACGCCGTCCAAATCAA
AAAGTGACTTTAAAG
AATCTGTTCAAATTAATAGTGATTCTTGTGAAGGTAGCAAAAAGC
ATGAAAGTAGCTCTG
AGGAAACATCGGACGATGCCGAAAGCCAGACAAGCTCATCGAAT
AGTGAAGATGAGAAAC
ACGAAGAAAGTGAAGAGTCTGAGATTAATGATCGGTGCTTTGATA
GGATGCCGGTTTTAT
CCGAATACAGCGAAAAATACGACAAATTATCTCAAGTATATTGTA
GAGATTTGAGATTCA
AGCCGAATTTTCTTGAAGGTTCCAGAATAGAGTTTATCGTGAATC
GAGGAAAACTAATTC
AAATAGGAGTTGGTGCAAGCGGAAATGTAATTCTCGGACGATTG
GATGGCGAGGTCATTG
TCGCCATAAAACTGGGTCGAAAAGAAAAGCCAATGAATGGATGT
TACTCCTCGGAGATTC
ATTGCAATTTTAGAGAAGCAACAATCCTCACACAGCTAAGCGCA
AAAGATCATTCCAATA
GTTTCAAAATAATGCCGACATTCTATGGCGTTGCCCATGTGACGG
AACACAAGCATT
>ConsensusfromContig1900 Average coverage: 16.64
AAGATGGTGAAATTAAAACCTCTGGTGAGGACGGTGGCCAGGAT

AACAACTGCCGTGAAT
GCTACGAACATATCCCAAAAGAAAGTCAGGAACCAGTACAGAAC
AGAGCCGACTCCGCAG
ATGAGCTGAAGTCGTTTCTCCTGATTCATCCTCTCAGCTATGACG
TAATATACATACGAG
GCGGGAACGAAAGTGAAAGCGCACATGATCGACAGCGACACGCC
ACTGTCTGACATCTTC
AAAAACAAATCAGCTCTCGTCATAGGCCAGCGAGAAGCGTTGAA
GGGTTGATTGTAAACT
GTTATTCCATATTCCGATCCTCTAGCCACATCGTCAGTCCAGACA
GCAGCCCGTAGAATC
ATGTTGTTGATGGCGTTATAGAAAGCAGGCATGGCATGCCAACCT
TTGTTGAAATACCAC
GCTTCCACTAGAGATTCGCCTGTTTCCTTGTCTTTGGTGAACACA
ATGCCTCCAAAACGA
CCAGATAAACTATTATCTTGGTACAAATGTTGACTTCTGAGCATG
TATTCCTGAGTTGTG
AATGGAACCGAGGTGATGTTCTGAATATGCAAGCCAGTCGGGAG
CACCATTTGGGGGACA
GGAGGCCCATCTGACCCCTCGGGGCAAATGGTCCGCCCCTCAGT
CAGGATACACGTGGGG
GAATCGTAGAAGGCCTGAGGCTCGGGGGGCGGATGGTCGCGTTG
GTCGGGAAATGAGACG
AATTGCTTGTTGCACTTATCGTTCGTATCAGAATCAGAGATGCAG
GTTGTGCCAAACCAT
GGGGAATATAACATGGTATTCATCAGTCGCTCAGAGGCAGGGGA
TGAGACATCTTCTCGG
AAAAAGGCAAATCCACCCGGTCCATAGATGTCCGGTGAAATATT
GAGAGGTTCGATGAAA
CCGGGTTCAGGCCGCAACCAGATGATGAGACATGTGAAGAAGAC
AAATGTTGAAGGGATG
ACGATTTGGAGGGCGTGGTTTCGGAAGTCCCGCCTATACCGATGG
GTTTTCTTGATGAGA
ATAGCCTTGGCCTGTTGCCACTTCAGGCTGCGACCCGTCAATCTG
TTATCGCTCGTCAGT
TTGTGTTTGGCCATGCTCGGCATTTTTTTGGAGCTATTTTTAGAAG
AGGACGATGTATTC
GTGCGTTTTGATTTAGCAGACGATCCTCCACTGGAACTGCTGAGA
GGCGCTTTCTTTCCT
GACGACCCTGTTTCATTGCTACTGCCCGCGCTCCCGGTGTCGTCT
GCACTTGATGTTGAA
TGTTTTGCCGCTTTGTCCAGGCTGTCACTTGAACTGGAAATGTTC

TCAGATCCAGATGAA
GGTCGATCGAATGAGACAGGTGTAGTATCTGACCCTGCTGACCCT
GAACCTATCGACCCT
GAATCGGGAGGCGAAGGTGAAGGATCAGAGGATTCTGCGGTCCC
GTCATTGCTGCTTCCG
ACATC
>ConsensusfromContig1901 Average coverage: 12.79
CGAAAATGCTCCGGCAAGCGAACTTGTACCATAAATATTCCAGA
CAAAGACTTTGAGCTG
GAAAACCCCTGCAACAGGGAACTCAGAAACTACCTCGACATAGC
GTTCGAATGTGTTAAC
GTGACAGAAAATGGTGATCATTGCACGAACTCAATACAGCCTGAA
TCAGCCAACTTCTCC
GGATGGATCTCCACCAATTTTGATCGGAACAAATGTCTGTCAAGC
AGTTGGATGATCCAA
GTGCTGCTTGGACAAGTCATAAACATCACCGTCTTCATATTCGAT
GTCCGTCGTCCCAGT
AACTATCTACTGCCATCTCACCAGCCTTACATTGACTACGAGAAA
GAATATTGTCAAAAA
GTTGCCATACTTCGGTCTGTATCAGTGGATTCTGCAGCAAGAATC
TTGTGTGGGCACGGT
CGCCGCCAGTCGCGCGCCCACCTCTCGAACTGGCACGCGGTTGAG
ATAACTTTTGATCAG
GATGCCCCTCCCGCTTTGCTCCATATACAAGCTGTTGGATGTCCT
GATGTCAAGCCGATA
GAAAACAGCTGGTTCCGTCGGGAAGGGACCACGGCATCAGCACA
ACTTTCGACAGAAGGA
CCGTCCCCAGATGCATGGATGGGTTGTCTCTATTCCTCACAACGG
TGGTATCTCATTTGT
AAGGACAACGC
>ConsensusfromContig1902 Average coverage: 9.09
GGCTCTCTTTCATGTTCGACTGAATTCATATTAACATCCTCTGTTT
CATTCACAATCTTA
CTTGAATTGATTTCAGACAAGACTTTCTTCCTATGATTGTCTCTCA
TTTCTCGAATCAGA
ATTCGGAGATATTTGGGTTCCATATTGAGTTTTTCTGATAAAGATT
TTTCTTGCTCAGTT
ACTACACATCCAATATGCATTATGTTGTTAGATACCCTTGCAAGA
ACAAGTATAATGAAC
GAAACAATGAGATTAACAAAAGGTGTTACATCGACGTCGTTATA
AGACGTGGGAAATTGA
GATGAAAAGTAGTCAAACATAATCCAAAACCCTCGAAATATATT

GACTACACCGATTACA
GCTATCAAACAAATTCCGTCTTCTACAGCCATTACTAGATAGTTG
CTGTACCGTCTGGCT
TTCCAGTGAATTTCCTCAGCAAGATCTTCGAGTTGTACGCAAAGA
AAGCAGACAACATAG
CCGAGAATCAATGAGAAAATGCAGGAGATGTTCGGAGCTGACGG
AAAGACGAGCATGTCC
AGGAGGAACCATAGGCCGATCCAGACGCAATTCACAGAGGCCTG
GGCGAGGCCAACCG
>ConsensusfromContig1903 Average coverage: 12.84
GTGGCTGTTCATCAGCTACATACCATGGCTTTACAATCGACATAA
TTTTCTCCAACATAG
CAAATGCATCGTGCTCGATGAATTGAGGGTCCATCACTTGGCAC
AAAAACGAATCGAGAT
TCTCAACTTCTTTGGCGTGCAGGAAAGCCTGATGGTCGCAATGG
AGCACAAATATAAGAG
GCGCCAACAGTTCATGCATTCCCTGATGATAATGGAAGGCTTCAT
GTTTTTTCGAGAAGA
AAAACAAAACGCTTGCAATTTGATCATGAATCTCCTCGCTCTTGA
AGAACGCAATTTCCG
GGTGAGTTCTTACAACGTCCTGCTTGATCGTGGCGCGTAATTCCT
TGTCTTTGAAAAACG
TGTTCCATGGACTCGCATCTTTTTGAGAGAGAGGGTTATTGGAAG
AAACGTCAGTCTCAT
GATGCAACCTCGGGTCATCAATGAACTTCATTTTTAACTGTTCATA
GCGTCCCCTTCTGT
CCTCCAGCGCTTCTAGCCATTTCGATCGATCGCACGGAAGAACAT
CCAAGAACAACTGCC
AGCAGACACCTCGAAAACGACAGCTCCTCAAACTTCCGTCTAAA
GCAGAGCGTCGAAGCC
TCAAAAGATAATCGGCTGGCGAGAACAGTCG
>ConsensusfromContig1904 Average coverage: 13.83
CTGCGACAGAGGCAGAAGCACGCTTCGAGGCAAGATCATTCTCA
ATGACGCATGTTATAA
CTGTACCTTTTTGAACCTTATGCAATATGATTTTATTCTCACTGAA
CAATTCCAGGTTTC
CAGAAGTCTTCGTTATTTCTTTTTCCACACCATGTTCATCGAATTT
CATCCATTTCGCAG
TGGGCTGTGGTACACCTTTCCCGGCGCATAAGAGTGTATGCGTAT
CTTCTACTGTCAAGT
TGACAGTCATCGGTTTCATATTCGGTGCAAAATCAGGATATTCAT
GTACCTCGATGTTGA

AAATTTTTTTTTCACCCTTGAATTCACAGTAATATCCATTTCCAGT
CATATCCAACATTA
CTGTTGGGACGCAAAGCTGTTCATTATTATATTTCATTACGCCTCC
TTTGCGTTTCAATT
CCGGATCCCATTTGACAATTTGATCAGAGAAACCTTTCGTCAGAC
ATTTCAAACATTTGT
TTTTATCACCTTCAATCCATGTTATTACACCACTCGGATATTCCAC
CAATTCTTGTTCAA
CGACAACATTATCATCACCATCTTTGGCGTCGAATAACAACTGAT
ATTTCACATTTAAAT
ATTCCGACCTGTCACCACCAACCAATTTTGATGCGCATTCCAATTT
GACTTGCCGTTCTC
CCCCTACCCATTTGAAGGTCATGCCTTCGGGTATTTCAAAGAGAT
TTCGTATGATCAAAG
TTCCATCATCGAGAACGAACACAGTATTCAATTTTTGATTCAGCA
AAGCACCATTTTTCC
ATACAAAATCATAC
>ConsensusfromContig1905 Average coverage: 11.44
ACACGACGCTCTTCCGATCTGGAGATGCCATATTTGAATTCAAAT
CATCCTGGATGTACA
GAGGAGTAGAGAGACACCCAACAATAAAGATGATCACAGCTCAA
ACAATGATAAGTGCAG
ACATAACTCCAGATGACTTCTACAATTCTGCCTTATCTTGCACCC
AACAATTGCTGGCCA
ACAATGGTGACTATGAGTTGTGCTTGCTTTTGATTCAGAGTTTTT
GCAGAAGGTTCCAAG
TTCTTTACGGTGTCGGACCAGGCGGAAGATATAGAATGATTCCA
GATCACTCACAAGGGA
TGCTTCCACCTTCGTTAGGTGGGTTTCCTGCATTGAACCTATCAG
ATATCTATCTGGGAC
TAGGGTCTTTCAGAATTATGAGGTGCCTGCAAATTCTCTGGAGGT
CTGAATGCGCTGCAT
CGAAGTTCTGTCATCATGATGCCATAATCAGGCTCATGAAACTGG
CAAAGTCTGAAGACA
GTTTCTACGTTCTGGGCTTCTACATGCCTAGAAGTTTCCGTCAGA
GAATGGTGATGCAGG
CTCATAGAGACAAAATTGTTAAAGATGATTCAGATGATCCTCTAA
TTTGGATCGACAAAT
GTGTCTTAGAAGCTCACTGCAGCGACAAGATGTGCGGAGCTACT
CAGAGGGTGGTCAATG
CTATGACATCTTATACCAGATCAAGTTTCCTTAATGTAGATCCTT
TGAAGAAAATGAGGA

AAGCAGTTCATCTTAGAGAAGGGCAGTTTGTGAAAGGCGTTGAT
GGAAAGGAGAGATGGT
CGGATTT
>ConsensusfromContig1906 Average coverage: 13.06
AGTTTTTATTAATCCCATCCACTACTGCGTGGTTGTCTGAATAAC
ATGTCAGACGACAGT
TTTTGAGTGATTTCCCCCATACCAGCACCCCTGCAATAATGGGGA
AAAGCTCCAATACCG
CAATATTCAAGTCTTTGAACTGATTCTCCCAGATACCATGAAGCC
ACTTGGGTCCCAACT
TTGCCCCATAACCTAACTGAGAAGAATCTGTGAACAAAAACTTAT
CTTGAAGGAACGCAT
CTCCGAAATAGAGCCTCCCATTGAAAGATTCCAGGAATTTAAGCC
AAATCCTCAAGTCCT
CTTTTGATCCCGGATTCAGGCGCCTCCTGTGGTGGGGCTTTGTTA
GGCCAATTGAAAGGT
CAATTAGCCGCCGCAGAAAGGGGCGACCCACAGATATGACTACA
GTCAGAAATTGCAGTG
AACCAATAAGTGATTGCAGCTGACGCAGGTGCAAATAGCGCTGC
TGCAAAGTAGACCTGA
TGAGAGATAATGAGTTAACCAACTTATCCTCAGGAATCCTAGTGA
CACTTGCAAAAGTGT
CAATCTCAATTCCCAGAAATGCCAGGCAATTTGTCGGTCCTTCTG
TCTTGGAATGGGCCA
GAGGAACACCTAATCGATCACAAAGTTCAAAGAAGATCATCAAT
GCAGCGCGACAGAGTT
CAGGTGAAGAAGCTATGAACAAAAAATCATCCAAGTAATGTAAG
GACTTCACCCCCTCAG
GAAGCTGGCGGTCGAGTCCCCATTGTAGCGCTGTACTGAAAGTTT
CAAAAATCTGACAGC
TTTGGGCAAGGCCCATTGGTAGACAGTTGTCATGATAAAACATAT
CTCGCCATTTGAAAC
CAAGTAACCTCCTGTGGGACGGCAAGATAGGAATAATCCTAAATG
CACTTTTTATGTCTG
CCTTGGCCAGATATGGCATACCTGGAAAGGACCTAATGATGCTG
ACAGCATCATCCAGCG
ATGCATACTGAACATGTCTGCTGCTAGTTGGTATGCTGTCATTCA
CAGATGACCCTGACG
GTGCACTCAAGTTTTGGATCACCCTGAATTCCCCTGCACACTTTT
TAGGTACCACCCCTA
TGGGACTTGTGACAAAATTCTCAAAAGGGGGTATTTCAAAAGGCC
CTGACATTCGTCCTA

AAGCTAGCTCCCCTTTAATATAATTGTCAATAATTTGAGGAAATT
CAGTTGCTGAAACAA
GATTTTTAATACATGACTCAGGATTACCCTGAGAATCAA
>ConsensusfromContig1907 Average coverage: 21.92
TAAGAATCAGATTTTTTGCCAAAGCGGCTGCCTTCGAAGAAATCG
TCTTGGTTTGATTGA
TGTGATTCCCACTTCAATATTACATCCTCGGGACGCCTCTCGAAT
GTAGAACAGAAAATT
TCGGATATTGTTGCTCCAGACTCCATGTCACCAAAACTTGTGAAA
ATGAAGCACGACATG
GCGATCATGAGATTGAGGAAAGCGAAAACGATAGTGGCAACAAG
ACTGGCTCGTGTGATG
GAGAAAACGGCCAGAATCCAGAAGGGGGCATCAGAGTTGCAA
TCAAGACAGAGGCAATA
GCACTGTTCCTTATTTGAGACCTCTTGCTTCCAGTCGATTCCAAAT
ATTTTGCACTTTTT
AGAATTGCAATTGCTCCAGAACACATGATCACTAGAGCAATGAA
GGGTGGACTTAGAAGG
TATGTTTTCATTTCATCATCCAGTGCAATCCAACAGTTTTGCCTCA
GAGAATCTTCATAT
TTCGCAGCGGTGAACATCACTCCCCAAAATATTGCTGAAAACCCC
CATCCAAAAATAGGC
AGGTAGAGGGTGATAATATGCGGCGACGAAGTAATAATATTCATT
TGCATGAGCATTTGC
ATTGTTTGCAGAATATTTACTGCGCTCGATACCCAAACAATGTAA
GTCATCACCATGGCA
ACCGAT
>ConsensusfromContig1908 Average coverage: 16.09
AGTTTTTCTATCCAATCGTGCCGTCTATATCCTGGCTTGGAACA
CAAGACTTGGATTCG
AACATTCTGGCCTGGAGTTTTGGCTTTGTAGTATAGCGTGCCATT
GCCCAAAAGCCCCAA
TATTCGTCGTTGGAACCCATGCAGATCAGATAACCAGACCGGAG
ATTCCCATAGGACAAC
TGAGGAAGCGATTTCCGAATATAACAGCCTTTTACACAGTCAGCT
CAATCACCGGCCAGG
ATATCGGGTCTCTCTATGAAAAATTGATGAGTGTGACACTGGAAC
AGCCCTATATTGGAG
AGCAGATTCCAGAGGCTTGGCATGACCTCGAGAAAAGAGTTATC
GCTAAGAGAAATGAAG
CAAGTTTAATATCTATGGAAAGATTTCGGCACATCGCCAAGTCAG
CAGGAGTTTATGGCG

AAGAGATCCAGCGGGCAGTGTCTTTCTTGCATGAGCTTGGAGCTA
TTCAACATTTCAACG
TCGATGTGTTGAGAGACAAAGTGGTGACGAACCCTCAATGGATT
GTGGACGCCATGGCAT
GTGTCGTATCTGTTCATAGCACGGTTATCGAGGACGGCAAGCTAT
ATCACCGAGACATCC
CCAAGATATGGTCCAACTACCCAAAAGATCTTCATAAATGGCTG
ATCAAACTGACAGAAA
TTTTTGACTTGACTTTCCCACTTCCAGATATTGAGGCCAACATAGT
ACCTTGTCTGCTAC
CACAGGAAGAAGTGCCGTACGAATGGCTTCCGAACCTTGAGAAT
GACGAGCGTTTAGCGA
AAATGAATTACAACTTTGATTACCTCCCCGCAGGACTGTTCAATA
GAACACAAGTACGCC
TGTATGAGTTTTCTGAGTATTCAAGAATGTGGAAATTTGGAAGTA
TTTTGAAGAAAAAGG
G
>ConsensusfromContig1909 Average coverage: 8.74
CAACAGTGACGGAGGTAGGGAAATTTTCGATATTTTATTGACTAT
ACAGACACCCAGGTT
TGAACGAATAACTCGTCTGCATAACTGACAAAGGGAACGGACAG
GAGGAAATTCGGAGTT
GAGTTTTTGAATAATACAAGACTGCTCGCTGTCCCCACTTCCTGAT
TGTGTTGCATTTTT
CGTCTCAGAGCTACCGAGCTGAACGTTTATGGTATAACCCAATTG
AGCGAACAATCTGAG
AGATTCGATGTGGCCCCGGTCAACCGCGACCCTTACCAGTGTTCG
CCGACCGTTGCCATC
ATAACCCAATTTCGCAGTGACGTCTAAATTGTAATTATAAAGTAG
AAACAGCCTCATCGT
TTCAGTTTTTCTGTTGGCAACCGCATCATATAACGCAAGCTCTCC
TTGATAATTGTAGAT
ATGGACGGACTTTCCTTTTCGTAATAGATGCTCGACAATTGGTATA
TAATTTTTTCTCAC
AGCGATATGCAGAGCTGTGTCACCAGACTTGTATTCCCTCAAATT
AATATCACAGCCAGC
GTTTTGAAGCAATGATGCAGCAGCCCTGTTGTTGTAAGAGACGGC
CTCGCTCAAAGGCGT
GTAACCCCACTGATTTTGAGCGTTCAAGGCACTACCTGCTTTTAT
CAACATATCAACAAG
AATGGGATTTCCATGTCTTGCCGCAAAATGGAGAGCGATATCTTT
ATTTTTGTCTCGCAT

ATTTAGATTGCATTTCGCTTTTATCAACTTCTTAATAGCAT
>ConsensusfromContig1910 Average coverage: 11.80
GACCACCAGCATCTTCTTTGGTCCAGTTCATATCAAGTCTTCAAA
CTACATTGAATTCAA
TGAAAAATAAACAGTGTATTATCATGGGGGACATGAACATAGAC
TTATCCAACAAAAATG
ACAGATATGTTCAGGAATATACATCATGTATATCAACAAGTGGGT
TTTGCTCTTGTATAA
GTGAACCAACCAGAATCACAGCTCATTCCAGCACAGTTATTGACC
ACATATTGACCAATA
TAGAGAACAAATATCTAGAATGTGGAAATATATATACATCAATCA
GTGACCATTTCACAA
ATTTTATATGTATGTTCAATGTTGATGAAAAGTTCAACTCAATGC
CACCTCAGAAAAGAT
ATATTTTCTCAAGATATAATGTAAATAGTTTCAGAACAAAACTTC
AACAAATTGACTGGT
CTCCTGTATTATCATGTGATGATGTTGATGCAGCCTATGAAATCTT
TTATGCTCTTTTTA
TGAAAATATGTGATGAATACATAACTATTGATGCATTGCAACAA
AATGCTAAATTGAGAA
AACCCTGGATGACCATTGGCTTGAATAAATGCATTAAAACCAGA
GATAAAATGTACAAGA
TATTAGTAAAACAACCATTCAACATTCATCTCAGGTCTAAATTCA
AAAAGTATAAAGCTG
TCTTAAAGAAAACAATCATAAATGCAGAAAAAGATTATTACATC
AAAAAATTTCAAGAGA
ACCCATCAGACTCCAGAAGTACATGGAAAACAATAAATGAGATC
TTAAACAATACAAAAT
CTAAAAATCAACATGTACCAAAAAAACTCATCATAAATGGGAAT
GTTTTGAATGAAACAA
CAGAAATTGCAAACTCATTCAACTATTTTTCTCAAAAATTGGAA
GTGATATATCTGAAT
CTCAACCACCATCAAATATATCCCCATATGATTTGATGAACACTA
ATAGAGAAAATAACT
TCAAATTCCAACCTCCCACTGCTCAAGAAATACAGAATATTATCA
AAAATATAAAAACTG
ATAAAGCAGTTGGGCCTGAT
>ConsensusfromContig1911 Average coverage: 7.03
TCCTGCACTCATCAAAAAGGTCATCTTTGAACGCGTCGAACGTCT
TCTCAATTGTCAGAT
GGATCAATACGCACGCGTTGATAGAATCAAGTCGGCTATCATTGC
TGGAGAGTATTTCGT

CAATCGATGATCTATCGGCAGAATTTGTTGAAATCTTCTCTAGAG
AATCATACGACTTTT
GAATCGAATTAAATTCAGAAATGATAAAGTCAACAATTTTTTGG
CCCAAAATTGAAAACG
ATTTGCCGCTTTGATTCAAGTACAATTCATAGATCGCCTCATGAA
CATATTTCTGAGCTT
CACAGATTCTTTTATATAAACTTTCAGCAAATTCCAGAGGATCGA
TATGTGGAACTTTGG
CGGCATCGACATGTGTATCTAGCACCATTTTGGAAGATTTTACAA
CAAATTCATTCCTCA
AATTTTGTTTTCGAGAATCTGAATCACAGCGAGACAGTTCATTAC
CATTTTCATATTGGG
TAAGGCAGTCAAAATATGAC
>ConsensusfromContig1912 Average coverage: 14.86
TTCGAGGTTTTCAATGCAACATTGTTAATTTATCAATATTCTTTTA
CTGTTAATATATAA
CATAAATATATTCTACGTTGCCATGTTATTTGTTATTTATTACTGA
GCATTGTCAGCTGA
ATCTTAATTGTTGTTGTTTGCTTTGGCTTAATTTATTTGTTGGTTTC
AATCCTAACATTT
TTCGGGACCATAAACCTCTGATTATCCACCCCATGTTTTTATGTAG
CTTATGTTATATTT
ATACAATAGTGATTCGTTTGAAATACCGTTGAGTATTATACAGCC
CGATTACATAGGATA
TCATGGTCTACATTCGACTCGCGTGTTTCCAGCACACGTTATTGAC
TTGTTATGTATTGT
TTCGTGAAAATTGTTCTTGTTTTGAATGAAATATTATAGGGTATA
GAGTATGTAATAAAC
ATATTGTCAATTAAGAATTGTGGGTTAGATTTGGTTAAAACACAA
ATTACACGTAGA
>ConsensusfromContig1913 Average coverage: 11.24
TTCTACACAAAAAAATACTGGGAAACCGTTGATTTGGATTGCCAG
ACAGGGTATGTGATT
GACAATGTCAAAAAAGAAACCTCTGCAGAATTCCGTTGTATGAG
GGACGGGAAATGGCTA
CCAAAAGACTGCGTTCCTCAGAAATGCCGACTCAAAACTACCAA
TGCTAGTCCACGCGAA
CTAATAGCAGAGTTTGGTCAACCAAAGGTTAAAATGACGTGCAA
AACAGGATTTGGCATT
ACAATATCGGGACATACGTTTCCTTCCTGGCACCACGGTTGCAAG
TGGGACGGGACATGG
GACCCTCCTATTATAGAATGCGTTGTACGGAGCTGTCCGGACAAA

ACTGTGGCATATGCT
GTGAATCGTTTCCATAGAACGATCAAAGGTAAAGTATATCAAGA
CAAACATGAAGTGATG
TGTAGAACGGGCTATGTATTCGAGCTCTACAGCACGAAACGTTCT
CTAATCTATGCCTGT
AACTCCAGCGGAGAATGGGATCCACCAATTTCCAACTGTGTCGAT
ATCAATGAATGCATC
TCCAATCCTTGCGTGAATGCAGGAACATGCATTAACGGAGCCAAC
AAGTATTCATGTAAT
TGTGCTCCTGAATGGGGAGGATCCAACTGCGAAAGACATAGATG
TCTTAACATGACAATG
ACCAATGCAATGCCAGCTTTGATCCCCGGAAAATTCCCTGGAGA
AAATGTAGAGACGCAG
TGCAAATCAGGCTTTGTTTTTGAAAACACCAACAAGAAAACTTTC
GAAAATCATAGATGT
GACTCAAGCGGAAAATGG
>ConsensusfromContig1914 Average coverage: 21.33
GAAGAGAAAGCAGACACTACATCTATTGAAGAGCAAATCAAGAT
GGAAGCTAGCACGATT
GAGAAAACGCCAAGCAAATCTTCTGCGATTTCTGTTGAGCCGTTC
ATTGAAGAATCGTTA
CAACCAGCGGAGGACAAACCAGTCGAAGAGTCTTCAGTGGAGAC
ATCTATCGAGAAGCCT
CAAGAAAAAGCAGAACGCAAGCCAAGCGTGACGATTCCAGAATT
CCTTGAGACATATCAG
GACACTACTGTTGAGGTGCGCAAAAATCTTGTGCTGAAGTCAAAA
ATTTCCGCAAAACCA
GCACCAGAAGTGAAGTGGTACAGAGACGGAGTTATAATCAAACC
AACCTTCAAGATCAAG
ATGACACAAGAAAATGAGACGTGCACATTGACTATTACGGGGGT
CACGGACAAAATGGGA
GGCGTTTACAAGTGCGAAGCCATAAACACCGCGGGGGCAATGCA
GCACACCGCCATGGTT
ACGGTTGTCGATAAAATTGAAATGCCAAAATTCACCAAGAAGCT
ACCAAGCAAAAAATCA
GACGAAGGATCAGATGTTAAACTCACTGCACAGCTCAAAGGGAA
ACCGAAACCAGAAATA
ACTTGCTATATCAACGAAAATGAAATCACATATACGGAGAAGAT
TACAGTAAAGATCACA
GAAACCAAGACAGACAACCTGGTCGAGTTGACCTTCAAAGAAAT
ATCGGCCGAATACAAC
GGAACAGTAAAAATGGA

>ConsensusfromContig1915 Average coverage: 24.53
CTTGTAGAGCCTCGGACTGGAACCGTCACCACCAACATGTACTTC
AGCAGTTACATGACT
GGATTTTTTCGGTTCAAGGTCATTGTGGATGACTCTATGAACAGC
CATCGCGACACCACA
ATGATCGAGATTTATATTCTGAACAAATACCAACGGGTGAAAGT
CATCATGAGAGGAACC
CCTGCATCTGTCAGAAGCCTCAAGGATGATTTTTCCATGTCACTG
TCTAATAAAACTGGA
GTCTCAATAAATGCCGATGAAGTTCGAACGCACATGTCGAATGG
GGGAAGCTCCGACGAC
AGGAGAGTGGATATGTTCATCCATGGAATCGAACCAGGGTCCGC
CAACGTCTTGGATGCC
AATCAGCTCATACGATTAATAGACGAGAATTATCTCTCACTAGTT
GACCTATTCAACGAT
TATAATGTGCTTGAGATTGTGCCTGCCGTGAGCGTAGCCTCGGAT
GCTTCGTGCGAAGAT
GATGAGTTAATCATCGTCCTCATCATCATTTCTGTGATTCTTTTCC
TCTTCCTCCTCCTC
ACATGTCTCTTCCTTTGGTGCTGTCGGTCCCGCTATAAACGACGG
ATAAAGGCTGCTACA
GCCATGGCTTTTGGCCCAAGAATAAACAGTTCGGCCGGCACAACC
GACGAGACTTTTGCC
AACGCGCACACAATATGGTCCGAGGCAAGCCTGAACGACCTTTC
AAAGGAAACCAACACA
GACAGTATAGACCGTGGCGCCGCCAATAACAATATAAACGTA
>ConsensusfromContig1916 Average coverage: 14.74
ACCCCGAAACGTTACCTGAAAAAGAACAAAGACGTTCCCCCACT
GACGCCAACGAAAACT
GAAAATTTCTTCGAGGCCATAAAAGCATCTGGGGTCAAGAGTGTT
GTTCTCGCAATGCAC
CCGGCATACTCCCAGTCTTTTTCTGATGTAACGGAAGACAGGCAA
CCCTCGCTTTCGGAG
CTTTACGATAGATCAGCGGTTGATTTTAACTTCGCAAAACTCTTG
GACAAATGTGAGGAG
GTGCTAGACAACATTGAGATTTCTACAACGACAGCCGAAGGAAT
CGAGTCCAGAACGAGA
AAACAGTCGAAGAGCAAGTCCTGGTTCAGGCATCGTGAAGGCAG
AGTAACCGCTTCAGTC
ATGAAATCCGTTCTGCATACAGACCCTTGTTCTCCATCTCTGAGC
CTTGTGCGGAAGATA
TGCTATCCGTCGCACTTTAAACTTAACACCGCATCAGTCAAATGG

GGGTGTACGCATGAG
CGAACTGCAAGAACCAAATACCTGTTGGAAGAAAAGGAGATTCA
TCAAGACCTCAGTGTA
TCAGATTGCGGATTCTACATATCTACGGACCACCCCTTCATCGGC
GCCTCTCCAGATGGC
ATTGTGGAGTGTTCATGTTGCGGTAGAGGTTGTCTCGAGGTCAAA
TGTCCTTCGTGTGTC
ACTCCAGACACAGCCGACTACTTGATTCGAGGGTCTGATGGAGCA
CTCAATCTAAATAGG
GATCATCAATATTATTATCAGGTGATCACCCAAATCCACGTGACC
GGACTCGAGTATTGT
GATTTTGTTGTATGGACGCCCACCGGCATGCATAATGAGCGAATC
ACACGAAACGATGAT
TTTTGGAAGAGATGTGTGACCGGTGCCAAAGAATTTTTTTGCAG
GCTGTATTACCAGAG
CTAGTGGCGAGATTCTACTCTCAGAGAAGGCCGGTTCTATCCGTC
CCGACTCTTGATGTC
TCCGGTGCTGTCTGTTACTGCCGAAGAGTAATTAGCGAAGATGCA
CTTAAGTGCACTAAT
CCAGATTGCGTTTTGAAGCTTTACCATCTAAGTTGTTTGAAATAT
AAAGCGATTCCTAAA
ACGAAAAAATGGTTTTGTCCGGAGTGTAGGTCAAAAAATTGATAT
ATTTTAATGTGTG
>ConsensusfromContig1917 Average coverage: 13.41
GAGATTCCGGAGTCAATTCTTTGATGAGCGAAGACGAAAACGAC
GGCACTGATTTTAGAA
AATTGGTCGTCAGAGACGCGGAAAAAATACTGAAAATAAAAAT
GGAATCGAAAACATCA
GCGACGTGGCATTTTTGTCAGATAACAAAATGGCCGTTTGCGAC
AAAGTGAAACAAACAC
TTTCTATCTTCACGCAAGAAGGAGAGCATCTAAAACCTCTAGTCA
AACAAATATACCCTA
TGGGAATTGCGTCTTACAGACCCGGTGGTGGCATCATGGCATTCA
CGGCCAGATATAGGG
GGGCAACGAGGGTATTCGTCATGAATGAATCAGGAGGACTCATG
GGTTCGTGGGGGGCTG
ATGAATCGGAGTGCAAAAACCCAACAGCTATTGCACTGACCAAC
AAAGGACAAGTTGTCG
TGGCTGATACTTTCCATAATTCCGTCGGAGTGTACACATTAGATG
GTCGCAAAATATGGT
CACACGGGTCGAGAAAGCCTTACATGAAGTACTGTTGTATAAAGT
CTTACACTAACACCG

AGTTCGATTCACC
>ConsensusfromContig1918 Average coverage: 12.24
GCTCGGTACATACTGATTCTTCTTGATACAGATGTGAGAATGAGG
AGTGGACGTTCTCCC
TTCTGAAGAATTTTCGATCAATATATGCAAATACGAGTAGGAGA
AATAAGACTCCGAGAA
TAATGGCGTATATTGTGCTGAATGCCCATCCGTCATTCGGGAAAT
GAAGTTCAAATTCAA
GTCTTGCCAGTTTCAAATATTCTGCGCTCTCCATGGATTCACCTT
GATACAAAGGTGTGA
TGCGGACAACGTAGTATCTGTCCTCGAGGACATCCCCAACATCTA
TCTGATTGGATGAGA
CTGTCTTATTTACCACTAGTCGGCTCATTGTTGTTGCGTCGTTTTT
TCGCCCTTTCCCAA
CCAGCACCGAATAGCCAATGGAATTGCAGAAAGCTGGTCGTTTG
GGGGCGTTCCAGACAA
GTTTCAAGACCCCCTCGATGAGCATCATACTTTTTTTACCGACTT
GCCAATCAGATCGGT
CTTCGCTGTCATACATTTCATCGAACAAAAGAGGAAGATTCACCC
CATCATACGTATCTT
TATTACTTAGATCGGAAGA
>ConsensusfromContig1919 Average coverage: 7.73
ACACGACGCTCTTCCGATCTCTCGGTATCGTCTGCTCAGGGTTGA
TCGTTGACTGTCCGT
CTGTCAGAAGAATTGCTATCTTTTTAACACTTGCGCTGTTTCTTCC
GAAGTTTCTGAAAT
GTTCTTGCATAGTTTGTATACCACTTGCTGTATGTGTGTTGCTGCC
AATATAAGCAATAT
TTAGGACTGCGTTCTGTATCTCGCCCTTGGAGAAAAATTGATCAA
GTCTGAATACGTTGG
CTCCAGAGTCTGAAAATGTCACTGCGCCTATACGAGTTCGATCAG
AACCAATGGCCAACG
AGTCGACCACTTGGTTGACAAATTGCAGCATAAGAAGCCAGTTA
TCGAAACTACCATCAG
ACGGGTTTGCATCACGTATACTGCCAGAGCTATCAAGGACAAAT
ATAATATCAGCAACAG
TATCTTGACATTCTTGAGCCTCGTTGACACACGTTTGCTGAGCAA
TATTCCCAGCAATAG
TGTTCAGTGTGGAGAAGTCTGTTGCAAGATAATAATTAACATCAA
GTTGTTGGGGTAGTG
AAGAAATTCTTTGTACTTCCTGTACATTAACAGCATCTGTTACTC
CGATCGATATAACTC

GAATTCCTTTTTGACGGGCATTTTCTGCCTCGGGAATTGTGAGAG
CAGCCTCTGTATTGG
ATTCACCGTCTGTTACGACAATTGCGACGTTTTGAGCGTTGGGTCT
ATCTCCATTGTTTG
AAGTGAATTGCTGTTCGTGCATAAATCGAAGACCTGAGGCGGTGT
TTGTGAAGCTTCCGA
TGTAGGAAATCTGATCTATTGCGTTTTTGATATCATTAATGGAGC
TATATCTGTTGAGGA
AAAAGTTGTTTATAGCATTTTCAGAATAGGTGACGGCACCGACTC
GAATAGAGATTGTCG
GAGATGAAGCTACCAAGATGTCGAGAATCTGTTTGCAGAAATCC
AACATGAATTGCCAAT
TATTTGCTCCTTTATCTCGAATACTTC
>ConsensusfromContig1920 Average coverage: 7.48
CAGAAATCAAAATTATAGAGCGGTCTTGGTTGAGAACATCAGGAC
GTGTCACTTTTTCTT
TTCCATCTTCGACACCCTCAAGAATCAAGTTCAGTCTCGATGTCA
GAACCTGGCAGCCTT
TTTCATCGCTATTCTGGTTTCGATGATTATTCACATTCTCGATTTT
GATCACGACACAAA
CCCTATCGATGAGCTGCTGTGGACGGCTCCGGTAACCGATCCGA
AAGACACGGCGAAAAG
TATCGGTATGTCTTTTGTATGTATAATTGCTGCCACATATCTTCCA
AACCTCATCTTCAA
AAAAGTCTCAAGGATAACAATTCTGAAGAAGTCTAGATTTATGA
TGAAGGAACGCCTTCA
AAGAGTCATAGACCGTTACCATGGGGATGCTAGAGAGGTCGGGC
CTACTGTACTCGAGAG
GGATCGCTGGGTGAAGTTTGCCGGAATAGACACAAGATATGTTC
CAGAAAAAGTCACAAA
AAGAAAAACCATAGATTCGCCAGTGCCGGCAGAGGACAAAAAC
GCATCAGAAGAGAGCGA
GGGAGAAGGATATTATAGCCAGTTCATGAACCGATTTGGGTTCA
CAACAAATGCACCAGG
ATTTGGCAGAAAAAAGCCACCCAAACATTCTTATCACAATAAAG
ATAACGTCTCAATCGA
AGCGGAAGTAAATTCTTCTGAAATTGATAACGACTACGGAAATGT
TACGTCCTCCAAAAT
AGATTATTTTACTGGAGAGATTTCGATTGGAGATGGAATTCATGG
ATGTGGAA
>ConsensusfromContig1921 Average coverage: 8.40
CAGCAATGGTGACATCTGACAGAGGAGCTCATTTCACTTCAGAAC

TTTTCACATCATTGA
CCAAACTTATCGGAACAACAAGAATCAAAACAACTGCTTACCAC
CCCAAAGGCGATGGAA
TGGTAGAACGTTTTCACCGTACTCTGAAAACAGCAATAAGAGCAA
ACTATTTCAAGTCCA
AGAGGACAGAAGCTTTACCCATGATTTTGCTGGGCATTCGGACG
GCAATCAAGGAAGATA
TTCAGCACTCATCAGCAGAACTCGTTTTCGGACAACCCTGTATCT
TTTAACCGATTTTT
TCAGCAATACTAGCGACAGCCAAGATATATCGTCATATGTCGGC
AGGTTGAAAAGAACGA
TGAACGACCTCAAACCAGTTCCCTGAACTAGACATGGCCAAAGC
AAAACAAGTTTCACGT
GAAAAGACTTATCTTTGGCGACACATGTTTTTGTAAGGGGCCCAC
CGATACGAAACGCCC
TAGACCCGCGATACGACGGACCTTACAAGGTTATAAGTCGACAAG
ACTACTTCATCGTAC
AACTCAACAATCGACAGGACAAATTATT
>ConsensusfromContig1922 Average coverage: 4.00
AAGAACAAGAGATTCTTGAGACCTATCCCACAGGAAGCAAATT
ATCATTCAATAAAGAA
GTACCGTCTGTACCGTCTATCAGATCTCCTGTGGAGAACAGAAGA
ACAAGAGATTCTTGA
GACCTATCCCACATGGAAGCAAATTATCATTCAATAAAGAAGTAC
CGTCTGTACCGTCTA
TCAGATCTCCTGTGGAGAACAGAAGAACAAGAGATTCTTGAGAC
CTATCCCACATGGAAG
CAAA
>ConsensusfromContig1923 Average coverage: 9.52
AGATATAATTGCCATGGAGAGCTCCTCGTCCATCCTCGCTTCTATC
TTTTCAAGATCCTC
TATCGAATTGATCGGGAGGAAATCGACGTCCTTGACATCCTCCGC
TACCGAATCCCCTTT
CATGAGGCGCATTATCAGTCTCGTGTTGTATTCGGTTTGTTGCCGT
ATTTTGTTCAGTTC
CGTCAAAACCTCGAGCTGAAATGCCTCATTCGAAGATGTTTTCTT
TCCATTTGCCGGTTG
AACAGAGCTCACGAAAGCTTGCAAAATCGGATTCAGTTGCCTGCT
GACGTCCCTCTGACC
TGTAGTCCGTGGGGTTTCCAGTGGAACCGTCAAGGTGGACGAAGT
ATTGATTGTTAGATA
GGA

>ConsensusfromContig1924 Average coverage: 58.45
TTCTACCAGAGTGAAGACACGATTGCATCGAGTATCTTCTTTGCT
GTTAGGGAATCAGGT
GGCACAATGGCGTATAAAGATATTGCCAACTATAAGGCGATAATT
CAATCTCCATTGGAG
ATTACCTACAAAAAGCACCAGACATTGACAAGTCGGGCGCCGAG
CGGAGGACCTCAAATG
ATGCAGATGATTTCTTTCGTGGCGAACAAGCCAATTTCTGCCAGC
AGCGGACCCAACAAA
GCATCCAAAGACTCGAAGGTGTCCAAACGGGAAGTTACTCCTTTA
ACAGCAGATCTTCCC
TTGGGCAATACTCCCGAGTTGAGCAGTGACAAGAATGATTCCAA
TGGAATTAACCAAGAC
ATCATCAGAGTTATTCAAATGTTGACGCAGACAGAATCACTTGG
GACTGACACGGACGAA
GGGGTGGTTAGTCTCAACCCATACAAACCCGCGGGTTCGACGCAT
TTTTCTATCATTGGT
GGGGACGGCATGCTCGTTAGCGTGACGCAGGGCCTGAATTCGCCG
TGGGGTTCCGGCTTG
ATGACCTACTCTGGTTTCATGCTCAACGATATGGTCACAGAAGAA
TCGATAATGCCGTCA
TCACGAGACGAGACGACTCGATTGGCATGGAACGTCGCTCCCTCC
GTTTTCCTGGACCCG
AGCTCTCCGTGCTCTCTTCGGGGGGCGTTTGGAGCGGCTGGGGGT
GAAGCTGTCCCGGCA
GTGTTAGCGCAAGTGATGATGGATATTCTCGATGATAGCGTTTCC
ATCAAGGAGGCAGTT
GTGTCTCCGAAGTTTTATGTGGACATGGGAAAACTTTATTATGAA
AGTTCGAGAAGTACT
TTTGACGCAAGTCAACTGGACAGGTTTGGCTACACAGTGGAGGCC
ACTCCCACATTGGGG
CGTGTCTTCGGTGTTCAAATGATAGAGGATCGCATGGATGCCTTC
TCCAATGTCAAGGAC
CTCAACGACGAGAATCTCATTGCTCTCAATTTTGTCTAATCTGGTC
TCTTATGTATTTCC
CTGATCGAATTTAATACCTTTATTGATACTGACACATAATAAACA
TCATGACATCTGACT
ATTGAAAGATCCGGCGTTGATGCATTTGATTATATTTTAATAAAT
CACTCTCTACATTCC
TCAAATGACATTTTATTTTTGTGATCACTGAAGGCGTTATGTATG
TATTGAAGAATCTCG
TTGAAATTAAGA

>ConsensusfromContig1925 Average coverage: 24.37
TCACAAACGGGTCTGGAGAAAACTCGTTGTAGTTGATTCTGGAAC
CGTTGAGAGCTATGT
CGAAAGCTGCTGAAAACCCGCCCACTTTTGTAGAGCCAACGATG
ACAATGATAACAAAAC
AAACAAAAACAATCACCATCTGAAGGGCGTCGTTCAGTACAGTGG
CTTTCATTCCACCCA
CCGCTGTGTAAAACGTACAAACTCCTCCTACTGCCAAAATAGCAA
CTTCCAAGGACATAT
CCGACACTTGACTAAGAGCTAAGGCTGGTGTGTATAGAACAACC
ATGAGGTACATCATCA
CCATGAGCGAGAAAGTAAGCGCACCGCAAACTCGCATTGTTCTG
TTGAATCGAAGCTCCA
AGTATTCATAGCCGCTGGTGACCTTAAGCCGATGCAAAACAGGA
AGATGCAAATGACCGG
CACCTCCGAATCCGAGATAGTAGGAGATGAAAATCCAAGTAAAC
GCAGTCCCGAATATGT
ATATCTCCGCAGGAATACTTATGACGAATATTG
>ConsensusfromContig1926 Average coverage: 8.30
CTGGAAGCCACACAAAGCTTGGAGAATCCTTCTCTTCTGGAGGCA
TCATTTTCTTCATTT
TGTAAAATACTTTGACCGTAAAATAGGTTTGCATATGAGAGCATG
ATATCCATTTGAGGA
GCTTCACTCCCATGCAATCGTGATGCCATTTCAAAACAGTGTTTG
ACGTCCTTGGGGAGA
CTTAATGACATCTTCGATATCCGAATCCAAATTTCCATCTCCACAA
TTTTCTCTGGCCAC
TTGTTTTTTTCAATCAATTGAATAAGCTCTGATGTCGAAATACTTT
CTTTGAACTCATAT
GTTTTGAATACAGTGGAAGATGTCATCTCAAGTTGAAACAAGACT
TTCACACATTCCAAT
GAATCTTTTACATCTGATGTATCGAAATTGAGAAATGTAGACTCC
AGAGAATTTATGGAA
ATCTGCTGTTGCAGCAGGCCTTTGATGATGACAAATATTTTGACC
AATGGCCATTTCACA
GACTCTGGAACTGAGCTTTGCAATGGGTTGAGGGCTGCGAAGGCA
TATTCACAAATCATC
AGGGCTGAATTCAAACGATCCTGTGATTTAGGGTCAACTGGCCCC
GTGGAACTTGTACTA
GAGCTCTGCATTTGGGCCCCCTTTTTTCCTTTAGCAGGATTCTTTG
GTTCTTCAGAGGTT
ATTTCTTTCTTGGGCTCTTGACTATAATGTGATTCCAATTTTTCTT

GACACAGTACCACG
CATGTTTTGATAAGCAGGCCTATGTTTAAAGTGTGTTCAAACGTC
TTGAGTACCTCAAAT
GCCGTTTGAAAATGTTCAGCAACCTTTTCATAACTTCCAGGAACC
ATTTTGTGACGAAGA
TAATTAAACAAGTAAGTGGCCGCATTGGTAGAGATCCATGAGACT
CTAATGCTCTCTCCA
ATTTTGAGAGCACGGATAAAGTCTACTATGATCGAATCTGAGAGT
TCATCACACCATTTG
CAAAACTTTACCCAACATTCGTTTGAATCAAGATCTTTT
>ConsensusfromContig1927 Average coverage: 12.43
CTTCCGATCTCTCCGAAAACACAAAAATACAGAGAAGGCTAATC
AACATAGAAAGCGTAC
GAAATGGGAATTTTTGAGTGTCTGTCCCATAGTCATAAGTTGGAT
ATTTTAAAACTGCCG
GTATACCAATCAATGGCTCTCCACCGAGAATTCGGAGGAGCATTC
CAACTGAATATGCGA
CCACTGACCCGTAGATATTCATCCTCTCGTAATAAAGGACACACG
TCAATTGGGGGAAAA
TTATGACGAATACCAAATCAGAGCAGAGAAAAAATAAGGCATAA
ATACTGTTGATAAAGA
TGGCGCAAATTGTTGCCGTAGCACCAGCACAGATGATAGCAAGC
CGCATGACGAGCAGCA
CTTCTTTGTCAGAGGCCTTCTTTCTAAATATGGCCTTGTATATATT
TCTTGCAAACATTG
AACCAGCGGAAAGTACAGCAGAGTCAG
>ConsensusfromContig1928 Average coverage: 76.39
TTCTTCGTCGTCGTCCGCAAGTTTAAAAATCCTATTTGAGAAATTA
CTTATAGCTATCTG
CTAAATATTATTTAAGTCAATTTCAAATTCACCGTCAAATGGATG
GAAACGGGTTTGAAA
AAATATCAAAGACAAGTCAGGCTGCTGATCAATTTGTTGAGTTAT
ATTATGAAACATTCA
ACAAAACACCGCAGGTTCTTGCAAAACTTTATTCAAGTTCAGCAT
CTGTCGTATGGAACG
GTAAGGTTGTTAAAGGAGTTGATGAGATCGGAACGTTTTTATCAA
TGCTCCCATGTGCGA
CGTTTGTAACAGAAGCTTTCAATGCTCAGCCAATAGAAGATATC
ACAGGAGAGAGTCGGA
CGAATATATTAGTCACCGTTTCAGGATATGTTTCTTTCGACAAAT
ACCCGCGGAAGTTTT
TCAATCAATCTTTCCTGCTGACGAGTGAGCAGACGTCTTGGAAGA

TTGTTTCAGACACTA
TACGTTTGATAGAATGATTTTGAAATGTTTATATGTATTTTTTCCT
GATTATAATCTCGT
TATGCAAAAAAAAAAAAA
>ConsensusfromContig1929 Average coverage: 11.74
TCTTCCGATCTCCGTGTTGTTATAGCGCCCGAAATTCTTTCAAAAA
ATTGTTTTTGCCCA
AATCTTCGGTGAGCAACTGTAGACGCTACTGTTTTTTCCTCGTGA
ATTAAATCGTGAACT
ATTTCATAAAACATGTTTTCTCTGCTAAGCTTAACACTGCATCCAA
ATTCTGATTTTATT
TGATCGAATACTGGCATCATCTTGAATTCGGCCAGCGCACCTGTC
ACACATCGAATTGGT
ACACATTCCTGTTCCATGTAGATTGAACAAGAGTTCAAGCATTTT
TTGAATTGACAGCAC
GAAAGGCATTTCTGAATTTCTTTCAGGTAACCCGATTCTATATCTT
TCAATGTGAATTTG
CAGCAAGGTTCATTTCTGCACACAGGCTCAGCTCTGCCTCTGTTT
ATCACGTCTTCAGCA
CATGAATTCAGACACATATGGCAGAATGTTCTCAGCCTCGAACAT
CTTTGTGTTCTGTCA
AGAAATGACGTTGAAAGACGTCCGAGTTGATTGTTTATTCCAAAC
GCCTTCCCCAAGGAA
TTCGGCTTCGTG
>ConsensusfromContig1930 Average coverage: 14.83
AATGGAAATTATTGGATCCGTGGGAGCTATCGGATCTGGGGTGTT
GTTCGACGGATCACG
TGGAATCCTCTTTGATGGGACTGACGGTGCTTTCATCGATCTTGG
ATTCCAGGAGACAAA
CATGATCGGAAATGAGACACAGAAAGACAAAGACCTCGAAATCG
CAGTCTGGATGTTTGT
GTCCACAAATGCCTACGGTGCATCTAATTTCATTCTTGGGAGTGG
GAGTAGGGCAAGACG
TTTGTACTATCAAAACAATATAATCAGATTCAAATTCTATGCAGG
AACATCTGGATGCTT
GAAATATCTCTTCATCAGCAGCAAAACGATAGATCCGCAAACAT
GGATTCACATCAAGGC
AACATACTATCATGAATTTGATAAGATCACTATTCATGTGAATGG
CAATTTAGAAAGCAC
TGACACCACTTATTCTGGTGATGATTGCTATGCACAACAACCGG
>ConsensusfromContig1931 Average coverage: 27.70
CGGACAGTAGGTAGTAGTAGTAAAGTAAAATGTCTTCAGCAGATT

TTAAACCTCGTTCAT
ATCAGGTGTCACTGGTGGAACGGGCTAAAGATGAAAATATAATA
GTTTACTTAGAGACAG
GCTCTGGGAAAACGTTTATCGCTGTTATGTTGATCAAAGAGCTCT
CAAGAGATTTATTCC
CACCTTTCTCGAGAAAGACTATTTTTTTAGTTCCAACCGTACCGC
TTGTGAAACAACAAG
CGACAGTTATCAGTCAACACACCAACTTGAAAGTTTCAGAATAT
ATTGGAGAAATGGGCG
TTGATTTTTGGTCCAATGAGAGATGGTACACTGAGATAGATTCTG
GGCAAGTGCTGATCA
TGACTGCCCAAATATTTCTCGATATTCTCAGTCATGGATTTTGTGC
ATTGTCTTTGGTGA
ATCTCATCATTTTTGATGAATGCCACCATTGCAAAGGAAATACTC
CCTACGCTCAAATAA
TGCAGAAATTCGATATTTGCGAGAAGCAGCCAAAGGTGCTGGGC
TTAACAGCCTCATTGA
TAAATACAAAAGGGGATAATCCACAAAGAATAAGAACCGAACTG
AAGACTCTTGAAACTC
TCATGCGTGCCAAAGCAGTTACTACACCAGAAGTTATACCGAAA
ACAGAAATGGCAAAAC
CATCTGAAGATGTGATAAGGTGTGAAACGCGATCAACTGACCGAT
ATGATGTCCTCATTG
ATGAATTAGATAAGGTCATCAGCGATTCCTTGACTTTCATTAATC
TGCGAATGCAAGCAT
TCGATGATCTGTACACCAAATCTAGTGATCCAAAATTTATGGAGG
AAGATGACAGCTTCG
ATTCAGATTCAGATTCCGAAGATTCTCTTGATACAGATTTGCTCA
AACCAGCCAAGATGT
TCAAAAATATACGCAACATACTTCAATTACTCGGACCATGGAGTT
TAGGTGAAGTATGCA
AGTCCTTTCGAATGGATTTACAGCGGACTATTGATAGGTGTTTTG
TAACGGAAAAGAAAC
ATCTTTTGCTGTGGATTTCAACACAACTTTGTCGCATGTCCATGC
TAGTTGAAAAGCATT
GCAGATTAGAATGCGTACAGGATTTGATAGACG
>ConsensusfromContig1932 Average coverage: 31.06
AATTTTCTGATATAACAAAAACGAAATAATAATGATAACAATAAT
ATAACATGATAATTA
TATAACAGTAATATAAAACTCATATTTTCTTTGTCAGGATGGCAT
CAAATGATTATATAG
ACTACTGTAAAATTGGCTTATGACTTAATAAGCCTCAGAATATTA

GACCTCTCTTAGAAT
TGGTACAACGTCATGTTGTTGTAGCCTGAAGCATTATACCCTTCG
CTGTACAACTTCTCG
GTCTTGTACCCCAGAACGTTATATCCTTCTCAGAATCGGTACAACT
TC
>ConsensusfromContig1933 Average coverage: 23.41
AAAATTTCTTTTTTTGGATATTGATACATAACATAGCTGTGTTCTT
CGAAGTTAATTGTT
TTTGTAGTATGAAGTTCATCGTTTTACAAGAACAGACGAATTTTC
ATGCATTCACATTTT
CCAAAGAAATGTGCGCTGTATTTTGTCATATGGATGGAGGTTTGC
CTGGGGACACTGTTA
TCAGACGAATAGAAAACATATTGAGTCATCCATTCTATCGGGGA
TACAGACGAGACTCAA
CCGATGGAGAAACAACATTTGCTGTTTTACCAAGAAAATGTCCG
GAATGTATGCCGACGG
ACCAACCTCAAAGGAAAAATACCCTCAAACTTAGTATGAAGAAT
TGTGCTCATGACCAAC
CTTTTATTATAAATCATAAGGATTATTTACTTGCTTCCAACAAGC
GGATACGAAAATTCC
TTGCGTACGCAGACCAGATATACGCTCTCTATTATGGCCAATTAC
AAGTGATCGACCCAT
CTGGGAACATAGGTCGATTAACTGATAGAGCCAACTATCAAACT
ATGGATATGATTGAAA
CGCGTGGCGGTACACATTTGATTCTTGGCTACCCAAGTTATTGGG
GTGCAAAAAAGAGTT
GGTACATAAATCAACACAGACTGGAGCTTGATCAAGGGGAAGAC
AGAGCCTTTATGTTCA
TGCAGTTCAAGGCTCAACCGGATGAAAGAAGTCTGGGTTTTCATA
TAAATGGACGTAAAT
CCATCCTGCTCACGAATTATGGTCAACTCTTCGTGGACAATTGGG
AAATACGGAACAATC
GTAATAAACTAGATCTGAGCACCCCCAAAAGGCGTCTCGCAACA
CAAAACAAAATACGTT
GTTTCACTCATGTAGCAGGTAGAAATTTAATATATGCAGCGAAT
AAAGACGACACTAGAC
TTATTTACGACATAGGCAGAAATCAGAAATTCACCAAAGATCCA
ATAGTAGGCAGCGACG
GGCGAACAAAAATTCATCGTTTTCTTTCGAACGTGGGCAATTATG
AAGCGCTTATTCATC
AACCCCGTGATATAAGAGGAATTGACGGAAGAGGAAGAGAATGG
ATTGATATTATCAAAA

CAAATTCAGATATTCGGTACGCAGTTGCGGTTGGTGCGAGATCGC
AAAATACAAATATCT
TGGCTCTTGCAACTGATGAGCACGACGTAACTCTCTATGATATCA
GAAACACGAGATACC
ATTACTCTTGTCTAAACAGGCACAGATTTCAGATACGCAAAGCC
GAACCGGATCACAAAG
TTGAAGCAATGACTTACGATGCATACACGGGGATCATATACTCAT
GTGTTTCTCTTGGAA
GTTATGGACCACACTGGGACAATAGAACCATTAACAAACAGATT
TGGAGCTGGAACATCG
GCAGAAACTGTCATGAGACCTACCATACAACTCAGAGAGCACAT
GGAAACATTTGCTATG
AGCGCTTTGGGGCTCATCGTCCATAATACTCCAATAGAACATGA
TGAATGAGAGGCATGA
TGAATGATACTTATCTTTTTTTTAATTTGAGCTTTGATCGAAGAA
AATAAATTGTGATGG
CGTAGTGAAAAATTTACTATTGGATAAT
>ConsensusfromContig1934 Average coverage: 10.71
CCGATCTGGGCAATGAAACTAGATGCTTTTATCACATTCACATTA
TTAATTAATTTCTCC
ATTTTTAAGCAATCCGATGTATATCTCGATTGAAATTGATGTTGA
AATAAAAAATATACG
TCACAAATGTGTTATATAATATTGAAAATTCTAGGATAAAAAACG
ACGCATCCCTGCGTT
CAATAATATTATTTGAGTCATTCATATGTCATCGTATTATTTCAAA
ACTTGTTTTTATGT
AATAATAATGTTTATACGATGCAGTGGTTCGTGATGATCAGACGT
CTCATATGAATTGTG
TTATCTTTCGATTTTTTAGTTTTTCATTTGTTATATTAAACTGTGAT
GTCATTTTAATTT
CAATTAATTTTCATGCTTATTGAAAAAAG
>ConsensusfromContig1935 Average coverage: 35.49
AAAGAAGAAGAAAAAGATGCTGAATCGGGATGCCATTCTCCAG
AGGAAGAAGAAGAAGTA
ACCGATGCGGATATGATGTCCGGCGAATATACTCTCAAATACCAA
ACAACTAAATCTATC
GGCAAAGGTGCATTCGGTTGTGTGAAGTTAGCTACAACCATAGC
CGAACCTAAAACAGAT
GTAATCGTCAAATTCATTAGTAAGTCGAAGGCCCATGACAACTCG
TGGATAGCGGACTCC
AAAACAGGCAACAAAATAACCATGGAGATTTTCTTTCTTAAACGA
CTCAAACATCCCAAC

ATCGTTCGAATGATTGACGTATTTGAGAACGCTACGCATTTCCAG
ATGGTCATGGCGAAG
CATGGGAGCGGTATGGATCTTTTCGAGTTTATTGATAACTCTCCT
AATATGGATGAAGCT
TTAGTAAGTTATATTTTTCGCCAAATTGTATCAGCAGTTGCCTTTG
TTCATTCCTTGGGA
ATTGTCCATCGCGACATCAAAGACGAAAATGTTATTTTGAATGA
GCGATATGAGATAAAA
CTCATCGATTTCGGCTCAGCTGCGTATTTCACGATGGGAAAGCTC
TTCAACACGTTTTGT
GGGACTGTAGAATATTGTAGTCCAGAGGTTTTGATGGGCAACAA
ATACGATGGGCCGGCT
TTGGAGCTTTGGTCAATGGGGGTCACTTTATACACCTTGGTATTT
GGACAAAATCCTTTT
TTCGGAGTCGAAGAAACAATAGCGTGTGTTCTAAAGCCACCTTTT
GACGTATCCCGTGAT
CTTTTCGAGGTGATAAGCTGGCTTCTTCATCCAAACCCCGCCGAA
AGAGCTACGATTTCG
GATATGGAGAATGACCCTTGGGTGTGGCAACCCATCGAACTAGAT
CAAACTTTCTATGAC
TGGGAGTGTGTGATGAATCCAAATGGCG
>ConsensusfromContig1936 Average coverage: 32.28
GTGGCATTTTTTAGTAGAACGCTCAGTCAAGGGGAAAAACATCAT
TGCTCTATGGAAAAG
GAAGCTTACGCTATCGTGGAGTCGGTGCGGAGGTGGAGACATTA
TTTAGCGCAACGTAAA
TTCATCCTTATAACCGACCAGCGCTCTGTATCATATATGTTTGAT
ATCAAACATAAAGGA
AAAATAAAAAGTGAAAAAATTTCCAGATGGAGGTTGGAATTAAT
GCCCTATTCTTACGAG
ATTCACCACCGTTCAGGGAATTCCAATGTTGTTCCAGATGCACTC
TCTAGAAGTGTATGT
GCGGCATCAGCTGACGATAAGGGCCTTTATCAAATTCACACAAAT
TTATGTCATCCTGGT
GTAACTAGGCTCATGCACTTTGTTAGAGATCGGAATCTACCATAT
TCTTTGGAAGACGTG
AAACGGATTTCAAATTCATGTTCGATATGCGCCAAAATAAAACCG
AACTTTTATAAACCA
AAATCAGGACAATTGATACAAGCAATGAAGCCTTTTGATCGAATC
AGCATTGATTTCAAA
GGTCCACTTCCATCGAAATCTGTTAATAAATATCTACTGATTTGT
GTGGACGAATATTCG

AGATTCCCTTTTGCATTCCCATGTAGGGATATCACCTCTCATTCTG
TTATAACATGTCTA
ATCACATTGTTTTCTATTTTTGGGTTGCCCGGATACGTTCACTCT
GACCGAGGATCAAGT
TTTATGTCAGCTGAATTAAA
>ConsensusfromContig1937 Average coverage: 26.67
CAATGTTGAGATCACTCTCGTGTCGCAGTCATATCCCACGGTCAG
AGTCGCAGTCCTATT
GATGTAATCAACGCGAACTTGATGCCATTGGCCATTTGAAACACC
GCCTTCTATGTGGGC
GGACACCTGCGTCGTCTTGGCCCCTCCCAGATTGAAGGAAAAAA
GAAGCTGAGCCTGATG
AACCTCAAGAGCGATGAAATCGTGACGGTCATTGAATCGGCCAT
TGTAAAGGAGAAGTGC
ATCTTCTCCCAAAGTCGAGAATTCTAATTGAATACTGAATCTGTG
ACGCCGCTTCAGGGA
TGGAAAGACCAGAAAAGATCCTGACTTGAAGGATCTGGTTGTCA
CGTGACAAAACTCTGA
GCGATACGGAAGAGAAGCGCAGTCAACACATTCGCCAGATGATG
ATGGTATAGATGACGC
TAACTGCTGAGTTGATGCTGATGACAACAGTGACGCTCGCAGGG
GAGCGGATGAGTCAAG
GCCACTGTTGGCCAATTCCTTCTTCGCGGCTGCACCACAAATTAC
GCAGACGAACTTATC
GGCTGCTTCTTCGTCTTCGTTCGATGAAAGCAGAAGACACTGTGC
GGGAGCAACACACAG
ATTTTGAGGACACGTAGCGTCTTTTCTGACCTCTCTCAAGTCCAT
TTCGCAAAAATCACC
ATGAAAATTGGGCGGGCAAATACATGTGAAGCCATTTTCTTTGCT
GATACAAGTGGAGCC
CGATTGACATGGAGAGGAATAGCAGAGATCCACTTCGGCCGTGC
AGACAGATTCCTCAGA
GATATCTGAAAATCCAGCAGGGCATGAACACTGGAAACCGTTTA
TGGGATGGATTGGCCG
AAATAGCATTGTGGACGAACTGGCGAAGGAAGAGGCGTTACCGA
ACTTGAGAATGGAGTT
ACATTCCTGGAAGTTGAGACACATTTCTCGGACGCAGAGAGTAT
CGTCGAATGGAAGTAC
CTTGAGCGTCGAAAGTTTGGAGAGCAGCGTCCGATGGAGATAAA
TACGCTCCTTGACCAC
CTCAGCTGGAAAGAAAATGTCTTTTGAGAACGCTCCGACGGGACT
GCTGCCATCTTTTGT

CGAGTGGCTGCGGATGGAGACGCTGACGTTGACAATGCTGTCGA
GGGGAACATCAGTGTC
GCGCTGGATGTTGATGACGAACACATTCTCTGCCGACGTCGGAAT
GACGCTGGCTAGCGC
CGA
>ConsensusfromContig1938 Average coverage: 2.77
CTTGACACACCATTTATATCCGACGGGTTACATGAATCATGTGTG
CAACGGGATGCTGAT
ACACAATTTGTAACTGATGGGTTATATGAATCAAATGTGCAACTG
GTTCCTGATACACCA
TTTATATCCGACGGGTTACATGAATCATGTGTGCAACGGGATGCT
GATACACAATTTGTA
ACTGATGGGTTATATGAATCAAATGTGCAAC
>ConsensusfromContig1939 Average coverage: 25.38
GTGAATGCGAAAAAGTGTACACGAATGCAGCAAACCTCCGCAGA
CACAAGAGTTCGCACA
CATTGCCAAAACCATACTCATGTGTTATTTGTGATATGGCTTTTA
CACGGAATTATAATC
TCATAGCACACCAACGTTTACATGGAATCATTACAAATAAAAGCA
ACTCTAATACTGCTG
TCTCAAATCCGTCCGATTCAGCCAATGATCCTCTGCATAAGCTAT
TTCCAACATGTAAAA
ATGGGCAACGTAAAAATCCACAAAACATAAAAATGTTCTTGTGC
GATATTTGTAAGAGAA
AATTTCGAAAATTGGCTAATCTCAAACTCCACCAAACATCTGAGT
GTACTCGAAAATGGT
ACAAGTGCAGTACCTGTCAAAAATCATTTGCAACAAGCCATAGTC
TGAAACATCATCAAT
TCAGACATACTGGTGAAAAACCATATTGCTGTGATATATGCCAGA
AATCATTTAGATTGC
CTTGCTCTCTTGCTTTTCACAAAAAATCACATGTTGAGAAAGAAT
ATCTGTGCCACGTTT
GCGCGAAGTCTTTTGCATGTTTGAGCCTTCTCCGTCAGCACATTG
TCATTCATTCCGAAG
AAAGGCCATATTGTTGTGGCTTGTGTCAAATGAGATTCAAAAGAT
TCAGCCATTTGCATT
CACACAAGCGTGTTCATGTTGCCAAAAAGTCATAAACATTCGAAT
TTCCATTACTCGTTC
ATCTTACAATTTATCA
>ConsensusfromContig1940 Average coverage: 17.26
GCCGAATCGATCTTAGCGAAGAAGACTATGGCGCGTGAGAGAAT
GCGATCGAGAAGCAGG

CCGTCCAGTTGTCCGTAGAGGGCGAAATGTTGAGCCATGGCCCGC
TCCGACACTTTGTCC
GAGACCTCGTCGAGCCATACGCATGACGTGGGCATGGACTTGCC
AAAACCAAGCTTGATC
TTATTCCCCGATATCGCCTGTCCGTCCATCCGCCGCATGGCTCGC
ACCACCGACTTGATA
TCGTCGTACTGTACAAAGGCGTATGCCGTGAGCGCAGCCGACTGC
TTCTTGATGTCGATG
TCGACGATGGCGCCAAAAGGACGAAAGGTGTCGCGCAGGTCGGA
GATGGTCGTGTCTTTT
GAGAGGTTGCCCACGAAAAGGGTCCGCGTTGCTTTGGAGTGGTAC
GCATCCAGCTCCTGA
ACATCACCACTGCTGCCACCTAGGCCTGACACGCCCCCTAGCGAT
CCTCCAGTTCCGGTT
TTGGGGTCGGTCTCATCGCATTCAAATCCCTCGTGAATCGTGACC
GAAATTCGCGACCCG
AAAAAGCATTTTGAGGTGGAGCTTTGACAAGCCTTTGTCGCATCT
TCGGGTTTTTTGAAG
CTAACGATGGCGTACCGATCGTCCCCTTGGCCGAAAACGTTCACT
GACGTCACTTTGCCA
TGTTTCTTGTATTCGTGGTAAAGACCGTCTCGCAGACTTGAGTCC
GAGGATCGGACAGGG
AGGCCATGGATTGAAATCCCAAGCTTCTCTCGCCCCTCGCAGGGA
ACACCGGTCGTCGAA
GATACCACGCTTGACGATCCGCATGTGTTGGGGTTGACAGAT
>ConsensusfromContig1941 Average coverage: 32.11
ATAGGTCCGCCTGGAAGCGGAAAGTCTACTTATTGCAAGGCGAT
GAAAATGTTCCTCGAA
GCTTCAGGGCGTGAAGTATGCATCATCAATTTGGACCCAGCAAAC
GACGACCTTCAATAC
GAGTGTGCGATCAACATCTTTGATTTGATATCTCTCGGGGATGTC
ATGGACACTTATAAA
CTAGGACCGAATGGTGGAATGATTTACTGCATCGAATATCTAGA
GAAAAATATCGAATGG
TTACGAACTCATTTGGATAATTCAAAAGGAAAATATTTCCTATTC
GACTGTCCAGGTCAA
ATTGAATTATATACTCATCATGATTCCATGCGAAATATTTTGGAT
CAAATGACTTCGAAA
TCGTGGGATATTCGGCTGGCGGCGGTGCATCTCGTTGATTCCCAT
TACTGTAATGATCCC
GCAAAATATCTTTCTGTGGTACTAGTCAGTCTCTCAAGCATGCTT
CAGTTATCATTACCG

CATATCAATATATTGTCCAAAGTTGATTTGATGGAATCTTCCGAC
AATTTGGAACAGAAT
CTCGAGTTTTTCACCGATGTTCTCGATCTTCAATATCTCACACGA
GACATGGATGATGAT
CGTTTTCTCTCAAAATTCAAGAAACTGAATTCTGCCATCGCCGAT
TTGATTCAAGATTAC
AGCCTGGTCTCTTTTATTCCGCTCGATGTTGAGAACAGAGATCTC
ATGTCGAGAGTGGCT
GAACGAGTCGACCAAGCCAACGGCTATGTTTTTGGCTCCGCAAC
TGAAGAACGTAACATC
CGTCACCTGATGTCTCGAGCGGTAGGGGCAGAATTCGCATCTGAG
AAACTGTCATCGATA
GATGAACGCCTCAGCTCCCCGAAGGATTCCACGTGATAAGATGTT
TCGTATAAATATATT
>ConsensusfromContig1942 Average coverage: 40.80
AACCAAAAACTTTTGTTTCTTGAAATCAGTTTGAGACCATCAATA
AAAGGTTGAGGGGTC
TCCGCAGCACTGGCTGATGGTGGGGTGGGCGGCCGAGAGGAACG
AAGGGCAATGGATGTT
AGAATGGCGATTAAAGCAGCTGGAGTGGCCAATGCAATATGATA
AATCATGAAACCATTT
TTCTCGTCAATTATAGCTGGCGCAACAGCATTTGCAAGTAAGCAT
CCCAAAGGGTTGGCC
ATTGACCCCAGCATATTAGCTATTGCCCTTTGATCGTCCTTGAAC
CACAAAGCAGCGACT
TTTGTCGGAGCAAACACGAAAAAAGGTTGAGCGAATGAGCACAC
TATTTGGCCGATTAGG
AGAGTGATTGAACGCCAGTTTTGGGGCATGAGAGATACGGCACT
GATAAATCGGATGAAA
CCACCAAGAAAATTTATCCAAACACCTTGAAGAATAGCTGACTTT
AATCCTTGTGAGTCC
AAGGCATGCACAGCATAGGGCCAAATGACAAGAGACGAAACTGC
GAAGCACATGGATAAC
CAATTGATGAATGTTGCCTCAACATCATAGAAAGAGGCGGTGTA
ATCAGCGACAGGTGAG
AACGATAGCCAGAACATTCCATTGAGAGCAGTTGAGGCACAAAC
AATGCTCAATATAAAC
CATCTTCTTGTATATGCAACAAAATGCACCTGGTCGTGGTGAGTT
TGTTGATTCGCAGAA
TCATCTTCTTGATACATTTTTGCTGAATATTTCTTGATAGTTGATG
TATTTTTTAGTTAG
AGATGTGTGTTTTTGTTTGTTCATTCATCATGAATCAGTTGAAAT

GAGACAGACAAAATG
AAAAATCATTTGATTGCCTGAATATTTGATATACTGAAACAAGCA
GAAAGTACTTGCCAT
ATGATTGTGATGAATCAGTCAAT
>ConsensusfromContig1943 Average coverage: 27.03
TACATTCTAATATTTTCTATCTTGTGAACAACTGCGAAATTGAAC
GGAATCTTTTGAACT
TAAAATCGAAATACTCAAAATAAATGTTACAATAAAATACTTGT
GGTAAAATAGATCGCG
TGAAAATAATAAAAGTAGAGCGCATGATAGAATATATCCCGATA
AATAACCAGACATTGT
TTGAAATGCAGCGTTGAGAGATTATGGGAATACACATCTCAGCA
TAACGAAAATGTGCAG
AATCAAATCGCTTATATAATTTGAAAATAACCGAACTGTTCACTA
ATTATTAAAATTAGA
GCAAATACATGCGAACACGTGAAAATTATTTTAAAATAAACTAC
TGCGTGAAAACATGAA
TCAGCGCAAAAATAACTCAAAAATGGCATATAATTTAATACAAA
CATGATTACGACATAA
AACTACATTATGCTTGCAATTAATAAAAAACATCTCGAAAACTAG
AAGCAAATTTTATTC
TACAGAAAATAAACGAGCACATACAATATACGCCATACAAAACA
ATTACTCAAATTATGA
CAA
>ConsensusfromContig1944 Average coverage: 6.10
ATTTTTAGACAATCGCGATACAAGATTTTGACATTCTAAAAAAAG
ATGGCACGAAATTCA
TATAACCACGAACAGCACGATGACTCTTGGACTATCATACAAAAT
GGTTCAGATACGCTT
TGCCTGGCCTGCTGCTTAGAATGTATTGTTAGCGAAAACACGTCT
TATACTAGAAGATCC
CACGCACTCTCGCAATTGGAACAATCATTTCATCAACACAGTGAT
GCCGCGAACTCAATT
ATCACCAAAAATCATCAAGTATATGTCTCACTGATGTCAAATCTA
TTGGAATTCATCGAG
GAATCCAATACCGAATACGGCACACTATCTGGGAATGTTCTTGTA
TTAATGATCGGAACG
ATAAAAGACTTGAAAATCAAACACATGTTTCTGAAAAATATTTGT
CAGAAGGTCTTATGC
GCTCGTGACAGCAAAACAGCTTTACCACACATACATTGCCTCGGA
CGTATAATGAGCGAT
AGCCTCGATGTCACGAATTGGATAGTCTTGTATCAAGAGCGCCTG

TGTTATCATTTGTTC
GACGGCTTTGG
>ConsensusfromContig1945 Average coverage: 32.57
GCTTTTAAATCTCCTGCACTCTTCCATCGAGTCGTCAATTCTTCGA
ATCTGGCATTATCC
CTCACTTGTGCTTTTTCAATCCGCTTCCTCTCCGTTCTTTCGCTAT
ATCTGCGACTGGGT
GTTTCTCGGCCGCTATGACCCTGAAAGTACAGAATTCATGCTAAA
GCGAGATGAACGATA
TTGCATTGCTAGAGATGATCATTATTGGACCAGAGCATATGCTCT
TAAAGAGGGTTCAAC
AGCCGAAAATGCTCTTCCAACGTTTCTCTCTTCGATTGCGAAAGT
TATATTTCTCGCTGG
AAAGAATCTGAATCTTCTTCGCCTATGTAAACAAGATCATTTCGC
TCTTCAAACAAGAAA
TCCTTCAGGCCTACTCAGGCTGGTCTTGACAGAGTCCAAAGTTTC
GAAGCTGGAGGAGAT
GTGGTCGGAGCATTTTGCAGCAGCACGCGAGATCGCGGTTCGACA
TCTTCTTTTGAAAAA
TGACAAAAGACAAATGGAGAACGACAAAATCAGCATGGCCCAAA
AGGAACATCGTCTCCA
AATCGAAAAAGATATTGCTCACTTGAGAGCAAAAAGAGAAGAAA
AACGACAGATCGAGAA
TGACAAAAAACGACAGCTTGCCGAAGACAGGGATGCTTTTATAG
CGGATCGAAATCAGTT
TTTGAAAGAAGAAAAGTTAAGGATGGAGGAAGCCGGAAAACCA
GATGAGAACAAAGTCGC
GTATACTGACATGATCGGATATTCTGGAAAAGTGGATCAACAGAT
TATACGTCAAGTCAG
GGAAGAACTGATCGAAGAGTATGCGAGTAAAACTGAGGAAGTGG
AAAAACGAAAAAGTCT
AGCATTGGATCGGATAAAGGTGTCTGAGAAAACATTAGAAAATC
ATCCACAGTCACCGAG
AATCAATCGATTCAAGAATTCTGATGAACAGAATATCGATAAAA
CAAAACAAAATATGCT
GAAATCGTCAAT
>ConsensusfromContig1946 Average coverage: 33.69
ACCGGCGGCTGGTGGCTTGAACGAGTTGAAGTAAATGAATATTAT
GGGTCTATCCACTAT
ACCAAATTCATTCCTAGTCCGAATTGGATAGAGCAAATTGAGACA
ACTTTATTGTATGAT
TTTAAGGTGCAGATATTGAAGCAAATACCACCTATAAACTGCGGA

TTGGCTTGCCTATAT
AATGGAATGTGCGGAGATCTGGGAAAAGGCATCGTTTGTATGTA
TGGGTCGCGACATCGC
ATGTGGGACGAACCAGAATGCACAAAAATGTGTCCGGGTGTGTAC
CCTGATATTTGGTGT
TATAAAAAACCGAACCGTATAAATACTGTTATTCGGTCTTACACT
ACGGCCTGCTGGAAG
AAGGGATCTAAAGAACCTAAAAGAGTATCCTACGTAGAAGATGA
TTGTGGAGAAT
>ConsensusfromContig1947 Average coverage: 14.53
GCTCTTCCGATCTGCCATGTTAGAACTTTCTCTTGACCTCTCCTTT
ATGCCTAGATATGC
ATTTATTCAGCATCGCAGAGCCATCCGAAATAGCCCTCATCACAG
ATGCAGTCGTGGCCG
AGGGGCGACTCTGACACCTCACAATCTCCATTCAGGCAGGGGCT
GCTCGTACAGGGGTCA
TTTGGGACGCATCTGGGAATTCCGTCTGTGTTCATGACACATCTC
TCTTGGAATTCACAT
GGTTGTACTTCGCAGGAAGCAGCTGTCCCAAAAACATCGCAGAG
CCACCCAACGTATCCG
TCAGCACAATCACATGTGAACCCGAGAGGGGCGTCAGAATTAGAG
CATGTTCCCTCATTG
TGACAAGGAAAGCTCGTGCAAGGCTGATCAGGGACGCACCTCGG
GTTGCCATCAGTGGTC
AGAACACATCTCTCTGTGGAATCGCAACCCAGCTCTTCGCAGGAG
CCTGCTTGACCCCTG
AAATCACTGCAAAAGTACCCGTTATAATCCTCGGTGCATTCGCAT
CTGAATCCCAGAGGA
CCTTCCGAATCGGTACAGGATCCCCCATTCATGCAGGGCTGGCTG
AGGCAGGGTCTTTTG
GGGACGCACATTGGAGAACCATCGAAGCTGATGACGCACTTTTG
TGAGCGATAACATTGC
TTCGTTTCGCATGAAGAGGCTTGAATGGAAAAAGAATCGCAGAG
CCACCCAGTGAAGCCT
TCTACGCACGCGCAGTCGTAACCGAGGGGGCTATCAGAGAGGGA
GCATGTCCCCCCATTC
TGACAGGGAACCTGTGCACAGCCGTCCTGAGCACCTCCATAATTT
ACAACCCCCAAGATC
AAAAACAGAGATAGAAAAGATCGGAAGA
>ConsensusfromContig1948 Average coverage: 14.30
AAATTTATCATTTTCACCCGCACCAACGTCTAAAAAAATCGGACT
CAGTGTGTCTCGAAA

TCTTCAAAAAACGGGGACTTTATTGAAGGAACCGAAGCAGAAGG
ATAAAATGGCCCCGCC
CAGAACTTATGCACGGCGGACTCCGTTGCAGAATGCGACCAACA
ACCGGGAGACGTATGA
TTCACTCATCAAACGTCTGCCGGAATTTCCTTTTCATGTCACAAA
CAATACTTATATGCG
GTTCATGTCCAAAAAACCAGTGAAACGTCACGAGTCTTTCGAAA
ATGGAAAATCATCGAA
AGCACAGACGACGTTTTGGGATCCTGAACACAGCAAACCGTTGA
AAACAAATAGAGCTGA
AATGATAATGCATCTACATGATCTGACTAATTCAAATCCGAAAG
CACAACGTGCAAAAAG
TGCTGCATCGGCCAGAATTTCGAAGTATAAAGAGAATGCGGATA
ATAGACCTGCGAGTTT
AATGTCAAAGAAAGACGACGTCTCAACTCCTTCGTACCCGAATG
AACCCGCGCGAAGAAT
GTGGAAGCCACGGATCGATTCTCGCTCAGCCCAAATATTTCATCC
CATAAAAACGAGACC
GCCAGCAGAAAGATGTGCATCTGTTAATGAATACGTGCGTCA
>ConsensusfromContig1949 Average coverage: 58.29
AATACCTAAAATTGTTTCCTTGTTTTATTTTTTATAATACATTTTT
GGATATATGGAACA
TGAAAAACACGATAATACTATTGTTTCATATTTTAATTAGTCACG
TAGCTATTATAGTGT
GCCATGAAACACTCCCGGGTCTTGCTGACTATGATATCATTCATT
CACCAACTGTTCACC
ATCATACAGTAAAGAGAAGTGTCTCAAGCAGTCATCCACATAGTC
GACACATAGTATTCA
ATGCACTGGGCAGGGACTTCGACTTGTTTCTCCAGAAAAGAAATG
GGTTGTTGCATAACA
C
>ConsensusfromContig1950 Average coverage: 55.01
TGTGGAATAGGAATTTTGAGTATTTCAAACGAAATAAATTCATAT
GAATAATTGAGTTCA
ATTTTTCGTTAAAATATATTCCAAAATTAAGTTTAAAATTAACGA
AAATATCTCAGCTGG
TTTTCATTCGTTTTGAGGTTAGGCTGTCTTCGGAGTCCGTCGAGCG
TTTTGAAGGTGATT
TCATCAGACCATTTGTGGGCGCTGTTGAATTCATTAGTGAGTGTGC
TTTTTCTCTCATTG
CACCGTCCTTGAGAAAAGAGTGTTGAAGGCATTCGTCTATAGTAA
GTCTCTTCGAAGGAT

CGACAGTCAAGAGTTGTTTCACGAGGTCGAGAGCCTGATTTGAGA
TCCCCTTCCAGTGTT
CGAGGGGGTATTCCACGTGCCCCCCAGTTATTTGCTGAGCGAGGT
CCATGTCTGTCCTTT
CTTGAGTAAATGGCGGATATCCCACAAGACAAACAAAGAGAATG
ACACCGAGGCTCCAGC
AATCGACAGCGGACGTATAAGAACCCGCGCCGGCCGTCAAAAGG
ACTTCGGGTGCCAGGT
AGGTCGGAGTCCCGCAGAACGTTCTCAGCATGGTTCCCACGTCGA
CGAATTTGGATAGGC
CGAAGTCTGTCACCTTTATTAAAGTTTCTTTTTCATCAGAAGCCA
GCAAAATATTTTCAG
GCTTCAAATCTCTATGCGAAATTCCACTGTCGTGTAAATACTTGA
CAGCAGACACCATTT
GGAAAAAGTAAAGCTTCGTCAGATCTTCGGAGAGTTGGCCAAAC
GAGACGACTCGATCAA
ACAGTTCTCCGCCCTCCATTAGTTCTAGAATGATGTAAATCATGT
CATCACTCTCCATAA
CATCTAAAATTTGGACTATACAGGGATGTGAGAGATGCTTCATAA
TTTCAACCTCGGAAT
GGACTTGGTTCAAATGGTTCGTATTCCCGGCACTGAATTTCTTTT
TGGAAATGATCTTCA
CGGCAACTTTCTTGCAAGAACCTTTCTCATAAGCCAACTTCACAA
TTCCGCAAGCACCGC
TCCCCAAAGTCCGTCCGATCGTGTATTTTACTCGGAGTGTTTCTGG
TAAATTCGGATCTT
CCCGAGCATTGTGGTCCATGAATACAAAAGCTCGATTTGACTTCA
TGGCGAGCGAAATCT
CATCGCAATTATTCAAGGCTTGCTTCTTCCCTTTTCCTATCTTCAT
TCCGTTGACGAATG
TACCATTACTGCTTTCATCTTTAATGAAAACATCATGGTCTTGAC
CAGACACAACCCTGG
AAAGCCTGAAGTGAATTTTACTGAAGGCCTGGAAACAGGAATTTT
TTCGAATCTCCTCTC
CAGAAAAATGATAGTCGCAAGAATCTGCTCTGCCGAAAGTGTAT
TCATCAGACTGCAAAT
CAAATGCTCGGAAACATGTATTCATAGGAAGAAGTCTTCCCCAAA
AATGACTTGATCCAT
TAGATATTCCAAGTTGATCTGTTTCTTCTGCAGAATCACATGGAA
TCGTTTGAGTTTCCA
TGCTACTCATATTTTCAAAATAAAAGATTACACGGGTCAGTCACT
GAGTGGGTGTGTAGA

AAATTCTAGGTATATTTACTAAATGAATCAAAAAAAATTTCTGTG
GAAATTCCAATTTGT
CATATGATCGACTTCAACCTTAATCACTGGATAGACTAGTCAGTC
ACTAGAAAACAAACA
AGACAGACTTCAGATTCAGTGTAAATAAATACATACTACTACTAC
TCTACTACTGCATTC
AATTCATCCATCATCATTCATTTCATTACATATGATATGATATAGT
TTGTTTTTTTTTCA
ATGTTGAGGAAAACAATAGTATGGCAAATGGTGTAGAAATAAAA
TGTGAGTTTTGAATTT
TGTTCACTGAGAATCTATTGCAAAATTGTGAATTATACTGTATTG
GAAT
>ConsensusfromContig1951 Average coverage: 27.44
CTCCACGATTTAACACAAAGCTATTGTATGATGATATTTCAGAGA
TAAAAAATGTTGTTA
GCAAATGACATTGCAGGATAAATGACATGAGGTAAATATGATCAA
ATGCTCGTTTCCTGA
GCAATAACCATGGGTTCGTCAAGATATGCAAGATGGTGGCCATCT
TTTAACCCGCCGTTC
ATGAACCGAGGATTACAGGCAGGGGTTCGCTGATGAGAACTGTA
ACTGAACTCCGGTCCG
TGATCCTCATCCGAACCGGATAGCAGCCGGATGACTTGGCGGGT
GCACCCGAACCCGAAG
CACGTGAACACGCCCTGAAGTGTGTTGAATCCGATAAAGGCGAA
CCACAGATCGTTGATG
CCGTTGGTATACGCCCCAAATGCCGCTGCCCATGTGAGCGTCAT
GATGAGGAACAGAGTG
AAGATGACGAACAGCTGGCACTTTCCGCTGCCACCTATGCTACCA
TTTTCTCTGTCA
>ConsensusfromContig1952 Average coverage: 21.40
AGAGTGAGGGATGTCAATGAAATGACGCACCACCCCGGCCCGGC
CATTGCGGCTCTCATG
AAGCTATCATTTGATGAAGAACACCGATATGCCATTTGTGTTTTG
GGTGGCCTTCATGCA
ATCGCTGAAATACTAGAGTTAGAGAGCATGATATGTAACGTCAT
AAGATCAATCGATGGC
ATCTCTGAGCTGGCTGAAATCGACGAAACTATTCGTCATCGCGAC
AACGCCTACAGCGCA
ACGATGCGGCGATACGCGTGCATGGCGCTGACGAATCTTACGTT
CGGCGACGGGACAAAC
AAGGCTTTGATCTGTTCGATGAGCGGAGCTCTGGCCGCCCTCGTC
CAGCAATTGTGTCGC

AGAAATTCTGCTCCGAGTGATATGAGCGAAGATTTGCGGCA
>ConsensusfromContig1953 Average coverage: 99.80
GCATTGCCGCCGGTGATGGTTCCCATCGGGGCGGGGCAAAGCAC
TCATTCGGGGGGCGGT
GGTCAGTCGTCGGGTGCGGGGCCATCGGCTCCAAGGCAATTCTCA
AACCTCGACGATTAT
GCCGTGGGAGTTCCTGACAACATAGATTTCTCAACGGGTCTGGTG
GATCAGGCTGCCAAC
ATCTCTGGACCAGGAACGCCCGGTTATATATCTGAGGATGGAAAT
AACAGCGGATCTCCT
CAAGCCATGGATGGAATTGACGACCCTTCAAGCCCTCCATCAGA
GATGGAACCGGTGAGC
TACACAGAGCCCGCGTGTTGGTGCTCGGTGTCGTACTACGAACTC
AACACTCGAGTCGGG
GAAGCTTTCAACGCGAGCAAACCGTCGCTGTGCATCGATGGGTTC
ACAGATCCTTCGAAC
GACGAGCGGTTTTGTCTCGGTCTTCTCTCAAACATCAACAGAAGT
CAGCAGGTGGAAACC
ACGCGACGACACATCGGCAAAGGAGTGCGCCTCTACTACATCGG
AGGCGAGGTATTCGCG
GAGTGTTTGGGCGAAAGCGCCGTTTTCGTTCAGAGCCCGAACTGT
AATAACCGGTACGGA
TGGCATCCGGCCACCGTTTGTAAAATACCTCCCGGCTGTAACTTG
AAGATCTTCAACAAT
CAGGAATTCGCGGCCCTTTTGGCCCAATCAGTGAACAACGGTTTC
GAATCCGTTTTCCGT
CTCACCCGAATGTGTACCATCAGAATGAGTTTCGTCAAGGGCTGG
GGAGCTGAATATCGG
CGCCAGACGGTTACGAGCACCCCTTGCTGGATCGAAATCCACCTC
CATGGACCTCTGCAG
TGGCTGGACAAGGTCCTCACCCACATGGACTCGCCCCGCCTCCCC
TGTTCGAGCATGTCT
TGAAGGAACAACACCTCAGAGTTTTATATCTTCTTTTTTGTATTGG
TGTCAATATGATTC
TATATCATCGAATCTGTCGATATACCTTCACTCATATGATGGTCG
CTGATTCGGTATTGA
TACTTGGAATTTCATCGCGGCACTACTACCTCAGATTTTTATATTT
CTGTTTTGTATTGG
TGTCAATTATATTGTCGAATCTGCCGAAATAGACCTCACTTATAT
GATGAACGTTATTCG
GTATCGATACTTATAATTTCATTTTGATTCAAATTGTTATTATGAA
ATGTTCCGAATCTT

TTGTTATGATTGTTTTGAAAGTATTTTCGCGAATTTTTCTTTGTTT
TTATGATTCAATCC
CAATAAGGTGATATGTACAAACAAATTTCTCTCACAGATTTCTAG
GATAGGACAGTGAAT
GGCTTAATATTATCCTGTTTTATTCGTTATAGTACATTTTATCATG
AAGAATAATTTCTA
ATATTGTTTTATTTATAAAATTGAATGCGTTTTCAGACACATTGC
TCGTAAGACAAAATG
GC
>ConsensusfromContig1954 Average coverage: 18.30
ATGAAAATTCAAAATTCTGCTACAGGATCAAATTGTATGCAAAA
ACAGTTGTTGAACGAG
GATACAAAAAGCCAGTCAATTATTGAAACACATGAGTAGTACATA
TTGCCATGTTTATAT
TGACAAATAATAATAACAAACAAATGAATCGAAGTAATCCAGAT
TTCTGAATGCATGATT
AATACTACGACTGTATTCCTTATAATACTCCAAATATATAAGCAT
GAAAATTCACTTATT
GAAAAAACATAATCATACAAAATTAATTTATTCAAAGTTT
>ConsensusfromContig1955 Average coverage: 19.69
ATCAAGTTCATTCGCACTATATCGAGTGCGGTATCAAAAGCATA
ATCAGGAATGAAAGAC
CGCAACTATCCAGTCCAGATTCTGTGGATACCATTTATTTCAATG
GCTCGTTTCATGGAA
AAGCGAGACATCAATATCATCGCATAAACCTGACACCAAACAGC
TTTCATAAGAAATTCC
CCACAAAAAGCGTTTCTAGGATACAATTTACGAATGTTTCAATCG
GGACAGTTAAAGCGA
AAGCGTTCGAAACCTTCACTATGCTGGTGGTGCTGGGATTCTCTG
ATTTGAAATCTTCAA
TTGAATTTCTTCCCTACGCATTCCATGGAATTCCTGTCATGGCTCA
TTATTTTTTCTGTG
GTCCGTCGTGCAAATCGTATTCAGTCCATCTCCATCAAAGTGTCT
TCAAGGGGGCTATCA
TAAGACCTAACTATATTTTCGAATTTCAAAACGTAACCTTGGCAA
ACACAGAACTGAAAT
TCTTGGAAGATGTGACTATTGATGAACCTGAGATCAGGATACAGT
TCAAAGATATGTTTC
TCAATGATTTGGATCCTTACCCTCTT
>ConsensusfromContig1956 Average coverage: 29.35
AGTGATTTCCATCCTGTTAAAGATATCATTCAGAAATATCCTGGC
GAGGAGATTCTTGTT

GGATATGATGCCGACTTCCAGTTTGGTCAGAATTTCTATATTGCA
ACCACAGAGGAGGCC
AAAGAATTATTTTTATACCCCGAAGTGACCGATGAAGGCGATGAT
ATGTCTGTAGACGGG
GGCCTGCCCCTTGAAGAAGAATTCATACCCAAGTACGTGCCCCCA
GAATCAAAACCTTGG
GTTTCCTTGGGCTCCGAGGTTGAAATTGACGAAGCGCTCTCACCA
GTACCTAGGACCAAG
ATAAAATCAAAAATATTACGGCTCCGGCGTCATTTTGGGGAACCG
ATTGAATTCAA
>ConsensusfromContig1957 Average coverage: 29.16
GTGACGCCAATGTGTTTCCAATCAAGAATCTCCTCTGGATTGAGG
GTCACATTGAATACA
TGTCTCTTGGTGCCCGTTCGAACTGTGAACCCTATATGATTTTGA
AGGGTTGATGCCCAC
ATAGATGTTCCCGTGGACCTTTTTGTGTGTCCTCCGTTGGAGTAA
ATGAACATACGTTTG
GTCCGATTAGCATTCAGTCTGAAAGCCGCGTATACGGTAAGGCCA
TCGTTTTGACACATG
TCGGCGTTCCCGAAACATTCCAATTTGTTTGCTCGGCTCATCGTGT
CGATCATGCCTGCT
CCATCGAAGTAGAGAGTCTCCTTGCTGCACGAGGTCGACTCATAC
CTTACTGGATCCAGG
CCACTTGCCGACGAGGACAGTTTTGTCTTTCCATCCGTGACGACT
CCACTTTTGTATGAT
T
>ConsensusfromContig1958 Average coverage: 10.45
CGTGCCCAGATTGTTTTAGTAAAGAATCCGAAGAAAACGCGAATG
GTTATTGATTATTCC
CGGACTATAAATCGGTTCACGATCCTTGACGCCTATCCACTACCT
CGCATAGAAGATATC
ACTCAAAAAATAGCCAAAAATATGGTATTTTCTACAATCGACATG
AAAGATGCTTATCAT
TCCATTCCCCTTCAGGAATCTGACAAAGAGTATACTGCATTCGAG
GCTGCAGGGAGGTTG
TATCAGTTCAAGAGACTGAGTTTTGGATTGACAAACGGGGTTTCT
TGCTTTCAACGCATT
ATGGACAGCATCATTTCGGAAAATTCTTTGGCCGAAACATACGCC
TATTTGGATGATATA
ACCATCTGTGGAAAGACACAAAAAGAACACGATGAAAACTTAAA
TAAATTCAAGGAAGTG
GTTAAA

>ConsensusfromContig1959 Average coverage: 69.43
CTTTGTGAGCTGTGACGTAACAGGTTTCATAAGATGCTGGTTCCG
ACGATGATGCAACCA
CATCCGAGTCGTTTTCAAGATCAAGCTCATTTATTTCAACTGATTC
TCCCGAATTCTTCT
TTTTCTCTGTATGACTTTCCTCCTCCTGGAGACCCATTGCCAATAG
CTTCATGAGCCGAT
CCGATGGCTGACAAGGAGGAGGGCCCACATTTGCAGCTCTGATCA
GACTCAACGCTACTT
GTTGATGACCATCATAGAAAAGCTGACTTATTATTAGTCTATACA
TATCAGATCTCGTCT
TCGAATCCATTTGGGACTTCACACTAGATAACATATTACTGGTAA
AATATTTTGTAATTC
ATGAAATTAAAATTCGAG
>ConsensusfromContig1960 Average coverage: 32.64
GCTGGACACAATAACAGAAAAAAGAACAGCATCGGGAATCCTTA
TAATCATCGATTGTTT
GATGGTGTTCAAACACGACCCTATTTGCAGCAAGAAAAAGTGGTT
GACAGGTATTGCTCC
TTCTACAATAAGTATGCTGATCTGGATCCTATGATAAGTGACTTT
GAGCAGCCAAATCCA
TGGGTAACAGACAATCCTGAGCTATGGACTTCAGACGAACACTCA
TGCATTATTCCGTCC
GCACGAATCCGAAAATGGGCTTTCTCAATCCAAGAGCTGCTGAG
CGACCCGGCGGGCCAG
GAGCACTTCCAGCAATTCCTTGAGAAGGAATTCAGTGCCGAGAAT
TTGAAGTTCTATCTG
GCATGCCGCGAGCTTCGGATATGTTCAATTGCAAGAATAAAAGA
AAAAATAGACCAAATT
TATAGACTTTTCATTTCCGAGGATGCCGAGTTGCCGATCAACGTG
GACAGCACTGTGACA
GAAGCAGTTAAAAACGCCATGAGAATGAACCCAAAGAACAGATT
TTGTTTTGATGAGGCT
GAGAGCCATATTTTGAAGCTGATGAAGAGTGACAGCTACAGTCGA
TATATTCGATCACCG
ATGTATATCGACTTGACTTCCAAAGCAAGGAGGAAGCATCAGCCG
ATGTTCCAGCTCAGC
TTTATGCCCAAAAACTCTGAAAACTGATATATGTGTGTTTACTTTA
GAATATCTCTATTC
AGCAGTTTATATCGAACTGAGAAAAACTTTCAAAACACGAGTTAT
AAACATATATTTTGG
ATCCATACTGC

>ConsensusfromContig1961 Average coverage: 13.03
TCTCTTGCAGTTTTTGGCCAGCTGGAGGAGACAGTTCATGTTGAG
TTTTTTGCAGAAGTT
GAGCTTCAGAATTTCCAAGTGTTTCATGTTTCCCTCAGAAAGTTT
CGACACTCCGCTATG
AGAGACGGAAGTTGAACTGATGTCGAGCAGACGTAAACCAGGAA
GTGACGCCGCCAGCCT
CGCTACGCATGCGTTGTTCACGTTCTTATTTCC
>ConsensusfromContig1962 Average coverage: 7.36
TTCCGATCTGACAGAGATGTAGGATACTATGTGATTCAGTGGTCC
CATGACGATGGCTCT
CAGTATGACGTCATCGTTGGCTCCAACCTGCTCAAAACCAACTTC
TTCGTCATCGCACGC
AACCTCAACGACTACAAGAACTCTTTGGAGAGTCTTGCACTTGCG
GAAATCAAAAATCTC
GGGTACTTTGGGCCGATGCTGAGGACCGCTCATCCTTCCAGATGC
ACATATTGAGATGTC
TTTCGGGAAGAGGATTGTTAAAAATTAGAAAAGTAGCAGAATCT
TAAAACCTTTTGAAAA
>ConsensusfromContig1963 Average coverage: 14.25
CCGGTTCAGTTCGTTTCCGAAGCCACGCCCCCTGGATCTCCAGCG
GACATATTATCGCCC
ATCGAGCCAATGACTATTGAAGAGGGAAAAGAAGCGTTGTTCAA
ATGCGAAATAACCGGA
ACTCCAAACCCCACCATTTCCTGGCTGAAGAACAACAATGTCCTA
GCAATGACACCGCGC
CACCAGTCTTCTGTCTCACAGGACAATCGTGTTCACACACTCACC
ATTTCCCCGGTTGTC
CTCGAAGACACAGATGAATACACTTTCGTCGCGCGCAATGATTA
TGGAGACGCGCGTGCA
TCGGCCAATCTGGAGGTGACGCCAAAACCCAAAAGCAAGCCGGG
AAAGTTCAAGACACAG
CCCCCCGAGTTCACGAAAACACTCACAGACCAGGTACTCCTGGC
AAAAAAGAACTTGACC
TTGATGACTGAG
>ConsensusfromContig1964 Average coverage: 30.40
GTTATATTTCTTGCTCAATTTCATCATGCAACGTACAGCTTTCGTC
ATACAATGCGAGCT
GGAAGACGAAAAAGCAATCAATACTGCTATTGAAAAGATAAAAA
CTTATTCTTCAACAGA
GTCTTCTTTCAAAATAATTACTTGCGATGGCGATTTGCTTCATGAT
AGTCTCATAGGTTA

TTTTATGAAAGCTTTTCGATCTCGAAGCTCAAAATCGAATGAAAG
ACAAACTATAATATC
CGAGGACGATGTCATGGTTGCAATTCAATTGTGTATTCTGAAAGA
GAAGAAAATCAGCTC
TGTGACAGGAGATGTTTCTCTGAGCGATGTTCTTCATTCGGCGGC
CATTATAGGAAAATA
TAAACTTGGTTTGTCTGACCCGACTGAGAAACCGGCCATTTTGGA
TATCTATGACTCATT
TCTCAAGTCATGCAACTGCTGTGATTTATACGACGTCTGCCAACG
TTTCTCAAATGATGA
AGAAGCAGCCGAACATTTGTTATCAGGGAATCTAATATTTGTTCA
GAATCGGAAGCTTTT
GGATTATGAAAACAAAATGGTTGAGATTTTGTGCCGAAAATGTGA
CGTCATGAATGTTAT
TATGGAAATTCCTCTGGAAGACAATGAGCCTTGTCGGGTGAAAA
TTGAAGGGGCTGCCAG
CATTAATGACGTCATTAATGAGATGGCCTAC
>ConsensusfromContig1965 Average coverage: 23.80
TGGCCATCTCCAATAATGCGATACGACAAAAGTCCGTTTCGTCTC
GAGTCCGGATCGACT
GCGCGAACGACCAAAGGCGATCCATTTTCATCTTGAACGAAGCT
ACCAACAGCTGCAGAT
TCGCTCAAACGTCCTTCGTAATGTTTCTCGATGAATTTCGGCAAGT
TATCATTAATATCC
ATGATGTGAACGAGAACGTGACATTCCGCATGTATCTGAGCGACA
CTCGTTGCTCTCACC
GTCAAGTTATAATGAACAGACATCTCGCTCTCGTAATCTAAGACG
GAATTGGTGAGTATA
ATACCCGAGAGTGGATTGATGAAAAACTGGTTCAGGAAATCTCC
GCCCACAATCGAATAA
GTGATGCTTGACCTTCCAGCCGCCGAAATCTGTGCGACGAAGCTT
CCTCGAGGTGAGTTC
TCAGCAACTTCTGCGGAAAAACTCGAGACTTCGAACAGCGGAACT
GAATCGTCGGATTCT
GTGACGTTTATTCTGATCTGGGCTGAAGT
>ConsensusfromContig1966 Average coverage: 38.52
ATGGAGATGCAGTGTCTGAGTGGGAGCGAATCTTCCGACATCGC
GAGTGTGGAGTGGTAC
GCCGGTCCGGAGTTTGAGCCCGATGCGCCGGTCCTCGTCGTCGCT
TTCGAAAACGGACGC
TGCCAGTTCATGCGCGACGAACAAGATACAGCCCCTATCCTGCT
GGAGACCAATATGCGG

GTCGTGCAGGCTCGCTGGAGTCACACCGGAGGCATTCTGGCAAT
TGTAGGGACGCAGAAG
CTTGAGGGGTCGGAAAAAATCGTCAACATCGTTCAGTTCTACTCC
CCTTATGCTGAACAT
TTACGGACGTTGAAAGTGCCTGGCACTCAGATTCAATCGTGTTCA
TGGGACGAGAGCAGC
CTTCGGTTGGCCATCGCTGTCGATTCTTTCATATATTTTGCGAATG
TCAGGCCTGATTAT
AAGTGGGGCTATTGCGCAGAGACTTTAGTCTACGCTTTTCGAAG
GCCCGACAGAGAGGAA
CACAGCGTGGTTTTTTGGGATGCCAAAAGTTCAGAAACATATGTG
AAGTACGTTCGTAAT
TTGGTACACTTGGTCGCTTCGGGAGAGTACTGCTGTCTTATTACA
AAATCCGAAGAGAAT
GAAAAACAGCATGCGGTCGTGCTCTGCAATGCCATCGGAACGCC
ACTGGACTCCAAGTAC
GTGGAGGGACTGGAGCCCCAATTCGCAGTCATGAGTGGCTCTCAT
GTTGTCGTCGCCTCA
AACAGGGCCGTCTACAC
>ConsensusfromContig1967 Average coverage: 27.95
ACGTTTTCGTGAATCCATGTGAGACATCATATGAGAAATGAAAT
CAGATTCAGAACGGAA
GGAATTCTCACAAGCGCCGCAAACAAAGCGCAACATACTCACAG
GGCGCATCGTCGAATC
ACCGACCGAACAATTCGTAGACGAACAAATTTTTTTAGAGGGCCG
TAAATCTGATTTTTT
TAGACGTGTTTTACCTTGTTTCGATTTTGAATATTTGTCCTTCTCA
AAGCATCTCATAAG
ACGTTTACATGCTCGAATTGTCCTATACTTTTTCAAGATTAATCGA
CTCGATGGAGTTTT
CAAATGTTTGTAAGTAATACGATTTTTACCGCATTTGGTCATTCT
ATGCAGGAGAAAAAT
TGTCGACCAATTTGTGGACTTGGAGCATGTCGAACAACGTAGCTT
CGTCAGAGATTTGGA
ACGCTCACGAGATTTACATGAAAGACAAATGAGATCATTCCGTGT
CACTTTTGACCAAGG
TCCCTTGAAAAGACGATGACATATACTACAAGCACTATCTAGAA
AGAAATGATGACGTAA
TTTGACATGAAATCGATTACGAGCAGTTCTCGAGCAACAACACTT
TATTTTGTTCGGTTT
>ConsensusfromContig1968 Average coverage: 28.69
TCCAAGCAAATGCTGAATGTCTTCACTGTTGCAAGGGGGACAATG

GAACATCTGTTTGCC
GCGGTACATCTCCATTAACAAATATCGTCTGCTATTGTGATGAAG
GGTGTTTGGCCAAAG
GAGACTGCTGTGAAAATTATAAAGAAAGATGTCAAGCGCGGAT
TGCGTGATGGGGGACT
GGTCCCCTTGGACTCCTTGCAGCACCACCTGTGGACACGGCAGAC
GCAGCCGACGTCGTA
CCATCGACCTTCACCCATCCCTCCACGGCAGAGCTTGCGACCAC
AAAAAAGAGGCTGAGA
AATGCAACAGAGGTCGCTGCAGGAGGATCTCTACAAGGAGTATA
CCAAACCGAAATTCAG
AACACATCATT
>ConsensusfromContig1969 Average coverage: 10.64
GCTCGTTGTGTTGGAGTCGTTCCGAGCACTTCCCCATCTAGAATT
CCACTAAAATCACCG
TATTCTTTCGAAGGCATGGACATTGGTCTTCGATTTGGGATTTCA
GAAAGTTCTCCGATG
CGCTTAGATAGTTCTTTGACACTTTTCCATTCGATATCAGGTGAA
CGAATATTAACCTTA
TCAGGCTTTTCAGACTCAGTAAGTGTTTCGTTGAATGAGCTGCTG
ATATTTCCCGCCGCA
TCCATCGATATCAAATCTCCCATTGATCGAGTCATTCTCTTCGACA
GTTTTTGTTCATCT
TCACTCAAATCCTGAGAGCTTGTCAAATTATCAATTATGCGTGAA
ATTCTTCTATGAACC
TTTTTTTCTTTCACGAAACTCTTTCTGAATACCTGAGCGGCTGTAA
GCTCTTTGCCACAT
TCAGGACTTTTTGCAATTCCTGATTTTGTAGCAGAAGATTCTTTTT
CGACATCTTTAGAT
GATAT
>ConsensusfromContig1970 Average coverage: 27.10
TGTGATACTGAGTTTGAGACATTATCGAGTGATGGTTTAGATCTT
GGGAAGTAACACTAG
TACAGTACTACTAGCATGATACCATACAATATCATAATACATATA
TGAGCTCTCTCGATT
GATATTGTTCTATTATACGGAGTGCCGCTATGTGATCATGAATAT
TCAGGAGTCTCCATT
CGCTTTCTATGATTGGCTTGAATACTTGGAGATGTGGCGAATGAG
ATGGGGCCGTTTCGT
CATCATGAATATCCAGGATGTCTCCACTGACATTGTATCATTGGA
TGAAATG
>ConsensusfromContig1971 Average coverage: 40.16

TGAGGAGACAAGCAGCAGCAGATGCATGAGAATAGCTCTCAGGT
ACTTTCCTCAACTCTA
TGGAATCATTTATATCTTCATCGGTCTCATTTTCTTGGCTGGATCC
CTCTGGCTCAGCTC
CATCACCTGGGACGCAAGAGGCTTCGAATCGATTCCAATGCCCGT
CATCGTGTTCTTTGG
ACTGGGGCTGGTATCCATTGCCGTCGGGATCATTGGAATCTGGGG
AGGAAGTTACCAGAT
TCCCTGCTGTTTGAAATTCTTCTTCTTTGGAATCCTTATCTTACTT
CTGGCGCAAGTTTG
TGGCGCAGCTCTGGTGATCCTGTTCAACGATGAGCAAATACTTGG
TGTTAGGTCGAGATT
GGAGTATATGATGGAGAACTATGACAAGAGGGATGTCTCGCTCAG
TCTTTTTGATGATAT
ACAGCTGGAGTTCTCTTGCTGTGGAATCGATGGTTGGAGCGACT
GGCGAGAGACAAGAGG
ATACCAGTGTATGAATCACGATGAACTCTGCTCGCTTCCAGACAC
CTGTTGCGAGACTGC
GTTGAGTGGATGCGGCTATCATAGTTATACTATCAGAGGAAACA
CCACAGAACTCACAGA
TGGAAATACAATAAATAAAAGAGGATGTATGGAGAAAATGGTGG
CAACACTGCATAGTAA
TATTCATGCTATCATCATTACCACAAGTTGTCTCATTTTTGTACAG
CTGATTGCAGTGTT
TGTAGGATTTTTTACTATTTGTTATCTACGAAAATT
>ConsensusfromContig1972 Average coverage: 32.58
TGTTTTTTGGAGCAATTTGTCGTAGAACGAGTTTTCCCTTATTGAC
CAGCTTGTTTCCAG
CAGTTTTGAGTCTATCGGCGTCATTTTGTGAATTTAATCGTACCC
TAGAATTTTGAAGAC
GTGAGCTGACTGGAGTTGAACTTGGTATTCCGGTCTGACCATTGG
CTGCCTTGGGCACAC
TTTTACTGTTGGGTGTGCTAAGCGGTGTTAACATCGCAGAGCATC
CGAATGAATTTGACA
CAGCTGTTGTCTGACTAGCATGTGTGGAGTCTGGATGCATCTCAG
AAAATCGAGTGGAAC
CAGGCGAGGTAGCTGGTGTAGGGTACAGATTCACAGTATCAGAA
GCGGACACGAAAGGAG
TATAAGGCAGAGGCTGATTAACATTCAGAGCAATAGAATTAGCG
TCAAAATATGGAGATT
CATTTGGATTTTCAACGCAAGATAGATATGCCATTTCAGCTATCG
AAGGTCGATTTAAAA

CCGGCGGAGAATTATTGGATATGGAAGAATCTTTTCTAGAAATAG
TATTATTTTTAGACT
TCGACAATTGTTTTAGCTTTCTACGTTTGACATGTAGTCCTTCTGT
TACTGCTGAATTTT
TCCCTTCTGACTTCTGCTTGGTGGCACTGGTCTCTATGAATCGCT
GCGCATTCAGGACCA
GCCGCTTATGCTTCAGAGTATGTCTTGAGAAGTCTGCTATGTCTG
AGAAGGATTCGTTGC
ACAAAAAACAGGATAACTCATCTTTGACAACTGAGAAATATCA
CCACACTGGTTTGGGA
TGCTGACCGGTGTCTCATTTGTCAACACTACAGAGGAACTGATA
TTGGGCGACGAAAAAA
CAT
>ConsensusfromContig1973 Average coverage: 10.18
ACCAGGAATTTTCTCCTCGCATGCCCCGCACATCGCCTGGCATAG
ATCAAAAATGGTAGG
ATTTACACAATCGGACTGATCAGGCCAACCAGGGCTCTTTCCGCA
ATTCTTATGCTTATC
AATACATTCTTCTGCACAGGAGAGTCCCATGAAACCAGCAGGAC
AAACGCAGCTGCAGGA
CTGGAAGTCGACAGTCGCGCAGTTCTGACAAACACTCTCGGCATT
GCAATAGCAGTTTTC
ATCGGAACTCGAGCAACCATCTTTCATGCACAGTCCATTTTGGCA
GTTGAGGTTTCCACC
CTCACATCTCGAACAGGCCGGGCCGGGGATATAAGGGGCCGTTTC
GTCTAGGTTCCCACC
AGGTCCATAGTTGCAAACGAGGTAGTAGCCGCTACCCCTCAAGC
CAGAAATTGTGTGACA
GTAGGCGATTGCACACCCCAGCTCGTGACTGGTAGCCCAAACAAC
TTGTGTGTAGTGACC
ACACATTTGACCACGTTCACAGGTTCCGTCTTCAAATGT
>ConsensusfromContig1974 Average coverage: 29.42
CGATCTAGAATGAGTGATGCAGAGCAGGAACATTATGGGGTCAA
CAAAGAAATTGTTGTT
GACCCCACAACAAAACCAGATTTTGCTGTGATTTGTTCTTTTTAT
GAACGTTATGCAGAG
CTCCTGGGATTGAATAAAATAACTTTTGAGAGTCTACAGAGACAT
ATTGAAAATTCAAAA
ATAGTAACAAAAGAATTTTCAGACATTCTCATCAAACTAATGCGA
AAATGGAAATCCTGT
GTGAGACCTGATAATTACGAACGTTATGTTATTCGGTTTGTTTAC
ACATACTCCGAGAAA

GACGGACGCGAATTGGAGCGATTGGGTCTTCCCAAGTCTTCTGTT
GATGTCAAACTCAGA
CTCCTCAAAACTTTGCTGGAGTGTCAATTTGACTTGAATGCCAAG
TTCAAGGAGAAGGTC
AAC
>ConsensusfromContig1975 Average coverage: 44.26
ATTAAGATCTGATGCAATCTGGCCATTCGCTTGATAAAAGAAGAG
TTGGGTTATCCTAGC
TCTTGTTTTTCTATAACGTAGAGTTAACGTGCCATGAGACAGGTG
AATATTGCAAATCAA
CATTTCGTATCCATGAATACATGGGTTCTGCTCCTCATTCATCAT
TAAAGCCAGCTTGTG
AGAGCGAACAGCATGTCAAAAAAACTGAAAGACCTGCTATTTGTC
ATCTCTATGCTTGTA
CTCTCTGAAACTACTGCGTCGACATGCGAATCATGCAAATCACTT
CCAGTATGGCCAGGA
ATAAGCTTCGAAGATTGCCTGAATGGAGTCGGTGTGGATTATATC
GAGTGTACCTATCCG
GGGCAGTGTGCAACCCTGCAGTGGGGAACCAGAGCAATTTACAG
AACTTGTTTCGAAAGT
CCTAACAGCTGCGTCTATGGTTTGGACAATTTGTTGAGCATAATG
CCAGTAGGGTTTCAA
ACAAGGTTAGCCTTGCAACTAATCGGAACGCCGAAGCTCACATG
CATTCTGCCCACAAGG
AGGACGACGAAAATTCCGAAGTCCTTCCGCCGCTATACAACAACC
TCACCGGCTATGATA
AGGACGACAAAGTCGGGGGCTTCTAAAAATAGATCGACTCTTGTA
AGCCTGATTTTCGCA
TTCGTAATTTTCAAATGGCTATGAGGACTTCTGAGTTATTCATAA
AAGATTTTAATATAT
TTTATTGTGTTTAGA
>ConsensusfromContig1976 Average coverage: 51.02
AGATGATGTCGGGAGGCCATTCTACTTTGAATCAGATAGTCTTGC
TCTGAAAAATAATAG
TGATTATCAAATGATATTGGGTACTCTCGTGGTGCTCGAAGCACA
GCGTACGCAGGCGAT
TAATGATTATGATAAACTCCTTGATGGCCAGAAAAACGCATTGTC
AAACCCTGCAGACTT
TGCCCGGAAAGTTGCTCGAGGGGAAAATCTTTGTCTTCCGAAACC
AATTGATATCGCTGA
GCTGCCAGAAATCGACTTGAATAAGTACTACATTCAGCAAGCCA
CGGGAAAATCCGTTGA

AAACTTTGTGTGTGATCAAACCAACCCCGCTGCACTTAGAACCC
GCAATAAAAAGATCGA
CTTCGCCAGTTTGTCTTCGGCCCAATCCGCGAGCAATGCAGATTC
TGAGAGCAGATACAA
ACTTAAACACCCAAAGACTGCTTCCTACAATCAAGCCTGGTCTGC
TGAGGAGCAGAGTCG
ATTGGAGGCGCTGCTTATCAAGTACCCTCCTGAAGACGTGGAGT
CGAAGCGATGGGCCAA
GATAGCTGAAGAACTTG
>ConsensusfromContig1977 Average coverage: 10.31
GGATTTATTGAGGTTCATCTTATAAAATTTGCATACAATTTTTTTG
TTATTTTAAAATAT
TTCTGTTATTGCACAATTAGGTTTTTATTGCATTTATTCTACGATT
TTTTCAAGTTTGGC
AAAATACTGAAAAATGTATGCAACGAAAAAAGGTGACTGGGTTAT
AATTTCTATGATATT
AACACAAGCTATGTGTCCTTATATTAGTTTCAATAAATTTATGATG
ATTTTTTAAAATTG
ATTCGTGTGACGATGGTTAAAACGAATTGCGTATGTATAGTATTG
ATTATCGTCGTTGTA
CCAAT
>ConsensusfromContig1978 Average coverage: 17.71
CCGGCTCATTAAGTATCTACATCAAGAAATTGATTCATCAAAGTA
TGACATCAACTCATC
ATAAATTGAAAACGGACAAATTAGACTATTGAGGAATCGATTCT
TCAAGATTCACAAAGT
ATGACATCAAATCATTGTATTCTAGTCGAGACAGATAATTGATTC
TATTGAGGAAATGAT
TCTTGAAGATCATCAAAGTATGATATCAACTTATTATATTTCGGT
TGATACAGATAATTG
A
>ConsensusfromContig1979 Average coverage: 8.08
GCGGGTTCCTTCAAAAGTGACACATTGGTTCCGAGACTTTGCATGC
TTTCTTTTCTTGAC
GCTCTTCTTGACTTTGATTTGACCTTTGTGATATCTGGAAGAGAA
TCGACGCGAGATGAT
TGGTCGTCCGCTGAACAATCCGATGTCAAGGACGATGAAGAAGC
AGACGACGATGACCTA
GATTCGAAAGATTCTTGAGAGAGGATCGTGTCAACAGATCGGAT
AGATGGCAGTACTCTA
TGATTCGACATGCGGCCGGATGGCGTGCGGCTAGGCACTGGTGA
TGGAGCTGAGAGATCG

TCTGCTTTTGAGCTCTTATTGCTTGAAATCGACGTCGCCGAAGATT
TCCGTCTGTTTTGG
CCAGCAGGAGAAGAAGCTGAGAGCCTTCTTGAATTCCGAGAGAC
ATTGAGGGGAGAAGAT
TTCCGTTTTGGTGAATCTCGGGAGAGGATGCGGCTCATTTCAGAT
TTGTTGGCTTTTGGG
GCGATGATTGTTGATTTAGGAAATGTTTTAAAATGATCGGTGTTT
TGAAACTGTTCACAA
TTGAGGCTGTTAATCGGATCTAAGTCCAGCCGAAGAGAGCTTGGA
CTGATGATTTCAGAT
CGATACATTTTTAATCTTGTGTTGAAATCGATGAACACGATTTAC
TATAATTAAT
>ConsensusfromContig1980 Average coverage: 52.43
CAACAGAAAGGTTGATACAAATGCGGTGCTTGGGAGACTCAAGG
ATAATTTCAGACTACG
GGTAGATGGGGCCCTAAAAGAGAATAATGACTTCTCCCCGAAAG
CCCCATCTCGTCGAGT
TATGTTCAACACCGAGTGTGTCCAATCCTCGATAGGGGGCACTGA
GACTGTTGTAGATAC
AAACTTTGATTCCTTTTGTAATGATTCTACGGCATCATTTCCATCC
TCATGTGAAGCAAC
TCCTGAACCTTCCCCAAGGCCATTAAAAGAGAAATGTGTAATCG
AAAACAGAATGATTGT
AACATCTCCCAAAGCATCTCTTAATCTGAGTGATTCAATTGAAAC
TACAATTGAAATCGA
AAGCTCAACGTCTATAGTCAGCAACATGGATAAAAACGTCCTCGA
AAAATCGTTTTTTCA
AAGCCCACCATCGAAAGAGTCTCCTAAAGCCTCGCCTAGGAAAAA
ATCTGTGTATCTCAG
TCCTCAGCGAAACTCGTATTCGCCAGAAAAAGCCTCCAAACATGT
TCTGGAAGTGACACC
CAATGCAAGAACGTCCGCGTTTAAACCTGGAAGTGCCCTGCGTCG
CCACACCTTTGGGAC
GCCTGAAGGTCACTCACCTGTCATTTTCGGGACCCCACCGCCCCT
GGTATTAGATTCTGA
CGTGAAGGGAGACGCCAGCGCAATTGTGGTCGCCGTGAGGGTCA
GGCCGTTTTCTCAAAA
AGAAGTGTCAGAAGAGAATAGCTGCGGGGTCAAGATGAAGAACG
GGCAGGTCACGGTCAT
CCAAGACAGTGTCTCTCATCGCTTCGATTTCGATCATTCTTTTAAC
TCTTTCCAGAAGAT
TAGTTCCACATTTGCCGACCAGAAGAATGTCTTCAATACTCTCGC

GAGACCTTTGCTACT
AAAAGCTGTAGAGGGATACAATGTCTGTCTCTTTGCGTACGGAC
AAACGGGCAGCGGGAA
AAGTTACAGTATGATGGGCCCCGCTTCGAATCCTGGGATCGTCCC
TATGTTCTGCCGGGA
GCTATTTAACACTCTCCAATCTTCGATGAGTGCGATTGTGGAAAT
CAGCTTCTTTGAGAT
TTACAACGAAAAAATTCACGATCTTCTGGAGACGAAAGAAAAGA
AACAAGGAAAAAAACC
AAGTTTAAAAGTGAGGGAGCACCCGGTCATGGGCCCTTTCGTGGA
AGGTCTTTCCACCTA
TGTGGTCAACTCGTATGAAGACATTGAAACGTGGCTGGCGGTGG
GGAACAAGCGGCGGGC
CACAGCATCAACCGTCGCCAACGATCAGAGCAGTCGATCTCATTC
GGTCTTTTCTCTCAA
CGTGACGCAAACAAAGGTTGAGAAATTTGAAGAAGAAGAGCATA
GACACACGATTTCCAG
TAAGGTGAACCTTATCGATCTGGCTGGAAGTGAGAGACAAACAA
CGGCTACGGGGGATAG
GCTGAGGGAGGGGACGTGTATCAACAAGTCATTATTATCTCTCGG
AAAAGTGATACTTAC
TCTCTCAGAACAAGGATCGACCAATAGGAAGAAAGCTTACATACC
CTACCGCGACTCTGT
CCTCACGTGGCTCCTCAAGGAATCTCTGGGGGGAAATTCCACGAC
GGCCATGCTCGCCAC
CATTGGTCCCTCTGGATCTACCGTCGATGAAACACTCAACACACT
CAGATATGCGACGCA
GGCAAGAAGTATTGTCAACAGAGCGCATGTCAACGAGGATCCGC
AGGACAAACGAATCAG
GGAACTAATGCAAGAGCTCCAGCTGATGAAGAGTTGCGAAAACG
CCGACAGCTTCGACGA
GATTCGCAGACTCCGACAGATGCTGACGCAGAAGGAAAGCGAAC
TGGAAACGTTGGCAAA
ATCTTGGACTGAGAAACTGGCTGATTCGGAGCAACGAAAGCTTG
AAGAATCGAAATTGCT
CGAGCAATGCGGCATATCTCTTCGAATGGAACCCGGTCAAGTCTA
CCTGGTCAACCTAAA
CGAAGATCCACAACTAGCAGAGACCCTCCTATACGTCCTCCACA
AAGACGGGGTCACCAG
TGTAGGGTGCGCCACACAGGACTCTGATTGCGCCATCAAGCTAAG
CGGGGTTCTCATTGC
GGATGACCATTGTACATTTGAGCACTTCGGTGGTCGGATTTCAAT

AACGCCATCTGGTGA
TGGGAGGGACACACCGTGCTATGTTAACGGCAATCTTATCACCCA
TCAAACCAAACTTCA
TCATGGCGATCGTATCATACTCGGAAGTGAATGCTTTTTCAGGTT
GAGCTATGTCCATCA
ATCCGACAAACGTGCTTCCAAATCTGGAATCCCAAAAGACTACG
AATTCGCAAAGCAAGA
ATTGCTCTTAGCCCAAAACAAAATAATGGAAGCGGAACTTGAGG
CAACGAAGAAGAAGTG
CATGGCGGAATACGAAGGAAGGATTGAAGAGCTTGAACGAGATC
TGGATTCATACAAGAA
GATCTTGGATGTCAAGAAAAAATCGGAGTTGGAGAAGATTGACT
CTGGGATGGAATGCAT
GAAAGGACCCGATCTCTCGTGGCTCCCGAGAATGCTGGAGAAGG
CACAAGACGAGATCGA
CGCTAGGCGGGAGGAAGTGCGGCTTCAGTCCGAGATCTACAACA
GTCGAGCGAAGGAACA
AGATGGACAGAAAAGGTCTGAGATATTCCGTGCGACGATGTTGA
TGGGAGAGGCCAATAA
AATCTGCCAATCTTTGGAATACCCCGTTGAGATAACTAGAGAATG
CATTTACTCGAACGG
AAATTACGAGTGGTTCTACCGGGTGGCAAATAGCCAACACTCTGT
CTACACACTCTGGTC
CTTCGATGAGCTGGAGAAGAGAGTGACAGAAATGAGGGATCTTA
AACA
>ConsensusfromContig1981 Average coverage: 33.90
GTATATGTGACTATAATGACCCTATTGTTTGAATTGTCTCAATGTT
ATATCTCAAACCAT
TTTGGAATTTAAAATGTTGTTTTATCATGACATATTCACCAACTCC
TAAACATTTTCTTG
ACCAGGATGGAATAATCATAGCCTAAGTCATCCTAATTTCAATAT
GTATCATCAATATAT
GTAGATGTACTTTCTGTTGATGTTTTTCATTGTACTGGAAAATTT
GTACAAAATTCAGAG
GTATTTATTCTGTAATAATTTGTTGTTTTAAGTTTGCAATTGATTT
GTATACGTTTTCCA
CACTTATGCTTTGTTCATATGTTAAAATTTGCCATATGCAAGAAG
TGACACTAGGGTTGA
AGTGAATTCTAGAGATCACACAGTGATGTGTATTATTGTTATTTA
TGTGATCATTTGGCA
TATCGATTGAACGTTATTCCATGAAATATTGTGACGCATTGTTTGT
TTGTATTTGAAATA

TGTGCATCATGAGATTTACAGTTTCA
>ConsensusfromContig1982 Average coverage: 16.52
AACGCTCTTGTTATAAAGAATGGACGCCATCCGATACTGAACCA
CATAAAAAGAGAAGAA
CTGGTGCCCAATCACATGGCAGCTTCGATCATAACAAGTTTCAAC
ATCATCCATGGACCT
AATATGAGTGGAAAATCAACGTACCTCCGTCAAATAGCGCTTCTC
CAGATCATGGCTCAA
ATCGGTTCATTCATCCCAGCCGAATACGCTTCTTTCCGTATCTGCG
ATCAACTTTTTTCT
AGAGTGGGAAGCGACGACGACATAGAGAAGAATGCATCGACATT
CATGGTTGAGATGCGT
GAGATGTCTTACATAATTCAAAACATGACTGATTCATCTATGATC
GTTATTGACGAGCTT
GGAAGAGGTACCTCTACTGACGAAGGTGTCGCTTTGTGTTTTTCA
ATTTGTGAGCATCTG
ATGAAAACCAAAGCCT
>ConsensusfromContig1983 Average coverage: 39.24
CATCACCATGGTCCACCTTCATCAACTCTTCGGCATCTTGCTTCTC
TTTGATCTGCTCCA
AACACCGGCACCCAACTTTGAGTATGCGGATGCTTTCTGTAAGAT
TTGCGTTCATGTCCT
TGAGAGAATCCAGCTCTGTTTTAAACTTTTTTTTGGCATTTTCAAT
GATTTGATCCCCAT
CTTCCTCGCACATGCGCACTCTGTTCTCGTCAACTTTCCGCTCCTC
ACTGCCAAGCTGAT
CTTCAAAATCCTCTATGAGCGAGAAATTGTCTTCGTCAAATCTGT
GAGAAACCGCCGAGG
CTGTGACATTGGTTTCTGCCGGTCCAAAGAGAGATTCTGCAGCAT
TGAAATCGGTCGTCT
GCTTGCCGGAGATTGGGATCGTCAATCCGAGGTCATCTGATGAAT
CCAAGCTCTCAGTTT
CCCAAGGGAACTTGAGACTATCTTGTTCATCCATCACCCATCGCT
CGCAGTATGTCATGT
T
>ConsensusfromContig1984 Average coverage: 16.08
TTTTGCTCAATTTTCAATAGATCAGCATGTGAAACCAATAATGAC
TGAATATATTCAAGT
ATATGGAACCCTGTTGCTATTTACAATGCTGCCTGCTGGGGTTCC
TTTTATTACAACATG
AAATTCTGCTCACATGTTATACTACTGGCGTCATTTATATTCCAAA
GTCATTTCTTATTT

GATCATGTATCATCACTTGAATGCTACGTCTGCACATACACCTAC
AACGGACCGAGTTTT
TCCACCTGTAGCGATCCGTGGTCCAGTGCCCGGTCGACCTTCCGA
AAAGTTTTCGGTCCT
CCGATACAGAACTGCCGCAAGGGGCAAGTGTGTCTCAAACTTGTC
GACTACTCATGGCCG
GATGACCCCAGCGTGGTGCGACTTTGTGGACAACCCTGTAATGA
AACGAATGAGGGCAGC
ACCGTTTTGTCTTGTTGTGACGACAAAAACCTGTGCAATGAATCT
CCATCACTCTCAATG
TTCACAAAATCTACTTGTTTTCTAATTGTTTTATTCCATGTTATGA
TGTATTGTATAGGT
TTATTCGATTTTAATTTTACGTAACCAGTGCATTGTAAATAGTAC
TATAATTTATAGAAA
ATAATTCAGAATTATTTATTCTTGTTTCGTGATATAAATCTCATTT
TTATAATTATCGGA
TTTATTTTTGTATTTATGA
>ConsensusfromContig1985 Average coverage: 38.49
CCGAGTCTCATCCAATCAGCGAGAAAAGCACATCGCATCACAAG
GTCCTTGACCCATGAC
CCCAGGGGCTTCAGTGATGGGTAAGCTGCATTCGCCCACAGTTG
GGGGACGATGTTGAGA
AGGAACGCGGTGTAGACACGCTCTAATTCTTCGCTCATGACCACC
ATACCTTTGATTGCT
TTTTGCAGTTCTACCAACGATTTCTTTATTACTCGTAATAATTTGT
TGAAGCGATCAACT
TCTTGGATGAGAACGGTTGTGAGAGAATTGATTCTCCCCTTGCTA
TCTTCCTTGAAGAGA
TCATGTCTTGCCGTTTCAATATCCAGTTTGTCCATCAATTTGTCCA
AAATACTCTCCGCC
AATTCGTAGACGATTTCGTCGGGTGATTTCCCGGTGGCTCCACCA
CCCACACTTGGTTGC
ATGTCCATGATACTTCCCACGAGCTTATTTGTTTCTTGGACCTGA
AAGACGATATTGGCA
TTGTCGTGCATCCCGAAGACTTCTGGTTCGTCAATGATAGGAAGA
TTGTCAATGAATTCT
TTATATGTTTCCAGTTTCTCGTATTCGGGGGCGAAATAGATACCG
GATTTCGAGTACTTA
TATCCGGTCTGAAGAGTGTCCGGTCTGAAGAATCGAGCCAGGATT
GTACGAAGACATCTC
TGATCCCATTGGTCAGTCACACGTCCGCCGTACGTTATCTCTCCA
GTGATGTACATGAGG

GCATCCCATGGAATAACTTCATCCTTACAGAACATCGCCAGGTTA
CTGAGTGCACACTCG
CGATCGCTGTCGCTGAATTCATACCGGATGTTCCATCCGAGAGGC
CCGAATTTTTTTCTC
TCTTGAACAATTGCGTGGAAGAAACATATACCGAAAACCAGGCG
GCGCCAGTTGTTGCCG
AGAATATGGTCTTCGAAATATGTCTGCGTCAGCTCATTGAATGCT
CGTTTCGTGTTCGCT
CTCAGACCTTTGGGGGGCTCATTCGTGACTTTGACACTATTCTGA
AGGACAGTGACGGGG
AATATTTTGGCTGGCATAGAGCTGAGAAACAAGCGGAAGTCGGT
GTGAACATCCTTGGGT
TTTTCGGACAAAGCTTTAATTGTCTCTTCCATCCGAATCATCCAA
GACGCCGCCAAGTGG
CAATTCTGTAAGAAAACCCAATCCCCAGTCTTCACTGCTGCATGA
ATCAGCTTCTCCGCA
ACAGGCCCTTGTCCCTGGCCCAGCGAAATGGCCTGGATCCTGTCA
CCATAATTCATTTCC
GCAGCAAATCGGAGAAAGGCGTTCATTGGATCAGATCCTGTGCT
CAGAACAAAAACGAGC
GGTGTGGTGTTTGCCATGTCCGCGTAAAGTGTGGCCAAGGAAGTG
TCGGGACTTTCAACA
AAAGACTTTCCCAGGCCGTGGGAAACAAATTCCGTCACACTTGCC
ACGACCCTGTCTTCA
AAAAAAGCTTTTATGATGAGCAGCTTCTGAAAACTGGAGAGCCGT
TTGTCCCAATGACCT
CTAACTTTTCCCTCAACGGGTGGTGGGGCCTCTTTCCCCCCTCTT
CGAATTGTGGGATCA
GGGGGCTCAGGGCCGTATTGATCCCAATTACCAGCCGAGGGATTA
AGTTCCGCCTTGATG
GCCCCCAGCTCAACCCAGACAGGGGTAGCAGTCAGTTCATTGCTG
AGCCCCTTCATATCA
GGAATAATAGCCCCCAAGTCGCATGCTGCTCGCCAGAAATTCAT
>ConsensusfromContig1986 Average coverage: 41.19
CGACCATTACTATGATTCCTCTTTTGCTTAAGTATAAATCTTCAGA
TATTGTCGATGTTG
AAATGAAGAAAATTGCAAGTTTGGAAGTTTTTTGTGTCAAAACTT
GGAACGACTTTTCTT
TTCGTCGTCTGTTCAAAATGAATGTCAAATCCTGAAGCCAAAATT
ACGTCCGCTTTTTTA
TATTTTATTTTTGGGTGTGACAACCGCGAATTTATTTATGATTTAT
TTATGTGTTGTGAG

CAGTATTTCAGTGATATTTTTATGAGCAATAAGAAACCGTTTATA
AATTTTAGGAAAATT
ATTTTTATTGATTTGGGAGACTACAAAAAGTAAGATCCAACTTTC
ACTGAGATGACATGT
GGATAACATTAAAATGTTACTATTTCGAATTGAGATAATTCCGAG
ACCA
>ConsensusfromContig1987 Average coverage: 44.61
AAACAATGGGCGTAAATAAATAATTTTAAAACGGTCTTTTGATTA
ATTTTAATTGAAGAG
TAGCAAATGTATTAAATACATTGATGTGATCATAAGTAAGAAAC
AAGATGGAGTCAGTTG
AAAGTGATGGTGCTCAATCTGGTCTCAGGAAATATGGTGTAACTC
ACGAACCACCAACTG
ATGAAGCTGTGACATACCCAGGCGATGAAAGATATTCTTTTTTCC
AGAAGGGTCGAGATT
ATGAAAGTGCCTTCACGAATCACAAATATACTGAAGATGAAAGG
AAAATTCTCGCCACAT
ATGAAAGTCTTGATTATCTCCCATCACACAGCACTGTTTACAAGA
AATGGCTCCAATCTC
AACCAACAAGGCTGGATTGGGACCGATGGGTGATGATGGGACTTA
TCGGTTTCGCCGTTG
GGTTAATCGGATTCTTTTTGCACCAAATGATTGACCTTTTGACTG
AGTTCAAATGGGACC
AAACCAAAGAATACTTGCAGAGAAATGGAACAGTAGAGGCTTGG
CTTGTTCTTTTGGGCA
TCAGCGGGTTTTTCACTCTTGGAAGCAGTGCTGTTATTGTGTTTC
TCCGACCGAGCGCCG
CAGGATCTGGTCTACCAGAACTCATCGGCTTTCTCAACGGGACCG
TCGTCCGACACATCT
TCAACCTCAAAACCCTCGCAGCGAAGTTCGTCTCGTGTGTCTTGG
CGGTCAGCAGCGGAC
TGCCGGTAGGGCCGGAGGGGCCCATGATCCATCTCGGAAGTCTAG
TCGGTGCTGGCCTTA
GTCAATTTAAATCGGACACTCTTTCATTCAGCTTTCCTTTCTTCGA
ACGATTCCGAAATT
CGGAAGACAGACGTAACTTCATTAGTGCTGGGGCGGCTGCAGGG
GTGGCGTCGGCCTTCG
GGGCTCCCGTTGGGGGGCTGCTGTTCGCCATGGAAGAAGTATCAT
CCTTCT
>ConsensusfromContig1988 Average coverage: 43.93
AATGAGGCCGGACGATGCGTCGGAAAATTCACTATGACAATGTC
GGACATTGTAAACGGA

AAGATCACAAGTATGAATGGTTTTTATGTTGGAACTGTGAAACC
GAGAGTGAGACTCACA
TCCAGGGGAAAATTCGGAAATGGGCTCCATTTTGACGGTTCCTAT
GTTGACTTGGGAGAA
CATGAAAATACCGTCCTAGGAAACGAGGCGATAAGAAATGCGGA
TTTGGTAATTGACTTT
TGGGTGAAACAAGAAAGAACATCAAAGAACACCTATGGGTGGTT
GTTCGGAAGTGGCAAA
AGCTCACGGGAAATGCATATGAATCCTGAACATGTTGCATTCAAC
TTTTGGACTGGAGAA
CCCTCTTGTTTAAAATATTTGTGTAACTATGAGCACAGGAACCTA
ACCGGATGGATCCAC
ATGAGGATTGAATTCAACCATCAAGACGTGACACTCAAAATGAT
TTTGGACGGGGAGCTT
GTGTCTAAGGACACCACTCTGGATTCAACGTGTCACAAGCAGCAA
TCCTCCCATCCAACA
CTGATCGGGGCCATAGTGGTTGGATGGAATAACAAATTTCACGGC
ACAATCGATGAACTT
TGTATTGCGGAGGATATCGGGCAATGCTAGATTGGCGAAAACATT
GTTCTGATCAATTTT
ATTCCAGATAGTCAAATTTCTTATTGGAATTTTTTGGGATTTTCAA
TATTGATTCAACTT
CCCACTTATACTGTTATATATGTATTGTTTTATTCAATTTTATCCC
AGA
>ConsensusfromContig1989 Average coverage: 21.92
TTTCTGTTCACCGATACGCAAGTCGTTGAGGAGGGATTTCTTGAA
GACATCAATAACATT
CTTAGTTCTGGTGAAGTACCTAACCTCTATCGTCAAGATGAGTTT
GAAGAGGTTCGAAAT
GCCCTTGCAGATGCTGCCAAGAAGGCGGGCGTCGACGACACTCC
ACAGACCATGTTCAAC
TTTCTTCTGGATCGCGTTCGAGCCAACCTCCACGTTGTCCTGTGC
ATGAGTCCCGTTGGA
GAGGCTTTCAGAAACCGGATTCGGATGTATCCGGCTTTCGTCAAT
TGCACCACAATCGAC
TGGTTCAGCGACTGGCCTCAAGACGCGCTGCTGGAGGTTGCTGAT
AGATATCTGGCAGAC
CTGCAGACGACGACTCCAGAAGAGGAGGCATGCAAGCCATCACT
TGCAAAGATATTTTCT
GTCATGCATCAGTCGGTTTGCGATTGCTCAAAGCGCATGCTCTTG
GAAATGCGACGTCAC
AACTATGTCACGCCGACAAATTACTTGGAACTTGTCAAAGGATAT

AAACTATTGCTGGCA
GAGAAACGCGAAGAGCTGGGCGGCGCAGCTGGGAAACTGCGCAA
CGG
>ConsensusfromContig1990 Average coverage: 56.68
AGTAGTCCGCACTGTGCGAATCAATCACTTGACTATTGCAAAACA
TTCATCGACCCGATG
GTACCGCAATTAATTGATGTCAGTGAAAAACAATTCCAATGTGAT
ATATGTCCTAAATCA
TTTTCTAGAATTAGTGATCTACGATGCCATTATCGCATCCACACT
GGAGAAAAGCCATAT
GTTTGCACTGAATGCCAGAAATCATTTGTGCAGTCATCTCACCTT
AAAGCACACCAAAGT
GTTCATGCCGGTGTGAAACCACATACGTGCAGTGACTGTAAGAGG
TCTTTCGTTTTATTA
TCACAACTGAAAGCACATCAAAGTACACATACTGGTATAAAACC
TTACACTTGTAACGAG
TGTATGAAGTCATTCACAAAATGGACGACACTACGGATTCACCAA
AATATTCACCTTGGC
GTGAAACCGTATGAATGTGATGTGTGTATGAAGCTATTCTCGCAG
TCATCAGGACTAACG
GTACACAAACGAATACATACCGGTGAAAGACCATACTCGTGTGA
AGAATGTCAGAAGACA
TTTGTGAGGGAAGCAGAATTGAAGAGGCACCGGCTTGTTCATACT
GGTGAACGGTCCTTC
GTGTGCAATGAATGTGGTATCGCGTTTGCAAGTATGGCTGATCTA
CGAAGCCATGAACGT
ATTCATAGTGATAATAAACCATATACGTGCAATGAATGTCAGAGA
TCATTTCTAAGGATG
GGCGACCTACGGAAACACCACCGAACTCATACCGGGGAGAAGCC
CTATACTTGTTATCAA
TGTCAAAAGTCCTTCTCGCAATCATCTGCACTCAAGATACATGAA
CGCATTCACACTGGC
GAAAAACCATATATCTGTCCAGACTGCCAGAAAACATTTACAAC
GATGAGTGAGCTACGA
TGTCATCACCGAATTCATACTGGTGAGAGGCCCTACGTGTGTAGT
GAGTGTCCAATGTCA
TTTGTGAGATCAGCGCAGCTTCGGGTACATCGACGTATACACGTA
TCTTGAATATGTTAT
GTTGAACATTCATTGGTCCTGTCAAACGATCTTCGAACGAAAAAA
TTATATGAAAATAAC
GCG
>ConsensusfromContig1991 Average coverage: 22.86

AAGAAGCTTCGATCATTAGAAACTGACAAATTGCTCTGATTATCG
TGAGTCAAGAGACCA
GAAAAAACGAGTGATTGCTCATTCGGCACTGGCGAAAGAATACT
GAAACCGCCTTCATCG
AGCGGGTCGGCCGGGAACAAGTTTGCGCGCTTGACACCATCTCTT
GGACCCGTGGAGCCT
TCAACAAGCGAAACGCTTTTCTCGTTCTTCGAGGACGAGCTACCA
CTGTCGCCACCTGCG
ATGGAGCCATTCGGGGTCTTGACCGGAGTATGTGTCGGCGTCCAG
CATTTGCTTAACACA
TCCGAGCATTGATCACGGACATAGCCTATGACGTAGGCAGTGAA
ATCAGCGGGATGAACA
TGTGCTGATATCTCAGGAAATGAAACATTCTCGTCGTATGCATTA
ATCCATTTTAAAAGC
AAGTCGTGTGACAGAGATTTGTTCAATAGCCTCTGCAACAAAAGA
TCCGCCATAAGTTTT
AAATTTTGTAAACAAATTCCGATCGCAG
>ConsensusfromContig1992 Average coverage: 25.80
TTTTTTTTTGCATAAGAAAAATATTATAAAGTCAAGTATGTTGAA
ACATAAAAATAAATT
TTTTGGCCGCAGGACTTCCATGTATGAGAAATTTTTGTTAATTTTC
TGCATCATGGTTTT
CATCCATTTATATCAATAGAGCATTCTCGAGGAGCATTTTCACCG
AGATTCTGCTAGAGT
AATGGTTACATACTCGTGGAGTACTCTTTTCTTATAGACTTTCAG
AAACGATTGATACAT
TGTAAAACTCAATATTCTTGGATCTGAGTTTTTTTCTGTCCTTTTC
TTTCTCATTAACAC
TGATTGTACAGTCTGATAACACAATTTTCAGCCTAGTTGTCTTCA
CAATAAGCATTCGAG
CTTTGATTTCATACCAGGTTGTGAGCAATGAATCATTTTTTGGGT
ACCCAAGATATATAA
CAAACACAAACGTGTCAAGTCCAGGTAGTCACCACACATTTGATG
AACTTGATAATTTTG
GTTCATTTACTTGCGTTCAGCTGAAATTGACCGATGATATCACAG
ATTTTGAAAGCAAAA
TGATGGAAGTCAGTTGAAAAGTTTAGGCATGTGTTTGACATTGTG
GTTCATCATTTGTGT
TGATTTATCTTTATTTATTTATTATTGTTTTGACTTTTTTTTATCTT
CCTCCAGTTCCAG
TGGAGGCTCCACTGCAGTAGAAATATCTCTTTGAGTTCAGTGTGG
TCCAAACTCTCAATT

CATTCAAATTTAATTATATTGTGTAAGAAATATCATCAAAGTTGA
TCCGCTTCCATCGTC
ATACAATCATTTATATTATCATTCTGTCGTCATACTTTCGTGGAA
AATTTAGATCGGAAG
A
>ConsensusfromContig1993 Average coverage: 9.24
CTCGGCGAGAAGATATTTGCTTTTATTATTTTTAATCAGAACAGAA
TCATTTATCTTTGC
TTGAAGGCCACTAATTATACCTTTCTATTCAGTCGAATGAATGTA
CGGATGAAACACATA
ATTTTAAATTAATGCTGATATGCGAATGGGCTTCAAGACTCATAT
TATACAGGGCAATGT
ATTATACTCATATTAGTATTAATTCCAGATTATTGAGAATGTTAG
TCATTGTCAAAAACA
AATGCAGTGTTTATGGATTCCCATCGACGCATGAACTGTATAACG
ATTCCATCCCGTCGC
TCAGTTTATTTTTTGT
>ConsensusfromContig1994 Average coverage: 7.82
CACAATTTTTCAATTTATCTAATTTTTGAAGATAGACATTTTCTGA
GTTCTTTTCTTTTT
TCATTTGAGTCTCAAACAACATATGAGAACTCATATAATATCTTC
AGTCTGGCAATCATG
TCTCAAATAACGTGATAAAAAGCCATCATTTTCTCCATTGTACAA
AAAAACTCACATTTT
TGTCCTCTCACAACCTAATGCATCATATGTGCAGCTGAGTCTCGA
ATTATATGTTGAAAC
ATTCTGTGATATCGAATTATATTGGTGACTTGTACACTGTGCTTCG
TTGTGTTATACCTG
TTTGTCACAACAGGGTTATTATTTTAGTTACATGTGAGTATGTAC
ACTATAAGCACGAAA
ACTAGAACTGATTCAATTATACGTACTTTTGACTGTTATGTTAAA
TATGTTCGAGGTTGT
ATTCAATTTTATATGTACGTCGATTTTCCTTATCTCGTGAAATAAA
GTATACTTGCCATA
TATTCGTCCATAAACGAATATTCAAAGAACATATCAGGCATTCGG
TTGAAATTTCT
>ConsensusfromContig1995 Average coverage: 6.82
TCTCCATCCTCAAAACAAAATTCGTAAGATTATCGCTGCACACAC
GCATCAAAGAGATCG
TCACTTATTCTGAATACTTTGGAGTTTTTGTGAGCTGTGAAGAAA
ATGGGCAAGGGGCAA
TAGTCGCATACAAGAATGACGTGATTACTGGAGTTTTTTACAAGG

GATACACATCCGTGT
TCAGCTTTTGGATACAGCCGTTCGAGTTGTTGCAGCCGTATATCT
CTACGAGCAATGTAG
AGGTCGAAAATAGAAAAGTCGGACGACCAGCAAGAAACAGTCTT
CCTGC
>ConsensusfromContig1996 Average coverage: 14.70
ATCGTGGAGCCTTCCAAAGTTTATGACGCGAGGTTTCTGAGAACC
ATTGATTCTCAAGTG
ATGAATAAAAATGGTATATCAACTTTAGAAGTGATTGGAGCCGA
AGACAAGCTTCCGGTT
CTCATAGATGGCGATGTTGGTTTGAGATACGATGAGAAGAACGA
CTTGATCGTGGTGGAG
CCCTACGAACCTGAGAAATACAAGCCAGAGAATAAGCCAGTACC
GAGGTTGCGTTGCAAA
GGAATCAAGTTCTATGCTCCTGAAGAGAAAACTGTTGAAGAAAA
AAAAGGTATGCTAAAA
GTAAAAGGTATGGTACTCAGTGACGAAGGTGTGGGAGCACCTTTC
AATTGCAAGTGTGTT
CATTTCTACGTGGGAGGAAAAACAGTCAACGTCGTTGTTTATGAT
TCAGATACCAAGCTC
GATGAGAAGCGTTTTAGAAAAACGAAGGGGCCTATGTGATTCAT
ATCAAATGATTCATCA
ATATTATCA
>ConsensusfromContig1997 Average coverage: 16.30
CAATAATATACAATATATCTAATAGTTCAACAATCCGTATCGGGT
TCACGTAGACGCATT
TGTTCCGATCCTCCTGATGCTGGTCTCATATCAATAGATGTCGCG
TCGAGCGTAATTTGT
CCAGGCAGGGGACTTTCTCTAGGAAGTGTTGGTGATTGGAGTTGC
AGTCTGCTACTTGTT
TCACTTGTGGATGCTAGTCCCATTGAGCTCGGAGTCAGAGATCCC
GGGCCGTTGCGCAAT
TCTCTGGATACGTCATCGGCGATTTTGTCCTTTAGAATAGTTGAT
AGATCATCATCTTGG
TATTCTTCGATCAAGTGAGCATCTCTGAACCTTGCACTATTGTCCA
GTTCAAAATTTTCA
TTGATCTCATCAAGTTCGAAAGATAGAACTGTAATTGGTCTTTCC
AATTCGGCTGGGTTT
TCAGATGGGTAAATACTCTTGAAATATGCGTCATCCATGTAAATA
TCTTCTGGATTTTGA
CCATAATTTCCCCATAATTTATTGATTCCCGGTCGCACTCTTTTCT
CCTTAGCCAATTTT

TCTCGACGACCGTTCAGGAATACACCAATGACAAAAAGTTTGAGA
AGCAATTTGCGCTTG
ACGGGAGTCACCAGGATGAAGTCGAACTCTCGCTGAGCCAACCG
TTGGACGAAGGTCGGC
ATTATCTTCATCAAAGTGACAGCTGTTGCGTGGGAGGAACGTTTG
CGATTCTCAGCGAAT
ATAGAATAAGGAAGAACTCTCGAGTGAGTCTCTATAAGTTCATC
AAGCAAGTCCCACATG
GTGTCTTGTATTGGATCAGCCATCAACTCAGCCTCTCGACAAAAT
GCATTCACGATTTGA
ATATATCTCGCCAATTCATGAGCGGGAGCGAACAAGGGTGGCATC
GCAAGGCTTTTTTCA
ACAAGTGTTCGTCCTTTAACTATAGTTTTGATATACTTTTGAGTTT
CTTGGTCCATTCGT
TCGAGACCATTGGCGTGCAATGCCGGCCAAAGAGGTCCATTACA
TAGATTGAGACAAATG
TCTCGTTGTCGAATAAGTAGATAAAATACAAACATCACAGCAAG
AACCAACCAGATGACA
TACATCATCGTTAACAATCTCAACAGATAGACTGGCGTTACCCAA
ATC
>ConsensusfromContig1998 Average coverage: 29.30
CAGATGTCATCACATGTGAGAGTGCAGAGCCAAGATTCTCAACCA
ATTGCGATATCACCG
GTTATGTCGCTGTAACGGGTCAGGTCTTAAACATTTGTGATGCAT
ACGAGGATCCTAGAT
TCGATAAAAGTAGTGACACTGATCCATCATTCAAAACGAAGAGCA
TCCTCTGCATCCCGA
TTCCAGACACAAAATCAAAAGTTCTGGGCGTGGCGCAACTCGTA
AACAAACTGGATGGAA
AACCTTTCAACAAAAACGACGAGAGTCTTTTTGAAGCTTTCGCAT
TGTTCAGTGGGATGG
GAATTAATAACACGAGAATGTACGAGAGGGCCACCCACGCCATC
GCCAAGAACCAAGTCA
CCATCGAGGTTCTCTCTTACCACAGGTCTCCCGCCACCACCGAGC
TCGACAGAATCAAAT
CTTTAACAGTGCCATCTGCAAAATCACTTCAACTCGAAGAGCCAT
CTTTCTCCGATTTTT
CTCTGAGTGAGGACAGTACAATTACCGCCGTCATACGGATGTTCA
CAGAATTCGACTTGC
TAGAGAAATTCAGCATACCTTATAAGGTTTTCTGCAATTGGATTC
TGTGTGTGAAAAGAA
ATTATCGTGACGTCACATATCACAACTGGCGTCACGCCTTCAATG

TCACCCAATCAATGT
TCTATATTCTCAAGAAATGCGACAGCTGTCGGAAATCTTATACCG
ATATCGATAAGTTTG
GATTTCTGATCGCATCCCTCTGCCATGATTTGGATCACAGAGGGA
CTACCAATTCGTTCC
AAAAGCAATCGATGTCACCACTAAGTCAGCTATATTCCACGTCAG
TGCTCGAGCACCATC
ACTTCGACCAATGCATCATGATTCTAAATACAAAGGGTTCAGAAA
TACTGAAGAATCTGG
AAAGACACGAATACGAGCACATCATTCGAAGTATCGAAGACAAC
ATTCT
>ConsensusfromContig1999 Average coverage: 9.96
CAAACGTATTGATTTTTTCAAGTAATATTCAAGTCATATTTTTGTT
TCCATCTGCATCAC
GATTGACGGCCTCAAGCAAATACATTCTTCATCATGTTTTCCGAT
CAGTCTTCGGCGCAA
GTCTATTTTAATGTGTCTTTTCTTTTAACTCTCATCTTATTCTTTAT
CTTGTTGATGACA
AACTCAGCAACAAAATCAAACAGTTCCGTCATAGTTAAGATCGC
TGCTCCGATGAACAAA
CCCACAGCACCGCGGAAATCCGATAGAAATTGGTCAAATTTGTAA
CTTGGTTTCTCCTTG
AAAACTTTGTAACTGAGATCGTTGAAATAAATTGCCACTTTTGCG
AAGTAATTTCTTAGA
AAGTCCTGTCTCTGGTCTTCCATCGTGTGCTCTCTCATATTTATAC
ATGTCGAACTGTTT
TCGTTGAAACACGCCAAGTCTTTCAAACTGCTCCACTCATTCTCG
TCGGGCCAAAGATCT
TGGCTAATCGTCAATGAATATTTCGTGCCATTGCATGATTGGTGG
CACCCACAAGGTGTT
TTTGAATGAATCGCTTTCTTTATTTCTCTGTTTGCACATTTCTTGT
CAACTTTTAAACAT
TCATTTTTCAATTTCGTCACGTTATGATGATAGTTTTCCATGTAGC

>ConsensusfromContig2000 Average coverage: 27.67
AAAGTCATCGGTCTCATGGCGGTTGACGACGCAGAAGAGGTACTG
CGGGTTTCACATCTG
ACTTTTTGGGGGAACAGGAATGATTTCTATGTTCCCATAGAACAC
GTTGTCCCTTTGACC
GACACCGCAAATAATCCACGGGATATTCTCCTCAAGTTTGAGAT
GTACAATAGCAAAAAA
AGCTATTATTTCTCACTCGTTCATGGTAATGTTTCGGACTGGGAC

GTCTTCAAGAGGGTT
TTTGGAAATCCAGAATAATGTTGAGAAATTGAGTAAAAATCTATT
TGAATATGAATGAAT
ACACATTTATAAGAGATATAAAAT
>ConsensusfromContig2001 Average coverage: 5.45
ATCGCCGGCAGGAAGATGTTCAATAATTGATCTCAAGATGACTTC
TATTCTAGTAAGTAG
GCTTCCTGACATCACTCCTTTTCCTGGTGTGCAGACATCTGGAACT
CTTATTCCCAAATT
CTCAGCTATTTTCCTACATCTTTCAAACAAAGCTTTGTCTTCACTA
GGAAATGGTTCAAT
TCCTGCTGCCTCTGATTTGCTAGGATCAATTATGTGATTGCAAAT
GAAACTTTCTTTGAA
TTTGTCTGGGAAATTTTCTGATCTCAATTCTCTCATCAACCATTCT
AATCTTTTGTCCCA
CTGAGACTGAATCTCTTCATCATCATGGAAATCAGAATTCAGCCA
GTTATAGGCTCTTTC
CCTCATGAGCTTGGAATCATGAGCTGCGAA
>ConsensusfromContig2002 Average coverage: 25.76
GAAGCACTCCCTCGAGGAGCACAATTTCATGAAGATTTCATAGAA
AATTGATATTCACTT
TATGAAATGGATAAAAAAATTGAGTCGGAGTGGGTCATGATCTT
GAGAAAAAAACTCAAA
TTAAAATCATTTAAACAAATTTTCTCTGCAATGATGGCAATACTA
GCAGTTGATATAGGT
GTAAAGCAGGCATTTTTATATGATATTGCAACAGTATCACCCCTC
CAATTAGTTGAGTTG
ATCGAAAAATTATTACACAGCAGTCTTCTATCAAATAAACTTCTT
GTGAAGTGTGTAGCT
GGCAACTGTTTCATTATTAATCCTGTTATCTTCAACAATAATTGG
TACAATATTAATATA
GTAGATGTAACATATACACATCAACTGCCAAGATTACTTACAAAG
GATGATCAAATATTT
ACGAAGTGTTATAAAACAATCAGATCATTGCGACAATCATCTTTG
GAAGATATGAATATG
TATCCTGATATAATTGGTGATTCCAATATCTCTACAATTTTTGGTG
CATTTCTTGGCTAT
CCATATGTGTATTGGTTCTCAACTGATGGTAATAAAGACTGTAAG
ACAAATCTTGATATG
CAGCCTCTTATTTTATATGAAGTAAATATGTGGCATACTGAGATA
TTTTCTGGTAAATCC
TGCATCATATACTCATTCTCTGTACCTCAAGAATTGGATGATACT

GCCTTTGTGGAAAAT
TGGTACAAAAGAGCCCTGTCAAATTGCACAACATTGAATAGAGAA
TATTTTGATTTCAGT
TTAACAAAAAAGAAGGTTGTTATTCAAAATGTTATTTTATGAAAA
AATGACATTATGTTA
TCAAGATTTTTCATGCACCATTTGATTCCATTTTATAATTAATTCT
GATTATTCTTTTTA
TGATGAC
>ConsensusfromContig2003 Average coverage: 57.00
TTTACATTGCTTTCGATTTTAAAATTTTGGTTTAATTCACAGAAAA
TGCCATGTGATTTG
TTAGGAATTATATAGCAAAATATAGAATTGCAGAATGATACGACA
ATTTTTAAAGTCGCA
ACGACAATTTCAAAGTATTCTTTTTCATTGGACATATCAGCGGTC
TTATCTAACAAATTC
AAGTAGTCTATTTGAAAGAATACGGAATGTCGAAGATATATTAC
AGGAAGACCCATTGGA
TTTAAAAACAGCGAAGGAACTTGAAGTTTCTGCACAAAAAGAAC
TAACGCGATTGAGTGA
AGATGATGAATTCAAGCTGAAGGCTTATAAAATGGAGCATACGGT
TCTTTATTCGTTAGA
AAAACAAGTTCCGAGTGAAGTCTCTGCGACTACATGGCTTAAATT
TCTAAGGGGATCATT
CTCGCAGTCAGGACGTGAAAAATACTTCAAATTCCTTCATGGAGT
TCAGAATCGACAAGA
CAAGGCTGCAAGGAAGAAGGAAGAACTCGCTCATGTACGTGAAG
AACACCGTAAGACAGT
AGACTTTAGTCACTTGAGAAATTCCATCCATCTTATGGTGCGCAC
AGACACCATGAAGAA
GCATTACTACCATAATTTAGCTCATGCGATGCTCATGGATCAAAC
TATTGTTTACGATAT
GAGTTATGAGGATATAATGAATGAGAAGGAAATTAAACGGACAC
TGGATCAACTTCTCAA
AGTCTACGCAGACAATAAGGTCTCCGTTGAGCCGTATCATTTGAT
GTTCACCGGTGTCAA
GCCTCATGGAACATTCATGAAACATTTATTAAAGGTATTTCCTTT
GGAAGTTCTTAAAAA
AATGCCAATAACACTAACCGATCAATGCTACCTGGATGTGTTTCC
GAAAAAGAACGTCGT
GTATTTATCGCCAAATGCTCCGAAAACGATGGACTTTTACGATGT
CAATGCAACTTACAT
AATTGGAGGGATAGTTGACACGAGGGGTCACCCCCCATTGACGC

AAGCAAGAGCGAAAAA
ACAAGGCATCAAGAGTATGCGCCTCCCTCTTGACAAACATCTAA
GATGGCGCGTTGGACA
TAAAAACCTGAGTCTGAATCAAATGATGGCTATCCTGCTGGAGC
TCAAACATGGGAAATC
CTGGTCTGCTGCATTGAAACACGTTCCTTCGAGAAAAGTAGAAAG
ATTATAGTTTTTCAT
TAGGCAATTCCATTCTTTTTGTTTTTTATAATGTGATATTTTTCATT
ATTTTACGATTTA
CACATTTCAATAATAAACTCCGAAATCTGAAATTAT
>ConsensusfromContig2004 Average coverage: 8.66
CTTCCGATCTCATGAATACATGGCAGTTGGGTCTGTGGTACTTGG
ACATGCAGCTCCCGG
TTCACAGAGAGTACAGGAAGTATTTCCTGCGATCGAATAAGATC
CCAGCGTGCAGGGGCT
GATTGCTGCTGTTGTCGGGCATGAGTAGCCCGGGGGGCAGGCGA
CACAAGCCAGCCAGTT
CACGCCGCTGTATTCATCGATTTCGCATGCTTCCGGCTGCAAAAC
TGGATCAGAGCAATA
GCTCCCAGCTGGGCAATCAGAACATTGTGTCTGTCCTTCCATTCCA
TTCGTATGTTGTCC
AGCAGCGCAAGGGATCGGATTAACGCC
>ConsensusfromContig2005 Average coverage: 13.50
CTTTAGGATCCTTCCTAATATTATGCACTCCAAGAACCACTCTATA
ATTCTTCATATAAT
TTCCTGTTGAATAACAATGCGCAACAGTTGCTACAATGCGAGACC
CAATCAGTGCCCCTC
CACATATATGTTGTTCGAAGAGTTGGATTGAGGCCATGTGCGGCC
ATTTCCCGTCAGGTA
GTGCTCTTTCGCCCCCCACAATGCGTCGCTCAATACATTCCATTC
GTCCAAAAATGACCA
CCATCAACAACCCGTTGAGAAACATATTAATATTCAACATCATAG
CGATAACTTGTTTCA
GAAGCCTC
>ConsensusfromContig2006 Average coverage: 8.34
CTTTAGAGTCTTGAATTTAGAATTCAAACAACATTAAATGTCGAT
ACAGCCATTAAATTG
TTTGAAATATACTTATTAATATTATTTATATTCTTATCCATCATTT
AAACGATACCTAGG
AGCTAATCGTGTCTGTTTGTTGAATATTTCCGGATATAAGTATGT
GATGTTTGAATATAT
TGCTATTCATACTTATATAGTTAAGTCATCAATTGGCTACTGAAT

GAATGACATTTTCAT
CTGGATTATTTTCCTATCTGTCGAGCCTACCACATACGCTTTCTGG
ATTATACTGAAATT
TTTCAAATAAAAGATCTTTGGTATTGAATACCTCTTACGGTATTTT
CCATATATCTCATT
CATCCATAATTTATATATTGAATTTGAGATGTCTACGCAAA
>ConsensusfromContig2007 Average coverage: 27.65
CGGCGGGTGCATTCCGCTGAGACATCCAAATAGTATGTGATAGCT
CTCGCTGTCACCTCA
AGTACGTTGTCAGGCGCGCACTCGTCGAGAAAGATTTTGCACAA
CGCCAAGAGGAAAGTT
CGAGGAGGGCAGCTTTCAAAACACCTGTCAATATTATCAGACAT
GAGGAGCAGCATGCAT
AGTTGCTCCAATGCAATGAGCTGCATGTCTCTCTCTTCCCCTTGG
CCCATCTGGAGCCAT
TCCAGAAGTGTGTCAGGATCAACATCAGACATTATTTTTTTGCTT
ATTCTCTGTAAAATG
TTTCAAATGAAGTTCAATACACTATTATGCATAAAAGTCATTATC
TATGATGACATCATC
TGGAATGAGCAGCACAAATTTATGATTGTTTATTTCCAGAATTTG
CTAAAATTTGTTTGT
TTTG
>ConsensusfromContig2008 Average coverage: 11.98
GAGTTTCAGTTAGGATTACAGAATTACGTGAAAAAATACAAATT
CCAGAACTCACAGATG
TCTGAATTGTGGAGCATATTTCAAGATGTATACAAAGGTCCCTTC
AATGTCATGAAAATC
ATGGACACATGGACTCGACAAATGGGGTTTCCTGTGGTCACCGT
CAAACAGGTCGATGAG
GGGGGCGATAAATATAAACTCGTTCAGGAACGGTTTCTTCTCAAC
CCCGATGATTCATAT
GATCCCCACACCAGCAAATATGGATACAAGTGGCGAATTCCGTTT
TCATATGTTGTGCAG
GGAAACCAATCCATTCATACTGTATTATTGGATTTATGGTCTGCA
ACAATTTATGATATT
CCGCAAAACAAATGGCTTCTCGGAAATTACGAGTACATGGGGTTT
TATCGAACGAATTAT
GACGTTAGGATCTGGAAGATGCTAACTGAACAGCTGATGCGCAA
CCATACGGAGATACCT
CCGTCTAACCGAGCTGGTCTTATAAGTGACGCATTCAGTTTGGCG
AGGGCCGGCATGCTG
AGTTATCACATAGCTTTCAATTTGACCTGGTATCTGTCTAAAGAG

CAACATTACGTTCCG
TGGAAAGCGTTCGTGGATTCCATGTCCTTCATAGATGGCATGCTA
TGTAACAGTGAAATA
TACGGTCATTTCAAAAAATACATGCAGAGTCTTATCGAACCTCGT
TATAGACAATATAGA
ATGAGGGTGGAGAAAGAGCTACTCAAAAGATATTTCGTT
>ConsensusfromContig2009 Average coverage: 7.42
TTGGTCTCTAGCTTTAAACTTGAAAATAAATATTTTATCTGGAATG
TATATTCTGGAGTT
GCAACACCTTTAGCTTCGACTGTAAAGCGGCAGAATTCTTCTTTA
AATCTAGGAACTGTT
TTTGTCATAAACCGTACTCTGCCTTCGCTGATATCTGTTTGCGTGA
ACTCCCGTACAATG
CGCCACCGATTGCTTGGAAGCACGAGTTTTTGGAAGAACCCATCC
ACCTGAGTACTCACG
ATATTATAAGTTATAATGAGCTCATCCGGTGCGTTTGTAGTGAAT
GTGAGGTTATGAATA
GAGATCAGCTCAGATGTGAGAGGTTTAAGCTCGAGTCCTGTATTG
CTTCTCATGATCAAC
TCTAGTTCTTCGGCTTGAATGTGAAGAACCCGCGCATCACTCATT
TTACCATCGTCGGCA
GTGACTCGTAGATTGACAGAGGATAATTTGTTGTTCCCACGATGA
ACATATTTAATTTCT
CCCTTATTGATGTTTTCCTGGGTGAAACTTTTGACAACAATTTTTC
TAACCCTGTTTTCG
AAAAACCCGTTTTCGCCCGAATGATCAACAGAGAATTCTAGTTTG
TCTGGGGCAACTCTT
GCATATTTTGCACGGATCATCTTACTAGTGATAACCACTTCGTGA
TTTCGAATCATAACC
AAGGTTGAGTCTTTCGGTAGAATTATAACAGGC
>ConsensusfromContig2010 Average coverage: 43.66
TTTCCCCATCCATGTTTCAATTTCACTTATAGTAAGATCTCTATA
AACAATAGATAACAC
TGTTATGTTGATGAAATATCAGACTTTCTTTGTTAATATTTCTAGA
AATGAGCGATTCTT
ACAAAACAGGGACTCTCCAAACATTCATTCATTATCCGAGAAAAT
TGTTCAAGTGTTTAA
TGCACGCACTCTATCGTGAGATATTCTTCAAAACCTCCAAAAGTG
TGAAGTTGGGTTTGG
GACGCAAATAAAAAACTTTATTCGATGCGATAAAAATTCACTTCT
ACATAACGAACGTCG
ATGGGGAAGGCCCATCGCTGTACTCCATGATTGAAGAATGAGAG

AGTTGAATAATCAGAC
ATACTTCCTACTTCGTCATTCGATTCTCATTATTCGGTGCATACTC
AATCTCTGTTGTGA
ATCGAATTGTTTTTGTGCTCATTTTCCAACCATTTTTTCTATGGGC
ACTATCGAAAATTG
TCACTATTATAAGCATCTGATTCAAACACGAGCTTCATGATTTCA
AATTTGACATAATTC
>ConsensusfromContig2011 Average coverage: 18.83
ACCGATGCCATAAACCGAGTGCGACCAATGTCGGCTTTATCCGTT
CTAATGTTTTTGATT
TGAATAAACCTATGTGTTTCTTGCCCGCCTCTGCACCCGCATATG
GAAGGTCTCAAAAAT
CAGATGAAGGACATCGTGAACCCAAGTATCACAGAGTCACACGA
ACCCAAATGGAGACGA
CATCAGGAAGGGGCTATATGCCCCCTATAAACCCTCAGAAAGCA
TGCCATAAACATAGTG
CCAATCATTATAGGGAGATAGCCAGGGGTGTTGTACCCGTGACAT
CCCTTTGGGACCCTA
GTGGACAACCGAGATTTTATCGCGAATCTATGTCCTTTGATCACG
ATTTCAACAGCCGGA
ACGATCCCCCATACCGCCCGACAGGGAAGCGTCAAGGAGCCTTT
GTATGGAACCAATGCA
GCCGAGAAGTGGATTTCAAAGTCGAAAAACAAATAACAGCCGCA
TAAGATTCATCGAGAG
TTCAATCACGGCTATATCTAACAATCTATCATGATGAGTATTTTC
AGAATACAATCACAT
TTCAAACTCT
>ConsensusfromContig2012 Average coverage: 37.55
AAAAAAGAAGGAATACTAATTTTATTGATAAAGATGTTAAAAAG
CAGAGGACTGAGGATC
GCACCTTGAGGCGTGCCAGAAATGAGTGATAAAGGACCAGACTC
TGCATTGGCCACCTTG
AAAAAGCATGTGCGATCAGATAGAAAAGAAGATATGATCCTGAT
TATTTTTGAAGGAAGT
GGAGTCTCTTTAGCCAATTTGTATCTTAGCCCATCGTGCCATACC
GTATCATAAGCCTTA
GCTAGGTCCAGCAGAACAACGGATGTGCATCTGCGAGGCTTTACT
GATGTGAGTCGACTG
TCGTGGATAAGCTTGTGAGAATTTTCATTACATTCTAATCCAGGT
ATGAAAGCCCGCTGG
TTGAGATTGAAGAAGTTGATACGGGTAAAAAAGTCCATGATCCTA
TGATTTAAAAGTCTT

TCAAAAATCTTCCCCAGAACCGAAAGAAGACTTATTGGCCGGTA AGTTGAAGGATCAGAT
GGTTTAAAAGAATTCTTCGGAATCATTTTAACAGATGCTATTTTC CAAATTTTTGGAAAG
TAACCTGAAGAAAAGAGAGATGTGTAAAGAACTGAGAGACGTTG GAAAAGGGAAGTTGGA
CTGAAAAGAATCATCTTGTACGTTATTTTGTCCGCTCCAGGTGACG TGCTTGACTTTTTG
CGGGATGTAACCGCAAGGCGAACTTCCTCTGGCGTGAACGAGTC ACACAACCCATCAATA
TCATCGGAATCCGAAGGAAGATCAGACAATTTAGGTTCGAATAGT TGCCTGTGAGATGAT
ACGAACTCTCTGACTATTTCAAACTGGGAATGAGAGAAGTAAGG AGGAGGTGAAGCATGG
ATGATATTGTGAGAAAAATTTCTGAAAGTGTTCGCAGCCTCTATA GCAGATGTGGCCGAA
GCCGAAAAGGAATGAAGTTTGACAGGTGGAATGACTGGAGCAGC TACCTCATCCTCGGAA
GTGAGTTTTGAGACCTGCTTCCAAAAATTTCGACCAGACGAAAAC CTAAGATTATCAAGA
TTTTGGCATTGCAACTCGAAAAAGGATTTCTGTTCAGCAGCAGCG AGTTTCCGAATGTCT
TTTTTCAGTTTGTTGAAAACCGGACGGAGAACACCTGCCAGATCA GGATTATGCTGGATG
TGGGATCGTAGTCGACGAGAATAACGGATCAAAGAAAGGAGATA CGGCGAAGATCGCCAG
TCATGAACAGGAATGAGACGAGAAGTAGTGGCGTCAAAAAATGA TTCTGTAATGGTGTTA
GAAATCAACTGGCATTCGTCGTCTGTATCTTCAATGGAATTCATC AATAGAGGAGGAAGA
ATTGATAGGCGGTCATTCAAGATGCTTGAATAAAGGGTCCAATCA GTCTTGCGAACGAGC
TTTCTTGGGGAATAGTGTCTTGTCGAAAATGGGCCAGATAACTTG ACTATAATTGAAACA
TGATCAGACGTGATCGAACTATCGTAACTATGAGAGGCAAATGG GAGTAAGGAAGAAGAA
ATGAGTATAAGATCAGGTTTCGAGAGATAGTTCGACCGAGGAGGG CTAATAGTGTTTGTT
TGATCTGTGTTCACAATTAATAGACAGGCAAGGTCGATTTCTGCA GAGAGCAAACTGCCT
CGATTCGTTGGAATGATAGAATTAGTGAGGTCTGGATGATAGGAG TTGAAGTCACCCATT

ATGATCGAATGGGGAGCTAGGGATGATATATATGGAAGTATTCC
AGGGCTGATAGTTTGG
GAAGGTGGCACATATATTGACGAAACAATTATTCTACGGTTGTTT
ATACGAACAACAGCA
GCCACCGCCTCGTCTGAGTAATGTTGGGGAATGGGACATTGGGT
AAAAGAAATGTCGTCG
CGGATTAATAAAAGAGCACCGCCCCCTAAAGAAGGACGATCGTG
CCTAATGGTATTGTAA
TGTTTGATGGAAAATTTACAGGTTGGAGAGAGAAAAGTTTCGTTA
ATCGACATGACCAAT
GGGGAATACCTCTCAAGAAAAACTATCAGTTCTGGTTTTTTGTTT
CGAACAGAATGAATA
TTCAAATGAATAAACATTGCAGTTGAAAAAATATAAAGTCAAAT
GAGAGATGAGGAAATA
AAAGGGAAGATAATTGCGAGAGAAGTCCCAACTTGTGATAGTTG
TGTTGTGGAGATGAAT
CCAGAACGCGTGACGGAGGATGAGGAAACGAAGGCCTTATCGG
GGGAAGATCAAGAAG
>ConsensusfromContig2013 Average coverage: 12.24
GGCTTCGTGTTAGGCCGCTGCTTCACAAATCCTCTCGGAGCTCCT
TTCCTCATCCCGAAA
CCTCCCTTGTGAGACACCTCAATTGGACTCGGCAATATGTCATTT
CCAATCGAACGCAGC
CCTGTTGTATTGCGTAGATTCGAGTCCGTATTGTTATTATTATCGA
TCCGTTTTTGAAAA
ATTCTGGGTTGATTCGATTCAGTGACCGGTTCCCAATCGGTATAC
AGGTCCGGATAAGCG
GTGTCGTCGAACGAGACCCGAGCTCCCCTAAGACCACAGTCGGTG
GGCTTATTGTCATTG
TTATTGTTTTTTCTCTTGTTACTGGATCTGCGATTGAGAGAGGCCG
TGGATCCCGCACTC
TCCGTGTTGCGCAGTTTAATTTTGGACAGGGCTTTGTTGAAGGTA
TTGTCAGCGTCAAAT
GCGGACATTTTCTTTTTGGTGAA
>ConsensusfromContig2014 Average coverage: 43.85
AAAATGTCTACTTGTAAATACTAAATTTTATAATTTTTTTTTTATT
ATGGATTCTAAAAA
TTTTCGATTGAAAGTTACCTCGCATATGAAACAGGGTGGACGTGC
ATATATGGAAGATGT
CTTAAGTGTTCACTTCAAATATAATACTAAAACAAATGACCTAGA
TTATGCATATTTCGC
TGTTTTCGATGGACATGGTGGCGACTCTGCATCAATTTTTGCCAA

ACAAAATCTATTATC
AGAGATAACAAAGAGAAAAGGCTTCTGGTCCTCAGATAGGGAAG
ATGTCAAACGAGCGAT
ACGTGATGGCTTTTTATCCACTCATAAACTCATGTGGCGCGCACG
AGGTATGTGGCCAAA
AACAAGACATGGGTTCCCGAGTACTGCCGGAACAACTGCAAGTG
TAGTATTCATCAAAGA
TGGTTTCATATATATTGCACATGTTGGAGATTCGAGAGTGGCAAT
GGGATATAAGAATAA
AAGTTCCATGTTACGTGGAAAGAATCTAACTGAGGATCACAAAC
CAGAGAGCCCCAAAGA
GCTGGAGCGGATACGAGCTGCCGGAG
>ConsensusfromContig2015 Average coverage: 40.38
TATCCTCTAGGTCTGCAACCTTCTTTTTATATTTTGTTTTTGGTTCT
TCTTCACTCAGTT
TTGCTTCAACTTTTCTCGCCTTCTCTTCTTTTTTGTAATTCTTTACC
TTTTCTTTATAAT
CAGCACATTTCCTCTCAGAATCTTCGAGTTTTGATTTCAAATCTGC
AATACATTTTTTAT
CTTCTGCATAGTGTTTAGGCCGCTTCACGTTCTGGTTTTCTTCCTTC
AATTTTTCTATTT
CTGCTGTGAGGTTTTTGATCTTGTCGTGTTTCTCAGGTGTTGATTC
TTCGGCATTTTCGT
TCTGAGCATCCAATGACCTTGTCCTTCCAAAAGAAGATCGTCTGC
CCGCAGCTGTTACGT
CTTTGCTGGATTCTTTTGCTTTCTCAAGGTCACGCGTCGTTTCTGC
GAGTTGTTTTCGGA
GATCATCAACTTCTTGGGCAAGACTCTTGATTCTCTGCTCGTAAG
ATCGACCACCATCAT
TCCCTGAAGCGATTGAATTCTCTATGGCAGCACTGTGTTTCACTA
GCTTTTCATGTAAAA
CCGATACTTCTTCCATCAATGTTTCATTCTCCTGTTCCATCTCATG
CAGCTTCCTCTTTA
GAACGACAACTTCCTGCTCAGCCAGTTCCATTTGAAGTTGATACTC
TTCTTTCTTCTTCG
AGTCATCATCGCTGCTTTCATCATCTGTTCTTTTAAAATATCTGCC
ACTCTTAGCGGATG
ACATTTTCTGCAGCTGTAGTGTCATACTTTCATTCTCTTCTTCGAA
GTCTTGCATTTTCG
ATCTGACCATTCTGTTCTCCTCTTCTGTATATTTCATTTGTTCTTTG
AGGTCGTTTTCTC
GTTCGAGGCTTGCAGCCAGATCTCTCCTGATGGCATCAATGTCTTC

GTCTTGACTTTTTT
CTTTTGGAAGACTACCGAGCGTCGGCTTTCGAGGAGATAATTTAT
CGCCGGAACGCGCGT
CATCAAGCTGATTCTGAAGAGTGAGAACTTCGTTATGGAGCACTT
TTCTTCTGCCTTCAG
AATCGGACAGGGAATCATGTAACCTCTGCAGTTCTTCTTCGTATTT
TATCCTTCGTTCTT
CCATTGTCTCCAGCTCATCATGAAGTCTAACTGAGACTTCCTTCGC
CATTTGCAGCTCCT
CTTCCAGCTCTATGATGGTGTTTTTATCTTCGGCTGACCCAGTGCT
GCCGTTCGCTCGAT
TGTTCCTCATTTGAAGCTGTCTTATTTTCTCTTCAAATTGCAGACG
TTCACTCTCCGCCT
GTTCATAACGCCTGTCCGCTTTACGCAGCTTGAATTGAAGAATCC
TGCAGTTTTTCGCAG
TCATTTCGAGATCTTTTTGGAGTTCTCTAAATTCTAGCATTTCATC
TTCTCGTAATTGAT
CTTGAATTTCATCCATTTCTAAATTTAAATCACTTATTTCATTGCG
AAGTTCTTTGTTGA
TATTATTGAGAT
>ConsensusfromContig2016 Average coverage: 48.51
TGTTCTACGTGGGACTGGTTTTGTTTGCTTTTGCATCTGCCACTGT
TGTATCTCTTCTGA
CGACTGCTATAGCTTTCCATGGCACTCCTGAGAATCAAGGAATAA
TTATGGGCATTTTTC
GCTCTCTGGGTTCTCTCGCCAGAGCTCTGGGTCCAATATTCGCAT
CTACAGTTTATTGGA
GCACTAGTGCCTCTACGTGCTACACCCTCGGGGCTATAGCATTCAT
TCCTCTTCTTCTCT
ACATGCGGGGACTGACTAAAGATCATACCGCTGAGCAATCGAAA
CTAGATTGAAAAACAT
TTACCAACTGTATGCGAGTTTATGCATCTCATTATATCTATGTGGA
TATTTCTCATATCA
ATAGAAACTTTTATTTGAGTTCTCATGTGAATGAAAATGAATGCC
GAA
>ConsensusfromContig2017 Average coverage: 44.06
GCCATCTCCAATTCGTGCATCTCTTTCGGGGTGCAGTATGGAAAT
TGAATGGGTGACAGA
TACCTGGCTGCGGCGATGATGTTGATTCTTGCAGCGACGAATAGA
TTTCTCGCCTCTGTA
GCCAGAACGTATCGGGCTGGTTGATTGCTCGGATGATACCTCAGA
ACATCCTTCCACTTC

TCGACACCAGTTTGCGATTGTCGCTTAGCAAATTCTATAAGATTCT
TTCCACTTGTCCAT
TTATATTTCGCAGCTTCGATTCGTTTCCTTTTGTCTTGCAAAACCT
CATCTCGCTGTTCT
AAGTTGAATTCTTCCTCACTGCCGTACGACTCACCAAATACTTGT
GTTAGTAAATCATCT
TGTTCATCGTAATAGTCCTCAAGATCTTCAGCTTGTGATTTCGAT
GCAGAGATAGACTTC
TCCAATTCGTCTTTTCTGTGCTTCAATGTATTGAACTGTGTTCGCG
CTATTTCTTCTACG
TTCAGTGCCTCCATATATTCAGCTTTTTCTTTATCCATCTCTTGGT
CATATTCTTGCTCG
TTCTTGAACATACTCCGAACGGAAGGAAGTTTGTCAACATCTTT
>ConsensusfromContig2018 Average coverage: 21.85
TTCATGTCGAGCAAAGTTATTTTTGTTAATATATGGTTATCGTAA
ATGATACATAATATG
ATTTGGTAATGACTTGTTTCTATATTCACCAACTCGAGTTATTTGT
AAACCTCTTTGCTA
TACATGATTTATCATGGGGTGTTTTGACGGGTATTGTAATTTTTCA
AATTTTTGTAAGTT
TGCTGGATAGTTGTGATAAATTATCGCTGTATACATGGATTATTC
ACCAATGTTCGTAAT
TCAAATATTCATTTATCCATTGATATGTTTTGCCAATGCATAAATT
TTATTTCTATGTAA
ATGTTTTTATTCATCATCATTGAGAA
>ConsensusfromContig2019 Average coverage: 73.33
ATCACCAGCTTTCTACACGATTGAGGGTCCGGTGACTGGAACAGT
TTTCAATTATGTAGA
CCACGTTGTTCATACACTCTCTCTGCATAAAATGGATGGGCTCGA
GGCGAGATCGCTCTA
TATACAGAATTTAGTTGATATGGGAAATGCTAAAGATCTTGAAGT
CGCTATGAGAGTCAC
AACAGATATCAACTCCGAACAACACTTCTTTACCGATCTTAATGG
ATATCAGTTAACGCG
GCGAACTCGGTATGATAAATTACCGTTACAAGGAAATTTCTATCC
AATGCCTACAATGGG
ATATATAGAAGATTCACGTCTTCGAGCGACGCTGTTATCAGGTCA
ATCTCTTGGATTTTC
CAGTCTACAGTCAGGCTGGATGGAATCGGTGCTTGATAGGCGTTT
ATCTAAAGATGATGG
ACGGGGTATGCAGGAGGCCGTTCTAGATAATGTCATCACACCCAA
TTTTTTCCAAATTCT

CTTAGAGCCAATCGAGAAATCTCCTGTTTCCAGCGTGAATTTATC
AGCCGGATACCCTTC
AATGACAGCAATCCTATCATCACGCGAGTTGCTCCATCCCCTTCA
TGTTCTTCCAGTGGC
ACAAAATCTGAATCTCGAAACAAAGGATTATTTGACGACATGGA
GTGGTGTCAAAGAATC
ATTGCCTTGTGAATTAGATGTGGTGAATATACGTTCCATGGATTT
CGTTGCGGAAAATTC
ACCAGTTGAGAAGGGCGATGATCATTGTGACGACACGGCTCTGAT
CTTATATCGCCCCGG
GTTCGATTGCGCTATTCCACCTCCTAGCGCTTCTTGCTCAACTGC
AGAAGGCTCGGTACA
TATAAAAGATATATTTACGGGTCTCAAAATTTCCTCCGCATCTTCG
GCTTCGCTGTCTTT
AGTGAGTGGACCCAAAATTGATCTCAACAAGCCTATTCATATTGC
TACTATGGAAATTTC
CGCTCATTTGCTCAGTTGAACATTGAATGCGCTGGATACTGGATT
TTACTGATTTATTAC
GTATATTATGCATTTTGTTTATTTATTATCATGAAAAGCATTTTAT
GCTAATTCTCACCA
AATCATGTTCGAAATATTTTATGGATGAATGAATTATGGACTTTT
AAAAAAAAA
>ConsensusfromContig2020 Average coverage: 15.73
TTTCCTAGATTCGCCCATCGAGACTGACGATGTTTGTGTTCTCAAT
TTATTCTGAAGTGA
CACTGAGCCCAATCCACAGAGAAAGCCGGTCATGTTGGCCCATTC
GTTTATCTGATCCTC
GAGTTCATGTTCTGTGCTCTGATGGTAAGTCCTCCGTTTGCCACC
CATTCGCACATGAGA
AGATTCGCCATAAGAACATTCGTCCTGTTTGCTTTTGGGAAAGTT
GACCAAATTTCGTGT
ACAGACTTCCCAGTTTCGGAAGGTTTCTTCCCAAGCCTGCATATT
CCCCGGCGTGGGATA
TACGATTCTTCTGAGAAGGGACATAATCTTCTTTTGAAGAGCCAT
CCGCCCTGTCGTCAA
TTCGGAGCTGCTCACGGCCAGTTCTGTGTAAGTCAGATGGTTCGG
CAGGATGAGTTGCAC
ACCCAGCTCATCGGTGCAGCAAATGATGTTTGCCTCTTCGCACA
GAAGATGGAAGCAGAA
CATGGCTGTGAGAACAGCGTCGATATCGATGGACCACAAATACA
TGAAGAGAACGACCTC
CAATTTGATGCGTGCCTGCTTGCAGATAGGCATATTGCTGCCCAC

GTAAGGATATTCTTT
ACGATGACGCAAAAAACTGTTTCTTCTCACCAAAATTTCACGGAG
CCATTTAAGGATTTC
CGTGTAATTAATGATTTGATGCTTGATCAACTTTTGAGATATCGA
GAATACGACTTGGGA
ACTGATCTCCCAAAACGTGTTTATCGGTGTCTCCGGATTCCACAA
CTCGATTATTTCCGC
CCGATGTAAACAAAGTAGAGCCTCCATAGCCTCCTGGGCAACATC
AGGCATCATGTGTTG
GTGTATCAGTAGAACCAATCCGTTGATCACCTCCATCGTCGAACT
CTGAATTTCTTGACC
TTTCTTGCCTTGGTTATGAAGCATAAGAACGGGGTCAGCATGAAT
CAGTCTGACGATCCA
CAAGAGAAGATACTTGCAGCTCGGTATCTCGTCAGACACTTTGTC
TTTGAATTTCAATGA
AGTCACCTTCTGTGTCAAGCTCATCTGAGTGTGTCTTGGAGGGCC
GTGGTTCATCTGCGT
GACTTTGTTCTGAGTGTCTGTGAATAAGT
>ConsensusfromContig2021 Average coverage: 19.62
TTTTTTTTTTTTTCAGTCTCTTCATTATCATGAATAAACTAACATT
GCAAGCAAGTTCGA
AAATAGGTAGGTATCAATATTATATATGCTGAAAAAGGTAGATG
GCAAATTGAAAACAAA
GAATATGAAGAGCCTTGGTCCTCAAATATGCGACATTATAGAAG
AATAACACGTGGTACG
AAAAAATTGGAACTTTCAACAATCTGCAGATATGAACGCTAAAA
ATTTCAAAAGAACGAA
AAAAAAGTTTATAAGCATCTACGTGTATACAGCAGGTCAAAATA
TTCATAAATGCGAAAT
AAAGATAAATAATATAAATTTGTGTTCATGAAACCGTATGAAAGC
ATCACAAAATTTAAC
ACATATATAGCGAATGTATAGATTCATAGGAAATGAATGAAAGG
CCATAATTATTGCTAA
TATGTTTTGAAGAAACTTGCAATTATTGGAAATTGAAATGAGCAG
TCCAAACCTTCGAAT
TTTCAGTTTAAATATTAGAAACTTTTGATCACTGATTTGTCAAAT
ATATCGGGAAATACT
GAATATATATAGTTTGGATTCAAT
>ConsensusfromContig2022 Average coverage: 13.49
CGCAAATGGCTTTAGTTTCTTCTATCAGATCGTTTATTACAAGTTT
GACTCGGCATTTCA
CGCAACGGTATTCAGGAATATGGATCTCTTGCATTCTTATTTTCGG

CCAATTTTCTTTGC
ATGCTTCACAGTCACATTTAAATGTGAACCTTTTCTTCAAATAAG
GATGTCGTTCTCGTA
TGCTTACCATATAATAAACAAAATCGTAGTCAATGGTTGCTTCTT
CGCCTTCTGCAATCT
CCCTCGTTGCCCTTACTATTATATCACTTGCATGATACTGCCTATC
AGTATTTGGGTCAC
ATGAATGATTCATCAGGGCGGCAGAAGGGTGAATAGCCATTGCA
ATACTGAGAGGTTTTG
GGTCATCGAATTTACTTGGATTTGTCAACGTGAATACAGATTTTG
CGTTGCACGCGGCTA
TTTGAAGTAATCTCACAAGAGCCACAGTAGTATCAATTTTTATCT
CATCGATTTTCTCAC
CGTCCTTATCTGCATCTTTGTCGAAATAAAGAAATTTTTCAAGAA
CCTTCATCAAAAATA
TAGCAAGAACATTAA
>ConsensusfromContig2023 Average coverage: 37.21
TCAGATTGATAGAAGAAGGCGGTATAGATCCTATAATCAGAGGT
CTGATTGGAAGAGGAA
CTAAAAAAACAAATCCGCAAAGTATGATGAATATCGAGCTGACA
GAGAAACTCTTTGAGC
TGGCACACAGGGTTGCATTAGATTTGGCTGCACTTAACATTCAA
AGAGGACGCGACCACG
GATTGAAATCTTATAACCACTACCGAGATTTTTGTGGCATGTCGA
AGGCTGAAAATTTCA
ACGATCTCGAAAATGAGATCCAGGGCTCAATCCTAGAAAAGCTT
GAAGATGTGTATGGCC
ACCCAGACAACGTTGATCTGTTTGTGGGCGGGCTGAGCGAGACGC
CCTATACCGGATCCC
GGGTCGGACCTACGTTCCTCTGCATTATCACCGAGCAATTCCGCA
GGCTGAGAGACGGAG
ACAGGTTTTATTACGAGAATGGTGACGTTTTTCGCTCCAACCAGC
TGGACCAAATTCGGA
AAAGTTCTCTTGGAAGGATAATTTGTGACAACTCTGATGGCATTG
ACCAAGTGCAGAGGA
ATGTTTTTGTTCTTTCAGAAAAAAGAATAGCTTGTTCAGAAATCC
CACAAATGGATCTCA
GATTCTGGAGTTCTTGTTTTGGCGATTCGCAATCATGTCATCTCA
CGGAAAATGAAGAAT
CTGGCGAAGTGAGACGAGAGATTTGAGGCAAAAGACATTGAGAC
AAGAATGAGCAGACTG
GAACAAACCATGTCAGCCCTTAGCATGCGGCTAGAAACCGCCTCC

AAAATGATGGATTCT
GTGAGTGGTGTGGACAAGAGTAT
>ConsensusfromContig2024 Average coverage: 98.71
ACTGGAGTTCAGACGTGTTCCCTTCCGATCTGTTGCCAGGGTAAA
CAATTGAGGAAAACA
AATCGACGAATTTTCACGCACGATTCCAAATAGACTATCCAATT
GAAAGAAATGGCCAAG
ATTCTCCAGCCGCCCCCCAAATTCGCCCACCCCGAATGGGCCGTT
TCAAACCAGCGCAAG
TACGCAAACGCGGAGGGGGAAAGGGCGGCGGCCGAGAGACTCAT
TGCGGAGTTCGGGTCG
GGGATGAAACTGACGAACGCACCAAAGACGCACAAAACGACGTC
GCAAAAAAACTAGATC
AAAGAATCAATGACATAAAATACTGGGAAAAAGAGCTTGACGAT
AAATTGGACAACTTGA
AGAAAGAAAACGACGCCGCGATTGCATTCCGAAACAGAATTGAG
AAAGCGATCGACGACT
ACAGGGAACCTCTGCACATTGCCCAGCAGTGCCTCAAGAACAGG
GAGAAACGATATGGTA
TTGACCTTGTACATGACGACCCTGAGAAGAACCTCATTAAAGAGG
TCGAGGTCATTCAAG
GTGTTTTGGCTCTGCTACAGAGAACACATGAACAGATGACGGAG
CAAATCAGACTGAACC
GAAAGGCAATATATAAGCTGGAAGGAGATTTGAAAAATAAATTC
GCCGCTTTCCACATTG
ACAAAGGCTGCCGAGAGCTCAACAGCAACACTCCAGGGATCCAG
TTCAAACAAGGCGTGG
CTAAAATTGACACAAATTCTTCTAACCCTGATGAGTGGGCGGGAT
TTAGCGATGACAACA
TTTCATTCGCCGAGCAGCAACGTCAAAGCTCCGCCTCCTTACGAT
CCATGGTTGACGACA
TTCTGCAGAGCACAGCCAACGATATGAACAAGCAAGTCTCGTGC
ACGAACAAAGCATTCG
AGAGGAGAATTGCTGAAACCAGGGATACTAAGGGCAAGCTGGAA
GACCATTTGAATAAGG
TGGTCTCACAAATAAAGGACATGATGGACAACATTGCAAAATTG
AAGAAGAATATTGAAG
CAAGAAAATCCGACTAAACTGAACAATACGAGATTGGATGCCAG
AAAAGAACGCCCAGGG
GTGGAGTTGTGCAGAGACCCAGTGCAGTATCGTCTACTCCAGGA
GGACCAACAGTTGAAA
ATGTCTGTGGCTGAACTTCAAACGAGATTGGCAAATTCGGAACAT

TCTTTGAAGGCCTTG
ACCCGA
>ConsensusfromContig2025 Average coverage: 16.31
TGTCTAGGCGGGTTAATTTAGTCGGTTGGAAAAGAGCTTGTTTGG
GGGTAGAGGAGTGTA
TAGGCCTAGGACTTCAGCGCATGCAGTTAAACCGCATGCCGATTA
TGTGTACTTAAGGTC
AGATATTTGTTACTGGTTTTGTGGTCAAATCTCCATCTCAAGACT
GGATTTTATGTACGG
AGATTCTTACTGGGCTTAGCTCAAACCTAGTAATGTATGGAGCTT
CGGTCTGGTCAACCG
CAGATTTAGCGAAATAGGAGGGTACATGCTCAACCCTCCCGCCCC
GCACCGTTTAATTTC
GGAATAACCTTCGTTCTGGACGCACCCAGAGATCG
>ConsensusfromContig2026 Average coverage: 12.47
CAAAACAATACTGGCGCACTGCTAAGAGCATATATAAATTTGCG
AAAAAAAGTGATTCTA
TCCCTAATTTAGAACACAACAACGTCATATTCGATACAGATATAG
ACAAAGCTAATCTAC
TAAATAGGACTTTCACGACATCAGAAAATTATCTATCAAGTTTCA
ATGATATCCAGACAA
ATGAAATTACAACAGAAATTTTAGATGACATTGAGTTCAATCCTG
CACTTATATTGAATA
AACTGAAAACATTAGATTGTGACTCAGCAACTGGTCCGGATAATA
TACCGAATTGCCTCA
TGAAATACTGTGCTGAAGAATTGTGTGAGCAACTTTCTTTCCTGTT
CAATTTATCTTTCA
GGACAGGCAAACTTCCGAATATTTGGAAACTCGCTAATGTGACA
CCAATATTCAAAAAAG
GTGAAACTTCTAACCCAAAAAATTATCGTCCAATTTCTATCACGT
CATGTGTTGGAAAAG
TAATGGAAAGCATCTGCAATGATCTTTTACAACATCATCTTATAT
CACACTCGCTCATCT
CAAAATCTCAGTTTGGTTTTCTCCCAAAGCATTCAACAATGGATC
AACTATTATTAGTAT
ACCATAAATGGGTTATGTCAATGAATTCTAAAAAAGACACTATAG
TTGCCTTCCTCGATT
TACAAAATGCATTTAATATGGTTCCACACAGAGCCCTTCGTATTA
AACTTCCTCAATTTG
GCATCAGAGGAAAATTCTACGCGTGGATATCGAATTTTTTACACG
ACCGCTCACAAAGGA
CAAAAATTAATGAAACACTATCCGAT

>ConsensusfromContig2027 Average coverage: 33.73
CCGGGTTTTCCTTCAATATCTGCAACAAATGAATTCATTTCGTATG
TGAATGTTCCCTGC
TTGCCCAAAAATGCTTGTGTTGAATGGCATTTGAATTATGGCCAG
ATTGGGAAGTCATCT
ACGATCAGTAGCATAGTATTCAAGAAGCATGGTATGGATTGCATT
GTTCGGCTCAAACTG
ATGGGGTCATTTCTCCGAATTAAGGGCGAAATAGTGGGAAATGA
TGAGGGTAGTGAAGAG
CATTGTCTAGCCGTTCTTTCTATTGCTCTTCGAAAGATTGCAGAG
AAAGTGGAATTATTT
AAGCAAGAAATTATTTTAAAAGATTTTCTTGATATAAATTACTCT
GAGATCAAATTACCA
GCTATTCAAGTTTTCCATACTATTCCGATCAGCGAGAACTGCAGG
GTTTCCGAACCAACG
CGAATATCTTTCATTCCTGTTTCATATTTGGATCTCGATTGTTTGA
TGAGTTTCATGAAC
TTATGATATAGTTGTATTCATTCTTAACTCTGCATTTGTCATGG
>ConsensusfromContig2028 Average coverage: 35.85
TGACCCACGTTCCCGGCCAGCGTGATTCTCGCATCCTTCGCGTGT
TCGGTGCAGAGCCAT
AACACTTTTCCGTTCGCCATCAGACATCTTTCGAGTGAAATTTCC
TGGTCTTTGAATGCA
TCGAACGCCTCTCTATATATCTTGAGAAAAGCAGGAGCCATGGTG
CTGTTCTCCTCACAC
AACCCTTCAATATAAGTAACGAAATCGCTGTTTCCAT
>ConsensusfromContig2029 Average coverage: 20.96
ACTCCATATAAAACTTGGGTTGATAAAGCAGTTCGTGACAGCTC
TAGACAGAGAAAAAGA
TTGCTTAAGCTATCTGTGTTCATCATTTCCCGCAGTGAGTTACGA
AAAAGTTAAAGCGAG
TTTTTTGATGGTCTGCAGATTCGAAAACTCATCAGAGATCCAAAC
TTTACGAATTATATG
ATTCCTGTGGAGCGAAGAGCGTTGAACAAGTTTGTTTCTGTTGTC
CAGAATTTCTTGGGT
AACAACAAATTGCCAAATTTTCGAGAGTTAATACGAACGATGCT
CAAAAATTTTCAAAAA
CTTGGATGTTATATGAGTATAAAAGTTCATTTCCTTTAAAATCAT
AAAGGTGAATTTCCG
CATAACTTAGGAGATCTTAGTGACGAGCAGGATGAACGCTTCCA
CAAGATTTACGCGTAA
TAGAGGATCGCTATCAGGGTAGATGGGACAATCACATGATTGCCG

ATTTTTGTTGCAGCA
TTTCTCGAAATACCTCAGACTTGCATTCACAAAAATCCAGAAAAA
GAAAGTTCATCACTC
AGCAATCAGTTTAACTTTGATCTGTGATATGATCCTCTGTTTTGTG
AATATTTACTATTA
ATTATTTCAATCGATAAAATTTTTTTTCA
>ConsensusfromContig2030 Average coverage: 45.91
AATATTTGCCGGTCTCTCATCATATTCTCGACTGTCCTCTCTTTCA
TTTTGATATCTAAT
AGACAAAGAGTGTGAAATCCACAATTCCGATTCTTCACTATTTTG
TCATAAAAACTCGCC
GGTTTCCATGTTTCTGTCCAAAAAACGATCGAAACTGTTTCTCCG
AAGCTGTAAAGTTGT
AAGCCACAACATCCAACGGCATTCATAATGGAGGCATTATGCAC
AATTTGGTATGGAATA
TCCTGCTCTATGAGGCGTAAAACTAGGTCAGCATGAGTGGTTGCT
CCAAATGGATCACCA
ACCACAAGAAAAGCTACATCATTCTCTTTAGCCTCTCTAAATAAG
TTTTCTGGAGTATTT
TCAACATCTTCTCTATCGGCAAGAGTGATAGGCTTCCCATATATTT
CTCCCATGGTTTCA
TTCCCATTTATCAATATTGATGTGTATGCCTCAAGATAAATTTTAT
CACACTTGTGTATA
ATTTTTAAGCCACGATTTGTGATATCATCTAC
>ConsensusfromContig2031 Average coverage: 20.56
GAATGTTCTTTTTTTAAAAAACTTTTCACTGGCATTACTTTCGATC
TCATCATCAGAATT
CTGTGGAAAACAGGTTGCTAAGATATCAAACGGAAGGCTAGGCAC
TATTATATTCGATGT
CACACCAGGACTCTGCGATGGCTGTTTGAAAGAAGGCTGCTGTGT
GTTTTTACACGAGAT
AATAACATCTGCCTGCCTTGATGGCATCACCATAGGAAAAGGTTT
CTTTGTTGAGTGGTC
TTTGATGCATTGTTCTTCATATGTTGCGGCACTCAGTATATGCTGT
CTCATATCTTTGCT
ATTGATGATGCCCTTTCGAGATGACATGGCCATGCCGGCTTCAAT
GATTGACTGATATCG
CCTTTGATCGCATACAGAATCTTGTTGATTTGGACTTTTGTAGTTT
TGTGATTGATCAAA
TTGCATCGGGTAGCCTGAATGTATATTTTGTGAATAATGGCTGTT
CGATAAAATCATTTG
ATAATTC

>ConsensusfromContig2032 Average coverage: 64.61
ATGACTATCGTCTCGGTTTTAGTCGCGATCTATTATAATATGATCA
TTGCCTGGGCCTTC
TACTATTTGTTTCATTCCTTCACATCGGAGCTTCCATGGGAGAAA
TGCTCTCAAGAATGG
TCTACGCCATGGTGTGCTGACTATATCGAAAAGGGTGTCTCCGCT
ACATACTGCATCAAT
CGAAACTGGACGGCAGCGGAGGATGGGGTTTGCTACCTCTATCA
CCACGACCACAATATA
TCTGAGCCCAAGGCGATATGGGATGATGCGTTGGCCGCAGAGAA
CGGAATCGAAACGGTG
ATTCCTGCGGAGGAGTTTTTTTTCAATAAGGTTCTGCGCATAAGT
GACACCGTGACTGAT
GTCGGTCCGATCCATTGGGATCTTGCTTTGTGCCTCCTCTTGGCCT
GGGTCGTCGTGTGC
GCTTCGCTCATCAAAGGAGTCAAATCATCAGGAAAGGTTGTCTAC
TTCACTGCAATCTTT
CCATATGTCGTTCTCATTATCCTCTTTGTTCGAGGCCTTATGCTC
GATGGATCAGGAAAG
GGCATCGAGTTTTACATACTCAAGGCCGATCTGAACAAGCTGAA
AGAACCGGAGGTTTGG
CGGGATGCCGCCGTTCAAATTTTCTTCTCTCTGTCGGCCTCGTGG
GGAGGTCTTATCGCT
CTTGCCAGTTATAACAAGTTTCATAACGATTGTATAAGAGACACC
CTGGTTGTCAGCATC
GGGAATTGTCTGACAAGTTTCTTCGCCGGTTTTGCCATTTTCGCCT
TCCTTGGAAATTTG
GCACATGTCAACAATATGGAGGTGGAAGAAGTGGTTTCATCTGGA
CCCGGCCTGGCTTTC
ATCGTGTACCCATGGGCAGTGACAACTATTGCCGGCTCACCATTT
TGGGCGGTCATCTTC
TTCATCATGCTCATAACTCTTGGACTGGATAGTGAGTTTGCGCTC
GTGGAGACTGTTGTC
ACTGTCATCACAGATCAGTTTCCCAGTCTGAGAAAGAAGAAGTG
GGCAG
>ConsensusfromContig2033 Average coverage: 17.88
GACTACTGGAATAAAGCAGCCTCTGAGATTGGAGAGCTTAATGCT
ACTTCAGTATGGCTC
GGTCTTAGATCAGGTCTCACACTATATCATGAGTTCGAGCCTGGC
AGTTTTGACATGTCC
AATCGATCAGTACCCATAACATCGGGGTACTACCTCCGCGCCGTC
GAAATGGGACTCAAT

GAGTATAACTACTACATAGACGTTGATGAATTCGAAAAGGTAAA
TAAAGGAATTAGACAG
CTCGCGGACGTGCCAGTCACCGTTAGCACGCCACTGGTGCTTGTT
GACAGCAATAAAAAA
TCAGTTTACGGAGTCATAGGATTCGAACTGCGGTACCCCGTCTTC
TCGGAGATCATCCGT
CAAACAATTTTGAGCACTGGAAATGAAACGACTTGCAACGAAAA
ACAGGAGGGTTGCTTC
GTCATAGATCAGAATGGATTCATCCTTTATGACCAAGGATCCCCC
TCTGAGATTGGAAAA
TTCTACGGAGAAGTAGACGGCACACTTATTCGCAAGATCGTGAA
AACACCAAGCGAGGAT
GGTCTAGGCGGTTTCGAGAACACCGATTCAGAACAAATCCTTTTT
GATGGGTACAAAATC
CGGGATTATCAAGCCGCATGTGATGTATGGATAAGACAGAATGT
CGACGGTGCCGCCTCC
TTTCTACGCAATCCGTATCGCAACGTCTTTCGATGGTTTTTGAAT
GCAATGGGGACATTT
ATGAGAAATGTGATTCGGTTCCTTGTATGGGGTGCTCTTCAAGTG
AACGACTGCTGGGCC
CAAGATGAAATGGTCACCGCTGCTGCTAACGAGAACGACATGAT
TCAAGCTGCTGTGGTG
GAACACATGGAAAAGTTGGGAAAACTCGGAGCAGGAAGAGAAGT
TCTGGAAGCGTTCAAC
GCATTTCGATCTAAGTATCATAGCTTTTCCATTGAAATGGTAAAA
GAATGGTACGAAACT
ACGAGGAGAGGACTGCTGGAAACTTCTTTCAAAAAAGAGTCCTG
TATAAAAGCGGCATTC
GCGGTTTTGAGCAAACCGAATCAACTGCCAATGGCTCATTTGGA
GATGTCGAAAACGGAT
GATTGTGAGGATAGATGTAGAAACAGTAAACCGGTCTACAACTTC
CACGCCATCC
>ConsensusfromContig2034 Average coverage: 284.66
TCTATAATTCTCATTCTAGTGCCACTCAATTGCTGAAATACATGA
ATTCATTTGAAACAA
AAATCCATGATTCACATAAAGGATAAACAAATAACTTGTCAGCAC
GCTTTTATGTCACAT
GTCGGTCCCCCGAAGCCTGAAGCACAGATGCACTGACCGGCTTTG
CAGGTCGAACCATTC
ATGCATTTGATTGGATAATTTCCGCACACGTCCTCAGAACAGTTA
TCACCGTGAAAACCA
GAAACACATCTGCAGCTGCCGTCAATGCATACTCCACCGTTGTTG

CAGCTGAATTCATTG
CTCTCATCGCATGGATTGACTTCACAGAATTTTCCACGATAAGGC
GGAACACAAACGCAA
TTTCCTTTGATGCATTGTCCACCGTTGCTGCAATTCATGAAATCAC
ACATTGTCAGCTCG
CAGAAGGGCCCGAACACTCCGGCTTCACAGTTGCATTTACCGTTC
ATGCAAACACCATCG
TTGAAGCAGGATATGTTATGCTTATGGCAAGCATCTATCTCACAG
AAGTATCCGTGAAAA
CCTGCAACACATACGCATTTGTCGTTGATACATTCGCCATGCTCA
CATGTTCTGTTGTCA
GTCTTACCACATGCTTCAAGACCACAGTTTGCTCCAAAAAAGCCT
TCAGCGCATCGACAT
TCTCCTGAAATACAAGACCCGTTGTTCTCGCAGGTGATGTAGTCA
CATGTGTCAACCGTA
CAATACGCACCTTCAAATCCAGAATGACATTCGCAGATACCTCCT
TTGCATTGACCGTTA
TTGCAATCAATTTCTTTCAAGCTGCAATCGTCGACTTCACAGTTC
TCACCAGAGTAACCC
GCATTACAGATGCATTCGCCCAGGTAACATTCACCGTTATGAGAG
CAGGTCTTGTTTTCC
GATTCACAGATATTATATTGGCATTGTTGACCTTTGTAGCCAGTA
GCACACTCGCAAAAT
CGAGAAGAGCATTGGCCGTCGTTATGACAATCGATGTTCGAGATA
CATTGCGTGCATTTA
AGTCTTGTAGCATATAGGTCAGATCTCCTGGCATAAGAATACATG
CAATTTTCAATATCT
GTATCAAGATCATCACATCTTTGATCATATTTATAGCTATACTCA
TGAGTGTAACTAATG
CGTTGAGATGACTTTATACCGAATACGTATCCCATCATTTGACAA
CTTTTTGTTGCTGCA
ACATCACTGAAACCGTCGTACACTACAAATCCAGCTCCTTCCGTG
AGGTTCACTGCAAGA
TATCCTTCATTCCCAGAGATGACTTGAAAATCGGAATTTACTAAA
GACAATGTCAAAGAC
CATTCCACCATCGACGCCAAAATATACAATTTTAGAAAGGTGATC
ATTGTTATTGAAAAA
ATGGAATCAAA
>ConsensusfromContig2035 Average coverage: 66.13
CCAGCGAGAATACTGCTTGAAAAGTGAGTTATAATATTATCTTTG
AAAATTCCCGTGTAT
ATGAGCATTTGCTTGTATTGGTCGTAGAAAGACAACTGACCAATC

GTGATAAACATTCCT
CTAACTGTCGCCATCGAAGCACCGTTGTACAATCGCCGGAAACCT
TCGTAGCGATAAACA
CGAATAATTCCATCTATCGCGTGTTTATAGTTTCTGCGTAAACCT
TCTGGCAACGAAACG
TCATTCTGCATCCTAACATTCATGAGGTCGGCTGGAGCACCCACA
ATCGCACCGAGAGAA
CCGGCGACACCTCCCGCCAATACCTTGTACCCGAATGCCATGTTC
CCTTTACTATCTTTG
GGTAAAACCCCTTTGAGACCTTCATAAATCGCGAAGCGGATGTC
GAATATGTCATCTGT
CGTAGTAGTGATGCAGATATGCCACTGTACAATGCCAAAAAACC
ATCAGCTCGAGCTATG
TTAAGCCCTGTCCTGAATATACCCATTTGGGGACCATGTTGAGTT
TGTAATATTACCTTG
ATGAGATCAAGAGGATGTGTACAACATGCAGCAGAAGCACTGGC
CAATCCACCAAAATAC
CATCTTGAAACTCTTTTAAAACTACTAGTAGTTTGCTTCTTATTTA
TAAAGTTACTTGGT
AAATTTGTGGAAGATTGATTAATTGTGTTACAAGTTGTGGTCACA
CTCATATTTTTTAGC
CTAACAACTGTTGATCATAAAAAGACAAAATAGGTTCTGGCTAAT
CATATCCTATCAGCA
AATTGAATTAGAGAGAACTTTTTAAACTTTTTGTTCACTCACACA
CACAGCCAACTGCTT
ATTTTATGAAATGTGATTTTGATGGCCTCATAAATTTAATAAATTT
TTTCAGGTTAATCA
GTACATCAAAT
>ConsensusfromContig2036 Average coverage: 23.77
TTCGTCGATGCCATGAATTTGATGATCGCTATGCCAGAGAGTTTG
ACCAAAATCCATATT
CACAAAAATGTCACGGACCTTTTCTGTTTTTGCGCATACCTTATC
GATGTCCAGAATAGA
CGTATTAGGGTCCTCGCAATCATAAATACGTAGATCATCCCTTGA
GCTTGATGTGAAGAT
TACGTGTTTACTTTCACGCATGAGTTTAAACTGAGCCACAGTTCTA
TTATTGATTTGATG
TTCGGAGATTGATTCACTACAACACATTATCAATCGATTTGGATT
GACTCGGATAGCAGT
AAGATATGCTATATCAGTTAAATGGATCAACGTGTCGTTGTCGTA
TTGTTGAACACCAAG
GCCTTCTCCTTTTCTGTTCGCATTGTATTCATATAGAGTGTAGAGA

AGTTGATCATTACA
TTTATCGAGTTCCATGCAGCGTATCGTACGTTCTGGAGGATTCCT
TCTAGGTGTCGGTGA
GTCCCAGCAAATGTGGGTTCGGTCATTCATTTTGATCAATTCAAT
GTAATTATCACTCTC
GTTATTAGGCCCATGATGCCAATCTTCCAGACATCTTGTAAGCAT
GCTCTCGCTCATTCG
CATCAGCTCTTTGTTCCGAGCACAATATATGACCCCATGGTACGA
TTTCAGCGACAGACA
GTCACCCCCTTCGTTATCAAGGGTTTTCAAAAATGTTTTACGTGAT
ATGTTGTGGCATTC
CGAACATTTTTGCACATCCATCTCATATTCAGATTTACCTGCTTCA
AGATTTCTCATGTA
TGGAAGAAATGACCCATTATTCAACACGATTTCTGTTCTTTTCAA
ATCAATCAACTCGCA
GCCTCCATCAATATGACAAAATACTTCGCAGATTTCCCGAGCAGT
TTTGTAAAGGTGTTT
TCGGGGATAATCAGGCAGTTCAGTCCAGAAATTAAATTTCAATTC
GTTACCATGTACATC
ATACGTTTTAAGCATCAGACACATATTTGATATCAATACCCAAGG
AATAAGATTGCCCTC
CAATGCTAA
>ConsensusfromContig2037 Average coverage: 10.72
TCCAGTGAGCAGACAGGAAGATTTGAACAAAATTTTTTAGATGA
TCCTGAAGTATTATA
TAATCATTCCCCGTACTCCGACTGGCCCAAGTACCCGGACGACAA
TGCGGATGGCCAGTA
TTCCTCCGGCTATAGTTCAATAGGCAGTTCAAAAATGTTAAGATC
GAAGAGCCAAGGGTC
ACTTCACCATATCAGTCAACCAAGCAATTGTAGCTATCAGTCTGC
CAGTGGTTTTGCCAG
AAAACAAAAGTCTCCAAAACAGATCGGAAGAGCGTCG
>ConsensusfromContig2038 Average coverage: 20.62
CCTTCGACACAGTCTCACATTGATCTGATGACGTCAAGAAAGATT
CTCCCTGTTCGTGTC
CTTGACCTACAAGTTTCCCCTATCGTTTTGGCAGATCGAGCGACC
GCGGATCGTTATTCA
TCAGACAGGTCCCTGGCCCTTCACGACAGCGTCGAGACATTTCTC
GCGCGGGTTGGAGTG
TGGAACCGCGAGATCCGTCAAGTGAGAAAGCATTACTTGGAGCA
GCACCGGAACCTGAAG
CAAGTTCAAGGAGAGCAGTCCAAATGGTCGCTGTGGGACCAAGC

CCTGGGTCTGGCCTTA
GAGTCGGTGCGCCGAATGCAAAAGTACATCAAACAAATTGAGAT
GGGAGGAGCTGCCTCC
GTGGGTGAACTGTGCATCACACCGAAGGAGTTTGCGGGGCGTCTC
GGAGATTTCGGGTCC
TACTGCCCGGTGAGTCTGGCTCTGAGAAACGAGTTCGTGGACTGT
GCTGATCTGTCTGGC
CACGACTTCTCCGTCGAATTCAGAGGTCATTACTATCGAACTGCT
GGAGCTGAGGAGGAA
AATGCATTTCTGGCAGACCCCGAGAGGTTTGTGCCCCCTCTCGCC
CCACGAAGGCTTCCC
CCCACCGAGGAACTCCCCCACAAGATAGATTCTGGGAAAGTGAA
GACCATGTTTCCGGCA
CCGATTCAATTACAGGGCTACTGCCCCGTCAGCTTTCTCGAAGG
GAAGAAAAGATACGAA
GCTATTATCGAAGGAGACACCGAGATTGCTGCTATATACAATTCG
AATATATTCTATTTC
ATCAATACGGAAAATCGAGATAAATTCATGAGGAATCCCGATCTT
TATTGCGGACTCAGT
CTGCCCCATAAATTGCCTCCTAAGAAAGTTCCCATTCCAATTACG
TCACTACCCATGCTG
GGATTCCTCGAGCAGGGTGTATCTCGTGACGTCACGAAAGCCTTG
ACAGCTGTTGGCCAA
TTCAAACCTAAGTATCCGTTCTTATCCGTTTCTCAGTCAGCCCTGA
TCTTCGTTGCATTG
CATCTCAAAGCCCACAACCCGACGGGATCTCCCTACAGCCGCAA
GAAGTATCGCGAGGAA
CTGGAGCGATTCAAAGAGCAGTGCGGTCTCATTGTTTACCTTGGC
GACAGAATGAGTCGA
CG
>ConsensusfromContig2039 Average coverage: 53.99
CCCCCAAAGGACCGTGATCATTGAGCCAGCCGGAGAGCGGTAA
AGTCAAAACTGTCCCG
AGTTGTGCCCCTGCGAGTGCGAAAGGAAGGGCGAAATTCCCCTCC
GATTTTGAAATCCAA
TTCGGCAGAACTCCGAATATACTCGGTATCAAGACACCCTGAGCC
AGACCTTGCAGTACT
CGAGAGGCGATCAACATTTCAGAACCAAGCCGTGTTGCAATCAA
AGGTGTTAACAAACAT
ACAGCATTGCTTATGAATACAGTTATCGAGACCAAAACTTTACTT
CCAAATCTCTCCGCT
ATATAACCACCAGGAACCTGAGAAATGACGTAACCATAAAAGAA

GGCACCGACAATACGA
TCTTGTAAAGCAATATCCCAATTCAAATCTCCACCATTTTTTGTTA
TAACCGTAGCATTC
GAAGTAGTCTCACACTGAGATGACGAAGTAGATGGCTCAATGTAA
CTACCATTCACCATC
TCTACATTAGCCATACTCAGGTCTACCCTGGCCATATATGCATTT
ACCACAGCAAAGTGG
CATAGGATGACGAAAAAGAATCGGAAGCTGAGGACGAAAC
>ConsensusfromContig2040 Average coverage: 28.73
ATTGAGTTCACAGATGAGTTGCTATTAGACGACGATATAATAGA
AGAACTACCTGTGGAA
TTGCTGTTGGATACAGAGAAGCCTTATGTATGTGATCAATGCGAT
ATAGGATTTGAACGA
TCTTATGAGCTCAACAATCATTATCTTATTCATACGGGCGAAAAT
TCATATATGTGCGAA
ATTTGTGCCAAAAGTTTTTCAGATTACAAATACCTCAGACGTCAT
AAGATAGTACATACA
GCGAGAAAACCAAAATGCGACCAATGTTCGCGTTCATTCACTCGA
CACACAGCCCTACGT
GATCACAAACGCACTCAACACGGTGGAGTCAGGGTGAAAGAAGA
ACAAATTTATGAATGT
GATAAATGCTCGACTAAATTTACTCGAGTCAGTAGTCTCCGGAGA
CATCTCCGCGGCGAG
CGATGCCACAAATCGAAAGGGCGCGCCCGTCGCTCACGTCTGTGT
CTCTCGTCAACTGTG
AGTGATTCAGCAAGGCTTTACCAATGCGACGAGTGTCAAAAATGC
TTTAGCAACTCTGGC
AATTTGAGTAAACATAAACTCATTCATTCTGGAGAGAAGCCGTTT
TTGTGCGAGTATTGC
TCGAAAGCGTTCAACCGAAGATCCAACCTTAAAAGTCATGTATTC
ACACATACTGGTGAG
CACCCTTATGATTGCGACGAATGTCCTAAGGCATACTCATGCCAT
GCTGCTCTACGAAGA
CATCAGATTAAAAAGCATA
>ConsensusfromContig2041 Average coverage: 7.28
TAGGTTTTATTGAGAAATTAATTGATTTAGAGGAAAAAATTAAA
ATATATGAAATGTTC
ATGGAAATATGTTTATCCACATATAGAGCCGATGGAAAATGTCT
CAGATATAGATATAG
ATAGTTTTATTGAGCAAATATAACATATGGATGAAATCATTGAGA
GATATGGAAGTATGA
TGAAAAGTATATAAAATTAGCTTTTTTGCATTTAGAACCAAAGAT

AAACATCTTAGATAA
GGATAGATTTTTTAAATTGAGAAAAGTATTGCCATTCTCAAGGGC
CGATTGCTTTTATAT
ATGAGGAGCTTAGTGACAGTATATATTATAACATTTTTCATCTGA
ATCACACATCGTTTT
TTTTAATTTATGAGCTTTCGTATTGAATTCGAAGTGTCACAGACG
TCAGGCGATGCTTTT
CAAAATTCTTGTTGAATTGTTGCGAAGTTCTAATGCCTCGCGTTA
AGGCATGCTTCAAAT
TAATTTAATGCAATTTTTCACGATGAATGCTTAATTTCCATCACT
TCGTAACTGCGAAAA
ATGATTTC
>ConsensusfromContig2042 Average coverage: 20.01
TCGCATTTTGTTTTCAACTGGCAGCACCACAGTGATGCCTTCAAT
AGAAAATAGGACACT
CCCGAAGAAGAGGGGAAGCGTTGACCAGGATTTGAACTCGGGAT
ATTTGGTGACAGGCTG
CAGCTCTTCGAAACACCACGACAAGATAATTCCAAGCGAGAGCA
AGATTAGGATATTTGA
TAGGAACGAGAATATAGCGATGATCCGAATTTTGCTGAAGAAAA
TCATTGGCAGGAGACA
AAGAAAAACAATCAATTCGTAGACGCGGATGGGCATGTTATAGCT
GCTCACCGTCCATAT
GACCTGACATATGTTCTGTGCAATAAAAAGAATATAGACGCAACA
AACTCCATACTGTGT
GAAGCATAATAATGTATTGAGGAGTTTCTCAGCTGGCGAGCATAA
TTTTTGAATAGATGA
GGGTCCAGTCCTGAATGCATGGTAGACAACCTCACCATAATCCAT
CGAACTTTTATTCAA
TCTACGGCAAAGAGTTTGAGAAGCGTGCACCAACTG
>ConsensusfromContig2043 Average coverage: 36.64
CGGCACACTGGTTGGCTGACATGTGCTCGAAAAGAGAGTCGCAG
AGGCGTGGGATGATTC
CCTTCTGGTCAACGGAGCCCATCATGGAATAGGATTTGCCGGAGC
CTGTCTGCCCATACG
CGAAGATGCACCCATTGTACCCTTCGTAGGCACGCTCCAGCACAT
CTCGACCCAACGAGT
CGAATACGGCATCTTGTCCTGCAAACCGCTCATCTCCTGGCTCGA
TGGACCAGAAGCAAT
GGTCGAAAGCGAAAGACTTTGGTTCTTTCTTGGGTCGAACTCTGC
CATCTGAGTCATTCA
CGACCCCCATTTTGAATCCTTCTGTCGGATGGTAGAGTAGTGTTTG

GCCCTCTTCCATGT
CGACCACGATTTTGGTCCCGAGGTTCGACTCTCTTTTACTCAAGG
GTCTAACACGAACGG
CAACCTTTACTTTATCGGACATGATTTTCAAAAGAAGAACAATCA
AAAAATACAAATCAT
CATTAAATAATTTTAAAAAAAGTAATACTATTTTGAAGTGGGCCA
AAAA
>ConsensusfromContig2044 Average coverage: 44.29
TCGTTATAGTGCCTATGAGCACAGAAAGGGGGAGAATATATCTCT
CGATTCGTCGCGAAT
CGCGATTTAACTGTTCTTCTTCTTCAAATGTCAATTCCATCGGATG
ATTTATTGTTTCAA
AATAAGTTTTTGAATTTGGAATGACTGTCCCCAAAACAATTGAGT
TTATCATGCTGACTG
TAGTGGCTATTGTATGATATCTGTGATTGTTGAAGTAGCCGGTTA
CATTGAAGTGTGTAT
TGTCTGTACATTGAGTCATATCCACTGCAGTTATCTGATTTTTGTT
CAAAGCATATACGT
TTTTTCTAGCCTCTTCAAGTAAATAGTTTTCCATCGATTCACCATC
TATCTTTATATATT
TCTGACCAAATTTTTCAACCAATTCGATAATTTTTTTTTCGAAATT
TTCTCCACACTTAT
TTTTTGGTACGGCATATGGGAAAATTATTTCTTTTCCATAGTGAT
TCGACGAAATATCCA
GAGGCGGCTCTGATGATGTCACACGTCCACCGAATATCATGAGA
AGAGCTTTCAACACAG
ACACACAACATAGTGCGGTTACAAAA
>ConsensusfromContig2045 Average coverage: 7.93
CACAGATTATTTGATAATAATTATGCCCAGAAAACCACTAGGAAA
TATTTTTCAGCCACA
CAAAATAAAGCTAATATCTCAAAATTGAGAAAAACATACTCCTG
AAAAATACTTGCCTAA
TAACAAACCCATGTCCCTGGAAAGGAGAATTCTTGATGTGTAAAC
CGTGAATATTTATGA
GCATCAGAAAGATTACGCCATGAAGATGGTTGATGGACCTTTTTT
CAGATCTGAGAGAGG
TTGACATGACACACATCCCATTCCCAAATTGATACCAGATTTGAA
AGCTATATCAACCTG
AGATGGATAATGTATGAAGCCAAAATTTTCCTTCTTTGTTTCTCC
CAAGCAGATGAGGAT
GCAAACTTGTATTGCATAGGATGTATACTTGATGAACCTTCTTTC
AGTTATTGACA

>ConsensusfromContig2046 Average coverage: 7.02
AAATAATGAGAAGAAAATGCTGAATTCTTCTTGAAATAAAAATTT
ATGCGTCTGGCTCTT
CTTGGAATGATGGAGCGAGACGGACGGTCGCGAGCTATTTTTGT
ACGAAAGCATGGCGGG
GGCTCTGTGATGCTCAAAATTTTGCGAGATTTTACTCGTATCGGC
TTCCGGAACAATCGA
GGTGTACAGAGATCAAAAAGCATGTCAGCTCAGACTTCACTCGAA
ACTAATTATTGTGAG
GGTGATACAAACGCAAATAGCTGCAGACTGCAGGAGAATTACAG
AATAGCAAATGGCTGT
TCAGAGCCAGCCAGAGAGCAGCCAATGTCCCATCAAGAATCTCA
GGAGCATCGACCGCTG
GAGTCCAGCCGTGCTGCCTTGAGTGTTCACAGTCAGGTCTCGAGA
TTCATTATTTTTCAC
TATTCCCCCTTCAAGACTGCCTGGGACTGGATAACTCTCATTCTT
GTGCTGTACACAGCG
GTTTTTACTCCTTATTTGGCTGCATTTCTCTTGAACGAGAAGGAT
ATGCAGATGCAGCTC
ATTGAGAAAGTAAAAACAGATCCTATTGTATTCATAGACATGTTC
GTTGACATAATCTTC
ATTGCTGATATTCTCATCAATTTTCGAACAACGTACATTGATAAT
AGCGAGGTCGTGACA
TGTCAACGCAAGATTGCGGCGAATTATTTGAAGAGTTGGTTCCTC
ATTGATGCCTTGGCT
GCAATTCCGTTTGATCTTCTTCTGTCAGGCTCCGAAACCAGAGAA
ACGATGACAGTCACT
GGCAGATCG
>ConsensusfromContig2047 Average coverage: 24.03
TCTGTTTATATTGATAACACTGACTTTTTTCCAGTGGAATTATGTT
TGAATTCGATTTAT
TTAACATTTCTTTTCTAATTTCCATGCTAGCAAGCAATTCTTTAC
AAGGAGAGGATAATT
CTTTTCATGTTTCTCGATGCCCGAGTTCTGTTCAATGTGATAAACT
CTCTAATGATTGTT
TGGATTGTGATTTTAATCATACAATTAATAACAAATCTGCATCAT
GCATATATGGAAAAC
AGTACTCAACAGTTTGTTGGCCAAAATCAGATATCAATTGTACGG
GAGATCAAATGTTTA
TGAATACATATTCTTGTAGGTATTGTTTTCAAACCCCATATTATC
AGCATATCTGCACAG
GATCAACAGATTGTGATGTTTATGCTTATCCACCTGAGTATACTA

CATCAAATTGTACTG
TATGGCCAAATGTAACATGTTTAGGACATAGGTACTTCAAAAAAC
GAACACGGTGTGATT
GGACATCTGGATATAGTCATTCATCTGCGGTATTGTATAGCATTG
TATTTGGAGGTTTTG
GTGGTGATCATTTCTATCTTCAACAATTTGGTGAAGCTTTTGGAA
AATTGTTAACGTTGG
GAGGTCTTGGTGTTTGGACACTTGTTGATATTGTCTTAGTGGCTG
CTGGATATATAACAC
CATCTGACGGATCTCTTTATATATGAATCAGTCGATAACTTATAA
AAACTTGTGATGATG
ATATTCTTGATGTTTTTTTCATAATCATAAAC
>ConsensusfromContig2048 Average coverage: 26.87
TAAATACAATTTTGTTTTCAAGTGATTTATTTCAATCTGCAATGA
ATGAACATCAAACAA
CCGAATCAGAGGATTCTAAATCTGAAGAAAGTTCAGAGCTTGCT
GAGAAGAAAACTTGTC
CGCCCCCAATTACTGCTCCTGTTATCTATGCAAATCAAAGAAATG
TCAACATTCCTCTTA
TTGAGAGAAGAAATTGGCTCATTCATATGCACTATATACGCAAAG
AACTAGATGTTTGTG
AGAAAATTATAAACCAGCAGCTTGCTGAAACAAATGAAATGTGT
GAATATGCTCTATATG
TCAAAGGTTTACTGAAAAGACAATATGGGGCAATACAAGAATCG
CTGCAATTGTTTCAGA
AATGTGCATCAATGAACCCGGC
>ConsensusfromContig2049 Average coverage: 46.39
ATTCTCAAGGATTGAAATTCAATAATAATATTATCGAAGACCTGT
ACATAAAGATATATC
AACGCATTCCGACCGAAACAGTATTCGATGAGCTACAACAGCTG
ATCGTTGAGAGACTAA
AAAGCAAACCTTACCTGCAAGCCTTCAAGAAATCCTTTCATTATA
TAAGACTTCTGGCGG
AGATGGATCTCTTGACAGATCGCTCTGTTAAATCCGACTTTTCGA
ATTCTCCCGATACGT
TATCAATCGCGTCCGAACCAGAAATTCTGACAATTGCTGAAGAA
AAAGGATCTTCTAAAG
CTGAAGGGAAATGTGAGAAGGAGCTGAACGTGTTGCCGGTCATT
AATGCGTATATTCAGA
GTAGTGGACTATGTAATAATCATGGCAAGCCGTTCACAACATATT
TGATATCTGTCACGA
TCGATCCATCCAACACTAGTCCATTCATTGTTTGTAGACGTTATTC

TCATTTCTGTGATC
TGCATTCGATTCTAATTGAAAAGTTCAACCTAAGAGGAATATCAC
TTCCTTCTAAAAAGG
CATTCAATAATCTGAAAAAAGATTTTCTTGAGAAGAGAAAGTTTT
CTTTGAATGCATATC
TTCAGGTCATACTGAATTTGCCTGAAATGAAAAACAATGTGGAAT
TTTACACTGTGATAT
ACGAATTCCTCAAGAAGAGCGATTGGGAAAAGGAGTCGTTCAAT
ATCAAAAAATCTTTCG
ATCTCATTGTTAACCCGTTGAAAACATCCGTCAAGAACGTTGGTA
ATATGGTCAAATCTG
CTCCGGACAATATCTTCGAAGGAGTGATCAAAGTTACGGATGGA
ATAAAGAACATTCCCG
GAAATGTGATTGATGGAGTGGAAAAAGTTCTCAGTCTAAGATCA
CAACAAACAAACATTC
CAAACTTTTCCGACACGACCAAAGTCTGCGATGCTTTAATCACC
GAAGAAGACAGCAATA
ACATTCCTCTGCGAATAATGCTTTTATTGTTGGACGAAGTCTTCG
ATCTGAAGTCACGGA
ACCAATGGCTCCGCAGACAAATCAGTTCCATTCTCCAGCAAATCA
TACGCCGTACGATGG
GTGATAAATATAATCGCCAAATCATCGAAACAGTTGAGTCCCTC
ACAAGCGCATCGCAAA
TGGCCGAATATATACGGAGGTTCAAAGTCTCCAACTGGCCCAATA
ACGCTCTTGCTGAGG
CCGGGATTCCTTGTGATTTTCACACGGAAATGAAAACGCGTGTCG
TTTGCAAAGCGAAAC
TAATCGGAGGTATATCTGACGAAATGCGACATGTGATTGGCTCT
GAGAATGCCAGACATG
GAGTGACGCGCATCTTCAACATGTTTCAGAAAGTCCAATTAAATA
AACGCCTTTTCTACG
TCATTATCGAAGGGTTTTTAGAGACACTATATCCGGACCATACAT
TCGCCGAATTTTTCA
AAGATTTTCACATGAAAAGTAAAAGCTGAAGAACGTCTTTTACCT
CTG
>ConsensusfromContig2050 Average coverage: 20.96
TTTTTTCTTTCCCTGTTGCTGTCATGGTATCTTTGGGCATATCCGT
ATTCAGTTTCAATA
GCTGAAGAGCATCTATTTTCCCTCTGAAGTAAGATTGTAGTACTT
GGGTGAGTGCAAGAA
CATCATCTTTCCTATCTGATCCAATTTCTTGTGTATCCTCCACAA
AAATGATGGGGTCAG

AAGGGGATTCTACATCGACTAGTTTATCAGAGACATTGAGTTCCT
TGAGTTCATTTTCAT
CAGACACGGCGAGCTTTTCATTCACAATATCTTCAATTTTGAGAT
CCTCTTCGGCGGACT
TCTCCACAATTTCAGACATTCCATCGATTGAGATATTCACATCCA
AAAGTATTTTTGCTG
TATATGCATATTTGTAGAGCTTAAAAAATTGAAAGAAGTTTCCGA
CGATATACATCTGGA
TTTCTTGAGCCATTTCGTATGAAAATATCAGTGCACTGAACGGAG
GTCTCTGCACGGAAT
ACTTGATGATACCTTCTTGCATGAATTCGTTACAGATTTCTAGATT
ACCGAATGGTGTTT
CAGTTGCTATGAGGAATAAATCTTTTAGTAAAGAATAAAAACAA
GCGAGCTGAACAGATG
TCATGCCAGCATTCTTAGCAAACATCAAAGCTTTCGTATAAAGAT
TGATGATCATTAAAT
CTTTTCCAGTAGTTTCGGGGTTCAATATCACCTTGACCAGCTTCT
TCACCGATGATGAGT
CATAAGGCAACTTTAAAATATCGTCTGCTTGATCTTTGTTCATGT
GACGCCATAATGAGA
ATTTTGAGACGTGAACCGGATTCAGAATTCCCTGATCTTCCATTA
TGAAAAGAAATAAGT
TTTTTTTTCTTTTTTCTTTTT
>ConsensusfromContig2051 Average coverage: 34.26
ATAAATGAAATAAGCTATACAATCATGCACTGCCCTAGATATTTTT
TTTATCTTCACTTT
CCTCAATGTTCATCGGAAGTTTTGAAACTTCGACAGATATAATGT
TTGAATCGTCCACGA
AGTTCTCAAGTCGCTGAGGATCATCATAGAATGATCTGCGGACAA
CTGTAGTTTCTGGAC
AACTATTCATACCAATGCCGACATTTTCAACATCTCCCATCAAGGT
CTCTTCTTCAAATT
GATCATTCCTGATAATCATTAGTTGGTCACTATCATCCAAGTCAC
TGCTCTTTGAGAAAG
ACCACTGCCCAGCAGCCCATCTTCTTACACTTTTTCGAACACTAT
GTCTCCACACCTGAT
TCACAACCTCCGGATCATAGGGTGATACACGTTCTCTATTCATGA
CATGTTTATTGAAGA
ACTTCTTTATGTTTATATCTTTTTTTTCAAGAGCTACTCTATCATA
ATATATTATACGGC
GCTTGATGGTATGGGGCCACAGAGGCGACCCCTTTTCCACATTAT
TTTTTCCGAATAGGC

CAATGTTGAATGCAACTCGTGCGCAGTGCAGCTCCCACGCCATCT
CCATTTCGACTTCAC
GTGGCGTTTTCAGACGTTGTGCCCGTGGGATGGTCAAAATATTCA
ACACTGGTCGTGCAT
CCCGCTTCCTCACTTGTTCTGGTTTCATACGCAAATCAAACCTTTC
TACAGCGGTCTGTG
GATCAATACAAACCTCTTCGTGCCAAAAAGAATCCCTTTTCAACA
TTGAGCCAGTAACAG
AGCCGGGATCAATCAATGCAGAGAAGTCCCCGATCTCGCTGTATA
TCAAATTTGAATTTT
TCTTGATAGACGATGAGTTATACGGTAA
>ConsensusfromContig2052 Average coverage: 50.94
GACCTCTCAGACTCGTATCCGGTGTTCCAAGTTTACAAGTTGTGC
TCAATGCGATTTTGC
GAGCTATGATTCCACTCTTACACATTGCCTTGTTGGTCATTTTTGT
TATATTTATATATG
CCATCATCGGTTTGGAGTTATTCTCAGGCAAATTGCATAGAACTTG
TTTTTCTCTAGATT
CAGGTGAAATAGCCCTCGATGACCCCCATCCGTGTGGACCCTCGG
GTTTTCAATGTGATG
AGGGTCAGGAATGTAGGGGTGAATGGGAAGGGCCGAATAATGGC
ATCACAAATTTTGATA
ACTTTGGTCTGGCTATGCTCACCGTTTTTCAGTGCATTAGTATGG
AAGGCTGGACCGACA
TTATGTACTGGGTAAACGATGCGGAGGGTAATGAGTGGCCTTGGT
TATATTTTGTCAGTC
TGATCATCTTAGGATCATTTTTTGTACTCAACCTTGTTCTTGGTGT
CTTGAGTGGAGAAT
TTTCTAAAGAGCGAGAGAAAGCGAGAGCTCGTGGAGACTTTCAG
AAACAGAGAGAAAAGC
AGCAGGTGGAGGAAGATCTGCGGGGTTATCTTGACTGGATAACA
CAAGCAGAGGATATAG
AAGTCGAAGGAGATGAGGATGAACTTGAAGAACAAAAACGTATT
CCTGAGCCGGAGGAAA
TGATGGAAGAGCCGACAGTTGAAGACATGGCAGCATCTTGGTTT
AATCGAACAAGTCGTA
ATTTAGGAAATACGAATCGTCGCTTCAGAAAGTGGTGTCGGAAA
ATTGTCAAAACGCAAA
CATTCTACTGGGGAGTCATTATCATGGTCTTCATCAACACTTGCA
TTTTGTCAACGGAAC
ATCATGGACAACCGATGTGGCTGGACAAATTTCAGGAAGTGGCAA
ATCTATTCTTCATCG

TCGTCTTCACGCTCGAGATGTGCGTCAAGATCTACAGCATGGGGG
CGCAGGGATACTTCG
TGTCGCTCTTCAACCGTTTTGATTGCTTCGTCGTCATCTGCTCTCT
TGTCCAGATGGCGC
TCGTACGAACTGGTCTCATGGCGCCCCTTGGCGTTTCTGTGCTCA
GATGCGCGAGGCTTT
TGCGGGTTTTCAAAGCTACTAGATATTGGGCATCTTTACGAAATC
TTGTCGCTTCCTTGC
TCAACTCGATGCGATCCATTGTGTCTCTGCTGCTGCTGTTGTTTCT
CTTCATCATAATTT
TTGCGCTTCTCGGAATGCAATTATTTGGTGGTCATTTCAACTTCA
ATCCTGAAGAGCCGA
AACCTCGCAGTAACTTCGATTCCTTTTGGCAATCCTTACTCACTGT
GTTCCAGATCTTAA
CCGGAGAGGACTGGAATGTTGTCATGTACGACGGTATAGCGGCA
TATGGTGGAATCAACA
AAGGATTCATCGTTATCGCGTACTTCGTATTCCTATTCATCGGCG
GAAATTATATTCTAC
TGAATGTCTTCTTGGCTATCGCCGTTGACAATTTGGCTGACGCTG
ATAGTTTGGGAGAAG
AACAGGCGAAAGAGGAGAAACCTTCAGAGTTTCCAGAAGTTAAT
GTCATGGAAAAACCGA
ATGGAGATCATGCAGTTACTGGTAATCACATCAAAAATAATCCA
AATGATGGATTAGAAG
GAATGGAATATGGCGAAGAAGCATTAGAACACACGACAATCAAC
ATGAATGCCATTCAGA
AGCCATATCAACGAAGAGATAATCAGTTATATGAAGAAGATGAT
GATGGCTACGATGATG
AGGAAGATGATTACGAAGATGATGATGAGGAAGATGAAGAGGAA
ATCACCCAACCAACTG
CAGTGCCTCGTAGGACCTCAGAAGTGAAGATCAACACCAAAATA
AAACCCATTCCTAAAT
TCAAAGCTTTTTTTATCTTCTCTGATAAAAATAAATTCCGCATCTT
TTGTCATTATATAT
GCAACCATCCTTACTTCGGCAACTTCATCTTGGCGTGTATTATGA
TAAGCAGCGCCATGT
TAGCAGCTGAAGATCCCTTAAACGCGAAGTCGGAGTTGAATCAGG
TTTTGAAGTACTTCG
ACTATTTCTTCACTACAGTGTTCACAATTGAAATATTTTTAAAAG
TAACAGCTTACGGTG
TGATATTACATAAAGGATCTTTTTGTCGGAGTTTGTTCAACCTTTT
AGACTTGCTGGTTG

TCGCCGTCGCTTTAGTATCATTTTTTCTCGACAATCAAGCCATATC
TGTCGTGAAGATAT
TGCGTGTATGTCGTGTTCTGCGTCCATTGCGTGCTATCAATCGCG
CGAAAGGCTTAAAGC
ATGTAGTTCAATGCGTCATCGTTGCTGTAAAAACTATCTGGAATA
TCATGATCGTCACAA
CTTTACTCGAGTTCATGTTTGCCACTATAGGTGTTCAACTTTTCAA
GGGTGCTTTTTATG
AGTGTACAGATCAATCTAAAAAGACGATAGAAGAATGCAAGGGC
GATTATTTTGAAAATG
GCGAGCTTAACACACGGTATTGGGTGAACAACGACTTCAATTACG
ATGACGTTTTACAAG
CTATGCTCACGCTGTTCACCGTTTCCACATTTGAGGGATGGCCGA
ATCTCCTGTACAAGT
CAATCGACTCCGTAGGAGAAGATATAGGTCCTGAAATCAACAAT
CGACCTGGCGTGGCAG
TCTTCTACTTTGTCTTCATTATTATCATTGCTTTCTTCATGGTCAAT
ATCTTTGTCGGTT
TCGTTATCGTCACTTTTCAAAATGAAGGAGAACAGGAGTACAAA
AATTGCGAGCTGGATA
AAAATCAGAGAAAATGTATAGAATTTGCTCTCAAAGCCAAAACT
CAGAGGCGATATATAC
CCAAAGACAGGATGCAATATAAAATATGGTGGTTTGTGACGTCAC
GACCATTTGAATATG
GCATATTTGTTCTAATTCTCATCAACACAGTTACACTTGCTATGA
AGTATTACGGTCAGG
GAGAGACGTACTCAACTATATTAGATTATATGAACATATTCTTTA
CTGGTATCTTCACTG
TTGAGTTTGTATTGAAATTAGCAGCCTTCAGATGTAAAAACTATT
T
>ConsensusfromContig2053 Average coverage: 38.18
CTCGCATCAGAAATGACCCGGAGAGGAGACATTTGATCATGTAG
GAACCTAGCAACAGGA
TTGTGACATCTCTGTCGCAACCTTGATGACTGGATGGCAACTCCT
GGGGAGACTTGTGAA
GAGATTTGCTTGACTTCTCTTGGAGATTCTGTAGTGGCGTCCTTCA
TCAATTCATTGACG
AGTTCCAGCTCTCTGTCTATCGCAACCTCACTAGCTCTTCTGTTC
ATTGGAGAGGTCAAG
GGCGTTCTGCTGTATGGAGATCCCAACCTCAATCGCTCAACTCGA
CATTTTCTTTGAGGC
GTCATTCGCGGGCTATTCATCTGGGGGTCATCGAGAACCTGCACC

AAATGTTGCGGAGAT
GCTAAGCCCTCTGCCGGCATGAATTTGCTCCTCGCTACCATTCTA
CTTCGCAGTCGAATC
AGATCAGCTTCTTCATCGGCTTTGATCTTTATTCTGGCGCGGATTT
CTTCCATCTTCTGT
>ConsensusfromContig2054 Average coverage: 10.61
GTAGGGATCACTGATTCAGCCGACTCTGTTTATTTGAATGGAGTT
CGTGTGGCTACTGCT
TGGTGGAAACAAAGGTTTCAAACAGAAGGTTATGCCCCACTCAC
AGTCGGATCCTATGGA
ATTGGGTCTTTGTTAAACAAACTCAAGTTTTTCGACATCCCCTTT
CTGCTGAAGATGTC
CGCTACTTATATAGGATGGATACATCATGTCCAGAGACTCCAACG
GAACAATATTATGAT
CTCGACCGAATCATCAAAAAAGGATATCTCGATCTGCCAATGTC
CAGTATCGACGGAAAA
AAAGTCCACGGGGACATCGACGCGCATGTTTTTAGTCGCAATGG
AGTGATCAGCACACGA
GCGACTAAAGGATTCATAAAGAGTCTTGTCCATGAACATTCATAC
CTTAGTTATGAGCAC
TTTGACCCTCTTCAGTGCTTCACAAACATTGAGCGATGTGGAAAT
TATGGAAGTTTGAAA
ACCGGGCATTCTATTTCCGTTGCTGTATACCCAGTGGATAGTGCC
AACCGACAATACCTC
ATCGATAGCGGTGGATCCTCTGACAATTCTTATGGCTTCGCTCTA
ACTATTGAATCTGGG
CTGAAACTCGAAATAACGTATCGGCTAAATTACACTCACACTCTT
GAAGATCAGTTGACA
GCAGGAAGTTGGGTTCATATCACATGTACATGGGAAG
>ConsensusfromContig2055 Average coverage: 19.02
GCAGGAATGAATTAAAACTCTTATGATATCTTTGACGACCAAGAA
GTACAATAATAAGTC
CGCTCAGAACAAAGCTTCAAACAAAAGTAAACTACTTTTTCCAAA
ATCCTTTGAAGTGTA
AAAAGGATGAATATGTCTCAAAGGCTTTTTCGATTTTCGACCGCA
ACATGAAAAAGCGGC
CTAGTTTTCATCAGGTTGTTCTCGAGTGAAAAAATGTGTATGAGT
ATTTCTTAATTAGAA
TAGCCGCGGGCATATCAGGTCCCGCGAGGATCTTCTTTGGTCATC
CAAAAAATGCAGGTC
GGAAAAAGTTTGTCCGTCGCACGTTTGTTGCCTTCGATCCGAATT
TGACCTCGGGCCGAA

CTGACCACTGGAGAGCCGGCGGCCGAAGGAACGCGTGTTATGCT
TCCGGGA
>ConsensusfromContig2056 Average coverage: 5.36
GTTATGAGTGAAATATTGAAACAATTCAATTGTTCAATTACTTTT
TATATGTAACCCATT
TTGAATAGTTTCGATAACTGATAAACTGTTGTTAATTTGTAACTT
AGAGTATAATTTGGT
TTTACGCCACGGATTATGTTTGTATGATAGAGGCTTATAATCAAA
AGATTGAAACAATAT
AATTGTTTAAAGACTTTTCATAGCCAGCTCACATCAGAT
>ConsensusfromContig2057 Average coverage: 64.61
GCAATCATATCACCATCGTAATAGTACCTCAGAGCCCTTGAGAGC
TTTTCGTAGTTCATA
GTAGGTTTGTTTTTACGAATGCCCCATAGGGATGCCACGCGTTCT
GGTTTACTCAACTTG
AATTCCCCTTCATCCCCGATCCAATGTATTGTGTCTTGATGCATTT
TATCCGTCAGCAGA
TCCAACAGAAACTGCCATAGTTGAATTTGGCCATTGTTACCTGTC
CTGTTTCCTGGATGT
ATTCGTTCACCGTCTGTGAGAGTCATTCGGGATCTGGAGGTACTT
TTCAATCTGTTGAGA
GGTCTCGATAAAGCGATTGTATACGTTCGTGGTGTAGACTTCTGG
ATTAAGGCCACAAAT
TTGCATTTTCGTAAAAGCTCTATATGTGTCCAAAACGGTAAACCG
CCTTCATTTTGAAAT
ACCTGTGAAAATGTGGAATGATCTAATGCACAAAGGGTGGCACC
ATCCATTTGTACACAG
TCTGGTGTTGTTGGGTCGAGATTGAAGTGAGGGACGGCCCACTGT
AACCAGTACTGAACG
TCCTCGACACTCCATAGAGCCGGATCTAAGGGCATATTTGATGTA
AGCTGATGATTTCTA
AACGATTGATCTACAATCCATTTTGTGACCTTTTGTGGTTCTTCG
GGAACCATCGTAGCG
TCCAAGCCGTTTGACGCTTCTTCAAAGCAAGAATTCACCGTTGAT
GCCTCTTGGGACTCC
TCCGACTCATCTTCGGATCTGACGGGTTTCAAAATATCTATGATA
TTCAGTCTTGGCCGA
CAACCAGGTATTGTTTTAATTTCTACATTTATCTGCACCATTCCGG
ATCCAGTTACGCAT
TGACTTGCTAGATCTTTAGAAGGCTCCAACAGTGTTTTGTCTTGC
AACCAATACTCGTGG
TGCTGAAGGTCGCACTCAAGTTTTGTCTCTAGCAGTTTCTTCAGG

CCGGAAAGAGGTTCG
CAAATATCCATGTTTTGTACAACGAGATTTTGATAAGACAGGAGA
TCATTTGTTTCACTC
TTCTGACCGACGTCAGTTTCAGATAAGCATTCGTCTACTGGAGCA
ACTATGATTGGTTCA
TGATGCTCATCCGGGTCGTAGTTAAGAGATATAACATCAGACTCT
TCAATATATGATGAA
CAAACTTCATTGTCGGTCGGTTGCACTAACAGTGCAGCCCCTTCG
CAATGACCAATGCCA
CCATCTTCAAGTGATAGAGTGTTCGCGTCATCCAAAGACAGTGGG
TTGTTCACCGCATAT
AATTCGGTGGAATCGCCAGTGTAAATCGAGGAACCGTCAGAAAA
TCCACTAAGGCCAAAA
CCGTCACTCCCAATCATTGAATCGGTCGTTTCAGTTTGAATGACA
TCTGCGTGGGAGTTT
TCCTCATCCAGACAATCATCCATTCCGACCAGATATGTAGAACTG
CAGTCATTTATTAAA
TTTGGCTCATCTGGAACCAGTTTCTTCAAACCATGCTGTTCATCT
GAATATGGACTCGCC
TTTCTTTTCATGTTTAGGGTTCTAGATTTTTTCAATTCAGTAAAAT
TTCCAAAATCAGTA
GTAGTAATTAATAAATGGCATGCAGTGTGTGTTGCTA
>ConsensusfromContig2058 Average coverage: 34.19
CTGAGTCCCAGGTAGACGTCGGGATGTCTTATGATGCGGGGGAC
AGTGGCGCCGGGAACA
TTCATTCAGACATGGACCAGAATCTCGTTTTCCATCAGAATGAGA
CGCACTCCTTTGTGA
GCTTGGACGATAGCACATGTTCCAATTTAGCGCCCGGGGAAGAA
AAGGCGCTACTAGATG
AGCTTGCAACATCACATAGAAACGAAGAGAATCCCAGCCTCAGG
GAAGAAGTCTTCTCTT
CATCAACGACAGCAACTACAGCACCTGAGCATAGGCCTATTCCCA
ATCCCACTTTCCGTG
GTTCAAATGACATATCTTCACACAAATACGTCACTACAAGCACCC
AGACAGTGGATGATG
AGCTGGAATATTCGCCAGTCCATCTCCCGCCCATCGATCAAAACG
GCCATGAGGATGATT
CGCCAGTGCCTGCCAGTCTGCATCACGGGTCATGGCCCGATGAAG
CCTTCGAAGCATCCG
ACAACAAGAACACGCAGGAGACGGGCAGCATGATGAGTTTCCAT
TCGACGAACCTCGCCA
>ConsensusfromContig2059 Average coverage: 9.76

ATCTGCCGTCGATACATCAGCCGAATTACCGGTAACACTAATTAC
CATCATCATTAGGCT
CGCCTGTACGTTGTTGTGCTGTTGCGATATTGTCTTATGATTGATT
TCGCCATGTTTAGC
GTTCTAATTGATATGTTCTCCAAGTCTTTTTTCGAAAATTGATTT
TCTGCATGAATGTA
GCAGATTTACCAGAGAACGAACCTCTCCAAGTTAGGGCGCATGCT
CCAACTTCAATGTCG
GCACCAAGGTTTGTCATGGCCCATGTCCTGATATCATCGGTGTTG
TAATACGTAACCTTC
CGGTCGTGGGCGTCATTGGGGTTAAAGTTGTCAGCGGCAATTGTC
ACATCGATGATTCTC
ACCTTGTCACCGACTTTCACTACTATGTCTGGTCGTCGAATTCCA
GCGGTAGTTGCAATG
GCCGGTTCCAATATGGGTGCATGTCCATCTTTTTTCAGCTTTGTCA
TGATGTACTCCACC
ACTTTATCATGACGCAGACAACTTGCGTCATGT
>ConsensusfromContig2060 Average coverage: 6.13
GGGATTTGGACGCAGAAATTTCTCGAATGATGGATGAAATCTGA
ATCCTCAACGCCAGAA
AAATGGATTGGGAGCAAACATCACCAAAGAGCCATTCGGTGTTCA
TATCTTTCGAGTTTT
CGTCAACAGAGACTTTCCTGGCCCCCGCACTATGAATCTCACGGT
AGATGGCGATACCTA
GAGGGTCATCGACCTCCAAAGTGGTCTTAAGGTCCCTTCGAATG
GCGCTGGCGAGAGAGA
GTTCGACACGGGGTAACCACTCGTCCGGCATCCCATCGATTAGAA
GAGGCTCTATCAAGC
GAAGTCTGTCACCAAACCTAGATCCCACGCATACAACGGTCAAG
AGATCGGAAGA
>ConsensusfromContig2061 Average coverage: 8.60
GAAAATATTACTGTGAATTTTACAGCAACCAGCGGGTAACTGTGT
TGCCAGCAATTTGCT
GTAAAATTTACAGTATAACGTTGATGTACTGTATTCTGATTTCGTC
TTGTTGGTACACTT
TTATGAATAGTGCAGGACAAATTAACAGTCAAATACCATTTCTTT
CCAGTCAGAGCTCAG
TGTAGTATACAGTAGTAGCGACCGAACATTCAGTGAATATGCAAA
AATTAGAATTTGAAT
GAATTTGAATTTCATTGACCAAATAGATCATTATTTAATAAAAAC
ACA
>ConsensusfromContig2062 Average coverage: 54.86

GGTGACAAACCGTACGTATGTGAAAAATGCGGTCGCTCATTTGCC
AATCCGAATTCGTTA
CGCTACCACAGCAAGTTACACGCCGATCAAAAGCCACATGGTTGT
AAACTTTGCCCAGCA
CGCTTCAATAGCCTGGGCGGCCTGCGCTACCATGAGCGAAATCA
CACCGGAGAGCGTCCT
TTCAAATGCCGGTATTGCGCCGCATCATTCAGTGTTTCGAGCAGT
CTCACCGTCCATGAA
AGAACACATACAGGCGAGCGGCCATATAGTTGTTTATTTTGTCCC
GCGACCTTCTCATCG
TCGACGCATCTGACAAAGCACGGAAGAACACACACTGGCGAGAA
ACCTTACAAGTGCGAG
GTATGCGACGCGGCTTTCGCCACATCCGGAGCACTCAAGAGTCA
CGAGCGAATACATACC
GGCGAGAAGCCTTGGACATGTGAGGTGTGCGGCATGGGATTCTC
GCGCATCAGCAACTAT
CGTCGGCATCAGAGGTTGCATACGTCGCAGACGCTTTATTTATGT
GAGTTGTGCGACGTG
ACGTTCACGACGATCACGGCGCTTCGGACACATCTACGTTCGCAC
GGTTTCAAAGGCAAG
AGTCTTCAGACAGCCATCAGCGGAGATAACCTGTTACCGTGTGAG
CAGTGCGATAAATTT
TTTGTCTCTGAAAAGAGTCTACGTCAACACGTCAAAATTCATGTC
AAACGCGATTATGCG
TGTTTTTGTGATAAGAGCTTCAACACTCTCGTCTCCCTGCAGAAG
CATGCGGCAAAATGC
AACGAAGGCGCCCGGACTTGCGAAGTCTGTCACCGAGTCTTCACG
ACGATTTCGCAGCTG
CAAATTCACTCGCGCGTTCACAGCGGAGAGCGGCCG
>ConsensusfromContig2063 Average coverage: 12.66
TTATTTGTTGATCAGCTCTAGTGTATCAAAAACCCAGTGGAACAA
CGTGAGGGTAGAGTT
TGACCATGCAGACATCTCGATCAAAATGTTTCTAAACGGAGGAC
ATGTTGGCACGGATAA
TACTCGTTCTCATGGCTGCTATGATCAGAAACCGTCGCACAACAC
GCTCTTAGGAGCATG
CGTGATTGGTGCCTGTCAATTCAAAGGATTGCTGGATGAGTTATG
CATATCTGAGGACCT
GAATCGGTGTTAAATGATGGGAAAATGAAATTTCAAACTATGCGT
CCACTCATTTATTTC
AGATTTCTACATCATAATTACCAATTTATAATGTATAGATGATTC
TGCAAAAGGTAGAGG

AGCC
>ConsensusfromContig2064 Average coverage: 12.31
TGATGGGAGCAAGACTATTGACGAGTGCAATCGACCTGCTGGAA
AACCTGTTGTCGGAAT
GGTATCCCGGTCTTCTGGAAGCCTCTCCTGCCGGATCTCAAATGA
TCACAGCTCTTGTTC
CGTGTCCGATCTGCCAGAGTCACGCAAATCCTCATTATTTTGATAT
GGATTCTCTCATTC
TGGCATCCGAAAACTCTGATTGTGTAGAATGTAAAATCAGTTCGT
CA
>ConsensusfromContig2065 Average coverage: 31.09
CGAAGAGTTTATTCCTAATTCTGGTAGAGTTTGAAAATATTCACA
TTGCTTGATTCCTAA
TCTGGTCCAGCTCCAAGGAATATCATAAAATTAACAGGTTTGCAG
TATAATGAGAATTTG
AGAATATATTCTAGTCACTCTTCATTTCGATTTTTGAAAATTTTCC
TGAATCAAATTTAA
CGTAATGATCGCGAAATTTTACTAATATGAAAATAATGTCAATTT
TAATATGCAGAACAT
ATTTTGACACAATTGAAACCTATAAGTCTGGAAAAAGCCTGTTCT
GGATCCTTTACAAAA
ACAATTTTTAATTCCTAAACCCGTCCAAATCCATCAAAAATTTCC
AACCCCGTTTCAATT
CCGGTCCCGTTCCTGTCCATGCGCAATGTAATAAAACAACTTCAG
TCCAACCTCAAAGTT
GAACTAAGATAACAATTCCAAGAGAAACTAAATTCAACCAGACA
TCTGTAGTCAATAATA
TATAGAATAACGCTTGCAAAAACAATATCAGTTCAGATCACTTCC
TGAATAATCAGAAAG
TCGCAATTAAGTATTGAACTTGGCCCCTATTATATACCATAACAT
ATCATATCTAGTTAT
CTTTTTCTGAATAACAACAAGGTTTTATTTTTTCACATTCATTGA
TCATGCAGTCTTAT
GTGATTTTCATCGCGTTTATCTTCAAAACAATTTTTTATTATCAA
ATAACCCGAATGAGA
ATATTTTCAGTG
>ConsensusfromContig2066 Average coverage: 8.12
TTTGGTCAAAATGGACAGTTATGGTTGGGAACTGGTCTATGGCAT
ATGACGCAATAGCTG
TAAAAAGTTCGCTTGATTACGCTGATCGTCCCAATTTTTCCCGATT
CGGTTTAGTCTATC
CGGACTACACCAAAAAATCAGGTATACTATTTGCGAGTTACAAC

AATGAATACAAAATTC
AGCGTAACATCACTTCTCGCCTATTCAAACTGCTCGGGGCTGGCA
AAAAAGTACTCCAAA
ATATCACAAATGAGGAATTTTCTCATTTCGAAGGGCATTTGAAGA
CATTCAAAGATGCAA
TACGTCTTGATGACAAAGTTATACAGGCTTTCACTAACAATATTA
GTCAGATAGTACTTG
GAAGGAGATTTGAATACGATGATGCGCAATTAATGTCAATTATTG
ATCCTGTGCGAGAAA
TTGGACAAAATATGTTTAAAGACATGTACTTAGATTACACTTGGC

>ConsensusfromContig2067 Average coverage: 5.99
TTCGTAACCATCCGCACAATGGCATGTGTATCCATTGACTTTATT
TTTGCAGGTGCCACG
ATTTTTGCAAGGATTTGATCCACACTCGTCTTTTTCTTTCTCGCAA
CGTTGGCCTTCGTA
ACCATCCGCACAATTGCATGTGTACCCATTGACTTTATTTTTGCAG
GTGCCACCATTTTT
GCAAGGATTTGATCCACACTCGTCTTTTTCT
>ConsensusfromContig2068 Average coverage: 21.56
GAGGAAAATCCATCAAATTCGTTCAGAGGTTTTCGGAGATGTTTC
AGTTCGAGAGATTTG
TTATTATTCGACGGTTTCGTGTCCATAGACTCTGCGTTCGATGTC
TGTGAAATGCTGGGA
TCCTTAGTGTGATTATCAGAGCCGACAGCATCTTTCGATTTCATT
CCACCCGAACTGAGT
AGATCGCAATTATCTTCCATCATACTATCCGAAAGAATTCCGGAT
ATCGAGAACGAAGTA
GACCCATACAGAAGAGCATGTCCGTCACTGTGCCTCGTCGTAGAT
TCGTGGATCGGAACC
GGGGGAAGGCTCACGTCTCCGGTTTTCGATGGAATAGAAGCGGA
CTGAGAATCATCAGGT
GATACAGCTTTCGCCGATGCATTCATCTTTCGATATGAAATTGGT
ACCACATTGGACTCA
TGAACATATATAACTAATAAGAATTAGGAGATTTCTCTCTAATTA
GTCACAGTTTTATAT
AAATCGATGAAATATTTGAATTCAGCGGTTCTTGTTC
>ConsensusfromContig2069 Average coverage: 16.95
CGGTGAAGAGCCGGAAGAACGGGTCAATGTTGGCGGCAATTTCC
TCCGTCTCCTGGAAGT
TGGAAATGCCGTGTTTGTAGAGTCTCTCCTCGTTGTTGATCCACG
CTATCGTGTCCACGG

CTTCCGCCAGCTGCTTCTTGAGTGCACGGATGTCCTGAACATATT
GGGAAACAAGATCCA
TTTCTCCTAAATCATTCAGCTCTTCCACTCGCTGCTTCAGTTTGG
CCAACTCAAGGGAAA
CCCTCTCCCGCTTTTCGATCAACTTCGCCTCGCCCTTTTCTTTCGA
TAACGCCATCAACT
GTTCGTTCTCATCGAAGATAGGATTGATATTTTTGGGCCAAGTCA
GAACTTCTGCATTCA
GCTCCAAATCTTCCGTCTCGAACATGTGGACGTTGAGTAAGTACT
GCATTCGGTTATTGG
CCGCAGCGATCCGTTTGTTGAGACTGACGATTCCGGCGCGCTGTA
TCGACTCTATGTAGG
CTGTTGTGTCAGCCATTTCTTGGGTGGATTCAGGTTCTTTGAGAC
AACGGTCTCGAATGC
CGTAGTACTCGCTGCAGATTTGTTTGCAGTCATCGGAGTGATCTT
TGACTACTCTCTCAA
TGAGAGTTCCTGCGAGGTCAGCTACTCTTGCTGCGAGCCCAGTCT
TGAGGTCTTCGCAAT
CCAGCTGAATCATGTCGTAGTGTTGCATGCTTTCCAGGAGCATTA
TCTCAGAACGCAGAT
CGCGGTATTTCTCGATCGTCTCGCAATATTCATCAAAGTTGTGTT
CCTCAGCCATAAAGT
CCTCGTTTTTCTTCGCAGCTTCTCCATTAACAATCCAGTCATATTG
CTCGTAAAACCAGT
TCAGATGAGAAACTGGATCCTCGAAAATTCGACCAAGTGCAATTT
TCAGCTTTTTGCCGA
TCTCCTCCATAACGCTTTCACTCACAGACGTGTCTGTGAATTCGA
CTGTGTTTGATCCGG
CTAGCCATGACTGAATTGTGGGAACCTGTTGTAGGGAGACCTTAA
TCTCATCGACAATCC
CAATGAACAGCTCTTCGACGTGTGAGCGAGTGGGGTAGAATTGCA
TTTTGTTCTCTTCGA
ATATCAGATCGACCTTCAATATCGGTAACTTGGCTCTGTTCGTCT
CATCAAACATATTCA
CCCATTCTTCGATTGTCCTCAACTTC
>ConsensusfromContig2070 Average coverage: 25.63
CCCCGATTTACTAAGGAAATACCAGAGAGATCCAACAAACACAG
AAGAACAAATCAAAGA
AAAGGAACACAATATGGCTAACTTGTGAAAAAAAATATCCAGAT
AAAGTTGAAATACGAA
GAGGTCATATATAGCTATTAAAAAAGAATAGGGGCAGTTTCTCAC
TTGCACCTATCTCTC

TGTTATCTCTCTGACAGATCTAAATCAAAGGGATCATTGAAGAAA
CCTTTCTAATTTGAG
CTCTTTATCGGCTAGCAGCTGTGTTCAAGACAAGAGGAAACCAC
CGTCAACTGGAAGGCA
GCTCCCCGTTATCATTCTTGATTCGTTGTCTAATAGATGCAGAAT
TGCAGAAACGACATC
AGAAATTTCCGCAAATTGTCCGAGAGGTGTCTTATCAAGCATTGG
CTTGGCTTTGATTGG
ATCGCTCCAAGCCATTTTTCCCATTTCTGTCATGACCACTGTTGGT
TGCACACTATTGAC
ACGTATCCCATGGGATCCAAGCTCCAATGCCATCACCCGTGTAA
TGGAGTCAAGAGCAGC
TTTTGTCGCGCAATACCCAACATGGTCTTTTAATGGTCGTACAGC
AGCAATAGATGAAAC
ATTCACCACGGAGCCTTTTATTTGATTCATAATCATCTTCTTACAC
ATGATCTGTGATAC
ATTGATGACTGCTTTTACATTGACATCAAACATCTTATCGAGATCT
GTTTTCTCAGCATC
CATAAATGAAGCAACTTTAGAAAAACCTGCGTTATTCACAACATA
ATCAACATATGGTAA
GAATTCAAGAGCAGATCTTGACGAATTCCAGTCTTCCAAGTCCAC
ACACAGAGTTTCGAT
CCCAGGATTAGCTGATTTCAATTTCTCTAAATTTTCTTCAGTTTTA
CTCAAGGCATACAC
TTTTACCCCTTTTCTATATAGTGCTTCAGCAACTGCATATCCAATC
CCTTTTCCAGAGCC
TGTCACCAAAGCAGACTTGCGACAAACAGATTCTTCTACGACTTC
ATTTTTGGGCGCGAG
AAAATCACACATTTTTTGGCGAGG
>ConsensusfromContig2071 Average coverage: 32.81
AGACAAATCAGTATCAATTCCGATTGATTTTAGCTCCAGTTTGTC
CAAATATAACGACCA
AATTAATATAAGGAACAATGTCGAACCATTGTTTGTTGAAACTAA
TGACAGTTCTTCTCG
ACCGTCAGCATTTTCTCAAAATATACCAACTTTGACTTTTGATCG
AGATGAGTCCTCCTC
CGGTGCGCAATTGAGTAAACAGATCCTTTATCAGGCAAAAGACC
AAGTACAAAAATCCAC
AGAAGAAAGGATAATGAACAGTGTGAAATCGAAATACAAAATGA
ATTTAAATGCAAATAC
TAGTTGCCCCACATCGACTGGGATACGATATAACTCAGCGGCACC
ATACCGTAACACCGA

ACATGTTACAAAGATAGCAGCTTTAGACAGTAGAGAGATTCCTA
TACGAATATTGAAGGA
CGACTCGCCCGAGAAAATGATGGGGGAAGTTGAAGCAAAAGTCA
AAAGAAGACCGCCGGG
GTTTT
>ConsensusfromContig2072 Average coverage: 24.57
GATATCCGGAGTGTGTCGGCGACTACAGAATGGAATCATTTTCG
GGTAGAGTATGAACAC
GGGAGGGTTGCTCTGACCATGTTCAGGAACGGGAAACATGTTGAA
ACTGATACCTCTCTT
GGCTCCAAGTGCTATCTGAAGCCACCAGCACACAATACGTACGTT
GGTGCGTACGCAATT
GGAAGTAACACTTTGAAAGGAATTGTTGACGAATT
>ConsensusfromContig2073 Average coverage: 17.80
CGCCGCTGTCTTCGCCATCGGGGTTGTTGTGCTGCTCATGAAGCT
ACAGAAAGTGTGCCA
GATCCGACAAGGTCGGCAGGAATTGCGAGGAGCAGTGGTTCTCA
TCACTGGGGCAAGCAG
TGGACTTGGGGAGGCACTTGCACAGATTTTCTACGAGGCAGGATC
CAAATTGATTCTTTG
TGCAAGGAGAAAGAAAGAGTTGGAAAGAGTGAAACAAAATTTGA
TAGAATCCATGTATCC
CAACCCTTTTGAGCCCTCTATCATACACCTGGACGTCCAAGACCA
GGAGAGCATCAAAAC
ATGCGTTACAGATGCTGAACGGATTCATGGAGTCATCGATATCCT
CGTCAACAATGCTGG
AATCAGTTTTAGAGGATCAGCTATTGAAACGGATCCGACTGTCTT
CCGACAACTCATGGA
GGTCAACTATATAGGCCAAATAGCCATGACTCAAGCTGTTGTGA
AGGGAATGTTGAAAAG
AAAACGTGGAAAAATCGTGATGCTCGGGAGTATACAATCTTTTG
TCGGCATACCAAGAAG
ATCAGCATATGGAGCTACAAAACACGCCCTGCAGGCGTACTCAG
ACTCACTCAGGGCTGA
AATATCTGGATCCGGTGTCAGCGTTTTCGTCTTCCAGCCCAGTTA
CATTCAGAGCAATCT
GGCACACAGCGCCCTCACCACCAGTGGAGCTATGAGCAGACAAG
ATGAGGAAGCAATCAG
ATGTGGGATGACGGCATTATATGCGGCTAGGAAAGCTGTAGACG
GGATTTGCGCTGGGAA
AAGGGAACTGATGATTGGCCCGTTCGTTCACATCTTCTTCACTTAC
TTGAGAGTTTTGTT

GCCATCCGTGTTTTTTAAAGAAAGCGCTAAAATGATGAAA
>ConsensusfromContig2074 Average coverage: 19.63
TCATCATCATCTTCTTCCTCTTTCGTGAATTCCTGGGAGTATGAT
GATGGGGAGGATATT
GTGGTTGCGGTAGGGGTCCTAGATTTTGACCTTTTCACGACCCAG
TTTCCATCAGAATCC
AAAACATAACTTTCATCTATATCTTCTCCGTAGTCTTCGTTAGATA
TATCCTCTTCTTCT
TCCTCCTCATCATCTTCTTCCATTTGTGTTCGCTTTTTGATCAACC
GGGCATGTTCTTCG
GCATTCCGAATCGCTACCGCCGCCCCTGCTTGTGCTTTTCTTTGCG
CACTTGCTTTCGTT
TCTCTTAGAACAATTGTTCCGGACTTTTTAATTTCGGGCATTTTAG
GCATCGTGGCTCTC
GCTGTTCGACGGCGGTGAGATGGTTTCGGACTTGGTGTGAAGACC
TCCTTCTTCGCTGAC
GGATCTGCCGCAGCTTTCTCTCGTTCACTGATTTCTCTTAATTCTT
CGGCATTTTCATCA
TCCTCATCTCCTTTTTCTGTTTCTTCTGTTGACTTTGATTCCTCTAA
TTCCTCTGCTTCA
ATTTTCGTTTTCTTGTGGGCGAATTTAGCCTTGGACACGGCAAGG
GTTTCGATTTCACTG
TGAAATGGCGGGGTTTCGATTTCACTGTGAAATGGCGAGAAGACG
CTGTCCTCCGTCGTT
TCGGATGGAGTTGGGCCAAAGACCTCTACTTTTGGCATTACTGCA
TGCACTTTCAAGGAG
GTGGTATGTCTTTGCAACTTGAGAAATGCTGGCTTCTCCTCCTCTT
CTTCTTCTGACAAC
GACGAGATGGAGCTTACTCTTCTACTCTCCGCAAACATCGGTATTA
TTTTTTTAGTTTGG
TTTCTACTGTCAAACGTCACTTTCCCACTCTCGTCCTCGATCTTCA
T
>ConsensusfromContig2075 Average coverage: 13.52
TTCGTGGTGATATAAACGTCAGCCTCACTTCTCGCCCAGGAGAAA
TGCACTCTGCGATTT
CCAGCTACACTGGTGAATCCGACTTCGCTCTGGTGACTGGATCCA
GCCATCTCAACTATG
GCTATCTTCACGATCTGGGATGCATCTATGACATCTCCTGGTGCA
GCGGCCATTCCATCT
CTATGTCTGTGTATCCGGTGGATGCTGATCATGAGCAATACCTTG
TGACAACTGGAGGGC
ACAATGAAGATGTGTTAGGTTTTTCTGTATCGATCAAACAGATCG

GAAGA
>ConsensusfromContig2076 Average coverage: 5.65
AGACACGCAATTGAAGTTCAACGTCATGTCAATGAGAGTACCCTC
CTATAGTCATGTATA
TGTGACCTTTTGTGTCACACTGTATGCTAGCAGCGATATCATCCA
CGAGACACGAAATTG
AAATTTAACGTCATGTCAATGAGAGTACCCTCCTATAGTCATGTA
TATGTGACCTTTTGT
GTCACACTGTATGCTAGCAGCGATATCATCCACGAG
>ConsensusfromContig2077 Average coverage: 18.83
ACTTCTTGACCTAAAAAAATTCCAGAATGGAATCGACTTAATTTC
TGGTCTGAAAAAAAA
TCAAGATAGGAATTTTGTCAGGTATATTCTCAACAACTTTGGGTC
AAACTGTTACCACGT
TTGAAAAAAAGCATAAACAAGCTGTATACAGAACGAAAACGACA
TGATTAAAATAATGAA
GAAAATTTGCCCCTTTTATCAGAAAGAGAGAGCCAAAATAAAGG
CGAAATGATACACAAA
AATAATGCACAATAGACACATTTCCGTACATGAGCACATGACCAG
TGTAAATAATATTGT
GCGGTAAAACTAAAAATATAAAAACGACGTGAAAATACCAAGCC
ACCTTTAAAAAACAAC
TAAGAATAACAAAAGAGCATCAGAGAGAATCCCACATTAAATAA
ACCCCATTTTAAAGAA
ACAGCTCATTCATATATCGAACGGAAACTAACAATATAAAACCGC
CCGTAGAAGTATATT
CATATATTACTCATAACGGCTCTTGAATCGGAGTTGACGAGGTCC
ATGAAGCTGTTGATA
TTGATGATATTACTTCCACCGCAATTGGCTTCCACCAAATGCTTC
TTGAACTGGCGGTTG
TATCTATAGCCTTTGCCACACTTTGAGCACTTAAAAGCACACATG
TTGGTATGGACGGTC
AGGTGCCCGATAAGGTTGGTCGTGCAGGAGAATCCTTTGCCGCA
AAAACTGCAGTGGTAA
GGAAAGATCTTGCAGTGAACGAGTTTCATGTGTTTCTGCAGTTCC
TTGGGAGTCTGGAAC
TGGCGACCGCAGGCCGGGCAATCGCTAGCTCCATCTGGTGCCACG
T
>ConsensusfromContig2078 Average coverage: 17.21
CGCATATTCTCTCAAAACTGGCTGAGGCAGTCCCCAGCTATTCAG
CGACAGTTGCTCATT
TGAAACAGCCTCTACTGGATTGTTCGAAGAAGATGAGGTTGATAC

TTCAATAACATCACT
TGTGATTTCTCTTAATGATTTATTCTGACGATTTTCAATATGAATA
TTTGAATTCTCTGA
TGCGATTTCTCCATTTGTATCATCATCTCCGAGAGACAAAGAAAC
ATCCATACTCAGCTC
GAATATATTTTGATCAGATCTCACTGTGGGGTTACGTGAGCTTTC
ACTAATGTGTTCTGC
ATTTGTATTACGAGATACGGATCGACGGCCACATCTGAGATCAGA
ATCCTTGAAGAAATT
ATCCTCCATTTCTGCAGCACATAACATTTCTTCAACAAGATCATC
TGCTGCAAATGAACT
GGAAAATGACCCATCCATCTTGTCGAAATATCTCAAGGTCACCGT
AAAAAACAACTACTT
TCTTCCATTCAGACTAGGCCTGGCCTGGTAAGTATTTTTTTATAG
CCTGAACTAATGGGA
TTTCCAGTTATCTGCATTTTGGCAAACTTTCAAAAATATTTTGGTT
CATATCATATGCAT
GGTGAAATAATTGTACAACAATATATTTTTTTCATTTTTAAATTAC
AATGTTT
>ConsensusfromContig2079 Average coverage: 14.47
CATATACACACTTTAGGATTATCTATTGGGAATTTGAGGCTAACT
GATTTCAATGTTGAT
GAAAACTTATGGATACAGCTCTCAATCTCAAAATTCATTCAAAAA
CAGGATACATTCCAA
AGCAAATTCATCAAAACAACGGATTCTAGATTAGAAGAGCCAAT
AGCAAATACTGAACAT
GATTCAAACCATGAAACTGAATTTCCCAAGAATGAAAATATTTAT
ACCAATAGCAATACA
ACTGATTTCAATTCTCATAGACAAAACCGTTCGAAATCAAATGAA
A
>ConsensusfromContig2080 Average coverage: 14.91
GACCCCGTCTCTGATGGAAACTCTGATACTCGAATCGAACTTCAT
AAGGGCCGTAGGGGT
GAAGGTTGCCTCGAGCTCCGCATGCCCCCCACAGGAACTACTCC
GTGAGTCGGACCAAT
TGAAAGTCCGGGCAATGGGTTAGGGTCCATGACCTGAAAAAATG
CATTGAAGATTCCAGT
GTTTTTCAGCAAAGCGGTTCGGATTGTCGTGAGCTGGAGTGGAAT
TTGCCCAAAATGAAT
TCTCCGATCGATGAAATGTACACTGGTTTTCCCCAACTCTGCAGT
GCATTTGAGTCTCTG
TATGGCTCCTGAGTGAACTTTCAAGTTGAAGGCACTTTCCTCGGG

GGCCATGTACGACGG
ATGGAAGACCACTTCGCAATCCAGGATCGAAAATGGCGCGACGA
ATCCTGTAGCCGGTCG
AATAGAAAATGAAGTGCC
>ConsensusfromContig2081 Average coverage: 20.94
TATAAGCCCGAGATGCCGCGCTTCAAGATCGATTATATCGGCGA
CCGGGAGTTGAAAGAA
GATGCATATAACATTAGTGTTTTAAGACGACATAAATCGCTGAAT
TCCTTGGAAGATAAC
ATCTCTGCGTCTACTATACGACCAAATGGCAACGGCACAAATGA
AATGAAACCTAGAGAG
AAAGTGTTCAAGCATAGAAGTCGAAATCATTCTGCTCCATGCCAC
GACCTGGATAAAGAT
GATGAGGCTATCATAATACTGGATGATGAAGATCACGTTATGAA
AGAAGTGGATGGCCAG
AAGCAAAGAGCTTTTGAGATCCATCGGCTCAAACAGCAACATCTT
CCGATTGTTGAGTCG
ATTTCATCTCCTGCAGATGCTGTTGAATTTACGCAAAATATGCAG
ACATTTTCAGGGGTG
AAGTTCGATCTGATTACAGTAGAGAAAACAAGATCTATCATCAGC
GTCGACACCGATCAT
CATAAAAAATTGACGCCGGCGGAAGAAGCTCTGGCAGCCGGGAG
GTCATACGTCGTATTG
CCTCCGAAAAAAAAGTTGAAAAGGCGACCGATGTCATTTTCAAG
ACTGAACGAAATTGAA
TCATTTCTAAAGAACTCTACAAAAATGATTTCAAATTCACAATCA
TGCGATAATCTTCAA
AAAAGTGTTTTTGGACCAATCAAAAGGAAAAAGATCAAGAGAAG
ACCACAGAGTCTAACA
AGCTCACTCGATTTTGAGAAATTCAAAACAATCCGATATTCATCA
TTTTCGGATTGCGAA
AATGAACGAGAGTTTGCCCGTGAGGTTTGGAATCAATGGTTCGAT
CAAGTTTTTCCACCA
GCTTCAGAAGATTTGAGTTGTGATGGAAATTGCGATTTAGACTCG
GAAGCGAGAGATCTG
GCCGAAAGTCCTGAACAGGAAAATCTTCAGAGGACAAATG
>ConsensusfromContig2082 Average coverage: 13.66
CATTCATTGAGTATTCTTGAACATTCTTTCTTGCAGATTTCAAATA
ATAGCGGACATTGT
CTACAGTTATTATTCCATCTTTGAGCTCTGATCGCGTTATGAGACC
TTCATTATACAGCT
TTTCAAACTCCATATACGACAGCGGTACAGCGTCTTCATATTTGG

CGAGCGGACTCGTGA
ATGTTACCGGCGTTCCAGGCTCTCTTGCACCGCTGAGAGTCATGG
CGACTTCGTTGTCAT
C
>ConsensusfromContig2083 Average coverage: 8.72
ACGATTGACAGGAAAGCGCTGGTAAAAAGTCTTTTAGAATACAA
TCTAGACAATAGAATA
ATAAACCTAATCGTGAACCTATATGGAAATGATGAGATGAACTT
AACTATAGGAGAAATG
AAAACTAAAATAGACATAAACAGAGGAATAAAACAGGGATGCAC
TCTGTCTCCAACGCTT
TTCAAACTAATCACCTTCAGTATCATTAGAAAACTACGAAAACTG
GATTTTGGTTTTAAA
GACCAATATTTGAAAATAAATTCACTATTCTATGCAGAC
>ConsensusfromContig2084 Average coverage: 59.83
CTGCGTAATAGCTGCATTCTGAATCAACAGTGTTTGTTACAACCGA
TTTTTGATGTTTTG
AATTATACATCGTAAATACAATAGTGCTGTAAATATTTCATTCCC
AGAGATGCATTTCTA
CCGCGAGCCGACGAGCACCAGGCGGCGCTCTACTTCACATCAAA
GACTCTCCGCAATCGA
TGAATACGCAATTGCTGCACAAGTGGACGAATTAGTGACGTCACA
TCCTTTGAGGGGCAC
CTTTGATCGCCCTGTTCCTATCTTAAATCATGTGTATCTTGGGTGT
CTGTCTCATGCTGA
GAACATCAAGCAGCTCAAACTCTTTGGAATCACCCACATTCTTTG
CATGGGAAATTTTTC
CATGTCTAAAGCAGTAAGTGTGTACGCCCAGCATGGAATAGAACT
ACTTCAAATCCCGGC
GTTGGACAGAGAAAGCTACAACATCACGCAGCATTTTTCGCAAGC
ATTCAAATATTTAGA
CTTTGTCAAAAGACAAGGTGGAATATGCTTAGTTCATTGTGCAAT
GGGCATCAACCGAAG
TGTTGCAATTTGCGTAGCCTACTTAATGATATCCAGAAGCATGCG
CCTGTTCCCAGCTTT
GGGAAAGATAAAGTTTGTGCGACCTCTAATGCTAACAAATCGAG
GGTTCCAGAGGCAGCT
AGTACTCTTTGCACATCAACAGAGCCTTCTTGATGCGCAACGTTC
ATCAAGCATTATCCC
ATCTCCACGAATTGAAAGATCAAAAAGCATTTGTAATACTCCCTG
CACATCATTATATGA
AAAAAACAGTTTTTCACAATCAGGACTACCTCGCATCAATTATT

CGAGAAAAAATGAGCA
TGATGATAGTTTCTTCTCATCATCGCTTCGTGGTGATTCGCGAGAT
AAATTTTTGCGATA
AATATCAGTGCATCACAATTTTCGACTTATATTTCTATTTCATCTG
TGTTTGTATAATCT
TACATCATTAACGATGCGCCTCAGAATTTTCGAAGATGGGGCGCA
ATACAAATGATTGTT
GTTATTATTATTATTATTAATTATTATTATATCATTCGAAGAATGA
CTGAATTTGCA
>ConsensusfromContig2085 Average coverage: 38.12
GCCAAATGAAAAAACTCGAATGCCACCTCGACAAGAATAAGAGT
AATAATCATAAGTTCA
AGTCTTGTGTGATGTTTGTCTGTCAACTGACTCATGAGGAGATCC
ACCAACTCTGAACAA
TGTGTCAACTTCTCGTTCATCACCTGTGTTCGCTTATAAATGCTCA
AATAGCGATTTGTC
TTCTTATACAACAGCTCGAGATGTGGGCGGTCCCAATAGACGTCG
GGTGTGTCTAGTAGA
TCGGAACTGAGATTTAGTAGATGTCGCAAAGTAAGAAGCTGTCCT
GTCTTCTGCAAAACT
TCAGATCGAGAAACGCTGATTTTTCTTCCTTGCCGAAGATCGTCC
AAAACACCAGCCATT
GAATCTACGTAAGTTTCGAGAGAAGACTCCCAGATGGAGAGCTT
GACAGAAGCCGAGAGA
GCATTTGAAAAAGCATACTTTTCCAGTCTTGAAGATTGACTATCG
TTATTCGACTGCAGA
TGAATTATTTCTCCTCGCAATTCTGTTTGTTCTTCTGTCTGTGAAT
AGTTCATGGTCTCA
CTCTCTTCTTCTACGATTCTCTGATTATAGGAGGAACTCTCGTGC
GATGAACATATATCA
AGGACGTCTCTTTGCTCGACATCACTCGTATTCCAAAATACAACA
GATCCCTCTCTGAAG
AAATAAATATCTCGGTCTCCATTGGTTGTTTCATATTTCGACTTC
ATGTGAAGAGCGTCC
TCGAAATCGATGGGTAGATGACAAAAATGATACAGCCCCTGTTG
TTGAAAGGAATCCCGT
AAGGATTCTAGATCAATAGATTCTGCGCGTGCATATGCCTTCACT
TCCAGAAGTTTCTCA
TCTTGCTCAACAGATATCTTGAGTCTTTTCCGACGAGTCAGATTT
TTAGCAGTCAATGGA
AATATATCTGCTTGCCCTCTTATTGGAATGGTGTTATTTGACATA
AATCGAATTGAACCT

CTCAGGTTGAGATATGTGTGTTGATGTAATATAGCATTTGAGAAA
CATAGTTCAGTCAAA
CCGAAAATTGACTTGACAAATAAC
>ConsensusfromContig2086 Average coverage: 19.15
AGATAATTCCTTGTAGACAAATTCGAATTACTTCAACTCCTAAAT
GTTGATGAACACATA
TTCTGTTGATCTTCAATCAGGTCTTTAACGCCGTAAAATTTCATA
AATTAACAGCAAATC
TGATTTAAAAACAATATTTGAGAAATAGTGATGCCTGTCGATGA
AAGGTATCAACAAAGA
TCTACAAACAGTGACAACTCGTGTACAATTCGTGAGATTTCCACT
TCACAGTACAATGAC
TGAAATGTTTATCGATTTGTTTCACAAAGATCCGATTTTGAGGCG
GACAGCGGTCTATTG
ATTTATGGACAGGTGTAATCCGAATAAGGTCCCAATATTAGGAGT
TTGACGATGCCGCCA
CTCGAATTCAGATTTCAATAATTCGCAGATTTGAATATTCTTGAT
GGAGTTGGAGAGAAA
CTAAGATATATCTTGAAGAAAATCCCGATTGACTGATCTTCTGTA
GCGAACTGGTCTTAC
AAATCTATGAACCAATTCAATAATAAAACAGATTCGACGCGACGT
TGATTTATAAGCGGT
GTAAACCCAGTTGTTAGTAAAGCAAGAAAACTATATACCACATT
GACGTAACACCGTATA
AAATTTATTGAATAATGGTTGAAACTG
>ConsensusfromContig2087 Average coverage: 41.71
TGCGTCGCTGAAGCCTGAGAAGCTTCATGTTAAAGTCTTGCTTGT
CTGCGATGATTTCTT
CGGTTAGGGAAGACGGGTGAGAGCTTTCGCCTTCAGTTTCGCGC
GAGGAGCTGTGAGGAT
GAAGGTCTTCCGTGTCTGACAAAGAACAGGTTCTTCGATCGCATG
CCCTTCGGTCTACCG
AATGGTGTTTCGCGTTTTGGCAGTAGTCGCTCATGCCCTCTAGAAC
TTCCACTTGGTTCT
CCAGCCAGAGGTACAGATGCACACGCAGCCTGCCACCAACAACA
TCGAATCCGGCCGCGA
GCGTTGACAGCTCTTCCATCATTATCTTAAGACAGGCGATGAACT
TGAGCTGTTGCGCCA
TAATGTCTCCACGCCGCTTCCGCGAAATAGCGCTAGAAACGACAT
TACTAGTGTCATTTC
CATTTTGGTTAGGTTCGATTGTTTTGACTTGAGGATAATTTCCATG
TCCATGTGCCGTGG

CATTGTTGTGGTTATTATTCTCAATTCTGTGAGTTGTTTCCCTCGTT
TTATTTTCGCCAT
CAGAATCGTCTTCAGAAGAAGAAGAAGATGACATTTTGTAA
GCTGGGATGTTCATCG
AGAAACTGGATGGCGCGTTCCAGTCTAGTGAGAAAGGCTCTTCAT
CTTTGGATTTGTTCT
CATCGGAATATTTGATGCCATTCACTAAAGGAGCAGCTTTATTTT
CACAAGATTTTGCTT
CTGAATCGTCGATGTTCATATCCGGCAGTTTGGCCAGGACTTCTA
GAGCCAGCATCGGAC
ATCCGTTCTTGAAGTGGTTATTG
>ConsensusfromContig2088 Average coverage: 2.34
CCGCATTGGAATCTCAGTAGTGATGAAAGAATAAAGAAGAACAT
TCAACCGAATCCATCA
AACATCATTTAAAACAGGACGACCGATATCATTTCCAGACGTTGT
AAACACCGCATTGGA
ATCTCAGTAGTGATGAAAAAAAAAAGAAGAAAATTCAACCGAAT
CCATCAAACATCATTT
AAAACAGGACGACCGATATCATTTCCAGACGTTGTAAACACCGC
ATTGGAATCTCAGTAG
TGATGAAAGAATAAAGAAG
>ConsensusfromContig2089 Average coverage: 11.08
GATCTTTGAGATCGAGTAAAAATGATCGAACATCCTTCTCAAGCA
TGGTTGCCTCGGACT
ACATGCGAAGTAGCTGCAGCTCGAAGAGGAACCCTACAGAAGAT
GATACCTTCGCTGAAG
GCTTGACAGACGACGAAGACTTCAGAAGGTCTCGAGATGGACTGC
CTTTTCCCATGTTCA
TGCCGATCACTTTCTACTGCCTCACTCAAACGATGCAGCCACGGC
TCATCTGCCTTCAGA
TGATCGTTAATCCGTGGTTCGAAAGAGTGAGTATGGGTGTCATCT
TGCTGAACTGCCTGA
CCTTGGGGATGTACCAACCATGCGAGGATTTACTCTGTGATACAA
AACGCTGCATAACGC
TCGAGTATTTCGACCACCTCATCTTCGCATTTTTCGCCATCGAGC
TGGTCATCAAAATCA
CTGCCATGGGTCTGTTCGGCAAAGACGCTTACATGGACGAGACGT
GGAATCGGCTGGATT
TCTTCATCGTCGTGGCTGGCTCTGTGGAATATATGATTCAAGACC
AGAATTTGAGCTTGT
CGGCGATACGCACTGTAAGAGTTTTGAGACCTCTGAGAGCAATTA
ATCGGATCCCAAGTC

TACGGATATTAGTTATGTTGCTACTGGATACCCTTCCGATGCTCG
GAAATGTGCTCCTCT
TGTGCTTCTTTGTTTTCTTCATTTTTGGGATAATTGGTGTTCAGCTT
TGGCT
>ConsensusfromContig2090 Average coverage: 15.46
GGAAGTTTGTGGAGGAGACGATAAGAGGTTCACATGTGAATGCCC
TTTGAGTTCTATTGG
AGATCTATGTGAGATTCCATGCGTAAAATCAGGAGAAATGTGTG
AAAATGGCGGAATTTG
CGTGAGAGGTTATGATCAGCACAAGTGTGAGGCTGAAGAAATGC
GGATTTGTGAGGGAGC
CCACCAATTCCAGTGTCATTGTCCACAAGCATACGAAGGGGAATT
TTGTGAAATTGAAAA
GTGCAGTGCAGCGGTAGATCCCTGCAAGAATGGAGCCACCTGCA
TCAATCAAAATGGCGT
GGAATGTGGGGCAGACGACAAGAGAATCTGTGGGATGAGTGGAA
GACAGTTCACTTGTGT
CTGTCCCGATGGATATGGCGGGGCACCCTGG
>ConsensusfromContig2091 Average coverage: 9.86
GATCAGCACATTGAATTGGTACTATATGGCAAATTTTCAGAACTG
TTAAGTATATTTTCA
TTATCATCCTTCTCAAATTCAGGCACAATGGTGATATTTTTATTCA
ATGAATTATTAACC
TTTTTACCTTGTTTCAATTTTTTTTTCAAATCTTTAAAAGCAGACA
CAGATACATATCGA
TTGTGAGAATTTTTCTTAATTTTGCCAAATGCAACTTCACTTTTCA
ATATCTTTTTTATA
ACCAAACTATCTACATTCACAGTTTCTTTGAAATGTTTCACAATTT
CTCTCATTCCGACC
CCTTTAATAGACATAACTGATAGGGAAACTGGAAGATTCTGTTTA
CCACCAATTGTAGTA
GTAGTAGTAACACCATTACTACTACTA
>ConsensusfromContig2092 Average coverage: 47.73
TCATCCAAAGACAAATCGAGGGAGTGTGTACGAGTGTACGATGG
GAACGGTTCTTATAAC
AAGAAACTCTACCGATGCATTTCCATACCAAAGTACGCCCCTGCA
AGAGTTGCTTTGGAA
GCAGCTTTGAGGACATTCCATATATCTGAAGACCCTAAAAACTAC
TATATTTCTGAGGTT
GTAGATGGAGACGCAATAGGGGAACTCGAATTAGACAACAGTCT
TCCACTTCGGACACAG
CTCACTTGCCATGACGGGAAAAGGCCATCGCTATTTCTACGCTAT

CGGAACAATAACGCT
CACGAGGCATGCATCCGTGTCTACACGGGGAAATTGAGCATCCC
ACGATCCAGAGAGGGC
AATACATGCATAAAACACATTCCCGTATTTCAGAGGACATCAGC
AAGGGAGGTGGTTGAA
ATGGCACTCACCAAATATCAAATACAGGACGTGAATCCAAAAGAC
TTCTCGTTGATGCTT
CAATCCTTAGACAGAGGAGTCACGGAGAGAGTCCTAGACCCTCA
AGAATACCCTCTAAAG
ATCATGCAAGATCTGCGGCAGATCAGTTTACGGAGCAGCGATCA
ACGCGGATTTATCTG
AAGCAGACTAAAGATCCTCACGGCCAGCAGATTGCTATGTTTGTA
GGAAATCTCCCAACG
GGATTAACGCAACGGCAGTACGAGAAAATCCTACATGATCTTATA
GGATCTTTTACGTGG
TCCAAATTCGACGTGATCTACTACGAGTACGGAGCGTTGGTGATG
CTGTTCGACAAGCCC
GACATGGCAGCCCATTCCAACGCCGTCCTCAAGACATCATCTTTT
GACAAAAAGGAGCTT
GTCGTTCTTTTATTGCCCAATATACAGCCCCACCTGATACCCCCA
GAAATCACACCTCTT
CTGCTGCTAGTGAATGTCAAGAGTGGGGGCCAGCAGGGTCTCCAG
CTCCTCCAGGCTTTC
AGAAAACTACTCAATCCGCTCCAGGTCTTTGACCTCATGGCTGGA
GGTCCTTTGCCAGGC
CTGTATGTATTTCGAAACGTGCCCGTCTACCGCATCATGGTGTGC
GGGGGCGATGGCTCC
GTGGGATGGGCTCTCTCCTGCCTGGACAACGTGGGGCAAGACGC
CAAGTGCAGCAGCCCC
CCAATGTCGATCATTCCCCTGGGAACGGGCAACGACCTGGCCAG
GGTCCTGCGATGGGGG
TCAGGATTCGCC
>ConsensusfromContig2093 Average coverage: 7.14
TGTTTTGATAACAATAAATATTGTCCAACTGATGACGAAATAGTC
TATGAAAATTCAAAT
CATGTTGAGAATTGCGTTGCATATTCAAGTTCTGCATCGAAAATT
CGATTGATAGGCTCA
TGTAAAGATGGTAGTTTTTGTGAAAGAATCTCCATGGTTACAAAT
CGGTTAGATCCTGAT
CTCAATATACCAGTTGTTGACGAAGAAACAAACAATATTTACATG
AATGAACGCTGTGCT
ATATGCAATGAAATCAATTCTTTTGAACCTTGGGTTGTCGTACTG

GAGTGTCAGAGCTGG
GTTGAAAGTTTTCTGATGGAGTCATCCACAAATTTGATAAAATTT
AACGCACTAACAAAT
ATGCTGCTTGAA
>ConsensusfromContig2094 Average coverage: 21.93
CAGAAGAAAACCGTCCAAAAAGCACATTTCAATGCGAATGAGAA
TTTGGTTCTGGCGCTC
GAAAAGCAACAAATTTCTGGCGTGGCTCAAACAAATATTCAAAG
ATTGAGGAAGAAATCA
GGGGAAGATTGGAAGGACGTGACTTCGCCGGCTGCTGCAAGCTG
CCTCGCGTCGGCAAAG
GACAAGTTGAAGAGGAGTCTCCTTCAAAAAATTCGTGGTCTTCTC
TCCGAAAAATCGAAT
ATGGTTCAATTGATGCGAAGAGAAGGAAGTCATGGTCAGATGGC
TGCCAAACGAGTGCAG
AGGAGGATGCGCGGCGTGACAAAAGCTGTGGGGGACGCTGTGGC
ATGCTACAACAGTCTG
CAGGGTCGAGCCATGCCAGCCAGTATCAGCACCGAAGATCTGGT
CGACGAGTCGGAGCTC
TGGAATCATCTGGAGACTTCGCCTACCTCGACCAATAAAAGAGC
AAAAATATCAATGCTA
AATGGGTTAGAGAGAGCGACGGAAGAGAAGGGGATGGTTTTGCG
TGAAATGGCTTCATGT
ATTTCATTTTATCACGATAGTGCAAATAAAATACATGAAGCCATT
TCTAATGAAGCCTGT
GAGATGGAAAAGAGGGTTTTAGAGCGTCAGGTGAAGATGTATAC
CGACAAAGCCGATGAA
CTCAAAGCTGTTTTCACACCATTTTTAGATGGAAGTGATGCTATG
AGTTCATCGGCTGAG
TCATCCAGTGATTTTGAGGGCAATGATTCCGAAAGTGACAGTTGT
GAGTCATC
>ConsensusfromContig2095 Average coverage: 41.73
CAGCCGTTTATTTGTGATCTTGCACAAGTAGCAGACCGTATCACT
CTTGTACTTTGAAGA
AGTGTCGAAGAAACACCTCTGCTGACACTGAGGACACGGCTTCG
GGTCACCAAACTGACC
GAGGAGCCCAGCGCAACGTCGACAGCGAGAGCCGATGAAAGCCG
CCAGCACGCCGCAGTG
CATACAGGCGCTGGGCTCCCCATGCTCCTTCAGGCTTCGATAGCA
CTTCTTACAGGCCGT
TGAGCTTCCCACTTGATCTGTGCTCGGAAACTCTGCTCTGCAATA
ACCACATGTTAGCTT

TTTAATTCCAGACTGACAGGCCTCACACAGTTGGCCAACTTCAGA
CAAGTCTTCTAGGGG
CCTTCTTTTATGACATTTGGTACATGCGAAAAGAGGAGCCGACAT
TGTTTGTTTTTAATT
TGTGATTTGAAGAT
>ConsensusfromContig2096 Average coverage: 13.85
CGGCCAATGCATACTCAAAACAAAAACACTCTTATACTTTATATA
GAATTTCTTTCGCAT
ATTATCATGAATTACGTAAAGCTTGACAAATACTCTGAATCATCG
GGGCTCATATTGAGA
AAGAGATAGGTTTGAACACAGGTGCTGAGAACAAGAAAAATCCA
GAAGAAGATGTAGTCT
ATACACTTGGCAATGACTCTCCACTCTGTCTCGAGGGTAGAGGCA
TCTTCAGGCGGATTC
AAGGCACTTGAGCTGGAAGACTTGTGATGGCAGTGTTGGCAACA
GGTGGAAGAGTGGAGA
GAGATATGCGCTGCCATTTCCTCTTCTGTTATAACCTTGATAAGTT
TTTCACCTCGTCGC
TTGTGAGCCAACTTCCCATTGGTCATAGGCTTGTTAGCTCCTCCCC
TTAGCAATTTCCCG
AGGCCAGGCGATAATTCATTTGGATACATCCTCCAGGCAATTCTG
ACCAATAAACGTGGT
GCTTGTGTTTTCGAATCTTTTTTGGCGTAAATATTGGTTACTATGA
CGGCCATCACCAAC
GCTATAGTTACTATTATCATAGTCGAGCCAAAAAACATGCCTAAG
ATTGGTATTGAGTCG
GGCGTGGGGGCATGAGCTCTCCGACCATAAGAAGGAAAACAAC
TAAAGCGAGTAAAATT
GTGATCTCCAGATTGACTTTTTCTCCCGACTCGACGGGAAGAAAG
AATCCTAGAAACGAG
ATGAGAAAGATGAGGAAGCAGGGGGCGATCAAATTGACGACATA
GAAGAGGGACCCCCTC
CTCATGTGGATAGTGAAAGTTACGTCGG
>ConsensusfromContig2097 Average coverage: 18.54
TCCAGGTCTTTGGTTATCCGTTGCTGATTTTGACTATCAATTTGTA
TACTCCGTTCATCC
GGTATTTTTTCTATTTTTGGAGCTCTTGATGTCGAAATGTCTTTAA
ACGCTGATTCACGA
TCAAGGTCAATTTCCTTTGCCATCACTTGAGTGGTTTTGTCCTCTC
CAACTGGATTTTTG
TTCCGCCTCCGTTTTCTCCAATACCATCCTACGACCAATACTATT
AGGGAAACAACAGAC

CCCGAAATTGCTAGTAGTAACATTGGAATGCTCTTTCCATCGCAT
TCAATTGATACCTGG
CTTGTCACGA
>ConsensusfromContig2098 Average coverage: 6.28
GGCGATTCTTCAATATCCGAGCAGGAATAGATGTCAGTAGCATGC
TTGACGCCGCTCGTG
TCTGCCAAAAAGCAACACATGCTGAAGTCAGAGAGAGAACAACT
AGATATGTGGCTCACC
AGATTGACCGTTACCTCCTCTGCCAACGGACTATCGTCAAGGCT
GTATGGTGAGATTCA
AACAGATTTTCGCTAGGCATTGCTTCATGTTTGGCGGAAAGCGTC
ACGGGAATTATCTGA
CAGTTATTTTTTTGCTCATGAAATTCTTGTACGGATTCAACGCTAT
TGGCCAACTATTTC
TGATGGACAAATTTTTAGGAATGGATTATACAATGTATGGGTTCT
ATGTTTTCACAAGAC
TATTAAGCGGAAAAGATTGGGAAGAAACGTCAAGATTTCCTCGTG
TCACCTTATGCAATT
TTGATATAAGATACAATGACCGCGTTCACAATTATGTTATTCAGT
GTGCATTAACAATTA
ATCTATTCAATGAAAAGATTTTCATCATGTTGTGGTTCTGGTATA
TAATTCTGGCCATCG
TCTGTTGTTTAAGTTTCTTTCAATGGTTTCTAAGATCTGTCTACTG
GCCAAACCAAGTTG
ACTACGTCAAGAAAAAACTCAGAGCATTCGATGCACCTCATCAC
GCCAGAGCAGCCATAT
CCAAATTCACTCAGTACTATTTACGTCGCGACGGTGTTCTTCTCAT
TCGCCTCCTCTCCC
TCAACGTCGGTGAGATGGTGGCGGCCGAAGCGCTGTGTGGTCTG
TGGGAAAACTACGGGC
CAGA
>ConsensusfromContig2099 Average coverage: 63.73
GATTTGCTCAAAATGCGAGCACCACTCGTGGGAATATTGCGGCCA
ATTCTTCGCAATCAC
CGGATAGTAATATATGCATAAGCCGGCTTGAATCTTGCTCCTCCT
CCCCGAAAGGTGACG
ATCGCGGCATAGAAGTTTCTTTTTCTAACTCTGATCGGGTTCCTG
GGATGGATTCCAACG
AGCATTTAGATGGAAATTCAAACTGCCTCAGACCCTCCTCGAATT
CTTCAGAAATCATCT
CATCAAATCGTTCATCCGACGACGTTCGAAACACCGAGAGCCATT
CGCGATCCTTCGCGT

CTAATTCTTTGAAAATATTGGCTGACATGCACGAGAGGAGAGAG
ATGGAGTCCGGAACGA
CGCCAACCGAGATTGTCAACAAATTGCGTGGCGGAGAGTTTTCCA
TCGCAGACCATACGC
GGATTTTAGTCTCTAGAGGTACGAAGAAATCATGTGGTTTATCAA
CGACGTCTCTGGTAG
GTCCACTTCCAGATGGCAGAGACGTGGGGGCAAGTGAAACCTCG
CATCCGAAGCAGTTGG
TCGTTTGTCGGAAATGCGGCGCCTTCTGCAATGAGACTGGCGGGG
ATATCTCACAGCTAT
GTGTTACTTGCCTCATCACTTCGTGATTCCTTCATGTATTCACACG
CGCTTAAATCATTA
GTTCTCATGAAAAGGGTTTATCGATGTTACGAGTTGTGTATAATG
ACTTGTGATCTATAA
TAACTAAGGGGGGGGTTTAACCATTGGGTAATGT
>ConsensusfromContig2100 Average coverage: 9.34
CCGATCTAAAATGACGCGTGGGGCGCACATCTCCTTATCGAAGCT
AAACGAATCATTGTA
AGAGTCTTAAGCAGGAAACTGGATCTTGACATTCATTGGACTAGT
GAATATAGTCTACAG
CCTCAATCATGGATGCACGTCGCTGTTGTTCTTACATCAGAGTAT
AAACTTCAAGTATAT
TTTGATGGTGAGCGACAATCAACGGTGAATGCCATTCGTGTTTTG
AAAATGAGGAAATTC
GACTATTCGGGAGGAGCAATCATCTTGGGAAGAAAATACAACTC
TGGAACTACAAGAAGT
TGGGGGATG
>ConsensusfromContig2101 Average coverage: 11.58
AAATGAGCTCTCAGATTCTGACACAGAGTTGGACTGCTCGCGCCA
CGCATGCCCCGCGGA
TATCGCTCCTCGAAACATTGTCTCGGCCAACGATGGAAAACATGC
TTCAAAAGATATCTG
GACTGAAGAAGAGGTTCCGGAAAATCCTTATTACAATGATGAAA
ATGATGCAAGAATTCA
ACCGGAATATGATATTATATACAAACAA
>ConsensusfromContig2102 Average coverage: 40.09
GCCGTGTGCTTCCCAACGGGTGTGTGTGGCTGTTTGATCTTTTCTA
CCACGTTCTTATGA
CCGAGGTATTTCAGACATGCTGAAGCCATTCAATAGAAGACCTG
AAGAGCCTGGAATGGC
CTTCAGATTGCTTCGTGGTGGCGAGAGTGACCAAGATAGCCATA
TGCCGAGGAAACCCAA

TGACGTCACGGCTCGATACATCCATTATTTGGTTTCCCATCAAAA
TCGAGGCTTCCAAAA
ATGCAGCGGGTACATCCGGGCTCACAGGGCTGTTTTCCTTGCAAA
TGAATGTCGTCAACC
TCTGCATGATGAGATTTATCTTCGCGACATGCTTCGTTTGATTGG
GATTCGAGGGTGAAG
GAGTGAGCAGTTTCGAGAGCCATTCGTTCAGTCTTTCTGGACCTA
GGTCCGCCAAACCGT
TGAGCGAGGCACACACAGACCTAACGTTTCCGTCTCGTGGGTCTT
TGTTAGCGTAATTGA
AGAGTGTATTGAAGAAGAGAAGGACTCTTGTGGAGTAGGCCGGT
CCCAAGTATTCATTAG
TTGCGGAGAGCAAAAGATCTGTCATGGCGTTCGGATGATTTTGA
ATACACCGCACAAAAA
TGCGTTTGGAATTGGAGAACTTTGTCTTCAGTTGGCATATCATTTT
TTCGAACTTTTCCG
CCTTCATTTTACTTTTCTCGTTGGCGTATTTCGTTAGAATGATGAA
CGATTTCTCCACCA
AGAGCTCCTCATGAACCTCCATCATCTTCTTATGCGCGCCCCTCG
GGTCCTTTTTCAGGA
TTCCCATCATCTTATCTACTTTATCGAAAAGATTTCCATCAAAGT
CCAAGGCCGTTACGG
GAGCGAAATGTCGCACAAGCTCATCGAATTCCACAAAGTCTTTGC
ACCATCCAATGGTTT
CTGAGGCAATGGAGACAAGCTGGTGTAGTACCATATAGAGGCAA
ACAATTGGTCTGTGAA
TGAGGTTGTCTTCAACTAGCACAGTACGTGGAGAGCCCGGGACTC
CCATTATAGCCCCCG
AGAATGTCTGAGCAAAGTATCCGAAATGATAGTCGAGGATCGCTG
TCGTATGTTTGCTCG
CTTTGTCCGCGTCCAGAAAGCACATCGGAATCTTTTCACTCTTTT
TCGACCCACACATCA
AGAC
>ConsensusfromContig2103 Average coverage: 18.56
CAGACGTGTGCTCTTCCGATCTGGCCTTTTTACCTACAGGCACCTA
AATTGTATTCGTTT
TTGAGGTAACGATATGAGTATGTCCGGTTATATTAGAGGTTGTCC
TTCTGATATATTTGT
TTTATTTCGTATATTGTCATAAACCCATTATGTGTTTAGTCCCAA
ATGAAACATCAAACA
CGGACATAATTTCAAGATTGGTCCTCTAATTAAACTCTCGTGAGT
TTCAGTACTCACCAT

ACGTTTTTAGAAATGTGAAATATAACTTTTTATTTTTTGTGCAAAC
CAGATTCCGTTATA
TATCATGAGCTTATCAAGAAAGGTTGTATATTTCGAAAATACTAG
TTTGTTCATGACAGC
AATATTTATATACTTCATATCGAAATTTTTGAAACTGATTTTTCAA
TCCAATATCACTCG
TTTTATTCACATTTTCCATATTTTTACATATATGGCACCATCCTGA
CCAATCTTATCGTG
TTTATCAAAATCTAACTCTGCAACCAATAATTCCTCACGTTCAAAT
CACTCGGTTGTTTT
AGAAACTTTTCGCAGTTATGATTTATGGTCGTGTTGATGGTTATTC
TGATCCCATTTTAT
ATTTTTTCATTTTTTAAAATCCACGTGTCTATTCTCCTATCTGTTG
GATAGAATTTCATG
CTATATATTCTTTCTATTTTTCTATATTTTCATAATGCTAGTCAAA
ATAGGCG
>ConsensusfromContig2104 Average coverage: 31.37
GTTGACCCTGCATCGTACGGGATTTACGTCATTCAACGACGCGCG
TGCCACGTGGAGGAA
ACCATTCAAGAGCTAGCACAGCACATGTTCGAATTCACGTGTCT
GAATCGGCGGCAGAGA
ATAATTCAACGGAATCGGACGGAGAGATTGAGTGAGCTGCTTGA
CTGGAAGAGCCTCGGA
TTTTTTTACCGCAAGGCGCGCCACCAGTCCCTCAAAAAAGCTTAT
CCGGGATTGGATGAT
GACGAGATATCAGAGATTTCGGAGAACATGCGACTGATGCGCCCC
CTGTCTGCCCCTGCA
TCCCCTTCAGCGTCCAGGCCCACCACGCCCACCTTTTCGCAATCA
AGCAGCAGGACAGAA
GAAGACTATGAAAACGACGATGACGAATCTAACAAAGCCTCGCT
GCCTAGAAACCGTAGC
GTCGCCAGTAACCTTGACGACGATGACGTCGATAGCGAGGACGA
GCGTGCGCAGTTGAGT
TCTACGGTGACGGATGTAGATTATGAGGAATCTGAGGCCTAGTT
CAAGGACACATGTGAA
CACAAACACCTTTTCTCTAAGAGGTTCACTCACTGAACCTATTTTT
CCGAAATTGTTTTG
ATTACTGACCACTTCATTTTGAACGAACTTTTTTCCCTATATGTTT
CTATTGTCTTAAGC
CTAGTGTTCACGAACATCACAGAGAGCTCTCAACAATTGATTAAC
ACATTTTCTTTAATG
GGTTGTGAATTTCACTTCATTCTCGGAATTTTCCTAA

>ConsensusfromContig2105 Average coverage: 5.47
GCCGGATCAATTGAAACAGGTAATGCATCGATAGACCAATTCAT
CCGCGTAATATTAAAA
ACAGGAATGTTCCTAGGAGGAGCTACTGGTTTCATTTTGGACAAC
ACTATTCCAGGAACA
TTGGAAGAAAGGGGAATTGAAAAATGGACCTCGCAAGCTGGAGC
TCATCCAAAAGACATG
CCGTCGAGCAAATATGACATCCCACTTGTGACGGATATTTGGAT
AGGTGGAAAGCCTTC
ATTCATGCACGAGATAAGGCATCGACCTATAATCTTCCATATATC
ACAAAATATCTTGAA
GAATGGCAGTGGACCTACTATATACCGA
>ConsensusfromContig2106 Average coverage: 23.93
TGTCTGTGCTCACTGTTACGGAGCGTTGCAATTCTGTCACTAGAA
GCACACTCGATATAT
AGGAAATACTGACCAAAAACCAAAGCGCGCAGTTGATGACTGTA
TCAATCCAAAGATCCG
ATGCAAAAAGCTCTTTGAATTGTCCTCGTTTCTCCGCTGGATTCAC
GATAATCCTTTCTA
TCGAAATGGGATTTCCGTTCGACTCTCCAATTTTTCGCAGCGTGG
AAATCGCTTTATCTG
TGCACCCGATCTCTGCATCGAATTTAGGAGTCTCGTCTAGAGCCA
TCACAGCGAGAAAAA
ATGAGGGAATGGAGAGAGCGGATACGGTTACAAGCCAAAGACGC
CATCCGGAACTATTCA
GAAAAATTATGGCCAAAATAGTACAGCAGGCTTCGCCGAATGAG
AAAAATGTATTTGCTA
ACATGATGAATAGATTGCGGAAAGCTGGAGGAGACAATTCATCA
ACACAGGTCGAAAAAA
TTGTTGTTAAAGCAAAACCGTGCCCGGCAATGAATCGAAAAAGC
ACCATGAAAGTGAAAA
TGGGCGAAAAAGCCGTCAACAAGTTGTAGAATGAAGTCAACACG
ATACACAACAGTATTA
TTTTTTTGCGACCGTAAATATCACACATTTTCCCGAAGTACAGGT
TACCGAAAACGGAAC
CAAATTGAGTAGCCATGGTCACCATAGCAACTTGGAAGTTACTTA
TGTTCCAATCGCATT
GCAAAACTGTTCCCAAGAACGACAATACTGATAATTCTGCTCCGT
CGCAAATCCAACCAG
ATCCAACAATGAGAGTGGATTTGACCTGATACCATCCCACACCCG
TTGCGATTAGAGCAT
CATTCACAGTCCATTATTAGGAAGTGATCGTATTTCTATTTCTGCA

CGGTGTCCCATTTC
CGAGAATCCTTTTTCATCGAAG
>ConsensusfromContig2107 Average coverage: 6.88
CAAGTGTTATTTTTCTTTGAATTGATTCATCAGGGTTGATGATGAA
AATCTTGCCTTTTT
TGACATTCAGGAGGAGATTTGTTCTTGCACCGGTGGTTTTTATATT
GCTCTTATCATCTT
CAGAAGGTCGTCCTTGCTTGTCGCGATGAGGGTTATGTCAACTGC
ATATCGTATTTTGTT
GACCAGTCTTTCGCCAATCTTACCCCCGCCCCGAAAAGATACATG
TAGAGAATTTCGCAT
TATCTGTTCAGCGTAAAAGTTGAACAGCGTCGGTGACATTATGCA
ACACAGTCTGACCCC
TCT
>ConsensusfromContig2108 Average coverage: 8.71
GATTCTCACCTGAGAGACGAGGTCATGGCCGTCATCCAGCTCGTT
CAAGATGCATGTGCT
ATGGCAGAGGAACTTGCCAAGGACGTGACCTTTTCTCTCGTCATC
ATGTCCCCGCAAGCA
ATGGGCAGAGAGTCCGGAAAAGATGAGGTTAATATTCGCATGAA
TGAGCTGGCCACCGAA
AATGAGTACTTGTGGGACAAAAACAAGTTTCTCATCCGCTTCAGT
CACATGCAGGACATG
TATCAAACATACGAAGAGACGGGTCATATTCCGCAGGTCACCAA
GGAGGCAGATCCGTTC
TGGGAGCCGCCTGAAAGTACAGAAGTACTGGTTGGTATATCATCG
CTGCCCTTAACCTTC
CTGAGTCATCTTCTCGAGTTCACGGATTCATCCTTGACCGTC
>ConsensusfromContig2109 Average coverage: 20.26
ATATTAATCCCAACCATTTGCAGAGCCATTTTTGCCGCAAAAAAT
CCGCTTCCCCAACTC
ATAGCAACGCCGTGCCCACCGTGGCCGTAGTTGTGTACCACCTTC
AGCTGCTTATTCCCG
GGTAGAGTCAATACTTCTTTCTCCAATCGAGTTGTTTGTCGATTT
GGTCTGAGCCCCGCC
CATGTCTCCAAAATTTCAGCATTCTTGATGCTGGGGATGCGTTGA
CTGGCTCTGCGAATG
ATGTCTGCCGTATCTTGGGGGCGAACCGAGTCGTCATACGTGTTG
GGGATTCGAAGCCCT
CCGAGCACGACACTTTCTTTACCAGGAAATATATAAATATCATCA
TCAATGTAGAGAAAT
TGCTTTATCCAAGGGGCACGAACTCGGACGACCTGACCTCTGATT

GGTGTGACTTCTGTA
TCTCCAAGAAGGTCAACATTTCCCAATCCAGCACAGTTAATAACC
ACATCGTAGTCTTTT
AACTCATTAAAACTGCCGATGCGCTTTCGAATAAACATCCCACCA
TGTTTTGCTATTAGC
TCTTGTAGCCATCTTGAATAATCTCTACATTCTACCATTGTGGTA
ACTTGAAAGTGACAA
TATTTTCTGTTGCAGTTTGGATATAAGTTTA
>ConsensusfromContig2110 Average coverage: 45.41
TGCGGCGAGCAGAGGTGAATGAATTACATCACGAAAATATCTCAA
ATATATTTGATTACT
CTTGGGATGATGGATTGGATTTGAATATTATCAAAACAATCAAGA
ACGTTCATAATGCCA
CTGTGAATGACGTCATGATGTCGGCGCTCAGCGGGGCTTTCCGGC
AGCATTTTATTACCA
AATATGGAACTTCTGACAGGAATTTCACAGGCGCAATACCGGTCA
ATATTCGTACAAACA
CCGAATCCACTACAAAAAATGCTTTTGTTATTGTTCCAGTTGCCA
TGGAAATTAAAATTG
AAGAACCACTCAACAGACTACAGAGAATGCACGAAAATTCTCAG
AGAAACATAAAAGACT
CGGTGAGCAACTTTATCAACGGAAAATATATAACAATTTGGCATC
AGTTTCTGCCTGAAA
ATCTCAGAAATATGTGGCTTGACATCATTACAGATCGCTTGTATA
GCTTGGTGCTATCAA
ATCTTCCAGGGCCTAGCTCCAAAATGACTGTCGGCCCTCACAGAG
TCAACACATCTGTTT
TTTGGCCGCCCACTGCTGCAAATATAGGAGTGCGGTGTTCGTTTC
TCAGCCATGGCGACA
AATACTCGATCGCTGTCGCAACAGATAAAACGACGAATCTCTCAC
CGGATCTCATCATCG
AAAAATACAAAGAAGAAGTCGAAATTTTGCTGAAGAAAAGCTCGT
GATTTTTTAGTTATA
CTGTTCAAATGTAAGACAAATTTTGATATTTTGCGTTTTTATGATT
TTTTCCATGTCATT
CAATTTTGTCTATTCATGTGTCCTCATATCTGATTGAACATGTCG
AATGATACGTGCGAA
ATTTGAAAAAAATAGATTTACGAAAAATCACGATGAAGATAATA
TGTCCTAAAAAT
>ConsensusfromContig2111 Average coverage: 14.76
CATTTGATTTGGCGGTGAGATGATTCCATCCCTCGGGGGTCGTGA
CCTCATATGCATCAC

TCGAGTTGATACGTTGGCCTGATACAGAAGTGTAGATGAAGAGA
TGCTCGTCGCAATGAG
AGGCCCATGTTTCTTTGACAGCTCTGCCTTTGCTCGACCAACCCT
TCACGGTGGTCAGAA
TGATGCAAAAAGACCTGACTGAGTGGGTTGCTCATCTTGACGAA
CGGTCTTTTCTTCTG
GCCGAGTAAGTATTTCCTCTTGACGAATGGCATCCCTTGCATTCA
ATATTACTGGATTGT
AGGAACATTTTTGTTTATTTCGCGTTGAAAGAAATATTGAAATAA
GACCAATATTTAATA
ACACGGAAAAGACAAGAGTAGTTCGGATACTTGTTGTTGACATC
AAATACCTGAATTGAA
TTCATTTTCAGTCCAAAGAT
>ConsensusfromContig2112 Average coverage: 18.49
GCCAATGTCATGCTGGGATTTCGCACCATTTTTCAAAGGGCTCGG
GGTTGGATGTGTTAT
CATTTGTAGTCTGAAACTTGTTTACTACACAATGATCATCGCATTC
ACTGTCTATTACTT
TTTTGCATCCTTGACATCGCGCCTTCCGTGGAGTGAATGCTCTCC
TGAATGGACCGATAA
GTTCAATTGCTACGTCAGGAGTGCTCTGCCTACAAAAAATGATCA
TCCCTGTTTCCAGAT
TGTAGACGCGGCGAAATCTTATGACAAATGTCAATATCTGAAAC
CAGAAAAGAGTATATA
CATCAGCTGCGTCAGTGATTACACTGCAATTATAACAGATTCTAC
TGGATATTTGTGGGG
AACTTGTACTGATGCTATGTCCAAAAAATGGAACTGTACGGAGG
GCCAGCAACTCAATCA
AACAGCAACTGAATTCTTTTGTGATGGATCCCCGATTAATAGTCC
GAGTAAAATATATTG
GGAGCAAGAAGTGTTGCGAATCTCCTCTGACATAACAGAAGGAG
CTCTCGATATGAACTG
GCATTTGATGCTCTGTCTGTTTATATCATGGTTCCTCTGCTTCATT
TGCCTGATCCGTGG
AGTTAAATCATCTGGAAAAGTCGTCTATGTAACAGCAACACTTCC
ATACGTTCTTTTAAC
CGTCTTACTCGTGAAAAATATCTCTCTAGAAGGCGCTTATGATGG
TATACAGTACTACAT
AACTCCGAACTGGACAAAGATAGGAGAAATACAGGTATGGCATG
CAGCATCTATACAAAT
ACTTTACACCCTTGGAATCGCACTCGGCGTTACCCACACACTTTC
AAGTTATAATAAATT

TCATAATAACATTTATAGGCATTCGCTCATTATAACCAGTCTGAA
TTGTTTGACAAGTGT
TTTTGCGGGCTTTGTGATTTTTGCTGTCATTGGATTTATGTCCAAT
CAGACAGGTCTACC
AGTTGACGAAGTGGCTGACGCAGGACTTGGACTGGCCTTTGTGGC
ATATACGGAGGGTTT
GGCACAGATGTCTGCGGCCCCTCTTTGGTCTACCATTTTCTTTTTT
ATGCTCTTCACACT
CGGGCTGGACAGTCAAAAGATCGGAAGAGCGTCGTGTA
>ConsensusfromContig2113 Average coverage: 12.80
TGATAGAAACCTGAGAGAAAAACCACTCGAGACTTTTCGCCAATA
TCGGTCTACTCGTGA
AGAAGAACGATATTGTAAAAATTATAGTATAGAATAGTTTTACAA
ATCAATTTTAAAAAT
CATTATGTAAATAATATTATTCAACATACATGCACTCGGCTGATA
ATAAGGCAAGTAGTA
ATTTGAGAGCATCATGAACAGAAGAAGCCAACCCTTGCAGCGGC
GTAGCCAACAAATTTT
CCAGGGGGGGGGCGAATCTTCAAAATCGCAGCAATTGATACCTCC
CTTAGTCAATTCACT
TTTAAAGTATAGGCTCATATCTGAAACCAGGGGGAGG
>ConsensusfromContig2114 Average coverage: 10.59
ACCGCACTGCGGTACATCAGTCACATAATAAATCGGAACATGCAT
AATGTGGTCTATATC
AACATACATCTTCTATTTTTATATATAGTGCGAGTTGTTCTTTATA
ATAGTTTCGTTCAT
CCTCAGATTTTCTTAACTTCTCCTTCAGCTCTTCTATCTCGTTGTC
TCTCAATGATATCA
TATCGGTTTTCTTCGCGAACTCCAATTTGAAAGCGTCGTCTTGTAC
CTTCTTCTTCATAC
GACACTTTGTCGCCGACTTTCTGTTGCTCGCTTTCCGTCGCTCTCG
TCTAATTTCATCGG
CAGGACTCAA
>ConsensusfromContig2115 Average coverage: 7.71
AATATGAGAATGCTTCTGAAATGAATTCAAGTTCATCAGATCTTT
TCTACAAAAATTTTA
TTGGATATAATGGTCGTCACAATAGCGTTGCTTTTGGGTTTTCCA
AAGGCGCAAAATGCA
GTAGATGGACAATCTATTTTGGAGGTGATATGCAGAACTTCAGA
AGTAAGATGATAGCAA
ACAAAATTGATATCAAGTACCTGCCATATAGTATAGATTCAATTT
GCTACAAAATTATGA

AATGGAAGGCCTCTAATGTAGTTGCTATTCTGCCGTCGTATTTCA
AAAGCATGTTTGCTG
TTTATTCTAACTTTGTGGAAAGTGATGATTATGGTAACCCATCTT
TCACTAAAAATATTG
ATGCCTGGAGACATTTGCAAAAAATAACAACGTCAGTCTTGTCAA
ACTATGATGAACCCA
GTGAAAATTCTATTGGTATAGACATTGTTGGATTC
>ConsensusfromContig2116 Average coverage: 10.02
TAAAACAATAACCAATCTGAATTAGCTGTAAGAGATCAATCAATA
ATACATCAGAATATT
CTAACTGCTTATACGATACAATTTTGTTATGGGCTTCTACTGATA
CAAATAGGGAAGGAA
CAAATATTATAAGAATGTATTAAAATATGTTAGAACATGTACGGT
TTTAATGTAGCTGGA
CCATTTAAACAACATGTCTTCATATATGTCATAAACAAAGACTAT
TCTAGTATCGATCAA
AGCTGAGTATATATAAAATAACATCATAGCTCGAATATTAGTATA
AAATATCCAACACAA
GAGAACAAACATGATTTTCCATGAAAGATCTGTAAAATTATCGA
TCAGACATGAGGGGAC
AATATTTCGAATAAAGTACACCTCCAAAAACCCAAACATTCAATA
TAATCATAGCAAATA
AGCATGATTAGGATTTCAAACACATAACAAGATAAACTTTGAAT
TTAAAGCTGAAAATCG
TTTGATGATGATACATGTAGAAAATCAATCAATAGTAAGGAAAA
CAACAAACATAAAATA
CTTCGAACGTATATGATTTCACCTTCAATTTTAATATGACATCGTA
TTGAACCTATTGTA
TCTACCATTT
>ConsensusfromContig2117 Average coverage: 35.63
CAAAGATTGGCATAATAGAAATGTTGATTCCAAATTTAACTTTGA
GATGTTTTGGAAGAA
TGTCATGGTGCTGATAAGTATATTGGAGTGATGAATTCTAGTTTA
ATTTCGGTTTATATA
GTGTATTTATATTTTTATGGTGTTGCTGGTTACAGAGATATAAGA
GATGAGTATTCATCA
CATGAAGTTTTTAATCCAAGTCTCTATTCATTGAAATGGAAGAAG
CTCTCATTGATGCAG
CTCGATGATCAATATTCATTGTTTATGACTTTCACTGCATTTCAGC
ACAATTTTACTCTA
AAACTAATACAGAACAAATATGTTTACTCAAAAGACTTGAAAAT
CAAGACATATGGCAAG

AACAACATTCCTAAAACTCAAACACTGGCAGATATGGGGCGACA
TGGGGGTTATCATTAT
CACGGAAGCGTGTCCGGCCAAAAACGAACGTCTGCTTCGTTTTCC
ATCTGGCCTCATCAA
ATCAGTGGATACATAAATTTACCCCACGA
>ConsensusfromContig2118 Average coverage: 9.99
TGCTCTTCCGATCTTAACAGCAGAAAAGAAGATAATTTCAACTC
CAGAAAAAACATTAAA
AAATCAAGGACAAAATGTATCAGCTCTACAATCTATATTCTATGA
TTATGTGAAATGAAT
CTCTTTAAGTACTTTTCGATTTTCACCGCATTGATCGATAACATAG
TTTCGTTTGGCCCA
GAGGACATTTTTCAATATGACGCAAAACCAGGAACACGACATGA
ATGTCCTAATAGCAAG
GAAATCTACGTGATACAATACGTTTCAACTTTCTGGAAAGATAAT
GAGGACTCGGAGACC
ATAAAACGAAGTCAAAGTTTCCACCCCGAACAAAACCCAAGACT
CTGTGAAATTGAACTC
AAACGCAATCTCGGGCAAGATGGTCCGAGGAGTCATCAAATCCT
GCATGAATCTAGTGTG
CGAAGACACATGAAGAGTCAGGATTGTGAATATTTCTC
>ConsensusfromContig2119 Average coverage: 15.00
GTCGTGAGGTTATTCTTAAAACATCAAATATTTGTCTTGAAGACA
TTTTTCTAGTAGTGG
CTTGGAACACACTTGTGATGTGTCCTAATAGAGCACGTAAATCCG
TCAAATGATTTTGAC
CAAGTTTTTTATTCGCATTCAATGTGTTGATGGTTTCTTTGAGATT
TTTTTTTGTATACA
ATAATGGAATATTTTTTTGACAGGCCCACTCTTTTGCAGTAATTT
TGTTATCTAGAAAAC
TATTCGTCACTGGATCTGTACAAGTGTAACAACACAATATATGGT
GTTTCCAAAATTCCG
CAGGTAATATTACTTTCATATCACACGCCAAAGAATTGAAATGAG
AATCTTTAGATAAAA
AATGAAATAATCTATTACAAACACTGTTAAATTCATTGTGCT
>ConsensusfromContig2120 Average coverage: 49.63
CGATGTTTTTCAAATCTCTCGGGGTGGATTCCGTCTATAGCACAT
GCACAGCAGGCCAGA
TAAGTATTGTGGAGAGCTGTAGAGAATTTTTGGAGCGATACGAG
TTGAGACAATCGGGTA
ACCACGATTCTCTACCTTTGCTTGCATCAGCATGTCCAGGTTGGA
TATGCTATGCTGAAA

AGACGCATGGCGATCTAATCCTTCCGTATATCTCACGTGTTAAAT
CGGCGCAGCAGATTA
TGGGCTCGTACGTCAAACAGTCATCGAGCGGGCTTTTCAAATCCA
CGTACCACGTGACTG
TCATGCCATGCTACGATAAGAAGCTGGAAGCGTCGAGGGAAGAT
TTCCAGAATGAAGACT
TGAAGGATGTTGATTGCGTGTTGACTTCAGGCGAAATTGTGGACC
TTTTAAACTCTAAAA
ACATCAAGTTGAATGATATACCTGGTTCTCCATCCTTCGACCAAT
TACTTTTTGACGACG
AAGTCAATGCAGAGAAAGGCGGAGGGTCGGACGGTTATCTTGAGT
ACATCTTTCGTTTTG
CATCAGAGAAAATATTCGGAATTGTTCCTCCCGAGAAGCTGGAG
TACAAGACTCTAAAGA
ACCAAGATTTCAAAGAAGTTATTTTGGAAAGGAAACGGGGAAGTC
TTGCTGCGATTTGCC
GCTGCTTACGGTTTCAGGAATATCCAGAATATCGTAAACAAGAT
AAAGCGCAAGAGAATG
AACTATGACTACGTTGAA
>ConsensusfromContig2121 Average coverage: 8.19
TCGATATTTTGGTGCGAAGAGTCGGTAATTCTTTAGAATCATCAT
CATTCTTAGAATTAC
CTCTTGTATCTATAATGCCACCTCCAACAGCGGTCGTCTTGACCT
TAGATTTAACGGGTT
TCCCAAAACCCACGGCGTTGCCAGCTGCGACATGGAATCGATAC
TCAGTCTGTGGAGAGA
GCCCGCATACTGGCCAAAGAGGGTCATCTGGTTTGAAGTAAAAA
TACTCAGACCAATCTG
TCGCCTTTTTATTGAGCATTAACGATATAGATGTCGCGTGTTTCA
CTCTGAATGAAGTGA
TTGGAGGAGTGTTTCCGTTGACCATGGAGTCGAGAACGCTGCGCT
GCACAGTTAGCCAAA
C
>ConsensusfromContig2122 Average coverage: 48.24
AAAATTACCTACGATATTCAGATATTTGAAAATCACAAAAACGAT
TCATATAAACTGTCA
AGAATGTTGATATTTGCCATAAAAGGTGATTATATTGATAGATAA
GGCGTTATGTTAGCA
CGGTACTCTGATTATTGGTAAAATTTCGAATTATTTTGATATACA
GACAGTGCAGGGCAA
ATGGTTTAAGAAATAAATTTTGTCATTCAATCATCGAAAATATTA
TAAATTATATTCCAG

GCAGATATCGATTTATCAAGCATGTGTTCGCAAAGGGTCTCTCGC
ATATAACAGTGTTAT
TTAAATTTAACTTTTAAATATTACAAATTAAAAAATAAGTCGATT
CTGAAATAAACTCTA
GAGAAACAATAGCATTCCGTTTCGCATATATAACCTATATTAGGT
TGAGAGGCATACCAT
TAATATCTAAAATGATTGTATCTGTAAGTAATTATATATTAGTTC
AATCAATCAATCAAT
AGCGCCGTTCGTCTACGGAAGACATAGCGCGGCTATAGTTTCTCT
TTCACTCAGATCAT
>ConsensusfromContig2123 Average coverage: 12.51
ATGGATTGTGCTATTGAGGCCGGTGATCCCGAGGAGGACCATAC
TCAAATGAAAGAAACC
ATCGATGAATTCAGAAATGCCATGAAAGACTTTCAAGAGATGCT
CAATAATAAAGACGAC
TTCGCTCACAGCACTCAAGCAGAAAATGAAAAGCAATCCACCAA
TCTTGAGAGGGCCGCT
AGTCGAACCAAAGCAAATCCATTCAACAAAAGCAAGACCAATGC
TGCTGCGAAAAAAGAT
GTAGGTCAAACTCCGCCCAATTCAGGCGGGGCTTCATCAACAAC
TACGGAGGGGTGTGAC
GATGT
>ConsensusfromContig2124 Average coverage: 17.34
GGCAATCGCCGATTTTTAATTACATTGTCCAGTGTTTATTTTGAC
GAAGAATTGTTTCAA
CATAAGTCAATTCCTTTGAACTTCATATTGGACGATCAAATAACC
CGATCAATCGATCCG
ATGTAATTTCGTGTGTGTCCGCGAACAACTCTGCAAATATACTTC
ATTATTCACCAAGAA
GAGTCAAACATGATTTCAAAAACAGGCAAATAATGGAAGCAAAC
ATATTATGTAGATTCT
TTAGTGTCAACTCGTCCATAACACAACCAGCCCCATCCTGAACTC
ATGACTAAAAGAGAA
CGCCAAGCCACGCCTCAATCCAGTTTTTCAGCCCCGCCTACTCAA
AATCCATCATGGCAA
AAGCCCTTGAAAGTATCTACAAAATTATGCAGGCTGGTGAACCGT
AGTATGATTTTGAGG
ATTAGTACGATTCCAGTTCTTGTTCTTCTGGTGATGATGACGTTTG
CTATATCCTCTGGA
AAGAAGGAAGTCTCCGCAGACGACACTGGCGAAAGTCGAAATTT
TAAGAGAGATAAGTCC
TTCAAATATAAATTGAATTCAAGAAACAAAACGAATAATGAATT

TCAGCAGAATACGAAA
GACTTCGGATCTGTATA
>ConsensusfromContig2125 Average coverage: 30.64
AACAACATTGTCGCTGATTGTAGTTTTCTGAACGCAGTGGAAAAC
AATTGCATATGGTCG
ATGAAGAACTTGGAATTGCATCAGTTTCTCGTAATTTTCCACATA
AACGACAAACAGGGA
AGCTTTTGGATGAAAAATCGGTACATAAATCATTAGATGAAAAAC
AATGAGATTTTCTTA
CATTTTGGCACATTTGTGAGTGACGGTTAACAGCAGATATATCAC
GCCTATGCAGAACCG
ACGGTTCGGGAGATTCAGAACATTTCTGCGAACAGATTTGGTATC
GACAACGGCTGGCGC
CGCGAGAACAACGGTTTTCGCCTATGAAATCCGGCGATTGATTTG
AACAATGACTCGTTG
CCAGACAAGAATTACAACGATAAGGCATACAAAACTCACTTCCGC
ATTCTCTTGCATAGT
GATAACATGTATTAGAACGTGAAAAATGATAACAGCAACATTTA
C
>ConsensusfromContig2126 Average coverage: 12.46
ATCGCCCGAAAATTATATAATGGATATTGATAGTTATGATAATGA
AATGGCTGTGATTGA
ACGTTTAATAGAAGAGAAAGAACATGAATTAGCTACATTGAGAA
AGAGACTTAAAAATAT
AAAGTTATCAAGACGACCTACTAGAAATCTCGAAAGATCAATAA
ATAAAGATGAAATATG
CAGATATAGTCGTCAATTGATCCTTCCAGAATTTCGAGTCAAGGG
TCAGAATTT
>ConsensusfromContig2127 Average coverage: 37.71
CGAAGTTCATATTAACACCCAACCAATCAGAAAGTTGCCATTGAA
TGTGAGACTGGACCC
TCATCACTTACATAACATATATCGTATTAACTTTTTTTTAATAGCA
TTTAGATATTTATT
ATTTCAGATTTATTGATAATGCAGTCGCATGTGATTTTTATCAGG
CTTATGTTCAAGAAG
ATTTTTGCATTATCAAATAACCCGACTGAGAATATTTTCTATTTCG
TATATTTTACAATG
TTTTAACTGATCGGTCATCCCATGAAAGTTTCC
>ConsensusfromContig2128 Average coverage: 12.00
CTTGCCATGTCATGAAAGTGATGAACTTGAATGCTGGAACTGGAT
ACAAAGAGCCTCGAT
ACAGAACAATCGATGCCGATGGACAGACTGAATCATTTTCTATAA

CACAGCTGAAGCCTC
CACCCACGAAAGTACACATTGGTGGTGTGTGGTACAAGACCTCC
GCGGAAATAACAAACT
TTCTGGATTGCCCTCCGTGATGGCTATATACTTGACATTATTTTCG
TTTGGATATTGTGT
TACATTGAGATAGTATGCTCTTATTTTTTCGGATGTTATCGGCATT
CTTGATGTATTCTA
TGATTTAGGAAAATTTGAAGAGTTGAGGTTGAGAGGACTTTTGTT
GAGAAGGGCTTGGCG
TCTATCCAAAACAAGAATCGAAAATATTTCATAAAATGCCATTTG
GATGAAATTTTCAAG
GGTATGCATATGTATCAGATTTATTGAATGTAGCATTTAACAG
>ConsensusfromContig2129 Average coverage: 28.02
GCTTTGTAAAACGAAGAAGTCGAACATTTGATACGAGTATTTCG
AAATAAAAAAATAATGA
AAATAAAGAAAATAAGAGGGGAGTTTGAAACCCGCTCCGCGAGG
GCGGTTTCGATGAAGA
AGACAGAGGAGATGATGGCTCATTGCCAGTTTGACGACCAGGTCT
CCCCTTCTTGTGAGC
AGTAGATGGAGCTGTGGTGTGCGGAATGTCTACATCTTCGTCATT
TGTTCTACCAGATCT
TGGCGTTACATTTTTAAGTTGGTCTGCAGACTGCCATGGTGTCTT
ATGAAGTCTGCAGTC
TGGTTTATAGTCGAACCAAAGATAATAAGGGTCTGACATGGCCAT
TTGCATCAAGGCTGA
AATTGCATCACTGCTTTCTTTCCCGCAGGAACTTGTGAAGACGTC
TGTATGGCAGTTTTG
GAGGTCTTGTAACCGGTCAGTAACTCGTGTGGTTTTGTCGGAAGA
GCTTTGGCAATAGAA
TTCGAAGGATGTGAGGTCTGTTAACAG
>ConsensusfromContig2130 Average coverage: 51.45
CCCGGTTCTAATGCCGCAGATATGTTGTAATCACGTAGAAACAAA
CTCAGTTTGACCGGT
AGTAGATTGTACACCTGCAACGGTGGCATTATGATGATGATGTGA
CCAGGTTCATTGACC
AAGTGGCCAGCCAATGGCCCTGTAGGGTACCGTTGGCAATAGAC
AGCCGCACAACATCTG
TATATATCTCTGTATCTCCTTCGCGGCTCTATGGCTTCGCATTGAA
TGACCGCAGTCTGC
GTGGAACCCGGGGTTCTATTCAGGCGCCAGTCCACGTCTTGCGTA
CTGTAACCGTACGTG
AGGGATAGAGGTCGGAGGCGAAAAGATTGCGCTACGCATTTGAG

AGGAACGGCTTCTTTT
GATTTGGCGGGGATGGTCATAGATTCATTGCTGTTGCCATAAACT
GAAGGGATCATTTGT
AGTTGGACATCGTGAGACATTCGGTTGCACACCAACACTCCCGA
ACGCAACGTAACCAGC
TTCTGTGAAGAGGTCTCCATTGATACGTCAAAGACAAGACGAGTT
GAAGGTAGCTCTAGC
ATAACATCTGAGCTCGAGGTGAAGGTCGGTTTTATTGGGCGAAAC
GCCACGCCCACTCTA
TCGACGGCCACGGGTGAAGC
>ConsensusfromContig2131 Average coverage: 41.29
GACGGATTTGAAAGTGCCGAGAATCGTTCATTCCTGTTGCGTCTT
CATTTACAATTATGG
CCTGTCTTGCGAAGGACTTTTTAGAAAATCTGCCAGGGAGGTCGC
TGTGAATAGATGGCG
CAAAAGCTTCGAAGATGAATACCAATCACCGGATGTTCGTAACA
ACGATCAAGGCCTTGA
CTTTGTTGACCCTTTGATCGCCAGTGAACTTCTCAAGCGGTACAT
CCGCAATCTGCAAGA
TCCTCTGATTCCCGAAAGTCTCTACGAAAGAGTGGTTCACATCGC
ATCACGGGCTATCTT
CGAGGACAAGGAAAATGCCGTGAGAGATCTAGCCATCATCATGA
GGGATGAAATGCCTTT
CCATAACTACAACGTCCTTCAATACATCATCAGACTGCTCTCTGA
GGTAAGCA
>ConsensusfromContig2132 Average coverage: 9.59
GCATATTCCCCAAATGGCCTCAAGAAAAGTTAATGAAAAAAGTT
TGCCCACAATAAAATC
GGACCACGATTTCGTCATTCTTCGTTTCATAAGAAGTATGATATC
AACCCCCAAGAACAA
CAAAAATGGTCCTGAGAATGCGAATTATAGCCCGTCAGTGAAAA
CCACGCCCAAACATGA
AGACAGTGGCGAGGAAACAACGGTCGAAGATGAGCCTCCTCCG
AAAAAGAAGACCAAAAG
CTATGAAACAAGATATTCGTCGAATAGGACGGAGGTAGGAAGCA
ATGCTGACACCATGAC
TTCACCAAACTTGGTCAAGGATAACAAATCAAACTTGCTAGCTGA
TCTCTTGAGCACTCC
AATCAACACTCCAACGAAGAGTCCAAAGCGAAAAGCGGAGACTC
CCACGAATGAATGCAG
TAGAAACGAAGGCGCCCTCAGTCCCTCGATGAAGGTGATGTCGT
CCAAGCGAGCTGGAAG

TGGCGGTGGCGGTGTTGTGGAAAAAGAAAAAGATAGCCCCCCA
CCCCGA
>ConsensusfromContig2133 Average coverage: 15.08
TCTGAGCCGGAATCATCGTATGATGTTATGATCGAAAAAATTAC
CAATCAGCGAACGAGA
TTGAAAAATTGCAAACTTGAAAAATCATTCGAATCGCATTTAAAA
GATACGTGTAACTTT
GAATCGGAACCCGCATATAATGAAATCGGAAAACATAGTTTAGC
AGAGCATAACATGATA
CCTGAATCTGAAACATTTTGTGTATCTGCGATTTGTTATGGTATT
GGAAATTTATCCACA
ACAGTCTCTCAAATTCAGTTTGCTGTTTTCTTGAATTTATGTGAAA
CCCTAAATGTTCCG
AATCGTTTGGTCTATGATCCAATTCATTGTGAATCAGAACTTAAA
ACCATTAGATTCTTT
GGAGTTGAAATTATTGCTAAAAATGAGCAATGCAAACGACAAGT
TGCCGAGCGGACATTG
TTTTATATGCCTCATTGTAGTAAACCAATGTACAATAATTTGTTA
TGGGCTAATTGGAAA
CCAGAATTGATTCAGAACATCTATATCATAGGAAATAGTTTTCGT
GCAATGAAGGATAAT
ATACTGTCTTCAAAGCTGTTGAAAGACATGTTTTATCTTGACAAG
TCGTGTGATTTCGTA
TCAGAACGATATATGGACTATGAGCAAGATCATAATGATCGGAAT
GATGTTTTCAATGAT
CTAGCTGTGCATTGTTTTTCAAAGGATAAAATTAACCTAGTACCT
ATAGAAATTTGGGAC
CACAAAACTGAACCATTTTATGAAAATGAGGATGAGTTGAAATA
AAACAACTTTTCCGAG
AACAGGTACACTGAGAATCCCACATATT
>ConsensusfromContig2134 Average coverage: 16.64
CATCATCGAATTCCTATTGGCTGAAAAATCGTCCCAGAGGCCGCA
AATGAGCTCGGCCGC
AATTAGGTCACTCGCGTTCATTGACACCATTCTGATGATGAAGAT
TCCATCTCGACGGAG
GTAGTTGGTTGCGAAGGCAGTTACAGAGGCGTGCGTTGCCATGCC
AAGTCTATCCATGGC
AATGAGTCGCTTCTTGATGTAGTGAATCTGGTTCCTAAATGGAAG
TGACCTAATTAACCA
CATCCAGAAACTACCTACAGTTGCTATGCCAACGATAATTATCC
AGAACCAAACAAAAAT
AAATATTTTCTCGTTGAATAAATTCACAGGAAGCGCA

>ConsensusfromContig2135 Average coverage: 21.52
ATCAAACATGTTAATAATATAACAGAATCGCAAAGGAAATTTTGT
CATGTTTTGAAGCTG
ACCAAAATTATTGAACTTCGCAGCTTCAGTCCAAATGAAAAAGT
CATAGAGCATAAGGA
CAACACAGAACGGGCGCTTATAACATTATAATGGAGAAGGGGGT
CCGTACAGAAGTCACT
GTAAGACTCTATCAAATATCTCTCAAAACACTTCCAAACAACGGG
ACATATGAAGCTACT
CTTCAGCATTATATCAAAGAAAATACGTTCAAAATGAAAGATAC
AGATATCAGTAGAACT
AATAAATATGGCGAACAACCTGCTTGTGTAATGAAAGAGTTCCCT
CATTTACGGCCATAT
TGCTATTGTAAATAGGTTTCGATTTTTGTTTGGATCAATTCTCAT
GAGAAACATGGTCAT
CATTTCCAG
>ConsensusfromContig2136 Average coverage: 11.22
TTTGATCGTTGATTCGATAAAATCTGGGGGCACATTATTTGACCG
TATTTGAGTCCTTAA
TAAGTCTTTGGTATCGGATAATGTGTACTTCGAATTCGATTTTAT
ATAAGCTTCATCGAA
CGCCCAAGTGAATCTACGCTTGTGTGTATCACATTTGTCGGCCTC
ATTCTGATCACTAGT
GTCCACTGAATTAATTTCCTCACATTTCTTCGTCCTGGACTTTCCT
CTCTCGAAATCTGC
ATCGATAGAATGCTTGCTGACATCTGTCGACAAATCTGCATCGAG
TTTGATCGCTATTGA
AACCATGCTGTGGGATTTCATGGAAGAACCACCGTATCTGGAACC
GGCTGTTTCAGACGC
CTTGCCTGAGTTATCCTGTCTAGATGGAAGTCGGTCAGCAGGATT
GGTTTTCACCAGAGT
ATTTTTCGTGCTGGAAGCTGGATTCAATTTCG
>ConsensusfromContig2137 Average coverage: 8.69
GTGAAATGCGGCAATCTCGAGATGCAATTGATCAGATGATTGTTG
CTTTACAATTGAAAG
CTTCTGTAGATTCTCAGTGGGATGAAAAGCGACGTGAAAACGGA
GGCAGACTGAAGGACC
TAGAAAGGAGATTTGATGATGTTCAAAATGATGTATATTCAGAC
CAACATGAGAAATCTA
TCGAAGATACTCGCGATGAACTCGAGTCTATTTTGCGAGAAACG
AGCGATCTGAAGAACG
AGCTATTCGCAATGCGAGATCTGATCGAGAACATGACTGTAACA

GGAGATGAGATCGACT
CATCTGAAGCCATGCGCGAACTCGAAGAATCCG
>ConsensusfromContig2138 Average coverage: 54.93
TTTATAAATAAACAAACTTTGGTGCAGCCTGTAGCAACTTTTAGA
TGTCTAGTACTGGTT
CGGAAACAGTAAATTTTTCATAAATTTGCAAAACGTTTCTATAAA
CCAATATATTCGCAT
GATTATGGGGCATGGAATACATCCACACTTTGAAAGAGACAGTG
CGGCAGGCGGCTTTGT
CAGGGGATTTTGAATTCCTCGAGAAAAAATGTCTTCAAAAAGCCT
CATCTGTTAAAAACG
ATGACTTTCTAGAGCATCTCCTTGATGAAGCACTATTTACATGTA
GTCGATTAGGTCACT
ATAATTGTGTGAAAATCCTGCTAAGTCATGGAGCAAAATTAAATC
ATCAAGATTTTGAGA
CAGATGAAAGTGTTCTCAGTATTGCCTGTGCCTCTGGATATGTTG
ACATTGTCAAATTGC
TTCTGGAAACAGGCTGCAATGTCAATCTCAAATCGATTGGCGATA
GATATACCGCCCTGC
ACAGAGCTGCTGCACAAAATCAGGTGGAGTGCTGTCGAGTTTTG
ATAAAAGCGAATGTTC
GCGTCGATTCAGAGGATTGCTATGGCAACTCTGCACTGTTAGTGG
CATGCAAACACGACA
ATGTCGGATGCGTTTCTTTGCTCCTTGATAATGGCTCAAGAATCA
CAAAACTGAATGTTT
GCCGACAAAATGCGCTCTCGATATGCGTGGAAAATTCGAGCCATA
ACAGTCTATGGGTTA
TCTTGAAACGTCATCCGCATCTAGTCACGAACCGGTCCCGTAATG
GGGATAGCCTATTTT
TGCAAAGCATTCGAGAGAGAAAAACGAATATTATCGACGTTTTCC
TCAGTTGTGGTTTTG
ATTCCAATCATATAGTTGATGGTTTACCACTCGTACAGATAGCTG
TGGAAATGAATGATG
AGAAATTACTGAAAGTGTTAGTTAAACATAAAGCTAATTTCAATC
AATCTCTCTATGCAA
TGCCCGGAACGACGCCCCTTATGCTGACAGTCACGAAAAATTTGC
CTGATCTCATCACAA
TTCTTGCTATGAACGACGATTGCGATTTGAATCGGTTTGATTTCA
AGGGCCGTACTGCTT
TATATTTTGCCGCAAAACATGATTTTCTTGATTGTGCCAAAAATC
TGATATCACTTGGAG
CCAATCCTGATGCATATGTTCAAGCTGAACCCGGACTTCTGTCCT

TCCAATGACAAGCTC
CCCGCCCCTCCTCCGATATACGCTGCAATCCGTAGAAACAATCCG
GATATCGCAGTAATG
TTGATCGCTTCAGGATGCGATATCGACTTTCCAGTCCTCCATCTC
ACCGAAGACGAGAGA
TTCGAGATGTTTGCACCAATTCAGATAGCGTGTGTGGAAGGTGTT
CGCTGGGCTGTAGAT
CTGATTCTAGAATGCAAATGGAAATTACCATATGCGACATTGGAA
TGGTGCGAAACCCAT
GCTGGGACTGACGAGATACGAGATATTTTAAAGATAGCCCTCGA
TGGCTGGGAATACAGA
CCGCCTCCTCTCAAGTGGTTATGCCGGCGCCAAGTTCGCAAATCC
ATTGGCCACAATATT
ATTGAAAAAATAGATACTCTCAGATGTGCAGCACCCACCAGGATT
ATTGATTATATCATG
TACAATGAGCTTCTCCAATATACAAAAGAATCGTCGGAGATCGG
AAGAGCACACGT
>ConsensusfromContig2139 Average coverage: 15.62
CCGATCTCAGGAATTGTAAAGTACAACGTTGATCGGTCCAAACGG
CTCTTCTTGGTATAT
CTGTATTTTGCGCTGCTTTACCAGGCCAAGCAAATCTTGGAACAT
TTTTGCAGCCATCAC
CTTCGAAGAAGTCAATGGACAGAGGTCTTCGAATGTCGTGGGTTC
AGTTATATCATCGAT
AGACAGCCTTCTTGATGCTCCTTGGAATTTCGTAGAGATATCAAG
TTGAGGTGACCCAAA
TTCGGCTGGTAATGTTGTGGATGCACAATGCTGGGGCGGTGATTG
ACCTGGGATGGTCAT
ATCGGCCATTTCAGCGACCGTTTCAGCCATGGCCGAAACGTCCCT
TGATCTCATTGATGT
TTCTGTCTGCCTTCGATTGGACGTGTCTCCTCGAGGAGATTGGAG
AACACTGAAGCTTTT
AGATTGGTCTGGGTCACGCGCCTGTTCGATGGTTTCATTTACTGG
AAACTCCATAACCTG
GTTTTGCTCCACGGGGAGCTGGTCCTCCTGTGGTTGTAATGCCTC
CAGTTCAATTGCCAA
ACTTCTTCTGGCTTTTGCTGTTTTTCTGCGTTTCTCCTGTGGCTTTT
CTGTATTCTCTTT
GTCTTCCTTACTTGTTGTCAGATATCGCTTCATCAGATCGGAAACT
GTATTCGTCATGAT
GAAATCACTTGAACCGAAACTGGTCAGCAATTTGTTTGCTGAATT
CATCTTTATATCAAC

CACTTGGAAGGGGGCCCTGGGTGCCTTGGGTCGACTCCTGATCTG
ACGCAGGACTCTTTT
GGGGATTTCTATTTCCGCGTCCACGATCAGGAATCTCGTGTCGGA
TCGCCGTACCCGGGG
TCGAGAGTCGGACGGGTCGATCGATTG
>ConsensusfromContig2140 Average coverage: 40.91
ACGAATGACAAGTTTTGGGACTATGATGATGATCTGTTTACTTTT
TTCCAATCAAAATTT
GACATTTTCATCGACAACCGGTCGTTATACTTGAACGAAATGAAA
AAACTACTAGATATT
GTTCAGAAAGAATTTAAATACGAGCTCCTATATAATCTTGATACA
ATGACGGATCTTAAA
ACAATGAAAAAAAAAAGTGAATATATGGAATGCATTATGCATCT
ATTGATAAGAAGCGAC
GAAGAGCTGAATCGAAACGCATACGATCTAACGCAACAAATACT
ATGGCATAATAGTGCA
GGGGCACGAGCAGTGATAGAATTGAAGAACATTAATCAAGAGAT
ACCACCAAAGGCTTTA
AAAATCACAAACATTGATCGCAGCACGTATATGGAGATGTGCTCG
CATCTTTTAGACAAA
TTTGATATTGAAAATTTCTACGATTTTATTGATCACGCACCTCGAT
ATGGCTGCACTCTG
TCATATAAGAAGCTTTTGAAAAAAATTTATTTGCAATATTGTGTC
GGCAAATATCGTTTC
AAAGTCATAGAATTTGTCAGAAAACATGGTTTCTACATGTTTAAA
GACCCTCGAAATAAT
GGCAAAATTCATTACCGCGGTAGTCCGTCTGAATTTGGACTACTT
CCACTTCAGTATCTC
TGTGAACATACATTGATTAAACATGTAAATTGGAACGATCTTCAT
AT
>ConsensusfromContig2141 Average coverage: 15.23
GTCCGAAACAAACAAACTACTAATTAATTTCAAAAAATTTCTGA
AAAATAACAAATAAAG
TCAAAAATATATTCAAAAAGAAACTGACGCATGACACATGAACG
TACTGAAATGTCATGT
CAGGTTAGTCATCTTAAAATTGGTCCCATTCAATCTAAATTTCGAT
TTTAATTTCCAAAC
AATGAATAATAGGTTTCGGGCATTGATGTCTGATCTCATAAACAA
TGTTAATTCCCATTA
TATATCTGGAGAGAGACAAGCGTATTCAGCAGCTCGCTCTCAAAC
GTGTGAAAATGGTCT
TACAATAAAATTAATAGTATTCGACTCTCAAATTTATAGTGTATA

CATAATCTGAACCGA
GGGACACGTCTGTAGTAATATTTGCATCGATTTTAGGTCTGATAA
ATCGAAGGTTGGAAT
TTCGCAATAAGAGAAATTAAAAAAAATGAAACATTGAGATAGAA
GTATTATTGAAATGAC
AATGGATTATTTCATCGTATTACCGCTTTTTGCGATTATCAGTGA
TACGTGAATAATAAT
TTACAGAAAAC
>ConsensusfromContig2142 Average coverage: 60.62
CATGAAATGATACGTAAACAGAAAATTGTAAAAAAAATAGGAGA
ATGGATTTATCTTCAT
TTTTGGAAGCTGATTTTGATCCAAAGGAATGGATCAACAAAACTT
TCTCAACAGACTATG
GCGGATCCACAGAGCAATATGCGTCAAAACTTGTTATGAAACTCC
AGAAATCTATTCAAG
ATAATAACGAAGCCATTGAAGAATGCTGCCATCAAACTTTGCAA
AGTCTTCCAAGAATAG
TGCGAGACCTCGAGTCGGTGAAGCAGGAGGCGTGTCTTCTGCAG
GAGCAAATGACGACAG
TGAAATATGACATAGAAAAGGTTGAAAAGCAGACCTCTCAGTCA
ATGAAAGTTCTTGTGG
AACT
>ConsensusfromContig2143 Average coverage: 38.98
AGATCCTCGAGCGTTTTTATTACTTCAGCCAAGCTGATGCTGTCC
CAGAGTTTGGTGGTC
CTCCCTTCGGTTTTCATACTCTCTAACACTCTTATGAATCTGGAG
ATAAGAGATGTACAT
TTGCGGATGACTCGAGCCGATTTATTTTCCTCTTCTTGTGCCCTC
GAAAGTGTTAAACCG
TCATCCATATGACCCTCAACCATACCGACCGCCTTTTTCTCTTCGA
CCCTTCCTACGCCT
CGCTTCTTTGTCTCAAAGGTTTGGTAGGAGAGCCAGAGACCGCTG
CTCATGTGTTGGATG
TAAATCACAGAGTCTCCATATTTGATGTCAGCCGTTCCCATAGCT
TCGTCTTCGCGAGTT
TCAGAAAACTTCTTGTCATCCTTCGACTGTCTGAGAAAGAAAGCG
GTCGTCTGCTCGGTG
GCGTCTGTTCGATGAATTGTAATCATCTGGTTCTCTTCGTTTATA
CCCAAGAAACGGCCG
GATGTGACGTGACGTAAACGACAGGGCTGAGCCCAACTCATGAAC
CCCTCTGCCCACTTG
ATTCGAATGTGTTCGATTTTCCAGAGAGATCTTGCGTGAGAGGAT

ACCGAACCAGTCTCG
TACATGACACACGTCAGTGGGTTTTCCGAATCTCTAGGGGGGACA
GTGAGGCACTCGTCC
CCGTGAAACAGTCGTAGTACGTGACCCCCCAATACGAAACCAAC
AGATTTGATGCGGG
>ConsensusfromContig2144 Average coverage: 26.90
GGAAATTGCAATGATGAACACATATTCTGCTCATAATTGTTTTCA
TATCCATTGTACACT
TTGAACAATTTTATAATATGAAAATAGATTCAAAGAGTGTTTGAC
ATAGTGATTAAAAAA
TTGGCTACGATGATATTGCTGTACGAGGAATTCACAGACATCTAG
GTAATATACACAGGG
AATAAAGTGCATTTCCGTGCAGGACATAATATAGAAATTAACAAA
GTTTGTCAAAGTTAA
TTTGAACAAATGTCACATAAGTCAAAAGCTTTAGAATATACAATA
ATCATAAGTGATTGG
AATGTTTTCGTTCAAATTAAAGCATTGATTCAATAAACACGATTT
GTTGATGAGGCCATC
TACAAACTGATTAAGACCATATACGAATTAAAATGTCGTGATAA
AAAGTCGTACGAGAAT
GCCGTACGAAAAACGTTTGGTACAAAAACACACAGGGAATTCGA
TATAAATGAATTTATC
ACCTTCGTTTTGATCGATACTTTTGTCACATCTAAAATCATGCTT
TTGAAGTAATACAGA
TGGCAACATATCAAACATATATATCAAACATTCATTATCATAAAT
ATCATACGCTTGTTA
TAATCTACATATAATGTTCAAATCCGACTATATTGACTATTCCAA
GAAGACTCCCATGAA
TATACCACAGATTCAGTGATTTGTTCATTGCCTAAAACAATAAAT
TTTTGATGATTTGAA
ATGCTGATAACAATGAGTTTTCTCGGTCACACATATTCAGATACG
AACATGTATGTTTAT
GAAATATGAAAAAACAAAGGGAATTCGTCTATGAATAAATAGCG
GACGGTTCATAAAATG
AAAACAATTCAAAATGATTTGGAATATACAGCGACTGAATGGGA
AAGCAGTATATGGCAT
TAATTCTTGGAAGAGAAGAGGGTCAACATTTCAAATTTTTAAAAA
TATTTAAATTTGTTC
TCACACCAGAGTTCGAACGTTCATAATATGTCATTT
>ConsensusfromContig2145 Average coverage: 11.33
GCACCACATTGGTGTAACACGGATAAATTAATTATGATATTCACG
ATAATATTTCGTATA

GTAACTAATCTTTTCTAACATCTTCATTCTTTGTAGAAGACATCTC
AGCTTGACTCCAAA
ATTATCACGATTGTAGTGATTTCGCTGCTAGTGAAAAAAGTAATT
TTTGTTGAAATAGTG
CATGCCATCTGTTTTTGAAAAAATTGCTTTATACGTACATTCTCAA
ATTTCTAGGTGTGA
GCAATTCTAACGTTAAATCTGATATGCCTGTAACTCATCTGATCA
TGCTGGCTACACGTT
TTCTGATTTTGCAAATGTGTGACTTGGTTTCTGCTTAATTGATTA
CAGAATGAAACGTCA
TATCATTATTATTTAGTAACTCCTCTCCTCTTTCCTTCTCCTTTTAG
TAATTCTATCAAA
TCGGACCGGTTAATGAATTAAACATTATCATTTGAG
>ConsensusfromContig2146 Average coverage: 26.29
GAAACTCAGTTTTCGATTCTCATACTTATACACCATCGATTTTCA
ATAAAGTAGAGAACA
CAGATTTTGATAAATCACCGAATCTACCACGTCACAACAGAGGTC
AGATGGTACGCTCCC
AAACATTGCCAGATTATGCTTTGGCAGGACCAAGCAGTTATTCGA
AGTACTCGAGTGGGG
GAGGTGGCAAGACAACTGGTATATATGCTAGTAAAGACAAGCCTT
TCCAAAGTCGATTTC
TTCAGAAGGTGCGGTCGGACCGGGCATCGGGTATCGCAGCTGAG
CCTGGTTCTCGGGGGG
ATAAACCATTCAAAAGCCGTTTTATGGGAAGTGGAATCGGTACAG
CATCTTTCGACACGC
CCTCATCCTTGGACAGGGTCGACCCGACATCTTCTACTGCAGGAT
CTTCACAAGAAAATT
AAAGGATGAAGATTGGATTCGTGCGAAACGCTTGCTGGAATCAG
GAGATACAAAAAATTG
GTTTGGATAGACAGTTAACGTTAGGGTTACTGATTAGTTTAGATT
CATTGCATGAATGTT
TTATAAGATGGAATTGAATCCAATAGGATTGATGTTCATAGCAAA
ATCACATGAAGCCTG
ATTAATTTTCGTATATTATTGCACTGT
>ConsensusfromContig2147 Average coverage: 41.30
TAGAGGAGTTGAAGCAGGAAGTTAATAGGCTTCAGCGAGAAAAT
GCGGATCTGCGTGTGT
TCATGGAGGAGAAAGATTCTGTCATCAAAGATCTCGAGAAATCA
CGGCAAGAAATGAACT
CGAAGATCATTTCCCTTGAGCAAGAAGTCGGACGACTCAAACAA
TCTCTTGCCGAAGCAA

ATTGCACCGGCGATGACCTTCACAAATCATCGCAGCAAATGCAG
GATAAGATCAACCAAT
TGGAGAATCAGAATCAGGAAATGCAGAACTCGCTCTCCACTGCAC
TTTCTACGAGTTCTA
CTCTTGAAACCTCGAAGCATGAAATGAAGGATCAACTGGAGGAC
TTGGAGAGGTC
>ConsensusfromContig2148 Average coverage: 8.50
TATCTGTCGTTTGAGAACAAAGCGAATCTCTGAACAAGGAAAAGC
TCCTGCAGTGTAGTT
TTGTGAACAATCCTGAAATTCTTCATCCTCTTTGGAAAACTGAGG
AATTGTCATTCCTTC
CTCGATCTCAACGGGTGTGTTCGAGACCCATCCGAAGTACATTAT
GTCCATAGTATACCC
AAACGACTCGAACTTGATGGGACATTTTTGTGTATCCATTGGATA
TTTCTGTAATTTCAT
TTGGCACGCAAGAGTAGCTGATATTTTTCGTACATACCACAAATA
ACCAGTGGCGTTGAG
ATTCAAAAGGACATTTGGAACAGAGACAACATGAAAATCTGCCCT
CTTTTCGTTTCTGAA
AAAAGTATCAGGAATCCAAATTTGCTCCAAGGTTTTTGTGCTCAA
GCGGATTTTGTCAAT
CCGGTTGTTTGTCGAATGGAACATGAGACGAGGATCTCGCCAGCT
TTGCCGAAGGTAAAT
GCTCAAGATCGGAAGAGCACACGTCT
>ConsensusfromContig2149 Average coverage: 19.01
GGAGGAACAAATCCCGACATCTATAATAAAAATAGTGAAATGAA
ATATGTGTAAAGAAAG
ATAAAGTCCTAATCCCTCGCCATGAAAAATATAGGCACAAGTAA
TCTGCAATAATGTAAA
GTGCAACTCTAACCAAGCATAACAGTTTCAATCATCATCCGAAAT
TTATTGCGAACTTTT
CATTTCGAAATTATATAATAGCCTTGAAATTTTTGAGCCATACTG
TGTTTCGGAACTGCA
ACAATTGGTTCATTACCCGTTTACTTGTCCGCAATGATTTATCGA
TCAAGTGACCTTAAA
TGATAATGAAGCTCTGTGCAGTCAAAAAGAGATTGCAGCCATAA
TTCTGAAAAAATAAAG
CCTGGTTAAACACTGACAGTTCATAACTGAGAAATCAACATGTC
TAAAAAAAAGCAAGAC
CAGCCAAGAACTCTGAAATCTGAAAAAGAAAAGAAGAAAAGGTC
ACATCTACCTAAATTC
GAATACGATCAAATATTAAGCACTTTTCCAATGCATAAATAATAC

TATTGATTGAAAAAT
TCTTCAGACTGGGGCTGC
>ConsensusfromContig2150 Average coverage: 10.50
GTCATGCTTTCGTAGCAATAGCTTTCGTATTCGATTTCGAGAACG
TAGGCAATTCGAAAA
CCATCTCGAGAATTTCCAAACTCCGAAGAAGCAGAAAAATCTTCG
TTTGACACTTTCGAA
TTTTTCACGAAATCACGAATTCGCCGCATAAACTCCGCCTTCTTT
CGAGTCACGTGATCT
AGACACGCCTCCGACACGCCCACCCGCCACAAGAGGCGTGGCTG
GGAGGGGCGAAAGGCG
GCGTTAGAATCAGCGGCGCTCACATGTTGCGCCGACGGCAAGCGA
TACTCGCGCCGTTGC
AGAGCGTTCTCCGCCACGCGCTCATGACATTCAATGGACGTCACG
TTTCTCAAAAACAGC
GGAACAAGTGGCGCCTCTCGTGCGAAAGATTCAAGAAGCATGCG
CATCCGATCATTTCCA
CAGCTATTATCGCTCAGTGGCGACGCTTTCGTGCGCAACGGGAAC
CGGAAGAGGGTGTTT
GGGTAGAGGCCATTCTCGCAAAAGGAGACCCGCTCCTGAGGTGT
AGAGAACGCGAAATCA
TCATTCATACAAAATGGCGACATTTGATCGGGACAGGAAAAGCA
AGATTCATCGAGAGGA
GGATTATTGTCTGACGTTTGATTCCGATGTAATCTTGAAGATGAT
GAAGATGAAAAAGAA
GATGAAGATGATGATATCCCCGAGTCCCCTCTTGATATGAGAAG
GTCGGGGAGCGCGTGT
CCGAAGGTGGTCTCTCTACCGCAGATCTGTTGGGGGTCGATATAG
GCCAGGCGCCCCCCA
CTCAAGATGGACGGCAAATCTGTTATGTGAAAAACGCTCTTGAA
ACCGAGGCCGAAACAT
CCGACCTTGAACTTGTCGCCATGTTTGATGCTTTGAGCGAGCATG
CGCACACCCTGCCAG
TCGGCAGACGAGAAAG
>ConsensusfromContig2151 Average coverage: 13.47
TAGAAATATCGCCAATTGACGATATCAGATTGACGGCGGTTTTCG
ACCGCTCTGTTCAGT
TGTGGGCAGATTTCACGCTTTCGTTCGATTCTGTGATCCAGCTCC
CATGTGCTGAACAGC
AATCTTCGCTCTCTCGATCCATATGGGTTGATGAAATGATCAGTT
GAGCTGCATTTCTCG
CTATCAAAAGAGCCTTGGCAGTCAAAATTGCCTTGAGCATCACAG

AGGCGCATTCTTGAA
AAGGCTTGTGAATCCAATGACTCCTTTTCTTCTGAGGTCCGAGTC
TGCTCCTGTAATGCT
TTCCTGTCAAACATAGCGCCGTGAAAGCTGTTGCGTTTGAGTTCA
TCAAACATCAGATCA
AACAGAATGAGAAGCTCTTGTTTTCTCCGTCCATCGGTCCTGACC
ACGTTTATGGAATGA
TCCTTTGTATTATAGAAGTAGTTACGAATCCTTCGCTTTGCAGCA
TCACGCATAAAATCT
GATTTCTTCTTGTATTTCAGTGAAAGATCCGAAAACCAGCACGGG
TCTTCCTCAAGCAGC
TCAGCCTCCACAGATTCATCTGATAGGCTCAAAACTTTCATCAAA
TCGCGGATACGCTCC
TCTCCTACT
>ConsensusfromContig2152 Average coverage: 63.39
CTAGCTGTTAAATCACACATATTCAACAATGATTACGAATAATTT
AGCATCCTCCGATAC
TCTCAGACATACAGAAGTCATCGACATCTCCGGTGAAATGACGA
TCACCAGGAGAAACTG
CCCCGATGATGGTCCTGGAAGCGGGTTTTACTGGACTGTCTAATT
GTGCCGAGGTATCCG
TGTCGACTAGTATATCCCCAACATACATTTTGAGAGACAGAGGAC
TGCGGACGTATGCAA
ATTTCAGTTTCAGCCACTGGCCTTCTGTTCTCGCATTGGCAACTCT
CAGGTACTTTTGTC
CATTGTCAACAAAGTAGAGTCTCATCCCAAATTCGTGATGCTGGA
ACACAGAAGTCTTCT
GGTTGTATCCATTACCGAAGATTCCCAATGTACTGGCGCTTGCCA
TGCTTGCCACTTTCA
CCCAAAGAGACAGCTCGAAAGCACTGGTACATCTGTCCGCATTTC
CAAAGCAATGATCGT
CCAAATACCCGAGGTCAATGTAGCTGTTTGCTGTCCCATCGAAAT
GCACTCCCCTGGAGT
TGACTGTTACTGCTGACTTCAGGCATCCCGCGAAGTTGGAGGAGC
TGCTGACAATGTAAT
TGGAGG
>ConsensusfromContig2153 Average coverage: 23.16
TTGACTTCTATTCTTTTGTATGCTCTCTTCACGGTCTTATCATTTTT
ATTGCTAATAATT
AGTACGATCAAAATCGTACTCCACTATGTTTTATCTATTTTCAAGA
TATGTGCTGTATAT
ATTCATGGGGTTTCGACGGGCTTTCTCGCGTTCATAACTAGAATA

GTGTCAAATAACCTT
AGTGGTGTTTATTCTAGTCTGGTTCTTATATGGGGAAATTTCACA
GATATATGTGTATAT
ATTCAGGAGTTATCCTCAGACATTCTCATTCTATCAATTCTCTTTC
TACAAACACATATG
CTTGAATTGTGGGAATATTCGATAGCGAATTTACATGTCTTGAAA
AATGTAGTGTGCAAT
TTTGGTAATTCATTACACAATTTTCTGGTGTATATATTATCATTCC
TCAGTGGTGCTGTT
ATATCCCCTGTCTATAATTTATCTAAAACTACTCTCGCACAATTCA
TTTTATGTATGCTC
ACATCATCCTTCTGTTTCGCTTGTCTTCAAAGCTTCATCACGAGG
TCTGAGAAAAGGCAG
ACAAATATCTTGGATTTCGACGAAAGTGATTATGGGCCGATAGAC
GACAGTGCTTCGGCA
CATAGCGACGACTCCTCTCAATCCTCTGGCGAGTACGAGCCTGAT
GATGGTTCATCCACA
GACAGCGACGAAGGTAGCAGCACTGGGCTCTCGGTAGACATAAT
ATTGGATAGAAATCGT
CCAGCCGATCCCAGCGTATTTCGACGCAGAATTCCCAGTTGTGAT
AATCTTTCTCTGGAT
TCAAATGATATATCGTCATCTTATCATAGTATGACTGCGTCAACG
ATATGCAACGGGATA
TCACCATCATTTGAGAATGGCATATCTAACACTCATATATCTGAT
ACAACAGAATCTCTT
TCAGCTGACAATGTTTGTGTCGTGTGCCAAACAGAACAGAAGACG
GTGTTAGTACTTCCC
TGCAGACATCTTTGTTTATGTACGGAATGTGCACAAAATATTGCA
ACTTGTCGCACTAGA
AGTCGAAAGATCTGTCCGTTATGTCGAGGAAGGATGGCATCAGTT
ATCAATGTTTATGTT
TAAAATCATGTTGTACTGTCTGGTGTTTTTGTGCTCATTTTTATAT
CACGAATGTGG
>ConsensusfromContig2154 Average coverage: 7.89
AGATCTACAATAATGACATGAGTTGTAGTTTCACAAAAGAATTCG
CTGACATGCCTCTTT
CCAAAATCGAATTGGGAAACAAATTGTCTGGCCAAAGACCAGGG
ACTTTAGTCACTGCAA
GTGGACTTTTTGATTATGAACAAGGATACTGTCCATCAGAGGTTG
CTATCAGCTTCATGG
ACAACATTATTGACTACGGCGTCAATGACGAATGTCCATACGATT
ATTACGACGATTGTA

AAACCCCATCTATGACGTTTCTGGTTTATGTGAGATATAGAAGTA
CCAATCAATTTCTTA
TTAATAAACTAGCATCTGGCAAAATAGGGTACGACGTCGTTTTA
AAATCGACGAAAGAGC
TGCAAGTTACCTACATGAATGATGGAAAACGAAAACGGGCGATT
TCGACAGCGCTGAAAG
AGAACCAATGGACTCATG
>ConsensusfromContig2155 Average coverage: 68.84
AATTTATTGACCTATTTACTATTTTCATTTATCAAGATGATATTCT
CAAAGCATCTTTGG
AAGAATATCACTTCGTCTCATCATAATTTGCTGAAATTATGGTAT
TCTTCTGGGAGCCCT
TCACCTCGAACCGGAGTGCTGATGCTCAATATGGGCGGCCCAGAA
ACTCTAGATGATGTG
CATGGCTTCTTATTGAACCTTTTTTCCGACACGGATCTGATGAAA
CTTCCGGCTCAAAAA
TACCTATCGCAATATATCGCCAAACGCCGCACACCCAAGATCAT
TGAGCAATATCGGGAG
ATCGGCGGAGGGTCCCCGATCCGAAAGTGGACTGAGGCACAAGG
CCGTGAAATGGTTCGG
CTTCTCGACCAAATTTCCCCAGAAACTGGTCCTCATAAGTTCTAC
GTCGGTTTTCGGTAC
GTAGAACCCTCAACTGAGTCATCAATTGATGAAATGGAAAGAGA
CGGTGTAGAGCGAGCA
GTGGCTTTCAGTCAATATCCACAGTACAGCTGTTCCACGACCGGG
AGTAGTATGAACGCT
CTAGCACGCCACTATCTCAAAAGGAAGGAGGAAAGCCAGATAAA
GTGGTCGGCCATTGAT
AGGTGGCCCGTGCACCCGGGTCTTATTCAGGCATTCACCGAAAC
AATAACGACGGAGCTG
AACAAATTTCCAGAAGAAGATAGAGAAGATGTTGTGATATTGTTC
TCTGCTCATTCCTTG
CCAATGTCGGTAGTCAACCGTGGTGATCCATATCCACAGGAAGTC
GCCGCTACTGTCCAG
GGAGTTATGCAGAACTTGAATTTTTCAAACATATATCGACTCGTG
TGGCAGTCACAGGTT
GGACCAAGGCCGTGGCTTGGCCTTCACACCGATGCTGCAATAGA
AGGGCTGGTGAAAAGA
GGGCGCAAAAATCTGCTCTTAGTGCCCATCGCATTCACGAGTGAT
CATATTGAGACTCTT
TATGAGATGGATATCGAGTATATGGGTCAATTGGGCGATAAGGTT
GGTGCTAAGATGATT

CGACGAGCTGCTGCTTTCAATGATAATCCAACATTCACAAAGACT
CAAGCAGAAATTGTT
CGAGATCATTTGGCGGCCAAGACCTGTCACAGTCGCCAGTTCGAG
CTGCGATGCCCGATG
TGTGTCAATCCGATGTGCGACTTGACAAGAAAGTACTTCGAGAAG
CATCGACAGGCGCTT
CGCGGCAATTGAAATAACGTGATCTTTTGATGCATATTTGATGTT
CTATTTTAACACATA
TTTTTATATATTTGATGATGATTCTAATGATGTTATACGCAATTGG
TATATTAGAATATT
TGGGAGAGGTAGTGTTGTGCCATAAATTCTGATAAGTTAACTTCG
ATCTGACGTTGTATT
TGACCAAAGCTATTGATAATTAACTTTGAAATCATTTAATTTTGTG
CATTTTATTGATAA
ATC
>ConsensusfromContig2156 Average coverage: 30.87
CCGTGTTTTCTAGCCGGTGATCATAGGGCTAACGAGCAATTAGGA
TTGTTATCTATGCAT
ACTTTATGGATGAGGGAACATAATCGAATAGCAGACATAATTGG
AGTGATTAATAGCCAT
TGGTCTTCTGACAAAGTGTATTATGAAACTAGAAAAATAGTCGGT
GCTGTACAGCAACAC
ATCACATATACGCAATGGCTTCCGGAGATACTCGGCAGTGTCGG
AGTGAAAAGTTTAGAG
AAATATACGGATATAAATCAGATGTTGATCCAACGATTCTTAAC
GAGTTTGCTGCAGCC
GCTTTCAGGTTTGGGCATACACTGATACACCCTATTTTATACAGA
CTTAATGAAACATAT
GGCACAACAAAAAAAGGTAATCTACCATTGCATAAAGCATTTTTT
GCTCCGTTCAGATTG
ATAGAAGAAGGCGG
>ConsensusfromContig2157 Average coverage: 10.15
TAAAAAACGTCTGTCGATCCCTACCTCGAGCCGGACAAGTTAGC
GTGGACGAACAGGTGA
TATCCTTCTCGGGGCGTGCACCTGCTCGTCAATTCATCCCGAGGA
AGCCACATCCGACCG
GTTTGAAGAACTTCGTGTTGGCTGCACCCAATGGACTGGCTCTAG
ATTTTATCATTTATC
AGGGGGCAAATACCTTCACTCATATAAAAATGAACGGTAAGGCG
GTTGGGCAGGGGGTTG
GGGCTGTGCTCCATCTGATCAGGGATTTGGAATCAGGCACTAGAA
TTTATTGTGATAGGT

TCTTCACAACTCTCGCTCTGGCCAATCATCTTTTGAGCAGGGACA
TGTATTTGACGGGAA
CGATGAAATCT
>ConsensusfromContig2158 Average coverage: 49.18
GCAGAGTCGACATGAACTTGCAGGCGGCTGAGAGCGAAGTCAAG
GCGTCAGACAACTGTT
GCAGGAGCTCAACAGACATCTCTCTGACGAGGCGGCGACAGAGG
CGTTCCAGGCAGTCGA
ACAGCTGGTGGGCGTCGCGGCTGCACATCCTCCTGACAAAGTCCT
GATGGCGCCCCACGA
CGTCGGCCGACAACTCGCAGCCAGGCGTAGAATCGTCGTTGGAGC
ATTCCTTGTAGATGG
ACTGGATCGAAAGTCGCAGTTCTTCCCAGCCTTGTTGCAGCACAA
CCGTCTCTTCCTCAT
TGGTCTTCTCCAATTCACGTCTTTCCAGGCAGGCTTCACAGTCGC
AGCGTCCTCCATCCG
CGGCGAGAGGCTCGAGGGCCGGCGCCATGTTCGGGAACAGAAGA
GGCTCGTCGGGATCAT
CCGTGCTCAGAATGGATTCCAACTTATCCACTTCTGATTCAGCCT
CCAAGCTGATGATTC
CATGACATGTGCCACACACAAAGACGGTTACCGGGATTTCATTAT
CAATCTCAACAATTT
GACTGACATCATCATCGTCTTCATGATCAACAGTATATGTTTTAA
ATTTATGATGTTCAC
AAAGAAGACATTTTGCAGTCAA
>ConsensusfromContig2159 Average coverage: 11.77
TTCAATCGTGTGCTATTCCAATCTCGTCAATATAACATTATCGGT
GAATAACAAACACAA
ATTAATAGAAACGGACATATATATTTTGATATCTTCAATATATAA
ATAGAATATTTGCTC
GAACAGATTGTTCAAAAAATTAAATTTGTTCCGGATAACACGACA
CTCTTGGTGAATAAC
AAATCAAACCAATAAACAATATATGCCAATGTCTCGAATATAACA
ATTATAATTGGTCTC
AATATAATGTTTAACATCAAAATTTCATTCAGTGTATACCCGAAC
ATTCTTGGTGAATGA
AAGAGCAATATTAATT
>ConsensusfromContig2160 Average coverage: 42.01
ACGCTCTTCCGATCTCAGTTATCAAAATTTGAAGATGAATGAAGG
CGTTGTATCAGAAGA
ACTCGATGAAATAAGCGAAAGACATCATTATAAGAAAATTTACAA
TGCATTTATGCTTTA

TAAAAAATATTCTTGTGCCTTGCTTGACAAAAAAGAGAAGGACT
TCAAGAAATTAACAAG
ATTTCACAGAGAACTCATTCCTGCTTACAAATCCAGACTGGATAA
GATTTGTGACCGCAT
TTCAATCAATTATGAGGTGATTCGTATGATAGCAGCTTCCTCTAC
AGACATGTTTGTCAA
TGAACAGCAAGATTCAATAGATCTGGAGGCACTAGAGTCGTCAGA
ATGGTCAAGTTTTTA
CATGAGCAAAGTGTTGACGACTCTCCGGCAGTTTGTTCGCGACT
GGAGCCAAGATGGACG
ATGCGAACGGGAGGAATGTTACGGGCCCATTTTGAAAGAACTGGA
ACATTTATTTCCTGA
ACGATCTACAAATCGGTCCGAATTGACGGTTCTTGTTCCCGGGGC
CGGCTTGGGTCGCCT
GGCCTTCGAAATAGCATCCATGGGTTTCACATGTCAAGGAAACGA
GTTCAGTCTCTTCAT
GCTCATTGCCTCCAATTTCATTCTCAATAAATCAATCAAAGAATC
TGCTTACATCATCCA
TCCATGGATTCATCAATACTCGAATGTGATGGACGAAGAGAGCCA
ATTGAAGGCTGTGTC
TATTCCTGATATTGAGCCGTCGCTTCTAAAAGAATCTTCCAATTT
TTCGATGGCTGCGGG
CGATTTTCTTGAGGTATATACCGATCCAAACTGTTGGAACTGCAT
CGTGACGTCATATTT
CATCGACACAGCCAAAAACGTCATGAGCTATATCGAAAAGATAC
ACGAAATTCTCGTTCC
TGGAGGCTATTGGATTAATATGGGCCCTCTACTGTATCACTTTGC
GGACGATCCCGATGA
AACATCAATCGAGCTCACGTATCAAGAGATACGTGACGTCATTGA
ACAGTTCGGATTTAA
ATTTCTGAAGGAAAAAACGAATCTTCGCTCTACGTACATACAAGA
TGAGAACTGTATGCA
AAAAAACTTCTACGATTGTGTATTTTTTGTTGTTCAGAAAACCTAC
TCATGATTCAATCT
TATATCAAATATTATTTACATCTATAGAAAACAATGAGAGGATAG
CATTTCAATCAGA
>ConsensusfromContig2161 Average coverage: 18.56
TGGAGTTCGAGAATCCGAATTGTGGGGAGCGGTATACCTTCCTGA
AAAAGCATTTCGACG
CCCTGACAAAAGGCGAAGACAATTTGTTGAATGGTCACGCTTTTC
TGCAGTATCTTGAAA
ATGACCGATCAATTTCCAAAGCTGCTGAGGCAATTACAAATAGA

ACAGGAGTGAATGTGA
GGGCTGAAGATCTTGATGTCATAGATACATTGGAGAAATGCGAA
TCCACAATCAAAACAG
ACTTATTCGAAGTTGTTTGTTATGAAAAGTGTTCTCTTTTCCTC
GAAACATGATGAAAG
TGGAAGAGTCCGACTCGGATGATTCAGATAGCT
>ConsensusfromContig2162 Average coverage: 17.23
TCTGAGTGGTAATCAAAATCGACTATTTTTTTGCGAAGAAAATTTG
TATTTTTTCCCATT
TATTGATTCGAATTGTATTGAATATAATATACCCACTTCGTTTGGA
GAATGTCTCATTTC
ATCTTCACTGAATGCCAGAACTTTAGTGTACCAATTTAAAATAAG
CCCGCGATTATTTGG
CTGTCTATCGTTAAGATGATAGGTTTGTATACCGCTGATATTAAA
AAAAAGAATTATTTT
AAGCATCCTGGGATGACTAAGATCAAGATATTGAGTATTCTGGCA
TATATGATCGGATTA
CGATGATTAATTTAGAGCTGGTTGAATTCAATATTTATATTTCGAT
ATAAACTCTTGTTT
ACC
>ConsensusfromContig2163 Average coverage: 5.73
CCCTCTGCTTCACCCTCGGCAGCCCCCTCAGCTTCGCCCTCTCCAG
TGCCATGTGCCTCA
CCCTCACCCTCTGCCTCGCCTTCAGCAGTTCCCTCTGCTTCACCCT
CGGCAGCCCCCTCA
GCTTCGCCCTCTCCAGTGCCATGTGCCTCACCC
>ConsensusfromContig2164 Average coverage: 36.31
AAAAAATGTAAACAAACAAATTGGATCATATGATAAATTAATTTT
CACTAATAAATATAG
ATCAAATGATAAACATCATAATCTGTCCTGTTTGTTAGGAATTCA
ATAAAATGCCTGACG
AAGGAATTAATCAAAATGATGCATCATACTCAACCGTGGTTGGTT
TCATTTATGTATTCA
ACCTGATTGTTGGAACTGGGGCATTGACTATGCCCAAAGCGTTCG
CTGCGGCTGGCTGGT
TGTTGAGTACGATTGCTATAATTGTTCTCTCCTTGATGAGTTACA
TGACAGCAACGTTCA
TCATTGAGGCTATGGCAATTTCGAATGCCGTTCGAAAATCACTGA
AACAGCAATTGGAGG
AATCAGAACAGGAAGAAGCTCGAATTGTGTCAAGTGAGGAATCG
AATGATGAACTGAAGC
CTCTGATCAAACGTACCGGTGTCTCCGTCTCGCGACGTTATATAA

TTCGCCAATACGATC
ATCTTGAAATAAAAGAACGTGTTGAAATGGGTCAAATGGCCTTCT
TATATTTCAACAAAA
TTGGGCTCAATTTATTCTACATCAGCATAATTTTGTATTTGTATGG
TGACCTATCTATAT
ACGCCGCCGCTGTGCCGGAGTCATTAAGGGATATTTCATGCACAT
ATCGCGTGCCACAAT
TGGCCAATGGAACCTGTAATGGAACACTTGAAGAGGGCGACCCC
TGCTGGCCGAGCTCTG
ACGTCACCCGGATGACGGCATATCGCATTTTCGTGATCATATTTGT
TTTTCTCCTCGGCC
CCTTCGTGTTTTTTGATGTTCAAAAAACAAAATATCTTCAGGTCGT
GACGACATTTCTTC
GCTGGATATCATTTCTAACGATGATTGTTTTGGCAATCAAAGTGA
TTGCTGGAGGCCCGG
CGAAGACACCAGTGGCGGCCGATTTCTCCGGAATACCGACTCTTT
TCGGTGTTTGTGTGT
ATTCGTTCATGTGCCACCATTCAATTCCTTCAATGATCACACCCA
TTCGGAACAAGCAGC
GTTTGCGTGGAATGTTCAGCGCAGATTATTTGCTCATTCTAGTCTT
CTATCTTGTTCTAT
CTCTGACGGCAGTATTTGCCTTTGACAAACTCCAAAATTTATACAC
ACTGAATTTTTTTC
AGCCAGACACATGTGATCCCGATATTGATTCACCAACCGATATCG
CATTTATTCAGTATT
TCCTTGCATTATTTCCTGTATTCACTCTTAGCACCAGTTTTCCAAT
TATCGGGGGAACGT
TACGTAATAATTTGAAAACGTTATGTCTTTCTGATAATAAAAGATA
TTCTTTTTTCGTAG
ATAAATTACTGTTTCCTATTCTCACCTTGATGGTACCTCTTGCCAT
AGCCTTATCAACAA
ACGAGTTAGATATGCTCGTCGGAATAACTGGGTCATATGCTGGCG
CTATTATTCAATATA
TTGTACCAGCTGCACTCGTTTATTTCGCTAGGAAAAATATGATAA
GGAATTTCGGAATTG
GATTTAAATTCAAATATAGTTCCCCATTCAGTCATAACGCTTGGG
TCTTTGTGGCGCTCA
TATGGGCTGTAATCTCGATAATATTTGTAACAGTTAATCATATTA
TTACAAAGAAGTAAT
ATTCACTTAGATTTCAGTATTGGTATTCAATCGCTACATTCGCA
>ConsensusfromContig2165 Average coverage: 27.70
CTCAAATCCTCTGGTCTTTCTGACGAAATGATTAAAGTTTTATTGT

CTACTTTGGATGCC
CAGCGAAATCCCACTAGTCCAGAAAATTCACCTCCAAAACCCGTT
TCTAATGCGGCCATG
AAACAAACGGGAGACATTTCACTCGAAGATGATCCAAACACCAA
GAATCAACAGCGACTC
AGGGCAGATCAGGAAGCTGCTTTCGTCAGCTGTGTGCCCGAAAAA
CAGCTTCCTGTAGAT
GGCTCCAAAAAGGCGGTTCATTGGGACGATGTGAACACGAAAAG
GTATTCGGACAGTTTT
GCTGAATGCAGTACAATGCGAGAACAATCGGATGAAGATGAATC
GGATGATGATGAAGAA
GAAGTCTGTTGCGAAGATGAATCAAGCGGAAGCGATTGTAGTGA
TTGCGAATCTCCCAAG
GCAAACCCAACCGAAATTGACTCGCAGACCTCCTTGCCGCAGAAT
CCATCTTTGGACGAC
ACCCCCGTCGCTGGACAACACAAAACTTTTGAGCAGTTACTGGA
AGCAGAACTTATGAAG
TCATCAGAAATTGAACAGCATTCAATACATAATGCTACAACTCCT
ACCCTCAAGGCAAAC
TCCGCCCATTCTGATGACAAGGCGACACCCCCCAAAAGAACGTT
TCTTCGGCGAGGGGAA
GGCATCTCGCGGTTCTCAAAACCCCCGACAAGAAAGGCTGTGAAC
TCTTCCAAACATACT
TCTTCAAGTGCATCAAGCACAACCTCAGCCGTAACCTCAAGACCC
TCAGTCGCTCCCAAA
AAACCTGCGACCAACACGAAATCCACGCTCCGCACTTTGAGTAA
GAAGACATCTGTGAGT
GAACCAAAAAGAAATACTATTCAAATTGAATCGAAATTCAAAGA
ATCGAATGATGATAAA
AACAATAAAAACAAAAATATAAACAAAGATGTTGCCTCGAAACC
TCCTACCCCCTCGAAC
AAATCACACCAGCACAAATCTTTCAAGAAAATGAGTGCATTGGA
ATTGCGGAGAAAGATT
AGAGGAATGTCTAGGAATGATATCGATGAAGATAAAGAGAACTC
AGCGAAATCGGAAGCA
GATTCCATGGCCGAATCTCTCGATAGATTG
>ConsensusfromContig2166 Average coverage: 27.10
AGATGAGGAGAAACGACGCGTTATGCTGGGCAAACGGTCTTCTT
CATATGGAGGTAAACG
TCGCATAATGCTTGGAAAAAAAGAAAACAACAGTCTGGAAAAAA
GACGAATCATGCTGGG
GAAAAAGTCAGAGGATAATGCTGAGAAACGTCGTATTATGCTCG

GCAAAAAGTCATATGG
GGAAAACTATGAAATTGAAGAGAATAGCGACAAAAGAAGAGTC
ATGCTCGGAAAAAGAAG
AGTTATGCTGGGCAAAAGAAACATGCAAGAAGACGGACAGACGT
ATTTGAGAGACTTTGG
TGATGAGGACATAGAAAAGAGGAGAATAATGCTCGGAAAAAGA
GATGCCCCCAAGAGGAG
ATTCATGCTGGGCAAGCGTAGAGTTATGTTGGGAAAAAGAAGAA
TAATGCTGGGAAAGAA
AAATGTCGAAGGAGACGATGACGAAGATGGCTTTGAACGAGAAT
CCAGGCGAATCATGCT
GGGGAAAAAGAAGATAATGCTCGGAAAAAAACCTTTCGAAATAG
TTTGATGGTCTAATCG
GCTATAAGCAATGTTGTCATGTTGTATAAATTATTGCATTCAAAA
TACAAGTAGTTGCCA
TTGATATTTCATATATATTCATGAAATAAAC
>ConsensusfromContig2167 Average coverage: 54.59
CTCGTATTCATTTTATATATAACTGTTATCAATTTTTGCAGTGAA
TATCAGAAAATAGCA
GTTAATTATATAATTTAGAATAAATATTATGATTATTTAGAACGA
CCGATACGGCCTGGT
CGTTTTGGACCGATATAATTTATAAGCAAGAAAACACAAGGCAG
AGGTTCCAAGAGCAAA
GACAATTCCACCAAAAAAGGAGAGTCCGTCATAATGGCGCCCAT
GCTTAGGGCATGGGTC
AACAGTAGGCGTGGTTTGCTCTGTGAATGGTTCCGGTGTCCCCGC
CCCATTCGCAGACGA
GGAGCTTATCGAAAATTCGGTGGTGGAGTTTTCTGGAGATACAAA
AATGGTTGTTGTCGA
AATCCCCGTGGTGAAGTTGTCTGCGGAACTCGTCTCTGCTTTTATG
TCACTAGTTTTAGC
AGATTCTGTTGACAATGATGAAGTCGTCTTGTTTGGTAGTCCATC
TGTTGTGAAATCTGT
TGAAATAGCAGTGAGGGTACTTGTACTTATGGTCCATGTTGTATTT
TCTCCATTGCATTT
CTCTACAAACAAAAATATAAAAACAAAAATGATATATCTCATCAT
TGTTGAATGGGATTT
GAATTCCTGATATCAGAATAAATTTTCACGGTATGTCACGAGATA
TGCGAGGGAGGGCGT
GTGTCAGATAAATTTGTACACGACCGGAGATAATACTAACTGCA
AAAGATCGGAAGAGCA
CA

>ConsensusfromContig2168 Average coverage: 33.62
TCGTCGTATGATTAGAAGAGTTCGCTGCACTAAAATTTGAGCAAG
GATCACAGAACGATT
CGCGACGGAACATGTCCACACAATCTGTATTGTTTCTTATACCTA
CAATGCCAAGCATTG
GAATGGCCTCGGCCAAACAAGCCACAGGAGAAATCGGCTGAACA
GGAATGTTCATGGCAG
ATCTATTTGGAGATCCTGTTGCATTTCTCAAAGGTAATGATGCTA
AATGCAGAGGATAAA
GTGTGAAAAGAGCATTTGCAATAAAAATCACATAACATACACAA
CACATGTATTGGGTAA
TGTAGACCGTCACATCGATAAGACTCTTGCACCAAGGCCCCAATG
TCGTATTTCCCAGCT
CGCCATAACCGAGCGACTTCTGTAGCTCTTCACGCCTCTCTGGTA
TTTCGCTTTCAGACA
AAGTTCCAGCCCTCTTCTCTTTCTCTATAAAATCTTCAACAAGCTC
ATTCTTGCAGCGCA
AAAGTAGTAAGATGCAGTAGGCCGTTAAAGTTCCCAATATGAAA
ACGCCTATGATTCCAA
GCACAAATCCACTTTGACTGAATGCATATGGCAGGGCGAGGTAA
TTTGAACCAATAAAAG
CCTTTAATGTATTAAAAAAGTCGCCAGCGACTGTGTACCATTTCA
CTTTTGGTTGAACCA
AGCTGCCCCCAAGAAGAGGCGATGATTCGGTGGACATCTAATTT
GACTTATGGCACAAAA
TCAACATATTTTTCAGAGAATGAAAGAGAAAGTGATACCGATCAA
CAAAAAGTTTTGTTT
GTTTATGTGATTAGGGCAATAACC
>ConsensusfromContig2169 Average coverage: 25.65
ATTTTTTTAAATACTCAAATTGATGCAATTCAAGCGATCTCTTCT
GAAGGCAGTATTCGG
TTGCGGTATCGTTTGCCTGGTTTTGTACCTGGCGTGGTTTTATTTT
CCCATTTTGTTGAA
TAACGCAAACTGGTTATCGGGTCAGATAGATCTTTATCGAAACAA
GCATTATTTGTACGA
ATGTGATAATTGCAATGAGTTCAATTTCGAATATTTAGAAACGAA
TCCAAATCTGTGTGG
GAACCATTCTATATTTTTACTGACTATCGTTTTGTCATTTCACGCT
AATGTGAGGTCAAG
AAATGCAATTCGTAAAACTTGGGGATCTTTCACAAACTATAGAG
GAATAAAATTGAGAAC
TCTATTCGTATTTGGTGTCAACAAAGACGAGAATCTTAATAAACA

AATATATTTCGAGAA
GAATAATTATCAGGATGTCATGCAAGTTGATCTCATTGAGTCATA
CCGGAATTTGACGCA
CAAAGCGGTATCTTCTATGATTTGGGCCACAAGATTCTGTTCCCA
AGCAAGATACGTATT
CAAAACCGACGAAGACAGCTTCAACAATCCCTATCGCCTTGTGGA
TTATTTGCTGGGAGA
AAAAGATCCATTATATGTTGGTGGCCATTGTTTCACTGTTCCTCC
TGACCACGATAAAGG
ATCAAAGTTTTACGTTTCTCTTGAAGACTTTCCTCATGCCATGTAC
CCCATCTATTGTGC
TGGTCCTGGATATGTCCTGAGCATGCCTGCCGTATTCAAAATCCT
GGGTGTTGTGAATAG
CACAAAATATATGCCGATGGAGGATGTTTTCATTACTGGGTTGT
GCAGAGAAGTGGCAGG
CCTGTCATTTTCGAAGATTCCGGGTATCATCATATCAGGCAAACA
ACTCAATAAATGTGA
TCTGACCTTCTGGGCAAAAACGTCACATCACGTTGGCGCCGAGTC
AATGACTCGCATATG
GGAATCATATATAAGTGAGAATCTTCTAATGTCATGTTTTTCGCA
TAGGACATACTTGTG
TTTGGCAATTCTATGTTTTTCATTTTCATGGATATATATTCTATTCG
ACGTTTT
>ConsensusfromContig2170 Average coverage: 45.88
CGCCGCCTTCCCGTATCCGTACTTCAGCCCATTCCCCAATCAATT
GGATCCTGCTGCGGC
CCATAACAGATACGCTGAGTCCAACTATTTTATAGAAGGAGGTC
CGCCTGAAAAGGACGT
TGAATTTCAGCCACATTTCCAGCAAAGGTCAACAAGGATGAATGA
TGCGGCTGCTGGGCT
GATAATGACTGAGAACCCCACTAGAGACGAACATACTGAATATG
ACACTCGACCCGATTT
CGTACCAGGTGGAACTTTGACCCAAAATTCCACCAAGGATCTTG
ATCAAGAGACTAACGA
AATCACTATTGTCAGTAAACATACTCACTTGTATAAATCAGTAGA
GAATGAGCCCCGAGC
ACAGCATGTGGAGGAGGTGAAACGAATAGAAAATGGATTTCAGC
AGTACGACGAGGAATT
TATGCGAACAGACGG
>ConsensusfromContig2171 Average coverage: 17.35
GTTCAGACGTGTGCTCTTCCGATCTGTGACGACCTCAACTCTCAA
TCAACTATCTTCAAT

GGAAACAACTTCCCAGAATAGAACTCCTAGACAAGTGAAAGACA
CAAAAGCATCACTAAT
CTAATAATGTTACCTATTGATACACTTGAGTCGAGAAGGTCATAA
AGTCACACTAGCCAA
AATTTGTTTTTCAAATATTCTAAACCTCAAGGCCATAATATTCAG
ATGAATTCCAACTGT
GAAGATTATCAAACATTAATGTGGGGTATGTATTGGCTCATCTCT
CAATTGTAAATTCGC
ATGATGATG
>ConsensusfromContig2172 Average coverage: 14.66
TTCATCAGACATTCTTTCCGCTATTGCCTCGCCTTTGTACCTTGC
AGGGCGGGCCAAACT
TCGGTTTATGTCGCGGGAGAAGTAGTCCAGCACCCAGAACCCTTC
ATCTATCACAGATCC
TATACCCTCATAATATTCCGGGTCCGCAGCCTTCACGTAGGGAAC
CGCTAGAAAGCTGAT
GTTGTTTGCCTTCTCCCGTATTTCATACAGCTTTGAAATTATTCCC
GGAGATAGTTTGTA
GCGCTCACTTCCCAGCGCTATGTTGAAGAGCAGTATTCCCATTTG
ACCTTCTGTTATCCG
TGTTCCAATAATCGCGTCAAGGTTTATAAATTGATAATTATCGAG
ATAGTAATCAAGAGC
TTTGTCTATCGAGTCGATCGCCGTGTTGAAAAGGTGATTATTGAA
CGTTCCATAG
>ConsensusfromContig2173 Average coverage: 7.71
CTCTTCCGATCTGTCGTTCATCCGATCCTCAAAAAGGAATCATTA
GACAAGAACATAATG
TCCAATTATAGACCAGTCTCTAATCTAGAATTCTTGTCTAAAATA
CTTGAACGCGTGATT
TATGATCAGCTATTAAATTTCTTGAATGCATCCAAAATTATTCCA
CAATTCCAATCTGCA
TACCGAAAACATCACTCGACAGAAACTGCTCTGCTCAAGATCAA
TAATGACTTGATAGAA
AGATATGCTCGTGGAGGCAGCACTGCTCTCATTCTCCTTGATCGC
TCTGCTGCTTTTGAC
ACTATTAAGCATGATTTACCTTTTTATGATCTT
>ConsensusfromContig2174 Average coverage: 48.39
GGAGAAATCACGTCTATAGCCACTATACAGGAGTTTCTCACCGA
GGAAACATCAGATAAT
ATGTATTTTGCATTGAGTATTGAGGCGATAGATCGAGGACGACCT
GCCTTATCGAGCCGA
ACGGAAATCGTTATACGAGTTGCCGCCCTGAATGTGTATGAACCG

ACGTTCTCACAACCG
GTATACATCGGAGCAGTACCCGATAGCGCGGAAGTTCAATACTTT
GTATTAACGGTTCAT
GGATTCGATGCGGATATTGAGAAGAGTGATGCAGCAATAAGTTAT
TCGATAATTTCCGGG
AATGGCACATCCTTGTTTGACATTGATAAAAGTACAGGTTCAGTA
ACTGTAGGTACCTCG
TTAGTGGGTCATACAGGCCGGACTTATCACATGGAGGTTGAGGCT
CGCGATTCTGGTCTT
CCGTCACGCGCCTCGTCCGTACTGGTCAAGGTCAC
>ConsensusfromContig2175 Average coverage: 15.18
AGCATTTTGATTTTCGAAATTTATTCTCCTGATCTTCAAGCTTTTC
AATCAGAAATCAAT
CGTTATGGAGAATCGTTCTTCTCTATTCTTTCTTATAATCACCATT
TTAGTGGCATCAAC
AACGGGGCCGCCGCCTTCGCTTCCACCTCTACCTGAATCTCCACC
TGAGAGCTACGAAGG
ACCTGAAATCGAATGTGCATGTACAACAGATAACTCACCAGACT
GTGAGGAAAACGGGAG
AAGAGGGTGTGAATCAGGAAAGTGTAAGACAGTGAAAGGCACTT
GCTTTGATCAAGTTGG
AGAAGAGTTTGCATATCTATGGAAAGGGTGCTCTGAAGAAAAGG
TTAAGTGCTCGGATGA
GTATCAAAAATATGAGAATTGCATCCTCAATTTCTGCGTCTGTAG
AGGAGAACTCTGTAA
TGAAGATTATGCGGTGAGACTTTCTCGTCGTACTCGCATAGGCCA
GTGGGGTTGGCGTTA
CGGAAACTAGGGTTGTATGAAAGTTTCGAGGGAATGGAATGCATA
TAAATGTTTATGGCT
TCATTGAGTTTCCAATGACCAGTCATATGTTTGAGTTATTAGAAA
CAATAATATACAATT
TTCTCTATT
>ConsensusfromContig2176 Average coverage: 18.54
GCAAACAATGATAAAATATATAAATAATCATTTGTTTATATGAGC
AATTTTACATGTAAA
CAAAATCATGTGAACGTCTGCTCGCCAGGTTTCCGTTTCAACGCG
TCCTTTATCATGAAT
CTCATTCCTAGGAATCTCAATATACAACAATAATCACAATCGATG
GACTTTGACCACTTT
TTCTCCAGAGTGCGCAATCGTTCGCCGATCCATTTTGTGTCACCTT
CTAGCCTCACTTTT
TCTGCCATCAATGACTTGTATTCCGTCCAGAGCTTTCCCTGGCCC

ATCTTCGAATACAGC
TTCTGCATGCTTCTATTCTCAGGAACAGTAAAAGTGTGGCAGATC
TGAGCTTGCCCCCGC
AGCGTTTGTCGAAATGATTCTACTGCCCCTGCTATCATTATCTTG
TAAGCCGGGTACCAC
AGACAAAACCACATGTGCTCAGAAACTTCGAAAAGATGCTCCAG
GAGACCTCGGATGGAA
TGCAGCTGCATCCTCAGACGCTGTATGCTCTCCATCTCGTGTAGC
ATTGCATCAATCCGT
CGCAACAGTGCGCTCCGACCGAGCTTCCTTCCCGAATGAATCAGA
TCTTGGAGTTTGGCT
TTCGTCTTTTCGGTGTTCCGCAAAATTTCTTCGTTCCGCTTGGCTT
CATTTTCCCTAAAA
AAATTGCTTAAGTCCTCAAACTTCATTTTGTAATATTCCTTTTTGT
TATTGTTTATATTT
GTT
>ConsensusfromContig2177 Average coverage: 6.99
GGCAAATATTTCGATTTTTTTCAACGATACTTGAAGAATTTGGAA
AACTCTTTCATCACT
CTTTCCGATTTTTTGGTCACTTATATTTTAACAAATATCATTCGAT
TTGAAGATAATCAA
CTTTGGTGTCAGACCAAAGATGACCAGTAATGATCATTTTCGTTG
TTCGATATATGTTAT
ATAAGAACAGAATCCACGAGAGACGATTCTTTATCACAAAATTCG
CTAGGTAACATTTGC
CCCCACATACAACAATGGCAGCAGTAAGGCTCATGTCCCTGCGAT
ATGATATGATGCATC
ATCTTTTTCAGCTTTCGAATCTTGGTTTCCAGGAATGATGGATAGA
TCAATTTTTCAATG
AGAAATTTTACATACTCATATTCAATATAGTCATATTCGGTTTGAT
TCAAGCTTTCACTG
TGCGCAGTATCTGATCATTCAGTGAAATTTAGTGCTAAAATCGTT
CCCAGAATAAAAACA
TGATTTTGTTCTCAATAATTCCAATTTTAAGTGAACATTTTTCCAT
TCGATTTTGAAAGA
AATGAAATTTTCACCACAAAGCACCATACAATACCAA
>ConsensusfromContig2178 Average coverage: 8.73
GCGGGGTCAACTGCTAGTAATAAATAATTGTGATCGATTACGAGC
AACTGAGGCCCTTTG
TTGTTAATTGGTGTTGTTTCCTGAATGACTCGGCCTTTATTATATG
TGTGTCCTTGGTGA
CATATTGTTTGGCAGCATGTGTTTGCGCGATACCATCTCTGATAG

CATGAGTGCAGTTCT
GGCTTCTTTGAATAATACTACTAGATGAGACGATTATTATCCATGT
TTATTTTGTATATA
TGAAGCTTGTCCTCTTGGTGATGTTGAATGTTGCCGTACATGT
>ConsensusfromContig2179 Average coverage: 17.03
TCAGTCTGAAGCATGGTGAAAGGTCAGTAGCAATCCCGTAGGCT
ACATGAGCTCGAATGT
GTTCTCTGTTTTTGTGTTACACAGTCTAAACTCAGATAAAACATC
ATCACACGATCACAT
TACATAACCATATGTAAAATCGACCATTTAATTTCGATAAAACAG
GACATTATCGTTATT
ATGACATAAGCTTATCATGAAATCTCTCCTTTTCGAAGTGTTGAA
CTAAGATCATTTTTG
TAGATTCTCATATGCCATCAAAATGATGGAGGGGCTCGTGAAGA
AAAGCGCTGGACGACC
AACAGTCATCATGTATGACATTGGATGCGCGGTCGATACCCACCT
AAATAATAATCCCCC
GCATTTCTCAA
>ConsensusfromContig2180 Average coverage: 35.55
TCGACTGGATGCGGAAGCTGGCGTTCAGGTACCGCCGCATCAAGG
AGCTCTACAGCTCAT
ACCGCAGTAACATTGGAGCATTACTTGGACCTCAAAAGCAAGAA
ATGTGGATTCAATTGA
GATCTGAGATCGAAGCTTTCACAGACTCATGGCACACTCTAGCTA
TCAAATCATTAGAAG
CCATCACTTCAAGGTCAACATGCGTCAATGTCCTAGTCACAACTA
CGCAACTGGTGCCCG
CCCTCTCCAAAGTACTTCTCTACGGACTAGGGGGCGTATTCAACA
TCGAAAATATATATA
GTGCTACGAAGATAGGCAAGGAAAGTTGCTTCGAACGAATTGTT
TCAAGATTCGGGAAAG
GCTGCACCTACGTCGTCGTTGGTGATGGTCGCGATGAGGAACTAG
CTTCGAAACAGATGA
ATTGGCCATTTTGGCGAATAACAAATCATTCTGACTTTATGGCAC
TACACCATGCCCTTG
AACTTGATTACCTGTGATGTC
>ConsensusfromContig2181 Average coverage: 49.55
GTTTTTGTCAAAAATGTATTCCGATTATTACCATCGAATAAAAGC
ATGGTTCAGTTCATG
TTATCTTATATATTTGTGCAATACCAATTTGTACATGCTCGAAAAT
TGGGAAATTTGTCT
TTTTAATACTTCACTTCTCAGTATAGTGCTTCTCACTGGATATGCA

GCTTATATGTTCTT
GCCTGGTTTTGCCTACAAGGTTGCAGTATTTTTTGGTCAGCATGC
AACAGATTATGTATC
CTCATCTAACAGTATACCATCCGGAGAATTGTGATATTGC
>ConsensusfromContig2182 Average coverage: 8.03
CACGCTATTGCAGGTGTAGTCATACAGGTGAACACATATTTCAGC
ACAAGAGTTCAAAGT
GATATCCTTGAATGTTGCCAGCTTCGTTTCCATGATATCCGTCCAT
CCCAATCCTTCGTA
GTAGGTATCGTATTCATACCACAACTCGAAGTTAAAATCGCTGTG
TTCATATTCTTCGTT
AAGAAAATGCTTAATAATGCGGTCATAATGTCCCTGGCGGAAGGC
TTCTGTAGATCTGAA
AAAGTTTTGACTGCAAATGCTGACAGCAGGAAATGTTAGATTGG
AGGTGTACTGAACATC
GACGCTGACAGAACGCGGGTAGGAGTAGTATAGTACCACTTGGA
ATGAGACGAACAATAT
GAAGCCCACCAATGCCGTCAAGCATGCACATATCCATATTATTCG
TCTTCCAGGAATCTT
TCCATCTTCGGTTATGTGTTTGATTCCATGCAAAGTAGTCTCTTG
TGAAAAATCGTAGGC
TAATTTCCTCCATGTCTCTGCTTGCAGATTCTCCTCCTGGACTACC
TTCTCAACAAATTC
CCTGTCG
>ConsensusfromContig2183 Average coverage: 14.82
CTGGATGATGATGATGCTGAAGGTCTTTTCAGTGTCAAGGATAAA
TCCCCATCGGTGATG
TTGAGCGCACTCATATCCGATAGAGATTTCCGTTTTGAGACATTC
AGTGATAGCGCTTGT
TTTAGAGATCCCCACTTATTGATACCGTCTTCGATGACTGTGGGT
TCGGGCTCGAATGGC
GTTTGAGACTGATAGTCCATCTCTTTGTATTCGGCTGATCGACAG
TATGTCAGGATTTCA
GTGAGCTTTGTGGAGGTCGAGGAGTCCACATCTTTGGAAATGAAG
TCATCGAGTGCCTTG
ATAAGTTTTTCGTTGGGTTCAAAATCCAGGCGATAAAACCCGCG
AAGCCAAGAGTGAACG
AGATCGATGACTCGCGTCTTAACTTTGACGGGAATTTCGGCGTCG
GGGGAAAGGCTAGAT
TTGGCCGCTCGGTACTTGCTCGTTACGAACTCGAAGACATCCCGA
GGAGTGCTGAAGTAA
CGGAAGCAGTAGAGGAACTCGTGGACAAAACCGTCCAGTAGAGC

ATACCTAGCGAACTGA
TGAGACATAAGACCCTGGGCTGTTCCCGCTTGCAGAATATTCTGT
GTTGCATCAAGTTGA
CTGGCTTTCACAGCTTGGTATTGCAGA
>ConsensusfromContig2184 Average coverage: 8.76
CGAGAATCGACAATTTGGTCAACAAAGATGTTGTTCCTCAAAACG
CTGTCAAGACTTATG
ATGATTGGGCTGGAACATTCGAAAAAGACCTGGTTGGGCCTTCA
GGAACTAAGGAAACGG
CAGATGAGGTTATGAGGGTTTTGGATGGAAAACTCGATGCAGAA
ATAATCAACTGCGGAG
CCGGCACTGGCATCCTTGGGGTGGAGTTAAACAGAAGAGGATTC
ACCAGCATCGATTACC
AGATCGGAAGAGCACACGTCTG
>ConsensusfromContig2185 Average coverage: 21.42
ATAATAGCATCTATCAGTTTATCCTTAGAATGAGATTCATGATGG
AAAACGATAATAAAA
GTGGAAAATACTCTTTTAGATACAAGATTATGGAGCTTGACTGCC
CGACGATGGCTACAA
ACTCGCGTCAGCATACAAGTTTCACCCTATACGATGTGTAATGTA
TATAAGTACCTAACA
GCAGAAGCACTATCAGAATCGAAATTTGAATGAATTATAAATAA
TATTCCGAAAAAATGA
ATACTCACAGAATTAAAAAACGATTTATCAATATATATACACAAG
TCATTAAATAAGTAG
ACACGAGCATCAAATCAATTTCATCCGTCTGATAATATCGATGAC
CAACAAGATCCAGAG
AATTATTCCGGATTCTACGGCCGCAAATATCCGCCGGGAAGCTGA
CAAAAACGGCCTCAT
CTCAATCTGAAACGACGCGCCAGCCTCGATCGATACTCGAACTGT
TGTTTGTGATTATAT
TATAACTAGTATAATAGCAGTTTCGTGTTGAAAACGTAACATTTC
AGTTAATATTCATTC
CGATTGTGATCGTATATATAACATATAATATATAGAAAATATTGC
ATGGCTATCTAGTCA
AATGGAAAGTGAATTTTTGCCATTTTCATGTCGTTAATTATTGTGC
CATTGTTGTTG
>ConsensusfromContig2186 Average coverage: 7.22
TTTCTTGTTTTGCTGGCCTTTCAAACTGAAAATCGTTTGCAAACTT
CTGCCGAGCTTCGT
CAGACTTGATTGTGATGCGATGGACACGATGCATACCGGCTTTGG
AGAGGCTTACGATAC

ATCGAAAGAGGCAGCTGAGGCCATGGACACAAATTTCGATGTTC
GCGTCAAATCTGAGCT
CGTGATCATCCCTCCTGAGGATCAACCGGGAATAGCAAAACTGA
GAG
>ConsensusfromContig2187 Average coverage: 5.37
AAGTTACGACTCTTGAGGAGCCTCCATACATGATGCAAATCAGT
ACCGAGGAACACCTCA
AGGGGAACAACCGCTTTGAGGGGTTCTGCGTGGATCTACTCGAG
GAGATTTCAAAGATAA
TCAATTTCAAATACGAGATAAATCTCGTGAAGGACGGGCAATAC
GGAGTGCAGAAAGAGA
ATGGAACATGGACAGGAATGATTGGAGACCTCGTGAGGAATGAG
GCAGACCTAGCTGTGG
C
>ConsensusfromContig2188 Average coverage: 9.79
ATTTTTATTGGCATCCGAAAATCCGTTGAGCAAGTTTTTCGTGAC
AGGATTGTTTTGCGA
ATGATCTGTTTGAGCGTTACCCGGTTGCCTTGGTGACGGCATTGG
AAAATTGTTCGAATG
TGGTCGAAGAGGAATGTCGCTAGGGGGCGCCTGATAAGTCGGAT
CTGAAAATCGCCGAGT
GGTGTACGGATCTCCGTAGGATGATTGGTTATTTGGTTCAGCCCC
GCCTCCAGGAACTGA
ACCAATAACAGATCCATATGAGTTTGAACGATCCGCATACGGATC
TATAAACGTGTTCTG
ATTGGTCGATTCCGCAACGTCTGCTTGAGGGGAACCAAAACCAG
ACAGAGATGGACTCAT
AAGAGATGATAAAAACCCGTTAGTGTTGTTCAGGAGAGGGTTTG
ATGAGAATCCGAATCC
AGGCTGAAATTGCTGTGAAGGATTTGTGATAATACCCGGATGAGA
TGGATTCGTTAAGTC
GGTGGAGAAGCTGGAATGTGGCATGCCAGCCTGTGAAGCACCTC
CGTACATGCCTGGATA
T
>ConsensusfromContig2189 Average coverage: 14.50
CTTGTACTGATGTTTCTTCAAAAACAGTGATAAAATGTTTAATTCA
GCTTTTTTCATTAT
TTGGGATGCCAGATTATATTCATTCAGATCGTGGATCGGGCTTTA
TGAGCAAGGAGCTGA
AAGAATTCTTAACAACAAAAGGTATTGCAGCCAGCCGAACTACA
CCATTCAATCCACGAG
GGAATGGACAGGCCGAACGATTCAACGGAACGATCTGGCGCGGT

ATATCTCTCGCGTTAA
AAACACGGCAACTTGCAGTGACTCGATGGCAAGACGTTCTTCCAG
ATGTGCTACATTCTT
TGAGATCAATGCTATGTACCGCGACAAACTGCACCCCCCATGAGA
GGCTATTCAACTATT
CGAGAAAGTCTACATCCGGCTCTACTCTTCCTCAATGGTTACTTG
AACCAGGAAAAGTGC
TGATGAAGAATCATTACA
>ConsensusfromContig2190 Average coverage: 8.39
TGATGAATGAAAATCGACGCAGTATGTTTTCCTCGTGAAGATCAA
TGACGGAGCAGTGTA
AATTGGACATGATCACTCCTGTTCTTCCTGTTATCCCAACAATTGT
TGCACTTATGAATG
TTATTTTGAGCGAAAGTTCCTTCTCTACCAGACTGAGTATCGCAT
AAAGGAGAAAGCCAA
ATATTTGACCCATACAACAAAACACCATTGGCGACTTTCGCCCCT
TCTTGTCACTCCAAA
TCCCACATAAACTAGTCACAAGAATTGCTGGTACGTTCATAAGAA
CCTGATTAACTGTGA
TGTAATACGCCGTCGTTTTTTCGATTTTTTCACTGGTTAATGTTGA
ATTCATTGTGGAAT
CATGCATTTGCGAGATTTCGATAACAGTTGTTTGTATATATGCCA
TAACGGCTGATGTAT
ATATATTCTCCGAGAATTTCAAGAGGAAATATATAATTTCCGGTA
CACCAATATTAATAA
CACCACAGTATCTATACATATGATACACCTTTTGATACACTTGAA
GTAGATGTTAAACCG
AACAAAAC
>ConsensusfromContig2191 Average coverage: 31.83
CATTCATTCGATGATTTTTTGAGAGGAAAATATAAGTGCGATGAT
CCTCAACTCAGAATC
GGGACTGATGAGCGATCTGAAGAAGGCCGTACGAGCGTTGTCTCT
TTCCTCATCCCACGT
CTCCAATGGAAGTTCTTGTCGAACCTTCAATATCACCTCGCTCAT
CAAAGCTCCACTTTC
ATGCTTGGACCAACGCGACACATTGCGATCGTCGACGCAGCAACT
TATACAGTGATAAAT
CAACCTTCCATCCGATCGGTATTTTCACTCAAGAAGCACTTGGCA
AAAAACATCCTCTTC
CAATTGATGTTCGACACAGCCTTGCTCACAACGATTCCACGCAAT
GAACTGTCACATATT
CCTCGACCTTCGAAACATAAACCCCCTTGGGAATCGAATGAGTTC

TATCTAATTCAAATG
ACCCCAAATAAAGATTTCATGAAGAACTTTATCGATAAAATGCCT
GATTTTCTTCAATTT
GCTCGGATGATCTGTCGTGATACCCGCATTCCTCTGATAGCTAAA
CTGGAGAGAATGACA
CCAGGAATTGGTCCTGAATTAATTGCTCTGAATTACACGATGGAG
ACAACAGTCGATGAT
ATAGATGCTGCAGAATTGCCCTTCATATTTGAGTTGATGAACACA
TTGAAGACTGATTCC
AAGAGCACTGAGAAGTATAGTACTTACATCAAGTCTGACGAATTT
TAGTATTCTGTATTA
TTTAACATATTACGTGTTCTGTTCAACTTTTACAGTACAATTATAG
GTGTAAATTTGATG
AATCCGATTAACCATTACTAAATTTAGC
>ConsensusfromContig2192 Average coverage: 7.34
GAGATGCGATACATTTTGATGCGGATATACTTGAGCAAAACCAGT
CGTGTGCAATAAACT
ACAATATCACGATCTTTAACACTAATACCAATGCTGATATGATAG
GACAAAACGAGTTAC
TTGAGAATGATTTATTCACGCTTATCAAAGGAGGTTTGTATATAT
CGCTGAGGGCAGAAA
ATGAGATCAAATTTAACAGGACCTTTAAACAGCAAAATTTGTCA
TGCAAATGGAACAATG
TTTCAGAAGTTGAAGCCCAAATGAACATAACGATTATTCCGGAGC
TCTCTGTAAGAGAAT
CATAAGAAANTACAAAGTTTCATTG
>ConsensusfromContig2193 Average coverage: 9.20
CAGCGTCAGTGGATGAACAGCTAACCGAAGAATTACTGACACCA
AATGAACGTTTACTGA
AACTATATGGCTTATCAGCATTTCCTGGCTGTAGCTCGTAGCGTC
GTGGGCTTTCTGGGG
GGTCGTAAAGTCCATGAAGAGATACATCAGGTCTTTGTAGCCGGT
TAAGTCTGAAGTCAT
ACGGCTGGTAATGATGATGATCTGGTCTCATGAAAGGTACATGGT
CAAAATGCTTAAAAC
TTCGTCTCTCGTTCAAATTATGTTTTCCATGGACATCTCCAAGAG
TCAAATCTAAGGAAC
CAACACTGGAAACAT
>ConsensusfromContig2194 Average coverage: 18.52
CCAGCTGTCTGTCCAAACTGATCACTGAGCGAATTCTACCGAAAT
CAAGTTTTGTTGATG
GCGTGCTTTACAGTAATTCTGATCAAGCTGCGTGGGTTTTAGGAT

ACAAGAACAGATGCT
TGGCAATCTATAGAGACTTTAGCGTTCTTCGAGATTGTTTTCCAA
GTGATCAAAGCTTGA
CAATGCGACAATTTCTATTCATGATGTCAGACTTGGGTCTGTGTA
ATGCGAATTTGAGTG
CGGCTGCAATTGTCAATATTTTCGCGGAAAACAACTGTAATATCA
TCACAGATGATGATT
GCTATAACCTTGAGTGGGAGTTGACATTTCTCGAGTTTTTTGAAA
CACTAATTTCTTGCG
CTTTGAAATCAATCTCCATTGAAGATTTGGCTAGAGGAGTTGATG
CTATTGAAACAATGA
CAGCAAACGGAATCTCGAGAACAGAGACATCCAATTCAGTCTTT
GAATCATCGAGTGCAT
TGGTGCAGCACAAGGCAGATTCCATCGAGAGCGGTAGTCGTCCG
GTAAGCTCAGGTCAAA
ACAGTCGGACATCCTTGAACTCAAAGCTCAAGGACGATACTACC
GGTGATGAGACTAAAG
GGGGCATTGAAGAGAATGATCTTATGTCTCCCACTGAGAAAATTG
ACCATTTGGAAGCAC
GTGATAATGACGTCACGCGATCACAACCCGGGACCCCTAAAAAG
GACCTCAGCGGACGCA
GCGCCACATCTGTTGTTTTTGCTCCTGAACCGATCCTCTTAGGGC
CAGAACAGATGAGCG
AGGAGGCCCTATGGCAATCCAGAGTGAAATTCTTTTT
>ConsensusfromContig2195 Average coverage: 36.77
AACAAAAAGTATTCTACATCAATTTCATTGCCGCAAGAGTGGTAT
GGAATAACCTAAACC
TCATAATCCTATAGAAGTGTCTATTCGAATAAAAATATTCCATGA
ATTTTACCTTTGCCA
TTCATTTCTCAATCATCTTCATTGAACCAATTCAGTAAAATTGTG
ATATCGATAAGGAAT
GAAGCTGAACCGTATAAAAAGAAAGTCTATAATACGATTCTTTTA
AGCACAAATTTATTA
GGGTTTGACATAGATATGGAATCAAACCATATTCCTAACAAGAAA
TTCAAATCAAATAAT
CTTCGAT
>ConsensusfromContig2196 Average coverage: 52.25
AGAAAAAACCATTAAAATCCGTAACAAAAGAGCCCACCCTTTGCG
AATATTTGTATTTTC
TTTCGTTTTGAGAAAAAAATCAACAATATGGGACTATTGTCTGTT
GGGGAACCATTATCA
TGGGAAGAGGCAAAGAAACTATCAAGACATGTTAGAAAACATGG

CATTCAACAATTTCTC
AGTTTATATAAAAGATTGAAAGACAGATCTAATGACTGTTTGAAG
TGGGGTGATGAGGTT
GAGTACACATTGATCCAATTCAACGATGATGAAAAAACTGTCAAG
CTTCTGCTGAAAGCC
GCAGACGTACTCACTCATTTCGACAGTAATAAAGATCCTGCAATT
GCGAACTTCGTTCCT
GAATATGGTAGTTTTTGTGTGGAAGGAATACCATCAAAGCCTTA
TGGACACAGGATGGCA
GATTTTAACACTGTGGAAGCCAATCTCAGAGCAAGACGAGAGAT
GGTGCAATCATATTTG
GAAGAGAATCAAGCCATCATCAGTCTAACCCACTTTCCCAGAATG
GGATGTCCCGAATCA
ACAATCCCACCCTCGCTACCGGACCCGCAGAACGGCGCCTCGCGG
TCCCTTTTCTTATCT
CACGAAGTCATCAATCAAACACATCCAAGATTCTCAACCATAAC
TCGGAACATCCGAGAG
CGCCGTGGACAGAAAGTCGCCATAAATATTCCAATATACAAAGA
CATAAAGACCCCATCG
CCTTTCGCGGAGGACTTCCGATCCCTTGGGGATATAGATGATGAG
TTGACGTCACAGGCA
CTTCCGGATCACGTGACCCTAGACGCCATGGGGTTCGGAATGGGA
AGTTGCTGTATTCAG
GTCACCTTTCAATCTACAAACATCAAAGAAGCTCGCGTTTTGTAC
GACCAACTCGCACCG
CTATGTCCGATTTTAATGGCGTTGAGTGCCAGCTCTCCGATTTTCC
GAGGTTTTCTGTGT
GACGTGGATTGTCGATGGGATGTCATCAGTGACTCGGTCGACGA
CAGAACCCCGCAGGAG
AGGGGCATTGGTGATTTGAGAGAAGGCGAGCGGCTGATAAGAAA
GTCTCGCTACGGAGCC
ATCAGCAGTTACCTTTCGCCAGGAAGTGGCCCCTACAACGACATC
CCCTTGGAAATGGAC
TCAGATACGTATGAGACACTTCGCAATAATGGTGTTGATGAGCTG
CTATCACGTCATTTG
GCACATCTCTTCATTCGAGATCCTCTCTCGATTTTTCGAGAGAAA
GTGGATCAGGACG
>ConsensusfromContig2197 Average coverage: 7.09
TCCGATCTGCCGGACTTCGAAGTTGAAGAGAGACTCATGTCTTCA
ATTCTTGAAAAGTAC
AACAATGTCATCCCGCCCACACGCAACATGACCGTGAACTTCCGA
GCGTCCTTGACAAGT

GTCTCCCCCATCCCGGAAGAAGGTGGCAGCGTTCGGGTCAAGAT
CATGATGAGGATGTGG
TGGCAAGATGTTCGTCTTTCATGGCGTCCTAGAGACTTCAAAGGA
ATCAGCTTTCTGACC
CTGCCCCCTGACCTTATCTGGAAACCGGATGTTGGGATGTACAAT
GGAATCATTCGTTCA
ACTGTTGACTCCATCGAGGCTGTTCTCGACAGCGATGGGAGCGTC
TCCTGGATGCCGACC
AATGAATTTGAAGTCGTCTGCACTTATGCCACAAGATCCGATTCC
TGGTACTGCCCGATG
ACTTTCAGATCATGGGCCCATGATATACGCACTCTTCGCTTGAGA
CCTGTTCCTCCTGGT
AAATTCGAGGGCGTGTCACAGATGGAAACTGATGAGCTCCAGCA
AAACCCGAAGTGGAGA
GTTCAAGAGGCCGAGGAGAAATTCCAGGAAATGAAGTTCGCCTA
CCACTCGCAACCCTTT
GACGTTGTCGA
>ConsensusfromContig2198 Average coverage: 18.87
TTGAAGCTGATTCAGTGAAGGATGATGGTAATACTGATATTGATA
TGAATACTTCGATGA
CGAATCGGCATGAGATACCATTGGGTGTTTCCTATGTACCATCAA
AAATGACATTGCTGC
ATGATCAAACGAAATCTTCTGCCAAACAAACGGAAGATCCAGACT
GTTCGAGAGTCATTT
CTGATACTACATCCGTAATTAAAATCGACGAAACAAACCAGCTTT
CGATTGTTTGTAAAA
AATCAGGAGCAGTAAATGCCCAAAAACATGTTGAGAGAATTGAT
GATATAAGTGAGTCCC
CATCCCAGTCATCTCTTAGTTCTAAAACAAAATCTAGAGATTATT
TTCCTAAAAAGCCTC
ACCGCAATAATAATGTTGCCAATATCGATAAAAAGTTGAGCAGTT
TATCAGATTCTGTGA
ACACC
>ConsensusfromContig2199 Average coverage: 32.58
GCCACAGGTTTTAACTTGGCAGGAGCAAATTGCTTTCCTCTGGGT
GTGATGTCGCCCAAT
CTGCTGGATTTTGCATCTATATAATGAAGTTGTGGAATACGAAGA
GGCAGTGTGTGGTAT
TCAGCTGGTGCTGACTCAGGATCAAAACAGCAGGGATTTCCCAT
GATGGACAGTTCGGCA
AGAAATTCCAGTTGACAAAGTTTCTCGACATAATTCCAGTGTTTG
ATTTCATTACAGCTG

ATGTTCAAATTGACTAGTTGAGGGAATGATGTTATAGCTATTCTT
GGATCGTCGATCAAG
TTATCTTTCAGGTATAATTCCTCGAGATTTTTAACCTTCCAATAC
TTTTCAGGCTTGTG
AGATTATTGTGGACAAGTCGAGATTCTTGAGACTGCCGAGGCA
ACGAAGCCCTTGAACG
TCTGTTATATTATTGCAGGAGAGATCACATTCTTCTAGATTGACG
CATTTTTGAAGGTTA
GGAACATGTCGTATTTTATTTTTTGTGGCAAAAAATTCCTTCAAC
GCAGCCAAATTTCCT
ACGGCAGAAATATCCTCGATAGAATTGTTGCTGATATCCAAATAT
GAAATTGAGCGTGTT
GTTTCAAGCTCCTTATTGGTCAATTTCGAAAGTTTGTTATTGTCT
AAACGGAGAAAGGTC
AGTTTCCGCAGGAACTTCAGTCCGATTCCGACTTGTGTTATTTTAT
TGTGCTGTAATTGC
AGAGCATCCAAATCCACAAGCCAATCTAAGCCTTCGATGACCAGC
AAATTGTTATCATAG
AGTTTGAGTTTTCAGTTTGCTCAGTAAATTCAAATTACAGATGTT
TCGGATGTAGTTGCC
GGAGAGATCCAATGATCTGATGTTCGTGAACTTGTCCAGATTTTC
GATTTTCTCGATTCT
TAACCCATGCAGAACAATTTCATAAACATTTTCTTCTTTTCTTACT
CTTGCGTGCTTGAG
TATGTCTTTTGGTTCTATCGTCCTCTTTTCACGCGGTGTTGATTTT
TTGTCAAAAGGCAA
GCGGTCTGTATCCAGAATTGGTTCAAGTCCAACATTGAGTTTTGC
AAGAAGTTCCTCCTC
AGTCCTGGCCCGTGACGACATCAGGTACTCGATCCCTGCCTTGTT
TTCGTGGGCGTGGCA
TTTCT
>ConsensusfromContig2200 Average coverage: 17.69
CTCCCAATTGACATTCTTCGGACCTCTCTTCTTTTCAATTTCCAAT
AAGGTCAAATATGC
TAACAAGAGCCCACTCAGTAAAAAGAACGTATCAACGGATTTAT
CAGCATTCCACATGAT
TTGAAACGGAAACCACCAAGTGTATCTCTCAAGAAAGCTCGCAA
CATTGACTCCGGCAAA
CGAAATGGCGAAAAAGAATGTGTGGCCCAGAAGGACCCACGTCA
TGCTCAAAAACCGAAT
CCCGTGAACCGCAGTCAGCATTCCTTTGGATTGCTTGCAATTGAA
TAGTTTGCGGAAGTT

TCTCTGTATGCTGAATGCCAGAAGAATTTGAACGAAAATATTTTG
TTTTCCATGAGGATT
TTCAATCGGGTGTTCATCAGCTGCGTTATTGTCAATCAATTCTTCT
CTGGTGTTTTTATT
CTGATTCATATTTATTTCTTCATAGAACAGATCGTAGAATGAGGC
AGCAAAATGAATGAT
CAAGGATGCGGAGAAGATGACGATAACTGCAATTGCTGTGGTTG
AAAAAGGGTAGGACTT
TGGCTTAGTGGTGACCTCTGTGGCCTCAATTGAATCAGATAACAC
TGAATTTAAGATCAA
AAACACCTCAGAATCTGTGCATGTACTTGGAAGACATAACCCCAA
TTTACAGCCAGCAAT
GCAAATGTTTGTATTTTTCAGTGTCACCGATACATATCTTGTGTC
AAATGAAGAGGCATC
ACTGTAACTAGAATTGAACAGAGGTTCAATTTTGTAGCATTGATC
AAAATCTCCGAGCCA
GATTGTGTTTCCGGTCAAAATTCCGCTTCCCGGTTTCCCCGCAGC
GTCTATCATTTGAAG
AGCCCATCCTTGGCCAGTCTTCGCTGAGTCCTCTATGTAGGCCAG
GCCTGTCGAGCATTC
TTGACTAACATTCCCAATAGTTGCCGATCGTGAATATATAGTATT
GATTGTACTTTCATC
GATAGAATCATAATAACCATTTCTGATCATTCTGCTAAACTCTTCG
TTATATTTCATTTG
AGTT
>ConsensusfromContig2201 Average coverage: 15.09
CTGGAATATAATAACATATTCTAAAATATCACGAAGTATTTCAAC
ATAGATCATTAAATT
CTATTTCATTCTATGGTGTTTTTCAGATATTTTGCATTATTTTATT
AGATAGTCGCAAGT
TACAATTTTGTATCGTTCATATATTTTTAAAACATGCACAAAGTT
ATGAGCGGTTAATTT
AAATACAATGTAGCGCAAACTCTCCTTCACACAACCAATGTAAAT
ATCTATAAATCAACC
GGAGATGAGAACAAACTTAAGATTATATTTTTGAAATAAGAATA
TCACAATCTCAAAGCG
GTGAGAGATATATTCTAATGGTGTATCGAACAATAAAATATCAA
GAAATAATCTATAGGG
CATCTGTAAA
>ConsensusfromContig2202 Average coverage: 43.43
GGTTATTCTATTTAATATATCCTAACCGGACTTAGATTGGTAATT
AACCAATTCTAATCC

AATGATTTTTTTTAATTTATCAGCATATGACACTCTCACAGTCGCA
TTCTATTTATTAGT
AAAATTTTACGAAAATCCTGTAAACATCGTAATCTTGAATTTTTT
GTCAAAATCGGCCGG
TAAATTTCGCCGTTATGTGAATTTTAGATATTTTGTCTCGACTTCA
GTAATTGATGAATT
AATTAACTAATTGAAAGATTGAAACCAATAAGATTCTTCTATAAA
AATCCTTTCTTAAAT
>ConsensusfromContig2203 Average coverage: 13.89
CGCCGAACTTTGGGGTATCACCGTACGTGTACATGAATATGTCAG
GTTCTTCGGGATCTT
CCACCCACAGTTTGCAACCAGCTCCATCGAAGCTCATCATCACAT
ATCTGCTGCCCTCCT
CATACGCGGCTCTGCATGCAGTGAAGTTTTTGTCGGCACCATCGA
TGTTATTATCAGTGG
AGATTTTGAGGCTACATTCTCTACTCTTTTTCAACACCAGCTCTGT
TGTCGTCATTCTCG
CTTCGAATGGCTGTGCAGTTTTTGTCCAGGTCATTTCGCCTCTGG
ATTCCTCGCAGACAG
>ConsensusfromContig2204 Average coverage: 17.16
TGCTCCATGTCAAGTTTTCAAACAAAATATGAGAACAAAAAATGT
TATTATATATAACTT
AAGCGAAATTTCTCCACGATGAAACGGGAAATAATGACGATTTCC
TCCTTTTTTGGAAAT
TTTTTGTTTCCTTTTGTTGGAATAAACAAAAATAATCAATCAGCAA
TATCTTCACGTTCT
ACTCATATATTCAATATAATATTGACTAAGATTTATATAAAATTT
GATAATTCACAAAAT
GACAACGATTAGTTTATAAACAAATATTAATTCCAGTTTTAACCG
AAGATCGGAATGTCA
AACTCCGATAGATCGGAAGAG
>ConsensusfromContig2205 Average coverage: 38.01
GGTGAAGACGTGGAAAGAGAGGGTGATATACTTGGTTAAGCGAA
ACCGACTGGTGAATGC
TCTCAGTCTCTGTCGCACTTTCTACCAAGGAGACGAAGCTAGTGC
CAAAGGCCTTTCACG
ATCTAAGCATTTTCGGCAAGCAGAAGTTGCAGAACAGATGCTTTC
TCTTCTCTCGAAATT
CCTTGAGTCGCTACTATGCTCGCCATCCTTCTCATCAGCCGAATC
TTTTCAAAAACTCGA
GCAATCGTGTCGAGTTCTCATAGAAAATTACTTACTCATTGGAAA
ACCGGATATTCTCTT

TCGTGATGTTTACATAAAAATCAAACAACTGGATAGCGGCGGAGA
TGCTTTTTTATCAGC
ACTAGCCGACTACCTAGCTTCGGACTCGTCTTGTGAAGTATTCGA
AAACCGCAACCGCGA
ACCCACCATAGTAATGCCAGATGTGTTGAATGACCTAGTGACCTA
TTTAGAAATTATCGC
CGAATTTGACAAACTTGAGATCTGCCTTCAACGCGTTGATATCTT
GCGAATGGATCTGGA
TAATGTGATAAAACTATGCAAAAAGCGGTATTTGTGGACGCCTT
TTTCCATATTTTTTG
CAACGGAATCGGTGATTTCACAACTCCTCTTGAACACTGCCTCGA
CGTTCTGGTAACGGC
GCTGAAGACAGATGGCCACATCCGTGAAGGAAATTTGCTGAAAT

>ConsensusfromContig2206 Average coverage: 14.14
ATTTGAATGCACATGGAAGTGCTCAATGTGGAAAATGTCCAAAT
GATGAATGCCTGCAGG
GGTGCAATGAAGCCATTCAGACACAGAACCTCGCCAACTGCCTC
GAGATGTGTCGAAAGA
GCAGCCGATTGGAGACATCTTGTACTCTCGGATGCGATATTGGTT
TTATCGGCACAAAAT
CTGCTCTTCGAAAAAGAATATCAAGTTTACCCGGGCTTACGCTGA
TGAATATAACCTCAA
CATCCGCCGTACTCTCGTTCCCATGGTTCAAGAAAACATCGCAGC
AATTCAGCGGCCTCC
TGAATACATCGCATGTGCATTTCAAGAGTGTCCAGGCCGTGGAGT
TGAGCAGGTCATCAG
TATGGAAGACAGTGGATGCATCCATAACAGAAGAACATGTGGAA
GTTTCCGATTTGAAAC
CTTGGACCAATTACAAGTTCAGAGGCATCCTTGAAGTACAAGAAA
TGTTCTCAGCATGCA
CGGGAGAAAGTGACATCATAACAACAGATCGAGAAAATATTGCT
CCATCGGATAGCCCAA
ATATAAAAGATGTATCGATGTCTCTGGTCAACTCCGGTGTAAGAG
TGGTTTGGAAGATGC
CTCTTTACCTCAATCACCCAGCTTCCAAAATACTGCTTTCTGCGA
GCACCGTACAGAGTA
ATATGATTCATCAAAGCATTGGATATATAGCCAAAAACAACATA
ACACTACTGAAAGAGC
TAGAGCCTGGATCAGAATTGTCGTACACTTTTCAACACGTCAAAC
AAAGAATGCAAATCG
GGGTCAACATTTCTGTATGCAATCAATTTGGATGCAGCCCGACCG

CCCGCCAAAATCTGA
CAACTGCCCCTCCTCAAATCATCTACAAAGAAAATGACCCTCCCT
ATTTAGTGGTC
>ConsensusfromContig2207 Average coverage: 2.60
GTGACCAGACATATGATCCATTGTATACACCAGCAGCCACTCAAC
TACACAGGGGATCTA
ATGAACAGGGGCGTCATGATCATGAGGAAAGCAAAGAGGGTATC
GCTTTTATGAGCACAA
TGTGACCAGACATATGATCCATTTTATATACCAGCAGCCATTCAA
CTACACAGGGGATCT
AATGAACAGGGGCGTCATGATCATGAGGAAAGCAAAGAGGGTTT
TATATTGCTTTTATGA
GCACAATGTGACCAGACATATTATCCATTTTATATACCAG
>ConsensusfromContig2208 Average coverage: 10.71
TCAGACGTGTGCTCTTCCGATCTCTACTAATGAGGCATACAACGC
CAGTAATATATCTTC
AAACAGCTTGGCAGGAAATCATAAAATGAAGAAAATCAAAATGA
AAAACAGAATTGAAAA
GTCCTGGAGAGCTCTGCTCGAAAAAATAGACCTCGCTGATCATTT
GATAAAAAATCTCAT
GCTGAAAAAGTCAGCTGCCGAAAGATTTGATGAAGGCTGCAAAT
CACACGAAGAGTGTAT
GGGCAAAGGGAAGACGTTCAAGAGTGTTGTTGTTCCACCAGAAG
CAATTCAGAGACGGGG
AAGTCCGAGGAACATTGCTCGGCTGTATGCCGGAAATCTTCATAG
AACGCATGCCTTCAT
TCCGTCTCATGAGGTCTTTCTTATGAATAAGCCACCTGTTGTAAGC
TCTAGACCTATGTT
CAGATGCATAAGGCTAAAAAACTCTGGGGTTCTCGTTACGAAATT
GGCTCCAAACGTCCG
AAGAAAAGTTAGTAATATGAAACCTCCATATGAGCCAACCGTAAC
CTGGAGTTTTCATGA
TGATCAGTCGACTGAAGTGACAAAGCCGGATGAATTTAAAACTG
ATGGGGGCACTGAAAC
GACTGAGTACCTCACTCGCGCAGACTCCTCATCCTCTTCAAAAAG
ATTGCTTTCGCTTCA
TACACGC
>ConsensusfromContig2209 Average coverage: 29.27
TGTGCTTATATTCCATTCATGAGCTCAGAACAACAAAAATTTCCG
AAATAATAAGGCAAC
ATAATATATGCCGAAAAAACAATATAAATTAAAGTATCTAAATG
AATGAAAACAGAATGT

GTGCAAAATTAGACCACACCAAAAAATTTGAATATCACTTTAATA
CCCATGCTGGTATAT
GCATGACGAAAAATGATTACATTGGATACCATATTGGGACCACA
TGAAAGGCATGAGAGA
ATGTATGCTGAATATTATAATATATATGCCGACAATTGTTTTAAT
TTGTCCATATCGATG
CCCCAACAGTTTCGCTCTATATTCAAAACAAAATCATCACTTGAT
TGCCTATGCTGATAT
ATGCATAAAACAAAAATAAATACGTTTCAAGAACATTTTGGGAC
CACATAGAAAGCATGA
GAAAATGTATAATATATCATGTCGACAATTTATTTAGGTTTTACC
CATATTAATTCCTAG
TAAGTTTCGCTCTGTATATTCTGAACAAATGATATCTATAGAAAC
ACCATTGTTTTATAA
TAAGCATAGAATTTTCAGATATAAAATCTGTCAT
>ConsensusfromContig2210 Average coverage: 20.89
AATCCTGCATGTGATCTTTTATATTTATCTATTCAGCATATGGAA
GATCTTACAGATCGA
ACCGCTGAACTAATTGGTACAAACATCAATGGAAATCCATACAA
ACTCGGTACCAAAAAA
AACCCAATTCATATGTTAGTTCCTCATGGTGAAATTACTCTCAAC
TATGACATAAATATT
CAAGACCCATCATCTGTTTTGCAATTTTTTGACAGTGATGAAGGT
GCTATCGAAGGATTG
ATTTGGCGATGTGAAAACTCAAAAATTTTCAAATTGCACAGACAT
CATCTGAAATTACAA
TGGCCAATTAAGAGTCCATTGTTATCATTAAGAAAAATCAAAGTC
TGTATTGACACTGTG
TATTCAAATTGTAATATAAATCATATAACAAAATTGATCCAAATT
AATGACAGCATTGTG
ACATTTAATGAGATTAACTCACACATTTTGGATCCTGGAA
>ConsensusfromContig2211 Average coverage: 23.40
ATGCGCTCTCAAAAGAAATGCGGCTCAGATCAGGAAAGCAATCT
AAAAGTGGTGCAACGT
CATCTAACTCAGCAAAGCGTTCGTCTGAAGTTACCAAAGATTCA
GAGAAGTCGCAGAACC
TCCACAAGTCATCTATCCGAACAAAAAAGTCTATAAGGCCGAAC
GACAAAAAGGCCTCTG
CTGATTCACTAGCAAGTAAACCCGAGGAAGATTCAGAAAAACGT
AAATCTTTCGAACGAT
TGAAAGCTTCTTTAGATACACTAACAATGAACCCTGAGAAAGAT
TCCAAGAAAATTGATA

AATCTCGTACACGATTGAAGTCTTCATCAGAGAAGCCATTAGATC
AATTACCTCGAAAAA
CTCCTCATTGGAACAAACATTTATCAGCCCCTTTCGTGTGCAGGA
CTAGGCGAGCATCTT
CTGTTCGGAGATCGCCAAATCGTAGAGTTGAACGCAAAAGTAAT
AAACTCGGCTCATGTA
AAGAACGAGGGAGAGATGATAGGTTTCCAAGGGGAGAGAAAGA
GCGACATAGAAGCAGAT
CACCTTCCCGAAAATATCCTCGTGAAATTTCGACTTCGCGAAAAA
CTGATCGAGAAAAAA
GGCGT
>ConsensusfromContig2212 Average coverage: 9.29
AAAATTTTCGTTAAAATATTTTGGTTAAAATTAGTTAGGTGATTA
ATTGACAGTTCATAG
TCGTGTTAGAGTACATACAGTCGAAAAACTTCAGAATTTAGGAG
CCAATTTCAAAAAAAA
TTCAGGCTTATGGAAATTATATCATTACGACACGGAAGGGGCAG
CGCAAGGGCGGCCAAC
TAAACATAATTATGCATATCTACAAAAGCGAATTTTGATGAGATC
CTCAAGCAGATTATG
ATCAGGCATATGATCCGTTCTGTAGTTTAGCAGCAGGTCACTCAG
CTACGCTCGGGCTGT
TATCCAGAACAAACTGAAATTCATCAGTATGTAGACCAGGGGCG
TAACCAGAAAATTTCA
AGAGGGGGGAATTTTCCAGGTGAGGGAAAATTATCAAAATCGGC
AGCATTTAATAACTCC
TTTAGCCAATCCACTGTTTAGGTATC
>ConsensusfromContig2213 Average coverage: 27.02
AAGAAAAGTCTCTCAGATACACCGATAGAAGAATTCACGATGCA
AGTGATTGCCAAAGAC
CAAGGAAAGCCAGCCAAGGCCAGCAACACAACTTTAGTGGTTGA
AGTTGACAGAAACAAG
TTTCCAACAGTCTTTGCCGGCACGCCTTACTCCAGAACTCTTTCA
GAGTCAATCACCAAC
GGAACGATCGTGTTCACAGCCACAGCAACTGATGATGATCTCAA
GGGACAAATCAAGTAT
GTCCTGACAGGTGATTCTGTAGCTCGAAATGTGTTCGCCATTCAG
CCCACGAACGGCCGG
ATATATCTCATCAAAAACCTCCGAAACGACTTGGCTCTATCATAT
AGGATCGATCTAGAG
GCGTATGACACGGCCAACCCCGATGAGCGGGCCCGAACCACGTT
GACGGTCGACGTGAAA

CGTAACGAGAACGCGCCGGTGTTCGGGAGTCTGCTTTACACGAG
CTCCATCAACAAAGAC
GCCCCTCTCGGTCTCGAAGTCATCACTGTGGAAGCCTCGGACGCT
GATCAGGCTGATCGG
TTGCAATATGGAATCAAAGGAGACTCAAGTTGTCAGAATTA
>ConsensusfromContig2214 Average coverage: 19.66
AGCGAAACTACCCTCTTTCGGTTGGATACTCAACATAGAGTCAAG
AATGCTTTTTGCTGT
CTCGCTCTCGTATGTGATATCCGCATTAGGATGAAGCCCAAACAC
CTCGGGGGAATCATA
AGCCGGCAGTCCGTCGATGTAATCGTGGAATTCTTCCAAGCGATG
AGCACGTGGAATGAT
GTATGACTTGAAGAACTTGAATTGCGGAACAAACATCTGCTCGTT
GAACCATACGCGGCA
ATATGTGTTCAGTAAATGCTTGTCGTAATCGTCCGTCACGCGGCC
GCCATACTGGATTTC
ACCCAAGATGTAACGAACGCAGTCCCAATTTATGCCCTTTTTGGC
ATCGATGTCATCCAA
GTGGTTCTGCACGAACTGGACACTGGCATTAAAGTCTGCTGTGTT
GAATTCGTAAGGAAC
GTTCCATCCCAAAGCCCCGAATTTACGCCGCTCCTGA
>ConsensusfromContig2215 Average coverage: 21.22
TCAGCCTTCATCGTCCAAATGATTTGCAGACCAAGCAGCCCGACC
TGAGCAGGAAGATTA
TCCTCAAATTCCTTCATCTGGAAATTGCCCGTATCTTGCAGCTGA
ACATTGGCTTGGCGA
ATGATGACGTGGACGGATCTACGCGTCGTCGCCAGAAGCTCTCC
GAGCCACAATTCGACA
TTGCCCTGTGCCATCACGGGGCTCTCAAGGTTTATCTTTTCACCTT
CTTGTGAAACTAGA
CAGATGATCTTGTCGTAAACCTTATCGTCGAAAACGACATTCTTT
GTGTTGTCGAAAACA
CTCAATAAATGATTTTGTATGGTGTGAGAGTCGCTGGCTTGACCA
AGAATCTCCAGCAAG
GCCGGATCAGAAACAAAGAAGAACCTTGGAAAGACCAATCTCTT
CTTCTCCAGATATCCG
GTGAGAGACTTCTGACAAATCTCCAGCTGCTCCAAGAGATGCGG
CAACAGCTGTCCAAGA
GTGTCGTCCCCAACACAGCATTGGATCACGTTGGTCATCTCATGG
GCTCTCTGCATGATC
TTCAGCCAGGACTTGTCGATGTTTCCGAACCTTTTCGCCTCTTGA
GGAAGCTGCTTGGCG

ATGTCTCCACCCACGAAGACAGCCTCAAGATATATCCAGAGGTTT
TGAACCACCAACCAG
TTCTCGATGACTTCGGTTGTTCCCGTGAGTTTCTGCACCCATCGCT
GTATCTCAGCTTTG
AATGGAGCGTTATATCTGTTTGTCACAAGACCAGCCAAAACCATC
AAGCTATCTTCCATC
AAAGATACAATTTCAGCAGTAACATCACCTTTGAGTAAAAGTTCA
CCACGATTTTTGAAT
GTCGAGAATTCGAGATTTTGGACGCTCCAATCGTTGATCACTTGT
TTGAGCTTAGC
>ConsensusfromContig2216 Average coverage: 15.78
CTGAAACTAGCTATTGCAAAACCCTCAATTATATCTTCAATCATG
AGTGGACTAGGTGAT
CTTTGACGTTTCCCTAATGATAGCTTTTTAGATGATTCAGAAACA
GCAGATAATTCCTCT
TTTTTTTGTTGTTCCTCCTCCTCATAATTTTGTTTCGGCACAGGTT
GAAAAAGCTGCTTA
GGGCCATTCTCTTGTTTCATATCCTTATCTAGATTAATTTTGCACTT
TGCTTTGCTTTTT
TTCTTTTTCGAGCCGACAAAGGCCGATGTATTATCTCCACTTTCC
ATTTTGCCGAGGAAA
AGTGGGCAGCTTTCTATTTTAAATTCTGCATTTCTCCGATCAAAA
CAAAGTCACAAGAAA
GGATTCTCATTAAAGATGCATGATACTTCATGGACAGACGTAAAA
TACGTGCATAACCAG
CTAAAACATGTATACAAAATATATTATTATTCGAAGAATTTGCTT
TTGAAGCTGGTATTA
TCCGATCGAATAAAACCTTTGTACAAATTCAAAACAAAACAAGA
GCGATGTTTCAAATGG
C
>ConsensusfromContig2217 Average coverage: 22.93
ATGAAGTCACGGAAGCTGATTGTACGTTCTGTCAAGATAGATGA
GAAATACATACAGGAG
ATTTTGAATGAAGGCCTATCCATTCTTGAAAATAATCAGGTTGGA
CCACAAAAGTTTCTG
GAATCTTATACAAAATACAATGATCTTTTAAATGGAAAGGCGGA
AGCTGCGGTGACTGAT
TTTTTGAAAGTTGATCATGTTATTGAAGATTTCAAAGACGAAATC
GGAGTCTATCAAAGT
ATTCATGACGAAATCGCCTTATTACGAACAAGGGTCAGACTGAGC
CTGGTCGCTCTCGAC
TGCACTTCACTCAATGACGGTCTTTGTGCACGAGCCGAAGGTCTA

AAGCAACGTATAATC
AAATACGAAGTGGACCACAACAGGACCTTTAACAGAAACATTTG
TAGGCAATACGAGGCG
ATGACTGACCGAATGACAGAACAACCCGAATCCACAGAAGCGCT
CGTCAATCTCCAAGAT
TTCATGAGAGAGTGTATCGAGACAACTCAAAATGATTTGAGAAT
GGAAATATACGAAGCG
GCTGCTCGACTGCAATTTCTTCTAACATATGCGATCTTGCCCCCT
GACGACATCAAGCTA
AATAGCAAGCTCTTCCACTGGCCCGATCAATTGCTTCAAGTTTAT
GATTTCAATTATGGC
CGGATCGCCACAAGACGGGGTCACGTGGAGGACGAGGTTCGCGC
CAAAATCCTGGCGTTC
GATCAGAAGATTCAAGAATACGCCAAGGAGGTGGAGAGCTTCCG
GAAAAGGGAGCTGATG
AGTGCAGATGAGATGCAGAACAACGTGGATCAGCTGCAGACTCT
TGGTGATAACCTCTCG
GCCGCCTTAGAAGAGCTGGACGGAATCAACACGGAGGAGAAATT
GCTGCAGTGGGATTCA
ACCCAATTTCCACTCTTGATGCAAACCATCGATGCGAAAGAGCCG
TACGACAAGCTCTGG
AGAACTGCTCTTAACTTTCATACCAGTAATGAAACCTGGCTCAAT
GAGCCTTTCAAAGAA
TTCAACGCCGAGCAAATTGACGAGCAAGTGAATGAGATGTGGAG
AACAATGTA
>ConsensusfromContig2218 Average coverage: 13.71
AAACCATTCGGCAATGTATTGTAGAATTCATAAACCTGAATATAT
TCATGTTTCTGTATA
ATTTGATGTTTCTATAAATGATGGCTGATATCGAAAAACCGCTTT
CACATATACATGCCT
ACACGGCAGATGTGAAATTTGACGTGCCCGCCCCCATGCATCCA
TGGATGATGACCCAA
CCAATGAAGTGTCGGTCATTGACTCGTTGAGTCTCGGAAGGGGCG
TGTCCCAATGGAATC
GTGACGTCAACGGACTAATGTCAACAAAGAAGTGGCTCCAAACC
TATGGACTGCGCAAAA
ACAGGCTTGATCTGTACAGCCTCTTGCCACAAATAGGCTTCAAGC
ATAGTAATGATTTTG
ATTACGCTCTCAAAAAACCGGTGTCCTCCAAGTACGGCGATGGCT
ATTTCCAAAAATATT
CACGGGCAGACGGAGCCACATTCAACATATCCTGTAGTCGTGAA
CGGATGGAGCAAATCG

AACGGCGACTGGAGGCAGCTCTGGAATTATACAAACGCCGTCAA
GATTGGTTGACCTCTG
AAAGCAGACGATCTTTTGGGGTCATAGAGGAAAAAAGTGCATGC
ATAG
>ConsensusfromContig2219 Average coverage: 31.92
TTTTCCCCGAATGCGTTCACCAAAAGTGCGAACTTGCGACGACCT
CTTCTGGTGAATTCC
TTGAACTTGACCGGGTTTTCGGCGATCCACTTCTCGGAAACTCCC
TCACAAAACTGTGCC
ACATGATCGAATTGTTTTTCGGGAACATCGCTTTCAGTTATACAA
GTATCTCTAGGCCAA
CCTGAAGCGACCATGTACATAACAAGTAAAATTTCGTTTATGGTC
AAATCACTCTTCTCA
AATATAGAGTCACAACGAACGCTGAACAGTTTTCGACAGACTTTA
CAAACCCATTGCACA
CCATCAATGCCGCCGCGATTTATAACACAAGGTTTATTACAATCA
TCACAATCTCGTTGA
TTTCTGAGTAACCTGGTTTGGACACACCATGAGATGGTCTTTTCG
TCACGTTCAAGCATG
GAAATATGACCAGCAATCATTTTCATATCGTGGAGAATCTCTTTA
CTGGACATTATTGCA
GCTGGAAACAATAATTCTGTGTCGCCTTCTGCCATTCT
>ConsensusfromContig2220 Average coverage: 17.48
AGAAAACTAGATTGTCATATTCTGTAACAGATGATGCTACCGTGC
TATCACCTCGAGGTA
ATCGTCTCAATCCTTCGAGAGTGTGTGAGCCAGTTGTCAAAAAAA
CTAGGTTGTCATGTT
GTCGTGATGCGACCGTGCTATCTCCTGCCGTAAACAGCAAACAAT
TCGAGATTGAAGACT
ATAAGCGCTCAAATTTCCCTAAAAATCGAACACACGGAGATGAA
AACTGCAGTCCATTCA
TGTCGATATATTCGAATTCTCCATCGGCAGATCAACCATGTGGTA
ATGCTAAGCCACCCC
CTTCCGTACCATTGCTCATTAATCCGTTCGATATTTTGAAGCAAG
AGGCAACAGATCCAC
CAGAAGTGTCTTCGAATATCGTTGACAACTCTGTGCCAGTGATGA
GCCAAT
>ConsensusfromContig2221 Average coverage: 28.72
CGATTTACATATTTTGTATCTTGATCCTTTTTGTGAAATGTGATGG
TTTTTAAACATAAG
TATCGTTTTTGAAATATATACTTTGATTGAATCTTATTTCGACCGT
TTAATATTAACAAG

GTGCAATACGAAAAAGTCATAATCGACTATTTTTTCATTCCATGT
CTAGTTGAATTTGTA
ACAATTTCTCAGTTAGTAAATCCAAAACAATGGGGAAATCTGCAT
TATATTCATCGAGTG
ACAAAGTATCAAAATTGGAGACAGTTCTAATCCATAACCGATTTT
ACAGTTTATATAATA
TGCTTCTGGTAAAGTTTCTACATCTGTAAAGCTATTTTAGCACGC
ACATTTCAAGTAAAT
GTTCAAGATTTGTGAAGTAGAGGGAGCTTAAGGTGATAAGGATGT
AATTTGTATGTATTC
TAGTTTTCAATGTTGTGGGAATGTCGATTTTGTATAACTAGTATAA
TTATTATTAATTAT
TAACGCGATTTTTGTAAAGTCAATAAAGTTAATAATTGACGGAAA
ATATTTTGAAGGCCT
ATGTCTTGTTTTGTGAAAAACCTGTAGATGGAAGCTGAATATATG
ATTTGTATATTTTGA
AATATTTCATTCAGAGAATAAATTTGACCCTCTTTGTTATGCTTA
ATATAGAATTGCGTG
CCATATTGAATTTCATATTTCTGCGGTTTTACATATTAGCGAAT
>ConsensusfromContig2222 Average coverage: 5.99
CGAAAAAGCTCAGTTCAACTCACATGTATTTCAATTCATTTTTGT
CATCGGAAATGGCTG
AGAACTTGAATGAAACTAAACTGAACATGCTCATCACACGATCCA
AAGATTTATCATTTC
GCTCGCAACATTTATATAAAAAACTTAATTACTCTCTCGTCCAATC
TTTTTTCCTGACTT
GGCGAAGCAAGTGGCCGAAAAACTCTTCAAGACTGAAATTCATA
CCAGAGACCATCGACC
GATTGAAGTCTTTGGAGGAATCCCAGATTAGATCGACTGCAAAT
ATCAACGAAACAAGAA
GAAAAATCGAGTTCGAGATTGCACTTCTGAAGCAT
>ConsensusfromContig2223 Average coverage: 13.28
CCCTTACAAGCAGTCTTATGAAAAATCAAAATCCTATCTCATACT
ATGCAATAATCCTTA
CCCAGTGACTATCAGTCCTACATTGACAAAGACTAAATTGCAACA
ATACTTTATTCAGCA
TCACGCTTATATCAGTCTCAAACTGAACCTAATAGAGTATAAATG
GATGTTTCACTTTAT
GTTGTGCCGATACACAGTTTGATGACATGATGAGCCAAACATACA
ATGAATTTGAATTGG
TCAAGCCGATGACCAGTAATGCTGATAAAAGATTTTGATATGCAA
CCATTCACTTGGGGG

CTCACTGACCGACAGTCTCGCATTGTTTAGAGAATACAATATGCC
ATTTTGTAAAAATTC
AGATTCACGCCGTTTACATGCTGGACATCAAATCATTTAACATGA
GATAGTTTCAATCAG
TTTTTAATAGATGTACAAATTAATAAAAAGCAGTTCATGTGCAGA
AACCAAGGTTACTAT
TAGTGTTACACTGATAGTCAATCTTACACTGAATAAATGAAACAA
ATATGTCACAATATT
TTCCATATCATGCTTCATGTGTCACTCTGGGACAGTCAAAGTGAT
CAACCATGCTAAACT
ATGTCACACTGATCATCATAAAAGAAACACAAATTCAATAACATT
TGATACAGCACTATG
ATGTTTAGCAACATCAGATTAATAATAGAATCATGTATGTTGTCC
ATAATACATATGCAA
TGGAGCTCCCATATATCATAAATTATTAACTCAGCGTCACAGATC
AAAATTATACCAATC
ATCAGTACTGCAATGATGCAATAAAATCATTCAGAGAAAATTAAT
TTAGCTCCTCATTGA
TAACTATCATGGCACTTTTTCAAACA
>ConsensusfromContig2224 Average coverage: 23.24
GGACAGGGATGCCGTCGGCCAAACGGTCCACGACCAGTCTGGAG
GTGGCGGTCGCTCGAG
ATCGGTCCGCTCCGCAATTCGTCGGCGAGGACCGAGTCTCGATC
CTGGAGACGGCGCCAG
TTGGGGACACTGTTCTCTTCGTCTACGCCCAAGACGATACTGAGG
TGATCTATACTCTAC
ATGGAGAGTATCCGGCGCCGAGCTTTTTTGAACTCAATTCGAAGA
CAGGACAAGTTTTAG
TGAAGCAGAGTTTGAAGGAAGATTCCACACTATCACAGATGTAC
ACACTTGATATCGGAG
CGGCGGAATTATCTTGGCCGGCAATTGTCTCACGACGACGGTATT
TTGTGACGGTGGAGC
GAAACCCGTCAGCTCCGCGCTTCACTCGCTCTCGCTTCGA
>ConsensusfromContig2225 Average coverage: 50.71
GAAAAAACACGAAAGTAACAATATCATGAAAGACACAGATAATT
ATCATAACTCATTAAC
AATTTTTAATTTGGTTTAAAAATACAAATATCACAACAATGGAGA
TTGCTAATACATCAA
TGGTGCAGTAACATATACTGTCTTTTCCGCATAAGCACGTATAAG
GTACAGAAAACGATT
ACAACTGATTTTATTTTATGAGATTCGCATTCAGAAGTATTTGTA
TGCAGATATACGCTT

TTCAAATTGCTTTAATGTATTAACTATGAGTAAATATTCAATATT
ACAAAATCAATAGCT
CGATGAATCAAGACGTTCTGACGGAGCTCGTAGCTTTAGCAGCT
ACTGCAGCAACAAGAG
CCGCCCCCTTTCCACTGCCATCTTCTGACAACATCAAGTCGAACT
TGATATTGGTTGGAA
TAAGCTCTTTGATCGCCTCGCTCATGACGCGTTTGAAATCTGGAT
GGTATTTGTAGAGGC
TCCCGTCAATGGCGACCGTTGCCTCCTTACGATCGATACGTCGAA
GCAGAGTCGCAATTC
CCGCCCCTGCTAATCTTGATGCTCGCTCGCTGATGGCGATGCATA
TCTGCCGCAGGTGTT
TGACGTCATCAGAGGAAGGCGAATCATAACCGATTGCTTTGAAGA
CAGCTCCAGTCTTTG
GTGACTCAGAATCATTCTTCTCCTCGCAAACCGTGGATACATCTT
TTGTCTCGAAGGCTA
GCGGGGTTGAGACAGTCGCATTCAGCTTTCCTTCGCACATCAGAC
CTTCTTCAATGCACT
CTATCATGACACGACGAACCAGCTCCCCCATGTACATTCCACTTA
TCATTTTCTCGTATA
TTTGCATTGAAGGATTGAGAGATGCTTTATCAATAGCGAAATCTG
CCTCAGTTCGAATGA
AATCCAAAGTGCCGTCACTGCCAAAAGCCCCCCATTCTGTGTTGA
TGATCACCTGTTTCG
GTTCTGTGATTTCTTCGTCCCACAATTCAACTTTCTCCAAGTTTTC
AATATAGCAAGCGT
TTGTGCCAGTTCCTAAAATGACCCCGATGGCACAGCTTCTGTTGG
AATGGGCGCATGACA
TCAAACATCCCACCGTATCATTGATCAGAGCCACACATCTAACAT
TGAGATTCTTCTTTC
TCTGAATGGCTTTGTGGAGCAGGTCCGTCACGTCTTCACCGACTA
CTCCAGCGCAGGCAA
AACCTTTCGTCCACATGGTCAAACGAGCACGAACGAGACTCTCT
TGGCGACACGGGAAAC
TGAAGGTGAAACCCAAGGGGAACGATTCTTCTGCTAAACCTTGT
GATTCGAGAAGATCCG
CAATGCAGCTTGCGATGTGGTCGAAAAGCTTGTCTCCATGTCCAA
TCATGACGTCATCGG
GGACCGGATAGGTCTTACTGTGAGTGATCAGCTCCTCGTTCAAAC
TCGGAAGTTTTATGA
GCAGAACGCGGAAGTTGGTTCCCCCGAGGTCAAGAGCTAGGAAC
CGACCTTCCTCTGATC

CGTCGGGCAGGAGTCGCACATAGGTGGGAAACATCTTGACAACG
GCCTTGGCATTGCTCA
GCTTCCCCAAGCCCTTGTCGAGCTCGGCGTTTAAGAGATCCCGAA
TCTTCGTCAAGTCAG
AATTGCCCAAACTGTATTTCGCCAATATAGAATCCATTCTTATTAT
GTTTATTTTTCCAT
AATAATAAATAACCAGAATTGGTCCTGGAAATATT
>ConsensusfromContig2226 Average coverage: 15.22
CTCCTTTTTTCGCTAGATATTCGACGGTTTGCCAGTGGCCAGAGC
GTGCCGCGGCATGCA
TGGGTGTACACCCCATTCTGTCTGCCATATTCCGACAAGCACCTG
ACTTTAAGAGATATC
GAACTATTCCAATTTGTTCATTAAATGCTGCATACCACAATGGTG
ATCTCAAATGATCGT
CAATTATGTTCACAGAGCAATTTCCAACAGTTATTAGTTTTTTCAA
AATGGATGTTTTCC
CACAATAGGCGGCGCAATGAATTGGTGTCCACATGAAAGTTTGAT
CGCTGAATGAGGCTT
TGCACGTCACGCGGGCAGTGGCGATCAATTTCATAACCATTCCAT
GATTACACAAAATGT
TTTCAATCTTCACCATATTGTTTTCTTGAATCCAGTCGCAAAATC
TGGCAGCTTGAGACA
CTAAGCATCTATCCGGTTCGATGGTAGACATCCTTCTTTGTCGCCG
TTCTTCCTGACTTC
CAGACTCATCATAATCACCCATTCCTCCTGGCTTGGAAAACAACT
CTCAGCGTTTCTCAT
GTAGACATGTTGTCATGGTGATTTCAGGCAGACGATTCTTGATAG
AGGATTGAGATCGGA
AGAGCG
>ConsensusfromContig2227 Average coverage: 28.71
GATCTGGGAATGTTGCGGTGGCCAGACACCAGTCATTGGTTTACA
AATATTATCATAGAA
TAAGGTTGGTGGGGTCGTTGCTCCTTTCATTCCAATCACAAGGAT
CGGTGCGGTTTTGTT
ATTGAGGAGATTTATATTCAAGAGATGATTCATCATAATCAAGC
TAAATACCAAGAAGGA
TGACTGAGTTCACACACGTGTATTTCTAATAGATGGAGCAGCAAT
GTCAGAAAATCGCCG
CATCTATACCAAGAGTATAAGTTTCAATGGAAAATCAAAGCAAC
CAATAGCGAAAGGATC
TGCCCTCAAAAACTTCCAGAAAAAGCTGATAGTCGTTGGCGACG
GAGGCTGTGGCAAAAC

ATCGCTTCTTTTCACTTTCAGCAAAAACAAGTTCCCAGAGTTTTA
CGTGCCCACAGTGTT
CGAAACGTACGTCTCTGACATCGTCCTACCCGAAGGTCATGTTCA
ATTGACTCTGTGGGA
TACGGCGGGTCAAGATGACTACAATAGACTCCGCCCCCTCTCATA
CCCAGATGCTGATGT
CGTCCTTATCTGTTTTTCTGTCGTTGAGCCCATCAGTGCTGACAA
CATCGAGTCGAGATG
GTGTGAAGAAATCCAACACTTCTGCCCAAACGCTCCTTTCTTATT
AATCGGACTCAAGAC
AGATCTTAGAAATGACCCGGAAATTAATGCCCAGCTGCACAAGG
CCAAGCAGCAAGTCTG
TTCTTCAGAGCTGGGAAATCGACTGGCGAAGCAGTTCGGAGCAG
TGGCGTATTACGAGTG
TTCTGCTGTTTCAAGAGATGGGGTTAAAAATGTATTCGAACAGGC
TGCGAGGATTTCTTT
GAAATCCGGGAAACCTAGACGAAGTGTCATTCAGAGGAATTGCC
AAATTCTGTAGATCAA
AGCTGGGGTAAGAGCTGAACTGAAACATATATATATAAGCGAAA
AACATACTGACATGTG
TTTTTCATCACATAAATTCGGCAAGTGATAGATGGCATTAGTGTT
TTTATTCAGTATAAA
TTGATAAAGCCGTGATGAAACCCTAACAAAAAATGAATTAACAT
ACTCTAACAAT
>ConsensusfromContig2228 Average coverage: 8.29
GAAAAAAACATCAGAATGGAGCACATATGAAGACAATAACCGAC
ATCCACTAGCGCTATC
GAGACATTTGGCGCAATGAGAACGCGCAAAATACAGATTGCGCTC
AAAATTTCTTCGCAT
CCTCCTCATACGTCAGCGCTGTTTTTTTTCGAAAGTTCAGAGTAA
TGCTCACTAATTGGA
TAGATCGCAATTTGAAAATAAGACTCATAGGACTTCTAAACCAC
CTGCAAAAGTGCAATG
TGATGAAAGAACGCTCCAAATAACGCCATCGAGTGAACAACAAG
ACAAGAAAACCATTTC
TATCACAGTTCGGTAGTTCAGCGCCAGATATTCATCGCCTTCTAT
TTTGAAGCGCTTGAA
TGCAACTTATGATTGAACAAAAT
>ConsensusfromContig2229 Average coverage: 19.39
CCTACATCTCGATTACAAGGGTAAAGAATCATCATTTAGTTTTGTC
TTACTATTTAGTAT
TTGAATTTGAAGCTTATTTTGAGGGAAGTCTGACATTTCCTATGA

TATGCGTATAAGGGT
GTTATCTCATATTGTTCACACTTTATTTTGGAGGTTGTTTTGCATT
GCTTTCATGTTTAT
TCAGGTTTGTAGAAATGAAAAATCTTGTTTAGTTTTTCGACATA
TAAAATTGATATCCG
AAGCTTATTTCTTGAGGGGGGTCTGACACATCACTTGAGATGCTT
AATAGGTCTTCGATT
TATGAAGTTCACGACTTTCTCGGATTTTGTCATGTGTTGCTATTAT
GTTGATTCAGGTTT
TTAAGACTCAGGAATTATATTGGCGGACTAGGGTGATTATGAACA
GCCAAACTTGTGAAT
TCAAGCAAATACTTGTGAAGCATTTACCAGCTATAAAATCGAGA
TCGGAAGA
>ConsensusfromContig2230 Average coverage: 9.81
TCCTGAAATTTTTTTCTCCCTCCTGCCTCGCTTCCTCTACCGCCTG
TTGTTTCTCCTTC
TCCCACTCAGTTCTCGCTTTTTGCACCGCTGCCTTTTTCGTCAGCA
GAGATTGGGATTTG
ACTTGGCTTATCATAGCCTTCATCTGCTGGCATTGGATCATCAGGT
TATTTACCATATCT
TCAAGCTGCCACCATGTTCGCTGTTCGCACATTTCATGACTGAGA
ATTG
>ConsensusfromContig2231 Average coverage: 22.96
TTGGAGTCAAATTTTTTATCATAATTATGTTAATTTTAACAGATA
AAAATATCAGTTTGA
TATCAAAGTTATGTGAAACTAAATCTTATCATGGTAGAAATAGAG
AGAAGTGAGGTTTTC
AAGATTCTTTGGCAATGGACTCTTCATTCCTGCAGCAGTTTTGTC
ACAGTGGACGCACAG
AGTTTTGGCATAATGGGTTTCTCAATAATTGTTATCGCCTGTTGCT
TTTTACTATTCTAC
CATATTCCATATTTTGTATTTCCAACGGATTTTTCATCGGAGCAAT
TCCTCCACAACCGC
AATACCTTGAAAGGCGCTTAGCATACTCGAAAGTTTGTTCATTTA
TTATGAGTCTGATAT
TGAGTCTTGTGCCTTTGATGAGGCTCGGATATTCCCAACTCTTTGG
ATCTGCGCCTTCAT
ATTCTTACAGCGCATCACTGGTGGTCACGAGTTTCTCTTGGGCTTT
GCACTCTTGCTATA
TATTTTCGAATCGAAAACGGGACTCAGGGAATAGGTTTTTGTATG
TTTTTGTATGGTTTC
TACTTATCTGTCAATCCCTCGATCTATGCAATACAACTTTGTTCT

GGGAAGATCAAATTT
TGATAGATAAAATCTATTCTATATCATGTTTCAGTCTCATTTTAAT
CTACCTCACAATTC
CAG
>ConsensusfromContig2232 Average coverage: 6.37
GAAATTACTTGCAATATTCAAGTCAGTGATCGCCGTGTTTTCTTG
AAGCATCAATGCGAG
GTGATGCGCCCCCAGGATACCTAATTCATTTCGCTCTATGTCCAA
AATCTTGACTGTATC
ATTATCATGAAGTGAGTAGAAGAGGGCGCTGAGTGCTTCTGGTCC
ACCGTCCCGCCCTCG
AAGATCGATCAGTGGTGTGCTTATTTGCCTAATGAGTTTAGAGAC
AGGAACAGCCCCGAT
GCGGGAACACATCTTGGAATGATGCTCAGC
>ConsensusfromContig2233 Average coverage: 11.83
ATATTTCCATCGATGCCTTATGATTTTATAAGCGATTTAAATGAA
TTGCTGTATAGATGG
CGCGCGAGAAATTCACTTCCCCGGACTCGTTCCACTGTTGAGGTA
GCGCCACTGATTGGA
GAGGTTGAGGGCATTCTCCGTCGTGCAGTGACCTCCCAAGGCCAG
CCTACATGATCGTTT
GTTGACTTTTGAAAAACGTTTGTTTATATCATTCAAATGAGCATT
TATAGCATCAATTGT
GTAGTTCATGTCTAGCTGATATGAGTCATTGTAATCGTCTCTGTC
TGCCATAAGTGGTTC
TAAGAGTTCTTTTATGGACATCAGTGATTCTTGAGTTTTCGAAAT
TCGATTTGACAACAG
CTGAAGATCGCCTTGTCTTATTTCAAC
>ConsensusfromContig2234 Average coverage: 14.82
TAGAGGATGAGGATCTAGATATCAATGATAAAGAATTGGATATT
GGACAGTATCTGGAAT
ATCCAGATAGTGAATATGTTGAGAATGATTATAGAGAAATTGATA
TTAAGCAGGAATTTG
AAGGTTTTGGCGGCATTGCAGATATAAAATCCGAGAATGATGTT
ATTGGCAGCATAAAAA
TAGAGGAAGATGACAATGAATATGAACGGTTAGAAGATTTTATA
AAAGAGGAATACATCG
AGAATAACCCATGCTTGGATGAATATCTTGATAATAAATCAAGAG
ATGATTTAATGAATG
ATATACAAATCGCAGGATCCTGTTCGGAAGATATATCCATAAAAT
ATGAAGATTTAATTG
ATGAAGACCATATCAAACAAGAATATAATGGAAACGACTCTATA

AACCATGTTGCGCCCA
CACTTTTGGTCAGTCCGGATAATAGGAGCGATGGACAGATGAAA
CTACCACAAGTGAATA
TACCTACGAATATCATAATTGCGAATGAGGTTAAGGTGAAAACA
GAAAATGTTGGATATT
TTTCTCCCATTAAAGAAGAATCGGAGGAAGCCGAAAGATCGGAA
G
>ConsensusfromContig2235 Average coverage: 15.14
TCCGATCTTCCAGATCCAGCTCGGACTGGGTCAGCAGCCCTACAG
CGTGCACGACGTCCT
CCTGTCCGATCCGCGAGTCCTGGTCGAAATCAAACATTTGAAAGG
CATACTTGACCTTGA
GGTCCACAGGGGCAGCCTCGCTCAGAATGGAAAACATGTCGAGG
AATTGATTGAAGCAGA
GGTTTCCCCGCCCATTGGAGCTGAACACTTCGCACATGCGCTCTT
TGTACGGGTTCTCCT
TCAGTTCAGCCATCTCGCAAATTCGACTTTTTTCAATAGTTATTTG
AGCAGCATTGGCTT
CGGACATCTGCGTTGGAATATTGAACGCGTCCAATTCCCTGAACC
GCTTGAAGACCCGAG
TTATTTCTTTCTTTGAGAAAAACGTGCAATCCTGATAAATATCAT
ACTGTTCGGGATTGA
AGATTGCTATGGAATTCCCCATACCTCTCAAAATACGGGTTCGTA
TATTTTATCAAAGTC
GCATGAATGCCATGTACTATTTCAGAGCGTAACAAAATGATGTTT
CAGATCG
>ConsensusfromContig2236 Average coverage: 8.38
CACAATCCTCGACTTTTTCAAATATGTTTCACTCAGAATTGATACA
TTCCATTCTTTATT
GTTGTTAAAGTGAATCAATGGTTTATGCTTTATTTATTCAATTGAT
GATATTTCGGAATT
TTAATAAGGTTATTGTGTCGATATGATTTTACCACGCAAATTACGG
TGCCATTATTTTGT
TCATTTTGATTATGTGCCCATTTTCATCATCTGTACATCATGCACT
TATCACACGAGTCA
CAAAGCTACACATGAGTTTTGTGTTGTCACATCCCATTTCAGGTA
TTTATAAGAGATCGG
AAGAG
>ConsensusfromContig2237 Average coverage: 66.88
CGATTAGTCTTTTTCTATTGATAAACCGAAAAAAGCAGCATATTC
AGAAATAAAAGGCAT
TCCTAGGATATTGAAATCTTCCGATTTTCGGTCTTTCAATCGGCGC

CTCTCCTTCACGAT
TGATCAACCGATCTTGACCGAGCAAAGGTGAAGGGTCATCAGGT
AGAAAGGGTCGTAGGT
CAAGGCCCACTCTCTCCTTGGGCTCTACGAGCATACATTTGACGA
GAGCTTTGAGCGCAG
CAGGATTTATGCTCTTGGAGGAATCTATCTGATCAGTATAAAGAG
GCATCCGTTCGCTCA
AAGGCCATAAAATATTTCTATAAGGATTTGTCGCTTCAGGTCGCC
ACATCTGTAACGTGT
TGAGCTGATAAACAAATGTCTGAATCCAGGAACCATCCGTTACCA
CGATTTGGGACGCCA
GAGGGTAGGTGAGATCGTGATACATACCGAATCCTTGATTTTGGG
CTTGAGAAGCCGTCG
AGGCAAATCCAGCCACGAGCGCGACGTGCTTCGATAATTCATCCA
GGTGGCGCTCCCCAT
GCTGCTCCAAAAATCTGTCATAGCGTTTATCGAGGATGTGGACCG
AAGTTTGGCAGAATT
CAAACGGGTCTCCAATCCAATGCCCTGAAATGTTTGTAGGTCTTG
CATGTTCCATAAATT
GATGTAGAGAGAGAGGTCCATAGTTTATATCAGGTACAAATGAG
TTAGTGCAGATATCAC
TGTCTCTACTCACAACCTCGGGAATTGGTTGACAAAATCTATATT
GCATTGAAGCATGTT
CTTTAATTTGTGCAATTATTGTTTTGTCATGCTTTAAACCTTTGAA
AATCTTGTCATCTT
TTGAAAATTTTTGAGTTCTCCAGAAGGTCTCTAATCGTACATTTTC
ATCTTTTTGATATC
CTCTCATGAAGCTATTTTCATCTGATGATAATTGTCTCATAACTGC
AGATACAATTTCGT
CAAAGATACTGTTAAGAGAAGTATATGCAATCGTATATCTATTTG
AAGCTGATGATTGAG
ATATATCTATCAAGTTCATTTGATCATCAATATTTGAAATCAATT
GATATCTATCAATGA
TACTTTCAGCATTCTCCTCATCGCAGCAT
>ConsensusfromContig2238 Average coverage: 36.04
CATTCCTTCCATCTCGACTCCGTCAAGATAACAGACACCTCCAGA
AGTGACAACCTCTCC
CGACAGACCGTCATCCCGCCAAAACGCTGTTTTATATGTGATGAT
AACTTTAATTGTGTT
CCCCATTATCATGGAGTGGTGGAGGTCCGAGAGATTTCGAGGCA
ACGGAGGTGTGAACTG
AATTTCGCGAGTACAGTTCGGTGGAGTGGTACAAATCAAATACTT

GCATCGTATTTTATA
GTCCTTCGTGCTTACAATAAT
>ConsensusfromContig2239 Average coverage: 20.01
CCGTCAGCTCTTCGTGCGGGACTCAGCCAGGTCTATTAGAGATG
TTTCAAAATAACAAC
TCTCTCCTGGATCAAATCATGAAATGTCTGGAGGCTTATCTCGAG
TCCAAAAGAGTCGTT
TTCCCGCGCTTTTACTTTTTATCAAATGACGAGCTTTTGGAGATT
TTGGCGCAAACCCGA
AATCCACTCGCTGTACAGCCCCATCTTCGAAAGTGCTTTGACGCT
ATCATCAAATTAGAT
TTTGGTTCTGGAGGAGGCGGCGGAGCTGCGGATGACGATGGAAA
AGCATTCAGTGGTGAT
ATACAAGCCATGATATCGCCAGAAGGGGAGAAAGTTTCGCTAGG
AAAGGGCCTCCGCGCA
AGAGGAAACGTTGAAGAATGGCTGGGGAAAGTTGAGGAAGCCATG
TTTTTTAATCTCTTT
CGATTAAGCAAAGCTGCGCTAGTAGATTACCAGGAGAAACCAAG
GGAGGAATGGACCCTC
GCAGGACACGCCTCTCAGGTGGTGCTCACTGTTTCTCAAATTATG
TGGTGTGCGGACGTG
ATTCAATGCTTGACTGCAGATGGCGACAGAATCGCTGCGGTTAAA
GACTTTGAAATCAAG
AGTTTTTCAAATTTGAACACTCTCGCTGCTGTGGTTCGCGGCTCG
CTGACGAAACTAAAC
CGTGCCATTCTGACGGCTCTTATCACGATCGATGTCCACGCTCGA
GACATTATAACTGGC
CTGGTTCATAAAGGAGTGGACTCAGAAGGGGACTTTGAGTGGCA
GAAGCAGCTCAGGTAC
TACTGGGACCAGGATATTGACAACTGTCTCGTCCACATGTC
>ConsensusfromContig2240 Average coverage: 16.85
CACGACGCTCTTCCGATCTGTCATTTCTGAAGCTGAGTAGAGATG
GGAAATTGTATTTTC
TGCTTCTGAAATTGCAACAAGACGATGGATTCGATATTATAGTGA
CTGATTCTAGACACA
TCTGGAAAGGCTCTGTGAGTGCAGAAAATCTTGCAAATTTCGCG
AAAGATACAGATAAAC
AGTTTCAAGAATATAGAGATATGACTCATGCTGCATTGAGGGGT
GAGAGACCCGATGACC
TTGAGGTTTCTTTGACGCCTGTCAAGGAATCCTTCGTTCTCGCCT
GGAAGCGATTTGGGG
ACTCAAAAGACGTCAAGTTCAAACTGGGAGTGTGTCGACTGCAC

TGGTTCAAGGACGAAT
CTCTCGAATTCTTTCTCCAAGAATGTGTTGAACAGAATGCCTGTC
TGGTTGAACAGATAA
AAGTTCTGGAAAAAAAGGAGAG
>ConsensusfromContig2241 Average coverage: 38.25
CAGCCACATGACCTTAGGCTCCTCCTCGTTGACCCAATCGTTGTAT
TGATTGAATCTCTT
CAGGAAATGTAGCATCCAATTTCCGAGGCTTTTGAGTGTGGCCGG
AGCCAGCTTTCGCCA
GATCCCAGGAATTTGCCCGTTGAATAAAGACCTGGCGACGTCATC
AAGCTCATTGCTCAT
CCCCACTTCTCCTGCGAGCGCCTTTTTCAGCGTGTCCAGAGAACG
CTTCATGCGGACGAT
AAGTTTGTTGAATCTTTCCAGCTCTTGTAGCAGCACAACCGTCGT
GGGTGTTATGTCAAT
GCCGAACGCCTTCTTGATGATGTCAAGGTCGAATGGGACCGAGT
TTGGCGAGGACGTCGC
ATGCCACTTTGTCGATGTATGCCTCGCGACTGATACCCACGCCGC
TGTCGCCCGTTTGAG
GTTGAAGGTCAACTAAGTGAGCCCACATCTCTTTGGCGGCATTCG
TGTAGTATCCGATTT
CGGCATTGGGGTGAAGGCCAAATACTTCGGGTGTGTTCACCAAAG
GCCATTCATCGATGC
ATTCAACATGAGCGTCCTTATTGGGCTCCGCGGGAATGTGATAGT
CAACTTCGGCCGAAT
GGTAGAAATGGAAAGGCTGGAAAGTGTCGAATATAAAGTCTCCCA
TATATTCGTCCATGT
ACGTGTTGAGTACCCTTCGATCAAAGTTATCGATGGCTCTGCCTC
CATACATGACCTCGC
CGATGAGGTACTTCAGGCTGTTCCATGGAATCTTCGTGTCTTTGT
TCTCGGAAGCTTTGG
TCAGGTAAGTTTTGAGGATTTCAAAACACACCTGGAAATCGCTTT
CATTGAAATCGTAAG
GAATGTTCCATCCGATCTTTCCATACTTTCTCCTCTCCAACACCA
CACCATGGAAGAACG
AAAGGACGAAAACGAGGGGCGGAAAGGCCTCATGGGGGCAGTCC
ACTAGGGATGTCTGAG
CGATCTTGAAGTAGGTTCCTCGTAGGTTCAGTTTAAGGCCGTTGG
GGGGCTCCGTAACAA
CCTTCAGTGACCTCTGTAGAATCCCTATTGGGAAACTAGGGGTGG
GGTCCGTCGTGAGCC
AGAGGCGGAAGTCCGGGTGGGGTTTGTTCATCTTTTCGAGCTCTT

TTTCCAAATCGCGTA
GCCACTTAACAAGAAGATGACAATTCAGCAACATCAGCCATTGTC
CTCTTTGAATTGCAG
TTTCGAGAAGTTGCAACGCCACCTTTTCTTGACCTTGACCAAGTG
AAAGGTACTTGAGCT
TGCTCGTTCCAAAGCCAGTTCGTTCCGCCAACTTTTGGAGGTCCG
TGGCGGGGTCAGA
>ConsensusfromContig2242 Average coverage: 28.06
GGATCATTTGCTATTGATGCTTTAGCAATTCTGACAAGAAGTCAA
GGTCATGCAATTTTG
TCAGAGGAAGCAAAGTTTTCACTCAGTTTTGATGTTTTGTCATAT
CTGAATCAACATATT
TTGGACACCAATACCGAGCTTTCAACATCTTTCGATGTCAAATCT
CAAAAAAGTCATCCG
CTGCCCATTGCTTACAACGTCCAGCTTCTCTATGAATTCATTCAG
AAAACACCTTCACTA
AAGTTACATGGCTCACCAAGACATCCCAAGACCTTCTCGAATGTT
GCCGTTTTTCGATCG
TTGGTATCATTGGAACTATGTCAAGTTGACATCACAGCTATCTAT
GGGATACAGAAACTT
CGACCTCGTCTCAAATACTTGTTTATTTCCGAATGCGATTTTCAAT
TGGAAGATGTTCTG
GAAAGATGTGGAGCGGATGGACCCCTCATATGCTTTGGCCATCG
CTTTCTTTGTTGTGC
ATTCGGTTTTGTCAAATAAAGAATTTGGATGGATCACTGCGGT
>ConsensusfromContig2243 Average coverage: 26.35
GATGATGGAAACGAGGAGAGATCAAGCGTTCTATTCCACATTTCA
ATGGATTTATGCTTG
GGTGTCTGAATTTCATTATTTGTTAGGGTGTATATCATAACCATA
GTTTACCTAATAGAA
CCCAATTATTGACTTGAAATTTTCACAGCCTTAGTTTACTATAAC
TACGATATTTAGAAT
AATTACACTATCCAATTGTTCCGTGTATTTTCATGATCATATTAT
CAAGACATATAGGTA
GAATTAATCAGATTCATTAGTTGTTCAAATTTCGGTGCTCACGAC
TAATATGCTACTAGA
TATTGACCAATATTATTCATCAGAGGTCGACTTATATAAGATGTC
AAAAAATAAGTACAT
ATTTATATTAAATTTCGTTCAAAATTTCAATTTTTTGTGAATGTTG
TTGAGAAGCG
>ConsensusfromContig2244 Average coverage: 21.81
TCAAAATACAACAATCCTTATGGCCTAAAGCAGAAAATGCTACG

GTGGTGGTCGCAGTGT
TATGTTTCCGACGTTTCAACCATTTTTGTCGGTATAAGAGATTCG
CAAAATATTGTGCGA
AGAATCAAAACATTTTCTGTAAAAGATTTGCCAAAGTTCAGTAAA
GGTATATGGGATCAG
GATGTATGTTTCGGTAATTTGAAAAGGATATTTGAATTCATACGA
GAAAACATCACAGTC
AATGATGCGAAGCAAGCTTTTTTGCTGCATTTCAATGGGCAAGAA
CGCAACAAAAGTTTT
GAGCTGCACAAGGCGGAGCCAAATTCGAAATACGCTTTCCTCTCC
GATTCATTCATACGA
AAATATGCCGAAGATGTCGCATGAAATTATGATAACTGAAGATTT
GTCTAGTAGATGGGT
GAGATC
>ConsensusfromContig2245 Average coverage: 48.49
GCACCTCGTGCTTACTCTCGCAAAAAATCAATGAAATGAAATTAT
CCAACATAGGAAATA
TTCAGCATTTTCATGATGTTGGTGATTCATGTCCAGTAAGATCTG
ATGAATGTAATAATA
TAGATATGAATGGTTCTTATTCTTCATGTCAAGTAGACGTGGATA
ATGAACGTTCGCCCG
AAATTTCTCTTTATAAAAGCTGTTTGCCTCTTGAGAATTTTGTGCA
GTCGAATAACTTTG
TGCTCAGCGATGGCTCTTCGCTGAGCACGAACGGATGTGCTGCTT
CACTTCTAGCCTCCC
CCAACGACGTGTCACCGCTCAACAGCAGTCCCCTGCCATTAGGGG
CCCCTCGAAAGCCAT
TAGACCATTATCGTACGACTGATGTGTGGTGATATACTAGTTATG
TTTTGAGCAGCATTT
TATGACTATAGTTATCTCTCTTCAAATATGCCAATATTTTATTGTT
TTTGTTTGTTAAGA
AATAGTGAATTTTATAAAGCTTGTCAACTGTTTTGTCTTAATCTGT
TATGTACGCTATTT
TAGATTTAAGCCGCTTATGACCAGTTGCATGGTTGTTGAGTCATC
GATGTATCGATATAT
ACATATGTTGAGTAATATGAAATTCATATAATCACATTTTTCTAA
GGTAGAATTTTAATT
TCATTTGACCACGCATATGTGGTTATGAGTATGGGTTTATATATG
TAGAATTCTCTTAAT
TTATGTATTGTAATGTATATCTTATACATTTTCAGAATAAAGTCT
TAACGAATGAATTGT
TGACATAATATGTGTGAAGATTCACACCTTTTGTATACCGTTTGA

ATTGAGTTTGATGGA
ATGCTATTGGCCTTACAATTCAATTAAAATCCTGCTCTAGAGCTCA
TCGAATATTTTTAT
ACTACATCATTACAGTTGTGTGACTATAGAGATAAAAACTAGCTG
TGAATTATTTTTATA
ATATAAATATATATGTTTACTTGTATGCGATAGTCACCCTTATAT
ATACTATGATCACAT
GAATACATCAGCATTGTTAAATTTTTTACGCTCGTTGTGACACTG
CTATTGAAAATACTG
ATATTCGGATACATGTTCTCAAAATTTTCAAATGAAGCTATATAA
AACAGTTTTCGCACA
CATTCAATATATATTTT
>ConsensusfromContig2246 Average coverage: 9.15
GTCAAAATAAAGATATTTTAGACGGTATAAATGACAATTCGGGTC
AAACTCAAGATTCTG
TCGATAGTATAGATGACATTTCGGGTCAAACTGAAGATTCTGTAG
GCAATTCAAATGACA
CTTCTGGTCAAACTCAAGATCCTGTAGACAGGTCAGATAACACTT
CGGGTCAAACTCAAG
ATCCTGTGAATAGTATAAATGACATTTCGGGTCAAGCTCAAGATC
ATGTAGACAGTATAA
ATGACACTTCGG
>ConsensusfromContig2247 Average coverage: 61.85
TGACTGGTTTCGTTTTTAAATCGTGATTACGAATCCGTAGAGAGT
TATGATGAAAAGTTG
ATAATCGTATGGTAATTACGTACTGAACCTTATGTTTAACGTCTC
ACCAGATGCTAAATA
CTAGAAATAAAACATTTTCTACTAATTTCTTATTGGTCAATCACTA
CTACATCTCAATTA
TTTTTTCATGTTTTTGTGCTCACATATTGAATGTTCATTGCTTCCT
TGATGAAAACATTC
AATTTGATGGGTCATCGGAAAAAACAAATTGTTTTTGTGAGCTGA
AAGGAAATGTCGATG
ACTGCTCATGTACAGTTGAAAATTTAGAATTATTCAACAATCTCA
ATGTATTTCCGATCC
TGAGTGACTTACTTCAAAGACCATACTTCAGATATTGGAAGG
>ConsensusfromContig2248 Average coverage: 45.94
GGCTTCAAATTCATGAGCGGAAACAAGACGTATTCCGTGGAATG
CCTCGCCAGTGGGAAC
TGGTCTGATATCGTTCCTAAATGCCACGAGGTGAAGTGCCCTCAG
CTCCCCTACTGGGAT
TTGGTTCTGAACACGCGGTCTAACGCCATCTGGACCCTGGTCAAC

TATTCCTGCCCGACT
GGTTACCAATTCGCCGGAGACAAGAAGAGTCGGCTGGCGCTTTG
CGATTCATACGGGGAA
TGGGTTCCTGAAATCGCGGCATGCAATGAGTACACAAACTCATC
GGCGAAGCAAGTGGTG
ACCGAGGAGGCACCCTCTTCCCAGCTCATCGGCATCGTCTTCGTA
GCGACCCTCGCCGTC
GGTTATGTGGCGCTCGTTGTCGTTGACGTTCTCTTCGTCTTTCCAA
ACCTTTTTTTCAAA
AAGTAGTGAATGACTCATGGAGGTCGGTAGCACCGAGTTCTATTG
GATTGA
>ConsensusfromContig2249 Average coverage: 18.59
TTCTATAAGAGAATATATTTGATACAACGTCGCAACTCCCCGTAA
CTTATACAGGTTAGA
AGAGACAGAAGCTTGCGAGATTTTTTGGACGAGGAATTTCAAGC
CCGGTCGGCTATCGAA
GTCCGAGGCAACAGATGCGGAAGTTTCGAGGCAAGAAAACAGAG
ACAGAATATTATGAAT
GGATAAGTATTTGATGATTCCAGGAGATGAAGCATCGGAGAATG
ACGTCGATGAAAATCC
GCTATGTTGATTGCTATCCGATCTATTATTTTGTGAAGGAACGTC
TACGATTGAATCAGA
AGAATACATGGATTTTTCGGGACTTTTTTGACTCATAAAGTGTGT
TGAATTTGAAATCAT
AGGTTTTTCGGCTGAAATCAAAACTTTGCTCCCATTAAGAAGCAT
CTCTCCGATGAATTG
AACAAGAAGTTGATGAGTTATGAGCCCGATGACGACCTCTTCGAA
CGACACTCTCGCGTG
GGGCTCACTTATTGATTCGACCTCTTCTGGCTTGAAGATAAGAAA
AGTGTACAGATGGTT
CTGCTCGTCTTGAAGAGGGA
>ConsensusfromContig2250 Average coverage: 10.77
ATTGGATTCAAACTACAGGAAAGTTGTGAATTCAAGCCCATACTG
ACTGATATATCATCT
AACGATTCATATGGCAAGGGGAGTGTTGTTCATAGAATCGTTGGC
AATATGGCCGTTTTC
ACTGTATATTCATCTAAATCAGCCGAATACTGTTTTGAAGTTCTG
GCATATAATAAGAAA
AGCAAATCTGGAAAACTTGAGCCAGTGTACAAATATTTTCTTGAA
TTCACAAACAACGCA
CAAAATAGCTTGATCGCAGATCCGAGTGGTTTGATGGGCCCAACG
CCCATATTTTCTGCG

CATAACTGTGAGGTGGTTTTTCCATCAGATCCGGTGATTGAAACA
TCCAACCAAGAATTT
ATTGTTCAACTTAAAGTCAGTCGGAGTGTCATGTCTTTGGCCACG
ATGGAATGTCTCACG
CAAGCCAAACAAGTCGATATGAAAGACTATGTGTTTCAACAAAG
AAATGGAGATACGCTC
AACATTCTCGCTCGGTTCAACCAGGCGGGTGTGTTTATATTGAAG
ATGTACTGCAAAGAT
AAAACGAATGATTCTCAACCCCCGACAAATGCGTACAAATGGCT
CATCAGATATAAAGGT
TTGAATAATGATGATATGAGAGTAAAATGTGAGCCATTCCCGAG
AAAATATGCACCGTTG
AATAAATATGAGTGCGAAGTATTAACACCATTAATGAGAGACCT
GCG
>ConsensusfromContig2251 Average coverage: 59.45
CCAAATTTAGAGCCAAGCTCTTCCAAAACTTTTAAGACCACAGTA
ACAGGGTCTCCGGAA
TCTGGTCGGATGACCAGAACGCCCCCTGCATTCCCACGACTGATG
ACAGACTCTTTCAAC
GCATCTCCCCAAATATTCGCACAAGCGTTAAACACATCGAAGCTG
TCACTGACGACTGCA
ACCGCCCCTGTTGGGAACTGCGAGAGCATGTTGGCGAAAGCAGCC
CTTTCGCCATCCCCT
CGACCCCAAGTCGTTATTGTGCTGTGTTCAGCGGCGGGTATCGAA
TTTCCGGCACACTGC
GAGCCGTAAAACCTGTGAGCTGTAACTAGGGCCGCCAGTGTATCT
GTACCTTTGAAATTT
ACGAGATGGGCGGCGCCACCGATCCCTGCACTTTCAACCGAGGA
GCAGCCGCGGAACCCA
AAGTCATGTAACATAAAGGGCAGAGAATCGATGTTGTCAGCTGT
AGAATCCAGAAAGTCT
CGAAGGAGAGCTTTTTGATAGAAGGAATTCGTCGCAACAGTCATC
GGATACCATGTTTGT
ACTAGAAGTGTCTCGAGGTAATTTGTAAGCCAGAAGCATTCAGGG
TCAGTGTTCTCAACG
GTGAACAGAACGTTCTTATACGGAACCAAAGAGCCCTCGGGCACC
GCTTTGATTCTCACT
GGCAATCTACCTGAGTGTTTCTCCAGTATATAGCGCCATCCATTT
GAATTGAAGACGTTT
TGTCCGAAATGTGACTGCATGAGGATTTCTGCCTCTTTGATCTTC
TCCTCGGTGACAACA
CATCCCGTTAGCCATTTTTTGAGGATGTATTGGAGGCCGAAGAAT

AAAATCTTGGGAAAC
TTCCCTCCTCTACTCTCGAAATACGAGTAGACATGCGTTGTGTTTT
TCGGGTATTGCTTG
TGATGTGTTATCTTGTAGGAATCGGTAGCAACTAGAATGTTATTT
GACATGATTGAATCA
GAATATCTGAAAACCCAAGTCTCCTAAAGT
>ConsensusfromContig2252 Average coverage: 25.75
ATGAATCTGGATTGTCTTTGCGATATCCGATGTGATATAGTTTTTT
TTGAAAGTCTTTTT
CTTTCTTCGAATTTTCTTTCATAATTTTTTCTGAAAGAACAGCACT
TTTATAAAACTAAT
CGTTTAAAGGTTTAATCATCTTCAGTATAAATTCGTTCACGATGT
ACATTCCATCATTAA
AAACAACTAGCTTGTTCCTATATCGAGCCATTCTGAACTCTTCTA
TTTGGGTTGAAAACT
TTGATGTGTGGAGTGTTTGTGTGCAACTGCAATATTTGTGATATTC
TCGTACAATCTTTC
AATTTACTTCTTTTTACTTCGTTTCCGAAGACATTTTCCGTATTTT
CCGTATTTTCCCAG
TCTGTCCTTTATGTCATTTATGTCATCTTTGTCATCTTTGTCTTCG
GAGGTGTTTATGCA
CATTCCTTTACGTATGTGCAGATCACCAATTAGTAACGAATTTTA
GATCAAATAAACTAT
TCTGATTTCTGATATTCTTGTTGTCTCTTGTTGTCTTTTAACCTCTA
TCCAGTTCAAATC
GACATCCATTTTGGGTCAGTGTATCGTCA
>ConsensusfromContig2253 Average coverage: 16.21
CATGAGACATCTCATACCCTTGATGCCAAAAACGAAGGGCCAGT
ATTGTATATACAGCAA
TTTTTAGCATTGTTGCACAAGAAACTGATACTTTCCTATAGAAAT
AAAATCACAACTTTG
ATCCAAGTATGTGTTCCTTTCGCATCCGTATTATGTGGTGTGTGTC
TGCTGGCAGCATTG
AAGATAATCTTAGGAGAAGAAGCAAAGCCATCGGAGTCGCCTCT
GAGTATTTCGTCGAAT
CACTATGGGAAAGAAATAGTGTTCCCATACGCCGTACCAGACAA
TGAATGTGGAAGGAAT
TTTGAAGAAAAAATTATTGATTTTGTTGAAAAATTTGGTCAGAAA
TGTAAAAAGATAGAT
GATGATTCGATGGAGAACTATTTACTAGAAAATGCTAGAAAAAGC
TATTATGATCTCAAC
AAACATCAGATAACTGCAGTGGATATGACTGAATGTACAGACGA

TACACACTTCAATGTA
ACTGGCTACTTCAACGATTACAAATTTCATACAATAGCCACTACA
GTC
>ConsensusfromContig2254 Average coverage: 7.24
GCACGCTCCGTTTACATATCACCGAAAGCCACGCCCACAAGTGAT
ATATGTTTGACTGCA
GTGAATCCACAAATTGAGGTGATGGCAAGGAGGATTCCGATGAG
AGCATGCTTCCAAATA
TCCTTTCTGCATATTCCAGTTGTCGGTCGAAAAATTAACACATCA
AACACTGCAAATATA
ATCACAGTCAACATTCTGAACAAGTTCAACAGTCCGTCAGACACA
GATGCCTGTATCAGC
TGACAACTAACCAAGCTGCAATCTCCTATGATTTGCGCAATGAAG
ACAATCAAAATCCCT
GTAATTATAAGTTTTGTATGTGAAACTTGCTCGTTGTTTCTTTCAT
TCGATTCATCATCA
CTTGAAACGGACTCGATAGGTTGCATGATTTCTGATTTTCATATA
TCTGAGCGGCTACTC
ATTTGAAACTATAATGCCATTCGATAGATCGGAAGAGCACACG
>ConsensusfromContig2255 Average coverage: 15.86
CTCGAACAGAAGAGTGAGTAGACCGAAGAAAATCCCGATCAAGA
TGACCAAAAACACACC
GCCGATATTCTTGATACTGATACCATCACTCTCGTCTTCCACCTTG
GGGCATTGCACTTT
GTTGTACTCCCACCAGAGTGCCTTGAGGTCCTCTAAGTACCGTTG
GTTCAGCAATTGTAG
TATTATTCCCGACAGCTCGTTTTTCAGAGGAGAACCCTCTTGTAC
CGCCAGTGCGTACGG
CTTACGACTGAACTCCTCTCCAACTTCCTCGAGCTCACAGTCTGTC
AGTGTTACGTACTT
GTTGGAGGTTGCATCGCCTATAAAAGCAAATTCCTGTCCATCGGG
AGGGTTTTTGATTCG
TTTGATTGCCTCGTTGAAGTCCTTTGGTAATCCTGAATCTAGCATG
CTTGCCCACATGTT
CGTGAATTTATCACTGACGGGATAATCCCAAACAGCCAGCTTGGC
TCGCTCGATAGGATC
CAAATCGTCATTCAAACTCATGTCTTTCCAAATTTCGTAGAACATT
TCTTCAATTTCTTT
>ConsensusfromContig2256 Average coverage: 16.80
AGATTGGCTCCATCGGCCTCCATCACGTCATGACAGTATTTATTT
TGAATTTTGAAAGTT
CCCTCCATCGGCTCGGCAATTATCGATTAATGTCTACAATGCAGT

GGCTAAACATAATTT
TGGGACATTATTGATTACTATATTGAGCCGTTCCCAGAAGGCACA
CATGAAACGTCGAGA
TTTGGAATGATGAAAAATTGATCCAGCTTACTTTTGTGCTATTGTG
TACTCGTGCGATTT
CGTACACAACATTCATGTAGGCGGCTTTCAAATGGATTCCAATAT
TACTTCTTTTGATGA
CATCAGTTCGTCGACGACTACATGCAGTAACTGTAGCAACAACAG
CTATGCAAACCCTGT
GCCTCCCAGTATAATTGTTTGGAAAGCCATTATACTGTCACTCAT
ATTCGCGATAATAAT
TGGCGGCACAATAGTCGGTAATGTACAGATCGGAAGAGCACACG
TCTGAA
>ConsensusfromContig2257 Average coverage: 18.56
CAAATAGTGTTAAAAACATCATCGCATGGGGAATAATCTAATGAG
ATTCTTGATTAATAC
GAGATATAGCGGTCAGTTATACGCAAATTTGTATTTTAAACTCTG
ATATTCAAATGATAA
TTCGCTAAAGTCAAAATGAAAGAACTTTTGGAATGATGAGATTAT
GGCCAAACTTTTGAA
AATCAATAATGGAGAGTGTAGAATAATTTGAACAGTGTACCAGA
ACGGACAATTAGTGTA
AGAAACTGATACGGTAGCCACACAGTGTGGAATGAAGAATGAAC
AACCCTTACAAACTTT
TGAAAAACGATTGAATAGTAGAAAAATAAACATTTCATCAACAT
GAAATAGAATTGAAAT
AATACTAAAATTGGTAAGTTCGAACCATTGCCTATTCAAAAAATG
AATATAATATAAACA
ACACGTTTTGTAAGAATAAACGAATAAGTTTTCATATGGCGAGAT

>ConsensusfromContig2258 Average coverage: 8.94
GAAGGAATCTGTTGATTATCGTATCAATATGAGATTCTCTCCTCG
AATATCCATTCGTAA
ATTTGGAGATGAGAATATTGAGCTGCAGTCGCCTCTCACATTGTA
TTGCGCGGTTTGTGC
AAACCCTGAAATAAACCGAGAAAGCATAACATGGGAATTCACAG
ATAGCACATCATACGC
CAATGCCATAAAGAAGAGCTTACCTAACAAGAGTCAAACCCTTC
ACATAGAAAGGCTGGA
AAAAAGTCACATTGGAGAATATTCATGTTCTGCATCGAATTCAGT
ATCTATGAGCATTAG
CAAGCCTTTTTCTGTACCGATAGAATACGGCCCGGATGTATCTGT

CAGCTACTTATCATC
TGACCACATGAGTGAAATGGCCGTAGAAGATCCAGACCAACCT
>ConsensusfromContig2259 Average coverage: 79.23
CTACACGACGCTCTTCCGATCTCAAATAGTTAGAAACCATCAACA
AAACAAAATGTGTTG
AAACAACACGTACGAGCTGCATTAGAACAAAACCTTTGATGCTTA
CTGTCATTTCAAAAT
CATCTCTGAGATAACAGCACTGACCAGAAGACAGAATGCAAAAC
AAAAGGCGGAGAGCAG
CTCGAAGAAATAGAGAGAAAATCTCTAAAGCCGACCAGTCGCTT
GGACCAAACAAAATGT
GCGCAAGTGCCAAAACGACACAAGGCTCAAGAAAGCCCAAGAGA
CAGACCCTTTTTTCTT
TCCAGATCATGCGGACCGCGTCTCCATATATAACGCAGAGTGGA
TTCCAGGAACTGGACA
AGGAGTATGTTCTTCAGCAGGATGTGCCAACAGCTGGAAAAACA
TTGCATCAAGAGGATG
AGCTGAGCACCTCAGCACTTTGCAATGTCTCTACTAATAGTGATG
CCACATGTGGCTCTC
AAGATCGAATTAATGTTCTGTATTCGAAAGTCTCAACGGAGAAAT
TATTGGATATGCTCC
CAGAATCGTGTGAAGAATTTGAGCAAGTTGATCCTTATATGAACA
AATTCACAGAAACCT
CACATCCAAAATCCCAATTTCTGCAGAACTCGAAAATTCTTCAAA
ATATTTGGTCAAAGA
ACAGCAATACTTCTATTTCATTGGCACCGGATTATTGCTCAAGTT
CTTGGGAAAAGAATG
ACGACTCTGGCAGGACCGAGAGTACGTACAATAAGGATGAAAAT
TCTTTGTCATGCAAAG
AAATTGAGGGGATATTAAAGATCGCTTCACTGACTTTACAGCAAG
ATAACGATATTGCGA
ATAGTGAAACTTATGCAGAGTGCTCGGGAAAGAAGAAAACGAAT
GAAAAAGACTCTACAC
TAGATAAAGAATTGCCTAAAAAAGATGACAATCAACGTGAACAT
GCTGAAAATCAAATTT
CCGTGAGTAATTCCCAAGTTTATCCGACATATAATCAGCTCCAGA
CGTCTTATTCCAACG
TCGGTTATTGCCAGACAGGTAACGGTCAGCCAATGATGGTCGCAG
TTCAGCAGCACTACG
TACAGCGGCCTGATATGGCCTCAGTCTTTTATCCTGTACAATTTTC
TGTTCCTTCTCGAG
GAAGTCAGTCTATAACGCGGACAAATAAGCAGCAAACAGCTAGA

CCTCTTCAAGCGGGAA
TGTTTCGACCGGTTTTTCATTCTTTGGTACAACCAACATATTTTAT
TACACCAAATATCA
AGTACAGATCATCAAAATAATTTCACCGATTATAGTTTTTCGATA
AAACATTCAGTTGTT
GTTTGAAAAAAATAGCTATAAATATACTTCAAATCTGGAATCATT
CAGTGTAAATTATTT
ATAACACTGAATACGCCTTTGTCTTTATACATGCATGCTCTCAGA
ACGTAGGCAATTTTT
CATGAAAAATCAGCATACTCAGATACTCACTTTATTCATTTCATA
TTATCCGTGTACCTC
TCCTTTGTTCTATGAAATT
>ConsensusfromContig2260 Average coverage: 56.90
GGCTGCTGCTGGATATTCATGACATATTCTCTTAGAGTCTTCAGTT
CGCGTCGCATGTCT
CTTATAAGTGATTTTTGTGATTCAATTTCCCGATTGCATGAAGCAA
TTTCATTCTGTTGA
GTAAGGAGAAGGCAACGAACATCCTTGACGCAATTGTGATCCTTG
AGTTCGTCTTTAGGC
ACAACAAGTCCACAACCTTTTTCACATGGCATTGGCTTCTTCGGG
TTGAAATCACATACT
GCTTCGTGACTGGCTAAACTGTCAAGTTGAAGCACGGTGTCACAT
CCCTCCCCCAAATTG
CCACAAGACAGTTTCAACCTAGAGAGCATGTTACGAAGGATTCT
CGGTGGGTGACGTAAT
TGCCCGATAGACAAAGGGCCTCTGTCCACAGGGCATGACTGTTGG
TGATTCAGCCACTCT
GTGATGCATGCGGTACAGTAAGCATGCTCGCAGTTGGGTGCCTGT
GAAGGATTCTCGAGT
ACACTACTACAGATCGGACAAATGAGTTCCTCATCAACTGTCCCT
TCGAATCTTGTCAGA
TCATATCCCATCTACAACATCGATATGTCCGTATATATAAAATGA
ACCGAGCACAATTTT
GTAAAAAATTACTTGTTTACAT
>ConsensusfromContig2261 Average coverage: 19.49
CAAATATATAATTCTAAAAAATATCGATTCAGATCTGGTTTAAAT
AATAATTCGGAAAGT
GAAGTTTATAAAAGAGAAATGAGTTTTCCGGCACGAGCTCTTCGA
TTTGTGAAAGTCCCG
CATATATGTCGGGCGTATACGAGCGGTAATCATTCAGTCTCAATC
CTTGGAGCACCTTTC
TCTAAAGGACAGGGCCATGCGGGACCAGAAGAATCGTGCTCACT

CTTGCGCGAAGCTGGC
CTCAACGAAAGACTAAAAAATATAGGATATACAGTGAACGACCA
CGGGGATCTGAAGTTC
GACACGATCGGCGATGATCCGACATCTTATCAGATAAAACACCC
ACGGACGATCGGAC
>ConsensusfromContig2262 Average coverage: 13.95
GACTGCGTCGACATGGACTGCGATGGACTCAAGAAGCTGATCGT
GACGGACCAGGATGGA
AGCTTCTTGGGCAAAATCGGGTCGGTCATTTCCGAGAGTGAGTA
CGAGTGGGACGGAGAT
TCCCGGAGAGGCTACGGGGACTATCGGCTGCCAAAGACGCTTCTT
TCCGACCTCGCTGGC
AACAGAATCCCTGTTGAGGATTTGGTGCCCGACAAAGGAATCAT
CCGAGATCAGAGTTGC
GAGTATATCGACAACTGGCAAGCCTATCACTGCGGCGAGTCCATG
GACTATGCACAGGTT
GAGCTTGAATCGATGGATTCTGACACAGAGACGCGGCGGGTGTCT
CCAGTCTCGATCAGC
AGCGGTTCATTCGTTGACCTCATCAACGGGCCTCAGGATCATGGA
TGGTGCTCGGGCTAT
ACGTGCCAGAAACGCCTCTCCACATTCCCACTTGTTCTCGCCATG
GGCCATGATTACGAC
TGCCACTTCACGGGCACATCTCCCCAGCGGCTGCGTGTTCATCTC
AATGATGATGATGAT
TCCAGAAGCTTCGTGTTTGGAATCTTCTATTCGTCACCACAGCGTC
TGGATGTGTTCGTT
GCCGGAAGTTACATACCTCCGACGAACGCCGACACGAATGAAAA
CAACCAGCAAATTCTG
CTCAGTCCGACGTACGACGGGCAATACCGACCGACGATAAACGA
CACGGAGGGTACAAAC
TACTTCCACAGCGCTTCTCAAACGCTTTATTTCACGGTGCGCGGT
GGTGGGTCGACTTTT
GTCGAAGTGAAAACGTCTCCACTTCTTGTGATATCATTCAACGTT
CCTGCTATAACCGTT
GACGAATTTTTCGGCGCAAATTTAGTCGCCAACATCGCTCTTCT
>ConsensusfromContig2263 Average coverage: 21.43
TTTCGGCCTCTTTTATTTTCTGCTGATGTCTGAGGAACTTGCGTCT
TGTCGCGTCTATGA
TTCCGAGTTCTTTTGACTTGAGCTCTCGCATCATACTGCGGAGTCT
ATTCCTTTGATGGG
TAAGTTTCTGTTCCTCTTGGAGCACCAATTGCCTTCTTTTTTCTTC
GGCATCCTGCAAAA

GATTTTGCTGTCTGAACCACGCCTCCTCCTCCTGTTGGCGTACCT
CAGCGGCCATTTGGC
GGTTAACGGCGGCGTCGCGTTCTTGCAAATATTCGACCTCTTCTT
GGCGAATTCTCTCCC
TTTCATGAATCTGGTAGTCAACAATATATTTGGGATACTTGTTGA
ATATGGGGTATTGGG
TCAAGGTCAGAGGTTCAAAGGCCAGCAG
>ConsensusfromContig2264 Average coverage: 10.82
CTTCGAATGAGCCATTTCCTTCGAATGAGCCGCGTCTTTCGGATG
ATCCGTGACCTTCAA
ATGAGCCGCGCCCTTCAAATGAACTTTGACCTTCGTATGAGCCTT
GACCTTTGAATGAGA
AGCGACCTTCGAATGAGCCTTGACCTTCGTTTGAACTATGACCCA
TGGATGAATCCTCGT
TTGGGGTCAGGGCGGAATTCGAGCTGGTGGCTCCATCATTCGTCG
TCGTCGATCTCAGCG
AATGCTTTTCCATGTGCTTCTGGAGATAAGCTCCTTGGGTGTAGG
ACTTTCCGCAGATCT
GACAGAGATGAGTTTTGAGGTGCTTGGTCAGCATGTGTTTG
>ConsensusfromContig2265 Average coverage: 37.13
CTCTTCCGATCTCGACCGTCTTTCCACCTTATCACCTTCATTCTTAA
ATCTTTTCTTTTG
AGAAGTTTCGCGTCCAATGAATAACCGATTCTAGGACATGCTATA
TCATCTGGATTCAAT
GTTAAAACAGGGCACAAGTCATTACTCCCGTCTCCAACATACAC
AATTGAATGGAAAGTG
CTCCCTCTTTTCTGCTGATGCTTTATATGAGAATTCAAAATTTGAC
CTTTACACATATTT
CTCGCACAGTTGTCACATGATTCTTGCTCTTGGCAATACTGAATA
TGTAGGCATTCATTC
TCATCGAAGTGAGCAGGATTTGAATATGTTTTTCTCACGCAATTA
GAAAGATCATTTGCT
TTTAAAATTGCATCTATGAAATAAGTGTTAGAGTCTGAGATAATT
ATGACTTCATTATGG
AGATAATGTAGCTTAGTCAACAGATCCTTCATACCCTCTACAAGA
GACAGTCCCCGAAGA
CATCTATCGTAATCATCACTGTTGATTCCATTTGAATGAAAGTAT
TGAAATATCAACCTC
ATATGATCTGTCCAGCATTGCTCTCTCGGACAAGAATCCAAATAT
TCAGGCAAAATTCCA
CCGGGACATTTTAAGGCAAGTGCTCGTATGTCAACATCACTGTTA
CTGTTTAAAATCGTA

TTGTCGAAATCGAATGCCCATAGGACATTCCTCATAATGTTAGAT
GGGTTTATTCACTAT
GAAAACCGCGCTGAATGATGTTCTCAAGTTGTATT
>ConsensusfromContig2266 Average coverage: 11.97
TACACGACGCTCTTCCGATCTCTCGAAGAAGCATTTTTGTCAACG
AAGAAGGCGCGCAGC
AGACCTCCAAAGAGAGCGGCTTCTGAACCTCTGACCATCACATCT
TCGATCGATGTTGGA
GCTTCAAGAGAGAAGGATGAGATGAAATTGGACAAACAGATCAA
AGCCATAGAGGACTCA
ATGTGGAAACACAAGCAAGAAGAAAGAGAGCTCAAGCGAACAG
AAGGGGACATCATCAAA
AACCAGTACAGCATTCGTTCATCCTTGAGAGACTATGAAAAAAA
GACTCTCAATCAAAGA
CTTTCAGAGACGAAAAAACTTAATGAAGGTCTCGAAAAGTACGT
GGTAGAACAACAAGAA
TTCGCCCACAAAAAAGAAAAGGATTTGAAAGCCAGAATTGA
>ConsensusfromContig2267 Average coverage: 7.02
GATCTTTGGATTCCTCCTTCCCTCTACTTGCCTTTCAGGTGCCTCC
CATGTTGTCCTATG
TGCCATTTTGTGTTTGTACATGGTGTTGGAAAGGAGTAGTTTGTTG
TTTTCACAGAATTT
TAGTAGGTGTTCACCATTTTCATTCATCTCTCCTTTCCCATACCTG
CCCATATTCTCTGG
ATAATCACGATAAGCTGAGCCTGTTTTGGCATTAAAGTCCCCTAG
TAATATAATTATTTC
TTGGGAGCTTATGATCGTAAAG
>ConsensusfromContig2268 Average coverage: 15.80
ACACCACTGCCGACAAATCTCTCGACAATAATGACACCAGTCGAT
TATAGTATTATTGCT
GAAATTCCCAGTAGTAGTGGCTTCATGTTTTATATCTTTTTGTGAC
ATTTTGTGTGACCA
GCAATAGAGTATCAATACCAGACTTTCAAGTTCTACATGGCTCTT
GTAGAAAAATGAGCC
ATATCTTATTGATTTTTGTGAATTACAGACTTTACAAGCCCATCGT
ACACCATCTTGTGA
TTCTTTCCAACTCCGTAAAGCAGAAAGTGTTCCACAACAAATCAT
TGAATTATTGATGAG
GCGATGTCTTGCGAGCCATTCTCTCGCTCTCTTCTTATCAACAAA
GTTATCCACTAATAG
ACGAATTGTCCAATTCTCAAAAATTATATCTGAATCTTTGAGGAT
TTCAACTTTGTCAAC

TATAGTAACTGGACCAGGAGCAGGCATGCTTAAAATACAAGTGT
ATCCCCAAAAACTTTA
ATCCCTTAGTTTTATAATATCAACAGTAGG
>ConsensusfromContig2269 Average coverage: 44.15
CTCTTCCGATCTAAATGAATGGCTAATTTTGGAGATCAAGTTAAA
TGCAAAACTGAATGG
GGACAGTGGTGGCAGACAATAGGGGAAGTATATGTAGAATATGT
CCATGGTTCCAATGAT
TTAAAAGCAAAAGATGTGCAGTGCAATTTTTCTGCTACAAATATA
GATTTGATGATAAAG
AGATCATGCATTTTTAAGGGAAAATTAGCTGGAATTATTTGTGTA
GATGAGAGTACATGG
ACCATAGAAGATGGAAAGATTGTATCAATCTGTTTATCCAAGGC
AAGAGAGGATGCTTCA
AATTCGTGGAGGTCATTGCTTGATGGGTTGTATGTTGCGGACCCT
ATTGTATTTGATGAG
ATGCAAAAAAAGCTGACCCTGGAGCGCTATCAAAATGAGAATCC
TGGCATGGATTTTAGC
GGAGCCGAAATAACAGGAAACTATCATGGGGGAGGACCGAAATT
TTAATGATGAGAAAAT
GAAATCGATATTCTTATGTAACATAAGTGTTGGAGCAGGATCTTT
GTCGAGGACGCATCA
TAATACTGCCATTTTACTACTTGATGCATTTTATAGAATTATTTGG
AATTGGAAG
>ConsensusfromContig2270 Average coverage: 16.14
TCGACAGTGTCCCCCTCGGGGGGTCACAACACTCCAGGGTCTGAT
CAGGATCCAAGTCAA
TTAGACGAGACATCACGATATGTTCCTGAAAAGTTGACCCACTTT
CGATGGATCATCCCC
CCCAACGGAGAGATCCCCCTCAGGCTCCGTTTCGAGTCCTCGGAT
ATCGGCCAATTCGAC
CAGACTCTCAATTTCGAGATAACGGGCACCAGGCGGCGGTACCA
GCTCTACTGTCGGGGC
ATGTGTGCTTTCCCCACCATCAGCCGAGAACCAAGAATCGTATTT
CCATCTCGAAAGAAG
GCGAAAGGAACGGATGAAATCATACACAAGAAATACGTGATGAG
CACAGAAACGTACGAG
TTTGGCCCGCTTTTGGTCGGCCGCTCTAGGGATAAGTATAAAGA
AGGGAAAATCACAGAA
AACATGGAGAAGTTCACAATATCGAACACGTCACCACTAGAGGC
GGAAGTGACGTTCTGT
TTTCGGCACGACACCAAGGCCGATACATATATCCTTGACCCCCAG

AATGCATGCTTGAAA
CCCGGAGAATCACAGGAACTGCGAGTGTGGGCGTTCCCAAGAGC
ACCAGGTACATACGCA
GACGCGATCGTCTGCTGCGTGAAAGAAAACCCTGAACCAATCACA
TTCAACTTATTATGT
CACGGGGTCAGACCTGAACTGGAACT
>ConsensusfromContig2271 Average coverage: 35.14
CATCATGATACTCCATTTGTGAAACGGATGGAAAACTGCATTCAT
AATTATAATGTATTA
TTTGCTGTGTGTTGACATACTGATCATCTTTCTTAAATTTAACAGC
AGAAGTTGTTGTTT
TATCCAGCGTTACTGTTTTCACATCATATTTATGTTTTATATCAGC
TTCCCCTTCATGTA
ATTTCTTATTATTCATAGCAAGTCGTTGCTCCGCTATGCTCTTCTT
CACTTCCTCCAGAT
TGTCCATTTTGTCCGTTTTGTCAGTTTTTTATAATATATAAATTTG
AACTTTAGTTTTCA
TTTACACAAATGGATAAATCTGTTTTTTTATACCTATTTATAACTTT
TTCCTTTCTTTTG
AAGAGTGACTAAACGGA
>ConsensusfromContig2272 Average coverage: 4.94
AGCTGTGAACAAGGGGTCTTCCTGACAATTAACCTGCAATCATGG
TTTTCTCTGTATAAC
TATGTCTCAATTTTTAAGATCTATGTGTAATTACACTACTTTCCTG
ACTGGCGTATTGCA
TCAGGCCAAACTGGGAAATAGGAGGGTTTTCAACAATCGATTCG
GTGAGCACCTCAAGTT
TTATCAGCTATCCTCCATATTGGCATCTGATGTTTTCGGTATTCCT
CGGTGAATCCCTTT
TTTTTCAATTTAGGGGGTCAGATAAGGGTTAGGGAGGAGAGTGTT
AGGATTAGGGTATTG
GTAGAATTAGAGTAGGGGTCAGGGGAGGTTAATGTCTGGGGTCA
GGGTTCATTCAGGGGT
AATATTAGGGTCAGGG
>ConsensusfromContig2273 Average coverage: 20.99
GTTGTTATTTCGGTTCGCTTTATTGAACCATCTTCCAAAGCGTCTT
CGGAGAAAGTCTTG
TTCCTAACTTTCAGACCAGACTCTGATTCTGGTTCTTCGACAATTT
TTTCAACAGATTCC
GCCGTTTCGCTGCCGATAATCGTTTCTGATACAATTTTATGTTTC
CATGAACCATCAGGA
AATACTTCCTTACATTCCCGGGATGTTTCAAACTTAGGTAAACTC

TCTGTATCAACAACA
CCAACGGGAACCAATACAACCTCTTCAGCGATTTGTATACCCAAG
ATCTTTTCGATTTCA
CGAGAACTTTGTACTCCATCTTGGTCAATAGTCTGAATAACAGGC
ATAATATGACGAGTC
GTTTTAATAAC
>ConsensusfromContig2274 Average coverage: 49.45
TGGAATTTCTCGGGGTTTAAATGGTTTATCTGTATCGTATGAATTT
ATTCAATCAATTTT
AAATCTCTTTTTTTAATTTGTGTAAATAGTGGAATCTATTATTAAT
TTTAGCATTTTTGA
TCTTAGCGTATTTTGATATGCGTCGTCGAATGGGTTTAATTGAAAT
CGATTGTTTTTATT
AATTTTACATTCGTTTTTAACAAAGGGTTATTCTATAGTTTATTT
TCAACGCGGCAAAAG
TTCGTTATTATCTTATATTGGATCCATATATGTGTTAATACGAACA
CAATCACGTTTCGG
ATTTCATCGTTGTAAATAGTCTTGTTTTGATTTGGCTACGTTTGGC
GCCTTTTCGGCCTG
CTACCGCCAGTATTCGATTAAGTTGAAGGTCGTATCGGCTTTACA
TTATCTGACTGTGAT
ACAATAATTGTTTTGTTTGTTTATGTTTCGCTCTAAATTGCTTTAT
TCGAAACCCGCTAT
TTCCTTCGCATAATCATCGCTTGCTCAGCCATAACACACGCCATG
CATAACTCCCATGAT
ATTTGCCCCCGTGGCATTTGTTCAATATATTATCTCGTCTTGTTTT
TGTATGATTGTGGC
AGATTCTAGGTTTGGAGTCCGCTTGTGAATATTTTTGGCCATATT
AAATCGCTTTCAGAT
CGGAAGAGCACACG
>ConsensusfromContig2275 Average coverage: 43.27
ATTTATCCGGTTGACAGAGGCGTTGAACATAGCATATCCCACAAT
ATTTCTCCTGGCAAT
AGTCTTGCAATTCCTCAGAGCATCACCGTATAATTTCTGCCATCC
TTCCCAACTGTCGTA
TAACGGATTTGTGGGCGTGATGTCAGCTCCGAACCATTGAACGTC
TGGTCCGCTAAAGTA
TTCATTGTTTGCAGAGGAGCAAACGGTTACTTTCTTCGTATCTTG
TAACCGCCAGGGGCG
TGGAGTAGGCGAAACCATGATTAGATAGATCCTGTCAGCCGTTGA
CTTTCGGACGACGTT
GATGATGCCTTCGGAATTGAGATCCACCATCTCTGATCTGTGTGG

AGCAGCAAACTCGAG
GACGAAAGCCATATCCCTTCCAGTCTTTTCGAATGTACATCCGGA
CGTCGGTAGAGCGGG
GAAGCTGACAGCAGCCTTTACTGCATGATTCTGAACCGGGGATGG
TTTGAGTTCACACTC
GTCCGTTTTATCTTCTTCCTCGACGTCGGATATGGAAAGCTCGAC
TGAGACCGCCCACTT
GAAGGTGAACCACGCCGTGACATGTTCGTACTTCCTGATGATGGT
TCGGTTCAATCTTCC
GTGCGTTTTTATTGGAGGAACATCGAAGAAGTAGGTGTGGTCTTT
AAAAAGCTTCCTCCC
ATTGGCGAATCGCATTGTCTGCTCGTAAGGGTGGAATATCCTGTT
CTTTGTTCCGTGAAA
GTGTTTTGTATCGATGTAGATTTTGTAGGAAGTGTCGATCTCAAA
AGGACTGTAGATGAA
GACAACCACATTCTGCGTCACATTTCTGTCCCCCTCTGACGTGATG
TTGATTGTCATATC
TTTCGAATCTGTTTCGCTCAGAGGCCCAGTGAATTCCAGTACATA
GACTTGATAGTCCAC
AAATTCCGTAGATCCCTTATCGGCTCGTCTGCCCAGTCTGTTTGCC
TTGCACCATTGGAT
GGGTTGTGACCCAATGGGTTTGTATCCGTCCATGTACACAGACTG
GAATCCTTTATCGAA
AGTACAATAGCGGTCCCAACGCCCTTTCGCCCCATCCTTCAAACT
ACCACCCTGAGCTTC
GGTCGCAGGAATGGCACAACACAATGTAATGATCCCGAAAATAA
CACAATGAGGAATATT
CTTCATAATATTCACATTATATATCCAATTTCTGATGATAAATCTT
CACATAGAAAGTTT
GTAATGACAAGTTCAACCCATTCCAAAAATCTGGCAAACGTTAAA
TATTT
>ConsensusfromContig2276 Average coverage: 23.15
ATGTATGCATAACTCTTTTCTGTCCCTCCTCGCAAAACACGATAA
AGGTCCATTTCCGGG
TATTTGAAAATGTTTATGTTTGGGTTGGTTCCTTTTTCTGCAGCAG
CAAAGTACAGTTTC
GATGGATGAATCGCGATGGCCCCTATACCTCGCCCCCCAGTTGAG
CGAAAATACGTGTGT
TCCTTCGTCTCCAGGTTGAGCATCTGGAGGTAATTTCCGGCGACG
AAGCAAGAGACCCGT
GGCGCCACCATGATGAGGTTGGACCGGTTCTCGCAGTCGTACCCG
TACGAGTGGTCCAAA

GTAAGAAAGTTATCCGGCAAAGCGGAATCTTCGGTGACGAAACT
TGTGGAAACCAATTCA
GCAAAATTGTAGACGAAATCGACCGGAATTCCTCCATCGTCTGCT
TGGCCTTCTTCGCCC
AGAATGTCAACATCGAAGG
>ConsensusfromContig2277 Average coverage: 13.10
GCTAAGTCATTCATGATCCGGATTATGTTATATGTACAAAAACGG
AACGTTATCAAAGAT
GGCTTGAAAATAAAGAAAAATATTCACAGAGGCACAGTACCATG
CCATCGATTAGAAGAG
GCCCAACGGAATATCACACAAGTTGCACAGTCATTATTTTATGCC
TTTTCACAGGCAGTA
TTTCTGTTACAATGACTCGCTACCCATGGATCATTATGCGGGCTTG
GATTATTCTCATAC
TTGTGACAGATTCAAAAGTACTCTGTAGATTCACATTCCAAATTC
GGCTTGGAACTTTCA
CGAACGATCTCGGTTTATCATCAGACGGTTTATGCTGTGAGGGCG
AAGGCACCGCAATAT
GCTCGCAGCCTTGTCGAACGTTTTTCCACATTTGCGTCTCATACT
ACGAGAAAAATTTCA
GAGAGCATTGCCATTTCGGACAGCATTATACAGATATTTTTCATA
CGAATTCTTTTGATG
CTTCTGAGGCCACATTTCCCATGTCGTCCAACAACGACTTCGCCT
GGCCGGGTTCTTTTA
CAATGTCAATTACTGCATATCATTCTAAAGTGAAGGATTTCAAAA
ACTTGACATCCAATA
CACCCCGCAAAGTAATACTGGAGACTTTAGAGTCGGGCATAAAC
AAAAGTCTTCCGATCG
AATGGACGGAGCGACAGGCCCACAACGACGTGGCAACTCTCTCAT
TCAACTACTCCGTTG
TGTGCCAGCCCCACAACTACGGGCCCCGATGCGAACACAACTGC
AAGGCCAGGGACGACT
CCTTGGGCCACTACACTTGCCATTACGACAAAGAACACGGCTTCC
GCAGGGAGTGCCTCA
ACGACGGTTGGACCGGAAAATACTGCGACAAACCCGTTTGCCTG
GAGGAGTGCAAAGGGA
CCTGCAACGCTCCCCATGAATGCATCTGCGA
>ConsensusfromContig2278 Average coverage: 13.14
GAAACATGAAGGCCACTCATTTCACACTTGGATCGCAGCCACTCG
GAAACTTTAGCACAA
TTCCAAGACTCGACACTTTCTTCGGCTGTTGCATTGATTGGAGGT
AAGGGTGGGGTAGAA

GACATCAATCCAAGTGTACGTTCTGACACAGGAGTTATGAGTGGT
TGGTCGACTTTCTGT
GGATTTTTGGGTGCCGTTTCCTTCCGTATTTCTCGTATCAATCCAT
TAATGGAATTTTCG
AAGCGTTCCTCCTGGCTGAAGTCAAAGAAGAGCTTTGTTCCGACC
ATGATGCCAAGCCAG
CCGTCGGCAGAATACCGATGCTGCACCCGCATGGGAATGAATGTC
TTCCGTAATTTATAA
GTGTATTCAGCCTCTGTTCTGCAGCTGGGGCTCTCTTTGTATTTC
TGGGACATGGCAACC
AAAACGACCGCCGCGTCTTCAACAGCCTTGGCCATTGCTTCCAAT
GTTGATCCCGTCATA
TCGTCGACATCCATCCATACACTGAAGCCGTGGGATACAAGAGC
GTCACGCACTTGAAGC
ATGACGGGTTTTGACCCCCAATTGTAACTGATCATAATGTGACCTT
TCTGTTCTTTCGAT
TTATCCTCATCTGCATTGACTTGTTCTTCTTGACCCTCAATCACCC
ATACAGCACCCGCA
GCCGCTCGCTTCACTCCATATGACTCCGATGTTGACAGGTGGCGC
AGCCTTTCAAGCGCA
TCTGTCATTGTCTTGATTTCACCCTTACAAGACTCGTTGAATGCC
AGCGTCCAGAAGCCC
TGAGCAGCCAATTTCTGTCTCTTCGTGCAATCGTCATGAAGGAGC
ATTGCATAGTAATCC
AATGCACCTGCATCCACAATTCTTTGTTTATTGGAATCATTGGCA
GCAATCAGATTGAGT
GCTTGTACAATTTCCCAGATCGGAAGAGCACATACTG
>ConsensusfromContig2279 Average coverage: 26.08
ATCTCATATTTTGCTTCTTCTATGACAACAGACGCACATGCCAAA
CAATGTCTAGCATTT
TTGAAGTCATTATTGATTATATAAAACTGTGATAAAGTTGCAGCG
TTTAATGACCAATCT
AGAGGATTGAATTTACCTGAAATGATCTGCCTCTTTAAAGTAGCA
TAGCAGTACTCTGCT
GATTTCGAATGATTATCAAGTTTTCCATAAATTTGAGCTTTATAA
TAGAGAGTGTGAGTG
AAAATATCTTCAAACTCCTCTTCTCGTTTAGAATCTGATTTTGAAC
AATCTTTATTGAAT
AGGTACATTTCGTCTACCATTAGCGGGGGATGATCTTTAGATTTG
AAGACATTGAATGTA
TCAGCAGCATTATCAAGATACTCCATTGATTTCTCATATTCTTCC
CTGACAGACCAGACA

ATACTCAATGAGTTCATGATATTAAGATATAGGCAGCATGAAAAT
TCATCAATTCCATAA
TACATTAATGTGTCAGCAGCAAGTGTCAATTTTTCTGAAGATTCA
CTCAGTTCCTCAGTT
TCTCTAAAGTTTTCACCAAGATAATAATCTATTAGAGCTAATAACT
GCTTGATTTTCTTC
TGTGCAGTACCTTTTTTATCATCATCACCAACATAAGTTTGTCTGT
TGTTAGCTATCTTA
TCCCTGAGTAGATTTATATCTGATCTTCCTTTATATTTTGATTTGA
AAGGTTCTGCCTCA
GGATCATTGCTGGAATTCCTCATAAGAATCTTGTAATTTTCTAAAG
ATTCTTGTATTTCT
ACATAAAATTCCTGATTTGAATTTGAATCGGTC
>ConsensusfromContig2280 Average coverage: 6.72
GCAGAAATAAGCAATTCTAGAAAAACAAGTGACATGGGCATATT
AAACGCTGAAATGAGA
ATTGTGCATCAAACAGAGAGTTTAGGTCAACCAAAAGTGACGTA
TGCATATCAACCGCAA
AAATGTTATTCAGCAAAAAAGCGAGTGTTATGTCAAACAGGAAA
GTGACATAAGCATAAA
AAGCGCAGAAAAATGTTATGCGGCGAAAAACGAGTGTTATGTCA
AACAGAAAATTGACGT
GAGGAAATCACACGTTAAAAACTGTCATGCAGCAAATAAAAGGC
TCGAATAAATTTTGTT
TTCAATCACAGAGTGTTAGGTCACACAAAATAAACAAAAAGTGA
CGTAATCATATCAAGC
GCAAAGATGAATGTTATGCAGCGAACAACGAGTGTTAAGTGAAG
CAAAGAGAACTGCGAA
ACCAAAACAAGGCATGTGAGCAAACAATACCAGCAAGAAGTGAA
AAG
>ConsensusfromContig2281 Average coverage: 15.63
AAAGGTTCTCCCACCAGCATGAACCCGTGACGTACGATCATCATC
TCGTACATCTGAATA
ATCTTTTCGTTGAAGAAAGTGACGTTTTGAAGATTTTGCGTCTCG
CAAACGTCTTTGACA
GCGTCTAGGAAAACCGTATAGTCGGCCTTCGGGAGAGTGACGCC
GGGGAAGAGATCTGAA
ATGATCCCTTCGAAGAGCGGGATATCGTGCGCCAAAAATTTGGG
AAGATTCACGTCCAAA
ATCGATCGAAGTAGCAGAATATTTTCATCCTCATCCGGGAATTTG
AGCTTGAGGTTTCCA
GCGGCCGAGAGAACGGCCTTGACCGCCCGCATCCCGTAATCGTA

GTGGAATTGGGATGAA
AGCTGCTCGGAGCAAAGTCGGTAGGTCGTGACGATCTTCACGGCC
AGAATCCTCGCGTTG
GTGAAGCCGAAGGAATAGAGCGATATTTCGCCAATCATGGCGTA
GTCCGGCACCATCATA
GCCACCGTTCTGAAGAGCACCTTCA
>ConsensusfromContig2282 Average coverage: 34.49
GGTATGGCTGAATCTCGAACGCCTGAAGCAATTCTTCTTGAGTTC
CAACTATATCGTGAA
GAATTTCATCACTCATTCTCGGAGCCGCAGCGATAAACATTTCAA
CTTTATAGAATTGAT
GAACGCGATAAAGACCACTTTCTTTTGCAGTGTCTGCGGCCTCCG
CCCTGAAGCATCGAC
TGCATGTTGCTATCTTGAGAGGCAATTCAGACGCCACCAGCTGCT
TATCTTTCAAATAGC
CTGCAATAGCTGCTTCACCTGTGCCTATAAGACAATCAGAAGGCG
AAACTCGATAGACTT
GACT
>ConsensusfromContig2283 Average coverage: 28.01
CAATTCGATTTCGTCTCTGGATAATTCTTTCTCCATGCTGAAGAT
ATATTGACGAGCATA
GCAATGAACAGCACAAGATGAGGAGTGATCGCTGTCCAAGGACT
GAACTCTTTCACTGTG
ATCTCTCCTCTTGAAACAACATCCCCAAAATATATTTGAGCGCTC
GCACCCATATAAACG
CCGTAACATGCAGCTATATGAAAAAGTTGGTGACTGGAAGGGTAT
AGATCGTGTATTTTC
GTGAACTTGCAGTATGATTTTTCAGGTAGATGAAGTATTTTAACG
ATAGCCGGAATGGCG
TCAGATATAAATAGCATACAAAAACGCCAAATTATGCCAAAATG
TGCATATATGTCTAGA
GTTTTTAAAGAAGCGAACAAACACAATGGAAAAATAAACATCTCT
TTCACAATTATCACA
GATCCAAGAATCATTTCTTTCTCCTGAGTCGAATAACTTGAAGTC
ATTCCGAACCATCGA
GGAAAAAGTATGGTTGCATGCCAAAATAACGTCGATGAGGTCGA
TAT
>ConsensusfromContig2284 Average coverage: 13.33
TGATGATCAAGATATTGAATATTAGTCATCAATTCTGTTCAAAGC
AGGTGAATTGAGTTC
GGAATTATAATACATATTCGAAACTTTCAAAACCGTTTTGTAGAT
GGAATTGAAATTCTA

CCAAAACCATCCCGATTTTATGTAGATCATTCTATTTGAAGCCGA
TCGATGTTCATGAAA
TTTGATACCCTTTTCATATAATTGATATTCTAGCCCCCCATGTTTT
ACATGATATTCGGA
AAGCTATGAATATCATGTAGATGATATTTCAACCCCAGCTCATGT
AGATGCTATTTGAAA
CTTGTGAATATCATGATGATTTTTAGACCCCGCCCATGTCC
>ConsensusfromContig2285 Average coverage: 13.39
GTGGAATACTTGTCAAAGTCTCTTATGCTGAAATTAATGAAAAAA
TCGAAGAAGCTGTTC
TTAAAGTGAGAGCACCTCGAGCAAATTCAAATAACAGAAACGCA
GAATATCGAGCCTCAT
TGGAGGGACGAGGGATGATGAAACGGAATGAAGATGGAATCTTC
ACGATGTACGCCGTAG
GGAAATACTGTGAGAAAGCCTCTGCAGCCCCTTCTATATGCAGGG
ACCATTACTTGCATA
TCGATGATGAAGAGGAGCACGAGAAGATTGGGGATGTCTCTTGG
TATTGAACGAACCTTT
CCGTGGTAAAAGTGAATAAAAGAGCTCCAATTCTCTGTTTTTCGT
TTCATTGATTGCTTA
TGGTACATTTATTATTTTTTTCGGGA
>ConsensusfromContig2286 Average coverage: 21.19
AAGATCTCCTGGTCCCGTCATCTATACAGGAGGATGGAAGCGCCC
ATGATGGTCTTGAAA
GAACAGGCTGAGATCACAAAGACTGACACATACAAGAAGACGGT
GAAGCTGTACAATCGA
ATGGCGACAGTTCTGATGGAGTTTGAGATCCTGTTCTTCAAGGGC
TGGTGCAACACAGTA
GAAGCCTCCGTCTCCAACCTCCACTCGACCCTCCTCGTACAGCAT
CCTGACAGCAAAGAA
ATCTTCGTCAATCTGGATCCTCAATTCCTCAGAGCATCTTCTGAG
ATCAAATACCTGGAA
AAACTGAACCTGGAGGTGCCAGGACATGCCAAACTCCTCATGAC
CTTCTCGGAAAAAGTC
AAAGACACCAAATCTAGACTTGACGATCTTCTGGAAGAGTACAA
CTCCATCACCGCCGAC
ATCCCCTCGGTGCTGGTTCCTCTCTTCAGACCCTACGAGGAACGG
ATCGATGAGGCCATA
GATCCCGGTCTCAACAAACTTACATGGATGGCCATCAATACAGAT
GATTATCTCGACGAC
GTAGCTTCGAACATTGATCAGCTAAAGGACTTGGTGAAGAACGC
CAAAGACATCCTGGAA

TGCAGAATCGAGTCTGCCCTTGAAGAGATGTCTGAAACGCGGCTC
TGCGAATTGCCTGAA
GACCAACCGATCGACGTTGAATCATTCGTGGAATTGACAAAGAA
GACCTGTGAAGATGCT
TTTGCCTCCATCACCAGACACAGTCAGCTAGTAGAGTGCTCAGTT
CAAGATCTGATCTTG
CT
>ConsensusfromContig2287 Average coverage: 11.43
TCGGCAGGATCCGAGGTACACAATCCATAGAAGAGACTTTCGTTC
GCTGATAACCTCCAC
GAGGCACAAAGGTCATTTTGCTCGGTCGTGTTGTCAGGGGAGGGG
CTTCCGTCGGCCAAG
GTCAAGTCATTGGAAGAGTCATGGTCGTAGAGGCCACACAACCCT
TTGGTCTGATTGAAA
TCCGATGGCGACGCTTGAATCATGACTTCGAAATTTGAGTAGGTG
TGTGAGCCAAATATC
TCTACCATGGCTCCATTGGGGAGAAACACCTTGAATCTTTGACCC
AAGTCATAATCGTAG
ATCTTTGTGCCCGCAGTGAGCGCACCATTCAAGTACAGTTTAGGG
CTGATGGGCAAGGCG
ACGTCCATGGAGCCATCAAGTGCACACTTATCCACGACAAAAAC
ATCGTCACCTGAACGG
ACTGTAACAGCGCAATTGCATGCACCTCGAGATTCCCCGTAATAA
TTTGTACACGACTTT
TGAATGGTCGTAACCATGATATTGTGTGAAGAGTGTTGATAAAGA
ACGAATTCGCCT
>ConsensusfromContig2288 Average coverage: 94.91
TCGACGTTCGAGCCACAAGCCGAGACATATAGGAATCTATGATTT
TCCTATGTATTTGCC
ATGGTGTGTCTGGCGAATCATGTTATATATTGCATCGAGAAAGG
AGGAAAAAAGCATGAC
CGACCGGAGAATATAAAGAATGTATCAACAAAAGGAAGACCGAT
GTCTCCCTTCTCACAT
GCTTCCTCTCAACATCTCTCCAGCTTTTTCTCATCTTGGCAAAATT
CGAAGTCTGCCACG
CCCTCACCATTTATTTCGTCATCAACAACCAAGAAAAATACCCAA
AAAACGTCATTCCCA
CGTGATAAACGTCATCGAAAACTGAAGACTAGAGGAAGATCAGA
AGCCTTGACGTCCTGT
TGCCAAAATGGCGGTCTCTGTGTTCTCAATTCCTTGTGCTTATGT
AAAAGTGAATATTTC
GGCCGACACTGCGAATACAAAAGAGAGGATTGTGGAACAATAGC

TGAAGGAACAATCATG
CATCTTGAGTGTGAGGTCTGCATCTGCACACAAAAAACGATCATC
TGCCAGCCTTTCCAG
TCTGTGAATTGTGCAGAGATTGTACAAAGTTCTGAAACGACGGA
TCAAAACGAGATCAGT
GAAATTCTACAGCGATATAGAGGGAGCGCAAGGTCCATCCACTCG
GATTGGCTCATCTCG
TTTTTGCTTTTATGTGTCTCGTATATTGCATAGTACTTTAGAGAAT
CCATGCACGCCTTG
CTTAATATATACTGTATGGGTTTTTGTTGTGTGTATTTGAAAATT
TCCTGGAAAAAAAA
AAAAAA
>ConsensusfromContig2289 Average coverage: 7.15
CACTCAACGTATCACATACGAAATAATAATACGCGAATGAGGAG
AGATAGCAAAGGCCCT
TGCGAACCGCAGATACTATCCCACCTTTAACATAATAAAATATA
AAAACATATAGCAGGA
CAACATAAAAACATTTTCACAACCATATCAACACAATATAAAGG
TAACAAATATATTAGA
TACACATGAATGGCATTCGACTACTCAAACCAAGAATAATATAAA
TGTTACTGACTGCAG
ACAAAACATCTAGGAATAACACATATATCACTG
>ConsensusfromContig2290 Average coverage: 23.83
CGCGGCCTTTTTACGGTCCGGGCTGATGTACCTCATTCCTTCATCA
GTCAGTCTGCCTTT
CCTCTGAGCTTCGTCGATACTTTCGATCGCCGATGTATATCGCTTG
TGTTCGGATGTTTC
AGTTTTAGGTCTTCTCTCCAGGGAGTCATCTTCTTCATCCGAATTT
CGAGGCCGCCTTTT
TATGAGCGGGTCAACGTCTACATCCTCGCACCACGAGCTCTCCCTT
TCTTTGGCTCCCTT
CTT
>ConsensusfromContig2291 Average coverage: 17.38
ATCTCGTGATACGTCACAGGGTCTTTTTAAGGATTTTCAGAAAAA
TACATCGAAGACTTC
TACCGTGTTTTCATCTGTCCAACCTAAAGATTCGTCTGGTTTTAGA
AATCCTTTAAATTT
TGGACTTTTGAGAGGCAGACGTCGTTCTCCTGCAAAGGCATTTGC
CACACAAGACCCGTT
GAGCGATAATCGAGTCTCGTCTGCTTTAATTTCATCATGTGAAAA
CCATTGTCTCAATTC
GACAGCTTCAGCTCCGAGTAACTTGAAAAAACACACTGCAGCTA

TAGTGAATAATGTTGA
TAAAACTAATCTGAGAAACCCAAACAACTCTCATATAAGCAGCA
GAAAGAAACCTACCTC
TAATTCGTGCTCCTCTGCTGATTTCTGTACAACAAAGTCGTATTCT
CACCATTCCACTAG
GCAGAAGTCGCTTGCTTCTAACGCAAACGGGTCACAGTTATATAC
CACTTTTCATAATGC
GACAAACACTACTGGTTTCATTTCCATTACATCACAATCTTCTCA
TGAACTATCCGTATC
AAGGCCGTCTGCATTTATTCCCAGTACATTACAGTCTTCTGCTTC
GAAGTTGACTACATC
GAGGCCGTCTGCTTTTAATTCGGACGCATCTTCTGCTTTAAAGTT
GACCACATCGAGGCC
GTCTGCTTT
>ConsensusfromContig2292 Average coverage: 5.06
ATTGATTTCGAAACTGGTGTATCATAATGATAATTTCATTATATT
ATATCCTCGAAGACT
ATAAAGTGAAATTGTCTCACAAACAAGTTATGATATTGATCATTC
CCTGGAGACCAAAGC
ATTCAAACATTTTCCGTTGTCTGTATAAACTAATATTGATTTCGA
AACTGGTGTATCATA
ATAATAATTTCATTATATTATATCCTTGAAGACTCTAAAGTGAAA
TTGTCTCACAAACAA
GTTATGATATTGATCATTCCCTGGAGACCAAA
>ConsensusfromContig2293 Average coverage: 19.64
CAGCTGGAATGGCGATGAGATCCTTGGTTAGAGTCTTTGCCTGGT
CTCAAGGCCTTGTCT
AGGATGTCCAGTTGCTCTGCCAGCTCACCGCTGTAGTCAAACTTTT
TTGTGTTTCTCGTC
TCATCACCAGAATTCAATTTTGTCTGTGGCTCTAACTTAACCAGT
GGCCATGATGTCCAC
GTTCTAGGGGCCCTCCGTGCTCCCGAAATCCACATGTGGATAATG
TGCAGCAAACCGTCT
TTGAAATCTTCATCGAAATGGCCCAATGAATCAGGAAATGTTCGT
GTGAACGAACAATAT
ACAGATTGGGCCAGAAGATCAGGAACGATCGAAAGTATTTTATCC
GAGTTTTCCAGTTTT
CGGGCCCAAACACACATATCGACGTAATTAGATGCAATTCGATC
GAACAGGTCGCTCTGG
TAGTGTTTATCGGGCTGCCAAATATTTTGAAACACCCACCAGTAA
GTATCCAGAATTATT
GCTTCCATGAAATCGGAATAAATGAGAACATATGCATCTCGTCCA

AATTTCGTCTGTTTA
CTCAAAGCATACTGGCTATTGTAAACAATATCAACTATATCTCTA
CACTTCACATCATTC
GGAAGTGTATTTGGACCTTTAGCGCTATATCCCGGGTACCTGTAA
AGCTCCACAAATTTC
GGTTTACCGTCATCAGCTTTTTCAATTGTTTGTTTGGATCCAGGC
TTATTTTCGAAATC
TGTCTCACTCTCTCACTTATTCCTGGCGTGAATGTTGCTTTTATGG
TATAAATTCCCGCA
AATTTATTTATTTCATTGAGTTCTCTGACTTTTTTATTATGATTTTC
CCTTCTCAATGAA
CTTGTTGGTCGGTTCTCTGAGCATGATTGTACACTGTCAGAATCA
GAGATGGTTTGGATG
CTGCCAAGATCTTCAAATATACTCAATTCTCCATCCAGACTTCCAA
TTTTCTGATTCAAC
TCATCTATTGATCCTATCTGAAATGGGGGTTTCTCAGTGATCAGA
GAGCTGGTGATTTCT
GAGTTAATTTCTTCAATTGATTCTCCATCAAGCCATTGAATAGGA
GTTGCTTGTCTCTGG
ATAATTAAAGCATTTTC
>ConsensusfromContig2294 Average coverage: 24.81
GCTTGGATCAATTTCATCATGTACATCCGAAGGCTTGACTTTATC
GGGATATATGTGATC
ATGTTTGTGACTGTTCTAGTTAGTTTATTAAAAGCGGTTGTTGTGT
ACCTGTTCTTTGTT
CTCGCCTTCGCTGGGTCATTCTTCATTCTTATGAATTCTCAGCAAG
CATTCAACAATTTA
TGGATAGCAATTCTAAATGTCTACGTGATGACGTTTGGCGAAATC
GAATTCGTCGACACT
TTCATTTCCAGCGATTTGAAAACGTTCAAAGCAGACGTATATTTTC
TCCTCGCAGTTTTT
CTCTTTCTTATGCCTCTTGTGTTAACAAACTTGATGATAGGTATT
GCGGTGGGGGATATA
GAAAAAATACAGAAAAAAGCATATATCAAAAGAATAGGATTACA
GGTGGACTTACTCTTT
CATATCGAAAACACCATGCCGACTTTTCTGCAGAAGAAAGT
>ConsensusfromContig2295 Average coverage: 7.75
CCGGAAGCTGGATACGTGTGGCTGCTGCTCTCTCGTCAGCCTCAG
CATGGGAAAGTCTCT
GACGTTCCAGCAGTCTGTTCGAGAAGCCATCGTCATGCAGCGAAG
CCATCTCTGCAAGAA
TCCAACGTGTCAGACAGAGTTGTGGAAGTACAAGCATAGACTGG

ACATCGCCCATATCAA
CGTTGAGAGACTCAAAGAGACGTTAAGATATATGAACCCTTCTGT
TCAAAATCGTCACCA
ACAAGAGAGCTTGGCTCTCCCCTATTCAAGTCACGTGACAAATAA
TGGCGACCGGATTCA
CTCACATTCAATCACTGAAACTGCGAAAAATAAATCTGATAAAA
AACGTAAAAA
>ConsensusfromContig2296 Average coverage: 52.63
TTTGGAATATCATACATAATAAGATGTAGTGAGGAAGACATGAAT
GAATTTGAATACCTA
ATCAAAGAATTAAACATTAAAATCAATGATATAAAAGCGTTCTTA
CAAGATATTCCACCG
ATTTTGTGGATGGAAATATTATACACGGTTAATTCAGTTACAATT
CAATCATAATCGAGA
GTAATATATTGTCGAACGTATTTCAATTTCAAATTAGAACATATG
TGATTAAACCTGATA
CTATGAATCCAGATATTTCATTGATTACCATGAACTATGATGATA
CACGAATTTCACACA
GATTCCATATCACTTTCAATCCCACGAGAATTTCAGCAGTATTTG
CACAAAGTGGAACGT
TCATATAATCAACGCACATTCCTGGCGGACAACCTAACACCTCAT
AAGTATAAATTGAAA
GACACGCCTTACCGTATTAGGAAATGATGTATACGGCAGTATTG
CAGGAGAAAGCATGGC
ACATGTTGCTAACATAATATGTTCCCCTAAACCTATACATAGACA
TTCATGCGAAAGAAA
GACCTTGTAGATTTACCCATTCAATTGAACAATTTCATGAAGAAA
TATACATCAACTCTA
CAGCAAAACACTTACACTCAAAGAACAAGTGAAAACCCACATTT
ATAAGGTAATATTGAC
TCATACGGTGACTTTTAAAAGATAAGCTTAAGAGCATAACACAG
AAAAGAATTACATCCC
ACCGAATCCACA
>ConsensusfromContig2297 Average coverage: 20.42
TTCTTACTGCAGATATTTGCGAAATAGTAAGAGGGGTGACTATAT
TTGATGATGTCAGTT
GATTACACGAATTTCGAGATATTTCTTGTTTTGCAGTTACCACAG
GAAATTTAGGCATCG
CGGGAGGTATAACCACATTCGATGTTGCAAGCAAAGTACCTGTAC
CAACCATCCCTGCTG
GTATTTTTACGGCAGACATTTGAGACATGGCTAAAGGAAGAGCTA
TATTTGAAGATGCGG

GCATATTACATGTAGTACCCATTCCTGGTGGGATTTTAACTGTAG
ACATTTGAGATATGG
CTAAAGGAAGAGTCATATTTGATGATGCGGGCAAAGTACATGTAC
TACCCATTCCTACTG
TAGACATTTGAGATATGGCTATAGGAATAACTATATTCGATTTCG
CAGCTGA
>ConsensusfromContig2298 Average coverage: 5.96
AACTCGTGATCAAACAGAAATATAATGAAAACATCAGCGACGGC
ATAAGATTTTCATTGA
GGCCATATAATTCAACACATATTCACGATTATTTATGCGCATTTG
AACTTTGATATGCGG
TAAATAGCAACTACAGATGATATCAGAATACACAATTCATGATTT
AAAAGTACATTTGGA
CGGTAAATGATCAATGGACCCATCCGATTAAGCTTTAAAACCCT
TAAAAAACCGAAACAG
TCGGAGCTGGAATTTGAATACACGAATGAAAGGAACGGAACGAC
GCGCGCGGAAAACATT
TCCGGAAGTTGATTGGCTCACTGTTTATAAACGGACGCTGTTATA
TCTTCTAAAGAAGGT
CAGTTTAGCAACTGGGCAGGGATAAGAATTTGGAGCACTCCAATT
TTTTGTAGCTGACTG
TACAACTCTGTTGAAATAATGTTGACTTTCTTTGGTGTGAAATGG
TCTTTCAATCAATCA
ATATACTATAATTGAAATATCAGAATTATTTTTCATATTAGAATA
AATACAAAATAATAT
ACA
>ConsensusfromContig2299 Average coverage: 44.35
CAGGCTGAACTTCTGGACAAACGAATACTCTATTTTGATTTACTG
AACTCCTCAATGATT
ATGATATCATAAAAATATAATGTCGTCATCAGCATCCGGACGTTT
CAGAAGTTCAAAATG
GGATCCAATTTTGATCATATTTCAAATGATCACAATGCAGTCGCT
GTTCTACCTAGGTTC
TTCTTTTTTAATTCTTATAGTTGACACACTGTTTGACATTCCAAAG
TCATTATCACAAGT
ATTTGGTTATAAGACCTTACAGTTAAATAACATGGAGGGGCGACT
ACTAGTCATCAGTTT
CATCTCGAATTCTTTCATTGGGTCGATAGTCTTATGGTATGCGAT
CAAGCGAACGAAACA
ATGCCTGGACTTCGCTGCCACGTGTCATGGCTTTCATCTTCTATTT
TGCTGGATTTACAA
CCGCGCGTTTCCCAACACGTTGTCATGGTGGCTAGTACAAGGGAT

CTGTGTCACCATAAC
AACGGTTTTAGCAGAGTTCTTGTGTATGCGTACAGAAATGAAGGC
TATTCCGGTTTCTTT
GAATTCAAGGGTTGATTTATAGCATATAAAAAGTTATCCGGTTCT
ATACTCATCCAGTAA
GCCAAATTTTGAATTTATTTCGTCCCCATCATTGATCGGTCATTCG
TGTATAAATGTTGT
ACTTCACAGAATATATTGTGATAATTCATATTCTAGTTATTAATT
AGGAAATGCGATTTC
ATGATAAGCATTTCAAATCTGA
>ConsensusfromContig2300 Average coverage: 23.39
TCTCGAACAATATCCGAACATAAACAGGACATCTGACTAATCTCA
TGGAATTTCTTGGGA
TTCATCATCACAACTGAATTACTACCATATTCATTAGCATCAGCA
TTTATGGATATCTGG
CTCTGTTGAGGCACAATCAATGACTGGAATTCGGTATAAGCCAC
AGCAAATGGATACAAA
TATCTCTCGAGGGGGTTATAACCACACTTATGCAAATCCATTATT
AGAAGTGTAGAGATA
CAGGGGACATCATCAATACTATAATTCTCAAAATAATTCTTGAAT
TCTTCACTGAAATTT
TGCCATAGGTTTTGAGAGAAAAATTCAGATGCTTTGATGTTTAGC
ATATAGTGATGAGAT
TCCAATAGATTTATAATTTGTCGTAAAATATTTCTGATTTTTTCTG
TATTCTCAGATAAC
ATCATATGAACCTTTCGATAATGTAGATGATTAGGAAGAATACTG
ATAGTTCGGATGCTC
ACATCCCAGCTGTTCCAACGTAAAATAACAATGAAAATTATGAA
>ConsensusfromContig2301 Average coverage: 37.46
CGTTTCGTGGCAGAACTTTCATGAATACATGTACATCTTGAAAAT
GTTTTTCAAGATGTT
CTTTAAGACATTCTTGAGGAGAGTCAGTGATTATGATTTCCTTTG
TTTTATGAAAATCTG
GGTCTATACCCAAGCTAACTCTTATACCAATCGGGTTATTCATCT
TAATATAAACCCATT
TCATTGGTTTGATCTTACATTCTTCAAAAGATTTTGTAAATGCTTC
GGGATTTGAAATAT
AATACTTTATTGTGCCAATATATGTTTTACCCTCTACTCCATCATA
TTGATTCGTGTGAC
TCGTGTCACTTGAAGTGTCATTAGATTGTAAAGAATTCGGGAACA
AGGAAATCAATATTT
CACTATTCTCTTTCAAGATTTCTGTATCCAAGATCTCTTTATTCAC

AAACACTTGTCTCC
ATATATTGTACAATGACTGGCATTGAACTGTAAATTCATCATCATT
TGATAATTCATTTT
TCTCAATTTCATCATTGACGAAAACATATGATCCCTTTGGAGTTG
TGAAAAGGCTATTCA
ATCTTTTTTCAACATCCT
>ConsensusfromContig2302 Average coverage: 30.73
CATTCGGGGGGCAGCTGGCGGATTTTCTTCGCAAGGCCGGTGTCC
TCACTACTACCCAAG
TTCGCAAGGTGTTCAATTGTGGGGGCTTCGGCATGGAAGCTGTTC
TGCTCCTCATCGTTG
GTGTTTCCAGAGACACGACTTCTGCAATAGCTTGTCTCACGTTAG
CCGTGGGATTCAGCG
GTTTCGCCATCTCTGGCTTCAACGTGAACCACCTGGACATTGCCC
CCCGATTCGCCAGCA
TCCTGATGGGGATGTCCAACGGCATCGGGACGCTGGCGGGGATGT
TCTGTCCGATCGTCA
CGGCCAAGCTCACCAAACACGATACTGTGGACGAGTGGCAGACA
GTTTTCGTGATCGCCA
GCTTGATTCATTTTGCGGGAGTTATTTTCTATGGGATTTTCGCTT
CGGGGGAACTCCAAG
AATGGGCGAAGGACCCAGGGGCGGACGAGTGGGTAATGCAGGA
CCGGGGGGACGACCCGG
AATTTCTCGACAAACAATACGGAGCTGTCACGAATGGTATTGAA
AAAAGCTCCATACAAA
GACAGAACTCCAAAGGTCAAGCTCCTCCGGGGGGCAGACCCCCT
CCCCCCACCAATATGC
CCCCGTCAGACTGTCAACAGAAGATGATACAGAAGCCCAACCAA
GAGGAGGCTGAGGCCC
AGCCGTTGGATGGAGGTGAGAGGCGCTCTGAGGTCACATCTCCC
CTGATGCAGGTTGCGA
GGCCAAGAGCACAGGTTCCGCACAGGAGGAAAAGCAGAGAGTCT
TCCA
>ConsensusfromContig2303 Average coverage: 27.36
TTCAACCCGGCAGACCCAACGTTGGTCGCCACTGCCGGCAGCAG
GTTCGGAATCGCCACG
TGGGACACTCGGCGACCTGGCTTATGCGTTCAGCGCTACAAGGG
ACGTTCACAAAGGGAA
AAGACGTTCGACGCCATGCACATCTCGTTCAACTCGACCGGAGAT
CGACTGGCAGCCATT
CGTCGACGTTTGCCTCCGATCGTTTTCGACGTTTTCGACGCCGATC
TTTATTGCGAATTC

GATCATCCGGGCTACTACAATTCGTGTACAAAAAAATCTTGCACC
TTTGCCGGCTATAGG
GACGAATTTATAATGACGGGCTCTGATGATTTCAACGTTTACATC
TGGGAAATTCCTGAT
CACTTGGCTGATGTAGGTGTGGTTGCGCGCTTCAAAGGTCTTG
CGCGGACATCGCTCG
ATCGTCAATCAATCTCGTTACGACGTGAATAGAGGGATTATCATT
TCTGCGGGGATCGAA
AAAGTCATTAAGATGTGGACCCCATATTGGAACCCGGGATGTAT
CGAAGAAAGTCCCTCG
GTGCAGGAAGTGAGCGACCCGAAAACGGTTCCGGCAGAAGGTCT
TTATGGGGGCAGGATG
GTATACACTCATGAGGAATACATCGACATGGTCCTGCGGACAGGC
GGCATTGTTGACCAC
AGCGAAGCCTCCACAGAAGAAGATCCTCGAATGCTGGCCTTTTTT
GATTCTTTGGTACAA
AGAGAGCTGGAACAGAGCAGTGATGATTCTAGCGATTCCGACAT
CGCTCAATGTGATTCA
ATTCCTATTTCTTCTGATACCGCTTCAGACGATGACAAGAAAGAA
CTTCCAGATGCGATT
CCGAAGTCTCTATCAATGGCCAAAAGCACGGTTGATACTGCAAA
CAAGAACGCGAGCTTT
TTAGATGCTCTTGAAGAATTCAAACGCAGAGAAGAGAGGAGGGA
AAGGGGACAAGCTGGA
AATATAGATTCTGGATCGGATGATGACGCAGATTTAGTTCCGGGT
GCTCCGGCGATCAGT
TCAGTTCTCAGCGCCAATAGGCTCAGAAAAATCAATCGTATGAA
CGCGTCGAATAATACA
CAAGAAGAACT
>ConsensusfromContig2304 Average coverage: 7.06
AGAGCAGAAAGACAGAACACTTTCGATTTGACCGTTTCTCCACAG
TTCATTAATGACCTC
AACTTGTTCTGTGACTTCCATCAGGCTCGGAGCATCGGAAAGATC
CGGAATCGGGATCGG
AAATGCGCTGTCATCCGACCCAAGTGCCAAAGCTTGGCTTATCAG
AGCCTGTAAGTCCCT
ATGAGCAGTATCCATGTGGCTCTAGTTACACCGATAGAACTTCCT
GGATCTTATATCCAA
AAATTAATAGTCTAAGAAGCTCGGATATTGAAGACGTTTCATAAA
AGTTTACTAAAAAAT
CAATTTCAAGCAATAGAATTATATGT
>ConsensusfromContig2305 Average coverage: 18.31

CAATAATATAAATGCTAAAAACAGAAACATGAAATAGTGAATTTA
TTATTTTAGGAAATC
ACTGATAGGTTGGAGGAGAGTTCATTGTTCAAATTGATTGAGGTA
ACCGGAATGTCTTTA
GAACGATATCTGCATGGTATGAATATTTCAATAAAAGCCTTTCTG
AATTTCTGGCCAGTG
AGGAAGTATAGGTAAAAATTTATAGCGTTGTTGAAGCAGGATATT
GCATGTAATATATCC
CATATCATCGCATTTGCATGAGTTAGGCTGTTGGACCATATCACA
TATGTCACCCAAATG
TTATGGGGAAATGTTAACACAACAAAAGCTAGAGACACTGATGC
AACTGTTATGACAGTT
TTTGTTGTTTGGTTTCTGTCATGAGATTGAAAGACGTTCATCTGCT
GCCTCCGTTTTTTC
ATTTGAATTATTTTCCATATTGTCAATCCATTGAAAATCAGAAGC
GGTATCGCGGGTATG
TAGATAAGGCATGCAGAGATCAATAAAATCCAGATTCTCACTGT
GATTGAATATACAAGC
CAATATTCCACGACAACGACAAAAATTGTTGATGCAAGTATGATG
CCGACAATAATCGCT
AATGATTTCTTCGCCGAGCACAGTTGTCGAGCCTTAAAAGGATAG
CAGACCACCATATAC
CTATCGAAAGCTATGGCTACCAGTAGCCAGGAACTCGCACATAA
AAAGACATGGCGAACG
TTATTTATAATAGGAGCTGAATATTGAATGTATGGATATACGCAT
GGTTTAACTTGCAAA
AGTAGATAAAGCAGTAGACAGCACGCATCTGATATTGTGAGAGC
GGCAATATGAATCCCC
AACGAGTTTTTCCATAATTGTTCTCTGAATATGATTGCTAGTGAA
AAAATGGTTCCAAAT
AACCCGAGAACATATATCACGATGTAATGTATGATGTGAAAAATT
TCATAAGCATTTGCA
TGTGTCTCAAATATACAAGGATACATAGTAAT
>ConsensusfromContig2306 Average coverage: 7.26
GCGGAAGTTTTGCATAGATGAGAACGAGATAGACATTCCAAAGA
TCACAAGGGGGCGATG
AAGGCCCCCCGAGCCGGTTGAACTTTCGCATATAAGATCAGATC
AGAAGATAAGCCAGAC
AGTCCGAGAGGGGGGCGAGGAAGCCCCCCCAAGCCGGTTGAAC
TTTCGCATACAAGATC
AGCCGAGGAGATAAGCAATGAAACGAGCAGGGCGAGGGGGCCCC
CTAGTTGTCGGTTGAA

CTTTAGCATACAAGATCAGCAGAAGATAAGCAATGACACGAGGG
GGGCTAGGAAGCCCCC
AGTTGTTGGTTGAAGTGCCCGCCGACGGGAGTTGGAGGAGTTGA
ATCTCACCCGGGTCAA
CATACAACGATGAGATAAAACTCTCTCAACTACCATTGAGGGGC
TGTCACGTGACAGAGC
AAACAATGAAGGTTACTCCTCCAAAGATAAAATCAAAAAG
>ConsensusfromContig2307 Average coverage: 29.07
GTTTGTGCGAAGAATTCTTGATTGTGGCTGTACTGCTGAGCTTTTA
TATTCATATGATTG
CTATCTATATAATAAAGAATATATTGAATTTCGTAAAAGCAATGC
AAGGACGTAAAATTC
TCGCTTTCAGCAATATGAAATTATATCTCAGGAAAGCTTCCATCA
ACTCTGTAAAATATT
CTTGATTCTTTCTTCTTAAACTCTTTTTTATTTATTTTTTTCTTCAG
GCTCCTATACGAA
GCTATTAAATACTTATTCTTTGATTACAATATTTTAAACAAG
>ConsensusfromContig2308 Average coverage: 8.27
CTTCCGATCTGTCGTTTTGAAATGACGTAACAAAGCCCCACAACG
ATAAGAATAACAACA
AGAAGGGCGCAACCAGACCCGATGATGACGTAAAAGACATGGGC
GGAGCTATCTCCCGCC
TTAACCTGTGTGAAATTGCCTTTGGTTTCGAGTATTGGTTTGGGG
GGCGCCGGATCTACA
TCTCTCTGATCATGCCAACATCTTTTGTGTCGTCGCAAGCGAAGTA
TATTTGGCTCTCCA
GATAAGGTGAAGGACACATTCAACTGAATGAGCACCTGAACATCA
TCCTCTTCAAGTCCA
GTGCAGAGTAGCGCAACACGAAAGAGTGATAGTTT
>ConsensusfromContig2309 Average coverage: 25.88
TTGAAATAATCAGATCCGAAGAATTGTTGTTTTCTTCCGATGATA
GGAATTTCGAATAGA
TATGTTGCTTGTTTGTTCCGTAGACACGTTTTCTAGAAGGTTCGAA
TGAATTGTTTGATG
GTTTTTTGACCATACTCAAAGCTTTCTGGAAAAAGCTCGAAGAGC
GGTTTGGTTTATTTG
TAGATACTCTGGATATATCAGAAAATTCAGAGCATTTATCGTGGA
ATGAATCATTCGCAC
TGACTATTTCAGCATCCGCCTTCATCCTCATGTCATCTCTACATGT
TAATGGAGTCGAGT
TCTGCGTTGGTGCAAACGATTTCTTCTTGTCCATTTTCGCAGGATT
TATGGAATCTGGCT

CATGCATAATATTGGAACCGAAAACTCCTATTCTTTTTGGTTGTAA
CTT
>ConsensusfromContig2310 Average coverage: 7.60
TATTTCGCTTCTATTTCGTCTGTCGATAGATTGAGCATAACTTGTG
CTGTGAAACCTTTG
AAGCAAAGGTTGAGAAATGCATTCCATCCGTCCCATATATCAACA
TCCACGACGCTTCCA
ACCAAAATTCCATAGGCGTAAACTTTATGACAATCAAACATTTCC
CTGCCGTCCTTGGTC
ACGAGTCTGAACATACCGATAGGGAGTCCTGTACGTCTAGTTGCA
ATCGCACGGAGCTGC
GAGGTGGAGACCTCGGGAAGAACTAGGTCATCATAGAGGGAGAT
AATTTCCTGCCGAAAC
TTGTTATTGAGGTAGAGCAGGGGCTTGGGTTCGTCTCTGTGGTAC
ACCTTGAGAGTGCTT
CCGTTCTTAAGGCCCAGATCTGTCAGGATCCATGAGTCATTCAGT
TCCGACCCGCAGTAC
CTGATACAGAGGAGCTGTGCCACCCGCCCCTCATGTCCCCCCTTG
GAGTTGTCATCCCCC
ATCGAGTATTTCTTTCGGATTCGCTCCTTGACATCCTGAACTGTTT
CGTACTCTTCAATG
TCGAAGTCGAATAAATCTTTGTTGTAGGAAAAAAACAGGCGCAT
TCCAAAGAAGTCC
>ConsensusfromContig2311 Average coverage: 8.47
GCATAGTTCCCCTCTCGTGTCCGCCTTATTCCTTCTTCGTTCGATT
TCGTATAAACAGAT
GGGCCCATGGAGTCCATTTTTCTTTTGATTATAGAATCAGGAGAC
TGAGTCGCTCTCTCA
AACTCGTACTTAATGACGCCTTTGTTTTGAGTTCCAAACGGAATG
TTGGACCGAATCAAG
TCCCCTATCGAATCCGTTTTGGAAATCCCGCCAAATATCGGTTTT
CGGATCAAAACTGTT
GCGTGGACTGTGATGAATGGCTCTGAGAAATCCACGACTTTATCT
CGATATTCCGTCACA
TATATTGGGGCAGCTGCCATGTCGACGATCTCTGTCATGACTTCA
CCAATCATGCCATTC
CACAGCATCGTACCCGAGATCCTTTGGCCATACTGACCATCTTTG
ACCAACCGGAGGGTG
AAATGAAGGCCTGTTTGATTGGCTATTTCCTGAATCAAGTCGGGT
ATGTATCCAATGAAC
GTCCGCTCAGAATTGTGGATCATTGTATATGGCTCCAAAACAATT
GTTGAAACAAGTATA

CTGTGGGTTCCTGATGCAATGGCTTTGAGCAAAAC
>ConsensusfromContig2312 Average coverage: 12.09
GGTCGGATTTAGTCAATTCGGGTATGAGTCACGATCATTGTCTAA
AAGAAAATCATGATG
AAGATGGTCTGCTGTTCTTTAATCTTAGAGACAATCCTGTCGGTC
GCATGCAGGATGAAT
TTGATGCAGTCGCAAATAGCTTTGTGAGTTCTAATGACAGCACAT
CACATTCGACATTTA
ACCATGGTGGAACAATTGTGGCAGAGATGTTTCCGGGACTGAGGA
ATGAGATGTCTGTTC
TATTTGCTCTCCATCATGAAAGAAAGGCTGTTTTAAGTCAGGTTC
CTGCTGGAGTCATTA
AACTAGACACATCTGATGACCATTTGACGAACTCAACAATAACG
AAGTGCGGGAATGACT
CAGTTAAAGGGAAGTTCTACATTCACAACTCCAAATTAAGGAGTG
TTCCCGGATCATACC
TGTTT
>ConsensusfromContig2313 Average coverage: 10.38
CAAAAACCGAAGGCGAAGAATTATACTGATCAGTTGATTCAGCT
AGGTCAAACTTCAATG
AGGGCAGAGGTCGCGAGCCCGTTGTCACATCCCCTACATCCGAGT
TGCTTGCTTCTGAAT
CTGTCGAGGACGTCTTCGACATCTTCTTGCGGAGATTCGCCTGGT
TCTGAAGTTGACCGC
TGAATTCGACTTTGGAGCTATTCGAAGAAGCGCCAGAGAATGAA
GTGTTCAAGGAAGAAT
GGCCAGTTGAAGAGGTCGAGCTGAGTTTTCTGATTCTTGAAATCT
TCGGAGTTGTTTTTC
CCGTTTTTTGAATGCGAAAGGATACCATGACATGCTGGACGTTGTT
TTTTTCCTCCCCTT
GCAAAAAGTCCGGTGTATCATGAACGATTTGAATGTTCTCGATGT
TCGAAAGATGGACAG
CTTCTTGCACTTTTTTCGGTGGATTTTCCGTCAGATAAATCCATTG
ATCTGAGAGAATTA
CAAATTTATATTGCTTTTCAACAATACAAGTTTCGTTCGCTCGAA
GTCTGTTGAAAATAT
ACTCATCAAAGTTCCGTCGCAAAAAAGCGTCTAGTTTTGTGTTTTT
CTTTTTTG
>ConsensusfromContig2314 Average coverage: 46.61
GCGAACAGCCTTTGCTGTCGCTGATGTTAGGGGGAGCTCCACTCT
CGAGCAGGAGCTTCG
TAATATCATGATGTCCATTTAAGGCTGCGAGATGTAACGGCGTAT

ACCCATTCTGATCCT
GGCTGCTCAAACTGATTAGCTTTCTTGTTATCTGGCTAAGAACAT
GTCGTCGACGATGAG
GTCTCAATAATTTTTCTACGATATCAAGATTTCCAAGCCGACACG
CCTCGCGTAATTCTG
AATCTTTACCAATCATTTATTGTGAAAATAAATGCATGATTTTTAT
TTATCACATCTTTC
TCTGAGATACTACGATATGTGCATAGGTACAATTGAATTCATAAT
ATGGAAATTTGCGAG
GATTTCGAAACAATTTATTGAAAAAAGTTAAATTGAGAAGTTTTT
GTTTAC
>ConsensusfromContig2315 Average coverage: 23.65
GCTTCCTTGGACCCTTATGTTGTCGTTTCACTCACATGTTCTGAAT
GTGGCTCTATGTGG
CCAGCTGGGGTTCTCCTGGAGCCCATGGACTGTTCTAAGTTATTA
TCTTGTCCGAAATGC
ATTCATGAAGGAGTCGAAGATGCCTGTCTCATCCGAAGATTTCTT
CTTCAGCTTGATATC
GAAGATGATTCGACATCACTCACAGTCTTACTGGCATCCAATGAG
GCCGTCCAGTTTTTG
GGCGGTCTCTCTCCAAGTGATGCTCTCAAAGACCCTCAAAAATTC
GAGCAAATATTCCGA
AGTCTCCTCAAATTGGCTGATTCGGGAGAGGCGGAAGAAACACCA
CACAGTGCTTGTCTC
ATGTCATATTCAGGAATGGACAGGACTTGCTTTCTAATTTTTGAC
ACTCGGTTATTCGAG
AAGTCATAGTCCTTTTATTAAATCTTTTATATTTAAAACGTCGTTT
CCATGTGTTAATTT
TTTT
>ConsensusfromContig2316 Average coverage: 20.55
GTACAGTCTCTCAGAAAAGTACAGGTGTCGGTTGACAGTGCGAG
CCCGGTGATATTGGAG
CCTCTGTTGAACCTGTTAAAATCGCCACACCTGGAAATTCAATAT
GAAGTGAGTCAATTC
ATTGTGGATTTGATGGACTACAACATTCAGAGCTCACTTCTGCAA
GGTCTGATAAAACTT
CTTGTGCCTGTTTATGAGAGCAGATCAACTTTTCAAGACTCAAAA
CAAAGCTCAGATCTG
TCCATGCCCGTCTCTCTTTTTCAACAACAAGCGGCTGCTGCAAAG
TGTATCAGAATACTG
TGTGATTCTCGATCGGATGTTTTCGAAAAACTCATGGAGCTTCGC
GCTGTTCACTTCTTG

GTCCTCGCTCTGTCGAATCGTAAAAGTCCCGACAGTCAGCGGCAA
GCGGCCCTTGCTCTT
GAGAAAATTGTCGATCTGTCCCCAAAAGTTGGTCAAGTTTTGGAG
CAAGCGTTTGGAGAA
ACTCTCTATCAAAGTTTCAAGTCCTGTCCTGAAACTTTGTACACT
CGTCTGGATCCGATG
CAAGCTGATATTTTTGCCACGAATAAAATCAATATTGTTGGTTCG
ACCCACGAAATATGA
TCCGAATACCAA
>ConsensusfromContig2317 Average coverage: 13.60
GCTTCCATTGACTTGTGACGAGGTCTTCCACTTGTTCTGCTACTG
ACACCCCCAAGTGAC
GTTTTCTTCTGCAGAGCCAACAGCACATAGCATAGTAGATAAAAG
TGAGGATAATTCTTG
TGTAAAAAAATCTGAAGGATCTTGCCATAATTCCACCAATATTGG
CTAAACTCATCAATG
TCAAGGGAATTCCGATAATAGCATATGGTATTGTGAGAAACTTCC
CAGCTACCGATTTCG
GTGTTATGTGGCCGTAACCAATAGTTGTTACTACAGTCATAGCGT
AGAGCGTTGACCCGA
AGAAGTCCCAATCCGTATCTTCATCGGGATTTTTCAACCAGCCTC
CTCCATACATTTCCA
CCAACTGCTCCGTAAAGTTTTTAGTCTGCTCTCTTCCTGATCGCA
TTTTTCTTCACAAT
AGTCTTTGACGTTTCTTACTCGGTTGAGCTGCAAACGCTCGTGACT
CGTCTCTACATTCT
GAAACATAGCGCCTCCCGCCGCTGTGTATAAGATGCAGACAACGA
TTCCT
>ConsensusfromContig2318 Average coverage: 12.94
GTAATCGCCAAAAAATCAGGTAAATTTTCATAATAAGTATATGTT
TGTGATAATGGATCT
GTTGTCATTGGAATATTTTCAACTAAATATGTACCAATTAAACCG
GCAACTGAATGAATT
GGAACGTTAATCATGATAAAAATCTTTTGTTTTTTAATTGATTGC
GCCACTACAATGTCT
TCTATATTATAATAAAAATTTACATCCTCAGAAATGAGTGATAATT
GATCTCCTCTTTTC
AATAAATCTGATTTAATAGTTTTAATCATTTCTTGCATAACTTCTA
TTGGTATAAATTGT
GGAGGGATTTTTCCTTCAATTAAAGTATACATACCCTCTTCGAAT
CTTTCAAATTGAGTT
ACGGCATATTCTACATCGTTCATAAATTGAAGCAATGATTGATAA

ATTCTGCTTGATAAT
TCTGTTCCTAATTCCAAGTTTGATAATTTTCCATATATATCATTGA
TTTCATTTCCTATG
GTATTTGCTACATCTATTAAACTTTGTCGAGTGTCCATTATGTTAT
TATTTATCGCCGTC
ATACCGTCTTGTAATGTTGACATGCGATTATTTATTTCCCCCGAA
AAAGACGTAAATTGT
TCCGTAAGATGAACGATTTGATCAGTGTTTGCCGCTGCCGCATCA
GCAACTTCACATATA
TGTGTAGCCAAAACTTGCAAATCCTTCTTATGTGGTAATCCGAAT
AAAGAACTAAATGCA
TTACCTGCTGCTCCGAATATGTTTGTGCTTGATGTTTGTTCGGTAT
CATCGCAAAGTGAT
TCATCACCAGATGCACGACGATTTCGATGATGGTGATAATTATGG
AATAAATTTAAATTT
GAAGTAGTTTCACCAACCACATCTCTTTTTTTTCGCGAAAATGATG
TTTTATCAACATTC
GGGAGAATTGGAAGTATTTGTTCTATTCGTTTTTGAATCATGTCTT
GTTTCATCCGTATA
TCCTTATTGAACAAAT
>ConsensusfromContig2319 Average coverage: 7.96
CCCCCTAAGCCCCTCTTCCCCATTGTAACATCAACAGTCCATTGT
ACTAAACTCATTGAT
ACTGTTTGTACTGGAGATAGCAGTTGTGTTTCTTCTTCCGTTTGTA
CGATTCCGCGGATT
TCACAAACATTATCGAGTTGTTCAAAAACTAATTCGTTAACGAAA
AGTGGAGTGTATTCG
TCTTATTCTCATTATGGATCATTATCATCGCCATCATCAACGAAA
AATTCGAATTTGATA
TACTCGCAACCATTTCGGCCATCGACTCTCCTAACGAGTTCTTCG
TGTCGGGTTTCAGAT
CCTAATTTATTGAAGCATTTGTCTCTTGAGTCAGACCAGGATTCA
GGTCGTGGGAAGGCG
TCGAGCTGTGAACGGAATTTAACCCGAACAAGTCCACTGGCGCAT
GCTCC
>ConsensusfromContig2320 Average coverage: 27.48
AACAAAAAGTCCTGAACCTTGTAATACCGATAATAAAATATATT
CAAGAGAATGCTCTGA
TAGTGTCGAATCATCATCTTCCAATCATGAATGCTGTATTGATTC
CATGTCAGAACAGTT
GCGGAAATCTTCCCTAAATTCATCAACAACACTGTCAAGCTATGA
CAATAATAATGCTTT

TCCACAATCATCCAAGGTTGAATTAGAATCTATAAAAAAGAGAA
CTTCAAATTACAGGAA
ACAAAACTCATTTTGTCAAGCATGTAATCCATTTCACGAGGAGAA
ATCAGGATATGTTAG
TTATGATGTTGGCGCAAAAGTGACATATATAACCAGCCATTCTTC
CAAACAACCTGCCAT
CAATTCTATGCTGAAGCAAGCGTGCATTCGAAGTCTCAGTTGTGA
GGTGTGTCCGGATAA
AGAGGGCCCGGTGTTTTTTGGTGATGAACAGCGAGGACATGTGAT
CAGCTATACTTTTTT
CGTGCAAGACAAGATGGCTCGAGGATTTCAAGCGTGGTACAGCAT
CATTGTCCTGATGCG
TGACAAAATCTACCTCCTCAATTCATGGCCCTTCTTGGTGGAGCA
AATACACCGGCTTAT
CACCACTATCCAATGTAGGGCGAGACTGGCCCAGACTGAATCAC
TGCACGGGCCAAAACA
TTCAGCTTATAAAAGTGATAGAACGTCTTTCTTGATCAAAAAGAG
TGGTTTGGCGCCTCC
TAGATCTATCGTGGATATTACCAAGGATGATTTAATTCTTATGAA
TATTCATCGATGGTT
TGTTTGGATTCTGAAGGCGGGGGCGTGCCGACTCTCAGAGAGAAT
ATTGGAAGCTCCTCC
CTCCGATGA
>ConsensusfromContig2321 Average coverage: 28.84
ATCGATTGATATCTTTCAACGTTATCGGTATATGAAATAATAGGT
AATAGATTTTCCAAT
ATTGAATAAAATAGTTCTAAAAAATACAATTTTTGAATGAAGACT
CTCATTCGTCACTTC
CGTCACTATCCACGATTCCTTTAAGGCGGGAAAACTTGAAGGCTT
ACGACCTTGACTTTT
TGTGCATCCCTGCTCCTGCTACTCCGACCCGAAGTGAAAATCACC
GCATCTTGATTCAGT
TCCGCAACCTCATCTCGCATTCGAATTTCCTCAGCCTTTTCCCGCG
TCGGCCGACCTTCC
ATACCCGCTTCTCTAAGAATCTGTTTGAGAACGGATATCTTTTGC
GAGACAGAGCTGCAG
TCTCCGAAGACGTTCGCATGCGCTTTTCCAACAGGCATCTTCTTA
CAGAGCCGGGCAATT
TTGCAGAGATTCTTAAAAGTTTTTTCTCCATCCCTTATATTCTTCG
ATTGAGGCTGGTCT
TCATCCCTTTTCGCCTTTTTGTTTTTCCTGGATTTCTTCTTCTTTTT
CACAACTGTGATT

ATTTTATCTTCTGCAAACTTATCTGATTTGATATTCGTCGACCGTT
CTTTCAGCATTCTT
TCCTCGTCTGATGATAAACTTGAGATGCTTATATCTCCATCTATAG
ATTTATTCTCCTCA
GCATTCGATTGTTTGGAAATCCCCTCATTACTTTCATTGAACTTAA
AAATATTTTCTTTC
CCTACAGAGACTCTACGT
>ConsensusfromContig2322 Average coverage: 17.72
CTGCTAAAGATACATCATTTAGATGTAACTTTTGTGTCGTAACAC
CGACATCATATGGCC
CATGATCACCGTTTGAAAACGACTTGGTGGTTAGCGCAGTGTTGT
AACTGATTTGTTGGG
GTTCATAATGTTTATGTATGGATTTTGTGTCATCGCTTGAGAGGT
AAGCAGAAAGAATCA
CATTGGAATGGTTATTATTATTCACATTGTTGAAATCGTTTTGAAT
TAATGTGTTTTGGA
ATTCTCCGGTGTAACCGAACTGAGAAACGAGCTGTAAACTCGGTT
GCATGTTCTGAGCTT
CAGAAGCCATCACAATGTTAGCTATGAGACTGTTTTCGTCGTGCA
GAGGTCGTTTTGAGG
GGTCGACGTGGGGGTTTGGATTCAGCATTGCCTGAAGCGTTGCG
TGGGAGGAGCCGATCA
TGGACTGGGTCGGGTTGAAAAGATTCTTCGTACTTCGAATATTTT
CATTCGTCATCGTAC
CAACGAGGGCAATGTTGTCGTCTAAGATTGGAGACTGGCTTTTGC
GAATTGATTTTGTTT
TGGTTGTCTTTGGCTTTCTTTTCCGTGTCTGAATTCCTTCTTTCTTC
ATTGAAATTGGTC
TGTTCACCTGATGTAGTTTGTAGTAGAGTCCACAGGCATTGCAAA
CAGGGTCTCCGTCGT
TGTTTCTTCTCCAAAGAGTCGTTGAACGCGTGTTGCAGTTGGCGC
ATTGCTGCCCACCTC
TCCTCATGGTTGATTGCTGATTGAGAGCTAGTCGCTTTGGGGACT
TGCTCATGAGTTCCC
TCTCCACATCCGCCCCTGCTCTTTCCAGCAGCCCACAAGCGTTGC
ACAAATAGTGGCCTG
TGTCATCCTTTCTCCAGAGGGGCGTTGAGATGGAGCCGCATTGGA
CACATTCTCTGTCCA
CATTCAAATAGGCCAATT
>ConsensusfromContig2323 Average coverage: 39.09
TGAGCAAGTTTGTACTGATGTTCCGGCAGTCTGATTGGGGAGCAG
CGTTTTATGGATGAA

ATATTTGACATACTTTTCTAGGATATGGATCTTTTTATTGTCTGTC
AAGTCAGAATCCGA
TAAAAGTTCACCGAAATGAGTATGGAAGGCTTGAATGGTTTTCA
CGAGATGCAATCGATG
TGGTAAGTTCAGACTGGGATGTTTTCGCTTGCTGTTCTGAGCAAC
AACAATCTCCATCCC
GCTTCTAGCAGCATTCAGAATATCATTCAAATCCTGTCGTTCAGG
AGCATCTTGAATCTC
CATCAAAATGTCAGTGAGAAACTCGGCACAATCGATGGCTAGGTT
TTGTTCTTCGTCCCG
TCAGATGGTGAACCTTTTGATTCATCGTCCTGTTTATCCACTAGC
GATTGTATGAGATCG
GGGGCTTCACACAACTTATTTGTTTTCAGAAGATGTCGAATATAC
AGCCGATTAATCATC
TTGGCTGATAGCATTCCGGTCTGCGAACCAATGAAGAGCGCATCT
GTGAGACTTTTCTTG
CTGTTACTTGCCAAAATCCCTTCAACAAACCAGGTGATGTTTCCA
TCATCTATCACGTGA
CCATAGAGCCCATAATCGTACAACACTTTGTAGTATCCTATTTTGC
CGTACTCTCCCTTC
AAATTACCGGCACTCGGATGGTTGATTTCCAATGACCGATGAACG
AGTGTCTCCATTGCA
TTCGATAATGGTATGTTGGCTTTTCCGAGCGCAAACACTATGGCT
TCTATTCGATAGTGC
GGGTCACTTATAGCATTAATGATGGCGATTGCTTTCGACTCCCAC
GGTGCTTCTCCGACG
AATTGTCTGATGCTATTTTTACACAAATCCTGAATGTAATTGAAG
AGAATCCTATCCGGC
AAAAGGTTCTGCTGGGAACAATACGGGCCGATGAACTGAGCCAC
TGTATGCTGCACGACC
TCAGGGGCCACGACGCGATCCAGCATGCGGAAAGCGAGCGATTC
CGTTGTTTCCTGGGAA
TACTGATCGATTGTCAGATGGCAATTATAATTCGTTTTCAGATCT
GAAATTGCCCGCAGC
TGGTCAAGAAGATGGCAAACACTTGTTGTGGCAG
>ConsensusfromContig2324 Average coverage: 11.38
CCTACACGACGCTCTTCCGATCTGCGCTACAATGAATACATCACC
AGCATCCTCGAGAAA
GTACATCCTGGTGGAAGCATTTCAGAAGAAGCCATTCGATGGCTG
AATTCCTTCGAGAAC
AACATCTTCAACAAAATTGCTACAAGAGCATCCCGCCGTACGGCT
TCGTCTTCTTCTTCC

TCTGATGAAGGTTTTAAAGGCCGGACAGCCATCATCACGATGCGC
GGAATTCATAGGGCT
GTAGAACATGTGTTTCCGGCTCAGCTAGCAAAGCACGCCCTCAG
TGAAGGCAAGAGGGCT
TGGACTATGCATATTGATACAGGAGCCCTGGAGTTCCCCATCGAG
CGCATCCACTCCCAA
CTCAGGCGTGGAGATCGGTATGCCTCGAAGATCGC
>ConsensusfromContig2325 Average coverage: 19.79
TCAATGGATTCAAACAATTACTTTAAAACTGGATTTCTTTAGTTTT
TTTGCATATTCTTT
AAATTTTTGTCCTTTGATTTCATCTTTATATTCTATTACACTTGAT
CCATAATATTCCGC
ATTTTCCTCTATATCCCGCAAAACATTGTTAATATTGTATGGGGT
CATCATTCTAGGGTG
GGATACTACGTGATCTGCTTCGTCTTCTTCTATCCAAGTAGTTGTT
TCAGTATTTAAATA
AATATTAGGTATCACTGACTTATACTTATGATTTACAATTTTATTT
CTCGACGCGCAATA
TTCTAAATAATTCATTATAGTTCTAATTTCTT
>ConsensusfromContig2326 Average coverage: 35.85
CGTCGAAAGATGACAAGAGAGGAGCATGCCCAAATCATTCTCTA
CAAGTCAAACCACGTC
TACGCAACGCAATCGGAAATCGCTCAGTATTTCTCGAAAGTTTTC
CAGATCAACATCGGC
CAGCGAGCTATTTCACACATTTTATCGAAGATTAGATCATGATCT
CCAATGGAGACCCGT
TAATATAAGATTTATCCATATTGTGAGATTCATAAGTGATCAATTT
TAATCTTTTTTAAT
AGCTTTTATGGAAGATTATTCTGTATAAGTTCATTCATATGGCC
>ConsensusfromContig2327 Average coverage: 4.61
CGCTCATGTTTGGATATGATTAAAAGGCAATTGGATCGTTGTCTA
TTGCAACAGTCTTTA
CAAGTCCTCCGTTATGGTCGCAATATGCCGTGAGATTCTTGTGTTG
TGGACTTCTGTTAT
TATTAGCATTGATAGTTTTTATAGTTGAAGTGTTGTTGAGATTAA
GGTCATCGATACAGT
CGTAATTATCAGCGTCAATGCTACGTGATGTTGTGGCAGCTGCCA
GAGATTCTGTGCTGG
TTTCTGTCCTTGCTGTAACGAATGAATCTTCAGAAACAGCTCGCC
TGATATGGACAGGAG
AGCTTGGTGTCTTCACTCGC
>ConsensusfromContig2328 Average coverage: 36.77

AAAACAAATAGTATTTTCATGATTACGCTGAAAAATCATTGTATT
TCGTACATATAACCT
TATTATAAGCAGGCACATTCATTAATGTAGAAGAATGAAAAAAG
AGAACGTATTCATATA
CGTGATACGAGTGACCGCATTGATATTGAATGTTATCGTTTACCC
CTGCATCCACGCCAT
CGCGGTCATTCCAGTCTTGCCTCTTTTGTATCTTGCCCCCGAATTA
TTTTTGCGGTATTC
CGGCTGGTGGTATTGTGTGGCTGGATATGGCTTGTCAACCATGAT
TCCTCTATCTAGATG
CAAGGTGTTTGTTTGCGGAGAATATCCTACGTCTCCAGGCGCGAC
CTCTAATCTACTCCT
CTGCAATCATCTTTCCTACATGGATCCTTTTGCGATGGCTCGCTC
TCTAGCGCTAAACGA
TTTCAATTTTACCAAGTCTTTGTGCTGGATAACCTGGCATCCTGTC
AGGTACACCTCTAT
ATACGCGCTGCACTTCCTCGCCAGTTTCTTCCTTAAAGTTGGTGG
TCGAAAAGTTCGCGA
TAAAGAAATGGAAAGATTATATGAATACGTTAAAACTCGGATGA
ATAATGGGAGAACCAG
ATCTGTGGTTATTTTTCCGGAAGGAGGTCTGAGAGAAAATATAT
GGGAACAGCACGAAAG
GTTCGTGAAGAAGAATGATTTGGAACCACTCGAGTATTGTGCCT
ACCCGAGAACGACAGC
TGTTCTAACCGTCCTCCGGGGTATTCGAGACTCCATCGATTCAGA
GTTCAATATCATAGA
CACGACTGTTTTTTACAAAGATAAAAAAGGGATTGATACCGTTGG
AAAATGCTTTCATAG
GATTGAGGCTGAAACAGAAGCAATTTATCTGCATTTCCGCAAAAT
TCATTTCCCTAGACT
TCCAGAAGGATTCCTCGATGATGAAAAATTACAGCAATTTCTCTT
TGATTTGACAAAAGA
CAAAGAGAAAATGATGAAAAGTTTCTACGAGACTGGTGTTATTT
GTCACCCCAAAGAGCA
ACATGAATATACTCCTTCTGAATATAGACCAGTAACCCGCTGGTT
TGACTTTTTGCTCGA
ATATGGTCTGTTATTCATTGTGACGTGTCTTATCCTTTGTAGGTTT
GTATTCAATATGTT
TTAGATATTTTAATTATAGATTTTGCTGAACTTTGATTTTTTATGT
TTTCGGGTTTGAAA
ATTCAGATCGGA
>ConsensusfromContig2329 Average coverage: 41.08

CACCGATAGCATGTATGAGCTGAAAAAATCATTAAGATAATCAA
AATCGATACTAGTTGA
TGGAGATTTTGTCTTTGATGAAGATTGTTCAAAAGAATTAGGTCT
ACTTTCTGATATTAC
AGTGTTGTTTTCAAAGTCAATTAACATATGACTCGAAGATTTTGC
ATATGTATCAGGCAC
TTCAGATGAGCTTTGTGTTGATGAGAGAGTATCGAGAGTTTCACT
TAGCTCCTGTACCAT
GAGTTTCCAGCATTCGAATGAAGATTGAGATATATATTTCACCCA
TGATTCCATATCTTC
TTTCGAGTCAGCAGCTAAAGAGTATATTCTTGACGATGTACTGTC
AAATATGATTTTAAA
GTTGAACTTTTCTAATTCATCATCTGGTTCAACTCTACATCCCTCC
AATATGACCAGACC
TAATGGTTCTGTGTCAGTATTTTTCTCAAAATAAAATAACATGTT
ACTTTTCAAAACAAA
CCACCTTTTGTGGTATGCCTTATTCACATCACCTCTTTTGTATAAA
TACCCTTCTTTGTC
AGTTGGGGCATCGCTTATAGAGTAATTTATCAAATGTTTTTCGTT
AAGTTTCATGATTAG
GAAAATGAACCGTATTTCAATCTTGAAGTTGAC
>ConsensusfromContig2330 Average coverage: 6.27
TCCGATCTGCGGATTTGACATGACACGTCCACGCTGACATTGCGA
AATTTAAAATTCTGA
AATCTTTAATTTTTTTTCCACCTCCCTGAGCTTTGGCAATTATTAT
CATCACAATCATTA
TATCACTATTTGAACTAGTAGAGTGTGTTTTCTGGTGTGAGTTAAC
TTTTATTCACTTAT
TAAGCTGTGTGTCGTCACTTATTTATTATTTGTTCTAATTCTTAAG
TGAGTAGTGAGGAA
AGTAGTTGTTATCATGTCAACTACTAGGCAAAGAGCCAAAAGTAA
TGCTAGTCAGTCTTG
GGAGTGTGGTATGTGCTCCACAGAGACCCTAGAAAATGGGGTTG
AATGTGGAAAATGCAG
AAAATGGGCATGTGGCTCATGTAGCCACATGTCAAATGATGAG
>ConsensusfromContig2331 Average coverage: 28.25
TCTTGTATGTAGCTGTTTCAATGCCCATCATAGTATGAATGAGCT
TCAAGTGTTTTGAAG
TTATAATATCTCCTACAGTAACACCAATTTTTTTCTGAAGAGTTTT
TGCTATGTTTGACA
TCACCATATTCTCTCTGAACTTATAAACTTCACTCATGGCTTCTTT
GTTGCCATAGATAT

CTTTTGTATATTTTATACAATTTTTGAAATATCGCAGTAAAGTATC
ATTTATGTGTAGAA
TATTAGATGCTATATTATATTTAGCTAGATTCATGATATTTCGACC
GAAGTTTGTTATAT
TCTCATTAAAAAATGAGTGCATGCCATGCTGGAAAGCAATTGCAC
TTTCCTTTGATCTCA
AATAGCTACTTGATTTGAAAGTTATTTCACCATGTAAACAAGTTT
TAAAAAGTTCTTTAT
AATCATTTGCGAATCGTTGGCCTATCTTAACTAGTTCAATTCTTC
CAGTTGGTGTTAAAA
GAGCACCTTCAATAGATATAAATGATGGTGACCATTTTTCAAGAC
TTTCATGAGCATTTC
TAATTTTATCCAATCCAATCTGATTATTGATAAAATCTGAGAATTT
CACAATATCCTTGT
CCAATGGATATCTTGTGCCATGTCTGAAAACACCAGTAATGTGTA
TCACTTTGCACTGTT
TGAAAAATTGATCGTCGATTATAGAATCATTAAAATTACTACTTT
CCTTCTCTTCACCAC
GATTACTTTCTTTTTCATCAAATGAGTTCTCGTAAGCAGTTTTTGT
TCCGAATTTTTTAC
GCAAGCCTGCTTCGCCGAACGAGAGGTGAACAAAAATATTGAAC
AGAATAAATAAATTGA
ATTTGAATGTATTGGTTTGTTTATAATTCTTCATGTTTCTTGATCA
AAAATAATAATTTG
TAGAGCAGTGCTCAAG
>ConsensusfromContig2332 Average coverage: 8.56
TGGCAACGGTCGGTATGACTGCCTTTGTGAAGCTGGGAAAAAGT
ATGGACTGATCTGCGG
CCAAGAGCAAATTGATTGTGATGCTGATGCGTGTGACATTGATAA
TGGCTTTGTGTGTCA
ATTAACCAATCATGACGGAAATTTTGTGCATGGATGCATGGAAAA
CCCATGTGCTTCTAA
TCCATGTTTAAACGGAGCCACGTGTACAGGAGACGGAAATGGGTA
CATCTGCAATTGTGT
TCCAGGGTCCTTCAAAAACATTGGAGGAAATTGCAGTCCAGTGGC
GAATAGGTTTATCTC
GTTGGATGAGCAAAAAAATGACTATGGCCCTACTATGTGCGGGA
TTTTAAGAGCTGACAA
TGCTGTTGTCATTCATCTGGGGACGAAATGTCGTAAGTACAGCAT
TAGTGAAGTGGCGAC
CGAAGAAAACCCATTCAACGGATACATTGGTGTCGCGAACGGAA
GATATACTGGTGTAAT

TGAACATGTAAAAGGAATCAGTGTTGAAGAGTGTGCTCTTAAAT
GCAAGGAATGCCTGAT
CTGGCAACGTGTTGTTTTTCAAGGCTCGCCTTACTGCATGACAGT
GCATGAGGGAGATGA
CAATCAGCCGAAGATCGGAAGAG
>ConsensusfromContig2333 Average coverage: 6.11
TATAAGCTAGGTTCCGATCACTAAGTTCACAAAATTTAAACAGCT
GTACAGGTGGCGTAT
CATTGGGGAGCGAATAGAAGACTATAAGGTAGGGTCCGATGACT
GAAAAAGATTATTTAT
GAGCTAGGTTCCGATCACTGAGTTCACAAAATTTAAACAGCTGTA
CAGGTGGCGTATCAT
TGGGGAGCGAATAGAAGACTATAAGG
>ConsensusfromContig2334 Average coverage: 11.69
CCGAAAATAAAATATTTCTCAAAACAAAATGAATCGGAATTCGG
CCGAAAGATAACGGCC
TGTCGAGCAGCATTCGAATTCGGAACATCGACATACAGGAGATTC
AGCTTGAGGGGTTTT
GAAAGGTCTATACTCCTGCGATTGAGGTTCAAGGGGCTTTGTTCT
TTCCGGGGGTATTTC
AAAAACACTTCATCCGTCATACTCTTCCGAGTTGATTCTGCCATA
GTTGAACCCGTTTCG
CACATTTTGGTCTTTCGTCTTTTCACATTGCAATGCTCTGAACTAA
CTTCAAGTTCGATT
TGGTTTTCCGAATTCTGTTCTTCATTTACCTTATCTCCCAGTATCG
CATCAGAATGCCTT
GTTCCCTCTTCGGTTCTACCTTTGTCATCCACGAGTGCTCTAGAGC
TGTAAATCATTTCT
ATAAAGCTGTCAGTCCTTTTCTCAGTACTTGAGGGTTCAACATCC
GGCATTATTTCGGTA
CTTCTTCTATAAGACGGTTCTTTATTATTTAAATCAATGCCATCAG
ATTTTGACCCCTCA
GAACCCATTTGAGTGAGATCCAACTCAGTGCTATCCATCGCAAAG
GACAGATTAGGCCTT
TTCACTTCCCAATTGCTACTACTTCTCTTCAGCTAGGAAGGGC
>ConsensusfromContig2335 Average coverage: 20.21
TTTACTAGTCAGCGGCCGCCGCCAGTTGCAGAGATTCTTAGATCG
TTTTGAATAAAGTGC
CGATGCTTCATCATCGCGGTGCGTAAAAAGAAATCAAAACCCAA
TTCGAGATGACAGAAG
TGGGAAACGTTCGGCATCTTCTCGGCAAACCTCACCAGATGCAGA
ATCACCGATATATAG

ACTACGACGAAAACATGACCTCAGACGGAGGGAATCAAAGCGAT
GAAAGCCAGTTCATTG
ATGAAGATGGCATTTCTCACAATGGGGGCAGTAAACTTGAAGAT
TTGTGCAGAAAACGTG
GTCGCAAACAGTCGAAACCCATACGAGTTCCTGTTCCTTTCGATT
CGGAAAACGACAGTC
TTTCACCACCGAACAACTACCGGAGAGCTTACGACTCTGCTTCCG
AACCAATCGTAGTGC
GTGGTCGAAGTGACTCCCAAGACCACCATGGATTAATCAGCAAT
GATCAAAAATACTATC
CGGATCCCGATTCACCACCAAATCGTGCAGATAATCGGTTGGATT
TGCAACATCAGCAGT
CAGTTTTTCATCGAGATCCATCCCCGTTTCCAA
>ConsensusfromContig2336 Average coverage: 9.77
CTCCATCGAAGTAGGTCGTCTTTCCCAGAATTGGGTCAATTTTGT
TGACAATATTCTGAA
TGGGCGTGTCTCCTACAATTTCATATCCACGCCGATTGGATGATC
GCGAGAATCGAAGAC
TATTCAATTTCACTTCGAACGAATCAAGAGTCTCGAGAGAAATGT
CTCCTGTATCATTTA
TCAGTATTGGCTGGACACTAGAATTGCTTAGATGTGCGATGAGCC
TGCCATCTGGTGAGA
TGAATGTTTGGTTGCCAGGGAACTGGTAATGTTTCAAGATGGTGT
CTGTGACGCTGTCGA
GAACCAATTTGTTCGAAACCTGCAGATCTGAATTTTCCGCGCAGT
CAACCAGT
>ConsensusfromContig2337 Average coverage: 36.53
AGCTGTGGAACGATGTTCATATGACCTCTCTTGAAGGGCGTTTTG
GGGTGGTCATCGCGG
ATGTGGGTTCTCAGCTGTTCGAGGGTATCGAACGGATGAGCGCAT
TGGTTACAAGTGAAC
AATTGTTGAGTGCATTCAATCTCATCTTGATGGCGCTTGACGTGG
TTTCTCAATCCACTG
GATGTTGAAAATTCTTGTTCACATTTCGTCATTTTACAACGGTAG
GGCCGTTCGCCGGTA
TGGACTCGGATATGCTTGCGGAGGTCAAACAAAGTCGTGAAAAAC
TTCGTACATCCATCT
GCGTCACAGTTGAAGGTCGTTCCCAGATGCAATCGCTTGTGTGCA
TTCAGTCTGAAGAGT
GTGTTGTACGACTTTCCGCATCCCTCGACGTCACACTCATACGGA
CGCTCCTTCGTGTGA
ACCCGAAGATGAACTTTGAGGCTATATGACGTCAGAAAGGCGCTT

CCGCATCCTTTCTCG
CTGCACACGAATGAGTATTGACCTTGATGAGCCTTCACGTGGGTT
TTCAGGTTCCCTTGG
GTCGAATAAACTCGCTCGCAACCTTCGTATTGACACTGAAATTTT
CGAACCCTGGCCTGC
TCCGTGACAGGATCTACAGTCTCGAGGGTGAGAGTGGCATGAGA
GGGGAACAATGTGAGT
CCGCC
>ConsensusfromContig2338 Average coverage: 49.54
CTAACATCATTTCTCGTCAAAACATCATCGTTCAGTCGTTCGGCG
CATTCAATCAAATTC
GTCGAACAAGAGAACTTTTTCGGATCAGCAGCAGCAGTGGTGTTA
TCCTCATTGAAACAT
TCCTCGTTCGGAGGCGTTCGATATTCGTCTTCAGGCGTCGAGGAA
TTGTCTTCAGAGTTC
GGTTCTTCTTCTTCACCGTCGACAAAGCCACCATCATCGCCGTCT
CCAACAGATGCCGTC
GCCTGACCTCGCCGCCAGGTGGAAGGAGACATCCCAGCTCTGGC
AGAAGATATTCCAGCT
CTAGACGAAGACATTGCAGTTCGGTTGGCTCCGAAAGAGCGTGG
CGCGGATATTGGACGG
GTTTGCTGCATTCCAGCTCGTTTCTTCATGCCAATTCCCTCCATGG
AAAGGCGGTCTTTG
GCTGCCTTCGTCCTGCCGGACTTTCGTTTATAGAACAACCGATCG
TCTTCAGGATCATCT
TCGTAATCATCCTCTCCGGTCATGCTCGCACTGACGTTCGATAAT
AAGGATGGCTCACGT
CGACTGAATGTATGCGAAGAGTTGTTTCTGTTGTCAATCTCTCTT
GACATGGAATTGGAT
GAAGAAGCTCGTCTATTCAAAAGAGTTTCGTGCCGAGGCGGAAG
ATTGAAAGTGGAAGAA
GGGTTTTCTCTAGCGTTTTCGGCATGTCTGAAACTCCTGCGCGTT
GCATGACTGATAACA
ACAGGTGCCTTCACGCCAGGAACGTGTCTCTCTTTTCGATTGACA
GCTTTCGTGGGGCGT
GGTGCACAAAGCGACCCTTCTAGCACGAGAAGAACATAGAGTTG
GGCGAGGAGAGTGATG
AAGTGAACTCTGAGTTCTTCCGAAGACTGAAGAATGTCTTCGAT
GCCGAGGAAGAAGCAA
GAAGCTCTCGATGGATGTGCGAAGGATGGACTGGTGAAGAAAGG
GATCTTGCTGCACCAC
TTGGAGACGAGCGCCAGATTGTGCATGCTGTCCGCGATACCGAG

GTCGTCCCGGATGGCG
ATTTCGTGTGTGGGAAAGGTGTGCGGACAATAGAACGAAATGAT
GAAGGCCAGAACGCGT
CCATCACAAAGGTCGGAGGTCAGATCGCTCACCATCGGCACCGTC
CCCATAAAATCGTAT
GCGGTGGAGCAAGTGCCGGCGGGGGAAAGACCAAGAGAAGAAC
CTTGGGAGGAGCTAACG
ACTCGCGCCCACAGGAACAGCGCATCTTCAGAGTCGTACGGCAGG
TCGTTTTTGGACGTT
GATAGCAATTCGAGGGTTCTCAGTTCTTGGACAACAGCAGAAGCT
TGTACAACATTCTTT
ATAAACGCAGTCATGAGTGACAGAAGCAAGGAAGAATGACAATC
AGTGAGGAACGGATCG
GTTTGAAGAAGCATCGAATCAGTCACCGGAACCTCATCCTCATCC
AAGATATAGAGCCCG
TGACGCGACAAGAGGTGGATAACATCCATCGGACATTTGAACGT
GAACACGGGGTCGGGG
AACAACGTACTGCACGCATCGCAATACAATAACCCCGATGCCAA
AAGGTCAACAAGTTCA
TGGCTGAAACAAAGCTCACCCTCTTCATTTTCAACGAAAGGTTTG
TCGATGGCATTTATC
AAACTCTTGGATTCAACTTTACTCAGAAGCCAACAGATCGATGCA
CATAAACTAGCCTTA
TCACAGTCATAGTTATCCAGTGAAGAAAGCTCTTGCACATCCATC
TGCAGTCACTCATAG
AATCCTTTATTCAATTGAAATGTCGCTGATATAGATCGGAAG
>ConsensusfromContig2339 Average coverage: 7.47
TGCCCAAGGAATACTGATCCAAAGCCCAAAGAATATGAAGGCGA
GCTGTATGATATCAGT
CTTGACTACAGAGTAGAGTCCGCCGAAAAGAGTATAGCTGACAG
CAACACATGCCGAGAC
AATGACTGCAATCTCAGTATTCAAACCCAAAACTATCGTCAATGT
CGTGCCCAAAGCAGA
AAGCACAGCAGATGACCAGAAGATGTCGCCCATCAGAGCCGGAA
TATACAAAAGTCCTGC
CATCAGCTTCCCGTATTTCAGTTTGAATGGATCGAGCATCGTAAC
GTAATTAGCAGAGCG
CATGGTTTTTGCGAAGAAGATTCCCCCGAGGATAATACTCAAGCT
GTACCCGAATGGGGG
CAAGCACCACACGAGACCAGAAGTGTAAATGATTTCTGCGGATCC
ATTGATATATCCACC
ACCCACCCACGTAGCTGTCATAGTGAATATTCCGACAAAAAGTC

CGATTGAGCGACCAGC
CACCATGGCGTCTTCGCTCTCTAACACCGAAGCGGGTCTGGC
>ConsensusfromContig2340 Average coverage: 21.03
CAACTTTATATAACAGGATAAAAGCACAGTGCGATTCATAAAAA
TGACGATAACCTTGAA
CATTGAAACAAAGTTTAGATCATTGCGATTCTATAACCCATACAA
CATGTTCATGATCTT
GGACTGCTCAAGAGCCTTGTACCCTCAGTGATGTCTTCCTCACTCG
ATTCCTTCTCTTCT
TCATAACGCGAAAATTCCGACTGCTGCCGACTCTGATTGAACCTA
TCAAATCCCGCCGCT
GAAGGCGTTGCTGACGACATTGCGTTCGCGGCACCGTCGTTGCGT
CGTCCACTGTGGCGC
GAGGAACGACCAGAGTTGTGGCGACTGCGGTCGTTGTTGCGATGT
CGGTCGCTGTTGCGA
GGTCGGTCGCCGTTGCGAGGTCGGTCGTTGTTGCGATGTCGGTCG
TTTCGACGCCGAGAG
GAGCGGTTGGAACGATCCGATTTTCGGCCACTGTGGCGGCTATTG
CGGCTGGTCCCACCG
CTTCTCCGGTTCCGTTCCGGGTGCGAGGAATTGTTACTTCCATCT
TCACTGTCGGGCAGT
TTGTAATGGACCCCCCGCCCACTACTGTAGTCGTCGCTGCTGTTG
AGGCTCGCTTGGCAA
ATATCGGAATAATCATCGTT
>ConsensusfromContig2341 Average coverage: 23.41
TTTTCCGTAGTGAACGTTTTTGCATCGAACCAAGAGTTGATAAAT
GATGTAATGCCAGTT
CCGAGGCTTTCGAACTGACTTTTGAATTCCGCAAAATCCGTATCA
AATTCACTGCGCCGA
TGGTCAAGGATGTCGTAACTCTTCTTTTTGGCGCTATCCACAATG
GATCTATAGCGCACA
ACCATGATGTCTATTCCCTCCACGCGGACGTCCAGCAGCCCAGAG
TAGTTTTGAATAAGA
TTCAGCATGTGGGTGATGTTCTGCAGCCGGCGACAGAACGTGTCA
AATTTCCCAAAGATG
AGGTTTTCGCTGAATTCGAATTTGTCGGCCTGTTGGCTTTGAATCG
CCCTTTTTGTGCGG
TGGAAGTGAGACTGATATTTCCCGTTGAGGTCGATGCTGTCTTTG
AGCCTCTTCTTCAGA
ACATCTTTGGGGTGGTCCCATACTTTGGACACGCCCTCCATTATG
AAGGATTTACAAGTC
GAGATCATCTGATTGGTTACCTTGACGAAAAGCGAGGTCATTCGC

TCCGAAGTGTTGTAA
TACGTTGAAATGTTGTAGATCATTGCGATGGCGTTCAAGAGACCC
GGAATATGTTCCATC
ATTTCATGCGGGGCGCATTTGGAGAGCGGAACAAAGAACTTGTC
GAGCGTGTAGAGGAAT
TTAACGTTGTCTTTGGCCTCGTTGGCGCTGTCGGTTATTCTTCCGT
CAAGTAGCCTCCAG
CTTTGTATAGATTTGGACTTAGCAGCAGCCAATATTCCAAGAACA
GATTTGCATCGCTGG
GACTTAATTTGGTTCAGGAGGGTGTTGAACTTGGCCATTCGCATC
TTCCAATAAGCGAGT
TCAGCACTCGGGCCAATGTCGTCTGC
>ConsensusfromContig2342 Average coverage: 108.75
ACTGGAGTTCAGACGTGTGCTCTTCCGATCTGCAACATTCTCTGT
GATGTAAACACTTGC
GATATAACGTGCGGACTACTTGTTAAATCCAGTTGGCGGAGGGAT
ACGGTATCTTCGAAA
AAAGTGTCGTCTATTATGATCTTTTGTCGAATATACGAGAGGCCA
ATTTCTTGCATATTT
ACAGCGCCTTTTAAACTCTTTGCGTACAAGAATTCTAAAGGATTT
CGTGACAAGCTGAGT
GTTTTGATATTTGTACAATTTATAAACGTATTTGGGTATAATACAG
TTAACCTATTGCTG
CTTAAATCTAAAGATTCAAGCAATCGTAAATTGCTTATGGCAGA
AGGAGATAAATAATCA
ACAATATTGTTCGAAATGGACAGTTTATTAAGCTTTGATCTGAAG
GGGAAAATATTGGGA
ATTGCTCTTATTCTATTCGAGTCTAAAAGCAGACGTGTTATGCTG
CTTGGAAGACCTTCT
GG
>ConsensusfromContig2343 Average coverage: 7.11
TGGGTTGTTAAATATTTGTGATTTCGACTAACAAAGATCAAGGAT
TGCAGACATGGCGCT
GGTCGCTTATGCTATCAAAGATATCGACATTGAAAACGGAGAGTT
CGCAAAGCTGTTCGC
GGTTGCAGAAGCACCGAGAGGGATTCCATGCTCTCTCTCACATTC
AACAAGTACATCAGG
GTTGTCTTCAAATCCCAGCAGGAATAAATATCCCCTGTCACACTC
AAAGTCATCTTCGGC
ACTGCTGGGCAAACTCAAACGGAATATATCAACAGCAAGTTCTTC
TGCAGGAGTGGGTTC
CAGACACAAAAAGAGGCCTCTGGTATCCTCTCAGTCTTCTTCATC

ACTCAGAACTGAGTT
TCTGAGGAAGAAAGTCTCGATCAGCTTCCAGGATGGCATTCCAG
AAAGTCGACAAGGCCA
AG
>ConsensusfromContig2344 Average coverage: 77.50
ACTTTATTCAGGTAATTATACAGGTAAACAAAACAAGATCAACA
ATTTCAAAAAATCGAA
TAATGAAACAAATGAAATGACATTTTATTTTCGATATACACTTTG
ATATTTCTGTCGAAT
TCATTTAAATGGATCCACATACTGCAGGAGACCCTTTAGAGTGTT
ATGGGATTTCATTGA
CTCTCCACAAAGACCCTGAAGTCGATGTACATGGCAACAATTTTT
GTGATTCTGATGGGA
AACCGAGATATAGATGTGGTTTCAGAGTTGGAGGTGGAATTGAT
CAAGATCCATCAAAAA
GCCCTCAGGGGTATCCAGATACTGGTGTTTATGTGACATACATTC
ATGAGGATGGCCCAG
CACGAAGAGCCGGCTTGCAGCTTCATGACAAAATTTTACAGGTC
AACAATAAAGACTTGA
CTGTTGTCACTCATAAGAAAGCTGTAGAATACATAAAACGAAAAC
CTGTTCTCAACATGC
TGGTATCTCGTAAAGGAGTTCCTTCACTCAACCAGGCTCCAGACG
CGGGACTTGAAACCC
ACCAGATACCCCCTCAAGACCCATCTTTCGGTCCCCAAGATCCAA
CGAGAATCTCTGGCC
AAATTCCATCCAATATGTCTCACATGTACGGACCTCCGTCACACA
TGATGAGTCTGAGCC
AACCGGGATTAGATGGTCCACAACCAGGACAGATGCCAATCGTT
CAACAGCATGGCCCCG
CCCAGAACATCCCCCCTGGCTACGATCGAGGGCCATACAACCATC
GAAACGATCCGTCTT
CACAGTTCCATCAGAGGGTTTTCTGAAGCAGAATTCCTTTCCATC
CGTTCAATATCCGTT
TACATTTTATTAAAAGTTTTATTATTCAATTAATTTTTTTGTATTT
CAATCATGGTGTGA
TATATTTTATTTTTATATATGATTTTTGACCAAC
>ConsensusfromContig2345 Average coverage: 11.01
GCACTTGATCGCATAATCCCAAACGGATTATTTATAATATAATAG
AATAACAAACTTTCA
AATCTATAACAGAAGTTCATGTGGCCAATTTATTTAGCTCATCAG
AAATCTGTATGTTTT
TCGCTCTTGTATTTCAGGATGATCTATCGAAAGCTAGAACAACCA

TGACTGGTGAATCAA
ATTCGGGCAAAATATTCAATTATTATACAATGACATAATCTTCAT
ATATTCTTCCATCAG
GTTCATCTGTTGTGCTTTCATTATTCGGATTGAAATCTTCCAAAC
AGACTCCAATGTAGG
ATTGCGAACAATTTTTGTTGTGAGAACGTTTCTCTCCTCCGTTTGC
CATCGGACTTCCGT
GAATTGTCAATGGTGATACTTGAAATCGCCTTGATCGTCGTGGGT
CTTTCAAGTCGAACA
GATAATCTGGGCTCCCTTCGCGATCTGCATCAGGGGTTTCAGAAC
CGTGGCCTGAGTCCA
GAAGAGAGTCGTGGTGAACGAAGTCGATTGCCAGATCATTTTCAT
CTGTCAACTGCAGGG
TTGGTATGATGTTTCCATTCATCGAACTACTATTAAATGTGTCCG
GGTGTGGTTTATAAA
TGGCCGAATGAGAGGCAGCTTTC
>ConsensusfromContig2346 Average coverage: 10.01
CCAGATTCGGAGGTAATGTTGCTGTTTACAACGGTTTTAGTAGGA
CATGGAGATTGACAA
TCGGTGACAGGTGGATTCCAAACACCCTTGCAAACTCCACCAGAA
TTACATTCAATGTCA
CACGTATGATCCCGGGTGCCCTTTTTCGGCTCATTCGTGAAATAT
TTGTCTAAATCACAA
GTGACAGTATTCTTTTCACCATACTTCTTCAGTGAAACATCATTAC
CGTTTTCAACTGCC
TCATTAAGACATTTGACAGGTGAACAGCTTCTGATTTTTGGATCC
CAAGTCTCACTAGCA
GTACAGATCAGATCGAACGTTTGTGCACGAGTGTCGGTGACATAA
CCTTTGTCACAATTT
CCGGTGATCTTGTCTTGATATACACCAGCCTGGTTTGATACATTG
CTGTACCTGACTGTG
ATGCTTTCACATTCGACTCGAGTACATGTAAGACTGTTTTCTTGA
GGGTTCAACTTTCCA
GCACTATTGCAAAAGTAAGGGTAACTTTTTCTACCAACTGCATTG
AAGATATATCCGGGG
TTACATGTAATATTTTTC
>ConsensusfromContig2347 Average coverage: 29.58
GGAGATATCAATAATTCACAAAATTTTAAAATTTTATATGCATAA
AAACAAAAACGTCGG
TATATTAGAAGAAAAGGCTGCAAAGCTATCAATCCTCAATTTTCT
TTCTAACAGCATTCT
AATGAAGATCACTCTTCAACTTCGAAAAATAGTGATTCTGATGAC

ACTGTGTTCCATCAT
CTGCCACATATACATCCCCATCCACGGCCTCTCGAAAACAATGAT
GGATTCTTTGAAGTC
CATAATAAACGACGTCGCAGCGTCTGTCGGTCTGGACCGGTGAT
TTTGGCGGATAGATG
GCTCTGGCCTTATTATACTCAATGGGGAATCACAACATCCGCATG
GGGGGCGTGGTTGCT
GATGCGATGGGCGACAAAGAGGCAGGAAAAAGCTGTATGTGACA
TCCGCCCTCGTGCCGC
AGTGTGCTGATGTTGACTGTTTGTATGAACCAGTTTGAGAGCTGG
CGGTTCTGATGTCCC
CTAAAAAGATATGAGCACGTTGACTTTGAGCAGCCTTTAGTCACA
TTCGAAGAATCATAG
CTCAAATATATGATACCGGTTTTTATATGAATTACTCTGTTGACTA
CCCTTATTTTATTT
CACTGTTATGATTTTTTAGTCACTTTCTAGGATCTGAG
>ConsensusfromContig2348 Average coverage: 30.62
CATCGTTTATGAGCGATAACGACCCATCTATGTTGAATTATATAA
ATCAAGAATACCAAA
GTGATTCCACAAACAAATTCCAGTCAAGTGAACAATTTCATTTGG
ATATTAAAAATGTCA
AGAATGATGGAGCAGGACATGTCAACAAACTCAGTGCGAGATCA
CGTACACCTAAAGTGG
CGTGGAGCAATATGCCGGTCAGTCAGACATATCTGACCGAAGAAT
CTCCTCTCAGGACAC
CGCACAGTGCTACGTCCCAGCAGCGGGTCAAATCGGCTCAGCGC
CGGATGCAGAGAGGGG
GTCAGACGCCTGGTGGACCCGCCCATTCGCGACGCGGAAGAAGG
CCTCCAAATGCCCAAT
CGTTCATAGTAGAAAGGGTTCCTCCGGACAAACTAGATTCTGACT
ACGATGTAATGAGCA
AAAATAATTCTTCTTCGAAATATGGAGGAGCTTTTCCTGTAACTA
AGATCTCCCCACGCA
ATACAGGTAGACGACCGACAAGTCTCTCGGCGCCTCCTCGTTCTG
TCAATCCGTTTGAAA
TTGCTTCAAAGTCGAATTCGCAGGATATCTCCCGGGGTATCACTT
ACGCACATCTCAAGA
ATGCTGGGCTTGTCAAAGGAGATGACGCGGAGATCCAAGAATCG
AACCATTTCATTGAGA
AGAATTCTGTCTCTGAAACAT
>ConsensusfromContig2349 Average coverage: 25.64
ATCATATCCAAATGTAATCAACACATTGAGTATTCTTCGAATAAA

AGTAAATACGCGTTC
AAAATATCAGACCATATTCAAGCTGCGAAAGCATTTATCACCGAA
TTCATCAAAAAATGT
CCATTGAATCAAAATGAATCCAGCGTGTAAGACTGACATCCCAT
AACAAATACGAAAGAC
TGAACCATAAATATAAATGATAATAAAACTGCACGAGAGTATGCT
ATATATAGTTATCAC
TGTCAGCTGCATATTAACGTTCATGATATCTCAACAATGATTCCA
AACGAAAAACTGCAA
TTTGACTAATTTTGCAAGATTTTAGATCTGATTGACCAGGTGAGT
TAGTACCATAAAAAA
TACATATTCCTACACACACA
>ConsensusfromContig2350 Average coverage: 14.11
CAACGGCAGTTCGCATTAGCCGATGTACCACAACGCCAGTTCTGG
ATCCAAATCCTTGTC
GCCACTGAGTATGTCCCACAGAACCGGGTGACCCCATGGCGATA
TCAAAAGGTCAAGGGC
GATGATGAAATAATTGCTCAGCCCATCCGGTAGGCACACCAGGGC
CGCTTTCTTTAGCGC
GTGCGCAAACTTGGTTCGAACCTTCCAGAAGGAGGCTGCTTCTTC
TGACACTGCTGTGCC
ATCTGTGAATAATGGTTCAACACTGTTTACTTTGAATCCGAGGAG
AACTTCGACTGTGAC
TAAGAAAAAGTTCTCGATTACATCTTTGACAATGTGCGGCGAAGA
TCTTGAAGGGTAAAT
TGCAGAAAAGGCCTGAGTTCCATATGTAAGAATTTCTAATTGACG
AGCACCGTTGATAGC
ATCTTTGGACGTTTCTTTTATGACCTTCACAAGCTGATTGTATACA
TTGAT
>ConsensusfromContig2351 Average coverage: 27.05
ATTCGAGAATGCGAATAAATGTAAAAACAAACAAAACATGGATTC
ATTCTGTTAATTCTT
TGATAACAGGATTCACTAGTTGACTCTCAAGAGTCTCGAAAACAG
TGTGTGGGTCTTGAT
CACCATTCACGCTCAGAGCTTCTGTGTAGAAGTCCCCAAGTTCCT
CCACGTAAGCCAAAT
ATTGCTGTAGCCTTGACTGCACCTCTTCTTGGCTGTCTTCTGGAGC
TCTTGTGACGCGCT
GCTTGATTTCCGTGGAAGGCGGAGGGTTGTAAAGGTCGTGGTATC
TTTCTCCTGTAAGTG
GGTCAACCGACCGGTGTACCAGTCTCTCGATGACAACATCAGATG
CGACATCTATCATGT

AGACACGGTTCGGTGAATGGCCGACGCCTTCCAGCTGCTCTGCCT
GTTCGCGAGTGGTCG
GATAGCCGTGGAGGACCCAGCCTCTCGTTATACAATCCCTTTGGC
CGAGTCGATCCTTCA
GCAGGCTCAACACCAAGGAATCTGGAACTAGCATCTTCTTGTCC
ACGTAGGATAGACACG
CTTGACCGATCTTCGTCTCACCCGCCACAGCTTGCTTTATCAATT
TCCCACAGGAAAGAT
TGACAAGATTGTACTTGCTAGC
>ConsensusfromContig2352 Average coverage: 22.67
GTTTTTTCAACGATTTTTAATTTTCTTTTTCTTTAATTTCCCTTCA
AGGGAGCTTTTCG
AGGTTTCTGATTCTTTGCTGGCTAGTTTGGGTGGTTTCGAGAGAG
AAATGCTTTCTGAAG
TTTCGGTAGTGGGAGCGCTCATTCTTCTTTTGCTGCTTGAACCTCT
CGATGTATGTTTTG
AGCCGAGAGTTTTATTGGCCTTGGTCTCGCCTACTTGTTTCTTTTC
ATCATGCTTGGCAG
CGCTCGAAATTGTCTTATCATTTCGATAACGGAAGGCTCGCACGG
CTCGCACTTGCATTT
GGCTTCGTTGTTTAGGATCTAGAGGAGGTTTCAAACCTGCTTTCC
TAATGTTCAAGGCTT
TTGAAAGAGATGATATCGAAATTTTCGACACTTTTGACATTAGAC
TTCTCGTTAGAGAAG
ATAGCGATATTTTCGAGGCTTTTGATGTGAGTTCTGGGGGAATGG
TAGTGGCTCCGTCTT
CCATAGTTTCTGAAACTTCTGAGGTATTCCAATCTTCCTCCGGAAT
TGTATCTTCCTCAG
AGTCCCCTGAGATGCTCATTGATCTCGCGGAATCAGGGCTAAGCT
TATTTTCCTCAGTGC
AAGTCGACGTCGTCGGTAGAATTTGATGTCTTTTCTCCTTGGTCA
CGATTATCCTCGGGA
GCTTTTTACCATAATATGGATTAAATAATCTAATGAAGTTTGAGTA
CCTTTCTCGTAATT
TTTCGGATTGCATCAGTAAACAAGAAACTGCCAACACTATTCCGA
TTCCCAATCCTATTC
CGATGGAAATTCCGATTCCAGCATTTGACCAAAAATCAGTTGAGA
CTTGATTGATGCATA
TGTCTTTCTCTGGCTCAGAGCAGCAAAGTTGCTTCCGAGGTCTTT
CGTCTGACCACGCGT
TTTGTG
>ConsensusfromContig2353 Average coverage: 4.68

TGCTAGCGAACAGAGATGGCTTATTTCATCCGAGTTACTCCGTGA
CCTACGAAAATTGAA
GCGTCTTGGACTTTTATATCAGTCGAGCTGCTAACAAAGCTGGCT
TATTTCATCCGAGTT
ACTCCGTGACCTACGAAAATTGAAGCGTCTTGGACTTTTATATCA
GTCGAGCTGCTAACA
AAGCTGGCTTATTTCATCCGAGTTACTCTGTGACCTACGAAAATT
>ConsensusfromContig2354 Average coverage: 63.06
TAGGAATATCAAATTCAGATATTTATATCTGGCGAGGAGACTTTC
ATAAATCATGCGATT
ATCTGGGGATGTCATGGATATTCAGACTCTGGAACTGATTGCACA
GTTGAGGGATCGGCT
CACTGATGTTACCGACCTGAAAGATAAAGATGATGTGGCACTGCT
TCGCTGGCTCAAAGC
CCGAAATATGAATCTGGATAAAGCCGAAATGATGTTCAGAAATA
GTGCCATTTACCGAAG
AAAAATAGACGCTTTAAATCTTCTAGAGAACTACATCCCACCCAA
AGTGCTCTCCCAATA
CATCGCTGGAGGTCTTTGTGGCTTCGACAAGGAAGATTCTCCTGT
TCTCATAGAGCTTTA
TGGCTTGATGGATCTCAAAGGAATTATGTATTCATCTAAACGAGT
CGACATAGAAAAGAC
CAAATTGCTATTAGGAGAAAAAGTTCTCAAAATCCTACGACGCC
AATCAGAAAAACTGGG
GAAACCCATCTTAGGAATGACGGTCGTGGTTGATATGTCGAACTG
TGGACTCAAGATGCT
CTGGAGTCCAGGTATCGCATTATATGCTCACATGGGGAAAATCTT
ACAAGACAATTTTCC
GGAGATAGTCAAGAAGACCTATATTGTGAATGCCCCGAAAATTTA
CCCGATGCTTTGGAA
ACTCTTCCGCCCGCTCGTAACAGAGGAGATGAAGCGAAAAATTCA
AATCTTCTCAGACCA
CGGAGAATTGTTAAGACACATCGATGCGGACCAGCTGCCGACCT
TTCTTGGCGGAACCAG
AACAGGACCCGGAAACGACCCCCACTGCACACACGAGATCTGTA
TGGGAGGGGAGGTCCC
AAAGTCGTTCTACCTCAAAGACATCACAGAACGCGAATCCATGCT
AGACCGCCTCATTCT
TGCCGGCGACAAACACAAACTCTCTTTTTCAGTCGACGTTCCGCA
GTCCATTTTAAGGTG
GGAATTCGTAACGGATGATTATGACATTGGTTTCGCTCTCTATTT
GAAAAACTCTACAAA

TTCTAAAACGCCGATTTTGCCAATGTCAAGAGTTCAATGTCACGT
CGTCCCAGAAGACGG
CAGTCATATTTGTGACGTGATCGGAGAATATGTAATAACTTTCGA
CAACTCATACAGCTG
GACGCGGCCAAAACGCGTCTTCTTCAACATTGATGTTATTCCCCC
GTGCGACGAAGATGC
CAAAAAAGATATTCCGATTCTCCAGGATTCGTTATCTAACATCAG
ATTATAACTTCATAG
GTATTCCAATTCTCAATGATTCGTTATCAAACACTATATTTAAAC
TTGGTATATATACCA
ATCATCAACGATTAGTTAACGAACTAGTTATAACTTGATACATGT
TTCAATCCTCGATAA
TTTATCACCAAACACCAGACTATAACTTCATAGATATTTCAATTCT
CCATGATTCGTTAT
CAAACATTACACTGTAACTTGCTATGTATTTAA
>ConsensusfromContig2355 Average coverage: 19.49
TTCTTCAGCTTCTCGAGTGGCTACGGAAACGAGCGATCCTCAGAA
TATTTATGAAACCAG
TTCTAAAATATTCCCAGACACAATTTCATCTTCTCTTCAATCATGC
GTTGATTTTCATCG
GGGGAAAACTATACCAGACTCTTCTGCTACTTCGAAAAAAATTT
CCGATGGCAAACAAAG
TGTACTTTATTCGTATAAATCTGCGAAACCGGTGAAATCAGGTCG
GGAACAATATGCGTT
TGTCAGTTGCTGTGCTTCAAAGCCCGAACCCACATGTGCCTTAAT
ATCAAAATCCAATGA
GAGGAACATAAAAGGAGATATAAAGGGAAGATATTCAGAAGAGA
ACAGTATAGCCAATCT
TTCTTGTGAATGTTATTCAAATAATTATAACACTTTTTTGAAGCAA
AAGCATTCTTCTAT
TTTTGACGGAATTGCCCCATTGAAGTATGATGAATTCTACAAAAA
CTACTCAAGTTCGAA
AACGGATTTATGTAGAAC
>ConsensusfromContig2356 Average coverage: 18.91
TCATTATCAAAATTTATGGGTTTATCGTACAGTTAGTGTGATTAT
CGTACACATAACCAT
AATTTATATAAGATTCATTTCAAAATTTGACGTTTAAGGATCGGA
TCATTACAGACGTTA
TTTGTAGTCATTTTAAATCGAGATCATGGTTTCACCATTTGTAGC
ATTCCAGGTCCATGA
ATGGTCAGTTGAGATTTAGTTCCCATCATTTTCATTGGTAGGTTTT
CCAAGATTTCCATT

GGTTGAAACATCGGCATGAGGTCATGGTGAGGTCAGGACCAGGT
CAGGATAAGATCACAG
CGAAGGTACATGGAGGGAGAGAAGACATAAAGGACACAAGAATT
AGAATGAGTTCGCTAA
ATATATCAATAAATTCAATATTTTATCACTTCGTTAAATAACGTTT
GTTCAGTATCTACT
GCTCATGTTGTTGAGCATTCTGGTCATAGGAAATTCCAATCTCGC
TCACTAGCTTTAGAA
AAATTACATACATGGACAAAACTTTTTATCGAGAAGATTTACAGA
TCAAAGGTAACCGGT
AAAAG
>ConsensusfromContig2357 Average coverage: 84.75
GGATCTGTTTGCATAAAGCGGAAGATGAACACCTCGCGAGTACA
TAACCACAACAGCTGC
TCGCTTACCTTATTTCTGACATCTTGCGGTGCATCTACGAGTTTTT
CTACCGTACTTCCG
GTTACGTATTCCGTGGTAAGAATCTTTTGAGTTGAGAGGTCATCG
ATTACTTCCGGGACG
AAAAGATGAGGGTCATCAGCGAGGAGTGATTTAAATCTGCGAGC
GCAATCAGCTTCTCTG
ACGTAGTCGCACTCCCAAGCAAGTTCTTTGCCGGCAACTCTCATA
AGATTCTCTACGAAA
GCTCCTTCGGGAATGAGATTCCACATCTTGAGGACACCTAAAAGG
TTCTTTATATCACTT
GTTATGCTATCAGCCACACCTGGGTACTGGACTTTCATGGCGACT
TTGCGACCATCAAGC
AGCGTGGCTCGATGGACTTGGCCTATGCTCGCAGCAGCAAAAGGC
TTTTCTTCGAAGTCA
GCCAATTTGCTCCGCCAGTCTTCACCTAGCTCTGATATCAGGACA
CGCTCCATTTGCCAT
TTTGGCATGAAATCCGCGCTTTGTCTCACACGTTCAAAGATTTTTT
GAATCTCCGGTCCT
AGTAATGTATCATCAGAAATACTGAGCATTTGTCCGATCTTTAAA
GCTGCGCCCCTGACA
CGACATAGAGTACTCACGACACGCTCCAAGTTGGCTTCATTCAAG
AGGGCGCTACTGGCA
CCAAATATAGTTCGATAAGTGTCACCGACTTCATTGGTGGAGGCT
TTTCCGATGACGCGG
CGGGAGAGTTCTGCTACAGCTCCGACCCCGAGACCGGCAGCTAA
GCTGCCGAAACTGGCT
AGACGGCCGATCCTCGAGCCGGGTACT
>ConsensusfromContig2358 Average coverage: 7.60

GGAACATCATAATTATTTTGTATTCAAACATGATTTGTGTGTTGG
AGATGCGATGCGACC
CTGAGCTGCAGTGACGCCCATCCAGGTTTTGGGGCCTCCCATCCC
AGATCGCATCTGACG
CCCGAATCCGGCTCGAGGACGCCTCGTTTTCAGAGTCCTATCAAT
ACCTGTCATTCTGGA
CGCTTTCCATTGGCGGCATCTTCTGAAAGCCGGGTGATCTGAGGA
ATAAGATGTTTGATA
TTCCTCACTTATGAATAAAGTATTGTAAGACGTATTATTAAATGG
ATACCAAAGAGAATA
TGACACAGAATGAAAAATGCGATTTTGAAGAGAGGGATGAAATA
AATGATCAAATTATAA
AACCAAATCTGGAATATCCCGAATCAAAT
>ConsensusfromContig2359 Average coverage: 12.08
TTCCGATCTCGCATTTCTTATATGGATCCTAAAAAGTCACAGTAG
CGAAAACTATGGGCG
ATAAAATCATGTTCGGCTTCTCCGGAGATAGTCGTCTGCCGGATG
TAGAGAATACGACTA
CGAATTTCGGATATTCGACGCCGAACAAAAATGATGATTACATCA
AAAAGATTTTGGATG
GAGATGAAGATAAAGAATCTTGGAAACAAGTTGCTTACGAGTTC
ACACAAGACACAACAC
TCCACGGCGTCAAGTACATCACTCAAGGAGGGAATTTCGCCCTGC
GGAGGGCTTTCTGGC
TGTTTGCTGTCGTTGGAAGTTTCTTCGGCTTCAGTGTGTTCGTTGC
TCTCCAAATCCAGC
TCTATCTC
>ConsensusfromContig2360 Average coverage: 35.53
ATGTGAATCTCACATCATGATATGAACTGCTAGAGTCGGTAGAAA
ACATCTTATTAGAGA
TAAGAAGATAATTTTGAATTAGAATAGCTCTACTGAATAACATCA
TTTCTCTGACCTCTT
TTTGAGAAATTTATTTCGAAAATCTACCTCAGACCTAGTTTATGG
GGGTATACTTCAATT
GTATACAACACAGCAAAGATTCCAACAAACGAATTAAATTATAA
ACTATATATGACATAT
ATAGCCCGTTCCTAAGCCTCATATCGAAAATAACATCAAATTCAA
TTACATATGGGCGAT
GAAAGACGGCTAGACTAAAATAAGTTCATATTCTAGATATATATC
TAATGATTCTACTGG
TGTCTACTCCTTAAGTAATCTTCTCATAGTGTAGCCTTGTAAACCG
ATACTCTTTTTTCA

CTCTATTGCCAATCAAACAGAATTTGTTTATAAATACATTCGGAG
AAATAAGATATTGCA
CAATTAAAAGTCCGGTCAACTCACAATTTGAATGAGACAGTATA
AGAAAAGATGATTATT
GAATTAAATGATGTCAACTATCACAAACGGCTAAAAATACTTCTC
ATTGAGGACAATATC
GAAAAAAAAACTTTCTCAAACGCACAAGTACTGAAAATATCATAT
TATAATACTTGATAG
TAAAAAGCATATAAATTCATAAACCCGCCATCTCTCTTCCCGAC
>ConsensusfromContig2361 Average coverage: 38.75
ACGAGATGCCCAATACCTCATGGTCATATCAAGGATGTGATATCG
GATGGAGAAAACTTT
TATGCCATGTCAGGACGGTATCAACTTTTTCAATACAGTGTGGAT
AATGGAATATGGATA
GAAATGAAATCATTGGAACATTCTCATCTTCCCTACTCGAAGTAT
TTGTTCATTGAGAAT
GACTTCATTTTTTGCGCGACCGACACCCATGAGGAATTTATGGAT
GTTTACGTCCTATAC
GATGGAAGATGGCAATCGGCAGGATTCGCAACTGCATGGCAGCC
CGCTGTTTTCAAACGA
AAGTTTTATGACTGATATACAATTTCATCGATATCACAATGGAGA
TTGTATCTTAATAAA
TGAACTGTTTTGAATGTCGAACATATGAAGTATGTATATTTTTTC
ATATGCGTTGGCAAA
GAAACCTAATTTTTGTATTGCTTTTTCTGATTCTGAAATCAAATTA
TATCAATATATGGG
TGAGGTCATGAATTTAAGGAAATACAGTTGGAC
>ConsensusfromContig2362 Average coverage: 14.02
ATCTCACGAATATAAAACATGATTATATGTATCGACTATTAACTG
ATCAGAGTCTGATGA
AGTTATTTGATTCGGCATGATTCATCATTCCGGATGTTTCGTAATT
CCGGTAGACATTTT
GATTGGATGGAACGGAAACGCTGGCCACGTGACGTTTGGGGTTG
GGAAGGGTTCTTGAAT
AAACCGGGTCATTCGTGGAGGGTCGGGATTTGGCAGAATTCATG
AACTTGAGATGAAGAG
GCTGTGAATGGGACGCTGGTGTGTGGTGGTATATATGATTGGAAT
CTCCTTCGATTGTAT
TATTATTGTTATAAGAACTGCTACCGTTCTCGA
>ConsensusfromContig2363 Average coverage: 23.00
ACTCTTTCCCTACACGACGCTCTTCCGATCTTAATGACACTGTTTC
GTCTTTTGTATAAT

TATCATCATGTTTTACCATCGTAACAGCCACATTTGTTTTACCAC
GAGGAGAAGTAAAAC
AAATAATTTGATTATACATGCAGGAATGTTGATTAAATGTAGATT
TAGTGTCGCTGTGGT
AATCAAAGTTGAATTTTTTTGACAAGATATCATGGAGATATACGT
TAGATTTTCGTGCTT
TAAAACCACGGACAGTGTAATCACTGCTGCAGTTAACCGAGTTTT
CAGTTTGATTATATC
GATGAAAAAAACAATTTTCAGTTGAAACTATTAAAATATAGGCGG
TTTGATATTCATA
>ConsensusfromContig2364 Average coverage: 47.15
TTACTACCACTTTCATCGAATGCTGCCCTTTCCACCCTTTGAAGGG
ACCTGGTCTGACTT
CTTCGAACTGATAAGAGCTGACCGAGTCATGATGGGAAACTGGTT
CGACTTTTATGAGAG
TTGGCTTGAAGTGGATATGAGCACAACTCTTGTGGTTAGATATGA
CGCTGTGAAAGCAGA
TCCAGCAGAAGAGATCAAAAGAATAACCGCCTTTCTAGGCGCAA
CTCTCACGGAAACACA
AGTGAAAGAGGTTGTGGAACTGACAACAATTGATAATATGAGAA
AAAGCAGCAAATTCAA
CAAAAGGATCATTGAAATGAAGGAACAAAATCAGAAAGAGGGAG
ACTTTTTCAGAAAAGG
AATTACAGGGGATTGGATGAACCATTTCACTGTTGCTCAGAAGG
AAGAGTTCAAAGCGAT
GTTTGGAGAAAGAATGAAGAAGTTTCCCAAAGAATTCCAGTATGA
TATTTGATCTGATAT
CATCAAGTTATTGTTTAATTCGCACAATTGAATCATTTCTAATTTT
GTTTTTGTATTGTC
ATGTCAAAGCATAAATATGATGAAAAACTTGCTAAATAAATTGTT
TTTGTTTTATATGGA
AGGATGAATAAATGAATACATAAAGGAATGAATAATGAATGAGC
TAATGATTTAATGAAT
AAATG
>ConsensusfromContig2365 Average coverage: 8.83
CGGCACTGAATATTTGATTGAAAGAAAACATTCCATGAAAAGCAT
AAATCGTTCGTGGTA
TCAAATATATTGAAAGCGCTTGGAATATTTATCAAGTATCCTCCC
GAGGCTTGCTACAGG
TTCCGTACAGAGGAGCTAAACTCTTCAGCTCTTCATCAACTATTC
TCGGACTCAAGTAGC
CGCCCTCCCGCACGGCGCCGTTACTCAATTTGAGCGACCCTCTGT

GTAGCACGAGAGGGC
CTGGAGTGAAGTTTCCTCCACGGGCGGAACAAACGATTTGACCCG
ATGCATCTAGTTCGA
TTGCAGCTGTCGATACGGATACCAAAACTGTTC
>ConsensusfromContig2366 Average coverage: 13.69
TTCCGATCTGTTTTTGTAAGGTTCGACGTAGGAGTGGATGAAGAA
AAACGTGGTGCACAT
GATCATCACGATAAAATACTTCACAAGAGCATTTGTTATCAGTGT
AATTAGGATGACAAA
CAAGACCCTACGACCAACGATGATTCCTTCCCAGCAGAACTTCTC
ATTCAATTCTGGTTT
GTACGGATGCATGAGTAGTTTTACTATTTCCTCTTTCGCATGCTTT
TTTGAGACATTCGG
GCTTGTATTCTGCAGATCGGAA
>ConsensusfromContig2367 Average coverage: 16.88
TCCCGTACTCTGGTTACGCCTTCAGTCGTTGTGTTCACGAACACA
CTTGGCCGCGTCGAT
GACATGTAGGTAAACATCGTCTCGTAAACTGCTATTTTCGACGAA
CTGAAAAAGGTCTGA
GTAGATCCAGAGGCCAGTGTTCCATATTGAATTTCCGTTTGACGG
GAAAGATCGTCTGCT
GACTCTATTGGTGTAAGCATTCGCTCGACGGTCAGGAACGCAGCA
AGATTAGCAGTGTAT
GAAGAAATTATGATTAGGGTGAAGAACCACCAGGCACTTCCCAC
TAGACGACCAGACAGC
GACCTCGGAGAAATATCACAGCCTTGCTGCATGA
>ConsensusfromContig2368 Average coverage: 6.99
CATCTGGTCATCTGTTCCGTATCTAATCAATTGGTGGATGGGCAA
GCGCTCAAAGGGTGG
GGGATCCCCACACCCCCCTTCGAAGACATCGAAGAAAAACAATG
AAGAAAATATCGTTGA
CGATGATGCAATGGAAGATCTTGATGTGAACGAGGGCACATCGA
ATAATTTATTCGATAA
TTTACGGGATTGCGTTGAAGACACTTGGCCAGGACTCCAAAATA
CGTCAGGTACAACG
>ConsensusfromContig2369 Average coverage: 10.04
AACTGGGAAACTATGTGGCGGCACCACCAAAGGAAACTTGGACT
CAAATCAGAGCTGAAT
ACTTCAGTGAGTCCCGAATGACCCGACTCACCGTCGGTGAACGC
ACCGTAGAAAATCGGG
AAGCCACGAAAATCCGCGGGGAGTGCAACTTCGATAGCTCCGAC
GACGAGGCATACGATG

AAACATGGGCACAGCCGCAAAAGGTGTACATTGGGATGTCCTAC
AGTTCTAATTTCGAGG
GCCAGATCGCGGAGCTTTGCATCTCCGAGAA
>ConsensusfromContig2370 Average coverage: 14.86
TTCCCATTCATCGAACTACTATTGAATTTGTCCGGGTGTTGTTTA
TAAATGGCCGAATGA
GAGGCAGCTTTCTTGGTAATGAAATTATTAGGCAATTCAAGTTGAG
TTTCTTCTTTTTTA
ATTTTTTCGAAATTCATTCTATTCCTTGTTGGTGTTTTGCATTCTT
TCTTTGGGCGGTCA
GATTCTTCATGATCGGATGGTTGGCATGGATGTTTACTGTATAATT
CTATGGTATTCTTG
ATTGTATGGCCATCGGGGTGAATCTCGAAACGGTTGATTATTTCAC
TCGGGTTTCTTTTT
TCTTCGAGACTTCTAAGATCGTGCGAAGGTAATTCTGAGGCGCTT
CTGGATGTTCCTTTT
CTATCCTTTGATGAATTCGAAGATAAGTCAGAAATAAGGTGTGTA
TCTAGTTTTGATGGC
AGGATCCTTAGATTTGAATAGGGGTTGACAGTGCCGGCTATACGT
T
>ConsensusfromContig2371 Average coverage: 9.41
CACGGCTAAAACTACAATAGTTATGAGAAGAGTGAAGATGATGG
TCGCATGCCACCATTT
CATGCCTCCGGTTTCTTCAAGCGAGGCGTCATAGGTGAATGCAGT
GACGTTGAAAATTAT
GTTGTTGTGGTTTATCTCACGCACGAGTGTAATGTCAGTAACCTTT
CCTTGAAGCTCTTT
GAAAATGACGTTTTTCATCTCAGCCGGATCAGTATATTTCTCATC
GACCGCGGGTTGATC
TCCTTTGTCGGTGACAATTTTCAAAAAATACGCTATTTTATTCTTT
CCGCATTTGACCGG
CTCTTTAGGAGCTTCGATCATCAATGAAAGTAGCTCCGGTTTATC
CTCCAATATTTTCGC
CTGCACCTTCCACTGATATTCATCTTTCTCCTCTTTTGTAATAACG
TATAAGGCACCATT
GAA
>ConsensusfromContig2372 Average coverage: 34.93
GCCGTGACTAAAGACCCGCGAAATCTTGTTTCCGCCGTTGGGATT
GTCGTATATATACTA
CTGTTCTATATATTCTCCTATTCTCCCGCTCACATCAATTGGCGAG
CAGTGGTTTGGGGC
ATCTGCCTTCAATTTGTGTTCGCCTTGATTGTTCTGAAATGGTCA

TGGGGATACCAAGCA
TTTGATTGGTTGGGAGAACGCTTCGTGGAGTTTTTGAACCACACG
GCTGCAGGCTCTTCC
TTTGTTTTCGGAAATGATCTCCAACTGACTTTTGCTTTTGGGATCC
TCACAGTCATAATA
TTCTTCAGTTCCGCTGTTTCCATTTTGTATTACCTTGGAGCAGTCC
AGCATCTTATCAAG
GCGGTTGCGTGGGTCCTTTGCAATGTGATGGGAACCACTGCAGG
AGAATCCATGAATGCA
GCTGCCAACATATTCCTCGGCCAAACCGAAGCGCCCCTCGTCATC
CGTCACTTCCTCCCC
AAAATGACGCGTTCGGAACTCCACGCAATTCTCACGGGAGGGTTC
GCCACTGTCGCTGGA
TCCGTCTTCGCCATCTATATCAGTTTTGGAGCTCCAGCCAATCACC
TTCTGTCGGCATCC
ATCATGTCAGCGCCTGCTGCTCTAGCAATGTCCAAGCTCATTTAT
CCAGAGACAGAACAG
TCCAAGACAACCATCGAGTCTTTGGACAATATGGAGAAAAGTCC
GGATGAAAACATCATC
CAAGCGGCTTCAAACGGAGCCATTTACTCCATCAAGATGATTGCT
GCGATTACCGTCAAT
CTCATCGCCTTCCTCGCCCTTCGCGAATTCGTGGACTCAACTTTGA
TCTGGTTTGGGGAT
CGTGTTGGAGTTGACGGATTTTCGTTATCATTCATATTTTCCTACG
TACTTTGGCCCTTC
GCTTGGATGATGGGAGTCGAACCGGATGATTGTCGGCAGGTGGC
TCGATTGATCGGAGTC
AAAACTGTTATAAATGAGTTCATCGCGTATAAGGATCTCGGAGTT
CTCATCGAAAACACA
GAGACCTTCAGAAAATATCCGAACGCAACCCTTGAAATCATCGG
AGAAGACTATATGTTG
ACCCTGCAGGATAGTTCAATTCTGACACTTGCTGGCGGAATCGTA
TCGGATCGAACCGTG
GTCATCGCGACCTACGCACTCTGCGGCTTTGCCAACATCGGCTCG
ATGGGAATCATGCTG
GCGGGACTGGGGACGATGGCGCCCAGCAGAAAGT
>ConsensusfromContig2373 Average coverage: 24.95
TGCAAGAGGAAGCTTCAGCAGAATCCTCGTTGGTGGTGAGACATC
TCTTGACTGCATCAG
TTAATGCGAAAGAACCCCATGTGAGAGTATTCCTCTCCAGACTGT
CCAGCTTTTTATCCA
AATCTTCATCATCTAGCTCTGATTCATCCAATTCATCATAAGCCAG

TCTGCATTTATTTA
TCAAACAATCCTGTAGCATGAGATAGAATAACATTCGATCTTTTT
CAGTCCCAACCACAT
ATTCCAAGGCACATTTCATTGAGTCGGAGCGGACTGATGGAACAA
GAATATTCAATATTT
CATCGTAGCAATGCTCAACATATAAGCCAATGAGCCGCTGAAGTT
CGTTCCGGGATTCCA
ATTTCATCAACTCCTCGCTGATCATATCAAAACTGATTTTTCTCC
CATGTGAAGACAAGC
AAAAGTTGTCTATTTTTTCTTCTGTGCGAGATCCCGTCATTATCGA
TTGACACTCGCTCA
AGTAGAAAATTGTCTCATCTGGGTTATGCTTCAGGACAGATATTC
TCGATATCAAATATA
AGACTCTCAAAGTTTTAATTTTGGATGTTCGAATTGGAGATTTGG
ATTCGTAGAGAAGAT
CTGACAGAAATTCGATGTCTTGACGCCGAAAACTCTCGCTGCACT
CTTCAGCTTTCATCT
GATGACATTTCCCAGTGCAATTTCTATCGGCCGAGTGGGATAAAA
CTTCATCAACGCTGA
GCTCATGATAAGTGAGTAGGATCAAACACACGTCAGACAAGCGA
CTCTCGTTGGAACGGT
AATCATAGATAGAAGGTGATTGAAGATGTGGTCTCAACACAGTCA
TTGCCTCAACAAACT
CCGCAGTGAAATCCATTTTCAATCCTCCATTCACTCGGTCTTCGA
TCATGACTGCCAACC
TCAGGCAAAACTGGAAGCACAGCTCAATGACGTCACAACAAGAT
GGGAGCTCTTGAAGAA
ATGTCTGAATTTCTGGATTGGACTCTTTTGGTGTCATGTCGAGAGG
C
>ConsensusfromContig2374 Average coverage: 10.63
CTTGACGAAGATAATCGGACATCTCGAAAGGGTCAATGTCGGAA
TAGGGTTGAAGGGCAA
AGGTCATGATCTCCCAGAAACAAACACCGAACGCCCAGACGTCT
GAGAAAGTGGTGAAAC
GTCTGTTGTCAAGCGCTTCCGGAGACATCCATTTGATTGGTCGGT
TTTCATTATCGCCCA
AACAATTGTAATCTGATGGGAAGAGGTCACGTGATAGGGCGGAG
TCTGTCAGCCGAACTT
CCAATTGGCTGCTCGATATGCAGTTCCTCACAGCAATGTCTCGAT
GAATCAAAGATCTTC
TATGGAGGTATTGAATGCCTTGGGCGATTTGCATGGCCATGTAGA
TCAGCTGTTGAGTAC

TCAGAGCCTGCTGCATTGCAAGATCAGAAGTGACGAGTTGCTGTA
GCAGCCTCTTGAGAT
TTCCTTGGTCCAGC
>ConsensusfromContig2375 Average coverage: 26.87
TATCCAGAATCATAATAATGCATAACAATTTGAAAGAATTTAGCA
ATTCTTTGATTTGAG
GTATCTGATATATTTGATGTTCTTTATACACGTATTTCATACCACG
CGTTTCACACCAAA
ACGTGATGTCATGTGTACATGTATTATTATTTTTGAATTTCGATTT
TGTTCGCTCTACAT
GTGATGCTATTTTGCTCTGTATATCTGATGCCATGTGGGGTATGC
CAGAACATAATTTCG
TGTACACACAATTTAAGAAATATGCATTATTATTCTTGAATTTAT
AAGAGTATATCTTGT
TGCTATAGTAGGTCTATTATGCTCTTATACTTCATGCGAAGTAGG
GCATGGCAAAACATA
ATTTCATGTACTCACGATTCGCGAAATATGTTTTATTAACCTCGAA
TCATCTTGTCGCTA
GGTTTTGAATTTGAAAGATATATTGTCATTTATGATTTTTAAGCAG
TACATAATTGTGTT
AGCTTCACAATATTTTATATATTTTGGCTCTTGTGTACCTTTTATT
TCATGCCACGTACT
GTATGCTATCTAATTGAATATCATTCAAGATTCG
>ConsensusfromContig2376 Average coverage: 11.17
TTTTGCTGTTGATAAATGTAATTTAGGATAATTGTACAAAATTTG
GGGAACTTGAATTTC
AGATGCCGGCATGTTTCACAAAATAGAAGCAGAATTCAGTGAAA
CAGGAATTGCACACAA
AAAACTGAATTATCAAAACATTGTGAGAACAAAAGCCATGAAAC
ATAGTTGAATCAGAAT
CCTTGACCTTCTCACTAGGTTTGCTTCGCTCGTTAATTCGCTTTCA
GTGGCGTTCGAAGA
AGGTTCGCCCACGGGCGTTAACGGTTTTCGTCTTTGTTTTCCATTG
GCCTTTTGTCTGTG
GCCGTTTTTGCCTTTTGCTGGAACGTCCTTCAAAACCTTGTTATTC
GCTTGGACGATTTC
ACCTTGAACTTTGGTCGTGCTCTGCTGGTTGACTACTGTCGCGGCT
GTCGTTGGTTTTAG
TTGCCCTTCATGCATGAGCTCTGCAGAAGTTGGTTTTCTGACATC
CCTGTTGCAAAGAGT
CCCGTTGCAGGAGCTGTGACATCTGACCGTGAACCCTTTGTTTTC
AGTCGTGCAGAAGTT

TCCGATCTCCTTGACCTCACAGCTTCGAACGACGCTCTCCATGTC
GTAGTCATCAGGGGT
CAAAGGTTGGACCGTTGCCCAGCTCTGTTGTCCCAGAGGCACCAA
CAGCCGCTTCTGAGC
GCTGTATACAATTCTGGTGGTGCAGGAGCGCTTGGGGCAGTAGG
TGCGGGGGGCGTTGGT
GTCACCGGCGGGGATGTCCGTACAAGCGCCGTCGAAGCGTGGCC
CCTTGGCGGAGCATGT
GTAGCACTCGTGAACGGTGTATGGCAGGCCCTGGCTGGAAACCA
TTGTCACATCAAACAG
AAAG
>ConsensusfromContig2377 Average coverage: 13.20
CAAAATCAGATCATTTTGCAGGACTATATGAAAACCGTAACTGGA
ATCATCACCTCCATT
GTCTACGAGCATCACATCACTCCCTAGTTTCCTGACATACAAGA
GGAAAGAAACAGCATG
ACCGTTGTTGCATTGTGTGATGTCTATTGGACATCCACTCAAAGG
TGTTCCGTATTCGAT
TGCGTTTTCTCTGAAGCTGAAGGCAGCATCGGTCGGGCAGAAACC
ACGCTCGAAAGTTAC
TGTGCCGTTGTTGGAGACTGTACCATCGATATCACCAAGAACTTT
GTTCAAGGTCATCGT
TGTCAGTGATAGATTCAGAAAGCCTGCTTCTGTGTCGCAAATTTG
CGCTTTTCGATGATT
CTCTTCTACCTCCAGTGGTTGAAGTCTTTTATTGAAAATTTTCAGT
TTGTTGATAGGCGA
TATGGTATTTTCATATCTG
>ConsensusfromContig2378 Average coverage: 12.93
TGTAAATCTGCGTCGGAGATAGTTGAATCATGTTTTGTTTACATC
CAACAATATTATTAC
TATTATTCCCATTTTTTGTTTTTCTGCTCAACTCGATGGCGGAAA
TGAGAAGAAGTAAAT
AAGCTTTCGGTATCGCCTCTTTCATCAGACACTGATTCCACATGA
CGCGTTTATCCATAA
ACTCCGATCGTCCATTTCCGCCGAATGTGTTCATGGGCCATTTCAG
ATATTTCCGATTCT
GTTCGAGGGCGAATGTTCCATTCACGTGAACAGGTAAACCAGTAG
CGTTTGATTGGGCGA
GATCGGGGAGCGGCAGAAAACAGAAAATTCGTCCATTTCTGAAA
TCGGACAAATCTGAAT
CGTCTTTTTCGCATTTTTCCGAAAGTCCATTGGACAGAGCTTGAC
TGGAATAACCGTTTG

GCAGTGTCCTTGAACCCTCCGTGAGATACGTACTTCCGTTCATAG
ATTGATGAGAGAGGT
CATCATCATCTGATTCCAATGGAAGGGCCACGCCCACCAGAGGA
AGACATGATATATCCG
GATCGTTTTGAAGCTTTCGAAATATCGCCGTACTTTGAGTGCCGT
TATGGAATTGAGTTA
TGAGCCATGAATAAGTCTGTCGGGTCTTTCGCACAGCGCTCTGGT
CTTGCATTGTCGGCG
AAGCGCTCTTGGCTCTCAGCGCTTTATCCGTCATGCTTTCGTAGCA
ATAGC
>ConsensusfromContig2379 Average coverage: 11.41
AAAGATAACGTCAGGTTTCTCAGCACTCTCGAGAGACATCTCAA
GAACATTACACACGGG
AGCAACTTTCAGGCTGTGCTAGAAACACTTCCTTCGTTGATGAAT
TCCTTGCGAATGGTT
TGGACTATATCCCGACACTACAACTGCGACGAGAGAATGGTTCC
CCTGATGCAGAGAATC
GCCTGGGTCTTGGCTCAAAGGGTCTCGCACGTTATCAATATTCGT
AGCATTTTCAAGGAG
GGGCCGGACGAAGTAAGAAGGAGAACGAATGAGGCCAAGAAGA
TGCTGGATCTCTGGAAA
TCTTCTTACTTCGATGTTCGCGCCAAAATCGAGAGTTCGGGACGC
GACAGCAGATGGGAA
TTCGATCGTCGAAAGCTTTTCGAACAGACAGACTACATGGCCAAG
ATATGCCAGAATGTC
TACGACGTAGCACAGATTCTGGAAGAATTCTACAACATTTTCGG
GCCAGAGCTCAAAGCG
GTGACTGGAGACCCGCAGCGAAT
>ConsensusfromContig2380 Average coverage: 8.22
GTCACGGTCAAGTAAACAAAGATGTTGCAACTTGATGTAGAAGGC
TATAGGTACTTGCCC
TGGAGATCTTTTGTGGATTATTTTCTTGAACATATATCAAGCGGT
AAAATGACCATCACA
TTATCTCTCCAGTGCCTATTTGGCCTTATTGATTTGCTGAATCAA
AATGGAATAGTCGAA
TTCGAAAAACCGGAGATTGATCAAATTCTTTCATTCAAAGAGGA
GGCTAAAAATACGAAA
CTTCCGGGAAACAATTGGACGTACAATATATTTGAGA
>ConsensusfromContig2381 Average coverage: 28.18
TCATTATAAAATTTGTGATAAATAATAACCATGCGTTTAAAAAAC
TTTAATAATTGTATT
CATTTGCTGTTATTATCTTCTGCTTCCTTGAATAAAAGAAGTAAA

ATGAAGATAATATTT
CCTGCTCCCATCACAAGAAATGCCAGAATTTGAGATTCGCGAAAT
AAAACTTGAATGATG
AAGGGGGAAACTATTCCCCCAATCGCCCGAAAGTCAATAGAAC
AGCACTCCCGACAGAC
CGTATCCGGGTAGGGTATATCCTATTTGTACGCACAAGCATCAGC
TCAAACATGACCGCT
GCGATAACTTTGAGAAATGTTGTTAGAACAAGAGTGAATCTGTT
ACGTAGGACATAAAGA
AGTATATATCCGGCGATTGACATCAAACTTGCAGTTATAAGCATT
TTCCTAAGCTCCACG
TAATGAGATATGATTGCTCCAAGCAAGGTACCTGGAATAACACA
TACATTGGCCACTAAA
ATGTTGTAGAGGGCGGGCCGGTCGAATGTATTACAAAACGTATAT
TGACATGTTAAGACT
TGAGAATCATCTACATTTTCCCGTTGCAATTCTGCCACTAGAAGA
GTACTGGCCAAATAA
GTATTGCTGACTAGAAACCATAAAATGCAACCAATGACTGTATC
AACCCAAAGACTCGAG
GTGAACAAATCTTTAATTTGACCTCTTTTTCCATTCTCATTTGTTT
CAAGCGATTTTATA
CAAATCGGATTTCCATTTGTTTTTCCAATTTTCTGAAGAGTTGAAA
TCGCTTTTTTCTCT
CTCCCGA
>ConsensusfromContig2382 Average coverage: 6.51
TCTGTCTGTTCGGGTGTGGTCTTTTATTTCTACACAATCTGTCTGT
TCGGGTGTGGTCTT
TTTATCACTTGATTCTTCTACATCATGTTCTTTCATAATAGTGCTC
TTCTTGCGAATTGC
TCTGTATGCCGTTGTTGAAGCCAAACAGTTCAACGTCCTACGAGG
TGATATAACTTGAGA
TTCCCTCTCATCTTTCTCATGTGTGTAAACATGTTTCAAAACCGGT
CTATCAGCCAATGA
AGTTAAATGATTAGTTTTAAGAAAGTGTGATACAATGTCTTCTAA
AATTTCCATCAGCTC
AGGCATATTTTTAAATTCCAAGTGAGTTTTGTTGGGAGCATATGC
AATGTCGCATTGGTC
GAACGGACAACTGATAAAAACGATGAATATTGGATGCTTATCTTG
CTGTCTTCCAGGACT
TGCAAAATTTGTTTCGCTCCGAGATTTAGTTATGGATGAGTTTGC
AAGAAGATTTGATAT
GAGTTTATGTACAGCTGTTCGTTCTACTCGACGTTTATTGATAAA

TATATATTGCAAATC
TTTGGAATAGTGCGGTTTTATGGAGAAATATCCTTTCACCTTAAA
AACCTTTAGGCTGCC
TTTTACGAATCTCAACATGTCTGCTCTGGCACTGCCAAATATTTGT
TTGAATAATTCAAT
GAAAGAATCCGCCTTAGATGTTTTCAGAATAATTTCCCCGGAAAA
ATCATCCCTTAATGT
GAACGATATGGAGCGATGACTGATTGCAACACATATCAACATCTT
TCTGATTTTAGAGAG
TTCAACACCTTTATCAATCCACTTTTGTCGAACGGGCTGGTTGTA
GAACAGGTTTTCAAC
TGTGATTGTTACTCCATGACTGGGCCTTGCCTGCTTCAATATAGTT
TGACTGCCCCGTTT
GCCTTTCTTGAATCGAACAGAAAATGTATCTTTGGAATTTTTTGGT
CGA
>ConsensusfromContig2383 Average coverage: 7.70
ATTTCATATCAAGTGTTCCATCATATACAGTCAATGTTTACGATAT
AAACGTATTTGTTC
ATCCAATGAAATTTTTAACATTGAGATTCGTCACTTAGTTTATGAT
TTTTTATCATGCGG
TTATCCAATGTCTCTCATTCGGAAATACGTCGATATTGTATCTGC
AATGAGCCGAGATTC
GATTCTTGAAAGAAGAAGCTCGAAAAATGAAAATATAATCCCTCT
AATCATTGACTTTGA
TACCGAACGAAGCGATCTGGAAAAATCATTAAAATCAGCATATG
ATAACTGCAAGTCGGT
TTCTCCATGGCTAAGCAAAGAGGTGTCAATTGCACGAAGGCAGCC
TCGATGTCTACTTCG
ACGTATCAGCAAAAAACATTCAAGCAATGATGCTGCTGTGACCGG
AAATCTCCCTTGCCG
CAAATCGAGATGTAAAGCTTGCTCCCATATACATGCGGG
>ConsensusfromContig2384 Average coverage: 17.57
AATGTGTCGATGAATTCCCGCGCGCCAACGTAGCCCCTTTTGTAT
TGGTTGTTCTGTAAG
GATATGACGAGCGACTCCATTGACGCACATACCTCGCTTATAGAT
CGGTGTGTGACAATG
AATTTTGCGTCTGGATGCTCGCTGACAATGGAGTCTAGCCAGGGA
GTGTAGTAAGGAGAT
TTAAGCACAAATGTTGGATTCACATCTCTTCCACATGTTTTCCTA
TCCTTGGCAACCAGG
CACTGGAGAATTCTTGTATGCAATTTCATCAACCAATCCGTATATT
TCTTGGATTCTCTC

TTGAATATGTACAAATCCAATTCTTGACCAAATGATTCAAATTGA
GTGATGTTGAAAAGG
CTGAATACAATTTCCATCACCACTGTGAATTCTTCTGGTTTCATGG
CGTGGGTGTTGTGT
TTTTTTGCTAAGTCTTCTGACCCTGCCTCTTGAATGAAAATATCG
TACACGAAGCGCTGA
AAGAGCCGAGATGGGAGGGAGCTGAAGGGGGATGGGTTCATCCA
CTCGGCCGGGCACACC
GTCCGAAAATCCGGATCCGTCGAAAGCATGTTGAAGAAGAAAGT
CGATCCGGACCTGGGC
GGGCCCACGATGAATACCGGCTCGTGAATTTTAATCTGGTCAATA
TTAGCATGCTGGATG
GCTTCGTTGAGAGACCGAATAAGCACAGTAGATCTCCAAATTCT
GTAATAATGAAAGCCG
AGCCCATACACACTGAAGTGGTTTTCCCGCCTGCCAAGCATCACC
AGTTTCCGTCTCAGC
TCAAGAAATATCAAATCATTGTTTGTCCATTTCATACACTCCTTTT
GTATCCATTTCTAC
AACCCG
>ConsensusfromContig2385 Average coverage: 26.42
ATATAAAGTCGCTTAAACGCCGTGAGAATGAAATGAACGAAGAA
ATCTCAGATCTTCAGC
ACCAGCTGTCGTTGAGTGATGAGGAAGCGGCAAAGTTGAGGGTT
TTGTTGGCAGACAATG
AACAAAAGTCTCAAATTGGCGAAGAAAACGAAATCAAACGTCTT
TCGCGAACCCTAGCGG
AGATCAAAGATGTAAATGAAAACACAGCAACCAATCAACGATCA
GAGATTGAGCGTCTTC
GCAACGAGATTGGCCAATTATCGAACAGTCTTCAGGCACGTGAC
ACAGCAATGGCGTCGG
CCAAGGAGGCAGCGATAACGTTTCAAAATCGGTTGCGTCAGCAG
ACAGATGTTCTTGAAA
GAAAAGAGAATGAAATTAAAATCGCTCATGAACAAATCTCAGCT
TTGAAAATCGAGAACC
GTGAGTTACAAAAGATGGCGCTCAATGTGGATATTTGCACGAAA
GATGGCAGAAGTGAGG
CAGGCGATCGAATCGATAAACTTGAAGAGGCCTACAAATGTTCT
TTGGGCAGCTTACAGA
AAGAAAATGGCCGATTATGCGCTGAGATGTCAAAATTGAATGAG
GAAATGTCGCGGATCC
GTTTAGCCAATCAAAATGCCTTACTCGAAACCACGCAACAATCA
AAACAAAGCCTCGACC

AATCGCAACAGGAATTTGTAGCGCGTGTTGCGAAGGAGAGAACC
AATGCCGAGCAGACGG
TGGCCGATATCCAATCAAAGCTGGGCTCGCAGTCACGTGAGTACG
AGCATACTGTTGAGC
ACATGCGCAGTGAGATGAATCGACTCGGGACTGGTTTGGAACAG
CAGAAGGAACTTGTAG
AACAACTCGGTCTCGAGAATTTCGTCATGAACAATTTCGTCGAGG
GCGTCGGAGATATAA
TGAACACCCGGGCGCCGGTCAGTGAATCACCGGGACGTAGCCGA
ACTGCT
>ConsensusfromContig2386 Average coverage: 16.93
GTCGAACAGATGCATTATTTGAAAAATATGGATTTGAAATAATAA
TGTGAGATATTATAG
TGATGAGTATTTTTGAACAACAGCAAAGTCACCAATAAGTTCTTA
TTGTATGAAATGTGA
TGGATTCGTTTATGATCAGCAAACTCCGAGGTGCTCCCCAATACA
TACTTCATCAATCAT
TCCAATGAAATGTTGTGTTCCAGAGTATCGACAGCCAATCAATGT
ATTTCGCAATGTTTT
CGAATTACAGCCACCATACATGTCATCAGACCCAACCAACGTATT
GTTCACGTATACACT
CAAAACATCCTTGATATGGTCCCATTCCATCTTAACTTTCATCCA
AACGTCCTTCACAAC
GACTTTACTTTTTCCCACATTCAAATATTGGTAACAACTTCCATG
CCAGGCCTTGAAATA
GACAGCAAGTGAAGAATCACAGAGATAAATATCCCTTCCGACATG
TCCGTTTGTTATCAA
GAACTGACAGCCTTTGATTTGTGAAGTTTTGAACCACATTGATAT
CACGAGATCGTGACT
GACCATCTCATCATCTCCAACCATCGCATGA
>ConsensusfromContig2387 Average coverage: 15.00
TATATGATAGACCAGAGGTAATCGATAGATATAGGAAATGTATTA
TTCATAGCTGTATGC
AATCGCTCAAGAATGTTGTTTCTTTTCTAGCGTAGTCGAATAGTT
TTTTGTATGAGCCAG
AATAGTTCATTCATTCGTATTCGTAACAAAATAACTAATTAATGGA
TTTGTTCGTTTTTT
CAAACTACAACATATTGTATATGAATTTGGTATGGAGACTACATG
GTATGGTTTGCAATA
TCAAAATGCATATGATAAACGCTCCTATAAAAAGATTGATGTCGA
GATC
>ConsensusfromContig2388 Average coverage: 21.89

CATCAGCGCTGAACTGTTCAAAGGTACATGTCATGGCTGGTATTA
ACGGTATAGATGAGT
CTTCAGCAGAAATTCGAAAAACATATATTCAAAATTTGAAGTTTG
CTGCAGATTTCTTCT
CAAAGCACAGTATTAAAACTCTCATCGAACCAATAAAAACAAAGC
CAGGTTATTTTTTGG
ATAACCCTCACACCGCTGCTGAAATTATCGAAGAAATCAATCACC
CTGATCTTCGACTTC
AATTGGACTTGTATCATGCTCAATTGGCGACTGGTGATGTATGCG
GTCTTCTACGAAAAT
ACATGCACATAATAGGCCATATCCAAATCGCTCAATACCCTGGAC
GAGGCGCTCCCGACT
CGAACGGTGAATTAGACTACCGTTTGATTCTTAGCGAGATTCGAG
GTTTGGGGTATACTG
GAACGATAGCATTGGAATACACGCCAGACGAAAAACAGCCTGAT
ATATCAAAGACCTTGA
AATGGATGCATAAAGCTTTGGGCATTTGAATTTTGTAGAGACGCT
GATATTCTATACACA
ACGTGTGAGAGGAAATCACAAAAACGCTCTTTTTTCAATCGCATT
CAGACGAGTAGAGCT
TATACAGACGATCGG
>ConsensusfromContig2389 Average coverage: 9.16
GGATTTTGAAAGACCTGTTGTATACGACCAACTAGCACAGGAACC
ATTCTACGATTTGAT
CGATGACAGTCAAATCCATTCACAGGACACGATGCCTGTAAGAT
ATATACAAGAACATCC
TACACAACTTGCTAGACCAAATGGCAGTGTACAAAATTCGATGCC
GCTTAGATACGTACA
TGATTCTCCACAAAGCAGTGTCAGGCTTTCTAGCCGAAATCAACA
GTTCCAAGAACCTAT
GAGTTTTCAGAATTCAATCCCTATGACTTATGTACAAGATTCTCC
TCAGAGCAGTATAAG
GGTTTCTAACAGGAATTTCAATCAGCGAATTCAAGATCCAGATGT
GACGCAGAATATTAT
TTTGAACAGTGGCGGACGAACGCAAGGCAGACAGATGAGACCCG
TTTATAGATTGGCGGG
CAGTAATTTATCTACAACCTCTGAACCAACTCATTACATTTAATAT
CTTATCATACATAT
GCAGCCACTGATACGACATTTAAATAAGACATTAATTTAACCTGA
AAAATGAT
>ConsensusfromContig2390 Average coverage: 8.96
TGAGTTCAATCATGCCAAATGTTGTTCGACGTTGCATAGCTCGGT

CGGGGTTCACGCCAT
CTTCTAAGAGCACGGAATGGATTGGTTATTGGGGAAAGCATATG
AAATCAGAAGCCTTTA
AGCACATCAAAGAGCATCAAAAGGTGAATCACATACCAGGCTCT
TTTCAAATCGGAAGAA
AGGATAGACTTTGGCGGAATCTCTCTCATCTACAGCCGTCCTTCG
GCAAAAAGGAATTCG
GGTTTTTCCCTCAAACCTTCGTTTTGCCGAGCGATACCAAAATGC
TAAAACGCGTTTGGG
AGGAGAACTCCACTAAACAAAAGTGGATTATCAAACCCCCCGCT
TCTGCAAGAGGAATCG
GGATCAAACTCATCAACA
>ConsensusfromContig2391 Average coverage: 14.48
ATCTGCGAGATAGCGGACTGTGTCACAGTTCCGTGCATGGAGAA
GAATCCAGTGCAGTGT
ACATGCGAAATCGAAGATAATGAACATGTACTCGATTGCAAGCC
AGGATTTTACGGAACA
GGTTGCATGCAAGTTATTAACTGCGATAGTGGCACTGGTCCATGT
CTGCACGGGGCGGAA
TGCAAAACAGGCCCTCCAAAATCCTGCGATTGCGCGGAGGCGAA
GGGATACAAAGGCGAT
ATATGCGAGATAGCTTTCTGCGACACCGTAGAATGCTACGAAAA
GCATACGAAACAATGC
ACATGCAGCATCAAAAATAATAAGCATATAATCGATTGCAAGCC
GGGATGGGGAGGAGAC
AAATGTTTGCAAGCAA
>ConsensusfromContig2392 Average coverage: 17.60
AATACACTCAACAACAAATTCAAACCACACAACTGCAAGAAATA
CTAGCTATGAAGATAG
CATTTCTACAGCATCCTCCGAAAACAATCGAATGGGAATCCCCTT
CATCGATATCGAAAC
AGCGTCCATGACAACAGAAGACCAATTTGAGCTGGAATCGAACT
TGAGAGCTTTACAGAT
GGCTGCGGATTCCAATGAAAACATTGCGAAAAGTGCAGAAATTG
AAGAAACAGCACCGGT
GACCCAAGAATCAGAAAACCGGAAAGTCAGCTTTGCATCCGAAC
TTGAAACAAAAATAGA
AAGACATCAGAACAGGATGGCTGAGGCAGAGTTGGAGTGCCAA
GAAGACAGAGAAAGCAG
AAGTGAGGTCAGCGAAGAGGCATCCCTGATGAGCGCAATCAATG
ATGAGCACAAAGGCAG
TTACAAACTGGTCGGATCCGTGTCTGAAACAATCGACGACGAGG

AATACATAGGAGTCAT
CGTCCAAGTATCATGCGATGCAGAATCAACCGTGACGAATGAATC
ATTCGTATCGTTTGT
CGGTCAATTGGGAAGCGCAGCAGCAAACTACTTCAAAAGAAAAG
ACGAAACGAAGTTGAC
CCTGGATTCGATACAAGCTGGTACGTTGGCCCTCAAAGACACCC
GGGCTGCAGCAGCCCA
TCTGCAGGAAATGAGAGAATCTTTCAGTGACCACATCTCAATTTC
CTCCATTTTCGGTCG
AC
>ConsensusfromContig2393 Average coverage: 7.29
GTTAACATTGACTTGAAATTTTTACGTTGGGAAAATCGAGGCTTG
ATTTCGTGTGACTAC
GATTCAGTCGAACTCGAGTTTATCATACAGACTAACGACTGGAG
TCGAGGAAAAAACGCC
AAAGGTGAAGCTCGAGATGTGCTCAGAACACCCATATGGTCGTG
CGAAACTATAGATGAT
GAATTCGACAACATGAACTCCGACCTCAAGGATATAAACATGAG
TATGGCGTATAGAAGT
AATACGAACAAGCTGTCTGTCGAAATATACAACACAACTCAACAT
TTCCTAGAAACTTTG
ACTTATACATTCCCAAAGAAAGCCCCCAAGAACAAAAATCCAGG
GAATGAATATATAAAT
GTCACAATGGAGGGAACGTCCATTTTGTATATGCAATACCAAGCT
CATTGTAACCAGGAT
TTCTATGGAATCAACTGCGAAGATCGGA
>ConsensusfromContig2394 Average coverage: 16.38
TGTATATTATATTTTGTTTCGTTGTTTCGGCGTTTGTCACTGTAGT
AGAATCGATCACAT
CTGAGTGTGTAAATATTGAACAATATGGATATCTATCGTGTGATC
TACAAAGTCTACTTT
GCCCTGCTCCGGCCCCAAAAATAAGCCAACTTTTCGTCTCGTTCG
ACGGGAAATATCATC
GTGATATAAATTATAATGTCAATCAATCGATTCGTGATATGAATA
CCAAGTTTTTCCTAA
GGCCAGAACTGTTTGAATATTTCCCACAGAATATCACGAAACTGA
GTTTCTGGACTTTAT
CCATTACAGCGGTGAAATCCAAAACATTTGAAAGATTCATTAATC
TGCAGAAGCTAAAGT
TTCATAATCAAAAGATTGAAATTAAATTTTTAGCCCAAGCACTTG
CAGGGATCTCACCAA
AAGAAATTTTTCTCGGAGAAACCTCATT

>ConsensusfromContig2395 Average coverage: 21.06
CTCTTCCGATCTGTGAATGTATGTGTATCTCTGTCATTCTTTTGTGT
CTTATTTGTTTCT
TGGTCAAAGTTTTTTGGAGGTATTATTATATCATTTGGGATGTTGG
AAGTAACTTCTTTT
TGGATGAACGAATCAATATATTTCCCGAAATCTGGACTTTCTGCC
TTATAGCAAACACCA
AAATCATCGAATAGCTGGTCTTCTCCTTTGGTGATTATCAATGGT
AGGCTAACGCTCTTC
TGAAAGCACGAATTTGTGTCGAAGTTATCTCGAATGAATGTCAAT
TGATGATAGCAGTAT
GGCAAAAACGGGTTGACCGGAGTTGAGGCTCGGGGTGTATTGCCG
GTTTTATTCATATCC
ATTGTTTATATCCATTAATGTGTTTATGAAGCTTTGGTCATATTTT
ATATCTTAATGTTG
AAGTTATGGTTTGAATGACCATTTTTT
>ConsensusfromContig2396 Average coverage: 13.10
TCTCTCTGGATAGCTGATGATGGTACGCCAACAAAATAGGTCATT
TTCATATAACATAGG
TTTACCTGATGTCCTCTCGTTTATTTTTCATGAGTGAGACATTTGC
GATCCTGATAAACC
ACTTTAAACCATGCTTATTTGCTGAGTTGATGAAGATCAGAAATA
TATAAGCTCGTAAAA
TGTATATGTTTTGTAACCTAGGAACTCCAAATCACTCCATTCTTTG
GAAATTTGTATTTA
TTATTGTGAATAATCTCACACCATATTTAGAAACAATAGAATTTC
TCATGTTTTGGCTTA
ACATGGTTCTTGATCAGCG
>ConsensusfromContig2397 Average coverage: 17.07
GAAAAAATAACGACAGGTTTACAAAAATCGAACTTTTTACAAGT
GGAATGAATATAAGAC
ATAGATGAAAAACATTATTTTTGCATAAATAAAATGGTTTCACTT
CTTAACGTACATCAT
GAGTACATTGTGAAGTGAAAAACGACTAGGTTGCTCGCTGTATC
CATCGGTAAAAATACA
ACATTGAGGAAGACAACCGTTTTCAAAACTCATTCTAAGTTCGGC
GTATACATAACGCTT
ACATACCGTTTACATATCACTGAATTAGTGGCATAGAAAAATGCT
GATAGATTAACGAAT
CTTATTTCAAAATATTAAAATTATTTCACAAACTAGAATAGTTCA
GTTCTGTAGCTTTCA
GTTGCTAACAACCCGTGGTTGATAGGATTTATACATATCAGCTAT

CAAAACAACCATCAA
TGATAGCAACTTTTGAAGAGTATACAACGCTGGTGTAAGTTTAGA
GTCAGATCAATCCGG
ATTGATATAACGTAATATCGGATCTAAAAATATGAATATTAAGTG
GATTGAAAAATTGAT
GAAAC
>ConsensusfromContig2398 Average coverage: 13.05
CTTCCGATCTGTCGTGGTTTTCAGCATTTGTTTTAAAATTCAATTG
TTCTATTATTTCCA
GTTGGCTAATTTTTTTTTACAAATATCGTCGCCGGCTGCTGTTATT
AATATGGGTTTTAT
CACAGTTCTTTCATCAGTTGTTCAAGTTTTCTCGATATCAGATCGA
ATATTTGTCCGATG
TTTCGCAACGTATATAGCATGACTCTGAATTAATTATTTTTAGAGA
TTAGTTTCTATATT
GATGAGCCATTGTTATTTGTTTGGAACATGTTATTCTTTATAGATA
ATAATTCGCCTATT
TATCATAGTGTATAAATCATTATTTATTCTTGATAATTTGAGAACA
GCTCTTGTAGTTGG
TTTATGTTCATGATTGTATTTGGATCACGAAGCTATACAATAAAA
AGCATCATACAAAAA
AA
>ConsensusfromContig2399 Average coverage: 10.59
CGCTATTCAAACAAAATAAAGATCAGCATTTAACCGTACAAAATC
AATCCTGAAATATAT
GCAATAATATACCCTGCCGGATCATTATTAAACAACACACAAAA
AACGAATCAAAGCGGT
ACACAACCTCCTGGTGCTTTAAGGCCTGTACGCCTACAAATGAAG
ATAAGGATCGGATGT
TAATGTGAGAAATAATATATACGGGAATACCGCACAACAGCTAT
AAACGACGAGCATGTA
GAGATTCTCAACTCTCAAAGGGACGGAGACTGTAAATAAGTTTTA
AAAGCGTTCGACTTC
CATCGACCCATCACACGAATCTGGGCATCAGATTTACCTTCTACC
ATTGCCAACGACGCG
CCCCCGATTCTGAAACTATGA
>ConsensusfromContig2400 Average coverage: 23.48
GAAGAAGCGTTTGATGATTTATCAGGACCTGGCAAACGGTGCGA
GACACTCGACACATAC
TTCTCGGCAGTCTGCGAAGTCAGGCCGGCAGCTCTAGCGCTTTTC
TCCAACGCACCGAGA
GCTTTGTTTGAAGAAGCTGCGGTTTGTGAAACGGGTGCACGGGC

CACGCGAGATGGGGGT
CGTAGTGATCCCACTGGCTTTTGACTCATCGCGTATTCATTACTTG
AACATTCCTTTGGA
AATTTGTCTTGGAAATATTTAAACCAAAAATATTAATTATTGTGA
ATCTCAAAAACAA
>ConsensusfromContig2401 Average coverage: 9.92
ATCAAAAAACAGATATACCGAGCGGATTGGAACCATCCAATCAA
ATGCCAGATTCTCTGA
TGCTCTCTGAGACAGATCAAAAGATTACGGATTTTGTGCCAACAG
ATAATGTCGATGGAC
CTATCTATCTGACAAATGAGGCCAAACTGAAGGGAGATGTCGTT
ATGCTAAAATCGCAAA
TTGAGATCCTCGCGAAAAAAAATCAAGCGCTTGAAGAGATGGTA
CAGCAACAAGCGGACA
TTATAAAAAGTCTTCGATTGGAGGTTTGTAGCCAATCCAACAGTT
AGAATGCTTCGCTTC
CTTTCCCGCATAAAGTTTTTAAACTCATCTTTTGATTACGCCAT
>ConsensusfromContig2402 Average coverage: 12.11
CGGAAGATGGCGCGTACAGTCCCGCCAACATATTTATAGTAGTGG
ATTTCCCAGCACCAT
TTTGACCCAGAAAAGCCGTCACTTGTTCCTGATAGAAGTCTACAC
TGAGTTTATCGACAG
CCCTGTGTTTTCCGCCAGCGTATGTCTTCGTGAGATGGTGGATGG
AAATACCAAGAGGGA
GGTTTATATCACCGTCCAGATTTCCAAGTCCAAGCGAATAAGCAG
ATGTGACGTCATTTC
TCGAGGATTTCACTAATTTCTGCCGTCTGTCAGGAAACCAAAAAG
ATTTCTGAAGAGGGA
AATACCAGGGCTTTGGTATTTGCTGATATCCTGGGAATAGATTTC
CAAAATACCATCCCA
GAATCCCATATATGACACCATCGAGAATCATGAAGAATATCAAA
TGGCCAAA
>ConsensusfromContig2403 Average coverage: 22.37
GACTCGGATGATTCAGATAGCTGCCTTGTGGATGCATATTGTTAC
CGCCGAGGAGTATAT
TCATGATCGTTTGAAAGCCTTATTAACAAACCCCGTGATCGATTT
ATGTGTTATATCGTT
TCTCATACATTTCAAGTTTTGATAACTCATATTTTTATGATGTGGA
AATCATGTATTTGA
ATAGGTTCTCTCGGGCTTGAATTGGGCACAGTGGTGATAACATAT
ACAAAATGTCATCAA
TCGTTGAGAACACGACTGTTCTGTTGAAAAGAATCAAGGATATGT

GTTCTGTCAATACGT
ACAGACTAATATTATCGTTTCGCGGAGAATAAAATGAGATAAAA
GCACTTAGAAAAAGTC
AACAATTTGCAACGCAAATACGGACGAAACGTACCGACAAATTG
CTGCGATAGAGCGAGG
CAAAAACTCCGCGAGGTTCGAAAAGGATGGATTACTCTCAATATG
AGAAATATGCTTTTT
GGTGAAAGAAGTCGCTTTCCACAGGAAA
>ConsensusfromContig2404 Average coverage: 25.14
AGTTCTCGAATAATAATCTTCGCGATTACTATAATATTCAGAACT
ATAATAAAACAACTC
TCCCCGATAATTTTCCATCTTATTTGGTAGAGGGGTGCTGTATTA
TATGTGGAACATTTA
GATCGCGTCGCAATCCAAAGATATCTGAAACGGATGGGTTTAGA
GAGCTCTTCATAGACG
CTATTCGATCTAAAGATGATAAACGATTATTATTGATACATGCAC
ATTTTGTTGAGAATA
AATTCAAGCTTCATTATCACAAGGCCTGCTACAGTAATTATACTC
GTCCTCATCCAGATG
TGAGGATAGAAAAACCTTCACAACGCAAAGAGTTCCCCGAAATT
ACAGTCCCGGATGTTC
TGCCGTACTATTGCAAAGCATGCGACTTGTTCATTGACTCGAAAT
CATCGACGAAGACTC
ATTTGCAATATCATACTGGTGAGCGGACTCACCTCTGCACTGTAT
GTGGTGCTGCCTATA
CCAGTCATGGACAATTGAAATTTCATTCTCATGTGTACCACCTAA
ATTTCAAGTGTTCAG
TTCACTCTATGTACTTTGCAAAAGATATTGAACTGCATGAGCATA
TCACAACGAGTGTAA
ATGAACATGTCTTCAATCGGCAATGCTCAATATGTAAACTTGTCT
GTACAAATGTCGAGG
AACTTCACAATCATGTGAGATGTATGCACAACAAGAATATTTTTC
TAGCTTTGGAGCTAT
ATGGATGCGAGTTTAGATGCACATGGTGCCATAGATGGTATTACA
GTTATGCTACTTTTT
ACTCGCACGAATTATTTTGTAGAAAAATCAATAATATCGACGAAA
TTCCGTACTGTCATG
ATTGCGACATATTTTGCTTTTCCGAAGAATC
>ConsensusfromContig2405 Average coverage: 4.37
ACATCCTATGCTGGCCATAACATGAAATGAGAGAGGCTCAACCAC
TTTAGGCCTTTTTAT
CATTACACCATTTAGCCTGTGTTATCCATGAGATATGGCAAATGA

TTTCGTCACATCCTA
TGGTGGACATGACATGAAATGAGAGAAGCTCAACCACTTTAGGCC
TTTTTATCATTACAC
CATTTAGCCTGTGTTATCCATGAGATATGGCAACTGATTTCGTCA
CATCCTATGGTGGCC
ATGACATGAAATGAGAGAGGCTCAACCACTTTAGGCCTTTTTA
>ConsensusfromContig2406 Average coverage: 6.74
ACGCTCTTCCGATCTCGACGATTCAATCATCTGCAGAGCATGAAA
ACTCATAAAATTCTA
CATTCAGGAGAAAAACCCTATCAATGCGAACACTGCGAGTTTAG
GTTTGCTGACCAAAGG
AATTATAAACGACATATGCGAATTCATGAAAATACGTTTCCGTAT
AAATGTATACACTGT
CAAAAAACTTTCAGACATACGAATTCCTTGAAAACACATTGTAAA
AAATTGCATAATCAA
TTTACTGAAATTCAAACAGATTCGAATGAAGATGTGGTGTCGGAG
AACATCGAGCATTTC
CCAAATGTCTCAAACATTGAAATCAGACAAAACATCGAGAGCTA
CCGTGCCACCATGGGA
GATGAGCTGGTTATTCCACAAACGTACGCGGTGCCATGCTTGCAA
ATGAATCCACCTTCA
AGTGCA
>ConsensusfromContig2407 Average coverage: 29.99
GTACTGTTGACGGAACAGGGGGAGCTGTCGCAGGGGTCGCCGAT
AACGCATCCCACCTGG
GCATGTAGCTGATCGCCTATCACCATGATCGCTCTTGGGTTGTTG
CCCACCCGTACGTCC
TTGACACATCCAACAAATGGTTCTGAAATGATAGTCTTTCCAGAA
TGAACATTGGCGCCG
ACGTAAACGAAGAGAGTCGTTTTATCTCTCACTAAGCTTGTGTCA
GCCTCGAATTTCTGA
ACATTGCCAAAATCTATAGAGAGATGGATAATTCCTTCTGGAAGC
CATCTTGCTTCGAAG
AAATGCCAAACTCCGTCATCGACCCGCCCTTCACTCGCGTTGTAT
CGTATCAGATTGGGC
GTTGTGCCAGCTTCCCCATCCGAGCCGTATTTGGGGTATGAGTAA
ATGATTCCGCCGTGA
TCCAAAGTGGCACTGACGGACTCCATCGGCGGATTGGCGATTCG
GATATGGAAGAGCGTG
CCCGAGAGAGAACGAGTTCGAAACCAGAGACCGGTGTACCAAGG
GAGGCTGATTTGGGAG
CTGCTTGGAGGACTGTACTGTAACCAGCCCCGGCCGGTAAGAGA

GTATGTCTCAGAGACA
ATCTCACTACAGTCTCTTCCACCAAATCCATTTGGACATCGACAG
ACGAAGCGATCCCAG
GCGTCTTCACATGTCCCTCCATTTTGACA
>ConsensusfromContig2408 Average coverage: 18.85
GGTGCCTGGTCCCCATATTTGGCAGAAATGGTCGGAATAGATCTT
CCACTGACAGTGTTC
AAACACATGTACATCACGACCGATGCTATTGAGGGAATGAAGAA
TATGCCGAATATAAGA
GAATATGATCTATCGATGTACATGAGAGTTCAAGGAGATGCGATC
ACAGTTGGAGGGTTT
GAGCCGAATCCAATCTTCGTGGAAAAGGTGGAGAAAGATTTTTCC
TTCGGACTATATGAC
ATGGATTGGGATGTTTTTGATCAAGTGACTCGGGGTGCGATCAAG
AGGATTCCAGCTATC
GGAGAAGTCGGTACCAAATCTACAATCTCTGGACCAGAATCTTTC
ACCCCGGATCATAAA
GCTCTCATGGGCGAATCCCCGGATGTCCGTGGTCTTTACCTTGGA
TGTGGATTCAATTCC
GGGAATGCAGTTGAGTGGCGGCTGTGGGGAACAATTGGCGAAAT
GGATGAAAGATGGTAC
TCCTGAATTGGACATGTTTTCATTTGACATCAG
>ConsensusfromContig2409 Average coverage: 10.71
TGTGTTAGATTGTATTAGGTACCACATACATAACAATATTTATCT
AATATTCACACATTG
GTTCCATGTTCAAAGAATATTTCATCGGTTAATGATAATGAATGA
TTGAAAGCATACTTG
TGCCACATAACAGCAGGCATGTCACATGATTCTGATTTTAGTTTC
CAAGCATTGCAGTTC
ATTAATGATGAACAGTACCTGTCATTTGTTTCCATGTTTGTTATCC
CTTCTCATACATTT
TTCATTTTTTTTGACAAATTATTTATCACACTTTATGTGTCTATTG
AGTGCATATAAACT
GTGTTGGATGTTCACTAGTACATGGATATTTTCAGTATTGATTCT
TGACTGAATATCCCA
GCGGTGTATAAATAAGAGATTTATAAGAACGGGTG
>ConsensusfromContig2410 Average coverage: 17.57
CAAAGACGTTGTCAATCGAAGCGATCATATCTTTAATTCACAAGG
TGAAATAATTGATGA
GAAAATCATCAGCCTACCGAATTATATTGAATCTTTGGCCGGAAT
TATCTCTTCAATGGA
AGATATCCGTCTTTTCGCCGTATCTGAAATCACCAGGCTGTGCGT

TCTCCTCGCGTCCCA
GCATGTCAGACTGAGTCAACGCCTTCAGATCATAGCTCCGGTGGC
TATTGCGAAATTGTT
GATTGCCTTATCTTCGAAACCAAGCGACTTCAACAATATTGCATC
AAATATAGTGCATGA
AAGCCTGTTGGTGAGTTGCTGCCATGAGAGATTGTCTTTGGATTC
CGAGGAAGAT
>ConsensusfromContig2411 Average coverage: 23.92
AACTTGAACCAGACTCTCTCCTCGTGTGTTGGGATATCATGCCGA
TTTGATAACCGACGA
CTTCTTCTATCAACGGGAACAGGCTTTGACAGATTTTCAGAATGA
TTTGAATCGCTTATA
GGCGATGCGCAATAAGAATTGAAAGGCTCTATTCCTGATATAGAT
AACGTTGAACATTGC
TCATCATCTTCGCATACATTATCATCATGGCTGTTAAATATTAGTG
TGACATTTGTACAT
ATCCCACTTTTGGGAAGAGTGAACGCTAAAAAGGCTTCCATGAT
TCATTATCGAGATGC
GAGTCAGCCTTTATGAACAAATATAAGGGAAGAACACGGTTGAGT
GGTTTAACCTTTTCG
TACACACCGCCAATGATGATATCACGGAGAGCATTCCGATGAGCC
TCCTCCTCATTCGAG
GGGAAGCAGTTACACATTGGTGGCAAGACGAGTCGACAAATTTC
GATTGGAGTCATTGGA
GTTGTGCCAAACGCCAATAAAACTTCTTCAATAGCTATTCCAGAA
GTGAACACTTCGGAA
AGACAATCTTTCACACGATTGAATGAGTGGAGAAACCTTTCTGCC
TTTATGTCTCCTTTA
TCGCCGAGAGGTCTCGTACCAGATTTATCCAGGAAAAATTTCTTA
TTGATAGTACTACTT
TCCTCTCCTTTTGTTGGTACGAGCTTTTCCAGCACATCAACATGA
CAAGGTATTTGTGAA
CGAATATATAAACAGTATTTTAATACATCAGTGACTATTGCTAAA
CGTGCTGAAATGGAA
ATGCTGTCTTTGCATTTCACATGAAATGTTGTCATTTCAAAATTTG
CGGTATTTATTGGT
AAAC
>ConsensusfromContig2412 Average coverage: 23.26
CAATGAGCAAACTAAAAAGAATTTAAACATCCCATGTTATGAAAT
TTCAGTGAGGCTTCG
AATTCAAAGCCCGCCATATTCTCATATATACAATATAACGATTAG
AATCTTTTTGTATAT

CGACGATACGTATGCGCTGGTAGCCACTCGTATCGCCAATTGTAG
TGAAATGATCGATGC
TATCATTTTTCGATATATTGACACTCTTCACTTTTATATTCTGGCC
ATTGAGTTTTAATT
TCGCGTTATAGAACGAATCATCGACAATTTTTCTTGATAAATTTA
GAGCAAATCCTGTAA
TTTTATCAACTCTCAGCGACTCCATACCTCTTTTGACCTCTTTGTA
TGTTGAAGGCATTT
TACTATCATCTGGATGATGACTAGCGAATGTATCATCTTCCTCAT
CGTCATCATCATCAT
CTGATGAATCACTTTCATCATCTTCAGTTATATTTGATTTCTTATTT
CTCAATTTTTGTT
TTTTATTCGAAT
>ConsensusfromContig2413 Average coverage: 13.63
GTTTATATAATCGAAAAGTCTTTCATTGTAGTTTAACGCAAAGCA
GAACTAACACCAAAT
ACAAAAGTACAATAGAAGAGAAATGATGATCAAATATACAGTTG
CGGTAATTGTCGTAAT
TCTTTCAGTCGGTACAGCATTTTCGTTTCATGTCGACTTGAACAA
CTACAAAAAACAAGA
CAACCGAATTGCTGATAAGAAAGACGATCTACTTGAAACAGTTTC
TAAAAGACTAATTTC
ATTACAGAAAGAGTCAAACAATGACCACAGATCCGATTATGATG
TCATGGATGATGAGCA
ACAGATGACCAAACGGAGAGGCTGGGGCAAGAGATCATCCAACG
TTGATTCTTTGGACGA
TTCCTCAGATGAGTTCGAGTCGATGGCAAAAAGGAGAGGATGGG
GAAAGCGAGCAGAGGA
GTCAGATGCCACAGAGCTCACGGTTGAAGAAAAAC
>ConsensusfromContig2414 Average coverage: 6.74
TTTATGATTTTTGGCTTTCCAGACAACCATGGGTCAGACTGGAAA
GCCAAAATCAAAGAG
GCAGAACAAGAGGTTAACTCACTCATCAATACCATCAATGATGA
GATCAAACCGGTCAAA
CACTTTAGGATCGGTCTGTATAATAAGGATAAAAACAGGCCGAT
CAAAGTCAAAATGAGC
AATAAAGCTGATAAAGACTCGATTATGAGCAATCTATTCAAGCT
GAAGGGATCAAGGTTT
GAAAAGATTTCTATCAACTTCGACATGAGCTTTGATGAGAGAAA
ACAATATAAAGATCTG
CTCAGTGAAGCGAAGACTTTAAATGACAGTGACAACGAATTTTT
TCATAGAGTGAGGGGA

ACCCCACAACAGTGGAAAATTGTGAAATCTCCTAAACAGGGAAA
CTAATTTCGCCACACA
GTAGTCCCCAGAATACAGAATACCCTAATAACAATATCAAAAATA
CTTTTCATTATGAAA
CTTCTAATAACAAAACAAATGATAGACACTTTCACAATAACTTTA
TGAAAATAGGCCTAC
TTAACATACAAGGAATAACCAAATACAAGCTCTTAGAAATAGAT
ATGCTAGCTAAAAAAT
TAAACATATTAGGACTAACAGAAACCCAAAGTAAAAACAATATA
GTAGATCGGAAGAGCA
AA
>ConsensusfromContig2415 Average coverage: 29.52
TACTGATCTTTTCACTTGTTTTATCGCCGTCTATTGAGTTGTTCGT
CGATTTTCCGTTAG
CAACCGCGTCTTTGTGTCTTTGATTTATGTTTTTAGAGGCTCTAA
AACAATTTGAGCCAA
TGGCCACGCAGGATAAAGGAGCGGCGTTCAAAACTCCCGACTCG
ACATGATTGGTCGGGG
CGAATTTGGATGATTGAGCATACGACATGGTTTGCTGATCTGATT
GGTTGAACGATGCAG
ATAACGCAGTCGATACTCGCGTTTTGATGGGCAAAGACGGCAGA
AATTTCCGACAATAAT
CATCGTGCATTCCACTGCAATTCGAGTTTGAAGCATCGGCGGCGG
TCAACTCATCCTGCT
GATCTGCACCGCACATCTCTCTCAGCCTCTGCGAGAGTGGTCCGA
TGTCAGAAGTCGTTG
AAGTGATTCCTGCTTTCCCATTGGTCGTCGAAATAGACAGGGGCG
GGTTTTTGGCAAGTT
CGC
>ConsensusfromContig2416 Average coverage: 17.52
TTTGAATAATGTGATAAGTAGTGATATGTTTATGATATACTCACA
GACAGGTGAGGTCAG
AACAACCCAAACATTAGACAGAGAAGAGATACAAACTCATTATT
TTCAAATTGTAGCAAA
AAATGCAGAATCATCTTCATATACAACCGCTCAGCTCAATATTCA
TGTTGATGATATCAA
TGACAATGATCCCAAATTTGAATATGAATTATATGAGGAGGAAAT
ATCAGAAAGTGTTTC
AATTGGTACTACTGTTACAAAAGTACTCGCACAAGATAATGATGC
TGGTTTGAATTCTGA
AATTGAATACAAAATTGTAAATCCATCAGATTTGAATAAAGTGTT
TGGAATATATTCACA

TTCTGGGGAAATATACATTGCATCACAACTTGACAGAGAAACTCA
ACATTTCTATAAACT
GAAACTTGAAGCATGCGATAAAGCTCCTACAAAATACAGAAGAA
CATCAACTGCTATAAT
TCAATTCACTATTATTGATGTGAATGACAATGAGCCTAGGTTCAT
TGGAGTTCCTTATGA
AGCAACTGTGAGAGAAAACATGGCCCATAATGACAATCCAATTA
TCCTCAATATAACAGC
TGTTGATGCAGACTCAGGAAGAAATGGAAGAGTTACTTACAGCA
TCAGGGGTGGCGATGA
TTTCAATACTTTTCTCATCGATTCCAGGACAGGTATTGTTAGACT
GAATGGAGATCTGGA
TTATGAAATCCAACCCGAATATAGACTGAAGATAAGAGCACAGG
ACAGTGGATCGCC
>ConsensusfromContig2417 Average coverage: 13.71
TCATAGGTTCCATGAGATGTGCCATTTTCAACGATGAGTTGAGCA
GATGTGATTATATCA
GCCTCTTCATTTTTGGCAACGCATTCATAAGTCCCCGCATCTGATT
TTTCCACTCTCTGG
ATATCCAGCGTCCCTCTTTCGGTTAGGACAAAGCGACTGTGTCCA
GTAAACTTGTTCCTC
CGTCCAGGAAATATCACTATGTTTCGTGGTGACATTCGCCAGAAG
ATATCTGGAGACGGA
TTTCCGGTCACTTCACAGTCCATGTGCACATTTTGTCCGACTTCC
CGCTGGTAATAGAAG
TTGGGTCGCCGGGAGAAACTTGGTGGATCTACTACATTCACAGTG
ACGTTGTTGGAGAGA
CCTCCCAGTCCGAGCGGTGATCTTCCGACGCATGAATAATTGCCC
GCGTCTTCTGCCTGG
ACCTGTTCAATGATGAGATTGTTGCTCTTGAAAGAAAACCGCCGT
ACTTCAGATCGTAAT
TCGTGATTGTCCTTGACCCAGTGAATAATGTTTGCGGGAGGATTG
CCATCTATTTTGCAT
GTGAGACTTCCGCGTAATCCCAAGGTCAAGTTCACAGGGGTTGTC
ATCACTTCCACCTCG
GGGAGATATGTAACATTAAGGTGAAAGGAGTGAAGAACTTTCAGG
TCTACCATATTCTCT
GCACAGCAATAATAGTTTCCCGTGTCTTTTCGCTGCATCCTGTTGA
TTTGTAGAATTCCT
TTTGGTGGAGAAGTTCTTATGTCTGTTGTAATCCGTGTTCGGAAA
GATTGGAGTGTATTC
AGATTTTTCTTGTTGCTGTTCATCCAATAATAGGTGTGGTTATTCA

CCGTCCCCCTGGCC
TGACACTCCAGCCGAACACTCTTCCCCTCGCGGATCGTCGCGTTC
TTCGGGGGTATTTCG
ATCACAGAATGTTGTTTGATGGCAAGCTGTGTTATGGCATAATCT
TGGCCGTGGGCGTTG
TCCAGTCGGCATTTATATTCTCCTGCATCGGAATTTTTCAGGTATA
CGATCTGAAGTCCT
TCGTTTGCGAACGTCACGCGATCAGGTTCTTCTATGCGACCTTCGT
CAACCAGTCTTTTC
ATCGACTCTTTGTTCTTGTACCAGATGACGCTTGGCGGAGGTTTG
CCGTCTCCACGACAG
AAGAAGTTAACACTCTTTCCTCTCTCCCGGATCATGACCTCCGAG
TCGAAAGCCTGGGTT
ACTTGGGGTGGATACTCAACATCCAATAGGACCCAGGTAGAGTT
CCCAGGCTGCTGGTGA
TCTTTGAATGTGACCAGGCACTCCCACCACCCGGCATCTTCTCTT
GTCACGTTCAGCACT
TTGATATTAGCTCTCGCGTGGTTCTGCGGGACGAACTTGATGCGG
TTTTGGTATGCAGGA
GCCAGGTGTATGCTATAATCTCTACTTTGAAGGAGTCCGACAAAT
ATAGGATCTTTTTCT
TCTTCTTTTTTCCACTTCAAAACCACTTCACTCGGGTCTTCATCCT
TCAAGTCTGGGTTG
CAGACCAAGGTCACTTCCCCACCGACCTTGGCCCGTATGCTAATC
TTAGTAGACCGGATG
TGTCGTTCCTGTGGGCTAGGCCAAGTCTCGTCATCAATGGAGAAT
GCGAATCTGTCTAGG
GCGGACAAGAGACCCAATATACAAAGAATCCATAGTGTCTGACA
AACATTCATAATATTC
CGAAATCGATGCATTCCTGTGCGATCGAAACATCTGAGATGAAT
CAAAATAAGCTGCAAT
AATAGACTTGAAGAT
>ConsensusfromContig2418 Average coverage: 10.19
GAATCATTCTTAGAAATGTTTCCCGTTGATGTCGGTAAATTGTTC
AGAGTCGATCGCTGC
TGTACACCAGGAGTCATGGAGCAATAATTCGCAATTGAGGCAGGG
GACTTTTTAATGTCG
GTGTGGATACTGGCTTGAGAATGTGAGGGGGTACCGCGACCCAC
AGAACAGGTTTTCATA
CCAGGGACTGACACGGGAAGATGCGCTGAGCCGGTGAGAAAGGC
GGAAGATGAATTCGGA
CCCATTAAAAATGGCGGAGGGATCTGCCCGTCAATGTACATATTT

GAGAGCATTTCACTC
TGACTGTGAGCTAGCGAAACTCTTGGAGCCTGACATGAGTTATAT
CCAGTTGAGCTCCGT
GGCAGAGGTGTGTGTTCCTGGTTGTAGTTCTGCATTGATGGGAGA
ATGTGTCCTGATTGA
TTGGCTTGATGCTGAAATGGCGGTATAACAGCTAAATCTTTTCCT
TGTAGGAACTGGTGT
CGTGAATCAGGACCACGACCGCCTGCATCAATGAATAATTTACCA
TGATCATTTCGATTG
TGACCTACCTGATGAGGAAAAGTACCATTTAAGAACGGCTGGTAT
TGTCTCTGTTGCTGA
GCATACGGCAGAGTAGACATTTGAGTGTGCTGATGTCTATGCTGA
TCAGGGTCGTATTCA
ATATTCATTGTATTGTATCTC
>ConsensusfromContig2419 Average coverage: 41.89
AAAATTATTATAGAAGATCCAGGCTGTATCTAAACATAATAAAAT
ACATTCAAATATTTT
ATGACAAAATTAGAAGTCTTAGCTCAGAGCCATTTGGAAATTAC
AAATGCGAAAATCAGG
TTTACAAGAGTCGATCTATCAGTAGAACCCCCCGACTTTTTGGCG
CTTCTCACAACCGGT
GAGGTTGTTGTATAGCGGCGGAAGGTCTTCGGAGTTTTCGTCGTC
CTCCTTACGGGCATA
ACGCATGTGAGTTTCGGCGTTCCGATCATATACAGGGCTATTCTA
GCTGTGAAGCCCATA
GGCAACATCTTGAGCAAATAAGGCAGACTGGAGCTGCAGCTCCT
AGGATTGTTGTAACAA
GTTCTGTAAATTGCTTTTTTTCTCCACTGCACAGTTGCACACAGT
CCCGGGTAGCGACAC
TCGACATATTCAACACCAACTCCATTCAGGCAATCTTCGAAGCTT
ATTCCTGGCAGAAGT
TTTCCAGCTTCCAACGCTTGTACCGCTTTGCATGACTCGCATGTCT
GCGCATTAGTTCCA
GAGAGTACAAGTATAGAAATGACAAATAACTTGTATTTTAGTTTT
TTTAACATGATGTAT
TCTCTTACAAACTGGTTTTGATGATATGAGGAACAGAACCAATGT
ATTCATGGATATGAA
AAGTTTATCTGCGATATTCACCTGTCTCATGGCACAATAACTCTC
ATAAGTTATAGGAAA
ACAGAAGCTAGGATAACCCAACTCCTGTTTTTTCAAGCGAATGAC
C
>ConsensusfromContig2420 Average coverage: 34.83

TCCCCAGGCTCCCTTAGAAAAGGTTCGGTTCCATCAAGATTGCCA
CCAGCAGCTGTTTCA
TCATACGATGATTTATTCTTCTGGGAGACCAAAGCTTCGAGCAAT
ACACTGAGACTAATG
ATGCCATCTGAGATCACATTGTGTTTTGATCCGCCATCGTAATAG
TGATTTGAGAACAGA
TCGTTGGACACTTTTGACAAACATTGACTTCCTTCATATGCTCCG
ATAGAGTCAATCTGA
TCCATAACAGAAAGGCTCTCAGTAATCACGGCGGCTGTTTGCTCC
AAGTCAAACAAATCA
TGGACGGGAAGGGATCGGAGAGATGCCAGAATTGTACTTCTTATC
TCAATAGATTTCTGC
CGGATCATCAATTGAGAATCTTGAATCGCAGCCCCGGACTGGGA
GATTGTTCAAATGCC
GCATCAGTTCTCGATTCATTAGATCTACGGAGGCTGAGCGCGTTC
AACTTGTGTGCAACC
ATGTATGAAAATTGGCGGATGCTTGGATATCCC
>ConsensusfromContig2421 Average coverage: 37.21
CAAAAATTAAAAATGAAGGGGGGCGCGATAATATTAATATTCTA
CTGAGTATATACAACG
CCCATAATTGCATATTGAATGAAGAGAAATAGATTAGGAGTCAGC
ACTAAATTGCATCCC
CGCACAATTCATTGAAAGAACCACGTAGTAGTAAAAGTTGAAAG
ATGGAGAGAAATAAAC
TTGGATTCTGTTCAGCACTAAATTGCATTCCTGCAATATCAATTG
AAAAGCCACGAAAAC
CCTCGATGATTGTGATTGAAAAATTGAATAACCTCAAGCAGGACT
ATAGTCACAAGTCTA
TAAATATCGAACGGAAGTCCAGCTTAGAGCTGTCCGCCCCTATGA
AAGAGCGCCTGATGT
CCGCCTGTCGACTCGCGAAGGAAGATATTGAGGCGTCGAGAAGA
CTTTCCAAGAGACAGG
AAGACTTATCGGCCAATGCCTTTATGGAAGATTCATTCACCTACG
TTCAGGCAGAAGAGC
CACTGTCATCCGATCGGCCAAAAAAGAGACCGACTCGTCCCATG
GTGGATTCGGCCCGAA
ATAAAAGGGCCCTGAAAGAAGTTCAAGATCTTGCGAGGAAAAAA
CGAGAAAACTCGGCGT
TGAATCAAGAAATGACGTCACAATCACACCTTAGAAGAAAAACC
AATGAAATCACGTGTC
TTCAGAAAGATATTGTCGCCTGCTTGCAAAATATAAACAAACTGA
AAAACTTGGCCAATC

GCGATATCTCTTATAGGCCGCAGATTTTGCCAAATACAGAGGATC
TTCCGGCTGAGGTTC
AGAAGTCTCGGCGGGAATCCAAAGCGAATCAACTCTCCGCCAGGG
TCGCTCGCCTCACCT
ACTCCACCGCTCGAAAGATCGATCAACTCGCTTTAAACATTTCTG
TCGCCACGGATTCTT
CAGAAATCTCAGTTTCAAAAAAGAGAGACGTCGAAAAGGAAAGT
CTAGTGGCGCTCCTGG
CGGCCTGCCATCGCAGCGTTGCTCAATCCTTGCAACTCTTCGTATC
TCTTCTCTCACTTG
TTGATCTGAGGTGTGGGTTGCCTCCTTCTTTCCCGGACATTTGCCG
CCTCGTTGGGTCGC
TCACAGAATGCTGTGTCAAGGTCGAAGAGCTCCTAGAGACAAGG
GCGGTGAAAGGTCAAG
AGTCAGGTCACGAGGCGGGAAATCTTCTGCGACAAATGCAGATTT
TCTCCCGATGTTTTG
AATCTCGGAATGAAGTAACGCCCATTGCAACGAGCGAGCGAATT
ACAAAATCCAAATTGA
CCGAGGCTCCTCCCACCCCATCCTCGGCGAATCAAGATTCGAATG
TTCCTTATCAAACTG
AAGTACAGCCAATCAAAGA
>ConsensusfromContig2422 Average coverage: 8.64
CGAGACTGGATCCTGCGGGCAATACACTGGCAGGGACATGTTCTG
TATGGGATTACATAT
ATTGTGAATTTGGAAAAGGATTCAAATGCAACTGTCCATTGGGAC
GGGCCGGAGACTTTT
GCGAGTTATCCTCCTGCAACAACAAACCATGCGTGGAAGGAGAA
TGTGTAGAGGGAAATG
AAGGTGAATCAGAAATCTGTGAATGCAACAAAAACTACATGGGC
CATACCTGTGAAGTCA
GATTCCTTGCATAAATGGCTA
>ConsensusfromContig2423 Average coverage: 16.30
GAAAAACGTAGGGGATGGGGGAAACGCTCAAGCTCTCCTTCAGA
GGAAAGGGTTCGCGAT
CGCAGGGGTTGGGGAAAACGATCTGACTCATCACCAGAAGAAGG
GGTTCGGATGCGTAGA
GGATGGGGCAAAAGATCTTCTGACACTGCTGACAATAATGAAGA
ATTGGAAACACTGCTC
AATTGCAAAAGCCTCTTTGAGACACTTGATACCGGCTCTCAAAAT
ATCCTCATAAAGAAA
TTCTTGATCAGCTTGGCTCGCTCATGCCTCACGCGCTATGAGAGA
AACTGATAGACTCCA

CCGCGACATTTCAGTCAGATATTCATAAGGAATGGAAGAATAAT
TATGCGGGATAAAGAA
TCAAGAATATAAGATCACAACTAATGAAATAGGGACGATAAATTT
ATTTTTAGAAAATGT
GTTTTTGCGATTCATCTCTTAATCACAAATTCTATATGCGCAA
>ConsensusfromContig2424 Average coverage: 12.10
CCTTTATTAAAACATGATCGTCCATCTTCTTGCAGTGTATATCCT
AGATTGCATGAGCAG
ATTGCAAGGGAATTTTTCACATCACAGATCTGCTCACATTTCATA
TTCACACAAGGGTCT
TCATAACAACTTTGGTCATCATCGCGGAGTAGAAAACCATCATTA
CATTTACATTGAATT
TTAGCATCTACTGTATTGCAAGTATGTTCACAACCACCGTTATCA
TCAATCCTGAGTGAG
ATAATATTTTTCTGGGATAAAATCCTGTTATGAATCTTCAAGGAA
TACATTCTAGAAATT
ACTTTTGCGCTAGCAGCACTGGCAGCAGTACTCG
>ConsensusfromContig2425 Average coverage: 25.27
TTGGCAACTAATAGTAGCTGTAGATTGAGCAACTTCCGTTTCAGC
AGGAACGTTTTTTCG
TTTCGCATTATATTCGGGTTTGATGACCAAATCCTGACATACAGC
TTTGCAATCCATTGT
GAACGTCCCCCAAGATCCATCATCATTGCATGTCACCGTCGATTC
AACACCCTGGAATCC
ATCTTGGCAACTAATAGTAGCTGTAGATTGAGCAACTTCCGTTTC
AGCAGGAACGTTTTT
TCGTTTCGCATTATATTCGGGTTTGATGAC
>ConsensusfromContig2426 Average coverage: 8.74
GCATGACCTAGTGAGATTCAGCGTCCCTTTAGAGCCGGGACAGG
TCGGCGCCCAGGGCTA
CTTTCGCTCATGGCGTGACTGACTGGATTGTGACTCGTAAACTCC
CCGACACTTGCAACG
TGTGAACTCTCGGTTTGGCCAACTGAACTGTGTCATCTCTCTCGCT
CTCAATTTTAATCC
ATACGGAAGAAATGGTGTAAACATTAGTTAATGTAAAACCAAAA
AACTGACCTAAAAAAG
CTTTTTTTGTTTTGGCGGACAGGCCATCAGGTAGCTGGGCCAAAA
TGGACTTTAATTTCA
AAATCTTGTTATTGTTTGTTATTGTGACTCTTCTTTTTAATGGATT
GTTTGCTGTCGAGA
TGACACAATGTCATCAAGCGGGAGACTACGTCTGGAAACGCAGTG
ACCCCCATTACTTCT

TCTTCTGCTACAAAGTAAGGAAAGCCATGTTGATGAGCTGTCGG
GGAGGGAGCTACATGT
GCAAGCAGACGATGAAGAACTTCCGGCCCTGCAGTTGCGGTGGG
GGTCAGAACAGAGAGG
TTACGACCGTTCCGTCTGTCCAAAATCCCGGCAATTCAAACGCGG
G
>ConsensusfromContig2427 Average coverage: 4.20
CTCTTCCGATCTTTTCCGCCTGACTCACAGGTGTGAGTGATAGAA
GTTGCATTGTTGATC
TCGTATACTTGGCCTGTGAGGCAACTGCGATAGAGCTTATCCTCG
AACACATACTCGTTA
GGGTGATTAATTTCTTCAGCTGGGCAGTGCTGTCGGGCACAGTCG
ACCATAGTTGGTTTC
CAAACCCCAGTATAGTGGCATCTGTACTTTCTGGAAGATGTATTG
CTGTTCTCGAAAACA
AAACCTACTGAGCTGCACCCCACATAAACC
>ConsensusfromContig2428 Average coverage: 119.27
TCGATTTCATAACACTGAACACAGTTAATATATAACAATGAATAG
TTCATCAAGATCTTT
TGATATATTTCCACATGAGCAAAATTCAGAAAAAAAATCAAGAA
ACAATACGAATTAGGG
ATATATTCGAAGCACTCTCAATATCAGTGCATGCGTGTATTTACA
CACGGACATAAATAT
TCATAAATGACAAAATTCACAGAAACGGGCTTATTGATAATTATC
AGAAAACCCGAATTT
GAGAATAAAATATTTTGTTGAATCAACGATGAATAAAATTACGAA
ATATGTTCTCTGTTC
CTTCTACACCCTGCTGTTCCTTGTTGCGGTGACGAATTCAGCTTCA
ACAG
>ConsensusfromContig2429 Average coverage: 23.93
TCATGATCAAGTTTTGAGTTAATGTTAATCCTTATAATAGATATTT
GAACGATTCTTTCT
GATTTTTTTTTCGTTTTTGATCTGCTACTGTAGGTTTTTATTTCACT
CTGCGTATGTATG
TCGAAGTTGATTCTATCTTCCAATTTAGCATATCGATATTGTTGTT
ATGCTTTGTGCTGT
CAGGTATGAAGCTCAACCTATAAAGGTGGGCAAATGTTGAAAGT
TTGCGATCGTTTAAAA
TTAACTTTAAAATTATATGATCTGATTATTTATTTATATAGAATTC
TATCGGAACAATCC
AATATTTATCCATCAATCCATATAATTTTTCTGCTTGTAATGTTAA
TATTCACGACATGG

CATTACCTGCCGTACACTTTTTTTTCTATTTCTACGTTTTCTAGCC
GTATACAAATTTTT
GTAAAACATTGACCTACTATTTCGCTTTGATTATTCGATTTTTTTC
TGTTATAATATTTA
TTCAGATCATGGCTAAAAATTAATGTCCTCTATACTTTTCTTGATC
ACCTAAATCTGTCT
GTATTGCTGTCTCCGAATGTTATTAACAGCCATATAACATCCATC
TCAATTGCATGTGTA
TCCATCTTCATTTTAATATTGTTTATCGTGTTCTCAAATTTTCAAC
ATTCCAAATATGTG
TTTCACAATGCTCAAATTGTTATTATTGTTTTTCTTCTGACATTCC
TAATAATTTTGAAT
ATAT
>ConsensusfromContig2430 Average coverage: 9.12
ATTTGTGACCAGAGATCACGGAGACAAATACCCATTCGATGGAC
CGGGAAGCATTCTGGC
CCACGCATTCTACCCATCATACGGAGGAGAGGCTCATCTGGATG
ATGACGAGACATGGAC
TCACGGAACCTTCGAAGGTACAAACCTTAAATGGGTCCTGACCCA
CCAAATCGGACACTC
TTTGGGTCTCAAGCATTCACAGGTCGAGGGGTCCATCATGTACCC
CCTATACCC
>ConsensusfromContig2431 Average coverage: 22.30
TTTCATTCGAAAATAGAGTGCTTAACATTTGGAATCACTCGAAAA
TGAAGTTGAAAATTA
TTGTAGTTGCTGCTTCCATTATTTTAATTATTGATCCTTGTTCGGC
AGCGGAGGAATATC
TCATTTCCACGAAAGGTCTAGAGGAAATAAATCATAAAAACATC
GAGAGCCATGCGAGAC
AATTTGCATGGGCTACAGCCAAAATATTCGATATAGAAGATTCTA
CGGCCGTCAATGGAT
TGATGGTGCTGATGAATTCATTGGTTCAATATTTGGCGCAGAATA
TGGATGAAAATGTTT
TCATATTGAAAGCTGAAGCGAGTGATGGGTGTAAGTTGTGCGTC
GAGGGGGCGAATGTTG
CTGCAGCTCTTTTGAACTTCAAAACCCACTCCTACAACGCCGCGA
AAATTGCGGAAGTTT
GTGAGCCTCTGATATATGTGAAAATGCGTCAAGTCTGTGCATCAT
TATTCGAGCGTCACG
TCGGATTTGGCAAAATAATGAGCCTCGATAATGTGAATGAAATGC
TGGTCGCTTTATGCA
ATGAGGTTGATAGTAAGACTGGTCATTGCGAGCTTTTCGTGGACA

AATCTGTTGACTTTC
TCAAACTTCTACTGTTCCTGAACCCAGTGTGTCTCGACATATGCC
AGAATACGTTAACCA
GGACAGCAGCTGAATGGATTGATGAATGGTTTGATGGGTTTGATA
CGACACCGGTTGGTG
CTGGCGATTCCCATGCTCCGCTGCTGATCAAATTCGAGTCTGTAG
AAAGCTCATCATCGA
GCTATGGGATCCTTAACAGTCC
>ConsensusfromContig2432 Average coverage: 57.03
AGCGCAGAGAGATGATGAAGAATGTGGTTGGCGCAATTCGTTTT
TCCAGAGCCAGACTCG
CCCGAAATGACGACGCACTGACTCTGAGAATTATTGAGCAGTTGA
TGAAAAGTTCGATTG
GCCACGGCGAAGATGTGCGGCGGCAGATCGACGAGCGAACGGTG
CTGATACATGTTGATA
TACGACGGATTGTAGATGGGAAGAAATGAAAACGGATTGATGGC
GATGAGGATCGAACCC
ACGTAGGTATATATTTGATCTTCGTCGAACCGCAGACGGAGATTT
TCGAGCAGCGTCACT
TCTGTTAGCTCTGGCAGGAGACAGCAATCCGCATACTGACGCGTA
CGAGTCTTCTGCAGG
AAAGACGTCAGAAACTCGTCCAT
>ConsensusfromContig2433 Average coverage: 13.50
CTCGTTGCGATAGTAGATTGTGATTTCTCCTTTCTCTAATAATTTT
TTCGTAAATCTCTA
CTCGTAATAATGTAATACAATTCATATCATTTTGATTTAGATGAC
GCGCGAACCAGTTGC
TCTTTGAGTTCTTCGTACCTTTTCCGGAGTGCCTTTTTTCTGGATA
TGAGCACTTTGATT
TGTGTTTGTTCATCGTTTTCGGGTTGCATTTTCAAAAGCTCGGCAT
CCAATATCCCGTGT
TCTTCTCGCATTTCGTTTATGCGATTTTCAACAGCATGAATTTCGA
CTTCAGGCATATCC
TCGCCTCGAGGCACCTCTGCCAGGCATTGTCTGAAGCTATATACTT
TTTTTGACCATTCC
TCGCTTATGGTTTTCAGCTTCTCGCGTAGAGAATCATATTCTAGA
TTGAGCCCAGAGACT
TTCTCATCTAACTTCTGCAATTCTTGTTCATCAACCTCCAGTCCAA
TTGCCATACTTTGC
AACTCTCGTTTTTTAAAACACAAAGCAATCATTTTATCCTCCAAA
CTTTGCGTCAACGCT
TTCTTCCTCTGCTGAGCATTGCTGAGCGCCGTCATTCCGCTCTGGA

GCAGTCGCTTCTCC
TCAAGAATTTTAGATGACAATTCACGTGTAGCTTCATCATTCGAT
TTAATTTGTTCAAAA
ATTTGTTTGAAGGTCTCCATTTTTATGATATCGTACGTCTGCTTAT
AAACATGAATAGAT
GGC
>ConsensusfromContig2434 Average coverage: 6.03
CAAAGCTCAAAAAGGCGTCCAGGTTTACGTGTTTGTTCCGCAGAG
CCGCTGAATCATTGG
GAAAACCGATGGAAGAGGCGATCTTACGGAGAAAGGCTTCGTTA
TCATGAAAGCTGGGTT
GGCTGGGGTGAACGTTTTGAGTCAGAGAAAAATTCGTGCATGGG
GCTTCTGCAGATATGA
ACTGTCTATGCTCGGAGGAATCTAGTTGTTTTTCTTCACCAACTT
TGTCAACGACAAGTC
CCAAAAAAATTTCTTTCAAATCTCGTCTCGCATACA
>ConsensusfromContig2435 Average coverage: 16.82
CAAGATTGCGTGTAATATCACACCTTCAGAATCCTTAGTATCGCT
TGTTGTCGCTGGTTT
GAATGTTCAAGGTGTGACGGATAATTGGCCAGTTGAATCTGTTG
AAAGACCATGGGAAA
AGAACTAATTCATGACCTCAATTTTCCCTTAATTGAAAATGGCCC
CCTCTTGAAAAAAGG
TCGACGCGATTATCGGTGCAAAGCGGAGAACGATGGATCAACAT
TTATATCAGACAAAAT
CACGGTTCACTTTGCCGGTGTGGCACACGAAGCAACAGGTTCAGG
TCGTTATAGATGGGG
TCATGATCTACGCATAGAATGCCTGACAAATGACGATGTCTTTCA
ATACAACTTTAAATG
GTTCAGTGGTCCAGAATCAGTTCCAGTTCAGCTCAGTAAAAATAC
AATTTTGGTTCTAGA
GGATGGAACCTTGCTCTTAC
>ConsensusfromContig2436 Average coverage: 19.69
CCTCCAGGGCTTGGCTGCACTCAGAAGTACGTCCTGGTTGCAGAG
GCGGACATAGGGTGT
CAGAGTGATGAATTCAAGATGGTCTTTGCTACCAGACTTGGCGG
AAAGAGAATGGGTTGT
AACATAGACGTGGCTCTTGAGAAACTGAACATCTCCGCAACTCTC
CAAGTGTCAATCAAT
ATGGATATGGAAATCGCATTTCCTCATCTTCCTTCAATCAGCATC
AGCTTCAAAGATAAA
CCGGAAGTCTGGTTCGCCATCCGGATGCTGAAATCGGTTCAGATG

ATGGAGATTCCGCTG
CTCAAACACTGGATCCACTCTCTGGTCATGGACTGCGTTTTTGAC
TCTATTGTTGACCCG
GGGAAACTGGACCTTCAGATACAGTCATCCGACGACTGCTATTTG
CCTACAAACTCAAAA
AGCGAACCAAAAGCTGTTGGAGTTTTAACAGCAACTGTCAGTCA
ACAACAGGGCAGTGGC
GGAGGCAAAATGTTTGACGATATCCGGTTCTGTACCCTGCGTCT
TGGGGGAGACCAGAAG
CTGCAGACTCACTACACGCAAGGAACAGCCCCGTGGAAAGACACA
TGCAGTTTCTTGGTT
GGGAATCCGGATGCTGATGTGTTGAACATAAAGTATAGATGTCGC
CGCTTTCTTGCCACT
CAGACCATTGCATCCTCATCGATTTCTTTATCCGAGTTTTCCTCC
CAAAATACGACAATA
ATTGATAAAAC
>ConsensusfromContig2437 Average coverage: 36.72
AAACGGAAAAACAGAAGCAGGACTTACTGGTCGATCGATTGACG
CGAACACTCGACAAAC
TCACTGAAGATATATCCCTCTACGACGCTCAAATCGCAGCACAA
AGAGCCGAAGTTAAGA
CAACGGTCGATGCTTTGGCAGAGGCGCAGCTTGAGTCGGAGGCC
GTGAGCATGGAGAAGC
GTCAGATCTTCGGTCAGTGGAACAGCAGCCTCATCGGCCTTCGA
CGGAGGGATGAAACGG
TGGCAGCCATGCAGCACGCTCTTCGAGAGGAGAGGGAACGAATA
CTTTCGGTTAACACGG
AGGTAGAAGGGTACAACAAAGCTATTCAGAAAGAACAAGAGCA
GAATGAAAAGCTGACGA
TGATACTCAACAAAACAGATTCAGATATGGCCATGGTTAAAAAA
CAGTTGCAGTCATGTG
CTGATGCCCATGAAGCACTAAA
>ConsensusfromContig2438 Average coverage: 34.10
AAAATCAATGGAATATGGAGAGCAACTCATCACTGATACCTATC
AAAGAAAAGTCGTCAG
CAAAATCGTCAAATTTGACAAAAAACCTGATTTTCAACGCTCAAT
AATTATTCAGCCTTG
TTCATTTCTTGAATTTTTATGAATAAAAACATCACAGAATTATCA
GAGGCAAATCAGCGT
GGCTGGTATCAGTATTTTCGAAGGTTGATATGTCCAACAAATCAC
AGTCATTGCCTTCAT
TCGCAATTCCTACAGTTGTACCTTGGTGGGAATTTTTGAAACAAA

TCTTCCGAATGAAAC
TCCAATCACAAAACCCACAGAAGCCATTCGAAGGTCCATAAATT
CTACAAAGTGATTGAA
AGAGAAATGTCGAAAATAGCCAACTCATCGACATGACGATCATC
AAAAACTGTCCGAGTT
TAGCATAGGCTGCCACACGGGCCGGAAGAATTGCGAAGCCGGCA
ATAAACGTTGTTGAAG
CCGCCAGAAAAACAGCCGGTCCCACCCTCTCGAATGAATCTCTCA
CGCGGTATTTGGATT
GTTTTTGTTTCGACAAGCGATATGCGACCCCGTAGTGAATCGTGA
AATCTACGCATAAGC
CAGCAACCAGCGAGATAATCATCGACTCAGTCATATTCAATCTCC
ATCCAAGCGCAATGA
TGATTGCAACGGTAGATGCCATTGCCAAAGTTATTGTCAGCATTG
CATAGAGAGTCAGCA
GAATGTTCAACGAAGTCAAAAACATGAGAAC
>ConsensusfromContig2439 Average coverage: 13.23
TGCTCTTCCGATCTCCGGAATTTAAATTTGTGAGTGTATCCACAG
CTTATGTTATTGAAA
CAATAAATGAACTAAAAAATAGCTCGGCCAATGATTGCGACGATT
TCAACATCAAATTAT
TGAATTCAGTTAAATACATAATAGCAGAACCTGTCAAACTTCTTA
TTAATATGATTTTAC
AAAAAGGTATATTTCCAAATGGCATGAAACTCGCAAAAGTTTCAC
CTGTTTTCAAAAAAG
GTGACGCGAACAGTATAACCAACTATCGGCCTATCTCTATATTAC
CGTGGTTATCTAAAA
TAGTAGAAAAGGTTATTCATGACCAGTTGTCCACTCATATAAACA
AATATGATATTTTGT
ACCCATATCAATACGGTTTTCGTAAAAGGCACTCAACGGAATTAG
CTGCTGTACATTTTG
TGGATACCATATCTGAGTATATTAATAATAATAAAATTCCTGTTG
TCATTTATATTGATC
TTTCCAAGGCTTTTGACACCATTGACCATCTTAACCTTATTT
>ConsensusfromContig2440 Average coverage: 7.89
CCCCCAGGCACCATCTGTATGCTTCCATCCTGAGTATTGAACTGG
AAACCCCCATAGCCC
ATGCTGTTGTCATTCTTCGGACCGTAGCTGTGAATCATGGCGAGC
TTCGGTCGGCTGTGA
GATTGTGCTTGTGGTTTTCGCTGAAAACTGTTTTGATGCTGCTGGA
AAGCTCTTTGCTGT
GGATAGATACTTTGTTGCGAAAATGCATTATGTTGCTGATACGCG

TTTTGCTGGTTATGC
GCTGCACTTGGGAACGATGATCCTGAAAACTTTTGCGATTGCTTC
GGGAATGAGTACGGA
GTCCCGGGTACTCCATCGGATTTCGCCGTGCCTAATCCGGCCCCA
GTGAGTTCTGCCAGG
CTGTTCACAAACATTTGTCGACGAGCATTTCGGTGGTTGTCTTTC
AAGAAGTTGATAGCG
CCGGGTTGTCCGTAATTGAAATAATATTGCCCAAAAGTTACATCA
GATTGTATCACAAGA
ATCAGGGATAAATAAAACGCCACCATATTCATAGTTAATAGATG
ATAAAATATTGAATAA
TTC
>ConsensusfromContig2441 Average coverage: 24.04
TTGAGATATCGGTACAATTTATGGGTGGTTCGTTTTGGTAGAGAA
AATTTGAAATAGGAT
ATTCCTCAATTCGTCGTATCAGGAAGTAATCCGAAACCGAGCCTG
TTGAGGTATCGGTAC
AATTTATGGGTGGTTCGTTTTGGTAGAGAAAATTTGAAATAGGAT
ATTCCTCAATTCGTC
>ConsensusfromContig2442 Average coverage: 22.32
GGGTTCACGTCACTGAGTCGAGATGATCAAATTCTTCTTCTCAAG
GGCTCGGCATGCGAA
GTGATGATGATCCGAACTGCGCGGCGCTACGACGTCAGCTCAGAC
ACGATCGTGTTCGCC
AACGGAACGCCATACACCCGCGAATCCATGCGCTACGCCGGCCT
CCACGATTACGTCGAC
GACATGTTCGCTTTCTGTCGACGAATGGGCATATTATGTGTGGAC
AACGCCGAGTATGCC
CTCCTGACCGCCATCTGTATATTCTCAGATCGGCCAGGTCTTCAT
TACCCCAAGAAAGTA
GAGGAGTTCCAAGAGACATATGTGGCCGCTCTCAAGACCTACCA
CAACATCCGCCGACCA
AAAGCCAGCAACTATTTCGCCAAAATCCTGATGAAACTCACGGA
GCTACGGACGCTGAGC
GGGGAGTATGCGGACGTCCTTTTCTCCATGAAGGTAGAACGCGGT
TCCCTCCCGCCCCTG
CTGAGCGAATACTTCGACGTTGTTGATTGACGTTTTTGTCACACA
CTTCGAATATATCAT
TCTCCCGTGCTCCTTGGATTTTTGTGAAGGTTTTTCTGTTTCATT
GGTATTCGCTCTGT
TTTCTTGTTTATTTATTGTTGTACACTATGCTATGATAAATTGTTA
CATATTGCTTGCGT

GACACTGAAAATGATACATTTTCGTTGCTTTTACGGGTTTCAAACG
TCTAACGTTTTATT
ACATCGGCCCTGATGTGGGCTTCCGATCTTGCCTCGCTTTCATTA
TCATTCCAGAAGCTG
TCTGGAAAACTGCGTCCTGATATTATATTGTAACGGGTCCTTAGA
ATTGTTTCACCGATT
TTACGT
>ConsensusfromContig2443 Average coverage: 77.71
CTCATGTTTTGTGGACATTTCGGTGATGAGACACAATTAGCAGGG
GCGGCCCTCGGAATC
AGTATGGTGAACGTGTTATGCATTTCCGTCGCGCAAGGTTTGGGA
ACGGCTTGCGATACG
TTCTTCTCACAGACCTTTGGAGGGGAAAATAAAAAAAACATCGG
CATATATGTTCAAAAA
GCTAGTATGTTATTTGGGCTTTCTTTTCTTTGCTGCTGCGCCCTCT
ATGTTAATTTGGAA
TGGATGCTTTTGTTAACCGGACAAGAGCCCGAAGTTGCGAGATTG
GCAGCAAATTATGTC
ATCATATTCATTCCTGGTGCATTGGCATTTTTCGCTTATGTCATCC
AATCAAAGTACATG
CAAAATCAGAACGTTGTTTATCCCAACGTGATTATTGGCGCAATT
GCAAACGGAATCAAT
ATAATTCTGCATTATTTTCTTCTTTTCCACACTGATTTGGGAATTT
ATGGCTCTTCCATA
GCCCAGCTTATCAGCTATTGTTGCATGTCACTGTTCACGTATCTC
TATATCATGAACGCC
CGTGTCTACACAGAGACGTGGCC
>ConsensusfromContig2444 Average coverage: 38.17
TTTTTTTAATTGTCCGAATTTGAGATAACAAGTGAAAATTGTATG
AAACTCCATGAGCAA
TCTACGGCGTTCTGTGCTAAGCTCTTTCAAAAACCTGCATCTCGC
AAGGAAGCATGTTTT
CAAGGACGACACTAATGCATTGAATGAACTCCGAAAAATTATCA
ATCAAGAGTATTTAAA
GAATAAAGATGAAAAAGAAGAAGCTAGAATTCTAGAGCTCATCA
AAGTCAGCGAAAATAC
AGCAACATACCTTCGCAAAGCTGTTGTGCAGCTTGTCTCGGAAGA
TCAAAAGAATTTCAA
AGTGCGAGTAACGAATGAAACACATTTACACAGAAATGACGCAG
TCAAAGGCAAACCAAC
ATAAATTCCATGATGTGCAAATTCGTTCATGCAGTATTATTTTTC
ACGTGGTTAATATGA

TTTGTTGAGATTCATTGCGAAATAGAATGTATTATGTATGAGCAT
TGGGTG
>ConsensusfromContig2445 Average coverage: 31.95
TCGCCCACTGCTTAAATATCATTTTATCAATATTTTGCAATGGGA
GATGTGGACTCAAAT
ACTCTGGGTAAAATATATTATGCCTTGAGGGAGAAATTATACAAT
CATGCTCAACAATAT
GCTCTCGAAGGCATAAAAAAATATGGAAATGATCATGTTCTAAAA
TTTTGGCTCTCATAT
GCTCTGGTCCTTGAAGGAAAAGTTCAAGATGGATTTCGAAGTTTA
TCAGAAATTCAAGAT
AAAAAAGGGGTTAACCTATGCACTATACTCGCACTGAAAGATGC
TAACAAACTGCAGCGA
AATCCAGATCGCAGCATGGTTGAGCTGTTGGATACGCGGTTGAG
AGAGGAACGGAAGATT
TCGCCACCTATTGCCTTGTACCACGCCGGCGTATATCTGCTTCTC
TCTGGGAAATACGCG
AAGGCTAAGGAATACATCGACCGAGCGCTGAAACAGAACCCGCA
ATCGAAAGACATCCTG
GCAGCGAAGGGATGGCTCGAATTGTTCACTTCTGCTCAGGACGG
CAGAGTGTCCAGCAAG
ACCGATACTTATTTCTCGCAATCTTTGGAGATGGACCCAAGCAAT
TTCGACGCGCTGATG
GGGAAGGTCAAGTACAACGAGATGCGAGGGGATGTAGTCTCCGC
CCACAAATCCATCAGT
GAACTCGTCATCAAACACGACTCGATGTTTCCGATTCCTCAAATC
GAGAAGATGAAAATC
CTTCTGGCGCTGGGGGAGTTTGACGCCTCGTTGGATGCTGCTCGA
AGCATTCTCTCAGAC
GATGACTACAACATCCAAGCACACCAACACATGATTCTGATGAC
CCTCACCAGAGAGCCG
GACGAGAATATGTATTCAGAGGCGGC
>ConsensusfromContig2446 Average coverage: 41.47
TCCAGTAAAGGAACATTTCGAAAGCCGGAAGCCACAGACATGAC
CGGAAGTGCGTACTGA
GCCAACAAATCGTTGCGCGAGAAGCTATCGTGATCGAAGACTCT
GAAGAGGAGCACAGCG
AGATTTGGACATCGCAACTCGAAACGGAAGGAGTTGGCGTCACC
CCATGTTGGATGGAAT
ATGTTGTTTTGTACATGTTTTGTATCAAAACTTTTCTCGTCTTCTT
CTATGCCAAGAACA
TCAACGCTCACAAATGGGTCAGATGTTGACCTACTTCTCTGTGCC

GGAAGATCGAACGC
>ConsensusfromContig2447 Average coverage: 25.62
GAGTTCAGACGTGTGCTCTTCCGATCTGCGAATTATTGCAGTTGG
GGGAAGTAGGAATCT
TTGAGTTGCGATTCGGAGTTAAAGGCGATTTATCGTCCGTTTATT
GTTGTCTTTGGTTG
TCAAGTAAACGTCAGAAGCGTTCTGATTAACTTGAGTTGTCAAGG
AAACGTTTGAAGCCT
TCACATTTCCATTTTGATGCTCAGCACTTGTTTTTCAGCTTGTGT
TGTTTTCGTGTATC
GAGAGAGACCATAGAATAAGCCAAGCACGGCTTTTAATTTACCTT
CGTGTAATTCTTTTG
CTGTGATGTTGTCAAGGGGAACTTCAAGCTCTGTCAATATCACCA
GACATGTTTTGAGAT
TCTTTATCATTTGCTGATTATTCTTCGGTTTTCCTATGATTCCGGG
AATAGCTCTATTAG
TGATTGACTGGATGACTAGAGCAAGTATAACTCCATCCTTGAGAT
CTGCTTGTAAGTCTT
TTATTCGTCGCTTATGACATCCATTTATCAAATATCGATTAGCCCA
ATCTGTATAAATCC
TCC
>ConsensusfromContig2448 Average coverage: 7.72
TCTTCCGATCTTGTTGTTGCTTTAGCTCAAGGGGCGTGTGCCGCA
TTGTACGCCGGAGAG
ATGGAAAACAAGTTCAGATGGCAGAAAACGATCGAAATTGAAGA
CCCGGACTTTCGCAAT
ATCCGTGGTATGACAATGATGAGGGGACACCTCTTTCTCGTCACG
GCGGATTTTCAATTG
TTCGAAACAAACGATTCATTCACAATCGTTAGACGACATCGGGT
TGAGAATCGACCAGGG
GGCGAAGTTAAAGGAAAGAGTGTTTTCGCCCGCCATAACTCTGTG
TACTGCGTTTCAACT
GATGG
>ConsensusfromContig2449 Average coverage: 20.94
TTTTCAGGCTGCAGAGTGGTGTCGCCATCAGTGATCAGGACAACA
ACATTGGCTGCATCT
CTGCGGTCTCCTCTGGACGTCTGGAAAATGCTGTTTCTCAGCATG
GCCAAGCCAGATCCT
GTGTTCGTCTTTCCAGCTTTGAATGTGATCATGTCAATTGCGTCCA
ACATGTTCTCCTTC
ATGTGGAATTCATTGAAGTAGAACTCGACGTGGCTGTTGTCCGAA
AATGTGCCGAAACT

>ConsensusfromContig2450 Average coverage: 5.52
CGACCATTTAGGATGGGTACATCTGAGAAAGATGAAAGCGTTTCA
TATGCCACATATATA
AATGATCACTTCAACAACCTGTCTATCAACCCCCTCAAAAATGGT
CATATAGTCTTTCTC
TTCTTATCCCCCCGAAACTGTCCCATGCTAGTATCTTAGAACTGC
CTCCCAATGCAACCA
GCAACAAATCTTTCATAACTAGAACCATCACCTCCTTCAAAAAAC
TCCGAT
>ConsensusfromContig2451 Average coverage: 10.03
TCGATATTAATATTTCTCATGAAATTTTAAAATATCCAAACACTA
TGGGGACACTAGTTG
TTACATTCAATGCGAAGATTCAAAATCTGAAAGAACTTCAAAATC
GTCTGGTGAATGGCA
TATCACCAATTCCTTCAGGGGTTGACATAGCAGCCACAATCAAAT
ATTTTTCTCAAACCC
TCTGCAGCATTTTGAAGGATATTCCTGTCATTCCACTGGAGAGTT
ATGGGGCTCAACAGC
AAGACAGTGTTCGTCTCTCTGTGTTTCCCAACCTCAACTACTCTGC
TCTCTACCCGTAAT
GTCTATTGCTGAGGTATCGCAGAGTGTTTCTGCAGGTCAACAAGT
TCTAGGAGAGACGAC
TTTGCTTCTATTCAACATCCTTCTGCCGTA
>ConsensusfromContig2452 Average coverage: 64.94
TTTTGATAAGATACTTGAAATAGCAATAGGTCTATAATTCGATGA
ATCATTGATACTCTT
GGATGCTGATTTCACTATCGATGTGATTAAAATATCCATTAAGCT
CTCAGGTATAAAACA
GTGATTGATAAATGATTGGAACAATAATGATAATAAATAATAAA
CAGAATCCGGTCCATT
TTTCAAATGTTCAGCCATGACTCCATCCCTGCCAGCTGATTTCTC
CAGTTCAAGATTACA
GACTGCCAATCTGACCTCCTCGAGATCGGAAGAGCACACGTCTGA
ACTCCA
>ConsensusfromContig2453 Average coverage: 16.51
TTATGCTCAATGTCTATTTCTTCAAACCGCTGAGAGAATTTTTCTC
CCAGTTGCTGATGT
GAAAAACTGAGATCTTGTTGAGCAGATACAAAGACACTTTTGAT
GAAGCAGATCGGAATT
GAAGATAAGCAGTCCTCGGCGTTCTCCAGTTGACATCGATATTCA
TGAATGACTTGCTTG
AGAAATTCATCGGATTGACTCAGATAACTATCGAGTTTTTTGATT

ATAGCATCTATGCAA
GTGTCGTATGAGTCTTGTATGAGGTGTCGTCTAGTGACCGCTCGA
TTCCCCTTGAACTTG
TAATAGTAATTCTCGGCTGTTTCCACGAGAGCGTTGAGCGCATCC
CTCAGAATCTTCTTG
ATGCGACCCAAAAAGAAGTTCTC
>ConsensusfromContig2454 Average coverage: 46.43
ATATCGTAATTCGTTTCTTCCGCCACCTCGCGTATGGCGCATTCAG
CAGGCTCCTCGTTT
TCATTGAGTTTTCCTTTTGGGAATCCCCAGCCTTTCGTTTGGTAGT
TTTGTACCATCAAA
CAATTAGACAGGGTTGGGTCCAGTATTATTGCGCCGTATGTGGGC
ACAAATTGCTTGAAG
TCATTGAAAGCATCGACAATCTCTTCACAACTCGTCACACGCTGA
GAGAGCGATGAACAT
CGACTGAGAAGTTTTTTTGTAAATTTCTTCATCGACATGGGCTTA
CAACCATACTCATCA
TCCATGCAATACTGGTCTAAGTAGAACCAGTACGCAGTCTCTATG
TTCGAGCATAGTCGG
ATATTGTCTGATCTGTCTTTCTCTGTGAGTGTTGAGATGAATCGC
GTGAGAAGATTATCT
AGGATGCTGTCTTGATTAGACTGTTTTGTAGAGCTAGTCATCTTT
CATATAGAAGGTTAC
AATCGATAAATTGTTAATTGATCCAATCTGGTGAGCTGAATAATT
TCTGTGATTAGTAAA
TTCCGTAGAAA
>ConsensusfromContig2455 Average coverage: 14.61
ATCTCGCTTCCATCTCCGCCATTTATATCGACATTCTGCCGCTCTT
TGAGAAGAATCCCT
TTGAAGTAAAACTGCGTTGCCAACGACTGGCTCTCTTCAGAAAA
CTGGCAGAATAGCCGA
GCCTCTGCACCACGCCGGACAATGACAGCTTTGGAGTAGGTCTCT
GTATTCAGGTAAGGT
GTTCCATCTGGTCCAGGCATATTGAGGAGGAAAGTGTTCTGGCTC
CGCAAAGTCCCTGCA
GCATTTGTTGCTTGGCACCCGTAGACGCCATTGTCACCAACAGCC
GCCTCTTTGATCTTC
AGTGTCGCAGAAGATTTCAGGAACTTGTATCGTTCCGATGAACTG
CTGATTTCTTCGTCA
TTTTTGAACCAGGAAATCTTTGGATCTTTATTCCCTCCTATCTCGC
ACTTGAGACTCAAT
TCTTGGCCGATTTGGGGTTCTTCTTTGGGCTGCCTGAGAACGACTG

AACCCCCTTTTTCT
TGAATCCACATAATCTGCAGCTGCGCTTCACTGCTGCGTCGGGC
GATGAAGGAGGATGAA
TTCTGTGCTATGCAGGCGAACCATCCGGAGTCGAGGGTTCTGTCA
ACAGTCTCTATGACG
AGGTTTGAGCCTTCTTGATATCTCCTAGAATCATTGCTCAGCCGC
TGACCATCCAGCTCC
CAATAGAAGAAGATGAAGCGGCGGTTGGACACGTCACATCTCAG
TGTCACAGAGGAGCCT
TCGACTACCTCCTGATTTCTTGGTGAAGGGTCAAAGTAGAAGTCA
TCCTGCTGCTTCGTG
TCTGCCACAAATTGAATAACAATAAATAATCCAACAAGAATGATA
TTATTCATGATAAAC
TTTGTTTTTATTCGACCTTTCAAATCAT
>ConsensusfromContig2456 Average coverage: 12.39
CCCTACACGACGCTCTTCCGATCTCCTGGGTACACTTCGTATGTC
GAGGTCGCATTCATA
CACAGGTCGCTTGAAAATTTGTCCAAGCCAACAATTTCTTCACAA
ATCCACGAAAAAGAC
AATCCAGTTTTATCGAATTTGCTTCCACTTGGGTCATAGGTCAAG
GAAATCGCGTCCACA
ATAGCAATAGTACCGAAGGATACAGATCTGCGAGTCCCATCGGC
GATGACAACGACAAGA
GCCGGGAGAGCTATGCTGAAAAAGGAGTCACTAGGTCCGATGTT
TTCCAAGTCAGACCCG
ATGTGACTAGCATTGGCGTTAACAATGGCAGTATAATCATTGAAG
TCAAGCAGACTCGCG
GGTACGGTCAGATCACTCCTGTATGAATAGTCGGCGGAATCCAAT
TGGTATGCGCGAGTG
GAGTAAAAAGATCG
>ConsensusfromContig2457 Average coverage: 62.10
GAAGGGTTGCAAGCCCTTATCGCCATGCACTGATAAAGTCGTTGT
AGAAATTGATTTTGA
ACTGTTTTTGGTTTCAAGTGCTCGTGGCCTGCTTGAGTATCCTGC
GAATCGCTAGTTCGA
TGGCTCGTCAGATCCATGGTGTTAATGAGATCATCCATAAGATTC
AATTGGAAATCTGTT
GGCTGCTTTCGTCTTGATATTTCTGATTTCTCATCATTTAAGGGA
AGAGGTGCAAATGAG
AACAGACGCACGAATTCACAATATGGCAACTCCGTGAAATATAG
GCATTCATAATCAGCC
TTGATGTAAGGGGACAAAAATCCAATTTTAGGCGACCCATTATTA

GCATAGACCTTTCGG
ACAACAGCTACCATATCCATGTCGTGAAGTGCATTGATGAGGGCG
GATAACGCTACTGTC
CCATTCTCATCATCTTTCTGAGCAACAAAGATCATGCAGCCGTCC
CCTATCTTCATATGT
CGAGGTGTATTCATTGACTTCGTGAAACAGAGGAGTTTGAGACAT
TTGGAAGCTCTAATT
TTCGTCGCAGCTTTATCTTCATCACTGAATGGTACAATTGTGCTA
CCATATGGATGACCT
TCAATGATCTGCTCGCGCTCCAAACCCGCATCGCCATCTTCGTCG
TCCACATTATGAAAT
GTTACAGACCTCGATATTATAGCCTCTGGATTTATCGCGCACACG
TTTTTGAAAGATTTT
GCCTTAACTTCTTTGACTTTGATGTATCCAGCAACGGGTATTGCA
AGATCGGAACCAATT
TCCAGAATGCATTTCCAGGC
>ConsensusfromContig2458 Average coverage: 23.95
GGGAAAATTGAGACTGACTCCCCTTTGAGAGTCGGTTTCATTACA
AACCCTGTTCCAAAT
CACATGATTGATTCAAAATTGTGTACGAAAATTCACAAGAACAA
CATTATTGAAAGCAAA
CCTTCGATAATTCATTTCGGAGGGTTTACGATTGGAGAATTCCTG
AGTAGATCTATTGAA
ATTATCAATGCCTCTGCTCATGTACAGAGGATTCATATAATTCCA
CCTCAAACAAAGTAT
TTTTCTATGAAATATACTAAAACTGAGAGCATTGTTCCAGGCTTA
AGTATAAAATCGACA
CTTTATTTCAAGCCGAGTGAAATGCGATATTACTACGATTGTATT
CGAATTCATTGTAGG
GGCGAGGAGAATCTGGTCATACCAATTCATGGTTATCCAGTGATG
AGCCGAAAATGCTTT
CCTGATCAATTAAGATTTCCCGATGTCCCACTTGGCCAATCGTTT
TCTAAGACGGTTCCA
CTTCACTCAGATGCGCCTGTTGGTTTTGAATTCCACATCACTTTCC
CTCGTCCCCACTCT
GCATTCGAAATTAG
>ConsensusfromContig2459 Average coverage: 20.02
GATGGCGATGATGAGTGGATCGTGTCTGAGCTGGTGAGAAGTGA
TGAGGAATTGGTTGCA
CTCATCGTCGGGATGAACCCGTCCCTGAGGCTCGGTCGTCGCTGG
GAAATATCATCGCAT
CCGGCATTCTCCACGACCTGGCTTATCATCAGACTCATCACCGCA

AAGGTTCCGATTGAA
ATGTGTCGAGATGTTCCGAGAAAAAAATAGGACAGAGAGCCAAG
GACGGAGACGTAGAGA
CCATATTCGGGAGGCACAGAAGCAAGCAGGGCATAGGCGATAGA
TTGAGGGATTTCAATA
GCGCCATGGGTCAATCCTCCAAGAACATCAGGAAGGAGATCTTTT
CGCCATTGGTACCAA
CCCCCTAGCTGCAGGAAGGGAAGCCGTCTCTTGATGAAGTCGACC
AGTTCTGATCTCGAA
CATTGTAACTTTTCTGCTAGCCTCGTGCAAAAATTACGTGTCTCTG
TCGTACGTTTAAAT
TCATCCAGTTCATACTCGAAATGTAGCCGAGAAAAGTCAGGCGAT
GCCTTTTGTTCGTCC
TTCGCATTCGTTAATAGCCTGCCATTCGTTAATAATATTTGTTTAT
CAGTCTCTGATTGT
TGAGGTACGAGTGAATATCCGTTTGTTTTCCACATGGACACCACC
GAGATGCTCGAATGA
AATGTGCTCCTGAATCCGTCAAATTATTCGAAAAGCACGAGCGC
TCTCCAGAAGGGGGCG
TAGCACGATCCGCAACCTCTGACCTAGATCCTGCTCCTAACTCAC
CTCCGTCTATTTGGA
TAACGCATTGATTGAATCATGAAGTTATGTCGAATAATGCCCCTT
TATCTTAGTCATGCT
ATAATATTTAACCAAAGTTTG
>ConsensusfromContig2460 Average coverage: 28.22
AGTTCAGACGTGTGCTCTTCCGATCTAAAACAATCATGAAACCTA
ATCTTCATCGATATA
TACTGACATAGTTTAATTTTATCATGGTATTATTTCATAAAGATCT
TTCCATACTCTATG
TGGAGTTTGTAGTAGCTGTCTGTACAGCATATATCATCTCAAGCT
GATTATACATATAAA
GTTCTGCTTGATGATAGATGAATAAATATATGAAAATTAAAAACG
CTTTTATCTTCTCCT
TCTCGTGAATTATATAGAAATTCGCTTGTTCACATGAAGCTCTCTT
TTATTATGATTTTA
TGGTCTATCCGATTAAATCAACATGTGCATGAGAAATTTGTAGAA
TATTTGTAATGAGTG
AGATTAAATTCATTGTTCCCCATTTTATGCAAGAGTATTGATTCAT
ATATTCAATCATTT
TTTATCACTTGGGTCTGATCGAGATTTTTAAAAAATATTAGGCATT
CATCTCTATCATTT
TAGTTTTTATGAGCAATATTTCGCATGCTTTGCCAAAATTCCACGT

ATATGATCATCATC
TGAGAGATACATTCCCAACCAAGATTTCATATATTTTCCATTATTT
ATTTTGTTTAAGTA
CGCATAACTTTCCCACGTACAAAATTTTGAGAAATCTTAAATTTAT
TTTTTTTATTTCCA
GTGCATTTATCAAGAAATAAATCAATTTAGAAATAATTTGTTCTT
TGAAGTTGTGTTTGT
GAACCTTCAATTTGTTATTCAGCCTAGTTTTATTTATAGAGATTAT
AGTTGACTGTTTAA
TAGAAGAATATCCCTGTCTTGTATCCAATTTTGATTGGGCTCAAT
GGAAATGTGTTTACC
TGATGTCAAAAGAAAATCATCATCTCAAAAGGTAAAACTTCATTT
CGGTTGAATCAATCT
CATATGTTTTCAGTTGACTCATGTGTCGGAATTGTGATAATTGTAT
TTAATCACTATTTA
TGTGTTGGGCTATGTGGTCGCATCAGCAGTTCATCGCCATTTTAA
TTTATATATAAACAA
TTTGATCCAAACTCGTTTTTTTCTGTTTGACTATTTGTTAACCATTT
CTTATTCTAAATT
TGAATGTATGTAATTGTTGAATTTTCAAATGGGCCATTTTTATGAT
ATGTGGA
>ConsensusfromContig2461 Average coverage: 3.63
CCGGAATAGACAGTCGCGAAGACGGTCGATCCTGTATCAACGCC
AAACCCACGATTGATG
ATCTATTGAAGGCGGTTTTCAAGCGGAAAAAGCATTTCCGGAAAC
GTACGAGCTGGTTTC
TGCGACCCGATGTATCACATGTTCAACATCTATATTATTCTCAAC
CTTATCCGGAATAGA
CAGTCGCAAAGACGGTCGATCCTGTATCAACGCCAAACCCACGAT
TGATGATCTATTGAA
GGCGGTTTTCAAGCGGAAGGAGCATTTCCGGAAACGTACGAGCTG
GTTTCTCATCTATAT
TATTCTCAACCTTATCCGGAATAGACAGTCGCAAAGACGGTCGAT
CCTGTATCAACGCCA
>ConsensusfromContig2462 Average coverage: 8.53
GCAGGAGCACAACACTGTTCAGCTAATGGATGATAATAAATTCCT
CTACGATTTGATGAA
TGTCGGTCGAGCCAATGAAAACGTATCAATCCGAGACTTCGTCCT
CATATCAGCAGCCCC
AGTAGCGACAGCGGCCAAAATGTCTCACGCCTTTCGCCAGTTGG
CTACCAAAGAGAAAGA
GCGATCCAAAGACATCTTCATTGCGGCAGACTTTTGTGAAGCCAT

GGCAACAGAATTGAA
CTCTCTGACTGCAAGTTCTGACAACGGGGCTGGAAGTGTTTTGAA
GGCAGTCGACAGTCG
CGGTATGCCTTTTCTTGACGTTCTCATAGAATGCGAGCAGAAAAT
GGTTGTTTCTCATCC
TTCGGTGCAGAAGTACTTATCGGATGTCTGGACCGGCCACTTGAG
TCATTGGGCCGGATG
GAAAATGATGATGCTTTTTGTTGCTCTTCTGTTCTGTCCGCTTTTG
TGGGTACTCTTTTC
TCTACCAAGCCGGAAAATTCAGTTTAATAAAGTGCCCGTAATAAA
ATTCATGTTGTATCT
CGTCTCTCATATATATCTGATAGTCCTCTTCGCCATCG
>ConsensusfromContig2463 Average coverage: 10.06
ATATACAATTTTATTTAAACTGGGATTACGTATTCATTTCCAATTT
CGTATTATTTTAAT
TTCATGATTCAATATTGAAAAACTGCCATCGATATTTTTGATTTA
AATTACTATATGATG
CGGTGTTGAACGGTGCCGAAATGAGATTATTTGGTTGAATATTTG
TATATACAATTTTAT
TTAAACTGGGATTACGTATTCATTTCCAATTTCGTATTATTTTAAT
TTCATGATTCAATA
TTGAAAAACTGCCATCGA
>ConsensusfromContig2464 Average coverage: 34.40
GTTTTTATTGCTCCCATATATGATAATTATATATTGATCAGTGATA
CTTCGATCTGATGC
TCGTTTCTCACGCTCATAAAAAAAGTCAATATTTCTCGTCAGTTTT
ATTTCTAAAACTCA
ATAATCTGTTTCACCCACTCCTAAAAGACTCGGGCATATCTTCAT
GATACTATAGTCATT
CGAATGTTTATCTCTTGTGATTATTTTCGATAACATGGTATGCTTA
TCATATCATATTGT
CATTTCATGATAACATGATCAAAAGGTATTCAGTGTTTTAAACAT
AACGTTTAATATCTC
TAGGCATTTTGTACGGACATGCTTCATCGTATTAGCTATCAAATA
GCCAGAGGAGTATGT
CGGTTTATGAATTGTAGATTTGTGTAGAATGTATACTATGTACAT
AAATTCGTGCCTGAT
ATTTTTTCCAAGACAATGTATTCATTGCTTACTTATGCATGAATTG
CATTGCATTAACAA
TTCCGATTTTAGTACATTATGATTTTTCATTGACGAACTTTTTACC
TTTGGC
>ConsensusfromContig2465 Average coverage: 23.29

TTATAATAATAGAAATAATAATTATTCATCAGCAGAATCATCACC
TGTAGAATGTTTCAG
CAAGAATACTACTATTTCAAAAAATATGGAAATATCCAATGATGA
TAATCATAATGATAT
ATCATTTGAGAATTTTCAAATTTCAAAATCTCAATCAAGAACTGA
TGTACCAGACGGCTA
TCAACATACAGGTGATGTAATCTCATATAGAAATGGTTGCATAA
AAATGAAAAAAGAAAA
TGAAATTTCTTCTTTATCACATAACAAGAAACATGTCTCATTTGCT
GATGATTTTGGATT
GCGTCTGGAGAATGTGAAAGTAATTTCAAGCTGTGTGCACGACAT
GCCTTCATTGAAAGA
ATACTTCAATAAAAGACTTGAGAATTCGAGAATAATGAAAAAAA
GTAAAAATCTTTCAAC
AAATTTTATTTTGAATTTGTGTTTCTTACAACCGGTTGCGAATTAT
GCTTCTTTCACTTT
CAATCTGAATACAAA
>ConsensusfromContig2466 Average coverage: 20.85
GCATAATGTGAGAATTATCAAGTAAATAATTTCGAAAATGGAAA
AAACAGAATTTTGTGT
GATATTCAGCATAATATTGCATTAAAGGTTTGTGTTGTTCAACAT
CATGTATAGTCGTTT
AGTAGAATTATATTCGGGAACATTTCCACTTAGAATGAAGAGAAA
TGGTTCCTGCATTCA
GAAATTTTTAAAAAAATTCAATGAGAACCACCGATAATTTTCCGG
GCTAGACAACATCAT
TATTATGTGACGAACATCAAATTGAACAGCAATAACATTCACGAA
TATTATTTATAAAAA
TGTTGGTATATCAGGGAGTTTTGGTTTCATATTGTGGTTGTATGT
TAAAGAATAGCAACA
GATTATAATCTCAGATAGATATAGAATAATTCAATT
>ConsensusfromContig2467 Average coverage: 5.11
TATAATATAATAGGTTTTGTCATTATTTTTATATCACTCACGTATA
TTTGGATACATTCT
GATGTATGCTAGTATTCAACTTTTCATATATTTAATTTGATGTACG
TATGTTTGTGTATA
ACAATTGAAACTAGTCTTTTCTTCATTTTCGAATTTTGAAGAATTT
AGGTGACGCATTCC
AAACTTTTAAAATGTGTAAGAATTGTTTTTGATTCTGGCAGATGT
TTGATGATAAATAAT
TTTCATAA
>ConsensusfromContig2468 Average coverage: 7.62

AATGGCCGCAAACCCCCAAATTTCAATTCGTTCCCCTTTTCTTTTC
TGCCATCACATTTG
ATGGGTCCACTTATGGATGATACATCGCATAGAATTTCAAACTCC
ACGCAAAAAACCCTG
TATAATTCGACATCCCCTGAAAAGTCTGTGGTGAAATCCAGGAAA
AAGAGATTCATTCGT
CGTGAGACGACTGCTCAAATTGAAGATGCTTCCACGGATAAAAC
CAGCAGCGATGATCAG
ATATATGCACCTGTTGATGTCTATTCGCAAATATACTACGCCCCG
ATTGCCACTGAAGAC
GCCCATCAAATTCTTGACGACCAACCCAACGAACTTAACTCTCTA
CCCTTCATCAAAAAG
CACTCTGCCGGGAATGGGAATGCAAAGTATAGTTTGGTCTCAAG
AAATAATGTTAAAAGC
TACTCAGGAGCCGAACGAAGCAAGCACAATGCTGGTTTTGCTGG
TAGAATAGAAAACACG
CCTGCGTATGCGACAGACTTTGAAGAGAAATCAAGGATGAGTCT
GAGCCTTCCATTGTT
GATATTTATACGAAACCAACTACAGGTGACATGTATACGT
>ConsensusfromContig2469 Average coverage: 13.11
TTCCAGGGCTGCTTTATTCTCCCTCTCTGAAATTTCGGAACATTGA
GCTTCTTCCAAGCG
TTTAACGTACTCTGAAATCTGCACATGCAACTCTTCGATATTTCT
ATCCTTCAAAACAAC
TTCATCTGATTTTTCAGATAGCATCATGTCTGATTTTTTCACTTTC
GACTCTGCGATTGA
CAAATCCCGAAGGAGAGATTCAACTTTTCGCTCATGCAGCATAAT
CATGGCCAAGCATTT
TCTGTTCATTTCATTTTGCCGCAGCTTCTCCTTCTGCATGGCGAAA
GCAACAGCCTCCGC
TGCATCTGGATGTGCTCCTTGTAACTTCTTCCAAAGGGCCCACAC
GAAATCCTTATCGTC
TTTACATTGTTGCAATTCATCATTCAATGATTTATTCTCAGCTATA
ATGACATCCATTAA
TTCTGAATCATATATTCAACTATGCCTTGTAATTGTTATTGAAATT
CGATCATATCTCG
>ConsensusfromContig2470 Average coverage: 11.77
TTTAGAGCTGGCTGATTTTCGATACCAGTGATGATAATCGATGAT
TCTTTGTCGCAAGAT
CTAGTTCCTTCCAGTGTATTCTTCGACATCAAAGATTTTTTGCTAT
CTTTGATGGAAATT
AAAGTTCTTCCACAGCGGTTTATAGAATGAGGAGAATTTCTTAAA

CTGCGTTTAGTTGCA
GTTTCATTCCTTGACTCTATATGATCCAAATTTTGATCATTTTTCT
GTTTTTTAAAATCA
GGTATGGATGACTCCATAGAGCTTGTTCGCG
>ConsensusfromContig2471 Average coverage: 40.38
TTCTTTGAAATTGAATTGGCGACCCAGATCAGAGAATGCTCGACG
GAAAAGTGGATCTAC
GGCAGTTTCAAAGGACCTTAACTTACCCTTTCCATAGAAAGCGCT
AACGGAATCACAACC
CGTGAAAACATGTATTCCAATCAAACTGTCCGTGATATCGCCTAG
CTTTGACGAAATTGC
CGACAAATTTAAGACTCACGAATCATGCACACCAGAATTGAAAT
GCAGCTGACAACTCAA
AAATATTTCAAAAGAAATTTCCAACATCGCAACGTCTATGTCAGG
TGATCGGATATAAAC
CGTA
>ConsensusfromContig2472 Average coverage: 7.45
AGGAGATTCAAAAGAAGTATGTTTCCGATCAGAAGGTAGGCCGC
AGTGAGCAGAGGAACA
AGAGCACTGTAATTCGGACAGGGTCGGCCGTTGAAGAGAATGGTT
TCTGGATTTGTTTCG
CAAATACCAGATCGACCTTCCATTTCATCGAGGTAAAGCTCTCCA
GAAATCTGCCAGTAT
GGATAATACACAATATCCCTGAAAATTCCGGTGTAAAATGTTTGT
GTTGGATACTGGATA
GCATTTGTGGATATTCCGTATGCCAGAAGAAATACAGCCAGAAT
CATGACATAGAGTATG
AGCTGCTCCATCATTTTGACAATCATCACCAATTTTGGCCCAAGA
AAGCTACTCGCAGCG
TACGATCTCAGAAATCTTATGAAGAGAAAAACGCAGTTGAT
>ConsensusfromContig2473 Average coverage: 4.91
TCATTTTTATAGTAATCGTATAAACGAGAGGGGAAATTGCTCTAG
AAAACCAGATTCTCC
CATCAAAGAATCGAGGGCCAAGGAAAGATCAAATAATAGACTGC
AAACTCTCGCAACATT
TTTCCAAAGTTTAGCCCCAAGAAGCAAAAATCGCAGAGCTAAAA
ACAATGGCCACAAAAC
ACCAACAGGCCAGTTGTCGTCTGCCAGTTCTTTAACCTCTGCATT
GTCTGAAAACAACCT
TCGTCTGCTACCCCCTATCAGCCCCGGATGTGGAAAGGGTCTGAG
CACCTCACCATCCTC
TTCATCTTATTTTGATTCCGGATCTCTCATGGGAAGGTCGAAATC

CAGTACAGAACTGAT
CAACAGAGGGAAAGATCGGAAGAGA
>ConsensusfromContig2474 Average coverage: 11.54
TCATCTATACAGCTGATTTGAGATATTTATTTGTCCGCTTTAATAA
CTTATTTCCAAAAA
AATTTGTATGGGCCCAAGCTAGTATCGCCACATGTAGATATATCG
TATGAGTATATTTTA
GATACAATATTTCTTTTAGTTTTACCTGCTGTGTGAAATCAATAA
AATAAAGTGATTGGT
TATGAATCATTTTTCAGCATGAGATACATGATTTCATGTCTGTGA
ATTATCCTATAACGT
TTTGTGATTTGGTATGTTAAAAGATAGAAAGTGATGTATATATCC
GTAACAAAGACAATC
TCTGTTACGGTCCTACTTTGTATAGAAATATGGATTAGATATATT
AGATTAATCAAGGTA
TGACTCTTGAATTGTTAATATCTGAAATGAATTGTAAAAGTGTTC
>ConsensusfromContig2475 Average coverage: 12.12
CTTGGGAAGACTGGAGCGTGGAATGCCGTAGAGCGGCAGCACAA
GCGCTCGGCAAGACTG
GACACGGTCGGCGCGTTCATGACGAGCTGCTGGTCAAACTGGTG
GAGGGAACTGAAAGAC
AGAGGCTGGAGGCACTGCACAAGCTCGCCCAGCTCGGCATCATG
ACGAGTCGTTTGATGA
CCCCATTTCTGAAGTGCTTCAAAGACAGCTACGTCACTGTCCGAG
TTCAAACCTGCAAAA
CATGTGCTGTTCTCAAGCTCTCCGGGTCTCTGATCCAGGAAGCCA
TCGTTGACCTCATTT
ATTTTGACCCAATTGCTGATGTCAAACATGAGGCTCTCAAATGCC
TTTCTGAAGTATCTT
CCGTCATACCTGGAGACGTGGATGAAGTATTACTATGGGCATCTC
GATTTGAA
>ConsensusfromContig2476 Average coverage: 23.33
TGTTTTCATTGCCTCAAAATGGCCAATAGAAGACGTGGATTATCA
TATTTTTTCATCAGC
GGCAAGAGGAACAGGTGACTATTTGGTCAATAAAGGTGTGGTTT
ATGCAAAAATAAACAA
AACAGTCAATAATGAATCTCGAATATATCATGTGATGGGCTCTCA
TTTGCAGGCACACGA
TGAGTATTTCCTCACTAGAATTGAACAAGCAGAGGAGTGGAAAGT
GTTTATTGAGTCGAA
AGAAATCCCTGTTACAGAGCCTGTTGTGATTGGTGCAGACTTCAA
TGAAGATTGTTCAAA

TGACAAATCAAAAATCGCACAAATATTTGACATTCTTGGTGCAGA
AGTTAACCTTACATC
TGTGAAAAACAAACCGACTTGGAATCCTAAAAGGAATTACTTGG
CTGAAATTGCAGGAAA
CTATCCTTCAGGGGTGTATGATTACATAGTCACGCTGAAGAGGCA
CCTACAACCCAGTGT
TTCAAGACATTCTGTGCACAGAATTAAATCCAGAAGAGCATTCCT
TTCATGCTGGAAGTC
GTCGTGGTTTTATTTTTACCATGTGTTTCCTAACGAAACATCTAAT
TGTTATGATATCAG
GGAGATCAATGATTTGTCTGATCATTTCGCTGTTTTGAATAAATT
CTCGTTCGAATAAGT
AGTTAAACTATGTTTTGATGCTTGATTTTACCGGAGACAGTGTTTG
TGTCTTTTTAATAT
TAAATCTGACAATATCCCATCTCTCACGTATATATTTCGATTTCC
AAAGATGTAGCCACG
AGTAATGTGTCCGATTATCCCCTTTATCAGTTCATTTGCTTTTGCT
ATTCAGCGACACTG
AATTTAATATTTTTACAAAATATAACGCTCTGATTTTCTTACTAAC
ATGTTCCGGTACAC
ACAATAAAGTATGCGACATGTTTTACTGTGG
>ConsensusfromContig2477 Average coverage: 90.18
TGAATTATGTTAAGAAAATAAAACCCAGACATTTGAAAACAATGA
CAATCAATATTATCT
GTATTATTACACCATTAGAGACTCGTGATAAAGTCGCCTTCGCCC
TCAGCCATTTTTTGA
GCATTGAATTTGCTATCAATAATTCTGAATGACTCTAGGAACTCA
TTGAAACAAATTTGC
CCATCTTTGTTTATGTCCATATTGCTAGCCATTTTCTTGATATCAC
CATCGTTGAGTTTT
CTTCCGCTTGACACACTTTTATTGAGAAGTTCGAACGCTTGTGTAA
ATTCTTTAATACTT
ATGTAACCTGAGTTATCCTCATCGATGGCTCTGAATATGACTTCTA
AGTTATCCTTTTGA
CGATACAGGGATTGTGAATAGACCGGTCCTGACTTCTGAAACTCA
AGATTCTCAAACGTG
GTTCTGTACCGAACAATATTAGAATCCTTTGATTCTACTAGTTTTG
TTTTCATGAGTCGC
CATGCAATATTGGAGTTTATGTGTTTAGTGAGTACTTCACACCAA
TCATGAACAGTTATA
TAACCGTTACGTTTTTTATCTTTTTTCTCGAATGCTTCCATCAATTC
ATCTTTTTTTGAT

ACAATTTTTTGCCTCAATGCACCCAACGCTCTGTTTTCGAGATGAT
TCATTGTTTTAGCA
TCATTCGAGGTAACTCCAGATTTTGACGCGGAAAACTGAACAAAA
CGAGGTTTATTTTTG
CCTTCGATTTTGATATAAGCACCAAGATTGCTTCCAACAGCATAG
TAATTTGATGCCGAG
AAAATTGTTATGACCTTGTTATTGTGAGTGAACTCGAA
>ConsensusfromContig2478 Average coverage: 15.69
AGGTCATCAAGCAAATCCGCGAAGAATTTCCACACCATTTCGAAA
TTCTGAGCCGATATC
CAGTGCATTTTATGTTGAGCGGCGGAAATGGGTCTGATGATGCTT
TCGAACACATAGCCT
CGCATAGGGTTATTCAACTGAAAGATGACGGGGCACCAGATAGC
ATCACGTTCAGTACGT
CCCTCCGTGCACCCTACCTGCCTTACGATCCTGAAACCCAAGGGA
ATATTCTAGAGGCCC
TGCAGACGTTTTCAGCTGTTGTGGACAGACCGGAAAACATTGTG
TCCAGAAAGCTAAAAA
ATGGTGAAGGCGTCTGCTTCTACAATTCACGTCTGCTGCACGGGC
GAGAGGCGCTCAATC
TGGATGTGGGTGATTTCAGACATCTTGTCGGATGTTACGTGGATC
AAAACCAAGTCAACT
CAAAGAGGCGTGTACTGCTTCGTGATTTAGACTTGTGTACAAGAA
TTATATCTTAAA
>ConsensusfromContig2479 Average coverage: 28.05
ATGAATTCGAATTCATAATCAACAAATTTGTATGGGGTGAATACA
ATTTTGAATATCTCT
CATACTTATATATCAATCAATGTATATAATTTAATTAACACATAG
AAATTATCATTTGCC
CTATTACAGATCAATTATAGCCATCTCCCAAACACTGTGATCAGA
TTGTAACAATTAAAA
TATAAATGCAAAATATTCATGAAATCAACGAAGAATAACAATAA
TCCCATAATATAACAT
TACAAACCAAGGGTGCAATAAAAACCTTTTATGAACGAAATGA
>ConsensusfromContig2480 Average coverage: 21.24
TTCGGGACTAAATCGTTCAAATATAATATGGCAGAATGATTAATT
TCTGAGAATATAATA
AAATAATGAAATGACTAGAAATGAGTGAAATGAGAAAGAAATGA
GATGCGAACTGTGGCT
CCATGTCACTTTCGCCGAATCTGGCTGCCTCCTCCTTCTTGGGTCT
ATGTTCTTCTTCGA
CGATAATTGGCTGATCGTAATTTGAGCCGAGTCCTCTCCCATCTG

ATTGGTGGCTCTGAG
AATATAAGTACCCATACTTTTCTCTCCCGTCTTTGGAATGTTGAGT
TTAGTCTCGAGTTT
TCGACTATCGTCAAAATCCTTTCTGATAGCAGACACGCCTATATC
TGGGTAGTGTCCAAC
CGGCTCATAGTCCCGCCCATTTATCGTCCACTTTAGGTATGTCAG
CATCGGATTGGTTCT
TATTTCGCAAAGAATTTCGAGACGCGATCCATGTCTGGCGAATAA
GTTGTACTGCTGACA
GCGGATCTCTGGTTTATAATTGGCTGAAACGAGTGACGTCACCGC
TTGTGCATCGAATCC
TGGCATAGAGCCGTAACAGTTGAGGGCCATGCCATCCATTGGGGG
TCCGACGTCTTCGGG
CTTGTATACAGCCTTGATCTCCGTTTCTTGTTGACTGAGTGCCAGC
GCCCCCAGTCTCTC
GATGTCGCTCCACTGATCGTCTATGTAGGGGGCGTATGTCGTCGA
GTTGAATTTAAACAA
ATGGTTGACGTTGTTGCCCCCTAGTCGAACTTGCAGGTTGGGAGA
TTTGTTAGTGCCTTT
CACCCGTGCATAGCATCCGAAACCGTCCTTATGCCCCTGCCGGAC
AACGAGAGGGCTGCC
ATCTATGAGCCGACTCTCTATGCGAGAGACGAGGCTCAGAAGTCC
CATCTTGCTCACTGG
TGAGTCGACTTCGAGTCTGATTTGGTCGCGGTGTTTGATGGATGA
GAGGCCGCATCCCAA
TGTACCGCTGTCTCCGCCGTTGAGATCTGTAATATGTAGAGTTAG
AACGGCCTTCCCATT
GCTATCCGTTCTCGAGGTCGCACTGTATCTCCCTGTCTGGGCGAA
AACTTTGTTGACATT
GCCCCCAAGAGTGACCTTGTACCATATGCCTTCGACAATTTTATC
CAATTTGAGAGGGTC
GAACATCTTGTAAGTGCCCGTGCATTGGAAAGTGAAGTTATCTCC
TGCTCTGACATGAAT
TTGATCGGATTTATGAGTGAACTTGGCCCGATCGTCAGCGATCTT
TGATTCAATGACTAT
CATAGAATTCCAAGCAACTAATAAGATGGAAAAAATAATTTCCA
AACCAGCCATACTTGT
GTTTTTACGAAC
>ConsensusfromContig2481 Average coverage: 9.34
TACGTATCCACTGATAAGCCATAAGGATCATATCTCGTCTTATGA
ACCGGTCTCCGCTTT
TTATAACGATTGTATGTACTGAATGGTTTGACATGAATTATTGGT

ACTTCAAAGTCATTG
TCGTCATATTTCGTTCTTCTCATATTCCTGTATGTTGTTTCTTTTT
CAGGCTGATAGTCG
AAATTTCCATAGCTTTTTCGTGGCTCTACATAATTATCGAGGTATT
TGCTTAAACTTCCA
TCGTTGTCTTCACTGAGAGATTTGCGATCGCCACTGTACATTCCT
GATCGTGAACTTCGA
AAATGTTGACTCAGTGGTGTTGGCTGTCTTTTCTGAGTATATTTGT
ACATCTGTGGCATA
ACTTCATTCATATCATTATTGTAGTATTTACCATCATTATCATCTT
GATCACCATAATTA
TAATCATTATCTCGGTCACCAGAATCTCTATAACCTCCTTCAGGTT
CATA
>ConsensusfromContig2482 Average coverage: 25.22
GATCAGCTTGTGAAGTGCAAAATAGGTATGAAATCATGTCAATCT
GTTTTCAAATCTCTA
TCTTCTATAGAAAAATCTGTTGAAAACTGCTGTATAATCATTGAT
TTAGAAAATGACAGT
TTAATATTCAAATTGAAATGTAGACATAGTGTTATCAAAACATTC
AACTTTGCCTTCATT
GAATGTGAATCATTACAAGCAATTTTTTCAACAGAGACTATGTCA
AACCACATTACAGCG
TATCCCAAATTACTTGGAGAAGTTGTCATGAATTTTCAAATAAAT
CAAGAAGAATTGACA
TTACTTGCAATGAACGAAAAATGTAAATTCAAAAACTATGTAGA
TGAAAATGAGAAGAAT
AAAGCTGTACACACTGAAATGGTTTTAAACTCCAATGAATTTGAA
GAATACAGTATAAAT
GATGAGGTGGAACTAACATTCTGTCTGAGAGAGCTAAGATCATTT
TTAGTTTTTGCGGAT
GCTCTCAATATATCTTTAACAATTCATTTTGAAAAACCAGGAAAG
CCTGTTATATTATCT
CTCGAAGAATCATCACTATATAGTGCAAACTTTGTACTGGCAACA
TTATCAGATATAGAT
TTCACAACACAGAATTCAGTGCAGCAAAGTCAATCTCAAAATATA
TCTAGTTTTCGAACT
CAAGGACACAGTAGCTCGAAAACAAATGGTAGAAACATTTCCAA
TAAAAATGGCAAGTCT
TCATCCAGATATCAAACTCAGCCAAAAACATCAATTGCGAAGGAT
TCAGAAGATTTTATG
GTCAGTCAAGAGATCCCGTTAATTGATATATCTGTTACTTCTCCT
ACAAAAGATGCAGAT

GAACCAATACCTGGCAGTCCCCCGAGCAAAAAACAATGCTTTTTG
TTTAAAAGCTATTCC
ACATCATCACAATCCAAAAACAAGAAAGATGCATCCATTATTCTT
GCTGAAGACTCTGAT
TAATT
>ConsensusfromContig2483 Average coverage: 12.36
GAAAATTCTTAACCAATAGGATCACAGCATAACCAAGTCGTTTTA
GAAATTTTGAAACAA
CTTCGTGCAAGATTGACCTTTCAAAATTACGTCATTTTTGTAAAC
AAATGAGAGGTCAAT
CTTGCATCCCACTTCGTTTTAGGACTAAAGAATAGGGTATTAGAG
TGGATATAAACTTTC
ATTCATATTTTTAATTTTATTGCTGGTATTGCGAATCGTCGAATAT
ATGTAGCTCGGATT
CATTATTCTGGTTGATTTTTTATATATGTACAATTTTTCACAATTT
TCGATGGCTGGT
>ConsensusfromContig2484 Average coverage: 17.63
AAGGAGATGGCGAGCAAAGGGATAGGGACAGATGGAGAGATCT
CAAAATTTCAAACAAAG
GAGATGGTACGAAGAGGTACAGAAAAATCGAAGGCAAGTGTCCC
AAGCTTCGAACATATG
GAGAACGGAAGAAGAGGAACATTGAAATCGGTTACAACTAATAT
CCCGAAATTCAAAGAC
ATGGAGAATGGCAAAAGAGGGCCTCAAAAGGAAATGCCCAAAAT
CAATGAGATGGAGGAT
CGACAAAAAAAGCCAGAGAAGCCGGTCCCAAAAAAGGTAGAGAG
GCATGTCCCAACAATT
CTAAAAACGGCCAAAAAAGAGATGGACAGTGCACTCAAATCTTT
TGATGTAGGCAAAACG
AAATCAGTAAAAGATGTTCAACCAGGAGCGAAAAAAGAAATCAC
GTGGATGAGTGTACCG
CCAAAACCTGGTCTCCCACCGGCGCTGGCATATTTGGCTCAAGTC
GATGAGCTTATTATC
AAGCAAGAAATTGATCTGATAGAGGTTATGACAGAGTGGGAATC
CAAAAACAGATATAGA
ATTTAC
>ConsensusfromContig2485 Average coverage: 31.45
ATGAGGACGGCGCTCAACGTACCCCGGTAGCGTTTTTCTAAGGTC
TGGGGTCTAGCAACC
TCAGGAAACTTGAGGCCACTGAATCTTGGGTTGCTGTAGAAGAGT
TCAAGTTGTTCGGGC
GGAAGTCGTGCAACAACTTTTTGGATGATGTACAGTTGATCAATC

TCGCTTTCACCGGGG
AAAAGAGGCTGACCGTCACTCAGCTCACCCAAGATGCAACCAAT
CGACCAGATATCCACA
TCTTTTCCGTACGATCCCCCCAGCAAGAGTTCTGGCGAGCGGTAC
CAGCGCGTTGCCACG
TAATCCGTGTACAAGCCGTTCCCTTGTCTCGCTACGCTTCTTGCG
AATCCGAAATCACAA
AGCTTCACCACATCATTCTTTGATATCAATAAATTCTCAGGCTTTA
TATCTCTATGAATG
ATGGAGCGAGAATGGCAGGATTGGACGGCCTTGCAGAGCTGGTA
TACGTAGGATCGAGCT
TTCTCCAGAGCCACTCCGCGTGGAAATTGCTCGAGCAGTTCAATC
ATGTTTCTCTCGACA
TACTCGAAGACTAGAAACAGCTTTCCTCGACGCCTAAATGCTTCT
CTTAATGCAACGATG
TTCTCCTGCTTCAAAGTTCTCAGCATCTTCAACTCTCGCAATGTTG
TACGCCTAACATCA
TCGTTCTCTTCACTGTCTTTGAACTTCTTTATGGCAACGACTTCTC
CAGTATCCTTCACA
CGACACTTAAGGACCACACCATATGCTCCCTCTCCCACGATTCCC
AACACCTCGTATTTA
TTCATATCATCATTGTTCAGATCATGTTTCACAATATTCCATCTAT
TTGAATGTTTACAA
ACAGCAAAAATTAAAAATTGTTTTCATAGGCAACGAAAGCCAGA
TCG
>ConsensusfromContig2486 Average coverage: 3.41
CGTGCATTTCTTATATATCCCCATTTTCATTCCGTTATCGTTCAATT
TTTTTTAGTTTTC
TGATATGAAAATCAAAATGTATTCCTATCGTCTCTCTATCGTTGTC
TTTTCAAAGTAATT
CTTACATCCGTATATGAATCCGGTACTTGCCAAAAATAAACATTT
TATTTTCTATACCAA
AGTTGATATATTTTCATATTTAATGAATTATGAAATCCGTATGGT
CCGTATGATAAGTGA
TATCGCGTTTTTGCACTACTGCAATTCATGTTTGATAATGTGATGA
CG
>ConsensusfromContig2487 Average coverage: 37.87
TCGCAAATGATTCTAGAATAGATGGATTGTGTGAGACCCCGGCG
GATTTGAAGGGCAAGT
ATGTGAGTGAGATTGAACTTGATCTTTCTGATTGTGAGAAAGACC
ATTGTAAAAAATTTA
TCACTGAATATTCCATATTGTGCAGTGGAATAGTTCTTTTTGGTT

CAAGAACAACTAATT
TCAAAGGATATACATTCGAGGATAGTGACATACCTCGCTTGACGA
AATCCAGTCTTGACG
TATTTACAAACACTGTTGAAGAATTCACTTTCCATAATTGCGGCA
TCAAAGTGATAGAGC
CTTATGCATTTGAGAAATTTAGGAAACTTAGATCATTGGAAATAT
GGTCAAATCAATTAT
CGGAAATAGAGGAATTTGCATTTAGTGGATTGGAATTGGAATATT
TCAGATTATTGGTTC
GGAATAACGTTACTCTTTATGAAAACTCATTTTTCAAAATGTTCC
AAAAAGATCAGCACG
TGGAATTTTTTCTCGTCGGCTTTGGTCTTGATCTAAACCATGTTGA
ATATTTAAAAGCAT
TTGCGAACTTGCATATCAGTGTCCATTTCTTGGCAAACTCTATCA
CCCATATCCCGAGAA
ACTTCACCTTACCACCCATGAGAAAATTGGAATCATTTGACATAC
GAGGGAATCCTATAC
ACTGTGATTGCAATGCCAAGAAGGCCGGAGAGGCTATTCCACAG
ATAAAGGCAGAGTGCT
TCACGCCACCTTCTCTCGGTGACCGGAGCATCACCAATATTGATG
ATAACCTTTTGAATT
GCAGTGTGCCAATTTAAATAACGATGTTTAAATTTATGATTTAAA
GTGTAAAACTTCAGT
ATATTCAAGTAATA
>ConsensusfromContig2488 Average coverage: 35.07
GATTAAAACCGTTTTCGTTCGGATATGGACCTGGAATTAAATTCC
TGCTTGGTGTGGCTC
CACTTTGCGCTGCGTAGGCGCTATTGAGCATGCGCCGATAGTAGT
CCGCCCAGCGAGAGG
TCAAGGTCGCGGAAGAGGAAGGTGAATTTGTTGACGACCTTGGG
TAATTACTAGAATCGG
GGGGTGGAGATGCCCGGAAGTGGTCCGGGGGTGGATTGAATCTC
CCTAAGGCCGGATGAT
TCAAATGACTAAAAGGATTTCCTCCTATGAAGAGCCCTGGGTCTC
TTTGACCGTTTGTCG
GCGGTGGGATACTATTCGACGCTGCTGCCGCAAAAGCCGCCGCG
TTCAGCTGATGGCAGA
TTTCGTTTATCGGCAAACTTGGATTCGGAGGCAGTGGATACGATC
CATGTTGATGAGGCA
GATGTGGGAGATACGGCCATCCGGAGGCTGCGAGGGCTGCTGCA
TAACGACTTGCTTCGA
ATGAGGAAGGTGGGGAGGAGAAAGG

>ConsensusfromContig2489 Average coverage: 7.39
CAAGAACGGATATATGTGTCCTCTATTCTAATCAGAACCAGTGAA
TAAATCTTGCAAGAA
ATTTTATAGCTGTCATGAGTGCCTAAAGTGCCATATGTAATCGAT
CTGATAATTTTAACT
GCCTGGTTTTGGAAAGATAACCCCTTGGCAATTGACACCAAATG
AAAAACAAGAGGCTTA
AAGCAAATTGCTTATGGGCCAATCACATGCTCGCTAATTTTAGAA
TTTGTGGGACGAGAA
CAAGATAATAATTGAGATTGTTTAACGGTCCTTTCATATAAGTTT
TACCATAACTCTTCG
AAATCAATAGTAAGCAGTCTACATATTATATTCAGTGCTCGAAAA
CCGATAGTGTTTTTA
TTTGAATTACAAGGTGTTGGTATCGTCTGTCTTCTTTTTTTTGGTG
CGCAGATTG
>ConsensusfromContig2490 Average coverage: 16.38
ATCTCTTGAAGAAGCTTTCCTCCTCATGGATACAAACTTTGGGGG
CTCAACACTAAAACT
CAAATGTCTATTAAGTCGTTTTCTAAGTTTAATATCTTCATCTGGT
GAACCCATTTTATT
GTAGTTGTTTTTCTAAATGAAAAAAATCTTCAGAAGAAATTCAAT
TGAAATTTTGAATTA
ATTAATAAACAACGAAACGAGGAAGGAGAGACAATGTATATCCT
CCCTCTGACGTGAAAG
CAACGTCTCTTCTGATAATTTGAAATAAATGCTTTAACATATTGA
ATCAACCATATAAAA
ATACGAAACGTCAAAATTTTACGCTTCACCCCCGC
>ConsensusfromContig2491 Average coverage: 15.45
ACGGGTACGATGGATGCGGCGGTCATCACCCACCCCAACCCGTT
TGCCCACTGAGGAAAC
GTGTAGTTGGCAGGCCGATACTTGAGCGGTTCGTAGCTTGCCACC
GATAGGGATAGCGCC
ATAATGAGGACTATTGGAGCGAGAATCGGAATGCTGAAAGTCCA
ATAGCAGCTCAGTTTG
AATCCAATCATCATTTCAATATCCGATCTGAAGCGTTTGTAACCA
TAGACGTACGACACT
GCCAAGACTTCAATAAAAGCAATGGCGAGTAAGGATAGACCAGA
CGAGTAGTAGTCGAAT
ATTGTCAGAAGAAACATGCCATTGCGTGCACAAAGAGGGGTTCC
AAGAAGAAAGAATACC
AGACATACACCTCCCGCAACAACAATTTTGCGATG
>ConsensusfromContig2492 Average coverage: 25.54

GTTTATTCAAGCGACAATCGCTAGAGAAGATGATGATGGAGACG
AGAGAGCCTTTGGAGT
GGCACTTTAAACAACTTGATCACTCAATGGGACTAAGCTTTAAGA
CGAACTTCCATTTCG
CCATAGTTGGACATCTTCTCAAAGGTTTTCAACACCCTCACCCGA
CTACCATATCACGTA
CTGTCCGTGTCCTCAACAAACTTCTCACGATTGTCAGTCAAACAG
AAAACAGGGACCGGT
TTGAAGTAACTCGTGAAAGTGTACCCTACCTGGCTGCTCTGGTGC
CGGTG
>ConsensusfromContig2493 Average coverage: 19.47
CTTGTACCACAACATCGAGTTCCTTGCGTATGCTTTCGGGGGAAT
CACTGCGGGGGTAAT
CCATTTAGAATACACTGAATCGATCTTTCTCGATGAGCAGGCTTT
TGTTGGTATTGGCAA
CACTTCAAAGGATGAAAATAAGTTCCGTGTCCTTGAGGGTAAAC
TGAAAATAACCGACCT
GAAGCTGTTTGAGCCATTCAAGAAAACAAACAAGGATATCAAACT
TGTTTTCCAATTTGC
TAATTTGACAATTCTTGACCCGAACCCTTTTATTGATTACTCGAA
GATGGTGATCATTGA
AAAAGGGAACAATTTTGTTTGCAACTGCAGTAACAAAAACTTGA
TCGAGGCTCACAATCG
AAAAGGCGGCATCAGAATTGTCTCCTTCAACGAACAAAAATGTAG
CTTTCCTTCGGAGCC
GGAGATGAAAATCCCTCTAAAGAACCTTTACGGAGCAAACTTAT
GCCCAAATGCGACATG
CAGCGTGGGCAAGCGCACATGTCGACGGGTATCTCATAACAAAC
GTGACGTCATGGTATG
TGAT
>ConsensusfromContig2494 Average coverage: 12.40
CGCTCTTCCGATCTATCCGTTTTTGACTCGGTTAGTTCACTCTCGT
TTGATTGGGCACAG
ACATCTTTATAGTAAGCTACGTCTTCCTCATCATCATCTTCTTCTT
CATCTTCAAAATAA
TAGGTGTTCGTGTTATCAAGAGGCGCCTCTTGTCCTGCTTGGTTC
ATCTTGGATTTGAGA
TTCTGGTAGAATGATCCGCGGAGCTTCTTGTTTTTGATGAGGAAC
CTTCCCTTGAGCTGA
TTGGGGCTGGGCAGAACCGGGTCGTCCGACCAGTCCGCTTCGAAG
AGGAATTCTTTGACG
AGCGCATCGCCCAGAACTTCAGTGAGTGTGAGAGCCATGTTTTGT

TGCTGAGGAATCGAA
CAATGGTTCTCCAGGGACAGTATCACAGGAAACGGAGAGGTCTTG
AACGCACTTTTCTTT
ATTGCTTTTACTGCACTCTTGAAAGATATTTTTGTGGTGAGCGTTC
TCCCATGGTAGATG
ATCGGCTGTCCTTCATCCCCATCCCAGCAGTCCAGCTCAACGCAG
CGGCATCCAGTTTCG
AGGACTCTTGAGTACATCTCCGCACTGGACTCTCCCCTCAGCTGG
TGTC
>ConsensusfromContig2495 Average coverage: 7.57
CTTGCCTTCATTGCGATTCTCGCGGCTCTACTATTTTGAGTTCTCG
CTGATGGAAATGGC
TCTGGCCTCTATTTATGCTTTGCTTCTAAATTGTATGTTGTGGATA
CGTTAGAACTAGTT
AAATCGAATAAGAGTATATAACATTAATTTTTGTAATAATAGCTT
TGGAGTCAGAATTAT
ATTAACACATATTATGCTCATGTAAGAAACCCTTCACCGCATGGG
CTGCCGCGGTGTTAT
TGAGCCGAGCTTGAATAGGTTTTGAGATTATATATCAGTATAGAT
TTCTTCTGAGCATGA
TTGTGAGATCCCCGTGATTTCGGTATGTGGTGCAGTGCATGTTTA
TCACATTCCATGATT
ATGGTAGAGGCGAGCCTAAATTTGTATTTAGATATGTTTTTTTTA
TTGTTCTATGGGAAA
ATGGAG
>ConsensusfromContig2496 Average coverage: 14.89
CAAAACTACTCACGATTTCTTTTCCGAAGCTTTTAATTTTGTCTGT
GATATTTTCCTTAG
TTTGTGAATGAGCCGGTTTGGCAAGAATGATTGAAGGCAGAGTC
AGTATCGTGAAAGCTG
TCATGACGAAGAACGTGTAGCCTGTTCCGATCTCTGCAATGATTC
TTTCGAATATCTGAG
GTATTGTCGTGATTCCGAGGCACATCCCGGTATTACAAATGCCGT
ATGCAAAGCCTCTGT
ATTCCTCTTTCATTTGGTGAGCAATCATGAATGCCACACACATGT
TGCACACCCCAATTC
CGGCCGCTGCCGACATTGCATATCCAAAAAGAAAAGTCCATGGA
GAAGTGATAAATCCGC
AGAGAAGATACCCGAAATAGATGAGTGATAAACCAAAGCCAAAT
GCACATCTCACTCCAA
ACACCCGAATAAACGAATTTCCAACCAAAAACATGCCAGAGGCG
AAAACATTAGCTGTAG

ATCCACTGATTGCAAATATGTGGGCGTGGTCCGGGAATTGTGCGG
CCAGTGGTCGGTAGA
GGACCATGAAGGTATAGAAACC
>ConsensusfromContig2497 Average coverage: 12.46
AAACATTGCAAGAATCTTTGGCCAACGACACAGCTCTACTTCGGA
AAGCGTCCTTGGATG
TAGAATTACCGCCTGAATCGCAAGAGGACGCCACACACGCCAGTC
TGCTCCTCTACCGCA
GCGCTAAAAGCTTCGATGAGCGCGAAATGGAAACTCGAGATGCG
ATAGCATGTGAATCCA
TTTTCAAGAAAGAAAGATGCTCCCCATCAGCTTCCTCCAGCACCT
CAAGTACCGAAGGAA
TGAAAAATTACAAGAATTCGAAGACGCGACATTCAAGAGATGCT
CATGGCGATTCGACAC
CGTCTGCTTTTCATATCGAAAACACCAAGAATTCGAATAAGCGAA
AATTTTATACAACCG
AATCCCTCGGGATTGTTTTGAATAAGACACAAAAGCGACAGGAAT
CTTTTGATAACGATT
CTTCCGTAGGATTTGTTAATTGCTCCGATGAACTCGATAATGGTG
TGCCAGAGATCGGAA
G
>ConsensusfromContig2498 Average coverage: 12.00
ATCGGTTTTGTTTTTATTTGCCATATTCTGGGGACAGTTTGTCAG
CTAGAAGAGAAAGCA
ACTTTGGCAGAAGATCAACAAGTGGAGACTGATGTGTCTTTTAA
CGATACTGGGAACAAC
GAATCATTTGACCCGGACAATTCAGAACACAACAATACCAGCTT
AGCCGAAAAAAGGAGT
ACCAAAGATGTCGGCAAAAACAACAAAGCAATGTATGAGTCATC
TGACAATGACATCGAA
TCCAGAATCCATACTTCAAAGGAAGCAAAA
>ConsensusfromContig2499 Average coverage: 20.13
CTCTTCCGATCTCGAAGATTGACCCCTTGGGGATTGACTAGTCGG
ATCTGCTTCCGATTG
CCTGTTGCGTGTGCAATGGGTAATATATCATCGAACACTCTAGAC
AAGACTTTTTTATTA
AGCACAGGGACGTATGTAGGATCGTGAGGTATCAGAGTCGGGGC
CAATGCATCCAGGATT
GTCAACTTCTGAGCTGCAAGACCGTTTTTGCGAAGCCACTGCCGC
GAATTCTGATGAACA
TTCGATGCATATGGTTTTTTCTTAAATCTTTTTTTCTCGAGAGCTG
GTTTGTCATCGAAT

ACTGAGTTGATTCTGAGCTGTCTGTTCCATTGTTTTTCGAAGATCT
CTTCTGTTTCTGGA
AGAAGCCAGTCTGTTCTGGATGCTGTCTTGGCGTGGCATAGAGTA
CGCAACAAACTTGAT
TTTGTGTGACCAGCTTTTAGCGGATTTCGCGGTTGCTCTATGATGC
CCCCGAGTGTCTTG
GAGCTAGTCACTTTCGTGGGATGCTGGGCCGAGTAGGGTCGAAT
GCGATGACGTGGAGTT
GTCATGACCCGATCCCCTCTCTTGATCTCCGCCAATTCCCGAACA
ATGCGGTTGCTAGGA
GCGCTGATAGGCCGCTCCAGGTT
>ConsensusfromContig2500 Average coverage: 8.76
GGATGAATCGCTGAATAATTATGATATATGTGAACATTCGAATGA
CATGATATGTCATGA
TATCATCATGAGTGGAGAAGTCACATCCTAGGCTTCACAATATAT
TCATAGGGTTTTGGA
TGAATCGCTGAATAATTATGATATATGTGAACATTCGAATGACAT
GATATGTCATGATAT
CATCATGAGTGGAGAAGTCACATCCTAG
>ConsensusfromContig2501 Average coverage: 450.22
TGGTTGGAAACAAAAGACCTAGTGTAGATGAGACAGAGGGCTTG
ACACCGTATTCCAATA
ACTTCGGAGAATGGCTCAAAAAAGAGAAGACGGCCATTAGTCTG
ATCGTCCCGATTTGCC
TTCGCAAAATTGTGCATCGAGCACACCAAATAAATGACCTCACGT
CACATGATCACATTA
GTCTGTACGGCGACCTGAGGCTGTTTCTCAATTTCATCAAAGACA
CGTGTGGTGGAACAT
TCCGTAAGGTGGCGCTTTCGGCCATGTCGGATTCATTGCGTCACA
TGACGACCATTTCCG
TCGGTCAGAAGCCCAGAGCGGTCCGGATTGTCAGACCCAAGAAC
TCGATGACGGATAGCT
GAGATCGGAAGAGCACACGTCTGAACTCCAGTCACAT
>ConsensusfromContig2502 Average coverage: 34.88
GTGCAGCTACGAGGAGACAAAGTGGGACTCTACGGACGAACTGC
GTTGATTGCCTCGACA
GAACTAACTCAATCCAAAGTAGAGCTGCTCTTCAGATATTGCCGG
AAATGCTGTCTGCCC
TTGACCTGGGCGGGGCTTCCCACCTGTCAAGGTTCACGCAGATTT
TCAAGGACATGTGGA
CAGCCAATGGGGATCATTGCAGTCGGATGTATTCAGGAACGGGC
GCACTTGGTGGGGGCA

AGTCAAAGGCGGGAGATGCTGCGCGATCGGCGACTCGAACGATT
CAGAACAATTTTCTCG
ACTCCAATAAGCAAGAAGCAATGGACATTCTTCTGCAAGGCAATT
GCGTTCCACCAAACT
TGGCTGAGAAAACCAGAGCTTTTCTCTC
>ConsensusfromContig2503 Average coverage: 17.33
ATCGATGGAAATATTGATATATCAGAAATGTCGAATGACTCCATT
GGCTTTCCTCATGTG
CACAGAGAAGGATTTGATAATGCTCGAAAATCTCTGCAACTTATA
CCAGGAGAATCTTCG
GCTACGAATAATCATAAAACCAATGAGAAGAGTCAAGTAATAAA
TATGAATGGATACGTT
AGGTCATCATCTCAAGTATACACTCCTCATTATAGCGATATCAGC
TTCGACGGCCCTCCT
TCTGTTTCCAATATAGATAGAAGTAATGTACATGATTGTGCAGAT
GTGGAAGAGAATCCA
AAAAGTGCTGAAACCAACTTGGAAAGCACCGAAAATACTGATCA
TGAAGTGGAGTTCGAT
CAACGGTTTTCAAATAACAGTGTTTCGAACAAAAAAGAGAGGCA
CAAAGAGTTCGATAAT
TCTTCAAAGTGTGCTCAGGGGCGAGGCCACCATAACACTGGGAA
TAAAATTGTAAACACC
GATAATATTTTGTATTCTGACAATAATAAAAATTCTGAAGTTGGA
TTAAATCAACAGTTT
TCAAATAACGGTGCTTTTACCAAACCTGAAACGAATAATAGATTT
GAAATTTTAGATAAT
CATTCAAGATTTGGTCAAAGCCTGGGCTCTTATGATGTTACTACC
TCCATCGAGAGTGAT
GAAGAT
>ConsensusfromContig2504 Average coverage: 18.63
GTCCCGGTGGGAAAAAACATGAAAAACAAAAAAAGGTAAAAAAA
TCTAAATCTACTTTCA
GCGATTCCTCTGAGAATAAATCACAAAATCTCAGTTCGAAGGAAT
CCTACGATCTGAGTT
CGACCATCATGAGAAGCAGCAAATCCGTCCTTGAAGTTGCCAAG
GACATCCCAGAAAAAC
TCTCCGGCAGCTCTTCTGATCTGAGGGTTTCTGATCCTAATGTGG
AGGAACAAATGTATG
AAATGTGCTCCGAGTTGGATACATCAAGAAAATGCGAAAAAACC
TTTGATAATTCACCCG
AGCATATTGGTGAAATATCAAAAACGGGTGACGATGTTGCCTTAT
CTGAAACTGATCTAA

ACGAATC
>ConsensusfromContig2505 Average coverage: 38.38
CTTTGACCATTCCATCCGAACCATTATATTTAATGCTATTTAAAT
GCTGCTGAACCTCAA
AGTATCATCAATTCATTGATATCAACCAGCTATACAAACAGTTAT
TTGAACAATTTGTAT
ATGAGGCCTGATTAAACATAGCTTGAGAAAATAGAAATTTAACAC
ATGCTTTTTCGTTTG
AAAATTGTTATAGGAATATGTCTTCAAAACATGAATATTCATCCG
TAGCAGCATGGGCAA
CACCAGCATCTTTTTTGCTGTTCTTCCTCCTCTTTTTTCTTTTTTTC
TGATAACCTGAGT
TTTTCTAGAATCAGAGGAGCTTTTTCTTTTTCTTCCTCAACTGTTG
GGGTCTCTCTTGTT
TTAGCCGTCTGTTTAAATTTCATATAGCTTCTTGAAACTCCTTGA
AAACTCATTGGAAAG
CATTTGGCATTTGTATTTGTCTGAGTTTTGAAATTATTTGCATTCA
TTCATGAAAATTAA
CCAGAAAACTCTCTAGTTTGAGATTTTTCAGCAGAGAGTTGCATA
TTTTAAATGATTAAT
GGTTCAAATCGATTGCCGATAAAATATCAGATGAAATTGGAATG
AAAGCAGCTATACACT
TATTCTATATATACCTTCATGACAAGCTCTCATATATAGATATGAA
ATCTTATTTCGAAT
AATTAATAAAATGTTCTACAATTAGAACATACCACATGTTATTAA
GTCTGACTTTCGGTG
GTTATTGAATTTATCAAATTAAAC
>ConsensusfromContig2506 Average coverage: 21.63
TTTTCGGAGAAATGGCAGTCTCAGCCCGGAGCTGAGTTTCGCTAT
TAAAATATGTTTTTG
TAATTATCGGATATTTTTTTATTGATGAATTACTCTCAAATTTTTC
GGGTTTCAAATATC
CACAATTTTCTTCGACCATTCCTGGCAAACACACATCTGGGTCAA
TTTTGGTTGTGAGGA
ATATCTTTGATTTGGATTCAGTGGAGTTATGATTCAAATGTTCCG
ATTTCTCAGGTGAAA
GAATTTCTCCATTTCCTTTCTCAGGAATATCATATTTGCAACGTGA
TGAAGTCATCATCC
CCTTATTTTGAATCACTTTATTGAAGCTTTTTTCATTAATATTCCA
AGCATGGTCAGAAT
CATATTTGTGAGATACATCTCTTGACAAATTATCAAAAATATTTA
AACCTGTATAAGTAT

TATCATTGCTTTTTGAACATAGGGAACCATGAGCCGTAGTACACA
TGTTATTTGGTTGAT
GAATTAATTTTTCTGCATTCTTTGATTTTATTGCAACATGACCCG
AATAATTCGGGATGA
GATTTCTTTGAACATTGTATGATGTCGGCAGAGTTTTTCCACCAAT
AGTTCTGTTCGATA
CTGTTCCGTAAGGCCTTTTTTGTTTTTCAGATGATTTAAAGTCAC
AGTGAGAGGATGGTG
AGCATAGTCCACGGTTAGTTTTAGATAAAGTGCATTTTTTTGAGT
CACTGTTTTCTAAAA
CACAAAATTGCGATGAATCAACCATGCCTGGTATAATATTAATTG
GATGCACGGAAAGCT
TCAATTCATCTTCATTACGCTGTATTTTTCCAAATGATAACGATTC
CGAATCAAGTGATT
TTGATAAAATACCAGTATGTATGAATGATCTTTTTGACCGCTGCTT
ATTTTTATGATATC
TAGCCTCTGATTCATTCTTGACTAACTTAGTTTTACCATTTTCATC
TACCGCAGTGATTT
CATATACTTTCATTGTTTTCTTTCTTTTAAAATCAAAATTCGACAA
AGATTCGTAAGCCT
TGCGTAAATTACCATGCTCGGGCTTCTGCAAAAATGTACCATTCT
TCCGCGCCATAGTTT
TCATACTGATTGAGTTCGATGTTTGCAGTCCATCTTCTCCCACAAA
AACACTGTTTTTCA
TATTTTTCTTCGTGCGAAATATCCTCTCAAAGAGAGAAGGGTGCT
GACATCTGTGTTCAA
AATTATGATTATTCACATTACCCCGAGTATGGTCATTCGTGGAGA
AACTTATATTTTTCG
AGACCTCCACTCTTCTTTTTTTCTGTAAGGAATCG
>ConsensusfromContig2507 Average coverage: 33.66
TCTGTTCATCTTGATCAAATGCATAAGTCTGATCAGGTCCACTAC
GATTAACGGTGACAG
CATTCTCGATTGCTCCTACTACGATTGAAACTAAAACGAGATCAG
GTTCATTATTGTTTT
CCAACTGACATTTAAACAGCGCTAACACACCATCGATACTTTGCA
ATGGAAAATGTTTTT
TGATACCAGCACTAATCATAATTGGAAAAAATATGGCTAGACAA
TATATAAATACAACAA
TAAACTTATGTCATGTTCAACAAGTGTCATTACAAATTCGTAAGT
CATTTTCATTTTAAA
CGTTGCAATATTTTTGTTTTGATTGCGCAGTTTTACGCACTCG
>ConsensusfromContig2508 Average coverage: 11.78

AAGCAATATACATCGTCGATATGACACACCTTCAACACATCCTCA
TTCGTATGACATGAA
TTCACCTCGAGAGAATTGCCTCTATCCCAATATTGGTCATTTAGC
TGATGACCTCTTTGA
ATATCAATATCACCAAGAAAGTGCACCAGAATACGGCAGAACCA
ATCGAAATAGTTCCTC
TCACACGAATAATCAAAATCAGAACTCAAATGTTGAACTTGATGA
GATACTCTCGCAACT
CTTATCGGCTGATGAATTCAGACATAGAATGCAAGAGACAGATGA
CACTATTCGAAACTT
GTCGGAGTCACATGTTGAAAGCAATGTTCGTCTGCTTCTGAAAGA
GAACTTTGGCCTCCG
GGGTCATCTGCAGGATGAACTTTTGAGAATATTGGCCATGCTGAC
CAGAAATGACTCAAA
TCTTGATAAATTGGACGCTTTTATGAAATGTAATGACAAAGTCGT
TTGGTTCATGCGAGA
TTTGAACCTCAAAACAAAGCAAGCTGAAAATCTGGCGCAGTCGA
GAGCCAACGAAGCCTC
GACGGATGCAATTGTTTTGGAGACTCTGCCAGAAGACGC
>ConsensusfromContig2509 Average coverage: 5.64
TCCGATCTCGGGGATGATATTCATTGAGATTTTGAATTTTTCATG
TCTTGAATAAAGAAA
AAGAGGCATATGCCGGTGTTATCACAGAATGTATTTGTATGTATT
CTATGAGTTCATCAA
TAAATTATAGCTTAAATAAGAACTCTATTCCAATACGTGTTTGAT
GATATTAAAAGCCAT
TTTTTCTATTAAGCGTATTTGTTTTATTTGCCATCATAATTTCCAT
AATTGACCATATTC
GATTTTTTTTATTTTTGTTTTGATGTTGTTTCGATTTTTTATTGAGA
TCATTATCGATTG
ACGATTATATGGTATAAATTATTAAAAAACTATGAGTAACATGC
ACGATAAAG
>ConsensusfromContig2510 Average coverage: 17.11
CCTCCAATTTCTGCCTCTCTTCCTCCCTCCTCTCTGCCTCCTCAAT
CGCTGCTAATCGCT
CTTGCTCTTGTCTGATTCGATCCTGTTCCATTTGCTGCAGACGCAT
ATCGTCCATTCGTC
GTTCGTACTCTTCTTTCATCCTCCTTTCTCGCTCAGCCTCTCTCTTC
ATCTTGTCTTCCA
TGCGAGCCTCTTTTTCTTTCGCATCTCTTCTTTCCTGTTCTTTTTGC
AGTTGAAGTATCC
TTCGTTTTCTCTCGGCCTCAGATCGCTCCTCCTCCAACTGCCTCTC

CATCTCTTCCCTTC
TCGCTTGCTCCTCTTTCTCGCGTTTGATTGCTTCTTCTCTCTCCATT
CTTTTTCTCGCAA
CCTCTTCTCTTTTCTTCGCCGCCCGCGCAGCTTGTTTGGCGGCTCT
TGCGTCCCTCATTG
ATATCGATCTACGCCTGGATTCATCCTCTGTGTCGGTCATTAGAC
TCTCAAGATCAGTAT
TCTTGTTATCGACGGACGCCCTGCTTTTTGAACTCTGAGTTCGCTC
CGTGATTGGCTCCT
CAACGACCTTGTC
>ConsensusfromContig2511 Average coverage: 11.37
TATTTTTCAATGAAATAATCAGACTTCAAAATGACTGAATCACAT
GGATTGACTCAATTC
CAATTTGGGTAAAAATCCATGCTAACTTCCAAGCCCAGAGGAAGC
GAGCCTTTGACTATG
ATAGGATTCCCATCAACTGCAGTCAGGCCACAACCTGACAACCAC
TGCACAGTGCTACGC
GGAGGACCTGGATATCGCAGCTTCTGCCATGTCCACTTCTGAAAT
CCAGCAGGAATTCCA
CGACTGCCTTCTGCAGGGACATCCTCAAATATCAGAGAATAGCAT
ATCCCGCTCTGACCA
AGACAGCACAAACTGTTGCTAAAGTCGAACACCGATTTGAATGCT
ACGATATCTGGGTTC
CGAAAATTCAGATAGCCTGGTTGGCATGCGACGTATTTGGTGCTG
ATAGTAGTATCCCGA
AAATCGAATCTCCGAACAGAAAAATCGGAATTAACAGCCGCAGC
GAAGTCACCAAAATGA
GCAAGGCCCTGGACGGTGATCTCATCGTCGCTGAAGTGCTGCCAA
GTTCCTGCTTCATTG
AATATGTTGAGCTTCACGTTCACATTCAAATCATGCGAAATAGAG
ATGGCCTTGCCGTCC
TGAATCGCAACATGGGTGTTGGCCGGCACATGATTGTCAAGGCTT
AGTGTCTCCCATCGT
GTAGATCTATCGCGCA
>ConsensusfromContig2512 Average coverage: 29.11
TGATAGTTTGAACTCTTCGAATAAATGTTCTAAATCGGTTCCATC
AGAAAACAGATCACT
TCAATCAACAAGTTCTTTCACAAATCCCATACAATTAATGGCAT
CTGTAGAAATAATTC
ATTCATAGATGCTATCTCGTTCAACAGTTCTAGTTCAAGATCATC
CGGAAATTTATCGAA
AACTCCTTTATTATCAGAAAGACAAAATCAAGGAGATAATCCGAA

TTCGTTAGACTGTTT
TCCGACCGTCATTCCGCTACCAAACACACGCGCTTGCAATGCCTC
CATCAACCGTCACAT
TGCCTCAGGTGAAGGTCAAGCAACAAAGGTAAACTCGTCGCTAG
AAAAAGTTGCACCCGT
TCCAAACAAGTGTTTCCCAACGCTTCCAAACCGCCTCGCTCACGG
AGAGCCCGATGAACA
GGGCCCACCTCACTCAGATGCTGATGATGGCCAGCCCGTATCTGA
TAAATGCGATGTCTA
CGAGTCTTTACCATGTTCTTCGAATGATCTCTCCAGTCTTTATGA
CAATGAAACTATAAA
AGATCCAAATCGTTTATTGAAAAAGTCCAGCGTTTCTTATAATTT
GAATGCTTCAATAAT
TCCCAATGATCGGCTTGATAGAGTGCACTTATCGAATGCTGATTC
GAATGATATTTACGC
ATCTGTTTCGCTAACACC
>ConsensusfromContig2513 Average coverage: 13.21
TCATTATTCAATTTGCGAACATCATTTCGTGGTGAAGTGTTATTGT
GGTTCACCGTTGTG
ATGTATATAATTCTTCTGGCAATATCCTTCGGCAGAGCCAATTTA
CTGGAGCTTCGGCGA
TTCTGCTCGCCATTCTTCTACAGCAAAGTGAGCGCACCGACCTGC
TGCACTCCTGCCATA
CTCTTCAGCCGTGAATTCTTAGAAAAACTCGTGAAACACCTCAGT
TCGATCAAGTGCAAA
AAAGGCTTTCCGAAAGATGTCGCCTTACACGATCTACGCTTTCAA
ACTGGAATGCCTGCT
TTTTACATACAGCCGAATCTGGTATATCATATTGGCATGCAAACA
ACACTGAGGGAAAAT
GAAATAATTCCTGATATGAATGTTCCTATTTGAGACACAAAATTT
TGCGTCTTTGATACT
TCAAGAGCATTCATGCCGGATATGGTATATCATATCGGCATGCAA
TTGACTCTTAGAGCA
AATAGA
>ConsensusfromContig2514 Average coverage: 13.01
CTGGCGTGAGAGCTTCTCAGATCTTTTAGATATGAGATTTCTGCA
GCATCGCCGTGTAGT
GTCACTGCCAGTTTTCGGCGATGCTCATCTCCAGGATTATTGAAT
GTGAATTCGTCTGAC
TCTTGGCTGTCTACAGTTTCGTTAGCAGCCCACTCCTCGACCGTAG
TGATTGAAGTTTCT
GGTCGGTCTGATATTGCTGCTTCCAAAGCCGACGAGGCGAACACT

TTCTCTTCACGTGCT
CTTTTGAGTTTGGCAGGAAGACTTACATGAAGTTTGTTCCTTTGC
TTCCGAGCAAAGTGA
ATAGTGGCGCAGGAAAGTAAGTCGATCCGGGTTTCGGGGCACATC
TCCTTAGTGATCTTA
TGGAAGCCACCAAGGTA
>ConsensusfromContig2515 Average coverage: 10.87
TGAGATCATCTGGAACACAGACATTGTCTTCAAAGACGAGGAGG
GAACGGGAGCAGACCG
TTACATGTCATCGAGGTGTAAGGACAAACTCAACACCTTGGCAA
TAGCTGTGATGAACAG
ATGGACAGATGTTCGTCTGCGCGTGACTGAGGGGTGGGATGAAG
ATGGAAATCACGCTCG
TGATTCGCTGCATTATGAAGGGCGGGCCGTAGATATCACAACAT
CTGACAGGGACCAATC
AAAATATGGGACACTGGCGCGAATGGCTGTGAATGCAGGATTCGA
TTGGGTGTTCTACTA
TAGCCGCACCCACATTCATGCCTCTGTTAAATCTGATAAAGCAGT
GAGTACGTTATACGG
TGGCTGCTTTCCATCTTCTTCACGAATCCAGCTTCAAGATGGCCG
CCACATCGCCATAGG
AGCACTCACACCTGGCGATCACGTGTTAGCGATGGACCAATCGGG
AGCGCCAGTTTATTC
CGCATTCATAATGCATATGCATGTTCAATCATCCAATGAGAAGTC
GTTTTCTCAGAATCA
TGTGATACAAATAAAAACAGAGCTAGATTTTACTTTAAATATTTC
TCCAAATCATTTAAT
TTTTGCCGTCAAAGATCAAAGTCATCATACGGCCATTTTGGAAAA
TTCCAAGTTCGCACA
AAATCTCGAAATCGGAGAGTGGATTCAAACATCGTGGAATACTTC
C
>ConsensusfromContig2516 Average coverage: 16.91
AAATGTTTTGCGTTTGTAAATTTCATCATTTTGCTTTTGTTTTGAT
GATAATGAAATGAT
CAGTATTTGATTTGGTGGACAACACTTTATATAATAATCAAACTG
CTTCGAAATCGAGTA
ACAAATCAAGGAAAAAACGATACTAGCTCGTGTTCTGTTTTTTTCT
TTCATTTGTTTGTT
GACGCAATTTTTTTTGCTTAATTTAATATAAAAATTAATTATC
>ConsensusfromContig2517 Average coverage: 7.26
CGCCTGTTAGGATTTTTTCACACCATGGGAATTTTTCCCCTTTCGG
ACGATTTTCTTTCT

GTTCTTGGGCGTTCTTGAAATTTTTCTTCTCCAGCATCATCACAAT
TTCTTCAAGATGAA
TCCAGGCTGTGAGGGATAATGTTGTGCTGGTCCACAACCAATCTG
TGATGATGCGACCCT
CCAGAAGGAACGGGATCAAATTGAATACATTATAGAGAGCAAAG
TTGACAGGATTCAAAG
AACGAGTGAATACGTTCCTCTTTGTTCTTTTTGGATAACCACATTT
TATTTGAATTGCCG
ACATCAAAAGGTATGCTACTTTGAAGCAGTACCAAATCTGTGCTA
CAAAGTTCCGACGAA
ACTCGTATCGGGCAAAATTTGTCAGGCCAAAAATCATCCAGCCGT
GTATAATCATCACCG
AAATAAACATGAAGATAAGCTTAACAAGAACTTTTCTTCTGAGAT
AAATCCATCTATCAA
ACATCATGAGACTTAGAGATACCACAAGGGCCCCTATGAAGGCAA
ATTTCACTTTATTGT
CA
>ConsensusfromContig2518 Average coverage: 65.44
CTGAATTACAACATAAACAAATCTAAAATTAATTTTTAGAAAATT
TATTTCAATCACATC
AGTTTCTTTATATTCAATCAAAATCTTTATTATGAGAGTTGATAA
AGATTTAGACCCACC
GGCACCTGCTGGGACGGTACAGTCTGGAATACCAACCTCCATTCT
GTATGGGGGTTCGAC
TTTCACAGGCCATCAAAAGAGCAAGGGAAATTGTTATGATGTTGA
AGTCATCCTTCAGAA
TGTTGACATCGCAGCAAGCTATGTCTGCGGCTATCTGAAGATCTA
CCATCTAACGGAAGA
ATTCCCGCTTCTCACAACCTTCTTCGACGGCGAAATAATCTCCAA
GAAATATCCGTTCCT
GACCCGGAAGTGGGACGCGGATGAAGATATAGACCGACGCCACT
GGGGGAAGTTTGTTCC
GTTCTACAGCTACAACAAGACATTCAACGATCACGATTTCGACTA
CGACGAGCTCGCCAA
GTCCGACCACGTCTTCATGCGATGGAAGGAGCACTTCCTCGTGCC
CGACTACCGAATCCG
GGACATCAGTGGCGCGTCCTTCGCCGGGTTTTACTACACGTGCTT
CCAGAAGTCGACAGC
GACGATCGACGGATACTACTACCACCGCAACTCAGAACAATATCA
ATCTCTCAACCTCAC
CCACGTACCTGAACACAACACAAGTGTGTACGAAATGCGATGAGA
GCCAATCTACTTTCT

TCATTATTTATATATACATTAATGCATTTGTTTGTTTCTGTGTTTTT
TAGAACGTTTTAT
GCGAAAATATATTCATTTCAACAAAAAAAAAAAAAAAA
>ConsensusfromContig2519 Average coverage: 21.77
TCAAGAGTCTTTCTGAAGCGCGAGCCGCATCTCTTTTTTTCCAACC
ACTCGAATGATTTT
TTGTCGCATGCTGTGATTGGCGCTCATCGGTGCGCGGGGCGTGTC
GTAAATGACGTCTTC
GTCTTGTAGCTCCTCCCTCTCTTCTACGTCGTCTCGCGCTTCAATA
GCGGCGTTGACGAT
AGCGCTTAATTCATCCTGGGAGCTGATATCTTCAAGGTCACTCCC
TTCCCCGTTATCATC
TGCTACAAGGTCTTCTGGAATATCCGCTGAATCAGGCTGGATGAG
TCTGTCTTTGTACAA
TTCGTCGTTTATTTCTTCGGGAATATCTTCCTCCATCTCTGAGTTT
TGCGAGAGGCAGCG
GCGCAGCAAAGGTGACTCCGCTTGGCTCTGTTTTGACCT
>ConsensusfromContig2520 Average coverage: 49.86
GAGATAAAACTGCCAGAGGCTGTCCAGAGAGTTGTCGTATTGAAC
AGACATTTCGTTTGT
CGCCAGTAACCAAGAAGCAATATGATCCCTACCCACTTCTTGCTT
TTCACCGCTCTCCTG
ATCGATTGTGACTGTCGATGTACCGGAAACTATATCTGAAAGAA
GACTGGATGCACGCAA
CTCCGAACCACAAGCATCAAACCCAATAAGACCGAGCGTGACAT
TCGCAAGTACGACAGG
GCCCGCTCCCGAATT
>ConsensusfromContig2521 Average coverage: 4.20
CCAGTGGTGGCATATTGCTTACAGGTGTACTCCAATCCGAATTGC
AACTAAGGACAGAAC
CTTTTCGATAACTTCTGTGAATGTCTTGAAATCCAGTGGTAGCAT
ATTGCTTACAGGTGT
ACTCCAATCCGAATTGCAAC
>ConsensusfromContig2522 Average coverage: 32.45
AAGAAAAAAATGATGAGGGTGATCATCTGACTGAGGATATACTT
CAAGGTTTACAAAGTG
TAGAACATCAGTTTTCTAGTTCTAATGATAGAAATATAGCAGGTG
CTATGATGAGTGAAA
AGTCATCATATGAGACGCCTTTACATGAAACCAGTGCAGTTGAAA
ATTACAATTCAGATT
GTGGAATAAGTCTACATGCAGTTATGCAATCACAAGTGCCTCATC
CATTTGAATCAAATA

ATATGAGTGATGACTCGAGAATGTATCTGAACATGCCACAAGGA
CAAGGTGGCTCAGTTT
TAAAAGT
>ConsensusfromContig2523 Average coverage: 46.79
TGCTCTTCCGATCTACGAAACGTTCTTTTAGTCATAATAGATTGA
TATGGTTGAGACCTC
TTATTCCGGAAGCTCCGAAACAACATGCAACTACAAGATCAAAT
TGATAAACTCGATAGA
TGCAGCATTCTACAGATGGGGTCTTTTTGTGGCATCACACCCGTG
GCCGATTATAGTGAT
ATCATTCCTCATCCCCCTGTGCATCGGGACTGGATTTATATACAT
CGAGTATGAGTACAG
AGACGAATATCTCTATCTCCCGCTGGGATCCCCCAGTATTGAGTA
TCGAGACATCGTCAA
GGAGCTCTTCCCCAATTCGATTGCGGTTGAATCCGCCCTATTTGT
AGCGGATGATGTTCT
CAATCCAGATATCCTCAACCAGCTTCATACTTATATTGAAGAAAT
TAAAAACGTGGAGTC
GAGGAATGGAATCAAATATGAGGATATATGTGTGAGATATAATG
GTGAATGCAGATATCA
GTCTATACTTGAGTACTGGAATAACAATGGCGACGTCATACGCAA
CCTGACTCGTGATGA
TATCTTATCTACGATCAACAGTATTTCAAATGCGAGTAATTATCT
GCACTACCGTACCCG
AGTGGCAGGTTTGAGTGTTGATGAAAATGGGGAAATAAAAGGAG
CAAGGCTGTTAGCTAT
TATATTCACTGAACCAATCATATTTGACGTGGTGGAGAAAGGTAT
TTATAAATTTCCTAA
TGTATCGCAATGGGAAGATGCTTTCATTGATTTCAACCTGGAGTA
TAGAAAGAAGAGTGT
GGCCGACTTTTATTTCTTTTCAAGAAAGAGTTTCTCACAGGAGGT
CCTCAAAGCCACTCT
CGCCGATTTCAAGTTGCTGGCCTTGGGGTTCTTACTACTGATAGTG
TTCTCTGCTCTGGT
TCTTGGGAAACTCAATTTGCTACACAACAGAGTTCATCTGACACT
ACTGGGAGTGATATG
CATCGGGCTCGCCGTCATCGCGGCCATGGGAATCTCCTGTTTCCT
CGGTGTTATATATGC
ACCTACCAACATCGTCTTCACTTTCCTCATTTTGGGTCTAGGTATG
AACGACATGTTTGT
CATTGTGGAATCCTTCGAAGCACAACCGTCGGAGCTCCGAAGAT
CCTCAACCGAAAAACG

AATAGCTTTGGCCGTGCAAGACGCTGGAACGTCCATCACAGTCAC
ATCGCTCACAGATCT
CCTTGC
>ConsensusfromContig2524 Average coverage: 6.98
TATGGATAGCATATTCAAGGAAAATCATATTATGACATGTCAAAT
TTATGAGGCATTCTA
CAAATGAATAACAACATGGTGATTCCTTCTTATACCCAATACATC
GAAACAATCATCATG
CCTCATCCGGTGAATTTTCCGCCAATTGGGCCAGATCAGTTTGGT
TGATCTTGAGCGTTT
TGGGAAGCTCCCCATGATTGAGATTCATTGGATTCCAAAGGCCGG
TGTCTGGTTGGTAGA
TCGGAAG
>ConsensusfromContig2525 Average coverage: 30.28
ATCATCCACTCGTAATCTACCAATTTTCAGTTTTCCATATCTCTCA
GTTTTGTCTTTTGT
TTCCCAAACTCCCGAGGCGCTGTAGCTTGGAATGCGATCCATGCC
TCATCCGACTCGACA
CTGTTTGACCTATATCTCCTATCCCCATCTGGCAGCACTCTTTAT
CCGGCGAGTGGAGCG
AACCCATGCTCATGGATGACATAGAAGAGGAGAGGTTGAGAGTG
GCAGTGATTCCCAAAG
AGCTCGGGTAGGTCATCTCAAGGTCGTCACCAGGGAGACACGTTT
CAACCATCGCTTCAA
GACAATTGACGAACAGCTCCGCGTTGTCTTGGTTCTGACATGTTT
TTGTGAATGGTCCAG
CGAACCGCCACAAACCTCCAAAACCA
>ConsensusfromContig2526 Average coverage: 6.95
CAGATATACAAAAAGTAATTTCAGATGCTAAAAATTTAATATTAG
CAAAGTCGAATCATA
ATTTATTCACCAAAGATCAGACAGGAATCTCAAATAATGAAAGG
GAATCCAAACGATTAT
TTAAACGACATATTAACGAAGTAAAATATAAAAAGCACGATTTAC
TTCCGAGATCAGATT
TTTATGCCAAACAGATATTGAACATTCATCCATCAGTGATGCGCC
ATCAAAAACGTATGA
ATCCTGCAACTGGTGAAAAAATGAAAGGTCTTTTCATGGATTTCA
ATATACCGATAAACA
AAAATGATAGTCATGAAGTTAGCATAAGAAATGGAATTTTAAAA
CTAACAACAGTGCCAA
TCAACACTCGCCGCCATATACATAAAAAAGTTTTACTCAATATTT
ATTCAGTGCAATCAA

AATTTAGCTCATATGGGTCGCTAAAACTCATCGGCTCATCATATG
TCAATTCTCGGAGAA
GATATGTCACCAAAATTAAATTGCGCTCTGTGGTCCAAAAATGGG
TAACGAATAGTCAGT
CAAATAAGGGGCTATTTATTCAATGCGCTAATTGCAATGTTCAAA
AATTACTAAACTTTG
GTTCAAAAAAACGAGAATATACATTCCCGGATAGTTTCTCAAACG
AAATTTCACAGAGTA
CTGATGTAGCACGGGGCTCGAGAGAAAAGTCATATCCTCACATA
CTCGAAATAACTAGTC
AATATCGACCGATGGAGGAACCAAGACCAAAACGAGAATTGGTCC
TTTGCTCTTCATTAT
CTGAATCAAACGTGGAATTGCAATGCTGCCGTTCCATGATTCGTG
TGACTGCAGAAGAGC
TGGGATTCGG
>ConsensusfromContig2527 Average coverage: 7.98
CTCATTTTGATTTCATCACAGCCAAAAAACAGGTGGTTGACGCGC
TGTTCTACGCATATA
ATCATATAACAAACGATGAAGATCGGGGTGGCATTACGCAGGCT
ATTCTCGACGCCATGC
ACCAACGCCCTCTTATTGACCTTGAAGCCCCGTTTTTCATCAACA
GCTACCAGCTGGCTT
CTGGAGCTCTGAAAATCCAGGCGGATCTTCTCGTCAAAATCGCGC
GTACTCAGATTGCGG
AACAACGTGATTATCAGAACAAGATAAATGAAAATAATGGCGAC
GAGAAAAATTGCGATT
TTCGACAACTCATCAATTTGGTTTCGCCTAAATGCGGTTTACCTG
CGGTGGACTTTATGG
AGTTTCAACCTTCGCTTTCTGTTCTAACGCAAATCCCATCCGTGT
TACAAATGGCGTATG
AGGAACTCATCCGGGTGAATAAACCTCGAAGCACGACGGATTAT
CTTCTTATAAGGAAGA
ATCTTGCAAGTGCCGCTTTAGAAGAA
>ConsensusfromContig2528 Average coverage: 23.62
GAAGAATAATCTATTCGGACTCCGAGGATGAAGACCTGCAGGAT
GAAGATAAAATCCAAC
CTAAAGCACGACCTTACAATGTATGGAAGCCAGAAGAAACTGCA
GTGGTGAGAAAGTTCT
TCGCCAAATACATTGATGAGGAGAAGGTTCCCAAAAAGGACATGC
TCATCCATTTTGTCC
GGGCAACTGACTGCCGCTTCGACTGGAAAATCGTTAGGAACAAAG
TGTTCAATGAATTTC

GAGAGAAAAAGAGAAAAGGAGGCCGATGGTTAGAAAATGTGGCT
GTATGAAGAGGTTTTG
AAATTCTCTTGTTTTCAAGATGTATTATTCTTTGTTTCAGTGTGAAC
TTTTTTTTATTTA
TTTCAAGGTTTTGATTGAATACTTACAATGGGATGGAGTATTAAC
ACATACCCG
>ConsensusfromContig2529 Average coverage: 19.67
TGAAAATTATAATTCTAGTTTTCATTTGCGGTCTTCCCGCAGTAC
AATGTCAGAAAGAAA
GCGAAAGTACTTTGCCAGGGGCCACTACCACATACCAAGGAAAA
TCTGAGTATGAAATTC
AGGTCAAAACGGGTGACGAATGGTACGCCGGGACGGATGCCACC
ATCTTGATCCTTCTCA
TCGGAAGCAACGGTCAGACGAGCAAGCGAAAGCTAGAAAATCCC
AACCACAATGGATTCG
AGCGATACAGTACAAACGAATACAGCTTCGTTGATAGAGATATC
GGAGATCTGGAGAAAA
TACGCTTGTTCCATGACAACTCCAAAGATTGCTGGTTCGTGGACT
TCGTCATCGTTCAAA
ACAAAAGGACTGGGGATGTGACAAAGTTTACCTGTAAGAAGAGA
GTGTGTGGTGACATGT
ATCATGAATTCAATCGTGAATGATTATGTTTATGAGATTCAGACT
TATTTTACAAATACC
CGAGATTCATTAAAAAAT
>ConsensusfromContig2530 Average coverage: 9.03
CACCATATTCCAACATAGGATATCAATCCAGCCCCATTTTGATAA
TAGATGTACGGTCCT
ACGAAGAAGATGTACTCTCCCGAATTCTGTGCTTTCAATATGGCT
GTTGTCCCGCAGGGA
AAGTTCGGCAAAACGTCGGTTGCCGGCCTCGGGTTCAGGGGACTG
CTGCTGTAAGTGTTG
TCGCTCTCCAACTCCACTTCGTAGACCAGATTGTCTTTGAAAAGG
CCGAATCGACGTGGC
TGGCTCTGGTCCCAGGAAATGGACGAGGCTGCGTCAGGATAATCT
GGAATGTTGCTCTCA
AAAATGTCCCGGTAAGAATATTGGGAACTTGAAGAGTGGCATTGA
GATTCTACCATGTAC
AGACTCATGGACTGTTTCCGGAAACACATCACCTCCGAGCCAAG
CAGCAAGAAAT
>ConsensusfromContig2531 Average coverage: 34.18
ACTGACTTGTAACAACGTAGCTTGCCAGAAAGGACGCGCATCACA
ATGCATTGGTATTAT

AGCCAGCACAGACTGCGAATGTTTTACCGGATGGAAAGGAACCA
TTTGTGATACACCAAA
TGACGCTCCCAGTGTCCAATTCCGACAATGCGGGAACAAGGAAA
ATTGTGGACTCAAGAC
AGAAGGATGCGAAGGAGATTTCCACAATCAGTGCAATTGCGCCG
AAAAATGGGGTGGCCC
TGAGTGTCAACAACATGACTGTTTCCATAGTAGCGAACATATGAT
CTGTGCACTCGGTCA
GGCAAAAGGTTGTGAAACGGGTGCGGCTAAATGCATATGCCATA
CGGGCTGGACTGGGGA
CGCCTGCTCTGATACCGATGGAGATCAAATACGACACTGTAATGT
AACAACGGATTGTGG
AACTCACACGAAGGCATGCAAGGGACCTAAGGGGGAAACGTGCG
ATTGTGAAGAGGGCTG
GGATGGCCAAAAATGTACAAACGAGAAAGAAAAA
>ConsensusfromContig2532 Average coverage: 15.85
GTGAATGAAATAAGATGAACATTTCAGAAATATGCCAAAACCGA
CTATTGAGTCGGGGCG
AAAAACAGATATGAATCGTGGAAGTGAATTTAGCGTTAATTCAAT
TCTCGCAATGTCTTC
ATTTGGCGTAAAATAATAACTGATAATACAGCCCTGAGCTATAAT
AGACACAAATTTTAA
ATTATACTTCAGGATATTCATCAAAATTGTCTCTCGAATAAGGATG
TGTACATTTTTTTA
AAATTCTCATACCGATGATGCTGAACTATTTTCTAATGAAATCTA
GCGCAACAAGTGCAG
CGCCATAAGATGCACACAGCGCTTCAGCAGGCGCAATATTCACT
GGTAGACCGTAAACGT
TTTCGAGATATGAAATTAAAAACGGTGCTCTATCGAACATAGTCC
CAGATACTTTCAATC
GCTCGATACCAGACATGATAAGATGCTCCGGAGGCATGATGGTTG
ACATATTATCTAAAA
GCGATTCTAATGTTCTTCTGAATATCCCTGATAAACTCAGGTTTT
TAGGACTCAGGCCTA
GCATAGAAACAGTTCCGTTTGGTAAATGTCGTTCGCCAAATAATT
GCGGTATTATTTGTA
GAGAAGTTTTCATATCGTTCGTCTCAATGTCATCAGCCTTCAAAA
GTCGGTCCCACAACT
TGCTCTCATCTATTTCAATTCCT
>ConsensusfromContig2533 Average coverage: 18.01
CTATTGCGAAGGCACCGGGAATATAGAGGTAACCGGAGAGTGTT
CTGAGGGGTATTATTG

TCCACACGGACAAGCGGTGTCTTCTCCGATGGAGTATTCATGTCC
AATTGGTCATTATTG
TCCATCGGGTTCTGGTTTGCCCCTCCCATGTCCTTCGGGTCAATA
TCAAGACGTTGCGTC
GATGAGCAATTGTTCTAACTGCCCACCGAGCAAGTATTGCGATGC
GGTTGAGACGGCTAT
AGCGCTGAACGTTTCTAGCCACGGTGTGGTGCAACCTCTGGACTG
CCATCCCGGCCACTT
TTGTCCAGAGGCCACAGA
>ConsensusfromContig2534 Average coverage: 13.70
CTGGGCTCAGTGGATCTCCCGATGATCCATATCTCTATTTTGAAT
CGAGTTTACCCGTAC
AGCCACAAGAGAGAGCCAGGATTCAGACGCCAAGACTATCAAGC
ATTGGGGAAATTTGCG
TAGTTTTTTATGCGCATCTATACGGGAAAGATTTAGGGACACTGC
GGGTCAACTTGGTTT
CAGCATCCAATAACAGAGCGGATGTTTCCAGTATCAGTGGGGAG
CAAGGAGACGAATGGA
AGTTTTATAAAATCTCAGCGGTGTTATCAACTAATTCTGACCGAA
TAGAATTCGAAGCAG
TAAGAGGTGTCAGTTTTCAATCAGATATCGGGTTGGAAAATATCA
TTATATTTCAAGATG
TGTGCGGTAATGTAAATGATGATGAGATTGCCACAACGACAAGT
ATGCCACTAGCTGAGA
TTCGTTTGCCGTTTTCTTGCGGCTTCGAAAAAGAAGACTTGTGTG
GAATGCTCCAAGTTC
CTGGAGACGACTTTGACTTTACTCGACATCGCGGCAGCACGCCAA
CCGCGCAGACCGGTC
CCAATCAGGGGGCTTTTAATGGAGACTACTATATATACATTGAAT
CAAGCTTACCAAGGC
AGCAAGGTCACATTGCTCGAATAGAATCACCTCCAATTGATCACA
CTGGAGCCGTGTGTA
TGACTACTTATATCTATTACTATGGAATTGATATGGGAGAGGGTG
CTGCGTACAGAGTCA
CGTATTCAGGTTTCTATCTGGAAGAGAAGAGTCTGACAGATAACC
TCATATCTCGCGAAT
CATGGGTACTGCGAACTGTTGAATTCGATATCAACAGAAACGATG
CTATCGGTGTTTTAG
CCGTTGTCGGATATGGTATACGCGGGGATATGGCTTTTGATAGTA
TAACTATCAGAGCTG
GAAAATGTGATTCAACCGGTCTACCATTCGAATGCAGCTTTGAAC
AAGATCTCTGTGGGT

TTCTGCAGGCCAGCAGGGACCAGTACGACTGGCGAAGGCATTCC
GGTCGTGTACCAACTC
GCA
>ConsensusfromContig2535 Average coverage: 7.27
GGAGCACCTGTCTCCTCATGGCAACGGCCCTGGGGATGCGCATTA
TTGTAGTAGTAATAA
AAATATTAATAATATGGATTCTCTTAAAATCATTATTGATTTTTCT
CTTGAGTTTAGGTA
CGATGAACAAAGACTACCGAAGAGCAACGGTCAGCTGGAGATGT
GTTTGGCAGAGCTTTA
ATTGAGATTTTAAACAAATTAATAACAAAGTCGCATTATATACTG
AGACATCATTTCGAT
CGCAATGTCGCTACGTTGTCTCTTTTTGTTTGTCCTTTTGTCAGTG
TGCGTGTTTGTTTG
TTTGTCCTCTATTTTCTCCTAAACTACTGAACGGA
>ConsensusfromContig2536 Average coverage: 14.23
GCTATCACATTCGACACAGAATACTTGTCTGAATTTTGTACCTGG
CACGATTCATCATCT
GTTGTTGTTTCGACACAAGACTCAAAATCATTCACGATTATTCTA
TTACTATTTCGATAA
CCGAGATGCATTATAGAGGATTTTGACATTTTGCCTGAAACAATT
TTTCGATGCCGCTTG
AGTCGGGCTTTGCGTTTAAGAGCGTTCTGGCGTCGGAAAGACCGC
TTAGAATTGATGCTG
ATGATGGTCGCCGTATCGTTAAGGCTTTTGACGAGGCGCTCAGCC
TCTTTAAGTGTCCTA
GAGTCTTCATCGAGGTCTGTTTCATCTTCCGTCGTCGTAGCTTCTT
TTTCAGAGTCGGAA
TAGAGACTCTTTTGTAAATCGCGCTTTGTGTAAGCGCTATATATA
TCAATTGGCGTTGAT
AGCGCTGGAGGTAAGTTGTAGAAGCAATCTGTATTTGGGGCGCCA
AAGCGTTTTTTACTG
AGAGGAGATCTACCGTGTTGAGGGTTTTGTGGGGGCTTGTGATA
CGTCGAGAGAAGAACG
GGCATTTTGCACTTTTCAATTGAGTGCTTTATAATTTCAAGTTTAT
TCGAAGCAGTTCGG
CTGTCCTCAATTACTCCACGGGTGTTCTCGCACTTTTTTACTCTTC
GTCGTTTAGCACTA
GAAGTACGTTCAGACACTTCTTTTGATTTTATGACAGAATCATTTA
TTCTTTTGGAATTC
GCGCGTGTTCGTACACTTGATGTAGACTCAGATGTCTCCGTGTCA
ATCAGATAGAACACT

CAATCCTGAAACATTCATGCCCAAAATATACATGAAAT
>ConsensusfromContig2537 Average coverage: 10.46
TCAGACACCCGGTCTTACATATCAAAGGGAAATATTCTCAACATT
ACTTTCACCAGCAAT
GGTCATGTTGATGATGAAGGATTTGTTCTTTTCTATTTCTCAGCTT
GGCACTGTCGTGAA
CAATTCGACTCCCCGGCTGAGATCGCATCTCTAAATTGGCCCCTC
AGTTATCCAGACTCC
ATCGAATGTTCTTGGAGAATCGTCTCTCCCAAAAGACAAAGAATC
CGAATTCAATTCACG
GATTTCGACCTGGAGCAGCACTCACTGGGTCATTGCCATCTGAAC
TTTGACCACGTGCTC
ATTCTGGATGGGGGCAACACCGATGCACCCATAATAGGGCTTTAT
TGCGGGTTTCTAGAA
CCCTTCACCGTTAGATCCACAGGTCAAGACATGAAAATTGAATTC
AAAAGCGACAGTTCG
GAATCCGGCCGTGGGTTTCACGCCTCTTTCGACTTCGAAAGCATC
ACACAACTACTCTCT
TCGACCAAAGATGAGGTCCGACGAACGGGTCCTTCCAT
>ConsensusfromContig2538 Average coverage: 8.16
GCGCTGTGCTCTCAATGTATCATTGCATATGATAGTTTGTACAAC
ATTGAAAAACTTTCA
ATAGTCATCAGTCTTGGAATATTTGCGGTTTTTCGAATTCTTACTT
CGACTACGATTTCT
TGTGGATGATGAAAATATATTGTGTCTGAGAAGAGCTTCGATGT
TGTACAAAGATGAGGA
CGTCTGCTCTTCCAGATGTCCCAAACGTTCATCAAAATGTGATATT
TGTCCCGCCGTTTC
ATCGCGCATCGTCATGAAATCCTCCAACTCCTGTGATGCAAATCC
AGCGTTTGCGTGGCC
TTGCTGAGTTGATTGAAGACTGGAGACTTCTTCCCCAAATCGGTTT
CCTTGTTTTAATAC
TTTTTGTTGTTTATTCTGTCTGTTTAGCAGTCTGTCTATGTCTTTAT
AATGCGATTTATA
CGGATCAATCTGATTCTTTTCATCTTCGTCTGAATCAGATGAGTAG
AGTTCTTTTGATTT
CGGTTTTCTTCGTTTTCCACATGTCTGTTCTTCGTATAACTCGTTCA
ACTGTTTCTGTTC
TGAACTGTCTCGAATGTGCGGTGTGTTCGGTGTGAAAGAATTCTT
GTTCTTTGTGAGAGC
ACTACTGAATGTCATGTCCGTATTCTCAGGTCTTATTTTTGGCTTT
GTACTAGTGGCGTA

CGTAGAATTGTCATCTTTGACAG
>ConsensusfromContig2539 Average coverage: 16.85
AAATACCCCCCTCATTAAAAAAAAAATAACCGGCTGAGTAAAAC
AACCACCACGGCCTCA
CAGCCTTTATTTGTCTCCATTTCCTAGCCTCCATATTGTATTCATA
TAGATCCCAATCAT
AATATATTGCGAAGAAGTTTTCACCATCTGATATCAGATCGTGGG
CTTTGAGGGACGTTT
TAGGACCATCCTCGATTATTGTCAAGTCATCATTTTCGATATCGA
GTAATAATGCTTCTC
CAGTGATGTTCAGAAATAGGACTGTTTTTCCTATCGTTGCCATTTT
GAAATCTATCATTG
AATAATCAAACTGAGATGATTTCCATTCCCATTTCTCAACATCGA
AGATATAATATTGAG
ACATCCCGCCATAAGCAATAATTGAATCGTCGAATTCTGCCACTT
CCCATTTTAATGAAT
CGTAATCAAAGGGTAGAGCTGTTTCATCCCATATTCCCAATGGAA
TATTATAAACATTCA
GACTTTCACTTTTAAACACATATACTTTATCGTTCACAGCCATCAT
TTCGTAAGTATATT
CTTTTCTGTTTGGTATTTCAAT
>ConsensusfromContig2540 Average coverage: 18.73
GAAAGCCAAATATCAGATGTCGTACTTCGCTGTTCATCGAAAAAA
TTGAATTCCATGTGT
TCGCTCAAATTTCTCAATTGTGCATTTCATATTCCTTCGGGTGGA
AATGGGTTTTGGAGA
GATAGCCTGGACAACATCGCTTCCAGACTGGATGGGAATGTTGAT
ATAAGAGACCTCTGC
GTCGCAATAAGTGATGAAACCCGTTGCGAGATGTCAGTTTCATCA
AATCGACGACATGTG
TTTGGCTGTCCGCTTTTCATTGGTCACCTGAAGGTAAATATGAAA
TCTTGCTCTACAAAT
CCCACGACACCTCATTTTGTAAACTGTGACTGCGAAAATATCTGT
CTTGAATGGAGTCCG
AAACTGACAAAAGTTTTCGAGCTCTTCATCAGACCTTCAGTGCCT
CCACAAATGCCTCGA
AAGCGTGCAGGCAGCTCGGA
>ConsensusfromContig2541 Average coverage: 75.72
TATTCAGTGGAAAACTGTCGACATATCTTCAAAGACTATGAAAA
ATAAGGAAGGCGAGAC
ATCTAACAGATTTGTGATTGAGCCTTTGACGGATTCTGGTAAAAT
ATGTGTGTATGCAAA

GCACACACAATTCATTGAAGTCCCTTCTAGCCGGATCTGCAATGC
GAAACCTGATGTTTT
CAAGGTCGCTGAAGCCACTGAAGGACTGAAGCCAGGAGCATTCG
TCTGCAATGTCCTTGA
CCCGAAATCATCCGATAAAACTCGCTCTTGTTTTCAAATGGAGTC
TACCAGCAGCGGATA
TTCTGAGCAAGTTCAAAAAGTGCCGGATCTTGATGAAGTGGTCAT
TAAACTTTCGAGAGA
ATTACTGAACGAAAAGGTAAATCCTGACTCGTTCGATTATGATAT
TCAGTGGAAAACTGT
CGACATATCTTCAAAGACTATGAAAAATAAGGAAGGCGAGACAT
CTAACAGATTTGTGAT
TGAGCCTTTGACGGATTCTGG
>ConsensusfromContig2542 Average coverage: 517.19
ATCTGTTATGCTCTTCCCTCGATTCCTCAGCTGTTTTTACATGTTC
TTCGTGATCTTCGA
GCAAGGTTAATTGAATGCCCAAGTCATGAACCACTCGCATAAAGA
TATTGCTTGCCTTGT
AACTCTTCCGCTCGTGCTGCTTCCAAATAGCTGGAGAATCCCCTG
TACTAGCGATCAGGG
AATTCAAACTTTGCCAGGCGCTTTTGGGGATAGGATGGCACACAT
CCTGATTTGTTAGAT
CGGAAGAGCACACGTCTGAACTCCAGTCACATT
>ConsensusfromContig2543 Average coverage: 37.51
CAACGACATATCCCCGCGACGCATTCCCCGTTGTCATGGCAATTT
GGAATCTCACACTCG
CCGACAGGAGTGCGGCAGTCGGCCCCTTTCCATTCGGCGTGACAT
TCACAGAAGCCCAGC
ATGAAGGTCCCTCGCCCATTGCAAGGCTCCTTGCATGTAGTCTGT
TCACACCCGAAGCCG
CGATGACTGCTGTAACAGAGGCATTCCCCTTTCTCGCAAATGCCA
TTCCCGCTGCAGCCC
TTGGGACACGACGTGAGGGGCTCGCCGAGATATTTATCCATCTTG
ATCGAGACATTCCTG
GGATTATCTCCATCATTGAAGAGGTACACAAACCAGAGGCCTTCC
CTCAGCCACCGGATC
TTGGTCGAGTTGCCTCCGTGGGAGAGAGACACTGTCAGCACAGGA
GACCTCTTCGTCCTG
CTCGGCGATTTCCTCTTGGCGTATCCAGACTTGACGAC
>ConsensusfromContig2544 Average coverage: 273.42
CAAATTGATGAGATGATCTGCAACCCGATTCCAATTCCTGGCCTC
AAAGGCGACCCAGGA

ATTCCTGGACCTATAGGGGACAGGGGACCAACAGGAAGTCGGGG
TACACCAGGAGTTGAC
GGCGGACCTGGTTCCCCTGGGATACCTGGCCCTACAGGCGTCAAG
GGTATATCTGGTGAG
GGAACAGGTCCCGCAGGGCGACCAGGCCGACCAGGTACTCCCGG
AATCCCGGGGACGCCT
GGGGACAAAGGCGGCACGGGCAGATCGGAAGAGCACACGTCTGA
ACTCCAGTCACA
>ConsensusfromContig2545 Average coverage: 16.21
ATCTGTGTGGAAGACTGATGATCGGACGATGAATGCTGATTTTCC
AGCGAGCTCTTTCGA
CTTCAGCATCTGAATCCAGCTGTCGTGTTTTTGTGGAACAATGAT
CTCGTCCAAGTCATA
AAAAGCCATGAAGTCAGCCTCATACAAGTACCGGTAAAGGCAGT
CGTTCTCTATCGGAAG
TTGCCCTCTCACAATCACATCATTGACAACTTCGGCTGGTAAGTG
ATGTTGGTGGAACTC
CAAAAAGCCATCACGTACATAACCATCAATGTAGTTGGAGAGATT
TGAATTGATGCTGTC
GTTATATATTATGAATTTGCTGACTCCCAGGATTTTGTGGGCCTC
TATGAAATTCACAAT
CAAGTTAGCATCGTGGATGTTAAAGAGTCGATGTAGACAAACATA
CATTGAGTCGTTATT
TCGGTTTGTTTTTTTTCTTCGGGAATTTGTATTTGGATGGAAGAA
TTGTTTATTTCGTT
TGGGCTAACGAGAAGAGAAACTTTTGAACTTTTTCCAATCTTGCT
TTCAGTTATATTACA
AATAATGAGAAAAGCTGAG
>ConsensusfromContig2546 Average coverage: 18.84
AGTCAATGTTTTGGTCGAAAAAGTGAGGCGGCGTTTCGGGAAAAT
TTTTTGGAGAGGGGT
ATCGAAATTGCCTCTAGCGATGGAATTTGACCCCATTCCTTATCA
TTAACAAGATAAGAG
AAATGCTGGTATATTTACGAGCCGATTGATATATTGTTTGTTTACA
TTTCGTCGCGCCAT
TTTGGAACTACGCATTATCTAAAAAAGGTTGCGC
>ConsensusfromContig2547 Average coverage: 6.29
TTTGGTTCCTCCGAAATCCAAGTCTTTTGTTGATTTTTCACATATT
ATGAATGGAGGATA
TGCAAATTTATTCACACTTCTCGATCCCATAATTGCTACCACTTTT
GATCACAATCCATT
GATTGAAGCCAGACTTTCTATATCATATCTTGTGAAACATTCAGC

AATATCATTGCATCT
GGGGATATGTGAGAACATGTTCTTGAATGCTCTTTTCCATTTGAA
AGAATCAGGCCCATT
AGACATAGCCAATTTCATAGCTACAGTGATACCTCCCAAATAACC
TGGCATCGAGTTTTG
AAGGAAACTTGAATACCCCATCCCCTTTTGGTGATGTCTTGCTAC
TGGATCAAAATGTTA
CATTGAAGGTTTCTTCCTCTTCATTGTGTTTCCCTCTCTCCCGACT
ATCATGGAATCTTG
TCCCCACATCACCTCTTCCAATCGTGCTCCAAAAAGGTCAAAGAA
AGCGACAGCTTTTGA
CATTTGTCCCCATTTGTCTTTGGAATCAGCCTCAATCTTTGCGTCA
AG
>ConsensusfromContig2548 Average coverage: 12.77
AATACGCCATGAGAAGTTGGGATAAGCTATATCATTAGTAGTATT
TTTTTGTTCACATGA
AATTCATTTTGATCTAGCATCTTCATAAGCCGGAATATCAATCTA
TTTATAAGGAATTTC
CACAAAAATCAATCATATTCGAGCTCTAAATATAAGGGTTAATCT
AAAGTTCGTAATCGA
ATAATCAAATTCATATTTGAATATGAAAATTGCAAAACTAGAGCC
ATTCTATTTGGGATT
TCCAAAAAATGCACCCATAATTAGTACCGATATATAAACCAACC
AACAATAAATCATATA
TTACATACACAAGAAATCTATTTTGAACACATTTGAACACTGAAC
AATACTCAAATCCAT
GACAAATTTCAGC
>ConsensusfromContig2549 Average coverage: 7.38
CCGCATGGAGAGGACATGATTTACATATCAACCGATGAAAGAGTC
TATCCTTTGGACGTT
GGGGAGTGTTTGCGATTGGGAGATTCTTTCGATCTGCCTGAAGGC
TATATCATGACTGTC
GCGAGAGGAAATACGATGCTAGCACTTCACCCAAGCAAACCAAT
GGCAATAGAGTGGAAT
GAAGAGGATTTGAAGTGGAATGCTATCAAGCAGTGGCCGACCCA
TGACCATGTTTTGAGA
CAGTCTAGGAAGGGATTCAAGAGTGAGCTGCGAGGCGTTGCCAG
CGATGGAGATGCGATA
TGGTGCGTCTTCGCCAATTTCCAGTGTCTCAAAGTCGAGGGCTTC
GTTGCTGAGATGATG
ACAGATAAGAATCCAATTCGAGATGATGTTGAAGGTCTGATCGA
AGTGAAAATGAAAAGT

GCCTACTTGTTCGAAGGGAAGATCTACTGCAGAACCGAGATAGAC
GGCTCTACCTATCTT
CTGAAAGAGAAGAAATGGATTCTCAGCGAATTCGAAGGAATTCG
TCGCACGAAAGAAAAG
TTGTCATAAGCACAAGTCCGTTGAATGAGATATGTATTGTTATAA
TTTTGTTTGATTTAG
TAAAATCATCATCGAATAGCACCATCTTTGACCCTAAGGTTTTTCA
TCGTGTTTTTTTCT
A
>ConsensusfromContig2550 Average coverage: 25.19
AACAGCATCGACCTTTTCCTGGATAAACACATGAAATGCTAGAA
TGGTGAGGAATTTGAA
GTACATGGGCATGTCACTTTCATTTTTCTTTGATTCAAGCAAACG
ATCGTAAATGGTCCA
AAAATGGTGACTGAATTTTTCGCGAGATATTGGCATGCCATTCAG
TCGTATACGCTCACG
GACAGATACTAAATGCGGGGAGCTGAAAAATCCAGTCGAAAAAC
CGTTATGTCTTAGAAT
ACTCTCCACAAAGGCACACGTGGAGCCCTTGCCCTTAGTGCCACT
CACATGGATGATCCG
TAGTTTGTCCAAATCAACATCCCCCAAACCTATCCGTTTGGCACA
CTGTCTTGTATCAAC
CAATGTGTTGCTCTCTGTATTATGTTGCTGCTTTCTATTCTCTCTT
AACGCCGCTGCATT
TGTTTGTAATGTGTTCAGTGCCAATACTGCTCTGCTATAAGAAGA
TGAATGGTCGTCTTC
GATTTTCATACCATGAGTATTTTTATGATCAGAGGTTTCCATAAA
GAGTGAAGTTAAAGG
TCTGGCTTGTATTTTATATGGGATATGACTACTATATTTTAGATC
>ConsensusfromContig2551 Average coverage: 7.75
TGCAGTTTTATTGTAGCTGTCGTTCAACGCCCGAGAAACATGCCC
AAATCTACATTGAAT
ATTTTTCAAATGAATGAAAAATTCTCTGAATCTAATCAAACAAAT
AAAGATGATGTTCAA
ACAGCTTTTCACATAGTATACCACGCGGGAGAACTTCCATCATGC
GAGCCGTTTATTCCA
TGCACCTGACCCCGATGACAAGATGTTTTGATTCAGATATAAGAA
TGCGAAAATAAATCG
GAGAAGAGACAATATTAGGAGTATTAAAAAGGTCATAATAAAAA
CAAAACATTGGATGGA
ACAGAAACAATTGAGATACGTAGAAATACACGGAAACATATTGA
AAAATTATGGTGTGAA

GAATTTGAGCTGAAGATGAACAATCAGGCTCATTCACTAATTCGC
TGG
>ConsensusfromContig2552 Average coverage: 18.22
CAATGCTTTTCCCATGTTTCTCTAACGATGTGACCTGCTTTGGGTC
ATTCACAATAGCAT
CTGTTGCATTCGAAGATTTTCCTTGTTTGGTTGCAGTATCAATGCC
ATTCTTCTTAATTT
CTTGAACGTAAGGGTTCTCTTCTTCCTCAGTATTGATGCTGGATTC
TATGTCACCGTTCA
TTGAGTCAAGCATGGGATCGTTATTCTGGCCAGGATCAGAACCAT
GGCTTTCATTCTCAT
TTTCTTGAATGTCAAAATGTACTCCTTCTCCAGTATTTATGCTGGA
TTCAATTTCACTTT
CCAATGATTCGTGCGTTGCTGCGTTGTGCTTTCCTGGAAAAGGGCT
GTTCTTTTC
>ConsensusfromContig2553 Average coverage: 24.86
TCCAGATGCATCGAGTCTTTGATGCCGATTTCGGAACAGATTTCG
TCCAGCAAATCATTG
TGAATGCGACCCTCGTTGTAATCAATCGGTGCGAAAGGAACTTGA
CTTTCATCTGCGCGT
TTAGATTTGGATTGAGAAAGATGAACGCCTAAAAGATCGTCGGAG
TCTTCTTCGTCATCG
TCGAACAAAGGACCCGAATCGTTTGCGTTTCGCCAAACATTTGGA
GACGCTCGGACCTGT
TTGAAAGCACCAGTTTGAGGGGGGAGCGACCCCTGCGACCTGAT
GGCGGTAAACTGACTA
CCCCTCAGCGCCGCGCCATCTCTTGTGGAGGCAGACTCCCCAAGC
CCAGCT
>ConsensusfromContig2554 Average coverage: 17.72
GTGCTCTTCCGATCTCGGAATATAGATGGAGCCTGACAACGAAT
CCACAGACACGATAGA
TGACGCTGATGTTGGCCTATCCCACACGTGACTTGGAGGGGCCAA
CATGGCCGCGGTAGC
GACTGAAGGCGGACGTGACATGTCCATTTCGTTTCCAAGATTTTC
CGCACTCGAGCCGGG
CCGCTGAGTGGCGGCGAATGTCACGGATCTAGAATGAGCCCCTG
GACCGGAGGGGATGTC
ACCAACAGTGTCCCCTCGAATCTGCCAGTTGTATGAGAGACGTAC
CTTGCTTTCGTTCTT
CAGTGTGAACTGGAAGACTCTGGACTGAAGCATGAGCGTATCTCT
GAAGTGAATTTTCTC
CACAGAGCAGCTGTACCGGCAGAAGTCTGCAACGGCAGAAATGC

AAGCTTCGACCGTCGA
TTCTGAATGCGGAATTACCGTGTGACTCGGTTCTGGCTCGATCTC
AATCACCTT
>ConsensusfromContig2555 Average coverage: 13.03
ATGTCGTCCAAGAGAATTGTTTTCACAGTCTGTGGACACGCCCCT
CGGCACATATCCACG
CCCATTTTGATACGTGTATCTCCTTGATTTGTGCCACTCTCGTCTA
TTGTGGCCAACCCG
CGAACGTCGCTCACTCCGTTTTGAAGCACTCTAATCGACGATTTC
AAGCGCTCTCTCTCG
GCAGATTTATGCAACAAAATGATGTTCCTGTGTAGAGGCTCAACC
GCTACAACTCTTCTC
CCCATTTTCGCAGCCAGCAGAGAATAAACACCAATGTTTGCTCCC
AAGTCTATCAAACCA
GCTTCGGGGTTTGATCTGAGACATTTTTGAACGTCGTTGATTATG
AGAGGTTCCCAAAAC
CCTGTCGTCTCAAGGTCATGAGAAATATAGATGTCGCTGAAAGGG
TCATGGACGCATATC
CGTAGCGAAGGTTTCGTTTTCATGGGGACACATTTTGTGTTGAAT
GTATCTATAAAGTTA
TGAATCGGATATTCGTTGACCCATTTCTCTTTGATAAAAGATCGG
TTCTTATCAATTGGA
TGATCAAAATAGGTTTTTAGCGGAATTGAAGAACGAATTTTTGAT
ATAATTTCAAGATTT
GAGAAAGATTTCTTGAATGAATCGATCGCGTGATA
>ConsensusfromContig2556 Average coverage: 21.25
AAATTTTCAATATAAAGCCAGAATCTGATAATAATAATAATTTTC
TCATTGATTACCAAG
TTTCAGAAAACAAGTTATGTGATGAAATTGTCGATAGAAACATAT
TCCTTAATAACACAA
CGAGAAGAAATATCCCAAATGAGTCTGTAAACTTACCAAATACA
GTTATAAACCTCAAGA
AGAGATCAAAAGATGTATCTAGTACAGAATCAAATAGAAATATTT
CAACTTCACTGCTCC
ATCAATGCCAGATTTGTAAAAGTCGTTTTAAATACCCGAGTGCTC
TTCGAAAACATGCTT
TAATTCACACAGGCGTTCGGGGTTATAAATGCGAGGATTGTGAAA
TGTCGTATATCTTCC
AATCACATCTATCTCGTCATCGAAGAATAAGTCATGGAGCTAAA
ACATATAAGTGTAAAA
TATGTGAAATTTCCTTTTCCTCCCAATCAGAACTGAATGATCATA
GAAAAAACCATCAGC

ACATTTG
>ConsensusfromContig2557 Average coverage: 22.03
GTAAAATTTGATATTCATCTTTACTATTAGGCACTTCCCATTTCT
TTCTGAAGCGAGAAG
GAGAAGTCGACGCCTACCCAGCGATGATTTGTAACATTTAGATTT
CTATTTCCTCGGGCT
CTCTCCTGACAAACGAATTATCGTAGATGTGTGGTGAAGTTGGCT
GAGTTAACTGAAATT
CGTGCATATTTATTGAATCATCTTTTGTTTTTTTTGTGGCCTTAGC
AGGCTTCGGTTTTA
CCGCAGGTTTGAATTTACCTTCTGACTCTGTTAAAAGAGTAGGATT
TTGGTTATTTCGTT
TGATACTCCATCCGCTTATTCTGGATATATTCCTATTGAATCTCTC
ACGATAGCTCCTTT
TCTGTGGCTGATTCAATGGGACGTTCTGGTATTCTGAATCTGCTAT
ATCTCGATCTCCCA
CATAGGATTTCCTCACAAACGTTCCGAATTTCCGTCGGTAGAGTG
TTCGTCTTTTCACAC
TCTCGGAGCCATCAATATTTGGAACATAAGATATTCTCGCAGTCG
GATATGGCTTTCCAG
TCTTCACATTCGTTTCTTGTTCTGGAATGAATTCCTCATATACCGA
TTCTATGCCGAAAT
AGCTCTCGATGTAATCAGGAAAATATTTTGAAATAATATGCAAAA
GGACGCCTTTCATTA
TTATAAGAAGACCATTGGCGAGATTGAGCCAGAAAGTCCATCCC
CAGTGGAATTGAAGCT
TTCCATCCTCAAAAGGAACTGCCAAGTCTAATGGGTTCCT
>ConsensusfromContig2558 Average coverage: 17.03
GCGAGAGGGTCATTGAATATCTCTGAAGGAATAGAGATGGCAGA
AGCATAATAGATTACA
TTACCAACATCAGACGCAAGCCACAAGGCCTTTTCTAAGACACAT
AACTCAATATTCGTG
ATGAGAATCCCAGTCTCTGATAATGAAGATACTGTTACATCGCCT
GTTTGCAAGTCATTG
CAAATGGTCGTATAATCACTTTCGTACGAAATGAGTTTGATGATA
TTAGCGTCTGAATGG
AAGTCATTGAGATAGCTCCCGCAGTCCTCCGAAGCCCAAACCGTC
GGTGAATTAATAAAT
TGAATACTGAGAAATAGGTAGATGGAATGTGAGACTGTTGGACA
CATTGTGGGAAAACAT
GTTGATAGAATGGCCATATTCATACAAATTTGTAGTGAATAAAAC
AAATATTGAAATTTC

T
>ConsensusfromContig2559 Average coverage: 15.76
CATTCACACGCTGGATTTTGTTCTGGTGGAGTGGGACGCGTCGCT
AAATACTGATTTGTG
AATATTTCAGCATTGTTGGGATTAACATAAACATCATTCGATGGT
CTCCAGTGACATGAC
GAGGGGTGAGAGTCAGACTCATGTGGAGCAGGAGAATAGAAGA
GGCGAGGGGCAGGGTGA
GCCACAGATCCAGGAGGAGGTCGCCGAGGTAGAGTGCTGTTGGC
TGGGACATGGCTCGTG
CACAGGTCCGAACTGTTGTCAGCTAACCCATAGTTAAGCATAATC
CTAGAGTCGTCAATG
GCTTCCCGAGGATAATCAATGTGAAAATCCTCTGGAATAGGCCTG
GCGTCATCAACGTCG
AAGCCAGGATTGCGTGTGGTTTGAGCCATCATTTCATTGAGTCGA
TAAAAGTTGCCATTT
TGCCTAAGTTTTTGGCTTTCGGACAAAAGCCACTCTGAGAACAAC
GG
>ConsensusfromContig2560 Average coverage: 22.42
TCTATCTGAAGTACAACGACCAGCTCTGAATCAACGCCAAATTGA
TTTGAATCTGTTGAT
CTTTTGTAAATTCTCGTCTGCTCTTTATTTGTGTGATATAATTCAT
TCCTGAGGTAATCC
AGCTCTAGTACTGTAGTATCAATGTTATTTGCAAGAACCACCGGG
GATATTTGCCGATCT
ATTATCAATGTTTGAAGATTTTGTTTTTTTATCTCCTTGAGATTTC
TTTCTGCCCAGAAC
AGATCTCCAGAGGCATGACTGTACGCCATCACTGTCCAGTTCTGA
TACTTGACCGTTTCA
CTGGAGTATACTTTCCGTGTCTTGTAGTCGAACCATTGGTCAGGA
CTTTTGGCTATGCCC
CTGATTTTGGTCCAATGATGAGAATGTTGACTCCATAATAGCAAC
GACTCGTTGAATATC
GAAGCTGATATTGCTCCGGTGAATGCGGTCAATGCGTAGAGAATC
CAACGAATCTTAATC
ATTTCTTAAGAGATGATTCATACATAAATAAATCCCGGAATAAAA
CAGAGAACATTCAAT
TTCAATATGAATATTTGTTATTCGAGCAAATGTGTGTGAGTCACA
GCCGGCCGATGTTGA
TTATCAGGCATTTTTGGTTTCCTTTTTCCAATCCCCATGTTGTCTA
TGATCGTACCTTCT
CGTCACGGATGGCCG

>ConsensusfromContig2561 Average coverage: 9.28
TACACGACGCTCTTCCGATCTGCGATGTCCAATCTCAGAGACCAT
AAGATCATCGCAAAA
GTCTAGAATGGTCGAGCTTGTCACTACAGGTGCATCATCACTTTC
TGGCAATAATTCGTC
CAAACTTTTCAAACCGGATTCGTTACGAGAGTCCACCACCCAATT
GTTCACTCTAGCTTG
GAACATTTTTGTTTTGATGGCTCAGATTTTAACTTTTATTCAGTTT
TTGGACATACTCCG
AAAATGTTTTATTGAATTTCCAATATGAATATTTC
>ConsensusfromContig2562 Average coverage: 5.96
CCAGGTCTTTATTTCGACATGCCAGGCAGCTGCGATTCCAGAATT
ATCATGTCGGATTCG
GATGCGTTGGATAAGACCGACATCTTCACATTCGACTTGAAATAT
ATTTGTTTTTCCCCT
GTCAAAATTTTGTTTTCTCTCGCCCAATCGCATTTCTTTCGAAACC
TGAGATGTTCCGTG
AAGTTTAATAAAGACTTTAGCGTTTGCACCGGCACCCAAGGGCTT
GCCTGTCACCACAGT
TACTCGATACCGCGCATTGGCGACCTCTTCTTCTGATTGCCTTCTA
TGATTCTGGTGCTC
CAATGCCTTCTTATCTTTTATCGGAAAAGACGTTTCATTATCTTC
CCAGCCACTGGATC
TATTTCAGAATCAGAGATCCAAGATCCACATGGGAATAGATGGCG
CCTCACCTCTT
>ConsensusfromContig2563 Average coverage: 9.92
ATCTTCGATGCAAAACAACAAAAATTCGATGAGTTCAGTGAACT
CGAGTACGCCAAACAG
ACACAGAAGTCGAAAAGAACAACACAAAGCAGACGTCACAGAA
AAAAAAAACCCACGGAC
GAGAAGTTCAAGCAGAGGAACGCAAACCCAAAAGATGTCACCAC
AAAAGCATTTAAAAGC
AGACGACACCAGCGATTCAGATGGTAGAGAAGACAGCAATGAAG
AAATGTCAGAGATACT
GCAAAAGGCTTTTGCTCGATCAAAGAATTCAGAAGAGAAAAGGA
GTGTTGAGGGGGTTCG
AATCGGTCATCCCACTCCTGCTCATAGTCTTGCACTGGGTGGCGA
TGTAACATTAAGCTA
TGACCCAGTACATTTCGTAAATGGCGTTTGTTCTCCAACAGCCAG
TTTTGAGAGATCGGA
AG
>ConsensusfromContig2564 Average coverage: 6.44

TTTTACTGTAAATATTAATTCAAAAAAAATATTTTTAATTAGTGA
TAATAAATATTATGG
GCAAATGCATTCTCGTGTTGTCAACAAATTCCTTTGGATTCCTGT
AGTAATTTACATGCC
ATTTTATCTTGAATATTTAGTTTTATCTGGAATTTACTCGCACGA
ACAAGAGTCTCAGCA
GCTGTATGTGTTTGAATATTTTGACAAATACACACCAGGGATCGG
TACAACTGCCACTCA
GGGCGGAGGACATGGAGGACAACATATCAAATCGCCTCAAATCG
TACAACAACATACATG
GAGATGTACTGGCGAGGGCAAGGCGAAAGGGAATCGGTTGCAGA
ATAACAGAACAGATCG
GAAG
>ConsensusfromContig2565 Average coverage: 6.57
ACGCTCTTCCGATCTGAACGATGAAATCGAATACTTTACCATCAA
CCGCCAAATTCACCC
CTGACAAGGAGATGGCGAAACAAACTAGGGGGACCTCGGAACAA
CTGGTATGCGATGGCC
TATCGGCCATTAAATGGTATCATGCTAAGGCAGTCATGATCTTGT
CGGCGGCACATGGGA
AGACCAACGAGGATATTTGCTCTCGTTGGTCTAAAAAAGAAAAT
AGATCACTAGACGTTA
AGAGGCCAGAGGTAATACGAGAGTATAACATAAACAGATCGGAA
GAGCG
>ConsensusfromContig2566 Average coverage: 24.39
CTTCCGATCTATTTTATTCATTTTTACATCACATGCATCAAATCC
AAAAGGGTGAAAGCC
TGGATAAATGCACTCGGGATTCCGGATCCGGTATCGATGATCGC
GGAATTCAAGCCAGGC
AAAAGGGGAGGGGAGTTCGCATGTCCATTCGGTGTCAATATTCA
TATCGAACCAGGAATA
TTCAACAAAAATGATGTCATAACCTGTTGTCACGTGACGCCATCA
CACCGATTCAATTAT
GTGGCTCCATCTTTAACAGAATACGATTTCATTTCAGAAGAAATT
TGTATATTTTCTGCA
TCGAACACGAAAGCATTACGTAAGAATGGCGCAGATTTATACATC
CCCTTCAAACGATGT
CGTCTGCCTTTTTACACTGTACGCCTGCTAACTAAAGGCGTTGAT
AGCGATGGTTGGAGA
GACGTGGAATATGAACAATTTGCTCAAGAGTCATCCGATGATGAC
TCATTCATTGCTCAC
ATCCGGCTCAACGTCCCCTCAGTGATTCTCCTCGTCCAATCACCG

CGCGTGGAGAGACTG
CCAATCAAAGCCACCGGATGTCTACTCAACACCAACCTTCACCGA
TTAGTCAGTCTGAGA
GTCCCCAAAAAAGCGTCGGTCGTCGACTATGACTGCGCGCTTTTT
ATATCAACCATTGAT
CCCAATAAACTGTCGATACTTTCACGTGAATTCGCTTCCGACTTC
GCCCACATCGTTTCC
ATTTCGCCACTTTATCACTTCGAGCCAGAAATATCATCAGAAATG
CAGCGTGCCGCGACT
GTACGTCTGCCCTTTCCGCCCACCCCGGACGGAACTGACGTGGAC
ACAGACGATGTTGTT
GTTCTTTCCATA
>ConsensusfromContig2567 Average coverage: 7.36
GAATCAATTTGTGAGTATCGCGTACAATTTCACAACATTCTATAT
GGCCGATCAGTCCGA
CTCCGCGTATCGCGAATTTCTTCGAGTCTGGTACGAAATGGTCGG
AGAAGCCCTCAAAAA
ATCATGGGAGAAGGCCAACAATCATTTTATAAATCGCGACGAAT
ACACATCTGAATGTGA
ATTGTTTCGCAAGTATATGGGCTTCTGCGGACATTTGTTGAGTCC
AAATTTTGTTCAGAA
TCACAAACAATTGTCCTTATTGGAAATGCGTTCTAAATGCGATTT
CTTGCAGAGTACTGG
AATCATAGATTTATGCGTAGATCAGCTGATCAAGATGAAAGACAT
TCTTGTAGATATTCA
AGATGAG
>ConsensusfromContig2568 Average coverage: 14.12
GAGATGGACCGCTCAGAAGCAGCCATTGATGTTCGGGTTGGGGA
GGGAGGTTTAGAGAAA
GCCAAATTGGCTTCATGAACCAGACGTCTGCGACCACGTGATAAT
GACGTCATCACTTTT
TCGGTTGCGCTTTGTTCAGAACCCAGATTTTCCGTGAAAAGTCCA
TCACCACCGGAAGGA
AGAAATACGCGTTCGGCGGGTCTTTGGTAACACCGTGGTGATCGA
AGATGCAAAGCTTTG
GCTATGAAATAGTTGACGTCTCCCACGAGCGGATATCTCATGAGC
GTAGACATACATGTC
GCATACTCATTCCCCAACAATAGAAGCCTGATATATTGCAACATG
GCGACACTGATGTAG
TTGACAAGGTCGAAGGTCATTCCGCACTCTTCAGCAAAGATGGC
GTCCCAGAGGGAGAGA
AGATCTTGCATCGGAAATTCACGCCCGAAAAGAAGGCGGAGCCA

ACGTATGCCATAGATC
TGGGGTTCGATTCCCAAATCCTTCAAATAGCTGTACAAGTCCAAA
TCAGCCTCTCTTAAA
AGAACGTCATGAATGAAGCAGAGCTGCCGCGAGATGGCGTTGTG
CAGAACAGGGTCAGTT
CGTCTGGTAAAGGGGGCGGTGTTGTCGAAACGTTTTCTATTTTGA
AGGGAATTCCGGGGC
CCT
>ConsensusfromContig2569 Average coverage: 18.15
GATTCATCATGACCAGCCAAACTACGATTGAGTCTCCAAACAGAT
GAATGACTCTTCTTC
GAAGGTTTTTCTGATGGCAAGCTTCTATTGAGTTCCTCACCCCAA
GATGATATCGCAGAG
TCCACAACTGATGATTTGTTCACTGGTTCAGTAACAAGCTTTAGC
CGTTTTAGAGCATTG
TAACTTTTATATGCATCTATAATATGTTGAGGGGCGCTATCCACAT
CCTCCTTGTTTGGC
TTTCTCAAATGGCAAATCTGGAAAGCATTCTCCCACTGTTTTAAAT
CACTCTTTATTTTA
TCTAGATCCATAGCGTGTCTATTATTGAAGACAATGACGGTCTAT
ATTAACTATAATCAG
ATAATATACTTATCGAAAAATTCAATTTGCGCGCCAGAACAAAAT

>ConsensusfromContig2570 Average coverage: 12.61
CTATAATATTCTATAACAACTGTTAGAAAGACACCAACAAAAAC
ATGGAAGAAATTACAC
TGAGGCTAAAAGACGAAGGTTATCATGGCTTCAAATCAGAAAGT
GAGAAACTGAATTTCT
TCTACGACCTGTTCATCAAGACTGAGACTGATTTGGAAAGATCCA
TCATGGACATTGAAG
AGATGAAAGAACGACAAGCTCAAGAGATGGAAGATGTCGAGAAA
TATATAGATGGCATCA
GGAATCTCTCCAATGAGAGAGAAGCGATCGCAGAGGAATTCGAA
AAAGAAAATGACGAAT
TGAAGGCAGAAAATGAGTACCTAAAGAACAGCGGCAGCGGGCAA
G
>ConsensusfromContig2571 Average coverage: 31.35
TTCCGTTCAATCCATTTGGATTTTATTTATCGAAAGTTGAGGGGAT
TAATGATCATTTCA
ATGAGACTTATGCCATGAGCATGAAAGATATACTATCGAAAAGA
AATGGAGACTTAATCG
AATCTGCTCAGTTCAGCTACATGGTTGACCTAGAATGGCTGATAA

ACCAATATCCTGAAC
AATTCAGGAATCAGCCGTTGCTATTGGTTACTGGTGATCAGATGA
ATGAAAAGCTGAAAC
TCGATAAGTCTGCTGCCGTTCATTCCCACATCACATGTTGTGAGG
CTCCTTTGGATATTA
TGTATGGAACACATCACACAAAAATGTCGTTCCTGAGGTATTGCG
ATGGACTTCGAATAT
GTATAACTACCGCAAATCTTGTTTCTCAAGACTGGACTCATAAAA
CTCAGGGGATTTGGC
TTAGTCCAAAATTACCGCGTTGCAGTGGACCGGGTTCTCATGATT
CGACAACCAAATTCA
AAGCTGATCTCATAGATTATCTCTCGGCCTACCGACGCAAGCCGT
TGCAGGA
>ConsensusfromContig2572 Average coverage: 468.11
AATGTGACTGGAGTTCAGACGTGTGCTCTTCCGATCTGCCTCGTC
CACCTCCTCCATTAC
TTACACCAACACCCCCACCCCCGCATTTACACCAGAGTTTGAGA
AGCAGGCCGGCAGCGA
TGAAAACACCTCCAACAGAGTACAGTACGATTTGGAGTATTGGAA
ACCTTGATTTTACGG
CACAACTGTCGTCTACGATTGAATCTCGATAGTCTTTCACAATTT
TCAGAATGCCTTCGT
CCCACCGATCTTCTGATAAAAGAGAGCGTGCATCTTCATGAATAT
CATCGATGCAGTCAT
TGGATAAGCGTTCGCGAGCCGCTGAGCCCGTAACAGTGTAATGCA
TTCTATCTTCTATCG
AGATGACCATCACTATATCATTCGAACAAAGAGATAATTCCCATG
AGTCTGTTCTCAGAA
ATTTCCCAAAGTTTTCGACCTCTTCTTCCTTATTGTCTGAAGTCAA
ATATGTTCTGACTC
CACCATTCACGAGCACGACGCGCTG
>ConsensusfromContig2573 Average coverage: 13.20
GATTTTTTGAAGGTCATCCATTTGTCGAAGAAAAAATGAAAACA
ATTGTATGAGAAACAG
TTAGGCTTTAAGCTCAAACATCACAAGAACCTTGGATAACATCGA
AAAATCATGTGATCA
CACGAAGAAAGCGAACAACAAAATATATGCCGCTTGATCTCTTA
TCAGCAGGAAAACAAA
TATAAATGGACGGATTAAAATGAGAGTAGTAAGTGGTGTCATTG
AACATGAATTAAATAT
AAAACATTTGCTTTAACCTCGAATAGAATTTGAAATTTAAAACAC
TCTAGATGTTAATCT

AGTTGAATATCTT
>ConsensusfromContig2574 Average coverage: 29.93
ATATTCTTATCCAAAATAGTTTAATGAAAGTATATTAATTGTTCT
AACTCACACTCGTGA
TGTTGAATCTTGATGAGATCATATATGTGTGTGTTCTGGTTTTGTC
TATTGTAATGAGCT
ACTTCATAAGAAGAATAACAATAAATCTCAGACCATCATTATGTG
CGACAACAGGGTTTC
TCATAATCTTTGCAACATGCAAAACACAAACTCTACATTCAGTTG
TTACAGTTTTCGGAA
ATATGCTCACTCTGTGTTTTCTCCCTTCACGATTTGTGTCATTCTT
CAGTTTTGTATGGA
ACTTTGGATATCTAGTATTTTTCCGTTGTAGTGATTCATTCGGTTT
CGAGAAACCCTGCA
GTTTCGCAAATGCTGTGCAGTTGATGTTGACATTGAAGATGATCA
GTTTTGTATGTGACT
GGAATGAGAAAAATGAAATGTATCATTCCAAATCGAATATTTTC
TCAAATATGACGGAGC
TGTGGCTATATGCTTATAGTTATATCGGAATTCTGACAGGCCCAT
TCTACAAATACTCGA
CTTTCGTCGACGCCATTCGTAA
>ConsensusfromContig2575 Average coverage: 13.85
GTTGGTCAAAGCTACCCTGATCCAAGGGGACACGATAATCCTGTT
CCCCTGGAGAGGGTA
TGTAACTAGTAAAATAGTCCCTAGTTGATTGGACAGACATATACA
ACGTGCTAAATTTAG
ACCCTGCGAACCCCGATTCAACTACTCTTCCCAGGTAATCCATAC
TGTACATCCTAATAT
TCCCCCCCTTAACTACTGGCCTGACCTTCTTGGTCTCCCTTTTTCT
CTGAACGACGCATT
GTTCCACATGCGGCTTCAACATCAAGTCCTTGATCTCGGCGTCTG
AATAGACGGCGCTCA
TAACTCCGCGGTATGCCGCGCTTTTCATCCGCTTCCCCTCAGCGT
CAATCACCAATTTAG
GCCCGTCACCAGCTTTTCCTTTGGCCCAGGCTCCGCATGCTACGT
ATTCATCCAACGTCT
CTACCTTAGCTGTGTGCGTATTATAATTATCCCTTATGACTTTCAT
TGTTTGCTGCTCTA
GTAGCTGTAAATACTTGAAATGGCCTATACAACCCCCCACTTCTT
TCCTCCTAACGAGCC
AATCCAAAATTTCTTGTTCCATCTTCTCGTCATCAGAATTAACTG
CATAAGACAGTCCAT

TCTCCCAATAGGAGAGTCCCTCCCAGAATACATTCAGATAATACC
CTCCTAACTTCAGAA
ACCCTGTACGTTTTGAGAGTACTGAACGGAGATCATGTGGGCAG
ATATCGGGCCACAGAC
TTTTCATCCATTCGTACCACAGAGAAAGC
>ConsensusfromContig2576 Average coverage: 23.23
TCTCGCCTCGTCGGTAGGATTCGGGTATTTCTGATACCACATTATT
CTTCTCATATACAC
GTATATATATAAAATAATAATAATATGCATCAATATGAATCAACG
ATAAATATCGAAGTA
ACATAGCGACCAGCGATTGATAATGAGCGAGAAAACAGTTCAAC
AAAGCATATATTAATA
AGCAGAAATAAATTAAAACATAACCATAAACCACATTTTCATTT
CGTGGACGAGAATAAA
ACTTCTCAACGACGATAAAACCAATAGTGCTATTTTTTGAAGTAT
ATCATAGCTTCTCGA
AACGATAAACCATTTCAGGTCCCTTTGTAATCAATACGAAGGGT
TGAGAAAAGCACAGCT
TCGCGAAATGATCTTGTTAATAATAGATCTCTTGTTGATGATTAC
TATTACAAATTGAAT
AATGTATATTGTATCTATTTTAGT
>ConsensusfromContig2577 Average coverage: 6.88
CTTGAGTGGGAGTCCCTGATCCAACAGAAAATAATCCCTCTTTGT
TGACGTTGGTCTTTG
ATTTCCATGGTTTGAATGCAGGATCAGCCCAACCAAGCAACAGA
TCACAAAACCGCCAAG
CATTATCATGATACACAACACTCCCTTCGAAAATAATTCGCAGCG
TTCCCCAGTGAATTC
AGCTCCGCACAAGCAGATGTATTCAGTGTTGTTGCCATTCAAACA
GGTTCCACCATATTT
GCACGGTGCGGATGAGCAAGCATTGTTATCTCCATCCTCCTGGCC
ATTGCAGGTGCAAAT
GAATAGAGAAAGTGCGAGAAGACCCACCGAATATGATACATTAC
CAAAACGGCGCGCCAT
AGTTGA
>ConsensusfromContig2578 Average coverage: 19.22
AAAAAAAAAAAAAAAGAGTCACGTAAAAACTGCGAAACTCGGTG
GATCACAAAATTTAGG
TCAAACTTAAATTCTAGGTCAACGATCAATAAATACCGCGTATTT
CATATTTAGCTCACT
TTGTCAATTTTTTATGAACGGTGATAAAGGCATTAGCCGAAATAT
TCGTTTTTAATCGTT

AATCTCCACATTTTACCATCATTTTTGTGCAAAAAAGACCGTTTCT
TCATGTTTTTTATT
ATACACATTGCACAATCGAAGTTATGAACAATATGCATCATTCTA
ACGGCGAAATACTTC
GAATAGTGGATGAGATTGAGAAATCAATAATAAAATTGGCGCAA
A
>ConsensusfromContig2579 Average coverage: 31.86
AACACTTGATTGTTGTCATCGAAAGAAACACTCTTCGCAGAAGAA
TGGCATTCATTGCTT
GCTCCAGTTGTTGCCGAGCTCAGAGAGGTATTCGCGGAGTTTGAA
AAGCCAATTATGTTA
TCACCACGAAGAGTTTTGTCTGCATTCTCATAAGACAAATCAAGA
CAAGAATCTTCATCA
ATGATTGGCAGAAAACGGCTCTGAGGCCCTCGGGCGTTATCGCCT
TCCACACAACCACTA
ACTGCCACATCCTGATGGGTCTCGTTATCCCCGTTGTATGGAGCT
GTACTGTGCAGACTG
CTAAGAGAGCTGGCGCGTGAAAAGTTTCTTGGCGTTCCCTCAGTA
CAATATGTCACGACG
TTATCAAATTCAAAACTGTTATTCACGGATTTGTATATATCAACC
ATATTGGCTGGATTT
AATGCCTGATAGTGTGAAATATTGTTCCTTTTCGAATAATCAACTT
CTATTTTGTTAG
>ConsensusfromContig2580 Average coverage: 12.39
AGATTCATATTGCGCGAGTGTTGTAAAGTATTGTATTTTGTATCT
TCAGCCGCCTGACAA
GCACCATTCATCATGTCATCACTCAACGTTGATTTGGACGTGTGC
CGTATGCCTTGTAGG
GTTGCGAATCTTGGAGCCTGCATTGAAATTGTAGACATCACGGTC
ATCGGCAGAATTTGT
GGATTTTGATTTGACTGAATGCTGGATTTATTGTTCATCTTAGATT
TCTTCGATGAAACT
GGCGTTTTAGGTACATGCATCGGCACTTGGGGCGGTGCAACGGA
AACGGGCGTCCGAGCT
TCACCGGATAGCGGAGATATAGATCCGGTTGGTGATGTCATGAG
TGTACGCGATATAACA
CCACTCCGCAAACGACCCAGTTGGGGCGAGTTAACTCGTTGAGG
GAGCTTCGGTGACAAT
GTTAAGTGTTCTCTCCCATGAATGGATGAGGTTGGCGTGACACA
>ConsensusfromContig2581 Average coverage: 11.43
CTCCGCGGTCTCGGATGACTGCAACGTCCACGTTTAGGATGGAAG
ACTTGGCTGGATATC

CTCCGTCCTGTGCTTCAATCCGAAGCCTGTATTGTGTTCGGGAGG
TGCCTAAAATAGATG
AGAGGACACTGAGTCTTCCTGATGTGGGGTTGACGTAAAAGTATT
GCGTCTCTTCGCCCT
CACCCACCAGGGCGTACCTCACGACGTTGTTGGGAGAATCGTCG
TCGGCATCAATGGCAG
ATACAGTCATAATTTCCGTTCCCGGAGCGGCGTTGTCAAAGATCG
AGACCCGCTGAGATG
GAGAGTCGAAGACGGGAGCGTGGAGGTTGTGTTCCACCGTTATAT
CTACGGTTGCCGTAC
TCGACTTTGCGGGAACACCGCCGTCTTTGGCTTCAAGGCGGAGTG
TATACACTTGATTGC
TGTCTCTATCCAAGCCACTGTCTCGCTTCACTTTGATCGTTCCTCT
CCCATCCACCTCGA
AGAATGAAGGGGCGTAGTCATCGCCGATGATCGAGATTGTGTAC
TCATTGAACGGCCGAA
TCTTGTCATTATCCCTGACATTGACGCTGAGGACATCTGAGCCAC
CAGGAAGAGTTGCCA
CAATGGCCCGC
>ConsensusfromContig2582 Average coverage: 7.41
TTATCTGTCTTTGATAATGATTTCGGTGCCATATCGATGTTCATCT
TTCCCACCTGTTTT
GTAAATTGAGTATATTTCTGGTCTATTTGTGTGTTTATTTTTTGTT
GTTGTTATGTGCAT
GGTTTCTATTCCTGCGCCTGGAAGATGAGTTTTTTATTTTTTTAT
TCCTAGGATGTATA
TGGAGTAGCTGGGGATGTCTCTTTTTCAGCAGGGAGTATTCGCAG
TGTCCTCTCAGGCTT
GAAATATTATAATAGGCTTCCCTAATATTAGAGAATGTTGC
>ConsensusfromContig2583 Average coverage: 7.01
AAATAGACAAAGATGACAAGGTAATTAGACAAAAACGAAGACAG
AAATTATCGACAGTTC
AACTGACGTTCGTAAAGTTCAGAAAATAGACAAAGATGACAAGG
TAATTAGACAAAAGAA
AGGAGAAGATGGGAGATACATAACCGCTCTAACACCACCGGTTG
TTACAGATGAACATAT
CCACGAATTCCATATCAAGGATCTCTCAAAAAATGAGGAATTGAA
TATTTTCAGATACAA
ATTCACACCAGCCTTGAGAAGTCGATTGTTTATGGACATAAAAA
>ConsensusfromContig2584 Average coverage: 26.54
CTTCCGATCCCGGGCATCCAGCATCAATATGAACTCATCGAATGT
GATGACACCATTCTT

GTCCAAATCCGCCTGTCGGAGCAGCTCCGCGATCTCCTCTTCCGT
CATCTTTTCTCCGAG
ATTGGACATTGCTAACTCAAATTCTTTTGCGCTGATGTATCCATTA
CCGTCTTTGTCAAA
GAGTCGAAAGACTTCTTTCAGTTCTGTTTCTGACGAGATCTGAGA
GAGCTTCCGCGCCAT
CAAATGCACAAACTCGCGAAAATCAATAGTGCCGTTACCATTGGC
ATCGACCTCATTAAC
GATATGCAACAGCTCTGTCTCTGTTGGGTTCTCCCCCAAAGTCCG
CATCACTTCGCCGAG
TTCCTCCGTGTTTATTGTGCCATTTCCGTCGCGGTCGAACAGAGA
GAATGCTTCCCGGAA
TTCGGCCATTTGTTCCTGAGACATGCTATCGATAAAAAAATCCGC
ATCTTCTTTGATGAG
TATCTGTTCGGGAGTGAGAAATGCTGGCTTGGACGATCCCTTGAC
CTCTTCAATATGGTC
ATCTATGTCGAACATTCCATCGCATGAAAAATGGTTAATGATTCC
TCGCACACAGGAATT
CGTGTCTATGAAGTGTTGGAACGCATCTTTAAAGGATACCATACT
CAATTCACATGATTT
AAAATGTTTTCTGAATTGTTATAAATACAGAGCTTATGTCGAT
>ConsensusfromContig2585 Average coverage: 22.12
GAATCGGTGCCCAGTGGAACTTGTATTCTTCAGGTATCTGCGACA
GACTCTGACTGTGGC
GCTGCAATCCAGTTGAAGTATTCCCTGGTGTTGTCGGAATCTACT
CTTCGTGCACGGTCG
CTGTTCCAGGTGGATTCTTCGGATGGTCGAATCTGTACAACCGCG
CCTCTTGATGCCGAG
TCCAGCAACAGCTTTCATTTTCTTGTTGCTGCAAACGATCAAGAT
GCTCTGGAAGCAACA
ACGATTGTCCGCATATCGGTGACCAACGTCAATGACAATGCCCCC
GTCTTTGATGTGTCC
CAGATGACGGTCTCGCTTTCTCACGATGCCGATCGAGGAAGTCGA
CTAGTACAGGTGCAC
GCCACTGATGCGGATATTGACGGGGGGCTAGGTTCAAAGGGGAC
GAGCATCAGCTATCGC
ATCGCCGGGGGAAGCGCCAGAGACTATTTCACGATAGATAGCGG
CACAGGAATCGTTCGG
TTGGACAGCGCCGTTCCGTTGTCTCTTCGAGGTCCGAATTTGATG
GTGGAGGCGAGAGAC
GAAGGCGGCCTTACGTCCCGCATCAATGCAGAAATACTCATAAAC
GTCCTCAGGGCTGGT

GAGTCTATTCCGACCTTTTGGCGGTCGTCGTATCAGTTTTCTGTG
TTCGAGGACGCAGCA
TTGGGACAGGTTGTTGGAACGGTTGCGGCTTTTTTATCCCCT
>ConsensusfromContig2586 Average coverage: 24.75
AGTCTCATCAGACTGTAGGCAAGATGACTCTCTGACGTCACGACC
AGCATCTTGCAGTTA
TGCCTGATGAACTCGAAGCAGGGGTCCATCAGCCACTCGAAAAGA
TCGTCGATCAATTCT
CGATGTTCATCGTCAAGATTCGCAGGGAGCTCGTGCTGCAGATAC
GAGTCCTTGAACGGT
CTCCATCCCAGTTTGTGAGGTTCGAGGTAGATCATACCACATCGA
GAGACCGTGGCGGGA
GAAGCTTGCTCCAAATCAGCCGGTTCGAAGATGAGGTTCATCCGA
CCGCTCATCTGGATA
ATCTCGCCGCTCATCAGACATAACTTTTTGTTGTCGTCGAGAACAG
TATTCATGTTTTCG
ATCCAGACAGCGTCCACAGGTCCATCAAAGAGGATCCATTTCCGA
TCTTCGTTCGTTGAG
GAGGCTTGTTCGCGGAAAACGTTCGACAGAACACCATCACTCCA
TTCGTGGGACACCGGG
TCAAACTGGCCATAGAGCTGACCCATCGTGATAGCCTTCGGGTTC
ATTATCTGATAAATC
ACTTTGTGCTCATCCATATGACCCCCAGCCTCCAGGTCACCGAGT
GCCCCTGC
>ConsensusfromContig2587 Average coverage: 31.01
CTTTCCCTACACGACGCTCTTCCGATCTCGATGGTGGAGCTAGGG
GGAAAGGGAATGACA
GAGAAACAGATTGGTATATTACATACTTTGAATAACAAAAATGA
AATAAAGGTGGAGACT
CCTATCGGACTAACAAATAAATTCAAAATGAAGGATATTATAAG
GCAAGGAACTGTATGG
GGACCATTACTATGTTCAATTGAGACGGCAAAAATAAATGAGAT
GGGTAGAAATGTGGGA
AGACGGTACGGGGAGGCAAAACTAGGAGCGTGCATATTCATGGA
CGACATTATGAGGGTA
GGCAGAAGGGAAGATGTATTGGATACGATAGAAAATTGCAGGG
AACTAGAAAGAAAAAAG
>ConsensusfromContig2588 Average coverage: 19.11
AATGCTGATATTTCATTGGCTGTTGCAATTTGATTCATCCGAATC
GTGACAGTATATCCG
CTCCCAAACTTATTTTTGATGGCTTGGCCACTCCCGAGACACATA
AGCGATCCGTTGACC

ATAATAGCCAATCGAGTGCAGAGCGCATCGCATTCCTCCATGCTA
TGAGATGTGAGAATG
ACCGACTGGTTCCCCTTCACGGCCCGGATTATGACGTCCCACACA
AGGCGACGGCTCGAG
GGGTCCATGCCTGCAGTAGGCTCGTCCAATAGAAGAAGTTGTGGT
TCGCCAATGAGTGCC
AATCCAATGGAAAGTTTTCTTCTATTTCCCAAGCTGAGGGTTCCG
ATGCAGCGGGTGATG
TACTCATGGAGACCCAATTTATCAACGATAGTATCTCGAATAGTT
GGTATTTCGTATTCG
TCCATTCCCCTGAGGCGACAATAGAAGAAGAT
>ConsensusfromContig2589 Average coverage: 24.05
CTTCGGCAGTACCACTCATGGCGACATCTGGAACATCGTTTGACA
GCCCCTTCACCACAC
ATAGCGCACCGAGGTTTCTCGTTCAAGACGTTTTGTAAAGAATCC
AGATCGAAAGTGGAG
GCAATTTTCTGAGCTTGTCTTTTAAGAACAGAACTTTCGGCATTGA
AATAGTTTTCTTCT
TGAAACTTAGCGATTTCTGACCATTTCCCTTTGTGACTTGCAATGA
TATTATCCCGTATT
TCAAAAACCTGTTCCATGATCAGAGTGTCGCTAGTTCTACAGGAC
TTGGCTTGCATTAGG
CTCATTTCTTCAAGAGTTCTCAAGAACGGACCGAGGCAAGGGAG
CTGGTCCAGGAGAGTT
TCTGTTAAATGTTTTCTCAGTTTAATTAGGCAATTCTTCCTTGACT
CGATAACTTCATAT
TTCTGTCTGAATGTATCCCGCATCATCAACGTATATAGCGATAGC
CATACCTGCCCCTCT
AACTTCCCCAGCTTGAAACGATCTTCTAATGGCCTCCATTGATGGT
CAATGTATTCTTCT
TCTTTCCCATCTTTCGATTTTCGTTTCCATGGTTTCAAGTCAATAA
TCTCTGTCAATAAC
ATCGGGAAATCGTATTTATTGAGAAGCATGTTGGCTATACCAAG
AGGAAGACTCTCAGAG
TGATCGATCAAATGATAGACAATGCAGAGACAGGTCATTTGAATC
CCGAATTCAGTTTCG
GACTTTTGTTGTTCAAGCTCCTCTTTCGAATTTGTCGTTTGGCAAT
CGTTATGTGAACGT
TTTTCGGACCTACTCCATGATGCAATTCCATGGACGAGCTTGCGG
TGGCAATAGTCAAGG
AGATCCATGGATACATCGTC
>ConsensusfromContig2590 Average coverage: 5.58

AATAATATGAAATTGACTAAAAGGATATCATAATACAATTAGTG
AGGATCACGGGAAGAA
ACAACGAGTCTGTTTCCATACCTGTGGAGAATAATATGAAATTGA
CTAAAAGGATATTAT
AATACAATTAGTGAGGATCACGGAAAGAAACAACGAGTCTGTTT
C
>ConsensusfromContig2591 Average coverage: 5.67
TTGCTGTCTCGTTTTATCTTATAATCACAACATTGCCCGTGACGAT
CACGTATTTGTTGA
CATTCACGTTCCAACCCGGTGACCCTGCAATGGATATCGAGCAAA
TAAGAGCTGATGCTC
CTTGGCAAAGGTTTCTTTCATATGAATGTGCAAAAATAATAATCA
CAGAGCTTGGGATGT
CACACTACGCCTGTAATTTCTTCATTTATGTGCTCACTGGAAAGT
TATTCAGACGTCAAC
TAAAAATGGCATTTTTGAAAATATTTTGCAAAACTAAATCAGAA
AAATACCGGAGAAAAT
TTCTTGCTGAGCAATGTTCGTTTCTACCGCAGAACAATTCAATGA
AACCAAATGGAAAG
>ConsensusfromContig2592 Average coverage: 12.16
CACCGGAAGTACATTCATTGTCGTCATCACATGTCTTTCCATCTG
CATTCAGCTTGTAGC
CAGACGGACATTCGCAGTGGTGAGAGCCGAAAGTATTGACGCAA
GTCCTGCTGCAGCCTC
CATTGTTCACCAGGCATTCGTTCAAGTCATTGCACTTGACGAGGT
TGTTGTCCAGCTGGA
AGCCAACATCGCAGGAGCAACGGTAGCTTCCCGGGGTGTTGATG
CACGTCTGGTTGCAGG
AAGACAAAGCTGCAACTCCACACTCGTTTATGTCATCACAAGTCC
TCGAATCTGGGTTCA
GAGAGAAGCCGGGCGAGCATGCGCAGTAGTAGCCCCCTTG
>ConsensusfromContig2593 Average coverage: 12.64
GCGCTAAGTCTGAGGATTTATCAAAAAAAGTATTGCTGCGTTTTT
CAGATAGGAACACCT
TCATTATTACAGCTAATATTCCGCAGCCGCTTTCGTATATATTGAA
TATTTTCGTTGAAG
ACCTGAATGATGAGGAGAGCCTTCAGTTAGCTGTTTCCTTCGCTG
TCAATTGCACAAAAA
CTTCTGACAGTTCTTCAGTTAATTTTCCGCCCTGTGGCGTGCTCGG
TCTCACCAAATACG
GAGATCAGAATTCCTTTGAGGTTTTGAGCCCTGCTGATAGTGTTG
TTACTTGTAAGCGAA

CGATGAGTGTTAGATATAAATGTAAAGACCATTCTTTGAACTATA
CGCACAGTCTCAAGT
TGTGGAATAAATCTTTTTGTGATATTTCTAGAAGCTATATAGTGA
CTCATGAAATTGAAA
AAGGTCGTGAATGTAGATTACAGTTTCCAGATAAAGGCGAATATA
TATTCTCCGTATATG
A
>ConsensusfromContig2594 Average coverage: 13.25
TTATATTTATGTATATAATATATCTGCGAATTGGGAATTTGCACTA
TTAATGAACATCTT
GACTTGTGCTTTTAGCTTAACTGAGGTTTTTCGAATATATTTCGTG
TATATGTATATTTT
TGTGTCCATTTAAAGCGGTTGCAGCACTGGTTTATATTCATTTTTT
TCATATTGGATCAT
ATAGTTTCATGCTGTTTTCCCTTTTCCAGATGCTTGCGTTTAAACA
AAATATCGTGTTTA
AACCTATCGGTAGTTCGAGTTTTTAGATGAAGAGAATCCTAAATT
ATAAAATTAGTTATC
TGCACCAAGCAAAATACACCGTGTTTTTATAAAGTAAGC
>ConsensusfromContig2595 Average coverage: 11.51
CAAATGAAAATAAATTTTTATCGAATCAGAGCGAATCTGAATTTG
AGAATGGTTTTTCAG
AGTGTACTTTTAATGAAGATATTAGAGAAAACACATTAGCGGAG
ACTGAAGACACATTGA
ATAAATGTTCGAAATTCGTTCCATCGAAATTGTTGCATAACTTAG
ACAATAAGGGTTTGT
CATACAAGACAGAAAGTATTTCCATGGAATCTGCATTAGAGAAA
CTCCGTAAATGTGGCA
TCCTTAGTTTTTTTCAAAAGTTCACGATTATGTTGAGATATGTCA
CACAAATGTGTAATA
GGAACAAAATACCACAACAGAAGTTCTTGGCGGTTTGTAGAGACG
TATCAAGTTTGTGTT
TGGAATCAAAGTTAACTGATTTATTCTTTCAGGACACTGTTGAAA
GCATCAAACATTTCA
TACCAAAAATTGAAAA
>ConsensusfromContig2596 Average coverage: 21.44
CATCCGAAACCATCAGCAAGTCATAAATGGATTCAGAAAACCGAT
CTCTCTCCGTAAGAC
GTCCAGTATATAGATCATTTTCTTTGTGTATAAGATTGAATCTTTT
GCCGTTTCCAAAAT
GACTAGGATCTCGCACATGTTCGGGAAATGATTCGGTAAAACTTA
GTGTGTCAAAAGTAG

CCTCAGTTATTTCTAAATTATTTTCTAAGACAGTTGGAAGTATTCT
CGATATTGCGGTCA
TTGTATCTTTTGATGAAACTGTATTTCTTCTGTAGAACAAACCTCG
CACACATACATTTT
CAGGATAAGAAGTAATATGATTTAAAGCAGAAATTGCATTAATTG
GTCCAATTTGAGCGT
CGCTGCCGCTAGAAAACCATACAGCAGGACCCATATGATAATAA
GGTGGGTTATATAACA
TTCCAATGATAAAAGATACAATCGCGTGTAGTTGAAGTAAAAAAA
CTGGTGTCTTTCCAT
CTTCTTCTACCATCATAATCGAGTGT
>ConsensusfromContig2597 Average coverage: 5.37
CCGATCTGTAATTGTCTCACCAGTTAACCTAGAGGACAAAGAGG
TGGGAGTGACAATAAC
AAAGGGTATATTATCGGTGAATCTGTGAAGTAGTATCTTTGCTGG
CGTTGCTCTGTACGG
TGCAATAGTGTCAAGGGCAAAGGGTTCGGTGAAATCCGTATCAA
CAGCGGCGGCAAAAGC
AAAAAGTTTCGCGTACATGGTACGGGGGTCTAGCAACCCGGCCT
GGGCACTATAAGCACC
AAACAGATTCCAGGCATGATATACTTTATTGCCCAAGTGAGCGGT
CTTCACCGAAGAAGC
CCGGAACGCCGTGTGACCGACGTCCC
>ConsensusfromContig2598 Average coverage: 12.45
TAAAGGCCGTTCTCGAGAAATTTGCGTTGAGTGTAGATCGCGCCA
TGATAGATAATACAG
AAGTGTATCTGCGGGCCTCAAAAAAGATGGCGCTCATCTGCGAA
GCATTGGCCAATCGCG
TTTCTCTCTTCACAAACTCCAGCGTCGATTTGTCGGATAATACGA
GGGACGGCTCATTTG
GGGAAAGAGTTGGCGGCCCCCAAATGCAGGAGACGATGATGACC
CTCACAGAAAACGTCA
TCAGGAAGATTCTGGACGATTTCCACGCGGACGAAGAGCGAAGC
GTCCGAACGGAGCTGA
CGAGTCTTTCAAACACGTGCAGCGTCCTTCAACTTGACGACCGGA
TATCCACGTACACCG
GAAGTGAAACGGAATACCCAACCACGGATGAGGATGGCAAAAGC
AGCAGGTTTTCATCTG
TGGATAAATTGGATCAGAAAATATCGCCGATTTCGAGCGCAGGCC
CTTCTCCCCTTCCTC
GAAAACGATATTCTGAGTCAGATAGACAGATATCATCATCCACA
CCGCAATCAGACAGAC

CGACAACCGAAATCAGACACAGAACCGACGGTCACCGCTCCTATT
CGCCAGATTCGTCTG
GTTACAACACGCAGACTCCATCCGAGAAAAGCACTTCCGGTATCA
ACGTCACTTCCGCGC
CATTCGATTGGTCCGCTGGTGGACAAATGAAAAACGAGCAAAGTG
CTCATCTCTCGAAAT
CAATTTTCGACCAAAACGGCCATTTTAACGATGATTATAAATACG
ATTCGATAGATTTGA
CACTTTTAGACGATAATGAAGAGGAAAACAAAGATTCTCCGATGG
TGTCGCCACGGGTTT
CCGTTTATGGTGAAATCGAATTGCAGCCAATCGAATGCCACACA
ACACAAAATCAGCCAA
TTGCGCCAGTGGAAGAAATTAGTAAACAAAAAACAAACAATTTG
AGCCACGCCCAAAAAT
CTCTGGACGAATTTAATTTGGACGATTTGGTCGCGGCGAATGATC
CAATGACCGAATTTA
CGACGAAGAGCGATAATGGAGCGAAGCATGACGTGATAAAAGA
TGAGAGAGAAATCGCGG
ATGAATCGGAAGAAAGTAGTTCCACACTAGAGGCCATTTTGAGT
GATAGAGAGAGTGGTA
AAGATGATGGAAATACGAAAACCAAAAATCGATATAAGAAAGGG

>ConsensusfromContig2599 Average coverage: 49.25
TTATTTTCTCTGTAATAATTTATAAAATCAATTCAGAAATATTTCA
GAACATGATTGATT
CGTTATAACCATATAATAACTGTATATGATATAGAAGTGTAAAAC
TACAACAGTTTCTCA
ACATTTATTCTTCGTCTGACTGCATTCGTGAACCCTTCCTTGAATC
GGAACCAAATTTCG
CCAGTTATCAGTCCACCTTTATCTGACTCTTTTTTCTGAGGTTTAT
TCTTAATCGCTTTT
CCGAAAAAATCCACAGCAATCTTTGTGGACTTGCAGGTCTCTTTG
ATGGGTTTGGGTTCC
AATGTCTGACGCAAATGACTTGGGAGACTTGCATCTGTAGCTGAG
TCAGTTGTTGGAGGC
TTTTCTGACCTTTGCAGATCAGAAGTGTTGGACGATGATACTGATT
TTAATTTCATATTG
TTCACCTCTCGTGCAATGAGTTGCTTG
>ConsensusfromContig2600 Average coverage: 15.74
ATTTTCGATGGTCACAAACTCTCGGATTTTGATGAACTCGAGAAT
CGCTCAAATCGAGCT
TGGAGCTTCATCCAAAGAAGCTGCTAATATGGTTTCTATCTACGG

TTTTGTGCGGATTTT
AGGATCTCTTCTATTCAGTTTTCTTGGAGATCTTCGAAGGGTAAAC
CGATTACTTCTTCT
GGGCTTCATTATGGGCATCAGCGGAGTGTCTGCGGAATGTTGTT
TTTGGATGAGTCATT
GTTAACATTCACGATTGACTCGGCTTTGAACGGATTCACTTTTGG
TGGATACCTCGTGGT
TTTCCCACTTATATTGATCGACATTGGCGGAGTCGACGCAGGTCT
GGGGACGTTTACAAT
TGCGATGCTATGTATCGTCATCGGATATTCGCTTAGCGAACCAAC
CGCAGTCATCTTCAA
AAATTGGACTGGGAGTTACAAGTACGACTACTTTTTCCACCTCAT
GGTAACGATCATATG
TGTCGGTCTTGCCATCATTAGTTATATCCTGCAAAAAAAGCAAGA
AGACTACAGCGTTGA
GAAAGCGGACACAACCGAAGAAGTAAAGAAGAACACTCGGAGAT
CCAGCGTTGCTTCGGA
TATTTTGGAAATTTCAAAACCTAACCTACGTCGGCTCAGCGTTGT
ATCAGTTGACTCTGT
TCACGGAGATCAAATAGTCTGAAGGTCAAAGACTCCGACTAAGA
AATCCTACTCATATGA
T
>ConsensusfromContig2601 Average coverage: 23.39
TCAGGAAAACCAGAAAGAAGTCTCGTTCCGATTCCAGATGCCTCA
TAGATCATTCCGACC
GTACTCGATAGAGAATTCCATCCACCAAAATGTCACAAGCCGAGG
AGTTATCTGTTTTCC
CTTCAGATTCAAAGCTCTCATTCCGAGCCACGAAATCCAGAGAG
CCGGTGTCGACAGAGG
AATGGGGTCGAGGAGATGAAGAGGCTGAATGCTCTCTTTCGAAA
ACCCCACTCGAGGGGC
GAGATGAGTCCTTCGAAACACTCTTGTAGACGAAAGGAGACGAA
GACCGTCGGATAGCAG
CTTTTTCAAGATCAAACATCGGGTTCGGCTTTGACATTCTGAGAA
TCTGTGAAGCAGGCT
TTCCGTTTTCGGGAACTCGGTTGTTCGTTGTCATACGACACCTTTC
TTCTTTAGCAGTGT
CTGTCAACTTATTTTTACCAGCCGCAGATTTCTTCGAAGAAGAAT
CATTTCTGCGAACGA
CATCGACGACGCTCCAGCTGTCTCTGCTGGCACGCCGCTGGGATT
GGCGTTTCTGCTGTT
CCCAGATGCACGCCATGCAAGGCACGACGCGAGTGATGAGGTAC

ATGCCGCGGGTATTCT
GCACGAATCGTGAACCCAGGTCTGGGTACCAGTCTTCCAGAAGAG
TGTCGATGTGCTCCG
TGATCTTGGCGAGTAGAGAAGCTACAGCTCGATGATCAGGGTTTA
TATTCCAGGTCTTTC
TACTCTTAGAATCACCACGATTTTCATCGAAGGTTTTCGTATCTTT
TATACCACTTTTCG
ACTGTTCTTGAGATGATTTATTAGGTTTATCAATTATATTCTGTTT
TATCTGTTCGGTAT
TTGCAATTTCTGTTTGTCCCAATTCACATTTATCTTGATTGACAAC
CTCTTCGTCAATGT
AAAACTTACAACCTTCTGATTTTCCATTTGACATTTTTTGTTCATC
GGCATTGCATTCTA
TATCTGTTCGATTCGATTCCTTTATAAACAAATCACTCAATTCTTC
AGACAGTTTCTCAA
GCGCTAATGACGCTTTTTCCTTGGCGCTATGTGACGGCACGGAAT
CGTCAGACTCCTGAC
CCATCGTTCCATCACTTTTGATTTCCTCATCGACAGAAATATCAA
GCGATAAATTAGGAA
GGGTGATTTCGAGAATGGAAGTGTTGGACAAGTTGAGTGGAGTCA
TCCCTCCGTACTCAT
CGTCCTGTTTCACCAGAAGCGTGCACGAGCGATAGTCTACGAGGG
CGGCGGGTATTTCGC
GGGAGGCGGGAGACCCGTGAGGGGTTGATGAAGAGGCCGGATGT
ATGACAGACAGATCCA
CGTCGCGAGAGACCTCTTTAATCCGGGCCACGGGAGCACCGAGA
ACAACAATTTCAAGTC
CTGTTCGCCAACATCGCCACCAGCTGTCTTCTTCTTTCAACTTTT
GCGCAGATACGCGAA
CGCCTTCATCTTCTGCCGTCGAAAACAACGCTGATAGGTCGTACA
AATCCTTGGCC
>ConsensusfromContig2602 Average coverage: 6.39
GCTGCTGGTCTTGTCACACATGTGCGGAAAATGAGATTTTGTTTG
ACGAAGAAACCTGTC
ACGCTTGCCCGCTTGGCTGGCTGCCAGATGAGAATCTCAAAGAA
TGTATGCGGATAGAGA
TCCAGCACATCCAATGGTCTGACACTCCAGCAGTGGTTGCTATAG
TAATAGCATGCCTTG
GAATCATGTCAACATCTTTTGTGGCCTTCACCTTTATCCGTCACC
ACAACACAGCTGTCG
TCAAGGCCTCAACACGCGAGCTCAGCTATATCATTCTTCTGGGGA
TATGTCTGTGTTTTG

TCACGTCTTTTCCAATCGTCGCAAAACCATCAACATTCACCTGCTA
TCTGTCTCGTTTTC
TCCCAGGCTTTTCTTTCTCTCTTATTTATGGTGCTCTTGTAACGAA
AACGAATAGAATTG
CCAGGATTTTGGCTGGAAGCAAAAAGAAAATTCTAACAAGAAAA
CCACGCTTCATGACTG
CGTTTTCCCAAGTCATTATAACGTTAATCATTATTGGAATAGAAA
CAATAATAAT
>ConsensusfromContig2603 Average coverage: 16.37
TGAGAGGCACCAACAATTTCTAAGGTCTTCAACTCGTTTACTGCT
TGATTAAAACTCTCA
GCTGCACTCCATTTTCTGGCAGTTACAATGTCAGGTGGAGCATTT
TTGATATAACGATCT
TCAGAGTCAATCAGCGTAGAATGAAGCCGGACTTTAGCTAATTTG
AAGTGTTCCGTAAGC
GAGCGAATTGGAAGCTCTAGAATGGAGCAGGAAGCGTACAACCC
GATTGAAGTGAATGTT
CTTGGTAAGCCGAACCATTTTCTGATGTAGCTATTTATTGTTCGTT
CCATGGTTTCGACT
GCAGTTAAAGAAATTTCGTAGATCTGCAACGGCCACATAATTTTT
GGCACTAGCACAAAC
TGTAGAATCCATACTTTGAATTTTCCTTGAATGGGTGCATTGTTGA
TCTTTTCAAGGCCA
TCTTTGAGCTGAGTTGATATTTC
>ConsensusfromContig2604 Average coverage: 39.57
AAATGCTTGAACATAAGAATAACATTATGAAAATACCCAAAATT
CAGAGTGCAAATGTGA
AGAAGATGGGAATCAATATCATATCGTGTCACAATACTGGAAATT
CCAAATTTTCAACAC
ATTATTAAAATGGTGGATATCAATAAGATATAAGGGGGGCCCAT
ATCCCCTGATCAAAT
AAGATGAATTTTGATATGAAATATATGTGAAAACATGAACGAATT
GTCTAAAACACTTGT
TAGCAGAGTCTTTCGAAATGATATTTCCTACCCATTAGCCTCTTCT
GTTCTTGCTACATT
GCGAGACCCCCCAAATTGACAAAATCCTTGAATTGTCTCTCGATGT
CTTTTCTGTTGTTT
TCCACCTCTTTTTGTAACTGTTTCACATTTTCCAGTTCTCTCTGTG
CCTCAATCAGATCA
CGCTGAGCATCTGACTCTTTCTCTTCTTTTGAACGAGATGTCTTTT
TACGATGTTGCGTC
TCTTTTAGAGACTGGAGCTTTTGTTTAGTGGCCTTTTCACGTTCTT

TTGTACGAGACCAT
CTTTTTTCTTCGATTTTGAATTTGCTTCGAGGAGAAAGTTGATGCC
TCAGCTCTTCGTGT
TTGTTTTTACTTCAAGTGCAATGAGCCGTTCTTCATTTGAAGCCA
TTCGCATATCTTTT
GGGTCTGTGGCAAAGGGAGCATCCGCCGCTGCATTTGGGACATCC
ACCATTCCTCTATTG
ATAGCTCTGATTTTCTCCACCTGCAACCGTCGACTCTCTTCCCGCC
AGTCTTTGACGGCA
TCTCGGTCTTTCCTTTTCTGCAAAATCATATCCTTTAACATATTTT
TCCTCGATGCTTCC
GCCGTCTCAAATTCGCGAAGATAATTCATCTCCACAGAATCCAGA
TGATCTTCTCTCATA
TCCATAGACTTCGTCTTTCTCGTGGATGAATGCCGTTTACTTCCTC
TTGATAAGGAGCTC
CTGTTCAGATTCCCATTATGTTCCTGACTTATGGTTGAGTTTGGGC
AAGCTTCGTTTCTT
GCATGAGAGTTAGCCAAGAAAATGAGAACATCACGTGTCCCATT
CTTGATGACGTGCTGA
GGCGGGTATCTCAGTGGATTGCCATAAAGAAGAAGGTTTCGTAGT
GACCGTATTTCACCC
AAAGCATCTGGGAGTGCTTCAATGTTGTTATTTGCCAAGAGGAGA
GTTGATAGATGGGTG
TGATTTTCTAGATGACATGTAGGCAATTCTTTGAGGCAGTTACTT
CTCAAATCCAACCAA
TTGAGATTGGGCAATGATTCGAAGAAATGTTCAGGAAGTGTGACT
ATCTTATTACTTTTT
AAATATAAGTTCTTTATATGACCTAAAAGAAATAAGCTTTCGGGT
AATTCTTTGATATGC
ATACCACACAGATCCATAGTAAAACTAATTTTTGATGAGATCAAT
GAATTTATTTTTTTT
TTTTTTTTTTTTTT
>ConsensusfromContig2605 Average coverage: 17.35
GCTCAATGTTCTGAGCTTCTTTTACAAATTTCGTTTCTGAGAAAAT
CGAATTTGTGGTCG
CAATCTCATGTTTATTTTGATAAAAATTAGATGACATCGTCAAAA
AACATATATTATGGT
CAGATAAGCCGGTATCTATGACACCAGATGAAGTGATAATTTCTT
CAGAAATGAAAATAT
TGTCAATAATTGATGACGATTTATTTGTTATACGAGTAGGTTGAA
GTATTGAGGGGATGA
ACGAATATGAAAGAAAAATGTTTATCAGTTCTGAACAAGGTTTGT

AATCTTTCGATGTAT
CAATGTTGAAATCACCGCAGAATACAAAATTATTTTTAGGTTTTA

>ConsensusfromContig2606 Average coverage: 12.76
TTCCATTGCTAAAACATACACTTGCATATCATAAGAAAAGCTTTT
CAACAAATAACACAT
TATAATTATTCTGAAACTTCAGTTCACAATTTCCAGATGAAAGGT
GGCCCTGGTAGTTGT
TTAGTGCTATATATGGCACATAATTTATATCTACAAGGACTGATT
TTCCAATTGTACCGA
ATGGCAGTCACCCACTTAGACTGAGCACAGTTTTCCCTGGTGAGA
GAAACATAGTTGGGA
GCCCTTGTACGAATCCAAGCTGGGACCCATTTGATAGATGCAGCT
GGGCAAATCTCAAAG
GGCGTCGAAGCACAGCAAATTTCAAATCCACCATCAATTTTGACA
ATAATATCCATATCA
TATGACACAAATATGTCACCTTTAATTGCGACATTCCGAGGCATT
GGACCTGCGTAATGT
GTGTCAATTCCAGATCCAAAC
>ConsensusfromContig2607 Average coverage: 15.60
ACCATCATTCGATCATATACAGCTTTCGAACAGGCAATAACTTTC
ATCTTTGAGCTTGCA
TCTAAGTAATCGATTTTTCTAAATCCTCGTTCATATAATTCCTCCC
CAAGATTCCCAGTG
CCGGCTCCAGCGTTGAGGATTTTTCCGTCCGTTTTTCCTCCTGAA
AGCCGAATAATTTCA
TCTGCAGTTTCCGCCATTCCATCAGAACCGTGCTCACGCAAGTCC
TGGTCAAAAGTGTCC
GCCCAGCCGTCATATATCTTGATCATGTCTTCACTACAAATACTG
GAGTCGTTGATTTGG
TTGACCTTCCTCGTGCCTTCACTTTCTTTGTGGACTCCATTCTCCA
TGATCCACCTGGTC
TGAAATTACTTAATTGACATGTGCAGAGATGCCGAGCGCGATAGT
GAAATATTTGATTGG
ATCCTAGAAGCTATATTTCAGAGGTGGTAACT
>ConsensusfromContig2608 Average coverage: 11.79
TGACAACAGACGACGCGGACGACTTATTTGCAATCGATGCGATA
TTTCAAAGAATTATA
ATCGCCAATTTATGAGGTAAACAAGAAAAATGGCCGAGATCACT
GCGGAACCCACAGGCA
CGGATGGGTTACATAGACCAGGCAAGAGAGTAACCATGTCGGCA
CCGGCTGAATTGTTTG

AACGAACTCCTGTAATATCGCCATACCCAACATTGGTCAAGCAAG
TGACCACAATTGATC
TCAAAGGGGATGTGACACGGGGGGATAAACCGATGACCCCCATG
CCAGATTATCTGCGCC
CGATTATAAAAAGCGCGGCCGATCGTTTTCAGAAACTCCATGAA
GACCACATGATGGAAA
AAATAAAGCTGAAATGGGAAGTGAAGAAGGAAGAAGAGCGTTAT
ATCAAAGGCCAGGCTC
GTAAAAAACTGCGTCGAATTCCCATCGAACTCCTAGCTCGTGATT
GGTTCACTGAGAACG
ATACTGATATGGAAACAAGAGTATATCTAATCGATAAAATCCTGC
CAACTTTGGTGACTG
GGGTGGAGAAGTTGCTGCAAGAGATTGAAAGGAAGAGTTTCGAC
AAAAGCCTCAGGAATC
CCGACCCCAACTTCAACCCGG
>ConsensusfromContig2609 Average coverage: 10.98
ATTTCAGTTGTATTGCTTTCGATTAGGTATAACTGTCCATCATTCA
AAATTATCGAAGTT
TCTGTATGCACGATTGTTGTTGAAACGGAGACGGAATATCTGCAG
TTTTGTGATAACCAA
GAAAGCTGTAAACCCTTTTTGTTTGGGTCTTTATTAACGAAAGCT
GAGACAACCCATTTT
CCACTTCTGGGCGTTTCCCACTCAATCCTTTTCAGCGATTCATTTA
TTTGAACTGTTGTT
TGGTCAAATTGGCCTTCTTTTTCATCGCAAATCCCCTCATAAATA
AGGCAGGGCAGAGAA
TCGTGGCTG
>ConsensusfromContig2610 Average coverage: 4.01
TCAAGTTCTTTGATGTCGATGTTTTCATTTGAATTTGTTCTCTCAT
ATTTGGCAGTGAAG
TCACTCTCTTTTTTCCCCTGCTTCTTTTTTCTTCTATCATTCCAGTT
ATAGCCAAGTTCT
CCAGACCACCATCCCTCAACTCCTATCCAATGAGTCCCATCTGCC
TAATCCTGATTAATC
AATTTTTGTTCTCGACAACACCTTTTCGTTACTCGGTCTATCCACA
CATACAACTTTCAG
CATCCTTCTCAAGAGCCACATTTCACAG
>ConsensusfromContig2611 Average coverage: 22.16
CAAAAATATACGAATTCAATGAAAACTACATACATCTACATGTGG
TGATGATGAGTTCCA
AACTTACTTCTCGCACTCCCTCATTAATAAAAAGTATGTCCACATT
ATCCATATTTCCTA

TAGTGCATTTTGGCCCACCAGGCCGGAGCCCCGCCATTTGTGCGT
AAGGATGTAGTTGCC
CACCTTGACCAGCGTGTCCATAGGGGCATGTCCCGAGACACATG
TTAACATCAAGGAAGT
CTGGTGCTATGATTTCTGTGATGTGCTGGAGGCTGATTCTGTAAG
GACGCACACAGCATT
TCTCATATTCATCTCCACAGTAAGTAACCGCCGCTCTTTTTTTGA
GGCGCCGAGGAGTCT
TCATTGCGATTTCAATGAAAGGATTCTCCTGACCGTCTTGGTCAC
ATCCAGCTACGACAG
CGCGTTCATCTTGATTCTCATTTGCAACACGAAGGCCCAAAACCG
TTTGATTTCCCTCGA
GCATTTCCCTCAGTATTGTCTTATTATTCAGTTGGAAGACGTACC
AGGAATTTTTCGAAT
TTGAGATCAAATTTTTAACCCCAAACAAACCATCTCCTTTCGTAC
TGTTGAGAATTTCAA
TTTTGACGGATTGTGGACTTCCGTGAAATTCAAGCCGTTTTCAT
TGACAACGAATCTCA
AAAATATGGAATCTATCTTGTCGATGGTCAGATTGCTGTTGAATT
TGAAACTGCAGATTG
CATCAAAAGAAGCGTCGTTGTGAGATGCTATCGATCCAAAGACGA
GCCGTTCTTTCAATA
TTGGATCCGTCGTAGCACCATCTACTTCTGTCTCATCATCAATAA
GCATATCACGAACCC
ACTCCGAGTGATTGAAGTTGGTGAGCTCTTCAATTGGAGACTCTG
TTATACCAAGATCTA
GAAGCATGTTTTTGATTGTATCTGTTCGATTCTTTTTCGCTGCTTC
TATGATCGACACAT
TGAATATGTTTTCTAACGGGATCTCTTCACTTTTGATTGAAAAAAA

>ConsensusfromContig2612 Average coverage: 5.03
GATCTTTCACCGATACGGTCGAGAGCATCTTCTTTGCGATCCTGA
ACTTTTTTGAAGAAA
CTATAATGAATGTGGCATTATAATTATTATTGTCATTATCATTTCT
ATTATTATTGTTGT
TGTTACAAAAATACAAGGCTGTGTTGATATAGCAAATGACTCAGT
TGTTTCATCTGGAAA
CACTTCTCTCGCTTCGTCTTGAGGATCGCATTTAGTAATCTCTGAT
ATATGTTAATTGAA
TGATATCGGATCTGGCTCAACTCTTTTAAAATGAATTTCAATACCT
AAGATGTATTTTTT
GTGTGTTAAGGTACCATTTTTTATTTGTGTCAATTATGAGGAAGCT

GCTTCTTGGTTTCA
AGAACTTTC
>ConsensusfromContig2613 Average coverage: 9.84
AAAATCCCTTCTGAACTGTCGTTGCTTTTCTGCGAAGACGACGAT
GTTTGCCGACTCAAG
AGCGATGACTGTCGACTGGACTTCGAGACGTGACGAGAAGACGT
GGAACCGACTCGAATC
ACTTCATTTGTCCATAGTTTCTCATTTGGGAGTTGACTCCACTGAA
GAACAGCTTCGCTT
TCGCCAGTCCACCCAGCGCAACTCACTATCTTCGTCTTTCCAACA
TTTGAATAGCATAAA
CACCTCAGAGGCATGTCCGAAACTTTAATTCGTGCATCAAGAATC
AAATC
>ConsensusfromContig2614 Average coverage: 24.38
TTTGTGAATTATTGATATCTCCTTTATCAAAGTAGCTGTGTACAG
GAAAATAGCAACCAT
TGTGAGAATAGGCTTCAGAGGTCGATCGAATTGAAACAATGTTG
ATGCAAAACTCCCGAA
AGATGGCATGAGTCCATATGACGTATGGCCAGCTGAATACCAAC
TGTGGTTGAAGAGAAC
AACAATCCCAAGTATGAAATTCACAAAAGTAAGCACAAGTGAAA
CCTTCATCCGCCAGCA
GTCTATCAATATATTCACAGGTACTCTAGTCATTAAATAGCTTAT
TCGATCGTATACACC
AAGAAATCTCGACCTTGGTGGTGTTTT
>ConsensusfromContig2615 Average coverage: 31.83
CAGCCTTCTCTGGTGACTTCAGCGAACTTTGACGAGCATTTATCG
CAGCGACGTCCCATG
ACTCCTTCGATGCAGGGACACTGGCCGGTCGTGTTGTGGCATCGG
GCTCCAGATGAAGAG
ATTCCGTAGCAGTCGCAGGGCAGACAGGAGTCGCTTCCCGGCGG
TCGGTAGTGATCGGGC
TTGCAATGACACACACCAGTCTTTTGATCGCATAAAGGATCAAAC
CCCAGAGCCTCTTCA
CAATTACACGGTCCGCACACCGGAGCGCCCCACCACTTGGAGAG
GCACGTTTCCCGAATG
AGGGTCTCACAGAAGACACCCTTGAATCCCG
>ConsensusfromContig2616 Average coverage: 21.93
GTAAACAAAAACAAACAGTTCTAGGTATAACCCAATTTGATGGA
AGATAATTTACCAACA
ATTATTCATGGTCTTCATGAATCAAGCCTAGAGCAATCAGAAACA
TCCTATCTGCGAATA

AAAATCAATTGTGGCATAAATCTTGCGAAAAAAGATATATTTGGT
GCTAGTGATCCCTAT
GTGCGAATTGAGCAGCTACACCCCGTGGCTCAAAATGCAACTGCA
ACAGTTTCAATTGAC
AAGGTTGAGACGAGAACAATTAAAAAGACATTAAATCCTGTCTG
GAATGAGGAATTTGTC
TTCCGAGTCGTGCCTACCAAAAGCCGTTTTGTCATATCCGTGTTC
GACGAAAATCGAATC
ACAAGGGATGACTTTTTGGGAGAAGTTCACTTTGACCTCTCTCAG
GTCCACATACGGACG
GAGAGGTCAGACCGAGAGATTCAGTCCCGTTTCTACATCTTGAGA
CCTCGATCAACGAGG
TCACGGGTTCGTGGTCATCTCAAGTTGTGCTTGTCTTATGTCAAT
ACCGCGGGGGCGGAA
CCAGAAATGCCCGACCAGGAACCGGAAACCGAGC
>ConsensusfromContig2617 Average coverage: 18.75
ATTTCGAAATTTATAAGCATTTGAATGATAAGCATGATGTACAGC
TGATTAATATACCAA
TAAGATATAAAACATATATGCCATTTAAAATTCAGCAATAAATGA
AAGCACTCATTCGCT
ATTTCATTCACAGAGAATCAATTTGAAAAGCAAATCATATTTACA
TAAAGATCATATTAA
AAAACCAGATCGTTCAATGTAACCACGATCATTCTAAAAATCGA
TAAAAGAATTATGTGG
GACCGGATTGTTTTTTGATGAGCG
>ConsensusfromContig2618 Average coverage: 22.52
CTTTATGCCATCACAATTTCGGGGGCAGATATTGGAAGGAATGAC
GTGCTGCATTTCGAG
CATCTATCACAGTCATTCCACAACCGTCCAATCACAGGAGTTGAT
ATATGCATAAGGAAA
CCGATAATAGCCACGTGCTCTTTGGATAAATCAGTTCGAATATGG
AATTTTGAGAGGTGC
AATTTGGAGCTTTTCAAAGAGTTCAGTGAAGAGGCTTATTCCGTG
GCTATTCATCCGTCT
GGGCTGTACATACTCGTAGGCTTCTCTGACAAACTGCGGCTCATG
AATCTCTTGATTGAT
GACATAAAACCGTTCAAGGAATTCACAATTCGTGGATGCAGAGAG
TGCTCTTTCAGCAAC
GGTGGACATCTTTTTGCGGCTGTTCACGGCAATGTCATTCAAGTA
TATTCCACAACAAGC
TTCGAAAATGTCAGCAATCTGAAGGGTCATAATGGCAAAGTCAG
ATCAGTGGTCTGGAGT

GCAGATGACTCTAAGATTGTCTCCTGCGGAATGGATGGCGCCGT
CTACGAATGGGAAACC
AGCACGAATAAGAGGATTGCAGAAATTGTTCTCAAGTCTTGCAGC
TACACAAGCATCACT
GTAACGCCAGATTCCAAAACGACATACGCCGTCGGAACAGATGA
AACTATAAAAGAACTT
TCCGATTCTAGAATACTGCGAGAGGTGTCGAGCAACGGTGTGGT
ATTGACAGAAGTGGC
>ConsensusfromContig2619 Average coverage: 9.91
GCGGTATCTAATATTTTAGAATGATAAATTTTAATATCGCACTTA
TATATCATTTCATGG
TAATTTACAGCTTATTTCCTTTATGTTCTACGGGTTTCTTAATCAG
AAGTTCTACCAATG
TAGGAAAGGCCATCCTCACATTTGCATTACAACTTGTAGATAACA
TTTTATTAACAGATT
AACATTTATTTGATGTATTGGAGCCCAAGATTTGAAAATAAAAAG

>ConsensusfromContig2620 Average coverage: 13.28
TTTCTGCGAGATTTCTCTCGGAGAAGCGTTGGAAAGTGGTGTTGT
TGATGATGCGAGGAA
TGTGTATAGAGATGGAAGCACGGGATCGGTGATGAGGATTGAAA
AGGCGATTGAAAGAGG
TTTCATTGATATCGATATGGGAACTGTAACGGATCGGGAAGAGTT
TGAAGAGATTAGTCT
TCTGCACGCTTTGTATGCTGGAATCATTCTTCCCATCAATCAGAA
TAGAAGAGAAAATGA
GTATGGAGGTGGAATCAATGTTATTGAACATGATACTGTTAATAC
AGGCGTTGATTTGGA
AACAGTTATTGACAATGATATAAACATAGAATTTAAAAATGAGT
CGTCGAGTACAGTCAA
CCTCGCTCCATTCATTGAAAATATGTTTGAAGATCGAAATATCAC
CAGCGGACCGATCCC
TCTTGATTCAGATTCTGGATTTGTGTTTAAAGCGGTGACTGACGA
TTCATCTGTTGAGAG
CGGTAGAAACGAGTTGGACAGAAATGGAAATAGAAACGAGCAAA
ATCAGATTCTGAGCGA
AAAGGTTGAACCTCTCTCATCTGTGACCAGGGAACAAAAACATA
ACTTGCACACGACAGA
TACAACCGAAATCCCACTAAATTATGATATCCACGATTCGTGTAA
AAGCCCTTCGGTTTT
CTTAGATGCCGAGAGCGATTATGATGTCCATACATCAGAAAAATT
ACAAAGAGAGTTTTC

GAATGTCAATATAAGCATGAAAGCTACACGTGGCCCGAACACCA
TCGATCATGATTCGGG
CGAAAATGGCAACATCTCAAATATAGAGAGAAAAGGCGAAGACA
GTGAAATGCCGTCGTC
TGTAATCAGGGATCAAAAAC
>ConsensusfromContig2621 Average coverage: 19.50
GGCTTATAAGCTTACAACATTATATTATACTGTAGACATTTCAAA
GTGTATTGTTGTGAA
GAGTAGACTGTACATTTTAACAAATATTTTATATCAACCTGAATT
ACAACTTGTTCTCAT
GATTTACACGTCGGTAGAATACTGCATTTCACCTCAAAATGTAAG
AGGCTGGCATAACTT
TATTGTGAGAGTACGAATTGTGTTATGCAACATTGATCTGAGCTT
CTTTGCGTAGGTTCA
ATGTGATATTTTCTGTTCACATCCACTACTTGTGTAGAGTCATAGT
ATTTTAGCCGAATG
TGG
>ConsensusfromContig2622 Average coverage: 17.45
GTTTCTGCCATGGACGTCCGAAGCGATGACTATTCGAACGTTTCG
AATTCTTTTTTGATG
GACGATCCGAAGCTTTCTTTTGTACTAAGAATACATCGTTGAATA
AGGTCTTCAATTCCG
ATTCCGAACATTTTTCTATTTCTTGGGCTGCCTCCTGCTCATGCGC
CTGAACGATTTCCG
GTTCCGGAGGAAAGTTGGGCATATTTAAAGCTTCTTGAGATTTCA
TTTGATCTGTGGAGG
GCGATATCCGTATTACATCGTGATTAGAATTCCGGGCAATGTCAT
TCTTTGCATTCCAAT
TGACTAGTGGCCCCTTTGATGGGGTCACAGGGTCAACTGCTGCCC
CCCTCAATGGAGTCA
CCAGGTCAACTCCTGTCCCCCTTAATGGGGCCATAGGGTCGACTT
CTGACCCCTTCGTTG
GGGTCATTGGAGCCTGACCTGATGTCCGGACAACGCCACTGTTTA
CAAGTGCCTGCTGCA
ATATGTCTGCGCTGATATTCTCGTTCTCTGCAAGCTGCTCAATTTG
TTCTTGAGT
>ConsensusfromContig2623 Average coverage: 4.71
TCTCGGTACTATCTGCAGTGGAGGCAGTTTCACTGGATGATCCCT
CTTGAAGGAACCTGT
TTGTGGGCGGGGCTTGATGTTACAAATGTCAAGTCGGCTAGCTGG
TTTCTGTTTGGAGAG
AGGGCGTGGTCTAAGGATATGCGGGTTGTATTTTTCAGTTTGAAA

AGAGTTATAGTTATT
TGAATTATTTCTGAATGAAAAAGTTCTGGACTCTATCTTGCTCTG
ACCAAAACTTCTATT
CAATTTTCCTGAGTATCCACCACATCTTTCCGCTAATGGGTACGT
GTTTGCGAGAAGTGG
GAGGGTGTGGCTGCTAGCATTTGGAGTGATGTTTTCGAAGAATAT
GGTGGAATCCTCGGC
AGAGAGGTTTGTGAGATTGCTGAAGCTTTGTTCCAGAGATGTCTT
CGGACGCGTTGATTT
CTTCTTCAGCCTTCTCTTTC
>ConsensusfromContig2624 Average coverage: 18.40
GACTTGGCGTTGAGGTCACGAGGAACCCTGATGGGCGTGTCATCA
TCTCGCATTTGAAAA
TTGGGGGTCCTGCTAGTAGAAACGGCAACATAAAAGTCGGGGAT
ATTGTGCTGATGCTGA
ACGATACAACCCTTTCGAATATGACCCAAACCGACCTAGATCACA
CGATGAGGTCGTGCC
CCCGCGGCCTTGTTACGCTGACCCTTGAGGTGTCCGGCTGTGGCA
GTACATCTGACACAG
GTGATTGCAAGAATGGCGATACTTCACAAAAAAAGCCTTCAAACC
TGAAGTCATTACTCA
ACCCAGTGTACAGACCCATGACCCTCACACACATGAAACCTTTC
AGCCCAATAAAAGCAG
TGGGAACACGTAAAGCGTCTTATCAATCTTCCTCTTCAAACACCA
CCAATTCGGGCAAAA
ATATTGATTTTCAAAATAATGAAATCAAGAATAATGATACATCGA
TAGATCCTATTAGCC
CCACTATTCGGAACTCTCAGGAAGATCATTCC
>ConsensusfromContig2625 Average coverage: 11.43
GCTGTAGATCGACGAAGCGCGATTTGTATGTAGTCTGATATGATT
TATACTGGATTCATA
TTATTGTATAAAAACAGATAAATATGCTAATTATTCAGGGAATGC
TATCAAATCAGTGAA
ATGTTTACACGGGTTCTGTGATTTGTGGGGATGTTTCGTAGAATG
ATCAACATTTCATAT
TGATATTTCACCAAGGAATGTAACAAACATCCTCACAACTCCAGT
TCTAGTGATGATTTA
TTATTTTTATATTTATGAGCATTCAATATTCACAAATATCTGTTGT
TATGTATGGTCGAT
TATTTTCATATCATCAGAGAATAATCATCCACCAATTATTATTGT
GGACCGACAATGTTG
ATTTGTTTACCAGGATTGCTTTCATACATAGGTTTAAGATAAATTT

AGTATTATTATGTG
AAAGAATGAAAAAGGAAACGATTAAAAAGAGG
>ConsensusfromContig2626 Average coverage: 8.58
GCCCTATCAGTGTATGGTTCAAGAAAAAATTGTGACAGAGAGCA
GGCAAATATCTGTAAA
TGATCAGACTATCATCTTGTTTCAAAATATGATTGGAATGGATTG
AAATGATGAATATGG
ATTAGGTATGCCTCACGATATAATTATGCTTATCAGTGGATGGTT
CAAGAAGAAATTGTG
GTGGAGGAGGCAAATCTCTGTTTATGTTGGGATTATCCTCATGTTC
TTCTTCCTTCGCGT
CACCTTCTTTGAGCCGCATCAATCGCTGCAACGTTTCTTTCTCAGA
CTCGCAAATTGTAT
CCAATTGACCTGGTCGAGTATACCCAGGAGGGTAAGTGTTTTCAA
GATGCTGATTTTGTG
GATTATTTTTTGAATCATGTTTAGAAATGTTTCCCATTGATGTCGG
TAAATTGTTCAGAG
TCGATCGCTG
>ConsensusfromContig2627 Average coverage: 18.26
CGAACAAATTGTTATGTCAAAAACACATTTTATTGCACATACTCA
ACCAAAATGTTTTCA
CCGGCGTTTATTCCGTTTTGATGCCGGACTGGGCTTGGCATAGCG
ACCGGCACATCTCCT
CTTGGTTGGAGTCAGGCAATCTCCGCCTGCTACGATTCGACCACG
CGTTTGATGTGTACT
CTCTTCATAAACACTCTCACTGGATTCACTATCATCGGTTGTATCG
CTTTCTTCAACCTG
TGAATCCTTCTCAATAGAAACACTTGGTCTATGGGACCTTTCAGG
ATTGATCTGTTCGAA
TAGATCGGAAGAGCGTCGTGTAGGG
>ConsensusfromContig2628 Average coverage: 12.01
CTATTTTCTTCGGAATTCATGAAATTTATCTTTATCAAAGTCATAA
ATTGTATTTTCAGA
AGAGTAAGGATTCAGCCGGCAGCACTGGAGAAAGAAGCTCTACC
GTTGGAGAGAATATCA
CAAAACCACATTCTGGTCCAATCACCGGTCCACCTGAGCAGGTTG
TCTTCATAGAAATTT
ATATTGTGTGCGAATTGTGGCATTATCCAACAGATTTGATAGAAG
ATCCATTCTCACATT
TGTTCAAAAGAAAGTGCTCTTTGAGCTCCCATCTGGTATTGAAAA
AAATGTTGTGTGAGT
CGAAACCAGGATCACAAACTGATGAAATTTGGTGGAAGAATCCC

TGGTTGGATGTTGGAT
ATAAAATGGGGAGCATTGAACTGTGCTACTAATAAAATAGTTGC
TTACACAATTGAGA
>ConsensusfromContig2629 Average coverage: 29.65
TATGGTCATTAAAAATAAAAGCATTATTCTTGACATTCACACATA
CACGGTCAAATGTAT
ACAAATATGTTGTTTGGTGAAAAATAAAACCTTGCAAAAACAATC
GTGCATTCTTAGAGA
GAATTTTAAGTTATATTCCGAGTATTTTATACATAGATCACAAAA
CACGTTGAAATCAGA
GTTGAAAATAGTAAATGTCCAATGTATTCAAATATTCCCACGATG
ATATCACAAGCACAA
CCGAAGATCAACAAGCCTTTCTATATACCCGATAATGCTGTATAA
AAATAGACAGACTGA
TTGTTAGATTCTGAGAGAAAAAATATGTCGTTATGTCTATGAAAT
TCAAAGCTTCACAAT
CTTTATGAGTTTCAAAAATTGATGGATACTAATACAGATTTATTG
AGTAAAAATGAGATT
CTCTATGACGTAAATAGATTTGAAATAAGCTGAAATTCGAAAATG
AGATTATCACACAGC
TATAATCCAATAACTCATTTATTGAAAATCCAGAAC
>ConsensusfromContig2630 Average coverage: 9.82
AAAAAATTCGTTTAGCCATAGCTTCGCCGAGTGGATCCGCCATTG
TCAACCAGAAACTGC
ACCCTTTCAAAACCACCGAAGTCCATATAAGGGTATATCAACAAC
GACATCGAATGTCCG
TTAAGACATAGGCTTATATCCATTTTGCAGTCAATGTCGGGTTTT
CCATAATCAATGTAC
GCGCCTTGAAGAGAAAGGCCAATATCATCATCGCAGAATCCGTTC
TTGATACTGATTTTT
TTGCCATTAAATGACACTCTGGCACTGATATCTCCTATCACAGTT
GATCCCACCAGGTGT
TTCAACGGTAGATCTAATGATTCTTTGGAGAACAGGTATGTTGCA
TCATATGCATACACA
GCATCTGGATCACATGTCTGAGCTTCTTGATAAGCAGTGGTCCAA
TCTCCAGCTGACAAC
TCTCTGGGAATAATTTTTATATCGTGCATCAAGTGTTTTCCTTCTC
CACCAAATTCAAAT
TTCATACTTTTTCCGTAATCAGTCCAGGATACAGCAGAAGCTACT
GCCTGTTTTCCCCTA
TTTATATATATTGTCAAATCGGAATTCCATAT
>ConsensusfromContig2631 Average coverage: 4.28

CCCACGTCCTGCCTGATCTTTCACCCGAAGATTTATTGCATGGTTT
TGATGATACTCGTT
GCAGATTCTTGAACTTTTTTGATGGACTTGGAACCCTTCTTTCTAC
GTGGATGGTGGTTG
CTTGTAATGTGGAGCTTTATATTGCATCATCCTCTTTTACCTTCAG
AAAGAAAGTTTATC
ACCGATCCAGACCTTATTATACACTCTTCGGTATCATCCTCACAA
GTATGGCCGCA
>ConsensusfromContig2632 Average coverage: 9.82
GCTTTTGAGAAGGCGCAGTTCCGTTGGTCCGGAAACCAATTGCGG
TTCCCATCAACTGCT
TTGCTGGAGTTGGTTTCCTGAGAGGATTGTAATTCATTCTTCATTT
TCGTTCTCTTCTTA
TACGCCAGATTAGGTCCCATGTGAAGATGCGGAACTGTTTTGTTG
ATGATGTGAGAATGC
AATTCATCGCCGTGAATAAAATTGGATAAAGAGACTTTCGGAGCT
TTGCATCTTTTCTGG
AACGGATCCCAGGTATATCTTCTGAGGCATCTGCAATGAAAGTCC
CGTTTCAAGATCGGC
CCCACACCAACCTGATTGCAGCTCCCTGCCACGGCTCCTTCGATC
GCGTCGCACGGCCTG
CTCCCACAAATGCCGACTGAATTTAACCTGCAGCGACGCCATAG
AGATCGGAA
>ConsensusfromContig2633 Average coverage: 51.96
CGTCCCCTGGTGGAGGCGCCGGTAGAGCTACGAAGACACTCGGA
TTTGAGTTCGGGGTCG
ACTCAAAAAGTTCTGCGCAACAGAACAGAGCGGGCTCATCGGCTT
CGGTTCCACTGATGT
ACCTCGCATCTGGCCACGTGACTTCGGGGTCTTGTCGCTGTCGGC
TCTATGGACCGAGTG
TCGTTTCCGGTCTTTGGCTTAATAATGTTAGATTGGCTCCCGTCTC
TCAGGATGGTCATT
TTGTATTCCTCTGCCGGCCTCTGGGGAGTGGATTGAATATGGTTC
GCATCGAAGTCCCTT
TGATCCAGTGCTGTAAAGACAATGTTCTGATTATTGGTGTATACG
AAACATGCTACGGCC
AGTGAAAGAAATATCGGTACAAAAACTTTCGCTTGGGCTCTTCAT
TAATAGAGATTATTC
CCTTCCAAACAAGAATCAAGTGGACGAATGTCATCCGCATATTTA
AAATTATACAGAGAT
TACATATTATTTATATGTAACGTAGCGATTGAATTCGGATGTCCA
ACATATGTTCCTAAT

TGTAGGACAATAACTAAAATGCGTATATATTACTCATCGCGATTA
ATTCTGAAGACACTT
TAGTAGTTACTTTTTCATAACGACAATGATACGATAATTACTGCAT
TCTTACCATTTTTA
AATTACTACAAAACTGTTTGAGA
>ConsensusfromContig2634 Average coverage: 9.55
TCTTTGGTGACATCTGGATTGAAGTAGTTCGGGTTCTGACTGACG
ACCTTGAGGTTGTCC
ATCCACACCGCCGTGTTGCGGGTGTCGCCCCCTCGCAGCCACGTC
GAGGCTGAGTGCCCG
CCCCCCTGCGACCGACCACCGATTTTCCGTCTTCTGTACCTCTTC
ACAAAAAGAAAGATG
GCAATCACAAAAATAATAATGACGATAGATGGCACAATGATGTA
TAACAGAGTTTTCATG
AAATCGTATTCATCGGATGGGAAGACCTCCATGGAATCTGGATT
GAAGAGATCACGTGAC
GGAAATGTCCGCTGTTCGAAGGTTATGACTTCCCCGTGTTGACCT
GGCATTCCGGGGCAC
CTCCCATTATCCAGCGGGGGAAGCTCGGTGTTCGTGCACATACAA
TACACTTC
>ConsensusfromContig2635 Average coverage: 7.10
CCAACTATGAAGCGACATTTACGTATAGTCTTGGAACAACAACA
CTAGCGGCGAACACAG
CTATTCCGGGATCACATGTCAATCACACGAGAGAATGGACCCCAA
GCAGCAGTCTGCCTT
CACCACGAAAACCAAAGCAGACGACCAAGAAGTTTGATGAAGTC
GAGGCTGCAACCGCGA
CAACAGAGGAATTTCCTAGCCTCGCAATGGACGGCAGACAAGCTG
TTGAATATTTCGCCA
AATGCTTTCACCTCGGCAAAATTGAGTCTCTCCATTTCAATATAG
CAGAGAATAGGCATT
ACAATCCCTACCACATCGTGTGTGTTCCAGAGAGTAAGACAAATG
TGGAGCATTATGCGA
TGTCGACATTCGGGATTCTGCACACGTTTGGTCCAGAGGAGTTGT
CGGAGACCATGTCGC
TGGCGGAGTGGCATCGTCAGG
>ConsensusfromContig2636 Average coverage: 9.36
CAAATAATTAGAAACTGTTTTCATATCATTCGTTGAATATGTACA
AAAGACTGTTATGGG
CGGAGTCAACATGGATATCACGTGTCAAGCACTTGAGAAAATAA
AGATGGATGGTTCTCA
GTGCAACGGAGATATAACGCATTCCGTTGGTGGAGTCGGGAGGA

ATATCGCAGATGCAAT
CACCAGAATGGGTCATCCAGCCCCATTCGTTTCGTGCGTGGGTGG
TGATGATTTCTCCGA
AACAATTCAGGCCAAATGCTCCCACATGGATTTCAATCACGTCCA
TAAACTTGAAAATGA
GAAAACCGCCACATATGCAGCAACCGTCGACTCGAACGGATCTC
TCGTCCAGGGGTTGGC
GTGCTACGACATCAACAAGAAAATGACACCTGCGCTTATTGGAAG
TCATGAAGATCTTTT
CAAGAAT
>ConsensusfromContig2637 Average coverage: 18.09
TCCCCCTTGGGCGCCTCCCCCAATGAAAAGGGGGGGGTGGGCA
TGATGATACAGCCGAA
GGCCGCCACGAATGAGAGCATCTGGGCCCGGGCACCGGATTTGG
AGGAGAGAACGCGTTG
AAAGTACACCTGCCACGGGATTCCGCCGAAGATGAGCAGGAGGC
CCGTATCTACCCATGT
CCCGTAGTCCCTTGGTTCCAGCGTCCCCACCCAGTCTGTCGTTCTG
TTTGATGTCGCCTG
GATGTACTCGAAGTCTACTTTATCGCTAGTCGCTGCGAATGGAAC
ACTCAACCACAATCC
TATGAAAATGCAGAACAGTTGAACGACATCGGTGTACGCCACAG
AATAAAGACCCCCGAA
GAGAGTGTAAAAGATGGCGATACAAGCGGACGCGATCACCGAAA
CATCATCCCCAAGATC
CAGGATGACCGACAATGTGGCACCGAGTGCAGACAGAATGGCAG
CTGACCAGAAGACCTC
TCCAAAGAGAGCGGGGATGTACAGCAGCCCTCCCATTCGCTCACC
AAATTTGTTCTGGAA
TGGGTCGAGCATCGTGACGTATCCCTCTTC
>ConsensusfromContig2638 Average coverage: 6.21
CTAGTGATCGAGATAGATTGCTTTATCTATCGCAGTTAGTCTATCA
TTCGTTTTATTTTA
TCGGTTTTCAGCAAATAATCACATAACACAATTTTCGTTATTCTA
TAGAATCGAATCAAA
TAACAGTTGGATTTTGATGCATTACAGGTATTGAACCTCTAGAGC
AGTACAAAAAATACT
TGAATGATTTTTTGATAGGATATAGGCTTCATCCCACATTCAATC
ATATGTTGCAAGAAT
GTATCTTCCGTTATTTGTAGGAGGAAACTTGATATATTGTTTCTCA
TATAATATGTATTT
GAATAGTTTGATATATCAGTCGGCTGTTATATGTTATGTTTTTCTA

TATTGTCAGTTGCC
ATTCGAGAATATTTG
>ConsensusfromContig2639 Average coverage: 21.48
GGTTTAGGCGGCCCCGCCCCTTCCTTAACTCCTCCCCCTTTCTGAT
TTTGTAATCTGTTT
TTGATTGTTGATGACTTTAGCTCTGGTGGTTCGCCTTCGATGACG
AAAATCAGTTTGACG
CCGAGTTGCTGAAGGTTGGAAACCCGGAAAAAGAGGTTCCTTAG
ATAAATTCTGTCATGA
ATGCCATGCTTTTCACTGAGAGTCTGATCTCCACAAACCCAGATG
CTCAAATCAACGGCC
AATGATAACCCTGAGAGGGACGTTAGAGGTTGGTGGCAACGGAC
GGGAGACACAAGGCTC
CACAAGTCTTTGACTCCCATTCAACAATTGATTCATGCATATGAA
ATCTGTTCAAAATAA
TGTATCTCAGTATAGTAAATGTGTTGAAAAATATTATAATACATC
TCGAAATTTATCCAA
AATAATTTATTTTCGAATAATCCG
>ConsensusfromContig2640 Average coverage: 7.20
CGAACGTCAAGTAGCCTCATACTAAATCTCATTGTACTTGTGAAC
GGTCTCACACCGTCA
AAAAGATTACTCGATATCATACGGGATCCCTGTTTCACAATAATA
AATCAGTCTCGCACT
GATGTAATGATAGATTTCACACGGACTCCATGTATCTCATACTAA
ATCAGCTTCACACTA
ATTTTGTGAACGGTCTCACTACGTAAAAAAATCACACAATATCAC
ACGGGATCCATGTAT
CACAATATTAAATCAGTCTCACACTATGAAATGATAGGTCTCACA
CGGACGTCATGTATC
CTCC
>ConsensusfromContig2641 Average coverage: 8.08
GAAATATTTTAAGCATTTACTACAGGCGGCTTGAAATGTGTCAGC
AGTATGCATACGTTC
GCGAACCTCTGCACTCTCGGATTCTTTACATGAGGATGGTTTTTC
ATCTGAATGAACATC
GCTCATGTGAGCTTCCAGTTGCTCTTGGTATACAAATGATAAAGA
ACATACCAAACAAGC
ATATGATATTGATTTCGCATGACGGTACAAATGCAAAT
>ConsensusfromContig2642 Average coverage: 15.44
TTTTCGGATTTATTCTATTCATAATTGGTTTGATTGGCAATGTGTG
GCTTATAATCGCCA
TCCTACAAACGAAAAAGCTACGGACTGTTTCAAATTCATTTATCG

TGAGCCAATCGTTGA
ACGACATTTGCATTATTTTGTTTGTTTGTTTTTTCCAGATCGTGTT
CTCATTGGGCAAGA
TCATCAACTATCATAACACCGTCTGTTTACTCCTAACATTAAGTT
GGTACACATTAATTG
TCCAATCGGGAATGTTTTGTGTCGTCACTGCCTTTTATCGCTACGT
TTCCGTCGTTCATC
CTGAGAGTTGTTTACCTCAAAGCAAATGGGGGTGTGGCCTAATGA
ACATATGTGTCATCG
TTATATCTTTTTTGTGCTTTTGTCTTCCGTGGATAATCCGTGTATT
TATATTAAAACAGG
CTCTATATAATGACGTTTCATTTGACGATCGAGCCCTCTTATGCG
TCGTCAGCAAATTAT
ATAAAAACAACATGATATCTATTCTATGCTCTATTCTAGTCGGGA
TTATTCTCATCAGCT
TCTTCTACACACAAATCCTTCAAGCTGTCCGCAAAAGTTCGCATA
AACTCTTCAGGGTTC
GATCGAATATTCGCGCCAAACGAAATCTGAAGCTGGTCCGAACAA
GCTCTGTGATTTTCC
TGCTGTATGTGATCAAGCAAGCGCCAATTCCAGGTCTGTGGCTGC
TGGACACCTACACCC
ACGTTCCTGGAGCTGTCTTCAGCGTGTC
>ConsensusfromContig2643 Average coverage: 14.19
GTACCACTGGGAGATGATAGTGCACATTTGCCTCAAGCACCTGA
TAGTGGACGATGCAAG
ACTCCAAGGAACGGACCAAGAGTTGCTTGTGTTGATCCAATGCC
AAGGCTGCTGGGCAAA
GAAGAGCCCTATGCATACACGCCCCCTGTTGTTCCTGCAGCACCA
GAAAAACCACCGGAT
GAAAGAATGAAAACTCCTCGATATAGAATGGACGCCCGAATTCA
CATGCCTGACCTACCA
GACCCATCCAAGATCCAAGACTGCCCAAAACCTTTCGGACCTGAT
GAATATGTACAAGTG
GACATAACCGAGTACAAAGATAGGTACATCCCGCAAGACGAAAA
CATGAAAAACATCGCC
TCGTTGTAGATGGCGTTGTAGTTGTTCGAATGGTTCGAAGCTAAG
CATCTCAAACTGGAT
TTC
>ConsensusfromContig2644 Average coverage: 6.00
CAGTAAACTCCAATCCACAATAACACAATAATGATTTTAATTTAA
GATTCTCGTTTTCGA
AATCAATCATTAGATTTTTTTCATTTTTTTGTTTACATTCACTGCA

TAGATCATGTTTTA
CAAAACAACTTTCTTGGCAACACTTCAATAATTCAGTATCGCTTT
CACGCTCACATTCTT
CACAAACACCATTTTTCTTTTCAACAATTTTTTTCTTCAATAAGTG
CAACTCAATAAGTG
TGAGGTGATCTTTGATATGAGTGGAATAATATTTAGATATTAAAC
ATCTCGGTGTAGCGA
TGAGCAATTTTTTAGCCGAAAATGCAATTTTTCTAATCTTATTAAC
TTGTTTTACAATAG
AGTTTGAAGAAATTCCTCTTGAAGAATTTAAAACTTCACATATCA
ATTGCATATTTGCAT
GTTGCGATGTTGCAATGGTATGTTCATCTTTCTTTGAAAGTGCAAC
ATTCATTTTATCCA
TGATTTTAACTATCAAAAAACATCAATCATAAATACCACCATACG
TCAAAGAACACAATT
TCAACAACTGATGAATTTACGATATTACAACAATACTATGTAATG
ATTAATCTTAATTGG
TTGAAAAAATTCAAGGCGTGACTATTGATAGGCGCC
>ConsensusfromContig2645 Average coverage: 6.00
GGGAGGGACTCTTGAAGCTGATTCCTGATCCGATCGGGCAGTCAG
TTGATGAGCTTTTTG
CATTGACCAGACCAACAATCGATTTCTCTATCGATAGTATTATGG
CACACAGCAACAATC
AGTTCGAGATGATTTCTGTGGATCAGGTCCACAGAATCATCGACC
ATGGATCCGCATCGA
AATCATCCGCTATAGACGACACTGAAATCATGGCAATGGGGTTT
GACGATGACGGAACCT
CCAAGAGAATTCGGATCAAAGGTCAAATGGTTTACATCCGTGATT
GGGATCATATCGTGT
TCCTCGCCACGCCCATGTTCAACCATCTGGATGAATTGATCGACA
TCGGGCTCTACGTGG
CGGATCTGAGCAGTACAGACTCCAGCAGAGAGACGGTTTTGGCA
GGCAACAACCAGACAC
ACG
>ConsensusfromContig2646 Average coverage: 17.37
ACCTATCTTCTTCAAAGTCATTTAGTTGTTTTGATAAGCTTGTTTT
GCTAGGCTCAATTC
GCCGAATGTTTCTACTGGCATAATGAGATGAATCTGGCCGATGCG
ATTCATACATATTCT
CTTCAAAGCTCTTCGTATTATTTAATGCGCGTGTCTCGCTAAGTTC
GATTTGCCAAATGT
TTTCACTAGCAGCATGAGATAGATATGGTTCATGTGGTTCATACT

CATCTTTTCCAAATG
AACTTGGCTCATTTGATGACCGTATCTTACCATGTTCCATTTGTGG
AATGCTTCTACTAT
CAACATGAGATAGATACTGTTGATGTGGGTCATACCTGTTTTTCT
CAAAGTCATTTGTTT
CTTTCGAAAAGCATGTCTTTCTAGGTTCGATTTGCTGAAGGTCTC
CACTATCAACGGAGA
TAAACGGTTGATGTAACTTATATCTATTTCCTTCAAAGTCACTTGG
TTCATTTGATAACC
GTGTCCTGTTAGGCTCAATCTGTTGATTTCTCCTACTAGCGGTAT
CCGATAGACATGGTC
GATGTGGTTCATACATGCTTTCTTCAAAGTTACTCCCGTTCGATAT
GACTCTCTTATTGG
GTTCAACTTTCTGACTCTGCCCACTATATTTTGTATGAGGATTCG
CATAAGCTAACCCAT
ACATCTCTTCGTTAAGCTGATTCGGGTGATCATGGTAAGATGTCG
TCTGTGATGCAGGGT
CGTGCTTAGGTTGACCATACGTACTTGAATATC
>ConsensusfromContig2647 Average coverage: 9.12
ACATGATTGATGAAGTTCATAATACTGTTGTGATTGGACATGGAG
ATAAACTCAAGAAAT
ATCCGCAGATCAGTGGGAAGAAGTGGCGAGAAATGATGATA
AAAATGTTCCCTGATG
AATCTGAAGCTATTGACAAGTTCATGCATGCGGCGAAAATCTGCA
AAATGTACTTCATGC
AAGATATCTTTTTCAAAATGATTCCAATCTGGCTTGTTGATATCAT
ACGATTTGTTCGTC
TTGATAAAATAATGCTGAAGGAATATACTAGATGGGGTCAAACTC
CCTTGAACGAGTTTT
GTGACCAGCTAACGAGCAACGACGAGCTTAAAACAGTTCTCAGT
TATAGTTGGGGGATT
TGGGAATGCTTCCGGGCCTGATGAGTACCAAGCTTTATCTCATGT
TATTCAACCATTTCT
TCGAGGGAGGGTACTATCCAAGAGGTGGAGCAAGCGAAATTCCG
TTTCATATGATACAAG
TAATCGAAAAATTTGGAGGAAAAGTCCTCGTCAGAGCTAAGGTAA
AAGATATTCTTCTTG
ATTCTAATAACTCAGCAGTTGGAGTTCGTGTCGAAAAAGGTGGCG
AATTCTACGAAATAA
GAGCGAAATGTGTTATTTCCAACGCTGGTGTTATGAACACATTTA
CTCGATTGATTCCGA
GAAAAGTGAGTCTGAATA

>ConsensusfromContig2648 Average coverage: 4.93
TCTTCCGATCTGGCAGAGTTGGCAAAAGCCGCTACAAGAATTGA
AAACCCGTATAAAACG
CATCCGTACACAGCTGCTTTTCCACATCCCAGGGCATTTCCGACA
GGGACCCAGATTATT
GCAGATATACAGGGGAGTGCATTGAGTAGACTCGAACAGAGCGC
AATTGCGGAGCCGTTT
GAGGTGAATGACTTCTGCAGTTCAATGAATAATAGACCGAAGCTG
TTGATTGTCCCGTTG
TAAACGAAGTTAACAAAATATGCAGTGAGAGTGATCAGTGCCAT
ACATCGCTTCCCACAG
GCTGGG
>ConsensusfromContig2649 Average coverage: 18.34
TCAAGGGTGTTGATGAAGGAACCGATCCATCGTTTTTCGCTTCGA
TCGTCAATAATAATG
CTAATGGTCTCATTACTTGCTACAAAAAAGCAGATATACTCTTCT
CTCGACTTGCTGCTA
CGAAAGATTCTTTTATGGAATGGGTGGCCATTGGTTTTATGGACC
TTGAGAAGTATGTGG
AGGAACATTTATCAGACACGCAGGATTGGGAACTGAATTTCAAA
GCATTGAAAGCGAAAG
GGCGAGATGCCGAAAAGTTACCAAATGAAATAAAGGTGGATTGT
ATATGTGTGAACATGG
CGCCCGTGAAAGCGGTGATCGACGACCACATCCAACAGATGTTCG
ACGCTCTCGTCCTCT
CTCTTCGCCGCTCCATTTCAACCGACGTCAACCAGATTGATTCCT
TTGTGGAGTCTGCCA
TCGAAGCGCTTTCGCAGCGACCCAGGACTGTAGAGGAAATCGGC
AAAGCAAATGCACAGC
ACCAAGTCTT
>ConsensusfromContig2650 Average coverage: 13.80
CCACTTCAGTAACGAAGACTCTTCGATATTGTACGAAGAAAATG
ACAACGAAACATGTTA
CGGTTCGGAGTTCGTTATCAGCATCGATAAGTATCTGTATGGCGT
TGGACTCCCAATGTA
TGACCATACACTCCTAAAAGAGACAAAGGAAACCACGGCTGATG
CTTGCCTGCAGCTCTG
CGCTGTCCATCTCAACTATCTCTGCAACTCCGTCCAGTTCTACAA
ACCATCAAAAGACTG
CATTCTCTCTACAGCCGTCCCCGGTAGCGGGAATACGTCGATAGT
TCAGGTCGATGGTAT
GCTCTCGAATTGGCGCCAGAGATGTGGAGATGAGGTAGCCATTTC

GTGCAGCTTCGACGG
GGGGAAGCTCTGTTCAACCCCCGACGGTTGTGGTGACCCCTCCTC
CAAGACATGCCCTCT
CTATTTGGAGTCTTCTTCATCCAACGCCTTCTTCAGGCTTCGGTC
ATGGCCAAGGGCATA
TCTCACGAATAAATATTACCTGACACTATGGAGCAATCGCGCCGC
ATCCTACAACAAAAA
ATCCTATCTGATAACGCGCTTCATCAACGCCACAGAGCCCCT
>ConsensusfromContig2651 Average coverage: 63.80
AGCATCATGCTGCGAATCTTTGCCGTCGACGGAGGCAGTTGCAGC
GTCGACCGCGGCTGT
TGCGGCGTCCACCAGAGGAACCAGCGGGATCTCCCGGGATATGG
AAATGGCTCCCGTTCC
TTTCTCGCGCACTAGTTCGATGATTTCATCCACTTTTTCTATGAAG
TGAGTCATCAGAGT
TTCCGATAAGAGCTTTTCTTTTATCAGACTGTCAAAGATCTCAAT
CGCATCATCACCCTC
TAAATTGAACTTGAATGTAACCGCAGCGTGTTTCGCTGTTTCCAT
CTGACACTCCACAAT
TTCACCGTCCTGCTCGAGACCGATAACGGTCAAACGAAGAACCGG
CTTCTCCATAGTCTT
TCGCCGCCGACCTTTCGGTCGGTCTTTTCGGCGTCGGTCGCCTGT
CGTTGCCGCAAATTC
CGCGTCACTGTCTCGATAAAACAATGCATTTGGGTCCTCTTGAGA
CCCCGTGACAGGGGC
CGTAGTAATCCCGGTGA
>ConsensusfromContig2652 Average coverage: 12.37
CACGATTTGCGTAGACAGAAACGCCATTTGCATCGGTGGAATATG
TAGCTGTATTTTGGG
TTTCAAGTCGAGGAGTGGTGTTTGTGTTGCGGCAACAGCCACGAG
TCGGGAAGGGTTCTT
GAATGGCCCTTGTCTGTCTACTGGAGCCTGCATTTCTGCCAACCT
GGTCTGTGCCAGCAC
CAAGATCTGCGTTTGTGCCGAAGACCATTTCGCCAAAGGCTCTGT
GTGCGTCCCCAAAAT
CGCACTGGGATCGACGTGTGACGACGGAGACAGTTGCTATGACA
ACAACGCCGGCTGCAT
CAGGTCCATGTGTCGATGCGAACCGGACTTTTTCGAGAAAAGCGG
CGCCTGTGTCAGCAA
AATCCTTCTTCGTGAGGCGTGTGTTCCTTCAGATATATGTCTGAG
TCCTGAAGCGGAGTG
CCTCGGTGGAATGTGTCAGTGCCGATCTTCTTTTTTCCCCCAAGCT

CAACTGTGTTTGCC
CAAGATCCTCCTCAATGAACGCTGTCTCCCCAGCGACATCTGCG
>ConsensusfromContig2653 Average coverage: 9.69
CTTGACTGTGAGTATTGGTCGAGCGCACATGTATTTATTCGGACT
TTTCATCATTGTAT
AATATTATTGTATTTTCTGTGTCTTGGAACACGGTGGTCATTTGTG
TATTTCATTTCTTG
ACTGTGAGTATTGGTCGAGCGCACATGTATTTATTCGGACTTTT
CATCATTGTATGATA
TTATTGTATTTTCTG
>ConsensusfromContig2654 Average coverage: 71.03
TATATGAAAAAGCATATGAAGGCATTTGTTTTTATATAATCGAT
GCTAACATATACAGC
TTAATTTGAGGGTCATAGTTTCAGATGAATTATATAATGTCGGTC
TGATCAATGTCATCC
AACACAAGCATTACCGAGGCCTCGTTGCGAGACTTCTATATTGGT
CTAGGATTGTCTGTC
TCGTCAAGTTTCTTTATCGGAGCAAGCTTCGTTTTGAAGAAGAAA
GGTCTTTTGCGGATC
GCAACAAGAGCTGGCGACGGTGGGTACGGCTACCTTCGAGAATG
GATCTGGTGGAGTGGC
TTGATAACAATGGCTCTTGGGGAATGCTTGAATTTTACTGCTTACA
TGTTTGCCCCTGCT
GCGTTGGTCACTCCTCTCGGAGCCCTCTCTGTGCTCTGTAGTGCGA
TTTTTGCGTCTCGG
TTTCTTGGCGAGCAGCTAAACGCTCTAGGGAAGCTCGGCTGTTTG
ATTTGCATTCTCGGG
TCAACTGTTATCGTCATCCATTCCCCCAAAGAGCAACAGCTTCAG
ACACTCGAGCAATTG
GAACATAAGTTGATTCAGCCTCCTTTTGTCGTCTACTGCGCCTTC
GTAGGAATCTCCTGC
TGCACTCTCATCTTCTACTTCGGCCCCAAATACGGGCAAACAAAT
CCGCTGATATTCATC
GGCATCTCTGGTCTCATTGGGTCTCTGTCGGTCATGGCCTGCAAG
GGCTTGGGAGTAGCC
ATCAAGGAGACATTCGAAGGGGTCAGCGAAGTGACAAACTGGCT
CGCCTGGTTCATGCTT
CTTTGTGTCATCGTATTCATCACCATTCAAATGAACTATCTCAAT
AAAGCTCTGGATATA
TTCGCCATTCAAATAGTCTCTCCGATTCTCTACGTTATCTTCACGA
CATTTGTCATTATC
GCCTCTGCTATCCTATTTCGCGAGTGGGGAGCGTTGGAGGCCGAG

GACATCTTGGGTAAC
ATTTGTGGTTTTATCATCATCATCGCTGGAATATTTCTTATGCAAA
CCTTCAAAGATGTT
GTCATTCACTTTAATTCGATAACGGACATCCAAATCGAACCATCG
GTTTTGGAGTCGACT
CCGACCGCAAACAATGTTGTCTCTATTCCTGCTAACAACGATTTT
CAGTACGATCGGATT
AAGAATTCAAAGAAAAAGAGAGTCAACGAGATTCGGATTTATTA
CACAGCGGAAGTAGTA
GTCTCTCCTCCTCGGCATCGGCTTCACCCGTTCATAGCGTTCAAA
ATGTAGGATAGATGA
AGTGTGTGATTGAGATGTTAAAGCAGATAATTTATTTTATTCATTT
TCGATGCGATGTTT
GAATCAATGG
>ConsensusfromContig2655 Average coverage: 4.05
TCTGCCATTTGTTACTCGGATCGACTATTTCGGCAAATTTTATCTC
ATTCATGCCGAGAA
CATTCATCAATCCAAATACCGTGAATGCGATGAAAACATTCAGAA
TCATTTTGAAAATTC
AATGCTGATGACAAATCCAGCCAACTCTCAGAATAAGCTTTGCAA
GACTCTGACAAGTGT
GTAATGCTTATCGAACGGTTGGGTTTATATGCACATAAAATTCAA
TATCATATGATCTGA
TAGATGGAACTTTTCGATCAGATTGGCGCGTTATCAAATTTATCA
AGTATCGAATTATAC
ATTTTCGATTTAAATTGTTTGTTCACTCAATTCATGCGGAGCTTTT
TTTGAAATACTATA
TGCTTGATATGTGA
>ConsensusfromContig2656 Average coverage: 8.84
CGGCAGTCTCTCGATGTTCCGAGACATTGTTACTTTGTCTTTGGTG
TGTTTGTATGGTAT
TCCCTGTTCCTTTATCCGACCTGGTGAATTTCTGAAGCTACCTCAA
GTGCGGTTTGCATA
TCATTTCTCATCTGTTAATCGTTCGAATTGCACTGGGAGGGGATA
AGCTACAGCCTCTTA
TCAGGAATTTGAATGATGTATGTTAGCTATCAAGGATAATCCTGA
AGGATCCTACCACCC
GTTTCGAAAATGGATTTGAGAAATTCTAGAAATTTCAACGAAAAC
AAGTTTTCATCAACA
TAAAATGATGTAGTATATAACAATTTACTGCTCAAGTTTTCGAGT
TGATAGCCTTACAAA
AATGAGATCTTCTGCCGACTTTTGACAACAGTGGAGTATGGTCGA

TATAGAATTTCCCTC
GGATGATTGTTGATGATGATACGTTATCATAATAGAACTGTTCGA
TATGATGAATTTAGA
GGATGATATGCTCACCTTCTTC
>ConsensusfromContig2657 Average coverage: 24.35
TAACAATAATATATCTAGTCTTAAATAATGACAGTAACCTAATAA
TATCATATCAGAGGG
TAGAGAGACACTTTCTCCTCAATAGTCCCGATTCGAATCCACCAA
GTTCGATGAATAACA
ATAAAATATTTGGCATCCTTTAAGTCTCAAATAAAGTAATATAAT
TATTCAGAGGGCTCT
TTGCTTGAGAATATCATATACATCTGGGTTGATATCCTCTGCCCT
GGGAAATTCTCCACT
CTTCTCCTCAGTTTCTTTCCAGTTCTGACCAAGCTGCTTTTCATAA
ATCTTGCCCGAGGT
CGACCGAGCATAGACTGTCTCCCCTCGTGAAAACACGCTGTTTGC
TTCAACTTCGAATCT
TTGATACTCTTCTCCATCGAGGTCGATGAGTTTGCAGGTAGATGT
TCTCGAATCCCATTC
GAAGATCTCCCAATTCTTCGCGACGCAGACAAGCCGTGTTTTGGT
CGCCACCATTCCGCG
GATTTCAGATACCTCTTTGATCTGTGGGATTTTCAATTCGCTCCA
CTGAAAGAGATTATT
ACCAAAATCTTCGCATTTGTATAACAGGGACATTTCT
>ConsensusfromContig2658 Average coverage: 12.61
TATGTATTATGAGATCTTGTAGAGTTTTTGTGAATTTTGAGAATCG
GATTTTAGTATGCC
TAATTTGGGGTTTGAGAGTCAGATCGAGTAATTCTGTTAGAGATT
CCTGGATGACATAAA
CATCCCATATTAATTATCCTAAAACTGGATATTTCTTGTAGGATA
TAAATATCCAGTTTC
ACCATTCAAATCCTATATAAATGAGGTATTAGAACTTGAAAAAGG
CGTCATTGTTCATTT
GTCGTTCTGTATCAACTCTGAAATTTCAGAATTAATAGGTCTCAA
CGCCAATTAATTTTC
CAGAATATTGTGAGAAAATCACCATTGGTAGATTCAGTGGTTACG
CCCCCGTATATAGAG
CAAGGTATCCCAAATATTAACAGAATTGAGAGAATTGAAAATTTT
GAATTAAGATTTGAG
AATATTGATAAAACAGTGAGAATTTATGTTTGCATTATTTTTGGT
GTTTATAGGTTTGCA
ACAGATTTACAATTTTGTGGAAAAAGTGATTTAAATTGATAAGCC

TTGATATGTCAAGGT
TGTGAATGATGGATTATTTTCATACTTGACTCC
>ConsensusfromContig2659 Average coverage: 11.80
TACGAATATAACCAATCTACCGATCTATCACCAACTGAAGTTCTC
ATGAAATGGGATGAT
TTGGAGCCTGAGATCACTTTAACGAGAGTTGAAAAGGAGAGATC
AACAGATTATGAATTC
AAAAACATAATTGTGCTGTCATTCACTTATGGTGTAAGGTGGAAC
AAAGTTCGTCAAACT
GGCACATATCCAGAAACCATCAAAATGAAACTGAACAAGAATAT
CATCAAAGAAATAGCT
ATCAATCGGTTCCAAAACAAAACACAAACTCTTTTGGGAGGTTTG
CTCCCCAATTTTGAC
CCAATTCGTGACAAGAGCAGTGATTTCAGATGGATGAATGCTAG
CAAAGTCTTAATGCAG
AAAATTGCCTTTGATATCATCGCCTTTGCGAAAGAAAATTTATTG
AACGTTGCTCTCACT
CCTACATTGATTGACGAGATACATTTATGTTTCGAAGGGTTGGTT
GAGATATTGAAAAAC
AATGGAATTCCCGCTGAAAAATTGAGATCGGAAG
>ConsensusfromContig2660 Average coverage: 7.72
CTCTGGAATCTGTTCAGTCAGGGTCAAATACCATTTGTGGTGAGT
GACGGAGTTTCGGTT
GGCGTCCTGGTGAGACCGCTCCTCCCGGGCACTTCTGGCTCGCAT
CGATCGGAGACCCCG
GTGGATACTACTGCCCCCAGTCGGCATGTGGGAGTATTTGTTTCG
AAGAACACGAAGACG
TTTTCTGTCATTCGAAGTAAGTTGTTCATGAAGTGCCAATGCATG
TTCCTCAAGCACCTC
TGCAAACTGCTGTCGCATCTCAAAAGTGAGGCTTCCTTTCTGGGC
GCTTGGGAGAGATTT
CTGGTATTCGCGGACTTCCAATGTGTATTTGGGTTTGACGTTGAT
ATCAGCTCTGAGCTG
TTTGGACGACATCCGTTCTTTGGGGTTGCTCAGAGCATGAAGGCG
AGGGTCAATACTGCT
CCTTTCTATCCAGTCTAGGCATTTGACAAAGTCTGTGTTGGGGGG
CGGTCTGATAGTGGA
ACTTCTCGTGATGTCGGTATCTGTCATGGTGGGAGCCCTTGCTTG
GATTTTCTGAGGCTG
GTTCGACATTTGAAAATATTCTTCTTTTAATGGCTTTTCATCTTTA
GGATTTTCTGATTG
GTTTCTGTCACCGCCACTCTTCGGCCGTTGGCTTTCAGTAGGCGCT

G
>ConsensusfromContig2661 Average coverage: 18.35
CTTCAACAAAGCTGAGTTGCTCCAGCAAACACAAAGCGTCTTCAG
GTTATTCACAAAGGA
GTCTTTGGTTAAAATCGAGCGCCTCATAGCCGAAGACAAAGCAG
CAAAAGCTCTAGCAAA
GGAGAATGCAGAGAAAGACAAAGACGGAGGGGAGCAAGACAAG
AAGAACGAGGAGAAACT
GCGCCCAGACCCAACGCTGGAGGCTGGGAAATTCCTTCCGCTCCG
GTACGGGGAATTCCC
GGCGGAGTTATATGGAAAACCAATCGAGGATCTGGACAGATCGG
AAGAGCACCGTCTGAA
CTCCAGTCACA
>ConsensusfromContig2662 Average coverage: 23.31
CTTTGATAATGCTATGATAGAACCCCATTTAATCTAAATCTCAAG
TTCTGAAAGTTAAAT
CATTTGAAAAAATGTTTACTGTCATTTGGGATAGTATATTGAGTT
ATTTATGTCTCAAGG
CTTAAACATTAAATGTATCTGATTCATTGGAATATGATTTCATAT
AATGACATTGGTTGT
GTCATTGAATATTGTTGACATTCCATGCAGTTATAAGCTCGCGGA
GTCATGACGAACTGT
CCAATATTCGTTTGATGTGATTTAAAGGTAAACGTATTTAGTTAA
TTTGAAAAAATAAAT
GAATAAGCATATGATATATTGATCAGATACATAAAATTATGAAA
CAAGTATAGTTATGAA
>ConsensusfromContig2663 Average coverage: 9.79
GACAAAATGAATTGCAGCTGCCCATTTGGATGACAACATAGAAA
TGCAGAAACTAAAAAG
AAATACCCATCACGTAGTTCGTATATGAATCTGCTCGCTAGCTGT
GTTGAACCACGTCTC
TCGCGATGGCCCGCTATGGCCGTGAACGTTACTGGCGATTGCATA
AATGCGATACATCCT
TCCGGCAGAGAGGCCAGAAAGGAGGTATTGACAGCGACCCCGGA
AAAGGTTTGTCAGTTC
TATGTGCGAGTAAGATGTTTCTGACAGACCTTTGTATAGAGCCTC
TTTCCAATAGAGCGT
AATTTTTTCAGTTCGAATTTTCTCGTTGAAACGAATAGCAATGAA
TGCTCGATCGGAAGA
GACGGAACATTCTTCTGGGAGGATTTGTGGCGGTGTAGGAGATTC
TG
>ConsensusfromContig2664 Average coverage: 12.10

CCGAGTGCTGAAGAAAAAGAAGAATACGCACAGCTGACCTCTAG
ATGCCGTACAGCTCAA
AAGCTCATCGGACACACCTACTGTGTTCTGATTCTGGGCCTGGAT
TTCCCCACTCAGCAT
CACATGGAGATGGGAGGGTCCAGGACATCAAAGACAAGAAAAGA
TCGAAATATGTATGAG
ACACTCTACCAATTCTGCATCCAGCTTGTATGGGTAACATTCAAA
AGAAAAGATCACGAT
CTAATCAAGAGAGAAGTTGGACGATTACTGCGATCTGAAACCTTC
AATCCTGCTTTGAGA
ATCAAACACGCCGACGAGAAGGAGACGATTCAAGACTCGTTGAA
CC
>ConsensusfromContig2665 Average coverage: 9.77
TTGAATCGAAAATCACAGTACTTTATGAGCATACACGGGGAAAAG
TATTATCTATAGAGT
TAGACATAAGCATTCTCATAATGAAAACGATACCGATTGGTAGAT
AAAATAAATACGTTT
ATATAGAAACTCAGATTCTGTGTATTTTGTTCACGTATAAAGTGA
TTATATCGCTTAGAA
CTTGAACAATAAAATAAGTGTTTTTAATACATTTGATGAAGAGTT
GTACCCGTTTATTGA
ACGCGGGCTCCCTCAATATTTTAGTATGTGATTATGAGAGTCAAG
TATTACATAGTATTA
TGTGATATTATTACAAAATCCGTATGTTTATACCAACTCATCAATT
ATTGTATTTGATTG
TTTATATCATATCGAAAATGCCATATTATTTTCATGTTAAGCGAA
AACATAAAGACCGTG
TTCTCGCCGATTTTCATAATTTTGTAAATAGATTTCTCTCGCATCA
TGAATTTTGTACTT
TCGCGTTCAATAACTTTGTTTTTTTCGCCATTATGCTATGAATATT
TTGTTACGTG
>ConsensusfromContig2666 Average coverage: 24.96
TCGGTGATGGAATGAAGCAATGTCGACGCATTGCTTTTTTCGCTGT
CTTTTCTTTTGTTA
TGTGAAACAGATAAACTATCTTTCACGGGCTGGGTGGGGAATAT
ATCCAGACGAGGGTTG
CTGTCATATTCTACAGCTTTTTGCGCACTTAATTTTCTACCACTTG
TTTTTTCCACGACC
ATTTTGTCAGTGTTTGTTGTGATATTGAGGATAGAAATTTCATTTG
TTTCTCCACTAGTC
AAATTTGCTGAATCATAATCTTCATATATCAAACTTTCTTGATCTG
TAGTTTCATTCGTC

AAGGACTTTGTGTTTTTGGAATTTGCTTGAGTAGGTGACCCTTTTA
TCTGCTTAGCATTT
GATGTAGAATTAGATATTGCCAATTCCTCATGATCTTTGCATGGA
ATAATTTCAGGTTTA
TCAGTTAGAGATTCATCATTTTTCGAATTGGTTCGAATAGCTAACT
CTTCAATTTTCTTT
GCATTTGATGTAAGATTCAAGTTTGG
>ConsensusfromContig2667 Average coverage: 4.99
GGCAACCTCAATTTTAAATGTTCAATCTATTATCATCAAATATCA
AGGATATACATACTT
TAACAAGGTTTCGCGGATTTTCAAGGGTTACCTCAGCTATAAATA
CTCATTTCATTATCA
GGATTATCAATTATCGACATATAAAAACTAGAGTCTCGCGGCCTC
TTCTTCAGCCGTGAG
TTTGAAAATATCTTCGTTCTGCAACGTCGGACAAATCCTCATTCTT
TTCTTGATTTCTTC
CACGTCTTTTATGACTTTACACCTCTGAGGGTTGACCAGAGGATG
CTGTTCCTTTATGAG
GGAATCCCAGGTCAAATCCTCGAGATCAATCCCTTCAGCTGCCTC
GAGG
>ConsensusfromContig2668 Average coverage: 21.10
CATTGATATAATATATCGTCAGTAGCACTTCTCATTAAAAAACTA
GGAGGTTCTAATTTC
TTGACGGCATCGTCTTTTCTCTCAGCGTCTTTAAATGAATATTGAC
TTTGTCTTGCGGTC
ACATCATCACCTTGTCTTTCTTTCTGAGCTGAACATCTATCGATAT
CTTGATCACAGCTT
TCGACTCCTACCGTGCTGACTGCGGGTCCACTTCCGCTGCTCGCA
TACGATTCCTCCAAG
ATTTTCTCCGCTGCCTCTTGTAAACGGTGTGTGTCTCTAAAGAGG
CTTTCATGCCGACGA
GCTAATTGAGGATTCACTCTGGAGAGTGATACAGAATAAGCCAAC
TCTTCGGCTTTCTTC
AAATAACTGGACGCAGCAATGCTCATTCCACTTCGCTCTCCGTCA
GCATTGCAAGTTGTT
CCAAAGGCATTTCCACCCGATGATATAACATTGTTTTCATTATCTG
CACATTCATTTTTA
TACGATGTGGCGTCACAAGCATATTCAGAGAAATCAGCATACCTA
TGGCTTGTCGAATTA
TTTAAAGAACTGCTGAAATTGTTCGTGCCGTCTCGCTTTTTGTCT
ATAGAATCCCTAGAG
GAAGCAGTAGTTGTGAGATAGTCTTCCTTCTCTGCGAGTGTTCGA

CGAACATTATCACCG
CCGCTAGCATCCACATCTAGTAGTTTAGCAGGATTTAACCACCAA
AACTCTTCTTTCGAA
AGTCCTTTCCTTTCCTCTCTGCTCCGAGCTTCACCATTGCGAAAGC
GATTCAAATACTTC
TCTGCTACAGATATCTGGGACATTGAGCTCAAAGATACAGCGTCG
TGTGACTTGTCTCCA
GTTAATGTGGAGTCATGCACAGATGTATCTGGCCATTCTTCAACA
TAAATCTCCTGTTTA
GGTACAATCCTTTCCACTTCTTCAGTTGAACATTCTGTATGACTG
TCATCATTGAACGAA
TTCTTGATTTCCTGAATTGTAGGATGGTAGAAAGACTTTTTGTTG
AGAGATTTAGAGAAC
ATATTTTTTCGGTTTTTATCATCAGCTTTGAAAAATATCGCGTCCA
TGGTATTTTTTGTT
TTCGGTGGAATCTACG
>ConsensusfromContig2669 Average coverage: 27.37
AAAGATGAGATGAACAGACGGCGATCTTTCGACAGGGAGCATCC
TCTGTCTGAGGGTCGG
AGCGCGCAGGAGCTGGACGAGGAGCAGACCATCTACACCCCCGA
CGACATGATCAGCCTC
TGCATCCAGTACTGCGGATATGAAAATGCTCCCCTGCCCTCAAAA
GGAATAATAAACTTC
AACGAACTGAAACTTCAATCGGACTGCGATCGGCGATATCTCTTA
TGCCCAGCCGCGTTC
ACCATCAGACTTCTCAAGAAGTTCGTGAGACTAAAGTTTGGCGTT
CCGACTAATTATCTC
ATCGAGTTCTTCCACACAGACGATTGCCTCAGCAATGACTTTTCT
CTCATGGATGTCGCC
TACATCTACGAATGGGCCAGGGCAAGTCCTCTACAGCTGTACTAT
GTAGTGTACGAGACT
AATCCCAAGACCCTCCCGTCGTCTTCTCCAGCTATGGTCGCAGAA
GGGTCAGGGCCAACC
AAGGCAACTTCCAGTTCTGACATTGCCACAGAAGAGAGCCACAA
GCGACGGAAGAGGAAA
TCAAAATCTTCGTTCACTCACAAAAAATCGAGCAAGCATTCAGAG
GAGCAAACACTTCTG
TCGCGAGAGGATGAAAAATCTGCACACAGAAAGTCTAGTCAAGA
CGGAAGCAAAAAGAAA
CACCACAAGTCCAAAAAGACGCCAAAGACTGAGGTCTTCAAAGA
CAAGGAGAAAAGACGC
AAATCCAGCTCTGCAGAATTTCCCAGAGTCGACTGTGATAAAAGA

ACCGGGCTGCTTGAC
TCTATTGAACGGAAACGTTCGTCATCTCTCGTCA
>ConsensusfromContig2670 Average coverage: 18.65
GCCCCTCAGAAGGTCCCTCTTCCGGAGGAGCATTACCCCCAGACT
GCTCATTGCGGCGTC
GATAGTTCGGTCATTATCACTATGAATGCGAAAGTTCTTTCTTGT
GGAAATAATCGATTC
AACAAACTGGCGCTGGATGTGAGCGCGACGCAGAAAGTTCTCGA
GTGCGACACCTTCCGT
CAGTTGCAGACAGCGCCTCTTGCGGATTTCTCATTTCGTGCGGTG
GCCGTCGGAACTTCT
CATACAGCCTTTGTGACAAATGCCGGAGAATGCTACACGATCGGG
TCAAATCAGTTCAAC
CAACTAGGTCAAGGCCATAAGCAAATTCAGGAGGATAGCAGGAA
GCCCTTCAAGGTCGCA
TCGTTCAAGGACCATGTCATCATTTGTGTCTCATGTGGCGACACCT
ATTCCATTGCTGTT
TCGGATTCCGGTGAAGTTTTTTCCTGGGGCAAATCTTCACGCGGC
CGACTTGGAAGAATA
GCCGAAAATGGAGATGTTCCGAAGCGCGTCAACATCTCTCAAAA
AGACGCCATCGTCAAA
GTTGTTTCGTTGTCGAGTAGTCACGGAGTCACAATCACCGCTTCT
GAAGCCTCGCCCACT
CCAAGCGCGTGTGATTAGCTACAACATGGAGAGACGAAATTTCTT
CGAAATATTGCATTA
ACCCTACTAAAAAGAGCTTTGGGGCGAAATCTTGCGAAATGGGG
CCCTTCGAATGGCCTG
TAGAGTGGAGGTTGATATTTCAAAAAACGAATGGAAGCATAGAG
CGGGAATATGACCTCA
AACATCGGATGTTCCATACGAAAAAATATCAGCAATCCGAATTTT
GT
>ConsensusfromContig2671 Average coverage: 18.85
TTTATTATGAGTTTTTTCGTTAACACTATTAATGAGATCATTTTGT
TCCAAAATATCTAA
AAATTGTTTACTATATGGTTTTCCATTTGGATTGTCTATCCAATAG
TTGAAGTCTCCACA
TATAATACTGTTTCTTCTTAGTGAATTTAAGTATGAGATAAATGA
ATCAAATTCAGATAG
AAATATTAAATCATCTCCAGAGGTCCCAGGTCGATACACAGATGA
GATACTCATATTTTG
AGAGTTGATAGATATATTTATGATTATGTGTTCAAATGATTTATA
TTCATCAATACTAAC

CACACATTTTGCTTTCTTAAACTTGTTTTTATTATTATTCCTACCC
CACCTCCTACATT
ATTTGTATTCCTGGGATTTTGATTAAAAATGAAGCCAGGCAAGAG
TTGACCAGTTACAAC
AGCAGAATCCCCATCCATCATCCAAGTTTCAGTAATAGTTAGAAT
ATCTAGCTCATATTC
TAATAGCAATTCATTTATAAATTCCATTTTATTTCTCAGAGATCT
AATATTAAGAAGCCC
TATTCTGGTTATAGAAGAGGAAGGAGGAGGAGAAGGAGGAGGA
GAAGAAGAAACTAATGA
GGAATGATGAGGAATGTTGTTCAAAACTAGTTTTTGGACTATAC
AGTATCCTTTTTTCC
ATGTTCTCTTTATATGAAATTACTTTTGGACAGAGATGGCTGAAG
GAGGAGTGGTCGTTA
TTGATTTGACAATTGCAACACTTTGGTTGCTGCTTGTTTTCACAGG
TTTTTGAGGAATGA
GGTCCTGTACAAAAGCCACATGTTGGACTTTGATTTTTTGTGGGA
CATTCTGACATGGTA
TGGTCAAACCTCTGGCAATGAAAACATTTGTGAACGAAAATGTG
GTCAAACACCCTGCAA
GAGGAAAGAGAGAGAAAAATTTTGGAATTGTTTTTCAGAATTGA
GTTGCGGATTTGTGAG
CTGATTTTGATGATTGCAATATTGCCCCTCATCAAAACAAGCTCA
AATACCTCTCCGTTT
TCTATCATGTCATCAATGAGATGATTTTTCTCCCTGATCAGAGAA
ATGAAATTTGTCGA
>ConsensusfromContig2672 Average coverage: 6.71
CGACGCTCTTCCGATCGGGGTTGTAATGAAATAACAGAAGCTGGT
TTGTGGTCCAGCCTC
AACTGTAACATCGTATATCTCAGTATAAGTGATTGTATCAACATA
GCAGACGATTCTGTT
GGTGCCATAGCTCAGCTTTTGCCGTCTTTGGAAGAGTTGAATCTT
CAAGCGTATCATGTA
ACAGATGCTGCTCTGTCGTTCTTTAGTGCCAAACAGCGGGAATGC
CTCACGGTACTCCGT
CTTTTATCTTGCTGGGAAGTGACAAATCATGGTATCATGAATATA
GTCAGTTGCCTACCC
GGCCTCACAGTTTTGAGTCTTTCCGGTTGTAGCAAAATAACCGAT
GATGGAGTAGAGCTG
ATTTCTGAAAATTTGAAGCAGTTGAAAATCTTTGACCTGTCGTGG
TGTCCGAGGCTCACG
GACGCCGCTCTCGAATACATTGCGTGTGACTTGAATGTTTTAGAA

GAATTGTGTCTCGAT
AGATGCTCACTGATCACGAATATAGGAATATATTACATCTCGACG
ATGAATTCTCTCTCA
AAGCTTTATCTTCGCTGGTGCTCGCAGTTGAGTGATTTTGGATTG
CAGCACATCTACACG
ATGTTGAACTTGCGCGTTCTTTCAGTTGCAGGATGTGGCTTCCTCA
CATCGTCCGCTCTG
ATGGAGCTTCATCAGTTGTCAACTCTCCATGAATTGGAGCTGACC
AACTCTCAAGGCGCT
ACTGGAGAAGTTGTCACGTTC
>ConsensusfromContig2673 Average coverage: 8.70
GCGTAATCAAACGATGAATTACACCCCAAATACATATTGGAATA
GTGCTAACTACAGAGG
TCAAGCACCACGTTTTACAAACGGCCCAAGGTATGGCAATAATA
CAAATCGGAATATTCA
ACCGATGACTTCTCAGAGAGAATCGAGACCTACTAATGGAACAG
CCCCCCAGATTAGACA
AGCCAGACCTCACGCACCAACCGCGCCTAATCGTAATACTCAAGT
ACGCCCAGCTTATAA
TAATACTGGAAGAAATAAAGCCACTGGAAATCGTTTAAACTTCA
CCGGTCGAAGTTAAAT
GGAACCGTAACTAGACCACTGAATAATATTAAGGTTTCAAAGTCT
ATCGGTTCCCCCACC
ATAAGTATTTTCGTAGGACAAAATAAAATCAGAGCTCTAATAGAT
ACAGGCGCTTCGATT
AGTGTAATGAGGAAATCTGTTTTTGAAAACCTATCGATGAAATTT
CCTATTACGAGATCT
GATCAC
>ConsensusfromContig2674 Average coverage: 38.38
TTGACATCTGTTTCGACATAGGTTAACAATCTCCAATTTTGAGTTT
TATTCAAAAAATTT
CTTATTCGTATTTGTGGTTCAATATATTGAACAACGACCTCATTT
AAAACTGTTAATGGA
TTAAACAATCCCTCAGACATAACATTCTCAAGTATAAAATCCAAC
CTTTCGTCGTCTCTT
TTTCGTTCATTTGACATAGATTTACTGTCTTTCTTAATGTTATTTCT
TATACCTTTTAAC
ACATCCACTGGATTTTTATTATTTTTTTTGTAACAATGAAAGAA
GAATAAGATGAATCA
GAATAATGAGTCTTGGTAGTTATCGGCATGGTCATTAGATAATGA
TCATATAGCATGTCA
ATTTGCTCTTTCGCTGTATTTACTCCTCCACTATTTATATATAAAG

ATAAAACAGCCTTA
TTGAATTTACTTAGATTTTGTTTATTAATAAATTCTTTA
>ConsensusfromContig2675 Average coverage: 18.86
GAAAAATCGTAGCAAGAAATACTACGCTAACAAATCAAACACTG
TAAACACTCCAGAGGG
ACTGATAAGTTCAAAAGAATCAATTGCGTGCTTGCTTTGATGGGA
ATCATAGAGTCATTG
AAGTTACAAAATAGGTGATGAAAAACAAACAAAACGTTGAAAAC
CCAGCTGTAATAAAAT
GTATTATTATCACTGATTAGTTTGCATAATACGAAAAAAATGGGG
GCCCAGATCTCAGAC
GTATGATTAACATCAATACACACCAACATTTCATGTAAGAGTAC
CAGAAAAAAGTGTGCC
AAAAAATATGATAATTTCATACATATTAAAAAAATATCAAATACA
CACTTCCTTTGAGAT
TTTACATCC
>ConsensusfromContig2676 Average coverage: 9.25
TCTTGTCTCGCCTTTCCAGGAAGAGCTCAATTTTAGTGATTTTGCC
CAGCGTCTTATTGG
TGAACACAATGAATAGGTCACTCTGGCCTGTCATAAACAAATCGC
CGATATTCCGCTTCA
GTTTGACAATGTCTGAGTTCGCACACGTGCCAATTAGCCTCATGT
TGACCTTGAGGCGAC
CGTGAGCCCCTGGCCACCACGCTGTCTCGACATTCACGGCGTACG
CCTTCGACGCCAGCC
CCCGCAAGTATAGGTCAGGGTTCACAAAGGTCGGGATGATCTTTT
TCAGATCGCTTTTGT
CATATCTACGAAGAAAATAAATAGAGAGAAGCATTGTTGTCCAC
AGAAGCACAACAATAG
CATTGATGGCAGGATTTTTGTAGAATTCGATCAACAACTCTTCCT
CTGATTTCGAAGCGG
CCTTCGTGGAATTGCCTTTCTTCTGAGGATTGCCTTTCTCCTGTTT
TGTGCTGAAGACTG
TGAGGTGATTACATGCGCATCCAAATTCTGACTTTCCAGGCCGTG
GTCCTACCATTTGAC
AGATCGGAAGA
>ConsensusfromContig2677 Average coverage: 24.92
TCGTTGGATAAGAAGAAAAACCTTGGAAAGTACAACCGTTTCTTC
TCGAGATAGTCATTG
AGACCTTTTTGAATTTCATCGAGTAAAACATTGGCTTCGTTCAAC
CTGTCGAGCATGTTG
GGTTGCCCAGTCGCGATAAGGCAATGAGTGTCTTCCACGGCGACC

TTCATGAGGTCCTTC
CAGGTCGTATCCACAATCCCAAATTTTCTCCCTTCCTCCGGCATT
TGAGCGAGGATATCT
TCGGAGCTGAAGATCGGTTCCAGGTAAAGCCATGTTGATTGACAC
TTCAACCACCCATCA
AGAATGTCTTGCATAGAAAGCAGCTTTGCTTCCCACTCCACCATC
TCCTCCTCAAAAGGC
TTGATAAAGGGGGAGCCACGCATCGTCTGGGCTTTAACGATATG
ATCGTCCAAGAGAAGC
TG
>ConsensusfromContig2678 Average coverage: 23.95
TTTGTTCTTGTCTTTTTTTCATTTTCTTACTCTCCTCTTGCATTTTC
AAATGTTGGTGAA
AATAATGATGATCTGGCTGAAATTTGTTTTGGTCAAGTCGAAGCG
AAAAATAGTTTTCAT
TTGTAAAATATTGCATTTTTCAGCTCCAAAAGAAAAAGTGTAATC
AAATCAATTTAGCAG
ACCGCATCTTTCACGGGATGCGCG
>ConsensusfromContig2679 Average coverage: 25.34
CTGAACATTTTAGTACACATTTAATACATATATGTCAGTTGGTTT
TATAAGATCAGCACA
ACGGTCGCTATTCGTATGAATAATATTCCTGGTATACATTCATTT
TGGGAATGAACGTCG
AATTGGACATAAATTGGACAATTATTATATAATAAGATGCAATGG
ATTGGGATGTTTAAT
TTCTTCTTTGGTTCTGTTCACAAGTCGATCTGTCTGTCTGACTATA
CTCCCCAGAAATGC
ATCCTTGTTCTCCCTGAATGTGGATTTTTTTATAGACTGTATTTTC
TGCTTGGTGCCTTC
AAAAGTCTTGTCTATATTTTCCTTGGCAGTCCGGCTCTTTTCGGAA
ATGTATGTTTTAAC
CTCATCTCCTTTGTTCCTCATGTACAATGATGTATTTTTCCCAGCA
CTAATCATGCCATT
TTTTGCACGTTGCTTATTCTCATCAAACGTAGAGTTTTTGAAAGGC
TCCATTTGATGTTT
CACGTTTACATAAGCAAGCGAAATTTTCCCCTTGAGGGATTCATT
AAAAGAAGATATCTT
TGCTCCGGCAGCAGCTGTTTTATTACTTGCATAATCTCTAATGAAT
TTTGAGTTTTCCAA
GATACTTGTTCTCAACGCATTGAAAGCGTTAGACTTCTCGACTTCG
TTCTCATCTTCAAC
TGTGTTTGATGTTATTTCTAACGGGGGCTTAGATAAACTTGAGAG

ACTGATGTATGCAGA
TGTATTTAGAATTAAAAATCCACTCAGACAAAATAAAGCAAATA
AAAATGATCGAGATTT
CAATAATGTCTTTAATTTGTATTTAAGATGAATTAGCTTCATGATT
GTGTTCAAATTACA
CAGCAAAATGATGTGGATGAGCCAAAGATTGTTT
>ConsensusfromContig2680 Average coverage: 17.81
CTGGGCTCAAATTTGCGTGATCAAAGAGTAAAGGTCGTCGACAAG
TTCGCCATCTATTGA
GATCAGCACATTTCTAGAACTCGGCGCTGTGTCAGATGAACTGA
AGAAGTCGCTGAAATG
AGCGAACAAGACACAGATAAACCTCAGGACGAACATGCCGTCGC
CATGGAAATCTTCGAC
GCCGTTTTGCACAACGCCATGGCAACCACAGAACATCTCTTTCTC
AACTGCAGCGACGGC
CTCTTGCGGAGAAATATATCACTACAGGAATTCCCGCTCTCGGAC
AACCTCCAAAAACTA
GGAACGAAAGGAATACTTCTGGAATCTAAAATGTCCGCCTACAA
CGTCAA
>ConsensusfromContig2681 Average coverage: 7.61
CCAGCAGCCTTAGCATCATCAAAAATTTTCTCGACTTCAGAGCGT
TCCTCGTTCTCCAAA
TTACCATCAGTGATGAAAATCGTAATCTCCGGAACTGATCGACTT
CCAGGCTGATGGAGC
ATTTGTTCCTCGATCAGTTGGACTGCTTTTCCTGGTCGGGTCGTGG
TGCCAGCGTATTTG
ATTGGGGTGGCCGAAGGATCTAAATATTGGAGCACTGTTGCTCTA
TCACCAGAGAGATCA
AG
>ConsensusfromContig2682 Average coverage: 35.65
GCATCAGATTGAAAATGCCACTGTGGATTCTGAGTACTACAGCA
GATATGAGAGCAGATG
GGAGATTCATCATCATTCAGTTGAAATGATACGTAAGCGCTCGGC
TCAGTTATATCGTAC
CTCTTGCGAGTTGATTCGTCTGTGGGGCGTTGGGCTGACGGAAC
GAGGGGCAAATGCAGA
GCTTCTTCAACGCTGATGTGCAGCCGCATCTCTTCGCGAGAGAGA
GCGGCTTTTGGAAGC
TTGTTACCTTTCTTTTCTGCATCAATTTGAGCGCAGCTCGTTTGTG
TCTTCAAACAACTC
GGTTGGTTTTCAACAGTGTCCAACTCATTTTTCAGATCAGAACAA
TTTGCACTCAATCTG

GCTCCGTTCTGCTGGTTCTGGAGCGATACTCCATCTATATGATAC
TCTGATCTCAGTGTG
ATTCGAACCGAAGAGGCGCCTAGCAAAGATGCATTTGATCTCAT
CAAAGGGTAGCTCCCA
CTGCATGACGCCATTGGATTTTCTTCGCTGACCCAGAGTTCGTTC
AAATCGACGAAGGCA
GACCCAAGTAAGTTATC
>ConsensusfromContig2683 Average coverage: 22.02
AAAACATGTCGGGAGGGTCAAAGTTGTGAATTATCGCAAGATTT
CAGCGCGGAATGCGTA
GAAGTATATGAACTCCCATGTGGACCGAATCATTGCAAGAACGG
TGGTAAATGCAAGAGG
AATGATTATTCTGAAAAGGGATATCGCTGTGAATGTCAGGATGA
TTACGGAGGACCCGCC
TGTGAAAAAGTTCTTCCCTGCCATGGAAACCCGTGTCTAAATGGT
GGGACGTGCTATGAG
AATCATTACAAAGCAAGAGGCTACAGATGTATCTGTGAATACCC
AGAACACTTTGGCGGT
ATCAACTGTGGGATAAGTGATCCATGCAGCACTAATCCTTGTATT
AAAGGAACCTGTGGG
GTGAACTCTAATACGAAAAACGGTTACTCTTGCCGATGTCCGAAG
AACATTGGAGGATTT
TTGTGTGGAATAGAGAACCCATGCGAAGGAATCGACTGCGGCTC
CGGTCGATGCAGGAGG
ACATCCGGTGAGACTGGCTACAGGTGCGTCTGCGATGAGCCATA
CGGGGGCGCTCGTTGC
GATCTCAATCCGTGTGAAGGTCATGCGTGCCAAAACGGGGCCACC
TGTGCGAGTGCCTAT
GGAGGAAACACATACGTGTGCCGGTGTGCGACAGGATTCAGAGG
TCGACTTTGTGATAT
>ConsensusfromContig2684 Average coverage: 11.23
CCCTCTTTCCCTACCCGACGCTCTTCCGATCTCCTGGACAGCTTCT
GCCGCGTCTTCGAG
GGACGCATGCGTCGTCTCATAACACGCGCCATGTTGGATATCGAA
AATCTCGTCAAGGAC
TCTTTTGTCGTCGAACTGCTACAACATCTCTTCATGTGTCTCACGA
GATGCGAGATATTT
CACGGGCGAAAGAAAGAGATTGAACGGATCCGGAAGTACGTCAT
TGCCGATATGACACGT
GATGTAAACAAAGGAAGAGCAGACGATACGGAGATGCTGGGTCT
TGTGGACATTGAAAAG
GGCAATCGAAGCTCAGGTGATGAGAATGTGTTATGTGAAGAACC

AGACGATAACGAAAAA
GTATGTAGCGTCAACGAGCCAATCGCTGAAGATTTTTTCATATCA
CGTGATGTTAACAAG
ATAAAAGCAGACGATAAAAAGACGTCAGGTTTTGTGGAAAATAA
ACAAGCAGACGATGCA
GATCAAAGCTCGGATGAACATATGTCATGTGAAAAACAAGAGGA
TATTGAAATTGCATGT
GATGTCAACGGCCGAATTGCAGAAGATTTTGTCATATCACGTGA
TGTAAACAAGTACGAA
ATGAGTTCAAAGTTGTCTTTCAGTGAAAGAATGAAATCGAAGAA
GAAAATTTCCCTAAAA
ATTTCAAATGTTGATCATAATATCAAAAGCCATTGCAGTAAAATC
AAAATACATTCTCTG
TCCGCCTCCAATTCAGTCGATGATATACGAGCACTTGCGAAAGAT
CTGTCGTCTGCCTCT
GATGATATGGCTCAGTACATGAATAAAGAATCGTCAATGGTAAC
AGCAAATAACAAAGTG
GAAG
>ConsensusfromContig2685 Average coverage: 15.42
GAGAGTCGCTCGCGTAGCGTTGTGGAGATGGGCAATACTTCAAT
AGAGTCAAGCACAAGG
GCAATAATTGGTTGCCCACTGCAAGATTGTGATCGAGAACATTT
GGTAGAGCCGATAAAA
AAGCTGTATAGACCGGAGAATATCTCAATTTGTAAGGTGGAGAGC
TGAAGGACTTTTCCG
AAGGATTGTAACCCAGGAGAACATTGATGTGACGCAATATACCCA
TTTGGGCGTTCTGTT
CACCTTCACTTCGCCCCACTCGTTGACTCAGAGCAGGATTGGCCA
TGAACTTCATAACTG
AAGTTATGAGGAGATGAACAGTATCTTTGTCAACGGTTTCAGCTT
CCATGGCACGATGTA
ACATAGCCGACCTATTATTCTCTTCAGAAAATTCTCGTAAATGAC
CATCTGGGAACATAT
AGTTAGTGAAAAGTGCCAAACCGAACTTCGACCCCTTGACCTCTC
GTCGACTATACACGG
GTGCTGACTGTTGCCGAGGATATTGTTTATCATGAGTTATGTTCC
TAAATTCTGGTAGAT
TCGTGGAGCTGTAGAGCAAGGTCTGAGGAACGGATGCCGCTCTT
CTATACGCCAGCGGAA
TCTTTTGCAATGTGATTCCTCCAGTATCTCCTTTGAGGTGCCTCGA
ACCCGTCGCTCTAT
GTTG

>ConsensusfromContig2686 Average coverage: 29.00
TTTTACACGATTGAGAAACGGCTGAATTTGAATTTATTATGTATTG
TTTTAGCTCATTTT
AAAATTGTTTTTGGTATATCATTTCTAGTACATGCTGATTATATGT
TGAAATTTGTTGAT
TTTTTTTCTGTTATAAGTATTTTTATTTGTCATGGAGTCTTCAGCT
ATGATAAAAACGAT
GTATACAAAATATGCAGTTTGAATTTATTCTCCCTAGTGAAAATG
TTGTTGTTACTTAGT
CATCCTCTCAACCATCTTATAGGTTCTGTTCAAATGCACGTTTTCC
TATATTGTTACTAG
AGATTATATGTTGCCATTTTCATTATTGTTTTAGGAAGTTCCATTA
TAGCCTTTCATGTT
TATTCATGTCCCAATTATTTCCATTCATC
>ConsensusfromContig2687 Average coverage: 51.48
AAAAATTGAGCTGTCAGTAATGAGAAATGTCTGCGTCCCGTCTGA
ATTATGGCATTCTAG
AAATGCTATTCACATATTTAAATCTTGATGATCTGAGAAACTGTA
TGACTGTATGTAAAT
TCTGGTATGATGTTATTGCTGATGAGAATAATGATATTTGGAGAC
ATCAATATGCTGTGA
AAGTTGGCTCAACAAATGGTGGCCCTGATATATTTCCACGAAGAA
ATGACATTTTAAATG
CAATGCCCACTCTCAAAAAAAAATTACGAGCTTATTTCTATAGCT
GGAATCCTGAGGATT
GCTCGAGAAACATTTTTGTCAAAAATAATGGTTTCACAATACATC
GCAATCCTGTTGCCC
AAAGTACTGATGGATGCAGGAGCCGTAAAGGATTCACTCATGGG
AGGCATTGCTGGGAAG
TATGGTGGGAAGGACCTTTGGGCACTGTTGCAATGGTAGGTGTTG
CCACGAAGTTGGCTC
CAATGCAAGGTCATGGCTATATTGCATTATTAGGCATTGATGAA
GAAAGCTGGGGCTGGA
ACTTAGTAGATAACAGTTTATTACATGGTGGCTGCAATATCGGAA
AGTTTCCTGATTTGA
ATAATGCTCCAAAGTATGAGATAGGTGAAAAAATTCGATTGGTA
TTAGACTGTGAGAAAG
GAATGCTCTCGTTTGAAAAAGATGA
>ConsensusfromContig2688 Average coverage: 11.11
TCCCCAAAAAATGTTGTAACAATAATGACAAAAATACACAATTCA
ATAATGAAATATTTC
GAATCATAAATGACAATAAATTCATGCCCGAAAAATATTGAATC

GAAACGAAAATAAATT
ATACGTCTCACATTGACCTCGGTTCATCTCCATACAATAAACAAA
ATAAATCGATCATGG
TTCGATAAAATGAAGTAATTTTAAGTATATCATCCAATAACTTTC
TGACTCTCAAATATA
TAGAATCATAACAACTTAT
>ConsensusfromContig2689 Average coverage: 7.98
CACGTTGAAGTAGACTTAGACGGTGAGGTCACAAATGGTACCGT
CAAGTACATTGGCTAC
ATCAATGGAAGGCAAGGGCAATGGGTGGGCGTTGAACTGGATCT
CAAGGTTGGGTACCAT
GATGGCTTATTGCATGGAAGAAGATACTTTCAGTGTCCCCCAGGT
CATGGAATTTTCACA
ACGGCTGAACGAATAAGATTTGTTTCAATGAAAAGAAGATCTTTT
AATACATACCAAGTT
GTGCATCAGCGTTCCAGCGTTGATGATTTGTTGTTCATCCAACGA
AGAGAACCCATTATG
ACTGAAAGGGACCGTCTGCGTCGATCATTTGCCAGAAAATCAAG
AGAAAAATATGTCTAC
ACTGATGAATGGATTTG
>ConsensusfromContig2690 Average coverage: 10.57
GACGAGTGGCTGTAGCAGCCTCTTGAGGTTCCCTTGGTCCAGCGC
AGGATAGACAAGCAT
TGGCCGGAAGGAGGCGTTGCTGTCGAACACCGCGGCCATGACCC
CGTTGACATTGGGGTG
ACACGAGCCGTACAGTCTGCAGCCTTCTGTCAACATCTTCTGCTC
TTGATCCCTTCTGGC
GTGAGGGGAAACTGTTTTGATGTAGACATGTGTAGGCTGTCCAA
CTGGAGACTCTGGAAG
AATAACTCCGTGATGAATTCGGCCGAATGTCCCTTCCATAAGAAC
ATTGCCGAGTGATAT
GCGTTCTGGCCGGATGTAAATCGCACTCAAAGTCGATCTGATGTC
GCTGGTGGTGCTACC
TATCATCGAGGAGTTGGAATTGTCCGGTAGGGGGCTGTAGCCGCG
TTTGGACGATGATGA
TATGACTGAATAACTATTTGTAGGCGTGTCGGGTCGAGGATAAAC
CACGCCTCCTCCCTG
CTTACTAGATGCCAATGAACTACTGACGTCACTAGTAGATGGCGT
GGGTCGTTTTGAAAT
GAAGTAACAGAGCCCCACAACGATAAGAATAACAACAAGAA
>ConsensusfromContig2691 Average coverage: 9.39
CGATCTTTTTGAAGATGTATCCTTATAAAAATACGCTGATGAAGA

AAATAGATACATTGG
AAATGAGCAAATGCTTTTGGAACCACTGCCTTATTATCTTGTGCA
AACCAGAGGATTTTG
AGTGTGGTGCTATGGTAACTGAGATGTTAATTGAGATTATTTCCC
AGCGACAATATGTTG
ATTGGCAGACGCATCACAAGATTCTCGAATGCTTACAATCCTTAA
TGCAAAGCCATCACC
CAGATCTTTTACCCCAGTCTATGTTGAAATTAGCTCTGGAGACAA
AGAAAAGATTAGAAT
CGAACGAATTCCGTGAAGAAGTTTCACAAATTGCGTTCAGAGTTT
GCAGTTTCCTGAGTA
AATGTGATGAAAAGGGCGATTGTTTTCGATTACTTGTTGCGCTTC
TCACTGCCTGGTTGA
TCGATTCTGTTAATAAAAGGGAAACCAACTTGACCCAAGCTGTG
ATATCAAATGGAGAGC
TAATAATGAAGCTAAAGAAAAGTTTGATGGGAGATTCCATGAGGG
TAGCTTGCTGTTCTG
CTTCTTGTCTTGCTGTCTTATCGTTCACACAGTGTAGTATACCTTT
TCAGT
>ConsensusfromContig2692 Average coverage: 87.23
CGACAAAATAGAAAATCATTGGTAGGTACTAAGGTTTATTTGTAA
TCAAATAATACATAG
AAATAATACATGGAATAAGAACGAAAGGACTAAAATCTTAAATT
TCCTATCGACGGAAGG
TCGGGTAGTTTCATATCTTTTAGTTTTTGAGGAAAGTTCAGTTCT
TTGACAGCGGACACA
GCTCTTGCCGGAGCTGCAACCAAGCCGGCCTCGCTTTTTTCTATCT
TGTTCTGGTCTTCT
ACTCGTGACATGATCTCTGCCATGTTCGTTTCCAGCACCATACCTC
CAACCACTATCTCT
GATAGAATATGATGTACTTTATCCATATGAAATATCAGGTCTAGC
TCACATACATTTTCA
AAACATTTATCCAACGTTTCAACGAACACCTGAATAAGATCGAGA
ATCCCGAGTTCACTC
TCACAGGAGTCAACGCAAAATACGAAATATAATGTTGCATAATGT
CTGTATATTAGCCTA
TAATCCGATCCGCCGATCAACATTCCTCCCTCTAGAAAATTGCAA
ACATTGTCATCTCTT
TTAGACACTAAGTGGAAAGTTTCTCTGATGATTTGTTGTTGGAGA
TCCTCAGGATAATGT
TGATAAAACTTTGACATTCTTGGTTTGCCATGATTGTTAAAAACA
AGAACAGCTTTTATC

ATATTATTTGGCAATAAATA
>ConsensusfromContig2693 Average coverage: 41.97
GGGTCCTCGCTCTTCGTACTGGCTCTCAATTACAAACAAATGGAA
TTATATCATGCTTTG
CCATTCTTCTTCTATTTATTGGGCCATTCTTTCAAACGAAACCATG
GTTTGCTCAGAGTT
CTTGGCATTGGCTTCATTGTGATTTGCACATTCGTAGTCACGTGGC
TTCCCTTTGGATTG
GATGGAGTTATACCAGTTTTTAAACGCTTGTTTCCGGTCTCTAGA
GGAATCTATGAAGAT
ATTGTTGCTAATTTTTGGTATAGTTTTTCCATCATTTGCAAATTCC
GAAACAACTTTTCG
TCGGACGCACTTATATTATCGAGTGCTTGTATGACATTGGTTTCAT
CCTTGCCGTCTTCT
ATTCATCTTCTTCTGAATCCAACAAAACGGAACTTTCGCTTATCA
ATGGTCGTTGTGTCC
ATTTCGTTCTTCTTGTTCTCCTATCATGTACATGAAAAGACAATTC
TTATTCCAACTACC
TCTGTTTTACTTTTATCGGCTGAGTTTCCACTTTCGGTCTTTTATTT
CTGCAATGTCTCT
CTGTTCAGCATGGTCCCACTGCTCTTTTATGAAGGATTGATAATT
CCAGCCATCTCAGCG
CTCATCGTATTTGATGCAGTATTTATACAATTTGTTGATCTGAAA
GTAAAACCTATGTTC
AGCAAGTCGACCTATATGACCTATTTGGGATTCGTGTCGACGATC
GGAATGTGTATCTTG
ACCCTACTGCTGAATTTCGTCCCACCCCCAAAATCTCTACCATACT
TCTACCACATGCTT
ATTTCAGTATTTGCTTTTCTACATTTTCTTGGATTCTGGGTCTGCT
TCAATATCTACCAA
ATTCTTTCGAAAAGTCGAATATCTGAAA
>ConsensusfromContig2694 Average coverage: 9.35
CGAAAATCCAGAAGAGTAATTTGACTGAAGTCGGCCGTTCAAAG
AATATTGACTCATCCG
GGGATGAGGACCTGCTTCTGAACTTGACTCATCTAATGAACAACA
ACGTTTGACGAAAAT
CCAGAAGAGTAATTTGACTGAAGTCGGCCGTTCAAAGAATCTTG
ACTCATCCGGGGATGA
GGACCTGCTTCTGAACTTGACTCATCTAATGAACAACAACGTTTG
AC
>ConsensusfromContig2695 Average coverage: 7.99
GCCAGGCATAACGAACAATGACCTCTTTGTCCAGCTCCTTGAGGT

TTTGTTGGTTGTCGG
CGTACTTCGAGTAATCATACCAACAGCACGCTTCGATATCCGTCT
CGTCAATCCCCCAAA
ATTCCATCTCATGTCTGAAAACGGTGCCGCACATAGAGTTGTCGA
TATGCAACTCATGCC
CACGATGGAATTGAAGCACTGGTTCGAATCCCCGGGCACTCCGA
TCGAAAAAGTACTCCC
GTCGCTCTCTATCCCAACTCGGGTCTTTTTCCTGGTCTAGGCTCA
ATTGGTGGAGCCGGG
TTCCTGTTAGGCTACGCAGAGTGCTGAGCATGGTTTCGAA
>ConsensusfromContig2696 Average coverage: 14.39
TTGTGATATTTTTGTTGATATGCAATCAGTGTGGGTTCCCCATAG
AAGATTCTCCTGAAT
GTGGAGACCTAAGAAGTTAAAATTTGTTACATGATCTATGACATT
CCCTTGCATTTCTAA
TGACAGATTAGGTAAATTTTTATTCCGATTGTGAAAGAACATATA
TTTTGTCTTAGAAGT
ATTCAAAGATAGTTTATTGGCACTAAGCCAATCAGATATAGACAT
AAGTTCTGTATTCAT
GACTATAGATATGTCTTCATTTGGATTTTTTCTAATTATGCTATTT
ATAGATGTTAAAAG
AGTCGTGTCATCTGCATACATTATAAATTTAAAAAGATCTGATAT
TTTGGATAGGTCATT
AACATAAACAATAAATAATAAGGGGCCTAGAATAGAGCCCTGTG
GCACCCCTGTTTGTAT
ATCAAGCTGATCAGAGCGGCAGTCACCCATGAGTGTGTAATGACT
CCGGTTTAGCAGATA
AGATTTAATTAAATTAAGAGAAGATTCCACAAAGCCGTATCTTTT
TAACTTATTCAAAAG
AACATCGTGATTTAAAGTATCGAATGCGCTAGAGAGATCTAAAAA
TATTGCTATTGGAAT
ATCATTGTTTTCAATTATAAGTTTGACTTTATCTATAAGATGCATG
GCTGCAAGTTCAGT
ACTATGAGATTTGCGGAATCCGTATTGGGATTGAGATAGAAGGTG
ATTATTTTCAAAATA
TTTAAAAATCTGCTCAAAAACTATCTTTTCAAAGACTTTTGAGAT
AGCTGGTAAAAGGGA
TATGGGTCTGTAATTGTTTTTATCATGTTTATCTAATTTTTTATAA
ATCGGAATAATTTT
AGCAATCTTCATTTGATCAGGGAATGAACCT
>ConsensusfromContig2697 Average coverage: 3.89
CTTTGATCTTACTGCTTGTAGTGTGCATGTATCAAATGTGAATCGT

TTTGCAATCATTTC
GCCTTTTTAGATTATGTCGCCTTGAAAGTAGACTCATAACAGACT
TATTGCACATTAGAA
GCCTGCTTCCAGTAATTTTTTTAATAACTGCACTAGCTTTCACCT
ATTGACGTGAGATG
TATCACTCGAAAGTGAGACCAATTCATCAGTATCATTAGGTACTT
ATATGCTGGTGAATA
CTATTTGTTAGTAATGAAAAAACGTCCCTCGAGATAAAATTGATT
GATAAAATGATGTCC
TGATGTCAACTTAGTCCCCACCTTTCTCTGTATATTACCCTATGGC
CAGTAGTTACTATC
AGTCATGCTGATTTTTTTAGATCAAGTTT
>ConsensusfromContig2698 Average coverage: 7.86
AACATTCCATTACTTGTCCTAGAGAAAATTTATCAGCTGCTGACA
TTCCGAGAACGATAT
TACTGTTCAATCACTTGTCAAAACTGGTACGCTGCTTTTTATTGCC
CTAACCTATGGTCG
ACCATTCACGTTAAACCTAAAAGCTTCACATACAAGCGCTTTCAC
CCACAGAAAGGTTAT
CAACGGCTCCTACGCCATGACTCTGTCAAAATATGCCTTGTCAGA
GTCGGATATTTCATT
CGTGAACTAATTATCGAACCGAATCCAGATTTCTACAACCTCTAT
GGGTTCCTCACGGTC
TTTGGCCGATTTTTACAGTTCTTCGACGAGTATCCTCTACCGCGAC
TTGAAGCATTTTCA
TTCACA
>ConsensusfromContig2699 Average coverage: 9.76
CAATGCATCACACACCTTTTCAGTCAACATACACTTCAATATTCG
TTGTTTCGGGTGTGT
TGCCGCAAATGGATCTACTTCGTTCCGAACCTCTATCCCAGTTTT
CACTGTCAAAACATA
GATGCCTCCATTTTCCTTGGCTTTTTCCTCCTCAACTCTTTATCTAT
CCTTCCCTCATTT
TCTTCCATCCATTATTGACATATATTCTTCAGCAGGATATCTTTTT
AGATGCCTCAGCAT
ACCAGTTGTGCTGAAATCTTTCTTTGAGGCACCTGTTTTCCCACGT
CTAACTTTGCTTGC
CTTGCATCACGCGACAAGGATCCATACTCTCATCAATATAAAAAT
TAAAATATAA
>ConsensusfromContig2700 Average coverage: 6.44
GGTTTCAGCCGTGTCCTCTTCCGATCTAACGACTGCCAGGATCAC
GTGGTATATGCTGTA

GGCCTCTTCAAGTTGCCCACATACAACAGGGTTTTCGTCTGAGTT
GTCATTTGTTCCGCA
GTTTGGAAGACTGTTACAGAGAAAAGAATGACCTATGCAGTAGC
GGCTTGTGTTGCAGAC
GAACCCAGCACATTGTCCTTGCGGATTTATCTGTGCCACCTCTGT
CCACGTGACCTGGAA
CCCTTGCGCCCCTATGGATCGTGGGGTGCTGACGAAGCTGAG
>ConsensusfromContig2701 Average coverage: 26.70
TAACAAATGAGATATCAATCATAATGATTAACGAAATATTTGATT
GAACACAAAATGATT
GATTAAAAACAATCAAAGGCATGACTAAACATACATCGGAATAC
AAAAAGGAGGATAATA
GGTTATTGTGGACATTATTATCAGAACCGCTCATTATCTAAACGC
ATCATAACTGAAACA
TGTACGTATATATTTTGTTATATATAAATGAAATAGTACTATATAA
C
>ConsensusfromContig2702 Average coverage: 19.60
ATCTGTAAGCTAGATATTGAAAATAGTGCAAAGTCTGCAATAATT
ATGAATCTTGAACTC
CCAAATAACGCTGAACCAGCCAGGAATGAAAGTACAATATCTCTT
TCACCTCATTTAGAA
AAAGCAGAAGATATTATAAGAAAACAAGAAAAAATAATGGAAAA
TTTACAACTGAACGGG
ATGAATGGATCATACAGTTGTGAAATGAGTAGTAATCATGACACA
ACTTCTGATAAAAAT
CATTCAACGAGTGAAGAATGCTCTGAAGTAGTATATCCAGACTCT
TCCCTGAAATTGGCA
GAACGATTGATAGAAAAACACGAATTATTGAAAAAAGAAATGCT
TGAATTTCAAAGTTAC
AATACAACAGAACAAAATAACACTTCATCTCAAGAAAATGATGTT
ATCTCGTTTATTTCT
CAACACGAAAGCCCTGTCTCGGTTAGAAAACAAGGTTCATCAAC
AAAGAAACCAAAACCA
GCGCCGTATGTTCGTTCTTCTAGTCTCCCGGGTGACCCAATTGCA
CCAGCCCGCTCAAAA
CGAAAGAAGAAAAAACGGGTACAGTTTTCAGAAAATCTAGACGA
AATAATCAAAGAAGAT
GAATCGTTCAAAGAGTATGGCAATGCTGTTTTTGAAGAAATCGAT
AATAACCCGATTACA
GAATCAATTTATGTATCTAAAGCCGATAATTGCACTAATAAGGCT
ACAGACAATAATGAT
AAACCAAATACAGAATCTATTGTTCATATCAATAATCACACTGAT

GTGATTCAAGAAAAC
TCGTACAATTCGCCAAAAAATAAAATCTATGGAAAGCCTGAACTA
GAATCCGCCGTCCAT
ATGACCAAAACAAGCGAGTTTATTGATGACATTCAAGAAAATATT
CATATTTTTTCAATT
AATAGAAACAATGAAAACACAGATACAGAATCTACCATTGATAG
CAAAACCGATAATCAT
ACTAATAGGACTGAAGATAACGTATTTGGGAATTTCAATCATAAT
ATATTCGTAAGTATG
TCCTCTGATATCAGTAAAAAAATAGTTAATGAGAATAATGAAACT
AATATAGACTCTGAT
AACGATGAGTATGAGCGGGTTAATTTACGCAAATCACCAATTCTA
TCTGAAGAAAATGAT
TTCGAAACAAGTAAGGAGCGAAAATCATTAGATCAGGATCGATT
CACTGACAATAAAAAA
GAAATGGTAGCTGATACTGAGGTAGAATGTATGAATGACAGAAT
CAATATAGTGAAAAAA
GTTTCATCTATTACT
>ConsensusfromContig2703 Average coverage: 10.41
CAAGATCATGATATCAGATATATATAAGATAATGTTCATCAATAT
TTATCTAGAGTTCTC
AACAGTTCATTTCTCTCGCAACCGTCTGGAAATGGAAGAATATGT
AATAAATGCAAAATA
TCAGTCCTCCCAACACTTAGAGACATGATTCCTTCACCATTGCAC
CATCTATCATTGATC
CCTTGCATACCGTCAGCTTCAAAAGAGTCTGCAAGAATCATGATG
ACTTCCTTGAGCTTG
AGATAAGACGGAACGTGATTTTGAGCGAGAACGTCCGGAGCTGC
ATTCATACCGGAGAAA
AAGCACTTGATTTCTGCTGCAAGTTTTCTCATCGCACCGTACAAC
ATGATGCAATCGCAC
CTTGACAATCTTTCGGTACGCTTTCCTTGTTCGATGTTAG
>ConsensusfromContig2704 Average coverage: 6.54
AACACAGGATAAACACAAAAAGCATTCCAAATGATGTACTTAAT
AACACTCAAACACTGT
AAAAACAATCCATGCAAATAATTAGAAACGTTTCAAATCTGGATT
GTGCTCTCATGTCTG
TTTTTGTCGTACAACAATGCCACGCCTTGAAAATGCAGATAATGT
GACTTGCAATCCATT
GAGAGCACAATATGAAACACAAATCTACTATTATCTAATAAGCGC
ATACATCTTATGGCA
ACACAAGGTACTGAAAGCACATCTTAGAGATGATGAAAGAATCTT

GTTCGAAAAGTTTTC
GAGTTTGGTCGAAATTCAGTATCGCTATCATATGTCAAAAAAGCT
CGTCATATTAGTGAA
AACAAAATATCAAAAACATTCAAATGGTTTGCGTTTTCAAAATTA
GAAATATGATTGGGG
AATTTTTGTTGATAGCCCGTAAAAAATTTAAATTTCAAAAAGAGG
AACTTAAAGGTTTTG
ACTTTGAAAGCCTCCAGGTGAAATATGAGTATTTCCGGAATCGTG
AAAGTCGATCTGAAT
GAGAGAAAGTTCATTAAGAGACTTGTG
>ConsensusfromContig2705 Average coverage: 6.04
CAACCAACGTAACATTCCACTCGAGGTGCATAGATGACAACATCT
ATGTTTTCGTCTATG
TGGATCACTCTTTGAGAAACGCCATTCTCACCGAATATATGAGCC
ACATTCTCCGACAGT
ATGAGGTACTCCCCATCTTTCTCATTGTGTGATTGAGCTGATG
GAAGCACGAAGGTCA
ATGGCCTTGAGGTGGTGAAGGCCGTGTCTGACAGCGTTCTTCTCCT
TGTTTTCTCTTGGC
GCTTCCGCAGTAGCATCAGTGAGATGGTATTTCAGAACTCGCCAA
AGAAACCTGCCTTTC
CTCTCGTCTGATTTTTGCTCTGGACGCTTGTTTGGTTTAACTGATG
ACCCGGGTCTTT
>ConsensusfromContig2706 Average coverage: 22.62
GACCTACTCCACAAACGCGACGTTGCAGTATACCTGGTCAGTGGC
GGCTTCACAATCATT
ATCTCGGAGGTAGCGAAAATTCTGAAAATCAGCCAGAATCACGTC
TTCGCAAACCGTCTT
GTATTCGATCAAAACGGTCGATTTCAAGGATTCGACAGAAACGCT
CTCACTTCAAAATCT
CATGGAAAGACCGAAGTGATCAGACACTTGCGAGAGAGTAAAGG
ATACCAATCCATTGTC
ATGGTTGGAGATGGGTCGACCGACTTGGAAACTCTCCCTGTTGCT
AACACTTTTATCGGT
TTCGGAGGAAACAAAATCCGAGAGAGCGTGCATGCCGGCAGCCC
CTGGTTCGTGACGTCA
TTTCAAGAGTTAATCGACGTGCTACAATAGAGTGAGAGTGAATG
CATGAAACAAAAGACT
GAATGCTCACTTGTCAATTTCGTTTGA
>ConsensusfromContig2707 Average coverage: 8.53
CTTCCGATCTCTGAAAGCCATTATTTGCTTTGGGCTCTGATCAGC
ATTGTTCCCCAAAGA

AACAATTGCCATTCTATGGCTGGTCAGGTTCAGCTTGCTGACAAC
TTCTCGCATTGCATT
TTGTGTTTCCATCAAGTCAAAGTAATGATGATCGACGAGAAAAAC
AACATCGCTATCAGC
ACCACAAGTCTTTTGAATACTGTCTACTGAGCTACAGAAACCAAG
TGTGTTCTCGATAAA
ATCAATGGCTGCAGGCATTGCTGAGAAAAACTCCATCTTCTCAGG
TTCACTGACGTCGTT
TCCGCAATTGGCCAGAATCTCGTTGAAGAATCCGATGTTTTGCAA
TGCGTTCGGCAGGTC
ATATTCTGATAATTCGC
>ConsensusfromContig2708 Average coverage: 33.03
TACTTTGATTGCGGGCCTATATCAAAATCACAGGTCGCCTATGCT
CTCGACTGGAATTGC
AAAGCTTTTTTACGACACTATTCTTGTCGATACAGCATGTCTTGA
TTCATCATGCGGTCG
GGCAACGGAGAAAGACCGGATTGTAGTCGAGATGTTAGAAATCA
TCATTGGAGAATGCGA
TCGATTGAAGTCATTTCAAAGCATCCAAGCAGCTAGAAATAATGT
TCAACATTTGACGGC
ATATCAACTTCTCAGAAAGGACATGAAATCTTTTAAAGCGTTTTT
TGGCGTTGTTCTCAT
GAGCTCAATTCCTATTACAATACAGGATTTTTTCACCCGCCAGAA
CGTCGACGCTGAAAC
TGATGATATTCTTCAGAGATATGGAGCATCAGCGATTGTTATTAT
GGCGTTTGTGCCTAA
TCCTGAAGAGCAGTCAACTCGTCAATTAGCAGTTCATGCTCATAA
GAGTTCTATTCATGA
TGCGATGAATATGTGTTTACTGAAGTTATCCCAAGGTGGGAATGA
AAGAAATTTAAAATT
GAAAGAAATCCATGTGATAGAAAGTCCTAAGTTTACGTGCTATT
CACAAGAAAATACGAG
CTTGTCGAGAAAATTTGTTATGCCTGGCCTAATGAGACATCTTGA
AAATATTTCGAAGGA
ATTCAAGTAACTAAATTATAATGAAAAAGAAAACCAACTTTCCA
ATGGAGATGCAGCAAG
TAACTACGAACAGTC
>ConsensusfromContig2709 Average coverage: 15.57
TGATAACCCTCACCTTCTATGTATTCTCGTTTCTCGTCGAGAAAG
GCAACGTCATCGCAG
TGGGCAAGTTGTAGCTTTTCATGAAAACTCGTTATGTCAGTGTCT
CCAGCTGGTATGGAG

AGAGCAACCTTGCTTGACCAAACCCATATATGTGTGAAAAACCC
AGCCATATATATAAAGAA
AGGGAAGCTACCTTATATCCATCAATCAATCCAAATTCTGAAATT
TTTGAACTTGTATGT
ATTGTAGATCGGA
>ConsensusfromContig2710 Average coverage: 12.58
CCTGCTGCAATTGATTCCGAGGATATGCTCAGGTTGAAAAATGA
GCCATATAAAAGAAGA
AGGTCGAAAAAATGGCATGAAAAGACTGAGTTCAAAGAATCAAA
TAATCATTTCAACCCT
GATATTGATCAAATACTATGCAGCTACTGCTCTCAAAAATTTGAA
AATTCCAGTAAATTT
GATCAACACAAAATATTATGTGAAAAATCACAAATTTCACCTAAT
GTTAAAAATTCAGAA
GATCGAAATATCTCAGATATGAAGAAAGAAAAATATTTCTGCTCT
AGATGTGAATTTGAA
GCTACCTCAATCGAGTCATTGACTTCACATTTCAAGGAAATTCAT
GGCAAAGATGTTGAT
ACTGTTATTTCTACGAATCACAATGAATATTCGAAATATGATTCA
AAATCAAATGGTGAA
GAAAACACATCGAGTCAACCAATAGAAAAACA
>ConsensusfromContig2711 Average coverage: 15.84
ATATGAGTAATATTCAGAAATCTCGGGTTGCTGCTTATGAAGCGC
CTGTGAAGGATATAG
CAGACTTCCAAGCTGAATGCCTACAAAGGGTTAAACTGAAGGAA
AAAGAAACGCAAAGGA
TTTTTCGAGAGAATGGAGAAGAAAACGATGAGAAAGTAACACAT
GAAAAACTGATTGGAA
GATTGAAGGCTGCTGAAGCGCGAAGTAGGATCCGCTACATAAGA
ATTGATTATGAAACCA
ATCGTGGTCAAGAGATAGATCAACTGATAATGAGTCAACCTATT
GCACAAAAAGCAGTTC
GACTACAAGCAATGTTGCCCCATAATTCTGAAACCTCATTTAAAG
AAGACCCTCTAAATA
AAAGACAGAGAAAACGTGTCGAGAATCTTCTTGCTGATGACAGA
AACCTAATAACTGGAC
GGACCTGAGCTGAGAAATCTAATGAAGGGCCTCCACGACAACAG
TTTAAAAAACACTATT
TGTGAAAATTTTAAAATAAATTCACTATCTCCATGACACATTTTC
ATAATAATTTAAAAA
TCCTGGAAAAATATATAAAACGCCATCACAACGACAGCTAAGTT
CTCAGATGAATACACT

ACCATGCCACAGTTCAATAATGACTTTGTGACTAGACG
>ConsensusfromContig2712 Average coverage: 33.31
GCTAGCTATACGTCAAAACATGGGATGAGCGATTGTTTTTGATT
TTTCAATAAGATGAA
ACACACTAAAGATATTTGTGAACAATTGCTAAGGTGTCATCTTTT
CAATTTGAACCAGTG
TTGCTGTTTTAATGATTTAAAATTCTCTGTTAAAGAAGTGCGAGG
AGCATGTTTAGAGTT
AACTGTTAAATTAATTTATTCTTGTTTACAATTACAACAAGATGA
TTGGTGGTTCAATGA
CCACCGAGCAAAACGGAAAATTCAGCTGGTTGTGCACGCCATATT
TCGAAACGCATTCCA
TTCTCTCGAGGCTTCAGATTCCCTAGCGCAATGTGATCTGAATTC
TATCGGATCCGATGT
CGAGCGTCAACAATTGTTCGACGGGGCGAGTTTGGC
>ConsensusfromContig2713 Average coverage: 18.36
TGTGTCGTGATTAGTATTCGTTTATTTAGGGCTCATATGCAGATGG
ATTTCAGAACTTTT
CAACATATAACAATCTGAGATACCATCTATCATAATTTAATGTAC
TCATTAGTTATCTTG
CCTTCTTGATTTATCATCAGATTTTGCTCAATCTATCATTTTAATT
CAAAATGAATATGA
TTCCTACATCATAGGTTTCACTATGTATGATTTGACGTGTTTAATT
TATAAACGATAATG
CGTTAACCAATTAGGTATAGGTTAATAGGTTAAGTATAAATAGCC
TTTCAATGATGTTTC
TTTGAAGCAGAATGGTAAGTATCATAATGATGAATGTTTTGTTAC
AACTCTGTAGGAAAT
TTTGAAAGATTATTTATGAGTTTATCGTCTCACCAAAATCAAAAT
AGCCTTTTAATTGGT
ACATACATCTGTCATGATTCACATTCGGGTGAAACAACTAAACTA
TCAATTAATAGACAG
AAAAAATTGAGTTC
>ConsensusfromContig2714 Average coverage: 7.25
TCTTGCTGAGAACGAGTTACATCGATGTAACCCGATACGCGGCCT
TTAATAAATATTAGA
ATATCGAAATAGCCAAATGAAATTTTGTAAATTGTTTTTATCAAA
TATCAACCATGTATA
CCATACACACTTGAGAACAAACATGTTAGTTGTCTTCACTCGCAT
GGGTCATTTTATGTT
TAATGAGGTAAGATGGCTTCACGAAAAACATTCCACATTTATTGC
AAGAGTACGATTTCT

CAC
>ConsensusfromContig2715 Average coverage: 26.34
GTATGATTGTGTCCATGAGGATGAATCTGAAATTGAGGTCATCGT
GAGAGGTCTTGTCTG
TGAGAAACGAATAGAGCAAAGCCATAAGAAAATACATGCCTCCG
ACCATATCGGATATGA
TGTTCCCGAAGAAAAAATAAACATCGGCGTTCTTATAAATTAAA
ACACAATCGTACACAG
CTTGAAGACATATTCCGGAAATGTAAATAAGGACAACGGCTTTTC
GTCCGATTTTAGAAC
TCAGATTTTGAAGATAGATGACGCTGAACATCGCGAATGGAATCC
CCGCTAGTGAAATGT
AAGAATTCCAATTGGATGTGACGCTTTGAACCTGTTGGCGGTTAA
TGTATTGAGGGTCGG
AGTGGTTGACAGAACAATCGTCCAACGATGAAATTGAATTGATGT
CTATATCAAGTCTTT
CTGCTACTCGATTGTAAATAAACTGTGCCTTGAGTGTTGGAATG
GAGAAGCTGAAGAAGA
CGAAAATGCAGAGGAAGATCTCGACGCGACCGGTTCTATTCCAC
CAGAGGCGAACCTTCT
GAAGGAGTGAGTCATCGATGACGATGACCGGGGAATACGTCATC
TCGTCTTCCGATGAGG
ATGCAGACAGTATGCCAGCGGTGTCCGTCATCATTTCCTGAGCAA
TATATCGTTGTGAAT
CAGTTCAATAATATTTTTATCAAATAAAATTTTAAGATTATTGAA
CTCAAAA
>ConsensusfromContig2716 Average coverage: 8.84
GAATAATTATAACAATATCTGTTTAAAATTAACTGATAACAGCCA
AAAATCCGACAGTGA
TCAACAAAACTTCCCCTCAAAAGTTTGCAAAGTAGATCCTGATCA
ATTGGTATCGTTGGA
CTCGAATGATCTCGCACGAACAAGTTATACACATTCCGATTACAA
TTGTCAAAACATAAT
ATCTCAATACCAATATGATCAGACAGTCTATCCCAGCCATGCCTC
GGAGTATATGTATGA
CCAAATGTTGAATAAAAATCTGAGCTATGAGCCTCTGGAGCATC
AGAATTTCCAAGACAG
CGTTCCGAATATGACACACCACGGCAAACTCACACACGGCTCAA
TTCAGCAGCCCGGAGA
TCGGAAGAGCACACG
>ConsensusfromContig2717 Average coverage: 26.00
GAAAAACTATGGTGAAATTAACTCTGGATTTAATTGCAAGAGGA

ACATCGGGCTATACAA
AAAAGAAAAGGGACGAGAATATACCAACGTATTTGAAAAGACTC
ACACATTTATATCTCG
AGAAAAAAAATATTGACCATATTGGAAATGACATTGGGCTCTGTA
GAAATCTCTCAGTCT
TATATTTGTACGACAATTTACTGAAGGATGTCCCAATACTCAAAA
GCAACTATAACATCA
CACAGTTATACTTGCAAAATAACCAAATTCAATATATCGAAAACT
TGGAATCATTGGAAA
AGTTGAATAAACTGTATTTGGGTGGAAATAGCATAGCAGTTCTG
GAAGGGTTGCATGGAA
ATCATCAATTACAGGAACTTCATATTGAAAATCAGAAATTGCCG
CCAGGAGAAAAGCTGA
TATTCGACCCGCGTTCAATCAACGCCATCACGGATTCATTGCAAG
TATTGAATATCTCAG
GGAATGCAGTAGACACGCTAGTGGATTTAATACCTCTTTCGCAAC
TCCGCCAATTCATAG
CGACAGACAACGACATGTCTGACATGAAGGAGGTTACACATTGT
CTGGCCAGCTGGCGTT
GCATCTGGAAGCTTGATCTCATTGGAAACCCGCTCTGTCACAAAT
GCAAATACAGAGACA
GAATCATCGTAATGTCTAAATCCTTAGAACAATTGGATGGTAAAG
AAATTCCACAGTTAT
CCCGCCAATTTTTACAAAATTGGAAGGCTAACAAGGATGCGAAT
CAGAAAAAACTACAGA
GCAGACAGAATAGTAAAATTTCTGCAGCGGACGCTAGAGAAATT
GAAGAGCGGTGTTCAA
GTCCTTCTTATATATCAACCAGTCAGTTTACACATGCTATGATTC
AAGGAAGACCTGCAT
CGCGTTCAATGAGTTTTTGCCCGTATGATGGAAGCGATTCACATA
GCATATCTCCAGGAA
GATATCTGAATACATATGGAACGATGCATCATGGTTGAGCGAAG
AACAAGGGAGCTGAGT
ATCAAATTCTTGAATTAATCAGAGATACTGAATTCATACTAGTCT
CAAAATTCTTAAT
>ConsensusfromContig2718 Average coverage: 17.94
GTTGACGTCACCAATGACATCTCCGTCCCTGTATTCGATCAACCA
ATCGGGTACCGCGGC
TCGGTCTCGGAGACCGCCCCCATCGGTCATCCAATCGGAACGGTC
TCTGCAGTATCTCCC
AACATCCAAAGTGCCACAATCTTGTACGATGTCGTCGGGACCTAT
CCGGCTCCAGACTTC

TTCTCTGTCGATAAGGAATCTGGTTTGATTTCTGTCAAGAAGGAT
TTGAAACTCGATAGT
CTACAGCTGTCATCCTATATCATCGAAGTAGAAGCTTACGAGAT
GAGCACACCCGACAAG
CGTGCGGCGACGACCATAGCCATGGAGGTCGTGAGGAACGAAGG
CGGCCCGGTCTTCGCT
AAAACCTTCTACCGGGCCGAGATCTCCGCCTCGGACCCTCCTGGC
ACGAGGATAACTCAA
GTGGCCGCAGAAGATTCCGATGGCGATGCTGTCCGGTATTCATTC
GTTGA
>ConsensusfromContig2719 Average coverage: 40.81
GCTGCCTTTATGAATTTTCATAATCCAAAAAAGATATATTGTTCA
ACAAAAAAATTATTC
ATTCTGATTTCCAGAGTCATCCATTTTGAAGATAGATGACGTTGA
TGAGGCTGCAGTACC
GACGTCAGATCCTGTCTGATAGAGACCAAATTGTCCGACAGCCAA
GTTTCCGATTGAAGG
AGCGGCCGCAGAAAATTCATTTCGTTCAAGAAGATGGCCAGAAG
AAATGGATGGATTGGA
AGAGATCGGCATTTTATGACTTCTGTTGCCAGGAGGAATAATAAT
CGGCTGTATCTCTGA
GCCTTCATTCGATGAAATCTGTTGGAGACCGGATGCCGGCAAGGG
GGCGATTCTTCGCAG
CTCCGTTTTCGGCGCGGTTTTTGGCGAGAAACTGTCAGTGGCCGA
AGTCGCATCAGATGC
CGGGGGCGTATTTTGACTCGGGAGATTCATGGCCGCTCCCTCAAC
GACTTGTCGATTGAT
GGGTATTGATATCTGATGAGTGGTGGTGCCTGTCGTGAGAATATG
ACTGGATTTGACCCC
AAATGAACCCTCAAAGACCGATTTATGAATGTCAGGGACGTTTTG
GGTTGCGACGGCGAT
TTTCAGTTGCTGGATCA
>ConsensusfromContig2720 Average coverage: 15.06
CTCGTGGATGGCCATTGTCGANTCGACATTGTGCAAGGAGAATTG
TCAAATTGCTGGTTT
CTTTCTGCCTGTGCTGCAGTCGCTCAGCAAAAATCTTTTATCAGA
CAGATAGTGCCTGCG
AAGCAGACGTTCGAACAACCCGGATACCGCGGAGCATTTCTGTTT
CGGTTCTGGAGGTAT
GGCAAGTGGGTGGAGGTTTATATAGACGATCGCCTGCCTTCACGT
AATGGTCAACTCATT
TATGCCAGATCAGCAGAACCGACGGAAATGTGGCCGTCCCTTTG

CGAGAAAGCATACGCC
AAACTTCACGGCTGCTATGAAGCAATAGAAGGTGGACAGCCCAT
GGACGCATTCGTTGAC
CTCACAGGGGGACTTGCCGAGAGGTTTGAGATAAACAACGCCAGG
CCAGATCTCTATTCT
CTCATGTTCAGAGCGAATAAATCGGGCTCATTCATAGCCTGCTC
GAAGAAAGGAGATTGG
AGAAGCGCCAACATAGCAGACGACACAGGTCTGGTTTCTG
>ConsensusfromContig2721 Average coverage: 18.41
CTCTCATCTTATTATATCACCCATACTGTTTACCATAATGGATAG
AGATAAGTTGAATAA
ATGTGTGACTATTCTCAAAAGCAAGTACGATGTGGAAAGTAGGGT
CAAATCCCTGTTGAG
CCTCACCAAGGTCTCCAAGGGGGACTTCGCCAGAATTATGGTGAC
CCTTTTCGACAAATA
TGACTCAGATAGTCGTGACAAAAGTGTTGTACCCGGTGCCGATG
ATAAGAACTCGGATGA
ATCCAGTAATCCGGAGGGGGTCAACTTGGATCAGTCAGTGATTTT
CATAAGGGAGGATCC
TCCCACCTCAGTTGAGTCTATGCCTCAAACTACCCCTAAGCCTGA
CAATCCCAGCCCACC
AACCCGAATCGATATCCGTAAAAATATCCCTTGCAGATACCATGC
TATAAATTCATGTAG
GAACGGTAACAAATGCCCCTTTAAACACCAAAGGATCTGCAGATA
TTTTCGTTCGGGTGC
TGGATGTAGTAATATTAAATGTTCCTACCTGCATCCGAATATATC
CATGATGAGAACGGA
GCGACCATCACATATAAAACCTCTCATGGGTCATAATTCATTTCC
GAAACGCAATAGCAT
GTCAATAAGGAACGATCAACCCGTCACGCTAGATCAAAACACAAA
TCGTTTCTACTCATA
TGAGAGGCGGTCACCCGTGACGATTC
>ConsensusfromContig2722 Average coverage: 4.96
TCGCATCATGCAAAAATTTTCATATTTGTTTTCATGTTTAAACAC
ACAAAAATCTCTGGA
GAATTTCAAAATTTTGAGTCGCTGCAAAACCGGAATCATTCTCAA
CAAATAAAAACTGCT
CACCAGCCAAAGTTCGAATAACCAAGGCATGTCACTTCACCTTTG
ATTTTTTAAACATTA
TAGAGCACCACCGACCTGTAAAACATACCTACGCGCTTATTTTGC
CATTATGACTCACTT
TCAGAGAGTTCATGACACGATCCAATGAAAATACATGACTGTCCT

GTTACCTGATATACT
TCGCCC
>ConsensusfromContig2723 Average coverage: 9.94
GGGTGCTTTACTGAATCAAAAACTGAAGCTCTTGAACACACTGTA
TCTTTTATGTGGAAT
AATCTGATAAAACCAACAGTAACATGGAATGATGAAAGAAATTT
GTTTAATGAAGCGTCG
TGAGCAGATGACCCTATCCAGTCTTTAGTGTCCAGGGACGCACCT
GCGTACTCAATGCGG
TAATTGCGTTGGCCTGTTTTTCTTTCGTTCTCTTCATTTTTTTGTGC
TTTCGATTCTCAC
AGGAGAGAATGACCATGTAGATACCCCAAACTAGACTCCAGAAC
CATCCGACCAGCATGA
GCATCACCGTTGACATTTGTGCGATACCGACGAAGAGGTTGACCC
ATACAGTCGTTGCTA
CCGGGCTTTCTCGCGTCTGCACTCGCGCTTCTCCGCAACAACCCA
CGCATACGCCTCCAA
GCATCGTTCCCAAACCCGGAATGATGATATTGAGCATGAGACATA
TTGTAGCAACACTTC
GGGGGAGATATGGGATAACTGATCGAATATAAGAATCGTCCGAGA
GAATATTTTTGTTCT
CTTCCTCTTCTTCCTCTTCAACACTAGTATTCTGCATTTCTTGCAA
TTGACCCAAAGCTA
GGCTTATTGAGGCCATTGACCCAGTCTTCGATCTTGATTCATCTCT
ATCCACATCGTTTT
CAGAGTGACTGCCGATTTGACTCACTGCCTTCGATGTTTTTCGCGA
CTTCTTCTGCTTGA
ATTTTCCTAAATTTCTGCGAACTTTGTCCCAGTTTTGAGCGGCAG
AAAAGTTAGGCTGTT
CGGCTATTATGTGATTTGTGAGAGAACTGCTTTCATTGATAATAT
CGAGGTCTGCCATTT
TCACCGACTGAATAATC
>ConsensusfromContig2724 Average coverage: 12.47
GAGAACCTGAAAAACTTCATGTGGTGTTGCTGGGGAACTATCATC
TCCAGTATACAGCGA
TCTGTCGACTTGCCAGGAAGCGTCAGTTCCCAAAGATCCAAATA
AGAACAAATTACGGCC
ACCCAGGATCGGTGGGTTATCCCCAGGCTCATCCGGGTCCATGA
ACTGGTGGGACAAGAA
GGTTTCCGAACCCATTGCATTCTTCCCCACTTTCAGTTTCCCATCC
TTAGCTTTGATCCA
GAATCGCTTCCAGTCCTTGCACGAGAGAATGTTGGTAGTGCTTCG

CATGACGAGTACGGC
AGAAGATAGATTATCTTTGATGACTGAAACTTTATTATCTTCAGC
GCCAATTATGATCTC
AAATTGGTGATTTTCATCAATGGTCACAAGAATGTGTGCGTTTTT
GCAAGATCTCACTCT
GAACGAAACTTTTGGCTTTGACTGAACAATTGTGTGACTGACGTA
GTCATTTATTACGCT
GTTCGGAGTTTGGAATCTGTAATTATTACCTTCGATTGAAGAGAC
ATACACATTGCCTTC
CTTTTTACTCGCAAATGAAAGAGACGAAACAATGAAGTCTTCGGA
GGGCGTATATTCAAC
AATCGTGTTTGAGCCCGGATACCCACCGAGCCCCATGGCTAGGGT
CCCCTTCCAGGATAG
CCATATCGCTTCAAATGCGTTACAATCCAGTATTCGCTCTTCTAC
ATTGGCCGAAACCAT
CACGCTTCCTTCAGTCCCATTGAGTACCTGGGCAACCCCGCCCCC
AAATTTTACAATCAT
CGAATCAATGGCTCCCAACGCCAATGGTTGGGGCGTTGCAGAAA
GGGCTACTGAGAATTG
ATCGCATGACTTGACACTGAAAACAAAACTGCTACGACCGTTCAC
ACCGAC
>ConsensusfromContig2725 Average coverage: 7.79
TTTGAAGCTGAGTTTTCTTTGGCTATAGTTCTCATGTCATCACTAG
AGAGTATCATCCTC
TCATCATAATAGCCAGAAACATATTCCTGGAAATCATTAGCATCT
CCAATCAGGGTTCCC
TTGCCTCCTCGATCAACCAGCTCTCGCCACACCAGCGGTGACTTT
GAATGGCTCCAGCCT
CTCTGTTTGCATTCGTTTGAGAGCCATGGATCCCATTCTGTTGAAT
TCTTCACAATTGTG
TGAGTTTTAAAATTTGGAAGACATCTAGAAAGTTTTTCTGCCAAT
AATTCTATTTTTGCG
AAATATGGACAATCAGCCTTCCCTGCGATAACAATTTTTGCCGCC
ATGGGTTTGTTT
>ConsensusfromContig2726 Average coverage: 12.08
CCGCTTAGGTATTTTCGTGAAGAACTGTTGCAGCCATCATAATTG
CAGCTTTCAAGGCAC
CCTGTTATCGATTCCATATCGGTGTCACCATCGTGTTCAATGAAC
AGGTCACCGCATTGG
CTCCCCCAATCGTCCGCCGCACATGTTCGTATCAAGATTTTCCCGC
CATTTTCTTTAATG
CCTTTGATCTTTATGCAATAAAATGCCCTGAACTGGAGTTCCCCG

ACTCTGCACTCTCGT
TTGACCAGATGCCGCGTGGAATTATCAACTGGAAAGGGGTCTTCG
CAATCTCTGTTATCC
CCATTGAGCGAATTGCAGAAGAAACAATCTATTGCCACAGATCCA
CTGATGTTGATTATG
AGAAGTATCTGCAGGATTTTCCACTTGGTTATAATCATCCTCGCA
TAATCTTGTGATTTG
AGCGGGATGAAACACACTCTACATTATTTCGAGACTTTGTCGTTG
ATTGTATTTGACGGG
AACATATGGTAGATGGTATTGATTGTAGACCACAATATTTAGATC
CGAA
>ConsensusfromContig2727 Average coverage: 69.48
TTTGAACCATCCGGCCGGCCGTCCCCTGATGACCATTGCCTGGA
GGACAAGGAAAACCAG
TGCGTCGTTTGTGGAGAGCGGCAGAATCTAACTCGAAAGTACATC
GTCCCCAACGAATAT
CGAAGATTTTTTCCCGTGAAACTCAAGGACCATCGGTCATTAGAC
GTCGTACTGCTTTGT
TCGTCTTGTCATTTGAAAAGTGATCTGTTTGATAAAGTTTTAAAA
AAAGAGCTCATCTGT
GAATTTGATGCACCTCTCGGATCTCATGCCAATTCAAAATATAAA
TTCGATTCGAATATC
AAACGAAAGAAGATGGCTGCTGGTGTCTTGCGCCGCGCTTTGAA
TCTTCCCAAAGAACGA
ATTCTTGAACTGAAAGCCATAGTCCTGGAGGACAGAGCCGACAC
TGAACTCACCGACGAA
TTACTTGACGGAATTCTTAACATGAATATGAGATGCCTGAATGAT
GATTACGTGTGTCAC
GCTGAAAAGGTGGTCAAATCGTGTGAGATACACGACCTTGAACG
GAGATGGAGGACTCAC
TTTTTCGAATACATGAAGCCAGCTTTTATTCCTCCGGATAGATTCT
TTGTCGATTAGAAC
ATTCCCTTATTGTTGATCTGTTATAGTATATTTTTTCGAATATACA
TTTGTTGTTTGTGG
A
>ConsensusfromContig2728 Average coverage: 7.75
CAGAAATCAAAAGTGCCGTCCATGAACCAATGCCTTATGAGCCGA
TTCCAGCTTCTATGA
GTGAGCACTCGACTACGCTTGTTCATCCACATTCAATCTACCACA
TTTCAAATATGGCAT
CTAACAATTCTACAGGAGTTACACCTCGAATCACTACAAGTGTTG
ATCCGCCATCAATTT

TACACTTTGGAGATACAGCATCGAATAACTCTGCAGACGTGGCA
CATGGAAACACTTCGA
TTGCATATCCGCCATCATCCCAAGACATTGAAGAAACAATTTTAA
TACACACTACGACAG
CGAGAGACGCATTGCCTCCTGGAAATCGCCCTGTGAGAGAATTG
CCTCAAGAATCAAGAA
AACCAAAAGAACATTCCTCCAAAAGCACACCTGTGCTCGAGATA
GTTCTGCCAGATCGGA
AGA
>ConsensusfromContig2729 Average coverage: 9.14
TCTGTGTCCTGAGCTTTTTTCACCATCATAGGACCAAGCAAAATG
AGTTCCTCTTGCATC
TGTCCGACGAGCGACTTCGCTTCAGACAAACGCGACAGACCGAC
TAGAAGACGTTTCCTA
TTATCTGCCACTTGAGTTTTTCGTTTCTTTAGCATATGCGTATAA
AGCCTGATGAGAGAC
ATATAACTGCTCGGCGTGACGTAAAACCGACGCCTGAGCTCTGA
GAAGAACTTCGTCGAA
GATTCAGCGACGGCTAGATGGATTGAGACGCACTGCTGGGCTACA
CTGTCTTTCAAAGTC
TCAATATCAATGCCTTGAGAGGCAATGAAGTCAGAATTTTCCCCA
AAGTAGATGTCCGCG
ACCCCTTTGAGGGCTTCGGGGCTCCATTCGGCGTACCAATCAATG
GTAGAACAGTTGATG
AGGGAGGGGTGCATCCGACAGCGCATGCGGAATCTGTTTCCGGC
GGGGCTGAGCGCTAGC
GCTATATGAAGGTTGATGCGCACACGCTCCATGAAGAAGGAAGA
TATGGAGCTTTTGGTG
TCTGGGATGCCCGCGGAAGATGCTGCCCCCTGGAGGTCCATCGCG
ATACCGTCAAGATCC
TCAGAGTCGAA
>ConsensusfromContig2730 Average coverage: 9.32
TTTGTCCTCTTCCAATTCTGCATCTCTTTCTGTATTCGGAGGCGTG
ATAAGTATCGGGGC
AGGACTTCTGCGCATTGGTTTCTCATCAATTGTTTTGTTTCTTACC
GACCTATTGGACGG
TTGTCGAGCAGACCCCAATGGTGTCTGTGTGAACGATCGCGTAGT
CTCGCTGTTGATCGG
AATTTGTTGGGCAATTTGAATGAATTCTGTGTCCTTGTTCGTTTTT
GTTCTTCCTGCACC
TATTTTCGCATGACGTTTCGAAACACATTTTGTATTTGATTTTCGC
CTCTCATTGTGGAA

AAATTGTGTATCGATTTTTGTATATGAATCTTGAAGCTCATTGAA
TTTCTTCGAGAGATC
GG
>ConsensusfromContig2731 Average coverage: 31.38
CTTGAAAGTAACTCATTGGTGTGGAACTACACGTCATCCGGACAA
ATATGGACCCGGCTT
GATGGCAAGGTCTGTAAGTTGGATAATGCTCAAACGTTGAGTGC
CATAAAGAGAGCTACT
TCAGAGCAATGCAAGAGCTCACTTGTTCAAACAGCATGCGATGA
TAAACACGGCACACTC
TATGTAGAGTCGGATACGCTTCCTAACCTTTGTCCAAATCGAGGT
AATCGCAAAGGAATT
CACGTTGGTTGCTTTCATGATACATCTGTGGA
>ConsensusfromContig2732 Average coverage: 11.23
CCCGGGAATCTAAGGGCGCGGCTTCGAAAGTCTCCTCCGACAGG
TGAGAAGCAAAGAACG
ACGTGGAGGTTGTGACGTGTCCGGGTAAGAAAATAATCCATGACG
TTCTCGTTGGTCGGT
GTTCGTCGCGGAAACTCTTTTTTCATGATTGGCGCCAGCTCCAAAA
TTATCTCATCTATC
TCGTCTCGCGCGAAAAGATTCGATATAATTCCAGAACTGAGGACA
TTGTTGAGATATTCC
AAGAATCCTTCCTCCTTCACATCAGAGTCAGTGAAGATGAAAGTG
ATGCCCCTCCCTTGT
TGACCGGCGACTCTATATAGGAACTTTAGGTCCTCCAGCAAGTTC
GAAATGCCGTATGAT
CTCGTGAGGGTTATCTGAAACGTTTTGAAGCCAGCAATAAAAGA
AGCGAGGCGAGTGATG
CTCTGCTTGCCCGAGCCCCCAACCCCGACCAAGAGTGCGTTTCCC
CGAGGAGTTCTCAGG
ATCCTCGAGATCTTAACGAGATGTATCATAGCGTCACGGAAAAA
GACTAGGTCGACAGAT
CGGAAGAGCGTC
>ConsensusfromContig2733 Average coverage: 45.82
AACATCAGCAGATGGAGTTCGGGCTGATTCTCATGATTTCGTTGG
ATCAGAAGAGAATTC
TGGCCAAAGCGAAGAATCCAAAATGAGTTATTTCTCGACGGGGCC
TCCAGATGTTGAGTC
GTGGTCTTTGAGTTCGGACTTCAGGAAAACCAGAATGTTGTCTCC
ATTGAGGAGAGACAC
TGTGGGCAACACCAACATCTCGATAAAGATCAGCAAAGAAGGCT
CTCTGGATGAGAAGAA

ATCTATTGGGGGACACAGCAGAGCCAGCAAGGCTAATAGGCAAA
ATAGTAAAACAAAAGC
AGGGCAAAAGAAAGAAAGCATGGCGTTGAGTGGATCGAAAAAAT
CATCCGATTCCGGAAC
ATCCGGCTTCAATAAAGGCGAAAAAGAATCCGCCAAGGCCGTGT
CCTTGAAAGAACGTGA
CCTCATGGACGCTGTGCTGAATAAACAGGTCAAGGACAAAACGG
CGAAACCTCGAGCGTC
ACTCAAAGGGGATCTTCAAAAGCGCCGGAGACACCAATCGAAAT
CAGTCGACTTGACGAA
TACCTCAAAACAAAGCACATATCCGAACAAATGCAGAGACACAT
GATAAGTGAATTCGCT
CAACTTCGGAAAATGATGAATAGAGGTTCATTCAAGAAAGCTTCG
ACGAAAGGTTTTCAT
ACGATGTACGACATGTTGCCCGGAATGGGCGTGTCTGCCACTGGA
TACGCCTTTGTCGTT
CTTGTTTCCCTCTTCCATTCTAACATGAATCAAA
>ConsensusfromContig2734 Average coverage: 5.78
AAGACAGACAGCCATAGCAATTGAGATACGGAGGTTAAAAAAGA
GAGTGAATGGCATCCG
TTTCTAATGTTCAGTAAAGATGACAGGACAGTGAGGTTCGAATA
ATGTATGAAGAGGGAA
AGACAGACAGTCATAGCAATTGAGATACGGAGGTTAAAAAAGAG
AGTGAATGGCATCCGT
TTCTAATGTTCAGTAAAGATGACAGGACAGTGAGGTTCGAATAA
TGTATGAAGAGGAAAA
G
>ConsensusfromContig2735 Average coverage: 23.28
CGGACATGGAAGTTCCGGAGGTGGTAGAAGATCTACCGGATCTC
TACAGACTGATGTCTA
CACTCGATGGGACAACAGGTCCACTCGTACCCACCGAGGAAGAT
GAGTTACCATCGATCT
TCGTCCAACCCGAGAATGTTCCCAACTACAGGAAGCTGAACGAG
TTGCGGCTTCAGATGG
AAGACGAGGGGATCGATCCCTACGCGGATTTGTTCGATGTTGATG
TCCAAGATGATGTCC
TCATGGTCGGCGATATTGCTTTGGAAGATATCCAGGAAGAGGAA
AAACGACTTCAAAAAG
AACACATCGCATACAGAGAACAAGAAGCTCGGCTGGGTAGAGAG
AGACAAGAGGTTATAA
CTCGCAAGCAGGAAATTGCCAAGAAGTATCTGGCTTCGTACGTA
AAGGATCGTAGGACCA

TTCTCAAGAGAAGAAACGACATGCTGCTCCAGCAAGAACGGCTC
ATGATGAACCGTCTCC
ACAAAGCATTCCGTCGGCAGAGAACCAGCTCCGCGCCATCATC
GAAGCCAGAAAGGGAG
AGGTCAAGACAATGTATGGAGACCTATCATTGGCCGATGGGCAT
TACGGCGGAAGCAAAG
GAAGAAGGTGGAAAGTTGATTGGACGAAAACACCACAGCCTATT
CAAATCAAGATAAATA
GTCTCAGGGGTGTCAAGGACAAATTACCAGA
>ConsensusfromContig2736 Average coverage: 4.91
CCACAATGGCGTTCGCCACATCAACGCTGACGTTCTTAAACTGCT
GGATTTGTAGCCGCC
AGATGGCACTCCGACCCTGACCTCCGTAGTCGACCTTTTGACCCC
TGCCAGGCTCTACGT
GAACCGAAAAGGCTTTTTCTAATCTTTCACGTTTGAAAGGCGTTT
CCGCCACGCCTTTGG
CATATGATTTGAGAAGCTTAGAAACAGAGTCATCCGATTCGACA
AATTGGACAGAAACTC
CCAACAAAAGTTGGGCATCGAGGGCAATCTGGTCGATTTGCTCTT
TCGAGACCTTTGGTA
GAGGAGATTTCTTTTTCCCAACGAGGTTTTTGGAGGACAACGTCT
TATCTCTGAAATAGT
TTTCCATTCCAGAAACAATAATTGTGATATTTTGAGAGAGA
>ConsensusfromContig2737 Average coverage: 17.11
CTGCACTTCGGGCAACGCCCATCTTCTAACCATGCTGTGATGACG
CATGGGGAGCAGCCG
AGCATGCTCCAGCAGGACCTGGATACCATGGGCCTCATAAAATC
ATTAAAAAAGAAAGAC
TTGATCGGACCTCTTTTCAATCAGGGGCCGTAATATCCCCAGCCA
CTCATCGAGGCCAAC
GACCTCTATATGGCCTTCCCTGCCATTTTCAGCCCTGCACTTCGGG
CAACGCCCATCTTC
TCCCCATGCTGTTATGACGCATGGGGAGCAGCCGAGCATGCTCC
AGCAGGACCTGGATAC
CATGGGCCTCATAAAATCATTAAAAAAGAAAGACTTGATCGGAC

>ConsensusfromContig2738 Average coverage: 60.42
CATGAATACATGACTATACCATGTCCTTGTCAGTGATGATATCCC
ACAACTTAATAACAC
CGTCATCACTACATGACGCCAATAAACCCCTTTGGTTCGGAGACC
AGGCCACGCAGTTAA
CATCAGTGAGGTGAGCTGATGCTATTCGATGCATGAACACATAAG

AAGGCTCTCCATCCG
AACTCCTCTCGGGGTCAACTCCGAAGATTCTGATACAGTTATCGC
CCGAACCAGTTGCTA
TTAAATCAGAGTTCGGACACCACGAAACGGTATAGATGGCTCTGT
CGTGATATCCGCCGA
GAGTGGACCCGAGATACCATTCGTCCGATCCGCGCTCCAGCTGGA
TGCGATCTGTCGTCG
AATCCCGAAGAGAATTTCGATGTTTCTGATGCCAGATTCTCAGCG
TTTTGTCATCACTCA
CGCTCGCCATCTTCCTCCCGGTTTTGTCGAAAGCTACGCACCAGA
CAGTGGACTCATGAG
AAGCCAATGAACAAAAACACGACCAATCATCGTCTTCTTCATGGT
AAAACTTTATTGTGT
TGTCATAACTACAAGACACCAGGACATCTTCCATTGGATGCCATA
GAACAAATTTGACAT
CTTGTGTGTGGTCAGAAATAACACTCACGCAATCATATTCCTCAT
CATCGTCGACCTCCC
AAATCCAAACCGATTTGTCTCGACTACAAGTTGCCAAATATTGTC
CTGAACTCGACCATG
CGACGGATTTCACTTCATTCTCATGACCTTCTAATGTGGCACCAC
ATTCAAAGTTCACTG
ATGGATTTGGTCTTGTCCATATACAGGTCGTTGCATCAAAACTAG
CAGAGGCAATTTTAT
TACCACAGAGAGACCATTGGATACTTCTTATAGTTCTACTATGAC
CTTCCTCTAAAACTG
ATGTGCATTTCCAAACTTCTTCAATTTTGTTCCAAATTTTGATTTT
TTTATCAGTACCAC
ATGAGGCAAAACACCATTTTTTTGGGTGCCATGCAACAGCCCATA
CAGTTTTTCATTCT
CTTGTCCACATAAATCAGAGATTAGATTGTGCTTGATTAAATTCA
TACTAGATAGCTAGA
TAGATGAAGAACGTTCACTTGGCTTTTTTATAACCACAACCTTGCC
CTTTTTACAAATC
>ConsensusfromContig2739 Average coverage: 18.04
TGATTATATTTCACGAAGATTTCCCCTTCTTCTAACCCTTTTTTTT
CAAATAAAAACGTA
ACCTCTATATACGAATTTGGTATAATATCCACTTTTCAAATATTTC
TACAATAGTCTTAT
CTAAATCTCGATTTTATCAATCCTTTATTATGATACTCTCTCCCAC
ACAAGTCTTTGTCT
CCATTTCCGTATATCCATGTTTCAATAATATCAATATTCATAATTG
AAATCATTTCCCTC

CATATTCATTCTATTTCTCAATTTATTTTGAACCTCAACATAAAC
AAAACCTCAGAAATC
AATAAACATCTTTGAACCACCCATCTTAAGTTTACGTCCTTTATCT
TCCAATCTTCACCT
CCTTCTCCATTTCTCACGAAACTAAACGTGAACATAACCTCAAAG
GTCAACTAACGCCTT
GAA
>ConsensusfromContig2740 Average coverage: 17.77
TCACAAGTGTCTTGATCAATGTCGTGAGAGATATGCCAAAATTCA
GCATTTCCTCTACAA
ATGAACGTATATACATCGATGAGAAAACTGGATTTTTCATTTCTG
ATGAAAATGAGCAAC
CAATACCATCAAATCTCAGGCTGAACTTCGTATGGTCAATCGATC
TTTTTCAACACCAAA
CACAATTAGCTTTCATCGAGCACAAATTTTCTTCACCTGGGAGAA
AACTGGTGTCTGTAA
CGGTCTCAGACATTTTTCGAAACGTGACGAAACATATATTTGTGG
ATGTTCTCGATAAAT
CTTCTGAAGACAGGATCCACATGGAAAATCCTTTGATGGTTATTG
AGACAGGAAAATTGC
AGACTTTCAGAGTATCTAACGGCTTCGAAAAAGGTCCTGGAATC
GTGGAGGAACCCGAAT
TCTCGGGCAGCGGGAGTGGAAGCGGCTTTTTGCAGATCTTCAGCG
GGTTTTCGTCGGGAT
TTTCCGGGGATTACTCTGAGGACGATCAGA
>ConsensusfromContig2741 Average coverage: 6.39
TGTAGCAAAACTTATATCTGCATCACCATCCCATAGGATGATATC
ACCATTTCGATAAGC
CCCCAGAAGCGTGCCGTAGTCCAACCAATGATCTACTTTCAAAGC
TTCAAGAGATCTACA
AACTGCAACGATAGATTTTCTAAGATCGTCTGCTTGTGTTTTGTT
TACAATACAATCATC
ATTGAAATACCCTATAGAGTTCGATAAAACGTGCAATACATAGA
CAAGTAGCACGAGAAT
TAAAGACAAAACACAATACTTGATTCTGACTTTCATAACCTTCAG
AAATCCTGTTTTTCA
CTGCTCGTGATATATTTTGCAATCTCTGATGACATTACGGATCATG
CTT
>ConsensusfromContig2742 Average coverage: 11.61
TCGCTCATGTAACCCTAGTGGAAGGTTTTATGTTTAAATATTCAT
GAATCAACTCTCATT
ATATGATATATGGCCCTCAAATTTTATATTCTAGGGGGCAAATGTT

TTTATTAGTGTCTC
AATTAAGTGTTGAAGTTTTTTACGGAATTTCGAAATCTACCTCTTG
GATACATTTTGTTT
GTAAACCATCCTTTTCACCAATGACCTGTTTTCATTTTTTTTCTAA
GAAATTCGTTTTCT
CAATAAATGTGTCAAACCTATTTTTGGTATCCGAAAATCTACCTC
TGAGACACATGTCCC
ACAACCATCGCACTCATGGATGACTCATCGTGTGTTAATATTCCA
GGAAAAGGTAACGTA
CATATTTGTTTTTTCAACGAAGTGTTGAAGTTATT
>ConsensusfromContig2743 Average coverage: 26.51
TCTTAACAAGACTGGCAACATTGCATGTTGGGTTGGAGAAATCAC
AATCTTTACAGGTCG
CAGTTTCTTTCTGGTCTGAATCATCGCATTTGACGAGTGGAATAC
AAATGAGAGTTTGCA
GCAGAAAAAAGAAAAGATTCATCGTTTTATATTGATCTTTTGGAA
TCACAATTCTTTCTG
TCTTCTTCTTTATTCAAATGTGAGGAAC
>ConsensusfromContig2744 Average coverage: 10.35
TACAATCACGACATGGGAGTTGACATGGTGACGTGCCTGTCTGAC
ACAACTCTCGAAAAT
GGCTGGAATCATTTCATTTGTCAGAGACCAGTCTACGCTCGATAC
ATCATTCTGAAATCG
GTTGTTGCGCAGGCATTGTACGTCAATGAGATAATGGTGCAGCGA
GTTAAATGCTTCAAC
TCAAAAAGAGAAGAATCTTTGACTGAAGTTTAATTGTAATTCAAA
ATGAATAATATAATG
GTGCGGCGAGTTAGACATTGTAGCTCAGAAATAATGAACTATATG
ACTGAATTTTAATTG
TAAATACTTCGAAAGCAAACAAATAGTGGTACACCAAGATTAAT
AATTCAACCCAGCAAA
TAAAAAATAGATAATTAGATTGAATTTTAATAATTTAAAATAAAC
TGAATAGTGGTACAG
TGAGCTCAATTAAACTCAGAAAGCGGAAAATCTATGAATGGTTTG
TATTTTTAATACAGT
GGTCGATATATCAACTAAGAAAAAGAGAAATCTATGATTCGGTTG
TATTCATAATAATCT
AAAATCAACCAACTAGTAGTTTAGTGAGACCGATGCTCTAACGC
AGAAAGAGGAAAATCT
ATGATTTTATTGTAGTTGTAAGTAATTCGAAATGAAAAAAGGACC
TTTGACAGGAGA
>ConsensusfromContig2745 Average coverage: 13.93

CGCGAGAGAAGTTCGCACGGATTTTGAGGAAGCGAATTCAGAGC
TGACGACGTCGCAATC
CAAATCTGCCGCTTTTCGTTTGACGGAGCTTCCTGTCCGATCCATG
CTCGGTTTTGAGTC
CAACTCGCTCATCTTCGACGACGACGTAACTGACGAGACCGAAG
AAGGCAAATTTCCGCC
GGCGTTCGACGAAGCGACGGCAGACGAAGCTGTTTGAACAGCCT
GCCTCATCAATTCCGA
GCGTGCGGCGTTGGGCGATAGAGTTCCCATCCGATGAAGATGTT
CAGCATACGACGGCAT
TG
>ConsensusfromContig2746 Average coverage: 26.83
CGACGGGGCCTGAGGGTCCTATTGGTCCTTGGTCTCCTTGAGGGC
CTGATGGGCCAGGTC
CACCCAAAGGTCCGGCAGGCCCTGGAGAGCCTTGAGGACCGCTT
GGTCCTACTGTGCCGG
GTCCCCCGAGCGGTCCAGGTTGTCCTATCGGGCCAGATGGTCCCA
CTTCTCCTTTACCTC
CTTGTGGGCCGGGTGGACCTGCAGGTCCTGATGGACCTGATGGCC
CTGATCGTCCGTCGG
GTCCAGCCTGTCCTGGGATGCCAGGCTCTCCGATAATTCCTGAAG
GTCCAGGGCCTCCGG
GCTGACCACGACTACCAGCTGGTCCCGGTAAGCCTGGCACTCCTT
GTTCTCCAAGACCTG
GTGGACCTTGTTCACCCTGAGGTCCTCTTTCTCCCCTGTTCGATTG
AGGTCCGGGTTGAC
CTGGTTGACCTGAAGGTCCAGAAGGACCGGATTCTCCTCGTTGTC
CTCGAGATCCAGGTG
GTCCTGCTTCGCCAAGTCCTCCTGCCGGGCCTGGAGGTCCAGGCT
CGCCTCTCGGACCTG
GATTGCCTCTACCGCCAAGACCTCCGACTGGACCTGCCTCTCCGA
TTTCCCCTCTAGGTC
CAGAAGGACCAGATGGACCAGCGGGTCCTGCCCCTCCGAGTGGC
CCAGCTGGTCCGACAT
CACCAGGTCCTCCTCGAGGGCCTGGGGAGCCTAGAGATCCTTTGG
GTCCCTGCGGTCCAG
CAGGACCACTTTCACCTTGAAGGCCTGATTCTCCTC
>ConsensusfromContig2747 Average coverage: 8.10
CGGACAAGGAACATCGATCCGTCGATTTTCCTTCTTCTCGAAACG
ATCGACAGAGCCTAT
GGGTGCAGCAGAGGAGTAGGAAGATACGACCGTCACAGCTCTGT
TGTCGAACCACTTGAC

AGCACGTATCTTCCAACCCCCCAAACTGATCTCTTGTTCATCATG
TGAACCCCGACCTCG
CCGTTTCAGATCCTTATCGGATTCGAAACTCACACCTTTCAGGCG
GTTTGAACGAGCAGT
CCCGAGATCGGA
>ConsensusfromContig2748 Average coverage: 22.94
GAGCGCAAATGCCATAAATTTGATAGAGACAAATCTGGTGAATCC
GGCTCTGTATGCTCA
AATCAGATGAATTCATCAACTGAACATTCAAATTCTGAAGTATCA
TGCAATGCAACTTTG
CCTCTAAGTGTTTCCTCAGATATTGAGTGCAATATTTTTGATAGA
AATAATAAATTGCGT
AATTCTGCATATATTTTAGACGCGTGTGGTGATAAAGCTTTTCTC
AAGGCCAATGATGAA
TTTAGTTATAATAAAACGCCTGCAAAAATATGTGATTCGAAAAAA
ATCAGGGAGATTTCA
GAAGATGACAAAATACATCTGAAGCAATGTGAAGAGAAAAATTG
CGAGCGTTGTTTTTA
TCGATGAATCGAAATTTCTTCAGTCCCTGTACTTCTCCGCAAATGA
TTTCACCATCTCCG
CAAATTGAGGCTTCTTTACCCTCGAGCATGGAAGCAATCAGAAAA
CTTCGAAGCCAATTG
GTCTCTGAACACAATACACCAGCTTCAGAGGAAATCAAATTACC
GTTCGTGGGGAAGATC
CGGCTACTCGATGTGGGGAGCTGCTTCAATCCATTCGCAGAGTTT
GAAGACTTCATCTCT
CTCGGTATCGATATATCACCCGCTGTTGAGTCTGTGCGTCAGTGTG
ATTTTCTCTTCCTC
GAGCTGACAGATCCCTTGGCGTCGGACTCAATCGAAACGTACCTC
CGGCAAATCCGCGGT
CGGGTCTCCCGTCTCCCTCGTCAATCGTTCCACGCTATAGTGTTCT
CACTTCTCCTCAGC
TACTTTCCGTGTCCATTTCAACGCTGGATCTGTTGTCAGAAGGCA
CATGAGCTTCTCATG
CTCAACGGACTTTTGCTAATAATTCTTCCAGATTCGAACAATAGC
TACAAGTCTTCGAAA
ATGGTGAAACGCTGGAAAATAGCCATCGAATCTCTGGGATTCCTT
CGGTGTTCTTACGAA
AAGCTGACACATCTGCACTGCATGGCGTTTCGGAAAACAACATC
TGCACTACAGGACAAC
CAGCTGTTGGAGGGAATTTCTTCCGACCTTCTGAAAATGCC
>ConsensusfromContig2749 Average coverage: 13.94

GACACCTATTCTTGGCTTATCGTTCGGTAACAAAAGAATCAGCAA
ATATCATGAAGTATG
ACCTCTGGTATTTGAAAGGAGACTTTATTGATACGGGAACATGGA
ACACGTACTCCATCA
TTTCGGTGAACAAATCAATTGTGTTTGCATGGCACGCTGTACCAA
GCCAGGTTACCAGAT
TCGATATGAGTTCAGGAAAAATGTTCACACATATGATAACCGGT
GGCATAGCACAGAGCT
CGGCGATAAGCACAATATATACGAGATCATCACCTCCGCCACAA
AAAGCAGAAGATGAGG
GGCGGGAATTCGCAACCAAAACAAATGGCAACGTTGAGGATAGA
ATGCCTTCGCACACGG
ATCATTCGACGCCATCAGCAGAATATTCG
>ConsensusfromContig2750 Average coverage: 10.50
ATTGGAATTCCACTGACGATTCTTACCATAACCCACATCGGGAAA
TCTTTAGCCGTGGGA
GTCATTTACAATCATTTATTATTTCTCAACTTCATGGCAAAATCC
TATGAAGTGACGAAA
GCCGATATAGCAAAAAGGAGAAAGTCCCTCAAAACACGAATAAG
CCGATATCCACTATTG
GGAGGACGTGGATCAGAGGTGTCCGCGAGGACTTCAACCACAAT
ATCGGATAGTGATTTC
ATCGAAGACAAAACTTTTGATACTAAAGTTATAAAGTATAATGAT
GACCAAAATTACGGT
GAAAAACCGAATGATAATCAGAGTCGACAACTAATAAAAGTACA
GAGTGTATCTCAATCG
GATACTCGCAAAAA
>ConsensusfromContig2751 Average coverage: 12.86
ATTTGGCACTTATGGACATCTTCTGTATAATGGATTATTTTGTTTT
TGATTATAACGTTA
TTCGATAGCATCCAAAACAAAATGGTAGCCCCCATATGCCCGAAA
AATAGTATATTCTAC
GAACGTTTTTTTGTTATAATCCTCTAGGAATTACAATTCGCCATT
GTCAAAAACAGGTCA
AACCTTCAACTAGAATTACTTGAAATCATGAATTTAGACCGTTGA
AATACTTCAATCACT
TACAGAAATCAAACCCGTAAGAAATTTATTGATCTCGAAACTGA
AGCACTCAAACAGGCA
GAGGACATACTGTCTGACAAAACATTCCAACATCACCAATGTGTT
TCTGATCAACTAGTA
CTAATGGTAGACGCAAGGATTGTCGCTACAGGAACGGTATTACA
CCAACATGAACTCTCA

CGAAAATTACGGATCAAGGAGATAGAGCACGTCTGTTCGAAAAGT
TCTAGCAATTTATTC
AGCTTCAGACATTTTAGATACATCTTAGAAGGTTCAGCCACGATC
GTATTCAAGGATCAA
AGACCGATGAATCGTGTCAGAAAGACATTCTGGCAAATGAGTAC
GGCATCTCGA
>ConsensusfromContig2752 Average coverage: 12.58
ATTGAATCAAGAGAGCGGCAGTGTACGTGGTGATGGACAGTCGA
CTACGATCTTGGGCAA
TTCGCAGGTTATTTTGGGAGTAGGGCTGTTTGCGAAACTGCGGAT
TGAAACGAGGTCATA
TGAAGTCGCTGGCTTGAAGTTCGACATAGTTTTAGTGAGCTGCCG
AAACGATTTCTGTCG
ATAAAACGTACATGAGTGATCTGTTTCATTTTCTCTGTTTCTTTTT
GGAGAGTTGGTTTT
GCTAACTGTGAAAGACCTTTCGGAGACGAAACTATCTACTCTTTT
CACGTGTTTGTAATT
TAATCTGACTTTGAGCAAATGATTTTCGTTCAATAGAGAATTGAA
T
>ConsensusfromContig2753 Average coverage: 27.29
CCTGGTGATACATAAACAATTTTAGATGATCAAGTCAAAAATTAT
TATTATCGGACCAGA
AAAGTCTGGGAAAACGGTCATATCCAACTATTTAGCTGACGCCA
AAGAAAATATCTACAA
TAAATACAGACCTACATCTGGTGTGAGAATCGTTGAATTTGAATG
CAACGATATCTCGTC
AAGACAAAAGAACGTTGAAGTTGAGTTATGGGACTGTAGCGGGA
ACACAAAATATGAATC
CACGTGGTCGACCATGGTAGATGACGTCACCGGAGTTGTGTTCGT
AGCAGATTCCTCCAC
GAGGTCCCAAGAGCTAGCAATGTGGGTTGATAATTTCGCGAAGAA
TTCGTCCCTCTCAAA
GCAGCAATACTGCCTGGTTATGAATCGGAAGGCTGATCATGATCA
CTTCAACAATCTGTC
TTTCGATGGAGCCGAAGAGCTGAACATCAACATCAGAGAAGATG
GAGCGAGGCTTCAGGA
GTTTTTTAAGAAGTTTCTGGCGAATGCCATTGAAGAGAAGAAAC
GGAGGTCGGAGAAGGA
GGAATTGAACGTCATTGGAGCGTGACATTTGGAACTATTCGACAA
TTGTTTTGTTTTGAT
TCGTATTCATTTATCGCTGCTTTGTTATATAAATATAATTCGTTC
AAGGTGAGGGGACCA

TTTCGAATTATCACATTTCAAACCAAATAT
>ConsensusfromContig2754 Average coverage: 7.07
CGCCAAGTGTTAATACGACTGCATTCAGAATCGACATGCTTGTTG
AATCCTCAATTACTT
CGACTGTAATCGATTGTTGTTCTGCATTCAGTATTTCTTTCTCTCC
TCTCAGATTCATCA
AGGGAATTCCTTTCTCGAACTCCTCGACCAGGGATTTGTTGACTA
ACACCATGGAGGGAA
TTTCGTTCTCAAATATCACATCGAAGATAGCGACAACAGACCCAG
GACTGAATGATTGAA
CCAGTGTCCCAACATATGATTCATTCTCACTCAAAGCCGCTAATG
TGACCGTGTTCAAGC
CGGACATGAGTAGTTTAGACTTCTCCGAGTCCAGATCATAATATT
CTTCTTCGAACTGAG
TTTCTATGATGATTGAGACTTCGAAAATCGCCTGTTCTGTCGTTC
TCAGAAGTA
>ConsensusfromContig2755 Average coverage: 10.02
CTTAACAACTGATAATCACCGGCGTTAAAATAATGTCACATTCGG
ATTTACAAAAAATCA
GCGCAGAAACTATGTGGGGAATTACAAGAGGATTAGAAAACTTT
CAAATTGTTACCGAGA
GCTCTCTTTCCGAATACGAACATTTGTTGTGTAAAAATCGGTACA
TGAATCATTGCGCTG
GACATTCCTCTCTATCCCATCAACGCCGTTCCCATCTGATGGCTG
TCAAAAACAATTCAA
AGAGAAACTTCTCAAGATTCTCATCGTGCTTAAACATACCAAAAA
TTCTGAATATAATCG
CAAAAGAGCGTTCAAAACTCGCGAGTGTACTCATCGACCAACCAG
TTTTGTCAGCGAAGT
TATTTGGGTCACCACCCGCAGTGAAGAGGACTCGTAGATTCAGA
TGGGGTGACGCTAATG
TCGATATAAATGTTGTATCAAGACGAAAAACTGAAATTAATGTTT
TTCCAATTTCGAGCG
AATCTTCGAATCGTGACGGAAACTTACCTGTGATTTCTCTGCCATA
TGTTGTTTCTTCTT
GTTTTGTGCACGATTTACACTCTGC
>ConsensusfromContig2756 Average coverage: 56.10
CATTGAATGCTTCTTCTTCAAGAGTCCATGAATTGAGAAGTCAGA
AAGTTTTTAATGATG
AGTCAGCTTCTACAAGCAACGTTGATATACAAGGAGAATCGACAT
CAAGTGGAACTCCTT
CCTATGTACCTCTGCGCCTGAGTGAAGGCAGCTCTGACCCGAATG

CAGACGTTTATCCTT
TGCCTCCGGGAACGAATGTTGTTTATCGCCTTTGGATAATGCGTG
ACCTTCCCCCCTTCA
TTGTCAGATCCAGAATTCATGGATGCATCAAACCCAAAAATAAGT
CAATTCCGTATTTCG
CCAGCTTGATTCCAAAGGAAGAGTATCACACACCCATTGGACTTG
AGGTCACATCTTATG
ATGATTTCTGGAAACAGTGGTGCCACCTTCAGTTTGGTGGAGAGG
ATAGAATCATCCGTG
GTCGCATAAACCCTTTCACGAATGAAGTTGTTCAAAGGACGAAG
TTCTTCACGATGAAT
TTCTCAAAAAATCAACAAATTGTTTACCCACCGCTGAGTTTTTAT
TGGAGGGTGTCATGA
AAAAGCTGATGAGCCTTGTTCCCGGAACATACTTACTAGGTCACG
TGCCTGGCAACGCCA
CGCTCACCATCAAGCGAAACACAGAGGGCGCCCTAGGAGCTTAT
GATCTTCACCAAGCGT
ATCACGACAAAAC
>ConsensusfromContig2757 Average coverage: 54.46
GTGGGCCGAAGTGACTGGCATTCATACAAAGACTAGAGATGGTG
CTTCGAGCCATACCGT
ATTATCAAAATAAACAAGTTATTCCGAGTAAATGTCTTCATTTTC
TGAAAGTAGTCTTAA
AAAACGCATACAGGGTTTGAATGCGTCCCAACAAAGTATACAAAC
GCTGTCGCTTTGGCT
TATACATCATAGAAAAAAATGCATCTAAAATTGTTGGAACCTGGGA
TGCCGAGATTCGTAG
AGTGAAACCAGCAAAAAAACTCACTTTCTTGTATCTGGCAAATGA
TGTTCTGCAAAACAG
TAGGAAGAAAACCAATGAGTTCTGCTCCAACTTTGAAATTGTTCT
ACCAATGGTTTTCAA
ACACGTTAAAAAACATTCTGATGACAAAACAAGCAAAAGTATGG
AGCGATTAATTGAAAT
CTGGGCTGAGCGGCTTATTTTTAATTCGGACTTTATTGAATCTCT
TCGAGCCCTCATGAA
TACGA
>ConsensusfromContig2758 Average coverage: 17.72
TCTGGCGTAGATTCAGATTGAAGCCTCCTGAGAGAAGGGGTCAAT
TTAGAATCTTCTTTA
GATTTAGAATTCAGAAAATATTGTGACTGTGGAACATCGTCCATG
AGAACCTCGGGTCTG
GAATTGGAGTAGAATTCTAATGTGTTCAAATTATTATCTGACTGC

TGAGATCTAACTTCG
ATGAATCGAACCATAACAGTAGCAAGTCCTGAGATTATCATTCC
TACAAGAATCAAAGAG
AAAACCAGGATTCGTTTTGCTTTGGGTCTTGAAGTATTGTCGTTCC
ATAATTTGTTTGAC
GTCAAACGAACGACAGTGTTTTGAAAATGATTTTCAATCATTCTG
ACGATTTTTTAGTTA
TTTTTGATGATCATGAAATTATAAAAATCGAGAAAGAGGTCAAAA
ATTTGTTTCGAATAT
GTTTTTATAACGATTGAATGTTTCAACTCACATGCTATATACTGA
TAATCGACAGAATCA
AAAAATTTTCAGTAATAGTTC
>ConsensusfromContig2759 Average coverage: 14.25
GCAATGGCCAGCTATTTCGCGCCATCTTTAATGATTCTCATAACA
ACAATGGGAATCCTC
GTCAGCGCTCTCCAGCGTCGCAGCAGGATGCGTCGTGCCCGAAGT
ACGGCGACTTCATCC
AGCTCTTCGTACTCTGTTGCCGAAAGCCAGATTTCCTCGCAAGTT
CTCTACCAACAAGCG
TGCTCCGTTGACTCTCATTACATCAGTATCGCTGATGCATTCAAT
ATGTCTAACGTCACC
GCGGCAACTGTATGCGTGAACTTGAGTGTCACTCTTATGTGGGCT
CCATTCTACACCGTT
AATCTGATGATCCCGTTTTGCCACGGTTTTTGTGTGAACCCTATTG
TCTGGACGACTTTT
CTCTGGATTGGATATACTGGCTCTTTTCTCGGCCCATTGCTCTGG
GTTATAGATAACACA
ATCCGCCACCAAGGCAAATACGTCATATCCACTATAACAGGGGC
GTTCAAAAAAGAAGCG
TACAACTTTCAAATGGATGAAGAAATTCCTGATGGATACAAGCCC
TACCTCTATCTGCGA
TGAAATATATGCTAATCGCCTGCCAAAATGTTCAAAATATTTGTA
TTTGTTATTTCAGAA
CTAAACAGTCTAATGAATGTACGTATGTATATTTAACATTGAAAG
TCCCCAACCCTAAAT
ATCGG
>ConsensusfromContig2760 Average coverage: 10.94
TACGTTAACACCAATTTGACAGCTATCATAGAGTCCTACTTCAAT
ATTCTCGCTAAAATG
TCCGAGCAACAAGCTACAACAATATCGTCATTTTCTACTGTTGTT
AAAAAATCATCGAAT
CATCATCCGATCCCCCAAGTTTCACCTTCATCTACGTCTGCACATA

ATAACATTTCTCCA
AACCAGCCTGCCAGTAGCCTAACAAAGCCCAAATTCAATGAAAAC
ACGCTTCTTTTGAAG
AAAAATGGTCTCAAAACAAAACCCAATAATGTTGATGGGAACAT
ACTCAAGAATAAACTA
TGCGACACGATAAAACCGGTCAAAAATGGCTTAAAAATTGCAGG
TTTAAGAAATATAAAC
GGGGGTATAGCCATTCAATTCGGCTCAAAATCATGTTTAGAAAAA
GCTAAGCTTAAAATA
CAATCAGAACCACTCGCAAACTGCGAATTAACACCACCAACCAAG
CGTCTTCCCACTCTG
AAGCTTTCTGGCATCAGCAAAGATTTCCAAGAAGATGAATTTTTG
GAAGCTTTGAAAAAT
CAAAACAATATTTCGATCCAGAATTCTCAAGAAATAAAAGTTAT
CAAGATCTCAAAAAAT
AAATTTGAATCATTTGATTGGATTGTTAGAGTACCCTCGGC
>ConsensusfromContig2761 Average coverage: 3.77
CTTGAATAGGGCCATGTGGCAGTATCGGCTCTTGGGCGGGGTTA
AGAAGGCGGGCACAGG
TTTGTTGATGTCATACATTCAGTTTTGTTGATTACTCGAATAGGG
CCATGTGGCAGTATC
GGCTCTTGGGCGGAGTTAAGAAGGCGG
>ConsensusfromContig2762 Average coverage: 13.76
ATAATAACAATAAAAATAATTCCACAGTGTATATTCCGATGATTA
TATACATGGAATGAA
CCCTGTAATAATCTTTTGTCAGTTATCATCAGTCTCATCAGCAATG
CTGCTGTACCTCAG
ATGAGCCTCACGCACTTTCTCATCAAGCTCATCTGCCAAGATTTC
CACCAGTCGGGAATT
CAGGATCGGTTTCTTGGTCGAGTTATTAAAAAAAATGTTTCTGTA
ATGATGAACCAAGGC
AATGTCTTCGTGAACTTTGATTTCTTGCTTTTTTTTACCAGGGACA
TATTCAAACGGAAA
GTGGATCCCTACTTTCACCACATCTTTGGGCACCACGATGTATTTA
GTTCTGATTTTCCA
TCTATATATCAAGTCTTGCCTGAGGGTATATTGTAGATATTGGATC
TCATACTTCTCAGT
CAATGGGGTTTTCTTAGATGATGCATATTTCGGATAATCTGTGTG
GAAATTCAG
>ConsensusfromContig2763 Average coverage: 20.51
CAAACATTCAGTATTGAAAAAAACTGAAATATTTGAGAGACACA
GTGAGAATGCATGTCG

TTCTAATTCTTCTACACCGTGACGTGCTGGGATGCGGTGACTACT
TCCAGATAGAGAATT
CTTATGCAAAAGGCGTCATCACGACCCCAGAGTACCCTCTCGAAT
ACCCCAATGCCACTC
AATGCATCTGGCTCATCGAAGCCCCTACAAACCACTCTGTCATGC
TCAATATCACTAGTT
TCAATATAGATGGCACGAGTGGTAACTGCGGTGACTATATTGAG
TTACGAGAGGGGAGTA
TCACATCCGACCTAATACTCAAAGGATGTGACTTCAGCAACACCA
GCATCTCTTCCAGCC
ACCGATGGCTTTGGTTGAGGTTTCTCTCAGATCGATCAGGGACTG
ATAGTGGATTTAGAG
CTGTTTATTCTGTATTCGAGTCAGGGGTCGGCGATGGATCTTCGC
CTATACTTGAATGTA
AGACATCTCATCGCTGTCGAAACAAAGAATGTATAAGCCACGCCT
ACATCTGCGATAACT
TTGAGGACTGTGGTTGTACTGGGAGTGGATGTGACGAGTATGGTT
GCACGGGCCTTTCTT
GGTCAACCTTGGAAAGATTCATATGTGGACTACTCGGTGGACTTT
TTATGTTCTCTCTAA
TCTTCTTCGGCGAGATCGGAAGAGCGTCGTGTAGGGAAAG
>ConsensusfromContig2764 Average coverage: 5.23
ATAGTATTGACCGAACTTTTTGTTGTGATGCGATTAACTAGTCAG
GACTTCATTGAAGAG
ATTCCCTAACATGATAAGAAATATATAATAAAATATAAAAGCTGA
ATTTGTTTATCGGAA
GCTTTAAGTTTGAATGGTGCTGGGCACTATAAAGAAATTGGAAAA
AATGTTGTGTTTCTA
TGTATTAATCCTTATTGTTATATAGAATTACTTCTGACGACAAGA
AATAGATCGGAA
>ConsensusfromContig2765 Average coverage: 30.64
ACGCTCTCATTTTGTGGGTCATACGCAGGGGCAGTTATAGGAATG
CCCCTGTCGGGAATT
CTGACGCAGTACATTGGATGGCAGGCCTGCTTTTACTTTTATGGC
GCGTTTGGCCTTATT
TGGTACTGTGCGTGGATGCTGATGTCGAGCGAGAAACCCTCAACA
CATCCAACGATTTCA
TTGGACGAGAAGCTGTACATCGAGACGGCTATTGGCCCCAGTAGT
CTCGCTCATAAACAA
ATGAAACCGCCCTGGAGTTCCTTCATGATGTCTCTACCAGTGTAT
GCCATCATCGTCGCG
AACTTCTGTCGCAGCTGGAGTTTCTACTTACTCATCATAACGCAG

CCAAAATACTTTCGA
GATGCATTTCATTTCGAAGTTTCTAAGAGCGGTATGCTCTCGGC
>ConsensusfromContig2766 Average coverage: 26.64
TGACGTGACCGACAAGCTGTACATTGCCCACGAGCGCCTTGCAG
ACCTTGAGACCAAGGC
TGAACGCAAACTTAGCGGGGATGGTGGATCACTCGAGGCGGCCT
CACACCACACTGCCGA
CGACATGACTTCACAAGATCGTTTTCAGCGCCTCGTCAATGTGGC
TTCGCTCATCAGCCG
AATTCGAGCTGAGACCCCTGCTAGTTCTGAAAATTCTCCCGCTTC
GCCTGCTGCTATGAA
TTCTGTCGC
>ConsensusfromContig2767 Average coverage: 9.73
GCAAAGTTTTAATGACTTTCTGAGTAAATACATATGTTCGGAATA
CATTTCAAGCACACG
GGCTGTGCATAATGACAAAATAATCAAAACTTTGCTGTATTTCAG
AGCCCTGTACAAAAC
ATTAACTTTGAAGCATTCTGATTGGCTCTTGCTGTAGGCGGAGTC
AAATGAGAACCAATT
GAGGACGGCCAACTCTACTCCGAAAGATATTCCATCAATCAGAA
CAAGGGCGGCAATAAA
CAAACCCGAAGCATTCCTGAAAAGCACCTTGAAAGCACAGAACA
CGAGCAGGGTTATGCA
ACAGACAAATATTCCGATGCAAATGACGGCGAAAGAACCGTAGC
GGTATATATCCTCAGC
TTGGGTCATGTAGAGCAAGTGGAATTCAGACCAGCTTGTTCCCGA
ATCGCCGATTTCAGC
CATTTTTATGAAGTGAATTAAGTTCCCAGTGGACTGATTAGATCG
GAAGAGCACACGTCT
G
>ConsensusfromContig2768 Average coverage: 55.16
TTCCATATTTTTTCTGGAGCTATTTTTATATCTTATATGGTTTATAT
TTTAACATTTTTC
AAATTATTCTGAAAATAGCTCAAGTGATTTTCTCAACATTTTTTTC
AGGAATATACGTTT
ACAAAAATTCATCTAATATTATATTGTATTTATTAATATCTTGGT
CAGTAGAATGTGCAG
AAATCTCAAGAATAGAGAAACTTTGTTGTATCAAGTAAAATTACT
CACTTGAAGAAGATC
ATGCTGAGATGAAATGACTAATATTATGTAGCATTAGCTGAAGGC
GTTCTACCGCACCAG
AATTATGTGTGTCTGAATATGTTCATACTATTTGGATGGCCCGTA

TATAATTTTAACATG
CCATATGTTTATTTTTGATGCGTATCTCATGCGTTTTCTTTTTACA
ACGTATCAAAATCA
TTTTCTAACAAAAGAGTTCAAACTATATCGCAACACGCTTGATTT
TAATAAAATGGTATT
TGAGTGTCGGTTTTTCAAAAATATGACATGTTCGTATATTGTATT
CGAGTATGTGGTATT
TTTCAATGAGATGAA
>ConsensusfromContig2769 Average coverage: 11.02
AAACTATAATATATGATTATAAATATTATGAATAACGGAGTGTGA
GAATTTTAATTCTTT
GATGATTGTTTGCTTCGGAAATATGTGACATTGAAGATAGATGGC
TCTTGCAGCAATAAA
CAATGGCGGCATTGCTAAGACTCGAGAATCCGGGGTTCCCCAGA
ATCGCGAGAGGAAACT
TATGACGTCATGCCTCGCCCAGATCCTGGCCAATGACGACATCAG
ATTTGAAGATGACGT
CATCAGAGACCGAAGCGCCAATCGGGTTCATTACAACAAGGCTGT
TCTAATTCCAAGTCT
GGAGGTTTTGTTGGAAGAATTTATGGCTAAAAAGAATTCTTCTAG
AAAGTTCGAGCACAG
AATCATTCCGAAGCCGAAATTCAATCACCACAAATGCCTTGCTTC
TCGGTCGAATGATGC
TTTTCCCGTTTTGCCTCCGATTTCACCGACTGAGATTGCTGTTGAT
CGGGACATGATGTG
GTCGAATGATGAGGATGAATCGGAGGAGGTTGAGCCGCTTTCTG
CGAGGGAGATCTCAGC
AGAGCAAAAAGCCAGGTGGAGCGGACACGTCATCCA
>ConsensusfromContig2770 Average coverage: 34.08
TAAGTGACGTTGTGAGCATAGAAAGACCTGATTTGGAGGAACAG
AGAAACCAGCTTATTG
TATCCATCAACAATGACAAGACTCAACTGAAAGCCATCGAAGAC
CGGATTCTGAAACTGC
TTTTTGAATCTGAGGGTAACATTCTCGACAACGAAGAGCTCATCA
ACACATTGAACGAAT
CCAAGGTCACCTCTGGTGCCATCATGAACCGTCTAGCGGAAGCG
GAAAGAACAGAGGAGA
AGATCAGCGTGGCGCGAGAAAAGTACCGAGACGTAGCAACGCGG
GGTTCGGTTCTCTACT
TCGTTGTTGCAGACATGGCCCTGATTGACCCCATGTATCAGTTCT
CGCTGAAATACTTCA
AGCATCTCTTCAACAGCACCATATCACGTTC

>ConsensusfromContig2771 Average coverage: 7.40
CTGTATTTGAATATTTTTGAGAATTTGAACCCGTATAATTTGGAA
TTATTGTATAAGGCA
AAGTGGAATTTGAAGAACTATATTTCTTTTTTTGTTTTAGTTTCCC
CTTTTTCCGTTTAT
TTTTTCTGTTCCTTTTTCTCTTCTTTCCTCTTGTTCTGAGACTTTCG
TTCAAATGTAAAT
TTGTTTCGAGAGCAGATGTGAATGATAGCATAGGTGAAGGTGTT
GGGAGAGAGAAAGGGC
CTTCTGGAGATGTATGTACATCGGTTCCACATGGACTGGGACAAT
CATGGCTGTCTTGAA
TGGAATTCCCCTCTTTTTCGACGAGTGGATCACAGCCAGACAGG
AATCCGATAAGGGAGA
GGATCCGAAAGGCAAACCGGAGTGGGATCATTTCATCCGGTCTAT
TATCCCCGTCGATCC
CGC
>ConsensusfromContig2772 Average coverage: 26.19
TTTGACTGTTTATCTATAAAATCTAGTTTCATCAATTGAAATATCG
ATATCAACAATTTC
GCATATCTCCTCGTTAGAGAAATAAAAAGCTTAAGAAATGAAAAA
TCCGTTAGTGGTCAT
ACAATGGGTGTTGTGCATGCAGTTTTTGTCAGGATATTCTTTGAC
CAGTCAAACAGGGAT
GCATATCTTGGATGAGCAAAATTTGAATGTTTATAACTCCAAAAG
TATCGCAAAACT
>ConsensusfromContig2773 Average coverage: 9.98
AAACAGACTCAGCAAACATTCGGCAATTCTCGAACTGATTTCCAG
GAATTCTCTATTTGG
CGAACATCTCAGAGATTGAATCAGACATTCTTTCATTCCTTCGTT
TTCTTGAGATGTCAG
ATCAATTCCGTCTGACCAATCAATTTTAAGAATGCAACAATCATG
GGCGTGTTCTCTGGG
ATGATTGACCAATGAGGAAACGGAACCGATTCCCAAAAAGTTCGC
GTCGTCTGCTATCAC
AAGATGAACGATTCTCGATTCCTGTAATTTCTTTTGGATGGCATC
AGACAGAATTCTCGA
TACTCTCCACTCCCCATTACTTCTAGAAACAGCAACATCTTCCTG
GCACCCTTTGACGAG
AGATCGGAAGAGCGTCGTGT
>ConsensusfromContig2774 Average coverage: 29.70
CTCTCGTATGTTTGACTAAAAACAACTTTGACGCAGCCACAATTA
TTATTCATAAAATAA

GAGTCGTCATTATTATGATCACAGACAATGTAAGTATTTCCTTGT
GTAGTAGTAGCCGAT
TGGTCATCAAGATCTAATGATCTCAAATTATTGGCACTTTCTGAA
TGTGCGGGCACTGAT
TGAATAGGATCAGCTGAACAGTTTTCTTCGCCATTTTTATAAATT
CATCATGATTACTT
TTTTTTTCAATTTTCGTTGTATCATTTTGTATAACTGCATGATCTTT
TTCAACCAAGTTT
GTCCATGTTTCTTGATCTGATGTGGTGTTTTCATTAATGGGAAAA
GGATGGTCAGTAAAA
ATAGGTTGGCATTTAAAGTGTTCACCCACCCACTCAATGCTTACA
TCTTGACTTGTTTCA
CTTATTACATTGTTTATACAATATAGTTTTTTGTCAATCGGTAGCA
ATATATCTGCTGAT
TTCTCTCTTTCATGAGATCCTGTTATATTATAGTCATCAAAGTATC
TACAAGTAGGTTTT
ATAAAAGCTAA
>ConsensusfromContig2775 Average coverage: 5.46
TGAAAGTTATGCGAAAGACCTATGCAAAAAAATACGTCAAGTTC
GACAGCGATACAGACA
CACAAACCCAATTTGAGAATGACTTTTCGACCACAAAAATTATC
GAGAGAGAAAGGAGAA
CTAAACAAAAGTTGACAGAAACATGGAAAGCTAATTTCGTTGAGG
TTCGTCGATCTATTT
TGCATAACATGCCTCCAGATTTGACACGTTTAATACTGAAAAAAG
ATATAACATACTCCA
ACGCCACAATTCTCTATATCGACGAAATGCCGCACTTAGAAGTAA
CCGACAAAAATTCCG
TCGTCAAGTATGTAGATTTCGGCACCTCAAAGAGCGTGGCGAAG
CTGCTGACAGATGCCG
TGGTTGAAATTTCATTGAGAGAAGGAGAGATCG
>ConsensusfromContig2776 Average coverage: 21.42
GATCGAGGATCCGCTTTTCTATCACGGGAGTTCAGAGAGTTCCTG
CACAACCACGGGATT
TCTTCTAGCCGCTCCACTCCCTACCATCCCGAAGGAAACGGGCAG
TGTGAGCGATATGTA
GGCATCATCTGGAAAACAATAGCACTTGGCCTCAAGTCTAAGCAG
TTATCTATACAAAGA
TGGGAAGATGTTATACCAGATGCTCTCCATTCAATCAGAAGCCTG
TTATGCACTTCCACA
AACTCAACACCACACGAGCGACTGTTTTCTTATCCAAGACGATCC
TCCAGAGGATAAGC

CTTCCAAAATGGCTGAAAGAACCGGGAGCTGTTATGTTACGCAG
ACATGTGAGGAACAAA
GGCGAACCTCTAGTGGATATGGTGGATCTACTAGAGGCAAACGA
GCATTATGCACATATA
AAGTTCAATTCTGGAAGAGAGGATACCGTTTCAGTGAGGGATTTG
GCTCCAGT
>ConsensusfromContig2777 Average coverage: 5.03
ATCTTCGATTTGTACTTTTCGGGTATAGTTTTTGAGCTTTTCAATT
CTCGCCATCTCTCT
TTTTTTGGGAATGTTCCACCCACCTCGTGTAACAAAGAGACCAAT
GCGGGGGAACCCATA
AGTGCGGCGATTTCAATCGGACGAACTGATTCCTCTTCGAAGTAC
CGTGTATCTTGATTG
ACATCAACGCCAGCACGGATCA
>ConsensusfromContig2778 Average coverage: 16.77
CGACATCATCGGCGCGGGAACGCCTTGAGCTTCGACAGTCAGTCG
GACTGGCTCACCTTC
TCGTACCGTCGCGTTCGCCATTCGAGAATGGAAGCGAGGAATTGA
TGCCTCTTGCTTTGG
CGAAACAGTGATGGTGAGTTGAATAGTGTCCTGGCCGCCTCTGTT
CTTGGCGACCACCGT
GTACACCCCAGCGTCTTGCGGTTCTGCGCTCTGGATGATGAGTGA
ATTGATGCCGTCCTC
ATTGATGACCATTTGATGGTTGAGATCAGGGGTCAAGGCTGTACC
GTCCCTGAACCAAAA
CAACTCGGGTCGAGGACGACCTGTGACGACACAATCGAGTCTGA
GCGTACTTCCGGCGAC
CACAGTCTGGTCCGGAGGACACTTACGGAAGAT
>ConsensusfromContig2779 Average coverage: 9.26
CTCGAATCCCTCGGGGGTCTCAACCGAGACTCAAAGACCAAAGGT
TGCCCCTGGTTCGAC
TCACCTGTCGGATCGCCAACTTATAGAACTTCCAAAAGACTTTCA
AGCTCTTTCACTTCA
TCATTTGATGACAAAACAGCAGCCAAAGAGTTTATTCCCAAATC
GAAGGACACAGTCATA
GATATTGTCATGACTGTGAAGGTGTTAAGGTTTGTCACACAAGTC
TCGGTGGTCTTGCCA
TATAAATTAAAATAAGATGTGATAACTGTGGTCATTCATCAGAAC
ATCTAATTATACAAT
GCCTAAGAATTCAAAACAGTGTGATATAAATGTGAGAGATGTACT
GTCCAAACTCCTTTG
TGGAGCCACACAAGATGATGCGGTCCGGTATGCAGAAGTCATGG

GTACCATCAACCCGTT
GATTTCTCATCAATGCAACACCTCAGTTAAAGATATAAAT
>ConsensusfromContig2780 Average coverage: 27.92
CCATAATGATATTCGATTTCCAGTGTCATATTTATCATAACGTATT
TTATCGATAGATTG
GAATACTCTGAAATTCATGGTATCCTTCAATATAAACTTGTTTGTT
TTTAACTGTTTCGT
TTGTTTATTCATGATGTGGTCGTTAGATTTATTTTTAAGATCTATC
TTGCTATGTAATGG
CCTTAGGCACTTATTACTTTATGGACTGGACCAACCCTTTATCTGT
TTTGGTGCTTGTTT
ATTTTAATGCCATGTGAAATAAAAAACACATTCCATATGAGAAC
AGAATGGGAAATAATG
CAATAGGATTTCTATGGATATGTATTTCCATCTTATGGTATTGAT
GAAAAATGTAATAAG
CAGATGCAACCACTGATTAT
>ConsensusfromContig2781 Average coverage: 11.66
CTCTTATGCTGCTGATATCACGAAATATGAATTCCACATGCTGAA
CGCTGAGAACGGGTC
TATCGGTGTTTATAGAAGAAGAAAGAGGTTTGAAAAACTGTCGAT
GTATCGCATCCATAT
TTGCATAATGTTCTCGGATGAATGAGCAGAGTTGACTCTCAGCTG
CAGGGAACGACTTGG
AGGTCCTGATATTATTCTGAATAGCATTTTTAACAGAGAACATCT
TCGTGTCTTTAGTAG
TTGACTCATCATAAATGAGTTGGCCAGCCAATTGGGCAGCGATGT
CGCGACGGACGTCTG
CCAGGCGGTGCTCTAGACAGTGACGTATTTCCCGGCATGCCTCAT
TGCCAGCTTCCGCTA
TGGCTAGCCACAATCTCAATTTCTC
>ConsensusfromContig2782 Average coverage: 22.63
GATGATAGCACATTTGAAAAGATCTTACCCATTCTCAGCGGACAT
CAGTTTGTGGAGCAA
GGCGGTAAATGTTTAGATTGCGATTCATCGGCTTCAATGCTCAAA
CGTTTATGGGCCCAC
AATCAAGTATTAAATCCAACGTTCTTTTCTGCTCTGGCAATTTTCT
TCACCGATAAACCT
ATGCTTTCTGTGCACGGGAATCTTTTCGACCAAAAACAAAAATAT
TTCCTCGGTGACACC
ATGAGTCCCACCGATTTCAACACCGTTAGCATGTTCACAAAGAGC
AG
>ConsensusfromContig2783 Average coverage: 7.39

ACTTTTGAAATAGTCTGTGATGCAGTGAGAGTCGGTTGGAGCTGG
TGGCTGTTTCTCTTC
TGCAGTCTCAGTTTTGAAGGCTCAATTGGTTCAGAGTGCTCAGTG
TTGATATTGTACAGA
GTATTTGTAGAATATTGAACACAGCTTCTTTTTCGTGTGTATGCAT
ATTTGTCTAGATTG
GGATTTGTAACAGACACAGTATTTCGCAAATTTGCTTGTGTATTT
CCAATCGGTTTCCTA
ATCTGAGGAGTGCTCTGGGTGAAGCGAGAGATGGGTTGAGTGCT
GGAAACGTCGCTATTT
TTCTTGCAGCAGGCGTGGCCACACCATGCTTTGTTCGCACATGTA
TGACGACAAGAGGGT
TTCGATATGTTGTTAGGAACTCCTGAATTCGGTTGACTTGGCATTG
GTTTTGTATTTTCA
GGCATTATCGTA
>ConsensusfromContig2784 Average coverage: 10.09
GCTCTTCCGATCTGGAAAATTTTATGGCTGACAAAATTGAGAAAA
ATCGTTACAAATCAA
CCGAAAACAAAGTCAGGATCATCGAAGTACGACCAATTAAAAAT
GAGAAATTTATTAAAG
TTGATCAAAAAAGTATCATGGCTAGAAAATCGAGTAAGACCCAT
ACTGAGAGAGGTTATG
CCTCTTATGATTGTGATTATTGCAATAAGAAATTTTCATGCGAGA
AAAATTTGAAAAAGC
ACAAAATCCGGCATCTAGCAAATAAGTCATGTATCTGCATACTTT
GCGGCAAAGGTTTTC
ATTTCGCTTATGAACTGAAACGTCATTCAAGACGGAAACATGTTT
CGACATCCGATCAGT
TGAAGAAAAATACCAACGGGGAAGAAATACGTGACATGGTGG
>ConsensusfromContig2785 Average coverage: 9.99
ACACTATTGAGCATGGTTTACTTTTTAATGATCTTATATCTTTTGG
GATTTCAGGAAATG
CCCTCAATATCTTGATATCTTATTTTACATGCCGAAAACAGTTCGT
TCAAATTGGTTCTA
CAACGTCAGATACGAATAACATCACATCCGGCGTACCCCAGGGTT
CAATATTGGGCCCGT
TATTGTTCGTTATATATTGCCATACGCTTTCTCATGTTCTTAGGTT
GAATAACATTCAAT
TCCATTTTTACGCGGATGATACGCAACTATATCTGCCTCTT
>ConsensusfromContig2786 Average coverage: 6.23
GTATATGTCTACTCTTGATGAGTTTCAATGAATGGAAGAATAGTG
AGTCATTTCTTATGA

TATTATCATTCGTTTCATCAATATTGCGTGCCTCTTTTATCATCGG
TTCAACCAAGATCT
GGGGCCTCTTTCTTGGCTTTGCTCTCGGCTCATATGCACCTCTCTT
TCTTGTCTTTTCAA
AAGTCATAGCCGGAAAACTCATAACTGCTGAAATCCGAGGCCGCG
TGTACGGCGTACTTT
CAAGTGCGGAACTGTTGGGATGCATCGCTGGATCTATTTCATACC
TCCAGGTATACTCTC
TAACACTCAGTTGGATGCCAAAATTCATTTATATAGCCTCTCTCA
TCACAATGTGTATGT
ATTCACTCGCCCTAACACTGTCAAGTAATAGTATAAGGGAAGTA
ACAAAACGGGCAGATC
AAAGTGATGAGGAAAAATATTTTGCTAAAAATGTCACATATTCTT
CTGTTAGAGATCTAA
TCGCACTTCCATTCAGTGGAATCCTGTATCAAGTTCAGGGTCAAC
CTATTCATGAAGTTC
AAGAACAATCAATCATTC
>ConsensusfromContig2787 Average coverage: 27.11
GTTTATACAAGCCTAATCCGGCATTAGAACAGACAATACTTATGT
GAATTGATTGTGAAG
GCTAATATATGATCGATATATGATCTCCCTATTGTGCGCTACGAG
AATACCATTTTTTTA
CATTCAGAGGTTTGGATTGTCGAGTATTGTAGCGATCAGTATATT
AATCTTAGTAATACA
TCTATGAAGTTATTTTGATCTAAATTATCTCTGTAATCAGTCAAT
AAATGTTCAAAACGA
TGTCGATCTCCTCGATATGATCTTTTTGAAAAGTCGAAAGTGCAG
TTCTAATGCAAATAA
GATCCATCAAACACTCTATGGCCGCAGAATGGCTCTCTCCATTTT
GGCCCAAGGAACCTC
GATGAAGAGTGTCAAGAGAAAAGCAGAAGCATAAGATACGACCA
AGCATGCTATGAACAC
CATTGAAATATATTGATAAGTGAGATGAATTGGTGTTCGAA
>ConsensusfromContig2788 Average coverage: 12.83
CTTGCCAATGTCCTTTTTCAACTGAGTATCTTCAAGTTTGAGCATT
TCATGAACTTCATT
TCTGGATAAGTCAGGAATACTGATGGGGATTGCACATCGGGACC
ACGCAGATGGGTCTCT
CTCCAGAATTTCTTGGGTTTCTTCGTCGTTACTGACAAAGACGAG
CTTGACGATATTTGC
ATCTGCCCAGAGTTTCGCTTTACTTTGAAGTTTCTCGAGAGCCCC
GGGCATTGTCTGAAT

AAGAACGTCGATGTTATCAATGATAATCACGACTGGTTTGCCATA
TTTTTCTCTGTACTT
>ConsensusfromContig2789 Average coverage: 7.25
AAACAATTGAAAAATATTGCCGAATGTTATATCGATTCGATATTG
ATACATAACTGCTCG
TGAAAGGCAAAAAATATGATATTGTACCACCTGACCTATTGGATG
GCTTGAGCACATTTT
GATTCCAAAAAACAAAATGACAACACATGATGGAACTACCGAAG
AAACCTCGTCCAAAAA
GTCAACCAAAGAATTTATAGTCGGGGGAACAACCCAAAAGTACC
GATTGGTGCGAAAAAT
TGGCGGCGGTTCTTTCGGAAACGTTTATCTAGGAGTCAATCTCGC
TAATGGGGAAGAGGT
TGGAGTTAAATTGGAAACACAAAAATCTCAACACCCTCAACTCCT
CTTTGAGAGCAAACT
TTGTCGTATTCTTCAAGGCGGTGTTGGTATACCCAGTTTGCGGTG
GTATGGCACTCACGA
>ConsensusfromContig2790 Average coverage: 41.55
TTCTTCTAACTTTTCTCGAAGTTCGAAGACGTATTGGTAACAATTC
TTGACCTCCGGCTC
TTCAAGATCTTTCGTCCATAGCTGTTTCAGAATCTCCATAGGACC
TCGAACAGATCGGCC
GTACAGAAGTTCGAATGGCGAAAATCCGGTAGATTCCTGAGGAA
CTTCACGATAAGCAAA
TAATACAGCATTTATAAATCGATGCCATTGTCTCGGTTTCTCTTG
GCAAAGCCGTTTAAT
CATCATTTTCAAAGTGCCGTTGAATTTTTCCACCAAACCATTACA
CATTGGGTGATAAGG
GCTAGTATTCAGTTGTTTCATACTTAGTAGCCTTGAGACTTCCTTC
ATACATTCAGACAC
AAATTGTGTACCCATGTCGCTCAAGATTTCTTCCGGTACACCCGT
TCTACTGAACATGTC
GACAAGCGCTTCTGCTACGGTTTCAGTATCTATATTTTTCAAAGC
TATAGCCTCGGGATA
ACGTGACGCATGATCAATCATTGTAAGAATAAATTGGTGCCCGCT
CTCTGAAGGTGGGTG
AATCTTCCCAATGATGTCAACTGATACTCTTTTAAAAGGCGTTTC
>ConsensusfromContig2791 Average coverage: 28.99
CTTAAATGTTTTCAAGTTGGAGAATAAGTCATTATAATCCGTGAT
CATGAAAAATATAAT
GTAACTATACATGATGATGAATGATCGAAAGTGGTGAATTTGCA
ACACAGATATGGCAGA

GATATTTCTCAGAGAACAGATAACCGGAGTCACATGAGCCTATGT
GATATGTGAGATATT
TTTTTCGAATCAATAAGATATCAGATTCTATATTAGCATTATAAA
GAAAATCACAACAGC
TCCAACGGGAAGCAGCTGTTTTTTAAACAAAAAATCAGTTGAA
TGATATGATAAAGGAA
CGTCATTGCGTGGTTATTTATGATCAAAGCATAAAACAATAAAAA
CTTCGAAT
>ConsensusfromContig2792 Average coverage: 29.16
TGCGCCTTCCTCTTCTCCCGTCCTGCTCTCTTCTGGTTCATCGGCG
CCGCTGACTAATAG
CTCTTCAATCGGGGGTACTTCTTCGGGGGGGTCTTCAAGGGGGGA
GATTTCTGGAGATTG
GGGGACCTCTGGAGGGTTGCTATCAACCCCTGATGGCTCTTCACT
TTGATCGTTTTCGCT
TCTTGCTTCGCTGGGATCGGACTCTGTCTCCACCGTATCTTCCAC
ACTGATCTGATGACG
GTATGCTCTATTCAGTCCCGAATCAACGGGTTCTTCCACATATCT
GCGTGGTCTGTGAAC
TGTATTACTTCTCGCCCAGCGGTTGTGGTAGGACACAGAGCCGTC
CGTATCCGTCGTTTC
TTCCCATCCTTCAGGCAATGGTTCTTGACTAGCTGTTTCTCCATCG
TCAGTGCTTGCCGA
GCCAGGA
>ConsensusfromContig2793 Average coverage: 11.09
TATTTTTTGAACAAACTAATTTCCCTGTTAAAATCTGTGGAAATG
CTAACATTGATATAA
TGTTCTGTTTGCCCATAAAAACATCCACGATTTCTAATGTCGTCC
CATTCTAGAATAGGG
CATGATATGTTCCGACATTCACCCAGTTCCTCAACAAAAATCTCT
TCTTCATTGCAACGT
TGTATTGGTGAGAATTGAGGCTGCTCATCTGGTATGAAGTTCAA
AAGAATCGAAAACGAA
AACACGCTGAAGTCAGGTTCTGGTCTCGGTGCTGGCTGGATTGGT
GGCTGGATTGGTGGT
TGAATGCCACCATTAATTCCGGGTCTTCCAGGAAAAATGGGTAA
CGTCGATGAGCAAGAA
AAAGCTTCATCATCATTACAGATCGCACAATATTGATTTCGGTAG
ATCTCTGTGTCATCA
ATAAAGTGTGAGTTTTCACAATAATGATGAGCCTTTTCATTATTG
CAATCTTCAGAGCAT
ATTGATATCTTCG

>ConsensusfromContig2794 Average coverage: 3.95
TTGCAAATACTAAGACAAGAGTGATAGTAGCAGATATCAAAGCT
ACAGTCAATACGACAG
TGAAGTTTCGGTTTTGTATGAGTGATGTGCTCTCGCCACTGTTTTC
GATAATGATATTTA
CTACAACTTCCACACGATTTTTTGAGCGACCGTTATCAAGAACGG
CAACCGTTATTTGAA
AAAGCTGATCGCCCAATCCAGTTAAGCTTTTTATCAAAATTATGG
AGCCTGTTGACTCTT
CGACAGTGAAATGATCCCATCCATCTCCTTCGACAGCAAG
>ConsensusfromContig2795 Average coverage: 10.64
CTCTTCCGATCAGTTTACAAATTTATAACTGGTACTTGCAAACCA
ATCCGGCAGTCTCTG
CAAATTGCATTCATTATTTCGACTTTTCACCAATATGCGAATTTCC
ACCAGTGCTTTTTC
AAGTCTCTCTTGTTCATGAGCATCCAATTGCGGATCATGGCACG
CTCATTTGCTACGCT
TTTTCGTTTGTAGACCCCTTGTCTGTTCATTTTTGCACATGCTATT
CTAGAATTGTTGAT
AGAAACATTCAGAAAGCCAATTGTTTTTCTCAAAATCTTCTCCAT
GTCGGTGTCCAATTG
CTCGAAAAAAATTATGCAAACAGGCGATGTTGAGGTCAACCAAA
ACTTTGTGAAATCTCG
CCATTTTGTTATCCAATCCCGGCGAAATTTGATGTAACCTGTGAAA
TTGTTTCTTTTGAG
TTTTTCGAAATCAGCACGTCCTACGTGACTGTTTCCTCTCTGTGCG
GCATAACATCGGTT
GAACTCTGCGAATATGGCGTTCAGCGGATTTCGCAGTAACAAGA
CCACCCTGGTGAAAGG
CACCATTGGTTTGATTATCTCATAAACCGGCTCGTGTGTCTTTGT
TACAAGGACTCTTGA
GTCGCTCACCCCTTCCCCAGGAAACTGATTTCTGAGAGAGCGATC
ATTGTATACGCTTCC
AGTCTTCAACCCTGTACTTTGCTGTAAAAGATGTCTCAACCATGT
ATTTCCCGATCCTGG
TAAACTTGCA
>ConsensusfromContig2796 Average coverage: 25.23
CAAACATTCTAAATTCATCAATGCAAAATTTATTAAAAATGTGTT
AACCTATCACGAGCC
ATTTACGTGATGTTCACGTCCAATCTAGATATTATAGCTTCATTCG
AATTTCCACTGGCT
CGTTTCAAGTTGAAGGCTATGGAAATGAGACAAGCACCAACGAT

CCCACAAATCGAAACA
AAAATGAAAGGGGTCGCGAATCCCCCTGAGATACGTAGAAGCAG
ATCAGCCAGAGGAAAG
TTGACAAGCCAGCCGAAGCCAAGACTGAAGACTTGCAGAGTGTAC
GCCTTCTTGGCTTTG
GTCGGGTCATCAAACATGTCGTGCATAAGCAAGAACTGATTTGG
GTAGAAGAAACCGTCT
GTTAGAGATATGCCTGCTATATATGCTATGAACCACGGCATAGTT
GTCACAAACAGACTG
GCAACATCAAAGGCAGATCTCATCATCACAACGGCAGACACAAG
GTAGACCCTATTTACT
TGTCTCATGTCTCCGATAGGTCCAAACAGCAATCTGGCGCAAGAG
CTCACGATGGCATAA
ATTGTGGTCAGGAGGGACAGATCTGATGTACTTATTCCGAGAGA
ATCAGCGCGGACGGCT
GTGTAAGTGTACGTTGTTAGGCCTTGCAAGGCAATCAAAAAGAAT
CCTATAACTGATAAA
ATTGCTATTGGCTCTTTCAATATATCCAATTGAATTTGACTCTTTA
AATCGTCTTTAAAT
CGCACATTTCGTTTTTTTGAAATATTGATATTTCTTGTGGAAGAGA
TTAGCTCTTGCACC
TTTTGGCGTTTACCGATCAGAGGAATGGAAGACATGCACAAGAA
GCCAGCGCAACCCTCG
AATATCAC
>ConsensusfromContig2797 Average coverage: 36.18
CAAAAGACAGAATTTGGACTGCATAATATGGTTTCGAAATTCAAG
TAACTTCCTATATAT
GCATGAGATCACACTGAAATCTACAGAACAGATAGAGAAAGATG
AAATCGTAAAAATGGA
CGGAAACGAAATCCTAATGATTCGGGATACTGTCTTTCAGCATA
TAATCAAGGAAGAAAC
TGATGATGCTAAAGAACTATTGCAAAAAAATAGTGATACCATCA
GAACATTATGTTGCGA
TGTCGACCAATGCGGCAATTCTTTACTATCTTTGGCTGTTTCTGAA
GGTAATTTAACCTT
ATTAGCTTTCATGTTTTCAAACGGATGCTCTCTCAATGATATTCC
CGATGGTGGCGACAA
TCCTATTCACATGGCAAGCATTTCAAATAACTGTGAAATTGTAGA
ATTTTTGGTGAACCA
GGGTGCGCTCTTGAACCAACGTGATGGACGAAATTTCACACCTCT
TCACTTGGCGGTGAA
AAACGATTGCTCGGAAGT

>ConsensusfromContig2798 Average coverage: 10.91
AGCTCCGAAGATGAAGCGATGGAGACCGAAGTTGGTTGTGTGGA
AATTGAAGAAGGTTTG
ATGGAAAATAAAACTTCAAATCCAGAAGATGTGAAGAATGATGA
AATAGGAGAGACATCG
AATGAAGTGATGGAAGTTTCTAAATCGAACCAAGTTCTGTCATC
GAGAGATGTGAAGAAG
AGTAATGATGATAGAAAAGAGACATCGAATGAATTAAAGGAATG
TTCTAAATCGAACGAA
GTTTTGTCGTCGAAATCTGATTCAAAGAATTGTTCTGAATCTGAT
GCAATAACGAAAAAT
ATTGATAGAGATGGAGCTGTTGAGAATTCGTCAGATCAAAATGAT
GCTTCAG
>ConsensusfromContig2799 Average coverage: 8.06
GAAGGCACAATTCGCCGGTTGGATCGGCTAGGAGCGTGAGACTCA
TACACCTGTGTATTT
TTCGGAAAGTCTCTCTCAAGAGCGCCTTTGTCGTAATCGTCTTCGT
CATCTTTACTTTGT
TGACAGCACCCGGCTTTGGCGCATGGAGAGCGATCGCACAGATT
AGCCCCCCGCCAGTAG
CAGAGGCAGGCAAAGCCAACGAGGAAGGTGACGCCCAGGAGGAT
GCCCCACAGCTGCAGT
TGCTGATTCCTGGGTCAGAGTGAATTAGAATTGGGTCAGATTGTG
AGATCGGA
>ConsensusfromContig2800 Average coverage: 32.79
CTCCCAGTTTGAGTTTCTCCGCGACTTCTGTGGCTTTGCTTTTTGT
TTCTTCGAGCGTGT
CAATCAACTCGACGTTGTCCAGCATGTTGCCGGTAGATGTCGCCA
GCTCTCGCAGGAGAG
AGTCTTCGAGGTCTTTGAGGAGGCGCTTGTTGTTGCTAGTTTCTTG
GATCAGTCTTTCTC
TCTGCTCTTCCAGCTCTTTCCGCTCGAATCCAACGATGACACTCA
GAAGCTGGTCTTCTA
GACCCTTCAACGTGACTGTATAATTGATGACCATCGATTTTCCGA
AGACATTCGGCGTGT
ACTTCGGATTGGCCAGTTTCGTGTTGAGGTATAACCGGAAGTTGG
GATCGTAATCCACTT
CTTTATCGCCCAACATGACAAATTCTCTGCCTTGACCACCTCGTAT
GTTTTTCTCCAGAA
CGTTGTCGACAACTGGGTCAATGTATTCGTCGACGTCCTTGAACA
GAACTGGAAAACCGA
ACTTTATCGCCATTTCCAGCTGTTTCAGGAAGTCAGGGTCGTTGA

AGGTCATGACCTT
>ConsensusfromContig2801 Average coverage: 12.42
TGAGATTCGAACTTGAGATCGCCGGATATAATTCTCGAATTGAGA
ATGAAACTGCCTGGT
CTTTCTGAATTCTTGATATGACGATAATCAAAGAGTTTTTCTAATT
TCTTTCCAGCTAGT
TTTTCGCTTTTGGCAAGAACAACAATTGTCCCCTGTGACGTCATA
AGGGCAACTCGATCA
TCGCTCAAATAAACCCGATCTACCTCAGACTCTGCCGGATCTGCG
CATGCCCAGATGTGC
GTGAGGTGCCA
>ConsensusfromContig2802 Average coverage: 40.47
AGAAGAGCAACCTCCTTCTCAACTTCGCGAACCATCTCGCGGTAT
GTTCCCCGTGCTTTC
TCTGTTAGCTCCATGAGTCTTTTGAAATGTTTCTCTGCACTGCTGT
AGCTAAAAATGCCC
ACAATGGAAACTGGAACGAGCTTTAGAAGGGCGCGGCCTAAGGC
TCGGCGGCCTAGAGAG
AAGACGACAGGCGTATTGGATTCCTCGCAGTCAGACATAATTTCC
CGTAGAGTGTCATCG
ATACCACCTTTGCACTGAATTCTTTCCAGATTCGGCGATATGATG
ACGAGTTTCAGTGTA
CCGGCTTTTCCATGTTTAGCAACTTCCCTCAATCCCATGACGAATC
GCCGTTTAAGTTTT
GCTTTAACAGGATTTTTCTCAAATGCCTTGTTCTGAAACGCCACT
ATATCCGTGAGTAAA
TCCGAACAGCATTCGTCTATATCTTTATCCAGAACATGGTTACAG
TACTCCCTGAATCTT
CTGCTGTGAATCCGTTT
>ConsensusfromContig2803 Average coverage: 13.21
CCAATGGGATACTTCTGCGATAACACCATGGATCCTGTTGTTTTG
TTCAACAATTCTTTG
TGTCCTTCAGGTCATTATTGTCCTTCTGCTACAAAGTATTCAACCG
AGTATCAATGTCCC
TTGGGTACCTTCAACAATGCAACCGGGAGGTCCAGCCTCTTGGAC
TGTCTTCCTTGTCTT
GGTGGATTCTACTGCGACCAAACTGGATTATCTTCTCCAACAGGG
ATGTGTGAATCAGGC
TATTATTGCGAATACGGTTCGAGTAGCGCCACACCAACCGGGAA
TTCTAGCGCGGACGAA
TGCCCCCCAGGTCACTATTGCCCCCTCGGTTCCAGTCGGCCTTCT
AGTTGCGAACCAGGG

ACCTTCAGTTCGGCCACTCGACTAGAGTCTCTTCAAAATTGCACC
CAGTGTCCATCTGGG
TACTACTGCCCCTACTGGAATATGACTGAGCCTGGCCCTCTGTGT
AATGAAGGGTATTAT
TGCGACGGTGGTGCGGATATCGGGGCCTTTAAAGTGTGCACAGA
AGGGAACTTTTGC
>ConsensusfromContig2804 Average coverage: 34.25
AGACAGCGATGCAGATGCACAGTGATACCACACCAGCCGCCAGG
GCAGAGACCACAATTG
TGATGAAATTTAAGTCATACGCAAAATCTTCTCTTGAACCCAAAA
GGTGCTTGATGCGAT
CCAAGTCTCCGTCGTGATATGGAAACGGTTCGACGCCGAGGATG
TGACACATGAGTGGGT
AAATATCGACCATTCGGACAGAGGGTACTTTCAGATTGGACGCA
AATGCCGGGCCCATGG
CCATGAAAAAGGGGTGCATCTCGGCAACCTCATTGCTCCAACCAT
GGCCACCCTTGTCTG
TATACTCCGAACCACGGTCCACAAGAATATATCCGGGGTCGGCT
ACCACAAGAATCGGTG
GGATCCGACGGTTGAACTTATATCTGAAGTGATCAGGTATGTCCA
CTTTCCTCCACGTGT
TTGAGTGTGGAATCTTGAGGAGATCTTCATATACCTCATTCTCTG
AGCCTTTAATTGGGT
ATACTTGCATGATCATTGCACTATTTATTAAGATTTTGCAATGGCT
TATATTTATGAAAT
CATTTATAACTAAAAATTTGGATGAATCAATTTTAGAC
>ConsensusfromContig2805 Average coverage: 14.30
GTCGAACCGCTTTCGTCAAAATCGTCCACAGGATGTTGATTGACC
GTTGGAACATTCGTC
TGCTTCCGGGCGTTTCCTATTGGTTCGTAAATGTTTTCTTCAAACT
CTTCGTAATTGCTT
GATTCGTTCCCTTCGTTGCGAAAAGCTCCTCGCGATGCGGAATTCT
GATTTTCTGTTTTT
GAGCCATGCGATTCTTCGATTTCATACTCGTTATGAGAATAATTTT
CTGAATATTCATCC
CCACCAGGGGGCGTGAAATAGTCCTTTTCAAGCGTTTCACTCCCT
CTAATAACAGAAGAT
AACTGTTCGGGGAAATTTGTCGGGACAGCAACGTTTCTTGT
>ConsensusfromContig2806 Average coverage: 16.08
TTCGGAGATCGTCTCATCCAATCAGGTTCCAGCAACAGTAGGATT
TGTGATTGCTGCCCA
GGGATTGGGCAATCTGATCGGACCGCCCATCAGTGGCTATCTAC

GCGACACAACGGGGAC
CTATAAATACGGGTTTATGATGTGCGGGGCACTCTTGGCTATTAC
CACGTCATCGTACGT
CATCAAGTATATGTGGAGCAAGCGTCAGAGTGGGTATCATGTGA
GCGCTGAAAGTCGTGA
GTGAAGTGAGGTTTTGGTTTTGTTTTGGTTTTAGCTACTCTAGGTA
TTGATTATTTTATG
ATTGACTCTCTTCTCAATATCGTATAATGTGGTGATTTGG
>ConsensusfromContig2807 Average coverage: 29.96
TCTTCCGATCTCTCTGAACCAGTGACTTTCGACAGTTCTGGTGTTT
CTTGTTACTGCGTA
GGAGATGGCCATGGTTTGGATTCATTTGACATGAATCATCAGAAT
CTCATCCCTTTTAAA
GCCAAACCAACACCTTCTGATATTGACAACATGCGCCCTCCGACG
ATTCAAGATCGCGTC
AGCAATTACTTCAAACGATGCCTTGACGCCGAGGCAAGTCATTCA
CAAGATTTCAGTTCC
CGGCTCAAGGGTTATGCGAAACAGTTACAGACCATTCAAGGGGA
GAAGCGAACGAAATAC
GAGCCATCTAATGATGTTGTCAATCTTCTGAGAGCATTGAATTTC
TACGACATCAGCACA
TGAATGGCGACCCACGATATAAATATTGCATATTCGAATATATTT
TATCATTATTATATG
TT
>ConsensusfromContig2808 Average coverage: 10.92
TCTTGATAATTCCATGAGCAGAGAGGACCAGAGCCTTGCATAAAT
GTGACTGTATATGTG
GAGGTATAACACCAGTCAATGAGCATTGGGGTTGTATTTCCAGTT
TCGAGATGAGCAATT
TTCTTTCGATTTTTAACATCCCATATGATAATAGTATTGTTCTTCC
GATCTCTCAGCAGT
ACATCTATGATAGTGGGATGAAAAGCAATCCCTGTTATGGTTTTT
GTGTGCTCAGACATC
ACAGAATGAAAATAAAAGTTGTTCTTCCCATCTCTTTTATAAATG
TAAATGGCCAGTGTC
GCACAGTATGCTAGCATGTCATGGTTAGTGGTACATACATCGATA
TTCCAGGGTT
>ConsensusfromContig2809 Average coverage: 11.13
CGAAAGTTCATGGCAAACGCTATGCGTACAAATTCGACTTCTGTG
GCCTAGCGCAAGCCA
TGCAACCATCCTCTTCAGACACGACGAGCGCTTACAAATACCAGC
AAGACCTTCTCATGT

CCAGCTACCACCACCACACAAGCACCTCGAAATTGGGCTTCATGG
GAGCGCATCCTTCAA
TCGCCACATCCTCCCCTAACTTTTTTCATAGCGCCACAACCCCCCC
TTCGACTTCGAGTA
TTTATCCCAACGCCATGTCCTATATGGATCACTCTAGAATATGGG
GCAGCCCCTCCTCGA
ATATGTACTCTAGTCACCCCCCTGTTCCCCACCATCCGGGACATG
TGAATCACCAGAAGA
TCGGAAG
>ConsensusfromContig2810 Average coverage: 37.02
ACTCCGTCACCTCCGAAGCGTAAACTCAGGTCTCCATCGAAGCGT
CGCCGTACGATGATG
CCGGGCGAAATGGTTGCAGCCACATCTCCTGAAGTGGTTGAGGA
GAAACCGAAAAGTAAA
CCTAAATCAAAATCTCCGGACAAGACGAAGAAGAACTTAAGTAG
TAACGTTGCAGTGAAT
TTAGTTGTCGTTAAAGAAAAAATCACATTGGTGGAGAAATCTAC
TGTTCAAAGCCAGGAG
TCTGTTCCATCAGTAAAAAAGCCTCGAGCTGCTGGGAAACGGAA
GCCCGAAGAAAATTCT
GTTGAGACTCCACCGCCGAAGAAAAGGGTTGT
>ConsensusfromContig2811 Average coverage: 25.89
ATGGACTGGATGGTCTTGGGGACTCTCATGATGTTGTTTTTGATA
TGGAAAATAGAGAAA
TGACAGAGAGTGCTGTCACGTACTTGATCAGAGCAGCTGAAAAT
AAAAAAGGATTGATGA
CAGTCATGTGTTTGGGCCCATTGACAAACATTGCTGCAGCAATGT
TAATAGATCCCGCCT
TCACTCAAAATCTTGCTCGAATGGTGATCATGGGAGGATCCTATA
TGGGTCAAGGAAACA
TAGTCCCGGGAGCAGAATTTAACTTTTGGCATGATCCGATTGCAG
CAAAACACGTCATCA
ACCACAGCAAATGTCCAATTTTGCTTATTCCTTGGGAAGTATGTC
TAGATACTCCAACAT
CAAAGATCTGGCTGGATGAAGCATTTGGCCTACCGACAAAAACAA
GTGATTTTCTCCAGA
AAATACACGAGCGTATTCTCCAGTACAAGTTCTACAGAAACAAAT
CTGTTTTCAAGGCTT
TCCATGCAGATGCAATCGCATGTCTTGCGGCAATCAGGGAAGATT
TTATCCTAGAGAAAG
TCCAGCGGAACTGTGACGTTGAGATCCATGGAGACTTAACTGCAG
GAGCTCTTGTTTGTG

AATCTGGAGATACGTCTGAGAATGGCAGAAATCGGATTGAAATT
GTCACCAAAGTTAATG
CCGATTTATTGGATTCCCTCTTGATGAATATGTTGAAACAGAGCC
TTTAATATGAAACGG
TATTCTACATGATATATAATTTCACGTGATTATATCGCATTCCATC
CTGATATTTGCAAT
TTTTAAACAGTTAAAGGTATCACATGTTCATGTTTATCATTCAAA
AATAGGCGGG
>ConsensusfromContig2812 Average coverage: 9.83
ATCGGGAAGACGTCGAAGAACCTACGCTTGCGCAGTTCGGAAAG
AGCGCGTTCCTGATCT
TTGGCAGAAGGGAAAGAAGACGTGACTAGTCGAACGAAATTGTG
GGCGGACTCCAATTTC
AGCCCCACCCAGATCTCACTGAGATCTCTCACTTCCAATTGGCTG
AGAGCGCGTTCTGAT
TTGGCCTGTGCTGCGAGAAAGAGAAGATGAATCCATAACCGCGGC
CGTTTCGTCGCCATC
GACCGGCAGCAGACCCTTGTAGAGGCGGTCTTGACGCCGGCTGAT
ATCGGTAGAGATTTT
CCGCTGTACAGACAGAGGTCGTGACCACCTGGACATTCGAAAGA
TCGGAAGGG
>ConsensusfromContig2813 Average coverage: 12.13
GTAAAAATCAGAAGCAGTTTGTTCTATTAAGATGAATCAAGTTTA
AATAGAAGTTTGATA
AAGCGGAGAAATATTTCGTATATGGCAATGTAAATGTCTATGTCA
GATTCGTTGAATTTC
TTGTTTGATGAAGGATATGCGAGAAATTAACATTAATCACAGCG
TAAATCAACAAACAGC
ATTCCATTTGATGATTAATTTCAACAAAAGGGCTTATATTTATCG
AGAGTCATCCGATAT
TTTGAAAAAATAAAAATAAATACTGCATTCAAAAGAATTTGATT
ATAAACATATAAAAG
>ConsensusfromContig2814 Average coverage: 18.16
TGGAAATACCTGTGAAACGAAACTTGATACCGAAGACAGTATTGA
ACTAATTTTCACAAT
CATTGCACCAGTGGTTTTTCTGTTCGTATTGATCTTCATGATTGTC
TTCTTCAAAAAGAG
AAAAGTTGACAAATCGAAGAAAATGAACTCAGCTGGAAAACGCT
CAGAAAGTATGTCGAA
AGCTGGTTTGGAACAGCTGGACATTTCGGCATTGGAGACAAGGAA
AATTCCTGATCTGCC
TTCTCCAAGAATCCCTGCCCTGAGAGCCCCTCTGCCAGAAGCCCT

AACACTGGGAACACC
TTCACCAGGAAAATTGTTACCAGAAACTCATTCAACAGAAATATT
TTTACCAGAAACACC
GTCACAAAGTACGCCTGTATCAGGAGAGCTTCTGACAGCGAGTTC
TTCATCAACAGCTGC
TGTTCCACATACTTCTGAAGAATCTTCCTTTCGATTGCTCGGATT
CAGAAATAGGATTGA
AGATATTCCATGCGATTCCAATAAAGATCACGAACACAAATATC
TGGAAGTATTAGCAAA
TAATATTGACAATAAAGACCACGAATACAAC
>ConsensusfromContig2815 Average coverage: 39.13
TCAAACCACCGATGTGCATCACAAAAGCTGATGTGGATTTTGGAG
TAGCAGTTTTGAGAA
AAGTTTTCGCTGATCACTCTTAATATTAGTTCTCCATAAAACAATC
TGCCCATATTATTA
TTTTTGTAATATTATATCCTATACTGACTCAATGAGCTAGTAGCT
CTAGAAACAAATGAA
AGAATTATCAATATAATATCAACCCCAGAATCAAGCAAATATCAA
CTGTCATAAATCGAT
CAGATATCAACTATCATAGATCAATCATATCAACAGCAAGAAGC
CAATCTGGACTCAACA
TTTATATATAAATCAAAAATGAACTGTCATAAATTGATCAAATAT
CAACTGAGGGGAATC
AATCATCAACTGTCATAAATAAGAGATATATAGCAACCGCCATG
AACCAATGAGATTTAG
ACTACCACGAATCGATCAGAAACGAC
>ConsensusfromContig2816 Average coverage: 7.25
AAAGCAGAAGATGACAAAGCCAACAGGAGGCGGAGAAAGGAAA
GAAAAGTTTCCCCAGAA
GACCATTCGAAGTGGAATCAAAAAGATGAATCAAAAATCATATC
TGAAGATAGAAAAAGT
CGAAGAAAACAAAGAGAAAAATCGCTAGAATTCCAAATGGATGA
TCCGGAAATCGAGTCC
CAACACCTCATGGAGAAAAAAAGTCATTCTCACAGCAGATTTCA
AAATTCACAGACAAAA
GATGGCGGCATATATACGCAACTGCTGGAATCCAGGGCACGTGGT
TGCCCCTTTCCTTAT
ATCCAGCTACCCTCAACCTTCGGACGAG
>ConsensusfromContig2817 Average coverage: 10.03
CTGACTACCTGAAGACGAGTTGAAGAACCGACTATATTTCAACGA
TGAATGGACTCACTG
TATATGTGGCATTGACAGTCATACTGGCACAAACTTTAGTGATGA

AAGCTGGTCCTACTA
CCCCTCTTACTCCTACTCCCGTCCCTGCTCTAATAAGCACTTCTGC
GTGCTCGGGTTCTG
GTAACACTGTATGTGATGAGGATAATGCTGGAGACTTCAAAAACT
GTTATACTGTCGGAA
GTGATAAACATACGTGCGCATGTCCTGAAAGTAAGACCGGTCTTT
CATGTGAAGCATCTA
CAGATACCTGTATTGGCCTTTGCAGATCGGAAGAGCGTCGTGTAG
G
>ConsensusfromContig2818 Average coverage: 21.64
TCATAATCTGGACTGCCTTTCGGATATCTTGCAGACAAGAATGTC
TTCAAACATTTCATC
AGATGATCCTCACTAACTGTCTTTAGTAAAACTGGTGTACCATTC
TTACTGACATCCCTT
AGCCATTCAAGAACATCTCGCAATGTGTATCCAGCAACTTTCTGA
CCTTGAATTTCTAAA
AGTATATCACCAGTATACAAACATCCACTATGATAATTTATTATA
TCCTGCTTGACATTG
TCAACCCAACACAGCTGACCATTGTCGGAACCTCCACGAATGAT
GAGATGAAGAAGACCA
TCATCAGGTAAGGTTGATATTATTGTTTCACAAACACATCTTGTC
CAATGTGAATCATCA
TCATATATAATCACAGATTCTGGAGTCTTCATTGCATTATTACTG
AGTTTTTGAAAAAAC
AAATAAAATTATTAAGTATTTGCATTCTAATGATGTATGGAATAA
AATCGTTACGAATGG
>ConsensusfromContig2819 Average coverage: 80.37
TCGTCTTCAAAAGAACCAATATTGCCTTGAACACGCAAACTATAT
TTGTCTTGATTTCGA
TTCCGTGGGATGGTGCAAGATCCAGATGATTCTCCTAAGTTAGCA
TAGTGATTAGAACTT
TCGCCATTTTCTTGAGATGATGCATTAGCCAGTGGAGATGAATTT
GTGTTGACCGGTGTA
TGGGAAGCAACTTCATGTAATCCGGAGACACCAGATGAGGTTATC
CCTCTGAAATTTGCA
GCACTGCAAGAAACTGCATTTCTTGTACACATATTGGCAGCAGG
AGCATCAGAACTGGAA
ACAGCCGGATCCATCAGCGTCTCATTAGCACGAGATGACTCTGCT
CCCTCTTCCTGACAC
TGCGGGTATGGAGATGGAGGGGGACCAACTAGTGGAAGGTCCGA
CATTGGTTAATATTTT
TTGTCTATTTCTGAGGAACAAAGTTACACAATCAAAAATAATTCG

TT
>ConsensusfromContig2820 Average coverage: 30.21
GAGATTAATTTTGATGCATTAGTATTCATGCCTTTGGATAGATATT
CCATACATTTTCTA
CATATATCGAAGACCACTGTTTCCCCATAGGCCTCAAACAGTTCTA
ATCTTTTTATATCT
GTTGAAGATATATTTGCACGCATTTTTACGTTTGATCAAAAATATT
GACACCCCTTTATT
ATTTGTTATGAAGACTTGACATTTTGTAAGAGTTGCGATTTGTA
>ConsensusfromContig2821 Average coverage: 7.97
TATTAATACAATACCCTATATACCCAACATGGTAAAATTAATCAG
CACGGCTCTATATTT
AGATTACTTTGTAACATACGGATCGGAGGTTTGTGTGAATTACAT
TTGAGGTTACTACCA
TTCTTAGATGTACTTATAAAGAACATGAATACAAAATGTATAAGG
TGATGGATGTTCCTA
TCGTTTTTCAGCAAAATTGACCTTTTATGAAATCAACATCTTTGG
TGCACATTCACACAA
ATCAAATACCGCACATCACTAGAGAATCATAATATTTCATTCATC
ATCATGAAACTCAAT
CAAAAATCCTTAACACCATGATTGAAAACACCATAGATTGAAAA
AAAAACAATCATGCAT
TGAATTTAAAATCAAAAATAAATTAGTGTAAG
>ConsensusfromContig2822 Average coverage: 10.53
TGCGAAATATAACAGCGAACGGAGAAAAATTTAAAATACATCAC
ATCTTCATCATCATAA
TTTTTGTAAACTATGTCGACATATCTATTTATTGAAATACTCTTG
AAGAAGTAATTTGAA
AACCCACAACACCTTCTGAAAAGTACATTTTGTTCTCTCGATTCAT
TGTCTATGATCTCC
TGTGTTTAGATACAAACATACCCGAAAGGTTGTTGTTGCTGGAAT
TATTTGTTATGTATT
GATTATCTGTCACATGCCGAATGTGTTATATGTACATTTCTATGA
AACTACTGTTATGAT
ATATTCTACCGATCAAATGTGTGTCATTTACACATCGTATGGCTTT
TTATCCCCG
>ConsensusfromContig2823 Average coverage: 37.73
ATAAGACAGAAAAAAGAAGTTCTTAATATATAATGTATCAAATTT
GATAATTTGTTCCCA
ATTCTCATACGAGACATGTCAATAGAAGAGTTATATTGCAAGGG
AGCACGAGTATGGATC
CCAGACTCCGAGCTCATATGGAAAGGAGCCACAGTTGTTGATGA

CTATATTGGACAGAAG
GATCTGCAATTGATAGTAGACGAAGATGATTCGGAATATTGTAT
AAAAATAAAGAACAAG
TCTGACTTCCCATTTCTGCGAAATCCGAAATCTTTGATCGGAGAA
AATGATTTGGTTGCT
CTCAGTTATTTACATGAGCCCGGCGTCCTTCATAATCTACAAATT
CGGTTCTGTGAGAGG
AACGACATCTATACTTCGTGCGGAATTGTTCTTGTTGCCATCAAT
CCGTACGAAACTGTT
CATATCTATGACGATCAAACGATCGGAATGTACCGAAACAGCGA
ATCTGGGAGTCTGGAG
CCTCACATCTACGGTGTTGCAGAAGATGCTTATAAGAACATGTCC
AGATTCAACCAAAAT
CAATCGATCATCGTCAGTGGCGAATCAGGGGCTGGTAAAACGGT
TTCGGCCAAACATGTG
ATGCGATACTTCGCCGTGGTTGGTGGATCTCACAATGACGACAC
ACATGTCGAACAGAAA
GTTATGGCGTCTAATCCAATCATGGAGGCTATCGGAAATGCGAA
AACGACCCGAAATGAT
AACAGCAGTCGGTTCGGAAAGTTCATCGAAATTTGGTTCGACGAT
CGAAACACAATAATT
GGAGCGAATATGAAGACTTATCTGCTGGAAAAGTCGAGAGTGAT
GTTTCAAAGCGATGGA
GAACGGAACTACCATATATTCTACCAACTTTGCAGCTGGGCCTCA
GAAGAATCTATGAAA
CCGCTTCTATTGGGCTCTTCCAATGAATATGTCTTGCTGGCTTCA
AATGGCCGTAGTGTC
GCGAT
>ConsensusfromContig2824 Average coverage: 15.47
AACTACTTTTTTATTCTTCTCTATATTCAACTATAGTTTGGTTAAA
GCTTATAACCATAA
TATTATTATTAACCGATTTAGACTGATGTTGTTATTATGATCTGCA
ATTTTTGTTGGCAT
TCAATACTTTTAGGTCAGGCTCCTAGATAGACATGCTTATTATAC
ATTTAGGCATAGTTT
TTGGTGATGTTTTGTTTGAAGTCCGCTCAAATATGTTGAGAATCG
ATTCCGATCGTTCCA
AAATGATTCATGTTTAGTACGTAAAAAATTCGACTTTCTGAAACG
TAACACCAGTACGGT
GATTTACTTAAACTGGGGGAACTATCTGAACGTTGTGGAATAACA
AATATAAAATCATAA
TACTGAGAGAAGATCG

>ConsensusfromContig2825 Average coverage: 9.29
ATTATTTTCAAACATTTATTTTTATAGGCCATTTTTTTGCATTTTCA
ATTCCTTCATTTC
GCAGTTGCTATATGTTCAGGTTGTATTCGGCATTTTAAAACCAATA
TTTTTTATATCCAA
TTCTGGTCAAGTTTTATAGGCCTGTGCAATTTGCTATGTTTTTTT
CAGAAAGGCTAGTG
CAATCATAGATTTTTTTGAATTAATATATTTATTATTACCGGATTA
TTCTTATGTTTGTT
TATCCTCACAACAAAGATAAGTGCTATACGTGTTGCCGACCTTAT
TATGTGATTTCATAT
CATCATAACTATAGCTCGAAAATTTATTCATATACAGTATTTTAAA
AATTGTTTTCTATA
CATCTACTACAATGAGGGAACCATAAAAAAAATTTCTCTTGTAGA
TTCACTTTTCTTTCA
TCCAATATTTGATAGAAGTCATTTTTGTGCGAAACCATACTTGTAT
TTTTTACTACAGAT
CACATATGGTTGTCAGGATATGTATGTTGTTAGAC
>ConsensusfromContig2826 Average coverage: 16.27
ATATGTATTCTATTATTTCCGGTGTCTGCTAGGGCAATGAAGATA
TCGTCTGGGTGAACT
GCAATTGCTTGTGGTCGAAAGAACTTGGTGCTGTCATTGTTCGTA
TTGCCACTCACGGAT
CTGACTGGCACATAGCTATCATTGGGTCCTGCATAATCCTCTGGG
AAATGCAATGGTTTT
GGTGGTCTTCTGTTAATGCAAAGCTCATGAAATTCTGAATAGAAA
TATTCATCTACACCG
TCGTCATGAACGTTATTCAAAATAGCCATATATGTACGAATATCA
TCTTCATCTTCGTTA
TTGCTCGTCTCCAGAGCATCTATGAGTT
>ConsensusfromContig2827 Average coverage: 16.86
GTAACTGCTCATTCTCGATCGATACCATACGAATGACTTGAATTT
TTATAACTGTTCTGA
TTTTATTAACTGGTAGCATACTACCATTGATGTAGCATAATTGGT
TGAAAATCATCTTGA
ATCTTTTCATTTTATACTTTTCGTCGAATAGTTCCGTATGATGTCA
AGTCTGTGTTAGTC
CATGAGGCTTTTAAATTTTTATTTCGAATATTCATGAGTAAACGA
GAGTTTAGTTGTAGT
CAGGCCTCTGACCACTCTAAATGAAGGACTGTTTAAGTGCTCTTG
ATCTTGAAAAACCAT
TCGAAGTTTCATATGGAATTTGAACAGATAAATCATACCTTTCAT

AACAATTACATAGTA
TCTGCATATCATATCATGGAGCTATAATACTATTTTCGGTTTATT
>ConsensusfromContig2828 Average coverage: 8.90
GGGGCGTGGCGTAGTTGGAAACCGGGTAGAGAGAGGCTCGCGGG
TAGTCCGTGTTGAGCT
TTCCTGATTGGAGAAAAGATCTTCCATCTCGCACGGGGAGGCTGC
GTATCCTCAGTTGCT
TGTTGAGCTCATTTGAGAGCTGCTCTTGCTGCTTCAAATGTATCC
ATGTTGAGGCAAGCT
TGCTGAGCTTCTGCTCCATCCGTCTGTTCTCGGCTCGCTTCTGAGC
AAGTTCGTCATTGT
GATGACCGTAGTGTTGACTTTGAACGCTCTGACTCTCCGACACAC
TTTGTTTGGCCTCAA
CTTCAATTCTCTGTGAGACTCTTTCAGAACAACTGCTCGAGACTT
TTGATCGTGATGGAC
TCTTTGGTTTGTTAGATGCGGAATAAACGTTGATCACAGAGTTAT
CACCATTGTGTGTTT
TCATTTTTTTCATACGTCATTTAG
>ConsensusfromContig2829 Average coverage: 42.72
TTCGGGCGTTGTCATGGCAACCTGAGCGGCGGCCGCTGCTTGAAC
TGCCGCTGCGGCCGC
GGCAACGGCGCCCGGAATCGCGAAAGACGTTGCGGCGACAGGAG
ACTTTTGCATGACTGG
ATTTCGCGATAGAACATGTGGAGATACATTTGAGTCCTGTGAAAG
CGCCTGTTTCAGATG
AGGATGCATAACTGAAGCTTTGTATGATGACGTCCTGGAATCGGA
GTGTTCTTGTGGAGC
ATTTGTGTTGACGGGCATGCTGTACCGCGTATCCACCGACGGACT
TGCAGGGGCGGCCAT
TGGCGTTGCAGGATTCGACCACTGCTGTTCTGGGGTCATTGG
>ConsensusfromContig2830 Average coverage: 11.59
GAAGACTTCAATACAATTCTGAGGGAGATCACAAGGGATATGCA
ACAGATGAAAGTCGGG
ATTCCATTTCCTCAAAGGAGGATGGAGGTGACAACGATTCGTTA
GGTAAATCAGAGCCCG
TAAACCTCATAACAAAAACTGATCCTATTATATCTGATCAAGAAG
AAAAGCATGTTCAAA
AAGAAATAAAAGACAATAGTTGCAAAACGGCATGCCTTTACTGC
AGCAAAGAGTTCACAT
ATATCACAAGTCTGCAGAAGCACCTGCAAAATAACTGTCCCGAA
CGCAATATCAGCTCAA
CCAATAAAATCTGGGAGCAGCAGATTCTCACCATGCGGGCAGGT

CGGCTCAACA
>ConsensusfromContig2831 Average coverage: 18.65
CAATGATCGAGTTGATTCTTGTACTGAAGGTGTGTTTGATGAAAA
TCGCTTCGAGATCGT
CGTGGAATTGGTGCCGATGTGTGATTTTTCTCCACCACCTCTTCTT
GGCATTTCTGATGT
TGTTTATGTAAACTCTTCGCAGATGGTCCGAATAGAAAGCGTCGC
TCTCTGGCGGACCTG
CTGCTTCTGTCGACAGCAGGCGTTCGTGAGCATGGTTGTGTTGTT
CAGAGTCGTTGACGA
TCTCCGAAGAGTGGCGTGGAACGTATGAATGCTTGCTATCCGCGG
CCTCTTCAGGAATAT
TCCCATTTCTGATATCTCCCGTTTGGACAGTACGACGAGGCGATT
GCGGAGGAAGACCGG
TTACGGAGTGTCTACTGTGTACCTGACTTCTCTCACTGCCGGAGA
ACTGACCAGAGAACG
ACATTATGCTGCAAGAAATTATGTGCAGTGTTTTGTATCAGGAAA
ATTTGAGAATATCGT
CTTTTAATGTAGATGCCTGATTCATCATGGAGGAATCGATGAACA
GCGGAAAAC
>ConsensusfromContig2832 Average coverage: 21.42
CGCTCTTCCGATCTGTCAGAGATAAAAAATAAATATTTCTGCACT
TCATTAGATCAGGAT
TTCTGCTATAAACTGTGGAATATTTTCAATAATTTTTGCAAAACAA
ATTCTTCTATTGCT
CAAATTTCAACACCCTGCTTTCAATGGATTCTCGGTCACTTAGGT
ATTTTGATGAGCACA
AGAATCGAGGACAAAACTGACAAAGGGTATTCATATTCAGAACT
CATCAAACTAGTCAGC
ATCTCTTCTACACCCGATAGTAATAAATTCAAAGTCCAAAAAATA
GCCATTGCGAACTTG
CACTCTTATCTGATAGATGACATACTAATCGAAGAAACATTATTT
CGAGATCAGAAACAA
GAAAAAAAGTGTGCATGTAAGCTGCACTCAGACAAACTCTATAC
AAACCCTAAATTTGAT
CCTATCAAAATCTTGCCAGGTTACAAAGTCGAAGCAACGAAAAA
CAACAGTTTCACGATC
TTTGACGAAGATGAAATGTTTGTTATTTGCCTTTATTGCCGAAGC
GAAGATTCGAAACAA
CGTTGGTTGACCGAATTGGACAATGCTCTCTTAGCCTGCCAGTAT
GGCATAACTGTTGGC
AAACTTATAGCTCGA

>ConsensusfromContig2833 Average coverage: 21.13
AAGGACCAGCTTCATGGCTGAATGTGTCTCGTTATACTCTTCAAG
GATCTCTTGAAACAG
AGCCTTAGCGGCCTCAAAATCCTGAATGTCTTCGTAGAACCGCGG
CTCCGATTCGTCCGT
CGCATTCCGATAATCTCCGAACAGAATCGGATCCCGACTGATGTA
GTCCGAATGGTCCGG
CATGTTTTCCTCAACCAACGCCTTCACCAGGTTCTGAACGATAGTC
CTGTCTCCCTCATT
GATGAGACGATCATAAAAGACCCTCAACGTTTCGTTTCTCCAGA
GCCGGACGAAAGACTC
TAGCTGCTGAATCTTATCAGAGTCCGAGAGACACAATCCCGCGTA
TACACGCGACAGGTC
TCGGAGGTTGAAGATGTAGTGGAATTTGGATGGGGTCGGCAGAA
GGTCACGGACCACGTT
TGTGTAGAGCTTAAGCGTCATATTCGTGATGGTGTCCACGGCGCC
GACTATCTCAGGCTG
AAATCCAGCACAATGGCCTTCAAGGATGGAGTTGTAGATCCGGT
AGAGGGCGCTCTCGTC
CGGGAAGGTCATGTTGAAGACGGAGAAGAGGGTTATGAATCTAG
GGTCAACCTCATTTCT
GCCGCCTCCGGGTTTGCCCATTGCGGCCAGCCAGCCCACGTCTTT
GATGTTCC
>ConsensusfromContig2834 Average coverage: 6.24
TATTGGTCTGGCTGTTAGTGACATAGAGTCTATTCGCAGTAAAGC
CAAATTACAGAGACT
TGCGATGGAGATCGATACGTACACCAACCTCGAAAAACGCTTCC
CAACGCGGATTCTGAA
GTATATATACAAAACTTGCTGGACTGTCTACCCGAATCGAAAAAG
ACACCTGGCTTGTAT
GATATTTGGATCAAAATCTAGAGAGGAGATGTACCAGCGAGATA
CCAGAACAGATTACAT
TTACAGAGATCTTCAAAATCAGCGAAACGAGACGACACGTCTAC
AAACTAAAATAGATTT
TCAAACGGAGATGATGCGGTTGATAATGCAAAAAATGGAGATCC
AGACAGAAGCAGACGA
CCTGGATGAAGGAAGCGATGAACTGGACTCGACAGAAGACAATG
GACCAATCGACTGCAA
AGACCCCAGGAGAAATTTTGTGGGCCGCAAATCTCTACAGCGGAT
GCGATGGATTGTCCC
TCTCATGAGGAAACACAATCATGAGAAGTCCTTTGAAAAACTCT
GAAGCACCAAAATGCG

AAATACCATGGTGATCAAGGCTATAAATCATCATAAGAGATATC
AACATATGGAATATCG
TTGGATGGGAC
>ConsensusfromContig2835 Average coverage: 16.04
AGATGTGTTGGGTTTTTCCGTATCGATCAAACCTGGACTTGAACT
ACACGTTCAGTACCG
ATACAGTATCAATTGGGCCCAATCTCTTGATTTGACCGAAAAGAC
TTGGACTCATTTGGT
TATCCGCTGGGCTCAGACTCCAGTGGATATTTATGTCAATGGTGT
GAAAGAGCATCTCCC
GTTTCCTTGGCCCTGGGATGATCATGTTTGGAACCCTCAGGAGCA
ACAACGACCTCTGAT
TGTCTTGCTTGATTCTGTCTCGCTGATCAGAGGATTGAAAATCTA
TCCGCGCTGGTTGCA
GCAAAGTGATGTCACTATTTTGGCCAATGAGCTCACTTCCGGGTC
ATCCAATTGAAAATG
GTTCTCGCAGCCTTACAGCGACTCGTTACCGTAAGGAAAGTCCGG
GGACTCGCTTGATTT
TATTGTTGTTTTATTTCTCGATTATCGTTTAATGCGAAAATATTCT
AATCCCATTCAATC
AGTTCACGAT
>ConsensusfromContig2836 Average coverage: 4.13
GGGAAGTTCTGGTGTCAACTTGGTAGTAAGGGAATTCGTGCGTTG
ACAGTTAGCTCCATT
TCGTGGATCCGGTGATACGTCGGCATACTTTGAGCTTAGTGGGAA
GTTCTGGTGTCAACT
TGGTAGTAAGGGAATTCGTGCGTTGACAGTTAGCTCCATTTCGTG
GATCCGGTGATACGT
CGGCCATAC
>ConsensusfromContig2837 Average coverage: 52.24
CCGTTTTCCACCAAAAAGCCCGATTCTCTAAATTTCCACAATCCA
TTCGAATTCGGTAGT
CTATAAAATATTTCCAACTCCAAAGATGGCCTTATGTAGATAATG
AATCCATACGTGTTA
ACAAAACCACCACCAGTATCAACAACAGCTTGCCATGTAGTGGAT
CTTCTCGCATTGATG
AATAAAGACATTGAATGACCGGTTGAGCCGCAATTGTTGATATCA
ATTGTACATCCGATA
TCTGGTCTCCCGTAGTCTATGTAGGTGTATTTCGGAATGATTCCA
AGATCTCCATCACAA
AATCCGCTTTCAATTGTGATTTCGTCTACGTTCTTGAGTGTACCAT
CAATATCTCCAATC

ACTTTTGATTCTTCTAATTGAACCATAGGCAGATCCAGTGAGTCT
TTTGCAGTGATTTGG
TCCACATTATATTCGCATCTGATGACTGCAACCATCAGAAGAAG
CCAGGGAGTGAACCAC
CCCATTATACCGGTCATTTCGAAAACAACGCCTTCAGAAAGAT
>ConsensusfromContig2838 Average coverage: 10.32
AAAGATTCGAAGAAATAGCTCTAGATACATCATGAATACATTTGT
CCAAGTATTTGCCAT
AGTTTTCGCATCGCTCATAGCCCTCGAGTCAAACTGGTGTTCTGC
GAACGAGAGCGGGAA
AATAGAAGATTATGTTAATAAACTGAAACAGCAATACGCACTGG
CCGGAAGACCCAGATT
TGGGAAACGAAATGATGAGAGCTCATACCCTGCGGTATCGGAGA
CCTGGGCTAAACGGCT
ATACGAGTCACGAGAAAACTTTAACGACATTGTCAAAGAATTGA
AACAACATTATGCCCT
GGCCGGACGCCCTCGATTCGGGAGATCGG
>ConsensusfromContig2839 Average coverage: 11.88
CGAAAATCTTGCAATTTCTTGCCGCTTTGCTACGCGCAACGTCCG
AGAAATTTCACCGCC
GCCGCTCCGACTCAGACTCAACCATTGCCCGCATAGAAACGTGTA
CGTTTTGTCACTATT
TTGGCCACAGACGACTTCGACCTTGCTCAACTTCCAGTCCCTGAA
CCTTGATGGTTCGCC
GCTGTTGTCATGCCAGATTTTCAGCATTATTGGTCGACCAAGATC
GATTGAAGACCGGAG
GATGAAATGTATTACGCTGCCTGCGTAGAAAACCTCATGATCCTG
GCCAGCCAGTTTGCG
AACCCCGCTATCCGCCTTTGAGCCACAAAGAACGATGTTTACTTT
GGATCGAGTCTCGGA
CACAGATCTTACAGCGGTCTCTACGCTGATGAGATAGGAATAGTT
CCCTCCGACTTCATC
GTTCTCGATGCATTCTATGGCGCCGTTAATCCGGGCCGCGTCCTC
CTTTTTGTCGCAGCG
CCGCGCCCAAATCAGCAGCAAGATGTAGAGGCAGAT
>ConsensusfromContig2840 Average coverage: 3.80
GAACTACGACCGCTTTTATTCACAGATCCAAGAATTAAAATGAGC
TGCAACTATTCACAT
CATGTTTTATTCATTTATTCGTATCATTCGATTGCTTCATATTTTT
TATTCTTAGCAGAA
CTACGACCGCTTTTATTCACAGATCCAAGAATTAAAATGAGCTGC
AACTATTCACATCAT

GTTTTATTAATTATTCGTATCATTCGATTGCTCCATACTTTTT
>ConsensusfromContig2841 Average coverage: 8.28
ATATGATTCGCCCAAAAAGATTTTTGAGAACATTCATTCAAATAT
TATCAATACGAAATA
TCATAGATTCATGATACGAGAATGAATACGAAGATAACAGTTTGT
GGACTAATATTACAT
TGAGATCAAAGATTCAAGAGAAAATACCCACAGTATTTTGGCACA
CTTTTGAATATGATA
CACGATATATAACATCACACATGTGACGTAATCGCAAAAATCGAG
CTTATCAGCATCCA
>ConsensusfromContig2842 Average coverage: 47.08
CTTAAAGAATTCTATGCGAGAGCGTATGATAAACATAAAATCTAG
CGTTGATCTTTGTAG
ATTGGAATTGTTTAAAATAGTTCTTCCGAATCGTTTCAAATTGCA
CAATCAAGATTCAAC
ACCAGCTTTAGATGTATATTTTAATCAACTACTTGGTGTTCATTC
TGGAGAAACTTTGGC
TTTTTATCAAATAATTAACTTTACTGATTTTACATTGCTAGAGAA
TACCAGTGATGTGAT
CGATAAAATTGAAGAGTTGGAGAACAAATTAGCAGAAAAAAATA
ACTCTCAAAATATACC
AGAATATTCGTTACTGCCAACTGAAAAAAACGTTGATAAGTGTAT
TTTTATAATGAAACG
ATTATGGGGTGGTCTGTCGTGTTTTATCATATTCTTGGAAATTGT
TAGAAACAGATTTGA
AACATTATCCTCACTCACTATTGGAGATATTGAAAAAATTGACGA
TAAACCTATACCAAA
TAACCATATTTATTATCATCAGGGTTATTAC
>ConsensusfromContig2843 Average coverage: 8.63
ATGGTCGACGATCAACCTCAGATGATGAACCTCATAGACATTGG
TAGTACGGAGGGTCTG
TCGCAGGATAGCAGATTTCGCCAGTTCTGCAAACATCCGAGTTGC
AAGCAGGTACGCATA
CTTTGCTCGCGTCAAGAACATAGCCAATGCTACACTCGCACATTC
CCGTTGTTGCAGACA
CAGTGCAGGACAGATTGTTGCACGATTCGCAATCAGCATTTTGTC
CGGTAGCAACTGGAT
TTGTGAAGAAGAGTTTATCTTTGCAGCTGTCACACTTGAACCATA
TGTCACACTTATCAT
CTCTGGGAGCCCATGCAATGAGAACATCATCCTCAACGCTCTTTC
CAATGCATTGTTCAA
TGCCTAAATTGTAGAAGGTTTCAATCATGACTCTTGGTGTTTGTTT

CGA
>ConsensusfromContig2844 Average coverage: 4.37
GTTTTATTATTTCATATTTGACCATGTATGATCATACATTTAAGAA
ATATCATACTAGGT
AAACAAATAATGATTCTATCAAGATATATAATAACCAGCCATTGG
AATCATGAAACAGTT
TGATTATATACAATTTTGAAACCTCTGCTTTGCGATTGAAACTGAT
CTTATTCGTGAATT
TATGATTGTGAATTTATGATATATTCGATTCTATATTGTACAGTAA
TTTTCTAAAAATCG
CCTATTTCAATGTGAACGAA
>ConsensusfromContig2845 Average coverage: 25.70
CTGCATGCTGGATGGAGCAGAGTAGGAACGTTGGCATATTCGTT
GTCAAGAAAAACAAAA
AAAGTTCAAGATTTAAAATGGCAACAACAGTTATATCGATGAAA
ACCCCACGAAATGTAG
AACCGAATAAATCGAATCTTGCATTCGGGGATCGTGCACTTCCAC
GACTGAATTTTGAAT
TGAACTCCGGTGAAGTACCGACAATCCAGAGTGCATGTAGTGCAT
TGTGTAATTATTTAC
ATGATCCAGAGCATATTGCATCATCTATTAAATCAGGTGTGATTT
TATCCCTAAAGCCCT
TGATATCTCATGAAGATGAAATGATCAGACTGAAATCTACAGAAT
GTCTACAAATTGTCT
CAAGTCATGCAATCGGACGACAGACTGTTACAGAATCAGGGATG
ATAGAATGTTTAGCAG
CTGCTCTGAATGATC
>ConsensusfromContig2846 Average coverage: 13.61
TCTGGGCAGAAATGTCCTACAGGGCAAGGAAGCCTATTTGACAG
TATCTCTCCACGAGGA
CAATAGAAACCCGCACTGCAATTTCCAGTGGAATCCGTGAGGTTG
TGGGATCCACAATAC
TGGCCCAGTTGGCAATCGAAGCAATGTTCAGAGACGTTATAGTTG
CCTTCGTTAGAGCTG
TAAGTGCCGAGAGGGCAAGCCTGGGGCCACGCTGATCCACGCGG
ACACACAAATCCAGCT
GGACATATATCCCCCGTGGATCCATCTGTGGGCGTGGCCTCCATG
GCTCCTCCTCCACAA
TAATATCCTTCCGCACACTCATCGGTGGGAGAGGAAAGACCCGA
AGTTTGGCAATAGAAT
CCTCCGGTACATACCGTCGGGGAGCTCGAACCTGCAGGGCAGAA
CGTTCCAGCTTGACAC

TCTTGACCGGAAGGCTGGTTCGTAGACGCCGACCCGGGACAGTA
CCATCCGCCGG
>ConsensusfromContig2847 Average coverage: 7.91
TGCAGATATTCGGGATAAATTCGATAAATGGATCATTGGGATGG
AACATTCAGCAAGCAT
AACTTTTAGTCGCTGTTACAAGCCACTACTTTTCTCAAATCAAAC
TGCAATGGAACTGCA
TCATTTTTCAGATGCAAGCTCGGAGGGCTACGGACAATGATCGTA
CGTCCGATTCGTCGA
CTCAAAAGGACAAATTCATGTCGAACCTGTTATGGGGAAATCCA
GGAAGATATAAATATT
AGATCTGTTCCTCGCTCTACCATAATTCCCATGTTCCGATTGGTC
CTGTAGACAAAGCGG
AAGCAAATGGCATCATTCTACTAGATCGGA
>ConsensusfromContig2848 Average coverage: 54.96
CCGCCACTGAGGATCTGACCGTTCCATCAGGAAGCATCTTGAGAC
ATGACCCAAAATCCG
CAAGCACGATGTGGCCCGAAACGTCGATAAGAATGTTGTCGGGTT
TGATGTCTCGATGAA
CATAGCCGAGTCGGTGAAGCGAATCGATGGCCAGAATCACCTCC
GCTATGTAAAACCGCC
ACATGTCCTCGGTGGGGGCAGGCCCGTCCTCTTCGTATTTGGAGA
GCAGGGTCAGCAGGT
CACCTCCGCAGTAGTAGTCCATCACAAGATACAGATATTCGTCGT
CCTGGAATGCATAGT
GGAGTTTGGTCATCCATCGGTTGTCCCCATAGACGAGCACGTCCC
GCTCCTCCTGGTAGC
ACGCCGTATCAGTTCGCTTCAGCATCTCCCATTTATTGAGGATCT
TCATGGCGAAAACGT
CTTCTGTCTCTTTCAACCGAACCACGGCGACCTCCCCAAAGGCAC
CTCTTCCAATGATCT
TGAGGAACTCAAAGTCTTCTCGTTGCAACCGCAGCTCCTTGACGC
CATTGACGATTGGTT
TTGCATAATTGATAAAATTTTCAAAATTTTGATCTCTTTTCAGTGA
CGAGTTGCAACATT
CATCATATAAACACACCAAAACATCAAGAAGAGTTTCAGTACTAA
CAGATTCCCCCTTAC
TATTCCTGACACCGCTTATATACAGACTTTCAAGTCTTTTGAGAC
GGTCATAGGGTAACA
TCGATGATGACATCTTCAGATGATGCCAAGTGTAGTAGTGATGAA
CTAATCCTCATTCAA
TGAATGATTCATCTCATGAATGATCCATTCCTCCATTATTACTAA

ATTAAATCAATCAGA
TCAATTGAATCATCATTATTAACGAAAGACTGAAACTTGATTCCA
TGCACATTTGAACGA
CATGAATCCAAGAGTCATATCACATTCTGAGGAATAACAATAATT
ACTTGGAATACCATA
GAACAGAAAAAAATTCAAAACAAACAACAGAAACCCGGAAAAAT
TGTTTTTGTTCAATCA
TTTCTCATTGCGATAGGTCATTTTTACATGGATAATGAATCTCGA
TCGGAAGAAAACTAT
GTAATTCGATTTAAATAGATTCAACAAAATATTATAGACATAGAG
CAGTGAACTTTAGAG
TGCTGGATAGTTGAATAACAAAAATTAAAATAAGGCATGAAAAC
AAATTCTTCTGATCAA
AACAAATTCTGATTCATTTTAGGCGAA
>ConsensusfromContig2849 Average coverage: 26.26
TTTTTTTGGTTATAACCGGTTCTTACGTGAGGGGTGCCCGAAACA
ACGAAATTGAGGTGA
AATCTGCTCAACAATACTTGGATTACAAGATTGGAATTTCAACAA
GTTTTGAATCCGGTT
CGGGTACGGACGACTGGATTGGAATCTCTCTACATGGAATACAT
GGCAGTACAAAAACAC
ATTGGCTGGATACTCCAAATCATGATGATTTTGAACAATATCAGT
TTGACGAATTCATCA
TTACGGATAAAAATGTCGGGCGTCTGGAGAAAATAGAGCTTTTC
AAAAAAAATCGTGGCG
AAAACAGACATCTCTGGATATTCGATTTCGCGATTGTCGGAATTG
CGAACATAAATAAGG
AATGGAAATTCACGTGTCATATTCATATGGAGGGAACAACACAA
CGATATTGTATCAGGG
ATTAGTTAACAGAATATCAACTTGAAATTTATATTGAAATATTGA
ATGGGGAAAACCATA
TTGCTTGGATGATTCTCACATCAAAAAAAAA
>ConsensusfromContig2850 Average coverage: 6.32
CGTAGGGTGGCTGTTCCCGTTTTGGTTAGAGATGGCAAACCGTGT
CAACCACAATTGGGT
AATGTTCTGAAGACACATGACCCACTCTCAATGCACGCATCAGGA
CTCACTGCTGGATTC
GGACACAACTCTTTCGGCTCTATGAATTTCCACGGTTCTCCAAGT
TCTTTTGGCACAATG
CTGAACGTTTCGACTCCGCTACCATCTATCTCATCATCTTGCATGG
CTTCAGCGCTCTCC
AATGGTATAGGAGGAGCTGTGAACATCAGCCCTCCGACGTATGG

CACTTACGGAGCCATT
CCCCCTCATCACATGAATGTCGGAATGGGAAACATGTTAAATCA
GATCGGAAGAGCG
>ConsensusfromContig2851 Average coverage: 5.03
TTATACACTCTGTGACGACATCGGCGATGAACGATGAAGCTATGC
GATCAGCATTCTTCA
AATTGTCAACTCCGACTTCATGGATGCTACGATCAGACATCAAAT
TCAAGGCTTTCTCTT
GTGCAGCCAAATAAATTAAGAAAGCCTTCTCGGTACAACCTATTT
CTTTCAGAAGTCTCA
TCGGTGTCCTTGACCCTTTTGGCGTACGGCGGAGGTTGCGTTGTG
GTGACCCCACTATTT
CAACCTCCAGCGCCGAGGTCAGTTCTTCTAAAAGTCGCGCGATTT
TGTCGAGAATGTCGC
GACCAGCCTCATCATTCAAGTCGTCCTCGTAATGCTCAGCTTCCCT
TTGGATTTTTTTCA
ACAACGTTACAGCTTTCTCGAATGATCTGTCATGAATATGAATT
>ConsensusfromContig2852 Average coverage: 5.81
ATAGCATCAAAATACAGATCATATGACGATAGAGACAAATGTGA
TTTATATACGCATAAT
GACCGGGGAGCTTTTCATGTATATGGGCAACTTATTAAAGTATCT
AAGGGCACAATAATT
GAGTCTTGATATCATTTGTTGTTGAGATGTTATGCGATGGTGAAC
AATTTTGACAAATTA
CTGTCGTGATATTAGGGATAGCTAATTCTATAACCAGGTAATACC
ACTGAAGATCCTTTG
GATATCTGCCGTTGCAAGAGAATGTGTATGCGTCATTTCAGTTCT
GTGTTGTGCGGGTCT
ATTCAATAATCTAAGATAGATGTCCGTCTCTCTCCTGTAGGTCCC
CGAATTTATGTGAAA
ACACGAATATTCGATAATGAATATACTCACAGAATTCCGTTCAAA
ATACAGAAAAGTTTG
CCCTATTCGCTTGGTCCCCTTGTAACTAAGCCCATTGTCATACCCT
TGACTATTGATATC
AGTTGATTCAACATATCCTGAAATGTCTTTGCAAATT
>ConsensusfromContig2853 Average coverage: 13.68
GCAGCGTGGAGGTCGCGCCTGAACGCGTGCCAATTTGTAAATTTG
AAAGAGCGTTTTACT
GTTTTGTTTCTATTTTCCTTTACAAACGAATTGTGGAGCTTCAGG
TGGACAGGAGTGTGG
TCGGAGCCTATGTCTCCTTGAACAAAAGTCCAAACGTGCTGCAGT
ATAGGGCCTTTAGCA

AAAAATAAATCAAGAATACTGGGAGAACAATTAAGACGAGAGGG
GATGTACGTTGCGGTC
CCGTCGTGATTAATAAGCTCAAAGCTTCGGGCTGCCAAATCGAGA
AAGAACTGGTCTGCT
CCTGGGGAGTTTGTCGTGTCTCCAGCTAGGGAGGAACGTTTATTA
AAGTCACCTCCGAAA
ATGACATTGGGTGAGTTAATCGAGTTGAGGATGCTTGTGTCAATG
AGTTGATTTTGTGGG
GGGTTATACAGCACTACAAATGAAGAAATATGATTGTTTATGCTT
ATTTCAACGGCAACA
ACTTCTGACTGAGAAGGAAT
>ConsensusfromContig2854 Average coverage: 8.86
AACTCGGCGTCAATACAGGCGCTATAGCACGAGAGACCTAGCGC
GGGCCGTTGAAGATGT
AGAGAAAGGTACTTCTATCTTAAGAGCATCTCATGTTTACGGCAT
TCCTTACTCAACTCT
AGAGTTTAAGGTTAAGCGGCAGGACAATAAATCTTGTCGCAAAA
ACCTCGATAGAATTTC
AAAATCAAAGTTGAATGACCTGAAGGATAAAAATACGAAAAAAA
TTCAAATAACCAAACG
AAAAAAAGTTACTGAAAATCACAAACCACCTCAGCAGGCAGGTG
CTTCGAGTGGAGGTAG
ATTTAAGAAGAGTTCCA
>ConsensusfromContig2855 Average coverage: 15.53
CGACCGACGATGCGATCAAACCAGTTTTTGAAATATAGCCCAGAA
TTTTCTCTTGTAGGT
CATCTTGTACACCATCCAAATCGAAAAGAACAGCGAAGTTGGTA
AGATGATCGCAATTGC
AAATTTGAAGTTCACCACGGATGCCACTGAGCCAACAGCCATGAT
CGTTCCATTCACCTT
GACCGTCCAGCGTTGTCGAATCCCAAAAAACACAAGTGCCGTCCT
TGCTATATAGACTCT
TATCAGTTGGCTTGAACAGCAGTTCTAATGGTTCATCCAGATGTT
TAAATTCTGTGATCG
AGCCAAATATATCGCCGATCACCACGGACATAACCCGTGAACCCA
ATCTGAATACTTCAC
TATCGGAATTGTTC
>ConsensusfromContig2856 Average coverage: 44.10
GTTCTCTTAGCTACGACTTGAAATAATAAAAATGTAGAAAAATAT
CAATTTCGAATGATT
TTTCTTCAGCCCTTCTCATTTAAGAAGTATCCCTGACTCTGTCAC
AATAATGCAATGCAG

CAGCAGTACGCATATCGCAAACCTGCATCGACGTGGAGAATCAT
TCAGGAAGTACTCCCG
TCCTATGAGGTAGAAGAATTCCGAATTCTACTTGGCGAAGATCTC
ATAAATCAGACATTG
GAGCTGCACGAAGAAGTTGATAATTCTCTCTATATATGGCGAGA
AATACAGTCCGAGGTG
AACAACAATTTGATGCATTATCGTCTTCCTGAGCCCCCGCATGTG
AGGGAGAATCTTATT
GAGGAAATTAAATTATTCGCAAGTACTCTGAACTCAAGATCTGG
AGGCCAAAAAAATATT
TTGGGCGATAATCATAACCCGGATGTTCTCGATTATGCTCTCAGT
ACAGACAATTCGTCG
ACCCGATTGTTCGGTTCGCACAGCATTCGGACATCACATCTTGGC
AGTGAACGATTAGAC
AGAACCGACTCCTGCCTGTCCAATGATGACTTGGCTTCGCTACGT
AATGGCCTTTCTTCC
ATCGAAGCAGAGAATTCCCTGGTAAATATAAGGAGTGCTCTTGA
AATCGAATGTGTGCAG
CTTCTCGAAGATATAGATTTCATACAGAATTGCATGCATACCACA
ACTGATCAACAAGAG
AGTGAGGCTCCATCTATAAA
>ConsensusfromContig2857 Average coverage: 19.83
AGAGATGTTGGGATCGAATATGGAAGAAGATAAATTTGCATTTG
TTGGAGAAATGTTGGG
TAGAAATATATGAGGAATAAAAGTTGCATTTGTTGAGGAAATGT
AGAAGAAGAAGGTGGG
ACATGCAAGAAATATAGAAGAAATTGGTGGGTTCAGGATTTTTTT
TTCCATAAATTGGAG
AAAATAAATAGTTTAGTAAGCATCTTTTCATTTGATTTTTTGAAA
ACCTCTCATAACTGC
TGCATTTCTCACTGACATTTTGTTTTGACGAGGAGTTAACTATTA
TTAATGATGGGAGCT
CTTTGTTAACGGTATGCTCGATTTTTTTTATTGAGGTGTCCTCTAT
TACAATAGTATTTT
TCATTCATGAAGTGTTGTTTCTTCGCATTCTCGGTTGAGTTTTTTG
ATAAAGTCTATATC
GAAATCATAATATATTGAGTCATATTATTATCACTACAGCAAATC
TATCATTTGCGTATA
CTCGGTGATATTATTCGGGCATTCCTCTGCTAATGTATGTAACACG
TCTATTTTCTGCTC
TACTCACCTGTACAGCTAACACATGTTTCGATGATTTTTGTATAT
AGACTTTGCACGTGA

AGATTCCTTTTTGTTGGATGTCTACTATTTTTACCATTTGATCG
>ConsensusfromContig2858 Average coverage: 7.58
ATCCGATAGACAGAGCCGAAATGTAATGCTTCTCTTTCGGTACCT
CAAACCCGGGTATGC
TGGAGAAATGTCTCCCATTCCTATAAAACTTCGCGAAATAAATAT
TTTCAGGATTCCAAA
CTACTGTTAAATGTGTCCATTGACCAGACTGGACTCCGTCATATA
TTTCCCCAAGCCAGG
CATAATTTTTCAATAGAATTCCCACATGAAGGAGATTTTGTTCGT
GTGGCCGAGCTGCTA
AAACAACGCCATAAGTAGACTTGGAATGACCTCCACTCTCGAAGA
AATACTGGGTTTGTC
CCTTGTGGAATTCGTACTTGATAGCCATGGAAAGGGTGAAGCCAT
TCACGCATAAAGCTA
AGTTGTGATAGCATGTATCACGGAGTAGGGAGCCGTAAAATATAC
CTGCTTTTGGGCCGA
ATCTCAAGGCCGTGTTTTGTCCACATTGATCAAGGTCGGTCGCCA
CGGAGGTTCCATTTG
GCATGATTGTTCCATCGACATCCCCGATCACGTGATCACCTATCA
GTTCTCTCATTGGTA
GATGGAGTATGTTGTCATCTCGACCAGGCTTTATACTTTCATCAA
TGCAGGTAACTCCAT
CCTTAGCTATATATTTTCCAACGGGGCAGTTGCAAAAGAATTTGC
TATCTTTCGTGATGC
ATTTGTGCTCACATACTACTTTTTCGCAATAGTTTTGTGTTATACA
TGGT
>ConsensusfromContig2859 Average coverage: 16.96
CCTACATCAAATGAGACGATTTGGCACAACACAACGGGACTGTA
AAAAATGTGAGATAAA
TTCGGCCCTATCATATTGAAATGAGAAAAAACACTACAAAATTGT
GGTGAAAATGTATAT
TGCTCTCACGAATAATGAAATATTTTCACACACGGATTTTATGTT
ACTATAATAATTATG
ACAAAGTTGCCTACACTTTGAAACACAATTGTTGATGAATATGCA
TTCATTATTGTTCTA
TATAATTCGCTTCAAATATATGAAAAAAACAACGGAAACATAGTT
AATTAAGGTGCTAAT
ATTCATACACACAACGCCATGACACACATTTTTCATAAATATAAT
TTTACATTATAATTT
GATTCAAAAAACACCAACCCAGTGATATACATTTACCCATCCAGA
TAACTTAGAAA
>ConsensusfromContig2860 Average coverage: 14.56

ATTTTCTGTCAAAAGGTGAAATACCTCAACACCAGAGTCGCAAA
AGGAGACAAATCAAAC
CCGATGAACGGAATGTTCATTCCAAATCATCAAATGGAAATAAT
AAAAAGAATGGACCAG
CAAAAACAGAACAGGAAAATATATTCAAAAATGAGATAAAACGA
ACAAAATATATGGACA
GACAAAATCAAAGGTCAAAATACACAATGAGACGAAAAATTGAA
GCGCATGGTCGTGGAA
CTCAATCTCGTTCGAGGCGATCGGCTGAGAAAGAAGGAGTTCTA
GAAGAGCCGGTTATGC
TGGGGATGGATGTGATGGCTCCAACAGACTTTGGCGGAGTTGAGG
AATTCTTCAACCCAA
CACAATGGATGTTTGATGAGGAAGAGGCCGGTGTCGATGTGAAC
TATGATTGGGCATGCA
AATGCGCGGCTGTCACTGGCTCTAACAGTTTTGCGCCAGGAACTT
CAACTGAAACAGAAA
ACCCAAAAGAAGGACCTGAGTTTCAACAAACAAAATACGAGATA
GAAAAACAGTATTTCC
TGAATAATCCACCCGCGCCAGCTTTTGAGGCTGATGAATTATCAA
AGTTTTTCGAGGACT
TGGGTCACCAGCGACGAAACATGATATATTCGTGTGAATGGAGTG
GAGCGGTTTGTTCTG
AGGCGGATTTTCAGTCATCCGTTCTGGA
>ConsensusfromContig2861 Average coverage: 38.38
AAAAAATCCGAGTCTGATCATGAATCATTCAGCTTCAAGTATACA
GGCCCCTCCACTGCC
GTTGTCCAGGATACGACTCCGAGAATCAATAGAAGATGGCTCAA
GGAACAGGTCGAGGTG
TTTTTTCCTGTTGAGGGCCAGAATGACCTTGGCCTTGATGCAGCG
AATTTGAGTGTTCAA
CTGCTGGATCTCTTGACGACGCAGAAGTCGAACGAGGAACTTCAA
AATGACCTCTTTGAA
TTTCTGGGGTTTGATCGTTTCGAACTAATTCAGCTGCTTTTGGATA
ACCGCAAAGCTCTT
TGTGAGAGTTTGAACGAGAGACCGCAAGTCATGGCATCTTCAAAA
TCAGTTTCTCGTCCG
GGTCCCTCCAGTTCCTCTGGAGCGTCATCTATATCATATTGCGAC
ATGGTTACTGTTCAG
ACGTCATCGGAAAAATCACTCAAGAAAATATTACGGAAAGAAGA
GCGAAAAGCGTCCAAA
TTGGGTCAAGAGTTCGAGGACGCGACCAGGGTCAAGAACAGCGG
ATATGACCCCGAGCAG

TTGAGAAGGGCAAGAGAGGAATCTCTGCGACCACGTGATCGTGA
CGTCACACCACCGACA
TTCATTTCTGGGCGATCCCAGAAGAAGGCTTATCCCTTCGTTTTC
GACCAGATGGCGGCA
GTGCGGGAATCAGGGGCTCTCATTGGCGGAAAGAAGTTGGCTCT
ACCGGATGGATTTACG
AGAAACAATGACACTCTGACAGAAGAGGTCAACATTCCCCCCTC
ACTGCCCCCACCGCCA
TCTATTGGAGAAACCAGGATCAAGATAGCAGATCTAGATCAGAT
CGGAAAACTAGCTTTT
GTCGGAACAAAGGAGCTGAACCGTATCCAATCAGTGATCT
>ConsensusfromContig2862 Average coverage: 7.18
AAGAGATATGATATAATAAGACGGAATATAAATAAAATACATTC
GAAACAACTGCACGTA
TTTTCGTGAAAAATTGCACATAATAAAATAGCTTGGATGCATGTA
AGGTTGCTCTATCGA
GTGATTCAACAAATCATGCATCGCTGGGTGTCTCTCTAGTATATG
CATAATCTAGCAAAT
AGGCCCCGAGCACATCTAGAGCGTATAAATTCATTATCAGATCTC
TTAGGAATGACGGGC
AATAAATACGAATGAAGCTTCAGGGCAGTGGCGTAATTGCATGA
TGAACCGGTGATGATA
TGAAGTTAATGTGACAGCATTTTCTTGTTTATGAATTCTCAGATCA
TTAGCTCCAATTAT
CTCATTGTTTTAATCACTCAATGACATGATTAGAATATACAGTAG
TCATGTATTCTTTGA
GTTTCAAGGACGAATAGTCGTTAAATCCTACAATTA
>ConsensusfromContig2863 Average coverage: 14.10
TTTTGAAATAGTATTTTATTTCGCCTGAAGAAGAATTAAAATTAC
TGTTCTGTGCTACAT
TCAAAGCGATTTGAAAGTGGTAATTAACAAGAAGAAAAAAAATC
TTCGATTGTTTGAAAA
TTTGTACTGGAATCAGACTGCATGCTGGTACATGAGAAGTGCAGT
GACTACACAAGTATT
CGAAGAAAAGTGAGAAAGAATTTTGTAGTGTCATCAGCCTCGTTC
AACTACCGTTGTTTT
TGGGGGAACTTGAATAATGAGAAGGGTTTGAAAGTATGAAGTAT
AAATATTACAAACATC
ATATATGCATGACACTGTTCTACTTGCAGAGTCAGCTGAAGATCT
CT
>ConsensusfromContig2864 Average coverage: 29.43
CGAACCCATTTGAGTGCCTCGAGTCCATCCCAAAGACCGATGTTT

CCAGGGATGGTTTTC
TCTTTGCTTTGCAGAAAACCTAGAGTATTCAATCTGTAGTTGAAA
GTGACGATGATTGAT
CCAGAGTATGTAGATAACTCTTCTCCTGGGTATAGATTACTCAAT
CCCATCATGAATATG
CCGCCATATATGTACATCAACACGGGGGTTTTTCCATTCTCAGAA
GATGATCTGTTATGT
GGCACATAGACGTTCACGAACAGGCAATCTTCACTCTGCTTTTCT
CTCACCCCTAAGACA
GGCAGAAGTGGACTCATTATTTGCTGGCAATAAGAACCAAAAGA
TGAGGCATCCAGCACA
CCTGCCCACCGCGTCATTTCTTGAGGTTTCTCCCATCTCAAGTCTC
CAACTGGAGGCTTC
GCGAAGGGTATCCCAAGGTACGCATCGTAATACTTTCGGTCGGGA
AAATTTCCTGTGTAT
CGGGTAATACCTCGTAGGTAGCCTGATGATATTTCGAGAAAAGGT
CGCTCTTTTTTATTG
CCATAGCAGAGTACAAAAAACAGACCATAAACGATGCAATATCG
CATTTCAAAATGTTTT
CTCGACTGTTTTTTATAT
>ConsensusfromContig2865 Average coverage: 43.98
CGGGAATTAGCAATCGATCTAAACAAAACAAAAGATTTCTTTGTT
TCATCATATTTAGCG
CTCCAATAATGAATCATAGACTCTATGATGAATGCTGATCCTCAA
CGAAGAGGTGTTAAG
GTGATTAAGACTGTCGTTCGACCGCAGAGAGTAGTGAACAAAACA
TTCATTAACTCTGTG
GCCAACATAATAGATGCTTATCAGCAGGGAAATTCAAAAGTTGAA
AGTGATTCAATTCTG
TGGGCTAAATTCGAAACCACAGATTTGAATGATGTTGTCCGGCA
CCCGGACCTCAGAGGA
GGGACCACACCCCCTCCTCTTGTCCTTGCCCTTGGCTTCAAAAAC
AAAGTTCAGGTTTGG
AGCATTCAGGCCAACGGCGAAGCACATGAAGTTTTCACTCTACGA
CAGTCTCTCGTCAGG
CATTTCAAAATCCTGCCTCGGCCAACGAATGTATACGACCGTCGA
GATGCATTTTCACTC
AAGCGACCCCTTGTCGCACTCGTAGACTCCAATTCAAATTCGTCG
AATCAATGCAGTGTT
CGTTTTGTGTCAACCAAGACCGGAAATGACGCACACAGCATTAGT
TTTTGTCACGTGATC
TCAGATATCAAGGCGAATGATCGAGTGCTTTTGGTGGCCTTCAGC

GAGAGAATAGCCGCT
TTCGATAGCTGCCAGCTGAAACAAGTCTTTTGCGTTGTTTCGTGT
TTCCCAGGAAAAGCG
CCGAATGTCAATCCGATCGCGATGGGTCACA
>ConsensusfromContig2866 Average coverage: 20.12
TTGCCCGATAGGAAAGTATTGCTTGGAAGGAACGTATTCTCCTTC
ATTTTGTCCGAGCGG
AACTTGGTCAAATGAAACTGGTCTTCAAGATGAGTCCAGCTGCAC
AGTCTGTCCTGCTGG
TTCTTACTGTCAGCAACCAGGTCTTACAGAGCCTGAAGGAGAATG
TCTGCAGGGGTACTA
TTGTCCAGTCGGATCATCGGTGATGAATGCGGTCATATGTCCAGC
TGGTCTGCATTGCCC
AACAGGGTCCGATGTGCCTTTGGCCTGTCCTGGTGGCTCTTACAC
CAACAGCACTGGCTC
CAGAAGTTGTCTCACATGTCCTCAAGGGTTCTATTGCGTAGCCGT
CAACCAGACCAATGC
GACAACGGTGGATGGTCCCGGCTACTTCGACTGCCCTGCCGGCTT
CTACTGCCCTCTAGG
GACCGGTGTCGACTGGATGCCTTGCCCGGCCGGAACATATAGCAG
CAATACCAATCTTTA
TAGAATTGAGCAGTGCATTGACTGTGACGGGGGAAGCTATTGTC
TCAACCCAGGCCAAAC
TAACACAACTGGCCTTTGTGCAGCAGGACATTTTTGTACTTCAGG
AGTGGATCGACCGAA
CCCAGACAACGCAGCGAGCAACAGCTCTTATCCACCCAGTTGCCC
GCTCATCGGTGGACA
CTCAGGTTTCGGGGATGTTTGTCCCAGGGGAGCAATGTGCCCAGA
AGGGAGTTCTGAACC
AATTTTGTGCACTGCGGGAACGTATCAGGATTCTGAGGGTCAGA
GAGAATGTCTGGAGTG
TGTTTCGGGTTTCTACTGTTTATCGAACGCGACCGACTTTATCGA
CAAGCCGTGCCCCTC
GGGTCACTACTGCCCCCCTGGGACCCCCCACTCGGCGTCGTTCCC
CTGTGGTGCCGGCAC
GTTCCAGCCATACACGATGCGAACGAATGAATCTTCATGTCTGGC
TTGTACACCGGGGAT
GTACTGTCTGGGAAGTGGGCTTAGCGCCC
>ConsensusfromContig2867 Average coverage: 37.82
CAGAGACACTAAAAGTGGCGGTCGGTTTTGCAACGATATCACATT
TAAACAATAATGTTT
TCTCTCCGTTTTTCAAAGCATTTGTGTAAATAACAGCTTCCTTCGG

ACAGTCCACCTCTG
GGGCGAAATCCTTGAGCCCGATTTTGAATGACGCAGGCAGTGAAG
GGTTTTTCTCATCGT
CCTCGAATTCAGGCACTTGACACTCGACGGTTTTCTCGAAGGATT
TCATGTCGACTTTGA
TGTTCTCCTTCAATGCCATTTCCACCTCGTACGTGCTTGTGTCATC
CAGTCTGTCGGTTT
CTGGTACCCCCGTACTTCCGACCTCCACAGAGCTATCAACATCAA
GATTAACCCCTCCCA
GTTTCAGCTTCCCAGATACTTTGCCACCACGCCCTTCAATCTTGC
AGGAGACGTTCAGAC
TCGACCCCTTCGTCAACAAGACTGTTGCCTCATCATCACCTGCGT
>ConsensusfromContig2868 Average coverage: 17.78
GATCTGTCGAATCCGAAGCTGTTGTTTTAGAATCCTTCAATGAAT
CCAAATCAGATTCAT
CATGACTGAATTTCTGATGGCCTTCGTCAATAGCCGCATGTTTTA
ACTCATCCAGCAACG
TTGAATTCAGGAGGAAGTTTTTCTGTTTTTCTTTATGGTCTTCATA
TACAGTCGAAACAC
TGGCAGCGCTTTGGGTTATTGTTTCTTTCACATCGAAACCATTCGT
TCTACATTCTGCGA
TAAATTCATTTCTATCAGACTTCAAAACAAATGCATGGGTTGACT
CGAATGGTAAGCAGC
ACGCGTTAGTATTGGAGTCTGATCTGGCACTTGGCAATTCTGAAC
AAAACGAATTGTGCT
CAGTTTCACAGTTTTTAACTGCATCTGGTTCATGTTTGTTCTCATT
ATCATGACTTTGGG
ACTTTGACTGAAAATTTTGGTGAGATTCATTTATTTTCTTCAAATT
TTTGTTTTCAACGT
TGTTTTGAGCAGTAATCTCATCACGAATTGGAAATTTTCCACACTG
TTTTGTGATACTTT
TCTTATTATCACAACTGGTTTTCATATCATGAGCCATTGATCTTTT
TTCATTATTATTCC
CCATTAAATCTCCTGAAAAAC
>ConsensusfromContig2869 Average coverage: 5.50
CCAAGTTGGGATTTTTAGCCAATGCAGCCCCTATATATGCCGGTC
CCATGATCTCGAATC
TGTTATAGGACAGGTTGAGATCGAGTAGCGACGCACATTCAGTGA
TGATGTTTTCAAAAT
AGATGCCACTTTTATCTTCAAAATTATTGTGTGATAAATCCAAAT
ACTCGATGACGTAGC
TGTTAGCAAGGGCGGTCCATAGATGCTTCGCTGCCTCCAGAGACA

ATTTATTCCCGGACA
GGTTCAAATGAGTTATGAGACTGCTTTTCGATAGGACGGTGCTCA
GTGGTTTCAAGCCTT
>ConsensusfromContig2870 Average coverage: 18.04
TCTTGTGTCTGTTCATGTTTACTTTTTGCCATCGTTTCTGCTATGA
CACGAATGCCAAAA
CTCTCACAAATAATTTCTTTGTAATATTTCCCGGCATTTGAAATC
ATACATAACAGATTC
AAGGCTTCAAATTTCTCAGTATCTTTCGCTTGGTTGAGAGATATG
AATTCTAGTGTTGTG
AGAATACAGCCGACTTCTGCGAATTCATCGATGAATTTATGCCCA
CTCACAGACCCAATG
AAAACCTGCATAGCCTGCAGGAGACTCGATATATATGCGTTATTC
ATATATGATATCCTG
AACCAGGCTGTGAGGCGAGTGAAAAATAAACTGGC
>ConsensusfromContig2871 Average coverage: 6.93
AATCAAGCCAAAAGTCTTGATAACAGTGTTATCACGAAAAAGAA
AAATAATCATTTTAAT
AATAATGAACACAGTAATCCGAGTATCCCCCATATAGGTTGTCAT
GATTATGTACACATA
AATATGTCTAATGGCAAGACATCAGCTGGCTCTGATTCATTCCTT
ATCTCAAATACCACT
GGAGAAAACTACAGCGATAATGAAAACAGGATGGAGACTGAATA
CAATCACGACCCGAGA
AATCATTCCTCTCACCGTATTCGTGCTATGTCAAATGATGAGAAA
AGAGGACTGTATCGA
GATCTATGGTCACTCCGAACAACTTATGAGGCTGATGAGAGCGA
CAAAGCCATACCAGTA
ATGTGCGTGATTCCAGAAATATCGGGGAATGATTGTGAATATACA
ACTCAGAATTCAGTT
TCGTTTGATGAGTCTAGTGTAGAGTCGACATATGACAAGGATCT
AACAATAAGATCGGAA
GAGCGTCGTGG
>ConsensusfromContig2872 Average coverage: 3.37
GGAAAATATCAGAGTTGGAGAGGCAAGGGAGCATATTGTGTTCA
TAATGAGTGACAATAC
ATGTTCAAGAATGGCTTTTTACAGGTTCAGAGGGAATGTGTTAAA
AGATGTATCTATACA
GGTCCACAGGTCATAGGAATGGTAAAAAAATATCAGAGTTGGAG
AGGCAAGGAAGCAAAT
TGTGTTCATAATGAGTGACAATACATGTTCAAGAATGGCTTTTTA
CAGGTTCAGAGGGAA

TGTGTTAAAAGATGTATCTATACAGGTCCACAGGTCATAGGAAT
GGTAAAGGAAAATATC
AGAGTTGGAGAGGCAAGGGAGCATAT
>ConsensusfromContig2873 Average coverage: 13.35
TGTGCTCTTCCGATCTGCATGTCTGGCGGGGTAACAAGCCTTAGA
GACCAGCATGGATAT
TTCTCCGCCAACTCTGAGACGGACTGCAGGACGCAAACCCAAGTC
TTTGACATCAAGGCG
GATCTAGGACAGCGTATCAACCTCACCGTTTTCAACTTCACATCT
GAGGGTTTCTCGACT
TTTGATATGTATCAAGACGCTGCCCCAAAACCTTCGATTGGGGAA
TCATACGATTGGATA
CCAGGGGTTAACTGCATACCTATGGCCAGAATCAAAGAGAATGG
AGACAATAAGACTTCA
AAAAACTTCACAGTTTGCCACATGCCTTTGCAGAGAAGACAGAG
TTATATCAGCAGAACC
AATGATCTCCAAATAGCGGTACTCCCCAGTGCACTCCTTGGGAGA
GTCACAGGACAATTT
TTTTTCGCATATCAGATTATTGGCTGCTCCAATCCCAAGCTGCCG
CCTGGGTCCCACATT
GTACGTGCAGAAAATGTTGCCACCATCCACTGTAACCAATCAAAG
TACTCTTGGACAATC
ACGTGTCAACAAAACCAATGGTTCGGCGAGAATGGGACATGCTC
AGGAGGAATAGTTCCT
CATCCTCAAAAACCAGCAAACGAAGAAACTCTTTCATATGGATCG
TCGATTATAATAATC
ATGTGTGTCGCTTTATCTATTGGAATATTTGTACTCATATTGGGTC
TAATCTTTCTGCAA
AAGCAAAGAATAGGATTATCATGTTTGCCAAATCCGGAACAATC
GGCGAGCGAAGTCCTC
GCGAACGGAGATGCCCCTTTATATCACGACTTTCAAGACCCCTAC
AAACACAATTCCACG
TGGACAATGCGGACCCCTCCATCCAACAAAAGTTGTGGAAAAAAT
ATTGACTGCATTTAC
GCGTCTAGGGCGTCATTAGGCCGCAGTCGCCGACCCCTGCTTATG
AATGATTCCGATTAT
ACATATGGTAGGACCGAGGAAGTTTTAAACACATGCGTCGATTGT
TCAATATATGCTACT
GCTTTGAAGCGTTCAAATCACTCCCAAAAATCATATCATCATAGA
ACTAAGTCATTGGAT
GGGAACAAAATATTAAACTCGAATAACCTAGATAGAGCCTGCTT
ACAGCCAGATATTATC

TGTAGTAAACCACGTATTTCGGCCAGAACAATACAAAATCGTGCA
AACTCCAGCAACTCT
TCAACGGTATACGATTCAACGCCACTCTGTTCAGAAAAACTAAAC
ATTATTCGAAATTAC
GCAAAATGTTCACCTTTTGAGGGACGTTTGTGCGAATGTTCTCGT
TTCGAAAGTAATTGT
G
>ConsensusfromContig2874 Average coverage: 21.71
CTAAGAAAGTTAAACAGAGTATTAAAATATTTTTCATACTTTTCA
AAGTCTATTTTCTAG
TAAGAGTTGATATTGCTATATCAAGTGTAATCAAGTCTACTATTC
ATAATAATGCTATTT
CTTCATCTACTTATAAGTGTGATAAATACTCATGCCCTTCAAGTA
GGGGCCAATGCAGCA
AATATAATACTTGTGAATGTAATAAAGAATGGAGTGGTAAAAACT
GTTCTTTATGCAACG
AAAGAATGATAATAATGAATGGATTTGAAACTCACGGTTATATTT
CTGATGGACCTTCAA
TGTACGAACATGGAGAGAAATGCAATTGGTTGATAAAAGCCGAA
CCTGGATTGGTAATTC
GGCTCACAATCCATCATTTCTCCACCGAGTGTGGCTGGGATCATC
TCTTCATTCATGATG
GCTCCAGAGTTTCCGATCCCATCGTTGCTGTTTATAGTGGGACTC
TCCACGACAGCACTC
CGGAAATCTCCGCATATTCAGG
>ConsensusfromContig2875 Average coverage: 6.61
CGAAAGTATAACGGAATCTATCTTTACTCATCACAAAATCTCAGT
CATCAAAAATTGCTT
GGTATTCAAAAAAATTTTAATCCTGTCTCAAAATGTTATATATGT
TCAATGGTCACGAAG
GCTTGTCCAAAAAGGAGTGAAACGACGGCAAAGCTCAAAATACTT
ATCCATCCCGATTTC
GGGGAGGCGGAACCAGAACCACGATTGAATCCCGTATTCGTAAAG
GTGTTGCCTCCTCCT
CCCGTACCCGTTCTTCCACCTCCAGTATTGGTCTTTCTTGATGCTC
TCGGTGTGTTCTTT
TTTGGTGCTCTTGAGTTGCATTCTTCCAGAAAGCAAATCAAGACA
ACCAAGACTGTTGCC
GTGATAATAATAGTTTTTGAAAAAGACATATTCACAAGATTGCCG
ATAGAAAAAATATCT
AGTCAAAGTACAAAATT
>ConsensusfromContig2876 Average coverage: 10.38

TTGGTCGGAGGCTGACTACGAGGAGAATTCGAATCATTTGGGGAG
AGTTTGGTTCAACAT
TTCGTACGAAAGAGCGACTGAGTGCCTCAGAATTGAGAATATAA
AGGCCAGGAATTTCAC
AAGTCGTTCTAACACAGTCAAGAATTTGTGTAACCCAAGGCTAAC
AATAACACTTCTTCC
TTTGGAGCGATCCAAGATTCGATCGAGAATCTATCGAAGAACATG
TCATCCTGTTCTTGA
CGAAAATTTCATAATGCAGGTCCCACTCGTACAATTCAAGAAAAG
AACGCTCAAATTTGA
CTGGCATGACTATAAATCATCTAAATCAAAATTATACCTTGGCTCT
TCTGAATTTGTTCT
CCAAGAACTAATGCAATGTGACTCACAAGAGACAGGTTATAGAG
TTTGGGCAGATTTGAA
AAAAGATCAACAACCTACTCTTGTAAATCCACAAATATTCTTAGA
AATGTCGTACTGTCA
AAACACTGAGCGACTGAATATTGTTTTCCTGGATCTCAAAGATAT
TAACAAAGACGATCA
TTTCAAATTAAAAATCAAGCTCAAGTCGGGTGTTTCCAAATGGAA
TTGGACGAGTAGTAA
TTTTAAAG
>ConsensusfromContig2877 Average coverage: 8.94
TCCTGGTAAGTTCATTCTCTTTGATACAATTTATCTTTCCATTTAT
GGAGTTAGACCTAG
GCAAAGAAGAGCTGTAGAATTTTCCACAATTTGTCTGTAAACTGC
AGGGCTGTGTTGCCG
GTTTCCATTGAGTAAATATCCGAGAATTGTCAAAGCTTTTTTCAG
TGAAGTTTCCTGCAT
CGGAGGGATTTTCCTGTACATTCTGAACTTTTTTCTGCATTTCAGG
ATCTAATATGGCGT
ATATTGGCTCTGTCAGATTAGCTTTGTCCCCTTTCGAGTTTTTATG
CAGATGATTCGAAT
ATTGCTCAGAATTATTTGGCTTTGAATAAACAGGACTTGTATCTT
GAATAGACGAATACT
GATCTGGAGAATCGGTTAAATATGCTGATTTGTCATGTCTGAAGA
AATTTATCTCTGAAT
AAATGGATTCAACATTGGTAGCATCAGTGGATTTTGTTTCGTTTT
TGAATGTTGAATTCA
TGGGAACGCTATCAAGAACCAAACTATTCAAGGCATTATCAAATT
CTTCAGTTGAGAAAT
ACT
>ConsensusfromContig2878 Average coverage: 14.59

TGTCTCTCGTAGATAGTTCCCAAGCGGTTAAGAACCTCTATTGTC
TCTGGATTGCAGCAG
CGGAACTTTCTCGAATGATTGAATGCGCTGTAGATAATCCTGACG
TCATCGGCGTTCTCC
TTCTCTGAATTCTCCAGAACCTTTTCCAAAATGGATCCTGAGGGG
GCATATAGGCCAAGG
CTCCCAAAGAAATCTGCCAGTTTCCGCTGAATCTTGTCCAGCCTT
GATTGGTCAATATCA
CTCTTAGATAATGATTGGAGGTAAGCTTCGCTGGCATTGTCGTAC
GATCCAAGCAGAGAC
CAAAATTTTATGAGCATAAAAGACCCTTCCAGATCGGAAGAGCA
CACGTCTGAACTC
>ConsensusfromContig2879 Average coverage: 16.14
GTTTCTGTTCGTTTATTTTGTTGTCTTGAAAGAGGTTGTTGTTGAG
CATGAATTCCGAAT
CGTTGGTTGAACATTGCGGAACTTCATAGGTGTCGCATAAATCAG
TCGCGTAATCGTTGT
TTGATCGCATTTCCAGGGGTGTAGCCTGCAGGGCCATCGATTCAG
GGCGCACCCTCTCTT
CCACGAACAATTCTTGACGGTTAGCCAGTTTGGTTCTGTAATGCT
GGAGTACCAGTAAGA
GGACAACCATCAGAACCGTTACCACCACGAACAGAACGATCATA
ACAGCCCACGTCGTCG
CTCGATAGCTGTGGTCCACCAAGTGGTCTCCCCCAAATAACGCCT
TTGTCTCCAAAGATG
TTGAGGCATCTTCCACTATTGGTACCAATGGAGCCAATGTTTGGG
AGGATCCTAAGGAAT
TCCGTTTCCCGGGATCCATGCTGTTTGGGGTCCTCTCAAGGGTAA
C
>ConsensusfromContig2880 Average coverage: 4.87
TGCGGGATTGATATTAAGTCCAAGGTGACCCAGCGTGCATGTTAC
TCTGTCCACTTGTCT
CTGCAGGCCATCAGCCGTTTCGGCTAATAACACAAGATCGTCGG
CGAAAGCCAGGTAGTT
TATTCTTTCACAACCAACAGACACCCCAATGGAGTGATCTGCTGA
TTGTAAAGCTCTGTC
TATCACGGTGTTGAACAGGACCGGAGATATAGGGTCGCCCTGACG
GACTCCCCTAGTCAC
TCCGATCCAGTCTGACTTTCTGTCGCCGATTCTAAGTTGCGTTCTT
GTACCCTCGTACAG
ATGTTTGGTGTACTTTATTATTTCGTGTGGGACGCCGGCTCTCGTC
GCAGCTTTTATTAT

TGATTCATGGTTAACGCTATCGAAGGCTTTGGCCACATCTAGGAA
GACAAGGAACGTTGA
TTTGAACTTGTTTTTGCGGGAATCTATTGTATTGTGAAGTAGATA
GGTGTTGTCAACAAT
>ConsensusfromContig2881 Average coverage: 5.24
TGCAATCGTAGCGGCGTCTACAAAGAACGAGGAACAGGGAAACG
AAGCCTGAAAAGTCAA
GGCTTGTGCGGAATTTGTGGATGTGTGTGCATATATACAAGAAAG
TTAGTAATGGTTCTG
ATGATATAACGGTTGATTATTGTCTTGACCATTATAGCCATGAAA
AGAATTCAAATCATT
TGCCCTTATCGAATCATATGGCATCACCAATAGCTTCAGGCGTGA
ATCCCTCCAACAAGA
TAATATTCGTGAAGAACTTTTTACAATTGATCTCGATACTTACGT
GAGATCGGAAGAGCG
TCGTGTAG
>ConsensusfromContig2882 Average coverage: 31.97
GAATATCGCCCACACAATTATAAATATTTTTGAAGTTTAGTATTT
ATTGTATTCAATATA
AATTTGAAGATGAAACAAATATCATTCATCGTGACGTCATGGATG
CGATATAACACTGAT
CGACCATAAGCTTCATATCATTATAATTGCTCATATATGAAATAC
GACGAAATACGTGTT
ATAATATATAATCCCCACATAAATGAAATTA
>ConsensusfromContig2883 Average coverage: 11.86
CGTAAATACTGGAACGCTTGCCGTTCTAAAGCGTTGGTATAAATA
AATTGGATAAATCAT
GGATGATCCACGAGTAGAGTGGATTCGCGATCGAGTTAACAATG
GACTTCACATAACAGA
GCCTGGTATATTCGAAGAATTGCTAGGAAATGAAAATGCAGATG
CCGAAGTTCAATTGGC
AAAATTCTTGAATGAACCCCCTGATGAAAACGCTACAGCTCTCCT
CTTCTTTTATCGTAC
AACAGAGGAGGAAGAGATCCAGTATGAAGTAGAATATGAACCGT
CTATTCCTGATATAAA
AGATGAGCCAGCCAGGTCGCCCAATAGTGATGATGGCTCTGATG
AAGAACGCTCTGAGCG
GGGAAGCGCAGGTCATGAATCTGCCAATGCATCCAGAGCATCTG
GTACAGCTGAAAAGAC
ACCGGTTACCAGCGCCACAAAGGTTTCAGGTACTCTTAACGCCGA
CAACAGCGCTTTGCC
TCGTCAAGAGGGTGTAGTCGAGGAGCCTTCTCTAACCAACGATG

CTGGAGAAGAGAACGA
TGAAGAGGAATCTGGAGAAAATGAAGATAATGATATAGAGGCCG
CAAACGAAGATGCAGA
AGAAATTCTCGAATTGCCGAAAAAGAATACGCGTACCGAAACGA
GGATAATCGAGCGGAC
CGTCCTCCATATTCATTACGAGGGGATTCCAGAAGCTTCCATCAT
CCAAGAACACGTCTG
CATCTACTTCCTACGCGTCAAAGACGGGATGGTGACGTTGCCCAA
CTCTTCTGAGGAGGC
CATGGATATTCTTCCGCGGGAGATCGAGACGGGCGTTCTCAACGG
GCATTCTCTGGTCAT
GCTTCAGCAGCTTCTGACACACATCTACATTCCTCTGCTATCATT
CAGTCAACAGAAGCA
GGCCATGAGTGTGGGTGCGGAGACAGCACGGACAGCTTCTAAAG
CAAGTGATCGCTCCTC
GGCAACTCCGGGCAAGGACACAGAAAAACAAGAAGACCAGCACC
GCCCACATTCCAGATG
GGGCAATCGGGAGGCGGAGCTGGCGAGGTCAGAATCTGTTCTTG
CGACTTCAGACGCAAA
GGCAAGGCCGGGTGCGGTGTTCAGGGACGAGTTTCTGGTGAACA
CTCAGAAGTTCGCGGC
AAATATCACACGGACTATAGCTCAAATTGAGGGAGAGGTTCAAC
TAGAGATTCCTGATTA
TTATATGGGCGAAACGACCGAAGAGAATGTGCACAATGAACCCT
TGATGGAACAAGTGGA
GGCGACGTGTCATGCGTGGAACAAGCAGCTGACAGAGGCGATTG
ATGAAATACAGCAACG
GACGCCACAGGGTGACGGACCAGTGGCTGAGATGGACTATTGGC
GGGAACGGAATGCGAC
TCTAAGCGCCCTCACAGAGCAACTGAAGCAACCAAAAGTCCAACA
GATTCTCGATCTTTA
TTCAGTTAACGATCAATCAATAGAATACGTCCAAGCAGATCTTCA
CAAGCTCTATACGGA
AGC
>ConsensusfromContig2884 Average coverage: 13.21
GTCAATTCCATTTCTCGAGATCCGTCATCCGGAACGAAGTAGTCA
TTCGTGGAGCATTCT
TCATAGCGATCAAAGCTATCATGATGATAGAAATCGTCACACATT
TGATCTTGGATGTTC
CACTCAATCCTATCATTGAGAAGTCTTCTCAAGTGAATTTTCTTC
GCGTTATCTCCAAGA
ATGTTGTTTCTGACGAATGCACTAGCACACGCAGGTTTGTAATCT

GATTCTCTGTCCGGT
TCTTCGCAGACGGTCTTTAGAGACTTGGCATGAGCTGAATGATA
GCAGGAGGCCGTGGCA
TTGGACTTGTAACGAATATCCAGCATGTCGTACTGATACAAATAA
TCGTGATAGAAACTT
GAGAAACCATGCTCGAAATCTCTGAAGTAATTCACTTGGTTGATG
AGGTCGATCATGAAA
TCAGCCGTCGAATTGATATCTGACGTATTGAACAGTCTTAGAATG
TCATCATATTCGTGA
GTTGAGCCGTTCAGCGATGGAACATACCACAGAAGACCGTTGTAG
AGATCCTCTTTAAAC
TGCTGCAATGTAGAATTCAAAACTTTGTCCAGTTTCGTTTGATATA
TATCTTTGAAATCC
ACACCTAACGATGTACCATTAACAAAGAAAAAATCTGCAACAAA
TTGATCTATGCAATCG
CTTTGTGAGATGTTTTTGGCTTCCAATTCGAGAAGACGCAGGATC
TGTATCCTTGAATAG
ATTCTATGACAAAGA
>ConsensusfromContig2885 Average coverage: 9.84
GTGGTTGACTACGTTTAATTTTCAGCTATTATTCAGAATGTATCAC
TTCAATATTTTTGG
GAGGTTCGTTGAATTGCCATATACAATATATTTGGAGAGTCGGTA
ATCTCCCCATAGCCT
GTTATGTAGTCTTGAACAGTTTTGATTAAGGTAGAAGTTGGACTG
TGGTATATTTCGCGT
TTTTGTTGTATATTATTGAATATTTACACAGTGGTTCAGTATCAA
AAAGGCCTCTAAGTA
CCATAGACAATTTGAAATTATGTTCGGTATATTTCAAGAGAATAT
TAACATTGGAAATGA
TTTTTTTCATGAGCTTCCACAGGTGTATAAATATTTGGTTTTAAAA
AAGCTCTCTGTTTT
GATATA
>ConsensusfromContig2886 Average coverage: 37.28
ATGCAACCAGTTGTTGCCATGTATGCGAAGATCTATGTGGAAATG
GAACATGTTCTGCAA
TCAACAACACTGCCAGCTGCGAGTGCAATGACGGTTTTCAAGTGT
GCCCTGATGATAAAA
CCAGTTGTTGCCTTGACGTGTGTGCTGATAAATGCGGAAATGGGA
CGTGTTCTGCATTCA
ACAACACCGCCACATGTATATGTGATACGGATTTTCATGTGTGCA
CTGATGATAAAACAA
GTTGCTGCCATGTGTGTGCAAATAAATGCGCAAATGGAACATGT

GAAGTGAATAACGACA
GTCCCACCTGTACATGTAATATGGGCTACCAAGTGTGCTCTGATG
ATATCACCACTTGCT
GCCTGGTTC
>ConsensusfromContig2887 Average coverage: 6.57
GCTTCTTTCAAATCATCACCCCCTGAGCACTGATGAATGCGAAGA
TTTGGACCCCGATAT
CGGACCCATTTCAATCGAACTATTGGATCCAGCCCATTCATGTTC
ATTATTCAGCGACCA
ATATTGGATTTCAATAGTACAATTATACAAACCATGAACTACAGA
ATGTAAAAAGTAATA
TTTAATGCGATCAAGTATTTGCCGGCAGTTCAATGTTAATAAAAA
GTGACTCTTAGACAC
AATGGGCATATCAAACAATCTTCATTGATGAACCAATAACAGGG
AAATTAATAAAAAAAA
A
>ConsensusfromContig2888 Average coverage: 10.74
TGTGCTCTTCCGATCTCTTCCACTGTCAAGCAGGCCCCTTCTAAA
ACACCCGTTAAGCCA
ATAAAACCTGGTGCACAAGCTGTGGCAAAAAAGTCTTCGCCTCC
CAAGTCAGAAAAACCG
GCTGAAAATATTGCTCCTGAAAAACCACAGAGAACAGAACAACC
TGGTATCATAAATCAA
ACCGGAAACGCCAAAACTCGTCTTCAACTCGCGCATGCCCTATCT
CTCAAGAACGAACCT
GACCTTGAAAGCATTTGCGCCATCTATAATGAAGTTATAACCTTG
ACACCTGAGGTAGCA
GAACCCTACATCGAACTTGCCCGCATCCAAGCCAAAACCGACCCC
CTCTCAGCTGTGGCC
ACGCTGAGCGCGTATCCCCTGCCGCAGCGTGATGCCGCAGAT
>ConsensusfromContig2889 Average coverage: 10.41
GATTCTCCGGATGTGCCCATTCCGCGATGTTCTCCAGATGTGCCTC
TTCTCACGAATCTT
CGTGTTTTCCGAGAGGTCGCCTTGAGCTCTGGTGAATCTTCCTCG
GTTCTTGCGGGAGAG
ACTTTTGAGGAGAGCCTCCGCGGTCGGCGACTCCGCAATTTGCTC
GTGTCTTCAGTGTTA
AGAATGTTATTATTCAGATCCTCTTGAAAAGTGTTTTGAACGATG
GCATGCTTAGCAATA
TCATTATTCGAATCCATTTGGAGATCATCACAGCTATTTTTTATAC
ATGGTATCTCATGG
ATATTTCGTCCGTTCGTCTGAGATGCCGGCTTGAGGGGTGG

>ConsensusfromContig2890 Average coverage: 11.74
AGGAAAATTTCAATAATAATGTTCCTTGCGGTGCATCTGATACAA
TGGACCATGTTGGGT
TTGAAACCAGGCAAACATCTCAGGTAATAGCAAATAGCATTCCC
AAAATAGATGAACCGA
ATTCTAATTTAAGAAAGAAGTCCAAATATAAATCGAAGGGATTTG
ATGAAGCTGATTTAA
ACCCAGTTTTGTCATCAGTTTCGAATAGAGAAAGTGGAAACTTG
GATGAGAATGAAACTA
GAATTTC
>ConsensusfromContig2891 Average coverage: 8.56
TCATGACTTCTTCTGTTTTTTCGTTCTGGATCTCAGTTCTTCGACA
GATTTTTGCTGCCT
TCAAGCTTGACTGGAAATCAAATCCGGACCAGAGAGTCTTATTTC
TGAAGTATCTTTATC
TGAAGATGTATTTCTATAAGGATCGAAAAAGGATTCCAACGGTG
GCTGATGCCGTAAAAG
CGATTGGTGGAGATAAATTACACGACTGGCGGATGTCAGGGGTC
AGCGGGGTCAACATAA
TCGGATCCGGCGAAGAAATGGATCGCCAGGCACAAGCGGATGCT
GATTACCTGAGTGCCA
TGCAAAACCAAACTTCCGGCTTGAGAAGAAAGCCCAGTTTAATCA
ACAGATCTCTCCGTT
TCGCTGATAAGTATATCAGCAACGCTCTTGGTTCTAAGGATGCTT
CAAGATACGGAGAAT
ACGCTTCGCGTCAAAAAATCATGCAACATAGATTCGCAAGTCAG
AGAGTGTCCGTCGAAG
GCAGCAATCGACTGACTCTGCTGAGTGATGAAGAGGAAGACACA
GAGACCAAGAAAAAGT
TCCTTCTGCCGTGCGACAACAAAATTCGATCGAGTTCATCCTTCA
AGCGGAAATCGCGTT
ACCATGTCAACTTGGGATCCAACGCCAACTTCAAGGAACACCTGC
CATATGCGTTCATAT
CGGACAACGAGAAGGGGACGGACTCAACTCTCGTTCACTGTGAT
TCATCGTCCGATAGAC
GCAAAAGTTTGAGATTGCAAGGCGGCGATGGTACATGGGAGAGG
ATCCTGAACGCGAATA
TTTGGAACGAAAGGACACTCGGGCCGAAAGACTTCTACCAATTGT
ATATCTATATAATGA
TGTATGCGAGACGAGAGATCGGAAGAGC
>ConsensusfromContig2892 Average coverage: 5.45
TCTCCTCTTTATATTTTTTTATTTGGATTCCAAATTTCGTTGGATTC

TCCTCATCCGTGT
CACTGTCCGATTCCACCGCCTCTTGCGGGGGTTGGGGCTCCGTTTG
CCTCCTGTGTTGTT
CGGCTTTTGCTGCTGCCTTTCTCTCATTTTCCTGTTCTTCTATTCG
GTTGAAAAGTACTT
TAAATAACCCTTGACCGAGGCTCACACGGTGAATCAAATGTCTTG
AATGGTCCAAATTCC
GATTCAGAGTCTGTAATCCTTTCCTGCTTTGACGCAATATGAAATC
TTCTCTTTTTTTCT
GCTCTATCGACTTCGATTTCTCATTGAGATTGTGATTTTTCACACC
GCATGCAGATTCAC
TTTTTGTGTCCCCATCCTCGTCCCATTGACCAAGAGCGTGAAGAA
TGAGACTCTCCTCGT
CCAGATAGAAAGGTTCCCCGATGCTGGTATAAGGTTGGTTCGCG
GGGGAGTGAAGCCCTT
GCTTTCGAATTTGTGTCACTCGACGATCATAATTTTCTTTGCGAA
CATTTTCGCGGGGAG
GGTATGTAACGTCATAGATTCCAGCACTTCTAGCTCTGACCTTTA
CAGTAGGGTCAATAG
ACGAAGGAAGAGGCACTGACTTTATGTAGTGACGTGGGTCTCGCA
TACTGTGGTATGATT
TAAATTAAATGATATGCATCTCATACTAATACAAACATTCAAAT
>ConsensusfromContig2893 Average coverage: 8.43
CGAAGCAAGTGCTTTCTCCAATTTTTTCTTCCCTTTATTTGAGGAT
TTATTGAGCATTTG
TTGATGTAGATTTCCAACCACATCTCCATGAATTTTTTCAAGTCGT
TTTGCTAACTTATC
AGCTGTCCCCAATGTTTCGTCCATAACATCTGCCAATGCATCAAA
GTCCACCGAAGATGA
CTGTCCACTCATCATTATATTCTCCAGAGAGTCACCAACATCGGT
GAAAAAATGATTGAC
TTCATTTATGAGCTTTAACCTATCGCTGCAGGCTTTCTCATAGTC
AACAATGAAAACAGT
GACTTGGTGAAGGTATTTTCGCTTTTTCACATCATCTTTGATTATT
TCTTCCAGTTCTTC
AATATCTCTGACGTATTCTGTATTAGCGTTCTTGGGAATTTCGTTG
TCCTTCTCGTATTC
ACCATAAATCTTCTCAACTCTTGCCAATACAGTCTTTAATTCTTCT
GATGTTTCTTTCTT
CAATCTGTTGGTTCTTGATTGGCGAACTTTAGCAAGAACATCTTG
AGCCGGTCCATCACC
TCGGTCGATCGGCGGAAGTCGCTCCAAAAAGTCCATAACAGCTTG

GTCAGAATACTTATG
AGTGTGCGGAAAAAAGCATAACAATCCACTTTCATATTATACCAA
ATATTAATAAAGTTT
ATTCATCCTTATTACGAATGAATGGCATCAAATAGAAGTGAGAG
ACAGG
>ConsensusfromContig2894 Average coverage: 13.74
ACAACGACATACGTATCGTCGTCTGCTTTGAGGAACCAATTAAAA
TCCTTATCAGAGTGA
GCGACCGCCCACTCAATCGCCGCTTTTGATTTGGCAGCGAGGTAA
TCGTAGCCTTCCGGG
ACGTCCAATGAAACGACGTCCTCACCAGATATTGTAACCGGGCC
TGCCGAATAGAAGAAG
TAGCTCCGGTGGCAATTGCGCGCCCAGGTCTCACGAACGGCGCGA
CCTTCAAGCGCCCAT
CTTTGGGGCGTGGTCAAGATGATGCACAGGATCTTCACAGGGAAG
CCAATGCCTTCATTA
AGCGCTTTCTCCATGGGCCATTCAACGCCGATATC
>ConsensusfromContig2895 Average coverage: 5.41
GAAAAATGTTTGTTCTAATCATATTTATTTAATCGGGCCTCATTTG
TGTTCTATGACAAT
ATTATAACCTAATCAAACCCTATATCTGAAGGTGTTCTCCAATGA
TATATTCTTGGACCT
TTGTTATTCCTCATATTTATTGATGACATGTGTTTTGCTTCTGGTG
TACTCACCTCTATC
ACATTATGCGAATGATACTTTTTTATTATATTGTCCAGATCG
>ConsensusfromContig2896 Average coverage: 7.94
CCGTTGCTCCAAGATTCCCAACAGCTCTGCAGCATCAACATCAGT
ATCTGGCAAAACAGT
AGATCCTGAGGATGCCCCCGCTTCTACGCAACACATGAGCCCCTC
TTGTACTGTTCACAC
TTACTTCTCTGGGGATGTTGCTAGCAATGTTGATGAACACTTTTC
ACGTGCTCTCAGTCA
AACTCCCCCGAATACAGCATCACCATCACATCGATCTTCCAACAA
AAGATATTCTCCAAT
GGCCCAGCGAAATTTTCCCCCGTCGTTCTGGAACAGCAATTATTA
CATAAATCTCAGCGC
TGCTCTGAGAGGACCGCACCACGACGCAGCAGCCTTATCATCCCT
TCATCACAGCTCTGA
TTCTTACCTCTCATCTCTTCAACAATCCATGTCTACACTTCACCA
AGACCCCTGGGCACG
ATATAGCTTACAGCAGGGGCCTCATCATTCGTATGGGCATCACTC
TACAATGCACGCACA

TGATCTGGCCGCGGCGGCTTATGGTC
>ConsensusfromContig2897 Average coverage: 9.20
CGGCCAATCCGTTCATGCAGATATAGTATAGATGGCACAAAACT
TCGTGCTGGAAAAACC
CATCATGTCGGCATACGAAAGTTTCCCCAGACATTCCAGAATTGC
TGAATGCATGTTCTG
AAATTAACTCAGGAAAAGAATGTATTTGATCGTTTTCCGAATGGA
AATTCATCTTCGCCT
GATCCCCGAAAGGAACTCTCATGGTTACGGTCGGAGGCTGGTCTT
GAATCCCCGGCCTGT
ATTCTCGGATCTCATCGACAAAGTTGCGGCGAATAGGTCTGTCAT
GCGACAAAGATGCAT
CGGG
>ConsensusfromContig2898 Average coverage: 8.53
ATTTAGTGTTATGTGAGACAGGATTAAGTATCACGTTCATGCTCT
ATCCAATACAATCTG
GTCAAGACCAGTATCTCATCTCTAATGGGGGTCACACTTCTGGCT
CATACGGCCTAGCGC
TATTGATCAGATCTATCAAATTATTGGTTTATTTCACGATTCCTG
GAAAGTCGTGGTTCG
TTGAGCATACATTGGTATTTGGTCGATGGTCTCAAATTGCTACGG
TTTGGAATAAGGATT
CTCTTATTTTCTTTGTCGATGGGATCGAAATCTCACGTAGTAAAA
GCAATTCGT
>ConsensusfromContig2899 Average coverage: 12.58
CTCTTTTCTAATGCTTACTGCAAGGTGCTGAAGGAAAGTCCCCAA
TTGATTTTCGCGGAC
ATTGAGGACATTTCGGAAGACATGGTGGACAGCGTTGCTCATTCA
TTCCACATTCCTCTC
ATCACAACGAAAGAGTTTACTAACGGGTCAACGCCCCCCAACCC
CTACATACTACGGATG
CGCCCGTTATTTGACCAGGCCCTCATCGACATCATGCAGCACTAT
GGATGGACCAGAGTC
TATTATATTCACAACAAAGAACAAGGAAGCTTGCGGATCCAGCG
AATGATGAGTATCATG
GAGACCAATGAGACAGAGGCGATCGATTTTGATATCATCCGACTC
CAGAATGTCTCTCAA
AGCTACCCCGAACTCCGACAACTCGACTCGAGGGATCCGAGATC
CAGTAAGGCCATCATT
TTGGATCTGGAGACGAATGAGGAATTCGAAACCATATTGAAACA
GATCAAAACCCTCGGC
ATGAGTAGAAAAGAGTACCATTACCTCTTGACATCTATGACATTC

AATGAGCTGGATCTT
CATCAATTTGAGCACAGTGGTGTGAATATCACCGGTTTTCAACTG
ATGAATAAAAAT
>ConsensusfromContig2900 Average coverage: 23.85
TTTTTTTTTGCTGAAAAATGGTTTAATTGTATCACAGATATTAAT
AACAAGCATTGCAAA
TAAATAAGCGACTAAATAATATGTGCAATTTTTATTGGAATTTGG
AATATACGAAATAGA
AAAACAATGATCTTCATCATACTCCGGATGAGGCAGAGTCAAACA
ACTATATTGGCACGT
CCATTCCTCCAATATTTACATTCGGTGATTTCCCAATTTTTGGGG
AAAGTGATGCAAATG
ATGACACATGATGTATATGAACAAATCGTAGAAAATGTAAATAAA
ATTTAATCTTGCATA
ATTTTTAAGTTCACGCCGCATTTGAAATATCACTTGTAGAATATAA
ATTTGTGTTTCGTC
TCGTAATAAATAATAATCTGACATAAGTAACATATTCTACTGAAT
AACAACACCCATAAC
TGCGAAAATGTCACCCGAAATATTCTGTTGGGGTAAATAAATGG
CAAAATCAGGCAACAA
TCCTACAATAAAAAATGCTAAAAATGAAAACCCATTCAAGGTCTTT
CATATAAATATAAAA
ATACATCTTCTCTGTTCGTGGATAAAATGCCAGTTGTGCATAT
>ConsensusfromContig2901 Average coverage: 19.36
ACGTGTGCTCTTCCGATCTCCACGAATTAGATAGCTGAGCTCTGT
GAAGGTTTACACTCC
CCGTCATATGAAAAAATAACAAAACACAGTATACAATCACAAAA
CACTGAGTATTTTATC
GCAGAGCCATTGTGAAAAAGACTTTTGCTTACATTCGCCCTCACA
TACTTTCATAGTAGA
GACCAAAGAGAAACCTTGCTTGCCATCTTCTTGCCGCTTCTCAAA
TGAACGGAAATTGGA
ATTGCATTCACAAATGGTTGTTTGAGGTTTATCAAGAAATTGTTC
GACGTCATCATACAC
TTCATCGGGCCAACAAGAATTCGGAGCGTCACTGAAGGTAACAA
TATTGGAACGACACTC
TCCAGCACAGGCTTTCGTGAGAGAAACAATTGTCCCACACTCGCC
ACCACAAGCCTTCCT
TGTTGAATGATACGCTCTACAAAAATTGCATTCATCATACGTGTC
AATATTTGCGTTACA
CTGACCTTCACATTGACGCCATTCTGTGACGTTCATTTGACAAGC
ACCGTCACAACCCCT

AATAGAAATATGCCCTTTGACCCCTTCAATATTATCATGCAAACA
CTCACCTCTATCCGA
TTGGTTGCTTG
>ConsensusfromContig2902 Average coverage: 8.28
TATAAAAATTAGCATGAACTTTTGATCTTGAAGTCAATCTTATTT
GAAGTCTTTCGAAAG
GGAATTTTTAATTTTTTTGCTGATCAAAAGCCGGGATTGATTTGG
TGAACAATAAAAACA
TTGTAAGTCTTGATATAAATTGAGTATATTTTTGATTACACGTGTT
TTGTCGGACGGTCT
TCGAAATCTTCGTATTGTGTGATATTAATTGAATACTGAACGAGC
AATACATTTGAAAAC
ATTTGCTGATGTAATATGGTAGTTACCCAGAGTGAAAAAAGTTGA
ATGTTGATAATTTTT
CGATTTCACGTCTACATCCTATATTTTATTTATCAAAGGACTCAT
GTAATGAACTTTAC
ATTTTTATGCATCAGACATGTTTCACATAATGATAACCGCCGTAT
CGTCTGAAAA
>ConsensusfromContig2903 Average coverage: 4.61
ACTGTTGCAATGTGGCTTTTATCAAAATATGGCGACGTGTTATTG
TTGAAGAGTGCAAAA
TCGTAGAGTGTGATGTTGATTCTCTGACCTTTTTTGACCTGAATC
AGCCATGGACATGAT
GTTGAATCTCTCGATGAGGATTTATTTTCAGTTGAGAACGGTGAA
ATAAATTGTGGCTCC
TTTTTTAATACGATTTTATTGACCGGGCCATCGGTGCATCCGGCC
CTCCGAACTGGAACA
CATGAATATGTGGCGTCCATATGCATAGTGAGTTCCCTGTTACAT
GGCGTCAGTT
>ConsensusfromContig2904 Average coverage: 13.45
TTCACAAATGCTCTTGAATGGACTCTCTACGCGACCACATCCGCC
TTTGCGGTTCCAATA
ACATTTCAAATGCTGGATACTTTTCAATGGCAGTGTGCCTCCGTCG
CCATTTTCCTGGCT
TGGTTCAATTTTCTTTTGTACCTTCAGAGATTTGAATTTTTTGGGA
TCTACATTGTGATG
TTCTTGGTGATTTTGAGAACTGTTCTGCGAGCGTTGGTAGTGTTTT
CAATCCTCATCATC
GCCTTTGCTCTCTCCTTCTTCATTCTGCATTCCTCTGAGCCTAGGA
GCTCACACAGCACA
CCTGCTCTTTCGCTGGTACGTACCTTCGTCATGATGCTGGGGGAA
ATCGAATACCTGCCG

ACCTACACGAATGTAATAACTGATTGGAAGAATGGCCACGAATAC
AATTTTCTGTCCT
>ConsensusfromContig2905 Average coverage: 4.84
AGAGAAAAGCGAGGTTTTGCCATTGACGGAGGGGCATATATTTC
ATGCTGGTAGACGTGG
TGTCGCCTTCTATGTGTTCTATTTCGGTAGTTCTGCCGGTAATATC
TATTCTTCTGCTTC
GGTTTAAAATTATTGGATCTGATGATATTGCTTTTCAGAACAATA
TTATCATCGTCATTA
TCTCTACGATTGTTATAATGCACGACCGGCTCAAATTCTT
>ConsensusfromContig2906 Average coverage: 17.03
GTTGAGCATTTCGTCAAGAGCTGTCCCCCTTGTATGACAAAGCGG
GACTCAATTCACTGT
ACGCGCATCAAGCGGAGCGCTTCAAAAGCCATGTTGGAGCGCTG
TCAGATTGACATCATC
GACATGCGGGAAAAACCGTGTAAGGGATTCCAGTTCATTGGCCAT
GTTTTGGATCAAGTG
ACGAAATATCATGTCCTGTTCGAGATGAAGGAACATTGTGGCGA
GGAATTGGTGGAAAAC
CTGTGTCACCGTTTTTTGGCCTACTTCGGAATGCCAAAGTCTATT
CATACACACATCATG
GATGAATCTTGGACGGCTGTACTTAGTCAGATGGTCCGCAATTGG
C
>ConsensusfromContig2907 Average coverage: 12.33
GTTGATCATCATTCGAATATAGGATCATCATGCTGAATATCATAA
TAAACTCGGAAGACA
GTGTGTCATATAATTCGAGGCTAATACTGTTCGTTTGTTGATTTA
TATCAATTAAATGAA
TGAAACGAAATTGTCACTGCATTCCCACTGAATAAATATTCCTAA
CACAGAACTAAATCC
GCATATCCTATTCTGATTAGTTCACTTTGCGGCTATTCAAATAAT
AAACCATTAGAGTTT
ATTTGTGAATTAATCTCATGAATGCCTCGGCCGCCGTATTGGTAT
GAAGACGTGAATATA
CCGCGTGCCATTGTATATCGCAACAACAACTGCCAAATTAACTAA
TGCAAATATTGCATT
ATTCACCATTAGAATTCAAAAATCATGCATTTTTTATGGCTACTT
CATGGATATCATGTG
AGCATTATAATGAACCCGGTGAAGATAGTAAAACTACATATACCA
CTTGGTTGTGACCGC
TACCATCATCATATATCAATCGATTTTTTTTCAATTATGATCATTA
TCATCATGGAAATT

GTTTCATCATTTTATATTCATGATGAAAATTGTGATCACAAACAT
GTCGTGTTCATGTAA
TCCGCTCAAGAATGATAACCATCGCTTTCGCTATCAAATGAATGG
TTTTCGAGCAAAAGA
GGAATGTTCGATAAGCTCACTACGGTGTTCATGTATTTTCTGTGA
TAGATAATACACAAA
CTAGTATTAAAAATTTATATTATTTGTATTCTTATTACAGGATAAC
AC
>ConsensusfromContig2908 Average coverage: 8.72
GTCATATGCTACGGCCACGAGTTCTGGTTTTCCCCCAAAGGAAAA
AGAGAAATATAAACT
GCTTGTTCGGAAAGGCTCAGACACACTGAACAAGCAAGAGGATG
AAACATATAAGAAGAA
AATCGCGTCCCTCGTTCGGAAAAACCGGGATATACAAATAAACTA
TATGTCGTCTGTGAA
AGGGGGTGTTATCATCGATTTTCCCAATCAAGATCGAATGGAAAA
ATGTCAAGCCCTGAT
ACAAAATGCAGATCGGAA
>ConsensusfromContig2909 Average coverage: 10.79
CTTGAAATACTTCGACATAAAAGAGGGATATTTATATCGTATCTG
CACAATAAGATCGCA
CATGCAATATATGACAATCAGAATAGTTTTGTTATCGGTACTATA
TCCACAAATTTTGTT
ACGAACGACAGACGCTGAGCCGGAACTGCCCTATGACGTGAGCA
GGGATTCCCAACATGG
CTGGTTGAAGGGGTGCGTCTCCATATCGAGCTCTGACGTCATCG
AAGAAGCAGCAAATGA
TCCAGTAGAATGCTCCAAATTTTGTGCCGCAAAAAACGTCCCCTT
GGCGGTCATCTCGGA
AGATCTGTTCTGTCGCTGCAGAAATGAAATCGATCAAAATCTGTG
GGTCTCAAAAGAAGC
ATGCAATTT
>ConsensusfromContig2910 Average coverage: 21.49
ATGAGAAAGAAAAAGATATCATCCAACAATTCAAAATCACCTAT
CAATTTGGAAAAAACG
GGAGGTCTGTGGGGGTAATCTTACCACCCGATGTAATACAACCCC
TGAAACTATTTCTCA
AGATGCGCAAACACATAAACATACCAGTTGAGAACAAATATGTA
TTCGCAAACATCGACA
GCATAAGTTATTCGATTGGATCGAGTGAAATCCATAATATTGCTC
TAAAAGCAAACTGCA
GGAATCCGGATTTGATAACGGCCACAAATTTTAGGCACAGGGTCG

CAACACTGTTTTCGC
TGAATGGAGCAGATCAACAGGCATGGAAAGTTCTATGCAATCAC
ATGGGACATTCAGAGA
ACATAAACCAAACAATCTATCAATGCCCTCCGGCAATTAAAGATA
TTTTGACATTAGGTC
CTTTTCTAGAGAAACTTAACCAGCCCGGTTAGATCATTATTTTCTC
AATGTATCTTTTTC
CGTCATAAATCATTGTATTTTATATAAAATGAAAATATTATCGAC
ATAAATATATAAAAA
ATGCTCTAGTGTTTAC
>ConsensusfromContig2911 Average coverage: 14.64
GTATTCGGGAAGTGCCATCTTGTTGTCGAAGATGTAGGTGACGGA
ATGCTGGCCGCTACA
GTCACAGGTGGAGGGGACCCCGGTCGTGCTAGCGAGCATCTCAC
CGGCCGCGCCAGCCGT
GGGGACTGCTGCTTTAATCTTCATTTCATGTCGCAAATGAAAGGC
TGAGTCATACTTGTC
GTAGCGAACGTTTTTGAAAGATGATTCATCTTTGAGAACCTTGCA
TGTTCGACCAATGTA
TGACTTTGTTAATAACACCCGGCCGAATCTGAACGGACAAAAATC
AAGGACGTTGGCTTT
TTTGGCGTTTTCCACCAGCTTTGCGATCCTGAATCTGTCCGCGAG
GCAGAGAGAGTTGGT
GAGAGGAACAGCCTGAGGAAGACCCAGCCTCTGTAAAGTGCTGTT
GTCCATGAATCCTTC
TTCGAACACCCGATTGGGGTGACCAGCTTCGCCAGGGAGATCCG
GATCCCAAACCCAGAA
CAAATATTCCGTCAATTTCTGAGGGCTTGGCACGCAATTAATGCT
GCCCAATTCTTCGTC
TTCTCTGTGTTTACTTGAGAATATATCGTCGAACTGATGACGTAG
CATCCGGTTATGAAT
GCGTACTACTTGCGTAACCCAGATCGGAAGAG
>ConsensusfromContig2912 Average coverage: 8.50
CCGATTGTTGTCTCTTTGGTTGGTTTCTGTTCATTGCTGCAAGTTC
TGGGAGTCTTCATT
TCCTGTCTTCCGTGATAGTCTCCGTCAACTTGCAATCTTGTTATTC
CATAGTCACTGGAG
TAGGTGGCATTCACATGATGCCATTCCCCGATGGTTGCGGCTATT
TCGACTTGGGAGTGA
TATCTGCAATCATTGCTTATGTAGTTGAACGAAAACACAAATTTT
TCATCATCGTAATAC
AAAGCTCGAGTTAGATCTCCA

>ConsensusfromContig2913 Average coverage: 39.63
ATTCAGAAAACTGTCGCTTCACCGCCTCTGACACGACCAGGACCT
GTGAATGTTCTGACG
GCTACATCGGTACACTCTGCGATGTTGTTGACCTCTGTCCAACTG
CGGACTGTGGGATA
ACTCCATCGGCTGTGTTTTCGAGGCAACAGATACAGACTACACTT
GTCAATGCACGGATG
ACTACTTCGGAGATCGCTGTGACATGCCGAAAGCATGTACCCTTG
AACCTTGTGAGAACG
GTGGGACGTGCAAGGACAAGAATGGTGGCATGTCATACTCCTGTG
TGTGTACCTCAGATT
ACGAAGGCGCGGATTGCACTGAGATAATCGAAAGGCCCGCAGAC
CAAACTGCATTCAGCA
GATCGGAAG
>ConsensusfromContig2914 Average coverage: 17.17
AGAATGCGACGATGAATCGATAGATGACGTACACCCACTGCCGA
CATGGCCACTGTGAAG
TATATAGAAAAGACCGTCTTGAAACTCTAAAACCGAAATTTTTCA
GTTTGAATTCTGCTT
TTAAATCAAAAACTTCTCCGAAAGCTTGAAAAATAGTCGGAGGA
GGTGGTGGCTGCCATT
CAGGAACCTCGGCTTGGGGATGATCCGAACTAAAGGTAACAGAA
GGCATCGGGGACTGAA
TTTCGGGAGATTCTTCTTTCTCGAGTCTACGTCTTATGGTGCTCAA
CCTGGCTGCGACAT
CCGACTCTGGCAAATTTTTGATGCCTTCATCGATCAACTCCTCCG
ATCGCAATTTTCGAG
CCAGTTTGGATTTCAGAGAATGGAGCTGGTAAAGCCATGCAAGAT
CTTCGGTACATGTTC
CCAGCTGAACATTGATGTAGTTGTGCAATTCCTGGCATTCACTATC
TTTTTTTGCCACAA
CTTCTTTGATAGAATCCAGTTCATGATGCAATTTGTCTTCCTGTGT
CATGATTTTATCCA
CAATCAAAGACAAAGTCTTTTCCGGGGTCAACTCTTCAATGCTTG
TCGGCCACGTCCGCT
CCGATTGCCCTTCGGATTCTTTGTGATCTAGAATCTCGAAGAATG
AAGGATTCAGCGGCC
GGGAATCGGCAGTCTTGGTGAAGCTATGCATATATCGGAGGGTCG
CAAGAGTTTCTTCGT
AGAAAGCGAAAGCCGGGGAGACACATCCCAGCATCAGGATACTG
GCGCTCTTACTCAGAG
GCTCCTGCAAAATCCTCGTCAATTTGGACGTTCTGTATGGCACAT

ATCCCCTGGTGAGCG
GGTCTGGGTTTCCGAGCAAAGCTGTCATGACGTTCTCCAAGGCC
AACAGCGAAAGATTGA
TCTTTACCAGCTCCGATTTCTGATCTTTGTCTTCGTTTTTTGGGAT
TGTGCTGCCTGCTA
GATCCACCAAAAAGAGTGTTGATCTTTTGGCGACGGCTAAATTTT
CAATATCATCCGAGT
CTGCGCAAATGATTTCTACTGCAAGGATGGTGTGGCCAAGGCCCG
TTCGAGGGTTGGCGT
CTGTCTCCTGCGTTTTTCTGTTGGGGAGGCCGGCTGCAAGAACTC
G
>ConsensusfromContig2915 Average coverage: 27.23
CAACAAGTAAACAATGTAGAATTTTTATATATATTTATAATTCAT
GCTTTAAATTAACAT
TTATGATGCATTGAATTGATGCAGCATGTCTCTTGAAACATACGA
GAGAAATCGTGTTGT
TGGAAAGGGGAGCTATGGAGAGGTTTGGCTATGCAGGCATACCA
AAGACAGAAAACAGTA
CGTTCTGAAAAAAATGGATCTGAAGAATGCCTCGAAGCGAGAGC
GCAAAGCTGCTGAGCA
GGAAGCCAAATTGCTAGCCAAACTGAGACATCCCAACATTGTCTC
TTACAAAGATTCTTT
CGATGGTGGGGACGGGTACCTCTACATCGCCATGGGTTTCTGTGA
GGGAGGCGACCTCTA
TTCACGGCTCAAGGAGCAGCGCACAAACCCACTAGAGGAGCGAC
AGATTGTGGAGTGGTT
CGTCCAGATTGCCATGGCTCTCCAATACATGCACGAGCGGAATAT
TTTGCATCGAGATCT
CAAAACGCAGAACATTTTTCTGACGCGAAGCAGAATCATCAAAG
T
>ConsensusfromContig2916 Average coverage: 16.15
GGGATATACTGCAGTTTTCATCGCTTGTTTTTGCGGTAATATCGA
AACTCTAAAAATATT
GCTTTCTCAAAAAGCTGATGTAAATATTTCCACGAAAGATGGCGT
ATCCTGTCTTCACGC
AGCAACAGAACTTGGCTACGATGAAATCGTCGGCTTTTTGCTTCG
CTCAGGTGCAAAGGT
TATGGCGAAAATGCCAAATGGCAAAATTGCTTTACATATTGCTGC
TCAAAAGGGTAGAAT
AAAAATTCTCACACGTCTCCTTGATGCTTGCAAGAAAATTGATGT
TCAGACACACATGGG
AATGACAGCTTTACACCTAGCATGCCG

>ConsensusfromContig2917 Average coverage: 5.26
GGTACATGCGTCACGGACGGATCCTGGCTTTATTGCATTTGCCCC
TTGGGGTATGGCGGT
ATTCGATGCGAAAATATTTTGGGACTCTCAGATCATACTGACATT
GGATTATTCACACCA
CTGGAAAATAATGAAGACGATACCATTAAAGATGAAAAATTATC
CGATACTACAACAACA
ATTGATGAGAATCAAATTCTCCAGTCTTCCTCCTCTTCTCCCACAA
CAGCTTCACTTTCT
GCCAAGCCATCATCATTAGATGATGCAGAATCTACAGGTGAGAC
ATTATAATGAGATAAT
TCAATTAAT
>ConsensusfromContig2918 Average coverage: 8.45
CAAAAATGACAAAAGTTGAAAGTGAACATTTTTCAAGATCTTCG
GTGAATAACGATGTGA
CCGGGGATATTTTGCCATCTGATCAAAAGGACCAAAGCAAAAAA
GACTTGCATAATGACG
AAGTATCAGTGAACATCAATTTGAACAGCAGCAACTCATCACCC
TGCGCTGCAACTAAAA
GGACAGTTGGTGTTGCGATTCCAAAAGACTCA
>ConsensusfromContig2919 Average coverage: 5.93
GAAAAACATTGATATATTTTACAACATGTCAAATATATAGACCTC
TGTATCATGAATGAA
CTGCTCTTCAGTGATAAGTTACCAACATTTTTTGACAATGAAGAG
AAATCGAGGGACAAA
GAGAATTTCAATTGTTACTTTCAAAAAGTTGAAGATATGCAGTTC
ATTCGAAACACAAAT
TCTCAGTCCATTAGCATGTATATAATAGGTCGCCTTTCCTGTGAC
CTAATGAGAGAGGCT
TGAATG
>ConsensusfromContig2920 Average coverage: 63.93
CTCAGTGGACGGGGGACGGCCATGCGGTTTTGAAGAGAACGATG
CATCTAAAGAAGGTTG
GGGAGGCATGTTACGCGAATTAACATCTATAATACTATTGACAG
AATTAAGGGCAGAAGA
AGGTGGCACTTGCAGAGAGGGGTCAGGATTCAAGCTGAAAGTAC
TCTCTGAGGGAACCGT
GTCTACTGGGAAGGTGGCCGCCGAAGAAGTCGTTACATATACCG
GCGATGCTGGGAGAGG
GATTTCCGAGCAGTTTGGACCGGCACTGCTGAACGATGACGGCTT
CGTTGAGTCACTGGC
ATTTTGGTTCAAAGTTAAGCCTAAGCGAGCGGATGCAGAAAAAG

GCGGTGCTGCCAGTCG
ATTGTCTATTGAATTCAACGCAATCGTCGAGGATATGTTTGGAAG
CGAAATGTCAGAAGA
CGTGGCTGAATTTGAGGAGGCCGTGCAAAAGTATCACTGCTATT
GATTCCATCTTCGCT
ATCACCTACACGTCCTACATCTTCTTCCTCATCGTCATCAGCCTCG
TCTTCTTCTTCTTC
TTCTTCTTCTTCAGATGAAGAGTTTTCTGAGCCTGAGCTTTCTTCA
TCTTCTTCTTCATC
ACTCCCACTAGAAGATGATTCCGTGTAGATAGAAGAGTTTTGAGA
TTCCGACGACGTCAC
CAAAGAAGATCCGTCATCGTCTGAAAGAGAACT
>ConsensusfromContig2921 Average coverage: 35.01
TCATAATATCGAAATTCATTTTATCGTGGTGTTTGATTTTTGAAAT
TATACATTTCAATT
CAAAGTACAATGCACAAGTCTTACCAATCTATTCTCCCCACTCAT
AATAAACTTCTACAA
AAAAGATGGGATCAGACTTACTATGATGAACACCGGAAAAAAGT
AGCAGAACAGCGGTCA
ATGGTGAACACATGCGCTCCGAAAACCTATATGCATCTCCATCTT
AAACTCAAGAAACTA
CAACTTGAGGAAGAAAGGCTTGCCGTCATTGAACGAGATAACAG
AATTCTGCTAGAGAAA
ATGTCTCATATAATGCGAACAAAGGGGAGAGTTGACAACGTCAA
TAACTACGAATATAAA
AGCCTTAATCGAGAGAAAATGAAGCGGGAGAAGCTGCGCGTGGC
TCAAGAGAACCAAGAA
GTGTCTACCAGAATTCTGAAGCGACAACCAGAATACAAATCCGA
GATTTGGAAGAAAGAT
TGGGAGAGCAACATGAAGTTTATGGATAATATAAGCCAGTATCC
AGGAAATTGGTGGAAG
GACCAGAACAAAAAGCCATCTAAAACTGGTGAAAGCCCCAGGGT
CGACAAATCGAAAGAT
GATGAAACCAAGAGCATGGCTTCAGAGGAAAACACAGCGCAGC
AGGAGGCTGAGGAAGAA
ACAGAAGATGTGCAGGAAAAAGAACAGAAAGAGGAGTAAAGTAT
CAGTATTCACTTATTA
ATTGAATATCCGATCGCTATAAAAGACTTTATAAATTGTTAATCA
TGGG
>ConsensusfromContig2922 Average coverage: 21.37
TGTAAGAGCAGCAGCTACGAAGATATTTATTGCGGAACATCATT
CGAACTTGAAAAAGCT

CAATCTATGAATCCACAAGACATAGAAGTTGGAACTCAACAACC
TGAACGAGACGCATTT
ATTCAGAATAAAAATGCGAACAAATCTTCAAGCTTTGAAAATCTA
TATACAGAACAACTT
ATACGCAACTCACTGGTAGATGAGTCAACTCCTGAAGATATAGGT
AATTATTTCTCGAAC
AAAAATGACCCATGTGAACGTGAATCTGAAAATAGGGCTATGAT
CCAGAAATACGACTTA
GAAGATATGCCCAGCCAATATCGACCGAATTCTTCAGATATTAAA
CCAGATCATAACTGG
AGCCCTTCCGTCGACTCTAGATGCATAGCATCAGCTGATGCATTC
ACAATTCAACAGGAA
CCACTCGACATAACTCTCGAGTCCGGCCTTGATCAGATTGGCGAG
CAATCAGAAGAGTGT
TCATCCAAGCATGATAAATTGTCAAGGTCTCAAACTGTGGAGTCA
AGTTCGTCGGAGAGT
CAAGTCATATCCGTTGTTCATCAGAGAAAGGTTTCCAGTCCTGAA
ATACTCTTTGATGAC
TCACATGAGAATCTCGACTCCATCACAG
>ConsensusfromContig2923 Average coverage: 64.42
GTTGTCCCGGCAACAATTAAGGAGTGTACATCATTGCGAGAGCTG
TTTTTGTATGGCAAC
AAGATCTGGCAATTACCAGCAGAACTGGGCAATCTCACCAACCTC
AAGACTCTTTCCCTG
AGCGAAAACTCATTGACGACGCTCCCCGACACACTCCGGAACCT
CCGGCAGCTGGAAGTG
CTCGATTTGAGACACAACAAGCTCTCGGAGATCCCCGAAGTTGTC
TACAGCATCACTTCT
CTAACGACCCTCTATCTTAGGTTCAACCGAATATCCAGAGTCAGC
GAAAGCATCGGTAAT
CTTAGAAATCTGTTCAACTTCAGCATCCGGGAAAACAAAATCCGC
GAACTTCCTTCAGCC
ATGGGCCACTTGAAGCAGCTGCAGACACTGGACGTCACCAACAAC
CATTTAGAATCTTTA
CCCGAAGAGCTCGGTGAATGCTGTCGGCTGACAAGTGTGGATGCC
AATTACAATGAACTT
GCAAGTATTCCAGAATCAATAGGAAAATTGAGAGAACTCACACG
ATTAGGAATAAGATAC
AACAAATTGATGGCCATCCCTTCGTCGTTACGAAATTGTGTTAAC
ATAGATGAATTCAAC
GTAGAAAATAACTCAATTGAACACTTACCCGAGGGGTTATTATCA
TCTCTCACCCGAGTG

GGCAATATCTCCTTATCCCGAAACCGGTTCACATCTTACCCCCAA
GGCGGCCCTTCTCAG
TTCGTCAACGTAGCTTCAATCAATATGGAACGAAACCAAATCAAC
AAAATTCCGTTCGGC
ATTTTCTCGAGCGCAACTTGTCTGACTGTACTTAATATGAATGAA
AATAAACTTACTACT
CTACCCGTTGACATCGGTTCATGGGTCAACATGGTGGACCTTAAC
CTTGGCAACAACGAC
TTATCCAAAATCCCCGATGACCTTGGCAATCTGGTCAACCTTGAG
ACTCTGGTCCTGTGC
TATAACGGTCTCCGGAAGTTGACTCCTTGCATCGGCCTTCTGAGC
AAACTCCGCGTCCTT
GACCTAGAGGCCAACAAATTGGAGGCTCTACCCAACGAAATAGG
TGATCTCAAAGAATTA
ACGAAACTGACCGTTCAGTCCAACAACCTCACATCTCTTCCTCGA
AAAATAGGCCTTTTA
ATAAATTTAGTACATCTCAGTGCAGCGGAAAACAACCTAACAAG
TATTCCAGAGGAAATA
AGCAGTCTATGCAACCTCGAGCATTTGTACATCAACGACAATCGA
AACTTGCAGTGGCTG
CCCAGCGAATTGACCCTCTGTGCCAAACTCCAGTATATGAGCAT
>ConsensusfromContig2924 Average coverage: 4.59
GGTTCAACTGCTTTCTCGATCATAACAAGTGTCATTGTTCTCAAC
ATACATCACCATGAT
CATAACATTCCTGTTCCGAACCTCTTCAAAAAGGTCATACTCACA
TGGCTAAGGAAATTG
ACATGTATGCGGGCTCTCCCTCTTGTAAAAAACGACCCAAGAAG
GAACAATTTGAGGGGG
ACAGTCCATGCAGAATCATACAGACTTCCAGACACCAACAGGGC
GTTCATAATCGAGGAA
AACAGATGTGAAATTGGAACAGTGACTGACTCACCAAAAGTTTT
TCGAAATCACAATGGA
AAGCTGCAAACCAGCGAACCCATGCTGGAAATTCTATCTCACTTG
CGCATCATATCGGCC
AGGCTCAAGCACG
>ConsensusfromContig2925 Average coverage: 12.02
GTGAATTTATTTCACCAGCTGCGAACTTTAAGATGAACGCGGAAG
GTTCTAAAAATCAAA
AAATTGAGCTCATTGATTCTATCACAGTTCGGGAAAAATCTCATT
ATAAAGATGAAGGCA
GATCTCAACCAGGGAATACGATGAATGAAGGCCTTTTGTCTGGT
ACAAAAAAAACCACGG

AACAAATGACAAATCGAGAAAACATTGATAACTCAGAATTCATG
TCACTCAAAGAAGAAC
TTGATGAAAACAAATCCCTACCAGAGAAGAGTGGTGATGAAGTT
GTTTTCACAGGCACTG
GCAAAACCGTAAAACGGTTGAAAAATCGAGAAAACATTGATAAC

>ConsensusfromContig2926 Average coverage: 28.16
GTTATCGCATTATTTATAAGTAATGCATTATCACAATCGAGAACT
TTATAGATATCATTA
TCCAGATACTCCGGACAAGTATAATATATATTATCAGAATATTTC
CCTGAGGTTCGAACA
GGTTTCCCAAGCTGCAGTTTTCGTCTCCGTAATTTGATCCTCTCTA
AAACCTCCTGGATT
TCGATAACATCGATTTCTGACAGTCTATTCTCATTCAGTTTGACCA
TTCGTACTATTTCC
TCAGAGGCTATAACTGGTTTCTGATATTTTGAAATATAATCTTTCT
TTTCTGTGCGTTCG
TCTAAGCACATGATCGGCCAAGTGTTGGATATTATATACACAATC
ATGACTATTGAAGTC
AATATAGCGCCAAGGACCAGTGTCTTTTTCTGCATTATGTTTGCTC
GATCTCCCACATCT
AATCCAATTGTTTACATTTCCATCAATAACATATTCAGCGCAGAT
>ConsensusfromContig2927 Average coverage: 21.51
GTTTAATTTCGGAAATTCAAAATAAACAAAGCTAAGAACTAGAG
CTATCAATTAAAATCA
TTATAAATACTAAATTTCCATGGTTTGGTGAATACCGGATTAAGG
ATGGATGGCAAAATA
TCCGCTATGGATTCTATCATGAGTCGCGAGGTTTTGCTTAATCAG
GCATTACATGGAAAA
AATAAGAACATTCTCTACAAATCATTACTATCCAAAGACGGTCTT
TTTGATGCTCTTCTG
GTGCTGTACTATGAATGCAAGAGTGAAAAGATTAGAAGGATCC
TTTGGTACAAGAATTT
ATCAATAAATATGAATCGACGATATTGGATCTGAAGTCTCTCCGA
GTTGGAATTAATGAT
TTTGAAATAAAAAATGTTATTGGCCAAGGCCATTTCGGCACTGTG
AAATTGGTGAAAGAA
AAGGCAACAAATACTCTTTATGCAATGAAGATAATGCGGAAAAC

>ConsensusfromContig2928 Average coverage: 31.43
GCCGCCGACAGACCCCGACTTGATGATCCTAGTTGGACCCTTCTC
AGTGGTCTCGTGATG

GTCGGCCCCCCCAAAGTAGAGCTCCTCAAACCAATCATCGTCAGT
CTACAGCACTGCGCC
TCCCTCAAACACGCTCATTGGCAACTAGCTCTTTTCGCCAGTAAC
TCAAAACCCACAGAA
CCAACAAACTGGCAGTGCGCAGCGGAGATCGGTAGAGAGGTCAT
CATGACACCGGTGTAC
GCCCAAGTGGACGCCACCGCCTGCCACGTCATGACGGAGACCCT
ATGCCGATACGTACTA
GCAGGACGCCCAGCCCCCGCCCCACAAATGCCTCCCGGGACCCAA
CTCGCCCCCAACTCC
CCTCTCGCTAACATCAGCAGACCTGTCAAGATTCTCCGCTTGGCG
GCGTTCGCCCCTTGT
GTGCCATCAGCGCTTGACCACAATGTCAGGATATACTTTCTCGAG
GATACGCACGATGCC
TTGCTTGGTGTCGTGGAGACAGAAGAGCAGCTAGGGGGGCGACT
CCTGGACGGCCCCCGA
CAGATCCTCCTCCAGTATGGGGCCGGTGATCTCTC
>ConsensusfromContig2929 Average coverage: 8.15
GATTTTTTGTGATAAATATAATTTCATCAGTTCCTTAATGACTCGA
ATTTCTCGAATCGT
GTATGATATTATACGCTTTAGATGGTTGTTTTCTCAGTATACCGGT
TATTCTCTTCATTT
TGTTTATCACACATTTTCGCTCAAATAAGGACCAAAATAGTCCAG
AGGGAATATATATAA
ATGTGGTTGGTTGTTTATTTTCATTCGATTCAGTCATGTTTAAAAT
TCAAATTATTTGTT
TTTTTTTAATGACAACCTGTCATTCAGTACACATTTGCTCATAATG
GTGCATGCCTTTTA
TGCAATTTTCATATTCCTGGATTTTTTTTAAACTTATGTAATATAT
ACATGATTAACATT
ATTCATTTGCTAACCAACGTTTGATGAAGCTGGATATATGCCATG
CCTATAAAAATAGAT
GTAGTAATTTTTCCGAAATTCGATTTACTAATTAAGATCTGTTCA
>ConsensusfromContig2930 Average coverage: 19.98
GATTATGATCTGTAGAAAAAGTGTTATCGATTCGTTTATTAGTTTC
ATTATTTTCTTCTG
CCAACACACCTGCAAAGGATGTTCTCCTGCTGGAATTATCAGCTG
ATGTCCTTTGTTGGC
TGAGTTGCATATCTTTGAGGAAGTAGCAATCATCAAATCCCGATC
GATTCTTCACCTTTT
CATCCTCAGTAAAACTGTTTTCCTCAAACGTCAATTGATTTTCTAT
TATTCCATTGTGTG

GTTGGTCTAATAGCGTGTGGTCAGAGAGCGTTATATGATCGTCGA
CTTTGGCGAAACTCA
CGACAGATGCACGTTTTCTCAAATCTGCGTCAGCGAAAAACTCAT
AATCACACCGCAACG
ACGATTTGCGTGGACTGGCATCCTTTTTAACTTCTAACGCTCTGG
ATACTTGTAATGGCT
CAACAACTGTTTTTTGCCGTTGATTTATTTGATTTTTATCATCATA
TCCTATGGATTGTC
GTTTATTATTATCATATTGCATGACATTATTTAACTGAGGTATGCT
CTGTGATCTTTCCA
GAGGTAATTTTTGCTCAATTTGAATTTCTTTTGAAGGGTCTTTGTT
TGTATATCGTTGCT
GCCCTGAAAAAATTTGGTACGGCCTGTTTTCGATATCAGACTTCT
TAGATACCCATGAGA
TGTTTTCGACAAAATCTGGACCGGCTTTCAAATCACTTATACGTT
TGTGTATCAAACTAC
GTGAATTTTCTTCATCAATATTATTTGATAATATTGAGATATT
>ConsensusfromContig2931 Average coverage: 5.11
CGTAATAAAATTGATCATGTAATTGTCTTTGTTTTGTCATATCACA
GTACCGACATTTTT
TGTTTTAACAGAAAAATGAATTTCGAATGACAATATAAATTCAAT
CGAAGTCTTCCTACC
GACGCATCTTGAAATGAAATCAACGCCTAGAACTATTGGAGCTGT
CATAGGTATTATATA
GATTTCAAAGAAGGTAATTATGATCAAGAAAGTTTGACCACCTAA
GTAGCTGGTTTACGA
ACTCGAAGGTTGAGTCTCATTTTCAATTTCTTTCTCAACATTCTAA
TATTTGCGACTTTG
TGGTAACACGGAAAAATAGCATATCCTGATCCACATGGGAACAG
GGACGTAACCAGACTT
TCATAAGGGGGGGAGCGCTTTTTAGGCCAGGTTTTTTTTCACACC
TCCCTTTTATGTTG
TAACCCATATGCACGCATGCATAGATCGGA
>ConsensusfromContig2932 Average coverage: 8.84
CGAAAAGTTGAATCTGCATCAGATCCAATATTTGCTATCAAACAT
TGATGTATTTTTCTG
TGAAACATGTTTTCCGCCATATCACAAGAATGAAAAATCAACGG
GAACAACTATCATAGC
TGTTTGTCAAAGTAATAGGAATGGAAATACGAGAAGTTTATTGA
AAAAAATTAGAAGATC
ATTAGTCGGTTCGAAAAACAAACAAAATAAAACACATAGAAAAA
CGAATGTTCCTTGCAC

AGATTGTGGGACATGCTTGAAAATAAAGAATGTACAAAGAATTT
TTAAAAATGGAGAAAA
GGAGATGGTGGAAGATGATCATGGTAGAGGTCAGATAAATTCCT
ACTGTATGCGCAACAG
CATGGCTTCTTTGTGTGAAAGTGTTGCATCCGAGTGCATCATCAT
ACACGAAAAATTTGA
CTCCTCTTTATTGGGAAATCAGGACACAAAATCATACAATACTTC
CAGAAAAAAATATAG
AATATCACAAAGTGTTCA
>ConsensusfromContig2933 Average coverage: 20.10
ATTTCAAACTCCGCCCACCTTGACAACCACTCGTAAACATGTATA
TTTTATCTGACGAAG
ATTTAATCAGTTTCGTAAGAGAAAGTCCAATCTTGTGGGACTCCA
CTAATATTAAGAACA
AAAAGCTGAACATGAAGGAAGTTGCATGGTCTAAAATAAAAGGG
GAAACTGTTGAGAATG
CAAAAAAAAGATGGAAGGTCATAAGAGACCGTTATAGAAGAGAA
GTGGCTATCCCTCCTT
CAGGGAGCAAATATAAAAAAAAGAGCTGGAAGATCGGA
>ConsensusfromContig2934 Average coverage: 10.32
ATTTTACCAATCATTGGTAAAATATTCGAAAAAATAATTAAGCTA
CGTCTCACTGTATTA
TCTGATACTAATAACTGGATCAATTCAAATCAAAACGGTTTCACT
CGGGGATTATCAACA
GAAAATGCCGTTGAAAATATTGTTACAATGATCGAAAACTCCTT
CAAGCAAAAAAGATTA
AGAGGATGCTTATTTTTGGATATTCAAGGAGCCTTCAACAACGCA
TGGCATCCTTCAATA
TTAAGATCACTTATAGCAAAAAACTGTCCGAAGTACCTTATTCGA
ATCATAAAATCATTC
CTGGATGAAAGAAAATTCACGTATACTTTTAAATCAATAATATGT
CAAAGATCTATATCA
AAATCGTGCCCACAAGGTGCTGTTCTTTCGCCTTTACTTTGGAAC
TTGCTATTGGATGAA
CTCATTGGGAACTTCAACATCCCAAATACCACTATCCAAGCATAT
GCCGATGATATCTTG
GTGACAACATCGGATAAATCATTTGCAAATATTGAGAAATCTTTA
TCACTGGCATATATC
GAGTGTTGTTCATGGGCCCGATCTGTCAAACTCACCTTCAATGCC
AACAAAACACTGCCA
GTTATTTTCAGAAGAGTACTCACTAATATTCCTGAGAATATACAT
TTGGATGGCCATGCT

TTCAAAATAGCAGA
>ConsensusfromContig2935 Average coverage: 42.42
ACTGCTCGCACCGGGAATGGTGACGCTGCCTGTCTGTTTCATATC
GTCCGAGTACGGATG
ACTGCTTTCCTGTACGACAGACTTCACGATTCGCTTGTCATACGT
ATCAACATCGTTCGC
CACATTCTCCAGCTCAGCGATGCACAGCTCGAGGACCATCTCGGT
GACAGCGGGATTGTT
GGCGGAAGCTTGGTCAACCAAAACCCGGCTCAAAATCTGATCCGC
TCGGTGTCCGACTCG
TCCGGCCACGGCGAGCTTGAGCAAATCAATTATGATCCGAGCTTC
ATTAACGCTCAGCGG
TTTTCGAAATTCTGATTGGTTGCAAAGCTCCTGGTTAATAACAGTA
TTACTGTTGTCCCT
TGATGATGATTGGCCATGAGAGCAGCTCTCAGATATCGCATCTGG
CGTCGGAAACTGAGC
GATATCAGCTTCCGATATAACAGTATCCGCGATGAGGCTTATCAC
GTGACGGTTTTTGAC
CAATCCACTCACATTACTTGTTTGAGCCATGTGCGTGTTTCGTGA
CATAAATATATCTGA
CGCAGAAGTAGATGGTGAAGACTCTGAGATCAATGAAGGAGAAA
CTGCCGAAGAAACAGC
AGAATCTCTAATGGACTCTGCTGCTTTATCAGTTGCTTCACTCGAA
TCAT
>ConsensusfromContig2936 Average coverage: 39.81
TTTTTTTTTTTTTTCCTTTTTTTCCCTCCCCACCCCCGCCGTGGCC
GTGGCGGTGGCAGT
GGCAGTAGCGGCCGCAGCAACCATGGCAGCGGCTGAGAGGGTGT
CCTGTTGACCAGGGGG
GACTGACCCCGGAGTGCTGGGGGTCGGGGGATAGCCATTTGGTC
GACCCGCGTAGGCCGG
GCGCTGATAAGCCATCGGGTTGTAGCCCATCTGCTTGGGGTGGGC
CTGCATAGGGTTGAC
CGGGTAGGGACCACTCTGTTGGGCCGGTGTGTAGGGGGATGTCGA
CATCGTCGTGTTGGT
GAAACCGGGACCAGCCGGATTGTGATTGAAGGGACCGCTTTGAG
TTGTCTGGGTGACGCC
CCATACCGTCGTGACGACTGAGAGGGCCTGCCCCCCCGGAGGACC
TTGGTTCCAGCCCCC
CATTCCCACGTGATTTGTGGGTGGCATGCCTTTTGAAAGAGCATG
TGTCATCTGCATGGT
CCGACAGCCGAGACCGGAG

>ConsensusfromContig2937 Average coverage: 41.52
GGCGTCTTGCTCCAACACTACCACCGTTCGCAGAGTTATACCCAC
CTGTCGCAGAAATTG
TTTCTCGTTTATCCTAAATTGGTCCACAGAAGTGAAAATGATGAA
AACCAGCGAGCCATG
GGCGTATTTGTCATGAGCCCCTCCTCCATTGACACAGTCAAGGCC
ATCGAAAGAACATGC
CGCACTGTTGCACTCGGCGTCGCACTGTCCATTTCCATAGGCCTT
GGCGCAAAATGGCTC
ATGCATGTCGCTGCAGGTCGCCCCTGTTGCGCGATTGGGGTCGCA
GTCGTAACCGTCGTA
GAGGCACGCCGCGTCGGAGCAGATCGAGTCGCAGTGGCCGTCAC
CCCATCGGCCCGGGCA
ATCAAAATCGTGGGCCCGCATCACCGTACAATTCTCCCAAGAGCG
GGCGTG
>ConsensusfromContig2938 Average coverage: 6.18
AATTTCAATCCAATTGATGGGTTGGATAGACGAAATAATACTATT
GCTCAGAGCCTCCCA
TGGACATGCCATTCTGAAATAACCCCAAATATCATTATCGAAATA
TAACCTTTTTGAACA
AACGCCATCAAAACAGGAGCAAAACACAATGGTGCACTATCTAA
AGGAAATTGACTCTAG
AAAGGAATTGAAACTTCATGCGATATTATGAAAACAGTTGTGGA
TTCATAGAACAAAACG
AAACAAAAAACATTTTTAACTGGGGGAAGGGGAGG
>ConsensusfromContig2939 Average coverage: 8.85
AAGAATTTGGTGTAGATGCCATACTTGCCTACAGATAATTTCTAG
AAAAATATCTTTTAT
AGCTATATCTTTGACCACGCCTATTTGAATTTTTGATCCATCTTAC
ATAACCATAACGAT
CGGGTGATCCATTGAACTACATGGCAAGATAACGAAATATGATAA
AGTATACTTGCATCT
TTCCTTTTATGGTATAAGGAAATCAAAAGATAAATTTTTATAACA
TATGGAGGTACATAT
TAGAAGATTTTTTTGTTTTCATCGCGTTATAAACTGATTGATTTGG
TTGACTTCGAAACC
CTACTAAAGA
>ConsensusfromContig2940 Average coverage: 10.01
TCTTGGTCTATTCCTCGGAGTTCTATTGCTTCTTTTCATTCTATAC
TGGGACAGGAGCAA
GAGGAAATTAAAGGAAACAGCTACACGGAGTGCCGCGAGGAACC
AGAGCAATTCAAATCT

ACATAAATTAGAGACAATGTCGTGTGTGGACAATCACGCTACCA
CCATCTATGGAAGAAT
AAGGCCTCCTGGTCTTCATGATTCCTGGATCATGTCCTCTCCAGA
GGACATCTACGATTG
CAAAACCAGCATAGCCGACATTCTCGCTTCAGAAAACAAT
>ConsensusfromContig2941 Average coverage: 6.31
GTATTTACAACCTGTGAGATGAGTATAAGCATTTTAAGTACATAA
TAAATTCAAAGAACG
AAATATCAACAATTGAAAATCATCAAAAAACAATTTTTCTGAGA
CTTATAATTTAGGTC
AACTCTGCATCACCGGTCAGTGTTTATGCAATATTTCGCTGTGAC
CACTCCTGTTCCGC
GTTTTTCGCATGGTGAGAGCATTTTCGCATGACCCAGTTTCATTGT
TTTGAATGTCATCT
TCATATTCAGGAGGCGAGTCTACATCATTTTCTGGTTTGATTGTT
ATCGGTTGAATAGTC
TTGTCATATGTTTCATTCGGGAGCTTTTGAGAAGAATCATCATTA
CTCATGGATAAATCT
TCAGAAGTTTCCCTCTTCGAATATTCAGTAAATTGATCATCATGA
ATGCCATTGGAGCAG
ACGTCATCATCGAACTCATTTTTGGACACCATGCGTGTCTCATCT
GTGATCATGTCATCG
TCGCAGCCATAAATTCCGAATATCTTCTGATGGAGATTGGCATTC
GCGCTGTGGCGGTCT
CTACTTCTCTTGGAGGGAAATGTCAGCTTGCATCCAGATATTTTA
CAAGGATGTTGCAGC
CTTAGGTGAACGTTCTTG
>ConsensusfromContig2942 Average coverage: 8.67
AGACAGTGTGTGGTTTTGTACCTGTGTAATCTCTGCAGTGAAAAC
CTCCGAAGGTTTCCA
AAGCAACTCATCAATGGCCGAATCCTCTACCCTGAAATAGAGTAT
GTGGATGACCATTGA
GATCAATATACACGCAAAATAACAGGAGATGTCCTCGCAGCGCG
ACTGCACGTGGCACCT
GAGATTATCAAATATCGAAAGCAAAAGGTGCTCGGTTCGCATCTC
TTCGATGAATTGGTA
ACTCATCATGTTGTATACAGTCATTGCCTTGTCGAGGATTACTCG
AATAGTCTCGGATTC
TTTGGAACACACCAATTTTCCTTTATGATTGAAGTTTATTTCGACT
TTTGCCATTATATC
AAAAACGCAAACGGCTGTCAGATTCCAGTCTAATTTAGGTCCATT
TCCAATCATGACAAC

TTTGACTGCTGTGATTT
>ConsensusfromContig2943 Average coverage: 5.87
ACGACGCTCTTCCGATCTCTGGTATCGGGATTGAAAGCGTTGCCT
CGTGTATTTTATCAT
AGTAGTACAGGCCGACGGGCTGACTGCCATCAGACATATGTTCTG
TATATTATTTCTCCG
ATAATTTCGATTTGAGTGTGGTAATCTTGCAACCTATCTTTTCAAC
TTCACCGAGTTGTG
GGCCTTGTTTTTGAGCCCATTGTCTGATGGTCGTTAGGTGGCTAAA
TGTTCCCGCTTTGG
TGATTGACAGTTCAGTTCGGTTCTCGATTGTTTTCGTTCTGCAAA
TTTCTAAAAAGATA
TTCAAGGTGTTGTATATCTAACTACTAACCAGAATTCAATCATTC
ATGTTAGAATGTTGC
TGACTATGTGATGAAATGCTCAATTGACATTAAATTCCGCCGTAC
ATTCTAGACCATCTC
TTTGCGAGGCTGTCTTTTCGATCGATACCAATGGATTGACTTGTGA
TTGTGGTTACCGTT
TGTGATATCACAAGCACCTGGCACATATCGTGTGGTTCTTTATTG
CACATGTCCACTGTT
CGTGTTTATCACCGTTATCAATTCAGTTATGTTATATGTTGATACT
CGATCTAAATTGTA
TCTGC
>ConsensusfromContig2944 Average coverage: 14.83
CACACATTGTGAGAATTCATCGTCAACTCTACCTTATTTCTTCTTT
AGAAAAGTCAATTT
TGTTCCAGTGCTCTATAAGCCTCAGCATGATATTGTATTCAATTT
TCCCGATGTCGCCAA
TTGACAGACCTTTACTATCATAGCAAAGCCAGTGCTGGTTGCACA
CATTGTGAGAATTCA
TCGTCAACTCTACCTTATTTCTTCTTTAGAAAAGTCAATTTTGTTC
CAGTGTTCTATAAG
CCTCAGCATGATATTGTAT
>ConsensusfromContig2945 Average coverage: 19.64
GATATGACGTATAATCTCAACATCCGGCGTGTGAAGTTCCGCTGG
AAGCAGTGCAATTTC
TTGGGCAAAGGTCAGTTTGGTCAAGTTTATGCAGCCATGAACCTC
GACACTGGTGAAATG
ATGGCCGTCAAACAAATTAGTTTTCGGCCGAATGACCACGGGCGT
GTGAAAGACATTGTT
GACGAAATAAAAAGGTTTGAAGGGATTTCCCATCCAAACATTGTC
AGATACTTTGGAGCT

GAAGTTCATCGCGATGAAATGCTTGTTTTCATGGAGTTTTGTGCT
CAAGGAACCCTCTCT
GAAGTCTCCAGACATGGACTCTACGAAGTGATGATTCGCCTCTAC
ACCCTTAACATCCTC
AGGGCTGTCCACAAGCTCCATTGCCACGGAATTGTTCATCGAGA
CATAAAGGGAGCAAAC
ATTTTCCTCACCCCCACCGGTCTCAAATTGGGAGACTTCGGAAGT
TCCATAAAAATCTGC
CTCAACTCAAGCTTATCCATGTCATGTGCCAAAGAGGAACAGCA
GAAGTTACCCGGGACC
CCTGCTTATATGGCTCCAGAGGTCATTACCCAACGACTGTCGGGA
TCACCGAGCGATATT
TGGAGCGTTGGTTGTGTCGTCATAGAAATGGTTTCTGGGAAACCC
CCTTGGCATACTCTG
GATAACCCAAACCAAATATTTTATCAGGTCGGCTGTGGTGTTGCA
CCAACTGTTCCAGAG
AATCTCGGAGAGGAAGGAAGAGACTTTATTGGTCATTGTCTTGAG
ATAGACCCAGACTCC
AGATGGACTGCAGAACAACTCATCGATCATTCCTTCGTCAAGGTT
TGTATTGAAGAGAAA
GAATCGAAGAACATGTGGAAGCTTTGATCAAATGTATTTTCTTTT
GATGAAAAATTTGAT
ATTTTTTCATTTTATGCAATCCATGTTTTGTTGTTTGATAATCATTG
AATTTCC
>ConsensusfromContig2946 Average coverage: 26.33
TCTCTTACAACTTCGAATACTTGAGCATTTCCCTTGAGTATGTTAT
TCACAAACTGATCC
ATCGAACCAATGATTATATGTTGCACACTTATAGAAATAATAATC
AAAAACTTCCATGGA
TGAGCAGCGTTTGATAACTTACACAAATGCAAAACGAAACAAGT
AGAAACTAATTCTGTG
AAAGTAAAATACATTTGATGATTCCATTGATAGTGAAACTCTTCG
TTGACGTACTGAATC
AGGTTCCACCATCCGTAGTAATGTGAATATAGACTGGAAAAGAC
GAGCGCAGCCATCGTA
TAATTCATTTGAGAGATCCAAACCAGAGAAGACATATATTTACAA
GCCTCGTAGGTGGCC
AGAATACCAACGAGAAGAAGAAACCAAATGAATGTCATTTGGTG
AGTCGCGTTGAAGTAA
AAGTGCTTGTAGGCTGCAGGCCAATGCTCATATCGACCTCGAAAA
ATTGTGTCAAAGCAG
CCACATGAACACTTGACTTTATCCACTGCTGGCTTTTCGGGGGTC

CAGACATAATAATAA
GCAGCTATGTGAAGAACAGGTACAATGAAAGAAACCATCAATCC
TGATATTGAAATTAGG
AATTTATATTTAAGACCCCAGCCTACGCTGTCATAATGTAAATTTG
TTAAACGTTTCTCT
AAATATTTATTCATTTTTAAATTTGC
>ConsensusfromContig2947 Average coverage: 6.33
GCCCGATAGAACGAATCTTGTTTGATCGAGACGACATATCGAAA
GCATGAGCCAAACTCT
CATTCCCTTCAAGCCGGAATTTCCATGCAAATTCTCTTCGAACAC
ATCAGAGGAGAACGA
AGGCTTTGACTGGAATCCGCTATGGATTGCGCACCGAGATGACT
TCGAGAAGGAGATAAC
AAATCTGGAGCAGCAGGTGGATGGGACCTTCGACAAACTGCATCA
GCTGCTCCCCGTTCC
GTCTCTATACTCAAACGGTTTTACTGATTCAGTGGACAGTGGAGA
GGCGAAAGGGTCCGA
GAACCCAGATTCAGATGAGGGGCCCTGCCCAAT
>ConsensusfromContig2948 Average coverage: 10.90
CAAGTCCGTCAGGACATCGGTTGACTCTGATCACGCCGTCCAGGC
ATTGGTAGAAACGCT
CGCAACTGGAAGAATCCGGAATGGCTGTTTCTTTCTCGCATCTTA
TGAGAGTTGAGATAG
AATTCGGTGGCGTATCTTCTATTTTGAACCCATTATTGCTGTCAA
CAGTCGGCAACGCTG
GAATTATGAGCGATTTGAATTCAATTTCATTCTCGTTCTTCTTCAT
GTGCTTGGAATCGT
CTTGAGTGAGGTTTGTGGCTGCAGGTTCAAGGGCTGATTTCCTGT
TGAAGACTTCTTCGA
GAGAGTTGAGGGTCTGTGTTGTGTCGCGAGATGTCCGGTCTTCTC
CGGAGTAACATTCCG
GCACGTTGTAATCGTAATCACATACAGTATTCCGAATGTTGAACA
CCAGGCCTTCTGCGC
ATGTGTGGCGGTTTATCTCTCCGGCTGAACAGTCGTAAAAGGCGT
GGCAATCATTTGGGT
CGGGAAAGGGTTTTCGCAACCCACAAAGTTCATGTTTCACTTCG
>ConsensusfromContig2949 Average coverage: 9.37
TTATACTCCACTGATTCAAAGAGGTTTTTTCAATGGCTCAATGGT
AGATGTCTATTTTGA
GTCAGTTTTCAGTTGATACCACGCATCGAGGTTATACTCCACTGA
TTCAAAGAGGTTTTT
TCAATGGCTCAATGGTAGATGTCTATTTTGAGTCAGTTTTCAGTTG

AT
>ConsensusfromContig2950 Average coverage: 28.53
GGAATGAATCAATCAGACTGTATATACCCTCGCAGATTGATTTGT
ATTTTTTCGGCTACA
TCACCCTTGAGATTGCAGAATATAGTTGCTTTACACAGCGTATTA
CAATCATGATTTTTC
GGAGGTGTCCATTTGATAACGATTGGTCTTTCGGTCCCAGCCTCC
ACCATACCCTTTACT
GGATCAACCTCAAATCCGAGATTCACGGCAGCCTGCAGGGCGTC
GAAAAACCATTCGCCA
TTCTTCTTTTGACTAACAGCCAATGTTCTCACACAGCCAACTGTTA
TGCTTACACTTGCT
GCTTTTCGATCTTCTTTATTGCCACACAAGGGAA
>ConsensusfromContig2951 Average coverage: 20.78
TCTCCTACTCATTGTTTCTCTCGTTTTTTGTCACCCAGACATTCTGT
TAATGTATTTTGC
TTTAGAGTCATTTCTCCTAGTGAAGTGGAGAAAATTATTATGAAT
ATGCCGAATAAAAAA
TCAGTTGGCCATGACAATATTTCGATCATTCATCTTAAAAATATC
AAATCTTGTATTTCA
CCAATTATTGCAGATATATTCAATCATAGCATATCTGAGGGGTCA
ATCCCAGAAGGTTCG
AAACTGGCTAAAGTTATACCTGTTCCAAAAGGTGGTGATCCTAAA
CAAATATCTAATTAC
AGACCAATCTCACTTTTACCTGTTATTTCGAAAATTATAGAGAGA
ATCATATATATACAG
TTATTCAACTATTTCGATATCAACTTGCTTTTATATAAATATCAAT
ATGGTTATCGCCCT
TATCATTCCACCCAGATGGCATCACTGCATCTCATTGACTGGTGT
AAAACCAACATGGAT
GCTGGTAGACTACCAGTATCCATATTTCTTGATTTTTCTAAAGCAT
TTG
>ConsensusfromContig2952 Average coverage: 24.86
AGGAGGTCAAACTTGTGGTCGAGTCTCGCCCATGCTTCAGCTATT
GCCGTTGTATTGCTG
AGCATACACACTGCCCTTTGGACTTTGGCCAGATCCCCTTCTGGT
ACAACCGTGGGTGGT
TGATAATTGATCCCCACTTTGAATCCGGTTGGACACCAGTCAACG
AACTGAATACTACTC
TTGGTTTTGATTGTGGCAATTGCTGCATTGACATCCTTCGGTACAA
CATCTCCTCTAAAC
AGGAGGCAACATGCCATATATTTGCCATGTCGAGGATCGCATTTT

ACCATTTGATTGTTT
GGCTCAAAGCACGTGTTTGTGACCTCAGCCACGCTCAACTGTTCG
TGATATGCTTTTGCT
GCTGACACAATTGGAGCGTATGTTACAAGAGGAAAATGAATTCT
GGGATAAGGAACAAGG
TTCGTTTGAAACTCGGTCAAATCAACATTGAGGGCTCCATCAAA
GCGTAGAGAGGCAGTG
ATAGAACTAACAACTTGAGCGATAAGTCTATTCAAATTACTGTAT
GATGGACATTCGACG
TCCAAATTTCTTCTACAAATGTCGTATATCGCTTCGTTGTCTACC
ATGAATGCACAGTCA
GAATGTTCCAAAGTCGTATGAGTTGTTAAAATTGAGTTATATGGT
TCTACAACTGCCGTC
GAAACTTGGGGCGCAGGATAAACAGCAAATTCCA
>ConsensusfromContig2953 Average coverage: 21.73
TCCGTGTTAATATAATTTAGAGACAATATTTATTTAGCCAATTCC
ATGATCAAATCTAGC
AAAACTTTTCTCTGATTTGCGAAGTATTCATTCGGTAGCGAAGTG
ATGTCCTGGCAAGGG
GCGTGTCCAAGTAATTGGGAGCAGATTATTTTGGTCAATGAAGTC
ACATATATTCCGCAG
TCAAAACCATTATTTTGTTGGGGGGTTTTTTCACAGACGAATTGAA
TTGACTTTCTTCTG
CCTTGTATAATTTCATAAAGCTTCGAAGCCAAAGTACGTGCATCA
TCTTGGTTGATATTG
CCAGAAGAATCGAAATGATGAAACGAATCAGAGTCTTTTCTGTA
GATTAGAAGACTCCAA
TGGTTTCCACCCGCCG
>ConsensusfromContig2954 Average coverage: 8.48
GAGACGGAGGTGCTGAGGCTCGAACTGTTTCTCCGGAGGATCTCT
CACCTTTCTTCCATC
ATCTGCTGATACTCTCGAACTCTTTCTGAGTGCTGTTGCAATTCC
TGACAAGCTGTCGCG
CGTGACACTCGACATACACGGGGCGATTGATTGAAGAAAGTCAA
GAGCGATATACCGATC
ATCTTCTGTGGCTTTTCTGACCCAGTCTCCAATTCTTGATTCTGCC
TCCGATGACGTCTT
TCGGTTTTGGTCATAGTTGTGGCGATTTAGTAACTGTCTTACTGCG
TATGTTTTTGGTTG
TCTTTCGTTAGGTATAAAATCAGGCCCTGGTCGTATTTCAGGCAG
CGTCTTGGACTTTTG
CAGGGCCTCCATATATTCG

>ConsensusfromContig2955 Average coverage: 13.46
TTAGCTCGATCGAAGACAAATCGTTTCTTATGCCTGGTTCGGTTG
CCTATTTGAGTGCTA
TGATGGGCAAATTGATGATAGATTTTCAAAAAGAAATAAATAATC
CTCAAATTGGGTTAG
AAATTGTGAAAACCGCTCAAAGAATTTTAAGAGTGTGCGGTAATC
TTTTTCATGTCATCT
TTGCTGCTATGATAAGCATACAAGATAAATTTAGTTCTTCGGTTGT
CTTCATCGATTTTG
ATAACATTTTCATTTTTAGACCAATGCTAGGATATATCACTCTCCA
GTTAATTTCCATCT
TATCTTGTGATGAAATTTGTACTATCGAAAACGTGAAATTGTTAA
TAAAAGATCTCATCA
AAATATCTGAATATTCTGCGAAGTATTTGTCAATAACTGAACGTC
TTGTGAGGATACAAT
TATCGAAAAGCCCGCCAGCTGAGTCTTCATCCAGACCAGATGCCG
ATAAAATTCGCATAG
TCGACCAACCAGTGAATTTTCTCAATGTCAAGGTCCCATGTCCAT
GGAAATCTGGAAAAT
ATCTCGAAAGTCCTCATCCCGTCACGGATGGATTCAAGTTCAAGG
AAACTGTGCGAATCC
CAGGAGTCCCATGTCTGTACCTACAATTTGACCCAAAGTGTTCCA
CTCAATATGACTATG
ATAAAGTTTCTGTCTATGCCGGTGATGAAATAAACGTCAAACGAA
AGGTTTGTGAATATG
GCGGAAACCCGTTCGGATTCGGTTCTAAACTCGTCATCGGCGGG
GGGGGGGG
>ConsensusfromContig2956 Average coverage: 24.82
GAATCATGCATTTGGCCAGAAACTGCATCCGAGGGGCTTTTCAAT
AAAGTTAACTGTACA
GTTAACAGCGTGCCCATAATAGCTCCCGTGGAAACAATACAGTTG
CTGTTCGGAGCCTCA
CTGGACATCACCATCTCGGCCCGCGATCCAGATGGCGATACGTTC
GAATTCTCTGTCGAC
ACTTCCATTCCAAGATTGACTATCGACGGAACTTCCGGACAGATG
ACTTTGAACTCGATC
CCGGAAATAACAACAAACTCTCCAATCACTGTTATCGCAACAGA
TGCAAAGAAAGGCGTC
TCCAGAAAACAGCTTAATGTGCAATACTGCTACTGCAGAAATGCA
GGGACATGTCGTTTC
GAGACCAGTCCATCATCGGCCTT
>ConsensusfromContig2957 Average coverage: 18.93

AAAAAATCTCTAAGTTTTTATTTGTTTGTTATTATTTGGAGTAGC
CTGGCCTTTCGCGT
TAAATAAAGATCAATTCGTAGATAAAATTGACTAAGATAGGAAA
TATCAGAGAATAGTTA
CCATCCAGAATTTATGAATCAACATATAGACGATGAAGACTTATT
AGATGAAATATATGA
TTCCCTGCGAAACATCAAGGATCCCGAAAGGCCAGAGACCCTTCA
TGCCTTGGGGGTCAT
CGGCGAAAATAGTGTGACCCTCACAACATCTAAATATGGAGATA
AACAAATAAAGGTTGT
TTTTAAACCTACAGTGCCTCATTGCTCCCTCGCTCTTATAATAGGG
CTATGTATTCGGAT
AAAATTACAGAATTCATTGCCATACAGATTCAAGTTGGACATTGT
TGTTGAGG
>ConsensusfromContig2958 Average coverage: 15.50
TTTTCTTCAGTCTTCGCGTTAATATTCTTGAAATAATTATTATTG
AGCGGCGAAACAAAT
TTACGGAAAATTGGTTTATCGGGTATTGGTAGATTATGGATGGAA
ATGCCTTTGTTTTTA
TTGGTGAACATGTCAGGATTTCGTGCAAGTTGGGCCCACTCCATG
AATCGTTGGCCTTGT
GGATAACCGCTTCTTGGAGTTTCCATCATGAAATGTTTGTGTATTT
TGACTGCTGCTGAT
ATCCAGACACTGTTTTCGACAGAACCTTCTATTTGAATCAAATTC
TGTTCCAAATTCATC
AAGGACTTCTCCAAATGGTGCAACAATTTTGACTTTAATATTCCA
ACATCGTATCGGAAT
TTTTGAATCAACGCTTCAAAATACTCATGTTCGTTAATTTCTTCTC
TCTTGACTTGTTCA
AATTCATCTCTAAACTTCTTGATTTCTTCTAAGAACATCTTTATTT
CCTTGTCACA
>ConsensusfromContig2959 Average coverage: 13.27
AACAAAATTCTGAACAAAAGGAATGATGTTCGTTAAAAAGTATTT
CGTATATATATATCA
CTCGTATTGCTGGATTGTATAACTCGATATTACTAAAAGTATTAC
ATTTGTATGCGTAGG
ATGCTGCGTTTTCCTACTTAAGATTTAGCTGGAGGCTGTAATATCG
TTTTGTTATTATTT
ATTGCCCATCCTCCTGAATAAATTCGTACAATTTTCCATTCAGTGT
TATATTTTTTCGCA
TTTTTCGTTTGTTTGTTGTTATTTCATATTTGCCATTTAGCCGGAT
AAACGGATAGATGA

TCGGATCACATTCCTATTTGGCATGCGATTTTGGATGTCAAAACG
TCTGATCTCAATCTA
TGGGGTTTTAGTATGGACATTTTCATTTCGAACTTATTTTACTGCT
TAAGTTAAACATAA
AACACAAATGGTTTCATATTGGAAATAGCTTAAACTGATATATGA
TCATTCGTAACTCCG
ATAATACAGTTTATATAATACTACAAATTATGTATAGTGAACTGA
AACACTGATGAAACA
TTTATGCGAAAAACACTCAAATTTACACAAAATGATGTATCATAT
ACGACAATGACCAAG
CGTAGAATAGTGAGAAATTCGGATATTGCTCTAATGTATCTATTT
CTCTGTGACTCTTCC
AAACGAAAAAACGCCAGATCGGAAGAGCG
>ConsensusfromContig2960 Average coverage: 4.27
ATATCTGTCATCACTAGTCCTGATTCTAAGAGCTCATCAATAAAA
TCTTTTAACAGAGAA
TCAGAAAAATCGTCAACTTTAAGAACATGGAGAAGTATGAATGA
AGATGGAACTGTGTAC
AAACATCATTTCAAAAACAGCAGAACCTTGAGTTTGCATATGAGG
AATATCGTTGGTTCT
TATAAAAAGAAGAAATCAGAGGAGGATGGTTTAATTGTCGATAC
GAAAACCATTGATGCA
GA
>ConsensusfromContig2961 Average coverage: 4.32
CGTCTATGTTCCTTATTTCATCCGCATCAATGACGTCATTTTTGTC
TTGGTCCATTGTTT
CAAACGCTTTTCTTTTCACTCTGAGATTGCACTGTACCCCCTCAA
CCACGGATGGATAGA
AACAAATTAGAGATGAGAGTGCGGCAGGATTGAGAATGCATGTG
TTTCCAATGTCAAATG
TAGGTTCTTTCCAAACATCATGAATGAGAGAGAATGA
>ConsensusfromContig2962 Average coverage: 8.74
CATCCCCGAGCAGACAGAGGAGAGCCCTGAAAGCGATGACTCGT
CGGAGGACAACTCAAA
ATACCACGTGATATCGACAGCCATCCTGATAAAACTCTTAGGGAT
CTGCAAGGAGTGCGC
TGCGGCAGCTTCTATCGAGTCCAAGAACAGCATCGGGACAATGTT
CAAGTGCCGCATATC
CTGTGTCGAGGGCCACACGTATGATTGGCAGTCCCAGGAACTCG
TTAACAAAACTCCGGA
GGGAAACCTGTCACTGATGGCTTCGACATATTTCAGTGGTGCCTC
TCCGGAGAAGGTCGA

CGTACTCTTCCGTCACTGGGGCATGTCATCATACAATTTGAC
>ConsensusfromContig2963 Average coverage: 29.85
ATCGAAAGCCTTACGCGTCATATGAGGCTTCACAGTGGGGAGCTG
CCCTATAAATGTGGA
ACCTGTGGAAAGATGTTTCGTTACGGAACTGCTTTGAAGCGTCAT
GTGAAAGCATGGCAT
ACAGAAATCGGCTCTATACGTGAGTCAAAGTCGCAAGATTCAAG
CTCCAAAAACATTGCA
CACGTGCAACAAAAGAGTATTCCGAAGATCTTCGAACACAGTGA
AACTATCGCAGAAAGT
GTCATGAGAAATGTGACAGATAAAAATGTGAAACCTCGAGAAAA
CATTCACCCAGGTCAA
AGAATGGATACCCATCAATACGATGACGCAAGGGAAAGGTTATC
GGATGTTCAAAATGCT
CCCGTTTCATCAACAATGTTTGATAGCAAC
>ConsensusfromContig2964 Average coverage: 5.92
GTATCATCATTTTCTGATATTCTTCATTATATAATCGTTTTTATCA
TTTTGATCGCTCAA
TAATTCAACATTCCAAATTTTCACTTTGATATTGCATCAGGTCGA
CATAAAAAAATATCT
CAGTAATTCTTCTGCTCTAATTAATGACATGAATCCGAAAATCAT
TTAACTTTATTTCAA
TTTTGACAACCGTGATTCTGTTCTATATTTTGTCGATGAGTACATG
ACAATAATATTGAT
ATTTACCATAAAACTGCTGCTCTTTTTTCATCATGTCATGTTCTTG
A
>ConsensusfromContig2965 Average coverage: 7.27
ACCACACCAACTGGATTATCAAAGAACTTGAGGAAGATAACCAA
AGCATTGGGGTGAACA
TCAAGAGGCGCCACAAATCTGTAGCCCAGAGCAAATTGGCCGAA
TTCCGTGCAAGAGCAT
GTGAGAAAGCCGGGGCGACCCAAAGAAGTTCGCCGACATTTAGA
TTTTGACCACGTGTTA
CTCGGAGATATTTGCTTCAAACAATCAAAAGCGGTTAACTCCAA
AAGAGTCTCAAAAACA
GTCACAAAGGGTTTTGGTGCTTCCTCAATTCTGCATGTCTTGTCT
GGAAAATGCTTCATA
AAGAAATAAAACTGTACAATTCCTACATATGTGATGGGACCATCA
ACGCTGATTCCATGT
ATAATAACATCTGTTATAGTTTTTGTTGTTGTTGTGTTTGAATCCA
GTCTCCTGGAAATG
ATGCGTAGAATATGCCTTTCGGGGTTCGAGTCTGGAAAACACGAT

TTGGCGACAGATAAT
TTCTTGACCCCTTCCTCAAATGGATGCGTCTCCCCACTAGCCGGG
TCAACAAAATATAAT
TGCCTGTGCATAAAATGCAGCATTCTTGTAGGCTCTTTGGAGACT
GTTATGAGGTACGGA
AG
>ConsensusfromContig2966 Average coverage: 10.88
GATCTCTGACGCTCAACTCTGCATCAGGATGGCAAGCCTCTGCAG
CAATCTCAACCGTCT
TGGTGTTGCTTCTCTCAGATGTCCGGAGTCGATATTGGGAGAGTC
TCAACTGCACAATCG
CACCTGAGATCACCTGGACAGACTGACAGGCCTCGTCGGACCCG
AAAGGAGTTTGAATGG
TCATGCAGAACAAAAACGTATCTACAGGAAGTTCGGATGTGGAG
AACGTGAGGTAGGTTT
CATCAGCCGAGCTGAGGGCAAGTTCACTCAGCCAAAGGTCGAGCA
CATCGCCATTTCGTC
GCCGACATGTTTGGTCAAATGTATTGTCCGGCACAGAACATGTCC
ATGTTATCGTAGGAA
TGCCTCCGTAACGAGAAGGATCGGAACTTCTTCTACCATCCAGCG
TCAAAGAAGTGTTAG
AGCCGATCTCCTGCGGAGCAAGCAGCGTGGCAGATGGACCACGA
GGCCGAATCATCAAGG
TGTGGCTGGCCGAAACAAACGTCAAATTCCCGCTCGTTTTTCCT
CAACGGTCACCTCCA
CCGTGAAAACGTATGTTGCGTTTGCTCTGACGTTCATGGCTGAAC
CTACGACCTTGATCT
GATCTCGTTGGCAAATCGGAACAAAGTCGAGAGAATCTAGTGTG
CAGCTCAACTCCCTGA
AGAAGTATCTGGAGCAAGCACTTTTCCTGACGTTGATTGCAATGG
TGAATGTCTCATCAA
CCTCAGCAAACTCGCTCCCAAC
>ConsensusfromContig2967 Average coverage: 17.24
GTTGGGTTGTTGGCACCAGCTGCCATTGGATCAGGAATTCCTCAG
ATGAAATGCATTCTT
TCGGGCGTGTCTAGCCTCAGCCAATCGCTGACGCTTAAAACACTG
ATCGCCAAGGTGATT
GGCTTGTGCGCCGCCTTGGGGGCTGGGCTTCCAGTCGGGAAGGAG
GGGCCATTTGTTCAT
TCCGCCTCCATCATAGCGAAATGTCTGACTAGAGCATTTGATTGG
TTGTGCGGTAGTCAC
GAAGATGAGGCTCATGAGGATAATACTCATCGAGAGGATCGCTTA

TTGGCTGCTGCTTGT
GCTGTGGGCGTGGCATGTACATTTGCGGCTCCTATTGGTGGTGTT
CTATTCAGTATTGAG
GTCACCGCGACCTACTTTGCGGTTCAAAACTATTGGCGGGGTTTC
TACGCGGCCGTTTGT
GGCGCATTCACGTTTCGCCTCCTCGC
>ConsensusfromContig2968 Average coverage: 25.18
TCGGATTTTGGCAACAACCCTCAAGCAGAAGTACATGGGTGAGC
AGGTCCCCGCCATGTG
GCTCAAGTTCGAAGAGTCCATCATGGGAGAGCGTTCAAAGAGTGA
CTTGCTACCGTGGTC
TGCAGTCGTTGAATTCGCTGGATCGTACGGAATCCTTGAAGATGA
GATTCTGGAAGCCGT
ATTGTTCTTGAACAATCTCGGCTCTCTTCACTACTTCAACAATGAA
TATTTGAAATC
>ConsensusfromContig2969 Average coverage: 4.98
CTCGCGTATCAATTTCTATACTCCTTTTATCCTGTATTTTTCTATT
CTTTGATCTTCTG
ATGTCGAAATATCTCCAGCCACATCTATGTGTATTTCAAATCCTTC
GGTCAACTGTTGTT
CGTTTTGTATACGTTTTCCATCCTGTGTTCTTTCTTTTTTTGGATC
TCCCAATGTCAGAA
TAATTTGAGACGCTGCTCTCGGATCGATATCTCTTTTCTCCGACA
TCACTTGAGTAGACC
TCTGTTTGGATGATTTGTCATTTTTCTTTTTAACAGGTTTTTTGATC
CGTTTCCTTTTCA
TGATTGACAATCCAATTACCAAGACTAGTAAGAACACAACAGAT
CCTGAGACTGATAGTG
CT
>ConsensusfromContig2970 Average coverage: 7.69
TGCGGTTTATTATTGGGAATATTCAAATATACCTATTCTATAACT
GATCTAAAATAACAC
CATAATATATCATCTGTAATTTGCTTGTTAAATGACCTCTGTTGAA
TAATCTTCGTGTTG
GTATTATTCTACATAAAAGGTGTAGTTCGTGAAATAAAACAATAT
CAATCAATAAAGTCA
ATACGAAAAAAAACAATTTTTGGCGGAGAATTCGAGAATCTTGAG
TAAAATTCT
>ConsensusfromContig2971 Average coverage: 6.53
TATCTCCATTTCCCTGAAGTCTCTCTTGTAGCTCCTCCAGCCATCA
GCGCGTTTTGTCCA
GTCAGCTCTCGAAGCATTCTTACCAGATCTACTTCAGAGTCAAAG

TCGATGATGTCAATT
GCCCATAGAATAAAAGGAGTTTGTATGCTTGGAATGCTTCTTCTA
GCTTCACTCATCGAG
TTAACAGTGATCACGTTTATTCCTTTGTGAATAGAAATAATTCTC
ATGATGATATTATTG
ATAATGTACTTACTTGAATTTTGAGATTTATCGAAGACTCGACCA
ACAAAAATCGTAATG
ATTTCAC
>ConsensusfromContig2972 Average coverage: 9.06
CCCATTTCTGGCATTCAGGCAACGTGATCCAGATCTGTCAGCAAG
GGCTTTGTGGAGCTT
GAACGGATTGTTAGCAAGGTCAGATGATAGCCCCTCCGCTATAGC
AGGCGCTATTCCTTT
TTTGATTATGGTGGTTTTAAGTGAAGAAGTCTTTTTGCTCTTCGT
AGGCTCGGCCTCAGA
GGCTGAGGTGCACAATGTGCCTCGAGTTTTCTTCCTTGGTTGTTT
TACAAATTCGAAGGT
ACTGTTTTGAGCACCATCCTGCGTTCCGGGGGAGTTTGAAGCAAG
TCTTTTCTTACTAGC
TTGTTCACAACTGGCATAGTTAGAAGACGAGGCAATATTGTCGCT
AGGGGCGCTGTCTTT
ATTATTGAACCACGAACTCGGTGAGTCACACGCTGAATGAGATTT
TTCATTAGTCAAAAT
TGTACTAAATTTGCTCAAAGGCTTGGTGGGCTTTTTAAAGGGTTC
TATATTGTCGGGGGT
CTCTTTGTGTATTTGTTCTGCATCAAACAGCAGCTTTCTTAGCTTT
CTACAGCAAGTCGG
GGACAAGTCCTCAGGACGAGTCTTGTCCGCAACTTCCTCCAGCTC
TCGATCCGGGGAGGT
CTCATCGACCAAGTCTACCGGATTAATGTCGGGTGTGGCCCTGGT
GTTTCGTCCACTGGC
CACTCTAAATGGC
>ConsensusfromContig2973 Average coverage: 12.29
ATTTCAAATATGTTTTTTTCACAATTGGATCATGATGCTAATTTTA
ATTTCAAAATCATA
TATTTTCACATTTGTTACATAATGATTCCTTGAATCGTGTAGGTGC
TATAGCGGTAATGT
GATTTGATATATGGTATGGTTGGTATCTGATGTTTTTGAATTATTT
ATATGCAAATATTT
AATGCAGTATATACTTTGTAATAGTTTGTTATTATATTTTGTTGCA
TGTGGGGTTTATCG
TGCTAGCTGGACTAGCTTACAGTATTTTATATGA

>ConsensusfromContig2974 Average coverage: 6.39
TAGCGCGGAGGGCTATGGAGGCTGAAGCGCTAACGGAGCGCGCC
ATCATCACGGCAGGTC
CAGATATCGTCACTGAGCAGCTGGAGGCCAGAAAGGCGATGTTG
TCGTCGCTGCAGAGCG
TTGAGGAAAGTCTCCAAGGAATGGAAACTCTGCAAGAATCGCTGA
GAGTTTTCTGCTCTC
CGTCTTGCGTGGCTGATATGGAGACAGCTCTTGAAGATACGCAGC
GGATGTATGCTTCCA
CCATGGCGACTCTCGATTCTGGCGTATCAAATCC
>ConsensusfromContig2975 Average coverage: 11.82
TAAAGCAACTCCTGGAAAAAACAACAGGTACTTATTCTTTTTCCC
TAACCCAAACGATCG
GGATGATTTCATGCGTGCATGCGAAGCCAATCCCCTCAATGAGGG
TTGTACCCTCCGCCT
ACCAACCGATAAGGCTAGGCAGAAACAAACTCTACCCGGATCTCT
GGATGTGGTTTTACA
TAATGTGCCACCTGAAACAGACACAGAATCGGTCAAAAGTTTACT
CGGGGTTGGGTTCAC
GGTTGTACGCATCAAATCGTACAAACGTGAAGAAAAAGGTCTTC
CGGCCGAAACCAATAT
GGTTCGAATCAC
>ConsensusfromContig2976 Average coverage: 5.73
ACGTGTGCTCTTCCGATCTGCCTCAGATTATGAACCTCTTCAGGGT
CCATTCCCTGCTCC
TGCAACCGAGAGTCCCGTCGTCCAAATTCTTGATGACGCATCGGT
TGATGAGGTTCGCCA
GGCTCTCAACACAGCTCCTCTTGTAGGAAGTCCCATCGTCCCTAT
TGATGATGATGCATC
AGTTAATGAGGTTCGCCAGGCTCTGCTCTCAGCTCCTCTCATCCA
GAGTCCAATCCTCCA
AATTGATATCGTGAGTTTCTGAATTTATATACTATATAAAATGAA
GACAATTCTG
>ConsensusfromContig2977 Average coverage: 22.24
CCGTAGGGGAGTCCGGCGTATGATTGGATGGATGGCAGTACTCCG
TTCATCAGACCGTTC
ATAGAAGCTATCATGAACATAAGAGCTGCGAATCTCAGGTTCCAC
AATGTACCATCCACA
ACAGCGATTCCATCTTCCTTCAAGTTGTTTTCCTCGGGTTCTGACG
AAGATGATTCTCTG
AGATTCACTCCATCCGTGTTTGTTCCGTAATTCGGGTCTACTTGA
GGCTCCAAACTATCG

CTGACAGCCGCAGGCTTTGATTCTTCGTGGCGAAACTCGCGTTT
GCAGAAAGGCAAAAAA
TGAAGAATGGTGAAAGATCCGATTGAGATTGCCAGCAAGAAGAT
CAGAAGACCGAAGAAA
GTCTCAACAGAAAACACAGGTTTCGAATATGTTGCATTGTAAGAC
ACTTCTGTCACATTC
TTATTCAATGTGCTCGAGTAAACAATTGTTGAAACATTCTGACAA
TCGGCGTCTCCCCCA
ACACCTTGGCCTAAACCAACCAGACCAGGAATGAGTCCGCACAG
GCCCTGGCCAATATAG
AATGCGGCGATATATTCCGGTTTGAAGCGTGCCATATATGGCAGA
TACGATA
>ConsensusfromContig2978 Average coverage: 6.96
GTTTCATGGCATGTCGAAAGTGACATTTTGATCTACATCATAAAT
AGGGGCAATGGAATA
AGTTAGAATAATTTATATAAAGGTTACAAAAACTGGTGTGGAAT
GGGGAACAGAAAAAAA
CTGAAGCACTCGAATCTTCACACATGACATTTAAAATGTGCAAAC
CAAACTAGACATGTT
ATACTCAAAACAGCTCAACCAAGATCCTCTCGG
>ConsensusfromContig2979 Average coverage: 20.63
TTTTTTTTTTAACTTGTAACTTTCTGATACATTCGACATATTGAGG
TAATTCAAACTGAA
CTTATTGATTCATATTGATTAATTATAGAAGGTAACACTGAAGAA
TCAAATTTGAGGGTC
GGTATCGCAGATTTGCGTGAAGAATTAAGTACATATCGAATAGC
AGGTGAAAAAAATCAT
TTATGCTAAAACATTGATTAGCAAATCTAGCGAAAAGTTTATAAA
AAATACAATTTGGGG
TTAACACATATAATGATTTGAGAGTTTTCAATAGAAACTGTGAGA
CAATTT
>ConsensusfromContig2980 Average coverage: 4.38
ATGTAATTCAGCAAATAAAAAAAAGATTTATCTTGGCAAAAAAAC
ATATTGTTACACTAA
ATGTGGGAAATCATCAACAAATTCACCTCACCCAGCGAAACACA
AAAGAATTCACTCTGA
AGACAGAGGGTATACCTGCAAGGTATGCCAGAAATCCTTCTTCA
AACCATCACAACTGGC
AGTACACGAAAGAAATCATTCGAGGAAAAAACCATTCAGTTGCA
ATGAATGTGAAAAATC
TTTCA
>ConsensusfromContig2981 Average coverage: 5.76

CTAAGATATGCGTTTTATTGGTGGGGGTCTGTGGTTATAAAGGTT
CGTAAATACGACATG
ATGAAAACAGGGCTGTAGGGAAGCTTAAGGCTTGTTACAAAATA
CTGGTGATATAATGGT
GATATCTCAGTTATATTCACATCACTTCATGTTCTTATGGCACTG
AAAGACTAAGATATG
CGTTTTATTGGTGGGGGTCTGTGGTTATAAAGGTTCGTAAATACG
ACATGATGAAAACAG
GGCCGTAGGGAAGCTTAAGGCTTGTTACAAAATACTGGTGATATA
AT
>ConsensusfromContig2982 Average coverage: 8.85
AAAGAATGTCCCCATATGACATGTGAGAAGTGGATTTCATTTTGT
AGATTTCATGTTTCT
TCATGATTGAATAATAATTGATCGTTTTAGTTTTTGATTTTTAGAT
TTATTTCAAATTTG
TTGTATATCATTCTCTCACTGATGGTTGTTTTTGTTTTACTCAGAT
CTGAGCTGTTAACA
CTTTTTTCAAATTCTTTAGGTATTTTCACGGTTTTCCTTTACAAAA
CTGTGTCATATTTT
AATTCGCTGCAGAATATTTCATCACACATTTACTGAAATATATCG
CTCATTCATATGGTG
CGAAAATTATTCAGTGGAAAGAGATCAATGTATATAGTTTATATT
CTATATGCGAAAAGC
>ConsensusfromContig2983 Average coverage: 6.73
TGACAAGTTATTTCACAGAAAAATCCAGATACTAATAAACATTTC
TAAATTTTTCAGTGG
CGTACTCAATATACGTGCCAAAATATTCATCTATATCTGATCGGC
TGGTACTGACATATT
CTGATTGTTTGTTTTGATAACCCAATGAAAATCGTTTGAATTGTTG
TCGATTTTGTTTGG
AATATGGGATATTCAATGAATCGAGAGCATGCAAAGCAGTCTCG
AAAGGATTATTTATAA
GATCATCAAATTCGATGAACACCATTTTGTGTGTTTTCTTCTGTTC
AAGCGAT
>ConsensusfromContig2984 Average coverage: 11.94
GCCGTTTCATTCGCGCATATCTTAGTTAACTCTTGTAATTCATGAA
CCAAACTTTTCGCG
GCAATGGACAAACTATTTCTGTTATTGGATTTATCACTAGGAGCT
ATAGATTTATCGATG
TCAAGACAGGTTCCTGATACATCGCCATGTACATCACCAACAGCA
TTCTCTCGTAAAATA
ACATCTGTAGTGCACATAGTGGGTTGTTCTCCCGCGTTTTCCATC

AAAACATCATCGAGT
TTTCGGATTTCAATTTCAGATTTGATGGATTCTGGATTATCTTTAT
TTCGAATTACATTC
GGATCAAATGTTGAAGCATGTGAAGTATAATCCGTACCACCAATC
CTATATATCTGCTCC
TTAGTTTGGATTTGGGTATCACTGTGAACAAGAGTTTTCAATACC
ATTGAATTCGTCCGA
TCGAGTTTTTGGTCGAAAGACGAATCCACGTCGAAAACAGAAACT
GTTTGGATCGCCTTC
GAATCAAGGAAATGATCTGTCATTTGGCCTGGATCAGAGTTTATT
AATCCCAATGACGAA
TTGACATCAAACACGATTTGTCCTCTTGATACT
>ConsensusfromContig2985 Average coverage: 13.13
TACGCCCACTCATATTGCTGAGAATCCTTTTAATTGCGATCAGTG
TGATAGATCATTTCC
GCAGTTATCCAATTTGAAAGCACATAAACACGTTCATGTTGTTAA
AAAACCATTGGAGAA
CAATGACTGTGAAAATTCAGTCTCGCAATCATCTCATCTAATACA
TCATAAGCCTTCACA
AGAGTCTTCTCGTTCAAAACCATACATATGTTGTATATGTGATAA
GTCATTCTCGTGTTC
GTCTCATTTGAAAGCGCATATACGTACACATACTGGCGAAAAACC
CTACTCCTGTGATGC
ATGTCAATTATCATTTGCACAGTTGTCCAATCTCAAAGCACACAA
ACGCATTCACACTGG
TGAGAAACCATTCAGCTGCGATGAATGTGGAAAATCTTTTGCTCT
GAAATCTGATTTAAT
TCGACATAAACCCATTCACTCCATTGAAAAGCCATACAGTTG
>ConsensusfromContig2986 Average coverage: 29.90
AGTTCGGATGCAGCTATCCGGAAACATTCTCATAGTGATGGGTCC
GGCAGTACAGGTCAT
AATTCGTCAAGACATGAAAAAACTACTTCTGATGAACAAACGTT
AAGAACAGTGGATAT
AGTCGTAAGTCTTTATCAGACATAGATTTCAAAATTCTGCCATCA
CTGAAAAGCAGCGAC
ACAGCAAAGACGGGTGGAATGACATCGAAAAGCCAGCTCTCTAC
GATGTACAGCATCGGG
GGTCGGGAGAGCTCTCCGACCCTAACGCCCTCTTCCGGCTATACG
AACCCAGGCGAACAT
ATTTTCGATGAGCATTCCATTTGGTCAAGACCGTTGCCACCAGGC
ATGGTGATATACAAG
GATGTGTTTCAAGCCCCAAGAGTTATCGGCAG

>ConsensusfromContig2987 Average coverage: 43.00
AAACAAACAATATTTTGACAAATTTTATGATCCTAGAACTGAATT
TCATCAACTAAAAAT
AAATTGGATCACTCAATAACTGGAAAACCTCTCACATACGCTATT
ATCTCACATATGAAT
TCAATGAATTTCAGTAAATTGGTGAAATGCCTGGTGAATTTATCT
GAGAAGGCTGCACAA
GTAGCTAGAGCCATAAGATCCATTGACACTCTTTTTGAACTATTG
GTTGAGGAAAAAAAC
CCAGCAAAGAAAGGTGATAGTTTTTTCAAAGATTTCAAAACACTT
GCCGATGTTCTTCTA
CAAGAAACAATATCACATGATTTACAAATTGCATTTCCTTCATTA
GTCAATCACATCCAC
GGCGAAGAAAACAACAAATTCACGAATGCCAAAGGAGAGAAAAT
TCAGGTTCAAATCCTA
GAAACCGTCGAGCAGACCGCGGCGCTCTTGAAAACCGTGCTTGA
TGGCAACGACAAAGCG
GCCCATCTCTGGCCGAGTTGGTTCACCCCGCGGTTGCCGTGGAGA
CCGATGACGTCAAAA
TAGGTCAACTCGAGGGAATCCCGGTTGCCCTTGATGACGTAGGCG
T
>ConsensusfromContig2988 Average coverage: 5.84
CTCCGAAGCTGGTTCTTGATGGGCACAAAGCGAGTCGTCGACGGA
AGTTCGAGATTTATG
AGTTTATCATCTTCCTGCAGATCCCACATCTCAAACATGAATGCT
TGCATACTTACCAGA
TATCTATCATCAGAAGACATGACAAGCGAAAAGAGTTCGTTTCTA
TTCCCTGAAAATTCT
TTATTGAATACCCATTTGTCATCCTGTCGATTGAATAGACAAACC
GAATTCCCCTTTCCA
CTAAAACACGTGAACAATTTTTGTCTCGATTTATTCATGACAGCT
GAGCTATGAAACTGA
AGCGGCTCG
>ConsensusfromContig2989 Average coverage: 6.56
CAAGGGACGAGATCTATATAAAGAGAGGCCAGAAGACAGAAACT
TGTTCAGAACACCCTG
AATGCTTTGACTGCGAAGACGACCTTAAGTCCTATGAATGCTCCG
CGATAGCCAACCCTC
TTAAACCGTTCGATCGCCATGGAGATGTAACAAAAATGGTGGAGA
CCCAGTGTGTTTCTT
TGGGGCAAAAAGAATCAGTGGATGAATGCGCTGCCTTCTCACTG
AAATTGGAGATGCCAT

CATTCGTGTGGAGCTCTCTTTTCAGAACTTGCAAAATTTGTGAAT
ACGGTATTGGGGATA
CTGCTCTTGACATTCGCGAGGAGATATTTATTAGGCCTGCATGTT
CCGAGATACCATTCG
CATGTGGC
>ConsensusfromContig2990 Average coverage: 13.42
TTGAATCCACCGATGTGCTCAAGAGTGGTCTTTTCAATCGTATCA
TTGGTCAATGTATCG
AGATATTCTGACCACGCCTTCCCGATTGACGCGGCACCAATGATA
TTCTCAAGTAACATA
TTCCAGCCAATAACAAACGCCCATAGTTCCCCAACGCTGACATAA
CTATAAATATAGGCG
GAGCCAGCTCTTGGAACCCTTGCTGCTAATTCGGCATAACAAGTA
GCCGCTAAGAAGCTA
GCGAAGGCTGCGATGATGAAAGAGATACATGCTGCCGGCCCTGCT
TGATATTTTGCAACT
TGTCCAACCAGAATATAGACACCGGCTCCCAGAGTACTGCCCAC
ACCTAGACTAATGAGG
TCGATTGTTGTCAAGCATCGTTTGAGATCTGTATCTCTCAACTGG
GAGACATCGATGGTT
TTTGTCCGACTCATAGCCTCAAAGACAGCCTTGCCACGATTGACA
ATCCGCGTTGTCATC
TTCATGGTCTTGCTTTGTTCTTCGGTCTACAGCATAGTTGATTCAA
TTGTTAATTAGTCT
TTTCTTCGCATACCTGTTTTTG
>ConsensusfromContig2991 Average coverage: 16.31
GTTGGGAAAAGTCCATGTCCGACGTACTTTCTTGCTGAAATGTTC
CCTCATTGGAGGTGA
AATTAACTGTTGGCCTATTTTTAATCGCCAATCTGGCTTCACCGT
TGGCCACAGAAATCC
TTGACCCCGGCAAGGAACCTGGGACTGGTGCCTCCCCATCCCTGT
ATGATTCCTCGCCCA
TCTCCACTCGGTCTGGCCAATCAAGAAGCCCGCTGGAAAACTGAA
GCTCCACCTCGTATT
CCTTTTGGCCAGCCAGGTATCTGGGTAGCTTGACGACTTCAAAAT
ACATATTGCAGAGCC
AATGATCCAGGTCTGAGGACATCAGCTCCTCTATTTTCTCGTACTT
TTGAATTCTTGGAT
CCATTTCACCTCGTAAGAATGGAGCTCGGAATTTGCCCTCGATGA
CATCAAAGCCGGCAT
TGGCAATGGGAAAACAGCCCCAACCAACGCATTTGTCTGTGGAAA
CAACTGCTCCCCTCA

GTAGATATATCTCGAACACCAGCGCCATCCCGGGCCTTATATTTG
GCATAGATGGCAGAA
CTGTGAACACGCTTTGGTCAACCTTCATTTCGATGTTGGAGAAAT
GTCCGCCATGTTGAA
TTGGAAGA
>ConsensusfromContig2992 Average coverage: 7.99
TGCATGAAAGCGGTTAGACATCAAAATTGATGTTTCAACACCAA
TGAAAGGTTGAACAAA
TACATATTCATACCACAAACAGTTCTAAGACTTTTTCAGGATGGC
TGATAACAAAATAGG
TTTCCAAAAACTGAGGAAACCGAAAAGTTTGCCTCGAAAGCTACA
GCAATTTGTCCATGC
AGACGAACGCATAAACGCCATGTTGCGACTGGGCAGCGTTCCTC
>ConsensusfromContig2993 Average coverage: 6.57
AAACATTCCAACTTAGTCTCATCGCATCCGACTATGAGAGTGCTA
GAAGCAGGTCCAGTT
CGTGCAGAAATAGAATTTGGTTCGGCCAGTTTCTCCGCAAAATCA
TATATTTCCCCCACG
TAGAGGTTTATTTTGAATTCATTTTTTTCATAGACCAAAACAATT
ACATGCCAGTTCGTG
TTCATAGTGTATTTCGGAGTGCGATACCCTTTGCCATTTTCAAAA
CTATACATGTTGAAA
TATAGTTTTCCATCATCCAGAATACATTTGAATCCTGCCTTGCCA
ATCCTATCGAGAATC
ACTT
>ConsensusfromContig2994 Average coverage: 16.26
ATTACTCGTAACTATGTTTTGTTAGCCTTTAATTTGAAACATTTA
AGAGTTCTGAACAAC
TTGTCATCTTTTAACTTTGTGGAGAATTTCTTTTCAAGAGTAGAT
ATTACAGTACAAAAA
CCCTTGCTCTCAGAGAAATGTAGTTGAAGAAAATCCGAAATAAAC
AATGTGTTATTTTAA
TCGATATCGACTTTCTCTCAGTTTTTTCACTTATAAACTATTCACT
TTATGAACCTATTT
ATTATTCGCATTTTCATGTCTAGTCCTTTCGCCATTGCATTATGAC
TAGTCTCATTATCA
GATCTCAGGCGTTCAATATTTGAGGTAATCTTTACCTTTTTGTGTT
TCTCAAACATTCCT
CAATAATTTATTTATATGTTCAATGTGCCAGATGTAAATATACGTG
T
>ConsensusfromContig2995 Average coverage: 25.07
ACTCCATTGAGTTGAAGAATGACGTGTAAGCAATCTGTGAAAGT

TGAGAAGATGAAGGTC
AGGGTCACGATACCGCTGGTGACGGCGATATGCCTCCAGAGAGG
GGGCGGCTGTTTGAAG
GAGAACACGGCCACCTCTATCACCTGTCTGCAAATGAAGCACTC
CAAAGGGTACGTCAGA
AGAACAGTGACGGAGAAAGCTATGCGGCTCAAGTTCATGAGGTC
ATCGTGGTAGCAATAG
TTCTCAAGGACATCGCCTTGAGTGGCGCCCGTGAATGTCACATAG
CCGATGACCCCAACC
AAGGTGAACAGAACAGTGGCGAAAGCAATCCCCGAATGTGTCAC
GACCCCCCATCTCTTT
TCAGAGGGCAGCACCATTGATTCGTAGATGAGAAACGATGTGTG
GTGACACATGAAACCA
AACACCATGATGCTAAAAGCTTTAGCAAACCCAATGT
>ConsensusfromContig2996 Average coverage: 11.14
CGAACAACAGATTATGATGATAATAAACAAAAAAGTGAAGAAAG
TAGAAATGTGACAGAA
AAATATACTGATTCATGTTTCGATAGTATAACAGATTCTGATCAC
AATGGTAATAACGAA
AATGAAGAATTGTGTGAGAATGGTAAAGAAAACCTATTGGCGAG
CACGAATAGATCTGCT
TCAAGAACTGCTCGGAATGTCAAAAAGATATCCAAATCAATAGA
ATCGGATGA
>ConsensusfromContig2997 Average coverage: 6.46
CTCAGGTGGAGGAATACCTGTACGCCAGGCACCTGAGTGGAGAG
ATCAGCGACCAGACAC
TGCTCGTAGAAATGCTCCACTCTAGGCATTGAAAGATTCCATCCT
CAGAAATAATAGCTC
TCTACCAAATTGGGAATTTTTTTGTATTTGAAAATCTTTAACTTTC
TCCCTGATATTCGT
TGTGCGAACTTGAAGCACTTAACGTATCTTTCAGGGAATTATCTC
AGGGGTCATTGATGA
ACTATTGTAAATATACCTATGTTACCTAACGTCAAATGAACGGTG
GAATCCGTCTTTTAT
TAAACCCGTCCCACGATGACAAACTTTAGACTTGTTAAGATCAGG
CGGTGGCGAGAATTG
GGCTTTTC
>ConsensusfromContig2998 Average coverage: 26.16
CTGATAGATGCTATTATATTAATAAAAATATGTATATATCCATGT
ATATACATTCGGCGT
GATTTACATATCTATATATTCATGTATATATGAATCTGACGTGAT
TCACATCATGTAACT

ATATGTGTCGCTTCATATGTAGCGACAGATGGTCGCTCCGTGAAA
AGGCCCGATCGCATA
GGTGACAGCGGAACGGCCGGTGACCGGTGTGTTTTCGGTAGTGG
CGGGTCAGCTCATCCG
ACCGCGCGAATTTCCATCCGCATCCTTTCCAGTTGCAGTGGTATGG
TTTCTCTCCCGTGT
GTGTTCTGACGTGAGCTTTCAAGTGGGAGCTTTTTGTGTAGATCT
TCGAACAGCCAGTGA
AGCTACATGCGTGTTGTGATACGCGCTTCTTTGATCCCTGCGCTTT
GATTTTCTTTTCTT
TATCGAGACCTGATCG
>ConsensusfromContig2999 Average coverage: 12.55
GAAGCTTCACTCAAAGGGGAAACACCAACCCCCGATCGAATCCC
CCGACGTCCGGCAAGG
AGCTGACTCCGATGATGACAGTCCCTGCCTCTGGGATTTCTGATC
GATATGTTGAATTGA
GCACATCGGAGCAGCACGAGACGAGAAAGCAGGATTTGGAAGCG
GAAATTGTGGCTCACG
AGCACGCCTTCCAGCCTCTGTATGAGAGCACGCTCGAGAGGCAA
CGGGGGGAGATGAAAA
GTCCGGAGAGGAAGCGACCGGGGGACAAGAACGTCAAAGGACG
AGAGAAGAAGGATGAAA
CTGTCTTGACACTAGAGCAACGACTGGCGCAGATGAATGAAAGA
TGGAAAACACTCAAGC
ACAGCAATCATGAAATCAGAAATCGTTTGGAGACCAATGCCCAA
CACTGGAACCAGCTGT
TGCTGATCCTGCGAGAACTGATAGACTGGATGGTTGGTCAGGAG
AAACTCATTGAGGAGC
AAAGGA
>ConsensusfromContig3000 Average coverage: 12.71
GCGGACATTGCAAAAAAGTGGATATTTCAATGATGGAGATAGCA
AGTTTTATTCAAGACA
GATTTCATCAAACAAAGGCAATCTGGATGGCATCGAGGCCACCC
CATGTGGAACTGAAGA
GGTACGACTCTGCTTTCGAAGAAATGAATAGACTGAGCTGTATGA
CCCTGAGCCCAATTG
TCCTTGAGGCTCTTAAACCTCATTGGGCAATCAGACAACAAACA
GAGGAAGACTTCAAAA
TATCTTCAAGGACAAGTTCATATGCACCAAGGAGATTTTGGAC
GACGTGGAAGCGGACG
ATATCTTGGTTCGAGTTGGCGAAAACAGCATTTACACAGTCATA
AAGGTCAACGGAAGAA

CAGAGATAGAAGAACACGATTTGGAAGGAATTTCGAAATCGCAT
CTCGTCAGGGCCGTCT
TTTCGAAATCCGAATATATTCTCACAATAGGGGCGTGTTATAATA
CCATATTCAATAGGA
ATACAG
>ConsensusfromContig3001 Average coverage: 7.90
AGACATGTGCTCTTCCGATCTAAGGCTTATCAATATTTTTTGACT
GTGATGGAGAAGAGG
AAGTGGAAATAGTGGCATTTTGAGAAATTTGACTGATTGGAATGA
GGGAATTGAGTTTTG
CAGATATAATTTGGTTGCTCTGAACCAGACCTGACACAGTAGCCT
TCAAATCATTTAGCG
TACTGTCTGATTCAGGCTTGGTTTCAGATTGATCAAGAAGGTCCA
CAGCGTTCGAAAGAA
TGGCAACTGCTTCAAGATACAGTTTTTCGTACCCTGTTCTTTTGTC
TTTTT
>ConsensusfromContig3002 Average coverage: 66.99
CTCTCAGCGATACGTCCAAGTCGCTTAGCTCAGCTTCTGCTAGAT
CCGAAGCGCTGTATA
TCGAAGCGCCATCTTCTCGAGTAACGCAATATTTGACATTCAGTG
GCGAGAAGTCGGTGG
ATTTGAGCAGAGAAGAGACGGCCGCAACCGTCTCTCGTGAAGCA
GTTCGATCACCGATAG
CAATTCGTTCACAGCTATGTTTCAGAATGATGTCCTTGAGTCGTT
GCAGCTCGGTCTTTA
ACACATCATCGGGGCCACAATGTAACTGAACAGTCCCAGTTTCAA
TGACGATACCATGTT
TCGAAACTAACGCAAATTTACATCCAGTCCGAAATCCAGGGTCGA
TTCCCAAAATACAAT
TTTGTCGAACAGGGGCCGCAAGCAGAATGTGGCGAAGATTATCA
CAGAACATTTTTATGG
CAGTTTTCTCAGCCTTCTTAGTGAGTTCATTTCTTATTCGACGACA
TATCCTCGGTTCAA
GAAGGCGGTCAAACGCGTCTTGGATAGATGTGTCGATCAGTTCCC
GGATGTCGTCATTCG
TGGAGGGATTTCGCGGATACCATTTAAAACGTGCTTTTTGTAAGA
AATCGTTTCTGAGTT
TTATAGGAAGAACGAGCTTGACCGACAAAATGCTGGAGGATTCA
CCGCGATTTAGTGCTA
GAATCTGATGTGGTTCTATAGACCGGATTGAGCGTGAGAAATCAG
TATACAGATTAAACT
TATCAGCGGATTGATCCTTCCCAGTCTCTTTTTCTTGTTTGACTGT

TGCTTTCGATTTTG
TACTCTTTATTTCACATGGATAATGGAGCACCATATTATTTAACT
CATTCATAATTGAAA
TATCTTTCGATATTTTGTCAGCTATGAGATACTTGAGACTCTTTTT
AACATCATTCACAT
CCGCCAAGCCTTTCGTGTTCCACTTGATTAAACTTTGTAGAGAAA
CCGTATGGGGAGAAC
TGAGGATTTTGGAAGCTGCTGGGCCCAATCCGAGATCGTTGGCTC
GCGCTGCTAATGAAC
GCTTAGATCCAGTCTTGAAAGGAGCATACAAGTGCTCAACATCCT
CCAAGTTCGTGCACT
GCTTCAGAGAACAAATTGTTTCAGAACAAACACGTTTACTTTTTT
GTAAGATTGAGATAA
CAGATGAAACACGCTTAT
>ConsensusfromContig3003 Average coverage: 34.11
TGAGGTACGGCTCTTGCGACGCGATCTCACTCTGCCTCTGTCGAT
CTTACGTCAAAAATA
AAGAAAACAGCCTTGTTCCATATGCTGATCGACTTGCACAGATTC
GAGATTTTGTGAATT
CTCATCATAACGTTACACGCCGTCATCACAAAATTCACCGATTCA
ACTCCTGTCGTTCAT
CCAGCTCCAACGACGCGTATACGGATGATGATGAGATCACGCCGT
GTGTCAGATATATTT
TGTTGGACTGGCTTGTTGAAGTGACCACGATGAAAGGGCTGAAG
ACGAGTACCCTACACG
CAGCAATCGATGTTGTGGATCGCTTTCTTCTGCAGCAGACAACCT
CAAAAGACAGTTTTC
AGCTGACTGGAATTTCTGCATTAGTCCTCTGCTCAAGGCTGCTCG
ATGACTCAATAATAA
CGGTCCGCGAGGCAGTCTGGATGACAGATAACTCTTACAAATAC
GAAGAGGTTATTCAGA
TGATGGGAGAAATCACGGCCGCTTTGCGAGGAAACCTTAAAACT
CTGAATGACTTCGATA
TGGTGGAATTCCT
>ConsensusfromContig3004 Average coverage: 14.52
TCGAAGTTCATTTCGCTGATATTTCAGAATGTTAAAAGATGTATCA
TTGTTATTCATTAC
TTTATTCATATTTGGCAATACATATGGACAAGACAGCCCTGAGCA
TGATGTGGGATGCGA
AGACTTCGGTTGCCTCAATCTTGGCGTGGCTAAAATGGACAATGC
TAGCGAGTGCTATTG
CCATTGCATATCATCACGACAAGGAGCAAAGTGCGAAGATTTGA

GATCTGAAGCAGAAAA
GGCTATACAAACAGATGTGACAGGAGACATGGCAAATTTCGAGG
TCTGCTGGAATGGTAT
CTCCCAATCGATCGCTGAAGCACTCAATAAAATTTGTCTTGAGAA
TCTTGCAGCTTGCTG
TGTGGGAGGAAATCCAGCCGACAGCCCTCCGACAAACGGTGAAA
TGATAAACGAGCTCTC
CGTTCGACTGGCCGATGGATTTCCAAAACACAAGGAGGGTGTGAC
CGTGCTGGCTTTCTG
GGCGCTGGTGGACCCCTTCTCTGGGTTTTGCGACTATA
>ConsensusfromContig3005 Average coverage: 9.17
CTTTTGCTGTTTTCCACATTGCAGTAATTAGATGCTCGGACTTGA
AACCCTATGACGATC
AACCGCGTTGGCGCTATTGTACAGATGGCGCACTTAGAACCATTC
TTAACAAAACACATT
TACTTTGTGAATGCAAAATGGATAGAGCTGGGAAATATTGTGAA
AACGTTAGACAATGTG
AAGAATGGTGTATCGAGGGAGTTTGTACGCATTATCAGCCCAGCA
GAGTTTTATATTGTG
CATGCGAAAGAGATTGGTTTGATAGAAGTTATGATTCGACATTAC
AATGCGATTTACACT
GCACTGCCTGGTGTAAACATGGGGGTACATGTGATTCAAAAAAA
CGTGTACCACACTGCA
TTTGTCGGCCAGGATATTTTGGTATAATTGGTGATTTGGAGAATC
AGTGCGAATACCATC
TGGACGAGTTGGAAACACTAACAGAAACAGATTTAAAGGACTAT
TCGAAAGTAACCCATG
CACTCAAATTATATCGTGGGGCCCACTCGGCGTGTGCATCCACCT
TTCGCCAGGAATGCC
ATAAAAAAGTGGAAAAAATGACATACAAGCTCCAACAAACCAAG
GATGATATGG
>ConsensusfromContig3006 Average coverage: 14.20
TTTACTGTGACCTAATGATCTTGAATTCCAATATGGTCAATGAGT
TACATATAAGTGTAT
GTATGAGCAATAATTGGGTGCTCTCATACTTCATATTGTTATACT
CAGCTCTGGTATGCA
ATCATGATAACGGGGAGCACTCATTCATTTTTTTGGCTGAAACCT
ATTATATTTTCAAAC
GAAAATTCTATTATGAGATTCCGCGTTAATTATGCATTTTTAAAT
GATTTTAAATACCAT
ACCTGTTGAATAATTGCTCATATCCTGATGAGATTTCGGGATTCTT
AATGATTCTACTGT

TTTAGGTCGTAAATTTGCTTAATACTGCGAAAATGTTTGAGTAAA
TATAATGTGTCAATA
GTTTAGATCGGAAGAGCACACGTCTGAA
>ConsensusfromContig3007 Average coverage: 15.22
TGATTGACAGTAAGGATTATCAGTGCATTCTGTTTCACAAGCAAT
TACGTCATTAGTCTG
AGCACGACAGTTTTCGTTCTTGCAAGGATCAGTTTTCGGACACAC
AAACAACGGTCCGCA
AGTTTCACCGCCAGTTCCGGTATCACAAACGCACACATAGTTTGT
TTCGGTTGAATAGCA
CAACGCACTCCCACCGCAATTAGCATTGGGACACGAGCCGAGTTT
AGGTATACCGGCATC
AATTGCTGGCTCTGTGAGGCATATGTACCGCTCTCGGTCATGTTC
CCTGGCATGGACCTC
TCCTTCCTTACACGCAGTGGAAGTATCTTCAACATCTCGGCAGTA
AGGGCTATCAGTACA
GTCTCCACCGCATTTTATTGGAGTACTATCGTCTGTAGCCTCACA
GGGAA
>ConsensusfromContig3008 Average coverage: 10.20
ATCATTGAGATGCCATCATGGCCAACCGAGTGCTTGTTTTCAAAT
TGTAGAATAGTTTTT
TCAACGAAAGCATTGTCTATGTGGCTAAATGAAAAGTGTAAAGA
AGTTGGAAACGATAAA
AATAGTTTAAAGTCATTATTATCTACAATGCTAAAAGGAAGAGT
CGTAAAATGAGATAAA
AAGGATTTGGCAATAAAATCATGATCACTTGTTAAGTCATCATTG
ATAACAAAATGATCT
GTACTCTTCTTGTGAGTGCCTGTTAAACCTTTTATCACGTCCCATA
CCTGTTTGGGGTTT
GTTTTAACCCTTTCAAATTCATGATAATAATAATTTCGTTTAGAAG
TTCTTTTTTTGTTC
TTCAAGA
>ConsensusfromContig3009 Average coverage: 24.97
GCTAGCTTCTCCTCCGCCCCATTCTTGCACTTCCATTTCTTCTACTT
CTTCTTCACCTTT
TTTCTCATGTTCTCCACCGTCTCCATATTCTCTATTCAGACGCTCA
AAAACTGGGAGATT
TTGCGGTTGTTCTTCTGGTCTTTGCGTACTGCGTTCCCATCTTGTT
TTAAAAAAGATCCA
AGTGAAACCATCTGTTGATTTGGCGTATAGGCGACCATTCGATTG
AAAAACTCCGTTCCA
TTCGACTTGAGCACTGGGTCTTTTCGCGGGTTCGAACCAATCTGC

AATGCCAGTCTTCGG
GTCGAAACAATATGTGGAAAAGTCAGAAGCGACAAGCCACAAAC
AGTGGTCTATCTCAAC
CATTCCACGGATCTCCTCCAATCCTCGGTTTTGGTAGGCAGTGGC
GGGAACAATCCATGT
CGTCCACTCTTTGTCAACATTGTCCCATTTGTGCAAATTCTGTTCA
TCTGTCCCCATTGC
ATAAATTTCTGTGCCTGTTTCGACAACGAGTAAACGTCTCATGTC
AAGTTGATCATTCGG
AGGAAATGGGCGAAACGTTCCGGGCTGCTGCTCCCAGGCACCGT
AAGAAATATCGTATAT
CATAGTACCAGCAGAGCCCAAAATCTTGATCTGTCCATCTTGGAT
GAGTAAATCAGCGAC
CAAGTTGCTCGACCCGAAATTGTTTCTGATTGAAATCTTGAAACT
ACCCAGATTCTCTCC
ATCAACATTTTCTGCTTCGAACTTCAATGTGC
>ConsensusfromContig3010 Average coverage: 14.00
CCAATCCTGAAACCATCAAATATACTTTCACTGAATGCGTATTCT
CTTTTGTCAAAAAAC
ATGATGGATTTTGAACATCATTTCCATTTATTATCAAGCCTGTGT
GGAAAGCTGCAGTAC
TCTTATTGAAATGAGCATCTTCAGATAGGGAAATTTTCTCATACC
TGCTATGAGCTTCGA
ATTTTAGGGCGGTGAACGTGTGTGAATGGAAGTCTTTGATACGAA
CACTCGTTAAACTGT
CTTTGATTGTCACTTCTTTTATAACATTATACTCTAATAAAATTAT
GTTGCCTTTGGTAT
ATGACAGTCCGAAAGTGTTACAGTAATAGACAA
>ConsensusfromContig3011 Average coverage: 39.66
GTTAAAAGGTACGGAGGACAAGGGGCAGAAAGCACCTTGCCATT
CGTCAAAGAGTTGCTG
AAAATATCATCTGCGGAATCTTATTCTGACGTTGTGATGTGCATG
GGTCACAGGGGACGG
CTTACTCTTCTGAGCTGCTTACTGAATACGCCTCCCGCGTATATC
ATGAGAAAGATGAAA
GGCCTTCGAGAATTCCCGTCAGAGGTTGTTGGAATCGGAGACGTT
TTGTCGCATTTGACT
TCATCAACCGATGTTGAAATTGACGGAAAAGCGATTCATGCCACA
ATGATTCCAAACCCT
TCCCATCTTGAAGCGGCGAGATCGGAAGA
>ConsensusfromContig3012 Average coverage: 8.93
CGAAAAGTTCTTCGTTTCTCGCGTCAAGAAAAATCTCAAACTGAT

TATATGTCTTTCTCA
TGATAACTCTTATCTGCGATCTGCGGCTAGATCATACCCGGGCCT
GGTGTGTAACTGTCA
CATAATCTGGACATGCGAATGGATCCCCAACGCTTTGCTGGATGA
AGCAAAATACTTCAT
CTCATCAAAATCCATCGCTGCATCCTGGAATGAGAATTTCAGTCA
ATCCGTGGCTTCATG
CTTCGCTGACATACATGCCTTTATGCTCAAAGATACTGGACAGAT
TTCCCTGGCAGGAAA
TATGGATGAAGAGGTGAGTATAATGCACGTTAAAGTGAATGAAA
GAAAGAAAGATCAACT
GAAGCAAGCTCTCATAAACGTTCCGAATTGGCCAGAATCGAAGAT
TTCGCTTCTCGAAAA
CATCAAAACTCTACAAATTGATGAAAGGAGCTCTCAGTC
>ConsensusfromContig3013 Average coverage: 45.69
CTTAGACCACAGGGGTAAAACGAATGCTTTTATGGCTAAATCAGC
TTCGCCTCTTGCAGC
TATATATGGGACTTCTCTCATGGAACATCATCACTTCAATCAAAC
AGTCACCATTCTTCA
ACAAGAAGGTCACAACATCTTCAATATCATATCATCAGATGATTA
CAAAACAGTTTTGGG
AGCCATCAAACATTGCATTTTAGCCACAGACCTAGCTTTATTTTT
CGGGAATCGTGCAAA
ATTGGACAGCCTTTTATCGAACAATGAGTTTTCGTGGAAAATTT
TGAGCACAGACAATT
GGTGATGGCTATCTGCATGACAGCGGCAGATCTTTGTTCAATGTT
CAAACCATGGGATAT
ACAAAGAAAACTCGTTCAAGTGATCATGGAAGAGTTCTGGCAAC
AGGGTGACGAAGAAAA
GAAGAATGGCATACAACCTATTCCAATGATGGATAGGGAAAAAG
CTGAACAACTCCCGAA
TAGCCAAGTTGGGTTCTTGGTGGGGATATGTCTACCGTGTTATGA
GCTTCTGAGCAAAAT
ATTTCCTGAATCGAAACCCATGGTTCTCGGTGCACAGGCAAACC
TCAAGCGATGGAGGGA
AATGGCAGACAAAAATATTTCGTAAAATTGCTTCCGATATTATTA
AGTTATAACTTTTTT
GTTTGCTGTTCATTGTTAAAGTTTTTTTAAATATATGAAATTGAAC
CACATGTTTTAATG
AGATGATATAATTTTAATGGATTTAAAAAGTTG
>ConsensusfromContig3014 Average coverage: 12.83
ATATCATGTCACTTATATATTATCCATAATTAGGAGAACATTTCT

GATACAAATTCGACA
CCCAAATCGATGCGAGAAATAGGCATCTTTGTTCCGCCTTTTCTA
GTAATTTCGATTGAC
CTTATAGAGAAAAAGGGCAAATTTTAATTCTAAAATAATCCCTC
AGCAGCATCAAGACGA
AGCTCAGCTGAATTATAATGAAATACGGCATATCTATAGAGGAT
CAGTATAAAAAATGAC
AACATACCCAAAACACCAAACGTTTTGGAAAAAATAAACTGAAA
ATGAATGAGATCTTGA
AAAGTATAGAAGGCGTGAAGCGGCGAAAAGAATACAAACGAAA
ATAAAAAGCTAGAAAAG
GATGGCAATGGAAGAGCCGTCACAGAATATAACAGGATGTGAAC
ATACTCTATTATATCC
ATTTGAATCTGGTCATCGGAACAAAATGATAAATCATAAATAGT
ATAGAAATGGACGAAA
AGAGACAACAATCAAGAACTTACATGTTCAATCATGAAACAAAT
GTTAACAAAAG
>ConsensusfromContig3015 Average coverage: 13.96
TCTGCCTCAACAGCAGAACAGTCTGTGAAGGTTCTTAAAGTGTCA
TATAATACAATGGAT
TGTTCCTGTGCTTCTTCATATTGTGTTGGAACTTTCGTATAGGGT
GCAAAACAAAGTGTG
CTTCCAATAGAATCTTCATAGAGACACCTATGAGCAGGCAGGAGT
GACACATTCGGTCTG
CCCTCTTTTCTGCTCTCGATCTCTCCTAGAGTGGTTGATACAATTT
CCAATGACATTTTG
AAATCATTCATCCATATACCAAAGACTACTCCATCTGAAACTGGG
TAGCTCATGAATTCC
CCGCTCTTGTACTGTAGTATGACACCAACACCGTCGTCTTCACAG
CTGATAAAGACCGCC
CCATAAACAGGCGATGCTACAATTTCTTTTGCTCTAGAGAGTAGA
GCTACAACAACTGAT
GAAGATGCCATCAATGGTGAGAAACTTCC
>ConsensusfromContig3016 Average coverage: 5.31
AAATTTATTATAAGGCTTTTTTGTTTTGCTCTTATTGCTTTCAATT
GATTGATAGCGATC
TCAACGTCCTTTCCATCTTGGAAATCAATGCTGTTGTAAACACGA
TTTTGGGACTGGATT
CTCTGCTCGAACTTGCCATTGAAGACTTTTTCTTGAGTTTCTGTGA
AAGTGCTGTATTTC
TTGAAGAGGTACATTATCATTATATTCGCTGCCAGAAAAACGACT
ACTGAAGGTGTGTAG

ATAACAATCATAAACAAAGCAGCAATCACACCAGCATAGCCCGTT
TCCACTATCGGCTGC
CGGGAA
>ConsensusfromContig3017 Average coverage: 13.26
TTCCGATCTGGGAAGGATTGTTGTTAAATCAACAAAATCTGAAC
AACATTCGAAAACAGG
TTTAATGAGAATCTATTCAGGTAGAGATTAGACACCTGCCTGGAA
AGTTGTTCAGATACG
TGGAATGGACATTTTACACTATGGTCAAATTCATAGCAATAGCCA
TATTTATGCAATTGG
GTATGGTAAAATTGCTTTGATCCAACGAAATAAAAATATATCCAT
CTGGAATATGGATTG
TCCCACAGATACACCATTAATACTTGAGGGTCACCACAATAACAT
TTTCTCTCTTTGCCT
TGGATTATCTACTGGCCTAGAAGTTGTTCTATTGAGTTGCTCCTCC
GATTATTTGATAAT
TTGGAATGTTTCTGAATTGTTTGAACAACACGAGCAAGGGTTCGA
AAGTCAACTCAAGGG
GCGTGTCCTTCTTTCTGGATTCGATTCTGCCGACAGATGCTGTTT
CAGCACTGAATGCAA
ACTGGTGGCTGTTTCCCAGAGTTCTTCTGTTAATATTATTGACGT
GGAGAAAAAAGAGCT
GGTCGCCGTTTTAGAAGGTCATACTGGCTCCATCACATGTCTCGG
ATTTTTCTCCCACAA
ACCACAAATCCTGATAACAGCAGCTGAAGATCGGACATTCAAAA
TATGGGACATTTCTGA
GCACCGTCTATTGCATCAGTCGAGTATCATATCTTCATATCCATTC
ATCTCTCTCGCATC
ACATTCAGATAAATTTGCTCTGGGAACATTTGATGGACAGATACG
TGTTTACAGATTTGA
TGGCCAGACAGAATGTAGATTTTTACATCAAATTGATATTCAGAA
AAAAGTTAAACAATT
TTCAAATGGAAAAACGAAAGCATTTGAAAATTTGAATGATAGCG
ATATTGTAGTGAGCAG
CGACCCACAGTGGCGACGTGCGCATGAGCAAGGCAACTCTGAGG
AAGCCATCGATTCTGA
CAGCTCTGCTTCTGT
>ConsensusfromContig3018 Average coverage: 5.36
TAGTTCATCTACGGCTTCTCATGCTATTCACCATATCTATCCATCT
CTTATAGCTTATCT
ACGACCACAAATGCTATCACCATCACCCATATATGCTGTCTATCTC
TTTTATCTCTAATA

GTTCATCTACGGCTTCTCATGCTATTCACCATATCTATCCATCTCT
TATAGCTCATCTAC
GACCACAAATGCTATCACCATCACCCATATATGCTGTCTATCTCTT
TTATCTCTTGTAGT
TCATCTACGG
>ConsensusfromContig3019 Average coverage: 9.77
CTACCAGCATTTCCACATTGAAATATCATGCAGAGTATCCTATTG
ATCAATCAAGAGAAG
TTAATCTTCGTCCTGAGAGCTTCAAAGAATTATATGAGGTTGGCT
CTATCGAAGTTATCC
AATTTATTCACATATGCATAAAAACTGTGGCGACAAATACTTTCA
ATAGATTCAAAGAGC
TGAAAGAAATACATTTTCAGCTTTCTTCTCAGAATATCTCGTTCTC
TCCCAGAGCTTTCG
AAGGTCTCCCAATATGGCATTTCAGACTCTCATCAACCTCCTCGC
CACAAACCACCTTCT
ATTTCGATGATGAAGTATTCACTGGAATGCCGCATATTATTCACA
GAGTGTCTTTCATTG
AATCAAAACTGGGAAATTC
>ConsensusfromContig3020 Average coverage: 5.57
CGGAGATGAATCTATAGCGTTTCAACACTATCCCCAATATTGTTC
ATCCGTGCATTTGAG
CATTCCATTTGATATTATTATTCTGGAAGCTTGCCCCGGGTTCAC
CGGAGATGAATCTAT
AGCGTTTCAACACTATTCCCAATATTGTTCATCCGTGCATTTGAGC
ATTCCATTTGATAT
TATTATTCTGGAAGCTTGCCCCGGGTTCACCGGAGATGAATCTAT
AGCGTTTCAAC
>ConsensusfromContig3021 Average coverage: 59.95
GACGAATCCGTGGCTGGTGTGCTGACCGTTCTCTGCAATCACTCC
TTCCATACAGGCTGC
CTTGCTCAATGGGTGGACACTACATGCCCCGTATGTCGTTATATC
CAAACGCCGGAATCG
GTGGGAGACCACAGGTGCTTCCAATGCGACGCCACCGATGGCCTC
TGGATCTGCCTCGTG
TGTGGTCACGTGGGCTGTGGAAGATACGTCGGAGGCCACGCTCAC
AATCATTTCGACGTC
ACTGGCCACGCCTACTCCTTGGAACTAGGTCAAGACCGCGTTTGG
GACTACGTCGGAGAC
AACTACGTTCATCGGCTGCTGCAGAACTCGGCAGACGGAAAGAT
CGTACCCCTGGGAGAC
CCGGATATGACGAACGCGGTCGTTGGCTCAGAGGAGACGGATAT

CAAACTGGATTCAATC
ACTTTAGAGTACACTTATATCTTGACGAGTCAATTGGAGGCGCAG
AGGCGATATTATGAA
GACAAGATTCATCTCATCGAGTCAGAAGCGCTTCAACAGGTAGA
CATAACAGATACGAAA
ACCAAGTCTGCATTGTCAGAGTGCAAGCGGTTGGAGGAGCAATG
TTCGAGCCTCGTGAAG
GAGAAACAGGTGCTGGATCGAAGAGTGACCCTCCTGCAAGTGAA
GGTGTCGAAACTGAGC
GCGAGTGTTGAGGAGAGCAAGAAGCGACTCGAAGAAGAGGGTCT
GATGAACAAATGTCTG
CGGGAGAACCAAAGCCATTGGTCAGACCAGGTGGAAGAACTGAA
GAAAGCTCTCGAGAAA
TCGGAAAATGAGCTGACAGAAGCGAAGGAACAACTGAGAGACAT
GATGTTCTACATCGAA
GCTCAGCAAACGTTGGCGGAAAGCTCGG
>ConsensusfromContig3022 Average coverage: 5.63
CCCATCAGAACTCCGACGAGATTGAATTCTTGATAGCTATCACAA
GTGCTTGAGCTGAAC
CAATATACTCCGGCTTTTTCATCATATGTAAACATGCCGTACTCT
GGTTGAAAGATTTGT
CGGATGAGGAGCAGGAACCACTCCTTGGTCAAACCTCCCATGTCC
AAACCAGGCTCTCCT
GCGAATGTCACTTTCAGTTTCTTCTTCAAATCGTGCTGTTTTCTAG
CAATCTCATTGAGA
GAATCTGACACAAGATGACCTCTTCGCACATTGAGACTTAGAAAC
AGCATCGTGATGTCA
GGCATTTGTCGTCGCTGCACTTTAGCCACCAGCGATCGCCGTGCC
ATGACGATCATCTGT
TGTTCCGAATCACGCTGAAGAATAATTCTCTTTGATGCTAAACTG
ATTATGAAGGGGTAT
TGACAGAAGCTGAAACCAGAATGTGCTGAGGGACTCTGCCACGA
ATAGTATTCAGCCATT
ACATCCAAACTGTCGATGTGGTGATTGTAGAATTCGCTATGAGCC
ATCTTAGGTCCTCGA
GACAAGTTATTACCAGCATTGAGTAGAGCCAAGAGTCGCACTGC
ACAGGGAATCCACCAA
GATGACTTTGACATAGGGGGCAGATCGGCAGGTGTTGGTGGAAA
GAGTCGAATGCTGATG
AAGTTCTGGAGAGTATTTACAACCAGTCGCATTGTTGGTGCTGTG
AAATTTTTAAACCAA
TGAACGAGAAAATGATGATCATGATCAGAGAGCGATGAAACTTG

TCTCAATAAATGCGCA
AATATCACAAATGCAGAGATTTCCATGAATTGTGGATTTAGTATC
AGAATACAAAAAGCT
CTTAGATCATCTTTGGAGTGCGGCCCTTTCTCCTTGAGAAGACCGT
TTATTATACATTTC
AGTACTGTTTTTCGAGCATCGTATGGCAGTTTTAATATCAATTCA
TAAGTATCACAAATC
AATCCGATTTTCAATCCAGATTCATTTATACTCTTGTATTCCTTAC
AAACGTCTTCCTTA
AAAGTGTAGTTAAGAGAATCAAATGAATCAAAAATCAGTCTGATC
CATTCATTCACTCCG
TCCCAGCATCCACCTCTGTCAGCATCGTCAATTTCCG
>ConsensusfromContig3023 Average coverage: 6.28
GTCGAATGGGTAGAGGGTTATATCCAGCTGACAAGACGTCGTGA
ATCGTCCCCCGGGAC
CCACTGGACACGTCCGTCATTCCGGACCGCCGCGGAATCTTGT
ACTCGCTTGTTCATC
GTATATCGTATCGGCACTATTTTCGAGACCAAAGTCGGGCAGCC
AGATGAGATGGGGCGG
GAGGATTATCTTAGTTATTCCACTGTAATCATTCGGATCCCATTG
GAGAAACTCGTCTTG
CCAATGCACCTCCAACCATCCGAAAGTTTTTAATATTTGATTTTTT
TCATCCAATTGAAG
AATTTTCGTGAGATATAAGGCAATAGAAACGTTGATGGGTGAATG
CACATCTCTGATGGG
TCTCGCATGGGTGCTGTACCCCTTTGTAATATTTCCAATAAGATCT
CTTTCAGTTGCTGG
TTGCGTGTTTGTCTCATTTGAAATATGCCCGTCGTAGAATATTGCG
ACGGTTCCTTTCAT
AAAAAGATAGTATAAAATAATGATCTGCGAA
>ConsensusfromContig3024 Average coverage: 11.61
GAAAAATCTAACCCTGTGTCAAAAACCAAATATTCAGAGATAAC
CTCGGATAACATCAAG
CATGAGTATGAAGTCAATGATACATCAAACTTATTTTGTGAACTT
TCTGACTCAACAAAA
TCATTAAATTCAAATTTGCCCAGAAACGATTATCTCAGGAATACC
CATCCGAAATTTTCT
TCATCTGCGGCTTCCGGTACCCAATTGCTGAGTGCAATAAAACCG
GATGAGAAATTGGAA
GAAAAATCGGGTAATTTCTATCCGATTTCTA
>ConsensusfromContig3025 Average coverage: 7.57
GGAATCATGAGCTGCGAATATGCTTATTATCTTGGAAGAATTACG

TATTTCATAGTAAAG
AGAAGATTTAACTTTAAGTATGTAACAATCCCTTGGGCAGACAAC
AACCTTTTTACTACA
ACAATTTGAACAATGACCTAGCTTCCTGAAGGTCCTATCGCCTTTC
ATGTCTTTTATTAT
CATAGACTGATATCCAAATTTCCGAAGTCTGGCTGAGTCATCAAC
ACTTTCTCTGTCCTT
ACATTTACATGCTGAGCAGCTCTCGCACTTCCCAATTGACATGAT
CACGCAGTTTATTCT
GAACTCCTCATATTCTCTCCAGATTTCAGAAATTATAAGGAACAT
GGACATATGTCTCTT
CTCTCCTGAGTTTATTCTATCTCTATAAAGAGCTCCATATGTGTCT
GGCGGTGATCTTTT
GCACTGTTCAATCAAATTCTCTTGAATCCTTCTGAAAGTTTTCTT
TATGACAAAATAAAG
ATCTATTGCCAATATCTGTCTGAAGTGAGGCTTTCTCAGAAACAA
CGTCATCCAATTAAG
TCCCACAATAGAAAGACAGCTGGCAACATCAAC
>ConsensusfromContig3026 Average coverage: 21.04
AACTCAGAGCCGAACCGCGAACACGTCGATTCATTTCGCAGCCG
CGGTGTGTGGCTGGCG
TGCCACGTATATTCTAGAAGGACGGCAATGACAGCACAGAAAAG
GCCAACCAGCAAAACG
ACGAACACGCCCCCTACATGAGTCATGGCGAGGGCGTTGGCGTTC
TTTGGTTTCTCGGTG
AGGGCACACGCCCCACCCCCACCCTTCTCTTTCCACCATTTGTTAT
ATAATACTTGTAAT
CTTTGATTTTCTTGTAATTTTAATATAGCGTTCGATATAAGGTCTC
TATATTTTGAATTT
CTCGGCGTTCCGATCCCATAACCCTTGGAGTCGAGCAGTCCGCCG
ACTTGTACAAGGTCA
CAGTCACGCTGGGTGATGTACTCGTTCATGGTTGACTCAAGCAGG
AAGGCGTACTTTCCA
GTCTTGATTCGGTTGATGCCTTCTGGGGTAGAAGACACGAAGGG
GGAAGGCTGGGTTGAT
GACATGAAGTTCCACATCCTCTCGTATGTGGGGATTTTGGATTGTT
T
>ConsensusfromContig3027 Average coverage: 7.89
TGTCTTCGGGCACACTTGCTTTCACTTCTAACTTTCTTAATTCGCT
ATAAGGACCAGTAT
ACACGTAGAATACAAGGTATCCCTCTTCTTGATCCTTGAAGTACA
AAGCACGCGTGACTG

GTCCGCTTCCGATTATGTATTCGTCCCCTGTTCTATTTCGGTCATG
TATATACCAAAGAT
CTACGACCAGATCTTGCGCTCTTATTGTCGGGTTTCCCAAAATGC
GATAGTTCTGATCAC
CAAGATCGATGTAGGAGTTTTCAGTCCCATCGAAACTGAGAGCTT
TTCCGATGTGTCCAT
TGTCCAGTATCAGGACACTGGATCCAACTT
>ConsensusfromContig3028 Average coverage: 4.28
CTCTTCCGATCTGCTTGATGTGCTATACTTCTTACCGCAGTCGCAG
CAGTCATATTCATC
GCAATTTTCACATCTGCTATAATTGGACCTCGGAATATCAGTTACT
ATAGTTTCATCTTC
ACTCATCACTTCTTTGGTATATGCTTTGTTTTGTAGTTTTGCTATA
GAAATTTCATCGAG
ATTGCATCCGTATTTTGGCATTGTTCCCCTGTACACCATGTTTGCG
TATGCATGTATATT
TGACATCGGACTTGTTTTAGGGACAGTATAGCTCTCCCCATAATC
TTGGTGTCGTTTGTA
GAGATGTG
>ConsensusfromContig3029 Average coverage: 22.35
TCTATTCTGAAAGCCACATTAGATGTATTGGGTAGGCGTGGTTCT
GAATTACAGTTGAAA
ATGATATCAGAAGAGAAGGTCGTCATTAATCTCGCTTCGAAGTGG
TCCCGCACGCACTTC
ATCTGTGATGCATCTTGACCCAATGAAGCCAGCTCAGCTGCCTTT
CCGAATCCAGCAATC
ATAGGCGTGTTTTCCGTCCCAGGTCGGAGGCCCCATTCTTGTCCC
CCGCCGTAGAAGAGC
GGGTGGAGATTCTCCCGACCCCGGGCGAAGAGAGCCCCTATCCG
GGGACCGTAGAATTTA
TGGCCGACGATCGTCAAGAAATCAACACCCAGATCCCTCGCATCC
ACATTCAGCTTCCCA
ATCGCCTGAGCCGCATCCGTGTGGAAAAGAACTCTGGCCGATCCT
TCCGATAGCCGCTTT
TTATTGAGGGCGAATAACTCTCGGCCGATTTCAGCGACAGGCTGT
ACAGCTCCTGTCTCA
TTATTGGCTAGCATCACCGTCACCAAGCACGTGTGTGACGTCACA
GCATTGATGACGTCG
GCAACGCGCACCCTACCGGACGAATCAGCAATGGGAACGAAAGT
GACATCGATTTTGTTT
TCCCGCCGCAGTGCCTCTAGCGGGAGCCGTATGGCGTCAT
>ConsensusfromContig3030 Average coverage: 10.30

TGCCGGTCCAGAGAGCTTCATAAACTTCTCCGAAATGGCCATGAC
CAAGTTTTAAAACAT
TCTCCAGCGATGACTTATCGATCTCCCATTGATCAAGAGTGTTGC
ACACAACATCCACAA
TTGGCGGTTTCTCTTCCTTTACGCAAGGTTTTGAGAGTTTGAGTCC
GTTGCTTTGTGAAT
AGTGTGAAATGAGGTTGAAGAGTGTGTCGAAAGTGGCACAGGAT
TCGATGAAGACCCCTT
CATTCTCCATTTCAAGGATTGGATAATGTTCGATCACGTGACCGT
GGGTTTTAACTGAAA
GTACATATTCGCCTGGATGATTTTCTCTGTCCCTAATAAGAAAGG
CTCCACTATCATTCA
TGCCTGCCTTTAAATATTCATCAGCTTCTGTATAAGTTATTGGACC
GAAGTACCACTCTT
CTGATTCCAAACTTCTGAGCCTTGTCACATAGTTCGATGGGATAA
AGCCTTTTCGACCCG
TAGTTTTTGACTCTGCATACCACCAAAGGCCTGCAGTATCATCCA
GAATTTCCAGGTGTT
CATTCTTCTTGAAACTGAGATCCTCTTCAATCTGTGCATCATAATC
ATA
>ConsensusfromContig3031 Average coverage: 15.45
TGTGCTCTTCCGATCTTTTTAATTGATGTGTTTGCTGCCTCTTTCC
GAACAACAGGTAGT
TCATGTAAGTGCGGATCGGGTGTTGGCGGTGGAGGAGCAGGCAT
GGAATCCATTCTGTCA
GTTAAATTCATTTGTGTTTCGTTGATAATTTCATTGTTTTCTGATT
CTATTTTGCAACCA
TTGTTTGCATTTTTTGATATGACTGCCTGATCATCTTCGTGGTAAA
CTTGTGCTCTTGGA
ATAAAGACTGTTGGTTTGGTTTTGTGCTTTGTAGGACTCGAGTCCT
TTTGGTTGTAATTT
TTTCTGATTTCCTGTTCATCAACGCACGGCCATGTATCCGGTTGTG
GTTGACTGAGCTTC
TCTACACGTTTAGACCTGGTTGATGTGTTGCTATGAGGTCGCGTT
GAGGCTGAGATGCTC
GCTTTGCTAACAGAGAACATCGATTCGATATCTTCACGTAAGAGG
CTTGATGCATTCCCA
GAAACGGATTTCGCTGAACAATTACTTGCTGGACCGTTCTTTTCTC
TGTTGATCGATATT
TGAGTGCATGGGGTGGATGTTCTGTATGTACTCTCGGCTCTGTCT
TGGCTTCTGTGAGTC
AGCATCTCGTCTTCGTTCTCGCTCTCTTTAATGGTACAAAGCTGAC

CTGGCGTAACTGTT
TCGCTGCGTGTCGCAACAAGGTCCCGCAGAACAGAATTCCCTCC
AGACTGCGGAGGCAGA
TTTCGAACAATCACACTTGG
>ConsensusfromContig3032 Average coverage: 4.94
CATATATTTTATTGATTTGATCGAATATGAAAGATGAAGCAAGCG
AATATAAGATACCAA
ACCACGTCAGAAGGCGATACGAACATATCTTCTTCAGCTTATCAC
CAATTGAAGGATTCT
TGGCTGGAGAAGATATACGAAGACTATTCTTGAACACTGGCCTAC
CATTAACAATTCTTA
GCCGGATATGGAACTTAGTGGACAATCCTCACGACAATATGCTG
GACGCATGGGAGTTTG
TCTCAATGGTACACATAACGAAATGTTTTTTGAGAGGGGTAAAAT
TGCCCCCCAAACCAC
CGTCATGTCTCTTGGAACAACCCTCGAACTATCAGATCGG
>ConsensusfromContig3033 Average coverage: 13.41
AAATAATTTTGAGAAATATAATGAAATTCATGTCAAAATATTTCT
CCGTTACTGATATAC
TCCGATGGGAAGGTCGTTTTCTACCACAGTTTGAAGGCAGATAA
GACAGCTAGTATGAGA
GATGCTCCGATTGTTGAATAAGCAGCATTGTGTGTTTCTGCCCCT
CCAAAGTAGTAGTAA
TATCCTTGTCGTTTTTCTATGGTATTTCCAAGACAAAAATTGAGA
ATTATTGCAAAAATA
GAAACAAATAGAATAAGTTTAGACATTATTAGAAAAAGTTGATTT
ATCTGTTCGGCTCCT
>ConsensusfromContig3034 Average coverage: 13.60
AACGAATAATATTTTCTCTCGTATGGTTGTTCACCTTTATTTTCTC
TACAAATTCAGGTT
ATTACTCATATGCAAGTCAAAATGTGATAATTTCCATGTGATTAC
GTTGAGCCAATTTCG
CAAATTTTATTTCATTTTATTTGGTCTCGTGTGATCTGATTTTTAT
CAAGATCGTATATC
GGTGCTGATATACTTGTCATTTTTTATTTCTTAACTTTATTTTTCTT
GCCTCATTTCATT
CGATAAAATATGACGGAGTTGATTCAACATT
>ConsensusfromContig3035 Average coverage: 12.26
TATTGTTTTAGACGCGGAGTGGTGGGCCCGGCATTATTCTGTGCC
ATACTATTGAATCTT
CTGTACTGGTTTTGAGCGTTCATGATGGACCCATGATTGTGAGGC
ACCCGATTCGCCCCA

CCGTCATCCAGAATCGTCATGCTTATATTCTCAATCCCAGACATG
TCGGCGCAACAACCA
CTATCGGTGTCTTTGTCGTTCGGCGGTGTCTGTGCCAACTCGATCC
CATTTCGCGGCACT
TGCAGCTTATGGTTGTGCTCACTCGACTCAGCAGCACTGGAAATA
GAGCAACGGGGATTG
AAGGAAGATACAGAGAATGTTGTGATGGGCAAATCTGAACCTGC
AGATTTGGCTGCATCT
TCTTGTGATGTGAAATCTGTTCGATGTGAGCAGTTAGCGCCTTGA
CTCATAAAATGATTG
TTGTTGAGGAAAGATGATCCCAGACCAGTATTATTACTCTCTCCG
TATGAGGGTAGGGTT
GAGCCAGTGAAGTTGGATTCGTGAAGCTGTGCGTTAGAGTACGGA
GGTGGTGGTTGCTTG
TCGACATTAAAAACATCCGTCGAATCAGCACGATTCGCGTTTGAG
CTGAATTTGTATTCT
TGACTGGAAGGTGCCAGCGTACCACCTCGGTGAGAGCTGCTCGG
ACTAGAATCGGGAGAT
TTTCCGTCTTTCCGCTTTATGATTATGATTGCAATTATAAGAATTA
TGACTAGTACAATT
GTTATCGCTATCACGCATATTATGATTACGGTATTTCTGCCGGTGA
TACCACTTCCACTT
TGATCATTTAAGTACCCATTGTTCGGATCTATAACTGTTGCAGGG
TAGTATGGGGTGTTT
TTACTTACATGGATATTAAGTTTTTGCACAGTCGATCTATCAGGTG
TGTTATCCTGAACT
AATATTGTTAAACTATACTGAATATCATTGGATTCGAATCTGGAA
T
>ConsensusfromContig3036 Average coverage: 7.67
AAAATCGTTCCTATTCAAATTTATGAAAGGCTGTACAATTATTGG
AGTAAAGTTTTGTTC
AAAATTCTGAAAATCGTCAATAAAATCTCGTTCACTAGAATCTAT
GTAAGGCTGTACAGT
AGTAGTAGAATTTTGTTCTAAATTCTGAAAATCGTTAATAAAATTT
GTCTCTTTCAAATT
TGAGAGAGGCTGTACAGGGGTAGAATTTATTTCGAAATTCTGAA
AATCGTCAATGGGAGT
ATTTTTCGTATCCAAAAGCGGCACTTGCACAGAAGTCACAAGAAA
TCGTCCTCTCTTCTC
ACCACCTCTGATCAGCGAATTCTCAATACCTTTCAAATTCTGCGC
CAAAGGCGACAAAGC
TACAGTAGAGCGCTTGGC

>ConsensusfromContig3037 Average coverage: 14.10
TTTGAAACAGCACAATGAAAATTCTTAATATCTTTATTGGTTACTT
CGTGATTTTCGCAC
ATCTGCTACAAGCCTTCGGATCGAAAGAACTGATAAAACGTTCA
AATGCGTGTGCCAAAA
CACCGCCACCGTGTCGTTTTCTCCCGTGCACCAGCCCATTTTTTGG
GTTTGGGTTTGTTT
ACGGCTGCGAGTGTGATCCTGAACTATACCAGTTGGTTGGCTTTG
GA
>ConsensusfromContig3038 Average coverage: 11.51
ACATGTCGTAGTCTGTCTTGCGGATGCACCAAATGCTTCAAAGTA
CCCACCTCAGGCATT
GCTTTTATGGGTTGCCGTTGAAGACAGGGTGGAAAGTGATTTGAG
CCAACACTTTCACGA
GGCTTCGGACTGGATCCAGTCTGCCACCAAAGGCGCTTCCCGGTC
TGGAAAGGTTCTAGT
TCATTGCATTGCTGGAATCAGTCGTTCTTCATCAATTATTCTCGCA
TTCCTCATGAAACA
TCGTGACATGTCTTTGAAGCAGGCATATTACTTCGTAAAAGGAAA
AAGATCTGTCATACG
ACCAAATAACGGATTCTTCAAGCAACTGATAGACTTTGAGAAGT
CAGTAAGAGGATCAAC
TTCTGTAACAATGGTTTATCATAGTGGTTGCTTGATTCCAGATAT
ATACAAAGACGAATT
GAAAGGAATGGTCCCGTTCTAAACATGAGAACAACATGCTTCTT
AGATGGCACCGGGAGG
TCAGGTGAGAGTTGGGAATCAAGGATTTCAATCACATCACACACG
ATGGTTCACGTATAA
GATCTCGAATGAACTTGAAGATGAAGTGCAGTCGAATCGCATTAT
TGACATCGTTCATTT
GTTTGTTCGTGACCTTATTTGTTTACACTTAACATGATCTTTAATA
TTTATTTTAAGATC
CATAATAAACGCG
>ConsensusfromContig3039 Average coverage: 18.66
TTTGATGTCTCATGCATATTCAACAGTTTCCGTCTCCATGTATCCA
GTTTTGATGTGGCG
GCAGAACGCTGCAATGGCGATCATGGCATGAATATTGATGAGGA
AAACGATGCCATAGGA
AGCGATTATGCCTACTCTCAGCTCATCTTCAAAAGTTTTCTCTTG
AAAGACGTAACCAAT
GACAACAGCCGCCAGCGCCATTAGAACCGTCACGATGAATGTGA
CGAAGATCCAAAACGC

CAGTAGCGCGCGACTTTTCAAACACACGCCCATGAAGAGGAGGA
TCCCTGCCACATTCCA
GAGGCCATAGGCGGCGATTGCCACGATGTTGACATTCTCAAGGA
CATCGGGGCTGAGCAA
GCATTCCGTGGTTGTACTGTTGCAAGGGTCAATGCGAGAATACTG
CAAAACATTCTTGAG
GCCAAGGTCATGCATGAGTTGGTCGATAGCGTCTGCTTCATTGAG
ACGACCTCGAATATC
AAGAAGAACATGAATGAAAGCTCCGATCGATGCTATGAGAATAT
TATACAGCCCGATGAG
GACACCTCCACACGCATGCGGCAAACAACAACAGCAGCGACACA
TGGTGGACGGTAGGCG
GCGACACTGTAGATTTATTTCAGAAACTCGAATTCATTATTTTGA
ATCCTAGTGGAGGTT
TGATATAATGCTGAGTAGAACAATAAGATTGGCGATAATGTTCAG
ATTATAAAATTGCGA
AAACGAAGGCAAGCCAGTGTTATCCTATCTGGGCGGATTATAGAT

>ConsensusfromContig3040 Average coverage: 5.98
CAAAGATGAAGGATCGTTCTGGTTGGTCTATTACATCCAGCCATT
CGATCTGTTTAAATC
ATCAGAAATGAATTACACGGCCCTGCAAGATGGAATCGATGAGG
TTGCGTGGGATTTAAC
TGAAGAGCAGAAGGAACGAATCATAAATCTTTACCAGCCAAATA
GATCTGCTAAGGGCTT
TATGAATCAACAAATTCTCGACGACATATCCGGCGACAGGGCGTT
TGTCTGCCCCACTGT
AGCGATGTCGGAGCTGTACAGAGAGATCGGAAGGCGGTCTTTCTT
CTACTATTTCCCATA
TCGGGCATCCAATGAGGTGTTCGGAGAATGGTTTGGTGTCATTCA
CGGAGCCGAGATACA
GTGGGTTTTCGGCATGCCGGTCAATCGGTCCCGTAGATATCTCGA
CGATGAAGTGGAGTT
CAGCAAATCCATCATGACCTACTGGTCTAATTTCGCCAAATATGG
TGATCCGAACAGCGG
GGGGATGCCCGAGTGGC
>ConsensusfromContig3041 Average coverage: 6.96
ATGATGGATCGGGATAAGTCATCAGAACTCCCAAAAATGCAAAT
AGGATTCATCGATGGT
ATATGTTTGCCTGTGTATAAGATGGTACATCAAGCTTTCCCTGAT
ACGGCGCCCATGCTA
CGGATGTGTGACGCGAATAAACAAAATTGGTTATCGATAGCTGA

TCGATATGAACAAGAA
AAAGAAAATCTACAAAACCAGCAGGATTGAATTCTTTTTGGCCA
GAGCTCTGAACACGGC
ACGGAAGAGGCTTTCCATCGCACTCAAAGTGAAGAACACACCAG
CCCCGAAAAACTACGG
CCAATCATCACCAAGAAAGGCAACACCATTCACATCATAGCCAC
CAACAACACCCACAAG
ATACAGTGCGCCCCCAACATCATCATTCATAAAACCACCAAGCTC
TGCACTCTCCAA
>ConsensusfromContig3042 Average coverage: 8.28
CTCGTGACGTATTTCTGACCGAAGAATCTCTTCTCGAGACCTTTA
CGCTGAAGGTGACTG
AAGACTGCCCTTGACCATGACCTTTTCCTTTTGATGCTCTCCTCTT
TTGGCATTGTTTGG
GTCGAAGACCACGTGTTGAAAGGATAATTCATTGAGGACATAGA
GCCGGTGACGTCAGCG
GGGGTTCCATAGATTCTGAAGATCCCAGGAGCGAGGGGGCGCCA
GGGTAAACTGGATGCA
CGCGGGGGCGGATTCGGGAGGATAGGTGCAGGGGCGGCTCTGGG
TTGAAATTTATCGGGT
GATACATCCTGCTTCTCTGTAGCCTCATCACTTTTCAATTTTTTGG
AGGGTTGATCTTTC
TTGAGGATATTTTCTATACTGAATTTCAAATTATTTTCCGGCTTTT
CTTTCACTCCACAT
TCGTATTTCTGTTTCATAACATTCTCGAATCCCAGAAAATTGTTGT
TGGCAAT
>ConsensusfromContig3043 Average coverage: 8.02
ATCAGGAGCGACTGTTATGCCCTTATTAAGACGTTGAAAGCAATA
CTTGCGATGAACGAA
GATGATGTCCAATCGCAGATATAAGACGGAGGCAGCGCTGAGGT
ATCAGGAGCGACTGTT
ATGCCTTTATTAAGACGTTGAAAGCAATACTTGCGATGAACGAAG
ATGATGTCCAATCGC
AGATATAAGACGGAGGCAGCGCTGAGGTATCAGGAGC
>ConsensusfromContig3044 Average coverage: 9.28
GATTTTGATCACTATTTCAAGCATTTTCAATCCCCATTAGCATTAA
CGCATGACTATTCA
TCGAATGTTGTTGAAGTAGATGATAAAGGTCACGCAATAGGAAC
ACCAATCTTCGACCCA
GAAGGAGATGATGTTGAAAATAAAAGCAAAATGAGTATTTGTAT
AGAAGATGAAAACTCG
ATACGTTCGATTCTTAAAAGTGCGCCAAATGAAGAGAATATCGA

GAGAAGTATGAGTTCA
AAACAAAATTTGAGAAATGATTCGAAATTTTCCGATAATAGAAG
TGTTGACGGGGG
>ConsensusfromContig3045 Average coverage: 35.15
GAGCGGTCTTCGACCCTTTCTAATCGACCCTTCCTCTCGAGCGAC
CATGTGGCTGAAAAA
CCACCTCAAAGATCAACGACTTGAAGTTGTGAACCAACAGGATG
CCAACTTCAGCACATC
CGTTGAACTGGCTGTTCGTTTTGGCAAAACCTTGGTCATTCAAGA
AATGGACGGAGTGGA
ACCGATTCTGTACCCACTCTTACGGCGAGAGCTCATATCACAAGG
TCCTCGATACGTCGT
GCAGATTGGGGAGAAGACGATTGACTATAACCCGGAGTTCCGAT
TGTTCATGACGACACG
AAACCCGAACCCAGTGATACCACCGGATGCTGCTTCTATTGTCAA
TGAGGTCAACTTCAC
CACGACCAGGGCTGGGCTCACTGGACAGCTTCTGGCCGCTACGA
TACAGCACGAAAAGCC
AGAATTGGAAGTTCGCAAAACAGACCTATTGAAGCAAGAAGAAA
GTCTGAAGATAAAACT
GGCCCAGCTCGAAGAATCATTATTGGAGGAATTGGCGAATGCGA
CCGGCAATATCTTGGA
GAACAAAGAACTGTTGGAGTCTTTGAATAAAACCAAATCCAGCA
GCACGACTATAACTGA
ATCTTTGACTGAATCGCAGGCTTTACAAGCATCCCTCGATCAAGA
GCGCGGTGCCTATTT
ACCGCTCGCTCAAAGCGGGAGCACTCTCTATTTCATCATCAAAGA
TTTATCAAAGATCAA
TAATATGTACGAGTCAAGTCTCTCTTCATTCCTGGGTCTTTTTCAG
CGCTGTTTAGACGA
AAAACTGATGGAAAGCTCGACCTCTGAGAGAATATC
>ConsensusfromContig3046 Average coverage: 4.41
CTATTGGACGTACCATTCCTATATCTTCAGACTGTCCTCTCCTCAC
ACATCCATCATCAA
TCGAGTCCATATTGAGATGTCTTTCTATCTGACTCTCCTCACTATT
GGACGTACCATTCC
TATATCTTCAGACTGTCCTCTCCTCACACATCCATCATCAATCGA
GTCCATATTGAGATG
TCTTTC
>ConsensusfromContig3047 Average coverage: 7.05
TTCCGATCTATTCTTTTTTGGAGTGAAGACCGCGACGTTTTAGCG
GATAAATCGGAGGTT

TTGAACATAGGCGCTGTTGTGCACGTGGGAGTTCTGCCTGCTTAC
GACAAATGGTTGCAA
GCATCACTATTGGTACGCGATTCACATGTGTTTTTTTCGGGTTCT
TTGCTTCGTTTTGA
AATGAACGTTATTTCTGTGATCGTGTGCTCCACTGCACTTGATTG
CGGATGGATCAATGA
ATAAAAAAGA
>ConsensusfromContig3048 Average coverage: 37.57
ACTGGAGTTCAGACGTGTGCTCTTCCGATCTCAGGCAGGCTTCGC
GGCGCCCCTTCTCAA
TTCGCCGCGCGACACCCCCTCTGCCAGAGCGGATTTCGACGCCTC
GGAGATGCACCTCCG
CTTCATCCTCCAACTCCTTCGGCTCAGCTGACTTCTACGGGAGTG
GAGACAGCGACGGGG
GTGGCATGATGACCCTACAGAGAGCTCTTGTCATGTGTGCAGGAT
GTGACCATGCCATCA
TGGACAGGTTTCTCATGAATGTCTTAGAGCGAACATGGCATGTCA
AGTGCGTCCAATGCA
CCGACTGCAAGGGACCTCTCACAGACAAGTGTTTTTCCCGAGATG
GGAAGCTTTATTGTA
AAGACGACTTTTTTGGGAGGTTCGGCACCAAATGTGGCGGTTGCT
GCCAGGGAATCTCAC
CAAACGATCTGGTACGGAGGGCGCGGAACCAGGTGTTTCATCTGC
GCTGCTTCACCTGCA
TGGTGTGCCAGAAGCAACTCTCCACTGGAGAGGAACTCTACATTC
T
>ConsensusfromContig3049 Average coverage: 24.41
CAATGCCCGGGGCTGTCTTCACCAAAGAATCCAACAATCGCATTC
GTGTCTTTTGCGAAG
CTACCAGCAAATCTTTCACCATCTTCTGCGAGGGTGGCATTTGGA
AAAACACACGGAACA
TGTGCCCGAGTTCAAACGAGCGGCAAGGCATGACAAATGATGGC
GGCCTCCCTGTTTCGG
ATGACACTTTCCCATTAGGCATATTGCTGGTCGTGGCCATAGGCG
TGGCTCTGGGGGTCT
TTGTCGGGGGCCTTCTTCTGGCTTGTGCATCCTTCCACTTTAAAA
GAAAGCGGCAATCGG
CAGAAGCAGAGAGGGTATCCGCACTCAGAGCTTCGGCGTATCAA
ATGGAGAAAGAATCCG
GCTATGGGACCGAGAGGCGAGATCCAGAATTCACAGCCATTCATT
CAGCCATCATTCCTC
TTGAGCATCAACAAATACCCAACAAAATGAGACATCCAGATAAC

ATCTTTTATAGACAAA
CTGCTAGTCCGCCTCTCCCGCCATATCCCAGCGATAGATTTTTCT
CTACAAATGAGCCAT
TGCCTACACATATTTACGAGTCAACGTAACACTCATTGTAGACAA
TTTTTGAAATATATT
TTGATCAGCTTCTTCTCAGACAGGCTCTGAATTATTTCAACAAACA
CTGCTTATATTCTC
TTCTCTGTGTCAAGGCTTCAATGTTTCTATACTGAGAGTACTCAAC
TTCCATCTTATTCT
TTGAATGTATGCAGGA
>ConsensusfromContig3050 Average coverage: 31.61
CAATGTGGATGTCAAATATCCCGTACACAATCAAAGTCTTATTAC
TTTGTATATTATGGT
ATACGAGTAGCTCTATGAATAATGTCATAGGAAAAATAATTTTAA
GCGAATTTCCATACC
CAAAGACTTTAACAATGATACAACTATTTTCAATATGTTTGTATG
CAATACCAATTCTCA
GACTATTTCTAACTAATGAGAAATTGTCAAAAGATGGTGTCGTGA
AATTTTATGATATTG
GTTATCATACTCTTCCTCCACGCTATTGGTTCAAATGCATTTTGCC
ATTAGCATTTGCCA
AATTTTTATCTTCAATTTTAAGTCATGTTAGCATTTGGAAAGTGTC
TGTCTCCTATGCTC
ACACTGTGAAAGCCACGATGCCATTATTCACGGTTATACTTGGAC
GTGTGCTATTTCAAG
AGAGACAAACGTGGCCTGTTCTCTTATCTCTACTTCCTATCATTT
GTGGTGTGGCAATCG
CCACGATAACGGAGCTCTCTTTCAACATCACCGGCCTTCTGAGTG
CACTTCTGGCCACCT
TCGGGTTCTCTGCCCAAAATTTATTCTCTAAGAAGTGCCTCAAAG
AAAGTGGGATTCATC
ATCTTTCACTTCTCCTAATTTTAGCCCGAGTTGCCACTATTTGCAT
GTTCCCGTACTGGT
TTTTCACTGACGCAGTTTTTATCTATAACGACTCTAGTCTGCTCA
AGAGCGGCCATCTGA
TGAATATTATACCTCTT
>ConsensusfromContig3051 Average coverage: 6.95
CAGGCATATGTTTGAGAGCGACAGCTGAATGACCGGCAGAACGA
AGAAGTAGATCAACAC
CCCGTAATCTCTTCTTATACAATTGATGCTCTTGTAGAAAACAGC
AAACACATGATACGG
ATTCGGAAAAATTGTTTGATCTTCTTCCGCAACGGCATCACCGG

CTGTCGGGCAATCGA
TTCCATTGAATCGCACTGACGTTTTCTTTTTTGAATTCTCTTCGGA
ATGTAGCTAGTGAG
AGCATCCTGCTTTTGACCCCTGACCTCATCCTCACTGTCACAATCT
TTGCTATCAGCGAT
GCAAAGACGCAAAAACACGTCCTCTAAAGTATTTCGGTTATAGCG
CTGTAGCAATTCATC
CGGAGTAGATTCCAC
>ConsensusfromContig3052 Average coverage: 7.62
CTCACTATCACATCTCCAACTCTCACTTCGTACTGTTATGTTTGTT
TTTGAGTCAAGGGG
GAACTGATCAAATGTCGTCATGTTTCGAACCGTTATATTAATTCTT
AATTTTGATTCTAT
TGCTTTGAGAGATTCGATCATGATTAAATTTGACAGATTGAGATG
TGTACATTTTATTTT
CAACATGAACGATGTAGGTTCTTGCATTTTCGAAAACGCATTTTC
AGCGAATGAAACATT
ATTTTGTATGTTCATAATATACAATCGTGTAGCTACAAACTGTTT
GAATGCATAATCAAG
GAATTTTATGTCATGTTTGAGTCTATAGAATTTTAATTTCTTTAGC
TTTTCGAATCGCTT
AAAAGTTCTTGCCCCCACACTTATGATTGATAACTCTTCAAAAAT
AAGTTCTGTGATATT
TCGAGGATGATTCCTAAATGACCCAGAATATAGTTTCAAAGGGAA
TTTCGAATCTTCGTT
TCGTCGATTGAGATCTGCCTCATGATAGTTAGAAGTGTGTGTGAG
TTTCAGATTATCTTT
ATCTGAATTATTTATAGATAATTTGAGTTTATTTAATGGACTGGA
GCTTTTGAAAATACT
TATGTCGCACTTGAATGAATTTTTAATATTGGGACTATCATATTC
ACAATTGAAATCTGA
CTCACATTTAGAAATTAATAACAATATTTGAAGTACCACTCTCGA
TATTAGAATTGTATT
>ConsensusfromContig3053 Average coverage: 14.07
GGGGGCGTGTTGACCCACATAATCTCGAAGCATCTTCCAGCCTGG
TTCGACACATGGGGC
ATCACGGATTTGTTCATCAAGATCTGGTCCTTTATGAATAAAGAC
ACCAGGGATGGAGCG
CAAACAGCTATATATTGCGCTGTTTCACCTGAGATGGAGTCCATA
TCGGGATTTTATTTG
GATGACTGCAAAATAACAAAACTTAAAGATCATGTTGAAAACGA
AACGAATCCAGAAACC

TTGTGGAGGCTCTGTGAGAAATTGTCGGGTTTCACCGCCCCTGAA
TTATATGTCTGACGT
CACATTACTTAAACACTATATATTTAATTTGTT
>ConsensusfromContig3054 Average coverage: 16.58
TCATACCCGCATTTTTAGCTACATCGGGACAAATGGTCGCGATTT
TAGGATTCTCAAAAA
TAGATTTGGATTCGAAGGAAAGCATCTCGTTCCTGACGTTAAGTG
CCATAATTATTCTGA
TGGATATACAGGCTTTATGCGTGACGACATCTCATATATACCGG
AGTAAAAGTGAAACAG
ATTATTCCGAGAGCTGACATTGCCTTATTTATTAATATCTCAGAA
ATACTATCTCAAGAA
GGAGAGTCAAAGATTGTTTCTTGTTTTTTT
>ConsensusfromContig3055 Average coverage: 10.96
TTTTGGCTTGCTTGACAACAATGGCAAAAATGGCCAAATTTCGAT
ATAAACAGGTCTATT
ATTTACTAAATATATACGTTCTTTTTCTTTGTTCCAAAAGTTCGGA
GTCAATTGATTATC
TCGAAAATATATGTACGTGTGATTTAACTCAATATACGTGCGATA
TAAACTGTTGCTGTG
ATGATGACTGTTCTGAGACTGACATATCAGTTTTCAGCCAGTGTG
ATGAAGTTTCAAGTT
CAACTCTTATTAATAATAGGCATTGTATTGATGATGGCTTGATTTT
CATTGATAATTCTA
AGTTCAGATCATCAATTACAGTCGATGGCTTGCTTTGCATTCATA
AGGACAATTATGTCG
ATAGAAACTATTATAAAAGTCCCGAATTGATAACAGATTTGGCTG
GTTTTGAATCAAAAT
TAGGTGACTCATCAAATTCTCCATCATATAATTATCAGACTGATT
CCTTCATATACACCA
AGAACTTATATTTTGTTGGAGACCCAATAAAAATATATGCCGAA
AATGGGACAAAAAGAG
ACTTTTTATTTTTACGAGGAGGTGACAGATTGCAAAGATGCAATA
CAAAGAATTATGTAC
AATATTTGATCGATTCAAATGTAAAATGCCATAGTGAAAAAGAT
ATCACAATAACTGAAA
TTTGTTCAGCAAGCGAATATGATGCAAATTATTATT
>ConsensusfromContig3056 Average coverage: 9.89
GTCAAATGGCAGTTTTTCACCTGGATTTCGATATTCTCATGCGATT
GACTGTCTTTTAGC
TTTTCGGTTGAAAGTTCTTTTGATTCATTATTTATATATGAATGTG
TAGTATTTGATTCA

ATTTTATCATTGCCTTCTGTTGAAAATGTTGTTTGATTTGAAATAC
TTTCGGGTGATTTT
CTTATTTCTTTGCGTGTTGTTTTTGTCATATTTTTTGTATTTGAATG
AATTGTAGTTGTC
TCTTGATTGATTTGAATCACAGACAGCTCATCATCCGATGTTATT
TTATGTGAATATCCA
TCTTTTATTTTTATTGAAGGACTTGTATTTGTCTCATCCGTAACCA
TCGAGTTTATCCCA
GTTTTCAAAATTGTTTGATTATCTTTCGATATCAGCATATCTCGAC
TAATATCCGAATGA
CTTTTAGATCGGAAGAGCACA
>ConsensusfromContig3057 Average coverage: 11.71
GGGTGAAGTGTGTCTGGACGACCATGCTGAATGCACGCGCGGAT
CGTGCGAATGTGTTGG
ACACTTCTTTCAGGCCAGAAATAACACATGTGTTTCAAAGGGCCG
TCTCCGGGAGCCATG
CTTGGAGAAGGAGGGTTGCCAGGACAATCAGCTGCTCTGCATTG
GAAACACGTGCTCCTG
CCGACCTGGCTTTAGACTCTTTGGAGATTCTTGTGAAGTTGAGTT
GGTGAATACGACGGA
GCCGACCTCTCCATCCATCGCCACAGTTCCTCTTCTCGGAAAATG
CTCGTTCAGCGCCAT
CTGCGAAGACCCCAACGCCAGATGCTACAACGGCACTTGTCTCTG
CCACTCCTCCTACTA
CATCAGCCAATCCAGATGCAGTTTGAAGGGGAACTTGAACGCTCC
TTGTCTATTCGGTTT
TGCCTGCTC
>ConsensusfromContig3058 Average coverage: 7.89
TTGGAGGATCGTCTTCAACTATCAACGACAGCTTACTGACGAGGC
TTGTTTGCCTGGAAG
CATTCAAAGCCCAATGTCCGGAAGATCTGACAGTTTCTTATCACG
AAATCGTCTACGCCA
ACCTCAACCATCAACCTGAACCCGGCTGGCTCTGGGTCTTCGCTC
CTCTCGTGGAAAAGT
ACGGATTTGTACCCAAGAAAATAATCACAATCGACAAAAAAGTC
GTCGAAATCAGCCACC
ACTCGGTCGAGAACAAAAATCTTCTCTCGGTCAAATGTTCTTCGT
CTGTAAAATCTTTCT
CTTCCGATAAAAGTGTCGCTCCTTTCGACAAAAATATTCATCGAG
CAGCTCCACCTAGTC
GCGGAGATCGGAAGAGCGGCGTG
>ConsensusfromContig3059 Average coverage: 18.49

AAGCTAAAATGCCACATCATCTCCATATCAAGGGTTTGAGTGACC
GAATTGAAATAGATG
ACAACTCTCTACCCTCATATAAAGACATCGACTGTATCAAAAGG
ATAGAAGATAAGACCA
CTGATAAGACCAGTGAAAAAAAAGTGGTAACGATCAAAGAAAAC
AAACTGAAGTGGACAG
GTGACACTGTCATTTCATATGTTGCAGGAAGAGACCAGATAAAAA
TAGCATCTCTGGATA
AACCAGATGATTTCGAACCACGGTATTGCAGCGGCTTCCGCATCC
ATGAATCGGATAAGT
TCCAGGTCTTTTCTGGAGATTCAAAAATACTTGAGTGCGACACCA
TGGATTTCTTCGTGC
AGGAGGAAACCATCAAATTTACATTCCGCGAAATAACTGCATCG
GATGAGAACAGCTCTC
AAAAAAGCAAATAATTATAATATGCCTTTATTACTCAACATAATA
TGTGAAC
>ConsensusfromContig3060 Average coverage: 16.01
TCAGGATTTGTAGCTGCCATGGGCCAAATTGGCGGAATGCTTTTC
CCTTGGGCTGGATAC
CTCACGAAGATAGACATCCCTGTCATTCGTACCCAGCTGCCGCTT
ATCGTTTTTGGCATT
CTTTCGATATTCGGCGGTCTTCTGGCTCTTCCACTACCAGAGACA
AAAAACAGACCGTTG
ACAGAGACTATAGAAGAGCTAGAAAGCTACGAGCCGCAATCAAA
AGAGGCTGAATCAGGG
CATTGCCGCTCACAGAGTTTCTATGCAGAAGCAGGACTCACATT
GAAAGGCGAAACTACA
GTCTGAATGATGTGAAATTTAAATTGATGATGATGATGATTTACT
TCCATTTCCAATGCG
CCTAATATCCACAATATAAGTGAATTCAA
>ConsensusfromContig3061 Average coverage: 19.36
GTGAGAACCTCCCATACAAGAACGCCATATGACCACGAATCAGAT
TTGACATCTATATTT
CGGGGCTCTTGAAGTGTCTCGGGTGCCATCCATCTGATGGGATAA
AGTTTCTCCTTGAAA
AGGGAATATTCTGCCGTGTAGAGGTCCTGTGAGAGGGCGCTATCT
CCAACTCGAACATTG
CAGTTCTTGTCAACCATACAGTTTCTGGTTGCCAAATCACCATGCA
CTATGTTTTGATTG
CCCAGGTATCGCATTCCATCGGCTACTTGAGCACATAACTCCATG
AGGCTTCCTATAGAG
AATGCTCTCAGGGATGATAGCAGATACCTCCTGAGATCACCGAG

TTGGGAATGCGGAAGA
ATAACAAATGGCTTGTTGTTGTGCAAAACAATCCCGCGCGTGGT
CAAAACGTGGGCGTGG
CCTAAGTGCTGAAAACGGGTAGCTTCGCGAAGAAAGTCAACCATC
TGTGATTGTGTGAAT
TCTCTGCCTTGCACATGAGTTTTTATCAGAACTTGCTCTTCGGTTC
CATGCTTCCTATCC
AATA
>ConsensusfromContig3062 Average coverage: 14.47
TTCTGTGGCATGTTTTTGAATATAGGCTTCACAAAAGAAACTGCA
ATCTCCTCGTTATGC
CCGAATCCTATGAATATATCACTCGCATGGAGACTCTCGAACGCA
TTAGCATGGATATTG
AGATCATGGTGCAAGTGATGGAATGACAGTTTTTTGAGGCTCCTG
AAGTTCGTGAAAGTT
CGTGCAGCAATTGATACGATCGATGAATCACTGAAAGCCAGCCTT
TGGATTTGTTTCGGA
AACTTTCGAAGTGATGATGATGTGAGTCTTATATGTTGTTTTTCAT
CAATCTCCATTTGA
TGGTACCTGCCATTGAAATGGATAGAACTCACATCATTGTTGAGC
TCTTTTGAAATTATA
TTCATCTTCATCGTTAAAGAGCTCAAGGCACATTCTATCGATC
>ConsensusfromContig3063 Average coverage: 12.88
CCTGATTTTGTCACGTGATCGATGTTATTCGATTCATGACATAATA
ATTTAACAACATTT
AAATGTCCGTTTTGAGAAGCAACATAAAGAGGAATTATCCCCGA
AGAAGTACAAGAGTTT
GCAATTGCGGCTTTGGACAATAAAATTTTGCATATCTCTGTTTCG
CCACGATATGCTGCC
AAATGCAAAGGGGACCAGCCTGAATCGTTCTCGATATTATGTTCT
CGGAGGCTCATACTG
GCTAGGGAGATATTGTCCAAGTCGTC
>ConsensusfromContig3064 Average coverage: 18.30
TCCGACTATCCTCCACGCAGCGAATCGGAACTGATTTCTCATAGG
CGTATGTAATGTACC
CTTCTCTGCAAGAAACTCTCTCCTCATCTCCAATCCTTGCCACTCT
ACTTCTATCCAGAT
CGAAGACAATCTGGGTTGCAGCGGCAGATTTCATTTTAACATCGA
TCTCATCCTGTCTCG
TCCATCCGAAGCTCAACGGTGAGCAACCGATGGTGAGAAATTCTG
TATCCTCTCTTCCTT
CCACATTGTAACAGTTGATACGATCCCTCGTGAAAAATTTCGTCT

GGGCAATCATGCAAT
TTCTGTTGATCGCGCATTGCAAGATGCATTGTTCCACAGTATCAA
TGTCCGGAAAGACAT
ACTTGTAGTAAGCTTTCTCAATGACACGGAATATCACAAGGTTGC
CGTAGGTTCCTTCCA
TCATTGAAGCGTCTTTTGAAACTGTTGTGGATTGTCGAAGCAAA
TGTCGTGCCCAAATG
CAAAAAACGCAGTGTTGTGTTGACATGGCACATCCGCGGTTTTG
TTGATGACATCGTTA
TAAGTGAGGTCATA
>ConsensusfromContig3065 Average coverage: 13.46
CATTGAGATTTTCATTTTCATCGTGAGTCCCTGCATTCATAACGG
ACCTGCCAATGCTGA
GAGCATCTTGCACATGGGCGGGCAGCGGAGACCCTTTCGTCTCT
GGGATGAAAAAGGAGA
GAAATCCAGCGATGATGGATGTTGCTCCAAAAACAGCTAGTGGA
AGCCATTGGTAGACAT
TCCCCAGCACACTTACATAGGGTGCTATCAGTCCACCAATCCTGG
CAGACGTGCTGTAGA
AGAACATTCCAGATCCTCTGATGTCAGTTGGGAGTAGCTCAGCAG
AAATTACGTACACGA
ATTGGAATGAAGCACTCACAGAAAACTTAATTGCCATAGAAAGA
ATCGTGAGAAATACTG
AGCTGTTCTTTGAAAGAAGGCACATTCCAGCAAAACATATACCG
GTGAAGATGTTCAGGA
AAATAAAAGGCATTTTTCTGTTTATGTGTTTTGCGGTGAAAGATA
C
>ConsensusfromContig3066 Average coverage: 10.00
ATGAAAATGATTGTTATGGTGGATATTGGTATGCTTGAACGTACA
GAAAAAACGATTTCA
TGGTGTGTTCAAACTAGACTACTGAAAAATCAACGAAACTGTGAT
GTTTGTATAGAAAAA
TGTGTTATAAATCGTGAAGGCATTGATGGTGTACAATGGGTTTGC
AAGATATGTCAAAAA
AAGTTCAGCATTCGTTGTGACTCAATATTCGAAATAAGCGATTTA
ACAATAAACGAAATT
TTGGGCGTGATGTACACATCTGTTCAGAGATGGCCGAACCATCTT
GCTTTACAAGAGAGC
AATGTATCTGAAAAACAATTCAACCTAGTTTCGCAGTTTTGTGGA
GAAGTATCCGAGAAG
TGGATTGCCAATAACCCGGCCAAGTTCAAGGAATTCATCGAAAGA
GGTCGTCGCAAATTT

GCCCTCTTGGTCAATGCCCTCGGGGAAAAGTACTCGACGAACTGA
TGTTTTTCACCCATT
TCAACTAATTTTATACATTAACTTGTCAATTAAAA
>ConsensusfromContig3067 Average coverage: 14.10
CTGATATTCAATCTTTGTGTGTGTCCAGATTTAAAAATAAACTTC
GATGAAAATACATCG
TCTCGCATTATAAGCATAATATTATTTTCTGCTGGAATTCACTCA
TAAAATCTCAGGAAA
TCCCTAACTAGTTCTTCCTTGACCTATATTCGACATGTGTACATTT
GATCAATAACTTTA
TATTTCAATCGACCCCAAACACCTCAGGATAGTATTTGATGAATC
GAGTGAGGGTCTTAA
TAGCGTCAGCGTCAAGATGAACGATTTTCCCCTCAATCAACCCCA
ACCCCCGCAGAATAG
TATTTTCTGAATTGAGTTAACTTCCAGATGGACCACCTC
>ConsensusfromContig3068 Average coverage: 6.27
CTGATTATTCGGTGTTCGCGACATTGGCTTTCAATATGGTCCGAG
ATGCAGGCAGAGAAC
AGCTTATAGATGGTAGGGAGCAATTGTATGTCCCTGAAGTTGTTT
GGATCGGTTGTGTCC
CCTTCTTTATGGATTGGAACTGATAGCCCCAATTTCCACCAGTCAG
GTATTTTTCTGTGT
TCAAGGCATTTGTTGAAAATTACCGTTAGGACTATGGCCTTGGGG
TCAGCCCGTCGCCAG
>ConsensusfromContig3069 Average coverage: 5.58
GCGTTTCCTTCTCCACTGCAAGCTCTATGGCTCCGTCGTATGTCAA
TGGCATTTCTTATT
CGACAAGAACATCAACAGCTCCTTCATGCGTCGATTCACTTCAGC
GAGTACCATCCACCG
CCTCACTGGAACCCGCTTATGCTAACACTCTTTCCCGTTACACCA
CAAGCAGCACCATGG
ATGGCACGGCTTACCACTCCGCTCTCGATCAAGAAGTGCAAGAA
ATCGAAAGCCGTATTG
ACAACGTACGAAAATCAGTGAACATGTCTCTTTCCACCCCTGGTC
GACACGTGGTGTACT
CGGGACCGGTTCATTGGGCGGATGCCTGTCGGTGGCAGAAAGGTC
ACATGATCCTCTTCA
ACGACGTTCTTCTGCTAACTTCTCTCCCAGATTCGAATGGAAAGA
CAACGCTACTCGCGC
CACCTGTCTTCCTTCACCA
>ConsensusfromContig3070 Average coverage: 36.87
TCCGGGATCCGTGCCCATTTGATACACATCATAACAATCTTCACT

GCAGAGTTGAGAGAA
ATCATATGGCCGATTGATACATAGACTGGCTTCACAGATTTATCT
GTCGTTCTTAGAGCC
AATCCCCAATGAGTACCTGTGTCTCCCTTTAGGTCGATTCCAGTC
CCAGTCTTGAAATCA
TTCATTCTGGTGTTCTTGTAAATTTCCAGCCCATCAATCATGTGCA
GATTTTTCGCAACT
CCAATGGTTCGAACATTTGCTACAACTCCAATATGACACGCAAGA
CCGAACCCGTTTGGG
TGTAAAATCCCATTTCCATCAACAAGAATAACCTGTGGAAAAATA
TTCGGATGATGGATT
CGGACTTTCTCGAGCTTTTCTATAAGACATGCTCCTTCCCTAAAA
GCCAAAAATCCGGGT
ATATATGGGATCTCAAGCTGTACTTCCGAAATATCTTGATATATA
ACCTCTAAAGAAGAT
ACATCGATTATAACAAGTGCAGCAACCGCAATGCAGGAATCATT
CTTGAAAAATGAGATG
TCCACGCCGCCAACATAGAAATTGGGATCTGGAATATCCAATTCA
TCTGGCCATGCGCCA
ATATCCTCGACAATAAGTCTGGACTTGAGTTCGTCCTGTTTCAGT
TTCAATGTAATTGTA
TCATACAAGCTCATCTCTTTGAAACACACATGTTATGATGGAGAT
AATTTGATCTTAATT
GTAAGGCATACATTAAAGAGGCATTCTACTCCAAAAAATGTTTAT
CCAATCTAAAATATA
TCCTTCCTTTCCTTCGAATCGAATCGAATCGTG
>ConsensusfromContig3071 Average coverage: 6.90
AAATTTTTAATACCAAAAGCTAGAATTTTGAGCAGACATTCGATT
GTGAAGAGAATCGTG
AAGGCGGTGTTCAGAAGATTCAATGAGGTTTCGTAGACAATTGGC
TGATCGTAGAACTTC
ATCATGAGGATTAGAGTGTTGCAGGCTATCATGACCATGATAAA
GTACTCGAAGGGTGGA
GACACGACAATTCTCCAAATCTTGTATTTTATAGACTGTCTGTTT
TTCGGCATGTACCGG
CAAACGGGTTCTGCGTTGATCACGAAGTCTATGCACTGCTTCTGA
TTTTTATCCAAGTCT
TGATCCATAAGTTCGTTTTCTCCCTGCTCCTGGAATGTGATGATG
ATTAAAGCGACGAAG
ATGTTGATGAAGAAAAAGGGGAAGACGATGAAGAAGACGACGTA
GAAGATGGCCATTTCC
ATCCGGTATCGGGGCAGTGGGCCCATGTCTTCGTAGGTCGCGTCC

ATCGAATGCTTCAAG
ACTGTAGGCCAGCCTTCTCCAGTTTGAACAGTGAAGAGGGTCAGA
ATTGCTTCAAAGACA
TCGTCGTAATGGAAGTCCCAGCGGC
>ConsensusfromContig3072 Average coverage: 5.84
TTCCGATCTTGAAAATATAGTATTACTCGTATCATTGTGTCACTCC
TGTGTATTTGAACC
ACCCTCTTCAATTGTGGAATTCTGAAAGCAGCAGCAGCGGGCAG
TGTGTCAGCAAGAGGT
TCCCCCACTGGAGCTGCTCTCCTTGTCTGACTCTTGACCTAGGTT
GAGGACCCTCAGCTC
ATTGACGAACTCAGTGCTGCCGATTCTTTGGCTTATCTGACTGTAT
ACACACTTTTTGAT
TATATCGCTGAAAGGATTGTCGTCAGCGTCTCGTAAAAG
>ConsensusfromContig3073 Average coverage: 22.23
GAAAATATTTTCAAGAATAATCATTGGAACGAAAACGAAACATG
AAAATGTTATATCATT
GATTATGATCTCCAGGCATACCACTATTGTTTGTCATTGGTTTATC
ATATTTATATTGGT
TGTTCGAATTTTATTTTTATTGTCAGTATTTACGAAAATTAATTCG
TTTATAGCTCACCA
AATCCTAGACAAAACTAGCCACACCCTCTCCAATTTAAACACATT
TTAATTCTTGATAGT
ACGGGGTCCACTTTTGTGAATTGGAATGACCTATGAGAGTGGGGG
TAGGTTCATGCAGGT
AGTAAATTTACAACATACATGACACTTGATGTTTAGATGTAGAAT
TCTCAGGACCATACA
ATTCTATGTCCTCTTTATACTGAAACTGATTCACGGACCACATAC
GGACATTAAATTCTC
CGCGTATTTTGGTATAAGCATTTTCAGTTTATACAATATTTACGCG
GAGTTTTCATCCAT
ACGTCGAGCGTATATCGGTCGTTGTTTGAGTCCATCTTATATGGT
TTCAGGCATTCTCAG
GAAAATCTCGTATTCACATTGTGAAATAATATAAATTAGAATACA
ATGTTTGTATTTTAT
TTCATGATTAAATGTTTTCTGGCAATTCAGTACCCGAAG
>ConsensusfromContig3074 Average coverage: 14.06
TGGGCACCGGGGGTGAATATCTTGTGTGCAGCCAACTCGAATATC
CGGAACTGAATTTGT
TGACACAGATGGTCGATGTTTTGGATGAGAATAAGACACTCATTG
GTGGAATATTTGGAA
ATGAAGATAGCAATCTTACCGTCACTGAATTTATGCGGAGTTGTC

ACGAAGACGAATCTC
TGTTCGATGTCCTCAAATTGGAAGAAAGTTTTAATATAGAGGAGA
GTGTCAGCTATGATA
AGATTTTCAACAACACGGAACTAGCGTATGATATAGAAGTGGATA
TTTTCAACGCTGATG
TGAACTCTGTCTTGGACGATTTTGAGGAAGTGTTGGCTTACGA
>ConsensusfromContig3075 Average coverage: 10.23
TTTTTTTTTGTTTTTCATATAATTAGAAATGGACATGACCACAGGA
TGCATATTTATCTA
CATGCAAATATCAAAAATAGAATTTTTACATCAACTTCGATAAAA
TATCGTTGAATAAAC
AAAAACAACACGAAACAGCGATGGGTACCAAATCTTAAAAAAG
AAGAAATAATAAAAAAC
ATCAATACCGTTTTAGTGGAAATTATATAAATTACAGCGTGATTC
GAGACAATGAATTAT
TTTGATACCAGACAAAATGAAACAACCCCTTAAAAT
>ConsensusfromContig3076 Average coverage: 7.25
TTTCGTACAAGAGCAGTAGGACAGAGAGAAGCTCAAGCGATATG
AATTCACAGATTGAGA
GTTTGAAACAGACCAGGAAATCGATTGGCGATAAGGCAGATGCC
AAGAAGGAGAGTTGCG
ACTGGAAATGGATCGTGCTGGATGGTCCTGTAGACACGGTCTGG
GTGGAAAACCTCAACA
CTGTTCTCGACGATTCTAAAGTCCTCTGTTTGGCCAATGGGGAAC
GCGTATCACTGGCAG
ACGACATGCGTCTGCTTTTTGAGGTCGA
>ConsensusfromContig3077 Average coverage: 8.38
TTAGACCTTTCAAGTGCCTTTGACACAATAAATCATAGCATCCTG
TTAGATAAATTGCAT
AAGTATGGCTTTTCAGACTCCTCCATTTCACTTCTACGGTCATAT
CTTAAAGATAGAGAA
CAATATACTTGTGTAGGTAAAAATCTCTCTACTAATCTTGAAATC
GAAACAGGAGTACCA
CAAGGCTCAATTCTTGGACCACTACTTTTTATTATTTATATAAATG
ACATGCAGTCTGTC
ACTAGCTGTTGTAAATTCATAATGTATGCGGACGATACTTCTCTT
TTGACGTCAGTCAAT
AGTCTTCGGAAAAAATATTCTAATTTAGATATATCTACCTCTATC
AATAAGGAACTTAAA
GTTATTTCAGACTGGTTGAGCGCCC
>ConsensusfromContig3078 Average coverage: 6.56
CCTACCGCATCACTACTGAAATTGTGGACGATTATCGCTGGAATG

TCTGGATTAGACTTC
ATGGTACCGAGACAAAGACACAAAAACATGAGTTGGACTCCATG
TCTACGGCTGTATATC
TGAATAGAAAAGGGCACAACCACTATCATTTCTATGATAGGGAC
ATTGGAAATCTGACAC
ATCTTAGATATGAGTTGCCTGGGAGCACTGATGGTTGGTTCCAGA
CACGCTTGCTCAAGA
TAAAATACGGTGGAAAGGAATATAAATTTAGAAGTCTCGAAGTT
GAGCCTGGAAAACATA
AAGATTTGTCCGTGTAAAACTCTGTGTTAGATTCATCGGTGGA
>ConsensusfromContig3079 Average coverage: 13.68
GTTTTATAGAGTATCAAATTATATTTGTGAAACAAATTTATTGTTA
ATTATCTTTATGGT
TTGATGACCCATTTGATTATCTGTTAACATGGAACTATATTATAA
ATGTTCAATAAACCG
ATCTATTTTATTTATTCATACTTGCCAACGGACGCAATACTGCTCG
TGGTTTTTTAACTT
TAAGCCCTTTATCTCTGATCTGAATATTTTTTTGAATTGTTCATAT
TCATCATGATCTGC
CCAGGTATCTCATTGAGCTCTATGACACCTCTATTATAATCTTTG
TATCAGTTAATAAAA
TTATCTGAACACAACGTGTGGCATGTTTACCCTCTTAACTGGTTC
GAAAATTTGAATTTC
AGCCTCTTGAATGTGTTCTTGCGACTTGATTAGAATTTTAGAATCC
AAAACTTCTTCTAT
TGTTCTAAGGAAGTGTAATGCGGCAATATAATGATCTGTCAGTAT
GGTTATATCAAAACT
TACATGCTCTTTGAGTTTTTATGAACGTTCTGTCATACGAAATACG
TTCAGGCTTCTATA
TACATTTACTTCTCATATAACTTACTAATTGTGG
>ConsensusfromContig3080 Average coverage: 24.22
GACTGGAGTCGCGACTTTGTCAATCGGAAGTGTGACTGAGAAAA
ACAGTGGGACCTACCG
GTGCATGGCTATGAATGCGGCTGGCACTGCAGACATGACAACCGT
TGTCACTGTTGTAGA
CAAATTTGAAGCCCCACAATTTACGACTAAGCCAAAGAACACAA
AAGTGACCCCTGGTGA
AAAAGTCATTTTATTAGCCAAGGTGAAGGGAAAACCTCTTCCCAA
AGTCACATGTAAACT
GGATGAGAAAACAGTTGAGTCGAACGAATCTGTGACCGTCACAT
CTGAGAAGGTGAAGGA
AGAGGTCAGGTTTACGGTCGCTTTCGAAAGCGTCTCTTGGACAGA

GACGAAAAGTTTCGC
TTTCGAAGCTGAGAACCAATCAGGAAAGGCCACGTGTAATGCGT
CAATCACCGTCGAGCA
GCCAAAGGAAACAAGGATCGAACCTGAGCCTCAGCAGACGACAG
AGACAGTGATCTCCGA
GCAGACAACGTCAACAATTTCCCAAATCACAGAAGAATCTTCCG
TGGTTATAACGCAAAC
TCTTGAGACCTCCTCAAAGCAGACGGCAGAAGTCAAAGGAACTC
AAGAGTTGTCCACGGG
AACAATCCCCGTGGAAAATTTCGCCCCAGAGGGCGTGCTACCGA
TCGAGACAAAAGAAAC
TATCAAAACTTTGGAAAAATCTGTTACGCAACAAATAACTGAAA
AAGCGGAGAAAATAGG
AAAAACGCCAGTTGAAGCTGGCTTGATAAAAGTGGAGCCATTTA
TTGAAGATCAAGTAGA
ATCTAGCCCTGCTTTGCGCACAGATGAAACCCGGATAGAGCCAG
TAGAAGAGCAAGCCGT
TTTGACAGCGGAAAAAGGCGAACCGGCACCCTCGCAGTCCGGAG
TAATTTCCGTAGAAAC
TTTTGTCGAGGATCAGACCGATTCAATCGCATTACAATCCAATGA
AGAACAAATTAAAAC
AGAAGAGATCGGAAGAGCACACGTCTGAACT
>ConsensusfromContig3081 Average coverage: 11.03
ATTTATCTTATAATAATGCAGATTTTTGCAATACCTCTAGCAACA
ACAATTGCTAAATTG
TTTCACTGTAATGCATCAAGTAAGGTAACTGTAATGAATAATTTG
AAATGCTTCGAACCA
CTTCACTGGATATACATCTCACCCTCATTGATTTGTGTGCTGATGT
TCTATTTCATTATT
CCGGCCAGGCTTGTCAGAAAAACTCGGTTAGAACTATTAAATCT
GAAAAGAAGCCGTCAT
GAAGCATATTTACAGCTTAAAGAAACGGAGTATATACTTGGACTC
GATATTTTGTACATT
ACGGGTGGCTTTCATTTGTTTTCATCATTTCAAAAATCTGCTTCAC
ATTTCAGGGCGATA
TTTTTTGTCGCGGTCTGGTTTTTTTTTGTGGATTTTTTTGTTCTCTT
CCCA
>ConsensusfromContig3082 Average coverage: 12.04
TGGGTTTTCAAAGTACATGTGTCGGCACAGTCCTATCTGGACTTA
TCGTACTATCGATTG
GACTGACACTTCAAGGAATTTCTTCATTCGGACCTGTGAAATATC
GGCACAGATATTTCC

CTCTACTAAAGGCTGTGTGTGGTATTTTTTCAGGGATTCTGATCA
TGATGTGCCCAATCC
ACTTTGGAAGCGATATCGGAAGGTATCATACAAACGATCCTATTT
TCAATTGGCTGAGGA
CTGTGGAAAGGAATGGTGTATCATATCGAGTACAACACCTTTCTG
GTGCAGAACGCCCTT
GCTACGATAGCGCAGGGTGGTGCTTCTACGTTATGATATGCTCTT
GTTTTTCGGCTCTGC
TCGCTGGTTTATTACAAGGTGCCGCTGTTTATGGAATCAAGTGAA
TTGAAGTATTACCGA
TAAGTCCTGGTTCTCAAAAATAATAAAAATCATTCATAGTGTTCA

>ConsensusfromContig3083 Average coverage: 5.04
TGAACAATTGAAATGAAAAAATAGAATCCCTAGGAATATAAAAA
AAGTAATTGAAAATAC
TATTCTGATTCTTCACTACTCTCTTCGGCGGGCAATGCGAGCCTT
AGAGAGCAGAGTGAT
GTGAAAAAGTTAGGTATACTTTCAACCTGAATTTCAGGAGAAAAC
GCCCTCGTTAATTCC
AAGAGCATCCTCTCACATTTGGCCCCATATATGATAAGGCGAGGT
GCACTTAAGAAAGCT
GAACATTCCTCGTATATATCAGTGACGATAGAATGCTTGTTCTGA
TAAAAATCAACAGTA
TTCTTCATGTCACTGATAACGAGCTCAACCTCCTCCTCACACCGT
TCTATCCTCTGGAGG
AGACCGACAACACGCCGCCGGTACGTTCTTTCTTCAGAGAGAAA
AACCGGATCCAGGTCA
GCCCAGAAGCCATCCGTGTTCAGACTCTCTTCCCTAACGTGAGCG
GGCATAGTCGATATA
GGGGCCGGGATCTTGTTGTAAGCCTCGACGTATGCCCGCAACTTT
TTTGAAGCCTTGGAT
ATCTGGATACTGATGCGTTTCGCCAGCGCTTGTCCTCGTGCATGA
CTCTGCCGGAGGTCC
CGAAGGTATTGGAGCTCGGCCGCACAGGAGTGAAGAGTGACAGC
CATCTGTCTTCGCTGT
TTATCTCTGGCAGAC
>ConsensusfromContig3084 Average coverage: 11.88
GAAGGAGTTCTGCCAATTCGAGCGTATGCCTTGACGATACGACGC
AAATAATACCTTTTA
GCTTCACTCACGTCTTTGGGCAACTGCCGATCTAGAATCACCGAA
AGTCGTCGGCTGTCA
TCGTCGATGGCTGATGATCTGGGGCCGCGTAGCTTGGAGACGAAA

CTCTCCAACTCATTG
AATACTGAATTCGATTCGAAATTGGAAACTGTCAATTCTGGTCCT
GAAGGAACGTTATCG
GTAGGAACATTATGATGAAGAACATCAGATGTCTTTATGATTTCT
TCTGATAT
>ConsensusfromContig3085 Average coverage: 11.00
ATAACTCAGACCTTCTAAGAAACACAAATGATCACAAGAACAAT
GATAGTTCAAGTGATA
TTTTCGAGAATGATAGTGATGATGTTGAAACTTATGCCCAGAAAT
CTGTTGATGAGCAGA
GTGGATCGTACTGTAGAACCCTGAGAGGGGATGGAAATGATGTA
GAAAAAGTTATGGAAA
ATGATATATATTCAGATCACCTACCAAGGATTGAAGGTGAACAC
ATAAGAGACATAGCTT
CGAATCACCACAACTTGCCATCATCCCAGAAGTCAACCGCAATT
TTGTACAACAAAGGAA
GTGAATGGTCAACAAGATCATTCGATGGAGGTGGTACACTTAATG
TTCGTCGAAATAATC
ATCAGAGCAGAAAAAACAACAAGATGAAAAAAATTAAAGAATTC
TTCGGAATAAAAAAGA
ATAATATGCGATTTAAACAAAAGCAAGAACAATGCGATTCTATCG
CAAACATTTCAAACT
CAAGT
>ConsensusfromContig3086 Average coverage: 19.32
TTCCTTCAATCGAGCGAAGGCGAAATGTACGCGCAAGCCGTCT
TATTTACTGAAGCGA
CGCCGACTAAGGGTCCTGGTGTCGCCCCGAGACGGAGTGCACAT
GACGGAGAACGTAGAA
GACGATGAATTGTACGAGGATGGCACGGGCGAGGACATCGAGGT
CGGTGGGATTGGCTTC
CCGAATGAGAAGACGAAAGATCGGTATGACTACACGAAACGAAT
GCTTCTCGTCCCGTGG
CTTTTTCGCGAACTCGAGAATAACACGACACCTGGGGTGCGTTG
GATCGACAGAGAAAAG
GAGATTTTTCGAATTCCCTGGAGACACGCCTCTAGTGGGCGGTTC
AACGCGGATTGCGAC
GCCACCCTCTTCAGACGTTGGGCTATGAATACTGGCAAGTACAA
CGACCACGGCAAAGAT
GGTCCGAAGCGTTGGAAGACCAATTTCCGATGCGCCATGCACGC
CTGCAACGAGGTCAGG
GAGATCCGGCCCCTTTCGAGGTCGACGGGGGACAAAGCATTCCGT
CTCTTCCAATTCGTC

GATGTCCAACACAAATGTGAATGCGGCAATCGACCGGATCCGAA
ACAACCGACACATGAC
GCATTGGGAAACCCTCTTTGCACATGCAAGATTTCATACACACGG
AAGTCTATTTTTCGT
AACGCAACATGCAAAGCGAAAATGCGATTGGAGCAGCGAGATAT
CGACTTTACACAGATT
GACGAGGAGGTTGTCGAAGTGCCCGAGGGTGT
>ConsensusfromContig3087 Average coverage: 15.61
CCGATCTTCGAAATCTCAGCCTGAAGAACATGGTCGGATAAATAC
TGTTGAAGAATATTC
AGTTGATGAAAACCCGAAAATCGAAGACGCTTCCATAGAGGACT
GTGGATCTGAATATCC
AAACGTAGAAGCTTTTGTTACAAAAAACGAAAACTCTGGCAAAG
AGAACCTCAGACCTGC
ATATTCACATAGAGAACCTTTGGTTACAAAAAAAGAAAACTCTC
GCATAGAGAACCATAC
ATTCAAATATCCGCACATCGAAGCTTTTGTTACGAAATTTAGAAA
ATTGAAACAAAATGA
ATCACAAGGAGAAAATTTCTTATCATCTTCGAATGAGGGCATGTC
TGCTCAGAATTCTGA
TCGAATCGATGCAGAAAATCTGTCCAAAAACAACACTGATTATC
AAAACAACGCCAGGGA
CGAGCACGCAGAAATCGCCCGTAGGATTTCGAGAACAGTAGATTA
CTTTTCTAAATCCTG
TCCTCGTCCCATTGTAGGGAAACTGTACCCGATCAATAACAATG
ACGAAATAGAGGTGGA
AGCGACATTCCCATCAAAATTCTC
>ConsensusfromContig3088 Average coverage: 11.51
ACGCTCTTCCGATCTTGTGCCGGACGTGGATTCGGAACTAAGCGA
GTTCCGGATTCCGCG
GAACACTCTTCTCTTGACCATCGTCCGTTACCACGCTTCGGCATC
GCGCAGAATCAAGAT
GCTTGGCAACAGGGTCAACGAATCCGGGGTCAAGATCCCCGAGA
CGATTAATGTCGCCAC
CCACCAGAGGTTGATTGACATCGTCCATTCCCTCTTGAAGGTAGC
GCCGTATGATGCCGG
CGTCATGTCATGCAGTGGGCTCCAAAGATATATGATGGAGATATT
TCCAGCCATTGACTG
GACTCTGAGCAGCATGCGCAATTCCTTGAATCTTATTCTCCGCAG
ACTTGAAAGGCTTTT
TACAAAAATTCACAAAAAAGTCATTCTGAAGCGATATATCAATT
GGGCCGCAGCTTCAAA

TATCATTAATGGTGTCACTTCAGCGGTCAAAAGATCTCCCCACAT
CGCCC
>ConsensusfromContig3089 Average coverage: 4.74
CTCAACATCACATGATTTGAATATATTTTTCAATTTTCTACCTAAT
TTAAGTGAAGCATG
GCCAAGATATGGGATTTTGAAAATAAATTTGTGCTCCTTTTTCTC
TCTAAAATCCCGAGT
ACTACACTTATCAGATAGAAATTTTGATATAATACTGTCTATAAA
GAATAATGGAAAAGA
GTTGTTCAATAGTATTTCCCTAATCTTCTGTATTTCTTTGTCTAAA
TGAAACCAACCAGA
ACATATATCATAAGCCCTTGATAACATACCTAAAACTAGCCCCTT
TTTCCATTCTAAAGG
ACAAATACTATTATAATTAAGAAGCAGAGAAGTGAAGGTACTTTT
TCTATAAACTTTAGT
ATATAAGTCATTGTTT
>ConsensusfromContig3090 Average coverage: 7.50
TGAAAATGGAATTTGATTCTATTACGCCTGGCAAGAAATCTGTAG
AACTGCTATATCTAA
AGAATAAAACGCGAAAAAGTGAAAACGCCGAGATGAAAACGCCG
GCTGTTTCCCCCCTGT
TCACCTAGGACGTTGGAATTTACGCATGCGTGATTTAGTGATTTA
CACAGGCACTTCACT
CAAACAGATTCTCACTGACAAGCAAATGTTGAAAAAAGTTCCTGC
TCACTCAAAACTTTT
CATTTCTCTATTGTGCAGGATGAAATATATTTTAGTGAAATAATA
AACAAGAAAATACAA
AGAGTGCATCCTCACACATGTTTAATCTGGCTAATTAAATCAGCC
GTCCAGTTTTCGAAA
AACCTTCAATACTTTGACTACGCCCCTGTTCTCATGTTCACGGAT
CATCAGAAGTCACGC
CCCCCAGGTTCTCAAAGAGGGTGGGATCGTTCTTATAAAATTCTG
CCGTTTTACAATGAA
ATCTTCTGGATATTATGACATTTGCCAGTTTCTTTATGCTGATTCT
CCATATTTCATAAA
TACTCACGTTACAGTTAAAGCTACTCTTACGTCGT
>ConsensusfromContig3091 Average coverage: 10.34
GGCTTATCGAAATTTGATGTCCAATTGAATATGCAGATAATGTAG
AAACCATCATTTACA
GATATTTTATAATTGTGTCAAGGGCTGCACACCAAGAATTTTCAA
ACCGGATCCAATCGT
ACGACGAGCACAATGAAAAAGAGATGCCCGCGCTTTAGCAACAT

CTTGTGGCTCAGATTT
TAGACGCAGTGTTGCATAGGCTTTGTTCACACATTTACATAAATC
GTATAAATATTCAAC
AATATACCGTGACTCTAGATTGCTGTATGCAAGGCGTAGAGTTTC
GTCGTAGTATGATAA
GATGTGTATCAAATCAAGAGCTTCTTGAGAAATATAAATGGGAG
ATAGATCACAATTCAG
ATCAATTGTGAATTCTAAATCGTGCTCTAATTTACACAGTCGTGC
ATGGGTGTACT
>ConsensusfromContig3092 Average coverage: 12.41
AAAGGATATGAGCCTGGCACCCTTTCTGCAATGATGGCGCTCAGG
CATTTGCCCCCTTGG
CTGCTTCCTGGCTATGGATCCGAAGGGCATCATTACCGAGGATCT
GATAATTCACGACAC
GCACATGATAACAAATACGAGAATGTCAGCTCTCACCACGCACT
GGAAATGGCTCCGGCC
GAGTACCTTGGAGACGAACACGACTGCTGCAAAAGCAGTTGCCA
GCGAGTCATCATCAAC
GTCAGCGGGGGACGATACGAGACCCAGCTCCGGACATTAAACCG
CTTTCCGGACACGCTC
CTCGGCAGCGCTATCTGCCGCAAGGCGCTCTGGGATGACCGTAGA
AACGAATTCTTCATA
GACAGACACAGACCCTCATTCCAAGCAATTTTGTATTACTACCAA
AGCGGTGGACGTCTA
CGACGGCCAATGGAGGTTCCCGAAGATATATTCTTAGAAGAACTT
GAGTTTTATAGAATA
GGAGACGAAACTATCAAAGAATTTAAACAGAACGAAGGGTTTGC
ATCAGATGACGATAAA
GAAATTTGTGTGCAACGTGCAGGAACATTATCTCGCATCTGGGAA
TTTGTTGAAGAGCCC
GATAGTTCAATACCCGCTAAGATCTTCGCTCTCATCTCCATACTCT
GTATTCTAGTATCA
ATATTTGTTTTCTGTCTTGAAACTATCCCAACGATACATAGAGAG
CGCTGCATAAATGTC
A
>ConsensusfromContig3093 Average coverage: 6.06
GGAGTTCGCTCATTGTACATGTATGGCTGGTCTTAGGGAGGGGTG
CTCACACATTGCTGC
AACACTATTCTGGATTCTTAGTGCCGTTCGCATCGAAAAGGACAA
ATCATGTACTTCGCA
GAAAAACTCGTGGCTGCCCCCCAGACAGACTGACGATGGCAGTGC
TGCTTTGCCCCTCTA

CAGAATCAACTTCACCAATACAACTCGTAGAGATCCAACACCCA
AACGTTTCACAAAGAA
AAGAACAGTAACACCTCTCACTCCACGCAAGAAAACTCGTTTCTT
CGACAATTTGGTGGC
GAGTGGCTTGAAGAGCAACGTTTTGGCCATGCATCCAGCATATTC
CGCTGACTACAAGAG
GTATGCTATGAAGCCAACTCTCGAGGACCTCTACGATGAGGATCT
CGAGAAACTTGATTT
TGGGGATCTTCTCTCAAAGGCCGAACAAACAATGGAGGCCATGTT
TGTACCTGATACCTC
CATAGTTGAGATTGAAGAGGAGACTCGCAAGCAATCCAAGAGTG
CGGAGTGGTTCAGGC
>ConsensusfromContig3094 Average coverage: 13.63
GAGTGTAACTGTTCGAATAACTGGTGAACGTGACAAATGGTATC
CCAAGTACATAAAAGC
ACGAAAAAAGCATGATAATTTAACCACATTTCATGGTGCAATATG
GTTTGGCGAATATGA
GCAAACTCTTTATCCTGGAAATGATTTGAATTATGTGTACTACAG
AATACAAGTTCACAC
AAGCCCTGGTGAAATGACAGGGACAAAGGACGAATTATATATTGT
TTTGTCGTCATCAAA
TGGTACCACCGCATGGCATCAACTGGATAACAGCAAAACCAATC
TATTGGGTGGTCAAAT
TGATAATTTTGTCTTTAAAGACATAGTTATTGGTGAAGTGAACAA
AATAACTATTTATAA
TTTGGACACATTAGACGACTGGAAGCCTGATAAAATAGTAGTGG
GAAGAGATGATATACC
CGGAGATCTCTATTTTACTGCTGGTTCCTGGATTGATGTTTATGA
GACAATTATGGTTGC
AGATAAATGGGCAGTCCCTATGGATTTGATTTCACCTGATAACTG
CGGATTGGCTTGTCC
AGAATTGGGAAAATGCGGAGATCTGGGTAACGGCATCGTTTGTGT
TTGTAATAATACCAG
ACAGTGTGATAAAAAAGAACCAGATTGCAAATATTTGTGCCCTGA
ACCGTTGAATCCTAA
AATCAGATGCGGGTGGCCACGCAAGGATAGTCTTTCTGGTCCTGA
GTATATTCAACCTGG
TTGTTATGATTTCACTGGGAAGTATCCAAAAAGGCTTCCATACGT
GCAAGAT
>ConsensusfromContig3095 Average coverage: 16.28
TAATGAATATGCCAGATGGAACACCATATTGGGGAATGCTAAGA
GAGTTGGGAATATGGC

CTATAAGAAATACATTAGAGTATAAGAAAAGTATGCTATATAGA
ACGCTGAGAAAAGATA
CAGGAAGATTAGGAAGTAGAGTGTGCGAGGAGGAGAAGAAGAA
AAACAACGATAACAGCT
TTTGGGGAGATATGAAAAAAATCATTGAAAAATACAATATTGAA
GAAAATTTATGGGATA
GAAAAAAATCAGGGTTTAAAAGAGAAATAAAAAGGAGGATAGG
GGACAATATAAAAGAAC
AGATGGAAGAGAAGTGTAGAAGTATGACGAAATTGAGGTGGTTG
AGGGGTGAATGGAGTG
GAGAAATGGCTAGATATATTAATAAATTAAATATGAGAGAGGTA
GTAAATTTTATGAGAA
TTAGGTTAAAT
>ConsensusfromContig3096 Average coverage: 16.81
CTCATATCTATATTCGGTTCCTGTGTCCAGCATGCAGCAACAGGA
GTGCCTCCCAAAAAC
AGGGAGTATGGAAGAAAGCAATTGACAACAAAAGAGAAGAGAA
CAGTGAAGGAAGACAAA
ATAAAAATGACAACCACGTTAATATCTGCGATGCCAAAACTCCT
GGAGAAATATTCAATG
GACGAAAAAAAAGTTGGTGTATTACTGGAGATTCCAACTCACTTC
CACTTACAGCTATAT
ACAACTTCTCGACAAGAAGCGGATCTTGAACTGTTACTCAGGCAG
ATAAAACGTATTGTG
GATAAAAATACGAATGATCAGGTTCTTGAAAAATGCAGTGAACTG
CTTCACGTGCTTTGT
GCCGAGAACATCTCCA
>ConsensusfromContig3097 Average coverage: 3.47
GAAAAAATGATCCAAAACATGGTGAATTTAATGAGAATCTGACA
TCCATAATAATTGACA
TAGGGAAAAGCATTGCACTTGATGTACTAGATATAAAAAGCGAA
AATCGTATGTCTGAAA
AAATGGTCCGAAACATGGTGAATTTGATGAGAATCTGATATCCAT
AATAATTGCCATAGG
GAAAAGCATTGCACTTGATGTACTAGATGTAAAGAGCGAAAATC
GTATGTCTGAAAAAAT
GGTCCGAAACATGGTGAATTTTATGAGAATCTGATATCCAT
>ConsensusfromContig3098 Average coverage: 18.92
CTGGAATGCCTGGTCTAACACACGGCACACGATGCGCGGGTGAG
ATCGCGATGGTGGCCA
ACAACGGGATCTGTGGTGTTGGGGTTGCGTTCAACTCCAAAATTG
GGGGAGTTCGAATGC

TGGACGGAGATGTGACCGACAGCTCGGAGGCCATGGCCTTGACC
CACGCAATGAACCACA
TCGACATCTTCAGTGCCTCATGGGGACCAAATGATGATGGCAAA
ACAGTGGAAGGCCCAG
GAACGCTCGCTGCCAAAGCACTGAAGATAGCCGTTGAAGAAGGT
CGGAGTGGGAAGGGGG
TTATCTTTGTGTGGGCTTCTGGCAACGGAGGGGTTCACCAAGACA
ACTGCAACTGCGATG
GATACAGCTCTAGCCCCTACACCATCGCCATCAGCTCCGTGACGC
AAGGCCTCCGGAGCC
CGTGGTACGCCGAGAAATGCTCCTCAACGCTGGCTTCTACTTACT
CCAGTGGCACAAGGG
CCGAGGGACAAATCGT
>ConsensusfromContig3099 Average coverage: 22.65
GCAATGTGACTGGAGTTCAGACGTGTGCTCTTCCGATGTGCGAT
GGGTCAAAAGACGCAG
ACCTCTTCCGCAGAAGAATCGTTCCAAATCAAAAGTGATTAGAA
CCATGCAAAATGATAC
AAAATCATCATCTGAGGAAGATTGGAAGGTGCTTGAGGAAGGAG
AAATAGTGTCGAATAG
CATCCTACCGGTGGTAAAACCTGACGTGGAGGATGAATGGAAGG
TTTTAGATGAAACAGA
AGTGATTCATACAGTCCCAACGTCCCCCAGCTCAAGATATATATC
CCTGCGCGCCATGAA
ACAAAACTGTAATAGTAAATATGATGCTGATGTCGAAAAAAGAA
TGCACCGCATCGTATA
TGACATCTCTAAGGCGGCTGATAACCAATATTCGGAACCACATA
ACGCAAGTACGAAATT
GAGTGAAGCAATCTCGAAAGATTCCTCGTCAGATGAGTGGCGAG
TTATAGAAGAGGAAGT
GGTATTCCATGATGATTGTATCGATTCAACGCCGTCGGATC
>ConsensusfromContig3100 Average coverage: 12.08
ATCTGCCATTTGACATCGGCGATCTGAACGGTCTCTTCGTGAAAC
TCTTCAGTCATGTCA
ATTTTCTCCTCTTGCTGCGTCTCTTCTCTGATCTCATGTCGCAGCT
GTTCCATGTTGGCG
ATGAAGGCTTGCGTTCTCTCCAGGAATTGCCGTTCTTCCTCGTCAT
CCGTCGACGTTGTC
CTCCGTGGCGCCATCTGCATCTTGGGTTGTTTGCGATAGATGTCA
CACGAGTCGATGGAG
ACGTGGGATTCGGCTGCAGAGGCTGACGCTTCTTCGCTGTCCGGG
ATGTACTCGTAAACA

TCGACGTAAAGGAACGCCGAGGACGTTGCCGTGCCGTATGCATT
GACGGCGACGCACGAA
TATTCTCCCGCGTCCTGAGGGAACACTTCGACGATGTTGAGCACG
CATTGCCCCGTTCGT
CGATCGTAGAGTGTTCGGAAGTCAGGATTGTCAGCCACCTGTTGG
CCCTTGT
>ConsensusfromContig3101 Average coverage: 15.06
TACGCCAGGGCAGAACGGAGATGCCAAGGGGAGCACGAAAGAG
GGCGCCCGAGGGGGTCT
GACGTCATTAGGGAGTCGCAATGCAAAGGCTGACATCAAAAGGC
GGAAATCAGTGGCGAC
ATTCGAAATGAGAAAATCTAATTTCATGAATCGGCCGACCGACCC
CCACTCGCTGGGCCC
AAGTGGACCCTCTTCCGGGGCCGTATCCCCCATCCCTCGCTCTCC
CCTCAATGGAATCAA
CGCTCATGATGATGTCATGGATGGGATGGCGCCACCCGTAGCCCC
GCTTGCCATGGCTGA
AGAGGGATTGGTTGATTTATCTCCGGTGAACACGAACATTCACG
CGAATAAGCACATACG
GCGACATTCGAAAGAGATCCCCCTCAAGAGGTCCGTGTCAGGGAC
CTCTATCCGCTTCTT
CAATCCGGGGGACGCCCCCAGCATGTTATCATTGATCTGACTGG
CAAAGAGCTCCCAGA
TCGGAACTTCAATTTCATCTGGCGCTGGTTCTCAAAGAACGAAG
AAGAGATCGGAAG
>ConsensusfromContig3102 Average coverage: 8.35
TCACACGTTACAATAGAGTTGTATTGAAGTTCATTCAGGTTCATT
CAATCATCCGGTTTT
GTATTAGCCTTATGCGATTGCTTGAAAATAATGTTCAATTATTTCT
TGTGGTAAATTTTC
ACATTTTTATTTAAAGTATATCCCGATATCACCTGGTATTTATCTC
AATAATCATTTTCA
AATATCTCTTCGAATGATCATTTTGTTGAAATTGCCACACCACGT
CACATTCACAATTAT
TTGAATGACCCTATGATTCTTGATGCTTCGTACAATCACTGTTTTT
TGCTAGTATATCCT
TCATGTAACATTCTTG
>ConsensusfromContig3103 Average coverage: 15.14
GACCTAGATCTTGATCTTCGTTTAGGTGACCTTGACCTATATCGC
CGTCGAGAATACTTT
TGAGGTCTGGAGCCGGATCTGAACGACCTTGACTCACGAGATCT
AGATCTCGAATGGCGA

TTGGTTGATCTTCGATAATGACGCCTTGAAGATCTCCTCTCATCCA
ATAGTCTTCCGTCT
TCGCTCATTTCAAAATGGCTGCTACGCGATGAATCC
>ConsensusfromContig3104 Average coverage: 9.59
CGTGTGCTCTTCCGATCTCCTTGGAGCACATGAGTCGAAGTAGTT
TCGCAACATGATGGG
TGAAACCATCGACAAAAAAGGTGTCTGAGTTTTCTCTCGTTCCAT
CATCAATGTTCCGAT
AAAGAACATCCATCTTTTTATTTAGTTTGTCTACTTCATAATAAC
GATAGCCTTCAGTAA
GGTGGGTGAAAATCATACTGTTATGATGATCGAATATCAAATCAG
CATCTTCAATAAGAT
AGCTGGAGTCAAAATTCATATCCGAAAGGCGAGAGCTTTTTTTGG
ATTTTCGTCTACTGT
CCCAGGTTGTATTTTTCCCAGAATTTCTTTTATCAATCTGATCCCG
GACGATTTGCAGGA
GTTTCAGATCAATTTGTTCTCCTGAATCCGAGATCCGTTTAGTTTT
GCATGATTTCATGA
ACGAATCCTCTTCACTTTCTTCTTGAGAGCTTTCTTCCTCATCATC
GTCTGGAGATGAGG
TTGACACTGAACAAGAAGCGGTATTCAGGTCATTTTTAGCCACGC
CCCCTTCATTTTCCT
CGATCAGGGC
>ConsensusfromContig3105 Average coverage: 8.08
GTCCTCCGACCAAAACTCGGATGCAATCATTACAGAATGACTGAG
TGCGTGAAACCCATC
AAAAGCTCATATAACGACCAATGCTCAAGGGTGGCAGGCACCCA
CAAACCCGCCTACAGG
ACCGTTCTTGACTGCGCCACAATGTGCGAGAAGAACCCGACTTGC
TTGGCGGTGGATTTC
GACTTCACAACCAACAAATGCTTCCACCACGAATCAGACACGGC
CTGTGGAGAAATGGAA
GAGAAGACAGATTGCTATCACTTCAAGTTTGTTGAATGTGCTGGT
CGTTCACATGAGTCA
ACTAACACTATGATTACTGACACGATCTTCGACAATCATGTTGGA
AACGGACAGTACCTG
AAGAGCCCCGCTTCTCTTCCCGACTGTATCCGCCTTTGCCAAGCA
AAGTCTTACATCGGA
GACTGCAGATCGGAAG
>ConsensusfromContig3106 Average coverage: 8.64
CGCAATTCGCATTCACCGCAGAGGCTGGTTCTAACAGAGCCCCAG
ATGCTTTTCTTAATG

CATTTCTTCGCGTCGGATCCAGTTTCAACGAAGGCAGCATTGCCG
AAGACCTGGATAAGC
TGTCTACTGCCGGAAACCTCGGTTTGATGCGAAGTGCCAGTGCTC
GCACCATCGACAGCT
TAGGAGTGGGCATCGATGCAGACTTCACCAGCTCAAATTCCGAC
CTTAGGTACGAAAGTA
TTCAGAAAATGAATCAAATGGGATTTTCTGCATTTGCCAATTGCG
TTCCTCAACGATTAA
GTCGTGCCTCTTCGTGTCCGGCCATAACATCTCAAATAAGACTCA
GGCAGGATACAATGT
TCAACAATTTACGGATAGATATTGTTAACAATTTTTCAGCGTTTG
AGCAAGAATTTTTTT
CTGATTCGTGTGATAAGAGGAATCTAGATGAATCTATCAAGAAA
AAAGACGATTCAAATA
TAAAATTCCACAAGCCATGCACGAAAGAAC
>ConsensusfromContig3107 Average coverage: 8.84
AAATAGCGACCCAATAGCAAATATTGGAACTATCAGAAATCATA
CAATAATCACAAAAAC
AATTTATTGACCAGTGAACATAATAAAAACTCACTAGCATATATC
AAGGTCTCGCATTAA
TGAAAACAATAGAATTATCACCAGAGAAATTCATCGACCAGTAA
ACAAATTAGCAGCCCT
TTAGCATCAATAAAACAATACAATGATTAACCAAAAAATTGATTG
ACCTGTTGATAAATT
ATTAGCTGACTGACATATGTTCAAAATCTAGCATCAATAGACAA
TATAATAAAAAAACAA
TTGATC
>ConsensusfromContig3108 Average coverage: 22.11
GGTTGGCGAAACCTGGCGGTAGAGGCGTTGAGAAAGTCCATTCC
GTGAAGATTAAAATCG
ATCATGAACTGTAAATGAAATGGGATGTGACTTTCGTGGGGTTGA
AATGCTTTGTTCCCG
ACCCGACCCTGGAGAAATGCATCTGCCAGTCGCTTGATGTGATGA
GGATTATATAGATAG
ATTTTCAAGAAATGTTGATCCTTTTCTTGGTAGCCATAAAAAGAT
CTCCCAACGCATAGC
GTAATTTTGAAGACGTGTTGAGCAGTCGAATTTTTTGAGGGATGC
CGCCCTAGAGACCCA
TCGATGGCTCTGTCCACGTCACAGGCGAATTGGCGGAGGTACTCC
TGGGTGATCAGGGCC
ACGGGATCCACAGGAACGTACATATAGGGGAAGACCCCATGTAC
GTGCATGCAACATTTC

TGACCTCGAGGGGTTATGCCGTATATGTCAATGACGGGAACTCTT
CTGATATCCGTCTGA
CGAAATTCTGACGTCAATAAATCCAGGCCTTTGATTGGTTCCGCT
AAACATGATTCGACT
TTGACTATCCGCACGGAGAGTAGCATTGGGAAAGACTTCACATTT
TTTCTGGATTTTTTC
ATACGAGTCTCTAAAATAATGAAAGATCGGAAGAGCACACGTCT
G
>ConsensusfromContig3109 Average coverage: 6.00
TCTTCCGATCTAAGCTTTCAGGGGAGCATCGAAGTTAATAATCGA
GGAGTATTCATTCCC
TCGAATTGTCCCGGAATCGGTTCAGTAGTCGACTGCGATAGCTAT
AAGTCATTCACATTG
ACACAAGCAGACGGCAGAATAACTTTGACCAGCGCAAATGGATG
CAGTGCCAGCGTTCCT
ATGAACTCATTTCTGGTGCAGACTATCCATTTGGGTAACTTGGAC
GACTTTGTTAGAGCT
GACTGGGATTTCAACAAAGATGATGCAAACGGTCTGACTCTTCAC
TCCTTCCCTGATTTC
GAACATACTCAGATCTGGATGGCCTTTTCGGCCAGACGGTCGATG
ATATTCAAAGTCAAG
TCCTGCCAAGATGCTAAAATCCTTTTTGGGACAGAGATGGCTGCT
TCTGCCTGGGAACTG
AATATCGGAAGTGAAATTAACACGAAGACTCATTTAGAGAAGAA
TGGTGTGAAAATCCAC
GAATCAATCACAACGACTTTATCTGTTCTCGGATGTACTTCACTC
AAGGAGTTTTGGCTC
ACATGGTCCGATGATGGCTGGATAGAATTGGGACGCGACTCGGTG
GTTGGTCTCCATCGT
ATCTTCGCCTACCAGGACGCTGCGCCTGTTGATGCCAATTTCGTC
TCGCTATCCGGGAAG
AATAAAGCTATGTGGGTGATGGCTTTTGACG
>ConsensusfromContig3110 Average coverage: 46.20
CGGGGATCTGTTCTGGAATGTTCATCTACAAAGCTGAATGAATTC
TGATGCATCCATCTG
CATTCTTGGTGCGGGAATCATCGGCTTATGCGCAGCAAGAAATGT
GCAACAGCTCCTGCC
GAATTCCACGGTCACCATCATTTACGACAAAATCCAGGAGCAGA
CGACGAGTTATGGTGC
AGCCGGTCATATAGTCATGAGGGAGGATTACATGGGTGATATGG
ACAAGAAATTAGCCAG
AGAAATTTTCAGAGAATCCTTCGATCATTTCCATCGTCTGCTTTC

TCACCCAGATGCAAA
CGAGATTGGCGTATGCATGTTGTATGGGTACAGATTCACCAATGA
TGCAGGTTCTATAGA
GCAGAATAAAATGATGAGTGATGTATACAACTGTCGGCCTGCGA
CCTCCAAGGAGATTGA
GATGTTCCCCCATTGCCGACGAAAGTATAATCACCAAGAAGTTTC
TCTCATTGTCGAACC
GCGGTTTCATATGAAATGGCTGCAAAAAGAAATTATACGGAGAG
GAGGGCAATTTTTTCG
GAAGCGAATCAATTCATTTGATGAGCTGAAGGGTTTCGACATCGT
CATAAACTGTGCTGG
ATTTGGAAACAGAGAATTAGTTGAAGACAAGAAAATATACCCGA
GCAGGGGACAAGTAGT
CAGGGTGAAGGCGCCCTGGATAAAACAGACTTTATGTGTTGATGA
TGACATCTATGTGTA
TCCTTGCAGAGATCACGTGGTTGTGGGCGGGATTCGTGTTGTTGA
TTCCATCGATTCGTC
CGTTAAACCTGAAGATACTAAACTTATTATCGAAAAAGTGTCCAA
GTATATTCCAAGTTT
GAAGCACGCTATTGTTGAAGAAACGTGGGCGGGTTTAAGACCCA
TGAGGCAGACTCCGAG
AATAGAGCCGGAAATAATAACCTTGAAAGATCAAACGAAACTGA
AGGTTGTGCACAATTA
CGGACATGGCAGTCAAGGAGTCGCCATGAGTTGGGGGAGTGGTG
TCCATGCTGCAAGACT
AGTGATCCAGCTGTTCAACTGCAGAACTTTTCCGAAGCATAAACT
CTGAAACAAATACTC
ATTATCGTTTCATGAATTGAATTTTCTCAAAGTTATTGCTTACTGA
TCCAGTTTCATAAT
TCTTGTACATTATGTGATTAATTAGGAAAAGGTTGTCTGTAAATT
ACTCCGAGCAGCATT
TATAGATATCATTATTATCATTACCCTAAATATGTTAGTCGTGTA
TCAAACTGATTGACT
CTATATTCGGACACTTTGAGGAATGAGA
>ConsensusfromContig3111 Average coverage: 13.68
TTTGATTCGACTGTATGGAGATATTTTGTTCTCATAAATAGAAGT
TGAATAAACAAAATG
AGCATGTTCAGAATCGAGTCATATATAGCTCCTATTCTAATGGGA
TATTTGAATAGATAT
GTTGAATTGAAGCAAGAAGATTTTCAACTGTCTGTATGGGGTGGA
GATGTAGTGTTGAAG
AAACTAGACCTCAGGAAGAATTTCATTGAGAAATATCTCAATCTG

CCAGTAACTCTTCAT
AGCGGCTATGTTCATGAGTTGCATCTCCATATTCCTTGGACACGA
CTAGGCTCTGAATCA
GTATGCATCACTTTGAAAACAGTTGAATGTGTTTTGAAAGTCAAT
CAAAAGGTTGATAAG
CATCATAAAAAATATGATGCTGCTGAAAATTCGAAAGTTAAACA
AG
>ConsensusfromContig3112 Average coverage: 32.74
CTTTGAATTAATTGATCACCTTGATATATATATTGCATATAGTCAA
TATTCTACACCCAT
ACACTCATACTGATAAATATAAATTATTAATCCGCTTATAAAATC
AAAAGCAGCAAAGTA
AACGTGTTGATTAAGATTGAATAAAGCTTTTATCAAGAGATTGCA
GTTGAAAAGATTCTA
AGATATGGAGCTTCAGCAGAAAAGGAGAGCTCAGGACGGAGC
ACCCCACACTGACCCC
TCCTTCCGAAACATCTGCTGATTTTGTGCGGAGAATATGACCACC
AGAGGCGCTGTGGAT
GATCGTATTTGAGTCACCGAGCAAAACGCCGAAAACGCCAAGTT
CTGAGACACACGAATG
TTTCGTGGACGGTTTTCCATTTCGACTGAGAAAGGCTGGGAAGCG
CGGAGGGTTGATCAG
ATCCATGGCGATATAGGATGACCACTGAGAACGGTCAAGGGACT
GTAGATGATCACTTAT
GTCTTTGCTATATATGTTGTTCATGCCTCCCTCTCGCTGTGGTTTG
ATGACAAACTTCTC
AGGATT
>ConsensusfromContig3113 Average coverage: 9.70
GCGAGATGGAGCAGACATGTCGTTGTTCTATGTGGAGTTCGTGTT
CAATGCTCAACCCAA
GCGTTTGAGCGACGATATCAAATTTTGCAATGAAGACCTCAGTTG
GATCGATGTACCTAC
AAGATCAGAATTTGCGAATTCGATGTACAACCTGACTACATACGG
CTTTGCAGACCAGTC
TGACCATGCCTACCGCGCCTATCTGGGGAGCTGGTATGACATCTT
TGGAGAAAGTCTCGC
GGACGCATGGGGAATGGCGAAAACACATTATATAGCCAATGAAG
AGTACGGTTTGACCTG
TCAAATGATGCGCGGAATTTTGAGCCTCGGCGGCTATTTTCTTCG
CCCAGATTTTGTTCA
TGTTCACAAGCTGATAACCGAATACGAGATGGGCATAAAATGTA
GATTTCTACAAAGTGG

TCAATTGATCGATTTTTGTGTGGATCAGCTCATAAAAATACAGGA
CGCTTTAATCGATGT
TGATGAAGAGGAATTACCTACATTTGGGCAATTCTTCATCACTCT
GTTTGGACTTCCCGA
TGAAGATTACGACAGTGGAGTATGGGAGGATCAATTCGTCAAAA
TTGTTACACGAGATGC
ACGTGTATTCACTCGTAAGCAGGTCCTTGACATAATGACCTACAC
GGAAAAATTCTATCA
ACTAACAACGATATTGTTTGCTGCTACTACTGAATCGTGCCAACC
TAAGAATTTATTCAC
GGCTACAAGCTGC
>ConsensusfromContig3114 Average coverage: 6.98
AAAATATTCATTTATATTTTTCAGATCTATATTTAACACTGTGTCA
ATATCATCAATTTT
ATCGAAGTAATAAAGTAAAGTTGTATCATCAGCATACGAAATAAA
ATTAGCTATTTTTGA
CGAATAGTGCATATCATTGATATATAGGTTGAAGAGTAAGGGGCC
CAGTATTGATCCCTG
AGGAACTCCTCGATCAACCCTTGTTAGACTGGATCTAGAATCAGT
ATGTTCAACAAATTG
ATAACGGTTATTGAGATAGCTCTTAATAAGCAAGACTGCATTGAG
ACATAATCCATAACC
TTCTAGTTTTAATATAAGATATGAGTGATCGATCATATCGAAGGC
TTTCGAAAAATCAAT
AAAAATTGC
>ConsensusfromContig3115 Average coverage: 5.07
CAAATTCTAAGAATCTCGCTTTCAAGTCGTCAATCCAAAATATTT
GACCTAATTTTGCGA
ATGCTGCTTTGGACATCGTACGATCATCATCAGTGGATGAAGGAT
GTGAGCGCCTAACGA
ATTCCCTATAGCATGATCGATGATACTTGGCCTCCATGCTGACCA
AGTCTTGATTTGCAA
TTAAACGAAGAATCCTTTCATCATTCTTATCCGAGGCTGCTCCT
>ConsensusfromContig3116 Average coverage: 14.50
TGTTTGGATGCTAAGCCCCGGATGGCGAGCGCCGCTAGACAACTC
ATGTCGGCTGTAACC
AGTGTTTTGGTTCTGGCAGATGGCGTTTTGCTGCACACGATATTG
GCTGCGAGAACAAGG
GTTCTGAACCGACTTGATGCCTTGGAGCAGAGTTCCAGCCTCTCA
GAATTCGTTCAGAAT
TTTACATCTCTCGGCAACGAACTCGTGGAGTTTGCCAAATTGACC
GGAAATAGGATGATT

GATTTGAAATCTGAGCGAGCTAGTGCTCTCATGCACGTAACCAGG
GACGCTATCCAACAA
TGCACGATGATGATTCTTTCTGCGTCGAAGACATCTATGCGTCAT
CCTGAATGCGAATTT
TCACGCAAAACTCGAGACTCGGTATTTCTGCTCATGCGCACTTTG
ATCGAGAACGTTGGA
CCAATCAGCGCCGGTGATTGCGTTGACGTCATTCAAGCACTTTCC
GTGACCACGCCCCTC
GGTGAAGGCGAAGAAGATTCTGGAATATCGCCCAGCGTGGGTTC
ATCACGCGTTGTGAAC
ACGTTTGAGATTCTCAGAAAAATACAGATCGAAACAAAACCAGT
CGAAAGCAATACAGAC
ACGCCCCTCAAGTGGGAGGAGCTGAAGAACCTGACGGATGACGT
CACGTCGGGGATTCAA
GAGGTCACGGATTCTGCGTACACAACCAACGGAACGCGGTTGGGT
CTTTTGGAATGTCTA
CAGAACCTGCAGCGGGAAGTTGAAGTCGGGATGCTTGAAGCTAG
GAACGACAAACATCAG
GAATCCATATCGAGGCTCAAGTTGACCCAGTTGGAAATCTATAG
ATGCTGCGAAGAGCTG
TTACACCAGATGACAAAAGCTGTCTTTGAACGCCTCACTTCCGGT
AGTGTCCTCGAAACG
GGGTTCCTGAACGCCCTAAAGATGGCGTCATGTCACACGGATGAC
GTCACGTCCGTTTCA
TCAAAGTATCAAGACAGCGTCGACAAACTCGTTGAGATCTGTCGA
ATGCTCTGGCACGTA
TCTGAGTCCCCGCCCCTTTCCACGCGATGCCTCCACGCGGAATTC
ATGGTTCGAAGGCTC
TCTCCCATTCTCCTCGATTCCGCTTTTTCCCGCGCTGCACACCAAC
ATTCCAAAATGGCC
GCCGATTGCTTTAGCGTCCTTGGCGACGCTTTCACATATCAGATT
CGAAACGTGGCGCTT
CTGGCCGTGGAGTGGTC
>ConsensusfromContig3117 Average coverage: 10.33
TAAGAAACTATCTGCTATCGAAAACTTGAATCAAAACTTTGCTGA
AAGTTTGTGTGAAAG
ACAATCTGTGAAAGAGGAACCTCATCGCTCAGGTAGCTTGGATG
AGAGCGCCAGCATAAG
AGTTGAAGAGAGAGGGACAGATGCAATTGATGTCAAGAACAAC
AAACGGAAGAAAGTAGA
CATCGCCGAAGAGCTCTCACAGCTCGTCATTTACACTCAAGCTAC
AAAATTCAGAGGACC

CATTCTTAATTCTTCGTCATGTGGAAAGTCAAATAACAAGCGGTT
CAAAAATTTTCTTAC
ATCGAACACAATGACCACAGACCGAACGGAATCAGAAGACTTGT
CCTCCAGCATGGTGTC
TGTTTTCTCAAGCGCCAGCAGCGGCATTTCCCTCATAAACTCCAA
AGGGAAGAAACGCAC
AAAGACCTTCGCTCACTCCTGCTACGGAATGTCTTCGTTGAATGA
GAACAAGGCGAAGCA
GAGCTGCCGACGACAGCCCGCTGGCATCGTCGTTC
>ConsensusfromContig3118 Average coverage: 5.96
CAATTGGGAATACTTCGGAGCACCCGCCTGGCCAGCCTCTTCGA
CACGAGGTCGAGAGCA
CGCAACGGTATCTGCCTTCGATTTCTTCTCAACTTTGAGAGATGA
AAGCGACTTGCTCGA
CTCCAGTCTTGGCTTTTTCTTCTTACGAGAAAAAATTCCACGACG
GAACATTAGAAACGA
TTTCACATAAAGTAAATTAGAGCTTGCAAAGTATCACGCTCGATA
TTTGAGTCGTTTGAA
GATCATTCATACCATTTAAAGACAAGACGACACCACAATGAGAAT
ATTTTCACACGATGT
TATTAGCACTATGATCTTATCAATATCAATTAATATTGATGTTAAT
GCTATTGATTTTT
>ConsensusfromContig3119 Average coverage: 13.97
CGATGTTATATTTAGAACAATGAAAAATTTATTAACTCATTGGAA
TAGTATGAATATTTT
CTATGTCTGCTTTTCACATATTACACGTCATTCTATTGAGTCGAA
AGACGTAGTTACATC
TCCAGCCCTGGGGGCAGCACTTGTATCCCCCACAACAAACTCCAT
CGCACGGCGACCCCC
GACACCTTGTTTTCCACTTAGGGGGTTGCGAACCTCTCATTCTCAT
GAATCGCCTCTTCA
CAATATCACTGGGGTAGCTGGATTTTTTTTCGTAGGACATTTCGA
TGGCGAATATGCATG
AAAACAGAAGAATGATGAAACAGCATTGATATATATTTGGACCC
ATCGAGGCGAA
>ConsensusfromContig3120 Average coverage: 11.53
CGGTACTAAAACCAGCAGTCTACATTGTGAGGAATCATCTGTAGC
ACTGAATATGGAAGT
CTTTTGTACTGAAGATCCGGTATGTTCATCAATATCCTCAGAATAC
TTACAGATCTTGTT
CGAAATTTAATTATGATGAAAATATCGGTCCTAAAACCAGCAGTC
TACATTGTGAGGAAT

CATCTGTAGCACTGAATATGGAAGTCTTTT
>ConsensusfromContig3121 Average coverage: 43.54
TGATCTGAACTTTTGCCGAACTTGAAATGGCCGTATTCATTGAAA
GGTATAGAAACTATT
CATTGTATAGTCCAGCATTGATTTAGGGACCCGTGTTTCTTTATG
GACAACTATTCGACA
GATATATGTGAATATTAGTGATTTTTCCTTAATCTCTAGAAACGG
GTGTTATATAATCTA
TATCTGTTGGGTAATAGAATCAAACTACCTAAGAATTGAGCTGCT
GTTTGATCAATATGT
ATTTCAATCAGACATACGCGTGGAATAGACATACTGGAATAATT
TGGTGATGGAACATGA
TGGCTTAGAGCCAAAAAATATTATGACACATTTTGAATAATATTT
TATAAGTGTTCGAAT
TATACAATGTAAAACATTATTATCGATAATTTTGGAGATCGTGAG
CAAAACTGCTTGGTC
GAAAACAAGGCTCGTTCGCAGCCATTGGGAATAAAAATAAACCA
ATCAAAAACACATTCA
AAACGGAGTGACCTTTTGGGAAAATTATCTTTGGCAATACAAATA
CAGCATTATGAAAAT
TGCTTTAATTTAATCTATACGGATATAGGTACAAGGTCAAAGAGT
GACAGTTCCAGTAGA
ACAAACTCAAATCAAAACTACGTATAAATCATGAATCCTTAGTGA
TTAGATTTTAAAAAT
CAAACTTGTTCTGGTCATATTTATGGCAACTGAGATGTGAGAAAA
TATTCTATTGTGAAC
ACTCAAGCTGTAGCCTCGTTTTAGCGGCCTCATGAAATCGAGAAT
ATAGTCTATTAAATA
TTTTGAAGAATAATATTAACACTATCTGAGATCGGAGTTGTAGAT
AATTGAACACAAATT
GAGAACGAATAGCAAAACACCATATAGCACAGACTTTCAATGAA
AATACAAACCAGCTGA
ATGAAAATCCGAGCGATAATCCAAAAATGCGATCACAGCAAAAA
CGATATTGGTACAAAT
TGAAAACAACAAAATAGAAAGTGTAAAAACCGCAATGGTCTATT
TCAACCGTTTGAACAA
CCTAAAATCAAAGTATCAAATCTTATCATTTTGGCAAACTGAATG
ATGGTAAAGACCGCT
TCGTTAAAACTAAGTATATTATTACCAGACTTAATAAATGTTTTA
TATTGAATATATATAAG
TGGTGTAGGTTAAACGACTTATGTGGCTGCAATTGGTCTGATCGG
ACTGGCATTCGTGAG

ACTTTTCTGTTGGGGAACGACCTCGCTAAAGGCCCCCAAACGGTC
GAATTCTACTTTTAT
TGAGGGCGGTCGTTTTTTTGTGGGCGTGGTCATACCTCTTGAAGA
TGGACTTAGTGCTGA
AGATGACGTCATATGATGACCGGGCAATTCAGCTGAGAGAGATG
GAGAAGAAGAATGCGG
AGTTGCCGACTGATAGAGAATAGAGGCTAGGGCGAGAGGGTGGT
GTGGCGGG
>ConsensusfromContig3122 Average coverage: 20.49
CGATCTCACATTTCCAGTTTTCTAGGCTTCAGTACCAAGAGCTGA
ATTGAAATTGAAATG
AAAGAAGTGAAAGTGCACACCAAAGATTTTGTTCAAGAGATGGA
TAATTTACATGGAAAT
ATACTGCTGCTCGGTGAAGGAAATTTTTCATTTTCTGCAAATCTT
TGTCAATACATGAAA
TCGAATTGGTCTATTATAGCATCATGTTTGCCAACTAAAGATGAA
ATACTGAAAATTGAT
TCTTCATCATCAGATAATATGGATGAAATAGAAAAAGCAGGGGG
CAAGGTCTTCTTCGAG
GTGGATGCAACAAAAATTCACCAAACTTTCGTTTCGCAAACTTTC
GACTTTATCATCTTC
AACTTTCCGCACGCTGGAGGTAAAAACAACCTCAAGAAAAATCG
AAAGCTTCTCTACGAC
TTCTTTCAGAGTGCATCTGAAGTCATCACGCCTACATCGGGATGTA
TTTTAGTATCTCTC
TGTCAAAACCAAGGAGGAACCCCAGTTGACCCCCAGCGACGAGA
ATGGAATGATTCGTGG
AAAATAGCAGATGCTGCTATGACGTCACGGATTCTCGTGACTGAC
GTCAT
>ConsensusfromContig3123 Average coverage: 7.08
TAGAAGATTTGCCAGTTCCATATTGCTTGTGATTATTTGAAATGT
GTTGAGCAGTTATTG
TAGAATCCATGTTGAGATATCTCTTGTGAACAAACATCGGGAGTT
CTTCATATCTTTTCA
TTTGGGTTGGATCGTGTTCGCCATATATGACTTGAGCCTGATATC
CTGCTAGCTGCAGAG
CTGTGGTTTCTGATATGTTATAGTCATTTGTATGAATACTGTAAT
GAAGAAGTTGAGCAT
AGAGTGCGCTGATTTCGTTGTCGTCTTCTCCAACGTCCCTCAAAA
CCCGCGAGAAAATTC
TCTTGCAAAACACGAGCTGGCCATGCTGATTGGTTGAGCGCTTCG
G

>ConsensusfromContig3124 Average coverage: 5.38
TGCTCTTCCGATCTGCCAGAAATAAGGATAATAATTGTACTTGCT
GGAGGGGTCATAACA
GGTCTTAATAATATTTAGAAAAAAAAGACATGAAGAAAACTACC
AAAATAAGGATTTTGG
AGGCCCTTGTTTTCCCAATTGTCATTTACAGTACTGAATATTAAA
TAGTGCACTGAAAAA
CAAAGAATAAAATTGATGCATTAGAAATTTTGTGCTGTTTCGAAC
AATCAGGCTTTCTTG
ATTATCAAATGAGAAGTTCCTCCATATTTTAAGATAGAAAAACTA
CAGCGGTAATTTATT
GAACACATTACAAAAAAATGAATCAATAGAGAAGGATGTGATGT
TAGGAATGATGTCACA
GTCAAAACAAAGAGGCTGTCCACACATCAGGTGGATGGATGGCA
TCAAAAACAGGAACTG
GCTTTAAAGAATCGAGGAGGCTCATATACAACCGTGGTGACTGG
AGGAGGTTCACACAAA
TAGCCACTGAGAGGTTGGTTCTTGACCAGAACTGGACATGGTGT
GAGCGATACAAAATCC
CTTTACACTGCCAATGTTCAGACTGGGAAGAAGTAATGTTCTTGA
CAATTAGTT
>ConsensusfromContig3125 Average coverage: 12.35
GGAAAATGTTTCTTGTTTTGAGATCTCGATGTAAAACCCGCCTGC
TGTGCATATATTGTA
CAGCTAATAAAAGCTGAATGACCCATTCAATAACTAATGGCTCTG
CGAATGATTTTTTAG
ATTTTTTGTAGGAGTTTATGAGCATATCCATATCACCGCCCTCAC
AATATTCTGTCCCAA
TACAGAAAAAATCTCCGTCCACGAAACTGTCATGGAACCTTACAA
TTCCAGGATGATCCA
AATTTCTTAGAAGATTTGCTTCATGCATCGCATCAACAGTTTCAT
CTGGGGCTAAGCTCC
CAACTGATATTTCTTTGAGTACTTTTAAGCGATCCCTCTGATCTG
AGCAGCGAAGATCTG
TAACAAGCCATGCTGTACCAAAGTTTCCACTCCCAAGCTTCTTTTC
TACTCTGTATCGAT
TAGCTAATATTCTTTTTTGATCATGTTCATCACTATCATCATTATT
ACAACTACTGGATT
TTATTTGATTTCGAGATGATAGAGAATGAGGAATTTCTTTAGGCA
ATGGCATCTCATTTG
AAACACAAAACCTAGTTTCTCATATATCACTAAACTGCTAAAA
>ConsensusfromContig3126 Average coverage: 20.92

CCAAATTATAAGTTAATATGATTGATGGCATTTATTTTAATCATG
ATTTTCGGATAAACC
CCGTTTATTGTCAGCAGTCTGTATGTAGACGATCTAATCCGTGCA
ATTGAATCTACTTTA
GATTTATGATATTGTATAACCTTTCATAGTTTAATAGTCTCGATG
GACAATAATCTGTGT
AATGGACGTCATATGTTGATAAGCTGTGAAGAGCTTATAATCTGG
GTTGGTATGGCTTAT
TAATGATTGAATGTCGAATTAAGACCTTTAGGAGAATCTAAATTA
GATATAATAATTTTT
TTCAGGAAT
>ConsensusfromContig3127 Average coverage: 9.38
GTGGACTCCAAGAACTTAATTTGGAGGGAACAACGGTGAGCAAA
GATTTTGCGAGACTGG
CCTTTCAGATGGAAGTTTCTCGAGGGATCATAATCAAATTTGGAG
ACGTTGTCAGATTCT
CACAGTATGGCATTTATAAGCAGTGTGACCCAATCACGCTCTTCG
TGGATATATGCCAGA
AGAAGCGGCTGCGAATGATCGACATCTTCAGAGATATAAACGCT
ACTAAAGGGATCCAAA
GCGCAGATGACCTTGACAGAAGA
>ConsensusfromContig3128 Average coverage: 7.37
CTTGGGATTCTCCCAGTTTCTGTATTCATACCGAATCGAGTCTAT
TGCAAAGCTATTCGC
AGTGATTGGATACCGAGGCCAGTATTTGAAGAGTTGATTAACGCT
CTCGTCGAATTGTTG
TGTGTTCATAGAGCTTCGTCCGCCTATTGTTAATATGTTATTCAG
CTCATACAAGATGTA
CCCAGAGCCTTCATTTGAATTGCTGCCCAGTAGAATAGGGCACTT
TTTAAAGTCGCCTTT
CTGTAGGGATTTTGAGGGTTCGTCGTCAAGAAAGTTACCATCCA
CAACAGGCAGGAACGG
GTATTGTAAGATCCCGAGATCGGAAGAGCACACGT
>ConsensusfromContig3129 Average coverage: 8.10
CATTAAAGATAATTCTCCCTCTATCACTATTAGCATAGTGAGAAA
GTGCCACTAATTCTT
CATCTGAAGATTCCCGAGAGGATTGATCTGATTCATTGATCGTGT
CCAGACCCACGTGAA
AACTGGCCTGAATGGGCTGGTAGACTGAAGTGTTAGAAGGCTTCG
GAAATTTTTCTGGCG
GTTCCTTCAAGTCAGAAGGACTGACGCCAACGCTTTTGCTGACCA
ACGCTGTTTGATAGG

TCATTGTTGATTGATCTGCTTTTCGAGATTCAGGTTCTGGCGAAGA
TGTTCTGTCTATCA
ATGCTTTTGGTTCTGTTTCTGTCGATCTGGAGAAGAACTTTAGATT
TTTCTTGGACGCGA
TTGTTTCTCGATATGCTGCGGAAACATCCTTTATCGGTTGGGTGAA
TTGATCTATCATTT
GAACTCTAGGGGTGTTAGTCGTCCTGTTCATTTTTTTCGGTTCCTC
GAAACTTGTACTTC
GACTGAGCACTGCCGGTTTTTGTTTCAGTTTTATTATTTCTGCTTG
ACAGTTCTTCAAAT
CATTGCTCAGTGAAACACACATTTGACATGAAAGAGCTTCGAAAT
TCTGCAATGATTCTA
CGATTTTGTGTTCATCAGTTGAAGTCGTATTGAATCTCTCGTCTGA
CAACAATAGATTTT
TTGTATGATTTTCTTTTTCTACTGAATGATCATACTTTAAATGTTC
TGAAGTCGTAGACG
CATCGGCGTAGTGAATTGGT
>ConsensusfromContig3130 Average coverage: 10.02
ACAGTTTACATTATCATCCTTAGATGATAACCTACTTCTGTTTAGT
AGATCCGTGGCTTC
AGACAGGAGATCTAAAGCCTCCCCAAATAATGATTGATAGCCAAC
CCTCCTATTTTTGTT
TTTCAGGGTGTCAAAAGACACCCTGAATTTTTCAATTTCCCCCTC
CAGACTGGCTGAGAA
GAAAACTGTATGTCAAGTAACAGTATACACAGAAGCCGATAAAT
AATATATAAACGAAAA
GAATTGGATCGATCTCACCAGATTATATGAATAAATTCAATAAAT
ATATAAATAAAATTA
TTTTTTCAAAATTTGATTGTTTATACTCGCTGTC
>ConsensusfromContig3131 Average coverage: 11.30
GTCGTTATCTAACAAATGAAACAATTTATTTTCACATAAGAGAAC
AGCCAGAATTTTGAC
TCAGGCCCTCTTCGAATATATCATCTTTTGGCTGCTTGGGAATAT
TGTCCAGAATCTTGA
GAAAAGGCAAGGCTTTGAAAATAGTTTGTCTGTAGTTAATCACAA
AAGCAATCGGATTGT
CGAATAGTATGAGAGACTCGACGCTCCTGATTCTATTGATCCCAC
GAAAATCCGAGATGC
AATTGTCGCTAATGTTGAGATGCTTGACTTTCGGAAGTCTAGGTA
AATTCTTGAACGAAG
AGATGAAGTTTTGCGATAGATTCATCGATGTACAACTCGACAGAT
TGATAGAACCCAAAT

CGGAAATTTC
>ConsensusfromContig3132 Average coverage: 13.26
CAGAGGACTCATGAAATTTATATGTAGAAAATATCGTGAAAAAA
AAGAAAAAGCTATTGA
GGTTGATGAAGGTGGGACTCTGACAGATCATACGACAAATGAAA
AATTTGACAAAAGAGT
CGCAGACAGCGAACATCAAATTGAACGAAAACTTAGCGGCGATA
ACAAAATTCAGCCTTC
AGTGAAAAGTGATAATCTTGATCCAAATCCAATTGAGTCAAAAA
ATGTGCACCACAAGCA
TCACAAGCATCACAAGCATCACAAGCACAATAAGGACTCTAATC
GCCGACAAAATAAGAA
AAAAGATAAAGAAGATGAAAAAGATGAACCATCGACTCGCAGGA
ATAGTATAGCGCTCAG
CACCAGATCAACCAAAGAATCTCAAGTGATGCGAAGAAATAGCC
AAGAGAGCAGGGTAGC
AAACGAAATGTATGCAAG
>ConsensusfromContig3133 Average coverage: 7.52
CGGGGCTCCGTACACAAAACCCTGTCGATGATGAATATTATCTTC
TATGAATTTCTGAAG
CTGTTCAGCTTTACTATATGAGGAGAGAACCAGCGATTTGTTCAC
TTGAATGTTCGAATT
TTGGCGACTCATGAAGCTGTTGATGAGGCTGGACTTTCCTGCAGA
GTTGGGTCCAACCAG
AAGAACGTTTTGCCCAGCAGATGCAGCTAGCTCCATCAGCATTCG
AATTCTGACTGTCTC
GATTGTTTCAACGTGAATAGATTCAATTAAATCGTTGCTCTTAGA
GAAAGAGGCGTTGGG
AAC
>ConsensusfromContig3134 Average coverage: 9.68
ACCTTTATAATTAGGAAGTTTCCCAAACTCAATATCTCAGATAAA
ACCATAACACGCAAA
AATGTATTATTCACAATATCAATATGAAATATTATCGGCGAGTAT
AACCAATATTATCAA
AAGGTCTCATTTATGTCATGATCTAGTTTTTTATAACAATTTTTAT
TTTGAAAGCCCCCA
TTGATGAGTTAATGTGGAATCTAAAAAAACCGCTACAAATCCTAC
TACGACTCCACTTCA
GGAAATGTAGGCGAATTGCTATATTCAGCAAAAAAACAGAATAA
GATCGACCCATTTCAT
TGGATATCAGTAAC
>ConsensusfromContig3135 Average coverage: 4.88

AGAAATTATAGTGACGTGTCATTCATCTTGGTGGGTGCAGTCAAA
GATATTCTCAGATCA
ATTGTTTCCACACTTCTTCAAACACACAAAGCCGACGAAGGATGA
CCCTGTACTTTTGAT
TTTTTCAAAAAAATATTGCCCTGATCGAGAAAGCCAGAGAAAGTT
ATGTACATATTCCCG
TCATACCTCCTGACACTTCGCACCGACTCCAACCTCTCGATGTCA
CTTTCACGGGTCCTC
TCATTTTGAGTAGGATTTTAGAACCTGGTTAAAACAATAATGGAC
AGCTCACCACAGATC
GGAAG
>ConsensusfromContig3136 Average coverage: 5.71
CTCTCTGTTGTCTCGCAATTCGGTGCACCAGATGAGAAAACATCA
AGTGCTATAGATACG
ACTCCTTGCTTACCCGATCCCAACCCTCCAAATCTCGATAACTAT
CGGCGAATCATCATC
CTCTACGTGATCTTTGTATTCTCCATCGCATTCCAAGCATATCTTC
TGAGAATACGTCAC
GTGATAATGGGCTTCTACGCCCCGAATCGCAGACGGTCGAGAGCA
GCTTGGCTTTACAAT
CATATCCTTAG
>ConsensusfromContig3137 Average coverage: 11.55
AGTTTTGCGAAGGATCCATCAGCTTTATGATGCGATAGGATAAGT
TTTCTAGATAGAATA
TCAAAGTCAATTTTTCTAGGATATCGTACAAAATCTTGTTGTGTG
AGAACATATGACATG
AAGTTGCCATCGGGAGACCATAATGACACGCGATTGTTGTTTGTA
TCTGCGATGAAAATG
TTGTCGTGACAGTCTGTCTGGATGCCGTTTGGATTCAGTAGCTGGC
CTTCTTCTGACCCT
CTTTTGCCGAACTCTCTCAGAATCTCTCCGTCGTTGTCATATATCC
TCACTGTGCATTTC
ATTCGATCAACTACTAATATTCGATCGTGAAAATCCACCGCGACA
TCAACGGGTGAATCG
AACTCGGTGTTAGCGTAAGA
>ConsensusfromContig3138 Average coverage: 15.83
GTTGATCAATATCAGTTGGTTCTATGTTTTTATAATTATTTGTGA
ATCAAAGAAATGACG
ATTTGTGCCCCGGCGGGCTAAAATCGACATGCGTGATCTGTTTCT
TCAGTCTATACTTGC
CATTTTTGCATTATGTAGTTTTCCATACTCTGATTGTTCGGCGAG
TGCCAGCCCCAAAGA

CTCAAATTTCGCTCATAATTTCAATACTTCTCGATCTGCATACGTT
CTCAGTCAAACCCC
GAATTTATTACAAGCTCTGAGAGACAGTGATTCCAATATTATTTC
AATGAAGATTCCCAA
ACCTCAAACGAGAACACAAAAATTTGCTCTGTCGGACGAAAAAG
GATCTGATTTAGTGCG
CATCACAGTGCGACCTTCTGGGGACATCAAAATAATTTTGATGAG
TGAAAAAATGGTCGT
TCCCGTTTCCTTTGACGAGGCCAGGATTATTAACCTATATTTCAAT
TCTACGAATTGCAT
CTTATTCGCCGGGAAGAATGAAATCCCACAAAAAATCATTCCATC
AGAATTTCAAACCTT
AAAAACAAATCTCAACACAATTTCGTTCAGCAATGG
>ConsensusfromContig3139 Average coverage: 29.69
TTTTTTTTTTTTTTTTGCCATTTTGATGTCATCAAACAGCAAGATA
TCCTGGCATAAATA
CACAAAATAATGTTCGTACAATCGCAAGCATCTTATCGATATCCA
AATTTAACATACACA
CCTTGTTTATAATATAAAAAAAAACAAAAATAGTTAATGAAAAA
GTCCTGGTATAAAACG
CGACAAAACATTTGCAAACAATGACCAATCCACTGGGTAGTTAC
AATTACGATAAAATAA
TCATATTGAATCACGTTTGAAAAAGCAGCATATCAGCATTTGAAA
ATGCAAGAAATATTA
TTTTGATTTAAAAGGAAATTAAAATTACGAAATCAATACGAATGG
ACTCTTACCAACAAA
AGTTGAAGATCAAAACAATTTATCAAAAGTAGTAAGCATCGAAA
GAATACACTGTCAAAA
CGGGGGACTAAAATTCCACTGTGCTCAACACTGGGACAATAAAA
ATACAGACATTTACAA
CCAAACATATATTTTATATTCAACAGTTTTTGTGATATGCCATGG
CACACGAATCT
>ConsensusfromContig3140 Average coverage: 10.72
GGCTTCCCTCTCCAAAGTGGAGCTGCCCCACCGAGTACTACCTTC
CCACGTATCTGTTGA
TGATATCTCGTTGCTATCAATTCAGATCTTCGCCAGTTGAGCGTTC
GGCTTCCCTCTCCC
AAGTGGAGCTGCCCCACCGAGTACTACCTTCCCACGTATCTGTTG
ATGATATCTCG
>ConsensusfromContig3141 Average coverage: 11.14
TGGTCAAGATGATGACCTTGATCAGAGAGATGCGATTTCGACATT
CAAACACGATTACAA

GCCTTGGGAGGTTGAGCAGACTGAGTCTGTCAAACCCAGAGAAG
ATGCGAGACTGATGCA
TAAGGACACGAGACCACATTTGGAGACGATACAAACGGAAACCT
CCGATTCGTTCATTGC
ACGACAACATCCAGTCGACAACGGAGTTTGTAACAAATACCTCA
CCAACATACGAATGCA
CAGAGACGATGATAAAATCCCGCCAGAGGTCTCCACCCAGAAGA
ACGACTTCGTACCCAT
GGAGTTGCCGGAGAGACTTGATCGAGGGCCGGACTGGAACCCGT
ACAAGAGCTGTGGGTT
GCCGGGCGACGACAGGGAAGGACCAGCCATATCAGATTATAAAG
AGAATTATCGGAGAAA
GCAGCTTGATGAACCAGCTATTGC
>ConsensusfromContig3142 Average coverage: 8.90
TTAGCACGTACTATTTGAATCAATATGATAGATATATTGTACATA
GACATTCTTTCAAAA
AACTGTTTCGTTGTGTCTTGGGCTGTCATCCTCTGTATTGGTAATG
AGCTCATACGCATT
GTCCTTTGGCACACGTGTGTACGATGGGCTTGATGAAGCTGTAGA
AAGGTCATCGCTGAC
GTTCACCCCTCCAGGTATATAGGGATCGTGAGAATTAATCTTTTT
ATTTCTGAAGATTTG
CTTTAAAGAATTTGCGGATCTTGATTTTTTAACTTTTGCCATCCTT
TTTTTGGACTTTCT
TCCAATCGGCATCGACTTTAACCGCTCGCTAAGTCCATCAGGTTT
CCTGTGTCTGCCTCC
ATCAGAGTTCTGTGATGTATCCACAGTCGGGAAGGAAGGATATGC
TCCTGATATGACTTC
ATCGGTTCCCGGGCTACTTGGATCGATCAGTGGCTCCTCGCATGG
TGAATCGAGCGGTAG
GGAGTGACCTCTGTTCATTACTCTTGAGGGTGTCAATTGGGTCTT
ATGAATCGCTGACGC
CTGACTAGGCAGAAGTCCGCCCCTCTCCATCCTATGGAGCGAATT
CTGGTCGACATCAAA
>ConsensusfromContig3143 Average coverage: 4.76
CTGCTTTCTGGGTCATACTCGATATTTGGTTATGGAGCAAGGCGA
CCATGTCTCTCAGCT
TGTCCCGCTCAACCTCGGCAACTCGGAGAAGTGTTGTTTCGGAAT
TACCATTCGTCTTTT
TGCAGTTGTCTTGTGAGAAACTTCTCTTGCCCGATGATGGGCGCG
TTGAAGGTGGGCGTG
TCAAGGTAGCAGACGAACATGAGCCGGAACGAGCAGACG

>ConsensusfromContig3144 Average coverage: 25.99
CGAGTTGCCTGAAGATCTCATCAGTGTACCCAACACTGAAAAT
AATAAATTAGACAGGA
ACATTCTAAAAAATTACAGGCCAATTTCGAATATCGCTTTTTTAT
CAAAAGTAATTGAAA
AAATAATCTTCAATGTATTATACAGCTATTTTGATAATAATGACT
TGATACCTGAATTCC
AGTCCGCCTATCGAAAATTCCATTCAACTGAGACTGCATTAATAA
ACGTTTATGATGATC
TGAACAGCATAAAAAGTAACAGAGGCTCCTCAGTCCTGATACTTC
TCGACTTATCTGCTG
CGTTCGATACAATAGACCACAGTCTTCTATTCAAAGACCTTGAAT
CATACGGAATTAAAG
ACAATGTTCTTAAAATAATAAAATCTTACTTCTCAGAGAGGTACC
AATTTGTATCTATAG
GTGGCGTTAAGTCGGATAAGCTGAAGCTAACATTTGGGATACCAC
AGGGTTCCATCCTAG
GTCCGCTATTTTTTATAATTTACACGAGAACACTATCATCACTCA
TTACCAGACATAACT
GCAACTTCCATCTATATGCTGACGACACTCAGATTTACTTACCAA
TTTCAGATATAAACA
CAGCTATAGCTTCTGTAACGAGCTTGATCAATGATTTGAAGTCAT
GGATGATTCATCGCA
AATTAAAACTAAATGAGGAAAAAACTCAGGTCATTATATTTCCAA
ATTCTCAAAATTATT
TAAATTCAACAAATTTTTTAGGTCTAAAATTAGGAAATGAGGAAC
TACAGCCTTTAACGG
AAGTGCGAAATCTTGGTGTTATTTTTGATTCTAAACTAACAATGC
AACCACAGATAAAAA
ATGTTGTGAAACGATGCAACATACACATCAGAAATCTGTATAAGA
TCAAACCCTTTCTAA
AAAGAGACCATTTAGTAACTTTAGTAAACTGCTTAATTTTTTCGC
AGATAGACTATTGTA
ATTCATTATATGCGAACATCCAAAACTATGTCATAAAGAAAC
>ConsensusfromContig3145 Average coverage: 41.92
CCGGGAGATGCGAAAGACGGCTACATCGAATGCTGTCCTGTGCC
CCCAGGGGAGGAGCAT
GTCGGGAAAATAGAGCTTGATAAAGCCGTTCAGGCAATACCGGA
TACAGAAGATTTAGCT
ATGAACACGGACTGGCGGCATCCGAAGACAATCGCCATCCAGTA
CGAGGCTCGGGATATG
GAGCAGAAGGTCAAGGACGAAATATGCGACAGCGAAGAAATGCT

TGAATTTGTGAATTCC
TCTATTCCGAGATTCGTCGTGTCAGTTCAGCAGAACGAGACGCTG
GATATTTTCAAGAAT
TATTGGGAGAATTTGGCAGATCAGGAAACAACAAGTTTTGGAAG
CAAAGCCGACACCCAT
CTCAAAGAATACCAATCTTTCACGGATCTGAGTTTCAGCAAAGAC
AGAAATGTGAGCCAT
TGTGAATGGCATCCGTATCTCAAGGGGGTAGTGGCCGTATCGGTC
ATAGAAAGATGCTGT
TTGGACGATCGAATTGATAACGCAACAAGAATACTTATGACTCCA
TCCTTGATTCTAATT
TGGAATTTCGCCGATCCAATTCATCCTCAGCTCCTTCTAGAAGCG
CCGGACGACATCCTT
GTCTTCAAATTCTGCCCAACAGATCCAAATATAATCGCGGGCGGC
TGCATCAATGGTCAA
ATTGTTCTTTGGGACATCACAGCATATTCTGAAAAACTCAAAACT
GTCCAAAATAGCAAC
AAAAACAAAAGCAATCTACAATTGCTGCCCGGTTTTGAAGATGG
GAGCTCAATGACGACC
CCTATCGTCCGCTACAATGCAGTGTCCAGCATCGAACACGGCCAT
CGTTGCCCTATCACG
GACCTTCTTTGGATGCCAGATCATATCGAGTTGTCTAAAGGAGG
AGAGCCACACGAAAAC
TTAACATTTTTGTGCAAACAGCTCATCACATGCTCAGGGGACT
>ConsensusfromContig3146 Average coverage: 11.18
AACCAAAGATGCCAGCTTCTTCTCATGCATCGAAACCTCATCGCG
AATTCTGTCTACATT
TGTCGAGATCTTGTCGCTTTTGTTGGTCAACACGTCGACGGCGTT
TCGTCGAACGGAAAT
CTGTTGGTTGTCGGCCAAGGATCGAAGTTGAGCCATTTTACTCCT
CAGCCTTTCAACATG
TTGCAGAATCTCAGAGCTGGGAGGAATGACTTCGAAGCAGACAG
AAGGTACCACGCCCTC
TGCCCCTTCCTGGCCTCGGACCCTCCACCTCTTATTGGGAAGGTCT
TCGCTCTCCAACGT
GTATTCGGCATCTCTCCGAATATTCACATCGGCATGATTATAATC
ACAAATCGCCCGAAC
TCTTCTGTAAGCGAGAGCGTTCCGAGCGAGGATATTCTGAGGGGG
AACGATGCTTTTCAT
TCGGTTCTCCAATTGACTCAAGTCCCGTTCTCGTAGTGAAATGCC
ATCCAGTGTTTCATG
CAGAGTCTTTTCATTCCTCTGTAAGTCTTGAACTTGAAATGTCTTT

TGTTCGAATCTTGA
AATCTTTTTCTGTTCTGAATCAATCCACGATTCCAGATCGCCACAA
TCTTTCTCGAACTG
TCTGTATGTGTACGCATCGTCGATCTGTTTCTGAAGACACGAGAC
GAGTTGCAGTATCCA
CGACCACTGTGACCGTAGTGCAGACATGAACGCATCAATGGCAT
GCGCCGCAGGGTGCTT
GTTTTTTGACAGCATTTCCCCTTGGTCAAGGACGTTCCGGATGTG
GACCTCACGCATGCG
TAGCTTTTCACTCAGACCCTGTGCGTTTTTCTG
>ConsensusfromContig3147 Average coverage: 7.89
GACATCACCTGATGGCTGCGGGAAATTTTATCGCCCGACAGGCTG
TTTCTTGCGCATAGT
TTTTTATGTTTGGGTGTTTTGAAAACCAACGTATCTGAACACGTCA
AATCATTGCTTCTT
GATTCTCCTTCTTCTTCTTCAAAGTCATCATTCAGTGAATTGCGGA
AATATGATTGCTCA
CTGGAGTCTAACACGTCCATCGTGAAGTTGCTGGACATTTTATATT
TTTTTTCTATCAAA
TCATTAGAAGTATCCTTTCATTCACACCATAGAATTATGAGGATA
TTCTAAACCATATTA
TGTGATAGCCATTTGAGACAAATTTTCCATATCTCGATCGATTTTT
GATATTTTGCCTGA
GTTTTATTCCTGTTCCCATAGCCACAAATTAAAC
>ConsensusfromContig3148 Average coverage: 24.01
TGCTTGAGCAGCGGGGCCGCCATGAGGTTCCGCAGCAGGAAAGA
CTCCGACTCCACATCG
AAGGTGTGTCCAGTGAGGTTTGAGATCCGATCCCAGTGTCTCTCC
ACCATCGACTTGTTG
GCCATCATTTCCAGCAAAGGACACGTTTCGTTGAAGTCGTCGATG
GTCTTTCTCAGGTCT
TCGTAGGCCTGCCACTCTTTGAGGGCCTTTGGTAGCTTTCGGCAT
TTATTCTGGAAGTCG
ACAAGTTCGTTGTTGATCTTCTCGATGTCGATATCTACCCAGGCG
ATGTCTTGATAACTG
CTGACTCTTTCAAGCACATTGTTGTAGAGACCGTACAGCTTCTGC
AAGAGGTTCAGTTCT
TTCTTGGCCTTCATCAGATCTGGGTATTCCGTAATTGGAAGCCCA
AACAGCTCTTCGCCA
CCGTTGTACGTCGTGTACTTGCGCCAAAGCTCTTCGTAGAGGGTT
TGGAAGACAGCCAGT
CTGTCTGAAGCCTCGCGTGGTGGTATGCCTTCCACCATTGGGCCTC

TTTCCGTATATTCG
AGTGCAAACTCGTCCACATCCTTTATAAACTGCTCGACACCGTTG
AGCAAGTTGAGTTTG
AAAGTCGGCTGAATCTCGAGTAGATGCTCTTGCACTTTCACAGAC
TGAGCCTTGAGCAGT
TTCCAGCCATAAACAAGCGAGTCGACTCTCTCTGCATTTCCGTCG
TTGATTGTCAGGTTG
TAGCGATTCAACATGGCGTAAGTTTCTTCGATCGGAAGAGTCGTC
ATTTCCGTTCTGATT
TCGTTCTCCCGTAAATCGCTCAGAGCAGCCATATGCGTCCGGACA
TCATCAAGATCCTTG
ACTGGTCGACTGAGCCGTTTTGTGAGGTTGTCGAAGAACTCGAGT
ATCTCATCCATCTCA
AGAGAGCATTTCTTATTGAGGTGGGATGCGTAGGCCTGTTTCCAT
GCTGTTATCTCAGCC
AGCAAGGCGGATTTGAGGGGGTCCGTGAAGAACATGACGGGCCC
CACTTGAATGCTCCCC
TCGCTCTCTTCTATTTCAGTCTCTAGATCCACATATTTTTTAAACC
TTGCTTCATAATCG
GATATGATTGGATTTGTTTTCATGAATTCCGTCACAGTCTCTTCAA
TATCCTTATCCCAC
AAGCTCCGGTACTTGAGAAACCGGTCGAGCATGTCCAGAAATTCC
GGTTTGCTTGTCGAA
ATGATGCTCTGGAGTTGAAGGACAATTTTGCTGACGTCCTTGTGC
T
>ConsensusfromContig3149 Average coverage: 44.31
TCTAGATCTAAAACAGTTTGAAACTCATGAACACAATCATTTTTT
AAATACTTCAGTCCT
GATAAGAATCCCAGCCAGCAGAATCATAAAAAATTAGAAATACC
AAAAAAATGGAATTCA
ATTCTTCTTGCTTGAGTGTATAGACCGCATCTTTTCAATACGTGT
TTTCACAGAGTGGAG
ATATATTTCAACACATTTCTAGGTTTTGAATAGAATGGACAATAA
TAATTACATAAATTT
GGAATGGGTTGCTGATGATCAAATATCTTTTGAAGATAGTGATCA
TTTTGAAGAAGATTC
TTTATGTTCATGGGTCAGCGAGCCAGAAAGTTTGTGCAATAACT
GGAGAGGATGGAAAAA
GCAAGTTGCACCTATAATCAATTGTGTTCCGAATTCCGTCGAAAA
AGCCTCATCCGTTCT
CCCTCTGGTGGAGTTGACTTCTCGACTCATCGCTCAAATTTTCCCT
TTCGAAATTGTGGA

GAAGTTTTATCTGCCGATCCCTGAAGAGTTGCAGCTTCGCATTGC
GTTCTGGAG
>ConsensusfromContig3150 Average coverage: 13.94
TGTCCAGATTTCGCTATTGTGTAGGAATATACTAGCTACATAGGTT
CTGTATATTTGTAT
TTTGCTATTTATTGATATTCTGTTGTTTTTGAAAATGTTTCTATGTT
TATTTATGGTCAT
CCATGCCATCCTTTTTCTGTTTGTTATATCTTTGTCTGTCTCAATG
TAGGACCCTAAGAT
TTTGAGTTCCTCTTTCCAGGAGTTCATTTCTGTATTAATTTTGAAG
TGTTCTGTTTTATC
TTGGTTTATTGTTAGGTTCCTCTCTGCTAGCCTCTGAGGTATTGTC
TGGGTAATGTGTGT
TATCCTGTTTTCGTTGTT
>ConsensusfromContig3151 Average coverage: 8.81
ATGTTAGAACGGAAATAATTTGACTCTTTTTATCACAACAAGAAT
ATTATATTCATTGAT
AAAAATTGATAAACAATTATTTTGGTGACTCAAATTTTGATCCGT
GTACCTGAATCAGCA
GATGAATCACGTTCCAGAACCAGAATCGTATAAAATAAAAAAGA
AGACGATTGATCGGAA
TATTTTTGATGGATAAAGAAAACTCTCAATATTCCTAGAAAAAA
TTCGAAAGGGGGGAAA
ATGTTAATAATTTTGTGAGCTTTTTCCGAATAAAACGTTCCGTAAT
ATATTCACGGTGTT
AAAATGCAGTAATCAGCAGTTGTTTTGATTTAACAATTTCATTTC
GATAAATAACTTTTT
ATTATATTAACAAAAAACATATATTGTGCGATCAATTATATAAAA
ACTATTTAACAGAAT
ATTACAATTTGTTGTGATAGTATTTCTCTACCAATCCGAGGTCAC
GAACTGCCCTACATC
TGACAGGTAAGATCACAGAGCGGTAAACAGAAATATTTTCAAAA
GTGCTGTTTTGAAAAT
T
>ConsensusfromContig3152 Average coverage: 8.37
AATTCACTAAAAAATTCTTAAATCCCAATAAAGACTGAGCCAATG
TGCTTCAGAAGTGAA
AATCGTTGTAAAGGAAGAATTGTTGTGTCTGATTACACCTAGCTC
ATATAAGATTCTAAG
ACGCATCTTCACTGTCAACGGCTAAGTAGAGTGGCAATACGTCAA
CAGAGCCTTTCAGGA
TAAGAAAAGTCATTCTCACATTCATTCTCCAAAATCACTGGTTTA

AAAAACATTGAGAAA
GACACAAATTCGCTGCATACTACGTCTACAACGATGATACTAGCA
GATTATATCTTTCAC
CAAATAAGTTTACTAATTACAAATACAGATTTATCGGACTTACTT
TCAATTTCTAGGCGT
ATACATTTGTGTATGAAGC
>ConsensusfromContig3153 Average coverage: 20.86
TGACATGCTTAACATTTTACAATGTGATAATAATAACAGAATATG
ATATATGAAGAGTAT
TATCCATCTTAGAGTATGAGAAAAACGAATTAAAAATCGAAGTT
AAAACAATTTGATTGA
CAAAAGCAAGGCACTACACAAGATACTATCATTTCAGCAATAAT
CTTGGTTCGACCAAAA
ATGTCTCATTCTAAAATAAAAATTACTTTCATACAAGATAGTCTC
ATCCCGAGAAGGAAT
ACGTATATATCTCTGCTTATAAATTCTTCAATATACTATCTGCAA
AATATACAACTTATA
TGATTCCTGGGCAGACCTTGCTTTCGCAAAACTATTCAGAATTAA
AAATAACCGTAGAAT
ATAACATATATTTTTTCAGATCCGGTTACACGATATTTAAAAATC
ATAAAAACAGAAAAG
TCAGAATGCACAG
>ConsensusfromContig3154 Average coverage: 5.64
ATTTTGTTCAATATTAAACTAACAATGGAGAAAGGTAAATTTTAA
TATAATGGAGAGATA
TGAATATTCAAGTAGCAGTTTCGTAATCTTCAGGATTTGATCTAG
ATAGTTTCGAGCCAG
AGATAAAGCGCAGAGCCCTTGAGCAAGGCTGACTTGAGTTTTTTC
GCTCCTGTCACTCGA
TTGTCTCTCTCCATCATGTAGTGCCAGTCCTTCTCTATCTACCTAG
GCATATAATGACGT
ATTTTTGTGAGTTTATTATTAATTATATACATAATATTTCTTATGC
ATACCGGTAGTGGT
ACTAAACTTGAAGGAGCCGTTGGCATAATTCGTAAGTAAAGTGTT
TTATTTTTT
>ConsensusfromContig3155 Average coverage: 11.10
TATGAATCAGAAGTACGGTCGGGGGTATCGAAGTGGGCGTTAAA
GTATGTGAACTGGACA
AAATCTTCAATATTTTTGTGCCAATATGACGTATCAAAACTCAAT
ATATTTAAAACTATT
CATTATTTTTATGATGATAGTGTTAATCGTACTACAATTCTTGAGA
TTCATTCGAACAAT

ATGAAAAACCCATGAAAAACAGGATGGTCCGCTATTTCAATGACA
ATGCATATACATTCT
TTTTCATATGTTGAAATGAGTAAAGTTTAACGAAACGACTATAAT
AAGTTTACCTTTTTG
TTTACGAGTTGTGAACGAGCGAATTTGACACGTAGTTATT
>ConsensusfromContig3156 Average coverage: 5.67
AGATGATAGCCCAACTGAATATCAGGATAAAAGATTCTAAAGACA
AACTTCCTTGGTCTA
ACAAAGGATCCAAATTTAAGATGAGATACGAACTAGTAGAGAAG
GGCTATATACAGTTAA
CCAACGGCCCTAAACAAAGACTTTGCAAAGGGTTTGAAGTCCAT
GGAAAAGCAGGACTCG
GCAAGAAAATTCAAGTTTCTTATTCTGATACAAAGAAGAATTTCA
AATGCGACGGCATGT
ATTTTTTCATTCATG
>ConsensusfromContig3157 Average coverage: 5.51
GAGATAAAGCGTTCCATCAACGCGCAAAACTTCGCCAATATCAC
CATGGGAACTAAGATG
CTGGGCAGTTCCGTCGTGAACGGGCTCAATGGCGGCGAACATCTC
ACATCTCCAACTCCC
CCACCAAGAGAAAACTTTCACAACAGAAGCATGGAGATGGTTTT
GTCAAACGGAAATCCT
TCAAGCCTGACTTCCCTCAGTGATACAGAAAGCAGTTCTACAGAT
TTTCAAGATGGCGAT
GAAAACAACTATCTCAGAGCTGTGAATGGGTCAGGAAGATCGGA
AGA
>ConsensusfromContig3158 Average coverage: 10.93
CAATAATCAACAATAAACTAGTGGTCGTATAAAAATTTCAAAAAT
GAATTTTTCAAATAA
TCAAAAATAAACTTAAAAGGTTCTACTATTATCTTTTGCGTACTA
TTTGATTGAAAGATT
CTCCCTGAGACGATCAAATTTTCTAGTTTAACCCAACCAAATAAA
TGGATAAACTTTTTG
AATTTCTAAGAATAGCTATCTAGGGATGATGCCCCCTCATAATGT
GCGAATAAAACCCAT
ATTTACTGTTACATTCTTCGCCACAGAGATTGCAGACAAACGCAC
CAAGCGGACTATGGC
ATCCCATGTGAATGAGATAGAGGACACTATCATCAAACACTATC
CCACAAGGGTCACATC
GCCTAGCCCTATTTTCATCATCAATGTTATCTCCCATGAATGGCA
GCCTCACCCCATGGC
CATGGATAGGCGTTATACCTGAACCATCTCCCCACGCTTTGAGTG

AGTCTTGATTATGCT
CTTCTGGC
>ConsensusfromContig3159 Average coverage: 12.78
GGCCGGAATAGTCGGACCCTGAAATTGTACCAATGATGGGAAT
TAGAAGGAATCACAGA
ATAGGGTCCTCCGGCGGGGGAGCAATGCTCCAAGGACATGCCAA
TGTGAGAGAATCATTC
TGGCTGTCAAGAGCCCCCTCCACGGCCGGAATAGTCGGGACCCT
GAAATCGTACCAATGA
TGGGAATTAGAAGGAATCAC
>ConsensusfromContig3160 Average coverage: 9.57
GGACAATTTACCTCACTCCCATTTCTTATTCCTCAAAACGCACAGT
TTTTGTTTTTTCCG
CAACAAGCAGGAATCATCAATCAAGAATATATCACACCAGTTCCT
ACAGTCAATGTTTGT
GTGTTACCCACACGACAAAACGCGTATACAAATAATCCACATATC
ATCACTCATCAAATG
CCTCAAGACAGCATAGAGATGATACAGAGGCCAGCAACGCAGAC
TACAGGATACAATAAT
GTCCGAGTAATAACTCCTCAATTCCAAACAAACTTCCCAGACAC
AGATAGTGAAGAATCA
AGGATGTTTTCAGTGGCGCAGTTATCGAAGAAAAGTCAGACTGT
GAAAACTAGTCGTGGA
AAATATGATGACTATGCAGAAACTTCACAAGTGCAAGAGAACTA
CCCACATGAGCGAAAT
GAACAGGCGAAGTTTTCAGGAAATCAAATTTCA
>ConsensusfromContig3161 Average coverage: 15.45
ACGCCTTATGGCGGTGTAATAGCAATGTAGAAGTCGCAGAAACA
GTTCAAGTTGAAGGTG
CGGTGGTGGACCAGTGTCACGGACTCAGGTGATACCGCGATGTT
AAAGTGACCGCCAATT
CTCTTTCCCGACAATGCGATGTGGTGACTGCTTGAGTTTTGCTCT
CACACATCTTCAACA
AGTTTATTAGGAACTCTCAGAAAGTGTACAAGTAGTTGTGAGTG
GTAACCAAGCAGAACT
CTTTCTAGAATCATGAACATTTCATAACCAGATCTCACTTGAAAT
GCAGATTAGGCGATC
AGCTTGTCATTGTCAATTTTGCAGTAAGTTTGATTTAACATTCCAG
TTCATATTTTCACC
TTATTCACAACATGCACTCAACATCAGAATATTCATGCTCCTCCC
AATACA
>ConsensusfromContig3162 Average coverage: 6.34

TTCGTGTCGCTCAAATCACTAAAAATTCTAAAATTGCGTTATAAT
CAAATTTTCCGCGTA
GAAGCTGATGCGTTTATTAACATCCCAGCTTTAGATACATTAGAT
TTGACTGGAAATATT
CTTTCTCATGTGCCTTCATCTGCCTTATCCCGCCTCACACAGCTCA
CTGATCTCTCACTG
CGCGATAACAATATTAGAGTGATCCCCGGATTCTCCTTCGACCGC
CTTCCTCTAGAGACG
TTGGACTTGGGGAAGAATGCCGCTCCACTCATCGTTGATAAACGA
GCATTTTGCGGCATC
TCAATCGGACCGGAATACAAAAGATCCTTTCTGAGATCGTGGCAC
AGTCTAGATAACCTC
AAATTGGACTATAATGGACTAGTGGCGCTTCATGTGTGCCTCAGC
AAATTGACCCCGGAT
GTCAGTCTCGTGGACTTGAGAGGTAATCC
>ConsensusfromContig3163 Average coverage: 10.22
TAATTATGTTCATTTTATTCACGAGCGAATTACGGCGTCTTATTG
GTTAATAAATTCAAA
CAAACGATGGAATTGGTTGAACTAAGAGGCTAATAAAATGCAGAT
ATCTACATGTTTTTG
TCTGAAACCAAGCTAATCCCGTAAATAGATTGTGCGGAATTCACT
TCAGTTCATTATGAT
AAATATATATGCAAACTTTGTGAATTCAACAATATAACTTGTATA
TAGAAACTTTTATGT
GCCCGTATTTTGAATTGAAGAGAATTAATTACTCGTGGGATTATA
ACAAGAATCACACGA
ATACGAATACAAAGCCATGAAACGATAAACATGGACAAAGTTAG
GTGTTTTTACAAAATA
ATCTACATAAATAGTATTAG
>ConsensusfromContig3164 Average coverage: 5.51
CAGATTCATATTCATTAATATACTTATATAAAATTTTATGAGTCTT
CATATCTGTAAAGT
GTCATGATACAGGTAACGGAATGATGAATACGGTGGATACTAGC
TGGGACAAGAATGGCA
AACTACACTTTAGGTGGTACGAAACGGCCCTACTATTCTCTTATT
CAATGACGACTTATG
AGAACAGGAGACTTTTGTTCAACAAGACCGAGCAACTCAGATTT
GAAGATATCGGACTTA
CATTGAACGGATTCCGAACGTATTTTTGCGCTGAAGAATTGTGTG
ATCGATATATATTAG
ACGGAACTGAATACAGGCAGTCAATGTATGACAACTACAATAAT
GAAGACGACGAATCTG

AATATATAAGATCAATGTATAACGAGTACATAAAAGAAAAAGGG
ACTAAAATTGTATATC
AAAATGGAATGACGTTGGACATGGACCCTATCATGGTGAGAAAA
GACATG
>ConsensusfromContig3165 Average coverage: 16.58
CTAAACACGGCCACGAGGATGTTGATCTATTGTTTCGAAAAATG
GATGAAATAATAGTAA
AGTCTTTGCAGTCGGTTCAAAAATCAATGATCAATGACAAGCATT
GCTTCGAACTATATG
GATATGATGTCCTCATTGACTCTAATTTGAAACCATGGCTGATTG
AAGTGAATGCGTCGC
CGTCGCTCACTGCCAGTAACAAGGACGACTATGACTTGAAGGTTG
GACTTCTCGAAGATG
TGCTGAATGTTATCGATCTGGAGAACAAACTTACCGGGAAGGAG
AAGCGCGTAGGGGGTT
TTGACCTCATGTGGGATGACGGACCTGTAATGGCGGATGAAGGG
GGCCTGGATTGTGTAG
GAAGCGCCACCTATCCCACAAACTCCTTCCTCGGCTGTTTCAGTG
ATCGGCGAAGGCAAC
TTTCTCGAATGTATCGAACCTTAGCTCTCAAGCGGAGAGCAGATT
CAGCTAGCTAGTCAT
CCGAACGTCCTGCCACAGTTATAAAAACATTCCTATTGATTAACG
TCGACCTCTTTTTGT
TTCAATCATTGATGTATTGCCATGTTACTGTTTCAAATTATATGCA
TTCTAATTTTCAGT
GTAAATAATGAGTCGGGAG
>ConsensusfromContig3166 Average coverage: 11.40
AAATCAGTATTTGAGATCATGAATAAATAGATGGCGATGCCCGTC
GATATCCCGCTCCGT
TGATCGTGGATGTCCGAAAAGGGGGTGGGCGGTTGTTGATGCTCA
GAATCTCAATCCCTT
CACTCATCCCAGAGTCAACGAGTAGGGCTTCTTCTTCAGCCATTG
GCATCTGACCACAGA
CTGTAGAGGTGGTCTGGAGGGGAACAGCGTCGTTGAGATCCCGA
TGCCGATGGTCGGGAG
AATGGTGGTTGACGTAGGAGATACGACAAATGACGACTAGAAGA
ACAGCAATGACACCCA
GCAGAACGGAGATGATCACGTAAGGAAGCCAGTGCCTGACGTTTT
CTTTGAGGTATTTCT
GGGCATTTATCCAGCCGAAGACGATCTTTGGCTCTTGCTTCTTTA
CAGCTCCTACGTCAG
GCGATCGCTCCTGATGCCCAGAGTCGCCATCATCAGAGTCCGGAG

TCCAGCTCGGACGTT
CTGTTGCTCTGTCGATAGGTAGCGCTTCAGTCTTGGGAATTGTGG
ATGTAGATGGCGT
>ConsensusfromContig3167 Average coverage: 11.44
TTTGTGGGTCAAATTCAATGATAAAAAACATAAATTGTTGATAGG
GGAATTTATCAAATG
CAAGAGAATAAAATGAATATGGTCAAAATGACAGAAAGTTATGA
GAAAATAAATCAAATT
ATTCGAATGCAGGACAAAGATACTAAGTTACTTATATGTGGAGAT
TTCAATATACACACA
GTAGGTAAGAAAGCAGAGAGAAAAGGAGGGAAGGTATTCAAAGA
ATTTATTAAAAGTAAT
GAAATGATGGTGGTAAATGAGTGGGAACATGCACAAGGGAAATG
GACTAGAGTTGTGGGA
GAACAAAAGTCAGTTCTAGACTATGTGCTGATCAGGAAGGAAGA
TGAAGGGAAGCTAGAT
ACTTTCAGAATTGATGAAGAGAAAGAGTG
>ConsensusfromContig3168 Average coverage: 9.18
CCTCTCATATATACACATTTGAACATCCGATGGTTCGTTTTATAC
TAGCTGGTGACCCAT
GTGTCTAAGGATGCTAAGTTCGCTACCTCCCCATTAAAAGGGCCT
CTCATATATACACAT
TTGAACATCCGATGGTTCGTTTTATACTAGCTGGTGACCCATGTG
TCTAAGGATGCTAAG
TTCGCTACCTCCCCATTAAAAG
>ConsensusfromContig3169 Average coverage: 66.17
GAAAAATAGCCATAATAATATATCCAGTCTAACATCTCTGGGATA
TTTTATTATCGAAAA
ACAATTTTAACATCTTTTGTTCGAAATCTACTGAATATGCTATAA
TAAATTAAATGATAA
TGATATCCTCATTCCGGTCTCTATTCATTATCAAATAACATTCATG
ACGTTTCAATAACT
AATCTTGTGAATGACAATAGCAGAAGTGATGGATATCATCATGA
CTCCGAACTGAGGCAG
AATGCGTCCCTGTCCATTGGCATTGTATTGATGTTGAACCATTGT
ATTGGATTGTACCAA
GCCACAGGAGCATTCCACGTAGGGCAGTCCTCTACGATTTGGCAT
ACAAAAGTCTTCGCA
TGTCCACGTCGATTCGCCATTACTTTCTATGGATTCCTTGCATTTT
GTTTTTCCTTCAGG
GCATGGAGTGCGCTTAAGAGATCCTGGGAACGATTTGGGGCAAGG
CTCCATTACGCCTCC

GCATTGATAACATTTGACCAAGTCGTTAGCAAGTTTTAATTCAAT
TTTGCAGATTATTTC
GAGGAAAATAAGGGCATTGAGATAGGATAGACGCATTCTCTTTGT
AACTTTTCACAGATG
AACAGATTAAAATCACTCTCTTGTTCTTGGACCCTTTTACTTGAAA
ACAACTTC
>ConsensusfromContig3170 Average coverage: 23.55
TCAAAATTGTTTTATAAGAATAATGTTGTCTGTGATAAAAAGCTC
ATTAATTTTGTTATC
ATTGTCATATATAACCTGTGAGGTGGAATATCGAAATCAACAAAA
TGTTACGTTTTGTGC
TTCAGGTTATGTTGAAGATTTCGATACTTGGTTGGCAGTCTGCGA
TATAAGTGTCTTATT
AGGCCACGAATTACCTGCTGAACTTCAAGGATTCAACGATTCATT
TCTTCAATCCACAAT
CAGAAAGCAAGATGAAATTACTCCGCTGCATCTGACTAGTGAATC
GTTCCAGTTTCATCC
TCCAGGTATAACTAGTCTTGGATTTGAAAAGTTATCGATTATCAG
CATAGCTGCCAAAAC
CTTTGAGCGATTCCAGAATCTTGAAGAATTGGGTTTTTTTGACCT
CAAAACTGAAATAAA
GTTTCTTGATTTTGCACTCGGGGGTTTTGCTGGGTCAAAGCTCAA
TGTTAGGG
>ConsensusfromContig3171 Average coverage: 12.20
TACTGCAGAGAAGAAATCTTACAAACTAGATATTAAAAATCTATC
CGAAGAACCTTCTTT
TGAAAGCGATTCATCCAGCGAAAATTCAGAAACTTCAGAATCAG
AATTAAGAACTCAGAA
AGAGGAGGAGGTAGAAGAGAGGTATGACAAATTCAATGCTCGGT
TCGGATCCAGTCACAA
TGCTGTCAGAAACGACGTAAATTTTACTGAATTAAAATTTTTAAA
GGGTTGTTTTGATAT
CGAAAACGAAACAAATATGGGAAGTGAGAGCATTCCACAAAGAG
AAAAATATATATTTGG
TCATAATAAAAGTGATTTTCCCTCACATAACTTTACATCATCTAA
TCACCGTCAAAAAAT
GCGAAATTCGAGCCCAGAAATCGAGATGGAGAATGGATGCATAA
AGAATGTACGAGTAAC
CCTTATGCAAAATATACACAGGAGATCGGAAGAGCACACGTCTG

>ConsensusfromContig3172 Average coverage: 4.81
CTTCTTTCCGGTTTCGGCTTGTCGTTGGTCTTCGTGAATTTGATCG

TTCTCTTCCATAAA
ATTGTACCAACGTCACACCAAAGCATACTTATGGGGATTGCAATG
TCATCAGGAGCAATC
AGCATCCCTATATTCTCAAAAGTCTCGGCTTATCTTATAGAAACT
TATAACTGGAAAACA
GTCATTCTATTTCAATGCTCTTTAACTCTGATTCCTACCATGTCTG
TGTTTTTCTTGAAG
AATAGCAAAAATCTCGAACCATTTGCATTGAGACTCAGAGCTTTC
AAAAACCGAC
>ConsensusfromContig3173 Average coverage: 5.94
GATCATGTGAATATAAAGTACCATATTTTAAAATTATAATTAAAT
CTCAAATAATTTTGT
ATAGATGGATAGGTCGTCTGTGAATTGATAGAACACGCCGCAATG
TTTTCTAATTTCAAT
CGTGGAACAGCCATCATTGCAGGGGGTTTTGTATGGAACGCGCTG
ATTCTTGGTATGCAA
AGAATGTGGCCCTATCTACAAACTGAATTGATTGTATATCTAGAA
GAACCTGCATCGACT
GTGGCTACAATAGAATTCGTACAAGGAATATTTTTGTCTATATTT
GTTTTTATTGGTGGA
ATCATCGGAGACAAAACATATACCCGGAGAGTCGTAATATCTTCT
GGCTTCTTTTGTGGA
ATTGCGTTGATTTTCTGTGCATTTATGTCAACATACATCAGTATTT
TGATATGTTATG
>ConsensusfromContig3174 Average coverage: 10.84
ATGGATTTGAATCACCTGAATTCACAGTAATTGAGAGGTATTTCG
GATACGGAGATTGCA
CCTTCAGTATCTCCGGATTGTACAGTGGTGCAAAGCCAGCCACTG
ACAGCAAGATCTGGA
TGGACGATCTAGCCTGCAAGGGCACCGAGGCGTCTCTGAAAAAC
TGTGCGTTCAATGGAT
GGGGTAAACATGACTGTAGACATACCGAAGATGTGGTTATTTATT
GTTCATAGAATTATT
TGAATACAACTCAGAGTAAAATTAATCAGTTAATGTTAAATATAA
T
>ConsensusfromContig3175 Average coverage: 17.36
CTTCCTCTTATTTCCGCCACTCAAATTTTTGGTCAATTTATGAACA
TGATTCGTCAGATC
AACAGATTTGATGGTTTCTGCTACCTTTGCCCCAATTTTGACACC
GGGAACACCCCTCAG
TCTGGAATACATATGAAGCATTTCGGATACGGTAAATTCGTCGAT
CAATCCACCGGATTG

GGGGCAGTAGCCCATACTTCTCCTTGCCTTGTTTATTTCACTAGA
AACGCTGTGACCATC
AACAAAAGCGACCCCGGATGTCATTGCAACATCCCCTGTGAGCAT
CTTGAAAGTGGTTGT
TTTTCCGGCTCCATTGACTCCGAGCAAGCCAAAACATTCCCCGCT
CTCGACACCCAGACA
GAGCTCATTGACGACTACTTTGCCCGAGTACATTTTTTTCATTCT
GACAAACCCGAGAGA
AAATGTCTTCATCAGATCGGGAATAGCCGTTGATAAAATCGTGTC
ATTCATCTGTTGAAC
TGCGGCATCTCTTTCCGTCAGCGGTTTATATATTTGAATCGAAGT
ACTTCGGGAGAAACC
TATAAGGTTTTGGATTTTTTTCACCAGGCCATTTTCTATGATCAT
TAGAATGAAGAAATA
CAAATATCCCTGGACGTTCAAAGCGACGATTTGTTTCCCGATACC
AGGATATTCCCATGA
AAAGACATGATCTGTATATGGCCTGCATGAGATGCATGATCCTGG
GCAACATTCGGATGT
CATATTGGTGCCCTTGATAGAATCACAGGTTTTCTGCAAATCTGTT
ATGCTGTTACATCT
TTTTCCATTACCGTAATTGAAGTACATGTTCATCGAACCGAGAAA
TAAACAAGAAGTTGG
TAACGCATAGTTGATTATTGTTCCGATTATCTCCGAAAAACCAGT
GATAATGAATACGCC
>ConsensusfromContig3176 Average coverage: 14.08
ACACATTCTCTTTTGATTAGTATTTCCATTTAATAAATCGTAATC
ACCGCGAACAACTAA
ATATGATGGTGTTTTCGAATGTCTGTCTTGTACATCTTTACAGATC
ATATCTCTGAAGTA
TCTTTCAATGATATATTCTAGATATTCCTCGTTGACGTTCGTATTA
CATAGAATATTGTT
TTTAATGTTTAAATTAATTGTATTGATTTTTATAATCTCTTGCATT
TGCTGTTTATGTGT
AAGATTGTCGAATCGATCATCGGGTTCAAAGTTATGAATTTTTAA
TAGAGCATTTGTGGT
AGATCTTCTGCTTTTTTGAGCAATCATTAAATTTATATTAAATGCA
TTATTATTACTGCA
TTTATCAGCTTTAATACAGCTCATCGTCCAATTATTCGTCCAAATA
TTATTCAATAATTT
ATTAATAAGTTTTGCAATAGATATTCTGTTGTGATTATTGTATCCG
TTTAATAAATCGTA
ATCACCTTGAACAATTAAATACGGTGGTGTTTTCTTCAAAACATG

TTCATCTTGAGAAAT
CATTGTTTTGAAAACATTTGTAATGAAATCTTCTAACTGGAATAG
CGATACATCTTCTTT
CATATAATAGAACACCATTTTTCCAATACTTTCGTTGACTTTTC
>ConsensusfromContig3177 Average coverage: 10.35
GGAGAAGAAATATTGAATATATTATCCAAAATGAAAGAGAAACG
TTTTTGTCGAGTTACA
ATACCCATGTTTAAAGCTCACACGGAAATAAATTTAAAGATGACT
CTAGCAAAAATGTAC
GAAAAATGGAACAAATGGGGGTAAAAACGTTATTTCGAAAGAA
TCATTCTCCTCAGATT
TACTCGGATTTCACAAGTGAAATCCTGAAAGAACTACAAAAATT
CACCGAATTTGAGCGA
AGATTCAAGATCGATTTCAAAATTAAAGTAAGCGAGAAAACCCG
CGAGAACCCGACAGAA
GAAACGCCACAGGTGGATGGTGGAGT
>ConsensusfromContig3178 Average coverage: 17.86
CGTAATACAAGTCTGTTGGGTTGATACCGAATAATATATCTTCCC
TTATCAAGTCGAATA
TATCATGTTTTTATTCAAAACACAATCGAATATCCGCCCTTATAA
AGTCGTAATACAAGT
CTGTTGGGTTGATACCGAATAATATATCTTCCCTTATCAAGTCGAA
TATATCATGTTTTT
ATTCAAAACACAATCGAATATCCGCCCTTATAAAGTCG
>ConsensusfromContig3179 Average coverage: 5.72
CTGGATTCTAATTTTGCCGAATGGACCATACCAGTTATTTCTGAA
CTCAATGGATCCATC
TTCGAAATTAATTTCGCGAATCGGTCTTGATTGAATTGGGCATGT
GGCCCTGTAGTATTT
CTCTATCCCACCAGTGATGTTCAGAAAGTCCTTTCGTTTGACCTC
TATAACATTAAATGG
GGAAGGGGTTTTCCGAGCATTCCGGAACTCATTTACCCAGTCCTT
CGGATGCTCAATATT
AGCTTTTGATTTATCACAACCATATCCCGATCACACTCCATGTAG
GAATGCCCTTTGATC
GGGAACGTCAAATTGATCTTTTGGAGATTCCTTACCTTCTGGACG
TAACTCAAAAATCGG
AACATTAACCAGTTCTTATTTTGCCCTG
>ConsensusfromContig3180 Average coverage: 13.20
GAAGTGGCGCGAAACGAGCGAACATCAGCTGACTCATCACCAGG
CACCAAAGTTTTCATC
CATCCTGCGTTGTCCAAGTTCTTAGTACCTGCTGGAGAACCTGCG

TTTTGGTCTCCGTGC
ATGAGAACCAATGAATACATGGCAAATGACTCATCGTCCCTTGA
ATATATGAAGAAGTAG
ATAGTATCCCAGATAACCAGTGGACCGATTCCTCTTGGCAGATTG
AAGGGCCA
>ConsensusfromContig3181 Average coverage: 5.02
TCTTCTGGAAGTTGTTTCAAAGTCCCAAACCATTCATTCGTTCTA
AGCGTTCTAATGGCG
TCAGAGGACTCTGTTGGCCGAAACTCCGCCCAATTTTCGGTTGTG
ATCACGTGGTCATTC
GTCAGCGGTCTCACGGATCCGTTCAGTATCAGATGAAACCCGTTT
TTGCCAAACACAAGC
GAGTTCGTTTCTTTCCATATCTCTAGTGAGGCTAGGCATGCTAGTT
TCTTCATAGCGTCA
TCACTGATCTGAGAGGTCAGAATTGGATATGTTCGGAGGATTTTG
TATATCCTCTCATTT
TCTTGCTGATTCCGCTGAAATGATAATTTCTTCAGAAGTCTCCGTA
ACTCATTGAGTTTG
TCGGATATTTCCACTTTTGGGTCTTTCAGTCGTCGCTTGCCATGA
ATGGCGGGCAAATAG
TCACGGATGTCATGAGAACGCTGTCTGAGTGTATCTTTGTACTCCT
TC
>ConsensusfromContig3182 Average coverage: 7.53
CACTACACTTACATTTCCATCTATATGTACTAGTCATATACGAAA
ATATGTTTGTCAATA
CATAATTTGATTTTGCTTCAGTTAACTTGGCACCATATCACATAA
ACAGTAATAAGAACA
ACAAAAAAGATGTACTACCATCATGTATAACAATTAGATAAGTG
GCAGCAATTAAATTCA
GAAAAAGCAGAATTAAGAGCGAATATGTACATGAAATAGGAAAA
AATTGAATCCCCACAA
AAACGAGCAGGCATTCGAATGAGAAGATGTATTCAAGAGAGATT
CTGACTTCATAACAAT
TGAATGAACATAAGAAATCATCAGTGTAAGACAAGAAGATCAAG
ATTTTATACCGAGTTT
CTTTTAATGTACAACCATTCATACTAAGTATACCAAAAGGCGGAT
TCAAATATCATTGAT
TATGTTTCAGATAAAATC
>ConsensusfromContig3183 Average coverage: 16.71
AGAATCTTGAATCTTGTGGATGCTTTCTGGACTCTTGGTTCGATC
GTCAATCAGTGGGTT
TTCGGGTTCATGGAGCCATCTCCAGTCAGGTATGAATGATTTTCTT

CTTCTTATACCATT
AGTCTCACAAACTCCGATCGATGACTTGGCTTTTAATTTTTCCTT
ATCATCAGGAACGGG
AGTATCTTCTTTATCATCTTGTTTGCAATAACCATTCGAGCCATTA
TTACCATCATCATT
ATTATTGAAAACATCTGATAGCTCGGCTTTTAAATATTCCTTGTT
GACAGAAACGCGATC
AACTATTTTATTATCGTGTTCAGAATAACCATTTGTCCTATGATTA
TCATTATTCTTATT
ATTCAAAACAGTTGAATTATCAATAACATCACTCATTTTCGGTTC
ACAGCTCCGTATAGC
GCTGGAAAAAACTCCCACAATTTGAAATATAAACTTTGTCATACA
GACCAATTTGAAAAA
TCAGACATATGAATAATTGTTTCACCGTTTTTTGTTTTTACTATA
AATCCAAGAGAATAG
AGTCCAGCAGGATCAACATATGATATGACATCACAACGTCATAA
CATTTGAAAACATATA
CGTATCACACTCTGAGCACTATAATAAAGAATAAGTCATTTTATC

>ConsensusfromContig3184 Average coverage: 14.08
ACTCCCGCAAATGGACCAAGCTCCCTGACCTTCCCGATGGAGTTG
ATTCCTTTTACCATA
TAGCAAACGTTGGCTCTTTGGTATTTGCCATTGATTTCGACTCCC
GCAAAATGTATTCCA
TGGATTCTAGTCGTGAAATATGGAAAAAAATGCCTGAATGTCCAG
CCAATGTCAGCATCA
GTTGTTTTCTATCCGACGAAACATCTCTGATTGTCATTTGTAGTGA
TTGTGAATGTTTCA
AATACAACACGAATACGAATAAATGGTCGCGAATAAAAGCAAAA
TGGCCGTACACAGGAT
CGACTCCCGTTTTTGATTCGGCGTTCATGAGAGGGCGACATTTAT
ATTGTCGGG
>ConsensusfromContig3185 Average coverage: 14.35
CTTCAACTGGTTTATGATTTTGCATTTCTGCACTCTTTCGCTTCGA
CATCTTTGGAGTCT
TCAACTGGTTTATGATTTTGCATTTCTGCACTCTTTCGGCTTTTTT
TAGGCTTTAAAAAA
CTTGTCAAACCATCCATAGATAGAGATCGTGATTTTTTATTGGTT
GTTGGGGAATTTTGA
ACGTTTTTGAATGGATCTGATTCTGCATTGTTTGTGCGAAACGAT
CCTAAACCTGGTGTT
CTTTCATCGCATGGTGGGTCTTTTTAGATCGGAAGAGCACACGTC

TGAACT
>ConsensusfromContig3186 Average coverage: 13.41
CCGATCGGGATCATCAAAGCGAAACAGCGGAATGCCAACCAGGG
ATCATCAAAGCGAAAG
ACGGCTGCAATTGCTGCTACCTCTGCCCGCGTCAACAAGGAGAAC
TATGTGACTCAAGGT
ATAAATGCGATGAAACTAAGAACCTTTTCTGCCACAGCGAAGGC
TTTTGTAGAGCGAAAG
ATGCACAGGCATGCAAATTGGATGGGTATACATACAAAGACGGT
GATCGCTTCAACATCA
CGTGTGATCAAATATGCACATGTCAAGACGGAATCATCGTATGCA
GTTCTCGTTGTGAGC
ATCAATACAGAAAGCCTTCCAGTAAATGCATCAATCCCCGACTTC
TATATATTGAAGGGA
GGTGTTGCCCTGAATGGGTTTGCAATAGAGATCCGTCAATGAGCG
ACCGGGCAATATTCT
ATGAGGAGCCATGCCCTCGACCTGATGGAGAGTGGACTCCTTGC
GACAAGCCATGTGGAA
TCGGTATCTCCAGAAGGGATACTTTCAAAGGGAAAAGTTGCGCT
TTGAGTGAGGAGACGA
GGCTTTGCCAGATTAGGATGTGTGGCACAAGTGATAGTCAAATG
ACATCGAAAAACTGCA
AAGCAACTTGGCTGGAAATGACTCCCATGTTTTTATTTTCCGAGG
AAGGCTGCAAAAGTC
GGATGAAATACCTGCCAAAATATTGTAGCGATTGCAAAAAAGGA
AAATGCTGCTATCCAG
TCAAAACAAAGACAAAGGTTGTTGATTTCATATGCGAGAATTCAG
CCTTGAATATACCAG
GCTTGAGCAAAGATAGGATTGACAAAAAAAATTATGAATGGATA
CAGGATTGTGCCTGCG
TGAAAAAGAACGCAGTGCCAGACAACCTCAAACATTTTTGCAGG
GATTGAATAAGCACGT
CTTGAACAGATCGGAAG
>ConsensusfromContig3187 Average coverage: 10.29
TGAGGCCATGGGTTAGTCGTGCCCCTCGAGCAATAATTCCAGACA
TCATGCATATAACAA
TTATTTAATTCTAGTATGCGGAGGAGACGGAACTGTGGGCTGGTT
GCTTGAGGCCATGGG
TTAGTCGTGCCCCTCGAGCAATAATTCCAGACATCATGCATATAA
CAATTATTTAATTCT
AGTATGCGGAGG
>ConsensusfromContig3188 Average coverage: 11.44

ATTTTTCCCAACATCATCCGCCCGATCGTCCTATCAAGCATGAAT
CACCATCCATACTCC
AGTGATGATTGATAGAACTGTTGCACCGGAAGTGGAAGCTGTGCT
CGTGGTTGTAGTAGA
TTTCTCGGTCACTGTCACAGCCTCTTCCGTATTGAGGATGCACACG
TATTCTTCTACGCT
AGGTTTTTTGAATGCAGTTTTCGTCCCTCCACAAGCGGCTAAAAC
TTCGGTAGCAGCTGC
TGCACAGTACGGACCAATGAGGTTAGTTGCACAAGCACATGTGTA
CGGACTTCCTGTGGC
TACCGCAGTGCAGGACCCACCGTTA
>ConsensusfromContig3189 Average coverage: 14.40
TCATATTTTATATATCGCCGTGTTTATCGGATAAGAGAGAATAAA
TTTTGTTTCACGAAT
AGGAAAATAGAGATTAATCATTCCATGGTCTGAAGAGTCTTGGA
ATAGAGCATGGGAAGG
CCGGCCACCGATCCGAGCTTATCCTCACTACTGTTCATGACGTCA
TCGAGACAGGGGCTG
TGAGGGGACGAGGGACTAGGGCTCATGCTTAGATCATCCGAGTC
CCCGGTGGGAGAGAGG
GCGTGGGAATGAGAATCTCCGGCCGATGATTGGTTGTCTCGATCA
TTCAGGTCGCTGCTT
GATTGGTCGTGTGAGCTGATCTCACCGTTTGATTGGCGGATCACG
TTTTTCCGATATTTG
GCGCGGGCGTTTTGAAACCAAACCTGTAGCACGCGTTTGCTGAGC
TGTGTTTTAGAAGAG
AGCTGCTTGAGATCTTTCGCATCCGGGTTGTGGTTGAGTGAGAAA
TAGGATTTCATCGTT
CGCAGCTGAAAATGCTTGAATGAAGTGCGCACACGCTTTGGCCTA
GAGTTGCCCCCACCA
CCGTACTCCCCTAGCATGCCCACAGCGTCCGCATCTGAAGGACA
TAGACGCATAGGATGG
CGTCCCTGTCCGCTGTTTAGCTGGTTTTTCCTTCTTCGCGACCGTC
CCTTCTGAGGTCCG
TTTGTGGAACCCACTTGGGGGATGTTAT
>ConsensusfromContig3190 Average coverage: 14.88
CGAGAAAATTCGATACAATGGAAAAAGTTAGCTGTTTAATGACTC
AATCATCATTGTATT
GTAGATATATATCTTTAAAGTGTTATAATCCAGCGTAGCAATTTT
GTGGCACAATTCTAT
CATATTTTATTGATGAGCACAATATATAGGGAAAAAATCTTGACA
AATCCAAGATTGAAC

TCACCTCGATATCTTCAACAAAAACAAAACAAACTTTATGGCGCC
AATCTGAGAATGAGT
AATGTAGGTCACCGCTTCGCGTGGGCATAAAAATTATTCGCCATC
TATCACCGTTTCTGC
GTGGATGGACTGCGTACTTTTGGCCGACTG
>ConsensusfromContig3191 Average coverage: 40.41
TCATAAAAGTCCACAAACTCAAATACGGTGAGCCGGTAATCTCGG
TCTTTATCGAACATG
TCGAGAAGTGATTCTGTTTTGTTCATGTCAAAGCCAAGCTCATAT
TCCAAAGTTGGGTGG
ATATCATCAGCTGTGAGAAATCCTCTTTTCTTCACGTCAAATTGA
TTGAAAATATCCCTA
TAGGGAGCAGCCCTTTTCTTTTTCGATGCCTGACTTTGCATTCCGA
TGCAGAATTCTTCG
AAATTAAGCTCGTCGGTTTCATGAAGGTCTTTTTTATTGAATATTT
CCCCAAGCTCAACT
TTGGAGAATGCATAGCCGAATATTCTGCCGAGCATGACAATGTCT
CTGAGACTTATCAAG
CCATCTTTATTTCGGTCGATTGCATCAAAACGCTTGCGGTATAAT
GCTTGAACATCCGGC
TGAACAAGAGCGGGGCTCGTCATTGTCTTTCCTTCGTATGAAAAA
ATAGACACTCTCCAC
TGTATACGTGAATATCAAAATATCTTCAACCGTGATATTTACAAA
AATACAAACGCAGGA
TTGAGATGTACCATAAGAGCTACAGTATTAGTTGCAAATATAATA
AATGAACCGACAACT
GAGATAGAGCATTGATCATTG
>ConsensusfromContig3192 Average coverage: 10.28
TGCGTTTGCCAACAGCATCAGAATTTGAAATTAGTTTGCATGGCC
TCAAAATTAGGTGAT
TATAAATCTTCACTCGAGAAAAATTGTATGTGATATTCCGAATTA
TGAATGTATGCTGAG
TGATTGTGATAATTGTCCAGATATTGCCATTCTTATGATTTTATTG
TATCACATTTTGAT
TTGAAGAGATAGTCATCAGGTCACATTAATGCATTGTTATGCCTG
CTGGTGCATTGAAAG
ACTTACTTTGTGAACAAATGATGAAAATTAAGGTTTGTCATTTAG
ATCGAAGAGGACAC
>ConsensusfromContig3193 Average coverage: 14.58
CCCACGTCTACCACAGTAATGAAACGGAGGCCGGAAGCCCAGGA
TGTCCATGGCAGTAAT
AATCGTCTGGCAGTAATTTTATCACATATCTTCGGAATTCCAGCA

TGGAGTATAGCTGGG
TTTGAGCTCCCTCCACAGAGTATTCGAGACAACCAGCATCATTCT
CGTATTGCATCCATA
CAGTGACTTTGCCATTGTGAAATGCAATGGCCATTTTTCACATCC
ATACAGTAACTGATT
CTACCGATGTCTGAGAAAACATAGCTTAAGATGGTTCAACATGGA
AAAGTTTCATACTGA
AGACATTCTATTTGGGGCTCTTTAAGCGACACTTATTTGAGATAT
CACGAGTTAATGAGT
TGTCCCAAGCTCCAGGTTTTTTCAAAATCTCCAACTTTGTATGTCA
TGGTACAGGTTTTC
TACGAATTCCGCAAAAAAACCCACCTGCTCATAGTTTCACGGATA
GAGTTCCCGAATAGA
GTTGCCCAGGTTTCAATATGCTTTTGCGAGGTCTTTCCTGCCACTT
GGTCATCTTGTCTT
TCAGTTACTCCATTAACTGTTCTCCATGTTTCTTCGTAACGTTTTC
CCACTTTTGTTTCA
GATGTTCTACTTGTCGGTATTTAAAAAAAATTTGTTTCTCTAATTT
GTACATG
>ConsensusfromContig3194 Average coverage: 6.75
GATTATGGTTATGATTAACTAGCCTGTTGACATTACATGTATGTT
ACCCTAGTGGTATAA
GAACAAGAGACTCACAATCCTTCTTTATGACGGAGGTTACGATTA
TGGTTATGATTAACT
AGCCTGTTAACATTACATGTATATTACTCTAGTGGAATAAGAACA
AGAGACTCACAATCC
TTCTTTATGACGGAG
>ConsensusfromContig3195 Average coverage: 9.32
TGGTGATTCATATGAATGTGAAAAAAATATTATATAGATATTTCT
TATATATTGTGATTT
TACATCTTTTACCTGCCTGAGCATCAAATCTGTTTAAAAAATTTT
GCTAAATTCCAGGAT
AAAAGTGGTTATAATATTGACTTTCAACCTTGTGATATCAGCTTA
CATATTGTTAGGTTT
TAAAAAGAAATGAAAACTGCAACTGAACATATAAACATTTTTGAT
AAATGTGTTTTAAAG
AAATTTTG
>ConsensusfromContig3196 Average coverage: 9.25
ATGAAATGCATAATAAGATAAAGAGAATAATGTGACCCTATAGT
ATTTATAAGGCTGGTC
TTATCTCGGGTTCAGCCGAGTCCCGATGGATTGCCGATAACGACC
AATTATGAGTGTCTC

GAAGCCTGCAAAGGTTCTCACTTCTCACGAAGATTTGTACTATAT
ATTAAGAACTAGCTG
ATCCCTCAGCTTCGAATCAATGCCTAAAATACCATGTCTCAATAC
ACATCAATTCCAATC
AATCCATCTCTGTGGGACTGCACTGTCCCACATTTCCTCCATCCAA
TATTTCGAAGTCTT
GAAACCAGTCTCATGGAGTCATACTCCAACAATCATATTCAGGTT
TCTCAATACTCGGGA
ACTTTCATTCTATTCCACGTTATAATTCTTAAAATACCCTACAGAT
CAATTCTCAAAATA
CCCATCCAACGTGAAATAGTGTTCCTCGATTCATCGGCAAAGTTC
ATTGATGTCACTGTG
TTCAATGTAATAGAGATTTCATTTGGCATAGCATTGATTCATTTA
AATGGATTTTTTGGA
CCAGTCATCCATCCGGAAGGATGTGCAGAGCCGATGCAGCCATG
CGGGCCACTAGTTATA
AAACTTTCTCGGAAGTTCACACGTGGAAATATAAACATCGG
>ConsensusfromContig3197 Average coverage: 25.36
GCATCTCAATCTCTCCAATCTGTTATCACGGATTTGAGCTCTAAA
GCTTGCCGCATTCAA
ACATCTAGTGAGATTATTGACAATGAGAAGCAGACGATCATCTC
CGAAAAACAGAAAAAT
TTGGAATCGAGAGAAGGACATCTCAAAAGAATGCAGCAGGATCT
GGAGCGGCAACTCCAC
GAGAGCGAGAAAGACCGATTGTCGCTGGAAGGAATCGTCTCTCA
GCTTCGCGAACAGCTG
ACTCAGCAAACGAGTGAATTGCAGCAGGAGAGATTCAGACTGCA
GCATCGAGAAGACGCG
CTTGATGTGAAGATGAGAGATGTTGAGGCTGAAAAAGAGAGAAT
AGCCAAGCAGTTCAGG
AATGAACAACGGATTCTGCAGCAAGCGAGGGATGATCTGATATA
CGAACAAAAATCTAAC
CTAGCTCCGTTGCAAGAAGAAAGAAAAAGATTAGCGGAAGAAAA
AGCCGAGTTCGAAATA
CGAAAAAAACTGCACCATGAGGAACAACAGCGCGAATTGGTCAA
CTTAGTTAAGTCGAAG
GCGGAAGTTGACGTCCAAATGCGTGTTCTCTCTGAAGAACAAAAC
AAACTATTCGCAATG
ACGGAAAAGCACAAAGCAGACGTACAAAAACTGACCTCGACTCA
AGAAAAACTCGACAGA
GACTGTCAGGAACTGGAGAAGGATCAGAAAGCACTGGAAGTCCA
GAAACGCGAACAAAGT

CAAAAAGAATATGAAATCGACGCCAAAATAAATAAAAATGCTCA
GATAAAAAAGGAAACG
GACGATGCCATGCTTCGATCTCAAACTTTGAAGATTGATCAGCA
AAAAAGACTGGATGAA
ATCAAGAGTCAGTTGCAAGACCTTCAGATCAAGGAAGGCAAGAT
CAATCAGGCTATGATG
GAAAATAGTCGGCAGAGCAGATGGATAGAGTCCGCTAAGAAAAG
TCTCACTTGCGTTCAA
TGTCAAAACAATTCCGTCAAAGATATTCCACAGATTGATGAA
>ConsensusfromContig3198 Average coverage: 4.75
TAATGCTATAAGTGTTTTGAAATCTCGGTTGATAGCTATGTCCAG
AGGTGTATGCCCTTC
GCTGTTTTGTATGTTTTGATCACAGCCAGCACGTATAAGTATCTT
TGAAATGTGTAACCG
ATTTCGATCAACAGCTAGATGTAAGGCTGTCATGCCTTGCCTCGA
TTCGGAGTTTTGCCT
TGAATCCAATTCACATCCATCATTGAGTAACGCACTTACAACACG
TTTTCCGCCAAACTG
AACGGCTATATGTAGTGGCCGGGTATCTGT
>ConsensusfromContig3199 Average coverage: 16.89
CGAATCTGTGCAGCACCCGACGCACGAGTTCCATTCCGATTGAGA
TGGGGGCAGAACTCT
GGACATCAGCTTCTGAATCTTCGGGAGATGGCCGTGATGTATAAG
ATCCTCGCATCCAAC
CGTCCCCCAGGGAATCCACGTCTGTAAGAAGCTGAACAAGAGGC
GATTCTGCGGACTGTG
ATAAACAAAAACCAGCTTCACTCAGAAGATCTCTACATGAGAGT
TGGTCGTAAACCGCGT
TCAGCCAGCTACAGGGCACAAAAAGCAGGAGACCACGACCAAGA
ATGGCGTCGAGAAATG
ATTTCCTGCTCGTTCTCGTAGATCCTTGGCCATTGGGGCAATGGT
GAAGTCGGAGATTCA
GCAAGAGGCTGAAAT
>ConsensusfromContig3200 Average coverage: 19.90
AATACGATAAACTTTAATATGAAAGCTATCTCCGAATGTTGACTG
CGCGAAATTGAGTTA
ATGAATACATTGAAACAATGAAATGGGTTGAAAACTAATATCGT
AATGGCATATTTAGGG
ATATTCACTGAGAACCGAACAAAGATGGGTTGTTGACTCCATGA
ATAAACAAGATGGAAG
AGAAGACAGAAATCAAACACAAATCAGAAGATATTCAAACTACG
ATAGAAATATTTGAAA

AATCTCCAGCAGAAATAGAGAGGTGCAATTATTATGAGGTGGCTT
TATATGACACTCGCC
TTCTAGTGTATAGCTATTGAAAACAAGAGCAAATGTAGATCACT
GTGAGGATGATGAAAA
AAATCTGACCTATGAAACATAATCATCATGCGTGTAGTCTTGATA
TTAAATAGAATGATG
AAATAGTTTTTGATTCGTCATAACCTATTACCTCATTTTACTAGAT
GGAACGCGTTTTCT
TACTTATTCACGATTTCAGTCTGTAAACATATCATATCACTCCTTT
TACCACTCTCATAT
ACTCGCATTTCACTTGTGATGAAACAAACACCAGTTCAAAAAGA
TCGGAAGAG
>ConsensusfromContig3201 Average coverage: 8.61
GGAAATTCAGGGAGTGGAGCAAACGATTCACGACCGGGAACGGG
AAAGGGTGGCATTCCC
AGAGGTGGCGTTAGTGTCGAGCTGCGGTTTGAAGAACTTCCCAA
ACTAAACGTCGATGAA
GGCGATTCAATACCAGTTGAACCAGTTGAATATTCAAATAACCCA
GTTGGATAATTGATT
GACCCAGTTTCGCTAGAGCAAGGTGAGTATGGTGGGCGGTTTTCG
CCTCCACCACTCCCA
TACCTAGTCTTCGTCGTCTTCTCGTGCGTAACAGGGATAGGTCTG
AATCTAGAAGACATT
ATAAGACCGTCAGATACTGGACGGGATTTTTGCGTCACCATAGCA
CCCGATGCCACGCCT
CCTCTCCGTACTGAAACAGCTTTGGCCACACCTCCTCCGCTTTGC
ACGAGAGTTGGTTTA
GGAATGGAAGAAAGCGTGACGCCATCCACAACAACCGACTGAAT
TCC
>ConsensusfromContig3202 Average coverage: 15.90
ATTCAATTGATAACTTTAAAATGATAAGCTCCAATACATTATGCA
CCCCTCATGGTGGTG
TTGTCATATATATTAAATCTTGTTTCAATTTCGAAGTTATTGCTAA
TATTTCTCATGAAT
ATTGGGACTGCCTGGTCGTTGATGTCGCCTATAATTCATTTTCAAA
ATTCCGTCTTATTA
ATATATATCGTCCCCCGAGGGATAATTACCAAGCACAATCTACTT
TTCTTGACGAGCTCG
ACGAACTCCTCACCAATCTAACTGTTCGATTCCCAATAATATTAT
GTGGTGACTTTAATA
TGAATTTACTCAAATTGAACATCAATATAAATAGGATTTTTATTG
ACAACTGCATTTTGA

ACGGTTATCTACCCACCATTTCAGCACCCACTCGAATCACAGACC
ATTCAGCCACTTTAA
TCGATAACATATTTATCAGAACCCCATGTACCATTCAAGAATTCA
AATCTGGAATTCTAT
TTCATCATATATCGGACCATTTTCCTTGTTTTCTATCTTTGAAAAT
GCATGACCATAAAG
CTGCAGTGAATGATATCAATAAAAATGCTTTGATCAGAAAAAAAG
TCACTGACTTTTATC
CTAAACTTTTGAATAGCATTCAATGTGCACTAACTAGCTTGGAAT
ATGACAATTTAGGTG
GCCATATCCAATATGATGATTTAATTAACCTGATCTCCAAATCAA
AAAACG
>ConsensusfromContig3203 Average coverage: 4.54
CAACGGAAGACTTTTTGAGCGAATTACATCCTGATGAGAAATTTC
TTTAGTAGAAACAAA
GACTTGATTTAAATTGTTGGCAATCTGATGGGCAGAACAATATCT
AGACTGTTGAACGCG
TTCGTCTTTCGTTTGACTACTAAGAATCGGATTTTGAGGGTTGTAA
ATTACTTTAGCTCG
TACTCCTGCATTATTTTGGAAAACATTATCACAGCTGCTTTCCTTC
TGCAATGATTCCGT
TGTATCTTGCGTTTCTGAACGGTCTGGTGGCATTCTGACGAGAGA
ATCGAAATAAGAATT
GATATGCTCTGTTTGTGGGGGGCTACTGGAATCCGTTGAGCTGGA
AAAGCAACGCTTGTT
TTGTGATGACTCATCCTCTTTACTGCCACAGCACTGGAATGAAGC
GGTTGAAGATATGCG
GCTGAGTAGAGTGACCATCTCGTACTGATGTTTTCGCACCTCCGT
TCTTTCGTCGTGAAT
CATCTCGAGAAGCAGCTCCATTTTTGAGTCTATGTCGTCGACCTGT
CGTTCTACTTTGAC
CACACGAGAAGCAAGGCTGATCTTACAGTCG
>ConsensusfromContig3204 Average coverage: 7.97
AAATGGCTTAGAAGTGTTGAGAGCCAAATTTTCATATTTGAAATT
CAATCGATCCTTGCG
AATAGACGCAAAACACACATTAACCACAGTTTACCGCAACTATG
CAATGCAGTTCAGGGA
GATTCTTTCTTGCACGGACAGCTACTATGGAGAGCGATGTGAAGT
CATGTTCGACAAGAG
TATTTATAAATTTTCCTTCTTCTTTCCAAATTGCTCTGAAATTGTT
TCTTGTTTTGAACG
AACAGACCGAGTTGGACATGCTTGCACATTGAACAGACAAGGAG

TAACCGATTCAGACTG
CAAGATATTGGGAACGACAGGTTTCATTGTTGGAGAAACCATAC
GTGATTACGGACAATG
TGTCCACAATAAATTTTATCCCAGCG
>ConsensusfromContig3205 Average coverage: 8.51
ATCTGGAGTTATAACATCAATAATGTTCATTATTTCACATTTTTCT
ATATATATTCCGTT
TATCTCTGTGTCCTTGTGAATTTATATGAATGACTAGAGAAATTTT
AATTTGTTTGGAAT
TTCATGTGAATGTGATCAAACGATATAGCGCGAATTTGTGAAGCA
ATGTGTTGATCGTTC
TCCTACCGGTATCAAATCATTGAAAGTTTAACTGTTCGAACTATA
TGATGATTGGTCATC
GTACATGACAAATGTGAAACACAAAATTGAACTAAAA
>ConsensusfromContig3206 Average coverage: 9.39
GTAGAGCCGCAGTAGAGCGTCTCTTTGGTCTGCAGACAGGTCCCA
TGCGATGACATCAAT
CCCCTTCTTCAAATTCTCGTATGAATATGCGGTGTCGTTTTCCGTA
TTGAATTCAGGAAT
GTAGTACGAAGTAAAGATGGCGGCATCGTCTTTGTTCACTCCGAT
TAGAATATCCTTCGA
ATTTTGATCCAAAGCAGCGAAGGTTTCGATAGCCTGTTCTGATAG
AAACCTGTCTTCGAA
CGTTGGTGCCCAGGGTGTGTACAGAAAATCTGTCTTGACCCAGTC
GTTCTCTGAAATTAT
TTCTGCCGGTAATTCATGAAGACACGTGAGAATGAGACCGCTGTC
AGAGCAGTTTACGGC
GTCGAAGAACTTTCTAGAATTGTTTATCATAGCGCCGGATGTTTT
TAAACTCCATTGTTC
GTCTGCTGCACCGCTCTGAAGTATCGCTCGGCGAAAATAATTTGT
TTGAAGGTCAAGGCT
GTAGTGAAGGCCAATGCTCGCTGCTCCAGCGTCATGTCCGAATAA
GGTTACTAGGTCTGG
GTCACCACCGAATGATGCTATGTTATCATGGACCCATTCGATAGC
CAATCGC
>ConsensusfromContig3207 Average coverage: 15.10
GATCTCGATAAAATTACAATATTCGATACAACAGATTTTGATGAA
GTCTATTATTATTTT
TTATCATTATTTCTATTTAACTCAAAAGCAATAGAAAATAGAAAC
TCTTATAGTTATGGA
TCAGAATCCCATTGGTGAATTGTATGAAGTGATGAGGTTAGGTGT
TGTTTTTTGTGTCGG

CATCAATTGCTTTTTAGTCGGATCTCCTCTCCCATCGAATCCGTTG
TCTTTCTCTCATTC
CACACATCTGTCTTTCATTCATGTCTTCCTAAATCCGCCAGATTTT
CTCATTTATTCCAT
ACTTAGCAATACTTGATAAAATTACCAATCTCTTCCTTTTTTCAGA
CCGCTTCTTTACAT
TTTATTGACAAGATTTTAATTCCATCTGGTGATTTGTTATTTTTTT
AGCTTATTCATAGT
TTTCCTGACATCATCCACACTAATACGCT
>ConsensusfromContig3208 Average coverage: 9.97
CTGGCATTGGCAAAACTGAATTCGCGTCTGATATTCAGTCTGTGC
ACCAAGCTCATCTTT
GGCTGTTATAAAGAGTTGCGAAGAACTGGTAAGCACGGGAATAC
CGCTCCATGTCAAAAC
TCCTGACGATTGGTCTATTGTGGGAGCTGAATCAATATCAACAGC
CCGAACATTATACGT
CAGTTTAGAGTCCTCATCAGGGTCAGTAGCTACGAACTCCATTGT
TGTGCTTTTCTCCAA
TTGCGCTTGAAGAGCTTTGGTTGGAATTTCGAAGGATGGAGCGGT
ATTCTCCAAGAGTTT
CTTTTCCG
>ConsensusfromContig3209 Average coverage: 33.30
CCCGCCACCAACATGAATAAAATGACGCCGAGGCTCCACAGATC
AACACAAGGTGCATCA
TATGCGTCTCCTAGTAAAATTTCTGGAGCTGAGTATGCCATTGAG
CCACATGATGTGTCT
AAAGTAGACCCAGGAGTAAATTTATTACTAAAACCAAAATCAGTT
AATTTCACTACACCA
AGTTTCTCAAAAAACACAACATTTTCAGGCTTCAAATCTCTATGA
ACAACATGTAGCTTA
TGACAGTAATGAATTGCAGTGACTATTTGATCAAAGTATAGTTTC
GACATATTTTCATCA
ACACCATTCTTGTATCGCATAATATAATCATACAAATCACCACCG
TCCCCATATTCCAGA
ATTAGAAATAACTTTGTTTGAGTATCGATAACTTCAAAAAGTCTT
ACAACATTAGGATGC
TGGACTAATTTCATACATTGCACCTCTTGACTCAAATGAGACCTG
GATACTTCATCTAAC
TTCGTCTTGTCAATAATTTTTGCAGCAACCTTCTCTTTTGTGAATA
CATGATGGGCCAGC
TTAACAACTGCATAATGACCTTTTCCAATAGTTTCAGCCAAATCA
TAATTGCCGGCAATC

TGATTACATGGTAGAGGCATTTTAACTAGCACACATAATAATATA
TAA
>ConsensusfromContig3210 Average coverage: 38.93
ACCCGAATGTTATCAAATACTTGGCTTCTTTCATTGAAGACAATG
AATTGAACATTGTCC
TCGAGCTTGCCGATGCTGGTGACCTTTCAAGAATGATCAAGCACT
TTAAAAAGAACCAGC
GCCTCATACCAGAAAAAACAATTTGGAAATACTTCTTGCAGATCT
GCGGAGCTCTTGCCC
ATATGCATTCAAGGAGAATTATGCACAGAGACATAAAGCCGGCG
AATGTGTTCATCACAG
CCAACGGAATCGTCAAACTCGGTGATTTGGGTTTGGGTCGCTTCT
TCAGCTCAAAGACAA
CAGCGGCTCATTCTCTTGTTGGAACTCCGTACTACATGTCTCCTG
AGAGAATTCATGAAA
CTGGCTACAATTTCAAATCCGATATTTGGTCTCTGGGCTGTCTTC
TATATGAGATGGCGG
CACTGCAATCTCCATTTTATGGTGACAAAATGAATCTCTATAGCT
TATGCAAGAAAATTG
AGCAGTGTGAATTTCCTCCCCTTTCTGCAGATATATACTCCACGA
CTCTTCGGGAACTGG
TGAACAGCTGTATAAATCCGTCCCCAGAAATGCGACCCGACATTT
CGTACATCCACGAAG
TCGCCAAGAAGATGAACGATCAATTTAGCGGGACATCGGCATGA
AAGAAGGTCAACGGAA
G
>ConsensusfromContig3211 Average coverage: 22.82
ATTTCTTGTCCAGACTCATCCAGCATCACGACAGTTTTACGACGTG
TGATTTTCCCTGTT
TTATTTTCATACGCTCGCTCTTTTAATATCACCCTCTTCTATGTCT
TCGTGGTGTCATTC
CAGGTGCATTCGAACCTATCAGTTCTTGTTGTTTCTGTTTTTCCTCT
TCTTCTTCCATGT
CTTCCGTTTTTTGCATTTCCCTCATTTCGTCTAAAATAGTTTGACG
TCTAAGAACAGCTT
GTCTTTTCATTCTCTCTTTCTGTTTCTCATTTAGTCCATCCCAAGC
TGCGATATCATAAT
CTTGTCCTTCTAATTTCTGCATTTCTTGCAATATCGTTTTTCGCCT
CTCAGCACGCCGTC
TTCGCTCCAGTGCCTGTTGGCCTCTGACTTTCAACGTCTCTGTGCC
CGAACTTCCCGTCG
TTTTCC

>ConsensusfromContig3212 Average coverage: 13.95
ATCTAATTTAGTCTGGATTTCTGTTCCCGCACTCTAGTATTTAGCA
TATCATGAAAACCC
TTACACTTGGTGTGTTTCTGCTGTTTTAAACTTTTCTAAAGGAG
TGAAAAAATCACCGC
CCACCATCACTCCTGATCGACCTGTTGTTGTCAACTACTTTGGGC
ATAATGAACTTGCTG
ATACAGCTATGGTGGAGGTCTTTGCTGACAGAGGCATCTATTATG
TATCAACTGAACTAG
ATTACAAGATTGCGTGTATATCACACCTTCAGAATCCT
>ConsensusfromContig3213 Average coverage: 5.47
TGCACGTTCTAATTAGATTATTTCACGTTTATATATAGATATTCAA
CTTGCCAAATTTGA
CTAGTTTCTTTCTTCGTCACTTGGGTACTTCCTTTCTTTCCCCTCTC
TCTCTGCTTTATT
TTTTAACTTAATTCAATTTTTTTTACATTTGCTCCTTTTTTTTTTAT
TTTCATTCTGTAT
TCTAATCTGATAATTCTAACGATCATTACGCCCGACATCACCATTC
TTCCCTCTCCTCCA
AAAGTTACATCCAACCCCAAAATTTAAAGTCGGAAACAAAAATG
AAGATTGTCAATTTTG
TATCCTTTGTTGTTATTACCACCCTCCTATTTGCCCCAATTCCC
>ConsensusfromContig3214 Average coverage: 17.35
TTCTTTATGAGTTTCGTCATTGGCTGACTACAGGCACATGATAAT
TCGATCCAATGAGGA
CGAGGTTTTCGAATTCATAGAATTTGAAGATTATTTGGAGAAATT
ATAAGTACAATATAG
CATTCAACACGGGCAATAATGAAGACAAATATACGATTACTTAC
AGTCAAATATCACAGA
TACACCAACGTTTAAAAACAAATTTTAACGAACGAATAACAGTT
CACCGACTCAAAATAG
GCATACATAAAAAGATGGTAATACCAGTACGACAGGATATAAAT
TGAATATATGAGAAAA
ACTGAAGTATTCCATTTTCCGCAAGTTATCGTTATATATATTCTTG
TCTATTTAAAAAAC
AGATAACACAGAGAAGTTTTTCTATATTAGGGATGACAGATAGT
AAGAGTATCCATGAAT
GAATCCATCTTATTGGGAATATTGGATATTAAATAGAGCCAGTTA
GTATAGATAACGTCA
GACATTCAATCATTTTTTAGATATGAAAATAAAGAAATTGGCAA
TGAAAACATAAGAATG
GAATCAGATGCAACAATGGATGGCAAAACGTAGAAATTATATAA

AACATTTGAATTATTT
CAATATAAA
>ConsensusfromContig3215 Average coverage: 13.85
TCTTGATTCATCATTTGAAATCATTCAACATGAAAATTCAATACGC
TTTCCTGCTCATAT
GCATTTTTGTATATATTCGCCCACTGGAGAGCAAACCAGAGACAA
CATGGTCAAAAGTAC
AACCTTGGATGGAACCTTGTGAAGACAATTGCAATGAAAATGGCT
CGTGCTACAACGCCT
ACTGCATCTGCCGAAGCCCGTATTATGGTAGAGCGTGCGATAAA
AAGGCTGAGGGCTGTA
GACCAACAATAGGACCTGTCCATCCGGCTCATAAGAAAATTGATT
GTTTTGGCAACGGAA
AATGCGTGAACAACCAATGTGTATGCGATCGGTATTTCAGCGATG
GATTGTGCTCATTCG
ATAGGCGCCTTATCCCGAGATATTGAGAAGCGCAAGAAAGACCCG
CACTTCTCAT
>ConsensusfromContig3216 Average coverage: 27.69
GAACCATGTATTATAGGAACATTATCAGTTTCAAATCCTATGTCA
CTCATAAGTTCAAGT
ATTTCAAGTTCAACTAACTCCACCATTTCATTATCAACAAGATCA
ATCTTATTTATGTAA
ACAATTATATGTGAGATACCGAGTTGCTTTGCGAGAAGCAAATGT
TCTCTGGTTTGGGGC
ATTGGACCATCGGTTGCTGCAACGACAAGTATAGCTCCATCCATT
TGTGATGTTCCTGAT
ATCATGTTTTTAATAAAATCTTCATGACCTGGACAGTCTGTATGT
GAATAATGTCGGGCG
GATGAAGAGTATCCAATATGAGCAGCATTCATCGTAATGCCTCTA
GCTTTCTCCTCTGGT
GCTCGGTCTATTTGATCATAATCAATATATTTAGTAGTATTGGTG
ACTGCTCCGCTACCA
GTAGTAGGTTTACTACTTTTTTTATCTGCAAGGACTTTTGTTATTG
CTGCTGTCAAAGTA
GTCTTCCCATGATCGATATGGCCAATAGTTCCAACATTGCAATTTT
TCACATTTCTGATA
TCTTTCGAATAATAAATACGAGATAACGAACATGTTAAGGTTTTC
AATCGATGTATATTT
CTAATATATTTTAAAAGCATATTTGTTTACTTTTTAATGGGCGCAG
CCATTTTTAAC
>ConsensusfromContig3217 Average coverage: 10.53
GTTCGGATGCTGTTTGGATGGATTCACTGCTGCGCTTGGGCCTGA

TAGGGCTGGATGTCC
GGAGAGATGCCATTGTAATCACATCGGTGCGTATAAGACGACATG
CGACCCTGACACTTC
GCAATGCTCCTGCCGGCCAGGAGTAGTTGGTCTCCAGTGCGATTC
GTGTGTCCGAGGCTT
CTGGGGCTTTAAAAAGATCGAGGCCGGAAATTCGGGTTGTTTACA
CTGCAGATGCAATCG
CCTCGGTTCCATGCGCGACGATTGCGAGCAGATGATTGGTCGGTG
TGTCTGCGTCCAAGG
GGTCACGGGTCAAAAGTGCCAACGGTGTGCGGATGGATCTCTCC
TGGGCGCAGATGGTTG
CGAGGGATTCCCCGAGAATAACATGGGCGACGTTCCTTGTTCGAA
GCGACTGTGCCCCTT
TCATGGCCAATGCTTTGAGGACGACATCGGACGTTCTCACTGCCG
GTGCAAAACCACTTG
TCCATCCGCCAACAAAGCGGACGGCGTCTGCGGGTCA
>ConsensusfromContig3218 Average coverage: 15.64
GTGAAATATCATCTTTGTCTCGATTCGCTATTTTCTCGGGAATAT
CGAACAATGGACACT
TTCGCCTGTAACTTTCGGAATTCTTGAAACAATCCTCATTCGAAG
GACTTGATATAGATT
TATCATTCATACGTGTGAGAGAAGCATCATTGACTTTCTCACGAC
AGTTATCAGGACTTA
TTCGAGCCTTGTCTAGTTCTTTTTGATCCATTAGACATTCTACCCA
TTCCAATTCTCTCT
CAATTTCAGCCTGGTATTTTGGATCTGATTTCTCTCCAGTTTTTCT
GCTCGTTTGAGCCT
CATCAGAATCTGAGCTGGAACTCGACGAGTAGGATGAGGAATTTG
AACTTTCATTTGAGT
CTTCTGTTTCAATGCTGACAATTGACGGAAGATTTGGACGCTTTC
TACTATACAGTTCCG
AAGATGCCTCATTTGGTTTATTTTCTTCAAACAAAGATTTTTGAGG
TTTCTCTT
>ConsensusfromContig3219 Average coverage: 6.29
TACAGTTTCATGATGAAAAAACAAACCCAATAATACAAACTATT
ATTGACAATGTCGAAA
AGCTGAAGTACGCAGGAGATTCGGTGAAGGTCATGGTGGACTTC
GAAAAGATATTTCCGA
ACACTAAAGATTACATGACATATGAAGGGTCTTCCACAATACCTC
CATGTCAGGAAAATG
TTCGATGGGTCATCCCAAACAAACCGATTTATATGAAACCATCTG
ATCTGAGGAAGTTAC

AAACCATATTTTGCACGGACATTACCGATCTCAATGGCATAACA
GCAAGTAAGAAAGCTA
GTATTGAAGAAGGAAAATACGGAGAAGAAGATGAACAATA
TATGATGAATAACAGAA
TTATGATGCTAGGAAACATCAGAAGCCCTCAGTCGGTGAACGAA
CGAACAATACGAACCA
ATATCAACTTCCAAG
>ConsensusfromContig3220 Average coverage: 10.16
ATGGAATGGCGACTAGGTCCCGGAAGGCTCTTTTGGCTGGCTATA
TCCTTGACCTTTCTT
CTGGGTGCAATATGCCTGGCGCGTGATCGACCAAAGAATGAATC
TTTGAAAAAGCTTGAA
GGTCGGAAATCGGCGAACGATGATGAGAGGAGCGGTGGATCCAG
ACTCGCGAACTCTATT
GCTGCAGGATTCGTTTTCACGGAGCCTCACTACAACGTTGAGCTT
CGTGAAGACGCAATG
CCATCTGGTAAAATTCTTCTCAAGCCAAGATCCAAAATGGGAATT
TA
>ConsensusfromContig3221 Average coverage: 12.83
GAAGCAGTATCAGATATACCGGTCACTAACGACGAGATTTATCAC
CATTATTCCGTTATG
GAAAAGAGAGGTGTGAATTACACAAGATCTTCTAAGTATGATCAT
TCTCATTTCACTTTC
ATGTTCACCCGGAACCCATTCGACCGATTGGTTTCTGCATTCAAA
GACAAAATAATTCAT
TCAGCGCAAAAGAGGGGTTTATTCACCCTTTTCAATAACCACGTG
GTCCATGCGTGTCAA
AATAGTAATATTTCTTTATTCGAATCCTTTGTATGTTGTATCTTCA
AAGTTTATGACTCA
AACGGAACACTTGAAGATCAT
>ConsensusfromContig3222 Average coverage: 12.52
CGAGAGGTTCCGCGGATTGAAGAACCCAATGAAAGTTTTCGCCCG
TTCAGTATTCTACGT
TGGGAAAGGAAGGGGCCCTCGCTCTTTCGCTCATTTTCATGAAGC
CTTAGCTGCCAAAGA
TGCAACATTGAATGAATGTTCTGCCAAAGTCCGCCGAATCAGGG
AGATCTGGGGGTCAGG
TATGGGCGTGGTTTCTCTTGAAGTGTTTCGAAACATCAGAGCTGT
GGAGGCGTACACCAA
AGAGGCTTGTATCATCGAGGCATTGGGATTGGAAAATGTTACAA
ACGAGAAAAGGGGGAG
CTGTTACGAAGGAGCACGGTGGTGGCCAATCAGCAAGAAGACTC

GCTTGGGGACACGCCT
TCTCGTGAGAGCCTACCGAATTCTGCTGA
>ConsensusfromContig3223 Average coverage: 23.82
TGTTTTCAGTAATTTTCGTACTTTATCCGACAAAATGAGTTTCTC
AATTGTACAAGATGG
CTTGCAATTTGTCGACGAATCTAATATAGTGCAAGATGATGGATT
TCACTCTGGAATTGT
CAATAATATGACCAAAAACCATATGGAATCCGATGATGATGAGGA
TTCTCTGAATTATTC
GGGTGATGGGCAGCCCAAGACTAAAAAAACCAAAGGCAGAGTTA
AAATCAAAATGGAATT
CATAGAGAATAAGCTCCGTAGATATACGACATTCAGCAAAAGAA
AAACCGGTATTATGAA
AAAGGCTTACGAACTATCGACACTGACGGGTACACAAGTGATGC
TAGTTGCGAGTGAGAC
GGGCCATGTTTACACTTTTGCCACCCGGAAGCTCCAGCCAATGAT
CACAAGCGACAACGG
AAAGGCCCTTATTCAGACGTGCCTCAACGCCCCCGATGAAGGGT
CGGACAGGTCAAGTGT
GTTGACGGCAGAGA
>ConsensusfromContig3224 Average coverage: 10.88
AAATCCCATTGAAAATCATATAAAGTATCTGCGATTGACTTTGAA
AGTATTTTTCCTGAA
TAATCAGAAGAATAACTCTTATTATGCTCGTCATCTAAAGAAGCG
CATGATGATGATTGC
TTTTTTTGCAATATATCCCTCAGATAATCACATAAATTATTAATT
TCAACTAATGAAAAT
TTTTTAAAATTACTAGTAGTTCTCATTTCTACATGTTCTTTTGTAT
TGAAGAACTTAAAA
CCCCATTTCAAAAGTTGAGTATCAACAAACGGGTAGTCTTCTTCA
TGTGGTAGGTTTTTG
GTTCGTACATAACTATTGTTTTTTTTCAATAATATACTGGAGAAA
AAACTCAGTAATTGA
ATTAAAATGAGCCTCAGTGATTCAATATTTTGAAGTGAATGTGTG
AAAAAGTGGAAGTCA
ATCAATATCGCGATTCCTGGCGAATAATCTATATGGACAGTAGA
CATAATGTCAAAAAAG
TTAAAATTTGCGAAAGTTTGTTTT
>ConsensusfromContig3225 Average coverage: 80.97
ACTGGAGTTCAGACGTGTGCTCTTCCGATCTCAATGCTGGCTCCG
ATATCTGAGGAGAAT
CGATTCAGAACCCGGCCGACGGTCGTGGACTCGAAAAAAGACAT

GGGCGCCCGGAGTATT
CCCCGTAGAAGGTTGCGATGAATTGATTTCGAAGCAGCTAATCTT
CCTTTGTTCATGAAG
AATGACATTACCAGAATCGATGAAGCTTGAACCAAGCTAGCAAT
CCAATAATACTGAAAA
TATGAATCCTTGACACTATTTATATTGGCATCTGCACCATCCTCCA
GTCCTGTCCCAGCT
GCCCATTTACTTATGAACAGATATGA
>ConsensusfromContig3226 Average coverage: 13.70
GCTTTGTTTGTTGGTTCTTCGGTAGTAAAAGTCATCTTAATACCG
GATTCATCAGGAACG
GTGAATGTTGTTCCATCTGCGTTGTTGTAGTGAATGTCTAGGACC
ATGACAGCAGAAGCA
CCGAACTTGAAGCCAGTGTCTTCAGGGAGATTGAAATGCTCTCCT
CCGATGGCCCAGACC
ATCACGACTTTTCTGCAGCTGCGAGGTGGCATGAATTCGTCTGCG
AAGCTTTTATCAAAA
CAGTCCACTTCCTTCTCTGGATAATCAGTGGTTTTAACATCCGGG
CCACAAATGTAAACT
ACGAGGTGATGGAGGAGATCTTTATTTCCAATTATGGGCTCAACC
TTTATCAAGTATTTG
GGTTGTCCAGCATAATCCGGCGAGTGAAACACTTTGCAATGATAG
TATGTC
>ConsensusfromContig3227 Average coverage: 12.09
GCAGAACTTTCTGTGGTGTTCTATGCAGGTTCATCATATTGATAG
AGAATGCCTTCTTAT
CATATTTGTATATTGTCTTTTTTCATTTTTTTATTCAGGTGTTCATG
TGGACTATGTTCG
AGTTCGAGCCATGGCCAAGAGGCTATTTGCTGCAGAAAAATGGCA
CATGTTCGTGATTTG
GCACCACCCCAATGCATCACACAGCAGCAAACATTCAAGGATAA
CTGCACTAACCCAAAT
GTCCTTTATGTAGACTATTGGCGACATATCCACAATGAAGGGCC
ACATGGCAAAGATGAA
CCAATCAATCGTTCATATCGAAATCTGGCATACAATGGCTTTGTG
AGCTGGATTTGGGGA
CGTTTAGGGCGTCGCAAAAGAAAAATTTTGCCGTCCTGTGTTGTT
GCTGCCATACGAGCT
GCATTTCCGTCGCCAGCATATGCAGGCTTCAAATATGCTGAGTAG
TGTAATGTGTTTTTT
ATAATGAAATGGTC
>ConsensusfromContig3228 Average coverage: 6.66

CGCCGACTCACCGATATTTTACATGATAATTGGTACAGATATGAT
ATATCCAACGAGATA
AAACTGACATGGAATAATTATCAATAATTGCTGGCATTCAAATAT
GACCTATAAACAGAA
GGACGTTGAGTTGGGGCAAATTGATGCACCAGCTGAGAGAAGAG
TGAGATAGCAGACACA
AGCAGACGGCGACTGGCACTTGAAATAAACTTTTGAAGAGAAAT
AAAGTGAGATCTGTTT
GGATCAAACATTCCAATCAAAGAATAAAATATTCACATTCATTCA
TCATTTAAATTCACA
CCAATTCATCAACAAAATCCTCCTAACACGCTCGATCCCAACGCC
ATAAAATCACTCACG
A
>ConsensusfromContig3229 Average coverage: 8.24
AGCGAACTCAGAAAGTCAGATAACTTATTCGAGCTTGATAATTCA
TGCTCCAAGTCAAGA
GCGTCTATCACACAACAATCAAATATATGCTCGTCCTGTAAATGT
CGGTTTAACGCAAAC
CGCGACTTCTCAGATGAAAGTAGCAATAAGAACACCTCCAAATC
CGACTCAGATTATACG
GAAGAATACTCGCGGGACTGGATTCGCGTCGCGGAAATTTTCGAC
CGTCTCTTCTTCTGG
CTTTTCCTTCTCGCCATACTGATATCCACATTGATTTTATTCCATC
CTCTCACAAATCCA
TTACAGCAAATAAAAAATGGGAATGAGTAGTGTGGATAATATGGT
TTCATAATGATTTGT
TTTCTGTTAAAAATGCC
>ConsensusfromContig3230 Average coverage: 4.99
GTCAATGATTTGTCGAATAAAAAAGTCATCCTTGATTGCAAACCA
GTCAAAGCCGCACCA
ACAAGAACAGCGTCAACTTGGGGAACAACAAAACCAGTGACCAT
GAAGAAAGAGAGATTA
ACATCGATAACAGCAACGAAATGGACAACACACGGAAAGACGAC
AGGAACACCTTCGAGG
ACATTACCATCAACAGGGTTCATCGAAAAACTAGCAAGAGCCAC
ACAAGAAAGAACAATT
CCAAGCGAACCTGAGATAGAACAACGATCTTGGGATTTCACAGA
AAAACAAGGAATAGCA
GACGAC
>ConsensusfromContig3231 Average coverage: 10.52
CGCTAATATCAAATGGAATCACTCGAATATCCATTCTCATATAAA
TATTTATTGTTGAAA

ATTATTTTGAAGATTGAGTCATTGAAATTTAATTTGACCAGAATC
AAAATTAATGTTTGC
TTAGTATTTATAGATAATTTGGTCGTACTCATTATATCTATTTGTT
AGTGCTGCTCCCGG
TAAAAGCTAGAATTTCAATTAATATTGTGAAGATACATTCAGCCC
TTGTTCATTTATATA
ATCAAACAAGACGTTGATTTTATAACTGGAAAGTCTTAATCTTCTT
TATTGCTTATCCTC
ATAGAATATATCACATCTGATACTTTGGCATAGTTTACTTTGAAT
ATATCATAACTCAAA
TATATTGAACAATTTGGCTTAAGTCTATTTAATGCATGGTGATCGA
TTTTTGATTCATAG
CTTTGATGAATGTAAAATTGGAGGTCAAACGGGGCTTCAATAGCA
TATTTGCAAATCATA
TATTTGATAAATATGTGTTCATGAAAGTTTATTGAGGAAGTTTCA
TCAGTTGTATTGCAT
>ConsensusfromContig3232 Average coverage: 21.20
TTGAGGGGACTGCTGCTTCAACGTCGCAGCAGCATTCATCGAAA
TTATCGGTGGAGAAAA
CGGAGGTGATTTCATGACGCCCGCCGAAAGATTTAATCGACAAT
GCGCGACACCTGGTGA
CGCCATCGGCGAAGAGCTCAGGGAGGAGGCCGCCGATTTCACCG
AAACCCGAAGTGGCAT
CGTTGCCGCAGAAAGACCGCTGCTTGGCAAAGTGATTTTTCTCAG
GGCGAATTTCGGCAG
AGGGTGATTGAAACTCGGTCTTCTCTGCCGTTTTCCGTATCGCCA
CGAGTCCGCAACGCC
GCCACTCTCTAGCGGTTGTGGTTTGTCATATTCATCAATCGAAGA
GAGCGTATTGGCGAA
ATCATCCAAAAACGCATCCGCCGAAACGGGTGGTAGACGAGGAT
CTGATGGAGGGTCGAG
GACATCTATCACTTCCAAACTGATCACCTTGGGAGCCAAGTTTGA
TGGTTTAGTCGAGCT
CTTCGAACGCCGTCGACTTTTGCCGCAATATTGAGATTTGCTGTG
AGGCCCGCCGGCGCT
GTTTGACGAAGAGTGAGACGAAATAGATGTTGTCGATGAAACGG
ATGACGAGAGAGAAGT
GACCGATGACATAAGTTTCGTGAGCGCGTGTGGTTTAGGTCCACT
TTCCGCACACACTTC
ACCTGACGATGTGGTAGATGCTGAATGAGTATTCGAATATTCATT
TGAAACTTTTGTGTT
TTTGTTATCAATATCACCGTCGCGCTTGTTACGGAGAAAACTAGA

ATCGTCAGACTTCAA
GGATTGAAGTCGATCATCTAAACTACTTTTAATTGAATCGTTGACC
TTAGTTTTATTGAT
TTCGTCTTTATTCATACATTCGTTCTTTTTCATGTCATTGCCTGCC
TTTGCATCGGAATT
TTCTGCTGATTGAGCGCAGTCCTCAACGCCTTTTAACGCGGGAGCA
TCTTCATCTTTTTC
CGATGAGCTCACACAACTCGCAACGTCCTGGCCGGCAAATCTGTT
TCGATAGACACGTCT
TCGTTGCTTCAACACAGACTCTTCATCTCGCTGTACGAATTTCGTA
GATTCTTTCTTGTC
CTCGGAGCCCCTATCATCTTCTGCAATGCCACCTATTTCATCTTCA
ATCG
>ConsensusfromContig3233 Average coverage: 7.54
AGGATATTCTCACTGAACATTCATTGGATTTGAAAAAAAGAGAAT
CTCTTGTGCATAACT
TCACATCATCTAATGACAGTCGATATCAGCAATATTCGAACGATG
ATTCCGAATCCGAAT
ATGGATGCTGTTCATGCGCACAGAAATGTTTGGCAAGAATACGT
GAAAATGTAACACGAA
ATATTAAAAGTCAGAAAAAATGCCTGGACTCTGATAGGATGGACG
TATATGATCATAGGGT
CAATTACCATATCAGTCTACATTGTCCTGGCCAGTGCTGAATTCA
ATCCAGCAAAAATAA
AAAGCTGGCTATTCAATTTTGGATTGTCTGTGGTTTTTTTCTTCTT
TATTCTTGAGCCAG
TCGTG
>ConsensusfromContig3234 Average coverage: 5.63
TTGAGGCAAAGATGTTCTCTGTATATTTTTCACGGTTCTTTCATCA
ATTTGGTCCATCTT
CGATTCTTCAATTTTGGGAAGCTTCACTTTTTTCGTCATCGATACG
TGGCTCTCAGTGAC
GAATGGCTCTATTTTCTTCTCAGCCATCAGTTCATACTCTTTAATT
GTTGCGTTGTTCAT
ATCAATCCTGTAGCAGTGCTTCAAGTAATCATCTTCATAATATTC
CTGATCATAGTAACT
CGGAATGTCTTCAATATGTCTGGCGAGAATTTCGTTATTTTTCTTG
ATCGTGTCTCTGAT
GTATTGTTTATATTCGGCATCACGGCTCTTAAAATTGATATCATT
CCAGTGATCTGTGAA
TCCGGGTCCCTGAAGAATCGATTGCAATTTGTCCATCAGAATTTC
ATTTTCATGCTGGAT

CTCAGCTTGCCTCTCTTCCTCAAGCTGTA
>ConsensusfromContig3235 Average coverage: 11.26
GAAATACGCAATTACGACTATCAGTAAACAGAACATGGCTTCAG
GTGCTGATGTAAAATC
TGAACATGGTAAACGTCCAATTGATGAAGATATGTTTGAAAATCT
CGAAAATAAACTTCG
TGAAAATTCTGATCAACAAACCAATTTTGCAGACGATACCGTGAT
TCAGCAAATAAAATC
CGAATGTTCGGCGCCTCTTGTTGAATCACATTCATGTACTCATTG
TGAAAAGTCATTCTC
ATGTTCTCTCGACTTGATTACTCATGAATGCCATGTTTTGCAG
>ConsensusfromContig3236 Average coverage: 9.88
CTGAATTAAAAATATGTTATTTTGGTAAAAAGAGTGTGTTTAACA
AAGTGAATTAATGTA
TACAATAAAGAGATACAATAGCTTGCTTATATCACCTAAGATATC
AAGATACAATCAATT
CCAATATAATATCCAATACATTTCTGAGTTATAACTACGTCAAAA
CGCACTACTATGATA
TATATTACCTGAATGCGTACTTGACATATTACAAGCAGCAACAAA
GAAAACTCTTATCAC
AGTGTCATAAATATCTTACACAAATTGATATAATGTCACTGAAGG
GAAGAAGCATTCAAT
ATATAGAGAAGATCGGAAGAG
>ConsensusfromContig3237 Average coverage: 4.40
AAACTAAACTACTGGGATATTGGTATATGGTTGTTGTAAACAGAG
TTACCCAACTGACAA
ATTTTGGATTTTTCGGAGAGTGCGTTACGGGCAACTTCACAGCGC
GTCCAAATGGCGGAT
TCTTCGGTGAGTATTTCTTCCGTTGGCCGACCAGTTTTGGGTGGAG
CATCTACTTATTGG
ACAGCACAAGAAGAGGTGAAGAAGACAACTACATCAACCACATC
GCCTTGGTGGATATTC
TGCACGTCAAATTCATATACGTGGAAGGGAAGGAC
>ConsensusfromContig3238 Average coverage: 49.59
CTGGAGTTCAGACGTGTGCTCTTCCGATCTCCAGGACTGATCGTTG
GTATTTTTCATAT
CAGAAGAGATAAAGCGGATCACGGAAAAATTTTCTCCGTCGATAT
CTTCTCCATTTTTAT
ATGATACTTCCTCTCCATCAATCACCATCTTCAACCCTGTAACTTC
ACCTTCCCGTCAAA
ACACCACGAATTTAATTTTCACCACCACAATCTCCAAAAAATTCA
CCATTCGCCAGTGAT

CGCCCCATCTAGAAGACATAGACATGCTGAAATAAAAAAGAATG
GAAGGACATCGTAGAA
GCAATTTTATGTCCATTCCATTAAACCCGAGAATACGTCCAGGCA
AATTCATCCCATGAA
CCCCAGCATAGTCGATTTCTTACAGAATCATGACAGGATATGG
>ConsensusfromContig3239 Average coverage: 7.43
AAAAGATTCTATACTTGATAGTCGAGATTTTCATTCCAGATTTGTG
ATGCTCAATCTTTC
AGTTGATGAATCCCATTTTTACTTATTCATACTGGATGTCCTAAG
CATTCATATATGGCA
CTTATTTTTTCAATTATCAAAATTAAATTTGTATCATTGATATGAT
AAGATCTCAAATAA
GAGTCACTATATATAATTCTCATCTATAATATAGAATCAGACTTGT
CTTATTTACTCGTA
TCATGTTGCATGAAAATGTCGAATTATAGGGACATTGTATCGCTA
TATATGCAAAC
>ConsensusfromContig3240 Average coverage: 6.07
TGTGCTCTTCCGATCTCTGCAACACATATACCAGGATATTTCTTA
TATTCGTGATGCAAA
ATCGATTTCTCGCAAATTGGTATTATTCAACTTCCAGCCTCATTT
TTCAATAAAATGAAG
AGAATCACATTTGAGCGAACCGCTGAATTTCGTGTTGACCATCAA
CATACTTTTATAGAA
ATGTTTTATTCATCATACATATTAAGTCAGAGGTCGTTTCTGAAG
AGGGTCCCATGCATT
GAGATCGAATTCTCCACAATTACCAAGAGCGGAAGCGTCGTCTGT
TTTTCTAAACCAGGT
AAGATGCTAGGAAAAATCGACAACAGTTTCGCAAGCTCAAATTTT
TAATTCATACAATCA
AACAACCAAGGATACAAGGCGATATCAAAATATACGTGGAAATC
AGAAATTTGAAGTTCT
CTAACCGATCAGTCGGATACATATTTTGAAAAATAAATTCTCACC
CTCTATTGACACTAC
GTATCTTTCGGAAA
>ConsensusfromContig3241 Average coverage: 10.10
AGCGAGATGATCTACAGAGCTGTGGCCAGAATGAAGATTTTTGC
TAGAAACAGAAGGATC
GATGAATTGCTGACTCAACCAACTAATACCAGTCAGAAATTGCTG
AGTGTTGATAAAACT
AGTCCGAAATCATTCAATTCCATCGCCAGCAACCGTTCGACTGC
AGGGCTGAGAAACCGA
CTGAATAACGTCTCCAGGAATGTATCTTCTGCCGATTGCGCTAAT

TTTGGCGAGGAATCA
AATATTTCGTCTGCCGATTTGGTGAATTATGGTGAAGAAAGAGAA
TCGCCAACGTTTTCG
AGTATCGGAATGAGTAAAAA
>ConsensusfromContig3242 Average coverage: 7.35
CCGATCCTCAATCTGGGCGAAAGTATAGATGCCGGCACATGTCT
GGTGGGAGGGGCACGC
CTTCACAGGGTCACTTTGACCAATAGAGGGGGACGAGGTCGCTTT
TGTATCATGCCCCGC
TCTTCATGGCCCAACACTAATTTCAAGCGAGTGGTGGGCCGAGGG
TTTGCGGAGATCGGT
CCATTCGAGGTCCGACCGGTTCACTTCGATCTCGCTCCTGGCACG
TCGGTTTCTCTCGAG
ATTCTTTTCTCTCCAACTGAGATCGAAGAGTACAAAGATGAGATG
ACTTTGATTTCTGAC
AATTGTCACGTCAAGCATATCACCTTGTATGGGCGTGGTCAAGTT
CCGGCCCTCGAAATC
GGAAATCCGG
>ConsensusfromContig3243 Average coverage: 9.01
AGGAAGGTCCATACGAGCATAAACGCTTTAAATTCGTTGTAGTCG
ACCTTGCCATCACAA
TTTGCATCTGCTTCCCTGATCATGTCCTGGACATCGTCATTGGAGA
GTTTTTCCCCAAGG
TTGGCCATCACATGTCTCAGTTCCTGGCAAGTTATGAACCCGTCG
TTGTTTCGGTCGAAT
ACTTTGAATGCCTCGATTATCTCTTCTTCTGTGTCTGTCTCTTTCAT
TTTTTTGGCCATC
ATCACAAGAAATTCGTCGAAGTCCACAATTCCATTACCATCCGTG
TCGACTTCTTCAATC
ATATCTTGCAGCTCTTTATCCGATGGGTTGTTACCCAGGCTCCGC
ATCACCGTACCCAGT
TCATCAACAGTGATACTTCCATCTCCGTCGTTATCAAAGACTGTA
AATGCCTCTCTGAAC
TCTGCGATTTGATCTTCTGTTAAGTCCAACGCTGAAACTGTCGAC
ATGATGAATTGATGT
TTTTTCACATTAGAATATTCCGAATAATAGAAAATAGATTCTAAA
AAAT
>ConsensusfromContig3244 Average coverage: 9.16
GTCAAGCTTTCCTCCGATCGTCGCAGTGGACTCGAAAGAATCGGC
ATATATTTCCGGGCT
ATCATAGTGACCTTGATCAACTCCAGAGTACTTGGGCAAATGCAG
TTGTTCTCCTTCCCC

CATCGTAGAATACTCAGGTAACTCGAATGATGATTCCCGGTTTGT
GCTGGTCGACTGATG
TCCATCCGCTCTGTTCATGGTAGCGTACCCAGAAG
>ConsensusfromContig3245 Average coverage: 7.07
GTTCAGGTAGATTTAGGTCGTCAGTCGATCTTTGTGTTTAATGAT
AATAATGATAATAAT
AATATTGATTCAATAATATAGTCCTCTGTTGTATGCCTTGCCAAC
TCACAACAATCTGTT
CAGTCGTCATGTATCATTATTATTATTATTTCCACTAATAATTACA
AATATTCGAACTAA
GATTCATTTTATTCTCGTATATATATATATGAATTTTAAGCTGTAT
TCACCGCGTAGAAA
GATCTATAAGGAGATGTATTAACAGAGGTGGCAGTGTTGTGCTTA
ATTTGCCTCTCGCAT
GATGATTATGCTGTTCAATTGACTCCGTTTCGATGATGATAGTGA
ATCAAAGAATTCCCT
TGCTTTTTTTCTTTCCGTTTTTTCACCTCGATTTCATTTTTGTTTTT
TTAGGAATGTTTG
CAATACACAAGGAACGTTATTTACGTCACAGCTTAATGTGGTTTC
TCGTATGATGAATTT
CATAACATTGTTATTATCATTTTGATGATTAAGCAGCACGTCTATG
CTTTATGGTATATC
TTTATTAAACTAATGCTCTCTTAAGTAACCTTTACTAGTCAATTTC
ATCTACAAATGGTA
AGGAATAATCAATGTCGACGATGTGTCGCGATGCCTCGCAATGGA
CTTGGCTTTCTGTCG
ATGATAAACTCGCCAAACAAGATGTGTATGTACTCATTCGTGGAA
CTCGGTATAAACA
>ConsensusfromContig3246 Average coverage: 7.80
CATGAAGTACTGCATCGCCAGAGCATCGCCAATTAGAGAATACG
AAACCTCATTTTGGGG
AGACTGTGGATCTGCATCTGTAGCGTGAACTTGAGTGACGTTAGC
GCCGAGCGCTTGAGA
ATAGAGAATACTGGGATTGAGCGAGGTACGCGAGAACCTCGGCG
CATTCAAATTCCTGTT
GACTTCCACTTTTACGATAGCGATGGCTTTCTTAGGGGGCGAACC
GTTGTCCGCTGCCTC
CACTCGGATCTGGTACGTTGTGACCGTATCGGCCGCCAGGTTAGA
CTTGACCGATATCTG
GCCGCTCGTCCGATCGATCTCGAAGTAGGTCGGCGCAGAGTCGTC
ACCGATTATCCGGTA
GCTGATCACTCCGTACCG

>ConsensusfromContig3247 Average coverage: 5.95
TCTGAGGAATTGTCCCCTTCCTGCTGTAAAAAACTTAAGAATCTT
ATAGATGCTGCTGAA
AAAATCCATTCTTCAGAAAGTGCCTCTGATAGGGAAACGGAAAGT
GATTCTCTTACTTCA
ATTGCAACATTTAAGAAACCATGTGCAAAATTTCCCCCTTTCACT
GTTCAAAAAGCTATA
GACTCAGTAACGTCACGCTCTAATCGTAGGAAAAAAACGAGTGC
CTGCGACTCCTCTGAG
TTCACTTCTTGTGAGAAGACAAGAAAACGACCCGCTTCGACTTCT
CCACAACAACCCGTC
TCCGAAACCTCTCAAATGTCAGCAATAAACCGACAAAAAAATCA
AGAACGATATCCGAAA
CAACGAGCGTAACAAACATGGCATGCTCATCAACAACAATATTG
AAGAAAGG
>ConsensusfromContig3248 Average coverage: 9.51
GACGCTTTCCGATCTTCTTGAAGCATATCTTCAATAGCTGCTTCA
TGTCTCAATCGACGA
CTTTCATTGATCCTATTGATAATGTCGGACTCTTTCTTAGCACCCT
CGACGATGTCTGGG
AGGCACTCCACTTCCTTATATGCGATAGCAGCCTCATTGTCCAAA
ATACTGCTTTTGTGA
TGCTTTGAATTGAATTTGGAGAGAACTGGGTTTTCAATGGAGCTT
ATATGTGGGAATTCA
TTCAACCAAGTCTTCTGACGACTATTCAGAAAGCTTTCATTTGTT
AGTGTTGAATCTTGA
ACAACAGGAATAGTCGATGACTTCAATGCAACATTTTTGCTGTCAT
TTATTCTCTCCTCT
TGATTTCTCACTGTCTGCTC
>ConsensusfromContig3249 Average coverage: 4.59
TTGAGTTGAGAAACGAACATGCTTTTACACTCCAGTACACTATAA
TGTATTCATATCCTC
AGTTGCTGTACATTTCTTCAGCAAAGATACACAAATTATTTATCA
TGAATGAAATTCATA
TGAATATTGTTACAGTGCTGAAATTCCTAGTACATTTTTAGAATA
GATCAACTCTAAATA
TTTTTTAAGGCTTCCAAGAATTGAGACAACAAAATGATTATGTTG
CTGTCATGAGTTGTT
ATGATGGACACCTCCAGAGCAGATCGGAAGAGCACA
>ConsensusfromContig3250 Average coverage: 8.13
GGAAAAGGGTCTCAAGTCCGAAGAGTTAGATGCATAGAATTTCT
CGAGAGGAACGAAGAA

GTTATCTCCAGGCTTGAGGTACCAAGAGAAAAGTGTGACATTCTA
ACAACCCCTCTTGAT
ACTCGGCTTTGCAGTATTTTTTGCCGGCACCAATGTGTTCTTTCCC
CGTGGTCACCGTGG
ACTTTTTGCGACAAAAATTGTTCTTCTATAAAAGGTCGTTCGCGG
ACTAGAAATCTTCTT
CGAGATGCTATGGGAACCGTGGATCATTTTGACTCCGATTCTTCC
AGCCAATGGTGTTCC
TCTGAGACGTCAACCGACCGCTGCATCCACGGCATCAATTGTTTC
ATCAGTGTTTGGAGC
TGGTCGGGCTGGTCAGGATGCTTCATGGAGGGATTGCTGAGTTG
CGGAGCAGGAAAGCAG
GTCCGATACCGTCAGTGTCTGCGAGGGGGGAGATG
>ConsensusfromContig3251 Average coverage: 10.63
ACACGACGCTCTTCCGATCTCAAATGCGGGCTGTTGTATTTGGCA
AGAGTGTGAAGAAGC
TGAAATCCGAAGAAACTAAAGGACTGACGGTGAACTTTCTGCCT
AATGAGGAGATAATTT
CTACCGACAAATATTGCCGAGAAGCGGATATTATCATTGTTCAAT
ACTCAGTTTTGAAAG
AGAATTCCTTTGCAGTTGGTGGGAGCAAGTGCCGAGAGCTGAAA
CAAAAGTTTTCCGGTA
AACCAATCATCTTGGTAGGAGTGAACATCGACCAGACCGGCAGG
AAAGTCAGGACGGAGC
TTGGGCAAATTTTCGCCGGAGCACTCGGGGTGCAATTTGCTGAA
CACAAAAAGCTGAACG
CGGACAAAATTGCTTCTCATGCAAAGAAGGCAGTTCAGATCAAC
AAACCGAGAC
>ConsensusfromContig3252 Average coverage: 4.79
GTTGAAATTGCTTTGTTTTTGCTTTTTGTTTGGTTTTTATCTGTCT
GTGATGAGTAGATA
GGTATTTGTTGATTTTTTTTTCGTATTTTGGGCTACCAACTTGCA
GCAAAATTGAAAAAA
ATTAAATACGATTTATTATTTGTTTCTACGGATCAGTATGAATGAA
TTTATATGTATATC
AATTTCAGCAGCATTATTATGAGACGAATATGTATGAAAGGTTTA
AAATTATTTTATTCG
AAATGCGTGTTTTGCTAGATTTGCGATGCCATTGTTGAATAATAA
TTTTTTGAGTATGCG
AAATTGTTTTTGATGGAACGCTATTGGAG
>ConsensusfromContig3253 Average coverage: 6.80
TTTTCGATACAATAATATTGCATTGAAAAAATCTGAGAGTGGACT

ATAAAATCATACGCA
CAAAACCAGTAAGGATGAATAAAATTTGAAGTGAATTAAGAATAA
TTTGATCCTCATTTG
CATGAAAAATGTGATCTTTCACTCAAGCAAATCATAGTTTATTAT
ATCAGCAATCAATTC
AGTAAATCTCTCACGAAATACTTAGAAGAGCGAGTTGATCAGTC
AATATGGATCAAGTAA
CAATCGAGATGATCCTGAACACCACAGAAGTAGCATTCCTCAAA
GATGCACCGCCACTGC
TGGAGTTCAGCACTTGGTACGAGCAGATCCACGGATACCTGGCCA
TTTGCGTCTGTCTTG
TCGGAATTGCAGCCAACGTCATGAACATCGTCGTACTGACCCGA
AAAAATATGATAACGC
CCACCAACTACATTCTGACGGCTCTGGCCGTAGCGGACATGTTGA
AAATGATTGCCTACC
TACCATTCGTCGTATATTTCTTTTGTTTAACTGGAATGGACCCCCT
CCTCTATGCCCACC
CTAAAGGATGGATATACTATCTTGTATTCCAAACAATGTTCAGTA
TCACTAGTCACACAG
TTGCTATGTG
>ConsensusfromContig3254 Average coverage: 16.79
AATTAGTGGGCGTGGCGTACTATGGTAAACAAAAGAAATAAAAA
GTTTCATATTATAGTC
TTTTTATATGGTTTAGCTCTTAATATACTCCATTTCATTCATTCTA
TATTGTGATCAGTA
GGATCTGCTGAGCAACACCAGTTAGTTAGAGTAGATTCGTGTCAG
TGTGTACCAAACTGC
ACAAGGTTCGACCTTCATTGAACCATCTGTGCAGTTTGTTCAATC
AGATACCAATGAGCG
GTAATGTTTGCGTGGATGAGATCGTCGCTTAAGCAATACTTTCCC
GCAAAGTCCAAGAAA
TGGGGCTATAAGTTGTATGTACTCGCTGATAGTTGCGGCATCGCA
TACAACTTCCGGGTG
CATGCTGGCAAAATCACAGCTGCACACGGATGGCCAGACATAGG
TGCAAG
>ConsensusfromContig3255 Average coverage: 41.38
AGTAAAGTTTGAACTTTTTCATAGATTGCATCATAACCTTATCTGT
TTCGATATCAACAT
CCTCTGAATGAAGAATTCCTTTCAGAGCATTATAAATTCTATGGC
GCATCAATGATGCTA
ATCTAATTGCGCAAAATTTATAATCCAAGCTGATGAAAAAGACTT
CTGTGTCTTGGCCCC

CCAAAACGACGCCACAATGGTACTGCGGTAGGATACATGCTGAC
AGGAGGTGAAGCAGAA
GCTGGGATTTTCCTGCTCCCTCTGGCCCACTTATCTCTACAAAACA
TTTGTCATGTAGAC
TATCCTTGAATATGTGTTGATCTATTAGCCTCAGTGAATGTCTTTT
GTTCAACCGAGTGA
AGAGCTGATATCCAGATTCGATTTCATACCGAAAATTTTTCATTAA
TTTTTTTCGTAAAC
ACGTTTGTGCTCCCATTCGAA
>ConsensusfromContig3256 Average coverage: 25.07
AAGCCGCTGACGACGGGTCTTCTCGATGCGATGCAACGGAATTC
TGTCGTGAGAAACCCG
TCAGCAATTGTGGATAAAGCGCTCTCAATTGATCTGAATCGCCGC
AAGAAGAAATCCCGT
GGCACGAAAAGAAAGAGAAAAGTGCGCCATCACAAAAAGTCCTC
TTCGCACAAATCACGA
AAAGATTCTCGAAAGAAGCAACGGAAAAGGGACAGGTCTTCCTC
TGTAACCTCTGAAAGT
CGAAGTCGGTCCGCGCGACGGAGATATCAACGGCGACGCAGAAC
GCCCTCTAGCGAGGAT
GACAGCAGCTGCCGATCGCGTTCTTCATCCCTTTCCTCATCCTAT
TCGTCCAGATCCAGA
AGGTCCAGCTCTAGTTCTAGCTTTTCGGGTTACGACCGGTCAAGG
TCACGAGACTACTAT
CGAAGTCCAGTGTCAAGCCGACGAAGTCGTAGCAGTGAGAGCGA
GGGAAGTCGACGTCGC
CAACGGAGACG
>ConsensusfromContig3257 Average coverage: 6.19
GAAACTTTTTCGAGTTGCCGTGAACTGGTGTAACCACCCCATTTT
GTTTAAAGTGGCACA
GCAATGTATAAAATCATGTCGAACTAACACAATGAATAGAACAG
AGATTTCCCAGCAAAT
GACATGACACAATGAATAGATGTTTCTTGACATGGCTAGGTACAT
CAACATCCTAATTAC
TCATTTTGGCTCATTATTTGTCGAAGGGTTGTTCGGGCATGGAAG
TGCCATGATTTACAG
CAATGTCAGGTCTTCTTTCGACATCTGCTATCACCAGCCCCAAAC
GTCATATTAATCTCT
GTGGTTGGAAGAATGGCCATCTGTTCGACCCGTGAAGGTTCAGC
TAAAAAAGAGACGAAC
CGATAAAGACTTTGAGATTGTTTTTTAAAATTATTTTTACTACTTC
AAAAACTCCACCAT

TATCGATTCCATCTTTAAACGGTGGACTTTTGTTAATAACTCCAGT
G
>ConsensusfromContig3258 Average coverage: 5.76
CTTCCGATCTGTCGCAACGGCGATGTAACCAGAGACTTGTGCAAC
GAGTTCTCTTCTTTT
GACTGTGCAGACTTTTGAACTGCTCTCATTTGAGCGGATTTAACG
CTACTACGTGATAAA
CTGATGTTAGATTGTGCAGAAGCCTGTCTTTGAGGAGATGGAACT
GGTCTAGTAACAGCG
GAGGATGGTTTGGGTGATCTCGATTTCTTATTCGCCTCGTTCAGTT
CATTTTGTAGGGAT
TCCTGATTATCTTGAAGCTGCATCAAAGTTTCTGCCATCTCTTGCA
GTCTCTGTTCGTAA
TGTTGCTGTAAAGTCTCCATCTTTGTATTCATTTCAACCCGAATCC
CGTCTATTTCTGTT
TTCGGAACCAATTCGTCCTCCTGCTTGTCTTTCTCTTTTGTCACTG
CCAATATCGGATAT
TGAA
>ConsensusfromContig3259 Average coverage: 18.02
CGCATATTATAAATTAAGTTTTACAAAAAAGTGTTTCATAAAATA
TACCAAATATGAACG
CCATTATGATTGAAAAGTATTACAGGATTCAGATCATAACTATGT
GACCCTAGTTTTGAA
ACCATAAACTAAATTTTCTACGCTTATTATCACAATTGCCTGAAT
TGATATACAAAATGT
CATGAATCATAAATAAGATTGCGAAATTTCACACGTCAGAACAAA
CTTTTGTGTCAGTAT
ATATATAGGAGAGCATTCAAAATTCTAACGACCATAACAGGATA
AAACTTGCATCGATAA
AAAATATGAATATGTATTTACACAAAATGAAATTTCCGTTTAAAA
TTACCCTAAATGATA
CATATTAGGAATTCAAAAATGCGCTTTAAGAC
>ConsensusfromContig3260 Average coverage: 14.08
GATGAAATCTGTTTTCTGTTGGTGATCCTTTCATTGTGTTGAATTC
ATTCGCATGTTCTC
GACGTTCAAGGTTATGACGTCAGAATTAACATATCAGAAAAATTT
CTTTTGCGCTCTTCT
TTCTAAATCACATCTCTTTTATTATTACAGTCCCCATATTCATCAA
TTCAGGATATACAT
ATTATCGCCAAAAATCTGTTTAGATATGCTTGTGATTTACATGCG
ATTTTGAAATGTTGT
GAAATTATGTCTCATACTTCGATGATTGTAATATATGACACCAA

>ConsensusfromContig3261 Average coverage: 8.99
CTCAACAAAAAAAAACCCTCCAAGCTAACTAATCCACCTCTCCTT
GCTATAACTGTTCGA
TGCTCCGGCAGCAGATATTATCAGGATCATGCTAGCTTCTCCTGA
CGCGAGGGTAACCAA
TGACATCAGAAGAGTGCATTGATGACTGAATTTCGCTGCGGAATA
TAACATATACAAACC
ATTTGTAGCTCAGGATTGTTCGATTTTGACAAGCTCTATTCGACT
GGAATAGGGGACTTC
TGGCAAGTTGTGTAATTTTCTTCATTCCAAATAAAGTATCCCAAG
TTATGCACAGAAAAT
ATTCCAGTGACATCGAGGGTAACTCAGGCTGACCACGGGGAGAG
CGACTGCAAACACAAA
ATTTCTGTTAATTATTCAAAAATGACATATTTTATTTTATATACAA
AATTATTGTCATGC
ATGGAACTACCTGTATAAAGACACTGAGCTCACTAAAC
>ConsensusfromContig3262 Average coverage: 5.68
TTGGGATTTTCAATACTCAATGAATTCGTTCATGAAAATCAGTAT
AATAAACTAAAAAAA
TGAAAATGGGATCCCAAATGGATCAAATCCCATCGAACACAAAA
GAGTTGATATAGGGGG
GTGACCTTTATGGTAATGTGAAATCTAATCAGGCTCTTCTTTAAT
GAGATTGAGTATCAG
AAATGATTGGAGAGTTGGATCTGAAATTGATCGAATTCTATCGAA
TCACTAATGCTTTGA
TGGAATCTTGTTTTTTGGTCACTTAAAAATCACATG
>ConsensusfromContig3263 Average coverage: 18.87
GAAAATTAATTTTTTTCATTGTATGATTTGATTTCAGTCCAGTCTC
TTTCAAAGCTTCAA
GTTTCAAACTCATCATTTCATTATAAATTTCAGCTACTTCGAATA
TTAGTTGCCGGCAAA
CTAATAAATAATGTTGAGGATTCAGGACATTCAACACATTGTCCA
ACAAATCAATTCTCC
GTTTATGCATTTTACATTGTCTGTCTATCTCACATTCAAAAAATGC
TAGATGCTTGAATG
CAGTGCTGTGATCCTGGCAAATTTCAATGTATTCGGTACAATATT
CATCTACAGTGAAGA
ATAATTTTGCATCATTGATTTGTTTTTGAATATATAAAAATACCTT
TTTTGCGGCTATGA
ATTCTGTCACATAGTCATATGAAATGGATTTCTCTATTTCTTCAAT
ATCCAAGCTGAATT
CTAATAATTCTTTACATTGAGTATTATCATCATTAGAATCACTTCC

ATTTTCATTATAAA
TTTTTGTTTTTGAGGTTTCTAGTAGTGATAAACCATACTTAACCCA
ACATCTATTTATAT
CTGCTAGAGTATGTGAAAACCGATATCTGAAATCCTTCTGTTCAT
CAGTATCTGTTTTCA
TGTC
>ConsensusfromContig3264 Average coverage: 28.27
TATTCTCTTCCAATTGACCCTTTTCGTGTATCTGTCCTAGCGCCTC
TGCCAGAACGCGGT
AGGCGCATGTCTTCCCCCCAAAGGGTTCTCCCACAATCATAAACC
CGTGCCGCACTATCA
TCATCTCGTAAATCTCTTGAATTTTCTTGAGGAAGAAATCCGTGC
ATTGAAGATTCATGA
TTTCGCATTGATTCACGATGGCTTCGTTGAGGATGTTGTAGTCGG
GTGTGGGGAGCGTGA
TCCCCGGAAATAGGTCAGAGGTTATGCCCTCGAAAAGGGGAAGG
TCGTGACTGAGGAACT
TGGGAAGGTTAACGTCGATGATGGACCTCAGCATGAGAATGTTTT
CGTCTTCGTC
>ConsensusfromContig3265 Average coverage: 13.91
ATGTCAAGCATCTGTTTTTGTCATTATTTATCCAATTAACGATCTT
TTCTGGGGCTTTGA
TGAGTTGGGGCTCAACAACATTCCCTGGATCACCGTCAAGAAGAT
CAAAGCTCAAATCGT
ATTGTAAATTCCTGCGATGTCTACCACTGCCGATAATTTGCGAAG
AGCAATCCAAAATGA
CGCTGCGGTAACCATCCGTCCTCATACTTTCTTCATTCGAAATCG
ACCAATCACGCGGTA
TCTCAAACAAATTGCGTATAACCAATGTTCCATCTGTCAGCATCA
TGATATCTCGATTAG
GCTGCAAGTCTGAATGTTTATTTTTCCAATTAAAGCCGTATTGGTA
ACTCTCCCTAATTA
AATGGCATGGTATAATCAGCTCCTGGCCCCATGTATAACGTCCCG
TACCTTTATCTTCGG
GTTTAGTGCCTATAAAGTTAACCGTTATTTTGTCCGTAATAAATGT
TTTTCCGCCGTTTT
CTGCACTGCACCAATATTCACGCGTTCCGCGTTCTACGTGTACTTT
CTTTCTCGATAACC
ACGGAAGCTCCAGGTCAAAAAAAACTTCCTTCCCCGACGGTCCTT
GTACAGAATTACTAG
TTTCCGGGTAAGCAATTTTTTCACGGCCTTCAACTGTTATTCCAA
CACCAGAAAGCAGCA

ACGTTGTTACTGATTCTGAAGGTGTTATATTACATGCGATCTTA
>ConsensusfromContig3266 Average coverage: 4.69
CATGAGAGAAATTGAGACATTGCGATAGTGATCAATCGAATCCTT
TGTTATGAGTCATAT
TAAGACAAATAAAATCAATTGCAGTTAATTGATTAATAAATTTCT
TGTATCATCATGAGA
GAAATTGAGACATTGCGATAGTGATCAATCGAATCCTTTGTTATG
AGTCAAATTAAGACA
AATAAAAT
>ConsensusfromContig3267 Average coverage: 4.99
CCCACATATGCCTAGGCTCCACCCTCTTTTGAGATTCTATCACATC
TATCTCCGCCCATA
CCCAAATAGAAAAGGTAGCGGTGAATAAGGAAAGCATAAAGCTG
TCATACCAGACCCAAT
TTTCCCTGAAATGCCAGCGCAGTCCAAATGTTGTAAGGTGCGAG
AAATACATGAGGAAAA
ACCCGAGCATATAGAGGGAAATCACGATTCCAATCCAAGTCCGG
TTGAATGGCGGGCCTC
TCTGATCTTTGTTCCTGTCGTCAAGGTAACTATCGAACATCAAAA
GTCCCCAAAACAAAA
TATACGAAGAGGTGAAGGTCATAAACTTGTGAACGGGGGTCGTGT
TTCTCTTGACGATAG
ACGAGCAAGGAGCAAAAATGTAGAGGAGA
>ConsensusfromContig3268 Average coverage: 16.87
AAATGTTTATTTACGCGGCACAGTTGGGAATCGCGAGGCAAAAC
AAGATGGAACCTGTAA
TACCCGAATCCCATGGGTTGAGTAAATGTTTCAATCTTGGTATGA
CTACTATGGGACCTG
AGATCAATGATATATCCACGATTGTTCTCAAAGGGATGCGACCTG
CTGCATACGACGAAC
AGTTCTCAAAATTGAATAATTCAAACAATATACAGATGCAAGG
>ConsensusfromContig3269 Average coverage: 8.88
CTTTGATTCTATGGCAGTGTTGTATTCCTTTTGAACAAACTCCAGT
TCCTTTCTGAGATG
GTCAATCGTATCGCGTAGTTCTCTCTCAGATAACCTTATTTTCTTC
ATTTCAGATTCTAG
TTTTTCTTTTTCTTCAAGCATTTCTTGAAGTATAGAATTTTTTTCG
GATGTTTCAGAAGC
CATAACTTCTTTGATCTTTGATTTCCAATGGGTCTCCTTTTGAACT
AATGCCGTCTCTGC
AGCTCTTTCAGAAATACGTCTTTCTTCATCGGCTTCAAGAGCCAG
GAGCTCTATCTGGC

>ConsensusfromContig3270 Average coverage: 7.17
CTCATCATAGGTTTAGCAATTGTAGCGTTTTTGATTATTATTCTTG
TGTCAGCCATCATA
TTTATACAATGTCGACAACGCCAAACTGCCCACAAACGCAATTAT
AATTTACGCATGGAG
GCTTCGAGAAGGGCAAATGGTGGAAATGAGATCCTGGATATTCA
TCAAAGCATCCCTGAC
CATTCTATTGACGAAAGTATGGTAACCAGTCCCCTGCATGGATCC
TGGGCAGACGTCAAC
GGTGTCGAGAGACCGCAGAAGAAAGCCGTGACATTCAGAATGGA
ACCAGCAACAGCTTCA
TTCAACGATTACGATGAGGGCTCATGTAACGGAGCCCCTGCATA
CCAGGAAGATGTTCGG
TGGAATGGCCGAGAACAAAGACCTCAGGAAATGCTGCAGCTGCT
TCCAGATGATTTGACC
TCAGAAGAAAGTGGGTCGTACACAGACTCCGGAAGGG
>ConsensusfromContig3271 Average coverage: 11.46
CTGGAGTGGCAGAGACTCGCTGGAGAACCAATGGCATCATTTGA
TAACGGGGAAATCGGA
CATTGAAGTAGAGAAGTGTTGCAGAAAGGGTCTTCCAAAGCTCTC
AGATGATCCTGTTGA
TGACCTCTGGGGTCTATGACATTTGGATTTATCAACTTGGTTCTG
TTTGGATGTAACAAA
AGTGCTTCTGTCCCTGATATTGTGTGGTATGAGCTGTGGGATGCG
ATTAGCCTCGAGCTC
TTTTTGCTGGTCACTCCAATATCGAGAGGTAGACTATTAGCGCGC
TTACGATCCAATCCA
CAATTCTGCATGTCCGCTCGGTTATTGTTATTTGATACAAAAACA
TCATTATTCAACAAA
TAACTCTGATTTTGCATCATCAGCTAGATATTGACTATGTTACCTC
CATCATATTGTTTA
CTTCAATAATTGTTTTCAGTTTTTCGAAAAACTGCTCGAATTCTT
ATTTGGAAAGCCAAG
ATC
>ConsensusfromContig3272 Average coverage: 7.87
TTCCGATCTGGGGAGATAATACACCCTTTGATAAATGATAGAAAC
CGTCAATCCATCTAT
CAGCAGCGTTCGAAATCTTCTCCGCATATGCATATAGACGAAATA
ATTCCATTCTTATAT
CTTTTCTTAAAAAATTCTGCATCTTAACGAGAAAAAAAGATCTTT
CGGTAAAGTCATCCT
TGAATTTCCCAAAATCTTTTTGCATACCTTCTATATCCCTCAGTAA

TTCACCAATTTTTT
TAACAGCGATTGTGCCTAAATTTTGAAGAGCATCCATTCTATTATT
TGTCGTTTGCATTA
TCTGTCCTAAATTATCACTGAGTGAAACTATACTTTCTGTGTTTG
AGTAAACACCCCCAG
CCAAAGTTTTTAAATTCCTTGAAAGCGCCTTCAGATCTCCAGCTC
CAGGTAAGCCAAACA
AACTACTACCAACTTCACCAATACTAGTATGCCATGAAGTTAAAC
CTTCTAGTTCAAGAT
CATCACTTTGGAGCCAATCTGGAAGATCTGCATTTCGCCGTCGTC
GTCTTTTTCGAAGAT
TATTTGTACTATTTTTAAATATTTCGTCATGCGCAAAAT
>ConsensusfromContig3273 Average coverage: 29.43
CACAAGTAGAAACAATATGACGTAATCATAACTGTGATGGTCAG
AATAAGTGAACTATTT
AGTTGCCAGATTACAAAACAATTTATAAAAAACTAGCTAATATA
AATAACATCACATGAA
AAATAACACAGGGCCTTCTTACACATTTGGCCAAAATTGTGAAAT
GGATTCAATAATATT
TTGAGATGAAATTCAACTGAAATCGACAAACAAATGAGATCTAC
TCGTGGAACAAATAAC
ATGTAATAAGCTTCAAAACATGAGTTTGAAATGCTTTATCGGATC
GACCAAGGTTCGTAC
CATGGATATCTCGGTCCAAGATATGTCTAGGATAGACAATCGAT
AATAACGAATTCGAAA
ACATCGAAGTCTAAGAACTACGCAAAACATTTTGAAAATAATTT
GCAAGAATAATTAAAT
TGAAACTTTGAGCCAAATATACAAAAACCCATAATTGCGTATTTC
ATAAAATCATCAAGC
TTTTGAAGACTCCCCCAAATAAACAATTCATGTTTTGGAAACGGA
AAGTGTTCATCAAG
>ConsensusfromContig3274 Average coverage: 15.94
GTCTATTCGAATATGATAGATAGATTTCTAAAATAATTTAAACGT
ATCGATCGAAGAAAG
CCTATTCCTGTTCCAAATGGAAAACGAATTTTCAATTCCTAATCC
GGTCCAAATTAAAAA
ATTCTACCGATCCGGTCCATGCTCAGCACTGATATCTAAAGCTCT
ACATCACACAGAGAC
ATCGGTGAAGTCTACAATCTGTAAAAATTGGTTATATAAAAATAT
TATGGCTGGATTTGA
GACATTATTTCATATCTATACAGATAATATATGTTTATCCATGAA
TAGAGCAGTAACACG

TTGCCGGTTTTATGATTGTTCCAGATGAACATGATTGATAGATGA
TGTAAGAGCGGATTT
GTTATAAAAACCGAAATGTTTGCCGATTTTTAGTTTCCGC
>ConsensusfromContig3275 Average coverage: 23.34
AGCCAAAATGTTTGAATACCCATAATCAAATAGTATAGCATAAAC
TGTTTTCGAAAATGT
TGGTCTTAATTTGTTCTCTGATGTGCTTGGTGAATATCGTGATGGG
TATTTTCCCAAGGA
TTCCAGTAAAGAGCATCAGGATAGAACCTCTTTCTGAGTCGCCCC
GCAAAGCAGTTTTGT
ACAATGACTCGTGTCCTGATGCAGGTGACACAAATCAGGAAATT
GGGAAAGTCAACATCA
CCATCACATTGGGGAACTACTCTTTGATACATCATTTCGAATTCG
TATCTCTGGGACATA
GAGCTTATTCGGTTTTTATCAACCGCGACAACAGAAAAACTTATA
TTGGTGCGACGCGAA
GAGCGTCTGGCAAACAACGTTTCACATTTTATGGAGATAGTAATC
ATTTCGTGAAAGA
>ConsensusfromContig3276 Average coverage: 9.77
GGTACACTCATTGAGGATAAACCTATTCCAACTCCGACTATAATC
CGTCCAATATATAGC
ATTATGACATTCAGAGCTGATGCCAGTAGTAGAGATCCAATTAGA
AAAAATATACTTGCA
ATGATGATGGTTGTTTTTCGACCTAAATTATCACTCATTAAACCT
CCAATAACTGCAAAT
AATGCAGCAGAACCAGCAGTTAAACTAACAAATACCTCTTGTTGG
AAATTTGATAGATTG
AAATAGCTCTTCAATAATAATGATGAACCAGAAACGACTCCAGTA
TCGTATCCAAATTGT
AAGCCTCCAATTGCTGAGATCGGAA
>ConsensusfromContig3277 Average coverage: 5.04
GACGTGTGCTCTTCCGATCTCTCGTGTCTTAACCATTCTCAGTTC
GCTGCGAAGTAACAA
AGTGATTAAAATAGGGAAAATATTATGAGTACTTTGAAAAATGTA
ATGATGGTGCTCTTC
CGATCTTAACGTCTGAATACATGGGTGTGGAGAATTTCATACACC
GTATCCATACATAAA
GGGCTGCAAGAAAGCACAACTCTGAGGGAAAAGCAATGTGTTCC
TTGAACTTCAAGAAAT
GTTATTTTGTTGGCGATGGTAAAGAAAATTGATGGTAAAAACAA
TGATGAATAGGGGACT
AATTGCGTCTCGGAGCTAGTTGGGCCAGCCCATATAATTCATGGA

ACCGATTATTATTTT
C
>ConsensusfromContig3278 Average coverage: 30.22
GCCACTCCCACAAACGAGTCATCGACAGGAATCGGTGGCCGTGC
CCCCCTGGGTACTAT
TGCCCCGAGCAAACGGAGGACCCTATTCCATGCCCAACGTCAAC
CTATCGGGATAGCATA
AGGGGAATAACCTCGATGGACTGCTCACCCTGCCCTCCTGGTCAG
TACTGCACCTCCCTG
GGTCTGTCCTCCGGAGAGGGGCCTTGTGATGCCGGGTTCTACTGT
CTGGAAGGAAACCAA
TTTCCGGACCCAGATGGATCTGATTCGAACTTGGGTGGTCCCTGC
CCTTCATCTCACTAC
TGTCCCCCTGGTACATCCGTTCCAGTACCTTGTCCTGCCGGTAGT
TACAGTAATCTGACT
GGACAAGCCAACTGTACCGATTGCCAAGCTGGTTTCTACTGTCCA
GGTGCAACATCGAGC
TTTGATGCCTTCCCCTGTCCAGTGGGATATTACTGTCCCCCGGGA
ACAACGTCCGCTTCA
GAATTCCCATGCCCCATTGGCCATTTCAGAAACATCACAAATGGA
ATGACCCAATCGGAT
TGCTTCGATTGCCCTGGTGGTTATTATTGCTCATCCGAAGGTCTC
AGCGCTCCAACTGGA
CTGTGTGACGAAGGCTACTACTGCGTTTCTGGGTCTCCAACCGCG
CAGCCCCGAGATCGG
AAGAGCACACGTCTGAACTCCAG
>ConsensusfromContig3279 Average coverage: 8.76
ATGGCAGCTGCAATCGTTGATGAGTTCCCAGAGTTGATGCGGCCT
CGCAGACAGATGCTG
ACCTTGGCTCTTTGCATCATCATGTACATCATGGGTATCCCCCTC
GTCACACAGGGAGGG
ATTTACGTTGTGACGCTGATGGATTGGTACACACTCCTATTTTCTT
TCTCAATCATCTCA
CTTTGTGAAATCATTGTCATAATTTACATATATGGGTACAATAAC
TTCAGTTCAGATATT
GAATTAATGATTGGGAAGAAACCTGGATTGTACTACAACGCCTGT
TGGATCGCGATATCG
CCTCTCGTCCTGGTTTGCGTGATAATCTTCTTGATCGTCAACTATA
TTCCGGCCTATTAC
GGTTCGTACCGGTACCCGGG
>ConsensusfromContig3280 Average coverage: 12.14
GGTTGAACTGCGTAAAGTTAAGGTATGAGAGTTAATGGATTTGAT

CACGATTTTGATATC
ACGGAGGAAAATCTAGCATTGAGTGGTATGGATTGAATAAATAAA
TATACATTTTTTAGG
GGTTTGTTCTTCTCGATACTCCATTATTTTGTCAGTATAACACAGC
ATTATAATATGAAT
GAGGGGCGCCATGGGAATTCCAGTATCAAGTCGGCATTCTTGTAC
AGACAAATTTTAACT
ACATTTTCACCCAATTCATATGATTTCTGTCGCTTCGTTTTTGATG
AATCGTTAATAGAA
CG
>ConsensusfromContig3281 Average coverage: 19.55
TTCAGACGTGTGCTCTTCCGATCTCGGCGATGATATAGCAATGGC
TGCAATGAGCGGCAA
TAAAGGCAGTCAAAAGAATGGTTCAATACCAATTGACTAATCAAT
GAGTGTATGGTCCTT
GAACCCAGGCGCGGAGGTCAGGGTCAGGCAGTTTCCCACAGTAAA
CTCTTTTGTTGTAGT
TTTCGTAATGTTTGATATCAAAAGCCCTGAAAACACGTTCGTAGT
CAAAGTCCGCATCTT
CACTATACGCACGAGAATACTTGTTTTCAGAAGAGGAAGCTCCG
AAGGTCTGTAGTAACG
CAAGCGGTTGAATCCATTCGATTCCGTCAGAATTCCGACAGATC
ACACGCGACAGAAGCG
TCTTCTTTATTTGAAGAATTTGCTTCGGTGTAAACCCCCCGTCCTT
CACCTCAGCCTCG
>ConsensusfromContig3282 Average coverage: 7.91
TCTTGGTTTGGTGGGTAGTGGTTAGGATAGTTGGGGCTCATTATG
TACCCGTCTTCCAGT
TTTCGTTGAATGTCACTGCAGATGTCTATCACCTTCTCGCGCTGA
ACACACTCGTACTCG
ACTTGAAGGTAATCGCTGAACTTCTGCCCCGAACAGTTGGGCATG
TAAGACTGCATGACT
GTCCTGATGCAGCTCTGATTTCCCATGCACGTGGACCAGTCGTAG
ACGAGGTTCATAGAG
TCGTCGATGCAATCTCCGGGACACTTCGTACAATTATTGAACAAA
TGACCAGGGCTGGGT
TCGCATCCGTCTGTCGCCGAGGACTGAGGGTCAGCCTCTTTTCCA
TTGGATGCGATTCCG
TGAAACGCCCGCCGAATTTTGACCATCTCGTCGTAATCACATCGG
AAATTGACGGTAGGG
ATCCCCGGACTTTTGAAGCACTGACTTGATTTTCGAATTTTTGAAA
CTGTATCGTATTGT

GCCGATATTTGTGAATAGCAGACGACCAGGACAAGGAGGCCAAA
CACTACCTCCCAACCC
TTCATTGTGTTTTTCAGTAAAAAAAACTTTTAAAGTTTGTTTATCT
TTGAAAGAAATTAT
TTTTATTCCAG
>ConsensusfromContig3283 Average coverage: 9.13
CCAAAACAAATTGAGTTGTTCATTATTGCCCATCATTGTTATCTTC
CATCATGTTCGTTC
CCTTCTGATTCATTTTGTTTTGAATAATTTTGAAGACTTCAAATCC
AAGTTTTACGTCTC
AAAAAGTTTCATATGTAAACAACATTTTATGTTCCATGTTCAACC
GATTATTGCGTTCCT
ACGAGCAGAATATATTGCCAATTGTACGTTGTAGTAATATATTAT
ATTTTGTTTTATATA
GCCTAGATATAGTTATCAATGCACTATATCGACTCTATAAGCAGT
ATTAGATCACAATAT
ATGACAATGGCTCAAATAGTCATATATCTTGTTTT
>ConsensusfromContig3284 Average coverage: 7.66
GACGTGTGCTCTTCCGATCTGGCGCATACAGTTAGATCACTGGCG
TCCTTGCAGAGATTC
CGAAAGTAGTTCGGGCCCTTTCTGACCTTGTGCAGGACCAGCTGA
CTCCAAATCAAGGAG
CGGTACTGCGGGGGAATGCCGAGTCTCAAGACGTGCTTCAGGTCT
TTCTTCAAAATAAGT
TCTCGTTGGCCTTGACTGATGATATTTTTCCAGCATCGCTCATTCT
CTGGGCAATTGAGA
CTTTGCGTGAGATAAAAGTGATAAAGTTGTTGACATATGAAATG
GAGCAGCAGATCATTG
TTCTTGATATTATTCCGGAATCCGTAATGATCAACATGAACGTCG
CTTCTCAACAACTTG
TCGTATGAAGGTAACGTTGGATTTACGGAGCGTGCCTCTCCAAGT
AATTGCCGAATTTTC
TCTTCATAATTGCTATGTCCACTGATTCGGAATTCTCTTCCATCC
GGGTCGCCGGAGCTG
GGAAGTGTCACGCAGTTTTGAAGGAGATAGACGTAATCTTTTT
>ConsensusfromContig3285 Average coverage: 42.80
ATTTCTCGGTTGTCTCAGATACGTATAAGAAGACTGTACATATTG
TACGTTTTGATTCAA
AATTGATCACGATGAATAAAATCGAATGAGATTGAAGATTTATCA
TTTCTCGGTTGTCTC
AGATACGAATCCGTGCCAGCTCGAATCATAATCCCATTGACCATT
TTCCAGTGCTAAATC

CATCTGTTCCACAACTGCTTGTGCCTCTCCATTGAAGATACCTTTA
TATATGTAACCATT
AATAAACATTTCATATCGTTGAAATCTTGAGATGAGTGAATCAAG
GTTGAACGTGGCGTT
CTTCGATATATATAAGAGAAATCGATCATATGTGAAAGTCATGCC
ACATTTCACATTATC
ACTCCGTGTTTTTATTTCTTGATACAATTCCGTTCTTGAAAGATCT
CCGTTATTGTCAGT
ATCCATTCCACCACAGTAGGCTCTCAGTTCATCAGCGTCAACTAG
ACGATCTCCTGAAGT
ATCCATTTTTATGAATTCCGCGTCTCTTTCAGTTAAAAAGCTGCTT
TGATCAATTGCAGA
GCAGTTTAATGATGGAACATCGGCGTTCGTTAGTACAAAGAGAA
AAGCCG
>ConsensusfromContig3286 Average coverage: 13.07
AGATTCTGAACATTTACGTTGTCTGCAATCGGCACCACCCCATTC
ATCGTGACACTTGCA
TTCATTGAGAAAATAATTCCCTTCGCATTCAATTGTGTTTGGTCCG
CATTCAGCGTTGTC
CCAGCATGTTCGATGTGAATTATTTTCTACGGTTCCACAAAGGGC
GCCTGACCATCCATT
GAGGCATAGGCAGTGTTTACTTTTGTCGTTGTCTATGCATTGTAG
CGCACGTCCAAGTTT
GCAGTCGCCGCTACAAGTCAGTGTTTGACACTTGACGTTGCCCCA
ATCAGTCATGCATTT
TTCACACTCAGTGCCTTGCCAGTTTATATCGCAAACACATGTATTG
TGATCATTTTCTCC
GCACTTTTTTGTCCCATATTTGCACTTCTTGTTTGTACCGCATGTA
CGATCTGCATCACC
CTTCTTTCGGATTGCACAATCGGCGCCTTCCCATCCAGCGAGGCA
GTTGCAGTCTCCAGC
AACATCTATTTTGCATCCGTCTGCGTGTCCAGC
>ConsensusfromContig3287 Average coverage: 10.64
CACAATCTATACGAGTTGTTTTTCTGAAATCAACGCAGTCCATGT
TGAAAGTAATTTCTA
TGAATAACCTTCAAAATCATCCTAAATTCGTATCAATAGTTTGGC
GAATAATATGTCCCA
ATTTGATATGGCTGATGAGCGCAGAAACATTCGATAAAAAGACA
TACACAAAAACGTGTA
ATCTTTCCACGCCACTGCAAACGGCTGTGAGCGAAAGCGACGAAA
CTTCATCGGTTGTTG
AGAGCGTCGGGCAGGGAGCGCGGCTGTCGGGAAATTTCTCAATT

TCCGAAGAACGGCATA
CAGTGGATCTGGTGTACAGCATTGTCGTGGATCTGGTGATTCTTG
TCGGCCATCTGTATT
CCTTGACGCCAGCCCTGCAGTCGGTTCTTCATCGAATGCTGCTGTT
CCCTCCTCCGTCTC
ACCGAGCAGATGCTGTAAAATCAATTGAATGTCTTTTATCGTCAC
CAGACTGTTTGCTCC
CCATCATAAGTCCACCATTAATTGATGAAATCAATTTAAGTGATA
GTGAAAGGGCTAATC
TTAAAAGTTGTGACAGAAGTAAACGAGTTGAAG
>ConsensusfromContig3288 Average coverage: 4.55
CCACGAATCAGAAAGGGGAGACAGGAATGTACCACGCCCTCTGA
GAAAGTAAATGTATAT
ACCATTCTTGTGTGTCTGAACAAGAACCACGAATCAGAAAGGGG
AGACAGGAATGTACCA
CGCCCTCTGAGAAAGTAAATGTATATACCATTCTTGTGTGTCTGA
ACAAGAACCACGAAT
CAGAAAGGGGAGACAGGAATGTACCACAACCTCTG
>ConsensusfromContig3289 Average coverage: 12.68
GATCTAGAAGATCCATCTCCCCGCACTCATATTGCAGCATATGTT
ATTCCAACAGAGTGA
AGAAAATGGATTGCAACTACATGAAACAGTCGAAATCGGATGAG
GAACTGGAAGAGTTTC
GTGCCATTTTAGAGCGGATGCCTAATTACGGAGTCACTCCAAATC
CTTCTCAAATCTATC
CTGGAGTTTATCTGGGCACATGCGCCAATGCATTGAATGGTCGTC
TGCTCGAAAAATTCG
GAATTACACATGTTCTCAATTGTGCAGGTCAAGCAGCTCTGAGGT
ACGTTCATCAGGAGC
GTTTCGGTCTGACGAACGTCACATACGAGGAAGTGCCAGCTGAG
GACGGAGAGAGCTATG
ACATTCTACATCTTCTTTCGAAAACAAACAGATACGTGGATATTT
CACGAAAACGGGGCG
GGAAAGTGCTTATCTACTGCACTGACGTTAACCGAAGCAGCGCCA
TTGCCATCGGATTTC
TTCTCAGCAAAGGCGTGTCTCTTATGACATCAGCAAAAATGCTG
AAGGAAAAACGAGTTG
CAGTTCTCTACAACAGAGGCTTCATGCGTCAATTATACCAGAAG
GCAAGAGACTATGGGC
TTCTACGAAATGACATCCCTCACATACAATC
>ConsensusfromContig3290 Average coverage: 16.68
TCTACACCGTGTTTAATATATAATCAATAATGAATGTGCTCCAAC

AACTAATTATTGACG
AATACCAATCAGATATAAAAGATGATAATGCATGTGAATGCAAA
TCGATCGGAGCGATTA
TACGAACATCAAAAATGCGAACATTTATCACAGACACAATCATT
GATATGACAAATACCA
AATAATGTTCCCAAAACAAAGCGAGAATGAGTGAACAAAGCACG
CTAATAACCATAAGAG
TACTTACAAAAACATAATCAAACAATGAAAAAATAATAATTCAG
CAAAATAATCTCTGAA
TACAATATCAAACAGATCGGA
>ConsensusfromContig3291 Average coverage: 19.79
TATTTTATGTAACATGAAGCAAAATAAAGTGGTTTATCATTTCTA
TGATTCCATATTGTG
TCATATTATGACGAAGTATTCTTCAGTACCTTTTGCATAGACATC
TAGATTTATCCAGAG
GTCCATGACCTTTTGACCTGAATCAGTTTTTCTCTTACGAAATGT
AGGTGCTTCAGCAAA
TATGAGATGATGCTCACCATACCCTACCTGCTCTTATAAAATAAG
TCTGGTATTTCGATT
TTGGTATTCCAATTCGATATTTGTAGATGCTTTCAAAAACTAAAC
ATTGATGATAAGCCT
TCATTTCCGAGAGAAAGGCTACAGAGCGATACACCATTTTTTAGA
TCCCAAAACTGAAAG
TTATCAATTTAATTTTCTATCTGAAATGCATAACTCGCGAATGATT
TATATTTTTAGTCA
TTCTTTTTTTATTAGCACG
>ConsensusfromContig3292 Average coverage: 20.47
TTCAGACGTGTGCTCTTCCGATCTCGATGACGAGACTCAATCTAG
TACAGAAGATGGCTT
TGTTATTGGATCTGGGAATAAATCTGGTCAAATATCTCAAAGCGA
TCAAGATGATTCAGT
GACAATGCAACAGGTTCTGCTCATCGTTTGTCTTGGTGTTGGGAT
ACCCATACTAATACT
CATAGTCATCATCATTATTCTGCTGTTGAGTCGACAAAAACGTGG
ATCAACCGCGCCAAT
AGATCGATGCAAGCCAATCGGGGAGGTTCAAGACTTTCAGCGAG
AAATGCAAGACAACAG
CACGAATGAAGATCTCAGAGTCAACAATAAAAACAAGTGCTTGG
ATGTCTCCAACAATAA
GTATATGTCTGGTCAAGAAAATAGCTCAATCCCTACTCAACCAGA
TCGGAAAGCGTCGTG
TAGGGA

>ConsensusfromContig3293 Average coverage: 31.85
AATTGAGAGCTTTGATGTTGTGGATGCTCAGCTCGAATTTACTGA
CGAAACTCAATGCCA
AGATTCAAATATAGACATCATGAGAACATCTGTTGATTCTTTGGA
ATTTGAAATTATTGG
TAAAACAGATTGCTGTATGGAAAAATCTGCAGATTCCTTGGAGCT
AGATCGTGCTCTCGC
AGAATCCCAATCGCAGGAAAGTCTCGATTGTGACTCATTAAGAGA
TGATGACAACTTCCA
CAGCTCCACTCCTGGGGAAGATATGGACATCAAATGTGAAACTGT
CCCGGCAGCAATGTT
CATGTCAATGGAATCGGGGGCATGGAGTCAAACAGGATCGACGC
TCTCACAAGACACCAT
GAGGTCGAGCGGAGAAAGTGAATCTGAAGTGACGGGAGATCCCG
ATGATACCTCTATAAG
CGAAATGCCAAACATTATGAGGGATTTAACAAGTATTAGAGATG
ACGATCTTGAAAGTTC
ATCTTTCAACACCTCTACAAAAAGAGACGAGTTTACATTTGAAAG
TGATGGGAATACTGA
AATCTCCGCTTTAAAATCCGAACGTTATGACGCTGAAGGAAACA
TAATCACAGAGAAATC
TACATCCGAAAGTTATGCTGAAGAAATCCTCAACGACAATACGG
TGTGCCGAACACAAAC
ATTCCAATCCTCATCAAAATCATCTGAGCAATTCATGTCCGAACA
TTCCTCCATGTCCGA
AACAAGAAAGACAATTTCGAAACCCGATCCCCTTGTGTTGGGGG
ATCTGAATCTGGGAAC
GGCTGCTTCGCCCGACTCTCCAGTTCCAGATTCCACATCATCTGAT
CTTTCTCATTCGGA
CGATTGCTACTG
>ConsensusfromContig3294 Average coverage: 18.23
TGTTTATAAGCCAAATTCCAGCCACATACACCTGTGCAAAGTCCA
TGTCCTGTAAGCTGA
TATCCCGTGTAACAGCTGCAGGTGTAGCTGCCTTCAGTGTTGCTA
CAGGAGTGCTGGCAC
GGGGATGGGATGAGCAGGCACTCGTCATCATCTTCACATCTGCCA
TTTTCTTTGAGCGTG
AAACCGGCATCGCATGCACAAGAGTAGCTTCCGACGCTGTTAAT
GCAGATGTGCTGGCAA
ACTGAAGGAGTCAGCGAGCATTC
>ConsensusfromContig3295 Average coverage: 12.09
TGGGAATGCGTGTGTGTTCGATGGATCTCTCGACTCTTACATAGA

CCTAGGGCAACATGA
GGACACGGTACTCGGTGGCGAACACATTACAGACTCGGATTTAG
AGATAAGTGTGTGGTT
CAAGACCGATCCAAAGCAAACCGGATGGGATTTCATTCTTGGGA
GTGGACAGTACTCGAG
GAGTATGAATGTTGGTGATGGTTATTTATATTCGAATTCTACAC
CATGAGCGAGGGGTG
CTTGAAACACCTGGCTTACGCAACCCGTTTATCTAGAGGGGAGT
GGCATCACGCAAAGGG
TATTTTTGACAGCCATGCAGTGGAAGCTCGCCTCTAC
>ConsensusfromContig3296 Average coverage: 12.62
ATAAAAGGATTCATGCTGGTGTGAGACCATTTCGCTGCGATGAAT
GTGGAAATACTTTTG
CACTCAAATCAGATTTAGCTCGACATAAACCTATTCACTCTACTG
AAAAACCGTTCACTT
GTGATGACTGCGGCAGGTCCTTCGCGCAATCTTCCTATCTAATCA
AACATAAATTGGTGC
ATGATGGTGGTAAACCATACACATGTGGCATATGTGGGAAATCAT
TCTCTTATTCTTCTC
ATTTGAAGACGCATATGAAAACTCATTCGGGCGAAAAACCACAC
CCTTGTGAAGAATGTG
GGAGATCTTTTCGGCAGTTTTCTGCGTTGAAAAAACATAAACGAA
TTCACACAGGCGAAA
GACCATACTCTTGCAATGAGTGTGGAAAGTCCTTCACGCAATCAT
CGAGTCTGAATGTAC
ATTCAGTAGTTCATACTGGCAAAAAATCATTTGTCTGTAATGAAT
GTGGAGAGACTTTTT
CACGTCCCTATCTTCTAACATCGCATAAGAAGATTCACACTGAGA
AACCTGTCGAATGAA
ATCAATATTACAAATATCATGAACAACTGTGTATTACCTCAATCA
CTCATCCAGAGAATT
TTCGAATAAGAAAAACATATTTACAATGGATGTCAATTATTTTA
CTATTGATGGATATT
TGATGAGAGCCATGAGTCGTCGAG
>ConsensusfromContig3297 Average coverage: 8.69
CACAGAGTGGCATTTTCTGGGCGTCACCTGGGATTCAGATAGGA
AAGAGATGCGGCCTTA
CATCAACGGAAATGAGTTTATTCCCTCTTCGCAAGGATATTCCGA
ATCGGATGTGTTCCC
GAATTCTTTGGATTCTTCGGATCACCAAAATCTGATGCTTGGACC
CGAATCGGCGGTCAG
TGGCCTCACTGTGAGCATGGACTCTATTACCATATATGGGTTCAA

GGCAAATCAAGAGTT
CATGACGAAATATGACAAGTACCACAACCCAGAGCCTGTGACGA
CGTTCAACTTCAATTA
TCCAGCCTTCGTATCGTCCCAACTCGATTCTCACAATTTCCCAAA
TGGTTATTCCGAAGC
AGAAGGTATTTTGGTTGGTGCTGTACTCTTCGATGGCAGTCAAGG
CCTTCAAATTGATCA
TTCCAGTTTCTGTTTTCCATCCAACCTCTGCTCTCTCGGCTTCACA
TTCAACCTCTGGAT
CAAAACTACCGCAACTGGAACACAGATCTTTAGACAATTGGCTTT
TGC
>ConsensusfromContig3298 Average coverage: 9.45
TGCACTGTTCAGAGCCATCCAATTTATGTTTTCAGTACACAAATT
GTTTGAACGAATCCC
AAAACTACCAGAATTCTTCGGAATTTTCAACTTGGAAATCGCCGT
ATTCTTCTCAAAGGT
TCAATCCGAGTTGGTACAGAAGCCTCAATACGAAACCTCCTGTAC
CGAGTCGAAATGTGC
TGCTGTCTCCAAGAAACCAGCACAGGTGTAGATACGCAATGTTCT
GTCACGATGAGGTAC
CTGAGCCAGAGATGGATTCAGATTCAGATGTTTCAGATTTCAAAA
ACTTCTTGCAGGAAA
ATTTGGATGAATTGGGGATTGTGTATGAAAATG
>ConsensusfromContig3299 Average coverage: 5.11
GAGAGATCATACAATGCGAGATTTATACGGACAAGAATGACCAT
TGAACGGTCATTCGGA
ATTTTAAAAACTCGTTTCAGGGTACTTCTACGCATCAATGGAGGC
ATCCGAATGAAGCCG
AAAAATGCTGCCCTGGTCACATGCGCTGCTGTTATTTTACATAAC
ATATGTGTAAATAAC
AATGTTCCACCAGTGGATGACTTGATGGTGGAAGAGCCTGATGT
TGACAATGAAGAACAA
ATAATGGTCGAGGCTAATGGTGCCGAAACCAGAACCCAACTTATT
CAATCGAGATTTTAA
AGATATCGTCATACTTTGTGCATGAGTTTGTTCTATGCAGTAATT
TATAAACAAAACAAA
ATTTATTTCTCGTTCATTTTGCAAAGTTCAAATTTTATGCCGCACA
TA
>ConsensusfromContig3300 Average coverage: 10.65
CGCGTATGAAAGAGTAAATCCATAATCTCGTTTCCAGCATGGCTC
GTCACATTCTTTTAA
TGAGGATTGTTCGGAATAATCAATCGTGTCTGTGAAAGCAGCCAG

GCATTCAAAATATTC
ATCAATGGTGCAGTAAGGGGGGTCAGTCGGATTGTTACCAGGCA
TATAAGAATCTCTGCA
TCCACAGTTTCTCATTGATATTAATGTTTCAATTTCCCTGCGGCAC
CCGCTGTACGTGTA
GGTATCGCTGAAATTGAGAGTTCTTTCAGGATCGCAGACACCCCG
TGGCTTTGGCAAGTT
GATTACCTTTAACAACAATCACAC
>ConsensusfromContig3301 Average coverage: 9.05
CGGCTATGGAGCAGGCGGGGCTAGAGACGAGAGCCCAATGAGCA
TCTTCGAAATGCTATT
CTTCGCTCTGTTTGGATTGGTTGACCCAGAAAACTTGCCACCGAT
CAATCGAGCCCCGCT
GTGGAGCATTTCATTGGCTAAAGTAGTCTTCGCTTTCTATATGAT
CATAACAATCATTGT
TCTGATCAATCTCCTGATCGCTATGATGTCCGACACGTACCAAAG
AATTCAGGCGGAATC
GGATACAGAGTGGAAATTTGGAAGGGCAAAATTGTTCAGGAATA
TGAACAAAACGTCCGC
AGCGCCACCTCCACTGAATCTCTTCACCAAACTGTACATCTATCT
GGCGATCCTCTGCAA
GCACGGCTCGAAAGCATGTGCAATAAATATCCAGAAATACATTG
ACGAAGAAGAACAACT
GAATTTTGATGGAGATGAAGAGATCGTTGGAGACAA
>ConsensusfromContig3302 Average coverage: 11.17
ATAACACTGAATTCTAATGAATCATGACCCTTACAGACTATATTC
ATTAAAATTCACAAT
CAGAATTTATTTTCATCACGTTCTTTAATGCGTTTTCTCATTGATC
CACATCACGAGTAA
ATATATTTCTACAACTTTTCAACCAATTCATGAAAATCCGCACATT
TTTATGACCTCTAT
GCAGACCTCATATGGATGCAGATATTTATTTTATATGTGAATAAT
CAAAAACATTCCTGA
GAACGTCCAGGAATTCT
>ConsensusfromContig3303 Average coverage: 13.03
ATAGGCGAAAACCGTCGCGTTGAAAATTTTGATTGATCGATCTTG
CTTCACAATAATGGA
TTCACCATCACCCTGGATTCATCCCGGATATGGAAGTATACCATC
CGCACTTCAGACGAA
CAACTCTCATGTTCCGATCTTACGTCAAAGGAGTTCGTACGGTGA
TGAGCACATCCCATC
ACCATTCGTATATCCTTCAGCCTATCCTGTCCTTAATTTTATGTGT

GCACAGAACCAATC
ACTCGAATCACAGATCCCTCTTACACAACAGACTACAAGCGATTA
TGAACCAGCGTATGA
>ConsensusfromContig3304 Average coverage: 5.60
CTTTTGACCTCAATATCTGTGATTGGCGGATTTTTGTTTGGGTAC
GATACGGGCGTGGTT
TCTGGAGCGATGCTTTTACTGCGCGAAGAGTTTTCCCTGAGTTCG
ACATGGCAGGAGATT
ACTGTTGGCATCACCATCGCTTTTGCTGCTTTCTTTGCCATTGCCT
CAGGACCATTCAAC
AACATAACAGGACGAAGACCTGTGATCATCATTTCAAGCTTCATG
TTTTTCACGGCGAGT
ATTGTGCTCGCATGTACCAGGAACAAAGCAATGCTTGTTCTGGG
CAGAGCGGCGGTGGGG
GGGGGCGCTGTGTGTTCTTGTGTTAAGATTGCACCCTGCACGAA
>ConsensusfromContig3305 Average coverage: 25.51
CGCATCATTCGACTTCTTCTAATCTTTATTAAACTAAGGAATTATC
ACAATGAATCTTCC
ACTATCATCTCAGTCATCTGGCTCAAAGCTCCAAAATTTGTCTCC
AGATTATTTATAATC
CGCCTTATCAAGCTCGGCCCTATTCTTTTTAAACCCAGAACTTAT
CAAAACTCCTCCATA
GATCCTTCGCTATCATCCCAGTCACATGGCTCAATCGATGCTCCA
AAATCCTTCTCCACA
TCATATTGAAAATCCGCATCACCCGGTTTCACCCGATTTTCTTCAA
ACTCTTTCCCCATA
GCTTCTTTCTTCAGATTCAACTCATCGTCATCCAGTTTATTTAGGT
CATCTTCTGGATCC
AAACTGACCGCATCAACGTTATCATCAATATCAGATATTTCTTCT
GCTATGGAATCTTCA
ACTGCATCTTCCCTATCGAATATTTTCCGAAACACAATCGGCTCT
TTTTCGAGAATACTA
TTTGTTTTACTGGAAGCGCTTTCTGTTTCAATTTGATGTGCTTGTC
GGTTTCTTGAGAGG
CTAAGTTTCAGCGAAATACCCTTTGATGTGTCAACAAGAATCCTG
ACGAGTTTTTCCAGC
TGCTGCAGCGACATCTTGTCAAGACAAAAACCATGCCGCTTACTC
GACTTGAGCTGGTTC
ACGACTTCGGAGACTGATGTGGAGTCTTCGATGTTTCTTACGGGC
ATTATCCGACGATGA
ATCTTGCCGGATGCTGTGTCTTCATAGGCAACGACAATGGCTGGA
GGTTTCATCCGAATG

CCACAGCGTCGTAGGGATACGTCCGTTCTCATTCTACTGATGAAA
TATCATGATAATAAT
AAGATACACGTCATAATTTTAAATAGTTCATTTCTAATGGTCTGCT
AATCATTCTTTTCA
GCATCTCACATTCCTTCAA
>ConsensusfromContig3306 Average coverage: 5.71
AGCCGGCTTTCAACGGAAGATCAAAGGGTTTCGGAATTCTCCCAT
AAGATGGCGGTGGCT
CCGAAGCAAATTCGCGTCCAGATTGACCACCAGATATAAAAGGT
GCTCTAGATGGAAATT
TCCCGCCGAGTCGGGAATTGCTCAAGTGAAGGTCGACATCAAGG
GTCAAGTTCACCAGTC
CTTTCTTCCGACGCCTACGCAGGCGTTGGGCGGAACTTGCTGAAG
ACATTCGATATTCCT
GAGTTTCACATTATTTCGTCAACTGATTCCCAATCACAAGCTGGA
TATAATTACACCGGC
GAGATC
>ConsensusfromContig3307 Average coverage: 7.91
TCAGGATAACAATCCCATTTCCTTTGTCACATTTTAGAACTTTAAT
ATTCTGATCTTTTT
TGAGGCCTTGTAGAGTTTTATGTAGGCTTCGATTCTGCTTTGTGTT
GATTTCGTTTTCTG
CCGAGTGTATGAAAGAATATGCCAAGTATCTCCATTCTTCCAGAA
ACTGTATTTCTGTTG
TTTTGTCCAGATTGACGTTGTGCGCAAATTTCTCAAATTCAGCCTT
TATTTTGAGCGTAT
TTAATTTGTATGGTAAGATTGAATTTTTCAGTCCTAATCTGAGGG
CATCCAGCTCTTCGA
TTGAGAGAACATGATCACTTTTATTCATCACACATTCTGGTGATT
TAATTCGCTGATCCA
AC
>ConsensusfromContig3308 Average coverage: 4.79
ACTCAACGGACTTTCTGCGATGGGACACAATCTAGATCGTGCAG
CGCCGAACAATGGAAT
AGGAGCTGAGATATTGAAATGTACGATTTGTGAAGAGAAACTGG
AAGATACGCATTTCGT
ACAATGTCCCTCTGTTACCGATCACAAGTTCTGCTTTCCCTGTTC
AAGGCACAGCATCCG
GCAGCAGGGTGCTGGGACGGAGGTGTACTGCCCCAGCGGCAAGA
AGTGTCCTCTCGTAGG
GTCGAACCTTCCGTGGGCTTTCATGAAAGGCGAGATCGAAACGA
TTTTGGGGGGCGAAGA

TCACAAAGAAGTTGCCATCAAGAAAGAGTGCGAGACATGATCTTT
GATCTTCTGTTC
>ConsensusfromContig3309 Average coverage: 22.92
GAAGCGAGTAAGTAGTCTTACTGATGTTACGTTCGGATTGGAGAA
TAGTAACTGTTCTAT
AACTGGTATAAATGATGGCTCTGAAAGTCATAATACCTCTGATAC
CAGTAATAGTAGTCG
AAATGACTCTTCTGATTATCCATCTTTCTCGTTAAATCCGACGAG
AATTCTGAAAGATAC
TGAAGATGACTGTGTATGCTCTTCTAAACAAAGTTCGAGGATAGT
GCCTAATGGTTCGTG
CCATTCATCGAATTGTCGATCAATAATACATGATTGCGGTGATGA
TAATTTTGATTGTGA
ATGTGATGGACAAGAATGTTCTTCTAAATCTATTTATTTGAATAA
CGAACATTGTCCATA
TACTGAAATAAAATGCCACAATATTTCTACAAGAAATGACAATAC
TGATAATTGGTGTGA
TGATACGAATGGTAAGCATATTGTTGATTGTTTGCCACAAAAACC
AAAACAATTCACGGC
GAATTCGAACCAGTGTCGTATGTCATATGAACACAATGTATCGA
ATGATATGCAACGAAA
AATTCTTCGAAATGATAATGAATCAAAATTCGAAAATAGCTCAGA
TGTAAATAATTCAGG
TTTACGTAACAGCGT
>ConsensusfromContig3310 Average coverage: 16.74
CTTCTAGCCTCATAACCTCTCATCAACTCCTTTTCCTTCTCCTTCA
ACACGAGTTTTTCG
AGATACCTCATCCATTTCTCCTCTTCGAACTCAGATTCCCTGGCA
GGTTCCTCGGCGAAA
ACGTTCTTGTGGTACATTTCCTCGTGTCGCACCAACTCGCCGAAA
ACGGCAGAGGAAAAT
CCGCAAAAGTGACAATCGTGGATCTCTGGTTTCTCATCGTTTGGA
TCCGGCTTCACTTCA
GGAACCTCGCTCTCTGAATCCGAGCCGCTGTCCGGATCACAATAA
CCAATCGTATCTTCT
TCTGAAGAAACGCTCTCCATAGCCTCGTAGAGTTCTTTGAATTTTC
TCCTCTTCGTATTC
AACATCTGAAGTTCATTATCGATGACGCCGAAGGGAATAAGGGT
CAGATCCGTCTGAATG
CCTACTTCCCCGGTTGAATCACCAGCAGAGCGGCCCTTGTCG
>ConsensusfromContig3311 Average coverage: 8.53
TATGCATTTCCACCATCACTCTAACAATACAGTACATCTTTTATTA

AATCTACAGCTTTT
ATCTTTTTTAATTCTTTAACAATATTAAATATTCCGAAATTAAAAT
TACCTAAAATCTAA
GATTCGCCCATAAAAAATTTATGCATTTCATACACAGAAGAACAT
TCCCAGACATAATTA
TCTAATACAAAAGTAGGTTCACATAAAAAATGTTATAATAATGTT
TTAATAGATGGTTAG
AAACTAGAATC
>ConsensusfromContig3312 Average coverage: 18.29
TAAAATTCCTCTCACATGAATGTGGATACATTATCTACAGATCAT
TGTTTCTAAAATGCT
GGTCGGTGAAACTCTGAAGGATTTTATATCAAAAATGTTTTTTG
TTCACCAATTGAAAG
AAAGTCTTACACAAAAAATCTGAAAGCTCACTATGGATGCATAAA
TGCAATTGAGTTTTC
TCCAAATGGTGGCGAGAACATTGCTTCTGGTGGTGATGATAACCG
TTTGCTTGTTTGGAA
CACCGCTGCTTGCCTCAGCAACAAACCGTCTTTTGATCAAATGAA
ACACAAACATAACAG
TAATATATTTTGTGTTGGTTTTGATTGCCAAATGCGATGCCTGTT
ATCCGGAGGAAATGA
CGACCAGGTTCTCATCCACGACCTCAAAACAGGCGAACTCATCG
ACGTCGTAGGGCATGA
AAACAACGTTTTTGAAATATCCTGTCACCCGACGGCCTCTGACAT
CTACGCCACTGCCGG
GGACGATGGGCGGGCGCTTATCTTCGACCGACGCGCCGCCTACGA
TGATGTCTTAGTGTT
GTCTAACAGCTTTGGCTCCATGCAGGCCGTGGCCTTCAACCCTGC
AGATCGGAAG
>ConsensusfromContig3313 Average coverage: 6.44
TCAAGATCAATTTGTAGTGTTTACTAGAAATATGATAGGACCTAT
TGTTAGTGTCAACTT
CACAGTTAAGAGGAATGGACTTAAAAACAATTGGAATCTGAAAA
CTGTTTGCGTATATGA
TATGGTGGACAAAACAGAGTGCAAATTTCAACAAGAAGGAACAA
TTATTTTGCCCACAGG
AACAAATTCAGTTTCCGTCAAAGTTACAAATAAAATGAAAAAAA
GAAATGAATTAAACAC
GATTTTGATGGAAAACTTCAGAACGTCTCATTTCCTAATTTCCATT
TTTGATTCCCTCTG
CAATGAAGTTAATTTGCGGTGCAAAAATATATCGGCTTTGTTTGT
CGCGATCCTGGTTAC

CATGATCATTAATATTATGGAGTTTGGTTTGCCAGCACAAGTGTT
TCTGAAAATTTGCGT
ATCCTTCTTTTGCATGAATGCAGCAACATACCTGCCGAGCTTGATT
TTTGGTTTAGTCTC
ACAAACAGTGATACTTAACAAATCGAAAGCTAAATTGGAAAACA
ACATAT
>ConsensusfromContig3314 Average coverage: 11.78
CAGCCACGTTGTCAACCTTGTTTTAAGTGTATTGAAGACGTCTTT
CGTCTTTTCGATGG
CCTTGTTTTTAATAGTCGCCACGCCATACTTCAAAAGTTCTTTACC
ACCTTCTTTCAGGA
GTTTAGAACCAGCAATCGAGGCAGCCGCTTTCGAACCTGTTGTTA
AGAGCCCTTTCACGA
CAGTTCCAGCAGCTGTTGAAGCTACTTTAGTTACAGTTGTTG
>ConsensusfromContig3315 Average coverage: 6.34
TCGAGTAGATGTTCAATGGAATGAAATTCATGTTCATTTGCCGTA
TAAACTTTGAGATAA
TTTTAAAACAATGCGGTGAAAGTTAAAAATAGCTGATCATTAAGA
TTCGTTACATCCAAA
ACAGATATGTTGTGACAAAGTATAACATTTCGATATCAAAAAGAG
ATAAAGATCTTTTTT
CATTGTGGCTAGATATCAAGTGCAACCCATTATCTCACTTCTCAA
CATTATGACCATATG
TATTTGTGATGTAAC
>ConsensusfromContig3316 Average coverage: 46.54
CTCGGATTCTGACGATGCTAGATGAATTATGTCATGACTAGAAAG
TCCAGAAGCTGTGAA
TTGCGGTGCCATGTGACGCCTGCGGTTGTTTCTGGGCGACGAGTG
GTTAGAGTCGCTGGC
ATTAGACCGCTGGCTACTGGAGCCGTGGCCGACCGTCGGGACTT
GCGGCGGATGAATAAA
AGGTTTCATGGGGATCCCGCCACTGATCGGATGAATATGATGACG
ATTTCGATGCTGCTG
GGAATGTAGGTAGGATTGGTCAAGCATCACATCTTGCGGAGAGT
GAGA
>ConsensusfromContig3317 Average coverage: 16.70
TGAAAAGTAAATAAATTTATACAAATATAAATCATGGATGATTCT
GCAATTATGCAAGCC
AGATGGGTGGCTGGGTACCAAGGAGAACCAGTATCTTTTATTGAT
GACAATACATATATG
TACAAATGTGGAAACACAATCAAGTTTGGAGAAAAAGCAGAAGA
TTCAGAATACATATAT

GCACCATCAATTGATGTTCTCGGAGTTCTCGCCGTTCACCCAACC
AAAAACATATTTGCT
TTCAGCAATGTTGAACCAAAGCCAACAATATTCATTTATAAATTT
CCTGAAATGGAAAAA
GTGAACTCTTTGGTTGATAGTGCTGAACTAGAATACAAGCATTTA
CGATTCGCAAAAGGC
GATATAATGATGAGTTTATCTGGAATGCCTGAATACACTTTAAGT
ATTTGGAATTACATG
ACAGACACGGTA
>ConsensusfromContig3318 Average coverage: 6.63
GTGACGATATATAATGAAAAATATACTAATATGTAGATAATCTTA
CAGTAAGTTCAGATT
CGTCCGTGCGTAAATGCTAGGCTCCCGATCACGTTGGATCTTTTA
ATCAAAATTGTTCGA
TGCCTTCCCTGGGCTTGTCTAAGATCTTACTGCCTTGTTCGCCTGT
CTTTTCTGGACTCG
CCCGAATGGTGTCACCCAATGGCTACTTGAAGCTCAGTCAAGCAA
GACCTCCCCAGACCG
GGTTAAGATAAGGCTCTGAAGCCTCGTTATTTCCCAG
>ConsensusfromContig3319 Average coverage: 6.64
AAAGTTTCGGGAGGGAAAACGATACCAATGAATTGCAACGCCTTT
TTCATTTACTCAAAT
AGCATAAGGACAATGATCGCTGTTCTCCAGGCTAGACCAAGATGT
TTTACTATCGTTCAA
AAGTTTCGGGAGGGAAAACGATACCAATGAATTGCAACGCCTTTT
TCATTTACTCAAATA
GCATAAGGACAATGATCGCTGT
>ConsensusfromContig3320 Average coverage: 5.00
CTCGATATTACTGGTAATACCATGGGCGGCACTGCCAATACTTGG
GAGTTCTTTTCCGGT
GTCAACTTCAATGCATGGGTGAACATCTTGGTATACAAGCATAGC
TGGAATCACCGCTGG
GAGATCTGGCATGCGAGCCCAGTTGTATTTTAGACATGTGCCATC
TATTCGGTGGTGTTC
ATATCGAGTAGTCAGTGTCGCTGTTGTTTTGTTAAGCAGCTTACG
GTCTACTATAGAACT
TCCACACTTGATACGTTAGGTTGCTTATCCACCATGTACATTAAT
GATACGGCTGGGAGC
GCAATATTGGCG
>ConsensusfromContig3321 Average coverage: 8.72
GTAACAATCAGTATGCCGTTGAAACACATGGATGAAATGATTCCT
ATGAATTCCGATCAT

AATTCATTCGATAACTTACTGATTGTTTAAAAAACGAAGAGCTCC
TTCCCATGGCGAGCA
TCAAAGCGAAACGATGAATCGGGTCAGACATTTCCACTTCGCCGC
TGTCCACCATTGCAT
TTATAGCATCTTTCAGGGGGTAGTTGACCTGGTTATTTACCTCTG
GCCACATTCGCTCAA
CACGATGATTTTGCAATGACATCACTATCCAACGGAAGTAGGGAT
GCGCGGCCATTCACT
GCCTTCTTTTTTTCTCTCAGGGATCAGCTGGTGTGCATAAAAAAA
AAAAAACGGAAG
>ConsensusfromContig3322 Average coverage: 12.81
GTATATTCTCCCTTTCTCATATTTTAAATCAAATGTATGACAGCA
TGCTAAAATTCAGAT
AATGATGGACATGATAATGCAAAGAAATATCATACTTAATCGAAT
CACAGTCTAATACAT
CATTTTCGTCATGAGCAAGCCTATATATATCAAGAAAACAAAGGA
GAAGATTTCCAAATT
CAAACAAACAGCTCATAGCTATGATGCTATTTTTCATTCATATAG
CTTTTTGAATGATAA
TGAAGAAATCTT
>ConsensusfromContig3323 Average coverage: 7.74
AAAGAATAGAGCAACAAATCGTATAGATAGACTGAGAAATATCT
GGCTTGAAAAGCTCTG
TAGGGCAGCCATGATTTGACAATTGGAGCCGGTTACGAAGGCTG
GACAATAACAGATGGG
TTGTCCCATTCGATCAAGGGCACATACGCCACTGCCGCAGTCTGT
TTCGATACAGGGGTG
GGTCGGCCTTGTGCAGAGGGCCCCGTCAAAGGCATCCGGACAGA
CGCAGAAAAATCTATC
CTTTTGATCATTCACGCAATATCCGTTTTTGCATGGTGAGCTGAA
GCAGGGATCGGGGTC
ATCCGTGCAGAATCTTCCCTGAAAGCCATCTACACAAGCGCAGA
TCGGAAGAGCA
>ConsensusfromContig3324 Average coverage: 5.24
CGAAAGGGGGTTTCCATTTTCCGTTTGAGTCACATTCGTGGGCTTG
ATTATTTTTCCAGT
GATTTTTTAAATGAAAGCCCGAGTTGCATTTAAATGTGAATTTAT
CCTTGAAGACTTTGT
TTTCAACTAAGCTTGGCTGAGCATTAGTAATAGTAATGTCAGGAC
AGGTTACTTTGCTGT
CACACGAAGATCCTTCCCATCCCATAGTGCAGGTACAAACATACT
TGTCGACCACATCTT

TACATGGTGCTCCATTTTTGCATGGGGACGATGCACATTCATTTA
TCCTCGTTGGAAATG
ACTGGCAGAAGTAGAAGTGGTTTTCA
>ConsensusfromContig3325 Average coverage: 13.54
ATCGATTTTGACAAATAAATTATACTGATAATAATATATAAATAT
CAGTTATGAATCAAA
TCTTGACCTGAAATCTCCTGAAATTGAAGGTAAGCATGAGATATC
ATCCAGTTGCTCTTT
TTTATTGTAACATTTGGACACTGTATTCGCAGATGCTGTCATATT
CTTCAAAATGTATGA
TAAACCTTTGATGTGATATCGAAACATTTTCAAAGCCTCCTCACC
ATCCATGATTGGTAT
CTCTTCTGGTTCATTATCGTAGTCCACATTTTCATCATCATCAGA
CACAGGACCACGGGT
CAATTCATATTTAACATCACACAATAGCTCATAATTTGTACATAA
ATCATTGTGTGGAAG
AATATTATTAGTTGAAGATCCTGAACATGAACCGTATGAAGAAAC
AGACATATCATCTCC
ATTTTCACTTGAAAAAGAACTTGAAGAATGAATACTTGTCATTGG
CTTTTTAGAACATTT
ACCTGTGGTATTCGTCTCAAAACTTGCTTGGTCATTTGTAGCAGA
TGACTCCACAATAAA
TTCAATATCTTGAAGCCGGCATGGTATCATAACAGTGCTCTTCAT
ATTATCAACAGCATT
AGAAAATTCACTCATTGAAGATAAAAGCGATTTTTTTCCACATTC
TTGTCTGAATTTTAT
TGTATTATTCATGATCCCGACCATTTGCGAATAGTCAAATGAATA
GTTTCGAAATTAATT
TTG
>ConsensusfromContig3326 Average coverage: 9.84
TGGTTATGTTGTTATGCATTTATCATGTGTTCATTATCTACAGCGA
TGTCTCACTCCTTA
GATCTGTTTTCGTTCATCTATATCGCCAATGTCGGGGTCAGGTGGT
ATCACTTCTCAATT
GCAGTTGTTGGTGCGTTTAGGTCAAACTGCTGATTACCACCAACA
CAAGAAATCACCATA
ATTATATGATTCGAAAGTTTGTGACTTTGGGAGCTCCACAAGTGT
GGAAACCCACATCTT
TTTTATCCATTCGACAGAAATAGCGTCTTATAAGTAGTCAAACCC
CCTGCCATCTAATTC
GCTATTTTGGAGTTATTTGTATTTCATCTTATTAGCACATTTCACT
CTGTCTATGAAATG

TGTGCCCCGGAA
>ConsensusfromContig3327 Average coverage: 5.72
GTCCACTCTCTGATAGTGTTGTTTTATGATATTTCGATAATATAAG
ATGCTTTTAAACGT
TGGATTTTTGTTTTTGTGTTTCTTACGATTTTGTATTCCCATAACG
GATGAAGAGATACA
TAGAAATGTTGCTTTTGCCCATGCCAACATAGGTTCCTATTTGAA
ATATTATGCTAAAGA
AAAGGGTTTGAAACCAATTCCGACTGACTACACCCAAAAAGTAA
GGGTCGGAGGGGTTGA
AGGCGTTCGAGGGGAAGATCTTGAAGAGCTGGATCAAAGTGAAG
TGGTCAAAGTATGCGG
ATTCAAAAACCCCAAAGAAACAATAGAGGGAGTAGTGATTGGAG
GCCGAATCGGAATTGA
AATCATGGGAAATAAAAACGGTTTTTTGATGGGTTTCTTTTCGAA
TGATCATGCTGCGAT
GGAGAAACGCATAGTTAAAAGAGCTCACACTCATGATTGGGGCC
AAGATGTCAGGTATGA
CTTCACACATGATGGTATGCAAATTGTTATCATTGAGCTCTCGAA
ACAAGAGATACAAGA
CTTTTTGAATGATGAATGGTACTATGTGGAGTGGGTCTATGGCA
AGAAGATCGGAAGACG
>ConsensusfromContig3328 Average coverage: 7.72
CACATATTGCTTTAAAACAGAAACAAAACAATGGCATAATTCGT
AACCACACAAAATGTT
ATACATTCAATAATTTAACAATAAATAAAGCATTAATTGAAATG
GCCTAGGAGAAAAGAT
GAAGCATCACATTGTTCATCATAACACACCAGTTTGATTGAAAG
CGATAAACATAACAAA
AGAATAGACTAGTAAGATGAAAAACTGACAATTTTCAAACATAAT
TGTATGATACATGTC
CGTACATATTCAGTAGAAGGATAATAAGGTTACACGTTTTCAAAT

>ConsensusfromContig3329 Average coverage: 10.04
CCGATCTATTGGTCACATTTTCGGGAATTGATGACTAAAGATTTA
TCAACCCTATGTCCC
ATGTTGAACTGTCCGGACGATATTGATAGAGCTGTTCAAAAGGTC
ACAGATATTACATGT
TCAAATCTTGATAAAACCTGTCCATTGCGCAGAAAAATCTCAAAT
GAGTTTTACAAACCC
TGGTGGTCACCTAAATTGTCTTCCCTCAGAGCAACAGTTAACTAC
AGACGGAAAATTTAT

CAACGTGATAGAGCTGATATAAACCACACGCGATACAAAGATGC
ACGAAATATATATTTG
ACCGAAATAAAAGCTTCAAAGCAAAGAAATTGGTACAATTTCTG
TGAAAATGCTAGCAGT
GACCCGTGGAATAGCATTTACAAAATTATGAAGTCAAATAGCAA
AATCGAGTTACCAAAT
CATATAATTAAATCGGATGGTCTTTATACAGAATCGAACGAAGA
AGCCGCGAAGTATATA
GCCGATTCATTCACATTTCAAGACGATTCCCTTTTAGCTCATCAC
AATCTTCTAGAGCCA
CAGGACGTGGATAGCAATAATCATATCCCGTTTATTAGTATACAT
GAAAATGAAATTTTT
TGGTCAGTTTCAAGTTTTGCACCTCTAAAAAGCTCCGGGTATGAC
GGGATTCGCCCCATT
GTTATTCAATCAGTTACAGATATAATGATGCCAATAATTAGATCA
ATATATA
>ConsensusfromContig3330 Average coverage: 5.84
CACGACGCTCTTCCGATCTCTCCCATTTCATCGTTTTGATCAATGA
ATATTTCACTCGTG
TTCATTTCGATTTTTGATTTATACTTCCTTCTTTTTTGAGGTGTTC
TAAAACATGCTGCT
TTCCCAATAGCCAGAATTGCAGCATTTTCTTGCCTAAATGGCATG
GCCGGTTTGAGTGCT
TTTTGCGTAACAGACTCTTCAACCTCCTTCACTTGAACTGGAATT
ATACTATCAGCAAAA
GTAGGCTTCACCGCTTTCATGATTGGAGTAGTTTCTTCAACTTGCA
TTATTGGTTCTTCA
CTGCTGCCATCTTCAGCAAAATACACTTGACTTGAAGAAATCTCA
ATAAAATCGGAGTTA
ATATTTGCATGTTGGAAAGATTTGTAAATATCTTGTAGAGCCACT
TGCGTATTTCTGCTG
TGGGTCTTGACATGTATTTGTAAATATCTTGTAGAGACACTTGCG
TATTTCTGCTGTGGG
TCTTGACATGTATTTGTGTTTCGACTTGTTTGGTATTCACCAATGT
CTGTGCGGCCTGGG
TGTGAACCTCAAGAAATGTTTGAGTTGTACAATCCGATAGATCTT
GACACACCTGTACAG
CAGTCTCAATTTTTGATGAAATCTCAGTTTCTT
>ConsensusfromContig3331 Average coverage: 57.81
CTTTTGGGCGAATGCAAATGCAAGCAAAACAATGATACAATGTG
AAGAAAGTGAATTAAA
ATCTTCTTCATTTATCGATTCTTTCAAATACTATAAGTCCAAAATA

TCGAGGCCATCTCA
CGATCATGTTGTCAAATTTTATGAAGATGTGAATAACTGCAAGAG
ACTTCCGTTTCACCA
ACCGAAATGCAAACTTTCGTGTGTTCGAGATCCAAGTCTCTGGGA
TATTTTTGAGCTCTA
TGAGCGACCTGGTCTCATATTCATTCAAAATCCATTTTTACACGGT
GCTTCTCTGTATTG
GCTGAATCGGTCATTGTGGGATTTCCCAGTTCACCCGCATCCAAC
AAACCTTTGTGAATT
CAACCCCGCTGATGCATGGCGAAATTTCGATCGCTTTAGACTATG
TGAGGCAAATGTCGT
GAAAAATTTGGACCTTTATAAATTGAGATGGACTACACTTGGTTA
TCATCATGACTGGAA
TACAAAGGTGTACGATGAGTCGATGAAATCTCCCTTTCCACGCGA
TGTCCGAACGCTGTG
CTCATATATAACCCAAGCGCTTGGATTTGCCAATTTCGAAGCACA
GGCTGCTATTGTGAA
CTACTATCACATGAATTCAACCCTGGCTCCACACGTGGACCACTC
TGAGCCTTTCATGGA
TGCCCCTCTGCTCTCAATTAGTTTCGGACAGCGTGCCATTTTCCA
ACTCGGCGGCGAATC
ACGTGAGGAGCCAGCAACGTCGATATTCGTACGCAGCG
>ConsensusfromContig3332 Average coverage: 7.21
GTGAGGGGTGCGGTGTTTATGCGATCTGCTCGAATGGGATCATGT
ATGACAGCAGACCCT
GTGGTGCTGGTCTTAAGTGGGACGATGATGCGAAGCGATGCATGT
GGACTTCTGGAACTT
GCGGAGATTCTGATTACTGAGAAGGTGGCGCCACCGAAAAGAGA
AGAATATAGATGGCAT
CATTGATTTGTTTTTCTAAGATTTCATCATTCTGATCAGTACGTAA
CGCACAATATATTC
AACAAATTAAGACTTAAGTTGCACCTTTGATCACACAAAATGTTC

>ConsensusfromContig3333 Average coverage: 16.20
GAATGTTCGATATCTGTGTATTATCGCCAACCCCAACAGGGCCTG
ACCCAGGTAAATACA
TTCCCAGCTGGATGCATCGATTCAGTCTGCAGAACTGGCAGTGAG
TTCGGGTGAATGTCG
TTATTTCGCACTTTTGAGAGCGATGACAAACAAGTTGATCGGCGA
TGTTTTGTTTGAGGC
AACGAACGAAGAACTTCTTGCATGCCTCGCAGACATAAGCGCCG
CAATGAAAGCCCGCCG

CGGAGTCGAGGCAAACCTGGCAAGCAACGCCGCTTTTACCCTCCG
TCGTGTGCGTGGAAC
TCGTGGCCTGAGTGACTGCGATTTCCGAGATAGAACCAGGGGGAT
G
>ConsensusfromContig3334 Average coverage: 12.13
ATTGCAAAATGGCTTCTGAACTCAATGTTGAGATGCTGCTCGCGG
TTCTTCTCTTGATAT
CTGAAAATTAAATTAGGCAATTGTCTGAAAAATTTATTCATATAA
ATGGAAATCGGAGTG
CTCACAGGATAAAAGCTTCACACATGACTACTTTGAATTATTCCTT
CTTCCCATATTTTG
TTGTATTTTACTCTAGGACTGTTGGAGCTTGTGGAATTAACATAA
AAATTAAAAAACAAG
TAAACAAATGAATGAGTCCGAATTGTAAAACATGTTTGTAAAATG
ATAATTAAATTTTAG
TCATTTTCTTCACAATGACAGCTATTGCCACCATGCATTCGAGCA
AAGTATTTGATGATG
CAGGTAATAATGGCACGTCGTATAAACGGGATATGTTACTTCGG
GCTCTTAAAAAAGAGG
TGAAGCAGATCATGGAGGAAGCTGTAACTCGCAAATTCGTTCATG
AGGATTCTAGCACTA
TAACGTCGCTTTGTGGTGCCATTGAGGCGTGTATCCTTGACGGAC
TGAAACGGCGTTCCC
TTGGGCTGTTTTTCCGAGGTGGTGCTGAGAACGGGGGCCAGGTGG
CATCCAGTTGCGCCC
TCTTTCATAAGATCTCCAAGAATTGCCCGGAGGCTGCTGTCACAC
TGAAGGGAATGCAGC
GGATACTGAAGGACATCGAGCCTCATCAGACACTACGATTATCTC
TTGAGAGTCTGACTC
GAGGAAGCTTGAACCGGTCGCCTCTATCAGCAACCCACTCGAAC
GGGTCAACCGAAGGCC
AGGGCAGCAGATCGGAGAG
>ConsensusfromContig3335 Average coverage: 6.41
CGATCATATCGAAGGCTTTTGAAAGATCAATAAAAATTGCAATAG
GTATTGATTTATTTT
TGATTTTTTCGGTTACGCAATCAACCAAATGAAGTGCTGCATGTT
CAGTTGAATGGGCAG
CTCTGAACCCGTACTGATAGGGAAATAAAATTTTATATTTTTCTA
TATGTGCACATAATT
GGTTTTGCATAGCTCTTTCAACGATCTTTGATAAAATAGGTAATA
TCGAAATTGGCCTAT
AATTTTTTATTAATTTAGGATCCCCTTTTTTAAAAACTGGTTTAAC

TCTGGCCACTTTCA
TCCCGTCAGGAAACAGTCCTGAATTGAGAATTTGATTTATCAGTA
GTGTTAATGGCGTGG
ACAGTAGATTTTTAGCCGTTTTCAAAAGCTTGATATTGAAATTAC
C
>ConsensusfromContig3336 Average coverage: 16.33
CTTGCTTATGTGATAATTTATGCTAAATTTTATGGATGATTCTCTT
CCTAGCCAATGACG
AAAACAGGATGTTGTCCGGAAAACAATCATCTTCGCTCTCTATGG
GGGTGTCCCCCAACC
TCTCAGACAAGTTTTCTTACTAGATCCATGATCTGTATCAAACCA
GTTTTGTAATCAAGC
TGTCTTCAAAGTGGACTGTCTCTCATAACCATCGAATTCCATCTA
GACGAGTACGCCGGG
GACTGGATGAGGAACTGCCTGAATTGA
>ConsensusfromContig3337 Average coverage: 12.39
GCTCTTCCGATCTGAAATACTGACAGAATGGAAGGCGAATTCCTG
TCAATATGATGTCAG
GCTCTTCAAAAGCGATTTTCCGTTCTCCGATGTGGTGGATTGGAA
AACTGTACATGAAAC
TGTCCATAAGCAAGAGTCTGATCTCATGATGGGCTCTATATCGGT
CGATGTAGGGAATTT
GGAGTTAAAAAACGTATCCAGCCAAGATGGAAGAAATGAAACAA
AAAGTTGTTTCGATTT
CTCTCTGCATAGTCTCTCGAATCTACCTGATGTGGTCTCATTCGT
GCAGGTTCGCGGTAG
GATTTTAAGGATTGGAAAGATGGACAGTATCGAAGACTTCCGTGT
CCTTCCAATCCTCAA
TAGCAATCACAAGAATCCAAGATTCGAATTCAAAATTCGAATCA
ATGATAAAGAACAACA
AGCTGACCGCGCTATGGTTGTTTTTCTACGTGTCTACGTCATGAC
GCATAGCTCTGAGAT
ATTCGTATTGGGCAACTGTCTTTTCAACTTTTTCACAACTAATGTG
AGTTGAAAGTTATT
GCAGGAACAAATTTATATATCATGAGTATATAGGGAATTTCTTTG
AAAATTT
>ConsensusfromContig3338 Average coverage: 19.75
GGTATAATGAATATTGAAAGTGGCCCACCTTTTCCAAGTTTTTCA
GGAGTCAAGACGGAG
TTAGCTACTCCGCAAGATTCACCTCTCGAAACTATATGTGCGACG
AATGTCTCTTCGGTT
TTCCATCAATCACAGATATATGAGTGTACGAGATGCCCAAAAAAG

TATTACTTCAAAACT
GATCTTGAAAGACATGCGAAAAAGCATGTACGATGCGATGTATG
TAATATTGAGTTCCAG
TCTAGATGGCAGTTGAAGAATCATGCAAGGAAACATTCAGAGGA
AAGACCTTATGTATGC
GATGAATGTCAAATTGCTTTCAAAGAATTGAGCAGTCTCCAACGA
CATAAAAAAGCTCAT
AGCTCCGGCAAAAAAAAAGTTTGTTTGCGAAGAATGTCCTGGTT
CATTCACAACAGAGCG
TGATCTAGATAACCATAAAGCCACACATTCTGAAGAAAAACCTC
ATACGTGTAGCAAATG
TGTCAAGTCGTTCAATTCCAAGTCCGCTCTCAGTCGCCACATGCA
CACGCACACAAATGA
GAAGCATCGTATGTGCGAAATCTGCCAAAAAACCCTCTCGAGCCC
ATTGCTGTTGCGAGA
CCATCTCCGCATACACACTGGCGAGAAACCTTACTCTTGCGATCA
ATGCGGAAAATCCTT
CGTGTCTTCGGCTTCACTTCGATACCACAAAAGTACACATTCGGA
AGTGAAAAAGCACGT
GTGTGATGTTTGTGAAAAGGGTTTTCATTCCACCTATAAGCTCAA
TATTCATAGGAGAGT
ACATAACAAATTGACAG
>ConsensusfromContig3339 Average coverage: 13.63
TTCGAGTGATGTTATTCCCGTTTAGGACTTGTGTATTATTTTCTCG
GTAGATGTCTTCAG
AATTCCTCATATCGGATTTCAAATTAAATGCACCTTCGTTTACAG
TTAGAGCTAGTCCAA
CAGTCCTTAACTTTGCATCATTAATCCCAAGGTTTGATGTGACAG
CGATCTCAAATTGAA
GTCCAACGCCTAAAGAAAGCACATTGGCTGATCCGCCATTGGATT
TATTGCTATTATTTT
TGATAACTTTCGCTTCGCATCCTGCGACATCTACATACAGTCTAG
ATGATTTGGAGGAGT
TGTGCCATTTGCACTTTGTGTCCCTCAGAGATATGTGAGTCATGC
ACGAGTGGTGAGGCT
TCCTGAGTGAAATGACTTGAAGCGAAGATCCGCATATTTCTATGT
ACTGTGTGAGACGAA
TATAAAAGGGAATTCTTTTTCCATCTTTTGCGACATAATCATATA
ACATACTAGAAAATT
TCCAGGAGGGTTTATCAGGATCACCAGGGTCTGTTCTGTTTTTAA
AATCTTCATTGTTAA
AATCTAACTGAATCCGTAGATTTTCGAAGCTCAATATCACAGGTT

TTTTCGAATCTCCAG
GAAAGCACTTTTGGAATGATATGCATACATTTTCGAAAGCTATCG
AGAACCTATTTTCAA
TTGAAAACGACAAATATCTGAGTCTTGTTAGAGATTCTATATCAA
TTTTCAGTCTCAATT
>ConsensusfromContig3340 Average coverage: 9.43
AGATTATTTTCCAAACAAAATCACAAGAAAAACACAATTGATACT
TTTGAGATCTTTTTT
GTGAGTTTTGAGGAACATAAATGAGTTTTGAGGAATGCGACAAC
AAACAGGGCACGAATA
AACAGCAGTCAACAGAATAGAATAAGAACAGTTTATATTTTTTCA
CGATATCACATGAGA
GTTCCACCATTCCAGAAAATGAACGACCCCGTAATAAAGGCTGG
CGGCTACGGGCCTGTG
AAGTCGGTTGGGTCGAATATTCTCCAAATGTCAACTGGGACCAAT
TATTTTGATGACAGT
TTTGAGCATCAC
>ConsensusfromContig3341 Average coverage: 22.03
CCAGTTATCAGACCTTCCAAACACTTCTCGGCAGAGTACAGCACG
CCTTCAACCCACGCT
GCACTCAGCGTCATAATTCCATCATCACTGGTCTACGCTTTCTCC
ACGCAATACCAAGTT
TCGTTCGTGAGTTTTGTGCCTCTTATCATCATCTGGAGTGTGATC
GTCAGCTTGAGTAGA
GTCTACATGGGAATGCACACATTCTTCGATATTTTAACAGGAATT
ATAGGCGGGCTGATT
TTCATCTCTCTCTACTGGATGTTACCTGTTCTAGAGATCATTTTTT
GTGGAATGGGGAGC
TATTCTCCGTTGATTGTGATGATGAGCACGGCTTTCGGATGCTTC
ATCTATCCGCAACCG
CCTGAACCTAACCCGGCTCGGAAAGATGCTATGGCCGTTCTCGCA
CTGCTCGCCAGTATC
TTGATTCAATCTTATTTGCACATACATGTATTTCATGTTGTTAAA
TACCCAACAGATAAA
CGACTACCGGTCAGCATTATCACACAAATACAGCTCAAATATTTG
TTGATTAGAATTTGT
ACGGGACTCTTAGTTTATACAACGATCTCGGAAATATT
>ConsensusfromContig3342 Average coverage: 16.33
TTCATGTATAAAGTTGTCCTATAACGATATAAAAAAATAAATGGC
CTTATATTATTTCAG
TTCATAATTGATTCTGATATTCTAGATTCACAACCTGAATTAATT
CTGAGGTAGATAATG

AAAACAAATATCATTCCCCTTCATTTTAATGGATTCACAGTCCAT
GGAAAAAAAATAATGA
CAGTGTGACGAAAATATGAATTTCTCCCTTTTTTCATTTCTAAAT
ATTGGGAAGCGAATT
TTTAATAAATAGCAGTTAACAACAGATAATTTTAAATATGGCTGT
GTAACATATATGATG
ATATATGAGAGGCTGAAAGAAGGAATGTTTATTTCAAAATCGAAT
CATAATTTTTGTAAA
GTTCATCACAGTTAATAATATATGCCCAATTTGTTTTTTTCGACA
TGCTCTCAGGAATC
CAACAGATATAATGGTTGGAAGTGCTCTTGAGAAACAACT
>ConsensusfromContig3343 Average coverage: 12.76
GTCGCCATATTTTCTGACCGACGAATCAAAATGATGCATGAAATT
CTCAACTCGATTCAC
GTCATCAAGATGTATTGCTGGGAGAAGCTGTACACGGCGGCCGT
GGGAAAGGCGCGCAGA
GATGAATATAACTCACTCAGAAAGAAGATGTTTCTGTCAACATCC
TCCGCATCCCTGGGT
TTTGTTGGGCAAAAGTGGATGCTGGGAGCCATGCTAGTAGTCGAA
GTGGGATTTTGTCAA
AGACCTCTACCGCCCCCTTCAGTCGTTTTTTCCACCCTGGCCATTT
GTGCCGTTGTGAAA
ATGAGCGTTATGATGAGGCTTCATCGCGCTTTGCTATTTG
>ConsensusfromContig3344 Average coverage: 2.29
CCCTTTTCGGGTCTCGGGATTGATTGATCCATCTTGTTCATTCGAA
ATCGTGTTATTATT
CTAACGCTTACTCTACTTGGTCTGAGATTTTCATTCAGGATCTTCC
CTTTTCGGGTCTCG
GGATTTTCATTCAGGATCTTCCCTTTTCGGGTCTCGGGATTGATTG
ATCCATCTTGTTCA
TTCGAAATCGTGTTATTAT
>ConsensusfromContig3345 Average coverage: 24.23
TCGTCGGCCCCCGCAACTGCAAACCCTCTCGTGTACGCGTTCGAG
GCCAGCCTCCGATAC
TTGGTGCCCTCTACATCTTCAGCTGCCTCCAGAAAAATTTCATAA
ACAATTTCGGGGTCA
TTTCGATCATCTCCATTTGGTCGTGCCACAACCGTGAATCCATAC
AGTTTCCGTTCGACC
TCATCAAAGTGGGCGTGGCTCGGGGGTAGGATTTCTTTCGTGGGA
GAATTCTGTTTCCAG
TTATCCAACTCAGTTGCTTCTCCTGTCCACGCCATGATTTTTTGTA
GTTTTCTGTCATAT

GCCATAGAAAATACTATGACGTTCCCACTAGAATGTATATTTTCA
ATGAAAGTTCCATCC
AACATATATTTCTGCAAGACATAATCGTTCTCAAGTTTGGCCGAA
ATGACCAGCTGATC
>ConsensusfromContig3346 Average coverage: 8.47
TGCCAATTGGGTCATATCAGGCCTGTGTGACGTGATCTCCGAAAT
GAGGGGGGCCAGTTG
AGATTTTCGTTCATTCAAATATGAAGAATAGTGCCACAGCTTCGT
GTGGGAATACCGAGG
CGAACACCATGGCAGACCTTTTTCTGCTTTGATGATATTCGGAAC
CCCAACATGTTGGAC
TTTCCCCCCAAGACCTTTTTGGGCTCGTGTAAGTGTTGACTGAATT
CCTGTTATTTCGTC
TCCGATTTGTTGATGAAGTTGCCAAAATTCACTCTGCTTTCGATAT
CCTTTGCCGTCTTC
GATGAGAGGAATCGTTTCGTCCATGAGATGGCGCTGCTCCTGCTT
CGAGATGTAATCGTC
GCAACTG
>ConsensusfromContig3347 Average coverage: 10.35
ATTTTTATTATGTGAATATATTGTACTTTAAATTATGACAAATTTT
GGTATATTCAATTT
TCATTCTCATCATAAACGTTTTTACATGCTGTCCAATCGAGATTT
CATAAGATATGTTA
TATCCGACTTGTCTCCGTTTTGGCTTAAATTTGAATGAGGTTTTCG
ATAAATATGTATCC
GAATTAAGAGAGTTAGATATTTTAATTATATACCTATGTGGATAT
ACGAAGTTTAAATAT
TTCTTGATATTTAAAAGTAACTGCCATAAAATTACATGACAATTC
ATCAGCATGATTTGA
TTTCATCGTTCTATCGCCTCATTGCCGCAATATGTACATTTTCTTA
TTTCTGTGAATATA
TCGAAC
>ConsensusfromContig3348 Average coverage: 7.50
CTCTTCCGATCTGAAACAAAAAAACTCATATGATTCATTTTCTTG
AAGCCTATCACAGTT
GTAAGATAATAATGATCACTGATGGTGTTCGGCTATTGGCCTATA
AAGTCGGGAAAATGT
GTTTATATTTACACAATTATTTGAACTACACTTAGAATTTGGTGT
CTCAATCCAAAGTAT
TAGTGACTTTTGATATTTTTAAACGTGGTATTTTATATGTAACTA
AAAGTTGTGAAAAAT
ATCAAAACTCACACTACTTCACCTTTTGCCATTT

>ConsensusfromContig3349 Average coverage: 10.14
CTTTTGTGTGCGTTTATAATTCAGAAATATTTATATTATATTATTT
CACACAGTTAAATT
TGTATATAATGAATATATACAGCTATCTCAATCCGATGCAAAGTA
GACAATCTGATTATT
ATGTACAGTTTTTTCGTATCCCTTTTAGCCACTCAGTAGAATATTT
CATGACGCATACCT
TATTCTTTATGCACACTCACTCATAAACAAACACCATTTCATTTTT
ATTGAGAACTAATC
GGTGGCCTTCTTCTGAGCTGTTCTTTTGGGCTTTATTT
>ConsensusfromContig3350 Average coverage: 19.29
CAACACACGGGGCTATGCATTCTCTTTCCTTTGGTTCATCGTCGT
GCCCATTCAACCAGA
CACCTACACTGGTGCCTTTTGAACAAGTGTACACTTCGCTCGATC
CTTCGAATTTATGCT
GAGTGGACGAACATGCCAAAGATGCTTTTGTTCCAATAGCAAGTT
GCGCCTCTTCAGCCA
CAGTTATCGCTTTACCAGCCCCGTCAGTATAGCTCACAGTGACGG
TGGAAAGTTTCGGGA
TTATGGCAACACACGGGGCTATGCATTCTCTTTCCTTTGGTTCATC
GTCGTGCCCATTCA
ACCAGACACCTACACTGGTGCCTTTTGAACAAGTGTACACTTCGC
TCGATCCTTCGAATT
TATGCTGAGTGGAC
>ConsensusfromContig3351 Average coverage: 11.15
TGTGCTCTTCCGATCTCAGCGCATAGTTTTCCTTTGATACACCCAG
CTTCATCAAATGTC
AAATAATATCCAGGCGGACAATAACATTCTCCTCCAAATTTGTTC
GGCCTGCATTTGTAT
ACACACGATTTGTTGTTGCAGAGAACATTGGACATAAGTGAAACT
TCCCATGTGTACCCT
TGTGAAACATCAGTTAACGTCTCAGCAACTTCGGATCTTGTCTTG
TTCCAGCTACATATT
CCCGATAATGGTTTTGCTTGCTTCAATTCTACTCTGACTTGCAAAT
CTTTCTGTTTGTCA
ATCAAGATGCCTCCAAACCAAAGTGTGTATGTCTTCAATTGCTTTT
TTGTTGTATTTGT
>ConsensusfromContig3352 Average coverage: 15.23
CTACATCTCAGGCATACAACCTCCATAGGAATGAATAATCGAAAC
TCCTGATATATCTAG
AAACACATGTTATAGTGCTTCTTTCATACCTCCCCTACATCTCAG
GCATACAACCTCCAT

AGGAATGAATAATCGAAACTCCTGATATATCTAGAAACACATGTT
ATAGTGCTTCTTTCA
TACCTCTCCTACATCCCAGGCATACAACC
>ConsensusfromContig3353 Average coverage: 10.91
GTATGCTCTTCCGATCTTAAAACTGCCCCTGCCACGGGTACTTGTG
GATTTCTTTTTTCG
CCACGCTCCATATTTTTTTCGACTCTTGAACTTCCCTTGGTATGGA
CTGGCATTAGCCTT
GGACGGGGAGTGCCAGAATCTAGAAACAAGCTCTTGTGATTGAG
GTGTCCCTTCAAAGTC
CATCTCATCGTCAATTTTTTCAGTCTTGTCGACAAACGGGGCAAT
GGCGCTCAGGAACCT
TGTTCCAAACTTCCCCATTTTGACGAGAGAGACGCCATCTATTTC
GGCCAGATCCTCAAT
CGTAGCTGGTCTTATTTCGGACATAGACCGAAGGACTTGATTGTC
GAAGACGGATGTGTA
CGACACGAAATGATTTTCGCGAGCAATTTCTTTGGCGAGAGCAA
TGAGGGCTACATAACA
CTCCTCTTGTGTGTCTTTGGTGGAGGCTTCTCCCGAAAGAGACGT
TTCCGCGACGACCGA
GGCGCGACGGTCTTTCTCTTTAACAGTTTCTCGTAGCTTCAGGTTC
ACCTTGTGCACACC
CCTGAGGAGTTCAACACCCCGTGGGCCGACCTTGATATAGGTTGC
CGTGTGTGTTTCGA
>ConsensusfromContig3354 Average coverage: 24.51
CCTAGAATTTAATTACTCATTCGCGCGAGTTTAATTTATTGAAGA
ATTACTTTGCGGAGT
GGTGTCGAAGTTTGTTGCTGGATTAACTTGATTCCGAAGGGACCT
CACAAAGCTCTGGGA
TAAGATTATCGATTTGGCAACAGAAATGCCTCAATTTGAAGTGA
CCAAAGCTGCAGATGA
CTCCGATCGAAAGATGTCGAACGATGAATTCACTACAACAGTCC
TGACGACGGAGATGGA
CCCGGCGGAATACGAGGAGAGCCTGATGGCGGAAAACCTGGACG
ATGCGGAGGCCCAGCT
GTACCTCTACAGGGACGACATCCGAAGCTTCCA
>ConsensusfromContig3355 Average coverage: 21.38
TCGTTCCAGAAATTAGATAGTTCATGGGAGTAAATGTAATCACAA
AATATGGCATTGTTT
CGAGACATAAATTCTATGGATCGAACATAATTTTTAGAAATATTT
GACTCATATTAGATA
TAGAAAGAATCGCGTATATTATAATAAAAAAACAATTGATCAAC

TGGATAGTAGCCCATC
AGCATTTATTGGAAATTTGACATCAATAAAACAATAGAGTAATA
ACCAGGAAAATTGGTG
GGCCTGTAAAAAATATGAGGCCACTGGCATAGGTTCAAAATCTA
GCATCAATGAAACAAT
GCATCGATCACCAAATCGACTGATATATTGTAGATCTTGTATCAA
TAGAAGCA
>ConsensusfromContig3356 Average coverage: 9.67
ATTCGATCGGGGGTTGGGGTTTCCCCTTTGAGTGAAGCTTCCCTG
GTGGGATGGAGTTGA
GGGGTTGATTGATCGGGGGTCGCAGATTCCTTGGTGTGCGGGAG
AAGCGCTGTGCTGGGA
GTGAACAGTTGTTGAAAGAATGTGAGTCGGATGCGCCGCTGTAAT
GGTTGTGCTCTGAGA
GGCTGAGACGTAAGGGTGTTCGAGAGCTGTCTTATTCGGTGAATG
ATGAGAGTTCTCTTT
AACGAAGTGGGGTCCCCCGTTGTACCGTTTAGGGACGCTGTAGGT
GTGGTGTTGGGGGGT
CGAAGATGCGCTCTTCGAATGGACCGATCCGTTCATCTTGGAGGG
TTTCAGTGGAGCCTG
GCGATGTTTCCCGATGCGACGATGCTGCTGTTCGAGAGATGATAT
CGTTTCCTGGATGGA
GTCCACAGCGCCCTGGAGAACAGTCTCTCCGTCTGCAGGGCTTTT
CCCTTGAGAGAGTTT
GTTGTGCTCTGAACAGAA
>ConsensusfromContig3357 Average coverage: 3.05
CAGCTGGGGTATGGATAATACCCATGATATTCTGATTATCTATCT
AACAGAGTAGACCCA
AAGAGTTACGAATAAAAAGCTGAGCGGTCGTCTGCGTTATAAAT
GATGACGATGATTTAA
TTTTCCATCTAACAGAGTCGCTAAAATTCAAAATGTGTACCTGTA
TCAGCTGGGGTATGG
ATAATACCGATGATATTCTGATTATATATCAAACAGAGTAGACCC
AAAGAGTTACGAATA
AAAAGCTGAGCGGTCGTCTGCGTTATAAATGATGACGATGATTTA
ATTTTCCATCTAACA
GAGTCGCTAGAATTCAAAATGTGTACCTGTATCAGCTGGGGTATG
GATAATACCGATGAT
ATTCTGATTATCTATCTAACAGAGTAGACCAAAAGAGTTACGAAT
GAAA
>ConsensusfromContig3358 Average coverage: 9.61
CACAGATAATATGAAGGAGATGCCATTGTTGATGCTTATTGATA

ATGATGAGGAACAAAA
AGCCTTCGTGATTTTCCTTCAGGATAATACCGTATGTGTTGGCAG
TGGTGTACTCACAAA
TGTCAGAAGCTATTGTGCCAATGCAGCATCTGTCAATCCAAATAA
TTGGAATTTCATTGC
CGTATCTCAGAATACTACAAGCGAAATTGTTACCATTTTTATCAA
CGGAAGCTCTGTATG
GCAATCAGAAAATTCAACTTACGCCACATTCATACAAAGTATTGA
TTATGTATTTTTCAA
TCTCAAAAGTGGATTCAACGAACAATTCAATGGTGTGGTGGATGA
GTTCAAATTGTTCGA
AAGACCGCTGGATGAGTCCGAGATTTCTACGATATACAGAAACGA
TATGATTTCTTGCAG
TCCAATTTCTAGTGAAATTCATTTCCGATTTGACGAAGAGCTCCAT
CCTAACGTATTTTT
CATTGACACTAATTCATCGTATATCTTGATGAATTCTTCTATAAT
AAATGAGCAACGTGG
ATTATGTGGATTGACT
>ConsensusfromContig3359 Average coverage: 7.59
TATGTCATACATCTCTGATGATAGGAAGATCACATCAGAAAACG
GAAAGATTGCAAACGT
TTCGTCCGGGGTGAAAATCACGCCAAAGGGTAGGTATGGAAGTGC
TACTGAGTCCTCTTG
CTATCAAAATAAATACATAAATCTTGGATACAATGAAGATTCAA
TGCTCGGGGACGAAAC
ATATAGTTTCAACGATTTTGCGATTTCTATGTGGATAAAATTCCC
TTCAGACTGCGGCCT
TGTTTGTGATATCTTATACAGTGGAGGAAATTCGAGAAGGATGG
AAAAGGGTGATAGATT
CATATGGTTTGATTTCTTTCATGGAGAAGATCGGAAGAGCG
>ConsensusfromContig3360 Average coverage: 10.31
GACGTGTGCTCTTCCGATCTCTTTTTCGAAATTCCCGTCCCCGTAA
TAATTGATGCTTCC
GGAGCTGTCTATGATCATGACGATGTCCAGGGCTTCAGCGCAGAT
TTTCTGACACGTGCC
ACTCAGAATTGACTGGACCATTCGTGGTGCAATCTCCTCCTTATC
CATCAAATCAAAGAT
GAAATTTTCCGGTGGAGAAGATGCCATTTTTCTTAGCAAACTTTC
ATCTCCATACTTAGA
CGTCTTCACCACATATATCTT
>ConsensusfromContig3361 Average coverage: 17.55
TAAATATGTAACAAATGAACAGTTTCGAATATATTAATCTCATGA

ATGCCATCTATATTC
TATGAAAATTCTACAGATCGCATACCATACCAATTTTTGAGCTAT
TTATAATTGGATTGT
TATCAAATGTCAAATTTATCCCAAAGGTTTTGAAATATGTAATCT
TACCACTAAACACTC
TTCATACGTTGATAAGACTCCGTAATTTGGATATCGAAATAAAAA
AAACATATGTAACGG
ACAAAAGTTTTGTGAAATAAATGCAAATATCAGAATGATGTAAA
TCAATTCGATGGACAA
GTAATACCAACATGCTATATATG
>ConsensusfromContig3362 Average coverage: 7.55
ATAAGATTGAGCAAGATGCTAAAAGGCGGATTGTGCACATGAAC
AAAAACATTGCCAGCA
CTTCTTCTGAACTTGCAGCAGAGAAAGCAAAGAGCGATAGACTTA
TGTATTCCATGCTGC
CGGCCAAAGTGGCTGATCAGCTGAGAGCGGGCAACTCTGTCCAG
TCCGTGAAGTATGACT
CTGTCAGTATCTTGTTTTGTGGCGTTTGCGAATTCAAGCAAATAT
GTGAGGTGAGGAGTG
CAGAAAGCATCGTTGATATGCTGAATAATCTCTTCCAAGTCTTCG
ATTCGGTTGCAGATA
GAGCAGATAAAGTGTACAAAGTTGAAACTGCGTCC
>ConsensusfromContig3363 Average coverage: 6.35
CTTTATAATTGCTTTGTCGTTGAAATGTTTCTCATATGGATCGTAG
TGTTTGTTTTTATG
TAAATCAGTTTCTTATAAATTCAAAAAATATATCTGATATATCGG
TGAAAAATGCTTTAC
TTGAACTGTGATCGATCCATATGTCGATTTGTCATGACATAATTG
GTTTTAAAATTTCAA
ACTGCTTATGTCATATATTTTGTTGTGGTTCTCACAATAATTACTG

>ConsensusfromContig3364 Average coverage: 20.67
TTCTTGAGGAGTATTCGCTCGATGCCGGAGATTTTGCATCTGAGG
AATCCGAATGTCTTG
ACTTGTTATTGTCGTCTCTCACAGAAACTCTGAGCTGCTCTTTTTT
GATACATTTGAATC
GTTTTTGTCTTCTAAGATAACAGCCGTTTTCAAACATGTTACCAG
AGTCCGGATGTAACG
TCCAGTAACTTCCTTTCCCTGGTTTATCTGGAGACCTCGGTACTT
TGAGAAAACAATCAT
TAAAAGATAAACTATGCCGGATTGAGTTCTGCCATCTCTGTTGAT
TTTGTCGATAAAATG

GGAAGAGGTCCATGATAAATTGATAAACTTCACTTAATGTACACA
TTTTGTTTTTGTTTT
GCTG
>ConsensusfromContig3365 Average coverage: 57.93
CTGGAGTTCAGACGTGTGCTCTTCCGATCTGGCCGATCGTGGAAC
TATGCTGGCCAGTCT
CAAGACAATGTTCGAACTGGCCCCCAGATCCGGCTCACCAACGA
CAGGGAGAATTTTCCC
GGTGTAGACTTCAAAGTTGTATGCAATACCGCAGACATCAGCCA
AAACGAATATCTTGAA
GCCCCACTTTTTTGGCTTTGCTGGCAAGTATTGCTTCAGACTACTA
ATCCCCTTGAATGG
TACCATTTGCTCATCAACACAAATGGCACCACTCATGGGGATTGC
TCTGAATTTTGCTTT
CAAATGATCGATAAGGGGCTTCAGTTTGAATAATCGATCATTTGA
AGCATGCT
>ConsensusfromContig3366 Average coverage: 52.37
TGGTCTTGAAAGAGGATCTGCCGCCATCAGAGACCAAATCAAGC
GTTTGAAATACAATGG
CCGACAGCTCGACATGAGAAGGTCTTCGGAGGGAATCTGGCCGCA
TCTCAATTCGTGCCA
GAGCTCTCCGTTTTGCGGAAGGTCGATGTCTGTTGCCATTTCGTA
GAAAGTGATTCCCAG
GCTGAATATATCAGCTGGTTTGCCGAAGTTCCCTTTCAGGAGTTC
TGGTGCCATATATCT
CGGATCTCCTTCGCTCCCCTCTATGAAACGAACAGGCTGAGATAA
ATCGACAACCAGACC
GAAATCGCCCAGTTTACACACTCC
>ConsensusfromContig3367 Average coverage: 11.77
CTTTCATATATACAATAAATCTGACCGACTTTTCATCACTTGATG
AACATGTTAAATCAT
ATAAATTTGCATCACTTGATAGAAATGAACAATGGCACAGACTGT
GATATTCATAAGCGA
GGAAGTCTTCTTTAATTCATCAATTAACGAGATGATGCAGCGTAG
AATAGAAAGTATGGC
CAGAGAGCTAATCGTGAACATATCGTTATGAAAGCTATATTCATT
ATACTTTAGAAATAA
AGAATAAAGCATGTTATTTGTGTGAGCGGAATTGGCGTTTAAAT
>ConsensusfromContig3368 Average coverage: 17.46
CGCTGCCATAAAACGTTTCATATACAAAACCCACGATGAAAACTC
TCATGAAACCAATAT
CGGTGACAATTCGAATACTTTTTCAGTGCCATTTTATGCTTCCTAC

AAATTACCTTTGTT
GTATTTAACACTACAAGATTTTCATTTATTTATTGAAAAAACAAA
TAAAATATCAAATAC
GGATGACGATTCAAATTCCAGTGACAAAAGCCCCTGCATCGACAC
CCTTCTATTCAGGGT
TGCTTCCCTCGAAATAAATCCTCAAGCTGAAAACCCATTGCCGCG
CGTTTTTCTTGATGG
ATCCTCTCCGTCGTTTAGTGGAGAAGGGAAACTTG
>ConsensusfromContig3369 Average coverage: 12.47
CTTCTGTCCACGCCAGAAATGCTGATTGAATCGGTTTTGGATGAT
AGCACAAAAGCCATC
GTCCGAAGTACCTTTTCACCGACAGCATCGTTGATTTGTTCTGAG
AGTGATTCCAGTGAC
GTTTCCTCATATTTATATTTCAAGCAGATAACATTTGATCGACAA
GCTGATTGTATGACA
GAGGAATTTTTGAATAAACTGGACAAAACCAACAGCTCTATGCAA
TCACCATTTGAGACT
ATTTGCGATGAGTCATCTCTTGAGTTTTTCCCACCAAAAAACTCA
ATATTACAGGCATCA
TCAAGACATCCATCAATGTTCATTTCTCAGCATAGCGAAGGTCAA
GGGATATTTTATACC
TCTAACCGATTCTATATACCTTTATTACACAAAATTCAGCGTTT
>ConsensusfromContig3370 Average coverage: 16.87
ATCCCCGCTACAACAGAGAAATATTTAGAAGCAAACACTCCACAT
GATATTTGTCACTAT
GAATTTTTCAATGCTGGATGCGAAGAAGGAGAAGTCATCAGAAT
GAAATATGCAAGATAC
GGCCGCATGAACCAGCAGTCAAAGTGTATCAAAGCGTTCCGGGA
TAAAGTCGGATGCTCA
CACGATGTTCTCAACCACTACGACTCGCTCTGCTCAGGTAAACAA
AAATGCAACATCTCA
GTTCCGGGCGACTTGTATCGGGACCATAAACCGTGTGGCGACGAT
ATCATCTCTTATTTA
TCCGCATCATATGAATGTATAAAAGTTGAACCATGGTATAAGGTC
GACCGGCCTTGTCAG
TCCTCAAACTCATACACCATCAGAGACAGTAGATTCGGATTCATT
GGGAGTTCCGAATAT
TACCACCTTGTCAGGGAATGTACCATCAACATCAAGGCCAGTCCC
GGGCAGTTTGTCAAC
ATATCTCTCTACGACTTTGCGACTCGCTCCCCAAATAACCGCCGC
TGTACTGAATATGCA
TCGATTTTCGATACT

>ConsensusfromContig3371 Average coverage: 76.49
CGCTCACAAACGATCCCTCAGCGGTAGCAGCTGCCAACTGTCTTG
CTGCAACGCCACTTT
CGACGTTCTGCTCGAGGAAATTCGACGTGGAAAACTTTGAAGG
CAACGCGAGTTCTGAT
GAGAGAAACGAGCGAAGTGAATGTGATGACCGGTGATTTGACTT
CAGGCATCGGCGAAAC
TGAATGCGAGAAGGGCAGGAATAGCTCGAAGACCGGAGGAGACC
GCAAGATTCTTCGGCC
AGACTTGGACGCCTTTTCGTGGAGCGTCGATGTTGATTCCGACGA
GGATGCTGAGGAATC
CAGCACCAAAGATAGACCTTTGACCTCTCTCGGCTCATTTCGGCT
AAAGAAGAAAAAACT
GAAGGCGGAAGCTATGAGACCGGAAGCCATAAAGGCGGAAGTAG
AAAGTTCAATCGTACG
CGACATCTATAGGCTCACATCTTTTGGAGAGGGAGGTATTGCTGA
CGACGGCTCAGACAC
CTTGCCAACGTTGAAAAGATTAAGCGCCGATGAAGAAGATCGAG
GCGACTCGAAATCTCT
GAAAAGCGACGATGACACTTTGGTACTGGATGAGATAATGATCG
ACGAATTGATCGGAAA
TGGCAACACGTCGACAACTCCAGATGTCGACGAGATGTGTGGTA
CGTACGATCCAAATGC
CAGTGTTTGCGACAGTGGCGTTGGAACCTTCGAGCGGGCAAAGTC
GAATAGGTCTTCTGG
GAAAGAACAGTCAGTCATCAGAAAATCTTCTGAAGGTTCCTGTA
GAGAAGACGATGGTGT
AGCTGTGGGAAATACTTCTTCGAGAAATTCAGTCAACCGAAAAAT
AATTCAAAATTTCTC
TGGCGATGAAATTTCCACTCAGAGATCCGGGATGAATTTAGATCT
GAATACGGATAGTTT
CAGGACCCCACCGCCCAAACCTCCTCGCAAGAGATTGGCCAATTC
GGTTTCGCCCCTGAC
CCCGCTCTCCCCGACTTTCATGCAGTTTTCTCCGGAGATGGATTT
ACGGGAACGAGGCGA
TGTTGCCAATCTGTTTGATGGACGTCTTCGGTTCCTGGATTCAGA
AAAGGATAAATCCCG
>ConsensusfromContig3372 Average coverage: 13.40
TCATGTCGACGGGCCTGGCACGGCGAGAAATTCCGTGTGTCGTCA
AGCTTGAAGATATCA
CCGATTTATATGAATAGTCTTCATTGATTTTTAGCTCAGAGTTATT
CTTGCCCGGACCGA

ATGCTGAGTCTGACTGCGAAGATTGTGCCGACATGAACGAATCC
AAGGAGATGTTACTGG
AGCGCCTGAAATCAGCAGCCGGGTGCTTGTCGTTCATCGATGCGA
TGCTTTCATTCGAAA
CAGGAGGAGACTTGTATGAGTCACGTGAACTGACCAACGAATCAC
TGCCCATCTCCTCAG
GAGCCGAGAAGAAATGTTCGGATATAGCCGAGATGCGATTGCTG
TCCTTAGAAGGAGAGT
TCTGGGAAATCCGACGGGGACTGACGAGGCCCGATGATCGACTG
CCCACCGAATTGAAAT
CTTCCACGTCACACCTATTCATGACGTCATCGCTCATCGGAGATT
CGATGGACTGAAATG
AGTCATGTGACCCTGCATAAGACCCTGAAGTGGCGGCAATAATAT
TATCAGGTGAAACTC
GCAAATTCGAACGCCTCTTATTCCGTTTACAAACATTTGCATCAG
TCCTAAAATCTGATT
CTGTCAGAG
>ConsensusfromContig3373 Average coverage: 11.95
CAAAATACTATGATACTTATATTTAAAATAATATTATTCATCTTTT
TCATTAATCCACAC
AATTCTGTCTTTATTCAGAGATGTGAATTAGTAAAACTATTGTAT
ACATATGAGATGCCA
GGATCGATACAAGATTGGGTATGCATGGTAAGAAGAACTAGTGT
CTATGATACAAATTTA
CGAACTGAGGATGGTGAAATCAAACAACATGGAATCTTCCAAATT
TCTTCTTCAGAGTGT
ATATCATCACCAGAAGAAATTAGAACCAATAAGACATGTCCCAA
TACTAGATGTAGATAT
CTGGAAGACAATGCAGTTGGTGACGACATGATTTGTGCTCAAAAA
TTATACGATAAATAT
GGTTTTGACAATTGGCCGCA
>ConsensusfromContig3374 Average coverage: 16.86
AAAATGATGAGATCATATGAAGACTGTCTGAGGAATTAAAAACA
AAAATATTCGAATTTT
AGCAGACTTGAAACAGTGTTTCAGCTTTATTCAAGAGATACATAT
CCAGGTGACGAATTC
ATCTATTAGCTAAATTCCACGGTGATAAATAGATAGTTGAAACCA
TGTATGGCAAGAAGT
GACAGAGTAATGAACTTACTGTGGTTGTGATCATTTTATTTCAAG
AAAATCCAAAACTAA
AACTGAGAAATTCGAGAACAATCATCGAACCGGATAAGATCATT
TGTGAACGAGGAAGCA

AAACAAGAAAAACACTGATTCATATACTATAAAAAAACATGATG
GGAACACACAACCAGC
TTCAATACTGGAAAAACCTGACCTACC
>ConsensusfromContig3375 Average coverage: 47.90
TTTTTTTTTTTTTTTTTTTTTTTTTTTTTTTTTTTTTTTTTTACTCACT
AAATTTCTATA
ATATCAAATCTTAAACATCAATAATACAATAGCAATAACCAAGA
AATCCACACATAGTAA
CTTTCAAAGCTCTCTCGAACAATCTCATTAAACAGTTATATTATG
AGCGAAATGGCAGTA
TAATTCAGTGAGCCGATATGTCATCAACTAAAAATCCTAGATATG
CGTTTAAAATAAATT
CCTGATCTTCAGATTGGGCATAAATATACATTCACTTAAATTAAG
TGAAAGTACTCAGAA
ATACATAATAACCACTTTCGTTGATGAAAAATCAATGACATAAAG
CGGTGAAACAGTATT
GCTT
>ConsensusfromContig3376 Average coverage: 10.11
TCCCTACACGACGCTCTTCCGATCTAAACCACTTCCCGGAACAGT
GAAAATGACAGGGGA
TCAGATGCCACAAAACTACCAAATATAACGAAAAATCCGTTGCA
ATACAGTGATAGAAGT
CATTCAACTCTCGAAATCAAAAATACTAAATTTCTAAACAACCGC
AGTTCTTCGTACTCG
CAATTTGATTGCACGAAACACTCACGTGATTCGGCGCATGAATA
CAAAAACAGAATGAAT
TCAAATAAAGTGTCGTCTGCTAGACCGAGTAAAAGCGATCATAA
ACATCAACACGAATGT
GATTTTGTTCGGCTGAGCAAACTCGGGATGCATCATAATAGTTTA
CCG
>ConsensusfromContig3377 Average coverage: 8.15
TTTGGAATAAACACGGCGAGCGAAATATTTCAAAAAACGATGAG
GGGAATATTGGAAGAT
ATTGAAGGAGTCATAAATGTCAGTGATGATGTTTTGATATATGCA
AATTCAGTAGAAGAA
CACGATGAAATATTGTTAAAAGTATTGAAGCGATTCAGAGAAAT
GGGAATCACACTAAAC
AAACAAAAATGTGAATGGAGACGCGATGAGATCAAATATCTTGG
TTATGTATTCTCCAAG
AATGGAGTGCGGCCTGATCAGGATAAAATAGAAGCCATAGAAAA
AATGGGAAGACCCGAG
AATGCCACAGCCGTAAGATCTTTTCTAGGAATGCTGCAATATGTT

GCACGATTCATCCCA
CGATTCGCTGAGGTGTCAACACCACTCAGAGAGCTCACTGCTAAA
GGAATACCTTGGCAT
TGGAATGATCGTCACGAAAACTCATTCAATTCGTTAAAGCAAATA
TTATCCTCGAGGATA
ACCAATTCATATTTTTCCATGAATGATACCACAGAGATAATTGTT
GACGCGAGTCCGGTA
GGAGTTTCAGCAATATTGACGCAAATGGACAAAGATAAAAATCA
TCACGTCATCGCATAC
GCAAGTCGCGCCCTTACTCCAAC
>ConsensusfromContig3378 Average coverage: 37.66
AACACAAAAAAAGTTAAAATTGTTAATAAGGATCTTTCAATTATA
CTTGTTCACAACTGG
CAACAAAAAAAACGAATTGGTGATGAATACTGGTCAAATAACAG
TGAAGATAAATCACAA
AACTGCTTGTAATTTTGAATAAACTGCGTCGAATATGATCAACAT
AAGCCCGCCGCCTGG
CCCGAGCCGCATGACTTTCGGCGCAAGTCCTTTGTATAAGGCACG
AATTCCTTCTTCAGC
ATAGATTGTCTGCAGGGTCCTCCAGCAATGCCGATACTTGATGAC
ACACATATCGGGTTG
GCGACCCTGGATTCTGCTTTTAGCGACATCAAAAGGAATGTTGA
GGCAGGAAGCGAGAGA
GCCCGAAGCGAACCCAATCCCGAAGCGTTGAAGGAGGTCCAACTT
TGGGTTCTCTTGTTT
AGGCACGATATTCTTGACAGAATGGTAGAAGCCAAAGTAGACCA
TGTTGTAGGTGGAGTG
ACGTAGAAGCGTTGCACTGAGACCAAGATTGAGGCCGAAAAGAC
CAAAGCCACGTTCGGC
AATGATTCCACGACAGACGGTGAACGCCGACAACTGCGGCGCAC
TCGAAAGAACAGTTTG
CTGATGAACTTTTACAACTTCGAATGGATTTATCACAAAGCCCTC
TGTTAATCCAGAAAG
ACCGCCTGCCAGGAGGTACGTCAAAGGTGTTGGCGATGAATTCC
CGAACAGCAAAACCTT
CTTGTATTGCTCAAACGTCACAAATTTGACAGCTCTTTTGGGGGT
TTCAGCGAGAATGGG
AGGCAGGATTCCTCGGTAGAAAGACATTGGCCCTTCATTTCTATA
CATTTTCTTGAAACA
ATCCAAAACGGAAGTGTAATACGTAGGGTCGTTCGTATATACCAT
TGGACCAGAACGTTG
TACTTGGAATCTCGTTTTGACCAGATCAAGGGGGTGCATGACACA

AACTTCTACGAAACC
AGCACTCCCACCAGCGACAATCTGCATACCCGCTAGCTTAATTGG
GCTATGATTTGATGA
TGACATTGCGTTTTTGATAGTATAAAATCAGATCTATTGAATAGT
TGATAATTCCGGCAA
TTTTTACATTTTTTCTAACACAGCTTTTTGATTTGATCTTTTTCTTA
TAT
>ConsensusfromContig3379 Average coverage: 6.69
GTGCGTTCGAAGATGGCGCTGTAGTTCGTCAGAGCGCGTGAAGC
GTTTGCCGCAGAAGAG
CCAGTTGCAGACGAAGGGTCTCTCGCCAGTGTGCCAGCGGAGGT
GGGCTTTAAGGTGGGA
GGTTTTCCCGTAGACTTTCCCGCATCCTGGGATATGGCAGCTGTG
GATGTTCCTCTTGCG
GAGATGAGCGCCAGCGGGGCCGAGACTGTCAGCTTCTTGACAGT
TTGGGCAATCGCACGT
TGCCCGCCCGGTGTATCTTCGCTGAGATCGTGGGCTTGCGACTG
GAGGGAGGGCGGGACG
AGGGAGATAGGCGTTTCCAAGGGAGGATGGGCTATACTCGCTCC
CCGCAGATGGCAGGAT
ATTCAGATCCGAA
>ConsensusfromContig3380 Average coverage: 9.07
GTTTTATCCTTCTTATTGGTTGGTAGCTTTTGATTTTCTGCAAATT
CGTAAGCACTATCA
TCATAATCTTCATTGTAATCATTTTCCCCTCTGATATTTCTCTGTG
GTTGCTGAATATTT
TCAACATTCGATAATTGTTCACGCTCATCGTTTATCTCCTTGAGAA
TTTTATATGCAGGA
CCTCGCTCTATATGCGGCTTCATCTCAGCTCCCTCATCGATGATT
AATGCGGTGTCATCA
GAATATAAAATGCTGGGTTTCGCAGAAGTTGATCGAGTAGCTGAT
TTTGTAGTGAAATCA
CGTTTTCTTAGTAATTGCGCGATCAACTTCACGCTATTTGTCGGCT
TTATGATATCATTC
G
>ConsensusfromContig3381 Average coverage: 3.09
CGGGTGTTCCTCTATCAGCGCTCTCAACACAGACAAATTGAAGGA
TTGCGAAAGTTGAAA
TACTTGTTGACAATTTCAGGCCTTTTATTATGTGGCCTGGTATTG
TATCACTTAGAGGAA
CGGGTGTTCCTCTATCAGCGCTCTCAACACAGACAAATTGAAGGA
TTGCGAA

>ConsensusfromContig3382 Average coverage: 7.52
ATTTTACAACATTATAATAAAATGAATAGTTCATAAATGACTCAC
ATGGCCATTCAAGCA
GTAACAACATCATCATCATTATTTTCAACTTTTTAAATGGCCATC
AGCAAATTCTATACA
AGGGAATCAGCATTTTATGTAAACAAAAAATCTTGGTGTAAAAT
CATATCAATGAAAATA
AAAATGTGGCCAAAAAGAAAGGAAGAATCATAATGTGTCTAAAA
TTTTCTTGGTACACAT
AGAATTTAGCCTGAA
>ConsensusfromContig3383 Average coverage: 10.99
CTGGATTCAGACGTGTGCTCTTCCGATCGTGTTGTGTCTCAATGA
TCATCACACGGAAGC
GAACATTATGACTCGGATGTGAAAATTATATTTTTGATGGAGTTTT
TATACTGTATTATT
TTATATTCATTCATATCAGTCATTATGATTCAATACATCAAAGCCT
CGTGTGATATCGTT
GCTGCAAATAATTGTCTAGACGATGCTGTTCATGATGCCCATAGA
TCCGTCTGCAGCAAA
ACAAATGTGATACCAGAACTGAGGTCATGCCTAGAATTTTCCTCT
TCAACATGTACAATC
ATAGCCCAATCGAGGCTTGAGGAATCGCTCTCAAATTTCGAGCAA
TTTCTTGCTGTCTCA
ATTAATACTACCGATTGCTCATTAGATTGCCTAGCAGAACAAAGT
ACGCTCCGTGCTGTT
AACGCTTGTGCAATAAATTCGGGAGAAGAAAGCCTTCTCTCTTAT
AGCAATAGAATCATT
AGATCTAAAGATGCAAGGGATATCTCAGTACTTAATCAAATATAT
GCAGATTGCTTATCC
GTCCAATCAATCATCCTCTGCATGACGAATAGAGCCGGAACCTGC
ACATCAGCCTTGAAT
TTCGCAGACGAGATTAAAGCTGGAGCAGGGTATTCAGAGACCAG
TGTCATTTGTGGGGAA
TCTGGGGTTGCTCTCGCAGAAGTCGACGATGGAAACGATGACGA
AAAAGAGACGTCATTT
CATACAAATATT
>ConsensusfromContig3384 Average coverage: 4.71
GTGTGCTCTTCCGATCTCAGGAGTGACTATAAACTATAAGACGTG
TTAGTGAGTAGGGGA
TAGAATTCAATGGATGATGCGACTCCACATCAAGTGGATCTGAAA
ATACACATTCCATTA
TATGAAACATAAAAATGCACAGGAGTGACTATAAACTATAAGTC

GTGTTAGTGAGTAGGG
CATAGAATTCAATGGATGATGCGACTCCACATCAAGTGGATCTGA
A
>ConsensusfromContig3385 Average coverage: 8.72
CAACGGTTACACCGGGAATGAATGCAACACAGTCATTTCGAGAC
AGTCTGATTGTATAAA
TGGTAATTATAGAAACAGTCAATGCGATTGCACACTTGGATTCAC
CGGCTCGATCTGTAA
TAAAGCCATATATTGTCGGGATATGATAGCAATCCATGGTAGCA
CAGTAGAACACAAGAC
CTCTGAATCAAAAAAAAGTACTATGCCCCATCCATTTGGTGCCAC
GGCAACTTTTGCATG
TGAAAAACCTGGATATGATCCAAATGTGGGTAGAGAGACGTTCA
CTTGTGGAGCTGGAGA
ACCCGGCAGAGAAACTGGTGAA
>ConsensusfromContig3386 Average coverage: 8.64
TCCACATCTTCAAGCCTAGTGGGACATGAGCGAATTCATTACAAT
GAGAGACCATGCTCA
TGTAATGTATGTCAGAAGTCATTTCGAGAGTCGACACATCTTGTG
AATCATCAAAGGACT
CATTCCAACGTAAAACCGTAGTCATGCATTATATGTCAAACATTA
TTTTTCACAACTCGG
AGTCTAGTGATCAACGAACTCATTCCAATGAGAAATCGTATCATT
AGCATATCAGCTGTA
AATATCAAAAGTCATTCATAACGGCTTCAAACATGATGACTCATT
AACGCACTTATACTG
TGGAAAACTATAGAACCTAGTTCAGATCAATGTGAAAAAACCTAT
AACTTTCTG
>ConsensusfromContig3387 Average coverage: 9.97
ACTACACTTTATAGCGTAGTAATAACGCAGGCACGAAATCAATTA
TTACTGTTATAGCAA
GTTTCTATCACACTAAAAAATAAACTGTATTAGAATACAAAACA
AACAAATTTAGTGGAA
ATGCAAAACAATTATATCAGCGATTCAAATGATCAGAGATCATA
AATAATATGGCAGCGA
GGTATGTTTAGTACAATATCGAGAAGTGCTTCAAATTCCAAATAA
TACAAACAGAATATT
ATTTCGAACGTCAGTCTTTTATAATTGAAATAATATGAGGTAGCG
TTCAAAAATTTCAAA
TATAGTAAATAGATGAATATTAATCATCGAAGTAGTGTTAAGAA
ACAACGACATAGA
>ConsensusfromContig3388 Average coverage: 8.70

GGGAAACATTCCCCACCCGTGCCAGTACAATCCACACCCTAGCCC
CAACCCAAGTGATCA
TAGGGAAACCGCCAGCCGGTCGAAAAAAGCCAATTGAAGACGAG
CGCATGTCATCTCTCG
ACTGGAATATCTGGATTGTTCTTGGTGCAGCAATTGGAATTTTCGT
CTTACTTCTCATTC
TTGTTTACGCCCTCTATAAATATCGAACACGCGAGGAGAGTTTAT
ATAAAGTAGAAAAGA
CAGAGGCCTATTCTTGTCAGCAACCCGAGACGTGTATCGGACCTA
TAGCACACCACAATG
TCGAGAGCTGCACCCTATTAGCACCGGATATGATGGGTACGGGT
CCATCCCCCCGAAGGA
AGCCTGTCAAAGAGTGGTATGTGTGATAATGGTTGAGCAAAAATT
GTTTTTTGTCATATT
TGGAGTATAGTAAAACATAATTGTGCCTAGGAATTGCAGCAATA
TACAGTGACAGGTGTG
TTTATCAATGTGAATTTATAGTCTGATTATCGTGTTACTTTGAACA
GATTATAGAAATTT
GAGGTAATTGAAGATGATTCAAGAATTCGAGGTTACATGTATTA
GAAAATAGATCGCAGT
TTTCACAAAAAAATAAAATAAGAATTCATCGCAATGGATATTTTA
CACTTACACCTACTG
TAC
>ConsensusfromContig3389 Average coverage: 14.65
TAATAGTGTGTGCGACATGTATTTGAACAAGCTCTGAAATCAGAC
ATTCTAGATTTTGCA
GCGAAATTTCATGAGCAGTTGAAACTATGAATATAATCACGCTCA
TTCTCGTCGTAATGC
TTATTGGTGGAGTCATATGTCCTATACCTATCGGACGATGTACAG
AAGGTCCAATAACAG
GGCAATGTCAACATGGAAGATGTGTAGCGAAATGGAAAAATGGC
TGTAATTTTGGGTGCA
AGGAAGAAGGAATGTACCCATTCACTTGCGTTTGTTCCAGAGGAT
ATGGTGGAACACTGT
GCAACGAAAAGAAGACCAGACGAAAGAGACCTCCGTGTAATCCG
CTC
>ConsensusfromContig3390 Average coverage: 20.41
TCATAATTATCTAAACACAAATAATGAATCAGATAGTGATAACA
CACCTGTAAGAGCCAA
TCATTGTGGAAAAATCTTGAAACTTCAAGAACGTCTTCGATTGAG
TTTTGATCTATGGTA
ATATGGCCGTGATAGAAATATTCTACCATAGCCTTCACTCCCAGT

GGACTAACATCGGAC
AATTCAATACTATCTTGTTCCTTTTCCCGCATATTCACAGAAAAC
ATCGCAGCAAAATAG
TCACAAAATTCTGCAAGAACATGTTTATGTGCTTTCATCACTTCTT
TTCCAGCTCTTACT
TTAATATCACATTTATGATCATACATCTTTGAAGATGGTTTCTTAA
AATTGCTGTTTGAG
TATTTTGTTCTTCCATCGGCTAACTTTAAGACCTCGATCGGCCCCA
TTATTCAATTTACC
CAATTATGTGTTTTTCGATTAAAAAATGCTCAGTAATTCCATTTAT
GATTTTAGTATGCT
GTTATTTCTGCAACAGGAATTTTTTTCGCGGCAGCTATTGTTACG
AGATCGGAA
>ConsensusfromContig3391 Average coverage: 9.52
AAGTTCTGGAAAAAGTAAAAGAGCCGTATCTGACTTTTGAAGCCC
TCTCCTTTCTGCGAA
CCACCCTGACATCCCACGAATTGCAGTTTTTCAGAACACTCGGGG
GTAACTGGACTGTTG
CAAGAGAGCAGTTAGAAGGGGAGTTGCCTCTTTTCAACTATCGG
AACGACAATGAGGGCG
TGAATCTTGCTAAACATCTCGAATACAGCTCCCCGGCTCATCTAC
AGGAAATATCAAAGT
CACAAAGAAAGGAATCGAGCCAATTTCGAAC
>ConsensusfromContig3392 Average coverage: 22.07
TTCCGATCTTATTAATCTAATTTGGAAGATTATTTGTATAAGAAA
CCTATATATTACTAG
TTTAACAATCTCTTTTTTACTTTTAACCACAAATAGAATTCGATAC
GTCAATTTATGAAG
CAATATGTCTAATTATCTATATTAGATGATATGAAGTTTGAGACC
AATCCGGTAATGGAT
AATTAGTATAATAACGATGAAAATGTATAATGCAGGTTTAGGTAC
AAACATATGTAGATT
CAAAGTGAATATTATGCTTTATATTGAAATTGTAATTTATTTGCT
ATTATTGAGCGAAGG
AAGCAATGTCGTACTGTTTATGAATTTTTCGCATCGTCGTTAATT
CAACTAATAATATAA
CATTAATTAATCTTAATTTGTATATTAATTGTATGGAAGAGCCAT
ACAAGTATGAACATT
CATTATTATCAATTCATCTATACTTCAGTCAATTGAGGGCATTTAT
CAATTGTTTTTGAC
GT
>ConsensusfromContig3393 Average coverage: 19.23

AATCCCTTGGAATTTTCATCGGATTCAATGTTATCACCAAACTCT
TCATCGATAGAACCT
TCATCATCGTGACCTTTCAGACTTTCATTCTCATCATCGACGCAAG
CCCCTTCCTCGTAA
ACTCTTTTCGCCGGTTTCCTGTTATACGAACTCTCATCCTCGGGAC
TTGCTCGTTTTATT
TGGTTCTGTTGTTTATTTGTTTGTTTATTTTTTGTCAGGCATGTAT
GTAGGCGAGGTGGC
AAGCACATATGGGCCGGACTCAGTCGGGTGTAAGTACCGTTTTGT
TCTTTGATAAGGGCG
GGGCAGACATTGCCTTTATTTCTCCGGCTGCATGACCAGAATACA
GTATCACCCCTCTTT
CTACTAATTCCAAATGAATATGTGCCTTCCTCGATGAGCAAAGGC
CCGCCCCGTTTTGAC
CCATTTTGAATCAAAACAAACTGCGGTGGCGCAATCCCACTTCCG
GTTGTGTCTACAAAT
AAATCGGCGAGGTTGATACAGTCTTCATTGAAAGCACTTTGGCCA
GGCATCATTGC
>ConsensusfromContig3394 Average coverage: 9.23
TTTCAATCTGATTGGCGATAAAAGAGTTCATTCTTGCTATCATGT
GGAAGATTTCTTCTA
AAAATGGTAAATTTGTTTCAGCTTGTGTATGGCTATTGCTGGGTT
TAACTGTACTGTGGC
CATTCGAATTGTTTGGAGTTTTAAAGTAATTTCTGCTATGAAAAA
TGATTAATCATTTTG
AAGGGATTCGGCAGTTGTGGACAAAATGTTATTCTCATTATGTAG
ATAAAGTTTATATCG
TTACGGTGGGTTGTCAAAACGGTATAGTTATTTTGTGATCTATCA
GGGTTAGGATTAATC
ACATGTGTATTGACCATGTTCGATCCTATTTCATGACGATTTTTGT
TTCGAGTGTTTCAA
AAATTCGTATGGTTTTGCATGACTGGGTGAAGGTGAAGTTATGTA
TTAGAAAGATCGGAA
GAG
>ConsensusfromContig3395 Average coverage: 8.60
ATGGAAACAAACTTATATACAAATGGATTACTCATCGTATTTTGA
TTTATTTCTATGTGC
TTGTATCAATACACAGGTACATCGTGTGCTTTTCAAAGACCATAT
TTATGCCATTCTAGA
GAAAAATCGATGTTTCATTGATGTATTATTTCATATTATAGATGT
ATAAAAATGTATTCA
CAGTTTTGATGAATCCATTATATAAAATCAATATCTTGGTTGAGA

AGGCACTATGGAGTA
GGTAAATCCAGTTGGAATGTGTTTTATTGATAAATCATACTATTTG
AGTCATTATATTCT
TTGTACCACACCGCACGTTTTTGGCGAATATATAACGAGTATTTA
T
>ConsensusfromContig3396 Average coverage: 6.37
GCACTGCATATCTCCTATGTGGCCTCCTAGTGGTGACCCAAATAT
TCCTGCTATCCCATA
AGCGGTTAAAACCACCGAAGCAGCCGATTTTGATTTCATGATTGT
TTCGGCCTTCCGAAC
GTAAAATGCACTGTTTTGAACATTAGTGATATGAAGGAAAGTGC
ACGACAACACGAAACA
TAGAGTGGCCGGATTTGCAATGGTAACCATTCCTTGGATCTGGTT
GACGTAACGTCTTTT
CAAGGTGCTGCAGACTGTCTCTTCCGGAGTGTCAGGATTTTCAAG
CCACGGGGACATGAG
AAAGAATCCAGCAATGAAGATAAAGATACCACTCACTGAGAGTA
GCATAAGGACAGTATT
ACAATATGTAGTTTGGTTTTTAG
>ConsensusfromContig3397 Average coverage: 9.14
TCACCGTCCATTGAGTCAAGCATCGCATCGCTATTCTGGCCAGAA
TCAGCGCCATGGCTT
TCATTGTCATTTTGTTGAATGTCAAAATGTCCTCCTTTCCCAGTAT
TTATGATGGATTCA
ATTTCACTTTCCAATGATTCGTGCGTTGCAGTGTTGTGCTTTCCTT
CATTTTCAGAATTG
TCTGTGTTATCTTTGGTGGTTTTGTTTTCGTCTGAATTCTTCGATT
CAGGAGGATTGTTG
CTTTCAGCCTCGTTCAGATGTTCATCATCTGGAAAAGGG
>ConsensusfromContig3398 Average coverage: 15.18
CCGAAATATATTTTTATGCAATTTGTTTTTTCATTGATGATTGGTA
TCTGTCAGCCATTC
TCTTTGTATTCAATGTTTTAGCGCCACGTCCTCTTTTGCCGAAGA
GATCAAAAAGCCCTT
CATATGCATCTTCGAACATATTGGGGGCGTTTTTCTTGAGAATTT
CATATCCAATCCGTC
GGCATCTATAACCACAGTAACTGTCTATTTGTATAATATGGGCCA
GTACAACCAGGAGCA
GCAAAACTGATCGCATCATACTGGTATCAAAGTGTCGTTTAAGCT
GTTGGAAATGAG
>ConsensusfromContig3399 Average coverage: 7.86
ATCTCGGGAATTTAATAATACTGCGAGTTTCTCTGAGCTGTTTGGC

TAAAGTTTTCTTCT
CCCTGTCGAACTTGGCTATTTGATTACTGTGCTGTGTTGATTTGAC
CTTTAAACTGTTGG
AAAGCAATCTTCCTTCTCTCTGATTTCTGAACCGCAATTCTTCTTT
GAGTCTGTGGTCCC
GTTCTCGCATGTAAATAATATCCATCGAAATGATTCTCAGTATTT
TGTTACCAGCCTCCG
TCTAAACAATGTGTAACGATCTTGGATGACTCAAAATGAATAGT
GGAGATCGGAAGAGCG
TCGTGT
>ConsensusfromContig3400 Average coverage: 7.76
GCAGTAAACAGCAGTATCATTCATCCCGAAATATTCACCCCTCGA
TAGGAGTTTGCGGCA
AATTGTGCACGAGAAGCAGCTCATGTGATAAATAAGATCCCTCGC
TCTCATCACGAGTTC
ATTGGCGGAGATGGGATGATGGCAGGCGGGGCACATCGTTTTAC
CACCAAATATTCGATA
GTAGTCAGCCTTGCAGTAGATGTAGCCGTTTTTGATGAAGCACTT
TTGTTCCGAGTCAAG
ACAGAGCCGGCACTCGACGCATTTGAGGCAGTCGAGGTGCCATC
TTTTGTCGGAAGTGGA
GAGATAATATCGGTCGAGGATCTGTCGCTTGCAGGCCGTGCAGAT
GCTCGGCTTCACATC
CGATGATCCGGGGTAAGACCGGAGATCGTC
>ConsensusfromContig3401 Average coverage: 19.73
AGATATCACATCCAGCACACAGTAAGCCATTAGCATATCTGTATA
TGACGGTGTGTTTTC
CGTTTTATCAAAGATATTCATAGCAGTGCTCTAGAAATCCACATC
TCAAGATCACCATTA
CGTTTCAGATTTTCCCCCGAAAGGGTACAAGACTCGTAATGTATG
TTGCAATCGGGAATG
CTGTGAGTCTGAGATTTGGTGTTTTCACTGTCATGTTGTTTCTGTT
ACTCTTCCAAATGT
TTTGCACCTTTTCACATCAACATAACGCGGTGAAATTAATTTGAA
TATCACATCCACCAT
ACACGACGATGTATCATCAACACACTGGACAAATTGTTAGAGATA
AATTAATGATCTCAG
ATATGTGCATTGTGGCAATATAATACAGTTACTC
>ConsensusfromContig3402 Average coverage: 6.50
ATATACTCGGTGGACACAGATGTTGCAATATTGGCAATGTCATGT
ACGGAGATTCCCTGC
AGTATGTTTGTCATGTCCGGAATCGGTAAAAAATTACTATCCATC

AATCTTTGCTCATCA
ACAATTTCGGCCGACGTTCTCGAGGCACTTCCGGGGCTTCACGTT
TTCACAGGTTGTGAT
AGCACAAGCTGCTTCAGAGGAAAGGAAAGAAGAAAATGTTTGA
AGCTATATGCTCAGCT
ACTAGGGACTCCGAAACGTTCAAATTGTTGGGCGGTGAGTGGTCT
GCGTCTTCTCTCCTG
GAGGAGCGTCTGGAGGAGTTTGTCTGTCGTATGTACGGTGTTATG
AATTGCTCAAATGCA
AATCAGGCCCGTTTAAATCTCTTCAGGCTTGGAACTTCGCTCACCT
ATCTTCCTCCTAAC
AGAGATGCTCTACGACAGCACATCAGCCGAGCCAATTACCAAAC
CGCCAAG
>ConsensusfromContig3403 Average coverage: 11.07
GCCATCCAAAACAAATGACCGTTCTTTGAATTGTGTGAACCCCCG
ACTCTGTCCAGTTTG
GTATATGTTCAAATCTGAATTTCTCTCAATCTTTTCTTCGAGATTG
TCATCGAACATAGA
GAATGTAACAATAGCGGTAGCAGAAGGATCATGGCCAAGATATG
TCGCAAATACTGATAG
GTATCCCTCACTTTTCTTTCTGTTATCCCCTTTCGGATGCAATTGA
ATACTCCATTTGGT
ATTCGAATTTT
>ConsensusfromContig3404 Average coverage: 5.74
CTCAACCGTCGTCGAAAGCGAGATGAGCGGCTTCAAATACAAAC
CAACCTTCAAGATATT
CACCCCAAAGTCGCAGGAGAAAATCGCCATTGCTGTAGAGGACC
ACAAGCAGCGCATGCA
TCGCAAGAAGAAAATCCAAGAAGGCAGGCTTGTAGACGGTCAAC
TCGTTTTCGGAGATGA
AGATGATGATGATGATTCACCAGGCAAAAAACGAGATCCTAGAC
TTGAGCAAGGCAACGA
ACTTCCAGAAGATCTCGATGCAATTTTTCCTTCTCGATTGCTTGG
TTCTCCGATCGAGGA
AATCGAC
>ConsensusfromContig3405 Average coverage: 10.80
CTTAACTGCTGACTTTGAGGACGATGAAGACGAAGATTCGTTCGG
TTTGATAATCAGAAT
TGCAAAAGTAGGTCAGCCAAAGGTCAAACCTAAACCGAAACCAA
AACAAAAGGTTGATCC
TCCCAAGCCCATCCCACAAAAGGTGGAGCCTAAACCATCACCTT
TCAAAGATAAAGTCAA

AATAGTTAGCACCGCCTCCAAAAGTGTAAGCTCCGCCCCCAAAC
CAGAACCCAAAGTCAA
GGAGCCGCCGAAGCCAGTGAAGAAGAAAGAAGACCCTTACTTCG
TGACGGAAGAATATGA
GGCTCAAGGGACTCTCAAACCATATGAATACTTTGACACCGATG
CAGACTGTGAAAAGCT
GAGAGCTGCTATGAAAGGGCTGGGAACGGATGAGGATGCAATCA
TGGACGTTTTGGGTTT
CAGATCCGGTCCTCAGCGTCAAGAGCTCGTCACTATGTTCAAGAC
TATGTTCGGAAAAGA
TCTCAAAAAAGAACTTAAATCTGAATTGAGTGGTAATCTCAAAGA
GACATGTTGTGCGCT
TTGTCTTTCTGGGGCCGATTAC
>ConsensusfromContig3406 Average coverage: 14.37
GTCGTATCAAAATTCCTGAAAATTCAGCGTAATGAAAGAGATATT
ACTACGAAAATTGTC
ATATGCAACGTTGAGGGCGACCCGCAAAATTTTGAAGAAGTGAGA
AAACTGTCTTCGTCT
GTAGAGGTATGGCAAAGATTCGACAAACGACATTCTAACACGGA
TTCAATATTCGAGAAA
GAAAAAAGCGATTATCTGTATTGCCTCAACAGATCCTTGCAGGTG
GACGCGAAATATATC
CTTATGATGGAGGATGACGCGTATCCTCATGAAGATATTTTCACG
GTTCTGAAACACTTT
CTAAAGCTTCCACATATGACCTCTTC
>ConsensusfromContig3407 Average coverage: 4.65
CAAGGGTCTTATCATATGACTAGAGGCCAACCCAGCTATGTTTTC
AATCATCCAGTCCAT
CAAACTTCTTCACCTTCTCCCACGACATTTCTATTATATCTTCACA
GACAATACTTGATA
AACAAAGGGTTTCAACTAGTGTGGTCAAGGGTCTTATCATAAGA
CTAGAGGCCAACCCAG
CTATGTTTTCAATCATCCAGTCCATCAAACTTCTTCACCTTCTCCC
ACGACATTTCTATT
ATATCTTCACAGACAATACTTGATAAACAAAGGGTTTC
>ConsensusfromContig3408 Average coverage: 12.91
AAAAAACCCTGAATAACCAAACAAGTATTCATTGAACGAGCATTA
TTATACATACATGTT
AAAGCTGTCCTGGAGATGCTTAGGACAAAATTAGAAAAACAATC
AATCAGTATACACTGG
TGAAAGACATAACGATTAGACATATATATCCTGTCTATAAAAGA
TATACGAGTATCAAAG

AATACGGTCGAAGAAATTCAGAACCGATAATGATTAATTGAATTG
TGATTTCATAAACTT
ATAACGAGACTGAATCATTTTATGAGAGATACTTATCTGAGAGAC
CCTAGATTAGAGTAT
GAAGAAACTCTCCATCTCATAGAAAAAGTTTCAATTATCAAACAT
TGGATTAGGTTGCTA
AATTCTTTAAAA
>ConsensusfromContig3409 Average coverage: 10.34
TACACGACGCTCTTCCGATCTTCCAGATCACACTCAGATTCTGCG
TCATCAATGCCTGGT
GTGAAAACCCAGCCCCTTTTCGTTCGCTCCACATCATCACATTCTA
TCCTAATTTTCACA
ACATTTTCGTGCCATTTCTCGAAGAATTCCAAGTCGGTTTGCCTC
CTGGCATCGGTCACA
ATCCACAGATCATCGTCTTTAGCACTCGCCGTGTTGGTCGCGAGG
TGACAAAAATAACTT
GAACACTCTTTCCGTTTTGCTTCACCCCACTCGATCATTGCAGATC
GGTATTTTTCTTTA
TATTCAGACGAATCCAGGAGTTTTTCATACTCGAGGCCATTGTCC
TTGGCATATTGTCTT
TTTAATGGTGAACTCAA
>ConsensusfromContig3410 Average coverage: 6.99
TGTTTTTGTCCATCGCTCGTAAGCATCGCAGAATCGTTATCCGTC
GAATCAATAGAATTT
TCTTCGGGTATTTCACAAGTATAATTGTTATCGTGATTCATTCGAT
CATATCGCGAGTCC
TCGGAATTATATGGCACCGTATATGGTGCAGCGACTATTCTATCA
TTCGAATTAGGTCGA
CACGCCTCATTTGTTTCATTACATTGTATCGAATCAGTGCGAGAA
GGGTTTGGTTGTTGT
GATAAATCGGACGGCGTTTGTTTGCTATTTGTCGCCTGATTGTTG
TGAGAAATGAGTTTT
CGCTTAGCACTTTTCGGCGAACCTGTTGCACAGGTATCTGAAAAA
CTAACACGTGTTTGC
TTATGCTGTTTGCTCAAGTTCTTCGTAGTGGGTTTAGAGGGATGG
GGGGTCACACCAATA
GCATCCCCCTCTAAGAGTACGGCTTCCTCGATCGAACCAATCGAA
ATATTTGGAGAATTC
GAAGCAAGAGAATCTGCATGATCTTCAGATGGGCACTGAGTTAA
G
>ConsensusfromContig3411 Average coverage: 17.36
ATCAAAATGACGTTACTGGCACAATTTGCACTGATCATTTTAATG

TCATTGACCTACTCT
TGGGAGATTGATGATCCAGTCGATCCTTATCCAAGCGAATTCTTC
AAAGTCATCCAAAGA
CAAAACTGGTGTGCCCAATTCAACGAATCGTGTCACCAAAATCCC
AAATATATTTTCGCT
GGTTGCTGTAAAGGCCTGACATGCATGTGTGGGAAGTTCTGGAAG
TCGAGTGAACGCTGT
AAATGCAAGTCCAGGGGATTTGGCTGAGAGTTCTCCCATTACTTC
GATTATACTAGTAGT
GAGATTATATTAGGCAGATCGGAAGAG
>ConsensusfromContig3412 Average coverage: 60.89
GGAGTTCAGACGTGTGCTCTTCCGATCTCTCGCTCCTGAAGAATT
TTTGGGATTATATTC
ATTAAAGCTTTCATGCATGATCATTTCACATTGTTCAAAGCTTCGC
TTTTCATATGTAAG
GCCATCTTTAGTTTTTTTATAAGTTTTCATCGCATGGTTTTATAAA
AGAAAGAATTTGGT
TATTGTTACATCTACGTAAATGGGTGAACTAGTAGGTCAGTTATA
TCATTGATGGACATT
CAACACTTTTTCAGGGGATTTTTGGAATATGTTTAGATCACCCATA
ATAATTTTA
>ConsensusfromContig3413 Average coverage: 8.09
TGAAATGAATACCATTTTTGACACAGTCTTCCTTTAGCACAATGTT
CCTCACATCTCCTA
CTACTGCTGCTTCTTTTCTGAGACCCAATTCTTCTACGTTGATAAT
TTGGTCAGTGAGAA
TGTTTCGGTTCGTGTCGATCTCGAGATCCATGATACGCTCTGGACC
ATGACTTTCCATTT
TACAATCGCCAGTTCCTAAGGCGAATCCACGCTTGCAGGAGACGC
AACTATTTTGATCAG
AGCCAGATATTTTTTTTCGACATTGGTGACAGTTTTTGTATCTTTC
GCAATCGGTCGTTT
TATCGCAGATTTTCCCACATTTGGGGAAAGTGAAGC
>ConsensusfromContig3414 Average coverage: 10.76
GGGCAAATGGGGCTGGAGACAGGGGTGAAACACGAAGAAGGGGC
AACTCCCCTCAGCGAC
ATGAGCTCTGAAGATACCATCCCAGCTGCAATCAGTAGGATCCAT
ATGCTCGGGACGCCG
GCTGTTCATGTTTAAAAGAATTCGAAAACTTCAACCCGAGTGCTTT
TTCTGTATTTTGTG
TATTTAGCCATGTCAATTTTCTTAATTTTTATATTTACTGAATC
>ConsensusfromContig3415 Average coverage: 7.34

GAAAAAAAGGGTAAGAAGAAAGATATTTTCGATAGTATTGAAAC
TGGAAGAAATTCTGAA
GAAGTTTCCTCTCTAAAGATGAAAGTGAAAGAAAATACTTCAGA
TATGAAATTACCGAAA
ATTAAATACCAACTGTCCTCATCACCAACTGAGGCTTCAGATAGC
TCTTGGAATGAAAAA
CAGATGAAAAAAACAGATGAAAACACTACAGAAATGAAAGCGA
ACAAAAAAAGTATCAAA
CATCGACGTGAAACTATAAAGTCTCTATCGCCTTCCATGAAAAG
CGTAAAGAATGGAAAA
AAAAGTACACAAGAGATGAGAAAAACAAATAAAAATACTACAG
ATATTAAAACAAAAAAA
GAGATAAAGAAAACAAACAAAAATACTGCAGAAATTAAAAATA
GCAAAATGATAAAGAAA
ATAGATAAAAATACTGCAGAGAAGAAATCTAACA
>ConsensusfromContig3416 Average coverage: 12.98
GCGGCACTAGAATAGGTCTGTAGTTAATATTTGATATATAAAATA
TATAAAAGTTACTTT
ATTAACATGTTCAGATTGCTCATGATAAGACATAATGTTGTTTAAT
TGCTTTATTATTCA
TTTTGATCCATTTTTCCGTTTAGTTTCGCTGTGACGAAAAACTTTA
TGATATTTTGTATG
ATACACGTTGTTGAATAATATTGTGAATTAATAAAAATTGTGAAT
CAATAACATGTTTTT
CTCATACA
>ConsensusfromContig3417 Average coverage: 2.59
GAAAGAACGGAATAGGGGTGAGAGATTCCAATGCGTAAGTCACT
AGAGCAAGGCAAATTC
TCGTTCATACACTCATACAAATACTACCTACGAAGGAGCCGACGG
TTTGGACCTGTATTG
AAAGAACGGAATAGGGGTGAGAGATTTAAATGCGTAAGTCACTA
GAGCAAGGCAAATTCT
CGTTCATACACTCATACAAATACTACCTACGAAGGAGCCGACGGT
TTGGACCTGTATTGA
AAGAACGGAATAGGGGTGAGAGATTTCAAT
>ConsensusfromContig3418 Average coverage: 13.03
TTTCCAGTCTTGTCACTCAGGAGTCTGCCAGTTTTGTCTTGGTAA
ATGGGCTTGATGATA
TCCTCAAGAAACACTGCTGCTAAGGCGTTCACACTTCCTGAAATT
GAGCTCAAGGCGGCG
CTAAAAGCAGCCCCTATAAAAAGACCAGGAATTCCTCGGTATTC
GCCTAGGACCTCCAGG

ACATAAAGTGGGAAAAGCTGATCAGTAGTTTCAACTCTTCCCTGC
TCAATAGGGTCACAA
TCCGCGTAGTAGGCATAACACACGATTGAAGCCATTGTTATGAGC
GCCATGAATATAGTT
TGGGATACTGCAGACATCCAGCAGGCCTTCTTGGCATCACTGATC
TTTTTGACACTCAGA
AATTTCTGAATGACAGTCTGGCTTGAACAGAAAAGTGTCATGC
>ConsensusfromContig3419 Average coverage: 5.29
TCGGGGTTTACTCTGATCATGTCCACGTATGATTGGCCAAATCCA
AACCACGCCCCTTGC
AGCTCCTCGGGGAATAAACATTGGGCCTCGACTCTGCCTAAAACT
TTGCAAAGACCACAA
GATCGTCGACATCTTCCTCTGTATGGCAGAGGCAGACTCGGACAG
CGCCCCTCTCTGGCA
AAATGCTCACATCTCTGGCTATCATCATGACATATTCCAGAGTCA
CTTATATTGCCATAT
ATATTAAAGTAGTCCATTTTTTGGTTATGACCACTTCCTGAGTTT
ATGATTGGATTGAAG
AATATCGTAGAATTCAGAACGGTACCCCCGTCATGTAATGGTGTT
CGCAGAATGAGC
>ConsensusfromContig3420 Average coverage: 5.78
TATCGAGGATGCTGTGTACAGACTGGACAGCAGCAAGATTGGTAC
GGACAGCCTCTTTGC
CCTGAGCGAGATGCAAGGAACCTCTGAAGAGCTGGACAAGATCA
GATCACACGTGCAATC
TAAATCGAAATTACCTCTGGACCAGCCAGAGCAATTCATCCTCCA
GATGGCCAACATGTG
CCACTTCTCAGAGCGGATTGAATGTATCGTTCTACAAAAGAGATT
CAACGAGACTTTATT
CAATATTGAGCAACAGATGGAGTCAGTTCAGTCAGCATGTCGTCA
AATCTG
>ConsensusfromContig3421 Average coverage: 8.31
GCCGCCAGTTTTTCATTATTTGCATCGGAGGCTTTTCTGACGATT
TTTATCCTCCCCAA
AGCAGTCAGATCTTCTTCCGGGCATTCGACAAGCAAGTAGACCGC
AGCAGCCATATTCTC
CGCCAATTTTCTCGATTTTGCAAATAATGTTGATCTATACAATGTT
CTGTTCACTGAAAC
GGTTGATTGAAACATTCCATTCTCCTCCTGATTCGTTTTATATATC
GGTTTTTCGTAGCC
TTTTTGTTGCGAAATATTGAAAACACTAGACTTAGGACTGCTGTG
GGAATCTGTGAGTCT

TGGAGGCCTGATGTAGCTGACAGATAGAGAAATTTCTGTTGCACC
ATTCTCTCCGAGTTT
CCTCCGCTTGTGATTGGCGATCTCAAAGGCGGTGGGTTCGAATTC
CAGTTGACCACCGAA
TAGTTCACTTCGCTTTCGTCTCACTTCCAATGCATCAGTTAGATA
ATCAGTGAGACCCCA
AACTTCACAGTAGTCTTCCATAGTATCAAGATCATCCACGCGCCG
ATTCTCGTCCTGGTT
TATTCTTTGAATTACGTATTTCGTGTTGATTGGGTCGTTGTCATAA
TCGATTGCAATTCT
GAGATATTCGCGAACGTCGTCTTCGAAAGGCTTGGGACCGTCTT
CAGCGAATATAGAAGG
GGACAGCTGGGCCCGTCGCGCAATCATGACTGACGATACGCCG
>ConsensusfromContig3422 Average coverage: 7.33
CTCCGCTTGACCACGCCTCCTTTGGCGGCGCATGTTTTGTAGCGA
ACCAAAGCCGTTGAG
GAAACCCAAGTCGGGAATCGAACACGGGTCTTTCACCAAACCAT
TATCAACGTTGGACGA
GTCTGATCTCTCTGCACTTGATAGTTTGGCAGTCATTGCACCCGA
TGACAGAGTCTTCGG
AATACTGTTATCGGATTGGGATTCGCCTGAAGACTGTCCAGACCT
TGATCGCAATGCGGG
CGGGGTGTAGATGGGCCGATCCTCG
>ConsensusfromContig3423 Average coverage: 36.70
AGAAGTCTCAGTACTTTTTTTTATTTAGAAAATAATTGTATTTAAA
ACCATTTGTTTTTT
TAATTTCTGTTTGACTGATTAGAGTATATAGTTGTATGAGACCCA
TAATGATAATAAATT
TGATAGGAATTTGAGAATGATTATAAGATGGTGCATTATTGTCTT
CAAAATCAACTATTC
TCAGTGTTCAGTTCGTATCAAAAGGCCTAATTGATTCATGATTGT
TACTAGTAATATTAT
CGTCCCATTTATGCTCATTTCTTTCCGTGATATATTTGATGCCATC
GTATATCCGTAATG
CGTTTATTTTCATTATTAAATGTTTAAACTCAAAAAAAAAAAAAA
AA
>ConsensusfromContig3424 Average coverage: 7.34
TTGGACACGCAGTAACGCTGGAACCAACTATACCAACAACCCCG
GGCATTAGAAAACCTG
ATCTGATCATAACATCAAACGGGACCACCACGGTCACCGATATCA
CCATCGTATCAGATC
AGGTAAATCCCAACGAGCCCCACGAAAGAAAAGTCCAATACTAT

GATACCCCTGAAATAA
GGGAGTGGGTTACGCGGAATATCCCGTCCGCAGGAACAATAAAC
TTCAGCGCTCTGGCAA
TTAACTGGCGGGGGACAATTGCAACCAAATCAGCTGAAAGCCTA
AGATCACTGGGCTTCA
ACACCAGTGATCTCGAACTCTTAAGTATACGCACGCTCGAAAATA
ACATCAATATAGTTA
GATCATATCGTACAAGCACAGCAGGTGAGAGGGAGATTTTATGA
AGTGGAGCTTCGGCCC
GGGAGTGCGACCGTTAATCTCTCTGCTCGGCAGTGTAGCCCTACT
GCTTAAACCATTACG
TTTTCATATATACACGTTTTCATCCAAG
>ConsensusfromContig3425 Average coverage: 8.37
GTGCTGGCTAAACCAGGCTTATTAGGTCACTTGGTCATCCAGGGA
CACCTATTGAGGTCA
CTGGGTCGACAAGATTGAAGCCACTCTCAGGACACTACAGGTAA
CTAGTGCTGGCTAAAC
CAGGCTTATTAGGTCACTTGGTCATCCAGGGACACCTATTGAGGT
AACTGGGTCGACAAG
ATTGAAGCCAC
>ConsensusfromContig3426 Average coverage: 17.15
GTTTAATATTTGTATAATCAATTATTATTTAATTCAACTGGAAGA
TGGCACATTATGAAT
CATTTTGATCATAAAAATAGGGTCACATGCTCAACAATTTCGAAT
CTCTCGGCATTTAAA
AAATCCAATAAGTTTGATTATGTTACCAAATTCACCTTCGGGTCC
AATCAATAATAGAAT
AACTGGAAATAAAACGAGTGAATTGACAAGAGAAAAGAAAATAA
CGCAGAATACGAATAT
TCCGAATTGTTTGATAAATAGAATGACAGCCAACGTCATGAATAT
CGTAGCACCGATAGT
AGTGCTCGCCCCCAATAGAACACACACCCCCACATCTCCGAGGGC
GGTTTTGACCCGGGT
ACTTCGGTCTTTACAC
>ConsensusfromContig3427 Average coverage: 4.20
CTATTTATTTCGATTTTATTTCCCGCTAGGCATTTCTTCATACTTCT
CTTGCTATTAATT
CTGATAAGAGCTTCCCACATTCTCTTGCTATTTATTTCGATTTTAT
TTCCCCTTAGGCAT
TTCTTCATACTTTTCTTGCTATTAATTCTGATAAGAGTTCCAACAT
TCTCTTGCTATTTA
TTTCGATTTTATTTCCAGTTAGGCATTTCTTCATACTTCCCTTGCT

ATTAATTCTGACCA
GAGCTTCCC
>ConsensusfromContig3428 Average coverage: 7.81
AATCTCAGTCCTTAGATCCTGATTCCGATGCTATAGAGAGAAACC
ACAGTCACGTCACCA
TGGTAATACTCACTCCAGCCGTGATCTTGTTTCTCAGTTGTGTTGT
GGTGCTAGCTACTC
TTCAATCGATATTGATAATACACAACACATGTGATTTTAAAGGTG
AAAACCGAAATTTCA
CAATGACGGATCAATTCAAATATCGAAAACTCTTTCTGACTTCGT
ATTTCATATATAAAG
TCTCGTATTCAATACTGATAACGTTTACAGTACTCGTATTAGCTA
TAAAGTACAAAACAA
TTGAATCTTGGGAAATATTGATTGGGGTGCGGAAACTTTTAACCA
AAGTGGAAATG
>ConsensusfromContig3429 Average coverage: 12.87
TTAATAACAAAATGGATATACAATATATAATTTGAATTGTGGAAT
AAATAGCTCATTGAG
AAAAGCAAACATTAATCATCAACACATGTTGAGTGCTTCTCTGCC
GCCATCCCTAGACGG
CGCTTCACTAAAGAGCTTTTCACGGTCGAGTGATTCGATAAACAT
CAGATTCTATTTCTA
TATTCGAAAGCATTCACGAACGTCAATCATAAATGATAGCTATA
TGAAATATACGCAGAG
TATTACATAGAATGACTTAACATATATATGAATGGTGGCTCGAAA
AATATGCGGTGAAAC
TTTCGCTTTCGTTCGAAACATATTACGATTCACAATAGAAATGAA
AAAAGAAAACAGTAC
GCGTTGAACGATTCATACACAAAGTGATAATACTATCAATAAAAA
C
>ConsensusfromContig3430 Average coverage: 6.34
ACGCTCTTCCGATCTGCGGGTTGTTTATGGCTCTCAAATCCTGGC
GAAGAATTATTACTC
AAAGATTGTTTACTTCTTGAGATTTTTTGATGCATTAAGTCCGGTC
TATTCAAGTCCCGC
CTACCGATGCCTTCATCATATGATATGTGGGCGGAGTTGCGTGCC
GAAGAACTAGCCAAT
GGCGAAGGATTATTAATCGTCGATTGTTTACCTCTTGAGATTTTC
CGCCTACCAGATCCT
TCATATGCTGACATATGGGCGGAGCTTTGTGCTGGGGAACCGGC
CAATGGTGAGGGAGAT
TTTGATGTCATGTTGCCGAGGAAAATGCTGTGAATTGCCGCTGCG

TGTCCGGCAAGGCAT
TGCTTTGGTCGGAAAGTGAGGTAATCGGTGGCTGGAGCGAACTG
CGAAATTCGCCGAATT
CCTTGGCGCCACAAATCTGCTTTTGCGGCAACAACCTCTTCGTCG
A
>ConsensusfromContig3431 Average coverage: 4.89
CATCATAGAGATGTTGAGCATTTTTATGAACCTTCATCATTTTTTT
TATTCTCATCGTGG
TGACAGGGTCATCCAAACGAGTGTTGGTAGACCGTGAGATATTTT
TAGTTGATTCACTGT
GTGGGGAAATTCTGACATTTGTAACGCCCAACATTTTGGATTTTT
CATATGCTACGGCAT
CATGAGGACGTTTTTTTCCTTCACGAGGTTTTTTTACACATGATAT
TTCAGTTATGGGTG
ATTCTGA
>ConsensusfromContig3432 Average coverage: 13.58
CGTAAATCGGATCGTACCGGAGCCTGGGGTCCTCCCACTCTTGCC
GTAAGTACATCGTTA
TTGAGTAATCCATTGTTCTCTCGTTTATGTCGAAAATACTGACGA
CAAAAATCCCAATCT
TAACTTTTGTCGGATGTTCCCGACTCGGTGGTTCGTTCTTTTCATA
ACCGTGGAATAGTG
AGTTTAATGTTTCTGTCGATTCATCTGTGCTTTTGCTGCATGCAAT
CAGTATGCTACAAA
TCAATAAAGATGGAAATAACATTCTTTGCGGGAACTGCGACATTT
TGTCGCGATTGTTGT
CCTTTTTC
>ConsensusfromContig3433 Average coverage: 9.34
AGTATCGATATTTTCTGAATTATTCTCGGTTTTATCGTTTTTGGAT
AACATCTTTTTTGA
TCTCATATTTTTCAAAGTAATTTTGTCAATGTTATCGTTACGATTG
TGTTGTATCATTTT
GGTTTTATTCTCAATAACTTTATCTACATTTTGAATCTCTTTGAAT
TCACTCAAATGAGC
ATTATCATCTCCAACATCCATATTTTCCTTATCAGATTCGCTATTC
CGATGATCATTGCT
TTTGAATGCCGCATCAGATCGGAAGA
>ConsensusfromContig3434 Average coverage: 11.83
CCCCATCAATGAACTCCTTATAGAGCCAATCCGACCGTGTTTCCA
ATTCACATCCGACAG
CTGTCGAGTCTACGTTCCAGGAGTGTCTAAGGCTCAATTCAATAG
CAGAAACCAATGCGC

CACCTGCATCATTAATTTCTGTCTTGCTGGCTTTGTACAATGTACA
TTCCCCAGAGTCAT
CAGATGGTCTCCAGCTAGCAGAAGTACAGTTCTTATTTTTGTTGC
ATTTATGCGTGCATC
TTTTTTCATCAACATCTCCCTGGAGTTTCATCACCTTTCTCCATCC
GAAAATGTTCGGCG
CCCCATAATTGTACATAACTTTCGTCAAATCGCCAAATATATGAT
GAACATAGCCAAAAC
AGGTTCCAGATATGTATTCCTTGTGAATCCAGTAGAATGTCTCAA
TCTCAGAGCATTTTT
TCGCTGATATTCTCATCTCCCAGGCCAAATGCAATCGTGACTCAA
TGGGTAAAACTTCAT
CCGTGTAAATGATGGCCTCCTGATTCACTTTATGGATGAGGGAAA
ACGATTGAGATTGTG
TGTAGGTAACACATTCGTTAAGAGCATAAATGAACTTGCAGAGAG
CAT
>ConsensusfromContig3435 Average coverage: 8.87
GCAGTTCCAACCATGCTGATGACAGGTCTTAGCGGAACTTGATCT
TTATGTATCTTAGCT
AGACCGTAAGCTTTCCCCGGTTGACTCCCATTTGGCCGAATCTTC
ATGTAATCTTCATAG
CTTATATGATTTTTCACTTTCTTGCATAAGAGATTGATGATTTTGT
TCTCATTCTTTAAG
ACTGGGTCATCCTTGAACGCTGTTGTTGGTGCTCCTATATTTTTGA
ATTTGGAAGTGTCT
AATATAATTTTACAGATCG
>ConsensusfromContig3436 Average coverage: 13.40
AGCAGGCCCCGGTCGTGTGTCAGATCCTCCGCGAATTTCAAGAAC
TGTGCCATCTACCGG
GCGAACCTGACGAACGGCATCCCATCAATCAGACGATTCTCATCA
ACAACGGTGTCTTCG
ATTTGATCGTTCAGCTCTTGAGCAGCCATATAGATGTGGACCTAA
TGAAACTATATGAGA
ACACAAGAGAGGTGTTACGAGAGTCTTTCGGGTTACTCAAGGTTC
TCATCCGTGGAAACC
AGCATGCTCAAGAGAAGGCTCTGGACTGCCTTGGTTTTCTGCTCA
ATATCAGAGGTGCTG
AGCAAGACCTTGCAATGGCCCTCGAGGAGATATTCACAGGGAAC
AAAGAAGCATGTCTTC
ACATCTCGACCCACCATCTAGACAGGATGATCGAACTGGCGTCT
GAACACGTGGAGGAAG
CTCCCGAATTCCTTGACCTTCTCTGTGCTGTTGTCAAAGTTGAAG

AGTTGAACTTGCCAA
TCAAACG
>ConsensusfromContig3437 Average coverage: 24.74
CTTTCTTTTTCCAAGCAGTAAACACATTCTCCAACAACGAGAAGA
TAAAAAATTTTCTTT
TTGAGGCCAGGAATCACAATATCCCTCATCCTCTGCGGTTCATTC
CAAGATTGAATAGAA
AGGTACTCTCCACCCGTTGAAAATATTAATGTCGAATCGGACGA
GCAAGCAGTACTGGAA
GCCTCTTGGAAAATTGAAACGGGATTTTGCTTATCATCATGTTCT
GATGGAATTTTGTAT
GATGCTTTTTCGGAATCTGACAAGGATTTCACTATTTCTCGGGCA
ACATGTAATTGCTCC
GAAGAGATCAAGCCGAAAATAAAACCTTCAAAAGCATATGGACC
ACACAAGACGATCTGG
GACCGGAATTTGGAGACTTTTTGTTTTCGAGTGGGTTGAGCGTTT
ATAAGTTCGACAGTG
TCTTCGGTAAATATTCGCTTCGATATCAAGGAATCCGTAAAATCC
TCGTTTATCCTTAAA
TTTTCACCCAAATAGACGGCATTGTCCATAATGATATTTTGATGG
ATATCATTCATGTTG
AGAAATAGCTTTGGAATTTGAAAACAAAATAAAGGCCTGTTGTG
AAAATATCGGGATTTC
GAACTGAA
>ConsensusfromContig3438 Average coverage: 7.87
TTCCTTGAGGTGAAGGTGAATGAAATACTTGAGGTGAAGGTGAAG
GTCTTCTGTATGACG
ATGCCTCTATAGGAGGTATCATTAAAGCACTCGAGTTGTTGGTTC
TATTGGCGGATTTTA
TCGCTGGAACTGCGTGAGGATGTTTGGAAAGTTTATTTGGTGACG
ATGGTAATGAGTAAG
ACCTCGAAAGCTGATGATTACTGCTCTTTTCTCTCACGGGAACAT
GATCGATTGTTGTCT
GCCTTACTCTCCCGGGAATATGGTCGATCGTCTGTTTTTCTCTCC
CGGAAACATAGTCTA
GTGTCGTCTGCCTTTCTTTCTCGCTGGAGCCTCGCGATAATTTGAA
TGTCTTGTTGACTT
TCTCCATGAGGCTTTTGTAATTTGTTAAGGGGCCATGCGTG
>ConsensusfromContig3439 Average coverage: 15.19
AATCAAGTAATTGAACTGGATGGAAGTAGGTCATTTGACCCGGA
TCTTGACCCAAATGAA
GTTCCAGATTTTTTGTACACGTGGACATGCTCTTATGAGATTTTG

CATACTCCTAACCGG
GATTCGAGAGGACTTTGTGAGTTACCCAAAGACAACGGAAGCAA
AGTTATCATTCCTGCA
AATTATCTGTTCCCAAGCAGACGATATTCAATCTTGTTGACAATC
AAATGTGCAGACGAC
AGAAAAGCCTACACTTCACAGAGGCTGGTTATTTGGAATACAAGT
GTAGCTGAAGTCTCC
ATCACTTGCATCTCCTGCAGAGAAAGTAACATCTACCTAATCAGC
CAGTACCTCACAACT
AAACTCCTTGGAGAATGCGTCAACTGCGTGGAAAGACAGAATA
CTACTGGGAAGTCATG
AGGGAAGATGGCGTTTATTTGATTCTTGATGGCAACACGACAAC
CACGGGGAACAAACAG
CCAAATTTGGTCATCAAATACAGCTCATTCAAAAATGATTACGCA
TATTCATTCAGTTTG
TTTGTAAAATCAGAAGCATTTGTCAATGAAGGGTATTCGAAT
>ConsensusfromContig3440 Average coverage: 10.59
CTTTCCCTACACGACGCTCTTCCGATCTTGTAAACAAAGCGGTGG
GTGACGTCATATGCA
CGCTCCCTATATCGGAAGAATAGTCTCTCGACATTTTATTACGCA
TATATCGGATGAATA
TCATCAGAAGAACGCTATTCATACGGGTTAATCGTGCCTAGTTCG
TTTTCTAACCGATTT
AACTTTAGCACTTTGTCTGGGCCATGAGCCGCTAAGACGAAATGA
CTGGAGCAGTGGTCT
TGGTTAGGCGCTTTTAATGAATTGTGTATATGTCAAGACAAGGTT
GATGACATTGTTTAG
TTAGTATCCAGACCAAAATTAAGGATATGGA
>ConsensusfromContig3441 Average coverage: 9.70
TTTTTGGAGCCCTTCGAAATGTTTAAATCATTGATCACCCCGACG
AAAGTTCTCCACAAC
ACAAAATTATTTCCAATCATAAAGTTTGCTATTCTCCCATTTTTTT
TGTAGTCATTCTTT
TGGTTTGTATTCTGGGATAGTCCATTCACATACAACTTTAGATCTT
CATCTGATGTCAGT
ACTGAAGTAACATGATACCATGTACCGATTTTAATTTCATAATTCG
TAACTCCCTCTTTC
AGTTTTTCCATGCCATTTTTCCATGCCTTCAATGTTATTTCGTTGC
GATGAATTTGTAGT
GAAGTTCCCCATACTCCATTATCAGAATTAATTTCATATCCATAA
GACGTAGTATTCCAA
AAAAAGCGGACTTTAAAGATAATCGAAATGGTAAAAC

>ConsensusfromContig3442 Average coverage: 5.81
TCTGGAAACATTTCTAGCAGCATTGTTGTTCCTATCACTCTGCTTT
TTCAGACTGATTTG
AACATTCTCATAAAGAACTCGTGTGGACACTGGGAATTGACTGT
TGTCAGATCTACATT
ACGGGGATCTACTCCGTTGAGAATACTCGCTTTTCCATTTATAACT
TTTATGTGAGTCTT
TGGGGATTGGGCATGTTCTGTATTATCTTTGTTTTGAGTGGATTTA
GCTTTGTTATCGTA
TTCATCATTAATATTTATGTAATTCTGTGAGTTATCATGATCTGTG
AGATCATTTGACCC
ATTTCCATCTCCACTGAAACTTCTATTGTGATAATCATCAAATGA
ATCGTCCGAGAACTC
ATCATAATCGTCTGTATGACCTTCATAATTTTGCGATGAATATTCA
GTGTTTGATTTATT
TTTGGGTTTGAGTTTTTTGTTTGGGGCGTGACTCACTGGTTCAG
>ConsensusfromContig3443 Average coverage: 5.55
CCCTCTTCCGATCTCGAACACACACAATTAGAAGTAAATCGATGA
CTCTGTGTACACCCA
ACCCAGTCGTTGGTCGTGCAAATTCCGTTGGAGTCACTGAGACAG
GATTTACTGCCAGTA
CAATTAATGGACCTGTCCTGGCAAAAGTTGGAGAGAAATCCACGG
TGTTTGATATAACAC
AGACAATCGAATTCAGATAACTTCAGAATACCTTCAGGTTGTGGT
TCACCCAGATCGG
>ConsensusfromContig3444 Average coverage: 4.34
ATTTTAAACACGTAAGCTCATGAAAAAAAGATCGCAAGAATGAA
ATGTTGAAACTCAAAT
TTATATTTGGAATATTTTCTCTTAAGCCTTAAAACTAGTTTATCAT
TTTTTAATTTTGTT
GTCGGATATAATTCATTTTATATTAGCGACGGATTATATTTTAAA
CACGGAAGCTCATGG
AAAAAAGATCGCAAGGATGAAATGTTGAAACTCAAATTTATATTT
GGAATATTTTGTCTT
AAGCCTTCAAACTAGTTTATCATTTTTTAATTTTGTTGTCG
>ConsensusfromContig3445 Average coverage: 6.22
AATTGCGGAGTCATGCAAGAACAAGAATGAAACATCATTTGAATT
CTTGGTTATGTTCGG
TAATGTTTTTCATTTATTCTTTCACTGTTATTGAGAATTTATTGAA
GAAGTTGAGCAATA
TATTTTCAGTGATTCAGATTTTAACTTTAAACTCTAAACTTTCATA
CAAATTTCTTTCTC

AGAATTGTTATACCTGATTCATATCTTAGGGCTGAATTTGAAGCA
GATATTTTCATCCAA
AGATATACTATTCATATTGATTTTGAGCTTTTCACTGTGATTGAA
TTAAACTATCAAGAC
CGTACTTAAGGGGAGAAGACTGGATGAGACCCAA
>ConsensusfromContig3446 Average coverage: 13.96
GTGAATCCAGCAAAATAGTCATGTACATTCTCTTGTTTCTGTCATT
TTTTGTTGTGATTT
TCGCCTCCGTCTTTCTTGTTATATGCATTTTAAAAGATAGAAATC
CACTGACGGATGAAT
CTGAAACTGTCACCTGTTGCCCACTTCAGATTGTTCCAGATGCCA
TTGCACAAAATCGAA
ATCCTCAGGATAGTAATTTATTTAATGATATATTCACATACCACC
GAGGCCCTACACTCT
ACGACAAAGATATAAAGTGTAATGGCAAGCAAAGACAACCAAAA
GATAGATGACCCCCAA
AATGTCTTGAAAACTTGCCAGTAAAGAGATTCGCACATTTAAAGA
TTTTT
>ConsensusfromContig3447 Average coverage: 25.94
TTTCACAGATTCTACACTTAGCAACGACAACCTTGCGTAATAATA
GCGTCAACAGCTTCC
GATGTTATTGATGCAGTACCTAGCATTGAAAAGGCTGAATACACT
TTGCGGAATACAAGA
ATCGTTGCTAAGAATCAGTTAGCGGAAAATGAAGACAACGATTTA
TCTGCGATATCTCAA
TACGAGAGCGCAAATACATCGGCGGTTGCCATGGAGTCGAATGA
AAACATTGGTGAGGAC
CGAACGGCCGCGATTTACTAGCCAGTGTTACGGGAGCCCTCCTTC
ATCGCCAACACCTTG
CAACGATGATGATGATTATGAAGAAAAAAAAAGATGAAGAAACT
GTGCGTTAGGTGAGAG
GAAATGGATTGGAAGAATAATTATGAAAT
>ConsensusfromContig3448 Average coverage: 25.27
CGACGACATACAAAACGGAAAATTTAAGCTGCCATATCGAATAA
GCTGTATAATTGATTG
AATTATATGAAACGTGTATCACAATGATAGATAGAAACATTAATG
AGTTAAGAATTACGT
AGAAAAACCTATGACGCGTCTAATATCAATGAGTTGAGATTTCA
GATGATAAAAGAAATG
AATAATCGTTAGTCTATGAACATCATGACATAGTCTCCATCAAAT

>ConsensusfromContig3449 Average coverage: 14.93

TTGCAAATGCAGCCGTGGGCGTCACCGCCTATTGCGCATTTGCCA
TTGGCATTACACTTT
GTATACCCAGTGCAGGTGGCTGTTCTACAAGTTACACCTTCCCAG
CCGGCTTCGCACTCG
CATTTATTCTTTTGATCGTCGCACGACCCCCCGTTGATACAGTCC
ACAGCTGTACAGTTG
ATTGTTTTGCAGAGAGGACCAGACCATCCGTCAAGGCAGTTGCAG
CTGTAATCTTCGCAT
GTTCCTCCATTTTCACAAATATCGGCCTTGTTGCAATCAACTCTA
TTGCAGATAGTACCA
GTCCAGCCTGCTACACAAGTGCAGAAATCAGCATCTCCATCATCA
TTCCATGTCCCCCCG
TTCTCACATGGTGTGTTTTTGTCGGGTGCCCACGTCAGTTCTGTC
TCGCAAAGAGAGCCG
TATTTTCCTCCTGGACATCGGCAGACATTGGGAATTACAGTGGAA
TCACATATTGAGGTA
GATTGACAAACGGTCGAGCCAGCCACACACTTAATTTCTTTTGAG
CAATCGAATCCATGC
CACCCGTCTCTACAAATGCATGTATGGACATTGTTTATGACTTTGC
ATATTTCTTTAGGT
TTACATCCCTCATACGTATCACAAGTGGCTTTATAGCAGGTTGAA
CAG
>ConsensusfromContig3450 Average coverage: 15.26
CAAAAATAATTATGTTGCACTTAATGCACTTAGAAATTCATTATC
TATTCTCAAGTATAA
TATCAAAAATAAAATAAGATTCGCCAAAAAATTGTTTTTCAAAA
TAAATTCAACGAAGT
TAAAAATGACATTAAAGCTACATGGCGGCTTATTAACTGCACATT
GGGACGTTCTTCTAA
TGAACCAATAGACTATATCATTGAAAATGGCAATATGATTACTCA
AAATAAACTTTTAGC
TAATATATTCAATACATTCTTCAACGGAATTCCCGTATCACGCCT
TTTAGCACTACCCCC
AACAAATAAAACTGTTTTTCATTTTGATCACAATATTCACACTAC
AACAGTTTTCGAATT
TACCCGGATTTGTAGCTCTGATGTTCTCAATGTCATAAATAATTT
TGAGAACAAAAGTAG
TAAAGGAGCCGATGATCTCTCTATCAATCAACTGAAACTTATATC
CAAAGAAATTTCACC
ATTTCTCACCCATATCATCAATTTAGCTTTTTCAGAAGGTTCATTC
CCTGATCAAATGAA
AATTGC

>ConsensusfromContig3451 Average coverage: 21.97
GGCGATGATCGAATGCGGGAAATCGATATTTTTATTATTTGTTGT
TCATGTAACAAATAA
TTATATCATATCTGATGTTCATATTAAAGTCTAGTGTCCATCTCCC
ATCTACATTTTTGA
AGCAAAATGAAGAGCAGGAAGCAGAATCTGCATATTCAAAAAAT
GTTGCAAATATTGAAA
AAAGGATTTGTGACATTTTAAGTCCTCATGAAACAAACCTTGGAA
AGTTTCAAAGTATAT
TGATCTGGACTGATGTACAGAAGTCTCTTACAATATTTTGCATTGC

>ConsensusfromContig3452 Average coverage: 12.54
AATTTTTAGAACGACTATTCATATTGAAAAGCATAGTCGATGATT
GTGGGTCCAGCTATG
TGATAATATGTGGGGATTTGAACACTAGTTTTAGCAGAAGCTCTT
CCAATTTTACAAACT
ACATGCAATCTTTCTGCAACGAAAGCAGTCTAATTTTATCATCTT
CCAATCGACTACCCC
CCAATAGTGCATCATATTATAGTGATAGATGGCTCACAGGATCAT
TGATTGATCACTTCA
TATGTTCTGCTGACAGTGATAATCTGAT
>ConsensusfromContig3453 Average coverage: 7.43
CGGGATCTCATAATTCGAGTGTAGAAAATGTTGTTGACGCGATCT
GCAATCAAAATATCA
CAAAATATGAATTGGATTCAGAGTTGAGAGAACTTTTCTATATTC
CAAAAATGAGTACAA
ATATGCAGCAACAAAGCATAGAGTTTGTAAGCACGAACATGAAG
TTACAGCAGATCTTAC
AAGATGTGGCAATCATGCAGTCTGTAACAGTTCTTCTTGGTACCG
TTGGGATCACGAAAT
ATGTCGAATTGGCCTTGAATGCGGGCCCTGCTTTCATCGAGTCAC
TCAAGATCGCCTTCC
AGGCCAACACAATCGTTGCCAAGGGATTGTTTAATGCAATCAAAG
AAGCTGTTTCCGATC
CTATCATGCTCGCCCACTATCTGGGTCTCATCAAACGAGATGTCA
GCACACTCGATGCCA
ACTCTCTCGGAGATTTCTTCAGAAACGTTTCGTCCATGAGCGATT
TTCTGGCAGACGTTC
AAGGAGTGGATTCATTGTTTGTGGATGAACTGTTAGGATCCAATG
TTTCACCGGAACAGT
TAATTAAGTTCATCTTGAATCCCGGTACTCTGGCGAACATTTTGT
GCTCTGATCAAAACT

TCACATCGACATTCAATCTCACCTCAAGCGAGACTTCCAGTGAAA
TAACGGACGCCATCT
GTACGGGGGATGTAGAGAACACAGTCGCCACCTTTATTGAAAAT
CTCGACGTAGATAAAA
TAGTCATGACTGAACTACTATCCACGATGCCAACGTTGATCTTCG
ACGCGGAATACTGGC
GTA
>ConsensusfromContig3454 Average coverage: 9.97
TCTCAAGTCATGTAATACGGGCATTCTTTCCGAAACACAATACAA
GTCATTACCCTGATC
CCGAAACAGATTATGGCGTAGAAAAAGAGGATAGTACACATGAA
AGATTTGGCAAATCAC
TGGCAAAGATGAAAGCGGGAATAATGTTTGCCGCAAAGGACAC
CAAAAACAGAAACCGG
ATCGACCTGTGAAAGCAAGCAGAAGAAATAGCAAGAGAAGCCAC
TCACAAGATATTAAAA
AAGAAGCAGCGATGAGAAATCTACTCAACAAACTTGTGACAGAT
GATAAACGGTACCTAA
AACATAAGAAGAGTGAGAGCGGCACATATGATGAAAAACGCGA
CATAAAAAAGAAGAAGA
CTGATAGCGACACAAATGTTGGCAGTCAGCTTTCGAATAATAAAG
ACATTCCGATCATCA
GAACTGATTCTCAGGAGAGCTTTGTGAAGAGTTTGGCTTCTGATA
CAATGTCCGGTCTTG
ATAGCAGTGTCATAAAACAAGATTACTCAAATTCTCCTACTCTCT
ACGACGATGTTTTCA
AGGTTAAACAGAAG
>ConsensusfromContig3455 Average coverage: 10.76
CCAGTCTGGACCCGAGGAAATTTCTTGTGTAGATGTTGGAGCTGT
ACGGGTGAATGTAGA
AGGCTTCGTTGAACGTTGGGAAACCACCGGCACAGCTGTAGCGG
CGGCAAGAGAGCGGTT
AGAAGGGATCTCGTCGAACGCCGACCGGGCGATTGAGCTGATGG
AGTCCGTGACGAATTG
GACGCAAGACTCGCTCCCACGCGTTGAAGCTGTCCTGCCACGTCC
ATCAGATATGTCACA
GGCTCTCAACTTGTTGCATGTCTATCAGAATAACTGCGAGGAATA
TGACGAAGACTGGAG
GGTCGCTCAGGAGTCTCTGGCACTTCTGCAGACCGCAGAAGCACC
TGTTTCGATTGTTGA
GCCTATCAAAAGAAGAGCAGAGGAAATTCAGTCGTTGAGGGATG
AACTGGCTGGCAAGAT

GTCAGAATTGGAGAAGATCATCTCGGATTTCAATATTCGACATG
AGGAGCTGCGAAATCG
CTTTGAGAGTTTGAACTTGACACTTGTTGAATGCAGTGAATCTGT
TGGGAAATGCGAAGA
GTTGAGTTGCAGGAAAGAAGATTTGGGAGATTGGTTTGAAGACA
TTCAGAACGCATCAGC
AACCATTGCGACGCTACACGGAGACATTTTCAAACTGAGCGAAG
ATGCGAAGTCTTTAGA
CGGCGAATTTGATGCCGTGGAAAGTCGA
>ConsensusfromContig3456 Average coverage: 13.72
TGTCATCTTGCATAACTTCCAAAGATCCTGTGACTATGAAGCACA
GCGCGTCAACACTCT
CACCCTGGTGATAAAGCCTGTCTCCGGGAGCGCAATGTATCGTTT
GGAAATGTGTGGCGA
GCGCGCGGAGACAACCCTCGCTGGCCAATCGAAAAGCAGTGTGTT
TCTGAAACACTTCTC
GATTGAGATGTACGTATATCTCTGAGCGGACGTCTTTTGGACAGT
AACTCATCACCTTTG
ATTCATCGATTCCCTTGTTGAGCGCCCACATAGACATGATGTAGT
CCATAACCCGTTCAA
CAAGATGCTTTGGAACTTCGTGGAACTTCATGAATTCGCGGACAT
TGGAGAGCACATCTT
GATATCTGGCGCTGGAGGAGTAGAGATGGGTCCAGATTGTCGTC
ACATTCCCAAAGATGC
TGGCGTACAGCAAGGATCCGAAAATCATCATGGCGATGGTGAAGA
ATTTTTCGCTTTCTG
TATTAGATGACACATTTCCAAATCCAACGCTTGTCATGCACGATA
GCGTATAATATAGAG
CAGTGGCGTAGTTTTCTC
>ConsensusfromContig3457 Average coverage: 7.20
TTTTTAAATCACCTTATATTTATTTTACAAATTTGCATACTATGGC
ACAGCCATCAAAAT
CAACGAATGACATTAAAAGAACAATAAATATAATATTTCTATCAA
CCTTTCATTAAGCAT
TCAACATTGCGATTTGATTTGCCAAGGGGACATTTCAGAACATAC
TCAATTAATCATAAT
AACAAAGCTCGAAAAATGTTTTATCAATATTAAAATTATACGTCT
CTGAAATTCATTAAA
AAGATCATAATAATAAAAGAAGAAATTAAAAATCACATTAAAGC
CATTTCTCCAAAATAA
AGAAGGGAATAGGATCAACTTCGTGTCTGTCACGTTGTCTGTCAT
GTTTGAATAGCTGAA

TAAAACTTGCATCAGTTTATCATATATA
>ConsensusfromContig3458 Average coverage: 13.85
GTTTGTCACAACTCATACTCCGCTTGAACGCATGTAGACCAATGG
GGTTCGATGGTTTCA
CGAAGGAACTTCTTTCGTTTTCCAATTTGAATGAGCCATTGCTTTT
TATTGGTCGATCGT
CTGACCCAGATAATCGAATGGGCGTGGCCATTGGCGTAAGAGGCA
TCGTTGAATCTTCGT
CGTCTTCCGTTATGGAGGGAAGGCGTTTCGGTGAGGAATTTAGGT
CAATATCTTCTGCTG
CATCATGATTTTCCCCCAAGTTGTAGGCGAGGTCATGCTGAATTT
CTTCCTCAAAAAGGC
GACAAAAGTCAGGATATGTTGAGAGGGTGTCTTTCAGTCCTTGCA
GAACAATTGCTTGCA
GATCACAATATGTAAGAGATCTGACGTCACAGTTCGACAGAGGC
CATGATTCAAAGTGTG
TCAGATCTGTTCCAAACAAATCACCCTTTCCCAAGATGGCCACCA
CGAGACCTTCTTTCA
GAATCTCCATAGAACCATTGCAGACAAAGTATATGTGTTGGACGG
GATCCCCCTTATGAA
TGATGTATTCGTTGGGGGCACAGAACATGGCTTTGGTGGCCATGG
CAACCGACAGTTTAC
AACCATGAGTGGCTTGTTCGAAGATGGGGCAGGCCAGAATTTCG
CGGTGGAGGTGGATAG
AGATGTCCCCCTGCATCTCCAAGGGAAAGTCCCGCATCACCTCCT
CCCTCGAAATACCGC
TGTTGAGTGACCACATCGTCTGGAAATACTCCTGCATCCTCCTTTT
CAGATCCTTTGGTA
TGTGATGGGTCCGAATGAAGTCCTTGAGATCCTGAGTTTTTGCCT
GGTAGATGGCGCGAC
GGGCGTACATTCGATTTATGATGGCCGTGACGTTCCCGAACACG
AGGGCGTGCATGAGAG
C
>ConsensusfromContig3459 Average coverage: 8.58
CTTTTCTATTCACGAACGTCAAATTACCTTCAATTCTGATTATTCT
CACATTAGATATTT
TAAGTGAGGTCGTTTTTGGATTGTACTTACACTCACTTGAACATA
CAAGATCTGATGTGC
CATATGGCATATTGGATTGGCCATACACTTTAATTTTCATTTGAG
GTATGATCAAGGTCA
GGATGGTCACGACTATTCCAACGAGAATTCCGATATCTATATTGA
GAACAGTGACGGCCA

GAAATGTTGAAGCCCACACGGCGAAGTCGGGTTTGCTTGAGCCC
AGCAGCATGAACATGA
CTTTAAGTTGTGTAAACAATGGAAGAA
>ConsensusfromContig3460 Average coverage: 5.92
CCAATATTTGCAGTGTTTTCTATTTCATTGCTGGTCTTATTCATCA
TCTCGACAAGGGGA
GTATCGCCCATAAACGGAAAACAATGGTGCTGTATGGATCAATG
GCCATATTATGTAGAA
TGGTATTCACCGAGATTATATGGAGGTATCTTGTGTTTATCAGTC
CCCCCAAAATATGTG
TTTTACTGGATGCGGGGAGTACCATACATAAATCAGGTATAATCA
TTGAATTGTTCTGGC
ATACATTGGTATTTTCTCTACTTCTCATACAAAATCGAATCCTCCG
AAGCCCTTATTTCC
GTCACATTCTTCTCCATGTAAAAGTTGAAGAGGACTTGGCTACCA
AGATAGCATATTTCA
GAGGGCACTCCTTTCGTGCTTTTTATAATCTTGTGACGATAAAAG
CGGGAGAAAAATGGC
AAGACAAGAAGAAAAGGCTTTGTGAACTTTGTGGAAAAATGCAA
GGCTCAAATTTGGGAT
GTGCGGAAACTCATAATACAGCGATTCTGTGCGGAGAATACAGCT
GTTTCACTAATGATG
AGGAGACGGAGGAAACTATTTCAAGTATCTTTACA
>ConsensusfromContig3461 Average coverage: 7.75
TGGACTTCAAAGTTATTCTTTTTAATTCGTTCATATTTTATTTATC
TTCATTTGGTGAAG
TTAAATTTAGTAAGGTATGCATGTGTATATATCATCCACCAATAC
CACCAATGAATGTCC
TCGACCGTTGCATCGCACAGTTGTAAGAGCTTCAGCCACATGCAC
GGAAGTGCCTTTCTC
TCCAATGGCTCGATTCATACAAATGAATGAAAAATGATACATAT
CGACTCGGCGCTAAGG
ATGAGAGGTGAAGGGCAGTGCTTCTACGACATTGATCAATATATC
TTGATTTTGTGAGCT
ATATAAATCCAGAACTGTTTGCTCTGCTAATTTGGTTCACAAGTA
AATGGCCATGTTTGC
TTTTTTGATATCACTCCGAAAATCTGTTCAAAATGGCG
>ConsensusfromContig3462 Average coverage: 7.93
ATTCATGTTCTTCAAGAGTAACCCCTGAAGCGGATGTGTTGTGTT
TATGTTTTCCGCCCA
AGATGCTTATATTTGGGAATATCCTAATCTCTATGGCTTGCTCAAT
TGTGATAATCTTTC

ATGAAATTTATCTAGAAGGTGGGATTGACATAAAGAATTATGTTC
TGTCAGCATTCTGCA
TTATTTGGCTAATCGTCTCTGGGCTTCTTCTCTTCGGCCTATATAA
GAATGCCGTCTTCC
TCTTCATACCATGGATAACTCTGTGCTGTCTCATGATACCGGTCCT
TCTTGTTGGAATCG
GATCCGGAGTGTTTGCTATATTGTCCTGTTCAAACAATATGACAG
ATTCACCATGTCATC
ATCACACCTACTTCTTTGGAAC
>ConsensusfromContig3463 Average coverage: 5.29
GCAGGCACTGAATACCATATATGATTTTTCAGCACTCTCCATACA
AATTTACGATGTTGA
GTGAATACAGAAACAAACAAATTACTATGTATCTAGCAGGAATA
ACCTAAGCATGACCAT
AATTTTCACGAACTCCTGGTATGCCACTGCATACAAGATAAAACA
TGTGTGTGATCATAA
TAAATCCAAATCTGCCTAATGATGGAATGCAACCTAATGTGCGTG
TTTATTTGCCTTTTA
TGTTCTTTCAAACCACTACTAAAGAGATATTTGTGATTTTCCATA
CTCGTCGAAGTCCGG
AGTGCCATAGACACAATTTCTAATATATTTATCAATTAA
>ConsensusfromContig3464 Average coverage: 16.73
GGCACTTTTAGCTCCCTCTGCTAGCAGTTCTTGCAAAACATCATT
CTTAGGTATAAGCTC
GGCGTCATAGATCCGCTTGTTCATGGATTGCGCGCAAAGCTTGAG
ATTCTTATGATGAAA
ATTCCTGTTGAATAATGCCTGATTAGAATATTCATATAAGCGATT
TTTGTCGAAGATCAT
GGTGATGCGATTCGCACAATCAAAACAACAAGCAACGTCAGATC
CTCCAGCCAGATTCTC
TCTGTTAATTTGATGTCTCGAAACATAAGATCCACACTTAACTTT
GAAACACATTTGAGT
GATAAAACTACGATTGGAATATGACTGAGGACCGTGGGTTCCGAT
AAGATGTTCTCGATG
AACATCCGTCAGAAATATCACGAGCGAACCATCCGTATAGACTG
GGGATTTACACAAAAC
ACCTAACTTCACGCCACAACTCGTTTCGATACTCTCTCTGTCAACT
TCGTCAATATTATT
GGCTGAAGTACTGGGCCTGTTAACAGAAACGCCCATATTCGTCAG
GGTCTGTTGCGCAAA
AGGTTTGACCAAAGACGAAGTGTTCGAAGGGAGAGCATCTTTCG
ATGAACTGCCGAACAA

CAACTGTGAAATATCATTCTTCCGCTTTGAAAACGAGTTGCCCGA
ATGACTCTTGGACA
>ConsensusfromContig3465 Average coverage: 7.79
TCTCTATATACCATTGGAGTGCATTGCGAAAGTCAATACATATCT
TTGATATCATAAAAC
AGTTCCATCATGTGGTATTAAAACAATCATTCGAGAATCGTCAAT
ATCCAACTCCACCAC
GAATCTCTCGGGCGCGCTCCCAAAATGATGGCACCGTACAGCTCT
GTTACCTTCCTCCCG
GAAACCATAAATCCATGGATGAGTTGGGTTTCGTATATACAGGA
ACAAAGTGATGCAGTC
GAATGTATTGTCCTCTATCAGATAAAAGGAGATCTGTCTGCATGC
AACTCAAGATTAGCA
ATCTCAAGAAAACTGATTTTTAATCTCAGAAGTATATTTGAAGGC
AGTGACTGCACAAAA
TCCTACATGGTAAACAACAGATCCTACAAAATGCTTATTCGCTCG
ACACGCTATGTAATT
TTGTTTGATGGAATTATCGACTACTTCATTATTGGACATTCGAAAA
CATTCTTGATTTTT
GC
>ConsensusfromContig3466 Average coverage: 6.80
GCCGCAAGACCGTTCACATGTGAGAGGCGCCATGGGGATTGCAG
TAGAAGGTCGGCATTT
CCATGATTCGAGAAAGTATAGCCATATAAATTGCCGCAAGACCG
TTCACATGTGAGAGGC
GCCATGGGGATTGCAGTAGAAGGTCGGCATTTCCATGATTC
>ConsensusfromContig3467 Average coverage: 10.90
TCGCAGCGCAACATTAATCAATGTTTCATCTACATTCGCATAGCA
ATGTTGATGGGAGTC
ACATCACTGTGTATCATCGTCTCCTATTTTGTTAATAAAGACCTGA
TGTGGTATATCTCC
GCCGCTCTACAAGGTCTTCAAGGGTTTTATATTTTTGTTGCACTG
TGTTTGACCAAAGAC
GTATTGAAATCTTTGAGATCCTCTAAATTCTACCGACGTGTGTTC
TGTCAAAGAGACCCA
AATGCTTCTGGTCATCGAAGTGCAGAAAATCCTAAAATAACTGA
GCTACAGAAGCACAAA
GACCTGCCCGAATCAACCGCATCAGATTCCGATATCAAGAACAA
GCCTGAACCAGGCTCT
GAAAACAAAAGCACAAAG
>ConsensusfromContig3468 Average coverage: 10.60
CCGATCTAACGAATTAGAACATGAGGCCGTTGATTTTGCTGAAAA

TGACGGTCAAAATTC
CACAGAAACATCAACCGGGAATGAGATCAATAAAAGAGCTGGAT
TCGTACGTATTGGAAA
GGCAGGCTTCGTAAGAATAGGAAAAGACTCTCCCAAAAGAGCTG
GTTTTGTCCGAATAGG
GAAGAACCCAACCAAAAAAGCAGGTTTTGTCAGAATAGGCAGAA
GTCCTTCAAAACAAGC
TCGAGTCGTGAGAATTGGCAAAACGCCGATCAAAAAAGCCGGCT
TTGTTAGAATAGGCAG
GGGTCCGTCGAAGAAGGCTGGTTTTGTACGAATTGGTAAAACAC
CGACCAAAAAAGCCGG
TTTTGTGAGAATAGGAAGAGACCCTTCAAAGAAAGCAGGTTTTG
TACGAATTGGTAGAAG
CTACCCAAAACGAGCAGGATTTGTAAGAATAGGGAGAGATCCAA
CTAAAAGAGCCATGTT
CGTCAGGATTGGAAAATCTAAAAACGCCAATGAAGATTCTGATA
ACCAAAACTCACAGCA
TGGCGAGTACTATGATGATGCTTTCAGCCATGCTGGATATAAAG
ATGACAGGGAAACTGA
GGAAG
>ConsensusfromContig3469 Average coverage: 9.47
GAACAATACTTATACCTACCCGAAAGGCGAAACAACGAAACACG
GATCATGGGGAAAAGA
AGTTCGTTATAAGCTGCAGATTCCCGAGAAAACTGATGAGGAAC
TGACGGATAGAAGTTG
GTTCGTTGAACGTGAATATCGGTGCAAGGCGGAGAACAGTGGAG
GATCGTATTTGTCTGA
AAGAATAACGATTTATTTTGCGGAGAAAAATGAAACGAGCGAAT
GGAAACATGTCAATTA
CACATGGGGACAAGAACTGATTCTTCCCTGTTATGAAAACAATAT
GGGCAACGCTATCTA
TTACGAATTCTATTGGGCTAAAGAATCACGTTTACGCGATGAAGA
AGATATTTATGTAGT
ACTTCAAAATGGTACATTGGTGCTCCGAAATATGTATGAATTGCC
ATTATTCGATTTCAT
CAGAAGGTACAATGACTCTCGAATTCCCCTTATAGTCTGCG
>ConsensusfromContig3470 Average coverage: 12.55
TTCTTGACTCACCCTTTGACCCAATTAATTTAATATCAACAAAGT
TGGAAGTCCAAGAGC
CAGGCATGAACCCTGTAATAAGTGTGACGAGAAAACAATGTGCGC
CTTTTCGTCGATTTG
ATGGAATATCCGTATAAATCGGCCCAAAATTAGTTTTCCCGAGTT

TTTCCATGTAAAATA
AAAATATAATGCCGAGAATGTAGAGAATCCATATACCAATAACG
AGAATCAATGGAAGTG
GGTTATTAAAAT
>ConsensusfromContig3471 Average coverage: 12.24
TCATATGCAATAGAGCAAACTTTGTTTTAGCTTTGATTTCGATTTG
TTTAATTATCCAGT
AATAATGTCCTTTCTTGCGAATTAATGCATAAGTTTGTAAATATT
GCATATTTTCGCCAA
TGAGATGAGCCCAGTTGCGGATAAGATCATAAGATGGCACAACTT
GTATATTCATTCTAT
ACGATTTTTTCGAAGTAGGCTTTACATATGTTACTTCAATATCAC
CGGTGGTGAGACGAC
TGTAACGTGGGACAAATATGACGAAGAAGACTTGTGAGGCCATA
AGTGGACTTTCGAACT
TATGTATTATTGATTGCTTGGCCTCCAAACGCGGATCTGTTTTAT
TGGCAATGTGAATGA
AATCCATGCACCCCGGAGGAACGGCAAATTTGTTATCTGGTAAGT
TTCGAGCAGTTCTGT
ACGGATAAAAGTCATCAGTAATCTCTCGGACACGAGTTATATTAT
AATGTAAACATTTGA
ACATCGACCTTACGGAGACATATGGAAGATGATCAATTTTTTTCT
GTTTGATCTCCATCC
ATTTTTCAACCATGTCGATTTGATAAAGTTTATTAACATTTTTCCC
GGTGGTAATTCCAG
GGTAATATTTCACATGAACTTGATCATTCGGACAAGGTTTATATC
CCATAATAGGCATTC
TGGCTTCCAAACTGTCATATATTCCAATACAAACTTCGAAATAAA
GAGCGTATTCCTTGC
CAATACAATCCGCCTCGATGTAGGCATTTATTTGGGAATAAGTGA
ATTCATGTCTTCTCA
CTTTCAGCTGGAAATCTCCAAATTTATTTCTTATATCTGCTTCGA
CATATAACAGAAGGA
AAATC
>ConsensusfromContig3472 Average coverage: 9.12
GAAAAACAAAATTAAATTTGTACTTCAAGATAAAAATTCTGACTT
GTTTGTGGATGCTCT
ATGGAAAGATATTTATAAACAGGATTTCGATTTCATAAACAAAAC
AACAACATTTGTCGA
GCTCGGCTGCGATTGGTTCCCTGCTTTCTTCAATCCGGGCTCCTG
GGTTCGGAATAATTA
TGGGCCCGCCCATTTCAAGCATGCTGAACATTATTCCAGATACAA

AAAAACCAAAACATT
GGTATACAAGAGCGGACATTTCAGCACATGTCATAAAAACGATG
GAACATGCTTGAAATA
TTTGTCCACTGATGGAA
>ConsensusfromContig3473 Average coverage: 7.64
AAAGTTGGTCATCATAGCACAGAATTTGAGCATGAAGATTTTAT
ACTCAGAAAAAATGAA
GAACCAGCTGACTGGCTCGATGCGCAAGAGGAGTGCGTTTGGCA
AGGACACAATCTTCCG
AGCTCCATAGGTATTAATGCTCTACAAAAACATTTTGGACACAAC
AGCCACCGTGTTTGT
GAGAAAAATCAAAAATCACATGTACCCTTCCAGGACCAGGACATT
GATTTTCATTGGATT
TATTTGACACGGAATGAAAACAGTTCATTCATCTGGGCTGATGGA
TCTAAAGCAACCGAG
>ConsensusfromContig3474 Average coverage: 11.44
CGAAACACCTTGTTTACGTCTTTAATATTGACTGGCGGCGAACTC
GTTTTCATCAACATC
CGAAAACAACAGTGGAGCGTCATCATCGTCATCATCATTTGCGCC
ATATTTATCATCATC
TGTCCCACCAATCGGTCCAATATCATCATTCATAGAATCCATCCT
TGAAATATGGTCCAA
GGTCGAAAGTTGGGGTAACCTCTCAAGGTCATCATTGTCTGACTT
GAAGGTCGTGAGCGC
TTGGTCCGCCATGTCCCACGTTTCTCTATCAGGTGCGTTGCTACG
GATGTAACCAAGGAC
GCGGTGGAGTTTCAAGTAGCGAGCGCGTTGCTTCAGTTCCAGCC
AATCAACGCAGTGGAA
TATGATCTTGATGACGATTTTCAGAATGAGAGAATTAGAGCTGCG
GCTACAGCGCTTTAG
TGCTTGGTAGATGTTGCGACATTCCAGCATCTTCTGACAACTATTT
TTCTCTTCCGCGAA
ATAGAGAAGAATTTTAGATCGGAAGAG
>ConsensusfromContig3475 Average coverage: 47.00
ATATAAGAACAAAATATACTCTCATAGCATTATTATTATCATGTG
CTTCATACCAAAGGA
AAGAATATCAATTAATAAAGCAACGGTCACCTACAAAATTTATA
AATGTAACCATCATGA
CATACATAATGTACAGCGGTCACCATTCATACAAATAATCAGTCG
CTGAAGCCCGAACTG
CTATCGGTGTCAGTGTCTGATGACTCCTCCTCCTTTTGGTTCCCCT
CTTCAGCGATTGGT

TTATTGGGATCGTCCAACACGTTCGGGTCTATGCCTTCCATTGCCA
TTTTCGCTTTAACA
GCGTGAGGAGGAACGCCCACTGAAAGCATTTTGAAGTATTTCATA
TATAGCGGATGGTTT
GATGCTTTGACCGAGTTCGAGTCCGCGACTTTGGTAGGCTCTGAA
TTCTCTTCGATTTGT
GTGTGATTGTCTGTCTGGTCTTGTGATTGGATAACTGGATTGGCGA
TGACCTCATTTTTG
GACTCGATCGAATCTTTTTTTATTTCTCTGATTTCGGGAACTGACG
ATAACTTGGCCTCT
AGAATTAACAAAAGCGATTCTAATTTCTGAATTCGAAGATCAACT
TCCTGCAGTTTTGAT
TGACAGTTGCGAGAAAATTTATTGAGAAAACTTGAAGTATGAAG
GAGAAAATGATTTATG
AAGTTGATAAGTGTACTCTGATCAATCGCGGGAATTCTTTTATAA
TCGAGTCCTGTTTCA
ATAATACTTTCACTTTCGATGGGTGTTTCTGAAGTCATTTTTGCTT
ATTT
>ConsensusfromContig3476 Average coverage: 15.59
TCGGAAATAATGTGAACTACCGCATCAGAGTTAAACCCAGCAGT
GATATGAATGCAGGGA
CAGATTGGCAGGAAATATATATCCGGATATTCGGCATCAATAGTT
TCACCAATAAACATC
GACTCAATAACCCTGACAACAGCTTTGAACATGGTCACACTTATG
ACTTCAGCTTTATCG
ACAAAAATGTCGGTATTCTGAAAAGACTTAATGTTCGGAAGATA
GGAGGAAGCAGCTGGA
AACTTGAAAACATCGAAGTAGGAATAATTGGATATCCTAAATGGA
CCACATTTATTGCTG
GAGTATGGATAAACGAAACTCAGACAACCTTCTATTCAACATATG
TTTTCACAGATCAAT
GTGATTCCTCACCATGTCTACACGAAGGAACATGTGTCGATGGAG
CTAATCGCTATACCT
GCATTTGTGCTAATGAATACTACGGTATACGTTGTGAAACAAGAT
ATGTGATATTTCCAT
TGCAATTTGGAGGAAAATATTTGGTAGATGGAGACATTCGAGGAA
ATGTTCACGGCTCTC
TCAAGAGATATACTGATGGCGATATTGAAGCAGTCTCATTCAAAC
CTGG
>ConsensusfromContig3477 Average coverage: 8.13
TTGCTATCAATTCTGCTGCCAATTACTGTGGTATACTTCTGTTTAC
TCTGATACTTCAAT

GGCTTATACAAGCAAAAACTCCGAGTAAATACTCATTGATAGCCG
CACTCTTGTGTATTC
TTTCAATAGTATTCAGTCTTCAACCTATTTTTCGAGATTTTTCCAA
CGGTTTTATGGAGA
ATGATATATTAACAGTTTTTGGTTTCGGCCTCACAATTATCAGCAG
TTTCGTATGCGCTT
GTTATGTCATCATTTTCACGAGTTATATCGCTGCTGAAGCATTTGA
TTCTACACTCAGCT
GGGTTTACTTGATATCAGTGTACATTTCCATAGTAGCTGTATTTG
TGTTAGAAACTCCAA
AATTTCCTACAAAATCTTCGGATATTTTATTAACATTACTACATA
GTTTCACGGGAGGAA
ATGCAGTTTTTCTGTATGTGCAATCCTTGAAAT
>ConsensusfromContig3478 Average coverage: 14.87
GCTTTCCACTCTTCTTGCTCGTCTTCGGATTGATGCCATCTGACTT
CTACTGCTCTGTTC
CCGTCATCCATCGGTTGGGAATAGATCTTCTGCTTCTCTATCCTA
TCTGCTGGAGACAGT
CCTTGCTCCATCAGCAGGATGATTTTCGCCCATTGGCGTAGCCACT
CATTTGCCGCTTCT
GCGATTTTGTTGTACGTGTCTCCCATCATTGCAATGAGAAGATTA
ATCAGAAGAAGGTTG
ACCAGTGTCATATAGACGAGAAAAATACCCTTGGGAACCACAGA
ATGTTCGAC
>ConsensusfromContig3479 Average coverage: 25.09
CTCAAATCAACTGGTCTGCCATCTGGCGGCTGAAGCATCTACATC
TTCTGGATATCAGCG
GGAACAGATTATCGCGGTTCGATCCTCCGGACGGCCATTCGAAAG
CTTTGCGGAATCTCA
AGTCTTTATATCTACACAATAATCGTCTATCGGATTTGAGCCCGG
GATCGTTCGCTCTGA
TGGAATCTCTCGAGACATTGAAACTGCAAAAGAACCGAATCTCTT
CAATTCCAAACGAAT
CTATAAAAGGACTCAAATCTCTCAAGCACTTGGATCTCAGCCGAA
ACCTCATAGAGACTA
TTGGACCATTGACCTTCACCGACCTTGACCGGCTTGAGACTCTTC
GTCTGAAGCGCAACA
ATATATCGCGCTTGG
>ConsensusfromContig3480 Average coverage: 28.07
TAGAAATGGAAGTGGCCTGGCTTGCTCCTCAACACCCTACAAGAA
TACGCTGATAGATTA
TCCGAATCAGGCCTATATGGAACCGAATCACCGTACGCCTATTTA

TCACACACCGCCCAG
TAAGCAGAGTGGGGGTTTCATGCAAAATTCGAGGACTTCTGAACC
AGGATTCAATTTATA
TACATCGCCCAATTCCTTTTACAATCAACCCCAACCTGAGGCAAG
ATCAGAATTCGGAAC
CACTGCTTATTTACCTGTGTCTATAATTCCGACGTCCCCGTACA
AAATAATGGAATGGT
ACCTGTTCAGCTAGTTGCTGTTCCACCTACCATATCTCCGGAGAC
GATCAATGACTCAAA
CCGTAACGCATCTCGTGGTCCCATTCGTTATTCCATCAGAGAATA
TGAAAGACTCGACAG
TGGACAGAATGATAAGCGCCATAGGAGGAGCAAACGTGATAGCG
ACCACGTGACATCAAT
GATACGTCAAAATCTATCAAGGAATTCGAGTAACACGAGCTGCG
ATGAGGAACATGACCT
GAATAACTCTCTGACCGAAGCGTGGCGGAGGGCCCGCAAAAT
>ConsensusfromContig3481 Average coverage: 7.08
GTTTAACATTAGAAATAAAGTATGCATGGGTATTCATAGAGTGAA
ACAGCCAATTTCACA
AAATATTTAATACTAGAATAAATGACTCAACATTGTGATTATTGA
AGTATGCTTAAACCC
TGAGAGGAGAACTGATAACACATTCGACACTGTTCAAATAGTGA
ATCGAAATATCTATAA
AATACTTATTGATTAAAACATCAAGCTGATATTGCTCTGACTATT
ATTTATACAAAGTTG
AATATGTATATCTGAATCATCAGACTAAAAAATGAATAAATTCAT
CACTAGTGATTCCAT
GGC
>ConsensusfromContig3482 Average coverage: 8.43
AATCCCCCGTGACGTCATCATCATTACGCAATGGAATTCCTCTCG
AAGGGGAAACCCCGT
CAATAGAAGAACCACGCCCTAGAGGTATGGGGCGTATCAACGAA
CGACTACGAGATCGGC
TGGATAAACTGGATTTCCGAGCTGCGGTCCGACCCCTCCTCGGAG
AGCGCGATCGATAAC
TGTTGGCGCTGCTGGCCCGGCTAGAAGCGCGGTAAAGGCGGTGAA
GCTGTTCCTGACTGC
CCAGGACCACCCGATACAGCTCTGCCACATTCGCCGTCGTTGAAG
ACTCATCGAACACAG
CCTGAACGCAAGCAGCTCTCGCCTCATCCAGCGTCGCCCGATAGA
GGGC
>ConsensusfromContig3483 Average coverage: 12.60

ATGATCATTCATTGGCGTCACCCAAAACAGAAGAAAATTACTCA
CCAAAGCCACAGTGCA
ATGATAATGAGAACCCCAATCGCGTGTTTGACGTTCATAAATCTC
TCTCAATAAAAATCG
AACCACGACCACTCTTGGATTGCATTTCACCCGTTTCTTCTGTGGT
GTCCAGTCCCGGTC
CATGTACGCCGAGTTTTAATGGGTATGAGAGCGGCGGAGATGTAT
CTGATTCGTCGAGAC
CTGGGAGTAACGACTCCCGTCACAACTCGACGTCGTCCGCCTCTG
GC
>ConsensusfromContig3484 Average coverage: 16.05
CTCGCGGTATATGAATGAAAAATGATGTGCTGCACAGTGCATTAT
ATTACGTTGATACGT
GAATATCAACAATCTAACGATCCCGGAATTTGAACAGTTTTTAA
AACAAGAAAACGAGAT
GGTTGTTGGATACGGGTGGGTTGTGGTATTTGCGTCACTAGTGAA
CCAAGCGATCGTCAT
GGGGTTCGTCCTGATGTTTGGTCTATTCCTGGATCCCCTTTGCCAG
CATTTTCAAGCTAA
AGAATCCACGGTTGTTCTCATTGGAACACTTCAAGCAGGCATTTG
CCATTTGACAGGTCC
ATTTGCAGGAGTATTCGCCAAAAAACTCGGTTTCCGGAATATCTC
AATGCTTGGAGGCGC
CTTGAGCGCTGTGGTATTCATCGTCGCAAGCTATCTTGATGATCT
GAATGCGCTGATATT
GATATGGGGTGTCATTGGAGGATTTAGCTTCAGCCTTATGTATCT
ACCAGCGGTGCTTGT
AGTCGACGAATACTTCGAAGAAAATCGGGGATTGGCCATTGG
>ConsensusfromContig3485 Average coverage: 8.44
CGAATGATGACGCTACATCTCCTTCAGTAACCTTCATTATTGTTAA
AGGTTTTTCATAAT
CGACTCCACCCTGTAGACGAAAGCCCCATGGAACAGAGCTGTCGT
GTCTTAAAAGTGTTG
TTTTGAATCGAACTTCTGCCATCTTGGAATGTATATTTTCTCAAA
GGATTCTAAAACTGC
TGTGAGGCCTTTTCATCATTTCACTCTCAAATTTTCACAGAAGGAT
TTGGTTATGTTTCG
AAAGTACACTCATTTGTAACGAAAATTCAGAAAATCTTATTCAC
ATCACAAAAAAAAGTA
TGCAAACACGTGAA
>ConsensusfromContig3486 Average coverage: 14.96
CGCAATAATTTATGAGAATCCAGATTCCAAACGTGGAGAAACCGC

GACTTGCCCCCTCCA
ATAACCGTGGAACCGTCCCTGGAAACAGCGAGTGACTTGAAACG
CGCAGGGGCCGATGTG
TTTGGGGTGTAGAGCTGGTAAAGACACTTGAAAGTATCAGCTTC
CCATGCAAAGATAGAA
TCATCTTTGAAACAGCTCAAAATCACGTTCCTTTTCGGCAGGAAT
TGCACCATCACAATG
CCGACATCGCCTTTCACAGTGAGTTTCCGTCGGCGCTCGAATGTA
TTCAAATCCCAGATG
GCGGCGGTGTCTATGGATGTCGTGATCGCATAGCGGCCGCTGGA
GTGAACGGAGATGTGT
GTGATGGC
>ConsensusfromContig3487 Average coverage: 7.88
CTTCCGATCTTTCGGATCTTTTCTCTACAGCTATCCCATCAAAGGC
GTTCTCGACCGTAC
GGATTGGATTCGAAAGTAGGTCATCATAATCAACATACGCAATTT
TGTGGGTTCTCTTTT
TTTCAAGAGCAACAATCTGCTTTGCGGTGTCGTTCATCATGGATG
AGAACATATGCTCTC
TATCCCGCCCAGGGACTGTGTTTTTGGGGTCCAATTGCTTTCTTAT
AGTATTGTTCAGGG
ATGTTATTGACCCCTTGACCTCGGTGATGCGTCTGTGAAGAACAA
TGAATTTTGCATCTG
GATGAGCATCCGCGATTGAATCTAGAAACCATGCGTAAGTGGGC
GCTTTGAGAATAAACT
GGCAATTACTCAGACTTTTCAAAGTCATGATACACTGCAGAATCT
GCTTATGTATATCAG
TGAACCAATCCG
>ConsensusfromContig3488 Average coverage: 12.52
CTCCGGTACGATCCGATTTACGGCCGGATCGATATGATCAAATTC
GACCAGGAATTTGCC
AACAAGATTTGGGTTCCAGACACATTTTTCAGAAACGAAAAAGA
GGCAACTGGTATACAT
GACATAACGGTAGAAAACAAGCTTTGTCGACTCTATAGCAATGGC
ATGATCTGGTATGTT
GTCAAAGTGACAACTAAACTACAGTGCACGATGAACTTGAGGAA
TTATCCTATGGACACA
CAAGCCTGCTCCATCATGTTTGAGAGCTTTGGATACACGATGAAT
TCCATGTACTTCGAC
TGGTGGCTCACCCCTGTACGCGTCGCTGGCGATGTGGAGATGCCA
CAATTCGCTCTCAAA
GACACAATTCTCTATGACTGCTCTGCTAACTATACATCAGGATCGT

TTCCTTGTCTTCAA
ATCACATTCATTCTCCAACGGAGTCTTGGCTACTTTCTCCTCAACC
TCTACATGCCAACC
ATTCTCATCGTGATTCTATCCTGGACGACATTCTGGATCAACATC
GACGCGGTCCCCGCC
CGCATATATCTCGGTCTCGTCACTGTCCTCACCATTTGCACTCAG
AGCGCATCGGAGGCT
GAGAGCCTACCCAAGGTGGCGTACATAAAATCTATGGATATTTGG
TTCACGACATGTCTG
CTGTACGTTTTCGGGGCCCTCATCGAATTCGCAGTTGTCAATGTT
GTCTCTAGACGCGAG
ATTCGAACCGAGAACCCTTTTCGTCGCCCTCTGCCAATCATGAAG
GCCATGCATGATCAG
CATAATCGCGGGAGAAACGGTCTGGCTGTCCAAAGACCCCGGAG
GCCAAAGGTGAAGGAC
CCTCAAGCCATGGCTCGAGCCTATAAGCTGGACAAAATGGCAAG
AACGATATTTCC
>ConsensusfromContig3489 Average coverage: 21.86
GGATCGAAAAAGCATTGTTGGGAAAGAAATAGTAGTTCTTCCTAC
TTTTGATATATCCAA
GGTCGATAATATGATACAATTACAATAAAATAGCTACGATTAAA
AGTGTGTGCAGGCCTA
TTATGAAAAAAATAGTTTTAGCAATCAATATCATTTCTGAGTGCA
GAATGTAATTATTAA
GAAAATGTTGAGTGACAAGTCTCGAATGCTTGTGTATTCCGATTC
AGTACAGTACTGTTT
AGCTGGCACCGTACTGTCAATTCACTGTAGTAAAACGTAGTGCTT
TGGTTCTCTCAGATC
GGAAGA
>ConsensusfromContig3490 Average coverage: 15.39
AACTGCGGTTACGAAAAAGGACACTTCAAGAGTGACGAGTACAC
GGAATTTGTGTTCACT
ATATGTGGGAACCTCAAAATATCAAACAGTGCTCGCTATCTCTCC
ATGGAGATCTTCGAC
AGTTTCATGTGCGTTCATCTGGAAGAACTGTTCAATCAAGTACAA
AATAGCGAGCATGAG
AATAAGCCCAGATATTGGCAGGTTGTTCTCGAGAGGGCTACGAAC
CAGGTGTGTTTGCGT
ATGATGACGTGTATTCAAATTGCTAGCAAGTTGACATCACATTAT
GCCCTTGTTTCACCG
CAAAACGTGAAGATCTTACTTCTAAAAATGGGGCACAACTACAG
TTGCAACAGCATCCTG

CAATCGGAATCGAGAGTCATAAAGACGCTTGGCTACAAAATGGT
CCAGCTGACACCTCTG
GATTTCATTGAATCGCTCTTGCAAATGATAACATGCAACGAGCC
GGCCAAGAAAGAGGTC
GTGACAGCCATGTACAAAACGTCTCTGGAGTTTCTTGACCTGGCC
TATACCAAATGGAGA
ACCATCCACGAGATCGGAAGAGCACACGTCTG
>ConsensusfromContig3491 Average coverage: 6.29
ACAGAAAAGGGAGGTTATAGCGCAACTCTTAATAATCTTCAATAT
CCGTTTGTTATTGAT
AAAAAAGGCAGTGAGCTCTTAATAACCGATTTGAGGAAAAAAAC
CGTTGTGTGTCGATAT
CATGATACAAAAGAGAAAAAGTACGAGCAATATTATAATGGTAGT
TCCTCTGATTTGAAT
TCTCTATCATCAGGTATCGTTACTCCTCTTTCAGAGAATCCTGACT
CATTCATCGATGAT
ACAAATTCACCGCTGTTACATGACAACGCGTCCAGCGGATGTATC
CGGCCAGACTTCCCG
AAAAAGTCTAAAATACCATTGCGGGCTAAAATAAGTAGATTCTTT
AATCCTCGCAGCAAA
GCAGACGAGTTTCGATTGAGAAAAAGAACAACCTCAGAGTCATT
TCAAAAGACAGATAAA
TACAAGATTGGGTCTCAGCTGGCTTCTGTCACGAAGAACTATAGA
TACGTGAATAACAAC
ACCAAGATGAACAACAGTTCTCTGTTGAATGATTTGTATATAGAC
AACACGGCTAAAGAT
AGTTTGATTGACAATTTCGATCAAAGAACACCTCGTGAAATGAAT
AGTGG
>ConsensusfromContig3492 Average coverage: 9.76
GTGCTCTTCCGATCTATAAGATTGTTTTAAATCGGATAGTCGGTC
CATTTGATGTCCCAC
CTTATCCTAATTTTCATGTTAGTTCCGTAGGCTTGATACCTAAAA
AAGCATCAGGTACTT
TCAGATTTATCCATAACTTGAGTCACCCAGATGGCGCATCAATCA
ATGATGGGATTGAGA
AAGTGCATTCGATGGTGAGATACTCATCTATTGGTGATGCTATCA
AAATTATAAAATCAA
CTCAATGTTTTGCCTACTTAGATGGACATCAAATCTGGCTCAGGA
TTATGCCTATACACC
CTGCTGATCGACATCTTTTGGGTTTCAAAGTACAAGGAAAATATT
ATTTTGACACTTGCT
TGCCTCAGGGTCTATCAATAAGCTGTAAGATTTTCCAAGACTTCA

GCAATGCTCTCACTT
GGATTGCACAGAATAAACTAGGTTGTATCAGCATCATTAATTACC
TTGATGATTTTTTAG
ATCGGAAGAGC
>ConsensusfromContig3493 Average coverage: 5.84
TGGGTGTTTAAACATTTGTGATCATTCATAACAGAATGTGAGTTC
ACCACCATTGATTGT
CATCGAAATTTACCGCGCTATCTAGTGGATTCTCTTCAACTATTTA
TTTTTTGTATATTC
ACGTTACTTATAGTAACATCATGGAAAGCTCGTCTTTAAAACCCG
CTTTAAATGACGTAG
AGGAAGACAATATCTACGACATTTCTCATCACGTTATAAACAAAA
ATCCATATTATCAGT
TTGTTTGGGGCAGTACAGATGA
>ConsensusfromContig3494 Average coverage: 11.12
ATTTATGTAAAAAATTATCTTCTGCCATAGGCAAAATAAATCAGA
TCAAATATACCGTTC
CGTTTTTTATTCGTATTATGTTGTACAATTCTTTGATAATGCCGCA
TATTCAATACGGCC
TGCTGGCATGGGGTAAATCCCTAAATAAGCATATAACAACATTG
CAGAAGAAGTCTATAA
GAGTAATATGTTTGAAGAAGTACTTCGCTCATACTGAGCCTCTTT
TCAAAAGAACTAAAT
TGTTAAAAGTGAATGACTTGTATAATGTTTCATCTTTAAAATTTCT
CTATAATTTGGTTA
AGAA
>ConsensusfromContig3495 Average coverage: 6.26
TGGATGAACTGAACTACTTTGGATGAACAAGTATTTGATCATGAA
CCACTGCAGCTGCAC
CATGATGAGAGTGAAAACAGTCGGTGGTATCATTACGAAAACTCT
TTCAACGGCTGGACT
TGTTCTTCCAGCCTTGATTGGGAGATATACCAGAATGACTATAAA
CATGATTGTCGCGAA
CAACGTGAGTGCGGTCAGTGCAAATCCTGCAAGCATGAAAGCAA
TCTGATAACTCGAGTA
TCGCATAGCTGATATGATCATCATGGAGGGATCGGTTGTGACCTT
ATAATGCCATGAATG
ATCACCCCTGTATATCATGATCATGTGGCGCTTGTATCGTATCCC
AGTATGAATTATGAA
GAATATATGAACCATGAACGAAAGCGCAAGAGATACCATCGCCG
TA
>ConsensusfromContig3496 Average coverage: 17.71

GTACTTTTTACGACTACCTGAAGTCTATTCTTGAAAATTAAAGCC
ATTAAGAATCCATAA
AGTGCATGTCTGTGTCGAGATCTGGCGACTGCTGTCAGTCGTCGT
CATCAGTGAAGTCGC
TACCTTGTCCAGGTCATATTATTGGGCGAATTTATTTCAAAAGCAC
TTTTTACAACTATC
TGAAGTCCTTTCATGAAAATTAAAGCCATTAAGAATT
>ConsensusfromContig3497 Average coverage: 3.73
CAGGAATTGGCGGATTGTGATTCGGTTCCTCCACCTTTGCGCTGC
CCCCTACGTGACGAC
ACCCAAGAAGACATACGCACAAATATGCTCACTGAGGATATTAT
TGTGCATCTCAAGGAA
TTGGCGGATTGTGATTCGGTTCCTCCACCTTTCCGCTGCCCCCTA
CGTGACGACACCCAA
GAAGATATACGCACAAATATGCTCACTGAGGATATTATTGTGCAT
CTCAAGGAATTGGCG
GATTGTGATTCGGTTCCTCCACCTCTGCGC
>ConsensusfromContig3498 Average coverage: 7.69
ATTGGTCATCTAATCTACAACAAATGGAGAGGAATGTACTTCGAT
GACTCATCGCGGAAT
CGACTAGAACCAATAGGAATGAATGAACTTTGGAATCCGATTCA
TCAGGGATACACAGAC
TATCAACTTAAGGTTATGTCAAAACTGAGCAGAATTTCCAATCGT
TCGTCAGCTCAATAT
GCCAAACTTCAATACGAGGCGTTCAAAGAAGTGGATAACAACAC
AATCAACCCACCTGAC
TACTATAAGAGGCGTCTGCCCCAGGCAATCATAGCTGGAGTCAA
GAAATGTG
>ConsensusfromContig3499 Average coverage: 4.66
TGTTTCACAATATAATACTGGATTGGTGAAACAACTGATCAATCT
GATCGTACGTACGAA
AATGTCAATATTTGCTGTTTGATTACAAATTCCATTGTGAAAGATT
TCACAATTCTACCT
ACAATTATTTGAGAACAAAGCTTCGTGATGGTTAGAAAAGGGCGA
TGACTCGGGTGATTT
CTTTCCTGTACTTTGAATAGTCACTCTAGCTTGATGACTCATCATA
AAAATTTATTAAAC
ACTTGTTG
>ConsensusfromContig3500 Average coverage: 7.76
AGCACTTGAGAGTAGTAGAATCGGATGAGGCAGAAAAATATTAT
AATGGCCACGATGACA
GCCGTTGAGCCGACAAAATCAGAAACCTTCGAAATCCCACTGGGC

AAATTTTTGACGGTT
TTCTTAATGATTTCGAATGGGTTTTCAAGAAACCTAAAAGGGCCA
CAACTCCGCGACGGT
CGAGCTTTGAACACGACGTACGTATTGAGTATGAAACAGAGCAC
ATAGGCGGCTATGAAG
ATATATACAAAAATTGCATGCGTCGTCGAAACACTGAAGGAATA
TGCATATGGCACATAG
TTGTTCATCGTAGTATATAATTTGGAGTAATATATGAGAACATTT
TTCAATGCCGCCAAT
GTGAAGC
>ConsensusfromContig3501 Average coverage: 6.20
TGAAAAACTAAGATATCATGAATTTGATTTTCAACTGGTGAAATA
CGAATATTCGATTCT
CATGAAAACAAAATTGTATCGTCTCTAGTGTTGCACATTTGATGA
AGAAAGTACCTCTGC
AAAAACAGCTGCCTACAAAAGCAAGATCACATGCCTCAATTCAA
GGTGCTTGACTGACAT
ATGATAAAATTAGTATTCGATTCTCATCAGAATAGATCGGTTTTTC
TCTATTTTTGTATA
TTCGAACAAAAAACTGACTCTGCAAGAATGTCTTCGCAAGTTGC
>ConsensusfromContig3502 Average coverage: 7.65
CGACATGCGATTAATAACATCCACGACTGCCATGTTATCCGAGT
GGCAAACAACGTGAGA
GTTGGCGAGCAATGACGACCAAGTTACGATCGCCGCAGTAATTG
GAAACAGTTCCAAGGT
AGCGATATTGCATAGCATTACTTCCGGCTCCCATTCCCCATAGAG
CCAATGAACGCCCAG
AGCGGCGCCGAAGCCCACGCCAGAAGCATCGGTGGTCAGATGCA
GGGGAGGAGATAACGT
CTTCGTTGGAAAAATTGTCCTCCCATTGAAACCTTCTAAAAATCG
CAACCACACATTCAA
ATCCTCTTTACAGCCCTTGGTCACTCTGCGCCTGTGCACAGGATG
CCGCAGACCCAAAGT
CATGTCCGTCAA
>ConsensusfromContig3503 Average coverage: 16.54
CGCTTTTGTAACATCATTCAAATTTAATAATACGAATGCATGACA
TGTTTTGAGTTTATC
GTCAACAGCTTTGAAATATTTGTTATCTTGGCCATACCCACTCGC
AATCTCGAGATTTCA
GAAAAAACGGGAAGGAAATAGTCTGACATGATTTTGGGCATAAT
TCTAGCCGCTTTGGTA
GCAGTCGCCTTGCTGATATTTGGAGTTATCATCATCATCCAGTGC

TGTCAAAATTCAGAG
GATGTCAACGAGGTGAAGGATGTGGACAGGATTTACGATGACCT
TGACAGTGTTGAGAGC
GATGAGCGTTTGAAGACGGGAAATTTGAACACATCAAACTGGCA
AATCTCACTCAGGCAA
AGTATACGTAGGTTCCGCGATAGTTCAAGGTCTCTAGCGAAAGGC
CACCATCATCCAAAC
GATTCAAGACACATCATCAATAGCAGTAGTGATTCTGTCAATATT
GGACAGGCAGACGAT
AGGCTAACTAATCGGTACACCTCAAGAGCAGACGTGAAAAATTC
AAGTGGAGAACTGGAA
CAGATCTCAACTGTTTCCCCGTCTTCTGGGTCACTGTCACCTGCT
AGCGATGCAGCACC
>ConsensusfromContig3504 Average coverage: 9.66
GTTGCCTTTATCACCTTGGTCTGTTCTGGTTTATAAAATTGTGGTT
TGCATTCGGCGCAT
ATGGCACATCGTGAAGTAACTTCTTTAACTTCATTCAGAGAATAG
GCTAGGTTTCTTGCT
TTGACGAAATGCCACATTCGTGTAACGCCAGGATGACATAACTCA
TTATGCAATTGAATT
AGTGAATCACTTGAATTATTTACGGAGCAGAGTCTTGAAATGTGG
TCAGCTGACACATTT
TCACAGCCTGGACGATATTTAATTTCAAAATCATAACAGGATAAT
TCCAACTTCCATCTG
AGAAACTTGTCGCTTTTAATTCTGTTTTTATGTTGTTTATCAAAGA
TATAACTCACTGAT
CTCTGATCCGTGACCAACTGGAACGGTCGCCCCGTCAAAAAATGC
CTCCAATTACGGACA
CACTCAATAATAGCCAGAGCCTCCTTTTCCAAGATCGGAAGA
>ConsensusfromContig3505 Average coverage: 17.40
CGGTGGTCCTCGCCAGATGCTCTCAAAGAATGTCCATCGATGAGA
AATGATCCATATGAC
TCCGTTTTATGGAATGCAATGATATATCCTCTGATAAACGTTACG
ATCACCGGAGCGATT
TGGTATCAAGGTGAAGAGAACGCTAACAGAAACTCTACGGATTAT
GACTGCACGTTTCCT
GCAATGATTGTCGACTGGAGATATAAGTTTAACCGTGGTTCTGGT
GGATTTACAAACTCG
ATCTTTCCATTTGGCTTTGTTCAGTTGGGCCCCTACCTTAATAGT
ACAAAGTTGGTGGAA
TACCCGATGCTTCGCCACAAACAAACTGCTGATTTCGGTTATGTA
CCAAATGCAGCACAA

GCCAACGTCTTCATGGCTGTAGCATTAGATCTTCCGGACTTTGAT
TCACCGTACAACGGC
GCTCACTACAGAGACAAG
>ConsensusfromContig3506 Average coverage: 14.85
TATGCATATGGGTCAGCAGTATCAGTACCAAATGATCCAGCAGGA
TCCGAATACTGGTGT
AGTTCAGCTGGTCCCCGTACCTCTTCCGCCTCACTTGTCATCACT
GCAGAGGTCAAATAG
CGAAGCAGTTGATAGTAAGAAATCTCCAAATTTTTTTCTCAGTCA
TCAACACCAGCGTTT
GATACACAGCGACTCAAGAGAATCTTCTGCTAGCTGCAATTCAGG
ACGCAATGACTCTGC
GAGCGAAAATTCACCTCATTCCTTCCATTCACCATGCTCTCCTAC
CTCACCGAGACTTCA
GGATGATTTGACTGTTGATACAAGAAGATATGTCCGAAAACAACT
GAACTCGTGTAGCTA
CAAACAAACCAAGGCGCGAAAAAATGTGAAGGGAAATACAAATA
GTAATTTATATCGAAC
AACTAGCATTCCAGAGAATATAATATCAAGTTCCCTTCATTCATT
TGGACCATCAAAACT
TCAAAGGTCGAATTCTTTAGAGATGATAGACCGTTCTGCTATGAG
GCGACGTGGTCGTTC
AAGACTATCGAGTAGAAGTGTAGAACAAACTACCAGTATAAAGC
GAACAAAAAGTGGATC
GTCTTTGGATTTCAATTCAAACAAAAAATCATCTCAAA
>ConsensusfromContig3507 Average coverage: 4.04
GAATATTTAAATATTGAGTTTTTCAGCACCATTTACGCTTTTCCAT
TATTGAAATCCCTA
CCTGTAAACAACTGATATACAATTGAACATGCGATGTTCGATGTT
TGGGTGCTCATATAT
TTTCCCATTCTGTCGACTTTTTCTACTGACCATACGTCATGTCGAT
AACTTTCATAATTA
TATGATTCCTACCTCTTCAGCCAAGATTGATTCTTCAATCAATAGA
ATTATAATGTTTCA
AACTGTTTTGCACAACTTTCCCGAAAAATAGTTAGTTTATGTGTC
GGCAAAATTGATTTG
TTCGATAGTTTTGACGGGGATAGAGGACAAACAAACATGCTAAT
GATCTAAATAATATAA
GAGCTTTTCTGTGAATAGCTGTTACATGGTGCAGAAATCTGTTTG
GTGAATGTAGAGTTT
TGTTGTAGAATAGTGTTCAATTTAATTTTGTTAGATGAATCAAGTT
ACCTTTACTAAGCT

CGC
>ConsensusfromContig3508 Average coverage: 14.03
CTGATATCATCATATCTCTCATGTAGAAAACAATATGTTTCTTAT
AATGATATTAATACC
GATCCAATCCTGATGACAAAGGGTATACCACAGGGATCAATATTG
GGTCCATTACTCTTT
CTTATTTATATAAACGATCTACCAAATGCATCTTCTTATTTTGAAC
CACTAATTTATGCT
GATGATTCTTCTCTACTATTTTGCCCAAGTAATATCAAAACAATC
AATTACAATAGCGTG
GTCAATACCGAATTGTCGAAAATTAATAATTGGGTTCTCAGCAAC
AAACTGACAATCAAT
AATAATAAATCAAAATATATGATATTTCATCATCCTCGAAAGCAA
ATACCAGAATTACAT
GTAAGTATGAATAACAAGGATATTCAAAGGGTTTCTAACTTCAAG
TTTTTAGGCCTGGAA
ATAGAAGAGAATTTATCATGGAAAAACCATATGTCGTATATCACT
AATAAATTGGCGCGT
GGTGCGGGAATACTAAATCGACTGAAGCATTTCCTACCCACTTAT
TCTTTATTACTCATA
TATCACTCTTTGATTTCGACTCATTTCGATTACAATATATTATCTT
GGGGCTGCGAGAGT
GACCGTATTTTTAAACTACAAAAAAAAGCAATTCGAAATATATC
TAAAGCAAAATATAAT
GCCCACACCGAGCCTCTATTTAAAAAATTATCTATACTGAAACTT
AATGATATGTATAAA
TTGAATGTTATAAAGTTTTATCACA
>ConsensusfromContig3509 Average coverage: 6.74
CTGATTGCAGAATCCTGGGAAGGCCGAAGTTGGCGAAAACATGGG
TCTTTAGACTCTCCG
CAACAGAGAGACCGCGTTTATCTTTCAGAGGGCACAGGATATGAT
ATTTGGACCAATGGT
TCATATAATGTCCAATATATTTGTAGTCGGCGTCGGGCTCATCCA
ACATACTAATCAGGT
CGATCTGGCATCGACTGTTGAATCCCCCAGCAAGAATTGGCTGCA
AGGGCGCTTTGGCTG
CTTGCTGCCGTTGTAATGCACATATCTGGCACCTGTTGACGAATT
CTTGTATGATGACCC
TGGGGATACCGTGGTACTGCTCGTTGACCCTTGCCAGAAGATGTT
TGTAGCCTCCATGGC
CAGCTTTTGCATGCTCCGTTGTTAGCACTTCATGTATTTCAGATC
GGAAGAGCAC

>ConsensusfromContig3510 Average coverage: 16.41
ATATAACCAACGGATTCTGCAAGAATACCGTTGGATCTTTCACTT
GTGAATGTAATACTG
GATTTACTATCATTGATGGTTTCACGTGTGACGATGACGATGAGT
GTGCTCTAGCATCAT
CGAATACATGCGACCAAACAAACGGAGTTTGCACCAACATTCCTG
GATCTTTCACTTGCG
GATGTGATTCTGGATTTACTCTGAGTAACGATGGCTCCACGTGCA
ATGACATCGACGAGT
GCATAATCGGAGAGTTCTGTCGCAACGGCGGAATTTGCGTCAAC
CAAATCGGAAGCGCAG
ACTGCCTTTGTGCGCCTGGATTCGAGGGAGCCAACTGCGAAATA
AACATAAACGATTGCA
TCAATGCATCTTGTCATTTCAACAACTCGTATTGCCTGGATCTGAT
TGACGGCTACTCCT
GCGTTTGTGACCATGGTTACGAGGGGCCAGCATGTGCAGAAGAT
ATTGATGAATAGATCG
GAAGAG
>ConsensusfromContig3511 Average coverage: 9.09
CGATCTCAACGATAGATACCCCATGGATCTGGATGAGTTATTCGT
GTGGTACAAATATCT
TCCTATTGCTGAAATGAAAGTCATGCACAAGCAGTATTTTGATAA
TTACAGTCCAGCCAT
TCGAATAATAACCTATAAGATCGCAATATATACCAGAGATCATCA
CCTGGCGGGTACTGG
TGGTCGTGTTTACATTACTTTTTTTGGAGAGCATGGAGAGACAGA
GGAACTTGGGCACTT
GGACAGCCCTAAAGACGATTTCGAGCAGAACGAAATAGACAGAT
ACACATTAGCTGCTAA
AGACGTTGGTGACCTGACCTCGATAAAGATTCGCAAATATGGAG
GGCTCGAATGGCA
>ConsensusfromContig3512 Average coverage: 17.94
TTTCTGCTTCTCGTTCTTAACAATAATGTGCATGCAATAGTGATAT
ATGATACTAATTCA
TTTTTCGAATAATGAAAACACATTACGGGTTGACGATACAGAAG
ACATAGGACGAGATGA
AGAGGCATGAGAAAAGATGGGAGAATTGTGTGAAGAAGAACAA
GAAGAAGATGAAGAAAT
AATATTATTATTGTAAATTATTGAAGAGGGCATTGAAGACGAAA
AAATAGGACAAAAAGT
AGGAGCAGAAAAAGAGGGAGAAGAAAATGAAGAGACATTGGAG
ACAGATGCTGAATAAGA

AGAATGAATAGAAGAGGAAGAGGAAGAAGAAGGAGAAGAAAA
AAGAGAAGAGGAAGAGGA
AGAGATCGGAAGAGCGTCG
>ConsensusfromContig3513 Average coverage: 10.23
GATCTTTTGGATAATTCATTATTTAAAACAAAGATATAGTCAATG
GGAATACTGTTATCA
TGACAGAACAATAGGGAGGTATCGTCAGCATAAACTATAGGAGT
GAACATTTTAGATGAC
TGGCTAATATCATTGACATAAATGAGGAAAAGCAGGGGACCGAG
AATTGAACCCTGTGGT
ACTCCTTTCACAAGTGGTTTAATGTCAGTGTTGTGATTTTTATAA
CATACAAACTGGCCT
CTATTGGATAAATATGAAGATATTAATGATATTGATATATTGTCA
GAT
>ConsensusfromContig3514 Average coverage: 5.36
GCCATCGACGGCGGTAACACTCGCTCCATGGTCTAATAACAACTG
TGCAATTCTGATGTT
CCCATTGGTCGCCGCGATAAGGATCAACGGGTTTCCATTGGCTGT
TTTCATGTTGACATC
AGCACCGCTCTTGATTATGGTCACAATCTCCTCATTGGATAGATT
ATTGATGACGCAAAA
CTCAAGTTCCTGTTGCTCGTCAGTTCCCATTTCTGGTTATTCCCAG
GAATGTAAACAACT
TCTCAGATCG
>ConsensusfromContig3515 Average coverage: 13.98
CTCAATCGGATACTCGCAAAAACGAAGTTGAATTGAGCAACTCAA
GTGGTAATTATGTTA
ATATCAAATTTTATCGTGCTTTTAAGCGATTGAATAGGTCCAATA
ATTCTGCAAGCCTGT
ATTCATCGTGTTCGCATAGAGAAAAGGGACACGTCTACAGCACTT
TTGCATTGCTCGTCA
TGGCTCTGTACATAACAATCGGTGCGGGGATTTTCAGCAGTTGGG
AAGATCATTGGGACT
TTTTGGATGGATTCTATTTCTCCTTCATAACTCTGACGACCGTTGG
TTTCGGGGATTTCG
TTCCCGGAAAAAGCCTCGCTCAATCAACTTTCCACTACTTCTTTTG
CTCTATTTACGTTG
CTTTCGGTCTCGCCCTCAATATTATGTGTTTTGAGTTGATGCAGGA
AG
>ConsensusfromContig3516 Average coverage: 9.85
GCATTTTTTCGAATGTCTCGGACATTTTGGTTTCGCTGCGTGAAA
TAATAAGAGAATTTC

TATGAAAATTATAAATATATCTGATTCACTTTCTGCTAATAATGAC
CACTCCTTATTTTC
ATCAATGGACACTTGATCATTTATCATGCGTAGGCATTCGATCGT
TCACCGAACTTCAAA
TAGAGTCAGGTGGGAATACAAATGTAATAAGCGTACATATAAGA
GTCTTATAATTACAGC
GAGTCTCTCAATTATGAATAAACTGCTGATTTATGAGAATTGCGA
TTGAATCATAGAAAC
GAGCCATGTCGGG
>ConsensusfromContig3517 Average coverage: 13.79
GTTGACGACTAGCAGAACCTATACGATCATAGGGAACAAGTTTC
CGTTTGAAAGCAAAGT
TGAGTGGGCCTAGCAGGATAAAACGGGGTGGAGCAAGAAATCCA
AGAAAGGGGGTTGACG
ACTAGCAGAACCTATACGATCATAGGGAACAAGTTTCCGTTTGA
AAGCAAAGTTGAGTGG
GCCTAGCAG
>ConsensusfromContig3518 Average coverage: 7.32
ATTTGATGATTTATCAGAAAATTTCTTTGGTGCATCCGCGTCATAC
TCATTCGAACTTTT
TGATAACTTATCAGCTCCTTTCATTGGTGTGTCCGCCGTATATCCA
TTAAATTTTTTTGT
TAACTTCACTGGTGTATCATTGTTATCGACATTTGCCAAAAATTCA
TTCGATTGTTTCAT
TGCACCATTGGTGGGATATGCATGGTTGTTCGTTAAAAATTTATT
AGGAGAAGCTCTAAT
ATCATTAGTTTTTTCGGTTATCAGCTTTGCATCACCTTTTTCGGG
AACAAGTGGCAAAGT
TAAACTTCTTTGAACACTTTTGTCCTGATTGCTGCTCCTGTAGTTA
TTCCCGGTTTTTAT
CGCAAAGCCGTTTTCTTTGGGGTGGTATTTTATTTTATCCCCGTCT
TTGATAGCCTTCAG
TGTCGGTTGTGAAATTGATGTGCTCGTATCTTCACGCCTAACATT
CGCTTGCTGATCGTA
CGGATAAGATGGTGATTGCTTTTTGAATTTGTCGCTTCTCACAAAA
ATATTTTTATCATA
GTCTTCAGTTGACGATGATTTTTTATTTGAGCTCTCTTCATTTATC
TCATCTGTGGAGGC
AGTAAGTGATTTGCAGACCGGAGAAATATTGCTCGCCATTT
>ConsensusfromContig3519 Average coverage: 12.47
CTCAAACTGCAGAGGGTTACATACAATTACACCCAATAACTCCA
AGATCAGGAATCACAT

ATTCGGTAATATAGCCGCTCTCTTCAGAATGTATCAGAATTGACT
GTGCGGCGTATTTTG
AAGGGGCACACCGGCGTAGGTTGTCTTCTGCTCCGGAGGCTGCTT
CGTTCCCTACCGAGG
AAACGTCAACTTCTCCAATAGTCGCATTGTTTTCTACGGGGCAAT
TGCCAACACAAATGT
TCGATTCATAGGATTTTGGTACTATCACAGATCGGTGATTGAAGT
CTTCAAAGTTGATTG
TAATCGGTTGAAGAAAGCACTGTTTATCATCATCACAATTCTTCG
AGTGTTTGGAACCAC
GTTTTGAAGGCTTTGATTCTTCTTTCATTAC
>ConsensusfromContig3520 Average coverage: 6.83
ATCGCACGTTGGCATTAATGAGATTATAGGTTGGCAAGATGATGA
CGGGTTTGTCGGACA
TGCTCTCAGTGTAAGCACCGGGGAACATGTGACAGGAAATTTTGT
GTTCAGCGATACTAA
AGAAAACATTTATCACATGAAAACCGTACCATTATATGCGGTCGG
GATGAATCGGTTATT
TCCAGTCCACAATTATTTCGAATTCAGCGTGGAAAGCTGTTCGCC
AATTTATGTCCAGCT
CTATACAAAGATCGAGTTTGTGCAAACAACTGCATACGAGCTGC
TTGTCGACACGAACAA
AATCGAGTTGCATTCTGTCGTGAATGGGAGAACAGCATTGATATC
GAGAAATATTTCCAA
CCTCATCAGCTGTACACACATGAGTCCGCTTTGGGTCAGCTGGTC
AGACGAGTTTTTGCG
TGCTGGAAAAGGCAGGGCGCCCAATCAAGTTCTTCTGGAGTACC
GCAATGCTGCTGAGGG
CATACAAAGTATATTTGAAGTGATGCTTATTTCGAATACAAACAT
TTTATCTCTTCCAGC
TTTATGGACATTCGACCAGTCTATGGTTGACAGAACAATAATCGC
CGCTGATGGGAACTT
AGACTACACAACGTTCTCCATGATGGTCCCCAAAGGCCAGTCGTA
CATGTATTTGAGACT
GTTGTCGTGTGGCAGCGCATCGATTCTGCTTGCAGAAGTTCCTGG
CTTATTCAATGAGTT
CGCAGAT
>ConsensusfromContig3521 Average coverage: 8.04
CTCACTTTCCCCACGATTCAAATTGGCAGTTGATAAAACTTTCAA
TTAATTTAAAGGAAT
TTCAGTATTTGCCATGTGTCAAGCCGCTATTTTTCGTGAATGACA
TATGTGTTGTGAATA

ACAAAGAATGTGAATTGGGAGATGATAAATGTCAATGTCTCAAT
ATAAAATGTGACGCTA
AAACAAAACTAACAGCATCTATTTGTAAAATAAAAGAAACAAAA
CAGTCGGGAGATTCTA
AAGAATTTTCAGATCGCCTAATCTGGCAAAATAATAAAGATTGTT
GGACATTCATTGTTC
GACCATTGTCTGACGATTTGCTTTGCCTGAATATATTTATCATCG
AGGGTAATAAGAATA
CAACCGAGAAACACGCGTATCCTCTTGTTTCGTACCGGATCGAAC
CTTCTGAAATCTCCA
AGAGTTTTCCTCAGGGAGGCAGCTACGGTCCCAGC
>ConsensusfromContig3522 Average coverage: 12.30
CCGCCTTATTCCATTTCTTGTCATGTTGCTCGCACCACGCCACCT
CCACACAATCCACAG
AGTCGATCGCTTTGTGGACAGTTTTGAAGGCGCCACAACCGAGCT
GCTCTCTAAATTTGA
GGTAGCGACCACAAGGGGAGCTACGGATCGCCATGTCATCATCT
TCAGCAGCTGGTCCGG
AAGAATTCTTGGAGATAACTTTGGTGGTTGATGAAGCTAACTCAT
TTTCGGAAGACTTGT
TCAAGCTTTCTGTGTTTTTTGTTGTAGGATCATGTTGGCTTCTAAA
TTTCAGAGACTGGC
CAAAAGAGCCTGTGGTGATTGTGTTGATGGTATCAATATGATATA
ATGTATCGTCAATAG
AATTTGAATCAATTTTATTATCCACTGGTTTCTCAGCTTTGTCAA
AAGAAGAATCACTGG
AATGAGGATTGAAATAAGAGCAATCGTGTTGCTCTTCGTTATACA
ATTTGGAGGGGTGTT
GACATGAAATATTTGCCGTAGAATTTTGTTCGAAATTCTGAAAAT
CGTTTCTATTCAAAT
TTATGAAAGGCTGTACAATTATTGGAGTAAAGTGTTGTTCAAAAT

>ConsensusfromContig3523 Average coverage: 9.38
CATTAGCTAATTTTCTTATCCCAGTCACACTGGATGATAATAACA
CAATGTCATCTGCAT
ATATAATATGATTAACATAACGTGCTCCAAGCATACAACCAGTTT
TAATATTATTTAGTT
TGATTGATAGATCATCGATATATACAGTAAATAAATATGGTGAGA
GAATTCCTCCTTGCT
TCACACCTCTACTAACTTTAAAACTATTTGATAGCTCATTTTCCCA
TTTGATATATAAAT
TTTGATGCGAATACCAATATCTGAGGAAATCTATTAAAACAATAT

TAATGTTTCTGGATT
CAAGTTTACTGAACAGTTTATTGTGGGAAATTTTATCGAATGCCT
TGCTGGCATCCAAAA
AACAAGCAAAAACATTACTCCTATTATTGTTATATTTGTTAATAC
ATTCCTTGAGTAAAT
AAATGGCCTGGGCTGTTCCATGACCTTTTTTAAATCCAAATTGAT
TATGACTGGTTTCTA
A
>ConsensusfromContig3524 Average coverage: 6.66
CCAGTTTTGAAGACGTTCGTAAACGAATCATGCAAGCAGGACCAA
GAAGTTGACCGTTTC
TCGCCATACAGTTTCGCATAAACCTTGCTCGAAGTTGATGCTTTT
GGCCACCAAGCTGTG
CAGACAGTAAGCATATAAGTATCGGCCCGGCGAGATCTCAATGTC
GGAAATTCTGTGTAT
ATCGGTCCGTAGTCGAACGCAGAATTCCTGTCGACTTTTATGGAG
TATATCGCAATCGAA
ATGTATATCAGCCACAGAACGAGCATGGACATGATGAGGATTGT
CTGAGGGGGCGGGCGG
AGGGCTGACTTATAGTCTTTGAGGATGCTTTCAATCGAAGCTCGC
AGACGGACCAGAATT
TTGCCATCAAAGAAGCTGCATGTGCATATGAGCTTTCTTTGAGTT
ACATCGAAGCTTGCC
TGACAAACTTCTTCTCCATTATCACATTTGAAAATATAATCCCTC
>ConsensusfromContig3525 Average coverage: 9.15
TCGATGTGTGGGTTAATGTTGATTTCAGAGCTACTGGATACAATT
CCATAATATGGAATG
GATATCGCAGCTTTGGATATAGCAGTGACAGATTTATATTCATAT
TTCATTATGGAGCCC
CCAATTGTCTGCAGAAACGGCGGGTCATATCGAAAGCCCCGGAA
TATCAATGGAACCATG
TTAGGGCGGAGTTTCACTATTCGATGATCGAAGCCAGAATGTACA
TCAATGGGAATCTCG
TTGGAACAGATTCCCTTATAGATGATTGCTGGCATGAAAGACCAC
GAGCACATCATAACA
CGATGATAGGCG
>ConsensusfromContig3526 Average coverage: 23.41
GCGGATGCATCTGAAGTATTATCAGTGTGTGTTACACTAGCAGCA
GTGACTTCAATTTCA
AAACATTCGAGTACTTCTAGATTAGCCACGATGTCACCTATTGTA
ACTTCCTCATGTATC
TCCGGCAGAAGTTTTGGAGATTTAAGATCGTCGAGTCGGAATCCA

GTTCCATCATTGGAA
TGCTCTCGAGAATTTGTTAATATCTCCTCATGTGTATCTGATGTC
ACCATGAATAACTCA
CATCCAATTACGGGGAATTCAAATA
>ConsensusfromContig3527 Average coverage: 9.25
CACTGTACTGGGCCCATTCGGCGTCAAGGGATGATGGCACGGTAT
GATCACTGCTCTCTT
TACTGGAAGAAAGCGTCCGAGATCTGGATGAGCTACTAGTCTCCT
GGGAATTCGGCTGGT
CTTCCAAGGGATACTGAAACCAGATCTGATCAGCATTCCAGTTTT
CCACAATCGATTCAC
GATTTCGCGTACTAGCCAGAAATCTAAGATCGGAAGAGCA
>ConsensusfromContig3528 Average coverage: 31.73
GACGACATTGAAACAGAGGCGGATATAATGAGAGCAGGAAAAAA
CGTCGTAAAAGGTTTA
CTGGCTTTGTTAGACCCAGACTATGTCAAGGATATGAATAAGGCA
AAGTTAATGGCTCTC
AGGATGAAGCGTCCCAGAGTGTCTGATTATAAGATACAAGCATCT
GTTGGCAACCTGTTA
AAGACACTGTTATCTAAACTTGATCCTTCGTATAGAGGAGGCAAA
TTGTCATTCGAAAGA
AGCACTAACTCTGAGGTGTGGTACCAACCCATCCGTCCTGACAT
GGCCGAAGTACGGACA
AGGAGAGGAAGGGTTGTCAAACCCATCATGGATCTGAGCTACTT
GTTCTACAAACCCAAA
GACAGGACGAAAACGGTCCCGAAGAGCATCGCGGAACAACCGGG
AAGGATACTTCGGAAA
AACAGCGTTTCCTCGAATTCGTCGTCAACCAATAAAATCATCGGA
CAGTGTCCTTCAAAT
GAAAGTCTTGCCAAGAGAAAGCTGCCTTCGTTTTGTATCTTTTGC
AAACAAATTATACAA
ACACTTCCGCGCCATATAAGGAGGTGTCATAGGAATGACCCGAG
GATGATTAAGATGCTG
AAAGATGCCAATGGAGACTCGAAGACGTTTTTGACCAGAATTAG
ACTAATGGGTATGATG
GAGTTGAACAAGAAATCGGGAAACAATATGGGCGAATCAGCACC
GATGAGGAGAGGAAGT
CGCACTTTCTACGTCGTCGAATGCACCCGCTGCGGGTACTTTGTT
TACCACAAGACCTTC
TCGCAGCACGTTTGCCTCGATGGAAAACCCATAGAAAAAGAATA
CGTCCGAAGTGCGCAA
CGACATCTCGTCGAATCCGAAACTTTGAGTACATCGAAGGAGA

>ConsensusfromContig3529 Average coverage: 5.02
GTTATGATAGGGAAATTGGAGGAAAACATTTTGCATCCGGTACCT
GTACTCTCATTATCG
GAGAAAAACGTATTTTAGTGGATACTGGTGGTCCATGGTACAAG
GATAAACTCATCAGTG
GTTTGAAAAACTGTGGAATCGAACCAGATCAAATTGACGTGGTC
GTTGGGACCCACGGCC
ACTCAGATCACATTGGCAACCTCAATCTCTTCCCAGATGCTCTCA
CGATTGTCTCCAATG
ATATATC
>ConsensusfromContig3530 Average coverage: 5.63
GTCGATTTGTCATCCAGGGTGGTCCACCACTGTAATAGTATGAGG
TGGGCTGTACCGGAT
GACTTTCGTGTGGGCTTAACAAAAGTTACCAACTATTTCGATATA
TTGAACCTGGAAGCC
AGCGAACTGGTTATTTACCACGAATCATCTGCGGTTGGACACCAC
CGTAAGTGCGGGGAC
ATGTGTAGAAAATACCCCTTGCAAACTTGTTCAGATGTGTGGTAG
TCGTGATTTGCTGGG
CCATTGCTGCCTTTAATGATCAGTTATTTGCTCATCTTACTAGATC
ATACCATCAGATAG
ATTTATATTTGAATGACCCCACGAAATTTGTCCGTTACCTCAGGAT
TATTCTTGTCTCCT
GGGTTGTTGATAAAACAATAAACATCGACGGCATTGTGCCAAAAA
TTCGTCCAATTCAAA
AATACGGAAAATATTGACATGATGATATCGAAGATTTAGAGGAT
TTCCAAGAGCCGAAAA
AGAGAAAACCAAAC
>ConsensusfromContig3531 Average coverage: 5.22
TTGAGTACATCGATACAAAGATCTATGAAATTTGATGCATGTAGA
AATAAACATCTTCTT
CTCATTTCGTTCAAAGATATCGTTCTGATGTGAACAGCTGAATCG
GCTCTCAACAAGTAC
CCCCAAATGTTCAGAATTCCACTAATAACTTCACAATCGGTACCA
TAAACTTCTGAGGAT
ATGCGAAATGTTTTGGCATGACTCCATGATTCTCTCATTCCTGAT
TCATAAGGCTTGTAC
CAGTCTTTTAGGCGCATCCAATAAGCCTCATCTCTTTGACTCACA
AAACCGATCGTTGTG
ATATTATACATCAAATTTGCGAATTCCTGAACTTCAATTAACGGA
CGACTCTTTTCACCG
AAATGCGGATCCCGTTGACTCTCACATAACAGCGGGCATCCATTT

GCGCGACTAAAAACT
GTATAACTTTCGAATATCAAATTAGTGACGTCATCACACGTCGAC
TTTGATATAGCCATG
TA
>ConsensusfromContig3532 Average coverage: 8.89
GAGAAATTCTGATGATTAGTTGTTATGATCGATCGTATTTGATAT
TAGGATTATGTGATA
TTTAAAATTATATGGAAATTACGTGGTTAGTGTATGGCCAATGCA
TTTCTGATAAGGTTG
TATGAGCGAATGAACATATGTAAAACATGTGTTTAAACTCTCTTTA
TTAATTTTTTCATT
CCACGTTCTCATTATCTTGCGATGTGTTCATTTTTTGTTTTTTTA
ATTGGAGTCATCTG
AGTGTGCCTGAAAATTGTATACACGTTTTTATTCTTACAAGATTTT
ACCCAGAATTT
>ConsensusfromContig3533 Average coverage: 11.87
CTGCCACTTATTTTTAATCACCTTTTCAAACACTTTGCTGACTGTA
GAAGTAATAAATAT
GCCCCTCTTATTTGCCATCTCTAATCTGCTTTTCTTATTTTTTTGAA
TTGCTTTTATTTT
CATTTTCTCCCATTCTGCAGGAATCTCACCTTCTCTAATCACATTA
TTAAATATTTCTAT
CAGCGGTTCGATCATTTCTTGGCCCCCTGCCTTGCACATTTCACTG
TTCCATCCATCTAT
ATCTGGCGCTTTTCTATTTTTCAATTCTCTTATTGCACATTCAATTT
CCATTCCTGTTAT
TTTCTCTGTGATCTGCCTTTCCGCTTTTTGTTTTATTACTTTAAATT
CCATATCTAGGAT
ATTTTCCACATCCGGATCTCCTTCCTTCTTACTATCCTGTAAAAGA
TCTTCATAAAAATC
TAGGTATACTTGTTTTATTCCTTCATCTGTTTCCACTACTTCGTGAT
GTTTATTCTTTAT
ACCCTCCCTTCTCATATCAGATCTTTTTCCAAGTCTTCTCTTAACT
TCCCAAAATTTCGA
GTTGTCTAGCCTTTTCTCTCCATTCACCCTTGTTATGATCTTTTCTG
CTTTGTTTGCCCT
ATCTTGTTCCTGAACCTTGAGTATTTGCTGGTTTATTATGCCTTTT
CTTTCAAAAAGTAT
ATCCCTCTTTTCTTTTTTCGTTTCATGCAATATCCTCTTGTTCACTT
CCTTTCTTATTTT
CATCCACATTCTCATTGATTTTGAACCATTGCTTTTCTTTTTCTTCT
TCTTGG

>ConsensusfromContig3534 Average coverage: 8.93
TCTCCGTTATAATTTCTTCTGCCATATCTTGAAGAGATAGACGAG
AATTGGGAAATTCAA
ATTTAGTATTTCTGCTGAAAGGACTAGCAGAACTACGACCACTAG
AACTACTACTCCAGA
AATTAGTTGGAGAAGAACTACTATTTGTTGAAGATTCACATGCAC
TAGAACTAACACCAG
GACTAGGACTAGGACTAGGGCTAGAGCTAAAACTAGGACTGAAA
CTAGAACCAACAAAAT
CAGAATTATTAGGAAGAGTTTTAGAGGAAACACTACTTAATGTTC
GTAATTGAGATCGC
>ConsensusfromContig3535 Average coverage: 6.24
CATCTCGTAGTACGTATCTGTTATTTTGTTGAGGTGTTCCATGACA
AAAAGTTTGTCATC
AATAGTTAAATTCCTTCCTTTTTTATCAAGGCATTGTTGTAACCGC
TTCGCAATTAATTT
CTTCGAATTCATAATATCCGATGACCCTTCCGATGACGTGGCTGG
GGATGATGACTGTTT
GCTAGGACCTGCAGGAAGTTCTGGATATTCCATATCCATAGAGTG
GTCCTGCTCACAATC
ACTATTCAAAATAGTGTCTAGACTGTCGTTTGAGTCATTCAATTTT
TTTGATTTCGGGGG
ATGATGAGGCGAACCTCTTTTAGAGCGTTTGCTCATCCCAGCAGA
GATGTGATGAATATC
ACATATACGGCCAAATGAATTCCGATATAGAAACTTGTTATGATT
GTCGGTGCCTGGACG
GTCAGGAAACAGTCAGCTACGAAACAGTGCGAATAAAAACCAGA
TGCGCCAGTCAAGGAC
TGACCTTAGAATCACAGATGTATTTGTTGTGATGGGCGTTTACTT
CCCGA
>ConsensusfromContig3536 Average coverage: 8.41
ATTTATATCAATAGTTCAGCTGCTATAATTAGAACAATACATGGA
TAAATCACGAGCAGA
CGAGATTTTGCTCTGTGGGCAAATCCATAGTTCAACTTACGAATG
GTCGAAGGAATGTCG
CTGATTGGTCGATGGGTCTTCTCGAACGAATTATAATTTCAAATC
GCGCGCACAATTCGT
CCTAAATAAAAATTGACGCGTCGAAACATTTTAAATGTTCTTCAA
ATCTTATTCACTTCT
TTCGCCAATAAAAAAAAGCCAAGCCGGTTTGCGGATGGGAAAAC
TCAGGGATTGTATAGA
AAATTCTGCTCTTTCAGATGATATCAAAAAACCTTTTGTTCTAATT

TTTTCCAGGTTCCC
TATATAACACTAACTAACTACCCCGGACTAAATCCGATTCTCTTC
TGAACACTTATCCAA
ACATCCAAAAAAAATACTAGATA
>ConsensusfromContig3537 Average coverage: 11.13
TCTGAGATGAGGGAGTCTCCTGGCAAGACAGTCTGGCTGAGAACC
GTACTTGTTTGTTCG
ACTTATGTCCCTTCGTGATACTTTGAACGTCAACAAAGTCTCATC
GTATGTCATTGTCGC
CTCGTAGTCTGCGTTATTCGGAATGGTGTTGATTGAAATGGAATA
TAAATTCACAACGTT
TTTAGAGCTTTTTCCAAAATTGACATTATGTTTAAAATTTTCCCAT
TTAGTATCAGTGAA
CTCG
>ConsensusfromContig3538 Average coverage: 9.44
TAGTCAGCATGGTATTTCGAATGTTATCTATCAGCAATGTGGCTG
CACAAACATTCAAAA
GGTTCAAAAACTTGGAGTTTCTGAAATTCACCGACTTGTATCTCG
ATACTGTTTTCCAAC
GAAATGCATTCAGAGGATTTGCAGGAAGAGAACTTCACTTTGAG
AGCAGTCAGCGTTCAA
ATGAATCGTACTTTTTTCACGATCAATCTTTCAACGGAATCCAGC
CAGCATTCGATGATC
TAATAATCATCAAATTCATGAAGACAACTTTGAAAAATACTGATC
TCAATTTCATCGCAC
CTTTATTCAATCTTCGTGGAAGAATTGAAATTCATTTGATTGATA
CGATGATAACCAAAT
TGAAGATCATCCTAACCGAGAACAATATGAAAAAGATAGATCTT
TACCAGGAAGGAAACC
TCTGGCATTGCAGTTGTGATACTCAAAGCTTGATCGGGTTTGTAC
GATATAAATATGTAA
GGGGTTATCTTGGGTATGACAATT
>ConsensusfromContig3539 Average coverage: 11.31
CTGACATGTGCGCGCGTATCCGTCCGTTGTGCGATTTCAGAGAAT
ACTTGGGAGAGAGCG
CGAGGGATAATCCCGCGATGCTGAAACCGCTCTGAGGTTCCTGTC
AGAGTGTGTGTCTTT
CCAGATCCGGTTTGACCAAAACAAATGAAAGTCGCATTGATTCCA
TCCATGGCTCGTGAA
ACTAGGTCAGAGTTCACGGCCTTCCAAACATCGTCTTGGCTGGTG
TCTCGAAGGATTCCG
TTGAA

>ConsensusfromContig3540 Average coverage: 7.63
AAATTATGTTGTATTTAATAATTTATTACGTGAAGATATAGCTGC
TTCTCCAAAAAGTTG
TTTCGATATTCAACAATGATCTCTGCATCACAGCAACTGTCACAT
TGGCATAACAGGCAA
CATCAAATGAGAAACCCAAATCAAACTTTCAACGGGTACAACGA
AAATATTTTCATAAAA
AAATATTGGCCAATAATATCGAGATCATTATAATATTGAAAACCA
ACAGCTAAAAATTTG
GTTCTACTAAATTTTTCAATGTATATGTACATAACCTATGAACGG
CGTATCAGACCTCGA
ATTTGT
>ConsensusfromContig3541 Average coverage: 3.28
TTTTCGGCTTCAAGTCTCACTTCGGTGGCGGACGCTCTACCGGTTT
CGGTCTCATCTACG
ACTCCGTAGAGAAGGCTGAGGCTTTCGAGCCCAAGCACAGGCTTC
GTCGTCACGGCCTCG
CTCCCGAGTTCCTCGCTAAGCGTCGTAGCGGAAAGGAGACCAAG
AACAAGTGCAAGAAGG
TCCGCGCCGGCGAGAAGGCCAAGCTTCG
>ConsensusfromContig3542 Average coverage: 27.21
AATTTGTAACAAATTACAAATTCAAGTGTCAAATTACATTTGTTGT
TTTTGTTCATGTGT
AGAGTAGAGAGAGGAGTAAATGTTAAATTAAAAAAAGAAGACTG
TTGTTTGTTTTTGTGA
GTGATGAGGAGACCTAAAACTTCCTCTCATCATCAATATGTTCGT
AAAAAAATCAAGATA
TAAGTTTGACATATGGCACGTTTTTTTAAGATTGCTGTTTATTTTT
CTGGTCATTCCATA
CATTTGTAGTGATGGAGATATTCCAATTAAACCAGGTGATGTAGA
TGAAGTCAACATGGA
CGGATTTTACGGAGATGATGTCACTGCCGGCAGTGACTTCGAAAA
TATACATATAGACAC
ATGTAGTGTTGACAAGTCATGCATGGATGCTCTCGACATGGCCCA
ACGAATGTACCGAGT
CGAAGCAGTCTTCGTAGATTCACCGCAGGATTTCATAACAGC
>ConsensusfromContig3543 Average coverage: 23.64
AGTTCACACGATATCAATTGTCCATGAAAATATAAGAAATTTGCA
TCCCTTATAACAAAC
AAATAAGAAGCAGAAATAATCAAATGCCGCATTGATTAATGCAA
TGAAGAATGGTGTTTC
TCTATACAAAATGATTCAATTCAATAGCCCTTGATACTGTAAAAC

AAAGTATGATAATCT
TGTTGTTTGTTTTAGTTATGGCTATCAGTCACTGGACCATGATAT
TCCAAGATCAATGCA
TGGATAATAAGAAAACAAATAATACCATCAACAAAAACAAACGA
ATAGTTTAGCAGGTCT
TAATACAGTCATGCTATTTATAAAATCAATATGCTCTAAATTGAT
GTTTATAAATTGTTG
ACTACAGTTTTTCACAAACATCAGAAACAAGA
>ConsensusfromContig3544 Average coverage: 23.35
GGAACTTTAATTTTCAACCTTACTCCAGCTAACCTGATTGTAAAC
AAAGAAGATTTTTAA
AATGCGATTTTATGTTGCTCACAAAATATAACTTTTTTCTCATTTC
ATGGTGTGATACAT
AATTTGAATTAACTTTCAATAAATATTCGTTGAAAGCCAAGATTC
TTAATTCAGGTTACC
ATCGCAATGTTGGCAGATGTTGGAGAAATAGTATGGCAAATCCTG
GCGATAAACTCTGCC
ATTGTACATCATCTACTGAAATTTGTATTAGAAGCCTGGAATACG
TTATTTTCGTTGATC
GCCGCCATATGGCTGGAAAGTGCTGTGCACTTCGAATCAATACTG
TTTTGTCTCATAGAA
ATTGGGAAGGTGGTTTTGCAAATCGGCAG
>ConsensusfromContig3545 Average coverage: 9.19
CCATGTACCGATACGTATGGGGTATGCTTCCAATCCGTCTTGCAT
GGTTGGAAATGCGGT
TGCCGAGGTGCCAGACACTGGTCCGAAGAGGAGCAAGCATGTGT
CGATGGCTACACGAGA
GATACATTCAGAAAAACAGCGGACTAACCCCAACAGTGAAATTT
CAGTCTGCAATAATGA
TTATAATTAAAAAGAGT
>ConsensusfromContig3546 Average coverage: 11.05
ATTATCATCATGTCAACATTACCATGTGCTTCAACATCTCCAAGA
AGCTTAATCGATGGT
GATCAGAGATTCTTTCAAAGGATGAGATGTAATTTAAAATCTCCT
CACAAACACAAATCT
CGTAGAACATCTTTAGATTCAAGTCAAATCGATCGAGCATCTGTC
AATATCACCCAGCGG
AAACGATCTAATAGTTACGGAGGAGATAATAGTTATAAACGATAT
TTCAAATCGGTGTCT
GACGCTGCCAATAAAATTCCTAATACTTTATATGGTGACCAAGAA
ATTGTTGACGATTTT
TCTGACCAATCAATTTTAAATGAGAAACCTGACACACAAATATCA

CCCGTATCAGAGGGT
AACGAAGATTTTACAGCAAATGAAGAAACGTGTTTTTCTTATGAT
AATGATTTGGGATCT
TCGTCAACTCCCCAACTCAGCTGGAATGAAAATGTAGACCTGTG
TTCGAGAGATCGGAAG
A
>ConsensusfromContig3547 Average coverage: 8.23
ATCCTGTAACGAAGACAACATAAGAGGCATCATTGATAATTTTTG
ACCAATTCAACTACA
AATCCCACCCCACAAAATGATGATACTACACATCTATTAATAAAT
CACTCAATGCAACGT
CCACCTTTCAATTCAGTGAACCCGTTAGATATACGATGAAACACC
TAAATATCTCCATAT
ACCGGAATCTAAGAGCAGTTCTTCTCATAGCGATTCTTTAATACT
AGAA
>ConsensusfromContig3548 Average coverage: 31.79
CGGCCGAAAAAGACCTTCATGGGGGTCGCAAGAAATGGGCGTTT
CCTGGGGTCGCTCAAG
GAAGTGCTGCGTATATTCATGGACAATCATTGAACACATGCGCA
ATGGAAGAGGAAGATG
GTGAATTGAAGAGTCAGGAAGAGAAGACATTTCGACAAACAGTC
GAGGCAGAAAAACCAA
GAATCTGCTGGAATATTGAGCGACGTTGTTTTTCGACAGTGCTG
CCCAAAAAATTTTCC
AAGACAAAGTTGC
>ConsensusfromContig3549 Average coverage: 9.90
ACGAAATCGTCTCCCACTTAACAGTTGAGCATGTGGGCGGTCCT
GAGCAAGCAAGCCACG
CCTGTTATTGGGAGGGTTGCTTACGAGAAGGAAGACCGTTCAAG
GCGAAATACAAATTGG
TGAATCACATCCGGGTCCATACTGGAGAGAAACCGTTTCCCTGTC
CGTTCCCCGGATGTG
GAAAGGTCTTTGCAAGATCTGAGAATCTCAAGATACACAAAAGG
ACTCATACAGGAGAGA
AGCCGTTCCGATGCGAGTACGAAGGCTGCGACCGACGATTCGCC
AACTCTTCGGACCGCA
AGAAGCACTCCCACGTCCA
>ConsensusfromContig3550 Average coverage: 6.11
ATCTTTAAAATATTTTGTAATGTTTGCAAACAAGGACATTTTTAA
TGTGATCTTAAATTA
CATTATCCACTCATTATTTCTCTCCGCCGTGTTTTGCACCTTATTT
GTGAATTTGAAACT

CGTAAGTTACGCAGCTCCTTGGTAAGGCTTGAAATACTGGTGAAA
ACTCAGTGAAATGTG
CCATTAGGTGCTTCAGACCAGTTCGGACACTATAAACGAAATTTT
GCTGAGAAGCTATTC
TTATTACGTTAGTGTATCCCATAAGTTAGTAATATCACATTTTAGT
GCTTTGATTTGATA
TAATTTTCACAGGGTGTTCAATCCCTTTTTTTACTCCGCGAGATCG
GAA
>ConsensusfromContig3551 Average coverage: 5.46
TGATCACATAATCGAAGAAAAAATCGACTGCTTGGAACTTGGAA
CCTCTAAAACCAGTGC
TGAAAAACCACATAAAAATTCGACAGCTACAGAAGCAGAAAACT
TTTCAAATTCAGACGT
GGGTGCAGACGACATTGTTCGTCTACGCAGGGAAAGAGACGAAT
ATGCAAAACAGTTATC
AGCGGCTAAAAATTCTATTCTTAAGGAGCAGCGGACATACAGAC
AAAATATTGACCGGTT
GAATGAGGAACTGGAAGATCGTCAGCAACGCATTGAAGAGTTAC
AGGACGGTGAGGTTGC
CGTCGAAAAAACACCACTGTCAGCAATGCTGGAAGAGGCAAAGA
AATTGGCAGAAGGAAG
GTCAGATATTGCACCGGTAAAATCGGATCCGCAAACAACAAAAG
AATCCAACAGAAACTT
TGGAGGGAGTCTTATGTTTGAAACTCGATTGCATCAGCAGAAGGA
G
>ConsensusfromContig3552 Average coverage: 9.28
TTTTTCTTCTGCTTTCCTCTTTGAATTTGACTGTTTAGTTGATTTAA
AGTTCGCTATGTT
ATATAGATATGGTAGATATTCTTTTCATTTCAAAGTGGCCTCTTAC
TTTCATGTGATTTT
AAAACTGGATTGTTGAAATTTCTTGATTTTAGTGTAAACTTCATAC
TTGGTTGGATTTAT
GTCTTATGATTATTAATCCTTCTCGATACTTTCAACGATTGCAATT
CCTTTCAGTTGATA
AATATGCTATTAAACGCCTAATAGTTGTATATGTTCAATTTACGGT
TATTCATT
>ConsensusfromContig3553 Average coverage: 5.82
TGTGCTCTTCCGATCTCTTTGATTTCTTGCGTAAGACATGCTGTAT
GAGTATCGGTGCTC
CATTCGAAAAACGCGCGATTGTCCAGAAAAAATTTCGGTTGAAGT
GGACCATAAGTCTTC
CTGTAATATCCGTCCCATGCCATATTAATTTTGCGGAATTCATCA

AGAATTTCCCGAAAA
GTCCATTTCTTGTCAATATCTTTTGAGAAAATCTGTATCATAAGC
CAAACGGGATTGCTT
CCCCCAAATGGAATGTTGAGATTGTTTTGATCACCTCCTTTCAAT
ATATTCTCACACATG
AGTAGATTAGGGAGGTTACTTTTCAGTTCTGTCGTATCACACGCA
CTATAAGATTTTCCT
TTCATCTGTTTATCATCAATGTATCTTGTTGTGTTTTTGGACGAAT
TTGAGTTTGTGCC
>ConsensusfromContig3554 Average coverage: 45.39
CCCTGGTTAGAACATATAATGCATCTTTACAGATCAAAAAACTCG
TTCAAAATATGGTAT
GTTAAATTAGTATAGCCTATCCAATGATGCATCTTTTCGGAATAT
ATCTTAAAACCTGAA
AAGATCGAAAGCCGTTACAACTTTTTCACTTCAATCGACTCATGA
GAGAAAGGATAATGC
CACAAAGAGTTGGAAAACAAGAACTATCTCAGAAGCATTGGATCT
ATATTTCTATTCAAC
CGGATCGATAACCGAGACTCAAACACTGAAATTCCGTTTTGATCC
ACAGAATTGAATTTT
TTCTGCATGAATATTGCATTTAACATCAATCAAGCACCTTAAGTA
ACAAACATGCGTAGA
TAAAATTTGAGTGATGAACATGATATGTTAGGAAAAAGTTTCCCA
TGATTTGGTATTAGA
CGACAACATATTCAACATATTGGAAGATGTCTTGTTGGACGATGG
CATATCAAACATGTT
AGCTGTTTTGTCTTGACTAGTGTTCGACAACTGTCTTTGAAAAGCT
GTTGGTTTGCTGTG
AAAGACGACAGGGGGTGATCGTTTGAGGGACGAATGATATCTGA
GAGGGTTTGGCACAGA
ACTGAGGGGATGGGACTGCAAGTTTACCGGAATAGGTGGAGAAC
TGATGGGCCGAGAGGG
AGAAATGAGGGGGCCAGAAGAAGCGGAGGGAAATTGATGTTTCA
TGTGAACTGGTGGAGA
ACTGAGGGGCCGAGAGGGAGAAATGAGGGCGCCAGAAGAAGCG
GAGGGAAATTGATGTTT
CATGTGGGCTGGTGGAGAACTGAGGGGCCGAGATGAGGAGCAGC
CAACATTTTGAACTGA
TGTAGAATTAGTCGCCTTGAGATTGTAGAATTCTGATAGCTCCTT
GACAGATTGACTCAG
AAACGGTTTTGAACTTTTCGTCATCTCGATATTTGTTTTCTTCTCT
GATGTGACTTCATA

TGAATCATTTTGGCAATCGCTAACGCTCTCTGGTATCAGTGGTCC
GTCCAATGGACACCC
AACAGATGGTATCTCAATATCAAGTGAATTGCATTGGTTCCAGGA
ATCCGTGTTGCGCCT
CTGAGCAATAGATTTCTCCGCAGAAAGTTCTGGCAAGCATTTGAA
GTCCAAATCCCACTG
CCTCAGGAGGTCTTCGTCGGCCTCGCTCGAGACAGGCCGTTGAGA
TTTGACGCTGCACTT
TCT
>ConsensusfromContig3555 Average coverage: 12.29
CGCCAGTATACCGGATCTGAAGGTAATTTTTATCGCCCTTGGTCG
TTGGCTTCCTTGCGT
GTATCTGCCTAGACGTGTGTGAGCCATGGGTGTCTCGCATGACAG
GGTCTTACATATGTT
AGTGATTACTTTTTTTTCTGTTTCCTCTCTTATTTTCGGGTTTTCTT
CCTGATCCTCTTT
GATATTAAAGACGATCAGGTTTCTCTCCCGATCTTCTTGCTCTCTG
TGTTTGACTAGCGT
TTGTTTTACTGCTTCAGCAGCATCTCCTATGTTGGTTTTCATAGCT
TCTGAGTAGCTCTT
TCTTACAGTCTCCACAATATTTTGTGTAATCAAAGAGTTATTCGCT
AGCTCTTCCTTGAT
GTCGGCTAGCTGTTTTGTGACATCAAGTTTCATCTCTGCTATTTCT
CTATTGCTTTCTTT
TTTTGCTTTCCACCCCTCCATACAACCATCACAGAAGAATGTGAGA
TTCTTTTTAATTCC
TATAAGTTTAAGTTCATTGTCCGTCATGTGGCTACAATTTTTGCAC
GCCCACTTTTCACA
TAGATCACAGGAGACACCATCTCCTGTGTTGACTTCGGAGCATAT
CCCACATTCCCATGT
GGATTGGGACATGCTTTTTGTCCTGAGTTGCATTTATTAACGAAT
TATAACAAATCACAA
ATATTTTAGTGGTAAAAATAAAATGAATAAATAATAACAAATAA
AAAG
>ConsensusfromContig3556 Average coverage: 10.28
GCCAGTTCTGCAGTCTATTGCAATTCTTAACTTTATGGTTTATTTG
AGAGCAGCTTCAAC
TTTTGACAAGGAAGCAATGTTACTAGAAGAGAGTGAATTGACGC
TCGATTTCGAAATGAA
AAACGCGACAGTATACATGGGGGACACCGTCCGGCTCAAATGCG
AGATCACGGGATCACC
CACCCCTACCTACCGATGGTATCACGACGATGAAGAGCTCATGA

GGGATGCTGATCCAAG
AATCGTCTTCAAAACTATCGCCTGGGGCTCTAAACTCCGCATCAC
TTCTGCTCGGGCCTC
GGATTCCGGGAAATACACTTGTTCGGGAAGTAACATCAAAGGAG
AACGGCTGTCGAGTGG
ATTTCTGGAGATTATGGCAGGTTCGAATCCCAACCCTCAACCATC
GAACCCTGGTGGACA
CAGCGATGATGATGACATCTATGATGAAAATGAACTCGTCATAC
AGTTCGAGGATGGCGT
GAACTT
>ConsensusfromContig3557 Average coverage: 5.39
TGTAACCCACATACCCGATTGGAAAAACTTATTCAAACTGAGGGT
TATTTAATTATACTT
TACGTATCACGATATGAACATGCACGAAGGAAATACGACCTTAAA
ATTTCATCATACCAT
TTTCTAAATCAACCTCTACATAAATTCTACCATAGCGCTTCTGAA
AGTCTTATCAACTGG
AACTACGTCTTAACTCTCACCTTTTTCTGATGAAAAATCGATAAC
AAAAACACATGCAGA
ATTAAATGAGAAATTGGTTTGGTGCGCTAGCTTTGTTTACATTTT
GAAAATCAAAACAAA
CCCCGCCCTCAGTTCCTTGAAAAAGTGTCATCTTCGCGTCTCGTT
CCACAAA
>ConsensusfromContig3558 Average coverage: 5.55
TTTTCGCGAGATATTTCTGTCTGGTTTGTGGTAATCGTTATGGTAA
TTATCTTGTTGCCT
TATACATGTTCACAAAAGTTTTATATTTTCTGAATCTATTAGTACA
ATTATTCCTTCTCA
ACGGTTTTCTAGGAACTGATTATCATTTATATGGCATTGAAATTT
TACGTGATATGTTGA
TGGGAGTTGACTGGTCAGCTTCGCGACGATTCCCTCGCGTCACTT
TGTGTGATTTTATGG
TTCGTCAAATGGGAAACATGCATAGACATACAGTTCAGTGCGTTT
TACCGATCAATTTAT
TCAATGAAAAAATATACATATTCCTGTGGTTTTGGTTCATATTCG
TGGGTGTAGCAACAG
TATTTGGTTTCATGCGATGGATGTGGTTACTAGGCTTCAGATACA
GTAGAATCCGATATA
TTCGTAAGCACCTGAAAATAATGGCTCGGCTTCCAGCTGATAACG

>ConsensusfromContig3559 Average coverage: 22.79
TGAGAGGGTCTCAGATTTCGTAGAGAAAAATGCTGAAGTTGTGA

AACAAAAAAGAAATGG
ACAGATGCCAAAAGAAATTTCCGAACATGGTTTGAATTCAACTA
AATACGGGGACGTGAC
CCAAAAAGTAGAATCTTTTGCGAAAAAAATAGATAACGAGATAG
AGATGAAAAGTGAGAA
ATTCAAAGCTAACATGATCGATGAAACTTATGCAAACAATTGGAA
TTTGTTGAGTGCGTC
ACAGGCAGCTGAGGAATTGTCGAAACGTGTCGAAAAAGATCGGA
AGAGCACACGTCTGAA
CTCC
>ConsensusfromContig3560 Average coverage: 4.36
CACGACATGGAATGGCAAAAGGAAATCTGGAGTATTCATCTACTA
TAATTAACAAGTATT
TATTTGGGGTACTAGAACTCAGGGGTCCCTTGAAATCCAGGCTGA
TTCTATCAAAAGGTT
TCATTGCCTTGATCAACTCTCCAGTATTTGGCTTGTGGAAATTTGG
TTTTATTTTTCTGC
ATATTGAGCAATTTGATACAGTATTTCTTACATCAGTTAGAGTGA
ATGGAAGATTCCGAA
TTTTGATACAATGCCACAACCTCACAACTCCCGGGTGACATAATT
CTTCATGTAGGTTTT
TAAGAGAGTCAATATCAGTTCTTATCATGCAAGCCCGAGAGAGTG
CATCAGCTGCCCCAT
TCAACTTTCCAGCCCGATGCTCTATTGAAAAAGAATATGGCATAA
GGTCCATTTTCCACC
TTAAAATTTTCTCATTTTTAATCTTCCC
>ConsensusfromContig3561 Average coverage: 21.06
AACAATTATCTAACATTGAATGAGCAAAATGTCGGGTTATTTATT
CATCTAGTTGAGGTT
CTTCATATAGGATCAACGCTTGCAACAGATTATGAATGCATTTTC
AACTTTATAGAAATG
ACAAAACTAATCAACATTAGTCGTGAAGATTATAATGACGTTATC
AATGGAATACATGGC
AAAATTACTGTTGACATTCTCTATAAACTGATCGAATGTCTACCG
AGATATGGATATATG
AAAGATTACAATTATTTTCTTGGTTGTGTAATAACAAAATCTCTA
AACAGGTCAATGGAA
TTTCTTAGTTTTGCTCAGAACCATGGCTACTATGCGCTGACACGC
ATTAACGACGATTAT
CATTACAAAATGTGTATATACCGCAAAAAAGGCGATCCATTCCA
AGTGGATTATCGAAAC
CAATTAAACCAATTTCGTCTTTCTAGAAATATACCACAATCTGAT

TATATCATTTCTGGA
TGGAAGGTTCCCGAAGGAACACAAAACAGGCATGAATCTCAGAT
TGGTTATTATGCATCG
CTAGTAATTAAAGACTGCCTATCTGAAGGAAATCTGGGATCTGGA
GTTAAAAGTTGTCCA
TGCTACTTCGAAACTCGGTCTCTTGAATATCTATGCGAGCAGACT
CTAATAAGACACATC
AATTGGAATCAATTACATATCGATGGGCATACTCCTCATAGAAAA
TCGCTAGTCCAATTT
CGGAGCACACTCTCGAAATATATGTTGACATAGCTATGAAGCGAT
AACAGTCATTTATTC
GAAAAAC
>ConsensusfromContig3562 Average coverage: 13.13
GAAATTTGGCGTGGAATTTGGTATCGACAAAGTACCAGATGAAT
TCGATGCCAACGCTGG
TACTATCATCTATATACATGGATTTGGTGGTGATGTAAATGCGAC
ATCTCACGATTATAC
CCATTATTTGAAAGCTATAAATAAGGGCTTCAACTTCATAGCTGT
AGATTGGGAAAAACT
GGCAAAGAGGCCGAATTATCTTCTAGCGAAGCGAAACACTGTTA
GAGTAGGAGCTGACGT
ATCTTTTTTTCTAAAGAACATTTCCGCATCAACACATCAACAAGC
CGGGGATATGTGGTG
TGTTGGATTTAGCCTGGGTGCACACGTTTGCGGCATAGCAGGAAT
GAGGACCACATTTCG
ACGAATAACAGGACTCGACCCAGCTGGCTTTTTGTATGGCGAAT
CAGACAAGAATACACT
GAATCGCAACGCAGCAAACTTTGTGGAAGT
>ConsensusfromContig3563 Average coverage: 9.67
CGATCTGAAGGAAAGATGGTTTCGACGATACAGAATACAATAAA
CAAATATCAATCCTCG
ATACAAATCAATTCTGTACCCAGGAGGTATTTCAATGACCAAAAA
GGTCTTCAGTATATC
AAAAACATTTGTATTCCATCATACAAAACTGTGTTGACAGAAATA
TCGACAAAATACTAC
TGCTTGTCGTCAGCTGCAGCCTTGATAAAATACATTGAGTTGATA
CAGCACGTAACATAT
GCTCCAAAATCAATAAAAATTCAATTCAAATCGTCGAGTGATACT
ATGCTTATTGATGTT
GCAACTGTCCGAAATCTAGAATTGCTTGAAAACAACAGCGATTA
CCGGAGCGACCAAACC
CTTTTCGGTGTCCTGAATGGAACGAAAACCGGAATGGGAACTCGA

CTTCTCCGAGCTAAC
ATTTTGCAGCCAAGTTGTTGCC
>ConsensusfromContig3564 Average coverage: 14.76
GAGAAAAAATCACTGATGATGAAGATTTCACCCAGTCGAATTCA
GGAGATTCTGAAATCA
TTGAAGCGACACCTCCACCGACTCAACCAAAAACAGAGGCGCCA
CAAAGTACAACTAGTA
AGCCAAAGAAGAAGAAAAAGAAGAAGAAAAAGAATAAAGACGA
TGAAACCACAATTGCAA
GAACAACGCCCAGCTCGAAGCCATCAACCTATCCAACTCCAACG
GCGAGGGAAACATTCC
CCACCCGCGCCAGTACAATCCACA
>ConsensusfromContig3565 Average coverage: 6.58
GGGGAGAAGAGCTGATTATCCCATGCATAACCAACCAGATTTCT
AACACAACCTACAGCT
ATAGATTCAAGTGGCTAGATTATCATGGAGATGAAATGAGTACAA
CTGAAAATGTTTTTC
TGGAACTTCAAAATGGTACTCTGGTGTTACGCAATATGTATCATC
TACCTGATTTCATGA
CGTACGAGAAAGATTCAAGTTATCCTGATCAGAGAGTGTCTCATT
TAACTTGTAAATCAG
ATGTGGAAGGATTTAGCAGCCGAAGGGAGCTTCTGACTTATACTA
TAATATTCGACGATA
AAGACGGTAATGAAAGCATGCTTCC
>ConsensusfromContig3566 Average coverage: 7.74
TGATTCAGCTACAATTAAAAAAATTCGTCCTGACAAAAAACAAT
ACAGTTGCAGCAAATG
CCAAAAACAGTTTTCTTGCCCATCACATCTACTGAAACAAAGAAG
GATGCATTATGGCAA
GGAACCTAGTTCCAATGAATGTGAGAAATCGCTCTTACGAAAATC
TAGTCTAACACAACA
CAAATTAGTACGCTCTGATGAAAAACCATACAGTTGCAAATCATG
TGAGAAATTATTGAC
ACAGTTTGAAAGAACTCATTCGGGAAAAAAACCATTCAATTGCG
AAGAATGCCAGAAATC
ATTCTCATCCTCTTCTCATCTAATTCAGCACAAAAGATCTCATTC
TGAAGAAAAACCATA
CAGTTGCAAAGAATGTCTGAGGTCATTTGAAAGTTCTTCCCTTCT
AGCAACACATAAAAG
AACTCATTCTGGTGAAAAACCATTCATTTGCGACATATGCCAGAA
ATCGTTCTCGC
>ConsensusfromContig3567 Average coverage: 11.14

GTAGATGACATAGAATGGACACATGGAGTAGAGATATATGGCAA
TCAACAGAGCTTGGGG
AATCCAAAAACATGTTCACGAGATCAATAGAGACAATATGAAAT
GGAAGTAGATGACATA
GAATGGACACATGGAGTAGAGATATATGGCAATCAACAGAG
>ConsensusfromContig3568 Average coverage: 5.43
AAAAAAACCAATTCCCGGAGTGAAGATATGCAAGATTCTCCTTTT
GAGCACTCTCTTCTA
TGACTCACACAATTGCCTCCGTCTTGGCAACGATTTCCTGTGTAC
CCTTTGACACAGTTG
CAAGACACACGCCCTTCTCGTATCTCGCATTCTGCGCCATTCTTGC
ATTTTGTTACACAT
GCTTTCAACCATTTAGCCTCGTTATTGAGAATGTGTTCCTTAAAG
CTCGGTCCTGCGTAG
CAGTCAAAGGTCGTCTTCGATGTTTTCTTGCAATCGAGTGGGAAA
TCAAATTCATCACAT
CTCTTCCCGGCGCAAATATC
>ConsensusfromContig3569 Average coverage: 56.59
CAATGTGACTGGAGTTCAGACGTGTGCTCTTCCGATCGCTTGATA
GCGCCGAGCGCTATG
GCGGAATGGGCTCGCCAGGTGGCGTCTGCGGCGAGTTTCCTTCAT
GGTCATCGTATTATT
CATCGAGACCTCAAAAGTCCAAACATTCTGATCGGCCGAGACGGC
TCTCTCAAGATATCA
GACTTCGGAAACAGCAAGCGGTTGGGGGACGCGTCTCAGCGGAT
GTCGTTTGCGGGACA
GTGGCTTGGATGGCGCCTGAGGTCATACGGAATGAACCCTGCTC
TGAGAAGGTCGATATA
TGGTCATTCGGGGTCGTGTTGTGGGAGATTTTGACCCGCCAAATC
CCATACGAAGCCGTT
GATTCGTCGGCCGTGAT
>ConsensusfromContig3570 Average coverage: 2.97
CGCCGACATGATATTTCAAATGACATTGAATAAATACTTATTCAT
CGATACTAGGCCATG
GCGAAGCACAATCTACACCAGTTCCCATAGCCGACATGATATTTC
AAATGACATTCGAAT
AAATACTTATTCATCGATACTAGGCCATGGCGGAGCACAATCTAC
ACCAGTTCCCATAGT
TGACATGATATTTCATATGAAATTCGAATAAATACTTATTCATCG
ATACTAGGCCATGGC
GAAGCA
>ConsensusfromContig3571 Average coverage: 32.06

CTCTTGCTTGGTTTTATTGTTTGAAAACGAAATTATTTGTTGATAT
TTTATATTTATAAA
TCATGTCGCTACCATTTTTACCTGGTAATAATTTTGATAAAAATC
TGGGTCGGGAGGCCT
TCCACAGACAGCAGATGTTCGATGTAAGAAACAACACCCAGATG
CTAGTGGGAGAGACAA
CTCCCCCTGCCAGGAGTTTCATCCCTCGCGGGGACGGCAGTGCAC
AGCCGGCATGGGTCG
CCTTCGACAAACAAGTTCTTTCGTTCGATGCCTACTTTCAGGATG
CTGTGCACGAGAAAA
GGGATGAACAGTACCGAATAAGAAAAGTTAAGGTTTACTTTTAT
CTGGAGGATGACAGCA
TTCAAGTTGTGGAACCACGGTCCTACAACACTGGAGTTCCACAAG
GGACAATCGTCCGGC
GCCACCGGATCCCGAAGGAACCGCCCCGAGACAGCGAGCTCCAC
AC
>ConsensusfromContig3572 Average coverage: 7.97
TAACATTTGAACAACCGTGTGAAACACCAAACAAATTCGTGACAA
ACAATTTGATCGTAC
AAAATTCATGTTCTGAGCATCCAGAAGATGAAGATCATGATAAC
GACGACGAATTCAACT
TTGAACCTAATGAGTTAACATTGCTAAAACCATTTGAAGCACTGA
ATAAAGATAGGTCAT
CCAAACAAGTTTCAAGCTCCAATCAACAAATAGATAAAAATCAA
GACAACATTCGTCAAT
TCAATTTTGAACCTAATGAGTTCACAT
>ConsensusfromContig3573 Average coverage: 251.16
GTGACTGGAGTTCAGACGTGTGCTCTTCCGATCTTCAGATTTGAT
ATTCCATGTTTGATG
ATGGTATCTGTTATGGGCCAGCATTTACTCGTCATGGCATGCATT
TCAGTGGTGAATTTC
CCCATCATTTGTTCTATTGGCGAGGCATAGAGGCTGAAAAATAGT
ACCACATTTTTAAAT
GAAATTTCGAGACCATGTTTGCCAGTGTGCTCATTGATTTTTGAT
AACATTTTCCAAACA
GACGGAGAAGGGCTTTCCAGCATTGCTGTTACCATGATCTCGATT
GATTTGATGCAGCCA
GTTGGCTCCAAGATGCTGTTCTCGGTCAAAAAAGCCTCCAGGCAT
TCTGAAACAGCAGTG
GCCGTTGGAGCTGCAAGCTGACCTATA
>ConsensusfromContig3574 Average coverage: 16.92
TGCGAGCTTGACGACTGCCGGAAATGTCTTTTTGTAGAGTCCTGT

TAAAGAGGCCGCTCT
AAAAGCTTCACTGCCCAGCATCAGGAGTGTGGAAGCGTGTAGCA
GCTCGCAAGCCGCCAT
CTTGGTGCTTCTATCTGAAGAGTTGACTGCGAGAGACACAATCCG
GGGCAGGAATCCATC
TAAATACAACGTTGGTTTCATGTCCACGAATGGAATGTCGAATTT
CAAAGTCAAATCTCC
GTGTGTCCACGATGTCTCCTTCTCCCGGAATTGCTGATTCATAGA
GATGATTTCATTGGA
TGACTCATTCAGCAACGCCTTCTTACCCCGCCCACCCAAGGAACC
AATGAAATGCAAACA
TTTCTCGCGAAGATATTGTAAGCTCTCGTTTTCTTTGTTCGACACT
TGATTCTCTGCCTT
TTTTTGCTTCATCATTTTCACAGGGATTTTTTTGCGGCCAACTCTT
CTACCGATATTGTC
AGATTGAGCCGTGAGAATGGTTGCTTCTTCTTCGAGGTCTCCGTCT
CTGCTTGAATTTCC
AGTGAGCAAAACATCGATATGAGGAAGAATGGATTGATAGAATG
GGTCAAGAGCTCCGTC
AGGAAGCTCAGCTGTCCAAAGGGTCAAGGCCTCAAGAGACTGGT
GAGCAACTGGAGTGAA
GCTGCTGCCAAGGTTCAAGGCTATCTGTAAAACAAGAACAGCTTC
CGATAACTGATCTTT
CATCACCGCCACAGGTGCTTCAAGAATAAACGATATGCACGCTAT
CTGTTGGTCGCCCTT
CAATTGTCGCATCCGCACAATTACCTCTTTAATGAATTTTTTCAG
AAGGGCAAAACATTC
TTCTTCATTTGTGCCCATGTTGATCTTATGTTTACCTTCTCCAGAC
TCATAGATAGATAT
ATACCTATTTTGCTCGGAATAGTCGGATGTTCTA
>ConsensusfromContig3575 Average coverage: 11.97
GAACTACGATTGTCAGATTGAATTTGCCTTCATTAGCGCAATCAT
CATCTGTCCATTGTT
GGATACGGTACGTTTTGTAGTTCTCGTTAAAGGATATCGTTTTGG
TGTAATGCTCATCCC
TGTACATCAACCAGCATTCCTGGTTTTTGCTGTGGTACCAAGAAG
CTAGAACACCATCTC
CCGTGGTTTCACCCAGGCATTCTTCTGCAGTTTTTTTCGGAGAATA
TTCGTCCCGGAATT
TGAAGGTTTGAGTGGTGAATCCAATTTTTGTGAGAAGGTCAGTTT
TGATCGCAAAGTTGG
GTCTGCTCCTTCCCCGAAGAAGTTTCAAATTCCGGCAAAAGTCAT

TTTCTCGGGTCATGG
TGAAATCCAGATCAGGAAGTACCCCACAAGTACCCTCTGTCTCTT
CTTCAATCAGAAATG
TTTCATAATTGTTATTGAAGAATAGTACCTTCTTCGATTCTGTTTT
GTCGTGCACAATCA
ACTGACATTCCATTGCATTGTTTGCAACATCGAAGAAAACAAAGA
TAATGTCGTCTGCTT
TATTGTTCTGAATGCAGTCAGCTGCC
>ConsensusfromContig3576 Average coverage: 7.40
GGGGAATGCAGGACAGTTTCGGATATTGAAAAAACAGATGAAGC
AAGATTTGAGAAATCT
TCAACCATTCTTCCAAAATGAACGCAAAGATCTTGCCTGGCTTCC
TTCAGAATCTTTGGC
GTATGATTATATTTATTCATGCATTGAAGTAAAATGGAAAGAATA
TTTCTGGAAACGTCC
ACCAAAAACGAAAACCAAGAGTCGTCGTTTTCATGTGACTTTGGT
TTCACCATTCCAAAA
CAAATGCGACTGATAATGTGTATGAGTGATCCTGCAATTTTCCATC
CATTTTTCATCTTA
CTGAAATCTTTCTGCAGAGATATGAAAAACATCCCCAAGACATTT
TGAGACCAAAGATTT
TCTTTCAAATCAGAGAGAATATTGAAATCAGAGCTGTTCTCATCG
AGCAGACTCTGAACA
GTTAATAGCACTTGCAAAGAGTCTTTT
>ConsensusfromContig3577 Average coverage: 4.34
GATCTCGACACCCTTTATCCACGTGATATGTGCTCCTGTACGACTC
GCAAATCTTCTTAT
GCTCGGCTGGATTCACCGCATCACTGACCAATAAAAATAGGTTCA
CAGTGATTGAAAACC
GCAGAGTGAAGTACATCATTATACTTCAATTCAGCTCAGAGCCAT
GATGTGAAAATTCAC
CAGCTGAAAAAATATGCAAGTATGGCTCACACTTGATGAAAAACT
TGCTTTGATGTCTCT
CGCA
>ConsensusfromContig3578 Average coverage: 11.25
CACGACGCTCTTCCGATCTCTCTGCTCATCCCAGTTGGTGAACGG
TGTTGTGACGATTTT
GACGACTCCTCAAAAGTCTGTCTTTTGTCCATTCCCGACTTCACCA
CTGTTGCTGTTTTG
ATTGCCTCTCGAGTAGCTTTTCTTTCCAATACCTTCATTTGCTCAG
CGTTTTTCTCCAGA
GCTTTTCTCCCCACGATCCGTTCGTTTTGTGCCACTCTAGAGTAGG

GTTTTTGTGACACA
ACCGCCCTGTTTCGATCGAAATAAAAAGCCTGTGGGGTTTGGTTC
GGGACCGCTTCGATA
ACAACCCCATCCACAACTGTTGGTCCCATTCCAGGAATAAGATGG
GCATGCCGAACTATC
CACACGGCTAAAACTACGATAGTTATGAGAAGAGTGAAAATGAT
GGTCGCAAGATCGGAA
GAGCGC
>ConsensusfromContig3579 Average coverage: 8.59
GTCACTTCATGAGGTTGCCATTGTTGATCCTTGCTCATAGTGTACA
TCTGATTGCCGTCA
TCTTTTATCAGAAAAGCATTCTTTCCGCTCAACTTCTTGCTGATGT
AAACTCCTGGTAGG
CTGGTCTCAAGTCTGTAGCTGGGGACGACAATGTGCTCAGCCTCC
ACGAACATGTCGGCG
AATGGCAGGTTTACATCCATTCTGTCGTTATGCGTGTTCCATTCCT
CTTTGTTGATATCG
AAACTCATGATGCACAAATTGGAATCCATCAGTATGATTTTCGGT
TCATCAAAATACATG
TGAG
>ConsensusfromContig3580 Average coverage: 22.73
TCTGCTCTTGGTGGTACTCAATTGTGTGAATACTGCAAGAAGATG
TGAGATCGACTTCAA
CCCGATGTGGTCGTCGGAAGTTGATTTGGATCCTGCCGTCGCCGC
TCCTTTGGTGTTCGA
CATCGATGCGGATGGCGTCAACGAAGTCATCAGCATCTCAACGC
GAGGCAAGCTCTCGGT
GATCGAGGGGTCAGGCGGCGAGCGGATCTCTCGAAGCCATGTTCT
ACCTCACAGTCTTCA
CGATAATTTTCTAAGCACACCTGTTCTCTATGACTTTGATAAAGA
TGGCGTCCAGGATTT
GATCATCACATCGAGTAGTGGGGTCGTGTCTGTGGTCACGTGGGA
CGGACGTATCCTCAG
CCGGATATTTATTCCCAACGCAATCGTGGATCCGCGTGAATGCA
GCGGCGATGCAAAGAC
GGTGATCTCGAACAGCCATGTTTTGCGGTTGAAGAAATCCAATG
AGAAATCCTCAAAAGA
TGACCTACAACCTCTTGTCTTCACTTCGCCTTTATTGATCGATTT
GAACTCGGATGGGAA
AGCGGATAAAATTCTCATATCTGTCACTTATGTTCCACACAAAAA
CAGTTCAAATTCGAA
CCACTGTCATGGAGCTGGTCTCGTTGTAATCAATCTCAAAAAAGA

TGGTGTGGTGGAGAA
ATTCATCCCGCTGGAAGTGTCGTCTGCTTCAACACGAAGACCGTC
AATGATAACCTCGCA
ACCAACTGCAAC
>ConsensusfromContig3581 Average coverage: 27.40
GGAGTTCAGACGTGTGCTCTTCCGATCTCTCGGGGTCGCGATCAG
TCGCTCTCATCTTAA
TGACATTGCTCCTGACTGATGCACTTTGCGTGATGGAAGCAGAGT
AAGCCGATTGTGAGA
ACACTGGAGGATTCATATTGCGAACGACATCAATGCTGACTGTGG
CGTTAACGCTGGACT
TGGCCGGCACGCCCTCGTCTACTGCCGCCACCAGTAACTTATACT
GCAGCGTGCCTTGCG
TATCTTTCGTGAGGGATTTTCGCACAAAAATAGCGCCAGATTCTG
GATCAACATTAAAGT
ACTGCGACGCCAATACATCTCCGGTTACCATGTAGGTGAACAGGT
TATTTGGTGCATTTT
GATCGCGGTCCCTGGCTGCCACACTCCGGAAGACAGCCCCCTCAG
GCTGCGTCTCCATGA
TCCGCACTTGGTAGTCCGTCTGTTGCCATTCGGGGGCGTGGAAGT
TCCGGATGACCTGGA
TGTAGACGATGGTGGTGGCGCTCCTGGGGGGCGTGCCGCTGTCC
GCCGAGACGACTCGAA
CCCTGTATATTTC
>ConsensusfromContig3582 Average coverage: 6.33
CTGAAAATGTAGCATCGGGATCTTGGGTACATGTGAAGGCTGTCT
TTGATCACGCGAATG
TGCAAGCAAGACTGTATGCAGATGAAGTGCTGGTGGACACACATT
CTACTTTCGATGCCA
GTTGTTATAACGAACAGCCACCACGGAATACTTACATTGGTGCA
AGGGAGATCGGCAAAT
ACCATTTCGAAGGGACCATTGATGAGGTGTGTATAGCATTTGATG
TAGACAAATGCTGAA
TTTTTGTTCATGGCTG
>ConsensusfromContig3583 Average coverage: 9.04
GCGACCTGCATAGGATTTCTCAGACTTCTCTTAGAGGTAAAACCT
CCAGAACTAATTCTG
AGAGTTATGAGAAGCCGTTTGGTAATTATGAAATGATGGAAGAAT
CTTCTGTGAATTCTT
CTTCATCCATGAAACCGAATGCAGTGAAGAAATCGGTTTCATCA
AAACTGGGAATGCCAG
AAGTGCTGTACGATGCGATCCTCGGGGAGTTACAACAACGGCAC

ATAAAACCGGCTGATT
TTATGTTGAACAAGATCGAGTCAATTTATGTGAGTCTGGAGAATC
GAGGATCGGTTTTGA
TCATGGGACCTTCGGGTGCTGGAAAAACGTTGGCCTTCTCAATCC
TCGCCAAAACAATGA
CTTACCTTCATTACAAAGGAGCTCTGGAAGAGAAATCGACTGAT
ACACTGAAAGAGAAAG
GGTTGAAGAGAAAAAGCATCGCGGAGCAGTCTCTCGCGTGGAAA
TGCTCACGTAGAATGA
GTGAACTGAAAGATATTGACGAACGACAAGATCATGTCCAGAGC
AAATCCAAATGGAAAA
AAGCTGGAGAAGC
>ConsensusfromContig3584 Average coverage: 11.97
GGGATACATAAATACCACAATTAAAGTCATGTAGTTTCAATAGGA
TCTTATAATTTGACA
AAATGTTTAAAATGCTGACTTTGTTGATCGTGGTGTCGCTAGGGC
TGGCCACAGTGCAAG
CGACGTGTGGCAAGCCCTGCCACAATGGTACCCACAACCCTACAT
GCACCGAATGTATTT
GCTACACTGGCTTTACTGGGCAAAATTGTGAAGAATCGTGTACCC
TAATTTGTGAGCACG
GACATCTCGACACGTCTGAATGTGCATGTAACTGCAACAATGGAT
TCATCGGGACTACTT
GTAGTGATGCTCTTGAGGTGAATCCAGCATATATGCAGCGTCCAG
CCACTAGGAAAAGTC
TAGCTCATGAGACAAGTATAAACTCTGATGGGAATCCAGTTCCTG
AGGAAAGCTTATCTG
ATGAGAGTTTTGCTCTCAAATCCTTCGGTGCTTTCAAAATAAGTG
CTGCTTCTGTCAAGA
AAAGTCCTCCGATCTATACAGATATAGAGATCTCCGTAAGCGTGT
CTTCTGGCTCCACTT
CCAGCCCCTGGAATCTGATGGGAATCGTTACTGCTGTCTTGCTTT
TCTTGATAGTTCAAA
GAGTGACAAATATCTAAGTTTGTATAGGATTCTTGACAAAATCTG
NAATTTGCCGGT
>ConsensusfromContig3585 Average coverage: 10.30
ATTCAGACAGCTATCTTTGGCGTTAGCCATGATGCATTTGCTGCC
ACCACATGTCAACTG
ATCGCATGCGTCTTCGGACCAAACCGACGAAGCAGGGGAAGTCTT
GCATTTGCATTTAGT
GTTACTTTCTTTGCAAGCAATCTCAGTCCCTGCCATGTGACATGG
GATAATATCGGCCGT

GTCGCCTGTTCCAGTATTACAATTCAGTTGTCCGCAGTCTGTTCC
AGTCCAGCCATCGGC
CGTTTTGCACGTGCATGCCGTGGCAGAGGTACACGCATTATTTGT
GCCAGCCATGTTACA
TTCTATGTAATTATTATTTTCTGGACCGCACTTTAGTTCATTGCAT
GTTTTGCCAAACCA
TTCAATCTTGTCAGTGGCGTCTACCTTGCAAACATCTTCCGATCT
CTCGATAGCTGTACA
GCCAGATTGACCTCCACCTGCATTATGGCCGTCCGCATAGCATTG
TGTTGTTTGACAGGA
AGCAGCCCTTGCACCATCCATAAGACCCCAAATAACTAAACCAAT
CACGCATACTTCTCC
AGATCTCACCATTCGTGCCATTTGAATCTTGAAATAAAAGTCAAC
TTAT
>ConsensusfromContig3586 Average coverage: 6.06
CTCTTCCGATCTCAATAGTGAGGAGAAGTATCACAGGCTGCTTAG
CATGAAATGACAAAT
AGGTTATCAACTCAAGATTTATTAATGACTTTATCAACGATTGGC
GCTATCTGTAGCCGT
TTCTTCTTGTTCTTGCTCTTCATGAAAACGTGCTTATACAGCTGCT
GAACCAGCTGTTCA
CTCCGTTTTTGCTTGGCAAGTTTCTTTGCAGTCTCATTCTCTTTGT
AGAGGCTCACAATT
CTGTAGACTTGCAGGACTAGATCGATTATAT
>ConsensusfromContig3587 Average coverage: 21.26
GGGGACAAAGATTGCACCGATGGGTCAGATGAAAAAAACTGCGA
TTGTGATGCAAATGAG
TTCCAGTGCCCGGATCGCGATTGTGTTCCGAACTGCTCAGTTTGC
GATGGGAAAAATGAT
TGCTCGGATGGGTCGGATGAACTCGACTGTGACGTAGATGAATG
CTCTCAAGAACCAGCC
CCATGTCAACAAGTATGTACCAACACAAATACGAGTTATGTCTGT
TCCTGCTTTCATGGC
TATCGGATGGACGTAAATAAGAGGTGCCAAGCATACGTGTTCTT
CGAGATGGAGAAATGG
GTCATTCGCCGACAGAAAGGTCAATCCTATCGGGAAATTGATGAT
TCGTTCAACCGGATG
AGAGGGGTCGTCCATCCTAATGTGAAAATCAATGAGAATGGTCA
CACTGGAAGTTGCATG
GAATTCGACGG
>ConsensusfromContig3588 Average coverage: 8.45
TTCCCTACACGACGCTCTTCCGATCTGCTGAATGGGTCGCACGCC

TACGTGTCAAGAGAA
GTATCAGACAGTGAGGAGAAGGAGACTCCCTCACTGGGATCAC
CTGGCCTCCCAGGTCC
GATCTTCCCAAATTAACGATCGCGGTCTACGTCTGTTCTGCAAGC
GACACCCAAACTGAG
ATGGAGTTGTTCAAAAAGAATCATTTTCAAGAGTTAGAACAAAA
GTGTCGGCGCCACCAT
CGCTTTCTGACCATCACACATATTCCAAATGATTCCACTCAGAAA
CTTCCTATTCAGGTC
GAAGAACTGCAGCAGGATGCGCGCAACAATCGCGTCGATCGCTGC
TCCATCTTTCTGTGC
TTTATCGCCGCACACACAAACAAATACACAGAGGAGGAAAGGAG
GCGGG
>ConsensusfromContig3589 Average coverage: 13.49
GTGCCTTCCGATCTCTCGTGGTGTGTTTGCAGTGACAGCCAGCAC
ATTGAGAGTCAGAAA
ATGAAGAATATCGTGGTGAGCCAATTGCTTTCAAGCGCTGTCATT
CATATCTTCATGGGT
CTACTCTGTGTCCTAAAATTCACTGCCTCTCAAATGGTTGCCACA
TGTGAGATAGCCCCC
AACAGTGATCAAGCTAACGCGAACAGCATTACAGGAACTGTAAC
GCTGACTGAGCTGACC
GCAACCTCGACAAGAGTTGTGACAAGACTCAGAGGCTTCCCAGC
GAC
>ConsensusfromContig3590 Average coverage: 5.95
GAAAACTATTTCGTGAATGATCTCATTTGATAGCTCCATAATAAC
AGTGATACCTTTAGA
AGTTCTAATCTCTGCGATTTTTTCCAGGATGGTCAAATAGAGAAG
ATAAGTACAGAACAA
ATTGCTGAAATAGAGGAAAACTATTTCGTGAATGATCTCATTTGA
TAGCTCCATAATAAC
AGTGATACCTTTAGAAGTTCTAATCTCTGCGATGGTCAAATAGA
GAAGATAAGTACAGAA
CAAATTGCTGAAAT
>ConsensusfromContig3591 Average coverage: 12.72
CGCGGAGTGAAGACATTAGCCGTTAGTGCATTCAAGCTGGTCTCT
TCGATCACATGTGCC
ACACTCGTGTTCATCCGAGAAATCGAAACAATCCGGCCATCCGTC
GCATCGGTAGGACTT
GTGGAGACAATAGGAACGGTCGCAGTTCACATAGTAATTCGATG
TACAATGGGTGAATAA
TTCATCCTCTTCATACGGACAATCTTCTTCGTCATCATCATACCA

TAGTGCCGGGAGCCT
CTCACCCGTATCGCATACGAAAATGTCCCTCGTGGGTTTGCAGCC
CGGATATTCGTCAGC
CGAATCTATGCAATCCACTTCTCCATCACAGAACCATTCGGAGGT
GATACAACTCGTACC
GCACGAGGAAGTACCGTACGGACAATTCGACGGGTCACAGACTTT
CTCATCAGAATTGTC
CTGGCAATCTTGGTATCCGTC
>ConsensusfromContig3592 Average coverage: 9.60
ATGATCACTATCAAAATAGCTGATTTCAGAATTATTTCTCTGCCG
ATACACGGGAAATTA
GAAGTGGATGGACGGAAAACGTTACAGTGGGTGATACGTTCAC
GCAGAAAGATGTAAAT
CAGAAGAAAGTGAAATATTCACATGATGGTTCAGAGACGAAGTTT
GATGCGCTGGTTTTA
TCTATCATTTTGCATGAGAAACAGTCATTCGATGAAGATATTGAT
GAGAGCTTTATAGAA
CCGAACCATTCCGATCCGTGGCATGCTTTTAGGACGTACGGAAAA
TGCGTTGTACTTGAT
ATTGAAATTAATCCCGTTGACGATCAGCCATTTAAAATGTTTCCT
GAAAATCCAGAATTT
ACAATTGTTCAA
>ConsensusfromContig3593 Average coverage: 12.25
ATCACTCATACAAGAAATCCCACAATCTACCGGAAAGCATGACT
TAATCACAAATTACAG
AGAAAAATCGAGTCTAAAAACTGATACTCAATCCACTTCACTGCA
GATAGACGTTTGTGA
GAATAAAGAACCTGATATAAAGAATAAACCCGATGACGATCCAA
TTTTTGGAAAACACGA
GAGCCAGTGTGATGACAATAGTGAAAATCCCGTGCATTCGATTCA
ATTTCACTCATGCCA
GAATGAATCAATTGTTGCAACGAATTCTGAAGGTATTCGAGATG
CTAAGAAAACTCAAAA
TGATTCTGATGAAGAGATGATTTCTGCACAATGTTGCGCTCCGCA
AAACATTCGCGTTGA
AATGATTCCTTTGATTGACAAGAATGATGAGGATCTTTCTTACAA
TGACACATCGGGTGA
CGATGACGAAGGAACTAGTGGCAGCCGCGCCGACAGCGTTACTG
TTCACTTCGACACGAC
TGACGATGATGATGATACGTTCTTCTGATTTTCAAAATTTATTTAT
TAAATTTTCATATG
TCAAAAAGTTATGAATTCAATTATAGACGCACTTTGAGAT

>ConsensusfromContig3594 Average coverage: 10.52
CCCAGGCACTCTTGAACCACATCTTAATTTGACTGATCGTCTTTC
AGTACTTGTAGAGAT
TGGCTTGAATTCCGAAGGTATCTCATACAAATTTCGTAGCATCAA
ACTTCCATTTGCGAG
CAATACAAGAGTGTTTTCAGCAAAATCCAAATGATTCACACCAGC
ATTCCATCTGAATTC
GTATGAGTGAGCTTCACGAATTGGTTCACATGGTAAGATCAAATG
CTGGCCCCATACATA
TGTATCATGTCTGTCAGGTCTATTCACTACACCCGCAAAGAACAC
CGTGATCGAGTCCGA
AACAAATGTGGATCCATCGTTCAAAGCTTCGCACCAATATTCATG
TTCTTCGCGTCTGAG
TTTCGTGGTGGGCTCACCATTGCTTGTGCTATATGGGTAATCGAT
GTCATGGACAAACAC
ATCCTGCCCCGATATTACTTCACTATTACCGATTTCATTGTAGATA
GCTGTTTTTTTCAC
ACCTTTCGAGTCTGTTTTAATAACCTCAAGATACGATACTGATTC
>ConsensusfromContig3595 Average coverage: 11.31
CTTAATACTAATGTCGTTAAGGAATGTTCTATCATCTCGAATATG
ACATGGCTAGTATTA
ATGACGAAAACTCAGAACTGCTTATACAAGCTATAATGTTATTCC
GTGAGAAAAATATTT
CATTTCGTTATAATAAACCTTGATTATATCAATGTGACAAATCCA
ATCATATCGAATGTG
ACATATGTGTGGCTCCAGTATTGATAACAAAATGACCCAATCCGA
TGACGTCAGAACTGC
CGAATGGCGACGCTGCTACCAGCGGCTTCGTCGCGCCTCGACTTT
CATTGGG
>ConsensusfromContig3596 Average coverage: 23.70
CGCTAGTTCTCTGGCTTCAATCCAGTTCACGATGGGGTCCTCCGTG
ACTTTTAATTCCTA
TACTCAAGATGCAGTATGGTTTCAAGCCGGGTTCCCTTTTTCCCA
AGAGAAAGTTCACAC
GGACAAAGACGCAGCTGCGTACACGGTTTTCGTCCGTTCTCACCC
CTTCACAAGAGCCGG
TCGACTCGAGGCGTTTGAATACTATGCAACAGACGCGGGAGATAT
CACGTTCTATATCCT
CTCAGTGACATGCACGACAGGGAATTTTTGCCCGGAGTCGGCCGC
CTGCACCACCACATC
CTGTGGGTCGACGTCGTCTACTTGTGCTCCCGGAA
>ConsensusfromContig3597 Average coverage: 4.86

ATTCGGGGTGCCTGTGAACAAGCATCAACTAATTCTCATTGGAAT
GTCTCTGGTAACATT
ACTCCTAGTAACACTTCTTGTCATGGCCATAATTCTAGTCAGAAG
AAACCAGCAAATTCA
AAACAACCAAGCTGCATGTCCCAAGCATGTACCAACCCAACCATT
CCTACGGGATGTTAA
ATTCTCGGGAATTTCTCGAAGTAATAGTGTGCATGAATCATCAAA
AAATGATACACTTTA
CGAATCAGGTCAACGAGGAAAATCTGTTGGGAAATACACATCTGT
ACCACCTTCATCTCC
TCATAATTGTTACGGAGCGACGCCTTTCAATGCCCCCAAAAATAA
TGACTGTACATCAAA
TGGAATCAAGCATCAGCCAGGAATTCTGCGGTGCAAAACTCTAG
AAACAGCATTCCAGCA
GCCTTGCCAACGTCAATGGATGAGTTGCTCTTCTGAACCAACCCG
TGTCTACAGTACCGT
CGTGACAGCGAAATGCAGAACGCTGAGAGGAGCTGGTCCCAACG
AAAACAATTCCAAAAA
GCTGCAGCGTAATTTTGACGGAATCGCGGACTCGGATACTACATC
AGCCTGCTCTGCGGA
TCTATCAGCGGCAGACAGCGGTCGAGGCTATAGCGACGATGGGG
AGCATAGATCTCACAT
TCACGGACCCCACACAGGCACACATCTCCGTC
>ConsensusfromContig3598 Average coverage: 11.68
AGTGCATTGTTTCACCTTTGTTATCCACTATGAGAGTAATTTGTTT
GTTTTTCTTATCAA
AAATGAATGCTACGCGGTGATAAATACTTTTGTCCACTTTAACGG
AAATCTCAGAGCAGG
AAAAACCAAGTTTGCTCTTGCCGTTTTTCCAGGTCACAGTTGCGT
AGAATGTGTTATCTA
TGAAATAAATTCCAAAGCCGGGTTTATTCAAGTCAAGACGTCCAG
TCTCCAAAATTGTTG
TTGGTT
>ConsensusfromContig3599 Average coverage: 5.77
GGAAGGGGGGGACGAAATGAGAGAATATCTGAAGAATATGGATC
ATGATATGGCACGAGA
CTTGTTAAAGATCATATTGAACATAGGGAAGGTTAAGAACAATA
TGAAGGGATAAAGGAG
TGTGTTGTATAAGCTCAGTGGTTAATTCTGGAAGGGGGGGATGA
AATGAGAGAATATCTG
AAGAATATGGATCATGATATGGCACGAGACTTGTTAAAGATCATA
TTG

>ConsensusfromContig3600 Average coverage: 3.24
GGATGCCACAAGCCTTGCAAAGAAACAGCCTTTCAATCGACATTC
TCATCTGCCGAATGG
CCCTCTGAAGTCTATTTCGAAACCCTATTGGATGAATCGAAACAA
AGGTTCGGAGACGAC
GAGGGTGTACGAATCGCCGAAAATAGTCTACTCATGCTCCATGTC
TACTTCGAGGATTTC
ACGACACAAAACATAGTCGAGAGAGCCGATTATACGCTGATATCT
TGCATGTCTGACATC
GGCGGAACCCTGGGCCTTCATCTTGGTTTCAGC
>ConsensusfromContig3601 Average coverage: 8.51
GCAAATATTTGTGCATTCACGTCCTCCAGTGTCAGGTTAAGTGAG
GCCACTCCATCCCTC
CCAGCATTCACATTCAAAGCTTTTCCCGTCGTCACGGATATAGCA
AGTACCTCCATTTAA
ACATGGTGACGGGTCACACAAATCAGGTTCTATTGGTATTCTAGA
CTCCCCACAGAATGT
ATAACCAGAGCGTGTTCTATTCCAGCACCCGCATTGTTGAAATCC
GTTGTAATTAACACA
TCTTGCTGGAGGAGGGCATTCTCCGCAATCAGGTGGTACGGTAGG
TCTCCTCTTGAGAGA
TCCTCCCCTGGCGTGAGAGAAAAAAGCGCGCTTTATTCGATCATC
GTAACAGGCCCACTT
ATACGGCGACCAATACAAATCCGACCTACAATGGACCTGCCCGC
TTAGCGGGTCAGGGCA
CATCGCGGAGCATTTTGGTTCGATTTTGG
>ConsensusfromContig3602 Average coverage: 5.97
CCGATCTGTTGGGATGATCGAATGATGCATTATGTTGTCTGTGGC
TCCTATCACCTGAGA
GACACATAAATATGTCCCGAATATACTTAGATACTTTCTTTTTGGA
TTTCTCTGTAATTT
TGTAGTTAGGGTCAGGGTTAGGGGTAGGGTTAGAGGTACTGGATG
AATCGTTTTCATCGT
CTCTGCCACGTGAGGACATTTTTCCGAGATTCGTTCCATTCGATA
TGGTCGGAGGTTCGA
GGAACTGCCTGCTACGTTGTTTTCTTTCGTCGGGCAATTTGTCGTC
CAGTCTTCTATGCT
GAGATTTTGGAGTAAGCAGAATTTCCACAGCTGGGTACTGAAATT
CACCACTTCCATGTT
TGGTGAAATAATGAACAGCAGAAAATTGAAGAACCGAAGCGAA
AACAAGAGCAAAGCATG
TCGCCACGTATCTGTCCAAAGCTGTTGTGTACGAGACTTTTGGGA

GATCTCCTCCTAGAT
CGATGCTCAATATGGTCATCGTCAGAACTGTTGTTACTCCCAGAG
CGATCCGATCCGCAG
TCGCCACTCTGTTGATCCAGAAAGACACCCAGGAGAGGACAACG
AGGAGGGCACAGGGGA
TGTACACGTTTATGAGAAAATGCCCCGTGTGGCGCCGAAGAGTG
AATTTGACCCACAGCG
TAGACGATTC
>ConsensusfromContig3603 Average coverage: 18.18
GGCATGAACTCAGTTCATTTCCTAGAAGCTCAGGTCAGAGAACAT
GAGTCCGAAATTGTT
GTTCATCGCTGCGGGGACGTTGAACAAAGCTCTTCGGAACCCGAA
AACTGGATGGCTCAT
TTAGGATTCCTGAGAACCAAGCATTCCTATGAAGTCACATTCTCA
CTCAAATCCGTCCCT
GATCTTCTATCGCCGGTCACTTGCTCTCACGATGCAGACCCTCAT
ATTTCTGATCTACAG
ATTCAAAGTCTGAATGCCAAAATGCAAATAAACACATTCGGAGA
GCATCAGTTATCGCAC
AAAGTCTTGCTGGAGTCAGAAGGAAAATCAATCA
>ConsensusfromContig3604 Average coverage: 6.07
GTTCAGTTTTGGTACAGATCGTTGGGAAGCACGCTGGACGAATTT
TCAGTGGTTATCTTC
ACGAATGGTTCTGAGAATACGCTGTTCTCCGTGAAGGGGATTTTG
CATGCTACGTGGATT
CCGGTCAGCTTGACCATTGCCCAGGACATCGGACAAGATGCCGA
GATAATGTTCACGGCC
ACAGCAAATGGACAACATTATCATAATCTGGGTGTTGATGACGTT
TCAATCACATCGGGA
GCTTGTGTAGCTAAAGTTTGTGAAACGAACGAAACTATGTGTCCC
AATTCCGATTACTGC
ATCTCTAATGACTGGCTATGTGA
>ConsensusfromContig3605 Average coverage: 7.93
AATGTGATGTTTCCCTAATGCATGCATCGATGAATTGGACCGGAG
ACAATGTTACAACAA
ATATTTTCCGCGCTGATGGCAAATGTGATGTGTCGAACAACAAA
ACTGGGGTCATGAGAA
GAGAAGGCTGTAACCATGGGTGGAAGTATGATTCAGGAGAATAC
AAATCCATTGTGACAG
AGTGGAACTTAGTTTGTGGCGATGCGTACTTGTACCGATTGAGCC
AGTCCCTCGGTTCTG
TAGGCACCATAATCGGTATAGTTGTATCACTTCTTCTGGTGGATT

CATACGGGAGACGCT
GGCTTTTCATTGCGGGACTTTTAGCCAGTGGAGTTGTGGGGGTTG
CAGTCGCCTTTGCTC
AAAATATTTGGTCATTCATCGCATTAAGAATAGTGACGCAATTCT
TCTGTGGCGTTTTTA
GCGTCCCTGGAAAACTGCTCATCATGGAGTTGTTTGATTCAAAAA
GAAGAGAATTCGCAG
GAATCGGTCTGAAATTTTTCTGGATTTCTGGTTTTCTGCTCTTGAC
TCTTTTCGCATACG
TCATCCCTAATTGGCGCAAATTACAGCTTTTACTCTCTTGCTGGCC
G
>ConsensusfromContig3606 Average coverage: 4.37
GTTCTATTCCCTCCTAGATCAAATCCAATTATTTCATCCAAATCG
TCGATCCTGGTGAAA
TGAAAGCAATTCAAGTCTGGGAACTTTCAGATATTGGATCCTGTA
GCTGTTTGGAAGGGT
GAAGCATCCATGGTTCGATTCCCTCCCAGATCAAATCCAATTATT
TCATCCAAATCGTCG
ATCCTGGTGAAATGAAAGCAATTCAAGTCTGGGAACTTTCAGATA
TTGGATCCTGTAGCT
GTTTGGAAGGGTGAAGC
>ConsensusfromContig3607 Average coverage: 18.68
TTTTTTTTTTTTTTAATTATTAGTATGGTTATCTAAATATAAAGTG
ATTAATAAACAGAA
AATATAAGCTTGAATTAAACATACTGCTATTTCAAATATGAAATA
ACCTATTTCTAATAA
TATAGTAAGAAATATAATAGAATTAAAAGTAAGTAATAATTTTGT
GAATAATGCTCTAAT
AAGAGATAATACAATATGTCCTGCTGTTATATTTGCTATTAATCG
AAATGATAAAGTTAA
TGGAACAATTGATAATCTAATTAGTTCAATAATTGCTAAAAAAAT
TCTGAG
>ConsensusfromContig3608 Average coverage: 7.75
GGAAAATCAAAGAAATTGAAATGAAGCGGTTGACAGAATTTGCA
AAAACGAGGATTCTTG
ATGGTCGAACAACATCTTTCAAAGTCTATCCCGAAGGTGCCCGAA
AATGTGTGGTTGAGG
AAGAGAATAAAAGGTTCACGGAAAAGAATGGATTGCGTCCTTTG
AAATGGTGCTCATACC
CTCGAACTGCAGCTGTTTCAGCAATCTTGGAAGGCATCAGTAAAT
CTGGAAATGAGGAAT
GCTGCATATACGACACCACAATAATGTTCGAGAACCACGATCCAC

CCTCCGTAACCGATC
TCCTCAAACGAAGGCGAAAAAACTCCCCGATTGTTCTCCACTACC
GCAGACTGAAAATCT
CTTCAGACATGAGAGAGGACGACCTCAAGGACTTCCTCTACGGA
TCGACCTACGACAAGG
ACGCTATGATGGACCACTACGCTCAAACGGGTCTAATCCAGCAC
CCTAAAGACCCAAGGT
CATGGACAGTCGATACCTGTAGATATGTAGAATTTTCTGGTACCA
AGCACATAATACTGC
AAATTGTTTATTTT
>ConsensusfromContig3609 Average coverage: 5.19
AAATCCATAGCCAGGAAATAAACAGTTAAAAAAAAGATAACTTC
ATGTTCTAATTGAATG
ATAGTGATGAGTTCACATGTTTCAGAAATTATAATTCATAAATTG
GATAAAATTTAATTA
CCAAGCAGCAGGAGTTTCTGACCCTTTTAAACAAACCAGGAAAAA
TATCCCAGTTATATT
CTGTCATGTATGAGCAAACGTTTTAAAAAAATGTATGTCGCTAAT
CAATAGATAATATGT
GCCAATGTGACTTGTTCGATACGCCCACTAAAAGGCCAAAACTTG
GTTTAAGAACCTGCA
GATCGG
>ConsensusfromContig3610 Average coverage: 11.68
CACATCTCATGTTCCTCGTTCTTTACTGACTTATGTGAGACAAAT
GAGTCAACAATGCAC
ACTAAACCGCTGCATCTTCTCATCCTAGTCGATTACTTGTTTGCTT
AAAATTAATCAGTC
TATATCAAACATCGTACCATTGCTTTTGCACTCCCATATCTCATGT
GCCTTAGTTTCTAC
TTGTTTTTGATATAATAATCAGTCAACAATGCATACCGCAACGTTT
CTTTTGCACTCTAT
CATCCCCTGTCCCTCGCTCTCTACCTATTT
>ConsensusfromContig3611 Average coverage: 5.05
CTCTTCCGATCTTTTATATTTTGTTAACGACAAGTGTATTTATCAG
TATGCTACATTTTC
AGCACACACCGTCACGTGGTTCAGGAGGAGATGGAGGACAATTA
CAAAAAGAAACGAACA
GAATTGAATACCGTTCACAAGCAACGAATCTGTAATGGAAAGTG
GGGGTCACATTATCAT
TTTCTGCAGATTTGTATCAGCTTTTCTCCCCTATCAAGGATCATGA
GCCTATACCAATCA
TTAACGCCCTATTTATTTTTTCTTACCCTCCATATTTTCAGGATCT

CACAAAGCTATTAC
TTGCGAAAATAGTATGGTATGTGGTTTTCCCGCCAGTTTATGAAT
GTATACAATTTATTT
CGTTTGTCTGCCTGTATGTGTGTAC
>ConsensusfromContig3612 Average coverage: 11.44
CGTACGCTCCCGATCAAGAAATTTTGATGCATGGCCCGACGACGA
AAATCCCCATCAATA
TGGATTTTAACGAGAAGTCATTCCAACCCAAAGTTCAGATTCCTT
ACAAGACCCCCCATG
GCCAATGCCCGAGAAAAATAGAAATTGAACGACGGAAGCGGAGT
TATGCGATGAAAAATC
TGGAAAGTATCCTCATGGAATTAGGAATCGCAACGAGTTCATTG
ATGCCAAAGAAACAAG
AAGAATCGATAAAACCAAAGTCGTTCACAGAAAGCGGGATTCCG
ATGTTGATGGACTACA
AGCCTTATCTTCCTCTGCACATTTTCGACAACACAGAATTCGACA
GTCGGAATCCGAAGG
AATGGATTGACCTCGGTGATGACAATGGCGTCAGAAAACCAGTC
CCAGGACAGGCACTTC
TTCCCGAATCAAATAATTCAGA
>ConsensusfromContig3613 Average coverage: 4.50
CTCGCAATCTCGGGATTATTGTCGACCGGGCTTGCAATTCTTTCGG
ATTTTGGGTTGTTG
AGTATTCTCAATGTGAAAGGTGTTCCGCAAATCGGTCTAGTTCCC
TTCTTGGTTCTTGGA
ATCGGCGTCGATAATGTCTTCATCCTCATCAGCGCATGGCGCAGG
AGCACCATACTTTTC
GACGCTCAATCATCTCCGCTGACTCCAGCTCTACGGACTGAGCTC
AGAATGGGTGCTGCC
ATGAAAGAAGCCGCACTTTCCATCACAATAACAAGCCTGACGGAT
ATAATGGCTTTCGGT
GTTGGTTCCATCACGCAATTTCCAGGAGTTCGGATTTTCTGCATCT
ATACTGGTCTCTCA
ATTTTCTTCTGTTATATTTTCTGCATAACCTTCTTTGCCGC
>ConsensusfromContig3614 Average coverage: 5.34
GATCTGTCGAAAAATGCATATCTATCTTGCATCGAGGATGCGCAA
AGCTCTTTCTCTGGA
GGACACAAGTAGAGCGACATTGAAGTCTCTTCAAGATGATAATG
AGTCTATTCGAGGACT
GCATGTAGACGTTTTCCGGCAGTGGGCAGAACAAGAGAGGGCCG
CTTATAAAGCGCATCT
TCATAACTCCTTTTCAATGTACACCAACGAAGAATCGTACGCCCT

CGCTGTGGAATCAAA
AGCCAGGACTGGAAAAGCCAAGGGAGTGCGATCGCTTCAGGTGG
CTGTCGGGGTTCGATG
GGAGTTGGGAGATGCCATGTGCCAAGCTCATCTTACGACAGCTC
GCCAGAAAAACAAGAG
GATGCTGCTGGAGAAGATGCGTTTGATGAATGGCG
>ConsensusfromContig3615 Average coverage: 8.58
GCGGAAACCACCGATAGATTATAGATTTGGAAGTCTATTTCATAA
CATATAAGAACTACG
TCCCGGTAATGACTGCAGTTAGTTTTTCCCCATCCCTTGAAATCA
CATTCTTTTAGAGAA
TTCTCGTTTCCAACGCATTCTAAATCATCCATCCAGATTTGACTC
GAAGTAGATGGCTTT
GCACCGCTGTATGACATTGAAGTTTGTGAGATGCACTTGGTGTAT
CCGAAATACCTCTCG
ATAACTCTGAGTTCAGGTTCTCCAAATCCATCAGCACAAACAGTT
CCCCACATATTGTTG
TGATAAACCTGGAGGACACCATTCTTTGGGGCAGATACAGACTCG
CTGTAGATGCGAGTT
TCTATTTCATCAGAACAAATAAGAATAATATCTTGTGCATGTGTA
CAGTTATTGTTTCCC
CAGTCATTGAACAAAC
>ConsensusfromContig3616 Average coverage: 12.63
TAACACTTGAAAAACAGCACAAACTGGATGTCCAGCTGGAGTTA
CGGGAGCGCAGCAACC
TTTTAGAACAGCAGAGCGAGTATTGCTCAAGTATGGGCTCAGCCT
TGGCTACAATTCTCT
GGCGAACATCCCGAACACAAGAAGCGATAGAACACATCCTTCAG
ACAGAAGAAGGAACGA
CATTCTTGTCCCTGACGGTTTCGACGGTGGAGAGCTTTGTGGAGG
CTTCAAGTGACCCGG
AAGAACCTCAGGGCGGGACTTCAGAACACGAATCTCAATTTGTGC
TCGCTCTCTGTGGAA
TCATCACCAACATGACGGCGTCTCCGCAAGTAAGAGACTATTTCG
CTTCGAATGAGATCG
GAAAAGCCATCATAGAATGC
>ConsensusfromContig3617 Average coverage: 39.98
AATAAACATGAATATTCAATTTAATAACACATTACTTCTGAATCA
AGCAAATTCATATTT
GAATCCCAAAATTATATCACTACACAAAAGTACATCGAAAATAA
CATCGGAAAACAATCG
ATAATTTTCTTCGCTCATCATATCCTGAGTCCGTCATAAATCATTT

TACCCTTCCACTCC
CCCACACCCTCCCTCTGTAGCATCACTCCATCAGCATCGCAACAT
CTGCTGGGGTCAGGG
TGATATTCTGCCCACCAGGCTCTCCTCCTTCCCCCATTTTCTGAAG
CTTTCTTTTGAGCC
TTAATTTTCGCATCCGGATCCGGGCTTTCTCTCTCGTTTCTTCGTC
CGATTTCCAGATTT
GAAGTGGGTGGAGGTCATGGGAATGATGGGGGGACTTTCCTCGAC
CTCCTCGCCTTGATG
CAGCTCCAAC
>ConsensusfromContig3618 Average coverage: 5.24
CGATCTCGTCTATCTTCAAAGTATATCCATCCAGTTCTCGCAGATA
CTGCTCATTGTCTC
TGAAAATTGAATCGACCAGGAGATCTGCAAAAGGGATGAGCAGA
AAGGTGAAGTCATGCT
CCAATAATATTCCAGAGACGATGTCCTTTACCGTTGCCACTCCTTT
TTGTGCGTCATAAT
CTTTCACTAACTCTGGAATAAAATCAACCCGATATCCGGTGCAGG
AAATTATAAAAATGA
TACTCAAATAAGTGAATAATCTAAGTTTGCTTTCAGAACACATTT
TGAAATGAC
>ConsensusfromContig3619 Average coverage: 12.85
CAGAATATATCATCGAGGTGAACGGCTCTAGCATGGCGCTTTTA
GGGGAGGGGCAGGAAG
AAGATCGAAGACTCATTGCGGAGATGTGTGTTCAAAAGATGACGT
CATTTTGCTCCCCCA
TTGTGGGCGGAACCGGCAGTACTGCACGAGCACCAAATGTGTCG
AACGCAGTATCGTCTC
TCTCACAGCAGAGCACCGAATCAGATCATGGTCATTTGACGTAC
GCTGACCAGGTGGCAG
CACAGGCTCAAAAGACTGCAGTTCAACACAATACATCAAATGCTG
GCCCACCTCGTATGC
AAATGCCCCCATCTTCTGGTAGAAACGCCCAGAATCAAAACCAA
CAACCAAGATCAGGAA
GCGCATCAAATACCACATCCCCCCGAGGCACCCCCAACACATCAG
CTTCTTCGCCTCCAC
CAA
>ConsensusfromContig3620 Average coverage: 5.75
GAAAGGATGTTTGAAGAGTTTATCCGACGATTCTGATATAGAAC
TTGATTAAGAAACAT
CTAATTTTGGATTAATAAGAGGAGCATGTTGCATGGGAATGCACG
GTACGTATGATGGTG

GGAGGTATCCTAGCATGTTCGACGATTCCATCCGATCGATCTTGC
CATACTGCATGAGCA
GCTAAGACGCCCCTGACTCAGGGGAATGTGCACCTTTCGTGATGT
GCGAGTAATTTAATT
AGAAAAGGATTTGAATCTCTGACAATGTTGTCGTGACCAAGATCC
CGGCTCTATATGGCA
CGATTCCCTTTTGGATCCAATCTGAACAGATGAATAGATATAGAG
TTTGCGATAGATCGG
A
>ConsensusfromContig3621 Average coverage: 15.82
TATAATTGAATTTTTATCAATGAGTTATCGCTATAATACTTCATA
TCAAAATTTCTAAAA
AATAATTCCTTCCGAGTAAAACCAGTTCGGGGTAATGCCGTTCAA
GACACAAATTTCTAT
TAATGTATATTCAATAATATATTTGTCGTTTATTCCTCAACCGCG
AGAAAACTCCGCTGT
TTCAATCAATATTCACTCTTTTCCTGAATTTTAAATCCATTTATCG
AACATCGCAAAACA
ACAAATGGAGGTGGGAAAGGTCTATTTCGAAAGATACATATCTTC
GTTATGCATCGTATG
TATGTTCTTTGTTTGACGAATACAAACTATTGTATGCTAACATCGT
CCATTAATTTTGAT
GAAAGATTATATCATAGTACCTATGAGGCAGCGTGATTCTAAAAA
TTAGGTTTCATCGTA
TTGCGCTTTGTGTGATGGATACAAATTAATATTACATATTCCTTTT
TTCTGATGAAAATT
TGTGATTATAGGTGATTATTTTGCTTTCTTTCGATACTAATCAGG
ATATATTGAGAGAAC
TTTGATGTGATACTCGCAGATC
>ConsensusfromContig3622 Average coverage: 7.26
CGTTGCCAAGATGTGTCTCTCACACTCATCGACTTGTGAAGAGAC
CTGCATTAGAAACTG
CGTGGGAGTGCCTGACGGGGACTTTCAGTCTTGCGAGGGGTGCGG
GGTGTATGCGTCATG
CAGCAATGGAATTCTGATCGACCGTCGCCCTTGTCCGGCAGATCT
CAGATGGGACGATGA
CCTGAAGAGATGCCAGAGGATATCAACGACATGTGACATGCCTCA
ATAATCGTATTCGAT
GCAGTCATTATAATGTCATAAACTGAAAGGCTTGATAAAGACTCT
ATGTTATAATGTTCT
AATGTGACATCTCATCAAAATATGTTGATGAATTGTGATGTCAGT
ATATGCAGA

>ConsensusfromContig3623 Average coverage: 17.28
GGGAGAGTATAAAACTGATAATAATAATCTCAGTCATTATAATTA
CAACAATTATATTGA
TCATAACAATCTATCGAAGGCTGGATATACAATTCCTGAAATTG
TAATCGAAGAGGCAAA
TTCAAACTGTAATAATAAAGGGAAGATAGAAAGTGATTTTTCGA
AGAAAACCACACCACT
GAACATCGATATCGATACAAATTCTGAGATCAATTCTTTGAATAG
TTTCTCTACGTTATC
GGACGACAAAGATCTGCCAATCACAAAACACGAATCATTCAAAA
ACAACATAACACAATT
TGAGCCATTCAGTTTTATTCCTTTTCAGCCGATGCCTAACATGAA
TAATACTTTTAAGGA
AAACTCGGACATGAACATGCGAATAATGCAGGAAAACATGTCCA
GAATGCAGCAACATAT
GCAGCAACAGTTCCAAAGTATGTCTTTTGGCCCTCAAAACTCTCT
GACGAACACAGGTCA
ATTTCAGAATAGAGATATCAAACAATCGATACCTATCATCCAAGT
GCCACCATCCCCATC
AC
>ConsensusfromContig3624 Average coverage: 15.39
TCCATTATTAGGTACATTTTCGTTGGCGATTCGATGATTTCCTTCA
GCTGTATGATGTGA
GGATGTTGAACTTTTTTGAGAATGGACACTTCGCGCTCCAGGAGT
TGGATATGATTACTG
CCGGCCTTCTCTTTGTTGACGACCTTGCAGGCCCATTTCTTCTTAG
TTTCGCGGTTGATG
ACTTCTCTGACATATCCGTAGCTCCCCTGACCCAGTTTCTTCCCA
AGCGAGTAAATCGTT
GACAGCATCTGTTCGTCATCTATTCTGGTGTGTGGGATGTGAAGG
GAGGCGTTGGTGGTC
TTGCTGCCGAAGCTGCTGACGCGGGTCCGGTCGCTCTTCGATTCA
GTTTGGCTCGCATCC
CGCTCGGACACACCTTCTGACATCTCTGTCAGCTTGCAACATAACT
TTTGTTGTGGTTTG
TTTTTGCGTTCGGTTCACG
>ConsensusfromContig3625 Average coverage: 11.38
CTTCCGATCTTCAAGTTATTGCTGGGTGAAGAAATATATGTTCAC
TGAATTATGTATATG
ATGTGTATGTTTTTCCACAATAATACAGGCTATATTATAGATGAT
AATATATTCAAATAA
GAGGTCATTATTTATAAGTGAATCTCATACGCGCTCGTCATTGAT

TTGGGAACTGAAATA
TATTCGTAGCTCGGTTACATAATGTTTCACATTCCATATGCTATTT
ACAGTGATTCCATG
TGAAAGGATATCAATGTAAGATTTATGTTTATTACGCAACAGTAT
AAAATTGCTTTAATG
TGTTATTAGTCAATCATGGAAGAACAAGCCAAAAAAAAAA
>ConsensusfromContig3626 Average coverage: 11.21
CGCTTTCAAAATTAGATCAAAAATGTACTATTATAATCCAAAATA
AATGTTTCAAAAAAC
GAGATTGTAGAAGTGAAATTTACCGAATCAAAGCATACCGAAAC
GAACGAGCATGTATAT
TTAAAGCTTTTTGAGAAGATTTGGCATCGACAAATATTATCATAC
AAGCCCATGTGAGAA
AGTCCAGAAGAATGAAAATATTTCGTAACGTGAATAAAATTTTCT
CGAACGTTTGAAATA
TATTCATGGAATGTTGAGGTGCTTTTGAATGCGCAAAATGTTTAA
AAAACCAACTAATTA
TCTAATAAGGCGACAATAGTCATTCGCTCGAATAAAAGAAATAAA
ACGAGAAATTTTTTT
ACCTGAGAATGATCACTAAAAGTATTTTAAATTAACAAGAAATA
AAACACACAAAGAGGT
ACGCAATCAATTATCAAAAATA
>ConsensusfromContig3627 Average coverage: 9.59
TTCTTAATTGAAGTTTTTCATGTTATTAGAGATGGTGAATTCAAC
GAAAAGAAAAAGTGT
CATGAGTGTCAAAACCAAAGCGTCATTGTGGGAGAACAAGGGGA
AAGAGGGAGCTACCCT
CCTGGCACCTCAGACTCCTCTTGTCAGAAGAAACAGCCTTCGAAT
ATCCAACGCCATCGC
AGATTTAACCAGCTCCAGCACTCTGAAAGAGGTGAAAGGATATA
AGATCAACACCGCAAC
GCCCCTGCTGCCATGCGCTGAGGGAACTTCATTCAATCGGTCATC
CTCCGCTCTACCTCC
GCTTCCCAAAGAGTATATCCCACATGAGGAAGCCTTGTACAAAGC
GGTCGTTGATCTGAG
ACCCCCAAAAACCTTCGACCCACGATCACAGGCACTAGATGTCG
ATGCCAAGCCAGGTCA
TATCGATGACAGACCGCGGCCTTTCGTCGATCCTCGGGTTTTCAA
AAATGTCGACCAGCA
CGTTCAACAAGTCTTGACAACCATTCAATCTAGTCCAGAAGAAAT
CTTATGGCAACTTGT
GTATTCTAAAGGTATAAAGAACGAGTTGGAAATAGTGAGAGCAA

TTTACATGTGGACATG
CAAAGTGGATTTGAGAAATTTCAAGTTTGAGCATCTCAAGCACCC
GCTGGCTGCTGCCGT
TGCCAATGTCACAAAAGAGGCGCTTCGCTTCGAGGATGCTCCAG
ATCGGAAG
>ConsensusfromContig3628 Average coverage: 12.84
TGAATCTGACAGCTCCAGGGTAGCTCGACAACATTTATTTATTGA
CAATGAATAGAATGA
CGTCATTGGATCAAGGACATGATTTTGTCCAATTATCATGGCTTT
ATTATTAACGTCAAC
ACGCCCAAGTGGGTTTCTAAAAGAAATGCATCTTCTAAGATCAAC
AAATAACATTCTACA
CTCTGAATGACCGAAACCTCATTTTGCTTACGAAAATGGCATCAC
CATTGATGCCATCTT
TATGCCTGCTCGAGACGTCTCATATTCTTCGGACTGGTGTACTAG
CCATACAGAGCATTT
GTTTGGTCGTCTGCTTATGTGTTGCGGTAGCCATTTTGAAGTTGA
GGAAAAGGAAGGTCA
TTCGAGTGAGTTTGTGGATACTCCTTCAAGTCGCGATTCTTGGGGT
ATTCATCTTCTATC
TCTCGGTCATCCTTCTGGTCTACCAGCCAACTACATCTATCTGCAT
AATTCTTCCTTGGC
TCCGATCTTTCGGATTCTGTCTCTTCTATGGTTATGCGATCTCCAA
GCTCTACAGGGTGT
CTGCCGAATTCCACGTTCGGAAAGCTCATCGTGTTCTCATCCGCG
AGAAAGACCTTCTTC
GCGGAATCGGCACAGCTTTGGC
>ConsensusfromContig3629 Average coverage: 34.67
CTGCTCAATGTGCAATAAAAATGAATTATTTTTACATGAGATCAT
TTTAGCGACATATTT
AAATAAATAACTGTCTCACCGATCCCCCTTTCTGCTGTGTCCATTG
GCTGAGCCATCTTT
CTCGTCTTCCTATTTAGAGACAGCAAACGACGCGAGAAAAAACA
AAAAGCATAATTCATT
AGTGATCAGTAAATGGTTGTGAGAATCATCATAAATGCATTTTAC
TTACCAAGAAGATTG
ATTAGCCACGTTGGTGGGACAAAAGAG
>ConsensusfromContig3630 Average coverage: 15.69
GATGATGTCATCGGATAAAGTCCATTTTGTTAATGCTATTGAATA
TGAATCAAAAAACAC
TTGAGAAGCCTAAGATTTTTTGAATATAAATAAGTGATATTTTAT
ATATTTATATCTGTA

AAAAACTTAGAAATATGATTCTTTGAAGAATTCGATATAGTGTTA
GGTCTTGCGCACCAA
CAATTCCCTAACATGGCAGATTCACTATTAATTTATCAATTTACG
TATCATGTAATATGC
ATAACGGATTCATTTGATTTCGTACATCTCAAACAGTGAAAAAAA
TGTCACAATTTCGAA
GTATTTATTGCATCTTCAAATATATTTCAT
>ConsensusfromContig3631 Average coverage: 17.87
ACCTCTTCCGATCTTTTTACTCTAATCTGAAATATTATAATTATTC
TCGTGGCGTCCCAA
GATGATCCTCGGCAACTGCTTGGGATGTATCAAAGTGATCATGTT
CGGTTTCAACTTCGT
CTTTTTTCTTCTGGGATGCGGAATGGTGACCCTCGGCCTCTGGGT
GCGACATGACCCCTC
CGTCGAGATGCCTCTATTCAGCGATAATCTCGTCAGCTATGTCTA
TACAGGGGCGTATAT
AATCCTGGTGGCGGGGACACTGATCATGATCATGGGCTTCATTGG
GTGTTGTGGGGCACT
CAGGGAAAGCAGGTGTCTTCTCGTCGTTTTCTTCTTTGGCATTCTT
CTTCTATTCGGAGC
ATTGAGTGGGGTCGCGGAGATCGGAAGAGCACACGTCTGAA
>ConsensusfromContig3632 Average coverage: 4.41
GTCGCAAGAGGAAAAAGTTCAGTTAAAGGTGGATTATAAGAGGG
ATATATTTTGTCAATT
ATGTTCCAAGCATGGTCATGGGGCCTCAACTTGTAAATCATTTGA
GAATCGTAATGATAG
TAATATTCAATCAATCACATCACGAAATAATCATTACAATTCCGG
GGGTTTTAACAGTCA
TAGAGCGCGTCAACATCAAAATTTGTCATCTCAATTTAGAACGTT
CAATGTCCCGAGAAA
TAATGAATTTGTTAACC
>ConsensusfromContig3633 Average coverage: 5.06
GGGAGATAGAAAGCAGCAACTGCTGTAATGACTGTAAGATATTG
ACTAGTATCTAGAAAC
TGGATTGAGCACTCGTGCTCCTTGACTGTACGTCTGCCTTCGATG
TAGGGCCACGCAAAT
ATCATCGGAGTCCACATCAGACCGGAAACAATCCAGGCAAGTGT
TATGATGAAGACCGCA
CGACGTGGAGTTCGTTTGGCTCGATAGGTCAGAGGTCGCGTAAC
AGAGAAATATCGATCG
AAACTAATAAGGAGTAAATTGGCTACAGAAGCATTGCTCATCGT
ATAGTCAATAGAAAGC

CACGTATCGCATACACAACAACCAGATCGGAAGAGCGTCGT
>ConsensusfromContig3634 Average coverage: 16.68
ACAATAGACTCTCACTGTAGAGATATGGATGACAAGCGCATTGA
TAATTCTAAAGCTGAG
CAAATCAAGATGGCTGGCATTTCAAATAATGATTTTACTGTGGGT
GAAGTACATGCGATT
TCGTCGAAGTGTTTCGAAGACATATTGTCAACTGATGAAGAACC
GAATAGTCGTCAAGAG
CTTGTCACGGCAATTGCTGCTACTGTAGACTTTATTCCTGGTGGT
TCTGAAGACACTATG
TCAGTTGATAAATCCGATGGAACATCTATACAAGGATCAATATTG
GAGAATGACTCTCTC
AACATTGATCTCAAAATTTGTGAAAAAGCAAATGTTATCGAAAG
CCATGAGAAGGATGAT
CCTGATAATGAAATTGAGACTTCTGCATTATCTTCGGAATCTATA
AACAACAGCTCCACT
CGAGAAAATATTCCCGTAGATAGTTTGGATGCGATGACAGATGA
AATATCTGAACCAATC
GATGATACTATGAGCATAGAGGCTGCTGAACGTGTATTTGATGGC
ATTGTCGATGATAAC
GATATGGCAAAATTGAGTTTCGTCCCAGAAGAATCTATTCATGGC
ACTAAAATGGATAAA
ACAGATAATTCGAATCCTCAAGATTCT
>ConsensusfromContig3635 Average coverage: 2.15
TTTCATTATCGTCACACTCTAAAATGAGGGGCTCAGTGTAATAGA
CTTATTTTCGGGAAT
TGATTTATCACATTTGGCTCAGTGTAATAGACTTATTTTCGGGAAT
TGATTTATCACATT
TGATATCAACTCGCATATTTAGAGCAGGATTTCATTATCGTCACA
CTCTAAAATGAG
>ConsensusfromContig3636 Average coverage: 14.59
CCCAGGAATCTTTTCAAATGCGGTTGGAGGAAGGACCAAGTCAG
CCATTGGTTACGTGGA
TACCAAAACCGAAAACTCGAAATCTCAGTATACAGGAATGGACA
GATGCCTTCATGATCC
TGATGGATATTGTTTTCGTCAATCGGCCAGAGGATATCCCGGCAA
TGTTGAAATATTCAG
CGATGATTCGCAGGATGGCCAGGGCTAACAAAGATTGGGTCAGG
TACGACCTGCAATTCC
GGAGGATAAAGGCGTCGCGTCCGATA
>ConsensusfromContig3637 Average coverage: 7.14
AATAGGGTAAGTTCCGTTGTTTGACGCAATGCAATAGCCTGGTGA

TGCCAGGATGGCATA
ACTGTTCATGTATTAATTTCAAATTATCATGACTGGTCGAACATG
ATCTCGACAGGGCAT
CTGCTACCACATTTAAATTCCCGGGGCGATGAACGATTGAAAATT
TATAGGGCATCAAAT
CCATCCTCCATCGTAAAATTTTTTCATTTTTGATTTTTCCGCTATG
TTTAATGTCAAACA
TATAAGCTACAGATTTCTGATCCGTTATTAAATTGAATTCGTTCC
TGGCTAGTAAGTGCC
TCCAATGACGGATTGATTCTATTATAGCATAAGCTTCTTTTTCAA
GAGAACAGTGATTCT
TCTCATGCTTAGTTAGCGTTCTACTGAAGAAAGAAACAGGGCGCC
CACCCTGGCTCAGGG
TTCCAGCAATAGCGCTCTGACTAGCATCAGTTTCAACTGTGAAAG
CAGCGTTTTCGTCTA
TATAGAATCTAGTCGCTTTCATAATACTTTCTTTTAATACATTTAT
AGCTTCACAAGCTT
CAGTACTTAGAGGAAAATTTTCCGTTATCAAAGGCCTAATTTTCT
CAGAGAACTTTTGAA
TCCATTTACTATGATATGAAAACATACCGACCAATCTTTGCAATTC
CTTCTTGTTCCCCG
>ConsensusfromContig3638 Average coverage: 7.48
CTGGAATTGAATTTCTGAATTCGATTATTATTCGTATCCGAAACA
TAAACATTGCCATGA
CTGTCTATCGCAATCCCGGACGGATGGTCGAATTCGCCTTGCTCT
TTTCCGAAAGAACCG
AATTCCGAAATATATGACCCTTTCATGGTGAAGATCTTGACTCCG
TGTTTTATACCGTCA
AGAACATAAATCTGTTGCGGCTCGCCTGTTCCAGGAGACAAAGCA
ACATCAATTGGTTTC
ATTTTATATGATGTTTTCTTTGCGTGATTCAGTGATGGGATACTCA
TTGTGACGTTGCCA
CTGTTCATATATCTTCCACGAACTTCCGGAGTAATTTCAGTACTTT
CAGTATTATTCCTA
ATGCCAGATGTAGCACTGTCATTATCAATGACAAATGTT
>ConsensusfromContig3639 Average coverage: 6.27
GCTGAGCTATCGAGAAGGATGACAATGTCAAGGGCACCGGCTCTA
CAAATTTTTGGCCTG
CAGTCTGTGCATTCTGATGTCCAGCGCCAGCTCGAGAAAGTTTCC
CTCTGTGTTCCCAGC
CAGACTTCCTCGAAATTTGATTGGTCCGCCGGTGCTGCAACTAAC
GAGGCGTTCTTGACA

ATTTCTGATATCAGTCTCGCTTCAGCTTCGTCCCTTGATGAAGCC
AGAGTACCTGGGAAG
TTGGACTCCTGGGCCTGCGCATTCGCTTCACATGTCGACTTTGCC
CAGCGCACAGAATAG
GCCATTTCTTGCATTTGATAGCACCTGGATCGGTGCACGACTCCG
TTTTGACATAATTCA
GTATTCGCAATCGGTATTGATAACAAAATCCAGAACAAACTAGAA
AATATTTTGGTGCTC
ATTTGTATT
>ConsensusfromContig3640 Average coverage: 10.84
CCACACTTTGGTAGTGTACATTCGGGGCCACCCCACTTTGTGTGG
CATTTGCATTCATTG
AAAGACTCTCCTTCACACCCAATAGTATGTTCCCCGCAATCAGGG
TTGTTCCCACATGTT
CGAGTTTCTTCACTTGACAAACTTTTGAGGTTTTGACACTGCTCGC
CATCCCATCCTTTG
AGGCAGTCACACTTATGAGAGTCATCTGACTTACATACAATTGCA
CGTCCCGCTTCGCAG
ACTGCACCATTACAAGTCAGCTCATTGCATGCGTTTTTGGTCCAAC
CCTTGCC
>ConsensusfromContig3641 Average coverage: 4.64
CATCCATACATGAACAATTTTTATGCCCTTCTCTTATGTACAGCTG
CTCTGTCATACCCA
AATTCACGTCAGAGTTCCAGTCATTGCAGAGTTTGCATTTTTTCG
TCAGAGTATCCCAAA
GGAAAGCCGGCATCGCGTTTTTGACGGCAGCTGTGCCGCATCCTT
GTTTCGTAGTCGTTT
TCGTGATCGGCACACATTGAGTCTTTTTGAGTTTTATTATGTTCT
TGAACAGCTTGAAGG
AGCTACATTCAGTGCCCAACTCTATGTCAAGCTTGTCTATGTTGT
CTTGGGGGGAACAGG
AAACCATAGACAAAG
>ConsensusfromContig3642 Average coverage: 9.03
ATGAAATAATGTAAATATAGATATGTGATACATTTTGTTTGTTCGT
GTGTGTTTTGTAAT
AATCAGTATTGTTTTTGTGTGTTTTCTTTCTGTTTGAGGTTTTAAAG
TTCAAATTTTTCT
CGCAATTATGGGTTGTAACCTGAATTAAAGATCACATTAAATTAC
ATATGAAATTCGTCA
CCAAATCATAACCTCATCCTTAATGGACACATAAAGTAAGTATTA
A
>ConsensusfromContig3643 Average coverage: 23.78

CTAATGAAAGTAGGATAAATTTGAGTTGATTATATCGTTTGAATA
AATATCAAATGTAGA
GTATAGTCTGATCCGCAACGTTATCTAAATCTTGTATTTCATTCC
CGACGATATAAAGCC
GAGTTCCATAAAAGAAATTTAGAGTCGGTGCTCAGTGAATTGTCT
TAATCGAGATCTTGT
AATAATACAAGTTACTTAGAACTTTTGAGTTGTCATAGAGTGGTA
TCGCAACCAAGTTTT
CATAGAATATATGAAAAGTCCGCGAATGAGTTGTGTGAATCCATC
GTACGTCACATAGCG
CATAAGAATCTGTAAGACGTCCTTGATCGAAACGTTTCAACTGAT
ATTTGTACTCATTCA
TGTTCTATAGATTCAATCGTTGAAATATTTCCCCATACTCATTCAA
GTTTTGGGAAATTT
TTGAGTAGTTTGATAGTGAATCGTATAAATCATCTTTTATACTTT
TAAAAGTTCTACAAA
ATTGTTAAGTGATTCAATGGCATATATCTTTCTTCAAACTCA
>ConsensusfromContig3644 Average coverage: 5.39
GTCTTTTCATCACTGCACATATTTTCTTGTTGGTATTTTTTGTCAC
ATGCTTTTAGGTTG
ACTGTTCGACCTGCTTTTTCGTGGGGGTGAATATTGTCTGTTTGA
GAAACCTGAATATCT
TCCATATTTGGAGTTTTCTCTTTTGCTGTCAGATCATCATCGACAT
CTGCATTCTGCTTT
GAATCAGTTTTGCCAACCTGATCACTGTTGAACAATTTTCTGACT
TTTTTAACAGACTGC
TTTGACCACGGGCT
>ConsensusfromContig3645 Average coverage: 6.65
CCCGTTCCATGTGACGCAGGATCATTTTCTTCGGCAGATGGGTTA
ACAGTTTGTAGTGCA
TGTCCGTATGGGTTCTATCAGAACACAACCGGTTCCTCATCCTGT
ATGCCATGCCCATCT
GGTCATACCTGCATAAATTCAACGTCAACAGAACAATGTCCAGCT
GGTTCATATTCAACT
GAGGGATTTTCTGAATGCAGTGCCTGTAAAGACGGATTTTATCAA
GATATGCCAGGACAA
AATTCATGCATTTCATGCCCAGAAGGACATCAATGTCAGAATTCT
TCGATTTCACCGATC
GCCTGTGGCAGTGGTTATTTTTCACCAAATGGATCTATCAGCTGC
TCAATATGTGCTCAA
GGTTATTATCAGTCTCTCACTGCAGCAGATCGGAAGAG
>ConsensusfromContig3646 Average coverage: 8.45

TCTTTGAATTTGGAGATCTCAGTGAGCTCGGCATTGATCGCAGAG
GATGATGGTGTTGTG
GCGGTATTGCAATCGCTCCGTTGGAATGATGCTGGTTGACCATGA
CACCAGGACTTGTGT
CGACGACGAACTGGACATCACTGCCTTCAGACTTCTCTGATCCAT
CTACGGAGAACGCCG
CGCCTCCCGACAGTGTGTGTCGCTGCATGTACTCAGAGCCATTGG
CGGGAAACGCCATGT
TTCCGGATAGCGTCTGTCGTTGCATATACTTGTCTTCGTTCATTTG
GTCGCCTTGGATCA
TATTTTGATTGCGA
>ConsensusfromContig3647 Average coverage: 8.98
GTGATACCAAAGAATTAAAGAATGGTGGACGGTGCCTTCATCGT
TATAAATCGAGATGAT
TCACCATTTAACGTTCCGTAGCAAAGCGCACGCCGTGTGAGTGGG
CGATCAGAAGTTTGA
TTCTCTCCATGGAGGCGTTTCTCGGTCGAGTGATACCAAAGAATT
AAAGAATGGTGGACG
GTGCCTTCATCGTTATAAATCGAGATGATTCACCATTTAACGTTT
CGTAGCAAAGCGCAC
GCCGTGTGAGTGGG
>ConsensusfromContig3648 Average coverage: 4.58
GAAACGTCCGAGCCTGGCATTCGATGTGCGGCTGCTGAGGCTTTT
AAGAGCTTTTGTCAG
AGAGAGCAAGATTTCAGCTCGGGCATTCTCTCGATCGCTTGAGGA
ATACTGCCACGGCTT
CCATGATGGTGCAAAAACGAAGAAAATGTGGTATCGCCTTCTACG
CGCCTCTGGGTCTTA
TGAAGAGTTTATGCGGCTCCAGCACATCATCGACCATCCCCATG
AAGTGGACGATGGAAC
GCAGTGCCCCCCATGCTACAAGGCACCGTCTCTGCAGTTCACTCT
GGATGCAGACTTCCA
GCTTGTAAGAAAGCTATCGTCCGGATTGTCATGGAGAGACCCGA
TCCACAAGGGAAAGTA
CTTCATGGACCAACATTTTGTCGATGCTTTTGTGGCAAATTACTC
GGCTACGAAAACAAA
GCCTGAGG
>ConsensusfromContig3649 Average coverage: 4.65
TTTCAACAACTTTGTCAATAGTAGTTCTCAACGTTCATTATACCA
AAGACAAGCCAATGC
AGCCGGTGCTCAAAAAATTTCTTCTGCATTGGCTGGCGAAGCTCT
TCTGTATCAAAACAA

AATATCACAATCCATATTTGAATATCATGTCACAAGATTCTGGTC
GGCATATATTAGCCA
GATTAAAGAAACCAGTTCAAATCAAAAACAAGAAGAATAACGAG
AGTCTATTCATCCTCG
G
>ConsensusfromContig3650 Average coverage: 8.73
TAGCACTAAATACAATATTATAAAAAAATATAAACTAAAGCCAT
CAAAATAATTAAAAAC
TCGATTAAATCCTGCATTATCGTTCTCATGGCGTTATTTTTTCGA
TAAAATGACAAAGGT
AGATACAAACTACAACCTAACCAGAACAAAAGAATCATACAATA
ACCCTTAAACGGTCTA
ATCAAGTTACCCATATAACCGATAACAAAAAAAGACTCTACCACG
ATATGAGCATATTTA
TTATGTCTGTATAAGATAGTCAGTTTTGAATTACATATTCT
>ConsensusfromContig3651 Average coverage: 19.14
CTGTCGTTCTAGAAATTTTATTGTTGGAATGCTTGAATTGTATATT
TTATATTACAATTG
ATTGTAGTATCATTGTTGTATGATTGTATGATGATGACTTATATT
AGGTGATAACCGCAG
CCCGAAAGGGCTATAAATCATCTCTCCTGTCTCAATATTTCATAA
TGTGTATTTACATGT
ATTGTGAATTTTTTGAGCAAATAAATTTTCCTTCCTTCCT
>ConsensusfromContig3652 Average coverage: 14.94
TCCATCAGTCCATGCGTATCCATCAGTGCATCCGTATCTTCCCCA
ATGAATCGGGGAGCC
CAATTCAACCCATTCGTAACGGTCATTATTTGCCCATTTAGAAGG
AATTCCATCAGAACA
TTCTGGAGCCGGTTTATTCCAAGAATAGATACGGTGTGATACGTT
TGTGGAACCATGTAA
GCTACAAGAGTTATCATCCCATTCAGCGATAAAACATCCAGAGTA
GTCGCTTACACATTT
ATGGAGACAATCGTCTTGGGAGGTATTGAATTTCAATCCTGGATA
TCTGGCTCGAATAGG
TGGGTCACCAAACACGAATATTGTCATTCCGTAAGTATCATCTTC
ATTATAGACATGACA
CAGAAAGTTCGAAACAAATATGACCAGATGAGCCGTTTCCAATTC
GGCCGAACAATTTTG
TTGCCCGATGCCGAAT
>ConsensusfromContig3653 Average coverage: 6.74
TTTTGTATATTTTAGATCGATGCCCCTAGTCATGAACTATTCTTCA
TCACCCACTATGCC

CCGCCTCCTTGACCTATTTCTATAAACAGCCATTTCTTACCCTTGA
CTTTTTGTATATTT
TAGATCAATGCCCCTACTCATGAACTATTCTTCATCACCCACTAT
GCCCCGCCTCCTTGA
CCTATTTCTATCAACAG
>ConsensusfromContig3654 Average coverage: 10.17
AGACGGAAGGCTGAAGTGCGAGCTTATGTATACTCTTGAAGTGG
AATGTGATGGAAGTCC
AAAAAAATTTAATATATGTAATAATCTTGGACACCCATTCTTATG
ACTATGCCAGATCGT
AGTCCTTCTCAGTGTAACCTTTGGCATTATAGATTCCAATGACTGC
ATCATCAATTCGTT
TAATTATGTATTGGATGCTTCTGTTCTGTCCCAAACACACGTTAAC
TATTTGATTATTCG
ATGCTATAACCATAACGAATTTTCTGTGCAATAATATGGTATTCA
TATTTGCATCTCTGA
ATTTACAACGATTCGTTATTGGTTTTTCGCATTTCCCCAGTCTGTC
ATGATAAAATCGAG
CCTTATGGGAAAT
>ConsensusfromContig3655 Average coverage: 6.15
TTCCTATAAAATATTTTGTGCCGGAGCTATCGAAAACAATCTTAT
AAAAAAAAGAAAAAT
CACAAGATTCAATAAAATCTTCAGGACATTCAGAAAGCTGGCCAA
GCTTCATGATGGTGT
TGATTTACAATCATTCCCGCCGATCCGCCTTCAGAGATGGCTTCA
AAAAGATTACCCAGT
ATTAACGTTCCAGACTCCGTCGAAATATCGAGCAGGGAAAATCA
TATATGTGGAGAATGT
GTCGGTGGAGGAAGTCGCCGATAATGTTACTGGCTTCTTATCAGA
TGGGTCGTCTCAAAC
ATTGAGCCAGTCACATGACGAGCCAAAAACATACTCCCTCCCATC
ATTATTTCAAGCCGG
CCTCCTGCTCAAAGAGGATATACAAAAATGTTCTCAATTTGCGCC
GTGGCCTCCTCAAGC
CAATGATTATAATGAAAACGTATTTAAATTATTTGTCCCTCATAAA
TTATTCAATTTTTT
>ConsensusfromContig3656 Average coverage: 5.76
TCTGATTTCAAATCTAAACAAACAGCCAACAAATGTTTCTCAGCT
TTGGATGTAGGGTGA
ATCCTCGAAATAAAATCGCCACCTACCCAAGTAAGATCTCCTCG
AAGAGAAGAATAAAAT
CTCTGCTTGTTCAATGAAACACGATATAGACATTCTGGGCATTCA

AGAAAGTTGTAGGGT
GTACCCGGGTGAACTTTTGTTCCTTAGGCTGAAAACTCACACCAA
ATTACATTGTCAACC
TGGAGAAATGAATCACAGGCTGTCAATGGCGGAGTCGGTATACT
CTTGAGCAAGAGAGCT
CGATTTGCTCTTCGGGATGTGATATCACACACTATTTCGAAATCG
AGAGCAACCCAGTTA
CCTCAATCATAGTCACCTGATCGCCGACTGCAACATGTTCTGACG
ACGAGATTTTTTCAT
AGAAACAGGTTTTATTATGTTTGCAATTTTCATAATTATGTATTCA
CGCGTGTATATTTT
CATACTATACCATAAATGCCATACTAAGCGAATATACAAATAGAA
GCTATAATTGATGTA
GTGTTCTCACAAGCCAACTTAAGTTATACCTGATATGATAGGCGA
GAGTGTGACATCGAT
ATCTGAATATCGGCAAAGTGTGAATAAG
>ConsensusfromContig3657 Average coverage: 6.32
AACGTATATTGCTTTGCGCCAATCCCCTGCAGTTTTGCCAGTTCA
TGCAACCAGAGCTAG
GCGGAGTTGGTGCTGTCCTGATTCTGATAATGAAGGTCAATCCAC
GCCAAGCTGTACCTG
TTCTCATCAGTGTAGTAACAGAGTGGCTGGATCAAGTCAAACTCT
TCAGAGCCAGATCAT
AAATCCTGCGATGCTGAATGGACAAAACCTGCAGAGATTGGTTTT
TCACAATGTGCCAAT
CCATAGTTCTATAGACAGGTCCTCGAGTTTGAGACAGAAGTGCCA
TCCATGAGAATTGTT
CAAAGCGTTACTTCACTGAAGGC
>ConsensusfromContig3658 Average coverage: 3.57
TCTAAATGTAACACAAGTTTATCACTATCTACGCTCTCGGCTGAC
CGCTGCTAATGTTGA
CATAACCTTGCTATGTTTCGATTCGGAGTTTCTCTAAATGTAACAC
AAGTTTATCACTAT
CTACGCTCTCGGCTGACCGCTGCTAATGTTGACATAACCTTGCTA
TGTTTCGATTCGGAG
TTTCTCGTAAAAGTAAACAATTGGAGGATTTGGTTTAA
>ConsensusfromContig3659 Average coverage: 14.80
ACTGAATTTTATGAAATGATCCATTCGATTGTTGTTTTTATGTCTA
GACAGTCTCATACG
ACAGTTTTGGTTCTCATTTTCAAATATAATCTGTTTAAAGGTTGAA
GTTTTTTTAATAAA
TAAATCTGCTAAAATCGTATCTAGTCTGACTTTCTTTTGAATTTAT

CAATTTGTATCCCG
AATCATAATTATTAACTTGAATTTAGATCTTTTAGTGTAGTGTTAT
TCCAAAACGTGTGT
TGAAAAATACGAAATTTTTCTATAGGGTTTATATCACTGTTTATG
CGTATAATAGCGTGA
TTCTCAAGGGTCGAGACTAGGTCCACTTTTGTTTGTATATTAATTT
AAAGTGCCTCATTT
TGAATATTGTTATTATTGTTAATATTTCTGCCATTCCATTTTATAT
TTGATCTGTGCTAT
GCAGTGTATTGTGATCATAATTAAAATTGATTATTTATCAAAATA
CCCACATTAAGGTGA
ATTTTCTTGTATCGATTGGACACATTGGTATTAAAGTTTGTTTCGT
GAACTTGTAAATAA
TCCCTTTTGCAACTTAAAAAAAA
>ConsensusfromContig3660 Average coverage: 11.92
AGGCTGCTCAGATAGAGCGTATGTTCTTAGTTTATACGATGTGCA
AGTTTGAATTATTTG
CGATTTTATCTGTGACTCAGTATCATTGAACTCACATTCGGCTGCT
AACTTCTTTAGACG
CGTATGAAAACTGTCGATATTCTCACCTTCTTCTTGTCTTGTTTCA
CGGAATTTGTACAC
GGCATATTCTACATTTTTGATTGGGCAAAAATGTTTTGTGAGACG
CGACACGGCCTCATC
AAATGTGTTTTCACTATCATTTTCGCTCTTGAGTGTTTCAAACACT
TCCTGAAAATCCTC
TCCACCAAGAATAAGTAACATTGCTAACTTTCTTTCGGATGATTT
TATGTCCATTGCACA
CATTAAATGATTGAAACGTTTTAAATATGATTTCCAACGTTTATC
AATATTAGATACATT
GTTTTCATTAGAACATTGAATGTCGA
>ConsensusfromContig3661 Average coverage: 6.04
GTGGGGCCATGGAGCAAGTGGAGTGGGTGTCTTCCTGAGAAATGC
AATGACCGTTTTTGG
TCCGCTCAGCATCTCAGTTACGCCAAGAGGACTCGGTTCATTATC
AGCAGCAGCGAGCAC
TCGAATTGCCCCTCCACCATAGAAGTTCGGTCATGTGACCCTCCG
CTGTGCTATGATTGG
ACTCTCATTCCAAACGGACAATGCACCACCGTGAAGAGATTCAG
GTCATGTGGAAGAGGC
CAAATAAATATGACTGTTATCTGCAAAGACTATGAAGGTAGAAC
CTGGCCAGAATCCTAC
TGCAGGAAACCCAAGCCCAAGAACCATCTCTCCTGCGACATCAGC

TGCCCGGATGATTGC
AAGA
>ConsensusfromContig3662 Average coverage: 7.43
CGGATCCGAATATTCATTATCCCGACAATCTTCTATTCGAGAAGA
CACGTTTCTCAATTG
GATCAATGTCAAGATTGAGCCATACGGTATTAGGGTGGAAACTT
TGCAGAATGAAATGTA
TAGTGGAGTGATTCTCTGCCGTCTTGTGGAGGAGATTCAGAAAC
GGAAACTGAAAAAAAT
GGAAAAATCTCCAAAAAATCTGAATCAATACCGAAAAAACATCG
GAATTGCTTTTGAAGC
ATTGCACAAGGATGGCATCC
>ConsensusfromContig3663 Average coverage: 8.77
GTTTTTCGTAATTAGTTTTTGTGGATCTTCCTTGAACACAAAGAGC
AAATCCTTTAAATT
AGTTCTCATCGCACTATGGAGTTCATTAATCGCTCTTCTTTCTTCT
TCAACATTTCCTTG
ATTTTCAATATCCGCTTGTTTTCTACGAATATAGTACTCAGAATCA
ACTTCTCTGTCAAT
TATCAACAAGACCAGTTCGTGATGGTATGGGATATACGAATTTCT
TTTTATTACAAAATA
CAAAGAAACTCTGATGATTATCATTATTGGTTCTAGTATAACAAA
AAACAGCACAGACAT
TCCGGATCGAAGGAGCCATATTTTCATTTCGGCGTGATCGAAGCT
TGCACTTGTCAGAAA
AATGTAGATTGAGATCAACACCGATCCTATGATCACATACGCCAA
TGCCATCATAGTCCA
TGTGCTCGACCACCTGTGTATATTTTG
>ConsensusfromContig3664 Average coverage: 5.35
CCCTAACTTGATAGACCTATTCCCCATATCATCAAACTTATCTAT
GACTCTTTTGAAAAG
AACGCCATTGCTTTTCATCCTTTGAAGTAGATCACAGTCTATTTG
AATACGCGCCAAGTT
CGACAACCGACCTTGCGACATTGTTGAACGGAGATAGGTCTTCA
GACGCTTCAAGGCAGA
GAACGATCGCTCCTCAGACGCTGTTGTTGGCTTTATCGTTAAAAT
TAATTTCAGAAATTT
TGTCGTCTCAGGAAGTCCATCCGTTAACTCCAAT
>ConsensusfromContig3665 Average coverage: 9.50
ATGTAAAGGACGATGAAGATAACAAGGAGTACGAGGAAAAACA
AACAGAGCCATATGATG
GGATCCCAAAAGGCTGTACCGTATCTTTGGCAGTCGCGGCCCGTA

AAGTAGATCGTACAT
TTGCAGGTGTAGCCGCGATGGAGCTCAGATCCTGGCACACATTTC
CCTTTATTCTGACAG
GCATGATGAAGACATGCATCCTCAGCTCCCAATATCTTCAGGATA
GCCACAATTAGAAAA
ACCTTCGCTTGAAAGCCGTATGAACTCATTCTGCGTCTCGATCAG
TGTCAACATCTAGGG
AACATTATTTTTTGAAATATTATTGAAACTTGGTTTGAGACAGTTT
ATTTTCTTTTCAAC
CGTTTAAAAATCTAAATGTGAGTTTTACGAATC
>ConsensusfromContig3666 Average coverage: 10.37
CGATTCGGAGAAGTAGCTCCACCACACACGCTTGGTACTCATAGT
CTCCTGTACTTATCA
TTGAGGGGCGAATATCCGCTATGTAGTCTTCTAGCACGGCCGTTG
CGTGGGATATAACTG
GACCAGAAGTGATATCCAGAAGCCAGTTCATGAGTTTCAGGCTCT
CGATTTTTATCTTGT
AATTCGTAGCGTCGGAGAGCGAAACTTTAAGAAGCCGCCGGAAT
AGAGTCAAGATGACCA
TTTTTCGACAAATTTTGGATAATCCGGCGATAGCTTTGATGCTCTC
GCAGATC
>ConsensusfromContig3667 Average coverage: 3.99
GACAATGTTTTATGAGTTATAACATGTCCATTTCGTGACAGAATT
GATATATAATCCGTA
TTTCATTTTGCAAAAGGAATTTGCGACCCCTTGATCGATGCCACT
GGTAATATTTGACCA
GGTGCAGACTTTGCAATCACATCATACATCCCTCTTAATATCTAAT
GTATATTCACCTTC
AGTTCTTAAGTTAGTGCAATGAATAAATATTGGAAATCAGTTCTA
TTAAAAAAGCATAAA
TCTACTTCGACTATTCGCAACTCATACGGCAGTATATGAATTAAA
TATTACTGTTCAGAT
AAGAAAGTATGTGATAAACCA
>ConsensusfromContig3668 Average coverage: 6.13
CTCTTCGCATGATGGTCTATTCAGTGAAGCCTATTGTTATTATAG
TGAGGCATAAGAAAC
TACTCCCTTATTGCTATGGTTTAAAAGCCTGCATCAGGAGCTTCA
ATCTGACACCCCTTG
TATCCCTAAATCTCCTCTCACAGTGAGAATGCTAAGTGGCATTTT
TATGGCCTTGAACAA
AATTTTTGAGCATCATTATGACGTGGTTTTCAAGCGGTTATTCCA
CTATATTTCACGGCA

TGGCACGGATGAGCGAAGTTTCGTTTCCTAGTTCTCAGATATAAG
TACTGTGAAGTAAAT
CATTTGGAATCAGGCGGAG
>ConsensusfromContig3669 Average coverage: 3.89
GCTCAAAGTAGTAGGTCAAAGTCGCGAAAATGAGCATTCCTATG
AAGAGTATGAGAAGGA
GCAGATAGATCTCTTTAGCACTTCGTTTGACCGACAAAAATATAA
CGTTTAAACCCGAAT
GTTTTCTCATGACCCGCATTATTCGCACAACTCTGATGAGCCTTG
AGATTCCGACCACAT
AGATCATTGCGCCATACGAATCGAAGATCGG
>ConsensusfromContig3670 Average coverage: 9.82
GATGAAGCGTATTCGTCCGTATCGTTCGAGTCCTCATCGAAATCA
AGCTCATCATATGGA
TTATATTCTTCACTCTCATTGGCCGAGTCATCCGCGTCCTTTTCGT
CGGAGCTTGATTCC
TCCATATCAGAATTCGTATCTATGAATATCGGTCGCACAGTTGCA
CTCTGTTGATCTTGA
AGAACAACTGGTTCCGTCCCGCTATGATTTGCAATGGTTTCTCGA
TTGACATCCATGCCC
GTATTGACAGCTGAAATATTCTCTCCTGGTAGGTAGAAAGGGAC
AATCGGTAGCAGCTGG
TCTCCACGCAGAGTTAGCCCTGGTTGTTTTTGTTTTAAGTATTGA
GAGTCAGAAGAAGGT
CTATTCTCGTTTTTAGTGATAATCCGTACTGGCTTTTCACGAAGGA
GTTTCAGTTCGAAT
TGGTTTTGAGATAACAAAGAGGCAAGATGGTCTAGTTTGCTGGA
CAGTATCAGAG
>ConsensusfromContig3671 Average coverage: 7.00
TATCATGACCGAGTAATCCAGCAAGGCAATTAGCTTTTTTTTCAA
AAACATTACTTTCTT
CCCGCCCATAATCGGATAGTATATCAACAGCAGAAACATCATGA
TGTTCACCCACAGAAC
AATAGTCGTCAATACAAATCCTGTCATCATGAAAGCTATCTGATA
GCTCGAAAAACGCAT
AGCTGATACCATCATGGTCGATGGAGAAGCTCTGTAATTGAAGTG
CCATGAGTGATCGCC
TCTGAACAACTTCATCATGTGTTTCTTATAACGGAATCCAGAATG
AA
>ConsensusfromContig3672 Average coverage: 10.42
ATTCGATACAACTTCCTTCCACGTTGCAACAATATACTCTTTGTT
TGAATTTGATGGAGA

TATGATCTAGAAACTCAGCTGTCTGCTTTTCCTTGCATTGATGGG
TAACGCAGAAGGCCG
TCCAGATGGCTTTGAATTCACACAGGACGATTTGCTACATTCAAA
TCAGGACCCTGGCAA
GAAAGCTGGCAAAAACGACAAGCGCAGCTCTATCAAATCCAAGA
ATACCCCCAAAAAATC
ATTAGTTGAGCCATGTACCTTCGAAAACATGCCTCATTTCTC
>ConsensusfromContig3673 Average coverage: 7.72
GTTTTCTCAAGTCATGTGATCGTAAAAAGACATTCCACAAGTTTT
ACAGGTATAGGATTT
CACACCAGTATGAATTTTGATATGTTTTTCTAGATGGGATGACAG
ACGAAAACATTCTTC
ACACTCATTGCAAAAGTAGGAATTCTCACAAATATGAATAAGTGT
CTGTGATTTTAAACA
AGAGGCCAATGAGAAAGACTTTTCACATGCATTACAGGTGTACGA
TTTCACATCAGTACG
GATTTTTATATGTTTTTCAAGATGCATTGACAGATGAAAACATTT
TACACATTCACTGCA
AGAGTATGGTTTCTCACAAGTATGACTTTTTACGTGTATTTTCAA
ACCATGTGATCGTAG
GAAAGACTTTACACATGTATTACAGGTG
>ConsensusfromContig3674 Average coverage: 7.18
TAGGTCGGGGCGAGATAAATGGGACGGTGTCGCCTGCTTCTGAAT
TCTTTGATCGTCACG
TGCTGGAGATCAACCAATCAGGGGGATTAGGTGATCTTGGTTCGA
TAAAATGGGATATTG
CCCTTTGCCTCGTCGCAGTGTACGCTTTAGTCTATTTCTCTCTAT
GGAAAGGAATAAAGA
GTTCAGGAAAGGCCGTGTGGGTGACCGCTACATTCCCCTATGTCG
TCCTCGTCATACTCC
TCATCCGCGGAATAACTCTCCCAGGCTCCTGTAACGGGATTCTCT
ACTATCTCAGCCCGC
AGTGGGACAAACTTCTCAAGTTTGAGGTTTGGATGATCGCGGCGA
CCCAGATCTTTTTCT
CCCTCGGACCCGGCTTCGGGGTTCTTCTAGCTCTCTCCAGCTACA
ACAAGTTTCACAACA
ACTGCTACAAAGATGCTTTGATCACAGCTTCAATAAACTGTTTCA
CGAGTTTTATTGCTG
GGTTCGTGGTATTCTCTGTTCTTGGGTACATGTCCCTCAAGCAGC
AGACCAGCATCGATA
AAGTCGCCACAGTCGGTCCAGGTCTTGTGTGCA
>ConsensusfromContig3675 Average coverage: 11.56

CCAAACCTAATCAATTCGTCACCTCCAACCAACATCATTGAAGGC
AATATCGAACATTTA
CTGAGCTCTATTTTTTCCAATACTTTAAAACCAAGGAAAATACGC
TACTTGAAGCTGAAC
CAATAATCGGACTGAAAGGTTTTTTTATCTCGGGAATCCATTAT
CTTAAATATCTAGAA
AAATTCATACAGAAAAACTCCCCATTGATATAATTGATGAAACTG
ACTTGAAGAGTTCAA
TACCTAGAGTTTTTTAACTTCCACAAAAATCGTACAAAAAGTGAG
CTCTCTAGTTGGGAC
AAATTTTATATAACGGAGAGAAAACTAATTGGAATAATTCAATTC
TTTCGATGTATTAAG
TTCCCACAAATTATACTATAAAACCC
>ConsensusfromContig3676 Average coverage: 5.11
GTAATTGGTTGAGATGAATAATATTTTGTGAAGTCAGAAACACT
AAATTGAGGGCAAAGG
AAGAGTGGATAAATGCGAGATGGATCACCAACAAGCTGGGCATC
TATCATGCATGAATAC
AACAAAACACGACATTTTGATGTTTGATAGCTCTAATAAAAGGCC
ATATTATTTAGGATC
ATCCGAATATATAATTCAGTCCGTTGGTGTGTGTGTGTGTTTGTTT
ATCCTCTATCACCA
CTGAACAGATACTAC
>ConsensusfromContig3677 Average coverage: 5.44
CAGAAAAGCAGGCCGATAATTCGGGACTAATGCGAACCTACATT
CAAGTATTCACAGGGG
CCGAAGAGGTTTACGAAGAAGGGGCGAATAAGACCAAAGCAATC
ATATCTGTTCTTTTTG
GCTTTGGTATATTTGCCTGTGTTGGAGGAATTTCATACACCTTTGG
TGTGATATATGCTG
ATCTTGAAAAGAGGTTTCCTGGCCAATCAAATGTTTTGGCGTTTG
CCGGAACTCTTCAAG
CCGCGCTCTCCTTTGCTTGCAGTTTTCTTCAAGTTCCTCTAGTCCT
CGCCTTAGGCACCC
GCAGACTGCTTTTTGCTTCAGGGCTCGGTGTCGCCGCTTGCTTCA
TTGCAAGTGCCTTCG
TCGAATCCGCATTACTCTTTACTTTCCTCTACGCATTCATTCCAGG
CTGGTTATTTGGTC
TGTCGAGAAGGTGCTGTTCTTTGAGTATCTACTACCAATATACAC
CAAGATCAAGAGGTG
CAGCTTTT
>ConsensusfromContig3678 Average coverage: 8.10

GAATTGGAATCCGAATACGATCCTGGTTCGCACGGAGTTGGATTT
TGCGAAGCATCAGAA
CATTCATATCCTGCAGGACAATCGAGACAAGCAGTAGACCCTGCG
CTACTCTGGTATTGG
CCTGTAGTGCAATTTGTGCATACAATAGAACCTGCATGTGAGTAT
GAGCCGGCCGGGCAA
CTGGAAGGTAGGTTCGAAGCATTGAAGCACTGGTAACCTGCATCA
CATTTATAACATGAT
TCTGAGGCTGTGTTGTTTTGATAGTAGCCATCAGCACAATTGAAA
CATTGAATTGATCCT
TCAGTTGAAAATGTTCCCGATGGGCATGATTGCGGATCTTCATTC
GCGTTATTGCATGAG
AAACCAGCATCGCAAACTGTGCAGGAGTTCGCTGCAGTGAGAGA
CTGATAAGATCGGAAG
>ConsensusfromContig3679 Average coverage: 8.96
GTTTAAAGGAGCATTATTTCAAGATGCACAATTTTTTGGCTATGG
TTTTGAGTATGATGT
ATTTATCAATGGGCGATTGCGAAAATTCAGAGACAAAAAAAGCT
GCAAATTGTGTGAAAA
AAAACATTAATACTTATACATGTGACTTGGACAGTTTGATCGGAA
TTGCAAATCCACCAG
TGATAAATGCATCGCAGTATGGACTGATTTTCAATGGTACTTTCA
ACGCAGAGAACACTG
AAAATAGCACTTTGGAAAAATACAGCCAAATCAATCTGACGCAA
AAAGTGT
>ConsensusfromContig3680 Average coverage: 3.85
ACTTCAGATCAAGACGCTTCTGTCCGAAGCGTTCCTCTAACAGTG
CAACTGTGAGTCCAT
TACAGACCGCTCTATCCGAGATACAGCTTTTGGTCTGATGAAAGA
CCTTAGCAAGGGTCT
CTGCTCTATCACTACCAGTAAAACTGGAATAGATATTAGCCATAT
CCTCTAGGGCCTCTA
TGATCATTCTAGCAGAATGTTCTGATGAGCCACTAGGGACACTAT
CTATACCAATAGAAA
ACTTATCCCCCTTTTCAGTATGCAAATTAACCTCCTGTATGTGTTC
CCCTTGTTTAGTGG
TAGAATCAAACCCTATAGTATTGAAAGGTGATCCAATGAAAATC
TCAGCAACCTGGATAC
TACACAATAAACCAAACTCCGTGCTCATCCGGCC
>ConsensusfromContig3681 Average coverage: 8.40
TGCGACTTATTTGACAGAAACTGCAGCTCGCGTCGTTTCATTGAT
GAACGATAACAAAGA

TTCATTCAAAGATGATATCTGCATTGCTGATTCGGCCACTGTTTC
AAAAGATGCGACTTA
TTTGACAGAAACTGCAGCTCGCGTTGTTTCATTGATGAACGATAA
CAAAGATTCATTCAA
AGATGATATCTGCATTGCTGATTCGGCCACTGTTT
>ConsensusfromContig3682 Average coverage: 6.97
TTTAAAACCACTCAATGTAATAGCAAACCCTTCACGGGATGTCCT
TTAATCCGCTCTCAC
CGGAGCCTTCACCCGAGCCCCCACCGGAACCGAATGTATCAGAA
TCATTGAAAGAATCCA
TGGGAGAAAACTCGTCTGGAGATGAGATTCGCAAATCCAAGCGC
CGAGGACTGGAGAGCG
CTAGATGGAAGCGAATTCCTTTTGCACCGACTGGAAGTCTTGATG
AGTTCTACACGTACA
TTACATCGGAATTCAGAAAATTCGGAATTGATCTGCCCAACGACA
GCCCTGTTTTCAAGT
TGCCTTCGCAGCAAGAGCTCTGCAGACACATGTTCAAATCCGAGC
TGAAGAGGCTGCACT
GCGAAATCATGACATTCAAGCAGAGTTGTGCGCTTGAG
>ConsensusfromContig3683 Average coverage: 8.15
ACCGAATGCAGCAGACGACAGGTTTTCGTATCGTTGTACATGTCT
CACTGATTTATTGTT
TATTTTTCAATCATTATGTCTCAAATGGATTATGTGTTGACATACC
AGCATGGCCTCTGC
TAAACCTTATTTATTGGAACAATTTTTTCGCCAAATTCAACTGAA
TACCTCACATGATTC
TGGGATCCCTAAAAAGTTTTATAACCCAGGTCAGAGAAGTGCCA
AAGATCGGAAGAG
>ConsensusfromContig3684 Average coverage: 6.26
TCCAGCCATGCCTACGTCTTGATCATCAGCTTGAACTAATAATAT
TTTCTGATCCTTGAG
AGTCTTGCTGACGGCAGTTATGAGAGATCCGTTTGTAGCAGGATA
CTTGAATACAGGAGG
ATTGTCATTGCGGTCCCCAACTATGAGTGTGAGGGTTACGGTACT
CTGAACATATTCGTC
GTCTTTGTTGTATGCGATAAGTCTCAAATCATATTTTGGTATTTTT
TCACGGTCAAGTAC
CCGATTTAGAATTATCCGCCCAGTGGCGACATCTACATTGATAAT
ATCCAAAAATTCTTC
TCTCACGAAAGTATCAATTCT
>ConsensusfromContig3685 Average coverage: 4.08
CTTTTGGACACCATATATATCGTTCCATGAACTCCATGGAAATAT

AATGTGCGAAGAATA
CTTCGATAATTTGCTGTATCCAGGATCTACGTCGTGAATAATTTCA
TTTTTGCGATCGTA
AAAAGAAATATCAATGAAAGGTCATCTCCATTTTATTTAAGTATT
TAATAAACCATTCTT
GCTGTAGAATTTCCAGCGGGCGCCTCTTGCATAGAGCCGCAACTC
AGAGTTTGATGAATT
CATTTTTCGAAGCAAAGATTCTTTGTTTTGTTTCCACAACATGAT
ATCAATGTCGTCAGG
GACAAAACCATGGTGTCTGTATGCTCCGATGAGACTCCCTCCATA
CAAAAAACATATGGC
ACCAATGTCATTCAGTACCCTGATGAATTCATCCAGAAGTATTAG
AGTTGAATTAGTT
>ConsensusfromContig3686 Average coverage: 6.55
CCGATCTTGAAATTTCTGAAACACTTAGGATTTGATAACAAAGAC
ATGATAAATGCTATT
GGTCATTCTGGAGAGACTCCGCTAACGACAGCTTTGTCTGGTCCT
TTTCGCGATGGTGAG
TTGGTGCGGTTCCTTCTCCGCAAATTGGGTGCAGATCCTAATGCA
AAAAACAGTGCCGGA
ATTTGTCCTTTGGATGTTCTGAACAAGCGGGATGAGAAGAAAGA
GGAGATTCGAGATTCG
TCGTCGTCGTTCACTAAACCTCGAAGAGACAGG
>ConsensusfromContig3687 Average coverage: 7.65
AAAAAATATGGTGACATGTTACTTATGAATTAAGACGAAATTATT
GAACATAAAATTCAA
CGGAAGTCTTGACTTGTCACGACTCTGGTGCTACTTCTGCTGCAAC
ATATTCTCTTCTGG
AAATGGTCAATTTCGTGAGTCTTGATGCGATGAAGACAAGAACTG
TTCCCACCAAAACAC
ATGACGAGAGTATTCCGAATGTGATCACATGGCTTTGAGACCGC
CCGAGGAGGAATGTTG
CCAAGGGATAATTGAGGATCCAGGCAAAACCTACGGCGGTCAAT
TGATACGTCGAGGCCT
TTCTTGCGAA
>ConsensusfromContig3688 Average coverage: 7.73
GAAAATGCTAGAGTCCTTTATTATCTCACAGAAAACAGTGCTAAA
ATGTATAAACACATG
TTCGAGGTCAAAAAAGAAACTGGCGAACTTATATTGAACGGTAC
TCTTGATTACGAAACG
TATACTTCGCCCATGACATTAGTAATTTCTGCGAGTAATCCGGGC
ACATCCATCCAATCA

TCTTTCACAAATGTTGTATTGCAGCTTGAAGACGTTAATGACAAT
CACCCAGTCATTACT
GTTAACACACTCAGAGATGACGACACCTTAACAATAGAGGAAAA
TTCAAAAAAAGATTCA
TTTGTTGCGATGGTGGTGGTAGATGATGCTGATGACGGAGATAAT
GGAAGATTCCAATGC
AATATGGTCAGCAACACATTTAGTCTGATTTCACGA
>ConsensusfromContig3689 Average coverage: 5.91
GTCCAGCACTCATGCCATTTTCGTTATCTGAACACGCTCTTATAGA
TGTTCTATGACCTG
TGGACTTCACCACGGCACGCCACCTTACACTATCAAGAGCTGTTC
TATAAGCTGATGCCG
AACCTCCTCCAGTTCATTATTCAATGGCGGTGGTGCAATTTCTGG
GAAGTCGGCCCCTTT
TTCCTCTTTCCATTGACATTCTCTGAATTATTAGAGACTCCAGACC
CTTTCGACGAATGG
GGTGTAAGCTTTTTAGAGACAATTTACTGACGTAGTACAGGGACG
TTGCTTATTGCTGTA
GAACGTGTTTGTCTGCTTTGCGTTTTCTCCAAGATATTCGGTACAG
GCCTTAATAGCTCC
CCATCTCGAAAGCATTCATTTGTTTCAGATCTCTTTCAGAAATGG
TATAAGACTAG
>ConsensusfromContig3690 Average coverage: 12.69
ATCAATAATAGCAATTTGTATTGACTTATTTTTATGTGCGAATGA
CCAGTAATATAATTT
CGAAATGTGATTGCCTGAATTTTGAAATGTAATGAAAATATTCTA
CCTCTAGATATATAA
CCGTCATTCACGACGTGAGGGTTGAGCGTCCCAAACAGTATACAG
CTCCCAGACTTTTAG
AAAGTAATGTTCTAATCGAAAGTAGAAACATAAAACACAAAAAA
CTCAGTGACCCGATAT
ATATTCAGATATTTAGGTATTAAATACTGTATAGTTTTTTTGGGTT
TATGATTA
>ConsensusfromContig3691 Average coverage: 12.82
TCTCGAACAGGATTTCGCATTTTGTGTAACCCATCTGCCGAGCTA
CCACTAGAAGTTCCG
CGTCATCAAAATAGTCGTTACACACAGTCCCCCAGTGAGAATTGT
GGAAAATTTGCAGGA
ACCCAAAATGCTGATACGGATTCAGCGGATAGTAAAGTCGAATTT
CATCTTTAAAGCCGG
TTTCTGCTTCCTTTGTAGTGACCTCAGATTCGTCTGAGTATTCATA
ATCTTCATATGAAC

ACTCCAAGACCACATCTTC
>ConsensusfromContig3692 Average coverage: 14.26
TGAATATTGATGTGATTTTGATCGGCTCCATTTAGAGAAAACTGA
GAATTAAAATACGAA
GCACTTTTAATTTCTGAGTTTATTTCATTTCGGACGCAATGACTTT
TGGCCAATTTCGGT
TTTTTGGCGAAATGACGATGGGACGATTTCGAAAGCTTTGGGCGA
TTCATACCTGGGCGA
ATTATGCATTCTGTTTGATCAATACGAGTTCATCTGGCCATCGAA
ATTCGACACAACATT
CTCGTTAAACGCCATCCCAGCATGTGAG
>ConsensusfromContig3693 Average coverage: 4.78
CCCTGCACGACGCTCTTCCGATCTTCGGGCCCTATTCCATAAATC
CAGAGAATCAGTAAG
TACTTTTAATGGAGAGGTTTGAGGGCTCCCATCAATATCATCAAA
GATAGAGACTGAGTA
TGTCATTGACGGGTTTTCTCTGTTAATGAATAGATAGCAATCCAA
GTTATACTCATCAAA
TCTCATTAGATCATAACTGCTCTCTTCGAAACTATTTCTACAATA
TGAAAAGTTCGAATT
TTC
>ConsensusfromContig3694 Average coverage: 13.43
TAAATAACAAATCTAAGATTTCACATCACCAATGTTAGGCACACA
ATCATTTGCAGTTTA
AAATCTATGTTATAAAAAGGTTAATAAGGTCAATACTAATTTCTA
ATCTTAGATTTTTTA
GGAAAATCTCATTTGCAGAGCTCATCCAAAACTTTTCTGCTGACT
ATTTCCACATCACTT
TCCTGAGCGACTGTGAATGCATTCGATGTTTGGGGGTCAGACGCC
ATTTCGGCCACACCC
ACATCCGCTCCAATTCCTACAGACATGATCCTGATGTTTTGAC
>ConsensusfromContig3695 Average coverage: 6.76
GGCGTATTTCAAAATTCAGAAACTGAAATAAAATAAACGAATAT
TATAAATTAGAAATGA
GTGATGATGGTGTCGTGGTCACGAAGAGACCAATCAAAACAGCTC
TCCATCAAGCTGTTT
TGGACTGCAGAAGGCATCAGGTACGTCTTTTGGTGGAGAGGCAC
AAGGTGAACGTAGACT
CCAAAGACATGTTTGGCAGAACGCCCCTGATGCTGGTTTGCCTA
ATGGATAATGAGGAAG
AAGCTGGTCACATGGCCCGAGTATTAATCAAGGCTGGAGCGTTTC
TCAATATAAGAGATT

CACAAGACAGGACTGCGTTGACTTATGCCTGCATCAGGGCAAGG
CAGGAGCTGGTGAAGC
TATTCACTAAGGAAGAATTGGTGGATCTGAACAATGCCG
>ConsensusfromContig3696 Average coverage: 4.57
ATAAAAAACATGACCTACCAGCAGCAAATCATGGACGCAGAATA
TCAAAACAGCCAAAAA
ACTTATTCAGAATCTCAACCACAAAATTACTGGAACCATTCAAAT
CTTCCAAAGCCGCCA
ATCAAAAGTCTCCCTCAGCCGCCTTTGCAAACAAGCATCATGATC
GAGCAGCCCCCGGCG
CCCATCCCCGCTACCCCTTTCGGTAGTGTCAATATCGGCCAGAAC
AACTGGTGTGCCAAG
TGCAACGCCTCTTTCCGAGATCGGAAAGCACAC
>ConsensusfromContig3697 Average coverage: 12.26
TAAAAGTTTGGGTAAAACAGTTCAATTCGTTTTGAAGAGTGTAAA
ATAATTTCAGATAAA
ATAACCAATTTTCTCAGCCAGCTAAAATGTTGGGTATATTCCTAA
ACTAACCGTGTTCAC
TTTGTGAGGGTGGCTAAAATGTACCAGCTTCGTTAGATCTTCAAT
AGTAATGTAGGCAGC
ACAATTTCGACAACGATCAATATACATTTCCCTAGTATTGTGCGG
CTTATAGGTTGAGAT
TTTACACCTAAATTTACTACTAGAGTCGATTTTTTAGACATATTCA
CAATTTTCTCCCTG
CTTCTGGTCCATACTAACATGCTGATATATTTGGGTTACATACTG
AAAATATAATATATA
TA
>ConsensusfromContig3698 Average coverage: 6.00
TCTTCATCTCCAGCCCATAATATAACCACTGTACTATGACACCGT
ACTATACTAAGATCT
CCAGCTCGAAGATTCTTCTTACCTTCATCTGAACATCATCCGTAG
AATCCTCGAGCACCG
TCTTCATCTCCAGCCCATAATATAACCACTGTACTATGACACCGT
ACTATACTAAGATCT
CCAGCTCGAAGATCCTTATTACCTTCATCTGAACATCATCC
>ConsensusfromContig3699 Average coverage: 6.11
TTCAAATTGTTTTACTGTTAGTCAGGTATCAAATGAGTCCCTCAT
CTGATGTCGCAGGGG
TGTACCCTTCAGACTGTTAGTTGAGTATCTAATGAACTGAGCCGA
TATGTTAAGGCACAA
TTCAAACGGCCATGTCCATGGTATTGTCGATATGTGCTTGGAACC
TCCAATCACAACAAG

TATTTTTGATATAAAATACAGTCATTCAATATATGAGCCATATTC
GAACGGTTCTGCAAC
TTTAAATGAGCGTCATTTAGGGTAGAAAGCTACAACCGTTTTCAA
CCGTTTGCCGCTTTC
TCGTTTTTGGCAATATTATATTTAAATATTGCTGCTAATTC
>ConsensusfromContig3700 Average coverage: 4.78
CTCCAATTTTTTCTCTTGATCAGTTTTTTCATTACTGTTTTCAGAT
ACATATGAATCATC
AATTCCAGCCGTTCTGCTCATAAAATGATCTTCATGCAGTGAACA
CGATGGCTCTCCAGA
AGCGATAATTGTTTTACAATCTTCCTTGTCTGAATAAGAATGGTCT
AGGACCTCATTTTT
CTGCCCGGAATTCTGTGAATTTTTCTCTGAGGTAGTTTTTATACAC
TGTCTATCAGATTC
TGGATCATTGGATTTATCTTTGATAATCGATGAATGAGTTTGATG
TATATTACTTTGCGA
GTTCGTATCAGACGATTTCGAAGTTTGAATGTTTTCAAGCGCAAT
GCTGAGCTCTTCGTG
ACCTTTCTTTTTGTCGGTTATTGACTCCGATATTGACTCACTTGCA
GGGTGATACGACGT
GTAATTATCATTGGATACCAGGCCGGTACAAGTTTCAGGGAAAAA
TGGAGATTCTGATAT
TTTTCTACTCAAGGACACCAAAGTTGGATTTGCATTA
>ConsensusfromContig3701 Average coverage: 12.52
TATATGATACTCTCCAAAAGTTACATATCACACAAAGGTCTACCA
AAATTCACGAAGGAA
TGACGGGCAATACTTTCAGAGATTCATAGCGAATAATCTCCAGAT
CGCTGAGTTTCGTTG
GCTAAGCATTGATTCCGACCCCACCATTCATAAATATCCTCGTAC
CACATAGGAGGTATA
TCCAGATGGAGCATTGCACAGAGTCTGCACAGGAAATTTGTATTT
ATGAATTCTCCGGTT
CCTGACCAAGACAGCATAGCATTATATGCGTCGTTGTCTTCATTC
AATTCCAACAAGTGC
CTGGCGAGGTCTGACGGTGAATTGAAATCGTCGACGTGTAGAAA
AGAGTTGCTAGATGGA
GCAGCCAAGAGGTAATCTTCTTTGGGAGGTCCCATAACAATAGGA
ACAACATTTTTCATC
AACCCGTTTTTGAAAAACTTTTCCGTGATGTAATCTCTGCAATTG
CCGCTTTCAAAGGCT
ATGACGAACTTGTAATCATCATAAAGCCGTTTCTCACATTTATCA
TCGCCTTTTGCGCAT

GGAATTTCATGATTACACTCACCGAATATATCAATAGGAATGTGT
TTCTTCAATTCCCTG
ACATAAGATGTTCTTGGCTCGACTCCGCAATTGGATACTACCCAG
ATGACGAGTTTGGAT
TTATTTTTGGCAAAGTCGGTATTTTTTTTAGTTTTTTG
>ConsensusfromContig3702 Average coverage: 8.75
GTTTATATATTAATGTCTAAAGAAGTGTCTTTATGTGGTCATTGG
TCATAGCCCATTGAA
GTGTTAGATTCCCGATTTGGTGGGGCACTCGCCTCGATCACGCCC
CGGGTCGGGATCGTG
TCGCCGGATTCTCAGAAACAGTGTTATTTTGGAAACTTTTTGCCG
CACCTACCTGTCCCC
ATTCCGATTCGACTACCGCGCCTAATAATGAGTGTCTCGTAAGCT
CTGTTCAACTAAGGA
ACATGGATACAGAATCGAGCCTGGACGGTCCGGTGGACCTATCC
AAGCCAACTGCCGATA
TCGATAACTACGGATGCCTTGATTTGAGACTCAACAAATCAGATC
ATTTGTTCAAACCTA
AGGATTTGAAAAGAGGAGATTCATCAGAAATTTATCAACATAAG
AAACCATGGAGTACCA
GCCTAAATGAAAAACCAAGATACG
>ConsensusfromContig3703 Average coverage: 9.37
AAGAGGGCGAGCAGAACAACAAGCAAACCAGCAAGAGTTCTGCC
AGGACTCGAGTCTTTC
TTGGCCCTCAAAACTTCGATCATGAAGGCAGCAATGACAGCAAA
CTGAGACCCAGCTGGT
CCCGCCTCTACGTGGTAAGGGAGAAACAAAGCGGAGACAATGGA
GCCGGACATTCCTCCT
CCGAGGTAGATGAAAGCCATTCGCCACCAACTAATCTTCTTCTCA
ATGTATCTCATGAAT
ACCAGCTGGAAGAGGACTGTCAGAATCACAGCAAATACTCCTGC
ATGTAGAAAAAGAGCA
GAGAAAAGTCTGTAGACCTGATCTGGTTTTCCGGGTTCCACGAAA
GGAAGCATGC
>ConsensusfromContig3704 Average coverage: 5.34
AAACTGTGGCAACGTTAATGATGCTTGAAAAAGATGTGCTGATCA
ATTCCAGCATGTCCA
TGAAACCCAGTTCCATCAACGCTCCGATATCATCTGTTAGAGAAA
TGATACCCTTCGCAG
CCGTGAAGGTGTTAGCCAAACAATCCCCAAACTTCCGCCTGACG
CACAGGGGTCGAATAC
CGTAAAAGACCAAATTCCGAACGAGGCTCAGATCGACGTAAGCA

TCACATCCTGATTGGT
TCATGGCCGCACGTGATTCATCCACAATCCGTATGAGTCCGTCAA
TAATCTGGCAGGGTT
TTGAGATCGCAGAGGCCCTGTCGAGTTTTCTTAGTGTTGTGTTTC
TTAGGAGAGATACGT
GACGGATAAAGCA
>ConsensusfromContig3705 Average coverage: 9.66
GATTGAAAGTAAATCATGAATAGATATGTTTTAAAATTAAATTCC
ACATGCATTGAAAAG
AATATTAAATTCTTCCAAAGTGATACTTCGCTCTTTATCAAAACA
TTCGTTATATGAATA
GGCATATAGGATAAATGTAACACTGTGTTCAGATTTCAATATAAG
GTGACATGATCACAG
TTAGATACGAAAAACGACCTTTAATCACAGATGATAATC
>ConsensusfromContig3706 Average coverage: 4.52
TGTCACAAACATTGCACAGCAACTCCACATTCAGTATTGTCAAGA
TTGGAGATTCACGAC
TCGATCTAGAGATACGTGTGAATGAGGAGAACGAGGATTGGGTG
TTCGGGGCCTACCGCT
GTGAATCTTCGAATAGGTTTGCGTCTCGGTATGAGCTCATCACTC
TCACACAAGCCCTTG
TACCTGCCAGACTACATGGCATTGATGCGGCGCGAGCGAACGAAT
CC
>ConsensusfromContig3707 Average coverage: 5.13
CGTTTCCATTCACTTTCTTGCCAATACAGTTGTAACGGTATGCTTA
CCGCTGTGGCTGCT
CATCATAGAAAAATTGCTCAACTTCCCATCCAGAATCGACAAGAC
TATATTAAGTCTCGA
GTCTTGGATCATCGCAAATGCGACTGTATACGCACTTGGCATCAT
CACCCGCCAATACAA
GTCCAACAGCGCCGAGGCCGTTCTAATGTGGCTGACTAAACCCCT
TTTGTTGCTCCATTG
TCTTGTTTTTCCCACCCTCGGGTCTTATATCAATATGTATGCTATC
TCTTCACTCAAATT
CCTATCAATTTTTGCCGCCATGCTCCTCCCGACGTTCGCGTATCTC
ATCAGCCATGCATC
ACTGAAGATA
>ConsensusfromContig3708 Average coverage: 8.85
GATACAAAATAGGACCATAATGAACAGTAGGAGAAGAAACAACA
ACAGGAAAGCCTATTT
TGAAGAATTTATCGACAATATCCTTCTGCTGGGTACAATCGTTGA
TAAGAAATCTTGATT

AAAACATACTTTTATTTTTGTCCTCCACATTCAGCTATGCGGCAAG
CATCTGTATTTGGT
TCTTATGGGATGGCCCGTCCCCGTCATGCCGTTTATGAACTCTTCC
TCCAATGTATTTCT
TTCTTGTATTGAAAAAATTCGCCTTTCCAGAT
>ConsensusfromContig3709 Average coverage: 14.22
TCCAACCCATCACGACTCCATCATCGAATCGGTTCCAGCCAAGAA
CTGGAGCATTGTGGC
CCACAGAGTCTTTCAAGATGTTTCTTCATCTCGGCCTTTGGGCGAA
AGTTTGTTGTTGTT
GTTTATACGCATTCCAATTATTTATCAAAGGGACTGAAGGTTGTG
ACCTATGACACGCCA
ACTTCCACAGAGCGGCTCTTTCATGAGGAGACGAGAGACTTGCTG
TACAGAGGATTTGTG
ATCGGCGACGAAGCCTCGTTTGTTTCTCTCAGCATTCACAATTCA
TTGCTGGGGAAAATC
CATGCATTCGGCGAGTCTTACATAATAGAACCAGACCCTGGTGC
AGGGGACGCAGACGAA
GGGCTGCAACGGACCCATATCATGTATAAAGTCACTCCAACAGT
CGCTGGCGCCATGACA
TCTCCACCAAAGCCTGGTTTCAGAACGAAGCTGGGCGACTTAGA
GAGGAGGGAGCTCAAG
AATCCTGGTCGAATTGATATCGCGGAGAATGGCGTTTGGAAGGAT
GATGATGATTCTGCT
GTGTTTGAGGATGATAAAAATCTGTTGAAGATGTTCGGAAAACT
CAATCTCAAAAAAGGA
AAAAGTCGGCAGAGGCGGTCCGTGTCCCGGGAGAGGTTCATCGA
GGTCATGGCGGTTGCA
GATCAGCCAATGTGGGAATTTCATGGGGATAAGCTGGAGCTTTAT
CTCACAACACTCTTC
AATATCGTTGACAGCATCTACCACGCCTCAAGCCTCG
>ConsensusfromContig3710 Average coverage: 11.72
GGAAATATGAGAACGTAGAAGAGCAACGCATGGACGAATGGCGA
TGCGATTCCACGAACA
GTTTCCGACCGCAAATATTTCAAGATTTGGATTTGACTGAAGTGT
GTCCCACAATGAAGA
GTGTGTGTACTAATTATAATTATCAAGGAAATATCCTCTGTCGGT
TCATCGATTTGGAAT
TTGGATCTAATATTGGAAAAAGAGAGTTCTTGGATCAAAATGAT
GAGGTGAGCCTTGAAC
CTAGACATT
>ConsensusfromContig3711 Average coverage: 7.93

GATTGAAGATCCTCGAAAATTTCTGAGTTTTTATGAATCTCCAAA
GCTGGATTCGTTAGA
GTCCAGATATGATTCATTGTATGTCAATTCAAAACTCAAGCAGAT
TCACCATAGATACGA
ACACCTTGCAGATCACGTTGTACTTGTCGATAAGCAGTCTTGTCTG
TTTACATCGTTTTT
AAGGCTTGAAAATGGCTCTTGTCTCGGTTCAGTGATGCCAGAGTT
TGTTATGTCTAGTAA
GTTACACGAAAGTGTTCCAGCGAAAGGTTCTGCAGTAGTGAATAG
CACTTCA
>ConsensusfromContig3712 Average coverage: 11.35
AAATAATATGATCGTTTTGCGGTTCTTATATCACGCTTAAGCACTT
GATTCATATTTCTT
AGATCCGATTTGGTTGTTTGGAAAGAATCATTAGTGATAGCTGTAG
TTCTTAATTTTTTT
AGCATACGATCTTTAATTTCTATTTTTTTAATCAAGTCATTGTCCA
TCCATGGCCTTAGA
AAATGTCTATGTCTTCTGAATTTTTTTTTAATGAGAGGATAATATG
CGTCATTTGTATTT
GTAAAAACTTTCAGCAATTCTTGGTACATGAGATCTGGGTTTTGT
TCAGCAGAAATTGTC
GCACTATTTAACAAAAAATCAAACATTTCAGCATAATTTGTGTCT
CCCAATAATGGTATT
TTTCTTTGATAAACAAAATGGGGCTGTTTAGTCTTGGTAGGATCA
GTAAGTGATGACTGA
AGAGATA
>ConsensusfromContig3713 Average coverage: 11.13
CGGATACTTACACAACAACTGGATCTGGTGACAGCACACGAGTT
TGGCCATAATTGGGGA
AGCCAACACGACCCGGACACTTCGGAATGCGCCCCAAGTTCCAG
GGACGACGGGAAATTT
GTGATGTACACATATTCTGTTGCGGGGTATGACAATAACAATGAC
AGATTCTCACAATGT
AGCATAAGAAGTATTAGATCTGTTCTCGATGCAAAATCAACCAAC
TGTTTTCAAGCCCGT
TCGGCATCTTTTTGCGGTAACCAAAAT
>ConsensusfromContig3714 Average coverage: 5.66
TTTCGATGACATTCGTCCAATCTCACTTTTAAATGTGTTAGGCAA
ATGTCTAGAAAAAGT
CTTACTAGTAAGATCGCTTTCTCTGAGTACTAATTGGATGGAAGA
TAACCAATTTGGATT
TACGCGAGGAAAAAATACCGGGGACGCGGTGGAGACGATTGTCA

CAGCGGCAGAAGATGC
TTTCAAAAAAGGCTTCATAATGCCATGTATATTTCTTGACATAAA
ATCGGCGTTCAATAA
TGCGTGGCATCCCTCCATACTCATGGAGCTCAT
>ConsensusfromContig3715 Average coverage: 22.52
ATAGGAGACATACCTCTGGTTTCATCAATTCGAAAAGGAGGTGAT
GATGGTGCACCAATT
GTCGTAGCATCACCTGAGAGTTCAGAAGCGAAAGAGTACACGAG
CATCGCAAAGGCAGTA
TATCACCATTTCTATGATCCGAAATGAATGAGATATGATTGTTGT
TATTACAAAAACTAT
AAATAGTGAATTCTTTTTTATCTGATAATACGAGGCATGTCAACG
CTATTCTCCAAACAT
GATTTGTAAACGATCTCCAGCATGTGATTTGTCTTTGTCTTGTTGT
ATTTGGTGTTTCAT
TTATATTCTCAGCCATCATATACCACACGGGAAGGATGGCACTGT
TTGATTTATGCACAC
TGAAATCGAACGCTTTCATAAGATC
>ConsensusfromContig3716 Average coverage: 6.61
TCCGATCTGTGCCGCTGGATTTGAGGGAGGAAACTGCGAAACAC
CAATCAATGAATGTGG
GTCAAATCCGTGCCTGAACGGTGCCACGTGCAACGATCTGATCAA
CCAATGGACCTGTAC
GTGTGCTGATGGATACGAGGGAACCTCTTGCGGAACCGACACCG
ACGAATGTGCATCAAG
CCCCTGTCAGAATGGAGGGACCTGCGCCGACCTCGTCAACCGATT
CATGTGTACCTGCGC
CCCTGGCTACCAAGGGACCTTCTGCGAACTCAACATCAACGAATG
TCTATCAAGCCCGTG
CCAGAATGGAGGAACCTGCAATAACCTCGTCAACCAATTCACAG
ATCGGAAGAGCGTCGT
GAAG
>ConsensusfromContig3717 Average coverage: 19.57
GCCAGTTGCAGCATCATCGCACCGCTCCATTTTGCAGAACAGATT
TTAGCAAACGTTTTT
TTTCGAAAATCAGCCCCAGTCAATTCTTCAAAGTCGATACTGATC
GAAGGTTTTGAAGAA
ACCAGATTCCAGAACTTAGGAAATAGCTCCAGTGACCTTGCACAC
TCATTACGTGTCACT
TTAGAAACCAACAGATTTGTCAATTTGACAGCGTTATCTTGATGA
ATGAAATCGGCATGC
ATTAGAAGAATACCGAGTAATTGAGAGCAAGTCTTTGATGAAATA

TCTTCACTATCGATG
CCAGTTATACACGACACGATAACTTCAAACTCTGTTTCATCGGAA
CATTGGGCAGCAGTT
TCTTGAGTATTCGTCTCCGAAGAAATGGCTTCCTGATTTCGAAGTC
CATTCAGTATTATT
TGTATGACAGCTATTGCATCGCTCTTCCCACGTACAAGCACATCA
GACACAAGAACTTTT
AGCTTTGTTCTTGAGTCATACAAAAGAGAATGAAGTTCTTCTTCTT
TCCCATTTCTTTGA
AGATCAATGAGTTGGTCACAGAGCTTCATATTATTTCCTTTTCAA
TGAAATTCATATCCC
ACTTTCACTATTTC
>ConsensusfromContig3718 Average coverage: 12.70
CATATTCAGGGGCAATCATTATGATAATTATGGAGATCACGCGCA
GCGTTATATAGAAAA
CACCTGCGCTTTTCCGACCATATTTGTCAGATAACACCCCCGATG
AAATGGTTCCAATCG
CACAACCTGCCATTAGAATGGACGCGATGAGTTCACTTTTCCACG
TCTCACCACAGACGA
GGTCCCATTGCGACGCGGCTGTACTCACGAAGATGCTCTGGTCAT
AACTCCAATCATCAC
ACTTAAGAATTTCCAAACTGTCATCAAGCATATGTGAGCGATCCC
AGTTCATGAAGTCTT
C
>ConsensusfromContig3719 Average coverage: 5.37
GCCATTGGGAGCATTTGTGTATTCTCAACACGAGTGATCTTCAAC
TGCATCTCGGACATC
CCTCCGATCGGAACCCGGTCGCTGCCTGTTGTGAAGAGCAGAAGC
TTTTTCTGGAGCTCT
GTTTGGAAGTTGAGAGCGACGCTCCAGAATAACTCTATTGTCGTT
GAACTCTCCGTATAA
CCGTCGTAAACTGTGACTTTCTTCAGCTGACCGAAGTCGAATGTT
GGACTCCCACAAACG
AGCATTTCAACTTCCTCTGGACGCAACATGATTAATGCATTCGAA
GCGCATACGCTATGA
AAGCCATGATAGAAACTTTGAAATTGGCGGTACATCGATTTGTTC
AATAGATAGTCCACG
TATCGATCGACGTATTCTTTTCGGTTTTCTTTGGTGACTGGAATAT
TTTGACCATTCGCT
TTCAAAGGCACAGTCGTAGTTATGCCATATTCACTCTTGGAAGCC
ACAAAATAAGCACAC
AAGTCTTCTTCTACATCACCATCATATTCAAGAAGTTCTTTAAAAC

>ConsensusfromContig3720 Average coverage: 8.73
CTTCCGATCTGCCGATTTGATTATGTGAAAGCGTTCGAAACTGAT
GACAGGTGTCTTCCT
AATTGCTGGATTGTAGTAAGAGTTGATGGCAAAGGCTTTCATAAA
TTTTCGGACTTGCAT
GCATTCGAGAAGCCTAATGATTCACGATGTTTAAAGTTAATGGCG
AAGTCAGCTGATTGC
GTCATGAAAGAATTCAAAGATATTATTTGGCGTATGGTGACAGT
GACGAGTTCAGCTTT
GTGTTCAAACGAAAAACGAAATGCTATAATAGACGATCGAGCAA
ACTCAGCTCGAATGTA
GTTAGTCTATTCAGTTCCTCGTTTGTTTACCATTGGGTGTCGTACT
TCGAGTCCTTGGCT
CTGCGTTATCCACCAGCCTTCGACAGTCGTACTGTTCTTTATCCC
ACAGATCAGAATCTT
AAAGACTACCTCAGCTGGAGACAGGTCGATTGTCACATAAACAA
CTTGTACAACACCGCA
TTCTGGGCCCTGGTCAACAAGGGTGGTTTTTCCAATCAAGATGC
GCAAAAAAAACTGAAG
GGAACTTTATCAGCTGAGAAGCACGAAATACTATTTTCTGATTTT
GGTATAAACTACAAT
AACGAGCCTGAGCTATACAAGAAGGGGACCACTGTTCTGCGGCC
AAGCACCCCTCTTGAT
TCAGTTCCAGCCAAAATCTGCAAAACTGCCGATAACCCCGAAGA
AGACCCAGAATGTATC
GCTGCTGCAATTCCTGTCGATTCCGGCGCTCCGAATTCTGATGGG
TTTTCAACGCTCCAA
ATGTCTCATTCTCACATCGATATTATCAAAGATACATTTTGGAAT
GCATATCCGACATTG
CTCGGATCTTGATATCAA
>ConsensusfromContig3721 Average coverage: 8.39
TCGTGCAAAATTTCAGCACGATTGATTTAGTTTGTTTAAGACAAT
AACAGTGTATACACG
ATAATCATTAATCAAATGATCGTGGCTGATACACGAAATATCTCG
ATTCTGATTGGCTGT
TAGACCCAACTACATCATATTTCGAGGTCAAGTTAGCACATATTT
TAACACAAATCTTAT
TGGCTGTCCATACACTGTTGTAAATCTTACCCGAACCCCGGGCAC
GGTTCATCCATTATT
GAAGTACCGTTAGGAATGCATCATTGCTGAATCATGACCGGGTCC
AAGAAATTTCAAAAT

CAAATCAAACTATTTTAACCCCCATAGCCGCTCCTGTGACTATGT
ACTGACCGTATTTGT
ATGTGTTATGATCTGTTTTACGAATATCTTTG
>ConsensusfromContig3722 Average coverage: 9.13
CCTACACGACGCTCTTCCGATCTCGTGATTGCCTAAGTCGACGAC
TGCGTCCTGGGAGCC
ATCGAAAATGAAACCGTTGCCGATTTTCCCATTTACGGTCACCCT
CACTCCTGCACCCAC
CAGCCCCACAAGCTTGTAATTAACGCTGAACGTTTTATTATTGGTT
ATTCTGCCCATTTT
GAGTGTCAATCTGTCTCTACATCTTCCATCAGCCATTGTGACGTA
GTAATCATTCTGGCA
GCTACAGGAGAAGTTTTGGGGGCCTTCGGAGCAGATGTGGAT
>ConsensusfromContig3723 Average coverage: 6.42
TTGCATTTGAAATCAGACACATTTAAAAGACAACCGTCTAAGCTC
TTGAAAGAAAATTTG
GAGTGTTTGAATTTGTCAAGACCAGGTCCACTGTACGTGTCTTGA
TCATATGGGCCTAAA
GCTGACTCAAAGTTAAAGTCAACTCAAACTGATAAGTTTGTCAG
GAAGACCTACTGAGGC
AATGTCCGTTGTGACTCGACCATCTGCACAGTATTGTTCTCATGG
ACAGAGCCCACTGCG
ATATTAAAGCAACATTTTTTCGGACAATTTATAAACAGTGATGAG
CCTAAAATAAATCCT
TGAAGTACACCTCTCTAATG
>ConsensusfromContig3724 Average coverage: 5.10
GTGCTCTTCCGATCTTTCCCATTGGCATCCGTACTGGTAAGTCATA
TCCTTTACTTTGCA
TGGTATTCTCAGTTGCTGTCCCCATTTGTATACAACAGTCAATTC
AATGTACTGGAAACC
AGCGAAAAACACTGTGATCGAGTCAGAAATATATGTTGATCCACC
GTTTACCGCTTTGCA
CCAATATTCACGTTTCCCCCTTTTCTTATGTTTATACTTTGTGCTG
TATTCACCATCGAT
TTTATCATATGGGAATTTTATCTCATGAGCAAACACATCTTGTCC
GGATTCTTCCACGGA
ATTACCAATTTCCGGGAAGGCATCATTCGTTTTTTCATCCCCTAT
GCCTAAACCGTTGAC
AATGAGAGATGAGATTG
>ConsensusfromContig3725 Average coverage: 8.09
TTCAATATCCCAAATTCGTTCAACAAGCAACGACCAATATCACTT
CAATCCACTAAGGCG

AGATAATATAATTACGGTAACGAACCATATGGAAAACCTCACTGC
CTCCTTATTACAATT
CTGCATAACCGATCAAAGAGTGAAAATACACACAACTGAAAACCT
TCACCTACCTTCTAA
TAATCACTATATTGACCAATAGGTGACAAAA
>ConsensusfromContig3726 Average coverage: 12.85
CTTTAAAAACTGTAGTGACCGGCAAGGTACAAGTTGAGCTCAAA
CTCAACAACGAATCAT
ACAGTGTGCAGTGTCTGATTATGCCAAACCTATGCACTGACTTTA
TTTTAGGTTTGCCAT
TTCTTGAGAGCCATAGCAGTCTAAAAATAGCATACGGGGGTTCTC
GCCCTGATCTTGAAA
TTTGCAGAATGTCTGCCCTATCGATAAGTCCTCCGTCCTTATTCA
CGCACTTATCAGAAG
ATTGCCATCCCATTGCGACAAAATCTAGAAATTTCAGCGAAAACG
ATAGCAAATTTATAC
AACAAGAGATCGATCGTCTAATATCTGAAGATGTAATAGAGCCTT
CGTATTCGCCATGGC
GCGCCCAAATAGTCTTAGTGAAACGCCCCAAGAAAACGCGCATGG
TCATCGATTATTCTA
GAACCATAAACAGATTCACCATTCTAGATGCTTATCCGTTACCAC
GTATAGAGGATATCA
CTCAAAAAATTGCCAGAAACAGAATTTTCTCGACCATCGACATGA
AGGATGCGTATCACT
CCGTTCCTCTTCAGGATAACGATAAACCTTTTACTGCATTTGAAG
CCGCG
>ConsensusfromContig3727 Average coverage: 11.15
TGGGAGAGAGATTATGGGTGAGAATGGAGAATGGGAAGAGAAA
CATAGTGATAGGGGTAA
TATATCAAATGCAGGAAGGGAAAATGACGAAGGAACAATGTAA
AAAAAGTGTGGAGAGAA
TTGAAGAACAAGTAAGGGACTGTAATCAAGGGGATAAAATTATA
ATAGTGGGAGATCTTA
ATTGTAGAATAGAAGAGGGTGGTGGCAGCAGTAAAATGGGGAAG
GAAATGAAAAAGATGT
GTGAAAAACAATGTCTTACAGTGGTGAATGAGAGTGAGAGGTGT
ACCGGAAAATGGACAT
GGGAAGCACGAGGGAAGAAGTCAGTACTAGACTATCTAATTA
>ConsensusfromContig3728 Average coverage: 7.84
TAGGCATCCAACATTATGTTCGTCATTGGATGTGACACATGGTAA
TATTTAGAGTGAATC
GCCAAGAAAATCACCATTTACCCAGAATCTGTAGGTTCAATGGTT

CTCCAGATAGGCATC
CTACATTATGTTTGTCATTGGATGTGACACATGGTAATATTTAGA
GTGAATCGCCAAGAA
AAT
>ConsensusfromContig3729 Average coverage: 6.10
AAGCCGTATTTTGTGAGGACGCGGACGGTAGTTGCGGTTGAGAT
CTCGGCCTGAGAGAGA
GCATCTGTAGTTGCCCACGATCACTCCATCAGGGTTTATCATTGG
CACGATTTTGAAGAC
AAATTTCTCTCGCAATTTCTTTGCTGTGTTACTAGGCCCAGAGAG
AAAATCCAAAATGCC
TTTCATCATCCAGCTGCTATTGCTCTCTCCTGGATGAACACGCGC
ACTGATGACCACTCC
CTTTTTGTTGCTTTCATTTATATCTGGATCAGTTATTGTAAGCACA
AAGCAGCTATTTCC
AGCTCTAGTCGAACACAAAATTTGCCGGTCAATATACTGAGAGCG
ACATTTATCGGCTTC
CAAAAGGTCAAGGTCGTCTTTGAGGTCAGTGAAGCAGTATGGAT
AGCAATGTGCCAGATA
GCACACGTCATCTTGATGGGGAAATTCCATTTGCCATTCAAGAAT
ATGATATGTTAATTC
TCTACTGAGGATTGGATTCTGTGCAAAGTCCGTGTTTTTGAAGTA
ACTTATATGATGGCC
CACCCGGAACCAGCCCTTCTTTTCGTTCTGAGCCCGCTTTTGAGA
GTACAAAAGCGG
>ConsensusfromContig3730 Average coverage: 13.33
TCAGACGTGTGCTCTTCCGATCTTCAAAAACGAGCTGTCTCAGAA
CTTTTTTATTTGCGG
TGTACTTATATCGACCGCCTTTTCTCTTTTTCTTCTGGTGACCCAG
TCAACAGTGTTAGT
AATGATAACATCAAGAATTTCAAGAATGGTCAGAACACTGCCGC
CAAGCAAGAGCCCCAT
GAAACCACCAATATCGCCAAAGAATTGACCAGTGTCGTAAGAAG
ATACTTGTTTGACACG
GTCCAGCCTCATCTCAGGAAAGAACACATCAACACTCAGGAAATT
TTTTATGAAAAATTC
ATTGTCCACTTTCAGACTTTTTTCATATTCCTCGACGAGCAATGTT
ACTTGTTGTAAGTA
TCCATCTAAATTTGCCTCAAGTGTGAGTATGTCTGCATTGGTTTG
GTCGATCAGCATGAA
GATTGGTTCGAAATAATTATCTAGAAATTCATCAACAGTTGGCTC
CATTGCCATCTCAAC

GAACTCATCGGGATTGC
>ConsensusfromContig3731 Average coverage: 4.43
AAATAAAGAAAACACACAACTCACTTTGATACAAATATCATCAA
AGAGAAGAAAAGCAGA
CAACTCACTTCATAAGAGATGTTATCAAAGAACATGAGCAGACA
TCATAGAAAAATAAAG
AAAACAAACGACTCACTTTGATACAAATATTATCAAAGAGAAGA
AAAGCAGACAACTCAC
TTCATAAGAGATGTTATCAAAGAAC
>ConsensusfromContig3732 Average coverage: 3.72
GCCGATTATTTCCCCTTAGGTCTTCGACTCTCGATTCAAAGTTTGT
TTGGAACCTTTCGC
TTTTCCCGCATGATCGAATGAAACATTCATAACATGTGCTTGTTC
GTGATGGCTGAGGAT
CCATCTAGCCATGGCGCTCCTCTTGATCGTCAGGCCGATTATTTCC
CCTTAGGTCTTCGA
CTCTCGATTCAAAGTTTGTTTGGAACCTTTCGCTTTTCCAGCATG
ATCGAATGAAACATT
CATAATATATGCTTGTTCGTGATGGCTGAGGATCCATCTAGC
>ConsensusfromContig3733 Average coverage: 8.36
CATTCAATTGTGATTTTTAGTTAACCAGTATATATCCAGGAGCAT
AAAACTGGGTATTGA
TTTTTCCCATGAGCGAACTGATCATAATGGGTACGTGGTTTGATTT
TTAGAATCTCGTTT
TGGAACCAAGAAAGACATTCGCCCCTTTCCTTCTCACCTTTCTTTC
CTTTTTCTGTGTAA
AACTGTTTACCTAGTATAGGGGGAAACTTTTTATTTTCGGTAATC
ATTCCAGGGATCCTG
GCAAAAACCATTTGAATATAATATACTCATAAATTCTACTCTTGA
ATCGAATGATTTCGG
ATTGGCTATAATATTTCTTAGTAAAATTTGGTGTGACGAATATAT
GAATGAA
>ConsensusfromContig3734 Average coverage: 7.94
AGTTGGACCACCACCATTTTCTTGCTTTCTGATATCTCTTAGAAGT
TCTAAATGAACATC
CGTTGAGCTCTCTATTATATGTATATAATATTTATTATATACTAA
AAGCATCCCAGTAAC
AGCTTCAAGCTGGAATATGCCTTGCCATTCCTTGAAAAGTTTCTC
ATAATGCTCTGATAT
AGTTTCACTTCCAGACAAATCCGAAATTCCTCCAATCACTGTCAT
CCGATGAAGAATGTT
TTTCTTATTCAATGCTTTATTCTTGTCTTCAATGATATCAAGTAGA

GATTGATTATGAGC
ACCAATGAGTGATTCATCATCTTCATTATCGGAAGGCATTTATAA
TATACTTAGAGAAAT
TT
>ConsensusfromContig3735 Average coverage: 7.34
CTGAATTCAAATGAACGTAAATGTGATTCTCTATAGACAAATTGA
GACAAATTGCAGCAC
AAGACCGATCTATCATTTGAAGTGATCAACAGTTCGATGAATCCG
GTTACAAGCATACAA
GACGCCGCGTAGAATCTGTGAGCTGGAGTATGCGGATTGCAAAAT
TCAAAATCGTTTCAT
CTCGAACCGATAATACGTATCGTTGTCGTTGAATGCCCCACATAA
TAATCAAGAG
>ConsensusfromContig3736 Average coverage: 8.01
CTGCTTCCGATGTATCCATGCAAGAGCATCAAGCTGCATAGCAAC
CAATTCAGCTTCCGA
GCCGACTGCCGTTTCGAAAATGGAAAGAGCCTGTCGATAGAGAG
CTTCAGCGGCACTGTA
TTTGGCCTGCGCTGCATGCAGAGAAGCCACCTGGCGGAGTGTTTC
GCCGACAATCACGTG
ATCTGGGTCAAGGACAGTCTCGCGGAGTTCAAGGCAACGTTGGGC
TGCGGTCATCGCATC
GTTTAGCATCGAGAATTCATGGAGGAAATGGCTGAGAGCTTCGTA
GATGTCTGCAACTCG
CTTTAGAGAGATGCCACCTGAATCATCACTGCACTGGGCCTCTCG
CTCCAAAACTCGTAA
ATGAGAGAAATAGTCAGAGACGAGATCAGTTTTGTTCCATTCCAC
ATCCTCGGACCATTC
GGTCATCAGTTCGAAACCACGCCCTCTGCTGAAGAGATTGATAA
AGACGTGAATATCAGA
TCGTGCCAGCTTGAAAGTGGTGTGTTCTTTGAGCTGACATGAAAA
CCAAATAACTGCCTC
GGAAATTCGATATGATATGAAG
>ConsensusfromContig3737 Average coverage: 4.94
CGATGGTGCAATATAAAATTAAATTATATAACGATCCAGAATCCC
AAGTTTTGGCGAGTT
GATCGTACTCCACATCTGGGCAATTTATTTTTTTCACACCCAAAT
AACTTTGTGAGACAG
TCGTTAATCTTCGATCAAATGGGTTGTGTGGATCATCCACCAGTG
GTTTAGTCGGCACGT
CATTTAATAAATCACGAGAACCCGTATTGTTCATTAACAGAGCAG
AAATATTCCACGAAT

CAATTGTGTGTGTTCTCTCTTTTAAATACTCACCACCATCATCACT
GTCATTCATAGCAT
CGTCATTCTCATTGATATCATCGTCCTTTCGAAGAAACGAAGTTAG
AGCATCATTTTTTT
TTTTACTATCATTTTTT
>ConsensusfromContig3738 Average coverage: 6.94
AAAATACAGTTGAAAATCCAGAAAGGAATGAGAATATATGTTTA
TTCTAAAATACCCGAA
CTCCTGGATACGGTCTTCATGGTTTACGAAAAAAACATGCACAA
ATATCGTTCTTACAC
GTTTTCCATCACGCTAGTGTTACTTTCATGTGCTGGACTCATGTG
AAATCTTCCGTAGGA
TTTGAATCAGGATGGGTCCAATAGATCGG
>ConsensusfromContig3739 Average coverage: 4.05
ATGCATATACCGAAGATACATTGAATTATGTCGCCGTTCTTTCCA
GGCTATCGATGAATG
ACTATGTACATTATGACGATGTTATTGATGGGGCTGTTGAATAGG
ATGAGGATAATCATG
ATGAATATGATAATGAAAAATTGGTGATGAAGATTAATGTGACTT
TCAACACCTGCTATC
ATTGAAATAATTGACCAGGACAGATCACAAGACGAGAGCTGTCA
AAATGATTTGTTATAT
GATTATGTATAAAAATAACTTCACACATGGCTTGTCAAGATGATA
GATCGTCTCGACGTT
GTTTGAAAAATGGAATAAAATTTATCATC
>ConsensusfromContig3740 Average coverage: 8.65
TCTCAATAGCTGCTGTAGTTTTGCCCATCAGCTGTGCTTCTTGGG
ATAAATAAAGATCTA
GAACAGGCGCTTTTCTTGAACGAATGTCTGTCTCTGTTAGTGAAT
TGACCATGAGCATGA
CCCATATCGGTCTTTTTCTTTCCCAATCACCTACAATGGCTTTGA
ATAGATAGTTGGGAA
ATAAGCTTTGACTGTTTGGTATAGACTCATCACCATGTTCTTCAA
TCAACCAAACTGGCA
TCATTTTCTTTATGTATGCTAAATGCTTTCTCAATCGCCTGTATAA
ATCTTTTGGAATGA
TATTCGAAAGAGTCTCACCGCGGGGCAGCATCTGGCATTTCGATA
AAGCAGTTAATGTAT
AGGGATCAGTGAGATCCAATTCGAAAAGTATTGTATCTGATAACT
CAAAAGCTTCTTTAA
CTTGGCTTGATACATATGGCCA
>ConsensusfromContig3741 Average coverage: 6.26

TACCTTCTTTCTTACTGTGGCACAATCAGTGTTGGTGTTTGATCCA
CTCAAGGTCATTGC
CTTGACACTCATTGCAACTATTCTGATGCGGAGCGCAGTTGGTGG
GCCAGAGAATGATGA
GGAAGAAGAAAAGTCCTATGACCAAGTAGAAGACCTCACAGAA
GAAACCAACGAAGAAAA
ATTAGAACGGGAAAGGCTGCTACAAACCCAAAAGAAAGCGGGGC
GAAAGATGATTCGCGA
AATTCTCATCTTGTCTGTCTTCATCGCTGTCGTTTACTGTCTTTTA
GATCGGGAGAGCAC
ACATCT
>ConsensusfromContig3742 Average coverage: 8.41
ATTTGCTGGTTTGAAACCTGTCATGTCCGATAATTTCGAGAAATA
TATAAAAGAATATAA
CTTGGGAGGTCACAATAAGATTTTACTGGACAAAATCCAGTTAGA
TTATGAATGCTGCGG
CGGTTATAGATTTTCCGAGTGGTTTAAGGAGCCATGGCTCCCCAA
TCGGATTGAATTACC
AACTTTGAGAGGAAAATGGAAAGTTTCAGTGCCTTTCAGTTGTTG
CAATCCACACTCATA
TCGCGCATGCGTTCACTTGGACATGTTCAACACGAGCGTCCATCA
TCATCACGGAATGCG
CATCGGTGAAGTGACGGTCAACGAGCGGGGTTGTGTCAAGGCCGT
TCACGAATATTTTTA
TGTTTATATCCTGATTCCATCGGTCGTTCTATCTTGCTTGCTTCTT
GTTTTAGGGGTGGC
AAACATGGGAATGATGCGCGCAGCTTTGAGAAAAAGCCGTTCATT
CCGCAGTGATTATGT
TTCACTTCAAAGTCAAGAGTCAGAGCAGCAGGAGCAGCTGCTCA
C
>ConsensusfromContig3743 Average coverage: 8.36
GACGTGTGCTCTTCCGATCTATCACGATGGTCCCGATGCCCCCTC
CCGCGAACGCGATTG
AGGAGGCCAAATCCCGGTAATTGGTGAACCATCTTGCTATCATCG
TGTTAATCGGACTGT
CCAATATCCCGAGGCAGCTCCCAAAAACTATTCCAAGAAAAACC
AAGAAGCAGACATAGG
CTGAACTCCAGGCACAGCAAAACAGCGATGCTCCGCCCAGAAGA
CCAAAGAAGATAATGA
AAGACCTCGAAAATGCTTTAT
>ConsensusfromContig3744 Average coverage: 7.75
GAGTTAACCCGCACGAATCACATACCAACATGATGACAGAATTTC

ACATGTATTTAATAA
TTAAACATTGTGAGTAAAGCACAAGTAGTCAAGCGAATTGTTATC
CAATCATGATTTATC
TTTGGTGTGACTTTGTATATGGTGAGTTAACCCGCACGAGTCGCT
GAAAGATTTCGCACA
AAAGTGACACATGTACGGCTTCTCACCCGTATGTATACGCTGATG
TATTCTCAGATAACC
AGAACTAGCGAATGATTTCGAGCAGACATCGCACGTGTATGGCTT
CTCATTAGAATGGCT
TTTAATGTGTTGAAGTAGACCACAATAATCAGAGAACAGCTTCCC
ACAAAGGT
>ConsensusfromContig3745 Average coverage: 57.33
AGAGCTGGCATAATGTGTTCTAGTGATAAAGAGTTTAGCGCGAC
ACCAATTGAAGAACGT
ATCACCGATACGCAAAAGTCGATTCTTGATTTTAAAGATTACGTT
GAAATTTTAAATCGA
GCCCTCTTGAAGAAGTTTTCGCATGTGGGAGAAGTCAAAGAAAA
GACAAAAGTAGGAGAA
ATGGGATTGTATGATCAGAATGAATTGATGAACGAAGAGGCTAG
TATGCAGAAGATTCAT
AAGTCGAACCATAAAGCCCTCTCCTGCGATGACTTGAGAATTAC
CAAGGATTTAGAAACA
GGGATCAGAGCCGAATCTTTACTAACTATTTGTGATTATTGCTGT
AAGAAGAATCACTCT
TCAGGTTCGCTTAGCTCTCTCACAACTGGCTCTGAACATTGCTCC
ATAAGCGGCCGTAGA
TTAAACACGAGAAGGGAGAGCTACCCCGCTCGTCGTCGGGTCTG
TCGGAAGCATCG
>ConsensusfromContig3746 Average coverage: 10.41
TCCGAATGAAAATAAGATCCAAAATCCACAGCGGCTCTTATTTGA
GAAAATCGCTCAGGT
AGAAAATCCTCGGCAGTCCCATATTTTGGGTTGCGCCTTCAACCC
CGGCCGGTACTAGCG
GAACTCCCTCTTGAGCAACGTCTAACAGCCATCTGGCGCCTTCAA
GGGTGTAGAAAGAGC
CGGCTTCTGTTTGGCCAATTATTGCTTTTCCGTGACTGAATAATCC
GTTTTTGTCCAATT
GGTTTTGGTAGAACTCCCCCCTTGCGACCTTTGTCCACTGTTTCCT
TTGTGGTTCCCATT
TGAAGCATGTCCATTTGTTAGTTACCGCAAAAAATCCGATTCCGT
TGTGAGCCACACCCC
GGATTTCCATATTCTGATCAATTTCACATTTCGGAATCTTCTGCCA

TTTCAGTGCGTCCA
ATGGAACCGAATCGTCAACCATCTCCAAACTGAATGCTGCTCCAT
CTTCATCAATGGCAA
TCGCTTGATAGCCTGAAGTTGCAGTGTAATATCTCGTGTGCTTAT
AATCTGTATAATGAG
GATCTGTGTTTCCCTCATTTGCGATCTTCGAAGTCTTCAGGACCA
GATCCGGTCCTTGCA
CCCAGGCTGGCTCGGGATGTGTGGTATCCAGAAAATACGAGAACA
TCTTCCCAGCCGTTT
CACTTCGTTGTTAGATCGGAAGAGCG
>ConsensusfromContig3747 Average coverage: 9.39
CGTTTTTCAGAGAGTCAGCCATGTCCCCGCGCCGGTAAGCATTTT
GACTTTGTTTTGACT
TCCATATGCCAAGAATTCCGATTGGACAGCAACAAAATAACGTC
ACAAGAACATTGGGAA
TAAGAATATCATATGAGATCCCGCCACCAACGATTATCGGATCG
GTTGAGGAGACAGTGG
AAGTCGGATGGTTTATTGAATGATCTGTTATATTCATTATTGAGC
CATCACTTGAAAATT
GCTCAGCTGGGTATGAGTAGGATTGTTGTGGTGAGAGCTCATGTG
TTCCGGGAAGACGAT
AGGGATGATATCCGATGGATGTAGAATAACTGGGAGGAAGTTCA
GCTGGATATTGCTCAT
TCTCATGCTCCATTATATGATGTATTTAGGAAGACTATTTCGCGA
CCAAAATACGTACGT
TTC
>ConsensusfromContig3748 Average coverage: 6.07
TCGAAATGTTATTATAGAGAACTCACTAGCATGCGAGTTTCGAAG
GTCCACTAGGTACTT
CACTTCCGCAGCGCAAGAATGAAGACTTGTGGCCAGTTGCCGACG
CTGCTTGTCCCTCGC
GCACCCTAAGACCTGAAGCGTGCGCTGGTCCATGATGGTCCCGA
CACGGAGGACATCTTG
CTCTTCGGGGGTCAGGTCTGCTGATGGTGTCTCGACAGACGCCGT
GTCCTGTAACACCCA
GTCTCTGGCATGTTCCATTGTGACACTCCTTTCGGCGAGAGAGCT
TTGAAGAGCGATTTC
CGCTACTTCTCTTTCATGAGATCGGAAG
>ConsensusfromContig3749 Average coverage: 5.14
GGGTTTTATTCATCTTATGGCAATTGTGATCGCTCTTATTGCTTTG
GAAATGCTATCTAT
CAGGAACTTTACCGAAACATTGAATACATACAATGGAAAGTCCG

TTACAGAAAAATCAAC
AGAAATTGAGTTCAGTGCACAGAATGCAGAGTTTGATAAAAATG
AAGATGTTGTGCAAGA
AGAATCAAGAGTCCGTAGATTCGTTGAGAAAAGCAGAGATTCAA
ACCGAAATTTTGAAGG
GACCGAAATTGGGTCAAGTGATTTTTTGGCAGTCGGGTTCGAATT
GACAAAAATATATGG
TGGCAG
>ConsensusfromContig3750 Average coverage: 27.31
GCCAGATATAAGTTTATTCTCAAAATTAATTTTATTTGTGTCTAC
GTATACAATTGTGAA
TGGATTAAAATTGGATCTTGATGCCGTGAACTCTAAACATTTAGA
ATTCACTATTAATCC
TGATGTCACATTGAGTACTATTACTACATTTCGCTTTCAAATATT
TCTCACAGATAGGAA
AATTTACAGAAAGGTCGTCGGAAAATGTGTTTTCGGATTCGATAT
TCCAGTCACCGGGTC
TCCTGTCATTGAAGAGCCGACATGTGAGCAGGCGACTGCAAAAT
GCAAAGAAGGTACTTC
CTGCAACCCTCCGGTTAATAACGGAGGCCTGGTTTATTCTGTTAC
GACCGAAAAACACAT
CTGCCAAAGTATCCAAGTTGTCATTGACTTATACGCGGCGCAGGA

>ConsensusfromContig3751 Average coverage: 5.53
TCAGAATTACCAGTGGGAACCGTCATAGGGTTCCTAATCTCTAAT
TTTTGAATTACAATA
ATTGAAAAAATGGGCCTCTCAAATTTATCGCCGTTTCTGCTAAGA
CTTTTGGTTTCGCAG
TTTCAACAATATAGCAATCATGAAATGAATCCTATGTCCAGCTTG
ATATTGTCACACATC
TACGGGCTTGTTATTTCATTAAACTAAATGAGCGTGTAATTTCGT
TAAACTAAATGAGCT
AAATATGGAAAATAGAATCTTGGCAAATATGATTGTTTGTTAGGG
GTGTCGAGTGCGGCA
GCTATTTCAAGCGGGAGCGAGGAAGCAGGTTTGAAAGGGAACCT
GAAGTGGGTCGGATGG
TTTGGGGCTCCTCCGAAAGAATAGGGGTCTTTCATGCTAAGATGA
GAACCGAAGCCATTG
GAGTGGAAGAATGGAGGAAGGCTGTGATTCTGTGCAGCGGGGAT
GGGACTATTGATGT
>ConsensusfromContig3752 Average coverage: 5.58
CAGGAAAGCGACGCATAAAAACTTATCAATGGTCTTTTGTTGAAA

AAGGAACGTCTGTGT
AGATCAACTGCATGTCTTTATGCACAATGGTTTCCAACATTGTAA
TAGTTTAATAAACTT
GAACATCACTGAATATAGTACTGAGATACAATCCGCCCGAAATG
CCGTGGCTATGAGGAA
GTATATTACAAAATTAGCTGATGAGTTGGCTTGTTCTGCTATCTC
GGTGATGAGAAAAAC
GAACCAGTACTGCTCAACATCTGACCAATACTTTTCACCAGCTAC
AATCACGAAATATTG
AGGGATGATAAGAA
>ConsensusfromContig3753 Average coverage: 7.35
CGCTCAGGACGTCGGTTAAAATTACTGTATTTCTTTTTTTGATCT
CTTACTGTTTTGCG
ATGAATCACATTTTGAAACATTTATCCGAGGAGTATCATTGTTAT
TATCACCAGTATCAT
TCAAAGTAGTCTCATTATCATCCGGAATTAATTTACTACGTTTAC
TACTAGATGATGATG
AAATATTCGAATCACAGTCCGTTGGGATCGAATCAGAATTACGGT
CCGTTACACACCTCT
CCGAATTACTAATAACAGTCCATGCTGATTTGGACAAGTTGTCCC
TCTCGACACATTTTT
CAGGTAACTGGGACACACTAGATTGACACACGCAAACAGTCGCA
TCGGTCGCTAATTGCT
GGCGATCACAATAGCGACACCTATTTTCGCCTTGAGAGGGACGGC
TACCTGAGATAGATT
GAACGCTTTGATCTCCCGCCCTGGATGAGCCAAAGGACGTCCCTG
ATTCGTTTTCTAACA
AATGTCCGCCAATGGGATCCCATGAATCGCTTTCGTTCACAATTG
ATACGCCATTTGA
>ConsensusfromContig3754 Average coverage: 5.89
GATCTTACTGCCATTTCAAGGTCGCTGAGGAACTGGTGACCTTCA
TCGCTCGAGAAAGAA
ACTTCTTTGAGGCGACTTCTCTCATTAACACTCCTAATTATGAAA
GCGAGACGGCGATGC
ATCTAGCAGCCGAAATCGTCAAAACACAAATCCACTACGAGTTT
GAAGATATTGACGTCA
TCAAACTGCTTTTGACGCATGGTGGAGACACGAGTACCCAAACG
CGGCTGACGCTCGAGA
CCCCGATGCATTATTGTGCTCGAGC
>ConsensusfromContig3755 Average coverage: 23.50
TCTCAACAAGTTCAATAAAATCAAAGGAGTCTTGTTGAATAAGAA
TCACCAATTCAATGA

TTTAAAACTTGTTTGAAATTATTACCTTCGATAATGCTAGGAGTC
CCACATAATTTAGAG
TGATCTTTTCGAGCTTTCGTTCTAGTCAAGTCGATCAAACAATTC
ACGCACCGACCGCAC
GGGACGATATCGATTCTATTTATTTATTTTATTTATTATTCTCATT
CTCGATTCTCACAT
ATTGATTTATTTAGTCGATTCTTGATATTTTATAAAATGAGAGTA
TGGACGTTCAAACAA
AATCTAAGTTGATTCGTGGGGCTGGAAATCGGGATCAGGTCTCTC
TGAACAACCCTCGCC
TTTCAAGAGATTCAGAATACCGGAGATCGTCAAATACAAAAGAC
TTGAAAATG
>ConsensusfromContig3756 Average coverage: 9.68
ACGCTGTCGATGCCCTTTCTCGTTCACAGATCGGCAAGTTTCGGG
ATGGGCATCCGGAGG
CTCTGGCGGATCCGTCCCAGATGGTGCGGACTCAGAAGCAAGTCT
TCACGCCGCGCTTGG
GGGCCTTCTGAAAGCTTCGCTGTCAACCAACACAAGAAAATTTTA
TTCTCGAGTGTTAAG
GGAATTCCGAAAATTTTTATTCCAACATCGCCGACCTCTAGTTCT
TCCAAATAGCCCTGC
CGATGTCGCACTTTACATAGCGCATCTTCACTCTGCAGGTCGAAA

>ConsensusfromContig3757 Average coverage: 4.88
AAGAAGTTTCTTGGGGGTCCACAGTTTTTGGACTTCCAAGAAAAA
GGCCCAATTAACGCC
GGTCAGCGAATTAATCATTCGGCATATATGATTTTTAACTCGGAT
GTGGCGAATGCCATT
CCTGTCGCCCAGGCTTACTTAGGACCTCCGCAACTGGCAGACCCC
CAGCCTCCTGGTCAA
GTAGGCCAAATGGTCAGACCTGTAGTGATGGCTACCCTTGGAGA
TGATGGTAGAAGGGCT
CGAGCACTGGAAGCGCTGCTGGAAGCTTGTATAAGCGGACCGGC
TCGCGCTCCTAGAGGT
ATAGAGAT
>ConsensusfromContig3758 Average coverage: 7.28
TTCTACAGATGCCTCAATAAGCAGAAGTACTTCTTCAGCTGTCCT
CAAGAACTTGTTTTT
GATGAAGACAAGCTGATGTGCAAGCCGGTTGAGGAAGTTCCGAG
ATGCAAGGATGAAAAC
AGCGTTCCTGCTGTAGATGGCGCTAAAGCAGCAAATGTCTACGA
GAACAGAGGCGGTGTG

GACTATCTCAATATTGTGCGGAGGATGAAAGAGCAGCGAATGTC
CAAAAACACCGAGTAC
ACATCAGACGAGAACTTCCAGTGTCCGATAGATGGTTATTTCCCA
CACATCGGCAGTTGT
TCTAGATTCTTTCACTGCGCAGACGGCGTCGCATACCCACGCATT
TGTCACGGCGCTTTG
GTCTTCAATGCAGACACCCGGATCTGTGATCGGCAGATCG
>ConsensusfromContig3759 Average coverage: 4.26
GATGAAATACTTGTAGCAAATTCATTTGGTGTTTCACATGGTTTA
AGCAAAGGTAAATCA
TTAGGTTTGAAACTCAATTTACCGTAATTATCGCGATTTTCAAAT
TCTGAATGATTGAAC
CATGAAATTTGTTTTGATGAACTTTTTGTAACAGATTTATTTTGTG
TTCGACATGGTTTG
AGATATGTTAACTCATTATGTGCAATAGTGAATTGACCGTCATCA
TAATGATCTTCAACT
TCTGAATGCTTAGACCATAGAATCTGTTTTGATGAACTGTTCGTA
ACAAATTTATTTCTG
GTTTCACTCGGGTTGACCAATGTCAACATTTTAGGTTC
>ConsensusfromContig3760 Average coverage: 3.79
CCTGTCTCGACATTTTTCTTACACGATTTGAAAAAGAGGAGGTTC
GGCCCAAATGAGTTT
TACGACAAATGCTTTTTGATCATGAAGAATGGATTGAAGCGGAC
ATAAGAAACATTTAAA
TCGAGAAAATCGGCTCAATAAAACTACTCAAACCTGCTAAGCATT
TGTATAACACATAGA
TACCATGAAGACCATACATATACCTTTGTTTTCATGTTATATCATG
CAGTTATATTGTT
>ConsensusfromContig3761 Average coverage: 4.75
CAGTGTTTCATCCCGGGAAAATGTTTTGTTCTTGTGCTGCAGATGA
TTTTTAAAACGTTC
TCAGCTTTTTAGAAACTAATAATATCTGATACTTTTGGTATAAAA
TTGACTGATAATGGA
CTCACGCATGTATTATGCAAGATCTGTGTTATCTCAATAGGATTT
GATTATCACCCACTT
CTTCAGTGTGGGAGCGATAGGGTCATAGATTTTTCAAACACGGG
>ConsensusfromContig3762 Average coverage: 9.22
CGAGCCAGGATCTCTTTCCATTCGTATATTCAGTAGAGGTGTACC
ACCGCTTCGATCAGC
ATCTTGTAGACTGGGTGGCTGGTGGATTACAGTTCTCATTTCTTCA
TAGTCATTCATATT
GGTAATTTTGGTTGTAAATCCATTTTCACTTCTTGTGACATTTTCA

GATTCAGTGGTGAT
ATTTGAGTTTCCTTTTCCACATAAGTGAGGCTTGATTAGCCTACCA
TACTTTGTTTTATG
CTTTGGTGGTATAAAGTCTTGTATATCATAAAACAATTGTTTCTGT
TCTGCAGTTTCAAA
TAATTTCAGTAAATGTTCAACAAATACATCTACCTTCTTGTGTTTT
C
>ConsensusfromContig3763 Average coverage: 7.10
ATACTACTGCTGATAAAAACACGGAAACATAAAGCTACTAATTT
TGAAGATATATAAAAA
CATAGTTTTTATGGAAAAAGATGGGATGCTAATGGCTTGGGGGC
GCATAGGGAACAAGGG
TGCATGGAAGTGTCAAAAGAAAGAATTGAGAATAAATCCAGATG
AGCATGAACTCTCTCT
AGCTAAAGACGCAGGAATAGTCGAGGAATATCGTGCGTTTCAAC
ACGACGCCCATGGAGC
ACATACGTTTGATATTGAGGTGAATGGTCCCCGACCCAAATGTAA
AAGCGC
>ConsensusfromContig3764 Average coverage: 4.73
CTGATATGTATGGTATTTGTTTTTAGATTATTCAGAGTAAACTGT
GCCCGATATGAAAAC
TTCTGAGAATTTTCCAGTGGGAAAAACCCTAATTGACACGAAGCT
TGATAGAAATCCCCG
GCAGAAATGACTCCTGATGGATTCCGAACACAATTCAGAGATTC
TATCAGCGAGACGTGC
GTTTCCAGCGTTTCTTGTGAAACCGAATGAAAAGAATATTGACAA
TAGTATATGCCATGA
TCACCCCTAACAACGTTGGGGATTCTGATGCGAATTACACTATAT
TCTGAATACTTTTCA
TCCTCCACTTGGACAATATTCCAAGTCTCTTTGGCTGCGGTAATG
TTTAAACCATTCGTG
GATAAATCTACCCGTTCATCATTATCAGACCGGTTACGATGTAAG
CTCAATGCTCC
>ConsensusfromContig3765 Average coverage: 5.26
GACGAATTCATTCGATCGGACCGAGTTACGATGCGGTTTTGAATA
TCTGTATGCATCGTG
GTTCTGGTTGTCGTTGGATGTGGCGTGTGAACCGCGATTTTTATGA
TATCCACCATTGTT
TCTTCGTCCATGACTCGAGGTAGGTTTATTTCTCATATGAGATCGA
TGATTTTCATCATA
ATTTCGATCATTCCAAGAGGCCTTTTTCGTTGGGTATGAATTGCGG
TCTCTATCTGCACT

CTCATAATAACTGTTCGTACTAGGACTGGAACGTGAA
>ConsensusfromContig3766 Average coverage: 7.57
TTGTCTTTAGTTGGACGGAACAAAGACTCTTTGGACTCGAACATC
CAATCATATTCTGAA
CCTTCATCGAACCCTTTGAAACGAAACCCTTTCGGTAGTTCTATAT
TGTCTTTTTTGCAT
TCTCTCGCGACTTCGACGATCGGTTTTGCTTTATGAGTCTTTGTCA
TTTCCCTCGTGACC
CTACGTTGTAACTGGTTTCCACTCTGCTTGATTTCCGCTTGACCCG
TATGAGATGTTGTC
TGATGAATCGACGACTCTTTCTCATGGAAATATACTGTGAAACCG
TTTTCGAAAGGTTCG
ATGCTTTTTATTCTTTTCATTTCTTCTGAGTCAGAATCAACTTCCA
CGTGATATTGCATA
TTTTTGTTGGTGTGAACATAAGAGTTCTTGCAGAGTTCGGTGAGA
TCAGGTGCCTTCAAA
GGTTCACAAAGACCCGACAAATTTTTCTCAACATTAATCGAAGAC
GCTTTCTCTGTG
>ConsensusfromContig3767 Average coverage: 9.09
ACGCTCTTCCGATCTATGGCTGACTTTGTCTCATGCTGACGTCTGC
GACTCTCGGATGTC
CCCAGAACCATTTCTCCCCTCATGGCCAGGTAACTCCTCCCCTTC
CGTCGCTTCTGCGCA
GAACAAGCACTTCCTCCGGCCCGTTGTCTATCGTGTTGCGTTGAA
GAGGTCATTCCCAAA
ACAGCAACTGAAAGTTTGATTTCC
>ConsensusfromContig3768 Average coverage: 7.85
AAGAGCTGACATGTTTTCAAAGATATGGTGGCGGAAAATGCGGG
TTGAATTTATGACTAA
TATGAGAAGCTGGTCTGGTGCTTGGACTGTGATTGGATGGACATA
TATGCTATCAGGGGC
GGTAGTAATTTCGGCTTACATCGTTCTCCAAAGTGCCGGATTCAA
TCCGGCGAAAATGGA
TGGCTGGATTTTTAATTTTTCTATGTCTGTAATTTTTTCTGCTATC
GTACTCGAGCCAGC
ATTGATTTTTTTTCGAGTTACGGCGACTTTTCTTGTGAGGAAAATC
CCATATTTCCCGTA
TCACCACGAGTTGATGCTGATGATTATAGCTAGGGAGGTGGATTT
TGAGTACGTAATTAT
GAAAAAACCAATTCGACAGAAAAAGCAGACTAAAGGCTTCCGTA
CACTTGCATTGAAATT
AAGTCGTGATGAAAAACTGAGAGAAAAATTGAAACGCCAAGAA

AAGAAGCAAAACAGGTA
TCCCAGAATAACAAAATGTAGGAATCCCAGAAAAAGACCCAAAT
CAATTT
>ConsensusfromContig3769 Average coverage: 9.99
CTCCGCGATCCATTGCGTCTTTGTAATATTGTTTTTCATGTCATTT
GTCTTCATATAAGT
ATTTTGAGGAGGTGTATTTTCTAACTTCTTTCATTTCCAACTATCG
TTTTATATATGTAC
TATCCGATTATGGTAATACCCATCATTTATAGCATCCTACTATTTT
TCGTCCGATCCGTT
CAGTAATAATAATTCTGAATATGAATCTTGTTTTATCTGTGAATAA
TGCGTCTTATATTT
GACCATATATCTCACTCTCGTTAAACGTTATTCTAATTTATAAGA
CGATCATCGGCTTCA
CTGAATATCATTATTGATCCTATTTCTCCGAAAGATATTTTCTATA
ATTGAGGTTTCACC
A
>ConsensusfromContig3770 Average coverage: 5.83
ATCAAGAGTGTTCAATTTGTCTGGCCCGTGCACAATGCTATGATT
GTATTCTCACCATGG
TCTCACGTATCAATCAACTTGCTTCTATCGCTCACCGTGTTCGCAA
TCTCTCTGATCACA
AGCCCATCACAAGAGGATAAAAAGAATTCGTGTCCCATCACAATT
CCGTGTGCATGCATA
GGAAATGATATTATGAATTGCAGCAGTCGCAGATTGGATTATATA
CCCTCGCGAATTGGA
TTTTGGAAGACGTGGCAGATCTTAGATTTGACAGAAAACATACTG
ACGGCACTCCCGGCA
GATCGGAAGAGCA
>ConsensusfromContig3771 Average coverage: 22.81
GTTTACTGTCATTCTATTTATAAGAGACTTCAATTCATTTTCGAG
AAAAATATATAATTG
ATCTCAATTTTTTTGTGCTAACCTTAGGTGTGATATATTTGTTGTG
AACAATTCTGGATT
ATTTCATTATACAAAGAGCGGAATTCAGATCAGATGCTGAGGTTA
ACACCATCTGCTCGG
ATGTGGACGACTGAATTGGCTTTATTCAGTACTTTGGTACTGGTG
CTGTACTTCTTACCA
AGCTCTTCAGCTGGTGCACCTCCGAGTCTTACCAGTTTCGTCCGC
AAAAACGAGCTGGCC
ACGGATGGAGACGCTTTTGTGAATCTGATTTGCCCTGTGGAAAAC
TCCCAGTTCGTCCGA

TGGTACAAAGTGGGCGAACCAACGGAGCTCATCGACCAGAGCTG
GCTCCGGTTCGCCTAC
AAGAGGCAGCGTCTGAAGATACATGACGTGAACACTGCTGACTC
TGGCATCTACAAATGC
AAAGCCACCAACGGCTTCGGCCAGGTCGAGGCTCAAGTCGCTCTC
TACGTTCGCCCTGAT
ACAAACGATTCCTCGGTGACGTTGCCCCCTGAGTTTCGTACAGAG
AATTCTGTTAAGC
>ConsensusfromContig3772 Average coverage: 5.29
ATCTAAAATATCAAATATTGGGCCATTAATTGAGAATTAAATAGT
CCCTCATTCGGCTAT
GATAATGTGACTTAAGCCAGGTTTACCATGAAAAATGCGAAATG
ATTCCCGAAAGGAACA
AGGCTATATAATATGGATTAAAAGGAGACTTCGCCACCTTAGAC
GGCGATTGAGTTGGCG
CCACCTGCGAACGGACGTGAATGCCCCTGTCAGGTTACTCCTCTA
CAGTCAATACGGCCG
CCATCTTTGGTATGTGACATACTGCAACCTGATAGTCATCTATTG
GTCAGTGTTCATCAT
CATCTTGTTTATCGGAGGAATGAGCACAGGAGAAAACAGTTCGT
TGGGAAATTGGGCGCT
GCTCGCTTATTTCATCACGAACACCTTGAGGGCCGCCGGCGTGAC
GTATTGTTATTTTGT
ATCCGATCATCA
>ConsensusfromContig3773 Average coverage: 13.55
GACGACTCGTAAATAAAACCAAGTATCACGTAGCTGATTGACAT
GGAAATTGATGATCGG
TTATGGTGCTTGCTTACAATTCATGAAAACATATAAAGCAAATTG
AGAAGGTAAGGTTTT
GAATATTTGGCTTCATTTGATTGTATATATCTGACATTCACATATT
GCTCTCACGAACTT
CCAACAGAATAGGACTACTTGGACATGAAAAATTGAGCTGATTGC
TGTCCTG
>ConsensusfromContig3774 Average coverage: 4.44
TTGAACACAGTCTGTGTGTACGATTTAATGGACAAACTGGAGTAC
AATTTTCATTATTGG
ACAGAAATAAAAATCCCCCCAGGAACAGACAGAGTTTTCGTCGA
AATCTCTAAATTAATG
CAGAGAAAAGATGAATACCAAACAATACTTGTGGAAAACATGAG
AACTTGCCATTTTTTC
ATATCCATTATCGACACCCTCAGCAATCAGATAATGTCACGATGC
CAGAACTTGGCGGTT

TTTTTCGTCGCTATTTTGGTGTCCATGGTCATTCATATTATAGAGT
TTGATGAAGATTCA
GATCCCATTGATGAAATATTATGGACAGCGCCGGTGACACAGCC
GATAGCAA
>ConsensusfromContig3775 Average coverage: 6.98
CTTCCGATCTGTCAAAATAAAGTACGAGATAGTTTTGGCCTCTCA
AGCACTCGAGCTTCC
TGCTGTAGAGCTCAGTCTCAAAATTCAGAAGATTCATCGCCACTG
AACTATTATCGCGGG
CACATTTCTCCACAATTGCATCTAGCTCATCAAATGTAGGGGCGA
AGTTACCCCTCAGAA
TTGACAAACACAGATCATCATTGGTTCTCAATAATAAAGCGCCAT
CTGATGCCAGAAAAG
AAACCATATCCGATGTACAGGTCTCCTCTTTTATATCCGAGGCCT
TCCAGCATTCCGTCG
TCAATCCGATCTTTAAAAATTCCTCGATTGGATTCGATAAATCCG
AATTTGTCGAATTAT
AGATGGCGCATGTCGAGACTGCAGCGAGCAGCTTTTGGTTAAGTT
GCAGTCCATCTCCAA
CTGTAAGGAATCTGCAAGCCGCTTGTGTGTCTTTCTCTGCCAACA
AATTAAGAACAATTT
GGAGATTTGGATCATTTAGAACTGCCTTCGCAAGATTATCGCATT
CCTCACCCTCCGAAA
CTGTTATCAATATGCATAGTGTTGAAAGCAGTCCAAATTTTATTTT
TGGCATT
>ConsensusfromContig3776 Average coverage: 11.36
CGGAATATTATTTTCATGCTGTGCTGCTAGTCATATTGGTTGTAGT
ATTTCCATAAGGAT
TGTGTTTTTATGACAGATATAATTTCGGTTGCCATATGATATAACA
ATGTATTATTTATT
ATCGGATATATTTGATTTCGTTGATAATTAAATGTAATATTACAT
CCATTACTTGGAGGC
TGCCTTTCAAATTCTGAATACTGAAGGCTAACCGAACTATTCTCCT
ATGATTATTTTTTC
CTCCACGATTTTTTTAGTTTGGCTATGGGAGTCATTCTCGGGCTTT
GCATATCTGTTTGG
TTTTATACATTTTTATATAATGCCATTCGAGTATATTCGAAGTACG
ATCGTGTTGGTATA
TTTAAGAATATGTTTTTTCTTCTCCTTATGTAGCCCACTCTTTGTT
GTGATTTCAAATCA
GGCAAGATACTTCTGAATATTCTACTATGTTGAACAATCAATGAT
CGTGATAAATGTTTT

CATTTATTTTGATCTCTTAAGCCACTTTTTGCATGTAAATCCACTG
GATGGATT
>ConsensusfromContig3777 Average coverage: 28.75
CGCTTTGTTTATAAACAATTTTCATTTCAAAATAATTAGATCTGTT
TTTTGACATAATTT
AAATCATAATTAATGAATTATATAGAATACCATCACTCATAACCT
ACAAGAAGCACTCTT
GGCTCCCAGTACTAACATTAAAATGGCTCCATTTTTTGGGAAATT
CAGTCCAAAAAAGTC
TCTCCCGCGCAAGTCGCCATCATTGTCCAATTTAAATTTGGAAGA
ATTGGAGAAAAATGC
CGACAGTGATTCTACCACGAATTCCCCCAGGGTTCGTCTTGGGTC
GTTGGATCTACTATT
TGACAAAGAGCAAGGACAATGGATATCGGAAGGAATGGTGCCTG
TCGGTGGAAATTCAAT
TTCACGTGAGGTAGTTAGGCTCAGAGGACAAAACCAGCAACTTA
ATGAAGAGAATAATCT
ACTTAAGCTCAAAATAGAACTTCTACTAGACATGTTGGCTCAGAC
CACAGCGGAAGTTCA
TTTACAGGAGAACGAGATTGAAACACTTAAAAAGAAATCAAGA
>ConsensusfromContig3778 Average coverage: 4.01
CTCTTCCGATCTGTTTTTCTTATTTCATTTAGATTGTGGTCTGAAT
CGTATGCCCCATAT
CACTTATATGTTATCAACTGCTTTTCTCGTGGTTCTTGACATTGAT
GGCCGATTGAGGGC
TAACCGGCCTGAAATGAAGGAAGTTAAGGCAGCTCTTAAGAGAA
AACATAGGCTCAAAAT
AGAAGCCAATAAGGTGAAAACGCAGTATTGGCGAAGAAAATTCC
GGTCTGGTGAAGATAA
GGTGAAAAAGCTCAGGAACCAGCTGCAGGAAGTGAAACTTCTCG
AGAGCAGGAAGTTA
>ConsensusfromContig3779 Average coverage: 4.82
GAAAAACAAGGACAGAAAAAAGCTGAGAGATCGATTCAATTACG
ATTGTCCACGTTTCGA
TTTTTCAAAAAGCAAAGCTGGAGAAACAAAAACGTTGGATGGGA
GGATTAACGGAACAGT
TGGGACTGCTGTTAAAATAGTCACTGGGAAAAGCGGCAAGTGCTT
CTATTTCGATGGCTC
GGACAACGCATATATCGATTTGGGAATGATGGATGATCATATGA
TCGGAGATAGATACAA
TATCACAGGCGATTTGGAGATCTCTGGGTGGGTGAAGCCTGATG
AGCAGGCAAAAAACGG

TGCATGGATGTACGTCG
>ConsensusfromContig3780 Average coverage: 10.83
GGAATTGCCAATTCTGCCCTGCACTTTCATCCCAATAAATCACAA
AATTCTTCAAGGATC
TCCCAAATCAATCATGAACCAGGTCTCGTCATCTTTCGAACCGTT
CATCAGCTCCATTCA
ATTCGTCATTCACGTCGCTTTGTTTTCATGCATTGTCACGGCAACG
CTCTTAGCTGCATC
TCAGGGTGTTGCTGTTTTGGTGTGGCATTGGATACGTTCCCGGTC
GTTTTTCCCCACGAG
ACGTATATTCATCTCTTCGTGTCCGCTCCGTCTTTATATCCACGG
CAACACGGGGTCGTA
CTCGAGTCGAAAAAAGGAAAAAGATACCGATTTCACAGAGGAGA
GCGATAATGAGGAAAC
TGCTGCAATGTCTTTAGACCGCGCAACCAACCAAAACATACATCA
TCATCAAGGGACTTG
TCATCATCATTCATCTCTGTGTCCAAAACATTCCCATGCCACCAC
TTCCTGCCCCAAACA
TCGCCCAAACTCAATAACGGCTGACTATAACTATACTGAAACAAA
AGTCTAGCATTTCCA
TGCATGTTATGAAAATTAGATGACCAA
>ConsensusfromContig3781 Average coverage: 8.97
CTTATTTTCAATCATCATCGGTAAAAACTGGCTTCCAGCATATTAC
TTAGCAGTTTGTTT
TAGCATTTTCGAGAAGAAACTGTTGATATCAGTTCTTATCGTTTT
CGCCAGATGCCTATC
TTCGTTATTAGCTACATCTTCCGACTTCAAAGCCTCATGAGTTTC
CATGATGACAGACAC
CATCAAATTCAACATGGTGAGACTTATGAAAAGCATGTAGACGG
TGAAGAAGATATAGGC
AAACACCTTATCGTAGTCAATCATATCATCGATTATTTCAAATGA
ACCAAGTCCAAACAG
AAGCAAAGTCATCATTGATCGTGAGAACGATTTGAAGTCAGAAA
GTGAGGAGCAATACCA
TAAAAAGGAAATGCTACTGAATGCAATGAAAACGACGTTGTACT
GAACAAAAAACATGAA
TATTGGCCTCGTCATCGCCCGAAGAGAAAGCCCAAGTCTGAGTAT
TCTTGGGTTGAGACG
GAGCAGAAAGAGAAATCTAACCGTACAGAGGCAACTTATCATAG
CCAGACCGTAGGAATA
CAGATAGTTGAAGTACACAACTCTGTCCATCTGAACGAACTTGTG
T

>ConsensusfromContig3782 Average coverage: 5.62
GAATTCTTGAATTTCCATTGTGTTGTCGAAGAAGGGTTCAGGATT
GGTTTCGTTTAGTGG
ATTATGAATATTACGATTTCTCCAATTTGTCCTTTTCCTCTGCTCT
CTCGTTTCAGCATG
TCTCCATACTTAATTTTTGTTGGCTTCTAAATTCATTTGCTTCAGT
GTTGTATACCAAAG
AACTCTTCCAGTTCATTTCGTTGTGTTTCTGAGCATTATGTAATTT
TTCCAAAATCTGTG
TTCGCGGTTTTCTTGTGATTCTTGATTATCCGGTACTTCTAACACT
TTTTTAATAGCAAA
GCTC
>ConsensusfromContig3783 Average coverage: 5.42
AAAATTCAATGCAAGCCTGTAATGGTTATGCATATCGAATATAAA
ATAATCAACTTTTAT
TATAAAAGCAGCCCCTAACAAAAGTAGTTTTTTGAAAAGGATGA
CGAGATATGGTAATAG
AACTAGATTTTCGCAATTGAACTCAAAAAACGAAATATTTATGCC
ATAGCCTTCTTCCAG
GACAATTCTCAAATATAAAACCGTATAGCATAAATCCAAAACTG
AAAGCACAACAAAAAA
TAATGAAATGGGATTCTGGACTAAACGTCTCTTTG
>ConsensusfromContig3784 Average coverage: 8.62
TGACTGAATAAATAATAAATCCATTGCTCTCTCATGCTTCATATTT
TAGACTGAAATTTT
CTGGCTGATTATAACACAGCTTAGAGAAGCCACATCCCTGAATCT
CATTTCGGTACATGG
AAGCGTGCCAGATTCCGTGAAATGTTAGAATCCTGGCCCATTTCG
CCTGGCTTTTCGAGC
ATAACATTTGATTTCGGGAATGAGTCACGTGACCGGATAAGATA
GGAAAGATGATGTGCC
CGCTGCATCCCTAGGAAAGGTCAATCCGAGAACAAGGGGGAAAG
CGCTGAATTATTTGCA
GATGAATTTTGTGCGCCGACAGATAAGATTCTTTGTTCATTCAAC
CTATGTGCACCCGAG
ATCGGTTAATGATGATATCATCTGGTTTGATTGTACGAGGTAAAG
AATATAATTGTTAAA
ATTGCAATATC
>ConsensusfromContig3785 Average coverage: 9.24
CGGCAGGAGACAGAAATTTTATGAAATCAGGCTTTTCCTCAGAA
AAATTAATAGAATTAA
TCGAAACTAGACTTCCATGTTTCTTGGCGTGAGCTATAGCTTCTTT

CCGCTTCAGAGCTT
TCTCTACTCTCTTCTGATCTTTGTAAAATTCGGCAAAATTACTCAC
AATTATGGGGATTG
GCAGAGCAACGACTAACACCCCCATTATACAACAAGCACTTCCAA
TTACTTTTCCAACCA
CAGTTTTAGGGTAAATAGATC
>ConsensusfromContig3786 Average coverage: 7.97
CCCATGCCATTTTTCATCTTCTGTCATTCAAATGATGAAGTTTGGA
TCACATGTTTGAAT
TCACATGTTACGCCATATGTCCTCCGACCACTTCAAGAAACAGAA
CCCATGCCATTTTTC
ATCTTCTGTCATTCAAATGATGAAGTTTGGATCACATGTTTGGAT
TCACATGTTACGCCA
TATGTCCTCCGACCACTTCAAGAAACAGAACCCATGCCATTTTTC

>ConsensusfromContig3787 Average coverage: 8.89
CTCTTCCGATCTGTGATTGAGTCCGGTTTTTGCAATTCTTGTTCCA
TCGTTTTCTCCCAT
TCCTTCCCGTTGTAATCAATATACTTGATTGACTTGATTGTAGAA
GAATCAATACAGTGG
GGCATGATCCCATATCCATCAGGGTTCGAACGGGGTCGATAAAA
GCTTTGAACACCGCAA
ACGGCACAGAAAATATGTTTAGCCTGTTTTGTATTGAAAGTGTAC
GTCTTCAGATTCTCT
TCACCCTTCAGCAGAGTGAATCGGGAGATAGGGATGATGAAGTGG
TCGTTCTGCTTCTTA
TCACAAATGCTACAATTGCATCTATAGACTGTGATATCCTCTGGG
GCGATGGCTTGGAAA
GAAACAGCTCCGCAATGACATGAGCCCAAATGTGAAACCATTGAT
GAATTCTCATTCATA
ATGAAGATAATGAAATGCTTTCAGATCCGAATACACTCCAGAATC
CCGAAATTTTTTTAT
TTGGGAAATAAACTTT
>ConsensusfromContig3788 Average coverage: 13.09
TCGAGAGCAACCAAAATCCCGAAGATAGCAGCTGTGAACAGTCT
GGTAAACATGCAAATC
ATCCTGATGAAAATTCTAGCAATGTTCAGTTTGACATTGATAGC
GAAAAAAGTAAAAAAC
GAATAGAAGAAAAAAACCGCACATACAGTTCGAAATCGTTGGAG
AAAAATAAGTCTAATG
AGCAGATAGATAACGTACCAACGAGCTCGGGATCATCATCCACG
AAAACTGCAAAATACG

GTTCATATGATCATTTGACAATATCCAGTTTTACTTTCCCAACCG
AAAAAGGTGCAGAGA
CTTCATCATCAGTATCTGGTGAAGAATTCTCAACAAAATCCATGA
CACGATTAGACGGTG
CCACACGTGACCGCAGAGTTGAGCGCAAGCACTTCTTAGCGACT
TCTGGAGACGATCAGG
ATGG
>ConsensusfromContig3789 Average coverage: 8.19
CAGGACATCGATGGCAGACCTGTTCAGAATGTGGTCAATTTCTTT
CTTGGATGGAGGAAA
CAGCCGTCTCTGACCGTCGCGAAGGTTTGAAATAATCGTAATTCC
TTGGCGAGCAAAATC
GCTGGAGATCTGAGAGCCGCAGACTGCATATCGCCTCAAGTGGA
GCAGCAGGTGGTTCAG
AACCAATGGACATGAAGGCACACGAACACTGAGGGTCACGAGAA
GAAACTGCCAGTACTT
CACGTTCACTTTCGATGTAGCACAAAAGTAGGGGTCATTTATCTG
TTTTATGAGCTGCAA
GAACAGCTCGTCACACAGCAACTCATATTCGAAGCATTTCCCTAA
AACCGTCTGCATCAG
TCGGCATTGTCGGTCTGTGTCTTCTGTGGTTGCCAGACTTGATGA
ATCCAAACCGGCGTA
AATGAGCATAGAATTGAACATATCCAGAGCAGACAACTCCAGATC
ATGATTTCGTATTGA
GGAAAGAGATTTTTTGAGTGGCAGCTTGCTGAAAGTCCAAAATCC
ATCACCGCTTATTTG
ATGACTCCCAGCGTTGCTGTTATCATTCAATGAACGCGCATGATC
CTCTTCGGTGTTTTG
AGCAGACGATATGTGGCCAGATGATTCATTTCGATTCAAGAGCAA
ACGGTTTCGAATTCT
CCTGAATGCTGTTTTGAAAAGTCCCAAACCTTGATCGCTAGGATT
TCGAATGACATCGGA
TTGCGTCAGAAGTTGTCTATTTGAGAGGA
>ConsensusfromContig3790 Average coverage: 9.08
CTTGAACGAATCATCAATCGCGAGGCATTTTCGTTATCGGTGCAC
CAAAAATGAGCGCTA
GTGATAATGATAAAACCATTCACAGAGATTTCAAAGATTTCGATT
ATATTAGATTTGCAT
GGATTGACATAAGTGGCATTATGAGGGGCAAACTGGCACCTACCC
GAAACGCTTCTCTTG
AAGGCATTTACGCACATTCAGCTGCCATTCTGTACTCGGCAAACA
CAGATCTAGTTATCC

ACCCTAGTTTCACTGAGACACATGGCCTCCATCCAAATGTCAATG
TCCGTCCGGTTAATG
GAGCAATTCATGCAATACCATGGAGCAGCAATCGAGAACACCAA
GTTGGCCAGGTATTCT
GTGAGACTTTTGGCTGGATGATGGAGAACCACAGA
>ConsensusfromContig3791 Average coverage: 7.65
TCAAATTTGATCAATGAATGAGCCCCACACTGTTAATAAATTCAT
CGTGTTCAATTACAG
TGAAAAATACAACACAGAAATTCCCATGATTCATATCACTTTCAA
TTGCTCACAATCCGA
ATATGGCACCCGCTTTAATAAATTTTTTTTTGATTGAATGCAAA
ATAAACATAAATCAA
ATATGTTCCTCAATCTGCCAACACCAATCTTGGGATAATAATATT
GTCAGTGACATAACA
CCTCCAGCCCTTGCCTGGTATCACCGTACATAAAC
>ConsensusfromContig3792 Average coverage: 9.54
CTTTTTATTCGTGTGGGACAAATACTGGCGACAAATGGGTCAGAA
TCGGTAAATGCAATA
CTGATCAAGAAATTCGGGGCATCACTTCAATTGGCAGTCATGTCT
TTGTAGTCACGTGTG
ATTTCCAAATTGGAGAATTCGCAACCGAAATCAACAAATGGAAC
TGGGTCAAGAATATCA
ATCGAGAAATGCAAGATGATGATAAAGTCGCATATAAAGGAGTA
TTCTCTCGCGGAACC
GTCTTTACTGTGTGACTCAGGAGAAAGCAGTTGAGTATCATTTCC
AGCCCGATGGATGCA
AGTGGATTGGGATGGGTCTCAAGCATGAAAGCTATCCGAAAGGT
TATACAGCGACCAAGG
GGACCATCGTCAAACTGTGGAAGTACGCA
>ConsensusfromContig3793 Average coverage: 9.09
AAAAGAGTGAAGGCTATCAAATGTATGATATGGATGCTTATAAT
AAAAACGAATCGTCCT
ACATGCATACAAGGCCCAACAGATGTTTGGGATCATCCACCTTTC
ACGTTATAATAATCA
CAGCTCTAACTGCTACTATGCTCTTCATTTACGACGGACTACAGTC
TACTTATGGTGCTT
ATGTCTATCTATATGGATTAAAAAGTGTTGCCGGACTCAATAAAA
CCGAAGGGGCGTATT
TGAATGCGTCATTCTGGGGTTTGTTCGCACTCGGGCGTTTGTTTTC
GATTCTCTTCGCAA
CGCGTCTGGCACCTTCCTTCATGCTGCTCTGCAATGTGACTGGAT
GTATTGTATCGATGG

TGACGATGATATCTCTGAGGCATGATCAGGCAACTCTAT
>ConsensusfromContig3794 Average coverage: 8.26
GAATTATCCCAATGCTCGATCTTATCAATATATGATATTTAACGA
CAACTTCACATAAAT
AATAATTTTAGACCGATTGACGTGTTGCCTGGACAAAGCGAAGTG
TTGTAAGGACCATAC
ACATAAGCGCTTTATTATCTTCTACGAGATGACACAAATTCGAAC
AGTTTTAACAGTATA
TGATGACAAAAGACACATCAAAAAAAATATTACAATGAGGCAAG
ATTGTACGAATGGGAC
AGGAATATATTATGAAGTAAATGAAATCATAAGGAATAAAGTGA
ATAAACGTATAAAAAT
ACAGATTATTACAAGTGTAAGAGTTGAAACTGTAATG
>ConsensusfromContig3795 Average coverage: 4.74
CCTGCGCCTTAGACACATTTCGGATTTGGCAAAAGAAGTTATCAA
TGGGTCACTTTTGAG
GATTGCTCAGTTTGCTGTTTTCATTGTTGCATTTCATATGGTCAAT
GTAGGATATCCCAC
TATTTGAAAAGTGCGAGCATTCAAGTCTAGTTTTCATCAGCAGAA
AGTGAGGTATGGGGA
TAACAAAATCATGCATGATTATTGAAGGATCATGATGATCACTTC
CAATTGACCGAAT
>ConsensusfromContig3796 Average coverage: 8.30
CCGGAAGTCGGAAATCACACAAAAATACTACATTTTTACGAAAGA
ACTTTATCCTTGGAA
ACAGAGGTCCATAACCGCAAGATATAATTGACGGGATAATTCAT
CCAACACGCATGAAAT
CCACATTTTGCGAACCCTGATAATTCCAAATTGGCCAATTATACA
AACCCTAATAATCCG
AAATTACAGAAAATTGGTCAGAAATCCTAACCCCGATGACCCTA
ACAAGCATCAAGTTGT
CATCACAAGGACATCGGAAATTGCACCACGATTTCCATACGTTTT
GAAATATTACGAGCA
AAACTATAAAAAGAGATCG
>ConsensusfromContig3797 Average coverage: 3.32
ATGGTGAAATGTGAGTTTGAATGAGAATAAATACACATTATCTGG
CATATATTTCGAAAT
ATGGAGTCTGCCAACCGTTCATAACCAAATGATTCATTGCTTGTA
CTAATGAATTTTAGC
GTTTTATCATCAGTTTAAAATTTTCAGGGTTTTATTTACATAACTG
TCACAAAATATATT
TTTCTGATAGCGGGGTAGAAAAATATTATCAATAAAGTTTCGAAT

GGCC
>ConsensusfromContig3798 Average coverage: 2.61
CCTCGTCTACATAGANCATAATGATATTGGACCTGGGGTAGATAT
GAATATAAGAATGAT
AGGGACCTCGAGTACACTGGGGTAGATATGAATATAAGAATGAT
AGGGACCTCGAGTACA
TAGGGATAGGACGACAAAGACCTCGTCTACATGGATCATAATGC
TATTGGACCTGGGGTA
GATATGAATATAAGAATGATAGGGACCTCGAGTACATAGGGATA
GG
>ConsensusfromContig3799 Average coverage: 6.68
TAAGAATTCAAAAGGAGTTATATATGATTTCAACCTCTTATCAAC
GTCCAATGATTTGAA
GGAAGCGGATGAAAAAAATTCCCTGGAAGCATTGAGTATGATGG
TCAAATACAATCGCGT
TGAATGTCTATCGCATCCACTGTGCGAGAGACTACTCAGTATGAA
ATGGAGAGCTTATGG
TTTCTGGATTCATATGATGCATATAGTGATATACACGGTATTTTTA
ATCGTTC
>ConsensusfromContig3800 Average coverage: 664.81
ATTTTATATTACATATGATATTCAGAGCGCTGAAAATCAGAAATT
TCAGTGTGGTTCTGT
TGTGTCATGTATCCTCAGGAGATTTTTGTATACAAATCACCATTCA
CTACTACGTCATTT
GATGAGACCTCATGCAACTAAGCTCAGCATTGTAGATTACGTTGA
TATGGTTTTGTTTTA
TGTTGATTAGGCGACTAGGCTTAGTTATTATGCTCGTTTTTATCAG
TTCCAGCGATTTAC
TATCCATATATTAGAATGAGTAATTCATATATCAGGCATTTTTGA
GCAGTGGTTATTGTT
GGTGACAGTCATTAATATTCACTATGCATCAACTAGATTTCGTTG
TTCGTAGTGACAGGC
ATTTACTATGCACTAACCAGGCATTGTCTCAGAGATCGGAAGAGC
ACACGTCTGAACTCC
AGTCACATTGC
>ConsensusfromContig3801 Average coverage: 4.83
GCAATATTCAATAATTCGCTTATTTCATCAATGATTTGCTCATTTA
TTCCATTTACGATT
CAATTACATTCACATTTAGATGGCAGAACCCGGTAATGCGAAAT
TGAAATACAACTGGCA
ACAATATTACTTTCATTTGGTCATGCCGCCACTGCTGTTTCCTCTA
TTTTTCGCGATCAC

TACGATCCAACACGCTCTCAAAAACAGACTCTGGACAGATCTCTT
CTGCATGCTGCTGTT
TCACATAAAATTAATTTCTCTCTACGGACCGATTCTAGGTTTACC
AAGAGCTTATG
>ConsensusfromContig3802 Average coverage: 10.04
CTTCATCTTTTTGCAGTGCATTGGTGTGTTATTTTCATTTGGTTTG
GCAATAAATTTATG
AATATTGCTCAGTTACTTTTTTATTAGGAGAAATTGGTAATATTGG
TATAATGTTTCCCA
GGGATATTTCATGTATTTATCATGGCTTTGTATCTAATAATATTG
CAAATATCAAATAGT
ATTCTCAGATGACACTTTCTCAAGTAAATATCTCAACTGTGTAATT
TTCTTATTTTATAT
AAAAAAATAAAAAATTGTTCAGCTAATTAGTATAAACTAGAATTT
TTGTAAAATGAACTA
AACATAAATTTCAACTATATGCTGATGTACGAATAAAGTGTAACA
TAACATCCAATAGTG
GTTCTTAATTCATTGTGAATTTCAATTAAATGGGCATAGATCAAA
TTATGTAATCTATTA
ATAATACACAAATGTTGGGCGTTTACAAAATTT
>ConsensusfromContig3803 Average coverage: 8.61
AGATAGTAATGTTTCTACTAATAGAGACTTATATTCTTCAAAATC
CCCTTCTATAATCAG
TCACATATTGAGATTAAAATCTCCAACAAGGAATACCGCAGAAGC
AAATCGACAGTCTTT
TGTTGAATCGGAAGTCCTCTCTTCTATACGCGAAGAGTTCGATGA
AATGGAGCTTGATAA
TTCAATGGATGATATCCATTATGACATCAGTTATATTTCCCCCAA
ACCGTATAATCTCAG
TTCTCTGAATGATGTACGCTATATCGACTGTATAACACCTGAAAA
ACCTAAGAATAAACA
ACCTAGTAATATACGAGCAACACC
>ConsensusfromContig3804 Average coverage: 7.16
GGATTTTGCATGCAATGTGAAACAGTTGCGTTGGTTGAGTTTTCT
GATTTCGGATCAATT
TTTGTTGTAGAGCCGAACTCATTCACAGAAAATGAAGGATTTTA
TTCGCTTGCTTGTTT
TGTTTTGATTTGCGTTCTTATTATTCAGATATCGTCAGCTGAGAG
GCGATATGGACGAAA
ACATAGAAATCGCTCTAAATCAGCGCATAAAGATGTGAAGTGCTA
CCAATGTGCTTACTC
G

>ConsensusfromContig3805 Average coverage: 7.07
CGATCTGTACTTTCTCTTTTATCTCCATCGATAATCTCCATCTTGT
AAATTTTCACAACA
CTTCCTGAACTTCAACCATCCTAGTTCTTGCTTTCACGGACACTCC
AAATCCACCACTAT
CATCAATCTCCCAGGTAACAAAAATTACCCATTGTTTAGATATAT
TTGCACAATTCCTCT
GTAGCCTCCTCTACAACAGTTGCCCCTGCTCTACAATTTCTGCAA
ACAGATCGGAAGAG
>ConsensusfromContig3806 Average coverage: 12.95
CCGATGTATCAAAACACAATAATGAGAAAAGAATAAAAGTGTAG
ATATAAAAAAATTTAT
CGGTTCCAGTCTATGAAAACTCGGACATATATTCAGATTACCATG
CACATCATTTCTATC
AATTATTTAAAATAGAGGAAACAGTTTACGTTGTATTTTGCTATA
AGATAACAATTCTAA
AAAGCAAGACCAAAATATATAATCATATTGTGAATGCCTTGAAAA
AAAGCCCCATTCTGT
GAATACACACCCTCTTGACATAAGATGATTGGAAGGATATGGAAC
GGATGTGGTGGTTTT
ATCACGATTTTATAATATCAATCAAGTGATTTTATTCTCCATGGGA
AATATGTTTGCGTT
ACATAAACTGGTGTTCGCAGTGTGAAGTGTAGTTTGGTGAGCTGT
TTACGAATCCGATGA
TTGTCAACACGTAACCTAAGAGATTTTTTTCGAAAACTTGAAGAA
ACATTATAGCCGACC
TTCAGATTTTTCAATTTAGATGTACAAATAATAGTTTAGACTGAT
AGATGAACTTCTGAA
GATGCCACTTTTCTCAGCAAATATGACCATATGAATTTTATCAA
>ConsensusfromContig3807 Average coverage: 27.52
AACAGATGGGTGAAGAATCTATTGAAAAACTCGTTAATGCACTAG
ATCAATATATTCCTG
ACAAAAAACATGATTTGTCACAAGATTTTTTCTTGCCACTTGAAA
GTGCATTTCTTGTGA
AAGGTCGTGGAACTGTAGCTGTTGGAACAATAAATAATGGAACT
TTAGCAGTTAAGGATC
AAGTTGAGTTAAAAGGGTTCGGTATTCACAAGACAGTGCAGGTTG
CTGATATTCAAATGT
TTCATAAGTCACAATCAAAAGCAGTTGCAGGAGATCATGTCGGTG
TCTTATTGAAGGGTA
TACAACCTGATATTATTATGCGAGGAATGTCTTTAAGTGGTCTTA
ATTCTGTCATTCAAA

CAGATAGCTTTGAATCTCAAATTTATGTAAAGATGTCAAGTGAA
GGAGGGAGAAAAAAGC
CTTTATTGACAAATTATATCAACCAGCTATTCGCTGATAATTGGG
ATATAGCTTCTTGTA
TTCAACTCAAAGATG
>ConsensusfromContig3808 Average coverage: 9.19
TCCGATCTGCTAAAGACAAGTTTTTTGAAAGTCAGCAACTCGGTG
AGGAATGCCATTCAA
TGCCATCGACCAGTTGTGGCTTTGGAGACAGCTATTCTGACCCAT
GGCCTCCCTCATCCA
ATCAACGAAAAGGTCGCTAATGATCTCGAATCCATAATCCGGGA
GAATGGGGCGGAGCCA
GCCACGATTGGAGTGATTGACGGGGATATACGTGTCGGTTTGAAT
GCGAGAGATATATCT
AGGCTTTGCAGCAAAGATGGAGCTTCGGTGAAGCTGAGCAGACG
TGATTTGCCAATGGCC
GGTGCAAAAGGAATCTCTGGTGGGACA
>ConsensusfromContig3809 Average coverage: 6.93
CGCCAAGCGTCTTGCTGATGTAATTTCCTGGGGGTGGTGGCTGGC
CGGTTGAGACGTTTC
CACTGCGTCTGTCAATCCAAAGACTGTAGATGGGGACGACGATG
TGCCCAGCCTCCACAA
ACATGTCGGCGAATGGCAGGTTTACATCCGCCTTTTTGTCGTTATG
CTTCTTCCATTCCT
CCGTTTTGATATTGAAACTCATGATGCACAGATTGGAATCCATGA
GTATGATGCTCGGTT
GATCCCAATACATGTGTGTAAATTCGCCCAGAACA
>ConsensusfromContig3810 Average coverage: 7.71
GACCGGAGCTACTTGCGAAGATCTAGACACGTGCACAAGCAACC
CCTGCAATCACGGGGC
TACATGTCTGAATTCAGCTACCGGCTGGGATTGCCAATGCACCAT
GTGGTACACCGGCCT
TAATTGCGATGATCGCCGGAATGAGTACTTCAAAGCCACAGACCG
GGCTGTTATGCGCCA
AAGCGACATCGGGACTTTCTTCATATTCTGGAAACCGTCCTATCG
CTTGAGTCTCTACGT
AGAAGGATCAACAGCACAGCTTTTCATCATAGCTCTTGACCGACA
GACGGTCCATCGGAT
CAATATTCCCGCTGATCCAAGTGAAATAAGTTCTCCGGCCGAAGC
AACTGTCGTCGTGAG
CGGGTTCTTCTCAACTGCACCCAGAAACATGAGAGCCATTCAGAG
CATTCAGAACTCGGA

CGGGGATTGCATCATGTACATAGCTGGAGAGATGGCAGATGAAT
ATGTTATAACAGCCAG
CAGCTGTGATGGTAATTATATCGGAACCCCTTACAAAAACTCTGC
ATCCGTTCCAACAGC
AATGGGATATGATGACCTGTGGAAGCACTCTGTGAGTACATGGTA
TGACGCTGCTGATAT
GGAGGCTTGGCTGAA
>ConsensusfromContig3811 Average coverage: 8.34
TCTTCCGATCTTGAAGCTGTTTCGTTCAGACAGCAATTCTTTTCAC
AAATTCTTGGATGC
AAAAAAACTATTCTTCGAATATGATCAAGTGTAGAAGAAAGTAC
CTCAACATCTGGTGGC
AGTACAGACAGATATACACAGAGGAGACGACTCAATGCATTACG
AGCAGAGTTACTCAAC
TTGAATCTCTCTACATGTAATATCAATGAGTTTATAATATCGTTT
GCAAGAGAAATTTGA
TGTGATGAGATACCGGAATGCAGACTTGAAGTCAAAGACAGCGC
GATATCTTCAATATCG
GAATTTTCCAAATCAGATTCAATTGCACTATCCATCATGTACCAA
AAATCAATATCAGAT
TTAGGGACCAGAGTCACATTAGCTAAGATCCACGGTCCTAATTCT
GCATTCGAAGAGACT
TCCAATATTTTGACTGAAAATCGTTTAGCAGATGCTGGAAAATCC
TTGACCGAGA
>ConsensusfromContig3812 Average coverage: 4.43
GCCATTATGTTTTAATGAAGTTTCAACAAAATAAACCTGACTGAA
AGTTTGTGTTGTCAC
ATATAAGCGCTCCTCTAACCGAATTCTAGTTTGATTTGTAACGAT
ATTTCCTACGAATCT
TCCGACCTGATATTGACATAAACGAATATAATTCCACGATATAAT
GGCTGAATAAGTATG
GGGTGCTTTAATATCAAGTTCTACTCGTTGAATAACAGAAACTGG
GGCTATTTCACATGT
GTTATCTGTTTTTTTAATGGTTGTCG
>ConsensusfromContig3813 Average coverage: 6.03
CTTGATCACAGTTCTCATGATAATTTTGCCGCTGCTGTTGCTTCTT
TCGATAGGCGTGTT
TGTGCCGAGCGATATAAAGATAAACGGAGAAAACGCCCGTAAGC
TCAGAACCGACAAAGG
CGGCGATGGTCATGAGGAAGGACGAGCCGTACTGGTATTCGAAA
GCCGGCTCGTCGTCTT
CGGTCATTTTCGGTGTATTGCCGACCTCACCCGTGATAGCGCCAA

TGAAGAGGATTATCC
CGAC
>ConsensusfromContig3814 Average coverage: 7.88
GGTATGTTGATCAAATAACCCTATGAAGAACCAAGAATTAGAGT
CATTTGTACAGAAGAT
CCTCGGTTCAATCCTTGTGTAGAGACGGAGAGTGCGTCCCTGATT
CATACATCTGTGACA
AAGATAATGATTGCATGGATGAATCGAATGCATTCAGCTGTGGGG
GTATGTTGATCAAAT
AACCCTTCGAAGAACCAAGAATTAGAGTCATTCGTACATAAGATC
CTCGGTTC
>ConsensusfromContig3815 Average coverage: 9.42
GTACGTTCGGCAGTAGTAAGAATATCACAGGTTGTTATTTGGATT
CTAATTCAATGAGTG
TAACATGTCGTCTTCCACTCTCGTATCACGAAGCCGAAACCTATA
ACTTGAGTGTAAATG
AAACATTAAAGACTTATAAGTGCTCAGAACTTGTGGCTGATGCTG
ATCAAACTGTAGGAT
CCTGTAATGGTAAAAATTACCATTTCGCCGAAGAAGAGTTCAGGC
GCGCGGTCACCATTG
CGAATCTATACGAAAGCGGGTTTAATCCTATCTGGGCTGTCATCA
TCATCGCCAGTTTCG
TTCTTATCGGAATTCTCGGAGTCATCATCAGAAATTGGCTTGTAC
GCAGAGACCGACCAT
GCAGCTTCTGGGAGGGCCTCTG
>ConsensusfromContig3816 Average coverage: 12.26
AATTATTTTAAACTTTCCATCCGTTGTTTCGATGTCCGATTGCGAA
CCGTTTGTGAACGT
CTTTGTGCAATGCAAGTCACTCTTAGAAAGCAAGTCAGCAATTCC
AAATAGCTTCAAAAT
CGCCAAAAGGCATAAGCCTAGCCGCTGTTGGACCATGAGAGCAG
ATATACCGACTGAATT
CTGCTTCATGCTCTCTTTATTGAGGATTCTTATACCAGGAATAACA
GATCCGTTATTTTT
GAAACTTTCGGGATCAGGAGCGGGTTGTGAATGCATCCGCGGCAT
CTCCCGACCCTTCTG
AGTAACCACCTCGAAGAGCGCGATGATGCCATCTATCGGAACAG
GTGTTGGCAGCTGACT
GGTATTCAGCAATAGACTATTGCATTTCAATGAGAGAATAAACA
GATCGGAAGAGCACAC
GTCTG
>ConsensusfromContig3817 Average coverage: 7.84

GCGGAAGCTACTGCGCATGTACAGAAGGATTCAACGCCACCTGT
GGGGCGAATATCGACA
TGGTCACGTGCTCGATTGGGGGTGACGTGGGGGCATCAGATATG
GACATAACAGATCAAC
TCATAGCTCATTCAAGGGAGGCTTTGCCTTGCCACGCACGCCACA
TGCTGGATGAACCCT
ACTACAACCTCAATTACACATTCGTGCCAACGCCAACAACCAACG
CGTCCCTAGAAACGT
GCACGCAGAACATCACGGATAGTCTGGAAAGGATGGATGTCTG
GACATTATACCGGCTG
CCGTTCTCGATTTCCATATCCAAAATTGCGCACAAGATCTTACGT
TAACTGGAGACATGG
TTTTCGTGAATGTTGCCATAAATGTCCTTCGGTTGTCTTGTACCA
ACGCCCTCAACAGAA
ATGCTTCTTCTTCTAAAACCCCTCACATATGATATTCCCTTTCCAA
TACATCATGATCTG
TCC
>ConsensusfromContig3818 Average coverage: 4.96
CGAGAATCTCATAACAATTGTTTTATTGAATAATACTGATTCGTA
TTCCTCGGACATCTT
TGACGTCAATACTTCTCTATGCACCGTTTCCATATCCGAGATACC
GCGGACGGACAATAT
CACATCTTCATCGAAGAATGGCATATAATCATTGAACTGCAATCT
GTCAATTTCGATGAA
GAAAGTGAACATCTTTGGAAGGCATCTTATTTTTAGGTGTATCTT
TGACGGGAATATCAC
ATCGGTTTTCTTTGAAATGGGGGTCATATCTCTCCAGAATTCTTT
TAGTTCGCAACATAA
TCCTAGTGATTCAAGATTCATTTCAGTGGATAATGAATTTTTT
>ConsensusfromContig3819 Average coverage: 5.43
CCAATGTAAGTATATTAGATCAACAAATGGCTAAAGACACCTAT
ACAGCTAAGCGTAGAG
GTATACTTAAATCACGTACAGCCCTGATACCTCGAACAAAATCGC
AAAACAATAATGCAT
TAGAGGTCAATTTAAAACTGCACAACTCTGAGAAAAACAGTTCA
GATAAAAAGGAGCTGT
ACGATGACATGCAAAACGCTGCAGTGAAAAGTGAAAATGATGAT
ATTGATCGAAAAAATT
ACATAACTCAAAATGCCGACTCAGCTCACAAATCAAATACTGATT
ATGCTAACATACAGG
ATATCAACACTAATGCAGATGAAATGAAAGATGGTCATATGAGT
GATCCAGAGAGTCACT

CGAAAATAAATATCAACCCCTGGAATCATGAAGATGGAAGCATT
ACA
>ConsensusfromContig3820 Average coverage: 8.89
AATCGATGGAGAAGAAGTTCTAAATGAGTCGAATTCTGAGAAAA
ATCTAACAAATGAAAT
GAATGAAACGATATAAAATAGTTTATTTTCCTCAGAACTGTCTAT
GGAATGGTGAGAAGA
GAAGAAATGTAAAAAAATGAATCGATTATTCAATCATACATGTTTT
TTATAATTACATTGC
AAGCTTGTTCATAATATAAACCAGTATCATGTTCTGAAGGTTCAT
CCGCAAAATCATTTT
CCTCAGTCACAAACTTAATCTATAATCTGTTCCTCAACATTTCTCT
TATGTCAAAATTTT
GAATTTTCATAATTCTCCACAATTTCTGC
>ConsensusfromContig3821 Average coverage: 6.93
ATACTCTCCGCGGAAAAATTCTGAAAAAAATATTCACGACGCCTT
TATTCAAAACAAGAA
ATAAAAATAGCAGGAAGACAAATTGAAGTCTAAGAAAATTATAT
CACCATGAATAAAGTT
TATGGGTAATCCATAGTATTATCTACGATATTCTTGGAAGGTTTT
CATGAATCACCCAGC
AATATACTAGTTTGGTGGAAAAACAGGACAAAACACGATTCAAT
ATCAAATTTGAATGTG
ATCTAAAGAAAAGGCATGGATCTCACCCAGATC
>ConsensusfromContig3822 Average coverage: 6.83
CTGGATTTTGTTCATTCTCATTAATGGATTTACATCAAACTAAAC
AATAACAAGTGATTG
AATCGAATAACTGAACGGTGGAGTCGAGATCAACATCCTGATAC
GGAGAGGCAAGAAGAA
TATATGAATTAACCAGAAAAGCCCTGCAAAGTCTATTGAAAAATC
AATGCACTTTATGCA
GAACTATATTGTGTGAAAGTGTAACATCTACTGATACGTTGTGTG
ATAATCCTCGAGCGT
GCATTCAGACGGACTGAGACTCGGTTTGCAATCTTCAAAAACAAT
TTCGGGAGAATTCAT
TCAACATCTCACATAACTGAGATTTGATTTTCGATTAAATCACAT
CCAAAAAATAATAAA
TAGTATCAACATATTGTCAATCGCCTATCAAAT
>ConsensusfromContig3823 Average coverage: 14.52
CTCACTTTTGTTATTCTTGATTCTGTTACACTGACTTTGCTATTTTT
TCATTTGTTTGAT
ATTAAAATCATGATATATTATTTATAAGAGAAGGATTAGCCATGA

GAGCTATGAAAAGCT
GATAATTGTGTGCTTATAAACTAGGAATTTGTTGCCATGTGAATT
TCCTGAATATTTAAA
TTAAGTGCCTTAATCTTAATTTTTTTAATTGATTTTGATGACTTTT
CTGATTTGAGTATT
TTCGATAAAATTGAATTTCG
>ConsensusfromContig3824 Average coverage: 5.75
CTTTTTTTCACAAAGCTATTGTCGCCGATCGCCACACCGTCTCTAA
AAACATCAGCTTTT
TTGTTTCTGTCAAAGGAGTTTTCGAAAAGGACAGCGAAATGATGC
CAGACACCATGGTCA
CATTCCTGAACAAGTTTCTTGGTTTGATTATATTGAACAGATTTG
AATGAAAGTTGACAG
TCTGAATTAATCCTGATCTGATAATGCTTGGAGTGCACCAGCAAA
GAATCATCAGTGATT
TTGGTTGTCTTCAGCCCTTCCGATCTTGTGAATCCATTCTTGCTTT
GTCCTTCAGCGAAT
GCTTTATGGTCGATGTTCGAGACATGAATGGCGGAATTGCCTTGA
CCTAATTTTGCTGAC
CACCAAATACACTCTGGAACAAACTCAACATTTCCAACAATGCCA
AGGTCATGTGGTCCG
CCAACGATCTGCTGC
>ConsensusfromContig3825 Average coverage: 13.86
TGTGCTCTTTCGATCTCAGATGTTATTCCCAGCAATCTTATTTAAA
TTATTTTTCATCGT
TGTATTTCAAATTCCTGCTAGAACATTAGGTCCATAATCGAGGTA
ATTATTCATTAATTT
CCAGATTGGAACACCGTCAATTGTGGTCACGATATGGTTTATCAA
ATTCCTGTTATTAAC
TGACTTTGTATATATCAGTGCTGTTATTTCTTCAAACTGGTTTTCA
AATAAGCCTCAAGA
AAACCATGTTAAGATTATTTCAAACGCTAATGTACCATGTATTTC
TTCCACCATTATAAG
TTGTTTCTGGTACTTTATTATAATCAAAACCGATAAAAATATCT
>ConsensusfromContig3826 Average coverage: 4.56
ACGACATGTTTCTCATAGCTCAGCCGACAAAACATCAGACCTGGA
TTGCTGATGCTGTCG
TCATCAAAGCAAAGAAAAACCTGGTCATTCTTCATATGCTCATTT
TCAAATGCCCGAATT
CATGTGACCCACTGTATTGTGAACATGGAGGAAATTGTACGTTGC
ATCCTAACCAGAACC
GAACATGTAAATGTCCTTCTATGTATGAAGGAAATCTTTGTGAA

ACAGAAAAGAAGTGTG
AAGAATGGTGCATACACGGCAGATGTATTGGAAAACGCTGGTTG
GCTGAGTGTGAATGCA
AAAATGGGTATTATACCCGATCACCGACCATCAGCAAGCAGCAA
CAATGCATCTCGAAAG
ATTTGTGTTTTGACGGATACTGCAAAAACACCGAGTTCTGTTCCA
GAATTGGAGATATGT
TTTCTTGCTCAACAACACCAATAACCACTCCTAAACCACGA
>ConsensusfromContig3827 Average coverage: 6.06
TGCAGGTCTCACAACAATAAAGCCGATCGTCAACAACTTGATAAC
AATTTCCTCCTACCA
TTTCGCACCAAGTGACGTTCGTATCTCCATATTCGCAACCCGCTC
TACCAAATTCGAGTT
TGAGCTTATTGCAGGTCTCACAACAATAAAGCCGATCGTCAACAA
CTTGATAACAATTTC
CTCCAACCATTTCGCAACAACCGACGTCCGTATCTCCATATTCGC
AACCCG
>ConsensusfromContig3828 Average coverage: 5.80
GAAATATGAGAGAATCGGCCACAAGCCGTGATGATTTCACCAAT
CCAATGACAACAAGAG
TTGAGTCTCCAGCGTGCTCCCGTGAAAGGGCGAGGGAAAACGTTT
TTTGGAGTGATTTCA
TATTGATTGTTCCATGATGTCGTATTTGCCCACCTGACATGAGAA
TATAGTCAATACTTG
GCAATGGTACGGACGTCCTCACAGTGAATACAATGTATTTGTTCA
CCTCTGGATTTTCAG
TGGACGAACTCAGTTGAAGATATTCTCTCAATTTGCTCTTTGTTG
GTATTGCCGCCATGC
TGATTTTTTCATTCATGTAAATTGCTGTTATATGAATCGATTCGCT
GTTCACATCGGTAT
CCAATCGATAGCTTGCAAATCCAAGCTCATCTATCAATATGATAT
CTTCAAATATTGTCC
CTGAAGTTTCTATTTTCAATTTGATGTTTCTATCGATGTTGATTTT
ACATGGTCGACCGT
CTTCCATGTATCTGAATAGTCGAATGTCAATTGTTGATCCAGGAA
TGAAC
>ConsensusfromContig3829 Average coverage: 14.99
CTCGGTATGACCTGGGATTTTCTCCAGATAGGTGTCGCCACGTAG
GTCATATAGATATAC
AGATTTATCCTCAGAGCCTGTCGCAATGAACCGCGAGCAGGGGC
TGAAGCGGAGTCCGCA
GGGGTGGGCCCTCGTGATATGACCGCTGTACCTTCTTACCACTCT

ATTTGATCGAAGATC
CCAGAGTTTGATACAGTCTGAAGATGCGGATGTGGCGAACAGGTC
GTAGGTTT
>ConsensusfromContig3830 Average coverage: 5.43
CTCATCTAGATGTGTCGACACATGCTGGCCATCTATTTTAAGAAT
ATTTAATTTGATAGA
TGGCGTAACTAAAATAATTCCCATTTGATTATTTTCATTGCTCAT
TACACGAGGGGCTTC
CGAGTGTCGTAGAGGGCGGGGGCCTGGAGAACAATCCCAAAACA
TCCAACAACACAAGCT
GTGGTGAAGATCCAGAGGAAGAGTCTGTCCAGGACCAAAGCTACG
AATTTCCAGTCTTCT
TTGAGATGGTTGATTGCTTCATCTTCTTTCTTGAGATGCTGTGCGA
TGTACTGAACCCCT
TCAACGGCTCTAAAAAAAGCCGGCAGATAGCACCTTGGCATTTGT
TGCATCAGGTCTTCG
AAATCCAGACCTTCAGCTGCCTCATAGATGGCAGCGCGGTCTGA
GCCAAAAACAGAAAAG
TCTCTCTTTAGATTTGGTGATGGTATTGATGAATTTGCTTTTTCGA
GATC
>ConsensusfromContig3831 Average coverage: 7.15
ATTAATTAATATTCTCATTTGGGTGGAAATATCATTTTGGAATAT
AAATATAACGCCTAA
TATGACCGACTCGAAAACTAAAAAATTAATATTCATGAGGCGGGG
TGCCACTGTTTGTCT
TCTGCTGGCATTTGTACTCGGATTATCGGCTTTCATCTTTCCCAGC
TGGTATGAATGTGA
AAAGTACACTGCTGGAATCATCAATTTAGCCCCTAAGGGTTTTGC
GACGGATCTACATCT
TTGGTATCCAGTGGAATCCAAACTCGAATTTCAGAAGATCTGCAC
GCTGACAGTCATACC
A
>ConsensusfromContig3832 Average coverage: 3.99
GTTGAAATGAATATTCGATTCCTAAAAATATACAATTTTAGTTGG
TCGTCATACATTTCA
CATTCATTGCCTCATTATATTGTGGTAGACAAATCAATCATATTTT
TATATCAGTCCCAA
TTTCGCTGCATAAGTAATCGATTGGTTGTATCTCTTGTATTTCATT
TATTTTCTCAAGCT
GTTTTATATTGCCATATTATTTAGAACTATTTTCAACAATGCAACA
CTCTGATCCAC
>ConsensusfromContig3833 Average coverage: 9.18

TGTCAATAACTGAAAGAAGGTTTATCAAATATGAATTCTATGTAA
TACAAGTTTACATCC
TCATCTGCTTGAAAAAACGAAAAGAAAATGTTGGCTGCATCTCAG
TTTGACCTAATCTAG
TCTTCATCAAGAAGTGACGTCTCCATTCCGAAGGTTGACCTCCAT
GGCTCTCCAATTTTT
CTTCCACTCTGCTTTTCTCTTCATTATTGCATATGCTGAGCACCTA
TTATCGATCTGAAT
GGGCTATGTGTGTTATGTCAACTACTTCATGGGCTTCATCA
>ConsensusfromContig3834 Average coverage: 9.39
CAGTCCCATCTGCGTCGGTGGCATTGAAAACGAAATTTGCCTCTA
CGTCAACTTTTACAT
CCAATGTGAACATCCCGCTGATGACCGGAGGCATGTTTTCCAGTT
CTGCTTTGATCGAAT
TTTGTTCTGCCTCCACACTTTTTGTCGCCTGTGCTAGCTCCTGATT
ACCAGTTGCAAGAA
AATCCACGATGCAGGCCTGGGCCGCAGCTCCCGTTCCACATACTG
TACCCAGCTCCGTCA
TCTCAGCATCTGTTAATTT
>ConsensusfromContig3835 Average coverage: 28.58
GGGTATTTGACTGAATAGTGAGTGACATAATGACAATTGTCTTCA
TCTGCATGCATTGTC
ACTTCAACTTGACCACTACATCCGATCTATTCTTCAAGATCAAATT
CTACTTTTTCAGGC
GGAAAAAGCATTTTATGCTGATATTGGAGCTCATATGTGAGAGAA
TATCCATGAATCCAG
TCTTGTGCATATCTTCAACATTTTATAATATTCTAATAAAATATCA
TCCTATCAACCGCA
CAGTGCCTTATTCCCATTCATGATGTTCGACCCGATATTTGTGGG
AGAGCGGAAAATCCA
CTATGCCTACCTGACGTTACTCTGGCTGCTTCAGATAATTAATCTC
ATTTGGTTCGCTGT
AATTCTCCGCATGATGACCGGCGTCATCATTGATGGAAAGGTATG
ACACCCGAATGACAC
CCTGTGATGCCCCCGTTATACTCCGACGTCCCGAAGATCGGAAGA
GCGTCGTGTAGGGAA
AGAGTG
>ConsensusfromContig3836 Average coverage: 11.71
TTCATTGATGATCACGTTGCAGACATTTTCTTTCTCGACTAAAAA
CTGAATGATTTCACC
ACTTTTGCAGCCGTCGAGATTTGAAGATTCTTGGGCGATTTTCAA
GGGGAAAATAGAATT

TCCCAAGACTTTTGATGAAGGTCCAATGAGACCTGACATTACAGA
GCTTCCGTGGCTATA
TTCATCAGATCCAATTTTATTATTTCCAGCTGGATTATATCCACT
GTAGTAGGACCAATA
TGATCTAGCCAAGTTCTCCAGATGGTACTCAATGGATTGTAAATT
TCGA
>ConsensusfromContig3837 Average coverage: 3.65
CACGACGCTCTTCCGATCTGGTTGCTATGAGTGGCTTATCTCGAT
GGGAAGCAGAACTTC
ATTGGTTTTCTTCACACTACGCCGATTTTTTCGTTCCCATCGTATC
AATACTTTGCATGA
TAATAACTGTATTCCAATCTAAAAAATTGCTGGCTGAGAAATGTG
AAGCTCCAAGTCCAC
CACTTGCTGTCAATTTGGTTGAAGAAGATGTGTATCTAACAGCAG
AGGAAATAGGATGTA
CGGAGCGTAAAATTCCGAACAAGGATGAAGATAAAGCGGAAGAT
CGGAAGAGCAC
>ConsensusfromContig3838 Average coverage: 5.07
ACATGCTCATCGATTATGTTTCCATTGTCTCTCCTGGCCACAATT
ATATCAAAAACACCG
TCTTTTTTAGAACCTGTATCAGGAGTGAAATATTGATTTGAGTAA
GTACTTGAGGAGATT
TTTATCGGGAAATAATGCCCGAATATCTCTCTTTCTGCTTTCGAG
AAACTTGATGTTGGC
GTGAACAAATATTCAGAATCTGTTTCTAACTCGAATCGACAGATA
CCAAGACCGAGTGCA
CTCGCAAATATTAACGGTATGATGAAAAATAGAAAAGGATGTCGT
CCGACCATCTTCCCA
TGCTGCTCGAAAGCACTTCTTAGTGGTCCTTCCAAACAATCTGTTC
TTATATCACCATTC
TCTTTT
>ConsensusfromContig3839 Average coverage: 4.83
CCCGACGCTCTTCCGATCTCGCCGATCTGTTCTTTTTGTACAGATT
CTGTACTTCCCGTC
CTGCGCTTGGCACGGGACGTCGCTCTTCGGGAAATATTGGGTTCA
GAAGTGATGTTCCTA
GCTGGGATGTTACGATGTGTGGTAGACAAGTTTCTCACTAGCCAG
TCGTAATCGAGGTTA
ATATCCTCTTTTTAGACGTCACTACCGTCGGCCCTTGAGTGAGGA
GCCAGGTACCCCCAA
CCAATTTAACCTCCTTAATCTAATTGAAAACAGATCTTGATAAAA
GTAAATTAAGAGAGT

CATA
>ConsensusfromContig3840 Average coverage: 15.09
AAAAGTTGATGCTATACTTATTTTAAAATCGATGATGATATTAAT
TTCATCATCATAACT
TTTACGCTGGTGCTTAAAGCTCATTCAACCTCATATTCAGTTCCC
GAAACTACTGGAACA
CTGCCTATCAAAATTGTATTATAGATAATAATTATATCATACGAA
GTATAAATATCATAC
TGAATCAGGCATTCTCTTGATTTATATATTATATAGTTCATTTGTC
TCAG
>ConsensusfromContig3841 Average coverage: 7.99
CACCGGCTACACAAAGGCTTTATAAGAATACTTTTCGCTTGTTTG
GGGAATTTGCTGCTA
CTAGACAAAATTGTTCTTCATCTATGCCATTTTCATATGAGGCTGT
GGCTCTGTTCGTTA
CGTACTTACATAATAGAGGTCTGCAACCATCCACCATAAGGTCTC
ATTTATCTGCAATCT
CGTATTATCATAAATTGGGCAGCTATGTAGACCCTACTAAGCATT
TTGTAGTTCTGAACC
TTGTTAGAGGGGCTTCCAAACTACGGCCCCCTTCCGATTCTAGAC
TCCCGATAACTGTCT
CGATTTTACAGAAAATTATCAGAGCCTTGCCTCTCTTGTGTCATT
CGAGTTATGACGTCG
CATTGTTCTCGTCGATTTTCGCAACCGCCTTCTATGGTTTAGCTA
GGATGGGTGAATTGG
TCTCTTCAGGTGAGGACAATACTGTCATTCAGTATGGGGATGTTA
ACTTTCGTGCAGGGT
CCGTGCTTGTCACCTTTCGTGTGTTCAAACACAGCTATTCAAAAG
AGCCTAAGACTATTG
AAATCAAAGGTTTGAACACGCGCTATTGCCCTGTCAAGTTAGCAT
TAGGCTATGCGGCGG
TCAGAGGTAATGCAG
>ConsensusfromContig3842 Average coverage: 4.96
AATTGCACAAACAAGGAATCTTGGGTTTTGTAGAGTATATGCCG
AATAGGTGCAGGCGAA
AACATCTGCGGCGCCCAGGCGTTCTACCGGACAGGGATGATTTA
TACACACATCGCGAGC
ACATCATTAATCATGAAAAGGGTCTGCAGCGATTTCATTGAATTG
AGAGATCGTATCGTT
ACAAGGTTGACATTATTCAATGCGAGGCGAGGTGATGAAAGATT
GGACAGAAGACAGACA
AACGCTTGAGTAGACCAGAATACATCCGACCCGATCGAGAAGGC

GTGTATCGACA
>ConsensusfromContig3843 Average coverage: 4.93
ACGCTCTTCCGATCTAGAAGATTCACTGACGGAATCATTCATACC
TCTCCGCCTACGATT
TGATTTAAGAGTAGAATTCTCCGAATTACACCCGTCCACTTCCAG
GCCGGAAGAACTAGG
TGTTGATGAAGGTCTGGACGCAGGGTCTTTTTCACTGAACGTCAC
AAAACTCATGGAAGC
ACCTTCGTTGTAGGAGATATCTTGCATACCATTCCATTTTCGAAT
CGCCGAAGATTCGTT
GTTTTTGCACGCTTTCGCAAAATAGCTTTCTCCGTTCGTTCTGGA
ATCAGATGTGAGCGT
TCCAAAAGGACTGGAACCTTCTTCTGTGGTTGTAGAGAACGTATC
GCTGAAATCGGATGA
GCGACGATTTTTCTCTGATCCATTTTGG
>ConsensusfromContig3844 Average coverage: 2.81
CCCAACTCCAGGATCATCCAGTCAGACCGACAGACCGCACTAATC
TCATCATCCTGCAGG
CCTGCCAGGATTTTCTTGGAGAACTCCATCTCGATTTTGTCAACCA
TTGGTTGACTGGTT
GCACAGTCTGCAGGTTCACTTTGGGGATACGGAACTGTTTTCTGA
TTTTTCGATGCACAA
ATCTTGATATGTCTCGAAAAGTTTGACCGCGAGAAGACATTCAGA
CATATCCTGCAATAT
TTT
>ConsensusfromContig3845 Average coverage: 8.63
CTGATTCAAACGGATGTTCACTGATTGGTACAGATTTAAATGATC
CGTACACGTCGTACA
AAACTCAAAGGTACATACAAAAATAAATATACAAAATTAATGGTG
ACCATACCTTCTTCA
ATATGGATATGAAATACATCAATCAATACTATGAAATACATCTGA
TTCAAACGGATGTTC
ACTGATTGGTACAGATTTAAATGATCCGTACACGTCGTACAAAAC
TCAAAGGTACATACA
AAAATAAATATACAAAATGAATGGTGA
>ConsensusfromContig3846 Average coverage: 11.30
GACAATCACAATGGAGGGGGTTTTCCGACAGGTCAATGTAATAT
AGCTCCAGAAACTGGC
CAATGAGTGATGTGTTGAATGATGTGATGTAATTTCCTTTTAGAA
ATAATTGTCCGATAT
GCGAGATTGGGAGAAAGAGTTCGAGAGAGTTAATTGACAGAGTA
CAATTTTCAAGATTAA

GATTCTCAATACTAACATTAGAAAATGAACTGCCGACCAGCCGTA
TTCCAGGATTACCAC
CGAGTCGTAGATCATGGAGTCGGAGTCCGTAAAAAGTTGATCCTG
CGATTGAAATTATTC
GGTTGTTTTTAAGATTCAATGTCTGAAGGTTCTTGAATCTTGAGAA
TGTTCCTCGCTCTA
TTACCTGGATTTGGCAATTGTCTAAAAATAAATACCTCAACTGAA
TTGTATAACTGCTGA
AAGTAGAAGTTGTTAGATTGTTCCATTGTAAAGGATTGTTGTTGA
ACATTGCGATGACAG
TAGTTGGCGGAATATTTTCTGGTAATTTTATAGCATGAACATTTG
AACAATTGAGAAATG
TTGCATTGATACCAGGAACACGAATAATGCTTTGTTTTTCTTTGC
AATAAAACTGGAAGA
AATCACTTAGTGAAGAAACTCGAGCCACAAGAAAG
>ConsensusfromContig3847 Average coverage: 3.00
GGGGAAGAGCCAATGATATTTCGACCAGTGGTCCATGTAATGCCC
AATGTACGGATATTT
GCCATCAGCATCCATATCAATTATGTCAATCTGGCAACGGGCATT
GAAACCGTTCGCAAT
AATCGGTTTCAGTGGAGGCTGTGTATGCTGGGCACGTTGCTCGGC
ACACACATAGCATCT
CTTCACGAATTCCTGAACTATTGGTCTCGTTATCCCGTGATATTT
CTCGTTAACAGCAAA
CAGCAGTTTTTTGTAATTGCTGTGTTTCAATAGTCTGTGTTCTCTT
GTCAGTATTTCAT
>ConsensusfromContig3848 Average coverage: 7.56
TACACGACGCTCTTCCGATCTCGTTTCTCTCTTTGTTGAGCAGGTT
GCTTCTATCTACGA
AACTCTCAGAGTCGGCCGAACAACTTGCGACCTTGGCCCTGTTTC
CGAGGTCCCTCTACA
CAAGGGCGTATCTTTCTTCCTGACATCTTCACCGGGCAGCCCCAT
CCCAAGCGGGATTCG
AACTCAGTTTCGTGTCATATCTCTTGTTCGACCAGATTCTGCTCT
GCTCCTCAAGGCGAA
ATGTTC
>ConsensusfromContig3849 Average coverage: 12.80
AGACGTGTGCTCTTCCGATCTGGTCATTCTCTGAAGCAAAATATG
GGACTCGTTTCCTTG
AACTTGAGCATGAACGGCCTTGGGAACAATGGAGCTGTTGCTAT
TGGCAACGCATTGGCA
AAAAATAACAATCTTCAGCACCTTGACCTTTCATTTAACAGAGTA

GGCTACGAGGGGGCA
AAAGGGCTCGCCTCAGGTCTTGGATTAAACACATCTCTGAAGACA
CTCAAGGTTGGAATA
AATCCATTGACAAACAAAGGTGCCATGGAAATGTTACAATCTAT
ACAAAAAAACATCGGA
AATGGTCTGGAACTTTTGGATTATATGGGCGGTGTTGTGAACAAT
GACGTCATGACAGTG
TACAATGAGATCCAGAGCTGTGAAGGTCATGAGGATTTCGAGTTC
AAGTTTGGGAATCTC
AGCGGTCAGGACAGCCTAGGTCAAGGTGACAATCCGTTCGCCAA
TAAGAATCCACTCGAG
GTCGTTCTGATCTACATGGCCCGCAATCACCTACGCCTGGTGGAT
TTGTTCAGGACTTTT
GATAAGGACCAGGATAGAAGAATCTCTACTGACGAATTCATCAA
AGGAATGCAGAGTGCT
GGAATGCCGCTCACCGACTCACAGCTGGAGAAACTGGTTGAGAT
GCTTGACAAAGATGCT
GACGGAGAAGTTGACTTGAGAGAACTGCTCGATATCGACAAAGA
CCACAAAAAGATAAAG
AGGCGAGCGAAGGCTTTCGTTGAAAGCGAAAGAGA
>ConsensusfromContig3850 Average coverage: 23.73
GTCCTTTAGGTGGCATGATCACACGCCCAACACTCTTCTTACAAC
CATCCTCATCATCGT
CTCGCTTCAATAATCTAGGGTCATGGACAGGCGTGCCATCTTTAT
TTAACAAAACGCCCA
GAGCACCATCTAAGTCATTTCTCTCTCTTCCGTGTTCACTAGTTGC
GGTGGATTTTCTCT
TCTTTCGAGGAGTTGGCGATGGATCCTTATACGACTCGTCATAAT
TATCCGGCTTCAGTT
CTCTCAAATTCATTCTCGCGTTCGCAACATTCACCGCATCGTTGA
AGCCTCGCAGTTTGC
CACTCATTCTGCTGATTGTTGTGATAGATTCTTCACTCAATTGTCT
CTCATATTTCGGGC
TTGGACCCATTTTAGAATTAAAATCTGCCTTCGCCATCATATTTGT
ATAGCTCAGATTTC
TTGAAGGCACTTTCATGCCGGAAAAATCTTTGATAATTTTGGATT
TATCTTTTTGCGACA
ATGTACTCATATCACTTTGTGTCAAAATATATTTCCCCATTTGCAT
ATCTTTCTGTAATT
TCCTTGGTTTGATTTCCTCAATACTCCTCGTGTTTCCATCGGGGTC
TCTTATATTACTTC
TGTTACTCTGCAATTTCATGGGAAGATCGATAAATGACTCTCGAT

AAATCTTATCAATTT
CCTTGACTACGTCAATATCTTTTCGTTTCTTTCCTTTT
>ConsensusfromContig3851 Average coverage: 9.71
GGAGTTTCCATATTGGAGCCAATGAAATTAGTATTCGCACCCCAA
TTGATGATCTCAGAT
ACACATGATATATAGCTCAGTTTGATAGCTTCAGTTAGTAGAGAA
TTCACGAGTTTATCA
TTCTTTTGAGATACGTTGAATAAATCTAATAGAACTTTGAGAAAA
AATGGTCTGTTCAAT
TCTATAGCCTTGAGAAGTAAATTTGAATCGCAAAGAACATCCTCC
TCTCCTATAGTTTTC
ATGTGTTTTAAGACGATTTGGAAACACTCGGGGTTGTTATATTCC
ATTGTATGACCAATT
ATTTCGACCAGCTCCATTCGATTGAATACAGTCGCCAGCATCAAC
TTGAAAATTCTTGTT
CTTTTGCCCCTGATAGCCTCACAGAATTTCATGATTCACTTATTTA
ATATGTATTCACAT
ATCCGGATTTCGAATCACATATTCATTTTATAAGTGTGAGTGCTAG
CTTTCTTCATTACC
ATGAATTGACAAGATCGGAA
>ConsensusfromContig3852 Average coverage: 17.42
GATAGACATCGCAAAAAGAGCAGCAACTTGTATTCAGAAATTGTT
TATGATATTGGCGGC
AGAATTGATGAGACATAAAGAGAATGGTTCACGGATAGACATCG
CAAAAAGAGCATCAAC
TTGTATTCAGAAATTGTTTATGATATTGGCGGCAGAATTGATGAG
ACATAAAGAGAATGG
TTCACGGATAGACATCGCAACA
>ConsensusfromContig3853 Average coverage: 13.35
AATTAATTCTTTATGTTTTTGGCTCATCGAAAATATTCTGATCAT
ATTAGACTCAGACAG
TCTAATGTAATAAAACCGTTATATTTGTTATATCTATATTAGTTT
GTTACGTACGAAAAC
AATGGTATGATATAAAAATTCTTTCATGAATGGTGTTACTAATAC
GATGTGTATCCGATC
ATTCGTGTAGAAGATTTTTTAGCACTCTCCTATTCATGAAATAAT
GTCTGCGAAATGTGA
TTTAAATGATGAATTGTTTCATCAATCTTCAAAAATGTATTATTTT
TCATTCTTGTCACG
CGATCAAGTTCGAGATATTGTTTTGAAATAAACTCATATAGAATA
TACTGGAAGAAGC
>ConsensusfromContig3854 Average coverage: 14.92

TCAGACGTGTGCTCTTCCGATCTTGTTTCTCCAAAAGGAAAAGCG
TCTAAGACTAATGAG
CGTCAGAAGAAGGAAGAAATGTTCTCAGAGGTATATCATCTCCT
GCAACAGCAGAACTTG
ATTGAGAAGTTGTCTGAAGAGCTTCATACTATTCCAGTATCTCAA
ACGCAAGAACAAAAT
ACACTACCTGCTATTTCCAGTTTTTCTAATAAGGGCGATCCATAT
TCTACCATGCAAGGG
GTTGCCCGTCCACAATCTCAATATGACCTAAAGACTCTTCAGCTA
CACCAACCGACGTCC
ACAATGCCTGGAAAGCACCAGGAGAGCGGACAGATCATGTCACA
GGGTGGTCAACTACCG
CTGAGGTCTAACCAGGGTGTATTCAAAGTTCCCCAAGTAGAAGGA
ACACTGTCC
>ConsensusfromContig3855 Average coverage: 6.09
CCGTGTTACGGACATCAGCCGATTTTCCTGGCTCTATTTTCTTCTC
ATCAGTTTTGCCGA
GGCAACAGCAGCAGTAGATTATTCCGATCAGAATAAGGATCAGT
AGGATAACAGCAACCA
CAATCAGAGCAATTTCCAATGTTGAAAGGGTATTTCCGCCAGAAG
TTGATGCCCCAGCAG
AAGGTGCATTTGTTGGAGGGATCGCTGCAGTCGTGGTGGCGCCAT
CTGTATTATCATTAG
CTGTGGTAGCAGAAGCGGTGATGGCAGAAGCAGTTGTGGTTTGC
ATTGAAAGCTCGG
>ConsensusfromContig3856 Average coverage: 7.27
CATCACTGGGAAGTACGGTGATCTTGGCTTTGTTGCGTTGAGTAT
CAATAATAGCCCCAT
CACTGGTGGAAATCAGCTCGATGGAAAATGAAGACTTATTTTCGG
GAATATCATCGTCTA
TGAAATTCAAGGGAAACGTGCAAAGTTGTTCACCAAATGGACAC
AAGAAGTTACTTGTTG
TACGAGTGAGTTGTTTTTCCAAATTCCGATGATTATATATAAGTTC
ATCATCCTCATTCA
ATAACATTGCTCTCCAAGAAATCGATACAGAGCCGAACCAAGAGT
TTTGCCGTTCCACAG
TAAGAAATGCCGGAGTGGATTCTTCAGCTATTATCTCAGTCGAAT
TCGATTCGAGAAAAC
CCACATAACCCGCCCACAAATCATTCCGTTGTATAGTTATCTCCA
GCCATGCCTCATGTC
CTTCATCAACATTGGCCATGGCGACGCGAACACCTTCCGTCACAC
TGACGACATAAACGA

AGAAGCTCTCGTCAAATTCCG
>ConsensusfromContig3857 Average coverage: 7.10
AACGCCTCCGCTATAAACAAATCGATGCTCTAACTGCTGATGATA
TTGATATCCGGAGAA
CGCTGGAAAATTTTGCTCATTACGTCAAAAAAGTTCGACACACG
GGTGGGAGGGTATTGA
TATACTCAGACGAGGTCAGCCAAAGCGGGGTGGTGGCCTTGACTT
ATCTTGTTTTGGTCA
AGCGTGTGTTTCTTCTTGAGGCAGCAAGGTTTCTCAAGGATTGTA
GGCGAGTCGTCCTCT
GTAATGAAAGATACATGAGAAGCTTGGTTGAACTGGCGAGAAAT
CATGACGGTCTCGATA
AAAACCCGAATTTGGTTCCAGCTCTGAAATACGGGACAAGTTTTG
ACCTCCATCGGTTGA
GAGATGTCCATCTTCCTGATGTTTATATTATCGCCTGATGCATAC
AAAAATGACACTGCA
TTCGTCGAAATGAATTGCTTTGTGAAGTTATCATGTTATTTTCCT
GCCGAAAAAATTAAC
CTCTTGTCTGAAATCATTTATATCTGTTATGACGGAGAACGATGA
ACTGTTTTCACTCAG
GAAAAAGCCGCAATGAAGCTATAAAACTTTGATCGGTGATCCAT
>ConsensusfromContig3858 Average coverage: 4.67
GCTATTATAGTGGGGAGTGAGCTGGGTCACGTGTGAAAGGACCCT
TTGCCCCGCCCTCTG
GGATTTCATCTCTGCCGCCTTTTTCCTGGTATGTCTTATATAGATA
GATAAATAGATATG
CTATTATAGTGGGGAGTGAGCTGGGTCACGTGTGAAAGGACTCTT
TGCCCCGCCCTCTGG
GATCTCATCTCTGCCGCATTTTTCCTGGTATGTCTTATATAGATAG
ATAAAT
>ConsensusfromContig3859 Average coverage: 5.75
CCTCAATTTGCTGAAGATTATGACTTTGCATTTGTTACTATTTAGA
CTAAGCCTATTTTT
TGAAATCCAATCAGAGCAATGCTGATAACCAGCAGACATTTTAGA
ATTCAATTGGTTGAA
ATGCTTATCTGTGGCTGAGATAACCAAATCGTCGGCATAGGCGAG
TATATTGTAGTCATT
TGAATTGTAGTTGAAAAGGCTAGTTACCAATATGTTCCAAAGAA
AGGGTGAAAGAATAGC
ACCTTGAGGACATCCTTTAGTATTTATCCACTGTTTGGTAATATCA
TTAAATTTCATGGT
TGACTCTCTGTTCTGCAAATATGATCTTATGAAATTCAGTAAGTA

CTCTGGGCATTTAAC
ATCTATTAAAGATTCCATTATGCGTTTATGAGAAGCATTATTGAA
AGCACCTTCAATGTC
CAAAAAGGCACATGCATGCATTTTTTTTGTCTTGAATGCATTTTC
AGCATTGGTTACAAT
TAAG
>ConsensusfromContig3860 Average coverage: 4.12
TATCTCTACACAACACAAAATGAAGAACACCCTACAAAACCACA
TAAATAATGAATACAA
AAATATGAGAACAAAATGGTCTGAGATGGACCACCACATGATAA
AAAACAGAAAGAATTC
ACCACAACTCCCGAATTCGCGGACTCCAGAAACAGGATAGAAAA
AGTCACAGTATTACTC
AAAAATAGAAACCCCATGATATATAAAGACAAAACAGAGCAATA
TCTTATATTGTCGAAA
AACATCAACCAAATCAAAAACATAAAGATGCAAGGCTCATATAT
CG
>ConsensusfromContig3861 Average coverage: 4.25
ACCTCTTTTTCTTCCGTCTTTCTCCGAATATGATAAACAGAGATAT
TTTGCGATCTTTGA
TTGTTATTTCTACTTTGCTCAACTGTTAATGTTTACTATCACCTCTT
TTTCCTCCGTCTT
TCTCCGAATATGATAAACAGAGATATTTTGCGATCTTTGATTGTTA
TTTCTACTTTGCTC
AACTGTTAATGTTTACTATCACCTCTTTTTCC
>ConsensusfromContig3862 Average coverage: 8.06
TTACATTTTAAAAATCATCATATAGTCTATGATTGAGTTATCTGT
CATTCCAAAAACTTG
AAGAGTTGTTGTGAAAATCGGGCTTCTTAATAGATATGTCGATTT
TATTCGATTTATGTT
CGATGGGGGCTTTACATAGCCTGTAGGACAAAATCATGGCAAAA
TGATCTGGAATATGAT
ATGATATACACCTAAATAAGTCCATCTTCAAGCACCATGACATTT
GAGCGGCACAATGAG
GAGATAATCCTGGTGAACTTGAAATAGATGGCATCAATGAATTA
CCAACAATAGAAT
>ConsensusfromContig3863 Average coverage: 2.97
GAAAATACCGCCATACAACACGCGATTAATAAAGAAATTGATCA
ATCTGTTCAAATTGAG
ATAATCACTCTTCTTATGAAGGCAAATGCTGAGATCAATCACAA
AAACTCGAAGAATAAA
ACAGCACTGGACATTGCAATCGCTCTTAGAAGATTTAAACTGTCT

GAATTTCTCGAGCAA
TCAATAGCTCACAAAATAGATGAAACAAATGCAAGGAAGCTTCA
TCACGC
>ConsensusfromContig3864 Average coverage: 8.76
TGTTCGTTTGGTTTTGTATAGACTGTTGAAAAAGGAGAATAGGAT
AGCGTTTGATTTATA
TTTTAAGGCCTTTTTGTAGTTTAAGTCCCTTGACTTCATGGCGGAT
AAGATATCACTGCT
CATCCATTCACATTTCTGATTCGTGTATCTGTTCGAGTGGGTTTTA
GGACCACACTTACT
CCTAATCATCACGTCCTTGAACGTTCTTTCAAATGCATTATACAT
TTTATCGCAGTCAAG
ATTGTCAAGAGATGTGAGAGGAGTCATTCTCGAAAAGTATTTGTT
GGATTTGGATATAAA
CTTTTTATGTGATATATTTATATTTGATTCTTTAGTTTCAGCATAGT
C
>ConsensusfromContig3865 Average coverage: 4.40
CACTGATAGTTAAACAAGACAAGGGCTGCCAAAAAGGACATTCA
GAAGCAGGAGCAAACT
GGAAAGACGGACTAAAACCAAGAAAAAAACTGAGAACTCTATGG
ACACTGATAGTTAAAC
AAGACAAGGGCTGCCAAAAAGGACATTCAGAAGCAGGAGCAAA
CTGAAAAGACGGACTAA
AACCAAGAAAAAAACTGAGAAC
>ConsensusfromContig3866 Average coverage: 2.74
CTCTTCTTATCCCTCTTGGTAATAACGACAGTGTGGATGGGGGTT
CCATCACAGTAGTCA
CCGTCTAATAGGATGATGTCAAGCTGCAACTTCTTCCTCTCATCTC
TTCTTATCCCTCTT
GGTATAACGACAGTGTGGATGGGGGTTCCATCACAGTAGTCACC
GTCTAATAGGATGATG
TCAAGCTGCGACTTCTTCCTCTCATCTCTTCTTATCCCTCTTGGTA
ATAACGACAGTTTG
GGTGGAGGTTCCATCACAGTAGTCATCGTC
>ConsensusfromContig3867 Average coverage: 14.25
ACAAAGTAAATGAATTTATTTTAAAAATTTGCCATATCTATATGG
TAATAGTGGAATGCT
ATATTTTTGCATGATACTTTATGATTTTCATGTCTCAGTGATTATA
TATCAAATATAATC
ACTTGTAAATATTACCCGTTATACCAAATCCACGGTTGCTTATTGT
TTTGTTACATATCG
ATATGTATTATTTCATTTATATTTCTCGCTTGAGATTGTATTTGGT

AGAAGAAATTTCAG
AGGGACCAAAATGTTATAATCTCAAATAAAATAGTTTCATTGTTT
ACACTATAAATTGCA
GAATTTTACAAAAACACAGTGACCATCACTGGTATATGGAACAAT
AATTTCACTTCCACA
TTCCGTATTCTTCGTCTCAATGTATTTATGCAAGTTTCTCATTCAT
AATTTACGTCTATA
AATTTTGTTAACTCGGGCATAATTATCTTATTTAGCCAGGATATT
GTACTTATAATCACA
TGATGAATGTGAAAATACATGAGGCCTGCTCATGGT
>ConsensusfromContig3868 Average coverage: 12.16
CAGACGTGTGCTCTTCCGATCTTTTTGGAGAACAAGATGAAGAAG
ACGATTTGGTGCAGA
GTATTGGAAACTCAGATGGCTTGGGTGATGAAAATTTCGATAAG
GGCCCAGGACTATCCG
ATAGGGAAAGAATGCAACTGAAAATAAAACGTTCAGTGACCAAT
CAAGTAGAGAGCACAC
AAAGATCTCTGGGTTATATAAGATCTTCGCACAGCATGGGTGTA
GCAACAGCAGAGGAAT
TATCCGTACAAGCTGAAAAACTTGATAATATTGAGCGATTGAATG
ATGACATTAATGCTA
TTACCAAAGAAACACAGAGAGATTTGAGATC
>ConsensusfromContig3869 Average coverage: 5.15
TGTTGTTGCTCTTTGCTCTTGTTGGTTTCTTGTCTCCTTCTCGTTTT
ATTATGTTTTTCC
CATTTGGGAACCCTGAATTTCGAATAGGCATATGGAATTCTGCTT
ACGTCATACTCTCAC
TTTATTTTGTAAGGGTGTTATTATTGAACCATTTCTAACAAGCTG
ATCTGGACAACATTC
TGAATTAACATTTTTCGATATTTGAATCTTTCAACCTTATCTTTCG
CACTCTATTTGTAA
GTGGGTCAGATAACGAATGTCATGATCTTTCACACATGTTTCTCT
>ConsensusfromContig3870 Average coverage: 4.63
ATAATATTTTCTATATCCGTAATTTATTTATATATGAATATTCATG
TATACTCATATACA
TGTATTTTTTCGAAAATTTTAGACATGCGTTATAAGAAATTTTTCA
TGGAGTATTCTATG
CTAATTTGGAAAATATTTGTTATAATTCGTGTACTATTATCTGATC
TGCTGATGCTAATA
GATTAGGGTAAAGAAGTTATTTTTTAAGATTTCAAAATTTCCGTT
TTATGACAGTTTATC
GTTTTTTATCATCTTGTTTCATAGTGTTTTACTTTCATCAGCCAAT

CTGTCAAAAGTTCA
CTGCACAACTGAAAACGCTTACCTATCATGCTGATGTGGTATATA
CGCTATTGGAAGGCA
GTTAATGATATTTAAGTATTGTACCAATGAGGGAACTGTTTATAT
TATTTTATAATCGTG
TTCCATGAAGTGTTTTACCACTGATCACTAAAGTGTATCAAGTAG
TCACTGGTTATACTT
TCAGTAGTCAATCTGTC
>ConsensusfromContig3871 Average coverage: 9.06
CCGATCTATTTATTCAGTATAATTCGAGTGATAGAATGGGAAATA
TCTCTATAGTTTCAG
AGATACAGAACGTGATGACTAGCACCGAAATGCAGATGCTGATG
GGAGGAATCGGAGCTG
TGGCTATTTTCGCTGGCATAGTTTTATACTGCCTCTACAGCGTTCA
CCGTGATGCCTCGA
GAGAGATCAAGGCATCTGGGATATACAAAACAACACGAATGCTC
GGTGGATCCTATACAA
ACATCCGGAAAGACCCGGGATCGCTGGTGCACAACGAACAATTT
TCAGACAACAAGTTA
ATTCTCACACCATATGTTACACAATTACTGAAGATAAAATCAAAA
ATTCATCAGACAACA
ATATCTCCAAGGACCATATTCTAAAAATCGATGATTTACAATCTG
CCAAATCTGATTTTT
CGCAAAAAGTACAATATCCCATTGAATCAGAATCGATTTCAATG
TATAGAAATCCAAAGA
AATCGATGGAATCGAAATCCACACGGTGTGCGTATAAATCTTGGT
ACAGTCATTTCCCTG
GCACCAGCTTGTCAATTATTGAAGAAGAAAGCGACTTGGAGGAC
AAGGAGCAGACGATAA
ATTCTAGAAAATCTATTTCAATTGACGCAGCACCATGTCTAGCCA
CAAGCTTTGTACTTT
CAACAAATGTGAACATATCTGTATTATGAATGCAAT
>ConsensusfromContig3872 Average coverage: 4.28
GTCGCTTTCCTGTTTGCCCTCATATCCGTTATATTAACTCTCGTTTC
TATCATACTTTTC
TGTGTTGAGACACTGCCAGTGTTTTCCCAGAGCCATTGTGTTGTT
GACGAAGCGCCAAAT
TTTCTTGACCCTTTTTTCCTAATTGAAACAACTTGTACAGGATGGT
TCACCTTTGAGCTA
GTTGTTAGGTTTTTTGTTTGCCCAAGCAAGCCGGTTTTTGTGAAA
GATTTTAAAAATATA
GTTGACTTAACCGCTGTTGTTCCATATTACGTCACGCTTTTCCAAG

TTTTATCAACAATG
AGTTGCGCAAGTGCAAAGTCAAGTGCATCATTAGCGTTTCTTCGT
GAGAT
>ConsensusfromContig3873 Average coverage: 8.64
TTTTTAATGGCTGAGAACAATAATCAGAAAATGTATTTTCTACGA
AAACGAGAATCCAAA
ACGCATTACATAACCGGAGATTTAGAAGAAGAGGCAGTGAGAGC
GAGGAATCTATCAAAT
TACAGAGAAAATACTAGGAGCTCTTTATTGGATGCCATAATCGA
GAGACTCCTGCGCGAA
CCATGGTTTGTGCATCTGGAAAAGCCACAGCTTTCGGATATTTTC
GAGAAAATCGAAGAA
ATTCTTAACGCCGCAGATGATGTGTCTTTGTACGAAGCAGTCTGC
AGTTACATCCTTGAA
ATACATGGAGAATTCCCCGTACCGCCATCCATACCAAGGATTCTG
C
>ConsensusfromContig3874 Average coverage: 13.85
TCCCTACACGACGCTCTTCCGATCTCCTGCATGTCGATTTTTGTG
GAACCGAATCAGTCC
GGATGAACTCATCGACCTCCTCTTGATTCGCAAACACTTCGCTGA
AAAGGGGAGGTCTCC
AGCTCTTTCCTCCTGTCTTCTTTCTTACGAGTTGGAAGTTGCCATC
CAATTGGATGAATT
GATAGGGATTCGTATAACATGATGGACATTCTGTCCCATCATCGA
CATCAACATTATTTT
TATCGTGATCAAAACGTCTGTATTGGTCAAAGGATCCAGCCATCA
GATCTCTATACAGAT
TGACACGTTTCGAACCCTCGAAATAGCCATTCGATATCTCGTCCA
GCGCCATCGAGATTC
CTTTTACTGCTGTGTGGCAGTTCATCTGCAGCAAGTGAATGGTCT
TCATGAGATCCATTG
T
>ConsensusfromContig3875 Average coverage: 5.00
ATGTTATTCGAATACCTTTTTAATTATCGGCAAAACTGCATATGT
TGTGCGACCTAACAT
CGATTATTTAGATAGAAGAGTGACTAAAACATATTTTAGAAAGTG
AAGGTGTATTTTTTA
TAAAAAATAATATTAGGTTCTTTTCATCACATGTTTCATTTGTTCA
CAAAAGTTTCCATT
CTGGGAAGCTACAAAATCATCATTTTAATGTTTTGTTTCCGATAA
CAATCTAGG
>ConsensusfromContig3876 Average coverage: 6.42

GATGACTTCATTTTCCGGTTTATTGACACATATTGCAAAATTGCA
ATATCTCAGGTAATA
ATTTAATGCATCATATCAATCAATACAGTTTTTCTGTATGAGAAA
TATACCTGGAGTGTC
TTAAAATCGGTATCAAAGATCGGTTATACCGTCTGTAAATGAGAT
ATTTTAAGTAGTGGT
GTTTGTTATCTCATTTATATAATGTCCTTTGCCAGTTCATTAAAAT
CATTCATTACGTTT
TCCTTTTCGATGGGAATGACATCATGGTTCACCAAACAAAATTCT
TCATGCATTAACAAA
ATAGTTAACTCGTTATTTTCGTATTCTTATATCACTCGAAACTAGA
TAATTTCTGAGATT
ACTTCCTCTACGAAAAGCAATAAGAGGTTGAGTCTTTGTGTCGCT
TCTTAATCTCAGATT
GTTCACATCTTCTGAAAAT
>ConsensusfromContig3877 Average coverage: 6.33
GCGAAATCATCAAAAAACATCAGAAGCGTTCAGTGCAAGACGAT
GTTGATAAAGAAAAAC
GAAAAATAACACAAAGTCGTGATAGTAAAAATACCCATCATCAA
AAACCTGGACTGTCAA
AAGATGTCAGAGATAAAGATAATAAAAAAATAGAAGACAACATT
AAAATCAGAGAAACAC
ACAAATCCTCACTGGATAGTTCCAGTAAAATTCAGCGAGAACAA
GCTCAAAACGATAAAA
ATAAAGAACATAATTCTCGAAAACAAATATCTAGTACGAAGAAA
AATGATGATATCAAGA
GGGAAAAAATAACGGGTCCTGATTCTATTAGTATAC
>ConsensusfromContig3878 Average coverage: 8.75
TGTGTCTTCCGATCTCGGAGGCTGAGACTATCTGAGCATGGCTGC
CAGGACTTCTGCAAT
AAACCTTGCTGTAAATGTATTTTCGGGAATTATGCTGTTAGAAGT
ACATTAGGTTTTGTT
GGTGGACTATTTTTGACAACGATGTTATTCGTAATACTGCGATTG
GAAACGAGCTATCCG
GAGAAGGTTGTGCTGTATTCGTGCATGGTCATCGGAACACTTCTA
ACACTCTTGATGGCA
TTCAGTTCAAAATTCAGATGCCTCATCGCTCTCATTCTACCCCAA
TTCGCCACAAAGGAG
GGGAGGACAGCTGTTTCGGCTTATATTATGGTTTTGATCCTCACT
GGTCCCG
>ConsensusfromContig3879 Average coverage: 4.66
AAGCACCATCTTGTGTTGCTTTATACGAAAATATCGGAATGCTCT

CATTGGCAGTGATTG
CATCAAAAACAACATTATGGAGCATCAGATTGCCAGAACTTTCCA
TGATATCACCAGATG
TTGGTAATAAACCTGTATAACCTTTGGCATTTCGAAAGCTCGAGT
TTTTAACAACTACAG
TCACTCCATCACTAACATAGAAAGTGCCACCATTGTCCTCCGCCG
AGTTGTTACTGAATA
TGCAATTCTCGATGTTGCCTGTAACAACCCCAAGATCGGAAGAGC
GTCGTGT
>ConsensusfromContig3880 Average coverage: 11.65
CCTACACGACGCTCTTCCGATCTGCTGCTAAGAAGTTGAACTGA
GCAGCAAAAATGAGAC
ACATTCTGTTGTTAGCAGCTCTCACCATGGTTTTATTGAATGGAA
GTCATACGAAACAAG
TTCTGAGAAATAAATGTAAAGCAAAGAATCTCGTCAAAAATGTG
ACCCAGGATCCTGTGA
AAAATAATAAAAACGTTGGATTTGGGATTTCTTTTCACGGGAAT
GCATCGAAAGGACAAC
TCCTTGGGAATGATTCAGAGGCAGAGGGAAAATCGGTTGCTGGA
GAATCGGTGTATCAAG
CTCTGAAAAATACATCTGAGGTCAGAAATATATCAGAGGCTCCA
GAGAAAGATCAAGCTC
TTGACAATAGAACTGAAGTCAACGATATATCAGAGGTATTACAG
ACAGGTCAAGTCTTGA
AAAACACAAGTGAGATCAAGAATATATCGAGCGTCC
>ConsensusfromContig3881 Average coverage: 6.38
CGTGACCAAATGGGAGAATAACACAAATCTTTACAACTGACCAA
TTCCGAACCACGCGAT
TGTCACATAATGTTTCCGTATAATCATTGTTCCCATTTATATATGA
ACTATTGAAATTAT
AATTCGGAGTCGAACATATAATTGGCCTATTGAATAACACAATG
AACAGAAAAAGACAAC
AATATAATATCAGGAGGCAAGATCGTAAATTGAAGCAGCACATC
CTCGCGAATCATGACA
GGAATCAGACTTCAAATATCTCACAGATTATCAAGTGCACATAAA
TTATGATAAATGATA
TTATACATCCTCAG
>ConsensusfromContig3882 Average coverage: 6.30
AAATTCTTCCCCTGAGGAATGCAATTCATGCACTAATTCATTATA
TGTTAGCTTCATAAA
TACACAGATAACGCACGAAGCTGTCATATTGAGAGTGTATGGAG
TAACAGCGAAAATCTC

AGTTAAATTAAAAGCAAGTATTTTACCCTTACAAAAACTGATGAA
TACAAATATTGTCAA
AGAGAGGTTTTTTGAAGAACATGCGACAAATGATCATTGGGGTG
ATAAAAAAGCAGACGA
CACCAAGGAATTTCTTCTGCAGGATTTACATTTTTGGAGACATTA
CTGTGTTCACAAATC
AGAATTGAATTCGACATGGAGAACAAGAGGGTGTCAAATGGTAG
GACCACG
>ConsensusfromContig3883 Average coverage: 8.57
ATTATTTAGCATTTAGTGTTTCTTTCTTGATCTTGTTCAATTATTG
GAAGATGTTTCGAA
GTTGGAAATTCTATAATTGAAGAACGGATGCAATCTAATTTTGG
CTAGAGAAGAAAATAG
GAAACTTACAATATGACTTTTCGAAATCGAGACTGATTTGTATAT
ATTAGTGTTATTTAT
TTAAAAGAAAATTATTGGCAAAGGTGGTTTTGGAAGTCAGATTTT
CGTTCATATTACTGT
CTAAAATCATTGTTCATTGCTTCTGAAATATTTATCGATTGCAATA
CTTAATACTTCAAT
CCGAATTTTCACGCCGTCTGGAGTAGTGTTTCCAAATATTTTATTT
GTGTATATCACACT
CATGTAACTTATTTCACGAATTAGACGTACT
>ConsensusfromContig3884 Average coverage: 4.99
GGATACGAGAGATTTTGATGGGAAAACTTCGCTGATGAAAGCAT
GTCTGAATAAGGATTA
TAAAACTCGGAATTCCTCCGTGGGCTTATTGATTTCGAAAGGATG
TGACGTCAATTCAGC
AGACGACACGGGGAAAACAGCGCTCATGTACGCATGCTCCGCAG
CAGACGTCGAAACTGT
CAACGTGTTGGTTCAAAACAATGTCGACCCAAATCTCACAGATA
AAAATGGGACCACCGC
CCTGATGCTTACAGCCCGCAGATCGGAAGAGCGCACG
>ConsensusfromContig3885 Average coverage: 6.27
GTCACCATCAATGTCAGTTGCCTTCACATAACCGATTTCATATCCA
GCTTTCTCGTTCTC
ATACACAGAAAACTCGTAATTATTCCTCAAAAACTTTGGTGGATT
GTCGTTTTGGTCGAC
TACGTTGATTTTCAATATCGTCGTCGCCGTTTTTGGCGGAATACCG
TTATCACTTGCTAT
TATTCTATATTCATAAATTTGATTTACCTCATAATCAAAGCGAAT
ATTAGCACGCACTGT
CCCGGAACGAGAGTTTATATCTACAGCTCCATCTGATTGGGTGTT

GAGCGAAACCAATGA
ATATTCAATTCTACTATTTTCACCAGAATCATTATCTGAAGCATG
CATAACAACAATTTC
ATGATTGATTTC
>ConsensusfromContig3886 Average coverage: 6.52
CGAAAAACGAACATATCAGACCGGTCGTATATAGGAACATGAGT
GAATTAGATATATATA
ATTTCACTAATCATTCTATTCTTTCGTGTGTGGATGGAATGAAGA
TCGGCGATTACTCAT
CAAACTTTGAACGTTCGAATATATTTGGATCTGTTTTTCTGAGCG
AAAAAGAATCCCCCA
AAATTCGAATGGAATCATTTGTTAATATTTCTATAAATCACGGTT
GGGGTGA
>ConsensusfromContig3887 Average coverage: 5.41
GTCCTCACATGTTGCTCGGTTTCCCCCGATGACAGCCAGGCAAAC
ATAAGAAATTGAGAG
GTATTGAACGATTTCATTATTGCAGCCACCAGCTAGATGAAATCC
AGAAAACGGCAATTC
AATTTCGCAGCGACGTCTGCCCGAACAAATCCTGTGGGCGGGTTC
GATGACATCCCTCTC
CCAACCGCCGAATCCCAAATCCATCTTGACGCATCTCCCGGTCCG
CATCCGTCCATAGAT
GGCACTGGTAATAAGGATAACCTGGTTTCCAGAACATTCGGCGTT
GAATGTTTTGAACTG
GCAAATTTCATGAATATCTCCTGTGCTCATCGAAACTCCTGATGC
GACACATTCTCCCCA
GAAGATGTAACTGATGCAGGTGGTGATAATCAGATAGATCGGAA

>ConsensusfromContig3888 Average coverage: 2.25
CTTCCGATCTAGCATATCGAAGAAGATGGAAGCGTGAACGTCAA
AAGACAAAATAGAAAG
AAATAGGACTCCAGGCAGAACGTCAAAAGACAAAATAGAAAGAA
ATAGGACTCCATGCAG
GGCAATGATGTTCGGATCATGAGAGCATATCGAAGAATATGGAA
GCGTGAACGTCAAAAG
ACAAAATAGAAAGAA
>ConsensusfromContig3889 Average coverage: 6.16
CTGTTTTTCAAAGGACCATTCTCAGAGATTTGTCTTAGGGATTCGT
TCTCATTTCTTAAT
TCGAATATTTGCTTATTTAAATCATCAGTTTTGATTTGAAGTTTTC
TGTTCTGACTCCGA
AGTTCTGCTATTATGTCTTTGGCGCGTTTGGAGAGTGAGACATCG

TCGGCTGAGCACTCT
TCGGTCATGTTGATTCGATGATATATGTTTGATAAGATGCCTTGG
ATCCGAATGACTTGC
TATTCAAAT
>ConsensusfromContig3890 Average coverage: 3.86
CGTAACGCCTTGAGCAATCATCAGGATAAATGCAACAGCGAAAC
ACAGGCAGGGAAATCC
CGAAAACCAAAACAAAAGATTCTTCGGGCACTTTCAACATCTTCT
TTTTTCTATTTATGG
TATAAATCACACAATCTTCAAATCAGCAAAAACAGAATAAAAAA
GACATTCCAAATTAAA
CTGATGATGACACATGCTAGAAAGGAAATAGGAACTTCAATGGA
TATGCTCACTGATGCC
AATCAGAAAACACTGGATAAAAATAACGGGAATACAAACATTGG
GTTTGATGCAGATTCT
GCC
>ConsensusfromContig3891 Average coverage: 2.32
TTTATCCCCCAGTTCGGAAAAGATATTTTATGGTTTGAACCGTGA
ACAATTTCTAAGAAC
TTTTGTTACTTTTCACCATTCATATAACCTGATTATTTTAATGAAA
GAATGTCTTTTCAA
GTCCAATGTGATTTATCCCCCAGTTCGGAAAAGATATTTTATGGT
TTGAACCGTGAACAA
TTTCTAAGAACTTTTGTTACTTTTGACCATTCATATAACCTGATTA
TTTTAATGAAAGAA
TGTCTTTCCAAGTCCAATGTGA
>ConsensusfromContig3892 Average coverage: 6.62
AGAAATTTATGTCGTCTTCAGAAGATAGCCTCCTGTTATCTGCAT
TTGAATTCATTCAGC
GAAAGCTGATCTATCAGTCCGTATACGAGAGATATGGAAAGAAG
GTGTACATGGAATCAA
TTGTCACTCACCTCAGTGCTGAAAGATTCGTGAAAATAACTAAAA
TGCCTGACATCGAGA
CGTTCAGGGAAATGAATAAGAAGAATCAAATGAAAGAAATGGAA
GAATTGAGTGATGCAA
GGGGATTCGGAAAAGTAATGGAAGT
>ConsensusfromContig3893 Average coverage: 2.97
TTGGGTTACATGACCAATTGACAGCACACAAATAATTACTGTTTC
ATTCGACTGAAACGG
ACAACGGCTCGTGTTCGGATATTTTGACAGGCTTTGTGAATAGTT
ATGATTGGGTTACAT
GACCAATTGACAGCACACAAATAATTACTGTTGTATTCGACTGA

AACGGACAACGGCTCG
TGTTCGGATATTTTGACAGGCTTTGTGAATAGTTATGATTGGGTT
ACATGACCAATTGAC
AGCACACAAA
>ConsensusfromContig3894 Average coverage: 4.29
CTGTCTTCCTGAACATATGGAGTCGGCGAAGCCAAGAACATTGAC
GTTGCAATTCGAGTC
CTGGCCGTAGTTATGAGAGATACACCGACCAGTGTGCATTCTCC
CGAAGAGGGCGCTGCG
GATGAGGATCACCCTCCCAGGGGGGCACGTGACATTGAAGTGTTC
CGTGATGCAGTATTC
CTGGGGGGTGGGTTCACGATTTTCTCTTCCAAATCTGAATAGTTC
AATCCTGTGTATCTC
CAATGCGAAGAATAGAAAAAATAAGATCGG
>ConsensusfromContig3895 Average coverage: 10.23
TCGATATAAGCGACACATGATGAAGATGTATCGAGGAGATCACTC
TTGGTATTTCGATTA
TAAAACTTCACCTTCAACTATGATAGTGTCCAGTATGCGTTTCTC
GGGCTATCAGATTGC
CTTCATGATGATAGGATTTTTTATGACAGTGTTGTGGCTTTTCATG
ATGATTATCACATT
TGTTTCGGTGATTTATTATCCGTTCAGTGTCGGAAAATTTGATAT
CATCAAAAACAGATT
ATTCGCCAT
>ConsensusfromContig3896 Average coverage: 6.53
CCGAGGTGAAATTTTTCTCTCATGTATATCAGCATTTCCATGTTCC
TGTGCAGATTGTAT
CTCAAGCGGTGGCGTTGATATCTCAATTTCTTCTTCTTTGACATTG
TCATTGTTGTCCTC
ACTTTTGACCTCTTCTTGCTCAGAGGTTGAATTCTTAGAGCTCAAC
TCTAATATATGGCC
AATATTGAATGCTGACTTGAATACTTTTATCTGTGATACAGAATCT
TTTTTGGGTGAATT
TTGGGGTTTCTCAGAATCCATTTTAGTGTTGGTAATCTTCTTCTGT
ATTCTCGTTTTGGT
ATTCTGATATGTCAATCCGGGCTTATCCTTTTTGGGTTGCGGCTTG
TACCCCCTAGCTGC
AATTTGGGCGATCAGCATTCCCGGTTCTTTACCGTATTTAGCGAG
AATGTCATCCATGAA
CACGCACTTATTCATCTGTTTATACTCTTTTCGTATGTCATATAGT
TCTTCCTGAGATTT
CTCCAGAAGCAGGTTT

>ConsensusfromContig3897 Average coverage: 3.14
ATGTTAGCTCTCTTTTAAGATGATTTTTTCAGTTTGCCTCCCTATC
GAAAATGGTATTTC
TATATCTAGTTTCGACTCACCAGTGAAATGAGGTACAGACGGGGG
AGGTTTATATTGTTA
TCATATTCGGCATTCAAGGCTTCCACGGTGGTTTCAGATTATCAA
GATGATATTTTAATG
ATAGCTCTCTTTTAAGATGATTTTTTCAGTTTGCCTCCCTATCGAA
AATGGTATTTCTAT
ATCTAGTTTCGACTCACCAGTGAAATGAAGTACAGACGGAGGAGG
TTTATATTGTTATCA
TATTCGGCATTCAAGGCTTTCACGGTGGTTTCAGATTATTAAGATG
ATATTTT
>ConsensusfromContig3898 Average coverage: 6.88
CTGGATTTAGCAGAACAGATGGATTTTCGAGATCTCAAGGTGCCA
GGTCTCTGATGGGTG
TGGGCAGTTGAAGGCCTCAGATACCGTTGGTAAAGTGCCTTCTTG
ACAATGCTAGGATCT
TTACTCAACTCTTTTGCTTCCACATTCAAACTTTTTTGAGCGGAGA
AATTTTCCGTTTCC
ACCAGTTCGATGACGTCACCATTAGATTTGTCTCCGTTCAAAAACG
TCTTTTCTATTTCC
TCAAACAGTTTGCCACTTTTTGAAGTTTCTTCAGCACTATAGGTA
ACTGTCGGATTCGGC
TGCTTTTCTTCAATGGTTGTATTTGATGTTTCGAAAACCACGTTAT
GCTCTTCCTTCGAT
TGCTCAGCTGTTTTT
>ConsensusfromContig3899 Average coverage: 3.93
CACGGTTTAATATGAATTAAATATGACAATGACTCATTGATATCG
CCAAAACTAGGATGG
ATGTATGATCTCATATGGGTTGGATGTCATCTGAATTCATATATG
CCTATATATCAAATA
ATAAGTTATTAGAACAAAATATTAAAATGAATCTCCGACGCGAA
GAGACCTCGGAAAATG
ACTCCGCGATGTCCTCGGGGAAGATCCAAAATCCTCCGA
>ConsensusfromContig3900 Average coverage: 2.22
ACCAATAATACATTGATTTCGAATGCATCACCCTAATATGCTCGC
TCATATAACGGGTTA
TATTGGGGGCTATGCGATGATATATGCGCTCATATAACGGGTTAT
ATTGGGGGCTATGCG
ATGATATATGCAATTCCATATTCAACATTGCTAAATTAATCATAC
ATGAAACGAAATAAC

CAATAATACATTGATTTCGAATGCA
>ConsensusfromContig3901 Average coverage: 8.75
ATACGCTCTATCTGAGCAGCCTACCCTTGAAAAATTGTTACAAA
AAGGGAGAATGCAAGA
GATAACTAAGGCGCAATGCAATGAGATGGAAAAATCTTCTTATA
AAGACGCTATTTGTAA
CGTGAAAACGAATAAAGGATCAACGCAAATATCGAGGAATGTTG
ACCTAAATACGCCCAA
ACAGCGCACACATGAAAATCCTTTTCGAATTAACAGCGGGAACTC
TTTTCAGATAAAGAG
ATCGAATAACGCCCCCTTGTGTTATTTTTGTAACAAATCTTTTCC
ACATATAGGTGGCAG
AAGAAGTTGCACGGCGTACGGAAAACAATGCACGAAATGTGGAA
AGTTTAATCACTTCGC
GATCGTATGCAAATCTCAGAGTACAATTAATCAAATAGATGAGCA
TGCCTATGTATCAGA
ACAAATTAACGAGGAAGAAACGAACGTTGATTACACATTTTCGA
TCGATTCACGGACAAG
TAACAGACCATTCACTAAACTTAAAATAGCGAATTCCGAGGTTGA
CATTTTGATCGACAC
CGGTTCCTCAATCAATATTATGGATGAAACAACGTTTAGAGCAT
TGAGAAACAAAGGCAA
ATTATCAAAGTCAAACACTCAAATTAGAGCGTACAGATCGGA
>ConsensusfromContig3902 Average coverage: 11.77
CTTGTGTATAGCGGCTTATGGGACGAAAATATAATAATCTTGGG
AAATGAGATGATGAGA
ATATTTACTAGCACCTTATCATAAGTAAAGAGAATCCATTCAAGA
CGTGTGCTTATAACG
AAGAGTGATTTCGAAATCTAGGTTGAGATATTATCTCTAATCACC
TAGAAAAATCGCCTG
TTTGTTATGAGATATCCCAATTGAGAAAATCAGTGATTGACAAAC
GAAAAAACTACAGTT
TAACAAAAAAATTTCTACAACAAACAAGTGGTAGAGTACAAAAC
ATATTTAGAATCCATC
GTACATTCCATCGAAATGATTGGATATAGAAAAGATGTTTTATAA
GTGTTAGATCGG
>ConsensusfromContig3903 Average coverage: 18.69
GTGACATACAATGAATCTAGTCGATACTTTCACCAAGAGTTTCAT
TCAGTTTCGTCTTTG
TGTCCCATCAGTTCAATTTATTATTCACAACAATCAATTTTCAAAT
GATTCATTCTCTAT
ATCTACAAATCATATCGTTTCAAAATTTCCTTCGACGCGGGTGAC

AAAGTGACATACAAT
GAATCTAGTCGATACATTCACCAAGAGTTTCATTCAGTTTCGTCTT
TGTGTCCCATCAGT
TCAATTTATTATTCACAACAATCAATTTTCAAATGATTCATTCTCT

>ConsensusfromContig3904 Average coverage: 3.91
AAACAATTTATTCATCAATAAATATCGTGAATGAGAACAATTCTA
GTTCATGTCACTTGA
GTAATGTCTTAAAATGTATGAATGTCACACTATCTGTCCAATAGT
TTTATGACGACCCTC
ACTATGTCTTCGCACTTTTGATAATACTGTTGAAAATGACAATTT
GTACGAAACTTATCC
TATGACCCTCTTGCTTTTGGCCATCGATGCTTTTAAAACATGGGT
TATTATCAATAATAG
CATATAAACTTCAGGGAGAATGTTCATAAAAG
>ConsensusfromContig3905 Average coverage: 4.95
GGCAGGACTAGAACGATTATATTTTTGGCAACGAAGAAGTAATTT
GTATTGGTGCTGATG
TCATACTGCACAATACATACGAGCCGAACGAGGAGAAAAGGCCC
ATCCTGCGACAGCATC
GCAATGAGAATCGACCAAATCTCCGTCTCCAGTGTTAGCTTCATG
CTGGTGAGGAACCTC
TGATAGCAAGTCTCGGTTCTTTTGACTTCCACGTG
>ConsensusfromContig3906 Average coverage: 9.27
GCTCTTCCGATCTTTGGGGTCTTCCGTGGCGTCCTCGAACACAGA
GCCTGGCTTGCACTT
CACGATAAATTTATCCTCAGATACTTTATATTCAAATGTACTTGTA
TTAGAATTTGCCAT
ATCATGGCCTGGACATTTCCGCTGTTGACATGTGATAGTCCCTTCA
TTTGACGGCTTTCC
TGTGTTCGAGCACTCGACCCAATATTGATTATGGAATTTTGCTTT
GCCGTTGATACGGAT
GGTGTATCCATGGTTACATCTCACAAGGCGGCCGTCGCCTGACAT
CCATTCACTGTTGCT
TGTCGCGTAGTTAGAATTTGGAATTATCAATTTACCGCACTTTCT
TTGGATGCAGTCGGT
GATGTGCGGTTCCCAGCGCCCGTTC
>ConsensusfromContig3907 Average coverage: 3.50
TTCCTCTCGATATTGTCATGATTGACAAATCTACAAAAACAAGTC
TGTTTATAAATCTTG
GGTGATATTGAAACGATTCTGGAGACAGATTTAACTTGAAAGTCG
TATCGACATGTAACT

GCTCATCTTTTCCTCCGTGGTATCCACCTTTGTGAATGAGTGTGTT
TCTAATCCTACGAA
ATTTTTCCGGGATTGTTTTGTTGAATAATGTCCACATGTCACATTT
TGTAATAA
>ConsensusfromContig3908 Average coverage: 2.30
CTGTCTTTCAATGTAAGCCCATGATCATGCATCAGCCTATAAATT
ACATCAAAAAAGTCA
CATTACATGTCAATGAGATGCTTCAGTTTCAGCTATCTTTCAATG
TAAGCCCATTATCAT
GCATCAGCCTATAAATTACATCAAAAAAGTCACATTACATGTCAA
TGAGATGCTTCAGTT
TCAGCTGTCTTTCAATGTAAGCCCATGATCATGCATCAGCCTATA
AACTACATCAAAAAA
GTCACATTACATGTCAATGA
>ConsensusfromContig3909 Average coverage: 11.83
CGATCTGGAAAAACACATTAAAAATGAGAATCTGAGGTGTGGAG
TTTAAAAGTCTGTTTG
ACGCTTACATACTACCGCCTATTCAAAAAAGCACCGAATGCTTGC
CCAGAATGTGACGGC
AATAAGGGAAGGGAGAAGAGATTAAATTGCATAGAGAGTCAAA
AACGAAAACGATCATGG
AAAAACACATGAAAAATGAGAATCTGAGGTGTGGTGTTTGAAAGT
CTGTTTGACGCTTAC
ATACTACCGCCTATTCAAAGAAGCACCAATGCTTGCCCAGAATG
TGACGGCAATAAGCGA
AGAATGGAAAGATAAGAATGACAAGATCGGAAGAGCACACGTCT
G
>ConsensusfromContig3910 Average coverage: 6.05
GCTAGGGTTTCTAACCTGAACAACCAGCTGAGGGGTACTAAGTTT
GTCCCTTCCCCATTC
CCTCGACCGTTTACGGCCATTCCTTGGAATAGTCTTACTGTCGAG
ATTGAGCAGGAAGTG
GGTACTGGCACCAGTCTCTTGGAAGTTTCCGTCGGTAATGTGAAG
GCAAAAGTTCAAGAA
AAATCTGGTAGCATCCCCAAGATCAAGGTTCTGCGAGCGGAAGT
GTGGTGTACTGCCAAC
AATCTTTCGTATCCGTCCCTCAAGGCCAAGTTCTATGATCTTGAGC
AGGTTTCTGGTTCC
ACCAATACTGCCGAAGCGAGGTCACAGCAGTCTGACAGGGGCAC
CTTGAATATGCCTGCC
AA
>ConsensusfromContig3911 Average coverage: 5.23

TGCAAATCACGGATAATGAAATAAAAAAGTTTTGTTCCGAGAGAT
AAATGACTTTTAAAT
ATTTTCGACAGATTTCAAAAGAAAGATGGAGGTTCTCTACAGATA
AACATCAAGTCTTCG
ATTTTCGATTTCAAAGAGAGGTACCTAGAGCCTTCACAGAGAAAA
CATTCTTCAGCTTTT
CAAAGTCTTCCATTTTGATCAGCCATTGATGGGCAAACCTTTTCGC
CTTCCACCCTTCCA
GAAGCGTCCATGTTGTTTGCGTCATTCTATAAATATTACCTTCCG
TCGACACAACAGCGA
TGCGTCC
>ConsensusfromContig3912 Average coverage: 3.92
GTCACTAGGTATATGATATAATCGAGAGGCAAGGAACTTATCAG
GTCCAGGAAAAACCAA
GACTTGAGATAATGCTTGGCGATTTGTCGCGGCTCAAGAATGATT
TCATCCGCGAAATTA
TCCGCAATAATACCTGTCCTGAAGTTAATGATGAGATCCAGAATG
AAAACTGTGTCTGAA
ATGCAGTTGAAGGCGATCCAGCCAGGTGTGAGGTCGTCGTTGAA
GAACGTGATGGCAACG
GGTAGAATGATG
>ConsensusfromContig3913 Average coverage: 21.84
TTTCCCTACACGACGCTCTTCCGATCTGCGGGATTTTGGTCTGGTC
CGATTTGCCGATAC
AACAGACATGATTCTGTTACATGAGGGCGAGATTGTTGCGGATA
ACGAATCGTGAAACTG
GCGTAATGACACTTTTCGTTAACGGTACAATTTGGGACACATTGA
TCAGCGGGGGTTGCA
TACAACAATTCCAACATAACCACCGCATTCGGAGGATTTGATGTG
AATATATTGATATCA
GGTTCATTGGTGTTCTTTTAGATCAGAAGAGCGT
>ConsensusfromContig3914 Average coverage: 6.01
TTGGTTATTGCCCATGTTTCGTATAATAAAGTCGCATAGAGTCAC
TCTAGGAAATCTTGT
CGAGCCAGACCATTCATCATTCACCAGAATGTCTCGTACTATCTC
CAAACCATACATGTG
ATAATCTGGTCCCAAAAACTCGTTCAGGAGAAAGAGTTGGAGCA
CTACGTTAACCATGTA
CAAAACTTTTGTTGTCATATACAGTGCAACTAGATAGTTGCCATA
TCTGTTGCCACAAAA
AAGGCAGAAATATTCTGCGAGGAAAATCTTCAAACCTACGCACCA
ACCTCCTCTGTATAC

TCTCTGATATTCGAGATAATGATCCAAGTGTCGAATCGTGTATTTG
ATTGTTTTATCCCG
ACGCTCTGGATACAAAGCGTTTTGAATTGTTTCAGCAGCCTCGAC
CAAGTTGTTGATGTT
TACACCAGATCGCTCACTCAAAGTCCTCCAGAACATACATGGTA
AGTGAAACAGCACAGA
TTGTATTAGAAGCACGATTGGAACCCATTGGTAATATCCAATGTG
CTGCTTGAGTGCTCC
AGATTGTGGTGC
>ConsensusfromContig3915 Average coverage: 17.69
GCTGGATTACTCGGTCATGATAAGAAGCAAGGAATCTCTTGATCC
GGGACATAAAGCTTA
CCTTGGAATGTTATATGTGGATGAGCAGCACAACCACCCCGTCAT
GAAGGGATTCGTGCA
TCTACTGCAAGAGGCTCTGAAAGAAAAAAGGCGGAGCGAAAACA
CTGATTCTCTAAACGA
GGAGAATATTGAATCCCAGAGCTTTCGGAAAAAATCCAACCGTA
GTCAGAGAAAATATAA
TCGTAGAATTGTGCTTAGATGGTATAAAATGATCACACTGATCCG
CAATCCGTCAATTCT
GCTCAATCGAATGAAGCAAAAGGGCCGAGATCGGAAGAGCGTCG
TGTAGGGAA
>ConsensusfromContig3916 Average coverage: 10.02
CACAACACAATCCCAAATTTGGGAAAACCTCTGAGAAGAGACCT
AACCATCAATACATTT
GTGGTATATCAGAATTATAATTGTTTCGTTCCCTCATATTTCATG
ATGAATAACGATCTA
ATAATATACACAACATTTTCAAAAATACAGCTTCCAATGGCGGAA
AAGTAAAATATCTGT
GAAAAAATATTCGAAACCCAATACTTAAACAATCATCTATACGTT
ATAAAATATGTGATG
AAAAAAATTTTCTTCACCATCAAATCCAGTCAATATTTGTGAAAA
GGATTTAGAATTAAA
CCCAATGTTCACG
>ConsensusfromContig3917 Average coverage: 4.25
GTGATGAGCGTTGACTGTTTAGTCTGAGAGAAATTGATTGAAAAT
GTGTTCAAAGATATC
TCTTAATGTGTGATGAATTTTAAATTCATTTGAGAATTCTCACAAT
TAGATTTTACTAGT
TTAGTTCTCCAGCAGATTTTTAAATTTCAAGTCATTCTAATGAAA
GGCAATTTCCTGCTA
TTTGAAAACATTCGCTTCATTAAGATTTGTTCCATCAATTTTTTTT

CATTAGAACAGTGT
ACGATTAATCTTTTC
>ConsensusfromContig3918 Average coverage: 4.91
TTCTGATGTTTTATCAACCTGCCTGTTTCCCATATCCCTGCCTTG
CAGAAAAAAGTATAA
ATTTCAAATTATTTTCTTGTTTCATGTTTGTTGATAATTTCAAATA
ATTCTTTCATGAAA
AATAAACAATTGATACATATGAAGAATATTTTCCACACTTATTGC
ACCACAGCTTTGTTC
TATATGCTGAAAATATTGCTTGAAAGAGTTTTCTCTATGTGCTCA
TAGATAAAAATTGTC
CATATTATTTTAGATTTAAATATCATATGACATTGTCT
>ConsensusfromContig3919 Average coverage: 5.71
AGTGAGAAAGGGCAGAATTGCTTTTATAAATAATAATAATTCCTC
ACATTTACATACTAT
TGCTATTGGATGCGCAATCACAACAAAATCTTTATCATCATTCGA
TCTTGATCATATATC
TCAAACTTTTCCTCTGTTAAGAACACTCATTCCGAGTTTTTGTAC
AACGGCCCAAACACA
ATTTCGTTATACTTTCTACCTTGCTTACGATAATAATGATACATA
CCTCAGTGACAAAAA
TAGATTGAATGA
>ConsensusfromContig3920 Average coverage: 5.22
GGAAAGTCCGCGCAGCTCAAAAACCCAACATGTAATGAAGGTGA
ACGGAGGGGCGTTGGC
CAGGAGAAACCTACGAGATACACAGTATGCGAGGGAGGCCAGAT
TCGGGAACAAGAATGC
CCCGGCCACTCCCTATGGCAACCGGAAATGGGGGCATGCGTAGA
TTCATCACCGAGCCGT
GAAACCAAGTTCGATTCGACGGCAAACGGAATTCCGCACAACCC
ATGCTCGCAGCAAACT
TTAGAAAGTGAGCCCAAGCAAGGGGACGAATTCCACGCATACCC

>ConsensusfromContig3921 Average coverage: 4.85
TCTTATTGATGAAACAATACCATTTACAGTTGAAACAGATGCAAG
TGATGCAGCTATAGC
AGGCACCTTAAGTCAGAATGGTAGGCCAGTGGCCTTTTTTAGTAG
GACTCTCAATCATTC
AGAACGCAGACATTGTGCCATTGAAAAGGAAGCATATGCAATAG
TAGAGTCCATACGACA
TTGGCGGCATATTCTTTGCAGAAAACCTTTTGCTCTGGTGACAGA
CCAAAAATCAGTATC

CTTCATGTTTAACACTACTCACAAGGGGAAGATTAAAAATGAGA
AAATTTTAAGGTGGAA
AA
>ConsensusfromContig3922 Average coverage: 5.13
TGATCAGATAATCAAAACGTAGGCAAGTTTAAGAGAACATTCGT
AGGTAACAACAAACGG
CGGAATCATATTCGAAGGGTTTGACCATCAACAATAAGCAGACT
GTGAATGCCAAGAAGC
ATGAAATAAAAGAGGCCAGGTGTATAATTCATCATCCACTGAAC
ACCAGTCGAAGCAAAA
CGGAATACTTGACGAAATCACTGAACTAAAGGGAGTAACGAATT
TCGCATTAGAAGTTGT
ACACTCTTCTACTAAGAAAGTCGAAGATCGGAAG
>ConsensusfromContig3923 Average coverage: 10.82
CATACGGGTAGCAGCTAAAAAAATACGAGATGATTGATCACAACT
TATTGAACTATAGAAC
TGAATATGTATGTAATTGCGCTGGATGTATATTGAATTGTGAGGC
TTTGCAACACTAATC
CGTACTAAACAACAAAACACCGATGATTCACATTGAATAAATAA
TAACGAAATGACGATT
CGGCGTAATCTCTGAGTCGCAATTTGAATTACAAGAGATAACATT
AATATTCAAATATAA
TATGACATTATCCCGAGATCCGGATGAATAACTTAATATTCTAAT
TATGTACATTTAGAG
CAGAATTCCAGACATCATGCGTATATATGAATGATTATCAGATAA
AGTGCCTGGAAAAGT
ATCAATGTATGATATGTGTAAATTATGATATATTTGAATGTTATT
CATGGTCATAG
>ConsensusfromContig3924 Average coverage: 8.69
CAGTTATGTAAAATATAGAATCTATTTGAACAGTTCTCTTTTGGA
AAAACTGCTACCAAC
ACTTGAGCATGACTCGAGTAGCTGATTGCGGTAAGGTGTCATCAG
GCCACGCTTGACTGG
CGATGGGGTCATGTGACCGCCCTTCAGTTTATATCTCCTTGGCTT
GGACGGCTGCAAAAG
ACGAGCAACTGTAGAATGGCCACAGGTGACCGCGCACTCCATGGG
TATCATTCCCATGTA
GCTGCGAATCCTCGTGTCAGCACCCGACTGCATGAGAAATATAAC
CACTTGGACACTACC
TTCCGTCGCCGCGAGGTGAAGGACGGTGAAACCTCTACTGTTTGT
CGCATTGACATTGCA
TCCTGCTTGGATCAGATCACTGAGCAGTGATATATCGAGATCGGA

AGAGCGTCG
>ConsensusfromContig3925 Average coverage: 2.21
GCCAAATGTAAACTGGATTTAAGAACTTGGATTCTGATAAGCTGG
TTCAATAACAATGTA
AAACATAAGGTTGCAATGCACCAAAGATCGTTCAGTCAAAATAA
GCTGGTTCAATAACAA
TGTAAAACATAAGGTTGCAATGCACCAAAGATCGTTCAGCCAAA
TGTAAACTGGATTTAA
GAACTTGGATTCGTATAAGCTGGTTCAATATCAATGTAAAACA
>ConsensusfromContig3926 Average coverage: 5.22
CCGAATACACCAACCAATATCGGAGTGCAGACAGATGGCTCTCAA
TTACAGTGGCTTAGT
GAATCTTCTATGATAATACCATTGGTCAATGCTGAGTTCTGCAAG
AGCGAAAAATACATA
GAAGAATTCAACATAGCATTAGCAAGGCATCGAAAATGCGTGGT
TGATAAAAAGCCTCCT
GTTATATACCCTTTACAACTGAGCGGGGTCCCAGAATCGCCTTTG
TTTATGTCTGCCATT
CCGTATAAGATTAATTTGGGCGATGAATGCTGGACAACATTTAGT
TCTGATGAATTCTGG
AAGTGCTCAGAGACAGAAAA
>ConsensusfromContig3927 Average coverage: 6.64
TTCCGATCTCGGAAATCAACCCGAAATGTCCATTGATGGAAGTTC
TTTGTCTGGAGATAA
CGTTACTCCGACGGTCATCGAGAAGCAATCCGGCGGCGTGTTTTT
TGATCCGATACCGGG
AGATCTTCTGAGGACGGTGGAGAAAAAATCACAAGTCGCCGTCA
AAATAAATCATATTCT
GTCGAAGTGTATCGGGGAATGTGCTTTCGAATGGAGTGCCGAAA
TGACGCCTGAAATCAT
TGATGTTATGCCTGAGGAAGGTTCGTCACGTGACGGCACGGAAG
TAAAAATCAATGGGAC
CGGGTTTTCAAACATAACAAGCGACAATCACGT
>ConsensusfromContig3928 Average coverage: 5.71
CACGACGCTCTTCCGATCTGCCATTATGAAACAATTTGAAGAGCT
ACACATTTGGGCAGA
TTACGCATTGAAAAACATTTCTCCATCTAATCGTGTGCGGTGATG
TGAACTGACGAACGA
TACAGCCAAAGCTTTGTTTTGGACATGTTTAGGTTTGGTCGGCCTC
TCTGACCATTTGCT
TGAAGTAGACGCGAAAGAAGCCCTTCTAGGCGCAGAGGTCGCCA
CGAATATGTTCTTCCG

GGTTTTTTCCAGCAGGATTATATTGAAAATCATTTTGGCCATTTC
GGGCAATCGGCCGGA
TGTCAATATTTTATAACTGTCCAACAGGTGATGCACACCCACAAT
ATTGACCGAGCAATC
CATAATCTTGGCTTGGATTTCGAAAT
>ConsensusfromContig3929 Average coverage: 6.63
TTCCGATCTCGCCATCATAATCTTTCTCATGGTTTTGAAAATACTT
GTTTTTGCGGTTAA
ATTCACTACCATAACCATTATAACTTCGCTCAGATTTCATTCTACG
TCGAATTTGGTCTC
TTCTAGTTTTGCTTGGAGAGTCATGATGTTGGTACGAGTAATCTC
TGAATTGCTTCGGAT
GTCTATAGGCTGCTTCTTTCCATGATGCCGTCTGCTTTGGTGCAAA
ACCG
>ConsensusfromContig3930 Average coverage: 5.66
ATCGACATGTGAATAAAGACCGATTCAATTCATATGATAAAAAC
AGCATCACAGATCGGT
TAATAAATATTTGGCATTCAACCTGAGGTAACAACATTCGACTAG
AGTATTATGTTATAT
AACAATCAACGACATACACTGTGGTTTATGTATATACATACACAA
GAACCTAAAAATGTA
CGCTACTATTTTGCATATGAGGTGTATAAAATTATTCAGAATCCA
CGAAAGTTTTAAATT
TAGAACCTAATAAAACGAATGAAGATAGTGAAAATTAAAAATCA
TTCTGTATACATACAC
AAGAACCTGAAAAATGTACGCTACTAGTTTGCATATGAGGTGTAT
GAAATTACTCAGAAT
CCACGAAAGTTTCAAATTTAGAACCTAATAAAACGAATGAAG
>ConsensusfromContig3931 Average coverage: 11.61
AAACAGAGTTAATGAAATTTGATAATAAGTCTAGAATTGTGAATA
AAAATAGCATTTCGT
CACCAAAAGAACCAAACTTTGATCTCGGATTTGATTTAGAGGATT
CAGACGAATTTGACG
ATGTAAATCCAAAAACAACTTTGAAAGCATTTGATACTGCTGAAA
AGTCGCCGTCTAAAC
CGGTTAGTCAAAACTTCAAATTAGATACAGGCAGCACATCAACAA
AACATTCGTTGTTCA
ATGATTCAGTTATAGATGTTGACCCATCAACGACTTTGAGACCAA
CTGACATTGCTATGA
AATCGCCGTCTGACCTGATTACCCAGAACTTCAAATTTGACACCA
ACAATACATCAACAA
AACATTCATTATTCAG

>ConsensusfromContig3932 Average coverage: 5.08
TCTAGCTGCCGTCGACATCGCTGTGGATGATGATGAATTGCTTAG
AGCCTCTGCTTCTCG
TCGAGCAGGATGCCAGACTTGTTTCACTGCCAAGGTCGATTGTTT
CTATCCGACTGATTT
GAATTCCAGGCAAATTTGCTCTCACCACTCTCGTTTTGGAACTGA
GAACGCTGTCGTATG
AAAGTTTCCGCGCTGTGTGTTCCCTCCAATTTGCTTGATCTATGCT
CCCGC
>ConsensusfromContig3933 Average coverage: 6.96
CTTTAATACTGGCCAAAAATCACTCATAAACAATATACATCAGGC
ATTCTCAAATTTGAA
AACGGATTCAGATTGGCTGGCGAATAATCTAGTAGTTGCGAAACG
TCAGCCACTTAATAT
ATCCCGCATTTTCTCCAAGCGAAAGAGAAATATTATTATCGAGCA
TGGTAATAAAAAATG
TGGGAAAGCAAGATGCACTACATGCAACTACATGATTCCAGGAA
AAAGCATTCATATTGA
ATCTCTGAATTCCACGATTTCACTTCCTGATGCGGATTGCGATTC
AGATAACGTTTTATA
TATGTTCATTTGTAATAGGAGCCACATGCTTTATGTAGGTGAG
>ConsensusfromContig3934 Average coverage: 7.35
ATTTTATGTCACCATTGCCGATACAGTTTGGTTCTTTATAGATAGT
AAATATATCTTCTT
TCACAGAATTATCTTTAGTATTATGCAGCGATCCATTCTCTGATTT
ATTTTGATCAATCT
TTGGAATCTTTTTTTTTGAACTTTTTTTCGATTTACCCTTAAAAAA
ATATTTCGCTTTAT
TCCAAGGTCCTTCTTCAGTTTTCTTCAGAACATAAAACCGTCTTGT
TTTCGGTTGAACAA
CATATATCCGAGAA
>ConsensusfromContig3935 Average coverage: 6.41
TTGGAAACATGCTCTTCCAGTCCTAACCATTTGCCATCAAATGAA
ACAACAAATCCGCGG
AAGAAGGAACCGCTCAAAGTATACCACAGGTTTATGGCAACCTTC
CATTCGTAATTATAC
AATCGGAACTTTTGTTCATAATCCGAGCTATCAAGCGATTCTACA
CATATTGTGTCACTG
TACCACTCTCCGCGGCTTGGAACCATATAAATATAGTTGGTACTC
ATGCATTTTCCAGTG
GGAGATTCATTCAGCCAAGCCTCCATATCAGCAGCGTCATACCAT
GTACTCACCGAGTGC

TTCCACAGATCGGAA
>ConsensusfromContig3936 Average coverage: 7.89
CGGTTATATGTTTCATGTGTGTTAACCTGGTAAAGAATGGGAACG
GAATAAATATTGAGA
AGCATATGAATTATGCATATTTTCATCATATAAGACCTATGAACC
AAACCACATGCAAGA
TATTGCACGATTCAACATGTTTGTGAAAAAATGAAAAAAATAAA
ACAGATTCACAAAGAG
AGAAACAAATCAAAAATCAGTGCAATTATTGCACGTAGAACTTCA
ATACATATTCATAAC
AATTTCACATTTTTTTGCATTTATATCTGCGATATCAGTATAAAA
AAATGTTGAAA
>ConsensusfromContig3937 Average coverage: 5.37
GTTAACATTACTGTTATTTGGTTTCTTTAGAGCGTTAAAGATGTTT
GGATTCGATGTGTG
GTCTGTTCTATTGTAAGTAACTCTCGTGGCTGGTACATCTCCAAAT
TTTAATTTCTGTAA
TAGAAAAGGAGTGTTTGTTTTGTTCAACTTTTTGTTGCTTTCTTCA
TTTTTTTTAGTCCG
TATTTGTTTTTTTCGTCCATAACTTTCGATTCCAATAAGAGAATTT
AAACAACTTGATTT
AGCCATAAGATAATCGTTGTAGGGTGCTATCATAACTTTAATTTT
ATCAACGGTGTTGAT
AAGAGCATTTACTTTTTTTTCTGATTTCCCATGGCCGTCAAATCTT
AAAGAATCTTCGTC
ACAAGCCGGTTTTTCAAGGCAAGAAGAGTATATTTCTCCTCCATC
ATCTCGAATTCTTCC
TCTTATCCATTTTGAAGTATTGGTGTTGTAGATGATTTCACTCAAA
ATCGTATGGTTATT
TCCTTCCCGCATACATTTTGAGTAGAGCGATGCGACTAAATCTTC
ATCTTCG
>ConsensusfromContig3938 Average coverage: 3.98
CAGAGATTGAAAGTTTTGAGAGAGGGTGTATAGAGTGAAACATT
CGTGTTCCTGGGACCG
TCAATGAGAGATCATACCGTTTCTTCATTTGTTTTGTCATTTGGAT
ATAATCTATCTCAG
TCGTAAACATGAATTTGACTCCCGGAATATTTATGACAGCCCAGT
CGTATAATTGTTTGG
GAGTGAGAATATGATCTTCCCCGATACGTTGAAGACTTGTTAAGA
T
>ConsensusfromContig3939 Average coverage: 6.97
AGTTTTCCACGCAGTGTGCATCGAAGTCGACAAGCATAATGGCGA

TTCTGTAACACGGGT
TTTGTGCGGATTCCGCATTTAGAGTCCATTCACATTTTGCATTTGT
TCCAGAGGTCTCTA
TGACAGTGTAATCTTCATTGGCAACATATGTGCCCCCGCAATTTC
CAGAATGATACGCTC
CGGGAGAGACCCGTGCATATGAAGCATTGAATTCAGATTCGGAGC
TTCGATATATTGATT
TGAAACTGATCGAAATTTCAGTGGCGTCCGAAATGAGAACATTTC
TTGGTCTTTCATAAC
CACAAAAACGTGGACCGGGTAACCCCAGGTCTCCCCTATATCGCA
CTTCAACCCAGTCGA
CGCAATATATCTGGGATGTAAATATTGAAAAATGTCCGACGAAT
CTCAAAACCAGCACAC
CACCGATTTGTGTAGTTATAAGCCAATGACATTTTTGATAGTTTT
CATGATAGGGTGATT
CAATGAATTGATCAATAGTCGAAGCTTCCAGGGATCCACCACACG
ATTCTGGATTTGAAG
GTTGGACATTCTCACAGTAATCCCCCCCAAAGCCGTCTGGACATT
TACATTTCCGGCAGG
AATTGGGGTCCTGGTAACCTCCTCTCTCACATGCATGCATCAGAG
TCGAAGGACGACATA
TATCTGAGCAGTATGCTTCATTTATGGATTTCACTGTCAAGAATGT
TAATTCAATTCTCT
GGCCCA
>ConsensusfromContig3940 Average coverage: 6.36
TGTGCTCTTCCGATCTCTGCCCTTCGTATAACTCGTCACATTTGGA
CAAGCAACTACTTC
TTGAGAACCTTTTGAGCGCTTTTTGTAGAGTCGAGCTCTCGTCCA
TCTCAATCAACACTT
CATCTGGAATCGTCATCGGAAGAGTATTTTCTGCGATGATTTTCT
TGAGGGATTCCTTCT
GCAATCTCTCCCATTCGAATTCTTCCGCAGACTTCTGTTTCTCGG
CCTCCACGAGCGAAG
TTTGAATGTCCGGATCTTCGCTTATCTCTGCTGCTGTATAACCTCC
TTGGTTCAGCAAAT
GAGGGTTTGCACCTCTCGCAAGAAGCAGCCGTACAATTCTTACC
TGGCCGAATGAAGCAG
CGATGTGGAGAGCCGTACTCCCAACATCATTCCGTCGGTCTAA
>ConsensusfromContig3941 Average coverage: 5.86
CAGAAATATAATTCAAACACAAAATTGCATAAATGGATAAATAA
TTCTCATAAACAGTAA
AGCGAGAGCTTTTCAACACAAAAATGGGATTAGTATATCATGCA

AAAAAGAAAATCTCCA
AAATACAATTGTATGGTTAATCCAGCAGCATTGTCAACATAATG
AAAGCGAAATGAAAAA
GTAGCGATGATCCAAGTAGCAATTAGAAAAATAAAAATCAGAAT
TTCCACAATGAAATAA
GTCTTACATCATTACATATCTAATCTTAAAA
>ConsensusfromContig3942 Average coverage: 2.53
TGTAGGAATGCTTAAATATCCAACAAATATACCTCTTATATCATT
CGAAATCAATTTTTA
TAGAAGTATTCAAGTGATCTGAGTTTTGGGTTTCAAATTACCTCT
CATATTCTGTAGGAA
TGCTTAAATATCCAACAAATATACCTCTTATATCATTCGAAATCA
ATTTTTATAGAAGTA
TTCAAGTGATCTGAGTTTTGGGTTTCAAATTACCTCTCATATTCT
GTAGGAATGCTTAAA
>ConsensusfromContig3943 Average coverage: 6.41
TTCTCGTCTTTATAGTGAATCATTACTCCATATCAAATAATGATA
GGCTAAATATCAGTG
AACATATTCAATTTTTACAGAACAGATAACTCTTAAGATATGGAT
AGCTATGATTATTTC
ACTTCATTCTATAGTTCTTCATATCTAAAATGTTTAGTGAATATCT
GCTATCGGAATTCA
GTTAATCGATTTTATGCTCCAAATATTCAGTTCATCGAGAAAAAC
AAAAAGAATACAATG
AAAGATTTCAACTGTTGGTAATCGTGAATCATGGAGGATGCCATA
TGAACTTGTTCAATC
CAAAACACCGGATCTGTCGTTCCATATTAGATCCAACCTGTAACA
GCCAGTGAGAAATC
>ConsensusfromContig3944 Average coverage: 3.64
TTTTATTCTCTATTTGAAAAACTGGTTCCATGATTTATACTCAATT
TGATATTGAACCGA
ACTTTATGATTCCTTGCGGACAATGTGGAGGCCATAGATATGAAA
GTGAATTGCATGTAA
TTGAGTGAATATCAGACTTGGAACCTTTTATTCTTTATTTGAAAA
ACTGGTTCCATGATA
TATACTCAATTTGATATTGAACCGAACTTTATGATTCCTTGCGGA
CAATGTGGAGGCCAT
AGATATATGTTGGCGCATCCAATCACTCTATGATACCAAATGCGA
AACTGAAAGTGAATT
GCATGTAATTGAG
>ConsensusfromContig3945 Average coverage: 4.33
CCCATTTTCTTAAAGTTCCGTCGTATGGGAATTATGTTCTCGAATG

TCTCTGTATCGACT
ACAAGGTGTGTCTCCTTGCCTTACTGATTGGCCGATACGCGAAGG
AACCAGCTTGCCCAT
ACGAGCGAGACCGTAAAATGCGGCAAAAAGTCCGAGGGAGTCCG
TTCATGAACTGATTGA
GATATGGAGGGTAATATGATTACAAAAGGATATCCTCCGTGATG
GGGAGG
>ConsensusfromContig3946 Average coverage: 5.74
CAGCCCTTTTTGCCATTTCCTGCCCGGGAACATGCTCGCTTCTAC
GACGCATTGCAGAAC
TTTGCCAACATTTTGTACAGCAAAGAGAACATATTAGAGTTCAA
GATGACTGAAGGAGAT
TTGATCGTATTTATGAACCACCGCCTCTTGCACGGGCGGACCGGT
TTTGAAACAAGCCAA
GACCAAGAAAGACACCTCAGCGGTTGCTATATTCATAGGGATGCC
CTCGCATCTACGCGC
AATGTCCTTCTGAGAAAGTTACGCCAATGATACACTGAAAATACT
CACTAATCCATAAAC
C
>ConsensusfromContig3947 Average coverage: 8.38
CTAGCCGAGTCGTGACCTTGAAACCACTGCAGAACTCCCAAGGTC
GTTTCAGGAATGTAG
CAGACGACAACTGCTATACAGAGGGTGAGAGCGACCTGGACAGT
TTCGATGTTGTGAACT
CTGTTGACTGAGCGGAGATTGTATTGCAGCTGCCTCAATGTCTGG
AGGACTCCGCCCAAC
TTGACGACCAGCAGAAGAATGAGAAGGAGTGGAATGAGATAGA
CGGCGGTGAAAAGGACG
CCTTGAATGTATATCCGGTCGTAGAGATAATTTTCCGACAGCGTA
TACCAGCTCCGATCC
CCGGCGATTATCCCCAAGTGATAATCCCGGACGTGAAGATACGAA
TGATCGCTCTTCGGC
ATCGTATGATGATCAATCGGAATTTTCAGAATCGTGTCGAATGT
CAGGGGAATAGAGAGC
AGGAAA
>ConsensusfromContig3948 Average coverage: 6.55
TCCGATCTGTCATTTTGTAATCATACAACCCTCTATGTCAGATATA
TAGATGACATCTTC
ATGATATGGAATGCCAATTCAGAAGCATTTGATCAATTTTCACAC
GATTTCAACTCCATT
CACCAGTCCATATGTTTAACTCATGAAACTTCAGCTGTTGAAATTG
ATTTTCTTGATGTC

ACGATAGCGATTCGAGATTCTGAAATTTCAACATCCGTAAAGAAG
AGTTCCTGCATTTCA
GGAAAATACATCCATTTCAACAGTTTTCATCCTTATCACATCAAA
AAATCAATTTTGA
>ConsensusfromContig3949 Average coverage: 5.20
ATCTGGAACTTGAAAGATCGGTCAGATATGGTATTCACGAGAGA
GAAAATTCAAAGAAAA
TTGATTATGATAGCACACCTCTTATTAAAACTTTAATATTCAAGA
ATTATTCCGGTAAAA
AGGTTCATGCTCCTCGTGCTGGTACCACAAAAATGGCAGCGAAA
CTGAAATCAGACAGAA
AAAGACCCAAGCCAAAACACGAAGGAAATATCACGAGAATTTTG
CCTTCAAAGAAATGTG
CCAAAAGTAAAAATATCGCATGGACGAAAAAGATTCGAAAAAG
AATGGCGAACGAGGG
>ConsensusfromContig3950 Average coverage: 4.18
GGAGATCAAAAACAGCTCAACAATTTTCGAGAACATGCGAGATGT
GTTTGGACCTTTTTG
GCTGCTGAATCTGGTCTTCCCAACTCATATAATCTTCAAGCAACA
ATCTAATGGCACATA
CTGGCCAAATTGCAAGATGATGAGAAGCTTGCCGAAGTAAATTG
CATCTGTTGAAAAACT
GCGAACCAGAGAATCCACTAAGGGGCAATAAATGTATTTTCTAAT
GTTTTCGCCTGAGAT
TATGTTGCCCGTGTTCTTGCGTTGCTTTGTATACAACTTGATTGAT
ATATTCAATATTTG
TGCACGCATGTCTTTTACGACACAATCCAGTTCTATACATTTTGTG
TTATATATTTCG
>ConsensusfromContig3951 Average coverage: 3.51
GCTCACAATCTGAAAGCAGCGCCGTCAAAAGATCAAGTACTGGG
ATGTCGACCCACCGCT
TGTTAGAAGTAAAAGCCAACCTACCCACACATCGAACGTCCCTTC
TCCATGCGTCACGGC
ATCTATTAATATCCTTCTCTTTCCTCTTGTGTTTTGAGAGGAGGGA
GTATAGGGCGATTC
GTGTTCTATAGTGTGCTAGATAACCCACTACATGGGACAGGGCAT
GATTTTTGAGCAAAA
GGTCATAAGCAGAAATTAAC
>ConsensusfromContig3952 Average coverage: 2.44
TCACTGTCATTATGTTATTTGTTTCAAATGAGATGCTGACGACAT
TCAGCGAATGTCATG
GAAGTACAAATAGAGTTTCTCATCAACATATTGCACGAGTTCCA

ACATTAGAGAATGAAG
CCATTTACATTCTTTTACACTGTCATTATGTTATTTGTTTCAAATG
AGATGCTGACGACA
TTCAGCGAATGTCATGGAAGTACAAATAGAGTTTCTCATCAACAT
ATTGCACGAGTTCCA
ACATTAGAGAATGAAGCCATTTCTATTCTTTTACACTGTCATTATG
TTATTTGTTCCAAA
TGAAATGCTGA
>ConsensusfromContig3953 Average coverage: 3.84
GATTCCGGAACTTCTTTCCGGCGAAAAATGCATCGCCACTCAACT
TCCAGCCCACAACCC
TCTGATTGCTCGATGTGGACTGATTGCGAAAGAGGCCTTGAAAAC
TTCTTCTGAAGACAA
CACTTGCAGAATCCTGGCCCTCCAGGCCAAATGCGAAGAGTCTCT
CATTCGGCCGGCCTG
CGGAAGCCTTGTCGCCGACTTTATGTTGACCCTTGGCTATCTCCA
AAAGACACCTCTTCT
CAGAAAAATTAAC
>ConsensusfromContig3954 Average coverage: 7.65
GCGCAACAAAATGAGGCATCTCTGAAGAAAGCAAGCTGGTCGAC
GGCGAAAATCTCTGTG
ATGTCATCAACTGAAGCGCGGATAGCCAATAGCGCTGCGAAGAA
AGAACCAGCGCTAACT
CCAGCGAACCGGAACGTGTGCCGAAGCAGATCGGCCGCTTGCAG
GACCTGCAATCCGCCT
ATGAACGCATTACATTTTGCTCCACCCCCCTCGAAAATCAAATTT
TCGAAGGGAGGACGT
TGGGGGTCATAGCTATAAGATCGGAAGAGCACACG
>ConsensusfromContig3955 Average coverage: 10.79
AAATGCGTGACAAAATAATAAATTATTTCTAAACGAAAAAGCAC
CGACATACAGTTACGA
CAACGATGGATATACAAACTATAGCTTTCGATTTTCAAGAACAG
GAAACAAACTAAAGAT
AATCTGTAGAATATATTCCTTTTGATTGGCTTAAATATTAGATCA
AGACCCTAGCAGTTA
TTTGATTGGCCAACATCTTCAACATAATCAAGCAGCTACAATGTT
CTTCTTCCGTTTGGC
TCGCAACACAGGGCAGCTGCGAGTCTCTCCCTTTGAGCAGTTACA
AAACTCTTCATCTAA
TTGGCCAACATCTTCAGGTAGCTACTATGCC
>ConsensusfromContig3956 Average coverage: 6.55
TCCGATCTCCGAGATAAACTACAGTGAGATGATGACGTAATTGAG

GATAAAGTCCCCTCA
CAACATCGAGTGCTTTATTGACAATGCAGCAACCGGAGCTATGA
GCAATAACAATATCGA
CATTGAGTAACAAACCAACAAGTTTTTCAACCGTGCGTTGTGAAG
GTTCTTCCATCACCA
GCATTGGGTTGTGCACTGTCATTATTTCCACATTTTCAGTTCCGGC
GAATATATGTCGTT
TCAAAAACTTCGCAGCTGCCGCAACTTCGCTTTTCGACTTGCAAC
TTCCATTGATGTATG
CGACTCGCGGTATTTCGATATTCTGCATGATCGTTTAGGATTCTTT
ATAATTATATATTT
TCGCTTATTGAACACCGTATTAAACTTT
>ConsensusfromContig3957 Average coverage: 3.60
GTGAGACATGTCAGTCTGCGTTCGATCACATCCGACTGTCTGAAA
AACAATTATGGGAGT
TAGTCAAACCAATTGGCTGAAGGACAGACGTCGGAAAGGCACAG
AGCGTTGCTCTCTCAC
TTCAATGATACCGCCTCGTTTGAATAAGGTGAGGGGGCTTGTTTC
CAATCCTCCTTATCA
TCTTCTAGAGTTTATTGAAATGTCTTGGAATAA
>ConsensusfromContig3958 Average coverage: 9.46
CTTTTATCGTCTGCTGATGAAGAATGCCAGTATTCGACTCCATCG
ACATACTGGCCAATG
GCGAATCACCTCTTCATCGTGGCAGGGGAATGCCCCAAATCTGCG
ACCTTCTGGGCATCT
TTCACCATATCCTCGATGATCTTGTTCAGTTTAGCCAAATCTTTCT
GATAATGCTTGTCG
TTCTCCAAGGTCACAGCCGGAGCCTCTAGTGATATAACTACAATG
TGATCAACATAAGAA
TATTTGGCGACTTAAACAATTTTAAATATCATGTAACTACC
>ConsensusfromContig3959 Average coverage: 4.83
ATTTTCCTTTTTCTTTTAGGTAATCAGCTAACTTTTCTATTGCTTC
AACACAAACACTCG
GGTACATCCCATCTTTATAGGTTCCCATCACTATCATGGAGGCTG
TTCTGACCAGAATGA
GGCCACTTTGACCATGTTTCGCGTAAAAAGAACTTTTGTCGGCAC
GGACAGCTTTGTAAT
ATTTGTCACGGAAATAAACCCCTTGCTCGCGAGTGATGACAGTGT
CTTTGAACGCATCAA
GGAAGCTTTGGGTTTGCTCTTCGCTCACTTTGAAACCAATCGTTG
AGACATTAACTGAGC
CATCCTTTCTTCTGATCAGGGCACCCGCCTCCA

>ConsensusfromContig3960 Average coverage: 5.49
GTTTTTCAATGGTTCCTACATGTGTGGATCTAGTCCCATTTCCTTC
AAGGCCTATGACTA
GCCTAACAGTAGATATGGACGAGATACATCGTTTTTCAATGGTTC
CTACATGTGTGGATC
TAGTCCCATTTCCTTCAAGGCCTATGACTAGCCT
>ConsensusfromContig3961 Average coverage: 6.27
GTTTGATAAAGCGCAAACTCCTCATCTGGTATCGGACATTATAG
TTGCGCGCGATAGTA
TAGAGCCGATTGATGATGAAAAACTCCCGGTCAAGGGCGGGAAG
CTCCGTCGCCATCTTG
CTGAGGAACCCGAGATGTTCAACGAACTCTTCTACGGTTGAAGGG
CTCATGTCCAGCTTT
TTTGTAGCGCTCTTAACTATGGTCAGCAATTTATTATTTTGCGAA
TATGCAATGACTGGC
AACACATCTTCGACGTCCTTGATGACCTTGAGGGTGAAGGGCAAA
CATGCGTCTCTGAAA
GGTGCCGTGGTCACGTGAATCAGTCCGCACCGCTGATAGGCTGGC
ATGCCTTTCATTTCG
TGTTTTTTTTTGGTTATTA
>ConsensusfromContig3962 Average coverage: 6.29
AACAAGATACTTTACTGAGCATACAATAATTCAGGATAAAGTGA
AAATTCGCTAGTGAAA
ACTTGTTCAAATTATATCAAAAACAAATTGAATCATAATTAATCA
ATGACATGCTGGAGC
AATATCATGATGATTTTATTCATTAATTAGGATCAGCAGCCTGAT
CAGAAATCATATTTT
TCATAGCTTGGAAGGTCAAAACCCACAGAATGTGCTTTAAAATGT
TTAATATCA
>ConsensusfromContig3963 Average coverage: 8.48
GATGAAATATATTTTGGATGAAGAAAAACTCATTGATTTCAGCCG
AATTTAGACCTGAAG
AGAATACAATTTTCAGAGCCTTAATGACACAAAAATAATGGTTTA
TTAATATTGAAAAAG
TTCTCAATGGCCAGAATATCTTGTGAGTAGTTGAGATATGAAAGA
CATCTCCCTACCATT
GATTTTGCCAATTGAAAAACAGCTATCCGACTACTTCGAAAACTA
TACTTACTGTACATT
GTTTCACGAATGGTACACCGGTGATAAGCTCACGAAAGTATGTGC
TTGTGAAATGCTCTC
ATGAATGAACATTTAAAAATTGTATAACATTGAATGTTTCAGTTC
AAAAAATTCGGCAGA

GATGCGATAAATGTGGCTATCTTTTATCATGTTACTGATTCTTTTT
GAAATTCCGACCGA
CATTTAATCAAGCCGGACTATCACTCGGTGCACTCGGAGAAGTC
GATAATCAAAAAAAT
TAATCCAATTCCGATGATCATGAAATAATTTAAAAATTTAAAAAT
GTTAACTAATTTAAA
ATTTTTTAGAAGAACCGAAATAATGTCATGGTAAGTGATTTTCAG
CCGTCTTGATAATTT
ATATTCCATTTTTCCGCCATGTTAAAACTTTAGCGAAAAAATACG
AAATTTCGTCCCCCC
CACTTTTGAAAAGCGATCTGCGCCCCTGATAATGTTCATAATCAT
CTTGCAAATTTTGTA
TAATCATGTTTTTTGAATTCTGAGAAAATTATTTTTATCGCACA
>ConsensusfromContig3964 Average coverage: 7.58
AATTATATCATATAAACTAGTTTAGATCTGATGTGGTCGTATGAT
ACTATTATTTCAACT
TTTTATTGAAACAGAGAGCATATGCGATAGCTCCTGGCTTATCAT
AATTATGTTAGACTA
ATTTTATTCTCATATCAATGTTTTGACCATTATACATCGATATCTT
CTATTAAGAATAGC
TAAAATTCGATTTTAATTCAAATTTGGCTCCACCAATTCCTTCCAG
ATTCTTGATTGTAG
GTTTTTTCTTAATGCGAACACGTCTATCTCCTACTTTTTTCTGTTT
CATAATAAAACTAT
TTACTTCGATCGCAATTCGTATGCTGCTCTTTCGATTACCG
>ConsensusfromContig3965 Average coverage: 10.40
CGATTAAAATATATAATAAGACATATATAAAACACGATTATCCCG
ATCAATTATTGAAAA
TTATTGAGATAAGATCCGTCAAAAATCTTCAGAATATTCATACAC
CATTGACTATAAACT
TCTTATGTAGGCACATATATGCATTCTTAAAAGTAAAACAGGGAT
TTATAAAAGATTGAT
AATTTACAGTGAAATTAATTACAAAGTGCGCATGATATACATTGG
AATATTTTCTTAAAA
TCAAGACAAATACTGCCACACGAGGTTTTGATAACCTTGTCATAG
CATGACATAACGAGA
AAAAACACTGTATGAAAATATGTGATGAAAAATGACAAACATGA
ATACACCAAAAAACC
>ConsensusfromContig3966 Average coverage: 2.61
GAAACAACCATGATAATCTCTTTAGGCAGTGATTCGCTATCATGT
TCTCACTATCCGCTA
TTATTCCCCATACAGTTTTCTATTCACTGAATTGGTGGGCAGGAA

ACAACCATGATAATC
TCTTTACGCAGTGATTCGCTATCATGTTCTCACTATCCGCTATTAT
TCCCCATAC
>ConsensusfromContig3967 Average coverage: 5.31
TCTTCCGATCTCGGACAGGTTCACCGGTGCACTGCGTTGAGATCT
TGTAGTGCGTCGTGT
GACCCTTCTTGTACATGACCTTGCAGGCAGATCGTCGGCTGTGGA
AGAAACAGTCCAAGT
CGACGTCATTGAAGTCGACGGCGAGGCAGCCAGGGGCACGACAG
GCGTCGATGCAAGCCT
TGGCAGTCTTGTACATTCCCATGTACTCTCCACCGTGGATGGGGT
ATCCCTTGAAAGACC
CGGTGACAGGAGATGGGTTGGCACAATCCATCATGCGAAGATGCT
TCCAGTTCGAGTCTG
AAGCAGGAGAGTGACAGGAGACCTCCTTGCGGTANTTT
>ConsensusfromContig3968 Average coverage: 4.79
TATATACGTCACGGGATGGATCAAAAGGATGATACAGTATGTGA
AATCATTCACAACTCA
GTAAAGGTTCCACAAAAGTCACTATCCAAGGGATTCATCTTCAGA
GATAGAGCTATATAT
ACGTCACGGGATGGATCAAAAGGGTGGTACAGTATGTGAAATCA
TTCACAAC
>ConsensusfromContig3969 Average coverage: 2.75
CCTAGCTTCATCTTTTTTGGGGTTTATGAGGAGTGTCTATTTATCC
AACCTGTCTTTTTT
CAGTGATGAGCCAGTCAATATCGATCTATATCCTAGCTTCATCTTT
TTTGGGGTCTTATG
AGGAGTGTCTATTTATCCAACCTGTCTTTTTTCAGTGATGAG
>ConsensusfromContig3970 Average coverage: 5.22
ATTTGAATCTCAAATCAACTTCATGGGTAATGACACCTTGAAATC
TGATGAGAAATTCGC
ATATTTATCATTTGATCTTGAAGTTGAGTCTGGAGACAAAACCAT
TCATAAAGAGTACAA
GTTTTCAAATTTTTACACCACACTATCTCCATTTGAGGCAAGTCT
AGAGAAGGCATACAG
AGTGAATGAAGGATCAAAGCTGACCATGGAATGTATCTACAGCG
GAGGCAATCCAGAACC
ATTCGAATTCCGATGGGAG
>ConsensusfromContig3971 Average coverage: 2.48
CTTCACACTTAGTCTTCAGTCACAATTCGGCATCAATTCCATGTGA
ATGCATCTTTCACT
CCATGTGTTTATTAACGTCGACTGACGCTGATAAATATAAAGTAT

ATGAGTTCCCCCTTC
ACACTTAGTCTTCAGTCACAATTCGACATCAATTCCATGTGAATG
CATCTTTCACTCCAT
GTGTTTATTAACAAGTCGACTGACGCTGATAAATATAAAGTATAT
GAGTTCCCCCTTCAC
ACTTAGTCTTCAGTCACAATTCGGCATCAATTCCATGTGTATGCAT
CTTTCACTCCATGT
GTTTATTAACAAGCCG
>ConsensusfromContig3972 Average coverage: 4.66
GACGCTCTTCCGATCTGTTTTCGACCATGTTCCATCCGCCTTACAA
GTCATATCGTAGGT
CTCCGGTGTGTCTTCATTTATGGACCATCCATCGATACATTCCAC
AGTCTGAGTACACAG
CCCGAAAGCTGTGTATATTCCGAGCTCGGACTGGGCAACAGAGT
GTGGCTGGCAATCCCG
TTCTACAAAAAAACTCAAGTCCATATTATTTCGTGTCGCAAAGAC
ATGATTTATGGGAAG
AATTGCTACTGGAGTATGTGACAGGAGCACAGCTTCGC
>ConsensusfromContig3973 Average coverage: 12.12
GTTCAAAACATTCACAAAGGAGAATTATTTATTTGAGAAATTGGT
GAGGATCGACTGATA
CCAAAGAATTAGGAAAGGCTTATATATGAGTTATTTTATTTCACA
TACAGATACAAAAGT
AAAGAAAAGGATAAGAGATATGAATGAATGAACCCTCCCTTTAG
CTGGAAAGTAGGTTGG
CTGTGTACCCGAGATTTCCCTGGTACACATAGACTTATATCATTA
TATATAGAAATTGGT
ATAAAATTAGATTGGTGTCAGAGAAAAATTATCAATTATTTATTA
TTTGTTGAAACCAGA
TTGGAACCAGCTGTTTTTGAAAATTTA
>ConsensusfromContig3974 Average coverage: 4.26
TGAAATCCTAAAAAGCTCTGTTGATCCTATTGATAAACAAAAATT
ATCCAGTCCCAAAAC
AAACGAACCCACGCCATCTAACAGTGAAAAAACCGAACCCAAAA
AGTCATCCTATCCGAA
CATTCCACTCATAAGAACGCCCTCATTTTTCGAAGCACCACGCTT
CATGTACCCGCCCCC
TACTCCCAGTGGTTCATCGTCAGCTTGTGTCAAAGACGCTGGATC
AATCGATGAAAGGTC
AGAGCCCCTCATACGTGTGACCCTTGACCCCACAGATTCCGAGAT
GACTCCATGTGGCAA
AGGAGGATT

>ConsensusfromContig3975 Average coverage: 4.69
ATTCACTCAGATCCAGATCGGCATGGTGCGATATTGCCCGCTCTG
TAGGTTCGAAATTAA
GAAAACATAAAGAAAACTCCAAAAAATATAATTCACTCAGATCC
AGATCGGCATGGTGCG
A
>ConsensusfromContig3976 Average coverage: 2.40
GCTCTTTCAACCATTATGTGATCTATGATCACATTGTGTATGGGT
ATTGCCAATACTATG
TTTTATTCTGACCATTGTGTGAAATTTTCTCACTTTGTATATGGAC
TCTGTTAATATCGT
GGTATCCCTGGACTATTGCGTGATCTACGATCACTTTGTGTATGG
ATTCTGTCCATATCA
TAGGCTCTTTCAACCATTATGTGATCTATGATCACATTGTGTATG
GGTATTGCCAATACT
ATGTTTTATTCTGACCATTGTGTGACATTTTCTCACTTTGTATATG
GACTCTGTTAATAT
CGTGGTATCC
>ConsensusfromContig3977 Average coverage: 2.59
TTTCGATGATCTCATGTTAGTAGACATTATCACCCCCATTGCTTCT
AAATCTAATGTCAA
ATCCAAATCAAGCATATTCGTGTCGATGTTTTTATTCTCCAGTCGC
CTTCCTATTTTCAT
GGAGGAGGTTTTGTTCTCGTGTTAGTAGACATTATCACCCCCATT
GCTTCTAAATCTAAT
GTCAAATCCAAATCAAGCATATTCGTGTCGATGTTTTTATTCTTCG
GTCGCCTTCCTATT
TTCATGGAGGAGGTTTTTCGATGTTCTCGTGTTAGTAGACATTATC
AC
>ConsensusfromContig3978 Average coverage: 3.50
GAAAATTCTGAATTTGGCCCCAAGAATCTCGAATGTATTTCGATTG
TTATTCTGTATCTT
GATAATCTGTAGTTGATTCGCGTTGATGGAGGTGTTGAAAATTCT
GAATTTGGCCCCAAG
AATATCGAATGTATTTCGATTGTTATTCTGTATCTTGATAATCTGT
AGTTGATTCGCGTT
GATGGAGGTGTTGAAAATTCTGAATTTTGCCCCAATAATCTCGAA
TGTATTTCGAGTGTT
>ConsensusfromContig3979 Average coverage: 6.11
GCAGAACAAAACCCAAAACAAGAAAGGCCCCCTTTGTCAAGAAA
ACAAATCTGCAACTAT
TGAAAATACAGGAGACACAAGCACGTCTAAAGATGATGATATAG

TATTTGACGATAGCTC
TGATGATGAGCCTCTCGACGATGAAGTTATTGAAGGGGACTTTGT
TGTTGTTAAAGTTCG
TGGCAAATCAAGGATTGTCAACTACATAGCCCGCATAGATGCAA
TGGAGGATGATGAATA
CGAAGGCGTATTTCTGAAGAAGGTTTCAGGGCGGGTCGGTTCTGA
GGATGACTTGGTCAT
TCCAAACGAAAGTGACAATGCCTCATTTCTCAAAGAG
>ConsensusfromContig3980 Average coverage: 4.43
GACAATGACGAGGATGGGTATGTCGACGAAGACTGCTTCTGCCG
CATGATCGTAGAAACG
AAGGCGGGAGACAGCATTGACAACGATTGTGACGGAGTCGTGGA
TGAAGAGATTTGTGAC
GGACTTGACAATGACGAGGATGGGTATGTCGACGAAGACTGCTT
CTGCCGCATGATCGTA
GAAACGAAGGCGAGAGACAGCATTGACAACGATTGTGACGGAGT
CGTG
>ConsensusfromContig3981 Average coverage: 2.56
GAACAAAACAAGACCGTACAAAAAGATGTGAATAAAATGGTGG
ATAAGGTGAGAGGAAAA
GCTGAAAATATCACTCCCTGAAAAATATATTACAGTTCAACATA
GAGGGGAGAACAAAAC
AAGGCCGTACAAAAAGATGTGAATAAAATGGTGGATAAGGTGAG
AGGAAAGGCTGGAAAT
ATCACTCCCTGAAAAATATATTACAGTTCAACATAGAGGGGAGA
ACAAAACAAGGCCGCA
CAAAAAAGATGTGAATAAAATGGTGGATAAGGTGATAGAAAAG
>ConsensusfromContig3982 Average coverage: 4.68
TTTGACCAAAGCAATGGAAAAACAGCACAAAGAATTTGAAGCAG
TCCATGATAAACGTCG
AAGAAGCTCTCTCATGAAAGACTGTGACCCCTTCCATCAGTTAT
ATGAAGAACAAAAAGA
GAAGAAATTCTGTAAAAACATCCCAAAGTCTGAATACGAGGCACA
ATTTGATAATTGGTT
AACAGAAAATACGGATTTTGATCATCATATTCCTGGCCGAGATCT
TTTTAACCTCACAAA
AGAAGGCTCGAAAGTAGATGCAGCTCGCCATTCAATCAGCAGTC
TGGATGGGCCCATATC
TCTCACATGCGGGTCCA
>ConsensusfromContig3983 Average coverage: 2.38
TACATTCGGGACATACGGTGACATTCATATATTCATGCAATCAGT
GAAACAATTTGATTT

ATTTTTTGCATCGTTATTCATTGGGGAGCAATACATTCGTGACAT
ACGGTGACATTCATA
TATTCATG
>ConsensusfromContig3984 Average coverage: 26.02
AGTCAATCAGCTTCTCATGTGGATACTAAATCAGATGAGATTGGT
CTACATATTCAACAA
TACAAAGCAATGATGCTGAAAAAGATGATATATACATTACGAAA
CCCTGCAGTCACCGCA
ACTCAGCTGTTCATCCCTATATTTTTTGCTTGCATTCCCAGTCTTT
ATTTTCACCTACTA
CCAATCAACGAAAAATCACCGCCAATTGTGCTCAACACGTATCA
ATATGGGAAAGATATC
GTCACACCTTTCGAAATGAACTCAAATGAATCTCAGCTTTCACTC
GATCTGCTCCAGGCA
TATAAAAAATCATTCAACGAGTTTGGACAAAGTATTACTAATTTA
TCAGGCTTAGGCATC
GTCACGCTTTCAGATTTTTTGTTAGATGTCGCTTCTAATGATATA
GGGATCTTCAACAGG
AAGTACATAACAGCTATCAACTTCGAGAATTCACGAACCGCCGA
TGTGAAGATTACAGCA
TTTTTCAA
>ConsensusfromContig3985 Average coverage: 17.77
ATCTCGAAAATCTGTGTCAGTTGTTGGTAAATTTTTTAAGCGGTT
TTCAGCAACGCAGTT
GAAGTGCATATTGAAAGCGTTAGCCATGTCTGAGTGATCATCTAT
ATATTCACCATTATA
CATTAGTGATTTCACTAAGTCTGACTTCCCGCGTCCTGTTAACTTA
TTTATCAACTTCCA
GGTATTTTGGGGGTCATCTTTTAATGCCTCAAACTTGGTTCGATAG
TATTCAAATTTAGT
TTTCTTAATCATTTTTTGGGTTGATTTCCGAGCTTCGCCATACTGG
TTTTTTAACAAATC
ATAGATTTCAGGTTTATCCCTATTTTTGATCATTTTTTTGTACAGA
GAATCCCTCTTTTT
CAGTTCATTGAGAATGTCTTCATTAATCCATTCCTTTATTTTATTG
GAATATTTGCCTGA
TTTTGGGCGGCGTTTACAACTACCTTTATCGAGAAGTAATTTAAT
TTCTCCCTCTAAAGA
TGTATACATGCTGTTTGGGCCAAGTTCCATGTAAGATGTCATGTT
TATATCTTTACATAG
ATATCTCTTGGCTCTAGCCACAAATTTCGCATGTGATAATGAACA
TTCATTACTGTGCCG

TACTTTTTCT
>ConsensusfromContig3986 Average coverage: 23.58
CAGCACGTATTTGATTGACGGCGCCACCTATGAGGTTGTCTGGAA
AACGACACATCCGGA
GAATGCGGAAACATACAGCACTCCTGCTTTGGGTTTTTTCAACCA
AGACGAGACTCCTGA
TGTTTTGGTACATTGGCATATCGGAGAAGGCTACCCAGTTTACTA
CTACGCCGAAACTCA
AATATTAGATGGCATCACCGGCAGACCAATGCCTGAGTTTTCCTA
TACGGAGCCAATAGG
AACCCTTTCGAGTCCATTGACCTTGGCAATGTCCGGCCAAGGAAA
CGACGCATTCATTAT
TTGGAGTTCAATTTGTGAAAACGTAGAAAGTGGACAGGAGCGTG
AACGTTGGACATTT
>ConsensusfromContig3987 Average coverage: 59.45
GTCGTTGTTGATATGTGACATTGATTGATTAAAAACATCACTTGC
GTTTGAAAATGATTG
ATCGTTGTTGACATTTGTCAATGAAGGGTTAGAAATATTGTTGCC
GTTTGACCTAATATT
CACATGCATTTCTTTTCTCGAGTTTGATACATTATTATTCTTCTCA
AATGTATCAGACTC
AGTAAATGAAGGCTCGTCCTTTTTTCTAAATTGTTGATCCCTATC
AGACGTATTTCGGGG
GATTTTTCCTGTAATTTTCAGAGCGGAATTCGGGAATTTTCCAGTC
TGACTTTCTACGGT
GTCCGAAACTTTATCTTTTTGTTCATTCTTATCAGGAGAAACTTTC
TCTTTCTTGACTAT
GAACGCATCATAAGTGTGGGCCTTGATCGGATTATCATAAGAATC
GTCGGGTTCTTCTTT
CACTCTGATCGGCGCATCATTTAATAATGGGTTCTCATCCAGAGG
GTCAACATTCGGGAT
ACATGCTTCTGAATTGATTGACTCTTCCCGTGCCTCCGTTTTTATT
GCACGAGTCAGCGA
TATGGCACTCCGGATTTTCGCCAAGCCTTTCAGTTTGCTGAAGTT
CAAGGACAGAGGTTT
TGAAAGAATGTTCTTAGAAGGTCCAGTTTCGCTCTTGGTAGATGA
AGGTTCTGCATTTGG
TTTGGTTTTGCTGATGATGCTGGAGAATTGTGAAAATATGTTATCT
TTAAATTCCGTTTT
TATATGAATCACATCATTGTCTTCAATAGGCATTCCGGCATCGCG
AACACCCGAGCTTTT
TTCTAAACATTCAACTTTGATATCCACATCAGCTGTGTCTTCAATT

ATTTCGTTTGTCTG
ACATTTTTC
>ConsensusfromContig3988 Average coverage: 23.62
CATCAGTACCGTCTTCAGTATCAAAATATACCCAATGGGATGTGA
TCGCATGCCAATCGT
ATTCTCTGATCCATTTTGAAATACAAGTGAGACGTACTCCGCGGA
AACCATCTTTTAGCT
CACTGGTGAACCCTTCGGGTATTTTGAACAGATTTCGTAACATCA
ACGATCCATCTCCCA
GTACTAATACAGTTGCAGATATTGTCACAGCATCACCGTTTTGCC
ATATGAATCCGTGCT
GGTAAGCATTAGGAACGTCTCCACATGGCAAGCTCAGTTGCTGG
CCCCAGTGATAACGAA
CAATCGTTTGAGTATCTGAATTCACTGTCCCTGAGAAATGAACCG
TGATTTTGTCAGAGA
TGAATGTTGATTTTCCGTTTACCGCTTTGCACCAATATTCACGTTT
AGCAGCTTTCAAAT
AGGTGGTTTTACCATGCACTGGAAACTTGACGTCATGAACAAACT
CTTTCCTCCCTAAAA
TCGTCTTAGTATTGCTGCTGTCTGGATGAATCGTTTCTGGAATTTT
GTCGTCGGTTGTTT
GGATATATCTAAGGGATGGTACTGAATCCGATGGCGTGATATTAC
ACGCCATCTTGAAAA
TGAACGTCTCATCAATCTTTGATACATAAAAAATTCCTCTGTCCG
CAAAAACCTTGGCCA
TTGGTACTTCCTCCAGTGCAGCTTCTGACATGTAGTTAAGAACTG
CAGGTCGATCAGGAG
TGATGTTTGGCGGCGATTTGCGGATACACTCAGATCGGTGAAT
>ConsensusfromContig3989 Average coverage: 103.62
AACAAGCTTTATTTTCATTAAATATGGCAAATGCGTTGTAATTTT
ATCGAGAATTCTTCA
CATATTTTTGCCATTTTAATAAAAATAAAGCTTGTTATGAATATA
AAAAATGTGAATAAA
AATGCCTCTTAACCGATTGAAACTGAATAACACTTCCATTGTTGA
TGACAATCCTGATTT
TTCTGCTGGAAACTATAATTTGCAGTCCGTTCATTGTTCTGTTCCC
GGCGGATGTTCGCG
AAGCCATGAACCGGTTCAGTTGAATAATTCTGGTTTTGCGGTTAA
AGTATTGTGTAACAA
TGTAAACTGTACTGAATCAAAATGGATGCATTCTGAGTGTTTCAC
TGAATGGGAAGAACG
TATTCTGACATATTTGCGCTCTTGTGGCCGAGCCAGAAGCTGGAG

TGAAAAGCAGCGCCA
TCAAAATTTATGGACCAAAAAAGGGTATGATTTAGCCTATAAGGC
GTGCGATTGTAAATG
TGGTCGCGGTCATATTCGTAAAGATTTGGATTTTGAAGCCCCAAT
TGTTGAAAATGATGA
TACTGGGAAAAAATCAAAACGACATAGACGGAAATCAGATAAGT
CTCTGGAAAATAATAA
TCATCAGCATTATTATAACAATAATAAAATCAACTATACAGCAAG
TGGCAACCATTTTAG
ACCAAACCCAAATAATTTTGGAAATTTCAGTCCTTCAAGTACAGC
TTTTGCAGCAGGATT
TCCATATGAAAATGAAACATCAATGGCTCCATACTCAGCGAAGT
TGCAGCAAGA
>ConsensusfromContig3990 Average coverage: 8.20
GACGCTCTTCCGATCTGGCGGTGCCAGCTCTCCATTTCCAGGAAG
TATTGAAAGTCACCA
GAAAGCAGTGAGTTCCCTTTTGACGATTTGTGGGAATGTTGGTGT
AGATCGGGCCAAACC
GAAGAATGGCACCATACTCTATGCACACAACAATAATGATGGCA
AACAAGAATGAGAGCC
AGCAGCTAATCACGAGAATATACACAACTGGATTTTCATAAAAC
CCTCGGAGAGCGTCCA
GTCCTGACAATAAATAGATAGAGTTAGGCAAATAGGCTCTGTTGC
CAAAAATGTATGTTG
TGTGTTTTTTACAGCTGCAAACCAAATGAGAAGAGTTAGACTCC
ATAGAAGGAATACAAG
AGGGCGTGTTCGTTGCAGTCATCAAACTATCTCGGCAAGAGAGGA
TGTTAAGATTTACAG
TTATGTTGAAAACCGCTCGGTGTCTTTCTACTGAACTCCATGCAG
TGAAGTATTTAGAGT
TGACTGCCTTGGCGTAGCGGAGGGGTGTTACTCCGAGATAAAACT
TTCTCATTTCAACAT
GTCGTTTTGTTATGTAAATGGTATAAATTTCGATGATTCCTTTTCC
TCCGTCCACCCGTG
ATCGAATTCTCTTCACCATATCCGTCTTCTTGCGTGTCGGACAACA
GTCGTGCTCTGTTC
TCGTTTTTCCAGA
>ConsensusfromContig3991 Average coverage: 23.97
TACCAATATAAATCAATTACAGAAAAATAATTCGGCTATTATTGT
CGAATATAATGGTGT
TGATCTCAACCCCTGTAACAGTGATTATTCGGAATTGGAGAAAAA
ATGGGAACAATTATT

TAGAGGCTATCCCTTGCATGGCGAAAACCATAAAAGAAAAGTACC
TCTTATTATATTACA
AAGAACCCAAACAAGACCATCTCTTCTAAACAAATGGAGTAAATT
GTTTCAAATTAAAAT
CCAATCGATTCAGCTCGAAAACACGTGCAATTTTCAAAATTTGAA
TGACATGAATATTCA
TATGGCGATGGAAATGAATATTCAAAATCCGTCACTCACCTTTCA
TTCTTATCTAAGAGA
CATATGGGCGTAATATAATCGAGGCGGTATCCCGTATAAACGCG
ATCCGAGTGTCGAATA
TTCTAAATTATTGTGTCGAATATTCTAAAATATTGTCATATTTTAA
GGCATTTATG
>ConsensusfromContig3992 Average coverage: 7.56
ACGTGTGCTCTTCCGATCTCCCATTTTGAAGTTCGCCATCCAAAT
AGAAATCAATGAGGT
AATTCACTGTGAAAACTGATTTGATGTGATACCATTTCCATAAAT
CTACTGAAGCTTCGA
CAGTCAATGTTTTTGTCATTTTGTCTTTCCAGAATTGTGTCATGAT
TCTTATTTTACGAC
CACCTATTTTCAAGATCTTCAATGTAGTTCCCCAGAAATTGTCAT
TTGAAGCGATCGTGT
AAAGATAAAATTCACTGGAATGGCCTATTTTGAGAGACATGGAA
ATGGTGAGATTATCGT
CAGGATAGTCTTGGTTGAAGAGACTAGAAGCATCTTTTTCGGCAG
TAATTTTCAAATGTA
CGTTTCCTTTGAAGTGATAAGCAACGCCAAAAGGACTGACTACTG
TTGCAATATTTTTTT
TTGTTCCAACA
>ConsensusfromContig3993 Average coverage: 51.38
GTCTCATAATAGGTGAAGAAGATATTCATGAACTAAAAAGAGTGA
TAAAATTTTATAATT
ACTACAAAAACATCAGTGGAGGTCGGGTGACCAGGCTTCACCAA
GTGGTGTTGAAAATGA
TGCGTTTTTTCTGAATACTGTATCACTGTTCCGAATTTTGGTTACA
CCTTGAGCACGACT
CGCGATATCCGACCAATAGGAAGCAGGCTGTTCCGTTGCATATTC
ATGAGTCCCTGTTGG
CGCCTTGACTGATGGCTGAAAGTTAAGGTTGTCGTAGAAGTCAT
GAGCCGTTACAGAGAA
AGGTGTTTTAGAGGTCGCAAGCTTCTCTTTTTCTTTTAGTTTTGCT
TCCATTTGATTTAT
TGCTTCCGCCATTAGAGCTTTTTGAAGAAATGTGTCCCTGGGCCC

TACAAAACGTACTCC
AGATCCAGTGGGCCTCTTGAAATGTGAAGTGTGTGTTGTTTCATA
GCAGATATCATTTTG
TAATGAGAGAATACCATTATGGCCATTCTTCATAATCTTCGCTTTA
CATACAGTCTCTTC
TTCCGATTTTCTGAATTCAGGGTCTATATGTGCCATGGATCTCTCC
TCCACCCAATTTTC
AAGAAGTATTCTTCCCTCAGCATTGTTAACGGGTATATTATTCAG
GTCTGCCATAATTCG
ATCTATATTATTTTTGACAATTATTTATGTTACTGGTAGCCATAA
CAACATAGCTCAAAA
CAAGTCGCAGG
>ConsensusfromContig3994 Average coverage: 5.79
CAACTCCAAAAAACAAATAATTGTAACAGATTCTTTCTCAGATCA
AATATGCGTTTTAGA
TAGCCAGAAAGGGAAATTAATTGCGAAATTTGGGGCTCCCGGAT
CTGGCAGTAATCAGTT
GAACGATCCTCACCGAGTTACAGTCGACATTCACGATCGAATCT
GCGTAACAGACAGCCG
CAACAACTGCATCAAGATATTTGGTGCAGACGGCACCTACATATG
CTCCAGCGATATGGC
AGAGGCAATAAACAGCAACGCTTCAGCGCTCGAGAACATGTACT
TC
>ConsensusfromContig3995 Average coverage: 12.85
CAGCAATTGTATAGATCAATTATCCCATTCCGCCAATCAGCCAAT
GCAGCAGCAAGGCCA
TATATTGACAAATAAAAATCAGATCAAAGATAGCACTAGTGATGA
ACCGTCACCTGTGCT
TTATGGACACGAGAATAGACCAAAATCTCTTCCGAATTCGCCCG
CAAGAAGGTACGCCAT
GAAGATGTCGCTGTCAAGACCAGATATAAGTTCAAGGCCACCAAA
TTATGATGAATCTGT
ACATCCCCGCCATTATATCCCACGTGCTGTCAGCTATTATCACCC
CAAAGAAGATCAACT
ACCAAGTGCAAGAGGGGGAATACC
>ConsensusfromContig3996 Average coverage: 9.29
TCCGATCTGAATAACTTTTTTTTTCTTTGAAGTTCTTTCTTCCCAT
GGTCAAGAAATTTA
TAGTATTAAGTTTTACAAAATCTTTCTCACTTTTTGCAAGGTTGAA
TTTTGTATTCTGTG
GTAATATATATTTATCATATTTTGCCGTATATGCGAGTTTTCTCAA
CATGTTAACTTTCT

TGCCGTCTCCTGTATCCTTGACAGTACTAGATGGTTTTGTTTGAGG
CGATTTGTGTTCAG
AATGACGTTTTGAATCAGCTATGGCGTTGCTATTATCATTTGCATA
AACCTCTCCTTCGT
TGAAAATATTGGACGTAACCATGGGCAGATCTCGCTTCACGATAC
TTCTCATAGACACAT
TTTCAAGATCGTGTTTTTCTGGGCGGTCTTTGAAATTTTTAGCAA
TGTTGTAGATAAGCA
TGATGATATTTATAAGACTTTTCATTTTTGAAGAGTTTTCGTCTG
CAAACGGAGTACATT
GCAG
>ConsensusfromContig3997 Average coverage: 80.37
TGCAGAATCGTAACCTTTGATAATCCGTTAAATTTTTCCGTAGTTG
ATTTTGAGATATTA
TTCTGAATGAGATCATTTGTCTCATCGTCCAATATTTCCATCCGAT
CATCGTCTAATATG
ACGATTGGTGTGATGGCGGCCTCCACTCCTATCCTCCCTTTATTTT
CCGTCTCATATTCA
GCCAGGACCTGATCGAGATTATAGCGCCGCCTATAATTGTCGAA
GAAGTTCCGAACGTGG
ATTTCCTGCTTGTCGCCAATCATTTCCGCGATCGCCTCGAAGTCTT
GGCCGTACTTTCTG
ACGCCTTGTACAGCTAACAGCAATTGGTTATTTGTCCATCTCTGAT
TCAACTTTTGCGAT
TCTTCTGAGGATCGAAACCGATCGAATTCTCTCTCTGAATTTTCG
TTCGTTAGAGGACTC
AACTCTTGCTTTTGGCCTTGAACCTCTCGTTTTGTGACCTCCATTT
GTTCTTCTGAGGCT
TGTAGGACTCCGCCCACTTCGCCCGGAGAATCATCGGATGAAGTT
GATTTTTGACTTGCA
CTGGCAACGGAGTCGAAGTGATCCCGCGTCACATTCATCCCCTTG
GGAAGCCGGCCTTTG
ATTCGGTGGGTGGCGTAGCGCCCTTTGGGTTGTTTTTTCCTAGCTC
CCTTGTGGCTACCA
GAAGACTTGGCATCTCCGTTCCCGTTTGGTTTTGATCCCTCAACG
TCGCTGTCACTTGAA
TCAGAATCCTCGCGCTGAGTGACCAATCTGCGAGCTAGTCTGTCG
ATGAGACTCGTCCGC
GATCTCGTTTTCTTCCAAGAGTAGTAATATCTAACGAGTGTTGCT
ATGGATTTGTCCGGA
AGCATTTGCCTGATCCTACTGAAATTCTTTCCGTGGAAACTGAAG
GCCTGTTCGAACAGG

ACCTTGTCCTCCACGGTCCACTCATCTGGGTACGGCGTGTAGTTT
GTGAGGTCGCTCAGC
GCCGCGTCGACGTCGTGGTTGTGCCAGTATAGCATTCCCAGAGCC
TGCTCGGGGTTGTAG
CCATACTTCTGTTTTGTGATAGAGATGAATTCATCGATTTTTAAAT
CTGAGAGGTTCTTA
TTTGGTTTCCAAAGCAGTATTTGATGGGAATTGTTTGGCGATGAA
TCTTTGGAAGTCCCC
TCTGGAATCACCGCCTGGTATCCATCCCCGACCCGCATCCCATTT
TCTTGATCAGAATCC
TCATTCCCACTGTCATCTGATCCATGTTCATTTGGTGTTGAGACTC
GGGAGTTTCTTCCA
TTTTTAATATCAGCTGATACCATATTAAAATGTTTTTATAATAAT
AAGGAGAGGATGTCT
TGGATAAAAACAAGCAGAAATTCGATTTTTCGTACAAAATAG
>ConsensusfromContig3998 Average coverage: 37.34
AAATCATGCAACTTAATAATTTCAACAGACAATAATAATAACAT
GAACAAAACATTTAAA
TGTGAACACTTAGAGAAATTGAAAATTACTGGGATTGAACCTCT
ACGGATAATATACGAG
CATTTTGTGAATCCTCAAACAAAATTAGAGCATAAAAGAAAGGC
AAGAGCTACATGTCAT
CAGTGTGGTCAATATGGACCGCGTCTTCATTGCTGTGTCTATTGCG
TTTTCTATGGGTGT
TACAACTTCAGGCACATTCATGATCATGCTGATAGTCAAGGCCAT
AATTTGGCTGGTGGT
GTTTAACATGGTGGAATGTATTGTTTTAAAAGCGGGGAATCGAA
>ConsensusfromContig3999 Average coverage: 13.35
TGCGGAAATTCGAGACTTGAAGGCACAAATGCTGTCCAAAGAGA
AAGAAATTGAGAAAGC
AAAGTCTGAGAATGCGCGCGTTGGAGTTTTCCTCAAGGAGGCAG
AGTTAAAGGTTTCGGC
AGTTGAGGCTTCTCAGGCAAAACAGAGACAACTCAATGATGAAA
CAAATAATAAACTGAA
GAAACAGCGAAGCGAGAAAGATGGTGAGATCAACGCCTTGAAGA
AAGATTTTTCAGCGAA
AGAAAAAAGTCG
>ConsensusfromContig4000 Average coverage: 6.80
ACCCTCAAAAAGCCAGGAATACAAAATGCTGACCCCCAAACATA
GATTTCACGCACATTC
TCGATGTCAATCTGCACCGGAAATAGACATTTGTGCACAGGCGTC
CCGGGGTTCTCGCTT

TCGGGGGCGTGGCAGAAGTGTGAACAACATCACAAGATCTTCTTT
AGGGGTTTCCAGTTC
AAGGCTTGGTTTGAGCAATTCCAGTCTAGCTAGTGTTCCTGAATCT
TTTGATGAATCTTT
TTTGAATGAGCCGAGAATTTCTTCAGCAATGAATCTCACAGCGTC
ACTAGATGTCGATGC
TGGGAGACTGAGTTCCTCAATAGATGGCGATGTTGAGAGACTGAG
C
>ConsensusfromContig4001 Average coverage: 46.33
GCTCACTTGGCGAAACCCCGACCCGCTTACGAATGAAAGCAATGA
GAAGTTTTCCATTCT
CTTCAAGACCGGCGACGACCTTCGTCAGGACATGTTGACGTTAGA
AGCCATCAGACTGAT
GGACTCTATCTGGGTTCAGGAAGGCATTGATCTGAAAATGCAATT
CTACGAATGTCTTGC
GACCGGTCGGAACGAAGGTCTAATCCAAGTAGTGCGTTCGGCAG
AAACTGTGATGTCAAT
CCAGAAGAGTCAAGGGGTGAGGGCAGCCATGCAGGTCGGCAGCA
AAGAATTGCTTCGCTG
GATCAAGAAGAAAAATCCCGTTCATAATGATTTCCTACGGGCGA
AAGACAACTTCAAAAA
ATCCTGTGCCGGCTTTTGTGTAGCTACTTTTCTTTTGGGAATCGG
AGACAGAAATCCGGA
TAATATTATGGTGAACGAAAACGGCTGTGTTTTCCACATCGATTT
TGGTCATTTTCTCGA
CCATCGGAAAAAGAAGATGGGAATAAACAGAGAAAGAGTTCCTT
TCGTTCTCACAGATGA
TTTTATGAATGTTATTATGGGAGGAAGTGATGATGCCAGTCTTCT
GACAGACTTCATGGA
GTTATGCGGGAAAGCCTATTTATGTCTCCGGCAACATTCTTCGGA
ATTCATAAACTTATT
TTCGATGATGATGTCGAGTGGAATTCCTGAACTGCAAACCTCTCA
AGACATCGAATATCT
GCGGAAAACATTTGCTGTTGGATTGCAGGAGCGAGAAGCTTTAG
AATACTTCCAAAGACA
ATTCCGCGAGGCGTATGGGGGCGCGTGGACTACAAAGATTGACTG
GTTCTTTCATTGGGT
GAAACACAGAAAGTGAAAAATTCACAT
>ConsensusfromContig4002 Average coverage: 10.23
GCTTCGTCATGTGACACGTATTCGCCGTATGATTCGACACTATCA
CACTCCGACACAAAG
CGCCCCCTGCACCCAGAAGACGACGACACGGAGCAGGGGCTGTG

GTACCCGGATGAGAGA
CCGTCGCAACTGTCATACGCAGAGTCGTTACTATCCACTGAGTCG
GTGGCGCACAGGAGC
GGTCGAAAACCAAGCACATTCTCCAAAGAGTTAATTTCGTATTTG
ATAGATACATTCGCC
ATTCTTTTTAATATTTTTCGAGTGTCTAGTCTTTTTGTCAAAAAAC
TGTAGTTCCTTCAG
GAATATCGTCGAATGAATAAAGAGCAAAAAAACTACAAATA
>ConsensusfromContig4003 Average coverage: 45.96
CTTTTTCGATTCGATATAAGCGTATAAATTCGAGTTCGAACATATC
TTCCTCTTCATGCT
CTTCTCGAGCTTGGAGTGGATATAGGTCCGGTCAAGGACGAGGAG
CAGGTTCTGCATCTG
GAGGCCATGACCTTCGAAACGCGTTCGATGTTCACGTCATCGAA
GACAAAAATGTGGAAT
TCGTGTTCAACTATTTGGGCCGTGTGTCGAGGTCTTACGCCCAGT
TTAAACCAAGGCCAC
CGTGTCTGAAGGCTTCCAGGCAATACCTTTATATGTATTCTCAGTT
TTGTCTGGCTTACC
TCAATAGAGTGCGACCGATCCCAAACCGCAAATTCAGATACTCGT
ACCCAGTGTGCCTGA
GTCAGTTTGGGATTTGACTTGCGATTTAGTATAGATGTGTGAATA
TATTTTGAAAATCAA
AAAAAA
>ConsensusfromContig4004 Average coverage: 17.38
CAGCCATGCACCCAACAAAACTCTGCACCAACGCAGCGACTCTA
TCCAGAGTGACAACAC
CGACTTCTTGATTTCTCACGACTGGAAGAGCCATTGCGTTCTCAT
GGAAGACTCTACACC
AGAGCTAACACCAACTGCGGACGGAGAAACGAAGCGGAAGAGT
TAATTGACAATAGTCA
TTGTTATGCGTCTGAGGAGAATTTTCATCCACTCGCCTCGAATAG
GAATGATCATCAAAC
GTCAAGAATGAAATACCAAGCCGCACAAAATATCAACAATCTCA
CAGAATGGCCTTCAGT
TGATGAGCTCAATTCCAGCGACTGCCAGCTCCGCCCCCTTGTTCC
CCACTCCGTCACAGA
TCTCAGCCATGGGGACCTCGTCAAGTTTTCTCGCAGCAGCGGAA
AAGTGGCTGAAGGAAT
CGTCCGATTTTTGGGGACTTTGCCTCCAAAAACTACCCCTTATGT
TGGCCTAGAACTCAA
GCAGCCAGTTGGATATCATAATGGAACTTATCAGGGACGGAGATA

CTTTTTAACACCAGA
AAATAGAGGAGTATTTGTGGCATTCACCAAAATCATCATGGCGT
GGACAAAACAACACTG
ACGAAAATACAATGAGTTTCGAGGAAGCTTTTCGTTCAAATTTAT
AAAATATATATGATA
AATCAAGATCTGAACGTATATTCTGATTTGAGAAAGATGGACGA
AGTCGGCGA
>ConsensusfromContig4005 Average coverage: 51.01
GTTTTTCCAATGAGCCTCGTGTTCTGATCGCTTTCTTGTCGTAGG
TGCGACAAAGATGAA
GAGTTGTCCGACTTTGAACCCTTATTAGAGCTCATATTAGAAACA
TCAGCATAAACAATG
TCCAAGCACTGACTCACTAAAGAACATATTTCTTCTGCCGATGTC
CGATTATCACAATGA
AAAACGGCTAGACGACAAAATGGCTTTTCTTTGTCATTATTTCCG
TATTTTAACGCTAAG
ACATGTGCTAGATCATCGCGAATGTAACATATAGAAGCAATCTCG
TGTATCGCAGTCCGT
AGTAAAACTTTTTCATACTTATCATAAGTGGATACCTTCAAATTA
TAGATACTGAGCGAC
AAAATTGCGTCTTGATCCTGATTGGACCACCACGACAAATGTTCA
GTCTTCTTTCCATAA
TCGATAACTGAGAGTACATCGCTTCTATTCGTTGTGTCAAGATCA
TAAGGAATGTGTGGA
ATGATGCCTGAAAACTTTATCTCTTTCTCAATAAAACTGGAAATA
AATGCTTTCTGGTCA
ATTTTATAGTCGGGAGGTATGAGCTTTTCGCAGTTGAGGGGCCTCT
TTTCCTTCATATTC
TATGACAACGCTGCTTTCAAATGTTGAAAAAGAACTTTGTCAGTC
ATCACATTCATCATA
ATATTGAATTACAGTAAACAAAAACAGTGAAATCTCCA
>ConsensusfromContig4006 Average coverage: 51.36
CGTCCAAACATCGATGACGAAGGCATGAAGGTGATAAATCAGAC
ACATTCGTGGGGGATG
AGATCATGAAGCATTTTGTACTGGTGTGATTCTCTCCAGCCATCA
TAGGTGAGCAGCGAT
GAGAAAGTCTGGCAATTTTACACGGCTGGATAAAAAAGTTGGAT
GAACAAGGCCCATAAA
ATGATCCATCGTGATTGCACACTTCACCTTCGGTATGACAAAAAA
ACTGCTCGTCGGATG
CGCAGAAATCTTTTGATCCAAATTGACAACCAGCACCTTTTCCCC
ATGTGTATTCTTGTG

TCAATGATCTGTTGACTTTATACCAGCCTGCATCTTCGAATGCAG
CGACGGTCATAGGAT
CAATTATGCTCCACTGAGATTCCGCATATGACGTCATAAGTGAGC
CGTGCATAAAGATGG
CGTCCCAATGAGAAGTTATCACAGAGTCTTCTTG
>ConsensusfromContig4007 Average coverage: 56.03
CCGAGAATGAGTAGTCTCTGCACGATCCCCAGCTGATAGCACCAG
CAAATAACTTCAATT
CTTGACCAACAGTTACTGTCACAGGATCGGGCCTGAAGTGGTGCA
GAAGTAGCTCTTTAT
CGGTGCAGTTGACATGGTCGAAAGGAATCGGATTCTTATTTTTGA
AATACATTCCCATCA
TGTCACCTTTCTCTACTGGAATCCGATGCTGATGATCAACTGAGA
TCCGTAAATGTCCAG
CTTTC
>ConsensusfromContig4008 Average coverage: 19.81
GAAAAAAAGTGACGTATACTCTACCGGACGATTACGATTGTTCC
GAGGACGATTATGACG
CGCATAGCAGACGATCATCGCAACAGATTCTGATGCGACGACCG
ACGCCTGCGCCAATCA
GAGATAAGTCCCCGAAAGGCCCGCCCACGACAGAAGGGAAGACC
ACGCCCAATGGCGATC
TGGCTAATCTCTCAGATATCGACATGTCCATTATCAGGAAGACAC
TGAAAGCGAGATTTG
ATATGGGTTCTATACATACATTCATTGGCGA
>ConsensusfromContig4009 Average coverage: 4.96
AGTTAGATGCCCTGATGCAATAAAAAATTTGTCGAGCGTACAGTT
GACAACATGTGAGAT
AAATGCAATTAGATTCGGTCTAAAAAATCATATTCTACCCAGGTT
TTTGAACATTGTTAA
TTTCAAAGTCGCTGCCGAGAAATGTTTCTTCAACATCAAGAAAAT
TAACAATCAAATAGA
TAGGAGAGTTGGTGATGATTTCAAGCATAGCTGCCTTACCTTCAT
GCACCAAGCTGAAAG
CATATTGAAGAATAAAAGCAACAAAAGATTACATAGCACTCTGA
AAAATCTGAGCAGGAG
AGAGG
>ConsensusfromContig4010 Average coverage: 8.82
CCACTGGAGAGATTGTATACATTTTTCGGATGTCTTGAAACTCAA
TTCGGAGAATCTTGT
TGAAAAAACCCGACAAAAATGTCATAAACGACCCAAAAACTTTA
TTGTCAGCAAAGGCAC

ACGAGTGCCATGGCCATACGTAAAAAGTAGAGGGGATTGGTGTT
TGTATCAAAAAACAGG
TTCCAAATATTTCGAAAGGGTGAATAACACATACGGCCGTGATAT
TGGATTATTGATCAA
GAAAGCATCATCAGACGCGGCGTCATATTTCGTCGTCACATCAAA
TCATCACGGAGTAAA
CTGTTTTGCCTGTTTTTTCGGTATTGTGATAGAAT
>ConsensusfromContig4011 Average coverage: 14.39
TCTTCCGATCTTGAGAACTCAATGATGATTCTCACTTCATGCCTA
AATTCGAAAGATAAG
TTCTCAAAACAAGCGATGCATATCCCATCTATCTACTCGAATGAA
ACAGCTCACTCACTC
AAAGACAATTCTGGGAGAATGGAGAATTCTATCATTGAGTCTAC
TGGAAAACAAAAACAT
TCGCTCTCATCAAGTGTACCACTAAAATACAAGGATTTCAACCA
AAGAACACAAGAACAG
GACAGTTATACGAATTACTCGTTGCCGGTGGATAAAATAACGAAG
ATTCACAAGCCTTTA
GCTTTATACGATGATATTCCTGGTTCTAGTCAACCTCCGATTCTTT
CAAATTTCGTACCC
AGAGAACATCCTCTGATAATAAGCTTCGACAGCAGAGCGATTGG
AGTCGATTGCGAAGCC
ATTCC
>ConsensusfromContig4012 Average coverage: 13.90
GCTTGAAACATTTATTATGAAATCACACACTTTATAATGTATATT
TTAGGAAATTTATCC
ATCTGGCTTTTGAAATAACAGTCGCAACAATAATACATCTTATGA
ATAATGAGCAAATCG
TTAGGAATGTTACAAGAAACAAATTCCAAAAAAGGGAAAAATCA
AAATTAGTTTGAATCA
GTTTTTCTTACCCTCAGACTTATCACACTGTATTTCAACTAATTTT
TTTATACACCTTAT
GATATGATTTATTTCTATAAAACAGCTGGAAATAACATTTGCCAT
TTTATATGCGAGTTA
GGCCCTCAAATCTGTCTCGAGGAAACGATTTCTATTATAATAATC
AATCTACAAATAACT
TGTTTTTTGCCTAAATATTGCTAGTCGAAACATTTCAAAACAAAC
GATTATTTGCACGAT
ATCCGTAACTATGGTAA
>ConsensusfromContig4013 Average coverage: 17.77
ATCCCACGCAACAATATATGAGACCAAGCGGCATTGATGAAGAA
AACTCTTCTATTGGAA

TGGACCATGGTACAGCAGAGTATGAAGATTCGTCCTTGGGCTTT
CAGGAGGGGTCGGAAG
TTGCCCAGGACCCAGCTATTCCAGGACTAGACCAGACACGACGCC
TTGTATCCGGTGTTT
ATGAATGTAAACTGTGTGGCTATACAACTCGGCGCATTCAGAATC
TACGCAGGCACATGT
GTTCCATTCATAGTTGTGGGGGAAAAGTCGTTTGTACTCAATGCA
ATTCTGTGTTTGCCA
CTGAATACGAATTGAAGAATCATACGTCTGTCAGACACGATAAAA
GGTTCAAATGCCCCT
GCCATTCCAGGTGCTTTGCGTCTGCCGAGACTCTGGGAAATCACA
TAACGAAATATTACA
CAAGTCAGGAATTTCTGGTCAATAATGGCTGAAGGAATCAACAC
TGCGAAAAGTTGTAT
>ConsensusfromContig4014 Average coverage: 14.07
TGGTAACAACCGCATCCAATTCAGACGTGACGTAGAGTCATAAC
CAAGCATGACAATGTT
GAACTTGTCCGAATTTCGTGTTTTAGATTCAGTTCTAACTTTTTTT
ATCCCGGCATGTAC
ATTCTCATATTTTGATCCATCCTTAGCAACACAATGCACCTTCAG
AAAATCAACCGGAAT
TTCTGTACCATTTTTGAATTCTACTTTCGTACCGTATCCAACACTG
AAATCACTCTTTCT
GTTTATGGTTTGATATGCGCATTTCACATCACCATGTCGCTCCTGG
GCACTTATATTGAT
GTAGATATAATCATGTATGACATAAATCCAATTGGTTTCGGATTT
ACATTCAAGATGCAG
CTTTGAATCTAATTTTTTCATTTCTGGTGGCCATAATTCCATTGTA
GGCAGCGAACATAC
CTGTTTGTTGTCGGTATTAAGTTCAGGAGATTTTATCACTTCATGT
GATTCGATTTTG
>ConsensusfromContig4015 Average coverage: 7.64
CACAGAACATGCATCTCATACCAAATATCTCACAGACCCACCAGT
TGTTGAATATTCAGA
TGAACTCAGTGAGTACGAAAAAAGACCAGTGAAATACACCCACT
CAAAAATCCCTTATGC
AACAATCGATGATGCAAACAGATCGTATCAAACCATGGATCAAA
CTTTCCCAGAGAGAAC
TTTGGGGTTGAATAGCATCAGAGGTGATTGGGGATATGGATGT
CCGCTAGGGGGAGTTC
AACAGAAGATGAGAGGATGGGAAAGTACGAGAAAAGACAAAAA
AGCGAGTCCCATCCAAA

TATTGCTGATGGACTCAAAAGAAAATTTTTTGCAATGGCAGGTTG
AACATTCCGGTATCA
AAAAACATCATCTTTGCACAAATATATCATATAAAACGAATCTGG
ATTACTTTATTTACA
TGTTTGTCAAGATTTAAATACTGCACAACTATATCATATGTGTAG
AGCAATGAAATTAGG
ATTGTTTCATTCACC
>ConsensusfromContig4016 Average coverage: 7.15
TATAACGGCTTCCATAAACAGTATCGAGAAAAAATGAAAACTGG
AATGAGATTATCAAAA
TATTTTTCAGGCTTCCAAAATCTAGGATCAAAAATTGATTTCAAA
AAAGAAACTATTGAT
ACAGTCGCTAAGAATTATCAGGATGAGATGAGTACTGAAGATGA
GAAAGTCGCTGAAATA
TCTGAAAGGGGACATTTAAAATACATTTTAGTAGGTAAACAAGA
GAAAACTGAAGGGACT
CAGGATGAGAACCAAAAAAATTATGGCAATAAACAGAAAACTAT
GTCCAAAGTTGCGTCG
AAACAAATGAATATGGTACCAAACTTGTTCAAACACAGCCCAGA
TATCAAGGATAATGTT
CAATCGGACAACCATGATAGGATCCAATCTCCCATGGCAAGAAT
GATGAAACAAACCAAA
GACAATTTTCAAACTACTAATGCTTTTGTGGAAGACTTGAATTCGT
C
>ConsensusfromContig4017 Average coverage: 45.86
ACCTGAATTTCGATGATGCGTTACAGTGCCGAACATGAAGGGGG
TAGGTACCAGGGGCTT
CATAGGTATCTCTAGCTGTCTAATACTTGACGATGGAGTGCCTAC
GTGAAATTTCATCTC
CAAAGGATTCGGAAGTGCAGAGGGCTGTTTGAAGAGTGCCAGTGT
CTCTTGTGTGCTGAA
AGGCCGAGTGGCTCCCTCTGTGAAACCGATCGCGCGCGATAGGC
AACACAGACATCTCCT
GTTGACAACCGGAACTGAGGTGGCGCAGTTGTAGGAAGATGAGA
CCACGTGATCAGCGAA
AGCGGTGTAGCGCGAGAATGATCCGCTATTGCACGTGTTGAGAA
GAATATTGAGTCCCAA
TGTTTTCAAAGATGAAAGGTGATCTTGCCATTGCATGTCATCAAA
CATCAGATCTTCTTT
GAACTTCGGGTCAAATGTCGTATCTAGAACTTCGAATCTCACAGA
GCCTTCATTGCGTCC
CCGAGTTTCGAGAGAACAATCGCTTTTCTCCTTGGCGTTCTTGAG

GAGCACAATTTTCTC
TGGAGTTGGGGAAGGGTTCGACAGAATTCCTTTCTTGTCCAGACA
ACAAAGAGTCGTTAT
ATCACCAAAGGTCTGCGCCATATTCGCATTCCGATGGAGAGAATC
GTCATTCCCACACCA
AACAAGCCACGCCTTTCGAATCAGATTGAGAGATTTGGGACCGAA
CGGTTTCAATATTTT
GAAAACTGGAGTTCCACGCATGATGTCAACTTCATTGCTGAGAAT
TGCTGTACCGTAGCA
GGAGAAACAATGCCAGGCCACTGGAAAGACGATGGGAAGCAGA
GGAAAGACGGAATGAAC
CGGGTTTGAAATAAGAGCGAAAGCCAAAACGGTAGAAGTTTCCG
ATGTCAGAAAATATCG
GGTCAGACTGAGAAATAGGGAAAACAGGAAAACAAAAGAAATGA
CAAATTTGTCGATCCA
ATATGTTGATATTGTTC
>ConsensusfromContig4018 Average coverage: 13.22
ATATTTGAGTAATCATCAATATGACTTGATTGATTCAGTTTATTTC
TCATTTCACTTGAT
GATGTTTGAAAGTATTTATCCATTATTCTTCTTTTGTGAGGTAGCT
TAGCGTATGTGTAG
ATATTTTCATCGAATTCTTCATTGTCGTCTGCTGAGCTTGTAGGTG
ATAATTTTGGAGAT
CCACATGGCGATGAGCAAGGCGTACTCGATGAACCAGTTGTGTT
GATGGGAAGTTCAGAT
TTGATAACTGGGCTTTCCCGTTTCGTTGCATCATACATTATGTTTC
CTTGCTGATAACAA
ATGTTCGTATCCTGACACGTGTCTTCGGTTTTGCATTTGTCGTTGT
CCAGACAATTATCA
AATCGAATTCTATGATTCGTTCTCAATAAATATTCTAAATTTATA
TTGGCAGTCATCAAA
ACAGACTTGTAA
>ConsensusfromContig4019 Average coverage: 38.66
GCGATTTGCTGATATCTTGATTCTATGGAAGTTGATAACTTCTCT
TGATGACTAGAAGCC
ATTAGGCAATAGTAGGACCAGATACGATTCATATCGTCATATTCG
AGGTTGGACGAATCG
CATTTATTCACGTCATAAACCACACGGGTTTGCCATTACCTTCAG
GTTTCTCAAGATTAG
GCATCATCCAGAAGGACTTCACACCGTCTTTGGCTCCACTGTACG
TACCTACTATGAATC
CGATGCATGGCTGATATATGTCGCCATCACCTTTCATTTCAAGCTG

ATATTTCATTTGAT
TCGAGCAATCCTCCGGAGACGGCGCCAGTACATCATCACACGAAT
TTGAGCTTGTACAAG
ATGAATGAGAATGATACCAGCCAACAACGGACAGATTGTCTTTCC
GCATCGCTTCACGAA
TCTCTGATTCGGTTCGAATGGTTTCATCTATCATTGAGGAAGAAC
ACAAACAAGGATAAG
CTTTCACAACGCTTAGATTTTGCAGAACAGGGTCCCACTGGCCAC
CCAGATATCCTACGA
CCTCGTCTTCGAACAGTTGACAATGAAAGTCCATTATCACCAAAG
AACTGGGTTCGACGG
AGATGGTGAACGGTTGAAC
>ConsensusfromContig4020 Average coverage: 4.93
CTCCAGGGGAGAGTTGATTCCCTTGTGAAAGGCAAAGAAACCTG
TTTGAAAGAAATAACG
GAAGAAAATTCTAGGATAATTAAAGAAATGAATGAAGAACATGA
GGAGATGATGAAAGAG
AAAATCAAAGAACATGACAGCATTGTCACATCAATGAGTAACCA
AATTGCTGAATTACAA
AATAGCGTTCAGGAATGGAAATCTAAATTTGAGATGAGAAAACA
AGATTTGGAACTTCAT
TGCTTAGAAGAATCTAAACGACAAGTAGACACAGCCGTGGCAGC
CGTGACTTCAACACTC
AGATCTCAACATGAGGAGAAGATGCGTGACGTAATAGAGAGAAT
GGAGGGAGACTGTCAG
AGATCGGAAGAG
>ConsensusfromContig4021 Average coverage: 38.26
GTCGCTCGAGCATTTGTGGCCGTAGTCTTTCACAAAGTAGTTCAA
CAAATCCTTTATAAC
CGTAGTCTGCATCAGGCACATTATAGTTGTCACAAAAGCCAGAA
GGAGAGTGTACATGAA
CCTGGTTGTATGAGATGCCCTGACAGCAGGGAGGCAGGAGGAAC
AGAGTGAACATGGCCG
AGGTCCACAACAGCATGCCACCTGGGCATCAAGAGTGGGACACA
CGTCCATGAAATAAAA
TGGTATCTATGTTTTAAAATCATAGAACAGACTTCTGTTCTTGAC
AGTCATGACGGAGTC
TAGCAGCACATTGATCAACAAACGGGATGACACGGTCGTGACCT
AAGCACTGTGCAGGAG
>ConsensusfromContig4022 Average coverage: 9.58
GGAATCCCTGTTATCCGACTAAATGTGATAAGGGGAAAACCTGCA
TTCCAGCTGCAGATG

GCAGTAGCTTCAGTTGTGAAGATAATCTGAAGCCCATCACTTCGA
GCCCGTGTGATTCCG
GACCATGTACACCTACAACACACAAGGATTGTGTTGTCGACGGCG
CAGGTTACCGTTGCG
AATGTAAACCGTACTACCTTGGACCAAACTGCGATATCGGGAATC
CTTGTTTTCCGACAA
AAT
>ConsensusfromContig4023 Average coverage: 16.49
TATTATTTCTCACTATATCCCAGGACATATCACTGTCACTCCTGG
GTTCAATAGAGCTTA
TACCTTCACTATCTGATGAATTTTCATGCGGCGAAGTAACTAATT
GAGCCCTTCTCGTGG
GTCTAGCGTTGACATCCGCTTCTACTCCTTTCCAAGTGTCTATTTT
AGCTAAACGCAGGT
GTCGCTTATGTACTTTCATAGTTTTTCCCGTTATTTGGTTCCTTATT
TCATATGTTACTG
GCGATAATTCTTTCGTTATTCTAAAATATGGCAACCATTTCTTATC
TAATTTATTTTGCG
TATAATTATTTTTCATATACACGGGACTTCCTACTTCTAAATCATC
GCTTTTCCTATTTT
TATTCGCTTTTTCATTTCGCAATTTTCTCGCTTCCGCTATATTTTTT
CTAACTACGTTGA
AGTACTTATGATTGTTTTCGAATATAATTTTATGCGCGTCTTCCCC
TACATATCTCCTAC
GTGGTTTGAGTACATTATCTACCGCGTACACTGGATCTCTTCCAA
ACATTAGAAAGTATG
GTGAGTATTTCGAGGATTC
>ConsensusfromContig4024 Average coverage: 8.94
TCTTCCGATCTCGATCATGATACTGGTACGCAGATTAGTTATGCT
TTGTTTAAAGTACAA
CATTTTATTCAGGGCTTGTCATGTGCCAGGTTCGAAAAATTTAGTT
GCTGATTCGTTGTC
ACGTTTGCAGATACAAAAGTTTCGTACGTTGTGGAGGCAGGCTTC
GGCCCACCCTACCCC
CATCAGTCCGGTTCTCCAAGAGGAGCTATTGTTGAATCGATGTCC
TGACTTTTAATTGTA
TCTTTGTCCCCTTCCTCTCATAGGTTATTCCGGCAAACTGACGTAT
CTGGAGGCAGTTTT
TACTCACAATGGTCTTTTCTTCTTCATGGTGAATAGTTGGCCATTT
GGACATATACATTG
TTACATCTTTCCTTGGTTTCGGGGCGGTATTCGGCGAACGGTGGTT
TGTTGGTACTTGGT

TGATCGGTATTGGTTCCAGTCGGCTAAAT
>ConsensusfromContig4025 Average coverage: 9.99
CTCTACCCTTGGAAATACTATTCAGAAAATGACTCAAAAACACT
GCCGGCAGTGTGGAAG
TTGTCAACAATCTCACCAATATACAAGAAAGGTAGTAAAAGCCT
CGCAATAAATTACAGA
CCCGTCGCTCTTACGTCAATATCATGCAAGCTGATGGAGTCGTTT
GTGAGAGAAGGTATT
CTTAGTCACCTACACAGGAACAATTTACTTTCAAATACCCAGTTT
GGGTTCATAAATGGC
AGATCCAATGTACTTCAACTTTTGAACTTTGTGGACACATGCACA
GAAATAGTCGCTAGA
AATGGCAAAGTAGATGCAATATACCTTGATTTTGCGAAGGCTTTT
GATACCGTACCTCAC
GAAAGACTGCTTCTAAAATTGCAG
>ConsensusfromContig4026 Average coverage: 20.94
GGCAGTTAACATTTCTTCGTTATTATCATTCTTCTTGTTTATGTGT
ATATGCTGAGAAAT
ATAATGCCTGTTTGTGGACTATGATTTCGAATAATTCCAAATCGTA
TTTAAATGTTCATT
CTTGATTATATGTACATATAATAAATTGAAATTCATCCTCATGCAT
ACGTTGTTTTTGAT
ATAAGTTTTACATGTTATGAAATGAGTGTTGATTTTGACTGTATC
CTAACATACGTAAAA
TCACCGATCCAAAATCATTATGTTCACGGACTAGAG
>ConsensusfromContig4027 Average coverage: 5.99
GTTCCGATGTTTCAAGGCCAAGAGAAAACTGTCATCACCCCATTG
CTCTCTACTGGAGAC
CAGGCCTTTTCCAAGCCCGTGATGATGCGAGCTTTGGTGCAAGCA
GCTGTTGAGTGGATC
GAGACTGGTTTGCCCATGAAATGCCTCAAGCTCGTTTTGTATTCC
CCAAGACCACAGAGT
TTGAGTGCTGAAGAGAAAACATGCGTCAACATTTTCGGAGAGAT
CAAAAGTAAAGCAGAA
AAGCGAGCACAGCGAAAGGAAAAGAAGGAAGTTTCAGTGCAGTA
TGATGCTTATATCTCA
TACGATCACGATGACGGGGGTCTCATGTCGGACTTGATGAGTAA
ATTGAAAAATCTCAAA
TCTGATCTGAACATCTTAACTGTCAAACACGAGATGGACGCGAGC
AAGGCTTTTCAAGAC
ACAATACGAGATTCCATGATTATCTGTTCCAGAGTCATGACACTT
TTGAGCCCCCGTTAT

CTGACATCTGTGCGATGTATCGAAGAGTATAATATCGCACTCTGC
TGTAACCGGGGCCTC
AATCGTACGCTACTCGCCACGATGTACATGGACACCATTCAAAAT
CTACCCGTGTACATG
TCTCTCATGCAGTACATTGATTGCAGAAATATACCAGAAAGACA
AGCGAGAATGTGGGAT
GCTCTAAAGAGTTTATG
>ConsensusfromContig4028 Average coverage: 7.65
ATTTTTGCAAATAACACAAGTTGTATTTTTTGAATGGCATTATTC
ATATTAATCAGCAGA
GAGTAGTATATATCAGCTGAACTATAGAAAATTCATGTTCTCTAA
TTCCAACAATCTAAT
TTTTCGGGTCGTTGAGTTATGTTGTAGAATAATGGATTAAAATTA
AAATTAACAACAGAT
TTGTAAACAGTACATATGACTTAACAAACAGAAGCTAAGAACATT
CTCAACAAATGTGCT
GAATAGACTGTTTCTCCAGGATTTATTGCCATCAGACATATATTT
ATGATCTCCTTGGAA
AATAAGTTACTTTCTCTTACTCAGGCAGGGATGAAGAGACTTGTC
AATAGTTCAAAACTT
TCTCTTAAACAAACCCTCATTACTGAATGAGGTAACTAAGTCTGT
AGTTATACAATAACC
TATAGATAAGAAGACAGCTTCATGCAATACATTTTCG
>ConsensusfromContig4029 Average coverage: 6.65
CAAAAACAGATGCACGTAGTGTTTTTATGAAAATAGAAGAAGCC
TCAGAAATCAATATTC
CTGAGAGTGACACTAGTGTCATTTCAAATATTGTTGACTTTCAAA
AGGGAATCCATACAG
AAAAATTGCCAACAGAGTCATTTGTCCAGCCATCGGTATGTCAG
CATGAACATGGAGAGA
TTTCTTCAGTTCCTGTTCAATTCGTTCCTGATGAGGGAAAAACAA
CAGAAATAGAAATAT
TTTTGCACGAAGAACCAGCGTCTCGTACAACTAAAACTGTATTTG
TTAAAGAACAAAGAT
TAAACGTAGATTCTGAAAGTGACATGTCAATAGAAGAACGTATTT
CTCAGAAACCTTCAC
TAATTGATGCGAAGCCTGAGAAAATAACGGCTGTTACTGTCAAGC
AAGAAAATTTAGTTC
TGGAACAAGAGACTAAACCTGACGAAAATATCTCAA
>ConsensusfromContig4030 Average coverage: 19.79
TTGCAGACGGCGAAGTTCCTCAGATCTACACAGCCTTCTCTTCAG
TAGACACAGCTGGAC

AAGATCACCAGAGCTACATCGTCCAGGAGCCTCCAGGCGAGGCT
GTTGTTATACAGCCGG
GAAGTGCCATTCCGGATGTGACGTCAAGTGAAGCCATTATTCTAC
ATCCGGTATTCGATG
GTCACCTTGAGGAGAAGCCGGATGTTGGAGGATTCACTTCCATCG
GCGCTCATAATGCGG
CCAATTCGTAGGGTCTTTGTTGGGGGCCGTTCATATGTATGATAA
ATCACTCCACTTGTT
TAACCTTCTAGTTATTTGGGACCTGTTCGGATTAGCTGATTTTCTA
CGACTGATTTTCTT
ACGATGTAACACTTTCAATATTTCGCGTCCTTCTTGGGTAACTCCT
TTATGGATTTTTTT
CTTAATTTTCTATCATGTAAAACTTCCCTTTTTATCTCTACGTTAT
GAAACATTCCCCAC
TATATCTATATAAATTTCGAGCAAATTTAACAATGTATGAATTAT
GGGATTTTCTACAAT
GTCGAGCTCCCTATAAAAACCTCCCACATCTTCAAACGTGAACAA
CCCCTCATCTATATT
CCTG
>ConsensusfromContig4031 Average coverage: 10.22
AGCACATGGAACAGGTGTGGAGATTCCAGTCGAAGGACACGAGA
ATCCAGCCGGGCAAAC
GGTGCAGATGTCCTGGGCAGGACCTGAGTATTCCCCGGGAGAAC
AAGCCACGTTCCCAGC
TGGTCCTGTTGAGTTTACGCACGCATATCCGGCCGGACACTCCGT
ACAAACCGTTGACCC
AAGCGCACTCTGGTAACCTGGGTTGCAAGGAATCGGTGCAGCAT
CCGCAGATGCGCATGC
GTGTTCAGCCGGGCAAATTGTACAGGAAGCTGCCGTTGGGTCTAT
GTTATAAGTCCCGAT
TCCGCACGCCACTGGTGAACTGCTTTTGCTCGAACATTGATGTCC
TAATGGGCACAGCGT
ACAGTTCGTGGCTAAATCAAGTGAGTAATATCCTGCGCTACATGT
GACCGCTGACGTCGG
GTCTCCGCATTGTTTTCC
>ConsensusfromContig4032 Average coverage: 61.55
TACCATATTGGCACCATATCGATGATTCAGCTCTTGAATCTCTTA
CGTTGCCATGCGCTA
AACTTGAAAAACTAAACATGTCTTGGTGTGGCAAATTCAGAAGC
ATAACGCCCGAACGCT
TTTCGGAATTTTATGAGGGTTTCTCCTCATCTATAGAGTTTCTCTC
ATTAGACTCTTGCG

ATTTCCTGGACCAAAGCTGTTTGCTGGCGATATCACTTTCATCGA
ATTTGAAACATCTAA
GTTTACACAGCTGCGTACATCTAAAGGCTGAGAACTTTGTTTGTT
TATCAAAATTATACA
ATTTGAAGTATTTGAACTTATATCGGACATTTGTTGATTTGAAAA
CTCTTATAACATGTA
CTTGTAACATGGAGAACTTGGAATATCTGAATCTGGGGTCAACTT
ATCTTGAGCAAGCCG
ACAATATTTGTGAATCACTTGGAAAACACAACAAATGCTTGAAAT
ATATTGATTTATGGC
GTTGTCAAACCCTAACCGGAAAAGGAATCCTACATTTAGCCAATG
GCTGTCCCAATCTTC
AAGAAATTGACGTTGGATGGTGTACTGGAATCAGATCTCACATCA
GTGCTGTACAAATTT
TGGCGCGGAATTGCAAGAAATTGCAGAAACTATTCTTGACAGCT
ATACGTGAAGTGACCG
AAGGAGACCTCATAACCATAGCAGATAACTGTCAATACTTGCAGC
AATTAGATTTACTAG
GAGCACGGTCAGTTCGTGAAGAATCAGTCAGATATTTACTCTCGA
AATGCATCAGATTAC
AATTATTAGATCTTTCGTTCTGCTCTGATATTTCTGACGAGGAAT
GCAATAATTTGTCAG
CAGTGTTTCCTAATGTTGAGATTAAACGTAGCTTTCAAGGCTCAT
GACAGCATGAACAGT
ATCACTTGTTTGTTAAATTTGTTAAGTTCGATATCATATCAATTC
ACAAGCAAAATTGTG
AGATGGCCAGAGCATTCTTACCAATAATAATTTTTTAGAATAAAT
ACGGATT
>ConsensusfromContig4033 Average coverage: 19.04
GCCACACTGACTTTGTCACCTCCATCCGAGATTTCGGTCTGTGTC
ACACATTCAATGGAG
GGCAGAATGAGACGGTTCGGGAGTCTGAACTCACAGGTTCAGAG
AAAGGATTGAGATTGA
TCATAAATGTGGAACAGACGGAGTACATGAATGGTCCTAGTTTA
GATGCAGGAGTTAAGA
TGATTCTGCACGACCAAGACGAAATCCCATTGGTCAAAGATCTCG
GGTTCGTGGTGGCGC
CAGGGGCACACGCCCTCGTTGTCGCTTCGGTTCATGAGGTGACGT
ATGAAGAGCAGCCAT
GGGGAGATTGCGACCCTACTTTGACCCTTCAATATTCCGAAACGT
ATTCCTACACAGCAT
GCAGAAGAGAAGAGGAAATTGAAATGTTGTTGTCCAAGTGTGGA

TGTGTTGACGCGTATA
TGATGGA
>ConsensusfromContig4034 Average coverage: 177.06
TACACGACGCTCTTCCGATCTGGTGCATATGGTCCGATTCAATGG
GATTTATTAAATTTG
ACAACCTGTATGCAATGCGTGCTATCAGAATGCTATGGAATTTAT
CCAATATACAAAGCT
TTAATAACGCATAAAGTTTGGCATGTAAGACCATACATTCACGA
TGAAAAACGATACACA
AGAAGAACAACACTTGCATATCCCAATAATACAGTCATTATAACA
ATAATCATGTACATC
AAACACGTGTACACACCTATGCTTCAAATGAGATAAGCACTGAA
TAACAAAGGATAACAG
AAAACGATAATGAATATCACAAACGTTAATCAAAACCAATAAAT
ACACATGGACACCCAC
AAAATATCAACTGATAACATTTAACAAACAGAAATATATTTAACG
GTGCGTCTAGACATT
AAAATGGATGTGCCGAGCAATTAAGAATTTCTCAGCAATAATGA
AATACTGGAAAAAGAT
ATGAACAATGTATCACCTTGGGTCGCTTAAAATACCACATCATTC
ATCATGACTTCGAAG
TAGGAAAATATATGACTTTTCTCAATAACCCACAAACAAACTTAT
CACACTGGGAATGGA
AGGCACTGTACTAATAAACCCCACATCAAAAAACGCAAGGCTAT
CTAACGATGGATCACA
AACAAGAAAATTGCTAGCAGCCCTGATACTAATATTATAAGTAC
GATGATGTAAAAAATA
ATCCACCAAAATAAATCTAGGATAACAAAATGAAATTGATTCCAC
ATGTCGATATTGGTA
ATTCATA
>ConsensusfromContig4035 Average coverage: 19.29
GGTAGATTGGCGCATATTTGACAATCACGCAGTGAGCATAACTAA
TTTATTCAACCTATA
TCGCGTGTTTAATCGGTTTCATTGTTTGATCTCAAAAATTCAAGTT
TCATTAATTCTGGC
TCATGATTTATCATTATGGACTGAACACAGAAACAAAATAATAGA
TATTCAGCTACATAT
TGGATAGCTCGAGCGGGTTATCCGTACTGAACTGTCATACAAACA
TCAATACGGCATCTC
AACGTTCTGCATTTGAACCAACTGCACCTGAAAAAAAAGAAATTC
ATTAACTGTTCTATA
GTGCCAATGATCCTGTTGTATTATTAAAAATCCGAGATTCGACTG

CCTTCCGATCGTCCT
TGATACAACCTCCGATCGTCGCCCATCCCAGGAGAAGTCAGTGAC
AATCATCGTTTCTTT
TCCTGCTAAGCCCTCTCCAGCTTATTTATGAGTCACAACTGTTTT
CAAACCACAAAAAAT
TCTTCAGATTTACATATATGAATCACAGGATAATCACGCGTTAAT
TATGTTATATAATTA
AGTAAATGTAACATCTCC
>ConsensusfromContig4036 Average coverage: 19.90
ATTTTTTCTGCTTTTTCTTTTATTTCATTAGAATCATCATTTAATT
CATGTAGTCATGAG
AACTAGAAAAATTATTTATTTTTAATTTAAATAATAAAAAATGAT
TAAATAAATAAAAAA
AAACTAACCCAAGGACTTGACCTGCTTGCTCACTCACTTGTGTAA
TACGTCTTTCAATTC
CAAAGCCTACCTACTCTACGCTTCTTGCCCAGTTCTTCGTTTCTTC
ATTCCAAATTTAAG
AGAATACTTTTTTTGGTGTGGTGTTCAATCGTTCTCATCTCACATA
AGAATTTTGAATTT
AGTTAAATTGGGTTAAGATACTTGAGAAAGGGTCAAACCCAGAA
GTTCGGCTACAATACA
CAGGAATTCCTTCACTCAATTCTGAATGAGCAAACTGAATTAAAT
TGTGTGGTTTTATTC
TAACTCAATTGGAAACCTTCAATAATATCTATCTATGTGTCAGAA
GTTCGGCTACAATAC
ACAGG
>ConsensusfromContig4037 Average coverage: 11.95
TCGCTGGCCGATGCACTGAAAAGAAGAAATTTCTCCCTTGTTTGC
GATGCTCTCGGCCTT
GTATCATACACAGAAGATGGTAAACTCAAGGCTGGAATCCGATT
GACAACGGGATTCGCA
TTGAAAAAGGCGATCAAGAGATTGGTAGCAATATATATGGAAGA
AGAGAACGATGTTGAA
GTAGAAGAAACTAACAAGTTCAAGTGGCTCTACGAAGAGCAATGG
GATTCTCTTTTCCAT
ACAGCCGAAAAAAAAATCAAGGAACGACGACAAGAGGTTACAA
GAAAACCGGAGAATCTT
CCTCTTCATTCGGATGTGCAGAAGCTTCTGAGATATGTTCAAAGA
AACTTAGACAGTTTA
GAGCAAAATTCTGCAAAAACCCGAAATAATTATGTTCGTTATAGG
GCGGTACTTGTATGC
TACCTGACATCGTACAATGCCAGAAGAGGGGGTGAAGCATGTAA

ACTCACATTGTCAGAA
CTGAATGATGCGAAAGAAGGGGTATGGATACCTGAAAATCCTGA
GAACAATTCGTTGAGC
CACCACATAACATATCAGAGGGTGAAAAATGGAAGGCTGGCAAG
CTTAAT
>ConsensusfromContig4038 Average coverage: 17.56
AGTGCGAATTCCCATTCAGTCTGAGTTTGGCAGCATCTTTTCTAGC
AACTTCCTTTCGCG
ACCGACGGTTCTCGGCATCGATAGATAGGGCTTGCAGGAATCCGG
CTTGATCATTGACAT
GCTCTGGTGGCGAATTCAAATCTATAGATTTGTTTATCGATGTTTT
TGAGTAACCAATTT
GGCTATCTGTAAGAGATGATCCATCACCAAATTCTTCCTTTAATG
AAGCCAAGTCATTCA
TAAGTATCTCGAATTTATCCATTGAAATTCTAATTTAAACAAAAT
GTGTAAGTTAAGTAA
ATTAAATCACTGATTTTGAGTGTTGATTGTTTA
>ConsensusfromContig4039 Average coverage: 20.99
AAAAAAAAAAAAAAAAACAAAAATAACTATAAATGACCACCCCC
CCCCAGCCATGAGAAA
TTGCGCCTGTTTTCCGGGGTCAGGCATACAACTCGGACCACTCTT
ATTGCTGTACGAACA
TATTTGGTTCTTACACAGCTCACGCGCTTCCACGACGGCGCATCC
ATGGTTCATACTCTG
CTTAGAGCCCAGAGCACACGTTGAATTTTTCTCAACAAGAAGATT
GGCGTATTTCAAAGA
TTGCTTCGATGGGAAACTTGCTTTCCATCTGGTTTCAATGAT
>ConsensusfromContig4040 Average coverage: 19.77
TGCTCTTCCGATCTATTGTTGTTGACAAAAAGTTTTTATTTTGCA
AAACACACAAGTGAT
CGAGATGATGTTACCGTGAGTATTGGTTTCAATATAGATGGGAGT
GACTTGAATCTTTTT
TCGGCTAACAATATAGATACAGATCTGAACTCTCCTTTATCTGCA
CCACTCTCTACTGCT
ACAACTGCTGCGGCTGAGGAGGATTCAAATCCTCTTCCCAACATC
GACGAAAGCTCATCT
TTCTCAGCTCCGTCGCCGGAAGAAGTCGAATCATTATTCTCAACT
TCAAGAACTTCTAAG
TTGAATACTTCCGAATGTGAACACAGGGACCTCCTCGAGGAAGCT
ATAATTGCATCTTCT
GCTGGATCAGAAGAAAAATCATTCTCTGGTGTTGGATGTAACGA
TAAAGGAAGAGATCTG

CTGCATGAAGCATTATTGTCAAGTGGTTCAGTATTAGAGCCTGCT
GACGATGAAAACACA
ACGGAAACTAAGGTTGATATAGGAAATGATGACTGCTTCGATCTG
TTTTCAACCATCCAA
GATAGGAAAGATGACGTTGCACAGCTTTTGGACAATGCCAATTTT
CTG
>ConsensusfromContig4041 Average coverage: 26.11
AAATAAACTATCACTTCTTGATTTAATTAAATAGCAAGACAGCGT
TTTCATCAAATGAAA
TTTCATTTTAACATAATAAGCATCTTGCTAAATAAGTCGTCAGCC
TAAATAACGAAGAAA
GTTCATTTTTTGTCAATCCATGAGATGAATGAATGATTCTTTTGTC
TCTCTCATCCATCC
CCCCGAAAAAATATCTGAATTGATGACGATATGAAGAATTGATTG
ATTGATTATTTATTA
GGAATTATTTTTAATATTATCAGCCTTCTGTACTAACCGTGACAC
CTCATATTAAAAATG
AGCATGAAGGGTAAAGAAGTCGTGAAAGTGAATATATATGATAT
GTTCTGGACAAATGAG
TATACAACATTCTTGGGATTTGGAGTTTATCATACTGGAGTGGAG
GTGTTTGGTCGAGAG
TACGTGTA
>ConsensusfromContig4042 Average coverage: 24.35
TATTAATCGAAATCCCGCAACTCTATCTAAATTCAATATCACCAA
ATCACACAAAGATTA
TTATCACAGTTGTTAGTATTCATTGATGATAAAAATCCAAAATCG
CATTGATATTTTCAC
AAACACCAGCTATATAATGAACAATCACAGGGAATATGACATCC
AGACTTCACAGTAGGT
TATGAATGTGAAATAATAAGAGCCAACCGCCAAATATAACATAAC
ATTTAATAGTGATCT
GTCGCATATGATATAACGTAAATTCGGGTGATCAATCACAAAATA
ATACTTCAGCGATTG
GTAGTTATATATAAGCGTCAATCGAATACATAAAATAGAAAAAT
TGGTAAAATATATGTA
TGAAACGCAAAATATGTGGATAGGATGAATTGAAAAATTATATC
GATTAAACGTATAATG
ATGTTGATAATTTAGTTTATATCAACGAAACACATTCAGTCAAGA
TTTTTCTGTTATAAT
GCTCATACAACAAACTCAAAGTAATTAAAAG
>ConsensusfromContig4043 Average coverage: 7.28
CGTTCATTTCGAGTTCGATCAACAGTATAGAGTTGCGAGAGATGT

ATGCTCTGAAAGATA
TACACACGAAAGCTTTAGACTCTGCCAACATTCAGTCATTAGTCA
TAACTGATTGTGCGT
TGGGGAGAATTGGTCATCACACATTTGCGGGTTATTCTACGGTGA
TGCTTCATTTGAAAA
TCTCAAACAATTCTGAAATTATTGAGTTTGATGAAAGTTCTTTCT
TAAATTCGTCAATAG
AAGTGTTAGAATTAAATGGTTTGAAACTGAAAAACTTGTCAATGT
TATCTCAAATGAATC
ATCTCAAGCATCTAGATGTTGGCAGCAATGATCTTGCAGAGTGGC
TTAAAACAAATAATC
ATGATTTCAGTACATTGGAATTACTACAAATATCAGA
>ConsensusfromContig4044 Average coverage: 11.13
CTGGATTCCATTCAGGTTTATAATCACGATTCCGAAGCTCTGAGT
TCACTCCTTCTAAGA
AGTTATTACCGTAAAAAAATCTACAGCTGAGCCTGAAATCCTGTT
GCAGTGCCTCGGCTA
GATTCATCCAGCTTGACTGGTCAAAGTGTCTCAGCGTTATCTTGA
GTGAAAGTGGACACA
TTCTTTTCAAATTACTCAGTTGTTTTTCGCAAATTCTGAACCGTT
GGATTGGGCTTCAT
GCATTCGCTCCAAAATTTCTTCCATTGATCCAGAAGCGAACAGTG
AATCAATTTCCGCTA
AATGGGACTTGAGAGAGAAGGCATCTGGTTCAAAGCAACTGATG
TGGTATTCGTTCAGAA
G
>ConsensusfromContig4045 Average coverage: 16.82
TTTTGAATGAAATATGAATAAATTATATCAAATCATAATTTGCGG
TATTCTGGTGAAAAT
AGGAGTGGACTTTAATGGCCAAAATCACTTCGCGTAACAAAATC
CTCGACGGGGCAAAGC
ACTGCGCAAGCAGATAGCAAATAACTGGAGAAATTCAGATGTGA
TTACAAAATTAGAAAA
TCTATGAAGACGTGTAGTGTTCTAAGTAAAAGATGTTGAAAGTCA
TTCTCAGACATGATC
CAAAATACTGATCCCAGCTTCCTAAGCAGAAATTGTTTTCAAGTC
TTTTGTATATATCAA
GACTTGGTGTGTCCCTCGTGAAATATGTTGATAGTCGTTCTCAGA
CACGGTCGGAGATCC
TGATCCCAGCTTCCCTAACTTAAATTGTTTTCTAGTCTTTTGTATA
TATCAAGATTTGGT
GTGTCC

>ConsensusfromContig4046 Average coverage: 22.52
GATCTATCATTTTCAATACTCGTATAGTGCCTTCTGTCGAGCATA
TATGAGGTGATGAAT
TTTATTGCCGAAGGCTTAAATCCAATTTTCTTCAATTTGAAGAAT
AACACAGTATGGTCA
ATGGTATCGAATGCGTTCGACAGGTCCATAAAGATTGCTATTGGA
ATGTCACCTGAGTCC
ATTATATTTTTGACACGATCAATGAAGTGAAGGCCCGCTAACTCT
GTGTTATGACCACTT
CTGAAGCCATATTGATTTTTTGATAATGATGTGTTTACACTTAAAA
ATTCATTGATTTGT
TTGCACATTATTTTTTCTAGTATTTTCGACATAGCCGGTAGTAAT
GAGATCGGCCCATAA
TTTTCGAGATCGGAA
>ConsensusfromContig4047 Average coverage: 11.35
TATAGGATCAGGATTGCGGGATGATACGACAAAGGATTCTATTTT
TTATTTGAAATGGCT
TAATCACAAATTCCGATCCGCTTAGTATGCGGACTGGTGTTATTC
ATGTAATCAGAATAT
GTCCCCATGAATTGATACATTCTAATCTTCCGATAGACTTCGGGC
TGCTCAAAAACTCGC
ATTTATTTCAATTTTTAATGTATAAATCAAGCAAACTGATACTGG
TCAATCGTCAGATGA
TTTTCGTTCATTTGATATGTGTGAATTGTTACCATTGTACGTAAAT
TTGTATATGACCAA
CGTGATGTATTTATTCAGAAAATAATATTAAGACCTATGCTTTTT
TGCAGATATAA
>ConsensusfromContig4048 Average coverage: 57.66
TCAGTATGTTTTTATAGGATAATAAACAATATGCCAGTATTGAAT
AATGATGAAGATCTG
TTCTTCAGATGATCAAAACTCTTCTTGATGTTTTTTCCGAATCTAT
ATTTATGGCTGATC
CAGAATCAAAATCCCCATCAGCGCTGTTTCGTTGGCCACGTAGAA
TGTAGTTTATATGCT
TTCTGGTCAGTGGAGGCATTCCAACAAAATCTTGGGTCACAGCGT
GAAAATTGCTTGCAT
CAATGTGATGCTCCAGCTTTCCATCAGCCTCAGTAGTTCTTCTGCG
AAACTTTATTTTGA
GAAGAACGGTGGCCACGACAAGTAGAAATACAACCATGCATGAC
ATTCCGATCAGAATGT
AGATCATCAGCTCTGTCATCGACGATTTCTCAATACAGTATGGAG
TTTCTGGAAGCCATG

AGCCATCTTGAGAGCAGATCACAATTTTGGGTCCGAGCAAAGTAT
GTTCATAAATACAGT
CCACGTGAACAACATTTCCCACATATCGCGAAGTCGAATTTGGTA
TCAAATTGCCAAACA
GTTCAGGGCACAGATTGGGCTGATATTCATTCATTCTCCTCAATT
ACTTGTGATAATCGC
GAAAAATGTATGGTATCTTCAGATCACATCGGAATCGAATTATGT
GTTCA
>ConsensusfromContig4049 Average coverage: 14.97
TTTGGAGCAACAGTATGACGCGATTTATCAGCTCGAGTACACAGC
ACTCGAGTTATACAA
TCTTCGTTAATAAGAATGGATGTTCCATGCCCATTATTTTGAGAT
TTAACATCAGATGTT
ATCGGTATAAAAGTTTGTTCAGGAATGATCACAGGCTGTTGGATC
AAATTATGATCATAA
ATATCACAGATTTCGGTCGATAATGTGATAAAACCCAGATTTGAT
TCAGACTTTCGGTGA
GGAATTTCCTCCATATTTGATTCAGAGCTTTCAACAAAATTGTTTT
CTTGATCGTTTACC
GCAAGCTTTTCGGAAATACAAGGATCTCTGGAACTCATCAAACTC
TGATAAG
>ConsensusfromContig4050 Average coverage: 7.19
GGTGTGATCACTGTCGCATATGCATAAGTAGGAGTTCAGAGAGAC
CTCTTTGCATGTTGC
ACCATTTCGACAATCGTTACCCCAACAAGGATCTCGTTGATCACA
GTCGGGTCCTTTGAA
GACAGGGATGCAGACACATTCGAATTGATCCTCATCATCGATGCT
CTTCAGTTGACACGT
AGAACGATCGGGACAGGTGACCGAAGCACACAAATCTACGAGAT
GACAGAACTGGCCCGT
GAATCCATTTTGACATCTGCATACGGAGGATTGCCCATCAGATTC
GCATGTCCCGCCGTT
TCCACATATGTCATGTGGGCAAGGAT
>ConsensusfromContig4051 Average coverage: 14.17
AAAATTGTTATCCAGTTTCTGGAAATTTCGAGCATATTAAAATAT
TGACAGAATACACGA
TATAAAAGGTTTGGGCATGGGCTTTATCACAATGACATGGGATT
CAGATAAGGATATAA
ATCGAGTTTGTTCTAAAGTGTTATAATGCAAAGGAAAAATAGATT
TGTGCTTTAAGTATT
TGATAACTTGACGGGAACCCAGTATTTTATAGATATTGGGCCAAA
CATAATTTCACATCA

ATTTGTACAAATACATGATGGTTTAAATTAATTATTATATTCACCC
TTTTTGACAAATTT
TTGACGGATTTAATAATAACGAAATCCTGGCTTCTGCATAGCATT
GGTTTCATACAAAAA
ACT
>ConsensusfromContig4052 Average coverage: 6.08
CTCTTCCGATCTGACAAATTGGAGAATATTCTTTTAACTTCTAAA
TCAGTACCCGTGAAA
ATGATTTCTAGTTCGCACACACTCCGCTTCATAACACGATCAACA
CTTTGTCCGAGTATT
TCCTTCTTACACAATTCATTAAGTTCGCGGTGTCATGAGCGCATTC
AGCTTATTTCAATT
ATTACAAATATCATAATTATGAAAACTTATGTGAAGGTCGCAAAT
AAGTACATTTTTCAT
AGTCATAAATCTCACAAATGATCCATGCACATGAAAATTCAGATT
CAAATTCTAATAAAT
TCCTCACTG
>ConsensusfromContig4053 Average coverage: 9.81
ATTTCGAGACGTGCCCGCCAATAAAAGTCATTGATAGTCAGAAT
TACACAAAACAATGTG
ATATAGAAAAAGATATACATCCGATTGAGATTACTTCGACGAATC
TCCGTAAAAAATCTA
CAGATTTCGGAATCACGAATATCTTCACCAGATTGAAATCCAATC
TCGGGATAAAGCGCA
CAATGCGAGATAATGAGAGTTTAAATAGTGACGTCACGGAGTCG
TCAGGATACAGCACAA
CAAGTACAGCAAATACTGTTTTCTACATTCCGTTGGAAGATCGGA
GTTCATATTCCTATA
TGGGAAATAAAACAACAACTACGAATTCTGCAGATAAAGTTGAC
AGTTCCTATACGAACA
CTCCTG
>ConsensusfromContig4054 Average coverage: 19.94
GTGGATATTGCTGTTTTTTTAAGCGGTTTGTAATACCAAACATGCT
ATGTCTTACAATGT
TGATTATTTTGATATTTAATATCATATGACAATGATATCGAAGAG
AGATCGTCATATAGA
TATTCTATAACAGTTTATTTAATAAATTATTCGTATGTCATATTTT
GTACGGTGTTATGA
AGTGCCTTTTTTAATCTTTGAAAGATAAATAACTTGTAGGCCATC
CGCCTGATATCATTG
AAATTTCAGAACGTTATTGTTCGGTATTCCTCAGAAATATGAACA
ATTCCAGATATCGAC

CGATTAGTATTGTGATATCACCATGAAAGTTATGCAATTGAAGTG
AACGATGTTCATTGA
ACTGTTTGATGTTTCTGATATGTGATTTTTTCAAAAGGATTGACT
GATGAAATTGTAAAA
AAATTTGATTGCTTTATCTGTTCCAAAGTTTAACTATTCTAAAAAA
AC
>ConsensusfromContig4055 Average coverage: 26.74
TATGTCCAACGTTGAACATAAAATCGGAAAATTTTCAGAGCTACC
CGTCATATATCACAG
AACTCAGGTTTCGATACTTCACCGAAATATTGTTGAATGAAAATT
CATTTTCAGAGCTGA
GGGGATTGGAATTTCTACATATTGCAAACTGTGACAAGATCACTG
TGCGTCCATACACTT
TTGGCAAAACTACAAATTTTAAAGGAATTGAATTCCGTAGAGGGA
TGATTGATTTATATG
ATGAAACTTTCATGGATATGGGTCAATACAACGATACGTTGATAC
AATTTGATGGATGTA
AAATACACATAACAGGAAGAAATTTGAAAATGTTCGATCCGTTC
AAAGGACATAATATAA
CATTGAATATTCTAATGCTTGAGGGCGAACTCCATTATTATGGCC
CAC
>ConsensusfromContig4056 Average coverage: 12.27
GTTCTATCGCTGTGGACTTGCCTGTTCCGTCAACTCCCACCGCCA
ACAAATGACCAGCAG
GTTGCCTGAAGACGCGAGCTGCTCTGACAATGTGCTGACTGGCT
TCATGAAAGAAGACAA
GCTGGGGAGCCTGTGGGTTGACGAGGCACTTGGTCATATAGAGCT
CTTCCAGAGCTTTCG
CAAGTTTTTGTTGTCCGAAAACTGGTCTGTACGTTCTGTTAGCTGT
AGTTTTTGTTGCCT
GAACGTCGACAAAGTCTGCAAGGACGACTGGAGACTCTTTCAACT
CAGTTCCCGTCGATT
TCACTTTGAAATGGTCATGCAAAACATCAGAGAGTATATCGTAG
AAAGTGGCGGCATCAC
CATCGTTGATGAGTCTGTCGTGGAAAACTCGCGTCGCCTCGTGAG
TGAAGAGCTCAACAC
AGCTGTCGACACTTACCGCAACGGTCGGGTCTGCTTGCATCAT
>ConsensusfromContig4057 Average coverage: 12.35
CTCTTCCGATCTTTTTTTCTTGACCATGAAACCTGAAATTTTTATC
ATTTCAAGTATTCA
AGCTTAAGACATAAACGGGTCACTCTAAATAAAACGACGAAAGA
TAAACAATTTTCAACA

TGTCAAGGTTCTTGTTTATGGGAACATGTGAACTGAATTGATTCTC
TCATCTCTCGAGTT
TTCGGGTTTTGGGGTCAACTTGCCTGCATATTTTCTAATCGCCATT
CACACCATGACTGC
TTCGTATGGGTTAACGCCCACCTTTTGAGTGTGATGAAGCAAAGC
TTTTTAGTTGGGCTT
CTCGTGATATCATATTTCGGTTTAAAAATGCTAGCGGAATTCAAG
ATATGCATATATTTA
TGCGATATTTGGATGAGGTTTTGTTCATAATAGTTTTTATGCATTA
TATATAGCGTCATT
TGTCGCTTGGATTTGGTTATCAAGAATGTGGG
>ConsensusfromContig4058 Average coverage: 19.52
ATAATATTAATTTCAATAAACACATATAACGAAACAAATGAGGC
GCAAAAATCAAATTTG
GAATGACTATTGTAACATAACACAGTAGATTTATAAAATTAGGAT
TCTGTAGGCATGAAC
AAGGATAAATTTTGTTTGAATGTATTTATTTCTCTGTCGTAGTGA
TTGACTTGATCAAAG
AGATAAAACAATTCGTTCAATTTTTCGTTTAGGAACTGAAATTGT
ATGTTCTGTTTACTG
ACGAGTATTGTGAATATGAGTTCCGACCATCTTTTCATTTTTTCA
CACAAAAGAGTCTTC
ATTTCTTCAATCTTTCTTGTGTCCACTTCTCGATTGGCATTCATTC
TATCAACGACATCC
TGTAGCTTAGATTTTAACATTGCCACCATTTCCACATCCCTAGAA
TCCATCGGAATTTCC
CGGTCATTGTTATTCCCCATATCTGAATAAGGTGAACATTCGGAA
GGACAAACACCACCA
TCAAAATAAAATTCCTGATTCGCATCCATTCCTCCAAATTTCCAAT
TTTGG
>ConsensusfromContig4059 Average coverage: 9.49
ATCACTTTTTCGAATATACCATTCTGAGTGAAGAGTTTCATATCA
CCCGGGCATCTCAAG
AGTATTGTGTTTAGAAGGGTCATTCCGGCTTTCATAGATTCGCGAT
CTTTGTTTTTGAAT
GCTTGATCAACCCCTCTCAGGATCACAGGCATTCCATATACGGCG
TGGTATTTAAGATAG
AAATCTTCGGATTCAGCAATGACACAGAGACACTCCATGATGGAT
TGTATAACCGGCGAT
ATGGTCGTCGAAA
>ConsensusfromContig4060 Average coverage: 15.57
CACTCGTGCACGGGGTCTATAAAATTAACTTTTTTTTCGATTCAC

AGAAGGTTCAATCTT
CCGAACGAAGGTTGAAAGAACTGAAAATAAAGCAGTGTACAGGA
CTGAATCCAAATGTGA
TCCTTCACCATGTGGAAACGGTGGCTCGTGTGAATATGGAAAATA
TGGCTATGTTTGCCA
TTGCCCAAATGATTACTATGGCATTTTCTGTGAAGCATATGTCCA
TGGATTCGTTGAATT
CAAAGTTGATAAACTTAGAGCTCTTCCCTTGAGCCATGAAGAATG
TCGAAATTATGAGGA
TGATGAATGGAATTCTGTCCTCAAAGTATACAACTTCCAGACCTG
TTTGTTTTACGTGAA
GAATTATGATGGCAAACCGACAAACATTTTTCTCACGAAGTCTGG
AGACACTTTTCTCCC
GGCAAACTACGATTTTGATGACTTCAGTGATCTTGATTTATCCGA
ATGTGTTAACAAATG
CAACGAAAAAGACGGCTGCTCGATTTTGACTTGGGAAACTCCCTA
CACAAATATAATTAA
CCAAGTTGAAACCATTGATTATTCTGTCTCTTCTTCTTTCTCGATT
TCTTCCACGGACGA
GGTTATCAGCACCGGATATGTCTGCTACTTGTATGAAAAGCCCTT
TGAATTCCC
>ConsensusfromContig4061 Average coverage: 17.22
GACGAGCAAAGAAGGCCGAACAATGAAAAATTGATGGTCAATCA
AAATAAAAAACGTGAT
GATCCTAACCTGTCTTTGAGTGGTACTCAAAACAATAATGGTATA
AAAAAGATCGCTTTA
AGGTCTTCACAAGAAATTAGAAAACAGAAAAGACGAAAGAAGAA
GAAATCAAAATTAAAT
GATCAAAATGAGAACAACTGTATCGGATTAGCTTCTGAGTTAGAC
GGTCTTCATGATGAC
GTCATCGACACAGGAATGTCAAGAACGCGTTCTATGGCTCCTGTC
AGTTTGGTGCCATCT
TTACTAAAGAGTCAACCGGCCGCTAGTCTATATATCGAACGAAAA
AGTGGTTTTAAAATG
ACCGACAAAGACAGATTTGAACAGCGACTAAAAGAGCAGCAACT
CAAAAAGAAAGTGACG
GACGTTGAAAACAGAGGAGCAGACGACAGTAGGGAGAGAATGCT
CCAGTTTGGGCTCAAA
AGTCAGAGATACTTTTCCTCAGTCTGTCTCTTCTGCCAAGGGCTTC
TCGCTGG
>ConsensusfromContig4062 Average coverage: 15.50
GTTTGTTATCATTTTAGAATCACCTTTTTTGAAAATTGGAACAACT

TTAGCAATTTTCAT
AGAATTCGGAAATGTATAATTGATTATACAGTTGTTTATCGTCGC
TGTCAAACTCGGACC
TATAACTTCCTTAATATTTTTAATGTCATTTGTCGAAATAGAATC
AAAACCGAAACTTTC
TGTTGATGATAATGAATCGATGGCATTGTTAATGGTTTCAACGCT
AACTTCAGTGAACGT
GAAAAATAGATTTGGAGGTTCAGTCAAAAAAGTTGTAAAATCAC
CAATTGGACGTGGAGA
AATTATGCTTGTGAAGTTAATATTCATGCAATTGGCTATTTTATT
GGAATCACTGATCAA
GGTGTCCTCGATTAAGAATTGTTTTGTGACTGCCAATGACTTATC
CCTAGAAAGAGTTGC
ATTTATCAATTTCCATGTGTCTTTCGAGCTATTTTGAACTTTTAGA
AATTTTGTATGATG
ATATGCTTGTTTCGCATT
>ConsensusfromContig4063 Average coverage: 11.63
CTCGTATTGGTAGTCTCAGCAGTCAATCCAGGTTCTGACTTTGAT
GATGTTGTGGATTCC
TGCCCTGCATTGGTTCTTACAATTGTTGACTCTGTATTCAAATCTG
AATATATTTTGGAT
TTCTCTTCTGTATTTATTGTTTCAGCAGTTGACTTAGTTCCTGACT
CTAAAGATGTTGTT
AATTCTTGTCCCGCATTGACTCTTTCAACAGTTGTTGACTCAGTTT
CTGACTCTGAAGAT
ACAGCAGATTCCTGTCCAGAATCGG
>ConsensusfromContig4064 Average coverage: 25.39
ATGGGAACATGAATATTCAACTTGTGACTTCATTGAGCATAAAAT
TTGAGAACTGCTGCC
TCTGAAATTTCATGACCTGGTAATTGACCATCTTCCGGACGGCGA
AATAAGAGCAAAATC
CCTCAGCGATAATAAACAGAGCCAAGATTGCGTTGACAGCCAGC
TCTAGTGGGAGGATTT
TGCCGTTCCAGTTAAGAAGCAGGAACAGGACAAGAGGGAGTTGA
AGAAGCACTGCCAACA
GGAATGATGCTGCCAGTTCTGGTACCTTCTCCATTAGGTTTCCGA
TATATGCTAAATATA
GCCGTCCAGCTTCGATCAGTATTATGAATATGATTGCAGTTATTT
GAACGATTTTGTATA
AGGTGGACAACTCTGAATATTTTATACACATGTTAACAATGATTG
ACACAATCCAGCATG
GCAGATAATAGAGATTGAAATACAGAAGCATTTGAAGTGGTAGA

CTGGTCAAATATTCCT
CTCCTATTAAATTTGATTCATTCTGTTGTATATTTAATGCAATTCT
TGCATTTGGAAATA
CAACTTCTGTGACAGAATCAACTGCGCGCTTAATTTTTTCAGCCAT
TTGTTTACA
>ConsensusfromContig4065 Average coverage: 7.60
GTTTTGCCGAAAATGGAAACGAAAACCGAACGCCGCGAACCTTA
CATCGGACAAAAATAT
AAACAAATTCGAAATGGCTATGGTACATATGTATATGAAAATAAA
TTCTTCAAATATGAG
GGACAATGGAAGAATGGTCGAAAGCATGGGCATGGGAAATTATC
CATGGCTGACGGTAGC
TTCTACGAAGGCCAATTCGTAGAGGGAGAGATAGATGGTTATGG
CTTCAGACAGGACTCC
AGTGGATGTACATATGAGGGGCAGTTTGAGAGGGGCGAGCGTCA
TGGCCGAGGGATCATG
ACTTATGGGAATGGGTTCGTATATGATGGAGAGTGGGCCAACAA
CAAACGACAGG
>ConsensusfromContig4066 Average coverage: 4.66
GGAGCCCCCCCAGCCACAACATCTAATGTCGATAAATGGCTGG
AGGCCTCCATGCAAGA
TACAACAATGATAAAGACTACCATAAAGACTGCAATAAGACTTA
ATAAACAACTTGTATC
TTGGCAACAAAAGACTCAACTTCCAGACTCCATGATACCTCCGA
GGTTCGACACAACAAA
CTATGGGAGCTCGGAGGTATTCAAAGACGAACTAAAGACTATAA
ATGAAGACTATCAAAC
AAAGACTATAAATTGCATAACAAGACACCTGAAGACTCAAATAA
AACTCAACAAAGACAT
AATTGAAAAACACTTCGAAACAATAGATCGGAA
>ConsensusfromContig4067 Average coverage: 12.36
ATTTGAGTCAATTGTTCACATTCCCAAAATAATTTTGAATGATGAC
CTTTTTGTTATACT
TTGTATACGTTTCATCATTTTTAAGACGTTATCACCGCAAGAGAA
ACTGAAAAAACTTTC
GAGTATCTCAAAATTGAAAGCCTCAAACTGCCTCATATATCTCCC
CACTGCCAATACTTT
TCTGTTATTTCCATGAAATGACCAACTTTAACAACCATTTCACCG
TACATGTAGAGTTAA
TGTAAATTAAGTGTGTTTACTTGATTAAACCTTGTCTTATCTTACA
TTTCATCAAGCTTA
AATTGTATTTTCGACGACTTCGGTGTGTGTTTTCTCAAATTATTTA

TCACATCATGAATA
GTCATAAGGGGAGAACATATAAAGCATCAATAATTTATTGATAT
ACTTAAAAACTAAGTA
CCCGGTGATCATTTTTACCCAACCGGTGTTTCAAACTGTGAAGGT
TCTCATTCTCAATAA
CTTGTGAAACATTGTCTGTGTATTTTTGTTCATTTTATGTTTATACT
TTCTCATCATATC
ACTGAAATATGCAAAAACAGCCATGCAATAGAGTACAATCATAT
ATTGCTTCAACAATGA
TATCCTTCACGCTAAAAAACCCCTCTGATAGTGTTCTTCTCCATAT
CTAAGCTTATATGT
TATTTGCAATTATGCG
>ConsensusfromContig4068 Average coverage: 13.95
GAGACAAGGATATCTGTTGAAATGGAAAATAAGACAAGCTCACA
GAATGTTGAAGCATCA
CTCACAACAAAAGAAGAAATTGGATCTTCTTCAAATAAGAATTCA
ATCTCTTTCCACGAA
CATCTATCGAAGAATGAGAGCCAGAAATCTGATGGAAACTGTAT
TGAAACGGGAACATCA
GAATTCACATCTAGAACCGATGAACAAAATTCTAAGACCAAAACA
TTCAATCCATTCGAT
ATTATAAATGAGTCAGACGAGTTTAAAGATTCTGAACACAAAGA
AGATATAAAAAATTGT
AATTTAACAAGAACCAATTTGACACTCAAAACTTTCGATGGTCAT
AAATTTATAT
>ConsensusfromContig4069 Average coverage: 13.12
TGGGCGGTTTATGAGTCATCGTCTGGATCATTATAAAGAACCACA
AGAGTTATCAGAATG
GTTATCGAAATTAAAAAGCCACCAACGACTGTTGCGGCTATCGCG
ATTTCTTTCGCAGTC
TGGCCAAGTCGCCCGGCTTTGGCCGTGTCGCCGCGACGCCAAGCG
TTGCGACTTTGAAGG
GATTTCCAAATTCCGAAAACTCCGATACAATAACAGCAGCAATA
AGATGAGAAAATACTC
CAAGCCAAATAGTCATCGGGAATATCAGTCCCTACGACGTGTGGG
TTGGCTATAATCACA
CTCGGGTCTGGTATTGGCTGTGCGGCTGGAAGGCGACCTGCTGGA
TTCACGAAGCCAGGC
GGCTGATGCATTCCTGGATTTTGAGCTCCTCTTCCTGTGTATGGGT
TACCTG
>ConsensusfromContig4070 Average coverage: 11.51
TTTATACACTGTTCGTGTACGAGCGACCGATGGAGGGATGCCCCC

AAAGACCGCCACTGC
GACCGTCGCGGTGGGCGTCACCCGGAACTTGAACGCGCCTGTGTT
CAGACCACAACGTGT
GGACAAAGACATCTTGGAGACGTACCCTCTTGGGGAGGTTCTTGT
AACTGCTTCCGCCGC
GGATGCCGATTCTAAGGCACCCTACAACGAAGTTCGATATGCAAT
TTCTGGCCCTGACGG
AATTGACGAATTTTTCCTCATAAATGAAATGACTGGCGATTTGTA
TCTTCGAAAGCCTCT
CAACGAAGACCCGGCAAAACCCACGACGTATACTATAAAAGTGC
AAGCCAGAGACCAGAG
TCCTATTGACATGAAAGTCTCCCAAATCGACGCCACAATGACCGT
TCGTGTGACGCGGAA
CAAGAACTGCCCACAGTTTCAGAGAGAGCCTTACAAGAAGACGG
TGCGGGAGACGACGGC
CATCGACAGCGAG
>ConsensusfromContig4071 Average coverage: 3.91
ACGACGTTGTGATAAATTGTGCCGGACTGGGAAACATCCAACTTG
CCGGTGATTCAAGTC
TTCACCCGGGCCGAGCACAAGTTGTGAGGGTCAAAGCGCCGTGGA
TCAAACAGTTTCTTT
ACATAGATGAGGATATCCATGTTATACCCGGTCGTGACAGTGTTG
TTCTTGGTGGAATAA
GAGGGCCGATCACATTTGATGCTTCAATAAAAGACACTGACACAA
TTTCCATCATAGCAA
AAACTGCGAAGCTCATCCCGAGCATAAAGAAAGCCCCTATTGAT
GAATTATGGGCGGGAC
TACGACCATCCAGG
>ConsensusfromContig4072 Average coverage: 23.42
TGAACATACGTCCGGTGTTCGACGTATGCAAACTCGCAATCGATC
GTTTCCATGAAGAAA
CCCTCCCTTGCATTGAGGATATACCGCCGAGTCGCAGTACGCTCA
TATTCCTTCGCTATG
GTAATTCGGGCCATGATGAACGCTGGCAGTTTGGCACGATGGCTG
TTCGCCCCTTTCCCA
CTATAAAGACCATTCAGAAACATTGTGTCGTTATATACAGTTACA
GACAAATATCCACTT
GAAAGAGTGGGCATGTAACGATCGTCTAGAACAGTCGTCCCCTT
CATGTATGGAAGACTG
CTGCTCTGAATAATATCTGGCTCAGGACTTTCATCACCTATATTTG
TTTGTCTTTTTGAT
CTTTTGGGTAATGGCTCATTTTTTATAGGTTTTGATGTCCATCCA

AATAAATTTGAAAAA
GTGTCTTTAATATCGAATATTGATGCGCTTGATTGAAGACTTATT
GGTATGAGAAAAATT
AAC
>ConsensusfromContig4073 Average coverage: 34.60
GTTTTTTTCAACATTTTTATAGTTTTTTTTGTATTAGGTCGTGCTA
TATCGAATATAGAA
TACACCAAAGACATTTCACATGCAATATTACATTTTATAGACAAC
AAACTTATGAACTCT
CACTTTGTTTTGATGCAATAGCAGTTATGCAAGAATTATCACTTT
GAGAAATCGTTTTAT
AAATGGATTTGGCCTTTATCATTTGATATTTAGAATGTCAAATCA
CCAACGGTATTTTGA
AACAACCTCCAAATTGTATCAATTATTTTTTGTACATTCGAGCAG
CAGGTTTGGCATACG
ATTTGATAAGAGCTTTTTCAAAATTGTGATTTCGTGACCATCATTT
GGTATACATTATTT
ATGATGTTATGATATTGTATTGTTAGTGAATCAATCGTGTAATTTA
TCTTTTCTCAAAAA
GCAAGCTAATTGTATTTTTGGGCAAAAATGCTCAAGTTTTT
>ConsensusfromContig4074 Average coverage: 8.05
CTTCCGATCTGGAAAAGTGGATCCTAGGCAACGACGGGAGAAAA
GTCAGTTCCGCGGTAC
AAAAAGACATACCTTAAACCGTCCGAAAGTTGGGTAAACAAATA
ATGAAAATCCCAAACC
CTCGCAGACTTTCAGAAGGGATTCTTCTGAAGTCAATTTCAAGTG
CCCATCAATAACAAA
CATAGAATGTGCTACCAACATGTTGGAAATATGATATGTGTCATT
AGAAATGCTCACTCA
TCCTCATGTGGTGAGGATACCATCAAAGTTGATGAAACAGAACA
TGCAGGTAATGGAGAT
GATCAC
>ConsensusfromContig4075 Average coverage: 11.87
TTGTGATAAGGAATCGAAAGGTGTCGAAGCATTATGCTGGGCTCC
TTGAATGCTGTACTC
AAAAAGAGTGAAGGCCTGTCTGCATTACACAGTTGTCCTGCTTCA
CAGTCAATTACTTGA
ATAATGAGGTGCTCCTCCTCGACTACTGGCGACATGTCAAGGAC
CATGGGCCAGTGGGGA
ACGACCAACCTATAAATGGACTATATAGGAACCTCGCTTATAGAC
GCTTTGTCAGCTGGA
TCTGGGGCCATCTTGGGCGACACAAGAGAAAAGTTCTGCCATCAT

GTGTTGTCAAAGCCA
TTTGAGAAGGGTTTCCTTCTCAAGAATATGCTTGGTTTAAATATC
CTCAATACAATAAAA
AAAAAAAAAAAA
>ConsensusfromContig4076 Average coverage: 35.17
TAGTATGTAAATATAAAATATAAAATGCTGCAGTATAAGGATGGA
GTTTCATATATCGCC
ATATTATGTACTTTTCTGCTTTGCCTATGTGGGATTCCAATTTGC
AGAGAAATAATAAAG
ATGAAATCATGTGGAAAGCTCTCATACTTCCCTTTCATTGCAATG
CTTTGCAATGGTCTC
CTATGGATTAAGTATGGAACGATGCTACACAATCTGAATATCTAC
GCTGGGAATCTGCCG
GGAGTGCCTCTAACATTTATTTACATCTGCATATTTTATTATTATT
CATTCCACAAAAGA
AGAATCCTCATCCAGCTTCTCCTCTCTCTCACTGCCGTTGTGTGTG
TCATCGCCTATGGA
ACTTACATAAGTCTCATGGACATGGATAGGGCCATTCATTTCATC
GGAGAATTCTGTGTC
TTCGTGAGCCTCGTTTCGATAGGGGCGCCACTCTCATCAATCAAA
TACGTTTTTCAAACG
CGCTCAACGGAAACGATGGCTTTCTCGATTTGTTTGGGGAACTTC
ATAGTTGCTCTGTTA
TGGACACACTACGCATTCCTGATCGACGATAACCACCTCAAGATT
CCCAATATAATCGGT
TCCTTTCTTGGCCTACTACAAGTTTCTCTGTTCGCCATATTCCCAT
CGAAAAAACAATTT
ACAATTACTACTGCGTAGGAGCTTGCTTAATATGAAGTCATATCC
GATTACTTACACATT
CTGTTTTTGTTTTGTTATTGGTGCATGCCTATTGGCAGTTTTCTCA
AATTATCGATATGT
TCGTTGCTATTGAATATTCAGCGATATATGTTATACCAGA
>ConsensusfromContig4077 Average coverage: 12.52
TTGGGGTTGAAGGTCACCCACAGATCCCGCTTTTCGCCGCAAACC
AATAACCCTTCTGTT
ATATAAGACTGCGACACATCCGCACGTGAAATCGTGATTCGCGCC
GCGTCTAATTTGATC
TTGTAGTAGAAGTAAGGTTTGTCGAATTCACCAGGAGTCGAACT
GAGACCGATCACAGCG
CCCCCACACGATGTCACCTGAAAGAGGAAGTAGCGTTTGACAAT
TGAGAAATAAGCATTC
GTGAGTACGCCATCCCCAATGCTTTGGATTAGTATGCGATTCCCT

GAGTCAAAATTATTC
GGTATATCTGGGTCAGCTGTGCTCGGACCTCCATCTGTAATTGAT
TCAGAAAACACAAAA
GCCCCATTGACATCATCTGAGGTAGAGAGGCTGACTCCGTGAACT
GGTCGAGGGACTCTG
TGTTTCTCCAGGAGCAAAGACTCCCCAGGAATCAGACCTCGACC
AAAAGCAACCGTCTCG
TTGGACCA
>ConsensusfromContig4078 Average coverage: 22.65
CCTGCCTAGTTCTTTGATCAAGTTATCTCCACGTATAACCGAACC
GATGCCATCATGGTG
GAACCTCTCTATAATGCTAGTTTGAAAACTTGCTTCGACTTCTTCA
TCCAATGTTCTCGT
GAGAACGGCTGGTTCTTCCGGTTCTGCTCCCGCACAAAATTTAGC
GTGTCTGCCGAAGTT
TTTCCGTAGCAATATCAACGAGCACTTGTCACAGATTACCTGTTTT
TTTGATATCTCACC
TGACTTCTGAATGTGAGCCTTATTAGATTTCTTCCGTAAATGAGAA
ATGATTTTATTTCT
CGTTTTCGCATCTGACATGCTAGCCTCAACAACTTCTGGCTCACA
TGAATGTTTTCGGAA
AAAATGACGTTGTAGATTTGTATGAGCTTCACTGCAGTAATAACA
TTTTCGAGGCGGTTG
TTTTTTACGTTTCGGTCTTGATTTTTTTGAGACGAGGGTTTCAACA
ACAATTACTGTGGC
GTCTGAGGATGTCGAGACAGCACGACCGCCATCCTCCTGTGAGAA
AAGTTTAAAAGTTAT
TATTGGCGCGTTATACTATTTCTAGCACCAATTTATCATTCACATT
TTAAAACGCATTTG
AGGTCAAAAAACAAAATTCCAACAAAAGATTCATCTTATCGAT
>ConsensusfromContig4079 Average coverage: 21.25
TTTTTCCTTAATGAGAAGCTGCAATTTTGCACATCACTCCATATT
CGTGTTGTGAAATAA
ATTTCAATCGTGAGAGACTGGTTTTCCATACCTTTAGTGTATGTGA
TTTCGCTCATCACA
AATGGCTGGTTATTCATGTCTTTGATGCATGTAATAGGCTCATGA
CTCCACATTTGTGTC
ACGTATAATAAATTTTACGACATGAAGTCATGCCTTTTTTGTATAT
GTTGCTCATATTAT
TTGGGGAATTAATTGTGATGAAATGCTACTTCAGTTGTTTCTGTAG
CTGTTCATTCACTC
ATGAGGTCATTTCGTCTATGAATACATTATGATTCACTTTATGAT

ACGCTGTCAATCAAT
TTTCCATTTATATACATGAGTTTTGGTATAGGCTCGCTTCATTTTT
TGCACTGCTTTTCA
AGCCTACCCATCAAAACGACTGCAATACTAGGGGCCAGGAGGCGT
CCTTTAATCTCT
>ConsensusfromContig4080 Average coverage: 24.20
AGCCTCGAGAGAAATAATTTTCAGATTCAGCCTGCTCATAAAGTC
GTGAAACCTGCAGTC
TACGCATATCAATCGAGAGGATGGTACTGAAGAAAAATATCTGC
AGAAAGAAATCTGTCA
GAACACCAATTGAAGCGAACGTACAAAACTCCTGTATGGCTGGA
ACTCCAGTAAAGTAGC
CACAGCCGAGAACCATAAGCTCCCAGAGTAGATTTTTGAAAATAA
ACCACCCTTCTTTCT
TCAGACCCATGGCAAGACGCTGTGTGACTTCGATATCGATCGGTG
TTGCGACTACTGCTT
TTGTAACCACAACGACGTTTTCCATCCCAACGACGACTACCAGGT
AAGGAAATGTCTCGC
TTCCTGAGAGACTTGGAGTGAGGTCGAGCATGGAACAAATGCCA
ACAGAAGAAAGAAGCG
AACACAATACAGAAACGACGGCACTCAAGGCGATTCCGGATTTCG
ATTTCACCATCTCGA
TTTTACCAATAGAAAACCAAAGATATAAGAATACTGCTATATAA
GCCGTGATGAGTGGAG
CATACTCTTGAAAATAGTTGACACGCTTGAAGTGAAAATGCTCCA
TCATCATA
>ConsensusfromContig4081 Average coverage: 13.14
CGCCGATGTTCTTCCAAAAACTCATTTCTTGTTGTGTAGTTCATG
TCAAAGTAGACTTGC
ATTTTAACTGTATCCAAAGACGGAACTTTAACTTCCATCAATCTT
CCAACGCATACCTGA
ATAAGTTTTTCGACGTCGGACTTGGTGAGCGTTCTATCAATATTG
AACAAGTTATCCGGA
TCTAAAACAACTGCTTTTACCATGAAAGCTACTAGAGTTTCTGACG
TTGTCTGTCCTTTT
GTAGCACACTCTTGTGCTATTTCTTTTATAATGTTTTTTATAACAG
TTTCTGCTTGAACT
CGTGACATATTGTATTCGGGTTTATAGTAAACACTCCTATGTATA
TAAAGATTTGAAGAT
CCAGATTT
>ConsensusfromContig4082 Average coverage: 739.77
AATGTGACTGGAGTTCAGACGTGTGCTCTTCCGATCTCTCCCGTCT

CTCCATTTGGTTTC
CGATTTAAGATTTTCTCTTATAATCAATCTTTCTGCCGGCTGTCTC
AATTTTTTTCTCAT
GCGTTTTTGATGTCTATCACCGGGATGTAGAAAACCTTTCTCGAA
AGAAGGTTTCGTGAC
TCTCGGAGCACTTTTTTCACTGCTTTCCATCATGTTGTAATATTCT
CTCAGTTTAGTCGC
ACGGAAATTGTTCACACTCTCGCCCATCCCGCCACCTAGTGTCGA
AGGAAAGCAGTGGCT
GTCAACCCTGAGATCGCCCGTTTTAAAG
>ConsensusfromContig4083 Average coverage: 28.65
AAAAAAATAAACAAATAATCACAATTATTCACGAAATAGAGTATG
CCTGCTTTATTCGTG
TGTGGTCGGCATCTCAAACTCGGAAGTGATGATTTAGTGTATCCC
TGTATATCAGCATTC
TTTGGAAGATTATTTTGGACAGCTCTGCTAATTTTGGTATATGGC
ATTCACTTCAATGAG
TTCGTATGCTCAGGCGGCCCGCTTCTACGTCACTTGTACATATCG
ATGTTCATCATGCAA
TCTTGCCAAATGCTACTAAGTTTAATAATTGGATTCGTAAGTATG
AGAGGCTCTATCATG
GACGACGTCAAACGACAAAGTATGCCCAAGCTGTTCATTGTCAAA
CTTATAGCTCTGGTA
GTAGAAGTCCTCTACAGTGTGGCATGCAGTGTTTTGGTTTTCGGC
GACGCCGGAAACTGC
CAAGGCCGTGTGGTTGTTACAGCGAGAGTTTTGGTTGTCATGGCT
GCATTCATTTTATTT
TGCATTCTCGCCTCTGTCTACCTGCTGGTCAATCCACGTGGCGCC
TCCTATCCGTCGGGA
GA
>ConsensusfromContig4084 Average coverage: 38.03
CCGCCGCTGTTCTCCAATTCTGCGCCTGGTAGGTCTGAGCAATTG
AAGATTTCTCTCACC
ATCGCCTGAACATTTGGGGTCACAATTGTTGAGATGTCCATTTTG
TGGTTGTTCGATGTA
GTCGACGCCCATTTTCGTTCGCTTTTTCGAACAACCCTGCTCAAA
TCTGACATCGGATAT
CCATACCGGCGTTCAACGAAGGGTGCCCCGTTTTGATCACGATA
ATAAGGATAGAATGCA
GACGTGAAACCCAGTCCATGTATCATTTCGTGAATAAGGATTCGA
TTGATATCTGCTTGC
TTTTGCTTGAGGCTTGTACTGCATATTCCGATAATGGCTGCGATT

GGCCTATCCAGTGTC
TCTTCTCGAAAACAAGGCATTGAAAACATCAGCGTTTTATTCGCA
CACATATTTCTATGA
GCTTTCACAACGAAGAATATAATATCAGCATCAACGCCCCCTTCC
GGATACGGATCTGGA
TCTTCCGAAAAAGGATCGAATTCATCGTAATACATAACTGCCACA
ACTTCACTATTTACA
TTTCCCGAAGTGTCTGAATGAGTTGGACCTTCCACTAAATTTGCA
TAATTGTTCGCATAT
TCAAATTGAGAATATGAATATTCATCGGTTGAATCTATTCTCGATT
CACTTGTTTTTGTT
CTTTCTGCATCAGTGGGCAATGAGGTCACGTATTGATGAGGATCT
GATGGAATTGCTTTT
GAATCTTTTATTTTTTCATATATATTACGGGATTTAGCTTTCAGTT
GAAATTCTTTGTGT
TCAGAATTGAATATTTGTGATTCGATTGTAGAAAAATT
>ConsensusfromContig4085 Average coverage: 15.55
GTAGTTCTTACATGGAACCAGATCAATCTTCTAGAAACAAACGAC
CCCCACCCCCACCTA
CAACAGCAGCCCCCGATGATGCGGGGATGGCCAAGGGTGTCGTT
GCAGCAATTGTTATCG
CAAGCATTCTTCTGCTAGTCATTCTCATAGTTCTTATTTTCTGCTT
GAGGCGGTATCGTC
GTCTTCAGATGAAGCAGCCCATGCGACCCATGCCCAGCGGTGCC
TTGAAGAAGCAATGGA
AAACGCAGCGACGGAGCGACGATCCAGACAGCGAATACCCGAGT
GGCAACACTGGCCCGA
TTCCTCGCAAGGGCAGACTCAACTTGCCCAAGTATAAGATCTACT
CGTATTTGTTTCTGA
GTAAACAGCAACGAGATAACAACGAAAGAGCAGCACATCAGAGA
CCCAATACTGTTGGGA
ATAACATGCCTGACCTATTAC
>ConsensusfromContig4086 Average coverage: 35.23
GAAGAAGATGAAGAAGATGAAGAAGATGAAGAAGATGGAGGTG
AAGATGAAGAACCTGGG
TACATTTGCAATTGCCAAATTCCGTACACCGGCGCCATGTGTGAA
ACTGAACACAGTGAT
GGGTGCAATACGAACCCGTGCAACGATGGGACATGCAAAATTGT
GGATCAAAGTTTCGAA
TGCAAATGTCCAGAAGGGTACACCGGCAACTTCTGCAACATCAA
AGACGACACGAAATCT
TCCGGGGAGAGAAACGGATCCGGACTTGCCTTCATTGCGATTCTC

>ConsensusfromContig4087 Average coverage: 13.75
GTTATATTGTATTTAAAAAAACACATGAGTGCGATATTCGTAATT
GTATCGTATGCAATA
TGATGCATGATTTTTTATACCTGTGATTGTATATTGATTTGTCTTT
ATCCGTGAATATTT
CGTTATGTATGTTTGATGTATGTCTGGTGGTCAGACTAGAGCGGCT
ATTCATAGTTTTTC
TGGCCACCAATCAATTTTATGTACCGTGCATAATTTTTGATGTTTT
CTTTCTTATACAAT
CTCACACCAGCCCAGTTGATATACAGTAGAGGCCCAGTTAGAAG
GAACGGCCGACTAGGA
AATAGTTTATCTCTGTAAAGATAATGGTCTTTGAAGAAATTTTGA
AATTAATAATAAAAC
ATTCTGAGATATATTCGTTATACAGAGAACACTTTTCGTTCTTACT
CTCGATTCACTGCC
TATTTTAACAATCGGTCGCTTGATTTTTAATTTTATTTAACATGAT
TCCTATTGAGAATA
TGTATTTTCATCTTGATGATAGTGATGTAAGGGTTCTAAAATT
>ConsensusfromContig4088 Average coverage: 6.59
GGAGGGCAAACTTGACATAATTATGCATATCTTGAAAAGTTACTA
GCGAATTTTTATGAA
ATTTTACTTATATATTTATATCCATCGCGGGAATCCCAAATACAC
AGGGTCGTTATTATC
ATAAAAAACAGAGTTGAGTCGTTTGTATTTTCATGAAAGTTTACA
CACAAACTTATGTTC
ATCAGAAAGTAAACAATTTTAGTGGCTTGGCATTGTTATACATTT
TAATAATGGTGTTAT
GACGACAGTAATACCCTGGCGACGCTGTGTAACCCGCTTTTTATA
TCATTGGTAAAATTA
TGTAGAGTCCGAAACCGACCCTTGAATCCGAATTAGAATCCGACT
CCGGTCCTAACTCCG
ATACTCTCGAAGTCTGATCGACATGTCTCCAAATCCATTTAATTAT
TTTCACTTCAACTT
CTCAAGGAATATAA
>ConsensusfromContig4089 Average coverage: 7.82
GATCTTTTCACTACAACAGTTTGAGATTCCTCGAAGAAGAGCGAC
ACCAGCCGATAGAAC
GGTAGGCCGGAGCCTACCGAGGTGTTCAATCGGTGATGCCACCCC
TCAACATGGTTGTTA
GTCCTCACAACCTCCCTGAATACAGACCAGTTGGCGATCGCCCAG
ACGCTGCTGTTTATC

CATGTTGACTCCACGTACTTGATGGTATTTCTGATCAACTCGTGTT
CTGGTGCATTCTCT
TCGAGCGTCATGAAAGCCCC
>ConsensusfromContig4090 Average coverage: 18.13
GACAGCTACCCCCATTCTTGCAAGTTGGGTCGCATGGGCTCTGAA
CCTCAGCAGTAATGT
CGGCATTCCTTTTGGCTTCTTGAGTTTCAGATTCTTTTGTCGCTGC
ATAATCGAGGATAT
ATTCGATTATGCTCGTCAATTCATCATGGGTTATCTCACTATTCC
CGAATTCGGAGCCAT
TGCTTCCATTCCTTTTGGCTTCTTGAATTTCAGGTTTTTTTGTCGC
TGCACTATCGAAGG
TAGATTCGTTTCTGTTCGCCAACTCATCATGATTTGTCTCAGTATT
CCCAGCCATGTTGG
CGCCTCTGACTTCCGAATGCATAACAAGTTCGAAAACGCACCAAA
TGAGAACCAATTTTT
TCATTTTTTCTTCAGTGGATACGAAATAAAAAAATCAATAAACAG
AATCGAG
>ConsensusfromContig4091 Average coverage: 38.78
TAATTTTCCTCTGTAATTATTTCAAAATTTAATTATCTGATTTTTT
ATTTGAAGGATTGA
TTGTGTTTCACTTTTGTGTTTGGTATCATGAATTTGATCACTTCAT
CCGATCATGGGAAA
GTTTCGATTTTTTTTATTTTTATTTATATATCTGGTATTTTGTTTTG
AATATTTTCGGTG
ATAAAGAAAAGTCCCCCCAACATGGCATCAAATCAAAAGAACATG
TGTTTATTATTAATT
TGTTAATAAAAATGAAATTTTTCTAAATTTAAATCGTATTTCTATT
TTGTAAAATGTCTA
TTCCCGCCAATGATCAATCAAAGTTTCAATCTCAAATGAACGAAT
GTTTGCCATTTTGTA
TGAAGCAAATTAATCAGTGCCTCTATTCAAGACGTGTTATGAATT
GTAT
>ConsensusfromContig4092 Average coverage: 14.79
TGTACAGTTATTCCATGTCAAAACGAAACAATCACATCAGAAAA
GAATATAAAAATAAAA
GACCAGAACGGTGAAAGCATAACGTATATATGCGATAATGAGTA
TGTGAGATCCGCCGGA
GACCTGACCCGGAAATGTGTTGTGACAAAAGGAATATCAAACTG
GGATGGAGCCACGCTA
ATTTGCACAAAGTGTGGAGTGCCAACAACACTCAAAAATGCGAT
TGTGGAAGTCAGCACT

GACACAGAACCTGCAACTGCTACATACACCTGCGCAAGTGGTTA
CAGGAGCAATAAAAGC
ACAAATCAGACCGTGAAGGAATGTAAAGATGGGAACTGGACTGT
CGTGGTTTCT
>ConsensusfromContig4093 Average coverage: 44.81
CTCCCGACAGATATCTCATCTAACATGGGTGAAATATTTCCAGAA
TCTTTCGATGCATCA
GATAAGGTTCTCGATGCAGAGCTGATTGAAACTATGGATCTAAAA
GATACAATTGTTGGG
ACAAGCAGTTTTAATCCTTTGGGAGATTTCTTATCATCATTCACA
CCTGAATGTGCGATA
TCTAATTCGTCAGTTCTTGGATGCGACAATAACAATTCTAATGAA
ATGAATGACGAATCC
AGTCAACAATTAAACCATTTATTTTCATCAACACAAGGTAATCCA
TGCAATATGTATATT
GAAGAAAAACCGAAGGAAAATTTTTCCAAAATAATGAGCAGCCG
CTTACATGCTGGAGAA
TATAATATTGTAAAAAACAGTGTGTGTACTGATTTGGGATTTTCG
AATCAAAAGTATTTT
ACTGATCAATCCTCTACAAATTATGTATCAATTTTGAGGAATGAT
TTGAATAATTCATCC
ATTCAAAATCATAATAAATTCAATCAAAGAGATCTACCAAATTC
CAACACAGAGAAATCA
AAAACCAACGCTGTGACGCTTAGTAATAGAAAGGTTAAAACAAG
TATAGAAACCAAGTTT
CGTCCAATACTATCGAAACCTGCAAATTCAACGGTACTTGTACCA
ATCCCACTGAGCTCA
GTTAGCAATTGGGTGACGAATGGGTCTGCAATGCATAATATTCAA
CTACAAGAGCTAGAT
ACAATGTCCAGCCGCGCTCGTTCCCTACTCTCCGAGACATTGCCA
TCAGTGCATGTTAAT
AACGATTCCACATCATCAGGCTACTCGAATTCTGCAACGACTGTT
TCTCCTATATTGGAT
TCATTATTGACTATTGAAAATAAATCACTCACAAGCTCTCTTTTGG
GTCT
>ConsensusfromContig4094 Average coverage: 41.36
CAATAGCAGTATGAATATTTCACGCTTCATATGATAACGAAATGT
CCAATACCTTCATAT
GAAAATTATGAAATCAAAGAGGTTTTAGTGCAAATTTATAAATT
CAAAATCCAGCAATAA
TCCAATGAGCAGGCCCAAATATGAAAAAAATCACAAATCTGGAG
GAATGATGAACATGAA

AACAAATGGAAGCTAATCTTAATGATAGCCATTCGTATGATGTGC
CTCGTGTCTTTTGTA
TTGACAAGACACTTATATTAATAGTAATAATTTTTTTCCTACAGC
TTCTACGATGAATAA
GAAATAGTTCCAGGCAAGAGATACATTTGTAGGAAATAGTCATT
CAGAAATAAGCTATGT
TAAAAAATAGTCGAAATTTAAGAGAAAAATACAAGTTTTCAGAA
ATAAATAACATATGTA
GT
>ConsensusfromContig4095 Average coverage: 8.52
CAATTTGTGCCGTTGCCTACCCAAGAATTCCGCTGGCAAACCAGA
AAAAAAATATCGCCA
GAAATATTGCAGACGACAGAAGCCACATCCGGGGAATAGTAGAT
TTCGTGATCCGGGTAA
GAGAGAGTCTGCGGACATCCTTTCGCAATGAACTCCAATGTAAAA
TATATCGGTGACTCG
GAGTATGTGATAATAATCACTTCCATCCTGTGATTGACTGATTTG
ACGAATGGAAAATCA
GGCCACGACTGCATGCACATCGAATGGTACTGTTGATGTGACTCA
TCGATTCCTTGCGTG
AAGATGAAGAGCGGGCAGTCCTGCGCTGTCTGGTTGAGTGCGCG
GATGTTTCTAAGGCCG
ACAGATGTGAAGTCGTTGAAGTAGCCGTGGATGGTCTGCCCTCTT
TCGGCCATAAGTCGG
ATCGGACAGATGTGGTTCGTGATGTAGCCGTAGACGCCCGAACC
TGAGACGATGAAGAAA
GTTTCAGTGTTCACCCGCTGACGACTCGGCTCGTCGCA
>ConsensusfromContig4096 Average coverage: 16.78
AAAGGGGAAAATGATGGATGGTATCTTGATCAGCATTGGACAAC
ACTCAAAATTGTACAG
CTGACAGAAAAACGTGAAACGCAGACGTTAAAAAAATCAGCAAA
CGTAATTTTGGACGAT
CTACAGGGCGTCAAGTTAATCGCCAGACAGCCTCATCGTGATAG
GCTAGACGAGAACACG
TGGTCAAGAAAGTTACGCTCTCGAGGTGGGGTTCTAGCAGGAGTG
AAGGACATTCATGTT
CTCAAGGACACATATCACCCGGGGACTTGGTCTGGCTTGATCAGA
CCTGTTGTCAAGAGT
CTTTTTAGCAGAAGCGATGTTATAGCGGTGAACAAGTACTATGAG
AGGTTTTATAACCTG
AGCCTCTCGTCAGCTGGAAACGTGTTGGAAGGGGAGCCGTATCTG
GTTAAAGCTGCTTGT

TTGCCTTAATCAGATTTCTATATTGAAA
>ConsensusfromContig4097 Average coverage: 79.70
CCCGTATCAAAATTATAAAAAATAACCATTATCATGAGTTTTTGC
TCTGAAAATCGTAGC
ACGAATAATGAATTCGTGCCGCCGCAACCTAAATATATAAAAAT
GGATCATAAGCTTGAA
CATGTTTGACTAAGAGACCGACTCTTGGCTTAATTCATGATACAC
AGCAGCAAAAATCAT
GTCTTATGTCAGATCTAATCTTTTTTTGTTTTTGCCGAAAGCATTG
CAAAAAGGTACATT
GAAGAATACATAAAAGTTAATTGAGAGCAACTTTAGCGAATTTAG
GGTATTACTGACATC
GATTCTAGAGATCGGAAGAGCGTCGTGTAGGGAAAGAGTGTA
>ConsensusfromContig4098 Average coverage: 10.62
TCTCTGTGGTATTATTTCATGATTTTGTTTGATGACGCGAGACGCT
ATGCGGCGCTTGCT
GTCCTTTAGATTTCACGTGCATAGTTGTAACAGTTTCCTTTTCATA
TCTTTTTCTCAGAT
AAACAGCGATGGAAATTCCAATAATCGTCACGATTGCACCGAAA
ATGGTAACGAGTGTTG
GGATCAACGATTTAGTGTCGCCACCTTCCGGAAGTCCGAACTTTG
TAAATAAGATGGCGC
CAGCTATCAGGATAGCGCAGCCA
>ConsensusfromContig4099 Average coverage: 17.20
TCTGGCATTAAGACGTCCCAGATGTAATTGGCATCGAGGCACAGC
TCTTTGCCTCGATAG
GCCAACAGATAGTCCCGGAATATATCGGCAACCGCAACGATACTT
CGCTCCTCATTTACG
GGACCGACGTTGTTATTTGGCCGAGTGCCGGCCATGAATCGTAGA
CTGAATTCTCGTTTC
GTCCGGCCACTCATGATGTCAAAACAACGCCTCTTGACAACAGGG
GTCTTTAGAAACGCA
>ConsensusfromContig4100 Average coverage: 15.79
TCAACGCACTCATCCATATTCGTGCTATATTTGTGAGATGTCGTTT
TCTACATCATCAAA
TTTATTAACTCATCAACAAACTCATTATGTTGTAGAGCCTCACGT
TTGTACTGTATGCGA
GAAATTTTTCAAAACATTAGAACAACTGGAAATCCATAAACGCAC
CCATTCTGATGAAAT
ACGTTATTCATGTAAAATTTGTGAGAGGTCATTTTCTACATCATC
AAATTTATCAGTTCA
GCAACACTCTGGCGAAAATCCTCACGTGTCTACTGTTTCTCAGAA

GTCTTTCGCAACTTT
GTCAAACAAGAGAAAACATCAACGCACTTATTCTAGTGAGAAACC
TAAAATTTTTACAAT
GTGCGATGAATATTTCAAAACATCACGAAACTCATGATCCATAAG
CTCACTCTTTCTGGT
GAAGAACAATATTTATGCGATATTCGTGGTAAGTCTTCTTCTACA
TAAACAAATTTATTG
GTTCATCAACGGACTCATCTTGTTGTAGAACCTCATGTTTGTACTG
TATGC
>ConsensusfromContig4101 Average coverage: 14.49
TCGTTGCTGAGGACAAGCGTGCGTCAATCCAATCCAGTTCTCAGA
GTGCCAGAAACGCCA
GTGGTCTCGCTGCGGCCAGATGCGATGAAAGTCTATGCAGGGGCG
GAGTTATCTTCTCGG
GTCCAAACGGAAACCGGCCGCACAAAATATCTGTGATGGAGGGG
CAATACGTGGGGACCG
GAAAAGCCTCCTCTCAAATGACGGGGGACACAGCGTACCTGTTC
AGAGGGTCTCGACATA
CATCATTCCCCCAAACGAAAGCTGCCAATATAGGGGCAATAGGA
TGGGGGATAGGAACAT
GAAAATATTTGATATGTAGTTCTATCTCGAATCGAATTCACGATT
TAAATCAGTGTATAA
TCTGGC
>ConsensusfromContig4102 Average coverage: 14.25
ATTTCAGTGGGCCTCGCTTCTGATTCTGTATCCGGAGCCATATCC
TTGAGATCAATCGGA
TCTTGTTCCTCAATGTCTCTAATATATTCCTCTTCGGTCTTCTCCA
TTTCATCAGAGGTA
TCCGTAACAGTTACTTCAAATGGGATTTCAACGGTTTTATGCGACT
GATTTTTTTGGACT
TCAGAAGAAGTGACCTCCTCTTCCTCATTCTCTTCATATATCGGT
TCGCATGAGCCCCGC
AAGCTTCTGGACATATTCAGGTTGGAGTTATCCGTAACATAACTC
ATGAATCGTTTAGAC
TCCTCCGTTTCTTCTGGTTCGTCGTCCTTTTCAATCATGGATGAAC
AGGAATCGCTATCA
TTTTGATCTTCCTCGTTGTCTGACGTGGGACTCTGCACTAATGGA
CTTGGAGAAATGACT
GAAATACCTGGAATGAGTTGCGAAACTTGTGTCGAGTCCTCAATA
TTCGAAATTTCGAGA
GGAAAAGTTTCTTCAACTTGAAGAGTAACTGAACCTGTAAAATTC
AAATCTTCGGACGTA

CATGTATTCGGA
>ConsensusfromContig4103 Average coverage: 10.28
CTTCCGATCTGTTTTATTGTTATTTCATCATATTTGGCAACGGCTT
GATAAGATTGACCT
CCGCTGTTGAGTGTTAGCGTCGTATTATCCCATGTGAAATATACG
AAAGTCCATTTTCCA
AACTCAACCTGTGCCTGTAGGAACCATTTTCGACCAACCGTCTTT
ACAGCAATTGTAAGA
TATCTCCAGACTATGGTGACATCGACACCTCCAGAATTTGGCGAG
TTCCCGCCAGTTGAT
ATCAAGTTTTGAGTATTTTTTGATTCAATTGGCTGTAACATGAAT
GCCATAGTAAATCCA
TCAGAACATTTAGAGACATTGGTCGCGCAAATATTCGTGAAATTG
GTATAGGTCAAGTAT
ACATCTTCCTTGAATCCCACTGCAGTATTTTCACCACAATTGTCAA
CTCTTCATCTACGG
TATTTTCACAGATCGGAAGAGCGT
>ConsensusfromContig4104 Average coverage: 10.72
AACACATCAATTTCAGAGCGAGCTACAACCAGACATATAATATG
AATGAGTTAACGATAT
TAAACTGTCAGAAACATTGAATTATTGCATGCACTTCCGAGCAAG
TCAAAAACATCACTC
TTGGGTTGACATATAACTGTAAACCCTATAAAGACACATATATGA
TAACCCATAAAAATG
AACTACAATCACACACCACCCGAGAATATAAAACTGTCTCGTATT
ATTCCCAAATTATCT
GTAGCGATGTCCATCATGAAAGAAAATGAGCATGCAATGAATTC
AAA
>ConsensusfromContig4105 Average coverage: 6.43
AATAGAACAACAACTCATTTTGAACTCAATGAATCCTTCCGCGTC
TGGCCTTGGGTTGTC
TTTTGTTCTGCCAAATAGTTCCTGTATTCAAACAATGAATCAGAA
TTTGATACCAAATGT
CCAGAGCGCTCTTTCCAAAGACCTTTCGAAATGCTCAGAGGACGG
CAAACCTTTGTCTCA
GTTATACCCAACACTTGCATCGAGTCTTTCAAATGCTCAAAACAA
AAATGCTACAGCATC
GAAGCAAAATAAGATTACGAATAATCCTAAAAA
>ConsensusfromContig4106 Average coverage: 43.46
AAACAATCATCAGTATTAGTGAAAATATTCATTCATATTCAAGTT
ATCACGTATTAACAT
CATTAAATATCGTGAAAATGGCTGCTCTTGCAATGTCCCCAAAAC

CAAGTGGGATGACTA
GGGCTAAGAAATGGTCAGAAGATGTTGAAAATGTTTACAGATTC
CAATTAGCAGGATATA
GAGACGAAACAGATTACATAGCTGCTCGGGGAGAGACAGAGGTT
CAAAGATGGCCGGAAA
CAGGGTTTGTGAAAAAACTTCAGCGGAAAGATGGCTGTTTTTACT
ATTTGACAAAACCCG
AGAGTGCAAGGATAAAGAAGTTCACAAGACTAAACTATATACCT
ACGCCTGAAATCCTGT
CATATTCATTATATCGCTTTATACTGATGAGTCAATATTATCACG
GTCATGTATGATAAC
ATGTTCGTCGTAACACTATATGTATGCTTTTCAATCATCTCTATCA
AATCATTGAATGTA
TCATCGTGTAACGATTCTGAGCACATATGTAAAAA
>ConsensusfromContig4107 Average coverage: 6.42
CGAGAATGGCCAGAGAGGAACCCCCGAGCATATGGATCCCTCCA
TTGGAACTGTGGGCCG
AGTGGTGTGGGGGCTGAAGAATCATGGCAGCAGCTGCCACATCCG
CTTTCATATCCATAG
AGCTTCCATGGCCGATGTGGTGGTTGCCGATCGAGTGGGGCGGC
CCCCCAGAAGACCCCC
CTAGCCCCGAAAGACCGTTCATGTTGACGTTGCCCAACTGCATCT
GACAACGGGCGATTA
TGTGCTGTTGCTGTCGGATGGCCTGTTGCCGCA
>ConsensusfromContig4108 Average coverage: 14.46
GTCTGGTACAAGTACATATCTGCTGCAGAAATAAAAAGCATGCAC
AAGGACTTTTTTCAC
AGAGTGAAGGCGAAGACATGCAATGGAACGTGCCTTAGGTCCTA
CATATTGAACTCTGGC
AAGCGAGCGGGATATTATGGTGAAAAAGTTGATGCCAACACGCT
AGAAGCCTGTGCTTGC
CATTGCAACTCAAGAGCGGATTGCGTAGGAGCCGATTTCGACGAA
AGTGATAACTCTTGT
ATGCTATATAATGATCCAGAAGATTTGAAAGAACCATGGTCTTAT
A
>ConsensusfromContig4109 Average coverage: 31.01
AGAAGGGATTCCTGAACGTGCAAAACTCCAGTTTCTGGAGTCATG
TAGAAAAATCTTCGG
TTTCGTGCATTTTCCTCCTTCACAGATAAACCATACTTGATGGAAT
CCCCATCTGCATCC
GTAGCGTGAACCCTGATGACATCAAGCCCCACATCGATATTCTCG
TTTATATTCGCAGTA

TAGGTGGCGTTGCTGAAAGATGGCGCGTTAACATTTCGGACGACC
TCGACCTTGAACGCA
GTTTCCACCTGGTTATAATCCGTCAGACTGTCGTGCACCGATACA
ACGGCCATATAGTGA
GGGGAGAAATCCGTCCGTAGGTCTTTCTTGATGGTCATCGCTCCT
GTCTCAATGTCAACG
CTGAAGTATTCGATCGCAAGTGCATCACCAGTTAAGGCATAAACG
AGTTTCCCCTTCTGG
TCCCTATCGCTGGCGCGGATGGTGGCGACGCGGAATCCGACGGGT
TTGTCTTCGTTGAGG
GCGGGAATCGTGATGGGAACGTCCGCGAACTCAGGCAAGAATTT
ATCGTGGTCGATCCTG
ATTCTCACCGCTGTTTGTCCGCTCTTTGGAGGAATCCCAGAATCT
GTGACTGTCACTTGC
AGCACGAATTCGTCAGCCTCGGTTTCAGTCAGAGGAGCTTTTGTA
GAGATGAGTCCAGTG
ACAGTATTGATGAAAAAGAACGAATCTGATTCTTCATTTCCGCTG
AACGAGAATTCCATG
CTGTCCCCGTCCTGATCCTGTGCAGATACAGCTATAACGGAGCCT
CCAACAGCAAAGATT
TCAGGAATTTCTATCAAATAAGTGGATTCCATGAATTGGGGGGCG
TTCGGATTCCGGTTC
ACGGAGACAGACACGAGGGCAGAGGCGGGGTTCAGGGGATATAG
ACTGTCATATGCCAAC
ACCTCGATCGTGTATTGAGCCAGACGGAAGCTGTCGGCCTGCAG
ACTCTGAAGCACAGTG
AGCTCTCCAGTCGCGGCGTTGATCTCGAAGAGGT
>ConsensusfromContig4110 Average coverage: 5.53
TGGAAAAGCTTGAGAAAACTCCCTACAGTTCTGAATGATTGGCAT
GGTGTCTCTGGATCG
ACGTATCTCAGAGTGGAAATGTTTTCATTTTGTTGAATGTGTACTT
TATTCTTGTTATAA
GCTATTGTTACGACATTGCTAAACAGTAATGATAAATCCGGACAC
GTCTGGTTAAGGAAG
AAATACTTTGAAAACCAAATATTAATATACTACCATGGCGATAAT
GATCCCAGGAATACC
GTGAAAGATCGGAAGAGCACACTC
>ConsensusfromContig4111 Average coverage: 13.28
ATCGGAAACTTGCAACAGTGTTTCACGAATGGTCATGTAGAATCA
TTCAATATTGGGAGT
ATAAGTACAATTGCAACGCAAATATGTGATGGATTCGTATACCTA
GGAAATCAATTCATT

ATCCATAGACGAATATGTACTGAAAATATATATTTAGACGAGTTT
AAAATTCCGAAAATA
GCAAATTTCCACTACGCAGCTCGATGCGAAGATGATTTATATAC
AGCTGAAGAACATGAA
GAGATACTTGTTGCTTGGGCCTCACCAGAAGTACTGAGAGACTTT
TCGTACAGTTCAAAA
TCTGATGTTTGGTCATTCGGTGTGTTGCTTTGGAGTATTTTCAATT
TCGGTGAAGAGCCA
TGGGGGGATTACAGTAATGCAGATATCATATCCAAATGCTTCAAA
GGCGAAATTCTTCCA
AGAACGGCCAATATTGACAGAT
>ConsensusfromContig4112 Average coverage: 30.98
ATTCGGAAACGCCCACTCGGCATTCGTCGACGTCCCTGCAGTTTC
CAGCCCCTTGAGGTT
GATAGCCCACGGCGCACAGACATAGACCGGTGTTGGGGTCACAG
CCTTGCTGGCAGAGAG
TATGCTCACACAGGCTTTGCACACCCTTCTTCAGTGGTCCGCTGT
CAACAACATATTTGG
AAATGGTGATTGGATGAGCCATCTGACGAGTGTTTATGGAGATAG
CGCCCTCCGCCGGCA
TGTAAACAACGGATCCGTTCGCGAAGAGACTCGAGGGCGCGAAG
ATTCTAATAGTCGCGT
GGCGTTCCTCAACTGCCGAAATACCTCCTGCTATGAACATGCTGA
GCACACACACAGTCA
TCAGCATGGACATCTGCAAATATTTGTTGCCAACCAAACCCCCGG
CTTTCG
>ConsensusfromContig4113 Average coverage: 55.60
AAAAAGTAGAGAGAATATCACGCATAAAAGCAAATTTGATAAAT
GACCTAACAATCTATC
TAAGACTATGTCAAATTGAACGATGTTTCTGAGTATACTGGGTTA
TCAAAGTTGTCCGCG
AGTGGTTTTGAGGCGACACCGCGCCCCTTCATGATAAGACCCCGG
TTGTAGAAGAGCAAT
CCACCGAAGAGAGCGATGCAAATGAAGCCCAGACTCCCGAAGAC
TACCGCTATTATTTCT
CCACTGCTTAGCCCACCATCTGTTGTACTATCTTCATCTATTTTTC
CTGTGGAACCATTA
TCAGTTGAAAGCTCATCTTGCGTTTTCGTGGTCTCAAATATCGGCT
CTTCCGTCCTTATT
GTGATACTTTCTGTGGTTATCGATTCCTCCTTCGGTGTTGTCGTA
ATAACTGGATCGAGA
TAGGACAAATCTTCGACACTGAACTCCCACTGAGCTTCCATTCCG

CCTCGGGAAATGAGC
GTGACGGAAGTGAACTGACGTCGAACGGGCATCTCGTACCTCAAG
ATTTCATTGGCCTGG
ATCGTGAGCCCCTGACCTACCCGGATCACCCCCGTC
>ConsensusfromContig4114 Average coverage: 32.74
AAAATATTCAAGTTTTGTTATAACAGTATTCCTGGTAACAGTATC
ATGAAAAAATTCAAG
TATATTCATGATGACATTTCTCCGTGAACTCAATCAATACATTGTA
TGTCCATTATGTGC
CGGGTATTTCATCAATGCCACAACTATCACAGAATGTTTACATAC
TTTTTGTAAATCATG
CATTGTCAAGCACTTTGGAAGTAGTAATGAATGTCCGGAGTGTAA
TGTTGTGGTCCATGA
GACGCAACCTCTGCTAACACTCAGGCTGGATAGAACTCTTCAGAT
TGTTGTCAACAAAAT
TGTTCCTGGCTTAGTCGCAGAAGAATATGAGCGAGAAAACAGTTT
TTTGAGGTCGCGAGG
ATTGGGCGAACTCATAAATAACGCTGTACCAACCCCTCAACAAT
CGAATATAGAAGAAAC
ATGTATTCTGTATCAAGAAGACGTTAGATGTCGCCTGCATGCCTT
CGGGGATGGGGGCGA
CGGCCTGAACGAACTTCTCTCCTCGGCTCACGGATGCACAGTGTG
GTGTCCAATTCTGGC
CAAGATTCATCAAATCTGCTACATGCTGCATTCAAGATTGAAGAT
CGACTCCAAATCATT
CAT
>ConsensusfromContig4115 Average coverage: 7.97
CGATCTTTTGGATACTCAAAAAGCTGACAGCCTTCAACGGCATTA
AAAGATGTTGCAGAA
GGAAAACTGAGCTCAGAATTTTCACCAGCAGCGCACAGATCCAAG
AGTAGATTTTCGTAT
AGACTGTCACCATGCTTTTGAAACAGAAAACAGCACGAAGTGAG
TGAATGATTGACAGAT
ATTCGCATTTCACATTCCAGATCCGAAAACCATAAAGCGACATCT
TTTGACGGACGGATC
GAATTGTGAAAAGAGATAGACTCTTCGAATCCACCCTTCATCGCC
GAAAGCCGTAGCTCA
TTCGAGCGAAGATCCTCGTCCAAACTTGTTGTGCTGACCAAACCT
TCATCAATAGTCTTT
GGGAGAGAGAAATCGACGTTGTGAATTCCATCAAAAAGCCGTCT
CACAAAGCCGGTATAT
TTTCTCGGGTCTCCAGAAATCGAGAGCAGTTCAACCATTTCATTG

TCACTCAGGAACCGC
AGTCGGGCAAATCCGGC
>ConsensusfromContig4116 Average coverage: 25.22
ATAAAGATGATTGGTATTCCACTGCCCGCGTTACAAGATCTCGGT
CGAGAAGATCGATGG
CGGAGAATATTCCACCGGACCTTATAACGCAGCTTATCGACGAAA
CCTCTTTGCCTTTTC
CTAAAACTGGGTCACGCAGAAAGAAAATGAGAAGCTCTATCGCC
ATATTCAGAGAACGTA
ACATTCCTACTGAAATTCCAGTCGAATACGAATCAGTTGTTTCAG
AAACCAATACAGAAA
TTGTCAATAATATTGCAGAGAATCCTTATCTTGATGTCAATCCAA
CCCAACAGTTGACGG
GTGGTTATTTAGTTGAATCAAATACTGACCATGTTAACTCTATAG
ATGTACAACCAACAA
ATAATTTTTCGAATTGCTCTAGTAACTTGATCGAACCAATAAATA
ATGAGAACATAACAG
GAGCACATTCGAATGAAGAGTTTTCAATGAGCTCTAGTAATTC
>ConsensusfromContig4117 Average coverage: 5.37
TGAAGCTATGCTCATGAGACCAGCAGTTCTTGACGTTGTGAGCCT
TGGCACAATCGTTGG
AAAGAACATTGAACAAATAACCAGCGCCGCAGAATCTTTTGGAGG
CTTTGCCATTTCTGC
ATTCGAAAAAAACTCTGCAATCAACGTTATGAGGAAATCTGCCAT
CGTGCGGGCATTTGA
ACTTCTCTGCAGATCCCGGTTGTGCTGCGGACTTTTGGTCATTTCT
TGGTTCGGATGATT
TTGCAGAATTCATGTATTTCGACAGTTGACCCATATTGTCATTACT
CAAACTCGGTTTGT
GATTAGTCGAACATCCCTTGACTCTACTCGCTTCCACACAGCCTTT
CA
>ConsensusfromContig4118 Average coverage: 20.44
GTTCAATAGAATGCAAAATCCGGAGAACAACGCAGAGGAAATCA
CGTTCCAGATCGTCAT
CGCCTCCGACTTCAAAAACACTTACATGATATCTCTCTATGAAAC
GAGTGTTTTCAACTG
GAACTTGGTGGGGAACGGCATGACATACAACGCCATAATCGGAG
TGGCGCAGTATCAGAG
TGAGAGCGCAACGGATGATGAAGAATACACACATTCAATGGCTT
CTTACACAGGAGATTC
CCTTCTCGGGATAAAAGCGCCCTATCGGTACACCAAGGCTGGCA
AAAAGGACTTTGGTGC

AGTTGGTCATTTCGTTTTCAAGCTGAGCGACAACCCAGACAGTTT
TGTGAATACCCAACT
GGAATGTCACAAATGGGTCGAGGCGAACAACGAATCTCGCAAAA
TTCACGAGGATATATA
CGGATCTTGTCCATCAACACTTTCTCAAAAAGATTTATC
>ConsensusfromContig4119 Average coverage: 15.47
CTGTGTTGCTATTTGCCATGACAGCGTTTGTAGGACCAACAGCGC
GAAGTCGAAAGACGG
AATCCTCGTCAGTCATGTCAATATCAACCTTACTCGGAAGTGAAC
CATCATTTCTTAGCA
CAACCGGAAGTGACTGTTGCCGACCAATGCGCACGCGTTGAAAC
AAAAGCAATGGCTGTC
CTCTGCTATTACGCATGCTTGGTTTCAAAATGCCGACGCGTGGCA
AATTTCCTTCACCGC
ATACTTCGAAAACGAGGCTCGGTCCTCGCACTTGATTGGCTGTTT
GACTTTCGATGGAGG
CTTCGAATATCCCACAAAAAGTCTGCAATGCATTCGGCTTGAAAT
TGAGAGTGGCATATA
GATGGCTATGAGCGGGGATTTGGGCCCTAGGTGGCTCCACTTCGA
ATGCTTCCACATTTT
TGGAGGCGTGCTTTGCGTTGACGGGTTTAACGGCAAACGCGACAT
CGCACGGAACCTTGT
TTGAGTTGCATATTTTGAAGCGAGCTTTAGCCTTGACCCCGACCA
AAACATTGCAAAACA
TGAATCGATTCTCCGTTTCCCCGTAAACACATCCCGCCGCCGACT
GCTGTGAGTGAAGCC
AAGCGTTTAGATTTTTCACGACCCTGTGCTCCTCAAAGA
>ConsensusfromContig4120 Average coverage: 18.30
TCTTCCGATCTCACAAATGGAGTATCATGATGTATATGAATACGT
ATTTAGAAAAGTGTG
TAATGTATTGCAGAATGATGGTAGATGGAAATCGAATTTAAGAA
CAAAATCTACAGAAAG
AAATTCTATAGTAAAAAAATCTAAAATTACAATGCATGTATCTTT
TGGACTTGCTTCATT
AGACAAAGTAATTCAAATTCTTAGTTCACCGAATGATTTTTTAAC
AGGTTTTGAATTTAA
TAAAGACACGCTTAAAGAAATTATTATACAAATTATAAACACAC
TCAAGACTCATGTAGA
TAATCAAAAGAGATATGATATGAAAACGGAAGATTTTGTAATTTA
TGTTAATTTATGTGG
TTTACCTGATGATTGTGATCCGAGTTCATTAAATACTTTAGTTGT
GCGACAAGATTCACA

AAAACGACGATATGGATAATATCTTAAAACTTCATGACTCGTCA
AAAACTAAAGATGTGG
AAAAAACAACAAAGAATTCAAATTCATATAAAGAATATCACGAA
CATGATGATTATACAA
CGTTTACATCATCAACAAATAACGATTGTATTTCAAATGACGCAG
TTTTAAATATATCAT
CACTTAAAAAATATTTTTCTTCAGCATCGTCCAATGAATTGGATG
AAAATTTATTAGTAA
ATGAAACAGCTTTGAGAAAAAACTTTATGCAAGATCCTCAATATA
TTAAAGAATTTATTA
ATAAACAA
>ConsensusfromContig4121 Average coverage: 16.35
AATCCAGGGGAAGAGTCAAGGTGATAACATTGGATTTATCTGGA
CCGCCCCAAGGATCTT
GAAAAGACAATTTAATAGATGGAATCACAAGTCTCTGAAAATTT
AAAATACTGCGGGGAG
CAAGTTCAAATGCCTCGTTCTCGTCATCATCAGGTTGATATCCGT
TAACGACTACGGCTG
TTTGTGGAAGTGGATATAATTCTATGAATTCCCCCAGGGACAAGG
TTCCATCAAAGTCAT
TTTGATATGAATGTGGCGAGTCTTTCACTGGCATCTTGTTTTAAA
ATGTCTAAATAATTA
CGTAAATTCAAAATCTCATGATTTTTTGTTTATTTATTTAGCTGCT
CATCCCCACAAGCC
TGCCTGAGCCATGATTCGAATAGTGAAATTTC
>ConsensusfromContig4122 Average coverage: 11.82
GCCTTACTCTGACATGGAAGCGTTTATGTTTGGTTAGACTTAATA
TTTCAGAGAAAGACG
CATTACATATGTCACAGGTATATGACATCCTACCAGAAATTGGAA
ACACTTGGGTTATAC
GTCGAGAACGAAAGAATTCAACAACAGAAAGAGTTTTATGTTGA
GAGTGAGATAATTCAT
CACATGAAAGAGGAGAAACATCTGTTTTACGCTTAGTAGAAGAT
GATTTATCACAAAAAA
GAGGTGACACATTGGTGTGGCGTTTAATATCAGATGATTTATGAA
CAGAAA
>ConsensusfromContig4123 Average coverage: 38.67
CACGGTGTAGCTTACCATGTGAGATATTCATTTTGTTAAATCAAA
GTTATTTCATTATGC
CATATTAACATAAATTTTATAAATGTCAAAATTTGTGGATTTGAA
AGACGAAAGGGTCCC
ACAAGCTCTTTATTGGACTACAGAGGATGTTTGTAAATGGATAAG

AGAAGATTTAGGGTT
CCCGCAGTATGAAGAAACTTTTGCCTCCAACTTTATCGACGGCCG
AAAGCTGATTCATGT
TCACTACTCTACATTGCCAAATATGGGCATCACCGATGAGCGCCA
TATTCAGTACATAAG
TACCAAAGTCAGAGAGCTCTTGTTCTTGGAAAATCCATTCTGGGA
TGTCAGCATGGCAGA
TATCCC
>ConsensusfromContig4124 Average coverage: 11.01
CATCAGCCCAGAATGGAATAGTGTTTCGTGGAGACTATCATTTCC
TTTTCAACTCCGACT
TGGAGAGGGCCTTCCTTGATAAATCTGAGCAAGTTGATCTGAAAC
CGACACTCTTCGAGG
ATTTTGATCATGCAAAACAAATTCGTTTCTTGATTTTTTATGGATT
GTCCATCAAAACTG
TTGCAGCAAACACTTTCAATAAATTTGAAAACTTGAATAAGTTGA
CGTTCATATATTTGC
GAGTCAACATCGAATTTATGGGATATGCATTCGGAGGTATCCCAG
CTTACAAATTCGAGT
TTATCGGTAATGGTTATACCGATAAATCAATCCTGTTCAAT
>ConsensusfromContig4125 Average coverage: 12.98
GACGAGAGTGAGTAGATTCGTTCAATACCAGAAGTTGTTTTTATT
TTTGAATTTAGCCAG
TTTAGTAGAGTCTCCCACTTATAATATGCTTTTGCCGAAACTGAA
ACCATGAGTCCCTTG
TCAAAACGATCTCCATTGCGGAAGAATGTCACCAATTTGCCACGG
TATGATTTATCGTCA
CCCTCGCTGACCGAATCGGACTCCATTCCCCGGTGTGTTAAGATT
CGATCTCCCTCGTCA
TCGTCCCGTACAAACCGCTCTTCATACTGATGAAGAGGCATAGTA
AATGATTCGTGAGGA
TGATCCTAATTCTCCTGTTGCTGATCAGTCATGCGTGTCACGGAC
AGTTTTAAGTGGTTC
TGGGACGCTGGAGAGATCGGAAGAGCACACGTCTG
>ConsensusfromContig4126 Average coverage: 34.13
GATGTATGGAAGAACATTTTATGTGGCCTCTACCAAGCTATCGGC
AGAAGATGAGATTTG
CATTGGAAATATAATGAGTTTCTAACATAAAGATTCGAATCCTCT
GAATCATGGACAATT
ATCATTTACTGGGTCTTATCGGAGAGGGATCTTTTGGAAAGGTTC
ATAAAGGATCAAGAA
AGTTCTCTGGACAGGTAGTGGCTTTGAAGTTCATCCCTAAAGTCG

GTAGAAGTCACAAAG
ATTTGAAAAATTTAAGACGAGAAATCGACATCATGCGCGGCCTT
CATCATAAAAACATAA
TTGAAATGCTGGACAGTTTTGAGACTGATAAAGAGGTAGTGGCA
GTGACTGACTACGCTG
AGGGAGAATTATTTCAAGTCTTGGAAGATGACGGAGTCTACCG
GAAGACCAGGTGCAAT
TGATTGCTGCCCAGCTTGTCTCAGCCCTTTTTTACCTGCACAGCCA
TCGCATTCTGCACA
GAGACATGAAACCTCAGAATATTCTCCTTGGGAAAGATGGCGTTG
TGAAGCTGTGTGATT
TCGGTTTCGCACGGGC
>ConsensusfromContig4127 Average coverage: 17.40
GAGTCGGATGAAAGAGAAGTTTCTAGTGATTTATTGGGAAATTTA
TTTGATTCAAACTTC
AGATGCGCGAATTCTGGAGATTCGAATATTAAAACTGGAGAAGA
TTTGGAGATTGAACCT
CATTGTGAGCCCGTTTTAAGAAGAAGCTCTTATGAATATGGTGAG
CTGAATTTACAAGCG
AATTCATGTGAGCCTGATAACGTAGAAAACAACGCGTATAATTC
ATCAGATGAACAGATT
GAAGATGATATTCTCAACTTGG
>ConsensusfromContig4128 Average coverage: 4.34
GTAAATTCAACAAAAGATATTGATATAAATTCAGAGAAGAAAGAT
TTTTTGAATGGATTA
GAAAAATCCACAATTGAAGAATCACAAGACGGAATTGTAGGATA
TAAAACTGTAACAGAA
CATGATGAAAATTATAAGAACGTTGTAGAACGTTGTGTTTCAAA
CAAAAATAAAAGTGAC
ATCATGAATATAACATCAAATGAGATAGAAGTACCTTCAAGTGTC
ATAAATGAATTCAAT
ACGGCAGGATGTCTTGATGAAAAAACCTCTGAGAATGTTG
>ConsensusfromContig4129 Average coverage: 14.34
CTTGTCTAGAAACAAACTTAGCTATTTACTTCGCGTTTATTAGTTA
TCCCCATTCGACCT
TTAATCATCTTGTTGATTTTTAATCGCTGGCATTCCATCTTTTTTC
ACTCCCATCTTCAA
TGGTATGATGATACATAATTGAGTGTTATTTAATTTTCTACGACT
AAGAAATTGCAAAAG
TATTATAGATCAAATTACCTATTCCATCGGTTAAACCAGAAAAGC
CGCTTATATTCTTCG
AAAGGCATTATCCAACTGACTTCACTTGGATATTTGATTAACATC

ATGGCCAACGAATAC
TCATATGTACAGCAAGGCTTCTTATTCATAGTTCTAAAATAATTAT
AATCTGATTATTTA
TATGAATTCATCATTTGACATGTCATGATTTTTTTCATCATAATTG
TCATCATATTTG
>ConsensusfromContig4130 Average coverage: 7.12
TTCGGATGTACGGCAAGGAGCTAACCCCAAAGGATTTGTCAATAC
TCTGCTTGATATTGG
ATTGAAAGAGGGAAAACTGCACAAGAATCAAAGGTCTAAGAAAT
CGAAATACAAGCCACT
CGGATTGAACGGAAACATGACGGAGGAGGAGCGAATAGCTGCG
GAGGAGGCGGAGATTAA
TGCCGTTCAGAAATTGAGCTTACTTCTGGTGGACGTCGTTCAGCG
AAGTCAAAAACGGGC
CAAGCTCCGGAAAATCGCCGCAGAAGAAAGGATTTGGGTTGTGC
AGATGAACCATTTGAA
AGCCACTGCTTCTCTGATGAACCTGCAATCCAAAATGGAGTTCAA
AAATCGAATGCTTGG
AATCGGGATCGACAAATATAGAAATAACACAATGGATCCCGAAT
GGTACGGCTTTCAACT
ATCAGGAGTTGCCTTTATCGATCATACATTAGACCTTGAACCGTC
CTTGCCGAGTCAAAG
CCAA
>ConsensusfromContig4131 Average coverage: 8.72
AAAAAAAGAAAGCCTTTATCGACATTCTTTTGGCCACGATCGCTT
CTTCAATAGTTTGCT
CAGAGTTGGGCTGTTTGATTGCTCTCGTTCGCAAAGGGTAATCAA
AATCCACTTATCTTT
TCTCAAGTAGCCAGAAATAGTTTCTTCCAACTCTGTGCTGTCGCC
AGATATGACACGAAT
ATTTTGTTCAGCTTCAAGTTTATCATTCCCATTCCCATTTGAATGT
GCAACGTCTTCAGC
TGAGCCTTGGTGAAATTTTGTGTAGCAAACATCGAGCCCTCTGGA
AACCAAATCATCCGG
ATCCAACAAAAGGGTTCCAAGACCGCCATTGGAAGGATGGAAAG
GATGAAGCCGGGCAGC
TGCTCTCAAAGCGATGAGACTCTGCATCGTGTTCCAGTCGGTGTT
TAATTCATACTCAGC
CATGGAGCT
>ConsensusfromContig4132 Average coverage: 40.04
CCGCACGAAATCAACCAGTCGTCCTTGAGCAACGAATGGTCATC
AGATGTGGCGAGACAC

CGATGACCTTGAGCTGTCCTTACTGTCGTGAGAATATTATGACAT
CTATTGAGCGTGTGA
GCGGAAGCTACGTGTGGCTGGCGGCCGCGATTCTGTGTCTTGTTG
GATGCGGTTGGGGAT
GCTGTTTGATTCCATTCTGTGTTGACGGGGCCAAGGATGTCGTTC
ACAAGTGCCCTAAAT
GTGATAAACTCATTGGGAGATGCAACAAGCTGTAAAGAGTTCGA
CGACCGGGAAGCAGTG
AAATTTGAATTGTAAAATCAAGATATACTCATGATTCATACTATT
CATATTGGAGAGTGT
TTGATTCAGTTTATATTGGGTTACAATAATTTGTATTCTAGTTAGC
ATGTCTTTAAAG
>ConsensusfromContig4133 Average coverage: 11.92
TTGTAGTAATAAACTAAAACCGAGGGTAGGGCATCTTATTGTTTC
ACAGCTCGATGGTTA
CAGCAGTTGTGATTGGTCAGGTAATGTTTTGCTTTGATAAATAGT
TACCAGAGGTGTGTC
GTGAATTTTCATGTTGGTATTACATATGAATATTATATTTATATGT
GTGTACGTTACCCC
TCATTCCCACTCGTGTGTCCTTAGTGCTTACTATCAGGTAAATTT
ATTTGAAGCACTGAA
ATAATTATTAATAAGCTAAGTTGTTACTCCGCACTTCTAGCATTGT
CAATATTATTGCTG
AATATATAGCATTGACGTATTTACATGAAAATAAAATTTCATTCG
CTTAATTTTCACTAT
CACTGTTCTAAATTGGTTCTGGATATCATCGATTGGT
>ConsensusfromContig4134 Average coverage: 18.34
GTGCCTCGTCGTTTGAAATTTTGTTTGGCGAATTGCGGGTACGAC
TACTCTTGGATGTGT
TCATATGAAAGGACGAAGATATATGCAATGCAGATAGAAATGTA
CCAATTGGATTCACAA
CTCGATCGCGATTCTCTTGATCAGAAAAAGAGGAAAGTATATTCA
CATCCGCACAGTGCA
ATCGGATAATTTTATCGGCCTCAAGATATACACTTCCGAATGGTG
AAAGAAGTTCTTTTA
TATAATCCACTTGGGATGAAAATTCAGCATCTTTCCCATTTCCCA
CGTCAATGAAGGTAT
CCAGCCATGCTATTATGAATCTCATTTCATGCAACTTCTCCACAA
GAGCAGAAGGTAGGT
AGAACTCAGGGTTAGATTTTTGTATATTCGTAGAAGGAGAAACAA
TGAATATAAGTCTTT
TTCTCTGTTTAAAATTTTTATTATTATTAGATATCATTGCTATATC

AATATCATCATTGA
TTAATCTTTTGTTTTTATAGGATTTTTTCGGCGATCTTTTACATGG
AGA
>ConsensusfromContig4135 Average coverage: 42.33
GCAGATTTTATGCGATGAGACCGAAAAAACTCGTAGACCTTCCCC
GCATTCGAGATCGAG
AAAGCGGACGTCCGGACGTTTGTCGCTCGATCACACTTCGACTCT
TTCACCATATGTTGC
TCCATCGATGATTAATATAATTTCTCAGGATTCTTCTCCAGATTG
CCATATACCGGGTAT
GACGCTTTGTACAACATCTAATTATTCAAGTACATCTGGCTTACT
TATGTCCTCTACGAA
AGCTTCCGAAAACACATCGTGCTGTGCTGTCTCAAGTGATGGTAT
AAATCGTATGAATTC
CTCCGATTCTCTCAATCCGATCTCTATCAAGTGCAATGATGGCCC
GATGTACTCCTCAAA
TGTATCCAACAAAATTCTGCCATCAGTCTCATCGACTTCTGTTAG
AAGTCACGAAGAGAT
CACGCGAAAGAGTTCAGCCGCGAGCGAGAACTCTGCGCTTCAAG
GCCGGAAAAGAACATT
GAACGCGGCTTACCAAGATG
>ConsensusfromContig4136 Average coverage: 37.27
ATCTGCTTCATCTCATAATGCTTCATATTTCAACGAGACTTAATA
ATCATGTAATCAAGT
TTGATTTCAGTTCCCAAAGAAATAAAATCAAAAAATTAAACTGAA
ATGTATGTTATTCCT
ACTCCTTAATCCATAACCCGCGGACCTGACATGAATGTCGGCCTT
TAGCAGAAGATAAGC
GATGATCCGACAATCATAAACAAAAAAACAACAGAAAATAACCG
CACATAAACACATAAC
AATCCAATAAGAGCATAATGGACTCATTATCACTATTTCGAAGAA
AGTACATCAAAGTCA
GAAGTGACACGAACGATATGAAAAAGTATTATGAAGCATGAGAA
TTTGCCACGAAATATG
ACATAATATCACTATCGGATTAATGATATTCAATGGCATAAAGGT
CTTCTACATAGCATG
AATATCTGTTGATACAACATAATTCAAATTAGAAATTTGGTCAAT
TATTATTCAAAGAAA
CTGATGACTGAATAAATGTTTTAAGTCATAATTAATTTATGTCTT
GAATGACGTTAAAGC
AAATATGATAATA
>ConsensusfromContig4137 Average coverage: 6.24

TCGTCGCCTATGTATGCTGGTATATCTCCACACTCATGAACAGTG
AGTCCATAAAAGTGC
AATAACCCAGCTGCCTGCATATCCGCCGCAAGTAGATATGATGTG
TTGATGAATTTTCGA
TATGATTCCGGGGAGAGGAATTTGAGTGGGGTCAGTAGAAATCT
CAGAATGAGGCGTGGT
ATTCCACAAGTGAGGCAAGAAAAAGTGGTCAACACTACACGGAA
TATATAAAAGAATATG
TTCTCCTCTGCCATGCTCGAAAATATGTTATTCCTCAGTCTTCAGT
TTTATTGTTTTGAT
TTGATCGGAGATTGATTCAGAATTCAATCTTTATCTCGATAGATA
CAAATCCGAGGCAGT
CAACCCGAATGGATTCAGAATTT
>ConsensusfromContig4138 Average coverage: 7.20
CGATATTGAGACGTCTAGCTAGTTTGAATCACAATATTTAGATAG
AGCATTTCACTGACA
CAAATCCATACTCAATCATGATCCATGCCACAATTTATTTGGCTC
TCTTCAGTCTGCACA
AGCTCTTCTGTCATTCTTGAAGACGAATTTTGTACTGTTCCCCTTC
TCCTCGCCAGACCG
ATGATCAAAGCAATGCACGCCAAAACCACGATTGCCGAAATACT
GGCAACTGCGAC
>ConsensusfromContig4139 Average coverage: 3.90
AAATAATGTTTCTAATCTATTTGAAAGTTTGATGGAATCAAAGCC
TATGTATGGTAATTT
TAAAATATGAAAATGCTCATTATCATTCCTAGCTAACCTGTCTTT
ATGGTGTGATTTATA
ATATTATTCATAGGGCTTATCATAGATGCAGCAATTCAGGTTTAT
TTGATATTGAGTTAA
ATAATATAAAACAATTATTTTTGAAAAACGGTTATTCTAGTGATTT
TATT
>ConsensusfromContig4140 Average coverage: 200.11
GAATCAACTCGAATGAAGTAGGAAATATAAGTAATCATGGAGAG
TTCATCAACATTCTTG
CTGCACATATTTTCAGGACTCAGTATTGGTGGAATTCGGAAAAGA
CGTTCCGCCAAAGTT
ATTCCCTTTTGACAATTTTCCAGCCAAGTTGTTCTGTTCATTGCCC
TCCAGTTAGAAACA
GATCCAGGTTTGCAGAAATCAATGAGGCCGTTTAATAGGATGCCA
TCGTTCCACTGTTCC
TCAAAATTCCATATGTCCATATCATTAAAAGCAGCTCTTAGCCAA
AGAAGCATGTAACTT

TTTGCATATCTGTCGCATCCTATTTGATAGCGCAGAATCACATCC
CACATCAGGGCTAAT
ATCGAATTCATATCCCCTTCCACGATTTCTTCAACATCCAGAGAT
CGGAAGAGCGTCGTG
TAGGGAAAGAGTGTAG
>ConsensusfromContig4141 Average coverage: 12.85
TGTGTATGTACGATGTCTCAAAGATGGATTGGAGGCACTTTCTCC
ATCGTGACCCGGATA
ACATGAACACCTTCTCCGCCATGGCATGCTTCGAGAACACCGCTA
CGACGTCAGGATTCG
TTGAATACGAGCAGGATACTGAAGGCGATAACATTGCTGAAGTC
TTCACGGGCCCAATAT
GTCGATTGGGGAAACTATCCGATGCTCCAGATATAGAGGAATAC
AAGAACAAAGGCATCG
GTGCAGGATATATGCTGACAGTTTCAACGGGGGATGTCGCGTTTT
CATACGATCCTGTGA
AGTACTGGCTTATTCAATGGAACGCGAAGCTGGA
>ConsensusfromContig4142 Average coverage: 12.00
TTCAACTATTCAATCACTCATTTGAAGTTCTTAACTCTATCAAGTT
TCTTGGGTTCATTC
TAGATAGTAAGCTAACATGGCGTCCGCACATTGAACAATTATCA
TCAAAGTGTCGAACAG
CAATACACGGTTTTGATCGCTTCATCTCAAGCAAATGGGGAATCA
GCTCTAGGGCTGCTG
CATGGATTTATACTGCCGCTATAAAGCCTAAATTTCTCTCTTCCTG
TCACGTCTGGCTCA
CATGCCTAAAGAAGAAATGGGCAATGACTGCGTTACAATCTGTTC
AGCGTCTGGCTCTCA
TTAAAATTTACCGTTCCTTTTCAACCACGTCTTTAGAGGCTCTATG
TGTTATTGCCAATG
AACTTCCAATATCGTTGGAGATTGAGAAGATTATCGCTTTCTATC
CTCTGAAAAAAGTCT
GCATGTCTGGCTCTTCTGGACGTGCTAGACACATCGCTCATTGGG
AGGTTTTTGATGGCA
TTGTTTCAAGTTTAAGTATTCCACCGGTTGACAATTC
>ConsensusfromContig4143 Average coverage: 38.83
TGGACTCAATAATAAACAATAATTTACAAAATCTGACAAAATCA
ATTCTGAGCAACGAAT
CATGAAATCAGACATATAACAGTACAAGATTACTACTGCACATA
TTTACAACAAAACAAA
CGCTCCTTATGAGCGATCATTGATGCATACTATAAAGAGTTGAAA
ATATTTGCGGTTGGC

AATCAAATACAAAATTCCTCACATGAATAATAGTACTTATCAATC
CTCGAAAAAACTCCA
CTGATATATCACTGATACCTAAAAAACCGTATCCCCTAGTCCCGG
ATTTACGTCAATAAG
AGTCTTTATAGAAATAGTGAAATTAGGAATAAACGTCTCACATTT
TTATTTAAAAACTAA
TCAACATTTCAAATCATCGTTCAACCATATATCTCATACAGCTGC
ATAAAAATGATATAA
CTTATG
>ConsensusfromContig4144 Average coverage: 9.78
TACGCTGGATTCTCCATTTTCTTGTTTACGATTACGGAAAAGGCTC
TGACTTGGTACTTA
CATCTGATGACGTCACTTCATGCATATGCATGGCCGGGGCTCTTC
TCGATTATAGTGCGA
CGAAGCGAGAATTCAACATCGATTATCTCAGTAATTTCGCTATTT
CGCTCTCAAAGAAGA
ATCGATGGAAAGAGTGTCTACAAACTGGAAAGCAATTTATGTCGA
GTTTGGATTCTATTC
GAAGTTCTGGATATGCTTCAAATAGCGATTCTAAAGAAGAAGAT
AAAAAATGTGGAGAGA
AAATCCAATGGGGTTCAACAGTCGGTGACCTTTTCTTGGCGCGCA
TCTTCGGGACTCTGT
TCAACGCATTTCTCTCAGATTCCTTGAAGAGTAGTAAATCTGAGG
GTTATCCGGACG
>ConsensusfromContig4145 Average coverage: 17.54
TAAAATTCGAAAAATTCAAGAATAAACATATTTTTCACACATCAC
ATATAAACCGATATT
TATAAAACTTTTCTTCCGATCTGCTCTCTTCGCTTCACCATTTTGA
TATTCATGATGATT
TTATTTGCTGCTAAACCAATGACAATAGTGAGTAGAATCCAGGAG
ACATTCATGACAATA
TCGAAGAACATATAATTCTGACTCGTGAGCCATTTTTGCCGTATG
ACTACAATGGTAGAA
AATAAGACCGTCGAGGAGAAAACGAGCAGAATCCATTCTGATTTT
CTTTCTCGGATTCTT
TCCAATTTTCGCTCTGGAACCATGAGTTCGTAACAAAGAATGATG
TTTTGAATATTTT
>ConsensusfromContig4146 Average coverage: 8.97
CCGCAGTTGAGAGGTGTCTTTCTTAATAAAAATTCTGTAAAAGCC
ATGAAACGAGAAATA
TATACATTGGATCATCAGTAAAATTGGCCAAATAAGCAAAATTGG
TGTCCAAGCTTTTGA

ATTTCTTCCTAGACCAGCGTATGTTTCGTTACCGGCTTTTCTATCG
GCATGTTCTTCCAC
GAGAGGTATCGCAGTAAGACTGAAAACAGTCATAAAAATGACAT
GAAAAAGCATCAGAAA
AGCATATACGAACTGATACGTGCTCCAATAGTCGTCGACGAGCT
GGCGGAAAGGTGTCAT
GTTCAATATCTGATTGGAAAGAATATTTGATTTGATGAAAATAAT
GTGATCAAGTAAACA
TATGCTTCTATCACTGTGAAGTACATCA
>ConsensusfromContig4147 Average coverage: 7.73
CTCCGGTGTTTATCGCAGGTTTTTGACACTCAATCACGGCATCAT
TTGCAATATTTATG
TCAGTCTGTTCAAATGTTAGAGCAGCTTGAATAAAAGTAAGAAT
GCTGTAGCCGGAAGCA
TAGTAGACATTTGTTGATCTTTTTTGATCGATGTAAATTTTCGCTT
GCTCGAATTCATTG
TTCATAAATAAATCCAATGCGTAAATGATCTCCTGAATATCGCCA
TACAGACCATTTATT
GGTTCTACTGCATCTTCGAATGTCTCTTCCTCGGAATCATTATCTC
TTGGGAGTGCCGAT
TCTGTATTCTCCATTTTAAAATACAAATCTAGGCTTTCA
>ConsensusfromContig4148 Average coverage: 15.21
CCATAACACCACCACCTGGGTGGGCCGAGCTGCCGCAGAGATAT
AGACCGCTGATGGGGG
TTCTACATCCGGAATGTTCCGTGGTCGGTCGGGTGAAGTATAGTT
GATCAAGTGACATTG
CTCCTTGGAAGATGTTGCCGCCGGTGAGCCCAAACACATTCTCAA
TGTCTCTCGGAGTCA
AGATATCTGCGCCGACGACACTCTGTTTGAAACCAGGGGCATACA
CTTCGATCCAATCGA
AAACGAGATTTTGTATGCTTCTTTCTGCTCTGTTGTCCATTCTTT
ACCATCCAATAGAA
TCGGCGTATATTGGGAAAATATTTGAACCACGTGACAGCCAGGC
GGTGCCACTGTGGGGT
CGAGACTTGAAGGTATTGTCATCTCGATAACAGGGCGCTTCGAGT
ACTGTCCCCCCAGGC
CGTCGACATATGCGTCGTGGATGAGCTGGCAGTGCTCCGAATTGA
GATGAATGGTGCCTC
TGTGCTGCCACCCGGCTTGGCCAGTCTTGTCATTTGGGTCTGCCA
CGAAGTTTGGTATTC
TGTTCACAGCAACATTTATTTTCGTAACGGGTGATGTGTAGTCGAT
ACTTCTGATTTTTT

CAACAAAATCAGAGCCAAGTGATCCCTCTTTGAGTAAGTGTAGGA
ATGTCGTGTCAGCCG
TTGCATTCGAGAGGACAATATTTGCTTTGATCTCTCGTCCGTCCT
CAAGCGACACACCTT
CCGTCTTTCCATTTCTATTTACGTTTATCCGCGCTACTTTCTGGTT
AACAAAAATTTGAG
CTCCATGTTGTGCAGCAGCGCTACCTATAGCATTCGAGACAGATC
CCATGCCACCTTCCA
CATAACCCCACGCCCCTTTCACTCCTTCCAATTCACCCATCACGT
GGTGCAGGAGAACGT
ATCCACTACCTGAAAGTCTCGGACTGGTCATAGCACCAATCACCA
AGTCTGTTGCGAGAG
TGGCCTTCAGAG
>ConsensusfromContig4149 Average coverage: 6.23
CGCGAATATGATTCTATAATGAAAAGAACTTCCCGGGAGAATACT
CGAGTATACGATACT
CAGTCCAATGAACTAGATGATACAGTTTCCAAAGAAACTTCACTG
ATATGTGCAAGAAAT
GATGGTTTTTCATCGGAAACAGTATCGGGCGAATATATTTCTTTA
ATCGAAGAAGATACA
AGCGAATATATACTATGTGATTCCTCTATGGAAGCAAACTGCGAA
GACATCGTCGTAGCT
CTTGAAGGTCTGCAGACCGACCTAATATCTTTAGGTTATCTAGAA
GACATTGGTCATGTT
TTGCCCGGAATAGGGTCGC
>ConsensusfromContig4150 Average coverage: 9.73
GCTTTAAAATTACAGACAGTGGTGGGGAATGGTCCATATAATGA
AGTGAATCTGAAAAAT
TTGAAAGACGCTTTTGTTAATGGGTTGCAAGATCAAAATGTGAAA
CTAGCAGTGCTTAGA
AGCAACGATAAGGATTTCGCAAACTTGGTCGATATTGCTAGTGG
GGAACAGAAAATTCAG
AACCGCATAAAAGATCGAAAATCTGGACCCTGCCAAATATGTAA
TGGGGATAACCATGCA
GCAAATGATTGTTTTACATTCGTAAATGCGATGAGAAGACAATA
CCAGAAAAACAATGGG
CAAGCAAACCAGATGAGAATTGGGAATTATCCTAGGAATATGGG
ACATAATTATCCTAAA
AATAATTATGCACCTATGAGGGATTCTAGAGGATATGGGGGCTAT
ATTAGGCCGTTCCAA
TACACACGACCACAATTCAATAGAGAGCATACTCCCAGATCTCA
AACGCAGTATAGACCA

GCCTTCACAGGACAAAGGGGGCAATTCAC
>ConsensusfromContig4151 Average coverage: 8.87
CCTGTCAATGCTCCAAAGGGTGGACGGGGATCGTTGTCTTGGTC
TGAGTAGCTCATTAG
AACGTAAATGCGACGACAAAACTTGTGGTCCCAGAACCAAAGAA
TGTGGAGGTACCGGCA
ATGAATGTGTTTGTGAGCCTGGTTGGGTTGGAGATACATGCAATC
AACTGATCTGTGGTA
CGGGTGAATCCACGATCACATGTTTCATGATCGGATCAGAAGATG
TCTGCAGAGAATCTG
TATGTGACTGCAAAGACAACTGGTTCGGAGAAGCGTGCAGCGAA
TTGAAATGTGACAAAA
ACATATGCCACATACCTGGAACTAAAAAAGACAGCAGTTGCAGT
TCCGAGTCGCCAAAAA
CGTGCAATTGTGAACAACACTGGGGCGAGCCAACCTGCAATACA
CGAACATGTGGTACAA
CGACAAACTGCGGTCCTGAAACAAAAGGATGCGACGTTAATAAA
TGCCAATGTCAAAGCG
GCTGGGGAGGAATCACTTGTGCACAACGAAAATGTTCCATAACG
GGTGGAGAATTATTAT
GTGAATTGGGACGTGCATCGGGGTGTCTACC
>ConsensusfromContig4152 Average coverage: 13.16
AGAAAATAGAACGGGCAAGAGCGCTTTCGAAATTGCACAGACGG
CAGCTGCACGATCTCG
AAATAATTAATGCTGGGAGGATAAGTGATCATTTGCGAAGAGAG
CAGACTGCTGCAGCTG
TTAAAATCCAAAGTTTCTGGCGGGCGAAGAAGAGCGAGATGATG
TTCGATCGTAGTTTGG
CCAAGCGACAGAGAGCTGCCCTCATCATACAAAAACATGTCCGC
AAATGGCTTCAGTCAG
TAAAAGACGAGAAAAATACTGAGCAAACTAATGCCAGAGTTGAA
CAATTTTTAGTCAAAT
CTGTGGAAAATAAGCAAATATTGGAGCTCCAGAAACAATTCGAT
CAAATGATAAAAGACC
GAAAGGCCAAGTCATTCCCACGTTCCGAAGTAGAAATTCGAGAA
CGTCACGAGGAAATGC
TTTCGAAA
>ConsensusfromContig4153 Average coverage: 10.72
ATGAGATCAATGTGAGAATATCTGAATTCATCCAGAATTTACCTC
TGAATTTACTGGTTT
TCGAAGACTTCTTAACTATATGAGTGCTTTCAGCCAGACAGCTTG
AAATTGTGCTTTCTA

ATAAGTTATATGAGTTATTGCAGTTCATTTGGATTATTTCAGACTG
TATATGTTGTATAC
GATGTGCTTTTTTCAAAAATTTCTCTTTAGAGAATATAAAATGAT
CTGAAATATCAGAGG
AATGGTATTGCGAAAAGTTGGATGAAATAGTAAGGAAACAAATG
TAGTGATCGGATAGTC
CCGAAATTATTGTTCCTGATAAGCTTATCAATTCATCAGAATGAA
AAATATTGTCAATTA
TTGCTGACGATCTACTAGTGACATGAGTTGGCCCGAGAATAGAT
GGTATAAATGAAAAGG
ATAGAAATATGTTTATCAGTTCGGTTGAATATTTGAATTCCTTGG
ATATATCAATATTAA
AATCGCCACAAAAAATATAGCGATCATTAGGCTGCACAGTTTTGA
GAAAGTTCTCGAATT
CT
>ConsensusfromContig4154 Average coverage: 15.31
ACTTGCCAATCCGCCGCCCTGAAATCAAAGACTTATATATATAGA
CTAGCGGGTTACCCA
GCTTCGCCAGGGTTTCAGTGTAGTCATGACACCACCATTAAAATT
TATAACAATCCTGAC
CCACTAAACATCTTCGACCATCTGATGAATTTCAGTTCGTTTGAA
AATTAACATGAAAAT
ACAAACGATTTGATCATAACTCTCCTGTATATAGAAGACGGTGCG
TAATTAAATAACCTG
CTAGTATACTACAAAACGCACTATATGTCTGATCATAGTCTGTCC
ATAAACTTCAATCAA
AGATATGCACAATTATGTACATTTCATCCCTTCGCGATACCCCCT
TCATGGAAACAACCT
GACCATACCCTCGACATTGTAATAAGGTAATTGCAGCATAACCTA
TAACTAGGTTTATAA
GTTC
>ConsensusfromContig4155 Average coverage: 22.99
CCATCTGATGGACAGGCGGTTTCTTTGTTAACATCTGCTTGACAG
TATGGATTATCAGTG
CAAGCTGGATCGCAGGTTATTGGAGAAGTGTCCTGACAGGAACC
GCCCTTGCATGGATCA
TTCGCTGGACATGTAGTTATCTGACTGCAGTCACTGCTGCCATAT
CCGGTCTCACATACG
CACACATGGCTTTTTAGGGAAGCGTAGCAAACACCATGATCACC
ACAATCATCAGGGGGG
CATGTAGCTCCGATTTTGACGATACCAGCATCTTCTGTAAGTGCG
GCTTGTATGTAGTGA

ATCACAATCGAGAACACGATGAGATTTGTGAAATTAATTGAGGGA
AAATACACTCTTTTA
ATGATTCCGAACTGCATGGTTGATATTTATAGTTAAATAATATAA
TAAAAGTATAGATAT
TTGTTGTTTG
>ConsensusfromContig4156 Average coverage: 16.20
CATCCTCCCTATCGACGGTCTTGATGACTGATATGACCCCGCTTT
TGGGATCGATGGCAA
AGTATGATGTGTCTGAGCTTATGGCATATTCGATTTCATTGGGAC
TTCCGCGGTCTCCAT
CCTCGGCAACCACTTGAAAGACAGATGCACCAATATTGGCGTCTT
CAGCTATACTTGCGT
GGTACGGAAGACGAAGAAAGACTGGTGATGAATCTTGAACGTCT
CTGATGCCCACGCTGA
GGTCTGTGCTAGTAGCCAGTCCGAAACTATCCGATGCGGTGATGG
CGATGGAGTAGAATT
GAGTCACTTCGAAGTCCAGAGGTCCGGCGAGGGTCAACAACCCC
GACACCGCGTCCAGCG
CGAATGTCCTCGGTTCGAATCCAGCCATTGAGTAAGAGATGAGCC
CGCCCTGTTCTCGAT
CAGGGTCCGTGGCGACGACCCGGAAAACCTCCGTCCCCACCGCTG
CATTCTCAGATCGGA
AGAGCGTCG
>ConsensusfromContig4157 Average coverage: 22.76
CTTCCGATCTCTCAAAGAAGGGGCATGTCCTTCTATATTCCCAAA
TGCTCCTGTGTATCC
AACTCTGCAAATGTGGACTAAAGTCTCAATGAGTGAAAATCCAA
GAATAGAGATAAAGGA
TTCTCCGTCCAAACCCAGGCCCTCGAGTTTTTTGAAAGAAGAAAA
TAATATTGAAAGTGA
TGAAGCAACAATTTCGGATGAAGAAGATCATCCATTTTCATATA
CTGACGAAACAAATGA
AACAAACGGGTCTTTGCATAAAGATAACAAATCTTTGACAGACA
TTACAAATAAAAGCAA
CTCTAATACTGCTGTCTCAAGTCCCTCCGATCAAGCCAATGATCC
TCTGCATATGCTATC
CCCAACATGTAACAAATGCAGAAAATCGTTTTCGAATTCTACTGA
TCTGAAGGAACATCA
ATTGGTGCACAAAATTACTTGCTTCATTTGTATGGCCTTATTCAA
AACACTGCGTGCCTT
GAAAAAACACATGTGGGCCCATGATATGATCTCAGTCACATGCCC
AAAGTGTCACATCGT

ATTTTCTGACTCGTATTTTTACCAAACACACAAATGTGAGAACAA
AGGATCGAAATATTT
TTGTGCCATTTGCAAAAAAGGATTCTGCGTGCTGGCTTGTTATGA
AGAGCACCAAAACAT
TCATAAAGGTGAAAAGCCTTATATTTGCAACATTTGTCAAAAAGC
TTACCATGGAACACC
GAAACTACGCAAGCATATCCAATTAGTACATACTGCTAAAATTAA
AAATCCGGTAACTTG
CGATGTTTGCAATAGGACATTTCGAGAAAAAAGATTTATGAAAG
CACATATGATCACCCA
TGCTGAAGAAAAACCACGCTTCAAATGCAGTGAATGCGAAAAAG
TGTACAC
>ConsensusfromContig4158 Average coverage: 70.93
TTCATTGCCTCGCGGGGATTTCTCGCTCTCCAACCCTCACGATTG
GCTACATCATGAGGC
TCATGGACATGACAACCGATGATGCGTACAGATTTGTGAAAGAT
AAGCGATCGAACGTTG
CGCCAAACTTCAACTTCCTCGGCCAACTCTTCGAGTACGAGCAG
CAACTGAAGGCAGAAC
GAGACGATTGCGGTTATTCATTGAGGTCGGACAACACCAAAGACT
GCCTGGAGGTTTCGT
CCAAACGCCAATGCGTCGGAGAGTCAAGACATGCATTCCAGAAG
GTCCCCGAACCCAGAA
GTTGCAGCACGATATTCCAGACTGAATTCCTCTCTCAAACCGAAA
CGACGTCGTCTGCCA
ACTCTTTCTTCTCGGGAACGCCTCCTCCTCCTCCTCACTCGCCTCT
CATAAAGCAGAAGT
ACAGCGCTTCCTCGACGTCACCCATTCCGAAGTTCCTGCAAAAGC
CCCAAATCCAGAGCA
GCACAGCGCAATCTCCGACCACGGCTCTCGCCAGACTCAACTTCA
GCGCTGCTTCTCCTG
ATGCAAGTCCCTGTTGTGAGCGAGACTTGTCTTTCCCTCAAATTC
CAGCTCGGGTTGTTC
ACAAACTGAATTTCACTTCATGTACGACTGATATGTCTGATGTTC
ATTCCATGAAAGCCA
TTCACTCGAGTAAACCTTCGCATTCAGGTGACACGAGTGCGATAG
ATCGGATAATAGAGT
TGGGTAGGGCTGTCAAAAGACCCTTAGATCTCGGCCCGTCTGAAT
CCAGTAGGTTTTCTT
TGGGTTCGCCATACCTCTCGAAGCAAATGATTGTAAATAGGAATA
TATTTGAAACAGGCG
ATTCTAGAACTGGTCTCGATCAAAGTATTGACGCACCAGCATCTT

TAGTTCTCCAATCTT
CACGGAATTCAACCGGAATTACAAATGTCTTTGATCCTCTAACTC
CTAGTCATTCCCAAG
TCAAACATCAGTCTGACATAGAGACTAGTCACAAACCTCAGGTT
ACCGAAAGACCAAAAC
TGGTTTCTATATCTCCAAATACTTCGCATTTCAAGTCGAAACCAC
AGAGTGCGGAGCGTC
CAAAAGCCATCACAATATCACCAATATCATGCAGATTGAAGTCA
GGGGAAAGCAAGACAG
GTTCTTCGCCTGTTGTAATCTTCAAACCATCTGAATGTAGACTAA
AGTCAGGGGAAAGCA
AGTCAATTTCTTCGCCTGCTGTAACCTTCAAACCATCTGAT
>ConsensusfromContig4159 Average coverage: 7.91
GCTGTGAGTCATGACCGCTGTTCAAGATTTTTTCAAAGAGATTAC
AAGACTGCTTGTAGT
CATCAATGGTTTTTTCAATGTCCAATCTGGTGTCTTGTATGCGCT
TATAATGAGATATAG
CTTGTGTTTTGATATCCCTTTCTGTTGCATTCTGCTCACTTCGAAC
TCTTGCAAGTTCCT
TCAGTGTTTTCATAAAGAGAGCTTTATAAGCAATGCTTTCTTTTTC
GAGGATATTTCTTT
CCATGCGTTTATATTGATCAATTTTGACTGAACCTTTTTCAATGCT
ATTTTTACAAAGTT
TGAGCTGC
>ConsensusfromContig4160 Average coverage: 35.71
GCTGGGATTCCTGATTCTTCTCTGAAATGACTCAGAAACTGTTGA
TCCGCGATGATCCTT
CTCTCTTCCCGATATGTGTTGCTGTTATTTCGAAGTTGGTCGAGGT
GGTACCTATAGTTG
ACCATGCAGCCAAGAGACGAGTTGAGGCCATTTTCCGACAGATCC
GCCGCCCAGTTCAGT
CTCCACATGGTAGCTCCATTATTCAAATGAAAGCGAGCGACAGG
ATTTATGGGAAAACCA
GTCTCATTCTTCATTTCGAACAGATACGATGCACACGATCTCAAT
ATAGCCGACTTCAAG
GATTCGCGCAGTCTCCGATTCTCAAACCATGAACCAGATGATAGC
GCCTCGTGAAGAGCT
TCATGAATAGATTCACGAGAGCATTCGAGTTCCTGACACAAAACC
TTTTCTTCCATCCCG
TTGATAAAACTAGAAGTGTGGTGTTCACCGGTACTGAAGTTTAGC
TGTTTCAAAAACCAC
GTTTTGAATCCTGGAATAGGCGACAGAGTAGAGAAAGACGTAAG

ACCATTTCCGACCTCA
GATTTGAGGTTTTTAACAGCAAGATTGATGAGAGATCTTCCTAGA
TCCATACCTTCGAGT
GCTTTATTTGTCAGGGATATCGAATAAAAAACGGCCG
>ConsensusfromContig4161 Average coverage: 24.79
ATTTTTCCACAATGATTGGCTCTTACAGGTGTGTTATCACTATCTG
ATTCATTATCTGTG
TTTAGATAATTATGAGAGTGTGCTTGATCTATCTGAAAAATATTT
TCTAGAGGATTTGAG
GCATAAAATAATCGAATACCTTGCGGAAAACTTCCACACACTTGC
GGTAAAATCGTCCTT
TCTGAATTTATCTGCAGATCTGTTTGAGGAACTGCTCGACGGTGA
CTTTTATATAAATGC
TAATGAATATGTTGTTTTTGATACATTATGGAAATGGCTCAAACA
TGAATCTCGAAATAA
AGAGCCCAGACAGCTGGACTTATTGAAGAAAATCAGGTTTAAAT
TATTGACAATTGACGA
CCTTAATAATCTACCTGCCGATGTTAGG
>ConsensusfromContig4162 Average coverage: 9.20
TCGCGAAGGTCAAGGTTGCTGATGCTGAGGTCAAGATTATCCGTG
CTCTCAACTATATCG
CCGTTGTGGAGGACGCGCTCAAGAAAACCCCCGGAAATCCTGAC
CTTGAGGACGAATTGC
GGAAAGCTAAGGAAGAACACCTGCTGGGGACGCGGAAAAAGA
AGAGGCAGAAGCCGAGC
AGAAGCTGTCTGAAGCCCAACAGCACCACGCCTTCTTCCTCAAA
GAACAATGGGAAGATC
GCGAGGAACGAATTGTCTACCGCAAGGGCACTTTCTGGCAGCACT
GCGCCACGTATCTTC
TGTACTTTGTCGGCCTCATGAATGTTTTTGTGACCACCTTCGGAA
TTTCGGGTGGGAAAC
ATAGTTCTATAATATCCCACAATGATACATTTACATGAGTTCGAA
TGCGG
>ConsensusfromContig4163 Average coverage: 26.42
CTCAATGGATTGTAAATTTCGAAGGATATCAACAGAGTATTGGA
AAAACCCCAGAAAAGA
TTCACTCATCCGCTCAACACATAGAATGAAGAACCAGTCTTCGCG
GATGACTTTGTCAGA
GTCGCATTTGTATGAAGGCACTTTTGGGTTCATGTTCTCTTGGTCA
TGGAAAATCTCCTT
ATTCTGCTCTGAACAATCCTGATCGCACAAACAGTCGATATCGCA
GAGTCCAGAATGTAC

ATCACAATTACAAGGACCGAAATTTACATAAGCCGGGTTCTTTAT
AATACTCTCCTTCTC
TATATCAGCCAAATCTCGTGCTTC
>ConsensusfromContig4164 Average coverage: 6.11
ATTTAATGTTGTTAATGTTTAATATGGCTGAGTTCAATATGTTAA
TTAAAGTGTACTAAT
TTGAAACAAATAAATGCCGTAATTTGCTTGTAGTTGAAAATCACA
ATCACATAAAACTTG
TGAGCATGAGATATGGGAGATACAAATTTATGATGTATTTCAGAC
ATCATTTATTGACAA
AAATCGGTTTATAACACTTTACATTAAGGACCCTTAAGGACCAAA
GGACAACGGTGTCCA
GAT
>ConsensusfromContig4165 Average coverage: 22.65
AGATGAGAGAGAAATTCTTCAGTATCAAATCGTTTACCAAGCATT
ACTCTTCTTTTTGAT
TCGTCGTACAATTCTGGGTATGATTCTTCTGCTCGTCTGCCGAGCA
TTATGCGTCGTTTG
TTTACATTTCCCCATGGTGTTTCGAAGCCTCCGTGAAGGTTGTCTT
CTGATTCGCCAGCA
TTCGGGTACGACCAGCGAAGATCCTCAGCAGCATAGTACGGGTT
CGACATTCCAGAATAC
ATTTTTGGAAACGACGATGATAATATGGATGGTGTCTTAGGTTGT
CCAGAAAAACCAGGA
GTGTGTTCGCATTTCACATGTTCTGACAGCACACATATGCAACTG
ATGATTCCATAGACC
GTCAGGGAATATTTTTTTGATCTCGACATTTTCTGTATATGTTTAT
GTATATCTTAATTT
AAAATAAATCTAC
>ConsensusfromContig4166 Average coverage: 17.15
CGACAGGATTCACGGCCGCCATGTGATGATTAAATTTCAATACAT
AAGCCGTGAAGTATG
AATGATTCACAAAACAGTCTACGATTTGATGGATATGAAGTTATA
TTGTCAAGACACTAT
ATATCCGAAATAGTGAAGAATGAAGCTGATTTCAAGAAGCATAT
AAAAAAATGTGATGAA
AAATCAAAAGAATTTGATGATCATGAAGAGTGTTTATCAAAATTG
ACATACTTTATTGAA
CACTCAGAATTTCATGAGCAGGATGTGTACAGACATATTTTAAGT
ATATGTGTGAAGCTT
TTGAGAATCGA
>ConsensusfromContig4167 Average coverage: 34.93

GGTGCAAAAAAGACAAATCTACCTTCTGTGGAATATGGCTCAGC
TCCGAAATTCAACAGC
ACTGAGCCAACCGTCAACATCAGTGCAGAAAAACTGGGCTGGAA
AATTTCTCCAAGAAAT
CCAGCTGCTGCTTCTGATCGATCTCCGACGATTAATGAGAATGA
AGAGCTGAAAAATGAG
CCACCGAAACGAATGTTCAAGGGATACGGATCACTGCCGAGAAA
AGGAAAGGCGTCACGA
GACATGAGTCCGGTTGGGGTCAGGTACGAACCCAAAGAAATGCC
CCAAGGAAATCCTTCT
CTCCTTTGTTTGAGAGAAAGCTATGTATGAACTGCTTTGAATTAT
ATTAATCATGTTGAA
TTTA
>ConsensusfromContig4168 Average coverage: 307.62
GCGGTTACTGCGGCGCATTGTATTGATGAGGGAGAACACACTGTG
AGTCGATACACCTTG
AAACTGGGAGCGCACAACATCGGTATCGAGGAGCCAGGCACCGT
AGAGGTTGCTGTCAGT
CACGTCATCCTACATCCCAACTTCACTTTTCGGACTTCGCCATAC
AGAGTATCGCATGAC
ATTGGATTAATAAAGTTGAACTCAAACGTGGGTCTGACAGATTAT
ATCCAGCCTATATGC
CTCCCCTCAGAACCGAATGATGGTCCTCCTAATGAAGAATGTTAT
GTGCTCGGTTGGGGT
GATAAGTTCGTTAATGATCGAGGCAGAAGTGGAATGCAGGAGAA
AAAAGCCAAGCTCTTA
TCGGCTGAGTTCTGCACAAGTCAATATTTTCACTTCTATCAATGG
TTATTGGACAGTGGA
TCAATTTGCCTTATACCGGATGATTCCAGCAAAATTTGTCAAGGG
GATTCCGGTGGGCCG
CTGTTATGTCAGAAAGATGGGGTTTGGCAATTGAGATCGGAAGA
GCACACGTCTGAACTC
CAGTCACATTG
>ConsensusfromContig4169 Average coverage: 28.32
TGCTCTGTCACGGCAGACACCGGTGCCGATGACTGTGTACCTTCG
CCTTGCTTGAATTAT
GGGACATGTATCGATACAATTGGTGGATATGAATGCATATGCCA
AGGAGGATACACCGGG
TCTCGATGCGAGACAATAAAACCGTATTTTGCATGTTTACAATCA
TTTGGAATGTATCCC
ATTGAGAACATCATGGAGTTCAAGACCAATGATACCTACACATAT
CGTTGTCTCATGCAA

GCTGCAAATCTACAAGTTGTAGGATACTTCAAAGGTGATGACACA
TTCAATATGACGGCA
GCAGTAGCAAACAATGCAACATTCCTGAGGAATGCAACAAGGGT
AGTCGGCACAAAAAAT
GAAACACATTGCCATGGTGCGTGTGGAAGTGACACCTCGTGCAT
GTTGGCTACATGGAAA
TTTGACCAGAACAAGAATAGGACCAGATGCTGTGAATACTTCAT
CAACGATGGAGTTGCC
CGAACCTACGAAGTGGCTG
>ConsensusfromContig4170 Average coverage: 9.42
TTGTGGAGAGGAATATCTTTGAGAATAGATTAAGTTTATAGTGGT
TATCAAGAAGAAACA
TCTCTAGAATATATTTTCCCGCATTCCTAGGAAATTTCTTGGTGG
GTAATGGATAAACAG
AGACTTTCGAAGACCTACACAACAAATCAGTACCTCTTACAATTA
TCTGAGTTTCAATGT
CTCCAAAGGTACTTTTGACGATTTTGAAAGATATGGCATCAGTTA
AAAAAGATAAATTGT
GGACAGTTTTGTAGGTATGCTTTTCGAATATTTGTTCGCAATTATT
TGTGAAATCATGAT
TGAATTTCGATATGTGAGCCAATTTCTTTTCAATCCTTGGTGTCTT
TTTTGGAGTTTCA
>ConsensusfromContig4171 Average coverage: 16.17
CCGGGGCAAATTGGGAAAGGTACGACAAGTCATTCAGGCGGTTG
AAAGCAACAAATGGTG
GTTTGTCATATGGTGATCGACATTGGGAGCTTTGGCACGAGGCCA
TGGCATTCAGGCGAC
TTGCAGACCCAAGAAGTTCAGCCCGGTATAACTCGAAAGAGGGG
GATAAACTTCCAAAAG
GCAGATGCTGGAGGAAACTCAGGGGGGAGCGATGTCCAGGATGT
AACTATGGGTGCCCAA
GACCCAATCTTCGTCATCAGTCGACAAATTCTCATCAGCAAGGAC
CACCTATGAAATCTG
CAAAGAATATTACTACAATCAATAAAGTTGGTAATCTGCACTCCA
TAGATCGG
>ConsensusfromContig4172 Average coverage: 19.49
TCGGGCTTTTGCACAAATAGGGGTCGGTTGTCGTATGTCACCATT
GGCCATTCAATCTCA
ATCTGTGCTGCGAGCGCTAAAGTGGCTGGCCTCACCGACAACTCA
TATACATGTGAAATG
TTCATACCGCCAGCTTCTTCAGCATGTGTTAACTTGGTCAATGAA
GGGTACGCGTTTCCT

CTGAATTCAATTTTTGGCATTCGTAAAACTTCGATCAGCTCAGAT
GCTGTTTCAACATGA
CCGGATAATATGGCATGAGCTTCTATTCCGATGTTTTGGATTTTTC
CATCATTGCGCTTT
GGTCGAATTTCCATCTGGATTTCAAATTGTTTTTCATTCAGAATTT
GCCCATTATCTTCT
GAAAATTCACAGTGCACTTCAGTATTGGCGACTATGAAGCATTCT
GGAATATCAATTAAA
GGACAGTCCGTGAAAGGCTTGATGCTCCCCGGCTCAATCGATGCA
TTCGCCACATTTATT
TGGATTTTCCAAAGTTTCATCGTACAATTTTTATTCGTAAGTTCC
ACAACGATGATCACA
TTTTTCGGTGATTCCTCACTTATATTTGATGGAGATTGGATGGTG
AGCACAACGTCCGGA
GTATATGGTTTCAAACAGTACCCGACAAAACTTGTATTGAAATCC
ATATAGAGAGGCCTG
ATCCTCGGGTGCATGGT
>ConsensusfromContig4173 Average coverage: 12.07
GGCCTATGCCCACAATGCCCGCTCTATTGTTCAGGCTACTGACGT
CGGTGCTTTTGTCCG
TTATGTGTGCGACGAAGGATACGTTCACGTCGAGGGTGACATGG
AGAGAATTTGTCAGGC
CAACGGACATTGGTCCGGCCAGACACCCATTTGTGCTAAAAACGT
GGATTGTGGTGAACC
AGGAGATGTCATGTACGCCAAGCGGAAAGGTCGAGCGACAACAT
ACGGGAGTCAAGTGGA
GTACGAGTGTAGCAAGGGTTATCGGATGGCCAGCAATGACCCAA
AGCTCACGTGTACCTC
GTCGGGGCAGTGGGAGGGAGTTCCCCCCGTCTGTGTTCGAGAGA
AGCATCGGTGTGGCGA
CGGGGAAGAAGTGCGGAATGCGCAGAAGCGACACATCGGGGATG
TCCTTGAATACGACTG
CGACCCTGGCTATCAGATTTACGCAGGGGACATGAAACACATTT
GCAAAGAGGCGGAGAC
CCACTGGACGGGGAA
>ConsensusfromContig4174 Average coverage: 8.34
CCGAAATTCTTCATAACAAAGGGTTACTTCTCGATTCTGATGAAT
TAATACATGGCACGA
TGAGAATGAATGGCACGATGAAATTTCTTAAAGGCACAAGCCACT
TAAATTCCTTCCACC
ACAACACGAACTACACAAGATTCGCGCATCCAAGACTGGTTTCGC
CGCTGAAAACTTCCA

CGGACCCTCTGATAGTACAGCGGTTGTCACTCCACTCCATGCTCA
CTGTACCGTCTTCGG
AAATGACTGCATCGACTTCTCCGTATGTCTGGATATGTCGCCCTC
CGCATACACTCTGGT
TCCTGATCTTATAGGTCCCCGCTTGCAAACCTTCCGCTGGCGTCC
ACGTGGCGTTGTTGT
ATTTGGTCTGTCTCAATGTTGCGATTCGTCTACTTATTTCTGTAGT
AGTCGTTACACCCG
CTTGTCGATCGCAACCATCAGGCTCTCTGAATGCACTGCATGCAT
AAGTAGCTCCATTCT
TCTTGATTAGAATCATGCTTTTCGCCCAGGCATCACTGCAGCCA
>ConsensusfromContig4175 Average coverage: 23.43
GACTTTTGACTCGTTTGTTTGGGAATTCAAATCAAATGAAACAGA
AATAGGGCCAGTGTC
TGACATGAGAAGTGAAGGACTGCTCATGTCGCCATAAGTCAATAT
TCAGCAATTTGAGCC
TGAAGCTATGCCAACTAAAGCACAATTTCATGTTGTTCACGTTTC
TGGCCAGGATGATAA
GTATAAAGCAACTGAGCTGAATCATCATGGACCAACTGCCCAGG
GATGGCAAACTGGAAG
ATATTGTTTATACCCTCAAGACATCGTTGTGAAATTAGAAAAAAA
GACACGATTACGAAA
GCTCCAGATTCTTTCTCATCAGTATCTTATTGGCTCGAAAATTGA
ACTCTACGTGGGCGA
TGTTCCCGATTCAAGGACGGTCAACGTCAACAATGCAAGCTTCTC
AAGACTCGGTTATGT
AGAGTTGTCGGACAATGAGAAGACCAACTTCAGGGCCCGAGAGC
TGAAGTCTGTTTACGT
GGATGCCGTTGGAGTGTTTGTGAAATACGTCATCCATAAAAACCA
TGTCAACAAACATAA
CCTTTACAATCAGGTAGGGGTGGTCGCACTCAACATCATCGGGGA
TATTCTCGACAAATC
AGAACCGGAGATTTCTAAAATAACCGATGAGACACGAGATCCCGC
GTTGGCTGGTTTTC
>ConsensusfromContig4176 Average coverage: 12.08
CTGGTATCTATATTTATATTATAATTAATATATTTGGAATGTTGTT
TTTCTTTTCTCACA
ATTGTGGCATGTTGTTGACATAAAACTTTATTGAGCGAATCTTTT
AAAGGATTTTTTGAT
AATGTATGTAGGAATTCTATACTTATATCTTCTCGTGCTGCTGATT
TTTTCAGGTCACTC
CAGTAGTCTTGTATATTGTTCGAGGGGTTGTTCATTTTTTTTAGAT

GTTTTAAGCATTTG
ATCAATTTGTTTAAATAGGTCGTCTGCTGCTAAATGATCATTTTTA
TATATCTTCTTATC
TATTCGAGAATCACTGCTGAGTGAATGTTTAGATTGATTAATTTG
AA
>ConsensusfromContig4177 Average coverage: 10.82
TGACTATAAATTTCTCCACACTCCTCGCACGTATGATTTCTTAGAG
ACAGTTTCGAATTT
GTATGACATGAACGTTTATGTGTTCTGAGAGAATCTGATTTAACA
TACTTCTTTCCACAA
TTCTCGCACTTCCAAGGTTTTTCCCAGTGTGGACACGCATATGA
GCCACGAATCTGGAT
ACGGCTCTGAATGACTCTGAACACATATCACATGTATAAGGTTTC
TCAATTTTATGGGAT
TTTTGGTGATTTGTGAGATCATGTAAGGTTTCGAATGTTTG
>ConsensusfromContig4178 Average coverage: 11.46
ATGAGACCAGAAGCGCTCCCAAAACCCTATTGGCGTATAGGGGG
CTGGCGAGCCTGCTCT
GAGAGCTGTGGCTGGGGGACGGAGAAGCGGCTTGTCTCCTGCTA
TCAAGCCCAGAGGGAA
GTCGAGAGGTCGCTGTGTGACGGAGAGAGTCAACCACCAGAGTA
TCAGAAATGCCGGATC
GCATACTGTGCAGAATGGAGGTTTGGCCTTTGGGACCAGTGCGC
AGGTGACTGCAAACGA
GCCGTTCGCCAGCGTCTCGTCAGATGTGTGAACGAAGGCAGTGTT
ATCCCTGACGAATA
>ConsensusfromContig4179 Average coverage: 5.65
CGGAAAGTGATCGAAGTGATTCCTGTGAAAGATGCAAGAGCCGG
TCAGACAAGTGCTGGA
TTCTACCGTATATTCATCTGTATTTTTCAAAGACGATTCGACCTCA
ACATCCATTATGAC
GTCATTCAAATTTAGGTTCTCAATGGCCGATTGATCAACAAACGA
AAGAATTTTCGCTGA
GCCATAAGACATTGTTTTATTAACAGGGGTTGATCTTTCATTTGAT
TCATATTGATTTTG
TT
>ConsensusfromContig4180 Average coverage: 6.61
ATGCTGCGAAAGTTGCGAACAAAATGGTTACTCGGGTCCACGTC
TTGAATGTGTGGGAAA
CTTGGATGCGTTATTCCCTTACAAAAACGGATATGAAACGGTTTA
TCTTGACTGCGAAGG
CATGGAAGCTATGGGAGAGTGCAACAAGAATGAGAATGTTGGCC

GTTATTGCTGTAAAAC
ATGTACTGATGACATAGACCTTCGCTGCGAAGACACGCCAGGAA
TAGGATTCGGTGCACC
CAAGCCGAAGAATTGCAGAGAGTCGGCAAGCGAGGGCTATTGTG
CTCCCATAAATGTTGA
CGTTGTCAAATACTGCTGTGCAAGCTGCCTGTTGCAATCAGCAAG
AGAATCATATAACAA
TAATTAAGACATTG
>ConsensusfromContig4181 Average coverage: 8.54
AAAACAAAATTGAATGAAAATGTTAACTTGTTAGACTAAATTGA
GCATACAAAATTCAGG
TATAAAAAGCTATACTCATCACTAGTTTCCATAAAACTCGTGTAG
GACAGAGATAAAATT
ACATTCGTTGTCATTGTAATAGATTATTAAAACTAGATCATGAAT
AAAGCATGACCTTGT
ATTCGTGATGCTATCATTCGAAATGTCTACAATACAAATTGAAAA
AAATTCTTAGATCGG
AGTGAAATATAATAATTTTCGAATAAAATACACGAAAAGCAAAC
ACGAGATGAATCAAAT
TTAAAGCCATTAAACCATATAATTACAGTATCAACAATAACAAAT
AAATAGTTTGGTATA
ACATAGAATTGTC
>ConsensusfromContig4182 Average coverage: 30.16
CATCAAACGAATAAATCTGCAGGAGTTCAAGAAGAAAATCCTCA
ACATTTCCAGACACTC
AAAGTCGCTCAAACCACGATAAATAGCCTCCAAGCTCGCCTGCA
GAGCAAAGACAAATTG
ATCGCCAACTATGAAAGCATGCTCAAGCATTCTAATGAGGAAAT
TTCAGCTCAGAAGAAA
AATTATCAAAACGAGATTTTGGATTTACGAAAACAGCTTCAAAG
CAAGCGTGGAACTGAC
TTCAATTCGCTCAGCAAACAGTTGAGAAGCAATTTGAAAGAAAG
CAAAAATCCACAGTCA
TTAACTCAGGAACAGTTGCGGCGATTGGGTGAAATGGAGGAGAT
TATCTCGGAGCAGGAC
CAATCGTTGGCCTCCCTTCAAATGAGATTAGCTTCGGCCAATTCT
GAAATTGCTGGGCTC
AAAACCAAGTTTGACCAAACACTACTCGACCACGCTTCCAAAATT
GATGAGCTTCATGAG
AATCAGAGCAAAACAGTGGCAGATTTAGAGACGGAGAAGGGTCA
ACTTGAAGAAGAAATT
TCAGAAAAGAATTCTG

>ConsensusfromContig4183 Average coverage: 8.95
GCACCAAATTTTTTTGAAGTTTTCAATACAACTGTAGTAATAAAA
AATAGTCAAAAATCA
ATAAAAAAGTGTTTAAAATAAGAAGAACATGATCAAATCACACG
AGTTCATCATCGGTTT
CATCATTCTCATCATGTTCTTCAATGACGGATTTTTCAGTTGCTGG
AAGATTATTGTAGA
ATTCCTTATATTCAGGAGGAATAACCCCAGTTTTCAGGAGAGATA
ATAAATCGTTCTTTT
TCAGCATCGATATCGGCAATCTACTCGTGTATGCGCGTTCCAATT
CATGACTGCGAGGAT
TCTTATTTCGCCCTCGTTCATTCTCAATTTCATGAAAACATCGCTG
CTTAAATGATATTT
ATATTGAATCGTGT
>ConsensusfromContig4184 Average coverage: 9.62
ACAATCCTCTCATTCAGCCATTCAAGAGGGATTATCCGGAACTTA
TGAATTAATTAACTT
ACGTAATGATTTATTACGCCCTTATGGCGATGTCTCAAATTTTTC
ACTGGATGTATATAA
TTTATTAGGTGAACCAAATATTCACATCAATGGTCCCGAAAATTCA
TTTTATTACTCTCT
CGATAATTATATTACTCATACCAGAAAAGAAAATAAACTCAATAT
TATATCTATGAATAT
TCAATCCGTAGGAGCTAAATTCAAT
>ConsensusfromContig4185 Average coverage: 11.40
TCCATTCCAGCATCAAAAGCACAAATGCAGATGAAGAGAAAGAG
TAGAAATAGTGAAAAA
TACTTCCTATTTTTCCTCGGATGAATCAGTGACATAATAATGATT
CAATATTGGATTCAA
TAATTAGCAATGATTTTAATTGATGTTTGGTTTCAAACGTATATT
CAACACATAAAACAA
AATCTACTATAAACAATATCCGATATAAAACGCTAGATTTGATGA
ATTCAATTTTCACAG
ATAAGATTGAAAATCATAGATTTCTGATTGTGGGAAAGTAGAAA
ATTTCTAATCGCACA
TCCACATCTAAAAATAAGTACAAAATGAATATATCTGATGAGAA
TTGAACATGAATATGA
CCATGAAGGTCATAAAAGATATGAGAATTGAGTACATTGATTCTG
CTATCTGAAAGTTAA
C
>ConsensusfromContig4186 Average coverage: 43.66
CAATAATTTTTGTGAAATATGAAAAAAGAGAGAGACTTTTAGTTT

TGTATGGATATAGAC
TGTTCAATTTTGTGTTTAAGTCTATATAATCAGGCGAATGGCGACT
CCTAGATCTTTATC
GGCCAAATATGAGAGTGAGTTCGCTTACCACACAGTCAAGAATCG
GTTGCCTGTCATAAT
AACAAAAGTCATTGATCATCTAGTCAGATTCTTGCCAGTCCTCGA
AGACACTGAAGATGC
GTCAAAATACAAGAAAATGATCGGCGAATTATCCCGATTGAGAA
ATGAGGTCATGACATC
TAAACCAATCGCTCCTCTCAAGGACATGGCTGACGATGTCGGAAT
TTGGAATTCAGAGCT
TAAAGAGCGGA
>ConsensusfromContig4187 Average coverage: 11.86
CGGCTATGTACAAAAAAGCGGGAATATATAAAAATCTCATCTAA
AATATAGTAATACGAA
ATAAAATAAATAAAATTCCAAAACAAAATAAATTATTTGATTGC
ATGAAACAGATCAATT
GTTCCTAGCCATTTTTTCAAGTACTTTGTTAACCCAATTTGATCG
ATAGAAGAGCAGATC
ACTTTGATATCGCTTCGATAGTTTCTGTTATCAGCAATTATTCTT
GAAACAGAGGATATT
AGCGAGTGCAGATGTGTGATTTTCGATTCTGTATCGCATCCAACT
GCATAGGAATCAGTG
AGTACCTCCAGAGCTTTTTCATAGCTGTGCGTAGACGACAAAAC
GAAGAAGAATGCGCTC
GATGAGTGCATGAAATCCATACGAGAAAATGGAAATATATCAGA
GTTCCAACCACTTAGA
AATGTGTGCACCGAAGAAAGTAAGGCATGCAGATCTGAATGATC
TGAAATCGAGGTGTAG
CTTGCCAAAGTCATCCGCTTATATAGGCAACTAGTGATGGTTTTC
TCAAGACTTCGTTCG
GATTTGTCATTCTTGCGGACCCAAGTAATCCTTTCACAATAAGGC
CACACAAAATCCGAT
ACATCATCGGTCTCTTGATATTCCAATGAAATCACAATTCGAAAG
TCAGCCGAAATATCA
ATACTTTTATCACATATAATTTTCATCAGAATCTTGTTCAATCTGT
TTTTCTG
>ConsensusfromContig4188 Average coverage: 6.02
ATATTTTCTAGGTCTTCAATCTTGGCCTCCTCGGCATTTGAGAGTT
GTTGTTTTTTCTCA
TTCAGCATCCTTTTCATATCCGCCATCAGTTGACCTACCTCCACG
ACAGACCGATGCAGC

CCACTCAGGGCCGTTTGCAAATGGGATGGCAGTCCTATTCCGTTG
GAATTAGCTGAGTCT
GGATGCCAGATCATCTGTGAAAAAATATCATCATGAATCATCCTA
GATGAAGTATTTGGC
AGACGATCCATTATTCGCGAAACGGCATAGCATTCGTCCATTATT
TTCACATGAACTTGT
TGCATCAGCAAATCGAATTCCATGCTGGAGTCACG
>ConsensusfromContig4189 Average coverage: 9.35
GATCTTAAAATTTATCAATAACAAAGTCGTATCAAAACAAACATA
TAGTTTGGCACTCAT
GCCGAAAATATACCGCTTCAGGCACTATATGGTTTAATAGAGAAT
TTTTAGGCACTTGTA
ACCAGCATGGTCAAAATAAGAATTTGTCGGACAACAAAATGATAT
GTTTTGAAATATATG
TACAAAAATTTTAACATGAGCAATTTGTTGACAAAATTTTAGAGA
TCCTGGTAAATTTC
>ConsensusfromContig4190 Average coverage: 12.51
GATCTGACACCGGTTTCGAGTCTTCTGTATCGCAGTATAAACACA
AGTTTGAATCCGACA
TGAAAGACGACTTAAGTCGGGCTCATTCCAGAATTCAGTCGCTT
CAAGAGGAGCTAATGG
AATGTACAACGACCTTGATGCGAATGAAAGGTCAAAAGGTCAGT
ACAGAGCACGAGGCCG
TGTGCAGCAAGAGCGAAACCGACCAATTGCTGGACACGATCAAA
GGTCTGCAAGAGAAAC
AGCGCCAGAATATTGAAGAGATGCGATCGGGTGAGCAAGAAAGA
AAACTGCTACAATCAT
ATCTCGAGGACTTGAAGGATCAGCTGAACTCTGCAAACGCCAAA
GAGAGCATCGC
>ConsensusfromContig4191 Average coverage: 12.98
GAAACGTCAATTACTTCATAACCCTTTTCAGCTAATTTATGTCAC
ATTCAAGGTCTGAGA
TTTGCGCAGAAGCTTCAACTCTAAGCATAAACTCTGATCATAATT
CGTTTGAATTTCACC
AACGTATAATCGAGATTATCTAGGCAATGAAGTTCGGTTTTGTTT
ACATTCACCATCTCG
TAAGAATTTTATTAAAATGCAAACGAATTCGAGCACGAGATGGGC
GTGTTCGAATATGTA
AATA
>ConsensusfromContig4192 Average coverage: 31.81
GCAGATTTGAAAAACGAGGTCAGACTCAAACAATTCGATTCGGA
ACGAACTCGGTTGGTG

CACGAAGAAACTGTTCGAAATCTTCAGATTTCTCAGGCTGAAAA
TGAAAAATTGAACGAA
AAGTTGCAGGTCCTGAACAAGGAATATTGGGCGCAGCAGATGTC
CCTCGAGAAGCGATTA
GCGGAAAGCGACACAGCGCTGGGCGAAGCCAAAGTAAAGCTGGA
GTCATACGAGCGCCTT
GAAGCGCAGCTGGACGACGTCGTGTTAGAGGCCGCTCAGATTGA
AGATGAAGGGGATTCT
GAGAGAGTTCTTTCTGCATACGGATTTGG
>ConsensusfromContig4193 Average coverage: 14.99
TGAAGCTAGAAGTTTTAATGGTAGATATTTAAGATGTTGTGTTTTA
ATTTATTACTCTTG
CTAAATTATGTCCTATTGGATTTCTCGCAAAACATTTCTCTTATA
AAGAAATACAAGTAT
CAGCAGCAAAAATGACGCCAATAAAGCTGGAAGGTTTAATGGTA
GATATTAATGATGTTA
TGATGCATGATCTTTCACATGAAATTAAATAAAAATGTGATAAAA
AATATTATTAATACG
TGATAAGCGTTATCATTATATTACACATGCTAT
>ConsensusfromContig4194 Average coverage: 3.52
TGGAGAATGCTCCACAAATGATTGTGCAAATATTAACTGAGATTT
GACCAGGCTTATCAA
TCTCTGAATAGAGCTGGTATATGTTTATCATACAATTCGACACTC
ATCAACATTGACAAA
ATATATTTCTCATTTGATGATGTGGTGAAGTCATGGAAGGAAAGT
ATGGAGGTAGACCAT
GTGGAGCATTTTTCATCATCAGGTTAATTCCTAAGATCGGAAGAG
CA
>ConsensusfromContig4195 Average coverage: 11.40
ATCTATTGGAAACGGCGATGATAAGACCACAGACACAGAATTGA
GAAGTGGAAGTGAAGA
AATAGTAAACCTCAGGAAATTATCACAACAAAGCTTAGACACTA
TGATATCAGGGAATAT
TTATTCATGCGATGATCATCATGATGACGAGAACGATGGACGTC
GTAAAAGCCATGAGAC
AGATCGTGTAATCTCTAAGGATTCGCCGAAAAAGAAACGTAATTT
CTTCGGAAAATCTTC
ATGCATTGAGGATTACCAAAGGACGCAATCTCACATGCCTTCAAA
GCGAGCCTCTTCGAG
TTGTCAACGAACTCCCAGTGGGATAAAAGCATCCAGCAAAGAAA
CTTCTGCGAAGGAAGA
GAAAAAAGGAACGAAAACATCCTTTTCAGAAATCTCATTGGATG

CCTCTGAGAGTGGGCG
TAGCCTTGAATCACGTGATACAAACAGTTTTGTTACGCAAAAAAT
AAATTGTTTGGCGGA
>ConsensusfromContig4196 Average coverage: 16.78
TTCATTCATACATTTACATTAAATATGGAAAACACCTCAATCCTC
TACGAAACCTTCATT
AGGACACATCATGGATATCGAGTTTTGCCTGACTGGAGGCGAAG
GGACACAACTACTGAT
GCTGTTGACTCTTAGCCGGACCTGACTTTCAGACCTCGGTCGTGC
GTTGGAGGCTGGTTC
GAAAGAGGCCTGTCTTGTTACAGTGCTGTTCGAAGAGTTTGATGA
TGAAGTCGATCTTGT
TGAATTCCTGGATGTGTTAGGTTCACGCTCCCGGGGAACTCGGAC
GATCGATCTAAAGCT
TTCTGGATCCACTGAGACATTGGACCTGCGATAATGAATCGTCAT
CAAGCCCCCGCCAAT
GCCCCCCGTTATGGTTGCGGCTATAGCGGCTAAATACGACCACCC
CAGCGTAGAAGTGTC
TGGATAGCCTTGCATGATTCCGTAGAGGGTCACTCCTACGATTTC
CAGTGCCACTGATAT
CACGAATGAAGAAGTGGTGATTAGGAAAACATGAGAGGCGAATT
TTGTTGACATTTGAAA
GACCATCTGCACCAACGCAAGAAGAAATGATGCTAGTTTTGCTG
CGAGGGAGAAAGTGTA
TAC
>ConsensusfromContig4197 Average coverage: 16.76
GGACGATTACAAAGTTTAGACCGCCTGATTGGCCGATTTTATAGG
CGTTACGTTGATGTC
AATCACATCTTCTGAGAATAATTAATTCAATAATAATTTTTCAGT
GATAGTTGGTTCAGT
AATGATGCTCTAAAACCAAGAATATTTCACTAGCTTGATAATTAA
TATCTTAATCATCCT
TAATTCGATGTAATGAAGGGTTTGAGACCTTATAATTAATGAGTC
AAAACTAGTTGGCTT
CACAAATCCCTACAATTGTCTGAAATCCTTTAGATTTTTAAATGG
TCCACATTTCAAATT
GTTTATCACTGCAAGA
>ConsensusfromContig4198 Average coverage: 13.42
CTGCCTAAGATCTTCGAATAGCTCTGTCAATCTGTTGAACATTGA
ACCCTCGAGGTCGCT
CTCTCTGGAGGATTTCATGAGTGCACAGACGGCGCAGATGGGCG
TGGCCAGGGACACATT

GAAATGTCTGAAGGAGAATATCACCCTGTATGTGCTTGATGCTTG
CCAGAAAGTTGCTGA
GATCCGAAACGTGGAATTCGACGAAAAGAAGCACGAAGAACCG
GAACGCAAAGTCAAAAC
AGAAAAAAAAAAAAAAAAAAAAAAAAAAAAAAAAAA
>ConsensusfromContig4199 Average coverage: 11.77
TTTCCGATAAAATAATCTGTAATAGAAATTGGTGTATCAAGTGAA
CAAGCGTTATTCATC
AAATCAACATTAATTTCAGCAGGAATCATTTGAATCCTAACTTTT
GGGTCATCTGCTCCT
CTTTGATGTGAAAAAAACCAGTATACTTTTGGATAACAATTTGGC
CAATGATTTTTAAAA
TATCCATTAAGATCACTAGTTTTATCGGTGTTATATACACAGGTA
TCTCGTCGAGTAGCA
CAAACATCATGAGTTTCATTACCCAAAGGAAAATAACTCCTATTC
TTTAAATCACATTCA
CCAGTCCATATTTGATTTAGGATGATATAACTCCCGAATGCATAA
TAAATATGTGGTAGC
TTTTTAATAGGCTGCACATTACTTAT
>ConsensusfromContig4200 Average coverage: 8.54
CCACTCGTCATAAGCCTTCTCAATGTTCGGAGAGATCGGCGCAGA
ATCCTCTCAGACTGT
TTGGCCAAACAGGACGCGCCACAATACTCGGATAAATTTCTGCCC
GTGATTTTCAGAATT
GCTGATATCTTGAATCTTTTATGTCTTGACTTCTTAGGACGTAAAG
GTCCTTTGGAGTTT
TTGCACTTAATACTTGCTTTGATCAATGAACAGCGGCCTTCACAT
GAACTCTGAGGGTCA
CTTGTCACTCTGAGAAGGAGAGCGCTAGTCAATTGAGAATTGAAT
TTACTTTTTGAAGAA
CACCTGGATGTCGTGAATGTGAGTTTAATACGCTTATAAGTGTCC
ATTCCTTGCACTAAA
CTAGTACAAGTGATGTTATCAAGATCGG
>ConsensusfromContig4201 Average coverage: 8.06
GACCCTAGCACGTCACCCTTCGAGGGCGCATTCGCCCGCTTCACA
ATCACCGTGACCCCT
GACATAGAAGAACAATATGACCCCCTCACACTGAAATTTACGGG
TCCTACTGGTGCGAAA
GTCTGCAAGATGGTCGTCAGTAGGCCGCCACCAACCCTGCAATGC
TTCCAGGAGCCTGAT
CCCCCGACAGGGCTCAATACCAATTCGGCCGTTCTGAAGGTGGGG
CCTGGCATCGTCAAC

AGCTTCGCCAA
>ConsensusfromContig4202 Average coverage: 19.46
TTTCCGAACGGGCAACTTTTCTTCAAAAACAGAAATTCTATCGAT
GATGTCAACTGTGAA
AAATGCGTCATAGTTCATAACAATTATGTCGTATCTAAATCCGCG
AAGATATATCGAAAA
GAGATGGGTATGTGGGTATACGACAAAAATGAGTACTATTCAAA
CGAGAACAGCAAGTAT
TTGATAGTTCAGCGTGAATTTTATGGGAAGAATATGAACGAAAAA
ATTATTGAAGCATTA
CACATCTCGCGAAAGATGAATAGGATTCTGATTCTGCCTAAAAA
ATGGTATGGAAAACTA
AATAAATTTCCTTATGAATTCATGAAACGATTGGATCGACAATAC
CATGATCTCTACAGA
GAAAGCTCATTTTTTATGAACCAGCTAGTACCGAACTCAATAAAA
TACGGTGGAACGTAC
AATATCACAATTCATCTCGAGTGGGGATCAAATAATGTGATCCT
AGAAACACAAAAGGAA
AAACGCGACACAACATTTATGAAAATGTCTGAAGATGAATCACT
CAAGAGATACCCCGTT
GTTCGAATGACTGTTTT
>ConsensusfromContig4203 Average coverage: 27.99
CTTGGATTTGATTGGACGTCTTCACACCACGTGGCTCTATCTCCGT
ATTCACAGCCTTCA
ATGCCGGTGTGTACTGATGCGCAATATCCGCAACAACTATCCGCA
GTGCTCTCTCCGTAG
CAATCACGAGCCGGTTTGCCATCGCACCAAGATGATTTGTCCCCA
TACAGACATTCTGGT
GTTTCTGGCCTTGATAACTCATTGCAGGTGGCGCAGCACGTCTCG
TAATATTGATCCGTG
TAACACTTCCAGCTGTCTACAACCAGGCTGTTACAGGACAAGCCG
TAATATGTCACGTGA
>ConsensusfromContig4204 Average coverage: 7.28
CCGTCGTAGCACTTTGCAGTTGTTCTTTGTACCATACAATCAAGTT
TTCTGAAATCCTTG
CAGTGATCAGGATTGCTTGTACAGAAATTTTGATGAACTGCCCAT
TTTGTAGGAGCCGGT
AATACTGGGATTTTACGATTTGGTCCATCTTTTCCATCCACTCCAG
CAGCAATTTTATTC
TGAATGGAATGTGGGTCAGCTTCATTCACGACATCAGAAACAACG
ACGGTCGACCCATTC
TTGTCTTGACTATCAGAAACCAATACACCCGATCCAGTCTTCTCA

TGATTTTCAGGAATG
ACTTCACCCGACTTTATCTTCTTTTTGCATTTATGAACGATCGCAC
TGGAATTCTTTTTG
CTCGATTCTGTCATACCT
>ConsensusfromContig4205 Average coverage: 15.24
GCAATGTGACTGGAGTTCAGACGTGTGCTCTTCCGCTCGGCACAA
TTCATAAATTCATTG
TCTAGAGTAGCCCCTGAGATTGTTAGAAATAAATGCAAATTCGA
CACAAAGAAAAAGAGA
TGATCCGACCTGCTCGATAGCAGTTCGATGAGGACAGCCTTCTTT
CAGTATCTACACGTA
ATTTATCAATATGGTTGGCTTGATAATATAACAAAATATTTGGTA
TAATACTTATTAAAG
ATTTTGTATGATTGGCATTTCGCAAGACCTTACTTCTAGATAACA
TTAGCATGAACATGT
GTCAATATCATGATATTGAAACAATACAGTCTCTTATCACGGTTA
GATTTTATGTGGATA
TTTATGTGATTTATGAATTTGTGTTCATTGAATCATTC
>ConsensusfromContig4206 Average coverage: 6.21
CTGTTCTTGATCTCATCAACGGACATCTACAATTTCGTGACCACG
CGTTATCAAAGAATA
CTGTCTGAAGAAGTATGTCCACAGCTTCCTCGAAATATTGAGTGC
AGCGAATTCGGGTTT
TGTGAAATATTTAGGTTCTCAAGTGGAATTTCGAGCCAACCAATT
TTGATTAATGTATCC
GACGATGGGGTTCTCGATATTGTGACCTCATCAGATGACGGAAG
GGTCACGGCCATAGAT
GGAATCTCGTATGACGTCATTTGGACTCGGCAACTAGAAGGCGCG
ACGTGCAGGTATTTC
GATTATTACAA
>ConsensusfromContig4207 Average coverage: 7.96
GAGTTACGGCGTTACAAATAATTCATATAATGGATCGAGCCGTTC
ATCTAATCCTGTTTT
GCGTAACTTGGAACAAGTAACGAATTCACCCCGATCTTCTTCTGA
TCGGTTAAACCAATC
ATCTAACATGAATCGCGTTCCCGGATCTATCAATCAAAATCAAAG
CTCTATTTTCGGTGC
GAATCCATTTGCTGAGCCTATTTTGGAATCAGATAGCACATACGA
AGATCCTTATGAATT
TTCTCTACCAGTTGAAATAAATCAAAATGAGGAGAACCAGAATAG
CTTTATCAGAGTACC
TGTGAGAGAATCCGTTGCATTTGAAGTTGAATTAAATCCACCGA

ATCAAAGAACTGAGAG
GTTCACTAGAGCTAACATAAATAGACAATCAAAAAACAAGAATA
CAAATAATAGAAAAAA
TCAAGCTGCCAAAAACATATGCTCAAATTCTTCGCAAAATTCCCA
GCCAGGGAGTTCTAT
TCAGCAAATAGCTCCTGAAAGTAGCCATGTCTCAAGTAGCTCGAC
CACTAATCACTGTTC
TGCTATTGTCAAAAAAG
>ConsensusfromContig4208 Average coverage: 36.63
GAATGCTGCTAACGGGGATAGTGGATAATCGAAATGAATGATT
TACATCACATTGTAAA
ATAGCGAAATTGTAAAGTCACTTGCGAATTATCATGAATTACAA
CATCGACAAATAGAGC
TCCAAAATCAGTAAGAGCTGGGAGTGAGTGCGTTGAGGTCCTTG
TTCGAAAATCTAGCAA
AATTGCTAGCTCTGCTCATGAACGTGGGCTGCTTTTTGGACCAGG
ATGCAGCAGCGTCAA
CTTGGTCGCGACCGTTCTCCTCTTCCATGGCCGAAATGAAACGTT
TTTGTGGGCGTAGCA
CCCCTGCAAGGCCGAAGCCGGTTTGTGGGGGCGAAGACTCCTTTT
GTACAGATAATACCG
AGCCGCCCTCAATGGCGTCGGAGAAGGTGCGGTTCTCGTCGACTG
GTGTCGCCCCCCGAG
AGTAGAAGCCCCGCATGAACCGCGTGGGGCTGTCTCTAGGGGAT
ACGATGCTGCTGTGTG
CGGGGGTGACAGGCATGGATTGAATCATTCGCGCCGCATTCTCGA
AGCTCTGGATCTCCA
TGTCTTCGCTGGCCGACGGTCCTGAATAATCGGCCACTTGCGAGA
GGCGTCGGCTGAAAC
GAGGCGAACTGACTACCGACTCGTACAGCCGGTTTTTCATGACTT
GTGCTGAGATCTGGC
GTGACTTGGC
>ConsensusfromContig4209 Average coverage: 7.36
TGGATGTAATTGCTAAGATAACTGTTATTTTTCGAGAAGGCGAAT
CTCGTTCTTTCGAAT
TTTGGGCGAAATTCGCAGCGCCATCTAAAGACTCGAATGAGGAGA
TTATTGAATATTCGG
ATTTGATTTCGAAGTGTGATAAAAATATGCTGTAATTCTTTCGAC
CATCGGTCAGTCTAA
TGTCGAATGTTTTGTCGGAACTTTGACCCCAATTTGGAACTCGGA
AGGATAAGTTGAACT
GTGGAGGACAGAGGATGATGGAATCAATCCGGAACAACTGCATC

CCCTCCAGTCGACTGT
TGATGGTGACGCCCAAATAGGCGGGGCAATCGCTCATTCGACCTG
ATATCCGGATGTACC
AAATGCGACCGCCCGATGCCAAGATCG
>ConsensusfromContig4210 Average coverage: 19.25
GATGACGTCATGGCATGGAAGCATTCTTTTGACAATCTCATGTCA
ACGCCCATTGGACAA
AGAACATTCCGTGAATTTCTCAAATCCGAATACAGCGAAGAGAAC
ATGCTTTTCTGGCTG
GCCTGCGAAAAATTCAAGGCGATAGATGGCTCGAACGCTGTTGA
AGAAATTGCCAGAACT
ATTTATGACGATTATATATCTGTTCTTTCACCAAAAGAGGTCAGTT
TAGATTCTCGTGTC
CGAGAGGTCATCAACCAAAACATGGTGTACCCGACCAAAGAAAC
TTTTGACGAGGCCCAG
CTCCAAATATATACGCTCATGCATCGAGATTCCTACCCGAGGTTT
CTCAACTCACAGTTT
GTGAAGAGCATCCTCAATAATTAGTTTTTTGTTTCTGATATCGAAT

>ConsensusfromContig4211 Average coverage: 15.47
TCGTTCACCATAATTAAAAAGCATTTTATCCGAAAATCATTCAAT
CCCAAATAAGATTAT
CTCATTTCATGATTTTGAGGCTGTTCACTTACGTTTGATTCAGTTT
GGATTGTAACAACA
TTCTCAATCGATCATATCATTAAATATTAATCATGAACAAGGGTT
ATATTTCAGAGAAAT
GATGAAAACAATTTTAATTTTAGACTGAGCCAGTTTGAAAAAATG
TTCTTTAATTATCAA
CATGAACAAATGAAAATTATTAATATAAAATTCACTCAACGTTAT
CGTACAAAT
>ConsensusfromContig4212 Average coverage: 12.07
TCTATGATGGAAAGACTTTTCAATTCGGTTGAAACTTGAAAACTT
TCACATATCTCGATA
ACACGCTTTGCCGCGTTTCTTATTGTGTTCCATGCAAATGTTACGT
CATCCTCATTTGCA
TTTTGGGCGCAAATAACAAATCGTATGACGTAATCGTCGTTAATG
AGTGCCGGGACCACG
TGGATTTTCGCGCCTTTATTGATAAGCCTCAACAACACCTGTGTCT
TCATGTTCGAATTC
TGTAGACCAAAGCAAACAAGTCCCGTCGAGACTTCTCCGAAGACT
TCGAAACGATTATCT
TGTCGAATGAGTTTCTCCATCATTTTAGCTAGCCGCATATGCTCT

CTGATATAGAACCGG
ATCCCGTTGCCTCCAAATGTCCGGAGTACAAACCAGATTTT
>ConsensusfromContig4213 Average coverage: 14.89
CTGAATTCTTCAATAACCCCGCCCAATTTCTCAGGTGATCCCGAA
AATAACGCTACCTCT
ACGTATTAACGCCCTCCCCTAGTGGCTTCGGCGCGTTTTGGCA
TCAGTCACTATTCGA
CTCCCCTGTGAGCCGCCTGTACCTGGCTTATCTCTTTGGCTTGTGA
TTCTCCTATGATCC
TATTATATCTTTGGGCATCTTCTCCCTGTTCGAAACCCTGGCCCC
TTTTCGTGTTCCCC
CAATACGATATCCCGCTGACTTCCGACGCAATCTTGCTCTTCCAA
CTCATTCGGCGTTTC
TTTGTTTATACAATTGTTCACTCATTAGCCTATTTCCTTAGTTTCA
AACAGATTCCCACC
TGGTTTCCGCTGTACTTCGGAATTTCCAGCTGTTATTTGCAAGATT
ACGGGCTTAATTAT
TTACTTCGAACATTTGTTCATTTAATGATCTTGATTCTTTGATTTG
TAGTAATTTGTGAT
ACAAACCCTA
>ConsensusfromContig4214 Average coverage: 24.35
TGGTCCCTATATTTCGGACATTTTTCAATGTACAGTTGGTGAAT
TGAGCACAATTTTGG
TTGATAGATCTCAATATATATAATATTTATTTGTTGTTACCGGATG
TTCTCGATTGTTTT
CCTCAACACAATTATGTTATTTTTCGCGTAGCACATTCATAGACGA
TTTGGTTTATCTTT
TATTATGATAATAATTATTTCGATTCGCGCAAATATTCACGATGA
CATGGTGGCACAATA
TACCTCATTGCTTACAGATTACATTCCGTTAATATTCGATGAATT
ATATCATCCGTACGG
TGATTTTGTTGTTCTTCATAATCTTATTTATTCGATTTGTGTGTTC
ACCGTCATTCCAAT
TCTTTCGTTTCTCAAACGAATATTTTGGTGATCGATGTAATTCGTA
GAAAATTGATATTT
TTCATAATCAAAATAGAAAACTTATCGAACAGTTTTTCGGTATTA
TATGAATCGATTTGC
ACAAGTTCTGGCATTATTCAACATGAATTTGAAAAAATTGTTCCC
TTTGTGCATTCCAAA
AAAACGCG
>ConsensusfromContig4215 Average coverage: 10.05
GGAAAACTCGCCGCTCAGTACATGTCAGGAATACTTGAATATATC

GGATTCAGCGTACAT
GAGTGCGGTGATGTACCTTCTTACTTGGGAGAAGAAGTATGCATG
GTCCTGCCCAATCAT
CAATGTATGCTGGATACTGCGGTGTTGTGCATGGCAATGGCATC
ACATAAGAAGCACAGC
CTGGATATCAACGCGTGGATAATATGGGATAAAATGATGATGAC
TCCCGCGGGATGACG
GCCTTTGACCACGCATTCTTTCTCAAGATTGGTGGTCAAAAAAAC
AAAAGAGAATGAGAT
GAAAAGATTGACAGAGTTTTCAAGACCAG
>ConsensusfromContig4216 Average coverage: 90.09
TGAATTGTCTGTTGAGGCGGCCAAACTCAATTCTATAGGTGTTAT
GAACCAAGTGCCATA
TGAGAAACTGGTAAAAGTTTTATCTATTCTACAAATTGCTATCAT
CGGTGGGTCTAATGC
AGCATCTTCCCATATTGACTCCGACGAGAGCGAACAAGTATGGCG
CGATAATGTTATGAG
GCAAGCGACGGGTTGTATCGATGCATCTCTGACAGCACTATCGAT
AATGACCTCATCAAA
TATGCCTAAAGAAGTCTTTATCGAAGATGTGATAGATTTGCTAAC
TTCATCCACGAAAAC
CTTACTTAGCCGGGTTGTATATCCAGAATATGATCCTGTTTATCG
GCTTACTCAAGATAA
AGTAGAAAGCAAGAAACACAAGAGCCGTGCTGGCATGGCGAAAA
GTAGACTTGCTTCGTC
GTTTCTTCTCAAAATTGTTAACATCGTTTCTCAAATGGCAGATCTT
GTTTGTGTCCAAGA
ATTGACGGATACAATAATTTTAAAAATGTCATCACTAACTGTTACT
CCTTTTTTTGTCTC
AAACGTCAGCGAGCTGCAACTTAGCGCTTTAAAACTTGTTACCAG
TATATTCGCTCGCTA
TAGTAATCACCGGCAACTCATTCTCGAAGATATTTTAACTTCCAT
AACTAGACTACCCTC
CAGCAAAAAGAATCTCAGGACTTATCGGTTGAATGCAAACGAAA
ACATTCAAATGGTATC
TGCACTGGCCCTCCAGCTCATCCAGTGTGTGGTCAAACTGCCATG
CCCCGATGATCGACA
TTCTCCCGAGTCAAATTCTAACAGGGACGGCACAAACACTTCGGA
TCGTAATCAGGTTGA
TGACGAAGTTACAATTCTGACGTCCTACGATACTGCATTGCGTAC
TGCACATAATTTTCT
CGGAGTCTTCTTGCAAAAGTGTACAGCGAAGGGAGAAGATGATTA

TCGCCAGCTTTTCGA
GAATTTTGTCTATGACATGCTGGCGACGGTGAACAAACCGGAGT
GGCCCGCAGCCGAGGC
AATTCTCACATTTCTTGGCAACCTTCTTATGCAACAATTCAGCTC
AAAATCGGTGGATAT
GTCCATGCGACTAGCATCCCTTGACTATATCGGCATCGTTGCTGC
TCGACTAAGAAAAGA
TGCAGTCTCAAGTACACTGAATGAAGAAACCGTTGATGAGATTA
TACAATTGATAAAAGA
GGATCCTCAGAGTCAGGAAAAACTTAGCGAGACAACTGATCGTG
TAAAACTGCTTCAGAC
ATCACTTTTGGACTATTTGGCGCACAATGTACCAAGCGATCCATC
GTTATTGTTTGCAAG
AAACTTTTTGATCGCGCAATGGTACCGTGACTGTTCAACGGAATT
TGATGCGA
>ConsensusfromContig4217 Average coverage: 8.25
ACTAGATCTCTCATTCAAATAACATAAAGATATACAAAAGTTTCA
AAACGAATAAATATT
TGCGATAACTGCAAGTAGCGTTCAAAATCGAAAATGGAATGGGA
ACAGTGTGTTTCATAA
TTATCCCCAAAAAAGTTGGAAATATCGCATCAAATGAAAGAGTA
AAAAGCATGAAAAACG
GAGCGAAACTCATGATCAAGAAAAAATATTAGATGGTAAGAAAA
TCGTATTATTCGAATG
ATTTTTCCAATCGCGTGGTATTCT
>ConsensusfromContig4218 Average coverage: 27.80
ATTTTCGACATGCTCAGACATGCAATAAATTAGTTTTTGGTTTTA
TAAAATTGATAAACT
TCGAATTCGCATGATGAATAGCAGTAAAATCTTAGCAGTTTGTTT
CGATGAGCTCGAATG
AATTAAAACATTATAAATCAATTTTGAAACTCCATTTTGAATGAC
TGAATCTTTAAAATA
GATTAAAATACAGAATAAAATGCATGCTAGATCTATCCATGTTCA
TCTGATTCTTTGTTA
TAGGGGGCCATTCGGGAAGGCCTCGTTTCCCTGATCGATCTGTGG
GGGCGGTTTTCCGGT
CCGGCGGCCCATGAGTCGTACGGTCCCGCCGCAATTCTGAATCGA
CTCAAT
>ConsensusfromContig4219 Average coverage: 10.84
GATCTGAGAAAATGTGTGTTCGCATTATCAAGACATTATTCTCAA
TTAAGAATTATAACT
TATATCCCAGTTCTTTTCAAATTGCTCCGGAACATATATGCAGGA

CATATAGATGAAGTG
TATGAATTCATGAAAATAGATGCCCCAGATATAAATGATACCATT
AAACTCAACGAACTG
ATGATCTATATTTGCAAGAGAATTAATACGATCACTTATTAGGAG
CTATCGTGTGTGAAA
GAGATAAAAGAAGTGTATCTAAGTGAAATTGTAAGAAAAGACAG
GATATAAAACCAAAAA
ACGTGTTATAGACGTGTGAGCTACTAGTTTCCGCAGTTTGATTAT
AAATCATTGAAAAAA
AGACCATGTGAGTTATAATAATTTAGAAAGATACACAAAAATCCT
CAGAATGTAGTTCCA
ATTACTACTTACAACCAGAAAATACGGAACATATCAAAATCTGA
ATTGAAGTTTGGAAGA
AATGATCGAACCGCCCTTTTTCTTCATTAGGGGCTCGCATCAGAA
ATGACCCGGA
>ConsensusfromContig4220 Average coverage: 8.43
AGTACAAACGCTGGTAATCATGCCAATTCAACTTACACTGCTCGG
AAAGACATTGCGACT
TCTCACGACGGAGTAGCTTACTCCCCGTTTCCTGTTGAATTTCAA
GATACTAAGCATCGG
GAGCGGGGCGAAATTGGACGGAATATTTCCGTTAGAAAATATTCT
TCATTGGAAAATAAT
CCCTCCGCTGATCCCGTTAATAAAATTCATATCTCGACTAATCTG
TTGACTGATGATGTT
GATGTAAATGAGGATGAGCAATTTGAAGAATTTCCATCGCCGCTT
ATACGTCCGGCAACA
ACCGAGAAGCATTTAAGAAATTCTGCTCGCTTGTTGCAATCGGCC
GTCGTGTCTACTAAT
TGCGATCGAAACGGAATTTTTTCGTCGGATAGAGTATCTGCTCCA
TCTATCGATAACGAA
ACTATAGATGG
>ConsensusfromContig4221 Average coverage: 11.20
TCAGACGTGTGCTCTTCCGATCTCTTAATTCTGTCAATCAAATGA
ATGGCAGCGTATTCC
GTTGAATGTTTCTTCCGAAATCCATATTGGAATGATGTAAGGAGA
GCATTTGATTCAAGA
TAGTCGGAAATTTGTTGAAACATAACTTTTTCAAAAATTTTGAAA
ATGGCAGTGAGTATT
GAGACCGGACGGTAATTTTCTATACAGCGTTTATTGGATTTTTTA
TGGATAGGAATAATT
TTTCCGATTTTCATCATATCAGGAAATGCTGATTCCGTCAAACAC
TGATTTATAAGTAAT

GTTAAATGTGGAATAATATCAGTGGAAATGTATTTTAAATGCATA
ATCGAAATGTTATCA
TGGCCACAGCTCGATTTATTACATAATGCGTCAAGGATAGTAGAA
ATATTCGATTCATTG
ATTTGATCGAAATGAAACTTGCATGTA
>ConsensusfromContig4222 Average coverage: 8.08
GCACTACCACTGTTTTGAAACACTTTTCGGAAAGATTAGTTGAAT
TTTGATTTCAGTATC
CAAATGGAACATATTATTATGAGAGCTGGAGAAGTGTGCGTGATT
GGTTTACTTATTTTG
GGACTCATGGATGGTGCAAGGGCTTGGACATTCCTATGTAGTGGC
CATGCGTGTTATTTG
GTCGGAAATAACGGATTTCAATGTAGTCCTTACGGTTGTTGTGAT
TGCAAAAAGGCCACT
GCCGAGGCTGAATGGTTCGGTGACTCATGCGATAAACTAAAGTG
CAAAAGCGCTGTTGAA
CCTGCAAATGCTTTCAAGAAATGCTACATGATTGGCACCAAAGA
CGCATGCAAGTACGGC
AGATGTTTATGTCGTGATGACTGGTGTGGCGAAAAATGCGATATA
AAGTTGTCCAGCGGA
TGTACTAATATAACGGTCAGTAATCCTGATACAATTCAGGTGACA
TATTCTGACGAAAGT
TCAGCTCCACACTGCATTGGGATTGATGCAACACTTAGTTGCAAA
AATGGAGAAAACCCT
GAAGGAATAACAGCATACACCTGTGAATATGCTGAAAATATGGA
AGGTGTTTGGAAAACA
GGCTCTCACTTCGTGGAAAACCTAGTATCAATCGATTGCAATGGG
AAAAATATCA
>ConsensusfromContig4223 Average coverage: 14.18
ATGAATTTTCGAGTGCCTATAAATATAGACCGTTCTTGGAGATGC
TTTGAGAAGATCAAT
TAATAATGTTTGTCTAAAATTAATTCTCATTTTTTCAATTTGTTTA
CAACTCGTTAACGG
AAGAGTCATAATGATGATAGGGTGACATCAACACGTTTTATTAGA
ATCCTCCATTTTTTT
CATTTTTTTCTCTTTTCTCTCTTTTCTCTCTTGGTTTAGTTGATCAA
ATATCTCCAAACA
ATTAGTCACAAATTGCCAACAAAATACAATACACATAGTACACC
AACAAAAACAAATCAG
CCCATCAATCTCTTGAGTGTTCATGTTGAACTGACAAAATTTTAT
ATACACTGATCG
>ConsensusfromContig4224 Average coverage: 10.87

AGGTCTGAGTTCTGGGATGGATAATCTGGGTGGTTTAAAATGGGA
TTTGACCCTCGCTTT
GGTCGGTGCGTGGATAATCGCGTTTTTCTGTATGATAAAAGGCAT
AAAATCTTCAGGAAA
GGTGGTCTACTTCACGGCCCTCTTCCCCTACGTTGTTCTTGTCATT
CTTCTATTCCGCGG
AGTGACTCTGCCAAATGCCATCGAAGGGATCAAGTTTTATATAAT
TCCAGATTTCAGTCG
ACTAGCAGATGCAGGGGTCTGGAACGATGCAGCTGTTCAGATTTT
CTTCTCTCTTTCCGC
TGGTTGGGGTGGGCTCATCGCACTCGCCAGCTACAATCGTTTTCA
TAACAACGCCAAAAG
AGATACTTTGATTGTTGCTTTTGCCAACTGCGGAACGAGTGTCTT
CGCCGGTTTCGTCAT
CTTCAGTTTTCTCGGCTTCATGGCAGGCGAACTCAACACAAAGGT
GGAGGACGTCGTCGA
CAGTGG
>ConsensusfromContig4225 Average coverage: 11.41
GCGGTTACGAGGCTCCTGTGAAGTTGATTAAGTCGAAAGATGAA
GTTGACCAAAACGAAA
ATATAATCGAAAATTTGGAGAATAAAATATCTTGCGAAATAGGA
ATGTCAACTTTGAGAG
GTGTCATAAAGAATGAATTTCTTCGATATCGAAATTTTGTTTCTC
AAGATACTTCAAGTA
TGAATAGTTCGTGCCATAACTCCAGTGATGAGCTTGATGTGCCAT
ACGATTTATACGCAT
CCATCAAAAGTCTTTCTATCAAACGCAATGATATTCCTCGAGAAT
ATGCACCTCCACTGC
CCGATACACCCAGAAGAACATCCAAGATCGGAAGAGCACAC
>ConsensusfromContig4226 Average coverage: 7.62
CTCCAAATGTATTGCCTCTCTCATTTCGCATCATCAGCAAAATACG
TATCATGTTATCAT
AGCATTGTTCGCCGAAAACCTTGAATATGGGAAAGCTATATTGAT
AAAAATCAGATGTGT
TCTGATCACGGATTCCAGGCTTGTTAGAGCAATATGATTTTGATT
GATATCGACCAATGA
ACTTCATCAATAGTTTCAATGAACCTCGGAAGGACGCTCTTGTGT
GTAAACAAGTATATT
CGAGTAATGCGATGAGTTTATATTGACTTATACAGTCGTTGTCAA
TTATTTCCGGACATC
ATGTCAATTATTTTGAAAAAAAATGTTTACAATACGATAATTACG
TTTGAAATTGATGCC

GCGTGGAA
>ConsensusfromContig4227 Average coverage: 25.97
CCATGCTGCTGAATTCTCTTGGTGACATGCACTCTTCGAGTCTTGT
CCATGTTCTGGTCA
CACGGTCAAATTGCCACGTGTCTCCTGAGGGGCATTGGCCCCCTG
CACCCCCACCAGCTA
AACATCCACCGAGGATGAGCATCCGATCCTTTGTCAAGAGCGTG
CCGGAGTGGAGACATC
GGGCGTGGGGATATCCCCAACCGTATGATGTCCTGTGAGGATCTT
CGAGTCGCCATTG
>ConsensusfromContig4228 Average coverage: 8.33
TCGGCAACTTGGCGTAGAATCAGTTCCTCTATAGCAACACCATCA
CCGAGACTATGCGAG
ATTCTGAACATAATATTCACAATATTATCTGATTGCGGAATCCAA
ATTATCTCCCATCGT
GGCTTCTTTTCGGAGAGTCTTCTGTTACAATATTTCTGTATAACAT
CCTCGATCTTGACT
GAAGAAGATTGCATAAGGTCTAGACAAACTTCGAAATGGTCGTT
AATGTTAAAACCCTCA
TATGGGATAAGGACCCATGTACCAAATTTCCAGATAATATTATAT
TTCATCTTTGAAACA
AGGCTCTCAGAATTCGAATCTGTTAGTAGTTTTTCCTCAATGATT
GCAACAAACTCGTCT
TTCGTTAACATTCCTTCCATTCTTAACATTGTATTTATCACTCCAG
GGTTTTCTTGTGAG
TTCTGCAAATAGCAGAGATTAAGTCCATGTAGGAATTTGGCTGAT
TTGTGAACTTTGTGG
CGTATCATGAACATTCGTAGTTTATTGAAGATGAGCACAATTAAC
AAATATGGCATCA
>ConsensusfromContig4229 Average coverage: 21.26
GATTACGACACAAACTGAACAAAAGATGACTTCCAAAAAAGTTG
ATGAATCATTGGAGAT
TGAGGCTCTCGTCTCACGAGTTCTCACCATGAATCCAAAACGTCA
AAAGGAGCTTATGCG
AGCATTGGCCAACCTTGAAAAACCCACAAATTCTGATCAGCAGGC
GTCGCTAGATGTCCT
GAAATCTGCTCTACTCTCAGAATCAACAAGCAGAGATCATTCAT
CCGCGAAGAATGGGCG
TGACCACACAAAATCGAGTAATAAT
>ConsensusfromContig4230 Average coverage: 16.59
CGTTGCCATTATACAGAGGGTGCGCCGATTTACTCTACGGTGAAC
CAAGCTCATAGACGA

ATTGCCTCTGAGCCCGGTAATTCGACTTTCCCCAGCTCTAATAAT
TATGTTGCAAATAGA
TCTCCGCAACAAGTGGAAAATGAGGTCATGAATCAACAATGCCAT
ACTCGTTCTCGTAGT
GAATCTGGGGATTCACGCCTGCATGTCAAACATAATCAATCACA
GCCCTCAGAAAACATG
TTCGAAAAACAATCACAGCCCTCAGAAAACATGTTCGA
>ConsensusfromContig4231 Average coverage: 54.22
GCCGACGTCAAAGATGTTTTGGAGAAACTCAAGACTAAGGCCGT
CACCAAAATACGGAA
TTCATTCTTCAGAAAGTCTACCAATGTAAAAAACCGATGTCCAAT
TTCCAAATTCCACAA
AATTCCATGTTAAAGCATCGGTTCTTCTATGAGTTTCTCATGTCGC
ATGATCGGATTGTC
GCCAAGGAGCTCCGTGACGAATATGTGGAGACTCTTAGTAAAGTT
CATTTCTCGTATTTC
AAGAGTTACATAACATCACTATCCAAGTTACAGCAAGAAGAAAA
TGCCGATAGAAGTGAT
TTGATGGGAGTTGAGGACACTGCAAAACGAACAATGTTCACATC
GAAGAATGCTCTCAAA
AACCGCGC
>ConsensusfromContig4232 Average coverage: 8.33
CGTAACTCTAGATATTCAAGAGAAAAACAAAGACTCATTTCAATA
AAAACTTCGAATTTC
ATACCCATTATACTACATATAATGGATCAATTATTGTTTTCGCTT
ATCAAGAACTTCGAC
ACACCTTCGGTTCAGGCAAAACTGCTCAAAGTAGATCTGGTTGTG
AATTTTTTCCTGGTG
ACCCCACTCATTGTCCTCTACCTGCATTCCGTCTCTGCCATAATTA
CAAATCTATTCGTT
CATAATCTTCATATATTTGGTGTATGGCTTTTGCTTCTCATCGGAT
TTTACATTGAAATG
GCAGCAATTTTCAGGAAAAATGATTTATCTAATATAATCGGAAAT
CTCAAAGACCCATCG
GAGAAATATATCATGAGTAAATTATATATATTTATTGTAGGGTTA
GGAAATTCGTGTCAT
TAT
>ConsensusfromContig4233 Average coverage: 40.73
GTAGATGCTGTGGTAGTTACACGGGGCATTGCGGAGGGGGTCCAG
TCCGGACTGTCCTCT
GCCGTGGCCCGCTGGAGTGGCGGAAGGGGCACTGGCCGGCGGGG
GGATTTAGCGCTCTGT

CTGTCCGGCTCATTTTCTTCTTGAATAGAATCTCCATGAATAATA
TTCTGAGAATGTATA
CCATCTAAGCTAGATATACTTTTTGATTCATCACTCTCAGATAAA
TGTGATTCAATCCTA
TATCTTCCTGATGAGGAGTCAGAATCATGATTGGGCATGAGAGAT
TGCTTTTTCAACAGT
AATAAATTAAGATAAATAGGGTCATCCTCTTCATCCGAGGATA
>ConsensusfromContig4234 Average coverage: 21.78
GGAACATTGGAATTTAAATTGTATAACATATAAATATATTTAAGG
CCCCACATTATTCAA
TTGTATTTCATAAAGTTTTTCTCATTATTCATAAGATGATATCACA
TTTTAAAATTTCAT
CCATTGCATTGTATTGTCCTATGTTCAGTATTCCCACTAAGCTTAT
TGAATCCGCCCCTA
TTTTCTCATTGAATCGAATGCCTCGCAGTTAATATAACTCGAGGG
GGCAGGTTATGTTCC
CGCTTGCAGCTCACTCCGTACGCACATGCAGCGAAGAAGGAAAG
CAGAGCGGCGACTGCA
CCGGTCCAAAAGGCGAAGCCAAAAGTCGCCAAAGGTACATCAGG
CCCCAAAACTTTGAAT
GCGAATATGAGCAATGCAGCTCCGATTTCAAGAGAAGAGAAAAT
GGCCAAGACCAAATTC
ACTGTATTTGCAGATCTGCTGCAAAACGAAACTGCAAGACTGAG
AATGAGCGTGAGGCAA
GCTAATGCCATGAGAATCTGGACTGATAGATACCATTCTGAAATT
TTGTTGAAATGCCAT
AGACCATCCTGCCACAGCCACGTGCATCCTTGATTCGGAACACAG
TATCCTAAAAGGCCA
GCATTCGCATCATTCATAATCATCCAATATGGGGCGACCATACTG
GTGATGGCCCCAGCT
GCCGATCCGAATAACAAGGCCGAGTGTATCATATTGAATTTTATA
GAAATTATTCTTCAC
TTTTTGAAAATTAATATAGAAAC
>ConsensusfromContig4235 Average coverage: 8.22
CCAGGATAGGTCTTTGCCGCGATTGTGGACGAGATTTCACACCG
CTACTTGATGATGAA
TTTACGCTGATCGTAGACACCATAGCACATGTTCCTGATGTTGGT
AAAACCGAAGACCTT
TCGTTGTTGAATTCGATGAGATTTTTGGTAGATTTCGCTTGTGTTT
GAATTGGTGGTTTT
GAACTTTCAGTTCCGAATTTCGAAGTCTCTTCGAATAACGTCATA
GCTTCATACGATCCA

TGGGATTCTGAAACGTTCTTTTTCAAAATTGTAGGAGCTGCATGTC
CGCTCAATTTTGTT
GAGATATCCAAGCAATCGGTATCTCTCAGATACACATATACGACG
TCATCACCATCGCAA
TTTGATCTTGATGGATCTAGCTTATACGTTTT
>ConsensusfromContig4236 Average coverage: 15.07
GCTGGCTGTAACTCTCTACATCTGTAACCTTTAGGGGCAAGAATC
ACCTCTCCTTTGCCG
CTCCTAGCAGCGGGTTGCATATTCATAGAAGTTGAAGCGACCGGT
TGTGCATTTGTTCTT
TTTGACACAACAAGTGAGTTTGATCTTGATGATTTGGTAATTTTTC
CAGTTGCATTTCTC
TCCTTTTGTTGATTCATTTTTTCCGAGGAGAGTCCTTTATTGATAG
TTTTTCCACTTGAT
GAAGCTTTTTGATAAGATTTATCCATGCCTTTTGTAATGATTAGC
AGAAGAAATCATGAA
AAAATGAAAAGTTGTTCCAGAATCACAGCATGGCTCTCGCTATA
CAAAATTGAGATGAAA
GAAGTATGCTTTTTGTATTTTAGATAACTATAAATTTGAAGTATTT
TTATG
>ConsensusfromContig4237 Average coverage: 10.30
CTTCTGGAGACGATCAGGATGGTTATGGATCCTCGAGGTCTTGTC
ATAGCGCTCCGGTGT
CTGGAGGCTCGGCAGTTTCATTATCTAGATCCATAGATAGCGCTA
AGGAACGAAACTTCC
TGGTGGGAACAATCGGAGGTACCAGCTCACTTCTTGGTCAAGAA
GAGCTGAACAGACATT
TACCCGAAAGCAAAGTCAACATTCTTGTAGCGACATGGAATATG
AACGCTACAATGGCAT
CCTTGGCATCTTTGAACGCAATTCTTTTCCCA
>ConsensusfromContig4238 Average coverage: 17.59
ACGAACGTTGACATCGTAAGGATTACTCAAAAAATGAGGGGAATT
ATCATTTTCATCTTC
GATAACAACAGATAACGTTGCAGTGGACGTCAATCCGCCAACGC
CAATTTCACCATCACA
CGCTTCAACATAGAGTTCATATGAGCTCTTCTCTTCACGATCAAG
CACGGAAACAACGCT
GATAAGGCCAGTATTTGTGTTTATCAGAAATTTATCCAGGTCATC
TCGAAGAGAGTAGCT
GATTTTACTCGAAATACCGTTATCAGGATCAAAGGCACTCATTTT
CGTTAGAACGGAGTT
CGGACCGTTATTCTCCCCTATGGATATCGACTCAAAATTTTCAAA

CTCTGGAGGCTGATT
ATTGACATCGATTATATCAATTCTGGCAACGATAGATTTGTATCT
CGGCGGTTGGCCAAG
ATCTGAGATTTTTATAACTAAATCATAGAAAGAATTCACATCAAA
ATCCAGTTTCGATTG
AACAACCAATC
>ConsensusfromContig4239 Average coverage: 11.91
TGTAAACTATCTGATTTCAGAACATTCATTGAAGGTTTTTCAGTG
GATTGTAAATAAGGA
ACATACTTATTCGCCGTTCGGTCATCAGATAAAATTATGCTATTA
ATGTCAATAGATCCC
AGGCCATTCGAAGCATTCGCAGACGGGTCCTCAAAATTGCCAGC
CATGGCTGAATAAAGG
CCATTCATATAGTGGAAATTCGTGAACGGAACATTGTTTTTCACC
GAAGTATCGCATATA
CTAGCATTGGCAATAGCTGTATTAAATGGAAAACCATTATCATC
>ConsensusfromContig4240 Average coverage: 11.73
CAGTATTATTATTATTGATGTCATCGTAACAATCTTCTGAAGAGA
TCTTGGATTTGACTG
TAGAACGTTGATTCAAACTGTTTTCAGAAGTTACTTTGGGCAAGA
AAACTTCTTGACATA
TCATGGAATATCTATCTTTATTGTGGGAACTGAAAATCTCGAGA
ACTTCGATATCTTGT
TACTCAGGTTTGTGATCTGCAGACCGGTGATTGGAAAAGACAAT
GATTGAAAGTAGGCCA
CTGTGGAACAGAGTCTTTTCTCATGATCCAAGTATTGAAATAGAA
TATTGCCATTCTTGA
TGTTTGTAGAGTCAATTGACGAATAATTTTCTCGGAGAAATATGA
ATGCTGGCGTAGTTT
TGATTCCAGGAAACACTCTTTTCCTAGGAGCCATTCTGATGTGTT
AGCTTTAGAGAGAGC
AAC
>ConsensusfromContig4241 Average coverage: 9.15
TCTTCCGATCTCTGGTTGTTTCGTATCTAGTGGAGTTGAGTTTAG
TATGAAGGGAAATGT
CTGTGCGATGTCAAATGCGATTGGCGAGGATTGATTGGCGGAGG
TGCGCCATAATGAATC
CGATTGGTGCTTGAAGAACGCCCTCTTGTCGGTGGGGCGGGGTTT
CCTCTGGCGTCCAAT
GGTCTGATCGGCGGAGTACTCGGTTCGCTTGTACTTGATATCTGG
TGTGTCTTTCCCAGT
CTCTTCTCTACTGGTCTTCCTTCAGACTTCGACCGGAACCGATTCG

AAACTTTCGACTTC
TGAATTCTCTCCGTGTCGCTGGTGGCGCCACCTCTGCCAAGTGTG
TTTAAAATGATCAGC
TTCC
>ConsensusfromContig4242 Average coverage: 23.51
GACGAAAAGCTTGTGTATCAACATGAATTGATATATCATGCCTTC
ATAGCTCCTCTTTCA
TTTCAATAAAGCTCACATTTGATCGAGAAGGGGCTCTAACGGTAT
GATTCTGCTTCATCC
AGCTATTGTATCGGAGATCCAATTTGTGGACACAAAAAGGATTA
TATGAAAATTAATAAA
ATTTAGCAATAACTTATCTGTATACACACAAAATATATGCATGTA
GGTATAAGTATTTAT
TTGAAAGTATATATTGAAACAATCTTATATATATCCCAAATAACC
CAAAATCAATATTTT
ATATATATTTTTAAAATTGATATCCTATATTGTTGAGGAATGAGA
CTCGAATGATTCTTT
TGATGCAATCTCGGTTGTGCCACTTTTCACATTTTATTTCCATTTT
GTTAGATGATAGAT
GGGGCTGCTATCTTCATCATCTTATTGAAGATCTCATTTTGATGTT
TTAATGTTATTTCA
TCATTTTAAACTGCTTTCTCATGTTTCTAATTAGTAGCATGGATTT
TTGTTTTGATATAT
CCTCTGATTTATTGAATTTGCGACTTCACAGAAAAGTGATTCTTCG
TAATATTTTGCGTT
CCATATATCCACGAAAAATTGTGATGTTAGACAAAAAATTATTT
CATTCAAAAAAAAAAA
>ConsensusfromContig4243 Average coverage: 11.72
GGAATTCTGAACCGTTATCAAGTGCTCATCAAGTTCAACAGCATG
ATATTGAGGGATTTG
CGTTCTTTAGTTGAACAGTTAGAAGACCTGAAACAAGAATTCTCA
GGTATGCATGACGAC
CACGTGGCCACACTATCTGCCCGCAGCACCGAACTTTCTTCGCG
AAGAGAACACAGGGAC
ACGTGCTCCAACAAAACAGAACAGCAAGATGTCAAGTTTTTTGC
GATAAAGGAGGGCTTC
AGAAAACAGAAAGCTGAGATAGCTAAAATCCTTCTGGCCATAGA
CAATATGGCTCTAAAG
TGTCAGACGTCAGAAGATGGAAACGTGGAATCGAAATCCTATAT
CACCAAATTGGACATC
GTAAAACGGTATATACTAGAGCGGGAGGATGTCATCAATAAAAT
CAGAACAGGTCAACTT

TCCAACATGGACCCTGCTTTACAGTTCCTCATCCTGCAGGCCAAA
GTGGAGAAGGAGGCA
CTCGCTGATGTCGAAACTATTCGTCGAGACATTGAGAAATAAAA
GTTATGAAACAGCAAG
AAAACGACTTGGTCATGTATTGCATATAAGATCGC
>ConsensusfromContig4244 Average coverage: 12.17
CGGTCGTGGACGGCAGCTCCATCTTTAGACCTCCTCAGCCGGTA
AAAAGGCGGGAACTGA
CTCATTTCTCGTGGAATATGGCGACTCTCCGGAACCACGCCCACC
TCCTGCCTGAACTGC
GTGTGGATGATGATTGCAATAGTATGGTTTCATCAACGGGGGAG
AGTGATAAAACCAAGT
TGTTTGCGGATAATCCTTTCATATGCAGAACACCAAGCGCAACTA
TCATCGATATCAAGG
ATGTCATGCATGCGAACGC
>ConsensusfromContig4245 Average coverage: 4.16
GAACATTCGAGCGTTCGGTACTGAAAGGAGATATTAATACATATA
TATTCTCAAAACAAT
TAGCCGAATCTCTTCTATGTGAGCGGTCTCGTGAAAAATCTAAAC
TGATAATTATTCGAC
CCGGTGTTATTGTGGGTTGCGTTGAAACACCTGAGCCTGGCCATT
GTAGTTTTGATTTTC
CAGCTGTGAGGCAATCATATGCGATCTGCACTGGGAAGCAAAAA
TTCGCCATGATGAATG
ATACACCAGCGACTTTCGTTCCTATGGACTATGTTGTGAATTCGA
TCCTCGCTTCGATGT
TTTACTACACCAAAAGCAACCTGCCTTCACCCCATGTCATGAATT
ATGCAGCAAATGGCC
AAACAACTTGGAGAGAATGGGCCCACAATACTGTGCTCGAATAT
ACTGGAAGAGAGTATA
AGTTGACGTTCATACAAAACAGATTTCTATTCTATATATATTCTG
CGTATCACCA
>ConsensusfromContig4246 Average coverage: 10.97
CCGATTTCTAAATATTGTTTTAATGAATTATGTTTTTGAACAGCT
GGGAGTGTTCAATAC
ATGGGAGATTTCATTACTGTTCATACTAACAAAAACCTGATTTTG
AAATAGGCTAACCAA
GTTTTCGGAATGCTGTTCCTCGTGCTCTCGGTGTTTGTTGCAACAG
GATTCTTTTGCTGC
AATGGAAAGCTGATGAAGTCGGCTGAAGAGCAATTGATGGATCT
TCTGTTCGAAGGCTAC
AACCCAACGGCGCGACCTGTACTCAACTCTTCCCAGACCGTCGGC

GTCACTCTACAATTC
TCTCTCCTGCAAATTCAAAACCTCAACGTACGCAGTCAAGTTCTA
TCGACAACTGGACTG
ATTATTATGAATTGGTACGACGAGCGACTAG
>ConsensusfromContig4247 Average coverage: 7.91
GCCAATCCTTGTGCAAGAATATGTGAAAATGACAGATATGAAACA
TTTGATCACACCTAC
CAGGTGGAATTGAACATGGAGTTCATGTCTGCTCCTCAACAGCAT
GACAATGATATACCG
TTCTTCACTAATGATTTGAAATGGTTAGACTGGCCAACAGAAGCT
GAATTCACAGATAAC
TTATACAACTTTACAACTTTTGGCTTCGCCGGGCAATCAGATTATA
TTTATCGGGCTTAT
CTCAGAAGCTGGTATGACACGGTTGGAACACCCTTCAAAGAGAT
GTGGGGTCAGGCTAAA
AACCACTTCATAAATCACGATGAGTATACTCTGAAATGCCGAGTA
ATCCGAGAATCTATC
AATTTTGCTGGTTACATGATGAGACCAGATCTGATCCCGTCTCTC
AAAATGATTACTGAT
TCTGAAATGAGAATGAAATGCGATTTCTTGCAGATACCAATCCTC
GTAGATCAGTGTGTG
GATAACTTGAGATACCTGAGGAATTTGTTCGACAAGGTCCATGA
TGAAAAAATCACATCA
ATTTGGGATTATTACACGATATGGAAAGTTAGGTATGAACTATCT
ACGGGGGCG
>ConsensusfromContig4248 Average coverage: 14.58
TGCGGAAATCATTCCGAAAAAACATTTGATCTCCGAAAGAAAAA
AAGAAGTGGCAGATAA
TCTTTCACGTTCCTTCAAATTGTTGAATATCGATGGAATTTATAA
ACAAACAAAGGATAA
ACAATTCATAAATCATAACAACAGCTCGAATAGGTTGCGGCCAAA
CAAAATAGTGTTTTT
ATCTCCCGAGAAGCTGGCACAGTATCGACCAGCAAATCTTGGATT
GCCTTTTTTAAGCTC
ACAAGATAAAGTCAATAACAAATCGAAGCAGCTTTTGCAAATAA
AAACAGAAAAAGAGAG
CATAGAATGTCCGAATCTTATAGATGTTGATAGCTTTTATCTGAA
AGACAGGCATACTGA
TGAGTCAAAATTTGTTAGATTGCCTAACAATGCTGAAAAATCGCA
TCGTTTACTTTCACA
TCACAAACAAAGTGTGAATGAAAA
>ConsensusfromContig4249 Average coverage: 5.06

CGGGGGAACAGATGATCTCCACAATGAACTTGAGGAGAATACAG
AGAGACCATATGTCAT
CATCCACAATATTAGAGGTGGGAGCAACAATAAGAGGGAAGAGT
CGAAGAAAGCACCAGT
TCTGAGCAGCATGCCCGCTGATCTTGCAATTTGCCTCATTGATTGT
GACTGGCTTGTTTG
AAGCTTCACCTTGCTGATAAGGGAATTCAATGATCATTTTATTGA
GTTGTCTAAGGGTGA
AATATTTTTTAACCTTGATGAAATGAGTGATGTATAAAGTCATAT
CACTGGAAACAATTC
CTTCAAATAGATCATGTGCAACACATGGTGGCAAGCCTGGTTGG
CAGACATGAAAAGTCT
CAAGTTCATTGAAAACTGAATCCATTTTAACACCTCGACTATTAC
CACCT
>ConsensusfromContig4250 Average coverage: 4.18
CGACAAGTTTGCGAGGGTTTCCATGGCGATAGATGTTGGTGATGA
CAATTTTGTAGCACA
ACCCAAAGTTCAGGTCCTGATGAGACATCATTGGTTTGAGGGAT
GGAAAGACCCAAACAT
GTCAACAACTGAACTCCTATCGCGACTCACTCTCCGAACAATTCT
CCTCCCGGCGCTTTC
TCTCCTCTACATTTTTGCTTCCTGCTCTTCTATCGTCAAGAGGAAC
ACGACCCCCGTTCA
CA
>ConsensusfromContig4251 Average coverage: 6.64
GTGCATTCTTACTCTCTCACGTATTTCTCATGAGAACAGACGCCG
TCGTTTTTCAAGAGC
CATTCATTTGTACACATTTCACTCCCGTATTCGATTATATTAAATT
GAAGACCATTTGGA
TAACCTTTGTTTCTGGTGCGTTTTTGCTTCCACTCTCCGTCATTTGC
GGACTTTCTTTTG
TTGTTTTGAGTAGAAAACACCAGCGACTACCCGCATACGATGCTC
GTCATCATATTGTTG
AAATTCGAGTTGATTCGGCGACGCAAGAGGATGTGAAAGTTGGT
AGAGAGTTGGTGTGGC
TGGTTTCTGGATCAATGGCTGCAAGAACAATTTGCTGGTTACCGA
TACAACTGTTTGTGC
TAATAGATATCAGAT
>ConsensusfromContig4252 Average coverage: 6.54
GAATGTCGCACATTCAGTACCACCACTTCAGTTGGAAAGAGTGTC
AGTATCTTTCACAAA
GGAAATCCGCCAATTTTCATTGAACATGAGTCCAAAGGATTTCAA

AATACCATAGATGAT
TGTAAAAATGATTGCACTCATACCAATTGCATTCTGGGATATAT
GAGACCGACACCAAC
AGCTGCAACTTGTATCATCAATCTGAGCATTTCACTCTCATTCAT
GACCCCTATTACCGA
CATCAATGCTCCGTGGAACACACCTACACCAGCAATCCCCAAGAC
CTCCAGACGTTCCTT
TGCAACTGTCCAACACCTTCTGAAGGAGTTCATTGCACTTCAAGA
GCAAATCGACAACTA
TTCGGATTATTGGATGAAAACGAGGATACTCTCATCAGTCCGACT
[truncated: 506,937 more chars]
